# Identification and Verification of an Alternative Polyadenylation-Related lncRNA Prognostic Signature for Glioma

**DOI:** 10.1155/2022/2164229

**Published:** 2022-09-07

**Authors:** Hui Wang, ZhiJun Jiang

**Affiliations:** Department of Pathology, The First People's Hospital of Fuyang, Hangzhou City, Zhejiang Province 31400, China

## Abstract

Due to the high mortality and modality of glioma, it was urgently needed to develop a glioma prognostic assessment system. Previous studies demonstrated that alternative polyadenylation- (APA-) related genes are important in immune response and oncogenesis. mRNA and lncRNA expression information of glioma samples were acquired from CGGA and TCGA databases, and lncRNAs associated with APA were selected through correlation analysis. The prognosis model of APA-related lncRNAs was built by the univariate Cox, random forest, and univariate Cox regression analyses. Glioma samples were assigned into high- and low-risk groups. Independence and effectiveness of the prognostic model were evaluated by Kaplan-Meier analysis, ROC curve, and Cox regression analyses. GO, KEGG enrichment, and GSEA analyses showed that the mainly involved signaling pathways were enriched in cellular immunity and immune signal transduction. We further analyzed expression differences of negative immune regulatory genes and immune cell infiltration degree between two groups. Immune checkpoints CTLA4 and LAG3 and immune suppressors TGFB, IL10, NOS3, and IDO1 and immune cell infiltration were notably upregulated in the high-risk group. The PD1/PDL1 expression was significantly correlated with risk score, showing that the prognostic model of APA-related lncRNA could effectively assess the tumor immune suppression. In conclusion, we established a risk assessment model of APA-related lncRNA in glioma, which could effectively evaluate prognosis of patients with glioma and tumor immune suppression and could provide guidance for clinical treatment.

## 1. Introduction

Glioma is a brain tumor produced by glial cells within the brain and spinal cord. It can be classified into astrocytomas, oligodendrogliomas, and ependymomas according to the morphological appearance [1]. Neurosurgical resection is a traditional treatment strategy, followed by concurrent radiation therapy and chemotherapy for a 4-8 weeks recovery phase [2–4], while the tumor recurrence near the resection area happens to the majority of patients during the recovery phase [5–7], which indicates a poor prognosis and survival rate in glioma patients.

Targeted therapy and immunotherapy of cancer cells are investigated to improve survival rate of glioma patients. Wang et al. found that anlotinib could effectively treat patients with FGFR3-TACC3 fusion and recurrent glioblastoma (GBM) [8]. Other studies showed that IDO, CTLA-4, and PD-L1 are several immune checkpoints, which can negatively modulate T-cell activation [9, 10]. Inhibiting the immune checkpoints is able to significantly improve the efficacy of tumor therapy. Zhang et al. [11] discussed a set of promising targets for glioma treatment, particularly for IDH-wild-type GBM, which can provide support to the development of biomarkers for guiding cancer targeted therapies. Despite advanced development in glioma therapy, the overall survival rate of glioma patients was still at a relatively low level, and the prognosis was not ideal. For the clinical aspect, histopathology was often used to predict prognosis of glioma patients, but due to individual differences and complexity of glioma classification, patients with the same pathology might experience different results [12, 13]. As a result, more biomarkers are needed for prognosis and treatment of glioma.

With the further analysis of clinical information and expression profiles in the public database by researchers, more and more biomarkers of glioma were excavated. For example, some research showed that the lncRNA HOXA11-AS was excavated on the basis of CGGA database mRNA microarray, which could be used as a biomarker for recognizing molecular subtypes of gliomas and a molecular target for glioma treatment [14]. Other research showed that ATP metabolism-related signatures constructed on the basis of the ATP metabolism-related gene expression could effectively evaluate the prognosis and immune microenvironment of glioma patients [15]. There is a great potential to develop prognostic risk models by using public databases; so, it is necessary to continue to excavate the genes associated with glioma prognosis. APA might control stability of mRNA, translation, and intracellular localization. The shortening of the APA structure at the end of mRNA has differential effects on oncogenes and tumor suppressors [16]. Recently, disorder of APA events in tumor cells was widely recognized as a driving factor of tumor genesis. For example, downregulation of APA-related CFlm25 gene in GBM significantly enhanced the size of tumors [17]. Increased CSTF2 facilitates cell progression in urothelial carcinoma of bladder (UCB) and could be a predictive indicator for unfavorable prognosis of UCB [18]. The enrichment of mRNA APA events in pancreatic ductal adenocarcinoma was related to the expression of oncogenes and an independent indicator for prognostic prediction of pancreatic ductal adenocarcinoma [19]. As a result, combining the data analysis to the public databases and associating APA-related genes with the prognosis of glioma was of great importance for excavating potential glioma targets and establishing effective prognostic models.

Herein, a prognostic model was established on the basis of APA-related lncRNAs in glioma from public databases. Further analysis of the correlation between risk score and negative immune regulatory genes revealed potential connection between prognostic models and immune suppression of tumors. The feature lncRNAs selected in this study were expected to become potential biomarkers for glioma patients, and the prognostic model of APA-related lncRNAs could offer guidance for glioma immune treatment.

## 2. Materials and Methods

### 2.1. Data Source

In this study, 12 APA-related genes (CPSF1, CPSF2, CPSF3, CPSF4, CSTF1, CSTF2, CSTF3, CFI, PCF11, CLP1, NUDT21, PABPN1) were obtained from the latest literature [20]. The glioma transcriptome data and clinical information were downloaded from the Chinese Glioma Genome Atlas database (CGGA; http://www.cgga.org.cn/) as a training set. The glioma transcriptome data and clinical information (including 5 normal samples and 698 tumor samples) as a certification set (TCGA-GBM_LGG) were accessed from The Cancer Genome Atlas database (TCGA; https://portal.gdc.cancer.gov/). The transcriptome data of the two datasets were annotated through Ensembl (http://uswest.ensembl.org/index.html), and finally, 4348 lncRNAs and 16,689 mRNAs were obtained from the CGGA dataset, and 8252 lncRNAs and 17,887 mRNAs were obtained from the TCGA-GBM_LGG dataset.

### 2.2. Screen for lncRNAs Related to APA

The lncRNA expression data were gained from TCGA-GBM_LGG and CGGA datasets, and the mRNA expression data of 12 known APA-related genes were standardized by log2. The lncRNAs and 12 APA-related genes in two datasets were analyzed by Pearson correlation analysis. The correlation coefficient between the lncRNAs and APA-related genes in the datasets was determined, with ∣*r* | >0.5 and *p* < 0.001 as the screening criteria to gain the lncRNAs that were related to APA.

### 2.3. The Establishment and Verification of the Prognostic Model

The samples that were followed up for less than 30 days were removed, and the *R* package survival (https://CRAN.R-project.org/package=survival) was utilized to perform univariate Cox regression analysis on APA-related lncRNAs in CGGA and TCGA-GBM_LGG datasets. *p* < 0.001 was used as the criteria to screen lncRNAs that were related to prognosis, and the screening results of the two datasets were overlapped to obtain lncRNAs related to APA and prognosis. To prevent the overfitting of the model, the *R* package randomForestSRC (https://cran.r-project.org/web/packages/randomForestSRC/index.html) was used to screen APA and prognosis-related lncRNAs in the CGGA dataset (ntree = 1000, nrep = 20) to obtain the candidate feature lncRNAs. *R* package survival was used to perform univariate Cox regression analysis on the selected candidate feature lncRNAs, and finally, the prognosis model of APA-related lncRNAs was obtained.

According to the expression level and risk parameter of each feature lncRNA, the risk score of the glioma samples in CGGA training set and TCGA-GBM_LGG certification set were calculated, and the samples were divided into two groups (high- and low-risk) according to the median of risk score. *R* package factoextra (https://cloud.r-project.org/package=factoextra/) was used to perform principal component analysis (PCA) dimensionality reduction on these two groups in the training set on the basis of the expression of 3 lncRNAs. *R* package survival was utilized to portray the survival curve, and the difference in survival between the two groups was measured by log rank. *R* package timeROC [21] was used to draw ROC curve. The AUC value of the predicted 1-year, 3-year, and 5-year OS of glioma patients was calculated [21].

### 2.4. Functional Enrichment

The *R* package limma [22] was used to analyze the differential expression of the high- and low-risk groups of the CGGA dataset, and the differentially expressed genes of the high-risk group compared with the low-risk group were excavated under the condition of logFC > 2 and *p*.adj < 0.05 [22]. In order to investigate the biological functions and signaling pathways involved in the differentially expressed genes in the high-risk group, Metascape (https://metascape.org/gp/index.html#/main/step1) was used to conduct GO and KEGG enrichment analyses. GSEA software [23] was applied to analyze differentially activated pathways between the two groups.

### 2.5. Correlation Analysis between Prognostic Model and Tumor Immunity

It was known that tumor immune suppression was closely related to tumor progression. The excavation of immune suppressive factors would provide guidance to find suitable immune checkpoint inhibitors in immune therapies. In order to predict the correlation between the prognostic model and tumor immunity in this study, genes related to tumor immune suppression were acquired from the Tracking Tumor Immunophenotype database (TIP; http://biocc.hrbmu.edu.cn/TIP/index.jsp). They were intersected with the mRNA data of the CGGA brain glioma dataset, and the immune suppressive factor genes related to brain glioma were finally obtained. The expression of these immune suppressive factors in the high- and low-risk groups of glioma was analyzed. The expression differences of immune checkpoint and immune suppression-related genes between the two groups were measured by *R* package anova (https://www.scribbr.com/statistics/anova-in-r). The ssGSEA algorithm was used to explore the immune infiltration of cells in the high- and low-risk groups of glioma by *R* package GSVA [24].

### 2.6. Evaluation of the Effectiveness of the Prognostic Model

The univariate and multivariate Cox regression analyses were conducted on risk score, gender, histology, IDH_mutation, X1p19q_deletion, and MGMTp_methy in the CGGA dataset, and nomogram was generated by using *R* package rms [25]. The calibration curve was plotted to measure the ability of the nomogram to predict the survival of patients with glioma.

### 2.7. The Statistical Analyses

All statistical analyses were performed by using *R* software (https://www.r-project.org/). The *t*-test was used to compare the significance between two groups. The log-rank test was introduced to test the significance for *K*-*M* analysis, and omnibus test was used in Cox regression analysis.

## 3. Results

### 3.1. Construction of a Prognostic Model of APA-Related lncRNAs

**Figure 1:**
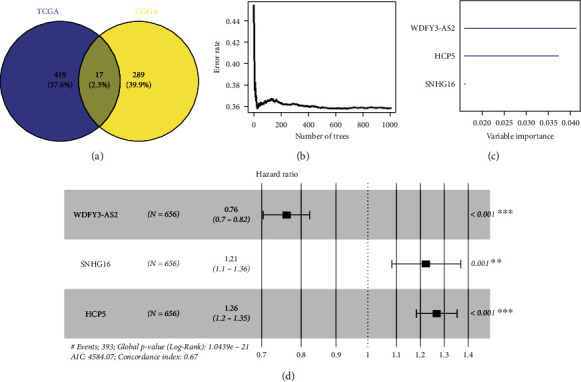
The construction of a prognostic model of APA-related lncRNAs. (a) The Venn diagram concerning the prognosis- and APA-related lncRNAs in TCGA-GBM_LGG and CGGA datasets. (b) The diagram of error rate and classification tree. (c) The importance ranking of the 3 feature lncRNAs. (d) Forest diagram of 3 feature lncRNAs,, ^∗∗^*p* < 0.01, ^∗∗∗^*p* < 0.001.

The mRNA expression of 12 APA-related genes was obtained from the CGGA and TCGA-GBM_LGG datasets, and Pearson correlation analysis was performed on the lncRNAs in the corresponding datasets. Finally, 1118 APA-related lncRNAs (Table S1) were obtained from the CGGA dataset. 500 APA-related lncRNAs were obtained from TCGA-GBM_LGG dataset (Table S2). Univariate Cox regression analysis was performed, 306 lncRNAs that were related to the prognosis were obtained from the CGGA dataset (Table S3), and 436 prognosis-related lncRNAs were obtained from TCGA-GBM_LGG dataset (Table S4). The results of the two datasets were intersected to identify 17 lncRNAs related to APA and prognosis (Figure 1(a)). In the CGGA dataset, random forest was used for feature selection of these 17 lncRNAs. In the random forest algorithm, 1000 decision trees were constructed, followed by ranking the diverse models in order of the number of decision trees involved (Figure 1(b)). Considering the relationship between the error rate and the number of trees, 3 lncRNAs were screened based on variable importance. The importance of the prognostic effect of 3 feature lncRNAs in glioma patients ranged from high to low: WDFY3-AS2, HCP5, and SNHG16 (Figure 1(c)). Afterwards, in the CGGA dataset, univariate Cox regression analysis was done on screened 3 lncRNAs, and all 3 lncRNAs were identified to be independent prognostic indicators for glioma (Figure 1(d)). An APA-related lncRNA prognostic model was finally obtained: risk score = −0.272∗WDFY3 − AS2 + 0.195∗SNHG16 + 0.231∗HCP5.

### 3.2. APA-Related lncRNA Prognostic Model Evaluation

**Figure 2:**
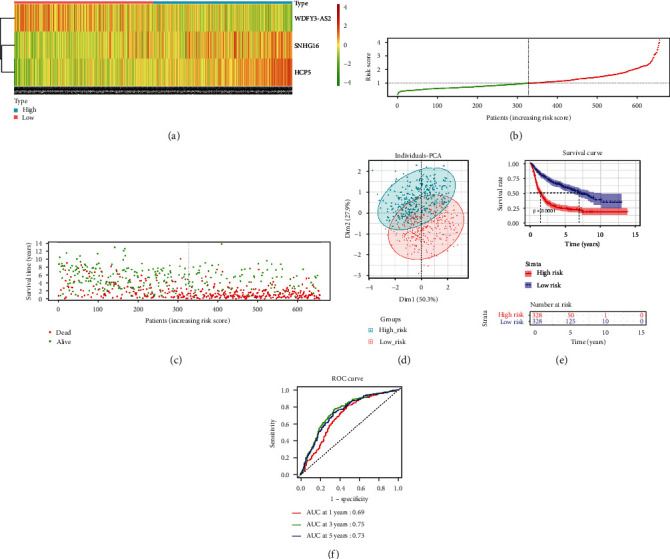
CGGA training collection evaluates the effectiveness of APA-related lncRNA prognostic models. (a) Heat map of expression levels of 3 lncRNAs in the high- and low-risk groups. (b) The risk score distribution map of patients with glioma, the green dots representing the samples in the low-risk group, and the red points representing the samples in the high-risk group. (c) Scatter plot of the survival status of patients with glioma. The green and red dots represent survival and death, respectively. (d) The PCA dimension reduction analysis is conducted on the samples of high- and low-risk groups. (e) *K*-*M* survival curve for high- and low-risk groups. (f) The ROC curve of the prognostic model to predict the overall survival rate of patients at 1, 3, and 5 years.

**Figure 3:**
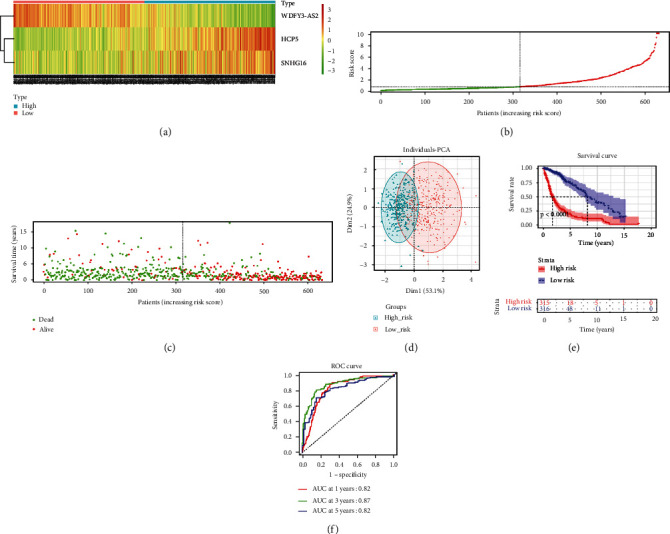
The TCGA-GBM_LGG certification set evaluates the effectiveness of the prognostic model of APA-related lncRNAs. (a) The heat map of the expression levels of 3 lncRNAs in the high- and low-risk groups. (b) The risk score distribution map of patients with glioma, the green representing the samples in the low-risk group, and the red points representing the samples in the high-risk group. (c) The scatter plot of the survival status of patients with glioma. The green and red dots refer to survival and death, respectively. (d) The PCA dimension reduction analysis of samples in the high- and low-risk groups. (e) The *K*-*M* survival curve for high- and low-risk groups. (f) The ROC curve of the prognostic model to predict the overall survival rate of patients at 1, 3, and 5 years.

In the CGGA training set, levels of 3 feature lncRNAs were remarkably different between high- and low-risk groups. The WDFY3-AS2 expression in the high-risk group was substantially downregulated, while SNHG16 and HCP5 levels in the high-risk group were notably upregulated (Figure 2(a)). Combining with risk score distribution and survival time of the high- and low-risk groups in the CGGA dataset, we noticed that, as the risk score increased, the number of deaths of patients increased and survival time declined (Figures 2(b) and 2(c)). According to the expression of the three feature lncRNAs, PCA dimensionality reduction was conducted on samples in the high- and low-risk groups, and samples in the two groups could be distinctly distinguished (Figure 2(d)). In addition, the survival curves of the CGGA dataset presented that patients with high-risk scores had a worse prognosis (Figure 2(e)). The results in TCGA-GBM_LGG certification collection were reflected in the same way (Figures 3(a)–3(e)). Finally, the ROC curve illustrated that the risk assessment model predicted the AUC values of the 1-year, 3-year, and 5-year survival time of samples in the CGGA dataset, which were 0.69, 0.75, and 0.73, respectively (Figure 2(f)). The AUC values predicted by the model for 1-year, 3-year and 5-year survival time of the samples in the TCGA-GBM_LGG dataset were 0.82, 0.87, and 0.82, respectively (Figure 3(f)). Thus, the risk assessment model on the basis of three features could predict prognosis of glioma patients.

### 3.3. Correlation Analysis between the Risk Score and Clinical Data

**Figure 4:**
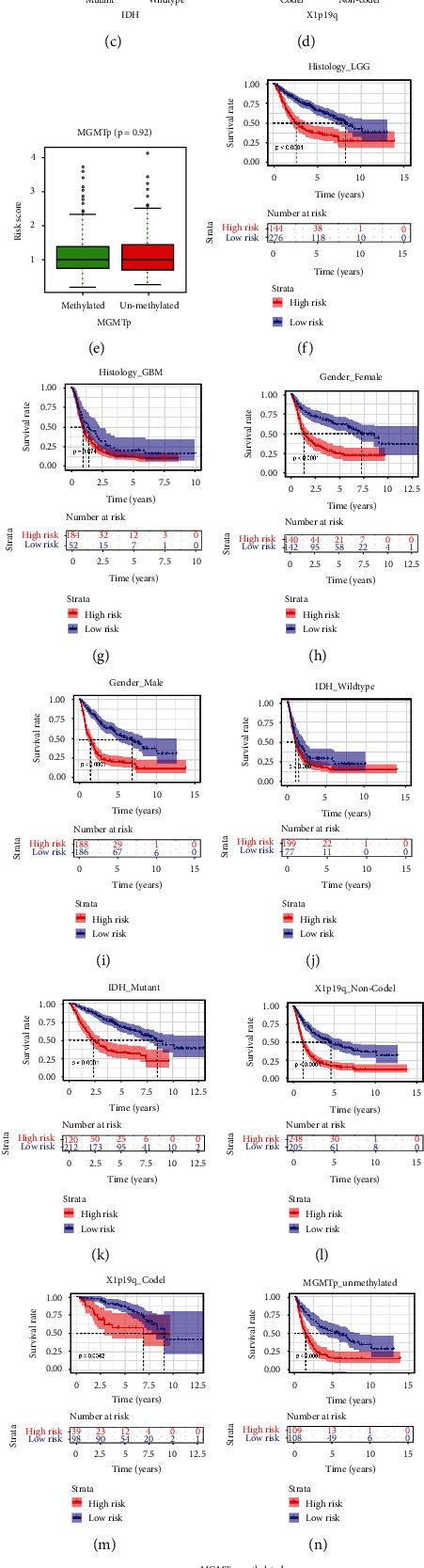
Correlation analysis between risk score and clinic pathologic features for patients with glioma. (a)–(e) Risk score box plots of clinical subgroups for various pathological features in the CGGA dataset. (f)–(o) The survival analysis of high- and low-risk groups in various clinical subgroups of the CGGA dataset.

To investigate the relationship between risk score of the prognostic model and clinical characteristics, samples in the CGGA dataset were classified by clinical factors and pathological characteristics to gain different clinical subgroups (Figures 4(a)–4(e)). GBM is the most malignant form of astrocytoma [26]. The analysis results presented that risk score of GBM group was notably higher than that of the LGG group (Figure 4(a)), while that in the male group was slightly higher than that in the female group (Figure 4(b)). Analysis results showed that the risk score of the IDH_Wild type group was clearly higher than that of the IDH_Mutant group (Figure 4(c)), and the risk score of the X1P19Q_Non-Codel group was clearly higher than that of the X1p19q_Codel group (Figure 4(d)). These results showed that mutations in isocitrate dehydrogenase and deletion of chromosomal X1p19q were closely associated with risk score, and that the risk score of the prognostic model could be utilized to evaluate pathological features illustrated above.

After the samples in each clinical subgroup were assigned into high- and low-risk groups according to the risk score, the results of KM survival analysis showed that, except for the prognostic survival rate of patients in the two groups of Histology_GBM and IDH_Wild type, the other 8 clinical subgroups had worse prognosis performance than the low-risk group (Figures 4(f)–4(o)). These results showed that the prognostic model could be utilized to measure survival rate of patients with different pathological characteristics.

### 3.4. The GSEA Enrichment Analysis

**Figure 5:**
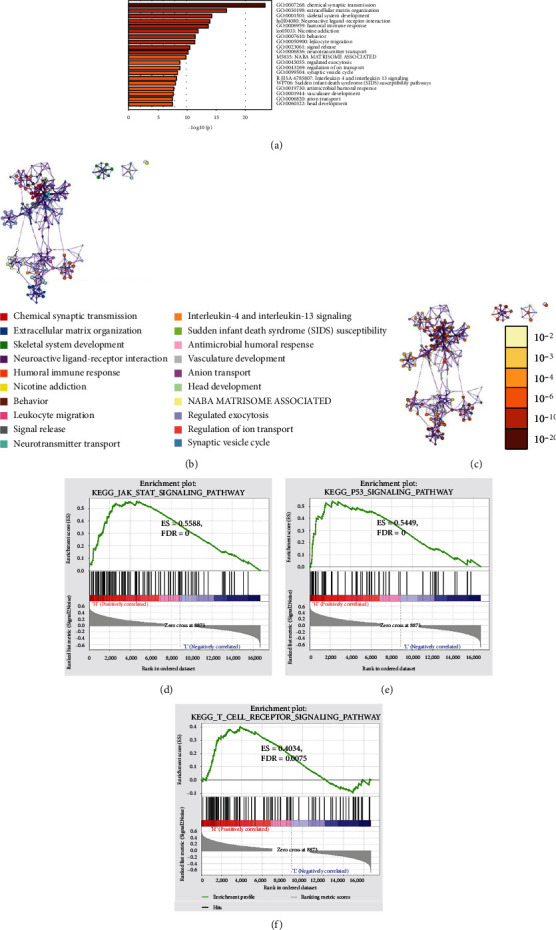
Enrichment analysis of functions in the high- and low-risk groups in CGGA dataset. (a) The top 20 pathways and *p* value distribution of the biological process enriched with differentially expressed genes in high- and low-risk groups. (b) Enrichment term network of differentially expressed genes is analyzed. The color of the node shows the function or path cluster of the node. (c) *p* value of differentially expressed genes is clustered as a network. The color of the node indicates the degree of significance. (d)–(f) Differential activation pathways gained by GSEA enrichment in the high- and low-risk group. (d) JAK-STAT signaling pathway: ES = 0.5588, FDR = 0. (e) P53 signaling pathway: ES = 0.5449, FDR = 0. (f) T cell receptor signaling pathway: ES = 0.4034, FDR = 0.0075.

Intergroup analysis was conducted on samples in the high- and low-risk groups of the CGGA dataset, and 335 differential expression genes in the high-risk group were obtained (Table S5). GO biological function enrichment, as well as KEGG analysis, was conducted on these genes, and results showed that these genes were mainly enriched in humoral immune response, leukocyte migration, interleukin-4 and interleukin-13 signaling, and biological functions and pathways related to the immune signal transduction (Figures 5(a)–5(c)). The GSEA enrichment analysis results exhibited that the two groups had significant differences in the activation of JAK-STAT signaling pathway, P53 signaling pathway, and T cell receptor signaling pathway (Figures 5(d)–5(f)). The differentially expressed genes were principally enriched in biological functions and pathways including activation, migration of immune cells, and immune signal transduction. These functions and pathways could be one of the main reasons contributing to the different prognosis in the high- and low-risk groups.

### 3.5. Correlation between Prognostic Model and Immune Suppression in Glioma

**Figure 6:**
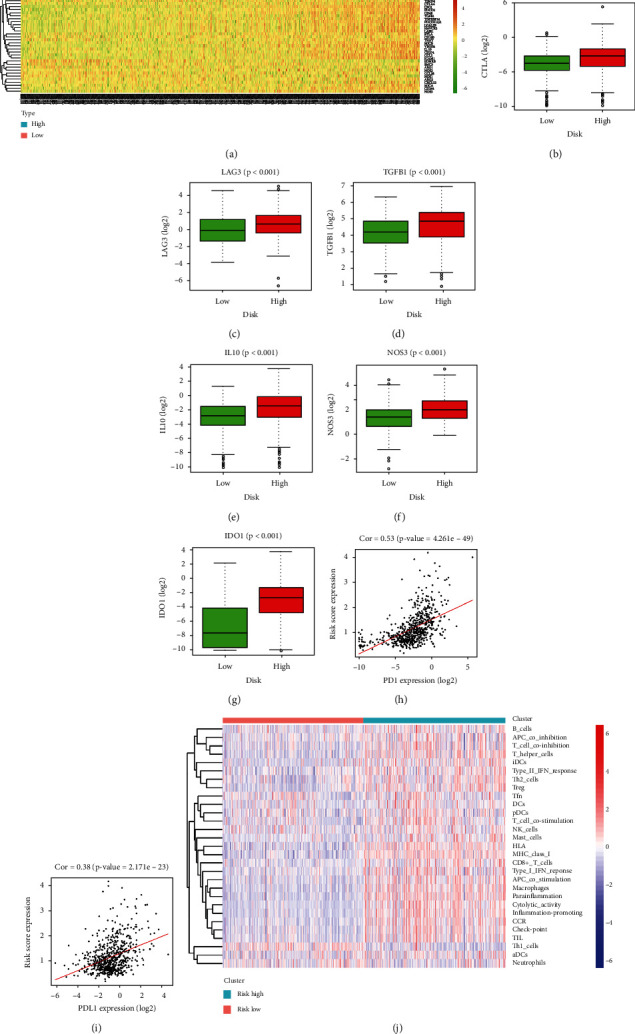
The correlation between prognosis model of APA-related lncRNAs and tumor immune suppression. (a) Heat map of negative immune regulatory gene expression in high- and low-risk groups. (b, c) The differences in the expression levels of CTLA4 (b) and LAG3 (c) in samples of high- and low-risk groups. (d)–(g) The differences in the expression levels of immune suppressive cytokines TGFB1 (d), IL10 (e), NOS3 (f), and IDO1 (g) in the high- and low-risk groups. (h, i) The correlation diagram between the risk score and PD1 (h) as well as PDL1 (i) concerning CGGA data collection samples. (j) Heat map of immune cell infiltration levels in high- and low-risk groups.

To investigate whether the prognostic model of APA-related lncRNAs could measure immune suppression in glioma, this study visualized the expression of immune suppressive genes in the high- and low-risk groups of the CGGA dataset. The result displayed that majority of the negative immune regulatory genes glioma were upregulated in the high-risk group (Figure 6(a)), and the expression of immune checkpoint CTLA4 and immune checkpoint receptor LAG3 was notably higher in the high-risk group (Figures 6(b) and 6(c)). Immune suppressive cytokines TGFB, IL10, NOS3, and IDO1 in the high-risk group were also notably upregulated (Figures 6(d)–6(g)). Pearson correlation analysis was carried out on the typical immune suppressive molecules PD1/PDL1 and the prognostic model. The results showed that risk score had a positive correlation with the expression of PD1 and PDL1 (Figures 6(h) and 6(i)). Also, ssGSEA was employed between high- and low- risk groups, where the results indicated that the infiltration of the immune cells performed a higher level in the high-risk group (Figure 6(j)). Interestingly, it seemed to conflict with the findings in Figures 6(h)–6(j), which showed a positive relationship between risk score and immunity suppression. The results above indicated that the 3-lncRNA prognostic model could be used to examine the expression levels of immune checkpoints including PD1 and PDL-1, revealing the tumor immunity status to some extent.

### 3.6. The Construction and Evaluation of Nomogram

**Figure 7:**
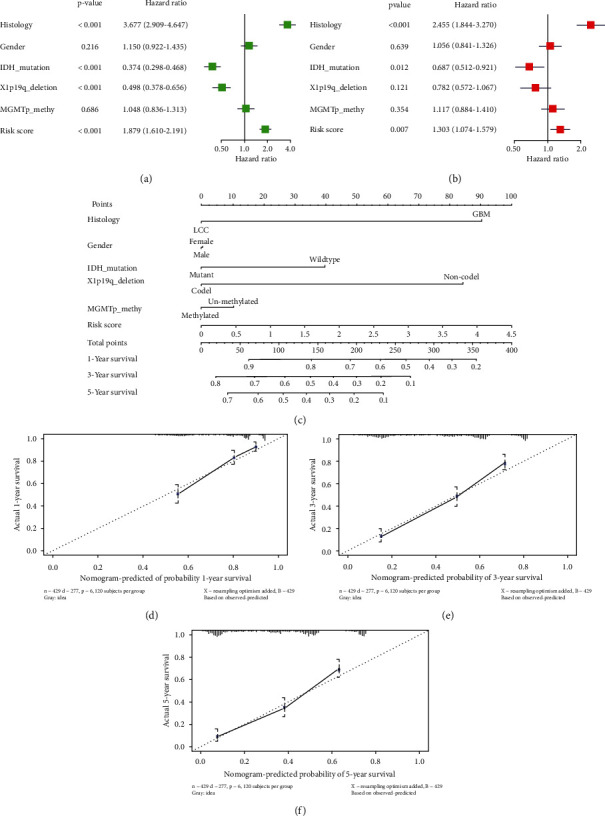
Construction and assessment of the nomogram. (a) Forest plot that combines the univariate Cox analysis of risk score and other pathological features. (b) Multivariate Cox analysis forest plot combining risk score and other pathological features. (c) A nomogram that combines the risk score of three feature lncRNAs and other clinical factors and pathological characteristics to predict the overall survival rate of patients diagnosed with glioma for 1, 3, and 5 years. (d)–(f) Calibration curve of the nomogram predicting the survival rate of patients at 1 year (d), 3 years (e), and 5 years (f).

Univariate Cox analysis of risk score and other pathological characteristics were performed in the CGGA dataset. The results presented that both histology and risk score had a substantial impact on prognosis. Among them, HR of risk score was 1.879 (*p* value =1.03*E*-15) (Figure 7(a)). The analysis results of multifactors presented that the HR of risk score was 1.303 (*p* value =0.007165) (Figure 7(b)), indicating that risk score could be used as a prognostic factor independent of clinical features. The nomogram drawn by combination of risk score, gender, histology, IDH_mutation, X1p19q_deletion, and MGMTp_methy was used to predict overall survival rate of patients with glioma at 1, 3, and 5 years (Figure 7(c)), and the corresponding calibration curve had relatively good fitting degree (Figures 7(d)–7(f)), which proved that the nomogram had relatively good predictive ability.

## 4. Discussion

RNA modifications, including 3′-terminal APA, editing of A-I, N1-methyladenosine modification (m1A), and N6-methyladenosine modification (m6A), are demonstrated to be relevant to tumor genesis and immune modulation. The studies of Zhang et al. demonstrated that the decrease of m6A RNA methylation would activate the oncogenic Wnt/PI3K-Akt pathway and thus lead to deterioration of stomach cancer [27]. Zhao et al. discovered that m1A regulatory gene in digestive tract cancer was relevant to the occurrence of cancer by modulating ErbB2 and mTOR [28].Other studies revealed that APA was relevant to NSCLC, and cleavage stimulation factor subunit 2 (CSTF2) may regulate 3′UTR length to serve as an oncogene in NSCLC [29]. Therefore, by conducting bioinformatic analyses on APA-related lncRNAs, a risk assessment model composed of 3 feature lncRNAs was finally developed. It was found by the stratified analysis of clinicopathological characteristics that the model was able to predict prognosis of patients with different pathological features. Functional enrichment and immune suppressor analysis manifested that the 3 feature lncRNAs could effectively evaluate the suppressive microenvironment of tumor immunity. Finally, Cox regression analysis of risk score and other pathological characteristics was conducted from perspective of single and multiple factors, and it was found that risk score could be utilized as a prognostic factor deemed to be independent and effective.

More and more researches show that lncRNA is an important prognostic biomarker and epigenetic regulator, playing a crucial role in progression of cancer [30–32]. In this study, three APA-related feature lncRNAs (WDFY3-AS2, HCP5, SNHG16) were dug up. WDFY3-AS2 was a prognostic factor beneficial for glioma, while remaining lncRNAs were regarded as unfavorable prognostic factors. These three lncRNAs were confirmed to be correlated with cancer development. Wu et al. found that WDFY3-AS2 could be an independent prognostic factor for glioma, and its expression was involved in synaptic transmission, glutamate receptor, and tumor necrosis factor (TNF) signaling [33]. Kong et al. pointed out that the low WDFY3-AS2 expression in the samples of esophageal tumor predicted an unfavorable prognosis, and the reexpression of this gene could inhibit the development of the esophageal tumor by inhibiting proliferation and migration [34]. However, in ovarian cancer, the function of WDFY3-AS2 was contrary to our results. Its expression was increased in tumor tissues and silencing of which could dramatically suppress progression of ovarian cancer cells [35]. So, we inferred that this gene might play different roles in different types of tumors. The HCP5 gene was principally expressed in immune system cells [36], which affects susceptibility to hepatitis C virus-associated hepatocellular carcinoma [37]. HCP5 could act as a tumor suppressor by modulating malignant phenotypes of glioma cells through binding to microRNA-139. Inhibiting the expression of HCP5 could induce the cell apoptosis and reduce the proliferation, migration, and invasion of glioma cells [38]. SNHG16, as a potential oncogenic factor in many cancers, was usually associated with the modulation of miRNA to influence the occurrence and development of tumor cells. For example, SNHG16 represses miR-216-5p level in the samples of the cervical cancer [39]. SNHG16 could inhibit the expression of miR-542-3p and upregulate the autophagy related gene 5 (ATG5) to foster proliferation, migration, invasion, and autophagy of neuroblastoma [40]. Therefore, the three distinctive lncRNAs found in this study were related to progression of tumors and might be potential biomarkers of glioma.

This study investigated the relationship between immune suppression and prognosis model of three distinctive lncRNAs and revealed that several immune-associated genes (CTLA4, LAG3, TGFB, IL10, NOS3, and IDO1) were remarkably upregulated in the high-risk group, and that PDL1/PD1 had positive correlations with risk score. Among them, CTLA4, PD1, and PDL1 were all typical immune checkpoint molecules, closely related to tumor immune suppression [41–43]. Studies showed that CTLA4 could regulate activation of T cells, and the high expression of this gene was notably associated with high levels of tumor-infiltrating lymphocytes. Also, CTLA4 can serve as a prognostic biomarker for clear cell renal cell carcinoma [44]. The mechanisms that influence infiltration and activation of immune cells in the immune microenvironment may vary from patient to patient. Referring to our findings in Figure 6(h)–6(j), the PD1/PDL1 expression showed positive associations with the risk score, while the risk score positively associated with immune cell infiltration. It was assumed as T-cell exhaustion [45], characterized as a significant upregulation of immune checkpoints and a minor change in cell infiltration. PD-1 was a membrane protein expressed by immune cells. Blocking PD-1 signal could enhance the anticancer effect of T cells [46]. Liu and other researchers found that PD-1/CTLA4 levels were relevant to the progression of various cancers and infiltration level of immune cells, and their expression levels were notably relevant to the survival rate of patients diagnosed with different cancers [42]. Thus, the prognostic model of APA-related lncRNAs could further predict immune suppression of tumor tissues by measuring the expression level of immune suppressive factors.

To sum up, the prognostic model of APA-related lncRNAs established in this research could be an alternative independent prognostic factor for glioma. The three feature lncRNAs had the potential to be prognostic predictors and therapeutic targets for glioma patients. For clinical aspect, the prognostic model of APA-related lncRNAs was suitable for samples of different pathological types and could be used to evaluate the expression of immune suppression factors such as CTLA4, PD-1, and IL1, thus predicting the immune suppression of tumor tissues. In conclusion, this prognostic feature could provide guidance for clinical treatment of glioma patients. However, certain limitations existed in this study. The prognostic model was gained on the basis of bioinformatic analysis of retrospective data; so, further clinical data are needed to support the results of this study.

## Data Availability

The datasets generated and analyzed during the current study are not publicly available but are available from the corresponding author on reasonable request.

## Supplementary Materials

Supplementary 1 Table S1: screening APA-related lncRNAs from the CGGA dataset based on Pearson analysis.

Supplementary 2 Table S2: screening APA-related lncRNAs from the TCGA dataset based on Pearson analysis.

Supplementary 3 Table S3: univariate Cox regression analysis of prognosis-related lncRNAs from the CGGA dataset.

Supplementary 4 Table S4: univariate Cox regression analysis of prognosis-related lncRNAs from the TCGA dataset.

Supplementary 5 Table S5: differentially expressed genes in the high- and low-risk groups of the CGGA dataset.
