# Supplementary material for: Identification and Verification of an Alternative Polyadenylation-Related lncRNA Prognostic Signature for Glioma
Source: Comput Math Methods Med. 2022 Sep 7;2022:2164229. doi: 10.1155/2022/2164229 (PMC11401696; doi:10.1155/2022/2164229)
Supplement: Supplementary 2 — Table S2: screening APA-related lncRNAs from the TCGA dataset based on Pearson analysis. [file 2164229.f2.pdf]

id	TCGA-06-	TCGA-06-	TCGA-06-	TCGA-06-	TCGA-06-	TCGA-28-	TCGA-06-	TCGA-28-
TOB1-AS1	108.7202	200.0188	154.22	225.6668	156.0271	52.91276	82.33448	82.19854
AC004492	24.1718	11.41233	34.19284	25.67534	32.88642	40.60554	14.83937	7.919062
AL122035	113.479	50.46462	89.37356	74.88305	138.8833	82.92836	104.2303	63.38154
NNT-AS1	1075.784	1154.376	1347.81	1377.594	2064.483	4526.885	2195.612	1835.173
LINC0100	47.12706	53.33148	40.27981	34.2474	115.6473	94.99306	154.0658	57.43855
AC004817	1.20515	0	1.778529	0.008694	1.215833	1.641391	16.6349	3.961362
LINC0112	8.406081	2.843796	9.685278	3.226706	2.503517	27.2227	138.4531	47.53939
AC005070	214.9538	313.3594	254.9988	327.2833	312.0012	58.92946	55.95193	70.31363
OIP5-AS1	15141.24	14638.4	19549.59	21542.98	8976.043	8899.082	6128.792	3838.713
AL731563	42.29312	44.75881	35.89995	31.03755	94.94305	19.78405	36.83149	26.73669
AC004908	7.285924	5.701546	8.748134	9.631153	15.77411	1.700901	13.32427	15.84435
AL157392	1198.9	1045.798	1313.646	1209.672	1055.455	295.3946	312.7948	649.687
KMT2E-AS	74.86682	76.1949	57.81434	52.42494	96.24947	168.6979	198.0905	114.8818
AC020905	2.78E-17	2.78E-17	2.78E-17	2.78E-17	2.78E-17	43.68999	18.22219	0.987127
AC079414	64.0317	84.76671	58.68532	35.32142	159.5219	19.79556	33.94387	42.58275
LEF1-AS1	3.661603	5.715343	4.366086	6.411293	3.697286	48.11674	61.44464	54.47361
LINC0127	1489.856	1319.157	1389.881	1517.699	932.3879	2579.77	1683.425	1938.174
AC068620	10.93244	11.41475	15.74841	9.650048	53.39168	9.249188	29.47959	16.83272
LINC0088	49.5412	66.67124	64.84431	74.86454	47.5771	127.9633	104.2274	123.7983
HCP5	237.8381	182.8663	186.6464	223.5379	169.443	1681.624	1134.224	660.5836
AP003486	1204.941	1274.394	1109.44	1359.406	1095.673	835.916	554.8978	548.668
AC109439	2.490291	6.658347	10.5242	9.625933	12.14837	0.163837	0.151526	0
AF106564	456.3999	587.672	552.0991	652.4194	195.0834	9.245322	14.87572	75.26635
DGCR9	565.0729	731.49	654.6186	1273.804	178.0289	40.88138	76.51429	102.0053
AL450326	97.83199	91.42637	152.4991	106.965	132.8411	64.88052	36.86885	70.31505
AC015961	32.63031	44.768	37.67377	34.23484	35.36782	9.23947	35.2165	8.909507
AC020915	163.0195	197.1589	184.9015	189.3147	158.4693	544.5566	524.8627	238.6822
AC015813	531.2734	702.9148	799.2329	650.2977	722.7194	918.7013	829.249	507.0719
LINC0131	38.7111	59.99316	88.50413	77.01776	158.3212	3.215954	8.997494	5.938234
AP006623	78.50854	123.8289	95.51778	104.814	37.86356	34.82561	36.85824	32.67891
NALT1	4.901862	15.24399	8.736574	7.505398	21.83593	13.76741	29.5113	17.8229
AP002360	47.14237	94.30053	89.39896	81.28516	37.85368	31.78797	39.74234	24.75595
AL008582	2.484663	5.707468	7.004095	2.163507	15.68151	6.213245	10.40661	7.919713
SNHG3	444.2828	396.2244	298.8127	272.752	352.2331	1121.895	1232.652	838.852
PDXDC2P	166.6447	153.3373	125.2836	118.7411	375.2335	104.0934	231.8831	124.785
AC009041	4414.057	4056.532	2613.228	2825.788	10965.99	1799.913	2933.928	5835.32
LINC0115	8.450093	8.57301	9.652754	4.297191	4.926596	31.56701	55.37955	22.77769
MANEA-D	39.83643	29.51883	28.02045	33.16286	37.78321	10.7576	13.40762	31.68876
LINC0261	13.35246	30.47804	36.82315	36.35644	19.55317	40.74132	135.8544	56.45225
L3MBTL4-	10.92856	16.18409	12.23531	19.25693	35.23595	54.14548	52.77836	47.53859
LINC0090	198.0507	287.6429	314.6148	435.2831	69.5612	445.0406	291.7624	171.3361
LINC0131	20.55646	35.25284	17.49926	16.06107	26.81073	40.76776	39.72747	23.76564
AC147651	12.1165	28.59539	13.1236	10.71311	13.44191	278.6333	252.7023	75.26995
AL117339	201.6102	80.94255	226.1199	186.102	114.6168	30.33495	29.55074	54.46745
RUSC1-AS	208.8778	168.578	230.4835	172.2094	193.7983	201.9171	211.4264	202.0356
AC096642	7.308415	12.37584	15.77633	18.17367	13.42509	15.17788	7.515871	13.86279
AL035461	10.94274	27.619	21.88657	21.40426	48.64171	112.6913	112.7641	85.17454
PWAR6	10064.36	10132.31	10340.91	11085.94	3308.949	546.9061	876.3532	679.3971
AC104083	953.8516	1713.485	1461.743	2263.155	522.9243	265.2374	208.5826	731.8932
AC116552	15.69055	13.33066	21.94955	7.507835	7.378933	4.716534	4.586933	10.89155
AC027682	9.702824	17.15008	14.01803	11.77407	11.00383	1.700837	8.955949	5.938598
AL133367	125.6041	135.2421	183.1609	146.5373	181.5855	132.6965	124.9066	167.3719
AC092119	33.85783	47.61885	36.77932	27.83026	93.71678	21.29162	51.46998	22.77486
MIR9-3HC	2484.667	1799.199	2164.585	1806.486	826.383	1994.171	1121.447	2595.785
AC005899	19.32436	11.41652	7.842999	14.9858	35.21954	12.20063	17.69188	11.88159
AL442128	37.44416	53.34742	38.55166	27.82432	28.07997	22.78481	30.97357	27.72726
AL358933	12.1336	13.32195	11.35432	11.78589	50.96781	10.71711	6.058348	16.8343

AC093010	992.4789	1147.711	773.7766	853.5252	2788.302	2756.192	1906.599	2663.131
AC020916	274.0704	413.3866	127.9036	350.8013	80.52717	194.4094	1002.93	425.8664
AC004253	12.15021	42.87181	34.18128	19.27061	35.33132	18.2295	35.2306	18.81431
LINC0059!	132.7915	106.6719	127.9524	119.7906	43.95864	75.16617	28.03251	138.6655
AL512785	3.517579	1.90798	0	1.078547	0.033272	98.47229	45.33869	19.80528
AC067750	100.2257	96.19378	90.24637	122.9942	86.56583	69.48357	167.3939	134.6891
AC092809	15.70141	10.46622	6.090262	5.373546	33.94192	21.12914	23.49194	4.947961
AC090241	59.19393	75.24354	70.09922	77.00842	59.7532	34.65129	30.8655	14.8523
AL356019	202.8458	195.2508	263.7987	260.9622	54.93567	48.34246	58.81016	36.6406
AC027307	72.50503	65.70391	39.38453	51.36537	383.5646	589.5843	368.0938	191.1427
AC124312	25.36378	29.52938	18.37687	32.07742	13.47433	0.154304	1.64753	1.976911
AC097359	9.653183	8.569922	4.344819	5.364862	12.14133	1.700251	8.984369	3.957532
AL353796	217.3459	226.6817	213.8128	284.4972	209.6531	111.6218	85.30495	95.07292
AC020558	33.79079	22.85083	28.91421	22.47333	26.83178	25.74508	42.5825	9.899776
AC127070	203.9978	139.0549	155.1101	151.8799	61.01998	16.78361	16.34444	43.5732
AC084824	13.34697	20.94634	13.10503	18.19815	55.8835	21.24933	20.70169	19.80432
NDUFA6-	363.399	280.018	331.2567	334.7721	199.9335	298.1393	173.2856	161.4296
AL021068	72.37541	33.32495	43.8043	70.57086	6.185625	28.71736	14.85183	20.79498
LINC0150:	305.4062	201.9176	200.6713	229.9552	89.05072	307.1966	771.2512	227.7862
AL591848	252.3694	271.4457	328.6291	331.5626	288.8614	84.53316	126.3544	161.43
SLC9A3-A	1872.543	1287.725	1111.186	912.3497	1518.542	516.7672	472.7578	385.2541
AL391425	22.96042	23.80779	10.47149	19.26518	41.34694	3.210414	39.75336	9.899695
GDNF-AS	71.23498	49.51788	35.01329	18.21145	143.6319	15.2665	55.97037	92.10159
AC126407	3.659152	2.845517	7.034071	1.093212	8.45368	1.694113	11.9391	54.46993
AL161421	83.19512	27.6077	24.49794	15.00162	68.18655	118.6301	80.62867	36.64136
WDFY3-A	795.6387	657.1932	619.5574	846.0164	669.1126	671.8542	458.0783	578.3789
LINC0034(	59.20968	96.20037	110.4387	121.9119	17.15601	117.4289	213.8737	306.0436
AC122129	181.1122	119.0453	108.6306	85.5892	369.1162	46.89155	57.41384	48.52479
PSMB8-A!	78.52841	83.8062	91.99429	141.174	149.8691	856.6019	802.5226	448.642
TGFB2-AS	61.53157	22.84334	56.97912	58.81428	12.27744	57.38683	120.3119	203.0347
AL391069	27.7932	42.87091	27.15067	35.2926	13.48432	343.1039	290.4365	232.7404
MORF4L2	25.38407	15.22389	30.66815	14.9967	54.7224	27.26687	25.10501	32.68008
AP003469	1239.959	1395.357	998.1331	1243.901	1623.336	152.3243	141.0907	411.9961
PSMA3-A:	1348.612	1326.777	1353.949	1281.335	1358.904	3041.956	2211.636	1160.724
AC013451	0.023891	0.950278	1.780333	1.070601	0.025084	1.611788	26.89031	8.924737
AL355987	44.70962	55.23967	64.85566	48.14024	54.85875	36.24991	32.40519	39.61362
AC015540	86.9639	123.8229	87.61405	122.9954	85.35091	6.232861	6.063008	15.842
SNHG16	339.2489	283.8308	271.656	263.1187	223.0723	1183.568	1501.049	1285.518
GUSBP11	64.02562	72.38189	84.12762	62.04759	95.04371	30.32533	60.29607	38.62124
LINC0178(	32.67097	82.86817	85.01855	51.35358	76.77596	42.32935	41.24442	18.81316
AC120114	31.43144	31.42195	25.37977	36.37349	69.36992	12.26353	20.7355	62.39322
AL136964	213.7018	220.972	169.1156	311.2171	45.18733	1.691792	6.061952	1.977142
AC114811	469.6681	234.2894	272.5058	256.7161	1045.485	76.95546	76.45317	77.24689
SNHG9	111.124	148.58	119.1578	110.1809	258.2802	293.7118	429.8911	392.1923
AC015967	48.35173	91.43962	74.47948	93.04575	62.19495	4.719765	7.50944	7.919552
AL121827	147.2653	116.1984	138.4741	110.1698	32.997	10.74746	16.31238	9.899833
AC132938	21.7128	21.9115	14.87945	11.78216	13.4487	0.15924	10.44704	10.89064
AC010542	29.01985	40.00166	31.52703	38.50838	46.29648	25.73073	33.81746	29.70953
AC025162	4.885254	5.703956	4.34481	1.095427	24.1084	9.22113	7.519201	15.84377
AL731533	70.0669	115.2542	107.7997	99.46813	40.29813	12.26319	16.33874	11.88046
AC002070	61.53568	39.04406	47.31908	29.96564	39.03145	43.40982	30.75573	25.74987
AC008124	1020.214	766.7226	791.3171	695.2291	1672.016	1144.703	1271.037	1397.426
AL138960	15.76289	31.42948	27.15118	31.02038	40.18422	18.27088	13.40274	21.78481
MIR22HG	2682.544	1053.416	956.0689	1056.733	418.1157	789.0545	1547.723	468.4484
PAXBP1-A	322.397	339.0695	360.1638	349.7545	548.386	322.3749	192.4187	288.1987
AC010655	12.03557	3.794327	5.225784	4.299374	15.74098	24.02475	45.03049	10.89146
AC078909	4.882569	2.840738	3.464824	4.298242	22.89428	1.700907	6.051629	0.986743
ENTPD1-A	89.33741	63.80645	67.45991	59.9097	98.69354	94.96117	82.2919	96.06544

AP001528	15.75778	25.71161	30.67363	41.6926	18.33677	46.44707	48.18585	19.80562
AC008610	61.59303	80.01173	71.8623	51.35174	45.14617	21.28296	10.47193	13.86126
LINC0092	19.32943	11.41596	21.03219	25.65838	14.66838	115.9917	57.3973	47.53463
AL590666	53.1334	56.19476	62.22891	51.34374	28.10153	126.7068	64.77751	102.9956
AC009126	147.3404	163.8157	201.5543	208.5649	226.6757	58.91097	77.90963	76.25664
AC010186	15.74237	30.48807	27.17812	24.59886	7.396841	45.36553	193.4387	136.6743
ADGRA1-.	433.4354	434.3231	466.2235	467.3922	125.6203	67.98742	88.24818	58.42831
AL355388	16.95869	22.85202	11.34759	4.307112	72.85339	39.25406	26.56862	17.82307
AC011374	29.00582	48.58746	28.90154	33.16087	20.7795	15.21409	39.50917	20.79608
AC245052	20.5431	19.9968	17.50572	8.581996	36.4708	71.99387	57.10225	53.48267
Z83843.1	193.2082	213.3465	279.5654	158.3118	265.675	108.617	113.1708	140.6312
AC087741	48.37118	127.6311	141.9821	72.74897	154.7373	37.86976	142.4718	43.5726
AC068152	327.2207	274.2982	347.8952	531.5545	364.4219	563.2006	510.7146	521.9311
LINC0064	8353.566	6992.98	8152.667	9428.125	4108.423	640.2791	1246.176	2264.999
AC120498	14.45421	7.609492	12.27322	5.369304	10.97751	6.228265	17.71596	7.919259
AL161457	26.60966	29.51736	42.94565	44.9163	35.36414	19.70749	17.746	40.60742
AL445309	45.90495	39.04121	44.67637	68.43975	47.5538	16.77648	14.87276	31.68896
AC017002	1.170817	0.957541	0	0.005378	0.015652	4.349235	16.1083	2.971821
AC061961	130.4189	122.8602	213.8626	218.1689	13.49805	1.700874	1.647532	2.967224
LINC0191	2.414621	2.862024	0	2.142538	2.43653	5.995229	4.451605	18.83461
AL136295	15.71138	17.14329	20.16831	10.71286	13.44082	12.1854	27.78499	11.88187
AC055713	92.945	51.42101	65.70847	43.87295	108.4139	96.48837	108.6701	55.45778
AC087289	29.0066	33.33299	25.38821	19.2731	54.76107	28.77006	45.55362	28.71807
AC005288	313.9351	308.5914	318.9756	345.4716	408.2656	716.7683	452.0911	614.0366
TMPO-AS	37.45026	28.56259	25.37958	25.68846	77.86705	194.1502	121.8678	158.4615
AC023830	3.695433	12.38554	7.864293	7.500987	16.98146	7.68103	4.5773	12.87388
AC005696	1116.784	1069.615	1071.772	1224.635	365.7078	149.3166	381.7533	71.30339
AP003352	59.19525	73.33736	57.81627	38.5267	116.9144	329.5121	247.9113	338.7165
AC011899	14.51912	14.27945	13.12103	28.84983	11.02512	130.9922	357.2878	236.7087
AC005776	16.9029	14.28201	22.81683	18.17879	1.302373	19.66034	68.34095	10.89085
ANKRD10	129.2192	114.2851	140.2008	130.4969	227.8428	732.8682	290.6144	137.6595
AP000766	227.0093	240.968	247.1185	337.9695	199.9176	95.06804	120.4936	129.737
AC107027	196.7946	158.1025	205.947	177.5508	120.7111	75.4639	83.78962	201.0499
AC067852	72.47733	67.61487	98.15167	57.7749	132.7802	24.30207	57.33571	40.60246
AL117379	18.11305	15.23381	13.12182	17.11642	17.0738	13.72678	22.10685	14.85257
AC138207	15.74391	31.44108	20.14068	17.12688	23.16507	52.74297	124.2998	89.13839
AC254562	108.673	113.3416	100.7667	120.8588	78.04997	64.90479	57.38751	42.58269
AL157394	8.431525	5.708506	7.007619	5.358815	4.916624	23.9188	26.20069	4.948274
LINC0215	7.000787	0	0	2.140416	0.039319	3.200231	33.1408	33.68611
AP002490	45.86163	22.84543	31.52854	33.16567	47.50589	45.30066	45.60524	48.52665
AL160313	33.84576	34.27971	49.9569	65.22266	24.44223	10.74711	8.999014	6.928607
AC010226	19.38477	29.51768	30.65184	44.91546	52.35605	97.64141	102.4689	87.15689
AL355488	101.4642	123.8138	128.8092	79.16901	239.9905	213.8726	180.5798	121.8139
AC002456	19.2519	15.24406	5.21551	7.505363	10.99411	788.3544	210.8164	129.7431
AC107375	317.5857	327.6351	271.6286	253.5081	1158.793	161.3496	234.9774	419.9203
AC011912	107.4461	50.46499	115.6897	126.1954	52.47489	36.25997	20.72095	39.6134
BX537318	35.06719	42.85226	28.87983	54.55036	93.73559	57.15832	46.97169	140.6501
AC073254	67.59653	50.47441	35.89608	55.6219	62.1546	61.78982	55.83552	62.39324
DDX11-AS	14.52819	18.09282	16.63453	12.85143	27.97337	40.74851	67.42182	58.43305
AC007608	7.310988	11.42042	14.89085	11.77757	25.50121	13.76909	45.67806	78.2375
ZNF32-AS	49.5383	52.37821	43.78842	40.66134	108.3598	42.30648	38.29932	39.61241
AL157700	94.2077	116.1982	89.36329	162.5559	85.35867	60.4604	66.24412	141.621
AC138028	305.3691	106.6613	133.1765	270.5784	65.89908	10.75756	14.87393	67.34547
POLH-AS	30.13991	12.368	18.38529	24.59737	17.10166	63.01045	45.43951	50.51156
AC093673	549.2462	320.0244	240.0919	216.0686	234.0479	2080.197	1546.212	554.6127
AL590999	581.9801	360.9665	363.6403	209.6624	2154.417	51.42055	91.19464	125.7742
AC010973	18.12281	18.09386	4.331692	6.44357	40.04386	51.24492	86.40548	24.75612
AL133355	109.9164	129.5274	177.0278	170.0585	149.9207	66.44354	67.67279	103.9879

TSC22D1-	365.762	205.7244	257.6435	257.7645	85.3996	57.40095	69.11299	40.60178
AC010245	37.44599	41.90764	24.50327	24.61905	59.65212	34.75725	32.40894	16.83265
CYTOR	462.3134	276.2124	195.3973	249.2139	107.3332	2864.913	2592.644	590.2691
RAB11B-A	125.6143	105.7089	95.48082	93.0749	448.256	249.9843	438.4936	234.7201
AC048341	24.13435	26.67588	14.87114	9.649517	21.93949	42.31312	57.31291	25.74615
AC135050	59.18829	34.27259	22.73528	9.655681	198.3337	3.215731	14.84896	13.86166
RNF139-A	101.4395	102.8611	113.0436	91.99325	119.4369	51.38645	76.43163	106.9603
AP001453	25.27505	8.558115	11.3672	11.77833	15.85116	72.0562	92.06627	52.4913
AL158163	37.46909	38.08933	52.58005	59.88848	45.11642	7.742902	14.86029	20.79466
MIR600HC	2005.396	2142.09	2339.859	2282.428	646.0187	271.2839	274.625	652.6598
AC025917	10.92887	12.3692	31.59192	24.5928	14.67049	15.23729	8.995895	3.957528
N4BP2L2-	49.54614	51.42339	45.53895	35.31819	138.7735	76.88093	44.18861	31.68836
RAP2C-AS	90.58207	65.70635	99.01457	111.2357	142.5528	219.8329	118.9996	230.76
RUNDC3A	135.27	184.7746	223.4776	257.7488	98.7903	24.31481	45.68656	31.68805
AL121890	9.707057	10.4673	4.33417	4.30484	38.7369	1.697585	7.456317	3.957971
AC009113	266.888	355.2583	422.3829	395.7475	863.9656	269.7532	397.8238	598.1902
AC127024	50.66022	36.19133	19.24034	20.34263	43.85345	22.68815	33.73543	18.81468
AP001486	1613.029	1781.103	1894.662	2209.695	1124.953	263.7656	349.4702	434.7738
CRNDE	20.53638	7.601856	2.578502	5.375708	61.83374	423.0824	586.742	570.4651
AL512625	30.23859	36.1843	59.60801	50.26956	46.32547	18.2889	35.40261	81.21002
AC093627	60.4112	104.778	65.70444	85.56573	69.49992	67.88031	126.1153	126.7708
DLEU1	72.45298	62.85568	58.69311	56.70007	97.46012	1233.912	592.698	366.4407
AL445524	29.00486	22.8475	21.87657	14.99848	70.52	183.0566	184.2833	105.9744
SGMS1-A:	166.6635	185.7209	271.6721	242.7925	279.078	108.6232	66.24076	180.247
LINC0029	3147.529	2663.082	2730.689	2929.516	2498.436	741.1145	854.26	825.9751
AL139089	20.58103	27.61422	22.75575	22.47745	62.02533	57.33739	89.49868	30.69813
AC018809	10.89604	17.15188	13.14007	7.506344	12.20621	10.63875	7.478226	5.938951
AC004803	214.9409	235.2528	287.4441	294.1255	209.6616	162.823	123.4612	249.5741
LINC0259	106.2266	74.28494	69.20842	46.01379	120.6084	18.27351	58.90661	64.37057
AC007406	530.0579	576.234	508.2576	667.4056	838.4575	819.3379	521.1431	655.6299
AC092171	16.97191	35.23965	20.9979	10.72335	77.80024	42.25293	33.8759	24.75619
AC245884	15.75248	30.48227	24.52784	12.85752	37.72153	4.727203	58.45879	10.89041
AC138207	28.9998	26.66144	29.78362	28.8861	37.76668	58.8285	175.6457	95.07791
AGAP2-AS	115.9465	112.3804	88.4737	102.695	302.0937	1457.182	941.9712	581.3535
AP000924	2.78E-17	2.78E-17	2.78E-17	2.78E-17	2.78E-17	55.8355	67.57086	258.5074
AC112491	4.854926	8.584447	2.592081	5.353168	4.901027	61.16869	55.32852	49.52627
AL158212	1581.62	1325.822	1488.906	1574.389	1400.35	277.3097	270.226	525.8899
AC010834	61.59583	44.75368	67.47303	82.34837	60.96081	52.85034	42.71716	63.38239
AL122125	12.11979	11.41788	21.92449	13.91583	21.90988	9.203915	10.41602	7.919584
AC026271	61.59157	50.47149	39.40031	49.21361	105.9401	173.1219	130.6527	126.7678
AC124312	467.2242	261.915	552.1282	530.4863	64.68807	24.31459	23.68333	43.57284
AL138724	19.28997	15.23742	9.608316	9.643599	17.05486	48.03065	74.38787	31.69176
AL139287	172.6586	157.1515	131.4276	119.8068	229.0804	116.0902	110.1957	140.6325
RFPL3S	10.90527	13.33011	11.37131	7.508191	24.2824	9.24529	36.72586	30.70013
VIM-AS1	21.73356	17.13735	22.78851	26.72782	7.394241	24.21812	57.05693	38.62495
SMIM25	28.83996	13.33006	8.730249	12.84256	3.741178	22.79175	276.1782	69.32639
LINC0150	0.043356	1.896402	2.62005	2.149152	5.976648	23.61267	18.80435	9.903531
AC009118	70.07343	114.2984	86.74258	98.40269	96.29434	40.87205	80.85637	95.07425
RNF219-A	330.8378	305.7325	466.2315	517.6499	165.8286	33.33814	20.72898	38.62089
FP671120.	68.83751	109.5476	13.09209	22.48916	133.9384	203.1471	854.8515	101.0169
OSMR-AS	14.3967	9.527118	5.228545	6.428848	2.515057	44.58503	52.11017	25.75294
FAM13A-/	141.2868	109.5212	121.7864	105.9045	291.159	30.33435	36.88184	57.43874
AC003092	1.144574	0	0	0.003069	0.008848	1.666615	22.45824	1.97773
AC007038	37.4857	78.10897	43.78964	41.72961	86.47595	24.28902	45.59671	15.84205
AC027601	61.60956	67.61913	59.56858	49.21796	120.572	49.83531	104.1135	43.57376
DICER1-A	276.5463	447.652	290.0335	343.3446	1015.044	199	232.061	300.0822
LINC0135	150.9533	140.0022	120.903	106.9769	364.254	153.789	330.2138	180.2464
AC012360	171.4732	170.4836	170.869	173.2781	272.9533	247.0723	215.8444	348.6146

ZFAS1	1168.736	1224.861	1189.186	1351.924	1722.047	6182.092	7754.542	2452.18
HAR1A	414.0965	392.4174	253.238	453.4789	104.9019	7.735656	17.81216	15.84196
PCED1B-A	126.7989	157.1565	117.4088	140.1172	149.9115	575.2474	1639.832	765.5683
AC017083	18.14878	25.71719	14.86536	4.306906	47.37892	4.727135	11.91771	12.87139
CTBP1-DT	772.7439	821.9661	835.1355	1078.116	1619.628	1859.852	1219.642	1725.244
SLC16A1-	146.0969	109.5235	87.599	88.79296	252.1678	337.0931	246.4892	238.6829
AL512353	16.91004	9.511012	15.76467	16.04803	19.48542	51.1006	39.62944	43.57737
ZKSCAN2-	78.52097	88.57172	64.81456	54.57048	213.1201	39.37179	104.3214	105.9683
LINC0060	32.59479	14.27086	2.578758	4.307167	86.21532	49.2216	33.58394	67.36199
LINC0142	12.04216	9.523667	5.224392	9.626987	6.142649	229.2264	229.308	116.8716
LINC0171	49.5527	74.29012	79.74868	55.63101	87.73053	124.98	88.1401	110.9222
AC084125	119.566	149.5336	242.7954	148.6731	63.45675	96.55123	85.28713	77.2461
LINC0228	4.854767	3.799772	6.134527	5.353106	6.095088	22.64493	56.81606	23.76838
FO393401	4.861608	6.667423	4.357493	6.418749	6.104579	15.024	28.94642	11.88378
SEMA6A-A	48.31388	29.51184	22.74007	18.20987	127.7326	36.31128	26.59722	28.71743
AL359643	3.70081	7.606627	9.615474	10.70601	29.06724	120.022	86.40112	119.8448
LPP-AS2	107.4987	111.4285	171.7724	207.4768	97.55528	741.7718	274.4565	261.4599
AL162741	8.492557	5.699879	6.977994	6.43719	26.6223	9.169708	7.484905	10.8922
AC008669	189.5881	231.4458	226.0887	269.5243	192.5963	74.00112	91.16938	132.7082
AC009148	16.94903	22.85544	18.37978	20.33146	34.07174	9.24045	16.29903	14.85232
FLJ37035	94.17697	71.42599	87.62607	88.77746	85.33052	30.31237	23.67197	28.71731
RFPL1S	4937.996	4125.116	4702.488	5692.161	720.3669	57.44083	155.7681	44.56319
LINC0114	6.048148	7.624235	7.015371	7.481348	0.057838	31.43147	108.3065	32.68679
AC027130	37.51092	101.9087	56.0445	48.15563	280.0558	10.75733	1.638927	10.89007
MCM3AP-	156.997	182.8667	206.8112	179.6952	260.7816	204.9596	202.6604	225.8045
AC132192	41.10694	64.76457	46.41759	28.90333	130.2549	70.9346	77.90872	65.36203
AC010300	68.85203	88.57865	85.00106	71.67011	80.45274	21.30086	47.12894	26.73623
AC120053	119.5251	100.9561	72.7047	73.81706	154.7273	147.664	102.8701	103.9872
AC110285	493.8389	337.1567	216.4081	132.6551	1759.537	77.02703	145.489	140.63
AC026801	38.70569	57.13731	44.65586	48.14973	182.5964	66.28535	87.96544	74.27898
AC012645	6.108588	17.14737	13.1317	5.373149	26.69744	1.700878	14.75723	6.929099
AL450384	89.37935	118.1051	101.6433	122.9973	92.65817	22.80853	73.52379	66.3523
FLJ46284	25.42501	46.665	47.30894	72.71418	54.84697	4.727119	8.993971	34.66343
MIR155HC	31.28239	23.82149	7.845113	11.78128	6.174821	85.62191	328.0178	31.68951
AC012213	67.60668	43.80254	85.03365	80.20507	12.28043	0.164219	7.535147	22.77538
CTBP1-AS	54.3937	94.2911	99.02429	71.67466	131.5753	66.39248	95.42772	63.38179
SOCS2-AS	82.13361	100.0061	87.61837	141.1646	63.43419	943.0025	604.086	45.55335
AC021739	33.68335	11.4175	8.723259	6.443176	23.12255	13.77082	16.344	26.73632
AL353751	64.0117	46.65849	42.90577	32.11153	147.2963	10.74913	27.99194	13.86156
AC009088	21.77159	25.71142	15.73666	21.40489	48.6475	3.216815	20.6465	14.85264
KIZ-AS1	14.52166	15.23281	19.27994	11.78221	23.12424	16.70573	17.73122	22.77728
AF111167	161.8496	318.1229	425.9387	403.2077	87.84008	111.5472	57.41641	94.08359
EBLN3P	2948.328	2384.007	3048.813	3188.345	2431.409	3817.709	2329.177	3698.083
AC008760	67.46481	19.03503	10.4667	45.97623	14.7027	70.82505	311.3718	157.4782
AL391422	91.76576	86.67052	70.96115	84.50132	92.6309	665.4911	809.8331	882.4364
LBX2-AS1	18.11753	11.41747	10.48147	20.31843	23.12289	286.8661	184.6172	169.3601
AC083799	86.93059	88.57923	38.51566	79.15229	95.04866	281.4736	290.4266	347.6292
AC018638	14.55634	40.01337	30.67039	21.40579	30.47265	27.25112	49.88636	12.87101
LINC0260	423.8059	545.7623	393.4575	602.1529	386.3809	403.3588	164.4798	463.5051
AC245060	43.51998	91.44555	58.69807	78.07412	50.01539	31.83528	16.34442	42.5827
AL441992	21.79607	25.7042	42.06413	52.39581	43.86879	52.66686	81.90977	94.09405
NCAM1-A	50.7233	39.99397	52.57477	43.8626	60.92395	42.30657	19.27143	48.52653
BX640514	2.3116	0.952901	0	0.007312	0.021448	10.53335	127.2427	7.921691
LINC0163	11.91581	1.889718	2.59467	7.472367	0.055552	8.933011	57.28406	58.49442
AC253576	8.491509	8.563102	10.50443	16.02848	9.778784	10.67336	16.18063	7.919829
AL354740	62.74839	52.38591	42.04845	39.5849	30.52736	37.73294	100.8639	66.35806
AC053513	39.84831	30.47017	48.21397	41.71248	22.00298	4.725273	7.523229	9.90022
AC009283	82.15168	127.629	94.62141	88.78868	186.3946	144.6549	157.0824	83.18864

AL118505	24.22193	50.47548	63.98048	39.58927	82.8072	66.47857	10.44456	509.0533
HOXA-AS	0.014378	0	1.816488	0.005186	0.015081	532.4456	9.001702	105.9715
AC099560	2.78E-17	2.78E-17	2.78E-17	2.78E-17	2.78E-17	0.067271	5.350622	0
SMCR5	14.55195	24.75946	20.13124	20.33518	46.20914	1.700838	3.123662	0
WAC-AS1	1626.309	1734.432	1849.09	1986.164	1471.05	1414.199	775.0491	934.9168
AC012593	15.77418	38.09385	14.84983	13.9317	111.8412	1.700483	0.146256	1.976924
NORAD	45707.37	40118.71	46722.51	50578.21	28422.14	25466.85	23161.69	25608.28
ZNF236-D	49.56896	62.85037	42.8965	72.74348	219.1592	310.8554	187.5434	111.9136
AL136295	143.7269	215.2541	160.3451	185.0433	320.4619	195.8869	220.209	91.11118
AL109811	222.1646	200.9636	212.9407	161.5193	285.1569	200.3784	250.9654	105.9672
SLC25A25	260.8368	307.6372	325.9832	223.5582	696.9917	555.6869	381.6583	232.7362
EMX2OS	4515.408	3038.352	3686.796	4037.568	1240.758	1209.381	129.3483	170.3413
AC110285	364.6659	336.2066	187.489	110.1944	1480.448	72.50465	19.2716	592.2553
AC060766	13.2751	4.745749	10.50203	17.09602	9.782527	83.55459	29.35397	40.60836
AC016773	18.12672	19.04691	12.23715	7.512177	32.80649	24.26896	48.46363	14.85178
ACAP2-IT	76.10097	90.47957	121.8286	143.2999	25.68536	9.224637	11.89624	13.86246
AC139887	70.03464	53.32863	66.59303	43.87075	93.79916	75.37763	63.21536	89.13378
ZFHX2-AS	194.4284	284.7859	255.8832	372.1899	169.465	43.8841	70.60768	35.64961
FOXN3-A:	65.14668	41.90317	37.66238	43.85753	29.30844	93.29335	79.24132	75.26877
AC009102	182.3306	120.949	202.4312	267.3757	173.0905	61.96198	60.37089	81.20733
AC095057	64.02944	111.4473	84.12426	96.25593	36.6417	22.80853	61.80285	73.28521
LINC0240	4.741112	0.940378	1.73374	2.145164	1.265666	46.10176	14.74439	23.77095
OTUD6B-/	2770.85	2294.476	2595.732	2905.984	2644.67	1947.346	1347.388	2469.02
AL139274	59.21256	64.75702	126.225	110.1562	59.77313	25.81903	44.21076	62.39084
AC022613	86.95999	103.8163	118.3131	140.0977	39.08461	159.3264	193.279	173.3239
ZBTB20-A	4.883307	5.70452	6.110744	7.494021	14.52961	4.726152	20.64146	20.79607
AC120036	604.8971	598.1418	772.9485	812.854	314.5104	326.8884	189.4847	176.2843
ZNF710-A	2287.947	2853.576	2437.979	3190.485	3578.184	837.5592	1282.85	1328.097
AC009120	268.0448	231.4409	256.7573	221.4108	342.4402	137.2412	397.6842	222.8332
BCRP3	30.20496	40.00796	47.35437	18.20385	19.56258	28.79178	39.7489	24.75589
LUCAT1	97.36423	20.94557	1.703838	3.237243	2.525408	4.725517	110.3353	44.5715
LINC0063	169.0194	92.37544	171.7705	175.4024	99.99224	99.46862	91.07783	111.9124
CAPN10-I	112.308	105.716	65.68507	108.0352	205.8758	113.0726	167.3194	102.0066
AC005785	59.17708	65.71926	51.68472	42.79917	75.5409	25.80091	98.23048	39.61222
AL583810	7.296948	11.42655	11.38446	5.370505	19.41498	4.711109	6.038675	4.948209
AC126118	12.13467	19.04352	7.840451	5.375878	57.01853	10.73284	29.37031	3.957524
AC018635	4.840983	1.890106	3.481815	5.34778	7.263994	1.690105	5.968648	0
AL021368	35.0387	38.09492	28.89262	35.30373	49.94036	6.233442	6.064125	16.83345
AL132711	7.123918	3.811267	0.837884	2.152809	1.279227	233.4791	39.16888	19.80852
LINC0010	61.60788	88.58281	99.92702	60.97633	41.50522	24.29045	39.75374	15.84204
AL136295	53.14929	45.70861	48.17711	49.21123	88.90477	43.79258	68.96661	28.71763
LANCL1-/	73.71011	95.23645	100.7576	152.94	176.6658	19.76344	9.002626	18.81363
AC132872	4.890527	10.47794	14.05438	9.628601	4.939	15.23524	19.21516	20.79541
ACVR2B-/	68.82679	52.37623	78.87886	97.31159	23.24305	21.30029	63.23033	124.7894
AC093788	10.89924	13.33195	6.972739	16.03816	15.8297	19.69228	32.26148	25.74897
RNF217-A	22.97683	16.17737	27.15526	22.47392	48.65605	1.700498	13.28249	9.901215
AC026356	37.42622	24.75348	25.38788	19.27322	55.97556	67.84036	73.42281	109.9346
BHLHE40-	97.65827	24.74793	42.04578	58.82085	13.49662	69.18895	77.64533	47.53723
KCNK4-TE	33.81418	32.38044	49.989	28.88743	12.27096	4.727142	13.37801	2.967215
AL133415	7.198465	2.845826	2.596408	7.469038	2.495861	386.6663	146.4187	335.7687
AC022400	148.4628	106.6723	141.9868	101.6151	24.46834	16.71449	14.82709	23.76759
BCDIN3D-	147.3464	198.1075	266.4245	278.0735	126.8136	82.98151	63.2763	121.8155
LINC0046	74.9031	64.75395	25.36139	25.69892	309.2298	4.723967	7.534812	6.928606
AC009227	50.71017	31.41904	41.1712	70.57062	31.74256	1.689554	3.112762	72.2955
LOXL1-AS	75.99343	40.94831	44.67974	33.17368	39.03958	105.2155	128.822	79.2315
LINC0032	42.29175	47.61818	58.71999	57.7543	39.04442	297.3923	203.6395	82.2001
AP000721	61.51287	18.07966	39.42584	32.09982	43.87637	18.22511	13.37922	36.64443
IL21-AS1	2.78E-17	2.78E-17	2.78E-17	2.78E-17	2.78E-17	0.131611	10.15374	26.76025

AL355974	58.0061	42.84441	42.02143	88.77628	185.0747	6148.757	2070.543	759.6195
LINC0199:	1.20515	0	1.778529	0.008694	1.215833	9.181794	126.4893	59.44236
ZNF529-A	100.2305	76.18575	124.4473	87.71578	126.7348	367.1018	403.1725	158.4596
MIR29B2C	444.3385	766.7431	772.9665	549.7561	123.1868	105.6103	127.8371	142.6119
AC022613	19.30827	9.510753	14.88303	16.04881	19.48857	34.46667	50.93616	34.6667
SNHG26	10.85636	7.612352	7.869862	5.366742	12.15791	30.0973	30.78978	22.77748
AC007292	57.98041	65.71705	89.4107	51.35233	51.23	19.7887	45.62381	61.40218
LINC0034:	398.476	547.661	587.1445	481.3128	855.497	476.0525	390.544	492.2165
AC232271	38.69101	44.7555	43.78954	33.17827	127.7964	46.8627	70.54907	62.39139
PAXIP1-A:	125.5932	118.0971	146.3436	183.9548	120.6909	922.5003	413.8086	293.1515
AC010883	10.87775	14.29367	9.624711	2.166058	14.59056	15.22417	25.0196	14.85253
LINC0152:	204.0787	195.2451	282.1856	287.7086	254.7286	126.6462	127.8094	258.4909
AL022313	1287.049	1770.634	1593.203	1884.546	397.4	16.75793	44.21962	71.30332
GAS5	1696.335	1732.525	1475.746	1610.761	2573.922	7713.733	7342.317	3059.283
AL162171	166.6524	143.8099	146.3195	108.0488	447.1025	30.30907	33.9198	51.49757
GABPB1-/	800.5234	819.1054	893.8452	820.3683	2776.09	942.9758	2402.529	1800.51
AC098613	6.113778	15.2338	14.00174	24.58299	20.70011	73.5634	125.5341	45.55738
FAM111A	45.93411	59.04509	86.77083	69.52577	75.56373	365.7262	230.4143	240.6628
TMEM72-	53.18577	80.00038	117.4479	134.7446	51.25545	19.77653	13.40435	14.8517
PVT1	141.1374	55.23229	72.72689	53.49417	37.85516	1101.794	445.6809	392.1982
LINC0113	115.89	49.51195	56.92511	63.12261	181.4652	174.653	161.4239	191.1452
AC017002	1.205371	1.916793	0	1.071259	0.025715	4.155895	1.482725	0.989098
AC004067	108.4805	34.27746	21.86422	31.036	36.6077	119.6248	157.2899	59.42639
AL357060	4.887175	8.568966	12.29309	11.75444	3.727937	66.06688	73.13411	41.59563
AF111169	24.19689	38.09849	37.6819	33.16263	34.14087	10.75408	14.86414	22.77544
AL049597	70.08476	120.9623	147.2452	145.4499	57.35486	87.2668	109.8742	104.9839
SLC25A21	78.52294	78.09133	70.95187	56.70932	226.5117	6.219491	7.521518	98.0445
LINCR-00	65.21182	66.66954	95.55181	80.21139	12.28084	27.31672	74.92501	62.39162
AL354919	10.65561	2.849593	1.71841	1.091217	1.283979	78.34185	331.909	92.10602
AL391807	706.3386	954.3654	1159.42	775.4403	995.7123	146.3045	434.576	324.841
AC104964	270.4766	411.4708	385.6033	358.2997	109.7748	167.3702	707.226	354.5544
LRRC75A-	3795.884	3718.413	3777.928	4159.503	3289.396	13686.73	17357.21	5036.083
AC004908	21.73249	18.09134	17.51061	17.12289	20.72595	6.232839	27.93052	28.72026
AL035563	29.05323	64.76304	63.08352	40.66377	130.2722	79.97012	96.96927	50.50564
LNCTAM3	3.6501	4.761907	6.155284	2.158073	3.685036	97.17286	93.30785	30.70078
AC106782	30.20625	19.98819	19.24304	14.99832	72.94019	3.216824	22.08312	18.81512
AC025178	10.89354	10.46989	9.616482	13.90346	14.61476	16.47987	7.460182	12.87479
AC073611	65.13056	45.7179	29.76647	26.75801	35.37862	55.64577	177.8553	39.61426
AC016876	43.48884	59.05729	47.31433	29.96701	49.97525	168.565	237.4434	129.7398
AC006033	10.90281	15.23985	14.89715	18.17222	6.167396	21.05052	42.11796	29.71542
AC022762	138.8496	171.4502	134.0749	126.2139	64.66932	22.79468	30.98996	27.72698
Z97832.2	44.68936	48.5741	28.00707	27.82885	74.26433	69.398	60.30646	67.34348
AC027348	2.469891	5.717407	7.037644	3.223047	3.691896	1.70029	1.6469	16.83405
AC005034	319.9399	260.9695	286.5588	269.5347	232.8169	338.968	388.9878	427.8435
AC103691	15.65103	10.4732	12.27103	8.568893	4.946711	30.19946	25.06017	29.70984
AL022328	36.24532	36.18779	36.79266	32.09942	51.15971	12.24715	11.92587	13.86166
AC069281	147.349	194.2962	107.7476	97.35526	505.5679	150.7896	305.32	132.7073
DNMBP-A	9.680655	16.20546	4.339084	14.95954	4.945187	4.720891	4.589874	7.918997
AC026401	51.99237	65.70473	25.36084	22.4907	406.6426	508.6564	612.94	338.7122
AL359878	16.97576	48.58303	56.12391	19.27469	35.36202	12.25278	11.93078	25.74725
AP000866	4.887668	4.748536	3.462882	10.69068	18.14199	3.210478	16.15108	6.92942
AL513217	25.41007	28.56306	18.35963	27.825	89.99264	6.235661	4.594497	7.918955
STX18-AS	118.3621	123.8113	161.244	136.9125	203.4941	167.2252	121.9324	190.1528
TMEM220	68.81986	46.66006	71.86362	51.35128	67.03615	135.2708	131.8501	82.2017
ITGB2-AS:	62.82082	89.53282	99.04101	65.2548	63.41461	24.30534	92.44837	33.6693
STXBP5-A	367.0366	343.8358	435.5584	416.0524	120.7434	19.79619	72.04595	42.58257
FGD5-AS1	8441.724	6927.256	7557.607	8174.622	12980.68	18779.96	19351.62	17308.89
DNAJC3-I	201.6545	241.9244	242.7454	266.3162	153.6179	278.7573	207.1	221.842

AL512770	25.39519	29.52044	27.14605	24.6145	51.11635	16.75081	36.73667	23.76637
LINC0112(	4.907279	12.37471	16.65326	8.576318	27.91095	12.24587	22.14378	21.78565
AL160270	6.044758	4.755087	8.793293	5.35477	2.504725	1.697004	0.134968	6.930118
AL159169	59.20596	95.24922	81.49367	67.39346	84.0999	42.3555	22.21332	31.68827
SNHG18	41.13031	59.03746	57.7986	28.90732	328.7121	1064.327	234.7759	264.4336
NRAV	596.407	402.8836	539.8312	574.3476	251.134	1070.363	799.6605	554.6135
AL135999	19.38596	42.86219	35.91897	25.6867	57.21576	7.727174	6.059069	1.976924
RAD51-A:	194.4206	190.4832	162.9683	171.1459	454.4613	146.236	237.7889	104.9767
AL035530	121.9703	121.9086	167.3919	157.2229	101.2051	39.36769	58.87777	102.0072
AP000757	54.3509	74.29844	56.95386	56.6916	35.40621	66.44711	75.00365	94.0836
AC107398	5064.792	4487.048	6704.962	6682.572	1535.696	155.3336	703.1624	148.5531
CARD8-A:	71.19611	48.57031	43.79675	46.00076	43.91067	302.3885	509.8978	206.0008
AC009690	18.17212	25.70833	39.44911	32.09	35.33908	164.9712	124.3788	56.45094
PIK3CD-A	0.054981	4.754372	7.901914	9.605874	6.10535	118.1867	93.45009	64.3788
AC018797	62.787	48.56725	44.66726	35.31605	94.9827	162.368	164.1028	163.4183
AP002360	22.99324	45.72534	42.07422	34.2308	21.99719	13.67866	11.87593	14.85363
AL133325	256.0112	265.7251	165.5831	83.45549	1101.431	268.206	154.2819	566.5
AL713998	2.78E-17	2.78E-17	2.78E-17	2.78E-17	2.78E-17	30.30704	4.163343	7.938918
ZNF433-A	48.30115	48.57407	44.68305	51.33822	30.52608	215.2097	312.2066	154.4994
AC125807	178.4284	46.65923	42.03026	36.38717	15.93602	176.0499	221.3227	234.727
BX324167	10.85751	10.47796	12.28628	4.300501	6.1454	1.700697	3.124021	9.900405
AL606970	2.78E-17	2.78E-17	2.78E-17	2.78E-17	2.78E-17	13.39193	4.5336	13.86888
AC114730	16.91323	14.28008	9.603808	8.578524	30.35837	9.238617	22.12124	11.88092
AC007375	188.2596	84.75883	105.1481	135.8287	32.99705	7.742775	7.534633	4.94788
AC053503	7.295328	13.33714	10.50421	14.96349	7.365866	48.41028	26.61763	45.55336
AC011477	59.20592	65.71156	60.44143	98.39297	103.5631	111.4504	161.278	90.12328
AL356056	84.49964	66.66769	56.06264	118.6913	14.71818	25.75387	9.002164	51.50028
AC084018	45.92664	62.85925	78.88371	24.62663	93.78384	25.82005	74.98772	41.59207
AC008764	50.72665	39.04047	49.06206	42.79479	73.08061	25.79677	51.45897	45.55512
AC245060	96.62887	100.9525	111.28	123.0038	168.1459	126.5931	117.5049	129.7383
LINC0033!	334.4511	315.2594	279.5319	317.6664	435.0645	838.6808	680.9079	746.7492
ASMTL-A:	143.7153	261.9382	222.6015	164.7209	101.2261	31.84554	154.1763	56.44771
AP000350	25.42838	68.58189	52.56893	60.96457	59.72093	12.24056	17.7582	14.85231
AP000892	7.306165	11.42256	8.732215	5.372479	31.49475	9.127488	8.894868	8.911591
HOTAIRM	8.444448	8.574374	11.42884	2.163846	3.719841	842.0822	95.56184	224.8165
H1FX-AS1	84.55198	109.5316	106.0311	94.12991	112.1276	87.53852	184.9725	156.4782
AC105219	21.76184	15.22589	22.77028	25.6727	35.29775	28.76567	19.25661	39.6135
C1RL-AS1	42.27228	31.42059	56.98197	64.15415	12.27705	69.06364	55.68383	65.36856
LINC0127(	14.52219	23.8196	19.27947	19.25223	7.390031	83.81447	38.14926	36.64399
ARHGAP2	195.6074	188.5824	184.8942	177.5564	237.6426	90.55216	189.3829	99.0346
AC060766	4.858713	7.625454	2.591	7.479638	4.90506	71.07984	11.84961	14.85439
AC018648	41.05709	47.62672	31.52563	34.23585	35.37113	30.28886	28.04775	14.85167
AL357033	3.693419	10.47713	8.750533	14.95284	7.352562	34.4905	43.74924	33.67555
TMEM254	230.629	210.4858	246.2422	267.3917	292.4906	226.0542	120.536	242.6407
AC093895	0.008449	0	0	0.003072	1.152003	24.08676	79.75618	115.9088
AC124312	41.06426	39.04616	48.20434	53.46883	18.3602	4.726425	11.93399	9.899746
AC009065	25.38505	39.05819	22.76026	16.0653	38.96706	46.76412	61.62943	49.51787
AL392172	68.79727	52.3809	40.28396	52.41491	52.42657	462.0294	330.1142	272.355
ZNF571-A	278.9022	194.2915	193.6441	289.8501	333.9096	83.04908	229.0741	85.16861
AC118344	45.90834	44.75838	67.49481	44.93202	49.98872	18.23993	20.68693	20.79511
DLGAP4-/	6.101322	14.28959	12.2629	5.371137	19.42592	21.06966	52.22911	18.81678
MIR4435-	808.807	406.6959	399.5982	377.5646	174.3647	4785.349	3601.517	863.6123
AL355922	9.475781	0.937472	0.836602	4.275231	1.282412	10.69142	233.1366	22.77948
BASP1-AS	181.1364	279.0777	247.133	275.9374	73.21333	0.165231	4.593067	26.73687
AC005562	126.7334	60.94473	87.62174	102.6774	74.38474	373.1395	239.1529	206.9898
USP30-AS	7.152031	3.807826	0.836065	3.21689	2.481339	26.83064	41.91817	18.81873
TRHDE-A:	380.3128	257.1543	460.1018	514.4377	80.53082	0.166887	4.588982	24.75555
LINC0096!	1.255017	2.857896	1.73118	3.203696	2.451346	12.01974	37.26971	15.84907

AC073389	22.98365	20.94234	27.15005	23.54437	53.52482	4.723228	17.80476	16.83245
DLGAP1-/	100.2218	70.47077	92.87946	58.84664	159.5751	186.861	293.4999	153.5062
AC010319	12.105	14.28391	10.48976	16.04256	15.84316	42.09658	67.17073	39.61611
ZNF252P-	15.7275	16.18519	16.63496	11.7837	29.18059	19.75688	22.16799	34.66162
AC147067	4.878889	8.57302	1.706532	6.427109	14.51162	20.93758	27.58438	26.7461
AC010864	29.00699	23.80033	37.68465	26.75196	45.0577	19.76811	28.02633	16.83266
AC108449	61.60498	37.13021	85.01402	79.14642	80.43158	22.80735	38.34427	103.9891
AL122035	103.8572	75.23174	71.82519	79.16465	225.3249	78.45582	111.5977	79.22778
AC040162	48.35477	77.14531	74.47601	64.18641	123.0241	46.85778	110.0356	58.4298
AC015922	74.86383	73.33699	91.15386	56.70023	53.67282	425.4461	271.227	140.6334
AC008764	64.00416	65.71728	66.59658	60.97185	56.09448	48.37024	121.7713	51.49655
AC018647	702.7002	667.6687	758.9053	732.654	1017.616	2701.327	1391.253	1727.227
AC007344	0.015094	0	0	1.060017	1.180299	1.699855	47.6563	22.78193
LINC0108!	334.4783	441.9395	432.0289	216.075	740.9007	226.1221	475.6695	302.0621
AL590644	2.78E-17	2.78E-17	2.78E-17	2.78E-17	2.78E-17	8.568478	4.333719	0
SUCLG2-/	35.00446	25.7092	33.30169	35.29207	15.91416	87.08174	67.40684	46.54715
SNHG12	298.2323	323.8355	248.8589	242.8034	402.1537	466.9832	923.0031	386.2457
AC026471	70.05105	63.80686	49.91807	52.42602	136.3913	27.32206	88.13295	66.35281
AL513320	32.64259	54.29521	29.76482	29.96482	63.32003	16.76127	9.002369	14.85186
AL589935	22.97344	27.61849	27.15784	25.6767	29.25503	10.75605	9.003639	12.8709
AC090796	0.008134	0	0.903867	0.002959	0.008526	8.617416	49.88861	4.95391
LINC0122(	16.9375	26.67518	19.26713	22.46103	13.46245	18.11494	21.99298	26.74238
LINC0068!	39.87493	55.24533	40.29312	17.14009	74.26833	18.27917	55.82072	33.66991
AC002398	22.9633	19.99297	17.49743	21.40169	38.93139	42.10802	55.57504	38.62533
AL731569	41.10764	58.09395	43.78533	43.86977	127.8274	21.29588	16.34317	37.63108
AC093726	59.17871	66.67178	53.43872	52.41934	67.03258	353.4734	165.8532	126.7669
HCG18	2902.375	1218.187	1892.904	1841.781	1391.844	1912.553	1228.448	1513.302
AL450263	26.60303	27.61268	44.71047	25.68433	41.42197	18.16124	8.976592	18.8157
SBF2-AS1	170.2488	167.6298	179.6474	214.9731	95.13149	336.7471	319.4335	244.6298
AC061992	17.89759	3.797005	2.587695	6.423105	1.297076	24.17999	85.92385	42.5888
TRG-AS1	12.09314	14.28735	13.13952	9.639969	12.20707	25.63344	62.63844	38.62759
AC007619	19.36955	12.36479	32.42744	45.9665	28.04792	3.215037	7.508597	12.87256
AC109460	37.48551	58.09653	56.07391	53.48663	81.61335	31.80496	87.98665	28.71747
ID2-AS1	100.2501	84.75658	163.0161	175.3937	93.89211	55.93353	51.56317	119.8334
AL022328	65.21273	61.90485	68.34909	51.35298	75.55661	40.81896	30.99207	42.58351
AC004148	167.8724	226.6811	218.1944	175.4236	389.9149	195.9495	368.3927	140.6303
AL117332	43.42787	29.52104	17.48785	22.47725	41.40831	82.7623	178.4286	68.3361

TCGA-32-	TCGA-26-	TCGA-41-	TCGA-08-	TCGA-06-	TCGA-R8-	TCGA-HT-	TCGA-HW	TCGA-E1-
127.5737	63.03371	46.27411	45.42494	12.87237	317.7585	203.0067	84.57163	54.13372
13.44029	24.73139	10.59502	18.6905	23.31623	3.681251	29.89465	30.68196	59.98012
64.44036	81.50606	47.73624	77.54221	46.61917	39.66822	132.3157	84.59358	61.43342
1438.257	2208.08	2810.513	1812.526	690.3065	2982.447	3634.769	1634.368	2999.329
51.09971	181.3802	127.8508	130.1625	144.33	195.4904	80.62468	146.8539	164.2708
6.014129	1.303149	0.106877	2.655183	0.038386	1.259101	1.787331	0	2.387196
82.42302	80.04543	19.48072	23.14998	5.888779	3.680922	38.05522	81.4423	52.63192
120.2914	74.13416	43.30381	58.80157	43.13548	245.8528	74.27592	131.0631	87.32523
10929.69	5824.389	6674.825	4656.617	6660.286	5026.79	7052.91	4926.435	9026.302
31.65496	51.87825	41.74294	38.30571	94.23564	49.2271	13.55292	88.81037	37.97138
7.368342	14.8595	13.49462	12.45252	46.41115	14.44018	28.11214	31.58459	16.9626
586.7614	388.8761	414.8533	264.7487	1421.183	680.2839	803.776	911.764	488.6259
89.95626	213.4885	283.6484	170.2849	59.42812	163.1725	160.3893	82.0767	280.0332
4.864177	31.17163	5.900593	0.854334	0.040911	0.049518	0	5.823463	4.850208
53.51718	43.28562	77.38593	32.05185	100.1131	176.2607	61.59386	124.4641	50.0979
36.45413	35.81016	54.85399	35.6543	9.379584	4.882091	25.3507	28.17411	29.90361
1580.351	1576.164	1608.223	2084.474	1684.301	1792.38	2740.35	1731.458	1190.123
4.947886	30.91598	23.94052	34.7481	5.888731	74.30807	21.71567	48.94574	45.29539
125.0959	183.7836	52.18525	249.7487	14.03794	10.88162	62.49976	136.0892	122.1848
959.5395	1240.256	4286.925	2162.248	149.0595	148.8488	872.6839	502.7359	355.1321
733.7141	912.1188	515.8889	628.5248	286.4063	805.0302	669.6556	735.8675	953.1048
6.163706	2.560662	0.157504	0	0.053496	39.61844	95.22046	0.767653	0
287.646	8.73626	7.629065	5.306341	98.96268	250.5725	67.9363	32.28493	1.548679
183.4785	91.43019	38.85227	95.353	11.70439	629.6793	419.5917	43.8977	78.40135
179.6351	39.58251	28.4384	65.95053	78.01204	37.2668	103.3083	43.91143	39.57466
21.91605	25.9586	29.77237	23.15685	74.33984	63.45078	28.98773	31.51625	31.54306
248.9922	375.0778	509.2555	254.9993	295.6085	99.64526	113.2443	168.3899	373.9072
543.0283	1226.782	1753.466	569.6799	1772.626	821.8176	539.1582	612.2402	1557.602
11.01799	2.561852	12.07969	30.29783	0.052952	14.47011	21.72253	9.055603	2.350628
38.94491	37.111	75.84647	78.44945	179.1103	73.2053	74.29773	39.76451	85.76107
31.65157	44.48217	23.96367	114.1878	48.91466	160.5073	102.4438	33.96347	20.15095
48.62253	24.77144	37.28884	48.12626	31.48012	105.4682	45.29126	36.45692	51.75727
12.19776	6.259131	19.3238	10.66835	32.50705	22.76974	20.84742	17.41069	21.04805
881.8652	808.44	995.7513	720.3695	1557.301	531.5219	598.0673	551.6745	748.3916
170.0966	225.8699	310.5004	170.2686	605.0391	227.9461	262.8058	326.9376	267.0234
2162.229	1960.059	2629.381	4032.511	1671.536	52531.83	21075.44	6094.58	922.2932
61.72549	32.05902	22.35274	7.981307	8.211784	6.079903	23.55251	37.38318	25.885
23.16497	21.07767	38.7925	86.4955	40.78738	95.93093	94.26385	38.9412	39.58782
123.6529	76.43909	41.68486	32.06839	11.70845	7.282675	54.37426	55.59431	102.0942
127.2109	49.35474	26.88274	17.79143	8.216996	9.681166	16.27579	117.2097	55.86064
468.524	230.7293	345.8532	504.8232	785.1085	15.68071	81.52679	301.2787	130.2356
7.378892	48.16453	71.26124	31.17194	58.19964	81.51914	55.27406	33.13759	99.6263
91.03832	182.3094	192.4026	20.46252	11.70967	12.08204	38.034	71.36046	52.564
59.59356	51.92473	29.93139	24.91711	122.2249	223.0286	75.1878	76.28089	31.47395
233.2511	203.6981	579.2435	335.2404	1318.249	267.5459	341.6474	228.9576	542.2289
4.945981	9.954497	17.91456	17.81327	2.393899	40.67885	19.01754	21.56036	45.44873
38.93081	54.3402	152.5837	43.66026	125.5979	98.30736	54.35814	60.55054	68.7739
3063.445	1309.607	734.4155	689.134	989.4451	2582.98	1879.423	1466.768	1690.954
720.2706	232.1235	209.7515	76.62763	78.05743	1035.215	620.748	208.1762	317.9174
6.154435	11.17033	12.00754	17.82524	31.35089	12.04518	31.76247	17.41075	18.60383
7.364703	18.52171	30.99606	17.82497	16.31375	44.1864	9.031662	15.74142	12.09016
94.82958	282.6303	114.6169	353.0865	178.1262	230.363	496.6608	135.1783	178.7664
43.78911	56.81639	63.96516	55.25634	31.48678	69.5954	77.93423	86.30059	66.32799
1048.365	406.1596	700.2328	2220.851	29.15719	15210.67	7519.639	880.189	523.4054
17.04622	19.77511	17.91607	2.630344	44.12161	78.78692	11.75119	33.2367	9.641678
34.07667	46.94479	26.92678	43.66289	33.80566	58.79799	36.22086	54.7388	46.89169
6.160077	2.562805	19.40142	14.23439	1.226241	31.16108	20.83038	13.22276	6.395348

1092.094	3826.171	3220.845	2934.11	241.0079	8321.037	4580.806	4419.537	4127.171
261.1841	597.1954	798.9592	400.3355	1754.56	63.67029	101.456	1140.348	349.5454
32.81028	43.16395	38.618	12.44041	48.84281	52.71581	38.06403	31.51173	55.90984
149.0978	42.00386	41.69972	85.6362	10.54527	25.26187	37.13285	58.91257	18.53432
13.43589	24.71896	79.63701	15.12161	0.052465	0.063816	2.680358	32.36014	8.013101
76.61053	177.7661	120.537	206.8359	250.2426	230.3407	241.9585	98.66774	71.12303
14.63068	30.80887	14.98165	26.75525	40.65295	37.10967	9.935259	23.22549	22.64618
44.9029	23.504	25.36221	15.11844	9.378382	33.6006	18.09458	46.49888	55.91119
99.58327	101.181	65.48217	65.06454	188.4077	57.63249	36.21363	120.3483	116.5578
130.0282	429.3158	626.3708	309.4116	247.9069	103.2407	100.5558	92.03032	203.0762
7.365466	0.072457	1.673419	2.630474	4.721175	4.877921	9.031191	3.248756	14.52968
9.797793	32.06405	22.35913	10.65928	12.85764	28.78786	7.21167	14.0514	7.204589
190.7397	133.3678	136.8782	54.339	395.6706	281.9069	83.33276	82.06774	79.21464
15.87315	39.51738	72.59958	38.32525	111.5413	76.67225	49.84733	44.79931	53.42526
82.63474	17.38007	13.58394	12.43555	75.69271	163.0798	79.72778	30.62684	241.308
37.67796	33.36412	35.73583	16.00704	47.71381	94.58953	26.25456	45.63463	72.91653
174.9636	134.6056	203.6929	127.458	169.9796	243.549	272.7679	92.85454	283.196
20.71697	21.05441	4.659681	37.43892	21.0024	4.882073	62.56583	16.53136	28.27825
995.551	463.9496	193.3193	651.8557	144.3853	94.85574	170.3396	404.9291	436.1866
113.0368	124.7288	49.25515	59.6892	160.6635	208.7622	161.283	131.8658	292.9322
475.0289	1330.523	1273.773	1696.66	454.0173	4866.781	1336.631	895.9788	1375.453
24.37602	13.67597	32.85697	5.304482	3.557283	217.9556	39.84693	67.20057	35.54136
24.38466	3.788616	35.88021	10.65578	285.1682	745.9085	536.5437	66.29984	132.6286
1.293561	13.67545	13.58114	61.51508	2.391326	55.20558	223.1329	13.20204	42.83941
63.16668	165.0738	108.2317	78.47582	79.12182	61.1919	30.77957	43.10372	42.02826
889.201	525.8717	564.9542	593.7441	181.6511	889.0247	634.3047	592.3114	587.3361
114.168	305.702	189.9257	305.0945	25.67656	26.47721	77.91553	19.83835	73.58768
62.02765	45.762	209.4143	51.66803	86.18098	45.66995	303.6547	234.0372	140.7734
729.9361	649.1412	1011.683	504.6304	72.23773	260.3951	892.6695	558.3779	510.5496
78.99684	284.7877	81.83162	264.9251	246.5913	13.28207	16.27106	107.8521	364.4873
145.8063	166.6539	291.1494	114.0882	47.7929	446.0927	251.9381	87.05171	126.9784
15.87723	11.20668	18.02382	34.74548	53.54289	49.19935	97.0246	23.17041	19.34549
550.2733	296.296	292.9595	123.8807	246.8247	571.0715	147.6697	546.7242	324.3869
1280.33	2166.061	1553.208	1295.419	3198.521	1402.488	1808.76	1704.92	2160.303
1.267836	15.43194	0.095245	5.396768	1.205651	0.041841	0.87936	5.040795	2.407108
35.27304	28.45639	19.50339	52.60292	76.76238	32.44221	30.78526	27.32626	27.45094
53.49033	5.032296	3.161614	3.523164	0.054131	200.0712	38.02998	44.75448	15.29128
782.267	829.4049	914.0096	627.6434	3476.31	446.3492	749.4168	351.711	582.5101
52.29813	79.0257	120.3119	49.00056	182.6334	137.9065	85.17236	97.89632	167.5872
15.88267	35.87596	117.2607	63.28282	31.48929	62.41948	152.304	79.64096	53.35383
20.73662	18.61027	21.00356	159.7068	37.29707	50.42616	76.1263	28.9761	14.48244
75.32668	46.9688	3.160505	6.196084	3.556387	196.5242	50.72207	16.52011	10.43655
98.43237	45.75973	49.23506	113.2149	18.69423	75.64057	165.8482	126.9268	51.7093
272.1151	477.5611	604.5608	267.4558	292.1701	203.9997	195.7129	291.195	237.8384
45.9606	6.261711	6.132604	2.630394	0.051193	16.82429	10.84588	9.067819	3.967015
19.50506	2.561577	0.156114	22.25739	1.225234	76.63941	106.1524	16.53157	3.158185
15.85313	21.02606	16.4838	41.93962	24.46199	58.6239	64.43236	27.37094	48.6453
23.14094	21.05558	63.71183	30.29281	60.47038	46.77103	35.33136	50.63574	51.00459
4.9466	7.496647	7.619068	16.91623	5.886262	80.01024	31.73476	16.55465	17.76257
80.13959	38.32795	15.06699	32.95243	18.69088	109.0755	65.24357	23.99417	22.57956
54.46972	13.64577	28.20777	16.02143	20.97273	8.473995	14.47288	16.55303	36.46886
790.8357	1315.734	912.7135	491.2076	1186.128	2794.007	2356.132	1103.395	1894.106
17.0915	37.08052	40.2231	36.52873	1.22442	119.7817	32.59718	22.33811	8.818521
1348.09	1058.838	2122.801	576.8369	1360.523	212.426	297.2004	975.7492	810.7599
274.5706	192.6217	234.9716	333.4292	114.1383	417.4972	448.571	361.7109	333.3192
37.50946	79.62161	26.63595	115.481	12.84148	2.479576	1.774947	7.404967	19.41439
1.296685	14.85811	0.149485	7.984632	7.045683	60.88016	12.66224	4.078321	16.15032
75.36652	39.58922	56.64422	76.647	62.90668	93.61014	142.2838	64.66012	62.23931

44.85092	27.16245	23.83764	30.31749	18.66142	13.26255	7.21143	42.37421	12.06961
38.92649	22.30699	57.99927	21.35486	17.52702	174.8614	27.15227	55.56994	78.51399
17.09796	58.08889	52.19535	23.13394	1.222521	36.07306	162.2304	25.64609	54.14141
37.74708	77.8576	157.7189	206.8113	12.8681	361.1136	631.6719	85.38135	119.6694
123.9063	70.42224	86.31578	79.32062	12.87323	111.5932	207.5629	122.781	115.6849
85.06283	76.57204	68.49227	47.21411	186.1453	73.22771	77.91365	79.61356	68.7245
154.3325	49.47099	53.71578	131.9135	3.551629	691.9981	223.814	63.80999	25.81023
17.09087	29.69078	43.17237	37.42259	118.5656	61.16678	68.8896	59.74703	59.88703
49.68874	23.48374	32.66226	8.872861	8.213224	21.63092	2.680297	29.86852	17.75046
57.02864	91.10166	126.9511	88.36615	55.82753	14.47264	9.929259	40.6506	54.25439
104.5422	146.9404	178.453	93.57242	265.382	357.4482	278.208	512.8683	156.1053
103.3217	141.9943	455.8495	104.2763	343.3007	142.8128	207.5143	262.194	171.4979
523.52	258.0281	215.6802	416.3488	204.9198	181.2319	666.0792	287.8418	272.6089
2787.733	1302.204	542.6921	1002.967	1782.097	2321.463	1679.149	2086.532	1169.07
6.160714	19.78936	25.2875	36.58357	10.53464	59.78669	41.72479	19.88173	10.44911
27.95858	13.65844	17.97335	27.6273	53.45984	6.08148	27.17527	24.86238	18.55648
18.30854	46.94929	12.09664	63.29651	65.18636	54.01419	23.52473	20.67279	17.72097
8.233686	3.655766	13.61463	2.676385	0.032303	0.038958	0.881873	0	0.757007
14.61415	2.562496	0.148459	3.521936	20.94595	0.061899	36.30765	0.76572	7.211868
4.871782	1.311659	4.506895	1.748092	20.61504	0.05043	0	2.440986	11.47406
20.6556	24.66	26.66828	14.24126	25.58491	33.50696	8.121865	19.90301	30.80814
66.8782	95.08496	139.6992	123.9204	178.0493	124.7811	107.8233	137.7236	295.5086
23.15565	53.07876	91.89137	29.38886	77.93415	65.95439	31.69059	54.756	63.94125
402.1051	530.732	297.4034	384.2403	721.6308	334.7715	550.9645	344.2603	507.2885
71.73713	72.89589	102.6525	177.439	132.7045	90.02947	358.9702	56.35199	139.9674
2.514268	6.250764	20.69038	17.84325	19.75498	16.784	11.76594	12.4228	12.10938
680.2694	187.7048	58.16382	184.5071	30.32725	3145.215	1581.358	133.5002	155.2636
248.9573	252.9556	345.964	112.3077	677.0488	248.2888	125.9397	115.2769	98.65014
351.897	140.6731	89.26367	33.83465	5.885535	22.88014	85.16325	249.0476	149.7183
44.82086	39.40213	75.02289	32.11574	49.9299	21.61722	12.6571	19.88499	17.76049
105.7633	230.8462	488.7666	155.0965	358.4882	126.0431	205.6838	736.9249	700.0649
133.6779	192.5721	87.876	81.98125	119.9423	130.8267	195.7263	215.6983	166.6343
106.9349	88.92844	38.8462	50.77636	78.03756	145.168	120.511	63.822	83.28097
24.38106	29.71181	47.69673	35.62401	43.11812	27.6718	125.9997	46.41113	75.2331
26.74536	23.49053	57.64273	11.54997	25.62833	28.80459	19.00597	29.86154	25.86779
139.4323	74.00389	57.96317	41.88305	89.555	12.08126	38.94566	84.68673	72.04555
81.42726	75.34318	89.25177	44.53754	2.389183	190.6459	129.595	41.41677	128.6637
16.99786	27.03441	10.51086	2.630992	17.45043	6.068846	6.31061	30.81731	19.44656
36.11206	15.99738	1.669857	56.49783	9.337532	0.058904	1.775532	0	6.418114
31.65548	56.80552	58.02215	61.50879	104.6932	26.46841	79.75527	62.21067	64.71772
19.5046	8.73709	15.04329	14.22364	10.5427	68.27409	18.99903	16.53185	7.201125
75.26948	81.39556	192.2735	196.3618	37.28876	8.482733	18.08609	66.38546	123.9598
99.67973	204.8971	298.61	178.2974	648.0596	120.0309	55.23817	260.5324	279.175
148.0846	161.5379	112.8676	66.84724	60.56431	37.26364	30.77367	63.01737	45.24749
213.8627	382.6528	227.5674	323.6115	437.671	756.9317	387.836	652.975	179.5447
36.489	27.22807	3.165256	12.43635	31.47307	22.86571	71.61151	21.50808	57.45275
77.63584	41.98513	66.73709	200.0213	72.10266	24.05736	34.41719	48.11992	12.05731
47.4199	39.56232	46.18584	48.12138	36.13438	44.4379	74.31232	43.09816	61.47474
28.00403	42.00008	46.1241	37.42312	45.41342	73.12716	134.2355	19.84709	68.00202
21.95696	9.971304	21.01701	20.45913	22.18628	505.7466	86.06681	77.11122	50.90154
41.3594	34.63568	59.50943	45.44303	216.2104	55.21848	102.4313	58.8836	32.29785
65.68583	24.78263	73.03506	24.9184	23.34778	594.921	189.3679	106.9565	84.87453
42.57179	21.07693	37.30669	46.33554	21.01812	54.02063	56.16992	33.95888	34.72843
24.34701	32.11905	79.84466	24.93783	14.02958	59.902	43.50529	23.18499	17.7355
538.1218	1216.751	2344.006	508.1805	145.5667	349.1684	309.8891	300.279	555.0302
181.0738	100.0745	154.7718	412.7768	82.71353	24.07413	470.3066	814.7887	231.333
30.43242	89.99487	162.7208	99.91959	151.0887	30.0539	36.22455	60.57124	121.5409
159.1148	122.2205	75.95516	65.93741	119.9132	131.9848	41.64602	92.04679	65.46889

125.1101	66.71904	52.19371	117.6831	86.16819	117.5772	57.96479	42.24526	56.57118
28.00343	62.91474	54.9814	30.28251	22.17431	77.9092	32.59876	56.42347	88.29103
1557.979	1427.567	1694.623	217.5052	223.5377	30.07523	148.5782	150.0987	414.2439
360.675	383.7327	350.577	243.3987	76.88843	346.6424	271.8682	237.2795	185.2525
11.02391	58.04568	139.3652	46.33375	143.0461	66.00034	49.81945	81.31711	168.494
19.50328	29.66145	26.85677	23.15144	19.83907	47.95592	13.55687	79.78897	22.60169
81.43191	96.3038	132.242	113.2239	212.9054	81.62398	98.76312	132.7572	99.49495
138.0933	43.20631	82.90745	36.53945	15.19532	33.62708	26.25382	14.0363	27.45954
15.87421	11.20527	7.635778	10.65337	67.45631	24.0564	25.34544	30.65709	23.40265
657.1686	243.2389	145.8636	389.579	2573.206	608.2781	292.6652	645.4495	519.3969
8.590997	9.968084	22.41491	10.65551	25.63962	9.678366	51.68419	15.70435	45.3461
42.58943	60.53651	93.64569	56.13838	165.1906	62.43242	30.77303	102.0543	131.937
138.5178	151.8478	70.04297	257.6893	83.86512	69.6604	233.8124	191.6472	83.2681
122.7132	28.48835	29.93407	24.91689	18.69405	392.0305	230.2116	62.99126	41.18427
2.512448	7.464818	3.155388	12.47988	3.553414	12.00588	2.681847	18.3049	4.787709
233.2998	360.4568	165.1762	412.7727	173.4997	583.0541	641.5934	379.1104	155.2664
29.1653	32.08892	28.27738	15.12126	27.9495	43.13576	10.83846	20.7019	29.11597
757.9948	592.4916	150.3229	192.5313	345.7642	802.6211	336.1618	434.6791	434.419
217.479	255.5236	647.6152	7.982663	357.33	3.671546	13.55555	543.5124	364.9182
23.16968	42.04763	10.60951	75.76551	15.20217	26.47623	109.6577	11.54227	68.73351
156.5945	72.86274	74.39706	73.98082	43.12505	10.88214	47.99739	122.8202	92.22762
336.4851	534.346	860.0109	162.2259	476.0328	314.3454	261.8705	144.2947	370.5637
122.559	74.04742	130.4626	65.96997	112.8297	32.46028	42.56604	33.12843	89.037
171.3254	54.40739	32.90695	120.3228	139.7295	293.9155	260.981	150.9461	154.4821
1719.947	940.5332	622.9061	633.8666	537.8173	1384.447	1194.358	1194.687	754.8325
35.30279	65.44982	98.02853	55.25215	80.31877	93.55836	64.32482	66.34765	59.02559
14.58207	11.14824	14.87007	12.46916	10.50796	25.09626	14.49632	11.58892	15.37478
202.9121	108.7075	40.33818	75.73648	240.9774	455.8361	338.0093	194.9189	128.573
32.88627	37.12582	81.95487	70.38652	565.6591	1175.424	321.6807	67.95746	52.50893
477.4425	803.5154	512.9092	233.544	522.6751	707.8611	694.1267	693.5554	878.6654
21.9432	46.92669	71.23626	32.95877	21.01292	65.95549	48.92654	56.4183	76.10971
36.43432	62.798	66.53104	21.3699	18.67229	35.98702	11.74437	42.33902	35.6027
178.3376	75.2986	137.939	40.97778	16.36548	42.05105	41.65579	119.5362	119.0104
428.8244	883.615	878.1814	177.3771	37.31504	38.4732	43.45807	295.3014	244.2775
46.2204	135.6459	140.9194	75.77639	3.556451	3.677857	16.27138	48.07247	164.4025
52.05249	45.52253	56.00845	15.12829	86.95728	14.45128	8.119562	4.077448	7.206041
795.6227	332.0951	301.8967	254.9437	570.382	758.22	501.1016	600.6373	265.3071
34.09239	62.99465	44.74505	53.46444	72.18975	16.88132	71.57569	39.76346	67.12086
9.786338	9.948122	47.07447	10.66602	33.67915	45.40195	11.75471	24.91334	19.40886
42.60215	132.0793	65.56474	57.01948	372.2525	76.84267	117.7925	79.59557	78.4243
51.1001	24.78576	10.60536	29.37508	25.67821	13.28109	136.8326	41.41254	71.9482
31.59176	54.19587	37.12176	47.28619	38.39475	21.64189	14.46646	15.70691	21.80083
98.45491	161.7045	196.16	164.0398	360.7	161.9763	114.1566	202.4488	262.2125
20.71705	28.43612	97.53704	36.54562	157.8852	64.69424	28.07199	37.32318	107.0609
63.05286	32.11578	31.27199	29.40466	63.93077	3.68111	13.55734	34.00288	54.27214
139.5233	61.73547	47.67146	46.33532	4.722629	2.476197	36.21786	53.89767	63.09268
20.49466	8.664205	10.40383	5.318753	12.77774	0.057459	9.054377	19.18967	18.72316
71.73702	71.66254	58.14474	98.04763	72.22009	292.5603	88.7823	123.5992	108.3843
139.7812	85.26358	31.40784	317.3616	19.85142	134.4525	279.9808	121.8851	47.65676
210.0612	82.75948	284.9601	121.241	93.15713	9.679959	5.400237	3.254078	3.16419
25.44384	57.5806	13.4576	24.99469	5.881055	1.276038	7.216853	18.25379	52.89793
94.78383	51.92374	81.86184	48.10333	124.5479	155.9264	57.96409	195.8715	104.349
9.618459	24.11051	4.514796	14.38463	9.243511	0.050919	0	0	0.741558
40.1415	66.64894	72.7941	36.52213	72.15666	79.15222	24.43151	79.67166	47.69921
61.98783	82.69592	142.4064	82.02235	165.1529	48.04428	63.41774	139.4729	146.5627
236.9455	334.5485	236.4997	200.5585	117.6326	1899.957	696.8729	545.8966	205.4342
116.6942	362.8485	457.6228	240.7017	699.3967	106.8538	132.2694	536.8699	524.3835
170.1227	175.3317	144.3373	228.2187	244.4628	275.9517	378.8022	270.447	282.3576

5313.004	3600.288	4504.831	1851.743	13447.12	6970.164	2833.65	1787.01	1474.909
299.7904	17.37999	13.58257	27.59207	3.554531	449.4479	174.9206	36.43415	4.779566
1443.907	741.6854	622.6511	190.7536	36.15171	115.2566	299.9251	664.5948	826.997
3.732442	8.734817	15.0288	21.37069	25.63569	45.5393	18.09572	36.51274	18.55315
993.663	1200.942	894.8571	1419.368	1195.426	1330.483	1507.909	948.2505	1386.781
211.352	254.2108	292.6332	228.2492	763.1661	87.65028	94.21177	301.2256	341.5397
46.13409	28.43344	37.18169	64.24424	11.70532	8.481125	27.16556	39.82261	46.95214
66.88797	143.1972	181.2977	119.4476	109.4533	243.4701	163.1126	185.8547	164.2423
35.13509	71.15865	16.43639	20.49914	1.22643	1.27587	4.492529	90.88508	31.61242
130.9939	158.9117	547.7274	28.49285	43.10233	9.682662	29.87322	47.26071	199.4028
105.6945	42.05489	59.60633	154.2662	36.14976	55.25405	182.1855	58.84998	54.9534
88.74656	84.01177	181.3484	213.9836	482.8412	185.9604	310.8698	107.8044	82.45873
48.45119	55.34033	35.55279	7.981437	5.886847	12.06365	23.55316	45.72512	33.20684
33.75912	14.79632	19.17822	7.996788	13.96445	1.275678	13.59316	18.29547	20.29735
25.59238	86.37048	134.9369	47.22579	54.73782	31.2643	78.84226	83.80938	149.0284
48.61018	104.7516	55.00484	127.6059	430.7963	37.23748	43.48182	42.28171	48.52543
283.0237	287.5756	313.5648	437.8047	105.9849	123.6406	196.624	165.0526	201.4257
3.728121	6.252519	20.70911	23.21614	25.53587	9.647418	10.8548	7.412299	12.10551
153.1034	88.9548	78.96584	37.39868	46.62985	365.8243	164.9073	132.694	113.2088
8.59072	23.50282	38.61106	21.36945	19.83396	70.62152	24.44778	60.66108	17.74007
71.68508	16.1449	21.00952	35.62442	39.62897	66.01033	78.83675	56.38036	17.71893
2011.222	160.5513	41.81338	109.6155	122.2887	1022.094	591.7298	133.5001	109.1452
57.98519	16.08015	17.88095	8.878792	1.226519	2.479648	22.66117	6.572057	56.0982
133.5188	6.26762	10.61043	16.89334	3.556326	109.1297	62.51009	42.25707	3.969308
148.2692	248.1278	405.6859	295.988	448.0938	356.3073	681.534	250.5218	395.6683
41.38558	76.5868	86.31467	88.241	36.15112	191.8767	118.7056	104.5125	140.7947
40.16664	114.7736	130.7145	35.61946	76.85544	428.953	43.46268	57.19348	76.01566
58.39169	175.2705	141.2758	101.6061	132.7256	256.6753	78.80514	105.3169	148.847
260.015	258.0339	295.9113	361.9506	170.007	714.9719	214.7331	889.4756	851.2569
71.66295	50.64835	49.14594	30.27487	18.69132	31.2614	41.65972	122.0613	61.47459
14.61447	24.64572	41.21124	25.87874	24.41754	41.8175	11.75605	37.45055	21.04352
55.95479	48.22679	23.98958	24.91699	262.9366	141.5613	77.90559	87.90093	84.90535
17.07296	11.19853	4.659629	16.01241	70.8593	21.64279	24.44925	29.8524	30.73215
52.23928	265.7641	164.1349	128.5077	18.68731	2.477419	12.64737	73.8843	43.66377
30.42664	6.269619	0.157868	2.631637	2.392031	49.19737	181.4424	4.906776	0
47.44745	102.4469	269.8494	93.60533	269.8232	103.1779	77.00883	149.3926	306.1561
153.1069	1033.611	727.4477	358.4463	30.33403	32.47817	47.98776	88.70618	379.545
2.510661	22.31501	34.37038	20.45979	23.34836	74.41155	174.0539	53.04835	37.14725
29.19819	11.20369	4.659565	2.631119	105.7178	77.84837	45.31401	43.1433	7.200918
15.85571	14.88519	10.5866	17.80218	22.14573	61.02361	8.117865	12.38404	16.12496
4.94766	9.962812	12.06087	6.196052	73.12795	21.63021	15.37709	48.20503	21.81228
280.4531	34.65855	83.37922	57.01831	67.56914	76.84636	157.6785	121.1021	78.42064
2724.564	1857.586	1626.117	3195.371	3008.884	2103.12	3063.843	2880.542	3407.925
435.1721	390.4037	121.6725	20.46074	7.052308	3.678039	92.443	151.9526	125.4967
318.2946	1217.857	316.6794	752.5357	807.6997	163.2376	327.1172	436.3897	525.9322
119.0143	123.3883	210.6264	322.9518	1048.398	20.48039	68.84952	105.3686	63.05969
253.8279	190.0829	197.6814	243.4139	139.7124	117.6267	153.1308	166.7384	154.5053
24.36115	57.97299	62.31407	33.85761	38.43421	94.61879	13.55481	41.46598	56.66229
633.7759	540.3597	850.5556	398.5831	203.7166	40.87641	45.26911	228.9747	320.4459
38.95436	33.4201	38.83376	15.10995	31.4952	587.0725	121.4347	55.53053	110.0322
75.20934	39.5219	25.4102	119.6118	86.02473	14.47573	28.97436	53.11412	29.89012
25.59103	33.40344	77.25853	63.29185	54.73413	15.68054	67.96093	69.68704	79.30627
13.31694	37.75184	7.511034	54.78806	1.225354	0.057335	7.23267	2.424334	42.57429
2.497217	4.972823	20.10482	0.852376	69.70685	0.053118	1.779496	0	10.59294
14.60506	30.73481	13.46035	6.200658	13.99315	38.21907	9.941666	14.07815	16.16698
99.4254	85.02649	62.32589	73.14937	14.03406	2.47789	23.53016	79.73009	32.31967
15.84929	21.01972	9.102194	1.740183	54.57853	0.063342	43.53937	19.87976	66.56946
126.3736	197.464	166.4936	87.33691	204.8453	285.4396	156.7619	139.3527	164.232

28.02212	34.65218	23.96861	74.84537	5.878359	2014.041	830.0762	104.4625	98.62734
4.944771	11.20786	138.0516	635.3872	471.9686	0.066191	0.872722	9.883465	39.57635
0.031497	2.416795	0.065053	0.874908	2.296745	0.029457	0.892099	0.801062	3.338076
4.945677	7.494192	34.01085	4.41323	3.558965	28.7567	35.37993	5.738895	5.586415
1470.996	785.0485	508.4944	687.3584	1424.718	2766.385	1193.443	1070.214	650.4439
9.79594	2.56279	3.169109	4.412777	0.051929	9.670947	9.933884	24.04988	0
27132.39	23696.01	28947.64	16050.84	19657.22	29688.9	22335.91	23840.92	23914.5
100.8231	160.3479	175.1065	156.96	109.4068	73.22209	56.15595	53.87426	82.50044
182.2533	238.2307	220.0369	152.4254	185.1095	478.5613	183.0296	231.453	245.1404
166.4629	250.5521	347.644	93.57252	161.8333	340.6618	139.526	292.887	276.7237
314.6588	280.2403	422.1543	546.5362	1026.485	646.5949	433.1516	461.2759	1101.372
2007.794	404.9244	628.8523	722.1347	331.8025	692.2904	1014.922	657.0284	2581.971
249.0384	30.95593	15.05878	14.22137	34.98983	418.6358	279.1025	462.1877	12.86927
35.1953	104.3908	28.25359	46.40979	74.28684	6.080742	19.91611	30.70235	89.33335
30.42787	40.7687	53.50659	52.6008	34.95767	77.91029	57.09473	43.94991	78.55165
23.09797	7.497756	6.141011	16.02054	22.13397	7.277419	17.1962	12.38916	22.63572
34.09428	76.5558	64.01912	67.73701	132.644	19.28053	117.8219	78.79546	198.3995
218.5765	44.52909	83.37921	75.74705	85.01953	363.2838	116.8825	67.14076	84.08799
106.828	74.06078	74.32573	164.1505	98.90231	30.06678	74.30553	78.81983	54.17044
79.042	56.87508	74.51321	147.9692	17.52766	21.67881	263.7048	43.06797	128.5845
63.23771	100.0181	53.68777	15.11025	207.1228	351.2466	56.14953	92.88265	158.6042
19.43085	13.61935	17.85411	8.881042	10.52089	1.27603	3.586678	0.765649	29.20653
2224.093	1302.182	1184.696	1039.528	1054.617	2082.69	2151.304	1540.628	1123.774
40.17096	55.62319	53.68011	77.53785	64.07116	87.62058	359.911	62.16803	117.3087
175.9619	167.6918	102.503	192.6809	4.721642	25.27587	25.33481	44.74591	281.9237
9.799713	2.562613	4.65877	11.55097	2.393682	19.24142	53.52525	9.059159	6.393661
402.0682	409.77	142.8734	78.41073	2.385777	494.2496	395.0969	273.7446	92.96414
1457.692	1589.762	1207.015	1848.189	712.4219	2658.547	1013.09	2144.621	523.4035
239.3427	377.6668	518.5333	228.2137	566.7802	490.6203	292.6885	472.1138	390.806
24.37513	24.77292	25.44725	48.12421	570.2814	109.0687	101.5338	20.67312	147.4534
78.62922	19.79388	7.624151	43.73338	2.393769	2.47966	6.304805	15.71548	33.19863
70.51311	55.62204	56.64492	124.8198	198.9598	36.07286	164.0451	58.0179	56.57093
116.6583	182.6573	202.0699	262.1645	299.045	137.9945	211.1591	173.3973	339.1633
59.55359	122.0796	165.9901	61.50269	117.4931	159.3821	19.89723	88.79271	63.08522
4.940664	11.15997	16.36425	19.62581	13.98772	17.98857	17.21813	16.58954	5.592114
12.22419	28.40147	25.32814	9.764848	19.82648	78.92284	23.54567	47.35963	22.61975
4.916151	4.99856	7.489223	1.74197	4.701553	32.00305	3.593778	4.095435	6.43757
34.00979	32.09799	31.2367	26.73157	11.70198	80.15116	11.74486	23.19474	34.79583
4.94115	148.9428	25.11194	9.777544	1.22659	0.061186	7.217418	7.409187	0.737371
21.94943	35.86446	75.75836	49.01517	4.722852	54.01547	57.9866	122.9002	53.37149
32.86609	54.33713	68.35973	51.69365	54.72781	97.10483	55.26644	42.27024	47.69914
47.38144	14.90474	15.05662	8.869564	21.00997	38.42279	39.85914	84.71838	25.83175
15.86367	13.66216	26.83309	25.83549	7.052139	129.1016	19.00233	31.5136	14.49549
64.43356	65.47319	23.9857	107.8822	36.14733	74.42098	152.2749	43.91012	8.819928
12.22277	25.94068	35.60291	16.01622	10.5374	31.18623	21.73139	41.53298	36.44036
27.84259	12.38667	3.164714	5.307997	57.88126	8.459685	16.30719	7.409194	5.591573
51.07313	140.5758	69.91223	51.68279	16.3657	95.95471	72.48638	87.94907	59.02493
59.51027	56.77296	52.04485	117.7853	33.79851	13.28002	28.06209	50.59672	18.53413
26.75578	2.562222	6.146602	7.087265	10.54026	100.4465	28.08015	17.36954	8.011969
300.6796	336.2246	290.0988	129.3357	97.74084	0.065909	4.492963	41.43108	47.68764
78.68122	19.80369	28.27079	22.26834	29.10704	34.77442	18.09853	14.87847	12.0668
150.6211	75.36399	65.56788	68.61301	79.20118	118.8002	125.9501	111.9725	91.37737
175.8106	16.14244	15.06587	28.49225	4.723054	46.82615	26.24547	29.80967	5.583982
12.23822	2.556414	1.6625	9.761589	1.22277	123.5525	203.0512	22.32749	2.355465
64.3894	208.1741	29.89658	48.12185	533.1953	1.272769	6.304079	77.17224	23.3889
132.3592	88.89144	189.9244	75.76177	93.13211	32.47263	85.16943	95.39804	111.6634
9.803471	2.562171	3.168782	10.6559	14.02658	31.2072	57.13726	15.70562	2.350307
10.86158	35.08211	5.997341	12.53666	25.29699	0.053987	0	2.431269	0

1429.662	1840.154	14579.34	1753.744	40.80326	160.8461	116.8593	1894.997	476.4855
13.39457	74.62543	3.164688	17.83293	24.39582	0.061177	1.774966	24.94382	0.737369
201.6325	416.9458	369.7169	269.2852	86.19207	54.06948	79.71063	136.8574	177.1794
242.9496	230.8228	363.9868	147.9682	64.08827	205.1801	251.0128	360.1183	279.1469
29.11664	18.54787	19.38175	37.49433	11.6908	13.25217	25.37827	17.39247	20.20817
19.4792	100.6839	25.302	18.69871	111.3159	9.673056	6.30478	66.56331	76.32925
30.4469	39.56957	69.88971	61.50204	43.115	154.5983	95.1665	38.93898	39.58526
434.9259	680.0954	902.1329	479.6309	1609.655	2003.264	875.387	1506.758	1144.925
36.52425	76.56605	112.9263	111.4511	131.4967	103.1761	131.4169	114.5043	115.7163
286.6963	589.8399	773.8591	1040.64	183.9602	62.47107	192.0847	343.4712	657.9283
11.0137	19.80618	16.49688	28.52171	19.82846	56.2668	20.82076	19.03706	15.3108
120.3168	96.35232	46.28238	192.5698	130.4089	115.2323	291.821	65.47299	83.2648
691.174	55.63826	16.53276	64.14693	4.713978	3215.817	1205.281	51.36777	5.59552
5757.608	4158.173	5148.414	3831.034	56743.5	5753.756	2926.982	2354.494	3482.305
80.16541	19.84534	26.94179	14.21855	33.8133	69.59846	60.70035	112.8921	29.05486
615.9375	3005.353	2577.208	1956.07	2040.483	4796.297	2887.137	2568.59	1915.088
262.6507	56.73565	9.121191	9.761572	3.558677	0.06494	57.10662	62.26863	48.55229
206.513	249.3008	254.1122	287.1046	26.84194	203.9657	311.7571	240.6048	260.548
63.15514	26.00108	18.03001	14.2196	10.54554	65.9685	31.68826	24.82816	12.05528
338.7817	474.9127	615.9218	147.0873	411.9068	51.67128	68.83363	108.6322	337.4953
180.9618	144.4177	255.3846	197.9503	61.75182	49.26819	67.02487	146.0111	172.353
0.031672	5.865362	2.818869	4.557196	2.297481	0.02962	0	0.800715	3.336619
50.97392	169.6458	68.10265	49.05386	16.35455	12.07638	11.74288	60.63181	52.63593
47.36035	71.4694	43.10799	58.8717	408.3857	16.86949	13.55566	41.47595	69.67086
14.66306	30.91713	35.76975	50.81865	57.021	74.31133	94.30836	52.27076	37.99362
126.1955	92.51545	108.3015	53.47475	24.50747	42.04419	38.93842	72.18058	38.77945
60.82209	2.553676	3.151123	5.307807	65.24852	416.1186	308.1457	89.54236	29.04474
42.58998	86.41626	65.50522	42.75681	28.00269	27.6752	107.8443	76.30152	82.50635
134.7295	298.156	111.3522	60.60504	1.223386	0.06604	8.116202	38.93536	31.48202
464.0827	369.1252	506.9663	554.5224	116.4683	1655.473	1129.13	1004.713	240.2207
205.3606	649.1493	604.8219	294.187	154.8753	419.9166	392.3688	337.6313	270.181
11031.79	8464.415	11418.91	6525.337	45731.03	17054.87	11352.81	4035.368	5707.31
15.85934	25.95137	10.59067	28.52081	106.7606	6.081488	35.34765	34.02164	31.55089
58.38455	74.12877	95.23547	57.01951	62.91323	244.6291	200.2966	119.448	127.0104
73.91426	52.98865	91.5262	1.740681	9.378509	8.480096	20.81663	19.03051	72.16374
25.5227	29.61323	32.64223	32.10742	41.84248	19.23721	16.28608	52.38466	26.69311
10.95609	7.466791	19.165	9.789296	30.1003	26.24354	6.314642	14.11241	25.21473
106.6575	73.94303	97.64717	32.0739	257.7002	19.26627	38.04863	41.47028	55.0462
184.5642	220.7989	221.1834	117.6784	252.4578	129.5697	100.5722	111.1546	93.81725
32.68114	8.715681	23.69844	13.35201	3.55803	0.061691	20.84833	35.79041	23.49522
69.25111	59.27951	46.20128	40.08713	34.97515	67.2	33.49635	34.78705	54.17156
43.80717	91.35775	173.6483	76.65333	75.69154	72.02663	112.3718	125.2998	145.6915
3.732362	9.960652	1.672811	33.00373	51.10409	39.52448	11.74807	9.89214	12.07127
295.2275	644.2158	563.2416	280.8116	435.3417	496.6759	422.2768	391.5703	328.4487
19.50267	40.72244	28.32945	10.65474	83.6522	130.3584	28.07416	37.32907	67.2651
9.805616	23.51275	53.37817	23.15123	136.9954	36.00421	63.47792	19.85781	77.82263
258.7728	244.4326	511.1244	452.0425	531.8794	236.3899	237.4003	455.5101	427.226
24.38116	6.267748	6.144451	8.869638	3.55642	28.8706	24.429	3.251683	0.740973
217.4572	334.4311	455.9986	210.3932	236.3006	62.46952	68.83131	179.9964	190.0995
17.08495	7.504121	7.635301	30.28979	31.46008	32.42904	58.92581	19.02198	35.57346
4.939785	8.706752	10.51476	7.096328	45.16315	40.55661	7.21856	21.60962	10.47409
6.163599	3.797744	32.82197	20.46559	8.217406	168.7797	106.1008	24.00134	6.391872
143.3741	93.88064	104.1885	159.5753	232.7728	154.792	139.5349	126.8953	173.1296
226.8103	194.7291	189.6885	55.25346	75.66632	3.67797	19.89692	170.2355	158.7343
146.8669	76.54692	112.8589	34.73044	19.85714	27.67354	86.99274	108.7123	100.3452
234.2835	63.02172	31.41575	78.42998	53.60188	63.64823	110.5473	68.81012	17.71771
11599.04	13190.23	10170.49	9521.014	12310.2	7459.78	13578.44	10659.37	7574.721
182.2848	419.6605	263.2124	277.2462	146.7279	694.5549	376.963	311.9086	339.7819

40.09428	19.82592	63.72442	24.93429	155.5885	37.20903	21.71934	48.13582	37.20096
17.07936	17.35895	10.59953	40.12317	14.02943	14.47067	49.86102	12.37785	32.34171
2.511977	1.322999	0.140142	6.209094	0.048509	25.03941	11.77751	5.754557	2.352038
60.79484	54.38004	49.21144	76.6539	64.06198	166.6663	38.02394	156.8697	46.05086
233.2022	467.5311	532.9201	254.1135	3036.86	58.86818	63.39529	364.3194	391.739
755.465	729.3651	947.923	689.2043	208.4135	157.243	327.1118	755.8691	922.4833
13.42697	3.798907	13.52311	8.874403	2.393844	25.1998	47.18079	24.88307	7.20547
216.2431	179.0168	197.7601	184.5309	261.8966	277.1248	268.2342	357.6259	339.0519
79.02005	103.7238	25.47601	73.07298	97.81181	257.8137	171.2824	62.99236	51.70747
32.88854	63.0324	287.9942	80.20658	18.694	272.2102	136.8272	55.52039	67.89623
1846.328	237.0737	591.7264	1478.198	51.27801	2071.869	2154.941	1599.55	1480.617
299.893	223.3388	310.2967	140.8541	681.6353	52.86768	111.4399	112.7935	237.1249
116.4643	83.87008	184.9728	49.01886	5.888221	62.38696	54.36212	85.49995	84.19416
76.383	124.2593	122.5528	17.79311	47.70603	2.478549	13.55617	10.71537	55.87669
148.0987	113.5168	186.8871	82.90505	52.42763	52.84566	91.5226	93.74798	119.7829
15.83175	3.798037	14.96612	8.877296	4.722288	44.23278	7.213807	23.23578	16.14722
117.9145	88.96594	221.6086	157.7649	58.26982	869.6177	379.6868	602.3782	75.16302
2.410419	9.39444	6.825295	9.136695	5.66376	0.034052	0	0	0
151.8395	119.7586	210.9127	129.2639	262.9664	139.1804	95.12594	142.69	143.1991
173.6374	272.5376	117.4396	32.05076	32.65999	12.08141	49.80571	43.9048	114.0691
0.067549	1.323539	14.98436	3.521403	9.371037	8.472657	16.29077	4.077585	5.585612
4.915159	42.4944	1.662902	8.016017	1.224664	0.056273	1.776949	0.766247	0
11.01522	28.41362	73.84382	35.66685	56.94687	31.20335	31.71262	44.01025	46.98427
37.68374	6.269721	1.669433	21.36095	1.224903	30.03971	107.9411	9.054083	6.39176
6.15572	19.84658	7.620462	7.981544	8.212183	567.2053	306.3183	13.20469	9.633036
81.42535	104.9216	74.42537	62.37766	69.88051	54.05298	121.4356	40.58711	52.52637
60.69233	5.034398	7.635908	40.10762	2.392346	37.22226	45.30569	6.564907	8.818973
71.73347	71.65868	136.7376	128.3802	129.2085	104.4077	158.5951	121.1146	169.135
29.23095	85.13081	117.1739	70.4316	60.5457	56.41667	72.49579	92.1273	119.8471
83.88274	103.7358	227.2608	72.17801	217.6251	105.6236	193.0306	155.9662	491.4487
678.9822	691.1369	658.3624	806.0081	434.1922	281.996	402.3306	519.3492	563.1268
104.5306	127.1848	264.4356	174.7363	24.51407	430.5009	112.3407	355.1977	130.2184
26.75962	27.19117	22.41247	20.47611	7.05218	56.29577	21.72467	17.36793	12.87356
2.512384	7.464426	16.25784	9.790931	4.712888	21.49445	4.497547	17.46774	12.1301
102.1076	227.0948	577.4752	241.6215	245.5855	1.269818	8.118801	101.1586	310.7536
75.39539	145.6941	110.1407	121.2206	61.75878	583.9668	184.8529	204.0853	99.45225
15.87725	44.46053	46.12211	39.20886	73.28403	39.62594	65.26153	15.69446	53.39994
80.0339	77.61981	90.25924	49.93691	323.7704	4.881956	20.81109	94.73734	106.2117
76.34094	76.32154	54.81415	81.23855	33.76597	6.082149	11.74368	66.47974	36.4066
184.6636	206.1361	243.728	134.5959	213.0175	208.7625	211.1373	293.732	302.6473
15.81333	47.83862	16.38998	11.56405	46.36262	6.073188	5.400821	31.61726	55.3311
25.58711	32.16466	41.72932	25.81573	10.5457	167.6807	32.59343	26.48731	264.3104
49.6302	22.23283	35.51186	28.54225	8.209985	1.27572	9.027318	26.55794	27.52494
272.139	137.0932	96.81376	189.8663	142.0696	550.6075	368.8149	262.1286	119.6676
27.90783	8.724624	31.09395	8.876329	0.051534	0.062659	0	0	213.4659
15.87526	12.43886	9.122019	2.631458	23.33365	58.75608	38.95282	36.47447	48.54411
41.34454	44.47398	87.51378	48.12963	62.84863	137.7469	71.60338	62.229	48.52042
318.2111	212.3191	151.7339	141.7262	571.3457	269.927	292.7099	222.3289	461.3062
122.7701	217.2931	288.4197	176.4924	336.404	355.1226	350.6914	153.4221	246.7276
19.5029	21.05131	15.04085	9.762554	21.00024	2.478788	32.61203	40.65766	45.33172
32.69542	27.09424	87.75502	24.98272	24.41866	14.43373	5.400122	30.76496	26.74629
2138.768	3103.51	2463.361	813.9977	448.1803	52.86938	174.8553	377.4328	1049.438
118.1211	58.88224	42.69135	3.521821	0.051054	6.075494	18.11612	13.23509	21.85388
91.02537	3.796914	1.667024	10.65243	37.29113	193.9836	121.5026	18.18306	21.77155
131.2373	204.8798	147.2309	137.2772	313.0366	81.65341	231.9968	105.3126	137.5046
47.01333	16.03921	90.07576	4.41629	3.556059	3.676843	10.8564	3.250599	36.6181
118.9852	9.973234	3.159746	2.633072	43.12148	115.1278	198.5526	17.34963	2.354509
15.72689	12.32424	13.30975	7.10891	1.225715	0.057996	1.775939	4.924163	8.876251

29.22904	17.37671	15.06739	38.30573	9.381658	105.4901	58.89291	38.11413	6.392605
337.6464	220.9614	184.4073	153.3172	92.0195	74.46497	207.5022	248.8833	503.3872
23.12803	57.89106	75.34795	52.64383	12.86502	20.44917	18.09501	32.34654	38.03868
18.29906	23.5243	20.97345	27.60935	19.84546	37.21906	52.56801	52.28313	29.07956
55.38545	13.59051	11.95326	9.783696	11.66302	3.676253	13.58742	19.95382	24.36427
17.0902	13.67308	13.57629	27.60508	15.1981	95.84617	45.30002	17.35562	19.34573
38.95532	35.88787	62.57222	78.43276	118.7251	14.48191	203.0424	64.66207	81.67803
70.5191	128.3698	161.9308	109.6476	96.64284	37.27359	98.75741	137.724	80.85785
87.47687	113.5257	203.2002	133.7632	201.2299	101.9717	67.03728	92.91224	69.54221
234.3492	249.2033	276.139	45.42521	34.98924	50.46747	27.14527	182.5454	154.5359
60.79711	118.4717	151.4516	130.1844	110.5763	123.5495	95.14175	91.24168	152.9792
770.1674	1601.985	1349.494	2409.092	313.1788	641.9032	1251.455	674.4556	3926.098
7.355528	34.34192	39.54432	21.42624	0.049838	0.060552	0.870724	7.413232	2.350215
443.4393	519.6904	939.3585	634.7647	613.4622	2359.608	1167.185	686.0805	1098.756
2.458115	11.95831	16.5779	22.73098	0.035869	0.043323	0	0	3.233098
47.38709	69.05941	32.81793	69.56976	40.76422	10.88135	37.13546	34.80703	20.15648
686.2995	961.3966	805.4907	598.2295	966.049	465.5289	318.0421	716.824	1059.188
38.95455	92.5998	108.5166	139.1017	95.46582	151.1134	100.5792	118.6445	63.05282
18.30073	34.60546	74.1189	31.17843	4.72392	26.45271	23.53029	40.63247	77.76181
23.1574	27.2311	31.35525	49.91722	46.58127	20.47149	33.50331	51.42516	54.20107
2.463928	4.89218	1.606511	0	0.036904	0.044592	0	0.77589	0
30.29195	25.87566	16.4156	16.9266	3.558473	4.878424	7.214778	26.58527	10.4603
29.22717	46.94746	105.2954	70.43897	2.391199	301.6678	23.52496	58.06088	43.6473
58.20266	106.9846	119.4139	51.74677	17.51246	24.0386	7.210272	34.00855	45.34596
28.02298	49.43513	31.39711	48.1131	51.25902	124.698	69.76453	66.34496	46.06067
139.7182	344.1571	397.7762	365.6439	65.24508	105.6259	38.02004	256.4284	321.3472
1073.826	2191.915	1137.07	1218.755	1783.188	927.42	1507.003	881.0437	1037.221
4.946379	6.264759	7.617777	13.34217	12.8526	6.078666	40.82663	24.89089	14.51345
279.2019	212.1934	215.3015	313.9443	85.01384	6.077751	23.52051	193.3591	159.4113
42.46072	117.9314	31.21951	24.05408	15.18491	2.479407	12.65286	47.35377	20.99319
58.11941	25.93141	29.70508	8.873533	14.0187	3.681264	25.3672	54.88804	29.94604
7.36735	6.261404	3.167634	5.306032	5.883934	1.275953	18.11486	21.5738	20.222
60.76068	173.756	117.1843	65.07537	288.2308	92.33774	96.98608	88.79988	121.4647
88.74843	63.04098	49.25244	117.6561	58.26652	54.07009	135.9079	104.4822	107.5508
30.44702	55.58697	146.7891	37.40977	204.6306	98.33569	87.00281	72.17048	132.8
188.3488	276.5173	573.5209	253.1779	574.9448	738.8716	359.7533	401.5538	436.9273
65.60289	91.27859	134.88	163.2788	71.00083	93.52481	57.98508	35.62119	82.55381

TCGA-HT	TCGA-DH	TCGA-S9-	TCGA-S9-	TCGA-DU	TCGA-S9-	TCGA-DU	TCGA-HT	TCGA-TM
103.8814	122.3707	150.216	79.88359	59.02505	183.4967	166.0108	70.90057	88.95238
7.662843	34.97533	55.31565	23.11423	54.74147	18.51217	35.85428	49.3745	61.96777
61.20718	102.7224	156.3053	64.53472	44.05962	91.73417	172.3295	173.934	112.9423
1941.377	2185.238	2747.173	1965.273	2620.814	2762.526	4182.956	2434.438	1672.537
215.723	179.3101	138.1338	128.7959	97.23563	86.16501	68.5115	154.6298	192.828
6.279908	0	0	1.673978	8.404839	0	0	0.017613	0
9.990303	7.171659	12.032	71.73081	66.7299	59.33469	235.5677	5.400293	14.39448
89.13269	227.4354	159.7193	76.82215	65.76515	138.0728	116.8638	206.1638	171.1878
3674.131	5774.661	6978.438	4969.747	8795.736	6175.596	11918.98	12649.99	5791.617
83.80565	129.8994	74.25572	30.84185	74.10349	53.73792	31.87916	135.2475	131.8894
27.97015	24.06668	16.38097	21.46696	11.14954	12.95502	15.54557	27.90505	46.37374
768.6085	1657.823	1270.862	515.2354	561.2028	1112.053	1159.704	1484.051	1601.175
208.6748	121.6276	88.01658	171.7006	112.942	129.7255	90.33561	66.60739	86.60794
0	1.366193	0	13.2211	9.890557	0	0	0.018719	0.434621
65.09213	158.9439	135.5695	67.58606	66.5387	106.5699	39.6624	108.4605	54.59012
21.65173	11.54934	16.35458	97.46456	27.63764	13.87259	8.480485	18.2804	6.226482
1056.345	1637.271	2002.959	1588.177	2138.863	2301.954	3713.811	2117.646	1407.064
88.55907	58.32522	51.80354	39.9328	24.61477	52.82506	26.43092	53.68936	43.03897
16.96718	29.76201	21.52491	330.2077	274.9636	154.7818	30.30343	24.73017	28.93425
381.5471	85.13713	201.1037	1466.48	816.5152	671.8639	283.6086	119.2301	59.20724
478.4924	1168.813	923.7725	545.873	877.0742	1105.571	631.2308	1255.325	791.7178
6.885306	46.62274	39.6948	0.17243	0	318.2386	35.02714	35.45172	0
58.09609	13.72867	32.75001	17.09044	3.644815	190.9329	3.811457	157.8423	51.07929
95.31156	929.5153	358.3028	213.1926	59.01293	266.8844	216.6159	397.3124	169.2903
48.01482	49.46103	139.0405	87.42393	64.29945	101.012	148.9443	65.52262	62.18801
85.59869	59.17584	31.07087	26.1475	36.68211	20.3681	24.89811	75.09875	49.03979
204.7551	108.4817	151.9192	471.6305	345.1133	88.01014	143.3499	164.3136	140.7864
1449.756	2053.642	1510.92	420.2025	1777.572	697.7993	349.0713	1842.682	2524.394
4.559554	133.2157	89.94352	7.885103	4.384658	25.00441	32.71156	32.22367	61.33163
46.46958	175.105	105.3494	58.37118	86.0602	46.31306	46.69481	84.83641	113.0392
74.48728	69.96917	31.89971	30.83383	41.8495	63.94256	58.43756	48.3364	60.52948
91.60316	53.89238	69.94157	20.13878	29.10101	64.87097	37.34957	95.54385	63.45929
17.82717	23.3577	29.40697	25.92975	4.388511	21.3195	12.4188	17.1875	24.5338
1200.749	754.871	695.8374	593.3514	784.2796	523.5717	462.0959	752.7814	896.5821
213.2871	397.2818	324.6618	141.167	139.8795	124.1569	148.0254	333.9437	520.9245
18517.72	68518.07	11500.67	2635.246	1410.594	25290.96	10420.93	17464.08	16142.11
49.00878	22.55738	19.83236	41.17826	19.41002	10.16693	10.83219	7.546345	12.08459
120.3361	87.47746	75.9772	43.06829	31.34176	58.37189	34.99764	55.85318	90.31014
79.20527	34.18396	40.55587	77.97532	56.14174	24.99701	18.62018	11.84336	12.63341
57.4422	32.00682	59.60351	83.9465	27.6248	29.63769	6.920535	8.621999	14.38912
57.30905	11.54596	18.93501	414.7208	233.5762	46.30519	7.705065	74.12189	12.63328
80.73227	78.76858	49.19536	30.82029	22.35717	48.17982	64.70482	50.4785	65.24161
67.48442	19.56399	17.21027	73.53349	95.13145	25.92079	9.25798	7.548238	6.809242
111.6691	189.5521	208.1328	26.28727	52.29341	192.784	113.7677	245.8588	316.0627
252.8361	279.071	284.9022	219.2974	293.3652	302.1097	301.6206	279.2051	286.4335
62.35632	28.45845	35.44902	16.962	42.81778	25.02418	13.97071	30.05334	32.72373
127.3628	50.22823	37.94748	70.50139	102.6273	35.19209	20.95407	37.60668	23.70721
1374.354	3604.029	3477.579	568.9296	1407.616	2747.693	2307.671	6794.215	3063.508
862.596	135.4166	696.7426	1406.545	216.9334	999.0377	440.3051	408.0807	141.8782
6.898083	30.72496	54.59676	1.738106	11.15836	11.10085	12.41885	58.93627	73.19646
21.73662	25.56618	21.59433	7.848215	2.890172	12.02938	11.63414	22.54269	35.7882
317.2677	255.7536	262.4557	245.2958	145.8582	240.0127	324.2527	222.2973	340.672
76.78928	105.0079	94.12935	35.43654	34.33838	43.53468	48.26622	100.9241	106.6719
5755.26	18774.8	10204.02	714.5516	237.8851	8429.405	5605.234	11584.68	10149.17
34.20208	41.69349	25.90902	15.45051	10.39171	22.23763	16.32087	25.76829	15.03681
67.48844	69.96963	45.73021	43.0408	33.59925	80.63796	54.53169	62.28546	100.9079
32.62208	23.29996	33.70254	7.869596	23.18279	30.59178	16.31426	40.77312	26.79449

5210.739	6641.872	7062.224	4060.709	5613.607	6621.367	4128.282	8510.274	4557.387
425.1116	274.6831	183.8551	341.8003	351.0119	247.4208	90.31542	155.7333	183.2746
48.92655	88.50067	45.78534	20.07884	20.88587	41.71056	17.85537	48.30446	75.47885
13.86804	2.080209	16.34832	29.28728	74.90496	32.41474	112.4144	19.35793	12.63205
48.1825	0.62892	0.812641	131.8459	50.2594	12.948	16.2996	0.023705	4.481304
313.4247	203.2777	259.8827	122.7927	96.44997	260.414	385.1326	273.8217	192.6607
25.60557	53.45985	34.57832	6.341873	16.41103	12.95259	5.365164	24.69763	40.38994
13.87914	36.43683	24.14392	15.51258	26.14704	40.78277	17.85569	47.23192	39.03476
63.55914	88.8996	55.21996	66.00733	23.84379	97.31048	64.64682	104.1538	87.32689
150.4278	77.86264	116.511	387.4645	190.0683	73.18126	148.813	84.86218	59.21
3.786332	13.05559	18.98931	7.850293	31.5443	12.02881	19.48101	31.11041	30.44283
61.49807	127.6676	20.6968	12.44109	5.133656	17.58993	14.74254	27.92005	77.4833
290.8967	436.6472	290.1043	98.30288	85.21628	211.2829	181.5527	383.3331	436.3325
68.34434	62.7449	61.33422	17.06002	27.62472	18.50776	40.50995	80.49368	64.76118
121.019	93.97208	62.1223	20.15592	9.622865	182.6026	103.6487	125.6324	138.6521
38.76209	73.7391	57.87629	39.8932	25.37475	50.97736	19.40756	44.02844	40.72975
137.2183	259.4052	273.6865	113.6236	141.3677	257.6251	377.2926	418.7677	253.8291
6.110031	19.58497	22.40705	14.0001	15.62249	38.91914	135.2752	31.15407	30.19859
235.7677	97.53469	96.64099	324.9109	485.0982	114.8852	183.0986	73.05329	72.01584
187.6743	202.5456	421.4066	124.3263	217.0187	175.1359	155.0442	250.2039	265.5283
2501.559	3065.752	1738.801	575.034	1503.497	1696.817	1917.296	2751.167	2290.538
88.47527	337.6519	75.98689	9.4233	46.34506	83.41681	57.64961	71.94509	104.9766
272.2495	301.6917	488.745	14.01151	70.24168	344.7519	306.3131	661.404	556.9475
10.76284	145.2967	143.4589	7.889024	19.35637	37.97548	28.76057	77.30586	104.4256
41.05423	17.37602	37.95022	90.27421	22.35366	51.88586	353.9902	23.65289	19.6261
498.6498	1079.121	1421.077	732.881	682.4419	1898.856	785.5422	1529.163	1314.008
50.34135	12.99955	25.84283	694.6034	32.07807	28.69763	31.08446	37.61359	18.44981
23.94764	258.1061	211.5637	63.04181	124.1993	184.4281	159.7804	318.866	341.0329
530.5558	133.9623	153.6209	802.9386	582.282	511.5487	498.8032	127.8198	87.73029
62.7627	19.557	5.125448	121.0592	229.9818	35.18564	27.9646	19.36121	38.84421
213.3062	61.09677	163.1525	167.1574	272.4731	254.8589	198.7329	75.19847	33.58595
63.63444	49.53505	47.47316	12.48459	42.62184	35.19814	62.377	91.23609	123.8885
502.5813	956.1258	513.6741	208.6859	359.8985	460.5617	233.7256	612.0996	1097.691
1994.172	2319.395	2407.904	2187.382	2040.842	2028.574	1857.229	2667.437	2045.271
0	0.645828	0	7.252131	6.927895	0	0	0.015968	0
58.19206	160.0691	69.96696	20.12448	31.36545	20.36057	18.6203	65.49098	92.83191
9.21186	111.5852	88.07746	3.273453	20.10164	44.46189	24.07203	134.1866	127.7279
811.3152	386.1022	556.8291	464.6204	457.1856	440.1655	1136.385	488.6247	698.0061
143.5702	195.4982	69.03877	61.45344	65.79877	60.21673	68.53675	114.8943	98.97019
43.36589	133.4768	100.1693	30.86023	71.06945	64.85835	55.2845	125.6116	151.6504
27.06344	37.07908	60.42083	26.25994	20.8525	54.66464	136.5615	40.82694	78.6344
12.31372	21.74679	35.3481	0.175407	4.388821	80.62173	165.403	83.76094	44.11779
52.65844	56.73681	79.38944	85.97745	122.7142	60.21025	180.0866	168.5828	126.3108
160.4836	394.2547	128.5877	141.216	446.8571	445.7697	131.6316	80.57048	179.7747
9.231485	15.98531	15.51554	4.810567	5.137001	25.03038	4.586166	27.90357	7.411805
16.20665	15.20041	37.11524	7.886454	8.12607	89.03602	46.78633	55.81969	106.5334
20.12005	35.01619	34.55239	7.876317	30.69288	35.2262	29.61826	28.99454	134.0981
41.88475	42.26495	36.24776	56.57449	45.66827	50.98118	15.50452	52.60461	41.3325
38.09408	30.64731	21.57006	10.91071	10.38882	23.1636	20.23384	19.34104	19.73298
45.7131	72.15162	35.35507	7.888761	15.60979	152.0575	42.0299	80.5286	41.22686
6.890569	12.2997	9.446262	87.81162	13.39484	13.87929	15.53054	17.19793	4.482656
445.1226	1631.477	1720.652	899.9738	1632.953	1770.946	2518.996	2046.76	1203.309
58.95715	81.70818	65.63205	20.13032	16.36227	65.8061	42.04774	54.76791	92.78832
275.2726	295.0195	202.8321	683.628	422.0306	274.2807	689.7598	263.1214	175.6497
201.5907	355.5601	443.7666	170.3482	294.0605	384.5806	567.4403	453.163	593.3923
23.29589	7.186773	12.91899	79.86984	7.392141	4.604074	0	3.251584	16.2486
34.23029	34.37264	27.65617	4.810774	25.47919	26.88817	9.27743	30.04603	36.91247
98.47445	97.60208	167.5284	76.77955	105.4959	110.2732	172.3101	135.3003	114.0885

18.56303	12.29321	8.579561	48.76441	36.72298	24.08628	9.266874	5.399878	8.567163
75.26538	83.85986	62.15754	38.46449	26.10134	58.37785	46.72024	81.59806	109.1037
46.45243	167.6658	269.4975	46.19058	11.11947	62.99281	127.8199	284.492	410.719
112.3759	934.5126	700.2767	145.8244	227.4417	551.422	271.1788	953.483	682.042
65.85717	65.49261	115.6773	81.3789	121.9736	145.5	187.1237	90.21967	80.81752
77.51765	69.15503	69.89725	130.2234	179.7383	61.1414	81.79372	76.25952	73.84403
139.5389	397.9269	344.4932	90.65757	19.35361	345.6729	183.8743	707.5826	382.0153
48.84849	62.69603	90.72398	35.38203	48.62518	33.34269	56.11924	92.31073	87.5305
18.56046	25.47704	13.76849	23.07381	11.88379	40.79626	5.363277	15.05673	13.24875
23.20513	42.99898	50.09562	29.23124	41.15968	10.16354	9.260136	37.58987	57.7645
317.278	263.0554	251.2326	190.1743	201.2761	276.1644	246.2744	645.29	297.5542
222.6092	665.7582	464.6078	135.0347	257.4739	260.4158	105.1403	330.7186	821.9296
230.2882	302.3269	551.6922	300.5867	514.1453	440.1823	1071.852	382.3044	303.2152
1694.7	4169.495	3232.373	729.8804	749.7706	2330.664	3293.608	4140.776	2506.279
33.39275	21.09152	20.69899	16.9893	20.91658	29.66022	33.55347	13.98234	20.3076
17.77477	20.33617	46.66683	10.93294	31.42642	28.72216	80.54905	28.99963	46.12925
89.25526	119.6736	117.498	27.78404	18.60597	33.33798	70.15028	42.97217	81.57538
0.713413	0	0	3.029475	0	1.843689	0	0.014921	0.456628
0	7.919442	5.125752	1.738162	44.40284	27.82286	6.932249	31.11061	6.829465
3.841684	0.63266	0.815582	1.698322	2.931963	1.830111	0.701609	0.019043	1.014207
42.83757	21.8637	27.65618	28.98883	11.9035	13.8839	13.97863	23.6196	26.85869
169.9077	202.6753	183.9306	70.67981	174.4272	110.2687	108.3002	172.8755	212.1059
52.73615	88.29817	67.36395	23.18289	56.88248	36.12468	31.11173	51.54864	148.4857
360.6163	402.8993	687.2475	538.0647	520.1086	417.9333	739.6729	529.4146	297.3715
145.0428	53.8171	84.56965	118.106	169.9129	86.16398	102.051	81.63468	66.22497
23.3548	29.3356	19.01635	7.820997	16.47635	12.03664	13.22818	16.10569	17.5062
318.6978	938.5194	1124.96	437.0595	195.2186	1571.739	1187.016	542.3189	1396.832
138.0216	132.5619	201.167	81.4335	75.49279	147.3358	118.4083	162.1597	127.4075
167.6067	64.03964	48.2969	220.3747	124.2368	83.38845	52.13908	18.2874	26.60244
32.62048	18.89639	18.96923	16.98138	20.16865	4.603192	15.53048	16.12588	13.84997
134.1043	499.2648	435.1679	176.4388	260.4042	242.7863	533.2112	558.3588	381.4041
151.1941	222.2316	181.2804	147.2943	115.1669	170.5005	344.5602	270.6038	200.8113
82.15103	129.6773	115.6669	98.2297	107.7178	121.3894	173.8267	111.6941	144.3742
13.08931	113.0204	124.3762	18.61947	31.33675	106.5879	166.9704	138.4876	185.5748
71.59595	36.46589	14.6318	15.49655	23.15498	41.72019	26.47447	41.86186	36.73117
72.17712	69.26454	60.43907	152.5413	72.64236	31.48652	10.03753	35.45622	37.74503
52.66633	161.122	130.3797	18.62367	52.29971	124.1838	94.2755	177.1542	112.9411
9.248214	3.534575	2.534896	24.32876	14.95527	33.42311	2.248824	4.323965	1.577977
1.462876	0	0	3.263196	0	0	4.599026	0.022012	0
55.81443	149.6371	107.978	36.9515	59.09464	58.37433	51.39809	114.8644	190.4225
37.21799	32.75428	62.21872	3.280268	15.62314	33.35266	14.72469	45.09568	36.65072
6.110539	9.356519	20.66723	166.274	106.4269	13.86988	8.478769	25.79864	10.30038
366.994	320.7082	455.1035	216.1503	222.2678	123.2309	64.5965	409.0885	248.6333
60.44824	41.44055	11.16505	143.8468	135.5536	26.84474	25.62823	22.58196	86.14811
518.9069	418.9516	566.3691	390.9722	186.2495	466.1317	437.9839	410.2229	898.0128
33.29405	29.79506	42.2824	12.48524	14.11492	32.41516	188.3062	55.83947	78.73941
39.52956	26.15195	23.26583	117.3868	40.38412	22.2164	59.27088	12.91648	7.390083
39.49334	73.60617	69.06662	46.10959	65.84673	73.21274	86.54992	80.5308	65.18539
49.62801	25.41289	31.90706	41.47357	144.8018	32.41555	38.14488	34.38131	31.90799
175.3555	282.9933	298.0054	37.00858	29.07942	107.4895	87.23945	91.29299	594.6498
69.02554	50.95262	114.026	29.31835	73.34524	65.79208	77.95097	138.4711	122.4995
390.1768	613.7308	375.5704	173.3831	47.78707	451.3294	236.8841	763.4292	497.3624
33.27652	55.3358	99.32636	53.74344	26.09701	83.41319	128.7393	74.09415	51.15104
40.33875	20.31975	44.90942	33.77434	27.64029	15.72764	24.88671	38.65934	16.73702
529.743	123.0281	126.8534	1083.395	423.5321	203.8475	841.0102	100.9745	164.0048
97.63316	1430.772	915.2277	236.254	1344.417	1063.925	120.7076	873.0076	793.1089
65.95395	151.9457	51.79112	41.49005	66.63575	30.55926	19.39791	38.67522	92.18739
98.45314	112.1667	151.0864	208.3123	115.9582	122.3162	306.4835	148.1929	119.2887

46.45304	172.051	152.8382	63.01701	41.80996	107.4919	159.8218	153.5473	199.32
72.97364	79.52918	49.20314	64.30004	51.6322	53.74962	33.45784	57.98355	75.23634
93.75546	26.12086	26.7077	459.8655	249.1346	115.808	149.5502	47.28128	2.762686
193.1032	164.6207	157.9609	205.4615	253.7122	204.7956	109.0351	98.82181	87.75019
88.45408	109.3995	91.53837	97.97776	89.09395	66.71757	78.72499	77.31576	58.73895
34.88542	107.5146	25.86905	24.65787	23.13177	25.93115	14.72553	25.78959	44.28512
124.107	139.9375	101.8599	63.01416	111.4947	50.01573	98.95154	168.5724	272.9157
16.97993	13.73607	12.03083	64.21143	23.12106	19.43517	107.8084	16.13598	14.38923
60.54811	51.02306	68.25184	12.48027	20.11727	34.27457	55.36373	105.1625	102.2896
676.3372	1002.713	799.4645	263.8675	650.3138	533.7702	342.0651	1017.997	852.9506
10.76946	32.77233	43.18736	4.818982	29.15317	37.99794	58.56049	79.39253	73.1242
43.3577	170.6779	156.3338	84.35794	107.7885	79.68802	38.88704	153.5267	196.5599
96.10548	116.5135	141.5628	127.3567	151.1324	200.171	222.923	154.6462	125.0695
44.11899	139.1586	122.5764	18.62299	58.27826	206.6771	304.9173	185.7647	206.2126
18.68986	19.78223	15.56067	1.73449	5.907748	12.04421	6.167505	15.02531	10.42862
703.5407	456.8344	563.7635	231.6651	227.4128	513.392	778.6479	391.974	530.3535
62.22403	71.69002	43.20449	20.05656	20.89732	27.79483	10.0454	44.00667	47.90383
662.3493	950.9153	901.3314	314.4544	378.5868	904.4683	599.2798	1086.734	899.4784
231.094	187.919	66.41883	459.6997	964.3437	38.89029	17.83546	44.06007	61.53161
9.212863	55.30039	114.8427	15.55728	24.59076	129.7577	210.641	108.4538	199.4689
62.77131	49.46202	34.4806	195.7776	119.0316	62.07126	28.74622	42.98039	18.45026
220.9948	246.9548	204.5723	374.028	342.7413	271.5122	388.9102	241.6357	143.0608
46.48551	18.10343	43.13145	53.74234	51.5902	31.4826	56.86251	11.84401	8.554307
241.9805	254.2916	269.3632	207.0314	158.5858	277.0875	546.5218	248.0657	238.6617
491.6692	1054.346	1125.792	1109.872	972.0992	1222.329	1608.654	1256.414	764.8883
84.54702	90.37212	79.42335	70.57134	38.08099	86.18601	61.52995	84.83366	81.50068
9.252951	17.51822	15.54269	3.271775	34.6827	11.10785	4.592561	21.45482	19.2956
126.3424	236.0296	457.6096	136.6265	223.705	278.0033	360.0755	440.2651	312.6105
488.6678	1215.099	1325.477	33.94661	37.3127	342.8711	1329.208	681.8677	599.7724
433.5035	901.3499	1112.871	778.7826	780.5252	934.1232	947.7085	1117.875	764.9426
56.62484	92.68619	42.28142	38.4339	52.37622	45.39944	37.36073	38.67411	48.28339
55.17167	56.97045	56.18256	17.03197	26.9018	28.71894	13.16739	46.15799	132.0254
173.1862	102.8016	97.58111	163.5593	118.3388	52.80558	22.51093	54.78263	28.94893
53.42368	29.76523	28.43522	800.012	677.315	30.55166	45.11069	33.31979	24.86717
11.5382	64.07992	13.75483	354.0274	74.82054	29.6265	119.32	4.32503	85.59346
6.113661	7.17866	11.17713	3.281133	18.6644	12.95152	17.09829	22.55687	8.573229
347.4056	767.2786	852.9939	381.8699	505.0923	783.9963	1074.026	1116.788	868.6593
55.01161	80.86188	133.0068	73.64837	89.80464	58.36526	113.8321	170.689	153.9385
19.37967	21.86912	13.78334	18.43844	10.39955	21.31537	6.148866	45.03387	23.32313
166.7867	106.3343	163.1851	102.8127	87.49276	94.50697	284.6439	152.4851	96.54763
20.06963	135.5226	145.9082	20.15685	91.24067	95.43465	223.7795	258.7499	143.805
23.22222	12.28545	12.89995	44.32915	50.98958	25.0083	26.46578	17.20451	15.57965
199.3543	250.0116	197.7151	138.0522	208.0758	118.6015	98.13313	233.0111	283.7138
25.53984	116.2852	49.23108	18.57481	35.14818	19.43676	15.50512	76.19304	50.13818
40.34309	18.85898	11.16881	59.54936	50.20258	20.36584	15.50808	21.4979	37.25433
13.86554	7.17201	12.02827	65.94656	14.86034	166.8899	6.142712	3.250723	2.746558
9.292931	10.19908	5.140356	34.28503	15.81161	7.400733	2.253309	1.101569	5.11684
173.7755	288.7735	154.5452	137.9822	130.9474	164.9618	63.8299	124.5751	158.967
310.9564	1128.877	1079.261	182.6347	314.9849	804.3999	36.53991	1419.562	226.8927
3.792171	114.3624	5.126492	12.48667	17.10455	9.236933	5.368627	8.621047	31.25962
7.680343	2.075782	2.53452	19.89062	44.45365	5.53232	2.248417	0.022862	1.577973
182.3521	241.3913	176.1645	79.84425	125.7258	103.7821	65.39727	293.0806	261.7768
0	0	0	0.129094	0.658955	0	0	0.019216	0
50.37829	69.23116	54.3731	55.249	82.36681	46.32024	37.34598	89.11093	61.10524
81.43757	139.321	69.05085	84.30657	78.56859	48.1684	46.69791	76.24856	119.4904
720.5969	878.1368	504.1775	259.2522	295.5255	734.8928	483.1743	588.4757	728.3839
626.1054	596.2293	670.075	185.6357	449.0975	183.4671	112.1414	1015.732	713.5964
335.859	315.51	327.2108	138.1515	224.4611	246.4932	338.2545	338.2618	314.9614

4081.403	3609.069	3019.102	2218.255	3330.999	3551.15	2787.75	2867.212	3285.263
19.29398	5.72103	21.52464	20.15693	9.624106	135.3019	177.7588	164.2851	519.8823
518.9083	330.0236	262.4152	642.1643	461.7358	191.8014	100.4432	136.4106	155.8585
40.36276	51.10998	35.40048	20.07043	16.38231	29.6478	13.16811	49.37249	40.8132
1116.881	1048.5	1422.794	1516.02	1125.52	1355.774	2475.393	1487.29	873.1785
196.2283	155.8883	139.832	246.7162	779.7012	50.9381	59.14076	134.2497	108.1579
46.56557	28.36439	19.81342	36.8204	43.42167	22.22	55.40018	30.08058	22.00177
197.0403	209.1774	160.5776	93.66355	83.73628	163.0993	151.9605	226.5641	263.3388
1.462562	4.259935	6.853734	21.47907	99.54912	9.241501	3.026389	11.83423	5.652787
29.39874	10.08508	3.39852	85.6936	76.37557	15.72355	22.5169	8.622286	2.745048
69.74588	69.14829	121.735	4.807544	71.77787	80.60782	249.6032	136.3712	42.92107
118.6135	239.0509	176.9772	63.06211	124.1688	136.2113	219.0233	242.6775	432.4218
65.42162	16.69272	18.96701	44.18779	23.17621	10.16707	12.39713	1.101746	3.900071
25.75063	10.15765	11.20806	6.296001	7.414669	9.252429	2.249971	6.465766	7.454005
31.72062	66.28569	88.07915	32.37918	114.6064	53.73481	92.00295	92.33885	124.2263
34.06817	18.10872	20.66821	87.15502	109.4523	45.39834	63.92795	18.28479	17.29742
175.2356	145.6471	143.2715	528.4732	270.9038	229.8142	250.9371	83.79118	220.0019
44.5363	41.14811	23.35605	10.84089	3.641416	10.1768	1.471141	16.10783	21.6478
231.1346	171.1834	229.6466	47.74915	118.1615	172.354	254.0843	316.7689	202.5578
55.16834	45.96768	45.78782	13.98804	14.87878	28.71848	18.6383	36.50944	39.03911
93.8782	82.34187	75.96798	20.15	64.32342	96.3882	99.79718	79.46667	97.28867
82.12149	53.07995	93.18267	78.40508	87.45216	490.2122	720.9053	998.6691	158.7593
50.68211	2.075662	1.672275	31.97416	36.82319	6.458888	0	1.101921	1.578323
12.31373	64.07591	14.61817	4.812196	11.86591	171.5032	55.28261	41.90444	35.94573
195.3957	428.5066	368.6561	230.057	368.2143	299.3205	578.4109	529.3799	702.4867
85.26757	177.1438	126.9099	84.43603	84.50691	114.9061	98.94278	107.3948	170.1016
75.96626	182.3173	168.4134	69.11074	19.34935	115.8427	25.6257	188.954	189.4685
339.8844	245.6308	240.9062	154.8844	119.68	159.387	78.63658	199.7328	225.9629
501.0502	1699.046	352.2134	141.25	300.0258	607.0027	242.3061	745.2342	2262.477
44.93281	25.39942	50.04735	85.75139	134.1493	44.46415	19.39341	30.09414	13.79391
21.73173	42.50684	25.06232	1.738167	20.96323	24.10536	7.714736	20.4023	22.15605
232.0392	407.0626	137.2716	59.97215	121.9643	94.50857	40.43654	253.3795	221.4364
15.4369	26.18874	37.99863	17.02875	29.1611	21.29622	39.77475	41.86798	77.87611
12.31571	9.356781	9.438569	58.22687	253.04	18.50562	10.03774	13.99069	12.04936
3.012173	3.532495	19.80569	3.27784	0.657455	62.09949	195.3779	32.23471	33.66617
129.5704	221.765	129.5258	81.32965	72.5385	118.6258	94.2863	174.9995	163.7802
68.93832	118.6814	48.28842	646.1474	194.539	54.64453	479.4796	29.02588	33.0042
14.64043	215.2506	277.3666	23.21664	35.82694	177.9817	20.16939	331.6552	168.5209
9.213465	43.72513	29.32498	24.67235	17.87158	51.90777	79.65035	32.22811	39.56772
43.50936	87.8769	20.69117	3.281494	10.38105	17.58756	9.265393	22.56394	60.29745
20.11944	54.83792	58.81802	4.817534	22.41073	25.9419	12.39271	81.50252	27.93086
82.92426	74.96218	106.1616	78.34943	111.4583	121.3876	204.2471	133.166	55.14526
2020.486	2059.885	2878.396	2956.966	3541.357	2596.637	6062.876	3179.687	2918.526
128.8661	14.45683	2.537319	248.8682	122.0914	20.35705	16.27248	4.3251	5.648943
422.7088	98.98225	307.3204	1329.669	270.0948	470.7703	235.2985	135.3363	113.3507
79.07846	87.41079	69.037	368.1918	161.7647	48.16415	20.94836	33.31901	34.76662
258.3354	85.88483	126.0138	289.536	336.9061	265.984	141.7991	83.7878	42.32038
44.19841	212.1603	61.32636	42.96172	17.86631	45.40591	12.37874	83.71696	70.01227
44.11412	39.23032	63.83022	352.386	476.9121	14.79729	1.480656	108.4857	20.20068
106.2513	91.0449	190.8745	104.2698	29.82992	88.02582	130.1782	179.3001	75.58912
40.31054	45.90147	30.18447	59.67503	155.4257	31.49187	14.72032	21.50211	30.75926
23.95637	151.0842	127.8621	32.37401	66.5925	75.99348	66.23387	147.0542	141.81
0.691783	0	0.812158	7.744899	21.15843	2.752957	0	0.021467	0
0	0	0	16.3278	0	0	0.699232	1.100186	0
35.84893	13.06546	13.79144	27.39614	6.643747	27.83014	3.027325	10.75762	25.74467
41.08257	23.95775	20.67163	33.83661	89.9738	35.20073	154.7287	31.15993	36.60427
5.336115	31.36018	45.8281	16.99405	23.17205	36.15884	28.06012	95.41557	59.783
182.2742	234.6905	213.2641	150.2919	146.6466	213.1527	159.75	175.041	158.3161

333.4423	1939.314	1655.158	159.6506	27.59126	1358.599	728.6934	1713.805	1014.266
2.241957	2.810082	0.814679	328.4066	13.3625	0.900536	0.701091	0.024516	1.591288
0.735202	0	0	4.206032	0	0	6.601313	0.011416	0.488862
4.561384	39.49931	28.51439	6.339613	5.135761	41.74866	30.44831	21.48064	17.98622
1825.882	1560.809	1247.519	1445.544	1101.564	1449.373	1834.663	1601.118	1156.747
3.009772	42.39236	13.77275	1.737817	3.637054	24.09025	1.471907	25.77355	2.158021
13911.06	25107.84	26052.45	21373	21895.39	28210.98	36822.99	30653.18	34092.49
79.8548	62.59368	61.26053	92.01609	90.53131	83.39396	52.14695	64.45013	58.09848
250.5159	361.485	290.0901	177.9449	180.2995	369.7773	183.1004	274.9074	521.3721
398.0049	545.3076	264.1887	125.8711	385.5197	182.5478	76.28772	406.9556	320.8749
446.7493	1570.066	679.4925	409.3533	466.9753	543.984	435.6453	617.4544	1589.034
68.16533	246.173	753.6634	1172.71	1116.552	2141.665	112.1361	2207.81	179.718
108.502	538.6596	480.9496	38.55218	73.23422	201.0794	258.727	576.6054	443.2043
31.0343	13.02354	18.96163	72.92911	122.6975	33.36873	6.923596	9.693067	2.158709
73.75078	80.99112	44.01351	24.70576	127.5173	29.63343	35.02003	49.40159	51.80516
3.784699	13.76297	23.29913	6.345405	20.16586	18.51963	14.74587	57.91685	33.26602
82.1949	61.87169	118.3034	56.86114	86.79497	62.9993	159.1089	119.1842	105.9932
69.7288	223.794	251.3013	72.2283	50.0402	200.1856	105.1651	222.2636	223.7056
73.67853	21.01922	29.30238	148.2863	37.33613	64.86155	14.7135	13.99173	33.61993
40.23579	455.5918	441.2517	147.3086	94.19876	224.2576	204.9425	464.9301	338.3604
229.7093	193.1822	146.7755	52.32035	78.50767	126.028	155.1147	201.858	169.4916
3.786872	2.075707	0	33.41152	45.18734	3.677131	30.499	0.022947	1.001139
1269.657	1447.801	1556.589	1270.917	2036.325	1743.133	2462.042	2179.929	2598.973
105.4601	64.03594	156.2907	33.94506	62.03284	127.8867	299.5352	159.9895	155.5196
62.00275	3.536409	39.66592	716.8398	130.3056	48.16667	20.16979	25.80283	3.908913
17.78127	24.0104	38.01606	1.737255	32.94901	25.94146	43.72485	35.42674	29.69233
173.6608	332.9619	249.4704	205.5831	83.70977	490.2398	457.5044	659.3136	377.232
1064.092	5678.012	4034.471	2868.035	2410.504	3990.442	1031.8	2789.879	1893.205
439.8259	567.7602	503.3754	219.3436	173.5401	397.5651	267.2836	500.394	396.508
37.16629	182.5828	108.8529	23.19849	18.60636	36.1201	51.40416	94.47447	97.96837
0	3.530209	3.396746	12.44227	3.636941	12.02192	56.29323	1.101705	1.003557
40.24351	66.95576	104.4499	107.3532	128.7289	72.26317	262.8559	112.7596	81.40974
152.7815	223.7628	159.7116	98.25824	157.1426	114.8953	153.5173	200.8022	231.2408
90.78052	214.6521	85.48296	49.18184	67.33176	71.35296	9.258213	64.43987	78.60345
18.62958	24.12881	31.16717	19.86832	6.646553	30.62595	10.07491	21.46361	28.74568
52.08773	47.47517	31.08074	32.17722	31.43444	24.08329	10.04608	47.21994	32.03091
11.67557	28.1273	6.885372	9.200568	1.399896	5.542972	1.474012	9.662512	15.94134
90.29417	89.27737	40.59837	3.281274	7.379036	19.44091	2.249205	59.01802	53.75793
0.691526	1.349887	0.811839	54.2593	7.39838	13.89065	0.696634	0.022801	1.000834
53.48541	83.11412	82.03945	43.05218	68.85318	64.86731	68.58575	89.11246	98.53823
104.8128	63.38424	70.80068	35.41827	21.60308	50.95703	52.96501	62.28725	51.16507
15.42308	33.45783	54.39952	10.9525	28.36703	24.99783	27.21253	60.1249	37.77145
62.18641	43.76843	33.66582	6.352555	38.93431	26.86254	28.80816	42.94253	110.22
114.8117	64.05256	98.41617	38.52547	80.03563	52.79917	183.2881	100.9442	77.35687
38.05215	32.07585	46.68024	26.10678	50.27622	23.1565	17.08525	63.29057	24.39922
14.71328	22.64758	16.39783	4.80224	9.659059	5.532603	16.35709	16.11183	10.37235
48.80288	21.01758	43.1244	157.5061	126.5773	54.65862	168.5279	37.6106	20.78332
22.41124	40.75433	28.44734	82.58079	82.41407	63.95072	39.70359	30.09013	11.46618
5.334904	87.79604	58.78457	44.33862	6.629727	26.86364	5.36259	57.9483	55.51106
13.86519	7.172405	6.849517	46.12948	11.11761	7.382849	0	3.250587	4.487305
15.44194	29.13451	22.42128	6.350165	20.14761	27.79582	33.52526	56.86585	30.85398
72.83555	101.9535	122.5774	70.69332	82.24653	120.4621	177.7266	186.8372	110.5396
20.07665	81.66604	31.03545	24.72481	10.36945	97.33403	24.07689	31.16609	55.26579
24.7248	143.6092	177.9156	23.22054	8.127576	84.31832	291.7946	260.8623	208.1443
6.111153	3.534404	3.398862	140.5729	204.7389	9.235818	2.252828	11.84398	1.588397
79.85244	45.80977	42.2535	175.9963	160.2569	38.89381	91.94493	48.34889	44.6785
8.439463	18.13391	37.99622	4.818869	7.378608	31.50315	82.0822	39.72517	24.95383
3.032814	0.629296	0	23.632	6.724044	0	3.049095	0.020297	0.429549

518.8208	298.6394	12.90065	1587.959	540.2407	1084.246	1013.93	25.80045	1923.175
3.011197	0.62728	0	6.321668	11.92249	2.750465	1.47112	4.324472	2.157675
163.634	136.9338	126.8796	203.856	250.7634	68.54797	85.65239	163.2347	123.3212
229.5683	327.2235	61.23942	141.1885	184.0489	349.389	185.4444	314.6287	359.9177
6.892404	8.643763	16.37579	44.09559	21.68832	24.0954	57.93627	11.83521	3.318866
24.80151	5.71648	6.851008	16.99836	16.39645	6.456915	3.026261	8.619703	5.06439
156.0909	69.20214	66.46367	46.13051	38.08518	83.40801	67.78481	110.5828	94.38406
955.6099	1520.912	1221.687	786.3985	679.4941	815.5044	893.9458	1361.613	1510.488
95.38946	112.2303	134.713	70.63103	112.2711	88.02936	131.7578	173.925	94.87017
72.0375	162.399	96.63785	606.7201	601.8381	258.5384	201.7926	172.9154	108.6998
35.69823	39.39478	30.21166	24.61066	24.65689	39.86196	17.86279	20.42089	54.96347
117.0539	117.9634	206.3361	122.7835	88.21625	159.3811	516.2074	227.6542	177.5173
13.87747	169.6635	157.9338	47.73904	22.35389	1022.181	1505.134	393.055	509.9054
6278.823	5607.846	4137.144	2377.694	2504.744	3872.714	2519.626	4290.065	2952.194
54.24758	43.64135	117.4697	32.3831	25.34477	56.51536	62.31793	93.41455	82.1053
2678.27	4884.422	5091.251	1684.791	2332.666	3259.252	2095.681	6702.926	9148.841
110.4012	26.886	11.16669	68.78101	53.1624	30.56469	6.141376	27.93962	9.137756
86.00989	71.29925	138.0989	312.5603	656.7399	219.6281	233.8131	142.8418	127.3771
68.27989	107.2887	58.70576	17.07772	7.376676	51.88888	39.69748	65.50007	54.70052
150.433	5.724586	20.66201	173.2734	342.8985	79.66967	10.82143	49.42868	6.241521
188.5167	67.66967	111.3449	292.3952	84.49117	110.2638	80.98523	30.09979	39.41312
0.734764	0	0	5.519775	0.700982	0	0	0.011477	0
21.65125	7.900858	8.576233	173.1635	84.79615	19.43714	56.96459	6.474245	5.062781
30.98075	10.81748	27.59347	155.1615	54.67873	30.56642	17.06477	9.695541	16.72802
101.0123	152.029	28.451	42.98128	18.61378	223.563	29.55484	37.59843	40.69394
31.72284	14.45755	41.40258	76.61772	70.34426	53.7365	66.23417	51.55939	26.62205
267.6523	265.3019	199.4322	35.48676	15.61048	307.6989	253.3381	231.9446	165.2892
42.58063	171.4031	114.8433	30.87122	32.08018	64.85293	120.061	124.5518	226.9427
14.64062	6.445205	0	52.25526	42.57691	2.751821	51.38015	0.024465	11.46414
455.9993	1719.101	1189.717	119.7952	387.567	950.8047	517.4329	1680.541	2233.764
343.5608	978.8266	617.3208	190.2799	175.0225	351.209	337.4184	489.6787	706.9295
11629.16	10454.94	13444.95	8659.366	9054.618	12284.49	13864.23	11745.83	6250.344
13.10531	26.92862	33.67424	17.02155	14.13232	16.65829	44.4862	44.00826	57.30983
191.6379	142.8152	114.8048	35.48132	100.2287	128.8067	106.7323	89.14936	201.5657
11.54655	10.09158	4.259586	35.26697	101.419	26.86174	2.249506	4.326169	2.739501
27.14158	37.22791	28.49161	35.15984	10.38394	21.30225	27.27294	19.34478	19.71078
11.61696	22.73653	12.07924	9.296692	8.929303	10.18304	6.166047	19.30466	24.73233
28.64212	41.51473	13.75872	29.25453	30.62662	14.7985	27.99991	25.79369	19.06218
122.5399	191.0089	100.1256	53.84609	99.49161	74.11575	85.67818	124.5701	83.14993
10.79755	10.85619	17.25765	21.41748	26.26262	9.244124	8.499707	12.9019	2.738414
48.03393	151.056	76.83904	44.59968	78.58608	92.68335	29.5336	89.12047	94.97767
159.87	169.1781	130.3887	78.27659	116.7648	92.66415	109.8956	162.1219	215.7676
35.72934	33.56279	28.49386	10.91966	24.67823	20.37479	13.96051	32.20657	52.11368
205.4621	383.2364	439.433	551.7948	430.2943	404.0365	567.4046	399.4847	136.0593
92.55881	45.20965	40.58138	71.68959	14.12406	23.14829	28.01499	57.96045	33.72757
42.67266	73.05103	48.37002	33.77936	14.12344	49.12886	57.75126	67.61167	61.89687
252.8095	1299.111	502.5079	288.2656	338.2526	227.9521	78.62127	427.3856	690.2044
2.241239	292.0781	18.07219	15.5561	5.883067	246.6313	34.99206	42.97738	168.61
352.9728	79.31356	161.4098	419.7646	336.8227	51.86417	51.34287	84.86443	36.49714
44.21024	45.91234	46.6255	7.887763	21.62124	28.71066	63.98297	53.68145	85.31232
10.028	14.55204	30.3055	10.84582	8.909427	13.89296	3.028158	19.32042	6.843158
20.85924	61.23805	51.7979	4.817594	11.11912	73.2312	98.35119	94.45365	48.29362
145.779	168.2938	202.8897	121.2371	198.3216	157.5304	172.2201	194.3688	178.1222
14.64078	9.356898	8.575521	190.9979	419.3329	56.51418	4.586738	12.91795	2.747898
157.5934	105.6943	42.25813	201.7786	241.3485	88.96394	21.72935	52.63919	76.22317
13.09021	64.03615	43.97727	81.3719	35.06953	102.8553	130.94	99.87953	63.90768
7087.738	10787.95	12745.63	11784.83	18277.05	11158.53	13887.61	13901.05	7934.09
194.6024	225.7955	415.2576	222.4514	198.2285	600.5236	482.4306	410.2206	338.7531

44.9958	94.27023	57.88148	27.71671	22.37461	31.49499	24.8795	36.51868	52.46973
13.87634	43.01347	30.19633	7.885481	24.63565	42.63409	45.23079	31.15191	133.643
9.271727	22.019	8.60729	1.733854	4.400705	12.04594	6.16961	23.57465	34.3658
189.4013	175.7548	200.3979	67.57721	69.54065	102.8635	45.12433	250.1223	112.3837
14.64382	38.50195	26.7048	618.3724	909.3112	32.40313	165.9726	21.50864	5.08003
270.6259	172.5854	244.2803	953.1134	861.5101	281.6976	441.8747	287.8158	198.9504
10.00134	26.96574	41.50167	6.345231	8.884629	42.66404	46.89838	54.70251	54.50713
253.6261	362.2256	259.8655	124.3458	318.8481	266.8931	283.7011	428.4321	401.3025
167.5612	139.1647	107.0291	59.9786	40.30776	154.7635	177.7308	83.78194	168.883
121.7406	286.5549	241.7981	43.14168	21.59421	198.3318	143.391	274.8609	122.1973
288.4408	861.9225	553.3505	132.0494	1025.953	4444.592	1122.231	1625.825	1420.485
129.4942	60.37379	119.1138	323.0413	167.6348	75.03779	112.9578	65.53308	43.48868
24.73706	53.89248	50.05399	53.70939	45.60244	39.83031	37.34962	52.62786	41.2371
8.437451	6.442836	12.03177	48.98537	16.37193	3.676622	4.583635	4.326096	5.06286
97.73383	53.84305	54.35185	101.1489	86.04512	70.41655	127.093	56.93436	28.93987
21.7143	21.857	33.72434	1.738204	9.643404	23.16998	10.84172	31.11978	46.35847
191.503	503.542	404.9031	75.34275	341.222	494.8713	288.3148	750.582	490.2475
0	0	0	0.082232	0	0.911826	0.728493	0.013122	0
262.2915	128.2119	119.9835	128.8279	96.47648	112.1179	119.9873	146.0477	107.6175
260.0378	38.50912	24.97834	205.1401	223.1748	23.13619	270.6532	29.02549	50.49589
10.00407	55.65919	43.24879	6.342556	2.889558	25.95085	15.53458	47.19584	64.61088
25.12775	1.352935	0	20.94976	12.03084	0	3.040168	0.021097	0
15.43737	106.8939	36.26779	13.98372	44.21284	22.22437	17.85917	38.65112	112.0436
14.64816	31.27288	36.23974	9.420051	5.880413	68.60384	83.53426	46.17618	89.97773
6.893678	816.0094	61.24048	30.88781	5.89054	239.0938	234.5934	169.6828	234.0485
84.50543	67.6937	116.5556	37.00087	75.52961	56.50581	130.9613	122.4148	91.34869
7.660852	18.84528	57.86498	9.420718	14.11757	63.96145	60.05257	56.90439	39.53667
145.0538	367.6455	96.66696	90.56459	42.55565	95.43582	163.6997	271.6255	280.9841
57.3647	99.90853	73.38615	59.84778	47.83852	53.73621	77.94977	90.18994	113.1341
102.3256	259.5265	430.134	58.45823	60.52131	126.0201	166.7845	359.6699	382.9628
802.0782	389.0479	401.4279	715.7632	765.6555	227.9435	342.082	432.7771	379.4926
249.021	388.5904	241.7586	110.5269	214.0485	144.5526	119.9628	201.8848	189.195
54.3867	26.18313	21.54835	17.03435	7.378384	35.21441	20.98384	35.43756	18.5024
24.19732	15.34074	19.91311	12.27835	5.90806	10.1842	12.46649	19.30206	9.835486
72.82027	123.7943	6.853673	381.3759	431.2652	37.9634	5.371352	18.28663	14.96352
369.3276	258.7139	164.0117	162.5841	335.3794	203.8725	140.2317	175.0493	217.1547
91.65346	110.9986	31.04279	32.3302	70.4085	55.60474	42.05283	40.81871	113.9199
8.436963	13.00644	10.30312	88.48523	284.9166	40.77062	14.7216	9.695611	9.138266
32.5573	13.74307	4.259605	56.50289	87.8474	25.00517	22.54474	7.547827	4.481232
235.0207	395.0944	196.8285	200.8603	217.7672	257.6319	141.0073	274.8967	708.7142
29.57367	7.922908	11.18886	75.25965	62.61813	12.95935	8.500972	6.470328	2.157439
34.8371	162.1206	75.12923	18.61018	40.35009	59.30564	22.51637	100.9087	62.28685
37.31809	6.448691	10.31255	51.65606	33.74618	23.16452	8.489666	7.545656	3.318694
295.4753	465.6493	701.1195	173.4089	354.7085	344.7286	376.4228	288.8822	293.9275
0.692084	0	0	3.280545	141.1132	0	0.697181	0.023308	0
24.74942	53.20957	57.86257	17.06601	48.64289	26.8531	39.7182	77.2843	79.38314
61.27955	67.79345	52.65257	52.16701	47.11055	46.32405	49.85362	60.13565	58.21157
443.7948	253.5719	149.3204	216.202	222.9913	122.301	143.3382	134.2553	155.333
478.6116	334.4336	344.4656	72.27641	151.0782	294.6821	500.4065	792.4398	503.1177
7.661846	54.73319	52.70028	15.52154	27.63986	26.85912	55.40566	122.2836	124.2177
10.01405	28.50224	15.51924	19.93298	10.40201	9.243227	7.714444	5.398316	25.70164
196.9168	76.39491	56.92414	1578.473	363.6235	106.5401	208.7776	102.0482	33.60085
31.11234	6.45367	2.534272	4.809354	42.11855	4.603938	5.367645	1.101923	0
8.436174	3.533669	8.57515	0.173918	7.376786	76.00226	41.25565	51.55429	75.16713
135.6864	109.9477	223.6187	138.0756	264.2395	104.6954	356.3108	172.8983	172.2865
16.29716	3.535356	1.672358	18.32659	15.71922	13.8957	17.95099	0.022544	1.578059
3.789291	15.18516	39.66613	20.1527	10.3698	304.1181	45.13264	93.42322	122.9642
2.239632	2.079751	2.537664	1.73145	4.405823	2.752392	1.472458	1.101678	0.427483

139.8061	70.686	79.44372	24.73015	13.36329	78.77833	56.08547	60.14269	94.43597
221.8011	119.4046	106.14	352.4508	312.0991	286.3558	457.6069	103.1185	143.0883
37.24004	29.84648	23.27953	47.38144	41.19259	27.79067	18.63859	22.56853	27.29855
56.65762	49.56168	46.62058	27.73403	18.61752	44.4796	24.87333	39.74048	83.51952
13.9521	9.40989	7.730451	34.70307	4.394368	8.320607	0.696493	6.467353	6.263021
70.64033	45.8801	52.66351	15.54294	30.61366	43.5462	51.43343	57.98317	60.59297
25.50014	61.85248	107.9099	67.59841	111.4981	131.5993	330.0188	89.14278	112.9323
93.02797	174.2115	165.7861	89.03282	59.78154	100.9984	123.1253	192.1961	146.1551
123.3668	139.9897	98.42013	67.5654	71.79526	59.28963	38.10568	94.5023	88.45899
48.77439	24.6572	42.24708	454.3725	328.7727	27.76966	17.05163	137.46	74.96354
136.5556	102.7306	101.8684	78.28204	120.5087	103.789	93.50142	76.25915	114.1244
764.7211	876.533	944.4797	993.3902	1198.897	732.0818	1344.423	1031.985	803.3284
11.59625	2.076262	0	3.272971	1.397005	2.750726	2.248948	1.101968	5.08256
1486.192	2549.934	691.5091	424.8092	532.0161	771.0097	1003.816	1519.482	2096.237
0.705784	0	0	3.09109	0	0	0	0.016503	0
23.96871	16.65175	42.28585	52.11944	60.64931	37.98145	78.0224	52.61855	20.22154
684.1116	1008.577	627.6451	548.8527	1128.686	417.0006	285.9474	504.7263	817.461
117.1248	144.3303	135.5645	46.1831	50.80137	101.0052	102.86	122.4159	173.6649
83.89693	114.7071	44.02083	35.35796	41.8834	38.91135	8.479137	39.74246	116.3414
19.30299	59.02682	51.79086	43.0147	101.9356	93.63419	19.39783	62.27933	23.13458
0	0	0	0.110963	0	0	0.708829	0.01696	0
17.81893	6.453657	9.452195	30.469	7.390956	9.242864	28.90311	5.398457	7.413999
62.04122	162.8323	66.47846	41.52053	11.11782	49.10267	28.75898	71.94347	37.72462
44.24914	22.52129	17.22229	48.91073	63.01286	12.01889	11.60412	30.07662	16.74404
87.6506	96.20891	109.6744	47.67509	64.31657	71.34796	82.60691	152.441	104.2815
47.22049	66.93529	26.7046	281.7795	245.5527	263.2169	43.55156	47.28039	39.41086
922.1765	744.612	1396.891	929.0675	1728.038	1174.132	2924.365	1791.181	851.0505
15.45852	16.7018	29.37317	10.90806	11.89383	22.23612	35.14304	37.55541	43.92318
93.80108	12.27284	82.8439	194.517	343.8191	101.9242	8.482587	32.2471	13.21304
13.10605	0.628974	4.259583	21.57511	57.78809	7.383498	79.77284	4.326154	3.899745
20.9037	21.0859	18.09851	35.15733	50.2999	36.15763	10.04913	8.619647	9.153329
6.117271	27.01729	18.11717	6.334858	13.4117	19.45702	12.41219	21.47619	13.29078
52.69971	142.3002	72.52001	23.20549	31.34221	59.29957	49.05036	69.80331	89.72897
92.99912	267.4888	145.0153	44.68164	129.4075	100.9877	223.6957	96.67189	227.0938
73.67661	192.71	99.31374	61.39542	53.07998	88.97205	88.0901	95.56053	141.7525
494.1256	855.7263	667.4452	279.0947	330.7545	382.7302	257.1389	571.2652	669.1797
54.25946	31.96856	37.94655	55.26271	134.9005	73.21272	56.0837	23.65375	34.213

TCGA-VM	TCGA-P5-	TCGA-P5-	TCGA-DU	TCGA-DU	TCGA-S9-	TCGA-TQ	TCGA-DU	TCGA-TM
188.8586	159.8012	223.926	214.9025	83.81599	163.2243	144.4919	42.63859	56.1799
1.831058	20.58524	32.30484	38.91092	32.37564	54.75445	41.94883	54.6556	61.41902
25.87346	125.6271	75.3855	158.1869	68.87689	48.57221	163.3929	182.9143	219.2833
1758.235	3145.418	3784.514	3215.137	1430.703	2056.758	2143.073	2441.811	2558.836
41.87568	49.31187	127.0538	104.4698	242.5728	212.7644	122.0864	165.8712	60.41612
1.756139	0.082273	0.018276	0	3.3043	0	2.100907	0.649275	1.078021
70.11643	13.7991	5.415607	18.36047	10.72545	6.021712	7.107963	13.16331	124.9226
94.63333	223.8771	226.078	137.6442	107.9124	173.6499	159.6859	92.79415	135.6299
4089.562	15987.81	8841.697	8234.723	3743.331	6443.817	6956.087	5689.954	7461.408
64.07818	43.79555	96.87718	52.20618	93.04914	221.6508	104.8857	34.55692	47.69268
6.633565	9.68186	12.93998	15.86983	21.589	50.49713	26.78164	13.2004	21.19233
477.3663	1088.558	1106.829	1571.964	554.6426	1210.287	1322.324	543.8524	1076.513
267.1619	84.80351	124.9111	128.6854	166.0681	149.3157	118.4355	111.964	44.5266
1.778671	0.088195	0.019426	0	1.606246	4.330217	0	25.11538	8.450931
76.98183	92.91646	128.1158	96.85412	113.7667	159.7965	102.5894	32.32227	58.29404
180.6167	8.346974	6.492377	6.220082	34.86096	14.71001	10.00129	90.93605	23.32644
957.7129	2244.016	2351.468	3630.874	1688.148	2018.572	1863.456	1222.683	1833.042
25.80584	16.53271	48.4529	26.02106	175.5852	66.88479	59.28653	34.585	19.09429
132.8185	178.7514	72.15649	27.893	215.1963	17.30726	44.6954	165.1939	81.59752
1016.525	734.789	304.728	126.0499	382.7577	223.9443	179.801	1131.499	315.769
480.5562	897.3688	1218.792	982.2365	760.6032	555.6124	877.8831	829.8778	697.2024
1.827929	141.4622	252.7295	6.219947	5.7411	0.82013	20.13091	1.374172	23.33011
73.81936	212.8604	120.5906	59.79476	50.59087	65.9428	46.86046	0.644661	57.2367
19.46092	196.7706	291.7852	466.7193	63.03609	128.4457	253.5792	102.3435	38.16977
9.85967	201.8316	202.3511	167.1971	51.43041	34.67669	81.61284	97.2844	81.59384
22.55432	24.65511	28.00376	42.12901	19.88267	47.79003	55.77136	19.82771	27.55682
343.9914	206.2572	100.1582	101.222	154.4157	133.6683	111.1842	287.507	153.6487
187.5966	357.9822	1092.824	458.8123	485.7211	1842.432	1187.215	935.3039	1392.239
16.22118	12.43404	31.23339	44.0234	17.38091	29.49853	45.56212	6.522167	32.85208
33.84926	30.19049	85.05953	98.83328	24.01104	120.7194	81.63552	62.59688	39.22435
40.18087	13.81232	29.09678	55.41808	133.011	122.514	44.74056	64.86097	61.45646
25.83661	51.94762	75.35688	47.1035	33.99469	115.5594	45.4672	26.43891	14.8588
8.208018	2.878044	8.638766	33.34704	9.079755	89.84811	29.73708	4.315723	4.262141
416.5097	408.5026	1000.23	552.5408	958.2755	1368.35	744.928	498.1661	917.576
38.68828	57.51858	149.6773	166.9301	200.1062	441.1472	264.4867	262.4142	303.0195
3297.036	1849.449	16873.71	19253.19	2177.206	26894.6	27049.44	4786.295	2266.418
50.82407	23.25493	25.84291	5.585931	13.23233	13.85097	25.27653	18.36998	32.83705
6.653906	51.97562	47.39261	51.55449	17.36661	100.7488	128.0569	60.40341	82.64039
149.5502	27.42801	26.94089	17.0669	26.51881	20.78859	38.24419	33.84156	61.44668
122.4241	30.13047	15.10339	5.582955	31.51943	24.27025	52.05456	27.20112	26.5052
362.8595	529.2151	59.24725	41.91613	377.1805	6.893256	36.73194	102.3816	16.97831
49.7011	13.81063	51.68715	58.64213	4.911837	106.0078	61.42778	58.96503	54.04079
72.01223	46.51136	11.8762	12.59306	19.02974	24.25964	11.44206	167.6569	66.75164
53.05909	110.6697	136.7356	148.5555	48.09738	330.0935	138.7456	54.44597	45.58426
149.1011	99.84862	209.968	258.7579	262.3894	280.4267	223.225	208.5313	336.9326
8.228095	12.40041	12.94203	13.28453	26.5875	45.24187	31.13549	12.45171	14.84817
78.39219	24.72426	57.07313	18.33469	61.43617	41.63952	42.56086	72.24147	26.51113
600.6952	5224.013	4243.179	4057.198	1015.496	3792.348	3448.809	1259.516	2565.197
101.1345	1353.101	231.5151	615.1157	145.2458	98.05012	361.2093	307.2666	95.38608
0.181922	2.87804	14.01085	32.69992	4.914796	57.51814	33.3926	29.58653	29.64398
20.7884	12.37742	12.93682	16.5356	10.74757	64.50584	29.73587	18.41635	20.13023
96.30394	151.7103	136.763	424.0474	304.7695	148.4243	298.4417	322.1298	189.673
32.23814	26.09301	68.91297	64.97742	35.64935	168.5661	82.38857	59.66047	36.04491
1779.129	1396.002	9009.622	6017.836	1295.334	11385.8	8759.773	1665.623	893.2324
28.73274	13.75611	5.414494	19.71694	11.57168	36.51832	10.74586	10.22837	14.84831
24.24308	27.44592	45.23626	47.74065	18.19909	116.4261	68.65689	87.78242	44.51401
6.642947	19.18347	14.01882	22.91981	14.06802	35.63508	17.28162	14.66888	11.67488

3995.022	4213.563	9733.147	7551.515	4363.662	7412.789	6774.075	3935.782	3292.061
160.3095	47.96186	95.85595	76.97261	420.2201	255.2349	194.2971	238.7581	439.6979
11.44161	23.30427	15.10057	42.75713	20.71289	20.8008	28.86427	13.90715	32.85004
187.6804	185.0002	22.63812	40.72711	103.0853	16.44266	27.36749	44.18476	49.80374
6.650561	0.116226	5.415369	2.401607	198.4768	1.68457	8.558578	5.048745	42.36665
72.29507	148.9594	192.7344	212.8779	224.2026	223.9958	230.4999	187.1764	184.3711
30.31229	12.40255	15.09218	24.22218	33.26331	43.49297	34.77519	6.528182	13.79051
17.80204	31.44528	59.17822	45.97129	9.896024	53.00948	28.13883	22.78504	34.96647
19.46815	111.9201	64.61505	118.6564	72.22199	62.48446	58.46395	66.28876	50.87544
494.0878	109.3808	128.1457	79.53709	234.1814	71.14044	113.3567	272.7637	174.8348
0.182408	15.08278	11.8631	29.45258	2.42201	22.58614	28.99799	7.274291	27.53163
13.00444	6.97604	19.39569	20.97429	24.06541	39.11006	45.66078	16.88504	10.61725
89.89734	219.9222	360.6594	212.2224	120.3502	296.9482	265.9205	107.5154	278.6575
9.856808	20.61117	43.07022	51.66698	38.17859	66.90019	38.26473	39.03708	63.55528
102.4998	113.3501	214.1977	160.7656	243.4818	169.3593	46.14425	7.998143	22.2762
51.16904	17.88739	33.39012	21.55423	42.34435	65.16383	47.70489	27.20355	32.85685
88.30261	199.4685	276.7035	385.1335	93.76773	203.1397	252.1673	191.5831	251.1169
0.192481	21.9617	48.44039	80.59867	22.3707	44.28618	31.7493	27.21128	36.03013
660.5922	258.16	82.936	220.4878	261.5628	86.76698	90.93108	129.6294	1418.521
115.4795	234.9145	271.3135	262.6503	120.3528	232.68	276.0598	202.6641	197.0841
226.025	448.1334	1295.255	1872.783	1783.697	1283.197	1823.072	1036.232	3506.008
181.6672	9.716214	83.9673	63.08511	21.52235	161.6409	147.6759	65.59068	158.8662
126.6978	24.73948	198.1239	663.9954	10.72771	369.0249	227.5745	10.21464	11.67891
0.1961	1.501043	136.6657	44.5436	9.061727	202.5447	66.48415	13.89244	19.09608
75.17947	34.25584	30.17279	55.41887	56.45142	34.68878	13.61081	97.39684	154.6246
635.8676	2770.897	2278.214	976.4429	476.5759	803.0557	1630.867	688.3178	536.1584
68.9925	84.7316	19.41322	25.98081	39.7941	19.91207	32.40278	373.5457	462.8409
3.427737	53.40885	124.9044	213.6516	93.79031	126.7431	245.8122	173.2356	177.9975
861.0325	293.7437	172.2988	386.8587	417.6691	211.7959	168.2481	586.7353	456.6714
25.87242	167.8422	38.79037	14.50419	202.7314	15.57093	49.76047	283.383	1650.991
265.6607	213.0571	269.1522	151.6362	227.5391	88.50974	90.22158	149.5698	64.65715
14.65495	12.44464	35.54784	77.89338	29.01318	105.1574	53.46949	48.62789	25.44909
694.8625	790.7304	731.0573	618.9199	368.6444	1234.695	721.1468	224.6964	386.7542
944.888	1282.576	1943.409	1920.514	1370.934	3977.371	2101.95	2189.79	1766.287
0.114785	0.07366	1.097025	0	6.765873	0	0	2.943478	0.012924
25.81117	23.3443	44.15231	29.85646	34.00609	64.27036	86.12863	39.01755	26.50768
43.40207	115.9435	358.2718	45.15768	35.64936	41.63477	174.4392	0	19.09693
582.9491	374.3581	670.7827	620.7741	489.0514	523.4959	428.3919	514.3911	1062.723
89.71046	45.19804	111.9679	89.20918	92.16328	103.3199	105.5055	89.16204	87.94848
14.66706	20.64004	75.37304	65.59871	75.55363	237.2327	151.9243	123.1857	109.1203
8.259051	19.27135	96.87863	134.1279	49.78932	94.67021	62.84206	36.77121	31.80719
17.86747	163.6113	31.2529	87.33589	21.51918	33.81158	49.05753	8.732903	37.10537
27.47756	77.95636	73.23787	140.2348	56.40401	138.9138	69.27645	50.7551	59.35653
404.9817	323.6971	313.3162	136.2737	368.7091	100.6584	228.2722	277.839	220.4013
27.10338	56.86372	11.86484	12.65398	5.745412	14.73274	17.30599	5.052851	19.07642
13.04357	49.11354	21.55673	222.0464	19.04243	38.19512	17.24337	5.048351	18.03296
3.445884	4.24801	20.47227	39.6176	19.06002	65.24884	36.17753	13.17832	13.79364
68.54911	26.04149	34.46325	29.88781	18.20949	87.80812	46.26581	36.08984	40.26482
33.48004	8.333293	12.94341	31.9427	14.90239	40.86902	28.2039	19.8623	12.733
43.37674	111.8057	69.98237	52.8494	27.34069	77.28447	78.06883	5.050513	77.34135
55.48815	50.26112	11.86875	12.63317	37.42528	6.892791	8.564411	6.526766	5.322235
1453.935	1573.435	2316.998	3399.592	731.5094	2002.968	1571.552	1626.648	1233.337
43.32663	15.17346	51.68501	32.40723	30.67436	127.768	94.08389	15.36999	21.21346
147.5636	372.9635	268.1211	185.3375	1095.387	81.55437	239.7856	510.0025	952.517
192.3488	315.5659	318.7108	527.2088	294.7519	497.5008	347.5174	251.2613	263.8451
14.51907	1.50625	7.564255	1.132594	25.77798	10.37932	4.94655	8.014932	13.78634
11.38242	6.966758	19.38545	22.31979	9.90929	95.03385	25.32602	5.791782	6.380414
43.46506	106.568	86.15035	125.5756	63.05571	106.777	142.3781	103.8943	184.3394

25.66442	46.26545	37.66605	11.34224	38.24002	2.550894	11.46124	37.65073	7.440933
76.77015	21.99515	43.0845	97.03108	26.5109	133.8216	78.80629	21.27056	34.98313
0.199397	4.239057	190.5407	156.2349	18.19526	357.9164	178.5661	89.8681	14.85932
1.811121	56.15677	454.346	386.2823	151.0678	743.2963	542.7468	274.1333	55.12286
144.0594	144.7459	152.8788	284.646	68.03869	99.82188	109.7976	65.51222	63.59313
83.36417	64.29455	102.2888	66.83193	34.80795	112.869	87.38901	75.12095	78.41924
85.11032	209.043	259.487	270.877	156.8907	272.6094	295.5276	86.12591	26.51419
21.03746	5.615517	30.16937	50.97577	25.6854	82.53282	65.07105	14.6326	28.62628
165.5954	42.22237	27.99672	14.5451	39.9031	26.03505	26.71573	11.69769	24.37712
13.04469	12.43748	23.70831	15.16082	39.85388	34.71326	36.82877	62.75464	39.20597
88.30114	195.3713	218.5743	251.7728	126.9938	533.1994	300.623	223.3009	242.641
35.48587	76.62907	171.2046	52.7507	155.2471	512.39	339.7228	106.784	136.6984
166.7603	461.6592	204.5975	515.6777	233.2813	356.8076	400.985	462.8535	414.2939
1282.762	5531.327	3262.346	4073.746	1324.405	2999.644	3487.814	1930.251	3937.31
28.79197	4.247502	14.01986	40.29587	8.237493	38.24331	18.7305	12.44299	13.79238
3.445883	9.704471	22.62585	61.45264	9.066043	35.60591	23.79444	31.68874	33.90392
27.43777	35.62225	38.78191	43.89614	54.78336	108.5906	70.09729	62.63595	77.34101
1.69405	0.068289	0.015479	0	0	0.833325	0.643706	2.1922	0.012081
0.182448	23.17699	4.337574	13.30712	1.593307	1.683893	13.67166	0.638991	6.379902
29.97817	0.089944	0.019762	0.508458	0	2.568807	0	12.01547	1.079841
33.37505	11.03572	32.27137	18.45004	44.15342	22.5782	20.22055	13.94401	13.78802
54.66202	49.31019	117.3659	137.6835	116.2363	495.193	138.012	110.5216	58.29703
52.85773	20.6244	75.34614	38.80835	48.14696	99.93022	70.87129	32.35825	49.80342
121.9473	411.1781	569.5641	563.4686	278.1236	365.483	283.8767	554.2543	440.7855
54.66916	41.12537	74.31552	174.0652	95.45414	141.5144	83.01477	185.7903	38.16904
6.601746	2.875124	10.78166	14.00044	5.752354	54.10638	32.76009	10.26244	10.6075
222.8192	631.0899	1436.263	1688.718	163.5126	151.879	873.5571	160.562	154.7213
158.5847	72.52598	131.3712	135.6974	121.1951	282.2194	117.7069	92.78146	98.55748
171.1274	38.38926	29.10278	30.44305	42.28349	35.54253	77.24639	131.95	62.53257
12.99383	5.61171	11.86877	8.785589	14.06764	20.81941	12.19329	18.37537	29.66128
77.11116	124.4256	265.9491	120.3323	177.6533	930.0758	313.59	487.3016	651.5751
56.29637	249.9279	215.3414	383.2641	175.1805	236.1518	222.5364	182.0072	143.0563
73.84995	131.1498	188.4038	236.6494	59.72484	111.9755	126.4177	60.34056	172.7009
0.197636	20.63992	43.09112	77.74753	24.84273	134.6379	55.57479	89.93624	146.1821
28.85615	28.70437	31.2241	24.16448	23.22087	118.3988	44.16256	13.91412	11.67717
51.28593	35.60428	36.62595	11.95669	38.99078	39.90975	32.43566	79.67845	21.21374
75.39562	19.27864	151.7928	173.5233	53.08677	105.9138	147.4638	85.45038	48.76106
39.34555	25.78805	2.1839	4.319646	7.41909	5.159849	3.501475	15.47081	308.3354
0.171686	0.10645	0.022849	0.502475	0	2.553052	2.057396	0	0.017772
5.04802	20.635	66.75665	72.03621	47.29482	125.1068	121.5572	57.4568	48.75196
44.78597	39.61395	28.00961	42.72176	17.37875	16.44849	41.91615	27.95296	52.96388
187.8193	123.9675	11.87603	7.49421	225.4551	6.889827	17.22832	68.57317	50.86528
83.49088	97.10261	282.0749	57.21399	182.663	690.4699	325.9766	252.0955	172.7175
48.21926	46.55306	90.44171	31.72885	163.6962	37.28471	47.60284	69.23802	57.23108
281.9824	385.2187	399.4601	714.6558	249.8908	496.6126	533.2623	237.2394	305.1686
1.826954	42.39749	50.60822	52.89614	8.231509	141.6966	41.13824	30.14191	149.3092
196.8692	77.67353	28.01468	27.30623	193.9452	15.57584	24.48006	58.26875	19.0938
16.26336	47.88194	108.7111	98.91613	40.64196	66.84767	61.39347	41.20292	65.69523
27.40833	9.715316	22.63766	50.33803	39.82797	36.43571	16.50762	58.98203	25.4492
16.26888	23.37616	187.3159	202.2202	78.01082	297.0707	307.4125	46.33168	8.50096
11.46392	30.17874	46.31566	76.50698	70.58077	78.1473	61.38907	50.80329	104.8747
104.3195	143.5405	394.0542	349.3155	167.6835	239.5995	334.5481	81.70059	57.24197
29.03986	30.17851	46.31556	181.5025	35.65204	58.1524	81.67905	44.15533	99.58031
14.63272	13.79655	38.76093	29.25866	12.38905	37.32878	16.521	16.12104	21.20861
549.2365	177.7049	244.4349	269.498	690.0717	127.5697	106.8188	740.0434	1177.12
3.414871	142.1974	761.1883	84.61794	601.2153	202.2418	1353.001	220.28	254.3141
36.97093	6.982985	38.77747	40.72164	112.2345	94.7025	57.80594	78.93978	42.39313
140.931	173.415	156.1166	195.1313	98.7762	105.0255	145.2279	71.40495	434.3198

33.87315	95.66381	116.2846	81.5048	41.45224	267.5384	133.6921	75.10981	95.36965
38.5427	21.98434	53.83362	41.37486	44.82164	113.859	62.17465	33.10049	56.15432
605.092	237.7655	12.94981	39.36588	211.6903	15.576	23.00944	344.1545	906.9328
535.7584	199.4514	146.4487	112.6985	272.3848	102.401	132.8696	171.68	173.7789
33.83091	11.08249	73.21547	57.94726	78.06196	176.3982	91.81382	112.1351	64.63879
54.26855	26.03328	32.31011	9.410383	46.5244	32.97673	35.38532	20.55692	18.03253
38.66851	60.21369	107.6741	73.84486	75.52231	222.3523	127.906	86.1823	79.48072
30.55415	9.713261	25.86181	41.40918	39.01141	16.44596	31.73611	14.63732	99.53945
3.443431	50.51636	58.12744	47.16818	44.0022	128.6897	63.65978	18.32888	51.91398
173.1836	798.9864	736.4524	1041.628	570.4449	393.2556	743.509	448.0506	438.6741
0.19113	15.15236	63.47806	45.32595	28.20755	93.9447	36.12821	16.1252	24.38278
43.43987	32.92282	97.97724	62.37885	59.74425	405.8403	138.8271	81.04473	75.23791
70.68415	184.399	161.5117	186.1111	220.8997	96.32773	139.3991	217.4512	169.535
40.28018	110.7002	136.7443	280.0731	40.61804	205.8011	88.79771	21.26063	53.00125
23.64483	5.58056	19.35209	14.04726	9.102947	25.27535	15.21009	8.046629	6.374986
254.7996	349.7446	641.6959	767.5327	364.4968	612.9594	445.0522	433.3362	234.1844
41.49501	28.70745	14.02303	17.74435	12.394	67.84196	21.61743	9.477115	12.73607
442.1273	1625.093	1208.019	799.2727	425.9294	920.2969	851.1584	529.1228	1175.038
32.2794	5.599118	53.86735	3.692297	371.1995	116.2881	79.36379	871.5677	1422.802
1.820999	24.73758	94.75003	172.9622	7.402975	379.7497	92.47578	60.36951	23.33542
72.16551	120.131	32.33093	34.91648	119.6029	20.78065	37.46812	117.9662	87.94806
333.0942	314.1791	455.4238	198.1217	215.0216	312.5383	140.7931	476.1858	403.6884
80.00393	36.99027	36.63119	34.30024	58.10493	16.44033	23.73512	39.72412	72.04947
225.8402	233.5914	192.7417	526.1422	140.2814	938.8214	268.7895	167.9779	115.5134
1439.411	1711.271	1306.015	1909.809	644.3243	1145.152	966.7362	379.4706	1139.031
46.6079	34.27799	80.75285	25.34949	60.58612	152.0249	86.71577	82.54735	47.69694
17.5489	9.64402	10.78106	9.475511	13.27206	19.12532	12.97957	8.775923	14.83472
193.9167	282.7691	320.8549	529.2059	135.2893	443.683	286.1005	128.1449	145.1811
30.67179	46.59397	714.8794	381.7699	119.5045	795.3614	1281.558	28.63599	15.91681
437.3291	964.2607	690.168	949.098	730.711	720.5879	897.4067	850.5281	943.0077
46.50332	32.87773	57.06259	54.81386	32.33842	153.0039	72.32049	46.40334	19.09512
52.60649	11.06889	23.70352	14.53153	27.37738	57.36798	42.67692	18.34655	16.97208
256.6173	65.60568	75.37099	25.98939	58.0948	32.94391	75.13392	46.36053	27.57175
1030.774	114.8858	63.55712	38.09425	163.5157	39.01458	34.56885	597.0124	423.8345
8.257666	1.49951	17.25955	0	109.6538	11.23017	25.17733	106.931	3.201947
31.91317	8.333757	5.414777	31.29474	20.7381	10.37193	9.290363	7.266031	10.61631
446.9046	1779.273	1021.755	1179.341	479.9173	555.6239	766.6276	309.4597	1006.569
8.256664	50.64053	62.46374	125.6812	33.98155	178.0886	80.90616	124.6391	105.948
28.65851	9.678507	17.23604	13.30037	15.74946	48.76208	25.33275	14.68947	7.438812
46.67504	76.59866	165.801	163.2095	39.78795	198.8568	134.3805	126.009	117.6192
1.817602	135.2288	91.53658	264.7815	12.38428	178.8765	180.6978	70.67071	128.2109
46.26637	16.50612	23.70234	30.57164	20.71673	18.19355	23.06312	37.61155	34.96389
200.1042	107.9991	206.7153	108.2604	185.1777	247.4753	218.2547	197.5442	246.8607
13.04413	5.616075	26.93469	20.92116	25.69987	77.36439	60.79867	32.39139	24.38569
55.82608	31.4579	16.17711	4.945953	31.5344	16.45004	10.00227	40.5502	51.90183
24.25241	2.873545	5.415007	28.54935	18.19769	9.49393	7.108407	4.315237	7.442014
12.71937	4.215615	2.182395	1.133295	4.095321	6.918883	6.439558	6.575107	10.5947
54.66898	94.32899	186.2496	135.1109	97.11623	121.533	171.2835	66.98055	76.30676
96.33105	564.1104	579.26	173.8613	631.9222	150.1435	1751.909	80.96138	22.27412
22.67539	6.975481	14.0294	4.321192	9.894339	5.157526	72.167	46.32851	12.73981
87.6384	13.71603	3.260935	4.316238	16.59846	1.683879	4.949023	38.56441	128.9302
53.05645	69.76474	130.277	115.9856	75.5191	297.9462	194.4866	77.32056	163.1589
0.144171	0.090886	0.019942	0	1.603663	0	0	0	0.015532
27.43693	45.15202	88.26879	55.41115	46.4664	160.7761	65.02545	23.48371	43.4559
27.45741	43.81882	87.20883	28.54103	99.67096	118.1185	130.1838	109.1446	133.476
514.0485	270.5596	412.3864	998.3646	703.3554	472.2825	752.2333	254.9215	299.8747
86.71584	187.2216	375.754	126.069	290.62	1380.834	476.2639	372.2326	410.0381
86.71454	148.9984	387.5931	420.1399	291.4541	377.6875	238.3949	283.0028	184.3801

5184.499	2494.062	3554.134	3892.002	2336.659	4538.122	2674.132	1801.248	3424.5
43.46961	170.6382	131.3547	270.5891	41.45109	148.4772	105.4563	1.378941	3.201265
2242.891	310.1325	299.3382	80.79347	239.0943	206.5848	405.3193	345.6182	425.9487
20.96756	24.65398	33.37861	27.35802	29.8786	84.37718	55.77393	7.260589	9.559947
877.606	1114.562	1373.849	1654.11	1267.152	821.2848	1048.381	1578.028	1435.702
157.0165	73.8958	176.5838	80.17685	160.2377	219.6626	148.0704	300.8101	227.8003
41.61421	26.03548	32.31075	4.946003	55.68683	34.71589	27.39828	27.95403	32.85377
91.44846	60.23779	136.749	217.437	84.64448	402.1352	153.1639	135.5793	87.96199
11.39036	13.74933	2.184134	1.13313	90.96377	10.37529	4.944357	17.65056	320.2737
71.98858	15.17635	7.569251	11.31805	43.14374	10.3621	15.05731	77.43602	40.27762
6.646962	60.2119	158.2521	108.9761	58.07143	105.0422	37.46148	38.95869	57.23587
6.638427	56.14884	174.4282	195.686	122.8546	281.3453	293.4721	85.40404	212.967
115.133	9.695635	10.79402	3.036315	19.06619	12.98105	26.0065	15.40628	25.43156
52.98674	6.934572	6.483536	2.40391	10.77242	10.39868	10.80185	5.809493	10.60398
8.258557	41.08258	51.69712	52.19139	48.12116	124.2224	59.93284	151.3148	75.22864
29.00983	28.79763	33.39808	52.8858	38.9916	9.494016	17.95335	75.24303	54.03978
241.8434	427.3528	237.9558	202.6229	280.6685	75.47865	270.9501	339.0789	209.8036
9.754056	0.109994	16.15007	5.603354	9.923716	40.97035	37.14068	8.027837	14.83699
182.6341	222.6452	441.3859	353.2626	95.43123	224.8597	202.2863	95.71808	205.5603
13.03213	11.06937	25.85438	29.91917	24.04479	100.9209	38.31267	35.37824	16.97225
81.65894	71.05548	94.73849	171.1599	77.21956	57.27472	63.5426	39.71424	46.63736
149.1687	1861.149	184.1451	715.1548	175.1391	69.40061	227.4929	24.21995	254.3177
12.95298	4.24418	1.105823	1.765547	7.410944	6.026052	1.33561	8.013286	22.24762
118.3052	62.89369	167.9008	141.0591	16.53506	27.73019	33.13408	10.94291	9.561324
57.90284	129.8918	204.5906	466.6821	203.3976	437.6028	272.3633	243.9079	328.4653
61.04766	30.20281	131.3545	114.0644	76.34883	231.8959	122.8244	108.3145	68.88957
21.07227	57.47594	80.76474	208.711	126.24	105.05	130.1037	73.64678	21.21671
163.3708	131.1915	152.8983	162.5075	168.5574	270.929	181.3588	137.0427	122.9225
233.9863	114.8851	305.7997	227.4234	180.9567	492.2644	1028.412	284.4179	691.8785
141.9945	80.54151	44.16295	12.59259	233.6914	13.83508	17.94837	38.987	38.16176
17.662	5.604755	25.8241	23.63213	7.411864	35.67655	28.26571	5.054102	13.78634
24.2763	120.2256	175.4831	128.0998	78.83826	203.2094	223.4091	45.59201	98.55079
16.20693	46.33903	32.30343	44.06099	21.54877	77.40841	31.05237	10.95282	41.31349
11.46028	6.98308	8.646057	6.220281	29.84362	14.70527	17.2308	83.39349	146.1342
0.194706	16.53454	10.79917	145.2417	3.252883	66.87895	31.71957	0.642094	72.03311
8.254478	45.2023	100.1343	101.9762	97.97656	137.207	165.5923	145.2751	151.4965
133.0821	68.44279	32.33356	27.25292	785.0392	45.08963	120.5751	432.0653	119.75
8.256054	4.241769	69.99818	32.36541	49.77084	166.7775	256.9232	79.57111	38.16599
30.5384	13.8008	26.93579	88.2818	23.20092	31.23128	20.86471	5.784929	13.79758
77.68355	27.34267	14.02228	36.38496	27.39075	22.5502	44.90016	13.9159	24.37801
9.834806	5.613797	35.51871	29.96354	14.89447	100.1197	18.7204	12.43792	13.79372
140.9463	338.3606	136.7455	137.6492	147.802	41.61882	115.5592	82.46779	54.06086
1079.422	2894.164	2212.595	3443.953	2515.218	2677.536	2331.658	3234.902	4486.148
76.87321	16.545	10.7996	15.7799	21.51978	18.17655	5.665832	41.93046	140.8843
1049.719	359.2652	244.4304	717.2462	421.8254	230.9001	228.2383	589.6928	565.7938
116.8254	45.2004	57.08662	29.80934	33.97845	78.11625	55.55343	81.03541	25.45443
136.2352	154.3976	107.6917	119.0954	225.0489	64.19406	89.4996	137.0319	114.4495
8.258341	13.8054	52.75191	24.7462	48.99516	31.22446	69.46197	70.85371	33.91781
276.9484	297.6888	24.79638	83.35658	409.4709	49.4309	31.67275	323.6265	11.67909
14.66747	54.75389	86.14764	89.82041	24.00861	334.484	77.25268	69.95097	83.71611
68.63732	46.44104	20.48366	37.55623	59.82291	38.18374	25.20657	32.37447	18.03456
0.196839	36.99108	50.61958	104.0255	79.73108	122.4921	154.8969	77.40507	32.86664
0.166146	0.103359	0.022282	0	4.095791	0	0.619081	11.07114	45.39091
0.151584	0.095132	0.020748	0.505482	0.768899	0	0.621655	5.122261	1.080818
11.35184	13.71817	23.67053	6.238743	14.09083	53.1697	11.49527	2.840527	14.84131
9.858728	31.50161	26.93953	22.1849	18.20447	16.44445	23.75364	39.76497	297.4525
0.187722	17.83998	16.1706	33.20038	12.3988	55.6734	29.64023	44.34728	38.12691
161.7696	95.72069	74.31932	168.8928	144.4618	204.9085	145.9172	106.7983	109.1506

67.50264	53.41797	1636.49	2109.696	322.1268	1010.618	1311.589	39.69411	47.70456
11.46353	1.498704	2.182156	3.046152	17.36516	3.421353	1.343377	34.53984	496.6968
0.077571	0.050918	0.01184	0	0	0.847255	0	0.687335	20.78373
0.184973	40.75646	18.31424	32.62217	1.593494	9.504265	27.4968	2.83963	3.20306
1207.379	1592.525	1565.494	1978.595	1272.13	992.3278	1090.288	623.4437	1701.646
0.187017	23.24816	170.8	3.672807	34.08363	11.24125	54.43282	1.370969	1.082046
30053.69	57502.56	44877.06	31554.55	18158.07	17055.05	25359.11	31225.84	39119.89
120.0153	75.18878	56.01046	47.0431	66.38972	58.13171	57.00038	137.1572	65.7073
449.6247	211.7605	311.1536	374.2604	148.5875	435.0325	274.5721	144.3787	158.9506
141.0803	174.9021	254.0968	149.0534	285.669	404.6473	330.9987	244.6922	161.0677
105.936	131.2713	291.8022	449.9831	201.7208	727.6036	614.9471	502.665	493.7549
414.9469	8769.255	222.9062	1058.684	2077.702	64.19419	1981.369	1422.511	28.6306
80.31054	292.2924	152.9135	433.5624	98.74791	523.607	286.846	63.27266	36.05063
20.93161	30.04296	30.14582	11.98137	38.23578	12.97816	15.08861	18.36135	10.6178
19.44161	9.715272	43.07783	17.06592	49.81449	80.79562	46.94186	61.94247	30.74383
0.187115	44.87388	22.61798	17.76888	17.39999	39.98934	18.00543	15.40748	44.46982
17.86976	57.46158	86.14008	85.38457	57.2511	161.5597	80.89612	125.3608	121.8367
35.48144	132.5233	212.0848	193.8398	99.60455	197.1095	139.4327	77.30392	40.28821
189.7903	96.90035	53.84992	53.46627	93.0301	12.96652	42.54966	36.76475	33.92671
9.843849	379.5539	200.2746	254.9627	33.14212	112.8202	351.9692	196.0107	380.3583
99.39007	133.8576	231.4446	140.8728	87.97911	240.5729	138.009	60.34448	136.6835
9.78307	2.877794	3.261064	1.132383	9.914963	8.63921	2.054922	12.47214	5.320899
1066.579	2659.172	2467.747	2178.662	1433.207	1182.457	1921.987	1618.504	2199.643
24.27536	67.03697	38.79162	131.9553	43.944	117.2008	136.5806	143.751	171.6318
107.1941	60.17878	21.56593	10.68068	169.521	5.155759	18.66793	205.9207	96.41682
0.188735	13.77815	14.02182	98.25193	9.067546	18.1991	22.35289	18.36059	5.322543
243.5469	553.0607	442.5128	656.6973	152.7254	226.5619	348.2409	215.1338	262.7855
3647.339	7236.928	4867.632	1502.265	3112.301	983.6271	4993.792	1710.615	2379.774
105.9245	147.6416	233.6582	347.3796	183.4613	617.3724	398.8773	350.8294	272.3166
24.24531	27.44762	54.91988	84.21495	20.6918	336.5009	54.15692	16.84248	34.98355
5.049199	0.115415	2.184029	2.401027	5.742375	5.154376	2.055599	1.371252	5.322437
11.46077	84.75926	79.69296	182.4358	102.1164	118.0717	108.3594	112.013	129.2641
32.28243	47.95503	161.506	186.7823	132.003	435.1437	201.6293	175.4194	130.336
86.40787	20.63803	44.16547	50.90951	103.0127	100.7436	98.328	63.35317	60.40377
8.191518	6.953801	10.78476	32.1185	7.416853	16.49269	22.46322	14.71622	5.32025
20.94471	8.341258	20.4742	19.03062	14.89212	87.02285	29.61618	19.09709	21.20372
4.96126	2.859084	6.474021	7.609095	14.18104	30.62965	16.78509	2.111628	7.42421
20.96354	15.14704	28.00268	14.53434	22.3826	75.66978	44.86909	7.260816	15.91285
3.438481	2.87696	0.023669	3.038567	64.32815	0.818515	0	17.69148	206.001
14.66364	19.27041	96.87595	30.46999	67.25991	116.4152	99.81545	57.46055	163.1013
94.27713	42.42954	62.45112	73.9689	35.65528	121.6371	61.40165	75.94294	39.2199
17.84144	58.6834	18.33259	56.76536	34.84103	36.43997	23.75246	30.14955	26.50701
14.62258	6.980826	19.40246	26.06877	22.37924	43.43275	25.96193	15.38681	29.67434
48.23971	52.01862	90.4485	330.8803	15.7042	67.68873	98.98082	52.98361	45.58202
9.836129	13.77877	37.66904	28.03009	21.55818	53.91281	41.99423	22.80594	24.37743
9.769905	27.1658	14.00702	15.26692	7.416085	8.641698	10.77089	8.764717	12.72528
166.0375	73.78559	37.71042	86.05701	78.04763	59.88014	17.22287	90.67234	190.6565
48.09648	49.20136	36.6251	33.68658	136.389	12.96778	28.09131	61.93216	63.5676
8.250331	11.06812	35.52863	22.22437	6.571943	9.49708	37.59244	9.475196	5.322727
29.04764	5.613066	1.104464	0	22.35112	13.83478	3.502327	51.53356	104.8798
1.831912	59.82643	29.07401	16.4641	30.72274	16.45654	28.16133	10.95604	16.97018
77.04679	165.2356	149.659	166.393	127.0302	111.975	135.0985	75.09342	159.9904
3.439646	13.81231	210.8757	41.34382	141.3346	73.81176	38.22073	19.05646	5.322455
1.819831	52.02445	213.1248	217.6451	2.427238	145.8907	114.8942	5.790027	16.97859
79.98902	87.33883	6.492178	14.50595	12.38316	5.15482	8.552265	16.8417	491.2839
131.1848	56.10992	130.2647	54.065	62.23253	67.68802	56.27505	72.17333	82.65465
9.84612	28.72696	36.60447	88.43171	7.402702	73.9183	23.05927	16.86699	11.67807
0.154545	0.096818	0.021065	0.504718	0	0	0.620951	2.118377	15.84302

1905.137	500.0204	81.8595	150.2726	648.4792	209.1808	184.1331	664.7356	995.9928
1.832315	0.110955	0.023666	0.502894	0.766575	4.28879	0.618936	10.25046	88.78769
139.4209	148.9337	91.54393	118.4624	122.0238	127.5953	174.8393	197.5312	194.9586
229.024	243.134	119.5383	263.2504	204.2482	157.9781	334.6046	97.92615	243.7016
16.13722	39.42179	11.86711	17.78691	19.91078	3.418363	10.74627	29.52998	31.77161
176.4133	25.97112	2.184025	5.58532	46.58789	6.891903	6.388605	88.93271	10.6174
59.34274	31.54707	94.73519	66.25242	89.70148	115.5221	114.2676	36.02592	39.22263
246.8276	226.8768	400.5512	1111.734	676.7415	1543.762	862.0211	699.425	774.5403
40.25469	45.20138	92.60086	119.2347	58.07743	148.5071	103.3242	103.9226	90.06831
43.49069	539.3439	106.6229	94.82188	617.9093	83.29159	185.6112	727.6943	553.0629
9.840984	8.34221	31.22518	33.15467	20.72006	48.67254	40.51871	14.65284	22.26289
13.05533	166.6867	189.5025	428.0021	30.65081	138.8812	153.8506	227.7589	126.1039
19.44406	622.8589	1536.361	2514.675	138.601	532.1822	469.5914	5.065789	146.2448
6193.264	2678.461	5474.923	6431.519	3074.045	7747.921	3365.625	2423.32	2595.936
70.49438	42.44903	46.31806	121.2671	34.81717	47.71702	59.92732	71.47721	83.70195
623.1101	521.8945	2564.66	2665.63	1191.544	9547.94	3372.232	2919.444	3971.205
192.041	26.05435	52.7496	11.32236	13.21742	11.23191	15.78865	33.11571	84.72497
272.1156	245.8266	155.0593	326.4739	206.7537	140.6148	127.0885	254.3018	192.8465
30.61023	53.29532	80.73049	61.83768	21.52508	68.60277	59.97303	20.53487	36.04069
302.3884	57.51523	43.10018	16.42004	75.50007	99.8004	22.28037	220.3935	304.0696
308.5342	133.8858	162.5763	93.60215	386.3469	58.98796	97.47562	95.74772	111.2654
0.078042	0.051212	1.085428	0	0	0.846979	0	2.263594	0.009306
114.3399	64.02414	4.338601	7.495311	49.02057	10.36438	3.497984	71.65802	105.8734
6.656061	45.06461	9.722139	12.60131	24.86378	9.494985	11.4468	114.6036	91.06934
5.051359	17.89576	34.47071	41.38305	25.68811	39.91734	103.5603	68.61433	42.38952
176.979	109.114	69.98563	41.9694	75.57201	24.25811	26.62934	47.10982	60.40148
153.8136	94.36527	150.749	318.8739	63.04061	264.835	214.6138	5.058699	218.2653
8.255593	24.73747	90.44547	75.79422	72.21264	214.5795	169.2438	37.4902	153.6088
24.26289	8.348468	1.104363	5.587397	3.254985	47.71339	3.503043	41.92623	1754.459
266.0448	487.7112	1392.123	899.3655	871.8933	2273.984	1381.63	540.9146	628.3322
49.89309	229.5806	405.9185	332.009	436.7694	872.6312	861.4638	134.0325	433.3644
9362.104	9482.825	14390.9	14307.38	4477.388	13210.65	7277.585	10079.83	11622.29
6.651461	12.42568	22.6259	31.22327	8.23457	86.13639	31.7886	25.75921	37.07835
38.6787	53.40706	87.23182	163.8487	108.7505	170.1854	119.1867	147.4116	48.76329
103.131	47.71576	6.492221	13.24918	9.895875	5.153768	10.00341	27.96663	50.84037
24.07856	15.13056	17.2462	30.61883	19.06252	45.20867	28.18008	24.29743	27.54947
8.146534	5.582563	15.06603	10.79308	5.757498	11.2741	11.53816	8.044071	11.65973
28.96943	19.2513	30.16487	17.07323	51.49858	46.01467	28.83325	20.54665	25.44669
102.5681	30.20299	89.38139	87.24177	100.4487	98.08398	120.6513	66.98749	134.5628
6.623832	19.12244	6.48857	10.73595	3.252189	8.638756	15.1348	15.44372	19.07228
43.40146	71.03958	102.2643	83.52746	36.48078	99.00623	80.21566	31.59782	64.63942
37.06142	38.38372	92.60194	115.393	60.56959	270.1859	132.2796	113.5174	63.58959
22.49244	16.48426	23.69445	19.04777	11.56616	59.161	42.74959	3.575587	13.79276
401.9753	568.1348	595.3929	486.3388	322.9799	380.2528	394.4784	451.7923	404.759
38.43527	5.615968	25.85763	29.89998	24.87078	42.55092	28.85308	16.12105	26.50178
6.655161	19.2392	14.026	63.27378	20.70793	54.73544	36.83657	25.73504	41.32097
318.6918	101.2222	565.2248	119.0497	507.4167	399.3835	633.1271	167.9553	225.7015
13.06598	54.71755	210.9288	52.17628	33.15272	32.94297	380.2616	6.523878	5.322083
390.4628	121.6833	95.85443	59.76119	224.1851	156.2407	70.69465	454.9097	95.3834
8.257267	31.48636	43.0691	40.13133	15.71242	98.23937	40.4453	16.11515	37.09152
9.758823	11.00394	3.260619	10.75548	9.087314	64.57696	19.5464	13.23247	20.12222
44.90068	23.34605	3.260877	86.22769	12.38468	32.95828	23.02418	11.68175	36.03808
75.48272	148.9361	233.629	200.7889	97.93029	130.2003	200.1526	133.3466	119.7458
91.21021	231.594	17.25948	5.586933	99.67635	8.62615	9.274935	238.5149	182.1812
248.8535	58.81498	49.54983	25.34708	78.04069	68.56669	109.8776	260.6181	245.7272
5.038872	147.4605	64.62518	142.816	153.6498	21.64824	139.4764	27.15815	25.45495
5921.295	18602.09	14928.16	13392.68	6559.184	7286.832	11949.02	14981.27	14821.08
161.9568	613.1666	723.4982	493.3641	271.4876	349.8626	298.3472	378.0656	173.7906

8.25652	12.43859	29.08689	37.57626	20.70457	57.33612	44.08275	28.68665	42.38269
0.191897	8.346484	43.06103	51.08069	19.87675	88.69824	56.45794	31.6594	57.19425
0.171689	5.577673	12.91796	26.45362	16.64964	7.783676	25.51622	0.638549	1.08211
49.83837	120.1532	119.504	84.72463	83.8443	90.27974	175.7312	28.63551	86.89128
797.4491	260.801	16.1823	45.10031	176.0222	19.91272	16.50242	212.2713	5.320534
432.3978	607.7452	346.7097	187.2562	1096.261	167.5105	254.971	665.6031	351.7872
6.644081	6.97447	26.91581	53.84924	9.070105	51.32437	32.56436	16.14911	7.440477
88.30409	98.47872	256.2522	256.2289	183.479	435.0394	284.7039	200.4309	200.2668
94.61553	88.875	147.5053	180.4496	85.48229	84.17845	118.4614	92.06392	97.49314
77.05222	26.10825	262.6675	135.0962	125.3649	714.9762	234.2215	65.5018	25.45515
844.0264	8348.02	2900.547	2362.311	2333.417	1113.005	1744.971	348.5097	356.0371
104.2326	86.16275	67.8594	52.12007	142.8072	58.98631	81.55681	108.281	129.2761
22.64626	16.54064	51.69072	21.52995	32.33161	96.42502	54.88801	36.77937	41.33676
90.68989	19.24379	2.183542	1.769798	9.894133	2.551698	6.385269	72.38413	11.67923
104.0379	62.91252	61.38948	54.07545	46.44567	43.36476	49.77011	92.86191	113.3644
5.042723	17.80484	24.75745	20.37631	8.241892	59.22013	23.85928	8.75067	12.73079
89.9246	164.0368	710.5707	522.0932	107.0479	711.1284	252.8184	230.6139	49.82486
0.091018	0.059257	0.013611	0	0	0	0.655745	0	0.010632
99.41971	72.51217	160.4236	66.16006	112.0693	177.997	90.9659	179.133	96.43504
29.07577	54.76224	37.71513	58.51771	28.99137	32.06825	32.39874	192.4789	755.1806
1.832977	8.332043	48.39158	26.79518	9.071837	63.5528	28.93871	20.60817	19.08105
3.398026	0.101278	1.105147	0	4.100003	0.819141	0.61952	20.14112	5.31224
3.445781	8.343661	25.85273	44.06444	24.8811	151.4826	78.33974	25.01293	39.19671
17.83447	42.36332	34.46741	140.2301	3.252399	24.26941	43.3403	21.28526	12.73895
29.07994	19.2752	540.4051	452.8553	14.04678	402.9294	293.4199	35.26409	31.81251
17.871	61.57015	156.0956	186.3157	67.21557	153.7102	88.10996	104.6471	47.70153
52.79705	107.5247	120.4909	103.6068	17.37354	19.05221	33.17919	19.06669	14.85751
5.037504	38.39378	181.9398	196.4455	94.62767	963.6143	238.6122	40.42969	78.42427
25.84916	30.17921	46.31586	82.90359	63.9257	128.578	78.77791	89.22974	51.9299
11.456	35.66758	176.5725	239.791	42.27822	641.0784	255.1812	156.9791	105.9706
725.2139	291.0352	590.0203	287.3533	470.8065	306.4362	291.8253	386.1518	346.4916
149.0186	68.43498	231.4777	54.02809	259.1184	337.805	254.3983	117.1167	325.2547
28.89996	28.73105	46.28002	24.78464	19.88096	39.07867	33.95112	10.95155	11.67823
12.81024	0.106916	7.555857	12.0984	2.424173	28.77935	15.94551	2.844934	22.21802
69.09383	27.47363	193.8096	4.961472	147.77	24.25326	30.94992	209.3105	848.5515
181.009	94.37202	175.5101	205.8661	214.2364	323.8905	213.1492	88.3478	121.8668
75.06931	32.87306	36.62362	31.77302	82.27997	79.92589	88.29617	7.258399	41.33221
40.07455	24.69042	7.569177	4.31007	49.83744	7.757979	17.23863	91.62002	177.8466
22.57053	40.94717	1.105309	3.037572	112.3993	12.1035	10.00281	26.4825	27.559
78.69531	94.3756	199.1938	135.6675	218.3844	229.2054	299.2032	165.0455	276.5358
31.70779	15.07138	19.37831	4.955441	18.26592	10.38199	6.399663	5.795622	4.261998
16.26022	56.03075	138.8171	61.17979	52.29246	25.12919	87.50907	25.70015	28.62865
41.3214	23.23462	6.490708	12.63741	29.08541	8.63261	18.74133	12.44853	14.8491
235.5417	447.9568	585.6879	859.569	960.1248	169.2521	361.2581	247.5779	193.9192
0.185218	5.609465	0.024196	0	0.766981	0	0	3.57607	1.082117
17.83967	74.96911	35.545	27.94376	12.38552	50.3584	69.45503	22.75989	60.38452
36.97962	39.69152	68.89887	46.47452	30.67119	61.64567	59.97325	44.91442	57.21801
797.8779	143.5182	185.2045	183.4999	157.7284	220.5101	273.862	151.7582	242.6417
57.90285	166.7591	136.7685	635.0524	162.6943	429.7741	398.8619	353.7668	128.2303
1.830163	24.67394	17.25329	36.95519	9.894843	42.55048	68.81127	58.32672	134.4366
19.23453	11.02922	16.16048	12.6614	14.9177	19.09569	22.42185	26.59583	10.61301
975.0298	267.8448	73.24682	130.5116	254.8598	79.81884	47.5753	916.9077	1447.319
89.51487	9.677274	3.261265	3.036998	2.421967	3.418753	7.848112	5.792809	10.61337
49.72301	70.98542	73.20454	205.3423	19.8617	15.57274	49.09235	2.109266	23.33277
62.68908	155.7604	107.6916	214.1873	88.79142	187.5261	84.43887	223.3469	287.1201
23.78186	6.946223	7.560117	8.827373	7.420855	3.420501	1.335275	3.580748	6.377483
37.04485	163.6508	21.5659	288.1853	19.02653	12.96643	95.39125	1.377257	668.2065
4.987442	2.86643	6.479042	0.502595	4.093367	5.168978	3.511694	8.810829	39.08059

94.29859	36.98683	63.52856	132.2175	55.61279	75.5434	68.64251	31.60318	16.97781
646.1841	365.9434	132.4575	182.8524	124.4987	118.0286	191.4198	192.3136	612.3629
9.846636	20.58558	25.85422	18.37445	49.04374	21.67186	36.13333	27.97035	24.38215
8.257984	11.07788	37.6942	58.71251	24.8605	99.09771	52.04798	29.41696	23.3292
84.02113	8.293247	7.55937	5.607938	13.27391	9.520366	10.05853	7.290134	8.49216
49.66447	24.70697	52.75772	52.26376	24.85484	112.9904	47.66893	34.57955	34.97908
19.47238	136.5378	97.98736	172.8709	28.99195	123.2896	130.806	51.49994	248.9409
206.3027	150.2095	108.7556	128.7431	97.95275	73.75797	185.7785	211.65	256.3693
86.52157	45.19804	114.12	70.04015	102.9717	170.2435	104.7814	72.17977	59.3514
646.7348	364.2544	114.1426	38.09097	82.98654	28.59387	71.4378	165.1096	247.9084
65.80793	84.73776	92.60295	112.1915	95.47909	145.8932	91.00975	83.24231	59.35309
424.5478	1200.554	958.2589	1155.604	782.1814	537.3728	786.0775	1919.381	642.1109
9.749353	0.109713	4.336154	1.131985	8.254822	0.818467	2.777908	19.2052	58.13998
648.6338	409.8804	1259.707	1264.672	621.0817	4186.844	1551.439	268.9109	571.12
0.119451	0.076438	0.017123	0	0	1.706272	0	3.70111	1.076359
9.859809	88.57443	31.24409	22.81992	25.68722	31.22043	39.6948	84.1511	42.39018
469.2763	221.4081	737.5228	304.5536	754.8333	1135.683	902.5458	514.411	868.8257
94.54486	31.56488	110.9001	180.5534	71.36994	207.5887	83.0412	97.9995	71.0057
44.86959	23.33902	60.28085	42.03257	50.65778	102.5674	99.95115	48.64367	22.27097
24.23153	35.60416	45.23219	20.89588	98.9134	39.04037	136.1605	41.22305	84.74282
0.123489	0.078829	1.099092	0	0	0	0	0	0.013721
52.09496	35.31164	8.639728	8.799668	38.31191	3.418751	12.2116	12.46339	33.87657
83.14298	17.90567	38.78146	72.05198	70.59152	59.89741	55.60609	34.56004	66.7517
55.78904	28.73382	24.77985	8.135044	53.20669	27.76162	20.15252	33.89248	42.37473
48.20847	35.64327	99.04581	103.9562	39.80087	232.0057	104.8182	47.83133	72.05678
123.4093	358.8755	62.47719	25.9766	607.3996	30.33019	65.64769	264.7121	135.6324
567.0425	937.0355	1288.795	1337.896	432.5618	1461.19	910.3549	1884.68	1366.833
0.186041	23.23361	17.24213	30.01717	14.07024	38.25711	27.48083	28.77737	33.88908
126.5363	187.0215	18.33658	225.1815	84.6541	23.38458	28.06046	207.209	185.4079
54.11362	8.342093	0.024628	3.036766	6.572415	0.819314	7.110439	16.13314	161.8906
14.59044	35.43978	20.47085	8.781883	9.900819	8.630499	11.46254	25.78185	25.43328
0.183453	17.79455	26.90156	37.83289	9.910036	19.09196	33.36168	22.12074	22.24842
19.4619	11.08247	82.89706	54.11366	58.93384	116.4012	102.6901	61.88244	76.28648
166.5991	172.1317	247.6237	141.4287	229.2033	84.16786	243.5458	80.9771	184.3671
29.04849	35.63605	42.01363	112.3095	39.80495	79.00805	116.4405	88.47214	111.2337
136.3366	101.2239	319.7847	330.1357	293.9264	945.6354	493.5635	290.3474	294.5677
92.71797	36.98838	62.45344	48.36652	81.39883	23.38973	29.52556	63.36812	59.34169

TCGA-DB	TCGA-TQ	TCGA-RY-	TCGA-HW	TCGA-DU	TCGA-HT-	TCGA-HT-	TCGA-WY	TCGA-HT-
101.5123	135.9764	232.3505	80.32033	109.5325	265.2268	172.8424	257.8242	117.0516
69.51508	40.79459	7.990681	42.04888	53.39449	15.95242	35.76129	11.09774	52.57777
209.1301	70.00311	119.0288	185.9401	161.0631	55.83594	103.0671	74.57474	159.7105
3309.731	1528.469	3183.265	2487.784	2302.34	3239.538	2480.228	1941.623	4092.893
80.35051	168.4387	113.3831	132.6544	100.1708	77.77196	159.7506	187.0552	86.29975
0	0	1.174206	0	0.915901	0.996169	0	1.214176	0
4.167189	9.41971	10.25774	7.445877	21.51539	6.978383	42.92032	5.801958	10.90845
158.2488	173.1972	172.3065	209.2778	202.2527	488.5782	148.9624	172.4536	179.5483
8997.422	3843.218	11115.36	6672.09	7042.805	22847.27	11414	9510.994	13026.62
92.28213	126.1052	30.65488	99.0482	43.05349	61.81921	38.02075	89.26659	32.73251
16.90197	37.98326	23.79787	28.97647	18.71974	7.975635	44.43536	10.46253	26.78467
1218.619	1408.821	1055.431	1526.51	1354.828	1061.898	1423.758	1171.368	2266.67
96.42648	154.5564	106.5991	77.51553	72.07105	122.6411	88.66132	124.1327	64.47527
4.216774	0.45774	0.03352	0.911754	0	0	0	0	0
171.8584	92.83194	115.6273	78.46005	65.5233	181.4714	83.95809	95.7639	81.34049
20.26206	12.42426	13.6551	19.59719	10.27311	3.987143	15.94293	5.802	22.81319
2940.781	1188.714	2204.942	1856.259	1792.996	1133.688	2544.668	1837.929	2968.984
35.5117	40.69198	62.32687	46.70674	18.70188	28.91472	62.64687	67.47586	22.81269
42.25418	43.59127	65.78874	45.75182	24.31645	32.90269	43.35018	32.89387	66.46013
754.8705	162.2151	201.8442	132.6287	325.8088	76.77449	187.5488	87.7217	262.8704
1176.313	885.5933	1095.096	871.6064	909.1366	1589.359	894.4527	854.1982	1321.313
52.47422	25.64152	225.1638	142.105	91.80057	6.978378	4.003799	82.76332	29.75713
42.25196	124.6334	205.122	43.88192	55.21889	679.0287	82.14277	302.3326	87.29213
324.9651	308.585	491.9438	429.7526	269.6459	449.6874	350.4382	559.795	194.425
142.2217	97.06287	102.0265	61.64052	116.1082	64.80999	88.16256	71.28393	135.9028
32.99914	35.965	20.44108	26.14683	23.39363	20.93809	31.5598	54.32687	17.8531
164.1453	85.53507	76.00718	142.9173	135.7459	215.3708	82.67884	173.0402	71.41898
816.6198	1411.284	445.5692	1744.204	763.0703	510.5079	793.7253	1011.487	948.3281
45.72086	46.8076	37.41213	38.30266	50.57576	8.972583	35.13762	39.65009	86.31026
117.6705	59.23286	70.30483	99.96691	56.16217	34.89698	206.0601	117.0258	89.27787
39.73457	120.7267	88.38628	36.41519	43.99201	14.95509	90.70913	44.8543	31.7406
49.05849	55.69145	74.80275	107.4702	35.56084	119.6526	90.71706	95.26975	26.78046
9.260107	24.64765	20.40093	19.61881	8.405227	4.984286	30.57017	43.27953	4.9567
718.4504	756.1598	589.5273	811.8201	729.3651	619.1907	450.3929	1002.26	617.0069
232.7207	450.3203	129.275	295.2227	235.9469	70.79202	187.0658	260.9943	108.1226
12154.8	22900.4	15596.66	26036.38	12015.47	9804.361	8733.31	9208.317	16853.66
11.79497	9.435442	18.16742	13.99313	29.96629	8.972674	5.791559	5.143685	12.89309
34.64466	61.67099	77.08402	89.69424	58.9774	57.83063	140.3404	111.789	118.0488
34.66141	58.15631	34.04084	62.60138	52.4325	42.87439	19.51304	27.64444	13.88435
21.10487	49.7628	29.50616	35.48979	60.87569	21.93498	12.94625	9.766951	6.940429
11.78366	62.16483	70.33422	7.44733	20.57105	280.1832	54.06784	143.3315	34.71569
55.84775	64.14411	63.47595	49.50566	30.87827	21.93483	77.5721	86.01121	41.66137
8.397005	22.61342	20.45912	17.72275	22.44618	10.96672	32.04769	5.803749	25.78838
206.5591	155.2548	111.1114	216.7711	125.4618	135.604	236.259	149.3483	151.7732
247.9362	357.1978	283.4148	309.2248	258.4119	83.75414	282.4604	303.2542	130.9379
17.74555	36.11443	23.80616	36.46115	3.718887	4.984247	31.68715	25.12034	7.932766
32.10609	33.4248	37.44979	46.69602	27.12904	48.85671	16.51611	26.29945	31.74049
3335.105	2281.721	4697.778	4743.922	3017.676	12199.37	5411.283	5673.352	3761.571
175.1121	196.3818	1896.32	392.3514	500.9111	1543.496	525.5813	540.4056	512.8505
55.24294	37.44544	9.111766	17.74604	46.88795	2.990078	25.72306	31.87975	25.7933
21.16593	38.05265	20.40129	11.19287	9.342862	15.95304	20.27379	37.2409	11.90172
313.1346	284.6436	268.674	204.58	223.7668	66.80363	442.3931	280.7945	450.3612
76.16288	76.7006	85.0121	90.62728	43.98801	39.8826	102.0097	111.1136	36.70043
6549.213	12398.75	4254.59	11521.62	6800.323	2261.393	4349.057	5672.496	8158.008
15.1964	30.03834	18.15975	36.46062	15.90311	29.91317	32.89232	33.13334	15.86985
62.61983	37.03869	65.74953	50.43708	21.50975	25.92321	144.6384	125.7924	55.54982
34.73734	25.76743	20.42426	27.09456	28.09317	26.92146	28.63899	21.76639	42.65906

7087.439	4344.83	6663.547	7812.784	4224.552	4156.857	3691.706	4070.963	6187.942
137.0387	170.0542	124.7511	155.0565	114.2032	43.87059	150.0382	153.1616	113.0821
17.72229	60.73839	31.75333	44.85393	14.95811	9.9697	30.94844	38.33454	8.924484
10.08957	16.01538	37.43998	7.445678	41.18551	71.79094	45.83625	42.22562	30.74889
0.78927	1.044644	0.042806	0	1.84741	0.995998	10.57098	0.536568	1.980768
392.7544	285.3033	316.2463	233.5527	247.1859	117.6554	221.6784	156.5043	286.6825
16.04493	40.35615	11.37961	41.14089	9.339985	20.93857	5.794243	39.81029	15.8698
35.54188	49.25045	58.88218	38.30572	23.39266	89.74155	43.5685	48.31887	17.85302
58.35639	44.81351	89.55641	70.05591	50.54194	222.3543	217.4278	152.1534	141.8564
93.88001	108.3286	74.87303	88.72548	130.1281	69.79496	85.66367	148.578	89.27485
63.75588	5.835349	23.78788	43.97141	48.76249	15.95301	19.05815	19.81919	19.83948
15.18833	63.94646	12.51482	42.06519	14.02471	10.96692	21.99182	46.42499	20.83012
233.5615	277.475	224.4702	297.0881	180.6931	504.5289	280.7279	296.6935	411.6742
26.19132	66.65794	27.24278	67.28733	23.3876	8.972559	60.2898	62.19853	31.74156
90.53121	42.99397	214.1514	48.55573	53.34795	129.6218	173.6102	111.6595	268.8355
32.97702	34.09118	37.42494	46.71289	20.57716	29.91199	28.50838	49.58005	25.78916
306.3655	227.0635	304.9336	338.1976	245.3064	155.5449	511.6326	319.8211	302.5534
38.92208	13.02418	32.89422	19.59675	29.01246	24.92643	81.97207	14.40454	62.49771
69.32784	108.3051	128.1485	64.43204	99.22226	229.3297	113.0763	119.4574	120.0261
315.6978	213.8962	178.0043	295.223	194.742	546.4073	136.3566	251.7359	466.2352
1640.904	1103.675	1479.409	1414.385	1290.208	697.9603	2063.829	1337.161	1280.639
98.21965	177.9263	36.31702	114.009	56.16989	59.825	30.84954	98.55956	32.73254
261.489	578.6212	267.5421	511.9928	307.1109	86.74544	699.5791	480.5173	313.4651
141.4816	86.39612	30.65252	124.3005	118.9542	26.92032	30.25755	50.82255	81.34365
21.94264	19.01192	40.84403	17.72288	31.81344	56.83374	39.22617	24.31671	67.4546
2525.341	666.7913	2209.408	1500.34	1509.313	2308.259	785.9197	710.1099	2174.414
31.24868	10.61527	57.85573	17.72154	23.38024	52.8447	117.4318	22.31732	81.34066
243.7984	216.4532	82.79594	314.8873	133.8849	2.990172	165.6987	87.77368	135.9
173.423	161.6278	355.9889	163.4599	342.6713	174.4892	218.5665	63.27676	149.785
33.78795	27.99445	21.5966	8.380472	38.36318	42.87367	26.04954	27.60506	24.79597
126.8984	132.9274	167.7975	150.3946	180.6991	117.6555	54.65605	116.1784	225.1789
74.51738	78.04138	52.15023	68.21078	48.6832	11.96383	255.877	52.18652	68.448
448.485	789.2385	693.7816	1037.002	367.0096	1186.536	466.5393	1055.267	609.0726
2010.71	2043.326	1578.053	2675.584	2065.469	1967.254	1962.402	1756.685	1431.419
2.525311	0.472206	0.028463	0	1.860855	0	1.078645	0	2.975793
73.67338	60.56863	37.43622	85.98077	33.69145	29.91181	53.64198	55.51178	35.70939
186.3658	122.4331	134.8208	51.36664	42.11455	301.1282	45.7788	95.86321	146.8184
562.7269	465.4707	556.6503	482.0317	511.2004	538.4266	520.7415	371.8358	691.4058
71.90187	109.6849	102.0262	76.59404	82.38594	54.83895	100.1167	93.79911	79.35685
91.40674	201.2191	66.90383	85.94837	103.9407	22.93181	104.3658	126.9884	32.7322
66.0014	61.68405	66.88971	36.41356	54.29501	13.95798	127.8	29.6082	88.28738
60.90117	55.03345	275.2056	49.49491	46.79617	277.1963	183.9512	116.3792	88.2861
87.12603	90.38645	155.2968	113.9649	95.48851	206.3983	316.2883	137.4174	212.2862
219.146	198.8328	260.7575	144.7787	186.3045	227.3353	84.45187	243.7256	140.8576
3.321756	10.0572	46.35422	11.19104	11.21677	185.4794	17.83278	55.95218	22.81582
35.52881	34.71238	49.85818	17.72703	14.95675	23.92931	66.34899	53.59535	20.82899
21.12779	22.70914	30.60395	37.38197	19.64836	16.9497	77.4378	49.73787	5.94853
19.41248	40.13562	36.28921	42.97476	24.32626	30.90919	47.11665	38.96267	31.74188
20.28965	22.74274	29.45854	22.41584	10.27678	23.92999	34.07523	15.10751	39.68254
53.29125	71.32224	125.7412	52.30506	36.49639	115.6638	97.27529	149.6556	189.4798
7.555655	5.826854	19.29477	5.578227	16.83876	47.86216	20.20174	21.09866	31.74456
2160.521	1099.452	2348.879	1916.06	1299.566	2440.871	2177.605	1109.173	2044.462
42.28667	37.66113	100.8083	98.13135	38.37442	44.86853	83.5864	80.71784	13.88432
305.4481	194.581	281.1908	180.2745	273.3755	730.8655	761.6696	334.854	530.7059
521.3223	261.1526	408.1158	417.5894	471.8983	392.8523	665.8432	319.704	333.3025
1.631312	6.438051	6.851432	7.449844	12.15591	2.990076	1.624753	5.813177	4.956681
22.00537	21.57324	22.66778	22.42298	5.592419	15.9529	59.59741	33.84391	9.917142
161.6727	88.6054	109.9739	87.80226	169.4882	74.78079	125.7482	81.18299	179.5504

12.64141	14.85813	26.08261	12.12173	21.52394	40.88152	14.17431	21.08023	34.72036
60.92437	73.14447	65.74956	67.2649	23.38302	29.91164	109.8808	147.6981	45.62925
354.829	184.7083	77.12253	258.8337	209.7648	9.969658	82.14347	69.9314	143.8373
606.8457	962.2918	341.2352	808.1537	511.236	50.8502	451.1872	228.502	148.7933
181.9926	61.58318	201.731	77.52113	125.4616	162.5258	141.2601	147.3611	123.9966
87.1415	80.21795	41.99545	100.89	92.68583	43.87077	61.26206	114.9661	92.25275
439.2667	373.3901	470.3996	778.2867	602.0928	344.9926	184.0388	575.7069	246.0089
49.07184	55.73318	60.07325	86.91205	40.24916	7.975461	117.789	58.15523	26.78074
19.42896	21.49748	33.99502	21.47454	14.02368	71.79374	11.17342	32.39597	22.81417
30.43732	37.12148	13.65573	30.81721	31.82382	16.94944	17.13716	20.37151	11.90047
192.0733	274.4608	250.5379	427.9021	213.4676	92.72801	231.1926	223.2735	168.6339
295.3837	318.9201	149.6712	397.0802	242.5049	27.91719	259.2824	280.1917	106.1387
647.4242	288.109	515.8043	441.8742	605.7939	298.1284	455.2684	398.3346	496.9796
3346.099	2998.421	4023.291	4620.599	4450.224	9126.346	4782.472	3323.802	5678.071
10.94686	28.17695	32.85403	24.28486	10.27605	2.990068	45.51039	44.43754	11.90099
40.64951	23.89684	28.35298	28.0199	40.27216	14.95534	53.84421	18.4039	33.72739
50.74918	88.78261	36.31665	112.1399	91.7749	71.79045	67.34071	55.4538	124.9945
0	0	1.163786	0.917412	0	0	0	0	0
25.41865	2.232262	41.82881	2.77579	12.15598	22.93332	16.64076	25.17082	25.79308
0	1.054958	1.17862	0.911399	0.914218	0.996086	1.047102	0	3.966693
13.50332	32.5148	19.28147	20.55053	19.65845	13.95856	17.22803	15.12914	12.89363
147.258	170.2533	104.3175	170.038	75.82119	38.88521	175.2935	183.0955	72.41173
49.07065	78.62929	41.96716	65.40351	52.43016	26.92042	30.87189	52.17908	35.7092
576.3026	339.0448	414.9397	405.432	473.7575	330.0351	437.3472	316.3587	693.3921
49.86843	77.17406	111.12	52.28993	98.29654	57.82999	144.2105	46.10943	129.9481
14.37922	18.63565	7.97445	14.94584	12.16293	4.984357	17.31736	23.92385	1.980758
697.2854	1087.005	1700.387	821.1593	559.8862	1154.628	1018.46	1799.229	1251.875
102.3507	162.346	171.1896	133.578	73.94337	94.72237	131.616	100.978	81.3391
35.47982	74.19825	45.39664	54.161	69.26779	11.96381	20.0868	14.38885	24.79594
12.64521	16.08002	12.51256	18.67188	11.21377	3.987145	13.58557	23.76479	2.972635
305.4947	255.2085	183.6882	606.3494	396.065	63.81234	294.3688	159.7715	224.1848
219.1719	149.7022	328.7168	229.813	217.2164	451.6828	483.055	198.8206	275.7701
190.4405	86.77341	132.6475	101.814	127.3293	248.2763	169.8822	119.5321	249.9821
88.86513	115.1703	52.1811	146.7114	113.3098	3.987189	180.9686	36.87872	56.54084
21.12292	32.36656	17.04356	31.76352	15.89725	18.94391	41.81701	35.71038	14.87701
26.1813	93.07315	38.574	58.85585	44.93227	14.95511	25.48408	17.70159	33.72496
171.8508	128.2668	189.249	142.0112	102.9877	124.6361	164.0175	159.9858	121.0213
1.631342	5.846626	6.846502	6.516814	3.71996	6.978647	8.222406	4.492585	9.917812
0	2.838551	0.039635	0	0.912233	0	0	0	2.972845
73.63068	162.8396	35.18554	133.6408	73.03319	14.95508	76.90613	76.66234	29.7564
31.28752	46.78573	37.41747	34.55888	42.13522	36.89206	27.92429	56.92422	68.4507
13.47527	20.81657	21.59038	20.52689	51.48971	26.92036	19.50548	12.40832	15.86829
171.765	238.5064	95.27444	448.4741	156.3479	140.5886	103.5532	391.9842	125.9785
19.39832	77.25441	30.66109	32.67178	51.47789	37.88827	27.84491	58.71721	30.74807
319.0002	495.5419	665.4172	664.2362	408.2157	516.4914	624.6087	415.5151	289.6545
49.07145	30.44354	75.91444	42.96355	56.17888	88.74222	106.988	40.23851	113.0922
18.56066	21.43324	22.71718	13.05197	34.63071	33.90039	11.15316	26.99021	25.78896
77.86866	51.45874	49.90679	81.28391	69.28535	70.79324	52.96946	51.46975	77.3747
29.57384	28.03897	26.11748	24.26675	29.94297	19.94065	31.4734	49.52999	29.75692
127.7828	369.1621	104.3142	377.5292	173.231	148.5664	48.71199	206.2873	39.67575
82.95117	78.52482	48.77602	121.4834	75.84252	21.93475	114.6112	60.74398	64.4778
166.6628	257.5974	426.2213	681.0941	228.4422	687.993	456.6028	241.0779	255.9283
84.64695	65.89027	117.8301	52.30325	89.89676	60.82203	46.98435	217.3061	74.39838
20.26307	19.04536	26.10348	23.33781	14.9572	28.91502	18.93876	17.72288	23.80535
123.4872	334.8421	232.4479	96.19458	198.469	342.9973	281.1419	293.8844	264.8546
2260.809	475.7401	396.8008	1624.702	1609.603	35.89394	108.2847	312.3993	518.8028
38.894	76.20013	21.5895	29.87421	45.86938	10.96672	29.67049	48.18955	14.87631
92.20183	81.97166	132.6481	101.8138	160.1094	150.5602	111.3655	118.8692	138.8761

155.7428	69.3927	133.7615	160.699	105.7949	245.2859	152.0357	148.0419	39.6758
38.05131	61.1634	57.80784	76.62712	29.94317	42.87436	37.46125	52.85075	43.64588
17.71041	20.2123	38.60042	11.18531	37.42492	265.2245	220.3589	77.15032	104.154
137.0498	42.37008	144.0089	145.7187	169.4564	204.4026	85.0602	135.9994	129.9463
81.25066	91.74912	79.34915	66.32326	58.97712	21.93474	41.59741	85.92487	31.74034
23.65481	48.0025	27.23539	55.13822	42.13643	29.91214	18.33877	42.96568	7.932437
91.36954	159.4907	70.32321	106.4938	72.07751	109.6794	171.763	117.5921	79.35614
5.858768	19.03184	10.25852	15.85624	22.45069	21.93498	12.94633	24.34318	24.79703
52.48064	89.57542	47.61239	59.80245	49.62599	58.8287	78.27886	24.33926	46.62276
566.1172	639.9088	859.2922	803.4188	499.0297	1413.872	725.2297	2057.806	883.8508
34.69217	29.30892	21.57439	63.56575	29.95384	7.97549	118.2014	4.481684	32.73465
137.9915	73.63597	66.91343	151.371	60.84321	40.87954	75.6211	105.0678	106.1419
220.0415	97.54117	183.6566	147.5931	239.7025	103.6962	157.2694	65.93178	226.1713
159.1013	128.1854	264.0599	115.8303	87.05722	185.4588	302.4682	149.3051	198.3969
14.39938	15.02907	14.724	14.95483	4.659021	11.96492	8.252892	14.567	7.933913
395.1679	525.4801	631.4323	482.0419	452.2203	669.046	874.3926	527.1876	578.3215
9.247523	40.83205	28.35147	40.18375	17.77155	49.85565	17.76307	44.36685	12.89281
835.2427	631.4776	1445.342	1818.949	832.3605	3246.523	1177.04	923.6046	1400.672
82.86813	20.80664	44.27007	60.69484	70.19469	17.94639	12.94984	30.90672	58.52306
215.1029	87.4527	99.75952	120.5238	142.3392	47.85924	73.82483	34.2219	90.26926
33.78986	55.61073	98.6275	70.05215	47.72993	114.6654	48.13722	60.03107	87.29314
302.0919	77.1252	251.7	241.0088	248.1031	231.3235	233.5004	138.6086	242.04
30.41047	26.81283	27.25758	23.3291	32.7486	40.87977	23.67759	27.62114	23.80425
192.915	146.0846	274.3421	231.6757	326.7764	237.3065	680.4768	203.4237	291.6411
891.0741	714.1389	1694.772	1034.155	834.2226	3073.027	1281.242	1855.256	993.9578
67.68007	59.84291	118.9881	84.07891	57.10023	48.85649	87.01912	111.7422	26.78013
7.570075	11.92816	11.35825	15.88366	14.98019	9.970243	18.53915	11.17339	9.917996
446.8589	264.1857	342.3619	403.5853	319.273	425.7569	394.5501	333.6276	291.6398
1226.46	989.1097	227.9057	1381.812	731.2787	320.0645	353.3258	467.7619	773.7454
936.8024	674.6272	824.1753	1149.082	940.0383	1360.028	719.2146	670.5025	1254.85
49.91798	67.77796	71.39067	33.61416	32.75306	21.93485	63.80979	56.15933	27.77275
34.69562	21.46982	13.6524	62.63322	15.89555	5.981303	18.35138	46.33036	7.932481
60.90473	83.30229	80.48845	101.8421	61.78523	28.91442	11.14884	19.67727	35.70832
32.94105	56.75391	32.93044	30.80199	92.66549	68.79778	32.01504	34.87554	38.6837
6.705949	34.00888	3.453424	16.78761	98.32002	3.987189	8.766934	1.85378	27.77215
14.34361	5.226489	19.29385	10.25292	13.08879	11.9641	5.19552	7.795058	2.972635
938.5176	688.4506	836.6275	731.4783	931.6205	1420.851	641.7576	940.1683	557.4884
150.7167	100.7024	51.05239	100.9	116.1146	50.85062	130.0532	72.62621	129.9515
6.711418	30.70234	22.66504	23.36067	11.21715	21.93603	25.08857	36.53428	15.87031
187.0563	79.57492	112.2527	142.9324	156.3646	49.85323	128.6864	58.67342	194.4292
271.7637	75.37682	125.8461	211.1555	222.8668	161.5285	254.7227	89.10354	199.3895
20.27008	16.65019	30.61727	16.79494	17.77044	23.92953	10.5674	14.41592	29.75859
193.7996	218.7722	99.80096	266.2723	175.0851	112.6701	122.67	258.4304	98.2031
28.74141	76.9824	17.05225	69.16683	38.38505	10.96677	29.72016	44.94873	21.82104
13.48163	52.85052	15.91828	17.72803	14.02055	27.91792	22.53744	21.04332	58.5299
8.397138	10.61346	13.66075	10.2481	6.527382	1.993069	53.562	8.443836	10.90829
1.632958	3.451322	5.703396	3.714074	5.600618	1.993062	4.034539	0.534448	8.927065
120.1489	123.993	149.639	146.6704	120.7741	206.3981	90.47727	158.5812	94.2357
3415.277	202.3709	578.1694	927.6891	913.8448	1529.536	300.2109	785.5887	894.7641
10.93712	3.442324	167.7621	5.579266	10.27278	5.98137	5.798699	17.03133	3.964823
0	2.831128	2.320764	2.775929	5.593544	4.984305	3.409987	1.844436	2.972678
139.6438	264.616	92.98402	199.0156	143.2592	95.71989	146.0487	105.0036	103.1643
0	0.455876	0.034438	0	0	0	0	0	0
70.24532	47.85881	49.90382	85.02652	37.43345	82.7588	75.72425	76.01208	41.66094
64.2905	117.5724	53.31414	81.27445	92.69828	23.92891	55.32819	62.70373	28.76414
598.2999	806.0339	765.1867	548.3732	370.7555	547.4008	852.3007	562.8643	756.8785
368.9809	474.7117	134.9533	769.8596	317.4018	177.4807	181.0412	531.3392	205.3368
334.2759	203.0319	329.8885	285.8615	247.1713	203.4051	402.9279	250.3386	519.799

3376.542	2848.559	3810.196	3504.196	2113.197	4496.865	5314.577	2111.376	6659.133
51.56434	58.58288	473.5454	54.15996	53.34542	481.6004	219.5267	473.8473	46.61972
230.1287	295.3005	387.7285	353.1161	645.1212	128.6231	73.12388	97.63154	189.4642
45.7368	32.94552	61.13386	41.11477	24.33117	32.9038	42.98429	58.99331	12.89271
1470.793	1097.082	1555.358	1140.654	1447.51	971.1625	1006.437	1027.262	1775.637
168.3847	206.728	65.80537	143.8538	132.9383	96.71649	77.91253	105.5982	136.8907
26.19845	36.52689	14.7874	23.33723	43.07307	14.95523	8.172505	6.46257	49.6002
165.864	170.7778	201.7694	208.3395	87.05523	69.79506	252.2363	181.7016	71.41931
1.631437	11.86535	2.321002	0.909427	8.403447	0.995987	9.99469	3.162471	0
9.243074	9.415369	9.126091	12.11665	18.70013	1.99306	11.74562	3.82578	15.86826
104.9232	152.8893	86.18245	70.04744	98.3032	64.80982	184.9201	42.81204	192.4472
175.1638	540.5129	130.3997	213.9379	242.5123	55.8357	341.1203	98.99102	122.0109
4.167254	19.09769	28.33575	20.54211	32.78081	6.978441	4.598057	7.793218	16.86164
6.724057	7.683432	10.22394	12.14012	17.80392	3.987258	2.220679	5.166429	5.949253
139.7402	55.65411	39.71856	90.62813	111.4432	4.984247	54.15135	38.87306	95.23125
31.26623	19.6179	32.91291	22.39663	51.49171	14.95511	11.74676	21.01234	19.8364
238.6262	122.6941	165.55	85.91995	252.7942	181.4692	140.5065	126.7279	182.5215
18.63498	25.95251	5.716383	23.37712	3.71997	8.972994	6.414251	20.54929	27.77973
458.7882	129.308	264.1292	261.5821	192.8679	572.3317	404.8642	151.2072	320.4101
40.63721	26.89865	53.22933	42.04845	19.64396	17.94667	45.97857	51.6526	22.81354
92.25736	77.88472	99.73801	87.81915	83.33131	57.83049	146.2654	46.8117	60.50905
88.79076	295.2589	2086.844	71.90583	293.9723	4421.099	1096.646	1280.55	594.1919
1.631339	2.830083	3.455264	3.709913	0	0.995985	1.62486	0.535002	5.948724
110.0479	50.82499	216.3498	86.88205	29.93668	55.83622	122.9024	90.52691	488.0879
389.2922	244.3955	253.9619	440.0244	396.0594	105.6901	411.249	301.2306	205.3367
123.5479	92.19772	122.4395	134.5273	76.75872	48.85618	86.91403	104.9994	77.37189
95.61394	163.1466	92.97265	335.502	55.22114	67.80121	131.7675	98.41645	74.39641
154.0002	212.1653	138.3286	250.3852	115.1479	225.3423	168.0251	184.3244	171.6113
677.035	864.8544	458.008	1269.661	389.486	103.6958	1291.861	485.563	318.4218
35.494	25.61278	46.50996	16.78808	49.61084	90.73562	38.01892	30.9326	26.78032
16.90893	38.64455	12.50216	23.36307	15.90823	5.981402	13.01902	19.81788	8.925121
108.2972	236.3201	103.1856	184.99	102.981	137.5981	77.35292	161.907	218.2383
29.60462	50.48232	52.09346	44.85744	30.89341	62.81847	42.38442	25.04376	51.58588
9.24324	14.81504	2.319302	11.18261	20.57451	8.972547	21.90098	5.142958	14.87634
21.10022	25.03384	79.30244	36.42019	10.27223	22.93199	203.661	35.6013	37.69349
80.36851	167.3672	54.45708	182.2087	130.1562	20.93759	63.65671	93.12454	35.70794
61.71158	35.17958	43.13649	53.22142	58.02308	74.78037	56.44032	66.58482	126.9703
271.9007	125.9375	75.97296	299.0718	124.5428	6.978407	29.63364	189.895	164.6727
31.28343	38.32056	57.77983	37.36242	45.88196	53.8433	105.3861	36.96524	26.78135
25.3699	50.54171	26.08726	37.37932	14.02326	22.93252	23.17546	33.72067	35.71213
48.31399	27.54607	19.30224	83.24652	56.22419	17.94684	20.7781	55.73982	14.87715
112.5221	92.76837	207.4233	80.32083	160.1075	342.0039	305.4369	277.6951	180.5404
3491.668	1995.875	3184.411	2717.597	3128.16	4105.01	3990.432	2070.446	3133.65
3.324358	10.01471	26.12681	8.379987	46.79721	3.987186	2.22842	5.80534	27.77218
202.2006	349.2705	269.8452	191.488	208.7714	101.7017	202.4724	292.5894	298.5826
59.19454	68.81816	39.72783	33.60518	43.04648	11.96381	43.3569	28.26781	52.57214
77.79736	106.5349	167.7962	100.8728	114.2095	95.71941	93.42192	77.82984	131.9308
25.34093	104.0515	47.61416	53.25494	48.68789	27.91765	44.67425	48.22935	20.82867
48.17018	49.5614	156.4766	46.6818	66.45025	556.3779	68.36355	279.4947	53.56322
128.6459	60.39702	146.2113	213.9806	114.228	93.72585	191.5161	92.44743	51.57993
16.86653	26.85201	14.79025	23.3341	22.45011	30.90904	19.52065	28.98281	23.80488
82.10343	82.13531	40.8492	146.7271	114.2579	8.972547	49.97377	61.40669	29.75635
0	0	0.038618	0	0	0	4.035654	0.534489	0.988966
0	0	0.035875	0.910489	0.913335	0	0.449	0.537185	4.958637
6.714393	14.32346	6.849572	27.11577	2.783026	23.93076	23.32372	42.64851	3.964683
27.8829	32.86649	18.18868	15.85554	19.63891	15.95225	39.27411	17.70831	25.7889
30.47703	26.35296	9.120204	57.04332	37.46901	3.987142	47.30648	27.74967	14.87729
140.4532	161.7528	176.8523	203.6623	132.0051	145.5743	196.0822	163.8186	153.7552

1057.858	1317.868	866.0988	1832.047	1181.627	910.3412	1427.505	618.3256	741.005
2.480202	1.648393	2.317643	0.910699	0	0	0	0.540968	0.989101
0	0	0.020197	0	0	0	0	0	0.990926
31.35302	33.69714	53.13978	38.33618	13.0907	4.984252	52.26429	15.78556	78.38259
1293.908	1115.651	1782.078	1231.273	1261.18	3172.735	1185.237	2350.12	3663.374
84.92105	20.31158	59.94346	39.26152	67.49647	19.94128	3.406651	36.43639	38.68993
32172.23	19455.81	33797.31	25090.91	25472.85	58815.2	26495.79	27774.48	41158.05
43.10332	34.59544	53.32339	62.57434	73.01803	57.83023	30.22678	77.89978	61.50029
197.9936	297.2313	277.7429	282.131	183.499	297.1322	255.0352	278.8027	208.3134
231.8645	322.4592	182.5433	412.0177	152.5989	294.1412	162.5896	289.4071	161.69
429.0356	571.0886	427.3993	784.762	389.4883	104.6929	347.3402	584.7298	500.9474
542.3938	385.1275	1618.826	695.0281	957.8167	2545.567	1257.394	2228.603	1475.068
407.0907	251.0199	315.1498	511.0471	268.7113	124.6349	422.64	318.4532	201.3691
7.553866	9.431045	15.90999	10.25136	10.27516	14.95541	11.17379	7.127904	18.84563
38.05098	69.60005	44.22873	54.18439	28.06929	5.981299	57.82877	65.46261	12.89233
17.738	20.31023	19.29619	23.34978	17.77582	101.7102	62.43141	44.44569	32.73659
131.2142	68.23029	75.97382	102.7662	114.2373	24.92597	98.93439	99.77098	92.25348
172.6485	197.2216	208.5561	226.1009	237.8453	207.3949	134.0428	215.4776	141.8519
30.40824	58.05456	43.11721	34.54279	42.11436	81.76131	20.09315	47.48054	37.69244
1115.846	156.8882	62.40724	497.0488	441.9688	41.87641	183.4665	147.893	709.2747
93.90056	194.2724	131.5078	223.3077	92.67876	281.1812	220.0908	113.5912	136.8925
2.476176	2.830705	1.184613	0.909335	9.343368	0	11.21872	0	2.972668
2512.551	1741.246	2869.229	1708.651	2054.227	5271.605	2415.926	1722.299	2605.92
136.2561	142.6507	102.0466	202.7544	238.8017	36.89104	197.434	81.17898	270.8169
8.39776	47.21314	64.64416	14.91898	24.31723	34.89697	10.55371	63.35387	26.78006
44.91243	11.8397	29.47556	32.70205	39.33996	7.975528	46.66271	9.778757	71.43156
464.6074	422.4457	463.6507	481.1231	291.1741	1162.609	547.7561	603.966	463.2531
2624.233	1977.912	4051.608	2758.707	2454.958	4462.965	844.2695	4250.187	3849.858
225.0652	369.7218	260.7643	535.3254	214.3942	98.71045	444.0416	350.8071	132.9216
48.20797	84.57544	88.38949	53.24074	46.80199	54.83946	182.9761	85.96549	48.60532
0.789049	2.829897	1.18419	0.909587	8.401587	0.995994	13.57659	1.189577	6.940568
177.7669	65.79027	91.84943	78.45693	157.3108	105.691	163.9833	65.30103	131.9331
173.4827	105.9551	166.6498	210.207	87.05482	36.89099	244.4623	269.7027	101.1792
29.56134	103.7726	60.10373	102.7798	27.12788	38.88549	34.42355	113.0998	32.73232
7.565582	13.72928	12.49364	35.55558	8.407105	3.987195	26.3868	33.28459	9.91765
23.67035	28.13682	27.21901	43.92854	14.023	11.96399	33.99668	28.38837	21.82186
8.454336	17.02764	5.698489	12.15899	5.602948	4.984664	10.77371	3.854446	0.988986
36.39819	185.6371	20.43989	148.7417	27.14402	65.81002	33.97096	62.33471	28.76647
0	0	5.717961	2.775986	10.2828	1.993011	0	0.534533	0.988931
134.6791	200.8217	35.18464	76.61105	76.78229	6.978385	81.70421	46.17217	21.82027
58.37788	50.86639	66.88501	74.7423	37.43343	74.78182	70.33586	107.1917	62.49407
21.94908	30.4554	46.48655	45.77194	58.05778	168.5139	83.04598	68.14251	42.65409
33.84546	35.9575	47.57831	28.95205	16.83248	15.95241	51.99293	35.67801	29.75836
104.9366	57.40573	136.006	83.13487	76.76447	84.75205	280.0846	90.47896	124.9901
21.12615	26.93668	19.30315	51.41854	28.08608	19.94111	30.39749	27.06468	16.86134
16.92177	14.33287	18.13497	18.6873	18.72909	40.88371	32.4501	17.17347	29.76355
42.26362	19.00453	56.71196	35.47618	42.11281	41.87674	43.97366	24.3056	47.61252
49.91696	18.41734	48.75932	26.13563	45.87023	35.89445	69.19957	58.8112	30.74887
53.38048	59.55584	66.7836	50.47087	42.14403	24.92665	18.35348	74.31934	17.85311
17.70638	5.825442	2.31827	3.710603	0.91336	0	0.453059	2.51095	8.924366
28.76447	30.55469	44.16964	24.27943	14.96015	75.78212	30.38577	81.72724	25.7904
170.1128	99.976	206.2848	108.355	131.0753	186.4559	142.4115	148.6466	166.6525
35.49759	37.63976	125.7308	79.41898	48.67643	124.6382	60.76775	92.60856	133.9238
293.0229	83.21978	125.8215	175.6612	183.5472	163.5234	174.1951	40.16943	251.9703
2.478987	9.415407	5.723609	6.511836	5.59133	24.92607	16.51584	15.05115	44.63696
109.171	50.19926	73.71485	37.34264	108.6115	69.79545	44.54894	29.58965	127.9661
66.11879	20.867	49.83667	35.50062	52.45732	3.98714	20.75054	14.41369	52.57783
0	0.452692	0.036441	0	2.788454	0	0	0	2.973432

43.09871	48.9762	70.3407	100.8665	108.5832	144.5764	191.1185	47.43353	140.857
0	1.041263	0.041105	2.775989	0.912187	0.995982	1.033464	0.53453	0
245.4454	98.74119	66.93782	126.1019	108.5915	152.5539	82.09179	167.1134	315.4521
180.2192	332.6442	175.7457	367.1632	224.704	62.81527	263.998	223.9289	138.874
11.79988	10.04787	20.41836	13.99587	10.27758	22.93295	18.41313	11.12099	17.85423
5.860117	13.0498	2.320866	6.512474	7.464698	1.993015	6.985691	3.162158	12.89301
45.65574	98.35238	79.35323	80.34329	56.16545	55.83633	58.92882	101.1607	47.61273
600.8116	1286.675	557.785	1497.573	694.7226	288.1571	1138.338	909.0951	231.1269
109.1723	97.05159	71.44871	125.1935	79.5744	19.9405	142.5427	79.88443	64.47632
236.9163	121.4809	223.3623	168.1346	312.7167	84.7512	115.4478	87.72438	127.9617
21.12166	33.56998	38.52401	30.82699	25.27071	6.978407	77.35239	32.3753	15.86907
305.5498	154.5177	319.6366	155.9997	335.222	105.6903	212.7438	145.2703	525.7569
469.6316	568.5765	1616.458	487.6386	307.0808	1928.371	1580.759	1349.944	423.571
3555.936	5124.438	4272.732	5097.022	3376.263	5023.327	6658.398	2793.849	6323.843
54.12799	52.64122	90.67561	63.51724	61.78604	226.3437	95.417	97.84394	63.48535
2588.688	3830.859	2037.189	4554.282	2453.085	786.7008	5819.003	2986.215	1927.406
16.86684	16.62477	51.00434	46.71059	66.49831	14.95517	2.22285	9.766661	17.85258
109.9617	136.5105	155.3395	93.39621	180.6951	109.6786	198.4044	38.17123	127.9624
49.91106	61.72804	47.6323	71.94385	56.17483	56.83388	47.61645	65.42939	123.0119
25.32138	51.96232	73.73875	50.41926	49.59661	49.85314	27.83366	48.08404	71.41907
93.04674	96.3698	82.79717	121.4365	132.9472	69.79511	52.28144	43.46307	100.1876
0.820723	0.510486	0.020307	0	0	0	0	0.590077	0
6.705014	9.420166	9.124636	7.445912	7.463509	43.87204	6.979506	16.39427	4.956511
9.244177	20.23812	12.52398	15.85656	26.19939	4.984217	17.13299	5.801941	17.85266
24.49081	116.6777	44.22511	52.31657	103.9828	3.987154	67.43891	45.55935	15.86841
24.48132	29.21491	36.31906	32.67464	44.92617	106.6895	48.18006	54.77929	43.64481
232.7384	120.3325	333.2217	173.7556	154.478	200.4146	378.7083	133.3752	277.7554
154.0893	308.1598	72.57747	155.1089	106.7419	22.93178	66.65569	91.15475	133.919
0.791326	19.00435	0.044235	0	13.08075	0	7.57681	0	2.97281
1455.622	1785.325	1135.897	1235.967	850.1497	606.2285	1172.258	1137.048	1069.35
469.6671	1289.468	351.4571	914.6337	358.5887	279.1837	371.7905	577.4657	364.0534
14667.36	9118.15	12338.6	11785.94	9219.686	12216.32	15320.88	6540.499	19854.39
44.04697	39.61405	31.74432	27.08437	30.89551	13.95822	69.5062	7.12598	28.76664
118.4554	163.622	119.0503	129.8494	59.89952	96.71682	373.6001	138.0659	154.7485
7.552015	8.822262	13.65316	7.446148	8.400287	0	21.34519	4.481686	5.948479
27.07598	29.37398	18.16945	18.66946	19.64933	12.96117	23.79383	35.07746	23.80654
5.874361	26.68282	3.4517	12.14118	14.98675	8.973201	10.06921	19.27436	5.949301
17.71434	34.08302	11.39168	36.42436	29.00939	45.86601	21.9157	30.97599	19.83669
90.51649	106.0053	81.65669	104.6215	84.25098	44.86779	107.2145	120.8819	41.65973
11.80999	9.46315	14.75786	11.19323	21.54041	7.975702	8.807112	3.163597	9.917374
62.60371	56.8546	85.01172	88.75781	59.91335	244.2921	106.7994	138.3058	68.44571
115.947	106.6591	62.38756	172.8607	90.81372	21.93468	220.257	160.6775	54.55609
27.07835	28.16968	29.46819	28.02697	9.338714	19.94121	25.00304	55.109	46.62718
511.1426	297.6999	343.5233	439.0711	531.8188	616.2006	362.8455	397.0113	634.8659
22.80726	28.68969	23.84073	53.26809	27.13945	8.972579	23.73296	27.01681	23.80535
35.53142	34.71902	24.97297	71.97682	52.44811	14.95523	86.20662	42.29922	23.8053
416.3735	573.6245	196.1619	490.4749	436.3223	122.6406	85.6413	523.3596	155.7372
34.64084	29.20528	77.09526	34.54175	61.78397	137.5996	44.57202	56.08067	30.74817
60.01841	61.54748	56.73975	45.74752	84.24075	86.74544	51.07506	147.8888	92.25054
43.15564	49.76967	43.08177	37.36076	43.06802	31.90624	76.51142	122.7351	106.1507
14.37313	15.56465	17.00189	17.75346	15.9155	2.990094	16.69018	14.50737	13.88681
47.37797	17.81906	44.22842	28.00601	13.08161	15.95223	192.8337	89.37264	41.66173
267.4599	135.9346	145.1315	168.15	199.4312	183.4639	153.0899	151.2363	288.6677
5.014801	12.41074	32.92458	5.578187	23.38132	39.88255	8.766778	32.90891	11.90026
34.63889	267.3499	48.78662	91.55321	204.1813	29.91147	40.38316	28.27224	21.82008
31.24665	74.19171	278.7405	71.91502	60.83797	492.5689	207.5942	210.9366	117.0526
16802.49	7083.785	14455.09	13365.69	13889.94	13291.18	8817.929	8395.018	16692.96
609.3376	149.0406	737.9454	397.0293	292.1072	651.099	659.2194	353.3936	670.5778

32.97976	76.35372	14.78866	60.74477	44.94455	10.96677	23.72059	50.2554	19.83682
58.45247	35.93184	47.59044	42.04331	46.82462	6.978387	131.3584	29.67514	26.78166
7.579714	13.81499	10.21802	20.58472	12.17112	10.96774	19.23547	17.94951	3.964977
79.5215	145.7274	64.65268	239.2271	90.81415	112.671	61.26784	195.7949	107.1334
26.16762	19.00463	87.33955	26.12938	62.70563	46.86187	48.69379	22.97761	15.86825
396.8667	182.6007	327.6587	217.6456	388.5501	425.7559	197.0971	356.6815	252.951
45.78724	32.42695	17.0355	43.94199	53.42154	10.96695	34.66069	28.42861	18.84601
352.9282	360.2371	205.2116	354.0809	255.6066	152.5536	528.327	243.1032	160.6978
88.81586	99.37921	149.6398	184.0511	174.1596	173.4936	259.4809	152.6225	343.2325
192.1279	221.8426	181.3667	305.5378	132.9471	87.74286	182.4075	149.3	89.27549
3079.606	2001.382	1284.451	1190.161	1481.208	5636.541	2981.625	1988.614	2362.887
128.6048	37.57866	103.1971	87.79459	119.8327	56.83283	135.8147	50.73224	90.26728
43.12556	60.50689	26.12217	61.65619	43.9925	15.95219	51.19507	42.20548	19.8363
13.47973	0.454742	0.043361	0.909948	1.847751	9.969668	17.73493	3.16388	11.90046
77.83514	42.40333	70.31082	47.62317	73.95706	70.79268	36.79541	43.48664	57.53242
18.60052	27.63602	33.95604	27.1025	10.27847	38.88811	21.44379	41.18964	11.90138
548.4047	784.0302	318.58	374.6096	328.6283	103.6959	1148.932	662.1262	232.1197
0	0	0.023279	0	0	0	0	0.575279	0
120.1439	81.36784	99.79341	141.0606	134.8203	69.79511	116.7318	105.6316	166.6523
44.79177	24.99425	28.39804	25.19528	112.3502	22.93176	75.57422	22.31592	91.26017
40.69861	31.8471	35.09999	35.52199	36.53908	4.984242	17.20146	18.44166	20.83064
0	0.451185	2.315958	1.843647	4.66378	0	0	0	0
27.90776	26.30401	9.123075	64.50813	30.89379	2.990073	27.35929	31.03139	7.932496
41.45475	47.94633	66.83983	47.64568	18.70276	41.87747	146.7675	89.44573	41.66237
222.562	216.898	251.6599	515.7511	378.2964	64.80948	364.901	66.58707	48.60334
139.6608	62.80004	75.98409	124.2544	116.102	88.74035	132.3506	70.60557	178.5593
144.9787	109.4835	62.32078	31.74789	115.2389	20.93782	57.26673	48.2288	84.32272
167.5848	116.2026	129.2415	216.7662	112.3471	25.92302	141.244	146.6849	56.53965
63.45579	81.53138	64.62804	78.47745	67.41009	21.93475	126.5835	95.21905	21.82021
331.0121	179.7861	119.0596	347.5841	229.4108	29.91137	227.7603	274.3521	96.21933
296.1408	317.4618	219.9783	365.2577	360.4571	952.2201	281.7408	450.5198	466.2272
232.7388	630.5328	108.8705	227.9527	134.8119	96.71651	163.2302	208.7794	49.59536
21.96391	38.37236	70.18135	28.95181	21.51827	81.7643	57.40383	77.62787	42.65611
5.02497	11.96785	6.8389	24.33441	5.597198	0.995985	10.68373	17.93789	7.933928
12.63097	32.78264	122.4734	44.8135	36.48786	1.99313	7.588409	22.31759	15.86826
153.14	171.9112	200.6641	229.8167	137.6191	112.67	239.5908	225.2926	120.0266
51.61761	106.3857	39.70147	71.01607	48.68315	27.91755	26.6874	30.95709	45.63002
18.56263	22.04176	17.05426	4.643548	25.2619	2.990086	21.91876	7.783596	18.84468
3.321652	5.82208	9.124001	11.18432	18.70591	22.93228	9.965728	11.75823	32.73451
184.4616	306.8973	180.2709	278.4033	224.7092	101.7019	319.5387	185.5985	112.0905
5.865238	9.465894	7.980515	1.842124	0.912208	3.987179	4.00687	3.825569	5.948818
73.64174	74.95328	140.4387	91.57426	68.35271	137.6009	60.17148	113.185	165.6708
10.94947	11.85541	24.93952	11.18859	15.90218	6.978461	11.18763	6.469768	13.88542
604.2866	538.792	410.3792	324.1597	419.4614	241.2944	480.9718	297.896	675.5394
0	1.042639	0.042061	0	0.912323	0	1.625381	0	0
114.4162	27.44839	57.79836	135.5593	84.303	36.89174	40.47324	37.60273	76.38559
37.19646	61.12616	63.47817	58.85417	38.37273	31.90592	58.39432	62.11224	22.81243
189.5323	119.704	163.2789	170.0098	252.7982	168.5071	111.2934	187.5628	227.1616
804.9319	210.2028	514.64	280.2491	331.4419	127.626	233.4974	410.9511	213.2723
93.26596	40.75839	15.91901	82.26948	59.01165	2.990079	62.16362	25.02377	46.62397
5.016059	17.94624	6.851578	13.06392	14.96983	6.978532	6.400049	5.812899	3.964641
40.55812	53.76294	76.01108	74.70851	81.43044	450.6827	194.697	170.3053	274.7739
5.01581	0.45157	5.720445	6.514318	2.782878	0	0.447125	0	0.988937
30.41494	25.01945	158.5356	5.577787	28.06685	147.5723	109.8986	142.4077	53.56584
247.134	96.93834	137.2006	144.789	225.6528	131.6149	174.5688	110.8909	241.0512
2.47688	5.24456	6.845294	4.646128	2.783385	0	4.012475	0	1.980758
19.39842	56.22492	194.8557	23.3277	39.30193	283.1784	195.2694	230.3701	99.19816
2.479653	1.041618	3.449034	4.649657	4.660809	2.990177	0.446142	0.534304	1.980853

55.83231	76.73305	61.22767	62.58738	42.11671	78.77022	178.7368	89.93011	138.8833
152.2805	225.8501	159.8818	109.2774	156.3428	120.6465	102.3444	114.8316	109.1143
27.90441	30.52225	24.96649	22.4051	19.64405	11.96393	15.35411	17.73143	18.84515
54.17876	63.62826	67.97167	42.03452	33.69446	19.94072	50.07971	28.98364	12.89241
1.631457	4.64214	12.48445	4.646425	10.28667	5.981529	1.033135	4.495593	2.972744
49.92258	57.54958	72.51565	54.18493	41.18704	43.87149	59.03115	74.09899	73.40854
231.9972	152.2892	91.84638	112.1022	171.3645	18.94341	114.4091	28.92586	193.4393
185.3742	95.79193	151.8966	229.8515	180.7213	79.7662	85.11588	107.6385	125.9804
81.22015	126.5156	39.72715	107.4373	90.81633	31.90563	99.51899	111.0199	38.68402
22.78241	27.39011	74.86533	38.27459	92.6758	111.6732	73.75869	65.94369	140.8599
98.15448	91.63313	139.41	124.2562	65.52355	97.71435	126.3855	114.3068	57.53209
990.944	883.1555	1044.104	745.4781	1440.965	914.3287	928.3864	612.9855	1341.151
0	13.13889	0.040702	7.453049	1.847226	0.995982	1.033205	0	0
1159.381	1101.958	875.1924	1631.15	572.0565	598.2516	1609.769	1509.729	830.282
0	0.468862	0.029443	0	0	0	0.464429	0.551768	0
66.88726	30.44976	31.77675	30.81101	83.36031	10.96673	60.23569	23.66782	55.55099
522.9602	943.8723	327.6689	630.5844	464.3877	362.9389	343.7199	553.589	395.7958
85.44442	119.8553	120.1619	100.8884	59.903	60.82144	143.6975	134.8207	63.48405
53.32419	65.41735	47.61595	58.8649	30.88202	39.88312	36.8786	118.667	25.78891
90.61894	52.10351	54.42056	108.415	116.1537	17.94641	23.09296	30.94916	24.79655
0	1.074602	0.030281	0	0	0	0	1.220789	0
12.65587	8.247134	3.455245	2.775757	9.342008	18.9445	11.20712	13.13379	13.88595
42.27556	68.32031	36.31594	106.5316	29.93956	18.94348	49.98973	106.5332	44.63711
17.72216	13.03093	20.44309	17.72886	19.64355	32.9037	5.788011	23.7061	19.83718
101.5721	88.09172	102.0092	115.8593	63.65655	47.85935	146.2328	119.0221	79.35757
54.09821	29.18754	199.5059	79.38482	72.0715	101.7021	44.52472	40.81686	50.58744
1766.159	1068.314	1241.359	1348.988	1544.888	1173.572	1678.772	840.2673	1061.412
44.10094	27.59927	13.64138	45.82079	33.72426	1.993011	40.72935	13.77893	29.76048
13.47486	19.6006	58.99802	26.12937	37.42518	170.5024	60.04371	93.73685	23.80394
0.789302	7.624142	0.04284	1.842426	4.654822	0.995999	0	3.162447	5.948503
10.94571	31.19234	20.42999	21.47628	29.02675	8.972662	15.37789	5.804826	16.86151
22.00748	15.50909	35.07514	27.10578	25.28918	5.981386	27.50455	12.46422	36.70768
66.84431	127.2708	48.77727	88.75995	55.23038	16.94926	73.89729	81.95203	32.73239
436.8181	206.7332	364.9466	393.3518	285.5903	124.6352	152.4873	69.23551	523.7739
61.75418	93.53774	82.75019	95.30007	69.28067	15.95216	120.5533	91.87823	51.58086
294.4698	431.4601	363.9081	608.1963	308.9674	161.5271	348.5848	426.1492	184.5047
21.9411	31.61993	51.03908	19.59132	39.30604	47.85957	20.09624	46.16822	16.86021

TCGA-VW	TCGA-HT	TCGA-FG	TCGA-DU	TCGA-FG	TCGA-S9	TCGA-DU	TCGA-DU	TCGA-S9-
130.0328	91.685	80.79592	168.9053	145.6431	246.043	150.2905	172.1628	222.6361
78.32848	12.44003	51.33093	109.0769	29.83014	37.36709	25.15884	56.87894	30.43939
46.52717	20.66064	173.0297	239.5695	155.9118	153.8567	95.70196	57.62739	98.93814
3313.639	1138.734	3483.887	6856.865	2956.229	3170.373	2246.894	2460.089	2599.15
254.9072	147.6085	75.583	91.61888	102.0859	83.31026	99.88657	55.03752	247.3872
0	0.082495	3.4663	1.373069	1.19107	0.494295	0	6.066714	0
8.736276	38.29043	19.94987	22.00647	6.942048	11.67425	5.809777	17.16604	5.687736
283.0217	69.84034	146.8916	265.4691	191.4795	141.8967	127.6045	153.214	254.0398
7605.251	3290.174	6256.682	16780.44	8407.714	8391.177	3992.511	8335.774	6252.421
82.61707	65.62285	35.59746	78.62542	89.41058	52.84988	112.5948	51.62226	86.58446
32.55777	9.689584	28.71534	14.24866	14.93752	20.52715	24.36367	17.19576	37.13004
1224.053	403.3687	1147.381	1582.047	1286.601	1457.33	1241.021	1981.594	995.1324
169.5865	471.051	66.8804	211.4281	134.1918	140.6246	88.11102	101.5315	204.5505
0	5.452649	0	0.07068	0.036715	0	0	1.677438	0
121.2711	60.25164	36.45555	98.02076	166.2151	112.052	121.7623	55.04465	220.759
3.464884	24.69589	14.72776	13.00694	1.201525	9.803278	3.292734	5.106038	44.71882
3077.286	928.2352	3607.359	3624.457	2199.403	2707.268	2070.581	2597.843	1508.868
67.69261	61.44926	19.07405	31.02038	40.15603	44.16319	29.34383	21.47011	61.84755
4.345832	120.2675	66.02495	28.48899	45.92259	52.1596	15.88432	24.90315	26.61786
120.3309	335.0054	418.063	790.3949	256.921	294.9229	83.89627	912.4571	112.241
1283.821	453.9297	937.0183	4566.259	1197.154	1021.502	759.858	1087.167	790.5834
75.61885	0.119148	121.8524	81.08993	93.92485	126.2381	18.41192	1.668089	92.31702
54.43694	32.95795	153.0058	23.33432	113.5353	69.60746	89.81125	120.5045	137.9527
314.5779	82.16651	113.8178	60.72866	369.2246	411.5517	297.2301	170.3913	228.3201
62.35764	26.12328	139.9862	215.0578	121.5282	100.8449	67.97663	44.71179	72.29498
33.38817	73.47381	23.44103	11.71551	64.11565	37.9999	44.53677	22.34764	48.53513
148.4744	248.7383	132.0867	161.2356	100.9621	92.61271	153.6222	163.5216	81.79935
1406.86	982.7759	371.9796	528.756	717.8716	568.8496	1089.046	600.7862	1119.772
110.9171	5.620581	24.30696	13.00519	14.96073	61.81197	74.85491	70.67953	49.48242
152.9691	205.9774	48.63842	46.50815	67.67979	128.9956	131.8898	45.57778	188.4188
51.82715	156.7459	12.11336	7.858982	22.99069	76.61957	62.13003	30.08024	249.3857
70.30666	39.73097	48.65542	25.89372	89.39737	50.99181	33.5372	42.14635	87.54065
25.51072	31.2692	6.034564	6.554324	13.78842	21.18979	24.38003	6.834847	39.99852
492.9213	1277.897	562.3571	710.5215	736.2105	622.4093	666.6571	730.7815	758.2393
336.5897	162.7336	178.17	76.19253	133.0609	295.1387	318.2756	294.4145	245.4564
6640.397	12244.36	14164.09	9614.053	14214.66	38439.49	32839.16	17354.19	27223.61
0.833433	32.74933	37.41434	21.95817	24.09249	9.818747	28.55443	76.82514	4.736679
79.96992	9.724019	70.40264	159.6111	49.34213	67.20345	94.07448	74.02671	90.38861
12.2512	58.74271	46.05773	56.71783	33.29055	16.03318	31.86518	84.42362	14.25068
6.978228	54.61965	28.65233	24.58481	47.01178	22.91275	52.076	30.95797	13.29966
3.469149	232.2511	36.45158	24.62431	55.10072	32.20642	14.2065	92.92342	13.29883
86.15851	91.41804	12.11374	15.59214	24.13396	63.51766	62.98305	44.73754	73.26504
4.344017	109.1576	24.28862	16.88263	12.67629	19.7667	22.60813	68.8667	10.44431
324.4006	17.92701	132.124	132.8076	181.1361	140.0816	157.0408	167.0213	156.0326
483.3556	211.9354	226.8474	158.6881	181.2191	321.8551	256.9201	203.1138	241.6428
42.25273	16.47598	13.00122	7.845822	10.36991	16.7337	35.32556	10.28195	20.9263
27.19534	325.4049	32.98753	18.17151	64.22269	24.13087	41.94127	25.77078	37.08906
4347.024	526.4011	1388.98	3651.646	5420.442	4088.595	1587.736	2449.75	3507.718
400.6663	117.7089	1174.437	270.8807	540.1114	1082.776	93.97165	111.8407	210.2346
18.44884	2.880585	28.73178	9.118008	10.36263	18.66506	24.38011	27.58875	27.60515
19.33103	20.49026	6.034508	5.270379	21.77543	19.29504	27.75956	9.424878	40.95163
269.765	60.29908	182.5088	185.7405	236.2363	315.0297	273.7298	286.6459	243.5483
140.6775	64.28402	46.03443	10.43888	61.94223	77.1919	109.2093	63.68112	82.77256
13421.61	2010.795	4898.961	15021.45	6171.605	7346.738	6951.544	6049.56	12435.46
33.42781	31.35294	12.12928	5.27469	20.65445	10.45405	54.74722	17.18969	37.12432
43.02953	91.46064	32.98956	43.90361	47.04272	65.9906	41.10402	62.83419	44.70437
34.30031	9.70094	22.589	21.94953	19.51793	33.70323	32.77957	43.11257	40.93135

6273.14	5330.783	4507.799	11990.17	9191.995	4575.257	5015.251	4200.572	7738.489
166.9178	797.7634	142.5077	59.43928	58.54607	109.4093	197.2781	248.7437	126.5146
50.12287	8.351889	22.56767	11.71708	32.11937	44.26162	65.59844	18.03246	33.29544
80.88433	73.72014	30.38433	161.9899	21.84169	24.76863	16.72851	18.88281	13.29891
0	34.14897	0.820683	12.99622	1.20203	5.443338	1.616371	6.828494	0
313.7363	80.78831	223.3896	341.5848	326.7635	191.6838	220.8257	226.3838	207.3963
43.13138	51.60812	8.643645	7.846613	35.49645	14.84537	37.00986	12.0075	46.65312
53.64815	27.39659	22.56794	25.84338	72.12268	55.56415	31.89442	25.79687	22.82128
132.7343	15.19263	80.83034	79.96434	94.02338	64.66912	36.04659	91.24611	21.86119
126.5013	422.0636	98.17759	253.977	206.4126	66.45296	74.66633	123.0517	145.5494
15.80002	0.11269	25.23324	38.47271	42.29756	9.842713	20.99764	30.18152	19.97896
21.95038	97.61593	2.554327	10.42122	18.38019	22.98717	18.43676	20.63445	66.6523
378.7779	72.59454	128.6049	342.8989	327.9221	164.2513	272.0615	388.276	296.8364
64.18446	20.62739	39.97317	7.858542	18.40049	56.09062	99.23641	29.23317	57.09178
154.6938	2.872579	112.1335	112.1739	94.04489	128.285	68.81191	86.05727	114.1667
51.85948	89.90495	30.3952	32.28953	56.15944	19.16486	36.92607	30.09708	60.90191
291.7432	98.55438	297.2896	219.2373	240.8187	379.8552	376.2216	173.845	259.7248
50.98741	2.880408	74.84012	110.4755	42.42501	24.80111	34.40764	39.59218	17.10743
144.9489	360.7735	155.5528	68.46294	99.8217	88.86293	168.7272	179.868	136.0302
101.0087	155.9053	353.8309	238.539	209.8591	215.362	216.6234	207.4368	305.403
1115.064	1366.889	634.489	631.9276	779.8127	2996.358	2440.119	768.6302	2327.082
53.58349	27.47107	53.00279	16.88317	76.81557	95.36023	71.37677	132.6691	206.5353
550.1778	56.19948	323.3716	126.4637	206.4339	498.2508	403.096	212.5904	409.1102
50.94805	1.502503	39.08138	59.32901	56.20173	70.99538	77.27379	124.9222	242.7232
51.82739	80.57492	81.73442	37.47332	59.63704	32.24861	15.04634	73.18041	21.8626
1487.669	333.6706	1442.924	3032.446	1665.006	1457.27	1307.339	935.6353	1787.643
12.2501	47.96703	126.057	77.42019	21.85032	22.87071	17.56358	49.87813	9.493268
159.931	13.82342	333.9178	58.13374	64.26887	244.8282	385.6176	167.002	97.97984
157.2409	867.7139	447.646	586.6423	212.1943	244.5178	110.7659	655.0921	147.4431
15.76454	34.31947	27.76032	182.9459	21.85049	40.31655	10.0082	587.6209	22.81215
77.28021	289.6595	178.1786	132.8823	321.0153	149.3243	137.6666	125.6367	151.2593
68.56559	27.45156	41.70281	41.30782	27.56676	61.66976	37.7522	47.33127	46.61284
1261.932	360.9579	215.5166	956.6422	670.8222	432.5475	614.624	527.6399	786.7951
1437.551	1071.732	1709.759	1328.181	1683.394	1653.291	1644.866	1269.614	1919.871
0.84363	0.073856	0	1.35825	1.184331	0	0	0	0
72.97043	60.1011	8.636565	11.72591	35.57988	42.9044	83.20121	30.95057	176.1062
19.28098	45.20586	39.94404	81.22073	159.2608	46.58827	66.31768	106.7798	80.8689
591.349	365.0832	1037.014	963.1719	853.1534	551.4388	532.3021	1215.469	539.4172
231.2365	188.3613	62.55133	67.11572	108.9283	79.62418	121.7767	77.44785	156.047
73.80172	43.85265	55.60082	25.90493	39.03785	156.5166	144.5158	92.97589	142.734
41.26634	5.618569	140.9239	64.48881	90.55791	89.09418	117.6389	102.4828	61.83513
94.91272	8.355353	69.52214	54.22574	60.80404	145.8881	15.04491	36.10309	105.612
67.62096	47.98393	135.5978	107.0689	125.0021	161.2734	98.20752	185.1042	96.07852
187.1312	988.9452	39.92797	201.2267	214.4702	249.5561	183.838	59.33325	391.0221
11.388	16.45869	7.775094	11.68653	35.47383	21.7931	10.02418	18.06171	28.55338
18.4117	8.354076	24.30371	11.72091	6.942027	214.3839	37.77603	47.35948	23.7711
14.9008	27.35416	4.291527	6.565367	13.80826	50.01136	42.03655	8.552562	61.88347
40.41779	74.94582	26.04414	10.43425	20.68767	42.33272	31.8806	42.17893	59.00054
21.95692	38.13012	15.6138	16.82587	25.22784	19.23666	25.18951	23.2333	48.55581
97.57898	37.0137	63.44766	32.33171	51.6265	68.47714	53.71465	44.7284	93.24887
14.02434	52.99994	31.31259	12.98351	18.3757	19.23216	9.176215	16.32135	6.640035
2307.527	865.3464	1833.205	1757.5	1880.607	2504.401	1310.673	1723.274	2223.37
64.1591	72.36279	26.03276	27.17254	51.61065	39.76412	66.35494	59.39976	105.6375
667.8279	261.205	510.2197	145.8296	184.6816	375.8827	318.1781	420.0281	122.7062
589.6539	127.2688	616.3303	355.9167	397.9166	592.1078	430.7455	516.4905	442.3905
36.10304	19.14763	8.648099	7.838392	3.49959	2.956364	7.498924	5.971548	15.21422
31.67713	52.87225	6.903907	2.695316	25.20947	22.42177	27.74318	11.14858	47.61634
110.7089	26.12709	207.8186	235.7472	100.9298	161.3163	122.5892	160.1372	120.8238

16.66271	13.78626	20.83725	15.55718	26.38303	22.35234	11.69817	16.31618	19.96894
148.6359	120.027	18.20027	23.32028	40.17204	59.73902	39.42223	36.97433	141.8035
326.1709	12.45703	235.6558	134.086	53.94801	86.44535	265.482	403.1195	167.4545
760.1931	30.2255	368.5519	161.2827	245.4313	1131.131	1306.871	618.1137	541.3494
74.65595	91.65592	125.1647	121.2223	110.1014	172.5236	113.338	109.3024	102.741
57.07817	45.2407	119.9618	108.3168	79.15227	67.12929	52.00445	324.7508	66.5832
137.9163	127.2503	326.8398	203.7932	640.8695	370.4547	466.0827	278.0248	410.0568
42.159	76.43099	35.60775	15.59044	81.36045	71.67749	118.5481	43.88054	59.9401
23.7086	57.13703	3.422692	42.45635	41.2383	15.44796	15.90631	35.31165	25.68257
33.37461	21.97993	9.50701	10.43407	16.10785	25.4256	54.6164	18.88999	19.0111
464.9203	125.8724	335.5536	63.30485	161.7237	241.5102	204.013	140.2651	165.5273
231.9842	61.66139	78.17928	109.6966	88.35181	129.9956	544.2978	256.5284	329.1941
243.3634	135.4729	1074.496	675.5796	475.8909	579.5884	423.1697	691.2484	406.2304
4969.161	486.7617	3085.749	8702.012	5451.418	3659.014	2171.309	3244.265	2375.57
52.81629	40.86119	12.12538	15.55123	11.5176	30.53963	45.42774	6.829839	35.21205
12.25722	1.506364	29.5455	36.08072	30.96614	46.81573	19.26974	56.03064	23.77554
111.6607	50.63705	76.50555	50.3385	97.41938	55.35434	79.78987	190.4537	175.1198
3.537845	0.068468	0.834634	0.05512	0.029041	0	0	0.823783	0
0.833054	0.112712	9.52001	24.45246	3.499574	18.02737	12.55669	2.525553	7.593563
1.718013	9.395926	4.331166	0.072037	0.037373	1.110668	0	11.29329	0
34.32532	27.26573	6.903909	7.841784	17.2204	16.1219	17.61318	30.16944	27.60004
257.5519	113.4939	90.36949	101.9165	94.06207	118.8606	152.8319	83.45903	155.079
81.76589	77.79555	23.42265	27.17145	34.4383	68.54324	84.87304	51.64011	75.17206
505.2496	52.0968	780.6047	857.3567	620.3633	502.9397	398.8028	640.4317	407.1783
61.46584	132.6115	159.9469	69.72666	91.77442	85.79887	107.4459	61.92565	105.593
12.28563	8.303791	6.039033	3.979544	9.210471	15.56482	10.88504	9.434638	12.36146
1683.685	278.9925	1135.228	258.0084	963.2427	1537.102	198.9291	660.1853	2339.486
216.1766	72.5826	126.0081	51.69786	142.2182	111.945	123.3913	154.9221	232.1438
26.30956	176.1923	147.7913	95.46093	45.92415	38.44249	95.6972	288.5263	17.10376
35.18135	63.80057	6.901366	9.13368	8.083685	10.44784	18.4417	23.2311	29.49755
493.0117	93.09793	417.2616	154.8279	223.6397	269.4972	271.1919	559.5929	191.2131
186.2684	99.91438	235.5599	421.4611	369.1772	274.563	164.5383	298.7176	170.2863
169.6038	65.73912	143.4186	347.8198	133.0308	126.3233	77.1977	148.0527	120.8178
476.8093	1.500952	135.6662	38.78071	67.67543	99.03151	110.8763	69.70709	58.02373
39.56563	63.93658	8.639633	6.566427	20.67406	17.94775	36.12292	27.53285	19.9672
19.28411	24.73306	37.34568	46.45946	20.69698	17.90083	70.55796	74.05689	17.10508
114.2321	94.36044	73.85262	69.70583	151.3303	188.8107	243.6511	105.8677	165.556
5.226989	5.59983	6.038038	47.2528	3.498069	7.347497	4.135386	5.112347	0.932949
32.67024	1.505715	16.54677	15.42421	1.202314	0	0	0	0
120.4547	57.45428	54.74157	41.34008	33.30343	67.84381	117.6402	88.68902	45.65456
41.30137	27.4166	57.4114	68.18305	65.29192	55.50999	31.88341	40.45661	83.76361
5.22176	111.8267	15.59189	15.59269	13.82213	19.14675	7.488689	16.29746	15.20177
135.2893	411.1729	66.87692	52.9899	236.2173	73.30248	205.7061	126.494	301.5987
50.93	614.4251	24.28399	59.38258	27.58061	22.25042	25.12334	50.74519	116.0778
392.765	214.7308	408.5189	419.1021	558.4392	896.6243	806.9855	361.4987	957.1179
121.3855	4.250211	47.79564	93.96311	41.30676	72.92778	51.21143	80.96521	38.04482
27.20572	136.1093	24.29712	37.43816	14.9655	15.41279	31.87048	34.40402	4.736588
69.41756	15.19037	73.88978	95.337	131.7664	70.96788	37.73635	72.30875	63.73891
47.44002	109.0418	55.63356	16.87752	26.42195	47.90152	25.13578	30.08704	35.18945
169.62	76.64521	88.63252	7.854597	229.2519	87.68646	185.6177	94.65907	235.0214
112.5298	6.987709	89.55513	25.89953	51.63067	89.0855	100.8091	80.92633	60.88263
330.3954	225.6141	331.1755	260.5101	446.0329	278.8319	212.3933	472.6136	282.5528
32.47065	19.28719	76.49869	58.06321	80.25685	84.71115	88.19216	98.16881	151.3136
43.06613	20.61323	35.63022	22.00194	45.84966	30.44463	27.67699	32.69371	28.53229
503.4883	996.2468	411.1163	564.7843	227.1057	152.3504	164.5055	379.5682	332.9665
906.0196	43.8943	827.561	375.2865	537.8099	1034.244	878.3612	104.0938	426.2082
49.19537	110.4413	21.68152	31.03277	34.44046	26.63929	71.40004	44.73881	50.4185
80.8046	65.73963	199.086	176.6144	212.0952	83.92544	146.9368	203.1816	119.866

152.9186	67.08611	57.32449	91.60164	74.57536	102.666	161.2524	177.3687	167.4555
47.44077	77.78134	22.55335	34.8822	59.6166	54.78636	67.20395	38.70866	44.70897
50.03446	964.6665	402.4407	37.51558	44.78453	26.61197	36.87726	62.77635	9.495361
101.0079	635.064	215.5589	149.6481	191.5225	121.2641	137.6579	163.5172	208.3463
107.2442	137.8014	20.80766	11.72857	34.45138	64.07793	54.54756	57.64979	93.24381
23.69344	64.06336	13.85771	2.694391	37.84597	30.44256	40.30598	36.14434	34.24492
159.9563	61.62539	72.11062	40.08725	86.03292	112.0284	147.8069	120.5087	126.5344
46.5741	80.41853	22.55821	23.29946	19.5455	16.6644	12.52948	9.409901	16.15481
80.91069	62.78391	46.06673	46.4234	85.90693	64.22125	68.90904	39.57988	52.32944
574.6559	300.8489	373.7243	447.5154	764.8606	760.6779	629.7201	578.4178	957.0908
79.20332	0.117464	40.86636	42.51371	29.83243	61.21217	31.89364	53.41978	14.25293
150.3134	82.05186	81.69286	42.65288	77.99682	62.15742	97.40081	152.4217	117.0267
99.2556	43.89639	467.8009	417.4996	178.8968	194.8382	124.2291	182.478	107.4907
399.985	79.39303	146.8959	71.01759	158.2428	332.163	132.6504	95.51101	172.2034
16.72164	5.585091	8.665571	12.89407	16.02866	15.62073	16.82868	10.31153	19.04453
561.4938	262.5666	504.1392	566.068	639.8566	591.9921	531.4914	550.892	1269.181
17.53939	73.41691	9.509689	3.987644	30.96428	14.81458	20.11288	12.8618	59.01832
1167.83	309.0588	704.0508	1467.249	1453.973	925.636	628.8647	809.1162	990.3817
68.48725	1353.509	31.23648	232.1577	25.28926	100.6891	209.8741	22.32236	66.57516
137.1164	1.499988	111.274	145.5966	88.30805	218.2599	136.0721	310.1457	158.9034
8.736126	87.51002	32.97888	33.63896	32.16646	44.68836	19.24322	69.69457	10.44445
667.898	267.9799	371.1586	558.2146	258.0432	171.053	254.3794	219.4592	162.6674
35.1091	136.4122	58.22129	60.63431	35.59526	40.97826	19.24579	25.76991	20.91045
140.5554	177.7806	301.632	131.6192	352.0092	417.2266	256.9272	214.3121	144.5937
1582.579	663.0489	1216.911	1522.79	1595.059	1626.631	868.1655	630.0422	1025.573
117.7866	69.75293	39.94101	18.17474	43.62059	51.56985	88.17064	67.98319	134.1674
27.31421	55.2315	0.819863	3.979069	14.90912	15.56982	9.196809	5.113507	33.34387
378.7338	113.5968	495.5048	381.6497	467.8193	321.1852	289.6624	397.6952	413.8549
449.9008	166.8987	398.0955	420.3693	653.5749	433.243	1175.744	35.23332	1299.658
753.9157	376.0269	892.6888	1459.525	1447.099	933.1003	650.6961	1859.384	636.4574
39.51806	151.18	19.07221	5.277844	32.14865	61.03177	38.59074	55.08889	99.92563
39.55371	21.95964	13.86021	3.987635	46.97732	26.07898	39.47897	16.30862	30.43951
19.28063	148.7319	66.04497	41.35241	51.63749	23.50044	101.6313	83.49974	16.15259
40.37024	511.2411	72.95855	56.85863	45.93033	50.88405	42.75531	24.90684	24.71628
10.49265	15.19225	27.76373	389.702	34.4545	2.344733	9.167978	64.53674	14.24972
5.222278	30.02389	3.422755	12.98241	13.8029	7.319795	2.453537	27.55308	17.11421
728.4433	313.1512	799.688	1518.745	1239.538	1164.808	684.3008	541.3971	880.9751
126.5727	6.985913	142.6139	145.5644	76.84562	107.7464	107.4991	135.1969	83.7183
25.5021	13.74986	14.75153	3.985143	20.64231	14.23842	17.61557	6.833399	12.35523
182.7957	16.55913	244.3244	199.779	185.7371	174.3532	212.4906	215.2468	100.8352
164.3332	4.241419	123.4176	224.2291	204.0657	223.0218	56.19693	203.1901	53.25833
24.58159	28.7424	20.8286	24.55269	20.67806	27.96707	12.53513	25.80087	15.20554
158.1562	259.5838	129.4883	112.2565	255.6719	138.7507	209.0914	188.5133	299.7077
43.06108	111.5485	19.07929	24.5755	32.13024	35.44524	88.31576	59.44169	28.53115
12.25448	70.82434	29.53353	33.54968	6.941793	6.064702	19.26189	26.6559	25.67648
21.91887	19.28734	32.98598	23.32444	2.349679	19.14044	18.40556	27.49259	0
6.121347	10.92258	13.06217	2.68507	0.04243	3.601324	0	8.592686	0
187.192	185.8173	86.01748	86.47133	147.9252	136.93	144.4192	112.7393	311.1453
70.24484	139.578	405.8963	1146.122	1222.29	457.4535	756.5506	115.2837	1169.271
8.737376	13.82387	4.295573	11.72601	11.52854	6.074309	5.814014	8.550765	14.24993
10.51278	32.57917	3.423881	10.39189	3.498971	6.710717	0.779325	15.48062	1.883318
217.1116	99.83702	95.59225	68.42772	75.72299	140.0891	157.885	206.6567	196.004
0.836079	0.091135	2.570987	0.072766	0.037727	0	0	0	0
87.02087	91.47496	35.5987	25.89623	71.08935	46.60865	41.10192	39.55815	98.96132
94.91307	79.29265	49.51038	11.72863	36.74645	91.53228	117.6024	115.3852	65.63738
401.5422	481.1951	524.1297	529.988	587.1242	800.6074	808.626	294.3388	1138.828
273.264	414.0908	145.9838	126.4748	434.5702	124.9758	346.7827	318.4833	443.3528
448.1807	179.1676	338.1344	270.8145	284.398	359.2095	307.3083	396.8434	313.9524

4303.931	3831.358	3720.28	5861.695	7528.106	2870.212	3435.845	6105.536	4756.871
105.4289	35.69068	74.71643	18.17665	186.8646	168.1569	21.76171	50.73357	241.6831
25.43342	897.7934	386.785	282.4705	444.938	111.2635	310.6309	762.7066	133.1719
30.74605	31.4597	16.4729	6.567435	30.97144	31.72699	50.43755	12.86	45.67815
1271.483	486.7512	2393.047	2206.114	1803.761	1426.111	1648.255	1459.015	1184.453
120.3496	244.6178	140.786	131.594	139.9321	81.40452	201.5142	200.558	176.9504
17.53224	55.93054	43.46957	100.2106	18.39667	16.67253	25.15012	43.0465	12.34859
258.3923	262.2773	96.44678	49.1176	129.6032	232.2988	190.6169	114.4523	156.0225
6.102808	25.93153	8.645119	6.55952	1.202556	8.577637	8.337854	5.969917	4.737027
10.49267	223.3318	48.65601	23.31925	14.9687	6.06573	15.88664	24.04961	0.933717
87.84929	9.721081	307.0175	100.6073	118.1121	90.82146	57.04321	142.0494	75.14646
364.7528	31.59669	171.2262	105.8209	208.6946	380.6404	358.6437	126.5008	376.7816
0.833416	69.22882	19.96976	9.135128	14.9485	14.8323	29.39922	40.51119	2.834203
10.52702	9.635377	20.03809	11.63216	4.6395	3.588461	8.35845	21.58063	5.692093
82.60776	11.09144	89.54925	68.35934	55.06965	91.56835	86.5025	130.0593	35.18442
17.52562	56.04673	113.105	32.31801	40.16482	29.13821	30.17872	29.22279	19.96007
76.3971	285.6755	307.714	466.6275	323.3594	272.6373	162.847	316.7774	156.9621
26.42012	56.63558	6.910166	10.3804	24.02776	12.39278	21.02715	13.75892	24.75521
388.4517	76.69231	165.1251	551.5562	311.8723	238.4121	170.4177	223.7954	355.8325
28.98224	68.07147	20.82642	16.85683	43.55003	24.82351	30.21115	30.11267	47.5814
55.33226	46.57711	114.7799	103.096	87.14285	96.53903	80.60563	38.68844	150.3507
706.4768	136.8458	237.2458	34.93099	286.7354	1090.101	76.3401	203.9457	179.7888
0.833091	16.45325	47.94679	18.07751	3.499756	1.097261	6.656126	216.4558	3.785628
100.1893	64.30564	63.4303	243.2351	119.2128	130.2589	23.44408	10.26984	47.55454
456.9601	94.4671	542.457	242.4746	248.8665	572.2659	341.7376	244.432	293.0176
186.3284	303.0284	52.10519	38.8011	88.32947	143.8254	118.3809	54.17835	175.0659
119.5142	35.68413	50.37065	60.68752	135.2846	87.71983	319.3437	87.78047	240.7477
196.8372	222.7424	115.5739	185.6782	247.6521	121.9194	229.2552	134.2545	334.9158
260.058	1220.222	77.30447	112.3056	349.7841	620.6631	628.921	290.9001	1311.052
10.49257	60.17909	44.29827	29.76008	10.3831	45.35174	27.64926	23.18605	38.04046
42.28577	29.93574	16.49979	6.555512	20.63781	29.39086	24.37545	30.18062	39.04266
168.7331	168.0645	57.32128	54.26453	165.1055	118.857	148.6278	151.5067	160.7881
29.8655	4.25204	47.84761	11.71509	32.11437	36.74801	38.63964	28.38929	59.9657
12.25091	94.1049	12.98367	6.568892	4.64739	22.8964	15.88835	18.88306	6.639037
131.9619	0.119406	127.0632	0.094358	32.14599	202.0283	15.889	86.14802	36.14182
132.7093	28.85601	56.4599	22.04392	67.69206	112.0649	153.7142	107.5989	65.63234
112.4327	53.46533	52.09732	178.0004	19.55568	83.26285	38.55452	222.0691	15.20198
96.66021	16.55967	111.2786	10.43825	80.28608	62.16065	557.4398	126.5707	124.6419
53.62498	39.6579	47.8192	51.53264	56.15389	108.1826	34.4042	11.99444	56.14273
46.62148	88.25615	6.030124	16.84505	30.95972	36.78541	33.59826	13.72552	60.92714
48.3904	4.25166	40.89254	9.138457	19.52673	21.72045	53.84401	32.72104	43.78145
28.9451	87.58639	118.1925	114.8136	184.6011	137.5371	122.565	94.64848	116.0588
3839.991	838.0411	3989.78	6301.234	1963.215	3438.033	1962.233	3495.627	2415.53
0	113.3303	200.9614	40.06519	12.67674	9.79811	37.73066	284.3768	0.933877
54.42783	242.0504	488.5111	282.4628	216.7795	466.2459	202.2997	683.5097	343.437
35.97984	72.51907	47.76291	90.28643	35.60561	51.54469	76.37959	115.3566	15.20104
110.6813	194.1068	239.9251	152.2042	142.2232	138.7326	72.14776	166.9732	99.87826
57.1334	21.99378	27.7791	32.29994	34.42995	36.66478	33.55323	27.50591	136.1232
5.226445	539.5458	25.1517	40.09797	60.83813	56.48501	25.12111	36.09225	51.35323
240.8803	41.14748	147.7986	34.93192	100.9233	120.1555	212.5467	149.8091	112.2615
16.6491	61.42398	16.46506	27.15802	21.83673	35.41752	23.45925	33.54305	27.57663
120.4495	1.501796	102.6127	46.48864	57.35666	96.58672	132.7766	103.3411	31.37823
3.472144	1.503295	1.687971	0.082381	0.042341	0.482443	0	0.808626	0
10.59174	0.095395	0	0.076049	0.039312	0	0	5.145294	0
28.16829	39.30691	4.293864	7.833391	27.46622	14.89301	18.47369	5.110381	17.12261
115.2477	12.45279	59.99815	127.2649	14.96589	26.65475	13.36878	96.51617	7.590333
43.10968	5.617525	41.77468	12.98818	11.51832	42.48739	31.92546	47.42142	8.543407
202.1131	161.3224	132.097	107.1037	146.8005	148.1006	249.4228	142.8672	217.8715

748.6557	186.0507	1101.348	569.9902	2348.229	639.2395	1108.39	1845.658	958.9913
0	23.39069	0	5.274237	0.048659	1.726559	1.620004	2.529232	0
0	0.051048	0.848468	0.041356	0.022065	0	0	0	0
14.91026	1.507564	9.515874	28.31955	8.081474	58.37142	15.92077	7.694208	31.40851
1528.957	1187.87	1936.662	1713.64	2012.455	1439.795	1253.582	1713.817	1544.079
51.05675	9.702269	15.61193	59.02095	37.79295	42.51125	9.175838	10.27917	32.35503
18288.63	12139.22	34170.28	48825.41	35845.1	31924.55	20425.46	19290.02	28213
53.56341	140.6847	88.64775	110.8761	66.54506	52.79167	66.29366	72.27633	48.5036
350.6312	218.7534	159.9009	149.6622	368.058	253.9529	204.8465	167.8146	386.2729
137.0422	540.9301	98.17322	99.39537	278.6414	149.9158	261.1359	149.7368	414.8237
981.6168	737.9734	274.6364	220.5965	297.04	751.4918	678.4844	386.4659	538.4799
344.4041	119.0726	455.4234	1010.968	978.177	2238.05	130.9179	57.61645	192.1564
237.2334	109.4919	260.7549	108.4254	287.8313	479.4932	459.3571	321.076	357.7231
4.342903	54.42882	33.04228	37.33636	25.2424	13.56913	9.173465	24.08425	6.63961
45.68026	187.8132	15.59311	14.30239	22.98606	38.5213	55.42112	25.77754	76.12571
116.3587	8.342156	22.58674	16.83114	27.51701	31.17458	10.01725	12.86742	8.543651
107.2137	28.85285	107.7966	112.1435	100.9052	116.4679	74.70442	155.8709	62.77925
135.3098	109.4257	119.9319	49.11538	207.5183	233.5819	162.0577	89.48118	269.2673
7.857123	99.69865	46.03398	83.79458	63.08825	40.34501	43.61619	27.49114	36.13582
284.7128	4.236376	199.0323	1686.301	415.0378	382.9798	363.6236	656.1008	274.9496
168.7332	114.8613	82.54125	73.58648	230.4108	116.9862	122.5764	129.1098	382.5273
52.01986	11.03039	20.87131	21.88793	3.499265	2.956839	1.61547	6.835439	0.932976
1901.52	698.6167	2230.437	4051.216	2316.363	2018.705	1764.931	1548.509	2172.936
115.9825	28.85999	222.6031	78.72949	48.21762	321.707	173.8554	237.6752	81.80641
3.467254	170.5913	27.76242	27.19539	5.793454	17.26741	7.489834	44.71519	24.7156
14.01981	0.116158	128.1653	36.05863	34.38456	34.28021	39.50379	62.09945	21.87294
284.6757	79.43712	488.5221	690.9859	534.3387	616.4049	534.0543	271.111	467.1286
1051.772	2258.119	1683.658	3796.026	4685.408	2962.435	4319.248	1187.822	4407.734
664.3913	143.6578	238.1379	80.06904	221.3545	373.5044	346.7732	290.9237	455.718
44.78774	124.1217	20.80922	32.32993	32.15596	127.9015	87.36409	24.91007	55.174
85.447	1.506981	26.94228	10.4217	3.500491	6.692172	2.453663	1.666566	2.834213
152.9179	13.82494	257.4097	146.9543	104.3677	136.9781	76.36717	161.8594	95.12943
261.9056	159.9443	83.40217	23.33464	123.8745	189.2628	109.1148	120.4794	136.039
78.20689	77.90803	19.93777	5.275879	79.12032	44.09026	83.97653	59.37141	151.3073
12.27968	6.95942	17.38606	2.69398	25.17668	21.8642	37.93982	11.15916	9.500365
42.21135	74.75033	14.73427	7.853653	33.24724	18.57604	31.06741	14.58755	37.11063
30.98193	21.50838	4.30691	3.958288	3.488748	8.062395	10.93564	5.128619	31.48843
21.05922	20.59595	8.638898	11.71451	24.10914	30.47684	45.38381	26.66415	43.77419
0	2.879499	6.908033	1.400313	1.202709	0.482622	0	0	0
139.8214	12.45785	45.16999	19.45938	34.44799	80.3529	147.9369	43.00442	117.0472
89.66076	53.3597	38.20915	50.33753	68.79976	57.23054	55.39785	68.86538	116.0966
53.60759	15.18185	58.25406	25.87826	51.5992	66.70224	31.02658	39.57528	48.5189
36.90967	61.29729	17.34269	11.71643	25.25616	34.22812	47.06081	11.13539	73.29825
72.90795	31.58657	92.99714	126.3179	116.9516	111.4452	117.5672	112.7704	141.7683
76.60653	65.25729	26.06509	12.99339	29.81504	19.20814	31.07174	20.62983	72.36166
1.70907	2.879497	30.49763	9.110311	16.06226	11.11701	22.7057	18.94536	14.26462
130.9821	54.76126	135.6639	51.65211	84.85595	54.6896	18.40423	65.39739	36.13491
29.83947	24.73092	44.31133	41.31462	32.14915	35.38843	38.59	11.99203	17.10522
7.858186	36.88624	14.73129	15.57044	19.53498	29.84472	86.68967	1.667043	161.8977
3.466739	2.87547	6.030295	18.17373	1.200034	2.963848	9.167824	44.72157	5.688169
25.4674	8.348139	24.31876	2.6953	24.10369	22.3377	27.69619	9.413582	35.20548
249.6189	27.49546	201.6955	234.5479	160.533	140.0408	108.2841	118.7664	135.0914
83.50495	20.64836	46.9136	121.0008	147.7673	86.00047	31.85498	46.45507	97.05937
88.73254	5.614219	173.9045	234.4086	166.2163	160.1108	186.4912	237.7077	113.2141
12.25004	52.00448	7.767555	16.88352	6.941903	6.066483	4.971871	4.247277	6.639194
27.18928	82.06968	88.64615	70.98392	69.98349	40.94244	58.72888	136.0338	21.86078
43.95926	8.351182	103.6597	27.12247	36.68954	46.15697	44.53509	60.33491	22.82179
8.803987	2.847913	2.564243	8.982825	0.039937	0	0	5.140631	0.933679

310.1341	1562.257	85.99915	1389.986	583.7284	323.5358	24.28963	289.1618	50.40346
1.709071	11.02052	1.686336	0.088191	1.202709	1.096634	0.779296	0.808305	0
85.19196	203.6509	57.31525	145.7575	90.64073	107.5786	161.1939	116.1671	152.2122
189.7781	302.0366	102.5195	77.48514	59.69198	413.5062	181.3309	401.1805	100.8271
18.4348	11.05346	14.74495	46.20245	14.94233	13.59214	4.131942	5.969367	7.592345
4.342976	42.23433	6.030561	6.564781	4.647612	3.576915	4.131364	14.59095	0
61.49198	84.72142	24.28585	76.08135	120.3415	80.93513	95.74901	55.92351	153.2101
777.6531	1709.635	801.4228	167.7516	610.0814	914.4603	1349.403	444.1178	847.674
165.2544	54.78818	103.4371	72.26976	61.96251	143.2824	111.6824	73.13725	75.14905
82.54709	201.0362	235.53	270.8308	188.1071	223.3775	120.8466	141.1153	102.7282
31.63252	138.3314	12.99165	11.71242	25.24913	24.21422	26.00844	15.44907	120.9437
79.9162	23.39368	394.7192	198.5895	224.7515	242.1842	369.542	373.6611	189.3191
2044.935	71.23293	861.4152	327.6215	1358.769	1646.841	70.46289	11.13868	1087.435
5690.542	5531.707	3783.72	4229.393	11274.39	3463.439	3057.978	6645.222	7140.07
50.05711	22.02004	133.0659	63.22152	106.6032	67.81546	66.31536	31.79784	79.91617
2866.358	982.9208	2347.766	2143.093	2588.149	4520.685	3101.694	4331.485	4811.122
2.587665	57.34356	56.52129	34.86433	14.96491	13.54207	11.68883	92.21568	11.39633
86.94618	232.3651	407.7503	297.7937	146.8159	167.3774	146.06	314.2235	108.4401
91.43649	12.45649	63.45939	52.89107	40.16779	82.27832	43.63206	116.3126	86.59194
28.06629	143.596	26.02035	161.2221	105.544	14.16012	13.36814	138.5545	114.1507
14.8866	405.4486	127.7597	183.0609	165.1204	118.8325	111.6425	188.5328	50.40331
0	2.634598	0	0.04159	0.022185	0.540807	0	0	0
6.099712	68.14269	26.91698	23.28913	10.38042	11.05087	10.00968	14.58058	8.542013
14.89113	31.50835	62.62767	14.29671	16.10919	12.9201	11.68949	69.78782	5.687734
14.88903	15.1826	24.29505	23.30809	40.15709	148.7781	73.10338	79.25169	39.95026
16.64438	77.88807	51.25872	42.62924	48.19481	48.47072	51.18733	34.38497	33.28161
300.5666	1.496551	176.4411	353.1285	252.2495	250.9336	264.5299	55.03164	204.5457
186.3773	17.92647	158.2641	54.24117	99.7623	107.0948	141.1177	274.8077	106.557
69.40261	42.49052	4.293647	50.3662	11.52993	1.725595	1.619632	76.59942	0.933915
1579.985	891.2204	797.9337	414.0133	668.5674	1621.787	1196.536	867.6523	2008.393
750.4707	168.27	474.5945	218.017	449.5223	422.631	653.2921	801.454	813.45
9399.569	6397.034	18172.13	19283.35	25843.4	7982.731	10734.14	20219.74	17041.02
64.24185	5.619586	45.23903	21.98268	30.96627	48.70047	49.60521	57.75822	26.6324
173.1237	94.4019	121.6764	76.16687	126.153	161.2551	188.1189	111.0169	195.0444
1.709989	50.4643	2.554797	10.43088	3.500359	5.442477	11.69252	8.550186	8.542278
29.88152	27.34539	12.99515	9.137002	19.52415	24.86877	36.98248	25.81513	19.017
25.56947	25.70866	15.66336	2.690595	11.4798	21.33908	20.21451	6.846194	19.04311
42.17068	46.47195	31.26369	11.72381	25.27065	20.41136	22.61892	31.81945	28.5289
138.8416	204.8661	69.49788	37.51277	142.1838	82.07265	114.1779	102.4108	136.9993
1.709056	9.678373	15.62986	18.06391	10.36211	3.579752	8.342722	50.10959	0
86.12583	109.2244	26.02566	41.34811	98.58422	70.32064	72.20438	40.41408	95.14669
121.2746	60.24787	67.76734	47.80991	105.4993	91.46685	105.7953	56.76864	93.23013
25.47622	80.05501	10.38275	15.55344	28.66377	14.82911	26.86613	49.15049	142.8915
485.9258	268.0173	494.59	1084.124	596.2697	493.0095	272.0002	355.4734	330.1154
30.73711	129.1133	36.50152	16.8631	19.54038	13.54969	190.3332	21.47909	50.43357
40.42242	51.85602	28.6598	24.57184	24.11882	58.65473	63.0484	37.86966	32.34044
567.7005	642.2046	158.1523	429.3456	165.1786	265.1062	840.6699	369.2736	673.6057
7.857312	19.29069	16.45936	168.6706	145.5443	284.0463	38.56954	18.01708	56.12019
63.21561	793.5061	124.2548	156.102	115.8707	70.80533	130.9334	182.4544	106.5357
66.8295	21.98706	63.49668	18.1561	28.70271	66.12689	44.5016	33.54664	39.00202
34.37318	16.40424	19.13804	7.826775	14.91685	15.54796	15.94993	14.62261	33.33688
88.81405	31.53683	18.20292	31.02605	17.25855	148.735	35.22868	13.7148	81.83848
166.0627	80.78004	197.3146	277.1365	272.8744	153.0723	217.4829	157.5022	198.8373
12.24986	124.2299	17.32889	19.46298	10.38304	20.38384	11.6863	13.71261	4.737153
10.49276	161.0519	57.33716	23.33023	32.16459	67.7871	57.05717	173.1235	32.32798
38.61407	24.761	86.89462	28.49064	49.36371	150.0743	54.52036	47.28964	69.43584
8615.753	5356.865	16629.23	23418.48	17407.09	12870	8587.126	10507.5	10357.6
549.2087	123.1727	465.0339	700.0568	497.6704	438.2091	474.404	429.5174	433.823

64.19348	24.70435	40.84953	6.568818	49.29214	36.06298	72.29862	67.2033	39.95542
51.87524	9.717909	30.40344	10.43263	24.11761	58.03778	20.10254	35.28295	40.91101
10.53273	5.582192	4.299411	1.399081	10.33627	14.99675	10.90298	2.527801	19.04655
72.02786	121.6095	53.85054	24.62167	71.12925	70.87863	83.10164	36.95908	279.7733
18.40166	1388.106	66.87772	28.4926	6.937469	93.86326	27.63928	11.99395	25.66613
228.4214	477.0653	423.2944	305.6789	290.1635	237.0351	212.3727	209.9773	170.2762
32.53359	4.250942	24.33147	9.134388	16.09086	51.33225	26.0281	7.692034	28.54418
269.7657	170.9481	168.5974	224.3946	212.1637	351.8012	271.2107	240.1469	254.9666
277.7616	46.62121	172.9935	370.9789	139.9049	156.8854	143.5778	143.7472	161.737
190.7014	78.02997	152.9823	43.96042	171.9959	266.0205	266.2632	158.3847	157.9285
414.7003	459.4222	2939.784	209.0123	1945.975	1097.965	214.0433	4194.709	1673.462
28.94497	125.8183	179.0662	152.1752	64.27191	78.30386	93.15389	191.9671	44.69418
66.78681	98.25579	32.99012	14.30538	19.55293	56.61736	39.42315	63.6979	60.88647
7.8572	210.5031	13.85669	6.568745	3.500083	1.71943	7.489051	3.385931	2.834476
117.7687	53.41466	73.86327	81.26594	57.37514	82.12988	43.60678	47.29741	46.6013
24.61329	17.81864	10.38835	2.695414	25.21371	18.63305	24.36106	16.33017	33.31644
456.9343	390.9523	289.4223	63.30792	366.9662	818.8463	535.7276	556.0915	1011.365
0	0.059411	0	0.047986	0.025447	0	0	0	0.941724
186.3054	121.7075	103.4081	141.8563	88.33921	93.8953	128.4473	170.4441	98.93084
50.92047	12.45696	185.1936	144.3856	40.19401	53.39771	29.31977	423.7964	7.591111
22.84051	9.698221	7.772542	6.561855	6.938062	63.36819	39.53919	35.33806	29.49971
0	21.4982	3.433181	0.080782	0.041579	0	1.618239	1.668656	0
94.20641	30.09985	21.69966	2.695038	3.500444	64.39148	54.65487	11.99817	41.86905
28.96714	1.504415	40.84242	60.5371	44.72599	68.61438	32.71422	32.68141	49.47372
300.5486	63.02869	194.6925	283.6236	435.6432	424.8024	189.7419	340.9214	570.8865
73.78329	27.49174	136.4899	280.7257	123.8344	130.769	99.06534	321.2962	69.43771
14.88977	0.119006	17.33473	43.85957	48.16223	47.3042	54.59342	16.30063	67.56262
161.6993	28.86116	91.23877	33.64743	167.396	196.2117	173.001	254.0266	154.127
143.3285	32.9303	37.33592	23.3246	61.93769	70.95874	73.89225	82.64963	52.31626
259.2736	31.59605	206.9001	50.40604	115.8495	227.9387	262.8857	184.217	175.0544
255.6622	1016.723	352.8717	658.8718	533.2318	209.0094	331.6178	482.8792	385.295
347.1628	299.2024	68.61721	23.33404	112.4212	168.027	363.6767	75.69518	645.1271
67.74894	39.60954	19.08419	43.79162	37.83512	34.85363	27.68401	19.75796	68.53512
18.49231	25.69108	8.665849	2.690011	10.33866	16.25882	15.13461	7.713116	17.13657
12.25249	386.6	9.509285	29.78205	14.96806	34.69545	10.85076	61.05718	20.90955
99.25209	831.4923	123.3921	86.50073	136.497	211.6353	277.1181	159.2168	363.4503
35.12035	34.26012	32.12678	52.86975	39.01574	55.41122	81.51234	47.33118	118.0191
20.16895	31.51171	19.07687	9.147245	5.795247	21.03891	11.68924	44.76038	2.834544
4.342819	40.97871	12.9885	20.71333	20.68275	11.67816	9.170142	18.89376	0.933345
304.0637	139.519	167.7356	74.90357	105.5488	268.3379	256.1073	228.1042	226.426
2.586786	95.70905	6.906532	32.0781	8.076075	7.335985	8.343612	12.01902	8.54658
15.76613	79.21234	45.17342	38.75894	92.83241	54.1151	148.7983	10.26974	64.69377
8.741803	28.66251	10.38531	19.384	1.202439	11.07791	10.86116	49.17427	2.8342
158.1223	263.8939	526.7816	411.3279	738.3871	365.9955	548.3381	110.1212	544.1984
0.83323	1.507533	2.55423	2.695515	0.046147	0	0	37.94103	0
55.3703	13.81712	41.70894	101.6114	65.32589	51.05506	35.23447	55.10231	64.70518
102.8796	126.7782	32.99239	40.03729	45.89242	54.13084	71.39414	35.25408	58.03273
190.6578	441.29	150.3394	263.0425	223.6246	103.192	208.2116	185.9002	256.8704
174.8215	35.69441	149.4577	187.0642	427.7127	492.4711	262.7768	362.3812	366.2771
53.6357	11.08203	44.34336	24.57051	14.96142	57.40558	71.4769	43.91208	24.72383
11.39009	36.67398	9.519662	5.271269	13.79081	14.87406	16.77482	15.47236	18.07262
108.0291	772.2976	622.344	103.2798	94.09385	50.26604	44.43632	220.3006	48.50008
2.586626	2.881047	27.84941	12.96308	9.222145	1.714902	2.453541	15.4709	2.834273
103.7519	13.82288	64.32549	2.691658	36.73494	70.37659	41.94684	15.43583	59.93503
140.5685	32.96359	380.8182	366.0049	223.5974	163.6642	143.5489	219.5077	176.9512
8.754709	35.17198	8.656892	14.19995	3.497653	9.87351	1.615575	12.03066	1.8834
17.52226	8.354879	104.3234	2.689246	33.31062	154.5988	186.5478	5.968221	247.4315
0	0.104953	1.687522	7.789467	4.634273	0.482271	0.779451	5.987742	0

59.74083	38.37867	53.87205	27.185	105.4366	71.59704	113.4363	55.93196	174.1648
470.1802	494.5681	168.5956	442.1364	141.093	140.5615	147.7301	165.2315	117.9528
28.10182	34.17986	27.79624	34.81744	35.54734	29.83914	20.10717	56.0148	26.63034
67.70313	15.17898	33.00448	32.29644	29.84997	61.09285	45.3379	36.13075	65.65999
7.873771	32.46895	9.531879	7.819146	12.62872	10.51098	10.04266	14.63164	3.786603
72.96825	65.54466	31.25676	28.4542	50.46077	51.03373	69.72998	54.2312	90.40789
108.074	5.613584	345.3043	258.8862	99.78074	203.152	73.84894	215.2881	61.82415
67.62144	42.52084	126.0311	197.1803	182.2887	159.4073	107.4521	73.12385	129.3851
130.9572	120.2279	31.23948	37.50347	57.37726	82.74461	117.5731	92.95678	170.3236
19.27945	294.9742	63.40535	154.7344	158.2455	37.18891	41.91577	67.95185	12.34749
118.6339	177.5087	49.50069	101.8797	96.3367	87.7182	73.85399	135.1674	87.51951
633.5124	429.3409	1645.479	1355.179	1051.556	931.1757	861.4513	919.2841	582.224
0.832912	5.598454	0.81987	14.20301	2.351678	2.336125	2.454306	5.112804	5.690781
848.8177	3360.43	474.552	519.7386	1564.068	1362.085	1065.521	1150.867	2685.791
0	0.076642	0	0.061518	0.032223	0	0	0.818555	0
32.48181	17.91093	93.96103	60.56904	28.71076	44.78113	24.29611	44.74629	4.736652
679.2426	1067.364	325.0465	301.831	478.2121	408.2488	1026.12	629.2189	956.145
216.2515	113.4534	84.29079	67.13074	143.3116	123.2736	126.8004	94.66804	130.3431
26.32511	140.1992	15.59433	24.59087	60.74947	49.17769	66.37645	36.12747	76.13105
23.68132	27.45863	56.49777	60.59298	44.74491	22.2688	92.43873	30.94613	44.70692
0	5.370194	1.705987	0.063388	0.033146	0	0	0.817211	0
14.03412	9.685014	12.13506	34.66423	6.935086	10.46745	5.814639	16.33534	6.641149
58.8642	136.3637	33.85881	13.01716	83.68043	35.99219	130.275	23.1871	145.6092
9.616473	85.69539	12.98902	28.41033	25.25753	2.337039	43.68704	25.79632	24.7254
151.2134	42.49172	73.00106	40.07034	75.69583	118.3845	120.9616	86.93974	90.38237
30.70243	391.8943	57.31691	126.4188	111.267	96.9985	41.91477	42.98003	37.08272
1644.079	259.8634	2402.607	2097.821	1439.133	1368.207	930.2964	1415.112	992.2713
57.24773	0.114685	47.90594	24.50065	9.227117	44.43676	26.87956	34.47308	12.35283
9.615632	162.6137	63.40808	18.17647	25.29104	28.47199	18.40327	14.57384	69.43438
10.49765	17.87245	19.96008	0.092285	1.202	4.198533	4.131224	10.27516	3.785296
4.342989	25.9896	25.19834	14.27235	40.08727	11.06842	25.17823	76.8135	0
26.38372	0.113265	26.97295	32.12209	33.19091	28.73729	15.08366	22.38635	13.30769
139.8052	46.56378	39.9455	14.30695	61.93906	104.0791	104.1697	56.78918	94.19653
138.81	181.8214	202.5297	220.4749	259.1265	212.9049	285.534	84.30527	232.1404
109.8777	111.9584	58.21512	25.90273	60.7987	100.9288	83.9746	76.60731	78.01262
701.2894	153.2277	321.5967	116.1684	402.4954	413.3459	669.2957	421.7879	480.4517
38.62777	101.0211	41.68778	34.90758	31.0124	36.61018	37.73634	39.55595	35.1853

TCGA-DU	TCGA-FG-	TCGA-S9-	TCGA-DU	TCGA-HT-	TCGA-HT-	TCGA-HW	TCGA-FG-	TCGA-HT-
125.7364	161.6745	115.9845	304.4882	131.5055	65.36088	176.2432	83.23496	52.64042
22.91574	33.86673	41.7868	22.22255	34.93033	73.95549	26.1602	26.68439	32.21516
87.64962	135.4443	142.0882	52.18221	196.2096	128.8704	118.2847	58.87286	117.0313
1565.548	2867.541	3057.162	3680.633	2976.258	2772.5	3323.759	1992.142	2291.271
96.55129	40.45674	134.7841	150.7837	59.59669	41.67026	51.51896	54.93062	113.8215
13.54826	0.02195	2.100267	0	5.132721	0.930792	0	3.925272	5.355147
72.20715	7.685628	12.55572	9.651627	54.44327	25.57046	12.61915	44.80897	20.42358
114.3214	332.0053	223.579	139.1802	148.9684	135.4924	212.0028	85.59463	46.19915
4884.38	24097.02	8651.166	7080.948	7629.168	9897.061	19197.24	5059.395	7461.095
66.03	37.16923	113.8628	71.52565	47.2644	74.85893	36.4527	37.65833	25.79649
8.980695	4.407135	29.24381	31.90788	7.201846	11.35489	9.455924	17.25916	11.83166
798.6608	1309.773	1469.017	1387.006	1012.978	1399.525	1432.89	932.8026	344.6856
219.6019	80.8696	95.09311	124.678	133.5616	83.36554	92.01782	123.3356	140.6651
5.078978	0.023342	0.01142	0	24.57771	0.929675	0	0	7.493705
102.8547	107.057	113.8852	184.6293	35.96847	117.4974	100.8022	73.0365	22.57911
29.2511	17.51026	41.79161	5.785202	9.257578	2.821384	3.883333	21.94211	34.36547
1210.05	1561.054	2020.699	3078.474	1698.229	1980.354	2683.15	1751.676	1293.821
35.60416	22.97175	79.38663	30.92103	18.50281	17.98319	26.92783	29.01759	33.30136
72.43556	131.0766	20.91583	47.34886	77.05793	173.4219	65.03505	63.59352	368.1288
1882.481	371.4435	520.332	183.6306	1967.349	447.2258	140.4174	581.5194	3086.633
760.5659	1560.998	1028.11	812.8641	1256.455	1149.37	1230.454	526.4658	608.8043
6.461697	61.1582	107.5739	6.75168	106.8193	30.3077	13.41035	21.93008	7.547095
55.95869	419.2872	48.079	95.68289	71.9229	53.04327	315.4154	165.0852	10.76788
26.77624	422.7339	331.2089	1667.435	184.9323	56.82961	394.5677	36.05793	114.9138
114.2396	96.13376	45.98777	70.55135	120.1969	88.11659	115.1273	83.27652	53.70703
17.85723	14.23395	37.61088	75.4235	10.28444	15.14259	26.95884	44.84307	7.546383
171.4461	276.3595	135.8394	118.8754	143.8369	143.0656	125.3637	200.3813	257.6777
723.7456	431.5295	1279.903	471.6656	374.9964	483.228	454.7893	875.445	624.9075
26.71689	43.68591	60.57928	29.95813	22.60916	21.77883	121.0519	50.34819	4.325434
45.79075	41.54204	68.96535	129.5304	14.39478	35.98737	111.1774	30.56549	25.79866
45.76107	4.407264	44.9369	93.76725	26.72121	33.14745	38.04734	25.85511	52.6206
17.88496	44.80693	55.37995	107.3056	47.26336	17.98182	59.52729	22.70796	25.79546
19.04846	2.221839	12.54811	24.16973	5.147552	5.66492	21.44875	6.994337	2.176926
827.8223	405.3115	837.9536	686.2454	634.9134	469.9633	573.8825	667.9528	849.2941
165.108	138.7595	249.7112	255.1718	102.7456	101.368	174.6046	117.812	154.6288
4598.737	2857.747	19985.43	30502.45	10317.77	3991.965	9286.899	6015.79	1627.749
45.56473	10.95671	21.94723	2.885946	100.6163	19.88893	4.676939	55.98126	18.26866
22.96168	34.98764	151.4611	82.15952	65.75319	76.75208	69.05226	55.75995	66.57439
117.7963	22.97251	36.57818	19.31837	86.2841	35.99534	14.99879	88.13432	59.04621
168.2144	33.88049	30.31004	23.18677	87.30505	83.41883	11.82241	87.3926	21.49822
322.2772	192.2505	14.6465	23.18276	41.10591	107.0593	258.8792	252.3546	316.6837
31.82098	9.870917	63.73155	74.43131	21.5849	27.46107	33.28208	43.18408	37.59699
92.59941	14.24023	14.6465	3.852699	34.93854	119.4245	9.437582	127.4753	53.69434
91.4698	61.2055	238.1901	142.0852	89.38614	126.9693	127.8025	73.81041	15.06324
208.2758	123.4744	264.3445	448.4937	166.44	158.2232	242.8841	230.2278	155.7082
19.08031	5.499338	28.20411	37.71069	12.33647	6.612032	10.24932	14.09007	2.176947
43.2345	41.53528	28.22656	61.85693	33.91167	22.72029	29.29811	88.85273	46.18511
1142.782	8706.264	3744.655	4861.764	2558.127	2926.947	8053.595	869.0863	1182.164
482.498	1109.806	308.238	480.3714	852.6979	1023.375	832.7885	948.6556	175.0422
7.71189	2.221838	33.40963	25.13756	16.44058	3.768862	8.663746	8.573208	6.469681
10.23573	6.58874	18.80798	38.68798	4.120463	16.10203	10.25929	15.68945	5.397201
119.4413	107.0894	214.196	173.9711	233.2131	163.9105	254.0148	139.0318	279.158
36.90835	15.33292	151.4635	153.7097	26.72195	43.57197	70.639	42.37455	34.38363
2898.671	1272.681	7754.691	14523.44	3004.005	1792.725	4955.822	1420.769	147.1242
21.6017	13.13489	27.161	39.64574	10.28291	21.79027	25.41214	12.51027	6.47137
31.82809	27.34284	52.24735	64.75901	38.01971	26.51211	34.86654	36.87566	51.54764
15.30557	24.03692	54.29074	14.48899	24.65739	25.58343	19.81366	10.13948	10.76251

5536.907	3407.209	6336.871	8265.955	4525.519	3020.741	4029.404	1313.104	3568.972
204.4774	243.6194	255.9887	70.54386	57.54396	808.3226	84.85256	200.3441	293.122
21.65415	6.593065	40.74397	64.78019	15.41993	19.88322	18.19336	23.52669	19.34827
68.51393	94.98636	25.09124	25.11884	110.9325	51.16406	53.97524	36.09941	55.83194
22.90293	128.6481	4.197955	2.885979	47.2479	4.715762	0.717669	1.488059	230.4016
129.581	87.42677	227.7723	135.308	347.2358	294.6968	142.8357	171.2827	199.7135
17.82246	13.1353	18.81363	16.42429	4.120705	0.927805	11.84233	21.99087	11.83378
16.59486	53.49231	26.12893	58.97658	21.58165	17.98678	62.0379	27.4712	13.98488
29.30818	224.9103	62.69856	52.18466	92.4613	182.9223	154.1271	50.23495	77.31239
239.9375	199.9131	147.3303	85.04398	168.491	85.25901	83.27362	194.8824	315.6378
6.449447	30.54993	19.85183	9.655037	34.91429	55.03395	16.64739	3.055533	7.542237
11.52105	8.774947	18.81648	21.25878	8.229637	13.24861	15.81951	6.198702	3.251355
85.17213	239.2366	226.73	302.5364	193.1476	150.6451	251.6455	133.5338	96.66
24.20418	24.06067	25.08887	83.14567	19.52942	8.505672	35.69109	51.88995	21.49824
101.5842	85.22102	58.52387	97.61916	203.3974	43.56743	103.1893	112.3852	6.472165
44.43708	26.24223	41.79492	40.59312	23.63745	20.82846	39.67577	34.54567	24.71605
77.55886	183.5418	272.6984	234.8694	218.8306	165.8064	327.095	130.3851	169.6612
17.86841	43.69158	36.57244	58.00367	40.06748	48.33145	55.62358	14.06307	21.49627
279.3235	288.3913	65.84324	116.9404	254.787	161.0667	134.8804	283.7006	169.6633
158.7639	303.6639	296.7211	234.8722	234.2376	219.8272	240.534	197.231	77.33525
684.4303	589.9289	1986.205	1733.029	766.4184	335.4066	1405.056	669.4881	709.7359
90.07687	16.42462	173.3817	87.96371	52.39967	15.13846	68.26952	49.46977	7.547004
97.86732	19.70168	375.0796	1104.862	96.58243	2.823635	343.767	203.5029	25.79998
5.189669	1.126158	155.6246	68.62694	42.12802	4.716115	63.50488	21.92094	11.84202
81.20339	61.17756	29.2705	48.32175	84.23694	36.93933	34.86664	41.60015	175.9753
824.0691	1982.666	1207.824	405.9376	1178.383	1901.729	1439.209	350.4089	1239.046
135.7929	91.77019	74.19199	27.04941	98.62782	48.30645	50.73588	168.2779	417.4683
125.7269	75.40532	159.8566	194.2841	68.8425	234.0654	137.3177	166.6217	108.4574
1068.756	199.9424	340.6227	148.8352	1027.332	472.8218	222.9934	514.7585	1012.423
42.00004	96.13946	26.13964	6.752182	357.4662	133.6116	21.34451	50.2219	54.78225
144.7953	152.9524	109.7207	165.2754	315.3877	71.9924	51.51119	120.9648	132.0824
29.28093	24.06443	48.06533	138.262	28.77423	26.51426	93.79602	33.7396	24.7198
449.5079	1329.361	748.09	502.6015	304.1074	817.7386	618.3837	348.8635	132.096
2017.455	1084.777	2245.329	1913.77	1332.49	1581.437	1607.463	1709.252	1942.311
4.987435	3.274247	0.009764	0	4.10448	0	0	0.721748	27.50725
46.99618	28.42959	94.00551	26.0862	28.77403	27.46264	42.84033	31.37831	33.30234
22.96248	132.1254	59.56206	94.72813	47.26549	104.2443	114.3917	13.26787	11.84219
449.5349	512.3581	558.9926	606.021	715.0445	439.6422	543.7194	512.3332	565.8524
119.3069	72.11609	65.83456	97.62103	49.32214	77.69	76.96732	93.5086	83.75662
50.85614	40.4491	136.8493	200.1158	48.2935	44.51841	69.03719	39.22146	32.23837
30.56617	62.27412	51.20492	48.32041	42.12899	74.85822	78.60693	21.91852	84.81011
9.001458	124.4982	34.49562	82.15626	68.83656	23.66693	208.2424	41.58172	192.1109
145.9973	389.8378	107.6242	67.64756	162.319	84.31727	176.2727	84.0319	125.6269
251.4326	256.7271	189.1263	156.57	91.44631	171.4884	138.8444	390.6115	369.3428
15.2862	76.26363	17.76718	9.654481	17.46847	17.04859	41.43225	14.88897	2.176956
6.46141	51.32469	21.95465	460.4198	16.44762	7.558202	71.56849	18.78897	0.023924
2.647825	6.592261	16.72953	173.1653	4.1208	1.87427	37.3527	6.198155	10.76419
50.74291	24.05828	40.74911	57.0358	15.42065	38.84644	28.53338	42.43937	15.05959
15.30392	10.9554	22.98899	38.67568	14.3905	14.19852	25.40339	12.50696	6.471719
36.89952	77.5501	57.47032	75.3938	40.0744	101.4073	96.91594	16.41347	33.30838
20.35327	41.46805	10.46412	15.45615	30.81692	17.04398	23.80328	45.71019	32.19745
968.817	1499.871	2458.464	1896.374	1235.918	1191.993	2000.453	1221.2	2430.819
52.0693	17.51514	54.33203	94.74044	24.66614	25.56569	51.58657	21.92516	51.54156
793.4689	890.272	390.7767	269.6557	454.0978	818.6872	438.9412	451.0493	817.0624
300.9593	346.3017	346.8884	644.7035	414.026	397.0168	667.701	333.1777	333.9295
10.23772	6.589076	4.197411	0.953898	1.037739	6.613032	6.271554	6.204672	36.45859
14.02661	2.221886	24.02723	31.90843	2.065682	2.821176	11.84924	11.72711	3.251158
97.80162	93.96148	147.3139	81.18249	152.0442	174.3645	117.4789	97.4166	100.9366

20.36685	43.65911	10.46492	14.48789	34.92587	21.78479	35.75948	12.50037	61.13318
90.06163	27.34284	73.1334	168.226	14.39456	12.29565	49.97839	25.85518	17.20949
35.66101	17.51815	229.8312	168.1888	132.5281	75.78925	90.45584	43.13879	80.54486
38.20464	48.10545	681.1963	712.3735	153.0865	129.7923	284.1541	54.918	97.73704
171.3368	197.6862	103.4445	219.4254	127.393	158.2476	165.1569	139.8965	105.2321
148.4761	17.51816	92.99471	85.05104	114.0362	78.63531	49.93926	116.3175	62.29546
17.88575	215.2199	783.556	508.4253	335.9436	135.48	246.8539	151.6051	46.2008
54.59463	10.96304	53.28696	143.096	17.47592	25.56602	52.38631	47.13162	21.50097
44.33519	40.39337	16.72973	13.52056	20.55277	39.81027	28.57241	18.80959	31.13442
29.25555	13.14509	33.44051	37.69331	14.39351	22.72566	7.851091	19.57599	22.56916
214.601	87.42832	333.2893	555.7999	179.7935	122.215	196.0399	162.6223	84.85118
313.5135	22.97982	244.4861	409.841	77.06268	116.5311	183.3473	204.3145	187.9044
370.7828	460.996	399.1301	341.1852	825.9815	548.6299	462.7888	394.4772	413.3804
1802.974	8327.229	5098.739	2245.29	4472.087	5714.64	6318.172	1762.645	823.5535
12.78322	5.499976	30.2959	59.95966	7.202465	2.821134	24.59611	14.87064	10.76317
25.43284	29.50065	28.21443	28.99402	22.60746	27.47305	33.34472	27.48274	21.49037
24.22637	55.72347	155.6295	37.68584	81.15707	90.97813	79.41042	19.55957	37.60054
12.09848	0.018569	1.055538	0	1.033967	0	0	0	0.014889
0.083269	82.76871	10.46143	0	10.28174	59.78271	72.70744	9.361627	0.022996
34.43426	1.123702	2.103115	0	3.088348	5.686816	0	16.83115	2.169179
25.35803	7.68074	12.54974	28.03751	6.17483	10.40642	11.05148	17.26078	6.470603
114.299	22.98024	146.2734	138.2171	69.86907	54.9379	75.35218	94.25904	51.56526
55.86216	27.33961	52.24312	67.66425	22.61179	27.46184	40.4469	33.7371	26.86632
422.8396	453.3581	547.4907	589.5968	616.4136	566.6285	466.7407	480.1438	594.8234
54.69763	44.82582	75.24176	125.6482	102.7416	55.88495	101.5654	180.0023	235.1029
5.176783	4.404757	9.414369	21.27331	6.173553	2.82164	9.475897	10.96214	7.538332
35.6564	968.9565	379.2892	1186.93	636.969	719.1729	1933.126	404.6458	180.4124
105.4592	60.11907	168.2207	235.8448	90.41675	66.30723	96.78076	73.79149	41.90555
561.5323	22.98007	33.45303	17.38349	298.9267	124.1288	11.81977	700.4106	59.07731
11.51709	7.682931	21.94606	17.39069	3.093304	17.99265	7.857473	32.26009	4.325203
341.5181	107.0916	701.0328	369.2266	287.6623	247.2997	238.1104	234.9368	202.9461
100.3994	352.8084	284.1862	409.8374	282.5197	271.002	291.3688	81.64612	153.5559
104.1673	180.231	157.7671	112.114	143.831	159.1892	230.2975	65.15126	58.00715
64.79331	30.62233	94.02996	183.6788	32.88561	66.32034	98.46316	73.85501	25.79825
33.00107	6.592601	49.08732	22.2241	14.39214	21.78265	15.81209	33.01462	15.05551
210.1247	16.42354	69.99625	32.85327	59.58619	54.00729	14.99718	81.80009	51.54296
54.68488	78.6726	130.5988	155.6239	67.81317	47.35783	127.8229	30.55967	53.70964
31.54298	13.12022	9.415031	9.657121	15.41123	14.20888	8.673601	12.54262	12.8941
123.4884	1.127398	1.061798	0	1.037714	0	0.716362	0	6.463002
45.76989	20.79292	79.40223	130.5082	48.29163	33.14641	64.28786	54.97917	24.72336
14.06894	32.78408	45.96786	13.51874	32.87905	45.48675	69.17931	33.76779	17.20497
24.22149	63.35537	16.73557	17.38432	27.74795	151.6745	22.1487	51.05878	79.43509
165.103	61.21275	363.5772	249.373	108.9091	88.10152	99.94918	189.3715	154.6275
43.25809	196.542	27.18374	22.21659	56.51174	11.34803	14.99379	88.79934	296.192
394.9009	623.7421	533.9049	303.4879	511.6245	293.7225	513.605	432.9931	526.1074
21.68277	105.8921	83.56766	61.86247	84.23185	53.06102	117.6827	33.73774	23.64715
76.0553	29.51835	17.77894	52.19584	27.74626	48.3247	43.64658	74.75137	53.67833
44.50347	63.36532	62.69299	88.92961	101.6988	64.42969	57.12844	61.27898	60.13421
19.14778	14.23917	25.09091	90.87407	31.85551	16.08705	38.06195	58.95519	31.15753
39.4685	17.51813	129.5587	168.1874	75.00491	113.7	40.40143	75.38368	6.471775
59.70551	29.52824	88.80224	133.4074	50.3462	53.05271	77.01183	95.1443	29.01667
64.86833	148.5984	392.8487	1029.439	318.4821	198.0215	366.7622	75.35202	64.45346
35.63666	103.7452	95.06793	73.45849	111.9699	35.98975	68.26171	46.31535	29.01663
20.39614	21.87417	51.18596	42.53043	31.85163	25.5715	19.78104	36.92774	23.64032
438.0772	377.9925	221.5206	208.7618	497.245	217.9138	221.3969	491.1426	866.444
481.2155	493.7691	258.0874	130.4701	520.8716	676.5537	305.5619	583.1304	278.1098
52.07385	16.42351	16.7354	29.95274	32.88318	32.20125	26.92073	47.125	42.96065
184.0521	227.1811	181.7928	174.9498	194.1647	176.2492	221.5536	146.1676	84.84308

71.17561	120.1653	185.961	128.5524	53.43238	59.67764	188.2229	57.29571	108.4493
41.9386	34.97815	67.90451	69.59941	25.69295	32.20256	33.28872	22.71428	26.86569
537.0237	154.0648	58.52972	46.38012	47.27023	49.24954	79.29259	289.933	615.2064
420.085	225.0379	135.8405	256.1377	180.8199	196.1339	134.8898	310.469	258.7555
36.90693	17.51723	74.18299	60.8883	29.80349	25.56306	30.08949	31.3578	50.47989
87.33536	34.96416	48.05474	31.89172	45.20084	21.77826	26.94594	55.07632	7.546776
78.78165	51.37583	145.224	137.2537	69.86807	65.36444	110.3284	76.96279	54.78384
31.7958	16.42093	11.51157	33.82342	44.17696	6.61056	10.23331	27.44969	44.02301
24.20698	31.69998	68.94258	56.06463	38.01601	70.13779	57.98452	38.47995	10.76769
312.432	2013.158	613.3201	405.9409	565.0525	708.7565	1624.351	576.7919	245.9055
14.06428	20.78096	35.52468	51.23792	32.87724	54.02989	47.67942	24.31517	17.20314
116.7646	44.81973	195.3447	89.88749	81.1645	86.22199	63.45746	51.80252	32.23993
125.7603	192.263	169.2664	86.01117	293.8137	363.8925	180.1857	157.1444	216.8799
26.77889	164.9463	140.0095	375.0637	57.54252	60.62299	246.9816	62.79129	46.19883
18.95276	12.02037	8.368335	18.37486	6.172461	8.51681	11.09511	7.014226	4.321274
401.2652	384.5453	557.9391	557.6987	421.2224	334.4666	716.8579	592.5503	337.1604
17.8508	37.13022	13.59833	24.15827	13.36526	9.454611	18.20094	21.95995	8.6192
515.5559	1838.431	1252.736	804.1666	1547.188	1083.991	1357.507	331.554	561.5616
113.1047	38.27369	179.7238	272.5632	113.0203	26.50902	29.28393	232.5743	636.6427
12.81178	18.61039	176.5467	55.08368	68.83872	41.67289	88.8936	8.554573	57.99959
242.1675	97.2245	47.03223	100.5217	59.5941	128.8777	60.27641	173.0225	106.2915
227.3383	176.9998	321.8115	489.0824	365.7404	168.6436	238.0947	176.7482	529.3072
87.55862	71.00609	30.31597	30.91782	60.6171	34.09402	23.73336	71.51487	45.1133
203.1916	190.0967	274.7894	457.1967	420.1781	433.0502	439.0842	361.5549	86.99891
1239.184	2352.964	1265.287	2256.915	903.0545	1029.02	2035.448	953.221	462.7906
57.19191	29.53046	119.0952	140.1682	50.34783	50.20566	49.16066	25.84749	51.55614
21.50248	12.02893	13.5859	42.57744	8.22649	12.3115	9.477272	17.31076	7.537987
227.3293	260.0066	263.304	400.156	327.7285	250.1403	394.5518	207.4125	74.11651
33.12345	122.3871	748.072	368.2515	310.2682	125.0534	215.8505	140.5841	155.7145
705.973	917.6173	952.8831	937.5533	1113.654	1025.24	997.838	568.9118	909.4216
41.94399	16.42321	61.64117	62.82906	13.36708	22.72196	31.69478	31.37386	27.93941
36.80968	14.23417	31.34812	25.12394	10.28448	11.35006	15.80664	29.05198	18.27511
276.1922	22.97812	59.56316	37.68423	118.1361	73.90513	11.81871	107.7217	50.48193
234.9841	123.4807	76.2918	37.68163	90.41947	142.1086	53.09841	621.6352	429.4909
245.8449	4.406675	16.73636	2.886669	658.3506	14.19057	0	24.27465	115.9358
14.0424	8.773912	6.287059	4.818945	7.20233	1.87418	25.40192	20.40361	20.41017
764.3362	1369.814	707.3522	747.1418	843.4608	910.5905	1094.737	1085.346	366.155
30.57731	53.5525	121.1881	75.38702	60.62032	98.54771	104.0144	52.59402	97.70157
12.76332	14.22044	31.32799	26.10294	19.52109	5.664535	16.64193	28.34819	7.542819
55.96617	64.48398	167.1674	120.8147	161.2932	87.15946	87.26472	77.73565	60.15377
34.39439	374.5618	149.4087	112.1148	169.5103	185.7301	400.4129	31.34328	42.97777
34.27758	14.23354	41.78514	14.48663	27.74208	14.19465	20.58669	22.74363	42.93272
130.8268	75.40926	184.9341	173.0112	144.8617	142.1226	126.1733	188.6141	109.5366
34.31167	2.221225	73.11001	46.39761	20.55583	15.14093	15.00478	30.6115	41.87307
123.8958	20.78247	24.04195	6.751776	17.47428	54.97527	9.440837	47.19097	41.86865
1046.26	18.60913	18.82548	4.819071	18.50384	71.06499	7.850701	119.5664	36.5289
47.44661	1.127061	16.69754	2.887322	1.037628	14.22685	0	18.19437	12.87208
85.14124	130.0027	115.9824	123.7148	75.00587	134.5476	147.6523	63.57942	68.74132
63.59728	599.7306	557.9421	161.3998	974.9524	519.2444	677.1379	369.2988	405.8745
182.7708	7.684188	12.55681	8.685256	7.202535	19.87568	15.78815	6.98658	5.397422
17.7781	9.857456	5.241379	2.8861	19.51826	37.94731	4.681427	18.86791	88.86116
156.119	98.3305	201.6306	96.64943	75.0047	121.2833	94.42765	137.5407	50.49098
2.612704	0.023968	1.060271	0	0.007173	0	0	0.709976	0.019174
55.8954	52.44893	91.93167	160.4873	35.96576	42.62653	73.04617	51.04657	31.1619
120.5233	32.80629	66.87532	120.8305	56.51082	69.16389	33.26416	116.3742	60.14118
478.6987	317.9169	412.7171	382.7454	304.1073	269.0825	511.9894	250.6138	271.6705
289.5038	59.02952	638.3551	288.0289	191.0965	113.684	129.3151	231.7872	154.637
154.9866	229.4219	300.9124	179.7686	284.5807	186.6505	242.8737	237.2938	181.4755

2698.059	1961.977	4651.569	4703.238	2248.897	2255.128	4683.56	1956.748	1902.61
26.77801	366.8986	76.28481	116.9501	46.2418	49.25211	293.9336	17.19686	1.099887
1130.964	157.3427	373.0114	117.9049	1062.263	405.5397	101.5201	708.9264	297.4332
31.75542	16.41594	41.78581	47.3707	19.52742	35.0598	18.99258	21.95342	10.76578
1240.474	1077.121	1580.822	836.0566	1837.94	1337.922	1031.13	1277.806	1213.283
185.3882	179.162	144.1956	156.5755	271.2161	136.4335	73.74504	236.571	229.7641
65.88463	22.96594	31.35155	8.684987	61.63004	52.12667	15.00562	40.0796	32.22082
101.6439	40.45789	237.1617	312.2191	70.89771	85.26165	128.5641	117.0492	82.69869
14.03154	26.20676	1.061809	0	19.52235	33.18257	6.268896	34.66624	46.10756
96.38008	8.778518	14.6464	6.751743	74.99325	4.716097	4.677836	92.81271	219.9431
95.26072	93.95968	58.52494	117.9191	149.9888	39.7759	115.0998	41.5676	66.59032
52.16806	52.47366	197.4719	544.2206	61.65259	97.57957	170.653	51.77781	94.50947
38.00447	12.04727	19.85953	6.752329	56.48027	46.45961	6.266123	47.28131	21.48386
31.46377	3.312755	12.54018	5.788299	34.89594	16.11516	7.085292	16.53697	5.3935
60.97906	39.35485	60.60615	67.65611	88.34928	65.37529	65.07339	151.0661	45.11477
45.7448	26.24884	13.60148	35.75402	60.61294	14.19126	23.74048	51.8534	96.59161
255.2194	204.2967	112.8581	133.3726	329.779	720.1991	215.8839	190.9179	248.0302
12.73363	6.585835	31.31207	9.657147	17.46348	4.717677	7.874874	11.7505	9.681568
134.6606	282.918	323.8841	243.5712	184.929	197.0816	339.0296	95.79661	103.101
25.44413	5.500692	54.31242	58.97716	18.50075	33.16152	14.21438	28.26157	21.4928
74.92468	73.19738	45.98526	124.6991	69.8632	138.3704	111.1937	61.26097	19.35749
170.2478	3442.938	121.2194	889.2305	452.0455	622.5262	2743.925	671.9002	17.20709
46.70577	2.221876	11.50557	0.953906	173.4341	6.612798	3.090444	39.4461	18.2588
58.4616	138.688	109.697	68.62049	55.48386	32.19651	129.4779	70.7025	5.398511
141.0301	178.0901	412.7003	504.5515	284.582	203.7064	338.1639	195.6198	284.5383
110.488	38.27172	202.6764	135.3181	52.40541	57.78151	70.58814	69.09101	85.91254
54.68097	69.93568	63.74678	150.7922	24.66823	47.35842	52.32384	144.6562	59.07514
143.5126	103.8047	223.5859	161.4108	99.66213	116.5342	139.6769	106.8181	133.1524
426.641	313.5441	845.2452	588.6315	123.2948	140.2137	494.5375	396.825	196.5145
87.55404	109.1987	20.91468	39.61903	48.29168	66.32574	76.22046	90.42322	77.30022
19.05392	14.21873	11.50509	34.81491	12.335	9.458656	12.6524	33.11432	5.397389
129.5163	76.49508	147.3185	120.8158	110.9586	57.78085	131.7671	180.0147	36.53637
26.70298	48.03684	36.56667	48.33816	27.74225	28.42005	50.88274	25.89837	2.176657
132.9891	17.51493	15.69062	11.58448	16.44875	54.95729	7.056702	32.16221	162.0028
3.918606	25.1556	36.57829	216.6017	23.63866	4.715902	177.4546	53.44149	0.024134
110.4501	34.99336	80.45923	55.08296	69.86674	89.06279	61.86106	158.0442	79.46612
58.51847	155.1442	61.66381	17.38401	172.6022	30.29865	62.62466	94.22273	2034.128
20.42891	9.870723	138.9455	42.51661	34.94065	37.8822	61.07464	25.84502	27.94606
38.10987	76.40926	31.35295	85.08359	26.71811	36.94907	69.16372	31.39613	10.76724
12.7905	33.85685	29.25644	16.42157	11.31107	31.2698	19.79787	20.38561	4.325409
17.84369	6.592293	120.0323	38.66995	15.41859	22.73284	19.80085	27.49575	9.691522
138.4136	325.4505	99.26972	76.34647	194.1658	401.8281	287.5058	143.8019	142.8059
2120.364	3681.367	3836.598	2580.686	3252.618	3504.006	3653.985	2058.934	1993.866
78.72093	21.88604	9.42252	9.651245	441.6472	72.95724	5.471868	283.404	54.77422
333.9688	265.4789	310.3239	129.5039	670.8557	508.8326	308.7492	440.0802	400.4948
273.856	59.01562	56.434	32.84931	78.08393	67.2629	36.43561	349.3859	138.4867
335.0434	109.2678	122.2568	115.9765	334.9046	216.0416	192.0968	224.7769	142.8163
49.51045	12.05454	50.15032	75.40591	13.3668	17.98357	36.48138	30.59697	25.79066
195.5653	558.1162	41.81253	46.37995	100.6913	90.94355	252.4383	143.7544	191.1293
71.16843	72.1212	153.5775	476.6074	90.4116	65.36536	82.5178	51.00689	23.65278
73.52029	19.69602	26.13361	10.61806	22.61085	45.48063	14.20615	50.30678	47.24229
30.56745	28.43645	75.22601	99.56476	49.31909	58.74068	53.94411	72.3012	22.57702
7.659868	1.127025	1.061636	0.954006	1.037619	4.721717	0	2.275655	53.42146
15.00302	0.024945	0.012184	1.921576	10.264	0	0	0.708113	1.099966
12.74933	4.406151	3.152595	22.2359	0.008501	7.562324	3.091112	3.842677	0.022836
55.83782	39.33932	27.17855	17.38509	63.69017	91.94817	18.97513	32.17079	125.5253
10.25595	10.957	31.34037	46.41228	20.55188	36.01852	23.79504	14.86904	13.98068
189.1705	85.23856	160.9074	254.213	119.1802	135.4885	157.9498	129.6252	149.2532

62.32561	72.13739	2011.235	3184.874	888.6635	41.67043	800.9641	21.92184	64.45259
12.81184	4.406314	4.197066	0	0.009087	2.822474	0.720622	2.276458	195.3479
3.650476	0.014182	2.083538	0	4.08884	0.940152	0	0	2.136348
10.24692	7.681662	27.15971	35.77649	7.202037	24.63862	63.81121	5.41408	4.324936
1399.168	1916.049	1616.347	1867.381	1768.082	1436.467	1989.36	2050.391	789.1882
36.73899	36.02334	6.287154	1.919773	15.41763	19.88973	23.80117	16.4512	3.251343
20904.65	53955.54	29873.68	28561.58	27016.45	37587.99	39359.43	22088.53	24319.13
80.0305	47.00496	45.98808	40.58265	103.7632	74.84554	45.17489	102.1613	159.9482
214.6125	676.0787	219.4203	301.5661	158.2211	289.949	320.729	197.2109	53.71651
232.3617	207.5676	238.2227	227.1368	127.4014	251.0975	198.4233	242.0409	96.66044
436.7835	152.9734	828.5247	418.5123	245.5479	224.5484	361.9419	335.5179	280.2557
403.8567	3734.763	784.6778	456.1987	629.7803	3970.281	4434.71	807.8186	1432.303
496.2916	193.3772	273.748	897.017	136.6483	133.5845	488.3019	478.6956	8.617996
38.03099	22.95427	29.2554	9.652622	31.84653	52.1466	6.265051	33.81582	85.77568
62.18057	20.78976	65.81643	28.98656	25.69298	17.98276	9.437949	43.1937	29.01162
5.189053	79.58719	15.68413	16.42313	20.55132	46.46064	37.3744	11.71558	26.84156
40.72511	49.18698	107.613	43.48324	101.7073	90.01395	59.48399	80.13395	153.5026
95.2942	128.9145	150.4564	183.6491	71.92453	125.0677	127.7783	90.31491	71.96277
100.2395	43.72221	32.40575	56.05362	53.42842	91.91901	58.70849	69.14085	57.99214
92.78851	232.6868	413.7294	79.24355	479.7569	202.7657	223.0445	142.9658	122.4258
57.23225	84.13892	177.6108	109.2152	92.46852	58.72856	86.47426	47.06775	34.38924
17.78605	2.221815	4.19726	2.88606	17.46652	1.87411	3.090905	10.15496	47.15295
1687.402	3818.968	2396.831	2377.716	2186.22	2807.57	3666.767	1460.099	1922.992
43.27393	64.48085	255.9445	194.2909	176.6973	129.8139	72.17888	69.87926	80.54519
52.13095	64.46975	23.00494	33.81657	25.69521	68.21385	38.82376	73.84411	81.60588
7.726284	8.775618	15.68576	25.12675	63.67077	25.57851	29.36986	5.411543	11.83703
155.0004	695.8077	503.5999	661.1361	320.5406	406.4932	775.7082	109.9319	182.5542
1113.577	5502.335	3051.938	1617.996	2651.615	8044.693	3257.865	1461.654	1737.255
345.3508	130.0311	336.4365	541.2819	211.6435	196.1249	250.8043	267.1577	227.6414
16.6174	22.97614	53.29228	112.1387	13.36729	49.26282	82.59702	20.34659	30.0886
48.09656	8.775059	3.152887	3.85232	18.49861	17.99089	1.506619	12.50145	12.90869
53.42106	125.6246	105.5323	111.1509	118.1472	89.05849	107.146	60.44161	100.9368
99.10905	72.13189	177.6198	244.5481	82.19794	98.52905	127.7673	162.6657	177.1582
87.57731	17.51736	47.02887	57.98731	23.64027	24.61511	33.26749	62.84204	49.40767
11.48357	2.22169	8.372388	31.91853	3.093074	5.66572	11.06701	15.70553	5.396149
16.58478	8.776016	64.73974	40.60289	9.257032	14.19559	29.3636	23.54099	2.176764
6.398333	3.308476	8.361343	23.23583	1.037543	0.927991	3.903329	2.277527	1.101094
15.32595	25.14106	54.30954	31.89492	15.41953	24.62648	26.16459	6.983393	17.20181
14.00191	0.028493	0.013864	0	16.43888	1.874131	0	0.705541	57.8367
82.48355	15.33251	64.78087	105.3686	46.23713	43.57411	42.81502	62.85794	20.42992
64.75787	27.34346	89.84319	37.68591	26.72138	24.61609	49.97472	66.01143	31.16192
43.19445	151.6804	45.97509	87.97637	44.17876	45.47839	110.5482	79.47211	18.2807
25.44416	7.685103	28.21662	35.76262	22.60843	7.558472	19.78691	22.73953	11.83902
45.80203	92.86104	122.2389	131.4576	87.32868	117.4999	142.1527	52.5848	42.97458
21.63412	4.407977	35.51794	29.96311	19.52608	6.611141	16.61161	28.28317	10.76446
17.77227	21.83143	32.36023	19.33351	13.35992	20.85312	28.67805	17.28764	10.75483
82.53038	50.275	34.49562	68.62075	54.45655	62.52827	45.18559	63.61929	62.28746
77.36964	53.5306	27.18017	28.98613	42.12644	66.33494	44.42391	21.13801	109.4594
7.728873	22.96077	9.421513	28.99287	5.148255	17.98738	28.55333	17.22187	45.0778
34.37415	4.406844	15.69151	4.819134	14.39473	8.505597	1.51079	10.91029	11.8422
22.90179	87.25061	15.68635	28.02762	23.63374	115.7254	65.27771	13.28522	15.05535
116.8498	232.6403	114.9385	135.3146	142.8039	145.9199	230.2956	93.46383	54.78679
41.96188	75.36379	40.75935	29.95176	50.3448	38.83523	77.03085	63.65341	55.83916
15.3515	51.37424	175.5102	180.7618	99.65588	31.24715	193.0221	17.19671	2.174818
68.56282	29.52759	8.377662	16.41731	24.66715	46.41767	27.7079	186.5652	444.127
159.8652	66.65927	66.88007	51.21613	104.7908	121.2909	48.35221	75.40122	102.0027
8.9969	29.50471	48.0492	64.78185	38.01051	85.34197	22.17732	29.84142	8.619715
5.12525	2.216358	4.190016	0	69.65987	0	0	0.707496	18.17193

410.2023	1415.737	280.0329	145.9348	680.1198	386.5749	285.6879	786.6042	4164.701
10.22677	1.127595	0.013862	0.953874	7.200865	3.769152	0	11.74289	0.022748
114.3425	194.4469	136.8816	86.01119	109.9353	88.10291	104.7217	180.7391	406.8623
279.3092	252.3466	218.3744	342.1667	75.00853	461.4841	362.8378	269.5578	121.3527
20.3406	19.67386	21.94369	10.62091	26.70882	13.25094	14.23594	19.62171	50.39681
21.62674	20.77198	9.420564	6.752208	19.52543	53.09904	3.883061	36.19544	15.05341
57.18342	27.34571	72.09545	171.1132	27.74924	39.78012	57.91237	52.60612	40.82304
857.0059	333.2157	817.056	2553.687	342.1206	548.6128	855.7419	656.9552	255.5691
87.63643	38.26933	202.6616	156.5945	75.00255	102.3335	97.63024	78.55001	50.48797
269.2111	356.1216	177.6353	81.17594	299.992	649.1	225.3935	233.353	863.1593
26.69381	8.776284	36.56432	38.66716	21.58048	17.04011	19.7939	16.43746	27.92267
120.6947	133.2963	234.0384	147.875	255.8084	104.2119	219.0975	193.3086	185.7562
17.87763	1444.083	362.5704	1967.942	418.1434	165.7969	1976.098	60.42215	2.172918
3212.269	2455.74	6408.965	8796.593	2505.738	2565.92	7298.448	3178.794	2850.678
112.9045	185.5997	62.69591	81.1912	99.64805	81.49004	151.78	109.3028	9.694642
1418.279	746.1456	6706.696	4421.017	1992.055	983.5255	3253.1	2998.107	2091.568
110.1485	16.42124	36.57556	13.51823	96.5474	34.10188	7.05673	85.8073	19.35274
241.2227	266.5338	145.2423	173.0067	274.3008	221.7228	215.91	161.8453	431.5714
38.15487	61.17279	44.93533	33.81974	78.07291	71.07287	108.8907	21.13595	20.42885
248.8038	85.24057	37.63317	108.2434	102.7447	17.03361	30.86962	91.87235	430.4816
445.2036	223.9094	118.0731	57.0136	169.5124	136.441	36.42808	145.376	316.6757
4.821335	0.014258	0.007044	0	0.004328	0	0	0.74736	0.011453
38.09981	42.5996	7.332656	8.684979	44.17448	39.79622	18.98353	32.19094	79.3951
112.6404	30.60605	29.26519	23.1871	44.1763	48.32845	9.439224	36.12364	213.4029
40.66727	20.789	67.90256	117.9558	45.20607	86.25481	39.65882	58.96481	33.30177
57.17241	101.5629	47.02768	33.81807	110.9429	54.94873	65.87508	45.52792	47.25953
133.3754	76.50299	376.1059	230.0426	183.8991	66.30678	309.6783	107.5986	0.024679
29.31129	38.26839	90.90232	92.78758	76.02887	38.82965	41.20434	17.19692	46.1936
59.72815	0.030599	41.80767	1.920543	251.6557	4.716501	3.09465	33.71321	30.09191
645.0413	407.4978	1115.871	1217.86	329.7931	386.5765	1266.987	307.9752	146.0542
133.4253	181.3736	374.0558	453.3103	260.9576	143.0568	202.3486	72.99304	252.3414
6088.013	6670.2	10200.65	22493.59	11993.39	7445.755	14475.38	7563.186	8354.419
25.43302	7.684667	60.569	43.50337	22.60747	16.09159	36.53477	32.21956	23.63482
81.33598	51.37871	150.4541	231.0213	66.78776	97.58468	141.2956	82.45481	65.52097
49.44134	25.14413	5.242934	7.718468	12.3388	21.77974	4.67622	21.94911	42.93757
30.45594	9.866302	26.12299	25.12778	15.41825	18.93941	13.4282	34.61477	9.691106
17.70986	5.492611	12.53943	13.5323	3.092657	2.822094	10.29036	13.36191	4.321551
50.76603	34.97216	32.39873	31.88917	15.42103	17.03596	26.93362	46.36717	38.66138
80.05838	57.9296	76.28492	112.1163	58.56904	63.46773	57.08182	84.82246	64.44577
52.93488	7.678977	8.373575	7.720748	39.01731	36.04251	3.88521	52.1724	10.7571
38.1755	122.3036	47.0287	104.3972	19.53121	59.68705	122.3489	51.82088	26.87082
77.49973	22.97974	105.528	151.7595	36.99563	49.2541	89.67812	105.3045	117.0273
15.31172	29.49009	32.3839	28.03038	19.52514	14.19715	23.79514	23.55463	12.90836
445.6648	556.0217	502.5615	525.8051	547.5802	543.8915	465.964	287.5688	503.5613
48.19909	16.41855	20.90986	76.3835	23.63634	29.36483	17.39345	31.40586	20.42254
39.36064	6.593266	36.57146	56.07058	17.47452	33.15785	41.28488	25.09571	26.85831
503.9705	108.1854	368.824	213.5983	139.7313	229.2911	153.1321	236.4949	226.5685
35.64603	14.24079	23.00468	9.651263	12.34005	27.45787	150.9626	12.48184	7.546733
222.2197	164.9758	101.3653	59.91182	129.4563	111.7906	38.80778	787.8151	423.0002
33.05843	31.69765	42.83917	53.16514	56.49919	24.62084	42.06431	56.62691	3.250988
16.50547	3.313982	25.05853	23.20724	17.46398	14.20822	7.075312	6.210654	3.250471
17.88006	25.15589	32.40133	408.0995	13.36701	12.29603	159.12	17.99141	27.93862
123.2241	196.6318	193.2937	85.04447	232.1794	248.2658	161.9131	156.3568	131.0076
21.69542	110.3061	14.64679	9.65125	97.59484	434.1889	19.75885	58.8999	244.6711
552.2309	64.46936	99.25496	47.35072	114.0311	97.60009	13.4062	117.9333	79.4593
35.6612	617.9591	43.9003	150.7869	50.35074	148.7708	525.3168	43.92492	30.0946
9151.619	13660.41	12639.27	11379.2	17668.51	15070.63	13236.21	11800.66	14625.83
170.2375	316.8166	393.9056	577.036	331.8428	457.6599	670.8448	213.6818	890.0392

46.95737	6.593362	30.30885	45.42942	35.96061	27.46615	16.59424	73.99719	12.91367
15.33352	9.869393	49.0979	55.10422	25.69014	16.08951	21.3735	17.21566	45.08624
0.079082	4.402089	7.324914	3.853916	1.037714	11.36763	22.35193	4.640597	0.021964
77.4983	66.65928	164.0181	46.38264	58.56749	70.10587	70.60331	62.81168	22.579
153.6776	234.8572	41.81242	243.5748	51.37954	70.09703	105.5116	310.5007	376.8183
356.8314	510.1525	161.9666	187.4977	489.0246	496.508	371.4649	377.1746	575.4953
20.35569	12.04689	20.90283	44.47934	10.28343	17.99227	33.38183	18.82018	9.690361
204.4568	202.1092	312.3974	323.8	155.1388	225.5086	255.6045	238.1026	177.1763
54.6979	86.32497	137.9193	237.788	141.7763	119.3829	233.4802	54.14272	41.90446
66.11859	31.71922	237.1571	321.8913	89.38799	35.98417	136.5189	28.98473	40.83121
952.3189	3551.317	596.6144	664.0073	2580.718	6863.153	5044.239	759.8589	33.312
277.913	98.34168	134.7893	58.94645	174.6504	174.3483	45.16153	143.0044	267.3156
81.19965	28.43422	52.24696	77.3296	28.77543	37.88756	26.91638	43.17633	49.4013
22.93199	10.96173	10.46672	1.920031	4.120777	3.768498	3.883466	112.6896	27.93244
93.95754	112.5047	76.27886	43.48323	102.7344	73.89965	59.48396	59.67181	70.87712
15.2909	19.67052	25.07175	28.03639	26.70733	6.612367	24.62453	5.414609	4.324829
281.9263	119.1099	324.9494	1319.402	122.2671	207.4934	475.5128	1574.608	61.23248
1.306942	0.016315	0.008043	0	0.004938	0	0	3.209069	0.013094
128.2676	91.78668	106.5821	113.0801	113.0147	76.73355	115.0667	106.8324	80.55014
132.034	61.20484	151.4932	9.651567	534.1452	53.99094	45.16779	43.92489	76.25184
6.455366	5.499548	34.46575	37.70914	7.202233	3.768475	12.63863	27.52421	13.97819
0.075416	9.826695	0.012846	0	44.0894	1.875371	0	0	1.101052
27.96473	1.127044	57.44087	80.26185	6.175595	1.874395	30.14939	21.95454	13.98411
5.190983	42.60902	54.32538	60.90061	22.61077	13.24431	98.62524	10.91279	9.694196
20.42828	16.42441	394.9184	1230.558	83.22668	0.929253	424.8392	56.49198	5.396465
54.68361	85.22274	120.1529	128.5548	100.6833	63.47	104.774	45.5016	88.05419
43.18796	102.5991	99.21956	47.36055	59.58194	11.3486	59.57348	47.14894	10.76776
55.96339	30.62652	121.2038	276.4617	55.48749	52.09475	87.26928	107.6316	70.88698
52.10731	26.25287	79.40359	124.7046	41.10213	49.26065	55.5341	77.81351	49.40582
96.56883	59.02552	310.2821	215.5483	94.52492	61.56989	126.1821	158.7381	31.16892
837.8696	505.7885	327.044	303.4864	530.119	458.6013	511.2032	532.8082	581.9416
329.9681	61.21193	146.2844	330.5777	84.25339	134.5386	115.0452	183.0939	79.48088
12.79771	56.76187	39.69969	42.53304	14.39285	22.72838	36.51955	17.21987	18.27548
8.938512	8.75743	18.79165	27.09225	6.172426	3.770574	6.28875	10.98253	6.463638
161.296	20.79478	24.05023	14.48443	14.39433	39.77394	1.514352	36.84305	363.9436
151.1461	122.3757	111.8109	244.5401	70.89877	80.52059	124.5704	124.8919	219.0337
47.00042	32.79569	47.02114	41.55646	56.50364	41.68363	34.08411	31.37643	37.59425
91.18576	28.42477	17.77841	3.852414	32.88047	48.32757	18.97874	44.00618	114.7875
40.61043	21.87269	7.332545	10.61862	33.90455	20.83099	25.35832	24.31271	61.16305
340.1645	107.0867	245.5325	380.8383	136.6463	239.7288	219.0863	229.4728	133.1589
29.08948	15.30481	20.8928	3.85271	16.43999	18.95131	2.297562	14.90259	73.89697
28.02642	76.45425	91.92954	83.13102	30.82977	20.82512	48.38812	27.42952	8.620912
115.9194	20.76575	28.20579	4.818983	35.94818	35.07552	12.63827	52.05855	15.05029
167.692	305.8874	317.6345	489.0798	327.7313	380.9084	384.201	224.6956	170.7441
25.37679	0.029134	0.014166	0	4.120678	2.821139	0	0.706069	122.12
16.60985	68.7925	66.85653	28.9875	86.28155	86.25792	48.42095	7.767509	21.4995
40.68715	20.79128	45.97962	98.60549	31.8564	24.61706	42.82614	41.60667	22.57522
229.8282	101.6277	218.3741	231.0029	424.2853	212.2419	159.5042	229.4562	183.6166
176.5736	229.4285	316.5884	284.1597	645.1663	104.2074	415.9751	433.822	296.3513
30.51261	16.41862	55.36162	42.53032	27.7441	48.33372	41.28687	38.5051	30.0752
15.27921	8.770168	10.46156	8.687651	9.255125	9.458559	11.05526	16.47685	7.542397
726.2596	302.6297	211.074	170.0985	569.1628	69.14823	96.75738	425.0887	1448.367
2.647635	1.127539	4.197479	1.919721	151.8872	6.612833	1.505845	1.486834	9.686833
12.81049	153.9134	9.42257	20.28433	25.69388	9.452984	137.5379	23.49534	1.100619
134.6436	180.2526	204.7856	197.1758	306.1416	166.7605	176.2098	172.8699	267.3307
95.25227	3.313637	7.327761	5.787471	17.46261	7.564307	3.888168	28.42962	63.15359
14.08171	165.9801	19.87075	57.01825	32.88596	68.21404	305.271	73.05784	0.024436
16.41266	2.220112	2.106874	0	17.45363	9.470591	3.896519	8.614798	4.319448

38.16797	36.07759	68.95861	78.294	55.48115	32.19866	82.58839	58.13061	34.38184
314.843	132.2111	182.8552	89.87578	180.8217	119.3712	87.23688	171.2648	112.765
30.49696	19.68936	21.95249	14.48635	40.06424	17.03877	18.19463	24.31744	28.99879
53.29704	8.778185	53.28153	76.37432	20.55666	35.04993	42.85413	31.38744	18.27986
73.98	5.494637	8.370625	5.787688	13.35756	21.81134	1.505842	22.87587	11.82059
53.3242	20.78959	83.56584	64.76447	31.85536	33.15076	26.12954	40.04309	17.20811
68.636	97.23505	123.2882	75.38273	184.9123	196.1686	90.46159	113.1588	77.3233
125.7094	133.2737	198.5008	56.04771	181.8353	144.0284	119.0549	80.88674	92.35384
85.09493	48.09614	68.96817	133.3927	43.15881	66.31571	59.4808	95.08277	46.19396
156.1663	179.1429	63.75095	37.6813	133.5595	350.6451	76.93309	290.925	363.8983
55.94955	73.21147	131.6412	126.6222	46.241	48.30609	65.83225	85.62734	90.19939
628.5604	960.2323	924.6804	1013.907	1148.589	961.744	943.8181	822.7575	1056.522
7.699737	3.313737	4.196626	0	9.253222	0.927665	0	3.057324	21.45441
523.1821	392.2062	842.1376	2391.281	422.2548	490.8074	747.7366	678.9397	525.0603
0.057568	0.020555	0.010087	0	0.006182	0.932097	0	0	2.160118
45.73107	64.4351	29.268	45.42478	44.17942	82.45995	56.3735	29.01526	40.81156
702.1098	322.2888	760.6309	606.0242	342.1195	427.3267	269.0277	731.6558	455.2632
72.43701	38.27051	172.3787	176.8932	51.37749	36.93303	102.3868	90.34295	59.07637
43.19074	25.15387	80.4289	47.36016	26.71938	33.15241	30.11246	51.08835	27.93671
20.41772	135.3525	69.99622	13.51769	50.34308	193.4074	30.10162	33.7338	54.7611
3.803293	0.021129	1.058074	0	1.035491	0	0	0.717522	5.348447
34.1456	10.9508	9.418077	4.819308	64.6743	19.89705	11.0539	15.68326	12.90199
55.89385	12.05577	33.44868	90.8655	18.50366	33.14713	29.29961	58.92376	22.57628
29.23758	21.87182	20.9088	11.58554	12.3388	24.62481	15.00934	25.8926	57.94468
72.39993	24.07073	177.5808	143.0674	57.53826	50.20512	81.75637	63.61695	31.16539
161.2577	193.3459	61.66225	40.5809	125.343	194.2506	75.33976	91.88124	356.4003
1377.579	1842.858	1531.716	1779.424	1501.999	1596.607	1428.078	829.8174	1056.529
17.82465	20.76552	18.81403	14.48945	11.30995	35.07577	20.61632	25.15121	15.05017
104.1597	229.3564	31.36394	26.08244	45.21477	111.802	106.3368	83.24388	134.2141
17.851	0.02966	3.15286	1.919863	21.58044	11.35071	3.090174	15.64917	18.27306
182.7131	9.866234	13.59704	2.88595	51.34993	24.63159	4.676773	59.92459	19.34167
17.80371	32.73326	25.06974	9.654574	29.78499	32.23738	30.23256	2.27034	8.615161
72.37266	24.06928	48.07255	98.59703	56.50916	31.25008	53.14665	47.10045	36.5293
58.51481	136.5705	187.0267	65.71201	419.138	270.0623	178.5918	95.01751	83.77508
78.71622	29.52955	50.16252	115.9989	32.8853	50.20695	72.22666	73.07621	38.67678
262.8782	135.4938	502.5525	638.9067	141.7862	198.0187	247.62	394.5074	152.4923
62.23401	17.51688	39.71619	31.88471	52.40008	40.7297	18.17214	63.64163	91.2467

TCGA-HT	TCGA-DH	TCGA-DU	TCGA-06-	TCGA-28-	TCGA-DB	TCGA-DU	TCGA-S9-	TCGA-06-
152.0556	133.676	123.5587	65.07709	48.05642	317.0734	85.78346	145.0091	91.00041
11.03686	58.72905	42.38765	17.85403	3.083046	19.60282	46.34846	34.87658	37.12484
78.86281	101.2696	165.9894	80.80891	85.66907	118.7505	67.04864	63.30514	79.45351
2212.507	1859.293	3584.668	2239.291	2991.277	4751.548	2222.59	2669.924	2454.852
114.5696	75.95525	64.3966	105.9915	87.15659	169.5474	104.5205	122.9799	168.9906
0.16403	3.039768	0	5.23859	5.702568	0	2.956179	0.891391	6.313001
16.3807	56.70669	3.482518	162.5165	63.63765	2.384311	69.03212	18.32817	28.23142
166.3085	145.8275	214.5273	83.96224	35.00872	126.0885	116.3531	111.0384	59.0133
6739.179	9233.664	9639.984	6229.917	7469.55	7105.051	4955.964	8512.752	9720.65
50.22643	80.00063	49.40823	22.05569	32.06688	49.92033	166.6619	72.51057	43.60823
10.96941	15.19281	11.44045	13.65322	10.29817	30.3539	30.57224	35.8356	10.33376
896.1616	1528.139	1826.363	356.7943	351.2469	949.122	1061.006	1552.051	444.4583
107.523	70.89272	69.68907	150.0609	110.3687	90.02405	101.5608	169.7928	208.6959
0.177717	2.029079	1.728627	4.202125	24.82134	0	55.33167	10.15113	39.49588
142.9029	87.09285	77.65347	47.2378	26.30229	133.5149	40.42335	92.68997	42.36402
11.04693	9.119439	28.22302	214.9137	113.8909	2.384205	0.980904	22.92262	40.97289
1616.963	1792.455	2611.041	1498.463	1484.145	2059.606	1716.738	1882.432	1618.676
46.5461	7.094177	35.28394	22.05326	24.80644	72.94781	46.34427	30.2662	20.59008
36.08553	84.05518	67.05319	40.94315	293.8667	141.7104	26.61788	10.97898	48.76111
263.2124	304.8228	549.9041	967.4732	1235.693	84.27191	285.954	198.2142	756.7911
749.5371	848.632	1827.262	570.8536	615.2097	1203.035	875.625	533.2253	517.4355
148.8744	73.92121	166.1903	0.016137	3.080607	109.9199	5.910522	4.554912	2.671808
219.5091	152.9134	51.15343	4.21652	95.82014	21.214	36.47871	280.0194	16.76777
429.0274	273.4264	272.7421	116.4954	59.67279	434.9056	111.42	170.6887	67.99008
62.79639	112.4069	127.1329	43.04039	53.81148	133.5327	70.99393	62.39058	104.9959
25.18371	46.58017	18.50193	23.09629	10.33223	19.60381	21.69004	71.66175	18.01269
102.2174	105.3236	126.1921	282.2614	542.0001	66.25921	86.76854	184.4708	359.6981
556.453	652.1723	526.0533	1514.171	415.0565	620.7062	1203.989	1121.581	2148.488
92.07507	69.86682	11.42836	9.464288	3.082616	33.56101	3.938483	25.68089	3.957175
64.53127	44.56219	37.03121	71.3614	32.08709	64.66257	63.10551	190.0685	46.18634
71.47872	46.58597	11.42494	27.30036	13.24649	109.0182	28.59106	25.66859	66.58267
48.42014	63.79914	34.39029	23.10421	30.61212	71.26374	21.68802	77.10993	23.15673
45.65381	11.14293	5.250033	14.69866	0.13749	56.79768	4.924808	17.43277	6.506886
611.8435	755.4648	801.4822	959.0909	853.0144	498.6769	4193.786	3190.52	1175.482
177.1951	391.9022	112.9466	387.1785	135.0394	216.1889	457.5458	244.1422	332.8489
20091.63	6741.45	13522.98	1787.04	3660.226	13285.58	12667	4768.029	2809.65
25.08189	28.35478	47.73603	32.52221	54.75915	5.657642	30.56933	17.41927	23.079
66.23567	87.08927	67.07712	39.88962	43.64306	87.65088	52.25866	112.0068	37.22573
30.61946	26.33385	75.95321	66.09842	119.9216	4.020079	73.95938	9.143197	51.22706
14.61553	16.2078	110.4789	53.50964	92.46291	15.48956	29.57872	22.91949	39.72244
66.46674	124.5625	49.38316	354.6282	382.4753	183.4558	40.4228	12.81504	316.0861
62.55938	29.37192	19.37326	43.03183	14.69322	45.01211	95.65542	62.41548	55.07617
16.42313	21.27143	28.20471	196.1408	205.1614	12.20658	111.4332	88.13169	146.9754
114.5335	151.9012	113.8576	9.4645	13.24703	145.7864	175.5235	254.3002	33.41203
291.5304	329.1216	162.382	334.733	155.3616	391.4976	371.7497	368.9787	312.4417
37.2193	14.18086	13.20768	11.55821	1.623876	27.04351	17.74684	31.22524	19.2442
48.4478	22.2841	28.20388	37.79055	84.0614	38.43773	57.1902	248.9231	92.12512
3605.553	4743.414	5471.924	984.2973	1114.294	3347.806	2104.261	1263.803	1088.582
1210.111	340.2659	364.525	82.91899	313.4839	1122.896	156.7786	332.2259	454.6415
2.092782	20.25332	11.44469	12.60316	5.97297	6.483464	33.53405	29.40205	6.506867
16.18734	9.118231	12.33019	12.60323	5.973183	15.53514	16.7618	17.43259	17.94328
164.749	293.6779	264.8087	170.0048	166.9435	347.269	203.1272	149.5799	253.5616
62.69782	45.57425	39.68502	70.30836	33.52328	85.1848	100.5821	66.99337	89.60217
4351.534	5201.152	8629.333	426.051	874.9494	3967.701	4218.388	3586.809	370.1957
58.1014	20.25502	8.783912	32.51602	3.083185	15.51847	41.42278	7.310633	14.15882
21.76876	43.54827	42.34236	57.71721	24.82914	97.52636	60.14934	63.32827	48.70877
5.68168	12.15632	22.05606	9.462166	20.37293	24.56247	33.52941	25.6989	3.956709

5317.249	3917.07	9198.679	2153.256	3201.764	8032.163	3661.26	1461.13	2929.994
184.4359	490.1341	80.27612	521.494	527.7995	235.8206	952.5647	439.6596	542.7903
39.29688	35.44414	12.31275	61.88285	11.77943	63.98885	41.4163	33.03652	82.93431
21.75187	102.2726	44.11792	34.64054	23.37134	36.81051	44.37098	25.67104	21.8727
0.246934	5.068725	5.247056	35.67256	87.80851	1.566909	142.0378	0	34.55491
120.0745	408.104	243.6313	105.9998	217.6163	278.4683	378.6568	195.4865	268.8779
26.76983	9.11878	13.20691	18.89537	11.74708	13.87264	24.65183	25.70253	25.59982
55.11792	19.24478	38.84598	16.80566	8.887512	41.78791	34.51193	56.94248	23.11594
73.42254	113.418	93.57161	65.06782	95.71575	67.12234	46.3408	40.35575	79.41449
239.5884	105.3234	179.1744	456.414	636.0588	67.07914	301.7407	387.3735	527.2341
5.666229	32.39987	48.68662	1.068075	4.530559	5.661679	6.897298	15.59095	6.507827
44.3543	12.15655	25.59175	17.85028	5.986161	13.86524	25.63695	25.69448	16.71936
234.321	313.9297	283.3605	99.70518	130.6994	269.4424	312.5859	739.9072	142.2206
32.34818	32.40842	21.1455	46.17029	5.991226	49.96805	68.04393	80.81905	77.9548
71.72296	169.1128	82.07097	6.316072	11.79657	153.1989	135.0936	58.71548	30.84869
48.23963	35.44576	32.63871	45.12082	24.78992	37.65176	55.22228	41.29773	29.51563
211.1518	259.2475	225.9597	161.6105	75.62108	303.0275	240.5993	186.3003	229.2443
3.896453	68.8558	54.75725	11.56262	19.00892	67.24386	43.38747	26.59737	14.19343
150.4769	74.94404	135.0136	228.762	417.6229	94.10506	233.6959	43.10169	192.1453
239.64	282.5371	243.6297	152.1654	146.6308	106.3988	180.4479	375.4282	73.10511
1386.137	871.9257	1018.61	1152.179	716.7849	2003.977	1308.508	1654.83	840.126
55.54532	97.21352	36.15508	30.4478	10.34644	46.64045	208.0852	115.6915	11.6446
182.6065	165.0711	167.6825	16.8097	5.972783	265.331	338.2232	438.757	6.50947
12.8517	45.57334	154.614	14.71145	26.27486	73.72499	9.854705	33.0158	19.32108
35.99691	91.13757	44.99352	77.64318	189.2769	49.92654	109.4612	42.20042	79.34495
1232.335	1302.314	2838.87	415.5572	580.4323	2067.063	481.1947	234.0076	700.5523
20.01059	54.68899	50.27454	696.4932	338.5778	13.844	92.68894	53.20694	130.5665
119.945	321.0092	86.47123	62.97812	24.85599	190.8485	134.1038	66.9717	112.7387
300.6689	295.7074	528.7465	579.2256	648.3268	305.4173	811.5401	304.6947	894.906
11.06291	606.5443	66.17106	85.00419	176.7648	7.295779	33.52058	39.43427	93.51113
78.99315	123.5511	223.3302	151.1137	460.8441	205.5515	75.9217	262.5127	268.851
32.39859	25.32135	23.79231	28.34704	29.14631	38.4549	43.38505	53.23303	19.31499
952.9568	429.3814	556.9733	264.4508	104.6445	312.773	480.2117	354.2531	147.3837
1434.53	1763.086	1299.307	1583.455	2009.178	2560.847	3235.29	2562.563	3265.265
0.144649	0.002786	0	9.381369	14.85468	0	2.957928	0	2.58404
44.79268	62.78457	22.02572	18.90681	8.894358	28.60947	48.31648	72.53221	34.63865
98.19558	50.63717	170.4829	3.167378	4.533172	43.35187	11.82682	89.03854	1.383126
357.9842	516.4728	717.6235	1162.649	1788.243	798.4549	1359.792	1570.445	1448.137
169.4672	93.16769	49.39298	65.07057	37.88783	95.80957	137.0672	99.12362	121.6067
60.95665	93.16595	25.55091	69.26217	37.87249	90.09341	94.66373	153.3316	89.62356
19.99387	51.64928	84.75768	25.20301	37.85251	70.42871	36.48011	45.87155	19.32347
124.8946	184.296	67.06845	31.49879	24.84436	209.8794	30.5627	21.07535	27.00337
184.0123	233.9232	90.89068	72.41953	200.0335	209.7219	335.2795	56.87455	223.9565
171.941	111.4004	275.3893	271.7839	426.3897	121.1325	205.0982	399.2646	587.5887
44.05503	22.27869	21.18418	3.16815	1.624029	23.76386	20.70709	1.802829	5.233667
60.51635	58.73144	28.22198	0.016028	5.991009	64.77954	24.64802	25.67868	5.239371
32.13808	31.39257	8.780764	8.414254	5.987234	96.99857	42.40564	71.68897	6.517438
51.72857	26.33294	17.61244	38.82799	20.45407	41.76538	28.59294	78.99127	49.9015
40.7664	12.15627	19.40108	13.65562	21.80272	22.91859	29.58373	27.53993	12.89068
94.54929	108.3513	34.38817	40.9371	20.49055	171.3945	58.17649	47.70979	19.32262
12.76419	11.14388	16.74425	13.65597	31.83893	9.760979	5.910711	10.9854	20.52824
1421.972	1349.912	1463.502	609.6837	1533.407	2618.199	1439.654	1911.817	1248.598
34.1854	39.49729	17.60726	27.29881	10.3452	74.56892	35.49495	20.16054	26.98366
350.7768	1665.849	267.4159	1653.683	1641.522	501.981	1413.048	492.8552	888.6105
407.7999	393.9352	476.6694	201.4892	191.6461	792.8592	263.276	298.2735	302.2668
9.195221	7.093461	2.600254	127.7762	7.414907	4.020811	3.938606	13.75046	16.67899
26.69177	11.14332	9.670852	7.363761	1.624024	22.93895	8.869761	20.18851	7.785571
71.759	140.7619	168.6297	77.6632	81.34216	118.7407	114.3829	152.3726	67.9522

16.31335	30.38005	22.0489	14.70581	11.76383	34.42164	2.952488	4.55481	28.17646
43.10121	22.28401	19.37202	34.64292	7.442974	63.05494	26.61874	35.77091	29.5482
14.64371	265.3117	173.9276	33.59931	79.89792	110.5406	164.6764	114.722	60.2794
150.5264	224.8195	264.7865	65.08027	46.6163	382.446	171.5707	184.4543	110.2431
103.8486	104.3084	105.0249	48.28839	92.93373	101.5191	67.04807	72.4833	75.63152
68.16646	65.82798	78.53677	136.4049	142.0671	53.99005	91.70243	183.6072	80.72764
380.7774	421.2729	244.4932	33.59998	20.49654	502.1087	575.8735	238.6209	69.26964
74.89246	17.22069	21.14126	65.05171	14.69093	73.75314	65.08237	25.67144	51.23468
35.65433	20.25638	26.47224	16.80306	16.08771	18.792	20.70458	16.49742	11.62592
11.04929	26.33289	21.14825	54.55364	113.9323	28.62479	29.57926	44.97814	93.19058
236.1282	178.2353	121.773	186.7912	143.7483	349.7365	254.4049	327.6783	197.2512
346.6142	222.7907	91.75782	174.1973	113.2964	281.751	360.9072	619.6558	78.22362
270.3008	502.2918	886.3076	326.3551	470.0349	378.3172	319.482	273.485	317.6461
2638.175	4781.897	8909.251	1660.066	818.3707	2157.844	3081.455	3389.496	1333.162
25.07806	20.25583	8.78189	15.75317	5.985371	31.9635	30.56942	14.66137	14.16932
9.253425	40.50506	25.58125	9.463631	17.53949	24.53792	28.59475	30.28406	35.8344
32.45161	87.08807	89.18221	35.69244	58.0711	12.20651	106.5016	114.7738	25.71601
0.132862	1.015777	0	3.14294	8.159107	0	19.7816	0	2.564604
7.433972	46.56987	14.10062	0.015416	0.137853	0	1.966555	4.555992	2.673707
0.18182	1.017036	4.400894	24.00781	12.6525	0	3.942133	2.732182	6.373839
16.2252	14.1804	4.365579	28.32049	4.532576	17.17309	43.39818	28.47214	34.44642
82.48044	83.04354	46.73689	144.8061	66.8789	128.565	183.412	109.2085	166.4247
39.50088	42.53482	28.2096	65.05227	26.261	81.96204	84.80754	41.28739	53.7888
347.1786	461.7863	842.1405	518.3753	744.0888	687.933	555.1548	706.734	567.2868
53.95756	47.60129	145.6449	80.81351	198.6178	67.09005	79.86716	205.6169	131.9177
16.07745	7.09284	0	14.69236	3.077175	55.26267	15.77726	15.60347	14.10286
1430.514	1160.536	1336.442	57.73377	169.9246	1099.845	181.4301	106.4314	93.60011
105.7518	82.03204	111.1879	222.4526	340.6111	148.1993	284.9787	223.959	216.3827
30.73431	83.04293	169.5157	193.0541	198.5083	20.3951	141.0092	40.35135	139.5472
14.52599	16.20604	6.131104	84.89952	8.872751	10.58222	38.46174	30.29825	62.42206
198.7244	439.5016	235.6597	347.3267	203.2052	493.0787	1428.855	608.5752	357.2479
255.7114	260.2592	308.9725	119.6403	172.7165	313.6968	208.0587	163.3536	183.1649
93.20322	171.1424	238.3851	81.86295	82.82134	100.6881	186.3691	90.84211	105.0634
30.70374	156.9569	68.83636	65.06634	10.34623	89.27396	93.67761	113.833	102.3944
26.90702	14.18186	12.31524	25.1908	10.32735	28.65146	14.78643	61.55726	23.10021
25.31305	28.3593	47.65129	120.6292	79.65573	33.52621	64.09542	25.67038	85.69126
132.2597	92.15615	93.5507	51.43468	20.50309	100.7127	64.09022	95.44299	52.59917
7.402235	9.117572	10.56664	12.59915	17.38482	5.666467	13.80357	8.235996	41.89834
0.221914	1.017485	0	87.87675	1.620982	0	12.81919	49.77028	3.94293
39.57096	67.85018	47.64038	71.35406	29.1752	43.35751	54.23145	87.20825	75.53568
19.92407	37.47003	39.72	14.70929	19.0073	47.52185	33.52457	53.25319	12.9153
21.76128	28.35942	28.20708	75.54258	135.8882	19.5828	17.74355	10.97875	88.25579
105.7884	158.9942	90.87473	253.9343	152.4201	165.3914	222.8509	762.8844	270.1524
12.8556	39.49923	47.62935	95.48791	105.8451	27.77035	81.84243	35.76421	42.35359
708.0014	443.5574	948.9874	293.828	294.6136	700.2425	416.118	312.9541	225.4766
19.97118	74.93512	50.307	34.64023	8.894676	37.63237	134.1221	78.04155	23.14962
69.48214	9.119534	31.75038	88.11111	53.63703	22.8703	11.82701	21.99974	26.9717
66.2062	62.78743	117.4631	44.08438	75.40943	55.66141	78.8866	66.99805	48.71946
25.30122	28.35907	27.32667	50.37051	59.44411	49.12437	45.35746	64.25898	35.91883
82.46702	57.72752	90.01042	12.61294	49.49355	40.87355	60.14525	76.15601	25.73154
55.56866	68.86299	46.75538	36.7423	37.85551	125.4062	142.0047	118.4411	95.96895
32.5288	157.9832	373.3932	27.30354	33.55798	410.3186	52.25544	97.25466	57.74672
76.86361	78.98848	66.19608	24.15408	23.38868	55.65922	104.528	73.42686	11.64478
14.59917	21.2701	21.15027	43.0191	33.41136	35.20151	25.63452	79.0009	53.70304
180.962	429.3811	353.0476	1979.995	1938.648	234.9657	841.1187	2025.798	2209.549
743.7833	933.6883	831.5544	91.3137	27.74576	478.2421	172.5559	172.5163	55.18397
39.51502	31.39699	25.55762	83.92925	26.26528	31.06537	72.97172	85.3881	74.21035
121.7261	168.1047	149.1755	108.0913	97.30391	85.93628	144.9513	108.2863	76.92251

77.10477	128.6106	70.58273	75.56504	61.07475	80.21245	51.27036	53.20407	70.51011
64.26701	32.40924	30.86197	39.88341	32.03391	112.3595	62.12385	68.85439	42.29913
48.62743	63.80469	46.73287	1811.021	2762.807	5.666237	217.9159	408.4217	1524.555
339.5563	87.09586	147.3803	151.1168	478.3473	126.8787	126.2121	117.4534	212.5951
23.56432	28.35997	28.20209	68.20999	65.32134	112.2657	53.24479	78.01705	67.88813
144.8014	23.29516	10.54456	38.82602	4.538205	14.67304	37.47	40.38533	21.85002
112.7145	86.08078	108.5635	124.8698	55.28055	105.626	124.2446	111.9693	96.08224
12.8358	29.37087	34.40574	126.8935	68.00726	12.20986	74.94821	37.62062	39.72206
30.58999	61.77083	85.69899	22.05253	7.443001	47.49889	62.12551	46.8081	19.3092
897.7341	1044.075	873.0016	131.1885	149.6146	290.652	449.6421	780.1445	212.6893
19.89969	65.81622	30.88255	7.365937	11.77956	50.0082	23.66238	11.89972	37.13071
84.13398	49.62559	37.91232	57.72678	32.09297	104.0147	161.7224	172.6021	84.54216
114.6828	147.8543	418.5127	150.0632	142.2522	193.2666	111.4215	137.6556	176.7364
148.4622	135.7009	99.7173	53.53544	43.70589	679.4776	58.1726	77.07018	41.09468
24.51083	6.080007	2.60202	5.263141	4.509948	10.62576	27.6222	18.38196	10.28083
538.3852	522.5466	521.6666	257.1049	538.2262	511.8172	542.3355	512.1343	437.9991
33.94566	15.1944	10.54714	32.52867	26.16785	20.43052	38.45872	32.1241	44.73332
806.7012	870.9102	1456.505	565.6056	574.5874	1093.297	1056.078	435.0166	464.934
36.10806	15.19538	46.73218	631.6628	435.0899	2.392211	105.5034	487.392	334.222
12.85532	115.4445	97.09581	15.76128	75.50884	50.71689	172.5701	368.2488	15.48717
80.58676	58.73919	105.0467	78.70735	55.25668	139.2766	59.16021	33.00887	73.0428
166.6103	168.1102	613.5358	343.1362	814.8376	327.5559	162.6959	248.709	673.3913
32.46229	35.44808	46.75573	81.84275	154.7817	22.85799	55.21749	89.04384	93.41368
205.8164	273.4251	373.4156	159.5129	165.4974	388.2342	140.0169	150.4974	137.1104
1741.815	981.2945	1109.54	683.1335	829.9012	1258.693	879.5676	1396.002	785.0573
62.73399	57.72575	35.2659	73.45777	55.22915	107.3203	90.71898	82.60291	108.7814
10.87416	15.19027	4.368693	14.69178	1.623047	14.7374	20.71108	18.3687	17.89274
259.4721	342.2878	330.1259	82.91857	139.4321	259.5701	344.137	340.509	94.87817
429.2677	223.8076	516.3928	10.51216	97.38897	303.7837	449.6461	338.66	24.44497
737.0007	889.1386	1341.739	544.62	919.7473	932.7592	1051.147	577.2841	459.8145
57.2161	23.2963	22.90787	22.05416	23.37111	31.06731	17.74366	44.96199	52.51404
39.27816	13.1696	10.54585	38.8215	14.66478	29.46163	12.81364	27.52241	35.8508
50.27548	47.59964	86.51154	98.62893	150.5464	37.60991	114.3883	25.66606	78.11917
146.9912	21.2714	48.49894	498.44	1026.799	35.13954	121.2801	73.38976	542.9701
3.886066	129.618	238.522	292.6525	162.1199	11.38733	70.00914	0.888329	16.76669
16.281	14.18116	20.28581	66.04292	110.3739	8.119347	24.65132	10.06657	72.55653
860.2371	1323.573	1116.65	258.1567	322.2186	613.3497	784.9081	975.6517	257.5051
25.36208	97.21708	129.7972	44.0883	65.36669	77.78064	83.81487	115.6627	85.80751
23.19213	14.18026	10.55665	8.411966	4.531906	21.29467	17.74775	21.11033	14.14517
62.87916	129.6248	186.2747	132.2198	139.2804	136.7534	161.7159	244.184	108.8972
32.52781	288.6045	355.8792	29.40259	30.65673	49.88401	76.90892	60.54626	32.13337
11.03314	21.26948	11.43003	39.86818	72.11716	14.67819	56.21304	21.0895	46.02298
177.0819	222.7887	153.5762	146.914	107.4747	117.0629	241.5896	455.3424	199.7476
41.13667	30.38283	14.07798	45.11794	8.891027	26.16207	60.15502	97.3827	72.81924
3.896922	25.31997	14.07929	58.74196	53.53556	1.567566	62.12908	33.95291	37.14123
3.888651	13.17033	7.894553	277.9461	89.85674	5.657334	24.64621	6.390335	29.5545
0.214816	4.054966	7.038526	20.94914	24.20861	3.210027	10.84716	2.722782	11.50476
176.9363	98.23335	95.30366	78.7152	37.90631	131.8352	224.829	177.1519	138.3113
366.8709	727.1069	1065.481	19.95661	82.88354	263.6297	244.539	75.2255	41.09378
3.875706	1.017172	8.77887	273.8395	189.9241	3.2072	15.7711	17.40319	73.08088
2.092035	15.19164	7.020168	74.36963	48.70124	1.565832	25.64205	4.556595	50.80474
151.9068	113.4218	82.94595	104.9398	24.85482	107.2599	182.427	89.01113	53.88433
0.184039	0.003334	1.726692	87.53738	1.59536	0	0	0	0.070632
66.18043	49.62384	34.38862	36.74125	45.07043	78.642	82.83209	87.21196	81.90725
73.39927	86.078	22.01868	77.65351	29.1885	89.27259	162.7117	87.19505	117.7224
915.4169	397.9885	356.5774	293.8309	385.9954	813.2447	333.286	475.4154	395.7609
136.217	254.1862	213.5847	385.1005	166.9737	174.3762	637.9953	469.9489	417.4056
234.4449	256.2113	282.4534	170.0078	168.4195	347.2383	343.1516	213.8322	239.5145

2967.308	1949.424	4464.697	5867.876	7625.94	1596.032	4706.493	3607.926	6571.702
221.3304	105.321	83.82796	3.166711	11.79486	167.1018	198.205	30.25301	34.69196
179.1586	246.0867	1098.197	902.3969	1267.296	94.91774	913.1064	203.7248	498.1379
48.0516	14.18209	22.92425	7.365825	20.4278	32.7515	17.74493	23.84639	10.35497
1166.231	1035.98	1679.789	1585.545	2165.701	1370.883	1473.182	1465.752	1046.272
130.7583	112.4119	83.81309	478.442	139.3662	48.23985	215.9489	353.4068	259.8972
12.82575	35.44503	60.95403	61.88985	47.80791	14.67261	32.53835	11.89876	28.22829
150.2994	83.04438	62.62661	74.52033	53.85644	203.9427	188.3398	121.1355	84.60762
30.21038	26.32829	6.132348	35.65556	28.92118	12.23197	58.19606	18.3459	26.85328
5.689901	23.29657	50.29732	263.2402	467.2191	13.026	40.42535	31.17926	121.4395
85.9988	91.14375	239.3114	59.82798	143.5443	181.8805	72.96527	89.9321	51.32193
123.6035	223.8021	243.6477	156.3581	53.86231	250.6336	240.6026	173.4607	119.1654
7.462722	18.23102	36.22172	39.85646	146.1425	17.15493	56.21811	7.309663	10.34556
3.865903	7.092414	17.67962	23.05961	22.98045	2.384689	36.50335	6.399254	6.488125
37.81001	142.7793	90.05285	62.96592	39.30749	96.6737	69.02377	92.71398	175.1216
28.86454	27.34669	37.92945	83.9288	191.9991	55.68415	42.39835	44.96078	107.366
179.0513	345.3237	277.165	314.7972	359.6781	396.4187	147.9055	229.4425	116.6341
9.150216	18.22716	6.137474	10.50424	4.522665	8.135137	40.44566	31.26579	10.30808
191.4834	143.8047	372.5516	77.67071	184.3095	198.9787	152.8368	284.5349	131.9807
32.24377	25.31958	9.662206	15.75686	18.99163	27.81701	14.78607	15.57464	14.18688
71.60927	68.86401	93.57933	24.15478	27.73903	83.53457	55.21662	30.25667	34.67512
895.9481	1479.524	500.4624	56.68479	117.7005	1405.411	194.2491	588.3064	187.0804
3.889947	31.38832	14.98445	42.98068	57.37045	0.751083	48.33226	10.07021	31.90002
163.9454	17.22107	48.5151	7.365976	8.893874	90.91055	21.68763	45.86602	28.28296
305.904	273.427	360.1446	275.9827	269.8974	348.0467	424.0107	281.7585	476.2851
107.4086	48.61374	63.51575	97.59691	59.63259	131.8501	147.9113	128.4936	119.1089
132.218	51.6512	30.84618	43.04111	21.95269	55.63021	76.91034	44.02498	62.82563
169.9562	116.462	155.3415	96.55439	140.7919	185.0787	166.6441	216.6153	105.0863
1881.541	112.4136	143.8308	153.2236	81.43359	542.9526	536.4197	381.7946	297.173
53.78315	22.28413	15.83903	78.69567	120.1471	40.89677	28.59084	17.40415	42.33222
24.90323	10.13069	8.787735	12.60374	1.624033	13.06225	16.76158	17.43126	6.508023
148.4042	160.0028	121.8024	66.12471	32.10607	73.64842	173.5503	350.7148	47.4909
33.98601	37.46847	53.89759	7.365816	8.885948	26.17687	36.48512	31.20071	6.519364
2.086478	10.13224	20.25767	137.4002	344.459	2.385525	32.53642	19.24245	61.44221
30.62085	99.23407	33.514	2.117816	0.146426	100.8639	84.80839	126.7562	2.67146
110.86	94.18075	56.45701	136.4027	40.78809	101.5424	156.7898	145.0388	131.8443
73.6528	77.98193	31.7265	476.3618	253.864	126.8764	368.7939	255.1541	2555.942
116.1174	120.5069	49.39471	6.316263	5.985782	204.9106	67.04967	13.73189	6.517842
30.55679	40.50788	32.64086	7.366179	13.23368	46.69178	23.66157	13.73496	21.85638
30.40735	8.106598	23.81537	5.267231	11.76846	23.72165	32.54086	18.33506	20.54949
18.08162	24.30597	13.20096	8.414284	5.987359	14.6841	46.35132	41.32709	15.4504
137.7835	143.8021	177.4346	65.07683	110.3436	131.0082	60.14475	72.47936	76.92535
1994.384	3448.193	3403.696	1976.962	2561.956	3527.16	3656.334	3143.521	3380.654
3.886724	69.87644	174.8916	114.361	182.2986	1.570363	274.1566	76.17594	144.5183
230.9748	404.0627	330.1061	962.1968	1041.108	563.4558	919.0248	589.2554	443.0817
28.93993	18.2338	60.87422	209.8247	269.199	24.49138	86.77222	55.96215	149.7252
171.7841	129.6269	220.6827	230.8488	303.0144	176.0533	287.9359	163.3599	189.5359
46.52089	35.44628	18.49316	38.8321	16.13207	54.8876	24.64731	45.88809	58.85849
209.3436	80.00725	46.73231	662.0591	321.9451	18.75961	38.45052	85.32449	29.57354
77.08114	71.90384	80.30165	18.90933	24.85327	101.5333	80.85459	187.2771	34.68878
34.12963	17.22044	23.79595	31.49108	53.62866	21.23036	22.67496	10.97951	61.40169
19.99545	92.15162	62.66092	27.30129	17.5961	63.86229	35.49386	48.62615	43.6124
2.074513	2.030199	0	74.2328	21.39628	0	9.860347	5.484659	6.464582
0.194146	7.088384	0	68.87039	1.604298	0.75326	3.941032	2.728032	2.647447
17.89858	7.093273	6.135152	13.6496	1.623904	42.78027	10.84316	28.48696	17.93176
25.28342	110.3696	26.44589	92.30669	53.6461	17.94778	90.72754	13.73376	53.76473
7.464715	58.72414	24.70706	10.51133	7.432288	17.15298	22.67781	67.0981	19.2646
225.2145	121.5252	121.7855	190.9777	169.7539	182.6214	177.4915	158.7742	183.1199

536.7366	733.1851	1558.054	16.80803	43.70395	930.3279	414.1432	131.212	19.31538
0.259545	0.004214	1.721886	437.4091	233.0044	0	9.854751	10.97872	10.36338
0.096226	0.002008	1.772207	1.055149	0.060992	0	6.92658	0	0.040211
35.45238	16.20545	7.016043	2.118452	0.139626	60.84805	1.966559	6.392204	10.33735
1790.216	1531.181	1687.728	752.3907	998.1774	1701.754	1332.173	1637.387	599.4064
42.55137	40.50214	40.65365	1.067955	0.141058	60.79652	0	8.228455	5.237021
22667.47	30366.38	32391.61	18817.75	26238.28	25300.16	16740.44	23556.69	22866.85
59.24384	55.70153	82.95728	134.3037	280.7609	31.8651	57.18772	119.3257	153.5594
361.092	144.818	236.5512	206.728	122.0164	284.1734	247.5015	254.2302	314.9873
307.4973	233.9311	158.856	280.169	150.9935	189.9602	319.4881	178.9572	321.3623
674.0618	394.9493	185.3216	287.5323	161.2072	651.9117	522.6153	724.1854	545.4999
1958.138	1779.285	564.0024	287.5393	185.8816	434.7807	112.4056	125.7059	1160.212
850.0829	167.0969	151.7862	47.24156	40.81389	445.567	129.1696	254.2255	39.81855
5.686787	3.043258	58.3508	56.62779	122.0521	4.837813	10.84156	10.06469	30.72496
55.42575	15.19544	12.30858	44.07833	17.58451	26.14692	67.05512	53.23287	58.88546
14.53118	42.52682	14.08866	7.364945	8.874081	22.91339	19.71893	13.7425	6.515677
37.85062	101.268	95.33114	54.57953	101.5276	76.95447	116.3581	122.087	74.31651
194.799	132.6632	131.5088	59.83086	35.00832	210.5148	47.32548	144.0924	71.80768
55.59551	42.53651	64.42338	78.69947	81.22021	58.93338	46.34139	43.11357	115.1409
75.44132	467.852	973.0352	41.99486	42.26495	62.16197	80.85176	117.4521	126.8669
93.17906	83.04361	90.00742	67.17385	49.49766	138.3998	178.4811	92.68085	65.4029
2.092532	19.24086	1.718613	52.39463	5.971511	4.841738	14.78897	5.475061	15.40149
2060.411	2123.602	3020.63	1179.469	1890.283	2941.65	1854.788	1451.048	1374.11
32.52271	164.0521	150.0755	31.50087	74.10921	117.0974	107.4797	188.184	65.3966
20.00661	52.66316	15.83814	110.1721	205.5116	24.4929	38.45158	41.27349	124.1325
26.88108	22.28123	107.9608	4.217845	11.76646	30.30324	13.80033	4.554766	2.67405
402.4396	478.9989	422.8137	151.1236	166.9963	555.2821	239.61	194.5488	222.9034
4520.997	3080.588	1970.16	855.2291	668.9524	2141.478	1262.159	2438.629	831.1954
368.4191	283.5538	132.3597	326.347	220.621	571.7013	414.1493	307.4598	505.7297
66.1744	38.48554	11.4249	51.42512	11.7969	97.52095	76.91487	144.1786	20.60089
7.46593	27.34275	6.130538	221.088	24.6997	0	330.4793	5.472805	20.53958
48.58367	149.8754	121.8112	96.54661	108.8402	63.8197	119.3136	130.3332	87.13528
136.0473	117.4743	59.0945	158.4529	101.6721	191.6447	282.0219	182.6487	177.9919
75.13131	28.36002	17.6044	91.28527	33.52397	60.57401	70.99597	75.25965	118.9701
12.65366	8.105475	11.45018	11.55299	5.965577	21.32463	13.80313	22.966	7.77359
32.17389	14.18183	11.43147	12.60959	7.436257	23.71998	42.4047	47.76077	18.00358
3.821978	4.054673	0	8.39757	4.481092	3.213052	5.913401	4.565483	2.660761
48.03551	8.106691	21.1561	14.70767	10.33101	21.24824	26.62177	14.6567	12.90808
0.233362	47.57835	5.251295	63.89813	15.98447	8.954317	0	1.802835	92.41662
37.78749	76.96285	3.483855	90.23054	11.79715	81.92132	109.4601	21.99497	38.49665
73.27288	62.78705	52.9449	47.23006	42.18097	77.00056	74.94228	62.4076	80.63111
125.9784	49.62169	46.77739	28.34607	13.24035	45.02809	66.0698	70.69793	10.36231
39.28562	22.28218	16.73277	35.67717	13.22252	50.83465	43.38925	34.87614	34.57988
78.82905	111.3947	112.9929	96.5416	50.92148	52.35348	88.74444	76.16335	47.47717
28.64128	11.14422	13.20051	22.04483	17.5281	17.14787	76.93147	57.8841	26.91158
16.1398	24.30081	16.768	3.167856	27.37391	20.49577	16.76272	10.07424	5.228494
57.40312	74.93971	53.81616	160.5115	170.794	76.1485	79.87083	133.1217	133.0474
25.3083	78.98546	42.34961	40.93332	43.59055	32.70753	32.53632	18.32393	94.60775
62.0958	30.3816	27.34716	3.168205	7.438862	69.76109	4.924491	5.472368	10.35503
0.258033	22.28426	7.894694	319.906	516.8776	0	26.61834	16.48575	214.7008
25.14394	45.56634	18.50581	11.56096	7.43636	17.14556	15.77274	22.01142	28.19106
150.2364	162.0289	157.1244	72.42066	62.54319	219.5398	144.9513	67.88979	64.12866
190.0388	57.72404	39.69131	6.316702	20.4883	149.2463	24.64643	10.06082	10.36393
41.43557	157.9747	178.3622	1.066952	3.072344	140.0737	59.15969	35.76193	5.233896
5.688521	21.27149	9.659511	426.8369	85.50986	6.47539	6.896598	15.56815	21.88122
46.77704	49.62587	168.6517	104.9339	231.6558	94.98298	56.20149	45.86165	184.2305
19.89133	27.3444	59.20547	0.015926	3.083077	9.754159	28.59416	37.63517	2.673565
0.198203	1.017285	0.839882	132.5077	18.36334	0	13.81375	0.887861	0.075061

619.0813	385.8383	510.1573	6950.589	4108.842	239.0502	343.1451	181.692	4691.251
3.882795	3.04317	0	109.9439	27.37322	2.38329	5.911243	1.802836	9.045989
146.7948	81.01947	125.3148	325.2675	486.8448	44.14498	145.9353	132.1473	391.6135
343.2082	291.6524	247.1506	327.3837	124.9104	230.9229	150.8641	134.8928	211.3313
5.677692	18.23031	9.668484	15.75086	53.22255	24.57289	22.6791	11.90631	20.51491
19.82966	6.081333	9.66513	10.51152	80.53438	1.566584	34.5146	8.227812	66.27856
92.88718	60.76307	67.07364	21.00712	42.20399	49.91109	60.14781	55.05245	29.55825
940.6885	566.094	304.4858	478.5116	254.053	1164.577	493.0291	1277.634	732.505
68.14966	101.2688	59.99044	92.34574	29.19912	167.1495	158.7624	101.8761	117.7843
114.7822	197.4774	270.9688	690.425	682.9274	284.9625	125.2249	101.8435	870.4304
26.91585	31.39344	12.31484	19.94959	7.43716	27.00499	33.5267	34.88233	24.37634
103.996	231.9042	283.3756	90.26087	211.8044	147.3685	167.629	189.981	99.9794
1076.444	1167.621	700.8552	2.115758	7.415927	1467.661	270.1766	14.65418	75.67248
3425.192	2016.261	8145.596	7901.486	11708	2514.883	5181.776	6953.393	5949.446
163.8259	83.03962	30.85099	38.84137	16.14863	58.93206	96.63697	116.5945	34.67277
1452.522	4483.153	1398.158	1278.109	2396.574	2858.893	6174.758	4630.422	1805.655
42.96688	36.45857	99.85701	58.7529	148.5535	1.568304	83.82465	31.18759	52.47575
84.36403	178.2347	308.0931	199.3787	378.3924	171.9436	92.68502	96.34077	220.2648
48.39624	93.16148	71.51591	17.85862	10.34581	62.24419	29.57748	9.14307	11.64383
43.26202	66.84231	137.675	496.2749	463.7254	139.9985	539.3981	618.7573	661.4815
123.5228	73.9303	121.7945	159.4982	315.86	183.4657	79.86692	62.38101	158.7848
0.096829	1.014313	0	13.44109	2.764967	0	9.89943	2.78158	10.68963
42.88814	30.3827	8.777953	124.7811	72.24857	10.57196	53.25098	6.38999	54.98579
5.692408	22.28305	29.98672	93.34506	579.1072	12.21036	22.67522	3.637456	142.8685
83.66553	52.65938	58.27031	32.54143	16.13541	160.8512	89.74043	53.23576	33.36
43.13289	77.97595	49.40622	83.94056	72.53034	61.4014	59.16222	44.03397	56.38656
278.7732	129.6268	242.7605	8.414303	7.43007	353.0707	150.8656	149.5891	12.92104
36.07073	174.1738	63.52638	72.41317	39.33419	58.09507	145.9432	145.9647	53.86624
5.684987	269.3499	2.60294	716.3347	245.8675	1.570578	637.0871	12.81411	161.1428
1037.299	1191.929	508.3982	438.6399	174.2766	976.1681	934.79	571.7768	536.6365
311.399	685.5841	232.1101	538.3062	281.5606	499.5507	690.2505	670.029	358.6094
8130.712	9074.676	21060.41	15559.55	19538.93	8419.419	15079.91	20484.79	18110.78
12.80203	30.38115	20.27315	25.19166	36.22957	32.75846	40.43125	39.47928	32.01699
159.1235	82.0312	67.04484	60.87944	72.6799	162.9838	100.5757	71.56269	128.0803
2.091445	11.14455	14.08025	35.67797	75.04263	4.837414	1.966641	77.17287	165.564
26.8552	26.33041	11.4334	31.47591	16.0808	34.42501	16.75947	33.97169	17.99405
22.83184	11.14084	2.601836	14.68687	11.64107	10.6232	15.77897	11.92638	17.85771
23.49595	21.27066	14.07636	65.04328	121.2344	20.4107	47.33142	50.48559	63.94488
96.72491	94.18224	85.59397	254.9503	186.9875	144.1454	231.7342	186.3438	209.8767
9.187394	7.093376	50.47125	26.22003	41.65228	4.841514	16.76193	11.91177	31.87294
87.54842	39.49868	45.86931	55.62402	26.28731	67.13721	56.20323	79.85304	57.67238
87.7377	56.71427	54.69023	62.9737	48.0288	104.8208	97.61964	85.34519	82.0015
26.84367	13.169	18.51123	7.365125	13.20019	27.02233	30.56915	17.41876	1.387408
366.7894	391.9112	720.3249	342.0943	480.1852	402.8906	323.4263	545.189	391.8922
88.6004	13.16981	13.19518	24.14713	13.22884	42.59675	35.49748	30.27524	30.77438
46.40372	50.63163	23.80211	25.19601	10.33778	63.1421	33.52472	72.56225	35.87477
521.9862	329.1238	150.8984	279.1319	74.17904	262.8412	316.5257	341.4232	217.7717
147.952	49.62501	83.85696	13.66255	17.5993	328.8781	3.938716	2.721685	3.952791
195.0956	59.75387	79.39482	335.7777	1002.73	74.44759	82.82387	1512.012	811.3622
23.48829	46.58311	37.05955	24.1493	7.442618	36.00883	45.35921	67.95221	14.1963
10.90025	6.080645	7.022212	6.31337	4.523736	41.16574	34.52426	38.63652	3.951121
189.5882	22.28362	10.54264	19.95594	4.536654	123.8597	14.7853	28.42748	7.802065
112.901	178.2335	196.8417	73.47247	179.9133	192.4458	110.4353	217.5288	112.7691
21.78674	30.38537	18.48705	66.11462	182.3109	4.839889	18.72937	4.555851	56.40296
103.6408	82.0283	41.44692	190.9325	24.84687	12.2062	147.9172	70.65962	85.80598
182.1537	114.4343	66.16602	30.45161	39.35007	90.04655	34.50652	69.72976	43.64886
9711.686	13771.51	22997.95	12843.87	20357.09	13321.6	11449.21	15204.57	15186.05
304.2427	353.4295	630.2766	172.1104	501.918	923.0774	205.0967	335.9037	344.5245

42.92297	53.66988	23.79921	29.39182	23.34232	37.65566	87.7719	69.79519	43.5353
21.68534	38.48212	10.54472	29.38962	11.78319	31.91604	48.3198	29.35642	14.19112
19.35939	7.092071	7.030959	0.014735	1.620983	28.85089	6.898754	3.640622	1.386846
112.6381	71.90345	53.80738	36.74605	26.30116	81.86455	102.5505	92.69216	51.31473
155.7438	22.28451	11.42733	43.04403	523.1154	11.39131	23.6596	79.81873	97.41842
309.6344	329.1261	503.1337	536.2108	878.875	271.0086	141.9876	169.7628	880.8751
37.3136	21.26806	22.05442	2.118411	0.141077	28.67447	1.966574	14.66193	3.956834
334.296	259.2477	206.533	230.8588	169.8406	352.1868	353.0151	209.2496	298.344
86.06752	169.1171	253.4034	62.9779	116.1221	126.0968	138.0486	111.0415	73.0831
148.4739	95.19576	112.0799	37.79699	52.4028	276.0949	123.2562	128.484	117.8592
2489.417	4457.827	3341.091	154.2732	1527.626	3639.475	183.4023	110.1043	393.251
62.89985	69.87994	150.0491	250.7727	524.3088	93.30446	171.5756	77.06805	345.5071
66.15117	44.56075	30.85612	148.9496	118.6369	30.2404	45.3563	55.97986	154.6055
5.692302	5.068747	4.364531	101.7263	175.6837	12.21115	24.6479	119.4473	91.92268
57.44305	60.76426	96.2146	113.3215	185.3398	104.8362	72.96654	40.3543	83.26265
21.48259	27.34038	7.900811	2.118456	10.30028	29.52583	14.78794	29.38976	6.511439
400.675	400.0115	101.4603	109.1519	62.57458	245.627	433.8689	1953.379	29.56926
0.113542	0.002301	0	3.134613	11.82103	0	0	0	2.529235
50.39338	98.23365	110.3138	158.4491	275.3442	105.6024	172.5627	179.9019	344.1808
21.80081	379.7346	115.6264	187.8122	223.1231	11.38858	1932.895	130.3318	125.496
10.99172	14.18102	10.55216	9.461812	1.623782	32.8027	2.952486	15.58333	12.88868
0.20907	4.054627	0.839028	9.442785	5.89656	0.751546	2.953323	1.804631	11.47605
25.17637	33.41874	5.246907	31.48255	14.6618	33.57524	53.25378	63.38688	29.47828
42.96776	77.96994	67.12563	5.267361	7.442912	21.23085	40.42741	49.56608	16.75349
348.4349	194.437	423.7776	6.314992	10.33472	272.7305	51.26946	573.7416	20.60757
57.48209	86.08036	180.1272	49.33623	191.2349	144.9914	83.81311	107.3817	170.2001
18.17766	61.7711	65.35187	10.51405	3.080878	11.38946	14.78543	21.08116	3.956312
100.3052	114.435	53.80072	89.2054	56.74085	157.2549	169.6058	140.4272	89.70541
60.89802	60.76257	34.3862	78.69698	37.85636	78.62941	103.5416	59.64787	97.2475
162.7703	120.512	76.75444	105.9953	62.55123	99.04137	175.5202	257.9421	112.7518
456.1904	323.0504	498.7133	912.8999	861.5287	228.4143	505.8505	404.7397	642.8165
273.4221	161.0188	23.78241	265.4699	52.41352	170.3179	221.8659	143.1628	157.5518
60.38126	26.33208	16.73258	10.513	7.439617	49.18868	20.70359	27.52164	20.56562
19.38598	9.116471	2.602057	4.215195	0.130851	23.03972	8.871909	13.77166	7.75071
16.42438	20.25909	42.31886	761.7096	226.2987	17.94033	70.00504	14.65104	138.3716
212.8528	183.2976	84.69472	69.27617	169.8024	98.20828	97.61561	257.9171	117.897
27.07557	33.4218	120.1692	10.51425	24.81287	51.58792	59.16503	110.2097	37.19448
2.08911	50.63304	20.26214	86.0087	53.60906	13.84982	51.27693	6.389917	67.7574
18.13649	31.39463	13.19598	94.3763	66.44908	31.09884	6.896611	9.144335	70.2301
268.1835	249.1199	114.7125	189.9363	104.606	321.8958	283.0033	175.2887	330.2897
12.68265	2.030448	22.96972	35.64259	102.645	2.383104	8.870184	2.720032	15.39996
53.74533	37.47284	195.2877	34.64282	19.04034	95.88617	38.45287	81.70303	37.21355
16.27224	14.18104	23.82987	28.3269	34.68135	1.566244	14.78739	10.98615	19.25047
357.7723	234.9466	501.3942	131.1869	265.5816	467.631	133.1133	147.7365	812.9197
0.240912	0.004015	0	156.0917	27.50627	0	45.3694	0.886924	3.956082
16.40174	68.85868	133.4605	13.66145	13.23964	29.43364	44.372	38.53594	10.36208
48.39581	36.45997	37.9273	56.66606	40.7167	91.79698	76.91619	71.60191	64.01339
112.9574	166.0835	434.3521	259.1862	275.6087	185.0424	400.3488	204.6606	201.0924
329.1685	391.9091	297.4509	154.2708	442.4053	424.2213	672.5055	681.0717	272.8154
16.37379	50.63147	25.57112	21.00139	7.441217	17.13487	14.78581	86.35658	21.84943
14.45459	15.19236	7.018057	28.31728	21.73634	7.304016	20.70764	7.31248	72.37176
120.1721	327.1021	112.9347	3907.556	4202.323	57.24991	5563.461	722.3139	3082.881
2.093218	25.31503	49.56542	105.8004	27.45393	0	80.89281	0	5.232907
83.8551	105.3123	39.69267	3.167625	5.989948	26.96075	16.75737	10.97869	9.083137
59.3441	194.4359	276.3193	189.9319	307.3541	142.4575	371.7566	268.9418	210.0023
3.87569	3.043101	4.368457	9.455985	20.21134	1.565927	15.77725	2.720509	21.68768
69.87471	59.75133	40.56362	1.067089	3.0746	30.22913	6.896678	4.556086	16.76728
3.845485	4.055108	17.70558	11.5412	21.44769	0.750994	4.925955	0	19.06051

69.7461	49.62404	38.8051	8.415656	33.51287	126.2383	58.17634	93.64007	18.04383
125.4698	241.0203	246.2638	301.1562	368.3551	47.4195	205.0992	424.0663	297.0734
21.66043	17.21971	14.96478	40.91825	44.87913	29.4609	30.56674	22.00728	85.46424
32.35728	33.42109	42.36174	32.5396	13.23773	58.99927	32.5373	22.00029	21.8628
9.130572	5.068046	22.1095	19.92537	15.93664	0.750762	5.911604	11.92085	9.032242
21.74515	58.73475	22.02536	14.71084	13.24213	46.6636	78.89057	71.61179	39.7459
23.58751	164.0512	209.2736	44.09095	36.44904	60.54364	92.68806	128.4996	69.22756
150.1726	108.3592	123.5702	90.25427	158.0679	148.2393	114.3821	179.9128	102.4928
114.3799	98.23066	37.02856	137.4487	53.81113	88.42961	96.63411	163.4132	147.1596
70.02703	133.6758	59.9792	240.2768	710.792	140.8435	165.6597	25.66328	212.4934
89.52702	64.81531	53.80635	83.95495	124.7103	133.5169	103.5364	71.57035	168.9114
615.5068	1044.08	2506.965	1270.746	3776.762	950.7503	815.4731	1011.422	1687.697
2.089856	39.47936	9.681389	26.2087	52.79565	0	21.6973	4.55767	10.30566
1192.871	772.6815	564.8913	674.7357	283.0711	859.0348	756.3097	1082.106	544.3263
0.150834	0.002877	0	25.99787	1.556823	0	0	0.893368	1.352448
28.8446	62.78456	64.45791	66.09823	118.4786	46.66541	19.7161	31.18389	47.39924
363.3135	564.0675	336.2696	2016.741	504.9213	295.5695	1775.924	1392.397	654.3756
132.2599	78.99219	53.80492	90.2504	63.96148	176.1476	122.2728	124.8284	90.96081
46.53094	30.38377	26.44608	19.95522	19.02465	21.22877	27.60588	27.51101	14.19909
35.96795	82.02336	110.4251	24.1524	8.894915	192.8251	16.75747	8.225319	25.70798
0.156206	0.002954	129.4886	41.58787	19.0141	0	0	0.89252	1.356438
9.200319	36.44913	22.07341	25.17616	34.58416	16.35349	16.76136	14.66946	26.83011
85.67043	31.39747	11.4249	97.56982	19.04167	132.8184	78.88731	44.9551	49.98919
14.58902	18.2323	23.8062	41.9676	62.12413	8.933719	60.15731	23.84458	57.496
71.63389	44.56212	40.56479	41.9896	26.2937	102.3931	94.66339	68.82467	57.6879
193.0888	66.84202	112.0747	287.4915	333.3078	211.3159	36.47846	21.99215	321.2203
751.4295	1180.793	1565.03	1288.59	1785.719	1026.083	1840.987	1504.301	892.6239
14.51379	27.3415	17.6318	1.067989	5.982865	14.69384	22.67874	54.23129	14.16184
71.79082	55.70243	29.07872	310.5531	367.8409	158.8908	71.97839	24.74553	254.6442
2.092527	12.15688	0	205.4185	52.00416	2.383456	262.3901	10.98247	14.18117
12.77927	15.19396	27.36169	40.90686	40.46819	17.97389	22.67769	7.309354	24.35628
7.440592	28.35196	29.16263	8.412042	5.976993	15.52804	18.73418	12.82886	11.60365
64.456	42.53632	18.48768	92.33191	29.17943	61.39885	74.94121	77.09959	69.16204
130.753	208.6129	484.7417	39.89607	77.05731	128.5272	144.9489	128.4737	62.86116
87.56563	71.90139	51.16918	66.11357	19.04719	94.20588	65.0787	143.2355	53.84292
439.9062	301.7824	149.1314	379.8612	168.4413	603.6301	425.9813	514.9144	515.9972
28.90025	15.19565	34.38715	71.35417	147.554	32.69626	47.32809	33.93222	92.13179

TCGA-28-	TCGA-28-	TCGA-19-	TCGA-02-	TCGA-15-	TCGA-06-	TCGA-06-	TCGA-26-	TCGA-06-
88.85761	49.8728	73.62847	144.0338	74.9449	105.9015	47.28903	122.7017	70.16923
12.01092	12.57265	10.69522	4.672132	15.0087	10.41627	44.54976	32.24549	21.03174
74.0447	67.77599	18.14068	66.8684	53.53876	107.0241	103.9107	92.02352	35.93471
2524.009	1943.244	1487.78	1793.978	1452.417	2330.227	1571.701	2919.74	1320.836
68.17422	113.3006	107.6054	100.2699	149.8385	59.89167	193.9351	126.6557	159.7464
1.607952	27.42649	0	1.198107	0.016777	11.33154	0.904662	0	7.077252
70.70003	53.77301	62.9962	38.03003	25.70459	37.98199	26.89002	10.92763	51.80597
54.89656	34.68961	28.07597	78.39812	39.63074	38.03771	54.71272	172.3169	62.26777
5365.941	7975.663	3970.814	4092.58	4395.736	3490.185	6382.445	6108.517	2767.213
35.63542	36.02534	47.15617	40.36798	58.8738	20.77376	38.94765	82.64372	32.43231
4.62751	9.792875	11.5409	8.12032	10.72484	5.813072	24.1288	18.86306	12.25346
415.6715	263.9465	269.0118	609.5483	264.3938	252.0723	963.0662	1250.207	476.5244
224.6545	328.4341	287.3833	226.9479	337.0931	201.3622	138.233	73.87894	181.6765
5.872226	8.142913	10.04916	6.879742	11.71092	22.62927	12.13071	0	6.160034
40.10623	42.95576	91.88216	117.5149	76.00632	36.8827	45.43666	120.3914	52.61856
67.75327	51.0205	40.57163	25.38471	57.77198	149.2276	36.17999	21.96783	59.72785
1795.087	2128.239	1530.022	1745.572	2019.659	1445.34	2059.75	2612.132	1839.558
23.81608	13.96544	26.44182	35.74781	47.09656	11.57014	36.17086	57.45685	41.23424
87.31465	220.6719	175.6019	18.5004	59.95834	215.0729	84.41849	14.857	163.2872
490.9945	1514.655	523.2363	894.0567	215.1587	2350.6	185.5331	253.2599	668.7723
434.8823	361.9893	520.7275	494.3352	556.5767	244.0169	662.4437	1037.761	487.937
7.594577	0.123721	2.415671	0.04966	0.022983	1.206528	0.901395	13.28758	0
10.55013	0.126653	0	86.44609	5.379372	5.813711	1.82916	22.72452	152.7328
164.2761	65.07447	25.59412	161.3537	31.06864	46.09419	94.60768	236.7819	137.7487
40.09883	60.86735	28.90775	70.31421	40.69716	51.82966	41.72558	58.94979	33.30255
23.75757	18.06879	38.92998	28.822	88.74201	12.71404	32.47051	50.42958	15.75676
412.1959	665.0076	333.7332	268.4338	495.4756	297.9972	429.6347	143.9286	192.195
514.7018	321.9413	707.0392	540.4163	1215.846	305.002	2583.146	1851.447	853.951
16.43046	0.12229	9.036196	8.12856	8.591552	6.967489	8.320255	48.03545	5.214426
20.90146	30.53169	146.641	43.83172	120.923	27.67767	95.56997	49.50572	80.73754
14.98624	26.37842	44.67325	47.26747	96.29977	43.76043	25.02418	46.37853	50.00727
29.73229	18.10877	33.89404	84.07108	67.42563	34.56629	55.66264	65.30708	70.22238
6.09426	2.914632	8.220847	8.117166	6.44808	3.513044	18.55413	18.08592	16.66375
1571.458	1723.282	775.7759	1788.053	1224.402	967.7467	999.2652	674.2318	700.3529
72.64539	132.6917	194.5489	150.9718	191.5924	219.7937	303.4133	362.0134	210.6297
4026.402	189.3458	269.0208	5674.519	4590.535	2858.448	6692.327	11350.07	4148.651
38.29575	33.08448	7.382593	29.94522	12.8663	63.12563	11.10833	26.74847	18.40481
40.06534	12.59222	35.54498	43.82403	11.80339	8.118755	42.65934	92.09262	38.57901
38.53088	48.35884	47.18104	43.80043	16.08255	122.9567	25.95569	23.53	74.64131
23.8032	38.70997	19.8109	55.2799	9.662165	93.07895	21.31576	21.96022	34.20783
88.86168	293.9258	274.9728	84.15759	87.78615	480.7481	204.1327	36.10044	279.1638
41.49637	53.88386	65.42335	57.61078	41.7555	16.17172	51.9519	65.32093	56.1665
98.87907	110.2808	64.57774	109.3768	171.1517	162.0648	64.94636	25.88461	113.2887
60.78516	20.88025	12.34637	36.93244	29.99824	13.87283	45.43478	97.52299	33.29965
213.0101	254.1533	447.1675	188.9941	312.5237	195.6569	393.4138	334.4037	273.8237
6.103713	11.17076	33.17011	13.8663	38.49694	5.814073	10.18131	15.68843	19.29305
91.54896	66.30014	81.99329	270.3543	251.3512	71.33998	129.9476	30.61044	49.12511
806.0368	319.1881	471.0319	1046.237	661.4745	502.9294	977.8902	4044.087	862.6884
553.0156	47.11676	54.57168	278.8782	93.14654	579.9638	198.5242	298.9078	148.2727
0.145783	4.294507	25.70788	9.265353	11.79078	1.208222	19.48437	3.846932	11.37762
6.094465	4.294602	16.54147	15.00161	33.14152	2.361898	30.64935	29.19106	13.13903
109.5929	175.4861	399.9689	182.0779	199.0883	132.3823	245.8671	317.1013	151.7996
31.22449	38.79046	86.95319	34.61764	74.92188	36.8727	67.72479	107.069	74.59898
2235.811	1313.553	346.8424	2658.121	2180.223	1542.025	790.4695	7976.448	556.3844
16.36323	5.676865	14.86123	5.822004	24.61528	1.208025	14.82744	34.69201	12.25021
38.57096	16.73023	27.26099	34.61105	38.5495	20.77266	38.94931	92.91358	42.97763
13.44936	0.119417	9.042952	13.87016	15.00288	11.55742	10.18019	32.29882	9.609405

2598.057	1613.24	3911.222	3404.721	3007.56	1192.209	3575.791	5271.412	2847.089
297.2191	426.6446	340.2993	192.4538	591.8241	124.3343	744.1936	346.9855	542.4653
7.590671	12.57403	35.60051	20.78091	33.18324	10.41665	69.64065	53.58142	35.10408
60.59199	112.926	111.0866	27.70097	35.33706	94.27563	16.67163	41.66207	47.38062
51.51354	201.0967	102.1745	17.32622	2.168604	58.58292	69.6584	47.28999	51.84423
94.80502	168.5598	111.7171	119.8737	102.7732	86.36141	184.6258	341.547	179.9018
38.23265	23.48648	50.65675	13.86737	36.3633	19.58184	25.05288	14.10515	17.53071
26.69804	23.56485	10.69486	12.73143	15.00897	5.816704	26.89438	45.67734	25.42809
34.18638	33.28946	28.08155	43.83187	50.32396	21.92682	84.42916	89.69564	26.28001
201.1106	296.8132	421.5681	346.7289	349.9514	211.7336	416.6469	76.23436	111.4244
1.662792	2.914856	0.763388	1.210223	2.168787	0.046831	7.396411	7.002038	5.217475
17.85501	19.41143	15.68318	12.72433	20.34835	8.114385	29.6937	53.65987	14.88426
102.198	106.4783	27.24856	279.9628	72.80918	76.00879	175.3431	295.0752	83.32911
13.49829	18.09241	34.74594	34.59052	79.16056	9.269054	78.90683	30.63737	60.58951
75.50918	12.59191	5.726122	88.7375	11.80321	12.72228	27.80426	208.6587	60.52272
44.35806	23.59228	35.57839	48.38121	48.15896	24.20922	33.38917	48.79629	23.66013
177.5378	139.6046	63.67939	114.1197	81.37125	93.26708	177.197	202.9552	128.9758
16.43724	22.20923	16.49671	10.43197	5.381013	23.05716	15.74621	19.5995	13.99532
171.6444	654.1967	195.3604	284.5869	183.038	569.4779	215.2414	58.9165	394.9851
108.1011	88.53295	202.8332	92.23094	172.3307	85.21138	123.3775	250.9958	128.102
446.733	1332.761	2120.446	593.4281	1754.21	464.952	1635.747	1976.441	1248.017
32.68664	11.21084	23.11541	35.76411	24.64374	20.77351	43.58998	227.0369	26.28414
63.78368	7.051029	26.42115	233.9075	13.94237	56.4502	28.7314	384.8046	131.6081
17.93816	9.829469	8.206169	85.22199	5.380643	28.8198	22.23963	42.43751	14.86874
62.11365	124.0072	49.64978	54.16945	74.91225	86.26447	99.30745	38.49523	55.28022
507.3428	316.4268	423.0148	328.4351	203.3872	670.919	246.7681	766.2608	437.0251
211.0452	70.52133	32.22036	171.5985	51.39753	140.3497	102.9863	38.47001	554.2925
35.68919	34.6887	50.43682	4.667514	36.41993	28.83294	141.0247	106.9587	69.29294
601.6948	700.061	544.8118	566.8497	467.7217	1017.093	195.7428	256.4245	611.7549
203.7142	211.0161	464.1634	50.74792	49.25827	108.1698	138.2589	51.85532	174.7115
243.9032	165.7819	197.0471	649.5369	433.406	549.8258	108.5321	131.3391	123.7169
47.35475	31.86618	27.26843	39.20204	24.64063	31.11088	26.88377	59.8139	36.83575
396.3866	149.3178	310.4259	214.3654	172.3475	96.72597	236.5657	1028.427	160.5599
1137.177	1132.582	827.892	2154.56	2894.061	2123.069	2804.816	1648.277	1189.196
2.968869	13.03182	6.747532	3.447363	0.015213	36.79841	3.719491	4.789229	14.33969
76.71301	52.47951	57.14084	86.3343	57.79059	19.61871	51.95748	50.34828	58.81467
12.03297	0.125396	4.897349	17.34689	3.238535	19.62494	5.537133	104.7033	18.3794
1088.133	899.0509	463.6038	1746.559	794.1638	1016.06	972.3619	526.3018	580.1072
74.01679	38.81246	48.79271	57.65163	95.25905	38.03069	112.2725	86.52495	48.23068
7.592567	38.79889	72.01509	27.71233	64.22927	33.42638	154.0676	76.30429	55.26439
26.79382	67.68242	18.97185	30.01066	20.36429	70.19396	26.87964	25.88248	42.09505
4.627262	5.679706	0.766519	6.97638	2.16718	2.358706	23.16563	66.05727	0.833869
54.88598	63.65968	91.86345	92.20882	33.20901	111.6404	128.9631	121.145	108.8114
582.2577	724.6625	322.0728	403.2339	608.9492	579.866	192.0383	198.2118	334.3907
9.033993	5.673622	5.727373	8.119803	4.309746	1.208152	0.900606	19.65782	3.460343
7.592833	0.122689	0	11.58302	4.310405	15.01583	5.536472	12.50364	13.99543
3.150801	9.812173	10.69937	19.62011	19.28116	11.56128	24.11454	24.36566	24.56095
16.4388	15.33453	31.43285	42.62854	69.53259	36.83583	49.18377	36.961	38.61026
23.67878	4.298895	13.19583	25.34749	25.68629	12.70427	7.394156	22.80233	9.609764
45.94128	56.66792	47.15761	26.55642	11.80332	16.17321	38.01974	73.18329	9.601907
33.89804	8.431321	13.19466	41.40787	28.8921	71.12353	17.61333	21.21685	18.40736
2071.455	676.867	520.7126	2057.769	1117.409	1148.454	1165.322	1287.13	1038.237
26.77241	8.447755	16.48944	19.6456	32.12864	8.119031	32.45361	48.76049	16.62611
677.1672	1007.988	723.6531	584.1608	656.0903	1146.012	1064.248	322.5119	806.5969
183.5174	174.1545	120.81	232.7851	225.8546	278.5095	215.2305	275.3216	308.0352
51.14684	138.794	119.1737	35.62592	40.61423	21.85397	29.71647	5.423172	36.94248
6.099552	12.52983	13.20472	15.00842	16.06629	5.812881	0.90061	29.96073	15.77628
72.58239	100.8669	57.07008	72.62881	55.6799	79.436	81.63129	124.3127	42.07947

16.39582	29.00053	20.66635	6.974864	34.2397	45.95904	21.3272	5.418491	29.84376
28.26044	41.5233	41.35615	10.43451	32.13151	16.17277	25.0242	74.76964	42.09895
85.86241	20.88015	10.69101	27.71711	21.4363	51.83733	120.6143	125.8854	31.54424
75.61504	179.6657	145.6552	86.47827	127.3933	72.56055	128.0103	471.3252	137.7454
94.72226	81.57986	88.55289	46.14638	65.31097	70.23968	61.21142	82.55845	73.68797
97.62801	110.4856	97.68414	200.3775	48.18833	133.4596	97.41462	196.8308	116.7338
26.81755	5.666854	8.213135	58.82677	103.8466	70.25811	35.22592	189.5612	10.48378
28.24125	34.61974	77.88496	39.2034	59.93161	27.66475	34.31134	68.49124	35.07704
22.25964	27.63973	9.869324	18.47223	11.79848	20.74316	13.8936	50.47197	10.48583
56.05891	83.92732	80.44311	52.96827	108.0045	60.93336	133.7462	24.33249	75.55968
136.1785	63.69031	115.8536	146.37	208.7175	81.76193	162.35	228.1489	204.477
71.16534	54.02312	207.8085	124.4795	87.79078	70.25481	162.3545	538.419	173.7577
245.6037	292.8906	225.9718	277.719	238.7001	438.4366	278.3242	285.5405	358.9392
1694.63	679.6479	484.2792	1722.553	653.9826	899.9255	899.9527	4551.569	1031.2
10.52125	13.92531	62.2825	17.31811	40.6439	9.262535	24.11802	24.37614	41.30035
28.13808	13.94219	14.01562	21.92422	9.660491	24.19116	27.82734	12.51035	28.9535
45.93994	13.97183	55.45155	73.72848	46.03797	8.118932	32.44983	105.5248	20.13667
1.561362	4.038815	5.058863	4.548707	4.257646	37.73708	4.670442	0.727958	6.242082
3.148619	0.116988	0.763391	0.047289	0.021941	0.046839	0	1.488954	4.338764
11.47646	0.093472	6.64833	0.03873	1.0942	2.344145	3.698564	5.510064	1.715547
9.034575	15.26433	11.54162	19.59633	39.55487	18.42623	27.85031	26.78929	8.73376
82.92765	106.4013	272.5403	138.2597	171.2339	57.59039	193.0096	196.7695	163.2631
25.29821	40.1226	67.09225	30.00167	64.20937	17.3207	86.3191	65.33204	39.47073
483.5542	407.4905	299.6658	841.0314	437.7637	426.9437	553.9054	340.6174	395.7984
118.3537	390.2911	189.6407	399.5762	221.5232	273.7635	102.9728	62.86582	139.5435
10.44852	5.658682	9.896378	9.255291	20.31971	10.38787	18.56674	17.32762	17.56507
68.21488	19.47855	48.77954	574.9784	47.12155	130.0966	198.5217	339.8045	221.9954
304.3552	158.8662	197.0587	596.5343	214.0526	317.5151	218.0456	299.8638	114.9411
85.85445	81.56767	52.92825	177.3786	49.25953	228.8816	42.65148	32.95608	201.0499
20.76494	19.39909	59.79781	48.28732	105.7678	17.29365	79.92101	29.92212	47.47443
195.2997	105.1133	110.8782	163.6573	114.5493	148.4943	288.5464	340.6872	293.1314
105.1494	54.02438	113.3718	163.6419	89.93189	64.50326	182.7682	191.9448	101.7644
133.1122	98.14531	46.29563	131.3616	41.77077	62.19376	110.397	128.223	61.39127
17.94582	16.73498	42.17102	13.89137	22.50534	13.87326	49.15472	109.4148	52.62935
23.73712	27.65349	47.26048	16.17691	52.40782	11.56309	46.41738	30.68088	30.71614
32.66506	36.00348	72.06442	67.95834	66.35072	86.24581	101.1758	39.29257	95.72909
32.72582	33.30171	10.69064	44.99177	50.32887	28.83131	87.20261	118.809	42.08062
22.05318	12.48624	19.06537	5.815418	9.650304	27.53692	11.11939	28.44874	11.38632
73.36832	315.5537	44.24846	78.78374	4.306123	4.654723	10.19773	7.819229	14.05406
43.00202	53.92219	80.33115	41.51928	104.8614	32.27124	104.872	90.52839	62.30438
13.49192	19.45855	12.35087	28.83406	24.63494	28.79743	27.81933	40.91437	11.36006
75.31703	112.975	81.1861	11.58636	83.46215	94.29129	111.3882	19.58514	70.22823
235.0792	120.2689	270.7688	197.0355	256.8637	112.8191	509.4503	317.9297	302.8264
84.30683	314.0502	302.559	232.5371	102.742	132.2795	45.43971	38.47537	97.42634
263.3413	136.8852	376.688	701.6297	572.6028	112.8352	493.5974	445.298	322.9483
37.06973	44.24563	7.378343	23.0977	8.592285	3.512956	30.59717	34.56562	11.35779
98.65416	85.38958	87.04769	27.69444	48.16339	66.69691	40.81623	21.16897	51.78882
63.61136	36.02678	25.60087	56.47739	38.55139	63.29998	56.5871	77.90923	25.40528
75.26303	89.56949	70.41634	102.4325	111.2495	73.60382	43.59768	25.10519	67.60331
100.6214	5.675136	8.208806	57.66232	113.4582	50.68821	76.9891	50.27348	108.8154
28.27019	33.27455	41.34948	69.13484	57.80581	42.61732	78.86974	110.2394	58.78745
195.3075	49.88377	63.67794	184.3933	71.74023	35.73697	151.2091	659.4966	73.67087
25.32048	25.00665	26.42902	28.86014	21.43441	17.32391	33.37704	14.85797	35.06647
28.18376	26.32339	38.08287	52.95884	68.45692	59.77516	51.97598	21.1803	31.57948
727.3916	1883.044	1093.017	853.7119	2122.24	861.8365	1395.519	315.432	606.4637
131.7986	47.1177	210.2354	46.15148	67.45922	72.5607	112.2347	728.616	377.3679
57.66509	169.2289	126.8502	41.50753	130.5017	26.51755	103.9627	64.5352	73.7497
96.22639	100.9042	87.71515	104.8801	67.45338	39.18765	76.98587	151.8497	61.39111

54.87543	85.70608	21.45249	46.14504	25.71727	40.33525	81.63128	133.7663	105.3064
29.70997	27.73873	34.73361	32.30077	57.79188	20.76866	59.3863	39.30012	46.50468
1052.235	1138.893	704.6627	1168.104	1989.467	2205.39	879.6256	69.93059	699.5457
422.5796	288.5831	236.7934	260.382	195.8738	352.0639	139.1523	121.8824	150.9266
26.79839	23.63008	31.40037	54.18129	126.2563	72.49873	119.7231	58.18847	111.5102
12.01754	31.81588	40.57332	18.48519	35.32509	15.01504	31.53573	40.1284	29.8199
74.05445	56.75248	168.1277	194.643	117.7341	61.0364	135.468	154.2545	105.3078
114.7134	372.9853	146.8859	141.437	105.8811	54.06227	38.03229	31.42672	50.91534
4.632925	2.91362	9.862885	18.49072	19.29071	3.513422	21.31514	38.52462	15.75012
436.3258	194.8917	348.5085	1440.113	202.316	181.878	510.2826	682.9058	496.7252
10.54	13.94977	13.18261	6.976592	4.310458	4.665705	20.39167	29.082	13.99769
45.99647	26.39775	36.36463	68.00964	44.97643	47.22897	118.7732	110.9595	86.00079
170.0842	251.2702	65.33987	142.8987	99.56025	107.062	154.0067	143.941	104.4039
29.77773	22.26146	19.79667	35.78213	31.06887	48.39095	62.13716	59.71517	68.41465
7.512976	5.64567	6.573662	4.661955	14.97612	3.508827	7.403948	13.38155	3.464022
312.1311	360.5573	326.9916	672.8536	588.6638	438.4506	323.7869	524.7658	296.6096
29.5963	12.56662	24.81261	23.07183	54.54576	9.26553	36.19212	33.84129	32.47516
423.0476	218.3714	187.0432	411.3816	163.7862	212.9477	369.244	985.8402	296.5992
511.3832	386.6483	568.9068	687.7103	148.7961	939.8917	308.0318	193.4885	681.1735
40.09721	31.91621	2.418543	50.7436	13.94409	85.16788	88.13717	63.67794	39.44946
118.2172	212.318	46.30717	116.3496	107.0256	213.8867	110.4166	25.87464	134.316
532.1409	455.6916	86.86157	670.4701	239.7644	891.6316	434.2267	298.9443	236.937
159.194	125.4433	185.6993	240.4836	107.0021	155.1988	67.72802	44.00495	63.18146
133.2327	153.4099	119.9922	161.3468	98.49483	77.1608	97.3926	223.417	114.051
573.8677	530.4716	630.015	1167.169	1031.779	628.344	921.3071	816.6208	622.2188
65.13205	51.19799	31.39709	58.79196	49.25287	78.25574	60.29427	87.33996	77.22744
16.24236	4.286839	23.251	6.961339	32.04804	11.53074	20.43016	18.12573	10.50785
167.2412	218.3043	110.0483	370.9926	149.867	202.5686	87.18427	705.1402	126.3356
56.39361	11.19422	44.63595	478.1462	101.7085	89.8213	99.24544	252.4938	82.44687
622.6084	377.1754	383.2781	740.8757	567.278	296.9473	762.6546	1967.129	471.2622
26.77137	30.49505	57.13239	34.60358	70.62521	25.36709	51.02569	50.33945	36.83382
12.01065	18.07016	56.38342	21.92874	29.97543	6.967072	93.8124	38.56821	35.98655
65.12194	42.92825	96.89804	102.5112	83.48114	97.78595	65.86589	45.57245	88.65332
805.6941	400.5925	955.5476	80.71843	894.7397	888.293	655.0455	60.49054	431.7859
187.2734	191.5451	238.7343	74.90204	54.60151	102.3869	66.79296	104.6849	122.9191
39.71881	28.96523	30.66009	138.797	50.24957	80.27403	44.57762	45.76712	14.88761
267.818	404.7769	282.2646	470.1312	339.3103	209.4946	291.3065	719.098	236.9175
26.80904	42.9403	38.85257	39.22785	43.90537	35.7282	72.35959	102.3026	43.84265
9.032001	5.672922	32.36195	6.970093	35.28274	5.812504	18.55074	24.41615	11.37471
103.5985	52.628	66.17658	289.0724	108.1129	153.0404	132.673	88.84973	93.00167
12.02917	12.5898	5.727527	9.280001	2.166418	8.116328	33.37074	86.48911	40.32172
41.32184	49.58185	306.8662	41.45263	58.82513	41.40059	42.69393	21.19074	45.6711
113.9746	139.5518	253.4058	141.7422	295.3677	79.45343	389.7519	245.5318	152.6981
44.33118	19.4601	29.77398	73.64308	29.98136	10.41829	73.34288	52.76002	32.45552
54.5499	83.8682	40.57806	58.69691	36.3924	55.18243	57.55339	29.07619	34.21993
68.03482	172.1835	187.3551	150.7965	42.83085	206.8833	36.1618	6.205033	78.99826
8.915016	9.684299	0.763517	10.36963	11.76686	27.42519	5.547285	2.278497	6.113671
85.89084	80.21537	111.7452	94.51524	194.7762	74.84466	102.9729	126.6507	123.7364
143.6294	0.128247	40.49863	44.99832	6.447233	100.1781	37.08307	399.6285	298.362
454.4817	653.3603	160.639	142.8702	43.91079	339.2857	42.65006	203.0593	140.4237
64.03028	31.54811	48.25777	9.262517	19.26209	18.40954	0	4.636242	55.50485
84.39034	51.24012	95.18436	64.5705	46.04999	25.38054	67.70854	160.546	70.17655
5.903002	13.44944	1.597925	3.488048	1.094431	12.53155	1.832253	3.897118	3.485087
22.36616	25.00276	34.72072	41.51687	59.94189	31.1202	71.44569	88.96087	42.0973
22.37683	58.08503	61.23647	62.24479	97.38855	46.07325	141.9965	85.76264	101.8286
297.3591	334.3343	577.9033	389.4744	814.4801	364.8199	401.7274	664.8396	226.3887
261.7847	204.4974	375.9082	223.5568	349.9849	85.21687	570.6498	579.9859	431.8368
314.9498	214.1534	257.469	438.9362	337.1416	222.1214	237.5079	250.1618	333.5137

4870.47	7302.885	3655.397	9792.199	5494.9	6238.004	3042.297	4292.661	1362.078
4.62168	2.904908	0	176.2414	5.379298	8.116731	5.538231	23.51126	11.35802
578.0776	810.4976	360.1259	603.7173	307.1942	1139.05	282.0353	304.4265	717.089
23.75618	1.525766	12.35447	38.0099	16.07763	18.45588	13.89138	37.78129	31.58886
1131.211	1541.263	1654.266	1374.557	1609.725	1230.139	1409.351	1256.461	1135.67
307.3638	396.0771	1106.03	366.2962	315.7085	180.6769	246.81	169.9169	216.7862
51.6471	93.49016	82.11543	26.53442	31.04957	35.68403	27.81962	52.76574	28.06029
121.3398	81.60994	289.0549	101.4326	190.5065	54.14448	211.5562	191.208	175.5351
25.09859	114.6175	59.84903	5.821364	16.06786	16.13847	33.42775	10.94749	53.67515
150.2568	99.26309	96.9441	78.31985	69.56393	101.1906	164.3234	42.4388	78.13413
87.32526	103.6147	42.1588	84.13952	64.23906	201.2906	36.15556	74.69059	85.98723
29.77897	71.96205	148.1746	182.0515	189.4444	52.99599	219.8996	210.8697	220.3044
26.61787	20.77425	14.85438	24.2061	5.380437	69.99171	9.250902	13.30841	36.89647
8.972541	15.16139	11.57485	11.53674	6.443388	30.91908	4.612956	8.606006	23.76624
31.22326	31.90059	31.40014	25.40757	19.29465	24.22503	153.1518	84.99278	70.20674
72.35471	92.34532	42.19461	72.55587	80.25032	106.9101	75.17071	24.31437	80.78408
235.1586	387.9737	577.2307	171.7154	345.6952	280.7778	313.6074	135.2537	262.4126
10.45609	15.20663	21.56961	8.110613	21.38925	9.245378	10.18925	16.52648	14.03202
142.0765	66.44972	27.24848	139.4561	49.26402	79.45984	83.47515	203.7526	107.9093
26.69516	9.820119	59.70616	20.78	49.21281	12.7146	44.54925	43.30857	33.346
25.32802	51.19474	17.31374	27.71187	29.99518	42.62324	25.02194	61.33195	14.86772
90.40083	16.71717	26.42694	547.3153	62.10693	47.24163	215.2235	255.6177	210.587
25.06474	19.35278	9.880676	37.92367	8.586628	19.56814	15.76137	1.48901	57.22703
14.99056	2.908594	4.070996	30.01561	10.73301	9.269623	4.609976	55.02327	20.13452
152.4659	225.206	229.303	245.4449	306.1115	250.8863	425.8806	308.3996	247.4753
77.01766	58.13789	128.3312	53.05603	145.5543	98.98723	101.1207	117.2147	98.27684
74.03424	15.35528	27.25015	61.1093	154.0997	13.87324	129.9053	207.8713	49.10655
93.306	76.09854	178.0051	197.0151	212.9815	57.59712	189.2758	214.029	101.772
103.7092	147.9336	290.5591	252.379	625.0464	191.0787	441.6313	655.4205	710.9334
165.0548	152.9218	78.67151	16.19449	73.84781	108.1029	45.4459	32.97314	65.81906
3.148662	28.87776	24.0358	9.266483	32.0768	9.254397	20.41298	26.01084	19.30548
62.26778	52.62495	89.37747	153.2274	149.8375	43.78806	208.7881	256.6399	57.88106
13.47985	7.062796	22.31514	24.22556	6.451144	4.665675	11.10575	27.50854	13.11995
82.61357	33.24526	146.7949	47.25445	187.1523	87.38439	169.9228	10.92432	40.34991
28.2366	0.123991	0.765481	81.73867	1.096163	0.04928	2.754705	40.09032	7.846828
44.53036	51.22536	120.0709	51.89779	114.5176	33.43141	180.0376	130.6437	170.3258
201.157	153.3985	221.8773	183.2248	1180.294	176.0913	630.0825	158.0889	262.4244
9.072443	1.519635	24.76682	4.669479	31.06658	4.663062	34.30145	171.6596	49.11067
17.91216	2.914331	10.69233	23.08845	11.80179	18.46403	7.392004	26.69751	21.90352
16.40424	18.05409	28.14117	18.47376	26.76346	5.816073	13.89313	15.67028	15.76062
31.03221	13.93395	18.1721	16.17401	20.3501	4.665406	15.75165	44.93349	21.9204
37.16777	66.42852	44.63843	101.4281	50.33226	131.1996	70.48894	78.60903	53.48892
3672.787	4364.065	1415.741	3102.816	2077.466	3719.108	3059.939	2730.887	2294.177
38.6012	37.41714	47.97544	38.07253	39.62379	55.26645	55.65396	37.69374	300.465
678.5017	713.8476	984.6003	898.5796	945.0126	311.8792	528.8634	139.1723	667.0634
208.078	234.371	326.5452	102.5444	238.5981	97.8169	45.43765	39.25828	65.79442
267.508	335.398	398.384	476.8255	286.8171	348.5799	134.5168	121.1061	148.3013
28.22293	16.71895	22.29691	26.54426	28.91656	19.61659	113.2759	54.30825	23.65738
610.0578	247.2118	353.5946	26.56526	208.7162	438.3292	347.0264	58.13068	318.6175
72.56937	15.35518	8.208143	44.9916	17.15508	20.7778	48.22047	108.5679	30.66713
70.78516	270.3757	81.23964	47.23889	38.53967	116.0362	51.03445	44.04985	54.42921
19.41863	15.35285	63.73847	13.89097	13.94386	21.92435	95.58357	99.19727	51.75826
38.84364	61.88894	30.07141	2.361214	26.66991	10.35522	7.41026	3.069188	30.02815
47.90565	22.80137	9.991669	15.98127	0.019038	9.170421	8.361706	0.709351	56.93575
10.47698	0.116012	14.88177	16.14263	22.46468	3.512659	19.48754	2.273622	2.583248
89.87771	84.03137	156.8127	33.44622	25.70874	24.21367	51.96062	31.41945	64.09718
4.631909	1.526752	3.240557	3.519111	2.168717	3.514139	24.11693	27.53708	14.00488
84.44479	67.81867	170.5496	169.3786	263.2693	122.011	223.6161	188.8315	105.2843

572.3168	7.042206	17.32182	106.0662	55.68478	201.4399	93.67843	627.028	121.9415
43.04494	48.45852	10.69	170.4249	65.30347	692.9657	14.81399	10.13737	40.3283
1.491775	2.655243	6.927562	2.276184	0.010881	3.374945	0	1.575482	0.858475
6.102634	4.297752	1.587673	3.518762	3.239679	3.513796	5.537749	16.48205	8.732223
1052.872	1107.695	950.4453	1537.007	970.7791	875.7422	628.1054	1232.062	538.8345
0.149628	0.11961	2.413735	0.048216	0.022349	0.047758	0.90076	33.08594	0.831943
16728.92	25100.83	11684.87	18296.58	24049.6	14731.54	30315.52	24221.8	15284.42
71.07581	314.199	99.34941	190.005	225.7631	93.21919	147.5471	63.67385	109.7129
130.2793	95.44399	126.6174	210.8741	438.7893	141.5866	514.9899	299.776	125.4626
241.0305	160.2998	178.8025	244.2661	402.3994	128.9291	373.0092	330.4967	198.3318
341.6637	395.0445	404.845	698.1737	314.6858	218.6908	810.0195	554.6946	589.8026
177.6388	1579.893	740.9674	87.62842	47.12077	143.9046	116.8744	550.6664	78.06152
720.992	5.666633	19.79963	116.4277	74.95072	71.4089	23.16516	136.0379	93.8597
39.79691	9.812588	40.61922	26.51083	20.35017	47.10961	44.56371	22.78362	17.52107
25.29503	42.86677	145.9748	33.45131	95.21273	34.55757	96.53961	36.93339	62.32864
3.150709	7.056689	5.724809	11.5734	5.380395	9.262164	13.89574	19.6325	22.80749
135.8746	88.40986	94.38486	81.82053	77.07125	80.56755	86.28179	76.2868	105.3282
44.55535	29.16663	44.63843	76.09404	65.31379	159.9476	76.05711	261.3265	65.78008
51.85766	148.8638	81.97557	184.1687	74.92236	93.18442	36.16056	38.48353	53.51154
44.56531	47.1212	66.99189	31.17438	19.29478	76.00943	97.39329	332.8568	91.22876
97.68507	15.35424	57.89431	175.0997	126.2997	28.83259	110.3999	101.4547	71.05166
81.42723	31.57094	81.64051	20.72878	8.585257	17.26972	11.11509	7.794151	32.54148
1694.559	1116.027	851.0714	2489.843	1372.141	1709.995	961.1927	1963.78	968.0144
63.73358	47.10181	42.98565	82.99259	28.92796	34.58418	44.50704	80.98592	68.42045
129.9529	348.4227	646.834	51.8909	141.2479	105.8468	135.4898	13.28343	171.2335
4.63268	4.300144	6.552504	3.519147	4.310402	9.264412	5.536837	13.30315	14.00258
94.83535	26.39748	183.7445	127.9539	132.7455	139.2974	179.0426	501.9965	111.4113
1421.09	1585.578	2995.542	1077.344	2452.062	1364.804	1293.352	3296.625	1011.893
260.3263	230.7318	618.5827	255.8141	534.0519	177.2605	748.8141	442.2072	600.3853
59.1799	19.48891	78.68059	41.51626	25.71328	16.173	37.09193	88.96332	50.0059
138.7651	54.95255	108.8992	41.42334	12.86688	27.61916	36.20045	10.93758	83.58381
84.38495	129.8067	65.35525	155.5139	44.97965	102.4327	74.20598	79.41266	77.20288
196.6056	55.39599	184.642	109.495	159.4787	107.056	237.5421	229.0058	237.0031
47.43791	47.05514	100.2227	57.63542	136.9537	29.97482	162.435	105.4901	65.81182
6.086113	1.527245	31.57479	5.816753	18.19201	4.661113	12.04777	18.89861	3.461383
23.73428	9.815007	14.84741	18.47472	51.33836	5.816166	33.40543	38.59106	19.27817
3.119469	0.105651	3.252527	8.077452	4.302155	2.356392	10.21109	13.44378	4.351866
23.75233	8.441046	12.35496	6.976179	10.73038	5.816512	34.33025	35.41313	13.12026
4.620041	20.66751	48.27054	1.210265	16.05843	28.6952	16.69847	1.488852	0
16.46421	40.15435	138.4157	13.89072	73.84618	25.37283	40.80473	87.37893	105.3723
38.5767	20.86802	41.35384	81.7787	74.91453	44.91164	71.44564	82.65038	41.21866
13.50242	5.683336	8.206268	24.24536	27.84808	17.31893	17.60078	36.93993	20.14157
14.95317	19.44479	72.1777	29.97295	116.5223	5.816671	24.10782	47.26108	42.14499
134.4458	85.67458	63.70749	242.9372	67.44568	72.52697	42.65317	73.12645	45.59504
16.40164	16.68015	22.32518	24.21569	18.21295	8.115415	9.249903	30.6884	33.36136
9.011813	11.13622	2.414019	20.71924	0.021707	5.809407	9.257331	10.17225	4.339872
78.38936	201.1841	172.365	89.86201	149.7971	46.07339	83.5032	34.54057	57.8994
82.62597	111.5584	50.49277	17.34339	32.12868	116.0899	50.09655	35.35219	50.01701
0.152294	1.525756	9.866455	6.97631	2.168508	3.514094	10.17727	7.779258	19.27389
617.741	635.3563	340.0001	122.0607	178.662	202.317	331.4487	161.4832	98.32333
4.633046	16.68471	16.50778	13.87761	17.14494	8.11591	24.11211	36.21757	19.27801
44.55374	49.86999	55.40673	97.97136	70.66356	78.29617	132.6716	152.6372	110.5623
7.594459	1.521965	10.68972	10.43451	3.238833	8.118983	3.682175	61.36102	6.092298
35.67769	0.126398	10.69051	17.3482	1.095418	11.57117	7.392814	101.4808	5.217
154.7581	119.9285	51.30219	38.06662	6.451204	59.8521	35.23423	23.51983	26.28374
122.6657	135.2625	54.59091	181.9549	105.9594	197.8158	62.14392	40.83232	72.81953
9.064906	0.121735	0.764502	10.42984	6.45119	1.207337	1.827252	17.24129	9.604808
35.50172	36.09298	69.15056	1.206796	0.019328	8.045051	16.76936	1.49505	18.5717

1601.219	8661.234	4819.142	700.5681	1158.067	4030.672	1443.692	1027.506	1599.121
36.55747	168.1762	50.77723	35.59407	29.93017	41.26175	15.76812	1.488852	92.62348
385.5248	299.5283	140.7187	202.7787	219.4046	272.6733	284.8663	265.9921	150.9378
108.1138	225.1541	175.4867	87.62619	324.288	154.2384	174.4128	450.1607	210.6207
32.3992	33.03388	39	19.60496	39.56464	26.45398	17.61614	14.1067	27.22072
32.47745	8.435186	33.97205	4.671709	19.28016	40.22667	131.9894	30.698	23.68319
43.01937	26.38934	81.97436	84.09975	98.45315	50.66725	52.86978	52.66715	43.84782
523.5481	274.9855	854.4498	381.4238	1008.211	275.0826	1311.037	887.4941	627.5041
81.39754	88.43031	119.2438	68.01459	112.3774	27.68002	119.6964	147.9842	115.861
314.9832	830.9703	201.1427	89.93421	251.5347	602.8929	496.4043	125.014	456.4091
25.20637	30.39775	23.15045	24.22103	34.24608	16.15693	19.46567	40.16625	50.08011
91.84556	116.1254	131.5994	212.0024	63.17654	105.9158	172.5638	103.7836	143.0302
32.72022	2.894898	0	109.5229	35.34812	6.958574	15.74564	368.9271	28.03682
8240.059	11263.67	6123.561	13021.74	2969.029	6277.163	3984.961	4410.651	2163.385
34.17582	1.520777	23.94121	51.8828	61.01787	27.67552	23.16609	51.87855	28.91614
1874.98	1146.428	1934.061	1115.365	2954.024	698.5505	3678.809	4632.652	1997.526
56.11225	26.34737	18.15163	19.64081	11.80225	56.36104	12.03191	43.26246	81.69699
233.6141	259.6043	233.4847	137.1505	125.2461	239.3455	137.2973	106.9288	229.9465
38.55805	12.59016	18.97442	23.10014	9.662654	19.62156	24.09686	122.9234	21.89491
479.9882	340.9149	391.7545	647.2271	494.3915	463.576	384.1694	37.67306	317.7678
248.1764	196.0354	308.1393	160.1494	127.3747	254.2321	86.2657	63.65065	90.36462
2.807342	2.657034	2.53084	1.167018	7.361915	8.850271	7.563714	0.748473	4.498224
78.00397	55.13567	143.6273	17.33572	82.35296	48.30783	85.4258	18.81172	48.28951
45.82158	41.44741	39.73021	50.67831	56.71023	94.21636	125.3709	13.28999	65.87233
12.02743	11.20789	18.14899	26.54702	29.9875	21.91678	39.88504	35.36021	20.14113
84.2176	58.05613	225.5295	99.04222	100.586	108.1074	58.44303	47.1572	75.48345
10.54354	4.286326	11.521	125.6264	4.307694	1.203708	0.902594	344.7315	34.17566
53.37226	29.15728	76.1448	30.01812	39.62689	20.7772	115.0589	148.001	103.569
419.637	616.3986	750.6356	76.0551	139.1027	78.25747	102.9995	146.4664	186.1867
273.7399	223.8958	542.259	443.6412	1511.231	403.958	699.5598	1260.467	549.379
418.4966	194.8735	578.7624	535.7527	819.8111	158.8601	851.7789	601.9216	627.552
11924.9	14526	6939.867	22219.63	7903.1	14639.43	8584.152	5536.494	5638.045
26.6742	15.31603	19.82707	13.87896	18.21489	10.41496	41.76728	56.77462	48.31728
88.84186	71.94005	60.3778	144.0236	170.1685	97.84524	102.973	139.2528	75.44019
29.63255	40.02201	43.07795	69.01975	111.1854	149.1723	53.8406	5.417461	50.94242
3.1508	11.18439	30.64527	24.21064	18.21139	6.96533	50.14656	31.48951	22.80312
4.604178	2.907948	14.91898	5.809745	21.37388	2.360262	13.92	2.275442	7.868607
53.17605	89.47929	60.48142	84.00853	73.8174	149.2986	69.61496	47.99974	61.4672
192.0437	140.8486	148.2204	275.2307	219.3732	185.2174	180.0174	118.001	202.7923
17.76796	26.13931	6.559553	15.0004	10.72229	50.43077	17.62452	9.374983	19.30915
16.46683	26.38825	24.77029	26.55913	67.43457	32.27401	52.87046	81.04856	49.11959
121.1972	59.49725	120.8983	87.58506	195.8167	42.63245	198.6038	140.886	158.9035
4.631899	0.120007	18.17638	25.3565	3.239775	17.29734	5.537037	33.86778	25.44553
356.4372	192.1126	254.1269	411.3461	388.5293	284.2709	468.5446	385.4876	238.6823
17.90321	22.20328	19.81718	38.02472	74.86873	19.60958	67.77301	41.71007	26.30301
22.31408	13.95526	28.11644	21.9352	9.66166	1.207053	42.6836	30.65092	41.25353
143.6048	298.3628	343.5922	161.359	541.545	145.0474	543.7281	413.8652	482.742
10.55365	8.446668	13.17325	10.43449	2.167192	12.72253	2.75553	75.51619	2.585845
453.6564	558.9801	412.3967	975.5253	699.8757	722.4265	548.4041	82.52497	363.3858
20.85905	20.8425	34.74782	42.63533	25.70657	5.816955	37.10421	49.58496	39.48478
7.545159	4.289886	7.395441	18.4224	11.78627	1.208237	13.90971	13.34831	14.03034
25.29455	11.20858	134.3504	3.518005	65.27645	8.118996	10.17534	67.70644	5.21474
137.6104	100.9393	139.0609	156.7185	141.2934	176.0721	112.2451	196.694	120.207
146.0691	19.49472	253.6783	19.65087	66.36753	218.4156	193.0709	13.28352	141.3771
12.03301	82.88491	71.17907	33.47093	26.78602	125.3814	53.79412	165.3714	75.46491
16.4698	26.4032	28.077	23.10909	13.94389	46.08681	38.01108	51.84926	30.66634
18269.38	19351.33	3847.438	15880.42	11026.22	18910.86	12153.48	12988.16	9588.412
310.6213	205.9161	97.62425	493.1286	188.399	425.7786	314.513	484.6574	429.166

23.79451	29.08437	28.94096	46.07844	16.08066	20.76171	112.3659	48.80281	44.76485
25.24717	16.70515	18.1579	19.63451	12.87082	15.01461	19.46142	48.03183	61.49238
0.13802	1.52526	1.588159	8.09785	4.306124	4.654726	7.404833	5.438636	0.831536
54.85767	31.9191	38.01936	92.18822	171.2128	47.23208	80.7081	82.57884	23.64472
1210.271	176.8204	354.4603	160.1783	144.5055	510.7343	145.6519	26.65836	416.9885
733.2462	1161.1	699.6624	238.5543	835.8715	1323.136	614.2231	178.5136	602.9636
1.662358	1.526919	3.240569	1.209893	5.38038	3.514087	4.609142	20.42385	6.093595
150.9554	185.1438	223.5276	124.4871	259.0137	97.86937	272.7795	298.9956	126.3416
152.2845	37.44893	42.15462	97.97011	34.27945	47.24006	116.8943	167.6059	74.56175
82.94833	20.88008	77.77184	71.48741	123.0948	67.94602	115.9642	144.7562	61.39037
214.6021	176.9382	716.9427	291.5667	56.75309	1918.25	268.108	1026.682	447.5542
174.4652	267.7507	75.2832	277.5967	127.3779	586.5334	183.7122	109.3104	177.2912
78.28141	106.1375	79.51681	53.01793	101.6448	80.51952	123.4557	67.67346	46.49332
105.8428	96.26292	68.80805	2596.242	150.7499	226.0753	73.34101	3.845365	27.17882
150.5995	88.40997	98.53008	143.9617	103.8146	141.4819	89.99503	62.89236	78.97398
14.88891	0.118094	9.047105	44.81245	14.99914	17.28338	6.46649	20.44437	16.65502
434.711	149.3063	102.5928	381.3849	977.1089	111.6835	115.019	255.6435	280.8209
8.097824	2.704571	4.240188	4.512784	3.190881	2.291931	4.689643	4.047689	2.652416
140.5006	161.5738	99.31219	206.201	141.2841	323.2158	192.0712	190.4374	142.1732
171.385	22.26113	79.44028	353.4793	79.21986	146.1236	229.219	420.5847	180.8393
7.576714	4.298603	4.068383	11.57091	4.31013	3.513966	16.68543	15.6853	6.094155
20.26949	0.105335	7.432039	17.1848	11.75928	68.19943	0	6.253944	54.93917
7.589192	19.43945	38.93297	11.57983	29.97404	4.665666	65.00071	43.31784	47.43116
6.114652	0.123392	1.589768	72.52031	2.168102	15.01851	8.319762	25.11359	6.969336
31.25735	2.900691	6.557148	49.60894	67.45775	31.13463	6.466879	106.9289	47.34087
54.86566	47.09419	22.28083	170.4602	34.27809	105.8729	113.1939	69.18111	59.6432
20.86728	34.60016	4.895637	4.671838	5.380957	17.31809	7.391882	47.99283	17.50674
37.16394	56.76285	63.6936	116.3867	81.36191	39.18662	149.3833	174.7095	104.4207
57.73337	41.53887	102.7229	40.37027	54.59723	28.82347	137.3702	98.40714	129.094
131.6677	54.01442	76.11212	84.158	61.03416	77.15	339.6433	227.4391	232.6173
688.9598	1332.31	737.7429	860.6182	839.0894	1112.632	552.9764	490.9259	567.8433
77.06865	138.1896	553.3363	49.60804	379.9045	48.39423	188.3434	258.8972	263.3269
32.55888	11.19728	10.69497	36.86596	13.93948	8.117256	27.82362	40.14566	14.87691
4.601849	5.645247	8.241534	3.513649	9.643734	3.508747	12.99027	24.53968	6.106196
125.8188	262.3495	172.1872	290.312	476.2147	680.9484	190.1951	25.87188	121.0803
238.026	280.2767	227.6915	174.0016	343.5344	192.1843	237.5254	114.0156	176.3921
35.59465	52.48645	37.22215	50.70175	52.44573	26.51494	41.74066	70.86297	18.38336
31.14923	114.1298	120.3015	9.281223	43.88329	40.28602	61.25633	28.27171	118.6562
36.9673	135.8331	101.2634	24.23091	70.58878	67.79315	58.48489	44.08932	44.77938
87.42014	71.97028	321.2981	138.3027	189.4521	118.5723	284.8525	380.9115	207.9959
22.1174	7.040536	26.5485	27.5983	12.85782	26.42055	30.65317	12.54308	15.78503
29.73318	11.21052	20.63054	38.06273	77.05069	19.62269	26.88082	51.89892	30.67761
23.67273	39.8726	29.00012	44.838	37.43298	39.05766	14.82648	8.57424	32.50205
152.4794	219.7058	149.7932	122.1932	172.3436	295.7641	264.4105	360.3265	168.4656
49.84086	500.4241	64.00226	2.365541	70.52009	107.6844	45.51722	0.707309	48.37604
7.594524	8.447142	20.63751	8.129679	7.52189	9.269426	5.536531	32.20853	17.5064
44.44309	19.48489	69.56924	79.46186	98.43129	35.71253	85.38322	64.52832	43.86043
316.3413	182.3802	270.7461	501.0801	254.7322	284.2174	98.32105	165.1704	143.8998
83.00729	69.21864	131.5765	582.9392	295.414	126.6396	380.4043	293.4325	112.2899
6.114114	18.08094	12.35146	4.67213	11.80122	5.816879	42.68432	34.60064	24.54422
27.9582	22.07229	51.553	17.29749	17.132	12.69369	33.43781	11.74532	40.46976
1677.8	2714.4	683.8684	1496.577	2282.839	5849.366	1923.447	169.0654	1306.891
59.86733	61.51801	62.39026	3.51832	9.655249	29.8682	7.396016	0.707044	41.34754
10.55335	1.522161	0	13.89022	2.167643	40.31131	6.464316	15.64659	5.215112
122.8499	240.2496	105.9246	197.0248	143.4341	296.829	154.9337	153.3859	178.1545
23.4766	16.55478	17.40244	2.364679	6.445429	12.6759	11.12073	3.850053	38.76534
22.37906	0.125872	0.766653	5.823042	3.238241	0.049929	5.537446	32.17554	2.586068
6.041073	15.09504	7.416747	14.94077	1.096781	14.92236	4.615545	2.277525	41.5283

38.5816	27.76065	43.01018	33.46301	39.62077	24.22337	22.23894	57.40949	36.82462
267.6383	408.6514	510.968	255.7918	406.6872	334.8352	153.9964	200.5875	149.1648
41.34023	56.44533	51.39404	28.82368	55.62383	34.52387	60.34884	33.03523	77.35448
28.21823	19.47053	26.44583	41.49058	34.26243	16.16805	31.52993	47.2083	35.96449
11.89223	23.32007	36.63102	25.2685	9.648268	16.1017	21.3628	1.488976	13.15537
35.59282	18.10134	46.34991	80.59056	49.23741	26.51457	25.02703	67.70786	34.19951
37.15748	30.54279	33.87526	25.41287	20.36598	24.22967	58.42893	64.45201	71.93582
75.54892	62.27911	43.81255	57.66365	114.5299	97.84056	139.1739	119.5718	44.71183
74.0168	115.9639	163.1943	88.7301	189.3912	46.08033	201.3972	162.1829	112.3517
140.5023	26.40483	87.71304	130.2143	154.1238	337.0139	334.0834	51.84243	120.2194
82.8827	107.7274	104.3165	86.4358	102.7518	64.48155	198.6012	115.6658	82.47893
1069.08	2232.945	856.8945	1147.578	1382.821	1358.991	1829.681	1006.259	1055.81
17.70253	58.51481	109.394	12.6934	8.5819	43.51141	51.16546	26.86529	57.32169
464.4518	595.353	578.6874	708.6247	876.584	538.5859	556.6675	1113.297	518.6557
7.086516	24.57814	3.310928	12.40849	8.496031	4.573117	9.359917	0.720608	26.94701
26.76375	81.31635	30.58737	27.69829	29.98797	55.22825	55.67253	21.1655	38.595
1506.218	1426.305	1533.548	766.1859	779.1709	584.5828	903.7109	593.2087	906.6512
68.14565	63.6411	129.177	80.68373	161.5927	52.9847	155.8934	127.4757	120.2435
25.28405	26.35374	43.86866	9.281685	34.26395	16.16898	46.38805	67.72584	78.16921
29.72075	11.20959	24.77776	4.67136	18.22302	10.42052	24.09735	30.61959	24.53096
5.758494	15.72778	0.773631	1.195454	0.016156	31.38319	0	0	31.40908
25.06272	41.13476	29.03097	34.48701	25.67463	26.43555	16.69143	12.53505	20.18438
38.575	23.62416	85.31775	92.12761	203.2286	13.87246	64.94629	120.5202	68.46066
41.35081	50.98054	110.4236	80.49863	131.4831	28.78924	105.8908	32.24163	35.9836
25.33055	33.28793	44.65589	35.77251	47.11395	25.37715	38.94342	120.4442	34.18344
103.6306	256.7312	114.2177	50.75827	168.0383	281.8494	208.7704	58.13698	197.4867
1366.259	935.0807	532.3072	2146.456	892.6483	1434.953	840.5812	1272.984	1503.43
7.576823	2.915949	12.36611	5.822345	1.096905	4.664661	26.91151	20.43135	27.21673
171.4376	306.2186	197.9361	43.84426	338.1298	377.2155	193.9352	37.67633	141.3035
33.986	132.8895	81.36333	91.93058	49.20429	58.58654	18.53678	18.03623	74.73709
19.32048	33.09577	10.70052	27.65382	10.72856	48.24525	23.18682	7.782375	29.84613
4.626625	12.52749	3.241018	1.210179	2.168802	5.812624	10.18323	49.00977	9.614096
22.37135	47.04863	74.51767	40.37102	105.9352	39.1702	162.4414	73.16979	89.54162
115.4684	116.1185	129.1181	49.60811	70.66734	52.99642	41.72068	151.023	98.25708
26.8012	36.03718	56.26767	20.80218	42.83271	23.07559	128.0759	55.81936	88.6561
201.243	96.8365	359.3205	332.9966	643.2138	209.4777	794.2725	301.3003	414.2595
87.14836	209.2591	130.9394	104.7886	215.002	92.0211	97.44284	24.30719	89.54747

TCGA-32-	TCGA-06-	TCGA-28-	TCGA-06-	TCGA-26-	TCGA-HT	TCGA-TM	TCGA-TM	TCGA-FG
103.0939	144.3244	112.2347	101.4172	85.52313	195.6376	135.2852	173.9931	153.5812
15.49625	3.318188	4.776778	13.25199	4.826051	33.59362	42.46533	38.40702	12.7322
28.09237	44.86071	49.9421	71.08272	36.87248	157.1048	76.74039	73.47349	75.21465
2179.616	2121.045	1606.999	2299.712	1778.207	2630.212	3401.957	2007.816	2342.884
122.4283	94.04626	90.73507	107.1092	96.18971	104.7321	77.45258	97.19714	86.86697
1.406744	3.289711	7.983844	0	0.043577	0	0	0.030616	5.285729
22.93239	4.412053	40.92657	28.42756	4.826056	13.8282	0.633624	10.22229	16.96779
73.38267	27.3732	22.03223	49.27209	27.38236	157.1012	172.6353	163.8298	149.3454
5373.021	6241.742	3226.667	6925.696	6266.32	6895.661	9017.923	10405	9332.309
31.0807	38.29317	17.70469	73.94262	41.59523	54.34145	39.42172	98.2563	62.49487
15.53593	9.870528	4.759775	17.05543	10.74118	16.79516	13.83388	21.46908	12.72561
503.8203	112.6512	144.7969	549.7919	396.5391	1136.268	563.8507	1293.695	1255.108
138.7382	376.014	268.7588	179.1619	259.8917	78.05239	109.645	128.8314	141.9371
4.434857	7.623616	2.440863	0	11.70572	0	0	0.032596	0.015035
68.95327	20.81306	32.77398	38.84554	124.6194	57.30367	83.33759	108.4699	119.6822
46.04171	117.9211	70.38114	62.58756	51.00314	3.947287	41.7005	2.311143	8.498039
1792.226	1154.569	1064.32	1728.099	1701.035	1871.382	1627.398	2433.745	2098.212
32.59274	16.43636	17.66863	29.37206	28.54154	43.47341	46.06373	31.66877	21.2044
104.627	144.2977	167.5542	90.0474	9.574453	39.51769	147.814	50.88961	60.39056
307.1533	806.8547	2596.675	2528.298	344.2982	148.2037	1414.831	163.8875	330.48
563.9469	176.064	323.5245	661.6542	359.7415	818.1109	976.4581	988.646	893.9424
3.604854	15.34354	0.368254	1.875771	0.060984	72.13248	8.662815	47.45911	37.0812
19.18405	40.48999	11.24987	10.40559	46.36559	82.00605	0.637103	236.0503	198.0369
351.051	30.65255	11.21923	34.10376	39.25249	316.1788	44.50114	642.7886	641.8045
45.92388	21.90599	37.05182	82.46479	48.72955	101.7702	99.4739	140.0644	101.6794
25.18527	6.598827	0.359613	38.87062	4.82603	10.86397	19.67404	36.15171	30.72045
311.7781	382.6056	335.5186	338.4385	195.8755	122.5157	75.23131	192.0921	128.176
690.877	336.7775	179.2577	1715.816	1026.768	629.3905	540.4527	946.8395	539.1334
3.603592	5.505733	0.362224	7.561744	7.20039	177.8905	10.13037	24.88727	43.41975
87.57184	7.692238	19.86809	76.77902	19.07178	63.23345	46.73009	179.5373	102.7315
34.80307	62.3213	41.20041	33.16184	24.99704	82.01134	77.57338	35.06478	60.3753
74.24667	31.73596	19.84017	32.21373	54.62174	54.34201	46.02959	73.42676	42.38001
2.862638	3.317748	4.750902	25.60678	13.09665	10.86536	1.356647	20.33353	21.17954
528.3306	2256.478	734.5662	1219.074	1330.581	391.267	351.7205	912.9383	1300.635
228.5749	182.5977	129.5616	264.4826	230.2839	152.1582	283.1112	155.9524	147.2389
3465.683	2327.703	493.6476	3555.729	3525.529	12273.67	1739.98	9188.169	17718.77
14.76821	55.67995	57.13467	43.63013	20.21149	4.935345	5.008058	13.59875	19.07669
86.11948	42.66539	13.41027	36.95318	42.78504	71.13988	94.41195	38.45619	50.8527
18.45413	90.69047	142.6067	186.8645	35.65123	20.74485	14.51881	18.12816	64.59878
25.15793	110.3127	87.44579	180.2488	11.94675	35.56831	5.737727	11.35232	45.54411
94.92023	408.7813	441.9906	445.6119	57.05069	19.75604	372.5874	127.697	130.2864
63.84104	35.0109	11.25199	52.13297	122.0956	30.62615	13.7845	70.03323	51.904
45.95893	306.9061	227.9349	72.04753	88.97642	19.75635	17.44316	5.703322	65.66854
43.68384	13.1594	6.921832	39.79262	57.04323	93.86326	51.10009	195.4169	108.0422
256.0137	135.603	146.8264	402.8928	487.7655	332.977	183.5225	258.7527	278.563
20.01031	12.05482	11.1353	20.85052	1.258251	19.75982	13.0899	24.8528	9.553343
202.303	81.98457	26.27297	169.7483	59.3689	33.59012	72.42396	26.03588	51.90912
929.7932	753.3325	250.3118	1103.39	1729.548	2386.16	1835.834	3910.478	3026.041
220.3161	131.2377	252.3979	177.2448	802.3727	940.6379	176.1664	342.3886	562.4191
11.80992	0.029025	6.873647	8.514152	7.189394	13.83057	15.32242	60.78104	12.72312
13.30522	14.22965	2.598256	13.26045	8.372036	18.77281	10.16069	27.07965	21.1797
251.5726	38.30643	67.25194	181.9942	210.132	286.5376	198.1702	270.0399	246.7893
87.60094	68.88536	19.85882	118.5162	61.75046	75.09232	30.62149	101.6556	77.31861
648.5188	512.8033	575.5275	1141.306	1120.628	4016.457	4474.177	4658.49	4957.961
15.52614	27.32302	4.765188	15.15484	11.92784	20.74816	2.814856	27.10426	20.13049
32.5721	14.25244	4.771426	65.40889	23.81133	53.35372	44.56102	64.40307	42.38037
14.77265	9.873534	4.768786	16.10248	10.74807	32.60833	18.97025	11.34254	34.93652

2378.461	703.0403	803.8246	3248.6	2353.931	4071.788	5380.453	5159.019	5153.907
263.4282	472.2745	161.895	285.3284	409.4795	98.80103	50.35246	66.71859	206.5515
31.89099	32.80538	9.078304	66.39142	37.97755	18.76948	24.81261	50.80377	31.77994
55.66017	54.66699	58.11576	20.83523	120.8944	8.887442	37.24305	19.25811	29.67453
20.7216	88.41482	21.77295	31.28208	3.638066	0	39.54982	0.041604	0.019
118.6611	63.45099	148.8796	84.34726	199.4367	169.944	102.3072	179.6698	174.7722
25.9887	26.23394	9.027502	18.9513	7.194877	8.888143	27.08662	16.9738	14.84307
8.056133	15.33892	0.360624	46.46104	2.448625	31.61689	33.63438	60.94154	41.30086
42.21605	25.18446	19.8683	69.19226	68.87604	47.42322	45.99716	98.28796	93.20411
265.7336	125.7569	198.2036	254.0573	108.0792	103.7422	180.6351	177.4064	115.4671
5.836618	6.594847	0.340317	4.718878	3.636644	11.85364	12.36928	22.58514	11.66629
4.344925	6.597854	4.771832	8.511165	13.1179	11.85258	17.48938	19.23449	11.67144
77.82014	71.10391	30.64782	83.3987	143.6817	196.622	89.86302	403.298	275.3778
35.58355	21.89674	2.597626	59.73306	19.05879	37.54477	48.28699	57.59762	55.06734
22.15349	104.9605	6.927366	15.14474	131.7292	70.1493	286.3733	191.9977	94.27146
25.9046	21.89611	13.37656	47.40001	38.00355	35.56847	23.32436	32.78957	47.65922
111.2275	135.6003	82.30716	221.8139	223.1799	224.2881	163.774	371.6857	282.7938
25.16677	7.692428	30.35737	28.42759	37.99478	26.6748	118.1965	29.40185	22.25991
501.8497	655.8826	1035.288	210.4349	85.54211	84.96816	245.7378	64.45847	308.2134
120.145	50.33247	24.18182	148.815	40.43981	141.2893	258.953	232.754	161.0058
579.5121	840.7854	532.416	1529.039	650.5551	1134.289	1499.447	1571.653	1502.957
10.27929	14.25264	11.25554	27.47143	9.57588	39.51905	3.549177	35.06665	75.19609
19.18808	44.86637	6.898978	109.9425	243.3541	214.407	17.4434	188.7169	401.4017
109.2418	13.15932	15.55292	54.97575	26.18225	33.59034	19.64081	94.86018	65.66712
42.98582	39.38343	70.98616	39.8005	52.25382	29.63771	85.64687	24.9056	52.96573
698.2698	799.238	842.3969	445.5143	1095.648	1634.251	1372.18	1537.748	520.0732
79.36355	75.45721	139.707	101.4287	583.2149	20.74411	31.34206	28.3009	62.50699
68.1869	17.53302	22.03254	120.3818	30.94288	283.5815	235.6439	231.5692	97.45761
178.7561	795.8821	1238.943	811.4721	297.9907	154.1324	681.7903	136.7688	358.0088
48.89056	76.55163	263.5412	48.32691	7.19879	20.7441	224.0513	14.74417	16.97216
267.2283	577.1227	352.5465	299.5698	174.5133	223.302	279.4835	129.9685	262.6609
57.15759	15.34422	34.73036	33.16496	40.39053	61.26094	37.9814	71.14942	57.19201
209.1795	80.94561	73.72278	283.4179	256.4662	832.9365	153.4877	1228.074	613.2588
1268.993	1098.807	913.5654	2387.887	934.2495	1733.054	1955.864	2179.52	1936.157
6.088719	6.49932	9.415091	0	0.039307	0	1.397414	0.027686	0.01284
61.6324	24.08439	26.20829	38.85663	96.02202	34.57934	21.11389	48.59122	23.32204
2.127699	13.15972	19.85881	5.666182	9.575776	61.25798	12.31896	143.4008	31.79635
814.8636	1471.541	693.6309	788.69	2062.805	378.4224	607.7744	395.5004	812.3816
71.93708	32.83649	32.76189	77.72347	53.47289	120.545	48.18572	77.98163	95.32704
43.70629	21.90506	30.59031	175.4181	32.11969	80.0324	81.9174	146.8039	51.91419
63.80691	35.016	21.99486	32.21244	20.25552	70.15217	56.28322	54.25468	68.84667
11.0214	13.15981	0.376645	6.613996	15.51133	87.93764	252.821	169.3721	68.85342
83.79332	28.46619	24.18593	161.1576	65.34925	375.4803	95.7615	170.5877	81.57202
312.4323	1395.876	529.2285	378.2373	876.9324	100.7771	429.4211	99.48212	345.2856
2.862253	10.96113	2.600132	3.770462	0.058321	16.79529	5.011197	36.08713	20.12654
5.086114	5.505762	2.599292	3.770275	1.256969	38.53382	13.79323	58.70957	62.4663
8.059828	12.0593	6.921545	30.33573	9.567944	20.74691	21.16184	35.00725	57.14967
49.75987	45.91252	19.76061	53.09562	24.97688	32.60412	25.53124	39.5508	29.66835
21.49573	15.32998	6.90864	14.20475	6.010623	15.80605	5.741343	30.48688	33.87818
19.93288	24.08881	17.70314	16.09324	14.32344	63.23519	21.83633	73.43381	80.48822
15.51836	65.48032	50.81584	26.54351	65.05184	6.911636	10.87801	18.10385	14.8444
1422.642	1450.839	676.7009	1018.08	1883.796	1212.346	1362.626	1727.582	2023.001
3.605583	19.71555	17.68354	21.78348	3.636568	38.53175	23.30939	57.62109	67.77734
460.0779	1492.261	1338.486	591.517	527.0588	252.9386	428.5645	240.7128	361.1924
259.6808	213.2263	226.4922	397.1875	157.9458	579.994	392.7683	434.9994	319.8784
31.28045	12.05001	21.48306	8.513813	13.09892	4.935535	6.480746	9.081436	7.435527
17.03318	7.687486	13.20384	8.513103	8.375511	11.85335	10.88968	33.83954	19.06959
100.8976	64.53446	52.10307	81.51143	97.36349	113.6261	100.904	99.44679	118.6282

19.23903	53.50858	32.2095	15.15162	2.449141	9.876059	37.36388	11.3454	31.77051
43.7296	24.08813	21.98329	53.07852	11.94981	83.98777	25.50108	80.19713	81.54387
44.42784	22.99979	28.48622	29.36427	32.12801	154.1393	74.53378	142.351	60.39215
80.78211	22.99807	106.0092	316.6088	199.4769	327.0464	582.3478	473.3902	217.1456
119.4679	116.9917	129.2335	116.5934	47.553	145.2456	162.4293	145.7428	163.1004
159.6501	91.84962	101.2951	147.8934	252.6318	48.41066	59.16471	48.6304	86.8606
16.96345	19.71832	9.05986	25.57255	30.94264	164.0142	162.2988	595.3348	461.77
25.88917	12.06565	21.954	85.33843	32.10031	50.39034	15.25034	50.85082	81.53292
7.315852	17.51765	28.0445	24.64056	16.67057	17.78196	10.87149	21.49165	43.4048
60.20368	22.98644	49.37479	66.38092	78.20977	12.84004	17.45928	21.50745	16.96788
160.9787	106.0856	47.87951	305.2466	103.3403	255.9072	157.1889	414.5995	143.0048
201.8445	73.28929	140.2794	396.2755	201.8062	219.349	144.0288	345.6838	201.2456
389.5835	279.9153	344.8354	340.3017	389.3716	579.004	393.4747	402.2531	328.3553
1204.389	445.0187	299.8502	1109.077	1272.559	3570.845	1422.55	3730.842	3478.302
21.48651	13.14926	2.602212	22.74516	23.75577	20.74738	6.473113	25.99035	25.42232
16.24606	20.79504	34.41228	16.09867	15.49188	28.65272	24.09047	29.38545	31.77588
25.13501	4.411217	6.938672	9.457226	19.06869	34.57832	53.35597	14.74324	94.24734
1.433606	9.687566	10.86725	0	1.227682	0	0.655407	0.025828	2.123484
10.31294	1.128823	4.753219	1.875084	10.73667	2.959312	0.631331	25.95861	10.6088
0.650982	13.02953	8.261485	10.45993	5.925084	0.983949	0	0.033176	7.400613
8.069797	33.85187	19.46103	51.24789	8.375507	4.935476	10.15377	27.09228	16.95506
233.9237	121.3653	64.99696	179.1747	169.709	77.06514	56.95453	220.2614	101.6905
38.53794	55.75838	17.6816	76.79944	43.9466	41.49641	32.84268	36.18619	33.90851
491.2694	205.5793	213.6591	510.9395	625.547	400.1613	490.7679	467.7853	580.4224
396.6856	161.8031	82.17617	148.8283	147.1903	67.18388	144.8223	79.13062	128.1645
20.09424	4.408766	4.72597	13.26635	9.540842	9.877873	7.229352	16.93949	11.66028
227.7262	126.8648	62.93447	171.5558	40.43337	767.7199	812.5951	1141.158	1651.207
210.7899	297.3382	324.5352	298.6277	295.4838	73.11185	44.50334	100.5988	177.9425
68.94538	107.1523	323.5282	653.3536	43.99179	13.82772	45.97885	17.00406	192.7399
30.4614	44.77263	9.038903	78.77026	56.79606	5.923482	10.14332	24.86091	27.53562
97.85706	28.46573	99.53779	346.9523	189.9742	390.2854	272.7992	271.1838	98.52655
194.4065	28.46635	58.63753	109.944	179.2728	203.5389	184.2797	235.0153	223.4867
97.90127	31.74608	62.87426	83.40409	155.4944	92.87447	45.97141	116.3976	142.9894
31.81492	3.316485	11.25664	60.65889	32.11951	141.2992	104.6495	76.83858	59.32498
27.43629	14.24435	15.43771	58.80902	22.58881	14.81698	10.13556	28.25612	24.3699
80.97356	43.74827	58.14661	372.8143	39.21253	29.63803	38.70612	17.00059	51.90348
30.31988	6.597782	17.7232	77.7201	79.57014	107.6979	78.93743	155.8867	123.919
5.097642	20.75375	41.637	41.7766	8.363586	2.959393	21.27441	2.311424	12.71826
14.12623	47.88336	16.94278	7.571186	37.67762	0	0	0.03853	0.017656
61.57568	13.15956	21.99412	56.87001	30.92767	70.15222	72.4199	81.33489	45.55805
29.63847	30.62833	9.08643	26.53056	19.05403	25.68664	36.55361	51.94638	30.7256
116.715	169.3169	238.1394	112.8429	9.575785	29.63791	134.1306	15.87182	28.61711
252.3519	134.5027	69.38624	639.0015	211.2977	121.5276	133.7804	223.7155	85.81492
47.41632	112.5946	211.9283	80.5711	100.8822	19.75609	109.7572	35.07493	13.79459
375.4747	120.3043	50.02717	323.2371	315.7959	775.6313	602.0122	641.7427	689.5006
16.96598	6.599269	0.37012	18.93879	46.31438	62.24892	17.4478	37.31401	55.07644
128.0147	53.56454	66.43118	49.29418	73.51808	28.65053	46.80684	20.38371	70.94227
50.41561	20.81073	13.40868	52.12786	43.96648	48.41236	43.08576	44.09742	122.8272
114.5297	47.02104	45.35842	68.26269	140.998	24.69742	39.44794	20.38636	43.43392
75.62598	26.27958	32.78845	7.562347	20.26008	125.4832	53.2964	166.0641	279.5627
61.57113	30.646	9.099339	59.71443	45.1535	101.7739	46.01545	68.9263	60.3792
150.5641	67.82572	17.69705	94.77312	87.9182	474.2723	22.56287	221.4855	397.1785
37.02637	2.222297	4.769009	23.67813	46.33844	52.3649	19.638	130.9779	55.0862
86.34702	100.4608	15.48998	47.4057	41.53715	14.81646	32.88507	21.50518	41.305
367.2906	811.2127	557.8686	572.5583	760.8421	169.9412	180.5551	149.1987	329.4184
142.382	74.38582	123.2547	203.7887	42.81152	2183.644	626.8757	468.9108	263.752
69.05783	27.36376	24.10046	77.74574	60.53111	21.73281	55.58262	42.95808	46.61124
77.84319	17.53303	56.43537	55.90892	74.84324	122.5177	65.73401	153.6612	166.2845

65.22877	20.81321	24.18337	67.2887	72.45975	53.35093	119.2182	194.2785	82.62832
25.14661	8.786155	24.08299	53.08479	15.50684	54.34338	28.4449	67.76605	34.96624
462.3529	3599.651	1195.852	360.2119	464.1244	19.7564	69.36981	67.84898	159.9574
155.7872	678.797	479.3124	250.2598	344.1718	279.6222	108.8909	125.4568	237.2537
69.00442	119.1271	32.70864	142.2306	34.48723	33.58987	57.74077	76.82896	30.73731
20.7015	22.98495	13.3611	36.96758	13.1296	126.4945	30.67916	21.50559	26.49219
150.7033	116.9867	47.80927	137.4578	121.0759	112.6381	63.55198	145.7334	88.98085
36.32892	186.6941	129.5473	33.16856	108.9859	9.875584	6.468785	14.73925	33.90361
9.538669	2.223159	6.940001	25.57915	10.76115	55.33282	9.394932	50.83718	40.25451
665.6713	96.25195	101.7235	303.3235	351.4238	383.3629	588.7621	1027.024	918.2889
16.23931	2.223645	6.933527	14.20025	6.01338	41.49978	38.78071	40.66361	22.25742
40.72454	27.37122	26.31931	111.8639	26.19197	77.06687	54.04821	125.3866	39.21088
170.6678	71.1015	92.99739	176.3144	144.8508	166.9807	248.7455	129.9657	141.9397
61.50192	57.98105	15.56352	40.73997	28.56918	220.3413	101.6083	242.8616	147.2257
15.62459	9.853798	0.317987	16.12448	2.446583	21.74343	5.765509	4.565314	7.429306
654.5893	544.4748	347.0617	255.9272	203.0545	720.297	514.9094	716.3185	622.7869
29.67313	21.88576	0.35728	35.0779	11.9391	14.81694	10.86866	40.6487	26.48607
264.0937	279.9267	153.4142	352.6163	350.2437	1042.403	386.1	1105.002	753.076
296.8364	579.3982	1102.416	410.4709	651.5226	31.61279	922.037	17.00084	123.9451
74.16927	15.34653	6.930195	35.05387	115.1172	82.00746	92.88155	225.8327	63.56421
60.04515	75.45418	131.0875	180.1449	83.11296	65.20911	100.9425	38.46438	144.0254
345.8202	497.4346	454.253	656.9588	687.1596	155.1208	291.8004	172.916	149.3647
66.78054	207.5739	194.2754	72.99282	111.4965	58.29393	32.82317	39.58349	79.43245
139.4393	67.82499	84.46351	185.786	112.8358	247.0138	83.27411	314.091	321.9787
1061.22	858.2738	269.7107	1082.549	491.4993	1261.751	771.5917	1746.756	1735.96
66.01237	87.46174	75.45992	97.64528	52.27595	96.83122	46.00151	90.38198	36.03263
5.099791	4.408582	10.97323	37.9806	7.180217	12.84355	14.62242	12.44408	5.31718
213.6688	152.0037	194.1701	122.2648	176.926	295.4283	182.7762	302.8185	525.3219
192.8629	59.07937	13.368	91.92838	97.41627	378.4252	31.33984	389.8197	526.398
488.9851	158.571	202.9455	536.5225	581.6919	813.171	692.6175	806.7476	759.4317
22.16951	32.8246	41.12712	121.3886	32.10145	38.5318	27.70864	92.58084	37.08413
34.13386	19.7059	4.776735	40.76808	24.96367	9.875792	3.545448	37.2798	20.14041
45.19627	107.1204	98.86719	326.2399	47.53151	39.51847	30.61927	45.23229	69.91042
710.2684	582.7364	420.2001	59.69837	64.17962	131.4067	3649.777	41.85964	111.2381
52.6283	47.03908	292.446	41.69302	243.0226	4.935446	4.280411	13.61421	4.26036
16.26614	92.71756	48.71573	59.77812	27.29213	2.95928	26.33838	1.179127	28.59228
482.3189	147.6378	73.71564	355.4616	404.8357	777.6019	533.1549	1252.976	868.5135
54.10695	20.81243	11.25608	49.27774	48.72421	68.17401	88.50004	132.1481	83.67732
17.78506	6.59543	8.997737	26.55282	11.92075	11.85345	11.62803	19.21524	8.493951
222.7495	29.55957	22.03259	176.3264	109.2392	106.7081	147.7528	192.0471	80.51418
31.05935	2.220942	4.749993	32.208	36.87612	111.6488	67.20134	220.2694	90.04397
53.55224	72.07219	67.98394	26.53485	80.50263	7.89955	35.11694	7.96246	22.25589
132.0502	45.95856	37.10769	386.8111	172.1266	175.8741	209.9525	198.8498	135.5841
34.10756	24.07815	11.22993	49.30099	111.3083	30.62773	10.12891	42.93118	28.60967
36.35646	45.90488	105.9472	50.25384	54.54095	6.91139	20.40032	10.22156	21.20023
76.45215	337.5147	914.5977	105.241	7.200683	3.947433	42.35005	5.703076	29.6781
4.369179	33.72483	60.00983	47.55359	5.981796	0.983577	8.007524	2.308584	7.424542
117.9643	48.14301	39.246	66.33823	52.30161	112.6368	111.1331	150.2709	141.9296
90.4288	321.4647	131.8739	248.3424	48.74634	342.8527	745.3671	822.522	600.5504
688.8843	1106.909	300.4577	310.0299	364.1664	0.983839	24.02117	6.831292	8.497501
17.80911	79.53477	2.596139	24.66066	14.27036	1.971343	37.53323	4.570481	14.8354
70.42705	43.76921	24.18428	102.371	74.83386	168.9611	51.8333	124.2886	97.45306
1.394748	8.713514	30.66485	20.00518	2.427435	0	0	2.297984	0.015431
48.18948	32.82967	21.98824	64.45909	52.25745	31.6139	37.22545	59.89304	60.37657
80.8865	32.83412	11.2566	161.1906	73.61175	79.04421	51.86334	79.09663	102.7289
267.8191	540.1057	342.7822	272.0423	260.0261	785.5092	442.4661	710.6776	731.8759
88.94832	167.3072	123.2051	630.4234	277.7855	239.1079	181.3157	336.7019	123.9452
472.8376	141.0707	101.6909	207.5877	550.6703	335.9404	238.3996	335.5692	324.1047

5246.207	13661.88	5709.226	4370.035	5265.605	1774.549	2373.436	2988.55	5322.302
21.41041	32.83866	9.086838	3.771758	10.76148	102.7563	7.935618	192.0304	184.2758
216.6115	1058.241	2332.834	877.8308	263.5774	208.476	56.93643	146.9375	317.7648
27.42338	15.33805	9.074022	22.73905	14.31015	38.53537	16.00259	76.69946	33.89386
1425.637	635.249	866.1164	1373.572	1666.564	1307.201	1594.547	1118.6	799.6888
460.394	284.2342	97.30786	495.8388	327.5305	137.3374	119.8803	129.9686	220.3038
60.21436	72.09898	72.4869	92.00705	66.37639	19.75736	21.86449	19.25015	15.90899
164.7493	67.82011	43.55738	171.5789	230.2299	185.7559	90.61616	245.1372	152.5243
26.00357	53.4683	36.09586	6.615298	17.83183	16.79496	9.415375	11.33853	9.552787
26.62478	231.5581	312.3645	66.35836	201.4312	3.947393	15.24759	11.35408	9.557744
118.7424	53.60472	60.66828	62.54844	86.68766	171.9265	178.5792	76.86263	79.45085
131.3002	41.58598	56.47069	187.692	132.9877	173.8974	111.1015	203.3736	323.0143
19.24689	85.10468	110.9761	45.52987	8.381477	15.80579	2.814894	1.179056	44.45473
5.104387	29.43484	63.21653	29.43376	8.353411	8.889956	4.288689	10.18962	13.76854
42.97091	19.71837	17.71074	103.3413	29.74472	101.7733	122.2845	70.05889	41.32495
65.33597	61.22064	81.46285	63.51679	48.68756	25.68542	56.318	17.00045	51.90305
192.1552	124.671	398.0817	275.8512	270.6487	169.9429	250.8591	206.7925	236.2016
12.57926	13.13119	0.330343	17.06524	21.3227	5.924088	7.963911	20.31345	8.490323
133.5089	90.78062	67.24293	106.1517	67.73784	121.5274	130.1148	186.45	200.1895
32.63983	12.06219	11.21293	17.99483	32.06215	43.47662	16.00066	33.90026	31.77928
16.21664	64.5192	45.56102	31.2627	11.9503	84.97344	40.14164	90.37949	111.1957
129.762	377.2242	62.94322	80.55318	17.87657	735.1153	1256.014	1472.131	994.5443
13.2988	18.59295	56.53009	60.7528	15.46281	0	2.085077	1.179347	2.142063
14.73177	84.18604	4.764525	50.22696	26.18987	284.5858	9.39425	63.29635	131.3113
281.9773	142.1652	125.3634	309.9745	289.6561	384.3558	214.9731	350.2609	276.4511
106.8349	127.9188	86.41305	136.5063	68.90483	172.9134	70.86899	204.448	136.6303
51.86349	7.691737	11.25331	67.29092	74.82234	111.6509	38.66197	146.848	154.6185
79.31597	62.35592	28.49547	95.72621	106.8849	115.5998	114.7665	158.1964	195.9433
663.5149	155.2892	58.64471	469.2286	79.61175	739.0714	225.9159	608.9913	295.5253
120.3759	299.2881	262.0682	72.04505	42.7814	86.95137	87.09105	42.96883	50.8512
17.79171	15.32099	0.340552	19.90668	7.190312	22.72651	15.31816	15.83647	16.95283
47.39512	53.60749	32.79246	116.5916	47.55446	99.79162	38.65484	126.5543	135.5742
16.24266	7.691729	6.931045	12.30373	15.49413	41.50038	24.08402	36.15062	38.12498
60.13121	236.967	507.5832	80.59447	26.17629	5.923331	27.70942	4.573038	21.2053
3.605199	4.411719	0.369399	0.928917	28.54402	76.08465	1.361198	20.38587	136.5522
68.95764	6.598002	17.72256	148.8443	65.3344	96.82903	87.73982	110.7234	59.33049
68.9112	1686.462	202.6068	250.2579	273.0026	49.39778	260.3997	36.21233	119.7057
11.02175	12.06638	2.583039	47.38058	14.32459	95.84181	162.5353	88.1369	70.97451
16.23107	48.09707	11.23335	9.457717	29.71401	132.4217	18.19124	14.73824	77.28063
31.92091	8.783548	9.061636	12.30472	9.568888	15.80532	17.4807	30.50661	37.06136
41.64174	4.412004	2.602182	9.459313	19.0357	24.70015	8.670637	48.51891	24.36784
64.47146	77.65568	114.3635	133.655	85.52156	120.5412	165.3163	168.3442	238.2878
2716.938	2492.77	2136.74	1973.612	3876.786	2963.188	2808.698	2654.094	2285.693
15.47439	48.13057	81.8224	205.775	27.37459	23.70856	5.740404	19.26243	24.38471
406.6681	179.3388	493.1463	251.1902	663.4821	158.0847	591.7905	73.49839	818.6986
196.86	178.155	188.7455	164.0194	51.10269	26.6726	36.46742	12.48415	96.38698
315.5233	200.0774	448.941	271.1265	114.0086	139.3137	88.40827	88.17665	166.2968
16.97014	12.065	6.940167	60.67893	14.31919	23.70957	43.86937	41.81748	28.61327
243.4177	902.8525	170.3924	54.01063	26.19453	97.81335	248.6896	246.3136	150.4173
34.77568	1.126656	2.579197	41.68965	41.61769	113.6267	33.53611	415.4838	52.97849
95.95636	236.9007	87.50782	38.85946	14.31877	20.7451	40.20147	27.15292	19.08639
54.8756	3.316831	11.25624	46.43696	52.26427	97.82117	48.94783	82.46658	49.79327
5.120467	36.97518	48.20703	3.77403	2.443559	0	0.631228	0.037543	4.255733
1.390147	3.305451	10.38095	0.929201	0.049833	0	0.633773	0.034881	1.080288
12.56401	6.593694	0.336487	8.514857	14.27152	14.81937	21.24329	32.68777	5.318593
47.49	79.76626	104.4737	53.08798	182.3508	29.63862	19.65052	27.15484	39.19762
11.78484	1.128555	2.602244	24.64274	9.566509	20.74723	19.69741	48.50734	19.07712
99.36846	83.12429	86.53826	166.8352	160.2652	176.8622	126.481	158.1956	204.414

671.6021	17.52839	32.76389	110.8872	141.3369	668.9143	91.31411	638.3999	1443.606
221.4462	216.3762	1096.955	201.0129	10.76273	0	705.3183	0.043083	3.200403
0	1.103394	9.992501	2.863145	0.027714	0	0	0.019655	0.009198
4.346224	0.029393	0.346299	5.666727	0.058653	34.5866	17.51121	18.09686	20.12934
865.2555	537.9472	605.6288	760.2361	574.5912	1307.2	932.5097	1688.028	2295.193
11.04212	28.42069	0.351095	6.614591	0.059172	78.07664	36.65248	5.702455	10.61261
20916.41	7546.082	10142.14	25166.11	21618.05	23937.7	38602.54	29469.38	24536.64
64.50039	239.3317	148.2165	122.2923	135.2762	44.45839	113.3921	63.30694	60.38889
125.3313	95.15516	75.86027	265.4236	66.55279	165.9907	177.6736	299.4092	347.395
120.1388	273.3232	73.70132	469.2701	218.4313	194.6456	302.8544	250.8339	199.1324
636.8059	302.8731	86.64889	415.1941	494.9914	564.1826	460.7884	804.4098	311.4104
664.1282	572.9301	254.6365	1072.12	55.8646	1166.896	807.4345	5171.109	2695.53
164.6765	272.2422	26.3306	126.0579	151.9996	1236.095	50.35248	460.9356	851.476
11.78154	16.42605	46.8633	31.28457	15.48716	14.81717	40.30418	7.961175	12.73035
86.2123	129.9921	49.59946	92.9294	43.94367	34.57921	26.97784	40.69728	28.61532
19.99524	3.318274	2.60217	14.20401	8.381177	19.75914	13.81667	37.24592	9.55444
118.7895	21.90581	34.90001	95.74261	105.6307	107.6995	99.48318	125.3853	32.85735
51.10427	60.16751	52.1461	123.2253	70.09972	162.042	81.10513	319.6415	198.052
66.76766	62.33151	126.4806	84.37095	90.18976	39.5186	59.93598	47.48819	67.79179
87.47005	15.3447	19.86455	63.49039	135.3786	128.4438	484.4097	497.0441	202.3097
71.90652	16.43972	4.750873	72.97582	77.21114	96.82728	62.81053	141.2325	67.8061
18.55045	40.36847	176.288	6.616465	68.429	2.959327	2.085126	3.442015	7.434938
1430.777	1166.595	1619.816	1461.722	2853.565	2431.613	1851.235	2245.064	1504.03
71.91332	108.2478	30.63697	31.26029	65.34515	152.1629	116.2825	123.1592	112.2765
115.0855	210.904	345.9511	60.6569	80.73206	4.935475	149.3543	47.49488	24.38535
37.1554	5.505289	2.602155	2.822553	9.568317	26.67673	13.80859	30.50418	28.59907
154.2649	103.9044	45.72221	83.39682	175.7476	346.8071	338.6183	568.2919	494.6228
1755.087	675.7073	594.917	875.8819	756.2114	3045.198	5082.194	3527.452	3854.292
532.9535	170.5885	153.3298	352.6355	322.8843	407.0814	220.8238	256.5079	227.7356
67.53886	18.62447	11.25507	6.613727	66.47244	75.0937	14.51494	67.79007	73.0778
26.70806	37.15015	148.4493	22.74393	4.825406	6.911559	0	1.178986	3.2018
137.3167	84.20568	99.23339	60.65151	153.092	85.9586	92.11303	106.2216	91.09924
207.8376	112.6337	125.1394	248.3799	193.4685	118.5644	114.0412	179.6469	158.8786
42.22564	30.64719	32.71518	77.73205	65.3064	29.63729	34.28406	90.37292	86.84592
23.07252	3.317271	0.333376	2.82282	9.547162	24.70539	14.60234	27.06052	18.0037
28.18463	9.876061	4.77484	36.02816	9.569273	31.61804	8.669237	29.38172	20.13786
2.128939	5.488792	0.296379	3.774996	3.621974	3.948476	2.828928	6.800282	5.310504
24.44266	27.34498	4.776273	14.20094	16.67737	23.71103	18.94165	24.8803	22.25582
0	3.317367	17.24568	1.875108	0.057345	0	0	1.179416	1.081678
111.4523	6.599	56.13993	114.7303	27.36998	65.21151	202.3411	54.25259	27.55993
50.42136	38.2922	15.55581	46.43854	53.44234	39.51912	52.62354	85.84224	83.66276
37.0605	14.2509	9.095877	27.4752	9.575157	136.3697	18.91666	29.41206	67.77071
19.21998	28.43887	26.05131	34.12474	14.31126	34.58186	8.666472	30.51925	58.22421
120.2563	40.48757	34.91304	41.69041	65.33338	87.9359	65.02976	115.2371	148.2637
31.17775	15.33493	6.923235	40.77602	21.40214	16.79365	6.471461	20.36533	15.90502
3.606304	15.31427	13.11152	2.822784	21.33706	16.79667	11.64528	10.20264	15.89119
143.3799	29.55566	83.9964	69.1936	97.3136	48.41153	60.65759	29.42722	85.79159
52.68026	62.3113	204.0548	37.90641	40.39479	33.59082	150.3323	29.41659	34.96738
8.80005	32.80248	9.074199	4.717928	7.199669	40.51199	67.50216	29.38997	15.90715
118.852	87.45594	258.2071	135.5882	122.1771	0.983749	3.550136	2.309282	1.080587
19.97675	27.34083	9.064142	23.69019	10.75405	27.66471	43.2345	22.62149	32.83186
129.8484	104.9777	101.4828	88.14463	129.4045	129.4345	97.94854	136.7237	130.2832
11.02155	89.62366	9.09956	10.40516	3.635948	177.8687	77.57342	68.91603	73.07668
20.66871	4.409952	0.379704	2.824047	40.43074	90.89985	9.395797	145.7228	91.09596
34.79801	86.35425	64.66804	50.23131	32.11288	6.911387	73.15498	18.13181	23.32486
80.85043	229.5038	414.7487	135.5678	78.37723	60.26819	42.32398	65.56603	68.85985
7.313488	2.223699	2.60113	3.770188	7.199794	28.65222	17.4699	33.89902	46.58834
7.425411	0.025578	52.39634	2.827254	9.459668	0	0.633079	0.03543	0.016291

337.5648	8175.299	3932.624	872.1003	7046.526	465.3711	2024.022	72.36532	155.723
21.56611	8.774609	106.7614	1.875108	35.46033	1.971347	2.085284	2.311646	7.434049
178.8395	421.9273	356.7295	298.6254	293.1196	111.6472	176.2577	101.7295	135.5858
120.8792	159.6487	138.197	311.8819	68.92593	352.7402	211.3475	413.4752	249.9656
28.23769	26.23236	19.52283	22.74927	34.36028	11.85302	41.85365	12.46698	14.84261
19.98733	14.24192	107.0423	27.48949	8.38254	5.923426	41.79117	5.702901	7.437952
45.1988	25.18328	13.41127	48.33207	57.01171	71.1395	76.79852	92.63105	158.8238
667.8851	238.3812	123.2619	971.6515	274.2775	958.4182	206.8828	999.9223	756.2495
86.79893	26.27868	17.72239	171.6053	77.19056	112.6394	66.49466	120.8813	93.21128
197.3319	150.9112	666.8245	639.898	243.381	126.4668	875.1355	175.1737	151.4823
22.95693	16.42833	28.0993	13.25284	20.22467	34.58274	10.13491	9.090807	38.12172
149.8574	70.00952	62.93132	136.4914	201.8025	247.016	120.6087	280.1789	175.8294
66.68319	43.77075	11.19311	23.67842	1.251134	468.3367	337.8282	1110.624	1936.08
5012.378	14530.01	6661.259	4394.675	9642.077	2441.489	3409.163	3089.118	6698.16
26.61576	16.43937	9.098575	63.50552	14.32422	159.0877	48.20579	58.77605	165.1751
3457.654	1521.922	993.313	1938.54	3322.457	5271.302	3082.29	3136.529	2594.963
13.25386	145.2373	156.9691	104.3283	10.76098	27.66247	6.468772	24.89602	20.14481
305.8265	289.7096	303.3896	242.6765	131.8129	143.2655	264.0788	111.9009	128.1766
8.053386	26.2721	21.97147	20.83468	7.201135	41.49597	14.51628	65.52377	55.08009
334.0959	461.2777	208.888	477.8254	800.7365	30.62465	341.7298	73.49233	40.27385
143.9605	190.2207	467.4681	118.4846	155.5002	95.8386	116.9831	63.3209	137.6966
2.278936	2.172224	0.12835	0	2.337417	0	0	1.143172	0.009246
90.81012	64.46149	32.46155	47.40422	28.5255	4.935287	32.14566	7.963544	29.66731
57.20442	45.91603	66.31098	53.09324	47.46974	12.83997	199.8249	18.12443	20.14409
23.66135	3.317592	0.368831	71.11168	15.50606	44.46153	46.06136	36.18202	40.25714
86.12735	164.9879	81.72228	63.50816	106.7602	63.23475	90.753	54.25609	70.96463
10.28541	114.8248	6.9079	18.93683	250.4275	301.3626	4.287065	211.2825	238.3044
90.52656	25.18521	6.93033	36.9502	62.95749	56.31599	150.7917	102.814	42.38767
80.88422	66.70618	156.429	13.24889	222.8977	2.959541	1.363734	3.44054	16.97193
453.3575	872.4717	114.6434	591.505	222.0533	1264.718	790.6474	1082.411	613.2724
644.2365	211.0432	65.1102	367.794	487.8681	262.82	550.796	342.3801	301.878
7646.185	35741.36	12396.72	8582.703	8713.965	6287.015	8630.92	10846.82	18291.78
42.36117	7.691457	13.32623	40.77166	34.41572	27.66441	18.21002	55.29222	24.37089
239.845	156.3388	84.31841	145.9839	92.6341	160.0664	101.6135	190.9178	187.4594
22.94374	48.08265	53.42656	112.9012	15.49644	8.887622	177.9729	15.86294	23.31563
25.21374	8.782776	4.772276	23.69294	11.93501	26.67714	21.16646	14.72733	31.76952
10.35402	6.587442	4.702082	15.1726	5.993996	9.878663	3.553974	7.940818	17.99046
43.03144	181.2448	125.3884	44.55219	80.60604	12.83991	16.72099	24.89582	29.67078
150.6826	216.4274	161.3284	326.1601	115.158	89.91075	57.68858	59.92828	114.3955
10.31593	20.76751	19.3796	64.565	9.553169	8.888684	3.547188	3.442091	23.29249
39.25252	13.15972	17.71138	32.21156	15.51084	28.64919	32.08679	137.7592	74.14278
100.9201	27.37191	49.92521	76.77352	51.10437	99.79336	56.97006	143.4597	94.27088
21.48329	2.223992	9.048527	5.666184	10.7505	13.82912	20.43343	30.49669	19.0771
329.4492	111.558	185.6617	324.1854	369.198	345.8181	487.1335	359.3258	472.3904
26.66196	1.128114	11.2246	52.15042	27.33935	28.65154	7.200859	78.98302	26.49192
25.91509	3.31804	2.599668	27.47966	49.81908	34.58097	18.92928	44.05467	30.72504
201.784	113.7427	146.883	464.5044	277.796	232.1908	280.8267	357.044	198.0822
5.08963	4.410695	47.7067	9.457319	4.82359	190.7083	0.635978	90.382	24.38495
498.9026	724.7265	196.2014	411.4317	669.2362	89.90861	280.1509	73.49607	119.7065
41.54726	12.06447	13.37721	25.58011	13.13215	77.07555	24.05772	46.3212	30.72825
14.82528	6.592057	4.734166	7.566874	18.97263	6.912378	5.7529	12.44947	15.88899
8.053406	5.5056	6.940557	6.613591	9.57545	84.97864	5.738127	24.90081	101.6365
221.9232	179.3098	165.9686	153.5592	269.4008	145.2424	176.2554	225.961	131.3503
158.2785	168.2866	330.5902	130.8423	10.76306	28.64908	212.4934	33.94304	73.08668
17.70073	127.8864	54.14716	276.8967	27.37705	72.12664	19.63443	55.39723	65.6793
48.14121	61.25645	79.97377	14.19694	25.00777	105.7209	202.0062	164.9311	113.3352
13274.64	19852.34	13764.22	15926.47	14728.54	13227.14	14361.82	10368.88	10893.53
223.2951	301.7781	336.2184	308.07	276.6292	452.5304	802.5348	582.9944	252.1001

21.44037	18.61874	19.76626	46.45272	20.24097	36.55696	15.25781	42.93492	30.72716
28.89838	3.318072	13.35849	15.1481	43.90173	52.37031	31.41636	16.99293	38.13073
5.108756	3.315085	2.576627	0.92828	3.6301	13.83379	7.986607	16.91448	9.541203
46.66395	37.20897	15.56824	54.96467	47.54586	57.30382	67.95857	118.6251	49.80077
356.3719	1780.315	489.7374	254.0581	26.1951	304.3263	308.7667	30.56289	133.4697
488.3099	1387.286	1280.077	685.377	407.1804	215.3924	479.8029	290.4174	259.5155
9.551827	5.504838	2.602156	18.0001	2.449255	40.51536	12.34672	63.12374	41.28162
228.5518	221.9539	116.7099	252.1518	115.2082	357.6805	176.9459	326.5117	294.4436
82.30174	125.741	56.43175	81.50804	147.1912	123.506	103.809	85.9068	162.0481
77.84079	24.09326	22.03241	110.8994	61.79489	76.07659	28.4099	115.2711	128.1667
353.8883	509.5231	271.866	2322.479	21.43449	1426.755	2493.535	2577.212	1430.944
152.8616	672.1346	478.4403	335.6156	232.6023	78.05253	97.20468	54.28631	92.16532
45.96347	97.26572	96.44891	131.8111	103.1778	46.43655	54.83177	28.29187	39.20427
110.1954	65.55738	40.94054	126.1679	75.84757	0	34.34398	12.48044	4.261225
83.09472	255.7006	139.5792	109.9676	198.0665	82.00764	59.90996	45.23871	58.26971
12.54523	14.23479	0.344896	18.95368	6.008787	14.81828	13.83185	39.46748	26.471
460.8775	196.8292	62.95747	275.8394	375.1186	614.5764	460.0846	422.577	815.516
2.243087	2.182896	16.83173	0.937584	3.494499	0	0	1.151246	0.010564
132.0726	303.8671	277.0913	266.4034	226.6535	83.98139	154.3319	61.06192	119.6954
167.0454	22.9998	34.9352	46.4294	168.5088	29.6367	18.16988	15.87401	74.15799
12.53755	2.22407	2.601806	6.61487	11.9293	44.47024	9.411118	68.72714	28.5905
7.391423	25.0208	20.50126	1.87634	2.441439	0	29.74275	0.036874	0.016928
19.96889	5.505594	11.20675	32.22841	28.50915	31.61732	19.67584	44.03579	33.89329
56.44416	9.878966	6.939834	14.19844	62.86184	21.73331	31.39293	82.40052	60.35907
7.32079	6.59588	9.068131	10.40654	21.44578	138.325	5.748672	253.0824	476.5716
78.61275	66.71756	41.36112	94.78878	83.12581	131.4133	78.93972	128.7918	69.92013
26.642	22.9903	11.24258	17.04311	26.1697	23.70957	60.0279	53.09449	49.77943
74.87853	15.34628	15.5657	162.106	111.6052	141.2927	83.31269	251.8735	171.5737
62.31408	19.71809	26.28271	81.52842	55.81991	65.21103	46.74783	77.95394	60.37943
146.1775	25.18658	22.03182	190.5428	102.1331	191.6847	46.7005	302.7204	122.8743
437.8161	1170.86	1404.967	755.5259	811.8651	239.1058	453.4494	309.6261	316.7086
302.8955	49.23866	86.5617	301.4677	98.58461	165.9924	355.647	159.3319	138.7636
16.2401	17.52275	15.47032	15.14892	29.69733	32.60522	10.86498	37.28163	37.06913
11.86209	13.11814	2.578932	18.02639	15.40477	19.76595	5.027925	11.30869	10.59826
184.0186	695.1697	2030.156	978.4467	81.97651	0	754.695	5.699029	23.32697
190.7041	110.4549	123.0993	195.2727	87.90938	243.0634	71.573	202.2557	258.4289
40.77483	10.97253	24.08376	29.37108	38.02185	69.16677	45.31986	76.78788	40.2585
152.6875	21.89637	102.1573	54.04106	49.83944	17.7807	13.05747	14.73905	9.557293
72.9312	81.90593	107.9716	32.22533	16.68176	13.82837	29.21789	16.99172	21.19978
140.9376	103.8975	75.84129	274.9119	141.3052	239.1105	184.2828	213.5542	229.8396
7.330127	19.67624	21.42405	24.65882	6.005295	4.935603	14.58955	4.570839	4.260278
19.93433	7.692733	9.099538	27.47202	3.635987	53.35376	56.29751	80.19624	66.72567
10.2998	18.60189	34.13938	56.93213	8.379592	10.86461	5.741664	7.958822	39.16268
149.8121	491.9764	220.0293	144.0675	71.30257	587.8992	383.2614	467.7542	431.0783
11.04873	22.96085	290.9478	13.25697	8.378097	0	13.82699	1.179233	1.081577
11.76654	6.599312	2.596319	17.99129	15.50524	32.60323	26.25098	55.35179	12.73435
95.85942	33.9192	17.69125	82.48666	83.03544	41.49597	40.16919	53.11463	52.96319
162.4622	478.8068	713.2748	241.7237	102.1542	93.86094	214.2772	132.2372	359.0398
207.72	119.2092	99.55633	127.952	783.2728	228.2383	184.9637	370.603	344.2337
25.17132	2.223508	9.084448	38.86551	14.31442	53.35847	10.86251	56.44975	7.439003
26.77969	21.86079	11.08769	52.20496	20.17951	5.923727	13.10503	9.081663	9.551326
787.3983	3450.225	1823.019	740.3425	885.5094	16.79249	223.7075	110.7841	230.9229
5.835694	26.221	179.1271	29.40297	6.007411	2.959304	0	0.040509	3.201603
28.11217	40.47482	4.772051	3.770733	16.69534	19.75642	0	76.80924	40.26254
191.462	137.7779	159.5454	155.4549	85.53317	111.6471	158.6815	109.6371	149.3532
8.088748	34.89363	41.53699	52.24069	11.89839	1.971367	2.08578	1.179382	10.60354
63.02872	17.53294	0.377437	8.509579	112.7305	22.72035	1.364015	138.9182	183.1846
11.13399	18.54023	66.21369	11.37645	1.25742	3.948081	0.631034	0.037959	5.313468

42.9788	40.47812	28.41338	33.16095	10.76306	82.99878	30.62682	51.99654	118.5923
114.1945	579.3703	486.0245	266.3716	173.3528	148.2052	223.7832	110.7762	137.7109
75.19614	67.71622	15.46767	86.32633	62.79603	27.66388	16.00092	22.62826	14.84912
42.2843	27.3582	11.24051	45.50045	38.0092	51.37993	21.11991	48.58098	74.11436
23.86518	16.3932	45.48138	24.67228	5.998662	5.924251	10.92533	1.179355	15.88478
43.00966	39.37591	36.85243	30.31969	28.5444	48.41413	21.84615	74.53121	75.18172
60.03077	3.315639	17.72327	63.49664	94.98799	100.7807	71.60969	100.5729	72.03888
67.45055	37.21143	52.12335	100.4729	43.99467	140.3046	104.5519	83.64451	100.6316
97.21466	45.95115	43.48115	90.99949	108.008	77.06666	63.56878	103.9449	97.44446
38.48203	168.3649	285.6608	221.8368	112.8035	47.42196	23.28968	177.3773	116.5189
115.7849	167.2376	62.78262	133.6694	147.1404	84.97118	63.56024	144.5916	109.0942
1277.964	704.1213	1216.875	1143.221	1227.385	1008.806	1864.538	801.1143	768.9704
5.844904	12.04078	551.3134	5.668962	3.634241	3.947572	2.085687	2.311338	0.018109
419.2105	829.8403	401.0328	1086.348	680.2109	870.4782	469.4874	1999.789	1642.742
0	2.202245	7.772011	2.839728	1.237032	0	0.646778	0.028638	0.013268
80.25997	48.11111	64.41124	53.08565	56.96463	45.4496	85.71549	38.43939	32.84881
365.7936	1857.403	747.2711	1378.358	570.9817	276.652	302.7195	498.3009	593.1395
97.93482	42.67488	17.7232	111.8567	108.0273	117.5792	58.4294	67.82809	128.154
21.432	6.599312	9.095184	57.83178	24.98595	27.66226	36.5247	83.53788	26.49672
16.22153	13.15892	26.23575	36.00891	14.32194	67.18947	69.52823	27.16036	28.61651
0	14.03437	101.6535	1.883981	1.239514	0	0.64459	0.029452	1.076565
10.31063	25.13144	38.02493	9.462501	36.68514	3.947372	6.479745	12.46131	34.92096
98.80499	20.81018	15.55515	93.86383	33.2961	85.96388	30.62938	94.86543	94.24615
58.00924	29.53136	19.71659	63.54382	52.16549	9.875758	31.42716	42.91732	22.25743
48.90763	42.66941	22.01496	54.96824	32.12055	86.94928	35.01067	135.5254	106.9647
80.06249	59.07577	279.4089	277.774	100.9489	74.10013	392.3417	56.54573	90.04767
1562.206	863.7444	932.8546	1156.487	1727.098	1390.198	800.1102	1359.25	1666.064
36.45933	5.504512	2.601821	13.25614	10.74649	35.57413	28.55751	29.35866	16.95883
294.1227	555.1515	460.7052	166.8461	14.32376	44.45783	321.395	36.21053	81.5724
37.13819	82.96654	211.4824	15.15019	59.2237	0.983597	1.358496	0.041637	9.555859
9.549446	53.50487	48.87396	99.65043	15.48444	12.8408	17.48853	3.443035	28.59667
12.54978	5.503488	11.10197	10.41128	1.258402	24.70295	16.04876	27.09	16.9545
86.86577	22.99695	11.25643	100.4973	47.52553	69.16361	53.34406	57.64333	68.84854
134.2668	48.14549	52.17326	43.58321	70.10802	326.0652	109.6341	233.8695	183.2401
38.50735	5.50488	13.41133	87.21571	39.23152	65.21048	43.07588	65.54764	66.73362
476.4928	177.1496	73.72531	495.7865	163.8777	302.344	220.0836	436.1221	369.652
257.402	107.1056	115.7015	84.3754	117.4136	30.62563	100.2981	24.90727	30.73654

TCGA-DU	TCGA-CS-	TCGA-DU	TCGA-HT-	TCGA-DU	TCGA-TM	TCGA-DB	TCGA-DB-	TCGA-DU
477.1258	75.86471	84.88778	104.2859	74.42013	282.4667	153.3083	110.8465	78.2236
38.062	86.56822	42.47974	43.26231	79.12712	32.66934	28.37927	28.87675	47.33982
150.0938	200.3739	130.0511	101.3497	109.3714	161.7643	200.785	143.104	51.83446
3861.256	3370.378	3199.681	2159.563	2577.178	3968.264	3283.639	2298.242	1484.968
139.9744	43.36896	101.7089	75.46501	164.531	120.6384	49.06517	94.68774	71.85529
0.623327	7.540011	0	0	2.743595	0	1.371537	0.036194	0.880906
12.08958	196.8853	18.53774	17.25476	24.79486	52.82171	57.87977	8.144795	1.784762
144.2625	203.6575	260.1366	154.1629	157.1667	140.7502	180.3231	176.5966	58.20031
11072.53	12761.07	9232.791	5964.713	6059.676	7652.382	7536.096	10783.47	9890.888
35.80341	31.44651	95.57349	54.53167	22.94588	42.69064	58.00263	106.1305	60.04659
14.29328	14.10296	18.56534	20.37874	20.21634	25.99722	2.692441	20.78257	34.61767
1515.4	905.7165	1510.96	1481.84	584.528	1490.633	1392.516	1280.11	1798.961
137.0573	49.87004	70.72638	115.0823	155.3216	65.29216	63.23541	49.69922	106.4365
0	3.266071	0.844966	2.287245	0	0	22.46401	0.038563	0
97.60028	46.61533	91.10138	57.46499	22.02391	72.85811	41.3327	70.45052	84.60806
9.932888	29.26646	19.42433	14.24498	40.4415	8.310779	27.11486	1.211971	203.2526
2515.283	2281.594	1924.037	1592.048	1283.973	2198.603	2806.975	1784.577	1948.138
39.43736	19.53166	38.01896	38.32671	24.78983	36.83675	23.28498	25.44345	63.7126
19.25935	243.6794	56.57837	59.86637	105.6936	40.99368	153.2191	56.61077	20.88945
205.1814	418.2067	343.1944	248.8009	3294.37	350.1825	524.3564	100.4915	151.0086
1084.643	1057.376	1183.634	1132.034	522.9473	1235.064	964.8912	931.5354	269.2995
27.91442	15.20057	191.3735	93.26952	0	36.83793	96.39628	31.20448	1.785123
42.9582	164.6535	85.78287	75.47962	0	175.1675	33.61373	121.2062	1200.96
384.0809	198.2796	274.2206	163.0445	92.79668	291.5733	175.2838	302.4336	20.89092
72.44585	97.50578	128.2947	129.6685	57.88118	131.5868	154.4627	146.5406	36.36226
25.07139	4.368234	31.83992	27.53931	28.48254	27.62773	14.27233	27.72437	15.43806
81.72289	278.4124	120.2732	136.6715	130.491	77.02128	170.0936	57.78016	425.0018
319.3186	430.1308	866.0331	917.2241	1271.147	647.6459	268.07	881.9002	831.6605
14.25111	11.94994	28.28751	123.1602	26.63773	15.02276	34.79987	13.90952	4.511908
79.65968	60.68685	51.27608	34.04418	68.00131	83.78294	33.60197	68.12824	397.9319
192.1471	9.78599	17.6449	40.69359	48.69897	144.2839	13.0043	48.50749	223.1286
71.08987	37.94051	55.72092	61.17891	31.22349	67.03731	46.42943	71.55235	22.71349
17.20595	0.02595	16.7972	14.936	12.84986	15.05765	1.398663	10.43285	1.783884
595.729	334.7959	856.308	533.1993	424.6012	356.0397	660.9102	959.2195	809.8257
137.7449	125.6972	199.9123	373.3315	289.5348	168.3832	88.99656	206.6332	212.9165
12682.55	1989.117	9253.11	8732.645	5419.045	27129.16	8132.584	18532.29	5979.083
2.754919	28.16557	26.53685	10.04997	27.57426	1.611755	57.70767	8.140446	2.692935
78.97443	43.35806	86.70754	74.38836	48.69398	52.75441	20.7296	61.19727	48.20463
12.80309	55.24903	55.73641	23.25717	56.98999	7.471919	68.20965	13.91499	0.877054
6.340496	60.64794	35.36702	23.87376	73.5683	6.63426	36.11147	12.75982	0
12.08531	365.0135	64.53386	52.0291	221.5267	14.17563	36.19339	40.46595	1.787266
59.58298	16.28475	22.95743	44.32475	47.78318	34.30886	16.86366	27.75458	60.96758
9.92851	53.09701	23.84054	10.62624	75.37563	1.614599	36.15505	12.76297	0
170.1917	70.44475	155.7103	138.5955	33.05393	194.4524	92.80933	125.8231	75.49844
336.6916	125.7019	219.3653	372.0198	453.1564	355.2889	107.0338	178.9579	319.3893
25.86057	4.367706	8.803978	19.75124	15.60683	11.67963	9.120766	27.68451	45.56217
28.61133	63.92333	21.18455	39.48135	58.81489	20.04325	37.44348	55.42735	86.4609
4269.695	4335.657	4063.968	2551.308	893.3413	2987.046	4672.267	3234.36	1261.121
601.5429	847.1722	452.9069	1149.126	236.1811	730.6654	481.8307	250.5397	133.7202
15.7565	15.17942	22.12714	23.45743	22.06659	20.95636	5.264961	33.38969	25.49869
11.41124	3.283942	23.01525	11.2917	14.69256	19.27013	3.980062	15.02946	9.982179
436.5802	279.5091	286.6226	230.9198	365.8286	396.3801	199.7261	229.7228	145.5605
70.33072	15.20284	51.28213	70.76674	64.3286	56.10835	16.86866	55.43587	56.39893
6446.112	1372.675	5822.577	3295.737	3024.767	10541.54	5339.668	9459.43	286.5897
15.72925	9.779543	8.803856	35.54444	9.161897	17.56702	14.24454	8.13802	2.692911
97.73925	10.86937	63.69162	34.67369	61.5777	38.49927	20.72563	25.45161	9.060759
5.626317	13.02608	34.53003	26.40393	19.28774	31.02658	10.40716	34.58921	42.81318

7190.516	2642.387	5779.227	3443.913	3240.759	4702.184	8802.268	4979.681	229.2652
60.89288	145.2015	162.7335	77.8168	443.9559	63.60908	52.9421	176.6538	142.8266
42.38873	2.199818	30.95026	22.69812	37.68617	55.36802	3.983452	26.5766	23.63738
37.2636	116.929	22.07307	22.65043	33.98609	20.0472	86.18674	33.51553	0.877132
2.755322	5.45164	0	2.236848	1.806284	3.284439	46.27189	1.212502	0
359.0432	100.7836	209.6482	132.4652	99.235	230.4192	469.9428	215.861	416.8068
10.67189	6.533372	24.77132	40.40234	7.321357	4.122244	11.68513	31.13363	10.88906
30.11716	26.01659	46.02497	37.80978	34.00408	35.18897	33.50728	33.48215	28.19438
70.30654	79.09279	74.29516	61.10521	36.73512	97.20703	94.02571	47.37307	105.5648
92.49838	187.4319	134.4331	202.75	187.4911	123.9611	148.2066	125.8618	463.2402
38.24957	47.57493	51.4581	24.66634	8.244072	19.26771	89.29476	10.43396	13.63034
22.93642	0.02658	30.97129	19.1135	32.17817	8.314955	9.127578	35.75068	0.876258
259.865	88.86937	290.1714	159.4767	67.98042	213.645	242.1882	348.5488	52.73687
58.20374	14.11699	31.82274	38.34913	52.39836	63.72925	2.690721	41.56459	71.01374
193.9939	131.0756	110.579	62.87705	6.400795	143.3182	241.8867	95.82047	111.0121
21.44281	20.61203	28.28002	35.94109	24.79283	30.12877	38.67264	32.34902	19.9876
517.0649	192.8567	205.2143	171.4763	179.2088	359.4999	132.779	353.1746	207.4494
57.5132	72.53532	30.94216	21.47408	26.63522	52.82188	27.11803	55.3657	111.1419
203.0889	717.0851	79.56786	50.81758	146.1129	56.0681	135.3597	68.16957	368.5406
119.0693	238.3415	230.8855	297.0371	118.5394	124.7953	337.4091	280.4711	227.4797
2255.518	789.8087	1327.805	1202.738	853.8296	2926.016	1433.798	1652.953	8549.138
17.82842	14.11929	69.88745	44.90032	29.38294	124.9557	14.29235	34.6777	13.61049
598.2351	10.86687	259.1896	150.4555	69.8182	678.0567	92.86591	451.2507	289.36
91.97875	16.2853	71.66473	29.25831	36.74175	117.4143	36.15216	144.1254	41.83548
52.36409	86.64799	24.72622	31.06282	57.89806	75.43053	31.01201	35.82817	58.22894
639.4861	1738.804	1675.491	1139.159	673.6809	1138.664	1870.409	1089.685	107.3331
50.87163	50.94647	27.37493	18.42399	64.31636	17.5256	50.33703	11.60869	27.26
224.789	168.9995	149.4999	338.1641	220.6159	287.5154	40.05519	165.0548	118.2763
163.5521	256.7862	344.0945	269.2373	962.3762	426.4696	486.9434	161.664	62.74571
14.95267	721.0837	33.56871	27.42658	65.23471	10.82372	304.8646	8.144043	61.84867
404.3603	69.36935	127.3553	141.4885	147.9606	263.1297	374.6792	50.85474	118.2644
109.3525	18.44968	37.12997	53.39028	54.22824	83.86098	7.851298	56.55456	74.64129
373.9088	304.4573	557.3004	705.5138	208.6055	183.4294	435.4807	586.3953	35.45015
2074.525	1320.657	1568.415	1520.077	2051.455	2074.606	1227.746	2105.443	3334.012
0	7.511802	0	0.47313	7.422148	0	1.356792	1.193715	0
37.99184	26.02769	33.58794	44.95279	20.1904	34.31576	11.71448	43.88103	50.04307
35.07699	42.27614	65.45011	63.54149	7.318826	15.01259	106.8326	25.45545	0
688.3592	804.952	657.2555	451.0021	383.2408	567.2114	870.8259	696.0637	1070.091
65.97431	47.6965	88.45234	122.4478	67.07676	52.73665	55.47792	109.6479	40.00333
145.8899	41.19545	37.11378	105.0617	155.3867	162.6764	36.17203	148.8071	56.39531
120.0385	110.4666	74.31309	34.66426	66.17252	116.5517	64.42818	64.64992	220.3716
186.2057	83.42062	98.20753	47.27641	1.807929	218.0725	77.31004	51.98323	9.970727
342.0439	173.3241	164.5563	350.8739	59.71327	158.3781	106.9681	158.1228	34.53913
142.7468	153.868	96.3761	268.1035	210.4565	147.4109	247.387	131.648	203.8
14.29564	17.34465	21.23044	21.60007	0.888631	11.68437	19.34222	23.07733	1.783906
88.56607	20.61031	26.51165	23.88735	6.399575	194.9512	16.84945	34.6455	10.88231
34.49337	13.02868	19.43607	15.47798	15.60099	24.27968	2.692524	34.6083	26.38165
23.6083	33.59616	24.73845	37.162	51.48417	21.73521	7.850159	21.97857	56.43932
23.67613	2.200188	28.31811	19.73756	20.20944	11.67711	7.840337	17.34641	22.74191
64.59481	69.33476	46.86055	50.32008	14.67148	75.42333	40.01286	35.8305	29.9961
12.11082	42.21095	10.57476	12.46959	11.92179	6.637613	20.65106	8.139614	5.422471
3003.539	1041.149	2093.027	1230.906	814.3034	3174.845	1077.027	1333.249	1822.58
60.31315	24.94653	43.32812	32.28278	24.78786	62.01616	19.43452	70.38271	36.37566
257.5954	741.0187	275.9606	365.8265	539.5095	160.8052	183.046	257.4666	403.9845
682.7921	391.1041	356.4889	494.9681	433.8214	708.1007	295.0627	402.8576	91.86357
0.61276	11.93847	5.262014	6.445906	11.92769	2.44754	7.830722	4.678773	9.069673
20.81089	7.614395	15.01569	13.09745	7.322558	19.25976	10.39611	9.286521	20.92661
145.7582	126.7534	145.9787	105.5411	108.448	176.0106	140.3852	123.5102	245.7543

2.755045	67.07804	26.53232	22.73887	83.77069	12.5126	47.51815	12.74953	7.242798
91.97568	17.36839	45.97747	28.05419	45.93949	67.87486	18.15265	28.91086	35.46056
170.2036	59.61377	251.3289	177.1002	124.9988	103.0391	137.8179	401.3949	0
660.5798	88.87253	344.111	331.7123	481.6322	1521.223	99.31374	316.296	8.155137
352.1504	162.4912	115.8757	169.2625	74.42473	320.2733	92.80986	114.2902	135.5811
103.3534	114.8357	115.8894	61.67257	110.293	98.85782	186.6313	101.5879	225.7395
226.0675	149.5331	568.0141	133.037	322.6129	607.6086	301.4397	472.0368	58.19617
50.95211	6.535456	33.58604	69.06961	87.35398	67.05883	11.71505	39.27602	24.53699
6.343206	19.51959	28.30418	11.25066	10.07936	8.313929	32.18774	18.50669	0.876331
14.24761	41.16883	27.39701	31.12617	49.64388	23.41492	20.70259	13.91133	69.20201
188.0174	75.87074	209.6399	265.7658	278.4955	311.7196	64.53274	337.019	223.833
114.0446	63.95375	193.7212	207.5457	148.8774	107.195	99.29392	324.303	508.747
876.6439	838.4873	547.5893	367.6652	433.8126	705.5528	700.6958	435.1862	406.7245
4365.868	4335.663	4612.439	3730.189	1092.789	3747.025	5508.288	3660.293	752.4688
64.20992	11.94527	15.89274	15.48825	17.44443	45.33253	3.983067	12.7472	6.332688
59.76986	50.882	37.16642	46.32396	14.67851	11.66938	30.92706	28.86961	68.32558
40.12251	45.52024	76.97447	70.80366	33.98135	37.65794	90.09997	104.9732	42.74434
2.888651	0.016799	0	0	0.898286	0	0.054907	0.030479	0
4.911426	56.21014	10.58185	11.89508	0	10.00503	2.692105	41.42369	3.602918
0.617902	5.417012	0	0	23.17697	0	0.071749	1.206048	0
12.12538	5.450061	18.5663	16.7378	22.98177	17.57557	3.981332	24.22638	42.83745
123.4464	42.28551	88.43435	93.49731	149.8209	104.7089	67.08177	110.8338	211.1419
60.31661	20.61557	53.07391	52.17514	66.18799	65.37733	28.43044	76.13906	36.37621
760.2504	708.5137	548.4612	405.4502	481.6044	644.3484	704.6033	498.6736	762.5401
303.1749	204.7325	78.69606	59.84246	172.8036	79.55007	101.8302	123.5281	1173.515
15.06988	0.025433	7.927639	15.60076	11.93639	10.86152	5.257323	13.8624	5.426876
1364.673	466.9641	1679.961	723.4035	169.081	1763.852	1673.229	1510.917	1.788854
206.0351	45.53726	130.0143	99.45878	86.36834	134.8669	123.7371	128.162	165.5981
32.18431	155.9876	82.24439	13.62608	267.5442	15.01221	117.2341	51.99874	4.514237
16.44282	14.10784	6.145341	7.640352	16.5249	11.67618	5.270559	10.44326	8.154435
240.4231	199.3627	528.1806	301.1337	1026.808	299.1175	163.6951	388.9737	223.8228
548.7344	248.0909	356.548	143.2683	144.2788	265.6235	252.4775	303.5489	61.83692
192.4398	171.1646	124.7177	104.2983	60.63138	160.8828	137.856	125.8368	171.9859
140.8527	45.52655	89.35321	120.7229	127.7909	132.4628	33.59922	117.6886	7.241658
15.70351	23.848	29.18667	26.9559	29.40785	30.15921	15.54933	27.71609	14.52891
15.67594	28.19495	35.355	43.12236	95.62973	17.53034	50.26733	24.29546	16.34188
193.9703	59.61191	81.36172	65.27421	57.87793	155.0527	68.35367	128.1144	69.13015
4.196046	19.48849	5.264734	7.065864	11.93471	10.85855	15.4671	3.523782	2.693502
0.612244	3.281599	2.608814	0	0	0	1.397406	0.045684	0
48.75374	20.61799	103.5445	87.06109	115.8507	56.95499	28.44564	76.17363	24.53307
16.40809	40.08555	49.55829	42.60871	15.59504	36.01642	39.93756	53.06151	2.693424
2.044085	56.33949	28.26922	31.06999	80.90322	9.985282	77.23066	19.68393	78.27432
107.5793	61.78728	167.1727	160.0933	308.8451	126.4735	73.54377	255.0849	214.7394
14.95266	66.10202	30.03128	32.8398	63.40157	14.1742	42.60872	36.99433	3.604276
488.8312	268.7036	631.6407	741.6211	308.7994	700.5193	452.1877	555.2094	274.7697
105.7352	42.26501	36.24306	34.69576	24.78827	62.01937	38.70438	65.7721	6.331399
14.96185	56.32503	14.99266	29.8941	48.71355	17.53407	32.2694	17.3731	4.512134
75.38789	45.52158	85.82805	58.74434	45.93665	88.00973	113.2214	115.3504	118.3402
44.4702	29.27592	39.78701	28.07152	41.3468	46.06677	43.83746	36.97073	55.50725
140.7109	8.701383	444.3298	134.3868	15.59014	35.96169	60.64438	178.872	107.3597
99.86355	29.28159	89.36814	135.267	64.33159	92.2025	32.30491	76.17724	152.0412
329.4799	43.37122	484.814	262.0952	36.72961	289.8926	426.337	136.2648	39.99776
126.5129	73.66797	55.71312	70.18229	56.05336	234.9358	68.28546	76.17684	47.29529
33.71068	27.10165	22.08302	16.65632	39.52244	48.62725	41.21155	19.67154	106.5941
200.8784	835.2587	118.4891	146.2049	912.7099	163.3194	371.0816	225.1485	193.7775
86.73403	202.6237	1495.188	2603.2	126.8026	292.3722	587.4087	855.2668	146.46
18.55155	21.69885	18.53132	43.72573	104.8324	21.72404	32.28808	30.05937	60.96884
93.96317	156.0042	138.8788	148.7711	72.58272	118.9553	198.3147	160.4408	223.8761

150.792	93.18476	102.6022	76.68488	98.33318	162.5898	90.22905	135.0424	112.8251
49.5151	16.28403	33.58695	34.09712	42.26725	36.83263	25.85837	53.09874	45.48672
68.07066	365.1107	24.72228	22.63393	368.5515	20.88187	101.893	11.60216	54.55654
137.0239	150.6106	87.53275	318.6491	141.5206	144.0724	166.2415	105.0965	194.7116
126.4947	20.61849	43.31426	32.85382	123.2027	50.23674	11.71712	48.51744	90.0965
10.6522	18.44508	24.74088	122.513	14.67552	9.987893	14.27879	39.24702	3.602502
98.30466	68.27683	94.63671	125.3902	56.9575	59.43666	46.4844	94.67653	121.9305
12.08713	44.4198	8.797432	28.69706	15.59364	10.825	60.47328	4.681357	8.151158
76.94826	23.86057	73.4739	57.65711	28.47119	53.64092	37.40018	71.50497	50.96097
887.2628	744.2833	643.1047	611.2357	247.2078	671.9669	467.7007	1255.806	550.4858
98.75911	20.60735	52.23221	42.04176	34.92422	66.30139	9.134927	30.03011	112.9967
50.15941	33.61727	117.6735	92.37103	79.0329	40.99802	36.17961	148.8392	79.15173
216.0875	280.5663	222.0514	165.5274	165.4307	246.3702	264.0062	222.7646	325.8014
377.9351	140.8439	107.0151	67.04684	44.08394	398.2212	61.94076	101.6142	5.424232
6.376175	4.363402	7.045576	19.34643	5.488146	9.187411	10.3423	18.4209	5.429844
962.0339	602.3563	499.7983	674.3325	357.5124	898.2811	546.2186	654.4765	609.652
7.780062	7.617773	22.97844	40.27312	18.36009	7.474143	5.27252	20.81281	1.784328
705.5709	910.0402	1257.074	765.4142	219.6326	748.2054	1827.761	1109.267	100.053
9.22262	215.6121	23.83654	61.61588	883.3833	5.804589	74.84006	124.7238	1180.383
341.5936	35.78353	152.2049	45.45539	11.9138	241.5124	118.4716	182.2724	142.8901
284.0038	131.0678	53.04219	40.0458	148.9282	19.2017	59.3352	33.53554	120.1247
352.4381	392.1806	244.1272	149.2244	370.4	324.2442	453.4088	317.4515	82.76436
51.62957	68.25545	19.41403	52.71861	48.69529	49.401	67.00014	20.84017	50.93801
353.2334	210.1891	194.5931	261.542	85.44433	598.4117	386.3724	255.1091	255.6842
1617.306	1407.305	1172.112	1128.964	773.8677	1463.794	917.2741	1162.399	498.6038
79.66935	28.20059	61.9027	52.69157	56.96724	72.88116	18.15723	66.97116	88.26652
7.088931	9.769095	7.92823	16.21735	30.39357	10.86283	3.974981	11.56693	5.427131
629.0054	237.278	317.5717	242.2753	90.03867	302.4611	749.4582	357.8304	464.9955
423.5046	30.36924	670.5873	295.6554	24.78289	309.9819	896.3606	189.3621	2104.056
1050.912	891.6256	1239.377	887.822	672.7708	889.8216	3377.07	967.3083	492.2397
65.35956	15.20143	45.9862	38.91137	79.07004	35.99056	16.86218	39.27729	57.32729
13.53504	10.86654	21.20112	28.74551	34.92605	15.86505	20.68803	6.990059	25.46122
20.69911	53.10467	48.62376	38.26006	90.08027	12.49856	99.14087	28.91687	12.70024
39.363	366.2031	39.75989	139.0055	393.3606	42.66421	45.20871	25.45651	58.1966
2.046248	36.86391	41.54012	38.25698	118.5926	1.615357	415.0131	10.45456	0
22.95094	3.284364	14.1213	1.639287	1.806045	11.67674	35.98109	8.13935	13.62248
1307.298	1064.941	1055.378	1024.706	252.7222	1000.462	1030.516	1110.397	336.6388
101.239	150.5448	107.9408	67.11685	102.0291	92.16961	59.32534	153.4354	105.5627
11.40482	16.2631	20.34408	20.99801	17.45399	20.94732	5.266802	16.18363	9.980522
246.3713	89.94203	207.9243	89.88066	181.999	163.4018	227.8856	130.4484	221.1484
325.4823	377.9795	230.0581	108.5139	41.32664	159.212	284.4581	191.5734	82.77782
28.68338	16.27757	23.86194	14.25661	26.64244	19.22624	34.77593	18.51342	26.37425
124.1209	96.44655	159.2207	293.5444	274.846	154.9904	157.1923	184.6879	128.2805
22.16885	8.701876	29.16969	48.04235	27.55537	27.61437	18.13415	50.7623	22.72206
2.041082	31.42813	15.88222	12.44127	29.39882	2.448393	25.82653	18.51853	120.2791
9.210964	20.61822	17.64404	22.03464	117.6872	1.614893	9.13995	3.524154	1.785844
1.325638	3.279876	2.610467	2.244147	26.75361	2.452053	19.14544	2.366429	1.785127
78.15657	76.94572	128.2596	113.9179	97.40707	74.51988	101.83	167.3574	83.68733
167.1341	516.7833	2767.444	1650.26	56.0314	156.6152	2364.471	1627.366	0.879078
3.481529	13.03575	7.030497	11.82948	3.645575	81.22807	50.35304	8.142881	11.79119
2.038867	16.25635	2.606995	10.08801	24.83842	2.447754	3.978809	2.370341	1.783894
72.41956	48.78351	117.6481	242.0578	75.34492	82.07339	32.32642	96.98738	72.76829
0	1.111947	0	0	0	0	0.072475	0.03963	0
73.23865	31.44587	35.3496	45.50531	48.69769	54.4413	25.87153	47.35729	54.58383
53.04916	48.77512	76.06963	144.2095	118.5915	58.61832	27.16707	68.12442	118.3192
838.4971	241.6248	382.1258	600.4878	200.3333	936.8196	353.0551	504.4518	508.6358
145.6179	121.3707	318.4595	381.5797	429.2425	123.1064	110.9019	425.9048	100.0547
333.796	258.9391	281.2985	336.5558	181.041	383.7743	212.6236	241.277	359.4278

3487.151	3045.394	3529.617	1574.578	1271.096	3224.982	5132.232	1936.977	2164.68
137.1153	114.8453	99.0579	25.62313	6.401236	215.4213	34.90197	168.4937	52.74233
51.56243	543.8508	379.482	221.8202	343.7293	35.1235	933.7545	161.6649	13.61412
40.23453	3.284316	43.3693	41.45557	19.27848	39.39833	6.560865	20.8171	13.61625
2178.699	1535.146	1192.451	1260.339	1270.201	1940.574	1888.472	1201.653	2427.722
93.21862	259.9942	99.9236	198.5542	254.6077	54.39434	85.12825	195.084	146.4794
8.49536	42.24846	17.6526	28.718	41.36184	9.987723	52.74799	15.06327	14.52477
171.5627	40.12042	118.515	124.1039	99.2414	150.806	31.04169	92.39066	95.5159
2.754879	136.1215	1.723302	39.83373	5.482062	0	12.95967	4.679859	0
5.624399	74.74135	11.45079	5.830635	94.7004	4.123228	15.57851	4.680466	56.40854
281.0003	123.5025	123.8507	63.46725	83.62249	216.2837	160.947	145.4156	91.88804
242.6778	102.9468	167.1812	115.0701	196.6899	225.4097	95.42333	426.9375	348.559
3.471531	22.75966	15.00619	9.4474	22.96972	3.284376	28.32939	6.987678	0.876218
3.479529	11.92383	11.48851	6.474995	11.94206	4.969928	14.16202	5.821883	4.516783
63.85584	61.76318	138.9651	181.6511	156.3211	56.9485	52.87596	85.40303	353.3594
28.62292	74.73342	48.64194	27.46338	55.14453	34.31029	43.84606	31.21169	81.01254
91.04961	596.8652	193.706	175.0648	301.4712	196.0293	324.602	202.0349	213.816
4.196103	4.365658	14.14452	9.495649	0.888506	8.331294	7.817489	18.45308	6.338112
287.1727	221.014	243.2717	137.2623	75.33421	385.5059	206.1444	266.6313	260.244
25.06823	15.19629	33.6114	30.55647	18.35749	20.90382	19.40659	21.97024	27.28354
148.7803	74.7581	95.55405	82.79068	30.29985	108.9672	114.5603	105.0075	68.23385
681.9088	1307.591	380.3485	533.2224	87.28058	532.8586	98.02936	793.0047	71.84564
2.75508	4.36721	14.12996	0.452517	32.20291	0	23.16406	2.370593	0
155.2396	26.03475	107.0617	176.7284	9.156749	129.1001	85.02319	11.60907	1.786165
874.6809	306.6033	431.716	286.7004	376.84	625.1673	153.4002	460.5285	414.9386
122.7364	42.28508	68.96487	80.88385	89.13609	118.1307	24.60012	106.2162	141.0439
124.9341	40.11688	83.13534	97.15325	96.50006	90.47219	32.32282	72.75596	128.3118
124.838	75.86715	162.7599	142.1098	133.255	120.6165	145.6127	200.8393	137.3821
348.0755	82.37299	242.3452	1473.348	717.8449	568.0903	90.30062	927.9719	2064.848
17.82782	98.561	60.1425	103.3322	32.14124	16.68949	88.82823	31.21976	99.21097
19.37511	6.531665	15.01941	23.4462	16.53445	28.53972	14.22511	17.32878	7.246445
28.59171	66.11401	163.6707	189.6837	75.3427	115.6087	81.23742	125.8295	56.38212
25.79446	40.07436	43.36976	47.51196	8.238711	33.51312	21.96766	54.18196	4.511898
10.64724	21.69822	10.56641	7.028064	61.58761	9.147537	7.851325	10.45411	5.421807
191.6068	22.78021	41.56009	11.8292	0	192.2593	3.981999	73.82775	1.785237
92.57393	49.86351	121.2056	145.8939	182.9507	137.4504	41.33068	88.89912	200.2495
23.56808	54.20472	38.87318	56.21885	918.3869	5.803491	145.6496	20.84249	126.4504
61.666	33.61703	119.4469	123.6739	111.223	90.48611	15.58236	123.4744	33.63226
109.4846	28.1868	14.99438	118.7936	6.399539	75.50949	29.68838	31.19461	13.61338
15.70584	21.68371	21.20741	22.72712	11.9191	17.55124	6.559447	15.05459	7.242442
14.98732	9.782318	30.96614	65.18944	27.56972	16.71275	10.41357	44.95517	14.53037
175.1747	284.8723	176.0503	73.65171	88.21109	346.2124	154.589	205.4287	10.88173
4229.493	3392.054	3114.745	2304.129	1604.739	3328.915	3256.615	2960.8	1501.344
2.046015	210.0584	41.54122	19.02776	105.7178	2.451231	119.6914	33.53035	4.513187
103.9635	429.0225	170.6871	166.6154	581.8122	907.5566	472.7728	178.9757	540.506
42.96704	39.03319	37.99446	34.6357	95.58308	11.6611	61.9116	12.76336	651.0562
82.44552	245.9133	162.7538	111.4615	124.9779	123.9641	253.7237	83.16669	153.7617
17.11973	20.61352	36.24979	37.73353	79.0848	17.53407	10.42628	58.84365	19.07542
32.89771	263.257	58.33573	159.4752	273.9011	12.50257	51.6528	50.85589	71.84679
164.4802	46.61583	84.90286	87.51944	77.18724	181.898	42.62101	100.4379	193.8648
14.96255	66.0601	21.19141	32.30947	29.39133	19.21277	30.98329	27.74487	47.3164
77.54169	32.53003	68.11134	116.5796	70.77052	68.70121	27.16105	79.63462	8.151063
0.612597	10.83046	0	0.451697	4.570372	0.777184	1.395726	0.044498	0
2.784012	0.022539	0	0	22.19919	0	2.664852	0.041302	0.877405
8.523216	2.200133	13.2485	17.99311	10.08771	23.49463	0.088213	23.057	3.603254
22.87544	66.06436	10.56685	25.6723	25.71023	19.21142	77.16522	15.06748	2.693808
38.12667	21.67973	40.73464	36.69381	56.13318	16.71626	11.6935	43.79365	54.66644
161.486	95.36347	111.4315	205.2008	149.8042	187.6886	121.1598	124.699	215.664

1113.428	44.45256	1348.222	593.8226	61.54641	1084.263	803.8517	1183.107	11.79572
0.618006	3.282464	1.726433	0	922.5668	0	7.849223	1.209931	615.5812
0	0.012842	0	0	1.844378	0	0.041199	0.023141	0
28.76487	2.20022	15.89959	14.9053	11.00346	65.60643	3.982039	21.93708	13.62534
2037.968	1092.063	1425.115	1301.127	459.5215	1870.178	1742.939	1341.319	2531.452
0.613555	7.616469	62.94085	87.18878	2.724226	15.87833	23.21143	24.24879	0.876196
38169.79	67573.4	33476.73	23654.01	22172.61	40431.33	34902.87	21411.19	26184.9
33.62505	119.1621	45.95982	52.06245	101.1007	34.28886	103.0586	50.83986	83.70075
210.2746	225.3538	235.2964	184.6766	202.1888	225.369	212.6036	182.4163	274.7965
104.6986	85.62004	215.8346	275.9783	179.2097	130.6582	137.9267	328.9418	106.4282
364.5998	243.788	397.1767	510.5155	306.9611	548.8239	149.5521	505.5855	351.2115
426.9915	2435.38	1523.326	2107.618	137.8319	786.7269	689.2897	486.0223	6397.078
501.1905	100.7875	252.9874	781.0133	90.95866	947.953	83.8544	217.0401	201.9823
14.98716	74.64652	32.74031	5.230882	28.49049	3.284384	18.10726	6.988723	180.5949
80.50733	10.86901	23.84481	38.31502	56.06835	29.27807	11.71484	39.27482	65.52959
22.22155	56.25972	38.07727	31.85552	27.57577	20.08284	6.556974	48.37995	30.94866
121.3703	66.10386	108.8201	103.8073	109.3827	77.90161	91.47326	95.80736	195.7119
197.4589	102.9405	143.3013	116.3081	141.5372	360.4701	83.82215	143.1434	42.72891
50.18096	82.33255	30.03406	37.66248	32.13963	35.97417	50.30837	57.7422	10.88047
215.3147	313.0822	451.228	420.7358	159.9044	522.9804	605.1274	251.6407	4.516004
231.2933	29.28692	156.5892	63.45123	77.18154	112.2546	99.24956	218.0923	160.1574
31.00532	7.612594	2.606896	1.043174	8.244951	4.123952	9.107296	0.047727	1.783884
2416.93	2438.68	2551.255	2276.613	1223.311	2324.291	2213.166	2587.934	906.2508
266.5747	93.18594	156.5987	130.7873	214.1978	245.626	54.20844	233.0631	67.30671
14.95268	70.43257	14.98888	15.42475	151.6973	21.71658	40.03559	39.30093	113.7583
53.29736	17.35638	31.85241	14.26813	8.239581	25.96015	41.13934	32.31077	4.512002
661.2465	471.262	369.761	263.8533	172.7628	580.7016	627.25	581.7231	40.90811
1541.801	3254.461	3377.493	2733.716	1365.77	1140.299	5083.139	3378.631	1303.89
302.1714	152.7865	216.7009	410.3724	329.9554	431.5346	109.6165	318.6011	235.6489
186.3637	15.20236	24.72575	50.92732	19.26841	81.30322	19.4399	38.13502	18.16143
2.039514	4.368082	7.030073	3.432023	20.20518	0	7.843533	0.049083	0
238.5345	255.603	101.7166	103.1346	99.25244	150.0074	105.6644	141.9624	517.1005
208.2124	47.70333	133.5588	118.0916	175.5506	185.1804	46.49625	103.929	241.1574
32.20034	14.11958	51.28178	62.93774	56.05048	45.20131	15.58118	24.30206	63.68366
38.32429	3.283517	15.02905	13.13645	13.77615	35.32697	12.92849	35.65259	55.67566
18.58983	4.368191	13.22914	29.98552	15.59964	20.91191	6.5597	13.90369	19.08575
10.06708	1.114373	4.388953	5.29849	10.11536	0.77748	1.394493	6.949391	0.876352
8.498141	0.026842	46.91956	29.35915	8.238788	13.34741	7.847445	55.32894	6.331746
1.323976	27.05038	0.841076	0	87.60246	0	16.7555	1.213209	0
64.59377	33.6117	32.6928	74.41527	190.3819	72.06502	6.561507	35.83074	92.83765
75.39889	67.16914	69.88892	65.98524	45.93822	89.6965	37.44132	55.42526	96.48327
53.85224	67.14783	44.22126	160.3462	39.50974	46.9139	30.99034	47.33283	31.82534
26.51111	11.94919	13.22639	20.88936	22.95887	34.34976	5.273012	36.93301	36.39878
158.7567	89.92865	120.3218	39.44135	76.27097	104.7359	96.6319	146.5477	212.0899
13.54377	11.94702	31.85174	47.57652	42.30457	25.11848	10.41428	40.36015	53.74241
19.40354	33.52546	28.36223	24.71957	2.724568	9.169429	16.75547	28.78244	67.55588
64.55836	76.92499	45.0807	46.07348	95.59578	60.29591	58.03107	51.98322	62.76885
23.58722	77.97818	58.38829	19.04031	65.26663	23.40252	68.229	36.97329	89.21458
33.73409	19.52403	32.727	36.00798	0.889087	42.76175	18.12144	36.92948	66.49237
1.331103	28.19972	1.725939	1.053073	29.38103	0.7798	32.30925	4.67969	26.3525
15.70417	31.41747	27.41355	19.69985	12.8389	31.00119	19.39431	40.36647	22.73166
213.2866	211.2324	122.947	131.3405	102.9226	152.4962	163.5853	171.9748	105.5325
86.21203	17.3684	131.022	65.39261	4.562357	112.3732	114.4718	32.36979	0.877318
165.9276	23.86991	182.2911	39.4375	0.890374	151.7029	150.6422	130.4163	5.423395
11.36403	161.3297	13.22001	20.23332	74.45236	0.779573	10.42864	4.680119	639.5008
59.49787	60.69274	116.7789	41.84386	79.94873	51.05715	105.6342	38.15155	97.35668
79.25807	29.26063	46.0281	27.53708	12.8373	46.96408	18.12268	61.08649	66.49009
0	9.73183	0	0	2.731144	0.778616	0.077037	0.041961	0.87708

149.9036	3525.18	141.4896	282.3805	1603.864	173.3716	552.748	41.61541	0
0	0.025723	1.723234	1.638012	26.6862	0	3.978192	0.0474	7.248619
69.51894	226.4159	96.38579	149.9089	139.6887	74.51005	180.3667	116.6265	181.0701
136.2975	230.7671	383.082	219.5144	344.6855	294.1112	46.50082	175.4898	66.38634
25.13643	17.34894	21.22255	19.14458	18.36994	32.72396	42.33468	6.985915	5.422903
5.625065	14.10988	6.144752	9.444511	67.18724	1.611853	11.69481	3.526388	1.784158
55.93554	44.44209	39.77149	66.54736	51.45153	146.7476	50.30938	72.72851	103.7546
725.6915	378.1284	379.4615	1004.886	556.9614	905.7602	268.0663	642.974	648.7608
123.505	50.9462	94.64644	100.7724	103.859	146.6809	20.73485	80.82594	135.5994
79.55741	1175.294	285.7157	260.2792	989.0875	120.5907	298.901	115.4926	417.6666
19.30809	6.534858	35.39361	13.65767	18.35991	27.63455	14.26797	18.51089	32.75869
454.6474	237.2535	168.0598	203.9495	139.6852	459.3151	391.4249	276.9989	1311.643
2657.284	414.9596	382.1178	145.6016	5.485812	1805.821	508.9152	975.3574	0.879139
3869.734	3569.752	4250.58	2309.462	1912.636	4181.009	4443.11	2622.632	3289.35
73.205	58.51705	81.3885	200.3035	40.41553	72.88782	49.02461	92.32334	29.99367
2887.088	891.6541	3468.611	3186.082	3285.824	3009.68	1284.456	2617.975	3322.141
0.615595	61.73155	63.72578	16.04246	54.23767	1.613386	125.8241	24.28749	4.512084
119.7879	401.8827	137.0846	121.0546	338.2624	201.9152	292.366	146.635	108.2503
17.11261	28.19568	38.89602	42.51305	21.10833	59.48892	41.28403	56.56718	37.28416
20.6963	197.1768	40.64274	51.42209	148.8805	66.12769	31.04206	137.3968	83.6799
64.50108	128.9323	76.9242	174.0059	176.4772	44.3408	320.5111	84.31082	22.70913
0	1.092766	0	0	2.785096	0	0.041433	0.023268	0.896271
1.327395	42.24879	7.913019	31.13203	93.83901	4.121817	33.52753	4.681512	78.32258
9.931345	35.76227	31.82466	23.27514	116.8417	12.50262	11.71132	26.58904	10.88184
58.89349	29.27456	54.85253	45.56087	79.99845	82.19042	23.28587	144.0468	74.64594
73.2225	98.56321	51.28529	52.11541	44.09642	61.98762	86.26654	39.29193	5.422303
298.0035	33.62086	190.1882	163.7175	1.809193	371.294	171.365	204.3108	164.6842
71.72988	33.61741	109.7033	79.13193	135.1331	49.38317	47.75876	137.3137	11.79046
6.34321	9.78587	0.843321	9.427538	43.17249	0	123.5658	1.21019	0.877644
1031.525	253.5457	760.7549	1296.48	980.69	1011.329	216.5445	1020.401	53.6487
323.672	114.8737	479.4614	352.6596	465.9851	206.06	199.781	562.1303	31.81003
20386.37	8812.213	9807.768	4908.224	4303.242	13904.69	26037.22	11850.09	15781.74
43.86256	14.11263	36.27931	23.92435	22.04128	31.83727	7.846804	20.81401	41.87609
181.6632	60.69954	78.69624	119.3277	78.09965	246.4285	25.88873	99.30411	157.4222
7.059487	103.871	6.143747	7.630683	21.1176	1.612565	5.273079	1.212197	6.331568
18.60009	17.35486	19.43782	35.4717	23.88832	29.33071	15.54059	3.526387	37.32886
7.099559	3.282088	9.71134	9.527387	7.331127	15.09835	3.970784	8.116672	10.90889
28.64185	20.6128	24.73485	22.0644	33.07228	15.8571	24.56272	10.45307	11.79185
94.69792	47.70083	113.2198	84.48821	115.8008	77.04041	78.659	108.5239	104.6282
6.35401	46.48917	14.13392	5.844219	17.45812	0.776958	18.04702	11.58168	0.875928
58.09714	48.77223	33.57517	36.46082	26.62277	60.30391	43.88003	76.18403	55.48846
102.6406	26.03622	69.85773	115.2036	67.99416	87.95724	29.74799	77.36806	95.53505
20.76989	8.699299	22.98692	19.11502	10.08021	14.19468	20.66023	23.10335	106.6891
481.6537	758.3298	596.2515	332.8518	431.9738	445.7416	633.7426	384.4083	596.9239
24.33579	8.701672	36.26359	48.66658	45.04563	23.4182	10.42258	39.24581	35.48121
71.9672	11.95031	28.28535	48.05552	71.74299	46.94365	10.42296	38.09654	66.47264
208.0871	160.3698	358.2685	414.5665	344.6621	172.5485	172.7198	260.9044	183.7752
15.67097	6.534838	192.0899	25.63381	1.807916	83.78887	73.4533	101.5533	0.877624
46.53607	122.4505	82.22265	226.718	215.9798	51.87863	144.3678	92.40517	142.8305
67.5899	16.28212	48.66195	43.7877	22.03274	43.56734	37.391	33.50118	84.68877
16.51054	6.529151	22.14446	13.14411	11.01213	16.75961	3.976996	11.57272	20.94588
94.20882	8.702457	18.5324	26.86813	14.67264	225.027	14.28846	13.91521	10.88096
234.7694	292.4803	221.1654	168.5288	121.3013	197.7405	239.5618	172.0059	592.5201
5.626126	269.5883	9.681585	40.06318	85.4805	5.798232	24.59248	83.10705	41.82829
15.67061	58.52208	37.99726	125.5043	74.43812	3.288104	83.74806	53.13985	4.513473
136.4036	107.2635	108.7967	130.1869	35.81188	112.2633	31.03873	117.7468	50.92245
13874.15	18713.16	16763.58	11852.18	10199.35	11009.78	21080.25	11875.49	12583.34
901.7941	393.2771	378.6001	322.0543	302.3667	1093.615	560.3383	428.2591	382.155

35.1383	11.9509	45.12032	66.1612	37.67669	29.29145	12.99653	28.89126	65.55213
64.03185	17.36246	32.71829	22.08421	68.06283	41.90375	21.98044	53.05475	83.79918
10.74305	4.362941	17.72838	8.927667	0	21.87805	0.084403	12.69487	0.875928
36.49837	26.03617	120.3204	122.4272	49.60456	70.3451	42.61737	87.7459	15.42989
35.05092	342.3053	42.41199	115.6609	181.9758	66.12711	40.05928	9.296063	20.88983
307.8665	1039.978	250.308	273.4253	534.9217	119.7477	591.2701	245.9203	343.9292
73.64705	10.86299	29.20165	37.32138	35.86738	111.0651	15.5343	26.54814	15.44481
328.0961	162.5288	236.184	397.2888	252.752	362.0111	105.7418	296.6431	394.0313
243.4911	113.7682	127.3739	109.1083	79.01875	162.5618	149.4308	121.2217	148.3179
135.6424	34.70372	147.7271	44.82712	140.618	237.1885	67.09051	233.1087	34.53859
1021.378	4112.423	3302.354	1639.479	468.7117	2494.417	2380.576	2710.257	3.608679
89.64069	134.3513	98.1603	73.04398	164.5187	59.42615	166.185	35.84965	102.7974
48.76647	15.20219	40.66461	43.7074	82.73873	72.91397	22.01128	35.8275	52.76482
4.905809	33.59616	3.491017	4.630496	31.23566	14.18126	7.850158	63.4248	18.16711
63.10284	121.3214	52.15807	74.32403	67.07825	75.38505	90.18791	42.76307	33.63211
22.2529	11.94124	24.77888	25.2353	16.53013	20.94052	7.835595	17.33746	2.692936
266.238	44.45419	509.5555	717.0843	334.5424	674.5663	72.26552	334.7741	1978.471
0	0.014767	0	0	0	0	0.047802	0.026698	0
127.0181	117.0194	94.62389	68.84717	121.3099	121.468	144.2958	88.92542	535.2337
31.46523	75.85886	22.06567	42.43401	198.5646	25.90477	501.6225	17.381	142.8669
27.30012	10.86183	18.55799	17.31844	20.21057	24.29731	21.92157	5.833806	9.977377
0	0.023792	0	1.044922	43.4512	1.614008	2.678077	0.043694	0
41.68286	7.618073	32.72821	21.49982	58.87042	44.44624	3.983509	36.9276	20.90565
119.5365	15.19993	64.61196	17.84807	25.71162	127.6012	27.13113	84.16368	28.18421
439.5222	19.53598	251.2401	7.641886	143.3604	521.3385	70.96929	136.2502	5.425254
193.9783	127.8302	119.4289	76.69907	79.94626	128.2131	141.6472	154.6319	157.4449
58.90903	73.63529	48.65487	64.29177	10.99546	137.6729	89.97359	85.32326	6.331326
241.3633	36.86963	182.261	109.7238	215.1082	65.30035	22.02411	128.1355	264.857
70.34122	39.02662	53.05591	53.31796	66.17092	82.96844	16.86792	63.50027	53.66938
130.6002	70.4498	84.00161	185.4011	148.8893	176.8002	49.07065	206.5927	189.2731
234.6238	760.5116	271.5388	363.4357	388.7648	197.6761	632.4969	444.4331	296.6063
124.1102	63.95299	85.76649	164.9196	354.8266	147.4369	82.55206	113.1668	191.9907
33.00516	29.26159	22.97293	16.66433	11.91718	28.46547	32.22517	26.57577	29.10609
8.555383	4.363338	10.60263	16.89048	6.409943	14.25713	7.797968	2.36857	5.429941
2.05175	54.20427	19.41282	10.03745	1513.314	3.291678	40.05953	33.54171	398.6057
220.372	100.783	160.979	158.8947	169.1028	139.8861	134.0511	85.47679	617.0708
19.99263	32.52306	68.13965	48.56492	38.58707	35.15353	69.49867	96.86096	55.50773
4.905983	61.72835	19.42134	24.47831	119.5997	4.959194	11.71177	15.06575	44.58597
4.905697	55.22097	25.62989	19.67615	31.24071	6.634724	43.76173	6.990378	1.784581
164.3244	114.8654	122.0416	293.4316	195.7612	212.8124	81.27092	258.5511	287.5525
3.473688	34.6131	12.35948	8.26509	18.38127	2.447666	19.3169	2.370425	64.79371
79.01107	11.95267	85.83849	46.71725	32.14302	75.43129	63.12588	57.7262	13.61068
7.786591	29.24018	15.89627	11.86945	19.28923	2.447478	9.122818	2.37064	7.243989
950.0032	331.525	602.47	508.7894	97.39114	556.4007	569.3033	384.3901	136.4526
0	0.026302	0	0	13.76538	0	0.090067	0.048517	0.876081
51.69419	88.78284	113.3577	46.77818	20.19127	43.55678	159.1734	56.54368	21.80705
67.50268	19.53368	36.23918	47.93596	65.26224	37.66451	20.72295	45.04433	70.07575
68.0746	135.4485	191.0564	110.2361	106.5869	175.9192	255.0719	112.023	446.8253
907.6589	275.1947	460.9044	444.571	135.0773	468.4036	820.3208	507.8558	184.6843
35.87431	24.9378	45.12861	75.28672	44.12479	57.03324	16.84719	46.15186	158.5733
8.517038	11.93869	10.58149	16.14354	10.08543	16.73899	9.111743	10.43453	10.8933
161.3884	861.2834	86.64328	103.0093	831.7943	85.39463	295.1149	70.47785	142.8189
1.32429	22.74483	14.13021	2.833821	6.402736	0.777052	29.53646	6.983316	0
71.0934	48.76491	30.92441	35.27928	1.80742	120.7788	7.851245	79.61505	15.43109
257.0416	224.2522	222.9335	114.4653	179.2199	159.1736	276.8826	187.0055	242.9639
1.323948	11.92961	3.492783	7.677082	59.01676	2.448359	16.73078	5.825698	1.784011
73.1811	67.1841	54.81631	22.62809	0.890167	73.71311	1.392908	50.83358	136.5258
0	17.30006	10.61258	1.043254	6.413459	0	7.782067	0.044998	0

91.23413	30.36373	61.91489	46.10184	22.02641	72.06333	41.29983	49.66489	64.60044
89.61452	215.6054	149.4657	155.2565	272.0554	67.79971	155.9583	128.1808	11.79303
19.29926	11.94914	25.63263	22.70161	34.92564	15.02516	24.535	35.78182	25.46092
53.14793	17.36535	38.02359	32.31061	28.47149	47.76193	36.11556	51.9311	30.00593
3.477329	9.768504	12.37178	5.253298	8.249933	0.776783	16.7226	3.522917	9.990338
99.98097	16.28398	38.01622	38.31704	32.1477	48.58738	38.70023	60.00889	112.9148
247.909	252.3477	153.0655	107.9561	108.45	181.0514	150.6609	108.514	43.64181
139.9821	164.66	170.7545	135.5762	155.3383	173.4765	182.8495	153.5074	39.99972
79.6378	40.11578	99.0766	118.837	93.74545	59.44575	34.89423	73.90362	75.5082
22.13043	291.3693	147.7269	90.47195	61.55007	29.25588	303.7941	218.1157	101.89
114.8627	58.52778	77.82353	52.05695	53.28172	60.27969	63.20351	95.82104	80.0565
1182.962	1999.877	848.3246	872.1656	1659.932	1282.801	1373.223	1239.725	636.0077
2.756991	3.283146	0.841037	2.236426	13.77991	4.966395	12.91651	2.369855	0
1271.306	230.7954	998.7297	701.7844	1023.885	460.7774	298.9869	1003.099	1376.734
0.62855	1.106675	0	0	1.820824	0	0.061277	0.03383	0
72.58556	139.6363	78.7772	47.96843	68.95308	51.10834	42.5488	46.18496	12.70141
439.238	219.9596	484.7475	469.6303	734.3721	299.0661	284.8027	431.7496	337.5518
136.427	75.85584	97.29551	76.09455	71.66999	164.28	54.20233	108.511	142.8749
36.5596	24.94383	55.74285	85.42453	27.5502	28.44338	15.57227	32.3558	27.27173
9.210957	32.52515	107.1194	54.57495	51.46385	19.20767	42.56349	43.88927	4.51245
0.626296	0.019106	0	0	0	0	0.063138	0.034802	0
4.910753	49.73979	8.806912	20.39387	9.164184	4.123194	33.35868	9.28516	1.783894
40.12427	26.03189	57.48957	61.77058	80.89543	61.15673	18.15324	30.06472	124.725
2.755934	24.93509	15.88343	27.52983	22.95821	4.121664	46.31527	6.990242	29.10539
99.09253	20.61928	82.26605	82.17322	34.89652	92.17594	16.86985	60.05582	79.15821
20.69537	301.1285	114.0891	82.65166	438.5231	42.66345	176.4815	40.46628	127.3728
2237.567	1843.896	1217.221	1648.546	1357.519	2124.924	1203.239	1587.163	1686.097
25.12807	17.3504	26.54505	54.99363	31.26587	33.55949	2.692649	20.79405	25.48008
12.80135	290.2711	37.10575	68.8564	123.1534	6.637981	27.17657	208.8698	61.84298
5.623941	19.52194	3.490649	6.431091	20.20088	0.777859	6.560067	0.049427	12.70647
15.71282	27.0865	17.6645	16.69225	34.94097	4.959695	28.3389	16.20201	1.784149
25.16382	17.3441	46.11031	33.77978	3.643277	15.05127	38.46254	20.77918	2.692975
88.33588	24.95033	51.28404	69.57272	79.04799	95.55625	15.58072	61.19648	71.88255
136.3227	295.7322	491.9897	245.4218	75.33589	113.0665	505.8998	281.6036	186.5289
120.7173	31.44856	64.5631	92.44035	114.9188	112.3323	23.30461	49.67293	101.022
337.3475	101.8727	456.4774	453.5671	452.2107	441.5787	118.6344	233.2122	529.5995
57.39001	65.00702	18.5292	25.64264	68.01243	38.4949	60.57411	26.60694	72.79656

TCGA-CS-	TCGA-DU	TCGA-VM	TCGA-HT	TCGA-DB	TCGA-06-	TCGA-06-	TCGA-32-	TCGA-76-
99.14008	55.14743	144.2948	166.8059	111.1196	110.074	183.7511	82.27177	69.69252
99.94184	28.17211	29.02221	36.99214	35.82898	8.161231	14.68648	25.13723	17.89806
158.4532	156.5488	90.6122	132.1098	78.87109	75.42267	40.68232	82.22359	74.07406
4365.532	2306.404	2769.048	3142.79	2089.018	2190.189	1548.723	2876.535	2681.783
78.09428	55.14304	122.2949	86.73238	161.221	92.7485	92.70389	78.08553	99.20366
0.054995	22.52592	0	1.436357	0.045226	8.141323	5.222281	0.087787	1.608668
7.530937	72.05486	26.36839	15.00234	8.441958	120.2297	23.36305	51.44214	23.79806
248.828	91.82899	172.4677	200.0557	151.6946	57.0807	72.75515	57.23754	54.91845
18259.86	7797.627	6185.691	16546.56	6989.568	6258.695	6782.648	8213.3	8502.335
43.40608	36.77397	76.56126	38.42085	87.15127	35.67675	37.22737	15.48061	25.32811
2.572912	4.983868	26.41568	11.24754	38.10791	8.160262	5.146233	5.723504	4.629494
970.9916	586.1468	1253.869	1308.006	1150.193	370.9838	426.357	242.4327	267.9367
63.25555	84.50008	102.0341	73.87529	57.4088	79.50163	164.6617	278.2918	252.7869
2.537172	22.67646	0	0	0.048245	13.22781	1.691241	8.20322	12.82193
89.20307	31.90373	146.0937	67.10785	132.5474	37.71664	33.74654	40.52769	38.64548
39.6329	378.6706	7.868581	8.954481	9.635173	22.42707	42.47357	43.13965	44.3377
1803.256	1815.764	2290.386	2267.265	1433.312	1718.315	1296.52	1558.091	1840.168
32.25794	4.987012	39.5775	26.34076	72.79101	33.63676	17.28195	21.02477	17.93278
284.5396	266.4743	114.3881	48.21575	90.80079	292.4742	107.4637	229.3985	74.07226
474.3899	1214.841	189.1286	268.5972	396.5926	1210.757	517.3853	885.4364	210.2408
1151.764	1581.944	857.8893	961.5361	1166.897	392.3858	372.6218	403.9212	674.623
32.25653	0.072832	179.7803	108.927	65.64379	3.065204	19.01738	7.126884	6.117174
59.52719	1.3035	128.4675	208.4408	95.58377	47.90801	346.0104	7.120406	3.139072
148.6988	17.22059	220.8298	409.1278	476.4833	62.17736	158.5595	25.22651	44.58312
87.9531	117.431	104.7122	123.0666	100.3285	60.13587	74.5108	22.44347	44.54295
7.529379	6.210909	43.13761	23.34401	34.63695	15.29402	32.92952	5.732402	14.95683
262.5254	282.5921	75.62388	150.1394	108.7536	358.7367	232.2749	624.5345	815.3559
199.4977	524.9673	791.895	402.9453	1227.792	586.0251	298.0845	356.5865	735.2396
19.88808	1.307517	43.12687	140.2121	56.06557	10.19944	22.49808	9.905532	0.153346
17.44047	58.78031	97.67928	39.91715	130.1142	37.71593	34.61778	14.09124	25.34165
16.19933	19.66641	99.48021	46.74764	53.77051	51.98005	13.81041	15.47927	32.69359
17.43642	8.659759	65.99608	61.12791	52.57672	29.56225	74.55222	18.25939	3.146706
1.329728	4.981984	22.00908	12.77651	22.67017	9.178578	4.279576	7.102725	3.149641
364.2006	583.6825	571.0263	395.401	693.9701	679.7864	627.4505	2015.668	467.5732
33.54216	133.4296	326.4817	109.3551	199.4827	105.9998	136.8991	214.4403	127.3534
1211.328	778.2613	23455.64	11827.66	13240.95	1474.74	4010.96	1254.643	2776.623
26.01505	56.09092	5.229599	5.936756	20.33737	71.31545	18.17129	48.48924	35.39257
66.89464	20.89169	58.93607	65.6429	152.7105	12.23819	26.81601	76.56846	23.85697
21.14072	124.5425	10.50794	18.77482	40.63957	96.80753	77.18399	116.5559	62.06029
14.9547	55.0242	22.83944	13.48681	39.43308	64.2013	57.22721	109.53	45.8523
221.6245	524.4669	119.6434	85.96421	75.31373	609.414	225.3753	218.4972	336.8401
13.72293	35.54104	61.59766	26.33164	57.33369	15.2954	18.14698	26.588	50.34046
22.38565	156.3542	21.06983	16.50198	17.99566	108.0217	80.62402	203.9515	182.6869
71.90214	61.25263	170.7299	122.2582	139.7327	44.85079	42.41416	18.26895	32.74331
88.02961	143.2282	346.698	243.7539	303.3693	184.4737	188.9044	201.9714	295.8341
1.329489	17.17048	16.69465	25.67642	25.07416	15.29189	8.617633	4.336394	7.577753
61.94026	58.75257	29.87444	32.37215	54.96877	37.71473	32.02238	83.4742	66.57321
2027.446	989.9341	2652.889	5224.492	4637.734	1118.031	1984.67	648.9897	812.2728
1869.632	239.8963	888.7506	711.7684	605.549	236.4534	366.573	222.921	213.1884
5.047378	1.309036	14.93993	13.53699	28.59672	6.122267	3.41228	2.941602	4.626009
7.515196	2.536745	26.42876	10.49611	17.92662	12.23454	16.45293	1.54304	6.097008
502.6498	128.5468	288.6145	264.914	511.0519	231.3524	90.08194	93.4399	140.6723
13.72514	36.77998	96.8139	56.56194	57.36423	30.582	60.6485	40.49689	31.23697
1371.092	67.37632	8440.073	4372.982	5501.339	1088.476	1189.9	137.9895	782.6931
8.756469	8.646854	29.05607	20.35357	25.07512	11.21701	9.486074	9.881961	17.83111
14.96173	12.33039	65.11361	33.13209	78.7968	26.50523	48.51132	7.126412	23.84808
24.77566	1.308642	29.93122	24.14169	14.38681	7.141761	6.012387	5.72804	1.663258

3617.646	2685.743	5912.066	4018.235	5504.928	2783.346	530.3541	894.0378	3354.915
31.06364	316.8963	594.0216	54.99008	120.7069	436.1969	193.2353	268.7603	298.8143
2.572262	14.76208	59.01365	12.73742	33.45286	15.29419	13.81782	9.903773	20.83925
50.79278	83.11478	10.50773	49.03766	22.76364	96.80931	90.20069	87.53975	72.37114
33.42807	317.3647	1.712519	0.664413	3.663202	156.8816	9.480835	177.924	157.9974
245.2048	83.28712	375.7813	172.0402	231.7086	95.80847	96.15346	69.77589	38.66631
2.573019	9.866554	16.69353	8.209252	8.433158	15.29205	9.485719	12.6472	10.5159
12.47537	13.5414	64.309	46.85155	39.40078	27.52051	29.45068	14.06538	19.36974
69.39042	45.34119	50.12017	92.08754	70.49628	31.60151	41.55596	30.78346	41.57785
213.0188	1069.753	71.22416	116.1572	106.3638	167.146	84.01821	183.8264	481.7038
35.7694	3.760759	6.994811	23.42874	34.52774	1.026434	3.412165	1.543045	1.663605
7.525911	11.0915	10.51482	9.719036	32.22597	20.38674	16.43181	2.943131	14.93134
79.35841	115.0889	205.0029	181.0944	244.8497	88.6748	102.2199	48.90046	93.37093
7.53145	24.53711	66.0347	25.59156	45.38719	18.35192	23.359	12.68945	13.50378
42.19757	6.208232	148.738	76.17892	112.2676	163.0588	40.68332	16.87648	9.074737
22.36749	9.881423	37.82337	27.86516	29.90221	39.74884	14.68158	47.31977	41.43979
242.7539	170.1255	272.7735	307.9709	246.052	109.0576	148.1643	200.5588	164.3104
33.46968	25.7495	33.42067	60.47002	33.46793	18.35152	29.44155	26.54637	12.02439
159.8325	743.5944	67.70131	126.7117	121.8971	1113.895	389.1804	290.9866	551.3559
177.1419	139.5435	215.5698	230.9524	207.8379	75.4259	107.423	98.9961	258.815
582.1741	325.5576	1921.731	1134.344	984.2204	909.1003	421.1527	161.6775	767.8494
3.810907	29.44368	109.166	32.37262	120.5112	26.50541	10.34308	5.731335	7.59738
47.16555	4.977601	564.1254	370.6511	178.0098	12.23766	83.14713	4.321843	9.061909
42.16307	6.211091	87.14415	64.15437	66.87902	8.161469	40.70157	16.86947	25.32214
44.63512	186.8315	27.23406	32.37595	20.3819	56.05585	80.62725	122.2365	119.4806
2915.145	955.6311	1562.719	1123.79	1498.94	1308.608	557.2282	1080.499	812.2021
52.09389	248.123	22.82617	76.18183	37.1017	78.47917	108.3377	171.0618	153.665
55.82294	97.93681	320.3978	322.5126	188.6757	88.67265	58.01736	28.01546	34.22517
223.0076	590.9296	263.0547	244.46	304.6169	633.9158	399.525	903.3284	709.8246
141.135	807.3806	12.26734	24.80222	32.32791	149.8119	51.08879	237.7061	141.8959
253.846	140.7564	117.8724	133.5311	80.09725	158.9929	286.9128	475.7382	401.9619
17.43236	8.659582	46.62377	55.85928	44.21614	10.19978	47.65712	14.08413	19.41095
621.719	341.4359	375.6851	606.0549	498.0889	160.017	246.0876	122.6942	304.8726
1579.091	1821.863	2137.289	1453.601	1848.919	1902.781	1072.914	1992.408	1943.653
22.50903	21.13795	0	0	0.040781	0.004202	2.589708	11.83506	11.06997
19.9044	18.43873	43.98168	35.41965	53.74338	39.75061	34.63702	18.25075	44.4264
79.25589	1.30508	44.8421	57.31829	60.94137	5.103665	14.67657	7.125354	1.655689
566.0365	1099.906	526.1512	379.5534	365.5512	871.3854	767.0071	1444.992	1356.167
59.5114	38.01418	109.9967	79.96846	132.528	39.75454	31.14681	36.35101	50.44552
16.20201	45.33773	124.1124	72.43257	101.4909	57.07739	28.54829	32.17015	22.38571
86.65093	18.44559	60.7016	79.27241	44.24057	18.35292	40.69707	36.3224	16.47171
39.71119	0.073886	102.0882	169.2701	91.95419	44.84909	44.1597	4.333234	0.157843
127.5764	91.81683	132.8618	491.1543	124.2304	89.6911	84.9003	173.9617	69.67541
226.6916	396.3896	235.7905	76.12482	109.9608	205.8761	484.5278	613.7259	1049.266
8.751915	4.983569	22.88362	40.17209	21.49818	8.160196	24.28036	1.543012	3.150812
7.530887	0.072278	51.93906	46.82143	26.32238	11.21859	36.39049	0.126275	1.660635
2.572853	9.87327	25.50651	11.98897	33.42253	10.19888	9.481953	0.123976	12.00169
21.12885	25.75103	41.35437	20.2986	37.04184	28.54073	23.3623	43.15338	28.20927
7.523685	2.537098	21.98686	15.03114	38.14471	4.084577	7.748212	5.727632	6.108531
17.43755	13.55385	166.4507	55.8209	87.1475	58.09435	50.24377	11.30563	40.06745
26.00719	45.14087	6.110361	14.27047	4.857376	12.23617	51.23918	62.20285	38.28498
1535.734	807.6138	2207.697	1827.252	1421.347	885.6606	1056.451	1801.659	1174.583
14.95962	6.211505	51.02653	40.71017	95.44174	34.65649	19.88247	15.47542	10.55663
201.9677	1482.666	545.5282	366.7398	224.6203	1834.464	1182.255	949.399	591.6919
528.7832	192.1736	505.9618	557.0653	318.9349	241.5476	399.5356	192.2768	149.5853
2.572696	1.309017	7.876615	2.918144	15.55598	13.25334	18.192	31.84886	64.1975
11.21668	8.642934	17.58325	12.00779	29.80386	12.23518	9.488842	8.49175	0.147363
151.0526	78.36165	110.859	123.7798	103.9333	78.48042	64.96009	110.0236	82.94145

64.16137	26.92805	14.92197	24.12465	22.72125	93.72581	26.86098	23.71698	26.65051
28.56781	12.33039	79.21203	27.83897	57.34603	14.2764	34.62721	29.37263	20.89762
70.66204	8.656969	113.4991	93.54526	176.7065	79.49962	32.87818	28.0133	31.26421
122.7046	153.0247	829.8739	305.642	280.7143	44.85154	91.81183	85.09939	97.82753
131.278	102.8113	107.3326	219.0111	109.9058	125.3579	112.6555	62.78521	54.90314
91.67637	126.0011	68.6016	41.4177	96.76281	98.86047	101.3939	93.32935	113.8867
258.8636	0.075009	472.5649	296.6162	706.8453	66.25394	135.1563	9.899417	26.82835
12.48449	23.32619	73.06319	76.30548	64.47044	42.80781	25.95633	23.80626	51.78941
27.26006	39.09989	16.68452	17.29325	27.48094	28.53713	19.03567	19.58025	19.33881
32.23708	29.40905	17.55563	13.48931	39.4217	33.63497	39.86373	87.36048	83.91448
28.58733	73.50486	367.8404	252.8383	236.4995	140.6505	157.7016	61.42888	103.7203
28.5879	71.05539	227.897	138.8113	292.5763	205.8723	75.34858	69.77481	59.36777
1193.601	562.8048	401.2191	643.8584	315.3661	258.8739	350.1129	321.7202	324.0547
6090.681	1348.424	2110.853	5604.884	3260.683	1015.096	2143.274	1024.908	1532.602
5.051957	9.870614	29.92647	35.53232	32.22042	6.122822	15.56425	5.729024	7.584327
53.13588	35.46766	32.55667	38.52641	32.24939	36.6881	25.11318	29.26609	14.95036
16.19991	14.7768	38.68203	31.61709	147.9089	38.73363	36.3608	23.82034	31.22448
1.288449	7.121526	0	0	0.037976	3.055841	1.712883	10.4411	1.562007
25.95584	0.069201	2.590722	72.25482	14.36842	3.065296	19.06291	2.941863	0.146355
2.541058	23.90396	0	2.190641	1.259112	14.24643	5.196994	22.74975	7.296733
1.329641	11.07857	14.93428	8.21376	27.43161	24.45805	20.79744	8.491741	9.038254
80.56627	80.81461	122.2974	66.33676	114.6833	99.88175	62.35519	90.59106	71.15026
16.19638	29.43237	77.46887	37.68445	56.13395	40.77006	32.03043	21.03096	32.67099
719.5065	875.9584	397.6904	486.0319	432.4011	271.1044	318.9003	342.6167	587.2254
87.9959	105.2684	69.47175	96.55045	112.3044	60.13777	110.0454	119.791	170.0482
3.807771	3.757357	9.650595	6.711452	23.81013	15.28762	6.020267	2.938308	4.615986
1500.932	217.886	1989.562	1017.408	851.6158	331.2362	586.7085	40.53381	183.6265
43.44833	66.15875	143.4036	76.89382	94.41956	169.1828	208.0142	428.4112	475.6681
68.18491	270.1679	39.54769	29.33147	60.97596	305.7241	134.3473	153.0742	131.615
5.051599	18.3961	6.991153	6.693639	8.434901	32.60959	29.48081	12.65411	30.99438
71.93373	172.5866	491.9183	323.0357	501.5512	139.6324	121.2849	182.5055	189.4656
226.6494	181.1215	160.1151	254.3632	226.9399	93.77036	90.9511	55.85989	102.2356
160.9884	225.0416	93.23896	123.7484	95.59864	74.40528	79.69398	64.18725	75.59208
16.20198	55.11043	128.5181	132.9539	84.7984	16.31483	44.16019	25.22004	22.38551
21.10606	17.19398	47.56243	17.28863	20.3497	42.79967	12.08479	16.82316	19.34922
31.03336	78.24565	43.09406	33.89673	32.30437	109.0368	32.89629	22.42229	40.0382
200.4887	58.80072	145.2083	97.33567	121.8187	40.77405	45.88477	47.48176	75.5503
7.506115	25.59689	23.7997	8.989893	2.465975	28.52541	21.70071	38.55347	9.004454
0.070119	3.752435	0	0	0.057362	4.083579	13.87815	11.16456	79.10304
14.9626	39.21752	82.72763	52.03429	80.00258	41.79088	48.5064	32.15652	19.42534
47.03339	19.64875	54.58605	33.93605	34.6568	34.65354	27.70557	30.69603	20.85567
227.2833	83.1346	42.21079	36.91954	37.07564	153.8684	73.69092	105.5779	101.7984
38.49611	100.4086	138.9936	100.2955	303.32	126.382	126.4972	169.9298	230.7418
241.1645	276.1213	9.627901	16.49892	21.58091	171.2059	97.07483	184.8449	205.1027
349.3019	257.0154	600.99	864.3282	501.6439	398.4964	271.2309	202.0332	220.5697
40.91148	24.54764	43.09729	87.66777	58.51527	18.35246	35.50246	16.8641	47.38013
42.12932	15.99307	31.64885	46.03205	13.21734	65.22132	48.53513	45.95949	183.6904
89.12049	74.62504	53.65444	58.08606	40.66293	54.0188	55.44826	40.48636	70.99606
23.61413	20.88294	31.64374	32.38925	23.9549	26.50448	80.65327	81.98749	67.94867
8.767514	7.432006	93.24591	45.94396	148.0837	7.141927	122.1963	15.48329	16.47635
16.20072	17.22322	93.29722	92.1326	63.31906	46.8861	32.02094	39.1015	25.33085
94.22319	53.93286	315.8804	215.7991	503.9506	29.56391	44.14306	41.93913	31.26656
27.33587	22.11339	64.22391	102.7294	66.89497	38.734	25.0821	14.08984	26.80605
10.00515	34.27845	28.13405	21.81769	28.70039	36.69077	25.97062	32.0723	56.04923
392.6536	1931.524	169.7712	221.0466	135.049	777.6184	727.1691	2381.187	2241.242
1386.768	90.63914	1675.559	1562.741	1683.717	92.75234	256.4932	126.8687	68.24974
19.90783	103.8556	43.09429	11.97191	51.37363	69.30027	26.82233	39.07049	67.97774
132.5378	248.2614	145.1806	191.0023	127.8205	96.82534	82.29502	75.31228	124.3114

60.76319	97.91566	150.4837	98.83769	186.245	58.09887	144.7539	57.21877	48.99082
28.55658	61.15295	55.43715	31.63338	65.65765	36.69402	19.01592	12.69392	32.66539
73.1738	1706.284	62.42092	62.53767	33.52407	954.9402	1572.455	1397.178	1831.391
281.1027	369.412	188.2807	276.2666	115.9202	269.0578	277.3547	460.5577	252.9207
12.48672	28.22567	58.93554	29.34401	66.90042	22.42939	44.16459	130.6415	43.03048
11.24198	7.43485	77.52558	79.45177	34.65398	37.70972	35.52458	16.84485	17.91157
45.9139	45.35601	82.68471	92.03831	88.42089	83.5756	76.23482	65.55684	65.22859
11.24459	137.8645	10.5087	22.56416	3.662488	96.80273	61.57107	92.94901	50.25472
14.95577	11.1047	46.63368	49.06565	71.58496	18.35204	19.01857	7.126757	14.98025
802.5266	521.2786	538.4745	1589.717	728.5843	319.0057	493.1096	104.5942	287.1463
10.00368	6.211157	55.48391	52.9206	44.1602	9.18032	13.81777	7.124489	6.116018
17.441	38.01295	131.1386	82.24123	107.4786	37.71627	36.35005	69.69215	66.66567
365.1799	264.2234	192.6971	172.0612	167.2345	187.5269	102.2269	174.0717	116.9851
81.81214	33.13118	197.1252	170.595	160.0408	149.8175	122.1834	12.69524	19.43519
2.569045	4.970291	13.20085	12.83081	8.402485	7.139284	9.51141	4.316559	4.604171
348.0769	387.9139	658.1782	498.1079	421.6562	335.3107	389.9769	225.7046	306.3408
2.572655	15.97571	22.85459	15.7721	47.7011	18.35021	25.98354	11.28563	25.21472
1011.824	487.0401	871.0956	1024.963	925.6531	402.5771	616.1797	440.1082	420.2577
777.4853	254.5325	17.54911	10.47072	47.85981	67.27316	400.4297	378.667	722.8359
94.13197	20.89542	133.7787	101.1413	74.08562	33.63997	72.77714	98.85381	17.95544
99.07798	117.4282	28.10743	79.96997	38.29322	211.9679	110.9476	118.2891	127.105
419.8236	907.5968	277.1466	221.073	195.9407	420.9135	352.7335	1284.262	1064.246
63.18189	123.4457	28.9929	54.30107	19.19035	79.49322	88.42613	170.7915	184.2477
225.4331	128.5478	232.2809	337.4164	353.4945	187.5305	113.4866	72.56528	57.89422
881.8508	785.5743	1336.571	2046.228	1018.844	827.5669	1013.997	603.0117	973.3936
32.28951	30.67377	132.0412	39.16422	69.29782	42.81096	77.99519	119.6064	54.84122
5.040914	4.976052	13.18807	13.56821	10.78555	8.158701	12.11592	5.706021	4.614974
215.556	47.81498	358.1232	270.9169	377.4127	236.4505	230.5071	144.9444	183.5668
55.83455	6.199887	674.9329	171.2344	1050.837	37.7172	83.14373	85.10159	87.47923
1100.983	1037.552	848.2139	795.4722	1282.724	600.2932	293.7509	504.1445	884.5853
17.43339	30.65367	54.5523	39.95433	48.98634	46.88371	35.50171	30.74499	48.85461
6.291846	8.656059	35.19658	20.31137	40.58633	23.44502	12.08258	18.21905	14.95809
49.60003	108.8331	32.51237	27.83003	51.40571	154.8984	41.5592	55.77477	78.4047
225.4932	1005.619	65.9409	98.01061	37.10636	404.6126	329.3008	801.8628	1620.441
21.1535	217.4767	20.18725	4.429424	22.77345	57.07732	64.11343	55.78023	81.36156
9.993004	17.17749	14.92643	8.964859	4.857272	30.57215	46.88949	117.0902	67.38965
823.5849	606.9159	832.3892	1293.001	849.2024	472.8979	532.9786	174.209	321.1627
181.8556	64.89121	88.86865	86.78904	119.3903	26.50611	19.01012	41.90283	37.15379
5.048779	4.983254	29.95565	17.33074	25.05445	20.38364	3.411973	9.870981	4.628367
216.642	124.8245	109.0865	111.662	90.82311	81.53881	96.17065	69.74746	128.7304
167.1634	56.36626	71.2331	276.4286	179.1198	22.42992	64.95507	33.58203	17.95573
18.64546	61.03357	22.85151	12.73988	15.59155	42.80108	37.27756	41.70143	66.19397
92.96217	132.1858	170.6949	185.6705	197.0628	148.8007	119.5706	154.5919	232.1332
8.768713	17.20869	39.59236	24.08547	46.56217	10.19955	16.41853	44.53085	42.88197
34.69251	63.50649	23.72747	10.46685	14.40449	57.06583	33.79003	91.4352	38.45587
6.291542	123.4488	8.747831	5.181954	12.02694	140.6297	146.5841	417.3052	50.39082
2.565189	21.8391	2.59449	1.414005	6.020088	13.24546	14.77395	39.48675	10.35576
96.65663	45.36151	150.4657	67.84341	115.8842	89.69152	103.108	105.892	133.1582
4652.743	94.30971	1007.542	540.371	1770.978	181.4193	180.2146	228.4944	71.20685
7.527106	7.431372	12.26804	5.94048	2.460602	55.04215	308.6693	436.477	389.785
21.02638	119.9826	3.470953	5.187908	1.268682	41.7656	26.04451	64.58401	1.663406
29.82452	41.68945	145.1967	122.2622	164.7809	67.2708	61.49025	34.97058	41.60803
0.060436	7.300447	0	0	0.049607	1.025791	10.47796	6.908751	8.711065
24.85967	75.83679	55.41936	43.71812	64.50172	24.46726	43.30253	40.48003	50.37404
19.91593	78.31725	100.3268	52.01511	134.8709	81.53358	52.83447	28.00081	47.4707
206.9209	129.7917	960.9056	555.4759	545.8517	281.2962	305.0285	303.6559	253.1177
31.06332	193.3821	170.6627	131.2341	472.9123	219.125	91.81278	143.5499	78.60097
305.9128	119.9929	291.239	225.6186	259.2091	140.6516	220.1082	213.1166	140.6933

3642.391	3631.496	3367.364	2520.035	2220.427	3890.154	4168.74	17071.65	5233.291
26.11036	1.303396	132.8668	243.2037	112.2919	54.02273	210.67	14.09013	1.65172
204.437	850.2346	167.1333	134.2422	281.9321	1122.081	420.3255	1287.366	761.5828
8.766195	7.433627	29.02385	21.07085	25.11744	21.4072	23.372	9.902008	1.661816
2035.99	1777.773	1419.277	1258.892	1055.878	1252.556	843.2439	829.9399	1933.188
154.8518	313.1347	99.38871	144.1053	127.8497	210.9669	117.8306	199.113	337.0107
29.76539	136.5639	9.629184	20.30138	22.74776	56.0482	20.75945	76.29208	78.03021
55.82661	73.49211	212.0797	118.4443	132.6069	54.02347	105.7013	48.89281	53.44396
5.049969	178.4125	17.58017	19.60138	4.856179	119.1682	26.88585	47.00149	51.28118
34.74705	291.6769	6.108872	10.4614	7.248353	121.2661	45.04142	113.9135	66.54358
153.5167	83.24675	137.2791	131.3462	59.78166	116.1851	176.8555	57.21524	54.89325
45.92508	79.61248	214.7051	134.2923	247.1979	97.84604	61.48055	43.32968	51.97182
13.69589	30.56591	6.990795	8.206122	14.38932	33.62883	47.74972	82.84217	35.38711
6.266731	17.08871	6.120968	9.767964	8.40629	24.44836	12.99663	23.45762	20.53123
68.13267	193.0196	105.626	147.3698	100.2787	47.90545	32.88702	50.21242	31.23574
97.70402	64.82524	25.47562	18.01619	22.76451	61.1489	32.89679	123.5378	154.6127
831.8548	770.4997	139.8646	165.9756	456.1626	452.5003	182.8372	147.7103	381.5051
1.32965	7.412264	12.30006	12.03556	13.16183	11.21456	12.11199	8.466229	10.46025
213.0374	74.72669	211.166	165.2371	240.0716	100.9043	154.2371	126.8227	71.19744
11.23929	9.877997	33.43151	23.34154	34.64031	12.23729	19.89605	5.732586	10.54333
117.5725	36.78335	92.4018	97.39899	90.75686	32.62034	26.81434	32.16857	16.47469
1524.373	35.57262	196.1673	2104.607	762.0211	479.0125	1172.677	93.45494	20.89311
1.329681	24.44209	1.711934	1.412457	10.8068	25.47593	16.4485	33.23664	14.8807
16.20214	3.758739	182.2542	163.9672	22.77388	23.44871	71.05219	9.912623	13.5176
333.1579	204.3932	359.8829	291.3041	269.9623	210.9723	172.4263	196.4312	193.9142
55.81676	33.12941	146.9554	83.71756	113.4846	74.4045	97.04479	83.63454	102.1366
29.82211	66.12976	143.4541	55.01833	113.4601	59.1173	38.94916	7.122052	47.50284
135.0393	122.4061	164.5318	104.0838	163.6475	100.9031	40.67698	43.3288	140.6041
40.96969	113.8856	1377.206	526.8086	618.6719	287.4105	210.5535	107.3785	353.6401
137.2828	62.41814	50.13085	64.89527	44.24043	122.2883	123.1508	104.2738	31.22881
5.047991	4.982535	24.65755	13.53372	40.45302	28.53055	16.45093	8.486533	14.87335
36.01569	64.92345	153.1125	85.22287	155.2525	43.83182	59.75351	37.75562	32.74493
11.23827	39.12956	29.90614	43.8278	39.39297	17.33176	12.95101	4.338958	7.59259
26.08697	209.8826	18.43097	7.442926	14.41264	134.5063	99.75659	260.4055	175.1047
0.077829	2.534736	67.77864	75.55764	23.95392	7.142282	36.37258	0.127623	4.634173
18.67967	78.34709	205.1104	69.37926	143.2728	64.21245	45.01966	33.57294	65.20779
81.83511	237.3756	28.98505	53.48161	64.57875	151.8607	229.6628	694.1593	1053.238
22.39316	6.209086	422.7383	55.02833	266.0413	15.29572	6.878244	8.518011	6.111673
24.83458	17.21025	60.75253	97.62623	28.70804	22.42745	45.9417	37.62579	9.074347
5.052804	4.986899	21.97499	11.22991	39.3724	6.12294	12.0855	9.896937	19.34391
3.813643	9.873455	34.331	18.81061	50.05745	8.16098	15.56059	12.66548	10.53232
277.2683	111.387	76.51185	189.4815	112.3106	229.3051	188.9581	78.09721	38.66091
3792.184	1802.339	2608.013	3231.079	2493.909	2661.042	2823.642	4732.355	3112.215
37.23494	169.8622	10.50739	12.72521	25.15991	313.8518	106.6365	44.66794	31.24084
257.6738	461.276	489.2301	190.106	250.8803	895.8312	534.7588	343.9752	1160.539
61.98753	243.226	38.66998	30.8447	41.87487	124.3351	66.70259	107.1975	361.3222
177.1243	205.5528	101.1496	144.1074	106.361	289.4364	163.7847	300.5916	323.7121
17.42897	25.76118	41.34343	20.29096	65.63695	38.73088	6.876846	7.126812	25.29163
485.2925	321.7392	76.50255	288.3445	50.24804	505.4891	550.4823	1790.914	198.2791
24.87115	39.24098	110.8651	151.7674	95.57354	12.23814	35.48012	23.83698	14.99705
58.16608	70.8771	35.17519	27.85984	28.71533	88.65362	65.04055	59.78977	62.01024
21.15081	19.66857	64.22362	71.70067	149.1248	26.50558	25.08203	11.30587	13.51638
1.327572	128.5631	2.594655	0	0.055838	62.08546	15.65122	25.99231	24.63626
0.063096	22.84639	0	0	0.051743	1.02603	5.181953	1.527152	38.17062
3.810492	2.536494	16.7111	25.73607	17.91861	2.045998	8.62445	8.478859	6.093125
31.02078	185.426	13.1492	18.77715	19.18182	151.8208	49.40114	55.65564	59.09644
8.761242	9.871454	36.1038	24.13063	33.41271	9.179805	5.14467	12.66009	11.99625
78.11	139.5214	169.8144	131.2775	139.7759	82.55906	88.35716	71.15623	149.4613

47.15999	35.57225	839.429	475.4229	886.222	23.44822	31.14772	25.21455	10.52994
1146.643	266.3832	0	0.667836	0.064434	2.045502	43.28808	101.6209	558.3985
0.034691	1.241358	0	0	0.028728	2.035946	0	2.672866	0.064253
0.074611	2.537081	45.84953	55.3792	20.32053	6.122608	7.749711	0.121874	3.151474
890.5341	1289.641	1521.347	1466.463	1245.774	1186.311	955.0493	958.0215	775.2504
30.93123	1.308592	36.99302	96.42647	64.26531	9.179677	18.17308	4.337622	0.149808
55723.72	29889.8	26231.65	37195.65	21262.46	33631.42	17988.86	18693.33	21658.2
129.9933	320.1536	73.88792	34.6213	49.03509	198.7226	97.06243	301.4757	286.2255
187.0635	79.62256	285.971	307.9581	267.5475	224.2189	117.8207	97.61619	250.0164
71.93058	199.4715	215.5627	171.2725	331.9934	223.1989	134.2945	112.9164	270.6682
95.4661	319.4002	669.6357	384.8801	527.9126	440.2811	359.6466	234.0451	198.3911
8239.662	1101.216	1583.838	2911.301	2193.991	1627.604	1352.892	190.9181	730.8629
44.68863	6.201837	489.2846	409.8955	247.264	53.0049	201.0394	76.74348	3.130982
92.4487	64.63038	14.03949	16.5355	22.72365	58.07746	14.69183	19.57938	35.4298
11.24665	36.75402	64.25071	17.26209	54.93849	24.46648	22.48609	27.96693	50.31415
13.695	14.74516	14.04295	35.53677	31.02957	12.23628	22.5201	7.117189	9.054712
83.00094	47.79021	102.9552	86.02314	83.62491	37.71624	52.82832	69.69041	85.82903
55.82418	20.89652	185.6764	155.477	307.9952	51.98504	245.3321	41.93326	31.2687
96.55525	97.83719	26.35004	42.94927	39.47593	186.484	64.98631	80.74113	57.77182
659.7828	188.4669	307.1038	285.3156	386.9024	121.2871	66.67846	41.93976	51.97795
52.10678	27.014	81.79799	82.20082	134.9692	40.77445	97.90814	29.40649	74.10514
8.745767	254.2507	0.83464	5.186794	3.661237	17.32689	26.90944	20.85658	6.095166
2952.473	1284.781	1971.849	2814.502	1625.598	1439.066	2046.229	2712.184	1871.228
125.0885	96.69695	135.5111	156.2769	142.1142	41.79339	64.09183	73.90205	38.65309
225.0986	875.3433	40.43366	36.13722	17.99884	210.9455	92.73759	72.45594	209.51
39.57188	7.431695	34.32996	36.2684	29.85903	11.21785	3.411463	12.66626	6.11295
190.8086	293.6967	544.6867	464.1893	536.2382	264.9877	217.4953	73.96335	269.3296
3841.697	2133.896	3203.731	2520.081	3340.678	2019.989	967.1656	630.889	2110.888
49.64246	241.0884	397.7251	288.2789	403.6849	300.656	251.3101	133.8138	156.9645
3.81107	24.55533	119.7461	37.66803	103.8235	14.27642	20.74709	14.08876	20.89892
0.075611	40.30325	6.990369	5.936322	1.26811	49.92734	33.82076	26.47045	20.79245
152.2907	106.4706	91.48786	140.4081	95.58219	69.3088	175.1135	121.1384	68.18429
73.15649	129.7349	233.2063	130.5287	188.7003	142.6855	181.1411	129.5558	103.6725
18.67705	35.55857	68.623	29.34285	88.36289	63.19066	22.4794	33.55362	72.50031
1.329714	3.758984	27.3311	21.93829	8.420004	5.103132	0.816406	1.542841	9.012621
0.076052	9.874956	40.5031	27.90838	42.94148	12.2369	19.90221	4.338728	4.63502
3.791347	9.780096	6.135011	5.220779	3.646263	3.064166	0.816999	4.292863	6.016107
16.1772	11.09799	23.73348	24.10487	64.35481	16.3128	9.479924	14.06089	1.661933
6.278351	245.5688	2.591003	0	1.268679	195.4941	2.545968	1.542896	258.4134
6.291806	46.54276	57.17996	29.34831	107.4057	25.48637	22.48114	12.69766	29.75143
23.62291	18.44475	69.51584	56.57942	62.11803	68.28375	25.95084	29.37562	43.01124
27.31673	19.65879	57.2062	118.7783	43.01758	20.39024	45.05782	19.63654	4.634541
5.053333	13.54095	43.13479	12.73806	29.88089	6.12303	10.3469	8.51427	28.17218
32.2966	25.78827	65.08227	95.08259	118.2247	53.00268	42.41852	55.81201	40.11795
1.328919	9.873954	44.03862	19.56739	34.61496	9.179993	11.21769	7.120804	19.34058
12.42956	18.34569	9.64558	29.56492	11.9833	15.28911	31.28163	4.32969	4.622001
54.5497	200.3974	29.87019	63.35669	34.70774	131.4637	33.75153	82.15364	141.7372
126.0765	83.11692	44.85805	32.3859	34.68667	101.9039	37.23702	73.69956	59.14902
22.34781	15.97993	93.4449	27.89368	19.16537	23.44487	19.89732	8.513505	17.89518
6.291168	61.20985	3.471964	0.667255	2.463184	223.1656	86.6804	452.0919	704.6849
43.2764	7.432414	30.79562	64.36661	35.81034	32.61263	43.37407	7.12176	13.4769
132.5378	52.69969	133.7353	205.3614	111.1136	107.0162	154.272	75.31232	90.359
64.40142	51.42072	133.852	124.7211	78.79715	43.82829	57.19013	58.50427	1.657028
11.24726	31.90385	102.0614	126.8237	165.944	5.103465	12.94317	4.330326	0.158785
168.1438	178.3353	7.868125	11.97077	12.02684	291.425	38.96242	134.751	113.6525
65.70009	129.6565	26.34653	57.28714	83.63575	119.2405	116.1454	327.858	193.4476
11.2389	0.071765	30.78658	55.20834	20.35708	7.142143	12.08263	1.541268	7.593112
0.064148	57.44231	0	0	0.052586	5.099188	1.685071	6.972799	11.64226

209.4061	8480.326	337.832	256.5087	112.3548	2657.964	1327.764	6861.08	9536.144
0.072887	106.6448	2.591008	2.164656	1.268679	39.72796	25.17759	8.474609	20.64771
309.5067	330.2299	67.70519	116.9198	108.7462	248.6722	177.6613	307.5214	308.9248
65.7402	162.7888	321.1843	328.3621	397.6518	363.8362	220.1217	115.702	170.2224
39.50864	56.03424	11.40047	21.87468	16.75905	48.90359	26.88023	16.78352	17.82966
12.46544	59.75172	6.990327	9.718505	9.628657	29.55514	18.16915	20.95302	48.54471
33.52335	39.22454	63.33714	90.60041	69.28974	21.41036	25.9479	18.26497	37.13863
78.12549	627.7169	990.7849	813.6114	661.7204	442.3236	514.7744	183.9551	322.6448
36.0092	79.56755	117.0378	118.5204	76.47704	43.83102	58.02868	44.69443	60.77979
1395.128	1282.968	212.9035	240.7093	249.6676	844.8654	252.1774	581.7914	581.0773
3.8137	8.65457	31.67546	16.52937	33.43688	12.237	12.08434	9.899251	28.14572
272.4161	102.8525	161.0007	346.5501	201.8604	115.1715	149.908	92.03444	91.88205
219.3096	9.870228	996.0748	1231.109	750.0802	88.67561	482.708	11.28337	4.606912
2599.643	4495.318	4981.994	3096.661	2351.816	3326.56	5126.422	19622.52	6020.257
73.08159	20.89264	110.9069	228.3465	66.90576	38.73447	56.30859	29.38686	10.55819
1092.427	1124.518	4936.315	2461.961	2833.09	1119.05	1365.847	1598.502	1224.938
21.13487	126.9163	10.50855	21.80602	17.98743	104.9538	84.15573	160.657	38.51619
366.476	503.874	195.3249	144.8496	191.1258	398.4821	177.6484	228.3437	248.4752
26.09152	31.87965	74.81445	56.59636	79.97382	29.56197	35.49807	14.08695	7.597898
79.35227	644.3735	91.46776	36.87639	47.85819	331.2189	116.0973	782.8553	683.8455
255.0035	284.9324	183.0356	166.8125	64.5678	262.9339	133.4555	342.1139	258.6013
0.034885	8.100564	0	0	0.028887	1.021597	2.640488	5.158007	4.101478
85.23652	123.1793	16.67528	14.24661	10.82839	91.7045	29.44276	68.01044	72.17764
77.87628	78.16678	21.95947	6.688531	28.71021	63.1817	48.54475	40.39979	47.30694
10.00841	35.52916	53.6783	49.80861	58.50433	9.180618	17.28171	8.519651	9.077134
66.8895	105.1428	24.59034	73.97103	38.27925	162.0269	140.5081	116.7586	172.4938
7.524629	3.753943	371.4014	200.7616	170.8191	128.4196	40.6766	12.69203	3.134341
36.00705	60.01161	152.2761	82.99549	90.78379	64.21189	61.501	36.34978	22.39033
1.325136	1252.194	0.836923	2.170866	3.659674	253.7385	69.31733	54.3957	643.3283
239.1273	176.2868	1321.645	812.079	1165.693	476.9756	783.4625	388.6045	472.0201
157.383	187.287	695.1609	247.4777	612.6844	436.2043	300.7037	211.7743	344.7509
8465.036	11807.17	13659.61	11348.39	7432.714	9862.462	12759.31	41373.43	16340.43
6.291289	20.85292	33.43882	37.76701	21.54239	20.38799	16.42549	20.97641	16.41929
126.3477	36.80026	177.7603	137.3537	94.40328	67.27147	91.83492	135.0765	50.48019
22.3525	134.0556	13.15334	8.20016	7.247282	177.2664	63.34738	118.9607	194.7983
7.526186	14.74935	36.98405	18.05612	21.53009	32.61108	16.43119	11.27789	11.99833
1.329132	11.03592	11.42814	14.35547	6.032877	14.26724	4.284501	1.541371	11.86622
8.769719	94.02285	22.83897	27.86147	15.60228	74.38979	99.79696	112.3058	69.335
58.29216	90.58993	110.8541	61.80628	142.1202	160.0057	166.4305	260.0454	190.6447
12.43849	34.1252	4.351194	10.49732	11.99023	31.58466	23.42194	44.14657	19.22931
26.10103	28.22628	77.4329	62.61329	82.40075	34.65819	37.2238	5.730356	38.61054
39.72222	42.90644	99.42274	163.1283	143.275	63.19346	85.78293	30.79184	51.92936
11.23036	1.308478	22.86335	9.719346	17.96022	13.25529	6.879298	1.542372	0.150331
1073.526	692.4547	361.6166	391.6772	281.9314	354.6733	148.148	330.0712	501.4409
3.813436	26.96442	29.01568	27.1204	34.65269	34.65313	15.55188	5.7331	12.02188
12.47841	30.62368	50.17841	37.72881	88.18825	15.29453	12.08054	15.45871	20.85317
111.5606	323.035	363.398	229.3803	611.4224	270.0823	139.485	140.7739	254.5231
3.809658	7.434637	132.0412	64.87006	211.151	11.21902	15.54311	9.912727	12.03805
210.5768	906.1595	113.4639	222.6216	68.16203	169.1862	201.9143	669.1942	1159.667
10.00707	9.881483	28.1274	33.92328	35.85808	15.29486	22.49177	5.733307	16.45032
0.072475	0.068	15.83501	10.51	16.7197	5.103048	6.018782	4.327741	11.91951
3.812219	7.435826	118.9097	123.2937	21.56958	13.2571	9.47674	8.519873	22.35844
357.7609	170.0946	147.8022	172.0596	181.5583	144.725	166.388	164.3414	85.96334
358.4054	226.0072	32.51214	33.11934	13.22154	277.1714	94.48522	323.3427	156.4272
24.86776	193.1029	36.03172	82.25296	87.19412	232.3433	94.47384	50.23703	165.3377
251.2148	62.47343	61.55349	274.9721	97.97052	109.0525	169.9058	54.44045	26.82988
25223.85	13570.26	12623.96	13762.11	11399.23	11139.47	15319.52	20653.48	17707.15
707.0802	204.412	549.952	464.1643	398.9486	448.4335	288.5696	293.8828	263.4381

2.571566	34.28993	43.99765	23.32523	58.47029	29.55978	25.96599	22.39624	29.68264
13.71365	11.10144	40.47931	35.45824	81.04508	10.19947	24.23407	12.68294	10.54737
5.033018	0.065808	17.6348	6.725427	17.85454	1.026418	4.285724	0.113924	0.138183
18.67966	25.78845	143.4574	66.35612	109.878	50.96461	32.88012	37.7444	60.78164
206.8287	685.953	128.434	126.732	23.97117	466.7577	188.927	317.2904	980.5358
848.2445	1096.096	261.291	330.516	259.2467	734.8124	544.2786	1615.782	1385.335
7.52471	1.308587	42.29177	48.46777	33.4042	6.122796	8.615647	2.943121	9.054395
75.64595	95.52322	411.8466	302.6793	336.7774	198.7406	153.365	135.1812	139.1934
169.6443	64.92658	173.3567	140.3744	103.9515	68.29062	107.4434	101.7236	68.20655
18.67948	12.32883	154.8621	117.6985	180.3292	58.09974	72.75594	44.71591	77.07406
923.9831	548.2383	2513.908	2653.769	1358.057	1766.214	1164.787	1105.614	250.189
209.2601	232.4082	67.70771	71.6122	105.1561	301.6607	273.9378	750.6067	280.792
14.96154	78.26711	44.85081	37.67129	59.72771	78.47226	72.81612	108.3786	106.2435
6.292604	93.98169	1.713175	1.415274	3.66274	101.8934	49.419	54.21309	228.7663
111.4337	71.00576	61.56398	80.73038	37.09881	77.45915	86.65902	208.4613	175.697
7.520043	3.761644	21.99665	20.36395	28.62437	10.19792	21.66469	2.942623	3.151194
24.86716	42.91933	846.5588	473.242	341.625	210.9736	294.6421	117.1196	112.6202
0.040169	0.03783	0	0	0.033202	2.038148	7.123282	11.49016	4.189272
136.2538	198.1661	99.39941	86.72419	88.44032	143.7033	167.2774	412.9544	161.2181
55.81539	89.36388	73.87842	41.41323	81.26552	289.4219	53.68635	22.44602	230.4299
5.051064	2.537103	19.34047	10.48187	38.13864	3.065354	6.880566	5.727003	0.149048
13.54269	10.98114	0.835188	0	3.645667	7.136708	23.56319	2.919647	21.69597
3.813677	14.7593	53.72912	13.49676	48.90371	12.23717	13.81931	11.2889	20.82898
33.48446	1.306832	67.79525	86.96936	34.67121	5.10385	25.96223	19.63109	7.597368
5.043938	4.978484	216.4506	314.8011	268.7121	9.180085	37.2095	18.2646	17.95067
130.0123	89.35121	58.91606	110.1893	91.99318	24.46814	58.89297	151.6575	75.54651
172.8391	26.98183	35.17379	62.70048	15.60332	13.25696	56.35127	15.46957	7.5976
8.767134	49.02843	201.5418	86.73462	255.4614	66.2521	77.09599	54.44662	53.43049
12.48662	53.87538	88.89107	64.89022	62.12769	39.75308	45.90141	22.43418	32.70664
29.82648	152.9566	168.0616	125.2449	250.744	48.9278	93.5628	94.79087	50.48764
450.8438	435.62	263.9287	421.102	296.2702	538.1202	949.0861	1005.023	501.4918
28.58812	89.39723	157.4837	58.76927	279.4275	90.71238	46.74401	29.40843	152.4385
17.41491	7.434019	26.37578	33.95543	16.78524	12.23731	46.83263	18.22101	32.57169
5.034421	7.39818	14.08698	16.65791	10.76956	4.083662	6.025612	0.114429	7.515198
1058.053	210.4574	21.94631	13.48453	10.82779	20.39143	84.88432	107.3393	939.3254
66.9751	62.49315	274.5521	125.9728	207.8315	128.4202	122.1629	114.2939	139.1636
13.72162	34.31295	79.23617	34.66022	60.89402	28.5424	14.67898	19.64068	25.30497
24.83825	148.816	21.95883	15.75564	23.94582	82.53933	43.33102	19.62813	61.98516
66.72664	112.177	17.55851	15.00649	37.0266	145.6906	20.76242	38.97153	175.8757
33.54217	160.3318	279.8276	215.0899	237.6796	191.6054	122.1608	96.21378	106.6666
33.29601	47.4393	12.29215	7.461415	6.044738	41.76687	6.885194	24.96636	11.94359
26.09462	13.55341	54.54124	39.18339	70.45438	30.5813	38.09824	12.69701	44.47552
25.99958	28.11954	7.872804	14.27391	15.57368	56.03435	23.39537	14.03066	17.83917
650.0865	157.9213	388.0323	459.6651	384.6008	250.7196	252.1702	204.7986	183.5868
2.573002	102.1127	0	1.412775	0.061007	18.34776	10.35537	77.20195	109.3752
86.54799	13.54988	36.05398	35.42627	68.02262	8.161453	7.743432	0.127286	10.55444
16.19802	69.71416	58.07094	37.67656	51.37819	64.20636	35.49811	40.46349	79.75378
100.4069	371.8806	121.3854	85.18816	127.8634	208.9315	221.8566	631.6325	371.1144
225.4693	201.954	257.7838	436.2691	478.9126	188.5519	131.683	139.3846	109.6584
29.76284	11.10165	30.77874	27.87766	42.9842	8.161334	9.478374	7.125497	9.072302
28.41296	17.15242	11.40593	8.216561	14.36936	49.91606	15.58002	14.0015	20.70074
115.2818	4819.717	81.77954	137.2576	43.07696	1924.162	2369.73	3778.85	3748.159
2.572785	36.57266	2.59068	1.412446	1.268619	69.26471	21.67197	15.3816	33.78943
18.67328	1.305772	43.08963	108.0727	35.88612	15.29548	174.4096	1.537648	1.657279
321.8893	281.3446	99.38949	135.7996	149.3337	112.1138	188.0637	320.0484	205.5996
0.071989	25.58504	2.591448	3.676636	3.658889	20.37889	9.501332	57.58008	3.144944
33.52942	1.304538	111.7701	156.3807	90.77186	31.60156	102.2826	5.728744	4.628442
2.5665	29.08103	0	2.168538	2.462188	14.26441	10.39518	11.13713	7.490478

28.57103	13.55397	65.10783	54.30526	91.91795	10.19984	21.61369	19.6523	19.42487
240.2874	368.2279	212.9166	111.6117	100.4039	290.4613	239.1914	557.9873	505.5548
21.1164	37.91754	22.84942	16.52337	15.59339	33.63307	42.48794	58.27689	28.17103
16.19181	14.77067	59.85896	49.82534	32.28916	28.54155	20.75439	18.24408	14.97967
2.570814	20.73691	8.768443	8.235047	6.037772	16.30537	10.37432	23.50303	29.2329
22.37733	20.88232	58.08191	29.36381	56.12804	13.25709	33.76817	11.30313	22.35782
373.5627	78.35818	92.37109	125.2991	86.03277	49.946	37.21362	34.96816	29.78464
152.311	132.147	130.2218	139.6334	131.3873	108.0343	58.01987	93.3702	121.3305
36.00779	73.45381	84.45717	55.77977	146.8379	86.63088	64.10206	47.47032	158.0512
284.6889	418.1248	50.10609	67.0846	87.24712	470.8201	175.9493	103.1233	136.1299
68.17678	58.79758	111.7495	56.52909	84.83213	69.30797	77.10763	53.03766	54.88377
2505.243	2154.526	702.1263	972.8309	762.0705	1439.059	938.5938	1702.702	1461.377
2.571318	24.38005	0.834547	0.662805	3.659145	17.32445	4.281769	53.51784	9.000756
74.40786	238.6862	1389.392	747.1485	1466.652	397.4817	625.7077	815.9681	477.9459
0.051277	2.476464	0	0	0.042222	0.004339	0.826329	2.818097	12.488
165.5028	151.3518	29.883	25.58193	44.21345	80.50587	20.7515	51.52008	32.66149
153.6735	578.7614	450.4808	228.5904	537.5052	516.7201	584.1291	1787.205	1270.292
53.33576	33.12733	124.0733	56.52583	108.6969	64.21298	48.48614	33.57617	40.12484
24.84443	7.435742	50.15642	21.80297	70.40318	17.33314	30.30008	16.85891	13.50702
127.3233	20.88531	35.16486	40.7069	133.5567	37.71358	12.07709	21.03363	17.94074
1.304081	7.209533	0	2.216958	0.043456	7.122943	4.348528	11.95479	3.003095
2.572786	42.63513	11.40511	15.81152	4.855104	48.89861	11.22896	51.04658	25.07242
13.72421	14.77662	74.80353	46.74508	105.0151	57.07511	27.68596	21.04052	43.00922
22.35237	63.49013	8.749495	8.200181	14.40279	59.10202	17.29019	29.29051	61.85781
11.24813	31.89706	89.75393	96.63078	78.8412	25.48692	51.09827	29.39212	23.86384
403.4712	513.4968	99.39488	104.0896	114.7059	424.9677	81.42305	171.2582	240.9584
1764.8	1524.53	1234.486	1729.155	825.3794	1037.514	1190.802	942.7021	980.8146
29.68792	6.207155	38.76871	42.40442	21.51605	14.27345	31.22707	2.943015	4.632189
221.5776	187.1443	91.4811	129.8028	32.3311	196.6929	210.6567	361.4713	201.0075
6.291134	212.9291	2.590861	1.413974	3.663198	44.83735	25.98337	25.11918	93.87645
19.86208	63.39402	18.45087	13.50663	16.7718	31.59235	21.64641	56.7635	25.18071
37.01546	14.7267	25.53563	39.4146	19.12281	8.160155	6.014523	5.722375	6.101211
7.531146	33.11266	88.00821	43.7093	105.0408	34.65803	45.9006	23.82478	62.17836
356.5344	79.61408	140.7587	182.6309	130.2358	164.0882	71.01566	61.42235	91.87633
17.43946	47.77607	110.0262	52.77813	108.6299	41.79156	26.81506	23.82736	40.08825
52.1193	159.1434	527.9725	221.8242	543.3889	245.6237	243.5032	153.3038	213.1486
86.65028	103.9158	36.91906	27.0793	26.34976	129.4207	78.01291	87.64109	121.0122

TCGA-06-	TCGA-TQ	TCGA-CS-	TCGA-32-	TCGA-DU	TCGA-S9-	TCGA-S9-	TCGA-DU	TCGA-TQ
40.56008	196.0256	254.4413	87.5899	114.2665	107.3469	150.3494	137.2648	220.1447
0.774703	49.5296	37.41935	9.502853	42.13236	24.21388	39.27965	48.20821	35.956
58.24213	110.139	122.9561	42.83171	66.58059	91.55777	76.97457	105.4507	97.27048
837.2994	3958.265	3533.749	3862.219	2456.823	3022.212	3549.48	2787.306	3571.46
65.48029	73.8228	124.0351	119.9746	98.73876	113.6559	171.1443	49.05589	126.4143
21.63504	0.024035	0.016522	6.684214	2.242604	0.012529	0	0	1.75477
94.18583	6.650882	16.0636	79.07006	24.43074	8.440338	2.119019	9.053039	12.24373
29.81685	142.084	219.1694	39.97308	145.3086	139.9611	181.4977	222.7581	146.8738
4250.173	7659.978	12476.66	2798.557	9434.587	8750.949	7851.41	18192.14	8332.214
26.08216	69.38546	42.78628	47.60087	88.70975	24.22321	70.3702	39.97523	67.55423
4.5638	20.92874	9.646055	8.552989	16.65479	19.99676	37.92532	8.148907	21.06521
298.9757	1755.528	1480.849	385.6302	1441.899	1249.08	1184.386	1521.861	1206.181
218.5047	66.12075	88.76764	308.5473	191.8808	109.4533	84.35163	84.51568	94.71415
0.459825	0.025567	0.017558	2.84567	7.728133	0.013307	0.65007	0	0
26.20255	98.02955	145.3966	59.97553	59.92798	62.10103	56.21051	97.26606	140.1574
83.86304	7.75205	3.235868	4.741084	5.591702	31.57671	10.26311	21.79184	7.276588
980.7196	2457.132	2514.792	2967.13	2898.237	2050.942	2391.731	2987.334	2368.337
11.81382	20.96218	35.29833	19.02651	28.86898	17.90917	46.64991	19.96689	54.56679
93.63641	52.8998	113.337	153.3206	35.53742	103.1291	22.8503	56.33485	63.11006
1909.039	239.0358	223.4943	2263.523	329.4607	306.2388	157.6578	288.152	193.2243
237.9807	1250.03	1476.565	216.1334	1037.074	964.9646	1145.142	1228.21	1536.053
0.801407	118.8285	181.603	0	7.810464	11.59715	34.75972	19.96713	129.4434
0.84379	81.52511	197.7734	0	97.62424	200.9648	111.0829	321.9447	135.7626
18.99724	296.2717	349.6288	81.87078	252.9068	937.512	686.1412	540.0583	336.5769
29.75315	118.9328	143.2518	189.5268	135.2892	90.50178	94.7991	108.1885	93.57378
8.196163	31.94495	43.82394	7.597932	17.78019	13.69885	48.22425	25.43858	23.47142
247.4368	99.15834	121.9115	448.536	143.1097	134.7088	110.2791	137.2507	116.4063
585.7135	676.2583	448.0258	648.4509	787.5261	1042.83	839.9261	673.6251	707.1372
4.604172	94.59105	41.6947	2.837274	19.9981	67.31546	28.84402	75.52804	46.54976
22.59594	92.51038	54.54767	53.31178	49.94373	102.067	196.5569	71.80735	151.4594
82.02887	173.8675	53.46928	25.69236	38.84986	119.926	123.1489	10.87034	170.3663
15.41187	45.17596	38.50993	12.35852	76.51585	52.62151	135.0508	58.17703	101.8331
8.02582	15.433	12.84572	7.600875	15.54299	15.79112	25.24129	9.972964	27.38933
1917.655	374.5002	484.3763	1084.586	1180.13	526.1661	496.1915	445.4304	413.9067
240.4041	276.4274	74.87304	109.4891	229.5993	128.3965	231.8381	154.5264	237.3956
639.8705	12142.52	11903.59	1773.014	24239.71	20350.46	38651.69	5579.133	22369.48
203.4264	4.447383	12.85299	24.7548	38.78539	3.178387	1.379803	19.98416	5.419906
15.43122	77.0924	36.37591	14.26289	44.39595	53.67744	68.87169	33.6055	76.24557
396.5271	11.05631	18.20339	28.5525	56.56137	24.22061	8.037	21.78584	13.47823
325.8872	11.05558	22.4757	56.18949	34.4026	7.388253	5.075644	27.24908	7.89567
533.2426	39.69203	202.0703	524.7658	87.65813	116.8163	45.0745	121.8029	22.76865
47.01745	48.47232	25.68537	36.17218	49.92414	67.34156	54.79963	12.68903	63.86674
403.8394	13.2594	9.652496	59.03404	25.55155	15.80646	5.07739	13.5979	4.183099
36.94695	138.7667	175.3329	27.59392	215.1215	139.9534	177.1001	200.0414	179.8607
172.9854	305.0743	173.2322	242.8103	187.4766	357.7742	451.198	194.5268	371.9695
21.69756	8.847772	17.12023	12.36462	21.08258	18.94856	32.66487	13.61194	39.24295
36.66277	23.16826	37.44305	74.2762	48.82502	30.53492	22.85957	15.41634	16.57525
306.162	1723.662	5323.593	297.0725	3964.154	3911.401	3555.379	6904.72	3875.159
320.1991	801.7622	1258.401	59.96941	245.1664	456.7077	432.5208	848.2249	599.9901
0.709271	40.66252	7.509042	13.32134	12.22667	9.486949	35.72401	37.33906	28.64869
11.42224	17.62846	20.31308	11.41416	12.22683	16.84169	20.00395	7.239955	16.69828
94.32591	465.8154	263.0284	128.5337	208.5413	231.5119	413.4223	260.0011	468.8139
36.73611	61.69005	45.99467	56.17223	69.88442	71.559	155.7711	31.78574	91.79158
280.9855	6048.671	3867.37	1624.469	9935.868	7047.26	19229.16	5214.61	12448.22
25.05182	14.34359	22.45639	10.45828	22.18874	10.5406	37.89363	18.16749	19.79004
22.51748	28.67058	55.60607	40.93375	34.41727	55.7771	66.66951	37.24789	93.10509
4.583181	25.33236	21.39229	12.3636	39.88388	10.54139	37.86983	34.5695	49.24456

948.6039	3769.985	8881.95	3949.801	7754.174	4418.628	7674.465	3820.938	6061.841
497.6761	240.1141	116.5713	272.3302	112.0689	62.1085	53.95838	141.7881	106.4573
25.6251	39.64168	28.87838	17.12433	16.67388	33.67616	58.64333	11.78297	34.07525
43.46539	39.66904	39.57475	225.8085	28.87221	102.0382	34.7477	60.91615	29.636
18.61594	1.139492	4.305661	89.58399	2.26192	10.54307	0.645287	0.875275	2.324312
37.00327	242.2949	300.4327	141.8686	249.5554	212.5678	337.1173	197.2664	339.1883
14.97925	2.243414	21.38992	14.27076	8.913332	12.64321	20.71392	15.43333	21.03965
8.201927	24.2519	39.55579	11.40794	38.8144	21.06009	51.19383	22.70554	34.07668
47.43137	82.6068	88.74463	18.07178	78.75945	84.18595	87.40102	143.6842	56.93193
208.2166	88.14617	91.97748	478.0648	74.35732	104.1953	36.18173	118.1563	86.63361
0.7127	9.94322	21.38075	0	29.90649	13.69059	8.056877	51.0262	19.2053
18.50775	12.14951	22.46267	8.551318	2.262075	9.490548	31.87809	15.42898	27.89295
51.35285	161.9239	226.6778	114.2501	385.9409	247.2909	225.8982	312.7505	197.0483
15.33392	52.85324	24.61247	26.64919	37.72491	58.91727	65.26896	21.78863	60.21652
15.44789	167.3634	150.7386	43.78451	325.9034	104.1796	232.829	139.1123	173.7318
18.83753	26.45952	24.61191	19.02764	26.64871	18.95983	25.85371	21.78924	32.78241
72.85147	415.1654	261.958	100.9173	221.8459	457.7196	375.6247	334.5695	441.5239
0.787612	79.22181	47.03809	2.837339	59.85658	71.52569	41.48114	45.4623	59.63614
1749.242	101.3648	112.2936	1007.561	87.66883	159.9652	91.74246	128.1518	46.32101
33.41264	114.5749	196.7434	71.39707	68.81335	149.4397	211.0825	287.2932	156.7289
585.9835	2462.594	1113.067	888.4084	875.1656	1950.958	2008.104	1089.996	2400.115
92.60216	49.58021	38.51149	5.693449	106.4312	52.62339	29.53658	27.2397	50.75829
37.00223	333.6951	234.1655	74.25322	207.4332	586.0818	838.2117	316.3789	529.0031
29.57548	70.47942	78.0412	10.454	116.39	67.34532	33.25113	56.35666	86.25784
113.2873	41.87567	56.67442	69.51492	86.48633	107.307	17.67001	34.51851	25.89282
241.5851	2448.262	2606.698	691.2966	813.0511	875.5288	2188.154	2240.985	1858.322
188.5852	15.46269	39.58737	147.6089	51.05802	61.04828	31.74439	46.3321	27.73273
11.82759	422.7631	96.24657	58.06738	140.87	152.5835	211.1808	138.1779	279.1842
1427.475	213.7	173.24	950.3552	374.9164	195.7477	178.4088	162.6932	178.3611
29.77372	37.48623	24.62143	137.1287	169.6562	57.89287	11.00029	59.97352	8.51849
129.8572	198.2483	207.4279	239.013	123.1476	115.7686	162.9148	110.8827	167.9195
22.44098	42.96714	29.95794	23.78898	83.14018	67.33715	106.8734	40.89472	85.71663
90.84622	190.5772	693.9167	209.4693	532.4142	376.7388	611.0701	778.2076	361.8642
1651.246	1971.438	1623.08	1249.301	1383.157	1513.22	2349.538	1222.719	1690.823
13.2092	0.021763	0.014983	4.775974	0.023393	0.011371	0	0.88313	0.499972
39.9025	89.15305	27.8205	57.13654	29.97805	84.15891	46.64426	30.88498	62.65902
0.825302	49.58534	91.9419	2.838037	67.66831	51.57442	26.56419	152.8046	45.76491
1172.769	585.9455	817.9516	970.3186	1187.884	846.0509	633.2545	540.8942	585.0293
51.06033	167.352	56.68833	60.92956	86.52588	77.87859	89.60504	56.3381	89.84544
22.58661	143.1186	36.37778	57.12288	67.67401	95.75378	120.0828	39.97029	107.3123
15.42539	113.3906	78.04705	71.41713	76.52543	54.72784	80.7657	55.44182	113.6148
0.829548	254.2341	163.5362	4.741776	37.74962	138.8766	79.98637	102.744	146.5064
29.80594	96.94079	120.8277	108.5459	192.9615	151.5278	147.4124	163.6513	171.1398
501.3458	121.1888	228.8267	346.6105	109.8512	162.0717	117.6704	82.68989	161.0278
0.721685	11.04383	29.91737	1.885341	17.75922	21.0466	22.22874	31.8524	16.67921
0.787086	130.8669	68.39279	0.934569	8.918849	69.42286	48.92502	85.53758	58.39005
15.12084	40.72023	12.85416	6.645909	5.591049	97.75791	32.61291	9.965506	27.25408
46.46245	38.55339	29.95145	33.32095	8.918976	30.52645	42.96572	25.43138	31.54635
4.581436	24.23284	18.18957	4.741374	17.76816	6.334871	22.19944	17.25396	29.80225
15.42094	118.8852	76.97651	33.3122	27.76885	120.9815	76.31682	91.84444	155.9934
28.54165	7.749107	26.72877	15.22292	17.76929	50.47086	7.303756	22.71992	7.285983
446.1292	2284.202	2345.844	618.9245	1545.084	1397.466	1533.223	1378.185	2204.142
18.9327	45.16969	69.48654	11.4064	76.50208	45.25612	80.08215	24.51398	96.91467
1507.816	801.7691	327.2019	491.3385	367.1654	822.8879	191.7353	443.6237	231.6653
151.7451	623.3326	613.7055	163.7638	434.7923	535.6114	629.6962	367.2687	559.7917
70.05079	2.243392	3.235787	57.20711	7.802801	6.333688	2.116594	1.781917	4.183016
0.722477	16.53608	23.51812	8.553112	12.23049	15.79374	38.67892	2.690159	27.97749
54.73523	170.6804	135.7851	131.4088	190.7293	126.2753	134.826	143.6498	142.604

28.72958	14.34934	20.32868	64.80008	8.915873	20.00437	12.50273	19.98175	14.75907
15.41415	39.67506	34.23597	31.40766	46.606	85.2236	103.8242	32.69891	80.02651
8.214693	88.12979	74.86384	12.35858	237.2818	99.97629	407.1656	140.0098	373.1752
11.76386	1162.845	161.4765	82.82267	432.5625	685.0127	1589.592	221.7963	1453.872
44.08268	179.4925	168.9206	134.264	140.8583	151.5253	94.0181	169.1137	126.4334
276.625	72.71357	75.9293	74.26244	109.8084	43.16399	56.21053	68.1592	53.17774
0.860912	583.6399	402.0061	56.1603	342.7205	653.4266	228.8343	310.007	164.7592
60.80588	38.56799	42.7791	21.88361	47.70411	29.48006	55.55596	31.79362	71.36905
18.5661	8.850775	20.32958	12.36209	31.05304	12.64576	10.27122	19.9806	6.039355
36.13035	47.34794	12.85798	75.2567	17.78491	20.01075	27.34675	19.97064	14.73038
169.2864	178.442	142.2263	53.30375	273.9593	274.6483	520.9367	125.4261	371.4064
243.8624	175.1318	75.94174	171.392	120.9323	237.8177	283.0028	179.99	255.4087
248.4792	718.0516	614.7865	410.4011	611.1327	426.1879	362.8944	557.2886	436.9392
349.2331	3488.013	5146.113	487.5162	2835.052	4432.289	4026.476	8690.228	2651.518
8.142718	22.03985	24.59617	19.98835	16.66452	30.51171	87.9102	10.87694	35.42034
11.70393	56.11115	39.54977	17.12565	41.01775	42.08045	17.7014	64.61919	31.60385
8.23929	64.9829	60.95004	19.97727	57.68794	43.15705	68.14689	86.38455	65.07186
21.59463	0.020319	0.014002	3.820655	1.134196	0.010632	0	0.885572	0
0.712987	24.21437	24.58037	0	24.38425	37.84628	14.02273	116.7655	8.547801
23.05399	1.135656	0.01786	1.88942	8.82908	3.168539	2.143408	0.877495	0
28.1624	13.24124	5.373681	18.08704	25.4962	9.488197	17.74619	14.52641	17.30497
118.6815	109.0493	79.1414	169.5042	156.3808	75.78118	198.6013	81.79479	187.2964
18.92776	35.26814	23.54728	44.74798	49.92021	46.30732	39.20553	26.3339	55.78643
291.5359	500.0275	698.1865	537.0502	715.393	539.8335	471.0526	568.1888	484.6561
90.3934	60.61195	112.2792	279.0363	115.3702	79.99069	153.3337	61.78606	79.22156
0.676506	17.61188	12.83837	9.510706	17.73542	11.58385	20.80548	3.600553	24.3379
40.50807	947.1739	2633.378	28.54807	290.6474	908.1402	1263.692	1313.677	1888.258
321.539	136.5885	184.9755	154.2534	127.5789	166.2684	219.279	91.7892	155.5261
793.6374	24.27345	17.13729	181.8934	182.9682	30.53876	7.30072	32.68987	21.52928
51.83612	8.849111	18.19034	42.87432	16.66291	20.0018	18.46796	7.236687	10.40116
97.95373	392.0665	194.6156	231.3805	431.4362	438.7946	301.4552	201.7982	412.8879
29.81864	238.9952	403.0554	72.34923	228.4921	274.6449	291.14	305.4786	170.9951
61.95186	140.9786	156.1034	66.63866	233.9855	252.5235	125.1479	194.5692	118.9496
8.234858	142.0175	63.09539	42.83494	85.40378	195.668	279.0504	105.4748	171.4075
28.86977	16.55076	32.07548	24.75077	24.41838	24.21116	24.40349	17.24676	46.62884
284.9714	15.46075	13.9287	61.89683	43.27661	27.37726	22.86611	25.42318	19.06443
11.84173	189.3813	130.4374	31.40344	56.60333	184.1277	110.3567	116.366	204.7833
68.44765	14.32541	7.506435	9.509986	10.00856	18.93503	0.643609	3.600278	2.324102
10.97722	0.030142	0.020638	133.7433	2.260732	4.227879	5.098117	0	0
26.08649	77.08746	29.96261	42.83758	83.17193	50.52052	102.3116	68.18114	110.5045
8.217288	45.14718	69.4596	6.645364	29.96693	46.29573	36.27537	91.00466	45.28063
50.54116	27.5685	31.02883	124.7834	16.6822	23.17014	9.518063	24.51273	11.61416
122.797	81.54002	82.35637	127.5838	199.6592	112.6133	126.5846	112.6993	107.7211
86.24468	22.0699	19.2754	144.7599	39.96857	61.0454	48.06551	31.78313	43.25961
112.3676	513.2339	606.2355	203.7574	410.4078	232.5771	487.3729	409.0835	416.4685
15.38415	41.86836	48.12077	12.35879	97.53829	91.52237	37.72069	122.8233	47.69092
101.7462	11.05591	18.2025	197.2437	34.4055	32.63276	19.90573	23.60733	13.4808
11.84366	57.28385	104.7561	29.50171	85.38784	63.14187	56.25732	86.38097	89.33461
18.91976	31.96675	28.88963	123.8406	29.97917	44.20338	48.86865	12.68939	53.30103
36.94676	122.2557	97.31097	11.40652	179.653	83.14478	419.0092	132.7303	143.8278
18.99542	93.59219	59.88432	48.55268	67.664	87.33258	94.87384	46.34243	111.1126
8.151837	124.4928	319.6977	23.78557	235.1663	384.0844	895.8913	312.7273	530.7474
18.9948	125.4936	85.52715	41.88456	61.01587	87.33242	78.53294	200.1443	92.43809
36.04004	29.75335	35.28889	47.61888	36.60751	24.21607	27.35337	14.51166	28.44123
796.0974	228.0206	306.8873	2966.345	221.8761	363.0585	250.2617	236.3325	210.5886
76.48917	981.2452	298.332	35.21234	327.2342	224.1597	422.1586	859.1452	1155.707
64.38585	36.36993	17.13537	135.2697	29.98125	25.27351	31.03178	18.14585	30.87647
72.63684	105.7517	223.4397	67.59095	149.7384	142.0631	129.5964	296.4407	158.6886

15.44646	197.0949	106.9282	42.83121	144.1779	256.7144	120.7303	133.6439	179.2646
25.95282	33.06655	51.32438	23.789	35.51813	45.25469	47.38388	20.87577	44.58249
2900.966	47.402	60.97535	378.9777	72.14351	139.9777	25.07781	64.5068	12.24976
211.9702	99.15973	193.5377	155.2004	135.3504	97.88346	119.168	87.23862	126.3247
43.77327	58.38779	43.85686	27.59619	47.72094	89.43798	60.70286	19.05323	105.4913
49.76058	18.75638	19.26815	16.17063	42.14307	13.69994	32.55914	10.87236	30.31035
83.10175	66.11192	99.44587	91.405	199.593	115.7549	184.5435	78.16098	110.3036
159.4312	7.752375	13.92734	255.4022	21.1107	35.78599	19.90877	9.962017	12.24034
8.230473	62.75222	38.50077	17.12184	59.87307	56.8164	71.21035	58.19849	43.98835
108.7914	777.5614	724.9322	306.597	785.2912	849.2047	702.9063	1765.576	677.4258
8.20309	71.50821	39.55624	7.597831	53.20042	43.1369	73.54849	37.27528	72.8339
58.11138	73.80813	69.51259	13.31055	98.71452	54.73477	82.93178	76.35107	102.9078
54.90156	347.9657	300.4202	154.2525	187.4531	167.3217	152.5436	182.7252	196.4833
15.4388	260.9643	266.1929	30.45048	103.1772	219.9131	262.3596	139.9963	303.4041
0.646783	11.0231	10.69973	1.885643	7.790498	2.125084	17.09798	6.33657	22.53015
255.7402	604.6451	453.3589	410.3971	595.6226	270.4599	595.5264	478.1761	706.0334
32.2902	6.650133	26.73814	16.17296	21.09865	17.90384	14.72778	23.62074	15.37259
90.8264	855.7657	1327.945	126.6246	988.2671	953.3863	1040.702	1333.682	895.6204
230.2395	31.98273	13.92744	710.4031	24.4474	223.0999	13.96961	79.0532	6.674343
15.44699	242.1729	141.113	16.16717	346.9222	296.6566	113.3568	44.51455	167.5672
314.4565	56.19844	85.54408	15.21495	104.2576	53.68313	28.03973	72.71141	46.35755
187.4741	194.9732	354.983	213.2835	502.4258	305.1751	428.8918	207.2481	243.4957
276.8422	34.17578	40.64983	205.7582	47.71887	31.58732	28.05036	38.15481	15.95399
90.7553	180.6471	302.5829	226.6225	306.1212	482.9743	222.916	240.9042	309.3262
553.6536	1013.27	2074.249	531.3211	1545.06	1433.234	1490.311	2027.336	1323.308
43.85813	85.90402	40.65293	23.78592	78.75552	90.49475	94.09392	26.32753	116.6444
4.490394	8.836993	4.303217	15.23568	16.63029	11.58339	17.80918	3.600677	6.692894
108.7168	473.5563	442.6406	209.4757	267.3275	284.1285	489.6879	483.6711	574.1552
94.42634	317.2131	235.2493	12.36042	944.9112	570.3373	621.5252	478.1922	413.3958
281.0131	843.6534	1242.415	513.2319	1136.886	753.4574	594.7132	890.0053	911.1128
22.45849	42.96913	23.54736	30.45701	37.73555	30.53211	74.88023	15.41756	63.26092
49.46089	27.54892	17.12958	17.12468	15.56556	20.00823	23.64611	10.87343	37.20683
521.1882	14.36112	28.89545	100.9449	70.99587	32.6405	14.70264	43.60981	17.19278
588.5884	57.31414	53.49027	975.0995	15.57041	64.21298	59.88552	64.50716	51.90316
75.53778	8.853811	4.304606	43.78749	57.69959	5.28285	3.600526	6.326013	22.77595
61.68193	6.648731	25.66088	23.80329	10.02156	9.489871	2.855683	9.057095	3.559997
202.0808	1231.281	1129.072	439.9113	962.7442	1133.314	740.6826	1529.177	725.7763
15.44447	89.21128	84.4714	48.54879	118.6547	46.31782	125.2567	128.2134	125.3185
4.557705	13.24025	16.04901	12.36647	24.38873	11.58992	28.96209	9.0603	13.54918
36.96843	172.8997	158.2398	96.16308	261.6933	107.3446	79.91806	108.1667	164.2875
4.560004	116.7576	272.5981	11.40663	293.8333	213.5969	165.9488	502.941	212.1246
66.2429	29.74528	17.12894	75.2738	22.20819	23.16166	8.782516	5.41644	16.6137
76.30809	126.6783	80.21582	110.4448	154.1871	133.6524	98.43402	91.7899	99.67613
18.79855	22.05738	21.40549	22.83955	21.108	10.54447	34.04095	25.43195	45.27523
90.41667	9.953558	7.51349	113.3958	16.67519	13.69968	7.29765	18.1523	4.797853
1397.173	9.955536	4.304863	180.9838	6.700706	10.5454	2.86078	20.87229	7.895802
70.27139	1.139386	1.095191	10.47479	4.471221	6.326021	0.643867	0.875004	0
26.22661	88.13608	148.6203	44.73492	104.2838	84.19904	127.376	66.33292	91.01584
22.54575	658.6131	686.4338	33.30828	218.5499	418.8292	271.0015	1045.513	489.5908
531.9931	5.54672	7.512495	191.4088	7.808446	10.54478	2.126454	20.87006	6.046568
30.97034	5.545262	6.44047	67.72032	2.262024	17.88949	0.643776	0.874766	4.808069
68.9659	107.9445	65.24413	52.35464	164.1326	71.57176	143.7212	81.79713	146.3203
9.714967	0.026255	0.018023	2.844247	0.028242	0.013656	0.648498	0	0.483905
50.67974	58.38131	55.60747	17.1201	61.01115	50.5194	91.9228	44.52546	73.16583
131.6337	89.20548	36.37784	83.79539	52.15869	108.3754	56.23495	23.59929	63.15599
245.0234	881.0561	748.4408	192.329	256.2584	408.3059	825.9471	291.7909	731.4364
198.1005	99.16424	103.7422	137.1022	424.7887	137.8709	188.0625	227.2556	183.9729
80.05056	323.8002	223.481	317.0888	315.0061	189.429	302.1934	284.5413	433.9755

5616.296	2576.114	4351.713	2984.248	3487.247	4267.09	2927.926	3840.04	3425.67
29.79979	78.22395	221.2888	2.838665	135.3158	162.0449	194.1639	352.8668	92.27556
3555.686	54.01028	150.788	415.1621	468.0753	103.1483	42.85077	211.7878	71.73357
18.68328	14.35339	45.95852	19.03074	22.20888	27.36718	34.81575	21.79658	34.08843
661.2952	1433.977	1866.839	1910.164	1348.759	907.0992	1613.258	1687.302	1941.974
183.2566	82.63991	134.7378	285.6804	189.6747	103.1427	95.46099	110.8827	77.95166
63.45408	20.9567	17.13171	126.7353	56.53321	13.70005	16.20167	28.16389	15.97676
90.51424	137.6839	146.4895	85.68448	161.9424	149.4315	220.7896	64.51174	232.5427
48.00384	2.243429	4.30515	91.5413	5.58952	13.69311	2.855774	0	2.322986
347.9954	2.241785	17.13592	19.9776	13.35621	21.06656	6.556675	17.23557	6.656816
44.04793	210.2961	149.6763	63.78428	210.673	268.2812	192.7127	134.5565	195.442
62.05096	285.2157	126.1843	115.2055	199.6488	234.6555	524.798	98.15369	237.4535
180.3079	4.447356	6.44339	17.12869	42.10056	10.5418	2.855717	7.236395	4.799425
73.09198	2.242523	4.302301	6.65116	13.31141	7.380474	2.860013	9.072109	3.571255
26.1143	158.5028	42.78835	21.88158	106.4451	51.57412	85.94882	83.64496	106.1106
46.98307	41.87042	37.43866	137.1764	65.42919	25.27343	35.48799	29.06265	17.20143
222.9264	231.3	347.4841	374.234	186.3667	159.9654	183.6299	151.792	183.9928
17.79021	13.22837	13.90615	7.602752	10.00847	7.382842	9.563253	8.154814	11.08042
22.62204	236.7938	289.7466	40.92474	279.497	246.2379	233.3151	278.1964	209.4634
8.200024	31.94652	33.14887	16.1717	36.59998	24.21405	36.29914	19.06433	49.07846
43.84131	126.6121	93.01449	42.83519	65.45621	103.1151	130.4879	97.28734	107.944
227.1742	1158.593	1408.112	33.30858	448.1355	1080.699	1285.252	2911.175	353.1637
150.5172	2.243412	0.021666	9.506621	23.28297	0.016386	2.856006	2.690192	1.090909
11.84798	16.56377	64.16496	9.501882	92.05465	224.067	196.5725	19.05258	229.2698
151.6601	406.3897	272.6609	181.8591	309.4673	299.9116	587.5202	345.4584	422.158
58.32097	100.2404	72.72692	94.26064	148.617	128.3815	211.9719	41.78223	228.3275
15.44789	57.30204	146.4638	39.02256	76.55494	67.36076	186.0664	74.52683	128.3554
62.03574	91.44628	155.0446	71.39845	127.5781	92.61964	224.474	119.9796	167.3237
76.48898	546.2754	216.0077	107.5802	357.1785	377.7879	356.215	101.78	738.3092
57.74817	13.25957	31.0314	42.83751	20.00962	27.3793	23.60049	46.34334	30.23628
31.26638	20.92272	19.24779	3.789426	12.2279	10.53833	22.2418	9.061166	17.32007
65.47	84.83137	80.21067	45.68753	135.3206	141.0079	85.8545	119.0832	120.8293
11.72216	18.75242	22.46929	9.502969	27.74347	21.05921	45.24657	25.43905	20.35361
157.392	19.86312	10.72149	74.28612	14.46416	13.70165	12.48273	7.234196	9.754515
0.805204	134.2328	97.25135	0.934748	14.4638	119.9067	293.6906	49.08701	104.4361
72.3411	127.742	80.20301	99.9812	61.03503	77.88005	117.0513	61.79377	123.3917
126.448	205.9677	56.6981	145.6763	59.94162	91.57059	43.58869	88.14728	23.39121
8.229668	601.923	57.75581	16.16718	35.53487	106.2778	132.6667	140.0362	331.2175
11.78338	59.443	76.93903	39.99109	37.7208	58.91371	42.21789	38.17547	50.25594
11.67959	25.34316	21.39814	10.45624	18.88299	12.64604	13.9868	19.06887	19.74573
4.593973	36.32729	11.78611	3.789038	26.62736	12.64567	49.76822	19.07011	38.51783
101.1156	123.3678	148.6237	65.68586	105.3957	123.1274	131.8151	344.6424	117.0782
2552.167	4065.107	3143.498	2698.587	3015.838	3849.317	3225.007	3500.05	3319.131
694.7294	9.95547	35.30836	31.40551	116.4237	5.282899	2.861504	9.052354	0.48533
434.3968	781.9108	420.207	942.7394	455.8704	420.9248	67.29095	218.1536	246.5697
321.7768	46.29246	18.20646	534.384	43.29705	42.11128	19.14651	33.60055	33.31798
431.8216	123.3794	147.5651	389.4946	106.5143	87.35991	75.45174	118.1579	66.78809
22.38475	35.26217	34.22856	37.12903	21.11211	14.75308	63.02103	41.81026	26.54137
233.3992	30.88189	153.985	468.5277	56.61433	97.88378	97.67504	302.7466	19.67415
15.44764	168.4702	169.9786	11.40637	159.6863	111.545	345.6315	99.99313	156.9256
81.08794	25.36145	25.68152	122.9021	17.78741	24.21918	30.30691	25.42789	27.16664
4.593159	64.9874	51.33603	19.97699	82.06698	61.0392	181.0683	77.27878	75.63254
10.72409	1.139346	1.095165	8.565076	0.031622	0.015235	0	0	0.478804
26.8576	0.027332	0.018748	3.797306	0.029405	0.014201	1.386717	0	0
11.37164	7.743771	12.84399	9.50815	3.371457	2.12554	16.27615	9.063183	9.181434
53.67857	35.26331	20.33997	113.3662	47.69692	57.86987	31.04532	30.88687	21.56081
4.589406	48.39989	20.32737	4.741242	14.45184	34.71597	52.77928	23.62763	39.17064
119.0577	156.4043	161.4578	119.0161	165.2743	153.6424	225.218	112.705	220.7141

44.12035	1106.84	851.0942	15.21758	709.879	792.3852	802.9154	1356.43	499.4731
47.48755	0.033636	2.164414	45.69065	7.80969	1.071082	0	0	211.1267
5.043862	0.015505	1.077065	0.945543	1.120581	0.008152	0	0	1.20927
0.731082	57.14778	35.25628	8.552535	11.12622	26.30226	30.43077	18.16904	72.5973
693.5979	1072.749	2013.32	1325.485	1639.35	1335.378	1702.883	1466.377	1468.327
4.585288	132.8923	35.26642	0.934285	4.481747	9.49012	34.87683	37.30215	19.77068
7821.345	44599.58	36324.46	17654.16	28470.15	28755.22	25007.54	44347	33572.58
237.6443	50.69588	72.72096	223.8185	59.92573	38.95507	37.67583	49.061	52.5632
101.4891	202.6697	297.2385	129.4856	224.0673	151.5473	248.8558	209.0778	231.767
119.3166	129.9931	155.0547	112.3451	205.2116	139.9719	125.8339	166.3441	133.7621
216.2736	576.0018	207.4536	239.9433	423.7157	663.9905	402.1619	317.2536	747.6446
284.6453	606.8861	5288.162	66.63609	668.8653	3271.547	1129.535	4396.601	307.2668
51.36318	248.921	128.3311	24.7376	443.6281	683.9412	784.7579	132.6965	429.6579
52.30954	6.649737	2.166068	24.75243	11.13169	17.90248	5.076206	6.326134	14.75655
50.36767	28.66591	21.40965	46.65435	37.7339	26.32449	44.41047	12.68942	57.66415
21.84735	83.51645	27.79712	8.551601	28.83137	42.06829	47.56867	46.41818	31.03983
43.94706	89.21546	49.20579	77.12323	131.9591	65.25423	84.41747	83.62908	111.6147
44.12987	231.2476	272.6083	65.68586	125.3517	106.2942	174.0864	179.1031	195.9363
134.8359	16.56344	93.011	135.2445	29.98827	28.4321	39.91679	50.88874	40.79144
4.532157	480.1208	227.7488	112.345	566.6738	451.4055	243.6805	658.3137	344.1002
26.22383	72.72134	119.759	20.92819	147.5142	63.15624	156.3131	77.24712	157.4735
50.44214	3.34504	14.9786	6.647981	3.371599	24.19227	0.643861	3.599496	0.479214
769.0714	1918.584	2247.494	1527.351	1919.985	1992.014	2956.193	2579.133	1876.76
19.04376	249.9273	133.6493	85.6892	73.2359	183.0824	153.3666	121.8171	267.4965
152.9032	24.272	51.34217	45.69131	14.46611	44.21386	22.11176	46.33552	33.32458
4.594597	35.22972	36.34191	7.598383	43.21971	38.92303	42.30414	33.64564	30.37761
47.76548	410.8099	609.4289	44.73385	339.4211	484.0534	739.3792	575.4881	360.0741
474.8666	3438.439	6019.629	826.5076	1366.534	1374.325	1823.537	5527.409	3304.285
255.3459	231.3109	312.218	251.3761	261.785	304.1215	542.2969	171.7897	541.2533
22.52562	91.3829	55.60705	16.16776	26.65997	62.08816	91.18283	28.14982	48.27501
345.1448	8.850175	5.374713	41.91572	1.150409	16.85038	4.335646	3.598697	2.941254
47.62465	192.6933	143.2668	92.35686	146.3953	179.9251	163.7597	220.9702	122.1001
65.58216	168.5106	94.11195	187.5903	143.0967	243.0655	248.2226	62.69282	232.5326
40.27058	38.58014	49.2008	74.27198	31.09674	43.15951	67.38058	31.78561	60.06378
4.520084	9.938714	8.574599	7.602075	4.479181	2.125496	20.77992	13.62423	26.18464
11.6869	19.84854	13.92316	13.31453	23.31102	23.15967	42.29149	18.15766	51.6439
0.586733	3.338287	3.231298	0.934416	4.468347	4.225001	9.637013	2.694475	9.299999
0.770871	11.05279	12.85601	5.693283	13.34945	11.59527	43.76011	15.42432	22.22741
7.974923	1.139988	4.30412	2.837336	1.150783	4.22986	0.643722	0.874753	4.186245
40.17562	77.08532	14.99828	57.12776	64.33624	99.94957	209.3511	14.50707	105.5352
4.596001	52.88023	82.31747	59.03367	55.47164	37.89778	47.35286	45.4353	60.09485
8.23384	30.86401	48.11682	11.40663	68.73746	38.94335	63.00999	86.41736	29.02618
35.87911	28.64883	13.92496	26.6541	23.31736	44.18702	55.66866	5.416379	50.32884
29.7621	95.8245	96.2334	98.07661	104.262	86.29528	120.023	150.0333	104.129
28.79406	16.5493	23.53288	11.40916	21.09578	9.491036	32.6079	13.60612	56.0499
11.33446	9.93942	20.3072	2.837336	6.693547	32.58653	14.78713	27.31151	14.2083
75.56155	66.09698	93.01633	276.2412	54.37544	57.88783	32.49357	34.51277	65.64209
64.33388	48.46982	63.07745	83.81384	23.33175	55.77234	39.20361	22.69444	27.76912
8.195358	85.77936	41.6885	4.741084	7.809233	23.16205	81.77535	60.96583	92.9037
197.5714	0.033502	1.094287	573.535	7.810187	13.70234	4.339111	16.32516	2.949482
15.16104	34.13518	35.27744	18.07925	14.45523	23.15976	48.2563	64.62572	32.86589
58.39189	198.2203	189.2377	100.9245	114.2631	187.3001	226.0168	300.9886	164.8986
8.239457	39.67512	88.72495	4.741449	745.3146	37.8972	39.18912	26.33089	73.79914
4.57267	107.9357	178.5256	2.838486	222.8521	142.0497	140.0439	163.6723	101.0048
96.14046	3.344275	17.13638	79.03934	12.2475	43.15764	17.66861	21.7821	17.19553
385.0593	30.87961	41.72521	59.97619	149.7023	59.99624	33.22699	55.42707	40.14403
4.602061	74.79782	36.35132	20.93636	105.1821	129.3082	72.81909	57.31934	72.8575
13.08895	1.137893	0.019033	2.841472	1.148491	1.07122	0	2.697726	0.480744

657.5968	138.8152	228.8424	5014.524	139.8024	413.576	74.70398	65.4184	96.53188
65.90048	2.243234	0.021372	77.27257	1.150783	3.177943	2.116817	0.874752	1.705651
346.4965	91.44754	64.18067	184.7289	183.018	87.35947	95.46465	154.534	100.9102
347.4965	292.9538	100.5327	84.72835	132.0267	336.7257	208.8389	185.4381	475.0312
44.90581	6.648079	21.38902	34.29611	13.34018	29.45514	13.26022	26.37141	17.28629
78.75376	5.548667	5.374725	92.45925	21.09288	11.59377	8.786934	9.055839	7.283091
33.21192	41.88226	75.91562	29.50082	64.34526	53.67856	71.09102	46.33976	81.20858
306.0719	571.6298	601.983	331.3547	606.7377	832.3724	1707.533	368.154	1253.416
51.089	125.5397	73.79002	41.88003	75.44464	136.7869	147.483	94.54035	156.9655
319.6249	141.0124	218.1385	913.2437	130.9243	197.8485	122.8535	162.6977	132.4944
18.63173	15.4517	14.9918	20.93788	21.09893	31.56967	46.01018	12.69459	41.61373
29.82108	276.4196	204.2231	177.1069	312.7468	214.67	194.0445	322.7675	297.0058
8.109001	1916.243	2492.208	2.83999	305.0629	1301.67	2082.485	1139.139	1232.995
9157.903	3153.229	5186.774	3776.495	7046.536	5247.838	4808.595	3885.485	5134.543
22.5727	71.59477	68.43609	12.35836	82.07462	93.64714	94.10897	120.0401	43.27567
643.5367	2326.092	2679.461	577.9772	2620.977	3857.73	5419.878	1952.731	4620.912
485.7162	2.242464	24.61287	16.16938	76.47671	6.335908	4.336002	36.3504	1.711319
165.6039	155.3149	179.6393	279.0083	298.3418	112.6137	290.4063	130.8845	174.7235
18.9527	116.6656	125.036	14.2633	83.15491	57.87796	41.42459	90.94738	79.43242
414.0948	55.11044	105.8742	454.2591	218.5005	89.46429	13.96555	42.68871	38.88396
228.743	110.1568	41.72835	183.7851	90.98228	74.73099	30.25678	56.32915	41.36853
2.927466	0.015589	0.010775	0.945437	0.01674	1.058552	0	0.895308	0.537336
39.52461	11.05468	22.47332	163.9139	7.809984	11.59634	15.45874	12.69159	12.24454
111.5378	12.15609	6.444522	40.94318	20.00198	22.11483	11.74491	18.14952	12.2414
22.41868	84.75205	40.6402	19.02641	49.91466	69.43767	31.78461	33.61579	53.31603
103.2099	46.28149	67.36372	99.9978	47.71913	40.00268	45.11798	77.27893	53.85817
29.82178	257.6973	269.4247	2.839111	45.52264	242.0218	281.5458	170.9011	277.169
15.44689	249.8739	83.40628	33.30892	154.1247	75.77415	157.1541	99.09292	120.9329
299.2468	3.343747	0.022929	298.1529	29.98987	22.12014	0	0	4.185779
202.0999	2176.187	659.7223	472.2853	1054.814	1361.664	1478.542	1051.836	1319.694
123.1211	654.1836	319.7126	262.7981	435.9132	384.0989	705.9746	281.7956	480.3503
13760.04	9428.776	19648.96	10104	16355.79	13140.1	17508	15660.22	14276.92
22.0809	52.81586	19.26418	14.26693	41.01789	36.82549	67.63267	25.44134	48.49103
51.24954	170.698	101.5901	44.73494	201.8361	108.3966	157.0433	39.05344	202.176
120.4731	23.15279	2.165851	63.82776	2.261682	18.95729	8.039555	12.69282	5.417287
15.09631	29.73281	18.19278	34.28759	32.15475	16.85043	25.16443	20.89349	27.26341
20.566	9.928368	13.89733	9.513714	6.686866	11.57958	11.83361	11.81203	9.855333
210.6297	9.954721	8.582967	56.1888	13.35402	39.99248	20.65045	27.2486	15.96826
277.5457	93.63605	100.5178	111.4045	47.73643	92.61338	75.47784	58.15055	87.92764
95.27873	3.345095	2.166171	20.952	22.17124	7.38468	4.339386	3.599438	2.32313
11.84652	58.3887	72.709	44.74129	76.5323	69.45531	123.0844	87.28389	68.15132
61.73573	63.90629	67.37796	90.45565	128.6468	51.58019	120.0173	59.97438	130.8467
11.63514	44.00776	36.33689	1.885444	31.04677	28.41104	47.55289	4.507671	50.45678
148.2112	701.5349	689.6237	263.7502	520.197	449.3371	305.1002	749.1371	361.9006
18.76838	11.0544	27.81312	20.93465	22.21387	28.42186	44.46888	15.42162	37.17625
8.215198	49.54238	35.28959	30.46341	23.32193	37.88462	37.02261	20.88184	60.27675
376.81	541.8325	223.4848	344.7014	206.3377	178.9089	317.7338	219.978	404.7683
57.96459	199.2268	382.5494	17.11958	37.74943	21.06792	16.18421	52.70537	83.6763
201.4321	47.40253	79.15048	683.7591	130.918	51.58547	37.66315	83.60054	42.60212
15.32685	49.55293	34.22603	39.98976	54.33136	44.19705	80.16256	51.82991	111.4313
14.57201	18.71356	11.7742	0.934118	8.904058	13.68588	21.53847	10.88952	26.83379
4.602723	90.25591	47.05144	3.789369	34.41033	103.086	88.27825	19.96603	118.1505
58.47965	232.3738	230.9419	117.11	224.0413	123.1328	146.6115	241.8369	188.4114
64.96549	6.650314	22.48204	131.4302	14.46597	27.38042	13.96176	22.69016	22.77662
338.0945	34.18041	20.34444	25.69035	49.94445	50.52559	19.14753	35.42124	24.01472
118.5577	101.3396	164.6431	76.16488	47.73578	152.5756	128.8856	408.3681	111.5349
10207.43	16058.94	14820.4	13372.99	11925.8	10631.42	9362.404	15783.86	12519.08
80.07929	659.686	671.4462	219.9463	500.2322	369.3669	505.1674	467.2769	614.3221

60.22588	30.85736	20.33786	37.13215	34.39913	17.90771	47.42586	39.08584	29.04921
8.212524	26.45435	27.81293	24.74625	45.46205	67.3168	98.85286	28.16523	67.16251
0.640517	21.969	7.50117	3.79125	3.368493	12.62696	34.44332	8.162648	25.09337
79.40903	87.0204	80.20298	67.59595	51.05798	68.41224	109.6306	91.81072	71.81506
463.9377	51.80695	57.76655	144.7272	21.12078	99.98642	91.75436	14.50789	38.26384
481.1224	360.1684	351.7886	1139.853	437.0274	251.5182	407.3408	401.8072	388.5522
0.746253	37.41403	33.13247	8.551629	35.46506	43.11855	26.66778	15.43059	55.50633
165.7586	200.4657	210.646	83.77601	348.2533	255.7112	325.211	186.347	250.3891
29.81173	127.7679	187.0986	67.59116	83.21934	189.4031	272.0169	191.8418	242.5466
29.81541	153.0907	180.6893	19.97603	129.786	210.4463	468.583	97.25078	312.0864
115.911	1918.573	2336.227	857.9338	2775.105	3041.131	1082.087	3791.031	3148.869
1109.532	113.4634	81.28366	165.6855	126.465	109.4522	39.88737	107.2513	44.46716
213.5638	37.47379	19.27374	67.60994	54.36051	52.62149	46.61644	19.96396	48.28238
53.32094	9.954205	5.375148	61.91213	91.95297	8.440373	2.857307	6.325266	12.24306
146.088	71.60646	69.5123	110.4631	88.73953	50.52666	51.77081	49.06278	76.8038
0.726743	12.14378	14.98379	6.646976	23.28897	14.74372	56.62474	19.99391	39.90583
108.7649	753.2722	177.5156	57.11252	614.4455	453.5412	777.1587	210.8813	301.7689
30.52585	0.017845	0.012317	3.830896	0.01917	0.00936	0	0	0
249.9743	79.33037	70.59231	130.4487	137.5462	170.4693	116.9854	66.33177	110.8714
79.60525	70.51682	41.72699	87.59413	117.5776	56.84258	17.66479	100.8973	24.00881
8.116251	37.40606	5.374294	3.789134	56.45248	87.22579	27.42779	23.63419	64.98392
4.294631	0.028868	0.019782	8.568239	2.258278	0.014976	0.644341	0.875262	0
22.12092	45.13034	16.06084	15.21935	16.67221	67.30559	37.79802	14.514	38.46724
0.796807	95.73034	75.87977	4.74115	38.83362	61.02112	48.1523	109.1952	72.68775
0.857215	292.9409	784.6545	8.551137	157.526	254.6531	451.2941	341.8577	251.0522
29.77776	98.03074	142.1919	50.45101	177.417	123.1158	91.81256	141.8381	118.3996
15.35156	80.34576	42.77424	2.837499	64.304	70.48551	50.37616	112.831	29.03138
51.22792	211.418	96.24388	37.11668	272.7664	224.114	145.9297	72.70014	230.1601
43.74443	81.49119	35.30676	49.50515	55.47627	86.2812	78.53081	28.14831	109.2416
51.2909	208.1384	118.697	87.58937	235.1092	189.4093	238.5925	194.5592	253.662
1292.951	155.3346	227.7692	936.056	533.5175	228.3694	255.4491	389.075	210.59
318.2272	131.0864	58.83542	188.5373	116.4937	229.3966	405.3819	69.05586	208.2679
0.775935	66.01258	20.33433	18.07731	31.06663	23.16282	42.25582	34.54388	72.84317
0.645903	5.539585	5.368862	4.744425	16.61447	15.77596	10.33769	6.336693	13.02184
211.8404	67.22451	43.86776	528.539	78.7937	33.69638	13.96625	1.78572	182.7981
101.3817	156.414	164.6717	142.8215	193.0055	137.865	180.6971	99.06034	187.1433
15.3791	23.16419	35.30006	29.50487	42.1647	50.51272	40.69523	50.90644	30.88501
53.47749	25.35992	4.305551	71.43894	23.326	25.27008	10.26128	32.7109	19.70213
103.7997	5.54933	12.85704	57.15324	15.56691	17.9061	3.595727	19.06307	13.49234
329.3409	300.6487	101.6002	100.9183	163.0706	419.8342	222.2015	89.05753	192.7166
31.07275	3.345	9.643448	10.46131	8.907727	10.53725	1.378707	4.509275	6.053721
18.97044	46.27658	87.65601	60.94041	20.00866	38.94885	50.32867	69.09711	48.90295
91.05147	5.548017	6.442979	24.75775	13.34163	17.8988	5.077781	33.66139	9.780566
130.248	517.6192	761.2327	266.6103	219.649	355.6833	637.8596	647.3238	526.3105
139.3219	0.031948	0.021849	24.75972	1.150619	1.072107	0	0.874952	0
4.604247	35.26318	50.25208	2.83752	56.55544	59.9726	43.68072	72.76229	58.31636
54.02062	46.27323	40.64532	40.9352	47.70983	45.25788	65.94205	26.33226	54.5197
699.644	118.9822	95.18706	376.1439	168.6202	93.67532	77.66696	115.4239	119.4933
44.17621	533.0287	402.0233	57.11246	1045.783	272.5572	597.1182	629.1454	225.5075
4.604589	53.9376	20.3363	13.31266	75.34216	64.16422	54.14717	40.91252	72.78202
11.44818	19.8258	22.44802	19.04254	15.5451	7.385123	9.546102	4.508805	13.55472
3237.584	60.61732	97.32858	1955.916	307.2822	448.297	97.66043	143.5987	43.23241
150.4378	1.139929	4.30486	7.60039	27.70158	3.178221	2.116587	9.060582	0.479504
4.598288	84.77719	76.97156	2.837797	46.60448	116.7688	155.8766	163.7645	64.46943
94.1932	183.934	217.047	253.3001	288.347	176.7916	149.5743	226.375	149.3068
46.22463	3.34403	2.165935	55.33262	12.21592	4.229297	1.378645	1.782041	2.945054
0.832243	272.9555	232.994	2.838233	64.35326	271.401	89.62432	173.7087	27.73811
376.5477	0.029702	0.020343	2.838672	9.989342	1.072006	1.379891	2.692772	0.478632

18.98736	52.88191	49.19763	57.1274	47.71733	35.79455	106.7735	59.08235	111.7559
518.5431	133.2987	179.6451	408.5206	200.7808	109.4591	53.95885	116.332	107.7047
35.87099	7.751523	20.33407	60.96976	53.19715	14.75068	13.97912	8.144305	17.23275
22.3679	53.95513	33.15942	35.22403	44.37083	57.86728	92.05246	19.9682	75.8055
42.89776	2.242859	5.370766	11.41939	11.10966	2.125346	4.345086	9.068881	2.324908
25.94891	50.66603	29.95775	39.03232	73.17335	56.82166	60.02205	41.80523	86.96815
33.35681	258.7186	222.3469	74.26115	247.2452	164.1434	137.8026	166.3948	162.5035
54.78464	112.3515	115.4829	86.64028	76.56432	98.92641	111.0711	90.88958	128.2866
72.27811	85.91655	65.2386	93.31523	44.40501	118.9028	75.50678	45.42372	76.79523
189.7992	35.28683	69.5234	61.87648	157.5016	53.68806	20.62757	90.88481	29.58688
51.11653	60.60469	70.58497	85.69254	97.61543	90.50448	102.9462	47.24096	108.4659
474.7393	762.1697	1031.803	2235.843	1089.216	810.2861	1093.247	1055.457	963.7525
24.17052	1.139977	3.235094	5.69617	1.150768	2.125416	1.378632	3.600434	0
592.9606	975.8141	700.3536	371.347	2047.447	1804.671	874.7309	539.9773	1244.017
13.503	0.022502	0.015484	7.655286	0.02419	3.15859	0	0	0
25.93876	67.16104	55.59601	43.79668	33.30156	53.66642	51.10422	60.01088	41.47303
1680.09	317.2275	225.6326	697.9776	544.6193	536.683	377.6784	291.7888	313.4946
72.42515	104.636	102.6502	89.50125	103.1609	114.7011	162.2973	85.43931	141.3857
8.232659	16.55979	17.13406	20.93198	55.44791	38.94272	31.04587	28.15685	35.87936
8.238493	82.56824	34.23355	18.07313	20.00729	33.68805	29.54664	56.36277	50.16977
117.9817	0.023133	0.015912	4.769673	1.14071	0.01207	0.657024	0	0
37.87592	9.944121	12.8473	31.44225	11.12294	17.89389	9.54474	12.70511	6.04928
26.06402	11.05691	54.53862	64.75027	29.98502	29.48261	78.55122	31.78859	67.56644
32.49409	2.242952	10.71969	53.34188	18.88899	7.387939	11.74992	27.25776	6.657304
40.34242	105.713	94.08617	58.07485	95.37965	90.49601	120.8165	51.7947	143.3829
129.6585	40.79348	150.7659	336.1714	49.95763	40.00979	33.96026	48.14468	59.35241
585.9865	1932.869	1478.728	936.9724	3003.543	2017.251	1783.64	1763.67	1623.942
8.116887	95.56316	19.25614	8.552052	18.87311	56.76874	63.29817	32.75006	48.01473
277.8602	34.18509	39.58965	516.2192	37.7573	53.68727	31.73939	82.70325	17.19111
149.4738	2.24315	7.512907	49.53592	4.48211	5.283354	2.856103	0.875294	0.481315
151.3449	11.05001	10.71736	14.26862	64.21648	11.5937	4.335654	9.966039	10.39586
0.720135	20.92649	32.04934	5.694448	21.07435	17.89456	16.2556	28.20492	26.72758
57.81238	137.5979	50.26813	86.65853	54.36904	85.23024	100.0674	24.50995	75.00381
33.41233	284.1197	281.1832	128.5378	99.86123	204.1466	134.7472	388.2553	149.3051
50.85376	96.9012	41.72043	43.78834	55.48027	95.75054	67.37826	32.69493	104.8506
230.4219	399.7938	294.0471	117.1034	283.9691	340.9514	534.8565	206.3377	451.2675
75.23472	34.17532	51.33518	174.3171	23.33549	34.74286	24.34221	15.41625	30.23634

TCGA-HT	TCGA-E1-	TCGA-DH	TCGA-TQ	TCGA-QH	TCGA-DB	TCGA-HT	TCGA-TQ	TCGA-E1-
257.5732	81.08425	125.9395	105.6815	186.6601	131.9238	192.6237	160.7299	131.6996
16.71244	24.04241	18.90436	31.51883	37.22976	32.68222	19.17442	28.46143	13.9314
77.15312	39.26948	110.1917	63.02674	130.5609	166.9123	157.7159	70.70021	86.41263
3449.364	1211.633	3868.194	1991.569	2822.968	3556.331	3308.14	2166.627	1514.756
198.058	229.7357	184.6881	106.6133	134.9423	108.9392	93.37093	219.5477	155.6112
0	5.378333	2.110517	1.835236	0.962641	1.272342	0	0	3.274426
24.15441	38.82666	12.61279	6.459481	19.84853	26.6705	11.29535	5.476369	90.73624
129.2249	72.83485	83.96995	115.8836	145.4788	146.4305	189.7683	99.17239	164.657
9708.405	3027.239	6048.936	5934.87	6960.476	12637.79	14087.79	5166.303	4049.592
31.58732	48.17406	108.0693	176.2613	48.78334	158.2892	39.86258	198.5201	54.30912
25.11084	17.00657	16.79852	27.83028	28.42537	30.19151	15.63812	32.17536	22.23076
1121.271	450.0701	1200.549	1250.772	1553.511	1258.448	1300.306	1137.968	1356.768
99.46192	222.5397	115.4496	88.98555	116.522	147.6543	114.7645	180.0177	162.1709
0	4.69216	1.065984	4.632344	0	17.77539	0.612792	0	6.620388
81.80477	64.03433	162.6436	76.01315	112.1907	154.822	79.82987	223.2544	147.4105
6.478541	58.41197	16.80838	9.241548	6.475326	26.66816	3.447678	9.150651	8.982168
1930.143	1285.818	2219.55	1438.967	2438.681	2460.04	2191.133	1559.523	1604.511
84.64468	55.94345	55.6162	51.92362	47.18606	71.32667	41.33263	93.7393	32.06427
136.6835	239.3272	19.96051	52.82454	76.54272	73.86355	99.83194	21.08941	65.81196
484.3819	938.8127	330.5879	296.6714	200.8615	692.1571	365.8131	178.1462	533.4467
1024.578	692.0152	856.3429	838.1613	1082.441	1110.825	1594.262	926.717	517.777
40.9	1.606529	1.067645	39.85866	89.70493	6.13863	194.7617	39.48194	10.62794
130.1661	2.200509	2.11712	36.13006	125.514	133.0977	197.7048	40.3794	45.21115
647.1631	55.14283	216.194	217.8715	286.5409	330.341	481.5688	340.7519	48.4959
80.87813	34.56166	94.45102	98.28005	98.85204	110.1003	164.9122	21.08967	72.41329
31.60709	63.2473	25.19545	40.80575	40.6008	33.88472	11.30053	75.39279	66.818
103.1775	185.336	205.6916	83.41832	97.03175	215.3967	113.3164	94.57156	191.8029
648.0156	1579.813	1546.84	1102.425	1136.907	1171.308	544.1335	2584.655	673.3933
5.54887	11.01609	9.465191	17.59207	35.52263	29.07569	68.65753	30.29751	23.83095
76.23243	75.28977	99.69101	107.5655	182.0068	56.92294	77.72183	191.1198	44.39999
91.13747	91.94682	61.91801	75.11055	140.9505	53.26879	37.00889	126.8117	71.63855
98.58365	41.68981	57.72203	84.39188	50.47504	54.47411	56.32437	137.8483	40.29663
17.66079	30.78819	29.36508	32.4889	30.74458	21.76324	9.899167	62.60839	27.22081
586.6521	638.4701	1005.352	1104.285	486.3737	750.2632	475.6564	1612.864	731.86
230.5762	221.2717	178.4117	297.6407	250.4997	235.9599	124.733	353.6399	335.9547
12840.71	3969.472	8915.984	21530.11	24659.05	8753.618	6900.73	16592.87	9460.954
27.89378	9.851299	41.95651	25.95885	5.368042	23.01321	9.878893	7.314967	49.51169
84.61423	63.51899	48.28555	76.03017	151.4477	113.6572	57.01455	102.8918	36.99011
37.17581	42.91005	60.86114	81.62386	15.92698	33.93144	23.43867	22.01387	87.36021
81.86278	20.45743	49.32025	52.85812	12.03506	31.5053	26.31155	24.77436	46.93033
158.9842	196.6275	82.92115	46.32882	74.82069	118.6276	164.7622	10.06957	84.74018
65.08792	67.13745	38.84026	83.47033	53.84551	54.46353	29.1516	137.8617	70.82885
23.21646	34.00588	31.49935	16.65891	4.814149	8.559631	19.14114	10.06814	65.86105
142.2558	46.92278	129.0805	127.95	202.3537	111.3476	121.2399	214.9618	111.9463
233.3557	358.4844	259.214	267.0232	440.0349	239.6198	200.3635	335.2458	339.2068
30.69701	37.85657	24.13629	22.24924	31.2072	14.57271	15.62787	48.74737	23.04814
37.16886	137.4709	57.72429	33.35469	30.39058	36.36567	43.43841	99.22212	57.60873
3871.468	498.3064	2598.401	3475.078	4108.208	1881.703	6732.024	3448.812	1041.362
1154.8	88.68881	82.92661	365.2905	1213.897	335.2471	2037.005	130.3859	171.9898
4.62062	15.83948	18.89071	57.60497	21.13324	12.15066	7.027503	38.63891	11.48005
12.06962	17.03223	19.93842	15.7527	14.92592	6.129762	8.462428	74.5943	13.13486
178.4947	377.3783	256.0632	255.8993	383.9153	350.8678	291.7424	276.4574	222.2599
109.734	90.10121	102.8311	86.23488	102.8645	130.5664	48.43106	154.3699	108.7299
7071.656	2453.437	9159.416	10203.68	10192.02	7254.26	3995.641	10566.92	2025.992
27.90106	11.04687	23.08863	10.17399	21.06869	19.38776	12.75487	31.24639	30.4975
132.0848	29.28511	64.01563	116.8729	114.1299	95.51555	21.99902	110.2615	55.96545
53.9877	7.487616	23.091	29.67805	31.73471	20.59942	24.23968	25.71688	13.11827

6051.713	3219.728	9570.793	6327.089	5926.433	8038.364	7671.211	8917.341	3118.483
173.8413	631.8366	396.6756	237.3455	186.4837	260.1897	122.5726	252.5641	440.5026
42.77801	38.86676	33.58425	54.73298	54.04444	20.62911	20.60492	80.90817	48.61971
17.63723	42.8967	39.88806	11.09528	15.36777	25.48261	57.06605	15.58047	79.09128
5.549091	15.16878	4.218291	6.459996	0.941033	0.067103	2.735232	0	17.23758
280.7955	135.8382	453.3138	224.3769	287.775	324.2262	249.6622	342.6174	251.9336
10.2029	24.725	24.13705	13.88796	18.25394	18.18595	9.884544	80.06743	31.32288
40.9161	43.02448	15.75846	36.1605	38.34388	26.65806	35.66209	35.81978	15.58081
71.581	28.08439	93.39727	64.88929	88.30549	89.54209	140.6495	46.81641	33.68733
94.80917	161.7746	241.3646	134.4228	64.79702	198.4612	99.75858	121.2128	209.1067
11.13756	2.185217	12.60481	20.39923	17.17522	3.714958	54.62317	20.20691	4.868777
23.23565	43.13885	75.48789	31.53127	13.18416	12.17199	9.161932	77.27291	38.74634
259.4017	83.40779	592.869	272.5972	236.5779	324.2439	223.2318	366.4965	166.2604
38.11149	46.50044	35.68811	44.50349	67.36747	23.05703	20.58669	75.35594	26.2957
91.10686	131.8979	184.6721	80.6516	180.7356	125.8213	224.9342	28.4381	43.56772
26.01384	43.54614	70.28916	46.36149	34.92321	30.29638	32.0425	90.99783	61.7988
249.1692	190.0161	200.4497	224.3703	312.7436	249.2798	276.0443	290.2393	109.43
35.32337	7.476157	12.61278	39.86723	35.50161	86.93183	41.37078	14.66433	9.80583
127.3505	329.0393	162.676	71.36212	102.0161	71.48238	126.86	56.90913	307.0899
212.9074	135.8359	154.2779	156.6773	179.3149	193.6347	366.751	250.7439	176.149
2037.09	2273.654	1065.184	1377.786	3236.604	1534.355	929.3055	2015.117	1454.707
52.05425	84.2353	53.52839	137.2843	105.1598	59.31209	24.85463	145.1937	122.7829
346.8097	74.5712	230.8805	562.864	570.7817	306.1258	125.4351	677.9266	184.3722
26.00727	22.79403	30.44993	44.48954	155.4928	135.3317	21.99936	167.2726	13.92069
40.89155	40.50604	32.54807	20.36894	48.79816	53.26836	36.29419	16.49834	28.75308
1025.496	463.5977	1002.217	992.0695	1478.438	2307.468	2619.316	842.2043	621.5049
21.35401	40.45288	82.91271	33.3492	29.80648	56.93644	25.55618	10.06856	88.89248
149.6884	161.2779	98.65834	228.1161	324.2148	89.60178	108.3593	190.137	167.9613
724.2999	678.7093	344.2235	296.679	219.7747	559.0216	293.075	148.7569	610.9014
12.98635	232.2625	113.3377	69.52033	8.145231	553.4149	39.11785	29.35665	96.30599
174.7842	223.0768	221.4276	70.4369	152.6465	154.9249	127.5994	126.7258	178.6314
71.60859	32.85182	17.85967	50.06435	72.31957	60.48283	32.02051	77.17848	26.28733
425.8071	241.7284	523.6729	377.3426	382.5343	455.0246	1127.85	734.7757	504.6365
2201.642	890.301	2325.535	2173.317	2242.653	1894.962	1513.494	3202.702	1791.416
0	6.135847	0.010755	0.906528	0	3.611822	0	0	0.77105
32.5234	71.92074	45.13037	44.49703	67.85795	60.47839	37.74827	157.1991	37.8368
115.3167	3.953602	87.09764	117.7833	68.83504	38.78849	120.6741	82.66619	26.27547
489.0263	391.8203	743.0018	636.9611	540.2735	615.9595	465.6758	1042.465	382.7578
110.6452	79.98143	130.1181	101.0632	113.3413	82.31072	95.5676	193.8577	98.7903
61.35021	100.1125	47.2386	118.7032	218.8862	89.53326	47.70724	143.3266	91.39512
74.38268	28.68779	35.69633	53.76457	79.46294	54.48569	80.63068	46.82286	25.45353
173.9249	4.541381	34.64923	30.56902	135.1731	49.66967	161.4147	58.76438	10.62677
186.8981	76.38718	118.5909	117.7442	153.8775	191.1264	196.9567	68.85785	86.39816
114.331	566.414	193.1091	218.7981	199.2628	133.1842	189.6472	428.0211	363.0758
19.52047	5.720866	2.118688	15.74938	17.72079	20.57653	53.85446	13.7581	13.95579
55.80449	4.534775	8.416159	15.73522	98.27368	21.84359	32.77082	8.231849	4.042723
42.79324	55.02467	11.56152	39.88888	57.55208	24.22607	12.74016	170.2789	18.06783
45.56208	37.04395	36.73441	36.15305	32.14062	35.11552	17.01474	65.24624	41.98704
31.6245	9.26388	22.04241	8.316814	24.4269	19.39382	24.24311	58.87036	18.90532
117.1893	26.92034	99.67774	36.13799	119.6829	93.11368	114.9965	67.04679	48.53823
10.20202	17.5686	7.365548	10.17286	5.368961	15.78357	18.49027	9.15408	8.16415
3044.025	707.2585	1499.644	1388.905	2261.584	1358.933	1927.965	1792.829	962.3452
62.2987	25.16474	78.69196	62.12695	95.21875	61.6976	29.15502	157.1834	55.15673
305.8628	145.1906	380.9569	281.8375	264.1974	435.6661	433.6022	187.3318	439.5879
462.0881	173.4643	259.2227	365.302	555.4846	569.8815	479.3409	374.7238	354.8073
1.83142	155.6306	22.03455	8.319541	5.935407	4.92344	2.735072	10.99856	7.344959
27.90785	41.50154	19.94169	15.74917	19.40876	18.17308	9.89178	56.1345	31.3454
123.6563	69.3273	128.0292	90.85039	143.8997	131.8854	114.8211	79.8863	46.03547

12.06211	26.46088	15.75568	18.52659	15.41942	30.24387	29.96583	10.99171	18.89512
90.20704	73.01858	34.64612	72.32671	58.84181	71.37697	46.30631	160.834	57.61512
80.87083	59.30265	223.4974	193.8126	112.152	244.267	58.38975	75.29166	44.38755
389.5656	124.604	219.3449	832.6854	1466.844	543.2401	159.6674	570.3971	158.0032
152.487	59.29858	118.5892	93.63086	101.0068	209.2394	148.3876	97.34186	107.0017
100.4077	79.37078	164.7416	107.5515	83.2293	88.36436	55.54209	100.1063	107.0205
259.3924	64.56121	116.5055	167.7986	558.4786	249.2967	263.17	414.2498	192.6019
81.84509	45.85884	36.74106	60.2724	53.86742	42.38481	22.00644	125.9183	71.66669
38.13464	19.32688	44.05771	23.16866	20.45691	33.85995	36.42547	37.6733	41.2159
17.64042	74.41803	29.39387	35.22516	19.84759	36.32032	11.29515	43.16994	27.95232
391.4522	149.9603	152.1812	587.9131	439.0057	370.2047	290.3264	230.5268	187.6715
380.3066	347.4194	281.2376	389.461	302.8072	391.9278	123.3097	1109.805	318.6652
378.3945	260.0015	295.9533	330.0596	514.8469	491.2858	439.3458	384.8208	367.9683
3789.645	505.369	2306.663	2020.307	2746.823	4387.639	6058.618	2539.52	2805.668
29.75723	50.90711	22.04422	13.88563	28.34169	26.62309	20.63929	39.52284	10.63896
24.16117	21.68462	16.80596	23.16548	37.25934	31.46624	24.20134	22.94537	28.80038
14.8462	18.66345	113.3115	42.63238	44.88483	58.10341	39.8651	55.09701	84.83177
0	3.62177	0.010057	0	0	0.040864	0	0	3.318861
7.412526	5.131862	0.015446	13.89324	9.858572	18.16403	54.62165	1.804349	0
1.837172	2.838087	0.012798	3.695888	0.396943	2.477587	0	0	1.587768
19.5202	20.58689	19.94169	13.89105	20.53564	19.37533	12.76445	22.96545	33.00287
130.1619	209.1033	157.409	131.6578	157.2231	146.4059	60.52641	222.3082	111.9416
41.82668	67.75176	47.22954	64.91223	61.6856	39.9719	25.58053	219.7311	80.74392
521.5824	293.5451	599.2239	434.8355	414.2248	596.5529	465.7221	752.2369	314.4351
109.6975	147.1332	56.68797	99.19212	78.72789	88.39057	87.65368	232.408	63.32836
11.14429	22.50431	20.97583	22.27601	17.82158	4.917977	14.25039	65.43754	21.45537
1359.308	309.3995	655.9064	320.7753	2121.975	525.2307	1937.453	685.1522	213.15
185.9481	73.99998	178.4055	186.3626	144.3236	186.3515	93.34334	229.6279	149.8085
107.845	113.5683	271.7505	129.8094	30.35824	54.52562	22.7007	28.4376	204.2694
11.13282	49.74045	16.80225	20.38748	14.31331	7.343118	11.31435	22.95504	21.3839
365.3978	205.886	581.3514	464.5444	424.9806	544.4047	148.974	403.2199	709.0183
309.6216	104.6122	235.0732	240.1404	209.8942	404.0406	303.9115	200.2144	115.1985
127.3675	45.14861	138.5277	136.2908	143.8297	169.3875	162.6425	150.6274	90.51231
82.74711	68.80783	69.26835	89.94106	159.7321	77.45419	74.87216	120.3468	50.9968
40.92478	54.38349	17.85406	38.02579	35.02661	25.43887	20.61927	91.98013	49.47513
70.67248	70.69349	129.0292	90.89676	28.73657	17.02561	19.14522	45.91113	78.25871
102.2663	55.7741	121.7319	104.7667	172.9005	119.7895	84.11128	228.7631	54.276
13.93976	16.48575	2.118558	9.252562	5.94858	4.919096	14.24273	4.560905	13.97972
0	2.189509	1.068148	106.1989	0	2.500984	1.313571	0.887674	0.756779
40.8895	56.44324	75.55609	155.8418	93.41991	65.35306	35.57245	161.7393	93.89967
31.6007	16.92084	19.95499	33.36961	73.60382	38.72844	40.65712	38.5725	24.65254
38.10227	86.06411	10.51539	28.7185	15.9216	25.48598	22.71644	17.41755	46.89901
162.6919	337.9835	380.9156	291.1518	142.0654	206.9321	73.351	659.593	307.1301
17.63503	120.7517	32.55151	55.61177	19.2479	66.59282	18.42192	96.44145	49.34282
379.3234	348.9085	380.9526	377.3494	764.3772	274.7476	447.9038	462.8973	510.426
85.5677	5.123892	29.39892	27.79246	40.46216	79.78942	97.18291	29.36615	22.16382
41.83201	63.07087	11.56429	24.08357	15.93136	27.88916	34.17988	17.41971	34.54311
87.40937	25.1483	67.16512	53.76469	57.7038	82.25643	67.7532	57.85279	46.06196
49.27208	57.10517	33.59426	31.5042	40.46546	39.96959	34.16613	46.83366	19.69119
100.4011	127.1151	125.9331	159.4877	55.3774	61.78372	136.9574	121.2323	110.2983
85.54667	48.16699	69.26383	92.73435	120.2023	77.43185	43.43329	75.31642	83.17094
597.8722	125.1991	274.9574	263.3085	369.3623	454.922	299.5492	524.4772	204.9474
46.47056	15.71539	53.52972	50.05271	72.20164	58.11093	66.31799	87.26701	74.9233
45.56623	24.02519	35.68373	33.37107	21.53075	17.01348	17.01867	39.49435	13.10378
144.0805	668.6048	585.584	105.667	156.9839	222.7321	279.5003	139.5707	441.238
614.5687	428.9772	39.89912	1432.599	976.647	161.0245	1148.625	699.8833	616.6457
35.31222	145.3141	41.9865	41.70933	44.91722	35.14612	18.43088	89.12568	125.3101
172.0104	37.49189	112.2981	138.1455	145.4973	104.1101	180.4988	157.0588	146.5379

109.7043	77.58264	99.70275	116.822	159.4948	127.0515	81.9594	192.9131	65.80737
66.02445	79.01768	60.86194	68.6272	58.3451	43.58939	35.5984	84.53567	71.67069
26.93373	191.7099	60.8885	15.73347	18.15043	50.91291	59.07645	14.66347	80.60328
155.2504	339.7172	138.539	114.0187	119.8166	125.9118	165.4151	180.922	350.7767
141.3725	169.939	56.67769	87.1641	88.92413	88.30579	34.85278	143.3408	84.81429
53.94493	38.24545	40.92653	38.94089	27.11958	15.80665	40.66237	96.53714	106.4573
151.5622	145.4311	137.4704	158.5656	103.2535	212.8401	81.96174	161.67	135.8557
13.91819	46.50145	43.02857	13.87868	10.36545	54.42147	12.72198	15.58229	105.5747
69.75697	33.46429	39.88386	63.06698	53.35925	36.33542	41.34379	68.91342	58.48377
624.7787	427.738	396.7011	548.8761	621.9577	684.9125	1653.615	533.6061	358.8834
41.84674	19.89079	40.92304	66.80661	80.98765	32.68593	17.7397	40.41924	11.45632
95.76317	55.20457	125.9203	80.65607	102.7652	128.2136	64.84209	234.3058	141.6666
149.6772	61.62886	75.5778	178.9413	177.6903	183.9386	198.2895	87.22479	84.73325
342.2202	58.69718	79.77227	102.9007	309.7273	69.04648	269.7931	143.2757	19.68215
20.48515	5.156199	16.7802	10.18924	12.20417	9.713326	7.776158	22.08672	12.34448
655.4766	292.9538	719.9016	540.5424	560.3605	700.5932	607.7075	548.3154	437.1231
20.43779	27.0291	46.158	25.95175	17.64466	10.97024	28.50941	106.708	39.5488
1149.176	116.9393	542.5706	962.4143	991.3598	1178.56	1744.123	669.5386	647.0484
14.84771	760.8401	294.8943	263.3088	13.15196	163.427	10.58684	160.703	96.2492
133.903	17.47831	81.86166	95.498	160.1726	120.9694	59.12401	77.13796	39.45146
105.9946	45.76466	56.68333	54.68222	87.15577	75.05933	63.41042	27.52003	163.0991
362.5983	85.7488	432.3606	173.3576	257.0404	444.0643	243.8658	160.7005	176.9419
33.44718	59.98347	31.50022	15.73123	23.14887	25.49201	37.00032	33.03681	93.8956
391.4464	61.03041	315.8733	485.8889	327.7385	515.3328	260.3298	405.9894	284.0317
1374.164	570.7249	1216.295	1043.994	1082.923	1253.633	1840.301	1188.477	1058.695
163.691	41.06284	121.7163	62.10815	113.4214	176.4732	66.29181	162.6309	119.4336
19.53923	12.90705	5.265059	32.51656	20.66799	16.92414	14.25375	22.07028	12.32717
344.9352	82.80716	395.6305	214.1599	355.4495	511.7756	410.1663	440.8769	231.2975
464.8741	136.9588	1355.778	598.0504	796.1348	844.4697	375.8549	982.8315	236.2223
1018.073	491.877	1353.753	843.7272	789.6829	1069.679	593.3574	1312.497	680.804
75.32859	63.01399	52.47361	53.77476	54.42021	68.9353	26.29518	147.9881	79.91723
12.05982	65.02262	52.45255	33.37609	14.28011	35.0915	7.726314	82.75382	51.10534
102.2868	52.8723	142.6914	82.52066	22.58725	35.1655	31.99083	42.22396	96.34934
35.30135	1616.738	21.00814	31.49442	50.35066	35.17825	62.64378	20.174	91.30568
15.77557	187.0015	141.645	92.72461	1.500024	327.3619	4.164254	28.44027	107.0646
10.2022	4.536207	10.51162	9.244832	9.835009	6.135024	6.302735	9.154255	13.11865
722.4059	222.2918	517.3824	718.5588	603.0567	638.9444	1169.135	1000.214	828.2083
94.83656	67.60853	107.0351	118.6968	105.0209	105.2491	77.00196	79.89899	50.16753
30.70635	14.03635	49.269	13.89182	20.54389	18.16987	14.92431	40.47223	20.57974
105.9774	59.87875	149.0186	120.5244	116.0084	148.8347	121.9337	80.80015	69.91847
94.81779	11.00965	60.88484	147.4235	156.0918	48.48539	246.2447	66.10101	32.85537
12.06018	38.88947	29.3886	22.23582	17.07769	12.17928	9.156046	20.18469	19.71064
90.16216	183.0321	194.1428	216.0431	166.5831	187.5559	99.77035	300.3777	228.0782
35.32331	74.42322	41.97656	42.65228	52.29728	37.52508	24.88959	128.7353	33.73173
19.5029	41.81559	60.8439	21.30444	9.813686	26.66383	10.58261	18.34266	40.34778
23.21569	52.89194	12.61433	2.753104	5.923051	3.713839	6.299134	2.72355	28.74921
3.694638	5.777055	12.58593	3.683465	4.86241	0.060381	0.606313	5.488353	3.229038
138.5292	88.16924	131.1828	143.712	108.217	108.9432	150.5042	202.085	117.7025
285.4068	27.49607	82.92655	967.0784	747.5888	510.6772	819.5973	917.566	213.9785
15.7756	112.3462	1.066936	2.754315	8.148744	3.710118	10.5793	12.82396	17.2126
5.551698	68.67794	0.015324	3.679359	3.149228	7.33259	5.596395	1.804359	21.42884
166.4411	167.2429	96.55614	226.2785	161.1506	148.8105	116.2436	253.5617	193.5411
0	5.283194	0.012913	0	0	0.053474	0	0	0
55.77648	57.04467	90.23772	83.46017	64.97035	46.02688	89.23243	96.46644	58.43614
92.04991	144.4312	71.36663	142.8273	98.36028	112.48	29.846	186.5311	173.8677
488.1053	706.2775	655.8931	523.8488	1043.241	845.7731	770.3757	788.972	604.2739
259.3878	509.8415	770.2276	752.9492	145.3498	346.0713	128.9918	1027.906	498.9699
226.8432	420.307	243.4768	197.4704	291.5409	346.066	296.7	258.0749	213.1848

3253.164	2577.642	4708.8	3094.914	3379.94	2650.09	2268.794	5077.241	2645.931
135.7448	3.959951	26.25704	43.54858	315.4391	133.1037	573.6532	78.04689	32.0327
266.815	516.1476	808.0386	481.2044	57.01196	232.4041	146.1	206.6228	855.4748
37.19411	41.25962	38.82422	38.02019	29.95076	45.92862	31.36616	54.22595	56.07026
1407.63	820.2855	1730.508	593.3684	1224.544	1401.262	1367.303	1164.591	1054.568
103.1788	207.1624	87.12081	178.0113	76.47052	124.6926	59.79552	186.4409	139.0921
22.29373	36.46258	47.21789	41.72533	9.255509	20.63571	17.73273	18.34168	54.38396
205.485	231.9996	173.1555	220.6861	201.666	177.8713	89.06853	360.1126	150.6435
2.76049	19.97765	0.015581	2.75175	7.606274	4.925095	5.590203	2.722014	37.1352
5.549176	26.92597	38.84163	12.02232	5.36756	29.11251	4.873929	2.723275	36.99876
145.0522	54.00241	98.65256	89.92418	132.2182	150.0046	179.1454	133.1839	50.15553
158.0471	234.8873	118.5989	294.8732	359.0808	150.0819	114.042	228.7066	150.6292
22.30575	10.44418	35.66848	23.17267	7.039566	14.5812	7.731922	9.153657	56.96671
13.94759	10.53421	22.01526	12.04799	2.041581	13.31339	3.454801	13.77781	28.13837
46.46921	73.5592	35.6972	118.7126	152.552	70.19395	46.28797	78.99028	72.44254
28.7998	44.66551	51.42572	32.43069	41.56973	19.44261	28.43845	33.04185	30.40735
170.1241	131.0993	147.9856	216.9478	168.7113	474.2321	212.4999	60.58307	210.7234
11.14303	18.28493	18.88373	22.27281	11.58192	28.91445	15.68701	24.83138	16.46827
227.7849	43.37257	157.4265	181.715	278.2976	279.4977	305.3367	286.5709	101.1976
31.606	46.59294	36.7288	60.30729	42.27338	38.70776	24.90679	80.91113	43.66408
116.2416	24.55108	35.69808	95.50909	84.42905	56.91642	134.2425	65.19895	55.11975
1045.982	39.85257	76.62968	43.54767	924.2099	873.6442	2702.48	44.05252	153.8743
11.13671	45.71829	101.6229	25.97458	0	12.15545	2.023252	3.640446	74.48916
115.3076	8.65308	28.3542	22.22199	145.764	78.66585	304.5137	40.3848	47.69816
259.3862	320.7244	267.6133	352.3293	425.4938	300.1162	310.9596	501.5062	226.3558
171.0903	114.728	165.7998	193.8091	147.2189	187.4864	104.0955	273.776	92.99408
59.48279	134.2599	173.1327	61.17292	116.6541	46.05774	73.40231	225.0954	76.52777
157.1191	193.6397	343.1296	202.1303	168.2487	148.8671	122.6143	309.5645	174.5248
695.4643	516.7128	380.9542	271.6402	1147.267	612.2711	354.4223	1186.729	1436.853
37.16837	122.073	23.10701	15.73129	20.36725	60.52355	82.77777	32.11817	82.35007
24.18397	40.9519	22.03456	15.75167	11.54371	13.35593	15.64987	52.46077	49.60952
119.9303	108.8153	154.2623	154.8457	154.9898	154.8692	82.66073	213.1172	275.1162
39.05669	11.61195	27.29211	49.16513	29.39149	38.70163	43.56855	33.06257	42.017
24.14888	105.6576	25.20314	19.44281	14.2552	6.138464	7.010513	4.558534	17.21812
89.29449	1.023448	49.32562	64.9154	140.032	80.98571	78.5691	41.31828	13.92265
127.386	193.9073	163.6899	124.2518	155.1138	81.1086	36.97755	160.7637	182.0453
58.54394	971.5703	117.5532	179.8587	70.90338	123.4953	61.93212	134.0698	92.13759
178.5622	22.18963	77.66431	208.6895	235.492	56.92889	107.7323	200.2999	32.86157
39.97582	10.42238	24.15088	48.2204	67.96409	23.05323	40.64612	57.88506	37.03045
20.43902	23.47669	27.28887	20.38197	37.28477	18.20566	25.64593	44.1139	21.36954
33.47794	28.83248	30.43231	39.88839	32.80525	30.24737	17.75767	87.39081	32.1193
169.2178	33.95816	106.0036	124.2315	89.28823	134.3383	321.2207	68.85546	92.98094
4013.721	1743.687	2998.23	2019.381	2865.147	4030.673	3412.969	2080.286	1850.653
11.12682	88.90253	152.131	19.4402	1.499694	9.769854	9.862914	3.641448	154.9108
275.1851	446.0924	821.6742	152.9556	568.7811	456.1961	193.9023	231.4246	437.1466
45.5328	155.5378	95.50101	29.63987	24.8037	32.75586	34.12261	61.51463	90.54371
214.7741	108.7519	115.4517	141.8434	149.8696	192.4079	156.8713	66.09621	181.1047
49.27771	71.36789	22.05479	38.93186	60.62418	33.92388	23.44448	53.27701	19.6946
50.1759	208.2918	10.51403	17.5861	20.91623	104.1416	201.101	40.37672	155.5526
213.8919	29.25358	62.98053	163.2085	142.2606	62.98519	71.25333	104.6992	55.92409
24.15151	108.7423	25.20116	25.93988	25.41494	21.85139	24.16161	23.85426	56.83306
52.05231	106.681	65.06818	110.3654	111.8272	129.3402	42.00343	84.5089	47.70906
0	3.980439	0.014403	0	0	0.060249	1.314843	0.887886	1.578073
0	11.99762	7.343243	0	0	1.283917	0	0	14.97434
12.07134	17.64651	37.73812	11.10809	25.68241	21.75273	10.6234	46.94565	10.65615
9.267666	97.43713	75.53848	34.29061	18.15888	97.84592	36.32441	11.906	94.80874
13.92445	13.40457	18.9001	38.03527	42.95651	14.58294	17.04692	24.79258	28.82007
196.1798	135.2857	205.6842	176.1621	168.2516	167.0023	177.5952	517.2217	158.0494

726.125	153.4275	1072.508	826.1187	796.4006	1022.46	371.5096	2835.461	200.8019
1.833346	614.2251	1.067179	4.606771	0	1.285949	6.301233	0.889344	4.868446
0	2.468	1.055255	0	0	1.22741	0	0.906433	0.793344
36.29097	5.718298	3.168539	12.03167	104.7094	14.57062	36.50535	34.9334	6.516309
1708.875	573.0675	2004.405	992.9921	1385.621	1675.926	2057.113	1252.764	1296.622
26.0324	1.60102	2.118642	18.52932	79.07241	1.289939	33.58304	2.722003	23.03636
33383.44	12869.85	28727.14	21238	28152.64	32205.7	37985.62	21851.94	18018.82
46.46279	63.45392	71.37196	64.88429	66.53713	56.93503	60.54788	35.7879	79.82836
282.6434	123.4461	309.5781	185.4195	322.1731	180.3494	264.6104	507.963	286.5012
187.7955	207.1071	349.4464	299.4892	205.4275	229.9266	181.8244	562.1796	226.384
712.2071	470.2129	504.777	443.1858	1189.019	388.4643	323.75	1352.088	321.0297
1626.134	507.154	29.40285	399.5849	1185.685	1016.489	3668.729	306.7298	665.1409
441.654	46.31484	301.1874	171.5073	435.567	227.5288	179.663	1382.549	498.1677
13.92327	13.39801	19.94981	38.03027	4.255655	13.38071	6.300242	29.39048	23.02506
60.44002	108.0448	49.3262	35.216	49.96179	55.6572	27.01287	101.089	70.84437
18.58099	5.714765	6.316662	25.96011	36.78728	33.83832	37.17811	7.31515	9.814047
69.71755	87.07499	93.4004	64.88653	125.0646	131.8352	76.99228	95.51935	88.07909
269.6674	65.76584	134.3324	166.8961	269.6193	130.7115	151.9184	326.1266	135.822
40.88731	84.77896	48.2863	37.06312	28.71029	32.74666	73.45607	27.52226	47.70424
125.4923	78.10606	230.8787	256.8291	273.2663	655.5699	208.9474	361.8986	106.96
188.7582	105.867	268.616	82.49936	156.1037	204.4209	119.8001	254.469	167.1512
0.903194	48.79593	5.266385	11.10727	1.487167	19.35751	8.464398	3.640741	11.48173
2597.714	837.9071	2100.966	1879.386	1724.921	3081.968	3186.943	1335.414	1392.921
142.2574	66.37568	40.94765	113.1104	205.7125	151.2267	91.24158	98.262	65.80591
25.0741	366.0366	27.30528	10.16797	27.58841	33.96132	51.27271	23.84594	64.1793
31.6146	3.946722	16.80466	25.95465	32.80158	29.0449	37.86214	29.39003	6.513018
382.1203	109.8814	295.9501	273.5008	510.4628	618.2629	535.0064	333.3879	207.4049
1998.936	1711.328	1831.272	1848.78	2924.607	2429.828	4839.63	3361.563	2111.644
467.6779	958.1332	497.416	330.0718	487.1872	252.9464	200.3418	600.7153	467.6563
69.73375	21.61247	30.45031	52.83905	103.5005	43.61052	26.99954	277.613	33.6982
3.689824	59.22453	4.218235	6.460412	0.940139	1.289785	4.159475	0.887975	25.50988
68.78048	29.25165	32.55306	81.57491	106.0307	142.7629	124.1079	66.10401	43.56413
224.0844	261.4732	144.8275	123.3004	217.2383	285.4649	86.92499	203.9084	228.088
53.91075	182.3353	104.9292	57.47238	76.6407	19.44855	30.56416	213.2076	146.6732
21.39684	29.05874	22.02781	12.03935	18.35091	15.74242	8.471294	34.96814	23.09692
25.09384	21.09914	51.3968	42.67105	36.15416	44.70179	20.6207	79.09316	43.68819
9.297782	7.605607	3.165312	5.545907	7.157925	7.288002	4.915061	25.83028	0.757428
36.26399	47.80391	40.91995	27.80661	26.5937	20.62424	27.7824	121.4236	20.53652
0	2.185574	33.54663	0.899637	0.937172	4.920949	2.735855	0	18.94561
73.45396	129.1912	55.6263	99.23448	109.0676	48.44411	52.02089	88.19042	128.5582
73.45504	35.77585	56.67468	32.42745	62.73786	56.89511	47.73214	81.75658	77.40818
53.92908	12.18718	17.85924	29.65017	43.83482	52.02705	100.808	37.64269	71.6831
31.60599	45.99887	48.26024	28.73308	78.75912	49.54859	25.62366	128.774	32.0957
122.7358	37.50659	105.9921	87.14643	70.43368	95.60759	74.12175	103.7854	40.2736
32.54555	37.74694	55.58615	64.04318	17.09377	44.69288	19.90769	31.23013	31.29002
15.80105	2.185575	12.60219	10.17996	22.30541	24.14289	21.44514	18.37205	22.26341
92.04973	39.88208	89.19949	23.14945	61.00674	36.37619	52.7093	45.89862	74.90336
16.70706	45.8521	24.15437	15.73233	31.52869	37.55895	44.89515	28.44641	18.04182
30.67624	32.9456	22.04979	21.30697	126.0104	18.21362	67.26898	25.70278	10.63275
1.833039	48.74534	59.82549	17.58563	0.948382	68.98989	0	9.149833	53.4749
40.9254	22.28523	19.9509	15.73946	34.46852	23.02822	76.6694	19.26727	22.19403
102.2564	85.80733	119.6425	105.6835	114.8883	124.6623	175.4984	130.413	100.398
39.03078	10.41675	19.95902	66.7588	91.22494	36.36199	106.4248	14.66094	20.51119
103.1979	13.94755	75.57001	93.63698	147.2865	197.1079	105.5519	125.8367	28.74027
12.98641	40.49517	57.72463	1.826779	8.695564	13.40006	14.8566	0.889006	19.68603
79.0159	85.87032	118.5813	62.10107	48.16414	90.77593	69.83317	45.89392	87.24423
38.12427	1.020181	26.24418	50.0921	37.23213	23.03863	25.62556	34.90136	15.58181
0	107.4738	3.163133	0	0	0.056712	0	0	5.740524

103.172	1198.82	407.1996	252.1617	503.5602	1276.612	284.4686	75.27994	1357.577
0	204.9323	1.068233	2.752062	0	3.713591	0.606234	1.804372	48.83446
74.35288	163.5632	223.5236	96.40294	60.35501	199.6565	82.63456	127.6463	144.0392
328.2132	321.4061	182.6118	382.0244	326.0866	93.25692	240.3505	146.0106	263.4451
20.44776	24.13563	14.70415	10.17408	7.603428	22.99565	24.25596	9.15522	9.817502
7.409622	25.87131	4.218239	12.02811	4.255867	2.50457	7.730531	0.88798	15.59165
66.00428	77.09465	81.8538	68.60519	70.50265	19.4487	59.1527	170.9151	45.23062
965.0824	777.4213	719.9159	1161.779	1209.758	538.5296	444.9856	2072.141	1113.099
121.8054	85.28281	50.38914	102.9162	186.3355	145.1435	56.9752	119.4082	83.12429
222.1909	467.3902	107.0624	163.1585	160.3473	193.6735	303.1044	73.44138	162.9453
41.85471	46.63844	30.43532	29.66594	40.6324	21.82577	13.45098	79.08726	23.01785
310.5593	58.68091	234.0202	120.5122	174.3272	203.2991	193.9862	146.0166	116.0268
967.8774	8.677895	112.3107	144.6086	1165.909	822.833	1685.719	88.13677	38.62188
4909.046	2086.768	6191.647	5319.232	4727.86	3282.954	2600.455	6860.889	3757.348
60.4222	18.65923	70.31557	50.05004	67.15546	65.36789	125.6733	71.63469	92.22983
3847.3	3985.63	2174.432	2747.233	5181.927	1944.617	1700.335	5357.416	1217.547
48.34886	17.50162	71.34059	27.79617	1.494274	13.39526	27.02536	13.74409	58.48681
249.1758	174.7226	131.1932	146.4764	168.7505	245.6322	171.8446	127.6414	140.7319
58.57278	11.00591	93.37762	52.84316	79.53137	72.57014	55.61964	44.07107	67.5247
22.28375	112.2867	97.61376	75.07358	49.2394	41.23255	88.34197	69.77053	94.6151
86.44557	137.0915	125.9386	79.71439	153.835	222.5801	58.37783	83.55477	196.7967
0.919413	0	0.007754	0	0.452852	0.031041	0	0	0.792986
18.57134	111.2659	15.75956	12.95197	7.58668	18.22202	13.44041	2.722441	36.21107
18.57005	83.28323	15.76024	34.29466	12.59327	30.29549	9.865463	8.231657	54.36751
68.8197	45.2829	25.2023	91.83754	107.0342	27.89328	31.30954	57.87073	48.56616
32.5169	49.93916	74.50828	34.28151	27.04519	49.65677	50.58342	25.68542	67.50026
323.5851	3.377771	296.9709	180.7946	320.6612	318.1495	302.5361	241.5664	162.1584
55.76465	121.9028	57.7323	164.1507	171.8845	96.80681	58.41003	86.32799	157.3298
5.549684	228.9559	74.51402	7.386942	0	37.58528	0	15.57878	191.1849
994.8276	760.9021	885.7243	1135.805	1619.154	1172.518	993.5759	1856.263	778.784
402.5701	661.6152	491.1345	614.7344	834.4229	436.85	382.2619	499.6414	815.1267
12585.03	4862.28	16090.93	11843.8	11480.7	8164.328	9483.482	14001.89	7922.253
33.47235	28.21159	17.85458	42.66753	49.61107	37.48826	24.20116	33.98564	34.58553
226.889	188.4225	161.6085	198.4372	219.5525	203.2254	113.3634	224.1386	110.2874
4.619288	16.33892	25.1965	4.605395	9.258367	6.138172	13.44475	5.476426	24.66016
28.82323	43.13181	47.19842	25.95701	33.94584	37.46025	18.47968	26.63209	42.88092
10.21696	8.740567	16.78143	15.77208	15.03986	6.115568	12.83377	31.31558	23.15184
15.77848	42.94207	24.15204	26.8683	17.60608	20.64299	12.00717	46.84218	32.07076
109.7015	112.9557	131.1791	97.34073	117.7058	84.75511	56.9587	177.2844	123.4821
42.83776	9.881869	20.98535	31.56007	4.261886	12.14973	10.61896	5.47903	16.4483
81.82146	63.51271	105.9775	104.7928	64.37472	66.57228	114.2355	221.4845	55.12513
182.2701	190.9478	97.60034	148.371	111.6451	98.02774	60.54482	184.6581	144.9439
26.03009	20.5276	46.14903	29.67382	31.70602	23.01511	12.0272	61.61903	27.99308
575.5204	231.1505	517.3686	348.6047	368.6881	375.1555	375.8391	343.4855	332.5591
36.25648	39.43476	35.68372	26.87296	37.19539	14.59879	24.17882	79.97576	20.52813
44.63423	42.98809	40.92674	38.0122	64.08453	43.5473	20.59657	56.0518	56.03828
338.4228	851.4724	367.3015	393.1281	254.2658	411.4028	186.782	756.8807	486.5942
92.05018	2.782215	23.10788	77.87966	207.1657	42.41872	121.3573	15.57881	17.21312
66.91189	263.6573	223.5348	99.18034	36.46185	106.5637	67.63861	281.0512	293.096
42.76622	31.10767	67.14412	31.50964	42.75109	62.8568	30.60949	64.32006	81.6221
30.72837	11.09556	14.69569	17.61995	23.46642	24.12974	9.193841	35.89569	13.97754
120.0103	19.85431	6.317442	49.13636	202.1606	13.39759	62.08868	40.39834	14.74643
127.358	118.1829	231.918	99.18477	176.5749	291.5454	187.577	216.7619	122.6212
45.53723	153.3196	11.56494	13.87644	13.69274	17.03013	22.70625	7.313436	44.40403
94.83701	125.4858	159.4823	195.7077	21.47167	58.13299	41.98547	70.70908	187.0421
131.096	25.12951	28.35573	43.54888	95.44815	71.45411	368.5272	22.00776	41.91629
11968.6	4979.399	14814.83	15456.12	12041.09	16086.5	15463.57	12433.16	8892.069
562.5048	175.222	358.9146	279.9882	661.0291	519.1033	604.1901	335.2198	204.9295

38.11412	32.29984	60.85092	39.86525	51.72049	19.43203	11.29445	50.52625	58.50197
69.77581	44.18242	23.10058	47.29819	49.52205	38.72234	23.46316	42.25439	31.2603
18.62062	5.75643	4.21539	7.399567	31.08021	3.707311	24.46496	5.484653	4.87936
58.55326	87.05042	319.9884	96.42212	166.2612	69.02314	90.55463	403.4118	149.8925
102.2486	741.9001	19.96022	26.85679	69.24389	32.76268	64.07642	10.07018	176.982
257.5157	438.4001	297.004	254.9501	226.9872	324.3525	392.2398	260.8148	267.504
52.12086	6.895669	9.463251	25.96035	44.67393	21.80589	37.89919	36.76276	23.86323
301.2442	297.2428	262.3587	382.9508	337.2041	260.167	175.3949	295.75	287.3302
165.501	70.48538	117.5436	117.7415	168.3262	121.0332	191.9343	151.5471	76.50821
212.0016	51.0373	113.3481	324.5794	303.5854	145.218	111.9257	236.077	57.5607
2873.869	165.2027	1794.531	1305.454	3292.524	3556.223	4223.512	640.1323	375.3332
148.7528	104.6512	149.0238	118.6639	35.90818	113.7934	74.79097	56.91195	153.1148
40.89193	86.63033	43.03755	51.91275	51.59121	36.36082	19.85648	55.09975	60.09159
19.50122	63.72999	13.66192	4.605445	3.149048	4.927843	3.447908	5.476369	13.92672
101.3452	53.43595	83.95901	48.19002	49.28814	82.30554	58.41222	67.03047	71.5929
26.97353	14.02629	16.799	25.0405	23.90568	14.56776	19.95104	34.93699	18.91708
460.2263	461.4227	484.8337	388.4827	526.5647	110.2045	224.5859	545.5791	750.0984
0	0	0.008855	0	0	0.035701	0.64373	0	0
114.3456	178.3703	179.4471	127.9417	98.19009	156.0974	48.3855	151.5439	120.9924
14.8458	87.60344	300.0796	24.07533	10.91978	141.5583	37.68559	52.32231	466.4004
36.28547	33.66116	8.414057	81.73744	21.62275	7.34203	13.46994	22.95765	17.25363
0.903592	67.64345	0.014158	1.826926	0	0.059127	0.606795	0	4.892444
33.47024	81.10885	19.95239	29.66345	52.39578	29.06232	16.31211	45.94502	42.84519
30.66551	9.241848	36.73735	56.5699	66.79633	45.98411	60.69423	32.12892	12.27635
209.1879	17.48327	289.6334	131.6389	415.7163	465.6934	63.36067	138.6652	49.32
115.2895	117.1293	97.60196	78.79523	84.89554	116.1611	105.5494	79.88934	54.27682
20.42949	29.31957	11.5643	67.70696	47.2003	38.7508	70.00066	24.77299	12.27589
212.9424	193.7586	150.0654	196.587	268.5988	108.9365	44.82072	139.6064	134.1892
126.4879	134.4743	66.1173	84.38261	101.7755	82.26256	34.85508	129.5549	84.81955
125.5032	157.7083	140.6296	185.4421	296.8527	145.2275	87.64173	290.2814	142.4061
330.0391	788.743	722.0002	461.7252	189.204	416.303	292.3442	567.6051	492.2935
168.2754	338.0417	172.1121	307.8546	207.164	88.40901	102.6178	395.9206	491.709
39.98532	18.70819	32.53546	12.0253	33.86226	41.1184	42.12137	50.54132	32.92108
12.08414	17.17015	10.50007	16.70518	13.91282	8.514132	3.456415	33.16776	34.82153
41.80811	1704.15	134.3405	90.83688	5.937758	32.76266	19.8497	31.19222	76.49403
263.1281	320.8969	206.7404	187.2826	234.3893	206.9301	148.2936	281.984	300.5425
62.30147	129.3813	45.1311	70.48334	64.49225	55.65696	40.60777	79.93721	54.3374
34.38928	153.9057	11.56406	15.73417	15.37821	23.05611	13.43691	9.15023	65.92629
2.760737	78.65265	16.80763	6.459605	15.95007	6.138279	33.50333	0.888283	48.61406
329.1528	345.037	222.4812	302.278	305.5659	347.2048	176.8404	614.5649	391.9736
11.13902	9.884881	27.26835	9.249441	4.262604	4.922263	5.595225	13.76271	26.39886
50.19558	34.00939	30.44996	57.47984	67.77519	25.48844	73.49784	88.19577	15.56803
17.65175	31.86949	20.9938	15.74484	7.042768	9.75526	13.46939	7.315683	37.11457
467.6694	139.318	483.7813	247.5378	637.7719	415.0486	731.9933	313.1805	277.4015
0.903318	11.04875	13.65572	0	0	1.290161	1.313822	0	0.757229
41.83111	19.86013	68.19833	55.63799	40.48736	6.138642	72.14423	65.23091	37.01685
64.15614	52.93369	58.76886	52.84321	72.82775	56.88047	42.02398	73.49072	37.00196
145.9494	209.4586	377.7779	250.3367	116.4725	233.5577	84.04934	439.0681	259.3283
425.8286	303.6185	629.634	277.2127	425.4523	583.1634	356.6149	462.915	363.8764
22.29423	23.43252	19.95459	52.8687	47.83643	47.16062	21.31363	41.33384	29.60793
9.274595	57.12961	12.60505	9.248075	13.2297	15.76156	10.61457	10.99837	18.9276
62.26344	215.228	450.2214	44.47497	64.79183	243.309	101.1592	77.11569	216.4446
4.620339	16.42259	53.45636	2.751814	2.592837	2.504529	2.023249	2.722082	31.35403
60.43162	5.712308	19.95885	11.09507	82.30356	39.98302	178.7784	19.25487	5.688892
162.6957	90.48931	224.5742	136.2793	130.4059	211.7528	134.0271	156.1247	127.5621
3.691359	17.69908	13.64651	3.680082	5.951893	7.325625	2.023878	4.561284	15.64366
64.1385	2.197286	14.7133	19.43979	274.0513	88.33704	129.9146	53.2484	13.91942
2.762655	20.87568	4.214468	5.541989	0.391098	8.500994	2.743286	0.887781	10.69904

110.6735	23.96944	90.23945	50.98176	66.6341	61.72906	90.65377	119.4476	55.95855
124.5612	174.6895	281.2473	172.4369	112.0209	332.7344	231.7763	141.4164	377.1062
12.99018	70.96375	17.85573	32.44722	29.38346	21.83355	24.90736	24.78203	28.79187
90.23848	39.97791	35.68878	63.99614	60.63716	52.01523	44.21106	56.03808	74.17589
15.80868	21.31891	19.92716	4.608611	2.596078	9.726362	8.483022	3.641947	39.75578
61.37137	19.26442	65.05637	40.78423	93.57716	67.71945	29.87502	66.14416	70.84641
79.9424	54.00256	60.88266	126.1001	109.3845	110.1263	98.39555	44.97351	77.34528
81.79874	51.04493	129.082	82.49982	142.1915	209.2501	146.2349	112.9604	165.5061
80.87823	126.6179	79.76395	89.93118	82.69593	96.80999	51.97844	144.2261	173.8151
37.15995	68.71212	18.91102	40.7654	59.81188	33.97157	97.6438	22.92615	83.91932
150.6394	163.1836	103.8956	116.8285	99.38287	127.0307	102.697	169.0317	82.29602
939.0295	1052.829	1087.217	753.7781	594.6561	1361.307	1097.682	798.119	860.2744
0	48.38243	0.015139	5.536664	0.936818	6.123103	1.313032	0.887605	34.74615
1405.791	1150.594	1108.199	1211.832	1637.43	1327.4	651.8442	1904.013	1097.416
0	3.546738	0.011111	0	0	0.045462	0	0	0.769022
30.66247	41.72764	17.85951	46.35317	30.98145	33.93111	48.487	13.74326	34.5368
571.7841	904.0766	792.3161	931.836	388.6323	447.7738	229.5553	1423.681	719.5394
121.7997	102.9615	138.5173	92.70806	138.915	99.24569	62.68123	180.0546	85.58813
47.4153	92.10497	40.93361	91.8425	33.78398	49.6108	27.73586	125.0177	99.76576
53.92184	31.06857	15.76175	118.7428	91.28711	49.63351	72.80991	33.96075	19.68952
0	7.967993	1.064017	0	0	0.0468	0	0	0
1.831424	20.00177	12.60544	6.462395	10.97767	16.96611	12.04982	8.237424	51.25768
56.70731	120.3352	82.89556	23.15133	64.41542	14.6093	46.30311	54.17859	84.83491
1.831907	44.20868	38.82635	11.09738	5.365128	20.62932	14.87602	42.25924	26.31181
102.2821	44.59964	56.68079	139.1132	105.5993	117.3164	55.56566	174.5768	79.84771
121.7822	195.4325	35.70195	77.85777	66.4767	77.51735	164.0395	39.45887	86.38564
1338.826	491.2563	1179.57	1362.023	1238.429	1494.428	1403.683	1038.757	982.9376
11.13357	20.55184	8.414071	27.82226	41.3337	16.9839	19.21536	13.75404	17.25347
39.95	173.1003	10.5149	24.07521	41.47059	46.06616	99.08448	12.82392	47.67953
2.76058	62.70559	19.95136	2.751846	0.394298	10.97031	2.735291	6.395228	93.32016
43.72837	27.65717	16.80371	30.60179	7.595544	20.60851	6.300984	6.395742	35.43527
12.99916	5.721321	11.55771	9.247356	31.82942	30.1841	20.68087	7.317302	9.821936
68.79785	121.453	97.58467	86.23736	117.3968	54.49046	47.72046	122.1982	101.3139
118.9878	72.22824	86.07133	194.7056	131.5165	229.8909	243.9794	199.304	125.0904
109.7322	102.5038	61.9237	96.43989	153.64	47.24772	39.85197	163.5599	130.1711
350.5079	599.8805	496.3717	408.8884	558.8524	289.2444	181.0689	712.7766	450.3451
56.705	135.0882	55.62705	29.64375	30.9455	38.78324	28.42561	44.06565	36.16853

TCGA-28-	TCGA-06-	TCGA-12-	TCGA-06-	TCGA-12-	TCGA-06-	TCGA-06-	TCGA-19-	TCGA-27-
103.7388	101.138	84.79127	31.2863	90.77019	87.2415	67.75565	86.95953	99.02456
28.7616	16.73436	45.21708	35.34884	26.65025	1.01975	9.50896	36.97693	14.45107
48.80561	69.30782	37.26566	94.78911	170.0633	83.18235	77.33594	70.79649	31.98702
3791.069	2289.397	2175.915	2641.386	3924.078	1169.592	1934.966	1952.33	1492.475
89.99907	123.2221	123.5036	89.18547	98.53548	77.0976	132.9929	231.9411	100.0541
3.758952	0.086089	0.22735	0.092254	3.190795	6.082093	12.34921	0.69391	4.125523
59.87088	30.51848	9.136471	81.6809	107.0306	50.71675	33.01435	22.26707	39.19504
33.83349	101.1371	80.47597	42.59304	82.97304	69.99771	49.4953	158.6534	72.21039
6030.877	6991.251	11568.82	7385.377	8376.49	3568.613	5580.686	4996.802	2709.554
48.76658	25.06078	58.57827	50.97921	23.6514	69.99246	54.74498	70.08803	47.45206
11.31618	9.81511	6.928285	7.224964	9.560262	6.091964	13.87762	18.46009	10.32426
224.9524	411.2344	351.1874	332.4621	190.8322	300.2642	538.0337	1405.6	333.1618
267.2187	175.79	252.9193	178.1673	248.221	290.1071	152.1078	281.2163	117.5894
6.212479	17.33882	8.234223	10.99492	23.50945	3.046203	8.755119	4.609487	17.47888
101.1955	41.66941	69.56805	55.27972	48.62585	47.68125	30.37023	109.3622	80.45677
28.7776	56.62672	17.71482	32.57563	56.06678	47.67406	53.92961	43.14291	106.1921
2684.359	2171.702	3859.333	1737.879	2607.241	1627.077	2592.178	2204.992	2174.236
35.02768	9.850527	4.802796	24.17385	14.28673	29.42112	36.48447	52.37785	15.48385
379.2143	165.9542	88.93784	49.63627	112.4816	171.4253	124.3151	53.06374	292.8804
6188.773	240.9896	9188.442	1937.96	3576.808	472.7069	599.7825	66.13658	1555.35
485.9993	343.4165	578.3695	811.7034	717.1693	592.4053	531.0822	429.6703	668.3682
0.081126	0.124705	0.371735	4.411886	0.181797	4.063462	3.423809	0.686826	2.075059
0.082903	1.522231	0.384464	0.13756	12.73538	72.02546	11.24534	9.146427	12.39037
42.57852	274.0957	19.96244	52.4979	126.748	90.28629	124.2563	67.67591	56.74177
41.31228	29.22352	118.9069	60.91123	40.82286	30.4374	59.94443	41.50546	35.08055
7.597652	26.36063	9.123422	21.31383	4.918858	15.22071	50.46413	25.37413	15.48197
357.1333	319.6024	296.2957	380.1315	503.9898	195.7777	309.4996	219.4952	167.0957
593.4067	824.9828	537.2432	478.0713	247.0651	549.8012	1208.308	1748.432	525.0028
1.342839	33.25369	4.804274	2.994314	1.776288	12.17826	12.9887	36.96318	13.42042
38.8104	49.93777	50.12178	43.96702	31.46022	37.5374	97.36896	83.16951	45.39234
27.56941	33.33855	30.69345	36.88078	28.3157	113.6024	59.10232	124.1713	34.0465
31.30894	29.19691	19.94466	34.06053	20.53029	42.60741	32.1215	74.73711	17.54704
12.54789	11.17487	13.22853	12.81657	3.348167	39.54926	13.0111	14.5954	10.32329
864.4103	766.8463	861.6654	998.254	701.515	1378.544	1024.883	867.2045	593.0742
172.4263	157.8756	225.1779	176.821	101.7427	108.5442	417.3347	376.7278	166.065
512.2434	8178.868	1463.951	1933.117	2167	6479.899	7604.344	14533.91	3232.485
36.16307	24.93626	17.58558	50.62141	48.08924	50.70935	28.69138	5.295647	31.96993
20.08887	54.05291	9.148623	18.55448	6.481558	9.135598	62.57289	43.06569	50.54644
26.3109	31.93429	54.09765	55.13095	79.44622	71.00307	46.93075	14.53919	57.7572
37.50477	22.26951	45.46529	31.19593	54.59328	104.4661	51.29937	17.62893	53.62967
482.9952	141.2216	321.6605	219.0671	224.7997	239.3899	144.2866	29.16691	357.8722
31.30347	45.73502	43.50512	43.90607	14.29292	62.89113	66.94774	86.34437	22.70292
63.71236	99.50373	64.96168	115.6972	550.5415	77.09148	73.89967	81.67967	40.23358
21.3428	40.29497	24.3126	46.82233	23.67468	49.71034	21.67523	202.6568	34.05003
206.1592	182.8029	218.8628	267.2741	87.71357	228.239	362.5093	356.6266	336.2396
18.77211	11.19601	6.939004	10.03952	3.351036	27.38678	16.48988	29.30678	22.69219
40.04074	118.8072	141.853	60.83547	53.20242	58.83581	101.7547	87.07713	43.32742
226.1971	1244.472	511.4171	793.431	609.4985	1273.059	1297.772	1184.433	564.2006
62.56381	198.0826	128.2289	142.9905	137.7133	197.8109	221.6138	69.98612	162.9776
13.78769	7.059095	2.613598	5.81353	3.348156	5.077598	16.50287	44.13595	8.26201
6.339012	2.921996	11.13302	12.81714	1.778802	9.134072	29.60648	32.46484	7.231279
93.78321	191.0867	160.518	255.9497	115.8042	128.8318	348.6075	214.0737	192.8819
66.22798	48.5386	32.8841	60.86148	23.65806	49.70829	93.90408	109.4422	24.76657
131.2541	2177.367	91.35805	1069.164	829.7671	938.3128	1292.55	1976.168	998.4286
11.32195	8.446508	15.41981	10.04006	8.021138	20.28919	16.4895	30.08126	7.232142
18.83798	36.09779	24.24925	19.96141	9.611581	52.7499	26.90032	107.9482	36.10878
7.592054	11.20292	2.617631	4.411194	1.778656	5.077851	10.38489	22.31408	8.263434

3110.422	2479.099	3886.221	3127.898	2268.513	1844.159	2845.08	999.5822	3842.048
313.5348	221.5397	182.1806	679.7326	120.503	434.1528	1395.595	291.8815	237.2323
25.03391	24.99337	34.67254	35.35624	8.041901	27.39078	51.32954	62.51803	15.48225
59.94475	40.213	24.2189	62.18088	56.23583	65.93308	19.07311	24.56538	151.5723
426.589	2.923195	170.6544	74.4747	9.592225	94.31319	39.14114	2.217695	38.15758
73.79917	99.7934	82.71642	141.507	136.0636	73.04156	222.5308	274.987	72.21215
13.80829	20.79926	13.31545	18.4483	18.83071	17.2473	23.46752	24.64857	10.32499
18.81218	16.73616	30.43263	17.10254	17.36454	19.27757	26.92513	43.9386	10.32678
21.33998	65.12361	45.82404	38.32426	28.34316	47.68044	52.12062	62.33731	42.29868
524.326	221.45	677.5248	397.043	348.0853	191.72	320.8127	308.9189	216.5981
2.598274	0.117884	4.77937	1.573136	1.778856	3.049013	0.820082	1.449235	2.075432
5.099205	18.08162	9.098041	18.47349	8.031084	55.77958	12.12505	34.70018	24.75651
61.31309	105.3315	93.5328	110.4372	84.58035	71.01293	97.30931	286.5324	42.30188
37.5049	33.28964	30.56517	32.6022	8.046702	27.39219	68.72446	98.80502	26.8254
2.592019	16.7746	0.383112	11.48745	15.86309	21.30827	20.80625	9.145503	11.35898
32.52295	27.77746	49.69122	48.05092	28.2549	43.61875	46.07511	29.21388	24.76309
126.2459	191.0816	195.0483	185.3186	173.5338	164.3341	104.2636	268.0234	141.3142
28.78059	26.38957	13.43758	18.52233	17.38135	3.048997	12.98749	9.916844	35.07153
234.868	850.5508	100.0402	318.1118	1915.21	343.8743	196.4273	99.26163	131.0012
121.2441	142.6648	205.7603	185.2939	123.5862	94.34317	139.0479	147.0451	100.0591
841.9912	985.5875	331.7278	712.7986	735.9805	1157.416	1725.515	3808.793	629.178
5.098765	12.62043	9.149051	11.4883	3.345322	46.66481	46.91292	26.0997	14.45321
15.09121	83.20419	4.759854	4.395039	9.593275	13.19282	15.59331	148.5736	43.33331
6.349876	63.67095	9.148991	18.54974	9.611557	2.034279	13.85297	22.24572	12.39042
51.2503	80.20757	67.07752	52.37269	124.5461	40.57899	22.55041	44.6216	25.79703
755.8014	423.6981	481.0619	724.0982	1059.258	553.8598	589.3183	253.2862	602.3608
42.56254	124.52	45.87478	103.2302	104.6722	134.9102	125.193	93.1706	116.5482
71.28496	40.29986	84.7689	68.01639	22.11304	19.27952	140.8153	171.7718	79.42914
1593.377	211.907	1631.964	988.0199	1501.762	492.9911	424.1928	271.8034	767.3617
146.0863	123.1535	48.03875	146.9498	160.704	97.38223	315.7587	49.21048	88.70654
166.1701	293.3075	369.446	229.0353	357.409	152.1605	214.7134	109.2929	148.5308
27.55841	48.47055	24.2091	25.58823	22.07236	19.27881	24.29489	131.2119	29.92023
168.7381	253.4382	266.7058	167.0268	211.1212	393.5853	264.2102	527.5382	236.2074
1034.363	1587.644	2426.638	1586.571	1521.67	1765.032	2000.192	1242.226	905.5981
1.309693	4.088247	4.147743	5.479561	12.93711	1.018314	3.486722	0	4.119906
17.58479	51.21832	11.30752	22.76832	45.3566	41.59186	46.06028	70.14614	33.01318
12.59792	7.080898	0.378972	10.0741	6.481222	44.63693	4.293492	9.143904	5.169969
973.0453	1462.78	976.2121	773.4822	1702.3	479.8085	675.4052	1080.602	797.2891
37.56865	67.90671	86.70805	42.56837	28.35021	111.5812	95.61542	57.69808	85.61117
48.78502	45.78962	150.7815	31.26478	17.42264	31.4514	90.41218	158.0537	61.89089
33.80879	31.96623	30.71478	34.07167	23.65261	14.20752	9.506053	78.57787	36.10956
0.082318	22.30507	0.380242	0.136492	3.342503	40.58018	5.162292	0.688334	5.169889
30.08568	62.41893	54.55545	65.18392	33.04376	73.0402	62.54205	37.64261	94.89748
681.7991	352.9276	402.4587	315.3331	488.743	605.578	271.1965	293.4179	292.9258
0.07745	4.306868	2.615628	10.03022	6.46298	13.19054	1.686481	7.616608	1.04333
0.080499	11.22798	0.367321	5.829026	8.04493	12.17837	13.8578	11.45937	14.45184
16.3089	19.46243	6.959232	12.86625	17.33134	56.79472	40.89541	11.46932	9.294969
47.4427	37.40051	47.51433	31.18043	22.04028	64.91447	43.4672	70.98311	34.04097
7.591584	22.17846	4.794652	10.04445	6.471817	9.134792	19.9747	16.89372	4.138658
25.07713	22.29714	9.149055	28.42827	31.4335	71.00648	13.85268	36.12828	6.201657
21.26568	33.1394	34.35989	24.05862	40.37696	39.5552	21.71736	10.70168	17.54082
870.7303	1623.602	704.0245	903.6814	892.1958	700.9458	1392.551	1418.62	690.0322
12.59579	31.94299	9.147714	11.48636	4.917358	43.62081	26.03403	56.22283	16.51542
800.6419	512.1755	234.2547	975.4759	779.428	556.8988	778.891	364.9963	347.598
323.5679	296.2826	229.8302	192.4487	250.1061	255.629	259.0081	375.8241	267.1458
34.83438	98.32682	11.14237	82.30998	108.6926	17.24591	23.4867	67.46983	14.44565
0.077489	19.40379	13.27168	1.573108	1.778947	13.19057	8.646699	13.035	10.32415
73.76596	130.0914	78.22351	72.22836	67.34184	35.50941	78.20196	87.75356	59.83277

34.93655	26.32152	42.8691	38.07834	25.04609	43.61287	29.55781	4.524782	39.18539
20.08567	19.53198	13.47726	27.01427	25.20286	60.86372	23.42032	63.92261	20.64089
2.591257	40.29336	26.47427	46.81995	40.84116	16.23636	72.98226	44.58044	14.45334
72.55773	90.13137	156.2956	99.15402	117.3924	37.53842	163.3763	192.4698	162.9756
114.9425	92.81375	63.16765	72.23653	70.46899	80.14022	80.80836	66.93176	81.49024
68.76604	121.7738	127.5941	216.0159	182.4827	27.39447	99.95694	83.13838	115.5181
8.840421	177.272	28.63812	11.47567	58.04413	93.32931	90.35064	244.8852	51.58467
27.5597	19.52418	19.92205	49.51787	37.61141	39.56376	43.44484	53.13734	35.07583
13.82213	9.835331	26.07799	49.27702	23.5126	37.52941	17.35041	13.01424	17.54244
53.65756	114.3069	91.92823	97.1039	74.62698	53.7592	100.9933	58.60632	34.04081
122.4991	106.7179	145.3835	148.587	25.23441	64.92675	180.7813	264.9441	129.9691
146.2073	135.745	82.71181	107.6019	34.61173	278.9532	208.619	348.222	134.0931
347.3117	476.1493	264.474	362.0631	453.0716	251.5722	452.8816	447.4526	390.9131
213.7046	2526.099	565.5388	1070.569	1165.62	1059.024	1091.751	1885.307	936.543
17.54472	20.82088	0.355183	14.26195	6.474451	39.55589	29.56455	26.95631	30.93919
13.82702	23.60086	11.25973	15.68668	34.34541	19.27712	26.93195	24.60755	25.78995
7.599947	59.54909	22.10486	32.6575	25.2067	24.35076	32.1196	42.30204	21.6723
0.047958	5.324212	2.251683	1.489658	3.081041	4.05332	1.718571	1.497376	3.09134
0.07702	4.305495	0.34352	1.573136	0.170968	2.034533	1.686438	3.756672	9.292895
7.437763	1.50737	6.388338	2.926863	6.20408	40.50149	10.51431	2.24239	7.21344
8.830132	7.064651	15.37193	36.54214	11.10205	11.16252	20.85984	19.23773	13.41605
133.6614	127.3581	147.1447	149.8539	82.94458	87.24068	155.6133	190.3034	90.77222
69.90151	47.10049	43.47135	48.11437	18.96339	57.81949	63.4713	83.27005	46.41748
414.7611	570.2593	508.8415	551.4722	551.4694	217.084	443.307	716.3034	324.9065
83.76321	196.444	263.3594	127.3	114.1286	78.1121	108.635	217.2758	96.96083
2.596642	12.50559	4.751781	7.199544	0.166104	4.063109	16.52254	30.98701	8.260249
15.08202	1100.343	30.76213	342.352	343.904	391.5582	387.6454	103.8742	122.7526
182.3803	189.624	265.9083	144.2975	313.7005	157.2315	161.6706	178.6702	180.4996
96.21937	113.515	76.0623	152.6282	89.14786	219.0977	168.6792	78.50254	122.7387
28.71064	34.50812	23.87924	36.63515	21.93016	45.63873	57.5021	44.8019	16.51014
81.29903	144.0826	184.3428	457.992	117.3813	44.63921	220.7669	513.8184	80.46409
63.81022	164.7925	149.6708	107.6086	106.4264	80.14218	137.3077	93.87455	62.92984
108.7224	91.45285	112.7759	176.6936	120.3674	65.94014	60.80069	120.8852	112.4303
37.55998	36.12264	26.44098	51.01107	14.29984	5.077779	64.3053	116.3679	17.54752
22.53187	30.45943	15.51807	12.8722	14.24716	22.31928	53.96884	49.39337	22.69713
43.76175	95.30623	26.37271	59.38013	28.30136	42.6068	114.8499	43.08904	54.66653
33.8285	44.43618	58.81993	70.80722	50.18892	80.13947	40.80487	125.5537	50.55069
10.05203	16.60249	64.20451	32.20503	23.25381	5.077319	18.26696	4.53203	14.44212
22.33615	25.97944	0.31858	60.84039	44.13103	1.019829	49.881	11.54238	8.257882
27.57297	52.66236	88.52418	62.24854	18.97703	27.39374	63.44813	83.21186	43.32765
10.09526	42.89121	13.43537	14.29692	17.37963	39.56127	17.33958	50.8761	12.38951
162.0248	58.14268	135.1782	107.2031	90.38617	238.342	103.5191	31.50626	128.8977
103.7638	240.8158	212.2045	114.6661	59.59876	157.2327	285.1484	484.6637	107.2783
87.44731	48.56145	108.0836	62.30626	620.1696	138.9649	98.23495	88.5645	187.6909
113.7783	256.187	119.5657	289.9896	92.41353	269.831	618.073	492.1333	119.6582
41.2641	31.94007	24.21486	24.18148	25.1849	3.048909	13.85405	49.27429	50.54137
48.71419	71.8272	39.12085	55.10179	82.47717	79.11472	19.07606	79.44669	116.5129
23.83083	70.58813	75.70244	76.32861	48.54592	25.36515	32.98844	43.84201	22.70371
48.73371	85.63791	49.85764	56.54597	74.81849	43.62051	41.7043	87.14158	29.92031
10.09683	92.81289	28.63804	25.62974	9.605641	25.36583	99.0748	43.8086	22.70482
37.55101	30.5874	35.01725	35.48396	17.41874	16.23624	59.09392	149.6202	53.63941
22.5879	81.82628	28.63461	27.04032	11.15493	87.24321	63.40006	83.8512	108.3118
17.59198	36.10939	45.74537	41.12226	22.09576	18.26493	38.20822	67.76596	21.67252
28.77486	60.73553	89.68329	33.97418	40.60154	29.41961	28.66219	72.55511	11.35821
1061.608	721.0689	1721.329	1005.13	3431.159	811.5045	482.4266	809.5115	629.1654
65.06272	56.91384	19.94281	89.26229	29.91136	85.21465	432.0085	325.7201	467.2366
49.98889	69.14822	101.0474	135.2744	57.80012	58.83413	106.1406	110.3067	81.47209
118.7054	69.33813	89.07886	77.90053	89.19873	67.96884	79.9326	150.9536	107.274

45.06466	99.70897	63.1495	35.52003	54.87337	78.11126	69.50386	326.8826	37.14394
33.78783	27.80061	43.45089	29.8131	40.70987	46.66303	53.02476	63.95834	21.67124
1017.789	1076.407	921.2656	1211.212	3344.322	509.2204	577.2211	219.4209	340.3746
308.4812	213.1861	182.0487	203.6616	321.6621	483.8496	224.2655	305.0359	164.0027
38.8007	34.73297	120.6672	55.22134	26.77301	53.76523	101.7412	43.06505	41.26605
15.08142	16.74406	15.57344	10.06674	11.16026	57.8148	37.37366	62.48923	24.76184
101.209	92.79896	91.12599	96.21205	47.07624	110.5689	118.2187	183.3954	118.6138
43.72645	48.42574	11.29847	74.7309	172.0055	77.08529	28.65384	17.62902	18.57687
13.84053	12.61371	36.97908	10.07096	4.918671	16.23572	17.33599	58.5747	15.48361
237.4378	519.1342	132.5643	380.504	292.3459	448.3625	545.8746	855.6765	606.4802
10.09307	7.08017	21.94636	8.653406	1.776704	3.049027	21.69758	29.23602	6.201713
35.0716	43.04189	67.35515	111.6584	25.22892	29.42301	73.86624	161.0888	29.92388
243.5347	112.2233	119.3756	162.6608	189.0402	91.29961	220.8073	161.707	207.3142
18.84407	66.57431	9.131333	14.31496	26.79974	45.65323	51.23503	98.52611	31.98752
0.07354	9.748285	2.594344	1.570985	1.774744	7.104569	10.41518	11.53717	5.167683
544.6361	467.8887	141.2134	244.7739	339.1548	195.7822	400.705	633.094	263.0233
13.82607	19.47487	15.52195	25.50887	11.14647	42.60052	38.26749	26.93129	16.51228
155.0013	413.9902	329.5227	503.5138	412.6209	445.3202	288.5441	320.2991	351.7265
1338.371	582.4747	784.4764	575.2476	1039.341	498.0574	474.667	345.8145	751.8713
32.57623	130.0125	19.97843	29.86166	43.93723	9.13553	7.768905	275.3113	22.70457
68.75488	85.85235	118.8919	135.625	75.08049	151.1374	39.93826	86.23774	109.3281
367.2515	500.9552	476.0424	617.7073	1111.317	340.8346	132.0776	465.2235	213.5132
93.62534	128.4738	109.9142	86.18907	135.4976	118.6754	54.74278	63.13499	78.38565
93.78383	85.97143	78.41742	83.59528	72.09707	123.7601	96.43768	264.9327	60.86729
472.2711	1007.709	835.9438	767.9221	726.5906	1030.618	845.7671	1216.083	537.3818
26.33154	70.63242	37.20651	46.78169	43.91418	89.26518	58.21392	142.6134	23.73556
14.99191	5.670726	6.859382	4.397166	1.777143	19.27054	13.90098	16.9631	22.6798
166.2187	249.2175	126.0147	363.3837	328.0829	112.6026	132.0793	138.5448	228.9827
6.337389	225.7369	22.11863	79.36404	58.04453	38.55274	78.17747	81.53815	43.33307
500.9832	660.3134	777.3667	766.4544	693.7293	315.4797	631.0583	596.0506	504.3746
27.56089	36.07775	22.07261	14.31055	14.29098	105.4856	30.38592	40.00459	37.13819
22.54308	24.98839	13.41126	31.14051	3.351755	32.46108	80.11257	49.36335	20.6368
81.19164	51.30592	60.81333	80.59687	81.22912	103.4641	79.10229	38.43215	59.82795
1152.751	28187.22	1416.579	868.0397	1380.326	415.901	659.7885	324.9436	373.382
121.0758	11.23686	50.09907	214.3634	64.13908	111.5785	78.22901	26.86439	214.4925
34.90707	61.83677	36.43412	43.60625	43.43596	39.55494	52.26675	19.21615	32.99858
164.9937	355.8568	136.8942	292.8603	239.2479	382.4283	349.3995	1031.305	282.6216
31.32555	33.36535	60.87618	35.5039	34.57936	19.27945	57.33954	115.575	44.36135
13.7954	23.49689	9.045052	11.42865	4.908827	19.27411	26.10189	11.4876	17.5376
43.82145	240.6369	69.66633	75.07322	111.011	22.3227	96.45854	151.73	111.3987
17.59485	40.29821	15.64816	25.63051	15.86154	4.063071	42.54074	43.80623	12.39028
44.90704	44.2031	38.84352	36.73809	57.49198	29.41848	37.38891	9.920454	41.25164
124.9684	139.8608	168.9288	209.2284	70.5113	169.4032	190.3739	305.8822	132.028
16.3292	51.1427	28.38624	49.42822	20.48612	56.80139	19.08023	53.96661	22.70034
66.05914	27.75332	45.30257	22.73292	45.22619	27.39115	39.99122	29.22971	51.56255
61.23426	98.16477	41.45765	124.1906	185.1448	679.5178	22.54862	87.83795	28.89131
8.768576	8.352103	18.81729	12.66478	22.92671	15.21101	12.18566	4.548181	10.3143
52.56032	98.35937	138.5992	132.9442	68.9267	105.4989	112.984	120.888	86.64792
10.08548	63.83386	80.60427	42.59184	31.47115	180.5666	89.47803	43.03398	335.2216
264.6431	248.9115	551.1873	864.7815	478.4147	269.8161	85.15291	78.48875	51.5832
22.44111	46.56979	44.13377	32.27359	55.28186	49.68418	42.73838	28.59823	29.89554
37.57597	52.73656	28.63693	55.29315	23.67444	53.76763	102.5558	248.1761	79.42737
1.332253	5.541511	2.478085	17.74862	20.77919	0.003779	4.32913	0.688611	0.008232
17.59082	41.62049	56.41605	81.94108	23.64885	50.72169	45.17325	46.16167	33.01561
50.03235	84.42963	90.85389	35.49823	25.22221	62.89441	146.1163	156.5082	95.91869
296.1295	347.5279	495.9284	200.9555	312.6214	589.3586	388.5303	599.9562	288.8086
149.9836	203.5674	337.7113	272.9565	84.59605	141.0048	368.5811	588.5546	207.3236
143.7385	293.4597	188.6699	134.4817	165.7715	113.6168	301.6304	419.7965	335.2108

4525.621	5309.447	4412.062	3945.109	9802.227	3648.747	3091.097	2376.674	2676.545
8.846572	45.82591	2.591934	2.983356	40.84368	71.01131	6.901217	2.993534	9.295807
357.3036	312.9109	299.0735	814.2599	1281.808	852.0757	513.7406	92.32055	655.9761
16.31985	20.86008	2.616907	7.240812	1.777086	17.24906	22.57129	26.92189	21.66746
1452.753	1107.371	1480.801	1283.961	1476.285	693.8447	1167.409	1305.415	1236.677
224.8285	267.0495	266.0248	382.905	175.0287	192.734	289.508	240.3212	182.5641
53.64878	26.38585	36.8711	64.84369	31.32872	39.56117	46.08591	12.23122	82.48643
103.7436	109.4387	65.38547	77.91274	50.22042	80.14132	238.2218	228.8034	153.6838
13.80244	9.817952	9.06169	39.35465	109.0028	23.33052	36.57184	9.161131	38.14579
387.2612	51.26301	333.0651	98.79432	150.8703	55.79238	31.25162	27.64565	42.29525
133.6338	110.7485	84.66162	53.87516	106.2717	23.33705	42.54358	107.8086	59.83247
97.51355	92.86564	145.2572	76.51161	29.92484	83.1848	199.0647	254.2101	129.9662
23.7535	7.073569	11.22367	28.26458	46.54466	38.54187	25.20332	5.295752	27.8475
10.03455	2.91589	15.07285	9.968086	26.16546	12.1736	16.53657	6.863771	8.258935
55.00853	37.49488	52.20192	60.85838	20.53952	20.29366	62.57173	128.7501	39.20373
88.58069	88.41852	101.0344	74.84434	156.9377	72.01852	59.1137	63.94459	123.74
335.9849	153.7551	229.6437	344.9524	295.2425	141.0043	276.4244	120.0627	290.8603
3.844546	7.046013	6.873457	7.203368	3.343272	18.25758	8.655704	19.28707	8.260614
159.9467	137.1381	145.3574	310.9857	140.7535	73.04164	67.74879	148.5834	69.11817
15.07651	9.842682	9.125614	19.91245	3.351705	29.41888	44.35763	41.61925	22.69871
55.01728	20.92163	50.09243	27.02927	34.56906	29.42271	25.15613	114.8289	40.23561
20.0819	860.8937	26.43506	19.95497	123.6579	499.0812	79.04756	91.55095	86.65286
16.27467	8.437546	19.53387	10.02724	79.85492	5.077674	26.10304	2.986054	61.83299
46.29251	4.305172	6.972238	12.90314	8.045898	55.79458	21.67705	20.69778	13.42196
271.1019	243.6861	307.5141	256.012	165.7825	156.2204	265.1053	368.1509	276.4254
47.56303	81.75995	63.16604	97.63881	29.92016	98.39786	102.5543	113.9645	78.39635
31.33055	52.72329	15.65133	58.09993	23.67184	43.62389	72.11945	108.5941	48.48773
53.81548	123.2755	117.1953	107.5805	84.55316	149.1167	128.6242	245.7425	117.5898
228.6828	492.775	188.8049	195.2942	33.03753	700.9372	365.9331	2976.147	265.0855
398.6191	43.00689	60.72872	27.02038	115.3131	130.8455	84.3394	26.86974	141.2782
19.98676	12.54855	15.33581	18.40557	12.62989	14.20414	13.00949	14.59206	11.35416
50.06178	69.33032	69.65082	39.76245	33.04385	71.01155	58.19371	210.3514	31.9874
3.850065	11.22091	2.616942	10.06166	17.35765	8.121002	8.639196	17.64343	15.48189
107.2322	117.29	43.46837	250.3435	357.9179	88.24464	92.21348	44.63949	34.04485
0.08129	38.82518	0.372896	0.134615	0.182233	6.092376	2.555992	2.988136	2.075019
91.21178	73.4396	93.19139	69.38337	18.9874	20.29389	171.3112	253.6604	71.17507
786.4809	376.3284	2595.522	286.9976	669.3632	136.9464	644.3074	665.7587	107.2791
17.59468	18.15788	9.144895	27.0357	11.17405	18.26511	57.33725	28.40274	28.8925
20.06859	22.26307	13.44281	26.96713	11.16391	50.71726	6.898766	13.77267	30.94852
7.595763	20.84261	17.63235	21.29339	6.478479	25.36131	26.06494	22.29323	8.263991
11.33256	15.34064	19.73631	8.646489	4.917353	14.20601	17.35075	75.005	17.54235
113.719	72.10639	48.09919	93.43636	36.16937	85.21271	73.8437	18.38525	106.2433
5323.691	4319.751	2273.374	1580.993	5083.131	1412.03	2285.284	3030.607	3424.315
81.19116	65.10443	54.37655	149.5897	219.4207	73.03666	138.2912	45.3744	317.5925
613.2641	884.1677	422.2328	528.7948	1364.397	371.2672	558.0914	1108.548	318.7158
86.2197	85.86175	245.4326	234.2796	96.88145	109.553	126.0664	43.04614	105.2045
258.5187	137.1143	125.8636	125.953	343.3677	181.5761	180.7977	89.25818	315.6007
21.3212	36.05358	62.52037	66.33808	23.61352	31.4493	39.97086	152.933	17.54604
1082.221	376.3228	391.3043	949.1389	472.9223	345.901	327.7477	190.194	307.3575
2.591753	15.39014	19.98228	25.62788	8.042033	20.29391	13.85223	174.1547	37.14368
53.68694	38.80285	24.17283	70.53968	93.28257	103.453	80.91738	34.6159	86.618
18.84021	47.1497	26.4193	70.70547	12.73674	16.23624	77.37245	113.3205	34.04737
28.38639	23.16544	6.732303	75.47725	95.82354	1.019781	6.928412	2.99616	1.043203
0.065821	20.19991	4.544164	2.948197	9.241879	10.139	0	0	0.008556
0.076423	9.798062	0.339545	7.213753	9.536512	6.091672	11.26867	6.848796	9.292199
108.4269	31.92546	28.46406	111.2733	101.0594	50.71898	73.9391	25.34404	115.4837
6.346988	5.693448	15.47649	11.45627	4.916362	1.019796	12.99645	26.95227	14.44929
122.4724	123.2739	112.8775	100.5181	82.99202	191.7184	147.7577	149.3823	78.39928

22.58068	153.8043	26.43328	87.84612	39.28199	68.98431	69.48625	293.3451	251.6794
198.3889	361.6697	32.92669	300.3968	261.6003	490.9066	73.86711	346.3518	240.2801
3.604153	0.05314	0.12838	10.16406	2.928489	0.002276	0.848518	0	1.035249
6.343608	4.308143	0.349484	4.409641	0.173291	4.063451	4.292489	8.387023	3.10713
945.6642	616.0801	805.7128	613.8345	771.9114	866.2896	636.2571	875.6148	710.6598
0.078623	0.120555	2.617726	1.572647	0.17517	11.16321	3.422911	6.06713	2.075402
23927.2	22018.64	19778.51	20446.53	27936.65	14283.57	26239.8	19538.42	17386.66
183.4665	154.8713	275.4355	175.1055	305.2288	131.8671	108.6636	164.154	80.45575
105.0225	124.7056	149.7308	203.6938	148.5806	160.277	208.602	147.8019	91.809
199.9	149.5918	279.1871	459.278	87.7048	368.2169	293.8286	429.8835	160.9095
273.6367	431.8877	251.5084	277.2692	173.6264	291.1328	713.713	902.0164	294.9951
1157.977	169.0291	50.25228	551.6152	115.8402	926.1372	358.0843	224.7897	240.3346
5.089053	109.4952	4.757344	2.976849	40.86032	124.7747	115.5631	539.2649	73.24423
83.30746	24.95519	120.2466	25.49122	15.78637	24.34698	45.25619	7.608293	62.88768
62.42653	49.84836	60.50917	19.95159	31.39706	154.1633	75.66811	108.7886	43.32392
10.08332	18.07393	9.090495	18.46495	9.579722	16.23377	10.3842	4.52519	13.41811
58.77412	105.1657	69.49867	82.0551	104.629	20.29385	96.4896	172.6686	51.58053
25.09043	85.93004	26.47981	52.48233	15.86053	79.12669	71.23483	69.2342	40.23861
117.3162	59.57853	95.05085	55.22725	74.99704	193.729	55.60808	17.61661	108.2892
35.08366	167.5705	17.80066	73.69942	26.79747	8.120685	109.4808	59.97511	83.55769
20.09363	81.76848	56.71304	65.18414	33.04382	92.31237	72.10852	107.0168	21.67341
6.337979	8.430362	2.612878	8.617547	20.28076	56.78151	14.75856	2.216985	23.71684
1777.56	1428.513	1476.735	1395.731	1860.65	1402.899	1126.531	969.561	1802.928
30.08473	52.73628	13.48126	10.0704	23.6744	53.76762	47.76009	111.6548	46.42601
165.9652	66.50869	112.3605	87.67782	172.9824	582.182	68.65026	23.00879	113.4501
2.598464	7.07644	4.800605	8.646899	4.917485	4.06352	9.511194	5.294959	21.66556
75.05637	264.4651	260.0894	169.8345	151.7519	222.1549	212.0596	122.3619	132.0346
1236.735	1719.188	1089.385	2443.45	1332.748	2984.322	1704.602	1487.895	1124.26
294.8289	139.9417	255.6935	281.4516	101.7762	221.1398	606.8233	476.0225	259.9241
18.83853	60.92318	28.55196	34.06509	14.29634	69.99197	18.20091	73.95691	21.67224
137.7749	49.59828	4.798937	123.1841	78.82021	7.106521	23.45539	31.59801	47.42845
154.8451	123.1887	108.3504	104.6824	100.0575	27.39455	82.55078	97.00614	103.1458
80.03037	146.76	117.1647	123.1022	78.30559	117.6719	249.5257	223.4004	128.9333
64.98273	71.99253	45.77541	69.31595	47.0119	88.24995	148.7457	170.4427	45.39106
1.344476	9.790466	8.990869	4.401998	11.06131	7.105594	18.26088	13.83375	7.230536
16.31332	13.97005	9.112289	8.648622	1.77775	21.30511	35.65442	46.29749	22.69696
5.061278	8.329475	16.67497	4.36294	1.765853	19.26179	0.820549	17.87253	0.00904
10.09137	26.35574	0.362251	5.827821	6.480822	65.92394	33.03048	34.66259	6.201657
2.597626	5.678752	21.46793	147.0495	280.817	9.133669	4.294519	1.449109	4.138126
54.99581	18.15187	62.84772	29.83886	3.345291	40.57928	68.67316	77.81187	87.66382
31.31201	67.81808	22.10391	45.33872	43.87196	24.35074	97.40521	56.19644	17.54716
18.83024	33.30587	4.80288	11.48434	14.28641	17.25012	30.39033	34.6092	14.4526
25.03196	15.35778	13.41346	14.28807	8.041359	53.75666	30.41152	115.982	16.51313
50.04832	84.48343	30.78134	56.68408	54.85035	37.53782	124.3248	56.15285	80.45586
13.82268	16.71746	19.74413	10.05532	4.91758	19.27669	12.99407	30.03735	27.8499
0.076105	13.8938	2.610472	2.992201	12.59977	3.048956	13.01711	34.05794	13.4133
63.74806	70.63415	35.05687	179.1872	118.5472	44.63736	113.9105	63.11394	118.6033
93.55309	62.25493	39.20742	97.32332	183.2205	84.18837	72.17792	14.53797	91.78058
5.100341	2.92288	2.61689	8.652027	6.481217	20.29154	15.6028	5.294297	16.51291
210.7322	197.4188	312.8529	249.4634	442.7117	66.95083	146.1328	87.82278	122.7244
20.04459	16.72207	15.51553	21.29719	6.479052	23.33332	12.99301	16.87659	12.38835
228.4895	101.1278	58.88776	97.6657	92.31643	80.14088	94.71749	84.65184	156.775
3.84752	5.695489	2.606612	10.07405	0.183579	112.5882	16.46172	11.45414	3.106715
1.338736	27.84365	2.595148	0.137278	3.338757	11.16434	5.163346	43.81388	18.57912
134.7136	107.7929	11.31644	115.7264	274.9959	65.93557	25.15878	8.373853	92.81961
185.9675	101.0536	149.0055	96.17957	171.5608	91.29611	115.6221	78.51599	103.1426
12.58477	15.35665	4.803624	7.241132	22.00727	5.077957	6.899408	41.62216	5.17027
0.066924	10.94503	2.527788	0.108915	4.796582	1.019602	35.17423	0	5.162469

904.4238	1119.787	5568.706	4758.485	1063.918	1107.71	1274.351	615.2897	803.4844
0.076096	58.75754	8.997089	10.00747	44.60316	34.47818	8.652732	9.175039	23.71445
223.5679	307.0985	278.877	236.0723	424.3589	207.9483	200.8042	110.0676	218.6583
182.4273	80.43598	89.22225	185.3203	37.73612	215.0516	263.3886	190.187	158.8472
20.0128	18.05504	25.9059	26.82727	21.90108	21.30311	35.69058	6.068792	24.7531
57.25473	46.86379	103.3989	26.88053	12.685	24.34663	62.71813	8.380979	61.85475
36.30904	76.13303	60.79579	32.67068	29.89183	65.93663	88.67932	63.12325	58.79633
383.572	299.1215	429.0241	364.9622	165.8362	591.3898	956.2095	1065.96	462.0859
60.0289	81.72246	63.09054	49.62882	20.54882	47.68108	123.4544	174.9538	78.39347
1249.823	214.643	1084.673	871.9281	969.2831	234.3265	581.6078	510.7003	791.0673
22.53409	23.5987	24.01212	15.68542	29.71137	56.79679	21.7036	65.66049	22.69742
53.81818	171.6871	84.86657	96.2976	101.7331	49.71103	80.79205	224.1262	91.80777
7.584846	562.0297	9.083114	5.80372	48.66017	289.1054	46.01753	0.691747	13.4202
3877.374	6137.185	4535.613	5308.195	13568.59	4338.529	2532.138	3353.257	2982.878
13.84703	84.40919	54.36571	36.90274	18.98137	35.50844	34.72518	31.49244	19.61013
996.9094	1990.456	1256.055	2466.073	775.0843	1046.851	3837.857	6648.348	1232.559
15.08714	9.849338	34.83821	31.19946	80.90172	60.85972	54.78168	12.99931	128.8796
344.6636	103.9435	608.8376	262.961	262.3906	190.7063	246.8796	174.0185	259.915
22.57794	15.384	26.38166	17.13594	15.85271	17.2504	21.68179	43.85718	11.35899
480.6273	530.9671	584.7677	394.1856	330.9022	238.3787	144.2723	519.4034	339.3204
104.9834	98.36891	164.484	331.897	176.4719	298.2185	121.6775	35.32967	232.0574
0.036308	1.402918	0.129226	2.708525	1.570144	0.002288	3.55404	3.191576	0.004963
54.89314	49.761	83.42013	81.66626	134.7591	62.88558	80.07819	13.00268	67.02583
27.54251	78.65165	49.68177	41.02573	49.93359	29.42037	100.9775	36.9426	43.31997
7.600209	22.27988	32.75293	29.80745	6.48416	11.16429	46.93276	22.25438	36.10604
127.2558	47.14938	35.01681	84.78368	88.943	75.06404	78.24314	12.99435	90.75864
1.336949	4.29667	4.767234	7.232027	6.466444	23.33705	6.034241	10.68893	4.137498
26.3352	62.37999	52.31088	55.2653	17.42492	21.30822	107.7993	157.9979	39.2056
46.29215	103.7444	6.972356	301.6473	264.5235	39.56591	117.3906	9.91403	67.04699
469.7592	622.9517	740.5952	172.6884	139.2795	1103.65	455.4526	1287.78	671.4617
168.7323	521.7993	344.4641	386.0882	181.4323	212.0118	587.6453	734.8308	285.7124
10020.61	16302.12	10653.54	9639.333	26583.39	10068.79	6413.435	10504.32	6540.247
15.07191	34.58558	11.25994	29.71934	17.34983	10.14949	53.09048	48.60896	36.09718
70.0348	124.6116	22.14928	35.52498	34.60633	56.811	134.7295	113.1785	70.14708
80.94048	24.99413	51.58496	53.5798	103.7741	56.79921	33.02408	8.376436	80.41918
22.51877	20.82939	11.23427	8.644624	9.584814	14.20585	39.15576	47.87673	17.54188
8.795877	4.290741	2.596207	9.963152	6.416368	6.090817	8.664608	15.431	8.258637
47.46227	64.93394	41.2231	55.08677	83.98949	62.88791	56.52467	36.16336	20.63919
116.1908	188.1107	91.17199	113.1621	176.3791	162.2991	174.7568	241.9922	109.3344
18.7412	11.17331	25.69787	18.39429	20.28698	22.31479	26.98707	2.21696	39.16813
17.59281	22.30192	39.33023	49.5878	28.33223	52.75108	56.47911	53.8656	30.9541
122.3883	63.77335	43.72255	122.9717	54.85427	111.582	176.5304	198.8663	172.2318
6.346975	13.95906	2.617832	5.824818	4.916348	10.14917	4.291866	22.30487	8.263689
415.9921	307.3754	532.5031	494.9038	470.2445	189.6956	286.822	337.283	463.1095
8.847051	11.22647	21.97555	39.58826	8.043981	37.53267	28.66221	29.2269	20.63787
7.599065	20.87774	21.98141	36.78597	11.16055	17.24955	35.6306	87.25744	18.57599
369.7445	276.8928	568.8631	223.5234	321.8653	408.7962	525.0826	608.5548	411.5341
12.59807	2.916106	17.81011	5.825255	3.342608	19.2794	0.822482	0	4.138234
281.0459	390.1819	510.0336	754.4125	859.6511	189.693	276.4314	437.5757	225.8846
25.05346	22.26751	13.44816	11.48131	12.72314	21.30681	16.46693	43.89723	19.60786
8.814529	11.15241	0.33454	5.804819	1.777929	5.077357	17.39046	9.179017	6.199662
22.57292	5.696617	2.610386	10.07273	3.348244	15.22163	19.07387	34.60318	15.48403
223.5708	189.6367	151.7339	142.8947	206.1921	91.29965	169.4942	160.1641	138.2169
251.8623	145.0865	110.088	232.6354	452.1656	280.953	191.3908	23.78149	111.3843
37.5639	27.83685	50.1294	56.66289	75.05076	333.6974	247.9431	51.53662	184.5965
41.32085	33.37918	24.31183	28.45609	36.16155	55.79627	70.37256	63.07944	48.48863
17292.97	16183.09	16048.08	15173.62	22207.84	7697.161	12954.74	10706.11	12792.71
212.4414	448.4749	227.7077	403.0394	442.1344	200.8536	199.0149	657.0214	165.0397

21.31426	23.64084	34.79012	26.96736	3.350465	26.37767	33.01168	87.22545	11.35846
17.57204	26.38078	34.72619	19.92347	29.77165	8.121096	22.56539	113.5973	24.76163
8.78948	4.287306	0.318586	0.120012	0.161055	8.11816	1.687109	4.54018	14.43629
40.06665	59.62802	37.25415	19.9735	34.59161	72.02453	62.55177	63.09187	82.51852
579.217	510.2711	41.63377	340.5687	860.7306	811.4708	235.584	56.89644	225.8794
3485.008	454.0353	1007.99	810.0557	1565.927	628.9172	643.2798	408.9214	1099.467
2.598638	1.531163	9.089731	8.641262	4.915473	13.19147	6.031373	15.34183	11.35639
294.7765	156.5126	214.4875	175.4339	114.2427	205.9226	328.6075	243.3588	149.5652
77.52382	113.5607	19.98311	32.69868	40.85002	60.8684	98.19748	159.439	62.92796
30.08659	105.2793	130.0326	79.31645	39.29137	58.83984	92.97644	127.8185	46.42679
752.0738	162.1046	28.57402	29.84763	81.45867	848.0314	258.1166	167.7997	501.2836
305.8749	232.4222	218.3976	212.0218	210.7926	288.0774	190.382	53.04854	153.6839
87.36711	78.82478	214.0487	84.73393	110.5809	82.16199	80.86952	73.19259	36.10862
94.67343	104.708	121.5054	127.9375	251.9929	40.57574	19.95028	12.23021	40.22629
210.8527	102.4059	84.53089	131.3833	169.9268	124.7661	122.5951	70.81371	114.4831
8.831724	12.5621	11.17572	4.408998	1.778942	14.20476	8.645884	20.78419	5.169777
122.5192	495.4787	95.75527	207.9989	75.22959	203.8964	442.4593	2159.638	233.1108
4.820541	1.429189	8.815203	6.579549	5.727256	6.070661	4.429066	0.716786	6.153644
119.9574	242.0507	110.6431	155.5407	245.0056	194.7592	193.8721	275.0953	106.2432
55.05041	131.4807	22.14814	163.9298	470.3957	11.1643	116.4746	34.56205	34.04997
2.598658	11.1993	4.793392	11.44622	9.573123	3.049056	7.772937	23.09539	5.169958
0.069834	88.41888	4.643231	7.125622	9.364404	41.54764	13.95786	2.999786	7.223954
32.4849	19.48283	36.73474	24.1183	4.918739	30.43259	53.08339	48.59774	10.3266
0.080926	26.40628	13.45301	4.412287	4.918757	11.16422	24.29953	18.39982	2.075104
27.58826	77.66342	147.4981	5.813743	12.72604	18.26498	5.166013	176.3308	57.77284
67.52026	226.6733	108.279	76.44767	93.79928	25.36576	81.68634	155.6429	40.23758
42.49177	22.27489	2.612262	31.20577	11.16833	20.29282	5.160724	23.02935	14.45248
52.55799	120.4534	56.71172	63.77161	9.604788	47.68177	128.6458	284.3984	51.58302
42.53862	81.62654	26.42035	45.35173	11.17465	33.4796	114.8048	148.0726	43.32805
72.53878	135.6961	102.0562	113.2127	42.41575	67.96914	167.7693	282.0101	123.7762
1242.652	693.3904	1010.305	870.8637	954.2117	761.8001	475.4734	539.1061	630.1961
128.7226	77.65625	128.0182	114.6534	23.67413	175.4901	191.2344	177.8894	217.6279
5.10043	31.86384	11.27343	19.91333	20.46293	28.40482	10.37893	21.50504	15.48218
1.343703	9.74737	0.320498	11.34329	6.411902	7.104546	19.1717	26.37425	6.197995
640.3926	743.8456	104.31	651.1482	360.5837	1046.796	243.4051	43.0301	672.4296
156.1928	157.8664	115.1037	86.40982	131.3775	258.6664	258.1902	411.4351	172.2521
71.13838	33.31563	37.04705	29.81349	26.73622	50.71984	64.34579	74.77699	45.38591
48.70049	13.99273	24.15685	88.75189	57.67953	46.6613	64.3703	29.98546	81.46152
130.6089	8.462955	89.56814	77.40532	79.15392	50.71528	18.21276	22.27461	77.32879
131.2316	77.66388	143.1856	134.4472	50.23135	231.28	314.712	234.1328	211.4433
47.15996	20.73288	70.86369	14.20581	9.539881	20.28685	27.86484	4.529038	40.19671
15.09393	31.95775	34.98093	14.31394	18.97282	43.6217	32.12122	42.30648	21.67212
62.14666	22.17399	50.9846	43.58925	24.99215	22.31747	23.46556	30.07323	25.78458
328.5579	178.6889	130.3639	140.1504	448.3094	361.1225	120.7747	182.4493	313.5571
96.74712	9.820758	131.5479	149.2524	93.8203	11.16284	1.686577	7.613667	9.293931
6.350521	15.37727	2.611922	7.244414	6.484196	5.077941	8.637134	15.31182	5.170247
45.01174	40.22492	26.38141	49.54059	15.85267	69.99077	53.8854	77.06774	30.95238
219.8762	232.563	119.4713	166.9542	317.0324	295.1848	124.2616	71.52982	189.7874
33.83223	329.4622	45.96411	76.53523	100.2174	136.9477	333.7869	62.28376	133.0657
11.34185	12.60673	9.133443	21.33094	4.919203	0.004671	30.40423	98.87752	13.42054
13.79179	15.28725	25.75241	29.55147	32.54692	25.35695	33.09645	27.7909	19.59763
1581.221	1596.985	1696.258	2184.05	3261.918	796.2931	1244.847	500.5485	290.873
8.8281	12.55251	11.15216	72.63854	175.6992	3.049024	21.73684	6.844625	30.93077
0.081723	20.91212	0.375974	1.567494	4.916209	38.55034	6.89884	2.989139	0.010347
217.3352	188.2597	248.7536	224.7909	444.6515	103.4719	179.059	138.5808	178.4384
42.12386	5.671898	209.2738	29.41006	20.18741	18.2571	18.27166	2.217705	41.21451
3.845433	4.304653	4.790078	1.565068	3.341619	53.76629	3.425794	0	6.201447
3.834067	2.909416	4.692149	14.06311	12.45127	6.09013	11.30272	0.684093	19.57911

12.59753	16.76981	6.976579	14.31521	9.611649	11.1644	30.37871	94.02549	25.7974
1295.744	214.6007	605.001	284.2071	530.719	901.7596	266.8623	128.5394	516.7192
54.85448	60.68303	85.23309	28.33631	34.37891	68.96709	80.98289	73.36959	48.46779
16.3342	33.29431	17.74825	41.0422	26.71407	27.39232	66.97769	40.79825	35.07371
6.328006	9.771942	11.01966	7.195921	20.16767	23.32482	21.7763	4.534092	15.47047
16.33805	47.09134	9.146645	22.76967	17.4039	42.60618	26.90613	73.23366	19.60886
38.8224	58.25872	52.36523	84.9215	53.3098	12.17874	92.99208	110.1226	90.77017
26.339	83.14793	56.70917	108.9369	134.354	84.1977	126.0372	80.80532	45.39498
91.20309	87.2354	71.67033	129.9931	71.97052	84.19564	130.4212	155.6762	94.89189
605.1774	291.776	192.4701	82.14085	310.4164	230.2601	189.5221	80.7953	133.0566
65.02259	128.6721	69.56307	89.13677	36.15323	109.5536	150.4222	154.8795	71.17549
1252.886	1490.649	1110.693	1110.031	1852.558	748.6205	1023.982	1233.037	1299.589
23.64168	15.23428	11.04599	39.10372	24.75878	6.091335	25.26838	0.68392	4.137853
393.5747	732.2874	541.6011	446.9775	184.5834	1734.592	585.8484	1516.537	316.6591
35.74623	10.56063	4.201229	39.24511	21.39953	0.003261	7.041768	3.070991	2.068636
97.25875	25.04023	71.12164	48.09996	67.05388	28.40705	71.31699	24.56871	63.94276
663.3002	820.7328	718.7157	1294.933	831.0048	1050.898	979.7095	812.5585	469.3035
61.28377	106.598	37.26627	69.39624	56.423	81.15377	90.38433	220.4431	57.76966
27.55199	18.13783	17.75672	36.83517	17.39806	61.87466	38.22785	53.15605	56.72446
26.3167	7.082354	56.30981	25.59531	4.917075	33.4787	39.95897	8.373652	40.23191
4.946849	8.025405	0.21552	2.873505	13.1169	0.003349	0	3.859623	1.040748
16.27407	16.65937	9.041734	44.8615	29.50346	43.6058	12.13566	11.48861	18.56773
31.31137	63.68015	22.10237	42.51955	18.97447	80.13414	108.7262	101.7607	66.01096
74.73198	35.98892	34.67492	49.37574	42.11189	61.86942	100.1635	41.61491	51.56076
38.80928	25.07068	41.51596	25.6199	22.10448	37.53734	39.94203	129.4808	38.17353
524.188	83.17556	461.5932	353.1395	494.2964	249.5338	228.651	201.0429	350.6559
1774.991	1193.173	823.0108	1405.544	1313.862	917.0084	1516.007	1129.027	1028.333
12.56699	13.94663	4.793493	4.410662	4.913972	7.106356	38.3036	51.02962	21.66247
275.8606	250.2821	233.1919	209.1133	462.8217	273.8728	185.1866	205.7194	338.2682
119.3166	35.95222	26.12559	49.31656	214.1387	36.51636	24.31832	11.46602	43.31118
23.76	29.05711	36.54623	40.86296	20.41119	50.71008	44.39188	13.01709	40.21504
6.34137	16.66378	13.2642	11.42967	6.462206	3.04903	13.00693	17.68871	9.293287
71.20654	65.08551	58.61828	55.21785	18.97916	25.36524	108.7081	129.5308	48.48402
62.55819	66.59166	39.46913	87.81644	100.1651	59.85466	72.09832	147.0579	162.9689
23.83396	56.82148	62.94156	53.82025	17.42113	51.73696	58.21795	137.2317	40.23525
193.6987	278.2886	175.7674	192.4424	83.03863	251.5712	485.0753	728.7401	162.976
79.91839	109.1758	122.6951	103.0693	195.9609	82.16335	119.164	23.01414	79.41623

TCGA-06-	TCGA-41-	TCGA-27-	TCGA-06-	TCGA-27-	TCGA-27-	TCGA-06-	TCGA-06-	TCGA-28-
88.85927	116.7601	68.86354	82.29844	108.0611	85.43539	61.67112	52.58102	96.50036
38.24226	15.31527	9.604438	17.2144	3.52747	16.63625	23.57319	15.35767	17.51378
43.36229	109.1063	50.32837	34.43876	46.01726	107.3547	50.7844	46.01021	83.08351
1884.563	1547.737	1727.14	1271.037	4499.547	3729.207	2101.623	1933.171	2553.528
179.3107	82.53146	79.91995	167.5017	57.46217	82.67128	128.8512	147.7742	109.8521
3.258414	3.816207	5.413681	6.716794	1.796622	1.48163	5.463329	5.454784	0.076646
16.34971	48.17158	57.18731	47.8611	76.41375	18.01925	103.5674	125.7158	29.52329
86.88069	34.3664	59.61901	49.75272	34.60875	78.56258	65.30188	56.9586	81.78775
5456.823	3824.829	3615.319	3430.293	4686.412	9400.255	5055.347	3713.081	5792.643
156.5397	50.79198	44.67259	37.31474	11.71562	40.01186	39.90174	23.02483	52.27888
27.07881	6.446552	5.888727	22.014	1.880288	9.756931	10.86076	16.43528	2.814479
1132.101	441.5643	220.8653	258.4033	131.0826	373.0803	477.2818	444.4632	432.6311
162.7412	234.6217	404.8792	203.8704	166.8151	181.6453	112.4996	121.5271	228.8996
1.871241	12.42283	7.20428	11.52256	27.37199	44.49785	0.875736	17.35035	28.29345
86.27166	44.49902	22.59836	97.62233	47.64423	75.76817	43.52278	33.97243	93.76801
11.7242	91.05271	169.8336	161.8703	68.31985	90.4315	41.7454	171.5782	52.14381
1459.528	1658.072	1491.79	1707.49	2070.093	1695.449	1657.883	1680.303	2233.479
75.25331	33.04515	7.768944	43.06581	13.34007	16.66026	28.10405	16.4569	28.2165
13.0331	161.0197	92.75595	148.3635	199.0932	171.8611	143.3951	186.0414	75.06302
573.2557	848.6305	330.1081	1064.332	2557.422	437.7034	931.0612	695.1028	986.7448
575.8354	337.5478	339.4248	645.086	186.6786	832.6079	671.4861	667.759	948.0442
5.786175	2.643047	0.274041	0	1.874969	1.516789	0.873603	0.030927	12.19129
22.25739	1.364779	4.032287	4.768727	1.865161	8.411195	4.50098	18.64986	20.23132
115.8456	19.13889	89.29401	66.97973	14.97107	40.07002	137.8983	44.92167	95.20917
62.51503	64.76174	72.42806	18.16678	28.05806	75.75131	32.63107	41.63103	72.37236
43.55898	12.78536	11.4431	10.51134	1.878391	19.37172	20.84723	30.6577	26.83228
151.4862	325.9252	931.3717	261.2985	359.4142	507.3827	250.4629	246.2888	293.1621
1722.088	801.7723	602.4196	664.2298	83.65939	1069.223	789.4632	932.6446	954.7248
24.96215	5.186384	4.052227	3.810701	1.877569	7.037544	5.405671	5.512971	30.84233
44.70141	45.75195	22.58891	50.71521	8.441244	40.03661	59.87114	48.19255	30.92868
75.16425	36.8643	22.56305	90.93976	29.64748	22.16402	69.87879	89.73266	61.60628
110.9121	17.86955	13.32854	66.99994	11.71463	33.14287	77.1478	39.43136	24.22895
23.77908	6.442835	11.3386	25.85264	1.879993	7.01655	7.226116	6.60302	20.07762
896.1924	819.5146	1724.412	1122.707	320.7306	946.7585	958.2652	713.7252	720.4384
476.7325	178.9125	102.2124	214.3918	49.32608	214.6968	198.7204	419.1883	301.201
22571.81	4008.785	752.6236	1450.972	737.6909	2097.368	6303.986	1865.321	11099.11
36.33073	30.42225	44.06286	28.71533	26.17845	8.402086	21.76616	33.91659	89.04961
145.2426	34.34242	4.043134	10.50993	10.08016	22.16992	45.34815	17.55417	30.91938
64.63899	76.0205	93.95768	52.64156	36.10671	24.89446	60.80981	60.19527	80.20034
55.4095	31.76918	51.79529	45.94194	21.46577	31.72792	32.65016	53.62515	69.48921
100.0718	214.3273	164.9125	213.4468	541.5684	213.2155	153.353	412.561	282.338
73.87218	22.93341	46.44677	80.4102	13.34596	42.72998	36.27423	32.86664	37.57403
38.12492	69.7602	330.3495	339.9432	187.0294	315.1928	78.9614	119.2547	120.285
148.9634	25.48679	9.616246	21.99517	14.98667	24.93184	47.15131	54.7667	42.98329
420.5374	196.6896	194.753	295.7495	80.38551	183.1205	272.2283	257.251	295.9202
49.05781	14.02413	7.731168	26.80336	3.526032	7.026195	20.86358	22.98964	29.39892
104.2579	39.4008	145.5828	87.10662	45.92513	129.0837	63.51623	88.64516	65.6221
799.1825	195.47	235.6744	489.0685	384.5241	1226.267	702.3222	1131.904	1130.26
167.2358	228.4441	213.4154	222.992	152.3332	747.1545	375.6591	313.0987	158.1458
35.80999	3.913356	4.0405	1.897086	0.191849	5.648583	16.32404	7.695705	5.486782
43.83587	10.22691	2.182106	22.01768	1.880009	7.016756	9.954089	18.61202	12.13248
296.5613	227.1043	68.91683	81.33742	85.28252	123.9806	91.61516	132.4836	159.4355
136.6253	30.54444	15.18434	91.88989	8.442684	41.39597	51.7039	78.81251	87.00062
8674.796	732.0801	304.2231	721.6482	454.827	1032.252	1172.379	1150.516	2395.614
16.40387	14.02473	11.38698	36.38767	5.160024	19.31445	19.04367	10.97748	12.15379
118.1866	21.67073	11.47799	53.59344	11.71484	29.02788	40.81216	26.30597	50.93252
25.70008	11.50672	7.739174	12.42888	8.41557	5.656902	9.948449	10.979	12.16015

3831.816	1953.702	1104.697	3369.042	1427.718	3243.625	1832.093	2108.329	5635.91
255.6323	397.0092	496.1881	1025.159	91.82841	781.1588	380.2335	726.7416	314.6694
76.09241	34.26109	15.12128	29.66535	5.167924	28.94992	42.6625	66.70912	40.14254
18.31658	69.72032	61.08652	81.37039	155.9872	71.47762	58.98786	75.50491	42.90273
1.840552	15.30842	107.5362	5.724797	103.3939	24.8245	36.30835	117.9923	45.41176
167.9734	140.8809	74.45776	145.4712	46.05661	99.21491	93.43265	87.60779	216.9325
33.70877	16.54729	20.47299	18.17806	11.64621	20.67969	30.87201	13.16292	24.10362
19.66995	12.78686	20.61533	30.62337	1.878093	12.52756	8.128597	30.65979	20.18054
44.70106	30.55036	55.79807	16.25259	3.516689	85.32852	27.18597	59.13231	41.62313
179.8586	298.0311	493.7751	365.6436	310.4449	418.0427	237.7581	274.736	218.2544
4.474143	2.64493	0.255455	0	0.192369	4.278355	3.591987	2.227266	1.469091
12.4049	16.56134	16.89159	20.09148	3.527418	8.403119	29.95083	9.887207	4.15641
196.3231	73.68711	131.8168	69.85179	36.24743	148.7214	120.6596	83.23088	99.21273
82.59522	36.82443	13.31008	37.32216	10.07406	16.65494	44.46319	45.97461	36.20175
34.78832	195.1853	39.23911	10.51014	1.866601	26.30475	47.15421	10.98661	10.85535
40.17426	33.02982	31.665	51.68966	11.70373	18.02477	29.9254	23.01668	42.85674
162.6771	85.10204	233.533	98.56669	237.1347	286.2066	104.3215	87.60961	237.0287
24.29036	10.25872	13.30069	5.724619	10.07037	8.4135	20.84226	24.10781	22.86021
90.14049	674.5595	1388.388	867.2273	1699.086	106.1055	276.769	907.2765	333.3681
110.5915	81.29385	65.20524	78.4666	34.61225	214.6956	110.677	70.09624	108.5757
1728.506	1009.843	328.3309	758.9802	265.1638	357.952	1325.768	1555.494	729.8756
125.4396	29.27199	15.18054	37.31495	16.61532	4.280067	27.18931	121.4456	21.55902
217.4097	22.94753	22.59328	53.58003	0.212632	2.88794	155.1463	49.30024	32.28231
122.164	3.913816	7.769175	5.724807	0.207052	15.29433	20.8346	10.98687	36.24958
38.12779	71.01945	57.50607	70.82988	49.158	89.33515	44.44518	176.0966	65.60714
300.3422	928.6529	300.5371	400.0574	1831.157	743.2194	561.6739	326.2457	512.9722
35.44905	117.9541	127.7175	183.7922	83.48581	225.3285	170.6449	167.4373	111.1259
129.7817	74.93314	28.16026	33.48078	13.34765	35.93856	97.07398	133.5551	87.13151
624.1212	463.0352	590.9253	735.0845	1454.102	323.4735	472.7773	366.7232	1350.523
72.4063	201.5208	100.1147	29.6528	184.4239	51.04483	107.0764	71.175	163.2312
208.235	135.8003	314.6398	228.7545	405.0016	249.0194	150.6181	168.5885	373.3606
147.4662	38.11004	17.0115	34.44597	24.74007	15.2895	39.91127	19.73944	21.54743
219.3009	175.1749	117.1032	169.3918	59.13435	262.9775	258.5873	197.0754	225.0872
2245.792	1420.852	1417.643	1449.068	951.8476	1879.797	1282.196	2393.982	2560.117
0	9.789772	9.991201	4.799118	3.257885	5.352744	4.562394	8.652537	1.417397
55.36834	50.74853	40.89315	32.53121	21.48423	34.48907	42.63791	30.67587	25.55088
15.66948	2.640457	2.170953	14.33841	0.208219	2.897747	12.66449	3.321222	4.152538
811.1809	1041.444	1892.745	441.2178	1364.99	1490.096	959.1791	930.4432	879.718
160.9545	29.28679	50.30166	71.77539	26.42545	51.03575	54.41893	55.85467	148.501
127.3228	41.94878	13.33368	36.35532	13.35203	13.92218	70.77056	125.8466	71.00405
101.6006	40.66884	17.03093	12.42417	24.77111	18.04553	50.79919	41.62223	24.23393
22.26285	3.911964	22.5863	2.854735	0.208773	11.16939	8.127944	14.27105	13.5353
146.3057	40.70421	48.50791	29.65228	46.03489	108.7703	41.70468	46.01285	42.98548
135.6222	452.7972	566.4748	570.4683	447.8341	400.3854	184.1843	359.0376	277.2126
2.495449	3.914483	4.044455	4.768232	3.524387	0.116192	5.407674	4.416956	12.14291
9.082611	2.644081	0.271166	0	0.203095	4.283421	17.20872	6.607769	20.19298
34.31035	11.51353	7.749545	20.09054	3.527604	7.032918	27.21884	8.794347	30.78466
66.05216	25.44065	40.78091	53.6077	11.70075	27.60799	29.01902	60.1737	50.82835
16.3959	11.50566	4.049218	13.38701	3.526732	24.77546	9.040005	3.322798	18.80417
20.94974	17.87091	22.56823	14.33857	44.29641	24.91295	24.46552	38.33969	32.24882
2.495579	36.7038	58.42751	46.92632	47.01287	81.8733	15.40247	22.99493	14.82115
1416.728	752.3594	1141.6	929.3476	489.1633	1424.34	631.545	606.4729	980.259
129.5338	12.79808	11.47467	57.42779	6.806369	15.29099	16.29646	18.64609	26.89171
308.2841	1130.117	2047.569	1592.701	492.2649	968.6236	941.057	2625.781	810.0251
364.3741	295.6332	94.8638	224.9093	338.5591	201.041	297.6225	225.5268	267.8877
27.10785	109.2913	138.0891	38.31543	18.03046	9.749483	117.4696	60.04747	17.43725
24.41574	3.914547	2.1829	18.18073	0.193839	4.279838	26.33114	1.130626	14.79817
123.227	90.11868	63.26776	44.01076	70.48994	108.7454	55.32184	72.27292	84.43119

3.810908	20.34962	16.90123	45.00325	212.0303	50.69174	19.03565	18.63093	69.23564
48.04419	15.33516	40.97242	41.1456	11.71484	19.41696	29.91435	23.02397	29.57355
398.679	25.4865	18.89522	7.639387	18.25912	29.05804	51.69048	23.0284	61.7055
253.627	71.15626	68.92699	62.19398	36.24316	56.58439	171.475	93.08526	172.8491
82.28498	81.25727	79.89842	81.34401	67.24018	71.67038	55.32067	54.76677	95.13451
172.8029	133.1581	83.52712	106.2388	93.27274	108.7185	106.1682	85.39779	107.129
260.2613	38.17322	11.45366	19.12461	44.42339	9.779574	57.1289	63.53035	80.48259
105.6966	25.46134	20.69889	50.72504	26.36886	24.89791	50.81226	31.77082	20.21288
11.0761	11.51418	18.7334	21.04825	32.65655	24.81269	12.67213	7.700737	36.09912
85.96687	59.53277	106.3471	52.6502	29.55579	75.44012	60.83231	48.15323	50.82546
425.8756	120.6055	80.02085	133.0258	21.52371	128.1016	279.4991	298.8272	148.7259
541.4348	218.1976	85.55611	138.7711	13.34067	49.70126	289.4966	105.1183	107.2341
524.5527	389.4918	304.0999	200.0216	341.8749	428.0109	363.8679	484.9309	356.2442
1402.814	640.7437	578.4754	343.5869	173.5783	1314.339	1249.515	1146.135	1000.383
18.37931	17.81961	13.23621	33.5069	3.527237	1.519983	19.03826	30.64066	18.81397
15.04283	21.62515	18.75992	15.29983	13.30476	19.36403	14.48768	48.12939	24.16105
46.71522	25.47225	4.044991	30.61306	6.805129	22.16593	39.90255	26.30668	21.55866
2.640559	10.90338	18.94518	6.739146	4.655958	14.11547	4.575565	10.76609	0.063729
6.459671	1.371415	0.255518	0	0.192413	0.1155	0.872543	4.416601	0.104309
2.54149	3.849071	43.54161	27.89269	0.149817	8.139645	18.30965	6.555831	23.27884
42.43863	14.01467	24.0191	22.97293	10.0139	12.48445	17.22906	18.61771	35.9786
239.4154	126.8708	100.2151	147.3987	42.76884	89.53625	202.3956	130.2664	87.11915
60.64981	40.64318	40.91655	62.21666	29.622	45.45906	60.80567	54.73312	36.23192
459.2518	454.1874	498.5109	279.4641	314.1312	624.7328	403.7937	498.074	550.3438
419.0008	176.2899	194.3403	75.59873	124.3383	167.8459	225.0857	112.7643	159.3208
21.85057	12.71726	0.247307	7.643963	1.878032	7.000493	10.87169	18.5954	12.09941
400.5256	24.20569	187.5132	58.36651	126.1787	177.6831	204.135	216.7811	190.2868
171.9679	182.6807	201.9234	217.2705	232.0746	228.3761	173.3162	169.6776	174.0914
251.376	202.8234	186.7722	138.7869	137.2839	48.30346	123.4139	188.2373	336.9008
47.0075	54.31145	96.19948	84.30957	3.526953	43.83383	48.15772	81.92343	41.35391
568.9105	126.9572	109.6579	129.195	65.67812	143.2482	341.2042	329.4867	243.7563
148.8397	81.29472	48.54248	75.59494	73.83789	125.3439	104.3232	91.98629	95.19616
84.24875	99.01073	63.30625	80.38481	44.40754	96.4197	62.58	95.25784	93.81668
88.97878	41.94837	18.88765	14.33835	11.71633	31.7919	45.34469	107.2543	34.93609
59.55269	32.97345	11.43196	38.29167	6.799681	22.09274	24.48701	41.57364	10.84033
69.24272	50.76619	51.9439	106.2693	16.60766	70.12187	85.33432	133.443	80.23917
66.45984	35.62842	31.85106	57.41318	41.12328	44.17749	7.221588	23.02822	87.09354
3.817767	22.75422	43.32321	30.65755	14.75401	35.42769	13.60196	44.74394	10.78366
0.531851	21.42164	72.42282	4.770891	5.112029	1.518457	21.8314	81.63174	0.098868
166.4611	22.94017	13.33112	37.3146	5.16402	18.04538	68.05661	54.74677	50.94586
46.16368	24.17355	35.28272	7.63888	8.438692	57.67427	6.312987	3.322567	44.16485
16.33354	84.9084	97.78177	145.5335	199.8163	71.50505	71.70153	50.36475	138.8844
776.3653	168.7665	116.9998	209.6067	31.34159	169.3277	307.6508	186.1018	220.9407
24.89926	97.66604	384.744	117.735	297.9482	66.12578	127.0705	88.66858	28.25573
537.0724	168.8272	113.3932	222.9931	47.68859	353.7428	123.373	211.3019	334.8329
23.60237	14.06506	5.911757	7.638703	13.34359	52.30525	26.2854	15.36364	12.19309
38.83302	25.4526	92.017	94.78864	121.8115	45.42737	23.56363	33.95309	88.1477
65.22085	24.20713	53.87164	30.6127	18.24842	48.24416	31.72872	40.52832	45.6063
44.76873	74.76586	108.6159	51.68302	63.68443	75.57058	101.7004	56.91796	101.5226
243.4034	38.16712	35.55844	40.18155	8.435148	11.16661	49.87442	5.510755	59.03403
132.0267	50.79692	20.72766	21.03914	11.71593	40.01667	52.61459	76.62078	50.95037
526.0154	57.20228	20.73275	60.27978	11.6947	67.59232	74.37071	30.69041	113.9504
31.51079	24.20793	5.908276	13.38131	5.16366	12.54544	12.66457	26.30762	26.90809
56.79145	55.72908	77.1519	37.32622	29.53809	67.21529	79.02501	25.19923	26.85149
144.8273	800.3977	2537.779	1049.984	3043.763	780.1734	494.5453	2317.123	611.9245
164.602	143.4613	46.68352	76.55076	5.133903	79.97856	364.7717	118.2628	415.1501
99.70522	81.10348	83.0885	97.64991	29.62934	529.5246	68.97961	62.38914	57.57778
84.24802	76.20035	54.06451	74.64116	42.77479	111.5343	64.39527	66.80688	71.08229

289.0198	30.55872	17.0415	64.11346	14.98718	40.05761	47.15211	85.40264	59.02966
40.79997	30.52099	20.69594	62.21792	16.60272	45.4519	33.55244	36.14415	42.8953
64.43049	748.3203	6756.557	1933.505	3264.794	1974.96	620.7171	3028.379	491.4009
195.6681	266.3772	756.1986	325.4319	525.8635	294.4241	152.4276	246.2941	186.1767
59.25712	35.6093	68.60914	92.8483	8.442853	49.62634	58.9701	72.2492	40.27226
36.22329	31.75075	22.47173	44.03106	10.06864	19.38579	13.57609	36.12913	41.49699
188.6179	81.24879	42.94719	121.5541	13.35065	60.67038	99.80737	121.5056	87.10165
18.32717	72.18385	432.0329	67.972	81.35348	35.83421	46.28099	84.22253	22.86986
37.51213	6.455897	9.619523	7.638742	1.875503	20.77187	26.28921	12.08011	29.54261
632.5624	464.3696	170.8339	324.4471	41.13644	225.8395	474.5689	283.5507	492.8417
30.27386	6.45497	7.761126	2.853909	0.201718	7.036903	17.21118	13.17125	6.83619
122.638	60.95848	46.60472	64.11699	8.439963	85.35171	50.78822	87.57845	92.40331
135.0148	135.7941	70.74073	67.93846	228.8342	192.6617	151.5283	178.4339	123.2746
104.0417	28.0244	33.71564	57.41068	19.89412	62.06403	65.30272	83.2217	57.70653
18.56227	1.370536	2.172231	3.812477	0.181941	8.338428	16.35192	7.685766	6.789726
365.6393	302.0048	139.3316	459.4098	274.9152	359.2663	300.3345	348.1242	310.7559
52.23007	15.30929	42.40701	57.45447	11.6802	28.92315	37.21675	28.46795	34.7919
275.3068	162.4938	241.2387	246.9182	152.3416	734.9101	439.171	414.9057	408.5213
765.4459	422.3755	377.8991	1212.772	1028.92	895.2909	416.5357	779.2792	285.2433
94.88205	55.89387	7.763117	5.725274	0.209577	23.55247	34.44678	18.64964	29.59586
94.88086	106.5446	294.9817	86.13656	80.20606	142.9852	125.2462	141.178	91.07271
402.645	192.9087	596.2402	431.6646	983.4523	946.2825	233.189	378.7484	634.5055
38.77733	146.9181	206.1248	79.4444	164.4482	90.74588	146.1792	89.74253	76.30639
394.84	154.8435	39.28012	129.1963	52.59806	107.479	111.5814	106.2167	100.557
903.3666	579.8327	704.37	663.2683	364.8955	824.395	1056.251	804.5923	877.1341
122.6946	53.34536	48.41224	84.22663	21.5201	74.34023	41.71265	73.3497	66.9979
13.81811	7.690515	9.463327	24.9057	5.13316	8.359207	25.45576	17.50419	17.37334
168.5821	105.4011	74.48275	158.8666	209.4639	140.5038	180.5523	120.4487	196.9326
291.1806	41.97714	87.45568	100.4789	3.496385	110.2496	45.33258	13.17248	64.41988
504.0009	482.1452	415.3604	384.7451	319.1022	791.322	471.84	594.4195	705.7267
44.10145	21.66487	26.22324	59.34342	5.166732	26.27114	22.65252	23.02165	41.56854
82.75942	19.10648	29.73674	85.2349	0.201329	33.04221	51.75857	15.35758	24.17256
55.94125	126.7329	167.84	196.2621	42.70572	56.49746	85.30415	96.31464	97.69178
338.6122	780.1011	1679.182	894.9229	325.5751	762.2835	625.2336	361.2588	713.6375
14.35042	139.3951	239.472	74.65209	335.0703	27.67047	286.9568	97.41115	71.00246
20.38145	39.21541	103.3399	41.17646	128.5026	69.63903	28.13775	38.27653	47.96893
350.4474	362.9058	211.5911	230.6475	113.0984	316.6503	308.4956	411.6185	357.6373
116.0531	40.68722	7.764771	24.86733	14.98761	45.53222	55.33001	37.25249	26.9179
30.42996	7.708542	16.78998	22.97383	3.52398	5.651329	8.134001	18.61621	12.14067
207.0276	114.2129	89.16345	34.43801	32.9724	154.1105	161.5324	101.8222	108.5213
31.48442	10.25982	2.161997	3.812057	6.793491	45.56314	20.8312	38.35218	17.55259
22.99094	46.85813	120.384	65.11605	55.27951	53.49837	39.03076	54.68662	36.13644
230.7065	129.4491	85.52437	155.0482	31.34154	167.9101	129.7457	157.639	242.2766
78.0062	22.91106	29.80628	59.35597	10.07043	19.38959	54.46953	20.82794	33.51916
26.95009	77.15881	51.65287	57.44399	53.75988	33.06199	49.93129	89.65441	22.85182
18.96824	225.3015	367.3172	254.6908	133.6366	22.16879	331.5261	160.8152	53.62066
29.49478	15.1364	18.08152	20.12952	12.97518	8.304139	8.154576	15.29328	13.32445
139.6894	45.77706	28.15971	147.3963	39.50687	119.7715	58.94959	55.86296	101.8365
153.3935	24.20908	33.69886	21.99685	19.86832	23.54249	184.1727	24.11972	353.6016
19.62152	956.2912	531.5379	462.3731	310.0481	413.5885	126.1267	32.87975	175.3526
0.532295	61.61416	63.07288	38.32282	92.53188	70.62142	45.48656	51.30483	16.09348
102.752	74.91946	39.25551	53.58306	16.62322	37.30925	88.91022	78.83904	107.1596
0	5.089938	18.96234	2.860264	3.404384	1.498688	62.48275	67.01284	1.450277
49.36085	30.53724	40.98326	90.93818	13.34932	72.90898	38.99463	68.96061	70.9522
210.6538	96.38105	59.4638	69.86449	8.441781	38.65944	98.92407	79.9126	99.04049
438.8015	471.954	222.6818	267.0203	153.9701	295.9934	299.4255	493.7006	292.0207
962.6612	242.3483	104.109	287.1309	41.15183	174.8849	360.261	449.8746	270.5265
407.9702	267.7013	94.84934	142.5953	117.9738	214.7617	205.9669	230.9897	186.2189

2223.242	3657.324	11310.9	2770.844	9272.168	10931.46	3692.378	5324.359	3494.88
6.452003	10.26049	41.10678	15.29549	8.435104	18.05137	13.57222	37.25707	25.58684
335.3353	801.6082	1774.984	755.1827	479.1111	201.0544	653.3812	922.7239	658.7135
42.23309	3.916416	4.052393	29.66657	0.201082	11.15426	12.66969	7.701644	6.835571
904.6687	1139.223	797.0149	810.6653	1053.146	1140.87	1290.376	1490.913	1226.582
277.5157	304.3566	229.6906	474.7776	166.8696	319.0974	377.5596	429.0171	306.5047
36.88286	40.5919	69.90909	34.45199	118.2992	35.80773	55.38123	59.07629	46.82336
278.2609	81.27835	39.27325	160.7941	8.430262	75.81842	136.1036	144.5037	93.83114
1.184382	63.028	49.22856	33.51487	110.6195	26.10435	17.22675	129.8533	33.3538
22.27311	91.24304	525.6678	159.895	271.1469	101.6497	132.5719	184.836	126.9228
86.92003	48.30424	33.7025	22.95237	63.96105	68.91097	157.9192	88.68327	69.72192
231.3454	83.82205	72.59018	83.25375	23.16402	60.70192	154.2517	130.2849	142.0002
25.0249	35.45561	146.6289	33.50705	32.6031	11.13985	21.76673	22.99697	62.56429
15.85669	20.20196	51.75425	21.07411	3.507689	9.700832	9.966185	18.58386	22.59691
105.5417	34.34335	26.27026	18.16722	10.08012	40.02196	58.06157	44.90602	70.98206
40.78895	108.8988	75.75774	66.04611	151.1723	118.016	111.6881	87.53227	84.23246
186.4057	301.909	215.0874	229.7021	596.2385	214.7416	267.6915	288.9867	237.0476
7.13632	12.72289	13.07713	16.27132	3.516119	13.7975	13.60208	15.32822	8.134582
103.3352	59.73645	109.6168	100.4817	65.6692	176.2138	74.37276	65.71843	99.21128
44.87877	20.37082	11.44566	33.49746	1.878196	19.37453	10.85274	25.19536	22.84279
37.44437	40.68063	35.50498	37.31289	54.10388	29.04352	29.91027	18.64902	29.58812
141.5296	333.7307	120.8098	79.42236	36.22829	77.22369	82.53765	197.0769	214.3838
32.43705	39.13969	43.72697	5.725853	22.84702	1.520555	24.51328	30.61626	14.79293
5.13235	6.453657	28.12976	21.99581	14.98714	16.67389	9.035162	23.0268	17.55144
494.9975	219.5304	142.9879	190.4535	153.9254	239.5268	332.1213	336.0597	322.7208
75.02595	66.05142	109.4191	133.9974	47.66208	90.90248	77.1075	70.0862	59.03398
56.56471	38.1616	72.457	58.37118	10.07693	108.7133	23.55404	81.02078	65.70135
225.423	77.48056	74.43251	144.518	55.85304	159.6681	78.00659	62.43256	85.81317
1398.792	246.1965	91.16307	274.6788	24.78175	150.1544	284.0009	407.2263	769.8223
11.05584	116.5658	77.75351	123.4946	195.2323	98.96026	112.5679	83.17995	62.95857
46.49395	7.70661	5.881706	33.52129	5.152453	5.649781	8.13474	25.15928	25.37587
218.2755	40.70428	35.56215	55.4969	23.16514	34.56139	77.10559	64.61651	36.29513
17.68764	17.84116	4.052388	22.0036	11.68661	4.283653	26.30127	27.37922	4.156948
29.55119	183.3886	705.0778	270.0627	149.4946	175.4205	96.24503	259.1076	108.1917
58.01457	12.79717	0.274795	0.941432	0.205555	0.122395	9.942572	3.322079	2.811773
177.4487	76.16651	70.60449	45.92641	11.71492	59.28244	102.539	148.8421	97.76699
18.30844	82.56455	663.9697	281.3965	571.5968	418.1375	273.1469	421.3875	392.1776
40.73425	17.87501	2.167727	21.99554	14.98786	8.413939	30.81622	19.74407	59.00268
11.06187	8.992103	26.15771	29.66178	36.04225	5.661713	10.85121	14.26715	18.86271
19.026	10.2509	5.906427	25.83763	3.527682	17.98898	12.67161	16.44739	16.16859
76.23777	11.51382	11.423	16.25877	10.0497	5.659245	29.94687	42.6588	22.81509
100.079	91.41178	42.97061	65.06834	119.4694	48.31681	64.39458	62.4302	127.2517
2744.557	3496.164	2427.459	1041.327	1743.222	4016.833	2000.89	2311.917	2973.971
257.0086	177.3251	347.5794	115.8263	23.14986	13.92197	175.2294	145.525	424.4326
204.8174	684.927	198.5692	711.1564	1511.197	597.1543	432.8461	1098.917	389.6929
42.70855	136.9375	195.7795	208.6881	122.5433	299.3675	112.5294	122.5871	135.1592
159.4177	246.0591	314.6198	249.8152	336.4956	277.8682	189.6513	262.6942	503.0374
58.04226	27.9821	17.00003	30.61735	11.70734	23.51543	42.64237	49.25759	82.82393
64.43501	315.8171	1421.02	558.0451	638.4609	595.4079	603.5647	330.5593	318.6104
79.00351	22.9493	17.04165	40.18222	0.210228	56.54328	14.47933	20.83907	26.92361
45.46038	146.6587	101.1049	92.87451	131.4688	50.89429	97.17769	63.46437	70.83442
111.5148	36.87281	24.41976	10.50993	8.443132	38.6445	59.88032	16.45963	66.96768
0	18.86321	191.7335	25.89303	6.672861	12.33328	46.55465	76.09167	6.764419
3.19756	11.29542	36.03618	98.13345	56.65161	39.81194	12.76209	33.6353	2.779801
31.15667	16.50937	27.48007	10.5169	1.879668	2.902173	10.86568	12.06181	6.815731
37.50457	64.63356	175.9406	67.01006	128.3102	74.16288	94.44323	168.3738	98.80875
45.64357	14.03645	4.050797	0.94104	0.198064	13.87724	13.58247	9.887096	5.494976
163.3963	140.8556	61.48437	246.9478	26.43593	111.5655	151.5316	167.4874	222.2218

767.6806	120.6309	16.99822	30.61077	28.04627	52.44837	128.8168	26.30858	259.9059
182.1299	8.992864	225.0734	455.7388	3.515459	60.64221	180.6493	249.4482	244.5843
0	3.648069	3.325925	0.951172	1.63372	0.052173	0	0.014515	0.04762
4.471597	1.371237	2.183199	4.768053	0.195118	1.520414	5.407162	4.417286	1.468919
654.8874	709.2252	793.3219	879.5782	876.6091	1070.708	644.2511	893.261	1052.544
4.470147	1.370944	4.050009	0	29.3792	0.118082	0	0.03006	12.16194
11432.31	16681.74	18538.44	13867.66	30766.11	37642.26	14286.78	29198.64	18948.42
75.0569	164.7871	337.3694	182.8362	268.9188	96.34778	127.9664	117.1181	112.4555
332.8306	96.51704	80.02713	184.7138	131.0242	264.2286	130.6405	194.8671	325.3355
386.2953	145.9622	96.67595	460.402	24.79671	218.8419	274.9611	303.204	398.872
1895.384	423.7343	311.5115	390.4969	52.59564	177.6677	679.7004	632.6888	522.2078
135.6013	265.2474	309.8006	91.86544	32.94495	159.7924	166.9297	644.7802	1160.962
307.7402	139.6356	28.15517	30.60942	6.780353	18.04385	229.5673	18.64801	134.0216
17.70358	25.39733	33.27459	43.08545	67.99683	15.25103	39.04274	81.95626	21.48605
94.44134	78.55445	22.53733	53.59852	6.806497	24.89649	72.62071	72.22169	50.89539
27.68837	10.24516	2.18322	3.810697	1.879986	5.657489	19.03898	12.07247	10.83089
97.53144	79.95177	61.35359	38.26883	75.31127	92.21085	79.84404	64.60541	68.35702
179.9365	30.56131	74.40286	76.55525	37.87603	42.81615	98.88824	39.44725	117.8921
21.60544	102.6855	114.5558	80.39921	419.2063	68.83531	42.62272	71.15749	54.9667
110.5838	49.59034	15.17165	8.597277	16.61287	74.46582	50.77686	39.44844	59.06139
106.0373	50.84601	17.04061	84.21502	6.794059	73.05064	58.04284	65.71077	81.77269
0	27.8144	56.06235	29.6892	57.78201	101.7394	29.07591	45.86599	12.1286
845.982	1602.26	817.4638	1125.556	1702.266	2567.809	934.6334	1261.064	1339.108
105.3935	47.0398	20.74842	28.69526	26.43307	45.55752	72.56922	41.63437	30.94029
20.94229	263.4129	256.1939	272.8546	154.9704	156.6552	139.7866	187.1007	353.9401
13.06255	7.719446	2.182753	2.853807	1.87943	13.8841	7.221726	8.794507	5.495909
105.2899	92.72102	63.36907	130.1505	153.9466	253.3079	96.14942	238.6609	234.4275
982.3837	1488.119	294.9756	1430.876	1000.939	1584.031	1592.537	1147.227	2761.062
704.7068	262.6453	120.7788	523.5586	39.51523	203.7752	292.1844	477.243	369.5617
106.2594	14.06776	29.93792	52.63533	19.87638	20.79157	58.97673	20.83607	112.2869
5.128958	80.76303	186.3921	52.67131	18.13338	19.34345	493.3802	325.2248	4.156464
82.29237	112.9201	87.26114	49.75423	93.31139	100.5075	90.72712	119.3193	85.76886
328.4172	131.9768	140.9358	194.2987	37.87902	148.6603	209.6343	165.2953	141.9797
77.09964	65.99302	92.51552	133.0646	31.29838	98.99819	41.71475	133.4909	104.3496
25.83052	12.73074	7.684802	5.726803	0.189156	12.45183	9.957716	7.693306	14.7608
50.24702	15.30756	15.09346	44.9993	3.527695	12.51886	19.03296	20.81901	21.49379
7.202053	3.890036	7.512515	6.692589	1.863847	2.883005	4.510385	4.406339	2.7969
56.85701	8.988232	13.27618	20.0882	0.200845	2.903699	13.57843	7.701547	13.51151
1.18352	16.5021	86.03143	52.71321	119.2925	58.43711	0	37.13437	39.82765
163.8318	34.33676	20.72327	14.33856	5.16436	24.91325	70.78334	61.30714	22.89589
95.6655	36.86714	40.98351	49.76251	54.04444	64.6867	20.83414	53.65094	41.59504
43.45941	16.59624	5.91232	9.553003	13.33976	13.91391	22.65444	15.36285	18.8728
52.84785	25.42264	20.61192	52.65761	0.201472	31.67901	34.48113	25.19537	29.49784
106.098	111.6202	35.53943	48.79845	44.37535	77.13122	68.03639	41.6317	81.73165
27.65853	26.66061	13.25692	53.6255	0.199255	17.98663	13.58066	19.72498	26.80523
5.139695	5.176272	11.30816	2.854012	11.58253	11.09602	9.957062	15.33301	5.482883
36.12163	102.7089	131.1779	153.1676	78.51626	126.4536	67.13769	304.0892	103.0459
5.786709	60.87487	42.75821	64.13141	130.1088	34.49945	34.45907	56.92084	120.1909
2.496865	2.644798	9.602791	2.853877	16.55523	11.15435	9.03679	4.418016	28.16188
57.92935	322.706	301.6381	168.495	257.0381	253.8213	85.30685	273.447	101.6831
17.6972	12.78058	2.182454	18.17369	5.166444	20.72513	14.48854	18.6337	20.16389
60.49833	74.93298	42.96798	89.00015	137.3906	88.17612	110.691	89.78688	93.81758
29.53688	2.641494	5.909881	5.724816	11.71486	7.038535	13.57239	8.797494	69.60945
69.76434	16.60696	7.760523	6.682316	31.3284	0.124764	16.29408	1.128501	10.85519
9.079437	114.0328	336.0391	122.5377	50.80573	135.9389	42.62605	393.6545	58.95274
73.73299	114.1554	265.7113	126.3459	433.1375	200.6358	127.9645	116.0259	93.75879
19.01033	3.9164	9.603948	1.897339	3.527495	8.410374	7.220916	2.226903	4.156945
1.18979	38.40524	21.06362	30.73695	9.678583	4.216753	34.76352	5.487095	4.09932

204.1313	1882.452	3474.737	1274.881	8219.568	3961.134	2430.207	4776.805	1604.067
8.46008	217.788	133.7943	104.5185	8.371777	4.275044	112.9833	222.2439	9.46817
144.9109	249.8501	578.4792	334.0588	514.1575	352.039	209.6247	237.523	191.4839
256.3306	222.0305	100.3815	252.6776	34.61162	117.1031	234.113	410.4511	187.5304
5.131379	37.93373	20.46298	47.89085	48.52666	27.47949	15.40545	57.90428	16.13909
11.07891	16.56273	40.48713	52.67098	75.95547	109.1435	34.4981	59.0276	32.10289
81.06352	38.14461	31.80893	63.16404	10.08002	41.39801	74.40746	25.21452	53.63269
1449.264	492.2811	328.2879	830.7772	54.22143	377.1904	646.992	686.3579	717.7496
171.5075	60.96585	55.84643	87.09257	8.438824	70.26664	85.28762	95.24058	100.4359
169.8979	336.1833	509.2649	297.6583	952.3499	432.0427	473.7092	772.7292	801.7115
66.19296	16.57306	18.75654	32.54246	19.77974	15.25915	20.84982	27.37587	26.81884
200.9677	97.77333	85.55197	54.53758	98.32673	125.3325	70.74357	69.00103	80.47076
51.91659	5.169702	22.56492	14.34067	0.213779	30.42398	20.83554	71.19244	61.73734
3273.649	4669.643	16361.67	4779.839	7230.343	5991.624	6019.08	3290.541	4450.935
37.44766	21.67606	7.767113	18.16713	14.986	18.04791	28.09512	40.53152	60.31006
5824.876	1529.98	702.6136	2510.514	808.0096	1841.348	1361.134	639.3154	2087.592
62.6939	45.67183	146.6323	47.85683	32.82867	22.14134	37.19237	52.53392	146.6529
171.929	178.9112	266.7179	267.9981	414.9023	247.6814	373.9134	194.8581	350.6808
53.34957	34.32666	9.62435	20.08267	8.443662	22.15998	16.29593	15.36429	24.22555
115.2204	220.7145	851.6833	417.3409	442.4861	464.7186	347.6044	704.7435	303.8236
71.70994	191.5069	268.1717	258.4446	718.9419	259.8837	165.159	131.3685	116.5542
0.587895	3.649859	16.31082	1.918119	4.408647	1.395133	1.821026	21.2354	0.047893
15.68967	94.83955	69.91534	137.9164	98.96828	158.6429	55.38071	71.09438	74.75194
54.75836	63.33665	29.83018	45.9431	27.94998	97.32569	76.27995	67.82781	68.14335
146.18	20.39513	9.621763	15.29664	1.874663	18.0335	16.29733	50.35502	37.5545
44.06265	64.71613	48.3646	101.4683	117.406	90.74811	75.32127	152.0678	56.28986
166.6753	168.752	7.7461	6.683084	0.212028	19.42537	24.4611	10.98497	9.508989
151.7115	28.0187	9.621873	18.1668	5.15874	73.00147	98.00396	58.04213	75.04054
7.763741	119.1609	1271.825	303.5057	98.033	72.97235	126.1703	376.259	33.60078
912.6504	331.2038	144.8973	532.1443	462.9529	280.8849	658.7833	1003.792	672.2593
920.1595	284.2364	104.1303	309.1379	50.96037	350.9877	556.2719	500.2539	461.9767
7193.185	6701.935	43506.34	6750.536	24864.67	24403.88	11061.74	10668.46	11672.2
80.81955	24.14964	4.05226	15.29981	1.87885	28.92777	42.67177	36.11573	18.83802
194.4838	85.06899	28.15964	133.9939	23.16533	96.41591	85.27403	139.0237	101.8359
9.085014	32.99989	69.77899	163.8042	118.1191	83.53731	15.39409	56.87965	53.43674
33.65654	15.30044	11.41666	21.04925	11.66698	20.70896	14.49089	36.10363	25.46441
11.16822	8.934374	4.012827	22.99465	3.50611	9.696497	18.17406	13.13613	10.75316
48.11517	62.08932	259.4438	128.3193	66.82103	71.39783	55.36794	167.2605	37.53681
209.7144	109.1298	334.2433	203.8849	127.5533	170.5528	161.5413	246.2279	218.0791
37.15438	20.28649	29.31456	22.97696	16.41377	8.381395	26.34153	40.41898	37.24401
30.84638	29.27757	15.18425	54.5473	16.61898	38.65051	38.99095	41.6247	58.97016
214.4496	62.23466	65.07568	156.9831	21.52757	57.91018	107.9865	143.374	115.136
64.2951	12.77374	2.183099	29.67301	11.66425	1.519896	10.85602	16.44435	6.831549
272.7097	217.0215	311.5063	284.2515	407.2265	679.6968	210.4934	435.6789	356.2453
38.21503	19.11454	9.611172	56.48473	6.804675	30.33254	13.57623	45.96278	20.189
74.70693	30.48855	26.13451	23.91675	3.526802	22.12519	17.20923	14.2662	33.51243
365.0641	390.7083	191.1193	330.2021	194.776	155.6386	392.9276	313.0801	321.3956
1.192672	10.26288	0.279492	9.552917	57.36486	4.278137	13.57187	2.225158	6.83561
245.115	303.1607	826.656	479.5439	374.2395	862.0767	349.3883	345.8873	512.5829
66.68817	26.71066	7.767243	21.99996	8.440906	9.789383	25.38248	17.54901	5.497692
30.53794	2.643717	4.031309	9.55997	0.188305	7.005677	11.77964	13.14849	6.809526
19.6395	24.19448	4.048864	14.33914	3.523825	9.791786	7.220218	8.797406	14.86701
193.731	123.1201	102.1789	95.69747	150.5374	155.5588	101.6044	124.8137	137.9895
11.71461	153.3007	211.9015	157.958	180.8407	234.6942	91.66097	67.87919	308.451
184.1517	81.20685	134.9227	141.6733	8.440988	8.414384	119.8068	93.04251	189.8233
26.21217	44.50462	24.45303	20.08094	19.89455	27.68275	42.61325	72.27376	48.33256
10809.65	14575.84	11297.24	6879.748	15801.61	16389.53	14421.11	12602.69	14127.91
287.876	142.1905	172.6527	289.9951	261.8094	418.3765	211.4016	232.0972	289.3182

87.9391	24.17849	24.32557	35.40818	14.95743	22.13336	39.92315	75.47285	48.17301
86.01013	22.90597	16.9685	6.681747	13.32279	23.49123	28.11343	18.6398	20.18842
11.85212	6.418954	0.238853	1.897421	0.1809	0.109363	2.686315	1.130504	9.42489
87.60073	44.49681	44.77566	43.05454	21.52735	31.80354	39.8924	82.11358	119.1401
15.01397	541.3133	853.6438	886.4182	1215.254	622.7317	114.31	223.3041	755.7458
192.9441	541.6777	1353.35	788.6808	1671.52	1141.823	617.0726	575.7801	757.7714
34.34447	10.24494	5.900453	3.8107	5.163042	1.520059	10.85681	8.793168	4.156067
404.7465	148.5018	65.21322	232.5755	42.78824	145.9817	264.9732	155.4666	242.3843
166.0918	83.80278	50.36423	65.06884	32.97268	81.30277	78.91934	78.84367	38.97309
277.643	22.95026	46.67045	30.60927	16.62133	64.8148	105.2428	47.10851	83.12275
102.0009	96.52014	311.6585	47.83944	26.39556	74.4597	395.6058	313.1106	184.9283
159.4544	303.016	487.7655	281.4153	189.6179	225.5882	245.0423	275.8039	310.4208
91.05331	68.48726	81.34613	134.0361	71.88289	67.41306	75.3309	175.0001	116.2656
106.5411	83.50812	51.73222	130.2468	134.4917	239.1667	69.01333	39.41133	56.15057
81.01499	116.6642	111.0733	143.5845	358.1535	145.7142	82.56817	78.82672	135.1319
33.74223	5.181885	2.183073	15.3051	0.194475	0.11659	14.49817	10.97587	9.48823
715.1527	261.3987	109.6828	276.5961	117.9943	196.9175	285.8227	236.473	242.4623
3.421656	21.28086	4.965334	7.725163	4.526824	1.420796	11.13582	7.520021	5.093772
177.2982	168.7029	450.686	138.7787	368.7909	277.7348	137.9239	160.911	172.7047
178.6908	229.4321	135.219	137.8278	29.70064	251.5214	124.3186	238.5643	196.6849
15.73508	5.183447	2.183295	2.853782	0.196229	9.765884	6.314895	0.029938	17.47228
12.61366	17.57912	14.4809	8.612622	0.171017	0.104022	35.60867	6.583812	44.44619
44.23011	24.15472	15.11043	18.17256	3.527592	23.47137	49.0339	28.47137	30.81902
50.10106	3.915572	2.178206	0.941353	1.875664	12.53813	8.127735	17.54968	16.19801
44.66213	11.52555	0.284812	22.95252	155.478	35.94219	23.55397	24.12277	33.62218
101.4561	52.10312	98.28316	23.9096	46.01554	166.3693	88.91569	66.79935	88.42656
28.24263	6.455901	20.68003	10.51028	5.167834	11.16522	22.65528	17.55021	16.19964
244.6941	90.13177	37.41186	38.26692	16.62266	64.80485	170.6166	95.25457	105.8373
84.39077	45.73492	29.95358	52.63338	21.51272	46.87756	79.86228	63.49794	44.27527
356.8265	100.2894	70.71851	116.7593	11.70805	96.4349	121.5798	90.88438	162.0289
473.0916	1014.693	1018.578	944.6964	908.8376	555.9666	788.5945	655.6858	779.2169
365.9549	145.9376	54.09036	153.1315	29.70653	119.8253	181.482	200.3213	162.0684
18.34593	10.25564	15.11941	23.91851	13.31498	23.47936	10.85268	5.512905	20.17992
17.89155	3.90401	4.009539	6.687648	0.181795	12.39341	7.235231	8.775816	9.429701
302.5628	111.7209	549.2291	1203.277	336.5715	189.9442	388.4429	222.2137	710.349
317.7407	275.2286	109.5997	267.0427	95.06411	114.3367	197.8158	111.6846	160.7462
58.01	36.84554	20.69638	24.87062	11.71098	19.40842	23.56132	33.9573	33.55977
53.42581	115.0829	110.1324	49.77363	26.33277	60.44927	276.3152	188.0058	94.75907
22.97796	44.36151	33.4142	95.76885	84.36197	68.55242	60.84521	291.6491	38.82076
419.3284	215.6713	100.3623	201.9477	8.424312	106.0918	215.0591	233.16	226.3002
9.784116	20.28136	22.1268	36.40227	40.30192	32.81038	24.52219	65.47765	17.42336
30.85893	17.86974	20.7176	54.55116	10.0799	29.02733	26.2823	39.43164	17.54608
17.72762	55.54643	34.9454	64.18366	21.31766	39.73328	35.42056	39.36441	36.037
196.2575	165.0139	105.9751	186.623	332.0136	129.5078	320.3135	125.9232	330.7829
1.184552	80.62514	117.3863	127.47	26.11053	28.83525	84.60227	289.0341	5.49199
15.01954	5.186104	0.273886	14.33945	1.875091	7.039047	15.38977	21.92554	16.20046
83.7951	48.2423	31.75727	68.91733	37.76421	56.4367	38.09007	62.39204	72.25384
177.1864	209.3519	318.5547	237.3623	754.3598	282.0819	241.3751	180.6372	281.173
380.8792	149.7929	154.1096	85.16502	64.0452	265.673	117.0224	194.8781	204.9739
74.04992	17.85039	9.611402	0	0.202656	15.27455	19.0264	14.26602	5.497639
20.43134	11.48952	56.19565	52.69829	30.81825	50.45869	36.3555	70.9487	25.37826
143.5063	1036.371	10410.68	2228.232	3157.413	3869.744	989.1274	5855.812	707.0364
1.838718	32.86206	230.7474	4.768327	3.523677	5.650829	14.50108	38.2496	0.104606
16.3325	7.724998	9.624711	0	1.872487	5.66034	8.127569	12.08138	9.51721
134.3508	178.8905	294.302	90.91118	181.5439	224.2762	176.0356	223.3007	231.6112
23.19141	7.691832	94.36659	64.243	90.48611	2.899728	19.07185	7.69098	60.75423
2.50482	0.088838	2.168516	1.898194	0.209123	2.896318	4.500091	16.46033	12.19605
5.835566	23.86783	18.14366	51.81801	11.43317	2.890648	91.34652	8.769585	12.03138

89.69836	53.32263	18.87774	28.69805	8.443367	13.92067	16.29507	20.8365	21.5596
148.1628	372.8869	425.8023	389.5605	619.0408	243.6068	375.7077	462.9848	295.9049
62.81518	39.30323	58.84825	49.78366	24.65489	71.24759	63.58053	55.78409	87.95124
64.01699	22.9207	9.619181	30.61783	24.71818	16.65641	22.65597	37.2315	21.53813
3.82	27.73304	27.26676	15.31472	14.72266	9.715009	12.69515	18.59233	12.09321
73.24566	27.99022	17.01069	38.27649	8.443169	19.4079	29.91926	30.67638	18.87567
98.80464	48.30415	15.18738	11.46724	24.7976	55.17268	76.20288	65.70672	63.0373
96.13976	96.4655	70.67714	60.28332	59.08952	127.9956	68.02836	100.7249	168.6532
130.5558	97.68476	39.22818	142.6248	34.58521	89.47691	65.31409	100.7073	113.7814
44.6687	177.5724	389.8677	359.9237	39.5097	456.2372	213.2757	225.4646	326.4102
143.7381	69.83716	118.5332	106.2391	26.42898	99.10648	78.0218	101.8071	72.385
1060.237	1055.483	791.4094	931.2672	1525.461	1179.356	1192.378	1089.185	1091.33
1.838976	112.6727	36.19626	25.86294	22.68977	17.85482	1.778044	10.9649	18.70164
1047.748	595.0398	491.3178	665.1854	167.0575	523.0521	924.6757	556.1118	740.5491
1.221766	6.211885	13.25701	20.2494	0.125315	7.950989	3.633993	26.89629	1.423492
18.31945	54.54002	57.3795	33.48879	68.52454	22.15103	53.54088	105.0078	40.22459
719.5997	915.8416	907.7231	705.3959	211.1896	553.2564	980.977	792.5158	954.618
174.7675	87.57632	24.45062	144.5332	34.59519	49.67419	75.29607	86.49369	61.6975
48.09991	21.65848	7.768656	49.77057	3.524839	23.51719	29.92175	14.2686	26.87934
26.24187	24.19877	9.623934	13.38165	24.75029	13.91766	19.0199	27.39763	101.5582
0	18.28831	48.02866	10.58163	0.129593	0.080878	19.3378	34.43877	0.073469
12.43386	57.95188	45.5096	17.22331	80.17917	40.99249	9.952717	21.88891	18.76752
30.19573	44.45971	44.6577	134.0329	16.61424	79.75652	66.24355	64.5859	69.61561
39.56171	38.04716	75.22388	86.19005	29.51334	113.5231	43.57147	55.78718	57.4225
92.27404	41.95078	24.43592	37.3124	14.98721	27.67212	57.14783	37.25157	26.91619
38.73306	126.907	227.6975	359.9152	435.7406	244.8263	164.244	341.4535	390.6288
992.3221	940.0802	1117.462	1108.334	1196.999	1206.915	1476.409	1271.993	1079.314
66.38141	10.2417	5.896906	5.72527	0.196262	1.520258	25.41051	20.80845	12.15668
17.64506	191.4786	321.4765	227.8153	744.6672	248.8424	274.1209	223.2638	208.7564
8.427712	171.4477	291.6495	111.1187	218.8853	48.01894	93.61983	107.0818	26.81806
40.31128	20.34659	42.28975	22.0074	58.31481	11.14297	29.95021	32.82766	69.21761
9.109479	3.914356	2.182785	1.897088	0.193489	8.388373	8.133865	8.789759	6.82272
115.4744	72.31179	37.32739	59.33544	13.3509	40.01926	46.25658	86.46465	64.30316
57.84768	126.9256	31.86804	44.96614	55.85807	68.95511	103.419	106.2106	65.74785
131.9834	28.01178	26.27425	83.27133	5.162392	22.17169	46.25456	70.06455	72.32499
752.1352	182.7668	224.4487	425.9196	32.97003	273.929	449.1942	445.5132	445.8596
55.30282	95.0685	202.4189	151.2662	193.6096	85.25765	87.13191	114.8896	74.96719

TCGA-27-	TCGA-06-	TCGA-06-	TCGA-TQ	TCGA-S9-	TCGA-VM	TCGA-DB	TCGA-DU	TCGA-DB
141.5016	92.7604	87.53523	189.5541	100.0293	167.7383	271.4242	60.59855	177.8138
13.30378	29.64425	20.27084	30.52324	42.73875	10.23708	32.2895	24.23195	27.75567
18.83738	118.5972	123.7282	52.3971	133.062	141.1422	103.9308	92.6891	71.95566
1557.552	1293.064	2422.562	2286.797	2289.185	2267.548	5156.141	1198.213	4316.909
145.9112	283.137	139.8923	215.4607	138.8855	143.1916	135.2104	84.00671	114.0938
1.137665	2.862464	2.748215	0	0	0.005495	0.002183	4.337061	2.062373
72.89185	33.46904	175.1922	4.607917	8.729024	3.077657	11.10301	20.75215	7.206605
71.88836	75.54242	119.7749	131.5432	205.9071	133.9893	143.283	70.99956	61.68081
2046.154	6030.219	6057.042	6452.426	6472.271	5052.632	11896.47	3884.126	13536.61
51.97232	72.68961	55.19779	114.4559	82.5588	84.88832	60.54263	61.50548	32.90007
5.568949	21.99794	16.20014	35.34605	21.36665	25.56712	37.32916	31.23475	10.28731
373.6703	623.5726	585.2281	1305.552	1418.001	1151.671	1502.444	973.8439	1818.232
155.8727	261.1183	92.9206	111.1527	85.45853	154.4449	86.77964	76.19685	229.202
4.429292	11.51474	4.07375	0	0	0.005831	1.012076	3.442318	0.007114
82.92408	60.24141	41.82877	148.859	97.12045	178.9785	114.0203	41.53742	53.45681
27.65713	26.7701	71.0765	3.043908	6.786471	7.169192	7.067019	294.5321	26.72998
1297.779	2042.91	2594.657	1773.825	1626.804	1576.138	3232.931	2137.457	2696.015
39.81026	40.1634	16.27678	53.26367	53.41886	111.4622	54.48713	18.14388	95.56875
349.1148	167.3899	127.7514	14.78669	34.95263	60.35211	34.31071	89.22135	53.45697
882.0949	1293.11	1568.022	154.1893	89.34068	78.76533	80.72634	1220.869	250.8034
708.59	915.29	786.9422	1034.554	1043.101	975.7511	1096.816	571.7986	900.3889
0.034434	0	8.216341	2.263881	11.64275	134.9759	37.33569	0.817698	12.34559
0.035121	3.808857	10.9055	97.86096	46.60801	38.87559	211.8914	767.4381	317.5654
81.8447	45.88923	48.57337	362.6259	218.5204	298.6583	256.295	63.19102	136.7125
30.99399	35.37058	100.8812	107.3042	105.8652	79.78162	100.903	51.07642	176.7709
34.26831	70.81231	18.93102	14.01907	31.07731	39.88656	20.18305	4.276213	42.13681
140.4135	211.367	279.7138	151.1014	109.7395	145.2429	81.73497	538.2096	175.7646
475.3608	2496.314	1390.72	1933.798	1202.387	992.1148	575.15	798.823	495.427
5.571282	3.807623	18.94048	10.87681	30.10394	56.24555	45.40509	12.07737	11.31731
75.17862	192.284	37.78531	80.65272	101.9826	97.16407	55.49892	103.1219	50.37192
13.30959	51.64052	13.59673	66.57349	33.98422	76.70669	53.47972	160.4571	21.59553
46.44672	37.28847	25.68258	81.49573	43.69921	78.75136	100.8988	24.20971	64.75342
7.774621	16.25434	13.51923	57.52715	17.48017	72.56506	13.1193	3.411124	4.122568
530.6252	1172.581	610.7519	1470.886	840.1123	964.4981	491.4006	1766.741	1400.931
107.2609	410.3313	144.0149	175.3901	198.1278	173.8799	165.4828	266.8728	145.9601
1559.775	5615.127	2947.251	53134.17	25925.06	14630.11	21899.99	16887.47	12421.42
61.79274	15.29059	86.78221	8.530356	26.22253	13.30358	3.030797	16.42791	8.233585
17.73079	47.81053	25.6909	59.48993	79.64242	65.46105	80.72185	33.74458	146.9583
99.40996	24.85246	118.0089	14.00761	43.70196	38.87043	11.10314	44.17262	20.56702
67.39041	18.1561	108.552	12.44197	43.70453	59.31705	11.10308	36.37107	17.48355
279.6095	93.71656	266.1612	16.35398	5.816112	20.46579	74.67141	199.2978	87.37487
42.026	83.22277	18.9674	80.729	80.62044	68.52463	61.55053	51.97449	33.92637
85.09308	90.87263	118.1481	13.22129	11.64246	18.41972	8.076082	70.18646	13.37372
30.99608	49.71733	35.11612	279.0136	186.4859	119.6688	212.9008	46.73497	141.8398
177.9988	505.9719	260.9772	291.3344	219.4928	318.089	240.1505	276.3828	119.2395
12.19124	26.78387	13.54445	29.8016	31.08603	23.52449	22.19907	9.483259	12.34236
96.13736	152.1155	57.87728	46.94304	25.24103	51.14395	35.31889	37.21637	52.4243
517.3766	1284.456	597.3725	4788.325	3651.827	2502.792	4342.865	2944.96	2469.9
285.2304	45.88982	618.7313	68.82447	142.7585	130.928	799.1497	153.3004	700.9814
4.463718	17.21219	20.1675	33.00994	39.84439	14.32313	17.15424	26.02636	7.204906
7.774691	39.24809	14.85062	40.1237	23.3136	38.84853	12.11055	6.882132	6.177586
127.1577	468.6722	137.3103	155.0043	293.3134	284.3371	389.4826	88.32149	534.4528
38.72274	102.3484	33.74789	122.2772	93.2429	94.09346	73.65947	42.41569	47.28766
755.039	1672.761	1513.377	15408.24	11194.42	6932.526	7708.991	4090.356	2516.157
6.673982	23.91058	22.87687	48.73766	23.30906	46.00916	18.164	5.143831	10.28787
25.46328	91.83073	35.0749	83.84827	81.58914	113.5167	50.45291	12.93947	74.00126
15.50262	10.50527	9.541551	28.20931	34	31.70283	30.26974	10.34963	73.96018

5790.126	5500.37	4764.377	8559.249	6334.364	6210.433	11599.81	2535.105	2945.793
138.2136	2631.429	317.4422	214.5378	222.4057	244.4526	86.7804	286.7766	56.54318
17.72014	69.85167	33.64411	43.89235	35.93543	69.53323	41.36927	26.83578	12.34472
118.1807	25.80863	73.8944	8.522128	8.728809	16.37399	34.30935	144.8754	51.39347
230.3978	1.895592	56.27874	0	0	14.32681	6.057943	3.410339	1.03827
86.26205	235.2803	134.6048	181.6618	223.3824	245.4704	287.5719	211.3998	186.0424
24.31867	28.69851	17.54944	34.52922	29.1412	26.59115	19.17285	13.82631	20.55965
30.96154	19.11541	26.96045	37.59944	37.87914	25.57439	17.15646	25.09923	13.37224
30.99235	64.07187	48.52525	47.70766	63.12429	41.94237	77.69613	97.91682	105.8631
237.654	580.6122	358.9515	75.88178	114.5963	207.6287	98.8881	64.92705	198.3743
2.253106	10.50789	4.17326	29.84188	31.09159	17.38956	13.11939	25.152	29.79693
6.675278	90.94527	18.90446	23.47147	74.82556	19.43775	4.039869	0.816634	30.83295
91.78931	102.321	87.56603	315.6617	265.1475	199.4488	256.2935	133.3886	311.4292
15.51644	88.98097	45.73247	50.13858	52.44967	51.13786	45.4064	32.89871	43.17024
8.887289	76.50466	21.66927	130.0371	50.49415	25.57962	225.0057	193.297	238.4304
42.00949	56.43781	22.9726	75.3044	49.53523	78.74131	28.25504	32.03232	20.56608
75.21136	70.75684	152.0929	251.3883	246.6912	228.0863	296.6541	199.2546	156.2389
18.82739	7.632987	10.90098	32.07672	34.96126	21.48572	76.68001	65.91174	127.4011
360.3406	207.535	344.2875	52.3777	68.94509	101.266	59.53669	815.5255	84.29416
160.3068	88.93051	268.9777	234.1665	264.1776	239.3344	265.3741	113.4571	260.0405
626.805	2310.729	882.4808	2424.771	1768.613	2596.873	2098.781	6027.199	1279.662
27.67357	22.937	41.7867	113.6757	123.3653	137.0359	74.6676	25.07543	10.29028
73.00112	56.40998	48.57313	417.5171	402.1001	218.8822	517.6259	152.4511	286.7658
7.782539	17.19759	9.562422	79.13864	172.9213	48.07547	82.73847	18.14026	60.64349
49.76009	156.9061	41.77972	37.53193	14.55632	45.00785	66.59579	32.88199	83.24863
432.2573	342.3799	625.5902	909.1994	1274.252	1622.156	2104.834	516.3365	785.2762
57.51283	176.0045	520.6321	19.48558	8.729032	18.4202	11.10317	168.1488	47.29027
36.52311	120.5026	86.18485	191.9197	181.6244	111.4893	204.8307	187.1718	58.59725
1148.393	430.3857	665.7183	121.298	149.5577	197.4086	101.916	411.5367	190.1605
114.9592	150.1702	80.77033	7.741772	14.55608	77.73737	16.14838	66.67903	66.81685
250.9104	126.2359	228.6394	129.1662	71.85969	130.9239	162.4553	228.7461	393.6411
8.888046	43.0329	31.03239	52.46862	47.58798	21.48709	77.69218	33.75747	44.20046
192.3842	514.548	367.3095	577.958	1074.196	1095.404	349.1273	203.5552	862.3484
871.099	3045.26	1689.48	1489.533	1977.424	1643.64	2232.984	2023.964	1424.589
3.312214	4.795824	5.247672	0	0	0.004994	0.001985	3.475278	1.035394
26.5629	67.9158	5.524177	53.25801	56.33299	64.4323	21.19292	71.95198	33.92521
28.78041	12.41476	6.869258	134.8385	36.89725	101.2513	62.56105	1.682742	14.40164
672.1028	747.9171	1162.058	827.7995	693.4546	441.8561	799.1525	1210.44	1374.205
75.18611	139.6508	64.64766	123.7788	105.8653	88.98533	79.71472	49.34248	35.98508
15.52085	48.76538	53.88675	116.7585	98.09802	133.9756	85.76742	56.28769	33.92885
58.59903	14.32775	32.39942	69.70012	73.81456	57.27971	124.1051	25.94184	119.2136
14.41552	17.19695	12.25348	15.57008	24.26898	78.75657	275.4471	70.16442	81.19992
66.35965	19.10959	76.77192	50.82203	70.89066	128.8738	154.3809	108.2823	113.0658
345.9879	292.6612	146.7365	186.3301	311.7638	380.4782	73.6631	560.6537	313.4914
11.08539	2.85145	5.516335	11.68584	13.59047	11.25747	35.31154	6.880442	14.39565
1.144674	0	8.214593	14.0133	24.27311	22.50817	49.44107	210.1873	19.53802
12.19639	38.26692	6.86568	28.97568	34.96845	89.96568	24.2178	13.81922	15.42587
24.34774	65.05549	51.05714	39.14802	39.81942	81.80621	17.15676	50.27265	15.42802
18.80996	13.37703	5.52026	32.94233	21.36402	39.87898	34.30461	7.745753	61.6372
3.35809	17.19741	14.9415	50.0831	27.18388	130.9008	114.0155	43.28863	63.72716
25.42567	16.24861	32.2202	9.317145	5.81562	7.168647	23.20835	66.89125	10.2883
690.9199	1201.254	626.951	1623.478	1674.399	1760.237	3084.601	1574.278	984.6772
13.30891	49.7303	13.59436	123.9589	76.73534	123.7357	86.77273	12.07347	41.11864
596.9239	1163.994	801.6027	285.7783	149.5571	319.1205	333.9919	1093.507	315.5553
228.8515	210.3981	236.8385	341.428	388.4902	440.8256	581.199	271.1573	466.6363
23.2016	22.00073	28.1439	3.827028	12.61917	14.32345	3.03077	11.2267	8.232327
2.253145	7.63526	6.856051	41.66878	24.28344	32.72054	31.27686	18.18085	11.3144
71.88078	65.02311	86.15789	111.9777	129.1746	88.98789	185.658	103.0887	158.2809

58.49885	15.28982	29.58544	10.09917	5.815467	47.03747	11.10262	18.16264	19.53485
19.93957	71.73514	35.07492	75.9958	41.75604	99.20179	40.36349	39.82125	77.08375
16.62647	51.63074	25.70463	283.7369	316.6588	96.14683	259.3128	0.81924	50.37437
60.84327	75.53834	115.8188	627.4333	748.8427	317.0701	475.2515	117.7793	121.2961
89.56098	55.45664	104.9662	106.481	75.74785	98.19268	113.0123	67.53881	129.5078
133.7375	67.89449	149.2121	80.62461	113.6345	52.17043	103.9305	67.54595	278.5077
9.989931	98.49383	72.78174	155.7813	658.5237	329.3393	275.4658	586.6769	319.656
24.35533	53.55892	59.16312	57.17836	61.19008	55.23094	33.30035	19.00966	38.03576
37.5629	31.56391	21.58468	57.33364	17.47451	40.90539	12.11157	12.95055	7.206215
22.13965	96.64685	76.43713	28.14686	35.93279	30.68802	19.1746	125.8672	28.78522
68.57948	206.5806	178.9705	443.3982	304.9703	121.7208	450.0221	181.0548	88.40507
48.68316	420.8571	127.8821	255.3351	339.9427	219.9019	106.9603	413.3786	123.3488
329.4359	258.2197	566.2485	343.7614	458.4189	244.4564	629.6332	272.8831	544.7521
778.2522	981.2688	1820.025	1975.852	4502.628	2747.241	4241.963	1342.037	4419.698
13.29794	44.01683	14.89358	21.11353	12.61661	39.88077	33.29643	17.29718	19.53404
11.09426	11.46057	8.210321	33.68193	33.99403	10.23689	33.29784	25.97429	25.69994
24.35929	47.81232	28.37033	60.28716	138.9113	47.05344	101.9084	33.74774	55.50671
4.383621	0.945437	6.445261	0	0.964042	0.004674	0.001859	0.831033	1.03456
9.980834	1.895412	0.106147	3.043551	1.930949	2.054732	19.17183	25.15172	26.71684
17.5175	3.818501	20.80975	0.706761	0	1.030607	0.002354	4.316618	0.007233
14.39296	38.28332	9.530096	31.40215	26.22784	27.61079	19.17225	36.46179	5.15036
124.9176	154.9445	78.11375	226.4493	144.7146	146.259	117.0485	104.8152	113.0656
36.5026	133.9514	48.45393	77.60107	61.18978	76.70306	37.33612	42.43313	31.87072
445.496	493.5084	706.1135	540.3721	473.9566	383.5547	766.8608	417.5902	985.6802
122.7127	109.024	149.3015	80.601	84.48886	74.67159	154.3813	81.40433	273.3851
11.07333	14.34419	14.80633	31.50247	12.6225	52.11757	7.066099	4.280599	9.257483
263.1314	152.0518	250.3342	814.4604	982.8846	533.9079	1821.294	123.8405	1929.23
176.8703	253.4629	185.6282	132.309	176.7625	170.809	234.0934	77.0623	176.7895
323.7304	76.50277	575.8303	24.18475	45.63688	52.17086	17.15741	203.6836	57.56826
23.22114	59.34415	36.21443	14.03413	12.61688	22.50364	13.12017	19.90685	23.64196
82.94999	362.4878	404.801	350.0896	453.5741	262.8621	303.7185	348.3114	108.9619
135.9953	138.6687	121.1692	197.3299	175.7873	283.3119	422.778	90.92354	292.9289
100.6159	135.8087	109.0184	181.7289	124.3139	76.71715	215.9294	70.13479	119.2332
15.52083	55.46249	53.88598	67.32276	111.6991	53.19108	170.5182	92.71887	35.98419
21.0259	40.17875	21.59353	39.19828	28.16357	26.59521	40.3593	22.50144	13.37158
165.6406	53.55721	207.6913	28.12298	43.70048	50.1193	7.067039	73.67393	30.84354
27.67973	47.80515	28.3922	127.6733	114.6051	88.98713	77.69736	51.93896	32.90227
13.27733	31.59424	26.7328	3.829734	1.930993	0.007011	9.083708	32.16103	4.122205
11.06154	8.599415	26.60087	0	0	0.006824	1.012447	1.680289	2.066548
37.61551	94.69762	57.88108	61.85194	101.9895	60.34734	76.68566	27.67616	45.2315
13.30589	16.24337	8.214478	23.43532	48.56581	45.00126	46.41442	19.88487	62.68616
139.1648	61.21144	108.7275	21.06145	15.52776	85.90749	15.13921	25.94551	98.65824
99.52387	368.2444	259.5622	442.6627	290.4044	180.0158	131.1765	245.207	117.1822
119.3591	69.81146	55.24019	34.37679	99.06727	181.0168	9.085093	22.47079	59.62118
199.0132	394.9945	335.0103	427.5809	679.8685	881.6388	654.8588	204.4259	1098.731
5.571118	10.50211	27.01302	57.96315	51.47392	10.23767	80.71911	22.47866	64.75
103.8073	59.30569	83.19536	14.79344	18.44268	17.39624	25.22852	23.34991	17.4838
66.32853	49.72515	71.28573	72.84046	62.15639	35.80513	93.83747	54.56395	72.97564
56.3724	43.98965	45.77055	35.9765	33.98593	28.64527	51.46087	23.34643	94.54427
47.57313	41.10849	24.36022	292.3481	219.5139	150.3488	188.6855	136.0327	45.23579
18.83564	65.03356	71.29238	53.21586	87.41542	63.41545	93.83772	62.36976	97.63688
132.6883	82.23362	188.4136	763.0386	384.6105	281.2722	379.3945	44.99601	207.6304
22.15047	26.76325	28.37429	50.07749	67.98502	68.52824	179.5958	46.75538	41.12135
38.69002	28.68471	37.67173	36.79965	17.47232	8.191918	46.41414	50.28161	38.03054
918.5493	872.2707	915.8665	115.8137	107.794	212.7511	214.927	332.6665	392.6408
157.0116	86.05883	279.8896	511.3952	757.5684	366.1671	146.3132	147.2358	131.5751
66.31253	146.3896	93.98358	50.88744	48.5583	47.05168	12.11218	47.63693	38.03639
72.99191	118.5895	118.421	151.9345	127.2279	120.6935	241.1539	117.8128	154.1752

29.89054	118.5949	66.01474	237.4645	177.7451	136.0305	128.1466	88.3488	29.81907
38.70922	109.0693	43.09001	77.60957	59.24735	46.02819	53.47862	34.62529	31.8704
747.2027	583.4252	933.2006	14.79525	10.67256	15.35092	82.74443	273.7529	82.23914
250.924	249.6269	246.1463	194.1943	101.9687	204.5622	73.66291	195.7938	111.0161
34.30363	93.7376	47.16413	75.18425	82.55677	74.66435	71.64145	36.34586	49.34266
13.30556	72.71745	44.35702	43.08854	30.10343	42.95607	20.18341	18.15	10.28976
93.97622	179.8238	71.38453	122.9591	154.4317	144.2113	129.1554	83.14782	225.0768
85.04192	29.63889	87.17392	16.3639	24.27214	24.55392	7.067038	28.55899	32.89606
15.51693	36.33699	22.97838	48.5604	67.02418	31.71182	31.282	25.08591	13.37318
570.4103	448.5486	422.4651	718.1462	529.3139	366.1697	840.5219	744.2524	1036.054
4.465544	20.07235	18.93553	77.73834	33.99182	12.28234	107.9508	28.57262	4.122929
34.30852	215.2395	43.16341	238.3529	102.9517	80.8038	87.78627	45.87554	39.06809
159.1929	108.0625	200.4137	138.5753	160.2492	73.65023	229.0488	154.203	183.9843
55.31118	55.4555	24.36084	173.8845	80.60318	104.3305	314.8095	55.39933	49.3474
9.963918	17.22617	6.821428	23.63272	15.54391	17.38184	24.21071	1.680157	5.148582
458.7606	430.3809	243.5891	716.6296	620.6172	824.3685	716.4102	310.1377	640.3412
22.12969	45.92291	18.92203	34.47218	32.05071	37.84039	12.11168	12.94864	21.59057
301.8191	361.5101	400.9648	706.3714	1478.225	795.7402	839.5142	535.4099	829.468
785.82	1284.588	437.0695	17.92415	59.23253	27.6252	3.030342	1001.826	35.98629
32.0988	29.6313	13.59882	115.937	135.9807	51.14706	202.8072	77.95831	134.6388
97.27727	65.02637	98.19432	23.40303	48.55208	27.62498	122.0911	84.89572	24.67981
249.8469	231.4421	302.6964	451.1326	167.0413	137.0639	273.449	452.2924	303.2162
101.6625	62.16328	104.7908	35.95622	23.29812	38.87313	31.28311	62.37037	127.4341
164.7342	65.97443	221.9883	525.6662	293.3129	429.5651	448.0049	155.0508	475.8723
547.2159	808.1632	1289.959	1427.713	1006.189	1217.132	1859.641	401.0986	903.4759
22.15189	112.8695	41.80869	141.8749	92.26912	141.1335	133.1877	73.63459	59.6201
12.17321	16.26133	5.502461	27.54621	12.62279	9.210766	16.14409	12.98352	11.31081
134.8996	98.49316	138.6737	422.9438	420.5473	356.9563	539.8273	305.8356	832.5152
28.78423	8.591398	24.35247	1888.06	947.9472	1094.367	408.658	3035.826	420.3872
677.6378	787.1274	692.7962	1025.948	1067.384	873.472	1234.043	603.8614	2416.412
24.35574	57.38623	17.62284	47.75108	73.82072	84.88285	32.29147	25.94781	31.87077
5.571175	54.53489	40.31468	40.75084	47.59814	48.06506	20.18309	21.62701	13.37213
214.2967	34.41561	256.243	32.8136	91.29861	85.91389	16.14834	71.90308	28.79018
403.4968	696.28	528.6485	15.57963	32.03883	24.55628	49.44632	70.98884	43.18086
384.3259	54.5058	293.7876	11.65422	25.24035	35.80604	10.09416	0	10.29027
25.42462	39.23124	33.54679	22.69519	14.56059	14.32552	8.075728	13.82416	12.3429
354.8737	433.2447	505.8461	773.7543	929.47	834.6034	969.6771	711.3201	694.8216
27.67778	84.16277	52.55461	77.5108	100.039	58.30502	134.1976	64.95564	107.9189
19.90096	21.99926	5.515735	42.46861	32.0622	32.71979	20.18084	4.277618	26.71813
108.3491	72.67374	157.3568	159.78	113.6294	93.08032	280.5036	78.80391	183.9775
8.886481	30.58677	10.90435	195.0691	164.1411	25.57982	179.6057	84.87301	162.3955
50.80773	41.13314	29.62208	14.8054	25.24703	12.28212	7.066969	19.02371	25.7005
112.7791	154.9352	184.2773	185.611	217.5596	212.7394	84.76167	186.2823	301.1421
9.992163	38.25461	28.31951	29.71909	52.4524	30.68793	30.27255	18.14878	12.34516
93.83028	38.25694	40.33836	25.01146	17.47248	47.04472	11.10295	47.67858	25.70202
152.4481	9.545581	242.7347	11.65429	22.32673	9.214909	3.030641	111.8272	27.76203
26.40402	7.644242	23.87295	2.266196	4.847445	9.206829	0.002647	6.031706	2.066279
140.3901	198.9476	78.12127	169.1883	187.4536	129.8973	80.72512	88.3394	61.68036
191.2791	39.19525	88.92629	194.918	841.0918	637.2039	356.1905	163.6972	619.7873
132.6547	102.3283	268.7703	48.4701	24.2683	5.12292	14.13027	53.66714	11.31786
16.58702	43.0876	30.75348	1.480202	0.960328	3.07761	3.030744	14.71264	2.066812
40.94235	118.5939	47.2104	283.7301	125.2883	145.235	86.77861	157.7134	48.31895
16.43661	1.898895	57.92714	0	0	0.005982	0.002375	0.81941	0.007298
38.71851	73.64768	20.31506	113.681	60.21421	80.79748	79.71209	77.12717	19.54023
36.51566	227.6929	96.80685	71.24437	88.38308	79.77911	46.41805	98.79023	46.2609
259.8095	1082.694	193.8346	633.5798	572.0527	1099.494	755.7624	323.9998	625.9533
142.6357	651.3567	333.5842	613.3655	491.4532	362.0693	140.2585	269.439	248.7418
292.9403	332.8351	161.5239	310.1253	224.348	317.0679	349.124	235.6415	542.6831

3378.146	4045.653	2668.769	3582.145	3944.162	3827.316	5096.613	3956.938	4131.911
12.20404	10.50237	6.86445	24.18437	44.66524	209.6626	161.4433	470.7827	149.0335
1394.861	233.3515	1833.883	196.4914	450.6484	385.599	33.30176	259.8842	126.4359
12.19925	26.77318	8.211625	44.68983	21.35992	64.42028	30.27167	12.9469	43.16382
1346.396	1227.081	2425.044	1286.709	1292.704	1126.106	2077.591	1113.328	3942.748
351.4599	210.4129	434.1482	178.539	164.1336	153.424	74.67177	168.9347	298.0634
73.99064	22.94197	85.76337	48.59062	19.41528	31.71006	17.15668	43.32909	57.54957
113.8809	262.0781	80.82181	232.662	172.8793	215.8057	206.8497	86.59963	95.59701
39.73239	59.35691	33.50195	24.28998	1.930957	6.145807	5.048814	83.48975	4.122758
66.32039	89.91801	139.5396	8.522099	9.700006	6.146349	10.09416	143.1026	29.81658
171.3013	71.71993	106.2888	132.3732	53.40764	60.35239	256.2849	39.80251	183.9708
71.89214	219.0242	109.0541	258.4978	412.7997	120.6962	247.2106	240.0225	173.7069
58.48559	12.41913	69.48928	12.45927	26.22275	19.43738	4.039864	12.95363	7.206002
17.66385	6.681867	29.28461	7.775032	18.46086	14.3183	5.048313	9.505294	3.094337
25.46572	65.03229	36.43112	57.13417	81.58509	48.07711	95.85602	85.78976	37.01123
82.8686	70.78298	104.6853	30.47773	33.98533	19.44205	34.30943	67.59936	92.49174
338.2404	132.9274	437.012	265.4796	158.3019	373.3162	221.9882	212.249	183.9898
17.67935	10.51101	13.48848	20.40432	16.51142	21.47325	9.083702	21.69451	6.176798
97.31494	132.9295	107.7287	329.7744	201.0412	158.5391	474.237	115.1894	114.0994
14.40798	59.32126	16.25621	51.76607	40.79489	31.70845	43.3868	19.88995	26.72845
48.66626	34.41547	33.75236	93.21962	121.4149	93.07226	103.9282	58.0237	56.53695
79.63457	63.10532	98.34159	61.77829	107.794	43.9897	1026.182	869.8997	472.8129
38.61827	19.12607	91.77384	6.180993	5.816109	0.007153	2.021679	10.35604	6.177786
1.143501	13.37115	14.94379	7.740326	63.12473	77.73418	56.50779	4.278522	69.89656
243.212	427.5273	211.2724	349.2899	511.8489	294.5686	451.0346	415.0357	356.6593
64.14814	148.2497	57.96005	187.2506	143.7443	309.8811	149.3355	90.94648	92.51102
46.46483	220.0139	67.34314	88.46865	93.23515	123.7562	70.63429	50.20684	126.4206
114.9896	310.8604	129.2021	218.5313	250.5852	287.3964	226.0213	105.6652	229.2028
73.00267	1703.467	175.0021	310.8524	526.4058	891.8688	199.7916	566.6439	204.5505
144.7153	67.90482	65.92483	52.43378	20.38411	15.35163	18.1663	92.74051	19.54035
8.878199	66.07805	14.85501	31.42053	15.53537	23.52151	13.11941	12.96596	6.177677
40.94326	87.02322	78.11549	196.645	91.28889	304.7709	84.76093	65.80312	314.4884
23.23612	23.90169	21.60504	53.34992	60.23368	47.04221	27.24509	8.60952	42.13652
66.30813	90.88259	53.80947	7.739109	7.757602	13.30589	9.08512	62.39587	4.122761
3.35872	6.67648	1.473668	0	22.32807	16.37387	97.86969	11.20708	66.80383
68.56056	209.4914	159.927	97.09884	133.0642	96.14461	66.59828	219.3246	41.12396
179.0972	388.3256	208.5322	24.96849	24.26849	53.19535	44.40126	300.6688	40.09777
8.887632	31.54461	12.25331	208.5404	80.609	84.89402	122.0907	120.4606	20.56861
16.61982	5.720137	9.558922	41.50319	43.70584	24.55348	55.49463	14.67768	15.4281
34.25814	49.75537	4.177546	29.7565	30.10787	38.86167	16.14718	14.68615	13.37141
14.40371	22.94754	6.865814	69.94914	57.32447	30.68326	31.27953	21.63671	12.34379
107.2475	36.3254	96.93601	48.4679	110.7143	106.3761	104.9412	41.53234	259.0005
2451.813	1818.142	2064.811	1473.05	2810.736	2054.809	4961.4	3924.913	3081.46
230.8565	87.03708	562.9241	2.266394	12.61364	5.123296	1.012289	17.27163	9.262383
823.4625	567.1643	755.7709	135.3971	194.2354	321.1637	435.9016	783.3199	276.4961
175.6999	97.55308	154.5503	32.80565	59.23689	46.03391	48.43652	200.2475	69.89915
560.2795	102.3229	293.1105	85.28563	91.28554	126.8329	82.74384	222.6801	142.8752
17.72625	90.89136	33.69775	78.42455	46.61838	31.71199	32.29098	13.80863	24.67678
517.2347	161.6246	414.1026	16.35648	18.44134	19.44275	70.63588	22.47027	308.3462
35.41565	16.24035	35.11213	317.5178	82.54899	105.3496	183.6392	75.34768	21.59657
113.7313	63.13512	63.1298	36.77561	20.38576	18.41876	16.14798	51.12743	12.34549
22.15051	101.3945	52.52319	93.24572	135.9932	63.41546	76.68588	29.4097	17.48491
4.454733	9.562405	46.07981	0	1.931459	0.00666	0.002642	12.14736	0.008128
3.344771	0.940734	42.89871	0	0	0.006216	1.012268	0	0.007584
7.773387	18.1718	12.17983	32.23754	14.56455	56.21443	26.23188	6.014821	14.39367
70.71066	103.3328	79.1959	11.65677	18.44257	17.39632	20.18387	34.62978	22.62185
4.465277	19.1195	13.55941	39.22779	44.69042	19.43767	37.33173	11.21609	13.37077
64.15503	275.4663	146.6653	218.5333	171.9063	217.8529	222.9943	140.3381	112.0417

288.5529	27.71906	126.5894	1427.835	1418.013	720.0523	1682.046	39.80132	620.819
4.463571	29.63144	360.9619	0	4.844597	1.030975	0.003	5.144537	0.009246
0.016099	1.916954	9.837373	0	0	0.003595	0.001432	0	0.00438
0.033184	1.895432	0.107438	5.393949	16.50549	11.25794	93.8155	7.747254	8.232999
813.614	450.4557	1179.714	1824.788	1009.101	2151.962	1862.669	1438.251	2894.373
0.033463	2.851403	6.863056	21.90371	5.815594	49.07867	28.25237	0	14.39769
32288.94	34989.68	24097.42	20947.18	26560.23	26200.01	39120.11	26868.63	42569.95
118.2674	119.5576	169.3064	71.221	53.40859	34.78417	58.52637	81.4235	141.8336
139.3156	587.283	230.0541	448.0759	246.6901	318.0879	211.8979	221.7849	232.2947
176.8877	181.7111	192.4087	453.589	248.6343	330.3588	137.2309	194.056	139.7943
211.1715	997.5812	226.0981	524.7232	650.7303	408.0999	348.1175	271.1491	291.9139
80.73815	142.4879	512.6146	85.27489	653.628	399.9237	1489.329	8200.463	2150.224
18.83538	31.54319	119.8433	684.7137	498.2558	425.4771	482.3124	61.45824	136.7122
19.92001	157.0258	130.7583	10.88372	12.61603	12.28158	4.039874	58.15496	4.122919
26.56365	101.4121	29.69232	68.17971	35.92907	69.54517	30.27349	45.03876	20.56713
13.29746	5.720622	10.88141	17.17967	30.11103	5.123402	54.47995	32.07863	5.150674
64.13752	68.85372	86.1118	76.71906	102.9519	52.16955	83.75036	105.7138	192.1825
54.20642	70.7599	87.53222	296.1947	172.8812	242.3933	126.1301	55.39886	77.09699
108.2967	35.37264	120.9033	40.65772	34.95437	82.84545	31.28325	23.33934	132.5749
80.73753	55.45362	126.5527	89.97825	409.8728	148.3123	241.1587	227.858	457.3701
71.88458	120.5048	70.05565	207.6238	113.6302	203.5285	219.9644	66.66999	122.3151
17.69118	0.939927	30.77358	2.261225	0	3.077643	4.039754	5.146098	5.150066
1311.044	1339.932	1528.11	1601.518	2211.49	3127.712	2559.91	1497.146	3122.564
55.30778	25.80463	23.01521	100.9964	148.6019	63.4209	179.6045	161.1825	231.2447
49.77449	199.9362	159.8518	16.35308	14.55607	7.169074	20.18445	218.4999	17.48517
11.09288	5.720401	6.86602	14.02604	33.02416	15.34891	53.4729	6.009436	89.37409
26.57335	328.0451	169.6108	397.0531	660.4494	476.622	745.6679	252.0926	753.3873
1382.901	1258.633	2169.707	1787.115	3487.69	2214.363	3528.579	3606.938	1712.386
192.3735	618.8292	258.3274	525.5796	293.3076	388.6622	433.8817	326.6318	150.0746
30.98679	121.4951	35.07734	63.42972	101.9915	116.585	68.61382	35.48309	11.31812
90.44232	14.33293	80.16646	0.70282	3.872978	1.031373	3.030796	34.68015	1.03831
74.09075	52.58741	86.15888	90.80619	77.69113	31.71633	229.0436	90.94951	124.3683
109.4619	241.0311	115.7575	238.144	177.7356	176.9439	187.6791	122.138	154.178
23.25617	198.0379	68.62698	96.37072	58.26892	134.9955	29.26529	80.58193	32.90073
5.566286	46.92675	6.844142	18.81124	11.64884	21.47459	7.066296	5.147472	48.26679
11.0936	57.41474	28.26896	30.54085	31.07938	38.86207	26.23577	13.81738	25.69927
5.551778	13.40361	4.139725	10.99252	9.713279	10.22637	6.056049	3.420564	4.119895
8.885941	66.02679	14.91343	46.26841	46.62747	42.95284	20.18297	4.276212	29.80956
1.145582	18.17317	47.9347	0	2.902063	43.95245	1.01246	8.623219	3.094729
19.94013	114.7944	51.17335	282.5623	68.95758	129.8785	69.62296	278.4981	18.51255
26.56859	82.25977	37.76119	93.26298	50.49906	114.5404	74.66747	62.37594	118.1841
21.0403	9.545731	36.38268	35.98339	33.98676	46.0273	79.70922	9.473947	41.11706
13.30391	58.36384	16.25622	56.48881	48.56964	76.68809	22.20089	32.04864	56.51812
43.14928	109.9901	40.48217	109.6507	168.0369	63.41953	171.5305	83.15733	379.2059
21.02373	33.47848	24.2568	63.63538	36.91173	32.72801	31.27965	52.9331	21.58982
7.772404	0.939912	9.512153	6.185809	21.37166	11.25597	24.21418	29.52856	28.76588
114.9324	81.29463	151.7699	32.81179	37.868	46.03271	133.1878	82.30803	161.3495
83.97015	47.81639	53.81462	8.522118	23.29917	34.78095	28.25572	92.77666	24.67782
3.359473	6.676715	1.475943	12.44895	25.24679	38.86417	43.38654	127.6851	25.70074
98.35941	295.6452	248.169	1.485558	6.786576	7.169156	6.057927	34.61049	7.206549
15.50878	15.28887	20.25597	14.02365	16.50234	28.63967	22.20042	10.3456	13.37153
148.1261	70.76028	98.27421	137.8227	124.3138	112.5119	207.8576	75.33542	164.4522
6.676757	8.5892	17.62779	108.9791	34.95568	115.5617	79.71181	1.682272	62.69873
34.3103	0	14.94409	280.6559	162.2058	71.60144	332.9642	32.86941	41.12418
5.570654	264.0827	150.3142	3.046744	4.844348	7.169203	11.1032	541.5363	10.29029
71.8747	104.2493	146.5118	95.5304	80.60728	60.35162	42.38262	64.07991	94.56324
11.09523	0.940218	5.524079	71.45277	39.8234	16.3723	92.81814	88.54776	17.48199
7.733729	1.897383	62.37414	0	0	0.006308	0.002503	0	2.065365

730.7044	703.9123	3966.032	52.38513	501.1438	429.5845	170.5301	25.07582	424.5082
53.98177	23.92234	43.96739	0.701823	0	0.00706	0.002799	94.96983	0.008617
102.8357	309.9007	213.8459	66.48278	97.11361	257.7396	73.66268	353.5894	227.1488
79.63255	432.3243	238.1071	206.7209	225.3223	328.3141	145.3031	212.2547	60.65435
23.21512	24.86846	16.21274	6.963248	13.58942	13.30271	20.18153	29.48246	14.39675
34.25112	8.5907	26.91368	15.60136	3.872977	9.213956	3.030796	5.142892	36.99571
61.91803	132.0104	40.46008	135.6174	101.986	71.59724	68.61485	56.29187	62.70194
342.7157	1509.258	680.669	1427.822	866.3374	1037.114	912.1632	540.6141	264.1657
53.09317	210.4491	96.86566	141.8124	108.7786	100.235	135.2081	103.1029	119.2263
486.35	631.2644	908.8259	54.72692	102.9384	194.3387	120.0782	308.4328	169.6027
25.43935	16.24562	12.23186	26.60189	39.82563	31.70695	29.26244	19.02601	20.56334
66.3674	58.32339	96.96772	304.7203	251.5522	135.0152	266.3824	153.3298	326.841
15.51546	66.93093	5.503275	33.58901	603.1291	502.1998	2343.962	12.08032	321.7236
3205.714	4845.218	4011.155	6151.737	6788.901	5411.63	10993.38	3081.823	6066.299
15.52067	21.97975	65.94852	37.51973	69.92634	59.32618	78.70435	33.74306	50.37077
549.4333	7030.693	835.4487	4139.07	5873.05	5170.234	2844.458	2065.522	2928.311
58.56578	30.59546	117.9189	11.65758	40.78917	13.3056	1.01236	27.69001	18.51123
308.3812	160.6693	205.8273	179.3094	147.6199	120.6974	299.68	136.8581	99.71027
12.20413	50.68549	20.31076	97.21966	88.39264	65.45756	94.8447	11.20653	22.62289
264.1683	251.5463	385.7878	217.7298	95.17071	34.78504	27.24773	438.5398	591.9879
204.4749	126.2415	305.0626	118.2204	49.52113	158.5335	33.30188	201.907	87.37435
6.470731	1.916773	5.030861	0	0	0.003613	2.017019	2.639756	0.004403
166.6189	39.21238	108.4568	5.390489	1.931192	5.123561	10.09402	85.03151	6.178764
72.90173	27.72551	52.40901	17.14973	22.32929	17.3959	18.16578	103.2558	12.34533
9.993226	26.76639	40.40375	50.90453	45.64557	23.53208	43.38929	52.8549	89.40524
110.4957	35.3734	80.67394	28.89734	31.06919	59.32513	39.35486	45.88789	93.52677
1.142423	4.76553	32.43182	218.5147	220.4711	299.6713	442.9553	94.39435	310.3952
32.09874	72.68005	56.59085	119.0761	181.6401	68.53225	139.2432	33.73809	27.76308
165.7283	696.5827	221.4628	3.047918	0.960891	8.191997	21.19343	68.42902	6.178517
423.4092	873.2078	265.129	1627.523	1275.231	1457.48	1041.319	529.3431	480.0096
183.5384	776.6329	343.0739	574.8664	793.5088	430.6005	856.6616	165.4332	161.3818
7554.387	7489.713	10404.38	19673.87	12427.86	12176.42	20201.8	14237.93	23176.7
7.781016	41.13474	30.94919	30.53402	44.68478	23.52862	42.37722	35.53246	23.6454
88.4606	221.9075	28.39549	165.2687	110.7153	236.2551	173.552	92.67403	191.1712
23.23858	24.85774	33.64514	5.390599	8.729217	12.28235	7.066994	126.7917	8.234231
32.04635	30.60815	28.24689	37.64667	14.55972	25.57146	28.25284	25.11608	22.61621
2.252077	5.724334	8.151602	22.83073	10.67996	25.55375	7.065729	14.74012	5.148719
71.80563	51.6505	56.43676	44.63494	21.35739	37.84644	24.21949	45.91983	12.34545
150.3241	242.0043	259.3255	119.8095	109.7456	107.3969	74.67086	111.754	106.8989
19.89463	5.721739	70.50229	6.183004	21.3694	9.212349	3.03076	13.83833	5.150105
37.61803	148.2791	31.0629	90.09367	86.44239	73.64206	69.62367	46.75227	22.62373
92.86431	193.2256	83.44965	145.7292	130.1496	119.6654	87.78682	57.14313	72.98258
6.675217	2.85141	16.23222	40.80428	23.30644	48.05824	38.34049	44.25032	54.45367
430.0143	250.5679	525.9193	503.5738	431.2232	254.6842	727.5078	296.2798	488.2235
15.51394	49.74287	41.68126	39.15857	23.30192	39.8888	27.24567	25.09452	22.62005
6.676798	52.6139	26.97617	39.15597	37.87702	29.66511	31.28127	26.83001	11.31742
179.1113	1166.916	316.1344	267.0155	468.1387	429.5727	173.5563	309.2969	146.9913
21.04681	2.852365	8.215545	180.3384	101.984	147.2689	107.9642	8.60761	34.95653
287.4001	357.7126	402.0449	100.1614	49.52025	98.19749	46.41933	104.7868	397.7625
18.82921	7.632905	25.65225	39.92513	41.76158	79.76385	69.61912	31.16393	102.7534
3.358148	21.04996	17.47254	34.64739	11.6493	27.60395	32.28227	21.6913	9.258076
3.358688	22.9387	5.524081	32.05212	18.44223	31.71288	122.083	10.34034	13.37344
120.5173	133.8891	119.8055	226.3572	162.1917	191.2639	269.4086	135.9994	362.8077
21.04661	143.4903	40.46387	2.266464	6.786607	15.35174	16.14835	147.381	32.90101
77.38892	78.42251	158.5063	47.70659	121.4117	114.5486	5.048806	69.29246	12.346
36.52179	53.5438	27.04934	39.85371	53.40726	68.53438	96.86842	19.86996	86.34448
10149.87	11558.33	13473.83	8786.235	13790.49	7459.278	15784.26	6862.024	22571.31
131.5877	160.6611	205.9245	248.1932	359.3499	531.8535	709.3451	285.0162	503.6397

19.93245	44.95337	60.42002	69.80999	45.64923	38.86784	27.24601	49.40154	58.57888
8.886934	23.89947	16.26282	32.86953	50.51054	38.86633	66.59088	40.72671	21.59256
4.458534	1.895752	2.821169	23.64456	10.68094	8.187193	11.09996	7.767098	5.148382
50.88379	108.0761	40.48296	110.4327	185.5226	132.9593	69.62522	90.96086	48.31797
362.5118	197.0204	207.1494	7.746069	10.67189	18.42002	45.41025	13.80748	42.15337
1355.091	392.1239	870.1051	279.5201	203.9473	197.409	262.3509	786.7608	250.802
2.253087	6.677345	14.89121	53.4349	34.97135	18.41493	27.2436	4.276554	13.37043
84.05374	451.4548	153.4393	366.5829	330.2234	369.2235	233.0869	272.0584	203.5166
76.30637	48.76004	60.65585	92.35789	175.7968	49.10341	199.7856	63.20084	399.7875
48.68062	41.1078	64.69061	327.5618	227.2782	256.7106	418.7344	20.73645	117.1784
868.8876	264.9094	661.9247	672.6497	933.3423	2756.435	3675.892	14.68137	2082.398
190.1208	108.0646	262.1445	72.75596	82.545	82.85424	87.78851	215.7669	56.54218
43.13392	91.83152	92.6879	69.71716	25.24139	63.41355	56.50643	92.75543	23.6508
131.3473	12.41602	43.03629	33.64633	2.902051	3.077649	15.13887	127.6019	18.51067
96.17033	77.46407	149.1535	50.05481	51.46646	77.73578	66.59792	58.88204	105.8652
12.18932	5.721121	10.86834	51.13481	19.42209	33.74342	17.15491	6.011914	12.34184
179.1151	209.4413	74.1304	869.4862	451.6225	287.4119	200.8002	4666.99	153.1588
4.359208	7.720638	17.28671	0	0	0.004121	0.00164	0	0.005023
75.2028	176.9422	149.3162	149.5775	101.9724	95.12627	102.9232	550.485	95.59619
37.62694	139.6407	271.3774	16.35294	41.75143	6.145941	30.27474	489.8791	74.01206
5.569835	13.37736	2.829446	292.077	34.97345	21.48077	32.28688	12.95673	5.150584
51.54146	6.687887	15.95694	0	0	0.006549	0.002598	0.81669	15.40378
9.990498	114.8615	13.57459	40.75737	47.59919	36.81931	37.33345	23.36563	7.206424
2.252149	7.632875	6.870754	17.9323	40.78914	24.55408	74.66394	67.62813	95.56481
14.41372	9.54685	40.50243	198.1177	394.3361	507.2849	580.1807	1.685262	167.5429
28.78479	125.2947	61.97674	107.2853	88.37752	60.35207	194.7376	75.34838	194.245
16.62177	6.676472	16.27482	72.92107	52.44827	20.46404	46.41565	16.41017	131.522
47.57394	196.0807	95.57482	180.9633	199.1123	113.5335	173.5514	153.3683	47.29151
29.88418	147.3261	36.42757	81.46927	73.8139	63.41555	85.76633	55.42919	99.69212
110.5654	158.764	135.9014	292.2511	231.1613	165.6936	217.9485	134.2766	59.62546
740.6014	680.9765	789.4771	340.6142	337.0086	407.0787	271.4323	387.2597	843.8452
86.25979	323.2911	162.8046	924.8564	165.1053	475.5749	85.77086	578.9901	79.15399
7.781565	20.07254	4.177712	35.24049	24.2746	60.33266	65.58092	13.81379	51.38246
6.662377	31.61244	6.820923	19.66499	10.68044	36.78635	8.0743	15.61654	5.148554
72.99912	99.45238	673.2711	14.79013	32.03811	40.92183	3.030403	288.5497	6.177747
148.1486	269.7179	113.0981	321.958	174.8171	208.6519	171.5366	806.0952	125.4043
30.98061	51.64552	16.27903	54.82491	64.10542	37.84813	21.19296	45.03887	102.763
59.66535	43.03766	63.11125	10.09024	13.58574	2.054509	5.048936	69.37132	26.73117
72.87499	43.044	63.03041	3.825452	4.844086	6.146379	8.076003	26.83386	11.31724
126.0484	420.8538	173.5793	192.6307	218.5254	221.9481	170.5279	343.1537	108.9603
19.89264	84.2993	69.15138	4.612414	10.67581	3.077636	3.030754	34.74623	6.177437
21.0443	21.98069	36.41534	29.68667	61.18654	78.75154	50.45287	25.94389	23.65084
46.35452	22.95187	37.52768	5.393034	8.730331	10.23595	4.03985	42.5297	5.150592
241.0081	71.71255	172.2981	351.617	528.3556	587.0786	499.4686	91.78341	341.2456
186.1238	8.591789	45.4768	0	0	0.007212	0.002859	46.89389	0.008804
4.465241	21.98267	4.176589	73.70193	76.73987	81.81186	46.41577	26.81975	45.22648
58.58828	82.26475	45.78975	65.80159	37.87088	74.66002	47.42575	60.65086	20.56758
312.8143	98.4946	197.7883	254.5233	268.06	443.8816	119.0687	157.6531	701.9722
59.73787	375.8722	123.8883	277.9795	174.8113	597.3045	233.0885	156.7729	1056.575
7.781988	32.51308	18.94289	39.94425	64.11559	30.68738	28.25457	52.01863	20.5652
33.11072	55.53562	21.50603	12.4776	11.64709	5.123041	10.09307	11.22637	7.205076
1470.127	987.0315	2317.063	40.63652	31.06797	41.94401	202.819	486.0254	120.2692
13.28904	4.764557	107.6511	5.395768	5.816124	4.100455	1.012452	3.410873	1.038395
4.46473	3.80804	1.472694	25.76571	35.92743	37.84955	91.81849	4.277422	42.14755
162.5096	153.9763	221.9152	132.303	183.5605	112.5146	194.7431	161.1353	237.4268
27.57255	4.765714	20.10284	0.701707	6.789181	1.031462	0.002769	7.757756	4.122097
2.250608	4.764546	1.47071	28.89027	50.49565	32.73831	121.0812	346.0492	70.92496
16.54367	0.940014	26.53212	0.701906	7.764533	3.076842	0.002669	4.286904	1.038324

35.40612	22.93691	13.5975	79.12319	67.01425	177.9361	87.78378	44.1551	117.1579
276.3523	491.6275	303.9671	84.49269	150.5319	249.5646	71.64498	146.3839	239.489
27.65085	96.663	54.99914	21.87434	26.21818	30.68604	20.18311	22.495	41.1101
25.4554	44.95052	28.33668	48.56213	81.60049	24.55412	66.59305	24.21859	95.56508
46.24478	7.638592	18.76825	5.40433	5.81741	4.099922	2.021646	32.17925	9.257122
18.83231	21.02524	14.93609	60.32548	54.38938	68.52247	75.67431	46.77545	121.2549
32.10064	56.41417	76.75319	97.86824	178.718	51.14806	193.7292	71.87775	138.7545
39.83793	209.4748	150.6231	66.49721	202.9983	233.1849	119.0664	92.67786	82.23445
84.02313	207.5822	68.67444	118.2872	71.86538	121.7093	72.65192	80.55875	48.31761
42.0495	119.5452	275.5305	104.1105	33.98072	92.05818	108.9772	277.3396	139.7881
33.20503	202.7916	70.02929	107.29	83.52099	106.3715	129.1547	104.833	182.94
1550.87	760.3421	1661.113	837.9312	1048.925	770.1736	1283.487	460.0182	2257.115
27.57514	31.596	5.504387	0	1.931004	1.031462	0.002773	1.679698	10.28449
268.6589	1310.302	492.4262	1452.821	1242.206	1231.447	1017.103	1189.607	487.205
0.023377	5.756761	5.272307	0	0	0.005158	0.00205	0	0.00629
17.72747	21.98213	77.88368	29.69884	47.58842	19.44176	57.51407	12.07392	34.95272
383.6081	1267.286	706.1732	986.0704	755.6187	1175.183	281.5229	921.0403	280.6102
79.61175	155.9086	70.03574	142.5753	151.519	98.19108	139.2448	83.15079	109.9796
25.45685	30.59453	29.68206	82.34951	87.42882	80.78938	18.16595	51.12307	62.69223
24.35655	38.24685	25.6761	19.49421	58.27398	126.8043	69.62171	12.94005	12.34581
16.33019	0	47.98018	0	0	0.005297	0.002105	0	0.00646
23.20419	61.28291	17.52179	3.043406	10.67496	6.145602	2.021678	32.98527	20.55683
66.32427	116.7102	88.68576	244.0889	58.27134	101.2475	40.36357	84.93857	30.84452
44.19888	76.55348	57.67934	13.23227	10.67206	34.77579	8.075991	39.86547	32.89261
32.09686	49.72165	35.09844	155.2084	93.23988	121.7056	139.2417	58.88784	53.45466
324.9133	347.2172	436.7015	19.4859	43.69321	176.9438	45.41016	160.2809	35.9863
1270.127	866.5039	1398.923	1535.779	1279.106	709.8302	2496.337	927.9027	1899.44
18.80875	21.03685	8.200806	19.5486	30.11282	7.168541	43.38294	32.08773	10.28805
218.8241	134.8538	220.4399	11.65623	19.41218	21.48863	52.47305	45.00021	30.84704
66.23254	42.09295	82.93443	3.043178	0	6.146288	0.002907	245.1828	13.37167
21.0206	9.547636	60.20939	28.19469	11.64466	26.59361	4.03987	34.68039	18.50732
12.18757	10.5072	10.86357	14.04974	19.42302	5.123118	18.16341	6.012587	14.39551
44.24428	157.8511	69.95737	101.0911	93.24399	86.93475	60.54296	61.50046	12.34598
100.6267	45.88951	83.52291	50.03033	315.6624	205.5824	106.9601	116.0623	164.4579
32.09514	86.08113	57.90123	83.81005	99.07138	68.52953	54.48953	84.05055	20.56832
205.6392	631.258	337.6568	687.7455	534.1843	618.783	446.9997	331.8239	217.91
128.1561	105.2235	84.68657	33.6041	45.64103	28.64666	24.22025	62.37189	75.03071

TCGA-FG	TCGA-DB	TCGA-DU	TCGA-P5-	TCGA-DB	TCGA-DB	TCGA-HT	TCGA-WY	TCGA-E1-
241.3458	151.9024	41.63892	204.434	88.15446	116.1785	142.8857	135.9605	136.9246
26.14702	35.05461	22.03989	29.06014	70.5996	19.02125	47.95747	21.05392	30.31188
78.43932	112.9537	59.65355	56.6068	161.5244	135.1267	126.9409	115.8581	104.3747
1460.157	2525.881	2315.592	3161.211	3178.052	2743.067	3413.622	2474.181	1963.92
52.29422	222.9989	75.19567	218.6657	55.66323	224.4377	84.85603	175.2308	92.05141
2.012612	0	6.613704	0	0	3.361852	0	0	0.028858
9.052895	15.5701	107.401	65.7608	23.18387	20.14141	2.457806	0.941802	61.67489
317.7703	177.2229	53.09419	187.2578	219.0683	183.1559	208.5016	148.4105	170.5722
24636.6	5446.126	9748.354	9756.619	14154.6	6549.414	13896.57	6034.17	9673.562
26.14815	74.00569	35.93333	46.93721	36.1764	132.8204	45.35432	143.6635	47.15988
8.04698	26.29494	13.04049	25.39179	16.69576	32.34699	19.32092	37.36608	9.020069
1152.437	992.2392	536.5132	1381.477	1461.88	994.0363	1256.68	1165.281	1215.364
95.53532	115.8688	80.91349	90.92011	51.01949	83.80605	68.86721	113.9322	185.1644
0.001441	0.964835	18.34371	0	0	0.029402	0	1.90219	18.83811
55.31074	67.18118	65.38628	94.70514	101.1658	184.2227	68.04576	120.6482	108.8572
8.04729	4.857695	65.52798	12.58015	15.75263	19.02529	6.657208	27.75878	19.11773
1664.297	2083.803	1408.464	2413.798	4173.092	1711.084	2033.354	2064.372	2291.582
11.06414	40.89361	40.05327	52.22053	16.67887	53.6051	42.01017	73.7429	28.09087
55.31077	107.1104	1463.896	83.4959	104.879	13.45258	82.34659	19.13171	203.0436
238.3334	270.6899	951.3268	137.1385	396.3067	572.976	196.6296	424.162	1817.835
1373.671	915.3139	1078.063	954.0113	1644.747	868.9483	1418.946	1055.168	836.0747
27.1533	167.5384	0	49.97734	26.89553	75.8998	127.1391	15.30308	234.1282
688.8236	106.1348	6.470031	108.879	174.5173	29.08858	250.6248	65.09677	181.7576
1261.028	479.0909	81.71812	495.4015	314.6481	452.2986	453.1264	554.4194	99.92763
77.43339	86.66034	55.56708	106.678	129.9599	80.43197	149.6856	78.50864	159.2985
28.15799	35.05494	4.013678	32.81127	13.89667	42.42169	23.50215	42.13706	20.2329
228.2749	111.9717	363.2618	146.1392	181.9113	155.2714	95.76884	164.6825	123.481
510.8551	767.3035	448.9914	565.3321	748.0909	1714.343	410.1453	1558.833	379.3583
64.35641	29.208	5.648745	23.80626	107.7799	55.80229	76.6242	22.01087	17.99579
39.22101	83.74081	23.645	79.04883	65.88739	120.5893	36.92812	156.0998	25.86095
11.06418	102.2549	37.57538	75.36949	4.611344	69.24053	27.68259	77.56411	12.39647
85.47593	76.92971	7.284275	127.0155	33.392	67.00824	54.62183	121.6346	26.9769
4.024735	17.52558	0	53.32809	6.470245	28.9958	13.41717	21.06761	5.658256
411.2992	927.0016	479.2626	449.7046	674.7651	911.375	289.0955	1063.791	544.3118
119.6702	209.3523	59.63355	134.9393	117.8542	466.7586	152.9482	320.7758	110.0195
4190.407	13073.44	353.2851	9280.651	12268.52	21924.32	7601.337	19456.46	8157.478
9.052693	16.54696	50.01703	2.878725	15.75954	55.7449	11.71333	35.43732	35.88173
32.18172	129.5234	50.67348	71.60157	64.96534	79.29266	105.9668	63.19064	80.79207
29.16451	28.23064	63.00985	23.78243	30.60986	53.6085	22.6396	42.12423	33.69602
12.0697	18.49159	123.7793	13.324	9.251406	44.67683	26.01347	39.25346	65.0484
263.4689	62.30938	265.1038	118.5503	95.58098	31.32315	126.0603	23.91916	296.1885
25.14241	43.81374	8.101813	98.60006	22.2491	51.38689	35.26226	67.98775	21.36874
31.176	9.726182	68.71335	6.605993	10.17911	41.35389	14.22228	29.6679	28.0991
143.8028	126.5857	47.37082	223.1763	138.3003	131.7912	145.4345	214.5026	92.04732
207.1582	184.0303	143.0809	401.3763	173.5476	287.0407	241.2263	242.2427	157.1489
13.0746	14.59961	17.14345	39.66693	15.76311	25.68131	11.71837	37.3614	14.61906
12.0698	62.3177	20.37494	41.705	23.17606	48.04985	27.68083	67.02448	23.61425
12595.34	3400.303	804.7864	4477.812	4858.058	4071.012	5716.051	2687.703	3612.431
1661.27	914.3543	242.0463	537.044	499.3465	316.1133	718.7246	168.5027	239.0791
9.052354	19.47488	12.2261	29.18763	12.97894	22.32514	10.88373	20.10862	4.537595
6.035844	7.780728	5.654797	32.20181	7.399604	25.6612	8.351779	49.84427	10.13604
114.6423	308.6787	294.4751	181.9468	134.561	226.7403	426.2498	188.6191	333.2782
24.13705	77.89964	31.83429	111.9808	35.24592	135.0699	56.28983	116.8331	31.46664
1833.242	11276.91	153.7046	5862.918	5309.118	11053.68	4022.152	13956.61	3282.539
26.14565	14.59955	4.832737	20.86377	12.04415	33.47111	10.03175	30.64905	12.38101
39.22052	54.52659	23.65119	105.3029	35.24914	70.3557	66.40942	118.7594	33.70441
10.05818	21.4193	16.3157	22.35472	28.77948	32.36715	62.3912	24.89412	22.45518

1364.624	4865.78	5442.636	8022.414	4039.372	6423.177	4766.918	9655.475	5668.357
132.7435	77.88811	379.5571	99.84979	244.0965	570.6492	75.58437	92.86018	48.30876
14.08068	26.28616	5.648853	34.30339	14.82507	51.33862	14.23188	60.34523	17.99409
107.5967	65.24399	152.4112	45.46798	105.8639	10.10157	74.85391	19.13355	169.2385
1.007719	0.96393	17.94457	0	0.901404	4.516151	4.136245	0	10.14732
158.8886	250.2533	96.45214	227.504	207.9049	264.6868	255.5512	206.8175	331.0125
12.06914	20.44575	4.014248	24.61849	10.18498	50.15934	8.345784	51.74412	11.262
72.39975	25.31205	10.56061	71.81549	27.83398	21.25176	39.51931	45.01075	13.51188
259.4395	69.1316	75.23234	64.1007	130.8992	67.03252	146.346	48.82014	85.292
39.22149	148.003	260.9568	61.05988	207.908	109.4917	69.70424	103.396	159.3779
55.30066	17.52518	14.69152	11.86945	69.76641	16.76579	40.47849	17.23113	25.78487
2.013473	28.23877	0.750313	51.64184	15.75895	51.29841	1.618261	38.31222	9.024749
225.2585	388.5348	72.72269	311.0996	312.8078	283.6743	213.4924	415.5798	265.9597
9.052918	32.12818	8.10224	40.25861	33.40092	55.82424	37.80717	32.54681	15.75813
116.6512	188.9215	17.91631	132.8163	98.38082	49.18329	126.1066	83.29569	87.55042
15.08642	18.49172	20.38737	33.52357	39.90611	50.24814	22.64598	45.96124	20.24134
345.9308	263.8839	80.90294	374.5371	443.705	218.9226	226.1007	291.0843	239.0416
62.34597	37.97447	50.74525	47.02016	66.86368	14.56389	85.88231	20.09387	81.82371
243.3598	135.3411	705.3087	96.86851	108.5683	148.5818	73.90403	187.6606	123.4883
265.4822	281.4154	241.3008	189.4294	323.953	329.4414	301.8061	235.546	193.0374
1155.455	1308.705	548.774	1892.439	804.6993	2087.418	783.3714	2226.208	626.2462
18.10342	44.78601	12.1913	77.60256	64.04058	149.5446	37.77979	104.3858	10.15222
13.07528	327.1809	12.19619	426.0334	261.7412	375.2377	205.9149	728.7213	102.1693
3.019074	100.3072	9.737446	70.88282	118.8559	74.81611	87.46604	210.737	57.24264
74.41498	26.28145	33.47952	31.99256	81.69353	26.84718	42.83344	35.41509	46.03517
841.7027	975.6829	665.7511	2663.122	1914.833	1254.266	2360.447	1050.373	2096.264
82.46145	16.54227	75.21488	16.30044	131.8133	43.60104	41.96977	17.21671	50.54095
61.34469	133.4002	76.01068	305.2968	134.5786	128.454	182.4315	99.57029	103.2713
141.7943	182.0795	673.2311	175.9379	175.3959	465.7424	241.1951	295.8571	741.7369
44.24921	29.20127	383.2062	22.26838	41.7403	10.10116	39.44551	39.24109	103.2504
47.26639	247.3341	134.9184	266.3489	104.8599	101.6747	96.6124	190.5404	153.7668
14.08095	49.6594	23.65731	62.68969	22.25024	50.26488	53.79777	62.24281	18.00322
1222.826	570.6101	215.0485	874.2892	917.9833	798.5475	617.8238	650.1446	439.9382
1445.072	1706.965	1647.33	1370.194	1517.542	2405.745	1763.527	2266.412	907.922
2.012152	0	16.91475	0	0	0.024998	0	0.945602	4.459104
50.28099	61.34909	25.29695	33.50512	28.75221	65.87292	21.798	79.49008	14.63864
126.7047	63.28995	25.28448	186.793	59.39153	112.7613	77.33496	64.14779	77.43228
403.2542	484.9155	711.5975	361.6785	1025.638	457.9566	524.4824	726.7401	686.817
83.46686	84.71255	27.73499	151.5248	66.81175	140.69	71.41776	118.7363	31.47142
50.28233	101.2733	40.01906	67.09718	29.67358	110.5421	42.81939	80.42902	11.27454
40.22625	91.53776	123.6227	57.40276	42.67692	50.28256	78.1876	44.03445	63.97468
279.5498	85.68954	4.833781	200.9526	124.4017	44.71093	211.1927	39.2432	59.50256
838.657	222.0257	50.64281	137.9943	167.0817	92.7276	259.8557	64.13812	172.799
96.54152	164.5537	228.9883	128.2021	161.4776	144.1192	110.8934	169.4645	62.8975
120.6503	15.57514	6.473031	32.17237	27.86121	26.7835	31.15677	6.686222	11.25805
90.49984	35.05206	3.197173	71.01253	27.82965	22.37164	45.40471	28.71653	10.15028
15.08596	42.85396	7.287302	39.60201	3.682849	15.67064	12.55356	32.5586	3.416939
33.18616	30.18097	43.35617	35.02904	18.53863	45.78413	19.27977	38.2975	25.84203
10.05817	27.26626	3.196454	18.60313	14.8317	25.68686	20.15209	26.81196	12.38284
100.5595	91.53877	13.82762	73.11489	69.61405	103.8208	116.092	51.69809	67.33404
25.14053	16.54742	32.75421	10.35213	47.38383	16.78005	29.4365	2.855092	30.28483
853.7705	1868.609	1002.754	2309.416	1806.215	1564.762	1957.725	1635.411	1309.659
17.09774	81.80362	4.831945	77.65111	21.32097	85.95097	49.58107	67.98946	57.23168
1843.286	310.6147	805.7221	224.4217	992.2454	539.4657	414.3695	216.3787	227.859
328.8376	375.8618	219.9713	455.0234	357.3365	373.0487	567.4349	434.7068	292.9268
7.041388	17.52509	4.015484	4.37221	2.755208	8.973534	3.296958	11.47862	4.537846
1.007743	29.21925	11.39803	57.03866	5.540376	35.68102	4.137511	47.91726	2.293547
73.41154	116.8479	67.83596	84.2333	246.9529	107.2316	141.2355	113.9404	117.8374

33.18472	18.49492	36.84614	29.09296	36.21242	13.44084	14.23968	11.4748	67.19912
23.13134	53.55258	17.1016	99.31554	25.0338	63.66343	48.72659	81.39618	28.09853
22.12599	245.4035	9.739313	114.8556	103.946	261.2442	194.2443	300.7066	219.8809
37.21022	367.1	82.53478	432.6634	229.2387	394.2541	224.3925	934.5778	92.07429
187.0428	81.78755	117.7716	114.1017	176.3714	48.07084	148.7994	61.2657	149.2429
22.12598	88.6067	91.5881	65.57147	109.5233	89.36651	57.10939	88.08406	158.1923
329.8418	803.3754	28.5506	244.6285	438.1225	477.9678	179.0011	597.5174	600.2853
4.024767	54.52946	13.82956	79.15659	16.67824	104.899	22.63835	88.11093	42.66315
33.18486	15.57199	24.5168	23.83517	23.19352	33.4954	20.98332	22.01515	33.65468
16.09193	21.4142	20.39031	21.5536	25.97091	21.25696	7.497809	59.37931	10.15041
246.3763	234.6699	116.0854	524.6026	110.4262	257.9993	212.6475	354.2885	65.14065
47.2664	222.0129	76.81555	214.0713	175.413	519.2161	133.6096	378.2377	31.47523
359.0063	493.6865	454.7695	415.446	740.7064	263.6202	661.5795	305.4321	607.1022
9155.127	4387.681	1603.938	3059.727	8427.85	2805.624	7073.627	2799.73	4284.633
2.013475	28.23941	10.56774	56.91971	3.682884	15.66764	26.05444	30.64436	4.539409
31.17428	44.79967	35.1831	41.82678	52.01121	29.04759	44.59628	29.68	40.37787
35.19832	111.0201	30.2012	23.02412	100.2699	101.5893	61.35229	136.0012	100.9521
0.001157	0	0	0	0	0.023329	0	0.946799	1.150002
190.0162	16.55056	26.21663	8.86635	118.1987	1.161404	68.41781	0	49.23222
1.007545	1.93931	1.57524	0	2.766111	1.156326	0	1.901677	2.279648
14.07989	11.6774	5.653102	8.112934	7.398511	33.45706	15.94368	20.10564	6.779954
37.21016	116.8462	47.36967	137.9967	106.7282	183.1374	78.12872	141.7115	72.98189
19.10889	27.25627	31.84938	32.00405	35.25294	64.76409	22.63798	48.82907	18.0037
329.8439	439.1527	613.4729	406.4733	836.3057	425.5531	519.4686	696.1112	709.2231
37.21018	135.3485	143.9567	66.29814	110.4395	94.9631	85.69496	93.82477	90.93162
5.030163	7.782094	5.660441	14.91252	7.403066	8.965329	8.361105	11.48294	0.037185
1320.373	1279.501	28.55759	1106.219	496.5466	464.6626	1086.044	232.6549	1670.922
59.33366	163.5859	68.63731	100.6206	188.4179	172.0075	84.002	200.1198	101.0376
16.09229	34.07008	145.6239	8.100057	60.30766	93.83647	52.90022	172.3647	206.4189
17.09674	18.49608	8.928658	19.35069	12.04267	17.89399	10.87206	18.18414	15.74202
80.45172	261.9329	110.3515	393.1447	375.9181	453.4145	228.6068	436.6335	168.3715
271.5159	187.928	204.4691	294.684	297.9559	167.5563	330.3974	242.2486	274.9305
205.1442	117.8187	168.5151	99.14809	240.4306	131.8022	193.3698	109.1472	177.2943
50.28232	87.63774	35.10649	89.52485	92.82314	65.91304	79.009	68.93501	56.13828
2.013463	15.57157	19.58585	32.07836	10.18199	39.06972	15.92158	32.55618	11.26803
13.07538	29.2039	59.71484	11.08044	23.17791	106.0247	27.68655	127.3905	24.73217
117.6571	92.50172	26.9151	139.5281	184.7405	132.8951	89.07632	59.35176	99.88954
2.013472	6.807356	17.18538	13.39785	12.05432	10.07952	5.826496	8.605414	25.75676
0.001684	0	5.668417	0	5.546182	4.508267	0.780563	0	3.412433
13.07542	52.57749	26.10546	77.59658	74.25681	69.24741	41.98675	75.64531	40.43405
73.40635	45.76837	6.466512	17.81419	50.13402	23.48644	46.24898	25.84236	53.83602
27.15358	47.70949	90.04578	14.81212	19.46309	26.84556	20.95278	6.684563	135.6605
46.26079	292.1284	140.6414	300.6726	60.30084	356.2297	62.13662	518.0564	78.60366
6.036004	87.63589	40.01539	36.45588	34.31495	48.06195	10.85938	45.94648	34.83502
363.0288	549.1914	210.1463	462.4515	263.577	390.9278	569.9288	445.2346	445.5341
128.7126	35.04832	30.21096	67.92471	207.161	27.95869	143.9617	49.78731	79.6348
33.18654	18.49133	99.13113	25.28361	23.1807	25.72331	32.74821	21.05025	32.57118
41.23182	62.31733	56.41365	51.42151	92.83487	67.01658	79.03008	91.93046	108.8021
17.09771	55.50376	22.01864	61.93923	24.10758	21.26374	32.74106	48.8297	28.09326
9.052875	263.9062	14.64513	240.3621	30.59904	77.09678	110.9397	441.4998	69.61523
21.12023	55.49903	25.28592	85.06992	65.89559	82.63611	58.82021	86.18161	33.70781
131.7379	353.4691	27.73338	520.7655	472.4613	517.0643	342.12	746.8968	89.82917
91.50993	91.53722	23.6486	60.39087	82.61523	91.55941	64.71366	70.85481	142.4228
23.13068	41.87224	14.65524	31.29348	39.91237	24.60001	24.33716	42.13255	31.43884
345.9338	208.3701	407.2987	158.7733	422.3015	243.5237	190.7473	98.60422	616.099
214.1985	832.5597	269.0461	1120.596	157.7588	502.603	1138.296	371.5016	551.0106
6.036061	46.73607	21.19761	27.51483	37.10954	46.92428	11.70046	51.702	13.51782
213.1889	110.0277	105.4777	105.8677	287.7862	129.5701	183.2732	98.61267	259.1622

310.7282	107.109	19.5521	112.6175	212.5929	138.4824	151.3326	149.3772	105.5017
17.09771	46.73721	21.19984	91.89475	18.53559	39.11242	22.63901	61.2848	23.60908
228.2768	42.83341	342.6884	35.69711	130.8418	26.85317	52.88785	33.49479	50.55268
96.5413	143.1328	190.5558	205.8469	109.4989	165.3237	78.11003	128.2923	167.2372
13.07542	96.40616	35.92952	93.28769	20.39004	101.6038	35.2509	71.81141	16.88499
19.10854	28.23341	13.01626	40.28339	18.53958	80.31682	20.12433	48.84019	16.87613
123.6908	131.4567	98.9485	132.045	127.1618	128.4359	109.2645	155.1247	87.55694
23.13094	24.33568	18.74754	23.04365	35.25927	23.49053	10.0201	34.46302	40.40964
46.25846	51.61005	29.40051	67.21588	31.54162	29.06871	58.02761	55.5407	41.53284
3037.953	994.195	934.1276	1021.968	1124.037	574.0989	972.5836	825.3688	906.7419
18.10287	21.41532	16.29725	60.55515	16.68304	24.59642	66.51349	31.5938	31.43351
61.34401	77.89562	42.46923	99.95899	72.38443	141.8011	53.75049	180.9983	37.07943
116.6531	193.7742	301.0842	119.2795	244.1218	129.5855	363.2438	109.1428	262.568
256.4292	132.4262	27.73158	370.2858	77.01481	151.8965	264.0373	126.385	71.86378
4.024532	10.70829	3.201433	16.46141	8.337817	18.94697	11.75923	23.96531	1.168604
291.6307	640.7233	363.123	521.3754	426.0153	528.2935	717.0436	575.4582	768.6939
37.20712	26.28782	4.831268	41.08102	17.61464	36.84367	9.182719	57.47733	14.62956
1993.127	866.6275	535.7044	978.6476	1279.035	1463.065	1603.918	946.0118	983.0659
12.06962	21.41142	1379.601	14.07075	95.57157	130.719	138.6378	131.1619	140.3223
29.16518	156.7847	19.5527	64.83553	186.6147	49.18052	112.6552	104.3696	394.6868
117.6564	68.15622	58.8431	33.46599	172.6827	53.64542	60.48019	66.05798	148.0856
100.564	137.2872	268.2507	375.9407	366.6246	367.4567	350.5137	391.6195	235.6985
29.16493	28.22877	50.67663	22.2755	40.81912	31.31363	21.79082	37.32946	51.64563
229.2813	277.5163	242.0977	255.0904	350.863	243.4899	311.8723	287.2521	299.6255
2854.941	994.1845	623.2158	1553.029	1385.756	932.6212	1462.623	1419.977	729.4828
25.1427	109.0652	25.2827	97.74816	35.24458	144.0091	63.85798	126.4076	33.71157
10.05737	11.68094	8.951106	25.49221	9.264221	14.52292	10.05283	23.9559	5.65419
304.7024	480.0622	211.8012	275.9523	395.4072	348.4667	402.6583	681.7759	458.9588
106.5978	967.9238	23.64639	211.7537	522.5673	1036.336	257.1721	1555.078	175.1133
527.9505	1070.143	965.9973	911.4968	1807.193	1039.812	860.741	1514.787	1428.548
4.024764	65.24405	19.56063	79.89985	19.46381	52.49819	20.11311	74.69641	19.12516
7.041662	37.00324	7.285451	23.81491	17.6127	54.67613	9.181405	93.89582	15.75247
5.030372	35.04542	80.1591	23.76588	55.67499	128.3853	32.72442	134.0733	51.65175
76.42931	51.59711	129.9759	31.97051	64.94081	61.47868	57.9319	41.15485	39.32866
3.019037	5.831632	136.6929	1.39781	25.95985	33.54929	17.58426	22.96253	374.4249
7.041549	4.857868	6.469999	2.134612	18.55028	4.515664	4.977533	14.35073	30.2833
1634.123	841.3126	509.5365	969.7282	1306.895	670.1443	1252.535	815.7917	1108.723
50.28248	98.34954	66.22016	102.2157	140.1849	74.8454	85.73332	79.46898	116.6839
14.07983	22.39736	13.04318	18.62888	19.48812	39.01195	9.192885	25.85989	5.659149
33.18775	75.94354	63.73542	117.0729	274.7899	90.49754	123.5477	146.4979	132.4309
570.1693	116.8455	39.18588	129.0257	445.6561	39.13945	371.7587	68.92601	252.4239
14.08062	12.6489	33.53509	10.34231	23.18912	4.516212	14.23383	15.30572	16.87174
88.49615	184.0371	161.1202	115.5532	121.5734	243.4504	79.7984	242.2587	65.13671
16.09193	14.59612	4.831154	47.7693	36.19243	71.41195	11.70371	83.33833	6.784829
13.07517	10.70056	173.1758	4.368843	12.03794	22.36906	5.816735	12.43097	30.31733
18.10345	3.884414	38.38838	2.883644	10.17909	23.50078	4.977954	15.30198	31.46522
4.024344	1.93742	9.809255	2.139512	3.687766	1.16072	1.619382	2.856803	3.409754
130.7306	138.2701	85.01585	162.631	116.0104	217.7468	108.406	190.5529	107.7562
389.1752	1715.769	364.7528	374.3782	1331.985	973.874	743.0918	578.3279	3118.222
5.03028	2.911373	7.287963	28.2353	25.95766	20.15431	3.301875	10.51484	12.3959
9.052252	10.70443	26.23611	1.391132	10.19036	5.628574	8.354484	1.898021	5.657242
88.49546	90.55262	46.55305	181.3428	94.66022	203.2165	105.0528	166.6156	39.32735
0.001478	0	0	0	0	0.0302	0	0	0.031553
52.29293	55.50008	40.85032	101.5493	42.67826	97.12715	33.57237	86.18467	25.85667
44.24886	69.13232	74.41877	58.12797	38.95867	110.5425	46.18498	83.30248	22.49511
401.2425	579.3744	245.3167	557.9263	391.6686	693.5736	561.4968	585.9894	372.6117
45.25519	485.9062	77.62746	288.645	212.5326	666.6806	93.23813	877.1325	132.4682
167.9398	304.7798	217.5338	298.3524	285.8697	387.5401	388.3734	397.3709	357.9796

1518.484	2243.49	1704.54	1587.957	4333.613	4708.771	2285.49	4188.112	5179.054
796.4209	247.3496	8.104733	209.7266	148.5143	6.748408	116.8286	35.40989	95.41206
112.6315	249.269	907.2271	102.8266	328.5542	603.0987	215.9719	564.9301	509.489
8.047249	35.05507	15.47943	45.56427	15.75484	51.33186	24.34561	51.72181	23.5923
841.7029	1224.962	1239.989	1041.991	2399.348	1398.343	1398.718	1577.965	1839.306
137.7708	120.7365	389.4772	87.17993	123.4268	172.0141	90.72613	184.7946	84.21199
8.04729	12.64829	112.3409	12.58009	64.07742	34.63454	18.43922	29.67502	39.27965
39.22145	116.8432	76.82401	319.4382	62.15954	185.3942	94.93911	261.4165	49.42817
1.00774	3.884172	204.249	6.613531	12.04526	4.515123	8.347474	1.898023	11.25989
5.030406	7.77877	26.92769	8.096613	4.6113	29.07852	9.178206	21.04837	14.64021
58.32761	74.97149	56.37744	126.0719	122.5199	67.04657	209.4045	98.6176	141.3824
139.7818	309.6619	73.54596	181.9909	55.66033	150.7979	147.915	223.1026	101.0388
12.06928	8.753587	54.95692	1.392188	9.254541	24.57827	13.39962	16.26672	35.88029
4.024569	1.937114	20.5177	3.631671	5.544817	15.624	9.214149	23.9621	7.88675
31.17615	63.29016	70.33497	85.80707	75.18148	36.89522	68.91785	38.28662	55.01184
12.06977	36.02191	74.47588	38.73205	82.63195	25.72842	30.21237	22.00672	60.59472
143.805	221.0349	334.5649	140.8944	191.1861	91.63153	214.3199	58.38928	507.1683
4.024664	24.35352	4.838319	9.632994	8.332481	34.51858	3.298391	11.48214	1.168824
297.6615	253.1737	189.7373	285.7231	241.3239	283.6718	274.0467	232.672	186.3087
15.08621	27.26059	12.19951	54.55918	17.61248	56.9055	18.44425	55.55399	19.11373
70.39372	96.40396	43.29576	102.9879	82.60763	74.83806	135.4184	75.64043	89.76875
6284.06	280.4271	212.5909	1759.145	1149.099	134.0767	3066.872	89.98685	273.8708
5.03033	5.832164	41.86307	0.651189	5.540598	56.79272	0.780758	14.35398	34.72568
110.6166	25.30657	28.55639	106.7151	88.17773	60.33523	246.5543	17.21688	30.3473
145.8165	288.224	195.4369	443.8743	331.3533	263.6043	341.2735	374.3857	216.6208
27.15406	108.082	49.82609	155.9351	38.02539	188.7115	95.79685	156.0793	39.32763
67.37776	87.63307	11.37377	216.5264	130.883	152.9752	21.78668	120.6488	49.42003
78.44014	195.723	162.7559	176.7734	143.8546	293.6852	80.63894	262.3695	150.3946
89.50234	404.0976	174.9678	643.7611	90.92918	748.2767	268.0928	405.9732	187.4599
74.41538	29.20275	342.563	48.43081	14.82017	41.3564	68.08375	27.75186	69.57861
6.035862	6.80639	2.37963	22.39933	9.258411	12.31436	11.72634	25.86165	3.416254
181.0097	157.7487	34.277	140.9815	73.30309	202.1132	106.728	179.0625	80.83418
55.3062	53.56735	35.99692	45.56535	70.60316	22.36443	52.18133	33.51198	24.71184
8.047324	10.70001	56.44214	6.605356	7.394807	31.30564	4.137286	13.38731	5.662262
95.52991	98.36627	0.751883	149.5876	27.82316	12.3345	70.65052	17.21832	18.00298
52.2939	95.42471	49.01485	83.5047	48.23995	127.3065	54.5876	155.131	37.08105
52.29449	73.01971	220.8307	34.94784	127.1359	91.62956	62.97612	36.36664	158.2644
9.052921	95.42672	18.73477	156.7767	45.45633	293.523	68.89745	68.9321	23.61799
34.19175	49.66397	31.04819	60.50128	28.75743	16.79594	66.47568	35.4225	51.60325
25.1411	27.26279	13.02391	26.0816	17.61565	43.52032	26.04188	24.88968	14.62829
4.024801	19.46895	10.56509	35.09457	19.47548	33.49465	12.55336	61.31753	10.14626
570.1712	188.9098	290.5008	149.9212	180.0727	110.5952	301.0501	137.8766	164.9622
3261.215	3853.096	3878.747	3103.004	4326.208	2583.365	4122.243	2764.304	3288.117
7.041667	33.09766	402.3012	7.352159	24.10326	156.2715	10.85918	29.66639	34.83206
106.5978	333.9879	530.051	302.7811	262.6506	373.0552	100.8011	303.518	586.8988
12.0698	20.43729	158.7605	16.30046	44.52647	80.43369	29.35469	80.42356	81.94022
110.6195	83.73206	292.0714	103.6012	95.57688	137.4026	154.64	156.0659	172.8358
18.1032	62.32531	11.37529	50.73253	34.32815	111.5592	19.27523	57.45632	44.89491
581.24	54.51821	143.908	121.5004	120.638	54.77734	196.6725	22.00454	193.0419
67.37786	117.8232	10.55638	833.2616	67.7367	142.9383	99.17348	193.4393	26.98512
20.11431	27.25751	81.08247	32.01946	22.25223	40.21978	19.27588	31.58815	57.21366
32.18168	74.97886	23.64852	77.59033	73.3263	54.74668	67.23899	97.67703	41.55638
0.001644	0.963892	4.853047	0	2.757874	1.160672	0	0	2.290316
1.007631	0.964301	0	0.653544	0	4.48985	0	0	0.032861
8.046795	17.52658	4.016384	35.24302	7.40043	20.09574	27.80174	23.94719	2.293379
40.2254	10.70011	23.66074	22.29056	42.68887	22.37742	45.38227	12.42994	52.73835
17.09687	33.11106	10.56696	47.1343	21.3368	19.01112	15.08458	25.85067	13.50532
117.6585	103.2089	82.54931	172.2951	145.7118	208.8448	130.2578	204.9092	71.86829

49.27754	1332.091	37.55235	742.9158	1181.583	929.2337	575.7653	2617.895	749.6535
0.001866	0	4.017626	0.655912	1.829608	0.038779	0.78386	1.899161	4.537584
0.000892	0	4.240743	0	2.810083	0.017775	0	0	0.01855
10.05807	27.26824	3.196599	60.0119	16.69385	13.43358	31.14339	21.06249	23.56486
2578.399	1999.095	880.8786	1535.089	1913.893	1503.328	1731.608	1879.582	1484.709
10.05821	43.83199	5.650625	35.8742	5.539441	26.80355	26.90136	6.685366	86.17193
48762.35	25018.32	25335.21	29313.08	41633.94	25470.49	37079.79	22739.72	27363.19
35.1988	61.33851	100.604	28.23931	82.59663	73.73753	44.49388	61.26854	116.6997
190.0628	243.4328	118.5371	244.6388	164.266	341.745	177.3234	285.3365	150.4151
242.3538	219.089	104.6306	213.3023	155.9133	450.03	123.5124	367.6957	102.1679
218.2209	446.9447	136.5213	593.7828	614.4588	859.9328	246.2372	795.7037	160.5277
4601.69	1010.738	1589.3	2407.231	3869.647	405.4762	2221.737	472.9893	1269.242
107.6032	353.4711	39.18278	209.5476	280.3025	443.3597	333.7224	626.2442	88.7062
7.041612	9.727313	124.0328	6.608193	14.82835	14.55627	8.342111	24.89047	10.1463
11.06416	35.04853	20.3806	40.9837	18.53554	44.6891	15.90669	67.99128	12.39558
118.6453	17.5215	17.95623	37.37569	37.14871	12.32387	73.369	14.35024	25.81331
43.24347	88.60895	62.12068	100.7083	115.1038	51.412	101.7176	97.66579	81.93515
230.2841	147.0337	37.54843	159.6298	106.7235	196.5482	182.4262	156.0727	53.9149
43.24314	31.15007	37.566	26.0071	49.17533	35.77985	53.76403	26.79351	97.60868
136.7656	452.8058	76.8126	101.3493	1047.177	307.1251	280.7653	377.2717	477.9807
133.7472	175.2787	53.9163	205.2244	116.9411	160.8167	114.2992	194.3864	83.07745
11.06325	3.884363	21.28333	2.879465	11.1194	1.16143	1.617686	1.898012	3.415999
3251.157	1979.612	2608.463	2541.358	3241.185	2119.849	3074.048	1714.879	2083.979
67.37805	120.7429	85.02568	166.4011	141.0876	60.35084	148.804	90.95434	85.31652
70.39405	29.20174	110.454	23.01693	39.88599	8.984392	31.88012	2.856116	66.23402
2.013468	31.16063	17.12731	19.3353	34.35047	8.980909	45.45679	28.72406	61.61612
560.1271	385.5997	46.54481	472.1882	796.421	500.3464	631.3399	407.8948	469.0795
3522.675	4536.669	3422.326	3564.029	2993.342	5578.74	2939.512	2841.865	4401.317
170.9568	231.7439	154.527	708.0792	136.4132	291.5223	292.5006	592.716	127.9815
33.18715	50.63023	13.82778	116.5203	44.53613	79.28096	41.1482	72.77337	15.76234
4.024798	0	45.89521	1.392379	7.396388	4.515953	1.618287	0	9.024905
142.7969	111.0045	104.6774	86.47323	201.4486	70.39705	190.0393	95.74339	136.9018
65.36723	149.9535	64.5482	196.9478	100.2218	163.07	131.1031	233.642	84.20743
56.31558	43.81087	24.46571	76.0814	22.24668	88.22081	17.58476	127.3701	16.88514
5.030233	10.70492	4.837097	3.627086	9.261399	13.4193	12.5811	29.70645	14.60103
2.013464	31.15977	13.02325	38.83557	13.89822	62.44996	18.45082	54.60315	5.661937
2.013333	8.762799	0.750136	10.46604	11.14809	15.57933	0.781181	8.615688	4.524017
42.23484	44.79864	6.467277	44.8189	16.68428	58.0112	29.40617	88.14818	4.539808
0.001741	0.963786	0.749512	0	0.900996	2.280475	0.780568	0	16.83659
42.23733	58.42178	79.36394	74.60983	11.10725	127.2417	36.93801	48.82432	7.907331
34.19274	80.82465	18.73817	36.47456	54.75272	54.74217	32.73073	67.02523	44.91623
160.8878	35.04927	38.41407	59.70972	61.27107	36.87841	66.44557	34.46104	33.69385
6.036058	30.18325	9.741714	20.06854	7.395371	51.33621	31.93101	39.26097	15.75279
80.45025	95.42496	53.10885	44.66405	68.66767	63.69285	119.38	106.2833	120.0641
8.047194	20.44302	27.80123	26.08437	10.18245	66.8947	24.35643	31.59929	5.661793
22.12249	17.52737	23.76879	23.93872	19.49738	16.75666	18.49934	24.9084	15.71961
56.3158	53.5493	99.81585	79.80364	36.173	55.87081	46.18483	42.11644	40.44056
60.3367	54.52892	203.2786	63.42749	60.33484	36.88416	62.21579	25.83815	103.1618
57.31721	74.02913	8.104265	26.81457	25.97671	44.64902	30.24746	70.89193	22.47256
3.019047	5.831586	19.55466	0.655314	31.53147	10.10182	1.621525	7.642097	12.39683
121.6652	29.21106	8.105503	53.10155	34.3476	23.47409	67.39845	22.9725	16.86898
320.7864	139.2435	147.2279	179.0565	228.3588	112.8257	249.7375	105.3164	164.9584
23.13134	45.76055	13.82793	64.89558	85.40925	40.23725	89.99094	18.17515	95.34351
52.29397	146.0675	19.55225	154.482	245.1092	113.92	128.6277	198.23	161.5568
34.19279	14.59496	109.6898	5.861172	36.17633	4.515067	13.3812	1.898872	3.415202
44.24917	51.59962	115.3434	38.68998	90.02461	81.55121	68.04848	72.76103	87.54854
20.11388	20.44148	4.013688	13.33272	85.47849	12.32992	49.64599	45.01186	41.50524
0.001558	0	7.36327	0.652867	2.761363	0.03194	0.782305	0.942042	0.033377

198.1089	63.28249	282.1148	60.31651	776.8577	63.70973	378.1961	33.4962	856.2845
2.013478	0	41.92114	2.880003	0.900995	6.742386	0.780567	1.898031	1.168835
113.6363	133.3967	166.0243	82.70477	204.1994	124.003	84.00092	137.8716	199.7539
428.3899	63.28197	326.3954	561.9177	155.9124	287.031	308.5139	136.9094	83.09416
18.10199	15.57398	40.17821	14.10645	19.48281	5.631522	20.15763	14.35177	43.68582
12.06934	15.57236	8.926599	2.878781	12.0413	17.89807	4.976984	8.600868	54.89681
64.36016	67.18554	24.46548	92.53019	28.74583	106.0708	32.72533	99.58936	42.68065
417.3328	595.9238	313.2046	1468.861	429.7185	881.2168	499.261	1596.187	251.4287
25.14279	68.15562	80.94813	107.4171	66.81049	103.8706	88.24322	121.608	42.6893
142.7997	249.2721	562.0153	199.8308	211.6025	112.8525	296.7055	89.02966	701.2927
6.036043	45.77441	33.54212	35.07569	10.18182	32.38876	10.86642	44.05719	25.82755
186.0397	294.0772	71.90704	149.129	309.1045	186.5307	244.6254	214.4803	273.7973
2527.104	1694.335	21.19643	1699.451	891.9769	111.739	1366.883	297.7669	876.4463
1924.753	3518.118	2450.514	2169.775	4358.664	6282.454	2677.197	5765.12	6759.143
183.0159	102.249	28.55853	55.89362	194.0781	98.26275	130.3756	66.06292	58.37729
960.3663	3298.062	1027.274	2248.149	3312.637	4664.046	3849.058	3695.008	1181.748
4.024784	21.41346	143.4736	20.79839	11.10814	41.33378	26.01242	41.16916	42.65168
78.44033	112.9452	508.133	190.9231	158.7011	167.5547	175.6545	159.8938	212.1077
93.51987	49.6576	17.10269	67.15551	81.69898	120.5258	82.42342	68.94496	59.47859
116.6531	147.0297	170.9305	79.71747	116.0003	43.60859	57.93405	21.04688	64.0163
54.30551	46.72882	283.1332	85.70481	143.862	68.17126	52.05264	90.95086	195.2432
0.000896	0	0	0	0	0.017871	0	0.95152	0.018651
31.175	12.64827	77.02199	9.59134	18.53924	14.56355	19.28122	11.47289	14.63494
16.09198	8.752608	56.47833	4.369221	30.61481	23.48941	19.2781	15.30373	20.24107
38.21442	39.91934	46.61147	58.2075	19.4647	53.60636	26.00785	27.75496	24.72887
108.6043	55.49905	127.7156	25.26268	55.67879	40.24145	62.18781	39.245	61.73456
37.21025	136.3178	14.64717	116.2907	189.3428	272.4876	314.4482	201.0752	141.4259
55.31054	84.71288	36.73851	101.4513	53.81244	63.69009	95.82364	90.00267	85.30049
2.013348	1.937617	76.05571	0.655583	57.53057	20.15311	1.621824	20.08962	14.64135
940.2519	1325.268	312.3833	1582.96	583.7978	1509.974	912.022	1059.957	444.4437
353.9782	557.9559	177.4241	530.36	160.5441	515.9959	348.8083	724.8447	235.7085
5140.715	9682.873	5093.312	7468.794	21460.2	13085.39	8668.881	12848.72	23793.32
12.06948	26.28754	20.40422	33.57246	25.04927	32.38989	23.50648	45.01507	20.23026
36.20457	114.8974	76.01239	172.3419	51.02109	123.9866	137.8488	134.0478	68.49716
6.036061	3.884062	116.5005	5.114168	17.61206	4.516229	10.02248	22.96977	6.784467
16.09141	24.34103	22.87908	20.09085	14.82923	30.15	18.45653	26.80858	16.86507
11.06238	6.808617	4.843412	12.67408	17.65258	11.18058	12.60401	17.24433	6.768636
45.25284	22.3875	29.40208	18.55541	39.9043	39.10352	22.64425	30.63045	20.24243
51.28856	87.63065	69.46954	80.49248	92.80211	158.5789	40.28311	121.6004	56.15511
0.001754	3.884343	48.48673	2.879379	12.04912	5.629379	19.33437	14.35547	24.6635
125.6992	74.00375	26.92187	138.9117	84.46916	97.144	94.174	69.89513	15.76312
16.09229	86.65938	68.66355	217.2803	72.38115	96.06052	48.69867	177.1604	55.02753
6.036007	34.08553	8.107507	20.84373	12.04166	42.39326	5.818167	41.18868	26.93543
310.737	368.0691	432.6782	452.008	891.0876	432.2447	427.8394	442.3624	641.8852
17.09743	35.05276	18.75351	48.53125	6.466861	51.34864	14.2298	65.13262	7.906668
18.103	37.97507	10.55896	57.52411	37.12339	51.35062	24.33621	40.21549	28.07983
93.52474	337.8863	143.0723	170.7272	214.3865	657.7687	138.6347	294.9045	150.4234
9.052934	25.30663	24.4642	76.06723	47.31603	29.0844	80.69195	8.599529	42.6831
37.21026	110.9968	316.5727	45.3905	123.4216	126.2473	42.80036	155.1034	129.0966
33.18634	58.43067	13.83279	75.47824	44.55203	21.25912	72.36875	59.37593	51.60799
17.09534	14.60408	7.301665	16.40955	4.613101	25.64	10.89299	45.06272	4.535943
10.05855	18.49092	1.566508	49.96448	20.39288	30.18903	49.586	44.03983	2.29206
151.8491	201.5647	158.6567	156.6048	315.6116	202.1538	203.424	224.0598	214.3372
72.40484	53.54968	102.2804	15.55529	19.46129	15.68629	23.47108	16.25938	113.3067
57.32153	54.52259	159.6162	47.66099	32.45823	141.7897	44.49915	38.2849	22.49567
656.6455	81.7879	65.37875	164.9076	280.3807	73.74671	266.6129	18.1742	106.6251
8996.249	12065.62	14297.79	10142.94	19929.65	12674.38	15149.82	10723.06	18784.87
294.6472	418.706	148.7927	764.6858	375.8968	359.6568	627.9524	370.5464	713.6876

28.15855	74.01962	18.74972	35.77489	49.20095	65.84672	24.33214	78.54384	22.48155
5.03044	58.43478	27.7752	48.53278	19.4689	27.94426	28.55103	37.34143	33.67812
5.029961	21.43655	4.023252	40.75783	2.756637	14.5057	5.834933	10.52836	6.766435
101.5675	179.183	35.91804	107.4149	55.66787	257.8558	40.28759	197.2757	35.95929
38.21587	27.25339	297.7935	21.52291	24.10148	27.97257	56.25214	8.600631	31.47526
534.9881	335.9338	2248.266	303.5142	433.4444	264.7395	376.5472	255.6387	583.5488
9.05267	19.47022	8.108417	42.63979	23.19827	16.78085	42.10313	17.2254	10.14417
159.8946	208.3764	160.2692	290.1782	157.7688	308.2457	251.3269	315.9817	158.2672
171.9598	141.1915	188.1672	174.5798	191.219	145.1961	244.694	154.1592	133.5533
57.32232	118.7919	13.82819	213.4055	62.1605	199.8964	147.095	310.2657	43.81738
5166.851	1950.403	927.4947	3768.571	4167.543	686.9281	2900.91	280.5297	2640.56
72.40643	81.78545	208.6092	57.33472	138.2874	100.5514	70.55057	134.0439	99.91175
23.13133	31.15117	20.37639	36.47866	22.24824	51.39228	28.52473	58.40516	12.39641
12.06966	26.28452	12.19598	2.880246	29.68719	6.750559	2.457865	7.64208	7.906956
58.32725	36.99228	64.57736	93.98171	104.888	74.84919	76.47002	87.12986	84.17764
44.24273	32.14225	3.19669	54.75761	13.90517	16.77244	24.38797	34.48792	10.1401
65.36753	815.0396	109.5281	490.8358	214.3829	494.7675	211.7731	532.3781	94.3195
4.019615	0	0	0	0	0.020472	0.80562	0	0.02137
41.23264	75.9431	58.00474	58.8306	125.2927	158.5956	94.10266	162.7768	121.2194
75.42278	14.59487	50.64539	14.80926	500.477	75.97931	26.83029	137.8832	39.32728
4.024785	13.62497	2.379603	48.6839	13.90269	22.34511	20.99851	20.10225	10.14267
1.007706	0.96397	2.386636	1.394189	0	1.160218	0	3.816518	0.034731
9.052825	37.97823	4.831157	30.56517	13.89694	49.10246	15.91855	51.7225	5.662205
81.45158	44.79137	4.014324	31.27233	45.47907	15.68168	68.14489	13.38786	76.24939
22.12596	698.2195	11.37768	167.7829	335.0945	221.1461	160.5175	217.3512	762.8585
52.29399	115.8757	40.00878	93.20814	147.5959	82.67101	93.28488	129.2675	118.9508
84.46842	81.80881	11.37527	57.4723	64.99092	25.72339	101.014	55.53993	37.05303
19.10914	130.4803	43.27783	131.2727	83.51609	222.2017	91.5868	153.204	69.6171
43.24302	58.42087	31.01738	113.4944	42.67617	73.71262	62.18702	78.51787	31.46534
52.29435	198.6468	22.00601	255.2099	134.5743	202.1371	152.1346	374.4279	116.737
275.5409	364.1723	620.833	228.9006	473.3558	572.9621	339.55	489.2786	579.0687
167.9387	149.9513	116.9156	229.769	42.66483	327.1878	81.47727	218.3131	33.71971
39.21864	37.97713	4.831108	61.31053	15.75423	45.76723	52.17315	35.42738	34.79112
1.007744	5.834182	6.490399	13.43577	6.476197	17.83711	10.06511	20.12546	5.650181
2.013249	33.09579	161.9196	32.71003	17.60501	15.68495	16.74488	39.23916	24.74139
177.9949	218.1177	198.7487	346.2215	55.65971	207.7452	96.61014	271.9382	86.45725
7.041699	63.29672	67.10687	32.75466	44.54349	30.18913	24.32237	59.36853	38.1797
39.21955	14.59582	117.2178	15.56636	11.10829	12.33319	17.59366	16.26144	14.6367
48.26834	7.77896	85.26273	12.58244	15.75361	14.56205	20.12705	1.898309	21.35596
169.9503	185.0069	141.4572	270.7996	102.0729	321.6268	173.1311	253.741	55.04105
4.024723	4.858358	68.30457	4.373322	5.541335	23.43311	4.980671	14.35599	9.015939
48.27057	85.69603	16.28318	87.34392	51.03897	153.9906	37.78285	118.7598	20.24871
22.12398	0.963853	29.4718	3.62388	17.62143	6.747644	15.09031	5.727724	32.51604
342.916	545.3016	362.3368	346.8232	485.442	434.4617	683.4797	304.478	520.6863
0.001778	0	13.85764	0	0	0.036814	0	0	0.038487
53.29734	51.60921	60.56029	61.96143	121.6797	60.2917	92.57842	46.91619	52.73806
30.17029	34.0735	35.94154	63.41415	25.96343	70.34817	39.47062	61.28087	25.8542
74.41797	209.3505	247.0139	87.91706	301.6631	307.1267	107.5358	376.3133	191.9231
296.6579	916.321	97.25896	369.2204	192.1073	205.5423	516.159	693.2593	814.6205
22.12515	29.20764	14.65513	32.04211	57.5725	30.17434	52.99956	33.50824	26.95908
13.07429	8.754829	20.45058	7.366145	12.97784	16.76663	6.664456	22.02532	10.13719
327.8332	110.0213	510.3532	80.45041	432.5035	129.6092	149.5542	75.62458	231.2293
3.019141	4.858159	31.97649	1.391348	4.61181	11.20202	4.979405	31.61475	12.37549
348.9248	86.67074	5.64948	97.07995	85.41167	8.984842	167.5058	15.30227	22.49129
97.5467	180.14	199.5784	97.63046	381.53	145.2175	144.5484	197.2447	218.826
1.00775	0	18.84152	0	4.613604	3.396624	1.617792	1.898095	4.535117
304.6899	109.0632	2.38439	209.1436	126.2536	27.96907	124.4541	14.34425	26.98298
4.024407	0.963853	55.42728	0.650858	3.687064	8.949215	4.148161	3.815034	5.645821

30.17047	118.8117	34.29523	79.84321	47.32113	90.43952	72.29562	118.755	50.52276
120.676	116.8393	748.6958	195.3779	218.1085	113.965	100.8079	113.9271	261.4835
6.036058	21.41555	31.06692	23.06499	20.40007	15.67617	11.7064	26.80309	20.2337
10.05853	67.19701	16.2889	61.97451	22.25234	29.06827	39.48985	58.41565	49.37291
8.046502	5.833357	11.42368	3.629528	4.613924	7.851495	3.299153	11.48368	27.9738
29.16453	59.4005	13.01133	44.72668	38.96989	75.909	38.6369	55.53655	44.90234
67.37793	91.52742	91.58138	87.97248	244.172	46.95251	174.9013	72.75912	155.9591
73.41172	128.5327	75.19765	88.70371	135.5123	192.0658	107.5708	220.2467	94.29277
35.19877	80.81689	44.9239	82.76535	52.88346	90.47562	40.28908	82.3398	41.56668
228.273	61.33582	414.144	37.18782	58.44708	55.88955	51.21168	82.33199	222.16
51.28836	93.47637	44.92067	118.6169	52.88176	150.7445	73.09437	87.12656	61.75756
604.3776	1253.204	960.2424	807.0185	1528.704	704.7871	1070.042	877.0628	1363.496
0.001725	0	4.018394	2.134996	4.613459	2.280206	4.983652	0.941395	4.535354
213.1932	1070.141	240.3992	1039.054	472.4117	1705.425	572.3863	1271.568	500.5543
0.001276	0	0.761982	0.666136	0	0.025852	0	0	0.026999
41.23111	44.78941	108.1304	26.02576	47.33171	8.984654	72.33743	10.51454	49.38276
224.2548	497.5762	380.2921	265.4493	474.2778	703.6349	210.9205	856.9719	245.8152
52.29401	96.39729	37.55305	109.6411	69.59354	118.387	71.40862	125.4357	56.15143
19.10879	40.89404	12.19367	60.46414	20.39397	66.98007	25.16777	55.53931	31.45159
40.22583	76.93223	110.5596	43.96694	27.82143	65.88375	107.7068	55.53393	86.36381
0.00131	0	0.760315	0	0	0.026582	0	0	1.15737
18.10157	12.65215	32.80023	7.365276	17.62808	12.31557	9.193377	16.27151	57.05471
13.07541	32.12484	15.46441	44.69894	30.6052	148.4231	16.74517	41.16185	25.85647
34.19119	27.26032	63.91156	12.58338	28.76304	30.16935	15.07394	32.55206	20.23471
22.12591	74.00167	27.73767	116.4331	55.6729	128.3959	90.78942	136.9429	42.68407
78.44006	77.89009	281.4674	149.1581	44.52183	59.24087	131.9446	27.74915	297.3178
2118.833	950.3642	1963.122	1341.136	2746.496	747.2327	2222.555	1261.025	1414.01
24.13488	24.34329	8.109879	33.63621	12.97305	14.55008	33.6672	18.18509	22.4521
152.8534	45.7553	230.756	36.44318	58.44837	30.20584	153.8375	17.21677	53.91302
2.013462	4.857721	132.202	0.652438	11.11074	14.55852	0.781544	0.941649	3.416914
3.019154	14.5982	39.31728	8.104007	11.11222	23.46771	18.45685	26.80873	21.34207
42.23145	33.11858	29.50061	23.14129	29.72336	16.76937	40.46339	14.35366	25.79144
24.13702	52.57684	19.55531	64.12559	33.38935	69.25163	54.6093	104.3817	15.76296
164.9219	304.7913	371.4733	161.8268	262.6877	172.0129	353.9811	237.4659	355.6705
30.1706	45.7586	16.28138	97.76419	44.53168	111.6489	35.24933	80.43111	25.85941
158.8895	307.6979	121.8002	423.6936	207.8871	574.0358	173.9436	726.7733	112.2733
17.09784	28.22886	72.80293	23.02285	30.6043	15.68568	17.5856	39.24532	35.94951

TCGA-06-	TCGA-27-	TCGA-27-	TCGA-WY	TCGA-FG-	TCGA-HT	TCGA-DU	TCGA-P5-	TCGA-HT
96.22828	61.23477	237.4412	98.72387	36.99188	115.5958	282.724	162.708	93.08146
27.34465	5.71501	3.3742	11.08392	46.21887	16.64754	10.38411	40.15902	26.70632
49.22802	23.76784	78.81445	131.9642	122.239	138.1351	55.61598	146.225	205.2991
1698.884	3554.105	2293.831	2236.355	1608.65	2991.879	2904.706	2292.336	2977.266
180.3606	148.5379	82.15069	175.2804	197.2196	51.91411	171.347	101.9558	53.40556
9.755506	0.086965	1.143269	0.001781	7.177892	5.883319	0	0.007172	0
110.2814	1.534837	22.227	9.069374	21.58009	84.26815	11.25271	19.57595	18.92263
62.3484	73.71597	64.40508	160.1713	103.7568	150.8666	122.6022	147.263	136.2167
6142.801	4599.712	4839.568	4332.687	6184.956	8967.799	3164.69	7275.531	8997.326
28.45739	41.7526	36.65452	97.71219	46.23146	39.18057	68.70364	54.58372	46.52955
9.869628	15.34738	5.592253	10.07606	37.99182	10.76994	32.22508	19.56828	13.76283
486.5151	84.85191	449.4825	951.9625	559.8493	1015.905	903.57	1931.886	1296.8
145.4105	547.692	341.7153	90.66538	182.8466	151.8449	148.6978	110.1963	62.88838
0.023546	29.1582	17.5621	2.016894	7.183495	0	0	3.09557	4.29935
50.32014	45.95647	55.52187	213.5561	67.80427	104.8227	106.0961	109.159	50.82398
34.99117	261.7523	28.87376	11.08402	36.98174	16.64697	19.0901	28.84001	13.74259
1663.893	588.8231	1863.253	1586.604	1575.776	2617.651	3481.354	2196.561	2857.441
22.98924	37.55403	17.79776	92.67276	43.14647	21.54531	55.66893	37.0782	24.96023
340.9183	153.9983	91.01333	104.7665	127.374	460.4969	13.85882	48.41122	41.33375
542.2506	6545.596	146.5319	94.69567	1584.987	379.1218	240.8476	421.1957	480.2291
742.3128	123.7307	522.719	1048.669	767.3458	845.4429	895.7493	878.4217	1300.262
5.505156	4.324243	10.03384	5.039911	0.008911	15.66648	88.7891	85.45617	31.8692
25.18401	2.922559	20.02253	28.20941	2.064504	90.12461	106.0853	40.17412	24.94526
41.58259	22.37513	149.8511	93.68814	61.64559	23.50331	1041.263	215.2346	240.5368
56.87398	29.31387	71.04497	75.55378	36.9907	126.3801	60.84264	174.0182	97.42845
15.33681	7.102332	6.704143	61.44481	13.36395	5.869419	27.81194	28.8378	12.88213
148.699	215.2052	135.4165	117.8642	288.6455	232.1797	84.3084	209.0506	163.7969
998.1059	352.7922	685.8371	1156.456	1504.88	277.235	795.7323	949.4752	656.9838
1.128014	4.325355	10.03224	78.56822	21.57947	6.849055	33.90703	43.24859	53.50068
40.48082	4.319271	11.1442	126.9256	95.5306	11.74719	83.48387	116.3597	86.22067
80.88016	52.80743	56.60605	44.32571	27.74485	29.3828	72.19424	26.78552	30.13142
51.39374	14.04677	55.49651	64.47139	35.9608	16.64578	142.7628	37.08095	22.36461
20.76659	15.32661	19.97302	45.32358	7.200434	4.890225	19.13149	16.47984	4.255449
676.7176	1374.303	2214.886	675.9444	929.6425	574.0751	592.2161	617.8857	632.8444
417.548	193.0284	136.5274	381.7882	334.8675	139.106	140.8521	179.1899	145.6809
1188.365	827.6133	7884.666	5759.118	2208.556	4536.793	41881.96	13489.33	7902.26
46.95497	48.35431	7.811423	10.07638	32.86711	16.64911	1.687769	12.36748	31.92026
27.36588	22.37016	89.8706	64.47216	61.63707	32.32158	66.95563	63.85049	49.1152
66.66701	116.2285	35.53536	34.25219	53.4156	49.96191	12.99089	22.66603	37.91169
62.28693	89.9339	29.98782	50.36775	30.82282	66.62416	5.163226	8.250986	45.6984
427.3201	445.0895	38.88725	23.17262	110.947	196.9144	38.21002	50.47228	30.98054
36.1	32.04632	86.51816	60.44184	48.2832	20.56499	58.26749	45.31586	35.31487
63.40926	480.7848	38.87045	26.19425	84.2271	72.49625	14.72934	21.63754	21.50106
32.83571	8.489298	34.44751	124.9137	46.23594	87.18503	174.8389	208.0051	114.6639
336.7003	111.2161	147.6298	276.0188	222.9135	143.0235	300.0391	290.4035	143.0836
31.67992	9.852503	3.374151	34.24799	15.41465	2.930643	31.33961	21.62758	18.95024
51.39734	144.0323	41.08923	55.40616	94.49712	37.22099	58.2542	26.78582	19.77497
1332.665	548.5656	1154.163	3469.371	1331.3	4005.829	3508.267	3121.321	2649.6
167.3008	188.9722	105.4721	227.6677	212.6472	89.14011	316.5211	278.0558	203.4307
2.223808	1.537528	3.373617	19.14004	18.49104	6.850134	13.01727	38.08006	36.30721
15.31837	9.835341	6.69897	43.30956	15.41226	1.951178	13.8902	17.50866	4.255419
106.077	66.80519	169.8165	205.504	323.5732	227.2786	213.9204	297.6094	420.8284
80.89396	83.275	39.98421	97.71299	60.61053	35.2608	73.04833	79.29366	38.75638
1073.578	229.2317	5579.727	1755.843	319.4796	2752.835	10559.66	7606.084	8142.044
15.32667	18.11129	15.56108	56.40431	20.5472	17.63032	15.62475	37.06013	13.75847
21.90072	45.89242	34.43483	52.38405	64.71542	39.18104	110.5234	59.72992	66.39432
3.317926	9.858161	6.702306	11.08355	4.120177	16.64952	28.71036	11.33785	26.73303

3434.911	1071.971	1655.764	10455.45	3663.108	8717.994	9696.053	5532.079	7325.495
427.4219	489.9156	211.9836	189.3869	899.809	415.3804	110.3971	362.4858	177.5781
29.52858	37.47473	42.15607	70.50998	44.16631	9.788353	46.12011	39.1305	11.15452
116.8895	107.9941	59.91943	28.2086	10.28387	273.3985	72.21233	28.8436	36.18092
127.6523	52.55371	6.703716	0.00237	86.24713	99.96353	4.29385	16.48574	15.47632
101.7019	83.45093	124.3229	360.6338	174.6336	139.1062	241.7748	270.8328	376.0026
16.41796	34.58418	7.810032	48.34774	19.52096	13.70963	25.22446	14.4249	11.16368
52.4412	12.64176	20.00636	38.27957	18.49849	30.36725	56.58768	47.3635	10.29139
30.64639	33.4667	23.34994	82.60441	155.0949	107.7659	70.42495	90.62167	95.71583
155.2547	205.4873	155.3834	120.8861	406.7627	212.5859	73.87074	96.81147	150.8611
0.029054	0.119154	3.373711	13.0974	11.30691	16.65194	18.25566	51.45127	55.38617
5.504542	4.324577	12.24426	97.69846	27.73526	5.869569	10.38807	50.44096	12.02378
80.93533	68.19068	198.6569	341.4951	123.2764	182.2129	270.478	540.6139	103.4169
19.71045	9.880303	34.42013	53.38949	52.38568	18.60619	37.37957	57.66271	12.87748
13.15849	20.99164	184.1537	214.5632	7.201804	81.3081	119.1545	45.32195	46.51082
64.46695	23.7177	43.28142	88.64225	39.03704	40.16465	53.06934	38.10551	18.92041
171.657	122.3072	305.1621	194.4231	172.581	178.2935	263.5115	246.1232	257.8038
52.4549	7.104441	51.02767	11.08403	13.36463	39.18568	32.1583	22.66419	31.01691
292.9796	1205.299	64.41137	122.9014	509.4881	393.8292	127.7963	63.86028	140.4976
145.4215	27.93412	96.5867	264.9367	147.9278	249.8142	136.5026	350.1194	359.6088
778.3998	445.8198	799.0405	496.6347	927.5958	522.1502	1953.314	1742.416	1233.826
30.64216	51.43457	18.91041	43.31857	33.90732	77.39518	62.60903	70.02564	30.12989
13.15619	12.65098	109.9041	207.5188	20.556	86.20175	538.4335	161.6866	325.9418
31.73326	5.714246	39.97783	38.2819	18.50121	4.889907	60.00162	60.7592	64.66807
79.78786	58.33706	128.6386	27.20153	53.41924	63.67766	83.51832	19.57816	37.90038
849.4533	774.8109	652.5589	2120.504	681.0623	2794.985	570.4575	1326.381	2169.423
75.45052	201.0234	241.802	19.14314	197.2088	140.0963	25.16535	75.18129	24.94616
54.69689	15.43685	43.32477	78.57691	156.1383	38.19813	111.294	302.7358	148.3008
364.0571	2583.481	182.0378	213.5643	686.1803	449.6625	356.5421	308.9469	563.0512
190.1649	51.50061	85.46674	84.62001	650.1091	110.7015	22.5556	85.47802	171.644
127.9305	459.1414	580.2154	127.9375	64.72653	168.4986	260.058	152.4157	108.5974
24.08274	8.49438	31.10268	79.57917	29.79753	14.68656	49.56436	53.54954	32.72897
182.6058	55.69863	160.9575	1211.857	309.2053	415.3705	375.6626	1468.459	287.0727
1553.475	1317.632	1754.494	2478.121	2179.78	1595.862	1090.53	1411.86	1514.906
3.276645	10.53006	0.02351	2.015934	0.005958	0	0	5.137724	0
28.45114	19.58223	28.8849	104.7601	21.58162	17.62594	55.66533	44.28456	46.54942
16.4378	2.927023	45.52846	165.201	8.229367	100.9099	72.1776	89.58859	97.46068
659.2235	892.6876	2299.194	365.6767	587.5811	529.0108	643.5365	381.0359	762.1982
79.81818	16.82691	68.82691	171.2481	107.8576	61.71327	193.1675	70.03249	62.90784
68.88425	30.69173	42.20476	97.71362	83.2057	43.09862	78.26456	90.62039	68.09775
39.38266	7.104396	48.85106	25.187	32.88052	32.32182	78.28172	82.38	86.24647
30.64578	4.319707	30.0051	19.14309	0.009024	195.9548	98.28966	27.81635	26.67378
71.08853	23.76939	113.2067	44.32709	132.5135	20.56405	82.5837	95.7776	137.9559
349.8242	1043.272	446.0802	607.4369	155.1209	265.4849	320.9076	179.1939	146.5299
18.59297	13.97027	2.263529	24.1761	5.147064	16.65119	19.99673	22.65429	5.117834
3.31767	0.12501	6.704604	20.14969	2.065158	9.788171	113.2558	15.45804	22.37772
18.60687	8.482906	4.484473	57.4142	11.30941	2.930645	26.95271	16.48509	18.93701
42.63421	60.95079	17.79429	61.4467	30.82181	38.20543	30.41422	36.04587	24.96803
3.317913	8.476329	25.52298	36.26277	14.38867	20.57027	30.45842	9.279147	18.94585
19.71578	11.27218	59.93457	54.39882	18.50135	124.4306	63.48015	74.14341	36.17173
17.5113	66.14077	29.95013	21.15562	33.89228	48.9974	8.647043	21.62912	5.116379
1136.968	391.6809	1360.549	1857.584	1675.413	1776.122	1872.41	1129.697	1919.321
31.72976	37.57211	68.78456	74.54328	17.47377	44.08161	80.05325	38.10904	28.40988
625.3199	1952.669	723.5316	212.5573	937.85	1039.429	259.9843	348.0807	220.6755
263.4891	125.1113	139.8701	365.6748	208.5366	403.6188	427.01	287.3205	406.1038
20.76871	35.8609	14.44661	15.1117	85.18251	5.870074	2.555857	6.190184	3.392469
15.32267	5.705667	2.263531	36.2613	12.33408	5.869931	19.12293	15.45251	5.117774
79.82713	47.35012	119.8531	37.27557	96.56377	95.02365	79.10851	145.1975	204.421

27.33372	99.13828	28.85281	27.19919	18.49652	59.77696	10.38738	30.89237	23.26198
22.99336	32.05668	23.34628	27.20154	40.06887	13.70661	84.38915	67.96554	17.18754
45.95093	12.65963	113.2003	12.09152	31.85557	9.788098	83.45871	170.9381	93.95833
87.49712	15.42647	193.1278	279.0419	93.48946	39.17763	513.1458	515.92	377.6562
125.7226	39.03218	51.08877	96.70855	54.4531	121.4766	116.5257	80.33206	125.881
139.9127	126.2982	139.8038	71.52481	100.6703	99.92379	230.5817	91.65573	72.39445
42.6759	20.98682	102.1386	174.2765	38.01929	95.01871	319.1772	434.564	209.4924
53.5689	37.56766	23.34312	38.28144	29.79769	11.74733	42.594	71.04923	30.13769
25.15378	38.7846	6.70338	44.32133	19.5235	26.44926	24.33475	33.98016	13.75009
16.43218	15.41653	23.33544	37.2731	74.97013	19.58646	24.31557	42.22136	37.92952
325.759	130.6291	78.83565	242.7757	174.6351	193.969	449.7012	229.6474	151.7138
141.0481	57.08578	109.8993	303.2153	190.0404	78.36475	375.772	266.7133	202.6141
306.1248	151.4887	437.2434	232.7042	391.3787	377.1653	279.1269	411.9231	873.5206
1088.881	248.6759	1216.308	3004.976	1285.075	3148.622	1927.156	4348.834	3974.009
31.6912	7.09641	16.67306	17.12698	8.22846	8.809128	38.30754	15.45509	15.48256
18.6103	15.39793	20.00268	21.1564	22.6046	24.48807	41.77248	23.69081	40.55035
20.80848	2.928169	8.924571	40.29663	43.15024	75.43558	45.19386	64.8781	157.0805
1.114811	12.94624	6.563972	0.001517	4.100667	0	0	0.006097	0
2.223827	0.119178	0.034176	1.010159	0.008519	2.930689	0.820637	4.131242	18.96422
17.34423	5.549637	2.251284	0.001921	1.036549	7.843078	0.824147	2.068615	0
14.23226	72.79439	16.6625	32.23295	19.5191	16.65113	19.99621	16.48143	11.16789
151.9491	91.71839	266.2488	177.2948	138.6757	50.93449	121.744	140.052	57.71933
29.54488	44.48033	63.24285	104.7608	60.60461	36.24266	51.30396	49.43259	49.13592
483.2121	169.5384	441.6907	423.0954	559.8407	471.2134	507.8782	418.1039	669.9671
120.2658	101.4323	297.3138	53.39328	114.0268	83.26516	137.4022	56.65017	105.1643
14.21688	2.928849	6.694506	20.14572	9.251863	6.850985	17.40514	19.5605	13.78565
85.31042	8.469656	243.0777	1145.375	65.75404	84.24205	1608.962	492.2535	365.5304
145.413	367.6071	436.0018	134.9886	156.1423	129.3105	230.4852	82.3945	82.72466
213.119	75.06996	78.81689	97.71557	133.5377	97.96316	32.9934	46.35219	85.33229
43.67879	71.62035	24.41847	10.07632	20.54842	3.910215	7.775961	18.54201	13.75498
277.6855	93.1794	92.15304	192.409	671.7827	135.1856	279.1534	343.9509	289.6987
133.3996	58.47518	85.492	244.79	305.0813	234.1383	213.9299	178.1604	191.3947
92.94731	83.41467	109.8838	124.9143	132.5149	169.483	122.607	113.283	125.0063
41.57217	1.530402	25.56803	47.34833	99.63716	24.48319	91.32536	121.5054	56.01399
27.3389	16.77465	20.00137	32.2356	41.0829	22.52828	20.84303	21.63197	16.34047
65.58363	48.63588	31.10531	59.43448	132.4831	58.77979	36.49407	45.31564	43.95055
51.41371	9.880906	79.92372	184.3445	43.15402	28.40154	147.8732	118.4262	122.4427
9.861645	33.00667	3.372684	1.010165	19.51331	67.65241	4.297595	10.3048	5.121599
5.495728	38.22441	72.77575	0.002208	8.22353	0	1.688073	1.041078	0
27.36498	23.75337	55.50238	53.39165	86.28242	25.46336	46.06257	91.64491	45.66566
8.784594	22.32551	34.41318	18.13511	24.66051	39.1859	32.15936	49.42538	36.2032
104.8929	164.6325	34.43247	30.22335	77.0364	129.3345	12.12003	23.69636	30.99712
223.0165	148.6431	135.4165	376.7511	344.1103	102.8572	118.235	210.0803	102.5571
168.291	329.514	54.40642	227.6554	78.0723	139.12	19.94698	35.02436	61.18692
223.0493	58.47802	316.3016	426.117	456.0921	507.4633	687.9472	419.1318	318.1259
26.26787	12.65712	28.88645	6.047301	38.01319	30.36352	51.30467	45.31478	42.22704
88.48736	48.58971	28.88193	29.21555	29.79655	78.38286	28.66447	28.84223	33.59851
44.84446	8.494397	51.06816	37.27481	55.47439	63.6765	88.73333	61.79043	84.51977
89.59473	70.71399	51.05196	16.12083	34.93227	16.64608	70.47401	10.31062	22.36829
20.81137	32.09563	23.35168	442.2181	40.07301	22.52337	257.5291	120.4881	58.58328
45.93776	12.66067	75.45769	63.46472	44.1779	24.48352	88.72995	71.05636	75.01806
47.04877	54.31426	253.0373	417.0484	60.61838	329.1652	865.5061	296.5844	157.7405
27.36537	2.927605	18.91086	35.26023	31.8536	48.97855	68.70024	85.46893	207.1617
21.89079	70.55586	49.91489	18.13506	45.19494	27.42636	53.08587	19.57556	23.24356
607.8276	1614.075	664.7201	134.9903	1111.444	518.2376	249.5482	244.0733	189.6336
144.3437	30.70274	322.9616	558.081	91.43531	148.8997	404.3745	1213.071	1399.582
69.95073	38.9589	65.46294	68.49988	46.22911	17.62571	22.56328	35.02091	30.99884
132.2875	16.82604	120.9761	60.44474	71.91523	292.934	45.16968	211.0993	232.8609

101.6808	20.99246	137.597	73.53976	61.64259	53.87404	106.9573	105.0426	138.8315
43.74056	44.47316	7.814689	90.6589	64.71115	25.46441	43.46655	48.40255	19.7791
795.8061	6762.678	542.6436	17.12815	786.8423	409.4958	46.90647	46.3537	22.3621
244.8791	600.6493	286.2943	222.6284	180.7972	278.2256	186.9592	141.0907	118.942
72.15472	83.26823	61.0495	57.4211	120.1727	34.28111	81.75828	73.1162	69.83359
22.9829	18.17821	22.22562	83.60467	44.1685	16.64702	31.2895	39.13254	14.60619
115.8838	98.62359	64.39796	124.9133	101.6989	53.87412	96.51505	85.47933	81.01803
51.37207	178.0793	82.04882	15.11337	17.47298	31.34483	21.699	21.63557	15.46669
28.44809	7.105233	28.88122	21.15723	12.33795	27.42486	25.18129	80.30767	44.83062
239.4525	39.03129	148.7516	602.4065	454.0432	240.0081	613.0995	831.046	549.2092
30.62004	1.535579	11.14054	5.039923	30.81971	23.50709	34.7834	29.86777	20.65516
116.9594	29.31253	28.89765	96.70721	134.5588	41.13838	73.90053	118.4218	72.40264
198.9643	101.4717	168.6901	135.9961	107.8672	289.9868	146.9517	126.6724	342.3878
89.66952	11.26799	93.24625	96.70904	31.85582	42.11688	254.8847	70.03658	55.129
3.315088	4.303373	14.41734	45.31612	11.30169	7.832395	13.04654	14.41468	10.3292
354.2271	266.698	441.692	573.1918	253.7355	435.9446	687.9285	371.7648	654.4412
20.7925	26.42457	29.96738	96.69432	17.47076	19.58806	25.20182	17.51514	11.15668
385.94	327.8039	593.7343	920.7334	630.7255	977.7	731.3731	1177.054	686.3042
777.1685	509.3546	564.7894	382.7985	606.0468	1532.256	5.169689	28.84699	137.9057
26.27595	1.529375	104.3099	22.1652	40.07195	27.42205	76.5107	121.5109	60.31987
55.78092	109.6483	108.7456	23.17255	70.88422	47.99657	16.4676	27.81685	27.53426
479.9026	1404.046	446.0969	247.814	372.8848	429.0927	428.7617	182.2843	183.608
43.75275	152.3508	137.523	26.19434	49.31279	68.57555	37.3557	43.25937	30.99149
127.9382	48.76054	187.5686	270.9817	93.48872	297.8176	271.3368	179.1921	270.7373
888.8066	347.2508	772.404	1058.744	735.5048	1741.838	2055.092	897.9912	875.1257
85.2694	167.6656	100.968	93.68443	98.61032	45.0582	89.58344	56.64547	42.20511
19.65815	13.9058	5.587508	20.14559	2.065276	3.910744	13.90748	3.101197	6.853364
223.0389	151.4683	240.8349	245.799	71.91784	325.2459	355.6987	232.7413	446.6637
20.80865	32.09402	75.50944	721.271	247.5705	11.74809	430.4836	590.0674	928.7397
819.9234	318.0882	443.9308	989.2341	907.0455	1107.017	879.229	838.2593	1138.154
49.20241	83.15049	54.3786	65.47785	48.28236	26.44405	46.0769	41.19727	32.72716
15.33707	26.44349	14.46541	93.67465	55.45799	10.76823	16.48087	28.83801	12.01836
84.17425	198.0465	35.54969	138.0049	120.1752	70.53368	37.35178	56.64495	98.31746
92.96397	323.6009	75.50928	42.31248	616.3369	174.3709	54.73381	102.9928	42.19141
143.1564	167.6607	444.5503	39.28977	57.53059	8.808308	2.558816	66.94096	92.26986
52.39545	146.8629	48.74877	7.054493	16.44199	15.66948	11.26168	1.041043	0
383.7508	101.5175	276.3667	491.5969	304.0705	739.6411	692.245	787.7968	1021.764
65.61071	16.82629	31.11539	32.23858	100.6662	45.05775	61.71841	79.29713	113.8412
6.594814	4.320797	13.34235	39.28224	20.54494	18.61198	15.6322	16.48103	6.845552
29.55691	26.54602	180.8661	133.9802	151.0021	81.30572	93.02273	140.0535	137.9503
44.85952	0.129304	23.35178	27.20208	40.07324	87.1843	78.23163	181.2384	188.8619
15.33634	85.57964	46.57709	16.12026	22.60506	48.98999	33.91765	4.131593	24.98015
171.6394	29.32335	61.07891	237.7368	271.1783	91.10165	167.8387	245.0843	131.8934
39.35871	12.64839	37.73735	44.32372	25.68751	23.50615	50.46326	41.19192	18.05905
46.992	66.41192	35.51671	21.15681	47.24766	37.22671	6.032684	30.89787	41.39561
132.2156	52.82885	27.78426	11.08419	40.06988	46.03903	27.78077	4.131215	31.85422
17.44544	29.97187	11.09221	6.046023	13.34856	8.814989	5.180372	10.29811	6.001071
78.73991	72.32101	36.66738	369.6939	131.4875	147.9287	200.9324	170.9428	114.6548
75.47218	43.20009	620.3432	1358.931	35.96419	142.0418	90.3893	1391.229	2597.405
201.1222	346.6144	130.9552	8.061932	2.064438	12.72698	140.8853	6.190645	9.427596
20.7612	54.87854	10.01782	3.02508	63.62987	30.38002	0	5.160497	4.256105
97.31173	55.67132	44.43216	165.2064	64.72417	53.87391	151.3418	112.2506	90.50599
11.95405	10.86908	0.028385	0.001938	3.088236	0	0.823819	0.007809	0
62.31787	99.81032	26.67385	104.7628	61.63531	51.91876	83.51352	37.08142	34.44597
100.5618	12.66125	77.68703	82.60415	95.52952	54.85613	77.3936	116.3582	51.69806
232.8909	370.795	474.9834	1061.76	310.2323	271.3583	761.8556	532.409	401.7634
170.5715	245.8094	125.4416	616.5033	458.1365	141.0635	178.2429	369.6953	210.3483
271.1285	72.36055	193.123	341.497	274.2734	141.0636	244.3556	290.4054	391.4727

2002.805	11875.39	7803.566	2316.946	3931.211	2281.619	7459.248	3147.071	3260.046
30.64961	14.04871	33.33801	49.36377	5.146886	2.931301	260.1399	74.15405	5.980446
522.555	1236.531	259.712	499.6539	645.0945	803.3301	63.42965	200.8219	403.5017
14.24477	5.714827	14.46498	56.40881	10.28309	17.62761	33.91615	31.92524	42.27297
1073.558	415.2784	1716.743	930.8088	1129.958	1160.893	1514.97	1445.838	1909.856
319.1788	403.7026	305.1348	178.3047	222.9073	78.36496	136.5081	183.3067	224.1871
80.82076	74.70153	62.09787	29.215	28.76766	65.6474	7.772159	27.81072	46.57682
161.7992	15.43581	55.53044	178.3036	207.4958	60.73094	166.9756	153.4421	76.69006
77.42407	78.31543	11.13141	2.017683	14.3876	36.25685	2.555751	2.071538	6.844284
134.3777	549.6165	169.6288	5.039879	110.9241	75.43678	1.68992	3.101224	19.77612
76.54764	55.66527	195.2555	32.2388	28.77394	70.52993	196.6153	41.20361	163.001
198.9641	25.15765	190.8756	162.1869	117.1116	62.68998	266.1569	179.1868	183.6479
20.78534	104.528	15.56571	12.09086	27.73454	34.29229	4.294113	15.45506	13.75359
10.94389	30.17664	6.691153	13.09594	21.55997	23.52303	3.428277	8.245585	11.19322
101.6439	27.91427	39.98377	31.23103	118.1191	27.42266	55.63538	63.85076	68.10624
54.66378	54.16047	45.51463	54.39806	43.14828	36.2424	33.01135	29.87332	49.1331
331.2336	248.5519	474.9064	167.2249	187.9895	715.1774	203.477	151.3897	296.609
7.68151	7.068282	8.907907	21.15303	13.35708	6.850807	13.02862	15.44781	15.51669
156.3522	136.1702	111.0108	228.6725	107.8687	298.7998	290.4917	144.1798	225.0344
28.43633	9.873855	16.68201	29.21453	21.57879	21.54728	38.27332	30.89663	29.29911
30.64534	22.37343	52.18405	42.3117	20.55598	83.27133	84.36159	79.2951	102.6322
300.6699	75.13663	21.12723	42.31241	62.67251	413.4098	1536.818	187.4361	136.1716
18.59131	41.34732	10.02201	5.039779	32.85922	28.41572	2.555813	2.071541	3.392332
18.62426	8.493323	383.6119	53.39229	6.174614	19.58457	123.5395	194.5997	72.41202
278.7836	144.5306	188.6876	345.5268	274.2745	122.4494	370.4866	305.8529	423.3753
86.38557	87.5532	185.2885	129.9503	130.4582	80.32678	146.1171	85.48028	123.2928
73.26627	2.923849	26.67975	183.3366	66.777	18.60475	122.6366	96.80341	20.63412
122.462	80.66255	63.29806	298.1769	176.6845	150.8648	154.7862	145.2062	124.129
236.1682	105.6827	99.92346	539.9486	388.2984	60.73009	707.9437	802.202	352.6167
65.59735	159.2447	36.65499	61.45002	35.96159	67.59594	39.96803	26.78594	62.06888
17.49952	5.703105	23.29155	26.18987	13.35986	9.79026	21.74985	20.59538	20.69671
120.2627	11.26902	79.93159	319.3271	64.72475	131.2735	192.2373	208.0075	96.54028
25.16068	7.10215	21.11238	18.1348	4.120248	35.26819	17.35296	14.42763	27.5728
73.22178	251.4062	21.1257	15.11352	136.5873	52.90102	19.95281	14.42947	11.15051
5.505092	0.126342	8.924438	17.12814	11.311	17.62591	169.841	22.66607	14.60149
78.72829	9.881642	52.19316	121.8905	152.0207	24.48285	120.028	159.6068	53.41379
165.0976	1907.84	218.6273	28.20943	545.432	352.6837	125.1904	78.2768	79.2689
35.01852	8.492496	31.11604	123.9044	34.93623	14.68613	138.3253	180.1926	110.3821
12.06302	48.55308	33.30864	19.14248	11.31066	42.12514	58.30465	16.48776	33.60644
16.42552	7.099842	6.703547	53.38606	18.4971	23.50864	22.58864	30.89368	31.04196
25.15337	12.62859	7.812225	38.27827	22.60335	4.889818	69.72169	19.57277	15.47881
132.2896	108.3739	101.0121	99.73113	19.52909	355.6382	58.21808	97.83846	203.5172
2617.173	2982.197	2561.276	1836.432	3389.858	3479.75	3351.721	2387.079	3661.883
55.77343	43.15537	5.593691	18.13572	111.9596	60.73543	1.690575	49.43797	21.49852
446.0353	490.0719	383.9809	142.0417	517.7216	1087.445	462.6651	299.6787	271.5623
65.61598	129.0419	52.19243	48.3561	140.7228	49.95588	61.71168	72.09211	61.1805
257.9796	533.964	116.553	162.1871	180.7947	345.8312	143.4693	75.18677	185.3695
47.0099	33.40094	16.6882	95.69388	62.65476	16.64636	42.60331	15.45872	13.73972
290.7834	834.9579	18.91137	30.22418	421.1488	480.0487	12.99211	51.50277	135.3279
60.1557	5.708663	10.03368	64.47359	81.15662	25.46247	206.1985	304.7772	85.33591
63.38182	167.1125	18.90527	22.16451	41.09165	43.10377	17.34427	27.81257	21.50891
41.5682	1.531302	43.30858	46.34064	90.39088	18.60506	99.18115	132.8234	48.25374
14.18654	17.84077	1.150847	0.002155	11.29759	1.951547	0	0.008699	0.810613
15.22153	132.4881	5.560163	0.002013	16.40746	0	0.822514	1.040464	0
17.49427	7.077748	4.48224	29.2103	20.54221	18.61378	16.5152	16.47879	0.810327
83.03367	66.5371	49.93693	15.11344	32.87747	51.92271	32.14742	29.87193	59.51503
65.48757	0.122273	4.484354	12.09091	31.84106	10.76891	18.23399	41.18072	24.1303
122.4616	88.98332	152.0467	255.8689	170.5216	170.4596	177.4099	144.1764	135.3439

71.09861	9.860324	584.8502	1728.634	44.18201	360.5073	949.6984	824.8686	570.764
445.7143	452.6378	65.49784	3.024942	87.31625	3.910617	0	2.070756	1.673411
1.103289	1.406008	1.121188	0.001169	0.004286	2.946993	0	0.004685	0
6.595812	0.120662	3.374102	12.09058	3.092842	12.72983	20.86357	41.17365	4.254331
806.8238	243.1297	1109.754	1401.248	628.6749	1372.5	2003.751	1701.226	1777.92
6.596868	1.537033	14.45773	25.18439	0.008672	70.56416	29.58083	62.78365	13.75437
22013.01	21476.38	13849.47	20575.47	29285.29	53602.06	21816.6	23443.37	32596.46
86.37517	264.6543	116.5115	74.54657	93.47985	69.55121	28.64524	74.15138	68.08358
130.1245	159.7702	154.285	415.0322	200.3151	209.6435	314.8348	243.0352	189.6593
241.6045	202.7555	140.9684	421.0754	269.1331	156.7401	160.8567	212.1421	151.7141
473.3674	93.18792	214.218	515.7717	415.0044	157.7168	670.5504	1158.491	283.6316
769.6516	348.6396	66.62828	240.7638	459.1826	53.87315	111.2631	2625.964	650.9335
45.95558	14.03936	174.2592	331.4232	22.61045	139.1045	533.1897	258.4827	143.9449
73.13074	30.53407	14.46123	11.08374	19.52344	53.89463	13.00067	18.5436	16.34308
61.21038	107.9691	48.8353	57.41953	61.63078	13.70681	46.95068	39.1379	24.95818
27.32824	0.121917	4.484244	3.025122	6.174468	8.809174	15.61977	27.80348	16.34811
57.96516	20.99007	37.77167	42.31199	120.1806	28.40182	62.58563	139.0123	101.7488
89.67029	15.43655	65.51402	230.6842	55.48104	30.36066	207.0148	170.9445	114.6501
61.23366	232.5558	239.4982	81.59639	22.61014	109.7285	52.15154	46.34887	56.88174
37.20979	5.700096	53.31438	81.59968	60.61817	131.2678	198.2642	601.3682	849.5777
57.9743	47.35672	41.10494	178.3023	76.02311	46.03588	181.7933	110.1928	62.03204
39.27637	49.46954	34.33238	0.002298	26.69963	15.67221	14.76548	3.101478	3.392743
1683.571	1364.852	1059.831	1784.048	1226.521	2702.882	1839.339	1989.574	2558.234
50.32264	79.23436	63.29021	66.48849	79.10363	47.01584	90.41997	125.6356	180.2508
311.3627	127.6036	54.40607	6.047224	47.26103	25.46276	6.903221	33.99466	15.46075
16.4248	0.122865	15.56914	17.12724	2.065305	21.54874	20.84613	20.60207	70.86074
245.9998	40.42765	177.5969	406.9763	123.2788	435.9487	674.0593	407.801	546.6825
1075.761	588.827	1179.685	2953.599	1342.599	3932.356	3109.954	4521.833	3752.432
446.0154	197.2621	129.8813	440.218	522.8503	173.3928	556.6472	246.1284	158.6004
80.88216	30.67465	33.32681	139.0098	20.5555	20.56469	51.29029	65.90718	37.89919
61.13101	38.76329	37.70272	0.002357	112.9225	9.788918	2.555852	5.161348	5.978633
83.10551	102.7852	135.3796	59.43709	64.72397	94.04381	87.81059	75.18318	195.7892
155.244	86.2042	208.6126	172.2598	181.8187	60.73088	150.4407	132.849	90.49045
87.44778	41.76626	48.85517	174.2666	60.61063	31.34166	92.20493	51.49665	20.63631
1.12873	1.537338	2.263276	30.21669	14.38414	4.890462	22.6423	20.59165	27.65543
19.70025	26.41898	8.921455	49.35759	30.81704	10.76853	31.30905	26.77762	16.34095
7.662526	5.6421	5.57427	27.18777	7.194349	1.951687	9.569601	10.29573	6.876121
8.783547	9.872026	16.68065	116.8386	36.97833	9.788479	18.22491	35.01231	6.840252
5.501168	272.1954	2.263318	1.010164	9.252924	22.53642	0.820527	2.071508	0.810294
119.1042	4.321752	44.41504	74.54462	55.47398	20.56463	88.73701	72.08462	44.80377
17.53005	79.08237	47.74001	59.43522	72.93148	51.91875	79.15839	46.34719	43.07822
21.89683	9.881771	24.44968	39.28842	21.5814	22.52518	57.41242	20.60671	43.96133
34.98378	12.64107	35.51123	21.15664	26.71255	4.889768	41.76166	14.42783	24.11284
32.83398	27.92817	244.0158	50.37078	61.64123	37.21916	138.3109	64.88491	119.0017
40.42436	38.7883	22.21375	52.37871	33.89595	4.88981	47.89296	13.39743	14.61392
8.77384	5.697069	7.803993	20.14654	6.173305	26.45859	5.167947	12.36326	28.5194
387.7794	146.9299	32.22342	33.24583	153.039	82.29103	107.8678	35.02393	55.15018
42.65102	141.1129	28.88721	38.28153	44.17495	138.1571	26.91686	29.87318	49.99821
5.505095	12.63935	2.263124	18.13481	1.0374	2.930678	78.40876	28.83771	32.75993
238.1505	62.5284	163.0292	11.08419	78.0693	22.52386	0.822904	16.48944	10.28769
41.51885	23.66542	20.001	18.13459	9.255833	29.38895	14.74187	38.09762	34.49987
137.7512	112.5253	154.2508	142.039	42.12771	192.9972	174.82	97.83802	200.9343
12.06509	4.322226	102.0448	131.9587	4.119977	35.2617	55.64614	32.96294	24.09015
7.690954	0.128804	41.10206	63.4662	0.009065	187.1261	117.4111	132.8396	144.886
89.62279	102.5916	64.37009	2.017539	48.28547	135.2089	3.426649	20.60804	0.812606
68.89475	317.2473	179.7143	63.46608	88.34498	125.3992	46.91662	63.85543	112.9585
4.411692	1.535766	22.22096	8.061976	7.201952	8.808612	44.37992	45.30446	47.46012
35.78406	4.255316	9.960801	2.017312	3.089976	0	0	0.008237	5.149656

1752.397	2420.983	3147.056	261.9185	1723.684	1083.501	163.442	240.9858	187.9052
92.56744	102.4381	24.38221	2.017677	27.72318	1.951191	0.820526	0.009223	0
97.3266	398.1179	194.2034	196.4365	238.3127	312.5211	54.73524	104.0188	118.0881
348.7127	84.84572	56.64347	171.2541	193.1244	188.0904	141.7168	424.2624	192.2499
43.66696	24.97586	42.10325	9.068959	9.254808	79.39343	17.37023	19.56972	34.53685
24.06033	216.9068	15.5675	8.061858	27.73556	28.4102	3.424651	9.279622	18.93892
48.12537	12.66108	65.48654	98.72042	47.25972	50.9375	71.30525	53.5559	72.41997
598.0088	150.1104	203.1276	1314.61	772.4787	182.2078	1083.64	865.0305	364.6715
95.11597	15.43857	68.8289	56.41476	102.7233	48.97606	106.1015	122.5422	87.93128
682.0971	426.1666	236.3984	122.9017	632.7565	465.3427	74.7356	245.0986	217.244
24.06657	37.4379	12.24721	44.32199	32.87087	36.24933	37.41203	20.60299	18.93234
108.2586	19.60121	258.5506	54.40092	81.16157	129.3095	206.9782	189.4856	437.2668
86.40424	2.91202	8.917851	442.2361	11.30959	479.0479	2168.297	176.1084	15.46807
2979.044	23164.93	10686.64	2621.171	3729.878	2512.818	17026.67	3085.286	3831.697
35.01527	11.27252	27.78565	48.35549	35.96234	37.22026	49.5391	41.20111	72.41945
1094.346	1108.051	2307.149	1015.429	2329.762	816.0475	4170.121	3750.521	2118.459
73.20321	157.454	20.01359	23.17178	39.03779	41.14403	1.689067	22.66521	40.51213
479.8374	202.7333	153.1683	137.0039	212.6382	349.7466	314.8587	173.0114	147.4065
19.71457	4.322874	12.2535	107.7835	31.85238	35.26219	46.94259	79.28709	62.94759
385.8364	528.4234	243.015	39.29049	466.3324	450.664	14.73009	16.4893	13.73847
95.13434	368.8465	156.4718	121.8925	86.29535	135.191	126.0848	106.0758	91.35769
2.17205	7.603998	0.016823	1.008321	2.051086	0	0	0.00471	0
110.2766	70.57375	14.468	7.054672	120.1393	123.4735	2.556572	8.250873	12.01607
55.73502	73.37619	29.9862	12.0914	43.144	53.88473	21.70009	21.63532	27.55715
21.89721	7.105386	17.79797	21.15735	35.95857	23.50493	51.31087	72.07651	32.73127
159.5139	139.9201	79.89136	46.34063	19.5286	138.1474	41.70817	33.99331	48.25386
12.06359	9.874561	328.4253	319.3318	1.036772	245.8977	374.9206	171.9796	295.7936
25.18295	9.882256	31.11646	72.53174	63.69474	18.60479	78.25192	151.3675	94.84148
104.932	92.99183	38.87817	4.032401	104.7726	128.3436	6.033897	5.16111	13.73633
595.8279	999.6067	487.208	1188.692	393.4392	608.3629	1116.673	962.8623	857.901
275.5181	143.1607	245.2866	687.0219	236.2721	125.3877	538.3357	459.2918	330.2005
4485.503	33412.87	63316.43	5228.236	7059.132	8660.181	20337.35	9108.575	12663.5
31.70493	5.714328	11.1391	18.13469	49.29616	13.7081	20.84129	32.95326	24.98275
78.73982	105.5894	75.49626	143.0462	137.6499	67.5895	156.5482	78.27378	72.38199
68.80518	169.4698	11.14056	7.05465	40.05986	668.4898	11.25432	11.33949	8.565266
20.7876	7.097807	12.24457	22.16314	27.73552	12.72876	39.17381	25.74678	26.72394
5.496971	2.925192	8.900265	12.08881	9.250223	12.73509	19.17584	14.41529	8.592215
67.74561	158.8247	66.5464	33.24451	77.02779	25.46522	26.05319	16.48813	13.74026
107.1476	242.6914	204.1421	164.1994	115.0522	80.3267	124.3579	81.36178	64.62166
52.34172	15.32415	3.37357	14.10442	26.70007	12.73115	4.295851	10.30651	34.57491
53.5866	5.712811	8.924457	100.7348	54.44854	27.42263	59.1173	71.05756	51.7033
53.59764	26.54166	31.11742	132.971	91.42631	24.48284	69.54275	95.77354	56.86465
10.96547	4.324511	18.88833	25.18462	7.201566	17.62906	36.55822	31.92032	24.99554
506.1582	268.0699	227.533	421.0801	519.7775	666.1734	505.281	425.31	469.9068
36.0812	4.325309	14.46748	20.1496	26.71376	11.74781	41.75093	55.5994	25.8356
24.07485	5.715613	17.79307	31.22946	53.40878	33.30611	33.03227	56.62913	31.8835
341.0889	98.73572	217.5377	681.9821	445.8135	129.307	211.293	680.6762	486.3288
10.97202	8.493477	48.85837	71.52379	2.064734	141.0822	24.29758	84.44323	32.71324
333.4131	391.3705	240.8196	129.9528	393.4188	86.2018	77.34699	96.81264	81.85514
36.08853	7.10499	88.68983	11.08408	15.41892	15.66676	53.94033	40.16425	50.02158
13.13117	4.314386	5.5889	19.13913	23.61762	4.890544	20.8974	13.3911	7.716303
21.89799	5.7153	6.704743	18.13547	8.229419	34.28317	66.9899	29.87273	37.04688
98.41986	72.34401	188.6581	168.231	123.2746	112.6549	209.5969	175.0683	269.0532
31.73796	228.463	17.80222	35.26044	132.4988	153.8215	3.427197	23.69752	17.18581
122.4137	79.16868	47.75188	79.58257	46.23389	96.00765	31.25739	31.93528	63.77717
53.60118	50.12495	27.7901	55.40778	31.85558	104.821	57.35321	79.30202	37.88165
11362.9	13959.82	18002.03	9997.108	10953.36	16582.69	9761.184	13298.82	16443.52
290.8205	234.7597	380.6541	401.9402	392.4054	775.9	373.9386	354.2563	291.3953

51.36715	16.80178	27.76895	62.45411	33.90251	17.62658	55.68906	125.5856	14.6046
21.8906	16.79468	42.16284	24.17863	39.03462	27.42642	47.85488	38.10294	32.74951
2.222675	0.112807	11.10483	20.14395	2.065095	27.44956	9.547262	27.77596	9.463684
98.39426	70.89342	26.67958	230.6796	65.74966	96.985	141.7912	71.06281	79.30056
616.4153	805.6262	59.97091	29.21682	361.569	490.8323	104.3181	29.87707	10.29107
940.1088	1569.561	616.9981	258.8957	690.2925	433.0064	247.8117	260.549	658.764
10.96466	5.711123	5.593596	15.1125	9.255266	8.809187	28.70777	41.17901	23.26897
236.1426	267.9503	233.0537	278.033	281.4599	157.7195	144.3261	270.8367	208.6371
35.02248	61.22893	139.8304	118.8703	104.7829	81.30565	159.1577	150.3502	126.733
37.20891	40.42494	208.6006	156.1418	109.9192	1.951913	241.8264	124.6099	81.00588
869.1398	52.90709	445.0491	1334.762	370.8418	6814.554	1664.54	1604.43	819.064
184.7475	586.4133	237.4482	87.64323	201.3332	184.1772	61.69629	95.77992	127.5851
113.6349	141.214	69.90485	30.22345	78.06473	32.32233	44.32636	38.11046	31.85833
49.18259	202.7193	73.17673	7.054678	46.22325	43.10544	2.556674	9.280618	5.977289
110.4042	206.4885	105.4163	91.67061	59.58628	120.5021	80.86491	75.17985	85.34931
19.68531	4.321927	8.916439	18.13352	9.254488	2.930654	47.07918	21.62636	8.572723
276.6073	212.5469	133.2127	729.3295	257.8423	200.823	905.4457	311.0053	105.1327
8.575629	6.490375	6.525763	0.001339	1.032241	0	0	0.005374	0
114.8049	234.4742	188.6364	140.0245	207.4934	59.7515	86.05904	131.8175	93.94577
82.01359	23.76886	221.8788	5.03974	421.1074	45.05641	32.12307	97.83564	200.1005
3.317889	1.537235	11.13367	45.32661	11.30847	5.869713	68.02772	23.68615	16.35198
3.310952	61.95018	60.50634	0.00212	14.37007	14.7038	0	2.070651	5.138445
17.5199	2.933117	6.704063	46.3368	45.19122	7.828878	26.94149	71.02942	12.88258
4.411489	1.534084	54.36301	12.09143	3.092688	22.52554	29.53715	33.98885	27.55427
17.53047	8.482677	28.90037	210.5401	61.6452	82.28342	286.1467	143.1497	117.2185
77.63836	50.11642	126.4993	98.7226	83.2106	50.93521	78.24301	63.85608	77.57065
17.52677	7.105291	48.82721	28.20827	10.28374	29.38451	60.90311	53.5471	51.74092
95.12971	5.70661	156.4607	202.478	107.8633	33.29979	113.0408	163.7337	33.56848
76.52119	5.713262	41.09125	73.53768	81.14868	10.76754	65.21618	49.43678	44.80054
160.7056	30.71126	64.40591	109.8047	146.8963	38.19802	160.8856	190.5096	98.25551
397.9514	1398.988	690.2375	418.0587	656.3973	447.7008	262.5954	356.3182	452.6461
278.7423	43.20539	156.4896	387.8305	142.7905	91.10119	275.7236	190.514	112.9111
20.79632	22.30908	33.29712	28.20733	26.71264	29.38728	33.9127	37.0719	24.97672
9.852686	2.924286	3.370727	6.046462	8.223861	6.851969	12.17132	13.38643	2.532457
606.5937	902.7168	104.352	49.36413	229.0716	46.03521	17.33875	27.81743	12.01502
114.817	72.34958	108.7897	224.6427	198.2574	76.40538	298.3336	182.2781	152.5843
17.5281	9.882498	34.42797	53.39046	43.14738	28.40394	37.36901	85.45926	101.8438
199.7647	26.48259	11.14258	7.054685	140.6796	45.0644	11.25187	18.54703	20.64701
149.5185	111.786	20.00756	8.062	38.00707	62.70923	15.60805	18.54578	2.531048
245.9697	70.96418	82.16306	325.3771	256.8053	150.8626	223.5021	227.586	151.7188
44.7173	46.73402	10.01896	11.08292	26.69932	25.47658	18.26107	15.45046	8.577555
29.54845	52.80554	27.7819	220.5973	38.01484	32.32228	64.35621	44.28763	37.03734
21.87186	12.61292	10.02622	8.061749	36.97004	29.39256	9.51924	13.39598	11.16298
196.8102	471.993	309.6339	468.4247	73.97238	244.9092	359.1631	428.3952	649.3208
69.80993	145.3102	7.809554	0.002332	68.77991	19.59091	0	0.009423	0
13.15677	4.324315	7.81462	78.57112	25.68909	62.70243	39.98788	63.84074	91.49211
59.03465	26.51318	47.73403	85.6237	46.22967	57.79937	62.62087	64.87547	36.17724
162.9131	811.4521	329.5677	220.6141	186.9612	609.3712	152.1563	101.9613	211.2264
73.28592	82.07981	431.6756	349.5568	241.406	210.6205	185.1965	324.39	394.0389
14.24688	0.124771	21.11732	4.032524	79.07412	16.64705	19.09075	108.0841	49.1733
27.30613	54.98383	19.97599	39.28182	20.54426	17.63211	12.1424	19.56681	6.846222
1260.44	7123.469	1080.853	52.38612	1435.024	587.7912	97.34704	106.0822	62.88459
9.868409	113.6585	1.151498	2.017684	35.93741	19.59248	0.820671	0.009343	15.4973
5.504825	7.105002	16.6912	7.054681	4.119997	60.7384	100.9456	21.63734	9.425543
139.9515	148.625	240.7954	146.0696	166.4147	160.6611	197.4136	119.4648	277.6773
47.94006	64.21013	11.11719	2.017664	11.30427	6.850983	3.426952	2.071446	4.25793
28.46097	4.31904	1.149314	38.28257	2.064691	281.2005	97.41156	39.14288	6.840555
14.19335	31.37271	1.151065	3.024839	8.222207	6.852934	0	0.00879	2.533849

24.08689	52.82082	35.5457	129.945	19.52851	51.91842	73.92908	53.5542	74.15901
348.7175	885.0038	267.4481	255.8716	304.0581	494.7409	89.52377	324.3827	293.1625
48.07575	47.10549	65.39901	26.19282	49.29874	29.38741	33.9137	27.8089	12.8817
28.44774	15.42301	52.14803	38.28087	19.52707	29.38474	66.13694	43.25315	39.64757
16.39176	15.26588	6.69363	16.11745	22.58868	21.55917	5.170675	10.30385	15.52418
33.91226	23.73533	75.42594	29.21579	29.79746	47.02197	75.70468	35.01999	39.63823
45.95009	5.708267	41.10301	19.14315	70.88619	92.08475	53.87465	131.8117	265.7107
40.48711	58.44946	79.93089	253.8511	145.8652	133.2333	58.2213	158.5852	174.1994
108.2228	27.92694	72.15379	136.9998	123.2628	53.87509	66.93524	121.5114	56.86745
134.4751	378.5461	16.69282	67.49621	248.5768	490.8479	21.68547	57.68018	94.80817
66.71064	152.5983	109.863	84.61998	150.9947	54.85435	88.68907	88.56672	68.08067
1327.162	797.0105	728.015	802.8731	1454.555	1215.756	790.4989	628.187	1477.009
4.408521	157.7853	14.4327	2.017666	155.9121	4.890656	0	6.189119	1.669638
789.3202	333.3622	1176.308	2047.971	506.4337	751.3941	743.5415	1146.165	287.063
5.433224	9.299647	5.508548	0.001673	1.03501	0.973741	0	0.00673	0
71.0333	55.51636	76.53171	11.08414	26.71649	89.16047	31.27362	28.84304	71.60411
698.5652	889.848	813.419	1101.048	1131.994	460.435	1106.285	549.917	534.5567
92.93612	29.31793	47.75834	108.7959	111.9686	50.93512	139.1686	87.53789	83.60965
19.71178	4.32433	15.57958	115.8387	51.36056	42.1233	22.56745	75.16285	32.73365
19.71413	33.42777	15.58129	37.27432	22.60912	94.05745	12.99019	116.3423	96.64352
2.204343	22.2239	0.024996	0.001718	6.152172	6.868623	0	0.006912	0
36.02242	83.65989	59.75197	9.068763	23.62345	22.5335	8.65191	10.30711	26.75924
98.35376	5.713995	37.76181	125.9154	40.06911	19.58494	41.71198	33.99268	21.50103
30.62008	36.09797	47.69201	43.31569	61.61864	24.48708	20.8363	26.78004	37.94284
43.75822	41.778	61.05717	84.61889	35.96288	37.21983	91.32165	94.73921	62.05364
379.2519	339.8332	68.84421	145.0617	261.9324	325.2616	59.08623	69.0076	74.10185
782.7727	1008.032	1378.294	550.0251	1161.802	977.6947	1349.721	1011.269	1822.764
20.78116	2.933465	4.484012	5.039855	13.36183	3.910234	23.47685	23.68619	18.0822
239.3614	282.8964	17.80289	24.17999	222.8961	199.8572	6.904897	64.88755	23.22045
84.04964	407.8143	56.52937	5.039913	23.63109	14.68812	3.424692	4.131591	8.566506
37.14672	42.88193	25.53075	14.10542	20.5495	11.7488	8.645742	15.45548	18.07457
22.95174	0.119769	1.151482	14.10472	19.51878	16.65132	18.25112	27.79758	18.95936
89.62822	18.21048	41.09181	116.8511	92.44533	20.56449	92.21069	64.87983	33.57958
54.69981	22.3798	92.1459	227.6642	72.94392	232.1813	46.90667	404.6863	708.196
62.32662	5.712578	34.44006	98.72045	110.9319	44.07882	85.23683	108.1199	44.79688
196.8095	186.1711	243.0599	638.6669	341.0431	152.8191	502.6978	325.4202	179.2947
59.0441	69.42011	98.73102	57.42084	69.85169	53.87796	81.76572	31.93396	37.89668

TCGA-FG-	TCGA-HT-	TCGA-DU-	TCGA-RY-	TCGA-HT-	TCGA-HT-	TCGA-E1-	TCGA-S9-	TCGA-S9-
123.8567	181.9544	138.4267	596.2894	63.7636	55.02766	135.0725	77.24514	175.7126
61.42017	14.7336	28.98829	21.688	42.48387	72.73916	29.33971	61.37825	29.12391
148.0209	103.8268	60.77858	25.00216	107.2991	159.2122	90.27394	54.33366	124.2868
3132.639	3104.572	2709.735	3210.941	2840.694	3496.657	1798.129	2699.974	2262.688
136.9461	52.60113	54.22647	208.1624	140.229	51.09677	167.4488	218.1741	119.9768
0.001682	0.051213	0	0	2.139192	6.886584	0	2.452976	0
6.044668	22.04943	56.1397	3.275577	70.07947	20.63249	13.84774	7.321783	11.94171
212.4659	247.8301	149.6545	147.9175	91.37894	107.1167	116.6965	180.8618	165.4213
8007.312	12070.18	7430.496	3968.058	7475.682	8316.102	5432.709	10644.26	8163.751
66.45927	37.9259	93.55379	50.95053	25.5198	56.01493	100.689	13.35228	111.4915
19.13274	13.48851	11.20488	29.29315	22.3081	12.77133	36.8554	19.31034	22.28657
1414.771	1013.711	1141.139	860.7872	569.4339	635.8396	1300.397	493.2787	1133.068
97.6768	130.723	110.3556	226.5196	124.3069	120.8754	166.6622	226.6638	122.532
1.009302	0.054675	0	0	6.364314	2.944463	4.385569	3.649349	0.807789
87.60617	83.08802	63.58623	253.4107	41.45493	30.45837	125.5853	86.85456	132.0125
7.051621	12.30058	15.87761	5.780762	23.38833	48.1578	14.58482	91.427	9.368585
1620.193	2105.56	1594.776	1760.05	1812.359	1760.118	1500.026	1233.677	1812.718
47.32723	37.89892	26.17011	87.85069	35.07013	22.59751	82.37027	38.62171	55.72162
75.52327	89.18941	76.68517	139.5984	277.1931	271.2619	24.11671	216.903	74.53314
699.8342	451.9408	314.2583	81.83198	202.9325	1267.77	65.23565	1628.911	134.5007
1057.303	1195.643	1099.051	1325.524	959.2954	975.8783	751.0934	842.9379	841.6397
249.6991	126.7259	188.1688	100.4269	0.020384	1.958835	14.57769	20.57372	23.9516
56.39195	197.752	106.6239	203.167	5.336403	9.820387	74.08687	8.525949	131.9966
279.9343	764.2776	155.2483	315.0739	37.20872	40.28564	312.7898	72.44091	738.1127
140.9716	161.1063	125.3506	78.53471	87.1177	127.7622	66.02115	48.30348	67.67798
22.15467	18.38553	11.19917	30.07184	5.337801	14.73536	55.19391	9.729428	36.00203
93.64941	119.7567	147.7718	91.03536	302.7493	313.5054	161.4849	183.3204	138.8113
660.5645	488.5987	595.8071	963.6098	1528.678	520.8566	1249.027	1210.667	1070.511
32.22334	32.99651	30.85833	35.09196	10.64852	13.75211	82.49225	6.115717	21.38661
58.40502	70.86654	22.42162	81.89485	43.57606	48.15006	89.58341	43.47838	125.1825
21.14882	62.29298	18.68089	171.5617	32.95213	50.11798	51.35972	45.86445	52.26304
28.19718	68.38126	37.39681	197.5404	27.64271	27.51116	67.56421	24.19465	70.29247
8.058035	12.2652	1.844889	52.88602	10.64089	4.906956	35.41528	10.90913	30.9196
714.9396	422.6508	708.0577	879.1992	680.9699	338.0623	949.405	584.9013	959.9441
178.2323	142.9518	143.0932	234.0088	130.6859	311.539	240.1082	267.682	228.8403
10782.48	12166.75	17945.84	24640.37	3088.244	3514.337	18189.86	1631.641	18015.16
28.19456	11.06975	23.37781	3.274929	19.13434	27.51741	6.499556	16.93257	12.80984
92.6386	47.68464	99.16504	61.82561	80.73364	51.09977	124.9705	67.54259	113.1951
51.35485	33.03017	29.91334	19.9965	44.62482	57.00109	25.61263	37.42261	17.94332
37.25824	18.39966	28.98053	14.97909	43.5578	57.98634	23.41376	21.77324	17.08826
12.08636	183.1798	41.12852	6.620835	145.5459	400.9865	20.44462	226.6461	71.94433
26.18325	28.16302	24.29608	117.9771	47.81333	16.69987	92.63897	60.28974	74.59636
10.07253	12.30734	12.13207	10.79429	51.00216	30.45966	16.04218	151.7157	59.12829
158.0909	105.0637	126.2721	200.6493	53.13984	38.32051	138.0522	53.13433	176.5996
146.0108	173.4877	154.3143	404.5488	184.8627	212.2725	239.3326	176.114	332.5641
11.07877	17.13911	11.20349	32.64068	18.0697	7.855523	40.53173	19.31998	25.72146
21.14886	31.82892	32.71616	50.95119	90.28349	29.47665	46.93535	75.95369	38.52786
4075.148	6353.107	3342.043	1760.027	1500.043	1863.305	2055.121	1044.374	2765.804
303.0959	2047.646	295.5529	1191.077	110.5103	261.4077	444.18	124.2943	266.5042
23.15901	6.189952	14.0186	19.22107	9.580238	56.0443	26.50569	21.68882	42.13109
20.13895	3.752994	5.587021	36.88766	12.76192	4.906951	50.27482	14.50534	39.54246
284.9682	290.6597	295.5786	213.0903	376.0483	360.6769	260.6552	335.1954	273.4124
46.32179	41.59054	21.48674	119.5854	35.07845	33.40772	108.0221	57.91695	75.42233
4818.284	8447.742	6709.35	9189.094	723.4793	1672.648	7256.735	382.3489	7010.146
13.0924	21.99579	20.57181	27.59843	7.460376	6.872535	45.71787	12.12139	20.55652
39.27308	80.56488	19.61654	95.33658	62.67927	68.79394	42.52562	85.5657	94.33146
31.21453	18.36177	45.87261	26.74872	25.49648	11.78741	24.21993	14.52733	22.27112

8069.741	5688.886	4937.76	9132.253	2657.992	3542.84	6589.259	7932.211	6998.998
102.7123	59.94031	104.7345	116.9408	877.3944	304.6547	286.3421	341.2516	247.6761
18.12738	17.17054	21.49624	40.96802	23.38598	8.83774	76.60848	24.16291	30.84065
46.32079	43.99994	39.27207	81.12567	25.5173	40.28981	7.963321	43.44332	24.80605
0.002238	0.070671	1.845172	17.50396	32.93037	208.4361	7.232665	90.11433	0.804076
287.9879	162.4793	318.9781	427.1839	158.3017	126.7708	243.0523	113.4198	294.8705
28.19393	13.49622	21.50829	17.52038	12.76647	7.855475	39.04185	31.30832	34.32869
20.14108	40.28347	30.86057	33.42183	24.44708	13.75228	33.02674	9.730096	35.99807
71.49479	125.7216	57.0415	14.13692	72.24702	93.36281	51.32426	91.64084	52.2435
123.8575	99.00258	115.9651	29.17989	262.3868	195.5678	121.0756	427.9153	95.08627
48.32581	14.69412	41.21073	17.53723	5.336184	25.5553	7.983334	6.107882	8.522338
22.15399	12.28794	14.01038	20.02703	12.76868	11.78712	82.68486	45.72057	16.24641
176.2186	265.0124	249.7419	305.9133	85.01167	55.02709	265.8175	139.9411	263.9908
27.18965	34.23464	42.0877	56.02345	43.55784	35.37664	40.36275	24.17953	56.59403
94.65448	131.8661	156.2274	96.09676	30.83349	9.820322	25.58631	8.526775	78.82466
49.34025	30.57928	19.62027	55.18957	15.95872	14.73466	53.64296	28.98875	40.27377
278.9264	390.7337	285.2893	273.2938	217.7869	167.0647	242.2906	207.4416	329.15
71.48975	33.00578	62.6957	23.35476	28.69585	61.9206	16.79229	25.37537	38.56265
86.60103	186.9129	71.99542	78.49145	158.3067	152.3223	68.17281	136.3332	77.93526
277.9187	304.0539	262.8427	193.8732	145.5562	83.52776	193.8125	188.145	224.5534
831.7475	1522.979	1011.11	2004.991	1335.365	1176.359	1821	1257.757	1721.903
118.8166	34.26717	48.62607	62.67159	56.31151	14.73408	155.2166	6.115115	92.60567
219.5173	127.0925	203.897	438.0104	8.522958	3.924682	244.4877	2.485605	354.8676
176.2067	64.72774	67.3485	29.19491	13.83675	7.854743	13.10351	6.115349	96.90908
20.14189	22.06973	44.88388	25.01117	46.75468	96.31706	20.45319	69.92792	20.51317
2589.885	2705.033	2035.369	2120.355	1239.753	997.4965	986.7902	1471.161	1409.911
31.21847	44.04766	40.19601	11.63031	123.2236	64.85756	18.24172	487.6907	23.08036
177.2233	68.47047	191.7623	115.3052	219.8816	324.327	196.1083	97.72582	128.5477
409.8317	426.2572	277.7837	220.5795	356.957	728.2312	179.0566	774.0725	162.7897
31.21849	28.18293	23.35636	8.289064	458.7491	266.3484	32.93309	148.2681	30.79602
145.0031	133.1773	191.7453	449.7936	75.45015	90.40764	82.87216	91.71944	207.4153
25.17621	24.50135	40.20935	60.18879	31.88681	33.40947	47.70606	33.81527	53.13861
570.9444	565.4869	505.085	727.951	234.8009	169.0272	883.3934	513.733	615.385
1323.141	1573.087	822.1579	1080.567	2392.377	1977.31	2249.058	1451.917	1793.873
0.00153	0.046127	2.803234	0	6.335547	9.85141	0	4.762225	0
32.22435	15.96543	32.72149	50.97748	29.76303	22.59739	68.3488	38.62512	56.57651
107.742	107.4056	46.75123	254.4074	3.211513	19.64799	28.53515	2.490685	131.2173
582.0218	481.261	799.731	746.3066	514.1881	498.2538	429.4562	424.5356	397.6401
85.59196	59.90386	60.78138	105.3101	78.6225	55.02937	126.346	76.00515	107.1434
70.48767	50.13293	49.55659	55.95785	93.48022	126.7829	77.08221	59.12953	131.1994
92.63821	44.02274	101.0411	61.83096	40.38634	52.08311	50.61253	62.72159	64.27277
87.60493	239.0222	41.13469	198.261	7.46247	98.27804	18.97788	3.698786	147.5065
109.7592	134.3492	109.4276	95.23957	86.06533	118.912	116.7173	106.151	113.117
141.9832	97.79603	176.7635	245.6801	264.5307	118.9071	451.6491	417.1895	314.5472
26.1797	74.03386	27.13832	18.37069	2.149685	0.976239	15.37015	12.11477	16.26188
22.15507	40.30547	37.41057	27.54366	2.149223	15.71778	21.21196	7.321745	46.29699
14.09961	15.94089	5.585284	75.43465	25.50168	22.60078	29.37137	8.522362	39.46387
36.25106	23.26846	14.00531	45.97789	38.24803	14.73479	44.06583	44.60772	31.68834
24.16714	34.13866	12.13904	35.99011	8.522289	13.75367	18.30766	6.112334	48.09987
55.38346	143.908	61.72963	140.5518	17.02339	27.51095	28.54073	9.734791	89.17194
26.18079	19.57729	14.01171	11.64545	13.82863	13.75355	12.39697	10.92398	16.2501
1653.422	1929.665	1704.224	2207.23	1514.902	1153.754	1193.842	1942.658	2165.025
64.44439	47.65494	28.04017	195.9291	37.19604	27.51165	152.3847	32.61539	95.21662
223.5469	450.7087	358.2245	294.9617	624.6552	670.2422	301.6909	324.4465	336.7916
396.7409	409.1462	366.6549	450.4738	275.1607	402.9303	359.0227	307.5387	377.1069
12.08522	3.753252	4.650827	11.65705	10.64145	12.77185	6.507309	16.90413	9.382025
19.13268	8.626659	6.522531	60.42228	14.8853	14.73783	44.28099	31.28268	26.59463
95.66188	107.496	183.3557	103.6136	75.4423	90.41058	99.82324	66.38561	111.4114

25.1745	24.44937	11.20091	10.80419	49.89393	39.31512	9.442746	28.94033	17.96378
24.16956	37.92106	13.06756	200.8844	37.19924	15.71692	83.02976	67.52222	97.7661
193.3325	67.23962	214.2347	147.9492	66.94637	113.016	113.7921	24.20608	230.6552
233.6155	280.9408	411.5637	595.1294	113.6957	228.9782	484.6729	115.8482	756.9322
99.68978	167.2727	110.3652	152.1257	92.43616	121.8612	90.99193	78.4375	101.971
70.48861	73.32987	105.6936	74.34429	83.9353	100.2402	64.54088	67.58306	68.53029
453.1271	432.2653	153.3786	124.466	52.08166	72.7168	159.2526	54.35382	655.8187
36.25198	29.37765	20.55366	81.96765	39.31849	23.58014	83.08048	22.98499	90.06828
41.28311	20.80643	14.9457	34.2845	12.76954	20.63443	28.62972	28.94465	31.71953
17.12073	11.08183	10.26228	25.86713	34.00268	61.9204	54.39831	66.22693	46.29502
129.8995	138.076	83.22106	247.3758	208.2268	175.91	327.5361	108.6037	211.6825
122.8507	74.58823	107.5449	177.9902	122.1875	97.28696	205.5776	49.52919	224.558
573.9639	404.2845	563.0936	275.747	436.6265	552.3118	380.3017	284.6461	284.5122
5616.795	4918.167	4055.729	2364.283	2059.892	1091.838	3983.375	2732.554	3019.503
16.11309	22.01137	5.585501	107.4099	17.01237	14.73643	14.6081	14.53026	27.42877
61.41932	28.11102	28.05468	14.15105	41.41831	37.34674	14.59514	28.95677	24.8326
139.9606	69.60943	96.36395	47.60438	24.4576	42.25431	69.76173	54.29261	76.29512
0.001433	1.268273	0	0	0.012636	0	0	1.250047	0
14.09872	41.33753	14.0178	2.440254	2.149673	7.855981	2.827728	2.494412	11.96284
4.028829	0.055692	0	0	21.11357	1.960175	0.640261	0.051938	0
10.0718	19.55493	13.07886	9.13693	7.459527	9.821939	23.51445	8.513654	14.54028
114.7937	37.95243	63.58246	175.5427	104.1196	57.97654	175.5467	109.7637	151.7165
30.21072	31.81577	43.01659	120.509	48.87273	39.30693	95.60976	378.6966	77.18184
703.8608	521.5158	528.4749	421.1845	466.3749	565.086	401.5838	520.9532	367.6554
97.6763	101.4164	119.7176	103.5987	72.25945	63.87317	68.19512	101.3379	71.09079
8.057658	7.396149	6.525859	37.799	4.273241	7.856967	22.11255	7.299378	24.05792
989.8374	2459.44	2251.483	2249.168	122.1959	544.4431	487.46	206.2942	1116.799
97.6769	105.0982	173.9732	155.4275	95.63051	51.09624	179.8837	125.459	127.674
95.66181	42.83203	71.06961	17.47969	51.01474	173.9542	39.54382	80.84053	14.50914
9.065208	2.531736	8.393413	11.64542	14.88963	20.63555	21.26101	18.1291	19.68993
307.1217	75.81582	205.7636	189.6692	387.743	460.9199	374.5078	174.9139	436.2993
335.314	233.2744	187.9977	208.0853	147.6811	200.4808	252.597	173.6856	175.6766
188.2993	133.1411	167.4353	132.8699	130.6734	97.28897	64.51956	242.2791	96.81639
89.61865	47.69356	61.72222	55.95825	110.4673	82.55163	100.6363	35.04436	64.25884
13.09287	8.63776	13.07226	42.66761	17.0156	9.820842	49.31327	18.14419	29.99344
23.16253	31.81891	16.81026	27.52509	39.32007	44.22131	27.81428	49.46218	11.93862
128.8897	152.6081	122.5354	144.6164	39.33104	54.04589	188.8421	174.7673	171.4732
2.016683	19.5084	4.652033	0.773871	20.17327	5.890372	5.77752	6.10176	3.371133
0.002085	0.065165	0	0	0.018598	0	0	0.060696	0
46.32155	31.82988	43.94584	56.80897	43.57159	51.10024	149.3074	30.2196	71.14023
53.36696	54.89895	68.31666	30.89582	23.38856	16.70074	45.54891	31.38471	46.29807
10.07252	52.53659	14.93882	4.946261	90.27425	94.35258	22.66186	83.14478	17.0834
133.927	63.60008	101.9318	238.1948	129.6229	89.42459	307.7321	306.234	276.0076
34.23913	19.63515	43.94049	43.4037	210.2742	152.3369	46.9087	281.7963	32.51396
646.4641	414.0553	692.185	539.0623	458.9347	558.2081	671.1782	259.33	753.4346
90.62239	85.39475	58.93129	41.76006	52.05687	24.56303	44.75544	31.41043	42.83258
13.09327	41.54618	14.00431	19.99902	81.7653	47.17216	21.93734	65.06197	16.22903
81.56254	90.32809	95.42495	95.32093	39.32446	35.37382	51.34897	44.66769	31.663
32.22443	35.46922	24.29717	58.51232	31.88698	28.49475	32.97286	48.25084	26.52347
288.9891	69.68244	43.93635	600.618	18.08663	15.71673	171.8824	12.14572	326.7362
59.41124	58.65193	62.6632	51.78443	71.17714	46.18549	83.7386	38.65004	70.27883
307.1219	223.5508	129.0553	892.8167	55.2687	34.38916	1115.902	86.90999	578.6203
67.46633	167.0709	85.127	49.27381	45.69577	42.25392	55.76231	14.55802	67.70417
13.09311	15.95623	44.90448	29.22272	28.69442	12.76914	14.5858	13.34364	21.38554
109.7612	323.716	335.7753	285.7689	1048.49	439.2898	168.0342	1045.372	217.6445
898.2007	362.781	782.9195	616.7966	235.8614	1446.646	739.4742	172.524	601.6873
44.30717	26.94303	30.84744	27.52524	48.87427	24.56285	55.06092	69.90775	41.97057
159.0987	239.2733	151.5286	140.3967	127.4875	118.9112	102.7377	107.3644	131.9786

119.8279	128.2314	96.33214	231.6204	74.38029	108.1019	155.7168	129.0219	218.6478
39.27254	23.28227	19.61807	67.72878	53.11721	39.3072	77.9266	55.46479	51.42132
16.11403	53.83335	24.29303	27.5119	342.0843	167.0622	11.64608	355.7677	50.50663
122.8508	185.6754	169.287	234.8415	228.405	164.1169	242.307	241.175	155.0982
33.23196	9.864821	14.00274	146.3849	64.80862	56.01422	46.19007	131.3149	77.99945
20.14127	13.51931	15.87776	20.00647	27.63344	21.61567	83.21367	18.15711	35.9887
56.39188	56.25581	133.7617	170.545	92.43376	56.99417	111.5924	67.58919	134.5749
18.12773	7.421267	38.3427	16.65284	18.08246	6.872007	7.229496	9.733137	29.10673
61.42271	39.1089	66.43086	30.04687	19.14465	27.51252	68.37853	38.61452	39.40932
625.3205	1488.615	704.3218	569.9465	370.7772	562.1345	539.6228	457.0853	857.0981
53.36609	6.199114	44.90905	20.01065	35.05853	15.71817	54.43977	20.55725	29.98107
94.65395	44.04357	105.7002	105.3146	64.81673	71.73825	81.47251	161.4769	149.1948
296.0421	173.4452	200.1681	85.18729	236.8915	336.1135	88.01791	136.3078	120.8122
98.68334	199.0223	108.4888	430.6707	19.14892	71.73552	82.891	25.41246	201.451
6.043879	6.173463	5.5912	24.34828	1.085974	7.857952	8.757829	7.288972	6.816526
576.9857	594.7779	721.1743	713.7531	386.7058	340.0296	660.8579	493.228	623.1092
21.14762	24.46136	15.88081	25.88683	12.77041	10.80375	49.30867	15.74139	41.1717
1026.087	1305.522	994.29	1455.935	626.7944	445.1837	715.12	729.5912	960.7954
49.34385	23.29718	17.74549	5.788819	735.0732	506.128	38.80094	1409.047	39.3643
106.7369	89.17125	100.0843	60.96715	36.1434	34.38982	50.57988	63.96065	169.7872
39.27385	69.66024	46.74626	35.87369	55.25976	95.3271	68.96464	115.738	66.82097
284.9697	266.2924	231.9524	254.0289	439.8005	330.2052	104.1535	399.1376	163.6523
47.32851	30.61135	39.26612	25.84508	72.23845	35.37374	43.98722	57.90959	24.79995
505.4869	250.3838	238.5107	182.1542	127.502	138.5634	356.1768	136.3316	620.6851
1138.868	1765.967	544.3557	1251.936	617.2402	766.5467	1127.789	571.6611	983.0521
48.33581	31.83741	38.32767	176.4953	58.44147	37.33883	110.9409	84.40984	99.44295
7.050885	8.607819	13.08913	15.88462	19.11054	8.840381	13.19362	18.06073	14.56667
513.5447	498.2196	571.5392	356.0303	120.0689	169.0287	242.2494	114.6409	386.5588
695.8033	428.6896	346.0726	562.4873	6.397015	38.32028	693.2543	33.85091	1251.571
1434.909	890.3538	1248.72	717.0372	718.1526	784.2388	1213.042	1005.699	723.355
30.21073	22.06478	15.87483	95.36926	36.13419	28.49461	93.39557	117.9847	50.55885
22.15471	8.639608	4.649718	46.84393	23.38524	12.7694	89.19221	44.57538	34.28138
68.47371	47.69097	31.77831	20.82513	31.89318	43.23619	42.50562	54.30994	30.80147
24.16971	47.72467	39.25825	44.22547	968.8198	248.6316	43.94392	592.0853	77.93289
55.38413	11.08638	33.64929	13.30124	115.7756	16.69953	37.35512	44.67943	7.653634
13.09253	2.531745	25.25431	13.32275	8.522372	0.976269	19.04551	7.316144	9.375223
1092.545	1000.219	940.0445	673.5889	424.9551	527.7371	809.8845	290.6975	839.0911
106.7363	83.06247	93.5377	57.62606	104.102	74.68751	58.67731	86.82926	85.70331
4.030656	14.69894	2.779679	19.21289	12.76357	17.68784	24.2615	14.5118	19.70729
145.0017	67.2484	169.308	102.7627	89.25281	118.9115	90.24493	82.05964	109.6821
247.7069	147.7735	218.9018	25.00126	82.87987	168.054	46.15157	31.44078	73.66436
14.09983	22.0346	14.00751	25.8818	12.77104	22.59964	14.59286	27.76067	25.68923
130.9056	92.89099	149.6491	70.9762	208.212	134.6347	225.4641	186.9084	173.9833
21.14822	24.48494	30.8558	54.36366	42.49206	17.68362	100.1804	36.19393	61.76615
7.051612	20.8267	14.94216	2.441123	21.26453	20.63286	25.64077	24.16734	10.22684
20.14197	18.41172	9.326001	6.61698	11.71241	30.45934	10.16558	4.907508	7.653251
4.030146	2.524739	6.53309	3.283756	15.91131	27.53949	6.549007	10.84676	0
164.1331	130.698	129.0741	178.0407	117.9282	88.44367	153.4694	141.0935	139.7003
2483.135	719.3362	1407.782	162.9004	92.45056	267.304	735.7536	326.8598	967.7077
14.10033	11.08411	5.586645	117.819	5.33617	7.855065	20.44423	10.93867	12.79591
1.009596	9.832569	4.651335	0.773956	32.89299	7.85632	10.20744	44.3758	3.370332
96.66892	94.08166	115.9805	131.2155	87.12532	77.63339	195.4262	63.97788	181.7507
0.00183	0.056239	0.912778	0	0.01625	0	0	0.052444	0
34.23868	37.92358	28.03761	91.98003	40.38527	78.62299	58.71764	81.96427	80.58863
52.36345	40.37657	63.59358	73.53096	133.8249	108.1073	133.7616	50.70128	72.83896
492.4024	864.6233	585.5326	679.4711	416.451	232.9071	831.2494	353.3821	862.2729
307.1219	61.16175	166.4731	204.7143	300.6463	149.3732	494.2578	338.8496	427.7179
275.9066	412.7567	407.8302	383.6302	288.9609	172.9602	304.7058	306.3012	293.1141

3963.378	3855.716	4776.887	3331.245	2335.04	2214.149	3018.472	1916.216	3630.6
52.36427	314.8317	111.301	477.6062	2.147672	16.69948	40.27632	6.111596	110.5485
369.554	224.8	459.2576	46.73167	316.5927	491.3789	374.4231	226.7775	186.7898
25.17515	13.51455	15.87962	56.91586	13.83276	15.71837	65.54418	16.94751	52.34065
1508.42	1719.589	1618.176	1463.383	1214.262	1532.121	866.348	1190.234	1285.615
100.6979	96.55952	133.7408	55.92614	156.1746	151.3415	113.7306	194.1561	171.3997
36.25088	29.35196	43.03006	7.452354	35.06269	35.37787	16.05719	40.99657	12.80012
118.8222	48.94382	52.3539	364.5439	110.4981	72.71793	196.8135	133.8834	192.8595
1.009586	3.754167	10.26765	2.440161	73.1823	16.70391	20.54278	19.31448	3.369387
12.08643	12.30707	14.93865	1.60936	48.87733	119.9093	3.563837	61.50346	3.370987
64.44725	134.3225	167.4504	101.9442	83.93737	68.78827	49.09831	36.25985	59.09267
160.1069	134.3925	336.7679	163.7867	121.1227	98.27028	187.2275	55.55564	258.019
17.11989	13.5027	28.06297	4.110289	14.89034	29.484	18.3005	7.31712	9.374127
7.050684	9.809524	6.527782	3.278983	7.453308	18.67711	16.93554	4.895945	11.98831
64.44588	51.34338	78.57231	26.68025	168.8467	124.8194	69.00408	54.30426	59.11739
33.23145	24.50441	71.09938	13.30294	93.45426	112.0481	14.57469	61.48835	25.66338
508.5082	239.4041	261.8967	178.8071	521.5661	558.2204	176.1523	241.1995	291.4113
16.11129	9.824419	12.14931	15.03607	5.334694	3.924262	16.89743	9.698053	36.13886
318.1962	301.621	131.8665	271.6332	121.1267	95.32112	157.0681	197.7902	267.4235
23.16158	12.29727	13.07091	77.04753	22.3243	15.71825	41.88902	32.57053	51.47527
90.62543	104.9842	82.31215	75.20772	49.94609	58.9622	73.40665	35.04362	51.38961
149.0325	2339.645	431.1819	1009.617	55.26765	146.4235	67.43895	54.35043	577.6507
2.016697	7.408832	3.715003	0.774125	12.76336	15.72136	0.635269	3.701061	0.803402
94.65326	35.49816	65.46467	545.7578	17.02405	8.837525	117.5597	15.76455	23.93959
285.9763	238.2051	319.8893	254.8709	202.9242	318.4126	296.6114	232.7819	353.9786
53.3712	52.5982	30.83947	331.174	94.56002	56.01107	235.874	80.84661	136.2819
100.6959	92.84294	101.0156	89.40397	65.88144	58.96034	208.7323	133.8158	119.1456
156.0789	163.6752	99.12838	196.407	127.4941	70.75206	310.0016	247.1519	186.8457
478.3046	147.8719	183.3048	902.6909	309.1586	203.4244	1546.043	309.9704	300.795
12.08644	80.58198	14.93832	50.11237	48.88051	51.10021	61.65471	85.58291	38.52682
16.11221	17.12034	21.51671	11.65704	9.580728	26.53861	35.4032	6.108011	18.85014
100.6969	106.2908	115.9771	70.98608	55.26477	54.04524	154.2167	68.80211	127.6978
44.3045	46.35034	58.02797	19.17567	15.95566	22.59956	21.96485	10.93296	36.00323
5.037673	7.421538	5.585043	5.781031	139.0852	35.37518	9.431814	39.83299	7.652907
19.1348	71.99546	6.520157	164.11	2.148887	2.941381	19.72483	1.282119	25.66607
89.61982	55.02829	69.2014	80.20347	130.6565	93.36058	152.0828	92.87098	164.6241
129.8995	86.80064	37.38635	50.90946	127.5006	224.0676	37.33169	438.8139	91.65298
182.2537	45.26242	99.15041	213.2886	37.20507	131.6949	107.2281	12.14684	183.5274
39.27167	42.74596	36.47241	46.81338	13.83481	23.58121	47.75017	4.909343	34.26384
33.22908	26.88727	24.31038	40.99439	18.07617	23.58354	34.53624	39.74722	24.83729
24.16779	12.29047	21.50156	35.96423	29.74499	26.53329	47.85714	3.702408	33.44078
158.092	284.4181	137.4922	71.81731	108.3718	180.8296	68.92618	50.72944	114.823
3025.902	2970.253	2558.2	2339.215	3587.513	4993.403	2116.067	4101.155	2233.538
16.11426	8.642768	38.32831	0	67.99635	65.84265	10.89974	25.40566	29.94382
200.3867	298.0555	290.8805	588.3973	570.465	450.1029	514.0011	416.0444	296.5166
49.34315	24.51974	34.58276	20.82315	38.26799	102.2068	43.96008	221.6832	26.50983
185.2803	203.9591	158.0657	87.69425	134.9314	163.1355	107.8539	138.7213	119.9529
41.28588	30.58664	18.68332	56.01504	69.03101	21.61478	37.40604	54.24521	31.67939
80.55925	269.8961	42.99851	1.61321	133.8736	181.8071	85.06871	224.308	101.9408
115.7998	44.05058	43.93757	142.1074	63.7585	42.25201	127.7847	41.07945	180.0544
17.12088	31.80261	13.06872	20.83679	32.94521	23.58073	38.14616	26.58761	23.95365
105.7278	56.21439	66.40699	71.03678	115.7646	72.72427	113.9338	37.44603	112.3423
0.002035	0.063408	0.911122	0	3.209275	4.908683	0	20.34918	0.803556
0.001901	0.058694	0	0	0.016901	0	0.637622	1.2783	0
4.030607	11.04699	4.651267	18.38712	12.76029	9.822665	24.29516	13.30139	26.61843
16.11402	34.24276	43.0207	26.69505	73.27772	58.96804	10.90199	33.80847	24.81025
19.13358	4.976517	28.0616	9.967658	33.98416	25.55082	30.86199	38.52665	25.70576
142.9888	173.4359	106.6129	243.243	110.4999	116.944	207.0863	184.4989	165.4074

1660.464	594.8229	792.2486	749.6522	34.01974	72.7168	717.3458	53.14406	1870.321
1.009448	1.301304	3.716049	3.279001	49.94926	60.92679	0.640175	103.6926	0
0.001104	0.032392	0	0	0.009679	0	0	1.223068	0
28.19362	32.90473	5.586084	37.68907	9.582654	9.821677	10.19006	7.313878	31.75271
1422.829	2059.087	1399.293	1621.345	718.1627	948.3572	1264.352	508.9642	1530.753
85.57774	47.49095	18.69476	34.30309	11.70667	5.889498	13.13382	2.494603	36.89854
32699.88	42175.55	28803.49	24882.61	34212.59	43605	19324.9	33910.46	28139.9
59.41235	78.20217	43.9387	48.41743	106.2327	73.70344	60.87027	115.7463	39.37191
186.2884	247.9442	205.7675	188.8418	156.1819	150.3568	256.241	172.4936	285.413
188.302	152.7242	199.2217	202.2258	257.0856	203.4285	351.789	385.7968	266.5589
325.2484	296.8413	260.9449	504.7887	224.1755	380.3245	733.6176	453.4266	654.8469
748.1702	3556.162	1044.787	24.17506	311.2919	1990.092	713.615	961.1283	2921.947
179.24	188.1399	199.2164	213.0804	91.38669	24.5617	454.6103	9.727241	457.7421
17.12012	18.37407	9.328454	4.945752	19.13648	71.7619	18.29052	42.13863	0.803956
22.15553	15.96603	18.68224	119.6803	56.30172	36.35829	98.57108	61.47759	63.44402
11.07893	28.08064	28.06364	11.64463	8.522667	23.5849	10.92028	7.316867	27.43062
103.716	65.99842	84.17673	77.70208	145.5148	114.9854	62.34869	61.5498	108.8647
197.3619	111.1843	106.6168	174.6848	48.89281	78.61511	88.76859	35.05837	187.7238
45.31491	81.81821	71.0837	20.82559	55.25381	37.33914	35.88855	25.4048	35.09145
548.7842	233.2862	377.9097	218.9473	148.745	421.6123	218.7802	95.34365	324.0078
140.9737	113.6107	95.39286	238.2835	53.14049	35.37206	147.5989	172.4019	151.7147
2.016695	2.531295	4.651098	1.6061	6.397168	7.85616	0.63506	10.90732	6.804474
2464.02	3001.94	3516.982	1813.54	2141.678	1698.203	2014.779	1840.201	1505.868
148.0216	48.93593	166.5098	98.59196	154.0288	269.2921	127.7623	85.66331	135.427
4.030581	70.87009	29.9059	38.38527	58.44359	71.73884	5.031947	156.6424	14.50893
57.39128	42.67262	32.74243	27.57125	9.585133	16.70206	21.981	7.318777	18.82157
417.8867	478.7329	318.9467	391.1193	107.3226	368.5328	302.4615	121.879	407.1126
4009.696	5296.701	3372.916	4090.18	2116.191	4165.919	2248.986	3177.48	3846.64
291.0112	229.6623	129.9899	682.9246	283.6539	290.8941	536.8458	314.7536	293.9618
74.51406	33.0463	13.06751	39.23569	19.14745	23.57957	162.6022	55.49387	115.7938
0.002225	2.531646	3.714614	1.606727	4.275211	8.838128	2.095306	0.065359	0.80387
111.7726	114.8155	111.3031	66.80979	78.62851	215.2345	53.50595	50.72237	101.9753
97.67672	85.56466	59.83758	279.219	162.5408	75.66626	210.7755	178.466	217.7281
44.308	33.05405	29.9074	118.7465	51.00654	28.49343	120.5382	45.87871	108.8965
15.10489	3.751453	10.27273	6.627982	8.517605	5.890217	28.03084	7.304045	18.0041
25.1748	17.1615	12.13623	54.42141	19.1377	10.80381	46.35965	42.15021	35.15452
10.06914	7.356061	5.597099	38.0266	3.208371	3.925588	12.54915	33.33807	11.16247
18.12725	25.68353	18.68874	23.36706	20.20068	9.820714	47.81249	34.96494	85.89514
8.05788	2.530946	7.461121	1.606096	30.77163	7.856438	0	81.34317	0
34.23871	35.48658	28.97312	92.81385	98.7755	140.5497	108.0602	38.64664	96.89809
61.4247	45.23733	52.37037	36.72437	61.61916	33.40826	80.81063	50.68157	94.32547
42.29291	91.45183	34.59469	30.88056	30.82377	59.95079	52.87712	20.57406	60.0172
25.17525	20.82157	35.54385	151.7565	35.05764	12.76937	39.67358	26.56446	50.61533
151.0409	81.86178	163.7175	52.59919	51.01271	120.8814	60.1343	69.98738	137.1688
25.17466	6.197734	23.37434	40.99762	23.38077	10.8039	19.76522	18.14079	36.87951
15.10498	27.99068	14.02187	7.466308	12.75908	13.75618	8.730122	2.493938	5.087394
49.34274	53.79175	38.32755	82.73671	100.9127	37.3388	29.26664	47.08888	25.6548
28.19697	46.43707	38.33582	24.17859	63.73406	172.0139	12.37013	96.35237	33.38805
19.13414	52.42679	47.72253	113.9932	7.462213	84.53827	16.80269	46.97214	86.7488
3.023587	1.301994	3.715677	0.776899	39.32628	11.78571	15.30568	86.80456	5.940321
15.10656	62.11007	24.30965	45.18704	10.64712	10.8038	19.02313	12.13334	28.2746
180.244	205.1185	179.5986	196.4385	94.56389	116.9455	50.55821	67.60116	164.5706
200.3713	53.76423	127.2606	61.83992	24.45721	52.08384	69.03375	25.39952	60.84713
218.5038	100.164	81.36421	104.4606	2.147869	46.18337	79.24671	2.48895	206.6587
7.05162	19.63143	2.780758	0	75.42196	26.52802	2.832276	86.78375	28.23174
96.66818	72.10595	68.26572	29.18298	61.63295	103.1893	65.28039	66.37514	60.81284
38.26383	26.90377	40.22776	24.20269	33.99603	17.68429	13.85442	13.34021	32.56246
0.001929	0.059664	1.847674	0	7.435529	0.976495	1.369758	0.055613	3.386873

196.3594	149.096	317.9951	71.80659	2444.392	2735.034	813.4928	1342.134	52.22523
1.009597	2.530938	3.715516	0.773926	66.77678	2.941313	0	3.699419	0
130.9058	88.01193	78.54626	70.97461	142.3652	312.5255	150.4797	284.5128	137.1043
175.2119	140.5194	118.7681	218.9447	226.2846	437.3367	259.1893	89.31683	125.9436
14.09918	18.35338	18.69798	5.783732	15.94844	19.65316	5.766197	10.92013	17.97566
11.07902	11.07136	15.88306	5.782089	33.98549	41.28194	8.706853	94.84484	7.656358
40.2804	62.3182	77.63478	80.2352	11.71244	24.56206	80.77585	19.38009	113.1866
429.9723	351.8201	190.7857	1342.281	525.8738	752.7915	1271.839	541.491	1038.814
93.6474	44.04711	61.71609	133.7545	90.3049	92.37784	80.72569	63.96531	101.9908
575.9752	177.1634	162.7301	70.96713	906.0921	1706.13	82.85737	814.9502	94.21941
20.14081	24.46208	11.19981	52.73505	11.70896	11.78668	34.52515	55.35269	73.8637
267.8489	336.983	315.2401	138.6934	156.1758	109.0806	93.15548	123.0587	315.4606
926.3982	1735.259	259.0689	2076.225	2.146271	66.82027	383.9289	21.7854	1326.852
3628.063	3530.88	5440.987	3738.243	2077.958	1763.062	4026.613	2874.882	3757.436
53.37016	141.5269	23.35777	23.33411	24.45879	34.3905	94.02579	13.35279	87.43547
3140.694	1343.524	3283.119	2711.142	2325.469	3047.534	2964.197	3692.365	3931.494
32.22403	26.92968	27.10781	0	62.66128	53.07079	27.09492	30.19514	11.08261
190.3155	161.26	239.454	27.50872	279.385	552.3309	96.82522	250.8095	107.0868
66.45851	100.0295	72.03263	97.02742	31.88903	28.49427	122.1151	15.76131	57.42051
39.27428	51.38866	175.8413	114.449	336.7284	177.8777	25.5842	100.1557	41.93568
86.60024	114.8444	176.7897	50.91463	120.0544	50.11375	63.04786	251.9265	37.65122
0.001109	0.032573	0	0	0.00973	0	0	0.030469	1.72006
5.037696	30.56955	13.06976	0.775456	56.28494	28.49658	10.9054	69.82101	10.22624
9.065523	23.27159	15.87663	7.452018	69.02397	266.41	12.37444	80.65514	11.08313
36.25179	37.90038	38.33855	61.87089	61.60612	101.2379	24.14196	19.37092	56.57889
29.20425	80.58649	55.17581	37.55774	71.17707	76.65603	33.68642	33.83353	62.55422
176.2179	290.5969	340.5069	164.6196	38.27114	19.64783	223.9698	8.522994	189.4094
57.39839	36.72351	102.892	65.15041	132.7751	71.73815	90.29847	31.43513	102.8557
8.058574	0.073167	16.80883	0	125.3325	11.78571	3.566007	4.906636	0.805998
857.9269	839.0674	985.8697	1892.25	842.441	692.8411	1439.977	934.5643	1218.804
233.6161	377.4238	418.0987	513.987	402.6347	193.5969	514.7221	176.139	548.5472
14916.03	13004.56	21752.77	13234.01	5456.193	5223.355	7888.063	6232.05	10761.57
28.1954	11.07614	17.75311	11.63852	32.93191	30.46475	24.18431	18.14639	26.55162
97.67628	159.9763	109.4258	208.9921	159.3465	134.6369	216.7085	139.8883	119.1163
13.09304	20.82323	8.391482	1.607462	101.888	40.29452	2.09648	13.34142	7.654073
20.14044	19.58598	20.56617	35.13141	24.43956	13.75314	39.73294	28.93733	37.74839
8.057313	6.174883	10.27973	12.5307	8.512474	11.79188	27.38897	1.284134	8.539392
21.14842	18.40026	14.00457	25.0237	30.82177	10.80316	20.46734	16.9598	26.52995
60.41973	55.03946	82.29531	108.6262	112.6144	51.09696	149.0811	49.5194	106.2592
20.13887	7.406049	8.396658	0.774022	20.18215	46.20933	7.24687	7.308405	5.0861
58.40456	115.9335	35.52184	81.9122	44.63532	17.68238	101.3959	54.30526	101.1727
73.50926	46.48843	59.84424	106.9752	88.18192	71.73748	193.2854	110.9353	113.9996
30.20819	25.65852	17.75676	62.01327	9.58452	10.80413	32.34139	6.113462	78.21739
688.7556	462.8903	409.6795	255.6852	403.6972	727.2475	300.2398	465.4796	385.6661
19.13439	25.69805	9.32686	50.18019	24.44918	32.42899	49.2479	13.34363	47.16209
35.244	28.1339	38.34819	25.03211	54.16191	19.64968	54.41079	21.7656	52.31694
462.1913	204.0256	190.793	373.5787	389.8758	380.3279	528.0212	379.8479	431.1328
502.4375	124.4883	92.60598	9.123316	11.71244	14.73398	183.8337	3.698828	144.0748
47.32994	58.71809	91.63973	35.8658	268.7714	201.4625	160.731	635.2544	75.36428
50.34712	31.79737	83.29252	49.32189	24.45197	24.56395	36.67994	1.282667	48.00501
18.12472	6.18473	3.71575	41.99307	8.516981	8.839933	26.55618	3.69868	18.00817
15.10717	28.15764	25.23322	59.35133	7.46282	9.82026	67.60543	18.16809	47.12773
235.6266	229.5802	210.4591	161.2769	171.0426	285.006	146.8034	238.7314	216.8534
27.19054	40.37471	16.80892	7.452589	144.4384	210.334	17.50901	152.9968	15.36631
43.30139	56.23608	34.58407	22.49617	204.9635	155.2861	72.65578	51.91027	62.53759
101.7035	424.5496	81.36065	31.68906	58.44996	144.4674	31.46094	54.33815	59.94798
17402.21	14175.79	15128.52	9499.872	14047.3	13273.14	8813.973	17440.58	11335.82
420.9081	735.1378	375.0693	780.6679	540.7244	729.2139	304.6504	466.6798	607.704

53.36735	15.95967	60.81951	35.08062	38.24887	25.54716	41.11111	15.75364	33.40474
28.19605	28.13162	18.6862	48.50416	37.18372	21.61576	36.70267	50.59963	50.60117
13.09015	15.8342	14.03638	21.82306	5.331881	5.891291	18.45173	0.060697	23.23596
102.7096	140.3951	101.0168	83.54997	35.0819	52.08057	204.3286	139.8306	114.0009
10.07236	46.50477	8.392603	32.52272	437.627	192.6196	81.40206	307.4241	29.07905
472.2628	431.1556	232.8815	210.5455	593.8423	641.7438	251.7596	505.2725	325.655
23.16056	18.36413	16.82124	29.26443	8.52263	7.855307	27.91352	3.702222	24.84991
245.6976	141.7409	231.0277	199.7136	365.4264	275.1728	376.0257	218.2911	245.9757
158.0917	186.816	248.8418	262.5282	105.184	52.07946	101.2699	117.0007	103.678
148.0227	92.88007	90.71186	634.794	78.63313	32.42362	205.658	21.79469	299.2322
1129.806	2962.817	2620.897	1037.108	325.1027	5682.339	926.5439	2271.849	1901.874
111.7737	61.15164	64.51526	47.56922	139.1744	243.73	113.7494	121.8345	48.79493
32.22478	22.06932	39.26864	51.79476	65.8633	52.08401	46.94451	73.53427	59.99036
8.058573	0.071639	5.584903	3.275631	10.64899	4.906566	4.294305	19.36287	1.658786
70.48818	48.92275	73.88302	48.42058	57.38302	118.9169	76.32438	69.97971	54.81299
18.12608	15.91869	7.458491	71.34738	10.6432	3.92396	31.65811	4.906544	36.06605
174.2059	149.0884	234.7555	555.8036	449.3682	130.6998	316.413	112.2338	370.2454
0.001264	0.03748	0	0	0.011118	0	0	0.035037	0
83.57944	67.25136	72.00057	81.01569	186.962	99.25435	110.0846	167.6053	99.38694
43.30183	34.28883	79.48934	11.63093	90.31118	46.18301	58.64972	26.61744	26.50841
41.28152	12.28151	12.13946	298.4807	15.94936	6.872483	15.35464	6.111953	22.27491
0.002002	0.06222	5.597466	1.608898	16.95838	14.74899	0	13.20067	0.803882
24.16828	13.51403	11.19933	54.40146	40.36	44.22811	44.11648	39.76759	36.00427
54.37473	101.1539	32.72457	20.00012	4.275147	15.71744	23.41216	4.909193	55.73198
956.5876	189.3321	101.9314	147.0504	19.14836	19.64789	39.53451	12.14231	305.1593
153.0553	67.23211	84.17095	82.70812	104.1127	98.27425	84.39282	100.1047	55.66402
41.28589	39.1114	118.8709	34.23297	12.77363	27.5124	29.30125	8.528153	76.34658
195.3471	84.32853	89.77923	141.2439	113.678	66.82187	100.5433	78.44097	179.1647
39.27335	39.14825	54.23963	121.2723	99.84141	68.79247	93.31004	83.18253	81.43675
181.2516	57.48905	81.3549	149.5854	118.9945	84.51168	182.8485	85.68291	302.6466
302.0889	370.1139	430.2537	238.1262	638.4611	566.0685	407.457	473.9384	300.7921
91.63539	75.80616	54.22384	399.62	184.8508	211.2937	468.0219	114.6178	348.9195
15.10677	52.43642	17.75142	77.88458	18.07914	12.76935	50.75052	16.94946	45.45528
6.04387	1.305323	2.780887	24.34957	7.45216	3.924663	19.18859	3.694053	10.26684
23.16296	31.85005	23.35633	231.5063	230.5257	239.7941	27.05281	609.8597	16.22497
129.8992	151.4912	185.1938	307.6047	175.295	167.066	345.2215	190.5494	272.5795
50.34809	23.28241	75.78495	40.08708	31.88683	55.03481	78.66288	39.83051	28.24027
12.08631	24.49102	10.26198	4.945462	145.4177	174.976	10.16827	41.01241	10.22531
33.23006	12.29869	14.00642	0.775273	59.46168	79.61961	7.230655	16.95146	9.36925
115.8022	151.496	98.18937	244.0433	219.9066	193.6013	370.9277	115.8315	165.389
12.08504	12.26201	7.460282	5.787408	15.9413	28.5068	6.509845	45.5846	0.80329
61.42451	39.13943	37.39663	76.07601	22.33324	19.64824	69.77217	50.67711	66.8574
9.065161	15.9284	8.393791	4.110557	10.64469	50.13484	2.827272	14.52484	12.81325
481.3239	606.9083	477.0419	527.3914	133.8797	338.0668	536.8071	246.0548	368.537
0.002202	0.069373	0	0	1.085955	4.906748	1.363882	2.494634	0
115.792	45.20154	42.08462	71.93332	22.33032	67.81538	74.26656	24.18381	36.83
30.21088	47.66292	34.59036	66.03643	43.56757	37.34049	49.16177	27.80455	58.27918
292.0165	205.2145	174.8977	121.9625	111.568	167.0647	208.4911	186.952	290.5649
221.5322	313.9078	283.402	107.7425	274.096	347.8952	480.2545	241.2285	329.1061
45.3122	17.1747	24.30315	25.87073	52.03908	69.78661	31.53701	26.57384	53.1784
11.07847	8.623964	8.395979	14.1756	17.00408	17.6882	19.08082	51.5912	12.8228
98.68466	195.505	158.9832	43.39178	617.2301	455.012	55.69305	741.6324	103.6456
13.09206	1.305913	1.844892	0	8.520229	7.855883	5.032791	1.284534	1.65727
45.31433	170.655	40.20491	103.7185	8.525247	9.820219	9.431483	2.491621	33.3831
212.4674	130.734	233.8479	90.20268	158.298	230.9501	110.0588	139.9257	163.6843
3.023635	3.749858	5.588968	0.773853	3.211687	12.77387	0.634752	2.493208	0.803176
64.44652	256.1183	25.2281	61.80986	7.462374	9.820255	35.14514	10.94069	109.7314
8.056822	0.064147	3.718462	0	3.209882	3.925022	4.31545	6.083841	0.803399

58.40419	78.1423	85.12929	169.8609	35.07688	32.42524	159.6292	38.6475	89.16917
105.733	200.3387	214.1871	70.96886	249.6541	202.445	153.381	335.2013	93.3656
25.17524	14.73372	33.67089	29.23152	32.93542	43.24429	38.93619	44.5768	21.39037
29.20345	28.14827	57.06772	89.54701	40.3749	31.44446	45.51892	22.97788	39.41018
8.057585	4.965832	4.652637	3.277837	3.211544	18.67554	16.91371	9.692265	4.230731
38.26565	56.17118	53.31572	96.21858	40.37922	9.820263	52.86575	21.78003	66.88104
214.4769	69.67577	121.5981	61.79383	90.30918	245.7051	63.79994	57.9498	82.25023
179.2364	94.08838	56.09797	45.06477	111.5536	155.2777	149.8089	94.10239	149.1435
55.38461	55.02468	75.75315	78.53494	128.5293	43.23524	149.1575	55.5308	95.13145
33.23252	95.32092	74.80714	10.79653	138.1093	146.4302	45.41447	431.3883	41.93753
58.40555	81.86708	59.8438	173.9125	132.7821	60.92598	106.4621	122.9804	78.82427
1173.104	944.1167	1195.388	569.0839	2021.601	1840.713	874.4434	1814.778	682.1976
6.044171	1.30587	6.525621	3.277275	111.2022	6.873635	2.095287	10.89348	2.513676
1011.991	636.3668	538.7463	1294.592	577.9314	287.941	867.8543	910.4628	1510.231
0.00158	0.047774	0	0	0.013968	0	0.650549	1.260474	0.813692
45.31366	41.55639	34.5936	17.48639	146.5057	134.6611	13.84128	57.86588	33.39184
363.5132	178.4056	469.5362	468.8151	490.8133	446.1681	912.0119	575.2303	661.6658
94.65471	66.01362	84.17034	195.6576	64.82066	44.21763	85.12563	116.9677	119.1389
40.2791	19.62157	27.10667	50.14735	31.88478	42.25692	143.6255	54.24848	72.90679
175.1979	56.18495	47.69543	44.26775	56.30509	135.6403	72.74424	65.09801	132.1463
0.001622	0.049187	0	0.783988	4.243618	0	0	0.04591	0
10.07173	17.12394	8.395706	0.774118	26.54536	23.58798	10.19715	9.712724	11.10101
52.36271	33.04633	23.35905	55.13976	35.07614	44.22025	125.7547	24.19592	102.9136
22.15479	13.51649	16.81501	8.289254	23.38606	24.56526	33.02526	22.96134	13.66041
64.44638	37.93828	54.23479	184.0208	25.52148	25.54471	105.78	27.81713	122.6113
181.2518	107.5324	50.48285	68.46961	325.0302	521.8756	37.33307	626.5665	34.22183
1367.446	1232.338	1656.527	1407.383	1185.58	1636.294	964.0135	875.527	1196.472
17.11965	11.06543	25.25588	20.87636	28.67573	48.16827	16.09332	16.92481	25.71712
27.1908	145.3313	19.61465	4.950111	167.8404	220.1457	32.92852	147.1105	44.51086
5.037686	1.305003	2.779708	3.275019	19.13832	20.6339	3.560504	7.319718	0.804105
13.0927	11.07109	43.05322	4.945955	20.19613	37.34912	10.91741	13.33112	2.512997
23.1595	30.45961	29.95186	11.6543	22.30659	11.78833	10.19561	10.91411	11.10007
55.38376	36.71048	54.23913	57.64143	81.79482	64.8606	94.0421	49.48687	94.31068
336.3195	188.0949	351.7339	198.068	109.4399	144.462	178.4007	127.8752	117.3805
64.44603	40.3725	54.23734	65.16869	76.48933	39.30483	112.4333	54.3076	97.73548
286.9837	197.9252	228.2095	584.2469	276.2212	221.1156	563.9987	208.6813	580.3006
32.22495	54.99254	28.03704	60.15703	49.94223	71.74168	25.59742	77.1607	34.23682

TCGA-HW	TCGA-P5-	TCGA-DB	TCGA-FG-	TCGA-E1-	TCGA-DH	TCGA-FG-	TCGA-HW	TCGA-P5-
191.7494	134.3015	178.3541	117.9232	102.2301	207.0711	180.4423	138.7897	214.5909
15.48369	31.00889	29.70176	10.36334	40.60776	15.84905	47.13674	41.93672	16.84565
89.08859	229.5592	103.6154	26.37883	106.3257	146.6346	121.7748	123.8126	143.3743
2575.963	1968.879	2651.765	1694.955	1823.079	3780.745	3175.792	3836.192	3513.786
49.37645	87.86399	185.6166	112.085	195.6822	213.0173	119.0906	110.8319	105.8707
0	0.066418	0	4.350891	0	0	0	6.990079	0
17.4202	63.13899	10.3621	47.95242	23.48738	75.304	15.06822	23.9633	16.84352
228.5549	246.4607	142.1725	96.13828	139.5985	153.5676	188.4466	217.6708	100.2437
16483.3	6355.771	7670.943	3173.064	5871.953	7737.841	13909.34	9567.513	6922.598
65.85067	45.25377	94.04436	22.0024	109.6706	78.27103	20.39792	86.86876	44.02394
9.673533	5.280006	33.82261	34.71116	22.73403	33.6897	24.00648	18.97114	23.42854
1310.259	1176.12	1025.831	598.0612	1844.323	1048.224	1402.591	1351.953	1355.858
99.7367	151.0835	97.94659	279.1182	66.48873	143.659	107.5182	48.92529	115.2364
0	5.161866	0	0.108055	0	0.987636	0	9.985854	0.91552
110.3985	111.0276	98.79381	145.3683	116.8958	145.6441	63.07513	108.8352	158.3756
4.828798	5.285643	5.540143	13.26871	5.602457	1.977897	5.286128	4.991806	0.913736
1759.592	1961.098	2045.252	1031.603	1275.159	2318.38	2517.171	2969.504	2219.768
15.48191	19.47305	60.2781	143.3045	60.90838	92.14692	69.36066	35.94527	26.21675
73.59146	149.6868	48.12875	19.10781	27.51895	60.43359	48.8459	64.9013	44.01567
234.3421	491.9419	137.2834	506.3558	90.01944	131.7672	70.16986	2354.438	151.768
1129.166	1080.581	742.2331	326.0576	823.0571	1296.908	1135.93	1034.434	765.5228
36.79384	43.91924	91.7137	0.147415	5.602617	116.9203	68.47212	22.96472	123.7644
514.3143	69.80101	95.5624	81.57721	31.5778	50.52538	222.2767	11.98122	46.82624
552.0114	112.4334	281.1469	51.11508	291.3959	444.8545	280.8601	214.6746	757.1759
132.6798	112.2934	115.7016	16.19786	61.64195	124.8376	104.8855	104.8413	116.1964
19.35922	14.3041	49.06637	108.2732	81.3847	18.82176	31.10116	23.9634	41.23737
176.2455	234.956	58.56004	370.7291	126.574	65.38641	113.7344	134.7955	108.6719
599.4362	366.7635	980.056	282.4177	1907.692	851.0626	678.1521	960.5455	813.3139
102.6968	20.74141	53.07878	13.26585	70.74957	41.61257	29.31367	12.97976	13.09391
56.15996	40.12159	77.10199	48.14259	78.72289	119.8847	92.44253	105.84	114.3295
10.63898	52.96919	98.08617	64.01416	39.72589	90.16207	47.97689	25.96014	65.59116
71.66521	15.6135	75.54071	24.89931	77.95776	60.43608	49.75751	53.91819	46.83888
4.829482	6.562837	25.7539	36.06925	35.02171	55.49686	21.33975	12.98002	31.88914
550.0474	389.9945	555.0464	819.0776	933.484	428.9963	444.3776	532.1937	488.1614
135.5683	112.4164	203.2231	58.38374	305.2527	195.1788	211.5337	135.794	162.0889
17398.43	4584.542	29119.96	7498.098	21519.65	24890.89	16001.61	14994.3	27875.08
16.45489	51.43449	17.62793	47.73841	14.55581	1.977839	3.509219	109.8378	14.97419
52.28762	38.82056	85.97948	27.81259	73.05283	73.31622	128.0566	67.8971	98.40162
15.48179	140.3047	12.77118	131.8029	24.28814	3.959257	7.95211	179.7311	17.77861
34.85758	135.0621	25.65057	76.8553	22.66821	11.88534	11.50886	121.8177	20.5927
202.4026	94.32837	40.88635	61.2757	16.96576	34.67266	115.5244	26.95852	21.52397
30.01075	38.79725	56.22697	66.8728	60.07082	77.2816	60.44289	32.94967	70.28498
19.35533	55.54695	15.18023	103.0882	10.47168	12.87596	3.510707	39.93913	14.96554
123.9551	112.3483	223.4388	75.77592	233.0856	142.6702	209.8181	246.6277	210.8579
163.6516	133.0772	508.6228	186.3265	271.9163	451.7909	303.0932	271.5887	350.4499
12.58004	16.83909	28.95194	10.33642	32.52163	25.76036	25.78311	9.984359	43.14296
40.66462	68.42469	38.50306	182.6511	51.91844	40.6187	23.06592	37.94211	51.52452
7727.933	1625.496	3018.083	376.9856	2648.622	3603.398	5564.131	2636.008	3732.105
864.8003	361.5633	964.0931	291.1069	115.9946	1876.519	879.0957	371.4372	535.0268
8.70527	11.69231	6.352264	5.990854	43.20676	8.913851	14.2016	9.98443	23.43464
13.55143	12.97304	24.13408	16.05688	43.2053	18.82424	13.3095	5.99035	19.67758
204.3287	242.7338	235.3564	48.20509	270.3038	435.9394	389.3464	180.726	371.0735
26.13466	40.11136	97.24368	46.6714	86.87366	97.09648	86.22714	58.91055	70.27185
3951.099	2864.877	7764.205	1172.753	7483.312	7023.505	5453.852	5999.915	10506.7
23.24122	15.55814	31.37609	24.72032	29.25348	30.71556	17.75756	10.98287	28.11844
41.634	43.95799	106.1407	26.35096	69.0061	52.50911	26.62464	57.9122	21.52641
15.48644	7.858787	38.63565	11.78739	20.26602	28.73286	49.85787	17.97248	27.17582

3845.541	8423.836	5708.432	4412.446	7209.747	6834.27	4800.551	12180.56	9237.044
105.5439	140.8244	465.1994	269.1849	349.055	235.7994	162.6203	106.8376	85.24122
20.32767	20.73636	75.68985	6.008384	74.02864	59.45037	15.07076	24.96189	42.17324
63.9184	95.37035	8.752568	27.78604	8.036871	12.87602	34.63624	32.9497	9.343215
0.956609	3.993072	0	1.628473	0.742093	4.949943	0.847508	13.97832	0.913524
162.6862	232.3807	300.4925	181.9143	297.9467	302.1844	268.4413	256.6116	399.2036
9.672871	12.99454	25.71532	37.63316	48.85699	22.78712	3.509344	21.96664	16.85347
52.30521	37.43073	46.63771	3.092865	62.61441	49.54073	55.15398	42.93521	43.11182
179.1826	76.1989	59.40222	19.10506	59.21341	84.21437	136.9286	49.92397	48.70583
65.83887	200.1231	97.13393	437.4937	67.29656	39.62643	40.83802	193.7066	73.06159
26.1543	38.5188	7.157664	18.92852	34.19666	2.968519	19.55284	16.97415	37.52385
12.57874	32.24205	28.11803	19.00751	100.2712	70.35579	14.18716	29.95473	25.29519
337.9837	200.1478	251.4442	68.56411	448.1981	301.1929	199.0825	389.4111	223.0033
40.67073	16.89232	65.94126	14.72561	51.15576	67.37519	44.4395	23.96325	79.68344
104.5872	91.69554	110.8618	20.56199	55.94938	215.9934	149.3557	38.94045	121.8166
26.13852	29.75789	61.11014	192.1131	57.67548	64.4027	29.30606	37.94239	35.59761
335.0761	153.7127	197.5841	131.0785	296.2973	239.7636	346.6714	259.6069	439.4911
34.85933	19.46098	32.10329	0.146183	17.79218	35.66653	90.77202	32.9499	44.04265
152.9989	280.1549	67.39551	478.3696	60.79807	52.50632	85.28198	154.7652	127.4109
458.0827	214.3266	160.6231	96.18246	273.5759	179.3262	192.8623	303.5407	182.7068
1098.169	914.1244	2485.591	224.2351	1035.715	2546.26	1730.561	991.4983	2628.342
41.63337	49.11461	104.5163	39.40522	214.6536	49.53631	66.66061	96.85385	104.0342
185.9274	27.23573	504.6269	56.93254	258.9321	402.2524	407.1273	392.4063	488.2215
30.01006	22.06028	89.2298	3.08807	88.53403	44.58232	74.67572	80.87784	42.15079
26.1355	25.9268	43.33584	77.03668	24.28067	32.69219	27.51418	56.91369	10.28013
1578.509	1962.26	1600.25	312.9743	1167.235	2367.924	1514.573	1380.909	1388.645
31.94462	50.44735	11.96579	104.7487	15.3415	20.80201	16.83978	55.91487	13.09108
81.33668	91.73985	271.6529	14.74048	315.1197	191.2187	316.5254	96.85297	199.5982
208.1964	379.6052	203.9734	875.4737	124.1138	425.0356	140.389	1163.24	205.1817
42.59807	123.9116	3.939613	155.5166	35.64038	17.82971	35.50862	129.8037	21.52409
86.17678	148.5254	105.1726	315.4985	78.66526	108.9808	54.17182	281.5741	280.1825
27.10545	16.89858	50.60171	4.550704	45.43621	108.9916	106.7502	24.96169	59.97407
1013.938	1667.594	492.4069	292.5698	1396.352	609.3176	540.3957	545.1743	548.1426
1173.702	1567.349	2599.609	1184.322	2191.662	2215.342	1872.757	2000.97	2135.442
0	2.604946	0	0.08972	0.755857	0	0	2.995444	0
26.13695	27.20101	40.93992	59.59909	72.29913	33.68367	40.86924	38.94077	25.2786
127.847	320.6134	36.08371	66.96065	108.02	118.8954	49.74814	134.7968	45.89657
531.6482	404.1883	604.8662	528.2023	495.9102	541.9439	465.7135	915.6137	545.323
82.31068	59.46605	140.6445	107.6291	133.1706	136.7276	108.444	72.88934	94.63516
49.38022	53.00377	115.7382	48.13539	127.5191	72.32465	93.33683	39.93902	102.1445
68.75648	77.43941	52.18106	17.64648	55.16871	87.18865	82.67597	36.94359	91.8416
123.968	7.868086	205.895	1.621449	37.27331	117.9034	169.8604	53.91799	69.33064
131.7024	210.3161	216.9879	16.1971	66.49838	124.8352	231.1561	80.87712	128.3667
221.7584	272.4411	139.7077	651.3656	221.5581	140.6847	119.0599	155.7636	143.3397
62.0285	7.85159	42.73284	17.51309	17.02039	14.85957	25.7929	19.96969	8.410713
44.5489	14.31205	55.48374	6.009191	25.92948	40.62116	70.28148	20.96778	101.2754
17.42309	18.14618	38.60725	42.03822	36.56278	71.34583	31.11421	10.98281	60.02214
28.07685	43.88478	30.4899	29.18879	59.31638	45.5756	24.85759	25.96031	29.97205
19.36319	19.41079	32.98153	164.9722	20.26778	37.65238	16.86349	9.984341	43.13719
91.03876	94.15685	112.5701	16.19266	68.99891	165.4684	83.5697	33.94811	46.83704
27.11653	19.4142	9.56738	23.30824	12.92898	9.904258	12.40796	15.97544	8.408884
1519.429	933.5029	2829.405	411.8996	1655.901	2378.819	2636.308	1613.557	2602.091
38.72966	27.20644	71.53738	11.82938	101.589	81.24562	48.87538	40.93773	34.65321
388.3235	209.2624	273.0604	378.3667	214.2198	192.2038	287.942	221.6638	119.9095
428.0366	311.1916	580.8619	176.196	319.7856	613.2842	613.3314	396.3997	388.854
3.860785	11.69534	6.351481	4.544623	21.11213	5.940996	2.621138	6.988858	2.785332
3.860697	6.566533	31.39828	33.27266	31.72506	35.67208	15.08829	26.95946	30.94206
88.11889	82.6912	107.6277	61.24857	85.1907	107.9921	162.6834	136.793	128.3719

28.0833	51.46034	15.20457	19.01372	8.855097	14.85861	20.42146	21.9665	5.595888
41.63399	46.5328	81.98003	40.84593	69.00607	138.7154	63.10506	22.96464	104.0385
85.21294	41.4285	73.85189	0.151046	138.0069	103.038	135.9978	111.8305	142.4315
150.0915	91.78639	377.5652	7.444871	580.4658	429.0005	600.9036	423.3591	664.3786
149.1382	114.927	134.159	49.63521	56.75342	106.0102	169.7919	94.85608	152.7401
89.08917	72.36601	56.97411	319.3009	108.7684	36.65448	55.96063	163.7526	75.88394
466.7872	72.42106	381.6234	51.11492	563.4752	154.5558	223.0765	647.0218	276.4138
32.91756	28.49193	79.59972	13.28159	77.98816	108.9913	66.68081	55.91536	53.4084
37.7744	32.25728	15.20291	46.35622	33.29766	38.64178	11.51438	49.92522	4.658741
17.42015	28.46408	24.04581	27.74184	60.94785	12.87623	18.62745	35.94543	21.53183
123.9457	120.1646	456.4149	61.29454	275.996	325.9623	420.4736	276.5813	470.4208
84.23945	156.2741	250.6568	160.1155	283.3311	281.378	192.8652	164.7503	160.2159
592.6705	482.8492	296.3738	246.0081	404.2124	268.4934	471.0751	416.3694	671.8515
6687.849	6054.741	2331.206	726.1347	3313.439	3384.439	5093.916	3047.385	2903.776
19.36223	20.70196	17.62854	8.901203	26.78622	34.67861	36.47172	11.98135	63.78756
33.89529	22.00981	31.32486	3.093273	30.83878	19.81287	37.34317	39.93978	25.28965
61.00758	34.9479	69.08989	35.0554	79.57472	22.78388	42.63639	149.7748	34.64955
0	0.055401	0	4.223618	0	0	0	0	0
78.52147	10.41367	2.329818	1.629745	0	1.977823	54.37723	4.991839	11.22681
0	1.386187	0.734156	3.014939	0	1.97867	0	19.97275	0
12.581	7.851872	31.39831	13.20486	11.3086	17.83263	14.19673	27.958	12.16386
45.50263	105.9102	184.8194	80.13877	222.5076	173.3851	109.3102	90.86207	157.4245
25.16784	52.94105	73.95725	95.74928	94.26639	52.50999	38.19625	52.91982	48.71903
451.2734	358.9791	493.2223	102.0328	401.7631	475.5641	473.732	839.729	416.9561
69.71474	58.20458	99.56881	110.6426	64.87131	79.25807	113.7524	53.91777	192.1015
7.737797	15.49216	20.12642	15.99268	40.83704	17.8347	8.858987	8.986001	17.80994
1707.322	433.8879	1887.162	81.66036	1386.481	2167.794	1143.936	238.638	2138.312
94.89377	72.40775	246.6677	254.4649	239.4968	146.6311	131.5232	118.8197	151.7863
38.72365	266.9446	22.40828	345.4973	37.26286	23.77429	10.6191	423.3624	31.83191
3.860551	15.56683	5.542228	31.92796	18.63437	9.904257	11.51733	11.98136	19.66694
176.2409	126.6293	489.3106	46.75056	685.2776	384.4167	246.1878	477.2779	133.9691
289.5593	246.5781	205.632	74.37864	211.0321	232.8286	267.5479	287.5648	252.0576
168.506	93.0284	113.2352	110.647	132.2947	123.844	167.1096	190.7115	152.7331
41.6314	34.9622	122.1787	10.37681	108.8159	91.15067	112.9124	31.95105	164.9647
9.671676	18.15343	37.78597	53.57645	42.2588	53.5062	15.07412	28.95607	22.47609
17.41863	67.09841	40.93027	61.08244	37.29424	33.68333	11.50755	107.8378	20.58997
180.1382	117.4787	90.74647	77.20612	132.3322	139.6988	108.4325	137.7916	228.6815
13.55434	42.22542	20.1183	45.87336	3.984308	22.7903	4.401037	22.9657	1.848911
0	0.085205	0	1.626639	1.54928	0	0	0	0
34.85254	28.50833	79.54988	14.74008	211.3831	77.28008	80.89671	50.92261	73.08871
53.27006	22.03251	44.19638	8.917012	37.32884	43.594	53.35675	34.94694	29.97305
48.41607	58.1035	20.81008	52.41427	7.225478	11.88521	17.73154	29.95416	3.722504
86.17634	98.2213	229.7549	86.00487	382.4295	109.9714	85.28523	230.6508	110.5461
30.00811	46.56907	15.98183	107.5931	61.64896	18.82051	17.7289	35.94501	36.51999
441.591	294.4361	793.7747	200.9315	741.1703	700.4707	809.7762	228.6532	711.2081
55.19942	33.6413	32.07926	1.624682	25.09934	44.58303	91.60904	60.9079	40.27979
35.82554	65.76056	23.23215	33.55358	10.47314	26.74788	11.50846	54.917	25.27998
66.8191	56.84382	56.20546	26.3573	72.24766	61.42636	67.54812	115.8254	58.08681
29.04279	25.91602	47.37908	102.9546	22.66089	37.64701	48.87804	16.9737	35.59179
90.05521	34.97887	159.0919	133.8315	446.859	60.43325	67.51607	115.8245	343.0339
51.3194	29.79863	131.8795	20.55225	104.7798	76.289	119.1618	48.92559	90.90244
276.9609	145.9894	154.9744	67.11666	505.7809	508.2642	329.7547	236.6414	576.297
90.06816	67.14669	49.76501	13.28658	69.80452	61.42623	56.86891	59.90911	71.21224
33.89134	25.88602	36.13922	86.8289	21.04902	41.6124	16.84897	34.94697	49.67431
253.7114	480.3035	109.9701	1352.468	143.5943	148.6102	172.3866	294.5535	257.6542
492.9176	644.1794	652.3315	33.64784	1113.85	611.3003	1124.469	995.4937	669.9715
18.38722	49.08952	29.66171	155.0023	65.76952	15.84839	15.06411	30.95268	31.83987
227.5887	179.4103	144.5927	132.4258	103.8599	153.5681	136.8722	122.8138	144.2971

162.7004	55.61514	101.9982	6.000661	143.6985	111.9554	158.236	103.8426	63.69665
30.98033	54.22013	86.85709	49.48724	108.1178	89.17341	37.30792	58.91091	56.22283
109.4166	28.52263	32.05099	353.5887	12.9147	13.86706	32.84021	106.8376	23.39959
146.2213	222.0724	235.3716	531.9819	124.1339	120.8698	96.84107	109.8332	142.4078
12.57576	62.00463	78.73358	61.15991	107.2112	114.9322	66.65343	52.91956	86.21187
21.29564	31.02768	68.3977	161.6742	109.0548	26.74854	30.20221	21.9663	24.34626
66.81083	59.48234	145.4326	38.00747	203.847	118.8912	160.0172	64.90127	80.56821
15.48231	23.32855	9.557055	84.06588	21.04155	14.8578	11.50887	14.97673	8.406409
42.60773	73.46199	53.03743	27.76381	71.50728	49.53837	45.32699	73.88879	48.72579
2174.126	565.5251	426.5069	182.043	669.6511	403.2367	1097.74	583.1168	594.0518
12.57726	1.400915	56.31863	1.627651	49.56852	56.47742	75.64993	25.9604	60.00123
58.09603	54.30733	202.6181	29.27987	169.7653	101.0579	84.42849	138.7904	54.32815
115.2318	140.779	157.4243	94.71259	89.22402	178.3363	266.6801	247.6254	199.5833
204.3424	62.07648	273.258	5.997877	159.9157	354.7028	244.4816	52.91927	220.2186
2.892959	7.815794	20.16584	24.42601	27.76816	20.8102	7.080541	13.97892	19.70173
584.9184	313.8085	701.3332	323.0985	431.801	865.9285	840.8704	371.4372	510.6629
31.95754	11.72701	31.32744	39.20201	65.91343	23.77667	29.32512	31.95162	18.72332
1414.856	1460.026	914.1784	232.9596	964.3265	1320.687	1081.716	958.5486	914.5167
41.62788	115.0159	32.85223	899.8261	49.43314	36.65418	21.28586	20.96762	22.46197
60.03302	59.4644	103.6366	7.461686	79.52296	78.26876	149.3688	145.7798	122.7598
125.8997	71.06334	35.27006	146.7604	39.70476	106.0119	57.74303	90.86232	35.58192
176.2394	158.9023	244.1631	166.0077	113.5637	250.6603	160.8377	440.3334	161.1424
42.60149	19.48504	24.8266	226.0771	28.34071	35.66443	26.62272	36.94359	24.33754
222.7296	209.1951	206.4203	177.5941	198.8354	316.0538	248.8644	259.6068	190.199
2104.365	1099.992	1409.041	864.2551	1138.825	1396.973	1590.13	1657.49	1450.49
48.41161	34.96247	102.8607	30.72376	70.59907	119.8852	43.51808	74.88649	55.26892
7.737952	9.10915	16.88564	15.98684	7.24692	13.87012	8.859656	3.993368	11.23348
324.4152	280.1925	320.5161	46.75024	298.6947	427.0196	453.3294	471.2868	476.018
541.345	281.518	540.6684	474.2433	892.2364	594.4588	199.9471	872.6798	516.2978
944.1969	693.3406	953.5432	267.8714	1285.812	1193.868	839.9314	1903.119	787.0768
30.01123	56.80067	73.15038	32.13119	65.77545	109.9819	61.33773	19.96918	67.47468
26.14156	32.29181	48.25651	52.2009	68.32977	22.78536	11.51164	39.93968	29.03974
31.9458	208.7447	57.80144	373.7918	65.72534	27.7377	14.17353	209.6846	29.02301
60.99615	67.25883	76.23061	266.3849	34.01491	39.62659	36.39603	27.95706	49.63469
47.44307	87.77703	0	36.52879	103.1239	9.903669	6.175757	311.5321	0
6.766211	3.992351	8.762205	23.30425	8.044245	8.913385	7.956317	8.985828	1.848932
1465.222	867.5559	721.3696	413.3066	830.3933	815.3961	892.3917	926.5971	904.2167
73.59485	64.60868	89.97182	27.8241	159.2142	74.30589	136.9251	106.8384	89.01495
11.61209	2.69905	29.78575	17.50807	30.09591	30.71684	15.08963	20.96825	29.06552
83.27405	80.13034	141.3805	94.67056	142.8598	133.7521	129.7599	140.7868	164.9193
189.8161	51.75348	56.15914	5.998743	92.49094	68.3594	334.3269	123.8124	187.4166
23.23519	36.13418	10.36553	115.4257	18.61536	10.89478	18.63343	32.95008	20.59807
147.1936	136.9006	167.8829	138.2837	273.6239	110.9628	121.7438	172.7384	70.25162
21.29527	19.46108	26.463	52.28002	79.69219	42.60295	29.30963	28.95585	30.91047
13.54573	45.14603	20.02358	69.53038	22.67834	6.931441	8.842156	19.9693	8.406821
9.67058	139.2078	7.146896	56.80424	8.848572	71.33472	5.287091	17.97215	7.46944
0.95655	14.12848	4.759407	14.42571	2.363243	2.968898	1.734729	24.96384	6.545023
159.7895	149.7542	179.1754	190.4714	183.4858	206.0812	113.7525	133.7973	111.4932
1279.293	1434.029	554.274	26.36734	568.1881	314.0681	395.4994	1338.974	98.3583
17.41777	14.32479	5.54557	59.81152	7.22943	4.950196	7.06584	16.97365	8.407319
8.70581	2.69823	0.729494	8.867584	7.239793	0.987256	2.621333	0.997914	0.913176
97.80405	96.87589	151.8575	40.91615	207.0841	100.0654	142.2224	125.8095	102.1249
0	0.073149	0	0.111474	0	0	0.849791	0	3.73627
65.85142	52.97434	107.742	75.60434	82.01661	76.28957	62.21249	67.89721	47.775
40.66273	65.88618	110.1033	46.68552	106.3745	50.52635	61.30844	88.86551	69.33076
493.8834	427.393	862.8314	513.5979	702.9691	1324.656	744.8551	538.1849	705.5725
161.7136	174.3796	273.9088	477.0154	699.8787	209.0482	159.9518	516.219	163.0183
245.9703	131.7922	328.5627	155.8144	250.792	358.6562	377.7666	227.655	299.8389

3699.314	2264.554	3737.045	4285.827	4396.278	3337.872	2530.463	4051.865	3830.477
414.5399	149.7267	182.4163	22.01778	51.88006	161.4957	204.481	29.954	277.4136
229.5017	899.6573	170.2267	2060.137	357.9309	62.41393	55.94833	1591.593	185.503
22.26603	27.15342	53.10344	39.23198	54.47191	34.6767	55.16029	15.97531	52.4983
1140.779	1331.074	1274.855	822.0931	844.9466	1567.384	1269.234	1705.418	1277.133
69.71282	174.3218	121.2475	266.1259	123.3304	59.44183	71.95136	120.8166	96.49016
17.42037	41.30654	8.754261	26.29043	23.48899	12.87627	11.50999	45.93062	12.15603
88.11521	121.4141	246.6918	254.4071	195.6468	353.71	206.2267	106.8378	203.3401
3.860647	11.70582	15.22123	14.65111	3.169159	7.922652	11.52106	0.997917	1.848866
5.797244	38.80528	7.14648	100.1679	3.981909	3.959295	0.848592	52.9197	14.96569
68.74812	34.97662	110.0452	11.83053	51.8835	124.8362	152.9034	153.7675	123.6869
168.4998	105.952	216.1103	38.02058	297.9809	235.8025	228.4433	141.785	290.4965
10.64118	32.2328	3.934649	63.50192	11.29851	1.977837	0.847283	85.87283	12.16009
3.861695	18.00688	6.365272	35.78083	14.62143	8.914866	2.622503	30.95465	1.849119
27.10328	23.35517	95.63647	1.622266	114.5304	74.30697	128.9441	53.91806	47.77182
39.69815	52.94759	27.24792	81.31917	29.97591	34.67422	21.28991	67.8974	29.02735
245.9724	436.2453	106.7667	245.9063	223.1937	211.0303	228.4128	139.7878	133.9704
13.55438	24.41885	14.44629	54.38369	26.06309	6.932255	6.182661	20.96858	5.59906
240.1652	173.0543	253.0555	129.6143	207.7823	238.7733	298.6674	418.3674	264.2407
34.86233	34.86114	57.93801	105.4294	41.42158	53.50474	34.66264	34.94707	42.17426
92.00181	94.21085	110.1092	56.83138	84.42398	118.8946	104.0165	46.92852	159.3408
3556.1	164.0859	397.5832	100.5775	163.0747	711.3657	1896.009	237.6395	852.6807
4.829334	2.699013	0.729703	158.6738	1.548669	0.987264	1.733326	130.8093	0
137.5294	73.61713	117.3432	13.28765	28.33574	281.395	184.9855	20.96761	79.64253
267.2754	165.3498	466.7844	67.11714	344.168	341.8123	478.2223	311.5282	347.6294
74.55844	117.504	134.1599	149.7914	116.8739	212.0275	141.3309	109.8335	119.9333
85.21511	24.65295	82.70967	113.4583	175.4252	107.9929	70.19075	68.8953	168.6891
136.5397	135.6126	214.5113	183.3076	350.8026	233.8214	111.9627	252.6179	133.9797
262.428	231.1993	880.5426	94.75896	1221.84	865.9293	1111.133	301.543	1304.389
60.03806	104.461	64.2542	24.90622	34.84331	46.56365	49.7522	22.96463	21.52581
14.5204	19.37229	42.7549	21.79427	33.37719	21.79732	12.41659	24.96247	21.55458
108.4568	85.28326	147.0175	58.35593	297.2719	120.872	116.4238	101.8455	179.9199
47.46137	27.15281	27.28571	17.59637	55.28837	25.75823	53.37842	62.90577	23.41188
5.797173	23.34158	14.37854	26.33561	6.414015	18.82087	6.174859	12.97971	2.785978
39.69933	13.03076	61.07609	1.624973	47.87913	101.0645	131.6881	10.98273	151.8978
68.74943	71.07193	152.7005	43.80895	268.9299	95.11236	81.7544	116.8232	101.1947
80.36512	50.47557	26.42543	328.6726	96.52341	117.8974	55.94853	116.8226	13.09251
65.84511	24.65072	267.0167	0.150261	294.1942	294.2732	279.2826	54.91642	115.2623
53.26834	29.75328	49.83078	13.27221	38.13663	37.64814	60.47612	30.95285	75.93689
33.89687	38.67273	28.91259	79.40398	47.1598	56.47965	9.733868	30.95315	40.3066
38.74385	9.150194	41.02878	1.628812	42.27253	18.82227	26.65755	16.97389	22.47806
322.5079	200.0467	77.05467	51.09936	74.61506	92.13821	206.2357	174.7355	98.36981
3136.673	2057.968	3006.836	922.524	2011.397	3365.615	3349.997	4108.779	3084.624
16.44947	76.18074	13.57198	323.1575	10.47169	1.978035	2.623498	296.5547	9.343235
140.4062	445.4135	186.2981	424.8278	228.0314	209.0473	243.5042	218.6684	145.2104
25.16529	111.0119	28.03469	90.23819	27.52008	38.63616	15.06218	45.92992	33.70715
91.98776	178.1877	95.52887	510.0372	65.67369	186.2625	105.7357	220.666	97.42759
9.670744	29.76716	49.81127	29.21365	110.599	83.22931	31.08264	86.86944	29.0307
329.2668	67.25499	80.25338	133.9787	38.88082	28.72809	138.6246	17.97215	26.2092
58.09442	82.68153	163.1416	27.83245	83.57209	107.0017	158.2451	53.91785	320.556
26.1379	37.4811	36.9235	46.5572	29.98807	27.73881	18.62467	24.96175	29.03121
74.56828	34.95298	81.1549	4.54788	193.465	48.54523	71.99521	54.91659	105.9052
0	0.082822	0	0.127424	0	0.987296	0	0	0
0	1.392376	0	7.25425	0.743412	0	0	0.997946	0.914304
9.674934	23.18243	16.05026	27.47208	32.58281	43.60379	7.962312	33.9496	13.10644
19.35654	31.05635	13.57611	75.4607	27.54439	32.69301	23.07239	28.95574	8.406298
24.2075	11.71963	48.31607	0.142822	61.06596	23.77737	52.52108	31.95179	48.76246
157.8473	142.0596	162.255	158.617	187.5105	154.5576	148.4191	183.7218	172.4064

1228.922	288.0132	999.3705	294.0447	1536.726	872.8607	933.2819	1811.259	907.0278
0	2.692639	0	190.2042	2.363642	0	0	2.994881	0
0	0.04156	0	6.581249	0	0	0	0	0.927984
14.51856	10.4241	48.3733	1.62965	21.91	47.56438	60.59067	5.990325	54.41641
1889.368	1533.747	1427.494	2074.422	1345.813	1806.158	1605.225	1569.623	1616.337
25.17767	37.3475	45.90514	7.45239	24.34196	15.84981	45.39368	51.92256	20.60491
37469.66	25102.7	25523.27	18190.74	18114.35	29164.04	32074.3	28333.1	28570.28
29.0392	91.68705	40.89635	93.13983	60.82727	75.29607	57.74139	108.8353	59.9508
238.2251	269.8304	343.8597	224.1	293.0393	369.5562	214.1897	254.6144	366.3855
210.1406	131.7766	260.2805	226.9834	349.0949	201.1231	184.855	222.6627	165.8351
279.8605	290.5641	690.1124	147.1218	800.445	463.6755	327.9508	323.5098	559.3979
6033.282	2922.603	1064.375	103.4808	687.467	1177.023	2067.461	244.6289	334.4847
211.1062	143.4028	775.4737	136.9094	290.5899	439.9011	375.9947	146.7772	482.5883
18.39199	42.5062	27.30289	20.45966	12.1091	8.913216	7.065116	106.8417	4.658747
17.41889	16.89866	51.40681	84.17458	38.92707	57.46461	35.52777	24.96169	47.7824
54.25758	10.43204	17.62992	3.093432	11.29904	10.89512	64.12654	14.9769	28.11306
56.15895	65.90483	90.76811	29.27944	77.90017	79.25972	112.8962	150.7724	81.51283
174.3161	109.7976	203.2869	56.91235	184.2861	187.2552	177.7771	246.6273	238.9632
67.78581	213.8626	34.47463	255.1107	29.96336	50.52664	31.06817	77.88208	57.14607
320.5491	262.0741	129.2718	0.152306	210.2112	202.1138	167.0731	763.846	156.4635
153.9795	103.3339	142.9967	393.5128	165.6194	158.5227	103.0842	151.7703	199.6044
3.860873	3.989095	1.528483	13.18235	7.238254	3.95932	0	13.97857	2.785402
3250.956	2016.608	2066.139	823.5873	1513.005	2847.448	3334.036	2088.837	1876.816
65.84184	32.3977	104.4075	40.91596	102.2509	124.8358	223.165	108.835	178.9887
62.9399	63.32046	28.84092	142.3617	8.038035	19.81129	22.17394	2.994879	27.1476
13.54717	32.25779	21.65385	0.143539	29.21983	23.77699	41.80947	40.93844	70.34683
758.2842	383.4542	450.6757	212.5463	409.9153	504.2986	513.7616	599.0934	519.113
5206.181	3295.965	2628.465	1281.831	4436.943	3860.997	2542.039	2377.4	2316.271
156.8708	136.9599	506.9613	174.7263	496.8293	509.2541	380.4231	232.6474	301.7087
30.97845	19.4831	78.75452	10.37691	110.4958	140.6967	89.80264	14.97668	66.5278
3.860519	5.283886	0	7.455103	1.549346	5.940756	1.733661	0.99792	0.913432
103.6159	45.2978	116.4735	16.19771	51.06959	58.45177	135.9989	120.817	120.8727
71.65061	87.88321	185.5783	56.91846	241.1388	240.7577	183.1032	70.8921	175.2207
40.66346	36.2463	86.77805	135.0179	174.7222	54.48999	41.74171	51.92104	91.83591
12.58384	28.26031	15.2487	14.5933	26.86776	27.74563	9.747349	7.987424	11.23023
9.671719	7.863836	55.55302	46.38158	28.39898	36.65939	24.87201	20.96792	35.61309
2.894031	10.30666	8.831213	0.125137	16.33963	8.91664	1.735443	5.99061	7.488597
37.77073	9.154087	24.06184	26.25504	81.39495	31.70396	6.175183	30.95304	23.41228
3.861011	19.34413	0	1.629484	0.740969	0.987255	0.846862	25.96123	16.86457
42.60202	47.82738	44.13614	27.80601	139.7806	54.49055	71.99983	12.9797	91.84367
48.41439	40.09911	81.16798	120.452	62.49369	67.37179	53.3139	42.93462	48.71255
94.92689	38.77671	55.4448	24.87667	29.98364	36.65644	68.47104	38.9408	21.52896
18.39012	11.73135	60.35925	21.93527	16.1711	71.34239	35.55346	14.97679	58.1262
108.4626	43.99968	91.56174	33.64155	60.01425	99.07574	115.5567	104.8413	114.3194
15.48493	15.57996	46.67841	6.0058	64.30273	25.75889	17.74728	54.91785	20.60106
16.46166	19.34408	17.67474	11.73415	14.59007	9.905119	27.6022	8.985945	17.80392
35.8198	58.15776	62.62441	139.4196	25.89907	70.34294	38.18132	43.93301	60.8934
36.79216	46.51231	23.22551	35.02898	25.91132	55.48252	32.85624	25.96018	30.90278
39.70834	3.993091	70.0556	4.552245	18.61434	107.019	39.11977	3.993324	43.11432
3.861007	31.09142	3.937653	33.62034	18.59033	0.987426	0	36.94356	3.722916
53.28012	22.00472	28.90976	0.143975	22.69367	21.79488	41.80374	6.988793	28.10587
227.5887	130.422	105.9985	62.72145	93.29941	141.6784	197.3467	115.8244	157.4192
78.44618	27.2155	101.3077	8.921526	60.87119	88.18032	63.99487	38.94064	29.96291
124.9275	78.81145	126.1419	1.61947	140.4658	160.5065	156.4692	235.6446	243.685
15.48117	15.61486	17.59109	42.30696	3.982379	2.968648	7.952234	5.990298	6.532612
67.78086	129.0537	44.91553	100.3975	109.5875	50.52572	29.28533	172.7392	39.33024
25.17264	9.154939	35.34812	3.092992	14.54428	9.903902	42.68227	38.94116	80.65104
0	0.07776	0	8.67468	0	0	0	16.97576	0

196.5731	732.0937	92.30008	682.434	827.0995	454.7556	292.3771	389.4097	39.3297
0	0.088668	1.528457	253.0472	2.358718	2.968553	4.400245	2.994854	2.785528
94.8935	178.1803	98.74576	248.6726	119.2735	96.10076	66.61815	134.7956	52.44603
235.3206	363.9827	217.6764	62.74947	254.0635	320.0173	335.1116	151.7698	203.3202
26.14937	16.8391	11.18298	29.02191	8.046013	11.88628	10.62885	9.984359	11.22381
4.828899	28.40162	9.564824	20.45113	4.791674	4.949965	5.286731	14.97687	9.345907
79.40993	52.9955	63.43698	188.572	82.80457	115.9226	57.75393	60.90754	80.58424
389.2891	366.7561	1086.942	531.1144	1631.704	1115.599	1022.17	487.2618	1359.627
41.62983	55.6031	143.0485	62.67775	84.39323	123.8464	138.6855	98.85026	100.2578
208.1988	455.6709	152.5607	75.84486	103.8224	269.4853	257.7384	414.3727	126.4712
12.57785	14.29967	35.36122	34.88637	46.33257	48.55095	21.30712	13.97831	29.98132
206.2699	131.7612	160.6298	68.55859	130.637	136.7227	207.0938	134.7955	305.4849
1919.424	288.0114	996.9689	10.3505	739.4704	1042.283	1367.975	453.3132	1646.38
4651.259	2020.569	4827.16	5161.627	5340.304	4831.939	2713.556	3695.404	4117.195
134.6271	32.38097	107.7033	39.42425	54.34583	67.37082	119.1491	76.88356	63.70817
1164.015	724.3889	4099.428	518.1016	5325.825	6996.764	4341.079	1498.73	4743.158
23.23177	78.59239	26.45451	79.75379	22.66722	3.959235	3.509784	433.3562	14.02991
120.0727	191.1079	150.9785	117.983	76.22648	148.6119	187.5279	408.3826	108.6714
56.16623	36.22507	83.60634	7.465576	119.4774	118.8988	95.15661	58.91078	47.77839
96.83014	49.18249	60.9721	361.9532	91.65864	99.07302	30.17196	35.94492	14.96582
80.36788	183.2867	117.2513	412.5088	55.12352	76.28554	41.72848	233.6468	43.07613
0.964039	0.041796	0	1.470641	0	0	0	0	0
16.4516	22.03215	11.97113	58.03774	4.791154	4.94993	7.952679	19.96928	6.532438
7.733901	19.46533	12.77389	33.53418	16.16346	7.922182	10.61981	21.96626	11.21814
28.07457	25.91212	93.31677	23.43039	50.32437	24.766	42.65068	22.96471	84.36307
87.16188	47.83388	46.54613	139.3083	28.34051	41.60939	66.65641	26.95861	21.52566
181.0899	169.1589	405.0406	126.6823	194.8078	451.7966	360.9358	208.684	333.6075
38.72457	36.25977	66.63281	19.10664	167.3225	57.46158	149.3695	81.87584	87.13658
2.892979	16.90744	1.533644	337.7321	18.58949	6.931477	0	89.86399	0
538.4254	400.3259	1352.858	214.0497	1492.833	1667.456	1681.728	608.0788	1085.996
268.2394	222.1588	596.0981	152.9384	986.3975	487.4542	467.5192	369.4403	416.9608
13265.25	7965.703	12324.61	11731.45	10782.07	12100.16	7333.694	13135.11	14481.45
19.35986	13.01443	47.46604	0.144349	27.57869	24.76752	48.92907	22.96493	30.91905
80.36849	69.8128	199.2811	32.19934	116.8612	278.4107	228.4794	34.94644	145.2358
3.860521	22.02207	7.147335	21.93892	3.169367	11.88554	6.174983	8.985772	4.658636
17.42349	10.43512	34.57617	13.24066	41.46582	37.65124	22.20461	27.95766	25.29466
9.6787	11.64215	12.0385	42.81592	12.98567	12.87974	7.972367	11.98176	6.540268
30.98205	11.73879	28.06538	92.73261	26.73489	23.7754	18.62505	35.94534	15.90481
84.24377	58.19852	114.0533	268.7399	171.3174	115.9182	59.5129	134.7959	83.37794
16.4599	29.57549	6.352581	7.433507	6.423154	2.968527	1.733304	46.93072	3.722464
133.6597	74.88313	84.36513	65.51099	112.0876	73.31605	79.10896	99.84917	74.02225
57.12644	72.36205	129.3669	72.83909	233.1513	137.7178	98.65366	53.91787	98.38202
10.64103	11.71958	27.31184	27.64071	40.6546	31.70536	18.64157	13.97837	17.78848
517.1307	400.2593	472.3477	429.2088	296.2267	528.0759	342.1748	760.8486	319.5038
14.51432	34.87978	43.39583	70.98768	49.5534	27.73949	30.20283	18.97079	96.58984
33.89103	33.59809	70.81631	55.14708	56.0687	51.52165	31.98282	19.96929	59.0553
395.1121	306.0094	392.8353	673.2597	630.0038	378.4708	369.7526	382.421	298.8965
360.3443	55.58031	92.39828	3.08524	490.3433	400.3001	379.896	57.91199	52.45662
81.33336	129.1996	100.3403	853.0674	90.83808	72.32167	63.94832	160.7562	35.57897
40.67116	14.31535	46.59948	11.82336	34.87561	39.6297	66.70526	18.97074	36.53532
10.64538	11.67411	37.14926	14.58385	21.14345	7.923216	23.1426	9.984507	15.92765
20.32476	10.45105	157.7862	13.28099	21.84827	146.6465	82.70906	7.987262	78.73187
148.1608	140.7803	151.7962	112.1498	116.8361	204.0969	183.0926	209.6826	109.6112
36.78862	190.7379	15.17881	17.64988	8.848867	9.903666	13.28465	32.94955	44.9579
31.94524	100.6733	46.53145	123.5234	51.08485	8.912942	9.729825	55.91495	44.01874
512.3765	96.87489	81.89197	7.4586	55.13021	102.0471	353.0394	42.93439	95.56389
12364.63	13229.14	12509.38	5726.202	7544.9	14196.61	12695.14	17667.25	12022.73
351.5261	417.0295	543.871	91.84881	265.3768	649.9417	586.6458	475.2803	262.3429

39.70311	33.60987	61.92451	33.52821	78.05057	35.66631	20.40656	37.94243	46.85479
21.2958	18.17051	45.00971	8.916021	60.14851	39.63058	45.34423	23.96333	41.23119
14.52993	7.812061	18.54798	8.811268	26.95831	32.70712	27.65999	1.996385	21.58601
119.1172	67.20522	112.4749	67.03299	246.1643	84.21334	83.53335	92.85926	120.8807
46.47004	45.31079	155.0054	212.3965	15.3441	51.51564	107.5128	13.97821	48.69746
468.7065	712.5721	212.8075	311.4531	320.5787	237.7793	335.0599	361.4523	259.5298
18.39343	30.94759	38.62993	4.550286	41.48166	30.71471	46.28539	32.95039	91.02393
122.0085	139.522	454.7952	141.2553	427.0566	377.4829	400.0171	146.7773	290.4763
132.6696	98.18488	133.3381	55.45528	125.7967	156.5406	166.2215	164.7507	245.5296
122.0145	85.29474	236.2584	104.8473	271.2285	211.0346	158.2126	190.7115	292.3916
3886.248	998.0553	2638.98	2170.503	1977.356	4425.739	3511.836	206.6864	967.9076
76.4932	109.8076	43.29667	132.457	71.36307	43.58959	30.17204	444.3293	51.50966
27.1042	25.92607	54.6065	30.69952	46.23277	49.53662	35.52106	54.9167	29.96317
2.892446	15.60078	2.331417	49.4022	9.662152	0	0	6.988778	3.722031
75.53108	81.36631	58.59136	88.76698	54.33133	95.11298	62.19144	119.8189	32.7705
35.84592	10.42172	24.11173	3.092638	37.43893	26.75191	32.03475	12.97995	27.18344
190.7651	65.96883	945.7065	115.1198	639.7048	480.5197	338.6256	131.7998	571.589
0	0.048226	0	0.071767	0	0	0	0	0.924335
47.43913	82.71508	78.6627	261.6108	98.98338	109.9726	60.39847	124.8108	118.9894
45.50294	82.69448	35.26481	899.2618	107.939	18.82047	18.61737	110.832	14.96509
11.61069	21.96915	37.83769	1.6295	30.88117	32.69738	19.53872	3.993328	178.4009
0.956653	22.90607	0	57.6078	0	0	0	3.993485	1.850312
12.57756	10.44222	73.29088	4.552206	85.47203	63.41516	58.72744	27.95748	58.13015
64.89297	18.18112	67.54861	17.62621	34.05773	37.64768	107.6708	44.93193	76.86722
198.5205	41.43887	137.3152	1.615259	158.2448	193.1973	230.2065	527.2034	300.7942
122.99	44.00374	110.8565	45.2663	78.69769	98.08445	111.9918	139.7886	129.3139
67.79847	41.3423	26.45215	13.27755	34.05457	32.6931	50.66736	58.91105	59.04209
119.1111	69.80845	278.9228	13.28533	277.769	176.3574	137.7685	107.8365	291.4684
43.56999	36.24184	86.789	37.96429	86.88294	98.08787	91.572	55.91509	69.33659
108.4541	51.75913	206.49	3.078135	256.5841	156.5398	220.4569	141.7851	216.4629
473.5472	387.3754	285.1153	1096.579	342.494	299.2068	217.7202	494.2514	207.0538
109.4206	86.60329	117.2303	205.12	419.831	153.5664	131.5201	40.93736	210.8286
39.70737	15.59229	28.08763	27.71065	28.38373	70.3513	27.53631	9.984272	21.53484
7.739623	6.538755	20.98023	17.344	20.37577	18.82762	9.760247	5.990461	5.601707
7.734872	38.85686	19.9997	180.4549	22.64768	11.88537	0.850106	157.7608	18.71356
150.0962	136.9238	322.2053	308.2734	328.0083	247.6911	177.7502	121.8151	273.6176
24.19938	42.64548	67.51828	61.05437	104.8597	84.21884	52.43913	26.9587	67.4769
14.51372	19.46658	3.133135	6.009411	14.53699	14.85781	7.952349	30.95281	10.28079
16.4521	43.85628	8.755046	10.36573	18.6102	15.84894	7.953035	9.984267	4.658637
153.9695	169.18	307.7233	165.9369	308.5021	285.3407	237.3189	149.7728	192.0785
12.58263	29.5666	9.581077	16.04665	8.869158	7.922954	4.399671	72.89341	1.848843
59.07111	31.0798	52.99544	48.08675	91.78826	68.36296	47.08754	35.94514	64.65389
14.51777	42.44258	3.934915	51.97264	0.74154	5.940824	2.621019	50.92418	14.03777
439.659	557.6403	442.6445	138.3858	413.1675	469.6217	462.2018	365.4465	514.4293
0	0.090933	0.729937	0.141048	0	0	0.847113	0.997917	0
40.6693	46.48851	30.47666	21.97764	51.14402	41.61095	48.88511	67.89763	59.97889
31.94792	42.6612	80.38468	79.89666	62.50872	69.35436	64.00137	38.94068	59.03079
151.0627	179.5155	107.5733	831.2488	164.7321	157.5285	95.95056	259.6069	127.4121
166.5548	136.9627	319.6991	484.3553	411.552	369.5535	260.4017	524.2067	403.8521
24.20223	14.31035	19.21663	0.145847	78.07754	23.77587	67.61515	22.96482	44.04507
2.892342	10.41454	18.46503	13.19438	16.20953	12.87774	7.960147	17.97267	16.8591
155.9	93.07922	47.31551	1114.248	59.98861	27.73768	63.06015	360.4537	50.57238
3.860746	43.63717	2.329784	96.00606	9.680067	3.959281	0.846981	95.85975	3.722278
155.9538	28.50195	78.76463	11.83101	14.53173	42.6007	170.821	10.98272	105.9167
121.0425	93.05398	122.053	100.5284	134.7027	136.7229	137.7441	281.5741	96.49055
1.924246	10.38881	0.729356	67.07751	3.171532	8.914432	5.292286	141.7968	3.723292
277.9916	19.48896	158.3773	4.545326	37.27082	44.58124	249.0437	2.994879	91.8277
0.956514	2.690533	1.529803	7.366501	1.5499	0.987281	0.847031	31.95389	1.849588

55.1948	38.81429	107.7333	35.05853	53.54407	112.9514	69.32901	43.93311	73.08929
150.0937	818.0074	109.1784	407.1713	210.2036	123.8419	135.0651	184.7199	223.936
15.48367	22.01921	11.16935	8.913262	33.27307	22.78533	21.30311	40.93819	21.53509
26.13796	37.48009	88.5086	13.27633	69.06645	70.34766	56.01275	26.95876	52.4781
6.768746	38.36521	5.553485	24.51462	17.0657	1.977853	1.73349	23.9644	4.661498
42.60558	27.2023	69.13152	37.91346	29.16678	67.37363	60.45186	34.94673	77.79445
56.15704	58.19141	59.38265	26.37951	128.2638	56.47027	191.1531	118.8201	146.1844
85.21191	242.5264	141.3911	88.84975	186.751	159.5137	106.6424	151.7703	113.3703
65.84437	53.02133	109.2658	146.7667	174.6341	104.0302	82.64723	78.88033	79.63646
107.4865	209.07	60.17501	170.1806	38.88304	34.67268	10.61991	53.91776	20.58697
53.25203	95.56248	113.2741	133.7632	92.51543	167.4425	96.87313	53.91786	120.8789
845.4129	763.0749	723.7398	705.7069	499.1378	964.9995	958.1379	806.778	796.4398
0.956429	3.985371	0.729366	51.5186	4.798928	0.987254	0	2.994861	2.785759
751.4779	743.6946	1555.306	955.836	1535.839	1532.71	973.2589	873.6769	1576.068
0	0.061835	0	0.093208	0.753727	0	0.855425	0	0
25.16825	38.7825	29.66742	10.37481	21.84909	26.74766	70.24693	29.95422	24.34104
390.2592	610.6817	402.4103	1108.333	789.012	367.5694	233.7175	530.1969	336.3644
71.65415	64.63556	143.0289	84.46198	116.8902	154.5612	105.7641	90.86219	149.9359
46.48239	74.7602	52.2256	35.00378	148.8794	59.44713	25.74208	42.93485	31.84342
19.3558	184.0572	22.42003	11.82988	82.8595	26.74742	58.66398	74.88695	21.52738
0	0.063715	0	0.096215	0	0	0	0	0
13.5507	85.69892	8.769347	47.53262	7.235591	15.85074	4.398988	17.97265	3.722277
25.16675	27.21677	106.9393	165.2651	77.95075	107.0067	46.19614	36.94362	74.96687
29.04793	34.86541	10.36415	138.5195	27.56783	8.913053	6.174987	34.94705	9.344339
38.72538	96.79955	110.9029	64.0914	110.4354	139.7019	114.6877	59.90897	127.4569
88.11504	310.9466	101.9682	61.27843	34.82286	210.0428	92.40414	193.7068	89.93289
1226	760.5092	1192.908	913.7304	778.3805	1020.482	1834.559	1509.714	1570.425
10.6417	10.42817	22.48114	0.141632	19.45443	9.904337	63.25151	21.96662	31.87198
98.77108	80.12695	52.94445	33.65256	21.02328	159.5134	74.62735	35.94494	11.2177
0.956616	11.72712	6.344422	73.70523	4.791266	0	0	5.990298	0
5.7974	48.88976	5.541513	34.8343	11.29689	9.90414	1.733658	66.90043	3.721884
30.03067	16.82308	22.50258	0.139796	23.55581	27.74345	38.29043	25.96094	19.6742
55.19378	52.98816	112.5484	29.26305	131.62	83.22483	88.01069	44.93157	98.40223
109.4205	519.8913	89.09921	65.64823	203.7422	149.6032	186.6472	149.7729	238.0079
44.53781	63.2993	107.7038	97.38529	182.8517	80.25181	69.31964	86.86859	113.4002
219.8193	178.2637	736.7893	285.2281	901.2154	517.1793	287.0701	364.4481	386.0455
28.07223	86.44694	25.63129	194.2436	31.59217	33.68282	15.95197	30.9526	63.71236

TCGA-TQ	TCGA-DU	TCGA-P5-	TCGA-DB	TCGA-QH	TCGA-TQ	TCGA-HT	TCGA-CS-	TCGA-HT
119.5734	240.8837	183.7329	157.3175	283.5898	134.137	128.3478	212.8518	149.0544
68.42104	35.45698	42.04639	42.14834	17.87328	46.70142	92.63742	10.16538	22.44201
78.15929	98.03325	95.20566	129.2322	88.24583	129.1704	94.89069	128.7384	196.0456
2434.457	2184.799	2380.94	3240.02	3818.369	2013.059	3358.764	3820.885	3087.412
148.7623	111.5923	123.9814	213.5299	167.0142	129.1693	291.9025	53.08836	82.96205
0	1.056209	0.025318	0	0.01209	0	0	0.138848	3.373376
39.0969	3.146008	27.69576	11.21118	6.323966	3.971809	3.284047	792.0001	5.653995
201.7064	168.9415	197.0081	173.2416	177.5217	96.37879	267.8878	244.1163	184.8947
5778.304	9423.278	6850.574	8822.954	7814.291	6825.129	6761.8	8441.305	6559.239
66.70291	29.21539	55.35959	73.98057	71.42924	115.2626	54.58898	54.58788	43.73546
28.42307	9.400265	7.787578	35.61495	33.59364	29.81015	31.5227	3.563453	13.47744
1539.73	1186.773	1183.266	1213.603	1214.317	1346.347	1722.515	1627.939	1044.463
102.68	131.4084	184.8489	88.93912	186.9784	115.2576	118.7586	193.1628	102.0148
0.685457	0.010923	2.243298	0	0.01284	0	0	8.026451	2.27434
69.71738	111.5857	154.9314	171.3928	185.9016	115.2593	105.1622	209.2339	142.2971
4.502688	5.232291	26.5894	4.655476	31.52086	10.92715	3.965211	15.10161	17.97159
2243.557	1778.083	1752.229	2305.467	2024.219	1550.035	2375.295	2069.088	1618.257
35.22319	15.65996	29.9148	82.43691	96.60379	51.668	62.20381	34.81387	52.66726
97.35565	46.9443	73.08076	58.03791	29.43367	11.92064	32.65088	49.76754	23.58547
378.7115	93.87599	791.4183	73.9499	293.0937	93.39689	268.2767	309.0195	567.0705
1055.073	859.3219	1241.918	936.4155	1086.162	795.8832	917.8509	1816.005	1535.258
83.70487	107.3734	76.33079	14.95721	11.57599	21.85728	331.5809	60.98708	78.39141
95.03889	324.271	111.8049	188.2455	203.7619	16.88868	63.4119	86.00817	79.59692
519.9868	892.6202	138.3999	1093.879	677.4915	188.7848	473.4036	71.28374	424.6988
102.7508	83.43395	67.54441	109.5655	44.1366	41.72944	61.38228	158.2231	116.5339
17.56313	38.58215	23.26805	52.46511	10.5244	29.80779	19.71729	13.44589	36.97566
81.18677	107.4289	124.0036	87.99963	118.7163	164.9386	77.73088	122.3893	135.6319
1400.193	816.5646	577.8279	1297.894	1095.614	2253.522	1030.575	257.8729	804.6496
102.3145	47.96324	53.09875	34.64342	33.61858	43.71968	82.25658	1.90003	24.68278
85.10583	163.6986	127.2549	123.6278	115.537	114.267	166.874	39.85443	44.86453
149.0099	117.8145	33.2402	149.8981	59.87826	71.54067	73.792	25.0106	17.97996
37.50825	67.78378	49.82634	72.11078	151.2057	49.67969	64.19934	23.36418	92.97101
30.77471	30.22493	8.889808	50.6506	36.73064	26.82937	44.03322	13.3422	2.289709
376.397	398.3877	914.2927	579.6285	720.6151	730.3049	440.3945	561.6005	660.0914
189.36	265.9247	129.538	339.0071	261.5571	289.1427	372.3583	25.04748	159.1601
37388.7	33349.55	6501.921	33529.55	22722.22	16319.11	13240.28	7052.765	22088.13
9.881198	6.274644	13.3164	0.9126	1.06947	11.92119	9.441214	13.41294	28.01372
82.83124	37.55657	37.66823	88.96955	30.48134	121.2241	126.5257	6.875576	96.349
14.47305	13.57486	39.86592	5.591805	13.67662	29.8066	19.00152	57.7467	78.39951
23.69815	8.360987	42.06772	6.528013	15.77612	22.85109	25.17708	64.19452	96.26569
20.60211	105.3395	78.62545	102.9899	47.29104	10.92711	92.78791	127.2549	6.771747
55.96632	72.9922	56.45444	38.37842	78.77029	72.53493	63.53377	10.18289	25.81717
9.101365	5.232014	42.08799	5.592063	48.32998	16.88877	9.425269	13.485	30.29887
252.4475	233.5728	83.04333	280.0512	165.9603	195.7444	187.2364	89.30996	160.2331
388.8097	326.4115	251.2759	435.4599	337.1884	385.5235	357.9168	160.3692	152.4478
24.54264	30.2342	21.03928	54.37947	29.40364	24.84055	34.94521	11.75944	10.12718
11.40143	37.55559	52.04185	30.87995	42.03096	36.76177	29.9381	41.45267	39.257
2398.403	3569.706	3973.728	4978.973	5073.645	2479.064	3138.809	2827.04	5448.581
1074.4	807.1609	724.9955	889.6255	757.3621	134.1351	1527.509	815.5289	267.8796
27.67811	23.97846	5.575818	25.29752	21.00605	86.46375	26.03248	1.902418	3.4108
20.71327	30.22511	7.785579	19.66523	12.61682	19.87235	28.79824	13.34314	14.58865
279.8768	451.5347	221.3922	410.1788	251.0597	257.3457	530.9065	194.9758	186.0553
89.74262	51.10979	40.9873	133.9469	71.4329	105.3249	141.5915	20.08432	49.33791
10899.01	14194.31	3535.442	11057.3	12219.82	9937.132	7911.351	3791.458	7720.186
25.31371	34.40007	14.41673	33.73099	37.79353	17.88397	25.97258	3.564886	19.06764
49.03413	86.54606	42.08701	114.283	65.12747	58.62277	78.59153	29.94213	39.25453
24.5292	23.98778	14.41936	47.80078	19.96614	45.71013	39.05496	19.91445	19.07208

5126.585	5360.301	6626.955	6322.715	13116.9	5330.735	10182.24	13090.58	8343.267
114.9244	162.6998	146.1467	117.028	337.1915	119.2312	229.4087	63.02955	45.99943
58.38797	54.212	15.53362	55.27517	49.35691	62.60137	45.13469	11.80956	30.27134
25.22333	42.76419	55.34523	16.83001	31.52779	3.971844	9.425616	65.96197	23.57675
1.442528	0.013771	11.10918	0.912719	0.016206	0	3.282863	21.60217	1.166094
236.9376	255.4968	210.3158	321.2126	241.601	254.3656	408.6078	234.4675	174.8429
20.67937	18.77797	16.62545	26.22358	37.79447	36.76622	28.72747	13.3925	19.06856
18.33014	40.66731	31.00318	52.46226	22.07216	10.92722	25.2054	47.72535	15.7307
79.7276	106.365	87.4452	99.26658	92.43767	68.55832	50.44815	97.37453	33.66521
55.10887	54.24681	171.5836	66.46	100.8602	166.926	53.13538	178.4156	99.77996
8.354243	28.14405	9.994554	6.53096	22.05558	36.7679	19.11384	13.34877	23.51821
19.12071	37.53169	64.08548	33.72112	28.36141	30.80251	30.75729	5.219279	20.19405
144.8545	164.7816	264.5413	283.7428	367.6371	216.6072	162.4671	376.2136	333.9226
44.46856	103.1979	26.59453	78.69634	60.91299	69.55557	51.93725	11.82429	30.28496
82.00384	90.73388	169.3071	147.0355	174.35	34.77391	126.3841	71.15793	59.43092
39.85514	19.82902	39.85584	41.19928	83.99931	28.81329	39.58678	51.1163	42.58851
495.5045	586.0419	194.8324	387.7024	472.6684	401.4225	341.5484	190.0212	178.2115
55.26894	35.46137	33.22103	46.82761	27.32298	47.69421	80.83804	28.20891	14.61438
105.7242	136.629	151.677	65.52224	95.61089	60.60767	92.07013	219.7226	254.3986
210.0779	173.1224	253.471	169.4801	200.6406	262.3146	195.2862	297.0874	211.8124
2228.311	3764.669	759.3633	3299.072	2459.075	1682.188	2227.887	827.4831	973.8827
157.4471	60.48925	45.40669	215.4986	17.87851	48.68581	62.81372	36.52329	51.5706
581.4326	469.2617	117.3691	722.0716	546.1909	499.7919	634.1338	63.02626	291.3653
48.26659	34.42722	12.2213	135.8405	102.9195	105.3263	171.8819	18.42761	61.63944
54.41475	24.00198	58.67219	43.99802	65.12741	15.89515	43.63891	59.48208	56.04418
1706.867	1057.467	2054.351	1119.95	1715.373	901.2058	2337.905	6038.285	1786.303
17.53493	24.00398	112.8979	9.337463	64.09074	25.83125	12.1572	59.63367	26.94639
382.1584	332.6287	63.13176	238.8168	84.05071	396.4608	454.7237	43.20126	66.16168
270.6007	94.9188	429.4827	152.6107	283.6351	116.25	276.5028	270.998	583.8436
19.83531	32.3462	132.8106	14.019	5.272415	65.57675	36.75319	39.88122	33.66871
85.79246	196.0593	127.3205	83.3178	184.8829	93.39743	125.5777	394.1658	298.0534
64.44433	81.3256	24.38802	108.6787	54.6226	94.39719	77.96897	11.83057	22.45624
377.1851	564.1907	1161.058	390.4706	867.6562	441.163	590.7311	1173.599	732.8974
2080.246	1683.18	1227.572	2482.462	2182.829	2702.63	2102.12	1291.451	1750.481
0	1.054413	0.022919	0	0.010973	0	0	0.123005	0.025781
74.45352	69.86052	47.60324	57.12603	51.47255	98.37231	121.9205	10.18105	34.76768
35.19172	29.21617	308.5268	64.60601	58.83324	38.74889	48.41084	415.0533	90.75624
515.2065	395.2584	410.6925	408.2582	1045.187	1001.563	533.9852	756.3275	670.1715
97.37467	110.5398	71.96888	116.1238	108.1932	122.2155	175.0273	33.2826	58.30781
158.1254	121.9991	24.39523	103.9546	100.8351	211.6481	87.42812	18.43898	26.94387
92.06799	93.84625	55.36054	98.34397	107.1248	105.3255	110.7831	43.10087	32.53999
161.1973	139.7204	37.67147	312.9209	224.723	32.78686	239.5434	21.73684	90.76633
103.4724	147.0412	105.1728	108.6151	238.422	86.44274	153.0055	69.56275	163.5994
269.8729	169.9996	282.2681	109.5358	107.166	220.5807	94.11519	94.3916	174.8598
13.748	30.22963	23.23809	30.9233	17.86325	12.91536	8.767453	27.92674	23.52516
109.2146	108.397	24.37984	117.1549	32.57098	20.8639	25.87483	6.876568	19.09123
73.90788	38.57553	21.05105	46.85082	28.36319	49.68418	52.10097	6.867953	12.36918
29.85842	26.08218	48.69033	41.20177	27.32336	35.76943	34.10634	34.75429	31.39916
25.30386	18.77884	16.62727	51.55637	44.08938	25.83405	21.82472	13.40039	19.07106
110.5324	79.25186	65.3095	140.5198	128.1179	45.7048	125.1989	69.34266	54.92843
6.038685	6.274511	23.25228	3.719345	14.72012	3.97181	8.757477	18.29046	21.3071
1938.401	1236.841	704.024	2796.18	3267.911	1778.568	2286.57	1606.736	2063.12
79.82063	74.03253	38.76374	145.2316	90.31416	60.6108	115.6969	41.39944	50.43843
180.8603	504.7501	584.4447	191.0037	512.6255	261.318	410.3752	269.3916	210.7321
466.2119	529.7645	358.6452	535.6455	515.7618	481.9032	690.6202	305.6124	282.434
9.124732	6.273485	7.786096	8.405998	8.420821	10.92794	6.709869	3.561614	0.037567
12.20457	29.18868	6.682281	35.61615	41.98001	22.8534	24.61981	13.36723	22.40961
129.5927	108.4611	107.3785	169.5108	105.0502	146.0621	176.9926	112.3283	113.1937

12.18695	15.65529	48.64552	12.15131	8.423512	3.971801	14.24184	52.42027	23.54892
72.86552	80.29222	42.08702	52.43114	67.22716	73.52806	77.90588	28.29863	23.57979
244.0151	197.0809	32.14473	309.0988	188.0103	217.6052	458.3381	1.887326	192.7018
996.3728	1025.065	155.0041	763.2388	518.9059	1347.355	753.5622	71.28662	236.4892
80.44984	136.6132	135.0384	99.24974	134.4564	97.37296	90.08058	161.7073	68.39784
49.75866	24.00406	71.97346	81.45687	86.14466	125.1959	98.31868	89.25805	89.66901
613.623	485.9505	209.2248	591.8705	160.7339	310.0076	174.7464	46.51814	864.9448
54.44001	52.14439	22.17662	75.86925	60.92167	92.40959	88.9421	13.47903	51.55521
16.03399	7.317453	64.09858	13.08824	10.52342	9.93369	12.18046	225.4643	28.02119
23.7047	26.08204	21.06433	18.70677	27.32318	69.55637	18.33003	8.526639	29.16142
433.3542	320.1497	191.5116	442.028	306.7234	271.2567	481.7074	48.16847	124.4334
264.5687	196.0613	80.84457	202.2626	132.3703	722.3698	154.2785	51.46689	94.17906
435.5029	665.333	412.8839	309.9389	397.0768	566.3603	590.7949	652.0914	508.777
4872.873	3779.322	4933.388	2527.386	4033.717	3252.096	3400.378	4937.097	5561.777
25.29347	63.56646	15.52543	44.98147	30.45819	24.83999	43.86434	1.902408	12.36705
36.82806	19.82523	16.6362	25.27371	27.31722	36.76432	62.38406	10.15993	31.38136
79.77654	59.4466	69.73142	140.5206	119.7193	69.55309	55.96371	16.7829	123.191
0	0.008736	1.131434	0	0.01026	0	0	0.113297	0.024057
19.93589	90.59509	21.02699	16.84884	4.222149	11.92173	8.771868	13.34932	7.88702
0	0.011109	1.143025	0	1.067654	0	0.570429	0.153257	1.161353
19.15177	9.400135	15.51614	17.78454	10.52022	27.82256	20.4782	13.3672	14.59385
139.5555	100.1225	62.02301	187.3026	170.1631	195.744	158.4824	38.24813	53.8367
40.59596	68.82077	39.86859	58.99765	48.32516	110.2963	47.77568	8.531015	42.60429
351.885	338.9412	500.3253	431.6787	699.5901	794.8927	459.5779	548.2422	616.3557
65.86042	50.07419	73.08965	98.30971	218.4737	141.0929	94.16908	59.68532	147.9264
18.43479	21.88701	12.19065	11.2258	14.70795	37.76468	12.24986	5.188278	7.880134
880.2303	3075.312	779.2684	1806.399	1472.709	517.6706	1707.555	771.244	1029.882
127.2307	112.6405	76.41518	206.9534	374.9687	147.0538	131.7418	125.6466	133.3832
72.7806	21.91853	50.95565	14.01907	15.77842	35.76747	34.69958	156.721	152.3853
9.113578	23.98798	13.31507	12.15278	11.57184	29.80911	8.071699	10.13903	7.891525
392.627	157.4859	162.7474	444.8197	232.1587	583.2555	545.1763	54.77449	189.4243
157.1327	164.781	230.2363	253.7731	388.6401	219.5882	307.3976	193.2863	304.7931
135.7018	130.3615	163.8126	153.5741	172.2678	133.1437	131.781	145.3262	188.2468
196.5637	186.6268	46.52043	152.6801	84.03546	255.3703	242.9817	6.874091	34.78393
19.10951	18.78267	13.31995	16.83681	67.18189	42.72712	23.85105	5.221606	17.96378
37.51642	12.53239	44.29309	14.01975	15.77742	51.66741	28.58138	23.35402	31.41423
203.3397	276.3064	109.5861	207.9274	252.0569	161.961	192.08	107.3644	142.3017
9.138529	9.396845	17.70308	6.533102	8.418226	0.991189	2.602738	8.442833	4.528834
0.679258	0.012807	7.772702	0	0.015065	0	0	0.185265	0.035823
92.83829	65.70201	43.1961	94.59594	48.33127	113.2751	132.7261	10.18402	66.12358
56.81355	60.46939	57.52371	39.32838	35.71944	40.73818	54.72154	26.56921	52.64346
32.13373	15.66062	27.70854	4.655873	12.62673	16.88882	8.742511	21.71418	22.45868
112.6392	95.95853	94.12476	162.925	147.0747	419.3095	157.0115	28.35216	242.0552
44.39564	35.47327	96.29537	19.63773	42.03519	67.56455	40.1822	29.98056	38.14603
466.1799	475.5461	298.8907	782.875	595.5978	479.915	648.1832	312.2609	768.723
53.67018	81.32684	38.76265	61.8098	32.57742	37.75596	40.92119	28.27326	62.74391
6.801685	27.12653	27.70197	14.02064	42.02249	18.87631	7.377184	46.25447	24.69202
46.72	51.10814	73.05153	80.53934	76.67918	62.59716	60.75219	89.03402	108.6512
40.59862	29.21281	17.75223	37.44355	25.22766	31.7939	30.64273	20.05951	28.0537
233.2482	493.1638	115.1259	262.2515	112.403	55.64006	681.4847	41.54294	442.4006
76.68832	76.12689	45.40907	98.34233	75.63036	140.1045	133.3966	10.18404	45.97634
394.1511	350.3992	150.5748	403.6076	1040.899	399.4336	547.8889	368.1814	569.225
78.22625	123.0327	77.47604	94.59418	86.12905	61.60339	60.74783	10.18404	77.31842
12.94425	34.41797	25.48327	29.95526	40.96571	36.76343	21.76343	21.65279	22.44649
190.8304	288.8866	578.9079	123.58	377.1239	133.1414	188.364	516.9143	160.305
1036.08	1441.17	354.2311	207.8611	35.7362	825.6954	1263.13	185.2006	738.48
45.97303	50.06126	45.39843	33.69374	21.02815	63.59175	37.48741	10.18267	21.33811
118.0462	180.4083	182.62	141.3966	163.8667	184.8132	200.8685	276.9652	199.447

184.1157	143.9092	77.50934	173.2581	280.4151	179.8463	176.9923	67.89465	89.67565
43.67575	77.15682	53.13134	82.43218	64.06981	39.74341	70.42094	26.62764	42.60219
12.94476	38.60394	384.1047	25.25736	63.04882	29.80575	14.2182	38.25403	38.15339
146.3909	115.7717	176.0159	126.397	134.4722	210.6455	102.3274	313.5859	234.22
54.39638	57.36428	58.68151	77.72472	117.6273	51.66654	153.942	11.83614	34.78127
31.4044	20.8701	29.9039	24.33085	11.57511	41.73203	22.44834	13.45891	15.73294
111.1676	85.52318	71.97698	154.5231	150.2047	129.1701	150.992	31.64566	87.43466
5.268965	8.360984	33.2264	11.21072	13.67599	27.81954	3.965846	38.05348	41.47187
59.85516	42.76017	49.80598	55.25686	40.97226	56.63692	62.22514	23.32582	30.28673
698.507	1917.765	686.2828	648.9317	537.8405	455.0731	666.522	469.1361	710.5059
98.48901	34.41585	18.84964	90.91381	54.60336	57.63255	191.8064	10.16706	12.37342
82.01853	55.28374	88.55806	139.5487	120.792	129.1714	199.7018	36.57397	73.9835
224.6888	129.3246	178.2168	195.7118	126.0661	134.1364	263.0192	163.5532	190.5146
243.944	287.798	122.88	343.7338	320.3403	124.2008	329.4898	102.526	90.80626
13.03766	6.270022	12.1796	23.45	22.03265	17.88684	18.54243	10.00081	19.00945
489.9364	616.3285	365.302	708.8805	913.8676	482.8953	568.2037	396.4508	377.6916
27.58179	28.1607	36.516	36.52727	22.06979	33.78314	19.72627	19.97389	32.49742
807.3794	799.8785	1469.907	1053.478	1293.088	670.6877	890.5455	3017.263	2167.224
13.70869	8.359296	437.1956	0.914425	212.203	296.0963	4.663576	221.4185	34.79218
195.7168	102.1991	62.01275	174.214	289.8377	116.2536	81.91538	39.86931	123.2493
25.97266	50.07096	46.52557	13.08258	66.1897	41.72946	23.08565	21.74257	43.74805
187.7814	148.1023	311.0506	310.8834	504.2022	157.9822	197.2716	605.701	294.7525
19.83995	25.04523	47.62055	21.51196	47.28174	35.76804	23.77801	28.31107	31.42065
262.2265	146.0136	114.0491	394.255	833.9647	284.1736	217.813	204.874	281.2833
850.286	1682.124	1238.619	1274.466	1797.304	1051.242	921.9078	1826.122	962.6704
65.1392	58.409	44.30835	112.3876	76.68572	82.46985	105.2374	31.61918	66.13656
15.33734	23.9673	13.29161	11.22633	14.70735	17.88585	12.2529	10.04616	7.879531
316.6599	304.5188	223.6183	500.0708	539.9079	648.8342	396.8266	368.2031	423.5886
561.3176	414.0179	95.23889	648.025	506.3115	699.5067	451.4274	599.2436	879.6218
802.7742	480.7726	812.4727	1036.62	972.7158	1167.497	865.9477	1164.054	1434.4
59.82099	70.90547	61.9769	58.99735	51.47469	59.61714	63.54805	15.12676	30.29344
36.04313	37.5407	14.42755	29.95903	42.01045	41.73267	26.58268	13.44731	30.26928
30.58279	28.17384	53.15537	12.14616	16.82865	61.60299	40.87628	80.90853	107.5514
32.10879	56.33273	142.8347	28.06691	42.03903	51.66518	33.3342	91.10318	80.7399
8.335619	4.188584	54.2628	2.784638	5.272821	5.959047	6.696942	199.0279	15.74085
9.114104	7.316911	10.00029	5.592477	10.52214	1.984676	2.601146	36.1256	11.24728
718.4399	1101.248	1096.902	481.3024	703.8057	777.0054	829.7825	1168.878	1061.236
98.92948	88.64369	74.17545	123.6257	61.98735	120.2289	149.7331	48.08521	78.45761
22.24695	19.81592	12.20396	35.61847	19.96015	11.92163	32.22946	11.73688	3.411085
132.6349	94.91051	105.1757	113.2967	171.2166	124.201	184.4595	99.22034	174.8048
127.2648	235.6638	87.47249	184.4892	76.6981	129.1693	172.8363	41.54891	121.0445
20.64329	17.74152	23.26702	14.0238	39.91027	22.85173	12.1734	29.77754	25.79466
144.1166	141.8358	100.7591	132.9595	104.009	278.2144	97.56364	67.95201	186.0282
19.86001	17.74357	8.899737	47.76506	35.71995	63.59398	46.47096	8.526423	39.22715
12.17664	3.146062	54.20302	18.70806	10.52487	11.92085	3.965024	60.7951	13.49366
14.46898	4.188744	56.46773	6.52831	43.08188	7.946201	3.288383	23.37499	39.2589
8.406609	0.012521	7.764977	0	1.069383	7.947974	3.990019	11.51364	6.749468
97.32569	129.3183	222.4412	196.6653	153.3637	75.5126	156.4107	145.316	134.4864
431.6359	365.0134	260.1597	284.6491	210.1101	436.1948	468.439	421.2309	2978.306
23.66876	5.231104	32.14549	3.721389	13.67722	2.978442	9.430124	54.73822	17.98243
9.902593	2.102867	4.469681	3.720068	8.419685	0.991191	2.601834	1.901981	2.289601
144.9434	125.1437	104.0619	187.3078	88.24838	214.624	174.2427	21.74667	127.7549
0	0.011208	1.143392	0	0.013176	0	0	0.155046	0.031157
59.79026	42.76763	61.99157	69.29687	76.67714	67.56576	85.43629	33.23416	61.64301
84.34541	109.4902	45.41462	63.66588	67.23577	128.1788	74.41543	11.83636	43.74315
1050.601	863.4749	450.5262	1097.524	1248.941	590.2061	1071.729	472.3753	466.2158
163.2441	108.4747	234.6834	263.1245	146.0303	779.9938	186.351	79.53834	310.4288
273.71	294.0893	235.7884	380.1953	307.7832	367.6376	351.7383	246.1492	282.4172

2248.851	2206.706	2770.581	3019.941	9122.055	2824.841	2621.056	7608.711	6697.017
198.6897	446.2508	161.5869	221.0309	135.5065	9.933472	92.13291	148.547	114.3182
100.3454	71.97596	252.4064	82.37731	45.19073	177.8543	163.7792	403.004	958.0736
23.72141	42.74938	24.37253	45.90088	46.20649	27.82034	43.08672	11.8054	33.62158
1561.909	1634.159	1103.593	1418.674	1854.033	1706.035	1424.61	1372.252	1539.785
72.75134	83.44469	102.9759	61.77766	106.1114	169.9071	80.46788	41.56212	112.1025
9.103857	7.318054	35.42936	13.08506	6.32395	27.81986	15.59103	44.53209	26.92238
190.1795	192.9246	111.8212	323.1127	213.2311	212.6345	288.3839	56.40216	106.4931
19.91711	3.146119	12.2065	2.783245	3.17199	2.978226	3.283143	1.902771	1.166532
6.802133	7.318159	14.43453	1.848609	2.120275	6.952596	12.8409	18.42673	24.69914
87.36849	115.7582	84.14539	124.5463	225.8088	148.0496	212.602	163.2845	117.67
431.1619	508.8558	176.0037	302.4917	184.881	271.2584	294.4781	67.9578	178.1927
6.038175	5.231983	18.83834	7.466036	2.121074	21.85868	9.441744	36.16013	32.48042
6.831866	1.059127	9.980091	7.474095	7.367012	11.92285	3.977318	3.545563	41.264
146.6346	64.66165	33.24415	124.5773	19.97943	133.1482	192.3311	11.83617	39.26048
45.20513	30.25596	35.44763	17.76659	10.52593	42.72426	19.68212	36.48555	42.6063
239.9705	117.8589	221.3959	136.6959	108.2155	56.6332	180.9008	537.8424	253.2793
13.00537	11.47941	6.677491	14.04084	16.80566	30.80652	7.410474	6.820171	21.2646
215.4439	177.294	204.7866	398.9492	367.6356	203.69	240.3971	470.0927	453.7781
33.72988	44.83441	27.68837	34.64656	46.20813	32.7889	52.01916	13.44853	36.97794
79.73999	101.1495	88.54459	118.9483	84.03476	65.5776	106.615	166.2181	86.28559
443.8908	2454.809	798.0679	672.3434	649.1833	35.76751	1101.695	221.541	173.7541
2.205299	1.059124	6.681808	0	0.015806	0	1.241134	0.197905	17.94166
42.09627	87.59938	46.52132	40.24471	244.6721	27.81864	51.13606	417.0615	15.74092
627.3688	460.9332	246.8589	449.4961	247.9161	546.4903	556.0701	148.8496	238.7269
105.7827	131.4	97.42543	212.5999	172.2609	147.0554	144.8114	117.2873	78.47836
111.9534	214.7943	110.6877	135.7932	82.99379	172.8918	181.837	44.82116	137.816
160.9986	116.8111	205.8733	305.3063	242.6416	150.0347	230.2116	198.1199	192.75
1095.14	1351.491	293.3593	796.917	567.2408	641.8751	539.5243	200.056	289.1676
35.19665	18.78918	60.88917	43.05894	17.87862	22.85058	23.77871	208.588	48.21411
13.75411	27.10317	13.30592	27.17301	16.81267	19.87223	26.71377	3.561619	15.70666
114.9865	106.3787	95.21588	177.9338	91.40079	147.0551	63.40447	81.09271	134.4818
53.77829	26.07844	46.46036	47.77678	28.36808	32.78906	45.83988	18.35517	9.014149
10.63548	9.403972	46.50119	6.528046	45.17563	15.89522	11.47558	15.12618	12.37787
106.0078	96.95685	17.75192	121.8046	74.56567	84.46018	124.6612	8.530681	20.21674
152.6679	149.1152	73.07774	102.0681	78.79267	169.911	90.11494	15.14035	80.70764
65.07687	39.64728	96.34025	25.25618	59.89705	89.42271	79.77635	122.4098	59.44636
540.0809	177.2555	49.84316	171.407	40.98541	222.5767	647.1764	5.214436	52.70625
87.61303	70.89205	35.43332	64.63852	86.09488	37.75678	93.19189	88.59722	62.71309
22.19343	37.53559	88.37864	23.40044	36.75802	12.91475	23.85595	16.69922	20.1988
36.84896	19.8236	13.31846	58.10797	22.06796	60.6159	69.33438	16.69214	43.66099
119.5767	209.6035	102.9661	121.7246	101.9056	98.36611	91.42637	170.0395	193.8521
3160.606	2524.771	2949.877	2921.626	4013.756	2763.238	4254.142	2973.984	2824.074
2.975712	5.231812	40.98869	0	0.016697	5.959041	11.47368	15.13854	78.44791
300.5158	58.4187	299.9941	299.6389	58.84697	162.9501	51.08441	422.77	410.1719
39.01659	12.53266	64.22701	22.44697	36.78525	55.64042	29.23586	135.2483	30.30711
104.2061	45.90416	93.01597	89.87388	141.8212	89.42294	111.2251	181.6904	230.8435
96.03643	70.89867	42.07149	148.0697	53.56866	24.83836	85.56718	15.11783	50.42549
25.20351	212.7478	190.4032	49.60281	43.09029	28.81204	15.57676	64.67329	124.4323
84.30168	308.623	81.93105	171.3902	160.7025	105.3227	365.3147	43.17961	76.23231
22.92728	29.21086	38.75451	23.3902	23.12611	24.8384	29.28701	77.28045	30.28655
153.5749	138.6677	46.515	131.1415	60.93151	111.2875	182.0804	23.37489	90.74926
0.679619	0.012496	35.2107	0	4.218131	0	0	0.179164	2.286609
0	1.058182	7.738276	0	1.068473	0	0	0.163166	0.032448
26.91961	22.93502	25.42778	35.63118	28.34125	26.82979	31.59383	8.463034	6.76724
29.84294	26.08448	46.49394	11.21041	32.57543	58.62409	27.22547	49.53678	13.49713
53.85192	38.57291	12.21195	44.97934	8.423289	44.71568	70.75873	3.566249	19.07595
196.3042	165.8178	161.6157	215.385	288.8502	319.9474	178.9256	107.5135	151.3043

1195.475	680.9927	401.8374	1942.211	1883.398	1682.193	1007.401	442.7388	1532.975
2.97681	0.014232	2.253998	0	1.068561	2.978379	0	5.214292	0.04003
0	0.006705	0.016317	0	0.007869	0	0	8.512198	0.018324
44.64616	25.02602	8.893461	74.0973	30.45101	26.82832	34.95561	6.855056	10.12649
1177.711	1599.747	1161.151	1655.593	2215.381	1231.086	958.0871	1588.526	1585.734
65.46422	37.52906	86.12844	56.24398	6.32315	7.946411	156.4831	10.14073	38.06142
25949.7	27774.48	34039.14	29544.07	31699.38	21327.91	25775.75	40817.44	31287.67
68.95568	74.05152	71.97053	77.71177	34.68475	44.71033	26.50183	48.10811	84.06563
302.889	239.862	369.6744	323.073	282.57	254.3646	303.9336	468.5959	199.5011
187.8147	164.7822	169.3793	261.2624	243.7057	398.4419	174.0815	147.1474	201.7367
612.6838	628.8365	314.3852	622.7307	804.6198	977.7205	575.7556	115.868	295.8879
1260.551	2168.085	920.959	1005.704	870.8345	290.1324	1078.323	934.7629	535.7207
424.0944	425.4733	90.80913	603.1069	446.422	1607.692	257.4351	92.73725	187.1817
5.269739	7.317436	18.84247	6.528734	6.323487	28.81469	11.49476	26.46756	21.31499
42.90616	72.98831	35.44505	58.99915	74.56668	49.68033	51.89628	11.83064	22.4563
33.01409	30.23847	25.4614	46.85962	17.86843	14.90243	55.60218	8.504097	17.95614
72.80347	119.9206	79.70916	87.08285	78.79006	125.1968	111.359	41.51238	72.86318
134.16	210.6461	163.8165	265.9795	218.4777	269.273	256.9519	125.5887	170.3323
39.79881	41.72723	134.976	15.89205	33.63213	23.84415	17.62226	180.8991	63.89231
361.9892	352.4741	203.6841	128.2689	97.71095	392.48	275.9275	173.5296	450.4307
115.7543	192.9158	195.8851	204.1636	162.8128	120.2266	168.0566	148.5792	248.7077
4.507014	0.01334	25.43139	3.719923	11.56734	5.959258	5.339296	0.195985	4.530489
1732.085	1767.654	2580.157	1978.65	2484.308	1430.802	2215.467	3850.409	2336.574
330.802	135.5696	48.74362	172.3192	64.09354	301.0724	316.6131	16.79185	84.0773
37.48679	88.64374	117.3062	33.68692	19.97996	14.90145	36.76266	44.79523	16.86162
76.98859	36.49283	12.21355	42.15924	42.00207	52.66541	61.05413	50.82916	39.19419
357.2874	454.6844	347.5779	535.6457	600.8397	244.4271	395.4108	630.5287	588.3087
3570.869	3387.204	5133.706	2555.485	1694.388	1948.474	3983.707	5336.484	3776.597
515.3463	395.2408	207.0195	679.8878	464.2871	400.4267	541.0194	92.74601	223.0426
87.46975	54.23414	47.61697	155.5186	87.17484	125.2	37.4717	31.58917	28.05947
7.573706	0.013693	26.56957	0	3.172063	1.984683	3.282692	6.866262	1.166265
92.73762	156.4203	66.44608	102.0618	136.5545	144.0747	166.7243	72.83511	113.1942
171.7538	168.9442	80.83971	205.0848	183.8263	207.6662	267.1684	43.20772	77.3679
73.60219	52.15242	54.25945	71.16447	73.53305	86.44495	57.99588	33.2553	40.38093
13.77151	19.80955	14.401	10.28468	34.62449	27.82419	22.61227	8.452833	15.69611
32.21066	50.03683	14.42485	59.04046	51.44603	45.70853	36.9239	10.15649	17.9633
12.32259	16.66092	16.54268	8.424216	5.26488	9.936453	24.30377	5.119432	7.856057
12.94933	55.25005	36.52093	68.41196	48.30386	46.70171	38.96494	13.44301	66.0255
1.440967	3.14583	5.574547	1.847456	0.015597	0	1.920244	0.194286	0.037145
98.22706	109.4795	57.5702	98.34625	46.23078	145.0736	87.48648	10.18396	19.10061
51.3356	54.23441	34.34714	75.85648	58.82946	72.53418	64.18778	41.44698	42.61448
42.14581	39.63496	46.49518	61.81489	65.11637	61.60513	28.59442	28.25648	45.95437
54.5387	50.04372	12.2166	61.84144	60.8973	60.61401	43.76482	8.521348	17.96803
70.49064	69.88129	54.27111	89.89002	204.7972	150.0378	101.7531	89.23649	100.8623
21.42493	16.69786	21.05217	43.09673	26.26553	34.77723	26.61037	19.95841	24.66916
11.44986	12.52224	8.887034	17.79276	10.51713	19.87289	14.30332	8.456294	23.50363
41.32929	22.96063	56.4755	51.48659	149.132	39.74239	53.87559	249.8684	40.38374
17.54246	49.01831	61.97784	35.56835	25.22812	14.90157	32.0089	52.86893	49.31955
100.8339	167.7486	13.32173	84.35514	50.40345	17.8831	180.173	3.566496	66.0296
2.975506	1.058366	15.54206	5.592302	4.222079	3.971896	1.248763	11.83628	0.039869
27.58493	68.78631	24.36758	31.8393	19.97044	19.87075	20.416	15.06905	22.43592
112.6733	183.5362	143.8997	161.0675	169.1176	138.1119	155.7194	219.3849	153.5287
43.6542	40.68218	52.03862	67.4243	126.0145	11.92064	148.547	80.789	68.35511
139.5972	100.1177	105.1592	122.6759	233.1534	128.1769	283.8469	156.6634	178.1268
5.27071	12.53263	38.77213	7.464462	25.23004	1.984796	1.927442	11.83558	3.409881
21.36937	37.55959	139.4421	33.68562	60.94022	27.81851	20.35188	171.4083	70.62897
46.06257	57.33569	8.898705	44.02421	61.94542	50.6765	29.33132	21.62867	29.15105
0	0.011827	0.029249	0.913266	0.013907	1.984983	0.566836	1.871955	0.032956

624.0715	76.14643	441.6923	60.8416	253.18	68.55671	304.4587	4126.852	52.71922
1.440967	2.102851	22.11735	1.847457	2.121062	0	0.564878	0.194252	1.166671
56.64448	73.0166	176.0037	62.71456	131.3175	146.06	83.88839	354.5867	187.1539
348.1701	452.5762	292.2111	173.2227	68.30042	148.0465	242.4266	100.975	115.4709
9.116854	14.61062	19.93571	12.15422	14.71861	8.940202	13.57261	44.14659	9.008849
6.037536	2.102831	11.10735	4.655623	4.222727	2.978222	6.014903	29.69994	9.011873
45.17439	129.2918	70.84809	86.15577	50.43355	103.3374	47.72401	69.40442	75.08732
1138.71	1317.113	522.4815	1349.412	1090.355	1351.317	1656.393	304.0886	461.7483
117.3368	112.6264	70.86478	146.1014	121.8457	171.8984	190.0715	28.34104	47.10958
284.4361	165.8295	247.9669	87.06003	55.69572	92.40335	180.8781	264.3238	158.0558
33.74674	21.90911	42.03693	60.91324	34.66184	18.87695	41.04385	18.34254	33.6156
193.9748	296.1596	111.8299	323.0903	217.4426	300.0732	157.7007	122.3818	215.1669
1447.053	2467.324	660.8271	1786.76	1591.386	11.92099	1884.632	330.4912	929.0095
3055.493	2567.536	2821.505	4380.563	13247.15	3470.69	3515.037	7798.787	7285.385
93.58029	88.63881	69.74264	98.33664	40.98308	103.3374	56.62381	75.97285	89.63957
5162.011	3918.014	994.0323	4988.345	3314.161	6521.091	5021.191	591.4361	2048.592
11.40458	8.361008	33.22752	2.783738	3.171753	15.89535	4.647578	23.32288	94.03404
216.2173	120.9851	198.1448	171.3534	137.6225	140.0978	187.084	209.7553	190.5273
92.8804	91.75371	67.51037	112.4159	50.4272	49.6799	174.6809	92.19584	47.08578
78.8888	45.90416	193.7102	35.55641	18.92949	12.91434	16.93986	58.06368	72.88987
145.6745	29.21858	67.55858	65.52636	60.94496	65.57617	69.54916	163.4526	59.44072
0	0.00674	3.269722	0	0.00791	0	0	0.083269	0.018423
8.336351	7.31806	27.69483	7.464563	10.5251	7.946224	10.79741	21.6588	11.25531
36.77826	9.403689	22.17144	12.14762	17.8758	7.946208	23.80939	5.223463	24.68876
140.6694	58.39567	15.53926	141.496	23.12712	65.57985	63.57218	8.530404	47.07473
29.05171	45.89625	45.40901	29.94253	33.63133	21.85692	27.8824	118.5526	51.57376
240.7985	202.3148	198.1356	367.1193	420.1339	233.4999	208.3051	275.5786	233.0834
187.2664	134.5165	59.80019	132.9894	63.03913	114.2663	270.9346	21.7423	42.62778
2.211396	0.014203	49.83951	0	1.068641	18.87594	0	34.91305	2.28706
1642.541	1352.577	972.9599	1794.224	1287.838	1376.157	2038.195	208.3411	771.0325
720.8449	654.9052	350.9069	766.0196	557.7835	297.0887	1108.072	79.5442	347.4297
8205.114	9397.218	8219.812	10795.05	36895.88	10346.5	10965.34	25660.26	21519.96
34.51514	28.16122	13.32065	44.96676	28.36661	59.62106	74.78333	3.566608	15.72797
186.3744	164.7677	70.87654	248.1876	192.2197	207.6673	194.7222	44.84829	93.0445
6.80267	3.146093	69.65474	2.783433	14.72435	7.946253	5.32945	13.45089	9.014666
31.45729	23.9902	21.04925	26.21663	34.65625	33.78379	29.37651	16.6832	22.43006
13.80953	9.393579	5.569634	12.17017	13.65465	19.87463	14.36182	6.7905	3.40792
17.54863	23.99895	37.64819	21.5164	37.82255	16.88901	25.1756	15.11472	13.49642
50.51843	79.26879	76.4055	99.24957	119.7545	115.2586	109.2327	64.61295	104.2391
8.356083	2.102889	14.40719	2.783384	0.015714	15.89708	10.15171	14.95703	19.05182
61.30925	82.38331	101.8063	100.2125	73.53268	86.44501	63.47646	52.97651	78.4435
96.59615	117.8399	52.05919	126.4255	80.89318	141.0947	139.3954	44.81943	66.14995
19.12159	22.94787	15.52625	35.59781	55.63251	38.75288	30.75959	15.04766	49.23438
363.4029	216.9302	437.2323	405.4614	527.3231	360.6804	423.3915	478.8885	486.3686
26.78752	75.05393	17.74636	40.26753	43.06458	54.6507	38.92737	8.524894	32.51324
49.88516	63.59417	16.64119	73.08382	55.6582	50.67562	54.7281	10.17201	30.27749
343.4991	489.0905	451.5942	258.4386	343.4994	454.0824	868.3906	178.5551	177.1048
145.8273	51.11126	77.48807	155.4908	36.78339	7.946218	246.4045	184.2822	137.778
40.53727	24.00395	180.4475	41.17487	106.1142	292.1228	42.88771	102.6258	90.82128
48.32085	53.17965	25.48834	61.8227	44.11923	68.56202	50.56989	11.82324	38.11477
19.19672	9.397146	11.09131	22.49103	39.85982	31.80022	35.80829	3.555365	14.57806
121.3979	74.0303	28.81109	170.551	237.2426	42.72448	124.6521	28.26873	19.09732
184.012	193.9726	147.236	146.0699	212.1879	273.2456	164.5516	148.7283	251.0026
12.93467	36.51492	68.63907	11.20978	11.57656	6.952625	16.93823	82.55527	22.46286
26.74186	33.38782	89.65835	6.528644	14.72797	60.609	14.20512	21.73943	57.18319
82.75575	336.7817	97.42388	156.3938	61.99304	32.78657	121.5553	49.78088	73.99694
10581.35	8630.717	12731.48	11174.3	10816.48	16196.89	13861.08	14275.2	17759.06
430.9051	398.3778	333.1976	659.2585	631.3073	267.2801	625.6526	554.7512	392.2468

54.48906	30.25145	26.59217	56.20045	31.52254	73.53105	62.94772	8.527398	23.56881
86.88466	47.96396	23.27275	77.77494	57.75557	46.7008	91.19013	21.6515	17.97077
30.16782	23.95556	5.568184	17.81208	33.54594	7.947493	59.77901	16.3691	14.55404
195.6885	83.43519	100.731	223.864	90.34388	159.9744	152.4071	46.46468	156.8463
23.66949	26.08998	66.45682	64.58708	72.50087	14.90154	20.35318	39.9116	31.43076
228.4119	412.9795	435.0235	220.9718	231.1169	205.6755	294.9375	569.6596	493.0975
47.68944	77.10159	12.21083	42.16853	12.62155	70.55553	34.21875	8.503714	12.36643
325.1482	276.3567	151.6751	387.7008	383.3963	338.8235	371.6204	102.6257	135.6385
200.191	182.4927	147.217	182.6135	264.6803	124.2009	176.931	74.51637	177.0459
339.1425	212.7312	107.3916	297.8295	700.4853	233.5017	632.6168	76.1732	182.6518
3529.585	2181.659	3245.351	2526.477	1425.468	828.6713	3784.453	2177.967	624.256
42.84045	43.81814	64.24052	43.98502	102.9574	78.49326	72.27776	58.05138	154.659
49.03596	53.19079	34.34558	49.62055	64.07705	64.58495	91.62478	23.36411	22.45965
2.971959	2.102553	19.9591	2.7836	12.62548	5.958986	1.24491	0.208452	5.654
63.58796	41.72956	54.26843	45.86339	54.6377	63.58979	47.70343	90.84552	76.22238
16.83089	35.43779	16.62142	33.7361	33.59485	20.8656	26.68151	6.852488	34.6891
622.6917	465.1136	209.2389	765.0941	258.4244	607.0997	448.7002	107.6094	221.9296
0	0.007695	0.018784	0	0.009034	0	0	0.097262	0.021107
75.83276	69.88658	99.64655	74.89241	173.3197	115.258	118.7796	168.3899	124.4104
9.104234	35.47496	84.14839	11.21024	22.08119	202.7002	19.66819	61.31246	44.87332
23.76434	22.94493	9.999599	38.42069	16.81769	43.72288	26.65435	11.76541	12.36437
0	0.012285	0.030425	0.912754	0.014448	0	0	0.175082	0.034292
71.52184	74.00134	19.95257	73.10123	76.63017	48.6892	79.5784	6.872905	11.25306
67.55768	125.0813	36.54339	52.44557	39.92211	48.68727	45.75371	24.96266	23.57172
306.7672	228.3866	129.5376	471.0752	678.5029	174.8749	531.6866	39.91244	264.4621
67.41262	101.1606	62.01767	106.7494	141.8006	97.37348	120.2141	136.9492	116.5456
53.69335	62.56189	49.80766	41.19593	59.86638	22.85099	56.72655	34.80064	75.03152
238.6041	185.6174	78.61987	292.2233	262.5743	160.9662	262.4895	13.48531	66.15914
88.9859	72.99991	53.15052	103.9644	76.68052	116.2559	114.8837	28.31264	39.25907
257.7343	315.9551	87.47794	391.4964	130.2636	341.8086	414.2682	21.74652	127.7754
163.9909	294.1004	573.372	220.0344	352.9643	435.2015	223.2023	751.1839	388.8967
213.9383	283.6442	148.3435	158.2453	138.6702	245.4236	181.6403	64.66165	89.69527
34.49915	33.37347	26.58397	42.14753	70.34033	24.83903	41.01226	13.44925	15.73055
16.92325	5.228604	7.773895	12.17145	13.6535	20.86886	12.2852	8.394536	3.407553
40.53751	10.44583	43.21501	14.02008	10.52478	11.92076	32.64372	63.01788	3.406488
185.5307	333.6976	109.6173	275.3197	325.62	136.1234	232.2137	99.30395	156.9167
75.21621	70.90386	55.34213	28.07311	36.77646	48.68663	51.21074	21.702	51.55372
57.55811	18.7867	36.54126	6.528017	6.323996	9.933462	13.53183	3.564742	6.774909
9.10489	14.61564	18.85046	13.08569	6.323887	6.952613	2.602722	24.91232	14.61246
316.7409	220.0451	192.6126	283.7461	164.9307	397.449	340.9073	67.97021	135.6335
6.045812	3.145933	17.7142	7.469146	4.221951	8.940511	4.653821	10.09112	24.62658
45.19208	37.55468	42.08672	88.04197	71.42619	43.71761	38.15982	52.92097	181.3551
8.345768	8.359078	27.66327	4.655866	27.30791	10.92759	6.702344	16.65221	17.95265
516.8483	484.9237	522.4305	488.8209	474.7966	302.0573	578.5588	597.5222	446.0144
0	0.013546	8.893732	0	1.06955	0	0	5.213981	0.037997
47.53395	40.67674	29.9138	41.19522	51.47026	62.59897	62.2122	26.61271	98.51883
57.50086	83.4157	33.23781	79.61189	84.02008	61.60417	61.47148	10.18312	22.45859
149.4529	67.80394	158.3141	150.7457	179.6376	246.4159	107.1057	336.7124	383.2205
444.7516	457.8094	245.7568	516.9209	498.9517	635.9159	552.6113	745.87	195.0337
44.49591	57.34195	27.69356	34.64232	11.57508	167.9396	49.92088	20.01637	5.653966
15.29779	13.5663	9.994918	17.78683	15.76412	6.952921	12.21315	5.207174	5.650315
31.34498	81.36139	822.4226	58.03153	136.5803	91.40968	50.40716	63.02152	80.73904
1.441161	0.013429	4.470642	4.656436	0.0158	2.978234	1.920183	3.562304	60.32171
147.4867	286.6451	76.35787	186.4575	108.1667	17.88244	157.4881	18.42639	56.04212
108.81	92.8293	158.302	114.2249	170.1784	261.3218	151.5583	227.8121	252.1251
2.973215	2.102758	7.780069	3.720713	3.171336	0.991189	6.035437	0.191321	2.289199
276.4646	152.2335	83.02288	334.4589	79.83815	20.86318	208.655	36.56575	60.54251
3.750583	0.012627	0.031307	0.912493	2.120266	1.984762	2.609458	0.181723	8.979692

42.87973	63.61688	37.66603	105.8424	111.3229	79.4898	108.7338	43.09671	56.04899
154.8183	109.5164	274.5103	108.6008	97.71151	170.8998	64.06126	682.8041	145.7234
31.41958	33.37315	31.00209	19.64671	32.56665	17.88305	16.97066	21.63089	13.49222
54.46928	52.13935	37.64891	69.31903	60.91439	49.68096	57.42223	20.04305	43.71141
1.441064	2.102719	17.69615	0.912346	3.171224	4.965725	3.28823	30.84166	16.79917
39.83157	46.93225	31.02222	66.49803	64.06952	45.70558	53.95653	20.05784	37.00668
161.0868	92.82107	79.72025	78.64469	40.98727	151.0306	165.3717	104.0872	63.91404
124.9691	118.8899	125.0852	125.4767	112.4043	145.0679	138.6425	146.9244	205.032
75.87029	114.7099	74.18115	84.27062	106.0931	96.38032	85.33462	44.81068	47.10839
48.21147	22.96145	104.0725	89.8783	55.69343	11.92069	18.30314	155.2238	431.2685
68.1829	102.2023	79.71647	107.6876	85.09424	104.3292	92.16191	56.348	50.47095
883.2657	641.3726	850.1182	697.6141	797.3034	1127.751	660.9915	1155.934	839.4022
0.679259	1.059158	9.986623	0.912345	0.01545	1.984684	2.602999	3.553368	3.409561
1267.495	1301.483	779.2738	1786.723	1859.265	1235.062	1324.285	431.2259	832.6701
0	0.009649	1.136474	0	0.011336	0.991765	0	0.128078	0.026664
39.06443	32.33952	26.59896	18.7041	25.22727	54.64895	45.04338	41.37653	47.07628
392.5165	403.5989	646.4312	308.0597	754.2191	855.5022	459.5479	569.7746	458.3782
129.606	126.1829	65.33728	129.2319	202.7052	182.8279	140.0569	56.35655	78.47253
39.072	45.88782	45.38836	35.572	44.12247	54.64928	46.42817	39.71737	51.54586
98.27767	27.12868	88.50661	58.99548	22.07825	42.72423	246.1236	98.70756	40.36855
0	0.009912	1.137801	0	0.011647	0	0	0.132471	0.027418
3.737939	6.273635	22.13237	5.593449	8.421114	0.991195	9.457356	19.83392	6.769366
51.33689	109.4778	33.24085	61.80073	86.12507	31.79352	72.41485	59.49305	57.16532
2.971335	10.44574	17.74442	14.96012	15.77414	14.90193	7.379201	54.24234	22.4433
95.86601	104.279	52.05218	134.8734	115.5359	110.2925	179.9044	61.2265	88.5295
108.8221	54.24606	147.2282	28.06506	30.48484	22.85035	72.27673	189.8485	67.28499
1327.251	1479.819	857.8732	1287.571	2026.303	1699.079	1749.742	976.0624	1009.742
44.62176	45.85672	13.31358	25.28453	22.0631	43.72286	70.84658	8.498104	9.009612
47.44707	54.24489	68.66264	52.41519	31.53498	14.90148	40.16276	41.54807	65.03962
2.206195	0.013782	8.897927	0.912733	4.222785	2.978227	3.282904	6.871169	3.411574
10.64923	3.146177	14.42211	5.592187	3.172063	11.92115	10.12551	19.94084	45.88745
26.88418	18.77473	13.30806	28.10827	13.66731	15.89677	21.86275	45.63573	17.94288
124.3463	131.3729	38.77401	102.0891	103.9781	100.3568	105.9666	10.18403	35.90052
121.0856	157.4787	338.6517	65.52383	130.268	155.9962	176.8527	924.2108	489.5825
119.7143	120.9531	45.41223	150.8124	100.8318	128.1794	86.76056	21.73322	48.2193
328.1471	430.6983	213.6629	603.0812	545.168	482.8972	739.174	134.0121	298.1166
42.87847	50.06569	48.72557	23.38549	44.13138	31.79344	29.93665	51.30988	47.0946

TCGA-VV	TCGA-P5-	TCGA-DU	TCGA-P5-	TCGA-TM	TCGA-HT	TCGA-HT	TCGA-P5-	TCGA-FG
223.8292	168.1565	117.1133	453.4671	106.8333	119.3874	167.2486	137.2518	169.8186
20.38729	19.6475	64.4088	18.47362	38.93133	34.03356	27.09212	17.10673	17.15774
98.37998	83.162	79.51413	99.84386	196.8601	130.018	143.8432	99.05597	81.08296
5578.539	2497.241	4456.18	4477.391	3097.406	2002.829	2216.167	2786.597	1908.042
143.5911	205.7576	88.11178	60.80018	126.8419	129.3368	113.1754	111.8073	54.36216
0.030794	0.860299	2.165708	0.020264	0.026632	0.489491	1.29753	1.529209	0.939373
30.55061	142.4442	15.06818	11.97107	10.05959	5.408896	35.68617	364.6043	22.88345
222.6984	112.6766	245.9837	164.9446	135.7466	107.6106	320.7897	154.1932	220.3937
13893.39	3719.009	12262.08	10866.91	7203.94	4627.132	15011	7382.365	12901.32
93.8079	139.6206	71.97604	45.59692	40.08393	67.45879	62.73586	32.67188	40.05636
20.35781	9.80374	16.12741	7.62779	23.35842	23.54228	11.13752	12.83838	14.30012
1745.673	778.1317	1164.544	2312.433	1107.016	1322.733	878.1272	1569.986	1086.634
170.7334	232.5762	159.0032	150.849	92.3795	92.10175	107.0565	123.1526	82.9845
0.032787	16.26703	0.018832	0.021545	0.02834	6.898064	0.056693	1.542951	0
150.3402	71.52912	165.4136	108.5186	47.88079	79.14988	179.4383	65.18102	52.45601
9.09942	7.110879	20.43482	5.46164	13.39444	22.79439	30.78187	19.934	10.47549
2497.602	2361.301	3145.539	4450.231	2018.222	1475.477	1404.51	2011.468	1839.357
44.10724	59.94172	41.90676	20.64904	41.17991	51.37749	39.38933	34.03948	19.06332
4.57406	47.36656	53.74016	4.374132	6.721289	41.3225	48.04549	84.94341	58.18108
222.7915	1410.607	1043.12	172.5789	51.22561	165.0957	81.26703	937.712	204.1426
835.5707	538.4141	2322.567	758.5529	963.4971	1078.214	1179.371	1530.346	903.4586
0.042657	0.855861	16.14376	0.027837	5.610098	46.40267	220.6461	5.831235	42.92672
74.65865	59.89144	156.8343	175.7717	169.0814	35.74139	385.7421	5.822859	402.6893
717.825	140.3882	235.2862	304.9367	3501.247	349.7323	758.5246	65.24629	594.3914
134.5131	134.1923	148.2259	254.903	70.11569	104.614	108.2105	79.27416	130.7124
11.35703	25.92052	10.77219	18.4732	71.108	39.03038	18.51513	19.91641	56.31029
131.1919	227.1867	156.8638	157.3678	253.6559	97.03826	153.7769	172.6374	152.6341
462.4814	826.4421	955.0578	452.5471	1305.023	1161.195	919.9142	451.3725	645.865
22.64768	2.640621	20.43382	11.97039	30.05602	116.4226	40.57332	15.70679	56.30455
44.1373	25.00072	66.61938	106.3334	44.53884	111.4896	75.04276	86.29058	128.8105
26.05275	63.50327	13.99736	44.51023	31.18913	50.69606	27.13821	24.2027	21.92464
77.99249	107.3903	52.6499	46.67811	134.5171	56.91456	60.26968	31.25329	74.42
21.47266	8.013433	9.691408	14.12254	12.26484	15.42119	1.317731	14.21814	14.30241
580.0567	1304.118	1085.035	641.3551	1016.884	768.69	1035.48	348.1269	339.6158
147.0177	133.2401	106.3818	212.6996	170.2454	292.2176	258.2427	99.16536	183.1661
11265.83	5677.815	17909.69	39913.62	5750.942	25072.59	12082.95	280.2288	4386.597
12.48024	21.45129	20.42417	0.027112	5.609136	16.61554	6.242485	99.46842	17.16146
65.58873	101.1005	29.0338	41.2614	128.9915	69.92511	61.51869	17.14831	72.50525
26.04682	51.87616	60.15288	10.88766	15.62131	27.11528	30.80777	100.1242	20.01741
15.87921	21.43131	30.09738	3.290082	13.39608	34.598	22.21084	70.54712	12.38358
23.80018	28.57608	149.3336	4.37326	24.52537	22.11719	200.4233	354.644	177.4514
99.42456	96.6533	51.57297	83.53024	504.2703	69.37496	35.72431	36.87998	36.2413
12.49281	68.87458	29.03216	202.7726	42.3051	10.97736	36.96072	86.18815	10.47462
276.8863	119.8476	146.0985	147.5752	222.4506	131.8388	81.22645	135.7803	143.1074
227.2821	211.065	172.9834	315.7808	240.3311	289.6629	140.2764	260.3044	251.8582
19.23782	19.66523	17.20241	19.54332	33.3429	26.02328	12.36642	24.04291	15.25378
49.77336	86.78145	50.50579	57.52391	90.09131	30.81555	25.91252	48.17764	45.78292
2430.926	1959.671	4348.762	2510.021	5571.715	2805.646	5820.573	3827.609	8136.951
73.54897	216.4116	334.1299	118.3208	161.3706	372.5105	998.4795	691.6391	1299.432
7.959597	18.7807	7.546397	32.51131	18.91584	45.60125	5.009464	5.815734	22.90261
7.959699	23.27006	9.691478	16.28708	23.34808	31.12176	12.35193	10.02435	21.94676
156.0668	150.2334	150.4243	259.3589	69.0272	360.9477	191.8974	140.1667	218.4659
59.94434	120.7972	45.14094	100.9001	116.7733	64.94867	38.19804	86.24521	61.05119
5215.704	1773.615	8916.684	17657.78	3181.998	7889.841	4445.817	6598.625	2967.963
22.61783	12.49063	13.98555	37.9425	20.03871	24.76877	22.14827	24.04483	42.9601
74.60769	102.9097	31.1792	57.52084	66.75168	84.26685	45.54951	46.75741	41.01189
18.11603	46.57819	62.22264	11.9647	11.16407	31.63204	11.14648	12.85839	20.98282

5907.655	4841.545	9655.8	5893.578	3168.649	4941.013	3988.342	10735.83	2338.302
131.2036	321.9967	209.5065	55.38086	85.71546	194.2495	99.70829	200.9636	95.38381
12.48697	25.02233	13.99311	48.80994	45.59328	35.2742	12.38587	14.29462	25.74982
19.27161	85.01465	34.39666	59.68014	17.84575	16.55733	54.11687	473.6539	86.83668
2.313515	9.797369	5.402497	0.027327	0.036084	0.480065	2.551863	22.70966	0
255.5156	341.7147	270.7139	136.7549	111.2949	346.7687	205.3961	202.3092	168.8543
15.85853	10.69759	9.695021	17.37876	26.69354	41.05815	28.25802	15.6547	18.11875
54.21953	47.43072	42.96122	24.97639	77.77228	26.54724	49.12808	21.32689	80.18519
54.30499	89.44426	58.02967	67.29491	57.88023	100.9301	147.4535	148.2959	220.4393
61.1093	100.1403	114.9732	111.7996	76.81155	68.54927	91.08938	494.6146	76.30418
15.84705	11.59978	18.26742	7.626573	45.49944	23.57128	26.996	8.625446	29.59162
3.445682	10.69477	16.13457	18.46682	12.27645	82.33985	5.014049	25.48581	4.751015
429.5679	236.1268	302.9403	379.7736	222.523	295.2964	340.5894	109.0635	292.8929
23.78362	39.34483	22.58409	108.4186	97.79988	52.0323	19.75712	38.23859	51.52192
76.91082	469.0054	204.0641	40.18402	55.66443	65.50667	143.831	49.65042	129.7544
56.50546	9.794526	44.04681	27.15114	25.62025	68.24594	34.47026	48.06799	20.97339
390.0239	147.5505	180.499	537.0857	379.3481	315.1037	269.3248	142.9899	316.7443
51.98388	24.12114	51.55231	47.73741	71.13928	21.54705	35.68603	21.34422	38.15872
100.6791	120.7101	63.41577	22.8245	95.72626	69.77912	146.4208	260.2943	142.1351
153.799	186.9215	345.8967	156.2842	183.5918	282.3032	234.8929	230.5672	223.2409
746.2735	1084.924	922.8465	3636.315	874.5165	2898.196	2099.227	461.2997	1165.813
72.35549	39.32561	35.47497	51.01793	168.9687	63.73444	50.46348	34.08046	9.520616
260.0545	113.5542	207.3532	352.6621	333.7533	805.9105	228.7725	19.976	153.5849
56.54344	136.9486	104.1701	57.52036	378.8621	104.7973	81.12072	0.135654	10.47463
68.96296	33.06088	65.53158	82.45494	51.1947	20.2727	40.64078	50.98296	20.9704
1704.999	846.9991	2520.265	532.8526	290.4308	1136.339	1945.493	1994.309	801.3695
69.0008	126.1302	84.88047	36.92917	165.7231	28.93213	51.72777	79.2855	67.72464
53.1917	75.99261	116.037	356.9229	145.7504	372.4682	38.22103	72.27872	167.9125
240.8714	791.6273	553.2578	227.9154	85.71727	265.4147	175.9527	567.1611	115.4176
21.53846	76.00402	7.550745	7.631342	191.2959	97.14165	33.29841	63.76919	18.10667
99.54109	341.7285	122.491	146.5158	180.2478	130.5019	113.2119	118.9365	80.12086
31.69341	61.72785	41.9091	87.85569	60.06892	45.76281	13.62288	31.23259	41.01567
904.4782	458.8226	790.6736	626.1464	440.6041	545.2522	1588.625	927.7674	1041.821
1052.678	1196.721	1434.21	1884.95	3592.433	1801.108	1545.915	1097.783	1342.305
0.027846	0.864086	0.016063	0.018362	0.024103	1.78169	1.286259	0.082532	0
45.23857	65.31353	31.17459	42.33313	37.84887	139.1888	51.65431	19.95707	51.51696
9.100745	25.00222	155.71	8.717535	14.51157	80.47834	103.242	15.73548	66.77771
936.1681	823.7702	1047.431	1397.657	1342.824	456.0489	455.1013	563.0682	327.2138
127.736	67.95351	88.09934	78.1457	153.4877	179.1215	102.0715	94.78844	82.04037
47.52456	50.95501	32.25693	238.6001	77.88585	125.1779	48.02741	19.97714	95.40759
167.1836	56.33445	59.09509	109.5647	38.97296	70.5613	38.19263	31.26294	58.19003
144.6474	21.42221	23.66521	71.63052	78.99789	26.46149	277.4682	32.68826	274.8524
65.62177	454.5878	134.2895	97.68297	102.3774	104.5367	170.9026	77.91785	224.2193
339.1918	92.97705	186.9501	143.2732	159.1357	274.1494	218.9595	251.8498	123.0518
16.97784	3.534354	11.83808	7.627529	15.59617	9.777787	102.5283	7.226528	61.12928
5.708156	7.110832	10.77335	17.39268	37.83346	38.98155	32.00951	2.995117	133.6553
11.3538	8.007502	5.402345	32.5482	5.609482	34.71432	14.82851	0.130881	5.705069
23.78032	25.01619	29.0216	41.23754	61.15151	42.0901	16.07231	22.7536	29.56566
13.60567	12.48901	34.35761	17.38031	11.16364	20.37521	18.48835	4.4127	10.47824
42.99687	41.11618	53.72599	47.7649	40.08319	81.13935	127.7346	41.12915	117.3725
16.98914	16.97039	17.2052	15.2149	26.69925	14.12607	27.04857	35.25074	35.31163
2564.275	937.3311	2615.902	1455.23	2566.674	1387.618	1871.772	1315.571	1184.89
72.33289	40.22891	41.91084	91.11235	43.40819	71.25774	50.43914	42.50494	51.51473
193.3949	611.7892	307.2754	163.8972	452.8402	296.3461	943.1757	581.3852	488.4544
782.3198	402.4902	337.3422	613.093	323.7784	324.8839	295.2044	322.5782	385.4237
0.040657	40.3342	9.691989	1.117574	24.45864	6.041416	3.782137	12.82599	3.797389
28.23614	8.907713	19.34261	36.84894	22.24877	22.9196	13.58233	10.03451	14.30039
175.2076	182.5044	176.1606	201.7984	139.061	142.4031	87.36324	90.60509	87.76127

10.22475	24.13791	13.9896	3.290525	3.384016	25.96997	28.28863	21.29027	22.89021
63.31833	65.29442	361.7173	62.94162	52.30609	50.07505	45.54953	39.71251	72.51028
159.3946	103.741	215.8914	186.6169	180.1985	300.6458	43.13383	38.35978	36.2341
229.5564	145.7525	340.5589	1107.813	1066.774	681.103	108.3188	79.39099	246.1281
146.9727	101.0537	104.2182	151.914	60.1178	82.22382	132.8177	121.6693	199.4107
166.1538	91.21879	93.47268	59.71027	55.66494	93.41849	59.10119	80.7051	20.96881
185.4603	84.92721	94.56877	539.2741	760.8489	329.3111	612.243	2.978206	532.3768
45.24188	47.39496	40.83648	87.85791	21.18062	65.0447	7.476152	32.64371	30.51562
47.4266	22.34315	55.81148	3.290521	6.7216	14.11175	34.40791	68.86118	21.93453
18.13565	18.74716	12.921	28.23283	47.82799	34.6169	20.97878	12.89374	21.92874
258.9185	316.6478	166.5349	519.725	649.5947	290.9437	102.1606	282.8699	231.8252
141.3639	211.9767	140.7523	274.5335	232.5244	452.7823	83.71529	107.6406	194.6173
561.9208	398.9016	603.7461	882.1916	423.9032	358.9153	289.0711	439.9464	422.6289
4137.039	1465.935	4430.414	1894.739	3242.052	2866.291	6041.953	6574.345	5693.653
19.24596	22.34846	8.62305	39.03713	31.13927	23.48638	8.696783	19.87598	20.98169
50.822	26.82024	25.79378	6.546417	24.49547	27.19077	20.96055	21.31172	17.15873
47.51376	33.05965	79.48831	31.49867	70.08885	118.4538	148.5753	66.48583	41.96557
0.025977	0	0.01501	1.105008	3.322337	0	0.044469	4.123255	0
1.180447	4.429508	18.26753	2.204512	5.606697	6.041549	51.35727	29.55673	134.7612
0.03337	0.857658	1.099552	8.648732	1.150594	1.730802	1.305847	4.293113	0
7.961934	25.05681	12.91054	22.78504	20.03171	24.17381	16.02647	26.80176	15.25563
79.18223	201.2869	69.85332	126.9697	101.2647	128.7251	29.61378	93.44768	77.26291
24.91913	47.39419	32.24947	60.76346	43.40732	63.17362	13.62335	34.05392	32.42438
800.4605	627.8986	713.3234	761.7699	591.8874	435.0549	412.0334	732.6253	328.172
66.75373	174.4293	131.0708	254.9646	125.7327	57.41893	65.26093	55.3236	57.22432
12.45391	14.30563	8.614563	20.59783	15.5755	19.88636	3.778547	5.802765	8.573694
1135.16	253.9739	446.9362	546.9528	688.7079	1177.913	1915.872	58.16544	1425.332
228.3709	116.2486	88.11828	314.6545	194.6983	80.32534	354.0212	96.31902	94.4337
62.22705	109.1132	87.03303	22.8253	28.97457	82.23624	7.472763	216.1524	10.47495
9.094022	26.83576	10.76894	14.13167	54.41622	15.99805	12.3717	5.825637	24.80334
244.2423	954.5464	247.1016	412.3463	955.5017	372.6562	132.904	126.0465	152.6288
458.9438	124.2929	215.9401	253.9264	287.0267	162.08	254.5599	236.2254	293.8485
204.6105	91.20418	282.4874	194.2265	143.5257	92.74385	161.0929	152.7609	156.4619
49.7836	63.48721	62.32255	155.1193	204.5731	111.512	19.77371	38.33274	75.36502
35.03929	55.51895	24.71986	29.30378	23.38362	35.31498	12.38097	38.12683	19.06875
13.62245	52.76483	129.9131	20.65092	27.85135	38.28763	9.936172	132.5148	20.97106
167.2896	64.36833	125.6883	177.9313	111.2603	157.3224	168.4004	68.00953	124.9805
14.70638	12.50643	8.615527	5.457094	29.96565	2.320255	8.673383	47.43743	11.43967
2.311953	290.3038	3.251002	1.117437	1.155229	0	0.067621	15.48844	0
39.61	48.27699	21.51591	139.9168	95.64922	100.4004	29.59757	24.20733	37.19308
37.31878	30.39265	22.58159	41.23543	25.61661	29.01719	45.48223	25.56209	50.57208
5.707763	67.09145	18.293	7.632343	7.835806	18.415	20.99557	22.7872	44.83114
99.54344	208.3971	133.2339	91.18607	454.9462	260.6241	102.1544	92.09542	104.9272
46.39826	150.3194	45.14489	4.374643	17.84939	64.91318	72.59246	41.16413	10.47469
322.2686	388.1655	228.8527	285.4257	387.1954	291.4149	1468.017	188.2897	623.9414
165.9746	23.21766	63.37523	108.449	72.28992	27.11079	87.20954	42.50011	68.69877
18.13922	19.63935	37.61191	40.16136	26.73429	16.56383	23.44091	59.33693	34.33596
91.55192	65.28832	137.4519	65.11477	146.7573	47.57552	89.72705	58.04519	39.10177
61.04252	50.08266	17.21827	82.43793	73.39882	62.55928	14.85157	15.72661	18.10854
88.21987	70.62828	55.89039	38.01604	199.1036	338.4734	146.3268	7.240138	82.03474
89.29739	107.3729	53.72823	125.8284	120.0975	112.1996	28.3707	45.36698	52.46287
1666.086	175.2757	276.1052	559.9024	101.2905	363.9675	327.1341	233.4803	220.3694
44.129	31.26795	40.84458	108.4828	95.65237	85.47063	93.41237	56.64141	94.45993
26.03413	44.73297	38.67547	72.64772	127.723	21.5528	23.42668	21.33818	20.02033
248.7915	826.4857	286.8641	174.7482	386.0914	164.4802	264.4922	413.1269	248.03
17.01075	128.753	278.2683	52.12472	65.69065	3744.361	223.911	114.754	557.1525
22.66119	33.95978	15.07092	21.73543	25.62818	86.18268	32.04167	25.60432	14.29126
220.4293	117.1539	215.9096	247.3755	43.43683	100.1816	148.81	252.9693	180.3179

258.7987	50.04929	170.7918	172.5159	141.2846	153.5712	196.6606	29.87953	197.5058
82.48175	50.97907	36.54227	68.34786	55.62544	50.73981	38.16925	43.90191	41.01572
87.1169	202.1039	51.59858	92.27664	714.2239	25.83953	104.6329	273.1142	77.25615
200.141	68.82751	156.866	244.163	141.3282	129.8633	119.3671	179.716	164.0825
73.49411	59.01679	26.88615	73.79269	84.54478	92.9127	23.45844	73.55978	31.46636
12.48846	22.33107	4.328117	30.39836	32.279	35.87434	32.00548	26.9639	41.02446
134.5365	138.6487	75.2202	77.06829	134.612	113.8701	202.7935	58.1327	105.8928
27.17014	75.1873	12.92167	32.57163	26.73215	14.70451	18.52987	34.01965	20.01866
35.07338	24.11664	29.02518	62.9185	26.73365	55.1341	89.60963	46.68276	42.92818
418.3813	412.3001	479.1588	229.0068	843.3253	642.4187	1207.577	374.9839	1987.299
82.39928	14.27252	34.38033	47.72701	53.36228	38.39226	6.245149	18.51612	21.93077
87.06976	44.68549	96.68742	79.2287	106.7986	187.2164	52.95041	51.04987	40.05239
208.034	173.5109	219.1513	107.4576	127.973	94.57168	137.7868	363.2366	113.5162
433.9591	40.20517	140.7381	440.4393	150.1992	137.3782	159.8682	42.60696	190.8141
5.69496	9.822776	9.680466	15.18172	11.13567	10.47651	8.655581	11.3485	6.665026
712.2891	603.7436	541.4532	595.7594	285.9693	475.9187	536.2141	278.7985	444.5685
23.76621	9.797214	12.91814	15.22002	27.82438	50.30746	56.43142	10.06223	35.30263
1253.861	457.0189	1867.08	457.972	739.8809	660.9535	1953.975	1649.071	1265.051
63.37312	663.7575	73.08455	4.371926	11.16986	100.1132	195.6039	395.9994	84.88895
382.9473	123.4521	248.0671	102.0041	106.8002	128.8319	75.05717	18.5661	84.9038
73.51587	136.8796	59.10671	14.14387	104.5774	45.67163	38.21054	197.6886	82.04067
767.6001	241.4703	574.7216	661.9038	687.496	144.0691	134.14	798.8092	94.42913
29.44426	51.85708	49.43359	77.0423	41.19671	34.53548	23.45719	43.95612	25.74108
414.8959	263.8542	227.7615	828.9198	129.0971	251.2687	216.4822	222.1346	236.594
1881.364	1244.148	1569.548	2581.563	976.863	777.2894	1855.723	1898.136	2442.339
131.0962	101.9831	44.06925	96.57037	121.2307	83.55824	34.51816	58.08144	44.82536
2.313061	4.431963	3.252194	18.43499	18.89417	6.057289	15.97942	4.398861	12.39679
414.9295	359.5644	523.1555	213.8014	244.7897	443.2486	392.2883	268.8228	243.2672
500.8679	58.98668	264.2994	405.8613	161.3675	1821.394	474.6999	28.46142	258.5281
1373.701	491.0084	1266.587	976.6569	1102.552	1177.295	933.439	1144.286	526.6086
97.15756	63.51589	18.29234	53.17623	33.40701	68.14965	19.76503	28.41975	27.65204
30.53935	19.64764	20.43184	31.47613	48.9192	30.91609	20.96691	25.53718	27.66012
38.48547	101.9872	97.74925	19.56887	57.87562	55.62498	18.54412	166.541	18.10704
44.15025	105.497	67.71319	32.59008	60.12735	41.92897	33.29923	70.90462	81.07228
32.83727	239.8828	238.3735	1.115963	12.2866	24.60256	29.60418	73.58802	10.47463
11.35033	5.323138	41.86091	11.96455	14.49436	14.12776	6.241547	26.86032	3.797076
1023.215	368.4687	709.0476	980.9841	516.2635	472.1483	1277.675	1296.909	1312.763
96.09931	91.23273	121.3765	100.9137	127.9096	92.84979	62.76891	39.75171	83.95195
5.70452	30.44687	23.62781	20.61919	10.04869	16.03314	11.13489	7.225339	20.0332
226.0734	179.7994	186.9128	252.7949	363.6679	146.6913	54.19998	106.1674	63.90347
105.1748	62.57278	219.1263	197.4761	233.5832	148.5585	93.52029	90.63153	435.1195
26.02554	19.64887	19.35794	41.22264	22.27696	20.32602	23.41376	35.34921	18.11292
71.28032	122.513	99.93257	115.05	503.8246	261.9435	97.22638	90.66424	138.3259
73.41042	47.41685	52.6252	27.14872	57.81887	46.45781	20.97828	18.52918	20.0197
19.26189	61.76745	39.7469	2.204042	22.28192	18.44477	12.38749	734.8664	11.43002
10.23168	42.90433	45.13937	9.802925	10.06126	6.647829	3.781083	66.50505	5.705095
5.687744	3.540574	7.532716	5.449312	5.5931	10.52719	2.544782	16.79217	2.845077
156.0237	100.1533	157.9183	79.24342	73.46636	219.8783	270.3994	121.6981	123.0641
5.70026	471.3451	456.5944	2.199985	20.07025	1698.31	1720.18	85.05032	695.4879
15.88498	1.750456	27.96389	6.545064	61.2321	2.33415	12.39478	31.29617	10.47517
2.313583	35.85896	5.399899	0.026349	7.825543	10.42086	5.007956	16.99142	10.48222
108.5565	85.84178	78.44341	80.32418	146.8469	162.8654	114.3899	151.2931	105.8914
0.033683	5.363717	0.019331	0.02212	0.029107	0	1.306746	35.09967	0
68.96668	49.17445	62.31323	51.0172	68.977	73.68421	54.14261	41.12703	38.14808
43.00591	66.17225	24.73922	117.1723	83.44359	115.8569	30.83308	43.97761	50.55121
487.3314	442.7221	490.968	565.3814	240.3591	734.1227	650.5652	253.3508	510.3982
114.2476	518.8174	189.0989	199.6951	857.6419	534.9562	184.5406	124.636	120.1894
357.2756	509.8751	472.6662	176.9089	219.2025	260.5237	273.0449	171.2838	207.9666

8909.437	2252.144	6759.49	6388.392	4564.899	2972.091	3401.713	3772.549	1796.415
193.2918	11.58306	58.03847	91.17239	16.73703	64.24546	315.7833	132.9585	456.1281
176.4332	673.5345	389.9842	59.72135	153.5816	246.2142	162.4285	880.9856	73.44005
31.66546	60.88642	27.94146	26.05818	13.39186	34.04008	20.96531	17.1042	33.39004
1550.122	1454.342	1871.424	3098.097	1526.454	1377.749	1046.623	1642.22	1146.732
67.89145	196.771	209.4886	80.33502	229.1816	166.4431	134.1054	80.78304	140.2316
18.13469	26.8095	81.58424	18.47648	15.61728	17.19672	13.61635	85.89632	11.42979
197.8476	212.0006	127.855	251.7276	192.4641	219.1937	44.36975	75.11501	132.6026
2.313811	16.07967	0.023392	3.290362	12.27047	3.555108	0.071817	8.635113	12.38932
13.62299	42.01442	12.92315	8.717661	22.29485	7.265087	7.475988	187.4158	4.751192
165.0351	183.4045	299.6329	135.6375	139.0572	32.02637	43.13207	96.24231	115.4363
87.10614	96.56399	84.8965	627.0621	159.1133	165.2163	366.3189	100.5615	146.9121
13.6079	22.34868	26.85753	5.4609	5.609075	17.86546	7.469888	28.27458	2.843246
5.696427	14.31504	4.324479	6.536937	6.709	6.693374	3.775784	12.74779	0.936622
30.57535	57.22588	70.90723	32.58534	144.5499	97.26022	21.00117	27.03475	38.14645
22.66101	41.1235	47.27842	93.2821	26.73953	35.18227	16.08115	38.28431	15.24549
221.6289	264.7457	156.8706	256.1077	125.7611	85.87705	145.1923	340.8371	151.676
2.313279	10.71062	10.75876	6.540144	3.382318	28.70155	35.448	7.206033	7.617788
493.9789	255.8125	436.1173	115.057	223.6334	96.41381	340.5831	188.1935	193.661
28.28448	38.46723	25.79698	30.39334	32.2718	49.63359	16.06489	22.73017	34.34396
178.5126	49.16573	81.64702	110.6641	23.41013	45.07111	77.48681	135.5725	76.31998
220.5315	78.66472	137.5432	192.112	3540.955	547.6887	2690.327	126.0718	4310.492
18.1018	35.83686	12.90958	1.117557	20.02905	7.285341	0.071342	42.09915	2.84331
31.70795	16.94902	280.2421	24.99372	2.267796	19.64466	35.74746	48.21208	93.4979
227.2923	454.3961	291.1451	704.205	522.8657	351.5639	185.7731	200.978	245.1753
125.5059	118.0578	83.8137	128.0516	89.03058	157.2733	39.44759	80.73336	80.1263
35.10241	34.84052	77.36465	62.96388	109.0321	106.4535	113.1359	41.17751	145.9784
220.4584	148.4605	151.4866	123.7293	178.0155	172.6666	140.2386	185.3014	108.7463
504.2782	555.4432	291.1592	1296.682	540.7126	433.8257	264.4872	362.2106	524.7133
3.443823	48.27681	110.6191	4.375243	8.948516	40.12397	48.01227	52.40954	63.91685
12.46865	25.06351	12.90863	47.65259	24.45866	27.96621	12.3542	26.77399	16.21256
101.783	115.369	177.2441	68.39381	120.169	193.8499	223.713	96.27418	106.8435
42.93976	19.64845	26.86853	52.05386	27.82889	35.91325	40.55234	17.10376	38.165
14.7521	35.75241	8.626033	4.375721	8.948466	10.35944	18.53671	28.41851	4.751059
2.312263	3.534762	4.327813	117.1121	86.72324	50.12109	38.16787	11.48959	83.9759
47.53285	101.9623	60.18225	182.2594	194.6234	181.5727	68.9243	51.05969	108.7599
115.3717	92.9802	47.30153	7.629255	61.23947	107.5594	76.3425	49.68676	122.1001
23.79845	26.78947	40.84997	6.546085	31.19678	621.6128	89.78545	14.32279	43.86955
59.88414	20.53746	39.75292	60.74198	18.95245	30.25285	32.01471	21.3485	54.38905
15.86858	16.06817	16.13667	7.630819	13.38931	40.32496	57.63818	25.50817	13.34063
55.31039	11.5895	16.13588	16.3018	28.92834	60.98988	35.62897	26.9009	5.705058
88.22876	58.09752	95.63256	60.80181	51.2235	89.01878	216.3789	86.4079	237.5716
5890.613	1863.965	4216.625	3017.874	2547.816	2126.609	3110.214	3072.334	2489.047
6.838366	339.304	18.29456	51.02399	43.42408	5.413316	3.780464	203.1425	14.29068
659.1274	429.3209	292.2295	84.68057	1536.234	210.3084	222.6742	389.0396	104.9232
27.1901	49.15838	87.02694	39.09851	30.08562	66.75468	18.54598	152.6104	14.29062
130.0571	149.3504	123.5646	71.65482	90.15833	72.26785	136.5616	497.3906	69.62577
27.17269	11.58366	52.63575	22.8168	31.17866	60.73292	52.86549	28.40163	36.24518
10.22782	344.3883	56.96985	7.629283	23.41196	33.87674	354.1095	48.27239	234.6887
209.0816	170.878	78.44019	572.6502	290.2326	87.82844	35.75727	44.00565	326.352
36.20176	39.34278	40.83053	15.22589	17.84313	19.66999	19.75864	48.08714	19.06388
37.3521	34.84811	40.84467	129.0807	86.76329	139.5587	27.14251	32.67573	63.9161
1.17959	18.84893	2.176674	4.367753	24.35794	0	0.065789	11.2882	0.936786
0.035086	1.75172	0.020112	2.198585	5.574549	0	2.53183	0.107102	0
29.33737	16.98909	8.617975	16.28397	18.91114	28.63597	6.233642	4.405883	6.661532
32.81836	94.88929	53.71031	26.06999	16.73229	25.25767	18.53314	133.7712	34.33546
23.75632	13.38279	17.20705	76.91901	86.57483	74.82455	3.78398	5.827061	40.08499
211.417	129.6709	140.7461	245.227	169.1181	168.9499	104.6003	189.5347	106.8381

1162.267	211.0412	1146.259	3118.57	101.2931	1082.634	851.0261	186.8929	442.6543
2.310522	231.7865	2.176507	0.028269	53.43635	5.415258	0.076146	168.0712	1.890918
0.019767	0	1.082645	0.013109	0.017148	0	0.033535	0.056767	0
7.963322	7.114593	30.06381	5.460185	7.830279	35.43733	3.783242	4.411361	19.07512
2153.857	1739.676	1826.308	1693.953	1122.612	1192.642	2254.178	1574.354	1958.625
6.836269	0.85494	9.696059	10.88152	2.270311	51.66861	62.46116	10.05104	15.25207
30988.89	19883.2	39129.33	36822.09	28243.84	19868.14	44902.9	31571.76	34812.44
55.44168	78.69215	94.54345	36.92891	56.7748	81.02341	57.86827	70.81555	49.59405
134.5911	127.8673	236.3548	195.3489	136.8838	201.7036	247.2114	318.2184	248.9975
121.0239	188.7057	152.5712	94.44292	245.8822	287.8475	335.683	126.0273	182.2099
306.4418	304.0781	497.4037	651.0856	537.3692	926.8233	204.2351	266.0575	482.7358
37.36197	47.36331	538.2541	105.2977	40.09644	375.5413	4221.532	1404.6	4124.339
175.2882	79.55992	256.767	1209.735	199.1778	393.1175	97.24832	14.31173	435.0517
18.12317	16.06921	34.36868	7.630606	6.721564	21.59435	3.784096	47.89297	9.522482
35.08067	67.10242	22.58685	58.59364	41.18351	63.80589	22.21917	25.59773	31.47035
6.836338	16.96945	34.36118	18.46504	47.76936	12.87645	62.46427	18.47157	53.4632
74.64344	168.2179	106.35	150.8002	191.2701	89.10869	45.58007	48.22612	74.40682
50.93167	90.30773	78.44822	171.4514	127.961	224.8088	240.9451	70.8695	175.5451
35.09507	31.26643	51.58334	143.1833	27.8576	15.31044	28.37331	179.1792	44.82649
171.889	82.24506	716.4438	367.8449	115.7473	512.1855	242.2836	174.0771	244.2291
296.1056	109.9996	99.92466	107.4457	276.9375	142.3657	211.4318	68.03237	87.75901
18.09431	16.08918	1.102576	1.117597	10.04563	13.5461	7.459181	51.74726	2.843391
2644.581	1620.688	3732.094	2491.554	1853.567	1581.326	3309.356	2516.383	2299.204
100.6472	94.79103	51.59391	277.7189	174.6412	268.9825	75.08151	41.18628	103.983
20.40711	106.4515	48.36624	21.73935	26.74766	11.59618	66.45254	42.5747	116.4014
29.39798	47.45396	27.93505	29.30055	3.384016	18.47406	23.3999	28.30443	15.25037
456.7754	186.8988	492.0216	1099.162	441.6887	329.2199	614.8245	208.0693	498.9623
1139.747	566.1294	2676.096	945.2208	560.7869	2941.85	5093.765	1532.045	3454.536
361.8086	340.7739	170.8395	403.6771	663.0173	459.9585	180.8577	304.1668	248.0369
100.5742	28.58453	26.88465	75.95262	44.528	122.8192	23.4549	32.66738	48.64696
4.576721	56.43493	6.476179	4.376009	16.71804	1.70436	1.317114	45.07156	3.797024
172.9492	100.1619	153.6117	163.8399	122.3844	74.79097	86.13554	92.01781	109.7092
203.5003	219.157	102.0794	193.1538	230.2746	155.9461	54.205	103.3719	155.5028
41.87304	59.91048	30.10813	52.10712	67.87669	92.90224	27.14482	43.96691	53.41584
2.313459	4.430701	10.7605	41.1408	4.494106	19.22134	17.21673	5.809232	18.12841
11.35508	32.2024	27.93713	21.7206	30.04216	37.81814	18.50733	26.91434	22.88847
11.28715	9.843402	4.31931	16.23139	8.90332	10.56011	12.24761	1.566242	8.584159
46.31952	25.02562	18.28484	3.290458	24.49745	39.03887	33.20749	11.47382	46.76092
0.040197	2.640501	0.022939	0.026277	12.26016	0.477808	0.070286	22.5466	0
35.09104	54.54551	26.88505	125.8213	64.53371	96.05909	20.999	18.55876	41.96537
35.09035	63.50079	76.2677	34.75196	75.64406	55.03866	34.50647	34.07865	63.91831
36.2058	20.53378	17.2176	27.15461	37.8467	36.44623	152.0911	39.66689	71.5672
17.00213	21.43908	26.86986	27.14299	28.94127	21.56528	17.29093	12.88525	25.7502
63.35097	61.68694	181.5126	211.536	62.33456	61.79078	98.39493	28.4617	74.40493
32.77992	17.86103	23.64536	37.96404	47.78863	31.58189	33.18698	29.70731	16.20506
13.58713	6.222514	9.689206	11.9543	10.043	14.81569	19.65721	11.40785	20.03933
94.96212	95.71515	39.77414	98.7396	133.4564	32.66147	9.936232	259.5075	34.32816
27.17838	19.63672	15.07078	15.22737	30.07338	28.97169	20.99328	122.6546	25.74309
3.445612	0	17.21217	1.117083	1.15481	212.9205	78.46516	0.132169	42.93977
9.100725	39.32149	5.402007	2.202743	6.72241	2.944796	5.012737	11.49299	0.937462
6.837723	23.23702	7.550468	29.3033	24.49316	22.83217	51.53493	19.90134	75.42244
226.0785	48.25682	227.7221	155.178	79.02792	111.3406	174.6063	292.4802	210.8543
102.828	8.899222	113.8305	16.31304	1.153731	65.61275	44.32261	17.14477	55.32883
395.4275	61.6847	183.667	559.6272	92.35751	149.2674	110.6845	42.59156	85.85498
3.44386	9.793563	19.36793	6.54671	125.6467	1.097154	29.59724	53.81626	65.82614
75.7794	67.95115	189.0297	68.38642	35.64685	46.90617	48.04216	108.9083	28.60169
63.2321	26.81615	68.69734	328.1119	43.37011	24.68243	17.2901	21.32306	47.71416
0.035639	46.08182	5.382979	0.023372	9.98545	1.094036	0.061888	2.955682	0

239.755	5886.616	210.5958	982.0959	405.0231	36.36998	71.42355	1015.676	127.8199
1.180491	49.35264	1.102591	4.374139	13.36834	0	0.070276	4.40441	1.889693
92.75755	212.8818	198.7452	90.09873	144.6554	149.7222	148.8464	106.213	124.012
65.63319	83.1393	111.7545	238.7442	329.3009	349.8016	56.67034	182.5605	252.8156
13.60313	16.9743	23.63484	13.04618	11.16203	6.035294	20.92555	54.72653	14.29869
4.576734	8.903252	12.9163	3.290525	1.155102	1.704392	14.82634	7.238663	8.568209
45.26229	21.4228	81.64439	139.9321	44.53482	81.09397	43.11076	60.8873	58.18754
503.1763	545.5771	372.8099	2329.739	1612.039	804.6148	211.626	449.9429	857.6701
67.87032	120.7606	102.0602	167.076	116.8106	77.29893	19.77567	58.11618	69.63344
62.24264	206.5837	131.0938	77.08401	76.8162	173.1776	171.0219	181.1909	203.1944
29.40464	23.23558	24.72061	28.22169	17.83318	27.81744	12.38177	24.1165	10.4765
254.3789	164.5577	276.0799	298.3971	176.9141	285.4306	191.8699	75.13187	255.6856
2427.265	5.328979	246.0457	439.5204	175.8353	300.044	1296.104	41.18961	2101.788
9754.067	3303.105	9628.945	8232.102	6074.661	3740.202	4266.275	3434.543	2100.749
11.36245	13.37114	39.77279	22.82336	58.98586	63.08032	86.07007	10.07781	118.3179
4043.177	1041.971	2305.468	3679.81	6328.226	5974.389	1992.355	1005.881	2129.378
19.26776	82.35335	31.17126	3.290048	7.835645	17.80764	8.705529	112.664	7.612712
261.1677	246.8685	197.6796	458.9583	67.91345	113.1414	120.594	448.0751	123.0553
94.91432	42.91211	61.23413	5.461328	20.07041	71.23344	47.99496	66.44863	62.96748
7.966211	304.1481	104.2311	9.800846	561.6775	46.87704	320.876	93.50072	69.62575
50.9316	129.6798	300.7477	73.82056	76.80462	61.13269	50.51454	79.34481	61.04045
0.019874	0.878058	1.082819	0.013179	2.206799	0	0.033722	0.057099	0
18.13484	44.73018	9.699414	3.290265	2.269639	4.790613	22.2031	146.1462	38.15932
28.29727	40.24314	27.94985	16.30951	20.06437	14.7061	11.16266	42.44545	17.15559
5.708057	23.21917	13.99631	68.34412	51.17858	50.75125	23.44431	11.48902	31.47111
28.31463	119.0152	30.10719	29.33069	73.42811	38.25886	77.46493	69.32147	98.27793
180.9146	134.1402	400.6546	257.1684	201.3767	196.1972	245.9255	361.847	133.553
83.68199	41.10569	41.9244	58.62228	279.0746	210.8108	39.43877	24.22051	69.63472
7.969442	470.0303	20.44303	7.631906	45.64958	2.945759	0.075969	75.00391	0.937518
777.9085	663.6442	547.9168	520.9099	1147.052	1047.894	1095.747	571.5788	798.5139
403.6603	191.3682	169.7694	217.0652	1291.543	1011.073	479.634	182.632	228.9505
22706.81	9309.221	20956.41	31765.11	17918	9041.493	9673.775	8904.426	6712.517
24.89523	25.92381	31.15701	37.97052	27.8257	31.55865	7.473165	24.11915	20.97782
182.0102	271.0874	122.4792	136.7359	106.8284	112.5867	120.5506	77.92475	84.89552
3.445547	40.25806	11.84618	3.290393	8.946738	13.47428	3.784027	47.99203	9.52154
15.86555	25.03583	35.43784	49.86644	35.58053	28.47462	7.470889	10.0556	32.44198
3.441613	7.126221	6.468091	5.45421	7.816069	12.36336	9.8756	9.966711	15.26947
32.81468	23.22182	19.36388	35.82399	36.73106	19.67143	28.34446	26.98832	28.60919
66.75025	114.4773	67.70461	132.3909	94.59097	157.8898	82.45578	94.8527	57.22525
9.086574	9.807101	11.83542	3.29001	5.606234	10.41353	2.551755	23.9725	9.526169
41.87262	23.21258	120.2877	59.69749	28.96931	106.5781	78.70261	52.42521	125.0011
58.83261	129.7097	70.92116	113.9392	130.1528	136.2488	21.00537	103.27	70.58728
41.78257	62.72062	27.93122	15.21665	10.05484	24.73062	31.94873	29.68235	14.29651
677.224	339.8606	823.9516	562.1119	364.9461	384.9228	440.294	750.9372	279.5163
20.39147	18.74866	18.28776	140.8761	58.922	30.89229	28.32866	38.19854	109.7844
32.80476	25.01822	30.09285	69.40035	61.14424	54.57736	25.8794	50.83503	27.65735
179.8172	257.5739	456.5657	473.1174	126.8782	730.048	209.1355	256.115	168.8467
37.35689	8.899501	44.06925	3.288851	2.267851	36.38315	111.8527	62.3116	55.32299
92.76496	328.2749	108.5322	181.243	323.7412	81.54515	88.63858	109.0702	84.88977
39.58325	69.81268	45.12011	50.99407	31.17507	50.79349	47.95016	18.53596	37.2017
18.08168	27.78228	9.687809	10.87023	24.43661	16.08671	9.895857	4.402328	20.04166
117.4576	2.641395	35.46889	213.5486	12.28543	10.97919	8.706171	43.90199	27.65252
186.5631	190.511	302.922	156.2778	153.5533	196.2037	166.0518	169.7837	151.6826
2.310998	113.6259	8.62588	2.202655	16.73658	6.648879	19.7734	48.20508	47.68875
27.18855	76.91033	26.88776	21.73931	41.2038	100.9245	21.00382	79.24734	38.1446
59.96851	15.16001	29.03779	163.8418	37.8735	74.16825	205.264	62.37377	624.1035
7153.674	7069.555	18229.84	11094.83	13825.97	11427.58	16511.47	19685.57	8066.283
776.6931	1397.28	716.5315	446.0111	571.8477	290.1836	391.1124	370.6691	419.7674

17.00729	33.07743	22.58282	46.65679	55.60315	65.14596	19.75398	26.97705	44.84088
77.90788	46.52616	27.94601	61.81531	77.78828	36.50186	19.74843	15.70813	16.20215
9.067412	0.854607	6.466833	7.616287	5.598269	9.85302	3.773441	8.566933	6.665511
83.68759	49.15777	77.36378	11.97324	57.88745	256.71	184.3338	66.58575	61.04477
48.67379	25.89296	13.99669	264.7655	56.78844	19.64413	35.76426	45.44207	54.36015
284.9648	972.3174	426.5095	360.2971	257.0429	200.392	308.7494	712.807	427.3972
9.09434	20.55621	31.14486	79.0727	53.31115	41.6417	6.242051	24.07182	41.99833
176.4123	129.6574	180.4997	265.8679	198.059	236.4057	158.7091	159.9517	203.1999
258.8391	84.04628	149.3276	122.635	133.517	175.8364	188.1133	140.0524	149.7826
218.175	95.67656	119.2601	261.4802	149.0889	191.941	61.57668	130.1842	75.35279
37.36071	131.4375	2990.829	133.5119	257.0538	1330.652	3122.39	8316.052	3786.568
70.1483	127.8853	153.6307	72.73727	114.6206	88.38964	123.026	185.273	46.72816
32.82956	50.0725	19.36732	55.35169	56.74998	54.42853	35.73049	32.66304	20.97047
20.39388	49.20759	16.14193	8.717052	16.72945	2.938806	2.550864	21.34632	6.658748
93.84626	89.43792	104.2027	77.05922	124.5825	52.49704	41.89486	99.00604	25.73961
27.11486	39.41861	16.1281	19.54027	33.33487	19.15118	19.69531	14.24124	37.23096
391.2162	157.3787	155.8032	737.8711	668.611	477.8519	177.179	184.0375	247.0795
0.02278	0	0.013199	1.099911	0.019746	0	0.038815	0.066185	0
133.4304	166.3697	151.4783	92.26304	175.776	91.49886	93.52769	101.9425	39.09569
46.40696	490.4259	104.2168	33.67593	134.6163	44.41511	40.67602	238.7491	22.8768
14.73207	8.90523	40.78569	204.5071	37.78151	39.16882	14.81639	11.4504	8.56901
0.037092	11.64799	0.021224	0.0243	5.58844	2.959078	0.064551	1.56586	0
28.2813	13.37819	16.13873	49.88698	43.36676	52.77702	6.244754	12.88233	28.61583
35.07238	19.64004	16.14312	48.82974	71.15816	38.32768	63.88461	14.30933	132.6832
197.8775	4.432533	273.9367	752.9372	69.02584	251.9382	195.5659	38.37141	178.3956
148.0837	79.58288	170.7839	186.6049	187.9646	84.10896	62.78783	45.41709	62.95234
58.77245	87.72412	82.67899	52.08336	13.39668	51.39116	40.60958	7.247103	118.3558
104.0424	161.0113	62.3354	487.0476	250.2539	223.632	55.4267	45.42921	144.0596
90.42727	125.2828	37.62369	71.62213	95.65356	76.76676	9.936497	35.49745	60.09814
110.8342	241.5334	76.3013	262.5733	582.7375	339.5106	51.74557	60.98396	151.6871
346.0155	486.5579	643.5022	219.2378	348.2669	293.876	538.6727	588.434	251.8465
126.6662	118.9302	64.48818	279.949	123.5255	280.4997	104.6068	63.8204	118.2865
54.21861	29.50356	48.3236	14.13808	24.50029	28.41813	49.12664	17.10816	40.07331
7.944966	17.91925	6.467473	19.50061	22.19005	13.00316	4.99713	7.181313	11.44514
15.88352	422.2506	23.6665	3.286884	228.0758	46.2567	27.15498	682.4436	4.752763
456.6662	205.7139	87.04646	250.6664	122.4166	219.1048	174.6681	123.184	163.1301
72.32667	41.12677	52.64187	20.64993	23.40339	30.21958	82.30106	34.04923	33.37937
7.969704	37.55411	8.625742	11.9717	6.722995	24.64671	12.39132	22.76155	25.74595
3.445509	17.85429	5.402693	5.461604	2.269805	2.321701	28.3233	49.40503	13.339
116.4996	196.7631	94.5662	225.7181	195.8262	251.935	140.2606	169.8196	157.4041
9.085756	9.807853	16.1215	7.625598	5.60592	14.80182	11.12684	33.69374	6.661372
65.57561	34.85146	33.32641	52.09957	55.63938	86.13535	130.1647	19.96839	39.10315
13.60491	9.801044	21.49284	11.96394	8.942166	9.14344	7.468033	36.62735	15.25303
255.5608	216.4196	487.7229	502.4206	73.47891	572.6457	452.554	275.9228	359.6639
1.180307	27.7417	8.621597	0.026866	13.38145	0	0.072009	68.63981	0
36.20455	62.6322	62.29457	24.98572	64.50261	94.36616	57.77139	29.81241	36.2447
44.11917	34.85374	27.95685	137.7239	78.96529	58.17327	39.40713	55.18937	51.51287
265.7015	153.8131	171.906	179.0722	154.6789	162.6854	199.2686	285.7013	95.38486
264.6012	898.129	450.1239	247.4406	223.6595	655.6813	343.1375	69.49089	310.0521
12.48836	35.77068	56.91218	81.31317	37.83044	79.54658	12.38827	17.117	35.29573
10.21536	12.49633	3.253127	9.793182	28.89078	14.78735	9.909553	8.626553	12.39102
109.73	507.1145	134.3203	186.6865	2003.613	99.4856	248.5175	457.011	187.9235
0.04075	79.86796	6.474145	0.026628	30.00123	5.418177	1.317689	19.8149	0
97.17915	9.793592	12.92313	10.88806	111.187	14.07454	147.3157	34.07103	220.4837
221.5982	298.7805	265.3348	182.3152	219.1701	163.9697	183.2581	165.551	61.0389
4.571918	74.5908	11.82874	3.289148	3.382034	3.561469	0.069416	16.94725	4.752835
50.91673	3.536219	22.59149	61.87312	1.15319	9.12221	170.7742	1.565762	287.2517
3.439018	11.6315	4.32209	2.202932	0.032955	4.20139	1.316266	2.980611	1.89025

89.29208	66.18476	50.50606	41.25937	32.30256	68.70189	55.37374	39.72213	49.60046
143.6341	292.4834	228.836	91.18874	140.2208	124.2744	80.03307	339.4109	103.9713
32.79543	33.98568	21.50505	45.55856	36.71167	17.83026	17.29066	24.13415	15.24848
74.56183	66.22194	41.90336	53.16487	54.50233	30.86016	32.0242	21.3576	41.97381
6.825351	10.71411	16.11037	4.37283	4.492492	12.96525	3.777842	41.83184	2.843784
63.29769	115.4849	80.54264	62.92826	31.1827	38.92357	28.35486	29.82302	29.56154
194.4046	56.31374	145.0182	157.3284	133.4983	110.1531	48.04735	84.95192	63.90589
96.13202	80.46994	72.00124	107.4449	222.4574	137.409	150.0198	92.03528	77.26296
41.88138	73.32369	61.25469	105.2586	121.2526	128.2046	86.11027	55.2879	48.64019
22.66951	236.1727	111.7424	10.88702	159.097	26.44947	105.8155	151.3627	83.94032
141.3021	222.8048	74.14338	137.8001	129.0434	98.38473	50.50101	76.46963	82.99252
1070.743	830.9016	1083.98	1547.444	1001.335	749.439	702.2934	903.9554	709.7822
0.039798	29.58784	1.102588	0.026024	8.930558	6.054716	1.317633	7.203929	0.936604
915.8432	1048.271	516.7652	2385.126	1248.297	1347.517	645.7164	513.6077	807.0981
0.028804	1.765458	0.016602	0.018981	0.024925	0	0.049513	0.085697	0
36.20816	37.54575	38.68823	100.8565	14.50936	40.79225	29.58066	81.85699	49.60769
476.0334	881.0364	420.0727	555.6215	967.9065	1005.303	696.0765	432.947	242.3046
100.6398	81.37186	131.0572	160.5793	66.78598	92.78967	61.56074	83.53502	86.80851
24.91533	43.81911	23.65895	19.5642	34.51257	81.28197	33.25503	21.36185	43.88176
10.23143	8.899245	25.80893	9.802827	5.609879	109.835	176.6796	45.32665	32.42372
0.029623	1.763301	0.017061	0.019509	1.145061	0	0.050982	12.11032	0
9.08874	9.805108	7.547151	6.543418	14.48546	11.03035	7.46255	26.78588	15.25649
24.92353	24.10933	53.72502	61.85891	26.74292	103.5357	8.706345	43.94399	55.32821
6.838529	24.12617	24.72475	9.801014	11.17008	17.82766	18.51808	26.94775	24.79498
140.1349	166.4458	109.5633	105.2473	61.21407	109.6344	29.60534	83.46517	89.67996
36.23699	93.88345	52.67116	18.48437	16.73646	55.55173	97.22229	127.3824	72.48931
1705.012	1313.014	2302.203	2494.771	1999.272	1019.317	1063.84	1324.021	1435.808
13.60473	19.66299	15.05887	22.79314	36.67329	41.6757	6.240974	7.233604	39.13591
9.098935	28.5764	26.88961	95.51418	71.24095	12.83633	105.8044	161.2088	105.8889
23.76636	78.83955	8.624348	6.54636	10.05706	1.705258	1.316753	33.9299	4.7509
9.095433	10.69462	30.07664	8.714517	8.944082	25.35133	7.470793	33.88534	6.659413
43.98277	2.640171	32.19954	13.04289	17.81316	13.52833	19.68785	25.40074	23.85499
41.87118	45.58911	39.77147	79.2128	83.4318	96.02695	39.42344	41.14046	65.82437
186.5656	155.6135	248.1516	93.35438	37.87628	351.7885	167.2844	469.1555	132.5987
50.91017	46.48197	51.58372	107.4074	95.66224	115.2674	39.42731	60.88806	67.73151
357.2957	289.7757	232.0703	615.2559	608.5307	482.2226	179.6334	176.9598	280.4744
47.51603	55.43928	33.32813	32.58399	42.30756	46.33341	36.96478	49.59099	40.05614

TCGA-HT	TCGA-DU	TCGA-P5-	TCGA-HT	TCGA-CS-	TCGA-CS-	TCGA-FG-	TCGA-FG-	TCGA-QH
262.8801	156.831	180.3259	73.20982	131.1264	168.1128	184.5838	133.2565	256.6915
19.10745	45.61426	41.24917	59.53569	30.52882	26.87921	17.54047	33.31247	15.78487
133.1636	76.11944	76.72364	149.0347	262.0755	70.09291	100.0306	89.78437	101.3555
2836.125	2003.713	3577.677	2437.571	5857.98	1453.576	2836.87	3369.971	2863.225
47.81214	238.3834	121.8094	80.54873	44.13241	33.92803	112.4068	166.6517	105.9987
0.828063	2.950505	0	0.753021	0.030702	1.217805	0.00751	0.632213	0
31.3075	13.34376	13.41229	19.47852	32.79559	9.409373	9.293914	1.34313	19.50526
232.4017	140.3829	139.0744	136.7591	189.8637	336.0817	180.4594	163.7203	186.923
25191.2	6146.431	8267.007	6535.227	13428.45	19667.3	7742.355	8219.832	11722.6
29.54218	64.21524	84.41306	49.62765	65.55937	49.0678	98.98595	100.0653	66.02212
16.51362	28.43722	31.66623	21.16442	5.702832	4.737554	19.59525	30.50799	33.50578
1392.413	1786.55	1667.036	1827.876	1680.635	1258.539	1631.37	2434.266	1131.713
120.0641	144.104	94.94428	69.94767	87.0726	74.78029	123.7524	85.38697	183.1953
0	0.658405	0	6.547598	0.032688	0.041762	0.007972	0.627936	0
89.61769	36.49431	132.3703	94.42292	61.07197	70.09078	175.2917	181.2435	182.3011
4.297022	8.856127	17.25065	8.878091	12.48409	3.571061	15.47968	15.10988	10.20035
2420.157	1386.812	3191.141	2403.406	8918.461	1674.208	2104.709	2162.342	2232.738
28.68143	34.29951	43.15803	31.70606	12.48672	11.74471	44.34491	73.26194	50.21541
60.00696	19.30842	18.20643	143.3299	20.40059	79.4238	39.19836	16.5423	59.49319
171.383	282.9574	336.6517	551.1571	1566.391	140.171	129.9465	286.7628	214.783
1551.697	576.4838	1011.914	1116.222	1869.35	1695.107	1027.089	1287.32	1200.536
19.09714	5.121437	9.574315	1.557219	19.26241	5.907795	81.4501	9.300049	70.69687
470.2551	9.603153	45.06472	97.66967	94.96355	578.5124	62.91414	36.10668	319.986
406.4319	148.534	792.3193	152.1851	183.1378	882.3605	118.6016	1412.75	454.7548
129.6958	40.98328	93.03642	126.2315	157.0462	168.0271	82.50106	47.71841	143.2313
14.74883	18.59618	60.45144	34.19334	10.22393	50.14398	53.60973	50.80883	33.47558
182.7343	241.991	142.9049	142.4318	131.1453	179.8166	141.2841	145.5396	131.0993
333.2521	1265.199	674.2811	672.4545	276.9657	460.0473	1068.336	1702.518	460.2867
68.83312	28.33541	38.36662	37.44898	37.30035	35.03433	34.03243	42.03651	41.85146
39.11574	37.99887	107.433	51.24059	42.99213	65.40907	136.1066	166.8178	79.96863
19.09277	73.96453	33.55796	20.27964	24.91407	1.228867	33.00866	42.67653	31.59446
73.98688	72.47006	128.5621	18.65018	41.84936	103.8217	33.00852	78.28463	43.69133
5.17025	2.883379	13.42003	11.34948	2.312741	3.570056	21.65294	14.4414	22.32564
337.6051	1866.576	1086.736	412.7141	241.9284	246.4099	920.8735	765.6668	511.436
91.32689	336.166	204.2976	151.389	114.1983	78.28826	172.2184	231.8088	109.7068
7749.498	8720.779	31040.41	2172.913	15865.3	3653.134	11089.68	33568.65	12366.08
2.557763	20.12058	3.819706	29.32069	9.091515	10.56766	11.35423	10.76706	12.06531
46.09	85.91669	81.53169	64.30086	18.13855	57.23366	71.15469	161.0892	100.4507
17.35399	58.27434	5.737787	79.04745	27.16629	17.57758	41.2532	10.74924	17.64115
15.61426	32.81266	9.574443	92.97367	28.28891	12.91001	31.97393	15.83075	20.43427
202.7893	63.38518	4.780059	131.8667	37.35388	285.9354	29.9187	3.517878	104.1334
12.1284	82.98108	83.46047	13.76268	15.87697	12.91282	72.17949	50.68187	47.41653
13.86881	34.2759	14.37013	25.98427	317.1406	4.738474	33.00884	6.403501	13.91907
118.3446	145.6559	261.8853	148.1962	136.7556	196.0707	107.2502	188.4428	147.8645
171.4019	316.6742	400.9409	166.849	258.8387	124.9817	200.0624	254.2396	238.0501
8.654638	16.37883	50.8733	37.53497	13.59821	10.56324	10.32271	22.44658	12.06763
38.2532	117.3996	30.67901	36.58088	38.46597	30.41287	39.19492	70.99935	34.38467
11779.07	1283.72	2477.506	1988.128	3137.58	16299.83	2461.513	4994.174	5972.928
1395.156	161.2111	1159.659	183.1101	119.8629	1324.918	362.9954	101.2969	1078.756
5.170256	49.61482	18.22233	43.33462	4.572844	19.84477	18.5628	32.74434	11.1403
6.914203	10.38232	36.47588	10.53096	10.21039	8.229267	26.8029	11.5191	16.73183
207.0971	105.2088	217.7239	419.3755	183.1294	99.30099	323.7963	264.4046	225.0325
23.44428	88.1564	138.1461	43.91236	30.56469	22.25045	62.90795	89.86327	90.21191
3037.116	4902.227	13889.66	5300.986	9131.227	1373.035	5872.761	11847.21	5198.254
17.38143	4.376097	35.50412	17.06219	22.60982	25.67565	26.80877	41.45055	22.31494
37.38453	64.97634	106.4881	52.08099	29.43099	31.5766	78.36716	57.93236	22.29086
24.36216	26.89486	36.46107	21.96372	21.48942	15.22109	18.56763	46.53907	29.76559

3403.493	3563.848	7455.567	6469.328	4634.045	1136.037	5203.507	13733.62	6884.213
63.47177	277.0542	147.6968	166.0305	48.65738	36.26421	170.1598	114.349	108.773
12.13335	17.84356	41.24826	18.66873	14.73973	15.23607	45.36736	44.2358	24.16249
110.6262	23.06069	14.37051	48.83314	144.4949	158.511	10.3254	8.575026	68.82662
0	5.868557	1.902155	16.2264	10.22255	0.053583	9.29328	0.62312	3.690585
100.032	260.674	209.0946	316.7648	156.0031	73.61907	348.5371	276.7684	265.9662
8.65421	12.62044	29.74086	12.15498	14.72605	3.571091	26.80914	29.74744	11.13602
50.51491	23.84218	77.73125	16.22028	16.99638	69.92084	30.93942	27.48328	33.47382
153.2409	90.37216	88.24282	107.5002	31.6972	409.2212	50.53829	101.4484	131.1458
74.79006	151.5566	76.71696	133.4746	115.3252	51.4406	105.1941	74.50537	79.01625
90.03875	14.14171	11.49906	37.58066	43.9542	25.6496	18.56332	28.33944	11.13972
6.909187	35.9036	26.85551	61.24462	2.312728	4.739196	62.87184	75.73923	13.927
207.9741	283.0809	380.8049	186.4107	142.4483	226.5022	411.4372	873.549	242.7086
17.35663	70.31118	80.59475	29.26537	12.48573	14.07627	35.06623	50.73189	26.01818
129.6856	48.45588	131.4115	71.60269	250.7711	168.0453	78.3783	152.9378	151.5987
19.09991	35.06463	66.19781	42.33027	19.2596	10.57674	60.83269	54.37775	29.74194
281.9669	168.7239	311.7333	234.4638	299.5021	305.86	221.7144	227.4374	264.0979
77.545	26.82747	80.60147	30.08992	118.4654	49.01039	8.262809	24.55699	50.22744
179.238	88.77095	50.8178	176.625	184.262	195.0065	153.6603	46.96658	98.54399
280.2396	105.9632	244.5877	374.6056	169.5622	340.8437	239.2409	427.5918	318.9834
1284.467	1652.77	1984.514	408.6301	758.4406	985.3993	1623.129	1572.694	976.3996
22.57524	19.31411	46.99027	20.27868	35.07839	25.74764	71.15267	180.0451	50.20303
85.23127	226.261	794.2543	174.9987	198.9488	26.92365	266.0537	564.561	241.7736
42.61265	112.9299	90.17419	19.46495	14.74797	10.57881	61.87356	61.56719	46.48284
26.05994	55.98927	37.39597	49.63397	112.9458	37.40588	12.38786	38.31945	31.59453
1770.107	641.4476	754.8474	1933.689	1409.42	1005.238	1904.642	2179.87	1950.997
75.6856	201.8357	50.82192	28.41819	37.34893	51.4247	25.79351	30.31159	33.44895
89.59979	106.7463	177.4502	247.6004	183.0795	87.60851	72.19543	214.5156	132.0423
161.8138	329.3004	181.2658	490.1267	324.4112	74.79204	184.5991	192.5892	363.5869
41.7225	145.6965	8.61542	151.4878	24.92087	31.59099	24.76241	60.76851	28.79824
111.3518	211.3592	211.9753	60.98549	172.9438	28.09211	114.474	83.20429	122.7303
31.29331	86.75825	124.7366	45.57332	24.90924	17.57801	39.19188	46.33565	40.90503
966.896	220.9642	465.1853	506.3799	142.4675	1521.035	1822.103	896.9043	776.4956
1164.352	2170.329	2127.421	1676.333	1717.994	986.5813	1508.671	2492.012	1646.881
0	0	0	1.587409	6.692117	0.035372	0.006823	0.640145	0.910067
33.03741	73.26923	38.35896	23.54685	12.48699	42.05245	55.68404	108.9084	31.59896
90.52369	51.47551	37.39394	244.6108	2.310216	135.3237	160.8406	250.4792	104.1709
367.1972	1454.995	1941.388	485.1867	823.9541	342.1355	499.1159	764.2315	656.5133
70.46328	81.37169	111.2661	111.5558	68.97316	86.41054	109.3065	166.7617	86.47575
39.1172	104.6065	135.262	32.49691	31.69613	35.08487	67.03325	86.21097	38.10268
99.2498	100.1608	64.26127	95.30815	128.7602	60.72556	44.34992	56.46561	59.5073
194.2086	2.888728	193.7956	44.72203	49.76642	335.7792	44.35179	68.78297	116.261
250.7173	274.2976	98.78547	83.80917	38.48249	232.2375	121.6859	48.42734	246.474
106.988	312.9176	137.1451	160.3281	152.6283	105.1414	512.4989	211.4602	215.7252
63.74472	5.874416	21.1012	9.708698	6.830969	120.7131	18.56465	25.39243	60.55228
57.47704	7.361178	178.5291	2.368075	41.81698	181.6031	20.63405	15.83537	68.85238
11.2659	16.35907	30.69462	9.698426	7.963857	12.89918	15.47748	18.76968	26.96387
19.97305	46.32405	58.52133	42.33787	19.25798	23.39751	37.1262	41.28854	31.6055
13.88698	10.36657	18.21647	14.60478	9.090182	9.40104	36.08234	34.12027	28.83482
93.15403	49.24356	42.19276	34.95078	18.13775	184.2142	139.1866	166.2309	100.4581
31.34917	13.36645	11.49618	12.15188	28.24765	58.20396	38.14362	10.76956	24.17474
1763.115	1664.69	2418.056	1020.088	1705.553	756.5923	2403.751	2296.429	2459.661
28.67739	11.09488	61.38915	34.14636	29.4264	29.23874	33.00731	67.41191	48.34899
530.8351	395.0137	210.9991	217.3071	663.4513	765.8426	284.6259	238.2245	372.8791
485.6111	300.9246	508.3615	471.4141	449.8338	422.6306	292.8714	350.5785	491.9367
1.688775	42.80058	9.578693	7.259361	4.573117	1.231407	3.106346	2.784244	3.6911
8.656812	32.96409	32.62752	18.7092	7.958987	3.570638	14.44366	29.0488	17.66002
124.446	183.8215	199.5259	109.897	160.465	73.59683	112.4043	142.0158	132.0525

34.83009	1.394674	0.943869	43.22242	67.66998	30.34812	24.75216	5.680328	28.82765
19.96357	29.03932	173.6677	36.58429	21.52587	43.23452	74.24386	59.38553	45.55198
46.9437	141.1766	281.0771	114.785	118.6815	18.75202	147.4594	199.3424	35.30802
88.70962	597.5949	1453.238	95.99232	604.6181	29.25759	278.4333	689.8299	297.5646
251.5999	85.07909	113.177	99.29587	213.5511	224.0607	131.9952	51.32959	190.6591
37.36928	109.0357	146.762	164.5321	98.34229	32.75799	63.94401	118.818	53.91231
280.2134	86.52866	302.1362	40.62733	171.8349	746.9715	427.9428	604.3948	276.1828
17.35338	70.25849	31.64102	24.36057	13.61702	10.57827	81.45385	101.6103	41.83506
26.09651	11.10903	8.616246	17.86353	11.34995	41.97665	50.51222	60.3574	31.6207
8.647953	68.09108	29.72612	12.13767	13.61373	11.74235	21.66501	28.19237	11.13046
105.2518	343.6129	869.0878	258.9039	280.2931	164.6567	166.033	242.6651	318.976
99.1623	298.8039	193.7467	104.1491	94.98797	99.2952	314.5099	469.6747	144.1209
601.3606	404.7531	489.1717	580.52	1104.118	638.5777	335.1515	320.1207	544.9388
9075.961	2880.523	4329.659	4826.39	3693.655	10435.88	4293.969	4388.506	5067.162
17.37475	18.61908	49.90403	8.884423	12.47547	9.402674	10.32346	23.15438	25.10454
45.29194	23.10283	64.29615	17.04134	81.21457	41.99265	10.3243	52.28936	43.72473
33.0271	139.1367	28.76031	65.94419	84.74303	39.73998	54.65847	192.4144	44.62017
0	1.444333	0	0	0.0259	0.032963	0.006384	0	0
165.3639	0.652363	7.658704	18.71772	31.58958	187.8014	3.106344	0.621732	54.03427
0	24.58216	0.94505	4.044212	0.03327	2.381973	2.071867	0.626857	0
10.40201	27.68722	25.90346	8.891172	10.21371	17.53321	17.53453	24.65951	14.86456
57.38916	187.5308	93.98899	56.10888	63.33853	33.92772	123.7478	132.5606	67.86142
14.74071	50.02097	33.55999	30.06885	17.00578	19.91111	45.37734	63.05253	37.18129
438.582	842.5293	635.9271	521.0498	795.6562	399.3158	464.0504	557.0632	437.981
67.8346	248.0924	54.65653	73.21401	152.58	25.75706	74.2574	87.57853	92.97462
6.91983	13.42094	25.9182	12.1866	4.570158	4.732595	4.136894	12.2777	14.87697
1727.517	103.7018	1163.468	422.4817	652.1849	1448.788	1422.033	1856.132	1843.154
56.51274	119.4307	367.3907	65.05941	140.1747	39.76652	178.4013	226.0562	158.0783
18.21909	194.3021	20.12474	287.6181	68.98237	17.58447	30.94951	32.48337	13.9188
9.5248	29.15018	22.05654	12.15184	9.090666	30.33371	20.62859	24.62051	8.342319
97.41442	519.9302	405.7338	364.7643	185.3975	101.639	243.3716	584.7875	108.773
319.4147	247.2109	271.4469	217.3688	350.3287	339.682	172.2187	265.8817	289.217
153.1653	118.7101	183.2068	144.916	171.7835	219.4257	134.0602	164.4607	173.9038
33.89212	87.38996	342.5374	135.2442	111.8598	51.41225	44.35167	78.22425	47.40608
2.557964	34.37205	24.93239	23.57909	19.24716	5.906975	28.87578	27.50344	12.06333
8.64705	53.01043	23.96432	73.31246	32.81415	14.07958	53.62475	27.43579	21.36163
104.4207	67.1452	91.11428	78.11835	81.40439	65.42712	117.5579	204.4581	127.4048
4.300686	5.132498	10.54177	5.63115	21.44382	4.733431	6.198233	2.785773	10.21287
0	12.70102	0	0.744273	0.03864	0.049534	0.009336	0	0.903455
26.92907	94.17014	79.61466	65.12369	56.53004	35.07806	41.25702	89.88738	43.68867
75.80294	32.07772	49.88353	23.55995	82.39802	55.99015	61.85994	36.92769	36.26175
46.10187	11.09441	17.24827	48.00907	21.52465	32.73924	20.63641	6.403063	32.52631
62.60366	492.4241	142.9049	131.0279	26.05179	91.12587	141.2841	299.9774	95.75657
46.08147	109.0759	20.12501	56.94401	14.74923	17.584	100.0255	25.96522	27.86925
635.3038	377.8543	372.1467	266.1719	377.5274	374.7943	984.7848	389.6849	685.3707
108.0138	80.75249	54.67209	37.41125	82.4568	143.3695	25.79075	28.16578	65.10437
43.50335	22.32001	30.68327	34.97405	37.31857	39.71513	37.12864	12.2007	39.04742
72.23336	117.3915	75.77621	25.98249	102.8049	44.4057	59.81364	99.33404	56.71576
19.96656	95.00375	76.74626	19.46885	28.29613	9.410969	17.54293	25.26181	37.18199
37.36705	85.82732	244.6156	70.77599	88.18848	18.75201	49.5101	93.39415	52.97968
38.25131	121.8767	127.5941	58.59519	49.75847	36.24542	55.69047	60.09364	57.6453
200.9918	91.00837	1078.179	187.2024	592.1679	106.309	395.9815	1145.896	320.8207
99.24687	88.17156	42.19194	65.12054	82.49348	201.711	63.93767	118.2222	146.0579
19.10367	94.40308	73.8848	11.32351	35.04727	9.40887	12.38682	34.75006	33.4701
203.5856	708.0372	163.0402	282.4499	260.0044	193.8704	274.3137	197.6523	229.6637
646.6113	176.1519	123.7142	1364.777	153.7682	344.4503	278.4377	810.7205	471.4632
14.74032	121.2204	23.0049	39.03899	15.87678	8.243545	41.25463	44.87073	17.64039
192.3519	107.4942	154.4256	184.8465	222.6101	182.1013	156.7436	192.0262	172.9731

213.2873	70.13037	90.15348	47.15095	230.4777	197.2245	71.16253	99.20551	101.3525
26.06515	34.29371	60.43112	48.83808	26.03808	36.22863	52.59227	66.6975	53.00506
76.52507	256.0907	32.59442	27.60485	61.09003	168.1831	23.73061	10.76089	39.027
154.8721	228.5236	104.534	131.8383	313.0485	120.3055	238.2106	178.879	134.8181
15.60915	296.4313	60.42159	30.87059	21.52792	21.08337	90.74176	52.82591	52.9914
33.92673	29.83109	34.52618	21.92892	21.51283	21.0647	45.36995	42.02877	32.53863
123.577	145.6734	116.0573	82.18921	47.51916	66.59631	201.0699	146.3748	93.91096
11.25944	46.30779	12.45245	8.062718	15.8734	16.40822	37.12767	31.81787	8.339625
52.22482	41.8022	70.99395	76.6226	32.80429	35.05434	43.31285	47.81328	40.90984
2390.803	1079.259	709.7764	843.4808	383.1979	2676.677	671.3233	902.6441	1099.189
33.06145	28.34219	72.93004	42.36009	71.10391	19.89557	19.60221	42.7778	36.26707
50.43377	87.36219	120.8621	108.2998	54.28977	38.58903	78.37647	245.9398	41.82151
160.9787	177.7304	194.7095	342.0605	361.5893	142.4654	130.9713	101.3299	189.7022
289.888	46.20061	243.6464	77.28546	161.6202	199.6004	125.812	139.7933	205.5314
4.304167	8.157805	13.42952	1.554966	9.066224	8.209804	21.63967	12.30745	10.21985
486.451	819.3616	534.25	455.0856	466.8124	399.3175	650.6933	521.5505	778.3617
30.45453	19.35292	40.29286	10.51222	7.964738	51.28988	35.05898	59.59402	31.61693
1611.766	316.5581	1071.387	644.7725	882.733	2149.164	1263.229	1004.038	1156.833
17.35208	901.0712	3.821767	51.2112	163.929	7.068241	223.7804	112.1735	14.85059
107.9197	102.3242	255.1929	37.38097	469.7261	18.75161	85.59369	112.3122	57.63572
110.5325	88.85499	56.5789	187.3935	184.1389	126.0534	62.91174	25.96349	81.82435
168.7818	195.5914	859.4525	158.6932	471.2899	93.47008	100.0409	465.1176	135.7411
34.76731	79.18576	23.00363	25.167	48.62908	11.74647	35.07141	20.89548	49.27095
244.5285	207.5775	191.8236	458.4732	868.9102	279.0273	288.7386	265.1258	241.7733
2168.715	1711.808	1641.131	632.5386	4476.649	4275.009	1339.547	1048.173	1927.75
23.44321	69.42569	92.08261	34.94177	33.95514	19.91774	59.81651	134.8759	35.31184
13.04155	16.44563	21.11321	12.18836	5.697005	5.895392	8.258371	10.0829	13.01225
277.5897	387.6319	517.963	469.0074	315.3485	275.5514	238.2171	661.5917	411.965
118.2986	222.4745	229.2263	304.4539	142.4644	19.91445	393.9265	929.6603	264.078
784.0719	1370.527	1033.018	1437.06	1054.517	569.7809	1038.431	3419.819	829.4814
17.35312	100.2412	46.03477	27.62176	23.78129	14.07917	62.90098	54.32595	67.89595
17.36378	20.84461	35.48936	26.83754	10.2241	21.05829	44.33595	55.90851	11.13162
9.517333	62.69559	38.35286	130.3587	28.30669	11.74658	91.77463	29.59543	26.0099
82.61685	94.73822	92.06166	56.09762	37.35219	70.1216	78.3858	20.17493	65.06426
0.823792	41.74194	2.861639	241.3027	62.18646	0.055384	77.34255	36.84849	4.621278
6.038863	21.63196	17.2562	4.807899	15.85538	3.57122	8.261671	15.14049	2.761067
1249.727	854.3916	916.9616	523.4587	1907.714	2512.122	1073.486	930.1668	832.2799
87.02095	139.7691	103.5939	118.9127	92.67814	74.73981	78.37523	99.99014	68.80241
7.785069	19.40522	11.49843	22.80857	3.443744	15.20755	18.56427	43.70481	26.05037
74.7998	380.4931	323.2791	104.9901	214.6993	53.76879	136.1218	112.9631	97.62568
717.5741	55.91787	124.6872	131.8834	277.9394	709.207	48.47939	144.1587	226.0035
15.62123	46.3762	18.21205	12.95824	23.76123	8.240878	16.50939	21.66682	20.44018
80.01634	306.3416	187.9963	124.5252	92.72244	131.9588	185.6182	198.5044	103.2008
13.00248	138.6939	31.64581	11.32263	11.35524	19.90154	40.21785	86.45458	22.29744
9.519297	60.61168	12.45319	73.41406	15.86964	9.4086	23.72568	42.03675	12.99193
10.38733	17.07061	12.4516	163.0301	99.42353	0.055214	9.294235	1.346264	6.480102
1.690312	11.21718	2.862399	13.8853	7.93092	1.230251	4.135153	2.066289	1.832876
100.9205	141.1457	188.9639	96.02701	94.97231	80.60633	191.7983	279.8289	135.7647
942.5221	74.57454	144.8157	2262.155	44.134	785.6914	948.7051	1049.079	455.6466
24.31071	2.892119	9.574742	7.252337	15.87919	10.57699	6.1997	6.409342	10.20003
5.171166	5.12921	2.860931	3.180941	1.179932	1.23145	5.168115	2.061265	2.761331
82.64221	164.3616	160.188	122.1183	57.68794	119.0916	187.6692	236.3511	118.0962
0	0	0	0	0.033582	0.042925	0.008178	0	0
53.93759	68.71456	129.5183	47.99937	33.94866	54.89299	35.07086	81.90553	59.50944
26.0551	181.7302	75.77087	34.12665	38.47305	40.9169	66.00236	84.03165	36.24205
481.2244	376.3386	614.8217	543.8412	340.2447	326.9487	946.6413	760.6736	782.0771
81.74787	555.7852	262.8039	250.7285	49.78762	98.13794	378.4523	644.2115	122.7224
221.8843	677.6171	264.7231	264.5776	169.5783	174.0066	323.8011	302.7891	403.5991

1924.935	4782.761	12938.19	2373.216	3992.057	1244.611	5174.628	7642.637	3042.684
471.1092	2.891562	107.4208	17.83128	124.3322	578.54	22.70018	12.19805	339.5183
91.31931	279.9894	82.47006	660.3281	133.4244	106.3136	586.7567	265.0466	157.128
11.26283	41.1136	33.56999	23.5716	12.48148	21.05712	39.18287	22.39299	47.44642
1260.969	1494.421	1068.497	1332.762	2349.734	1118.484	1402.452	1419.099	1255.384
113.9634	324.9905	152.499	137.55	79.16702	135.4682	128.9097	181.8015	99.47804
13.87411	35.07913	10.53408	51.33164	39.56004	10.57559	22.69548	15.10979	19.50571
65.21925	114.9568	241.7223	82.16832	54.30445	57.27324	124.7827	144.127	138.549
10.40047	18.641	0.943758	9.706458	14.72266	10.56139	3.106416	0.622054	19.52184
2.559862	14.83028	0.944326	21.09516	14.74787	0.055028	7.231702	4.233413	5.550303
100.9345	82.84628	209.1225	19.4595	219.1778	176.226	36.10516	94.13252	139.4975
121.8011	249.4984	342.4456	57.72892	109.6723	163.4679	61.88544	87.55654	106.9199
0.821811	25.38306	2.86075	32.59607	10.21956	5.905822	28.87227	15.86435	7.411409
6.924065	8.907525	5.741465	16.31006	3.440577	3.566115	12.37638	6.434192	9.285254
56.54337	127.1028	82.49073	131.1913	45.24468	42.07806	45.38168	73.88559	66.01814
15.61112	52.26314	45.07453	56.1747	49.74308	15.24617	25.79105	18.72579	40.90284
257.5828	144.0577	60.40954	361.5243	611.341	178.6685	182.5326	153.4896	153.4154
3.429138	31.55674	4.780266	9.723773	2.312316	9.383169	31.9438	32.82299	7.416968
275.0117	101.4782	406.7073	175.8235	247.5219	256.8384	180.4679	216.5777	334.7932
13.8766	25.34379	50.84922	14.58863	10.22425	15.23546	27.84737	35.4965	35.33688
107.0716	39.49895	119.9103	76.52266	111.8571	98.04317	51.56842	63.70539	110.68
4943.664	178.3858	171.6722	319.0818	1255.644	7430.782	220.6927	39.00602	2544.406
1.688794	37.50228	1.901989	40.03047	5.702246	8.231229	3.10637	3.508348	0
209.8912	27.53197	62.33782	31.68139	126.5384	66.57264	167.0319	68.7808	252.1289
351.5797	373.4326	597.5811	324.0257	339.0779	150.6659	257.8092	364.3856	332.9092
53.90783	86.57512	117.0146	72.40563	72.37427	61.93303	148.4915	146.356	140.4222
49.55924	101.5581	287.8068	56.11861	22.66069	122.5698	77.34725	213.9223	137.6428
107.0034	204.6534	163.0537	135.1151	85.94373	91.1191	218.6131	261.5999	162.7299
212.2907	1024.847	455.5965	162.7554	221.5775	223.0519	355.7762	552.7246	296.6235
68.74751	11.84074	34.51654	83.07032	19.2678	71.2148	30.94731	26.69935	71.605
13.02438	17.90583	10.53881	8.076478	9.08456	5.901937	29.89364	25.40891	14.86677
126.177	295.2423	171.6973	129.4423	46.39195	99.26947	162.9276	427.9111	120.8832
51.39511	2.883372	38.37012	32.56013	37.28967	58.28127	23.72433	53.00017	35.33871
11.2582	24.55817	22.04578	27.62229	9.097364	7.075737	10.32539	18.00145	5.550132
67.03567	69.51787	265.8409	14.57881	72.29884	68.84271	15.4808	37.61501	69.7614
36.49938	240.0072	98.79157	87.90667	59.94069	42.09165	100.029	97.05372	37.16951
81.75088	136.595	19.1661	35.74235	82.56047	241.6745	95.91475	108.5622	86.45493
50.43457	49.96012	68.09246	49.60607	21.52996	4.736904	127.862	405.7779	81.82615
71.42947	23.82624	87.31863	30.08663	47.45985	61.81886	36.09588	32.55315	97.717
20.85857	17.10599	23.97305	22.76455	6.835	11.73579	29.90563	34.07105	33.48218
12.13759	40.40122	102.719	12.96315	30.51333	4.739409	35.05685	54.51845	15.78807
486.7102	59.65087	31.63473	175.8757	237.2992	521.5165	110.3463	67.9959	151.5754
3105.899	2851.405	5117.142	3165.447	5278.207	3218.764	2197.524	3338.068	3203.576
6.037936	84.40314	7.656091	47.98578	5.70478	4.737672	18.57502	6.404675	9.269363
282.795	548.9607	73.83761	368.7865	560.584	71.28956	360.9298	191.1416	297.5573
28.66441	46.96339	43.14745	96.87662	48.64467	14.08174	76.3155	50.61809	30.65879
136.5991	164.2697	142.9065	184.8018	229.4282	71.28238	134.0651	86.82942	173.8878
27.81196	83.79666	42.1995	25.18203	13.61577	12.91081	30.94387	47.80957	28.80899
416.9191	48.43493	23.0025	37.3706	7.962478	563.7145	114.4755	14.37505	158.9992
66.97304	82.10276	587.166	65.8946	93.82739	164.5575	64.97539	110.8282	153.4557
15.61369	29.06241	21.08753	20.28871	30.54737	44.37147	26.82032	18.00755	21.36432
34.76706	66.45471	126.6344	95.30339	61.04758	25.74908	57.75231	126.2155	33.45344
0	0.652315	0.943845	4.008629	0.03765	0.048238	0.009111	0	0.903644
0	6.721794	0	0	4.545324	0.044748	0.0085	0	0
10.40801	14.90548	23.02757	12.99144	0.04023	9.389529	20.62144	29.83382	20.46422
28.68245	49.29128	10.53358	26.81232	72.28885	35.05766	14.44957	21.63617	23.2247
14.75601	32.14493	78.71527	58.79037	35.0118	22.20466	8.261957	37.01592	26.96658
121.8042	211.3842	207.1827	109.8612	146.9512	89.95173	184.5872	323.2567	185.0546

388.9555	220.211	1550.034	376.0772	985.5582	75.95802	1182.791	2154.777	539.3375
0.824016	229.5829	1.903096	2.371867	1.177163	1.227898	2.074117	2.791424	0.904887
0	0	0	0	0.019709	0.024997	1.035018	0.671511	0
24.37219	3.62919	14.3775	1.554677	28.23493	4.738046	19.59627	26.83621	19.52098
1728.314	2172.624	1820.492	1625.879	1964.353	2298.724	2320.22	1616.153	2026.312
19.12171	8.865516	66.23365	59.62684	101.3959	16.38571	51.53673	4.956128	26.96902
46018.64	19172.05	38042.26	24331.15	40153.22	61375.75	32429.6	26209.04	38858.63
42.59459	80.61896	72.88786	89.53939	35.08907	60.75547	107.2454	59.32315	53.91353
242.7865	232.9843	346.2649	267.8536	160.5324	200.8402	296.9879	350.6691	276.1862
153.1281	206.0926	164.9658	168.4876	74.65023	172.8254	282.55	339.8184	189.6912
186.1821	712.5647	742.4056	306.074	300.6825	155.3443	558.9155	870.898	281.7452
7454.198	104.451	543.8289	456.6691	64.47687	5166.872	1204.46	180.2576	2990.695
278.4702	188.8848	426.8396	86.22367	1266.63	185.6747	392.8854	472.4353	531.9576
7.779749	17.85921	16.29409	43.217	12.47802	5.906577	16.50819	29.70233	9.271897
14.741	94.25596	33.56042	20.28426	14.74647	7.07575	31.97596	32.52621	39.04353
54.06433	15.61681	35.49944	19.50703	18.11118	135.9671	13.41536	21.69825	42.80996
77.43407	136.0078	95.91609	52.05047	84.77972	68.9144	55.69424	94.89513	82.75604
144.4532	227.877	181.2863	90.31917	109.6614	198.4383	202.1109	292.8583	191.5757
53.92835	25.29206	63.29942	102.6317	80.24395	51.40744	45.38194	27.42083	52.99045
315.9218	34.99161	232.114	326.5169	442.9813	169.3249	367.1015	660.3102	154.3479
130.5312	174.0636	176.4943	106.6247	40.74241	45.59874	134.0585	223.2532	88.3252
4.299073	5.877744	1.901992	0.744403	11.33538	4.735926	1.043069	2.061192	1.831843
3174.692	1424.902	2441.066	2088.306	3005.306	2999.235	3351.433	2044.252	3082.706
100.9296	226.455	136.2031	138.4199	126.5883	67.76536	56.72767	189.9036	127.3994
51.30731	12.5873	22.04351	10.50467	4.57311	73.57391	20.6375	10.74811	52.98616
24.35073	13.35765	51.81867	63.67798	75.55908	7.073174	24.75273	31.88876	36.27842
708.4511	108.1845	627.3071	287.3571	531.1917	1017.845	560.9722	753.561	662.1357
3413.985	1433.845	1017.654	2015.817	2032.219	4300.963	8018.672	7063.288	3783.862
193.1545	436.9414	687.7491	269.4535	228.3433	164.6739	245.4358	450.6337	187.8219
27.80132	28.28987	48.90985	17.83476	36.20642	28.07931	74.24451	108.8038	46.48177
1.689204	17.86369	1.902088	8.067659	22.62242	2.402213	14.44649	0	4.620419
181.0583	140.4312	189.9312	120.4918	258.7086	184.3981	66.00724	87.59715	124.6096
52.16159	359.4567	203.3474	52.03009	68.99388	100.4511	163.9643	136.8713	114.3638
21.70283	60.45573	88.24731	17.01847	22.65795	40.91304	74.2482	101.4849	60.43426
7.790159	8.887571	27.835	13.81688	5.699395	4.73446	23.7091	18.8547	16.73751
16.49607	30.61801	35.49326	15.41042	11.35092	9.405648	23.72293	47.92741	28.824
0.821886	15.05146	9.59265	2.373475	1.178602	2.395849	10.30974	19.79558	7.430807
14.74951	7.362938	18.21208	15.40671	9.094525	22.22008	55.66955	44.25447	21.37129
0.821314	2.88406	0	5.629048	0.040075	0.051416	1.043077	0	0.903409
26.05869	100.9193	63.30253	51.26006	41.85281	19.91526	39.19486	47.7543	20.42992
43.4816	47.74787	76.73742	31.69021	26.04413	43.23691	84.55321	70.27798	60.44001
126.3529	86.78155	33.5615	33.33846	35.06448	95.61071	42.28324	41.25672	82.79918
16.49159	39.6046	28.76951	16.2208	7.965593	4.739769	19.60201	34.03951	38.13067
93.10608	144.2156	59.45633	102.5818	92.69109	78.25094	116.5244	78.9132	92.98654
19.98706	20.10947	75.82606	12.96247	16.98982	4.739464	32.99651	34.80482	7.410792
21.77921	8.886046	13.42198	29.40648	13.58227	27.94458	16.50064	22.51472	7.415696
46.9555	112.8399	83.44676	66.734	141.2101	30.41898	30.94833	49.18359	57.63997
45.23499	10.34772	19.16733	96.17712	58.76867	39.72811	25.79093	14.37365	26.01412
20.85247	15.59814	12.45376	19.48783	18.1231	50.14303	27.84688	185.8479	67.00801
1.691843	124.8464	4.779183	11.31841	1.177553	0.055304	3.10587	12.19634	2.762515
102.0752	17.10427	24.93258	34.20808	26.01068	81.49697	17.53938	23.1329	60.49796
266.3764	120.9522	216.7845	148.9895	153.7122	295.2408	57.75979	131.0915	193.4389
195.1647	6.614574	45.0721	13.76184	19.26686	46.73144	13.41902	68.10526	79.05516
107.9074	22.29636	270.5334	121.3222	335.4529	21.08672	119.6191	187.7746	101.3568
27.80016	20.06128	0.944381	5.621789	12.48836	89.85841	9.294241	7.127403	23.22047
46.94774	102.3108	24.92089	158.8352	46.38622	22.25377	82.50195	46.9898	40.89017
21.72386	10.35471	95.01663	25.20391	210.7443	42.00435	31.9695	20.208	32.54383
0	0.654127	0.944305	3.200652	1.177086	0.045468	0.008627	0.623468	0

424.6278	255.3092	164.958	103.3197	764.0843	54.93905	526.9617	200.5406	51.11769
0.821313	64.81632	0	0	0.04007	0.051409	0.00966	1.339695	0
86.97884	135.8711	69.04361	94.38054	148.0851	121.4616	220.6767	241.2774	75.29706
219.2859	69.34944	213.8873	389.2383	247.5309	407.38	220.6834	181.0415	173.8787
21.74871	19.38645	9.57721	17.06274	29.36371	9.399245	12.38385	7.136228	7.412313
7.780224	11.86079	6.697444	39.95257	10.22063	5.906261	7.231106	13.67542	11.13382
52.18555	50.72522	79.61104	30.05429	36.2121	89.87698	43.32006	95.66999	51.12932
370.6779	990.339	2396.049	587.783	539.1623	360.8143	760.007	1064.931	635.1232
58.26849	76.12958	126.6158	63.45536	74.6224	39.75787	71.1606	86.8955	90.19537
242.7693	134.3337	110.2868	165.2102	448.6842	169.3425	110.3526	96.95492	240.8336
17.36697	32.86559	29.73189	18.67543	15.86461	3.571385	21.66223	29.6863	12.0631
281.9904	437.1184	134.2721	159.5412	409.0539	339.6613	267.0786	177.4445	201.7894
2121.852	2.89889	1634.448	161.1232	2387.982	2034.696	276.3775	36.10917	1391.206
1914.487	6852.275	27191.39	2772.15	5578.922	1995.301	6431.678	13836.48	3463.006
187.2565	88.89973	103.5996	33.31409	103.95	148.1482	84.55766	52.82048	113.4751
1537.682	4015.817	2938.868	1809.827	2421.015	867.5118	2629.598	4255.979	2126.714
8.647466	47.05335	7.655967	78.26096	1.178573	10.57718	25.78934	14.37827	20.43396
147.0392	105.9635	145.7822	222.2603	496.0535	124.9719	90.75853	104.2182	159.9314
86.19871	15.57857	24.92349	61.06457	58.77489	115.4542	87.64187	147.3971	67.89119
52.15997	173.9865	51.77745	62.61453	230.5582	43.26897	18.57508	10.03025	66.92623
96.56376	92.5389	78.63805	96.83728	84.80795	68.94076	77.35121	50.59789	79.95093
0	4.733604	0	0	0.019816	0.025134	0.004931	0	0
20.84598	32.07827	10.53406	17.84755	45.19835	35.04003	23.7263	10.02776	20.43635
10.38895	26.07177	21.08819	36.61555	35.05603	21.06945	26.81957	10.75164	11.13016
29.55233	60.52863	42.19802	32.52101	32.80859	22.2408	27.85199	41.98067	23.2241
67.87398	58.21963	26.84104	20.27775	45.24238	132.9769	53.6286	17.99482	74.39555
195.8019	14.83101	184.1562	205.1699	198.9259	63.11225	390.8059	194.8575	372.0166
55.65855	201.1219	176.5123	99.32926	71.23054	51.42125	51.57033	155.8778	58.56614
2.560829	82.89468	0.944492	17.83219	0.043113	1.22818	0.010345	6.405095	1.833593
623.0627	745.3009	1398.47	819.8358	479.2697	563.9442	788.883	926.5034	981.996
294.1071	836.6008	708.8338	330.5059	206.8835	210.2084	331.0268	844.8163	300.3457
7151.635	13572.3	44645.19	6976.476	15040.23	5426.539	11948.68	21947.49	10375.11
15.62243	35.11642	58.53486	38.29011	18.12092	12.90264	16.50908	31.14138	25.09694
86.98929	174.0506	135.2395	80.54671	67.85951	66.60464	163.9601	175.3489	170.1843
6.907454	9.605586	0.943997	8.879146	16.99669	0.053958	7.23154	2.063088	10.20091
16.49999	30.63648	31.65597	12.14829	18.11451	12.89784	24.75179	14.40349	17.6514
13.05279	8.910257	11.50648	10.55997	21.41513	5.89076	15.46447	18.92208	16.75199
15.614	43.30532	43.16031	10.50625	15.87371	47.86259	14.44936	24.54666	19.50348
27.79221	195.7698	161.1461	37.37455	71.24506	35.09447	122.716	160.1397	54.83983
4.298947	20.92817	4.779585	64.694	15.83881	3.56997	7.229675	4.234726	3.691226
102.7241	47.73411	75.77356	75.71606	35.08211	165.6183	58.78417	185.0602	98.5875
33.01679	79.86678	114.1422	123.7743	45.2569	32.75745	76.31603	110.8415	62.28518
13.8837	59.99019	18.21493	8.068013	5.704793	4.739157	56.69018	41.39767	27.89822
662.2896	442.8543	605.2372	481.9776	794.5039	430.8194	467.1413	471.5773	423.1062
18.23202	28.33257	47.00511	36.62899	21.51239	12.90727	31.97157	41.30296	26.02214
20.84652	59.09664	33.56621	44.7965	33.92085	14.07339	23.72614	41.29941	23.22905
194.024	396.5847	161.1244	319.9463	208.0042	98.13931	599.1196	676.7843	211.0718
159.352	7.361997	7.656127	137.6901	93.79871	21.08473	261.8693	73.14057	153.485
59.99127	1161.376	74.79756	70.75378	87.08161	28.09141	116.5383	106.3847	107.8448
27.81546	89.08096	62.35803	42.32958	37.31319	32.72014	78.3533	90.78573	41.84335
7.791304	23.98455	21.10982	7.266116	2.312392	3.568667	10.31951	17.39668	23.26924
68.77778	8.107111	87.30421	6.434495	75.68482	38.55909	50.53071	51.42531	73.48402
228.0192	221.8342	142.907	198.6554	183.1078	134.2981	217.5838	218.7928	182.2604
38.24719	15.57504	5.738144	16.20369	5.704735	37.41659	14.45038	7.128287	15.77867
24.31306	58.19305	16.28819	25.97697	10.22766	32.75379	129.9219	47.72667	31.59008
650.581	14.08151	139.0814	71.59252	277.9098	493.4026	64.97634	45.53081	245.5538
13581.05	13806.81	7350.038	21541.12	19036.77	8379.094	13230.47	15609.55	12619.02
517.8031	287.464	1192.289	383.4421	533.4676	296.5853	347.5251	453.4667	382.1871

12.13092	103.3659	71.95923	48.05249	24.90103	14.07505	42.28003	88.62094	21.36596
13.00341	62.85835	57.56497	23.56264	27.15242	4.739781	17.54121	35.4792	9.270218
13.93523	5.143527	12.46929	14.67912	5.691758	11.68133	10.31553	13.04988	8.35504
61.75139	119.5189	126.6154	62.6396	45.25661	96.91169	196.9397	76.7344	130.2013
104.3869	63.37755	23.00235	12.13627	41.87548	59.61126	90.75801	23.06265	129.2406
494.2914	343.4831	377.9009	703.4785	422.7336	580.231	199.0363	218.6686	316.1536
21.74061	17.87032	48.94502	18.68971	35.00572	31.49811	18.56797	29.72684	33.49134
190.5577	284.5579	203.3352	286.5943	236.2334	155.3228	271.2089	355.0302	226.893
183.646	96.28189	341.507	199.52	85.93543	193.7623	204.171	186.9535	167.3928
79.1507	110.4807	426.8886	112.3177	83.67826	32.76149	123.7502	298.6644	99.48422
5243.418	161.2101	1505.876	1978.41	2605.193	8471.929	1994.367	253.4229	5170.461
63.47817	97.76399	81.515	150.604	78.03221	24.58965	95.91193	96.26648	61.34757
25.18927	82.95879	36.43665	25.98556	37.33376	17.5809	33.00851	21.62443	26.01234
5.166828	4.374976	3.819769	6.434499	9.096338	8.242651	10.32501	4.231143	0
68.72384	80.62967	77.68652	74.0587	108.4912	47.92097	79.40729	62.95881	68.80039
26.12385	26.17213	45.11401	7.256565	9.087387	27.98809	7.230355	21.72789	29.77512
147.8899	681.9759	568.7918	145.6601	698.4329	154.1742	390.8325	539.7314	314.2992
0	0	0	0	0.022713	1.193857	0.005626	0	0
61.73897	280.9788	117.0105	62.62137	59.95248	46.76788	125.8123	128.9104	50.18757
53.90853	409.0256	70.00669	198.7399	67.85467	29.2583	62.91418	110.0839	8.340403
6.039182	43.46949	19.17713	6.438604	13.59974	4.738524	9.292266	56.79962	12.06694
1.691242	15.05652	0	4.013111	0.036979	0.04736	0.008958	0.622041	0.903833
8.649437	41.11674	39.33047	8.880048	7.965261	4.739726	23.7242	32.59099	9.270915
96.71266	36.55741	54.67754	14.58108	59.87489	111.8808	42.2818	23.09283	60.46087
97.42038	281.6014	527.5835	81.34313	1195.287	9.406391	287.7026	1051.309	159.9315
98.32568	240.7237	94.9523	76.48991	236.103	67.75904	72.19259	108.6536	83.68022
68.79228	17.82852	67.15363	42.3201	84.68988	140.9872	58.77405	53.62908	71.63045
39.97789	304.971	177.4539	65.07	58.81979	51.43339	136.1205	155.7749	63.21049
26.92833	66.45369	71.93689	46.36202	27.1752	49.07121	53.62872	55.735	48.34005
59.99642	242.8127	191.8367	152.2354	107.4057	86.44692	148.4974	231.168	113.4347
308.8966	647.5296	202.3668	593.5243	264.5242	399.3174	480.5496	488.2172	326.3815
77.40256	197.9084	165.9296	123.7035	79.16647	85.28724	140.2517	152.8024	81.80708
46.15189	20.84302	42.20866	14.58839	9.095104	53.63911	41.24494	61.00585	76.31911
3.431512	5.141938	23.04447	3.184825	12.43521	16.31243	13.40475	12.30839	5.556795
6.04177	126.1427	8.616283	38.99901	3.438823	0.056103	4.136527	21.61462	5.552737
158.3589	212.8421	171.6833	144.8765	106.2871	102.7962	168.0931	191.2165	150.6317
23.45129	30.549	43.15666	34.96536	22.6513	38.5593	27.8524	39.79333	30.66773
24.32857	22.32448	6.696832	34.98059	20.38863	16.40789	13.41826	21.64225	14.85139
31.31451	4.374718	2.860886	8.878842	19.25409	21.06165	17.5409	4.954523	13.92274
139.204	247.9628	284.8778	175.8264	120.9778	123.8052	223.7743	291.9902	166.4419
2.557932	14.1492	11.49995	12.98962	4.572487	3.569785	13.41155	13.71542	7.414792
46.09791	96.44313	80.57755	65.95095	31.68904	60.71573	190.7206	289.2074	68.81798
7.782002	13.36966	18.21684	67.02712	22.6129	8.235843	6.199843	7.134965	11.13567
295.858	131.3412	234.9828	569.1546	276.9406	375.9409	430.015	449.877	489.1488
0	2.137569	0	1.554707	0.040999	1.231242	2.074932	0	0
114.146	33.55323	47.95731	92.13199	55.37012	46.7056	42.28286	76.90992	34.39273
38.25931	89.7201	81.54012	25.17241	55.38933	11.74589	34.03889	64.48806	50.20771
88.71349	115.6705	155.3729	183.1473	333.3958	23.42073	283.5817	258.6113	170.1588
395.9638	661.0991	166.8793	245.8299	524.3998	308.2408	387.7363	366.5391	342.2049
20.84698	82.38168	43.16528	71.77028	41.81298	45.51206	18.57211	60.97022	16.71398
8.658425	12.6355	18.22133	12.16539	6.830002	4.736746	12.38225	10.05681	15.79847
140.0529	1239.85	56.57349	51.21527	179.766	421.5169	41.26121	60.7347	115.2797
4.298408	12.6335	0	26.08944	0.040625	3.570423	10.32168	7.869158	5.551852
243.1368	12.58841	169.8318	24.35566	54.26447	339.0447	21.6676	7.850897	177.7267
148.7879	230.0532	184.1563	133.4789	275.7437	103.9594	173.2471	199.9342	145.0539
1.688885	16.44198	4.780462	4.814945	7.949823	0.050802	7.22832	2.061829	3.692131
389.3988	0	169.8014	2.371593	292.4735	296.1988	19.60633	17.99202	319.1239
0.821483	7.417061	1.902513	8.11066	1.179308	1.230547	5.165566	2.792447	3.694717

52.19266	50.73856	58.50438	74.91451	29.43285	22.24869	79.39993	86.9838	101.3872
160.0883	357.0454	52.73616	381.0813	59.95927	80.62576	156.7535	116.5291	143.1856
12.13374	23.84397	24.93011	17.85354	13.61065	8.241326	25.78604	22.39009	19.50822
24.32696	139.3478	67.15508	8.062607	28.29021	15.2429	25.78941	20.91299	34.39434
6.047424	9.652607	8.621849	23.69285	9.07362	2.401123	14.43765	4.968983	3.69234
27.80804	80.0126	48.91489	17.02374	32.81036	25.73932	56.7152	53.60805	67.89911
153.1926	109.0238	168.826	192.235	346.7658	61.9289	74.25527	68.74155	103.2147
130.5329	103.0205	104.5419	134.3346	162.7366	61.93472	180.4545	139.0906	111.5815
31.2765	123.2732	142.9287	44.71388	42.99598	56.08798	69.09798	115.2121	51.12341
86.11566	37.23431	39.30837	113.947	30.57288	143.611	88.69338	98.44983	62.27857
56.52513	109.0378	98.7907	41.45164	66.71893	70.09005	93.84364	113.74	88.33288
805.8143	1546.744	805.6849	1590.089	1725.856	812.6123	906.4432	1184.39	940.1369
0.821267	30.80916	0	8.08684	1.179911	0.050894	1.043078	13.74042	0
499.4756	1443.704	923.6675	430.6206	509.7875	335.1371	1501.438	1417.729	621.1669
0	2.972903	0	0.756958	0.028718	0.036607	1.040119	0	0
40.88183	22.31549	32.60123	85.58114	183.939	39.72239	30.9448	7.126611	29.7377
184.4373	1278.015	773.087	557.6724	255.4871	184.5334	687.8199	569.347	365.4369
51.29905	115.0131	198.5704	57.74544	64.46213	44.42748	95.90659	123.1637	87.40072
28.68269	38.04933	27.80412	41.50195	6.836567	26.90116	72.17349	55.80775	34.39281
31.29019	8.853759	21.08601	152.5097	17.00636	21.07838	37.13118	27.43584	37.18005
0	0.665259	0	0	0.029534	0.037665	0.007238	0	0
14.76989	17.14839	3.819974	38.39109	24.84628	24.49466	12.38249	1.339994	5.551849
28.67232	47.74913	72.89935	56.15707	27.1732	29.24548	58.78168	73.91384	59.50988
14.74767	37.34799	9.574996	21.93434	11.35357	33.8648	23.72506	34.03556	12.06187
39.98722	86.63467	128.5435	45.53595	66.70532	24.58582	109.3022	90.56173	105.0929
83.50027	37.23323	46.02263	56.91686	107.4073	66.61007	49.51116	48.42077	157.1519
2593.403	1658.744	3247.758	1108.035	2320.351	2125.942	850.7606	1355.342	1520.42
28.73386	32.17198	70.08214	28.51699	45.12496	30.32639	8.261534	23.89971	23.24523
181.9105	91.05322	63.29037	186.4894	141.2811	135.4333	25.79382	14.37055	82.7436
0.8221	20.85331	0	19.49253	4.575205	1.230663	2.07483	3.507192	1.832171
6.038147	20.86771	4.778817	49.7776	22.6223	9.40362	13.41583	3.507118	10.20307
41.87307	14.13546	23.02253	42.48617	23.72371	33.78052	14.44343	28.32237	20.45678
29.54064	141.3448	100.7236	22.72166	35.08104	32.74782	57.75267	71.71131	45.54824
224.5332	72.34441	63.28803	434.1247	116.453	135.467	280.4809	129.6004	223.186
26.92692	147.3115	85.36816	70.00381	47.50463	21.08395	73.2176	97.12192	66.01663
147.0213	545.2666	775.0345	228.726	205.7468	128.4911	345.4596	617.3175	214.7904
32.1548	56.72575	30.67882	23.53757	33.95026	3.569109	53.62822	36.86002	30.66278

TCGA-S9-	TCGA-HT-	TCGA-DU	TCGA-CS-	TCGA-DB-	TCGA-P5-	TCGA-DU	TCGA-HW	TCGA-R8-
288.2055	148.1171	130.5015	164.0437	176.7041	73.16269	93.0121	157.6032	145.8133
31.78948	35.36763	4.870198	45.37673	22.78265	31.21777	58.76545	10.03995	46.62828
132.7799	151.5572	91.00186	76.62277	64.4535	92.68016	119.238	73.28137	72.44586
3670.598	3357.807	1930.833	4335.214	2939.913	2444.889	2763.176	3511.437	3407.44
131.6678	90.66951	152.0096	85.68375	138.8569	125.8525	95.7279	224.8587	183.8209
0.031793	0	0.045814	0	0.060205	0	1.780695	1.00505	2.562444
17.07319	21.92052	13.24421	1.580692	12.7266	10.72178	37.93304	6.024669	7.707814
178.1853	128.0708	162.7919	150.8446	188.0491	175.6125	173.4387	229.8784	227.84
9238.979	9369.346	5800.783	12337.79	5140.185	6923.939	9051.926	11005.11	8192.824
71.5022	26.56664	95.70229	47.774	56.83674	115.1357	89.44818	74.28405	96.68277
22.69636	19.32535	26.30494	17.28639	25.21681	25.36889	7.189712	49.18433	32.84134
1313.193	1518.82	1104.852	1777.33	1046.47	1976.611	1204.096	857.2813	1753.383
133.9555	81.29256	167.5921	69.17711	117.4569	96.57849	139.092	344.3147	100.0511
0.033855	0	0.048875	0	0.064351	1.943951	9.083382	1.005138	0
170.1937	58.61858	251.1612	66.72385	160.2204	201.9689	65.91439	218.8343	99.21829
10.27032	3.864848	16.82772	5.696032	7.6836	25.36008	17.12872	28.10833	7.707929
2849.986	2515.015	1302.41	2913.216	2318.856	2176.597	2819.202	1939.422	2660.205
38.61241	53.39677	95.61844	36.23648	49.23494	60.49178	32.49552	164.6224	67.33794
48.8536	108.0884	24.02447	22.18132	64.4527	55.60208	37.90007	42.16281	64.67517
227.0566	100.615	148.515	128.5189	281.5926	378.5313	195.0714	368.411	197.5371
768.4274	1069.661	940.869	814.4093	1016.163	900.4906	2333.313	878.3617	1695.583
104.2988	176.2498	8.462504	36.23773	280.3548	27.3101	2.671313	2.009174	137.4024
220.1098	130.1285	79.05271	284.5815	209.4025	57.5529	106.5782	44.17053	42.22562
417.6515	361.7011	94.64533	175.5296	357.2094	339.5153	302.6002	704.6928	327.0253
91.94415	108.788	51.53384	141.016	85.86418	91.70631	98.4578	7.028475	105.2735
30.65605	28.6537	91.89421	42.89696	27.80621	35.12244	13.51418	35.13441	40.57203
110.1405	71.26479	124.5477	88.13725	82.15123	151.2149	178.8386	78.30114	103.4957
440.4318	864.1011	1517.732	587.6984	778.8834	1063.422	689.1939	789.0199	1002.641
20.47018	55.50216	26.37523	21.38265	70.51074	28.28855	20.74747	32.12335	69.98353
82.86137	55.97013	61.08906	49.40904	102.2145	107.3218	55.0767	107.4104	84.55625
70.36181	109.621	53.88896	23.83874	75.70473	35.1149	18.92992	134.5113	85.45693
72.62627	48.65731	82.549	64.29918	73.18411	134.6576	56.90033	208.7912	49.16116
19.29586	13.95135	46.4624	14.81022	32.68363	24.39494	16.24864	50.18709	30.25808
491.5005	388.2387	1766.639	313.1755	632.4711	850.7355	740.6894	563.1561	415.8496
97.6601	210.8382	170.0137	121.121	193.1654	151.2145	187.872	244.9372	294.2621
42038.26	11034.83	24092.2	16924.34	22710.01	15373.75	28243.43	42041.88	15863.65
6.864464	5.199463	15.61463	3.224559	17.73186	16.57932	42.49721	1.005286	7.711185
74.90978	122.2862	47.92776	133.656	64.4025	44.87188	40.61966	50.19292	100.1286
14.80998	5.864669	16.83593	4.049244	24.07255	76.10657	39.72874	6.024667	15.47605
11.40576	7.86575	13.24633	5.696032	31.61191	35.1183	46.06895	2.009181	41.40835
62.47708	55.25193	20.43401	27.1267	65.73785	48.77075	26.15366	46.1783	83.65297
49.95467	47.33205	67.01189	55.22266	53.03496	32.18815	30.6823	107.4084	74.2336
22.75269	2.538507	37.16395	5.696956	12.73453	15.59961	29.77542	33.128	8.570046
205.3747	200.3277	49.15258	309.3297	122.4556	125.8538	159.9001	133.5108	161.3794
329.1409	290.9328	365.0531	166.4636	284.0258	335.6143	174.3095	315.206	270.9292
13.65644	28.74573	26.3188	20.5924	20.22341	21.46194	18.95608	38.14417	35.42891
22.75332	23.2244	124.3591	25.48755	46.76213	94.64193	37.91013	103.3941	54.34008
3263.156	3028.113	2001.462	5171.097	3842.429	2642.939	2815.562	3843.708	3731.008
1056.606	711.3777	349.5826	772.4393	1217.89	291.701	353.1623	768.9416	679.9478
7.991514	18.00022	26.28319	27.25588	3.892109	11.70125	7.191364	11.04329	20.71516
15.90688	4.536428	59.51286	7.355719	8.921007	28.30109	15.34226	34.12839	22.44932
245.1712	453.2706	210.7053	230.7939	208.3229	164.8726	157.1464	271.0372	308.055
39.76727	60.0053	116.0298	55.19632	75.7402	74.14624	57.79446	90.3452	115.6787
6491.108	8713.536	9786.68	5696.117	6118.383	4950.271	12464.67	11505.02	7684.647
14.78821	23.35024	56.04313	20.59172	28.992	20.48555	20.76717	46.17386	23.29492
64.69536	92.19695	58.66667	78.33989	46.75513	77.07721	64.13355	193.7347	86.32156
24.97296	31.41751	23.94978	53.73638	23.99614	33.17621	19.85763	43.16315	31.9505

6411.664	5913.47	5416.506	6027.499	7031.008	7727.856	10129.61	12550.02	11530.47
145.3281	44.56341	301.6548	25.48015	231.0519	147.3096	292.6623	426.6316	146.6385
36.3197	21.26396	59.74912	16.42737	37.8599	73.1916	17.13094	61.23221	44.89383
9.137486	29.9278	13.24934	32.92566	31.63187	22.43026	60.52694	24.09355	26.70333
2.323985	0.545539	0.062965	2.401757	12.71643	3.892326	2.670481	0.001236	6.846
236.0788	375.8551	216.6673	206.0698	232.2612	118.0418	248.4126	173.6652	265.7772
29.48876	14.5951	31.08121	9.829938	13.9559	23.41421	20.76633	47.17765	18.96275
39.71479	34.69108	41.87123	28.82766	41.62518	41.95453	35.22729	38.1458	53.5496
65.85446	106.1499	81.4081	84.90111	84.58247	79.02302	95.75552	69.26564	123.4315
71.55945	65.9254	264.5049	79.06787	101.0735	173.6562	199.6234	62.23976	67.24725
13.64779	29.48151	14.40009	59.64585	12.68407	7.796294	28.93831	5.020733	19.84541
31.76636	9.88402	144.0896	5.697481	21.49969	38.05578	16.23342	67.25287	23.28173
290.544	105.3108	398.5074	212.6596	310.4743	641.9892	186.9657	342.3088	312.383
60.12262	54.09709	50.25967	48.64426	51.72828	47.80604	24.36002	71.27104	58.70449
191.7328	294.7679	94.58278	239.2335	162.7345	125.8573	87.60629	109.4187	75.90282
38.60273	21.91363	35.93654	18.8976	27.83549	60.49498	21.64783	82.31234	61.30332
280.3404	425.8959	180.7917	320.6945	296.6157	159.9945	265.5699	256.9835	235.55
45.39005	79.66119	3.67177	151.2717	20.28055	18.5284	75.94297	5.020819	38.82317
116.9557	119.3227	80.28183	169.7643	156.6039	97.5519	82.1599	223.8572	76.73686
244.0221	185.457	217.8658	321.5416	234.787	288.7868	294.4944	260.9984	401.3103
1648.027	1698.265	1935.533	1668.455	1412.536	817.5583	1401.909	2062.893	1308.94
121.3601	50.65471	186.4374	56.03256	94.59321	43.89687	79.5024	103.3941	86.31413
463.0076	429.2169	371.0192	418.0098	527.4406	520.0214	341.4645	605.3124	232.9569
53.36133	88.84977	100.4601	93.21232	48.01301	61.46308	74.08178	51.19645	31.0179
31.82389	64.72778	21.6234	103.1253	36.68105	25.35698	42.43254	57.21927	28.42643
1734.269	2316.326	1456.759	845.7157	1781.055	835.1203	1235.701	1069.092	3377.396
27.29673	44.58771	24.02394	21.35717	17.78514	39.01527	61.39702	62.23923	24.10169
124.868	267.7911	58.72789	212.7333	167.865	148.2932	163.5024	38.14759	296.0695
200.9462	176.0338	135.3448	168.9183	337.0907	319.9962	293.5454	270.0345	257.9523
8.000933	12.53584	8.460931	4.875975	16.52285	18.52618	155.3978	7.028465	39.63834
185.0172	177.463	100.6147	122.7769	186.8402	171.7053	131.8572	121.4659	149.2475
40.88254	95.63312	15.6407	139.5531	75.65467	53.65888	48.77175	92.35118	60.41313
478.9982	276.1359	1391.931	220.0263	834.3217	760.9837	836.4751	1392.324	1230.541
2014.643	1690.192	2147.435	1348.563	1830.361	1808.789	2139.008	2516.63	2102.788
0.028744	0	0.041307	0	0.054131	0	0	0.000846	0.822941
32.9486	33.28323	90.85656	13.1163	41.69325	61.467	39.72892	77.29435	34.48284
95.31535	37.92753	106.474	33.736	117.2834	175.6376	70.45158	52.20059	192.5961
743.4429	633.8621	462.1067	963.6611	539.0691	628.292	841.8717	392.5033	546.1559
78.33647	75.34533	155.5252	51.87769	111.0616	129.7624	57.78373	129.4947	120.8216
79.45558	59.9906	74.23083	70.87327	61.90032	49.74981	82.19873	49.18928	69.01162
79.43752	99.53387	31.19091	261.7447	40.46714	35.11433	108.4371	22.08605	64.70646
324.263	241.4341	28.80544	343.4167	156.3418	5.84366	23.44582	110.4216	103.5671
73.81695	211.0016	53.93961	182.2203	199.3529	143.4161	101.151	84.32369	84.52407
258.8024	113.3068	383.0189	91.42735	202.0371	276.0964	253.8107	276.0568	147.5006
28.34273	26.07833	26.3013	17.2883	41.46241	8.772062	8.999728	37.13994	31.97742
79.34737	100.5034	20.41146	217.5053	66.77095	3.892369	14.41584	14.05526	49.20404
29.50942	11.22199	40.64047	19.74605	42.82558	5.843584	10.80372	29.11141	33.66675
28.40446	20.57879	74.09381	24.68264	32.85907	53.66406	28.8852	71.2706	46.60581
18.18533	35.46854	51.30905	28.87002	21.48618	29.2713	28.00986	70.26315	26.75453
142.8867	90.16851	102.8629	35.39185	127.3047	45.84855	23.44806	126.4813	134.7177
18.18678	11.90101	15.61146	7.349217	11.44892	11.69926	18.95189	20.07741	11.17013
2918.044	2465.71	1593.244	4205.105	2085.299	1652.691	1675.608	3007.507	1822.356
73.74318	85.55014	81.3281	68.44863	44.2196	40.97132	41.53424	141.5367	92.39653
222.515	242.0976	253.8405	144.1823	204.5993	250.724	335.0928	506.9405	161.2963
576.5471	478.4747	215.5262	630.6802	416.5648	362.9264	437.1972	551.1081	437.4718
3.459767	1.86693	3.670663	3.225232	0.081057	7.796271	13.52904	6.024528	1.671073
30.60256	12.59065	35.80991	14.80206	16.45115	29.27503	13.52634	50.18791	28.50755
130.5202	191.6586	92.20493	218.5585	95.97625	41.94185	121.9442	60.23175	113.8896

15.92847	22.63923	12.03888	7.347606	16.48166	21.45935	15.32744	15.05877	24.14477
69.22838	45.3073	94.49262	40.34801	80.73753	43.89738	43.3366	248.9428	85.45719
65.87248	106.0612	33.59851	151.7048	95.9787	99.5092	180.6927	43.16669	144.1127
708.1453	648.1344	214.3218	425.3729	446.8164	308.2914	410.1035	370.4175	537.6084
145.2702	169.5662	91.01472	140.9704	106.0693	99.50865	111.9985	104.4001	112.1573
123.7042	80.67851	73.06365	106.3334	68.23229	123.9054	76.75967	50.1934	58.63214
590.094	423.8376	241.8235	303.3558	364.7606	75.11207	273.6929	424.6236	151.819
46.54956	31.26897	100.4154	44.49091	41.69914	61.46606	61.43313	94.35885	41.39415
27.24798	9.211286	51.35837	4.04825	44.08313	26.33971	36.14759	45.17131	44.05703
22.7395	11.20772	33.54271	13.11991	6.421732	18.52835	17.12803	9.036178	22.39369
244.0322	280.95	165.2362	410.601	329.4045	368.7911	201.42	672.5684	427.1884
264.4378	87.95677	459.4771	74.9441	304.1401	367.82	153.5395	361.381	158.7384
501.6735	812.2563	211.943	1268.056	325.7372	389.2668	510.3639	322.234	465.9367
3627.491	3955.018	2771.122	6872.568	3513.002	2872.208	4296.088	2794.695	4203.86
14.79363	36.11935	16.80629	34.65964	30.27189	24.38878	12.61456	47.17828	36.27593
38.57159	37.40561	8.45877	91.77437	21.51783	19.50597	33.42402	19.07401	37.1174
61.29928	54.67317	53.89335	89.07142	35.42495	46.82446	66.84337	127.4851	43.10964
0.026811	0.570452	0.038463	0	0.050316	0	0	2.008091	0
20.42806	15.29571	0.061076	14.80789	10.17705	0	25.31127	1.005297	9.447664
1.180435	1.217697	0.049774	0.762671	2.594076	0	3.595228	0.001002	0.814914
12.52076	4.534486	32.24523	4.874675	27.71527	16.58173	17.14944	20.07701	16.37245
101.0405	83.32301	141.2428	104.6609	119.9422	140.4891	111.0925	196.7516	134.6043
44.28423	26.58179	53.86941	37.0556	76.92137	64.39364	39.72633	59.22644	40.52908
607.2245	469.7245	305.2997	798.0053	488.5407	531.7091	569.0756	414.5873	399.474
49.99377	116.0578	83.84731	117.0294	68.25412	66.33336	89.40074	214.8207	83.6579
6.853886	7.908896	16.74693	14.83323	18.89351	19.51691	17.1702	32.1197	24.21819
1175.851	2116.947	751.7591	1461.603	2009.364	217.5517	531.1081	1317.039	949.1391
333.6086	125.3722	190.3246	164.8495	157.8196	120.9703	102.043	288.1009	143.2108
26.16289	13.20328	49.15004	37.84819	117.4	73.16469	93.92753	8.032314	24.96402
11.39571	7.876821	20.37775	15.61727	12.70536	21.46068	21.66827	25.09612	10.30529
231.5679	198.0978	118.5803	338.8079	198.2494	530.7481	491.4351	147.566	313.2156
354.0708	208.83	165.23	394.1229	237.3144	227.3176	229.4344	257.987	240.7383
167.9692	195.5934	95.80957	282.049	137.6071	148.2934	196.0384	188.7214	158.7719
88.52424	86.7579	27.60911	69.2228	55.60063	73.16907	103.8976	34.13201	85.42575
38.56761	9.209197	45.41966	25.53147	25.28149	36.10044	31.61577	145.5444	26.73433
13.67655	10.5343	82.52862	14.76496	63.09769	109.2866	45.14998	20.07832	21.5196
148.6558	151.5557	162.7299	73.32207	156.4523	62.43206	115.6222	252.9642	157.943
17.02012	15.33659	4.862246	5.706362	5.146478	9.750115	6.289241	24.09079	11.19008
0.040036	0	1.271686	0	0.076976	0.966396	2.67223	2.009185	72.19727
56.76837	52.65841	58.67617	48.5988	61.87456	60.48582	67.74504	60.23087	105.3237
53.31293	60.85394	20.411	25.51116	29.08317	9.746069	11.70394	26.10079	54.39616
14.81136	21.22454	9.659988	6.520361	20.29921	33.16379	17.12316	12.04773	15.4745
171.4091	71.93238	335.0866	96.38337	111.1688	359.0383	164.3817	159.6116	163.0538
29.56406	23.88322	74.24116	9.817184	24.09283	28.28301	85.80878	73.28096	23.2397
658.2752	629.9767	862.9364	469.0331	414.0719	391.2178	312.5148	540.0665	425.3723
100.9239	86.90154	31.17798	174.2704	20.29579	27.30943	81.33325	33.12778	77.7017
9.137086	11.20439	32.36294	13.11736	31.61805	34.14171	30.68864	39.15032	18.06858
98.70282	62.69963	39.55782	156.8058	64.39298	60.48578	44.23752	67.25749	63.84269
51.08076	34.61861	63.41608	51.92789	41.69766	53.65874	35.20588	51.19606	53.49624
180.426	57.2687	118.5176	188.8338	92.20161	206.842	109.2875	235.9002	175.1969
65.83884	70.05863	37.1689	111.3611	74.46926	68.29219	59.60577	55.2119	75.07308
136.2511	387.0656	524.2017	281.0764	465.7088	229.2643	467.0345	1160.432	444.4074
21.6197	112.9237	73.01527	73.3684	51.80476	33.16265	36.10097	39.15096	32.74162
31.79777	9.203511	28.7638	37.08849	37.87506	32.19232	42.46108	52.19877	27.58415
204.3556	206.0585	239.477	74.93783	276.5361	197.0645	317.9299	530.0286	128.5066
237.2653	1156.055	278.9673	207.664	753.5164	1063.44	317.0297	40.15527	1302.218
28.41879	13.87349	96.85094	5.696492	41.70588	67.32056	43.34159	30.11643	31.02181
140.7479	108.7024	107.7705	209.4334	123.7397	107.3128	191.5188	137.5265	181.2212

125.984	142.1703	67.09191	149.232	123.7054	58.52873	187.022	285.0867	99.20957
61.27582	33.95015	84.89651	22.19375	65.59501	106.3619	36.11056	91.34739	50.90386
28.43098	50.57024	34.79609	24.65817	40.50508	37.0631	36.09111	85.32819	28.41765
180.4903	140.7043	228.637	144.2103	219.6578	161.9467	134.5627	211.8109	165.6391
65.84226	52.64754	83.7735	55.19806	89.58465	40.96863	34.29216	148.5654	126.0528
22.73739	11.87808	151.5227	8.994423	41.64578	45.85685	26.17376	22.08561	49.20837
78.34807	105.4001	196.2117	106.3232	88.41062	112.1945	116.5225	113.4343	117.3456
19.34271	9.869141	39.51962	20.54835	31.61165	33.16643	25.26444	21.08196	7.707507
27.27756	47.37785	50.26554	70.13443	52.99345	52.68531	56.9225	38.14649	95.02807
488.0889	762.6995	502.7946	817.7409	747.2938	683.9042	749.7324	673.578	774.8451
40.84751	42.75166	12.04609	98.35262	20.27104	48.78726	71.43694	15.05898	65.66687
150.8963	64.65088	117.2758	82.41416	64.43756	186.3612	128.2923	131.5022	179.5706
259.883	318.4625	81.46903	360.3535	145.213	189.2688	228.546	72.27807	197.5951
254.1698	233.6901	75.47643	353.8345	182.9949	96.57983	131.8715	266.0157	182.083
15.86803	12.6759	28.54301	16.52247	7.638535	21.47567	12.64514	38.13987	26.86388
682.1245	753.4368	274.184	829.3327	634.9241	496.5855	460.6658	969.7088	522.8806
23.85825	19.26537	125.175	6.521388	26.53886	49.76704	19.8486	32.1229	23.27215
1120.238	992.9272	1006.682	1295.894	1427.566	794.148	1326.97	901.4498	1592.046
9.132127	23.88302	268.1582	36.19492	7.673547	257.5586	83.96555	7.028344	30.14168
205.3146	180.3934	2.468392	325.1467	41.75029	81.94877	100.267	33.12828	59.50074
17.08322	43.92402	33.59393	34.55322	66.96045	55.60302	57.7841	97.37267	60.36405
328.0312	328.9388	132.9462	329.715	248.7365	455.6162	560.08	485.8584	411.5918
29.55906	26.56492	29.99603	34.56404	29.12846	36.09002	33.38934	24.0937	24.10618
334.8053	377.781	274.1169	608.5368	229.7707	189.2641	377.606	291.1139	222.5986
1948.765	875.3959	720.6639	1627.254	1502.122	1290.738	1571.746	1883.205	1234.74
94.19284	80.73337	88.57025	58.49117	69.45824	67.31413	44.23187	129.4939	129.4888
3.457853	7.910616	8.432476	19.823	6.398674	24.40147	22.61749	20.07593	12.06154
522.0502	489.8949	496.6939	784.9511	377.4123	400.9809	329.6993	278.0646	394.3401
215.6975	408.3543	319.6433	170.5689	468.3085	204.8707	533.8612	177.6814	150.0823
1088.464	954.2076	1325.043	1392.35	872.276	822.4409	1118.29	677.5938	1025.942
52.21646	35.95419	133.8316	42.83705	44.21835	49.75455	39.72606	110.4194	56.0866
20.46657	11.88282	95.47278	16.42858	12.72113	38.05055	32.51374	80.3032	17.21338
32.96439	13.20238	100.5112	5.697783	68.19269	130.7448	63.21625	29.11288	37.91973
61.34853	29.89241	49.16098	41.1431	104.8855	137.5513	23.44717	119.4588	34.45786
5.730431	3.205355	9.659841	0.761577	2.624996	70.24165	254.0022	1.005225	0.813776
15.92299	3.197959	17.99372	11.48185	11.4481	14.6276	24.38603	28.10728	3.394633
514.194	956.2599	796.0196	1301.699	902.5292	694.6355	910.5299	634.4284	825.7552
72.65943	114.1729	35.98332	153.4243	77.02853	86.82907	84.90548	51.19702	88.87259
14.78117	17.98489	23.9202	6.527098	22.7059	28.29923	15.33893	58.21711	31.1134
182.7108	128.7569	22.82844	296.9099	95.99699	216.5959	183.3879	133.5112	106.1052
82.8936	171.5429	25.22239	461.1498	59.42395	68.28491	152.6618	22.08617	124.2385
23.8619	21.27183	27.55652	16.43013	31.56999	13.64987	37.04196	16.06272	22.40356
81.7682	97.98861	241.7485	117.8361	126.2873	166.8286	198.731	102.393	123.3576
40.8611	19.24035	124.1459	4.048489	37.88414	36.09565	28.88593	83.31562	46.60782
12.53748	23.26968	37.11123	5.696091	15.24227	44.88154	31.60384	22.08556	12.88991
22.75411	3.204068	8.462614	17.23702	34.1697	6.81907	8.0886	6.024647	5.120659
4.58214	0	3.658806	0.758539	6.371911	9.755228	5.392778	6.024004	6.005683
147.5507	140.1188	281.1487	117.0296	200.631	163.9056	76.74998	225.8627	197.6317
461.9765	445.0054	496.7943	166.4401	617.2777	455.6073	490.4726	136.5241	627.2937
12.54197	7.205535	46.76236	4.877308	8.943575	6.819522	1.77139	3.012968	8.572393
2.324043	3.868736	3.669731	0.758294	6.406653	2.91697	7.192583	9.035738	0.811185
101.0357	130.1257	255.9955	113.7438	112.3677	192.2061	125.557	183.7016	192.473
0.034782	0.548519	0.050257	0	1.349616	0	0	0.001011	0
77.16498	56.01369	67.03244	42.82262	100.8792	86.8354	46.95165	120.4585	81.99521
40.90462	35.24606	80.20636	27.95777	89.61047	75.12055	68.638	59.22742	81.96918
803.5572	882.9254	1072.41	450.8738	814.1256	506.3411	420.9138	1264.837	482.315
130.5771	121.3163	500.2739	112.0387	174.2807	533.6735	319.7636	199.7655	191.5152
345.0364	433.1557	271.7452	450.1382	323.1507	369.7609	246.5834	328.2561	295.9515

6466.112	2632.338	5993.412	3380.541	4199.713	4142.461	4368.332	10543.34	2653.247
143.0017	273.2242	51.54518	199.5617	198.0744	15.59938	164.4191	211.8086	65.53288
112.4241	81.93092	228.6989	31.2519	181.8745	465.3671	771.4552	32.12452	195.8166
51.02484	34.0296	47.82166	32.14093	29.06118	59.52673	34.32524	18.0703	48.36303
1383.587	1746.318	1319.13	1832.511	1098.258	1282.932	1614.193	1553.947	1003.471
63.61559	53.90986	221.4379	96.38632	97.28686	260.4942	227.6376	88.33943	94.00369
13.67204	21.92308	26.3793	7.344749	15.24456	44.88046	58.74913	12.04763	14.61686
141.8922	134.0743	180.7398	175.5866	165.3672	231.2278	162.5902	274.0468	188.9775
4.594942	7.882673	4.867747	7.351901	5.154642	18.53373	0.866941	2.009221	8.578944
18.21501	13.87197	8.462804	8.168354	3.892564	9.745735	8.991639	4.016926	5.982457
178.1429	201.04	24.02476	190.5056	98.49182	79.01927	140.0241	137.5258	74.17095
105.5969	170.126	149.6596	161.5466	230.9658	139.5081	158.0635	225.8639	184.6458
3.460525	6.536803	10.84279	4.048441	15.21987	7.795298	45.21663	4.016958	12.03394
3.456636	2.535857	13.17111	1.579143	6.392207	10.72847	15.36507	5.020555	12.07006
37.49883	126.9678	50.31919	90.70344	35.43262	73.17064	65.02778	12.04774	101.8546
32.95286	48.67668	8.462792	84.97304	58.0632	41.94699	38.82063	21.08213	24.11105
156.6721	302.9605	63.52638	239.0343	73.32456	79.99031	313.4502	114.4394	197.5646
2.323724	10.60402	27.42698	6.534204	15.15839	17.56263	23.51867	29.10903	9.456167
514.0296	314.3706	204.7108	481.556	282.7213	214.6335	295.3947	527.0129	309.7966
38.58159	23.95006	50.21175	45.37545	30.32185	56.59711	28.8931	55.20961	31.05098
100.9914	141.6632	44.34833	174.1005	60.63756	54.62897	80.39227	33.12817	110.4836
315.5831	501.7156	189.2125	1305.009	952.9971	70.23393	291.7272	596.2825	409.8143
2.324244	0.544	1.272256	0.758476	2.630042	9.748335	24.40142	0.001207	0.81132
56.78131	118.2095	61.0865	169.1305	390.4458	202.9566	48.75053	174.6651	155.4064
350.7178	608.0978	244.2324	520.2205	280.2692	398.0537	334.2162	306.172	304.5759
114.6478	116.0811	99.38515	98.88976	130.018	111.2176	82.17568	167.6407	157.9253
72.67123	62.631	179.4486	64.24974	83.3551	160.0101	121.9539	314.196	118.2206
174.7975	140.0719	356.5601	145.0547	167.9052	203.9054	123.7293	311.1887	172.5643
290.6044	306.853	1071.171	404.7134	584.4345	554.1498	286.3143	3351.808	1312.572
47.69988	39.27355	50.31301	31.26346	30.38781	52.67901	13.50813	16.06308	26.69701
29.46273	13.27283	55.96751	18.1238	21.44637	27.32382	7.190884	46.17266	29.38538
60.2036	85.99405	400.6726	73.31105	103.5558	510.3063	89.40282	188.7211	191.5954
40.84091	27.31249	44.24604	35.45093	25.29249	21.45757	59.67477	27.10427	30.18874
10.27235	6.531421	6.067735	5.696363	20.29592	22.43032	9.895067	31.12018	14.61204
74.86681	91.61532	14.44462	192.4853	99.5333	13.64855	19.83619	30.11632	49.17617
94.21668	68.65051	98.16584	77.45404	84.61198	96.58436	71.33869	87.33469	104.4043
31.83863	27.21565	62.32829	14.76579	93.51149	34.13597	83.9679	71.27434	34.45482
76.06459	253.384	171.0438	12.2895	111.049	95.61047	38.80634	13.05159	267.7046
47.65891	64.19026	19.21931	90.01239	26.57395	30.23924	31.5981	54.20666	31.03727
26.11872	21.28582	47.79416	13.12845	51.61237	25.36316	23.4705	42.16022	97.76473
39.6899	34.07052	20.38955	46.24049	31.54417	41.95939	21.66188	46.17501	42.32605
153.2287	138.7673	77.87017	79.07905	104.8312	68.2844	136.3885	112.431	64.66405
3745.521	3209.626	1617.233	5453.854	2729.14	3286.848	2057.682	2968.359	3453.159
4.594522	1.875775	14.44848	1.583138	12.73537	27.30768	32.48322	2.009134	25.83107
528.9012	511.8308	188.0048	751.8752	255.0649	161.9425	265.541	75.28978	238.1046
35.23838	15.87106	56.31852	21.35731	95.9489	103.4153	71.33975	66.25447	39.63974
123.7519	124.6948	61.12843	94.73775	193.1428	119.0183	180.6512	167.642	168.2401
51.06946	27.26439	84.86757	17.24386	65.56715	57.5649	37.01964	180.682	78.58893
45.45893	57.24404	77.88593	18.06184	44.29445	95.60106	50.54279	69.26666	49.98647
104.4294	125.474	61.10779	190.515	78.31979	90.72868	116.5265	212.8117	260.7324
17.07628	5.864347	37.13644	13.94289	16.51035	42.92554	33.40278	32.12375	27.57413
57.90411	86.12958	13.25124	74.19372	34.16961	76.09905	129.2339	34.13189	96.67659
1.185577	0	0.056579	0.758575	1.360236	0	0	0.001124	0
0.036234	0	0.052424	0	0.069177	0	0.868368	0.00105	0
29.44805	7.222738	15.58094	20.6244	48.8722	3.892664	13.53291	82.30321	16.3846
17.07717	13.87722	13.24771	11.46708	22.80964	28.28604	47.87259	7.028519	21.52355
6.864633	25.33783	8.455768	60.34626	22.75323	26.34083	20.75961	10.03984	28.47748
178.1997	176.1525	264.4721	141.7561	189.3403	123.8977	187.887	351.3416	221.776

2157.463	729.3137	794.8231	2850.1	788.9494	1576.613	1652.21	2315.856	761.0327
0.044784	0.549878	8.461633	1.583749	1.357256	3.892852	4.477864	0.001276	0.814012
0.020393	0	0.029089	0	0.037837	0.972337	0	0.00061	0
53.20222	72.00204	16.79379	69.54379	61.49121	13.65227	5.380562	47.17734	51.91085
1777.415	1688.908	1589.638	1764.902	1895.948	1484.885	2044.178	2124.128	1273.557
40.80316	44.89321	29.90577	4.873001	62.81621	12.67522	0.8671	7.028427	53.61204
36799.27	34754.21	17080.26	38685.01	25857.09	21044.02	23924.68	21557.5	31173.33
74.93666	46.59405	86.20879	40.32627	98.46956	53.6512	80.378	43.16658	56.04441
305.3077	231.5075	497.8299	184.6091	301.6743	249.7558	210.4513	504.9297	423.7207
122.6264	162.7348	540.8647	124.4154	270.1222	305.3713	193.2886	273.0447	192.3933
502.8107	308.1962	856.9529	320.6197	364.8586	721.9635	436.2817	679.5997	683.4201
2020.268	1108.312	888.2334	398.0782	881.1547	501.4569	1456.116	740.8362	638.4672
384.741	235.4974	418.8974	395.7189	464.4188	649.7833	377.5954	146.5621	268.3367
5.730908	17.93175	19.19746	6.522034	8.938687	24.38748	18.94649	19.07388	21.54655
25.01551	45.33868	107.5691	28.79879	68.11113	57.56261	32.49332	120.457	34.48137
18.1881	26.69374	17.99683	27.2065	12.70679	15.60342	18.95087	18.06995	39.74428
108.9472	100.7724	50.33558	94.79633	71.99724	79.99757	93.03728	69.2658	81.09341
211.0754	178.8677	242.9074	173.948	214.5164	193.1762	78.55541	122.4693	221.7993
43.16946	49.29782	24.01933	23.83477	48.03409	51.70214	31.58009	35.13577	26.69508
78.37089	407.8675	191.5579	396.5787	188.1332	93.64963	300.8099	75.28969	176.856
143.0081	121.4158	250.0462	172.3178	113.6413	114.1439	144.5304	349.3318	148.4188
13.6447	3.199609	0.060792	0.758346	7.66429	2.91694	5.382161	1.005299	0.811223
2728.547	2079.898	2185.759	2510.093	2097.972	2117.084	2067.636	1863.13	2795.679
94.23019	239.7997	62.3094	339.0551	80.85225	62.4314	120.1345	112.4306	112.1599
42.04125	59.31015	15.64576	9.817156	16.52238	5.843708	15.3144	8.032343	35.3259
36.29821	79.2077	13.23367	101.764	6.419144	16.57858	71.47315	12.04746	30.20167
627.6084	449.7652	288.5241	649.6473	639.8814	210.725	511.2808	527.016	478.8997
1411.999	2569.007	5232.035	1631.295	3848.715	2469.28	1961.031	1699.505	7230.048
406.321	342.3034	228.6794	394.0413	339.5723	427.323	276.3923	535.046	506.5408
141.7474	55.34616	137.4802	60.16468	79.48519	65.36604	35.19918	133.5077	71.62571
4.595953	2.532437	1.271747	4.048333	5.157342	3.89234	16.23309	3.0131	0
149.7999	146.1806	41.97332	263.9523	63.19878	82.92177	124.6551	75.28912	94.02815
120.3399	138.079	97.016	142.5863	150.2403	189.271	71.32479	183.7028	220.9203
62.44361	31.90893	167.3883	15.58723	65.66752	89.75968	26.15764	146.558	71.61021
24.9248	13.97226	33.37165	9.845399	15.16808	15.60838	9.004906	17.06531	6.855209
18.19625	17.92579	65.65741	24.70524	36.57269	62.45934	25.27988	44.16778	31.92857
6.831667	5.934048	13.11053	4.066925	17.50329	6.825395	1.769934	36.12926	18.21832
10.26824	19.92999	29.94086	12.2991	37.84565	19.50567	31.61111	67.25428	11.16411
0.041527	6.553111	1.272321	1.578752	2.629189	0	0	0.001191	1.671064
36.36119	52.66259	39.55605	32.08994	82.00953	26.33252	35.19835	37.14327	42.24512
69.23213	50.65741	93.30665	92.37706	55.5729	58.53475	55.99316	108.413	65.57524
44.27677	43.34124	31.17201	71.77539	58.03566	59.51594	30.68712	39.15041	42.26471
17.06879	44.77446	35.90862	28.00181	39.11169	21.45725	28.89309	79.29955	30.18582
63.59601	98.07602	52.7317	282.181	116.1101	99.51194	117.4375	122.4682	72.45109
23.85403	29.35613	27.54251	21.39891	36.56407	27.31573	8.090714	97.36514	44.92155
13.63982	24.79322	23.88781	20.63097	10.16681	18.53772	10.81618	7.028221	18.12353
80.58991	47.28103	25.21718	90.68646	68.19975	52.6771	44.23166	90.34552	58.64572
35.21897	75.49138	39.54141	26.31964	41.70284	40.9713	11.7019	22.08593	60.40764
48.76232	55.54562	71.64719	13.12451	56.67075	11.69804	3.573109	13.05135	31.91874
3.458302	5.867238	1.268766	0	0.086494	0.96682	12.60461	2.009136	5.120916
17.06405	42.12815	18.00874	9.823248	31.55543	28.29127	14.42068	13.05129	43.18321
124.8678	167.516	73.08332	166.5283	166.6039	123.9	150.8503	179.6871	172.5865
97.55675	71.42557	84.94058	51.9084	81.99622	85.86033	98.49923	285.0789	70.764
174.7302	115.4491	20.43399	256.5612	121.1643	97.55936	288.2841	132.5065	112.1722
10.27276	7.865697	15.64423	0.761188	16.51987	15.59955	16.2186	24.09369	9.432763
42.04548	32.56202	30.00646	71.67775	58.14381	85.85089	130.0916	79.30415	58.63398
40.84349	56.88404	1.27119	151.3814	41.61901	42.93126	51.52336	18.07032	21.5369
0.036807	0.545458	0.05328	0	0.070344	0	0	1.005235	0

305.3754	104.6228	93.44982	96.37266	113.7192	165.844	1798.493	120.4626	388.2296
1.186519	1.203344	0.060365	1.578753	1.362735	0	0	2.009218	0
68.15365	100.6543	156.8368	72.47341	106.1129	167.8034	115.5953	124.4773	71.56417
71.56215	233.521	240.6131	262.9603	212.1046	214.6321	147.2087	268.0256	231.2333
6.862858	16.61623	9.645077	6.524786	22.72925	8.771616	19.86178	9.035962	14.63472
9.132029	3.864311	6.065842	1.579469	1.36207	11.69882	5.379588	2.009214	5.120003
66.97911	45.95188	126.7845	75.00928	79.52126	69.26676	60.50568	106.4061	56.92302
801.3236	645.2055	987.5144	708.9015	650.1325	906.3479	804.8321	1426.457	1038.04
82.87561	142.2249	55.12348	154.2155	102.2513	115.1245	121.9571	179.6854	122.5436
137.3876	244.8197	75.49694	96.37277	128.8576	171.7001	162.5594	46.17837	173.3885
36.30729	25.30864	68.04992	18.9128	29.05055	21.45818	29.80392	77.29129	35.38758
207.7107	325.7699	101.8131	350.4285	133.8721	137.5557	266.4896	61.23592	97.45498
2489.95	1611.785	98.24065	2228.447	1426.23	47.79509	382.0618	1697.492	791.2409
6537.65	3269.591	8430.516	4729.151	6858.083	4824.415	4633.89	13383.21	3294.349
87.38406	92.12987	87.36506	56.84539	93.37244	101.4696	62.31347	37.14342	54.3312
4231.276	3373.815	2760.33	6245.276	1766.04	3991.249	3628.553	3936.061	3796.599
8.002145	9.868813	19.22268	2.403095	10.20795	20.47948	62.35281	2.00918	13.75056
154.3923	291.6706	67.11417	324.0172	103.6011	79.99085	340.5789	67.25896	217.434
61.28836	84.18273	93.28064	27.14258	50.52151	74.15117	55.99973	84.32147	92.38445
54.53702	142.7292	95.82823	50.20959	49.34128	39.9897	67.70855	32.12459	34.45478
72.68672	73.2882	85.04718	37.84466	135.0922	56.57626	145.4258	44.17061	54.30617
0.020504	0	0.02925	0	0.038051	0	0.888492	0.000613	0
11.40447	5.197454	32.34643	2.402547	21.53731	14.62503	8.992608	9.036173	7.707898
17.07477	17.89644	15.63607	14.76965	15.24813	19.50392	21.64808	18.07052	22.39147
71.46446	50.04079	62.21302	59.37268	138.4709	33.16516	37.01672	32.12388	22.38641
52.2359	31.24491	25.21353	49.4223	59.36063	45.84799	46.04467	31.12044	23.24265
346.1003	412.6706	223.8264	238.2547	384.8081	169.7542	211.3742	600.2905	377.1663
82.87074	126.8596	107.7169	146.7999	45.53222	155.1342	88.51612	105.4031	147.6036
0.044686	2.540682	6.06607	2.405682	2.624849	14.62376	89.43006	3.013017	0
1058.958	1016.28	1052.167	1043.608	1017.414	1469.286	756.0421	2014.706	2367.845
509.6171	581.2473	907.2032	460.7906	321.9516	809.7733	355.8792	1064.068	702.4108
18977.33	12620.32	13049.87	16691.05	17402.11	10215.66	17011.78	20228.41	9803.758
24.99155	40.76809	9.653938	71.06362	19.00497	22.4341	20.75275	41.15673	51.83519
136.2079	253.7683	154.4066	229.2439	209.453	119.9976	86.69	235.9008	194.1782
4.596314	11.21204	9.655892	2.402177	8.943379	6.819081	11.70509	14.05519	1.672141
21.58667	14.57904	50.15394	19.74878	30.28156	49.7711	33.4352	47.17853	20.68398
11.36251	14.02801	39.18875	16.51811	12.63093	16.5899	9.013629	10.03901	11.20356
29.54272	11.87353	37.13447	11.46777	25.32268	24.38299	12.60665	43.16536	25.84644
96.50107	62.61277	126.8889	65.06461	122.4572	81.94547	83.07897	128.4918	96.61451
5.726785	0.543773	16.77511	5.702369	20.1875	18.53613	14.4369	7.028282	5.12307
60.17549	61.34438	118.4178	68.40643	100.9188	102.4461	53.27446	105.4022	192.5971
72.67043	71.99192	144.7848	100.5604	87.13389	184.4056	116.5315	82.31562	120.8133
28.37252	29.37651	44.19785	27.20124	22.75311	26.34085	22.57034	99.37157	30.20957
575.4347	411.0041	397.4381	736.1923	477.1609	444.8787	487.7778	423.6215	348.5654
21.60404	17.23457	119.3511	37.08853	36.61867	14.62515	22.55581	60.22886	35.36798
45.38615	33.32681	44.27295	30.47206	29.08089	41.95247	38.8402	27.10454	31.0422
231.5766	119.3094	612.7673	83.18182	460.6927	477.0814	465.2163	377.4443	429.7196
394.5156	192.5466	26.41316	25.48314	68.19864	28.28327	82.19879	33.12819	75.05859
87.44998	29.2174	244.2037	23.831	108.6554	318.0542	105.6493	123.4738	61.20417
43.1336	60.14494	41.9041	83.38119	31.60955	99.53778	73.21619	60.22945	37.08669
24.91914	19.39366	16.75621	18.14781	33.86756	28.30613	19.8868	31.11646	17.26169
66.93982	82.21721	50.28318	19.71738	98.28204	20.47884	27.97247	378.4258	80.29978
220.1806	186.1575	165.2116	234.959	94.761	271.2282	180.6529	105.4045	185.508
6.866306	15.2046	15.64534	10.64098	34.17641	33.16203	12.60458	16.0631	63.83089
11.40782	11.20101	190.1408	8.169303	59.38878	90.73244	77.6741	18.07079	67.27893
125.9866	144.1685	39.58103	125.2968	93.45863	24.37989	56.8739	115.442	203.7017
14096.83	15108.66	11077.21	20986.67	12995.46	12434.22	12650.65	11206.89	15833.45
774.0108	734.8196	203.5643	1207.048	724.4583	323.8988	324.2612	691.6454	557.4223

23.87382	23.92701	55.02004	24.68137	31.60456	99.53984	33.40727	39.15005	45.73845
44.25144	54.82338	13.24277	43.7059	41.64227	31.21638	75.95201	28.10828	59.59456
15.86362	29.04502	6.043764	9.868947	13.86436	11.7065	3.576967	55.20002	23.38929
116.8955	108.1052	308.4986	65.07645	116.1132	137.5674	61.39707	221.8454	125.1333
49.99792	26.54748	110.1871	25.47842	75.84037	57.55113	35.18655	101.3893	20.65009
265.6369	350.9115	177.2374	166.4428	174.3041	210.7237	429.9547	119.4588	345.9722
29.49855	67.83433	9.648625	67.82606	46.54777	33.17563	18.95109	12.04737	21.5544
254.2472	256.8946	274.1129	421.3138	223.4593	296.5891	323.3954	323.2363	298.5608
191.7856	272.4833	108.9647	294.431	184.247	106.3373	86.6896	161.6182	190.7224
388.0006	126.7392	51.55022	216.8523	224.5997	172.6859	237.607	259.9929	199.3495
2288.163	3939.259	527.9665	3669.217	1710.449	312.1863	546.4534	742.8441	4178.95
51.13066	41.89787	80.26674	40.31739	113.6675	118.0443	141.8058	87.33543	52.57855
60.16077	29.24726	29.99111	35.39522	32.90138	32.18759	37.00845	31.12033	37.92904
1.185327	5.864281	1.27066	4.048529	0.084817	59.52048	20.74495	10.04001	2.533519
80.6012	56.62809	43.16091	68.38436	54.35434	86.82809	102.077	61.23527	94.04942
29.47785	16.62597	10.83482	29.7199	18.9612	42.94501	21.67737	81.30234	25.03533
505.0644	463.1079	403.4063	421.2266	599.5209	580.4973	153.5221	278.0651	373.6022
0.023507	0	0.033623	0	0.043855	0	0	0.000699	0
104.4515	93.99814	102.9891	60.10967	142.6568	153.1713	200.5532	64.24726	77.61258
30.70233	27.8836	21.63127	17.23511	46.80583	27.3068	136.4032	22.08617	7.709515
40.79166	20.64924	21.56315	97.73097	45.27006	12.67564	70.60967	11.04354	19.82762
0.038311	0	0.055533	0.758944	0.073423	1.942107	0	0.001106	1.673616
41.97131	24.62796	13.23868	18.08304	35.33653	33.17056	19.84651	66.25057	51.82797
118.9976	88.325	27.58402	79.23435	46.70474	14.62457	34.30779	62.23718	21.52532
485.6584	156.738	91.04701	497.2426	440.3468	322.9364	350.5189	853.2558	96.59003
96.48993	79.33843	56.32341	145.9463	80.83923	140.4932	152.6836	115.4417	98.35306
73.71982	38.65628	15.63851	108.9965	35.39253	19.50346	47.87412	20.0782	50.91418
201.9819	111.3911	88.62697	137.6629	145.1547	160.0039	134.5894	68.26247	200.2322
45.43417	71.39611	51.51077	80.79962	36.69002	72.1955	58.70129	93.3564	98.40405
139.6225	159.4689	67.10607	225.9141	109.8825	265.3788	205.9684	105.4043	197.6131
240.672	228.7525	666.7168	181.2807	354.7782	509.2689	670.2559	446.7101	219.113
121.4824	110.6695	439.109	55.98122	341.9374	109.2615	106.5588	394.5066	156.1551
53.29391	31.33401	34.71727	29.65526	31.57891	37.07409	26.17808	79.2996	37.10694
10.23256	8.606392	17.8991	9.866177	13.87053	12.68287	19.9121	36.13266	9.468736
6.862438	8.54279	24.02471	25.47859	54.39042	53.64848	46.02638	4.0168	37.04331
297.3362	174.1108	370.9661	198.6503	214.5979	124.8717	156.2504	431.6488	220.8914
43.14888	45.33897	78.93047	16.41618	58.0502	50.73105	52.38953	25.09732	56.9549
4.596146	9.869339	12.05043	9.818215	12.72884	10.72164	11.7031	9.03619	5.119463
6.8666	15.8981	3.672002	8.170002	8.943818	5.843429	17.13025	10.03997	10.29878
157.7965	174.1019	336.2919	130.1925	271.3629	233.1721	188.7756	276.0559	317.5707
3.459419	11.9339	9.634411	3.225594	11.42523	21.46612	9.002624	14.05441	12.04872
89.62401	69.41822	157.7611	71.73236	113.4444	58.5354	41.52863	81.31037	69.90072
9.12953	5.200762	10.83967	12.3102	29.00053	12.6756	22.57647	15.05859	1.671304
412.0083	870.4544	446.4736	604.2998	400.156	285.8502	236.636	330.2644	418.4879
0.042488	0	0.061825	0	0.082068	0	0	0.001216	0.811479
61.26549	70.17539	28.78198	99.89296	44.19979	42.92487	44.25455	43.16552	51.77677
47.68971	40.63156	92.08665	40.35324	69.39567	48.77759	37.01047	108.4124	57.80801
135.1089	104.6395	222.6659	91.43147	154.0737	220.4863	649.594	180.6923	120.7528
182.7825	339.6177	270.5668	694.2002	414.0228	585.3793	150.8138	372.4253	327.8706
14.80517	38.69949	9.657025	52.80367	30.33627	46.83308	84.09773	3.013086	22.39609
10.25576	5.87535	14.40124	4.875418	8.923022	14.62981	11.71723	26.09905	18.11036
71.5629	95.94511	149.7127	46.91471	69.53783	79.99005	155.3248	181.697	64.65657
4.594281	0	0.061237	1.578833	1.362711	9.748366	18.05769	0.001206	6.851122
238.0114	135.1131	14.44685	108.9162	31.6408	3.892577	27.96866	45.1736	83.7328
152.1149	209.5364	76.684	263.8273	123.7747	109.2615	269.2072	69.26658	143.2065
2.323573	0.54331	1.27223	3.227124	12.65464	4.868963	4.479549	7.028122	0.811106
460.2912	134.2543	25.21835	157.5545	121.1107	15.59936	27.05959	30.11676	128.6122
2.321385	0.543332	0.05723	0.758401	0.075748	0.966434	7.205767	0.001136	3.40133

30.69219	102.2206	127.9436	79.15502	83.27132	70.24462	39.7179	171.652	65.57216
91.98983	142.6928	254.9761	36.19438	107.3953	132.6755	216.7758	86.33193	293.3773
38.5811	15.90187	32.33109	18.08103	30.32117	24.38533	30.70327	22.08546	29.32107
64.65589	61.47031	50.26392	66.82916	46.70576	50.73353	33.40307	56.21454	68.2147
6.85277	1.867539	6.051739	2.402401	10.15149	5.845261	23.52749	9.035513	5.127157
57.87661	52.71368	82.50825	53.58255	53.01736	31.21307	46.96337	106.404	52.6334
144.1239	236.4948	14.44857	401.826	70.75991	76.09207	121.947	11.04388	121.6656
157.7501	120.7511	161.569	55.16522	106.0747	120.9743	155.3772	151.5797	82.79812
82.87238	57.2919	188.9847	30.42885	112.3214	125.8592	74.05264	128.4909	99.22764
38.64741	20.54197	65.90724	31.24879	59.42809	55.60057	34.28381	106.408	20.64935
72.67167	81.34889	160.3277	84.05291	109.8201	106.3416	73.14497	166.6361	124.2655
864.9081	1010.885	641.6643	1370.869	761.2425	1037.076	1220.349	847.2431	894.7544
0.041114	0.543337	2.472121	6.534995	3.888546	0	3.574871	6.024396	1.671153
1141.816	854.7389	1965.369	825.9476	1438.964	1047.81	1101.118	2094.01	1141.565
0.029734	0	3.584736	0	0.056096	0	0	0.000873	0
62.4059	61.43415	25.20176	80.03345	19.03396	22.43056	34.30285	16.063	29.29782
365.5158	192.7057	853.4386	211.7805	536.5238	611.7086	578.097	594.2746	421.9004
104.43	90.70073	100.5672	159.1483	117.3907	152.2021	114.7183	270.0289	144.9876
42.00913	23.24452	83.68015	22.19686	31.62002	61.46845	60.53793	61.23361	47.45392
35.22033	94.92548	24.01102	80.01245	68.12929	37.06752	37.91598	19.0745	209.9984
1.173652	0	0.044019	0	0.057782	0	0	0.000896	0
6.860664	6.545107	1.272261	1.578834	2.630026	5.844162	6.285781	6.02454	5.986497
38.62746	40.61847	158.9685	27.96435	100.8759	129.7762	42.43153	204.7766	24.10762
8.001003	5.86471	25.17762	4.048262	15.23974	44.88271	27.98711	15.05898	8.571864
96.46359	84.07226	92.15977	106.3703	83.31835	71.21694	119.2642	149.5701	134.6676
79.49726	73.94866	69.50001	26.30237	114.9304	105.36	79.45664	70.27032	116.4548
1620.789	2049.982	689.5502	3209.275	1109.619	1378.543	1648.519	1241.752	1237.318
15.92127	39.52561	2.473328	61.21609	18.97533	32.20087	19.85971	3.0131	47.55867
53.39679	29.8849	35.99324	12.2908	82.12301	34.13622	30.67065	23.09002	106.971
0.043276	3.198213	21.58874	6.52137	2.629972	2.916877	3.573064	4.016963	0.811995
10.26502	4.531414	101.2977	40.45149	41.55995	40.00814	20.75884	13.0512	10.30343
19.30317	31.48703	20.35424	34.70912	17.7011	18.53466	18.0564	5.020749	40.65463
103.2434	47.966	98.10445	41.99106	122.3043	81.95379	50.56387	56.21575	108.7718
102.1947	218.8876	141.2897	95.56236	272.589	198.0494	110.1727	47.18219	302.9161
86.25031	59.33232	122.0076	50.24168	80.78112	77.07338	54.17738	76.29202	80.24849
373.4244	254.1529	506.2956	314.8677	347.1585	504.3983	345.0496	632.4183	525.5147
36.36227	47.97272	39.55785	30.4382	24.0859	23.40518	45.14155	39.15093	31.01501

TCGA-DH	TCGA-HT	TCGA-06-	TCGA-14-	TCGA-32-	TCGA-HT	TCGA-HT	TCGA-DU	TCGA-DU
417.7248	106.5874	66.89663	96.65382	102.5551	166.6384	99.51318	259.518	244.6313
15.88468	22.17251	21.01814	2.775264	10.40629	3.591958	49.19962	52.80779	20.68751
76.73403	311.1997	49.69093	79.43824	54.91986	66.93983	119.6604	106.3681	83.78424
2123.415	3918.977	2077.154	2799.128	2611.111	1712.243	3427.702	2486.396	1866.614
220.5895	28.51397	119.4822	133.6189	29.62018	188.8941	123.0797	119.3327	52.92439
0	1.072326	4.783864	2.701606	0.366709	0.040547	0	0	1.371464
5.220707	67.50448	79.37268	10.71424	65.93534	9.460266	18.5686	18.98109	23.26788
124.6481	170.9387	51.60068	26.59624	16.86112	117.3865	136.2155	235.2863	280.6418
8515.889	10372.55	5127.416	7062.653	4836.766	3754.215	10720.14	15229.02	23165.84
67.66133	36.94848	28.66096	38.45583	7.269878	78.60924	96.22963	25.02853	56.7155
13.62709	4.243274	6.674251	8.053843	10.24473	15.2901	38.92049	10.33474	11.67654
1179.198	1289.442	337.4132	146.9745	173.1008	1201.699	1314.108	848.3814	1131.008
156.5951	67.55352	174.9281	296.1459	191.1772	242.8752	129.2721	689.627	101.8477
0	3.1766	1.898483	21.71671	8.697031	0.043226	0	6.950523	0.070385
225.2501	184.6292	40.13064	53.02093	16.86785	154.8529	58.00018	40.59549	101.7815
22.74119	5.299145	61.19561	31.79395	108.4461	17.66272	6.093708	52.78809	6.561656
1599.578	2779.372	1433.833	894.3576	1240.779	1017.52	2241.531	2188.902	1366.815
50.94024	14.79497	27.70807	23.91778	7.270726	42.26745	59.52065	32.83209	23.28392
9.023012	472.566	145.3058	113.7631	58.07207	22.37171	14.39123	133.2031	74.78025
329.8793	403.1	1351.671	2323.551	3730.523	389.6669	75.25376	184.1535	167.5837
1069.717	1284.162	538.1556	191.9512	310.133	514.0679	845.1153	1080.186	1613.938
0.673403	2.131885	1.894732	2.773327	4.042506	14.15274	2.642838	11.18616	25.85179
66.83089	147.7199	3.806561	25.27229	48.63727	10.63462	4.719752	353.0677	612.1205
231.8392	65.44587	98.43932	80.82942	55.14108	213.6239	43.43809	299.2278	754.5239
50.10094	136.1048	22.92279	26.59091	32.74169	63.41438	114.8423	135.8168	136.4643
28.09932	3.188181	12.41004	13.34604	0.661134	88.95938	72.20359	15.52123	12.98786
112.4437	230.0312	284.8664	330.5863	305.423	130.3116	222.0075	303.596	194.5395
716.1368	100.2671	649.9991	646.9428	233.655	1128.927	1663.514	336.3684	508.9146
10.54664	9.5194	7.628681	0.103777	0.667706	21.1734	17.87978	25.04938	59.13191
182.6834	13.74124	38.22026	9.396336	20.03962	99.73323	159.9713	55.31589	64.46126
162.2491	38.00204	59.26292	29.21312	10.47419	76.25663	56.68559	10.32019	19.43839
187.4374	6.35433	51.61256	30.5315	32.55844	52.83006	55.30089	46.67645	69.54056
37.42867	2.132508	11.45934	4.097356	4.009411	15.28075	4.023853	3.404135	18.0485
468.2562	389.3866	953.0303	1374.291	599.8467	1233.341	973.1243	313.8829	423.903
236.4494	59.11419	211.25	211.6754	51.93933	298.0417	191.5388	63.07664	99.2923
9631.941	304.972	3944.966	1442.025	1559.839	34351.62	4046.538	2757.913	4583.38
3.701431	30.59551	36.33531	27.77436	55.59222	62.01527	6.790543	6.863926	7.842195
47.84003	66.48137	43.00307	76.71643	29.48921	63.38923	93.42991	89.96507	37.4483
9.781571	45.37865	65.96547	62.12878	128.0782	92.60293	17.1689	29.36568	30.99343
4.461435	107.5666	37.27437	75.23468	87.64729	53.95875	8.860307	12.05227	9.137975
50.08047	836.5582	148.1605	268.3745	399.054	39.97393	11.62922	242.2041	243.3553
78.37112	6.354396	26.75005	46.34656	13.64802	99.65362	60.8712	64.00726	25.86329
21.19242	25.34529	96.56153	274.2214	163.0668	72.74637	12.31711	14.64508	18.15237
158.1773	57.00005	30.56995	18.65873	16.86707	125.5736	137.6449	135.7838	148.1088
437.254	113.9819	251.3961	150.9059	96.51318	213.6176	340.3134	236.0818	168.8309
58.83541	4.243449	21.98339	12.00374	7.187137	19.96892	76.66972	5.999467	14.24325
79.85944	24.29074	83.17016	79.33178	66.98708	137.1606	80.26792	44.94011	28.44332
3642.519	2576.793	558.2243	1177.448	794.7304	1984.502	2015.988	5976.124	9777.912
499.4775	109.7642	438.7485	76.86439	294.0047	232.4261	46.20849	1229.925	1196.449
22.07192	2.132508	10.5023	0.099166	10.18462	3.59063	6.801761	15.54907	6.547506
29.7466	2.13251	3.805684	14.60472	7.133184	36.26387	9.586856	12.9432	9.109037
418.2604	369.3018	86.01285	92.72592	153.6485	154.9566	199.8217	429.8898	127.6253
90.50688	15.8514	36.30934	92.54669	13.66911	106.7385	110.0742	27.62291	45.16378
11610.45	1421.37	922.4209	734.312	389.839	10061.82	1313.945	1210.716	2330.384
35.05871	9.5163	15.28404	13.31811	4.029186	68.96323	10.96423	7.731768	15.52334
132.4983	16.90595	33.44267	21.29223	16.82844	84.4537	58.76783	99.5145	54.1356
41.9333	36.91355	4.761247	0.101415	13.39098	11.7925	24.89168	59.82124	16.81118

5407.857	6410.3	1300.956	1366.624	2197.331	9737.939	4642.101	3309.698	1873.098
113.9485	327.1088	341.2576	860.6781	144.2169	359.0855	561.791	161.6816	172.7
83.89568	3.188153	51.63305	48.85816	4.04489	51.57854	21.35805	4.267647	14.27392
54.737	179.2753	73.61487	33.15719	88.27175	34.07746	64.35639	83.94308	86.1829
0	1.075908	60.25392	514.8814	83.11855	1.239054	0.578563	7.728198	0.091803
308.7398	200.4918	81.23396	100.6497	83.72848	328.5308	149.3164	209.2826	113.4521
43.48635	8.46216	32.51178	18.57033	13.35589	26.97722	18.62575	6.86525	23.19556
34.96864	23.22717	14.32251	10.71033	4.044842	15.31652	41.53349	29.38755	70.62331
107.2391	125.5498	28.65933	58.2792	26.38083	43.47742	114.881	142.7655	232.7487
129.9441	96.04202	230.3735	179.9427	226.2558	359.0079	456.7633	180.736	51.64873
7.516252	76.8927	0	0.099397	13.22	15.28292	21.45749	24.23684	20.60392
23.54325	3.188252	36.33418	4.101249	4.038239	70.19284	45.10495	4.267833	18.1002
265.3518	92.87818	101.3088	107.2646	32.81935	354.3484	282.215	160.8278	274.3528
64.70202	7.409717	41.10047	23.90909	7.267774	66.82882	104.7706	23.30852	16.85408
110.243	124.5069	58.29627	34.52317	7.252731	54.03912	39.99546	96.85118	82.49103
43.33208	30.61219	21.97153	18.63389	19.86305	72.67181	53.99709	18.9789	30.97466
285.121	176.2299	88.88085	124.4554	156.8006	185.4546	336.1949	277.6193	292.3866
31.13152	47.47737	21.97245	15.99212	19.82607	8.28742	36.63213	53.65145	43.78376
86.57389	131.9182	335.5259	301.5905	299.5749	254.671	53.12085	223.973	278.2462
152.7599	341.8621	95.57342	46.43846	93.26124	157.293	307.1566	397.0392	198.4068
3230.656	153.0268	1011.328	599.3652	230.4841	867.2959	1001.391	787.825	1719.664
11.30134	9.520309	17.18775	23.93551	13.66215	68.06517	11.62514	4.269139	15.57901
833.7056	6.352155	138.5908	26.59423	20.03309	436.4728	55.19619	52.69661	73.54571
66.15051	3.187511	24.8369	22.61232	13.65709	15.32859	80.98247	18.97169	19.43807
50.14131	42.22062	38.22397	71.40705	41.95513	27.05354	28.24824	167.0989	28.43978
338.9828	4948.73	491.3135	792.4693	899.5247	1002.236	2373.818	626.0959	1181.271
55.42587	151.9291	156.7837	104.5073	374.4025	22.37135	92.64895	133.2119	59.339
131.5058	46.45097	84.10592	17.335	23.25114	38.79973	474.3467	72.60126	73.52038
205.9492	463.2318	641.418	1130.648	1011.733	641.8808	235.048	276.7098	162.4228
13.58204	259.5142	125.2269	53.02074	102.1362	47.00385	316.5757	219.7804	54.19699
330.0762	70.71985	167.2758	263.1763	396.8364	317.9549	120.9472	86.43452	61.94967
111.9779	21.12417	36.31444	26.56004	10.46468	14.15379	61.58885	21.57167	19.43173
616.5809	686.9282	189.2493	100.6759	118.9026	1401.03	534.5852	847.5766	1312.377
2469.432	1179.722	1134.636	1672.1	718.143	1877.672	2828.821	995.3849	1227.691
0	0.01199	5.757695	10.12929	5.561969	0.036595	3.481981	2.583907	0.059099
80.69257	11.6303	43.96628	46.32546	35.53894	75.04783	81.74872	21.57228	51.52945
18.90641	270.0042	8.584334	8.072998	4.029192	36.43744	5.405502	59.65305	113.2486
402.8678	608.8576	1040.979	1042.332	860.7024	645.4751	737.2387	470.4326	394.2748
101.8806	38.00787	42.99959	59.61477	7.256692	148.9726	135.6426	36.27111	76.05
108.014	10.57552	47.78233	12.04358	13.67226	68.08808	105.1875	35.40991	65.73935
72.2316	82.298	49.69774	21.29528	10.47605	16.50244	78.17908	105.564	31.01629
171.2696	24.29204	5.717259	5.422635	0.704704	27.06056	1.958072	128.0521	103.0035
223.6417	54.89065	41.08549	58.32131	26.43585	79.84956	233.2478	158.2803	148.1229
313.9887	262.7511	207.4185	432.4847	188.7073	282.84	143.0552	187.6314	155.9643
21.28743	8.461039	4.761628	9.367555	4.019759	4.763959	9.580018	58.13771	93.18451
20.45173	1.075702	9.540652	1.444318	4.045092	1.238404	1.95281	47.58285	73.24209
35.77316	3.188247	14.32445	19.9108	4.04028	30.50669	28.34991	5.997774	21.94732
55.56044	43.26163	33.45021	38.38148	10.43793	62.13245	42.19062	25.91109	45.06956
31.97949	8.462479	6.673614	13.3235	4.033032	32.82051	16.52904	50.26798	7.839969
61.56688	112.8763	21.96813	8.073493	4.033631	71.57874	12.31694	96.90742	70.83918
25.08426	15.84173	36.33757	13.32567	25.53487	16.47057	6.098271	25.07745	20.65014
2336.396	1192.382	584.9908	875.8233	912.4587	1522.1	1525.677	1400.152	888.9243
108.905	19.01499	19.10076	25.24453	10.4678	71.54957	10.24257	17.24261	30.99968
209.7414	360.8921	821.1256	1499.706	656.6419	363.8463	216.3546	423.7405	622.1824
418.9119	375.6537	282.9352	176.0553	144.3252	238.2793	358.2238	329.482	322.0888
4.464445	24.26102	113.9389	62.91627	10.20161	23.44958	6.105268	4.269351	2.69198
54.28135	1.076137	28.68784	0.100046	0.621417	21.1259	17.94755	4.26887	9.115147
98.04138	157.2121	74.54821	95.29559	83.37127	63.42589	129.3443	108.9584	86.36622

9.792117	59.04397	22.93539	36.97109	31.73154	8.28339	0.578227	99.74718	37.29313
209.5543	23.23541	35.35516	23.93307	10.47414	195.6721	40.03552	26.7612	29.72535
127.7303	27.45862	72.63575	38.49301	29.60924	43.48898	165.3734	35.40251	33.61211
521.6278	35.90016	107.998	34.531	58.33047	225.366	192.8641	41.45404	121.2029
126.9613	134.0087	64.98659	78.13447	80.26764	72.8104	55.2179	141.8419	150.6817
83.58964	84.42473	101.3223	91.31581	183.745	107.9688	100.2637	91.65631	28.46003
74.40579	7.407467	36.305	25.27018	23.22687	423.5894	228.8755	257.7088	319.4472
78.39036	5.299004	34.40136	23.92336	10.46603	57.49673	40.05729	14.64663	14.28825
23.53483	33.76061	16.23754	34.35639	19.6111	51.53504	8.868875	23.33095	50.08308
18.92586	8.464695	59.28019	22.58266	44.52409	36.39482	42.88814	16.38255	12.99498
204.4804	51.72835	196.9074	138.9954	36.00932	143.2229	317.5094	97.67522	164.9548
296.5719	38.01093	204.5592	82.13997	29.63001	149.0789	410.3457	66.53712	87.70277
385.4237	710.1268	336.4657	447.1412	344.7636	227.7269	538.0991	481.7121	333.6984
2381.142	7138.342	1035.218	543.8248	587.533	1792.051	2269.125	6017.622	7154.19
63.37109	5.298733	22.937	8.062799	0.641993	30.49538	8.872366	22.47042	14.25477
28.10792	20.06248	14.32337	27.81187	7.241888	9.45716	27.63285	38.07022	41.16354
41.75442	46.44024	22.92428	9.397132	23.15455	76.26283	182.352	37.14844	56.71127
0.693306	0.01121	13.49919	7.586843	22.28522	2.355436	0	0	0.054897
2.942564	48.47493	0.939221	0.099416	0.613927	1.239787	0.577021	126.0594	135.0225
0.675655	2.126873	9.581374	12.92505	26.09152	10.45527	0	0	0.071735
22.05272	10.5691	16.24305	47.31178	7.162314	46.77812	27.73137	5.134531	11.67533
180.2462	50.67046	191.1932	132.2908	83.45791	133.7861	159.8041	66.54793	54.20989
66.943	20.06969	57.355	60.82648	32.4686	83.25246	53.94093	58.81543	27.14439
605.1929	483.2852	359.4058	230.2934	233.5297	430.7196	637.699	493.8092	407.1165
256.3605	90.76095	264.8124	119.0985	181.3223	103.3072	162.5554	262.1331	36.19009
23.66392	6.349866	9.548408	2.772654	0.584965	15.25765	27.85505	7.742979	7.814094
1003.572	25.34588	206.4546	119.1947	35.92932	258.252	38.60792	921.9113	1573.991
196.9198	40.12109	186.3993	346.3613	213.3831	249.9205	86.34546	38.8595	52.93408
12.82241	98.14043	112.7936	67.55856	202.8358	335.3629	45.52942	40.59388	13.00417
19.72878	4.243585	90.90635	72.27112	16.45076	22.31384	26.28518	17.26715	14.25145
202.9353	71.77686	284.8537	116.5343	64.69989	213.6236	170.7355	274.1403	96.72979
350.5792	223.7036	104.177	58.3416	118.6651	149.0837	213.6871	305.3177	312.9519
133.7906	141.3983	55.42505	46.42995	74.03462	133.797	51.05786	177.3109	162.29
127.8298	22.18196	48.7386	18.65614	16.85682	18.85001	94.09107	37.14103	36.17006
48.7608	10.57307	16.23679	15.97343	16.58088	41.03413	15.11582	9.459783	5.27218
50.91833	32.7267	84.13457	45.02619	57.40049	68.04228	26.17567	80.47111	14.28937
186.3884	65.43781	4.762027	29.23765	32.77132	213.4621	91.94141	86.46033	128.7899
21.34449	45.29193	18.16595	28.90891	53.33736	5.929425	7.509382	4.27148	12.91965
0.670367	2.131906	26.80464	95.94774	12.7353	7.085929	0.576419	0.813904	0.084385
40.22769	16.90623	45.87277	30.53833	7.269641	49.32369	146.9072	25.02831	38.72872
18.16482	74.88098	18.14698	33.11177	13.57722	39.90214	58.89685	51.05225	65.55571
18.15	221.4579	76.48085	121.43	100.8498	51.65325	98.36646	48.4124	54.12352
255.4795	46.45232	233.24	96.68259	74.19316	220.6276	370.8716	76.91717	79.97823
13.58174	73.87108	120.4538	113.7033	67.34651	58.71898	19.92544	120.2457	43.89296
439.4178	251.1544	110.865	105.9661	153.9176	682.9537	658.4916	689.3139	283.48
44.06325	36.94398	19.10089	6.750055	19.94473	7.114626	33.81057	67.4811	129.7723
44.08181	66.46104	26.75237	37.09067	66.32696	30.55428	6.784831	170.6794	27.13308
54.70336	48.55052	23.88017	18.65287	29.46642	59.86851	62.22101	48.40238	64.4247
96.70904	10.57532	119.5318	60.82014	41.78735	25.87494	85.21059	43.22305	11.71437
450.617	2.130743	47.77785	33.20632	13.66815	97.43451	88.45901	12.91511	51.63362
88.23408	17.96135	66.91053	50.3341	10.47634	38.77886	192.7185	17.23995	31.01766
261.4995	54.8941	83.14428	94.05537	45.57523	443.553	199.1024	87.29175	197.1675
70.7037	54.87912	13.36369	8.073291	13.66572	72.75756	32.39971	118.5539	209.4859
46.4109	15.84775	82.24714	44.94062	25.98875	24.68658	31.77446	22.44843	21.98004
255.3669	231.1085	475.0738	904.6885	882.2536	782.7104	328.4259	1489.424	215.2352
65.28032	662.6524	330.7285	70.25016	51.93768	99.82629	331.8958	117.5598	342.7227
24.24321	12.68557	52.57153	22.6071	26.23968	120.7171	112.9778	24.16709	9.139469
203.821	241.6127	58.29304	31.88631	77.20282	142.0058	89.82669	151.3471	170.0092

112.5062	15.85164	30.57009	37.17067	13.67003	97.42957	51.75965	137.5211	191.8076
71.52882	14.79525	26.75105	51.59878	16.79157	83.24511	74.79056	20.70597	23.28744
97.2123	147.7495	1002.779	1750.604	1814.417	118.6009	109.8357	440.1982	64.53311
206.772	265.9044	201.689	424.4262	201.0975	258.1689	107.7844	129.6847	73.54256
182.7625	19.01651	29.61647	80.67424	13.66811	155.9243	296.8469	30.21977	40.02008
11.30746	3.18808	17.19083	23.89452	10.42861	104.2038	49.85807	4.267728	25.82953
130.7829	20.07251	106.1006	117.7385	26.4266	81.01348	187.5649	50.97663	67.0689
14.35034	5.299123	68.84236	104.1557	102.9318	138.1696	13.01572	31.1054	11.7115
55.53196	50.64642	14.32081	13.35877	4.043469	34.06577	54.67421	29.37052	52.78972
799.8188	396.7707	341.2392	101.9983	80.63547	307.5327	384.4383	1293.006	2571.856
38.02313	13.73788	19.10468	12.02976	10.41215	7.113606	47.10112	11.18952	18.12453
83.60724	20.07227	35.35123	39.80265	39.0535	62.23965	123.8663	24.15867	36.17797
72.13411	509.582	111.8272	83.45495	86.84344	49.36194	210.9614	163.4383	117.3003
357.6063	38.01029	70.72121	63.61359	16.86289	23.54583	26.83955	83.84903	257.4717
16.00794	1.076119	9.551732	11.92143	3.935128	23.36478	8.93162	6.011886	12.88153
995.3427	442.136	303.0051	539.7352	274.9111	516.3727	580.9575	461.8032	304.0966
28.87488	5.299014	25.80342	18.60628	13.49576	66.73741	27.63627	22.45902	33.48173
665.1939	1945.73	351.7528	399.614	246.3965	482.3811	441.1462	1069.817	1903.66
12.82957	1369.481	457.8877	632.0696	582.0326	111.5554	811.657	187.6309	9.128087
85.12788	12.68599	20.05494	18.65798	48.52785	7.112857	68.40527	63.10025	47.75695
47.05696	66.48964	132.8813	42.44583	242.7729	79.82459	77.41383	85.60584	109.4708
333.7419	421.0178	282.9374	757.7133	404.7598	372.0145	292.5047	153.0261	116.0521
59.27302	39.0585	194.1126	91.20685	175.823	73.92844	34.47924	56.19433	31.01722
368.0393	517.0047	95.57226	26.59418	96.4945	211.2656	306.4142	157.362	148.2259
1555.561	975.0101	992.2122	496.1875	485.4217	1082.028	822.9449	1940.745	2712.617
101.918	24.292	52.56329	161.1384	16.85717	109.0981	62.88419	40.60298	19.44381
20.59026	7.403178	14.3368	5.406307	10.02417	2.415716	3.334876	1.675955	10.36337
456.9877	423.1235	178.7386	125.7946	153.7937	190.1654	331.2751	257.6974	296.3069
672.1811	3.185236	42.04064	46.43753	45.56378	1670.692	249.5827	37.13094	59.38025
697.8879	1159.651	395.7246	337.4507	462.9993	746.4011	1116.304	776.6045	609.3739
93.64591	9.520231	35.35741	48.97243	16.80064	97.29918	87.2833	39.75615	29.7143
31.91679	1.075825	41.10924	27.82525	7.250204	56.24769	33.88233	12.056	16.83914
51.64122	34.84091	58.30133	66.18184	98.45507	222.7046	15.77508	27.62199	25.87771
62.23987	32.73362	687.2952	359.8958	1149.108	51.70943	98.76714	373.5765	109.6187
2.189376	136.0916	218.0065	91.25126	170.2501	76.29013	2.646906	21.56462	3.978727
8.270763	12.6795	38.25272	119.3153	43.4737	16.46962	3.329706	3.403378	3.982584
824.1443	948.6129	259.9859	136.3925	163.532	596.1914	386.5099	1156.333	1321.508
63.81482	82.30873	43.95672	19.97937	29.5508	43.4785	205.0506	78.68771	67.03616
30.49412	7.406698	9.544521	14.61488	4.017693	28.12096	37.5392	12.93952	14.22976
104.1103	113.9696	151.9888	33.20831	80.34708	115.0332	201.3523	92.50397	58.07445
88.13032	142.4514	11.45228	21.30466	13.66443	9.459801	107.1467	357.3533	377.0204
25.81083	20.06326	41.11068	30.4478	71.38285	52.73372	16.49992	18.12164	19.40145
149.7435	65.44355	116.6084	53.04643	61.43639	214.7353	240.7583	71.73075	96.7013
34.18422	1.075695	31.53748	13.35411	13.58177	34.05359	97.87541	8.591632	15.56463
11.30829	90.68306	41.1061	40.99016	22.88019	50.41969	90.96093	11.18878	14.27656
2.188566	29.56548	437.0579	91.20987	135.5228	21.19414	2.645773	2.541998	28.44593
2.188382	21.06137	10.51531	14.46298	23.53426	7.074935	20.98763	0.814171	1.395802
108.6766	143.5073	61.16132	91.35783	32.80459	181.8665	205.5086	86.44671	127.5547
177.805	767.1185	248.5162	141.6822	45.54101	227.7351	311.8164	427.1984	690.4395
3.709608	23.23814	217.9627	515.1468	9694.006	8.285451	22.68978	16.37395	72.2296
8.285345	20.0444	61.26669	122.6444	39.47124	5.932282	6.109368	9.473753	6.544453
145.239	50.66984	83.15253	54.352	26.43081	131.4324	231.9004	52.7058	78.65205
0.67515	0.014404	73.0913	9.131455	3.602257	0.044434	0	0	0.072461
88.25194	20.07097	31.52983	62.18943	10.47508	64.549	49.74189	38.88039	65.69968
56.96837	28.5119	82.20645	30.54873	26.36961	112.6137	67.04335	17.23904	31.02547
1200.666	175.1852	258.0758	449.8268	188.9855	678.2947	652.2068	211.8316	356.9027
276.7123	44.34208	284.8524	220.9884	80.62667	421.2655	574.9177	98.53588	96.73106
311.7083	231.0985	201.6828	251.3895	118.7978	284.012	408.1179	284.52	145.6628

4229.421	2273.961	5963.847	20320.36	4890.231	6623.4	2713.135	1312.769	2106.239
73.67779	6.353316	21.96655	54.35358	7.243218	17.67781	5.410327	111.5499	617.312
56.15738	651.0424	776.2068	612.4193	1116.719	916.4179	142.345	158.2104	204.9242
38.79743	9.518946	10.49745	9.389423	4.04433	44.55563	13.72082	26.78961	23.2489
1746.389	1192.378	1111.699	545.1337	1527.027	832.092	1556.147	1612.065	837.3894
103.3199	117.1425	363.2678	234.11	317.8658	158.4568	327.2795	180.7387	108.2955
11.30712	100.1737	35.36449	62.03554	68.91405	66.8004	36.63699	32.84685	10.42268
222.8204	32.73484	94.62079	31.88731	35.99867	176.0262	170.8313	75.19329	64.51743
2.183245	20.05295	16.24204	150.4396	22.34448	2.416884	4.71449	16.40741	6.55269
2.945765	32.72812	137.6928	367.5943	301.6261	23.53626	10.93373	23.29934	3.980693
190.9478	126.6205	173.9894	104.5317	105.3813	34.10399	137.6698	65.6879	137.7978
278.3428	31.67976	135.7279	51.72579	36.00664	195.9809	220.6565	71.72941	139.179
2.183566	51.66378	27.72206	40.87881	106.0175	51.50878	6.097581	7.729984	9.125778
2.184949	13.71709	7.635464	17.13734	21.44444	19.89152	6.125669	3.407015	6.528075
47.8386	24.29142	50.65265	43.74305	57.74057	20.022	320.4499	43.20383	38.73509
22.72023	45.38131	88.91773	39.75204	79.06444	42.27863	78.24113	59.67825	16.86219
71.36459	897.8652	233.2328	197.1723	394.6636	138.5357	260.0274	804.5144	116.0412
20.57609	4.242174	12.42003	11.95957	3.98208	29.2307	12.40265	10.34743	6.538781
251.6611	218.4289	72.63007	187.8925	80.5664	151.4304	159.6927	191.9747	252.4683
38.79114	11.62852	16.23546	13.34734	7.251327	34.03756	27.61805	14.65421	16.83994
76.01615	178.2728	25.79154	29.22656	26.36287	63.39829	57.34128	44.93177	88.86378
494.1216	122.4266	55.42355	219.7249	71.06383	77.52793	352.6175	2554.145	4351.425
3.702745	5.297872	23.90228	18.54328	54.5449	28.11931	5.409735	3.403863	11.67217
231.4817	26.40209	0.94006	46.39551	4.024815	30.57892	14.39112	40.60224	88.87422
613.695	168.8495	277.2034	189.2631	141.0808	165.5279	427.4738	172.0583	206.1848
184.0658	34.84443	71.67914	101.9079	73.94781	160.7437	145.2669	181.658	63.21509
207.7375	4.242465	26.74651	21.30365	20.05533	211.1015	91.25848	55.30666	90.20595
229.6483	112.9204	90.79559	94.02485	48.73134	314.4108	301.7017	109.7974	106.9987
373.2446	42.23098	195.9415	143.0022	29.58028	665.368	542.9191	127.0734	194.6248
30.32637	223.5943	139.597	187.3772	85.71362	21.19369	7.476905	40.60981	66.9937
35.88108	2.132517	12.41594	22.46069	10.20173	25.78156	18.65934	6.868304	6.548707
54.65048	29.56908	64.02993	50.39224	35.97663	256.8834	166.0351	77.79613	122.3996
25.04573	31.65873	16.23583	17.297	0.660372	23.50709	3.330015	24.18883	53.9731
15.10722	8.465034	136.7489	63.45906	128.2885	43.44645	22.0188	38.89065	13.00166
140.2328	3.187765	4.761147	1.442999	4.0412	4.765983	0	30.23152	69.49076
130.8114	27.45812	69.77016	22.62576	16.8673	64.59018	190.3965	28.48342	61.91134
37.14956	53.83864	281.0375	1359.837	482.8965	154.9524	255.9032	661.7942	101.8713
20.42523	5.298289	24.83491	8.071621	4.020492	30.58125	128.0338	12.91447	9.138382
84.58322	27.44818	20.05919	15.99416	13.5913	37.5681	10.24593	31.9757	24.55468
29.6463	15.84461	11.4545	9.385673	7.233701	60.88481	26.25174	19.86018	23.2335
33.47672	7.40874	30.58995	8.065578	0.649214	23.49477	22.77596	5.132567	24.50832
141.3949	576.0017	53.51277	111.1833	99.36953	77.51035	69.05279	244.8102	378.3688
1514.394	3478.973	1939.501	3158.938	1231.39	1699.341	3200.804	2450.928	2976.956
2.947282	8.464909	161.5838	23.94168	101.5708	147.7459	29.62197	38.87329	46.45372
62.23884	706.9573	383.3088	328.1297	649.6665	309.8547	210.8358	567.3566	180.4515
80.55248	34.84318	133.8356	49.05296	186.6566	102.0994	66.32049	292.5324	13.00483
105.6034	208.9273	198.8257	203.7161	406.2414	120.9219	130.6406	122.7709	65.81186
47.89681	11.63005	35.36013	19.95782	19.89824	90.23962	55.36427	33.70112	30.98441
91.14087	412.5513	618.5218	807.5989	321.4852	32.9342	357.6746	537.1979	430.0727
84.34722	13.74116	15.27536	14.68953	7.250269	184.1604	84.32105	38.86443	85.06996
29.5916	77.00137	90.84356	77.87998	63.2015	36.407	26.88665	36.30178	34.83178
49.36674	25.34601	58.30377	12.0433	4.03147	27.05689	58.05399	26.75868	25.87366
1.427363	3.185334	40.25396	50.52858	15.24993	1.238486	1.263053	0.814203	0.082028
0.673159	0.01498	12.45263	13.0221	29.67831	1.234802	2.668966	0.815918	0.075728
16.71217	5.297226	6.67531	21.13005	0.604516	5.932566	17.27905	2.539561	1.398579
22.72737	75.95058	59.27227	79.21572	38.58699	31.72699	20.63826	46.69608	34.83796
14.37301	7.408461	11.45529	0.101906	4.037876	7.110995	66.75879	18.99847	25.77672
262.3775	86.54465	128.0808	80.80719	67.78495	296.8195	206.1307	89.0334	97.98852

294.8949	89.71527	64.02648	30.55295	55.10265	1059.667	7.496438	52.69909	126.3651
0.675304	3.186944	203.6478	235.0612	120.7075	12.98334	30.30731	173.9232	1.392564
0	1.061625	0.949769	2.562613	4.717077	3.429137	0	0	1.307197
40.43631	4.243397	4.761445	1.446477	4.026571	2.416891	60.58904	5.999762	21.90897
1998.821	1325.331	578.3001	554.395	536.4499	2395.126	1446.844	1655.304	1791.803
6.745479	48.50204	0.939337	6.743249	0.639952	3.591925	0	8.59648	23.21039
23833.24	44243.21	12809	14198.07	16804.21	16390.87	18162.54	37662.03	42080.15
46.29333	66.49065	171.1286	125.617	133.4153	139.6044	67.01292	55.30843	67.05335
326.9529	300.7269	110.8675	129.748	128.2697	337.9505	257.2655	262.0404	195.8593
212.0889	55.94899	219.8517	277.7736	58.31404	273.4255	430.3595	134.8729	194.5604
691.1622	70.722	469.3426	275.2475	35.97634	463.5595	803.8058	108.0465	264.1635
1367.742	66.49958	208.3662	242.219	93.36025	200.7508	1401.916	1665.701	6258.204
180.8809	10.57361	185.4322	60.98984	20.02601	232.3901	77.33077	67.39934	188.146
15.89566	48.51114	50.68747	93.32134	16.5417	18.81777	4.711794	13.79303	10.41317
99.00081	4.243462	92.74771	50.28262	13.63623	48.12804	45.61399	11.18572	10.42697
12.84837	11.62584	15.28242	4.100971	10.32537	1.239414	24.18829	86.74948	58.96788
108.7442	84.42031	63.07887	50.36741	70.53748	14.15702	206.4012	110.7151	37.46412
248.7247	39.06549	79.32524	49.07536	48.69993	191.2576	165.3095	185.9596	224.0419
24.23273	298.4762	66.90855	125.5075	126.4135	92.68316	44.86965	178.3068	50.3061
132.2124	123.4762	30.56966	21.30129	77.40364	83.39113	185.9856	93.35008	233.1765
318.8233	42.23022	51.60152	33.20747	35.9765	242.8161	186.1283	64.81692	190.5628
2.183305	6.351699	37.31374	70.67693	19.12573	4.762462	0.576878	2.539486	2.691665
1458.156	3821.884	936.762	1596.73	1314.069	1567.888	1886.695	1980.466	3058.017
112.5017	45.39476	51.60217	82.0933	83.39759	42.31627	125.18	215.4214	41.33611
34.12166	148.7538	157.7495	43.76008	232.9667	71.61152	2.647609	83.01636	96.5976
32.70983	40.08285	2.849645	4.101509	7.229973	16.47879	17.90238	5.997681	10.41349
283.5298	437.9041	72.62893	197.2128	128.4121	287.5557	181.0928	670.3139	839.6412
1525.044	2943.992	1496.919	656.2642	730.9703	2479.711	2167.474	2611.8	4270.149
683.6692	70.72191	262.863	205.1325	67.89211	296.9279	354.1041	100.2648	220.3514
60.80855	7.409699	42.04865	13.36513	29.44123	61.03371	94.857	6.861629	47.71709
2.183703	22.16704	419.2954	40.89644	37.71899	14.13618	0.57814	0.814482	5.271258
116.3119	112.9094	119.4859	95.29667	118.0337	31.75836	80.84616	134.0582	121.092
222.8144	28.51415	196.9191	117.8039	96.28274	178.3747	247.0106	77.78808	88.97461
120.9938	7.409562	48.73981	26.58231	35.80078	106.7399	78.15645	80.43448	46.44991
46.71776	3.187728	6.675801	5.411696	7.087173	21.09494	48.1843	6.871802	7.821309
38.05448	5.298982	17.19355	13.33962	0.653363	59.72219	10.25497	9.459962	12.98239
19.20001	2.130855	3.809424	1.441656	0.512577	26.73778	6.867469	4.283489	12.80603
45.68191	2.132303	14.32303	33.07528	4.044039	57.4072	49.91169	7.727618	45.01257
0	22.14847	0	54.98469	36.47363	10.60481	0	204.6206	0.087823
53.94481	7.409677	51.61096	5.424266	10.47551	30.57196	76.10523	12.04986	38.72647
89.01421	39.05735	21.9682	64.82613	38.85551	56.34931	53.21041	75.25369	24.58443
54.75634	28.50581	23.88284	17.32118	16.77561	20.01349	26.18664	70.09341	151.4971
60.19508	4.24375	25.80107	18.61677	7.251345	58.58787	16.49633	30.25555	15.55664
180.3282	91.80669	85.06897	72.82317	95.7721	55.20971	30.99683	83.87049	83.77068
51.83778	5.298931	8.585514	9.385002	16.55131	48.03371	17.20596	5.99764	6.558865
7.522156	29.51661	11.46102	17.20096	3.995365	12.93853	31.31151	14.68772	16.75472
56.96804	118.1643	62.12524	37.15226	129.7748	31.75115	127.3838	85.6208	32.31201
27.29172	94.93701	43.00814	106.9123	60.45629	18.84438	30.33909	53.6155	46.40914
22.75438	1.075839	4.761002	8.069283	0.660791	12.97292	17.88806	116.1696	18.12061
3.705546	2.131342	85.07804	103.0983	368.585	41.12676	19.92815	8.590932	5.270222
27.34963	33.76286	11.45435	25.17656	7.236673	7.112412	15.1165	49.35734	60.32752
136.8341	239.503	100.359	71.54175	105.656	88.06376	161.8557	238.7588	302.4756
82.16007	8.465042	3.805769	56.90956	7.271065	11.80931	17.85516	33.68688	46.42957
31.83297	48.55848	8.584692	0.107146	4.013689	97.41953	9.552799	25.02275	38.75734
23.47463	43.27687	67.86796	79.3352	321.0746	9.462268	8.167898	178.3392	20.72688
60.75458	150.8743	159.6525	249.6416	246.2083	120.856	62.85357	95.98797	49.0482
56.37647	27.44319	5.71685	1.445058	13.53107	16.48641	6.786457	51.07373	20.68685
0	0.015206	21.09272	46.2133	27.50056	2.404894	0	3.420401	0.077022

127.6242	2097.697	3199.443	13369.4	4260.817	728.8177	208.0429	673.6674	78.69682
0	0.017057	99.59925	1.446594	47.99875	2.416386	0.576702	0.813883	2.691106
111.6926	245.8492	303.0386	576.1989	150.192	258.1353	204.0377	209.2981	140.4659
206.759	176.2301	162.4909	129.7442	121.8943	312.1376	194.9796	132.2764	296.2502
21.27088	60.07613	21.02628	34.28552	43.26675	4.76491	10.9647	40.72314	16.80156
5.982868	35.86552	34.41941	26.47117	25.65132	19.98407	46.49459	7.72933	11.69427
100.4105	33.78571	53.52075	25.26185	20.02434	136.0256	105.9043	38.00864	29.73579
1767.867	86.54959	569.7041	292.4744	115.715	977.5395	1101.127	266.3152	467.6882
130.0527	27.45808	80.28808	30.55712	42.24236	50.51969	138.4013	56.1735	36.18114
120.7889	958.0356	439.72	669.1225	284.0536	72.83447	426.7736	642.6631	186.8737
46.45994	16.89964	21.01966	19.9224	13.49845	49.21699	26.93892	0.814712	11.70054
212.1149	212.0947	51.59964	55.6949	150.2947	91.59887	228.9398	426.4756	193.2459
1808.974	35.89884	21.96854	47.75521	10.36282	14.14751	9.569648	1156.34	1706.523
5058.876	2304.565	8386.093	14548.13	5983.779	5789.075	3610.169	1538.491	2417.98
42.50401	29.56627	15.27549	23.94097	35.80892	51.67582	31.00853	96.02047	128.6673
3395.432	556.1112	1167.133	1565.014	998.704	2120.622	4115.911	1051.59	1266.365
5.220897	69.62131	52.57863	75.24361	44.69679	55.1321	19.94835	7.726133	15.56965
218.1904	293.3324	310.6782	275.1024	308.6602	109.198	215.77	191.9777	108.3035
16.62777	46.43721	17.18818	9.396994	32.52291	51.65285	81.00339	49.27884	113.1495
12.06604	196.2677	376.654	494.3443	507.2717	47.01564	71.11542	159.9744	79.97407
131.5014	151.949	163.4599	179.8678	395.5997	207.6717	79.43764	86.44396	45.20411
0	0.008639	0.949672	2.564115	2.715059	0.025985	0	0.842963	0.041426
14.35404	81.20423	40.14753	62.03762	87.20451	42.24138	34.55007	89.21446	28.39706
15.87485	29.55757	80.3248	72.58921	59.97492	25.86455	12.3237	110.0068	3.982703
35.68362	11.63024	16.23276	6.750136	7.270977	35.24293	58.12715	9.455776	18.14436
49.36695	109.7171	73.60484	83.29965	141.721	38.77878	19.92989	155.8035	111.942
247.8924	382.9948	15.2759	5.414832	0.724143	66.96109	186.7192	33.67004	87.69784
17.38311	13.74124	89.85157	42.44519	20.04935	43.48172	269.51	31.94514	61.90339
4.464949	4.242839	86.03109	96.53376	98.56744	134.8756	0	7.726698	0.095182
961.7489	354.5668	497.0518	523.9423	150.7851	663.0896	1865.523	581.9988	912.033
191.4994	69.6668	442.5765	478.8745	99.77089	483.5019	1241.165	146.1019	363.3152
14563.17	8210.48	17761.52	47769.46	15018.29	21111.91	12401.4	6540.729	6202.582
28.87187	7.409095	35.37101	10.70671	19.6818	16.48326	45.04637	17.25647	6.559967
235.0443	45.39571	67.85357	265.6724	42.33647	115.033	148.7022	57.89375	58.07437
12.07153	381.4631	19.10465	113.1714	110.9445	9.458829	0.579116	31.98787	7.848198
44.96752	6.353775	9.542166	9.382642	25.64916	28.16517	33.23867	17.26271	10.41124
16.77275	6.347945	15.29925	1.445549	3.941571	12.91162	7.526873	3.407341	11.61649
25.01821	8.464882	79.36471	255.2663	148.825	35.23464	28.27713	18.97781	15.56945
117.8251	40.11982	186.4142	181.1332	168.4932	154.8839	125.1719	21.5625	34.90054
5.227483	20.04695	37.31267	18.52762	22.10228	25.77417	5.411956	7.736374	6.547023
101.9405	25.34642	20.05551	16.01092	7.267371	51.67384	97.58511	98.62232	133.792
161.2759	19.01726	91.76152	42.4505	29.58675	88.03746	155.0319	36.26989	40.04255
73.32128	4.243656	9.542302	4.101206	4.037839	34.00284	17.21486	3.40332	11.69292
345.1154	540.256	208.3692	173.4187	421.0596	387.2959	269.6373	503.3367	351.727
63.98461	7.40953	17.19094	8.071345	7.259461	194.1743	32.46982	13.78591	10.4221
74.67832	10.57442	11.45303	2.774689	13.57195	51.59644	119.4961	13.78559	25.83017
183.1486	101.3217	281.026	125.7975	112.4719	557.3614	444.0663	120.1581	179.1503
18.90528	207.8028	4.761626	10.72026	13.67224	14.15688	3.336339	7.726647	46.45765
121.5613	83.38277	355.6048	486.5845	248.7735	329.7334	361.8022	239.55	61.95468
73.87244	15.84894	37.27488	9.394702	10.44656	46.93495	61.64529	35.4391	27.12567
9.822875	5.296558	8.59021	10.65498	10.08376	16.43014	11.69943	5.137937	7.818809
156.2584	23.2336	8.584287	27.87866	13.63559	20.01532	22.71379	20.70596	25.85722
124.6254	216.3109	111.8271	148.2145	115.376	143.2054	253.8918	141.8067	158.4845
22.70964	316.4126	99.42083	195.3921	64.0826	28.23244	110.0532	136.7193	81.15395
18.90458	25.34735	105.1544	54.32134	57.9063	198.1515	27.53994	13.77922	81.17387
95.75429	265.8579	60.20666	58.31531	26.43031	24.71892	48.29645	224.9441	640.4
9247.231	23781.93	15944.36	21054.08	17294.2	8298.144	7217.641	12317.89	9864.774
730.7282	480.1135	181.6034	461.679	185.7306	239.4589	394.1824	449.7094	302.7904

41.04821	8.464756	25.79768	6.749822	19.84611	75.00214	54.00787	6.861423	24.55482
56.3447	9.519464	24.84277	5.426147	16.68694	20.00352	38.73036	57.12886	6.561495
3.711826	14.76537	1.894499	0.094275	0.556138	10.57848	8.936618	3.408189	17.94229
72.93552	30.62327	30.57058	43.77095	4.015852	213.4374	117.601	32.80922	60.62509
67.5682	116.0878	174.9236	473.2324	485.3311	35.28144	13.70468	137.4771	59.37483
183.8925	1495.124	1008.503	968.1018	576.9029	338.0235	421.8398	686.7108	466.3416
39.62764	5.298681	9.542601	1.446145	4.035503	22.31554	24.18925	15.53145	30.87972
283.5953	106.5952	214.1145	334.614	55.13372	262.8751	373.571	157.3632	176.545
153.5835	136.1232	68.80948	100.6064	83.51609	157.246	157.0119	89.04221	99.25326
101.8204	16.9067	86.01758	49.07522	58.2156	160.7712	99.51695	34.5357	74.8096
1403.429	2939.748	238.955	174.7513	99.71497	159.6747	416.2215	4006.917	4208.132
82.79105	179.3808	367.1074	510.0425	373.9677	136.1553	89.81489	66.54177	55.50616
52.42971	12.68578	76.47852	125.4108	119.5876	43.45832	46.28066	15.51053	29.72431
11.30623	9.51971	104.2482	123.8016	139.0722	5.940646	0.579685	10.32217	3.982882
51.62649	120.2814	111.8454	246.9323	98.7832	76.30523	71.18115	80.41433	59.32928
45.05296	2.132526	20.07084	0.100327	0.624779	5.936226	19.33775	7.733045	41.03135
227.2476	28.51312	169.1769	267.2983	64.70678	165.5357	232.288	72.58759	117.3435
0	0.009868	3.84405	0.052979	11.08591	2.334563	0	0	0.047793
144.4404	55.94671	142.4258	396.4183	112.0059	218.2236	115.4444	48.37613	50.35371
49.32814	17.9621	148.1697	21.30451	133.8047	256.8562	26.14901	44.05379	54.20587
9.798092	0.017489	5.717449	6.741452	4.031349	14.12941	17.9262	4.268201	11.6857
0.671152	14.74843	31.63157	18.28325	9.529021	14.01699	1.264284	28.77026	1.394187
25.81052	6.354216	24.84533	14.66243	4.044127	18.82604	49.90971	2.539714	21.96563
100.5883	12.68483	7.628463	21.27432	10.45167	21.18253	50.50784	73.57332	120.6966
212.8648	11.62947	17.18769	8.06338	45.57033	164.3304	8.177985	50.10256	45.21
149.0748	50.66853	90.80404	79.43608	32.76763	85.69769	179.9685	72.61369	61.91714
30.35193	60.13577	15.27688	21.27687	23.02463	27.04099	12.32141	67.49916	96.37688
189.3803	16.90682	120.4389	59.64249	16.86598	136.1321	298.392	26.75174	60.64512
102.7165	21.12634	51.60968	26.5795	10.47641	69.24436	102.46	33.68297	27.16009
195.4223	16.90662	117.5661	46.43323	32.81443	78.6861	208.9276	49.24023	131.4363
339.7775	440.0256	1100.269	1327.775	1082.367	739.2946	498.624	491.2125	515.2887
215.1708	24.29324	187.3533	39.82425	26.43999	222.959	210.9539	48.37346	145.6182
63.25141	19.00992	11.45353	9.390433	7.251975	34.03836	19.96934	57.14324	46.30907
28.35813	1.076115	3.807158	1.445333	3.933844	19.88019	10.33607	2.541341	3.96947
8.26837	21.12736	457.9137	388.7152	254.7954	37.62854	33.75534	131.4183	6.552022
281.3543	106.593	258.0977	138.9791	118.6205	436.4156	88.41224	88.16309	144.3423
13.58546	35.88894	27.70731	13.36188	10.46475	108.9917	51.16968	13.78145	43.83439
13.58912	35.88426	212.3605	18.63455	32.29467	8.287898	4.7117	16.38084	48.9307
15.1194	63.28097	76.50983	77.75975	95.965	17.65985	19.27074	31.1188	39.91764
229.602	61.22445	185.4375	121.8017	48.75549	258.1645	404.7836	56.15701	198.4073
10.57875	25.31031	10.50281	38.09999	13.16847	17.61015	1.949653	5.136084	5.26367
79.112	8.465044	21.01231	8.073637	10.47392	48.1451	49.05452	22.43346	55.41868
6.746682	41.12429	31.55357	22.50876	70.05298	44.48617	1.950065	10.33098	14.24743
405.9876	671.0749	281.9799	250.1009	71.08269	384.9289	237.8299	211.8426	280.8816
0.671101	0.017434	158.9162	116.5802	384.7763	0.054517	0	0.814196	0.090049
22.72754	182.4149	9.540298	6.750137	16.76997	18.84102	49.10468	54.49569	36.12099
78.36583	15.85068	36.31252	37.12114	41.8905	49.31029	67.80939	28.49481	23.29355
141.3393	291.2288	315.4529	394.073	204.3672	339.1126	185.9835	133.1419	101.8732
587.7961	138.252	115.6453	214.3948	83.8252	312.1856	241.9878	176.38	155.9744
18.16653	12.68389	24.8426	2.774754	7.259751	3.591763	95.81836	22.44826	20.69723
9.043884	16.88922	28.69078	40.74937	31.03615	10.61258	17.95895	10.33745	10.39093
208.9751	105.5433	1269.449	2652.976	2934.31	232.4334	170.6963	625.2541	186.9069
0	17.94321	22.94508	11.99052	60.35276	12.95028	5.409873	3.403883	20.61093
45.57169	150.8269	8.584259	12.04243	7.271457	4.765395	60.16197	148.9099	255.5372
165.7102	195.2123	179.705	191.8229	166.0527	103.3268	237.2666	142.6707	94.13403
2.184013	3.187443	11.46335	23.68496	70.07194	16.42222	1.261324	4.27204	2.689884
74.47819	14.79644	0	17.33482	4.022704	2.41285	6.097278	169.6071	302.1094
0	13.70831	133.3192	10.5918	72.71273	2.412797	4.042141	6.887787	1.396428

155.3497	33.7842	16.23169	45.05214	13.66328	104.375	69.16237	27.62512	20.72676
95.70048	680.531	430.1728	531.5335	413.5815	313.3182	561.1674	347.6915	154.6639
14.35934	22.17257	55.46172	40.96951	16.6429	34.03703	37.361	22.45334	11.70436
36.45673	34.8311	19.10202	47.61796	29.23127	57.47358	53.28683	30.23737	21.99424
11.36725	15.82554	20.08496	5.405377	27.3752	19.90828	6.81731	6.875783	0.086337
74.58273	24.28821	18.1449	33.15161	16.79006	79.73263	33.81424	39.75908	38.69849
51.61393	381.8708	49.69064	12.04229	29.60222	12.98289	135.5912	122.8108	91.50549
91.17924	323.8794	57.33782	87.38564	61.32829	123.2336	144.5627	104.6234	113.3924
99.59566	25.3478	78.37673	47.72985	42.22494	123.1912	107.2172	25.8886	47.7581
91.92753	433.5992	214.1335	171.9446	269.6961	36.45355	199.2513	70.86974	166.1594
115.5726	31.67847	82.1994	211.4047	10.46913	214.6195	177.2083	61.36428	49.05036
865.9052	995.0567	1132.734	732.9421	1240.161	703.0022	1202.712	1300.719	623.5645
11.36103	0.016898	7.633603	135.3177	27.51922	11.76392	21.52379	2.540074	0.086867
1688.687	272.2635	888.0218	554.3712	287.8366	1817.719	1711.206	300.9073	409.7451
0	0.012389	2.864361	7.689802	0.329339	0.037877	0.593391	2.578774	0.061265
32.63197	131.8278	81.27049	56.86106	57.23793	7.114642	19.24842	76.15403	29.70885
567.1362	211.0615	997.9707	1051.523	395.9403	1194.563	729.6678	173.7751	332.4425
211.5231	29.56857	53.51517	31.88149	45.43995	119.6959	100.9491	82.99831	63.20524
26.53857	9.520071	17.18918	8.073346	0.682299	78.5448	63.69947	31.10116	33.55375
17.38976	84.39564	24.83773	37.11678	13.6462	73.89607	14.3953	34.55756	43.8456
0	0.012729	22.1455	11.46024	17.28247	1.222499	0	0	0.063126
3.702786	42.1621	16.24395	53.81121	19.24048	16.4532	4.716068	113.8467	23.16192
103.5057	14.796	42.04864	25.25457	26.29808	140.6619	91.38539	34.55179	23.29793
18.17133	14.79243	55.46028	15.98429	22.84159	87.8087	36.65892	25.9192	30.94509
124.771	27.45711	37.26433	35.83313	23.20901	95.04311	68.42574	37.14005	55.46012
62.24977	399.8568	143.379	199.7101	127.9179	35.28086	129.9703	262.9671	109.5654
1390.535	834.6753	1483.55	801.7387	1157.608	848.5209	1267.698	1547.197	1205.761
23.56137	7.407858	25.8107	2.775906	4.031485	1.239538	18.62301	19.87347	18.08546
116.2928	443.0761	382.4245	446.4939	219.0055	24.71927	10.24575	211.9397	166.1332
0	14.79099	162.6677	57.99587	110.1819	8.284969	4.711456	6.862581	3.983302
11.31863	32.70432	40.16215	39.58496	46.72319	49.18605	6.789822	5.132751	3.983034
6.750779	26.36979	1.894158	1.446611	4.018546	2.416829	27.73708	19.01728	24.44251
95.08915	9.520291	37.26595	25.25957	26.33412	70.41779	103.8424	17.23979	40.01859
106.3641	1102.446	95.57462	46.43702	61.46108	82.21212	53.12486	258.615	213.8237
127.0873	25.34658	28.66004	6.748388	13.67005	92.68516	128.8021	32.81521	51.5928
531.4991	53.83886	377.5774	162.8277	77.45529	538.6063	466.192	73.45256	166.2777
73.75657	45.38648	71.69298	79.3381	194.3226	85.63909	31.01514	63.9896	25.87253

TCGA-S9-	TCGA-HT	TCGA-VM	TCGA-DU	TCGA-HT	TCGA-12-	TCGA-76-	TCGA-S9-	TCGA-02-
164.3518	101.9116	207.1085	114.2566	67.45364	88.10777	51.2827	119.4755	51.78044
87.32796	31.68797	6.967536	51.63533	42.06433	20.62513	21.99439	32.38615	20.94269
84.01391	119.9433	73.68692	135.4792	68.97883	41.22867	73.18861	146.9493	44.07195
3021.961	3510.784	2170.412	2930.537	1995.508	1999.216	1507.869	2126.941	2020.08
131.9731	86.48942	135.7971	31.29926	114.7924	145.2465	117.0427	156.2712	145.3098
1.259063	0	0.035394	3.682023	5.298653	2.31821	684.7353	0	6.568147
12.07649	39.40396	86.17355	11.02662	28.46684	29.7632	128.7996	5.641685	7.748336
209.894	150.7732	163.4096	233.1811	119.2837	83.53494	63.46537	172.8258	127.7131
11018.92	8883.14	4849.461	17308.66	5295.42	3921.79	5995.897	6465.898	6445.489
32.458	74.54148	44.91633	34.07201	17.15689	69.76457	53.66652	85.01393	119.9326
28.74528	20.55516	17.28289	7.342885	14.20287	13.75849	13.4617	36.14457	12.13739
1433.566	1227.66	857.3637	1180.51	509.4968	346.5996	341.3869	1283.853	408.4545
129.6032	129.3338	274.9744	68.165	76.46025	163.5584	233.9808	81.98732	160.7318
0.049511	0.807267	0.037707	0.894732	0.663368	1.190975	16.7095	1.345706	6.586644
98.3889	95.07935	223.1251	44.20544	47.94281	70.9443	34.21812	118.8219	96.88378
8.48089	11.07898	39.12918	11.94886	104.6617	144.9159	412.4258	11.40744	68.19622
2028.657	2334.513	1497.21	2045.84	1327.553	1431.947	1780.872	1813.736	1732.754
50.38656	59.13075	67.85678	2.73335	46.51645	19.49608	18.36668	40.28254	18.75238
76.82304	42.7806	75.98605	157.6094	310.332	234.33	224.1033	71.93442	191.4915
156.0428	186.7006	569.6604	316.9865	1815.787	1061.294	1772.017	142.4464	437.0631
1433.546	1343.34	764.1514	1012.784	895.3129	500.9882	765.5211	1172.248	494.3155
79.0853	29.94684	18.46928	18.40146	10.40142	1.19689	1.300237	57.63644	2.241089
119.9739	35.06537	151.8802	378.8974	16.40329	211.5051	1.297212	47.41587	9.949739
371.8723	127.595	109.3845	340.0566	37.41699	52.67257	68.35357	293.7592	142.0451
54.04544	84.79749	32.2823	172.3759	45.69399	19.50446	64.65548	100.8062	33.06552
33.59481	23.09925	16.16073	11.95023	14.92313	22.90618	9.838749	17.91536	35.22579
124.8243	146.4619	120.8785	177.8481	147.7889	346.4775	411.8423	102.1483	389.6395
1341.174	1216.541	423.5442	341.8649	399.9204	563.8846	583.9216	1095.922	706.7653
30.02107	38.55273	26.4991	74.70683	10.40541	1.197477	9.840547	47.57496	22.04461
160.6204	84.80983	104.6997	33.14618	49.46064	19.50381	40.29914	87.12303	102.3663
30.05904	22.21841	80.52692	11.94669	79.59638	22.92925	42.71235	43.86104	18.75541
74.35401	55.67288	64.4426	63.58714	35.9525	59.47902	30.54503	48.1955	39.65594
29.91076	11.09855	57.29476	2.732701	13.45889	8.055127	7.391356	20.89653	25.29724
453.5399	980.932	475.3224	307.7673	450.9651	925.2496	1003.151	557.9568	1061.205
188.3755	193.597	125.4818	139.1331	116.9961	177.2976	143.889	215.9964	224.576
23486.99	13821.17	4156.703	1894.678	2988.362	1661.854	1663.914	10772.47	1655.72
1.275495	19.67773	19.59143	18.41437	51.2104	13.76846	20.75642	28.81429	4.445122
70.7872	70.24396	43.77094	45.13554	63.76691	53.78115	14.72019	92.93925	64.95911
14.47742	32.51838	40.30183	42.38084	92.46419	43.47789	109.5061	51.11758	35.24954
9.680921	33.3852	29.955	38.69693	152.1157	24.06108	87.59758	59.83079	24.24894
111.6142	32.49282	111.6603	282.0398	380.7763	339.5523	747.6989	35.16157	257.554
67.15979	50.5313	105.7743	10.1036	27.68497	20.64187	47.56684	35.20765	60.5443
20.47427	16.21735	33.42061	25.77433	109.7046	90.31124	331.9422	15.00248	52.85491
251.7765	147.3668	93.24364	127.1728	46.43352	30.94074	40.31289	194.5199	5.544231
307.1086	182.4404	249.7373	145.5796	173.2951	185.3153	232.8456	288.0111	172.8612
22.79913	20.54774	23.01885	11.03308	19.48261	21.73858	23.16303	17.23	12.13983
30.06158	41.94125	67.89746	32.22802	63.02709	94.88396	57.31449	27.25816	118.8319
5904.64	5986.727	1957.53	7963.231	1104.628	638.2457	681.4687	3285.536	810.2676
614.2239	675.0969	591.5041	1451.545	338.4016	194.4863	195.1144	379.3922	116.7337
12.04489	16.26162	8.106991	4.576339	15.72859	2.345033	4.961731	23.80779	2.242137
18.00563	16.26129	37.86486	7.34422	12.70231	17.16722	7.391488	28.90376	15.42597
219.5659	155.0223	189.9109	160.3287	183.8161	81.26143	154.8647	211.6487	178.361
68.39625	34.21832	93.18858	27.61644	46.46523	89.19032	63.41014	90.76489	37.46174
6351.026	8203.827	3924.215	1539.882	1122.632	977.9239	491.3159	5300.915	2018.996
18.02776	21.40737	27.60178	15.64877	10.42214	10.34202	20.74135	34.65845	24.21762
36.0477	29.93608	117.2801	19.3209	52.5018	85.74095	20.80825	73.47324	61.65082
33.54547	52.37856	23.02668	31.34553	5.901657	6.919379	13.47314	23.74655	6.645518

7500.284	11768.63	7751.663	1410.864	2523.072	3228.702	3508.1	6907.372	4332.959
321.5089	53.05317	346.3762	166.7766	148.5127	213.9038	377.8316	138.1475	1720.2
43.15897	11.93824	34.52826	8.261457	12.66326	33.17546	23.21106	48.31522	38.52473
34.84034	44.52827	46.05	166.0148	52.52416	53.75758	46.34493	9.960966	24.25399
7.279784	0	2.361889	12.87385	179.7098	62.7879	30.47781	0	4.445353
323.8445	241.5961	134.6837	106.8733	207.1194	112.1298	68.34907	266.4501	66.09258
26.38	5.081348	13.85039	8.264385	19.47971	13.76367	20.74322	38.28786	26.41381
63.46043	44.57464	26.49492	50.71032	24.71415	16.06055	14.70489	46.86782	18.74499
172.5944	83.09402	52.97642	177.926	45.7021	38.93608	25.68658	67.6379	60.56836
90.04773	139.6081	411.8663	116.0919	157.5573	316.7497	598.1435	143.9444	313.7048
14.43222	47.27841	2.362097	59.10857	3.65007	0.044667	0.067775	10.72004	2.242157
34.75361	11.94436	24.18026	4.575101	17.95375	21.7516	11.0484	33.16176	31.9138
263.9162	204.7344	147.3415	121.6192	52.42714	124.7093	118.3082	278.6833	66.09318
62.32638	29.09332	23.06305	19.32487	35.98731	26.34462	39.02749	43.91522	56.12565
80.41415	258.8929	92.07958	147.4702	7.401789	11.49803	17.15908	34.44918	5.544761
32.42774	23.08698	60.94839	17.48077	23.18901	35.47465	32.94933	50.4315	53.92523
262.727	246.7239	119.7341	251.5815	165.7967	121.2814	114.656	179.949	224.5836
61.1077	44.55741	4.665959	91.31326	23.94778	34.32752	22.00562	24.40674	15.4494
64.86752	90.75249	112.8335	219.3169	313.7268	85.83617	421.6598	128.7895	256.5048
157.2011	349.5922	158.8416	227.6223	123.7547	209.3071	101.2481	319.7804	53.98464
1196.1	1158.236	559.3409	1270.811	744.4072	504.4284	803.3342	1494.797	594.4965
127.0232	53.09353	62.15184	17.47687	27.67723	3.488301	28.11412	72.01634	7.748625
426.9914	162.7343	64.50837	34.98504	11.90792	19.50276	30.56699	315.4103	0.033839
189.2061	53.95631	5.816732	7.339163	7.39885	49.20212	3.744628	109.6072	164.9908
49.21923	41.08676	81.67504	67.27591	59.27467	96.01525	105.9331	17.16503	71.54686
1954.194	2641.321	1742.261	1060.694	950.0715	694.2712	306.0422	2057.982	396.3474
38.46625	16.2155	62.18404	112.433	508.2205	390.7947	550.0851	16.43995	47.37168
112.8044	117.3454	31.13601	144.6812	89.24024	53.80974	35.44158	209.6164	83.6943
88.86221	317.8027	321.1043	218.3806	1117.769	492.9313	726.3849	143.8926	614.252
25.27776	84.79006	63.33597	140.0923	78.75224	160.0667	108.4864	14.28023	234.3857
60.06579	129.3254	105.9205	81.98881	197.3712	371.608	153.6232	96.38933	263.0818
83.88767	47.96458	27.66563	29.46673	29.94793	26.3514	20.8025	59.06469	40.74813
1192.327	814.7541	863.0485	812.8261	223.5489	191.0567	164.6463	1105.428	511.909
1456.436	1714.245	1378.676	1232.095	1187.213	1105.999	1818.638	2003.148	2319.476
0.041836	1.683636	0.031977	0	10.12375	0.030527	16.43989	0	1.127298
16.87397	21.36568	34.55803	29.46733	26.94016	35.48588	37.82789	45.3361	55.03854
91.14565	79.6778	195.4321	46.97894	65.26701	6.922265	7.405347	144.9343	6.647161
615.4673	711.9035	364.8535	408.2217	398.4279	926.3838	1597.834	346.2357	889.4812
61.2352	107.9528	108.165	49.73839	76.50902	21.79144	46.39341	74.1132	126.5772
52.8349	75.37962	21.92761	52.50886	66.75855	28.64864	32.99187	93.63054	34.16311
55.21476	53.94896	27.67467	44.21498	50.98628	22.9308	13.50129	35.19214	13.25321
157.0149	32.50094	54.12339	214.8266	11.15028	4.632618	11.06375	56.82091	5.545481
108.0011	232.2452	230.0763	423.1361	65.95974	64.09622	63.45898	107.2414	71.58688
298.73	220.997	506.2796	128.9879	303.9373	487.1589	448.4969	320.4133	391.8867
21.59265	11.95412	25.29729	44.30177	6.658222	3.489613	6.179476	25.23865	4.444249
32.41955	13.65158	12.71814	73.77509	9.652631	1.19733	0.070793	18.62629	30.8407
19.23969	11.08453	29.91801	5.496664	11.91911	4.634403	18.337	17.20643	60.45961
34.81397	29.95682	72.41199	27.62871	44.28983	29.76435	53.58607	44.65624	40.7334
24.00038	27.42345	59.67256	7.34152	8.912361	6.919154	4.965237	41.17977	13.24016
89.92484	60.81525	151.7469	70.96228	26.92568	27.50114	17.15584	50.35281	9.950706
24.00352	11.08779	84.86296	60.90985	36.09867	52.49162	41.34239	8.533016	22.02731
2437.671	2054.386	1498.343	1045.018	956.0522	1510.842	1170.212	1683.438	998.4998
88.68385	37.66295	157.4226	33.15469	39.72629	28.63751	28.10475	124.8542	27.55384
356.3593	240.6786	779.0546	895.7757	291.1058	1045.262	1054.241	115.8045	1347.341
524.2188	367.5111	345.2563	401.7938	315.1622	280.2396	192.6651	432.0271	147.5535
7.270497	1.656326	5.812212	5.498492	3.650021	76.27971	32.80454	8.544971	37.36086
35.88756	19.69587	33.31002	0.891994	14.96001	8.057736	3.745678	25.96425	13.23499
133.1511	154.2341	43.7885	132.7081	68.97275	37.8003	40.31151	131.7818	72.68367

21.62758	25.69034	36.7939	44.26716	35.31559	32.01183	24.40141	18.65619	31.91663
98.29049	37.65542	80.52665	28.54079	25.42443	11.49824	26.89482	55.41483	38.55664
76.83091	102.7857	5.814101	30.37805	44.93278	62.94971	15.93989	116.6319	38.5706
1264.019	409.5141	48.39839	74.6118	80.19534	57.24739	108.574	339.8403	76.00216
98.40767	118.2147	151.8856	205.542	89.25404	90.38288	57.36653	111.5762	47.37548
48.05759	62.49791	56.43646	29.45697	92.28114	146.3573	113.3553	79.14894	71.5794
174.0075	96.74964	122.039	321.6267	45.67161	61.82166	14.71549	455.9047	252.1055
53.9868	25.65316	51.79107	23.01084	25.43286	44.6227	41.47964	70.62687	30.85243
21.63039	32.56499	41.38313	15.64297	18.70195	35.434	64.39373	31.69596	16.54118
14.47228	17.08269	18.46423	14.71495	21.68815	38.8922	110.6112	28.74341	85.77797
400.5994	264.7241	202.5612	140.0524	147.7764	149.8646	199.9368	252.0097	147.5425
201.558	167.8888	97.87034	46.96541	101.23	38.94803	101.2461	229.6994	58.38736
373.132	512.311	311.9015	429.4358	505.0857	312.2659	284.0702	367.8888	293.9544
4679.858	3580.157	2270.54	8738.259	1524.914	1347.331	709.5039	3895.43	2018.986
13.25902	15.38133	31.05443	10.10831	20.22143	14.90872	17.11679	29.5416	14.34069
26.41841	21.3851	6.967023	48.87296	30.00648	12.63287	18.34484	28.77933	18.74193
54.01287	41.08441	49.51145	55.28323	15.65515	8.066801	13.50102	109.5867	17.65533
0.038952	0	0.029814	0	0	8.942995	3.614616	0	7.594022
16.81676	4.22533	0.046861	144.2148	3.650049	0.044674	0.067787	15.07528	0.031425
0.050423	0	4.616219	0.894315	20.01325	2.328279	4.888592	0	4.414175
13.24534	19.69588	28.73219	6.42042	8.919254	10.33862	15.88384	19.42257	63.68323
148.7455	89.06294	132.3439	34.06435	71.97017	147.5284	119.4736	169.2609	169.512
38.43085	41.09628	42.60317	18.40031	25.43215	44.62366	29.32029	49.66235	36.35128
530.2645	601.4099	225.611	429.4296	438.2469	671.3318	631.3818	420.4503	532.8161
64.8525	96.77382	93.25115	70.01165	128.3117	226.3899	126.7889	94.25677	128.8083
12.02571	10.24974	10.3885	3.655327	4.406895	5.769142	7.381123	11.47378	14.31609
1838.949	628.7834	1273.887	740.9144	346.63	69.82564	73.22682	553.6279	558.1597
304.6174	105.3322	155.3756	63.55594	95.98317	253.8625	430.0398	179.9997	215.7556
13.28146	50.49337	41.48759	29.45653	268.9362	46.94555	213.1738	62.55541	96.88889
12.06173	17.10209	9.262254	11.03156	10.41807	16.04712	36.49995	7.810449	59.3394
178.8089	231.282	27.68274	138.2048	249.8764	110.9953	301.0811	320.4188	244.405
526.449	260.4474	141.588	234.0733	115.493	139.5711	126.8347	226.0817	155.2438
323.7147	213.3668	133.5032	195.3877	77.97318	74.38716	114.6144	123.8119	45.17595
128.2783	58.2262	9.270264	49.74283	21.66025	18.36008	46.38238	104.4611	71.5652
38.35684	17.09234	55.14794	15.64172	17.94283	11.49041	25.62518	25.88729	24.23444
15.67755	37.6612	27.66862	14.71227	66.07025	44.62706	77.952	68.44263	47.34937
133.1394	83.92976	140.3706	54.34516	33.66988	44.65814	31.78243	114.4853	56.17646
3.677368	2.512251	40.09829	7.347148	8.180831	14.87578	29.12012	2.76649	12.12571
2.476277	4.232017	4.65502	1.812298	1.402671	13.71394	12.19169	0.615271	1.139056
56.41152	41.08255	12.72301	32.22767	21.66403	61.77163	45.15214	92.9568	135.3267
80.21835	13.65174	24.20621	55.31523	5.148092	12.63731	4.967008	35.98004	22.04649
22.86841	29.08081	46.0561	58.97823	157.9523	169.0422	103.4813	29.43042	71.54081
94.84616	168.7452	104.7731	58.94639	183.0877	267.604	202.3558	290.9607	179.4527
40.85818	60.7931	62.1755	17.47584	133.6793	129.1912	221.5763	29.40964	92.47042
539.8419	617.7054	651.3128	385.1954	222.8077	101.8513	154.8921	552.2877	154.1602
45.61111	84.87551	24.21883	124.4932	19.41685	10.35419	26.88694	42.43827	28.65304
24.05677	23.94187	47.18447	41.46217	60.08663	20.63707	87.61727	28.72709	30.84796
119.8525	108.006	24.22513	53.43707	43.46519	66.34002	39.06874	59.73389	39.65931
36.03491	43.67256	18.47104	12.86895	48.01189	50.32977	49.98733	21.49839	107.8007
34.87362	101.0681	18.4776	8.261728	29.91346	68.66633	42.74935	234.9171	187.1096
111.4821	51.3739	17.325	38.6816	42.71081	42.35596	45.15457	66.22764	35.25999
439.0291	568.0758	235.9421	54.33747	113.9761	124.7183	24.46984	360.0365	157.4556
32.45967	45.3698	42.61971	171.5017	28.42651	27.50253	25.68132	64.06212	5.545886
20.4549	25.66824	29.94467	16.56033	11.9086	65.11511	37.79162	15.01736	44.02452
393.5389	211.5489	502.9119	389.7977	304.6199	1244.214	2672.38	115.8047	675.9119
157.2393	939.8879	35.73701	566.7574	1072.66	253.9501	78.10586	970.795	244.4238
32.44804	38.51942	29.967	7.33909	32.95158	74.30853	63.36745	35.20945	90.22445
114.0028	158.4953	90.95244	190.7775	92.24535	74.38741	108.5263	120.2061	41.87411

316.4419	70.20857	90.93981	188.9516	76.4864	98.37862	79.28269	110.8656	79.28559
62.3579	29.08546	42.60054	13.79085	31.45302	34.34536	30.53431	63.40283	41.84752
40.8735	17.9338	145.0605	216.538	210.8021	1473.944	2457.651	22.2079	754.0296
109.2392	203.0223	260.0685	153.88	164.3036	377.3542	214.5463	195.8136	249.8896
51.62814	27.35964	83.99365	17.47642	35.18929	97.18132	70.70599	59.00155	73.75796
20.45549	22.23369	51.74992	16.56017	11.15644	24.05454	14.7085	72.19083	36.33376
128.3554	114.7927	98.98717	112.4254	70.47701	67.52011	86.58622	123.1305	106.7909
14.47456	14.50738	43.73394	33.16141	6.648365	32.05226	358.1233	7.801336	25.34841
63.52988	88.34134	23.0645	41.46302	26.19502	8.066663	12.27897	94.55516	19.85111
622.6569	423.1671	452.3019	1233.074	928.3975	147.5995	449.8424	498.1976	353.4078
82.56511	40.27705	1.207357	22.09725	24.7133	14.9191	11.05651	47.59002	9.948312
111.5463	71.93721	77.12446	30.37966	60.72717	51.51031	50.0442	141.229	78.17407
254.2836	281.9046	86.3624	198.1344	215.4104	266.4411	156.0531	149.7214	68.29226
297.361	105.3435	133.5051	147.4463	42.67457	28.65445	22.03504	87.04446	49.57861
14.37991	11.98748	25.20762	2.733994	3.65785	10.3117	4.950364	22.4793	11.01795
519.4733	774.4942	650.1824	421.134	333.8978	440.3491	359.6379	557.2997	322.5789
21.63669	13.65743	44.83397	22.10118	16.43451	43.42107	28.05346	55.60877	36.31768
1620.639	1231.969	1139.257	1725.198	556.7964	358.0328	202.4347	1178.754	297.2703
3.671447	104.4595	31.13546	145.5774	1249.458	1748.127	1338.972	44.52089	563.5766
119.9338	83.94154	4.663202	45.12913	14.90223	5.776795	14.72106	58.24672	7.747882
82.80053	34.21193	56.43151	99.52796	211.1261	137.2005	95.08194	44.54507	28.66338
227.9908	369.2356	281.9701	197.1875	345.9617	402.575	576.4561	169.1104	626.3295
18.07814	47.08551	26.52526	58.9697	60.0113	78.90384	259.1115	37.35553	99.04596
219.568	400.9888	142.7451	255.266	185.3159	85.83573	98.81747	271.4522	81.50429
1847.431	1375.008	1481.052	1838.52	658.1004	1336.984	766.7616	1274.458	670.45
69.60441	53.08026	148.3562	25.77184	66.00667	67.49765	76.80447	69.08944	138.6533
12.02392	19.73536	17.24747	8.272199	7.428619	14.87012	15.83951	18.03906	12.12268
236.3788	320.3983	173.8179	234.0567	156.766	77.83311	141.4753	329.7676	164.0607
247.1917	379.5023	42.64342	87.51347	110.2164	49.24083	20.8097	947.8316	96.91817
828.9939	1157.423	506.3944	611.8906	586.0675	726.2749	635.1089	992.215	542.7486
52.7928	36.80544	114.9458	12.86883	23.17568	51.47414	42.69741	65.5614	37.45116
26.42615	14.51305	33.3779	4.574899	14.92247	28.61069	21.99404	39.62956	27.53605
8.482019	80.53036	79.40547	27.61592	84.81649	83.48566	147.3393	116.7468	30.86156
27.67296	96.74486	77.16817	160.3173	297.8656	151.0289	1905.944	92.04299	6295.86
4.879389	51.36548	4.663916	0	169.8297	222.811	182.616	15.00044	148.5495
15.64546	8.512388	21.87974	4.575282	2.15017	75.24692	100.6041	10.70298	49.46282
568.6855	1012.647	685.8869	1621.997	413.4414	356.8852	336.5018	730.0971	343.5017
80.39379	88.23774	35.73035	95.84498	42.69423	51.50728	34.21238	88.56061	35.26481
18.01405	11.09527	21.86027	7.343345	12.69567	10.33765	4.962973	41.25831	24.20731
173.9231	92.48797	71.39855	71.85533	56.94366	214.9617	81.73138	90.65285	165.1175
248.2115	269.9664	11.571	650.8762	44.92875	4.630514	11.06153	110.1206	26.46388
26.42259	15.37244	16.15991	11.95049	23.21299	59.38601	45.04485	12.85732	34.12561
104.4285	129.3325	87.51052	126.2357	127.5312	117.836	128.0322	196.5856	182.7413
18.06391	35.10998	35.68854	23.01643	9.652513	12.63758	19.57441	25.12946	84.67802
7.28151	26.52839	34.53365	11.94927	51.09872	42.3052	106.9269	12.85232	33.03491
12.08203	8.505988	25.3756	15.63311	59.25769	40.07121	285.8547	9.240665	31.95973
0.057451	3.375573	1.207311	0.892119	18.15021	4.619375	16.96512	8.61048	9.910555
167.9315	106.2035	187.5456	86.60443	120.7982	108.6773	48.84364	145.4463	189.3232
399.5419	774.4794	273.9422	485.6442	531.3171	1407.75	15.92971	1096.777	154.1619
4.876361	12.78996	3.509547	11.94721	11.15291	145.2483	227.8338	4.211929	140.91
9.654348	2.511868	1.208342	9.19203	13.46715	43.29911	88.23156	2.765535	24.19363
133.1565	168.8065	105.8906	75.54593	86.2514	32.08398	53.71139	193.0856	36.36957
0.050912	0	1.201927	0	0	0.036969	17.93968	0	0.026125
97.10229	83.98497	90.87014	31.30642	43.46973	44.63659	53.66265	73.4649	33.05802
48.0431	46.21966	19.62696	35.91258	99.85195	48.07585	69.50491	85.68919	56.16601
615.4322	412.0399	834.2809	285.654	199.5303	273.3951	171.9588	704.2253	319.2785
124.8422	268.1274	93.27657	82.90643	165.0267	266.5023	366.8775	555.3013	260.9169
303.5252	407.819	263.5503	231.2941	201.0682	221.9087	182.9009	254.131	373.1759

8708.25	3084.95	4062.292	1606.235	1380.056	5512.604	4545.343	2796.556	3836.471
159.5221	77.06381	227.7645	161.2858	21.65633	14.92953	8.62243	49.57631	16.55608
38.47324	475.4631	377.4851	232.2041	811.4065	368.2981	1176.005	331.8718	105.725
31.20428	35.98655	57.45945	11.95031	13.41778	18.34174	4.966889	56.30438	18.7437
1383.232	1309.025	830.9138	1207.222	1013.86	1345.001	696.0774	1190.901	747.5098
70.85941	162.7514	76.01017	143.7455	212.3943	458.475	263.2402	174.9379	342.3108
13.27375	65.18017	27.65041	16.55993	51.83978	26.33794	156.6713	32.36426	31.93884
175.1469	60.77005	228.9655	72.77447	121.5306	125.8329	104.8834	181.4643	151.9249
7.273842	16.25072	9.258758	30.43161	57.35572	8.05884	50.97223	4.925599	5.544639
8.482506	19.64677	16.17352	15.63365	130.8091	321.9715	266.2977	18.60737	19.85547
196.6397	134.5167	23.08052	113.3484	38.92614	180.6445	58.57942	69.76667	134.2935
191.9497	169.6132	52.99917	80.14619	95.98101	43.52203	100.0209	179.2726	71.59412
0.063222	18.81896	17.29681	11.03101	42.1381	8.062017	314.6222	23.01381	9.944684
7.253579	7.672409	9.236569	5.502794	24.92579	5.765653	31.46642	13.68294	5.538064
73.18406	76.24769	11.57241	75.56746	53.98671	48.0704	68.27423	74.88279	46.26171
13.27988	36.80386	21.92112	29.46525	91.6802	68.60022	127.7602	22.9387	123.1981
212.3779	264.7126	214.0734	180.6045	436.8919	209.3241	349.7922	169.8522	203.6775
15.60011	14.55466	5.80795	7.347189	4.405841	12.60125	12.22366	18.75426	17.60703
603.1722	429.3002	271.5709	219.3245	93.71763	136.1416	68.35005	247.6998	60.58933
45.54454	39.42022	63.1992	10.10559	23.96177	18.34302	15.91995	51.21932	36.32617
129.4707	105.4066	66.76506	113.3749	39.69418	14.92959	23.24901	75.58923	25.36005
663.4386	295.5072	545.5048	3390.636	304.6062	323.7193	14.70926	300.1497	83.7079
0.062079	0	1.208273	5.498245	30.10503	14.89471	50.93516	1.329584	9.939479
134.2717	222.942	190.8734	153.9508	53.97365	24.07667	7.404848	66.19946	1.137288
437.8398	295.5461	133.5487	175.0695	185.2948	275.6528	276.7265	235.3896	479.9404
105.5978	149.0818	196.7233	42.36003	84.74633	60.66494	73.19849	143.3089	124.3993
256.4971	60.78412	77.13298	48.81499	35.17341	37.79818	34.21767	53.19096	37.46839
171.5638	126.7604	291.0804	81.06871	73.45502	174.9919	138.9952	219.6511	147.5285
561.441	319.5096	831.9559	195.3384	423.9892	353.4376	180.4875	1076.685	319.2744
22.87266	63.386	73.6448	85.71768	171.4202	120.0059	31.76701	43.13021	37.45938
13.24006	10.2373	12.69403	2.732661	11.18831	13.75369	13.45464	12.17058	7.741745
97.21452	253.6815	75.99689	91.21532	53.9417	50.37819	41.53252	141.8524	100.1968
24.03325	48.87935	20.75285	74.72227	8.904035	1.197704	11.05511	25.15109	11.04766
30.05048	17.93421	11.57143	11.02537	54.03241	37.77263	245.5263	11.40186	81.42493
64.74786	11.93361	12.72138	52.52825	0.660318	0.046991	0.071475	24.38672	2.240961
88.7986	47.06837	29.98255	36.83142	84.01879	61.79955	32.99899	108.0099	49.57236
46.87355	46.19973	71.41133	136.366	248.4114	672.2839	89.06654	37.32036	1261.189
32.4693	51.35906	49.53065	12.86803	55.46763	6.92153	7.404301	179.496	57.27087
61.11674	42.83541	23.06032	71.92378	16.41844	10.35263	6.186632	43.92833	17.6496
35.96375	14.51737	59.72487	15.64236	15.68396	17.19557	8.619448	35.31481	16.54204
19.24018	32.56589	9.264815	7.340485	11.91882	4.63442	19.55116	46.9326	6.646442
99.62199	119.0572	141.5575	282.9685	206.4475	33.22887	51.282	108.6664	60.58474
3098.754	2723.436	1515.639	3797.727	2198.139	2010.659	1648.046	2476.164	3126.415
3.67794	28.21557	10.42145	31.30357	272.2151	58.35556	40.29415	15.72091	37.46287
171.6299	174.712	379.7799	346.4895	456.2961	1215.55	673.9835	225.9987	1128.247
28.87439	21.35617	66.78184	24.84878	138.9086	88.07991	293.406	53.19467	117.7801
93.64295	145.6108	166.8836	76.45885	283.0132	289.3076	453.2084	123.7726	424.8352
111.3498	38.53161	54.07324	4.575092	12.65558	26.34713	69.40332	76.45455	28.64894
62.46722	90.75488	163.4472	398.1489	343.0507	730.5759	311.9993	84.85362	133.2336
160.6919	119.086	64.48755	68.1737	33.67003	8.065239	54.92406	97.89648	7.74748
16.87124	21.36874	26.51094	28.54772	58.58483	36.62049	119.1698	20.06206	25.34953
56.41467	67.67326	2.359405	39.60368	47.22299	16.07246	18.37477	102.3383	75.95442
0.057326	0	1.207246	5.508172	1.403943	11.42566	111.4072	0	4.435149
0.053111	0	0.04038	0	3.689742	5.731484	38.18969	0	4.423171
17.99735	5.08427	9.251551	1.811778	14.2226	1.198531	6.175388	9.276056	7.740019
28.84643	38.52975	13.87063	70.06357	42.00147	50.32065	85.19691	28.72472	22.05163
34.75211	25.69413	2.362095	18.41382	17.20006	5.777499	8.617176	34.61517	11.04441
238.6878	180.7622	278.4286	74.61675	111.7575	180.7062	158.481	188.6556	198.1469

989.695	507.9869	469.5644	28.53723	83.19779	32.08067	22.02491	1259.468	36.36854
1.271776	0.805678	0.050008	0	72.75611	560.8882	289.71	0	764.3964
0.029453	1.71984	2.273757	0	1.498499	3.358144	3.535004	0	2.185346
38.28795	7.655884	21.87045	11.95669	5.903545	2.345165	1.302449	13.60734	4.444605
1905.045	1743.397	1529.401	1616.403	801.4462	941.3035	853.3092	1133.284	433.7768
31.16436	19.67989	15.00146	23.95551	30.80194	0.045546	0.069173	54.97065	1.139008
37905.86	34764.99	23021.51	50973.89	17922.49	19994.15	22983.91	24096.09	15458.15
82.80557	29.06868	54.13387	57.11307	88.5322	141.7773	277.594	66.17493	97.97959
389.8286	345.2777	464.8353	117.0086	97.46615	112.1358	158.5219	285.8615	228.9893
237.5446	205.5885	223.2664	126.227	189.079	296.2002	188.972	258.4966	293.915
265.1896	242.3981	202.595	160.3183	228.0624	266.5266	297.4767	481.6951	450.2556
2527.563	868.6593	2108.177	4060.496	1508.511	1569.138	196.3408	912.2242	592.293
182.4037	192.7219	70.26251	283.8342	82.44977	14.92614	23.25116	255.5812	8.84581
12.06719	5.080316	13.85863	27.64312	40.59832	140.187	25.6173	17.92954	17.63979
42.01868	19.64972	56.38307	15.63463	43.49539	67.45026	24.45279	46.77688	47.34502
43.09107	19.67943	6.965037	96.02155	20.97865	8.061803	11.04635	26.64303	7.745564
95.97348	73.65296	40.3319	94.91929	55.46663	67.50757	29.3425	84.94145	40.76756
236.254	95.91302	96.70517	213.8226	54.68813	74.38878	40.31622	211.7684	98.00118
43.24573	45.36597	159.825	50.66711	63.00909	73.20207	186.2308	38.07107	55.06237
211.1674	414.7145	16.17319	275.5484	193.5838	37.80439	8.617946	155.4535	52.88424
209.8601	109.6366	127.746	75.54379	44.17866	77.81321	48.84206	156.2756	60.58244
2.479345	0	9.252831	0.891918	41.5168	76.25037	9.815663	2.765254	17.6183
2448.515	3175.835	2156.583	3575.658	1607.505	1823.076	1376.217	2000.24	1319.947
122.3715	113.9307	55.29128	107.8132	97.52352	41.23056	43.9668	111.5816	49.57599
25.27524	11.93231	40.33012	124.4325	218.6958	228.5513	152.2442	20.04189	89.16989
43.11943	70.41219	9.264987	11.02961	17.19357	12.63051	8.618905	24.44714	4.445288
525.4172	396.6463	468.358	566.7758	177.7761	137.3016	203.6324	414.7385	95.81711
2804.835	1841.871	4908.029	2478.967	3006.479	2557.324	769.2287	2427.213	1748.176
465.4267	255.2677	287.7188	111.4758	219.8285	279.0856	321.8115	439.9907	357.7767
46.82705	19.64607	129.9192	12.86829	31.43767	61.76654	98.64751	56.85518	16.55474
4.881189	7.652787	3.514387	5.496776	30.03231	24.03212	92.20763	1.330747	1.13893
133.1522	108.7884	40.33778	235.054	65.96695	162.3706	71.97725	81.29853	35.26874
181.1437	101.0506	177.2191	80.14808	132.7958	205.8454	136.552	163.4365	180.5363
98.33037	33.36066	65.61079	11.94651	34.43601	50.3564	80.44128	93.65136	112.2503
17.98814	10.24426	8.102852	6.422878	6.666424	5.771388	3.743333	15.82663	4.442718
29.99661	16.23408	36.8046	11.02908	14.17583	18.33721	13.4832	37.48542	25.33234
17.86002	9.425773	10.34529	2.736431	2.157345	3.47876	2.516602	5.696354	10.9957
61.05408	20.5242	26.49026	14.71783	5.898882	16.05848	8.621273	54.13636	9.947645
4.875544	0	4.661721	33.23072	13.47098	3.488504	113.4907	4.208394	1.13928
52.81681	24.78954	38.01962	34.9944	77.32626	40.06861	42.71681	74.18264	20.95662
64.78667	52.23685	93.16764	34.99481	23.16821	46.92099	22.02672	57.5754	42.95739
57.56232	74.58754	41.44683	123.5852	19.42025	34.34169	14.71508	75.71594	5.546521
34.79304	17.08763	31.08428	6.417919	26.97704	22.90759	18.3505	28.7651	34.12898
119.9444	143.9684	43.78439	80.16227	22.40815	115.502	41.5243	101.5199	48.47169
37.15406	78.15391	15.00717	5.496605	11.91803	19.47592	12.26646	30.2439	23.13344
12.03686	12.8255	18.40719	37.85608	7.420363	5.771813	9.810742	17.27883	1.13928
62.41863	55.65258	26.5285	43.28832	53.97504	131.4609	155.8685	50.32763	51.76592
14.47847	39.37897	29.96594	76.51077	89.42604	68.59792	88.88025	36.65637	62.73966
14.46605	5.936647	9.267151	36.86623	29.24322	4.63467	7.404041	38.18494	35.22543
2.475798	8.50613	3.511918	6.418164	7.399641	236.4855	702.7825	4.925576	88.05749
32.38548	24.82434	10.41481	56.265	24.73099	17.19654	17.12761	33.85974	19.83934
147.5625	129.3472	130.054	247.9385	137.3241	85.81855	91.47677	123.8108	48.47786
351.8683	137.2064	41.46471	66.35331	33.69501	284.3322	8.625426	33.03363	47.35514
133.1335	109.6562	32.28388	35.90914	13.40177	0.047779	8.623396	118.8204	1.136927
21.67386	11.07567	41.4686	58.97101	38.20185	35.49979	585.9158	7.081911	433.2287
38.4662	126.8136	97.82521	57.11258	72.74424	106.3634	140.1203	93.58169	68.27694
50.31866	49.73542	4.665994	45.17346	7.400636	1.197661	1.301421	46.15098	9.947999
0.053979	0.804344	0.041023	0	2.92288	2.335726	4.916881	0.6175	3.331834

127.2504	286.9335	1303.839	642.2968	1319.376	1339.264	7038.288	46.69168	1299
1.275993	1.656312	0.04633	2.73289	45.35519	10.33001	161.6459	0.615487	7.739057
50.47008	144.7565	127.7731	132.6862	179.353	277.8707	350.9048	148.2803	396.2188
230.355	155.8801	390.0597	152.9556	290.4598	122.4251	195.067	99.97839	127.7321
20.41347	17.10751	44.77505	23.03728	27.80002	27.43241	64.29081	14.32954	28.60684
2.479582	11.94392	6.965787	7.340726	87.50737	61.62156	43.7884	25.18023	53.86868
148.6099	65.9504	82.84951	37.75784	31.4291	38.93186	32.98903	73.43244	41.86265
885.3501	625.3651	532.8531	412.8293	438.9594	307.7108	1004.358	1073.634	405.1466
130.7256	70.21726	107.0207	65.41037	62.22337	56.08482	87.78719	133.2665	119.9813
158.4271	173.8626	99.03066	341.8932	776.2571	519.1879	516.7441	155.428	334.6631
26.41709	23.10392	59.73711	8.26206	19.44874	30.88367	21.98641	34.57803	14.34514
159.5925	230.46	58.75311	322.5743	88.46636	62.96262	58.59943	154.753	50.68203
1957.6	295.507	858.4766	1236.76	84.6984	40.08787	7.392524	266.3029	71.59813
12689.91	4753.817	3799.936	2216.304	1705.02	4928.212	7850.987	3250.172	4602.665
85.16343	120.8569	87.44662	138.2817	20.91025	20.64616	17.15753	97.97937	73.76113
2107.86	3193.79	1311.951	803.5609	1660.769	703.4356	1487.15	4545.718	2501.141
0.06493	39.39294	24.2127	22.09139	63.86427	26.34553	68.17977	25.84067	12.15071
102.0425	254.4513	143.887	194.4386	321.2882	367.0589	232.8119	159.0681	133.2313
104.2524	56.53643	65.58434	67.28052	17.91098	17.21385	17.15377	70.60151	20.95507
27.67871	118.1845	159.9825	114.2496	141.0395	171.5712	589.5903	45.24239	428.1368
67.25284	99.34197	105.9058	86.60283	190.6682	187.5442	179.156	63.98058	144.2168
1.217099	0	0.022767	0.909869	1.497589	0.021754	4.675848	0	5.386761
19.25988	22.23277	21.90915	33.1662	66.17042	66.26145	51.1501	10.6859	41.82948
8.481588	24.8038	17.31687	56.23144	88.76758	77.68296	179.7889	14.29079	162.6825
21.66483	29.08736	16.17073	11.02558	96.99556	6.92295	20.80079	56.18325	44.04399
42.04316	53.94741	44.91856	133.6805	102.9025	138.2781	96.2368	41.68454	42.96004
341.7941	225.3246	475.1014	103.1888	6.656904	122.4141	7.39948	227.5583	1.136259
121.1306	89.08745	28.83122	46.97304	53.96173	41.22495	29.34297	152.7731	154.0728
0.06603	27.35726	27.67877	1.813738	5.900929	4.632582	1229.297	4.207747	51.76626
1260.809	779.5767	1367.09	596.2234	535.0263	452.9543	549.7961	1538.128	598.8888
547.0336	393.2047	160.0203	149.2593	203.2916	390.0231	262.1352	664.6701	980.7741
21751.1	11527.76	9269.623	5903.438	4858.603	12691.62	9864.733	8523.75	14370.48
41.94497	45.44883	8.116891	20.25476	14.17332	16.05719	15.91552	32.40127	36.31937
118.7938	136.2085	233.5371	81.07352	54.69066	60.66838	64.67962	117.3271	124.4067
13.27095	12.79606	33.3813	27.63381	69.22923	16.06086	43.84684	9.245538	7.747857
27.5955	18.81592	27.62114	15.64415	18.70687	17.19181	19.54731	35.33501	15.44082
7.25202	13.71115	5.802393	13.82588	8.196801	9.177217	8.58005	12.95763	8.82771
30.03843	12.79213	54.06785	41.46414	17.16745	66.28613	245.3436	19.34115	59.42391
113.9879	65.0625	79.44449	23.9265	73.47541	208.0861	291.0967	94.26979	176.1093
1.275984	26.599	11.54579	11.96135	62.77235	4.63176	33.99972	25.99549	16.52224
103.1168	70.24146	121.9112	66.34453	26.9211	34.36052	31.77072	87.87734	24.25891
56.44599	100.2274	86.32976	28.53544	101.3069	116.6476	95.0932	112.3364	102.3822
35.94494	31.71377	44.80769	1.811972	8.909368	19.47103	9.832851	30.9872	5.545795
441.4951	506.312	322.2571	608.2378	413.4911	508.9437	409.5844	315.3064	257.6307
37.19659	19.65908	25.35249	11.02697	32.24091	17.20487	28.07684	34.53907	11.04907
31.22089	21.37506	40.27602	14.71547	38.26879	5.778845	26.86366	38.15432	17.64811
115.2495	165.2929	151.9595	112.3973	264.1237	305.3856	485.076	388.8313	296.1429
222.8501	79.66847	268.9867	21.16289	15.65323	54.9309	18.37653	71.97625	5.545502
48.07334	95.03782	77.16509	93.96755	186.0698	742.0357	727.363	85.57151	584.4492
33.62451	32.52815	28.80529	35.92993	42.77091	18.35064	20.79449	31.62632	6.647681
32.24382	7.664624	17.25705	10.118	5.158915	6.910567	4.958234	24.58419	16.51269
74.31933	32.5174	96.56796	33.15604	14.90782	14.9266	3.745515	46.77761	2.240931
159.5836	251.9005	120.8723	169.5575	163.5766	157.8511	48.84933	161.2515	64.99045
12.08219	25.64362	39.1765	100.4636	213.4924	158.8603	41.51214	71.98124	217.8209
7.281014	66.79721	58.72553	58.96107	533.2799	21.79068	104.7989	81.34554	55.06857
111.5863	71.06462	240.4023	293.1418	93.01508	92.66638	429.8059	94.2751	31.96702
11464.06	15324.69	5834.544	12792.07	10895.64	18671.97	14745.22	12489.06	13737.49
764.0944	242.4001	536.2539	321.6038	319.6525	478.0066	184.1398	352.7663	192.6862

37.20812	29.95544	18.4652	14.71462	19.42807	20.63328	31.73055	72.15726	28.64444
46.75663	24.81003	18.46194	9.183034	28.47345	27.47714	13.49156	45.39805	19.84651
8.437413	4.232015	57.07791	2.734193	4.41423	0.042582	3.736967	13.70176	5.536175
54.0484	70.21656	108.1716	24.84868	32.92089	13.78601	32.99897	110.1745	74.87814
44.47358	81.3324	77.161	57.10346	214.6446	132.7052	680.9368	27.96027	96.9095
212.4164	340.0745	277.3876	492.1041	927.7584	1240.752	939.6507	204.384	453.5613
33.55025	26.55868	9.262633	20.26191	10.41729	1.198178	1.302191	30.9984	3.343978
292.7048	316.143	254.3294	154.7987	176.3077	90.40924	195.0681	268.5755	186.0648
208.6776	124.2053	79.45014	143.7606	89.99346	114.3928	41.53375	144.7235	41.87394
214.682	145.6322	63.34894	32.22061	20.90597	80.10445	33.00461	146.1548	47.37761
2546.849	4120.012	3972.386	6729.35	901.2572	1389.622	61.0313	539.9122	614.3146
30.07807	91.62351	71.40356	115.1766	247.0019	256.1305	314.3275	100.008	214.6451
34.84912	44.51911	31.12024	13.79009	41.21803	68.61337	276.0229	39.53134	34.15659
3.680773	4.223758	15.01732	8.260939	32.23163	29.76435	115.4681	3.484521	45.12905
42.05874	79.65588	92.06749	69.1012	72.75562	147.4757	112.1125	66.90516	83.67383
39.4691	17.97209	41.3271	21.19312	9.67172	5.775511	3.745912	14.33614	7.743424
166.8303	336.6598	302.6897	211.9308	111.7179	20.6445	263.3454	420.4935	354.4874
0.034047	0	0.026119	0	0	14.34884	7.020455	0	4.344052
146.3684	79.62764	87.50365	54.34083	134.3136	172.6866	233.9444	98.57546	255.3448
13.28128	77.06539	23.08072	40.5168	14.90273	53.8055	113.3759	40.92997	56.17871
7.275812	36.0322	3.5142	7.341733	6.654579	5.776573	1.302334	17.95041	15.4362
0.056265	0	0.042715	0.892348	91.32284	18.19425	14.53531	0.616064	42.62759
26.42285	14.514	9.266956	11.95046	14.9242	26.32701	19.56238	40.36232	36.32272
113.7255	22.2271	37.99534	84.835	2.152895	2.343874	1.30047	30.89838	15.45118
476.0896	41.0595	196.8006	7.341851	47.92403	0.048154	36.66247	192.9375	19.85789
230.163	133.6657	34.5847	105.053	35.17236	101.7993	45.18088	82.03123	155.1901
25.25402	26.51589	24.21426	114.363	23.18343	2.343765	7.406028	20.78263	1.13815
194.2817	78.77574	24.23162	39.59442	77.22823	74.38381	50.06005	153.3924	82.59077
97.1204	39.36559	67.90323	32.22705	53.23933	61.77472	50.02185	72.72497	61.6582
201.5134	107.9096	26.53338	71.85292	94.48988	38.94691	218.1348	253.5684	162.9277
205.222	436.8921	377.4949	318.8335	451.7569	782.2515	937.2403	318.8927	486.5875
129.6131	68.47927	241.6427	65.39869	144.796	114.4113	158.4927	234.7673	62.78935
45.54588	19.66281	78.106	67.32944	19.43999	10.35059	12.27264	25.86923	14.34728
28.6016	11.12494	9.232858	8.276779	6.683238	3.485126	8.577365	13.69494	6.633841
0.066872	15.36104	39.19212	31.29878	355.104	874.5413	1087.689	5.652949	1804.68
144.0079	137.8923	159.9885	88.43981	146.2883	160.1454	154.848	176.3692	231.1748
43.21524	72.0008	77.05151	26.69992	28.44308	10.3541	35.39869	46.05449	41.84768
10.87954	13.65002	9.269538	31.31675	70.66002	34.33415	111.8638	10.68387	40.73797
16.86266	13.65347	15.01439	23.01913	72.9933	75.36403	126.3079	7.082333	30.83578
163.1965	185.8836	206.0036	108.7164	162.054	141.8565	207.2334	208.7902	198.1637
1.275993	4.225889	16.12513	11.03838	21.03641	71.70304	13.44828	12.90426	7.740467
45.62691	48.8082	52.95474	46.06244	23.92134	22.92907	28.11157	110.3291	30.85697
9.668723	5.938695	12.70426	17.49422	40.65133	8.06067	36.48899	12.15243	22.02471
325.1397	535.4728	201.4375	329.9048	488.6084	218.4906	86.63748	418.3468	169.5678
1.275776	0	0.047419	0	2.898917	35.39878	179.0353	0	6.644684
47.99033	87.47348	39.14823	103.2852	28.44978	3.489372	13.49722	48.96181	15.45184
44.42337	32.51207	56.39494	36.84198	32.19633	45.77147	47.56929	36.6496	38.55386
179.9933	400.1409	319.8926	74.61323	210.8544	228.7526	316.8823	161.9365	298.3191
338.3248	441.2166	96.73007	305.9437	119.2266	115.5726	76.88648	259.1501	315.9569
20.45511	81.51355	5.817251	42.3961	18.68046	9.208958	14.70827	51.91211	18.74734
20.39306	15.39801	18.42172	12.88349	38.45313	23.99814	24.34589	14.34755	13.23261
120.0537	47.0598	408.5795	602.6799	284.3379	2880.657	3445.244	43.08831	1530.111
0.062053	9.376659	20.71378	2.73263	11.18607	14.89438	76.28372	3.484719	0.031503
67.17085	41.94623	87.41357	172.4493	8.148886	5.778369	1.299434	30.14927	4.444619
122.4193	215.0391	62.20387	164.9476	132.0277	211.579	198.686	126.6559	205.8573
6.067203	5.946617	6.953247	8.271762	21.84036	4.62886	11.01077	2.046953	24.17649
101.9516	14.50222	77.11808	225.8824	9.65017	3.487268	1.298227	21.48289	31.96364
0.057987	5.095989	2.358834	7.356308	12.78602	4.621303	15.77988	3.498029	6.629313

58.80487	91.70346	118.4441	12.8682	20.91299	32.0721	11.06372	100.1847	25.35791
163.2102	311.8528	311.8492	163.093	273.9278	372.8069	685.9714	234.7014	377.5626
19.25267	32.54685	6.967552	17.48464	29.99373	49.13589	35.3483	25.14706	61.58782
49.17897	32.52502	46.03326	17.48007	29.95981	8.066635	22.01308	41.0178	26.44889
4.871809	4.228648	17.24521	7.348687	24.13229	13.73201	35.11441	7.839891	7.73509
36.03355	48.82368	75.90173	31.31156	10.40086	26.35109	24.45226	46.05607	9.950383
119.965	252.0062	28.83331	140.0869	64.46765	27.50971	29.34615	80.58278	39.6705
80.43246	112.2099	124.2946	107.8101	73.47288	62.95258	102.427	230.5705	103.4968
79.20766	55.64361	79.42639	21.16219	59.9725	105.2149	88.99857	79.16279	108.9774
68.45197	44.4892	149.6073	174.1801	264.3135	430.9538	179.1568	52.45152	93.59942
127.1404	49.63909	158.755	37.75288	84.01455	91.51268	97.53058	69.77493	116.685
619.0957	1130.834	584.6522	960.2439	1378.672	1135.704	1491.926	784.769	1207.635
2.478471	0.80265	2.361433	3.655221	11.96886	17.14659	21.87859	3.488237	11.02788
1761.014	690.4655	935.6055	269.06	463.7148	710.2709	486.4249	1148.473	507.526
0.043317	0.813184	0.033086	0	1.436258	5.662149	65.23439	0	1.128827
27.65291	43.67516	10.42076	70.9812	109.7999	66.30517	66.99352	32.33064	24.25257
409.1428	575.6852	523.6349	189.8069	592.8627	779.9895	965.3162	576.7158	699.0391
101.9883	58.20983	127.7249	46.04856	45.6873	84.66015	56.14219	141.1765	119.9893
39.61597	37.67182	85.06965	11.94757	23.9348	12.63982	19.58241	67.76631	22.0515
37.2374	35.08737	32.26549	37.76534	241.6334	26.35399	18.3705	167.5123	62.74197
0.044586	0	0.034035	0	0.670161	2.313148	10.69842	0	0.023018
15.62791	25.72705	10.40285	25.81793	17.9901	25.13864	47.31001	9.267649	7.742249
59.99683	38.51201	70.19121	17.47702	21.66557	34.35549	25.6786	74.91201	48.45599
10.87603	23.95536	19.60754	16.56161	22.45244	61.67965	55.97712	24.42037	61.59154
100.7463	79.66556	89.75657	30.3808	24.66389	41.22085	25.68607	111.6706	17.6566
63.65931	71.05357	191.0189	85.67873	341.6777	347.563	264.4125	94.96158	134.3196
1831.864	1611.461	781.4329	2437.544	959.0674	937.8703	1211.636	942.4574	1504.862
25.19004	22.26463	2.36219	24.88246	27.03546	5.776592	2.525989	24.48018	11.04213
24.07961	47.06164	96.69877	111.4962	223.0136	349.7881	358.0698	44.52937	176.1164
1.275098	4.223564	4.665892	3.653647	36.04749	11.49076	99.55236	2.047605	9.947067
8.475686	26.55237	23.03431	6.418688	57.24772	18.33188	29.24744	37.51348	20.9334
25.16535	36.92172	2.362162	30.43734	24.04115	12.61737	4.9631	23.06017	6.643543
98.32084	28.2174	38.02301	21.16365	41.95695	37.7871	35.42079	73.44375	51.76043
114.0269	270.7546	186.4369	145.5897	320.5761	67.53554	36.66226	533.9443	28.66574
111.5024	49.65346	74.80587	35.91376	59.24692	25.21849	50.02872	89.31515	53.96298
393.4928	294.6792	342.9496	105.9456	213.8146	308.8212	220.6886	440.6869	293.9452
45.63446	50.51781	50.66345	27.61753	55.50011	72.04999	116.8986	33.74935	100.1434

TCGA-E1-	TCGA-DU	TCGA-QH	TCGA-HT-	TCGA-VW	TCGA-19-	TCGA-06-	TCGA-06-	TCGA-TQ
111.964	75.84885	356.7994	139.152	109.3612	110.0378	39.39708	235.6596	216.9076
42.42114	77.65254	7.765397	70.13923	28.57078	24.97657	51.48736	25.94979	32.43608
51.1267	75.17672	35.81918	208.4614	53.1993	88.48185	223.5363	37.44328	38.52233
4752.395	2736.983	2791.63	4007.144	1438.482	1850.897	2957.194	3243.267	2529.957
44.06878	90.64524	108.445	84.77191	355.7001	116.8296	56.59686	124.0024	254.4056
0.092695	2.76306	1.355221	0	4.928091	5.640779	6.107088	6.526182	1.017875
34.03789	21.00463	9.041464	23.00727	35.46812	138.0836	113.4924	4.676618	2.032546
203.8384	144.1418	98.26961	170.4705	114.2878	199.5775	92.57195	80.93034	123.6619
17356.01	8169.489	3440.846	5632.617	4063.229	5973.371	10694.68	5828.658	6567.143
67.98031	37.88714	40.88388	38.65291	38.42106	36.33258	26.83743	108.0481	144.9329
14.25469	11.87015	33.05069	27.20737	34.49084	14.77163	8.035706	21.85764	47.62643
1611.449	719.3352	575.2477	1735.161	333.9988	763.2854	869.4016	586.9299	1129.155
58.22913	111.0345	339.0268	69.10612	298.5433	159.92	62.87436	156.7786	342.588
0.099503	0.599166	0.067389	0.762342	6.900183	4.544508	4.723766	0.466183	0.003485
195.1701	46.306	178.4059	115.2938	109.3643	72.61431	51.87972	51.85042	269.6019
8.669445	28.06953	34.45986	13.10687	42.36705	24.98361	42.26769	0.800016	11.15515
2407.442	1646.219	2162.911	3578.959	1409.911	1924.595	2968.032	1758.685	2374.874
19.97823	57.69705	68.78953	21.34791	305.5026	7.995608	15.88657	54.46463	130.7365
63.84706	562.9925	9.041694	74.07216	85.71556	87.34797	101.8524	22.95564	12.16904
226.7524	839.7083	418.3721	180.2692	343.8527	349.349	634.398	241.3036	177.3865
1212.175	973.9676	887.6354	1117.482	653.2271	714.5171	1251.399	419.6156	1336.941
24.20938	0	0.089592	68.40063	1.964819	6.861342	1.77967	82.38294	0.004453
147.2265	25.18879	374.4573	104.5615	5.905695	28.40977	50.33171	4.637905	48.65812
137.5163	43.47184	330.2944	362.3528	70.93278	175.8266	177.1609	66.6573	139.8822
80.77837	92.8109	23.07185	116.1328	14.77264	49.94413	103.3646	22.94301	89.19826
12.89899	9.725576	31.89753	38.71964	96.57854	24.97552	17.40548	22.44634	28.3826
119.0849	240.5234	220.6291	106.1703	185.228	208.6825	499.0623	178.7538	215.898
742.137	695.4395	431.1481	714.7532	1421.743	796.1621	598.483	848.4572	1901.511
42.45033	16.06949	14.13075	18.88403	34.48328	9.128106	8.065659	4.67558	12.16858
24.24592	49.84494	239.3434	60.07637	92.61553	54.47139	51.84256	22.92236	180.4096
15.751	125.4057	213.6872	27.11173	114.2999	9.129993	9.638051	26.42505	62.84373
31.28984	24.50237	89.18449	65.05864	101.4893	36.32919	22.1468	51.32278	86.1526
2.998715	4.803667	40.573	12.31088	32.52137	13.63537	1.78451	0.721652	57.75302
998.355	648.325	672.1305	545.1015	531.0539	530.7996	374.557	1211.515	1653.179
89.37546	216.5813	173.4897	142.4169	219.7135	110.0539	117.656	175.1961	432.7993
5981.963	2168.656	48908.15	12709.71	8076.216	6848.914	1450.644	6567.87	8169.652
22.70535	38.06592	0.086967	12.29333	11.81815	31.73839	31.26452	8.262955	10.14103
45.42463	50.56843	40.89026	102.1644	84.73439	62.3911	42.44997	115.3468	43.5887
19.98087	68.98905	29.41055	13.10244	76.85604	58.96126	57.91061	26.30743	21.29062
17.14851	54.19198	6.492328	13.10442	60.1048	67.99478	39.21334	26.20217	18.24982
119.0386	240.613	20.52309	60.04622	102.4638	227.9162	383.1141	30.25025	26.3596
10.08951	24.50667	108.2112	22.16785	94.59266	39.72312	12.76791	33.49644	135.8079
14.33687	37.89138	30.6999	8.982893	120.2122	89.55753	103.1254	12.01509	14.19622
200.914	80.79589	79.14106	259.5444	33.49283	63.55425	45.64407	69.96347	204.7418
112.0345	230.616	211.7529	218.1923	259.1235	205.3016	119.2495	316.5541	263.5381
17.07103	18.94707	10.29539	26.36609	37.44664	11.38368	6.486805	15.16565	45.60222
32.70927	34.3636	147.6686	36.17945	217.772	75.97428	34.63824	65.61191	55.75001
6294.477	1414.806	647.9686	564.8363	1000.038	1577.588	1494.458	1427.927	3507.069
439.0815	141.2239	420.9021	416.649	141.8719	324.3947	305.6096	415.5322	106.4343
8.633488	37.48945	2.660954	10.65571	9.84821	3.456546	11.11489	8.138677	13.17969
5.823069	11.172	33.01214	11.48297	40.40841	12.50599	11.11562	4.614067	42.55852
138.9147	200.3689	219.3873	359.9108	124.1383	250.6446	230.3074	117.427	156.0989
22.82665	48.4501	112.1249	74.93493	84.73402	48.7993	8.069335	80.00181	213.8492
7933.141	2570.229	11134.37	6225.586	1324.19	1812.357	388.6805	4961.895	8642.996
11.46094	11.86258	55.813	21.39813	46.31849	19.29726	6.487139	15.17155	21.2867
27.05573	23.09263	40.8771	50.2054	86.70726	44.2589	12.76992	69.03769	96.28711
46.46979	16.10234	21.70948	36.28872	17.73175	31.73183	15.80346	18.67931	46.61706

9024.948	5104.77	9193.47	9061.742	4373.59	4127.043	5500.91	3386.18	11674.68
116.2852	125.7682	309.9009	43.56824	325.1378	148.6138	338.2943	77.56182	609.1656
10.0796	8.315447	14.12823	23.84127	37.44049	23.8465	8.064124	4.674246	46.62278
34.09488	79.56471	39.58874	37.84196	28.56809	73.6839	70.37179	29.90336	22.30426
1.57486	17.49646	0.087744	3.220944	28.57175	3.457474	76.08506	184.8809	1.018698
230.8294	119.4574	289.4348	281.6648	82.75657	139.5305	217.7369	128.1773	289.8879
10.05692	9.739884	20.43425	17.25957	41.38928	7.989632	11.14844	22.01933	23.31325
46.63928	11.8396	5.21598	42.84706	31.52723	24.97774	18.96487	8.322884	41.55617
114.6122	38.57327	15.42153	89.76255	25.61087	79.39718	101.7449	62.41509	68.92717
68.14322	106.0915	262.6629	85.57694	198.0375	201.8768	469.3224	113.6604	153.0569
47.68243	19.68849	2.661076	11.48169	2.949793	3.456669	1.784576	14.99234	9.12686
1.575418	5.502263	14.11269	14.7705	45.32942	5.725698	4.93051	18.76168	28.38072
263.3822	87.78868	201.5323	215.7381	100.4916	121.3935	102.0176	189.7222	343.6075
10.08618	16.06058	38.28973	59.33197	36.45277	29.51832	25.22412	33.24794	49.66569
241.7323	15.34312	136.4028	188.6844	10.83167	44.27939	23.73537	355.0909	325.343
22.78702	23.11578	71.28482	31.25715	127.123	30.6489	25.21938	36.72308	84.11924
363.8231	120.8544	190.0718	270.9322	93.59445	111.191	167.7345	84.78327	247.3199
126.7028	59.88068	1.378647	57.69923	8.861227	19.32415	18.98445	64.3882	13.18219
141.7503	480.4795	119.9775	110.2807	176.3587	224.573	560.2767	52.10097	49.67251
293.0588	62.46372	126.3371	359.1184	98.5212	139.5313	147.3743	157.1199	233.1289
582.1967	1255.943	1930.739	1147.088	1854.272	675.9753	611.0729	503.4142	1744.411
29.88441	27.31724	81.57718	68.35028	42.36269	44.26178	6.500698	12.01682	31.42634
130.4259	28.00232	316.2397	364.0279	55.16849	82.84468	36.27003	189.8517	199.6826
121.4833	28.02541	7.76817	59.28187	14.77278	43.12573	17.46112	37.12526	52.7091
42.57994	95.75403	110.7913	57.63083	82.7655	43.12615	117.0983	12.01341	37.5071
3275.063	1987.452	825.1901	2702.83	486.7153	619.2696	806.8083	823.4163	1460.603
41.22407	44.19616	77.8439	44.40445	71.92112	98.67408	298.2682	66.19024	5.073461
179.7911	88.52551	16.69645	310.6112	23.63999	79.42915	73.80334	73.67259	95.2822
170.1014	604.8429	395.3766	267.5815	901.5285	436.6404	330.6148	183.0191	400.3752
52.53687	397.3238	9.041884	19.68757	66.99415	88.47925	1150.573	73.40775	145.9556
168.5774	170.8493	502.1284	154.7844	202.9644	174.6677	100.4321	376.8148	108.4599
50.99607	41.44428	16.68821	58.47973	43.3497	43.11441	9.636666	51.06021	58.78805
577.8184	190.455	260.262	239.5673	393.117	544.3857	313.4494	230.3637	989.2715
1035.338	1397.269	1870.87	2188.02	2672.043	1269.099	1046.474	1114.699	2275.54
0.082827	1.331802	0.056634	0	2.955505	1.168708	15.74043	0.352478	0.002987
19.9806	35.80157	29.41021	19.69748	59.11736	28.39234	17.44953	19.17844	80.06903
46.84062	89.34924	12.87066	31.22815	12.80213	12.53262	15.90294	12.02167	447.95
1057.78	727.1185	596.8931	718.8871	226.6048	861.9185	859.8937	1124.132	583.8388
69.47915	67.44433	156.7371	100.466	184.2548	56.7438	36.24282	26.5645	125.6842
17.17075	67.47034	52.35621	65.02808	92.61614	31.8055	28.41509	44.47978	122.6411
73.62532	117.6016	23.06391	117.0298	30.53803	60.12149	47.11606	58.55803	51.6963
75.0884	24.49186	79.08038	166.4878	22.655	44.27147	28.4156	8.358582	9.128209
104.8659	76.56524	85.51566	82.3009	71.91975	146.294	65.9747	84.41587	99.33586
69.57054	373.4513	365.9843	120.161	557.6687	121.4011	127.0851	197.2797	442.9412
29.62052	9.747626	19.1492	18.92645	2.949775	18.15945	9.583564	8.180879	21.28572
10.08358	11.13071	30.65407	46.13244	3.93496	12.52828	6.501658	4.67655	6.087362
7.24856	3.392523	25.5303	4.867085	71.94226	10.25739	4.931292	0.770936	31.42099
18.55254	17.47514	34.46699	31.26188	89.67303	21.58936	18.98682	22.60826	45.61129
12.87161	1.98958	81.09888	26.35729	52.23173	22.69161	9.601531	4.651269	50.66924
27.05949	50.5808	155.2864	58.45035	27.58206	46.52709	84.4749	12.01665	33.45334
25.49536	19.64246	26.77448	7.341299	10.83279	19.30269	15.80475	32.31036	10.14092
1774.441	1409.241	1516.356	2022.504	970.4818	822.2742	1497.462	1110.966	2322.164
31.27525	26.62433	89.14247	44.44268	97.55002	34.05818	17.45421	12.00063	96.28457
342.8337	822.8597	549.6767	243.6857	228.5758	500.1607	1100.765	244.8933	196.6447
378.1454	303.7393	311.2234	729.7147	200.0038	319.8415	189.723	95.77525	348.6815
0.127561	15.42577	0.085012	2.398494	24.6341	48.61137	87.61422	8.153028	16.21941
15.65246	8.332306	34.30832	8.171143	55.19388	5.723444	0.17342	18.46402	32.43009
198.058	105.4548	104.604	221.634	31.5223	83.95331	92.51126	138.0947	92.24036

21.31498	74.23352	11.57543	16.42114	53.21428	19.30899	57.50207	11.81998	13.18137
8.673267	12.53149	81.56348	49.3804	122.1837	31.79839	9.638047	8.363252	90.20655
195.2438	39.96505	17.97268	43.57565	20.68421	38.61399	29.99906	238.3111	145.9573
172.9134	83.55774	260.2178	396.93	113.2995	78.31123	149.0109	146.6006	528.0832
137.3517	167.4344	96.97239	97.13968	57.14016	94.15712	123.7617	48.32738	118.5926
111.8727	80.11201	57.4743	62.53426	55.17009	61.28123	97.16131	30.20291	36.49515
112.0351	106.0728	526.522	335.1769	144.8291	147.4778	30.00167	48.46297	47.6453
24.21828	20.98641	45.94151	32.068	77.84084	44.24804	8.070423	54.61255	34.46589
11.47948	23.87037	15.38663	13.11572	54.19929	46.43968	29.75916	11.83364	64.85746
14.32059	40.07736	21.76346	12.28153	52.22194	32.90834	70.17487	33.13273	28.38381
249.2518	177.1518	113.5985	466.2131	230.551	97.58433	64.45818	84.77737	249.3469
66.72946	85.68244	137.8028	129.2381	234.4938	88.51041	64.45226	59.34312	422.6627
774.4465	371.2563	248.7719	870.5365	244.3409	406.0279	593.5468	281.0642	291.9217
12607.03	1680.636	1531.727	4202.285	1069.992	3455.627	1594.704	1689.972	1750.499
8.65605	33.81221	62.19471	13.94591	20.68826	18.17447	9.60721	11.78739	18.24777
78.81176	47.22102	7.764138	63.54519	11.81768	22.70916	22.04919	25.85302	27.36879
69.38221	23.79528	114.621	60.92607	70.93969	35.19867	39.31321	104.4365	92.23406
0.076693	2.082611	1.331434	0	0	3.382454	3.088249	5.835357	0.002796
58.80666	4.803057	0.08499	12.30908	0	7.985605	9.57482	4.618396	2.032657
1.548769	7.091682	0.068674	0	5.912402	5.667119	3.252726	4.022664	0.003543
22.64327	11.87146	34.30828	18.92579	31.53368	10.24954	9.58432	15.07239	15.20668
127.4739	70.93384	153.0103	75.70891	122.1718	106.6268	48.77525	95.19832	200.6885
19.9842	42.14527	53.56865	20.52048	59.11667	30.65978	36.15682	33.44594	110.4717
710.8301	298.0789	297.2306	718.1165	185.2239	577.2598	812.7277	466.3064	578.7678
82.25798	108.948	216.6961	135.8659	144.8339	157.6335	64.419	127.6599	88.1872
8.609118	19.0373	11.52311	10.67033	12.80678	13.62	3.351619	4.561139	14.19121
1520.765	413.3948	4040.618	1111.726	484.7457	453.6893	263.3785	62.975	352.7395
70.96658	79.3578	254.9839	135.0168	165.5232	145.1877	97.29071	358.4382	264.5464
69.50882	184.3728	39.64373	55.11276	86.70046	219.9265	158.0623	30.21883	34.46814
12.87435	21.05932	52.06484	11.46917	25.61679	36.25099	40.48106	42.43475	10.14092
276.1893	227.0861	58.78292	463.6882	247.2993	204.1722	163.0769	323.9492	217.9278
332.6652	194.054	130.1626	207.5025	137.933	193.9501	350.6409	214.9644	261.5088
261.7341	131.479	116.0956	186.9657	37.43386	100.9656	178.4768	55.60805	180.4194
73.67386	35.75947	12.87086	177.2176	10.83161	53.3355	45.5902	30.12113	41.56214
5.836477	26.69194	30.61234	13.11359	62.08331	31.75405	8.059162	22.34958	39.52794
12.91969	67.54847	23.05604	22.99278	15.7582	81.61286	47.06355	33.48477	47.64108
90.69592	37.85652	268.7735	70.77422	78.81803	36.34537	53.44724	41.05602	176.3613
5.812731	5.516286	3.932907	13.15418	5.906317	27.14966	15.67544	11.41203	6.086435
7.18575	0	0.080702	0.757082	0	51.81083	176.2854	17.50273	3.046015
59.52223	39.29592	34.5219	65.87178	125.1382	48.79431	53.3479	47.88712	96.28838
38.2526	15.36083	31.92259	65.96705	17.72968	22.72017	26.75528	22.58245	7.100963
17.16269	103.5484	9.044054	22.16714	84.7375	56.71096	177.5983	33.51711	13.18259
102.1073	129.3115	58.77918	105.3464	450.2787	184.8763	89.49075	287.0109	308.1316
55.33962	225.4095	34.53699	23.80746	162.5781	52.20695	59.65325	66.03071	91.22438
229.5623	298.0896	479.5226	510.563	440.4121	643.0114	357.237	266.5655	284.8267
114.3355	19.57674	5.215429	44.4449	11.81697	57.83407	40.82392	15.60664	44.60036
41.11139	57.00345	59.88817	8.159794	54.19097	53.29242	109.0458	8.354393	10.14176
101.8152	71.03011	33.24928	85.67284	21.66992	57.85599	56.46465	83.358	44.6019
50.99832	11.12588	51.02282	41.97053	56.16056	61.22841	20.57251	61.61991	31.42546
82.24081	22.37515	89.33028	82.30469	180.308	66.95474	4.919874	62.75721	91.22719
66.58151	32.95064	28.15854	89.79387	37.43551	69.18425	26.84105	76.3332	72.97934
438.9036	91.29665	595.3841	434.8439	230.5489	100.9905	45.66843	421.7286	268.607
18.58232	45.63774	94.30485	55.97009	24.62589	49.92859	56.47155	4.665237	47.64249
28.40026	11.13186	69.96741	27.13874	19.70069	43.08595	36.05662	53.86778	18.24939
232.4068	337.4666	619.7872	169.565	1044.39	487.6844	911.3604	339.3134	226.0389
607.4907	678.6816	37.09815	399.359	198.0326	466.1317	106.7545	458.9667	390.2402
11.50481	41.43373	25.60107	18.04628	150.766	69.16024	36.16577	33.48159	54.73511
185.4424	169.508	102.0848	65.81633	56.15448	90.76447	201.9046	95.30426	204.7442

90.71041	105.4547	82.9559	80.66019	112.3193	52.21723	103.4419	55.51513	218.9298
18.57006	28.74382	45.93811	60.95671	82.76866	39.71752	23.69	47.53679	125.6714
58.24233	125.755	185.0204	27.92579	710.3827	186.0435	427.6338	244.7141	14.19576
182.7537	217.9834	470.3633	173.7218	295.5822	244.9651	108.2733	128.217	219.9529
35.54234	28.0169	72.69761	30.4045	218.7537	42.00062	15.9025	69.31075	155.0693
17.13777	20.30206	33.18843	23.00963	59.12162	36.29915	12.7504	22.56315	27.37012
144.3858	75.17049	61.30205	74.89208	76.84705	69.21861	47.20112	166.6786	254.402
14.32524	54.19261	45.90849	10.63169	28.56906	35.17823	74.89285	26.2003	9.128166
84.70473	28.05	16.68392	60.15129	28.56885	21.59342	15.88207	29.76514	70.94696
1008.204	702.512	237.3176	677.7139	174.3856	628.314	427.7729	241.3334	479.4383
83.10642	40.81621	3.939321	57.72688	6.890682	15.92349	22.07247	32.93692	32.43632
113.2388	48.42758	47.27622	92.22452	97.54134	49.94283	44.05156	58.93303	306.0823
266.1198	238.4468	79.17022	423.4464	61.08047	163.3275	234.8609	102.7474	130.758
102.0578	95.56478	189.9674	130.9164	38.41913	56.75676	59.73214	26.60878	129.7428
8.582922	4.109707	22.7974	19.83432	24.64188	12.47787	4.891052	4.503523	23.30459
608.9332	371.2346	489.7459	709.0517	399.0292	613.5474	289.9539	346.5527	577.7545
14.30141	2.691153	28.07946	13.11313	83.76778	21.57684	14.28697	11.86392	41.55465
1696.284	535.076	641.5358	1256.686	508.3923	647.6052	606.3045	466.83	982.182
121.9496	168.6818	30.72414	41.09803	1392.224	159.9539	172.4721	428.9418	247.3218
161.2286	23.78465	24.34618	87.27518	5.905589	56.74309	132.9829	69.69744	100.3466
46.87257	176.7027	108.3806	113.6611	42.36114	147.3821	114.2751	22.94121	11.15544
628.5482	369.8972	411.9021	533.6774	267.9892	238.1965	357.1433	382.213	510.8527
31.30092	49.86847	48.52127	36.17745	93.60375	89.56832	75.16104	122.2752	24.33182
264.8313	197.5513	193.9012	336.0136	74.874	212.0997	155.229	171.764	244.2797
1234.909	724.2432	1813.394	1275.576	617.7562	1236.185	1027.596	670.6712	1196.054
24.24411	45.62216	143.947	67.50213	122.1783	60.13362	28.41502	51.63499	157.0988
10.00246	10.48987	10.25895	8.185291	43.37402	6.847653	3.35094	14.64709	14.19105
520.9764	161.6427	391.4885	447.1939	134.9758	153.152	113.0021	237.1951	218.9419
431.9394	26.60083	1047.98	415.8513	93.59384	51.09262	45.66534	73.94918	862.5667
1550.492	684.1864	505.1045	1252.563	502.4806	796.1616	1118.288	732.1711	642.6282
11.5043	21.6903	77.70838	27.11763	137.9557	20.46467	9.637099	29.90141	175.3278
17.12641	11.84053	12.85545	11.46019	82.77902	26.1077	18.96094	15.44097	45.60907
32.72311	68.18219	94.33521	38.64553	46.30335	124.6927	81.4422	48.02413	36.49442
58.24021	109.576	136.5744	38.63061	7894.135	3562.708	648.4091	132.1803	36.49537
21.41524	82.27717	2.656015	10.62972	51.23002	23.87107	167.1189	22.91083	50.68374
8.653139	1.290296	19.17531	13.94872	6.890979	40.76602	34.3141	65.81496	19.26072
1363.528	558.3045	392.8833	1015.394	202.9584	846.0406	391.7688	386.7651	377.0654
137.1961	101.9981	20.52119	154.093	40.39075	40.8742	76.81031	69.59144	73.99485
5.826095	15.41943	17.88152	19.75695	36.46329	28.31766	11.12751	4.62633	40.53412
264.5442	28.00131	107.1762	196.8614	59.11047	138.3664	175.341	113.2692	124.6747
570.9813	105.431	6.486147	44.3968	17.72844	54.48798	81.60969	51.97474	68.9296
10.07699	8.316646	58.50461	7.337936	26.60001	23.84279	49.91533	22.42415	48.6484
80.86774	139.1903	161.9859	121.0115	243.3643	111.1782	167.6314	196.4386	201.7062
14.32013	12.53988	47.15626	22.18185	77.84659	30.64434	22.09597	29.62215	40.54431
32.61365	31.61044	23.02657	20.535	30.5412	38.55851	53.08528	71.0224	79.04915
17.16804	18.16001	19.24306	16.39513	27.5819	305.8099	142.0813	12.01965	10.14186
5.772091	22.77549	2.651847	4.058261	15.76973	6.832412	9.43202	7.700122	3.045764
94.982	108.9483	259.9857	81.47344	337.9624	76.02728	40.95834	88.07139	254.4064
913.2835	423.2775	58.78066	533.5977	174.3858	247.2866	105.1894	401.1151	256.4469
56.80107	2.701747	17.97238	1.58302	18.71369	149.6978	72.2324	689.5419	3.045928
11.42608	53.22517	2.660529	3.222104	30.55101	18.14533	11.10137	0.711389	5.073292
121.8025	91.36243	49.83951	154.845	73.89077	94.15535	44.0785	22.96306	286.8355
0.102603	1.302186	0.069365	0	0	4.551297	17.92219	6.988925	0.003575
42.5859	55.52021	65.0454	76.60392	102.4738	21.60116	23.71203	19.2541	123.6502
24.24428	83.68479	49.81087	32.04956	118.2366	49.93685	33.10207	33.72097	96.29086
412.1974	231.2503	1509.611	650.5612	532.0417	365.2199	268.0435	321.1826	526.062
271.9563	143.3534	48.58073	144.8707	777.3911	195.1039	91.0828	363.8489	581.8004
491.2312	407.234	205.391	328.5739	374.4026	285.8063	148.995	251.5946	310.1632

6405.126	1013.941	11365.08	4147.073	2473.011	2233.102	3071.668	7854.197	3914.54
73.75992	23.78179	107.1601	113.6248	2.950234	49.95144	45.64419	0.861199	67.91573
236.6395	466.9327	72.81147	161.3322	282.7668	242.744	534.0881	259.2993	208.8074
15.71632	13.25249	43.30626	17.23788	51.23941	27.23766	9.623331	36.30861	87.15235
1458.649	1228.514	1954.966	1828.171	573.4188	1375.681	2206.731	867.1432	1116.994
168.5776	158.1786	251.1883	64.98397	280.8047	235.8803	177.053	214.7084	168.2601
66.34462	51.40003	6.491976	18.88183	18.71514	44.22004	43.81875	15.5053	53.71749
86.51797	75.84544	153.0551	127.6098	181.2896	130.4443	83.20083	77.35485	400.3578
5.828884	87.24253	0.085844	3.22109	32.51865	47.51403	54.17873	8.202701	0.004295
11.50583	40.71692	11.59466	6.513404	28.56764	61.24456	356.2033	29.98593	7.10098
106.243	59.67959	42.19157	163.927	38.41945	87.35096	33.1262	288.0155	57.77992
95.02012	133.55	174.7367	111.1201	212.8183	66.9653	62.8801	55.68875	171.3003
17.09207	43.74685	14.10994	5.691538	19.70268	28.34725	32.80087	0.761675	12.16778
0.122125	13.359	3.928379	6.533893	18.72409	10.22815	18.64633	4.521741	3.046112
86.33981	106.2842	19.24394	109.587	52.21596	52.19718	114.1113	51.53141	37.50773
35.50876	61.19723	25.60062	60.9468	64.04357	39.72165	101.4574	33.47516	31.42571
307.2828	665.5976	137.8241	297.2839	194.094	257.4524	535.2732	200.7663	232.1172
16.97891	6.933084	16.5736	3.223017	32.52546	9.108721	11.0771	14.73053	30.39882
424.5835	185.6066	285.6262	406.079	98.5211	130.4628	169.2782	142.6901	423.678
25.56902	20.31179	34.43856	12.28485	32.5129	23.84551	15.85443	32.90483	72.96773
132.9086	56.90047	61.26191	145.8709	17.72852	49.93553	23.72498	40.88103	39.53504
637.323	274.8489	673.3831	437.2204	87.68226	655.5295	877.0817	30.165	114.5432
4.414805	60.25039	1.380708	3.221371	25.61944	29.44479	35.71294	15.03512	10.14028
60.97657	30.82808	82.89999	69.14928	23.64029	44.272	8.068252	15.66778	53.72441
235.175	203.8557	134.0105	471.9075	207.887	182.6333	170.9153	135.6849	305.0961
45.47945	75.86685	462.2974	80.65643	97.53867	79.42273	44.08011	44.71198	356.7679
55.36195	39.97061	175.8536	146.6292	206.9167	209.7065	61.24924	80.56085	98.32053
119.0585	123.7015	263.8944	181.1642	299.528	133.8508	112.9242	196.463	218.9366
82.31781	187.6465	470.6115	147.3303	1203.024	257.488	75.42887	135.8082	903.114
58.11276	107.0158	35.79492	17.21927	67.98298	89.56597	89.16257	97.50224	31.42643
14.24028	8.336239	59.48726	11.48138	31.53492	15.89607	8.027594	0.725844	19.25884
135.9567	64.59653	116.084	149.8856	124.1423	80.55911	45.64844	152.6952	175.3507
118.0654	13.25269	11.58286	40.37473	12.80299	29.49968	17.40385	15.4281	31.42251
17.15901	35.7953	10.31887	14.7501	83.75361	38.58696	166.5949	19.19728	12.16897
5.838297	2.694251	12.86713	70.04099	0	22.72928	3.358313	4.674888	9.128219
56.77233	72.36875	38.36484	71.60423	112.3209	87.34303	79.97912	41.02342	109.4689
44.08107	364.3829	88.10339	27.09933	899.5818	90.78068	349.1138	211.4377	66.90357
100.5281	20.26707	77.82802	40.28758	93.60007	7.99476	12.77229	19.30884	122.643
46.70469	18.1794	24.30758	46.12579	21.67123	23.85456	8.068041	26.14795	20.27656
1.575011	11.8468	98.93971	23.8549	43.35642	20.44322	17.38499	8.292163	65.87137
26.93191	12.55502	12.8468	50.33165	25.61557	29.48662	4.93138	25.72051	11.15463
222.1988	313.8924	68.96321	146.5725	59.1103	72.62878	108.1968	59.24218	59.8078
4969.107	2306.555	1615.889	4350.531	1785.296	2574.444	5062.079	4283.985	2935.398
25.65625	353.9641	0.090829	13.09991	54.18634	219.8134	280.6646	8.36015	10.14185
233.8003	275.5869	404.2951	457.8569	449.2806	379.9438	726.5147	266.4962	553.4259
44.04953	102.6714	103.2988	54.29562	146.8094	70.34398	72.16791	26.57007	66.90112
168.5732	190.5634	125.0506	168.7935	199.0234	255.1477	238.006	211.0642	88.18841
25.61573	25.93125	21.77371	24.64957	91.64051	24.99162	29.90202	22.70119	48.65289
62.48654	110.3034	37.10294	17.21978	39.40421	130.4641	680.4819	44.8191	20.27781
104.8203	110.4009	152.9628	190.3209	53.19931	91.88133	25.30158	26.58554	85.14516
18.56194	48.53014	28.12935	15.57719	33.49612	69.1317	68.71665	19.13403	31.42477
91.96126	52.68776	6.491152	120.3248	45.31854	103.1582	53.35608	62.14414	60.81774
0.117026	0.592642	2.651461	0.757421	0.979739	23.68225	60.30721	4.41014	0.003978
0.107503	0	0.072474	0	3.93764	19.09912	6.290343	0.529844	0.003714
1.57603	1.9892	19.12092	3.222022	50.26974	7.983275	1.784282	14.89367	19.25797
31.25687	53.46453	7.768128	19.6992	18.71454	55.5587	101.321	22.71556	24.33087
28.31575	16.8023	3.939101	57.8009	7.876278	15.91383	9.60922	0.764799	17.23459
52.56946	79.35944	275.3544	159.7399	337.9562	96.44089	125.4292	117.1394	208.801

768.9895	39.96421	871.0135	1111.758	72.90337	191.7215	81.69062	888.0621	604.1105
1.567613	5.504959	1.374992	2.403344	456.2298	80.53672	25.29594	12.02276	1.018481
0.056961	0	0.03953	0	0	1.147391	2.934458	2.946053	0.002153
3.000064	8.328854	12.82966	39.62631	8.862086	4.591098	1.784637	0.744005	11.15395
927.6967	945.7715	3179.083	1929.46	558.6396	1076.298	1051.13	990.838	1298.429
7.245	13.26974	6.487212	37.11223	5.905566	14.78067	1.784305	4.655336	0.004335
41340.09	26960	21264.29	39227.75	9728.504	25247.5	40775.55	16527.41	18374.62
89.25906	104.7848	82.93311	70.78153	63.05311	66.94457	162.6393	58.99237	46.63051
233.7125	124.3686	456.4117	252.7993	435.494	209.833	424.2311	88.42883	651.7317
177.1073	106.7837	160.7565	159.7118	504.4679	156.5419	91.06696	146.3612	473.3435
185.6772	568.9455	316.3438	288.1697	502.4852	218.9309	233.5765	248.4122	517.9515
2989.08	2240.664	145.5019	778.1451	676.8724	1545.775	780.187	117.6054	130.7607
68.15364	104.6648	419.4902	200.8897	97.53531	400.307	28.43416	88.45261	156.0994
12.88692	23.16336	17.92408	32.12765	19.70207	20.44134	62.14665	4.66469	11.15463
11.50372	52.73899	109.4402	16.39898	106.4205	22.72975	11.20163	40.4912	59.80149
21.29778	30.97996	6.487341	51.18823	4.920231	13.64981	26.62562	11.7783	14.19443
90.65053	103.3932	46.0021	96.34869	44.33191	97.53158	104.9035	62.50657	56.76553
172.7385	76.5548	254.9197	139.1551	86.69899	94.1669	59.73446	55.62523	105.4179
31.30723	57.61309	110.8565	54.31425	65.02586	138.2747	115.6845	40.84204	22.30484
324.2147	505.867	33.27665	466.2126	14.77295	180.3509	302.2226	30.2487	117.5828
114.7594	32.92607	165.747	103.7284	248.2964	121.3615	45.64838	91.61696	186.4995
7.227504	4.804322	3.935778	1.57675	4.920521	18.14943	15.7274	8.125594	3.046332
3448.165	2208.857	1159.332	2512.474	857.1751	1537.88	1928.198	1827.809	2652.6
73.75595	94.8845	43.46828	246.361	59.1109	132.686	61.27396	69.94198	37.50892
18.58718	37.86742	53.63709	18.86479	101.4837	71.46867	159.4142	26.5427	13.1827
56.34961	25.99393	1.379892	132.3071	6.890813	13.65372	1.783733	22.28669	9.127763
641.3436	171.4797	323.9704	618.5049	87.68225	233.6659	400.9973	248.2696	159.141
2117.231	3367.829	2611.829	1487.986	2414.883	2069.774	2387.141	1766.255	1748.471
168.6645	282.6552	270.4114	440.596	482.7839	226.8555	197.5255	240.8643	382.1281
25.6452	28.72808	169.2465	32.88272	80.79425	22.7343	29.95373	33.58616	66.89775
3.000493	3.392511	1.380068	4.043777	2.949756	67.88964	123.5925	15.30394	0.004351
226.3025	117.4291	61.30387	184.5299	14.77266	90.75365	126.8617	141.6909	70.95615
66.71656	104.7028	151.7854	123.4876	199.0252	123.6446	61.30729	189.1229	184.4753
24.24075	21.67827	93.05291	40.29634	240.4324	64.6594	54.93272	51.5543	100.3436
5.81634	1.28911	9.006398	23.93318	21.67868	12.49878	17.22822	4.587008	10.13951
12.89122	5.501198	36.94515	18.89518	33.49987	15.91855	11.17454	22.33622	95.2546
1.56847	3.415156	16.42633	1.579466	13.79881	5.700889	3.320286	4.361511	13.17283
8.665126	9.021387	15.39544	11.46107	59.12475	19.31672	15.84805	39.71678	67.90027
1.575931	13.31475	0.083966	7.351353	56.18743	17.01423	49.28453	11.48744	0.004215
15.7522	128.2037	33.24742	41.95254	71.92498	21.60146	12.77077	15.64622	42.57479
46.81841	18.16159	93.01291	62.57692	61.08553	68.04471	56.45	30.0205	85.13989
94.5841	27.33857	19.23152	45.2795	25.61229	74.79975	34.57826	15.58404	31.42511
10.07892	23.84516	69.92473	38.71603	40.3974	26.10845	6.499887	25.95834	42.56933
113.2657	39.26837	88.02622	122.7234	45.31698	71.4778	103.3808	141.2546	58.79296
21.32245	17.50122	33.13643	22.20335	25.61542	29.48825	11.17137	25.74487	26.35484
12.81799	11.18344	9.008039	25.58916	5.906138	20.39815	20.29929	0.706786	8.113181
45.4417	195.8348	85.44163	98.01721	31.5229	91.85348	117.2995	62.35878	20.2778
46.78229	118.417	7.768319	42.79215	33.49508	14.79862	104.5483	12.0008	20.27727
14.30792	16.07616	45.84149	13.93595	13.78841	1.184474	12.73971	32.85748	13.18185
0.136918	6.205317	1.375869	4.86881	11.81686	176.7753	673.9124	221.458	42.57538
29.75283	62.83306	14.1203	19.72113	6.890774	12.52311	15.83701	25.79961	15.2084
178.3772	122.3232	112.2753	177.8976	50.24267	60.15755	100.3739	106.1097	157.1082
73.5969	94.34092	59.95334	27.11174	65.02757	26.1334	12.76996	153.5031	40.54756
130.2326	26.59727	35.81835	253.0015	6.890828	32.94387	15.90603	37.43455	125.6856
17.16711	53.39832	12.8702	2.401959	54.18734	96.35847	160.7013	8.362946	2.032392
79.3772	61.09821	71.47771	24.62975	81.77466	59.0127	64.36754	137.7236	302.0319
66.26027	27.38214	30.63095	80.06771	4.920151	24.97616	11.1834	22.45636	22.30254
0.109451	8.471715	0.073704	0	1.965343	12.41017	49.043	4.225714	2.031961

1023.935	2232.259	76.63123	236.2644	1378.387	1426.694	2358.461	2342.477	581.8134
0.12585	10.47359	2.660293	0.757107	18.72078	14.75742	9.554751	4.59166	1.018771
79.45805	209.5808	107.2069	107.8245	207.8917	242.6739	230.171	210.9807	183.4629
170.0397	441.7951	89.37998	326.1332	121.1825	231.37	139.5774	37.53825	61.83565
12.86426	38.11456	29.28451	12.29936	11.81862	20.4267	58.83433	21.98783	10.1407
17.09983	11.85103	6.48851	4.867228	46.31481	35.13312	65.18093	4.661565	8.114225
39.78333	36.46815	188.4107	46.89197	103.4566	52.19903	34.65548	30.09502	127.7071
338.6163	694.0548	594.3455	1044.213	1166.558	646.4642	283.7292	495.8195	971.0313
123.1493	63.91691	107.1199	116.951	57.14099	106.6042	70.6101	55.40855	186.4944
245.0856	1890.805	47.30517	259.3645	263.0631	364.047	768.5115	132.0668	50.68611
3.000262	23.1511	39.48697	20.54537	85.73891	15.91971	12.73315	29.29247	64.85939
235.0533	142.6879	139.074	255.306	47.28641	231.3506	177.0663	73.84354	90.21572
250.8272	94.8085	2666.043	1353.919	19.69965	257.4962	163.1411	11.89178	210.8354
6382.57	1496.363	9878.284	5521.479	6589.456	2929.442	3372.431	11672.22	6147.508
73.65789	35.76324	76.52229	102.1541	35.46447	113.3635	33.09496	44.42896	25.34551
2583.103	2051.98	1918.111	3213.259	2206.992	2193.389	1209.403	2024.585	3177.648
22.79207	71.15115	5.215982	8.15991	22.65633	37.44369	40.77342	19.12759	8.114593
162.9428	541.1308	224.4577	275.8948	156.6539	187.1459	284.9671	272.6601	216.9119
101.7307	25.21022	39.59723	84.87658	13.78752	29.53011	37.73166	40.617	59.80266
117.6582	157.4771	53.67786	20.51181	380.323	242.6795	388.0218	153.3526	24.33236
160.0171	167.3867	158.1337	58.40015	150.7452	163.3067	131.6338	73.69224	230.083
0.057294	0.644313	0.039754	0	0.983283	2.245062	2.937181	0.214584	0.002164
10.08322	37.9626	23.03176	13.10671	105.4436	95.11262	162.9202	19.05175	4.060059
14.32361	76.83151	30.6633	17.22844	113.3253	43.09789	74.86662	11.96057	19.26319
12.91682	58.39987	28.13688	71.69819	90.65354	32.92118	12.76377	11.98859	7.100998
59.5288	104.1844	31.97789	29.5815	25.61122	70.31668	48.67983	65.6911	19.2641
287.3414	18.86119	326.3398	387.9971	8.8615	53.35931	11.19406	131.6811	65.88972
135.8255	95.63484	33.26588	275.324	51.22922	56.74287	79.95626	94.71743	115.549
10.0897	16.75044	6.490133	0	25.61093	147.3541	137.555	30.1293	9.128207
713.8214	388.0748	562.4843	1343.159	1441.449	305.1296	122.4175	615.883	634.5195
334.3058	260.8081	219.4483	1007.279	727.1313	302.8421	332.1877	480.58	586.8748
22045.02	3288.915	37828.64	19964.71	4674.093	6225.159	8144.971	21574.63	9416.384
35.37746	20.32075	7.76416	41.21	5.905433	31.75724	37.52807	39.63843	40.54165
79.42943	92.75363	170.8539	165.539	145.8192	121.3657	50.34438	102.4721	250.3523
7.253368	45.06173	7.765801	4.04371	119.2466	64.56063	57.69302	18.98326	6.087339
11.47578	15.38542	52.1025	26.3435	21.67352	15.91476	18.91855	0.767113	40.53963
5.797474	9.083575	15.27398	20.661	12.80816	7.971311	4.893562	14.43323	11.15103
22.79151	22.40693	44.64212	19.7014	39.40908	31.78331	37.66477	11.97097	44.59892
46.89453	44.88997	178.4648	71.59044	174.3957	113.4241	83.15589	84.36842	115.5521
17.02607	41.77148	5.208165	4.870717	8.862632	11.37545	37.18024	8.132917	12.16643
66.59668	39.99296	89.23499	42.77046	128.0926	37.46913	39.33442	12.02158	170.2713
107.626	41.37931	33.26867	122.7193	117.2476	83.94437	45.62569	105.5986	278.7222
29.71639	12.55918	76.11075	39.58614	54.20099	26.0896	4.930358	11.79951	56.75021
949.9255	500.0014	399.2068	615.1772	380.3099	294.9049	618.5891	360.9007	294.9625
32.61948	30.19281	238.4367	13.93225	73.90552	34.03537	20.53262	8.334865	24.32988
22.77338	13.24606	16.67592	48.61565	34.48292	18.19076	9.62963	11.93636	36.49053
103.5524	296.02	485.8203	57.56735	540.9161	183.7693	256.9958	135.7092	709.5125
190.7288	15.34363	10.31956	121.1154	1.964986	13.66682	1.774714	69.48164	98.31776
100.7059	141.2585	154.3885	62.5097	720.2508	286.9217	305.3696	247.6823	112.515
35.46302	25.23261	20.49723	66.77124	9.846488	30.64922	28.33066	50.71072	27.37078
15.59373	11.90132	55.56833	14.81067	20.69328	18.13642	11.08248	11.43292	42.55398
4.418725	11.82981	114.516	64.25902	11.81699	9.129795	6.502276	19.18705	43.58674
415.9625	208.1696	153.0796	261.9138	68.96278	139.5227	175.4738	99.13946	221.978
36.96256	111.1974	17.97061	10.62969	140.9017	61.26468	293.1353	12.02291	8.114574
17.1716	151.361	47.27057	19.68858	139.9143	47.67345	59.64907	8.356216	36.49473
83.64932	54.04264	34.54723	88.90038	36.44875	61.28621	65.96167	44.70189	37.50891
21654.17	10770.03	6779.552	18576.3	7085.038	10102.65	16219.07	12919.38	11269.25
300.3308	512.674	389.0028	793.1119	295.5757	267.6889	349.3967	161.2235	382.1307

34.04664	30.88616	31.93023	35.38867	35.46779	27.25092	29.87538	43.65562	35.47777
15.7267	57.77847	11.58694	36.22512	33.4976	17.05777	17.42114	19.02873	54.73033
5.791614	1.991554	14.00405	12.35217	1.964762	2.320189	0.162182	11.11796	11.15054
55.35904	29.41305	95.66634	110.353	254.2161	59.01271	22.16965	48.22538	169.2653
25.66981	361.6372	95.74277	22.15857	468.0163	266.4877	233.3349	62.96	22.30511
546.6296	930.5597	376.2696	368.0605	221.6793	310.7834	795.4705	342.8403	448.0146
53.47131	16.09967	16.64464	64.44377	12.80377	17.04253	3.361683	0.759611	14.19441
167.2118	260.8956	164.5847	205.8433	334.0078	186.0219	122.3683	178.9736	443.9513
121.8413	45.58979	90.61777	196.0352	73.89012	106.6321	131.6153	134.8605	52.7127
83.67976	14.6419	290.5715	249.6021	89.65496	59.02462	28.43772	77.3029	142.919
4255.116	4802.78	864.747	5013.637	565.5362	1541.272	1596.146	66.56048	3230.349
56.81143	225.8149	107.1939	53.45527	59.11013	137.2455	198.849	131.474	63.86225
24.23001	50.59063	59.95016	18.86873	89.66387	66.90707	70.43858	54.86376	72.97797
41.07434	3.393743	38.27575	0.758846	41.38102	62.32499	34.52836	43.61587	19.26305
123.1146	118.1966	76.55707	70.78771	47.28793	99.79748	131.4076	148.1241	50.68439
15.65873	8.33056	29.26794	29.68965	19.70415	7.988036	3.360228	15.10658	35.46995
215.3927	250.2667	304.8542	695.1067	432.5313	217.7926	55.06584	66.66788	475.3795
0.066415	0	0.045841	0	0	2.260646	7.088784	22.42075	0.002467
114.7848	130.7679	156.8598	94.65446	176.364	98.7007	106.6324	88.12436	211.8394
73.75566	20.26541	52.38743	48.51905	14.77267	138.3517	265.7513	12.01335	11.15537
33.871	16.10737	17.90407	12.29757	6.891027	5.724804	3.361249	4.648432	15.20742
0.114612	36.59855	1.376659	0.757791	62.14544	5.700127	0.153566	10.68016	0.003912
10.0771	16.07725	3.939388	11.46094	60.11026	6.860375	11.18128	11.88698	19.26226
53.76583	11.83217	39.56323	57.67625	12.80245	27.25496	14.32078	4.676643	9.128177
203.9608	16.75366	634.6732	334.3994	16.74336	40.886	3.33988	26.60531	63.86272
171.1832	56.86687	51.10703	127.6583	29.5518	100.9458	114.3353	123.5856	113.5236
39.704	47.81831	23.04568	75.84066	3.934999	26.12416	23.67658	43.8608	49.66631
123.237	63.18906	53.66566	151.5352	64.03722	86.22609	44.08383	116.778	132.7825
32.71453	56.91768	49.79521	51.84522	47.28928	68.05227	58.03268	30.06178	116.5578
165.6933	84.29258	46.02511	213.3208	72.90438	91.90269	81.63539	99.00895	238.1924
691.0241	406.4077	382.6539	230.5102	452.2344	528.5035	673.449	542.5424	566.605
42.66325	199.0127	195.1271	86.40267	611.8763	72.635	45.66481	73.8199	201.7072
22.75774	25.96496	87.66003	19.71294	36.45505	23.84588	12.7428	18.972	62.8352
5.794594	13.37136	24.04914	7.365865	26.61439	2.320409	3.343182	4.501679	11.15079
12.91535	44.17563	17.96974	62.51175	2620.968	162.2027	102.0058	30.25327	19.26423
110.5948	130.0167	305.9873	136.6533	486.7361	111.1858	50.36374	102.8275	486.5161
19.98229	47.09117	59.91492	41.1459	86.71057	39.71772	29.92257	40.48934	70.94891
8.671299	134.1294	5.216052	6.512997	56.16295	31.78153	45.41806	8.349174	13.18232
14.31372	43.63926	3.939273	14.75865	41.38237	43.08036	43.78303	19.0009	4.060069
75.22335	189.8347	114.8669	183.6113	310.3624	128.194	108.2694	113.7185	230.0882
10.03139	9.757804	26.69439	14.79824	11.81972	13.63312	15.72427	4.606048	4.059876
49.63084	23.09292	51.05111	70.00868	75.86748	47.65632	12.7698	33.56454	73.99151
21.28269	40.22872	9.028052	8.992997	5.905621	52.05009	52.74253	18.63779	12.16752
414.9286	359.3274	799.3911	574.0272	224.6361	144.0843	283.6237	404.0987	430.7812
0.129291	28.17417	0.08608	0	5.90568	21.55546	78.7415	8.216041	2.032654
111.437	53.46526	15.41248	24.64855	31.52495	26.12491	23.67928	29.80145	65.88072
19.9887	49.89485	113.3009	34.53732	73.89752	46.51927	19.01976	29.95333	78.04434
219.5512	147.5937	369.7448	196.783	244.3451	116.8601	144.2688	197.0315	631.4587
137.5295	141.9389	232.178	237.9419	506.4299	167.8959	170.9287	664.6213	787.5585
38.24376	61.31307	9.040778	51.09698	7.875944	30.64113	29.85272	11.93299	27.37008
7.231903	9.751695	26.71607	8.999745	40.40754	21.54172	12.66485	11.60519	9.126901
96.47791	434.5067	364.8326	104.5065	778.3588	365.2304	1223.081	506.7016	33.45435
18.44054	14.71194	0.085224	9.825649	0.979689	14.76844	23.44069	15.02961	2.032658
48.216	15.34691	39.60201	105.5035	0.979905	20.46683	17.46018	4.670228	18.2504
321.2682	113.8382	57.50216	273.4471	64.03629	108.915	208.3063	286.7107	144.9481
0.124148	29.03426	6.468426	3.223416	8.86351	20.38324	52.14491	0.688003	7.099563
68.04593	22.37881	34.53627	157.3941	4.920378	18.20299	23.72943	0.848208	21.29143
4.38529	8.393953	1.37876	0.757246	1.964839	10.21361	15.50552	4.444574	2.03245

19.99507	32.24875	110.818	82.37472	48.27496	40.86414	20.59029	33.61489	85.14032
151.6531	174.3275	140.3711	261.8619	560.6286	216.6354	213.1116	254.9775	352.7313
7.252912	30.20948	16.66887	17.23665	48.28215	46.46621	23.61911	32.89776	37.50272
25.61221	18.1744	48.4541	38.68252	45.32187	45.36724	18.99942	26.22427	78.04028
9.999488	11.91612	16.55783	3.223821	14.7789	28.26082	26.30421	4.550803	11.15186
17.15737	18.1687	118.3206	34.54501	20.68512	30.65807	15.88955	36.94623	91.21654
279.9139	185.7898	29.44757	256.2781	20.68421	132.6784	276.5935	220.2068	53.72573
42.65241	57.55818	54.93931	141.6454	94.58236	140.626	262.704	44.72616	81.09201
38.3926	48.42503	124.9267	34.51728	124.1461	55.61054	65.91694	37.39621	158.1159
151.5382	194.8526	189.9739	108.663	254.2059	201.841	496.9894	37.5111	18.25071
63.84218	75.88683	130.0406	41.93125	75.86231	87.34543	78.42687	109.2164	179.4009
1251.884	1920.66	395.4536	1129.811	1190.2	1483.396	2341.245	965.1259	776.4243
1.57552	6.934561	0.083059	2.399234	19.70797	49.66166	23.29755	8.024633	2.032624
872.4086	630.7195	1351.761	714.7459	1322.228	522.8685	199.1678	641.4183	1016.646
0.086006	0	0.058701	0	0.980987	10.03909	15.85945	8.733475	0.003084
65.06612	163.7067	12.86697	50.22781	28.56836	55.56505	135.502	36.92843	23.31758
286.2163	371.2172	754.9578	226.388	885.7566	601.0904	682.9219	590.0214	1456.537
70.91506	68.83729	131.3259	94.67884	161.5882	100.9474	89.36895	51.87308	187.5098
24.20789	20.99225	15.41241	13.92756	108.394	55.55806	6.502408	26.26049	109.4553
15.74774	181.9973	9.043977	126.1683	33.49495	23.86455	34.60889	19.21378	44.60074
0.088744	1.321285	0.060474	0	0	3.407026	3.173072	0.390247	0.003167
5.825525	23.94493	15.35024	11.47971	8.862396	11.37858	35.70927	21.76483	16.21961
31.29389	34.36644	131.1326	64.22837	114.2994	89.55775	19.02545	33.58662	30.41275
12.90188	20.30975	35.71097	11.45949	70.95057	58.90749	83.96301	36.4042	42.56959
53.91823	30.12359	82.90151	83.99275	45.31767	46.53863	28.41714	80.21944	294.9292
61.05703	654.7659	43.47612	73.22657	369.4886	149.7152	262.9057	73.75077	34.4684
1376.53	1038.614	1665.517	2273.719	586.2272	1145.472	1517.761	1027.158	1641.024
24.08176	40.23043	6.485844	76.07512	5.905625	18.16929	4.927945	8.23378	8.114015
27.08585	199.1127	5.208916	22.98125	153.7024	200.6921	526.4375	66.41189	18.25073
1.574781	14.66882	30.61532	4.043644	17.73058	14.78775	45.2446	25.83205	15.20848
22.71282	50.115	7.761062	15.59629	21.67356	74.66443	48.23887	11.80797	18.248
39.37234	18.96688	1.38069	28.0418	5.905813	17.02908	12.6736	11.63791	11.15362
27.06503	47.74965	58.70268	39.47415	85.71993	35.20177	48.68546	22.88157	87.16804
188.3712	199.7149	58.77717	200.1053	121.1836	128.1879	84.78747	102.775	112.5141
39.78372	42.10484	59.98358	65.03455	121.1941	52.19923	51.8178	8.360799	69.93971
232.3649	160.931	350.7295	404.335	663.0916	317.5692	197.5405	342.45	526.057
31.2993	43.52531	114.6365	20.51535	63.05592	61.25387	124.9461	19.26478	45.61539

TCGA-FG-	TCGA-DU	TCGA-HW	TCGA-28-	TCGA-28-	TCGA-06-	TCGA-27-	TCGA-06-	TCGA-14-
60.24701	95.87765	90.02768	133.4643	158.2103	131.5875	60.11825	139.8954	57.25143
24.5648	78.42749	46.82642	18.43046	13.82595	10.63542	14.30174	8.780876	2.647959
78.53791	95.2927	153.6663	51.49389	42.49595	58.38128	74.44285	76.52155	47.08551
1761.305	2895.905	2902.29	2099.528	3144.01	2150.911	1508.15	2014.168	1943.035
137.3169	97.17799	111.2354	99.20543	52.05232	132.6432	161.3213	81.99073	105.4509
1.527611	0.517805	0	0.051849	0.014777	3.190513	7.661265	65.5639	8.699126
10.23369	66.07613	9.66393	2.537423	11.70479	16.9998	132.7689	106.9746	7.732635
90.42924	168.9319	120.9454	63.74132	32.94262	70.05592	109.7616	15.3395	35.67294
3044.001	7683.149	8936.445	6947.303	9141.102	4712.472	2958.085	5269.813	6197.247
35.65488	36.36009	72.40694	40.45966	23.38397	52.00561	50.5819	10.96834	29.30429
11.84741	17.8715	20.30251	14.73765	7.455747	13.80906	18.13095	2.223012	5.187434
404.7379	657.0104	1733.299	456.7065	228.3155	389.457	616.6153	277.627	83.92754
367.6929	211.1946	143.0225	110.2357	156.0917	222.8249	153.6753	759.2632	284.3354
3.943313	25.42352	0	4.901513	20.04108	0.015528	3.81188	7.620648	18.53612
41.99123	52.34538	113.0208	133.4014	117.8577	50.95492	72.53393	18.6187	59.76748
43.67097	66.73462	12.31502	100.1001	531.2042	31.84238	30.5377	184.3882	396.3954
1634.317	2025.698	2384.88	1445.823	1256.137	1539.705	1484.29	1449.16	764.5977
46.82118	22.26905	37.94718	34.32591	24.44175	47.75303	213.9496	5.50323	14.07872
232.754	249.2619	42.33743	209.1809	467.0183	165.5132	161.3314	162.8048	280.3106
2167.355	331.3134	387.5846	1242.411	1654.225	465.8479	626.1709	644.7954	1828.305
626.2229	619.2643	1146.091	910.8177	450.2288	464.7941	641.4352	404.3948	147.4277
0	1.779432	43.25421	2.536576	2.147698	2.146064	0	0.030345	0.087852
0.721134	14.56162	67.95253	64.95265	19.13923	24.43216	0	8.781295	2.640654
165.8314	44.63219	223.3645	88.23514	21.2618	79.61025	74.43425	10.96506	34.40357
37.22802	62.61263	148.3816	45.37258	67.9694	57.3181	40.07361	119.109	17.89475
52.47149	6.8868	26.47005	13.54739	8.519771	16.99744	41.05133	3.316671	5.192122
352.5261	216.2561	78.53752	257.0545	247.3964	206.9227	194.7207	287.4027	365.5979
1938.534	500.8771	1427.788	405.2894	268.6633	565.5969	1672.37	221.8912	483.9279
11.82481	15.86766	66.2909	3.764716	3.210659	3.208329	9.526657	0.03005	5.192339
45.97432	26.72713	113.0448	42.92001	11.70636	22.30917	57.26132	7.688904	3.916245
26.91359	15.20437	45.00665	42.89769	20.19905	26.55026	41.98876	34.99933	20.42606
30.09417	22.89589	59.15247	72.20439	22.32183	41.39824	15.2535	7.689403	12.81351
13.45261	9.485002	11.44813	1.309937	4.272041	4.269035	7.620223	8.773283	6.450041
1324.866	498.9662	846.7533	385.7007	878.1166	1096.132	818.0328	1126.725	1485.663
173.0028	152.8361	218.0856	122.4888	92.40043	250.422	387.5654	138.819	153.7232
3838.555	1171.394	36421.41	1744.564	1723.341	10987.95	5826.463	617.505	1235.742
22.99744	22.35081	21.17357	37.88299	47.74447	28.64792	31.50501	70.9111	75.78818
31.67696	82.56974	67.98085	51.46301	31.87618	74.27868	29.5738	24.07984	26.7717
50.79608	51.82381	14.96275	53.86005	72.18502	67.89825	48.67889	177.9402	82.42321
26.93261	38.99383	17.61596	53.8337	84.90134	39.26753	50.5941	149.5358	135.4194
203.2391	150.3374	10.54729	468.505	360.9532	195.2387	144.1304	458.859	328.6877
61.12624	46.65664	51.20112	14.78319	29.75013	43.517	21.93715	80.84469	25.4929
96.1191	83.89368	7.014208	66.10609	52.03778	68.97109	63.95276	174.7266	553.0025
32.45724	50.4114	225.2242	44.15933	5.333477	31.85956	62.02946	13.15382	9.000752
253.1896	148.9663	224.2523	199.5926	142.3029	188.8951	304.4977	208.7558	73.76945
11.84255	13.98178	24.72656	13.52697	8.517289	8.511574	24.81944	4.409519	12.77693
146.248	58.18671	28.21229	74.66058	60.52822	49.88433	48.6722	32.81664	79.9708
792.8816	1125.973	4000.713	986.788	382.2775	985.8097	858.1074	455.7644	325.2139
425.4168	184.7668	103.2554	532.5378	156.1118	250.4475	281.5723	238.2788	125.8381
5.475585	13.36339	22.97252	4.984822	3.210502	5.329498	19.09017	4.40859	0.08315
19.84686	5.617493	11.44799	2.538306	1.085305	7.449651	11.44257	6.591714	3.917984
162.6662	369.292	289.6176	95.57442	48.86997	178.2831	276.8159	193.4552	34.4044
39.62384	42.1233	89.19382	44.13338	30.8151	74.27997	47.71525	25.17257	50.85805
768.2735	1884.822	12592.02	896.2031	678.5218	1642.646	1019.423	229.5383	430.6147
11.04512	13.98097	19.41029	8.651627	3.21076	18.0491	25.77506	9.868083	5.188937
93.73733	46.63938	60.03543	58.77548	38.24176	30.79287	36.25925	22.98582	11.54413
11.8384	17.1971	44.21409	9.874339	6.395773	8.512271	7.618436	41.48476	7.72442

6382.663	4474.972	5584.721	4518.516	1535.4	1559.878	3882.081	1631.684	617.2948
596.2674	111.1903	296.6693	115.1607	47.80822	213.2998	1990.438	286.3368	448.1918
53.25834	14.5864	23.81344	8.661304	12.7651	40.31744	38.18332	9.873258	26.7159
46.80862	55.66566	3.484404	52.64921	58.39729	46.6965	41.03671	68.8339	47.0169
71.65665	5.606936	0	24.51757	160.1017	21.23569	31.4997	115.6248	72.09826
194.4498	105.4493	282.5688	101.6866	163.5307	87.03546	146.9872	197.8173	66.14707
30.20086	6.895182	22.06681	12.31007	10.63893	23.34629	23.8629	7.685681	20.34826
18.19226	17.158	35.31714	29.4086	12.76505	11.69611	33.40649	6.596051	7.731223
45.17936	61.99053	98.01842	89.36363	36.12402	30.7964	68.71942	25.17394	20.4326
574.1935	215.6272	90.90257	254.599	152.9127	179.3372	287.327	383.5348	181.6278
3.884513	41.25798	33.61444	2.538375	3.210548	1.084354	3.799638	1.128203	0.083337
36.56194	6.249499	30.91314	20.84912	4.272858	30.76791	31.50404	1.127927	14.05218
82.46915	73.43343	344.3915	101.6899	76.4753	77.48686	104.0278	44.84766	44.56404
30.11605	44.14098	55.6461	17.22203	5.33497	32.90644	37.22103	3.316286	2.646767
15.78626	25.44058	67.07476	52.7142	3.209153	211.1161	16.20748	7.6884	3.914727
50.82338	19.06852	35.30182	33.09215	18.07299	27.60452	34.35616	18.61162	33.06496
110.258	184.1991	234.8587	160.4275	197.507	108.2571	105.9365	109.3203	132.1584
5.469164	40.94896	54.77333	55.03064	38.22817	28.66286	14.3006	29.52509	12.8055
409.6549	302.6622	52.04388	168.999	170.9676	335.3067	147.9386	417.4388	734.9069
135.676	217.5283	196.0041	83.33211	72.22806	74.30337	171.8079	57.96108	22.97352
905.6271	529.6441	3891.365	783.5521	355.7321	739.6241	1334.435	490.7332	122.0259
34.86079	16.48445	85.67052	7.439638	3.209897	11.6985	44.85264	30.63217	7.733447
57.06199	13.29084	378.8258	8.656551	28.6953	53.08267	132.6652	17.5242	17.89048
11.02437	31.87616	119.2824	9.889032	19.13749	49.88225	16.20817	38.27496	7.733615
111.2479	114.7598	29.09742	92.96077	85.99047	18.06412	30.52995	280.58	74.89217
449.1868	790.7376	1832.173	646.4481	1542.801	837.2434	627.1133	892.8919	1189.939
776.7825	83.12415	29.97239	337.4407	367.2485	109.2944	170.884	130.0346	185.2437
62.63189	184.9753	262.3095	17.23462	28.69548	83.84685	68.71072	30.64011	11.5417
1062.267	527.8932	186.2603	685.4701	844.099	375.6476	609.0024	902.6377	1844.486
81.72015	249.2818	29.97201	121.1783	66.91024	91.26427	76.35309	150.7886	22.97384
196.8457	151.574	92.66976	298.6236	429.9762	337.4065	126.9412	291.7646	213.34
22.15107	54.3905	83.04916	55.08391	37.17615	45.63476	51.54362	16.43006	29.28737
265.0412	202.6827	724.9227	213.0985	136.9999	191.0275	387.5289	101.6768	100.4409
2975.104	1795.999	1875.403	1732.249	938.6593	1426.164	1594.066	1436.041	786.18
0	2.528588	0	3.651499	4.234621	12.61148	3.824142	21.4785	9.79829
19.76677	37.68151	92.78404	29.44434	17.01324	42.45303	73.51349	12.06032	14.07947
3.886	13.92168	83.88983	25.79587	14.8917	6.392186	4.754159	4.408936	17.89233
815.1906	1190.86	711.6534	787.1632	617.9836	1205.416	741.6695	1049.131	1232.987
186.6809	47.86754	131.5896	25.80161	15.95373	42.46693	62.03308	44.83974	22.97279
40.41382	50.4518	146.6433	22.12838	11.70638	49.88926	63.94634	25.17348	7.732526
20.55384	78.09053	69.75327	53.90075	47.79483	41.40049	51.53647	74.31076	25.50144
14.99282	6.887425	17.60928	55.14095	10.64449	26.5526	0	42.65121	2.643077
33.25059	154.2326	71.48294	67.40434	47.80563	99.75755	36.25327	106.023	33.13209
450.1252	286.6156	194.2193	403.9412	1171.029	621.7729	282.5378	587.8993	869.5004
0.716667	3.049926	6.134893	15.9522	1.08527	5.329997	0.937369	5.501161	8.981642
1.507908	9.447154	25.57691	9.885851	0.020073	22.30146	12.39086	1.127435	0.087165
26.17912	18.46829	22.93976	9.878704	2.14829	15.93452	20.03571	0.029674	12.79107
62.78777	23.57173	19.38593	19.66119	30.8036	31.8438	40.08906	30.61722	34.32175
24.60306	9.465236	33.58148	6.211053	4.272729	9.572317	10.48401	15.32359	7.723893
16.58258	13.28258	66.22009	25.79201	10.64451	36.09677	18.11736	14.2466	5.190555
27.79293	5.609661	9.669638	48.81029	51.97862	46.65081	33.41861	81.79309	132.336
1333.484	1022.358	2120.004	1394.387	1575.729	1503.621	821.8384	1002.183	829.3488
37.26001	22.26171	46.78316	29.4491	35.05514	15.94173	23.84722	6.596364	21.68864
1224.147	282.0423	258.6643	730.8122	465.0831	942.2539	1405.123	1451.218	1451.21
142.0012	376.1953	452.0815	281.6154	271.838	263.1746	160.3444	172.7068	248.9787
6.271046	14.00547	4.367424	8.64459	4.272151	73.07853	83.16979	9.864449	73.04357
44.62841	7.543547	10.55902	15.95286	6.394778	6.390416	12.39678	0.029109	5.187163
56.28601	120.2834	162.4938	68.62017	67.97398	85.96385	61.07541	74.33945	38.2066

18.20694	57.88059	9.667797	52.49398	245.8926	33.9479	23.85801	33.8667	113.5904
65.08991	20.97174	60.91952	31.90123	48.85361	17.00326	30.52993	16.43153	14.08288
32.45775	135.0266	382.5346	44.15819	18.0774	49.89557	37.20825	6.594665	20.43507
108.6603	165.5909	835.4048	166.5685	74.3533	140.0919	141.2537	35.0112	16.61715
51.51744	80.526	71.48477	135.8787	190.0416	70.05286	83.98794	120.2206	30.5925
57.88055	135.0669	108.6025	93.06388	96.63017	117.7819	72.53394	133.3148	167.5188
20.55193	32.47681	599.6302	68.6504	6.394229	175.1024	57.25237	20.80308	3.90848
22.15034	33.82002	71.54768	27.00416	14.89062	32.91094	23.84747	30.62679	11.5428
41.3377	10.74222	27.36544	51.2875	42.45197	13.8147	20.03544	22.9644	48.1263
39.68176	19.07373	22.92313	55.03287	63.68264	44.56391	44.86585	62.25705	9.001741
145.1989	133.6129	236.6266	122.4944	62.67292	214.3544	165.1234	77.63133	45.83403
115.8247	178.4642	338.2228	116.368	56.30192	129.4743	303.555	101.6685	40.75417
323.0121	1071.444	534.1978	378.3121	301.5699	452.043	553.634	250.2958	236.2957
664.2903	3383.453	4169.355	1693.128	485.273	1662.803	1613.147	549.7507	294.7346
26.19029	17.8353	18.51793	13.53528	3.21081	21.23129	22.90414	12.05264	1.372745
11.82918	46.83124	38.86745	28.17709	17.00754	15.93629	14.30247	13.14751	19.12051
22.14383	33.15462	90.09261	39.23579	2.147283	19.12521	30.52949	12.06114	3.918077
3.200312	1.196586	0.852226	0.043446	2.128942	2.12753	2.865909	9.684325	9.723283
0	3.050369	20.30956	12.29666	5.333338	9.569387	1.890888	0.028949	0.083353
8.001688	1.801978	0	1.298436	2.142012	1.081937	9.568373	10.8662	11.23661
25.42805	9.476309	14.10123	11.08458	12.75766	31.81162	24.82299	15.31768	21.58799
76.93529	88.21079	126.2569	82.08068	87.08346	194.1654	141.2723	79.80481	82.61625
53.17647	38.95742	46.78417	24.56124	37.17698	39.27372	47.72231	40.4531	30.55648
455.5898	1047.663	600.4173	528.8622	328.1163	370.3473	546.9467	395.6366	215.9893
106.3245	160.625	69.71446	138.3429	169.8819	178.2589	129.8129	257.8584	52.17163
26.3042	7.567498	15.00808	8.62981	4.270918	12.74061	13.35937	4.406801	1.373087
195.9701	578.3137	1121.372	1696.593	267.6015	342.7673	63.93494	50.30949	14.06876
76.12467	93.29818	141.2528	275.3614	284.5396	193.1237	167.0397	278.6453	264.0585
133.3684	202.3862	50.28617	133.419	64.78907	114.6037	130.774	247.9982	82.60332
61.35273	19.13205	6.13303	14.75157	15.94248	57.24055	51.58102	21.86721	40.52048
253.9745	228.9915	437.9832	144.5323	147.6125	89.1599	206.1685	62.33342	57.26339
105.4976	215.6007	233.0997	178.7709	71.16649	97.64596	101.1642	59.05398	39.48451
93.61161	215.7513	172.1906	128.5627	83.90085	65.81134	130.767	121.3204	80.0872
53.92681	101.1545	112.1679	24.57486	7.458551	47.76778	37.21105	4.408728	5.189214
33.34728	16.52738	15.85686	2.538332	5.33482	21.23568	16.21321	18.60322	6.460963
65.90399	33.1706	31.75221	30.67267	27.62742	70.02446	78.28426	110.3104	45.75642
72.18046	94.00941	177.5259	53.94018	48.86538	35.04192	39.11799	29.54623	6.45879
29.50084	4.337688	1.719313	13.49067	7.452327	19.09231	20.05315	26.1854	31.54962
0.716279	0	0	1.309153	1.085214	46.54965	17.19427	19.63194	65.05037
41.21959	51.12024	86.55152	50.23512	35.05941	48.82434	50.58167	24.07914	10.27444
16.595	23.57578	57.43033	19.65933	21.25489	25.48197	23.85116	9.874051	15.33884
66.69283	88.43074	9.662536	203.9639	339.5079	89.11139	78.28233	100.4937	133.068
287.3874	120.1794	243.7048	124.9332	93.46166	197.3745	196.63	51.40427	87.72577
115.137	155.6864	21.14148	403.2747	228.2092	76.4081	134.6064	555.8155	352.3357
422.2565	632.2278	460.0141	149.4436	159.2963	236.6518	404.7159	250.2964	31.85945
29.3071	55.67017	54.74343	37.99668	14.89064	12.75907	13.34453	14.24554	2.64572
81.87329	84.67906	11.42968	63.60285	228.0761	137.8478	24.8038	157.1874	62.16618
45.19509	58.1821	117.4976	47.79193	45.67228	38.2186	30.52911	38.27771	20.42825
55.56787	88.48148	23.79922	79.48968	89.15979	47.75564	42.94755	45.91012	72.31503
69.78826	28.6386	91.80391	116.3154	36.12709	57.3223	20.9801	8.781205	38.20808
47.57841	38.92228	111.304	23.34837	17.0149	57.30945	64.90516	20.80225	17.8914
64.99921	40.79382	656.1346	68.65133	118.9466	92.34333	134.5725	10.96495	68.69199
28.49931	46.623	79.47601	11.11348	5.334499	29.73357	40.07729	26.26413	1.368831
30.13052	35.80975	26.46346	151.2254	6.396864	64.69692	18.12099	26.24913	41.8931
2136.495	1409.98	161.5326	628.0016	573.3785	582.5588	903.9624	3576.499	877.4142
107.8624	78.54334	1047.282	558.2251	275.0282	89.16107	228.1172	42.66079	40.75055
65.10874	55.65723	47.66562	41.66504	28.6886	57.30096	105.9844	29.53634	19.15435
106.322	109.3433	173.9569	149.3526	65.85403	44.59228	83.031	107.1193	10.27075

41.19382	96.56045	119.1968	106.5264	94.51105	44.59084	37.20832	19.71163	19.16511
57.16157	22.90671	39.71246	39.21512	20.19749	25.48717	40.08259	19.70663	14.07993
676.3627	442.1171	22.90951	285.2942	327.0494	914.639	576.5476	1675.137	2265.803
312.7914	158.5959	142.1245	213.0273	495.8089	125.2319	116.4381	202.1903	991.0238
119.9577	72.93499	25.56004	40.46533	37.18321	63.67397	53.44497	27.35705	43.25241
32.51893	6.885423	30.00135	7.439021	13.82707	32.90198	46.77847	9.873901	19.13586
80.12247	102.3345	136.8706	122.4168	57.3584	118.8467	116.452	36.10239	72.45772
32.50432	12.64984	22.92009	19.66518	48.83973	48.80836	23.84952	201.8879	79.84103
7.849879	50.56599	78.64536	25.77378	27.62384	19.12237	24.80403	4.40991	3.920102
265.8286	534.8263	635.716	552.1548	104.084	338.5206	566.9848	107.141	31.85559
15.0082	14.58625	65.41492	18.43217	18.07019	4.269939	13.3465	3.316626	0.086677
82.52951	47.87057	134.2454	42.92506	32.94034	31.858	51.53097	22.98934	20.43367
84.85886	347.0203	206.6175	187.3082	158.2179	178.273	205.2265	116.9616	118.1745
19.75506	47.20164	119.1814	88.20481	41.43627	57.32406	21.93463	12.06035	24.24508
7.088	4.994052	14.13553	7.402582	1.085243	8.501895	12.40791	0.027755	3.908687
299.9764	693.6354	673.7121	650.0258	574.439	534.8107	458.1695	351.9272	265.507
39.72638	13.30937	22.93486	19.64312	7.458024	33.95308	30.54377	16.4213	21.64617
511.9138	396.5675	1033.071	1056.457	502.2545	239.8401	209.978	227.3553	119.4891
254.7643	825.8597	66.1714	1684.836	1502.253	482.7892	262.4903	784.5663	553.5354
14.99254	24.80226	211.14	31.91826	18.07729	42.46674	22.88957	53.5781	5.187973
103.9907	100.4585	39.68996	152.9265	152.8736	106.1092	110.73	125.6601	271.3535
505.6811	504.2861	324.9173	236.3259	840.8877	254.6837	185.1646	389.0576	1177.974
67.46168	65.24127	22.91037	69.78244	100.8518	112.4512	99.28545	179.1097	215.408
76.90915	175.2266	211.8915	153.0896	158.2269	80.67065	125.9828	94.02304	43.29437
619.8576	603.8808	1171.677	984.2901	584.0165	720.5223	640.4772	551.9304	605.845
60.28631	52.37632	98.0241	44.13947	65.84129	63.67761	38.16579	30.63566	124.3476
9.476527	7.569245	16.78503	26.80266	5.331321	10.62298	14.31628	1.12822	2.646102
104.6921	248.1856	330.2231	342.7748	136.9964	237.7052	129.7992	120.2516	110.5919
68.17383	19.6904	1510.11	62.53099	12.7656	78.5498	79.20658	25.17387	12.80351
595.267	998.7916	1112.542	892.449	530.9228	428.7162	789.3935	374.8877	194.4143
57.95114	37.0292	40.59443	28.2269	27.62682	44.57573	31.48673	33.90283	39.42116
55.65876	14.58844	19.39156	17.20994	6.396714	23.3583	40.09532	10.96504	17.86213
102.4394	33.78177	41.46252	55.13651	45.6753	81.70576	89.73029	121.2703	110.4038
1103.481	5612.568	23.79326	3277.393	493.7478	4866.935	295.8909	456.8317	566.3903
193.913	60.71776	24.6753	30.69002	183.6295	56.25302	469.8325	64.49342	543.3509
7.857195	13.3286	21.17746	79.17074	43.50011	42.4135	41.06646	39.30458	71.97239
311.0762	449.6985	863.5401	380.7997	184.7811	286.5277	315.9336	203.3121	62.33933
54.71511	135.1473	131.6032	42.92159	37.18579	38.22244	50.57713	17.52558	9.003176
39.03475	10.76889	22.07865	8.646553	1.085279	21.22189	22.91192	3.31642	3.918845
86.46364	406.219	165.125	96.76289	34.00414	194.1702	106.8993	52.49349	12.81275
37.2203	129.8632	234.0448	16.01028	10.64381	32.92087	18.11658	13.15362	1.366179
46.09565	39.07224	17.6237	45.23759	24.43315	50.91002	45.82999	65.50159	58.26197
255.6556	160.5668	272.8802	96.7804	53.11589	165.5398	173.7235	66.70062	19.16393
71.56058	26.78923	39.73033	13.55316	21.25518	12.75767	32.44751	10.96647	33.05476
46.07068	22.9401	25.5801	59.88519	31.86131	43.4997	48.69081	55.70291	79.76348
80.98312	54.96653	11.42828	74.66951	18.07653	36.0978	89.73478	252.2435	69.85146
6.301485	5.6619	3.490451	13.42083	22.24466	19.06257	18.16118	30.45846	32.56106
127.7767	110.63	156.2898	133.4512	73.28476	78.54184	106.8993	40.47452	49.63345
221.3779	228.9206	1271.557	689.2079	1797.545	212.2495	61.0708	21.89373	36.93829
8.646793	12.00513	6.134127	363.2869	304.6803	66.87167	44.84473	376.9065	282.9727
19.05854	15.31947	0	12.28807	51.93279	24.39015	21.96139	42.52075	74.20133
55.49015	31.1997	167.7914	41.7118	25.50994	66.86981	104.9931	53.58391	28.05325
0	0	0	0.056943	1.083052	26.35693	13.4074	25.97426	6.330038
84.97571	39.57252	55.61261	11.11334	35.05866	45.64156	46.76284	38.27633	64.77182
63.46599	56.86628	66.20467	42.91729	26.56994	74.28374	89.72861	45.92779	9.003533
438.1143	220.6028	607.4744	419.9475	197.5214	385.2041	495.3967	285.2697	290.9035
257.1456	132.3115	461.8238	110.2668	149.7366	270.595	424.7775	72.16896	122.0166
267.4725	349.3737	315.2147	249.7707	212.3762	406.3985	283.4927	251.3733	129.6321

2175.635	1710.15	5165.329	3513.459	2560.037	3690.6	2583.912	3817.384	17525.44
1.512622	9.447542	37.91917	62.50857	4.271217	53.07873	8.572075	33.91768	12.81348
374.6185	300.6256	364.6398	579.0163	766.5996	643.0284	713.0521	2697.895	1599.571
19.78748	18.44874	33.55062	11.1046	15.94759	16.99733	17.16733	27.33463	10.26591
1200.932	1519.616	1712.071	1207.083	871.762	973.0662	1093.888	1768.242	369.6607
259.6008	188.0881	80.30517	132.2677	208.1142	213.2847	440.0835	153.018	172.7411
36.50001	45.46466	37.96287	51.36836	47.77251	31.84245	76.39609	132.05	63.3732
150.7982	100.9964	140.3762	95.55179	48.86867	97.64077	170.8619	38.28989	21.70483
27.81707	4.32977	3.483583	4.987196	8.516811	11.69063	38.20577	76.31118	48.0138
113.6428	28.66924	8.779614	99.05772	251.4807	68.9688	85.92091	315.4909	616.0993
39.6063	113.2394	79.43992	155.4181	67.97324	114.603	105.9498	39.37996	31.86019
113.4502	147.1	302.907	77.2078	52.05448	118.8621	173.7216	77.628	11.53964
22.20097	14.61217	18.51812	25.71462	47.74343	20.17159	20.99304	98.1422	90.87809
7.884704	8.879627	7.029954	11.04489	13.80275	18.02473	12.40638	39.20125	26.47349
108.8175	94.12512	96.26766	96.66229	32.93778	53.06866	62.99397	30.63409	16.62301
135.9741	135.4234	22.91413	72.18236	48.84955	78.50556	105.9849	116.8564	62.20838
188.073	838.802	129.7543	392.8996	221.9278	216.48	205.2158	779.0722	317.4576
10.2716	6.917399	28.3226	2.537315	3.210027	5.328185	17.18376	7.678939	5.179472
123.7616	115.6902	274.6134	423.4291	138.0526	99.76807	64.88931	62.33233	144.8429
17.39686	17.15914	31.77809	28.18795	9.581265	23.35858	23.85344	15.33224	2.647931
27.70068	49.17195	88.30328	41.69314	55.22765	39.28182	16.20765	22.98826	33.11651
15.0038	173.2412	120.9146	439.5492	67.98194	775.6823	103.07	123.534	67.42006
16.64165	18.52926	4.367199	13.51641	23.35418	56.15323	61.16454	63.22615	55.50389
6.264628	8.165468	116.5842	18.45801	12.76823	6.392023	3.800108	10.96833	26.77645
269.0521	473.5815	461.8179	221.6341	152.9224	310.9144	325.494	179.2592	128.3659
109.5158	88.85871	102.4057	57.61572	92.38975	79.60034	38.16273	120.2202	66.12186
314.6745	17.1199	149.2546	27.02635	17.01557	40.34607	171.8374	17.52584	0.089594
223.8738	138.7809	184.5382	138.3735	82.84273	91.2768	129.8072	54.68134	50.90907
423.0425	252.6119	1280.428	274.2911	34.0034	209.0648	461.035	36.10306	61.07228
77.0102	55.61357	24.67758	124.7258	464.7374	106.0877	73.50078	143.085	344.4341
12.65141	5.616497	12.33273	3.76302	2.1484	8.509759	16.22127	2.222982	3.918335
161.1594	46.56521	193.4047	46.60797	53.11378	61.5667	132.6788	37.1961	54.70717
2.298139	30.04185	39.74735	23.30725	7.458263	16.99727	14.30198	10.96478	10.2658
198.1273	47.31233	11.42894	47.76439	233.415	91.22459	73.51093	107.0287	212.6494
1.509013	2.416323	17.61342	31.88756	0.020247	9.57606	4.753773	1.12707	0.088032
112.7242	60.68247	116.5596	29.47309	29.75612	42.46757	114.5469	41.56347	1.36725
1610.835	714.646	31.73596	336.5922	709.1884	72.18176	293.0479	52.49746	625.7208
13.40516	49.79596	426.8503	20.90641	25.50904	14.88204	20.98042	0.030814	3.915705
6.262638	16.50252	24.69066	24.5458	48.83658	21.24192	14.30036	21.88607	53.28628
25.37651	16.53061	48.61707	28.16847	4.272931	11.69467	25.76755	7.68773	8.995722
11.03658	26.20599	37.10722	8.657581	9.580082	12.75448	19.08017	1.127863	0.085715
36.42522	109.9776	101.5123	181.1534	192.1765	78.54298	49.61744	194.5125	101.6581
1938.312	4439.936	3789.679	3712.943	1489.738	3971.779	2558.145	1978.108	4880.188
53.13495	110.1582	13.19411	25.79712	141.1821	114.5807	113.6046	151.8396	44.52566
410.3573	620.0964	135.9275	554.5251	216.6301	267.4208	764.6057	743.1027	299.7665
550.129	108.787	29.08941	106.4992	106.1795	64.74399	96.40578	150.7814	73.70868
130.1264	307.2696	67.94132	257.0339	334.4372	185.7007	216.6815	361.6785	281.8396
43.64323	23.55733	22.91835	14.77942	9.582342	21.24364	45.81607	13.15223	15.34529
669.4484	437.2345	11.43143	320.6867	1514.895	698.1224	22.88951	451.2901	1083.664
119.8658	86.95757	135.1092	17.23519	19.1392	11.69833	21.93474	19.71157	2.64122
48.42389	36.41423	14.96404	47.74117	49.90226	64.71098	58.23475	37.17097	164.4934
30.88346	50.4734	92.7369	19.67924	11.70635	49.88568	55.35567	4.409081	12.81435
2.302465	12.86279	2.605007	1.307983	0.017987	35.95415	21.03584	139.8976	18.88222
3.920405	72.1202	0	2.518965	0.016759	4.25725	15.31406	29.26569	28.53539
0.716407	3.692065	24.75347	9.857041	2.148356	9.567971	21.96089	5.499758	7.710569
36.47612	32.54924	34.41138	26.99782	18.0741	37.14978	65.87458	140.8306	60.9083
8.650998	23.63835	35.34299	17.19355	4.27285	12.75362	14.30428	3.316756	1.372675
229.437	171.4589	207.5071	145.7116	112.5678	132.6524	153.6747	90.73812	47.10052

37.22378	28.01115	2992.636	115.1712	87.09482	166.623	194.7056	13.14891	33.1254
127.0549	400.9587	0	766.0209	475.4596	140.046	173.7557	71.05277	112.9854
2.433144	0.563882	0	0.032779	4.198733	0.009495	2.880685	5.355986	2.476253
0	7.540029	25.61525	0.070188	1.085231	2.146765	2.844989	1.128108	1.373105
961.9849	781.7629	1410.077	1051.631	829.2916	691.8751	784.6123	890.7113	459.8218
0.717126	0.507586	13.20816	6.211671	0.019657	0.01944	0.937465	2.222951	1.372811
14368.64	25775.89	27196.95	22086.83	12520.86	17551.01	20415.58	17524.09	8177.097
94.44452	49.14651	54.70748	123.6102	144.3868	113.5359	100.225	213.0265	194.1035
332.618	147.6915	212.7741	154.314	96.64887	160.2471	232.9011	127.8971	72.49885
354.8757	157.9469	309.9385	224.0464	172.0268	146.4526	254.8607	98.39291	99.15541
417.4928	175.1731	816.796	194.7314	108.3311	434.0064	514.4945	125.7188	82.66206
877.057	358.7974	281.6138	1328.25	283.5294	486.019	366.5221	796.7238	99.16646
7.065271	40.79336	422.0907	36.82241	20.19998	162.3709	221.4433	14.24461	43.29427
24.58299	35.88956	13.20489	43.98642	91.20445	45.60255	53.48332	36.04919	46.86393
46.01734	49.2462	49.43912	48.98173	32.93192	64.71913	27.66736	29.53446	41.94841
3.882876	10.74923	24.7175	51.24895	1.085144	21.23074	4.75383	8.778288	10.25638
101.6108	162.7223	151.9239	60.04179	30.81713	93.38015	72.53686	67.77637	19.16411
85.66466	54.24448	194.2729	90.65304	63.73126	118.8563	75.39305	95.10374	15.35355
67.45125	85.76864	24.67617	62.46382	149.6671	128.3635	84.00205	221.6994	67.33028
34.04244	158.5862	666.7974	56.4089	29.75723	100.8298	52.47977	21.8965	0.090468
130.9626	70.90757	123.6053	110.2096	21.26287	32.92078	83.98698	19.71163	20.43512
32.66033	13.36769	2.601238	13.50784	14.87408	12.74635	56.39286	118.6762	47.92053
1009.593	3204.42	2866.111	2018.708	2977.296	1762.538	1414.608	1489.596	2312.519
66.61206	205.5684	192.5337	61.28384	73.28213	83.84301	60.12036	150.8018	63.58292
56.30467	124.8755	1.722375	259.1628	407.5337	55.19446	126.9674	420.4377	85.09629
3.882672	28.78499	53.93647	9.879179	18.06647	24.41338	0.937534	17.51043	14.05665
130.8868	237.2747	685.2363	137.1994	151.8626	170.8637	128.8435	141.0165	97.89908
1576.349	2240.011	3544.22	1989.358	1311.355	742.813	1334.423	409.8641	458.5572
469.1619	163.6728	260.4504	222.8607	156.108	307.7327	476.324	88.56143	152.4881
16.58289	17.7664	106.0116	34.34673	38.24226	20.18594	25.75553	21.89381	14.08315
7.059936	3.688205	4.365998	4.989312	5.334619	67.84053	117.5366	215.7993	41.80697
41.9879	165.1717	121.8471	105.3045	61.60494	214.3109	71.57729	88.54038	107.9681
118.2252	134.3081	154.5086	90.66012	78.59531	183.572	193.7744	62.32932	83.9044
174.0538	20.32495	36.16199	17.23337	15.95342	49.88741	86.86702	57.93778	26.77309
8.669456	5.623094	17.66185	3.761186	1.085329	4.268492	25.78962	1.128243	0.082176
31.754	19.10449	16.74194	19.64099	4.27294	20.17548	21.94535	5.502978	10.26279
9.529755	4.371076	7.936247	1.307109	2.146509	3.204323	2.847419	2.219943	3.89445
30.94491	4.327172	74.28484	7.437583	4.272962	18.05717	23.85442	2.222675	25.44338
77.61528	105.946	1.719252	2.537836	0.019096	7.448413	36.31081	165.3787	2.647421
36.45073	37.64491	100.7015	23.34688	8.520749	24.42933	71.5918	43.73694	1.369047
114.4203	39.57241	47.65674	24.56896	14.8914	73.21326	74.45756	52.47146	24.23147
14.20378	29.97653	45.90677	24.55622	6.396951	25.48619	20.98352	12.06011	6.463195
39.70661	25.52532	22.92959	15.99008	14.88706	26.53857	33.40686	8.780924	5.192197
52.32189	90.18201	129.8171	69.82951	36.12566	60.50118	162.291	56.85666	66.10263
37.34511	16.53264	27.36472	11.10033	5.334743	23.35391	10.4827	4.410042	6.460347
5.477983	8.849008	24.75894	14.71426	2.148323	7.448411	7.621155	19.66343	25.30967
96.86274	151.8789	33.50922	71.02787	46.7376	82.76844	80.17934	90.70227	43.26191
56.35764	82.02918	28.21748	125.8591	62.6414	88.04594	57.27271	67.74314	129.2371
3.090086	2.412829	53.91246	2.538078	50.94384	7.453215	3.799261	7.68843	1.371933
7.850326	10.08285	7.89714	318.8739	86.00131	94.42984	249.223	371.2454	64.79707
14.21868	12.66791	30.90296	31.82557	35.03404	24.41492	15.25808	6.595561	8.996306
57.86661	186.8993	155.4037	108.9962	108.3165	124.1593	111.6723	138.7991	58.51775
7.056471	6.885434	82.1396	24.56778	42.48664	6.392391	7.616982	9.875412	19.15777
0	2.42314	334.8495	18.45938	1.083887	21.24876	13.344	9.87481	6.459036
13.40582	54.33162	7.896938	49.01269	115.7039	27.61174	74.45618	357.0056	98.96351
88.87785	103.6485	93.58404	217.7076	178.3494	124.1435	53.43937	227.226	414.5437
1.50727	24.23973	68.07785	6.214491	1.084922	12.75636	2.845088	1.127647	0.086529
15.24491	0.508025	0.840237	1.30414	4.261698	1.083219	27.78145	7.647106	10.09096

2479.881	1575.35	436.1361	1685.65	1481.214	1157.691	770.2972	2009.732	2765.607
2.297538	68.73727	3.484623	1.309942	1.085328	28.62087	44.9212	7.680677	7.70803
207.9759	234.2358	168.6338	219.1092	331.2491	164.4813	159.4013	307.052	626.8055
202.3753	175.2322	203.9449	170.2168	83.90783	128.4164	268.2247	191.2691	130.8898
27.80795	26.26199	7.017874	36.6326	38.19602	24.40458	26.73118	74.14677	32.93938
69.30678	131.0736	2.601108	25.72209	40.32861	57.25018	58.26568	62.20221	160.1216
57.90795	41.47983	61.79022	47.80127	18.0768	58.3725	65.85788	24.08033	14.08442
701.66	266.6659	817.6177	270.6367	251.674	326.8496	729.2595	285.2745	113.1398
64.24123	79.92008	86.51568	36.81286	26.57111	45.64998	71.58022	38.28587	14.08484
386.5746	1065.321	127.1004	828.5243	1174.233	449.9047	670.1159	757.2673	374.6166
21.38593	22.32091	26.47468	37.91976	15.94656	22.29608	24.81086	7.688051	31.75
53.88855	230.36	317.0265	58.85462	65.85732	132.6565	103.0744	315.8034	25.51444
5.482755	43.36144	269.2565	565.6191	3.207052	191.0284	1.893352	35.00897	1.362527
2922.568	2028.786	6359.087	5889.573	3514.601	4627.573	3282.631	4390.05	24434.97
21.34631	46.6111	55.60313	28.24238	14.89174	61.5544	26.70916	15.33973	6.46151
1908.948	1895.11	5938.886	1404.221	1194.557	963.5257	1305.787	940.9932	1102.392
15.79522	28.70139	19.38335	35.53786	111.4171	47.74935	47.72764	172.4475	145.5381
165.0616	314.9032	149.1919	204.4583	475.6349	189.9491	158.4428	221.8541	350.3856
9.436903	17.12849	106.9125	9.888894	5.33474	6.392462	35.30543	9.875351	20.4236
484.439	232.9401	15.8442	215.4478	251.6373	197.3706	305.4691	568.1434	489.8626
220.7338	172.7858	90.02894	133.4599	300.4441	143.2554	149.8606	326.6807	279.2068
5.839651	0	0	0.032962	0.009649	3.156141	4.817913	5.356957	7.150614
188.0204	62.8629	4.365892	70.8674	76.40541	14.87863	62.06395	61.16291	65.90071
86.68446	89.24345	20.26875	106.2371	111.4093	49.86669	95.49772	165.8848	86.14322
19.76745	37.68494	52.09613	8.664267	13.82881	45.63312	28.62297	30.62482	1.370285
45.98824	69.09278	24.67721	101.5352	196.3416	76.39805	85.91489	408.3186	123.0267
3.891385	11.36974	322.3363	33.15026	9.581117	95.52194	5.709681	154.1095	16.62232
59.47779	125.4862	156.3398	34.36452	9.582386	44.58831	89.72343	13.15421	15.35497
73.80057	9.443839	8.779988	12.33791	2.146964	21.24808	232.9753	495.7323	48.33359
295.1949	936.0693	1467.526	892.4496	190.0904	751.2865	447.6608	631.6981	462.3421
415.9076	388.3259	422.0423	233.8964	94.52773	432.9445	591.8181	67.79788	221.0608
8204.281	10531.05	17497.65	9431.196	8405.287	11755.14	6385.821	8326.551	35878.58
11.82914	44.8939	29.12916	18.42518	14.88593	30.77493	38.18727	14.23904	7.729762
53.8966	152.9341	166.0087	95.53971	100.885	57.32361	98.30683	43.75248	109.2606
30.13883	22.94676	4.365814	95.17881	13.82626	20.17879	27.67414	154.9126	65.85968
15.81739	23.63387	17.63059	24.50424	6.396148	18.05351	30.54786	17.50872	2.648555
10.28956	4.992311	13.24476	9.830135	4.269778	12.73495	15.27756	4.404998	1.37253
47.63052	15.21478	21.15144	29.43471	15.95092	83.78876	54.41452	180.0808	197.3063
160.3747	63.86445	95.33723	101.6437	133.7871	113.5464	227.2061	201.0478	128.2668
6.272174	30.23107	18.53949	30.5049	18.05325	25.45232	24.82771	34.91269	25.33528
58.70536	30.57958	94.49833	44.13313	9.582623	15.94286	32.43794	3.315168	3.917244
111.9276	96.59011	120.0934	72.27705	27.63287	77.47382	142.2373	26.26784	43.27648
3.882777	3.68824	14.97643	9.876854	15.94382	6.391689	2.844982	4.409917	2.648585
565.1798	536.2044	460.8986	388.1035	366.3353	236.6515	462.9467	431.6903	129.6454
124.2196	19.72185	23.80989	20.87788	11.70448	11.69669	16.21091	17.51742	2.647525
27.73968	26.79451	38.84846	11.108	8.520251	10.63611	28.62741	7.688996	0.087058
364.3346	262.9005	592.5232	186.1494	117.8857	262.1102	459.1395	213.1348	54.72371
7.057509	11.36217	36.1599	18.45781	8.52058	12.75973	5.70852	19.71068	86.34756
851.3177	369.2951	50.27801	748.848	317.4717	459.431	321.6852	287.4179	491.271
3.883352	35.78143	36.18599	15.99944	13.82801	29.72517	20.98468	19.70357	3.920354
5.479574	9.502342	13.22907	3.760634	3.210103	10.62504	11.44545	6.589212	6.443212
5.469603	12.00504	37.05988	6.215138	5.33486	4.269635	22.89276	8.78237	3.919479
108.684	220.7757	271.1028	160.3991	169.8956	147.5073	101.1662	86.36928	156.2402
282.245	393.5177	16.7263	223.6842	544.3667	122.0049	65.85683	40.46584	344.612
53.92087	69.6859	30.85778	80.81254	105.1109	90.19533	102.14	347.2879	41.99836
43.57585	27.99889	64.4188	71.06798	34.0036	65.80884	56.30159	124.5875	55.97122
8032.033	17069.68	16896.35	16623.22	14101.87	17937.23	10135.24	13109.99	24648.91
356.3618	424.8217	401.733	654.8775	414.1114	297.1308	173.7075	181.451	193.1281

25.34527	14.57668	44.15079	8.6632	14.88899	31.84444	44.86475	25.16088	15.34114
23.76023	40.96455	53.89554	31.85691	9.581687	19.11975	34.35956	27.34014	9.000813
0.716279	1.138062	18.58483	0.065996	2.147701	3.206543	2.846039	7.671703	2.643124
49.93752	59.40044	81.21321	51.48978	10.64427	59.44068	99.26952	32.8234	49.61802
679.8727	53.59425	14.96158	505.3207	954.3615	296.0342	279.6902	479.666	1443.625
761.2864	666.138	277.2123	855.6011	1613.838	582.5535	417.1243	575.9342	1698.627
7.061004	12.03711	39.7791	4.988827	3.210805	5.330761	10.48365	2.22295	1.372806
203.1683	279.6219	374.4123	156.7584	108.3273	159.1853	153.6671	95.11543	217.192
120.6249	79.87079	264.9643	83.30973	56.29927	85.96816	108.8085	59.04914	111.7989
70.5731	40.15818	202.2248	38.0447	90.27107	97.63897	43.88975	3.313502	34.40309
2450.387	731.837	1113.38	2584.205	732.6712	394.7647	909.6558	889.6232	26.76775
115.0496	148.4092	102.392	320.5734	338.6656	292.8344	170.8618	576.832	1317.649
52.36275	24.81982	42.35564	63.65754	107.2085	49.88204	53.44881	143.0675	58.43186
21.36732	112.4917	9.663815	86.72065	17.01117	223.6508	60.15104	79.7111	81.06991
127.8491	97.90072	61.77894	129.7027	142.2587	153.8335	74.44651	148.5889	321.9906
3.883655	4.329983	16.75695	8.649071	9.577257	18.04694	3.799457	2.223013	6.454352
36.42589	200.784	200.3913	78.44551	43.56056	387.3158	426.6759	33.91779	108.0577
0	0	0	9.338056	8.384901	9.422338	0.945539	6.446881	4.884716
200.0739	115.1051	150.1002	190.9345	173.0695	168.715	102.1245	171.5725	465.584
53.10734	339.0011	72.36942	91.85703	13.82988	95.51174	172.7837	87.44891	3.913663
13.43486	12.68766	41.56204	4.988195	3.210779	10.63207	7.618655	3.316687	0.084723
11.9515	75.91577	0	7.371664	79.2186	44.3713	33.50656	1.127322	7.64662
34.13305	18.45018	53.91478	11.10423	5.334908	10.63517	47.74098	5.503159	0.086392
5.469268	22.91818	48.56661	16.00077	9.582266	63.65017	5.708038	3.316248	3.920173
31.6611	15.20671	536.0899	18.45697	9.580868	38.22701	57.2528	13.15232	7.724829
96.0233	74.77804	184.5988	68.61356	75.40239	125.2076	61.07652	30.63874	85.13086
3.883659	10.72559	160.0552	23.33256	22.31874	19.1226	20.98388	6.596426	17.88016
41.19134	46.56553	146.5771	56.39491	36.12734	73.23634	119.3124	31.73255	11.54239
61.89221	52.39802	50.30427	30.68475	17.01492	39.27984	68.72481	26.26426	19.16035
55.4811	120.2186	231.3707	68.63696	37.18971	116.7365	80.16565	44.84623	26.78503
762.0612	604.6562	450.2906	755.2736	1058.581	759.7509	597.5393	834.9163	1418.165
192.0831	82.40617	206.6147	58.85342	24.44821	112.4972	212.863	111.4997	17.89288
15.80458	16.51554	29.12281	18.43133	12.765	14.87734	9.526885	48.06148	17.86345
19.12529	6.938787	11.47057	3.755449	3.209028	2.146156	8.581457	3.313854	0.07949
342.9955	874.9926	11.43101	1643.899	1184.723	541.0979	450.5794	381.3551	340.223
146.7985	142.5965	141.2448	170.1987	128.4961	109.3158	193.7664	42.66183	144.8343
34.08149	44.74668	79.51074	37.99448	14.89053	20.18428	65.87075	12.06044	5.191755
114.5748	59.59627	14.08101	26.99081	9.582172	46.68762	116.5151	90.62995	20.40995
18.19031	33.88893	6.131366	29.4129	49.88891	28.65999	78.31307	109.1319	102.4602
261.1671	143.2298	317.8994	148.1824	57.36384	174.0353	342.695	165.0412	80.11132
31.06132	19.20249	4.367936	22.0072	85.81146	14.86411	35.34935	21.84611	62.95721
63.49969	15.84513	67.10909	50.22568	14.89127	30.79277	48.67365	8.782456	24.22972
38.98593	28.18978	10.55522	22.05246	15.94145	15.93105	45.84827	55.6419	43.0223
125.3302	189.2679	405.2778	301.1922	1092.51	243.0143	210.9377	96.21168	268.0167
3.883361	3.688998	1.719361	2.538655	29.71834	47.69996	154.8714	268.9918	194.9697
39.66022	55.05835	97.21932	12.33442	6.396955	6.392565	2.845386	10.96751	9.003354
50.77861	49.86485	59.15733	34.34005	20.19849	75.32781	54.40548	45.91534	12.81291
207.1418	177.1552	218.9606	255.8588	507.4955	256.7929	334.0969	492.8131	937.8319
286.5107	134.2238	646.391	102.9261	45.68442	272.7216	308.308	55.7764	184.2291
22.16724	37.09689	92.84482	4.990312	7.458601	19.11991	12.39106	6.596246	1.371419
39.84719	14.00424	5.251299	36.58471	20.17439	19.10252	25.78168	8.774057	25.34838
2405.444	717.8616	61.75814	509.322	455.5343	2506.267	1340.189	3459.736	6220.877
3.884211	1.138483	11.44585	4.985875	21.23488	75.20067	21.00035	98.04822	63.03082
0.719492	11.36281	16.72791	18.45347	1.084379	26.54989	4.753934	10.96823	1.369408
185.7284	355.3349	268.4501	144.4987	175.2042	182.5177	150.8083	144.2765	158.7806
22.29279	21.21363	3.485472	9.844662	21.21944	8.505792	22.92505	41.40294	35.28575
0	15.84072	40.57566	41.69857	0.020498	9.576047	3.800173	2.220758	0.089283
15.94109	1.777086	1.720371	9.809909	21.19385	8.497901	63.18447	118.3016	30.11423

29.29553	24.8151	48.53897	31.90489	45.67192	33.97581	37.2132	21.89429	22.96455
334.2066	348.7837	192.4604	396.561	736.8053	282.2583	163.2128	544.1715	861.7661
40.5047	38.41422	23.81452	33.06313	63.66874	50.91312	34.36241	26.2449	35.5562
20.56622	53.78702	40.60525	17.22296	12.76689	44.56931	22.89414	22.98001	11.54064
17.48847	8.866971	7.027069	20.74766	10.62944	13.79767	27.71142	57.696	25.25822
18.97129	12.64581	45.90266	4.989718	23.38125	20.18419	17.16367	47.00077	22.95314
63.43822	183.1512	211.9862	47.82602	15.95372	83.84121	92.58283	32.82436	15.35482
139.7064	85.00694	170.4342	78.41134	57.35998	55.201	108.8105	66.6952	92.76309
87.29486	54.91907	86.51866	31.91867	43.55602	84.89663	131.7369	41.56254	24.24206
445.6361	171.5026	24.67219	737.4068	451.1659	64.75094	137.4489	329.9583	270.3325
149.2862	80.55332	81.21127	42.93027	59.47977	125.206	78.26284	38.28646	69.91151
1169.2	3207.983	1184.044	1423.712	1564.032	1704.15	1582.627	1939.794	1027.4
23.09071	15.35056	0.838753	3.759871	2.148204	12.74119	5.710901	25.09468	117.7803
604.7863	345.3666	1399.518	434.6709	901.4826	542.2546	610.8887	365.0535	497.9012
0.72989	3.191505	0	2.47781	0.013851	3.185098	11.52636	109.2072	14.64405
53.18481	79.48787	25.5679	44.09559	87.03547	73.19864	54.40983	83.01444	81.15737
728.6809	371.6347	683.4094	383.2383	537.285	573.0151	786.5407	602.1772	1630.264
105.5581	57.46888	117.4355	78.39717	52.05001	77.47578	83.0357	62.32117	25.5132
33.2932	37.69264	35.29595	29.43962	28.68566	21.24388	29.57864	15.33693	3.919926
27.71367	46.01704	71.54103	24.56327	36.11694	18.06348	26.7116	30.62836	26.7592
0.72806	7.266839	0	0.049794	2.135987	8.438587	15.3729	47.24636	14.69261
22.23698	23.71348	14.98919	13.51599	11.69669	23.339	28.64921	81.72584	41.69014
102.4854	26.09998	47.65732	12.33731	11.70628	48.82281	187.1611	28.44731	9.004154
84.36781	30.67399	30.89164	33.06685	23.37428	42.43714	57.29283	31.70114	29.24397
32.46617	27.36863	73.27331	25.79883	34.00078	39.28258	48.66823	13.15423	28.04529
316.8528	332.3519	39.68326	424.5127	582.8007	167.6591	282.5655	345.2677	233.5908
818.3061	1920.225	1738.561	1084.677	991.7436	1199.08	923.0226	993.4355	819.1794
7.85802	16.55797	45.99421	11.09214	1.085192	22.28769	13.35037	2.222989	2.648637
232.6813	224.7559	12.31207	253.2605	590.1832	165.5273	168.0048	218.5318	645.4859
15.01372	36.50918	0.839498	2.538292	8.519406	14.8759	53.47805	131.9767	96.04995
32.5688	17.1848	16.74575	35.45767	37.14826	41.3617	34.37083	44.7646	36.76518
3.884027	16.58407	14.1021	12.3009	8.516077	5.329939	20.99961	2.223005	0.08374
79.38857	36.35397	67.09759	57.57103	27.63036	63.67334	88.77887	29.5414	17.89177
82.47495	132.3601	282.583	47.83882	105.1385	55.20401	71.57226	228.4	53.44979
88.92298	45.32829	88.30753	18.45709	22.32326	18.06491	58.21854	37.1883	5.189638
417.5272	195.6718	347.8758	143.3174	88.15661	330.0172	431.4525	82.00488	99.16799
100.8791	69.09904	21.14374	106.4126	160.2659	75.33642	63.95095	184.5633	60.98107

TCGA-06-	TCGA-06-	TCGA-E1-	TCGA-DU	TCGA-DU	TCGA-S9-	TCGA-WY	TCGA-TQ	TCGA-HT-
156.7909	81.8977	123.7066	140.9379	113.5552	152.5447	163.3059	157.1094	47.74718
10.39395	21.79978	46.07205	29.33649	5.226044	46.37372	31.01031	56.70075	51.99504
66.90539	46.12003	37.15552	110.8547	57.30765	30.49674	131.2614	100.5503	104.7476
2432.773	2261.288	1821.591	3526.299	1909.469	2167.702	2586.03	2511.19	2041.348
70.7738	93.37748	124.8209	138.0212	188.539	232.9331	167.4358	124.2168	90.73446
1.367634	8.749446	5.595307	0	4.166411	1.863722	1.04407	0.54158	0.405923
16.80867	28.18072	318.5275	17.52663	33.3403	20.75311	15.51624	1.837132	18.79878
30.9658	53.79734	72.00628	84.34794	147.9255	81.38343	185.0076	156.4525	70.62222
2871.078	7562.216	3640.913	8095.035	3892.894	6324.593	6119.857	9726.757	5072.55
24.52706	57.55469	71.95901	30.00749	76.04187	92.01587	91.98036	87.50296	85.34892
9.093795	11.5852	18.00544	20.54647	21.87587	32.73117	18.60788	50.99392	25.69483
389.5059	326.0723	546.5098	941.1814	1430.263	2368.226	2272.824	1512.736	2481.037
553.3616	311.7291	119.2172	285.7213	226.0433	218.4638	74.43033	91.92517	86.23523
28.56328	6.349685	2.282806	2.125242	0.010633	2.516862	0.008573	0	0
41.23263	75.47817	82.10503	51.30169	130.2084	173.5798	112.6602	114.3943	54.47183
30.88773	70.13753	35.99028	6.49489	25.01143	15.51872	4.148279	11.03611	13.76194
1587.188	1940.431	1524.741	1647.443	1270.901	1901.723	1637.204	2549.383	1590.48
18.10023	53.67432	52.84353	48.44792	61.45422	50.85464	34.11564	32.1286	45.10868
156.7	85.6912	314.6445	40.27639	28.1438	29.84451	82.69305	18.2587	55.58593
727.373	3115.51	4318.276	224.5508	190.6509	101.5542	205.6971	114.8938	31.58867
232.7299	731.2236	720.7795	546.1449	593.7881	475.809	980.8727	888.0947	714.3788
0.09277	0.090551	1.170432	27.82239	8.3514	3.118231	119.8558	5.771313	72.61002
131.074	9.062927	37.15675	103.4852	144.7933	84.67637	138.4977	212.5278	14.85708
87.50751	60.19883	75.38917	287.7785	639.5764	106.7823	451.6651	332.6493	244.5872
87.415	44.83612	48.39127	118.9743	57.30604	39.63983	100.2577	78.83469	117.6512
25.75192	24.3423	37.09921	55.92683	52.06927	70.70913	46.50247	44.77939	30.05462
316.0478	296.4862	130.4674	257.7271	173.9726	249.078	163.3126	103.0918	190.5536
870.0537	299.2272	1230.091	991.1406	479.2016	517.5465	1215.489	1274.754	1511.065
14.24219	1.383055	38.23006	23.42615	12.51676	5.068423	35.14373	62.6262	52.51617
38.65372	52.48338	69.73209	28.52697	115.6199	63.82598	80.62155	129.6346	32.15427
48.88302	53.72029	79.81157	52.82652	44.80238	83.52912	57.88322	133.7545	42.81175
45.03632	42.2458	15.79369	53.56534	105.1911	118.9046	42.38453	70.36815	36.09954
7.809621	5.221451	17.99704	5.77028	11.46961	24.18544	7.247173	27.05389	13.28432
1022.93	696.6979	575.7336	3327.21	2313.587	1474.257	818.6008	410.0908	714.4093
122.1758	148.3473	256.3583	137.2178	134.3927	524.546	272.8618	418.3231	353.5198
3252.765	2547.691	2075.731	11107.35	19067.49	13622.05	14955.95	39610.26	35176.63
3.972553	26.84577	34.83076	12.39253	16.67782	8.338873	14.48004	1.181586	15.48282
16.82678	19.28933	87.68759	198.6138	45.84602	139.772	44.45307	99.35593	122.8427
33.48218	43.49443	203.1981	26.34587	48.9625	16.15953	27.9169	8.399572	19.8972
9.115471	23.09574	69.66229	27.09379	44.79411	6.371459	16.55004	7.08528	27.75136
81.05683	466.123	318.1159	59.36495	65.64346	25.27544	75.46327	25.48953	5.955981
37.33797	32.03851	38.25938	104.4215	108.3094	85.53197	48.58251	57.84696	36.11189
70.66707	141.6215	252.6765	9.430388	22.93385	13.54276	11.38377	9.713777	17.65051
50.22727	26.96268	34.90886	129.9488	127.0897	263.689	281.1021	364.0742	137.675
183.8454	237.7947	362.0407	264.2844	206.2677	386.045	294.5714	318.1974	327.739
12.93239	10.31999	11.28397	34.59954	29.16089	45.23713	18.60938	41.61916	29.0549
69.39404	98.33147	19.16609	96.99805	207.2266	196.809	39.28563	22.87358	22.11626
595.1356	862.9428	756.7871	2493.224	1724.047	2726.452	3711.596	5363.153	2722.981
108.0742	156.0649	170.9632	723.947	201.067	221.5327	304.9179	693.5923	134.1226
0.087762	6.49462	10.15623	7.245497	8.347752	76.51899	27.89583	13.07511	74.66113
6.531447	14.11131	5.670101	8.722085	22.91241	48.67235	24.79934	28.38579	23.47385
147.8744	315.6938	281.1007	472.2409	185.4338	441.5572	188.1193	326.7786	327.7746
46.33931	52.46834	50.62739	64.57835	46.88784	116.8471	96.11667	94.06725	78.59075
3670.378	1435.603	445.3221	2568.798	4745.004	5748.986	9503.784	10374.5	7383.963
3.971791	10.32037	23.61373	13.13974	9.39028	8.344667	31.00006	29.64441	26.22625
32.2193	40.9725	46.12557	120.5943	63.54492	69.12631	65.11556	79.60531	43.37179
20.60053	14.13978	22.49913	27.9156	14.59544	17.52973	26.87208	24.97426	29.5874

1319.943	5321.416	3484.608	4590.043	6327.358	3555.044	5181.358	4681.22	3847.594
402.1836	254.413	363.1734	101.9435	97.93796	116.5662	103.3719	172.7902	206.1001
16.80087	19.25648	46.07522	7.230108	18.76352	39.13958	36.17537	48.07656	23.30305
41.17536	46.05155	75.30249	39.59284	28.13944	7.674768	24.81762	32.7762	16.54009
42.33476	33.22358	9.044804	9.438922	4.184201	1.1696	0.010794	2.489412	14.9025
214.6282	124.0752	98.99336	254.7832	227.0935	196.2044	174.6813	246.5415	321.7134
10.37816	14.13455	5.672375	16.83249	48.92646	37.30679	36.16283	15.69863	32.43788
6.544516	17.98283	14.66183	19.00885	22.92763	21.42297	35.14234	61.99344	16.01063
75.85089	30.78646	25.91235	35.87726	26.0598	76.89923	60.98856	89.40848	74.63148
77.21816	528.8473	133.8376	156.3295	338.537	130.2996	98.20208	76.78095	62.79394
1.394363	1.38485	5.67038	17.5992	3.142104	25.49867	25.83229	28.37733	33.10839
3.97265	5.227546	11.28823	13.12853	42.69305	12.26858	35.13528	42.21001	30.12217
117.0424	124.0811	48.40379	331.9214	273.9668	350.2191	594.2697	259.0248	169.8857
10.3995	16.72201	29.26457	16.04994	14.60039	86.96866	34.11407	50.64764	74.34555
95.12495	10.34337	24.79004	280.0841	109.3796	121.9483	90.95931	132.1918	121.5338
20.65694	29.46261	21.40444	24.14965	86.42989	43.67769	41.34498	28.84495	41.21911
138.8814	115.1422	120.3558	190.1011	120.853	136.8024	260.4633	482.1046	325.5548
36.00845	30.72636	28.13689	39.63895	11.47571	39.10564	20.68224	63.91645	23.84302
69.51767	560.9282	279.9814	79.18052	102.104	47.44166	51.69376	155.041	62.22881
74.65197	81.91764	63.01947	390.7307	157.3079	215.1278	272.8616	243.2461	225.1449
605.3998	847.5689	1217.764	696.6428	1350.063	1286.277	1059.431	3177.475	1035.949
32.223	6.507531	14.67009	37.36481	73.95884	109.0454	73.38246	78.92854	60.71337
66.94741	51.25093	27.03916	323.8106	545.8223	542.0657	396.8841	690.7139	117.434
29.65405	16.73231	6.798878	110.2823	8.351447	120.2115	63.04896	275.8314	98.83651
147.4799	125.0547	58.47806	35.89763	50.00871	31.83337	26.88525	31.44273	7.613419
124.7783	493.5173	829.8547	672.419	796.9217	261.9366	1931.757	922.2386	1056.037
406.6475	126.5068	594.2723	94.6885	25.01875	17.45248	13.451	45.89529	8.730956
15.54278	10.34098	166.4046	50.55258	10.43439	158.4387	142.636	304.6621	492.4469
493.4818	1115.607	938.7909	345.0111	347.94	93.73119	135.4135	97.80266	56.65462
23.25395	314.0026	195.5706	1959.765	23.97718	55.30421	20.68612	9.059585	32.14232
96.4817	344.98	41.65716	409.1539	316.6629	230.8239	268.724	64.28476	130.2997
15.53658	28.20645	28.14846	35.17906	23.97374	115.7338	59.94489	133.2265	65.27948
74.66028	104.9385	399.2005	342.0455	531.2783	263.2635	637.7212	553.4886	365.4713
688.9387	1535.237	1628.175	1746.585	1923.002	1576.385	1964.843	2466.585	2421.043
0.058518	8.645446	15.41418	0.653161	1.052962	0	0.007336	0	1.572072
53.96851	46.04107	25.90152	83.82385	86.44041	82.3055	44.44766	110.0987	71.46225
1.389387	5.226619	20.29107	18.97713	55.21875	30.51358	98.1831	44.60784	24.90299
938.1137	1066.01	339.6207	1048.459	857.3285	640.1662	458.9213	624.4581	398.8872
27.10574	37.17563	112.4304	49.1028	81.25915	65.11308	90.95762	104.5361	170.7916
41.21651	34.6138	103.4239	36.61527	43.76482	195.332	157.0805	295.9521	103.7427
187.219	34.6032	48.37651	108.7742	37.51428	119.5056	53.75187	156.8142	56.78616
0.094164	6.506269	3.422046	8.69726	68.75843	18.7588	175.6788	164.6186	56.74747
29.67975	126.565	59.63864	105.6775	59.39254	51.37136	77.52856	131.4655	92.41334
258.3522	361.7251	124.8579	514.7826	711.4478	203.9744	231.5296	179.3647	222.8281
3.970748	2.668459	15.7635	8.717029	13.55208	4.420717	22.73731	26.35484	2.047904
2.68387	5.229243	6.798902	22.68308	4.184165	10.9408	46.5078	188.4912	50.23575
5.258247	14.14962	22.50786	15.33786	28.12736	57.64563	39.2675	48.16356	10.98247
28.33463	45.99733	35.99372	43.32338	32.29966	100.1576	23.78185	40.09042	37.86744
6.539288	19.21866	9.041466	44.9486	43.72806	37.9484	38.2294	19.00732	18.87358
51.45272	28.22182	66.34265	24.86063	81.24727	79.58514	183.9282	72.33008	71.91386
18.0488	17.95198	9.041909	14.60979	30.20455	3.115009	18.61079	8.413876	10.99247
1080.865	1666.867	549.8918	1711.36	1385.484	2083.705	1775.697	2013.614	2108.935
27.08089	21.83328	36.01131	9.430662	51.04661	46.24601	34.11701	84.94206	63.01748
818.5553	1000.744	892.7468	259.0671	278.152	130.2414	179.8575	193.1323	202.1261
325.1833	282.5597	272.1399	255.4225	340.6458	386.5894	311.115	629.2537	299.1866
25.64444	24.24492	20.23867	11.67672	13.55102	16.25493	12.41116	5.121945	7.080524
2.684965	7.771057	3.423977	10.93186	46.83758	75.072	45.44778	33.67599	18.91243
102.8436	60.16979	74.24484	127.019	67.72368	116.6873	108.5292	164.4369	85.17473

27.00013	34.47266	61.72727	19.02914	22.92348	4.417506	24.80923	6.432056	9.302158
16.82444	35.87203	50.61764	22.65658	43.76106	63.8896	55.81677	80.26539	64.6492
43.8056	47.40129	21.41765	123.3387	47.93455	101.0055	182.9322	209.2353	202.5244
82.37035	120.2687	38.28355	254.7015	75.02133	484.4877	458.9052	1816.965	441.4557
122.1009	155.9074	102.3414	71.13532	64.59981	100.9975	101.2966	115.0129	69.52807
69.46836	149.4804	87.72294	159.4092	133.3326	412.9031	56.85771	50.50073	138.8583
208.2493	41.02496	33.78612	128.3858	486.4581	123.7449	787.5424	390.5733	163.1605
59.09843	38.40415	76.42199	60.95998	46.88125	136.05	52.71362	51.2613	78.73448
11.66853	42.09407	31.47822	10.17712	32.29039	11.60883	28.94092	20.30712	5.945063
74.3565	82.86111	38.23722	25.62905	79.13948	62.07122	22.74862	24.23014	43.48649
187.6818	118.9738	222.647	125.4564	166.684	448.7616	326.6054	589.4004	404.8158
108.045	152.1737	694.6548	94.61293	271.8798	331.9847	176.7477	365.6878	393.7444
308.503	416.7451	229.4244	358.2271	179.1908	335.0289	309.0502	456.2077	502.7015
790.4728	526.7641	882.7216	2844.169	1381.326	1844.284	3995.832	3785.92	1914.239
6.540654	20.49707	10.16514	19.77538	11.47323	70.88911	24.80787	27.61699	22.23977
16.79326	43.39036	21.39207	44.12355	4.184206	33.91346	24.81141	31.54799	36.82713
19.39278	44.80223	36.01896	98.4767	84.37063	40.32864	86.81357	147.5982	22.11718
7.41235	3.77279	0.024781	0	1.051698	0	0.006864	0	1.000877
1.394362	0.085892	6.792737	10.93755	0.013039	4.422009	2.080626	27.71012	5.397007
1.378463	3.878941	45.369	0.640152	4.172302	1.181109	0.008718	0	0.399572
18.02314	16.65934	47.10664	6.504275	26.03639	18.87793	13.44467	11.07248	14.39462
82.32393	107.4159	134.9314	88.77209	123.9666	226.4157	162.2678	169.0119	142.6874
25.79794	21.83257	66.32199	57.27304	22.93266	107.1897	41.3492	67.77003	53.49195
388.1708	779.6598	360.9699	582.2066	430.2345	676.7826	378.2995	443.025	406.7495
324.8433	150.8292	58.51606	312.2784	282.2721	359.011	58.92673	85.36472	90.16943
16.69113	6.484099	10.14681	14.6747	15.62537	35.56758	16.53571	30.50025	21.87209
272.5653	1274.362	124.8645	159.935	220.8609	678.6144	2081.606	2203.929	1068.399
332.6697	281.1096	110.2278	107.1132	380.1897	129.0115	139.54	257.7942	105.76
27.10887	107.3963	239.3996	52.03165	108.3412	65.0905	26.8875	13.65828	15.97095
37.17482	28.10479	11.28645	22.73719	16.67709	32.66627	8.281594	6.434359	35.7972
83.65293	148.3735	364.298	200.3636	109.3963	246.385	211.8924	401.0767	535.8261
54.09708	177.7279	119.2288	227.5866	123.9769	194.238	217.0548	291.9405	130.2787
39.95785	101.0449	55.14437	103.4623	159.38	115.3403	112.6665	163.7139	70.62893
16.82804	28.23075	78.71411	15.30351	8.351311	122.6997	77.52042	270.8769	168.1289
16.79066	21.78745	52.78141	24.9243	17.72078	33.26499	41.33538	34.20663	33.46013
15.53844	44.78284	156.1123	32.96298	35.42897	33.15228	34.11736	7.742255	36.11422
25.82366	43.56629	67.49943	61.59001	106.257	57.91247	142.6284	240.9194	111.4528
28.11311	26.70718	30.27571	5.038585	9.386236	3.118648	3.114157	4.468208	2.048428
14.1096	2.66291	3.419818	6.53255	1.057504	3.124966	0.010043	0	0
42.48349	67.75397	91.04888	63.85709	88.537	149.6317	77.51603	146.2531	134.6513
16.80778	15.44176	21.40141	24.89454	28.13482	25.34021	77.4939	87.76466	33.94631
148.7178	145.388	55.10409	32.2237	20.85041	8.978255	15.51767	9.713947	20.44736
208.2049	140.6792	164.1885	338.5647	275.0048	691.7246	349.3363	120.8553	260.3202
55.33551	191.4843	42.76996	66.76047	163.522	65.78037	50.65545	53.81206	40.52878
79.80172	163.7298	220.4308	699.0158	502.1058	584.8472	464.0813	888.9999	836.76
29.64256	7.787366	18.03953	44.75054	6.267855	37.08529	46.51488	86.27396	28.84479
115.3265	57.48132	111.1811	13.84156	25.01393	13.54925	7.249322	8.399995	11.51838
18.10992	89.41932	39.3904	77.10887	81.24853	73.03061	81.64892	72.32117	67.4231
92.36678	53.68821	2.297284	106.6691	101.0166	109.8267	21.7175	40.03962	51.81746
33.53271	83.15535	38.28123	150.5379	197.9081	324.4712	234.6015	113.0381	234.9211
60.43314	39.70754	38.26804	77.83935	45.84553	138.481	68.21779	106.6278	146.3978
123.4817	103.6523	32.66128	344.3363	350.0138	164.1792	678.0018	480.0141	205.5337
3.969455	34.60458	33.77424	55.02022	65.63042	40.97507	41.35273	125.7713	64.61715
79.43767	49.79469	7.922943	47.76625	56.23832	121.2503	22.74798	36.79974	9.848661
1767.839	1808.242	501.5059	669.5931	528.1519	177.8413	148.8504	160.2587	172.026
37.38734	80.65148	627.4108	221.6235	55.22864	164.8031	651.1526	133.9616	3753.295
33.49167	60.06959	68.57171	102.2175	90.61005	120.9308	44.44944	26.83751	56.28152
66.92662	126.5799	101.2258	122.5723	153.1312	106.198	88.89744	111.0355	85.14094

60.49424	94.63679	71.99733	38.06946	59.39148	109.5011	117.8296	238.8992	100.8164
37.32715	42.22342	49.48111	30.02517	76.03131	46.25589	53.74623	64.47776	60.79304
1327.059	823.0202	154.0951	87.25177	82.31341	27.23897	34.12234	22.21254	15.43089
168.4103	350.142	177.6809	198.1948	162.5164	118.5423	204.6532	83.35333	112.4263
41.20773	60.11686	74.21243	56.48629	78.12707	281.212	65.11891	74.2832	92.03482
9.113638	25.63012	60.66659	13.11172	54.15718	24.68812	33.0785	19.61258	38.44661
72.044	53.78437	71.9965	144.6732	133.3359	176.8087	124.0294	169.7154	186.3347
114.0119	28.19155	14.66626	32.9883	137.4325	25.32636	14.48341	11.03293	22.709
27.06702	16.72353	31.51163	39.61536	29.17853	39.7305	64.07303	75.10221	42.88401
376.6365	434.6907	269.9098	1238.685	373.9888	1244.107	737.9802	875.6742	589.0042
6.544579	14.16194	23.6414	16.06085	2.099923	45.70685	25.84607	152.8557	63.80494
25.82084	80.5638	93.33011	66.01796	40.64184	133.1018	100.2568	142.7822	124.9379
206.893	79.3551	151.8164	300.3924	60.43629	113.3428	113.7037	168.2565	174.962
66.92743	92.10914	25.91544	28.51802	54.18531	106.8487	188.1063	294.116	91.83788
5.235215	5.205749	10.13675	3.569541	18.73702	9.062422	19.62519	17.17575	5.425607
598.863	522.8307	293.5139	818.6919	719.8159	764.1789	575.7067	519.9663	450.2366
12.95249	21.78904	14.65877	10.91092	55.18664	39.83519	54.7609	24.92974	30.0777
162.0497	446.2084	410.4578	466.1148	625.0374	376.0495	951.9308	996.6124	280.1459
513.922	774.3052	602.604	565.4718	582.2911	1175.641	13.44996	5.788293	91.77201
93.82404	62.70324	29.28557	152.826	10.43484	127.8644	109.5571	160.5767	65.10319
24.537	194.0746	267.4279	52.04441	46.89106	25.93287	65.12362	20.2321	42.75542
567.9107	472.903	127.109	306.8502	392.7244	158.2989	113.708	257.6205	115.1826
112.9587	116.1821	52.87006	74.15978	80.20798	46.86004	36.18608	16.2897	31.05326
206.9544	107.4787	136.0954	248.1335	316.6757	132.8836	191.2201	340.5912	515.2802
827.6939	1225.87	638.7122	795.0416	1035.469	1311.736	1130.745	1247.706	377.117
56.60813	65.23404	66.35872	139.6382	61.46867	131.2029	111.6183	84.14792	50.03979
5.243166	14.07473	5.6659	2.829606	15.62484	14.33041	19.631	17.7922	17.32223
109.3511	281.255	150.7167	194.4794	306.267	597.401	313.1783	479.9979	192.142
36.10355	81.92955	74.2658	175.368	432.3119	612.9401	746.2297	552.266	309.2125
293.1255	1011.099	735.3923	800.2185	928.1675	1885.732	1023.247	896.656	1192.724
19.38666	40.95488	50.60691	63.16646	79.1564	103.2527	67.17767	63.80478	43.963
15.51861	21.79935	21.39563	18.27363	18.76317	58.86434	63.02718	42.78755	19.93981
14.2583	61.40219	120.2638	31.47068	105.2029	28.55149	55.82054	9.057229	41.10151
605.2867	1354.645	274.4009	50.54552	50.01987	22.02863	29.98758	59.01813	39.38498
109.1657	72.88503	58.49501	15.30353	99.99783	26.59117	7.249136	0	46.12782
34.62224	73.69628	5.672765	1.364006	26.04218	7.686674	9.31464	5.775574	1.4953
187.7502	369.5148	317.1333	698.9094	562.5338	428.8802	818.5984	717.8379	471.919
32.23819	66.52069	60.74652	96.92589	50.01444	83.43135	90.95523	178.4293	152.3925
10.36862	5.223187	23.60144	10.19502	21.87462	38.03724	22.73677	20.3731	17.22388
132.3874	136.7872	52.89584	167.4219	90.63971	262.9678	100.2649	174.2583	120.8821
12.97147	14.17892	20.29325	120.3751	17.72705	134.2882	136.4342	279.0044	179.5428
24.47047	57.36897	16.90529	12.38142	94.73174	34.5599	16.54805	15.66326	11.53317
106.745	235.1393	188.8997	352.6091	241.667	339.263	231.516	189.3361	180.5644
16.80869	29.45371	59.55122	38.90112	59.36333	75.20796	38.2443	36.12691	41.24228
66.6567	40.88961	87.57053	44.82	39.58389	17.48576	23.78075	9.719771	26.09989
18.11046	97.07302	239.2444	8.696638	6.267669	4.421513	6.215501	1.841644	18.20601
5.221442	2.65882	44.63684	7.293102	4.180538	3.790957	4.145403	1.837968	4.312967
50.23208	132.9579	66.38392	133.6033	324.9712	168.2448	154.0027	137.3786	117.5309
23.24465	193.132	454.2913	331.0291	107.3145	128.9371	1145.19	164.202	525.4865
323.5467	138.0598	6.796352	10.90152	6.266882	72.25541	38.25663	8.405662	4.842321
37.03423	64.58372	4.546558	3.562738	6.265595	1.814836	1.046346	3.146479	1.493618
33.53227	33.35108	179.8667	65.99251	116.6743	198.3512	172.5992	181.5545	168.979
1.37964	8.846457	1.16679	4.371193	0.01091	0	0.008796	0	0
16.82511	40.97609	56.23494	44.72515	64.58718	89.40979	80.61427	60.4564	57.35878
73.2779	53.75445	127.0086	43.23306	54.17904	239.1813	86.82017	70.96041	104.3007
271.2631	438.5012	494.7628	433.1085	567.736	223.486	716.2698	888.9053	607.4633
174.8659	184.1523	348.5658	516.2442	325.0151	734.4622	580.8537	235.9326	439.8309
235.232	230.1429	147.3421	573.5625	407.3011	436.9091	272.8699	262.9097	438.7305

5660.918	5275.352	2487.253	2066.619	6097.132	2489.076	3753.981	5278.87	1575.909
66.91678	42.29391	34.90888	32.19148	21.89398	26.58059	72.36077	126.8688	4.285488
122.2082	553.4667	404.8085	254.6738	557.3129	151.7635	296.6479	36.01309	259.5672
10.39323	12.88525	9.045759	44.84674	53.10959	49.01188	54.76432	20.28774	34.55601
1125.82	1596.537	1069.348	2059.443	1261.519	998.0675	1010.853	1482.405	806.296
403.3042	265.8173	217.0085	483.3926	160.4304	261.5063	86.83327	83.36102	140.3444
14.24433	35.81241	64.03354	35.95536	64.56612	24.03091	15.5161	6.42838	26.65157
48.95219	136.8124	120.338	162.2447	117.7223	326.2466	134.3709	242.0347	175.0069
51.11419	9.045486	29.20796	10.92844	3.142208	3.767148	2.080627	1.833572	3.716647
118.0285	193.7892	37.1396	11.63316	23.97502	19.41882	11.38374	1.18716	4.83182
297.7778	111.2215	43.89997	201.3153	76.05518	138.9127	50.65765	162.4743	65.07393
59.23207	70.41238	127.0891	83.60056	118.7663	283.7271	165.3771	767.437	251.4472
10.38361	16.68556	21.38119	15.34488	27.08424	4.417946	15.51282	3.800954	8.185438
3.961312	12.79341	23.5444	8.75113	9.383534	7.731904	10.34098	2.492152	9.387853
48.90237	34.60788	65.22828	80.77389	33.34936	124.0532	60.98597	137.6258	281.9856
59.10866	122.4491	25.90418	93.37512	62.49985	17.46292	20.68448	21.56488	60.20757
515.1478	226.2941	415.9906	290.7371	207.3089	73.52433	179.8521	161.6179	193.288
6.522689	14.08447	2.29834	19.86794	34.33945	22.26532	19.63284	23.11986	13.32495
109.3345	244.1445	46.155	118.8497	143.7676	190.3206	292.4986	370.917	92.34603
18.0797	16.70854	20.27392	35.9779	27.09124	88.45359	34.10916	27.55807	45.23568
45.06294	34.61251	55.12407	61.6256	78.12971	51.41806	93.01899	122.4104	58.43116
309.8216	65.30903	416.0734	247.3101	172.9422	36.36918	209.8316	1406.382	70.03311
0.088261	22.98738	26.95864	19.07411	31.23542	0.523164	1.046322	0.530544	0.94261
23.24992	7.786004	19.16782	21.91416	22.93496	27.89575	201.51	65.02441	28.80571
221.1125	317.0274	209.1756	220.1865	327.0996	329.2249	251.1675	426.0464	355.5724
90.01938	109.9615	95.59873	142.4494	146.8774	87.93682	143.6655	107.7695	84.0474
105.3863	46.11726	49.51731	110.8651	84.38525	169.6664	222.1912	144.7145	174.6563
88.76916	171.3027	131.5823	158.5545	331.2377	149.9028	212.9145	230.8079	197.31
204.4442	294.085	315.997	361.8879	676.0637	805.3016	522.9945	913.9585	506.0168
48.89381	141.6523	92.17222	51.34319	14.60169	8.326873	31.01923	18.26425	29.37811
14.19078	22.97968	20.23869	9.45916	32.27367	22.85445	21.70327	17.0577	30.835
109.2783	37.18554	51.77082	234.3519	286.4336	325.0761	280.0726	138.7092	367.3485
41.08146	6.506965	19.15028	35.24667	27.09049	26.67785	41.33812	46.77125	46.37807
20.66882	19.28086	70.81111	10.89963	17.72559	20.73176	10.3501	16.95472	9.284681
46.28938	14.17473	9.048161	39.5998	16.68394	106.5623	63.04368	399.2448	12.07442
52.78225	48.66925	165.234	64.53786	46.89169	216.1011	115.7591	120.9966	139.4375
179.9737	894.1355	66.39297	34.39106	63.56209	28.53628	44.45868	30.74919	125.2513
0.094394	10.3441	36.02839	72.64004	15.64363	105.6476	128.1552	169.1687	356.6783
53.91883	20.54271	4.549422	25.62627	43.75118	22.05906	34.11297	71.17992	35.61664
5.258579	12.87937	2.298593	35.2715	68.71006	23.41552	35.13809	30.90089	22.21141
10.38775	17.96593	12.41192	11.65091	30.20886	53.03128	81.60276	61.4411	52.10178
229.9315	80.61847	136.0666	146.8233	88.55761	38.97035	106.4666	77.45974	70.62434
4004.397	4109.556	2291.592	5831.01	2256.358	2115.523	2129.191	3300.75	2330.596
6.543794	141.7128	106.7876	64.57141	61.4678	12.23733	14.48456	0	13.74209
1964.103	926.5091	321.6107	890.0249	636.4743	201.9825	182.957	640.3975	94.55171
215.6411	148.183	216.8982	23.38058	76.05266	53.35128	21.71961	25.49368	31.02907
313.4464	276.0264	259.7141	220.2701	218.7572	110.7283	109.5701	68.23203	78.41355
11.68409	67.66083	46.105	29.29823	48.95997	62.66869	58.90861	57.2418	66.45095
259.5853	789.4225	286.7122	108.5637	76.06203	76.78938	77.53243	46.52604	32.13239
87.43543	26.96117	45.02314	74.82358	40.64298	136.9645	114.7277	161.1711	136.0493
73.12839	71.47007	37.12414	21.2001	25.01367	16.81782	19.65007	26.19669	40.08021
30.94389	24.39574	67.47076	32.94709	6.26767	105.7465	81.64972	125.1092	162.0896
3.949685	15.24328	4.536318	2.099817	1.057354	1.168191	0.009801	0	0
33.7988	6.40041	0.033441	5.101298	0.011342	0.52531	0.009142	0.532714	0
5.24977	15.36912	6.791083	22.06544	32.26857	24.86779	24.79722	32.41763	13.86209
34.75568	91.84079	75.28476	57.29818	29.17916	19.43059	20.68364	17.61829	26.05905
14.22263	5.227467	21.383	12.39151	1.057425	155.3494	23.77566	82.75993	179.5101
69.50529	227.4795	185.5283	110.7897	184.383	170.7952	195.3458	197.8938	138.1363

438.3147	107.4946	19.16261	354.5125	2105.244	730.1666	1743.624	1380.708	834.862
3.967328	109.899	357.2564	2.102239	8.351186	2.47474	0.011153	0.536553	0
1.304141	7.179949	7.6285	0	0.006528	1.28216	0.005273	0	0
2.685133	2.668636	9.039631	2.826969	2.100144	18.86382	30.99891	56.95161	23.41261
1240.19	1127.483	1104.206	1936.07	2789.68	1661.854	2463.016	1340.375	1713.206
0.089848	0.08771	18.01658	10.1821	4.184108	9.65024	72.30148	14.36394	40.30958
11472.39	25563.93	19082.54	26655.08	18410.19	12582.06	26609.62	29201.91	15444.17
198.9775	168.5903	71.989	111.6105	85.42564	66.41502	59.95727	71.58241	99.7471
77.22612	157.3065	165.3235	212.1331	499.9944	314.932	280.101	233.3336	199.4311
77.22403	204.5611	168.6918	222.4312	296.8828	217.065	289.4001	175.4486	218.9789
178.7442	364.3595	386.8212	263.4859	363.5659	485.0461	552.9651	526.5778	740.274
111.9261	434.7215	1123.311	798.7111	453.1616	201.304	2514.686	850.5725	154.7423
117.0545	19.28874	196.8035	147.4811	359.385	250.3078	407.2232	445.1781	225.0734
11.66825	67.45407	88.61597	18.2885	17.71989	8.335883	15.51379	7.090393	11.54257
10.40142	52.41363	39.3775	30.02478	61.45653	81.6378	42.38188	47.9633	27.72795
9.102935	42.05654	18.01693	10.1818	4.184113	27.39145	17.57834	58.18622	45.95751
80.99453	81.838	62.99654	269.865	33.35108	166.4579	79.59003	85.43351	145.0822
51.51871	37.18762	101.2278	32.18941	161.4645	306.0452	376.1841	166.9937	229.2188
63.00808	91.99428	92.18398	36.62002	143.7268	13.54145	38.25346	34.06416	22.11089
28.39507	94.69866	179.933	346.6046	38.56147	179.868	371.0448	285.3352	373.0015
63.06963	74.22389	36.03355	243.9126	266.6464	204.8477	227.371	190.7442	111.9596
9.084838	3.945833	15.75581	7.246729	18.75105	4.423251	0.010459	1.833277	7.084666
2295.232	1533.977	1190.799	2442.622	2020.926	916.5246	1767.434	2379.755	2098.814
74.6156	34.62856	109.0812	177.755	61.47485	166.9874	111.63	269.8548	232.704
187.3737	428.5279	121.4043	35.87602	41.68275	21.36733	39.28808	27.47125	10.95626
12.94858	6.505305	6.797466	20.50201	16.67909	29.33529	39.26806	52.13898	42.50251
66.95043	336.2231	81.01212	159.2148	292.7301	108.078	309.0479	642.4122	305.8796
246.864	1394.677	1535.996	2802.346	3001.173	1968.184	3786.011	1689.39	2260.947
187.7179	274.8732	296.8626	213.5726	335.4336	591.5073	314.2127	518.7985	394.0512
19.3923	24.39244	31.52499	17.51008	56.25689	50.79661	98.17764	121.8531	52.8826
23.16675	88.96214	7.920314	3.560025	3.142263	10.95691	1.046221	3.800823	7.623496
109.26	136.7524	57.3879	138.7837	51.05916	161.115	62.02588	138.74	98.02159
65.64991	150.8614	151.808	185.0258	123.9721	288.3523	151.9399	179.4755	174.4405
56.59779	32.05687	83.19954	28.53168	103.1186	102.447	102.3163	58.45001	167.0723
3.967331	5.217821	17.98637	12.43355	44.73897	34.8383	28.92277	33.12034	31.49944
11.67074	25.59901	14.65802	12.3852	22.92506	32.61098	23.77779	38.18773	31.77484
3.944204	8.953838	7.882517	1.365916	16.64329	13.14355	10.3332	7.184078	6.610651
10.39266	5.228866	16.9052	12.38154	76.00178	33.90374	50.63255	32.20145	29.49918
2.683847	55.73715	0.038294	0	8.346773	0	3.114277	0	0
12.9725	23.11733	129.2168	18.24439	15.64325	88.09321	86.81398	105.9911	73.03207
42.4789	38.42571	53.98899	82.27478	70.83461	97.26646	40.31864	44.62503	51.20056
23.22798	12.89707	42.74101	68.35284	39.59121	79.04555	44.44685	49.29984	64.74688
10.39416	19.25502	23.64039	33.76339	22.92742	38.48794	34.10916	24.91218	31.17235
210.519	98.43771	85.47189	154.2782	95.84002	75.56049	80.62492	92.00516	74.59245
18.06494	21.78144	28.11585	14.59864	67.66812	24.73278	25.84279	38.19994	34.0414
28.1427	5.218509	13.51174	23.56189	6.265396	11.00104	18.60302	27.09547	18.40851
30.95169	123.8877	55.12565	68.9788	65.63432	93.25871	29.98691	39.32738	27.68981
23.23468	80.43719	48.36236	52.11277	13.55966	7.023224	32.05056	9.056753	50.12414
5.259246	11.61083	48.31157	2.827658	29.17237	7.025501	48.56785	116.4173	82.44322
262.8409	689.3647	92.18436	21.91613	40.63964	17.45521	4.147823	1.842215	7.058321
15.51076	23.05764	19.1464	12.38503	10.43311	16.84896	40.30237	28.9078	37.40666
100.3051	87.00023	137.1873	65.98167	88.55708	144.0716	117.8338	131.4459	73.41885
11.68741	6.507766	29.27757	31.4827	7.309628	26.60404	51.68392	37.37576	42.81178
7.828229	16.73712	2.295286	91.73883	32.3102	52.03848	134.3606	172.386	124.3199
34.78935	65.2034	162.8986	5.029415	11.47668	1.823055	17.58491	9.056673	2.05835
145.1362	99.71609	104.5721	52.03967	145.8274	44.8599	69.25802	24.1776	49.45058
21.9169	6.507157	9.045927	21.22281	14.59869	33.89472	32.04307	56.70385	18.25674
7.700919	21.33096	4.524553	1.369567	2.097886	0	0.009279	0	0

1228.584	4171.668	2940.215	317.0521	371.9085	67.66161	65.12945	177.346	290.7281
11.62714	21.6826	4.546212	93.54189	1.057573	0.522678	1.046349	1.179754	0.394283
206.8923	304.1018	101.2369	291.5731	247.9189	210.6033	155.0425	63.62916	167.7061
64.37724	290.1446	476.6713	70.36947	114.6033	87.8754	193.2865	448.5343	448.3322
23.13721	19.20981	25.85358	19.05327	7.308107	10.96905	13.44579	7.757265	11.5634
122.5233	33.19922	159.1374	19.76956	9.39136	21.45798	4.148273	0.531682	5.387604
20.67995	71.59883	41.64118	38.82577	82.29351	123.3831	91.98434	107.9153	29.36997
361.2232	421.9175	880.4172	412.5192	870.8709	1340.589	1002.572	1389.895	1191.676
56.63112	58.88034	41.64962	72.6276	38.55933	181.453	68.22449	118.3641	108.1301
258.3657	773.0752	517.1891	118.8304	64.60429	136.1265	169.5192	72.82196	82.85015
39.78792	29.41636	25.8782	13.1204	29.17061	37.20324	26.87696	39.50307	14.90041
241.5775	83.19225	92.24718	385.6155	194.8032	180.5518	210.8517	216.9396	215.122
52.80867	248.0955	120.367	74.03848	122.9402	301.0727	864.0735	1898.96	34.93265
9215.492	4499.524	2383.814	3764.066	11766.11	3291.642	3917.291	7974.878	2212.925
5.256741	34.61019	58.49169	98.43246	30.22526	42.92627	65.1198	82.84596	37.19195
719.7903	1687.385	1869.941	2207.624	3053.259	8351.49	2605.668	7309.928	5376.692
12.96667	43.47374	58.44547	27.09193	51.04086	12.24383	44.44515	2.492139	13.19533
239.0256	296.4953	219.2664	72.57579	178.1396	126.3757	104.4037	125.4577	105.1784
27.08526	5.228109	38.26044	50.62904	28.14019	38.38142	71.31247	100.1075	128.6061
380.1928	680.7276	192.2809	115.9236	154.1806	44.8358	53.76046	32.06322	89.57034
322.3108	302.7302	169.779	194.6081	162.5058	39.62274	33.08909	101.1556	53.32716
9.547999	3.671668	1.137306	0	0.006563	0	1.037737	0.585868	0.455413
21.93049	89.19928	29.2581	17.52775	31.25783	11.59466	4.148276	10.37711	8.172641
56.48364	54.91272	153.77	12.37304	28.13622	16.82161	18.61636	7.742973	19.9124
36.04104	9.065507	41.61987	73.50799	128.0775	67.23548	84.73639	116.7257	118.0796
148.8202	74.1325	45.00791	110.9763	62.5066	36.39917	36.18614	23.53013	33.84749
57.94843	38.46804	42.78141	177.6473	272.9173	138.1388	177.7794	236.0458	161.557
75.86766	51.21683	51.76156	36.60757	42.72496	195.2227	146.7557	295.1184	227.8834
106.6095	280.6463	76.46946	8.697307	32.30881	8.979287	1.045686	3.152372	3.724603
228.874	306.896	439.6918	428.6647	687.539	1690.733	1667.152	1879.075	1061.15
372.736	308.1327	124.8633	187.8478	557.3122	796.2126	715.2283	1080.433	747.5372
14969.09	11496.71	3374.433	8880.558	19222.7	10191.37	11727.05	14929.99	5986.514
20.63038	19.2474	21.39213	25.65779	12.51579	58.90261	23.77853	62.70928	40.20575
100.3012	106.1487	92.23348	107.8755	157.2962	168.8991	125.0671	160.4284	142.6649
7.828754	70.09488	55.04645	7.230062	11.47517	1.170539	4.148304	3.801039	7.616334
20.61349	11.60202	52.7616	19.03135	62.46135	53.05444	35.13572	24.95343	31.80903
9.052161	10.26605	11.25626	8.753933	12.50203	17.68902	22.72079	24.54852	12.24015
73.12143	42.1999	36.00046	19.72896	80.1873	20.74325	24.81602	21.57921	17.10957
96.43749	150.8041	94.47563	85.83568	179.1627	181.348	104.3969	108.426	107.5037
2.684472	26.76258	50.43296	6.508112	11.46943	5.732579	3.114381	4.463255	11.02759
23.24702	29.50376	52.87378	38.82732	83.33406	61.89003	102.3159	88.79063	27.69569
72.03077	100.9942	45.02167	114.5458	63.55578	206.2865	69.25818	117.0393	164.0385
5.257687	10.32775	46.03631	27.16192	40.61173	111.7786	34.10238	30.92807	33.51018
224.9965	547.0756	493.6211	427.9982	384.3987	524.1882	482.6845	353.6053	393.9541
7.829659	20.53615	29.25625	28.58437	49.99336	56.84373	32.0453	18.95327	49.13051
11.67974	15.44088	32.62363	27.10781	39.58516	52.24081	33.0786	87.11476	52.49687
90.07992	235.2694	531.8072	166.5652	373.9745	677.6245	386.5593	279.3307	372.2872
2.679446	6.506321	67.48202	5.76372	20.85172	7.676652	99.21924	111.8467	85.83575
475.3132	1042.319	86.63032	633.1744	480.202	577.3265	49.62658	23.52064	90.66622
23.21941	19.27093	21.40461	42.57379	45.83449	171.7053	50.64222	49.33143	79.41763
9.07434	5.216569	9.029857	10.21457	12.50716	42.83098	21.69757	21.77467	16.72459
28.35895	12.89821	84.27481	8.696575	33.34526	14.19939	29.98355	134.5504	20.45505
138.8524	176.422	131.5857	265.8439	155.2215	199.5016	206.7157	182.7348	231.9007
120.6918	137.895	121.3884	31.4702	32.30848	10.28205	39.28725	1.842579	6.502559
20.6824	88.20345	105.6786	34.40627	130.1992	52.71315	71.32214	18.26008	117.7006
30.96321	18.01548	150.654	37.33412	39.60191	35.71376	57.89227	120.2898	48.87664
8185.023	14073.64	6324.898	13987.57	7673.265	8963.061	12695.51	10105.98	10910.48
420.2617	452.5167	256.4059	392.7501	269.8157	278.2881	341.0895	499.6252	345.4469

28.33713	14.16886	33.75078	23.41595	68.73306	121.8197	76.46398	32.15359	119.391
33.441	42.16513	24.76705	39.64994	6.267835	96.27308	34.11114	99.05917	45.75943
7.775765	2.662912	3.419819	4.309697	2.099701	9.068195	14.46627	48.83893	27.73838
39.94688	38.45483	107.9424	133.6744	178.1098	52.69587	211.8567	171.7504	119.867
571.4529	816.0757	155.1929	68.9061	40.64481	19.4108	14.48411	43.23985	17.64697
1124.221	1024.956	540.8451	447.082	242.7336	248.2763	255.3066	205.6321	260.1175
3.972414	5.226953	9.042223	5.028674	14.59576	66.94824	44.42611	55.52936	67.4565
63.09256	177.7424	202.4168	140.145	187.5169	311.6809	248.0619	342.5748	441.0694
149.0725	41.01971	100.1009	154.186	167.7114	93.14282	101.2985	190.7202	142.6611
79.76836	52.51898	29.28859	79.94089	132.3021	204.1467	176.7395	415.3085	375.6074
96.49984	1925.063	1613.557	1206.92	955.264	447.7471	1979.313	3780.255	395.5015
161.9357	224.9041	282.1628	121.8209	119.8055	99.65318	76.49694	34.03638	82.33511
29.65362	99.57934	138.1814	16.04103	34.38895	76.33265	51.68361	60.46789	57.93269
411.3399	246.7069	9.04728	36.6857	19.80629	0.525995	6.215673	4.457739	3.716694
172.0222	103.5228	85.46569	77.78715	83.34094	37.68373	46.52236	47.87863	81.31951
15.47844	7.772481	9.03882	8.715022	21.87646	49.23979	15.51045	40.31989	12.13415
656.5856	181.6154	178.8287	129.1056	623.9719	2001.371	1217.51	1581.174	578.0434
7.30772	11.9936	1.145021	0	0.007492	0	0.006048	0	0
209.3983	194.2425	105.7229	168.1428	181.2527	164.3082	89.93126	59.03456	94.62621
65.6307	488.8468	75.3698	303.5341	162.498	358.4856	13.4509	14.3156	25.44579
18.04246	2.668703	9.04097	11.66094	34.36405	33.34345	29.96855	54.2426	36.38837
3.943236	30.17354	0.035346	194.4977	2.098868	1.822245	0.009636	0	0
6.543998	17.97846	16.90537	15.32791	11.47482	137.2117	60.96005	101.8313	53.14243
39.86769	11.61897	4.549299	36.66977	13.55906	44.97698	39.27987	153.8693	32.79253
43.81748	9.057862	7.919639	17.50882	466.6513	111.3689	399.9768	668.4829	53.31283
54.07017	97.17557	36.0313	151.3115	48.9753	162.459	89.92662	134.157	94.69251
42.43354	5.228997	10.17203	38.87537	35.42583	16.16295	74.40373	96.92061	181.1075
96.44338	43.57291	91.10676	90.24127	59.39251	373.4415	199.4698	311.9715	314.2516
29.66135	38.43248	61.85596	49.13166	57.30029	131.2751	54.78563	111.245	158.7214
109.3033	62.74113	87.74348	131.3771	55.22741	275.9613	197.41	301.9675	264.9313
673.3717	953.4403	803.9229	945.0199	1153.142	490.8849	298.7165	203.6553	346.5275
38.67684	104.9049	340.6295	85.80271	157.3051	241.2769	219.1176	235.3885	167.6974
24.47725	24.34604	33.73739	37.45296	35.41843	17.49136	34.10932	33.51228	31.73268
7.778896	3.936404	7.900122	2.096607	5.222492	12.38043	4.14655	17.17754	13.96233
14.25389	173.8865	756.464	31.45432	52.1034	12.89635	17.58503	0	5.959768
72.08124	187.9344	86.62703	199.6777	278.1288	293.4711	205.6849	225.4896	192.2315
15.53661	38.40188	39.37741	59.49021	67.70297	75.08378	76.47408	102.149	43.97183
65.43969	199.8105	197.5118	10.16712	19.80693	11.59158	5.182001	13.00921	18.79073
12.9596	71.37651	111.1044	11.64188	8.351003	7.677192	14.48245	8.403064	12.08743
95.20421	118.967	295.6961	126.9328	156.2663	247.7614	263.5605	339.9956	294.9108
3.968927	41.90792	42.60072	8.724655	17.71064	2.464938	14.47521	1.179919	9.902556
15.54043	48.62061	20.28907	63.13651	84.36854	36.41086	125.0374	41.99299	27.70911
18.04328	45.83126	145.6171	6.500616	16.67634	6.378842	11.38046	12.38391	4.831488
546.103	407.7663	194.5666	408.2022	275.0215	229.3876	427.9026	402.9751	545.1677
7.817998	160.8242	0.039174	0	0.013184	0	0.010619	0.530959	0
14.25119	21.8263	9.047979	52.87567	17.72497	41.688	74.40455	80.37456	69.80893
23.23789	33.31529	49.48968	68.30676	64.58374	90.10914	23.78477	48.60141	50.66735
281.4253	207.1192	152.9556	447.2695	646.85	297.3304	194.3198	72.82743	123.5736
538.3784	110.046	208.0555	677.8108	368.7676	701.7425	561.224	411.542	513.9576
18.0869	10.33914	29.2565	19.00245	8.35111	142.9461	37.21025	54.00546	161.7566
25.64654	21.71577	24.717	7.243835	9.388847	22.1919	9.313132	9.090008	8.204323
2672.494	1572.057	289.0249	235.5604	376.0728	76.78285	40.32336	55.73549	42.73296
3.970331	19.1875	2.298797	21.29758	8.348249	0.523147	2.080629	1.180246	4.835781
24.52274	3.946984	14.66961	43.26031	17.72614	16.807	31.01834	248.0997	15.97742
191.4942	282.4082	79.8803	194.5501	108.3508	132.9181	146.7753	145.8739	170.4884
15.42231	29.21137	174.2061	3.565195	8.345384	3.773844	4.147283	1.83368	3.16478
0.094294	3.944456	61.86966	13.10069	86.46329	38.34098	33.08752	388.2527	135.6065
2.676619	5.196772	13.47424	4.314938	6.261014	0	0.009903	0	1.495949

133.4345	15.45637	15.79426	80.05754	128.1015	59.94756	66.15039	61.10899	80.30807
269.8817	186.6889	327.1889	470.7593	202.1002	205.956	100.2707	68.88044	201.6628
43.64375	47.22432	40.46557	31.54998	48.94812	22.08079	23.77973	27.55887	40.17271
6.545641	16.72311	42.73563	33.72147	41.67174	63.33514	36.1807	83.04526	83.31912
3.964081	16.59566	24.67622	5.041459	8.344913	5.087688	4.14716	1.179611	4.850165
68.05059	20.55511	33.76288	101.5147	62.49721	82.95375	50.64674	53.90979	47.89799
33.53069	33.34955	73.11934	150.5623	27.10225	92.52443	87.86069	250.1282	180.1967
68.20357	80.60636	41.65404	93.18284	113.5516	66.38494	154.0006	108.4197	133.7498
61.75942	90.77547	65.2458	92.49165	129.163	251.4573	95.09096	103.877	122.1263
236.3469	600.0621	154.0469	56.42854	199.9998	7.682917	158.1381	24.17481	40.49762
64.3345	74.20058	77.60996	134.4012	120.8354	165.0867	71.32525	92.65422	252.9581
1350.652	1753.691	891.6904	1575.595	2033.403	724.2264	633.5979	679.6345	889.9371
71.1016	16.6071	6.787621	6.517035	5.223924	18.29578	2.080534	1.833606	0.941417
640.0705	396.3689	583.6117	1013.152	1117.757	1878.502	1955.519	1562.732	1365.506
7.502071	6.253595	0.02747	0	0.009393	1.205195	0.007578	0	0
24.51236	49.86153	51.72425	31.4997	10.43476	25.31255	50.64637	26.84598	46.78128
696.5376	981.6151	735.3652	444.1115	1190.653	895.2165	581.9118	406.1699	473.0604
145.1615	80.58802	89.97268	60.85489	185.4043	131.0807	57.89149	147.3296	82.39519
9.11613	32.02129	34.88152	59.51133	60.41202	86.92745	44.44637	43.35941	65.88027
2.681859	3.947495	123.5683	33.69914	14.60128	18.76901	31.01759	67.09546	81.50994
0.062502	5.052978	3.378124	0.648268	0.009649	0	0.007783	0	0
40.9039	17.92074	19.12146	25.00346	13.55152	1.167324	7.247488	11.07654	16.097
32.22111	30.77204	67.46333	18.98001	50.00927	53.41351	69.24895	55.18045	58.48131
33.42873	68.82439	13.53917	55.17225	35.41886	30.60467	23.78009	4.45737	17.69163
51.48126	34.61528	50.63289	54.26296	43.76515	147.5448	80.62108	121.7287	112.1243
72.07081	434.2455	327.1142	43.93853	84.39249	31.14322	88.89886	26.1469	19.31569
633.6706	1599.095	891.6993	1320.025	1144.849	1502.109	990.1815	1921.615	1361.482
19.3186	3.948652	15.77099	37.55005	8.349447	48.51521	9.314457	46.25626	85.09774
950.684	311.6102	263.0251	29.25287	118.7599	11.5888	26.88766	91.29472	15.97191
42.33933	38.30694	35.96993	32.30621	22.9256	3.115377	4.148307	5.772313	1.497145
24.44296	14.14668	132.2648	19.03192	38.5316	15.54798	10.34824	4.457954	14.91607
9.09192	6.497522	6.793649	7.979701	6.266419	22.83957	19.63971	28.35598	31.37408
24.52941	44.81166	59.61086	43.24382	46.88724	154.1812	85.78324	142.9202	97.64259
149.1278	64.02567	73.13563	192.3437	50.01987	106.1602	276.991	116.9177	61.68068
47.62393	29.50491	89.93934	25.59071	60.42594	100.4787	54.78684	154.765	74.6659
119.6334	191.8321	305.8623	173.171	480.2227	710.8624	418.6001	525.3403	640.5735
52.73908	204.0573	37.14374	35.15546	152.0491	57.32773	26.88575	20.89735	22.67363

TCGA-DU	TCGA-S9-	TCGA-S9-	TCGA-FG-	TCGA-VM	TCGA-E1-	TCGA-DU	TCGA-DU	TCGA-HT-
150.4399	130.9732	278.6696	126.0512	155.2104	173.2142	73.9011	72.49208	96.43459
25.22971	44.34833	16.29019	29.9229	47.73311	49.40124	49.59814	28.8239	34.90768
120.3542	149.9633	73.05146	98.48178	92.26828	92.51798	199.4129	88.24648	306.0235
2576.913	3715.612	2072.172	2538.208	3057.486	4510.386	2644.091	3003.697	2987.399
110.6427	64.44607	70.46097	148.1566	137.8435	69.1313	74.91292	82.98853	106.5475
2.907337	1.073607	0	0	0.02031	0.068879	0.002968	1.713314	0
18.43116	17.97443	7.679321	8.579152	17.39602	141.3233	55.6724	41.07457	11.90951
131.0258	156.3149	261.4612	137.883	132.4258	218.7316	232.8213	161.6777	179.1409
6254.474	6008.591	20742.81	6159.161	7273.078	12184.68	10874.98	6728.362	9153.273
116.487	20.09018	53.28733	49.60437	106.3299	31.36337	19.23847	36.66662	32.12468
29.12502	21.12473	9.410545	33.18142	33.6114	7.925099	12.15085	5.192694	9.158313
1131.707	1757.451	1139.279	1163.126	1336.081	1553.786	1046.704	1088.037	1246.591
163.057	72.89684	100.5596	122.8855	94.45381	115.9948	100.2199	99.59065	90.9186
1.932375	0.014283	0	0	2.194633	0.073706	10.11473	4.359698	0.890804
90.26097	65.49816	92.84595	88.22834	131.3177	74.3099	44.54511	59.3889	112.0741
18.43134	3.191412	7.679436	9.368135	7.633437	6.639275	66.80434	16.55967	5.477505
1244.287	2660.498	1936.331	1750.168	2310.753	3616.869	2290.803	3909.158	1737.123
81.54385	28.53267	23.16801	69.40747	79.19387	49.49915	27.33536	23.55382	33.05192
46.57754	98.22969	73.91246	71.65739	32.59621	39.19311	86.04637	82.12514	58.76706
264.9597	205.9773	158.1493	278.0931	199.7437	566.6249	636.7286	253.3903	202.0657
1066.678	1313.87	1298.376	1191.516	1062.577	1437.858	959.6476	838.9541	1157.485
36.87717	311.2915	54.1788	129.5266	65.10213	80.60601	10.12804	0.827923	252.9331
51.43045	95.06542	683.2978	77.9599	29.34125	199.1399	109.328	500.2311	46.8192
260.1141	222.8647	1105.2	222.1781	133.5318	233.2028	339.1152	456.2339	140.5221
77.64248	127.7854	110.0689	95.34319	91.17684	126.2816	98.19192	133.7437	105.6461
43.68237	24.30324	30.08147	27.55122	44.48176	9.239371	17.21273	34.08201	18.34837
119.3735	90.85183	153.8799	95.2744	135.6941	183.6899	214.605	343.5081	140.5304
957.0006	699.1993	534.7742	619.3335	2399.649	252.8108	354.3048	817.982	686.2023
31.05507	69.6703	45.59173	74.23737	66.15775	31.29752	29.35823	36.70165	111.2891
19.3993	65.49281	85.98125	62.21126	199.6163	26.17254	39.48341	133.7635	35.79695
15.51711	78.14805	64.48733	99.38138	40.18032	19.65877	21.26285	12.18119	29.3684
92.21639	39.0929	83.43769	47.24378	60.78348	33.95596	52.64035	83.92835	33.04561
11.63961	13.74102	8.551585	25.26389	29.27252	7.919115	1.016946	2.570411	5.480054
806.5576	360.1767	404.9325	359.2598	945.3551	256.7161	428.2011	661.5405	374.7728
169.8476	152.1026	130.6538	178.8467	367.9009	75.66585	131.6007	80.35169	135.0163
17701.62	10247.02	11649.02	7490.119	12125.76	13752.96	10914.47	5223.43	8988.818
41.74755	11.63725	3.381241	13.32941	16.30254	5.333973	38.46297	21.83139	31.24259
84.44435	104.5434	41.23118	91.44858	194.1625	50.86208	41.50727	25.29458	76.24829
54.35379	13.7534	16.28091	22.77835	45.59353	20.95012	50.6146	61.19079	37.64825
50.47304	35.91817	7.679007	27.52558	21.73543	31.32344	89.07155	45.44422	51.45143
26.19318	173.2108	211.5491	27.48897	25.00047	56.12674	156.9048	153.8048	28.44241
53.38053	30.64635	23.16384	52.78251	92.2178	24.85471	30.37238	13.93004	23.8549
29.10742	11.64191	5.100993	23.55911	16.31619	26.16111	137.6598	79.54715	35.80246
110.644	176.3657	123.8109	166.3184	211.6044	115.9458	100.2183	110.9755	168.1283
199.9349	358.0339	220.0927	308.9138	338.6258	190.2365	128.5647	200.1019	171.7618
22.32237	22.18219	14.58241	29.98676	20.62967	7.928714	11.13911	28.85989	22.04326
32.99035	12.69808	21.43971	45.65742	31.50479	20.96124	124.503	66.41583	37.64004
3274.769	836.5142	9168.633	2477.514	3227.893	4134.038	1986.108	2091.281	2861.537
541.5873	1163.859	1129.93	601.2665	159.5853	1912.743	307.7387	375.7584	1536.071
6.783276	28.49707	15.4579	20.4972	30.35404	5.327323	8.102493	8.697684	9.160396
11.63958	15.84969	12.00335	14.14618	26.02787	5.327439	7.090467	7.821217	4.56054
226.144	273.5487	171.9322	218.2541	188.8722	172.0031	237.8882	228.9558	244.3511
53.37634	68.65548	48.97656	77.22814	130.196	26.16779	40.49518	26.16855	44.99085
7870.521	3610.019	4484.016	3346.686	6825.881	4326.914	2090.375	4664.146	2987.373
30.09396	17.96427	17.17056	22.84654	26.04329	9.225574	13.16319	18.33286	26.6473
32.9908	39.0932	63.62633	64.61833	47.77165	20.95924	45.55534	13.0553	39.4802
40.77809	46.43601	9.406347	39.48891	44.45152	19.58585	40.48598	17.45188	38.61394

9907.796	5391.776	1719.611	7708.515	8723.089	8143.061	2885.018	6808.8	6510.367
259.1436	117.2571	88.50454	327.824	403.7396	67.85954	87.06171	156.3919	140.5223
40.76759	30.63499	23.18233	22.01047	36.90119	14.43615	4.05425	9.561885	27.54585
14.5467	35.92336	83.45866	36.19911	7.633827	43.03015	28.34787	76.9429	19.25978
2.899743	4.248069	0.807832	3.851576	11.97035	99.72598	11.13977	6.939237	1.805145
208.6749	212.2941	127.2142	195.4081	227.9279	238.3455	99.20839	110.073	217.7135
46.6099	6.359463	11.99394	4.640473	36.86859	6.631651	16.19924	11.32006	13.7572
33.96953	35.90991	75.79341	23.59243	50.98483	35.17158	27.33372	41.96455	38.58715
57.25779	77.10584	182.4099	66.9464	58.62951	88.56833	61.75197	48.02849	64.28908
199.94	64.44967	36.05219	104.7372	107.4793	122.5203	211.5678	165.1521	138.6941
25.24108	7.413486	13.72892	13.35102	8.711483	34.99152	22.26925	6.068966	52.47163
50.48964	13.74791	13.71314	22.82661	39.04726	4.031871	21.25996	5.191147	10.99437
304.7706	153.1598	255.3748	141.7844	384.1826	204.5274	216.6302	238.5799	159.8228
32.02352	50.6917	37.81602	38.59044	33.66177	11.84618	15.18907	54.20162	13.74697
75.69982	222.8037	135.8807	86.65221	115.0456	117.2065	19.23872	98.74556	59.68705
60.18462	23.25295	15.42312	42.54723	66.17486	19.64129	12.15243	33.18752	37.65536
214.4965	434.0578	388.6993	209.5844	211.659	209.7436	181.2018	249.9395	179.1158
26.19884	47.52134	52.47937	39.39502	35.82496	106.3974	52.63624	41.95055	10.07135
156.2562	75.01202	216.6549	61.37475	89.03502	86.08791	372.5181	378.4446	81.72476
187.3197	296.773	245.0586	272.685	278.927	311.2141	151.8458	237.711	520.0328
873.5223	2038.403	1766.977	1226.925	1626.966	1518.702	817.9306	1185.902	733.0427
61.14502	80.26161	49.84453	40.1317	86.81151	37.85981	25.3118	75.16792	37.64024
328.0639	483.6924	160.7503	137.0476	240.9596	164.1949	98.1965	175.629	119.3935
80.56478	22.20145	24.02247	44.08444	91.14279	133.8818	90.08952	15.678	10.98882
47.55356	61.26015	17.13872	26.7163	45.60267	54.73686	48.59181	112.8135	27.53012
1306.413	2797.758	1115.186	1668.284	1764.794	2681.584	682.2846	690.3632	2392.172
43.6658	45.43559	48.96263	37.74291	44.53102	70.40372	190.3017	107.4946	67.95911
111.6124	292.5168	82.50208	200.212	206.1961	130.279	107.3048	51.512	106.5449
371.7311	410.858	108.2789	323.8304	202.9954	388.1834	793.6255	487.6538	228.7124
40.7535	35.93236	25.73555	40.10753	22.82932	17.06251	125.5222	202.8383	47.74001
259.1531	134.1478	97.11353	108.6814	93.37168	230.5146	133.6247	231.6026	134.1009
39.78896	78.13683	33.4985	49.63442	53.18361	39.13066	53.65113	16.55427	15.58383
1210.347	336.9372	971.6717	758.8991	810.7538	317.9059	361.3896	637.9684	1560.861
1788.794	1780.715	1225.227	1550.799	2168.565	935.2899	1846.41	1795.915	1635.158
0	0.0122	0	0	0.018404	0.061826	6.066767	6.193223	0
28.13832	27.47769	54.17333	30.67529	95.45554	18.35084	28.34764	53.31154	34.88983
55.31799	45.43066	104.9406	145.1535	70.55383	145.6538	10.12811	12.18087	237.1587
789.0881	533.3823	373.1186	427.0416	636.0417	634.3636	746.0556	1304.833	350.8885
88.32115	68.6635	79.08392	77.98141	126.9708	61.29574	63.77702	48.02412	97.37355
49.49209	103.4949	94.59576	51.16654	94.41889	19.66464	35.4344	16.5507	51.42252
47.55241	152.0345	44.67702	50.39228	57.53557	100.191	113.37	97.9188	85.44636
45.6089	140.441	229.7866	74.05609	30.42318	236.5755	21.26314	14.80262	80.83747
180.5367	81.34093	229.6509	218.3817	113.9746	196.5751	161.9641	68.12519	184.6668
252.3486	248.2106	202.8848	166.9936	184.5378	104.3213	299.6373	190.4813	196.5658
18.43845	19.0154	106.2593	23.65729	17.3767	23.42429	8.102708	13.07763	43.24209
46.59139	105.542	93.01147	41.769	16.3115	53.33188	37.45529	5.19047	14.6673
16.49182	27.46296	30.09722	30.74097	32.55408	6.635834	7.091008	2.570395	10.07409
21.34381	24.30739	29.20567	34.64755	83.50431	22.23299	25.31034	26.18659	38.57807
30.09242	15.85617	14.57935	26.8024	32.54281	16.99724	15.18743	20.08286	17.4374
38.81531	62.31758	131.6635	126.2337	91.14835	52.1482	11.1404	30.54404	63.38194
18.43557	4.247912	22.34252	11.74978	8.715305	15.70632	16.19955	28.85029	18.35696
2069.3	2095.444	1135.806	1594.157	2069.792	2533.242	1828.188	1410.503	1280.56
74.74292	43.31209	50.72057	63.06167	94.38068	41.73316	33.40872	24.42581	23.85559
308.6378	279.9061	734.3169	507.4592	311.5293	306.1851	267.2479	343.4164	232.383
350.3792	485.8342	366.2755	478.3518	490.5773	703.1816	527.3986	763.0234	414.3026
5.812118	8.468529	3.381918	3.85338	13.04363	0.093276	11.13855	6.06889	4.560394
18.43834	10.5792	11.99872	19.68838	33.60991	10.51395	19.23432	0	17.44368
95.11306	186.9198	111.769	109.5185	129.156	141.9363	100.2179	131.9701	142.3979

10.66562	20.07864	22.33434	17.28166	14.13678	18.30673	39.47569	10.44014	29.39758
71.82612	34.87056	51.57027	48.82166	95.48046	36.55566	24.29949	25.29721	33.04528
91.22983	43.32522	97.13896	66.12854	195.3286	67.82062	110.3402	153.8335	78.98211
308.6439	890.2881	245.0198	169.3515	868.1989	270.9754	199.4241	192.2245	91.82883
95.11217	173.1986	247.7356	156.8491	70.57494	248.5508	140.707	80.36864	160.7752
84.4364	61.27467	42.07879	66.92588	105.284	54.80283	71.87522	111.864	74.39108
396.0082	275.6646	736.1975	99.20651	321.2645	135.5638	299.6365	767.4838	120.3109
53.38195	35.92289	16.28033	43.31073	80.28604	36.53728	44.54216	20.05256	22.93679
28.14605	13.74879	28.37028	19.65333	53.12966	26.07532	17.21217	15.69318	21.11288
20.37294	19.03004	18.86887	17.26095	29.32198	5.336307	23.28606	27.06253	15.58644
267.8837	309.4504	171.0748	361.7721	332.1039	264.3985	193.3487	154.6516	163.4967
117.4322	79.23504	102.2719	107.1005	700.985	86.07848	56.69319	82.10077	186.4733
391.1432	666.4312	357.6636	482.2669	616.4744	791.767	719.7308	456.1836	650.4127
3691.153	3616.342	6773.02	3168.632	3008.657	5888.388	3817.329	6950.46	6192.562
15.52146	15.85746	13.71436	24.41503	18.46905	11.82468	11.13945	3.443456	13.75463
25.23084	39.06999	31.81189	25.97567	34.72755	64.88097	41.50115	35.84074	23.8699
62.11609	66.5395	36.93154	57.50383	100.9059	41.7564	73.89596	134.6895	78.09373
0	0.011406	0	0	1.105249	0.057409	0.002526	4.417666	0
0.959461	22.17525	194.6303	9.388271	0.026624	15.67337	12.15049	4.318575	11.00081
3.877679	4.231715	0.810818	0	1.114329	0.075129	3.039725	0	0.890374
27.18223	9.524297	18.90479	20.48181	13.04586	7.924417	8.102721	13.95421	14.68127
99.96523	85.56437	67.88132	79.53143	247.4075	53.5152	68.83938	67.25126	57.84459
90.27967	41.2007	31.77474	33.83038	91.12701	19.65302	23.28695	21.80178	32.12978
552.2647	511.193	323.2569	346.6743	605.6331	692.8748	819.9472	950.0288	424.3941
91.22789	84.50988	37.77378	115.0191	83.59798	88.64981	322.9067	147.6946	74.38328
10.67066	9.519059	6.832806	23.72557	19.52061	11.76943	9.113697	9.583174	5.482403
545.4626	1115.315	2261.52	743.0707	696.8254	1953.484	1095.292	1458.636	932.4103
197.0306	175.328	110.0195	145.7502	94.45497	100.3848	52.64397	125.8168	114.8077
51.43068	52.82863	12.83772	16.45707	73.82754	69.11779	244.9626	199.3221	56.00889
7.753202	5.303943	9.406187	13.33209	14.13428	4.031503	16.19956	22.71146	8.236282
239.7305	297.8439	79.9052	222.1786	625.1045	213.6766	187.2763	294.5073	191.9727
249.4426	163.7193	251.9376	264.0059	189.9522	309.9211	242.9484	181.7562	248.0337
95.11049	152.0885	193.4945	100.0308	130.2525	175.7959	138.6838	146.8183	101.9505
40.75516	115.1062	41.22667	47.22107	134.5436	79.46146	46.5687	58.52759	44.06956
18.43334	12.69416	15.43244	18.06642	67.21371	18.31616	15.18823	13.93993	14.67133
34.9335	21.14484	10.2582	14.8845	64.03005	18.35467	27.3358	31.42405	37.64487
110.6464	177.413	200.4263	128.4668	118.3036	67.81225	81.99759	93.49365	97.36626
6.78422	13.73588	13.74346	9.401189	4.374403	29.76281	10.12574	8.703718	12.84993
2.900437	0.016767	0	0	1.117708	0.088478	0.003681	16.63562	0
74.73693	90.81806	27.46272	43.28738	100.9097	27.46411	37.45828	69.91445	31.20534
27.17003	69.67526	61.96636	28.32587	47.74486	53.32917	40.49142	29.69068	39.49988
48.52548	17.97763	36.0758	24.3514	14.14557	31.35295	78.95534	44.54776	77.18289
204.7925	67.61818	79.90986	100.793	413.4666	69.15496	161.9681	177.3889	170.8523
95.11868	14.8106	20.57649	22.76365	40.18871	19.66587	125.5198	82.14016	30.28269
720.1872	452.0474	377.4417	700.6172	434.1573	653.7821	496.0203	201.8304	512.5982
22.31289	29.58964	107.59	54.3725	32.58398	115.6438	75.91777	132.1138	46.84194
25.22635	19.03172	33.50459	23.57218	11.97439	46.89535	59.72262	16.5559	37.65168
32.99019	82.37416	57.59127	44.0766	69.46424	115.7634	95.15144	100.5449	48.6706
37.84699	36.97814	20.58364	28.3039	23.90772	41.72741	79.96595	125.9874	10.98916
135.8835	78.17203	47.23671	157.6391	73.82975	39.19786	127.5482	104.854	100.1178
74.73626	73.93107	35.20748	69.34074	113.9242	43.06274	42.51933	90.91424	66.13694
651.2891	340.0874	149.562	303.3758	552.4079	224.0964	102.2459	96.95403	293.9559
36.87319	72.87537	169.5499	51.17987	90.06899	48.25929	54.66546	60.28689	45.91198
21.34439	23.25072	18.00954	17.26356	15.22642	30.00547	44.5393	40.20345	22.94394
326.1092	148.9445	281.9777	198.4972	244.2401	231.9578	386.6962	2158.903	199.311
662.9178	1969.631	358.5182	1894.893	366.8744	548.345	376.5729	65.49015	1677.578
39.78798	19.03321	17.14007	44.09484	47.7666	7.943024	29.36015	52.42763	25.69362
109.671	115.1324	189.1911	147.3571	153.0363	252.5172	97.18241	174.8012	169.0361

93.17156	106.6791	235.7005	83.48364	192.0724	83.42638	122.4866	59.38525	39.4686
52.41142	51.75387	30.05412	50.42497	91.12296	26.14763	13.16482	16.5543	39.48661
80.54615	13.75209	60.9874	22.76678	27.16937	28.77964	262.1856	680.8388	14.66636
285.3576	121.4782	129.7924	256.1188	108.5668	201.9203	263.1934	193.1208	370.245
38.81443	36.98432	28.32164	95.39585	87.90374	28.76785	21.26303	11.30697	33.96147
44.64997	23.25092	11.9819	74.22525	42.32496	18.33537	30.37052	14.80981	79.98939
116.47	75.00281	95.42071	101.631	145.4264	56.11011	65.80226	71.62783	78.06428
27.16874	9.529155	11.11969	6.214264	6.548285	26.13342	13.16467	141.8105	12.82788
51.44317	57.02472	41.25942	50.44417	62.92985	50.78065	25.31082	21.80575	77.20617
377.5495	631.6002	3570.503	471.9703	665.3412	523.6689	987.9883	936.8718	443.6768
16.49013	34.85522	21.45868	38.6239	57.48526	57.17254	14.17647	27.94607	22.9462
92.20498	65.4953	33.47866	120.6078	181.1958	39.18571	54.66697	73.38867	97.37554
103.844	353.7814	109.1577	224.6107	197.537	229.1988	261.1665	216.7446	238.8586
65.01891	186.9333	274.3787	131.5789	160.6316	234.3191	89.08457	74.24221	110.2203
26.22936	6.353735	6.839399	24.57623	24.90294	0.088914	9.112832	3.447633	14.70635
561.9705	790.0035	484.929	661.1735	665.3234	596.5358	713.6594	996.3514	473.0853
39.79966	21.13603	28.36375	30.72769	43.39142	9.237166	16.20037	13.06412	34.91381
1135.594	1265.283	1570.991	1012.622	1395.755	1336.258	820.965	784.7722	2134.073
55.31082	248.211	8.544501	32.21846	27.17028	79.58055	546.6278	60.24637	49.57086
47.54915	115.1159	89.41335	51.15534	82.49809	130.1736	84.02102	207.2378	16.50035
41.72475	44.37889	92.85573	31.43586	55.37872	35.2842	132.6062	190.613	56.0133
280.495	251.3827	102.2615	163.0449	347.3238	380.3258	362.3987	395.8942	173.5936
61.14414	46.48494	36.92916	49.60127	17.40167	45.66046	70.86007	76.038	60.62113
242.6449	460.4613	223.5382	254.5207	238.7896	368.5231	173.104	264.7972	275.5904
1047.262	980.141	2887.46	1178.866	1082.126	1939.299	1745.178	2411.253	1228.209
99.97516	55.98989	45.52948	114.3248	134.5441	40.47661	43.5321	24.41855	46.82681
20.38971	7.410329	12.01856	15.76647	5.457362	1.418101	9.11362	6.951144	9.166276
359.1189	401.3397	380.0569	354.6148	313.6781	332.144	374.545	472.8318	307.7357
895.8785	259.835	221.791	139.3939	1163.4	333.4926	167.0319	99.57371	780.8877
1253.037	937.8853	542.5136	936.166	1184.126	1323.246	1284.588	932.4776	962.7272
61.14932	28.53426	36.07993	57.53126	62.9431	18.35329	24.29915	26.17579	27.53285
52.42316	7.416574	12.84503	27.54977	54.2321	7.93942	19.23698	19.1899	11.911
81.52988	24.31473	10.25802	40.9102	72.7256	45.67297	63.77578	35.78721	94.6304
69.86974	23.25819	70.44578	71.61851	36.93791	144.7048	3167.391	663.3246	98.25841
51.43375	2.133593	3.384086	9.365932	46.69549	4.027493	47.58089	11.30699	15.5819
11.63734	9.526107	4.240962	14.12446	8.715139	10.5253	15.18748	53.42123	4.559476
712.41	850.2192	1342.274	894.4232	802.0902	950.7791	784.5218	657.177	982.951
62.11142	111.9443	65.32044	81.94024	134.553	79.47815	75.92284	92.63882	66.12647
36.89984	11.63354	11.13706	19.69207	35.77056	11.80491	8.102664	23.60778	11.92018
131.9981	116.1874	54.9759	100.8217	224.6351	183.5913	213.5878	109.2187	80.81573
66.96069	61.27828	328.612	92.14757	125.9104	269.394	160.9523	117.9647	111.1419
41.74054	24.30267	20.60023	10.16016	13.05562	20.91522	14.17632	14.81345	19.26875
179.5586	97.18544	85.07552	102.381	215.9772	57.43196	178.1626	158.1686	147.8888
40.76457	30.6389	17.14679	29.90545	44.49511	18.33816	18.22537	17.4341	6.396034
17.46074	7.416761	5.099976	18.05484	16.31038	23.52085	30.3703	21.81326	12.82944
12.60507	5.303998	3.383556	6.215505	13.06093	193.63	152.8436	26.16959	16.50106
5.815925	7.403145	1.665422	3.074277	4.368854	9.140672	7.088381	7.846208	5.489463
111.6129	139.4171	118.6381	155.2504	149.7797	88.64959	91.10886	102.2233	203.9619
468.7891	1415.218	680.1339	536.6186	1050.596	325.7204	219.6709	96.95125	2571.479
20.36967	6.359439	6.822352	4.645581	4.374451	35.29485	7.090945	19.17203	12.8265
4.841444	2.134827	0.807032	5.434672	6.543086	9.20731	9.114271	6.946688	1.804782
154.3285	147.8576	97.13786	153.6985	183.398	82.12987	78.96144	79.4955	161.6966
0	0.014658	0	0	0.022171	1.402444	0.003229	1.706109	0
69.88346	34.87112	52.42876	81.20138	68.37688	28.76159	22.27512	36.66788	71.65786
64.05405	78.15972	53.27521	68.53071	107.4332	23.56891	43.53212	63.77572	54.1799
568.7636	858.6535	581.2409	716.3387	795.5584	420.7719	202.4621	313.6997	507.0751
340.6774	107.7525	67.86632	167.7806	766.1769	92.60278	163.9943	184.3616	272.8237
333.8836	311.5731	216.6463	270.2675	291.9707	311.303	316.8456	443.9932	303.1455

5606.131	2863.305	1946.614	2646.916	3838.966	5002.838	2058.994	2465.316	2566.64
106.761	123.5747	716.8407	185.2558	19.57353	115.9461	42.52091	85.61538	31.19907
916.2564	194.3575	77.32406	204.8078	175.8637	122.5627	379.6093	453.5599	455.6367
27.17229	13.75048	24.04712	52.88705	62.89474	22.21284	22.27314	10.43726	27.54793
1376.294	1664.528	1109.16	1161.513	1367.573	1753.114	1391.894	2828.125	1331.093
122.2862	102.4674	81.63167	114.2005	156.3082	66.54868	270.2774	241.2202	159.8291
37.85182	12.69618	10.26041	20.42256	36.90744	18.33642	26.32225	20.93586	35.8206
137.8198	104.5759	83.35782	154.439	284.3203	50.92066	100.2197	70.7423	89.08218
1.92942	5.303514	1.663938	16.51065	8.713531	5.330993	19.23467	26.23278	9.157765
14.54648	14.81001	5.100842	10.94161	3.290129	2.722902	66.81031	36.67024	7.31457
46.57731	252.3726	73.91003	90.58761	77.08168	123.7268	151.8402	320.9194	56.92817
122.2868	235.5182	219.2707	156.787	122.6685	78.26179	75.92607	68.11692	93.67339
48.5483	17.9672	4.240808	18.0789	7.631361	17.00439	23.2837	25.33906	17.43537
15.53243	12.67655	4.247112	7.825358	8.702003	2.722099	6.077641	4.32306	11.01365
62.11407	66.54374	37.78778	89.07614	61.87695	75.54388	85.03082	63.78304	75.32797
34.93362	57.03354	24.88602	31.45888	20.65459	40.43767	55.67595	52.42825	22.01692
138.7845	326.3522	177.9495	193.8066	111.8254	467.4392	400.8613	307.6299	201.1639
13.58519	4.246473	9.421977	3.856102	20.60488	7.906682	12.1494	6.949477	19.3029
199.9379	234.4718	238.1732	204.0754	265.9089	410.1093	166.0175	225.468	238.8445
34.94094	15.8617	15.42854	49.71131	50.9836	18.32708	17.21281	7.812922	30.30671
53.37553	191.1036	97.1817	94.58867	60.79589	105.4335	76.93423	78.65035	117.6134
161.1052	555.5593	6018.052	395.5202	86.86644	666.906	610.4102	1067.104	240.6489
2.899763	3.19142	3.38178	0.708757	6.544723	0.093553	6.078481	47.32305	5.479559
142.6926	67.60343	70.48907	209.8797	85.74399	209.3251	71.87367	10.43324	139.674
336.7938	488.9912	235.5631	304.9462	299.5711	182.4432	306.7237	488.5663	248.0147
75.69778	99.28983	82.50923	138.7028	221.3671	60.01816	53.65564	53.26339	100.1179
88.31975	79.22369	73.05448	66.13841	115.0455	19.6673	56.69183	87.37546	83.58252
222.2695	110.9136	97.11747	148.1182	256.122	110.7935	101.2323	151.173	172.699
393.083	279.9043	317.2394	636.751	435.246	186.376	271.2966	142.3973	571.3932
37.84418	30.6488	63.61837	67.76499	19.57168	62.54299	58.71399	13.05501	116.71
9.696677	10.57812	9.413221	15.73006	19.53816	5.328187	6.078434	16.59003	22.05311
165.9753	85.5647	102.2945	118.9701	148.6912	50.9133	87.05967	148.5747	143.3089
35.9129	38.01769	58.54788	41.00894	35.8143	44.21809	26.32137	44.59946	54.23672
13.57602	10.58557	5.959733	11.73071	11.975	2.723822	25.3113	14.80494	4.559488
27.16736	184.7056	104.1521	29.88483	27.1608	89.70254	9.115794	50.68432	14.66492
76.67093	92.94836	69.6138	69.29624	125.8907	50.89757	71.875	124.1144	46.82175
33.95789	71.84316	116.0294	29.06558	115.0788	113.4221	1874.675	34.0286	27.52407
65.99394	345.235	59.29316	80.35546	269.0347	14.45719	38.47137	20.04653	104.7307
33.96646	68.6245	58.50861	71.03403	24.98667	58.52636	32.39519	6.064154	79.97862
27.17427	16.91476	23.19271	29.1504	32.55694	15.72177	25.30859	44.61529	22.03202
27.17493	42.22836	11.12634	19.65399	52.04598	6.635942	19.23623	12.19057	17.43261
100.9342	171.0965	478.3234	126.0544	93.36505	112.074	129.5741	259.6241	175.4665
2799.177	3889.884	3144.409	2751.758	3033.616	4649.786	1847.425	4386.283	2401.294
26.19426	6.36036	0.809617	16.45799	27.16774	2.72123	121.4692	124.1533	56.0199
181.4891	253.499	116.878	128.3594	441.7492	449.3668	583.0742	2540.175	233.3065
64.05139	24.31579	8.539627	25.91569	87.92411	109.3957	92.11896	86.50438	32.11935
129.0811	203.8417	73.89143	149.6855	103.1376	359.2967	139.6981	270.0765	172.6943
54.35585	47.52839	36.95032	57.55712	32.57975	40.41138	31.38373	24.4304	33.05355
127.1379	19.03469	408.4946	132.323	36.93965	103.0073	551.683	932.8005	155.2284
48.51891	127.7921	42.93836	113.4746	201.8255	97.71809	61.75314	27.03838	70.71391
17.45955	14.80868	23.1704	20.41443	19.56717	26.13692	40.49265	25.30633	28.4564
43.66882	100.3193	34.34679	54.3376	98.74401	49.55889	54.66549	73.41198	48.66966
0.959559	0.016357	0.807329	0	1.11737	1.415264	4.053203	3.451232	0
0.960015	3.184324	0.80909	0	0.023071	0.07934	1.016689	6.994621	0
35.93405	8.467419	16.32666	43.57872	21.69602	6.621356	3.04183	0.826407	12.84512
17.45935	17.97625	24.02983	35.42023	27.15914	45.6036	29.35956	25.30466	34.89185
13.57898	43.27854	25.78859	25.20398	21.7198	17.00754	19.23591	15.69594	14.67411
197.0313	135.2008	102.279	192.2854	329.8969	83.4643	98.19548	92.59582	166.2671

915.2686	1556.76	226.0778	317.4937	718.5231	962.4987	420.1025	261.2538	323.3286
9.693433	0.018651	1.667751	0.712786	1.116064	0.100139	660.9521	4.319438	1.807011
0	0.008743	0	0	2.157499	0.043026	1.013836	0.854051	0.906638
15.52314	27.45276	25.80716	23.64471	10.88115	29.89146	12.15105	7.817796	9.157445
1874.212	1295.934	2254.531	1297.06	1184.155	1531.736	1455.668	1966.363	1525.848
18.43536	58.03444	37.01631	70.43323	37.95921	10.52701	13.16352	6.940747	82.8271
26662.1	41919.55	49125.87	21757.35	28496.4	41301.76	26084.58	30190.78	29840.92
74.72971	68.66431	70.47563	34.5891	43.44568	73.00111	131.5946	216.8499	49.57909
362.0369	237.6452	210.6355	264.7691	286.5358	217.5621	202.4595	232.4504	270.0768
249.441	142.6001	195.1591	204.8566	348.3794	88.68829	143.7481	137.1684	219.5453
279.521	419.3085	296.6036	360.0843	1189.468	160.3289	211.5722	398.4923	297.6148
603.6963	1662.41	3868.619	1501.203	287.6601	730.7893	1028.485	509.4521	4090.821
413.4794	393.9404	202.8886	428.7539	563.2474	216.2743	265.22	68.11238	229.6444
20.37633	13.74872	3.381235	6.216199	16.30486	18.30945	24.29635	16.56931	22.95356
41.73079	26.4225	11.11852	34.62254	43.42628	4.0306	28.34774	22.67726	25.69493
12.60819	30.62262	67.24704	23.625	21.7179	26.05328	16.19965	11.31759	11.91502
82.49716	110.8917	56.71084	89.82503	126.9669	82.08808	137.6667	131.1255	63.36692
103.8463	158.4258	163.3729	139.4635	173.6523	132.8869	93.13363	59.37921	116.6519
47.55133	33.81733	50.69759	67.75161	29.33713	88.53571	39.48302	75.15553	45.90977
164.0224	416.1048	168.4943	82.65711	575.1565	151.1724	196.3855	274.4201	258.1364
150.4433	79.22911	98.85302	116.6036	169.3033	86.04172	93.13306	180.9334	155.2566
7.754571	4.247166	5.103895	2.277791	2.204948	10.50313	18.22145	3.444373	0.887902
2428.416	2260.215	3114.358	2584.741	2198.964	3326.45	1611.562	1893.788	2653.024
115.4982	335.7832	64.44279	141.0744	159.5325	153.6448	164.9997	95.23623	109.3093
14.54619	33.81895	95.44729	31.43818	13.0611	17.06179	99.20312	34.0354	29.36386
35.91637	81.24325	9.403557	32.3227	36.88729	54.50471	33.40431	49.88118	34.91863
434.8248	508.0114	791.1492	384.5423	545.9219	643.3023	335.0684	230.6794	352.7458
2408.999	3133.669	4055.018	4143.5	3251.765	1956.409	2927.531	3119.058	5613.832
203.8153	320.0257	228.6808	307.3068	482.9674	157.709	162.9823	195.7219	261.795
18.42914	85.53748	28.32477	38.55481	99.81975	46.94975	22.2751	24.42211	23.85331
2.899719	2.134795	1.664308	1.492783	3.291196	14.41932	16.19991	9.565232	6.397041
57.25471	179.5298	135.0034	88.21636	106.375	196.5338	145.7675	161.7067	73.46854
79.57819	118.304	137.5527	148.1261	261.5399	54.82668	90.0973	113.5809	81.73081
72.79306	35.92892	42.95099	58.27791	97.66576	15.75841	23.28754	37.53765	48.66745
25.24571	26.38166	8.555846	10.98393	33.5827	6.618083	14.17341	4.319927	8.242355
45.62833	16.91507	15.43288	33.8994	62.88074	14.42687	8.103259	15.69156	22.95149
8.734277	1.077072	5.122665	10.26035	8.686288	1.413944	1.01686	0.826943	1.806485
39.79813	13.75029	36.98296	7.792486	99.71064	10.53795	13.16418	1.698396	10.99203
5.812595	7.412154	1.663819	12.56947	0.026338	1.418403	5.066151	6.071108	1.804795
22.31193	110.8702	33.48799	80.40753	27.16566	28.76277	26.32401	6.064764	68.89824
81.53444	42.26087	52.4287	51.18697	95.48406	40.45658	65.79872	89.17333	45.9143
77.65984	38.03171	135.1978	98.67094	43.423	78.01767	61.74729	41.93398	112.1572
29.11405	23.24868	26.6311	25.17536	53.15012	15.73281	18.22492	15.68755	14.66925
98.02824	91.89207	90.26897	62.98562	60.80447	70.40181	95.15565	69.88583	56.93101
34.94486	21.13455	9.403436	24.40182	49.88255	9.235192	18.22427	13.06569	18.35217
10.66925	15.84624	13.7374	22.10553	15.20276	27.22698	14.17356	35.9358	20.21902
40.75507	62.32445	26.59866	39.32955	45.61112	117.1314	94.14148	97.02078	30.28358
18.42963	80.25058	69.67216	49.6279	18.48484	50.81789	22.2748	12.18177	36.72637
53.39508	27.46835	78.39351	17.27003	32.56436	14.43346	11.13992	4.316879	22.02788
2.900298	10.58578	0.809559	0	3.289685	0.099686	9.1158	6.065133	0.889366
14.54916	30.62976	149.2345	28.35553	11.97022	31.25922	15.18821	16.56719	27.55243
95.11041	149.9771	297.6164	144.2017	118.3184	174.4974	109.3292	180.9226	88.16626
53.37881	20.0898	47.26486	122.2988	53.19384	248.0005	73.89554	7.811741	68.90154
109.6763	142.5715	47.24044	68.50319	180.1291	219.876	41.5084	26.16442	51.41566
15.517	10.58577	32.62667	7.790167	2.203423	23.56285	55.67742	26.17032	1.806466
90.26174	46.49149	52.40429	59.04001	47.7859	74.30403	101.229	249.2157	87.26007
23.28762	69.66227	34.39186	23.59475	17.39284	39.04869	73.88565	116.49	20.1877
2.902515	0.015474	0.808626	0	0.023426	0.080705	5.063617	5.222585	0

508.578	725.6082	137.5131	299.3589	192.1478	1434.009	777.4387	299.7039	128.5739
1.929431	0.017359	0	0	1.117885	0.092098	13.16171	6.947437	0
164.9969	105.6345	99.69601	100.0116	164.9859	67.84831	178.1631	177.3991	165.3451
129.0787	275.6598	239.8843	508.4393	302.8089	165.4929	132.6134	90.83878	160.7394
13.58028	21.12773	15.44517	14.92158	11.96521	23.44733	15.18715	14.82626	21.12246
9.694747	3.191597	4.240689	4.639509	7.631763	4.031931	8.103157	84.98771	11.91389
73.76369	48.59822	85.13342	52.75099	68.3858	52.16748	60.73904	45.40949	30.28456
504.6985	700.2512	366.2388	836.8794	1272.021	394.7583	506.1462	457.0263	549.3233
36.87096	88.72481	45.52151	150.5863	166.0216	86.00257	67.82608	53.26959	75.31217
148.4894	354.8752	174.4997	295.4825	123.766	336.0562	459.575	162.5068	229.6385
19.40423	22.19124	13.70855	21.22911	23.89292	14.42908	15.18829	10.43825	19.27018
164.9953	269.3138	268.2946	141.7946	128.0971	259.1486	438.3091	494.7832	188.3125
399.8735	1067.777	2873.907	356.9014	35.85162	1166.912	304.7027	1010.291	364.6698
6568.953	3489.617	2496.908	2962.906	5347.627	4774.963	2447.711	5176.272	3491.706
85.41389	33.81749	169.5245	144.3551	66.21699	158.6462	149.8091	109.2802	189.3371
2100.35	3410.383	1150.405	2591.005	3976.776	1380.742	2146.048	2208.396	1304.431
103.8834	14.80859	3.382052	16.46737	18.48242	5.336181	50.61347	34.06019	88.24842
157.229	283.0451	125.4935	185.1558	142.2058	251.3603	290.5236	141.5435	162.5814
37.84573	72.86689	106.708	59.88983	64.03256	96.23875	56.68848	14.80419	38.56312
53.37007	151.0432	82.49215	48.76735	35.85422	41.80983	217.6408	266.5786	49.5717
125.2019	30.65328	68.7372	92.92941	118.3199	363.0511	117.4273	111.8381	219.5797
0	0.008789	0	0	1.094946	0.043271	0.001955	3.57054	0
10.66448	11.64048	12.84237	14.10328	10.88839	11.84301	46.56372	20.93568	16.50623
22.31436	17.97515	5.100038	14.10093	27.1558	10.54481	28.34691	27.93521	12.82814
37.84771	61.24987	35.22347	57.54538	27.16009	4.030876	23.28676	47.18505	45.92569
40.7564	31.70486	104.0895	39.33857	14.14619	70.33892	71.87228	97.04058	57.86282
356.2247	410.8037	122.9191	379.9817	268.065	304.6608	29.36161	8.688961	293.992
46.57845	82.38806	51.54673	67.72502	228.9163	54.79245	79.97218	2.573727	72.55733
42.69652	1.076438	0	0	30.42329	4.027301	15.18956	34.91124	0.889463
529.9329	1589.516	1018.907	1422.459	1823.363	706.0092	516.2696	212.3104	918.6298
271.7563	238.7144	286.285	446.0063	937.6971	187.6741	273.3208	26.16648	454.7188
17175.56	9862.571	6211.396	7816.362	11183.67	18708.85	7670.093	14201.36	7662.304
19.40409	48.56265	18.87833	39.43506	53.14076	26.09092	19.23668	15.69007	20.18974
181.5058	147.864	79.06243	193.1195	159.5435	100.3569	78.962	68.99781	127.6827
13.57733	35.91032	3.381458	5.426702	3.291064	9.240471	35.4302	26.19418	4.559084
23.29063	24.29742	18.88574	26.78543	40.13128	27.35883	19.23602	30.59535	27.55773
21.36643	16.88769	6.838327	13.39886	11.94449	4.018504	10.1247	7.833927	14.70462
26.19763	9.529194	32.64571	18.836	16.31318	10.54568	10.128	41.06472	18.34294
149.4738	67.61297	51.53752	103.9877	77.08494	47.00695	53.65566	97.85741	52.33186
19.41204	9.522775	3.382118	5.433293	14.12464	14.37742	35.42192	14.83781	9.160636
41.72665	52.82055	154.0293	69.33248	108.5087	53.46396	55.67801	62.90734	71.64995
93.17386	55.99472	61.86873	103.2257	174.7012	50.89833	66.81398	79.5049	41.30792
25.23358	25.35138	14.57552	29.95837	37.96372	11.82618	31.37962	11.31656	15.5942
478.4998	535.4776	352.5032	266.2871	383.1499	864.6711	600.2835	309.3383	446.4498
20.37342	12.696	12.8429	23.58561	50.99263	7.940963	15.18879	18.31073	34.90198
36.88091	52.79465	16.28661	56.01702	36.9069	22.22819	23.28589	34.94651	25.70265
309.6146	212.3072	220.9392	321.4938	522.0356	100.4183	136.6633	119.6775	133.1702
37.84286	410.6462	58.43979	89.05742	47.78043	396.2046	9.115787	2.57323	18.33811
135.8731	29.59703	25.73525	116.552	171.5095	125.1421	327.9784	549.8136	112.9625
49.50247	44.35956	38.67943	34.64037	37.99686	50.76608	24.29847	105.015	26.61883
19.41551	11.62862	12.01291	19.73067	22.76906	9.199486	7.090093	7.825748	12.84903
20.3714	117.1796	73.12575	52.00547	69.44423	30.04359	11.14033	28.802	33.96961
156.2605	207.0084	189.1589	170.9805	196.4526	240.9099	200.4325	166.0319	149.7234
15.51684	21.14687	44.67008	63.00881	2.203124	13.15437	90.09241	23.54457	128.6469
98.0316	106.6656	30.89916	49.58323	40.18838	10.54854	50.61782	42.78045	95.54191
53.3718	143.634	762.4903	171.0598	36.93762	149.7438	134.6334	41.02421	122.1763
11950.87	16858.65	10178.69	12434.24	11697.05	17776.29	17567.2	24407.18	16291.07
426.0864	745.6347	343.0457	611.5519	489.5011	908.9137	394.793	513.8768	298.535

47.56133	50.68863	21.45153	22.78909	45.58147	22.23482	18.22547	53.33324	21.10253
4.840617	39.0782	20.59394	37.82139	26.06766	17.03637	39.47899	34.07239	13.74857
8.729839	23.19905	11.16881	22.1909	5.454133	27.07841	2.029374	9.595003	11.01771
130.0642	59.16215	90.2678	109.541	218.0838	30.08157	23.28781	149.4828	121.2687
79.57712	45.43964	42.93125	65.31984	23.91482	54.83165	288.4979	59.37484	51.40897
254.2845	277.7918	549.4363	315.9391	195.3999	423.3547	512.217	283.9878	395.9161
13.57935	33.78577	28.38248	40.27579	18.46824	19.58937	13.16353	6.940728	22.0381
246.5281	146.8251	172.7928	293.9494	291.9587	200.6364	217.6431	123.1805	206.6796
160.1491	206.9926	218.4512	117.3848	160.6293	110.7647	96.17008	96.1021	176.3898
198.9771	202.7725	93.68423	96.08394	206.1991	138.0891	52.64373	215.8985	50.49222
1329.701	2735.475	3313.022	1498.001	797.7789	3680.609	722.7768	1031.197	1286.987
150.4389	73.95226	34.33297	43.25197	80.3463	118.59	107.3054	103.0901	90.00099
32.99093	24.31303	20.58046	21.98228	49.93983	28.75839	30.37264	30.54594	23.8538
3.870199	7.41691	11.98108	3.065507	2.204361	2.725482	22.27397	135.7066	0.888662
109.6798	70.77399	52.40812	66.93793	66.2258	121.0707	79.97204	66.39189	66.12426
16.49496	17.9623	35.31641	29.20634	20.62638	16.9803	20.24648	20.96928	12.83906
195.0781	837.5044	157.2949	413.7038	396.1665	254.0709	476.7858	602.1739	336.2065
0	0.01004	0	0	0.015109	0.04995	0.002229	0	0
157.2357	91.90264	35.19334	74.00296	158.463	110.7722	151.8431	159.9316	60.59923
223.254	26.42844	57.56037	11.73061	84.67829	53.51108	58.71677	159.0801	36.71191
3.870208	30.61894	22.34679	11.7523	22.79717	6.632077	17.2113	38.50777	7.317219
7.762012	0.016078	0	7.063	0.024356	1.413701	35.40136	0.826991	1.806576
12.60674	34.8526	15.42991	32.3021	44.48002	1.41704	9.115539	5.190586	4.559093
57.26992	60.18941	140.3992	28.31402	16.31327	36.51489	36.44414	93.61516	16.50431
125.1972	176.3905	173.6621	97.63829	224.6733	468.6337	165.005	236.8374	71.62037
123.2669	84.50386	85.09945	70.08002	78.16476	165.3048	131.5956	140.7266	48.65859
28.13906	64.41225	84.34804	15.67703	29.32713	36.52042	20.25009	22.68019	29.37523
84.43362	123.5766	53.25689	117.3932	97.70057	54.81692	88.07187	103.1014	41.3053
55.31877	54.93054	50.7014	93.03355	96.57591	33.96623	36.44621	36.66499	33.04296
83.46114	232.3414	125.5111	111.8511	327.714	61.33356	139.6969	138.9393	64.27356
471.7025	258.7828	321.5361	262.3378	350.5978	284.0433	493.9968	715.7698	328.8472
55.31134	196.4497	145.2845	237.2238	98.79692	39.20521	66.81577	52.38269	132.264
39.79668	31.68966	54.22634	25.9657	53.1508	18.32765	25.30955	30.57494	33.98674
14.56168	11.62132	5.111459	9.42074	31.37835	4.017306	6.077473	9.593096	14.70669
35.89905	10.58436	4.246576	3.074106	22.82926	5.324825	697.4414	15.67822	22.93104
160.1416	193.2851	196.8909	184.3726	130.268	71.75831	173.1028	156.4077	148.8004
51.44038	51.75399	36.94323	51.21479	70.52846	33.93882	45.55417	41.05417	36.72796
14.54731	32.75177	40.40239	22.7863	18.48187	14.44582	26.32275	11.309	3.641089
21.34485	27.47055	17.14979	18.05611	4.377027	31.29327	20.2493	33.19978	5.477538
213.5279	215.4628	194.3059	224.5816	327.7544	70.45804	87.06129	83.84816	194.7408
8.725891	2.134847	1.663809	2.277812	6.543539	15.66307	19.23318	11.3296	25.74447
83.47755	42.25994	65.34946	51.19166	56.44656	40.45189	41.50663	69.92291	128.6765
12.60869	10.58102	11.13102	8.58845	17.38277	26.03884	23.28308	29.73227	11.91629
247.4925	606.2229	408.4209	315.9636	306.0898	385.5498	176.1421	360.0477	657.7819
0.959501	1.07745	0	0	0.026928	1.41823	2.02949	0	0
84.45819	14.80894	28.33562	46.48513	58.59775	65.04999	26.32311	13.93163	102.0406
50.46739	27.47963	30.0495	41.7214	48.85275	33.95044	19.2383	55.92547	32.12774
325.1531	123.5915	49.80967	171.7396	298.4676	218.8533	185.2508	381.9536	180.9536
522.1853	597.7703	152.9979	293.109	295.2351	307.4316	149.8229	115.3068	236.9862
13.57699	40.13369	24.90127	28.32888	81.32773	28.70992	48.58751	24.43864	21.10448
13.58201	13.74194	5.964437	15.72923	10.87812	2.726415	6.078445	12.20328	17.44653
190.2229	35.93246	196.8421	33.01265	127.0255	65.25174	323.9361	1687.665	43.14267
14.55316	0.017589	1.66385	3.064874	7.628508	1.418416	2.029493	18.34298	11.92057
14.54648	84.47888	354.0078	99.39008	19.57076	15.75539	19.23836	227.5195	35.8038
230.0329	188.002	96.25425	123.6643	195.3688	233.1098	283.4361	219.3645	135.0207
6.784485	6.356079	0	3.856791	5.45761	6.612264	13.16093	12.21764	2.723289
8.722889	279.7813	453.6657	38.53758	118.2845	62.58261	49.60562	15.67648	17.41916
5.81537	3.189163	1.665046	2.281759	3.287241	1.416102	8.100336	13.12548	3.645422

65.02827	58.09595	27.46301	74.08594	96.57146	35.26233	29.36067	96.17086	79.93086
190.2292	162.6665	71.30707	326.2709	175.8514	205.8481	169.055	214.0922	203.9216
37.85458	19.02766	8.540582	18.05907	26.06404	22.21593	29.35772	33.20409	26.62677
53.38553	47.52722	30.9221	42.53967	67.26399	42.99969	26.32292	16.5564	26.61752
24.2786	6.355658	0.806946	7.025763	17.35604	11.76408	11.13715	13.97767	15.61956
100.9629	34.86676	50.72567	28.3049	59.68753	31.34132	54.66317	62.06495	29.37262
71.81585	271.3753	66.16633	122.1488	115.0511	280.9829	122.4863	110.1086	116.6656
125.2043	173.2008	91.11064	130.8061	166.0473	79.5357	182.2082	86.48778	317.0212
89.29194	51.77019	70.47771	73.24668	78.1599	26.17579	44.54487	67.26421	50.4988
68.90146	22.20395	61.85566	115.801	39.10937	80.84967	78.96224	88.2303	101.03
69.87524	51.77147	73.91454	82.70633	113.9613	56.10258	73.8996	84.75111	63.36296
978.3495	932.613	864.1021	719.4051	850.9502	1447.034	2030.639	2627.144	1072.037
14.55735	1.077546	0.806956	1.49188	2.204688	2.724322	2.029462	15.73076	0.887848
1085.119	710.8191	466.8394	985.8045	954.0485	554.9571	525.3804	627.4453	508.8954
0	0.012606	0	0	0.019024	0.064105	0.002786	2.607621	0
23.284	110.8462	54.17333	29.88547	28.24502	54.69435	49.60246	27.05276	54.20163
726.9725	239.774	247.5773	320.6527	772.777	216.3365	334.0584	455.2869	329.7619
115.5002	109.8441	73.05122	109.528	164.9471	92.51874	91.10753	120.6068	59.68572
52.41332	26.4211	45.56406	35.42077	82.44187	20.94629	49.6019	20.92975	36.73127
43.67156	115.0772	28.32905	202.2076	77.0394	50.82307	18.22603	6.064389	218.8393
0	0.012952	0	0	0.019554	0.066063	0.002861	0	0
9.696464	16.90566	16.31719	5.431844	10.87866	19.54858	6.078474	8.696056	11.00001
30.0782	53.87227	23.16122	69.35387	31.50409	14.45492	37.45807	37.54312	45.91468
31.05595	8.472579	11.1224	11.7376	20.64574	14.43631	28.34583	22.69115	15.58846
79.58675	80.27202	58.43797	89.05241	126.9563	56.07841	27.33663	58.52567	66.12846
111.6107	134.1431	79.91643	227.0028	46.70658	156.3199	199.4189	39.27295	134.1076
1694.652	1905.329	1329.29	1230.865	1240.59	2093.012	2041.777	2238.179	1064.68
16.49364	67.51347	7.684164	31.5654	44.44638	13.11329	32.39027	16.57751	25.72432
34.92881	129.9127	222.7625	69.27662	20.65892	56.11942	213.5872	270.14	85.41139
5.811251	6.360393	0.807861	6.215501	4.37701	0.096405	13.16406	11.31346	5.477731
29.11849	11.63762	6.821479	10.16522	17.38703	26.06502	83.99882	21.8292	40.44734
24.26793	32.71795	19.7701	27.62961	17.37614	36.3023	15.18656	13.95513	46.00891
51.43518	82.37658	68.78017	71.70644	77.05829	24.86567	29.36081	36.66445	32.12357
176.6445	530.1215	139.2625	530.6371	308.2102	205.7909	144.7593	127.5616	838.9475
78.6179	81.32377	61.02669	68.53952	126.9456	46.96994	27.33649	48.03357	38.55621
365.9106	331.6456	200.296	314.3904	627.3043	149.9019	178.1666	312.8476	242.4979
68.91151	34.87184	30.0444	29.08028	22.82658	41.76071	50.61664	47.16413	23.85265

TCGA-HW	TCGA-FG-	TCGA-DU	TCGA-S9-	TCGA-HT-	TCGA-KT-	TCGA-DU	TCGA-DH	TCGA-HT-
197.4606	136.3341	32.04653	313.338	140.3511	47.22279	159.0358	260.1051	31.59605
73.65405	57.89664	34.4406	18.99659	22.68957	54.21698	35.16423	7.036487	28.99723
137.9224	171.8892	42.77405	128.9399	92.8534	67.87378	139.0913	30.26741	113.3457
3402.15	2552.292	1491.732	2832.932	1619.373	2215.283	2810.728	3553.125	1312.358
200.0258	114.758	81.83433	141.9069	105.8115	166.7908	50.2705	112.6925	108.3486
0	0	0.687516	0	0.018952	2.552033	0.732922	0.037835	2.567486
10.49899	9.148633	6.789316	11.1938	23.77232	21.44824	15.10889	5.875586	66.62045
172.9788	176.4375	36.64009	114.1903	229.9203	78.18137	146.2381	69.74989	35.37331
7972.897	7138.436	8085.136	7797.916	12756.12	7657.713	12264.36	3447.511	5046.382
77.7404	81.6935	32.06824	25.90753	46.43908	61.02311	47.10833	60.41461	25.28622
29.5525	20.79331	15.26208	21.63136	19.43816	22.35335	24.7948	25.534	7.651943
1588.053	1391.367	369.6038	1054.771	867.9851	670.4843	1541.682	590.114	589.4698
137.8021	59.22845	122.4157	84.75199	59.40791	146.1209	81.44697	510.8161	186.3804
0.49504	0	3.794433	0	0.020147	10.41563	1.532885	0.040321	11.12873
82.02414	90.87714	77.25866	173.9835	116.59	51.53098	147.1016	300.5975	34.10795
3.604628	5.295742	70.56238	8.596426	5.431246	72.30131	10.3069	32.5441	49.07164
2101.775	1737.198	2158.479	1739.195	1115.218	1526.699	2133.814	1697.04	1904.205
57.692	36.93645	23.64957	42.38354	28.09169	33.4943	42.33387	17.48537	16.47354
68.20539	39.19994	551.9546	71.78493	69.11313	160.7999	55.87729	7.035228	105.8002
154.6228	148.5867	1155.909	95.98781	192.1961	1532.029	261.256	1382.103	1333.484
975.6815	808.1053	406.35	782.1913	1047.179	703.1583	1165.229	635.4055	585.685
89.22447	99.5697	0.680381	58.85776	86.31844	0	7.107199	16.32481	0.08444
207.6183	43.05149	0	18.97799	499.6228	0.810089	110.2695	56.97152	1.352262
421.2045	288.8814	98.64368	410.1737	850.5825	109.1193	123.7977	714.177	50.48766
112.2042	81.63117	50.44238	103.8373	105.7943	54.97558	101.4981	44.19534	108.291
50.89592	19.96736	36.75119	35.47921	29.15697	30.07483	19.92452	45.26238	17.71094
112.0497	95.44584	218.9066	100.3255	136.0465	472.0767	137.4073	123.1714	322.3095
1011.425	899.0334	320.617	392.7877	592.7034	1516.475	453.0683	1298.563	588.1986
26.87149	54.00124	1.440188	32.0003	39.94122	5.958249	35.95518	17.47726	20.2276
102.8197	108.6672	20.56711	117.714	100.3911	40.3553	47.09105	215.8451	96.9517
94.13197	49.26712	52.78371	220.9374	36.72782	38.64657	32.70285	149.6596	12.70381
72.73252	49.2691	16.74204	96.10078	80.95083	30.04073	44.71354	56.9276	13.96238
16.25194	10.72126	2.200771	29.47229	16.20241	18.04574	9.527839	13.96824	6.393666
578.309	708.7261	695.7294	378.9444	377.8773	814.074	475.4523	1224.219	1482.149
293.3486	184.8604	140.7748	134.9461	197.5694	231.2438	103.8233	119.69	297.1571
33132.94	18605.85	221.1198	15540.49	9892.078	3291.526	12468.08	40786.02	762.0263
7.998483	38.59363	28.32818	4.270297	14.05836	11.98743	23.15605	16.30313	48.95157
121.1338	103.303	106.4737	62.28495	55.07213	28.31553	81.53177	37.22097	24.03094
16.77144	53.93228	59.72721	5.135293	27.01388	36.9364	51.1442	25.60626	135.6619
8.614522	39.26784	49.00037	10.32703	24.85334	18.00118	50.36464	33.71339	116.7773
103.3024	3.764823	181.4215	16.38298	247.1896	307.8524	120.6387	27.94687	167.4949
66.46285	49.27923	22.10894	93.51421	37.80382	49.84452	25.50337	92.84857	50.4009
9.23964	18.39511	19.80603	6.865366	3.270298	113.5547	18.2987	46.49439	106.9189
269.796	195.7715	41.23815	190.4113	120.9157	28.30825	119.8659	33.75235	40.4049
363.5275	247.2699	289.3265	307.1798	253.706	411.8147	206.148	444.8036	197.7803
45.39155	36.30218	44.55817	8.60307	10.82184	17.16717	15.94055	25.54496	2.62326
45.66112	41.54096	58.14681	26.77369	33.49249	149.7179	14.29989	328.1473	30.31525
3422.795	3536.889	367.3015	3445.56	7573.068	1126.08	3836.115	1149.988	772.1066
1597.277	708.0274	381.8888	977.8419	1374.196	305.9896	435.5192	171.9747	1152.119
21.96879	63.61627	8.340145	13.81907	8.662753	9.412163	20.78907	4.710189	15.15048
22.60315	27.03008	6.033274	17.2953	17.27925	17.18158	21.59362	7.027967	12.65216
480.885	231.0944	590.3776	443.0952	185.7025	202.8519	282.1195	175.4312	195.2529
119.2311	70.09206	40.49561	96.93945	38.88956	43.80236	39.09673	43.0234	45.40739
11434.26	5427.416	175.9556	5315.426	3260.312	1833.569	4197.286	6481.374	395.5512
16.8469	21.55573	3.729706	19.0177	24.82773	14.57881	11.12312	11.66606	22.67786
142.0003	80.93603	24.40514	104.7663	34.57009	62.75135	57.52574	27.93386	16.47939
46.61357	41.71253	8.32926	33.77777	25.90914	13.71313	31.2001	18.61437	6.400986

6207.654	4606.148	2508.203	5758.454	1917.341	5055.564	4295.584	5049.23	2211.558
105.7554	184.0595	132.3322	74.35675	68.04692	662.9166	121.3998	185.8912	1978.415
36.97971	25.37705	7.555995	45.88768	37.78208	55.07495	19.92178	20.95162	11.4356
13.00271	14.54243	65.85838	21.582	125.1468	16.27638	40.72116	39.52453	41.60482
0.490494	0.685724	27.53483	0.815284	2.190849	62.86769	0.724984	4.713341	17.70377
319.0841	161.7359	143.0743	254.4047	145.7611	138.3656	221.3698	219.5357	166.2867
13.0512	32.41497	59.98971	16.41181	11.90013	22.34327	15.93879	19.76642	7.655537
46.45625	37.75819	12.1563	39.81393	44.24832	15.42471	19.92212	3.550972	10.17872
84.58722	35.35333	62.71775	32.82943	165.1166	110.9221	83.90735	63.91553	78.10258
125.2267	67.70164	217.3822	86.47826	68.04482	436.8224	104.6268	155.6773	327.3277
22.59622	56.59142	14.50091	10.34342	9.7408	3.381109	44.95806	1.223181	7.648242
11.14586	34.71022	2.96474	20.74366	10.82438	24.05693	19.94155	2.387932	0.082118
255.0584	198.7299	49.65105	185.1469	187.8578	101.3866	234.9538	63.9527	70.63222
88.01597	44.67971	18.28473	37.18986	31.32422	64.51873	41.54506	42.98356	29.02821
114.6929	29.9501	307.3093	167.9233	142.4836	7.678691	114.2911	98.74175	13.96424
30.62784	28.44963	18.2859	26.7886	37.7931	49.01079	27.12074	69.62011	18.98152
525.4514	334.4002	121.6215	424.056	306.5864	189.9547	198.1634	182.3959	148.6809
61.55755	33.86913	56.70987	37.19809	37.78939	14.56047	65.62777	3.550707	26.50807
252.512	113.1621	294.6944	116.7636	173.831	119.4397	131.7967	270.6442	1232.117
195.4735	265.04	243.4126	108.9797	260.1682	204.5836	500.4866	132.4619	107.1361
2158.32	1794.277	894.7127	3371.28	1718.662	807.1639	1114.826	2160.411	696.5237
48.80646	113.3711	9.084673	13.78714	36.72905	22.29528	43.10659	91.71802	49.16506
881.8238	346.7252	38.93462	474.2563	144.6865	13.69745	127.8011	579.4551	17.73845
133.1873	214.651	10.61537	107.3685	10.82962	42.09019	31.10251	4.712353	15.22094
43.78429	16.08233	102.6776	51.03203	42.12168	109.2536	45.51311	55.76941	539.6556
849.019	1328.152	345.8711	809.0018	1144.35	360.1324	2304.162	306.716	614.6657
40.59146	9.148116	55.80328	24.17065	36.73638	117.7833	51.08116	47.67926	687.5039
297.3349	267.4712	99.44983	182.5961	114.4476	174.5246	191.0463	11.68283	63.06303
160.9009	129.3296	504.4214	252.6204	142.5355	833.081	166.1511	631.8382	706.4117
9.868702	28.40825	276.6216	9.461487	29.18147	123.8019	193.5262	4.71003	220.176
211.8154	125.513	116.2756	159.1899	64.80624	168.4718	69.44917	321.6749	55.52095
137.7394	58.56589	61.25905	114.3444	21.62026	41.24056	39.12284	22.12732	16.47525
237.9848	601.6676	95.57554	388.4761	715.745	377.3459	686.5117	197.5277	170.1245
2866.444	2209.506	1344.027	1698.535	1511.406	3161.843	1296.23	1727.226	2655.953
0.509174	0	1.475917	0	0.017178	6.09922	0	0.034166	23.98575
39.41569	63.98171	45.90498	35.44721	53.97642	40.38117	29.51271	119.4647	31.55229
28.05663	43.07606	5.261329	38.89505	96.06571	1.666673	272.9871	11.68455	0.085504
521.9071	664.0452	466.0725	511.3464	295.8324	666.205	564.9788	700.4277	1328.516
113.462	109.4118	58.10654	38.8872	128.451	76.48519	95.09563	150.9352	78.12139
144.351	129.5268	49.68903	144.582	64.78651	101.4612	39.89233	39.54645	49.18644
141.94	74.73909	108.0245	159.3561	52.91241	22.29474	90.35215	17.49049	12.70434
204.7472	42.29802	13.67658	140.2482	411.0259	4.241898	114.3397	29.1015	22.77591
134.0957	157.9555	68.0456	254.4864	206.1661	45.50522	86.26088	17.49254	109.6036
96.97765	131.6519	284.715	160.8982	106.909	543.372	125.3958	288.0758	238.0709
10.53665	17.69232	2.200716	15.55082	79.70006	2.521616	14.34229	8.188502	2.623083
91.24904	57.85363	1.44051	188.2111	105.6903	5.95816	34.34265	31.3873	0.083784
39.57976	34.69957	6.792212	52.86874	14.05991	14.56999	10.31363	153.8556	2.623127
32.52846	32.32038	29.0374	43.26744	22.69432	58.49891	27.1251	62.66082	31.52833
21.89886	12.25329	5.261112	32.91133	15.1344	10.26617	11.92269	48.6658	8.911992
67.04923	45.40168	12.14623	85.69029	161.8412	9.396057	46.30935	27.9354	8.925832
9.890194	14.5736	14.47728	4.270432	57.13885	14.57498	22.35841	40.58334	46.43513
2978.686	1819.671	1336.384	2881.457	1158.394	1158.768	1367.372	2201.09	1294.687
99.24363	52.37575	11.38169	79.65001	63.68639	17.13659	54.34002	22.12867	6.405834
288.1316	223.3241	231.8321	108.967	848.5169	709.2218	226.0911	284.6337	6735.077
664.424	593.2753	201.2264	664.5986	380.0134	265.5948	529.8987	415.8337	228.0259
4.862569	6.074392	12.95874	0.814611	7.585458	12.86236	2.315711	8.186626	22.64478
35.91378	28.56085	19.11777	34.67475	10.81993	12.85872	7.918396	12.81837	1.3586
157.3705	168.0112	64.98835	126.3338	92.85644	79.05322	144.6793	92.95103	122.1609

9.88404	19.20932	22.16057	5.1355	21.60473	32.68056	22.34665	4.713092	27.71313
68.3217	44.63442	22.87212	107.3668	58.30211	30.90079	51.91943	180.9476	21.51105
308.1553	262.9156	16.73808	79.57256	90.6989	112.6032	90.26876	3.546455	12.70375
799.347	779.8503	51.94673	548.64	144.692	153.825	194.1389	152.2214	36.63261
120.9234	124.7958	92.56831	225.9183	172.7109	58.40673	160.6713	108.0434	64.31311
101.4955	105.5361	140.1204	63.12723	30.26085	67.0152	106.2858	65.09062	217.6636
736.7971	1035.927	2.976753	234.4713	578.5846	160.7111	150.9815	730.4051	15.21761
84.76165	87.16326	36.68811	77.92026	25.93594	38.65602	44.72884	59.22846	25.27479
9.253236	19.20655	6.025682	6.866861	49.61811	11.98494	23.94905	18.62388	11.42841
24.33842	40.05482	45.94404	25.92501	42.10224	74.01789	24.72148	25.59469	50.33364
453.3057	379.1208	255.65	282.9578	159.7962	203.7154	131.0021	133.6279	352.5485
138.3948	312.0826	127.7587	134.0827	136.0462	103.9692	183.7876	271.7727	231.7165
733.3279	568.5759	388.798	410.9926	292.5844	517.5113	576.2374	278.8168	253.2172
4109.015	4658.624	703.3212	2720.426	5804.787	1314.344	5627.957	417.0625	1245.622
31.37857	21.53968	10.6315	44.19676	10.82392	15.43529	15.93086	46.37043	12.67775
31.32361	39.32638	32.14618	17.26555	32.38713	20.59772	39.99419	23.26164	32.74226
26.18025	111.0564	9.084662	31.10444	61.5402	32.62045	48.712	16.32911	35.34209
0	0	3.085099	0.829569	1.098162	2.578598	0.74412	2.331512	9.659715
12.44284	13.04524	32.25848	4.271778	83.98331	2.521693	35.28393	0.050174	3.882499
0	0	0.682647	0.8182	0.020496	6.027354	0	4.659459	130.894
15.59849	16.91579	9.873645	17.28786	16.20654	41.36555	11.9316	35.92449	23.90751
133.4712	113.2198	146.208	158.3631	63.72148	149.5885	70.26481	162.592	83.18689
28.08161	54.69622	14.44595	70.1155	42.11623	58.4619	43.12523	55.75295	34.06924
715.0844	556.9897	428.5944	608.2999	346.562	643.888	465.0988	441.4115	790.8087
195.6131	80.82309	483.4636	134.9761	52.92957	220.1252	89.45584	362.1964	78.16071
14.39617	17.74342	3.734439	21.67188	11.88723	18.93494	9.542017	3.547819	6.384081
1487.829	843.5566	40.47363	1396.611	1254.439	158.9733	306.8053	2508.71	46.70162
222.5127	102.3928	58.07646	244.9059	73.4405	111.7154	97.437	219.5113	109.6384
50.62353	54.61818	162.3324	19.84328	24.86412	97.12042	97.47303	141.6867	369.7823
14.93846	13.02561	29.10746	8.601367	24.83232	18.02427	7.112395	8.192965	65.16226
460.724	441.5058	235.7071	240.5253	156.5633	841.7953	234.124	39.56219	440.7019
251.3038	191.796	98.6534	369.5354	243.9805	280.2636	313.3302	164.9731	119.7244
191.8376	82.3638	77.99953	139.3036	140.3473	73.88357	99.05236	117.3403	149.8654
199.0945	140.3395	32.82603	192.2429	43.2074	93.71495	67.90475	12.84621	79.34909
28.1724	18.4279	20.61291	44.17115	17.29589	30.08336	13.51647	25.57466	18.95742
23.04891	24.57088	58.17315	16.3854	20.54229	66.20802	36.71336	41.84613	237.2905
181.2757	110.1592	26.69006	186.1028	99.3279	46.36904	77.476	143.9996	30.33312
10.57093	2.218244	20.71489	8.614428	6.50469	16.33453	4.718052	17.41432	71.0238
0.487825	0	2.203362	0	0.023703	0	0	2.385541	78.13521
95.35766	95.59466	26.70166	64.02281	37.80859	99.76602	99.96607	16.32944	50.42469
66.61698	46.24811	12.9198	30.26162	45.33471	19.72656	60.81695	18.63873	22.74213
21.16183	33.83145	85.03745	15.51919	49.66985	90.3182	23.10133	8.19939	64.21525
149.0575	261.1923	297.0432	155.7223	107.9832	339.6179	143.0045	123.1715	75.66542
33.69639	26.099	125.5979	14.65178	28.10084	104.0319	36.68842	45.35236	50.45121
572.7973	469.9269	201.2177	461.1863	376.7832	336.0886	510.6798	235.8483	336.3134
70.26596	84.84267	13.67991	47.57788	102.5025	49.85033	67.9674	2.386067	20.24784
12.37849	9.918049	116.6263	15.52197	45.34511	43.83298	29.51836	45.30693	54.1292
116.7582	73.96738	48.93851	55.35825	38.88755	43.80666	26.29716	87.08553	47.91211
61.45091	39.24695	134.2455	80.52322	14.06717	61.04858	13.50261	242.2455	16.47547
225.8188	208.1165	8.323007	123.7304	161.9201	137.5491	82.26585	257.7305	10.18306
144.443	104.8563	50.4698	102.1477	48.59805	45.52658	61.51659	32.57863	20.25607
219.2411	390.6198	40.46591	358.2346	221.3295	147.8095	285.2883	477.3375	24.03635
48.79765	108.7195	25.9343	157.6172	887.5652	30.89751	71.12642	75.49374	75.55207
37.58933	13.00744	29.81327	31.99849	19.45737	43.85471	25.52751	118.1943	30.26522
143.971	173.2432	221.8815	152.2326	250.4885	632.7046	206.9094	641.1623	1121.916
599.1111	1129.607	557.2318	376.3692	470.6985	138.3436	887.1996	29.10442	119.752
34.99516	47.73664	45.89189	25.91211	27.01577	59.31866	35.91282	65.03073	105.6122
105.1997	153.3111	64.97686	87.3557	111.2099	72.16323	231.8502	52.33271	134.773

162.3958	150.2654	45.06952	120.2719	201.8379	117.7669	83.07031	20.9774	41.66173
64.60455	59.33919	17.51228	44.11284	32.40774	67.07963	33.51577	34.88392	27.78611
28.0453	16.85788	199.6908	0.818983	145.7737	703.2493	72.63841	365.909	3440.216
163.4743	174.0647	102.4818	179.0894	141.4451	209.7407	153.394	452.8818	209.0803
86.52249	57.74279	30.53121	82.21179	43.20487	90.28488	40.69858	62.74174	66.76697
30.01791	27.68776	19.05928	29.39606	47.48886	11.97918	17.51306	33.70129	84.13724
149.2111	88.55238	125.5359	107.2841	121.9904	74.75303	87.07213	51.16596	76.88582
11.12438	18.40627	6.023889	25.92077	19.46013	35.22369	23.91453	53.40959	54.1159
73.48207	74.83407	16.74939	49.32543	49.6571	12.83726	57.57447	15.16356	22.75337
725.0194	620.133	1114.534	372.026	1640.866	339.5099	817.5739	356.6489	194.0537
106.5317	60.98612	26.74817	61.50999	37.78223	21.4539	59.24087	3.55096	17.714
155.5923	134.8896	60.40878	36.29063	63.71295	139.3111	75.89197	33.74765	89.43002
325.4338	218.0409	409.7161	160.9253	99.34563	97.95402	210.1967	47.69165	202.7525
337.5228	154.8513	28.98467	405.146	261.2091	30.02708	79.8558	147.5198	17.74236
9.334755	15.44074	0.677386	7.754743	12.95439	6.83783	10.36424	12.77768	1.357852
967.1244	408.2599	309.9228	944.9553	421.0465	314.5873	517.8549	459.9947	175.1597
14.91643	29.2618	10.62542	13.79797	44.23871	27.49526	17.52425	17.46961	10.17548
825.893	967.616	186.6611	777.8709	1623.622	387.6481	1172.44	559.9074	380.428
28.66773	36.10972	1377.683	12.92574	29.18203	871.8817	374.8384	386.7718	267.0229
269.399	148.0071	42.0141	242.4479	65.87157	47.23377	98.29944	11.68433	44.16825
82.67433	17.62008	95.67188	25.90222	30.26007	37.77139	51.08471	54.64	230.1735
370.9733	302.7315	362.8024	261.2863	85.31987	513.2476	224.5159	823.371	273.3348
38.11263	36.9072	59.67561	38.03152	20.54421	68.76795	39.90113	49.97943	106.9449
425.036	271.1763	199.7302	466.4641	211.6079	101.384	351.6854	116.2126	261.9586
1158.646	1013.798	538.7692	1485.701	2591.977	865.629	1112.441	1410.104	629.7756
111.6425	71.62326	41.25655	98.66084	53.99734	68.75409	47.89447	62.75114	46.672
12.48957	17.74696	16.85787	28.64639	9.73404	13.74549	3.116452	11.63994	17.60241
494.5563	396.7778	241.0581	445.6504	292.5714	87.62442	322.8606	356.5894	151.2174
446.1745	794.4192	12.92123	902.6028	153.33	43.78194	287.6569	594.6683	671.1451
887.9419	1056.222	694.1891	851.4248	535.4889	898.3208	839.9223	758.512	591.9756
55.14342	49.28655	23.64469	79.65142	28.09388	32.62829	23.90462	74.29687	17.7338
26.25574	36.2143	11.38986	34.61021	29.15755	18.00939	14.3135	18.63272	20.22081
23.03303	48.47405	77.30137	18.11384	49.68037	30.03435	69.50991	136.9695	261.4294
25.54294	43.81606	288.4864	66.56864	89.63863	605.1937	63.04924	63.95422	688.8433
4.235981	8.37702	68.85972	4.27226	4.350177	155.6972	28.69223	8.198811	21.51721
4.230443	4.526857	3.729444	12.07038	6.50935	7.681854	5.51165	23.24082	3.884489
1213.346	976.1223	313.7335	496.6362	1356.972	437.5165	1132.506	470.4698	471.0796
125.4442	138.7686	91.0865	75.2635	60.47369	114.3615	119.9287	56.95578	41.64785
16.87096	6.84628	10.64476	21.63537	14.05135	13.72302	19.97338	18.59703	8.903581
221.362	202.6831	111.7125	175.6731	45.37402	112.5908	158.2511	106.8921	175.014
201.2762	280.6073	28.21948	96.88176	212.6437	51.52434	263.0711	9.358808	36.63074
11.13465	16.10334	32.13966	32.01045	8.668894	40.42393	10.30996	24.42259	86.55695
140.3084	211.8852	190.5967	156.6019	125.2471	259.659	119.0303	148.699	141.0923
25.59936	35.41559	14.45321	22.4579	17.30119	50.74379	19.9144	61.49959	25.25344
7.989521	10.69261	76.73651	5.134956	12.98536	31.79067	42.37353	19.79546	226.7458
18.01652	7.60659	27.46694	6.865577	3.270124	5.959037	32.69754	10.52288	719.2987
8.095174	14.70616	13.83317	3.413134	6.495274	5.116904	0.724033	4.695756	85.24697
107.7153	167.2	107.8811	84.75979	113.3672	150.4408	97.45445	301.8569	50.47694
421.0214	1330.672	627.6399	221.4588	553.8259	61.83454	981.482	12.83783	13.95142
1.119694	6.071699	6.794473	4.274335	6.509166	11.976	2.32315	10.52097	45.44191
0.488361	4.531091	13.74055	3.406478	6.506223	32.75502	3.914921	2.387705	58.70184
144.1678	207.3522	119.395	94.29132	126.3097	46.36672	115.8687	85.99002	123.4246
0	0	0	0	0.020683	0	1.529979	0.041441	40.29919
81.52789	63.93551	33.60368	88.29228	70.16885	54.99979	26.29872	116.0579	40.36821
102.8361	132.6144	59.65613	45.81993	55.07643	74.77823	36.69174	80.15151	113.2731
955.18	457.5705	269.344	616.9453	421.0487	390.2433	526.6404	439.0956	114.7157
146.5042	553.2653	326.8314	195.5163	116.6245	442.7535	210.9345	138.2825	161.2865
389.2108	232.6137	316.8811	271.6832	214.8521	331.826	268.5018	253.2407	221.7063

3446.531	2917.422	996.4784	4706.274	1657.165	1966.819	2212.078	14407.44	3235.385
144.1596	49.21769	2.972775	172.2246	347.5054	3.384951	103.8649	454.962	11.44337
80.67572	421.3777	435.5014	70.89459	115.5477	166.715	346.0033	309.0122	1547.292
46.47354	40.86353	23.67924	35.4799	25.92288	12.84257	39.9839	20.94887	3.885019
1622.426	1053.849	1121.299	979.4669	889.5848	1299.77	1342.614	1492.581	1292.146
79.44327	237.3065	529.2226	67.43794	148.9958	359.4162	122.22	151.0309	469.4668
9.871699	24.59406	67.48461	9.462187	17.30078	20.58806	43.16741	29.06872	35.28449
253.3149	152.5209	120.8896	299.4722	113.3726	150.4264	119.037	142.8878	84.46243
6.117359	6.844161	54.61854	4.270997	8.665087	16.30742	5.513168	9.348246	53.8607
10.49289	9.146417	24.40605	4.27128	6.510839	109.2582	9.504235	9.361146	204.7624
235.9295	97.03975	67.28973	120.2756	116.5942	37.76798	128.6816	114.9962	31.59238
391.973	345.2805	87.17557	458.7553	150.0728	114.2938	159.0111	151.0262	93.28084
12.40971	20.76545	20.62969	6.001648	2.190995	11.98779	9.514886	29.02899	48.94712
3.615255	12.31241	12.23164	3.408968	5.424789	5.108762	5.526311	12.78322	39.89375
97.215	97.89297	92.6572	81.34455	41.04718	135.916	71.92001	19.81315	47.9198
33.73814	42.33047	113.4727	38.04194	12.98819	84.29754	33.51095	39.52631	78.00828
121.4353	180.2138	241.0807	179.9445	140.3699	224.3457	375.6655	176.5963	324.8924
9.936259	11.51353	9.122065	5.141185	9.735748	8.556287	9.5391	25.4838	17.61454
248.1641	240.3627	110.9053	260.4586	256.9326	75.59191	204.5699	329.83	102.103
35.09038	29.24715	25.98185	43.28656	20.53343	14.56393	19.92292	37.16357	8.921277
162.6114	80.88994	45.09098	100.3966	90.6769	24.87297	103.1358	87.10736	12.70481
741.9446	287.2678	67.25756	700.0034	5040.072	25.73433	1122.925	426.3339	83.23017
1.107154	0.684638	22.20936	2.540852	7.585906	13.72354	2.315678	27.83616	36.37108
47.51674	57.72868	7.555603	105.589	57.23489	4.241954	82.31088	9.360811	15.22356
616.236	444.5658	167.5485	464.6917	193.266	374.8133	262.8969	120.864	199.0556
128.4604	105.5129	44.30179	165.297	93.93769	97.11496	87.06553	128.9322	46.69732
73.23516	57.70913	23.62786	72.65322	79.90255	116.0594	75.88084	52.32291	47.9473
127.7568	134.7748	78.75359	159.1981	124.1682	212.3478	128.6261	163.791	74.39943
1330.85	1203.59	192.0285	344.3484	423.2027	400.5686	198.9191	437.9236	69.37793
27.43382	21.47844	147.9437	15.51803	77.72232	43.80659	32.69877	26.77597	25.28679
25.77233	19.25379	9.108043	18.16233	20.51044	18.9068	14.34786	19.74786	6.394685
99.56468	158.7263	59.61886	50.13424	105.8108	85.92848	143.859	25.62353	55.50862
35.10015	53.2572	22.14244	32.87708	33.46777	7.678582	50.42248	22.107	8.920597
9.866863	6.066075	34.38524	0.816334	4.350974	67.07574	7.107227	15.16599	461.5774
106.8473	50.83997	3.730932	256.6058	37.80026	0.808584	33.51677	27.92593	0.084612
117.8427	101.6852	45.84181	77.85053	99.32372	209.0074	55.08102	153.2664	128.4141
24.90653	49.20581	454.8172	18.11469	213.7627	1215.187	51.8604	34.9162	138.6082
75.77215	211.3265	9.085788	87.38589	48.60559	69.60535	91.10141	27.94282	12.70478
108.2713	57.06831	17.52035	87.50957	47.49434	8.537298	25.52081	80.03231	12.69719
17.44331	13.78981	11.39447	18.13554	33.46092	16.29179	19.93369	68.36638	21.46074
70.62045	37.79848	15.23714	24.21147	18.37213	11.98514	21.54174	3.5511	13.93715
92.64237	122.457	145.4177	140.1654	328.115	41.20414	245.4412	52.3335	89.4896
4571.554	3401.292	3951.907	3754.475	2744.267	2872.923	2996.931	2262.715	4174.771
7.988181	12.9979	74.23361	0.817005	6.510462	12.83435	8.705652	24.45748	130.8429
58.74364	227.9603	376.555	331.3759	114.4679	582.0071	222.1056	94.15464	876.3349
24.28112	51.54212	73.43355	25.90185	17.30732	215.0387	62.28302	103.377	343.2701
110.8054	77.72246	294.0049	89.94177	103.6638	210.6169	101.4312	310.0634	424.1719
127.7402	47.76315	12.91584	56.26043	48.57996	81.7447	14.3045	42.98933	31.54429
26.15927	53.0583	195.1467	32.82239	397.2418	312.0793	142.1976	14.00446	172.5892
186.3063	180.3787	55.79855	153.1908	84.22076	45.50911	74.27636	70.8953	54.2401
19.29142	19.9488	129.7105	19.85364	35.63903	35.22113	18.30706	16.32366	45.34447
133.7402	104.8562	22.8689	68.35244	34.57241	66.18461	100.7602	2.385074	15.22237
1.107699	0	2.975269	0	2.189166	9.449383	0	1.222012	34.76539
0.490823	0	0.680181	0	0.02152	6.008405	0	2.375183	152.4533
18.80768	12.27756	16.82659	11.21564	14.04657	45.73271	3.114776	34.73352	1.358692
19.28828	19.94676	12.14914	13.78962	38.87613	33.4957	31.91989	47.62723	75.45487
66.867	79.71937	31.40614	29.4261	20.5255	34.41166	23.95721	2.387942	20.19668
179.8437	155.594	77.22231	179.1109	118.7715	146.1193	112.6325	159.1467	139.8343

1356.905	2094.239	4.509566	2205.805	391.9094	24.01555	596.9529	1704.994	21.50917
1.117412	2.224267	684.1632	0	0.02641	486.2395	3.916507	2.38404	98.22593
0	0.728732	3.175384	0	0.012272	0.837055	0	0.024162	7.105573
105.2148	22.33648	1.43821	45.09707	18.36359	5.10061	14.33629	4.711873	5.142097
1470.108	895.1295	802.8509	1432.897	1737.017	610.2949	1611.957	1748.107	1015.12
25.68714	70.42971	1.438623	7.734441	21.60105	2.521636	22.35652	7.033653	3.884626
26715.53	23376.03	22653.4	32468.89	41568.66	21998.32	28211.25	18389.53	32105.17
63.82211	51.5419	143.9709	35.4236	63.71502	181.4712	55.08228	61.60539	95.72685
250.635	197.1742	78.74099	229.283	181.3861	195.1105	174.9709	401.8355	132.3217
192.9452	229.5604	232.673	108.977	224.5567	253.5949	126.2054	332.1625	156.2304
545.8365	629.4538	236.4347	410.1253	292.5851	582.8556	225.2991	289.27	290.9896
1218.811	652.4454	1151.932	483.64	4492.992	806.3087	2968.372	80.21271	249.4687
702.8974	520.1482	20.56885	357.3795	270.9775	24.01103	250.1191	526.0816	34.1146
5.483262	27.72457	107.7066	5.135411	5.430854	17.15508	10.31339	16.3075	83.97229
57.67283	29.20695	44.36575	47.57959	27.01438	65.35617	17.50287	84.71872	27.78644
30.75097	29.29298	21.40117	12.0692	117.4284	24.06122	44.05958	9.35258	12.67666
114.1051	161.1385	113.3148	44.08325	49.68494	98.86246	81.49527	36.06901	103.2539
428.5718	390.8858	44.29728	181.7257	164.0913	101.4044	125.4436	83.68033	75.64873
40.61756	38.44563	40.49496	38.02864	44.2846	29.17495	83.92765	94.05783	69.28468
413.1481	310.5051	62.66484	115.9007	149.0026	102.2454	214.1574	22.13753	95.81204
203.1742	144.8417	42.76837	95.15196	102.5733	124.6384	94.25964	145.1868	35.37139
1.106782	0.684461	23.77256	0.814554	6.506761	12.00228	3.91441	18.58655	169.3391
2019.045	2012.246	1371.571	1833.514	2354.533	1280.831	3131.264	1434.544	1891.613
404.9223	296.0926	95.63714	109.8779	62.64083	133.2504	173.481	50.00651	81.92185
24.28417	15.30893	125.6003	19.84415	51.84168	93.70506	31.88933	34.90678	85.64767
75.68362	26.94883	10.62771	31.15494	10.82524	8.540544	45.63697	2.387832	3.884973
481.31	379.0162	32.04917	674.12	653.1026	234.6418	515.5087	501.762	85.74954
1906.816	2793.435	1380.74	1830.903	3257.046	1102.012	3174.373	902.5828	657.5043
483.8763	320.4646	187.4542	335.7226	302.2879	438.4424	168.5593	175.4464	254.447
46.9246	72.43346	10.61529	64.02784	29.17603	57.58335	35.10304	72.00478	16.4798
2.35372	2.218075	21.39383	2.540902	3.271394	2.52171	6.310728	3.551076	350.953
192.5483	139.4629	360.1418	174.8306	127.3872	69.59053	118.2726	81.34574	132.2214
272.1377	123.2164	106.332	217.2119	106.8978	171.928	55.06264	103.421	100.8227
38.73303	23.01856	19.80305	65.74743	58.31004	154.854	24.69461	139.2784	44.15107
23.28607	11.50749	7.576728	31.23596	4.349402	5.966004	6.321166	40.47793	8.891239
28.80725	17.6553	19.07642	31.15053	14.06104	13.70642	15.92203	39.45971	11.43035
4.911644	8.462218	0.678126	3.415591	6.491776	11.19571	1.521386	13.88207	0.07283
10.50581	42.41646	5.259107	55.44653	43.16584	11.12105	7.908855	0.051872	3.885032
7.392684	2.21793	0	2.541194	0.024556	106.3815	0.723781	3.549061	7.641833
82.77965	92.51104	170.2244	114.2907	85.27044	109.2426	31.8998	48.81621	71.77411
62.01929	55.44056	17.50729	90.02555	73.4047	60.1651	34.30193	82.44072	55.44383
56.43366	59.35185	27.48628	56.25549	90.63185	28.3284	59.16673	87.02061	26.52544
67.32394	9.150751	18.29813	51.09762	18.37688	23.17818	24.73393	73.0374	8.92128
181.9559	83.94098	58.1034	147.1414	75.58489	47.23189	105.4941	103.3782	45.42927
63.00236	21.52765	32.16053	46.78415	5.430891	19.74025	24.75076	15.14991	28.97166
15.63976	41.08023	20.6975	15.56504	39.8629	8.553104	23.2248	0.049607	26.34956
72.02684	34.58401	68.09088	38.89182	27.02088	87.6883	43.89282	112.627	113.2739
35.62767	50.83026	206.4616	30.24456	31.33038	51.57044	51.13489	14.00551	79.25807
24.36605	47.05867	0	36.34748	99.1919	5.958502	11.91104	12.83676	2.622682
1.116061	0.688412	17.50527	1.680076	0.026306	40.3598	30.29426	9.361017	40.38027
19.33339	17.65503	14.46531	13.79861	83.01505	11.9837	26.35162	5.874747	11.43049
137.8404	140.1997	95.61931	212.9032	193.225	60.98295	209.4479	139.3938	76.90445
44.41265	101.7969	0.681173	78.76311	47.51549	14.55471	19.89891	16.3287	0.08514
261.1167	289.987	16.73794	143.6671	96.08926	0.809906	79.87908	46.52015	11.44429
2.985098	4.528338	98.81753	6.86548	106.843	74.79743	14.2998	16.32937	229.9321
50.63427	86.25388	103.3279	29.36385	32.41921	36.04985	123.8999	76.69254	135.9553
61.0079	67.9706	0	59.7805	31.31324	5.098828	43.99451	11.67754	3.885002
0	0	5.320621	0	1.108289	4.265231	0	0.043887	25.93854

152.742	42.28147	40.47555	175.5938	109.0699	2385.62	608.9117	309.0384	1153.603
0	2.217935	151.7103	0.814493	1.109864	12.00667	0	7.024963	12.64046
83.83737	125.518	222.7627	79.55624	84.23468	337.0643	101.4333	65.1106	215.333
275.7399	170.9718	113.1978	186.8735	472.7983	163.2963	238.9423	61.63058	300.9629
11.15858	12.25714	22.95761	15.54481	14.05505	28.38872	16.74383	25.54497	50.14323
6.739692	9.15588	54.52009	3.405275	3.271394	81.8932	8.712368	0.051386	121.3972
93.42849	51.56362	25.16546	138.5307	94.98848	42.08053	48.69973	205.3643	13.96398
1360.018	976.1134	508.9414	1084.227	592.6988	893.1768	555.3855	779.4038	445.8998
211.5008	110.1754	110.2301	109.0298	41.0532	139.2988	67.08266	45.3574	80.64173
295.1064	250.3295	643.1074	72.62547	182.4722	602.6895	227.7177	47.69317	463.3906
43.97185	31.58375	32.14825	45.03636	18.3745	14.56682	12.71433	45.24951	6.404187
304.0478	336.7653	89.46839	327.9981	265.5592	104.8301	228.5759	83.69188	123.4927
1686.69	671.759	4.509439	1209.724	1304.073	14.56124	380.3451	907.1436	8.910124
4485.526	4089.969	1728.226	6198.889	2407.462	2329.588	2874.603	14754.84	4260.512
36.21881	100.2007	16.73937	75.27581	134.8992	54.98859	73.51617	41.86437	55.46451
8113.215	4681.782	1483.31	3750.16	2171.023	4466.773	1392.911	1044.288	2185.063
7.987353	26.90048	81.27607	6.865061	6.511013	17.14	59.98361	14.00297	46.59632
159.0895	131.6713	324.6138	212.8517	115.5394	235.545	174.1875	269.4568	199.0071
47.57175	94.09189	6.789302	12.92224	72.3185	26.60027	63.94482	8.199391	36.589
81.95283	54.60172	101.7247	16.38372	58.32949	544.3604	33.4786	39.56213	406.5624
24.90421	29.17568	139.2885	73.50339	61.56501	181.4027	83.85336	164.9318	104.5867
0	0	0.722138	0	0.012338	2.620743	0	0.024295	10.57139
11.12769	9.148874	29.04144	5.134955	15.14352	77.47323	7.107696	22.11616	102.9284
26.85019	17.63559	106.6766	13.79118	7.590566	99.00614	11.10563	25.59788	96.71349
113.8061	91.05055	30.55433	74.46199	27.01335	57.60933	11.90362	115.9833	51.63293
58.85792	50.02783	98.04074	41.49599	117.632	57.57669	68.72371	37.21952	91.87867
479.8289	117.8058	106.3204	351.3856	139.2813	1.669068	285.3739	299.6158	6.397482
165.0165	137.9738	47.37843	66.59711	25.94271	164.269	117.5107	23.29898	51.71416
1.739586	0.68866	16.73874	2.544054	3.269786	69.61335	1.523849	20.97543	233.8484
1716.631	1513.921	241.7733	1252.957	1123.82	594.837	969.4088	48.8502	89.52592
662.4712	499.2157	195.0941	278.5815	235.3724	834.794	432.3403	334.56	194.0415
16085.01	10914.13	2939.12	18508.43	5685.024	6418.069	9344.032	29175.41	9621.666
64.2143	41.65002	21.37853	29.41059	19.45295	34.3918	43.2062	7.035931	28.98542
321.2267	83.90777	131.6357	226.7637	86.38707	57.54376	83.85747	149.8356	54.25238
7.362782	9.921952	63.67177	3.405484	5.431194	50.76231	11.10903	18.63427	122.8765
20.61198	19.21125	20.62399	38.10823	18.37084	35.27176	19.13765	32.50514	32.71351
8.056427	6.867567	2.202705	9.494035	8.653625	10.29581	12.78289	10.47549	16.32912
16.77774	25.35586	24.42238	16.38885	35.63827	41.25231	18.30772	77.73551	183.1772
113.3871	111.682	42.76967	90.82583	51.8489	85.9313	54.27111	157.9448	210.1911
10.54813	16.15382	19.13535	0.814568	10.81731	4.241594	31.26508	3.549757	87.34777
72.67951	73.95292	19.80322	45.82418	99.30146	27.45469	75.12143	63.90322	29.06294
112.1845	92.42465	120.193	172.2572	52.92465	106.5956	88.68499	43.0366	73.10018
26.94034	24.63557	6.026483	28.55782	12.98066	4.239719	15.1269	33.65844	1.358078
461.1962	508.4626	371.9526	344.3533	351.955	565.661	430.7407	614.4261	362.7495
36.95846	27.68889	31.3502	18.12598	20.53581	28.34212	21.51986	44.12308	44.0537
65.36351	67.15051	12.15344	84.05632	44.2555	31.78748	35.94989	36.01843	42.80401
350.9116	500.8191	290.0585	192.9156	188.9496	222.6104	258.8939	378.6595	335.0116
51.9173	112.5396	2.206762	46.68617	34.57496	6.818596	218.4581	7.036526	17.74141
59.99861	113.9352	386.6211	50.12798	56.17172	1050.006	105.4171	281.0865	387.7942
82.97935	50.09675	27.49562	84.8967	35.6368	20.5843	67.21138	25.5987	27.77098
23.30104	23.18077	10.66311	25.14608	5.42729	10.28237	9.537479	4.707846	13.88068
55.78429	13.77195	2.967348	156.8428	42.11487	6.817578	20.70424	177.3851	18.98979
235.0489	177.1729	119.3429	78.6904	136.0426	85.05451	230.9901	155.6709	133.5518
17.38707	17.62133	90.33988	7.730746	32.41631	53.26531	30.29274	5.874158	123.3118
34.32516	108.6626	117.1652	23.30639	107.9443	30.03216	19.09474	37.22783	140.9317
129.0965	65.41117	28.22043	67.45081	347.4989	27.44863	153.4764	33.75201	50.47117
13753.18	13432.58	14674.68	10475.96	9097.522	12190.63	17986.52	7580.256	10486.84
752.154	399.0354	384.9734	636.0165	353.0331	368.7647	416.3557	192.876	134.8611

40.7198	43.91699	19.8223	50.20392	22.69482	33.50512	47.16726	11.68098	60.36792
77.38175	60.18984	62.87946	108.3672	16.22166	30.06582	39.16147	5.875509	11.43764
15.09823	9.98641	3.739625	19.08959	20.4736	1.664059	9.56087	18.52703	1.357658
49.37873	118.6616	49.67301	48.40946	85.29669	54.11318	48.68142	48.8399	44.17201
36.18378	43.04297	63.43389	30.22647	116.6167	318.1261	19.89418	101.1081	513.5035
251.7871	337.3738	1278.532	150.503	337.9244	508.0448	408.3493	442.5675	449.6272
152.6792	62.6635	6.793915	47.67691	15.13552	12.8502	19.94676	9.352466	2.623266
424.4221	259.6177	237.2615	250.9259	180.3054	210.592	225.3475	92.98682	157.4933
226.3811	120.9219	97.15352	216.3703	156.5336	81.6248	128.65	118.4999	68.09524
264.0314	228.8918	8.324314	345.3863	118.7657	113.4464	175.84	218.3189	29.07748
812.6338	700.198	565.5495	1495.186	4998.283	445.2364	1784.568	180.1075	65.58927
55.62258	120.9055	169.9234	57.05509	59.40735	211.4992	104.641	111.5459	529.7081
63.92056	61.6265	30.53943	55.36573	29.17534	45.53388	19.89928	27.93334	84.31278
2.356488	5.295758	33.64653	0.815817	7.590481	40.39984	3.114633	20.95873	37.7992
51.27188	62.34717	35.88549	44.94911	42.1311	71.32545	73.49203	90.60675	96.96985
24.46683	18.46289	6.030036	18.15412	26.97684	10.2699	18.35741	17.44658	1.358552
2327.041	726.6027	192.8026	301.9543	220.2574	174.4554	138.1774	1486.424	108.4164
0	0	0.710731	0	0.014109	0	1.581374	0.027885	0.043404
108.3325	84.67418	111.7054	128.9105	75.596	177.9619	71.85768	286.7883	245.4578
33.05608	16.07936	156.9548	14.65205	31.34096	126.3677	57.47174	22.13893	498.0155
21.27157	37.06812	5.261414	17.27907	15.13367	9.4052	3.912506	158.3185	3.884299
0.488544	0	6.076496	0	0.022714	8.59163	0	1.221335	1.354955
52.80231	41.6408	17.53296	41.55646	17.2976	88.73566	13.51357	27.89674	7.662936
89.26492	46.99576	0	211.5668	115.414	6.817572	31.12344	53.41308	1.356176
531.8204	566.5282	3.739925	244.0156	46.45568	30.88645	91.03182	265.9738	6.39656
158.663	193.5023	80.32242	153.1927	74.50852	165.1035	91.8798	81.33912	35.36691
70.31881	61.68028	20.58345	52.79166	63.67496	9.396662	45.5455	3.55026	47.85782
239.6077	300.6834	71.11024	141.9094	134.947	74.74693	82.26181	66.2616	79.41366
104.1535	56.97479	47.40127	84.81522	45.36172	78.23626	47.905	32.57887	69.27372
298.5269	356.1397	109.3995	181.7179	139.2737	208.9207	133.4348	62.78491	99.56119
206.0223	363.5664	832.8575	240.4988	407.0133	385.948	322.811	541.2866	1003.569
161.6235	153.2691	230.417	254.4201	190.0071	222.6591	79.04378	328.634	172.5616
41.40416	24.60474	8.322367	43.2857	55.02428	7.678286	27.13944	39.47974	7.663617
13.16821	11.54128	0.677389	14.72415	4.346766	9.432642	6.333593	7.012897	6.373016
26.15843	32.25686	644.1132	4.275841	11.90757	1063.882	12.70473	351.8708	368.8576
302.1552	156.343	423.4569	323.6655	151.1567	257.0516	102.2267	429.6428	98.32247
45.07711	76.34479	38.22497	56.24825	21.62027	42.10162	54.34664	131.0566	5.145161
21.18241	9.918747	121.2798	5.999962	17.30243	86.93176	20.71128	8.198877	260.7775
9.245588	4.525857	75.97695	0	16.22097	56.79435	16.71456	19.79435	41.53715
255.0752	278.9173	130.0528	178.2258	234.2655	195.9832	179.7843	216.055	385.2305
2.353348	6.076326	45.43264	6.873443	8.662076	13.72981	7.923508	23.20276	51.26402
105.4713	92.5271	29.00536	41.50154	57.22313	39.50775	59.92949	54.60939	7.665866
6.743478	6.069918	14.48065	8.602193	11.90057	16.30188	30.403	7.03277	189.7992
556.5741	520.8343	316.8467	700.9572	371.3762	113.4146	421.1748	228.8709	220.4696
0	0	65.41456	0	1.109734	9.406438	0	0.050775	37.66038
50.13781	84.87705	23.6511	38.05127	27.01239	41.24704	87.23645	3.550198	20.24307
64.56729	68.59017	26.70849	59.70423	42.11923	48.12081	27.10337	114.8711	35.33265
139.6293	107	184.4186	113.3038	38.89892	146.9586	178.9737	1470.954	445.6685
638.7584	291.9392	122.3723	574.5999	460.963	194.2344	327.6437	368.214	139.8914
43.89912	75.67196	25.20318	22.45995	31.31852	92.14051	62.43007	3.550812	31.51967
9.277729	14.59556	33.80069	8.606963	7.585562	19.7699	11.93572	12.81474	20.15051
60.62902	30.72485	512.0053	23.31049	397.3073	1213.011	149.3603	817.7269	3631.225
4.233266	11.49206	8.33732	2.540858	0.024882	1.663398	7.117693	2.387972	31.38264
98.55495	29.96929	34.37487	135.1142	323.5601	1.666087	71.94912	9.361143	8.92587
222.491	261.2159	211.2645	160.9231	93.94948	219.2187	200.5945	166.1208	177.5944
1.729134	2.218463	11.44173	1.67699	1.109854	32.79	0	12.79544	32.52164
142.4254	89.36471	1.444265	38.8899	185.6147	0.809561	116.7275	53.47446	0.085806
0.487938	6.102012	6.83443	0.814662	2.18959	3.387253	4.730189	0.047077	87.82101

90.32722	39.99661	21.33746	120.3553	45.36026	20.57461	87.95266	67.3722	12.70424
62.50525	65.38541	444.0583	114.1677	71.28495	209.7293	387.6678	162.6605	272.0286
19.31775	30.02151	19.83471	17.26252	6.510683	42.1431	11.91036	32.53204	17.71233
55.1963	46.22169	24.42155	82.28403	25.93257	19.722	38.33552	30.23821	13.9568
3.612128	15.41366	14.53943	3.407958	2.19066	5.969302	12.76796	7.019503	39.94986
79.10202	48.52056	22.11301	67.51992	21.62014	43.82465	47.13581	42.99857	29.04178
194.4564	206.6048	101.7773	96.02822	36.73742	87.65836	162.2928	22.13876	156.0983
143.5191	203.4744	49.66189	142.7766	106.8891	123.7798	167.0649	36.07522	127.2092
80.78715	103.238	80.33637	83.91679	85.2942	104.0212	75.08825	146.2953	93.20683
61.8991	80.81878	185.2649	29.36139	96.10215	292.3802	93.45026	76.71482	273.1265
158.048	81.62088	79.55928	100.3647	119.8262	105.733	57.47912	102.2233	56.75298
1062.133	874.3463	1161.129	1132.644	816.1717	1482.906	1083.673	608.7015	1007.538
2.354496	2.989018	12.985	0.814445	2.190775	17.20217	3.116051	1.223179	151.5621
1459.577	1435.3	479.0704	1326.501	688.7832	360.1353	640.8909	1681.84	399.322
0	0	5.444675	0	0.017755	0	0	0.035357	2.550289
50.75126	34.61538	117.3542	36.31387	30.24984	66.2213	84.81314	4.713006	81.74412
323.2247	714.1583	444.6515	433.4717	342.2488	685.1356	403.5316	1157.969	1153.449
138.549	87.01518	35.87999	167.9138	64.79697	78.19687	74.2758	114.9926	58.01436
21.80373	31.5312	39.00297	57.12527	44.26777	24.88557	59.97274	36.03718	17.72957
46.32051	85.60059	68.15441	28.51083	36.72467	42.95741	75.97131	10.52248	46.63197
0	0	3.04206	0	0.018248	0.81571	1.545137	0.036376	2.557522
2.978715	9.942531	9.106493	8.606367	9.741486	12.86079	21.58474	23.21641	26.39413
48.81126	56.21421	26.70386	50.16373	60.46064	32.62107	51.91678	88.23544	27.79903
11.76038	12.23769	32.89811	9.46312	21.61223	46.45102	29.5443	26.7441	29.00122
132.3844	76.25017	18.27002	144.5762	56.15619	40.35599	67.90094	105.672	20.25924
110.1987	91.60587	194.4391	34.5532	118.7695	289.7776	84.64736	51.17403	164.9869
2814.561	1589.328	1169.525	1412.999	1476.849	910.3264	1543.228	1965.291	1915.457
73.31947	41.72554	52.26137	12.93917	16.2113	26.65733	41.66588	1.222942	10.16529
65.67128	40.73624	192.9542	79.56706	116.603	93.66912	65.46456	14.00707	132.246
0.490563	1.451232	34.45761	4.270054	1.109448	12.84431	7.909547	8.196232	243.94
22.50907	20.76037	33.71373	11.20042	8.66752	24.05623	18.33546	10.51303	108.908
25.11856	37.89222	16.03629	9.473463	21.59004	12.85964	19.16783	2.387998	8.904018
111.0688	111.0293	41.26507	90.87895	69.09574	55.85343	23.09586	74.33731	52.94382
169.1511	381.5107	250.3343	97.73177	111.2192	56.67822	684.5299	68.59504	46.70752
99.08854	95.56798	39.72751	132.4639	79.88658	79.94949	56.70403	81.30297	75.56869
550.3159	404.4596	121.6061	504.488	265.5915	455.628	208.5246	386.796	182.6989
31.83098	36.90839	104.1875	39.76465	32.41396	102.3487	25.49706	134.6165	116.9825

TCGA-HT	TCGA-S9-	TCGA-S9-	TCGA-HT	TCGA-E1-	TCGA-16-	TCGA-CS-	TCGA-HT	TCGA-DU
212.7537	140.5119	199.9783	94.02576	124.4913	56.15799	111.0512	94.78936	77.32027
28.31384	11.30448	16.54837	47.04378	41.55776	17.44705	33.98971	39.15896	37.19633
143.7182	101.0858	100.4027	186.2903	64.56103	49.47598	177.804	85.23249	37.18698
3757.69	1846.082	2012.618	3701.062	2831.128	1927.675	2952.293	2600.29	2011.485
65.69185	119.5193	108.2517	61.74866	69.31233	106.7753	37.44067	87.83239	142.909
2.292313	2.470195	0.83196	0	10.5489	1.429046	0.031306	0	6.86064
146.8398	102.851	38.41329	18.41403	40.83216	81.10924	13.64617	26.93867	49.92359
161.8526	57.73039	160.6626	176.1144	108.1377	29.49161	241.2506	134.8284	79.27808
11149.76	4018.468	4515.098	10930.73	4880.216	5662.81	13083.62	7008.353	5459.893
45.30443	105.0828	78.59802	64.53869	66.68704	81.32804	81.54949	74.82434	44.04206
7.970983	21.62066	7.811647	17.50828	20.40279	6.797124	7.978626	25.24367	18.59559
1680.739	841.2976	929.0302	1274.258	1112.029	448.3426	2384.599	1324.089	731.1787
105.298	221.3766	142.3071	109.7015	187.8456	185.4152	75.9513	52.14376	94.93968
0.033079	20.54754	4.355763	8.330844	14.72825	1.439465	0.033334	3.457266	11.76499
236.4293	43.10823	101.2793	66.36555	49.56948	81.43413	106.4947	97.4239	50.89175
11.37146	6.840917	10.42601	21.18332	3.271005	42.6712	10.25032	8.645151	48.94492
4543.11	1441.615	1407.509	2483.965	2136.947	1990.326	2195.532	1908.669	1385.035
46.41328	41.92938	70.76577	23.02434	31.90776	12.14427	26.09744	25.18722	36.21251
93.95914	40.55971	61.95686	97.72787	49.56781	77.4435	61.21582	33.87524	117.4598
536.5159	2351.889	421.7044	515.4024	647.1948	2200.428	203.974	399.2724	1200.054
1676.195	534.0843	770.1081	1187.588	869.7187	524.3616	1238.223	1172.713	694.9624
223.6832	8.11189	29.65382	76.56189	19.61869	0.108547	15.91278	19.96158	0.971055
109.801	10.65781	112.6286	151.2256	21.63976	13.48182	108.7699	127.0145	34.25015
77.02145	149.3246	212.1498	205.5946	79.49013	33.49248	760.007	158.2895	54.80512
183.2695	114.5104	87.3046	116.1856	133.4719	73.43119	150.6208	124.4277	62.63957
18.14919	37.54459	30.55044	23.03353	38.82127	6.808433	13.6427	25.20486	32.30031
129.0675	362.0392	146.6617	80.18659	159.1869	208.1179	167.6903	113.9226	262.3285
346.3959	1058.882	932.535	629.73	1534.127	700.4062	599.3308	1223.183	859.4083
23.80138	24.0773	100.5904	42.42004	10.7597	1.460056	17.03852	40.02273	9.779605
45.31506	41.21398	66.33885	44.23268	25.04943	33.47512	51.0167	82.64027	100.8216
49.82219	38.05799	72.48424	20.25358	18.24419	24.13752	30.63209	32.14655	30.33654
36.25618	31.04285	61.99245	48.85523	59.19302	42.76145	38.55281	46.08304	60.68659
16.98894	8.783356	58.7173	7.348302	8.741785	9.441373	5.715541	9.529177	5.86483
329.4176	1931.104	970.9665	756.0588	1201.272	684.3882	552.8822	579.359	738.0329
87.20247	169.7301	194.7007	153.0393	226.6202	232.1133	142.7782	235.7593	254.4965
8476.402	3596.806	12491.5	14644.68	12233.08	2508.018	18286.96	23502.83	1940.027
28.29209	39.55714	24.43985	31.35807	16.93575	50.46445	30.57564	18.24093	10.75952
49.83058	73.76915	67.22512	74.68515	76.91218	20.14718	53.27138	53.04178	36.20968
41.89678	87.31914	54.13922	74.71047	38.73438	138.2672	30.62419	58.29786	63.62717
35.10797	45.79053	55.90449	68.26102	25.08782	102.3694	69.05215	69.65069	90.06906
52.11809	18.92206	49.71851	38.69224	34.57117	535.5929	100.8617	51.2746	142.9067
22.68582	93.65843	104.8608	28.55764	69.46196	29.45359	26.10228	22.57038	17.61036
15.90097	76.99617	27.02201	9.187029	15.51831	77.32779	28.36912	39.98383	136.0756
103.0174	115.0766	109.1308	155.8354	147.714	21.48766	123.4895	127.0112	85.15295
178.8662	417.9431	262.8129	185.3092	417.2903	294.8084	113.3387	220.9462	210.4436
12.48914	30.60526	20.0695	5.500543	16.26742	24.00883	7.980241	26.10829	11.73934
48.69546	27.84889	28.76891	23.02025	29.83212	26.80323	24.97413	38.24042	116.4924
3806.372	984.3071	2139.225	5208.608	3239.47	429.6851	3565.175	2346.247	1127.601
1132.873	403.115	536.9885	436.108	113.5151	282.9152	480.36	191.3381	564.7827
9.096827	10.7091	16.57759	24.91112	19.74044	8.117399	18.13223	41.00317	14.67899
11.35303	22.94153	31.49358	2.734338	16.9862	12.0864	15.87696	11.27492	16.63796
338.3949	293.1989	311.7352	328.2573	321.9536	130.8408	303.5999	240.0977	125.2826
34.00116	81.41994	78.58755	37.77813	48.24052	57.41884	43.08768	43.46084	50.89421
5346.83	3137.054	7161.729	7063.721	9463.389	285.6063	5549.995	8328.146	785.0103
15.87464	17.1022	27.95726	11.96239	17.63893	16.079	9.110969	21.74093	14.67716
29.4729	90.41959	118.8376	50.70019	59.18989	30.79198	56.65576	29.53415	42.0846
21.51976	13.24444	34.96107	41.52876	31.35824	8.13195	40.72403	33.08628	9.780578

10221.59	2229.516	5788.212	7923.985	7804.086	3736.55	4891.805	5857.419	3582.502
243.3679	545.8643	123.0684	96.78173	474.479	344.1441	63.4989	186.1347	416.0068
21.53965	49.06909	53.31005	22.10902	34.70012	13.46341	19.29841	19.10003	27.40368
57.72061	35.52478	20.03428	23.02275	86.57116	66.64987	24.96917	12.1245	50.89769
3.449747	34.36658	6.932803	0.892996	161.142	137.7216	1.182951	2.556983	39.15697
168.6688	356.2983	208.6785	340.2612	511.4394	121.4947	182.4133	179.1912	69.48808
7.972955	20.31043	19.19181	7.345915	18.32295	6.800834	14.76083	23.48613	34.26559
42.99059	22.80654	41.92788	38.73	38.12635	33.36617	20.42883	33.9233	42.0923
119.9384	61.61193	59.34688	90.36078	42.08818	40.13409	83.83816	86.12348	84.17844
105.3048	414.9257	174.6162	94.01981	76.77894	184.1229	115.5945	121.7565	279.9496
24.87675	7.499544	10.44067	59.13872	6.685214	6.793257	30.53454	26.12863	6.843923
13.62377	49.19021	30.56995	10.11339	9.405675	4.139242	24.93309	14.75071	15.65533
182.2508	163.9924	208.6726	267.3951	136.6986	64.17134	400.9729	274.0476	174.2269
27.19914	45.1506	40.15495	36.871	45.59804	30.75449	24.96222	48.72428	35.23446
115.442	99.18547	61.95853	233.3498	106.844	41.47936	140.4476	36.48671	26.4194
19.28719	41.95904	40.15708	22.10403	40.13161	26.76656	28.35398	37.39382	30.33913
233.1702	183.0785	247.974	301.5142	131.9257	197.4865	193.7473	260.1164	102.7689
54.29626	12.57387	9.552049	56.26619	45.62389	24.10161	47.56557	47.86363	24.46507
77.02046	225.7108	104.7309	90.32784	148.9434	460.0531	113.3379	140.0203	355.3185
181.1155	100.9866	213.9182	331.9589	228.6651	110.8324	325.0955	346.2938	195.7631
769.6958	2214.522	1357.762	1213.386	2245.294	1096.594	1567.907	1422.373	658.7431
33.99749	252.0922	65.48403	60.8484	127.4944	22.80837	62.31657	103.5788	37.18918
83.81017	344.1246	265.4396	162.2567	229.9936	1.450965	196.015	307.9773	83.19166
30.60334	23.39128	90.85089	137.458	31.20148	9.479855	72.49123	89.64629	4.885405
36.25674	80.83728	20.03198	57.15983	42.12005	42.76327	57.78688	37.3721	115.5152
3388.571	621.1993	805.023	2117.947	826.7962	879.1733	2442.411	1184.868	323.9828
53.239	92.81665	22.64809	36.85088	286.9918	171.9161	47.62729	26.04388	319.1353
216.1373	68.55648	86.40717	149.3689	273.7989	53.48926	134.8236	249.7462	67.53166
387.1087	1341.801	488.1007	499.7449	518.6068	846.8665	159.7897	294.8811	894.6632
69.07576	384.2944	34.87556	40.53958	1153.873	344.8708	22.71454	26.04359	83.19666
203.7334	193.3121	206.0665	214.8362	379.9736	121.4845	64.62826	132.2013	132.1365
20.42232	22.76489	62.88452	40.5564	26.43885	22.79565	41.93678	76.59976	37.19103
785.4754	300.6821	768.4055	325.4531	261.8983	172.2231	762.4104	514.1263	353.3504
1508.77	1397.114	1716.63	1823.779	1772.156	1364.732	1737.855	2162.72	1715.882
1.165898	3.189426	0	2.762162	1.278757	1.41142	2.282447	0	0
10.24232	41.2836	38.39611	24.86914	18.25174	16.14054	23.8357	52.19728	47.96128
253.2613	7.477366	19.15656	152.1997	20.28409	0.109977	39.69222	80.91168	6.842925
441.4598	876.9932	530.8528	818.7652	676.4343	900.4153	998.0504	844.7288	797.7435
65.6778	100.4816	103.0362	55.29907	115.051	52.12913	60.07838	135.7493	47.95548
31.74129	85.2233	91.69163	45.15626	113.7439	69.40703	45.35504	113.1302	51.87245
101.8195	76.3381	43.62604	70.99725	77.60916	60.06356	106.437	55.65824	53.83204
90.53998	25.29016	51.48375	70.98794	9.391564	8.144044	116.6469	17.3436	11.73685
73.6102	248.8973	152.814	219.481	45.47006	57.48255	106.5125	88.70385	83.19465
199.2392	403.8974	474.1744	136.4326	225.1971	308.1626	218.6768	128.7047	320.0762
22.63136	2.394035	11.31403	13.81269	3.270538	1.462054	10.23797	15.63612	13.69868
21.54478	1.764662	46.29041	18.41417	11.43983	1.459714	27.21884	39.14424	1.949363
6.844877	26.04647	19.18085	10.11282	6.67362	22.72829	11.37688	18.23713	6.84312
14.76418	44.53403	38.41182	22.10526	38.77526	37.37688	10.25074	22.58033	41.11047
19.26239	17.73473	24.44584	10.11467	29.30209	9.459129	20.40926	18.24502	5.864349
111.9836	64.85601	53.24403	52.54371	34.60789	8.145099	79.2848	39.11176	43.06321
10.23361	7.486885	11.30797	9.191226	27.23496	16.08935	14.76378	9.521531	51.89493
1943.366	746.4762	1167.391	1762.928	1352.901	1028.592	1840.933	1490.22	850.5937
69.01946	50.21301	89.99691	30.40395	38.04159	5.474696	56.64327	40.86364	25.44227
224.1565	1006.894	165.848	188.985	1148.328	1092.323	222.0972	183.5071	1656.206
448.2042	209.7451	476.7602	444.4233	161.8518	289.5465	497.3181	528.0884	296.5802
0.041025	29.38278	5.18862	11.96654	8.054373	30.55006	1.183531	3.426583	40.14873
12.48521	15.83514	30.6012	8.270351	11.47648	9.449148	13.6259	13.88951	14.67802
126.7636	126.5623	96.02843	174.29	121.1361	73.45899	128.0119	164.4558	82.21666

56.49039	33.75246	12.18014	17.49882	22.41041	74.2629	12.50717	7.777298	9.780215
19.29454	63.5916	95.22244	28.55591	29.15412	45.42297	20.44479	50.43761	16.63127
19.29823	56.473	116.9979	141.0786	146.3614	38.82039	200.4565	233.2405	38.16565
123.4237	111.1421	354.5006	208.3569	582.0251	117.5254	171.1113	656.899	69.48755
135.8175	99.78372	95.15083	133.6962	86.35687	116.0835	105.3766	93.05923	80.25831
98.48084	58.39535	99.53159	124.482	97.97443	130.6838	158.5583	95.68257	104.7337
72.49398	114.9697	233.1157	370.6727	144.1727	4.124415	595.7846	323.6326	32.2922
28.3368	45.10814	82.1278	19.33277	40.77779	45.39498	48.72422	57.42211	26.42141
18.14274	12.59194	24.43325	26.73363	8.720255	18.75585	21.54921	19.98086	19.57179
14.76401	26.62312	35.7875	12.87759	44.25234	67.86566	17.04078	40.01471	41.11061
149.4402	216.1827	152.7693	215.747	221.1504	234.8009	143.915	230.5288	342.5951
67.96436	318.1014	238.381	91.2527	245.7083	333.361	207.3242	389.821	147.798
796.7527	248.5394	313.4344	844.6282	319.1053	365.5715	664.9669	573.3169	295.6001
5213.22	964.5897	3211.493	4346.483	1693.811	348.3104	6349.724	3873.056	1546.54
14.75166	28.63502	25.31649	13.80675	19.67856	25.35944	4.58643	17.3689	15.65555
26.05034	23.46207	13.05192	28.57652	16.91701	33.34315	29.46365	37.42284	30.34266
47.56396	76.98995	68.108	38.70359	36.65534	14.81451	185.5907	62.63034	59.70675
0.026205	1.860717	0	0	4.192518	5.162378	0.026404	0	8.839804
18.11791	0.501296	6.06328	11.04265	7.369637	0.102787	27.15392	7.783403	0.970813
0.033668	1.788753	0	1.819214	28.9863	7.908975	2.303806	2.574587	8.818094
14.74104	26.77644	13.06566	12.88858	16.28177	12.09726	14.75473	24.37141	12.71926
57.77156	132.2701	96.0221	79.27313	185.202	140.0612	79.33895	105.2437	88.08934
31.72842	88.57218	77.7518	40.5552	145.4069	26.78842	28.36354	36.50838	27.40036
565.9226	512.5507	468.8732	707.2145	427.3355	460.269	638.9344	630.7286	607.8527
80.4002	64.73862	78.54613	73.73757	137.4585	73.47675	81.60664	92.18258	125.2877
5.705898	27.56542	24.50429	9.200721	19.8019	10.73697	10.22444	14.7855	13.70197
1720.321	134.6639	1174.426	721.0161	69.28379	177.5642	922.1733	601.1056	247.6343
84.93434	174.8671	141.4303	98.63324	183.0635	164.1112	98.60247	96.52367	125.285
60.03049	139.9538	106.5153	135.5462	86.36808	500.6087	70.2758	169.683	180.1125
11.36304	30.57474	11.30795	8.26823	16.94158	45.16145	19.28179	13.88129	29.36689
173.2117	914.4695	219.1371	400.1785	491.5057	182.8499	257.1827	327.9767	315.1822
256.9205	78.07626	192.0788	305.2104	189.1493	101.5027	233.3759	159.1724	264.2851
159.5834	69.19395	100.3853	141.9903	101.3325	82.80524	177.8436	144.4094	143.887
55.49014	52.69424	66.34253	81.13769	91.9014	44.12307	40.82709	123.584	80.26331
15.88667	41.42495	52.45664	15.65007	36.79093	26.71449	23.81246	13.87489	20.55053
37.38214	73.20894	59.37776	59.01138	74.25093	162.2242	20.44295	60.9021	124.3313
88.30529	99.17272	79.43099	89.42509	56.38171	64.12841	65.745	66.95045	15.65212
46.20474	9.444974	8.69583	1.813004	6.69654	25.20686	3.451192	9.536338	45.05706
0.039107	151.3732	0	0	20.57448	4.120846	0.039412	0	46.05264
47.56608	65.48685	75.9737	55.31066	173.2674	37.45194	43.0836	80.05035	75.37095
41.87487	26.62675	32.28934	44.26305	42.20507	9.475625	78.06891	37.40114	15.65345
13.63751	23.39482	37.51418	22.0992	12.79509	101.2001	22.70755	26.92371	39.1481
73.62259	216.8514	162.3843	121.685	215.7291	161.4765	124.6563	188.7658	220.2359
55.49496	136.236	34.87877	31.3186	34.58764	161.2037	32.9048	45.19549	62.64038
567.0419	472.4943	675.8625	424.1264	453.9093	293.5697	746.5206	448.8864	315.1769
57.71889	23.40108	38.39333	86.70715	29.85012	8.145264	89.43109	80.08289	51.87702
30.59179	17.03094	34.02811	21.17973	8.030515	78.53785	40.7981	32.15931	28.38028
66.78321	41.8755	35.75978	93.14536	31.19468	40.11323	91.73185	53.9164	70.47566
23.81446	36.8066	30.52473	25.79122	46.92907	30.77533	34.01932	34.76719	51.87724
13.63824	71.75473	234.965	47.91581	43.42892	22.82155	111.0367	238.4569	81.23727
40.78388	79.52329	67.22776	65.4594	101.5067	66.71636	82.68548	121.8668	49.91575
106.4465	192.591	638.3806	280.2895	378.4254	50.83621	362.5138	307.0872	64.59343
70.17628	68.03372	65.4795	69.15058	92.6285	25.47408	109.8337	38.23864	13.69454
14.7628	17.68297	12.17418	34.1083	49.06245	20.1123	23.82459	20.8401	51.88314
213.97	894.2922	194.6647	181.6087	600.2546	626.9588	145.0657	177.4175	688.1179
1028.747	392.3128	819.9544	1041.037	866.5098	248.2356	448.6382	1322.496	186.9476
21.55462	91.74919	28.7739	24.86745	60.58233	42.74675	13.65012	20.82929	46.98096
257.9839	144.9871	148.4366	189.0323	113.5984	90.79976	177.8445	180.1004	111.5824

45.32432	61.56959	178.1704	79.27603	68.64311	48.14817	151.7827	77.39338	86.13239
32.85718	55.97677	54.13745	31.32792	40.78117	29.44482	20.44137	36.5102	81.25132
9.10439	139.1243	31.38114	25.78503	106.0307	682.8682	46.50511	43.44236	1666.014
224.1114	96.52951	247.1085	164.105	127.8472	182.8111	177.8869	170.4852	176.185
37.39314	43.14284	46.24528	20.25253	59.84433	81.35103	28.3721	19.95509	45.02043
12.50202	32.3881	74.30949	17.49187	45.63492	33.38564	32.86918	48.74056	38.17376
63.4241	114.4575	87.292	63.59623	83.64116	96.09488	104.2384	99.1584	129.2063
7.978579	121.3532	20.03892	5.499354	29.1879	44.02869	26.09317	11.25557	65.58873
44.14815	17.0319	21.78637	55.33203	53.79748	20.12722	61.14602	54.82285	23.48509
556.8931	300.6678	476.7148	471.1386	275.502	205.5731	821.335	496.713	528.562
74.5874	13.85648	20.92127	59.97276	52.51525	18.77725	32.86221	38.28371	22.50777
69.06859	71.15956	66.33311	74.67223	70.71846	53.45739	58.9448	107.0149	104.7358
358.704	169.7644	91.63909	315.3741	234.8424	129.4736	277.5127	195.7401	98.85473
194.6491	41.82119	112.6149	114.3185	33.21054	37.49244	108.7834	135.7027	100.8143
4.573848	8.831516	21.01573	11.05728	15.70369	1.460731	6.834344	15.67947	15.66449
537.6335	587.6216	863.6083	625.1431	658.1328	358.9277	705.7654	514.1327	385.6529
14.75755	17.70282	38.43879	17.49619	18.28725	8.138823	11.3787	17.36136	41.11537
1326.48	207.8	802.4241	1746.386	372.827	244.2591	1582.567	1071.799	907.3721
57.78127	419.8059	32.25293	46.98988	539.1685	871.943	29.51134	97.38153	510.9571
32.87558	64.78224	88.18005	178.0041	101.4106	26.81773	107.6148	60.8635	45.01877
98.47091	83.26852	47.10845	45.15282	129.3827	314.1745	56.68181	44.32247	92.98765
345.2207	135.9519	192.9288	291.35	197.9431	393.5136	260.592	416.719	303.4334
47.56727	72.50314	33.13732	33.16685	42.79018	86.65426	36.29427	40.85129	56.76893
361.0255	187.5264	546.7067	389.1242	167.3337	181.4987	357.9596	297.5334	143.881
1376.319	1090.611	1307.126	1015.146	724.0199	672.4332	1315.28	1084.818	1111.945
48.70567	62.89676	91.69172	37.77649	41.41074	33.47224	40.82725	38.23353	58.72531
4.576833	20.44355	15.72022	7.352328	17.04233	13.36666	6.839947	14.78707	16.6415
370.106	127.0497	340.5327	408.4718	170.0348	216.1898	376.1088	347.1128	165.4147
37.40652	819.9903	850.5563	937.7799	123.0478	54.8374	410.1126	828.2887	39.14411
1240.473	776.4445	460.9879	1329.595	1076.671	593.7094	1540.661	865.5954	768.3761
15.89932	58.52198	76.87614	17.48761	20.29516	17.47418	22.70575	22.57158	24.46331
10.23905	29.85239	71.70797	11.03382	13.49368	37.33551	19.29724	19.97288	23.4872
35.13347	133.1078	102.1871	94.05769	87.81519	211.6305	90.61773	85.26185	122.3622
57.78039	38.00612	65.43273	26.7076	42.74155	473.6083	57.83389	57.36151	436.5531
5.713284	246.7476	65.46862	103.2826	617.2382	405.6489	10.25261	35.62195	28.3777
9.103682	6.848982	20.06446	12.88449	5.312265	51.75068	5.718485	5.166083	3.906549
1209.89	539.203	633.0243	1047.448	457.9241	325.6155	1353.725	939.5678	592.1864
87.1554	61.60798	49.73293	130.0337	93.24445	42.79914	142.6819	93.08823	75.36719
13.61187	28.07517	20.07927	15.66155	14.22495	9.447198	6.84738	10.39935	11.74009
141.4836	136.0755	107.3757	244.3815	159.2728	84.13502	201.6138	149.6348	146.8241
158.4472	17.01405	36.6196	378.1457	148.3715	14.81576	233.3061	133.0968	52.84854
11.369	34.34617	36.68093	14.72566	56.65779	36.00171	21.55648	22.59016	36.21772
71.35578	215.637	246.2666	176.1062	247.1307	132.129	132.5731	177.468	195.7665
22.67501	50.30161	27.03904	10.11017	69.59335	26.75732	22.69608	37.39972	7.821702
21.54204	68.91996	20.04445	24.87691	42.22054	42.65808	30.60597	31.30229	75.38654
7.978661	76.33073	9.550571	10.109	12.7935	418.6784	17.04906	110.5396	216.3581
2.313247	17.35161	13.12102	15.70333	1.916939	37.96922	1.182711	22.7369	21.55136
141.4837	114.4152	110.8703	117.0872	117.6924	64.14918	101.9874	129.6127	163.466
535.3784	188.0914	548.3335	1960.385	47.50541	48.16314	614.028	1720.962	60.67826
4.578446	8.118618	9.5524	4.579888	2.602802	77.47159	32.91017	8.646262	6.843292
3.448452	3.030274	6.940287	4.579373	6.689973	39.70308	6.844469	4.297918	35.25354
79.26266	174.9941	141.4634	80.19741	157.2675	82.78624	109.9028	217.5641	119.416
0.033984	11.72288	0	0	0	6.634792	0.034246	0	3.911693
36.25822	48.26276	68.10873	45.16249	22.33276	37.44744	43.08137	64.37336	71.45548
38.5277	119.6816	71.58628	39.62131	141.0523	82.71003	51.01456	76.54798	77.32616
656.4638	480.0853	729.1093	449.9375	628.8407	265.5824	542.6637	543.7107	194.778
65.70395	704.9793	309.967	274.7563	459.474	261.5109	201.6888	368.0018	353.3576
260.3422	270.2373	248.8314	297.8126	353.2339	156.1868	242.4605	307.9601	256.4496

2522.917	3041.045	5452.061	2870.273	1872.788	2526.652	4874.791	3930.45	3236.977
149.3889	10.65817	195.6392	140.1535	8.035441	5.467772	51.02955	70.4271	66.55351
217.3613	732.113	440.9367	431.5038	829.0907	954.8801	610.6027	345.3443	539.3349
32.82759	42.03452	53.3178	32.26788	18.27984	14.7892	22.68759	19.97373	27.40422
1781.504	1500.211	1554.236	1638.459	1434.61	1123.27	1561.111	1345.812	1452.579
54.38383	158.9283	113.4735	171.4887	176.9122	300.0264	124.6529	126.1093	274.077
35.09873	59.92038	41.91619	35.95364	40.83823	85.06722	27.21785	32.17057	24.46525
71.35394	215.0277	264.626	105.0918	138.7929	169.4156	79.34709	117.4164	134.0962
10.23001	24.18534	4.313687	5.500801	26.5837	27.95575	11.36944	10.39691	25.45146
4.581347	66.15275	15.66403	16.56434	28.47362	240.6011	14.78318	9.513891	87.12192
55.50503	45.65122	57.58695	116.1751	55.69778	77.4497	113.2927	80.00694	74.38553
136.9804	123.9201	166.7611	160.4239	255.9683	112.1491	295.6281	185.2958	70.46734
23.77914	27.35151	25.31681	23.04326	15.56677	30.64717	32.8317	32.2077	40.13997
2.315365	20.48874	10.45925	5.505855	22.61926	30.36157	13.59442	17.42573	15.66354
69.05042	59.7265	54.11155	124.5155	236.0771	111.9266	67.98239	98.33572	45.02038
41.90244	56.59738	15.66492	35.01721	57.1673	65.32937	45.33458	30.4091	41.10666
416.471	134.698	197.3078	331.9417	431.6048	400.0893	335.3163	149.5917	195.7608
9.090082	30.78446	8.695916	11.97342	18.40818	8.103195	19.24069	18.28163	14.68114
198.0891	84.43889	184.2169	292.2979	64.51943	204.1348	309.2475	230.5346	245.686
13.63041	51.0002	54.18858	29.49602	39.49992	12.13309	21.5588	21.7159	27.40387
93.92789	54.60993	62.8475	117.1251	50.27931	38.79663	114.3794	93.09926	29.35667
503.7022	41.82417	268.0114	189.9058	167.9609	177.5622	670.6805	145.2262	240.7829
21.50162	134.7637	7.812674	7.347492	99.07995	63.45452	0.041457	6.910638	34.26957
161.7565	13.19806	287.5401	110.662	12.79303	26.81331	57.80636	49.55134	29.35654
307.8726	271.4947	288.1286	264.6105	287.1563	262.8517	320.6139	397.584	239.8081
78.13256	124.6355	197.3875	70.05094	72.7267	69.46887	80.46898	54.76018	73.40592
46.45273	49.47867	107.3983	41.46208	98.66028	20.15303	91.77827	90.46	150.7458
110.9563	165.3189	192.0996	118.001	108.8012	73.49351	197.1156	137.4294	179.1248
319.2166	337.5967	612.1004	156.7135	272.8009	144.2101	598.1528	336.6393	170.3073
74.69527	11.92665	54.99022	50.69698	14.83634	109.2385	58.92501	23.43765	61.66429
4.580116	27.44708	16.57537	20.28547	21.79851	33.18281	13.62222	4.297052	21.53533
122.2504	104.2329	105.6317	110.6329	155.8787	60.1478	229.9045	174.0169	92.00488
22.66639	20.89361	27.04989	23.9572	21.7009	8.140431	38.50574	30.43846	32.30047
2.314992	21.48749	10.42404	13.79812	17.56769	77.26613	3.452189	17.34739	201.689
21.55265	61.73432	38.39555	34.09676	59.92203	8.145173	18.1775	17.34802	0.971074
57.76336	169.3388	95.16567	78.35875	220.1099	94.73796	63.4771	95.68611	116.482
88.33547	63.44128	35.74473	58.05484	296.7693	290.7669	44.24009	33.00204	110.6
52.10377	8.749439	36.62532	70.98296	46.85475	10.81372	54.41589	74.79691	4.885624
38.49194	43.24842	66.41586	44.25972	18.26266	12.14009	50.96057	54.83535	28.38123
38.457	17.70802	21.80485	10.11238	6.672893	8.13767	19.29099	19.97938	33.28154
6.844928	18.99359	47.21069	19.3451	17.61136	10.7951	33.97147	36.56175	30.34412
195.7835	66.64376	138.8227	204.7102	145.6236	146.7531	216.341	89.56841	57.74266
3218.964	2496.056	2064.13	3725.026	1840.145	2357.204	3661.468	3288.436	3941.74
64.53309	151.6252	13.04297	26.70824	117.8565	465.3635	49.88048	13.86345	216.3521
478.767	669.7853	436.5766	276.5889	741.9647	432.2267	175.6478	150.45	582.4063
35.13898	54.57989	62.83456	58.98803	25.7268	234.4206	47.62652	66.95557	124.3144
359.8429	156.385	105.6131	147.5105	189.1797	335.9857	165.4187	130.4617	223.1742
31.7218	36.18701	32.27893	17.48907	31.23058	29.43326	19.30732	38.25949	41.10856
50.98978	17.01921	168.4927	47.91204	18.2415	386.7038	113.3344	56.49151	243.7277
44.19142	55.20712	84.67408	134.6276	65.24291	40.14936	84.99004	163.5962	131.1652
32.8505	52.81737	21.78648	19.33466	20.30457	74.54957	28.35691	19.96294	55.79609
23.82067	21.47456	77.71932	65.45937	208.7836	32.13112	80.42299	124.4807	41.10476
1.181226	6.274797	0	0	2.60119	32.76743	0.038401	0.821234	152.9709
0.0354	7.665257	0	0	4.010239	4.075524	0.035674	0	27.45768
3.448526	22.31729	34.13906	8.272859	11.49379	5.462709	10.23226	7.785596	14.67962
20.42041	79.6723	30.52866	30.40728	72.25566	97.11638	19.30789	25.18805	70.48261
13.6235	26.70197	16.55733	33.20495	52.63552	17.42185	19.28564	29.58535	23.48967
150.5522	194.6178	190.3534	118.9236	135.3727	112.142	156.3522	171.375	171.2937

307.9119	338.1932	1122.054	1190.374	430.0149	28.1448	1686.745	1229.301	15.65327
4.579925	3.672109	3.443096	2.73622	4.63556	173.1956	0.044079	0	19.56749
0.019938	21.08893	0	0	16.73941	7.395054	0.020088	0.849333	1.959882
69.94789	1.760926	34.97663	22.12662	5.996582	1.461934	11.37018	16.50466	0
1992.019	1367.261	1693.946	1650.443	1210.664	795.1597	2537.596	1505.889	1025.806
74.49788	2.394344	17.43521	15.65428	0	5.472084	7.981922	15.62526	9.780499
40698.75	18338.93	22676.09	34568.93	20199.7	18132.81	35053.64	21203.44	43920.97
71.33437	95.37198	78.56336	51.60868	72.07244	90.74042	121.2014	44.32124	93.96598
323.6904	281.7314	261.9443	205.5994	214.3252	105.5115	214.1365	538.6051	147.7964
117.7571	265.8401	241.8619	139.2037	251.8047	212.1428	162.0354	190.4973	230.0229
659.8337	441.9533	469.7558	275.6646	342.2503	260.2322	278.7291	551.5609	572.616
1488.365	233.2365	542.188	939.5359	112.1577	157.5534	5060.263	387.0766	279.9354
98.52344	48.17405	422.6507	218.5067	71.32243	28.1554	258.312	671.7399	95.91648
10.23636	26.04459	6.933268	7.344527	27.20524	88.80374	13.63842	27.83349	47.97289
13.63664	43.83383	57.6358	13.79825	44.88007	41.40641	11.38479	29.54047	38.17007
22.64999	2.394368	6.93425	29.51165	16.93881	31.96478	26.05779	20.86024	11.73878
92.81428	115.1635	64.58586	111.5758	143.7249	56.11917	86.10898	87.85768	92.00913
77.00863	81.28754	108.2454	118.9292	84.97378	76.14726	158.6025	164.4266	59.70042
191.115	57.80818	54.1102	48.84854	51.6507	73.38074	64.58975	53.03952	41.10431
211.6714	104.7977	258.4573	751.5907	47.50071	45.50001	429.2904	307.1141	126.2617
122.2503	92.76661	148.4449	121.7017	185.1968	52.15222	173.3054	113.0773	170.3197
4.579578	17.148	2.56786	3.656509	25.95044	62.056	2.319022	3.426849	66.60586
3635.419	1308.687	1769.012	2651.779	1994.694	1576.834	2751.754	2147.046	2168.101
130.1594	90.86954	121.3659	142.0003	194.7773	25.48878	149.5225	171.4172	89.06891
38.52984	17.6503	20.02886	12.87497	123.9544	262.2388	17.05019	5.166952	62.64046
44.093	3.028621	28.81175	28.58096	14.18812	12.12373	79.11624	51.40237	14.67573
499.1244	238.3767	350.997	479.4659	294.6159	158.8713	643.4219	394.9541	153.6677
14089.85	900.9973	2762.688	2122.507	906.3775	519.062	3982.05	1809.475	1745.243
184.5326	384.7697	359.7436	188.0704	239.4834	349.5102	173.3758	369.7338	466.9054
59.99646	22.11505	136.3257	18.40869	20.28818	28.13218	35.1597	64.37433	31.31541
2.316543	144.4686	11.30588	6.422252	56.75003	82.16265	1.183139	8.648669	113.6011
93.96539	82.59068	44.48225	154.9168	106.1346	81.45081	155.1792	107.8624	110.6058
91.72011	263.4349	116.102	111.5471	104.0396	144.1105	95.2019	117.4151	155.6319
23.82168	90.9918	47.992	13.79709	81.68199	38.79178	18.18203	60.00688	72.43231
13.6001	27.51439	23.61094	11.97127	12.87871	5.460104	23.75287	24.39981	13.70077
12.49735	47.84999	46.32592	23.03723	25.13894	12.12635	13.63994	24.33955	23.48835
1.180783	10.20177	14.01704	2.738054	4.673227	5.416341	1.182208	8.69473	7.82858
13.62925	36.27005	59.45241	12.87982	16.91375	5.474787	40.76309	41.78135	21.52916
1.182138	7.508515	5.190292	12.89505	1.912133	30.49329	1.183577	0	6.844365
17.0326	48.25947	74.22753	23.0203	202.6802	87.96799	34.02909	75.69706	87.12015
63.38819	103.1816	70.73185	43.31708	70.10946	20.14409	51.00171	33.01626	22.50473
48.67218	22.76989	45.39592	37.7901	29.85927	10.81166	58.89271	73.12269	34.2544
13.63042	31.77129	41.92929	23.03275	37.44438	21.42973	11.38057	30.43566	17.6122
105.2606	88.35755	163.3392	124.4861	134.8234	78.76205	105.3572	82.62672	43.06068
6.845063	33.73989	30.56274	14.72786	23.08933	25.38089	31.71506	37.4325	36.21991
10.22079	12.65326	12.19943	18.44368	3.954727	9.431991	27.13445	20.02248	12.72098
80.36517	46.31715	36.62812	59.91674	44.13774	56.1028	17.04993	60.00125	133.1304
59.98115	100.7146	32.27165	27.63581	20.97673	40.08488	23.83773	14.73552	36.21155
13.62974	16.41588	30.55082	20.26393	1.914244	8.140555	15.90449	13.00126	1.949294
3.447539	151.6458	9.550644	11.95298	65.98083	186.357	4.585263	6.905116	56.76807
24.9187	14.5064	12.17825	35.04445	21.02825	38.62613	12.50923	17.36225	6.843043
187.861	43.09433	66.31568	165.0497	158.5832	110.7821	116.7082	154.8549	93.96201
104.0655	34.23037	111.8399	81.15424	10.07133	8.145222	24.97292	45.21097	9.779223
157.2835	50.11357	102.1527	285.9363	97.29117	18.81937	195.9066	207.1417	3.907051
15.90131	8.112059	10.42363	4.578307	17.56112	41.44248	7.986451	4.296859	73.41297
110.9159	104.9302	87.30071	106.9566	152.5573	174.5761	82.72093	80.01379	116.482
7.977288	11.30478	20.92276	79.37712	24.43558	2.802108	63.3552	59.22868	27.40403
0.035958	40.14765	0.826849	0	1.926495	5.38729	0.036237	0	3.909859

1715.837	516.8956	846.068	173.3086	307.4777	493.6977	179.0533	454.0666	1460.413
0.040556	51.46698	0	0.892551	23.90705	18.66027	1.183577	0.820917	86.21036
113.2214	265.9319	103.8676	209.306	115.6078	268.0319	112.1932	104.3549	150.736
422.1138	177.9849	309.9905	167.7908	294.0299	333.4265	309.2598	120.0087	327.9111
41.79971	22.88586	18.31706	23.04936	31.38361	30.61001	17.01812	8.65164	29.36855
7.975214	22.2053	5.18618	8.26747	18.30116	63.68912	4.58658	10.39127	24.46871
65.66161	66.10001	105.6883	41.46782	90.55427	25.47766	53.2742	64.3608	48.93607
292.0678	887.8021	1037.341	484.0454	821.4289	616.3625	490.5763	1168.39	1177.536
108.6532	122.7873	79.43662	94.04179	140.9629	96.06625	70.26863	98.29937	87.1131
667.6933	336.4027	130.0523	202.8249	272.851	768.048	207.3552	153.9355	513.8916
20.4047	35.64354	58.58583	29.50069	29.92977	12.12816	27.20324	23.46523	13.69622
129.0658	166.561	163.2601	321.8205	229.3643	116.1577	381.6979	207.9161	72.42479
835.2937	180.4547	663.5914	882.3942	32.53922	73.51115	213.0378	160.8857	124.3018
2254.681	3311.941	6346.172	4029.273	2641.829	3797.913	6976.277	5486.81	3513.984
39.65639	22.74541	87.32754	70.99139	74.85162	12.14858	95.14042	125.3363	74.3901
1093.411	2103.658	2645.679	2280.177	4527.905	1584.868	1822.844	2576.798	1160.881
43.01703	69.46048	39.27865	47.94764	13.48195	116.9612	52.10065	25.1903	46.98389
235.4215	203.4735	122.2038	241.5756	221.8537	268.0886	174.487	181.8012	301.4838
53.20723	82.14286	53.25296	75.6224	63.98893	17.47673	125.6292	64.38397	9.779235
28.35313	88.9037	49.71658	64.51086	275.0342	222.751	26.11367	36.4818	438.5352
271.5625	123.9579	61.07396	122.6187	127.9045	193.365	78.21189	110.4581	138.0129
0.020047	2.663171	0	0	2.090244	6.201414	0.020197	0	2.944682
4.582279	21.51343	21.79169	21.18316	60.69647	58.57824	15.90874	10.38609	85.17737
14.76503	25.33746	13.0466	21.18138	35.34583	124.8649	18.17357	36.52285	73.42336
18.15993	41.28756	43.64498	39.63522	80.45062	24.12247	57.76445	31.28486	33.27515
26.0828	79.52375	77.71954	52.54148	70.09241	89.31436	72.50357	24.30778	60.68496
268.2135	74.90227	289.0605	217.6044	250.5049	12.14253	270.7242	285.3877	27.398
36.26932	104.3138	54.09883	112.4969	179.8871	77.42031	77.05614	137.4942	120.3994
0.04364	471.5425	24.39664	40.54353	478.4804	125.2689	4.584935	2.559854	216.349
1009.602	425.3218	860.0558	650.9379	801.6582	623.0515	722.8046	1264.075	596.1001
189.0681	664.032	426.9649	304.2508	650.6944	301.5677	321.771	423.655	647.9887
10073.43	10182.19	11776.37	18379.85	6697.986	8528.196	16815.36	12098.48	7931.432
27.17924	26.02415	24.42718	23.03558	36.09593	37.31533	23.81459	42.65861	15.6544
127.9101	154.5527	157.177	65.43717	97.92854	49.48911	81.60642	83.47876	152.6978
24.92816	3.029535	6.932053	6.42145	3.949584	30.71712	14.77495	11.25813	103.7895
17.01146	41.46899	42.83586	23.04106	50.56287	20.07393	12.5066	31.32901	17.61371
2.315209	11.41504	8.704609	2.735496	12.2349	10.7129	2.317692	18.3055	15.66401
9.110251	24.05426	18.29004	8.265264	31.91945	50.66748	10.25147	19.96344	93.00618
44.1941	132.2797	91.65571	59.90525	200.218	197.2962	75.94062	77.3903	114.5208
9.096446	15.85645	29.74054	26.76191	3.271427	58.13087	19.2587	17.39085	31.3327
81.48586	32.94051	51.48871	36.85569	22.3282	30.80359	64.58875	56.52388	59.70525
66.81218	154.6648	116.1391	86.66068	95.9352	129.3427	57.8171	73.91859	90.0497
19.26776	31.19713	37.58091	17.50003	32.71119	1.461446	29.44851	13.87898	3.906525
615.6989	249.8111	283.7421	591.9571	529.4912	317.5697	497.3362	444.5374	433.6184
19.28332	38.79165	35.79226	22.10691	37.42147	34.70977	18.17029	31.30054	54.8209
57.67791	50.31432	24.41643	26.72189	41.52516	24.09766	29.47836	25.19722	22.50708
235.4562	291.2112	561.4977	129.0541	345.0285	281.5259	258.3254	366.2506	181.076
507.5572	8.748473	96.93683	65.45259	9.391525	32.14049	341.7545	52.16457	3.906884
81.54656	142.3414	134.4264	100.4719	153.035	264.1305	64.63085	146.1137	602.9803
22.67805	54.75503	55.03189	51.64315	90.78707	16.1338	24.96135	31.29211	31.3182
4.578023	6.229417	18.34661	5.503327	17.02163	8.105248	4.582573	19.15406	9.782895
54.32695	16.38867	18.28719	14.72064	20.97935	30.77428	21.5731	14.73603	6.842819
269.3413	99.72716	151.9099	303.3826	142.8558	144.1331	269.5885	191.3873	110.6013
31.74065	8.748285	28.76535	15.64121	54.3731	93.34048	23.84462	8.644372	42.08297
62.27907	71.17375	130.1467	72.83032	66.6378	162.5239	88.36635	144.4765	107.6742
645.7717	35.46256	54.09073	26.70559	51.60518	37.48778	88.39258	58.24244	52.84898
22885.79	8235.507	9323.632	19146.99	11013.49	13309.09	16527.61	12536.07	10426.46
890.6676	293.7142	464.5206	532.9421	167.292	237.5578	387.4647	414.0861	262.3193

11.37221	33.01221	35.78525	36.87379	83.96045	41.3614	44.17929	53.96311	49.92283
14.76266	41.99518	37.54315	34.10861	60.02644	16.12598	45.29886	27.8136	36.2156
42.74542	3.678738	10.46518	5.50732	2.596191	2.79487	7.959487	8.675038	5.866485
41.92717	217.8185	110.8965	65.44438	50.93586	29.48527	72.53327	89.59123	74.38652
35.1443	14.47547	95.13072	27.62731	25.7234	734.2706	61.2305	60.84323	132.1363
465.2005	235.809	177.2016	319.9246	294.5872	1098.904	287.7932	229.6215	474.7294
13.62185	12.60167	20.93827	35.0567	16.25404	1.461596	22.67046	33.08318	3.906537
189.0447	234.6346	309.1157	269.2344	324.6808	193.4912	229.9887	245.3199	237.8533
86.05685	159.0092	99.51234	102.3292	159.2701	33.49166	219.7279	124.389	84.17308
89.4531	34.18543	241.9227	129.0751	149.7139	48.15866	120.1073	345.5067	59.70044
8334.49	160.7424	274.9919	2947.776	385.0596	1398.074	2008.599	863.821	1546.544
66.82815	131.584	51.46494	92.17987	142.8811	389.1548	131.4358	109.5829	244.7142
14.76929	50.18676	45.37968	39.62784	61.24227	167.6114	35.15812	41.72774	56.77036
19.28554	14.4866	5.185574	4.577565	4.629498	25.4317	6.853297	2.557638	61.6739
141.431	118.9905	71.57704	75.59513	89.81924	98.70734	49.8883	71.31313	81.24034
11.35824	8.773236	24.45773	18.43159	14.90336	10.77658	14.7566	8.653008	20.55425
155.1143	167.7529	334.4007	376.1818	379.7216	218.8814	218.6899	646.4265	341.6073
0.022979	0.542827	0	0	0	6.297938	0.023153	0	0
108.6845	166.6391	142.318	106.0168	162.6649	205.3496	97.46166	142.6649	174.2332
57.76949	701.1234	58.4586	146.6128	310.0538	150.691	82.7319	29.52326	350.4529
3.449607	8.127846	43.73581	15.65616	19.69302	6.801329	9.111637	30.47147	0.970851
3.439101	18.7317	0.825943	1.814673	2.605273	10.63505	0.037716	0	2.929055
13.62938	42.03919	21.79923	6.421599	21.70205	25.40241	19.29585	13.00159	19.57076
52.05319	15.75713	76.90441	48.87071	82.54733	4.138912	72.44639	13.86709	6.84282
69.09646	34.18332	93.38168	135.5175	44.09814	10.80585	108.8019	172.2283	37.18624
81.51812	53.29732	83.80106	161.3825	89.10609	62.79458	176.6696	148.7949	89.07013
21.55029	11.29463	40.15064	69.17886	5.98946	6.810537	75.84332	41.74551	18.58987
95.10297	67.29102	78.54864	134.6157	150.4264	78.79961	109.9087	101.7612	76.34229
30.60691	110.1708	80.34168	53.46386	61.2155	57.40959	40.82187	45.20497	70.47516
35.14375	159.6116	171.1424	155.8198	241.7114	189.3919	134.8316	197.4979	97.87683
272.8194	533.5415	385.036	471.1486	368.1029	685.6202	532.4639	441.9155	622.5351
100.7769	162.7536	260.2317	58.97806	374.5345	258.7189	72.55621	184.4225	271.1408
35.08869	38.17629	36.67558	16.57072	34.70265	12.13339	15.90557	27.81894	8.800754
3.44522	10.12645	12.22064	3.658818	12.93379	2.795631	13.58898	8.673321	15.66459
24.95716	58.99081	44.47665	22.09555	36.61152	479.904	32.91123	28.65233	65.57272
81.54349	77.44332	272.4504	83.8754	177.5818	105.4965	172.2191	95.64868	136.0515
46.41815	30.42259	53.26248	60.86214	25.07361	12.14536	47.59177	60.91021	29.3586
15.89604	40.03757	8.678044	9.187512	49.02279	91.75805	14.77929	19.96428	140.0116
46.38112	34.9621	13.92343	25.80089	12.80786	82.37245	12.51288	19.97046	93.01533
134.7265	418.697	227.8941	127.2173	358.1135	277.4182	171.0901	304.5161	209.4675
15.85935	16.50597	15.70329	8.272472	10.80412	48.91827	4.584039	9.53049	21.5365
40.77855	25.30343	59.3685	46.08664	29.15461	12.14758	160.6985	55.66503	48.93788
9.103085	23.51781	15.68677	13.80867	14.89031	76.77548	9.11177	23.48418	81.28418
352.0167	237.744	460.1699	465.6354	104.6713	133.532	459.9382	329.6954	138.985
6.84248	3.66405	0	0.89272	5.996197	47.74721	0.041823	0	27.4098
55.44785	22.77159	43.6481	181.8289	26.4464	10.81136	69.06438	57.43352	41.1083
48.68659	59.77936	43.63455	38.70688	55.10723	66.67076	26.10286	35.63335	53.83397
200.3579	447.2878	228.7579	194.5352	230.0022	316.0984	224.3244	187.0149	163.4588
406.3266	515.8537	316.9425	1200.635	717.5384	104.1902	988.8271	434.9906	147.7948
19.28346	22.1556	26.1667	77.51287	23.05301	24.09618	57.72851	81.01967	40.13228
13.60999	10.06302	13.06863	10.11855	7.369074	18.69299	7.976898	11.27346	24.47395
74.7577	459.0494	116.0758	116.1424	375.5578	1593.85	78.22562	67.80169	3812.603
16.99336	106.943	15.69679	16.58634	32.12276	45.04016	2.31913	2.556786	14.6784
119.8768	4.936248	30.51935	7.343204	15.51928	1.457407	23.84031	13.86435	0
129.0625	142.3789	125.7033	296.9242	149.6655	145.4687	261.6654	217.4967	169.3347
6.833943	51.59146	8.6975	5.504068	21.18458	40.91229	0.0404	2.557583	24.4799
158.3763	5.574274	7.805275	22.09604	7.352404	2.795618	71.39093	38.2315	2.928299
1.1815	6.912328	2.570963	9.212061	8.113585	23.72357	9.078223	2.560079	9.786712

62.26069	57.82985	112.7026	43.31612	46.20633	22.80902	56.66101	56.53053	13.69456
570.3318	389.9563	187.7022	195.4552	330.8001	365.4271	146.1833	129.5791	210.4442
35.08764	32.41293	19.17256	11.03376	61.43964	42.63559	17.0361	16.48615	25.44552
32.85012	80.97999	62.02643	23.9486	49.01182	24.11475	66.7961	40.87634	22.5061
13.58994	21.09872	19.23634	11.05139	12.21226	21.24096	7.967547	22.66925	21.54113
28.33576	74.52078	104.8813	35.0194	125.5916	21.46475	61.16067	35.63928	18.58956
234.1844	87.69551	52.34528	216.7312	170.9329	117.3923	199.314	142.6933	105.7117
121.1177	147.5644	155.4371	159.5222	87.71507	66.8084	215.1858	101.7619	81.23694
35.13846	100.4816	77.69167	32.24002	125.2851	74.76187	63.47438	79.14678	72.42915
46.45957	251.3795	73.30272	106.0166	31.16804	343.828	58.96059	44.31395	207.5167
75.86136	138.0756	90.79377	56.21947	48.88877	72.11244	63.47858	42.57911	75.36516
1643.406	979.3086	1003.248	1272.407	1248.145	1445.997	2350.64	1224.025	1110.967
1.182113	180.4219	5.191962	5.503856	17.033	46.16279	1.183551	4.299442	13.70175
957.5508	450.7572	757.8805	670.2985	661.4208	414.9943	934.6424	644.6021	395.438
0.029058	3.847615	0	0	0.578534	5.23347	0.02928	0	3.917231
74.6578	31.70264	18.28758	57.17156	37.36853	75.9193	89.42218	29.54157	30.33781
147.1965	1603.007	683.6863	318.9967	865.7334	847.0208	385.2199	637.6698	717.4808
73.60203	101.0847	136.2316	87.58018	100.0125	70.78728	114.4218	107.8687	62.6386
15.8976	87.99531	79.52056	28.56169	37.37827	29.43549	27.22758	31.28696	29.35921
133.4123	31.05241	47.13449	98.70206	28.48026	21.46944	27.23348	71.35973	21.52621
0.029885	3.828815	0	0	0	20.28912	0.030113	0	6.863231
10.22715	43.57002	22.71151	4.578636	25.23333	26.60918	3.452653	12.14548	21.53486
43.04112	71.8865	57.61723	26.71048	57.81903	89.28629	12.5183	57.40488	46.00047
9.108617	38.81285	22.67106	25.80183	27.85255	41.31859	20.4292	25.20217	42.09214
55.49199	114.5656	155.5071	65.45139	78.92609	42.79502	32.90358	61.74153	45.0195
412.9657	127.7599	102.999	67.27913	105.4027	297.2813	71.42031	39.09246	189.894
1094.519	1100.764	967.4351	2086.586	1440.735	1776.817	1702.712	1594.634	1260.727
27.15462	6.85009	11.30933	26.74379	23.12548	10.78438	19.27861	45.32395	19.57359
141.48	18.92123	31.37976	38.69302	157.2378	261.2246	64.62011	81.73948	164.4457
11.36797	206.6525	3.440101	7.344126	37.4722	38.63373	3.453289	10.38903	27.40507
3.449749	24.13105	26.18904	48.91348	4.630002	50.48115	50.89427	42.68014	35.24182
30.51574	8.776906	15.69539	37.85166	17.6593	8.122491	48.5743	18.25713	15.65761
40.78482	76.32478	54.98715	40.5466	87.83977	65.39154	38.55897	58.2681	48.93655
492.191	70.44726	139.6789	486.9273	50.22608	60.16611	455.2794	441.1965	82.21364
74.70586	103.1148	75.08862	39.62271	78.26509	48.10906	39.69303	81.78046	44.04115
183.404	349.1064	405.1505	264.6059	538.4136	298.8693	175.644	433.2473	323.989
24.95129	68.67786	76.84774	37.78001	38.01607	53.41441	31.76636	19.95579	82.22427

TCGA-FG-	TCGA-HT-	TCGA-06-	TCGA-06-	TCGA-19-	TCGA-14-	TCGA-14-	TCGA-14-	TCGA-06-
117.9398	106.4602	33.70492	56.95335	71.78308	106.8496	71.68953	44.42611	80.03879
34.89049	61.67668	19.40671	22.58348	14.52348	38.40253	15.46817	43.87991	18.31398
197.1857	214.823	22.47297	47.13305	125.6128	122.3869	73.88297	179.6024	29.88347
3089.497	3540.529	600.4081	1577.207	2016.977	3296.451	1888.371	2656.272	1544.093
129.9442	114.3925	208.2937	206.2423	78.11224	245.9839	100.3355	55.87752	138.8822
0.047166	0	3.06604	1.959656	0.127312	0.09417	11.99425	8.927332	11.61733
2.48673	6.9898	32.67779	13.7421	36.6294	12.97615	344.3082	387.0148	108.069
132.3639	255.2767	30.6417	86.41717	54.31295	28.64984	46.34139	59.71696	53.9958
7789.946	8357.114	5064.596	4786.629	4679.649	10905.66	4491.056	9607.859	3523.168
70.97756	136.507	31.66089	79.55012	36.76214	39.97154	24.29154	34.76189	45.32146
26.4365	32.60758	11.23827	26.51886	9.737833	14.32752	3.348874	6.096817	13.49378
1337.798	1291.069	527.9092	405.5899	436.0436	341.7797	355.0025	421.6712	392.5221
87.89177	101.1728	152.1443	274.0064	124.1914	378.2196	113.569	168.5693	231.4789
0.050327	0	3.067684	10.8165	7.763471	23.09841	9.857716	14.22235	7.727594
111.8847	73.89835	31.66237	60.88334	135.0994	85.4692	30.90843	46.29221	52.06896
9.708216	12.27332	142.9042	41.24756	39.7675	14.39291	180.4604	32.67336	83.9403
2019.986	1687.066	1677.65	1506.499	1762.454	3888.366	1608.368	1869.235	1007.851
46.93049	58.97956	30.6381	36.33333	33.5356	35.66869	18.77865	11.86808	28.92393
53.00434	14.02957	212.3709	33.38322	87.57647	207.522	305.2038	95.8556	170.7241
148.0663	265.7343	2016.612	370.2367	232.4609	458.4221	1618.154	1680.345	1256.717
1757.585	1724.995	321.651	498.8881	851.0372	465.5867	703.3304	636.0484	567.0931
32.53029	36.94154	0.006902	0.975494	0.190855	0.137323	0.033442	0.293616	0.951428
20.53995	21.06977	128.6566	12.75935	32.05895	14.41415	2.242637	2.249048	21.20282
110.7633	240.2373	63.31651	407.565	40.0194	77.0413	44.1389	21.44441	40.49204
122.6796	187.5399	82.71068	20.61591	52.67155	62.73471	63.96218	97.68775	42.42355
20.506	23.73663	18.38584	70.72467	17.67756	17.21321	12.16474	23.18098	20.24415
110.7497	152.225	337.9712	332.9304	194.1074	676.7568	454.0851	632.4224	178.4204
1117.64	1108.895	553.4353	2755.731	1267.56	801.4069	760.6468	863.7841	1596.203
62.45826	66.95746	1.028928	4.902858	31.87939	4.444941	11.06458	0.289237	2.878956
42.1751	74.79295	76.58346	140.4453	117.5482	130.7854	36.41508	55.76242	39.53064
18.12916	43.97723	59.22431	76.60436	58.88946	27.19494	134.3966	17.60917	105.1442
37.35076	37.81066	15.32513	70.71091	16.14993	62.63239	14.37379	11.87645	22.16887
9.686499	1.712303	16.33966	9.815328	1.818186	10.08779	9.951817	2.268204	14.46094
699.0215	708.4353	2769.149	1332.683	542.5465	1602.431	836.6988	768.0466	1712.912
209.3508	119.6509	665.7141	471.4114	286.2004	375.5572	134.5172	191.6248	372.2996
12840.46	27888.56	207.2884	16756.17	2557.895	545.3123	951.3808	933.0181	2465.151
18.09073	10.51736	87.77293	26.51574	11.33841	5.865581	45.144	80.8247	52.10708
87.7966	139.1366	19.40941	28.47346	58.93684	37.14275	26.49611	23.34484	23.13284
37.33546	14.91235	290.9198	44.19138	28.80276	12.98516	121.1474	127.2148	181.3965
25.32297	24.60634	214.3564	37.31659	47.69706	8.717687	59.50358	66.69973	216.1622
26.5556	13.1507	451.2887	147.3098	111.4735	328.4494	481.5605	208.5621	564.2874
34.94354	36.93309	54.11877	218.0645	14.55952	39.94558	35.3026	40.40994	37.60547
13.32002	4.351335	132.7237	104.1082	65.21933	28.61597	73.84923	403.4731	156.2841
163.5789	120.5626	15.32534	52.04332	28.8817	25.80205	51.8491	25.28792	26.9897
150.4496	190.0654	322.6619	346.6749	176.713	226.3826	278.9016	128.6357	369.3962
16.8818	14.04982	29.60765	36.34408	8.171775	5.861992	11.05682	11.77104	17.35398
36.15444	74.81395	77.6005	132.5939	35.1773	37.13332	22.08786	17.61308	49.18067
2832.067	5733.71	168.4879	1026.261	3111.189	697.626	1100.198	860.2531	615.3116
217.8356	454.9753	3548.174	176.7661	133.8615	37.1823	233.7392	172.7344	279.6821
10.88327	5.233172	7.155234	16.69357	28.42846	5.854219	8.852175	0.272378	4.808177
7.29123	9.643764	8.175898	38.31453	6.579466	18.51878	13.24962	0.272511	14.46088
197.343	265.779	72.506	164.9839	270.3963	445.2857	145.5429	166.8479	68.46181
62.58611	47.49315	41.8708	115.8922	103.2274	105.2045	57.34063	21.43909	98.38303
5138.359	6666.526	168.4875	2218.501	851.2129	505.4702	357.2076	142.0661	723.3315
22.86451	18.46313	12.25941	34.37861	20.73124	0.131686	20.9535	8.000307	17.35387
63.76493	73.05749	27.57683	39.27833	17.73767	49.88448	28.69693	17.60902	30.85103
18.08609	50.25546	3.071913	11.77898	12.90578	12.94007	11.05865	0.280536	7.701791

9599.254	10937.56	2545.577	1887.541	2510.137	2826.923	2331.534	3504.83	2534.595
98.73499	58.91245	826.0296	2015.311	777.0488	375.7051	540.0984	568.1785	718.5631
25.30545	18.4443	29.61414	73.67086	19.26166	17.21771	29.77621	17.52764	45.3366
15.72261	24.60016	28.59702	13.74159	17.72935	45.60166	121.1599	25.20748	72.34278
0.064889	2.591396	2.050552	15.70758	5.014861	8.70871	364.9151	55.00206	54.99542
327.1676	392.5804	91.90565	94.27263	135.3605	170.8776	94.8406	174.4246	94.50429
18.08062	17.57944	31.64888	61.89384	26.99221	17.16186	27.54887	9.891701	17.35363
32.49668	24.61662	6.135373	14.72474	20.83741	7.292135	12.16526	26.96599	9.630206
83.0211	85.36369	39.82968	81.51124	57.40049	137.8757	49.6358	93.79621	53.03597
142.0103	217.3778	314.4869	727.7564	267.1044	1153.996	365.9252	891.6661	237.2606
31.19334	28.19943	1.029086	6.867873	3.416508	7.26938	0.031889	6.088237	1.914709
39.63626	36.10957	11.23944	105.1258	6.600036	1.585418	13.26134	17.45638	6.736767
325.9826	216.486	79.65326	184.627	90.89016	57.11481	100.3528	111.393	86.78769
28.92319	26.36898	15.32449	91.34774	27.19579	39.89135	27.58567	9.953711	27.96033
98.66614	95.03632	97.0049	21.59796	12.97611	15.83871	14.37467	15.71197	14.45165
42.11512	41.35738	37.78241	75.62987	49.26014	10.13893	31.98796	28.93794	28.92561
250.2469	389.9166	90.88504	110.968	78.18039	109.7525	157.6653	130.523	61.71044
25.31602	68.70923	13.28238	2.938962	11.3715	20.06288	19.8757	15.65626	21.20722
101.1378	58.91276	1652.02	330.9616	194.1788	261.9208	1714.776	684.4801	587.3898
405.3133	251.7068	58.21087	99.18312	178.2416	69.91763	69.4908	138.1347	62.67518
1721.585	996.2074	1094.609	2209.675	682.5643	775.8643	1032.936	481.055	1084.049
32.55027	63.35895	24.51416	16.68778	43.09056	8.719469	46.32144	32.85574	122.5099
250.2527	419.8438	113.3484	7.849669	11.36859	7.279011	14.37231	2.239214	45.31429
16.92687	240.5573	5.114153	16.68785	11.3844	5.869015	5.554336	15.69947	3.843474
20.53298	41.33421	44.93218	62.85249	82.58307	69.71877	83.75594	89.73124	156.286
2160.635	2386.814	434.9915	343.7187	1022.847	239.3078	669.168	576.7715	649.0711
28.958	25.47125	123.5489	50.07979	161.9908	79.78117	176.3231	582.1192	101.2674
167.2117	117.0298	30.64167	73.6497	76.53679	48.56396	74.994	52.06623	50.13789
207.0026	250.7814	939.3831	309.3493	337.3249	336.0007	574.3155	878.3947	748.4388
21.74249	27.23152	36.76748	72.66937	141.4334	166.3623	154.2988	251.7978	31.81266
145.6144	126.6982	121.5152	159.0932	116.2841	81.29011	241.4044	145.7342	173.5991
36.13704	35.17495	31.65938	48.12037	30.38752	49.83881	18.77953	17.5921	15.41698
1023.72	1130.963	101.0968	340.7745	462.971	125.4387	111.3831	289.4955	232.4216
1547.191	1889.499	2080.974	2715.443	1794.213	918.2105	1889.453	1467.119	2189.332
4.75222	0.845395	1.026828	0.977109	1.707374	8.131706	32.38185	40.28827	0.954541
43.33528	73.0826	22.47133	103.1322	33.54348	63.97969	18.77918	13.77834	28.92355
70.99209	82.73373	0.006976	3.921123	12.9763	5.867465	3.347423	4.192254	13.48724
600.3762	902.0758	815.8488	1034.128	466.2127	1199.742	932.5896	1435.77	1001.117
83.03716	99.44695	68.41741	164.0157	52.6711	40.01032	62.86038	38.64396	87.76291
60.19318	88.01148	33.70361	68.743	57.38733	65.53854	45.22753	32.89458	57.86024
45.76482	124.1641	46.97474	4.903023	47.84561	105.1629	14.37421	13.79205	19.27461
34.96273	48.36994	51.05989	4.903108	5.009236	11.56752	6.656647	15.70967	10.59389
116.7213	82.69395	120.4902	35.34717	59.06302	58.51018	103.64	32.94271	31.81213
323.6294	133.7259	287.9483	874.0731	338.7761	269.0677	254.6617	340.8535	648.1481
21.65359	22.00428	1.029076	2.9389	11.30529	4.439885	3.348826	2.269197	2.878864
3.69248	12.27307	0.006856	22.58256	9.785641	5.870345	1.140488	0.290455	0
21.6908	13.16065	12.26041	119.8626	8.185974	39.74005	11.06108	2.269498	9.63096
31.31513	21.08384	107.1862	65.80708	39.78594	28.55763	27.58241	21.34882	87.79816
28.85293	28.17229	3.071911	12.76147	19.18239	15.75963	9.957791	0.280073	10.59675
27.7445	66.88397	15.3252	18.65206	19.32694	49.89468	30.90115	25.24039	19.27472
9.698622	0.83522	43.89527	33.39444	20.75711	4.443101	35.24774	21.17972	31.83332
1701.161	2218.671	737.2325	831.8114	1119.916	956.6251	641.6148	1262.224	670.2877
62.54281	37.81637	13.28295	34.36801	24.06638	18.66888	18.78016	9.960111	49.18445
200.9962	241.9729	1565.299	673.7045	1073.294	435.6366	1725.039	897.8559	3484.758
395.8227	447.9592	183.8032	134.537	178.3595	301.8366	106.9725	159.302	209.2766
0.062952	12.29138	15.3196	24.55483	25.3371	325.3175	46.19152	96.5186	14.46043
15.67641	9.640933	46.94993	24.55372	8.163026	5.858594	7.754036	4.192735	1.914713
153.955	139.0623	55.14605	64.8114	47.93844	76.9685	57.35769	105.4242	75.21875

16.89701	5.230417	25.52883	3.920856	34.90109	5.866775	33.05973	17.46722	48.24195
31.34643	43.97732	40.84867	42.22504	24.08096	71.13573	20.98535	19.51673	30.85103
143.1446	102.0719	7.156186	90.34742	14.56696	44.28875	11.0667	9.954518	10.59416
308.0133	386.3528	22.47259	141.412	79.78292	129.6921	78.31187	109.5363	44.34992
122.7234	87.97968	70.46139	43.20416	100.3036	91.1836	81.6009	122.6079	72.32418
68.62746	192.8072	226.6628	117.8499	68.54609	162.1071	130.0651	145.3244	79.07888
45.80519	514.0309	1.02841	94.27195	92.49338	24.37527	4.447234	8.010362	21.20334
37.339	29.887	59.22172	51.06707	17.72792	31.43145	44.1083	21.40105	35.67682
48.02538	21.98071	26.5498	14.7255	41.1826	15.78163	22.06495	24.99604	45.34426
18.11645	32.54459	56.15407	91.35083	28.75256	86.44932	63.89381	49.65951	54.98318
241.8275	192.7136	213.4097	100.1649	90.89376	178.0095	149.9498	96.10418	261.3698
228.5806	67.71617	180.7352	440.9664	167.1137	179.4011	287.6931	138.1203	453.3284
477.6277	513.9624	183.8038	194.4441	280.1129	604.8543	300.9722	914.7664	220.8493
5202.035	6143.82	407.4227	1279.637	2196.737	612.2263	881.9331	1187.71	618.205
14.49597	9.635938	17.36338	29.46348	9.76047	15.76907	20.96028	9.908066	25.07431
36.07592	41.38797	19.40616	22.58407	14.51397	45.41945	14.36557	34.43325	16.3848
66.17187	161.1944	8.177654	57.94065	60.48517	27.19813	15.47614	29.04786	13.48735
0.039581	0	15.25104	2.947407	4.551679	2.83241	22.6315	3.665833	4.839019
2.487686	14.05782	0.006599	1.956976	0.179334	1.586117	0.031894	0.273277	0
0.051255	0.838221	43.78741	3.92553	12.23333	0.103159	1.136989	7.461593	26.14385
12.08654	8.758733	33.68675	42.24291	17.56958	22.75669	15.45207	11.74691	36.66898
95.0858	97.66371	168.4755	208.2074	65.41069	160.7819	79.39866	57.7816	145.6356
28.93612	36.9359	26.55519	127.6895	69.86747	37.10132	34.19921	19.49986	66.55339
554.6238	793.8571	339.0066	283.8138	324.6482	658.9647	479.5396	675.8949	429.1795
68.64452	530.9653	210.3367	68.73916	65.42046	230.3904	66.17739	61.61353	89.68568
4.887601	4.354252	5.1132	14.73056	6.556678	8.653654	9.944263	7.924452	11.56774
1039.451	768.2797	15.32413	225.8696	199.0775	132.5559	24.2912	36.74834	85.82207
184.075	186.573	171.544	285.7912	235.294	113.9817	251.3123	202.9481	282.6015
72.23939	21.06982	467.6025	63.82935	105.0336	21.53244	196.169	183.4704	350.1592
14.49356	11.40022	53.07871	88.42421	17.6204	64.94794	47.33471	30.52481	83.01106
303.1827	491.1299	192.991	341.7631	191.0282	190.8439	246.9454	140.1248	192.883
286.301	403.1385	43.91587	120.7895	143.3103	142.4501	73.8999	155.339	53.0302
129.9519	227.1068	77.60923	65.79269	103.5134	44.29728	80.50357	118.8688	99.33142
52.9851	45.72811	18.38851	46.15229	73.21701	42.83316	47.43058	50.03432	9.629486
24.09389	10.51469	29.61238	47.14652	14.50975	83.39088	30.86748	17.49329	43.41111
45.74665	101.2805	545.0847	119.8291	65.15824	37.10891	191.6015	110.3987	265.3447
103.4795	127.6196	39.83065	39.27595	25.6999	17.26232	29.80661	61.56031	21.20286
7.283846	6.118003	75.4928	8.8337	15.91279	1.585614	43.95119	22.716	34.75291
0.059884	0	3.071314	5.88698	119.451	23.9037	9.934351	162.184	25.10384
85.38422	72.16846	40.84927	159.1155	62.07976	59.8243	39.71409	48.07147	34.7092
43.30046	50.18558	4.093095	14.72437	19.27948	28.55141	8.86154	15.65394	9.629904
14.52164	11.38976	64.32739	68.74717	90.43034	78.18773	83.74842	49.90254	87.77885
200.9294	244.6669	318.5721	390.8768	156.001	224.8892	121.2909	67.40166	192.8889
93.83605	55.41261	114.3576	98.20851	55.82395	88.25866	265.4982	69.09857	526.7578
631.5782	930.313	92.92807	260.2442	450.1827	189.4693	198.4639	268.3953	144.6546
36.13898	79.24776	6.135402	7.848975	17.72821	18.66763	9.964719	29.00275	9.629496
24.12589	19.31779	84.73733	29.45765	42.99079	37.07461	89.22064	110.0959	45.32824
56.57221	122.402	54.12095	26.50928	32.01275	14.41282	28.6986	46.17262	19.27462
22.93043	30.76855	108.2178	49.10259	16.14198	100.761	23.18568	32.79356	30.85282
59.02125	128.4885	9.198516	296.6061	28.88168	3.005661	5.552404	9.953626	25.06072
58.97667	77.45282	46.97494	37.31328	51.0163	44.23282	32.00407	36.67401	53.03897
312.8095	727.9363	55.14786	89.36131	43.20095	24.37382	37.52438	31.02959	100.2895
49.3694	111.822	33.70294	26.50921	30.43108	34.30058	25.39383	25.24783	6.736322
15.71318	36.07623	22.46973	26.51216	20.85285	65.25088	35.28263	21.33105	30.85763
182.9511	183.0048	1182.402	1534.025	493.1551	724.4215	1612.605	1184.69	688.6301
1041.71	435.6163	32.68347	148.2858	307.152	45.72643	91.54062	78.91562	124.4004
28.93839	35.17162	147.0085	204.3124	54.10579	83.82789	80.44098	34.71154	61.72748
184.0366	205.9701	121.5122	55.9714	124.1396	78.41712	120.1723	219.9021	73.28738

89.06698	65.08794	23.49407	57.93636	59.04816	25.80134	36.41982	38.6693	86.79434
57.73579	34.29379	57.17978	73.66087	55.65282	37.09532	38.60219	63.07551	53.04524
48.21025	14.03438	1623.48	1813.941	324.5995	623.3128	4157.288	1235.793	1322.337
221.378	152.2217	152.1472	301.5006	224.2997	197.8998	317.4545	266.1206	267.1591
76.99272	28.99741	80.66464	134.5559	43.11058	54.16827	47.42682	13.79354	94.52449
46.89172	22.84884	38.80156	76.61553	60.20207	12.9741	26.47927	19.44099	73.32369
141.9363	154.918	115.3835	200.3528	71.73635	106.7874	55.15342	67.29503	127.3105
14.51675	16.67601	278.6543	49.10483	30.3523	20.07261	562.1394	96.81907	368.6702
62.51011	66.04188	18.38734	23.56409	27.20309	17.2398	12.16809	8.042507	30.85453
443.9944	758.6154	182.7836	392.8242	323.1037	199.4488	242.5614	214.8709	158.1565
28.90205	38.72922	24.51063	8.831305	20.83893	34.17732	12.16537	6.122321	14.4541
133.4817	105.6162	113.3379	193.4837	87.52299	72.66499	66.16399	51.98347	84.86953
269.4297	187.4504	139.8931	51.06037	133.7379	183.6325	114.6741	147.6199	102.2219
84.27567	100.2994	34.72595	33.38288	71.77602	21.53285	52.9536	4.17701	94.50814
20.36581	14.08913	3.071379	14.73295	12.72999	1.583862	0.030571	0.25668	6.741169
641.2263	711.9941	262.4267	169.8916	515.3986	940.6307	281.1368	266.5144	281.6111
24.09524	21.09473	19.40602	61.88433	22.38559	20.02983	16.56644	28.78928	23.14053
1340.165	865.9777	60.25285	359.4326	747.6621	106.9341	228.2327	435.0408	256.5321
380.1495	103.7992	644.2891	349.6195	43.20101	8.702435	458.5514	208.9727	1105.349
55.40027	78.30856	11.24095	16.68756	54.25378	91.11805	6.656228	74.84212	4.808081
38.57442	20.19017	269.5353	35.34776	41.56771	86.86222	199.4412	194.6084	271.0677
380.1723	425.9638	308.371	370.242	178.3454	316.0355	273.4058	591.3454	362.6367
28.94798	51.02033	99.03905	95.26593	54.17866	59.82959	156.4331	123.9379	141.805
316.386	426.0056	180.7373	55.97054	106.7914	64.23156	126.8069	99.93823	147.5519
1145.352	1113.27	886.3083	809.225	771.5977	861.2342	761.762	1063.004	871.8654
71.0033	59.82107	51.05983	145.3579	32.03262	98.15529	27.59954	44.32522	105.1335
10.86477	17.61507	13.27589	20.62826	8.11782	7.246781	19.82003	2.26296	30.89139
283.9529	1168.106	271.6123	103.1105	116.3473	92.69571	142.2425	109.5299	54.9589
772.2579	1177.698	62.29546	170.8742	89.32777	38.60974	63.9815	78.91547	27.95473
1266.796	3231.853	382.9155	421.3037	609.3434	553.8087	573.2571	919.2808	525.6211
28.93646	24.60022	24.51342	118.848	16.14366	64.00348	31.99702	9.959932	44.36024
25.30276	28.14589	32.67578	109.0431	30.27723	65.18323	20.97164	40.12068	48.23336
78.20387	55.41879	375.6878	126.696	41.53889	24.3677	161.9607	131.6716	202.5836
48.2089	29.87352	705.5677	634.4216	388.2385	488.2625	752.891	1249.671	325.9778
34.96215	136.4728	9.198738	27.49102	32.03194	83.97402	197.2027	65.24962	153.3743
15.69043	12.28263	54.0985	42.23837	17.61671	113.986	52.82523	89.93947	62.73294
1081.516	1080.752	217.5004	285.7768	369.2204	264.9148	286.6545	440.7467	303.7919
93.83443	104.7424	28.59889	57.93807	35.21271	57.03955	30.90627	78.6042	20.23862
18.06519	7.877246	20.42209	23.57143	19.12274	12.91344	7.753601	4.191568	14.45975
141.9677	161.942	51.0626	62.84638	52.71742	132.4013	104.7446	149.362	49.17349
114.3213	257.9389	6.134905	8.831321	74.9405	22.95638	15.47678	29.11767	5.772708
12.10702	20.21013	65.33369	25.53096	27.12025	56.70918	55.06762	79.41695	28.93031
158.8261	177.7708	208.2996	274.006	106.7337	166.5612	120.1817	97.949	92.57694
36.10996	17.55918	68.40176	110.018	6.608829	24.31009	11.06526	27.02015	38.57691
10.90904	19.32404	129.6335	30.44183	25.57878	25.71245	61.68251	49.58624	122.5554
13.3205	6.11042	394.0506	16.68772	47.85171	64.08465	131.1127	445.5245	573.1327
6.065254	0.835096	63.21482	13.75314	6.4939	1.580676	6.636422	4.123163	29.95129
149.179	159.3	63.31491	205.2593	125.716	34.34015	62.87134	36.77078	80.03974
2837.422	592.2774	5.112986	84.45068	41.59943	28.63695	27.60003	13.75126	41.45716
24.1493	14.03008	57.1887	286.7809	122.5362	27.22594	243.5639	40.59353	67.50034
2.487361	1.712305	13.27779	13.74622	31.47974	100.7543	83.45445	54.04533	39.57666
155.1631	87.10052	146.0131	96.24028	106.6375	45.7112	84.90528	59.67699	131.1677
0.051754	0	12.23605	0	12.26272	4.326243	38.11614	210.8472	5.78739
67.37116	47.49979	38.80713	108.0371	76.28522	48.47419	39.71257	30.94945	63.6533
56.58977	40.44341	122.5228	199.3823	109.6102	79.72325	90.38777	42.42216	130.2181
697.7672	525.3863	229.7527	612.8146	478.8607	316.1213	317.5132	176.5674	336.5865
326.0377	459.4306	412.5152	433.0993	294.3073	149.6009	251.3564	149.6968	324.0586
262.2985	330.9073	259.359	230.7841	125.8783	250.5781	157.6718	134.39	185.1666

4865.24	4128.385	2965.241	6762.575	4736.721	5075.653	4251.817	4889.146	5354.7
33.77128	72.13003	88.8392	29.45461	14.56657	14.41389	5.552397	2.248512	13.48734
347.7336	161.0037	1360.056	593.1757	415.2157	99.81818	1316.077	1566.727	1304.967
50.45595	22.85505	16.34426	14.72489	16.09911	29.92867	16.56844	9.935543	24.10444
1182.658	2135.081	1209.991	632.4498	930.6421	1014.95	1404.415	1375.089	731.0506
143.2098	125.8177	362.4729	392.8433	273.4321	256.1271	254.6277	315.6056	357.8367
20.51519	50.18665	81.66906	28.47666	42.91579	46.91753	107.8841	53.394	83.94016
123.9554	106.4578	79.65194	205.2566	76.55325	258.873	104.7509	75.01025	98.36518
8.494853	0.835044	46.95178	23.57037	42.53867	46.61316	175.7264	37.83243	29.90692
16.92665	8.749447	158.2413	42.22521	22.49389	216.854	69.44106	459.8053	281.7366
51.80426	88.86568	53.10388	22.58001	33.645	112.4614	60.6624	82.54033	46.28087
127.5746	598.6658	95.9891	208.2008	186.1184	69.90915	129.0003	52.08931	112.8321
26.46972	6.111311	79.6095	17.67333	17.62878	0.132849	26.45771	54.77995	108.1151
12.04592	8.774211	34.69082	15.7152	14.29508	4.42267	26.38302	40.67459	29.93177
102.2056	69.51857	40.84978	37.31305	57.35917	55.5879	29.80164	48.09956	35.67322
42.14454	25.48045	75.55521	36.3324	49.36533	58.35137	96.9508	161.3449	32.78177
123.9899	258.7311	293.0516	73.64806	246.5995	387.0369	415.5542	283.44	356.8578
8.479798	25.57497	14.29681	25.54182	28.29533	15.66545	5.549093	6.061609	11.56707
318.7639	388.1693	41.87371	36.329	125.8399	37.19079	48.54811	84.62143	72.32012
34.89153	23.73539	12.26109	51.074	33.42631	44.0474	8.860745	15.63117	26.03408
50.58105	77.44149	33.70352	19.63396	19.33462	25.78945	12.16987	9.961702	28.92012
81.89655	46.59412	86.80147	58.91697	92.50851	149.636	24.29191	138.2832	181.3037
2.487768	1.712328	153.0379	36.34724	3.417271	0.130131	42.90521	74.58663	44.39773
68.60363	15.78972	3.071463	22.58028	27.27493	4.438824	4.450488	8.040961	30.84913
261.1041	521.9273	417.6213	340.7797	219.6486	358.6769	252.4608	208.991	250.7532
53.01011	101.1888	66.37736	202.3154	51.11893	183.4768	59.56285	46.31651	93.54589
65.02074	94.15571	18.38865	326.0806	92.31686	81.20588	62.8633	93.92772	82.93787
245.377	190.0966	116.4092	259.2734	106.736	82.70332	94.8358	136.1413	107.0453
231.0648	307.9885	141.94	3727.077	338.9451	277.6904	120.2015	94.23641	325.0141
30.14886	2.593461	111.2886	30.43801	88.9514	71.16333	126.7045	63.26245	101.2809
13.27809	7.878154	32.66462	18.65858	20.66562	10.09127	18.74473	7.9758	42.46808
185.2209	172.5157	86.79772	133.5617	78.10904	34.3389	55.1562	25.28907	105.1204
28.89582	74.04055	11.24011	8.831375	16.09855	14.3816	23.17163	17.51512	24.1045
9.710789	6.109846	368.4972	122.7779	73.01885	456.7297	52.91622	229.1018	240.2607
26.53204	35.17569	6.13541	2.939057	0.19132	11.56396	4.451667	2.262874	4.807531
63.81723	43.95973	128.6535	181.6943	82.80089	30.06442	67.2694	71.0637	101.2674
44.60195	31.63056	2237.034	952.6587	176.6758	31.49646	382.4769	216.4953	201.5676
233.1433	49.24631	10.21983	53.02685	12.97682	20.10702	4.450193	2.253447	10.59395
55.29626	51.06151	12.26154	21.60003	16.12425	4.444645	19.87665	8.040215	23.13661
28.88154	45.81001	16.34359	66.79858	53.72667	15.78495	11.06176	2.269267	16.38543
24.08694	32.57189	5.114209	57.95641	6.602036	5.867178	8.859083	4.200993	13.49066
129.9573	139.9213	164.3942	31.41868	63.84027	42.87665	66.17877	59.71379	153.349
3134.026	3993.764	3586.056	2035.837	1991.558	7215.985	3185.83	4540.373	2554.855
6.099203	1.71522	122.5215	56.95679	62.12509	25.78855	77.16804	680.7585	187.1461
172.1187	176.8476	373.7208	329.9736	483.5189	360.1774	899.4484	625.9843	381.9225
83.04059	33.39461	418.5865	90.34929	198.3733	353.4953	220.3749	327.6301	207.3893
126.3754	166.317	415.5639	168.9147	162.3237	277.4334	297.6001	275.517	407.9975
34.92742	102.1993	37.78337	68.75121	42.99083	34.24379	33.09254	32.75343	47.25819
67.45726	15.79165	674.9066	655.0697	427.5168	479.343	238.1134	749.0711	693.5045
97.47076	80.94195	20.43082	36.32957	20.93087	28.64475	25.39792	27.19638	23.13185
15.71897	5.22998	122.5016	70.71644	46.1358	87.95138	146.4257	57.30489	93.58159
98.59402	71.28464	4.092849	74.6382	73.15945	3.011471	34.20731	25.24831	4.807775
0.058286	1.713874	5.111487	0.975352	9.569256	80.03319	14.30212	223.597	15.44436
1.274537	0	3.069345	3.924121	0.149118	0.10926	37.11153	9.357275	9.654865
19.24197	22.01942	8.175614	13.74612	6.574033	0.128578	8.850715	2.267301	0.951002
20.52486	14.03237	37.78363	39.28045	87.11984	65.36949	146.4392	132.7377	103.2296
24.07961	46.70865	15.32227	9.814043	11.34031	11.53383	11.06006	28.70223	7.701538
181.6666	164.5634	100.0729	222.934	133.7209	112.556	137.8133	130.4102	155.2745

1093.545	2176.579	33.70428	107.0384	32.04605	0.142562	12.16388	25.25206	85.82189
0.067308	0.837318	18.38861	8.831126	3.408762	4.437563	778.6499	126.1694	78.11684
0.029919	0.861326	9.136765	0	0.076455	0.05781	2.188057	4.905426	0.959595
10.89282	10.52126	0.006665	1.956982	9.744631	12.92703	1.141229	2.269655	2.878836
1454.535	1797.108	879.1621	425.231	574.4282	754.5224	835.6228	1086.052	761.9133
43.20963	1.712587	1.029033	1.957001	8.179507	1.585634	0.0325	0.281117	3.843125
28989.35	27154.47	17401.37	13030.17	20308.54	23942.27	24248.67	31114.08	19397.26
61.41221	65.09482	187.8659	288.758	109.7241	128.0257	176.3198	158.5667	217.0366
231.0156	315.0763	139.8957	362.39	267.2332	229.2133	183.0161	143.9192	189.0268
270.6833	209.4405	240.9774	604.9794	149.6779	64.22992	179.7061	157.274	368.4372
480.0365	352.0012	619.7961	1649.922	512.1619	333.1654	329.631	264.5684	483.1944
477.7022	1325.378	151.1299	188.5506	254.7485	2695.494	413.4279	2165.524	627.8527
359.6923	172.4584	19.40937	210.1602	184.664	22.95078	17.67976	9.930991	108.9704
27.67965	9.634437	60.22635	10.79617	20.79365	15.78095	33.06183	49.31693	38.58674
36.13726	18.43486	64.32532	389.0097	54.07702	65.40498	35.29921	11.87095	70.41424
7.298994	14.92782	7.156029	4.902958	5.011985	12.94324	3.349143	2.269822	10.59643
80.62939	95.92731	51.06078	68.74173	44.73596	145.0106	59.55369	128.0732	26.02575
149.1889	153.1296	67.39929	62.84615	59.07606	106.8445	60.66851	27.2052	59.78318
42.16793	43.96931	33.70329	47.13494	46.28387	62.68383	196.0851	53.81191	77.15699
454.6356	536.0705	0.007075	46.14975	63.87298	88.41527	39.72949	42.528	14.45241
119.1253	134.6478	50.04142	143.3834	79.69564	44.29394	39.72723	38.67926	66.53604
0.062641	3.471447	27.56212	9.815464	9.717483	4.436728	132.8185	13.57355	20.25553
3350.5	2884.003	843.4259	1222.678	1533.451	1002.194	1474.986	1524.614	1571.101
113.1054	168.1232	18.38866	38.29362	52.70363	48.55392	40.82843	34.84972	33.74135
10.91427	6.111113	235.8418	73.65326	60.57513	105.2763	396.5308	272.2427	61.71784
32.46954	76.72502	5.114214	5.885011	1.817475	7.287828	5.55427	6.116281	3.843086
442.7255	421.5519	10.21893	138.4654	211.7384	173.8053	160.9839	513.0889	135.9749
3479.277	2807.409	948.5982	1710.77	2600.646	703.3179	1222.557	1229.814	1071.504
255.0943	275.4408	442.127	568.6298	338.8198	360.1101	190.7405	214.7386	415.6874
25.34051	34.28535	12.26204	130.6304	25.66809	27.19671	56.23135	17.61073	40.49824
0.064476	0	18.38436	7.849455	47.40313	66.42432	92.39643	203.4061	245.2369
72.24119	211.2847	68.41905	60.88277	84.42999	227.4468	67.27487	137.8091	57.85566
89.0923	77.40374	151.1228	294.6327	141.6394	131.0221	110.2617	139.9278	150.4527
70.9931	39.56534	71.47717	301.5446	107.9742	72.60876	83.77281	69.01145	95.48831
15.65199	9.648253	11.23658	24.55764	12.82169	28.26092	6.65002	11.6764	5.773631
31.27904	15.80417	26.55038	36.33903	17.65911	24.26037	15.46517	8.025057	20.24555
6.057213	3.480049	3.070595	8.838176	3.375564	1.578239	2.242377	7.754796	3.846498
32.48836	33.44194	3.071887	54.02259	20.82473	10.12992	5.554606	6.120612	14.45458
1.280714	0	2.050602	8.833288	57.72197	74.25473	86.72944	79.55185	20.25772
38.55638	35.16523	55.14152	31.42031	65.23217	28.61858	37.51037	30.95346	54.00472
44.55984	35.16586	25.53507	92.32036	66.80489	178.7331	47.42202	36.65516	32.78016
50.52839	48.40059	8.177649	10.79528	33.53405	14.40424	5.554724	9.957313	13.4879
14.50921	12.27511	24.51045	57.95139	36.5702	59.55457	41.87596	11.8391	74.29551
116.6806	172.5564	18.38864	53.02634	117.6463	221.6447	44.13133	88.19524	29.88372
21.69301	32.57021	69.41097	75.64265	8.187217	4.444416	23.16539	8.02238	48.24006
25.19879	26.44805	22.45957	7.850746	17.49758	4.434242	23.12564	11.68404	16.39433
34.96272	37.80129	58.20641	79.54737	51.05344	78.30593	244.5534	122.2404	117.6755
51.74399	16.67283	145.9866	36.33252	43.04157	69.66777	79.33736	47.97229	76.2023
45.66673	44.9112	4.093119	11.77804	14.52097	3.017355	2.245439	8.031431	10.59508
3.689706	5.23116	235.8331	84.45967	204.3505	253.9236	629.8343	481.8667	108.9956
9.7038	20.21373	23.48852	21.60198	30.23409	11.5428	18.7666	2.269127	38.58482
99.90249	197.1637	69.44115	35.34708	63.83602	51.40778	108.0517	48.24386	60.74797
40.95459	48.38276	0.00695	5.884961	41.49703	5.868932	2.244115	2.259668	32.78047
134.7123	375.1634	1.028608	1.957512	11.38347	3.007146	4.449734	6.111448	13.48724
4.895337	6.11032	1006.5	13.74141	32.01142	88.16031	521.8699	314.9202	188.1228
62.6154	78.30421	227.6819	99.18876	66.95075	180.524	98.11567	133.8693	201.5992
30.09667	34.32114	0.006813	5.884926	9.778733	0.135068	0.032988	6.121546	3.843093
1.275643	11.48872	1.02873	2.940622	13.94489	4.365373	12.07969	61.42726	6.749656

220.2517	547.3586	137.8558	2406.097	1142.076	7218.769	2988.384	3367.289	2507.631
1.280713	2.591568	5.113518	20.62589	4.995383	32.45932	311.3832	77.72153	25.08751
123.9676	95.88779	165.4184	484.1859	187.7052	528.8565	224.8702	479.5379	256.5557
90.31128	106.4435	243.0199	147.306	270.3878	200.765	294.3219	361.6844	620.1904
15.68484	14.04981	44.91291	8.832135	25.41981	15.74953	47.32041	30.45324	24.112
4.896252	12.27981	177.5653	33.39296	28.62556	139.4066	85.80614	71.61528	97.48648
55.38199	82.73191	24.51453	116.874	35.19575	47.08227	44.1237	34.78779	38.56699
576.3201	515.6871	512.5913	1243.314	684.0314	303.3406	637.1837	325.9117	896.9514
51.79836	73.02057	63.31297	130.6194	131.8996	177.6802	60.65852	48.18913	84.86793
158.8782	138.125	394.1379	170.875	492.9045	711.3294	641.5084	950.4738	425.333
22.89755	14.92148	68.39331	31.42615	20.81391	17.20453	19.86799	4.20184	36.65331
227.3737	374.1005	90.88439	48.114	92.46566	132.4827	88.22672	119.0064	29.88294
50.61349	587.8635	0.007104	37.31161	55.91684	44.29753	3.342943	21.41849	35.6708
5010.843	6846.056	5205.502	9390.61	4964.253	5878.459	4834.974	5636.098	6197.637
91.40825	67.75361	16.34636	23.56254	41.53483	21.52436	13.27221	13.79436	27.9557
2731.001	1982.763	2709.966	5055.732	3297.22	2997.587	827.9168	1775.527	1507.443
20.52239	29.0123	174.5538	24.54645	22.45955	14.40095	93.61562	89.32659	86.82666
140.8138	218.2517	360.4342	75.61259	192.5305	455.131	249.1279	413.0645	302.8498
61.35352	64.25131	16.34605	20.61661	39.89781	1.580685	15.47544	21.4143	18.31052
45.80383	4.355015	561.5653	420.3441	94.04705	106.8837	486.0202	405.2427	510.2468
110.7241	71.24396	277.7221	125.7031	81.30389	173.6132	338.324	147.4976	154.3137
0.030085	0	9.137148	2.95321	0.076918	3.986136	19.20974	3.40966	2.906779
12.11147	6.989849	134.7401	60.8958	57.07285	25.72184	70.49003	115.3655	74.28775
20.51985	12.27223	117.3949	30.44061	58.70038	63.90213	67.20163	68.54366	103.2377
32.53258	73.96713	43.90887	41.24471	28.7984	27.17153	24.28585	11.86878	21.20541
38.55963	37.80606	68.41354	50.08211	63.67115	56.99557	117.9001	72.76458	106.1042
312.7119	374.9929	15.32512	8.831515	17.7452	5.856224	6.654065	8.020079	48.208
96.24906	75.66651	12.26207	50.08015	131.8599	61.31118	19.8863	53.89227	28.91936
9.710868	11.38942	266.4638	22.58028	360.8897	143.5236	1092.307	1495.246	299.0639
703.8417	868.6156	60.25283	745.3918	734.9526	384.4661	543.4952	122.9609	519.8341
638.7892	521.8843	104.16	925.1275	628.1558	232.1518	115.7918	306.6499	249.7842
13926.83	17599.61	6018.293	17408.27	7787.618	16421.18	8656.875	12043.98	9765.175
31.28554	27.26866	27.57157	27.49618	30.25048	53.87039	13.26475	15.61355	18.31482
98.6977	111.7487	95.98711	111.9536	92.40054	211.9289	65.07527	48.24048	93.54416
8.505119	5.229914	114.3223	37.31924	20.83936	17.21819	214.4904	17.52867	84.91244
20.48959	13.16165	21.44624	27.49779	27.05739	21.41923	26.46116	8.017511	26.03857
6.078748	7.006779	11.23402	17.68171	9.644659	15.60444	6.644199	7.882925	19.3012
18.1206	10.51063	160.2645	82.50564	39.82223	18.65643	169.5202	93.08362	159.2132
84.26516	84.45718	208.2919	258.3001	109.8199	62.76942	195.079	137.8538	298.0571
15.66507	14.94349	127.5267	17.67638	11.28607	4.437168	34.11181	57.84897	55.02954
33.75698	71.27985	35.74513	100.176	58.94482	54.17168	25.39465	42.39701	26.99121
77.03653	65.97404	70.45991	175.8007	47.92272	120.9439	88.20183	61.54011	41.45854
9.700614	16.69061	48.99926	9.814045	11.34022	1.585454	12.16065	2.269729	12.52617
481.2369	496.3572	213.4148	286.7608	410.4443	195.1595	609.589	740.7816	395.4243
32.50691	29.02116	83.70927	62.8614	17.69511	12.97352	37.48328	9.945405	70.42865
30.11064	41.36684	56.15305	15.70667	16.11689	15.80988	15.47054	19.44228	14.45355
338.078	300.0865	574.8625	1196.203	338.8297	266.2508	330.7167	226.2203	532.3956
89.01781	92.41669	23.49366	0.975626	12.9768	3.009989	7.759506	4.190361	51.10727
114.3656	109.084	340.0175	1113.726	229.104	620.1043	276.6903	287.2104	236.2903
21.721	41.35695	25.53304	8.831121	5.015966	20.07095	14.37088	11.86044	18.31202
13.26028	14.95358	12.25658	8.833585	9.693881	8.661645	3.347812	0.266739	9.635425
14.51999	16.67345	7.156551	19.63471	16.1414	28.59407	14.37269	2.262668	24.09937
251.4009	263.171	89.86297	147.308	109.9237	95.50594	86.02078	113.2504	105.1154
8.5073	21.07163	46.97587	93.29917	205.9954	397.0011	245.644	194.2546	135.0438
43.37784	30.75533	766.6789	99.19101	62.15695	32.89888	67.26197	42.43754	311.6003
66.23244	22.82997	40.85192	50.07915	122.4883	28.64701	70.58111	55.85857	88.7232
15324.79	18402.24	8784.415	10157.6	11670.1	16059.65	13652.76	12978.49	8916.447
443.9476	443.5468	79.65395	208.1935	297.591	818.1874	207.281	220.5509	148.5127

33.71601	33.4244	60.23748	51.07051	30.33738	15.81509	46.29304	34.59351	64.63316
19.31348	23.73115	47.98733	22.58285	17.6943	44.08195	7.759364	9.944996	19.27797
10.84441	7.893712	0.006313	7.852555	1.812932	0.122631	1.141143	0.255044	0.951029
120.2869	205.1488	106.193	158.1211	81.21592	45.69784	92.60767	23.36843	95.47926
69.8596	11.39203	861.7371	490.0758	376.585	1007.654	466.1939	136.1917	717.6372
268.3528	161.0029	571.8076	437.021	218.128	485.3946	720.9177	796.3209	639.4437
32.44845	20.22271	3.071914	0.975335	5.011876	1.585627	6.655388	2.269829	3.843124
210.5694	205.9162	227.7046	223.911	213.2223	355.7291	166.4829	109.4962	183.2401
197.2518	252.6486	62.29393	107.0427	57.48243	48.56285	36.4212	97.88038	53.03164
121.5427	190.1146	15.32527	109.0066	25.70409	8.711794	17.68165	11.86978	19.27395
72.26507	1265.508	24.51382	209.1743	396.3084	373.1026	1007.592	197.6446	930.6943
101.1139	64.19914	511.527	289.7224	157.4992	163.6978	294.2698	452.5508	477.4653
36.14979	36.04867	228.6743	93.30372	87.3111	98.03021	132.1917	91.60979	259.5403
1.27901	5.229883	79.62937	30.44099	9.786529	37.04194	74.89593	91.04226	55.94796
60.20495	104.7362	90.87755	101.1545	57.41902	72.66309	170.8011	101.4608	77.15206
4.894235	14.93538	5.113952	4.9031	5.00696	10.102	1.141259	6.098531	5.772607
170.9139	205.8947	27.57806	103.1102	384.9729	126.854	175.3139	73.17044	194.8088
0.034591	0	3.057915	0.978622	8.524909	9.143958	9.699426	9.657053	12.64918
90.28782	151.3691	200.1283	274.0101	144.7729	297.1627	165.3496	191.347	178.4295
62.62627	318.7414	161.3272	91.3295	51.11605	67.02793	340.4793	761.6313	56.89089
10.89579	26.40841	5.11407	9.814383	17.60768	1.585842	0.032367	8.003885	3.843165
1.278117	0	0.006058	3.922698	4.925104	0.116509	13.19637	0.239614	6.745614
22.90233	9.632619	27.57206	56.96999	25.54808	3.017418	25.37182	15.62229	22.1745
26.52458	53.69793	2.050398	13.74185	14.5478	7.294899	2.244857	6.124968	1.915118
65.05049	571.3169	3.070984	5.885589	12.96498	5.854793	5.550781	4.168771	27.95408
126.3049	115.2908	33.70446	92.31274	82.81935	140.8314	32.01068	160.5627	42.42283
57.71788	51.93074	18.38741	7.848992	9.790439	7.295053	6.657436	13.7723	13.48802
72.24776	69.48675	158.2661	153.2052	76.52092	30.07111	66.17587	36.76702	101.262
54.17403	37.80578	54.1213	119.8226	33.60146	113.6768	46.32405	68.9747	59.79229
104.7195	107.3388	234.8433	233.7394	55.90473	89.80051	89.32275	90.28597	88.71957
394.6554	478.7408	1284.506	563.7106	402.5436	1044.469	874.1328	924.5527	844.8816
97.51706	114.3741	287.9394	253.3789	152.7967	88.3984	191.8149	73.1271	374.2365
30.0991	20.20796	15.32364	22.58339	19.25972	27.11317	7.758996	8.03332	7.700913
6.077219	8.777025	4.092047	15.71609	8.085933	3.006966	3.345887	9.716059	8.672996
20.53848	7.873457	289.9836	212.1276	57.50903	8.707269	728.452	151.4923	492.8771
133.5959	185.6803	188.9033	263.1987	176.6384	116.8462	103.6569	42.52621	193.8538
86.52285	65.14449	35.74279	76.60783	36.70946	31.42891	14.37271	21.39818	42.43117
15.71763	25.48825	151.0768	59.9112	57.13863	87.91726	175.0087	62.91567	55.9455
20.51079	9.631678	115.3444	16.68918	25.57285	22.88045	150.7435	49.56397	162.1378
179.2917	159.2659	421.6948	474.3579	283.0245	158.0863	286.5959	134.314	406.0603
9.684431	4.352288	37.7633	14.72859	11.28108	1.586059	24.22992	54.12167	25.08496
190.9381	71.29554	16.34615	73.65764	19.32385	17.25411	15.47587	21.42245	18.31029
9.69712	6.993029	77.56424	11.77914	16.04076	17.16587	67.0979	60.21768	70.46103
499.2433	444.4415	243.0244	145.3401	135.4354	104.084	138.9384	111.4572	187.0934
0.063709	0	34.70923	1.956984	5.008682	48.04025	81.35663	452.5118	36.665
121.2594	113.6606	8.177643	26.51069	22.46748	12.98325	15.47362	15.6788	21.20569
48.15107	66.01421	57.18155	107.0576	19.31951	31.4433	49.61869	17.60306	69.44557
211.7692	258.7389	359.4163	221.947	132.2077	106.9076	299.831	239.4402	705.075
381.3779	703.2547	64.33764	510.6835	405.5753	424.129	112.4833	228.1465	82.92846
32.50737	46.66061	6.135405	11.77776	11.36899	17.22567	12.16637	28.89021	5.771795
12.08237	6.114201	26.54337	56.98753	19.10962	12.90792	27.53152	4.18988	29.91123
62.64529	54.51436	2812.028	2384.508	532.9892	1107.263	7644.045	2260.336	1792.006
3.691498	8.759713	10.21737	10.79754	8.158837	5.856919	142.7476	245.1792	18.32207
10.91418	14.03045	1.028772	9.813092	12.97343	5.869165	4.451295	2.260171	11.55839
214.1387	337.131	73.52641	123.7368	127.3942	317.2138	186.305	191.5424	72.32084
3.688264	0.834771	99.96283	3.921319	8.120894	8.653717	18.72485	35.46926	56.98399
19.33559	40.44148	1.028666	13.74134	8.199306	7.292248	8.862093	2.254303	5.772228
7.260989	2.59437	34.68076	3.922048	6.50617	1.581793	12.11928	24.09686	30.91335

66.17547	85.3882	33.70275	20.61632	20.91462	68.32443	16.57833	23.33706	26.99175
202.1558	116.1251	319.5977	270.0703	159.2258	180.8672	616.1142	275.7755	414.7329
43.27646	19.3267	46.96469	80.54923	16.10273	38.40348	28.67468	49.51306	46.30262
50.51739	41.35328	24.51252	31.42244	66.61156	21.4927	22.08114	30.84965	23.13571
10.86312	5.237016	51.01169	9.816887	6.552236	3.011347	50.50513	29.94346	29.92609
34.93604	40.46328	23.49231	26.51031	20.89386	48.42108	12.16882	13.77942	39.53669
138.3281	178.7022	34.72559	26.50839	46.34724	59.9163	36.41943	173.9338	20.23837
235.6822	201.5812	44.93622	92.31109	157.3969	123.8597	114.6572	227.3978	59.78411
68.61938	66.85783	132.736	238.6654	154.0335	159.2162	85.99466	101.4907	120.5633
71.05208	21.06979	172.5621	139.4532	322.363	236.1057	265.6114	479.0132	409.9465
105.8758	87.99003	47.99852	249.4665	82.81063	254.3039	65.06712	92.02792	93.54915
985.3539	1658.074	1245.726	792.5299	719.1119	3149.347	1660.134	2079.304	659.681
1.280638	7.884562	18.37706	10.79922	45.23173	5.842104	14.3369	22.69285	8.67038
755.5764	1015.591	798.4939	1372.945	493.2847	206.5703	338.467	212.9699	969.2819
0.044022	0	17.29891	0	4.654775	23.84726	49.66065	10.35267	2.890842
40.93534	58.09439	63.30402	39.27992	49.33216	88.01752	90.33393	78.16623	55.94066
635.2389	421.5199	1218.144	1181.446	682.3673	674.6813	595.2866	1492.878	1425.507
72.23579	99.43586	111.299	132.5822	124.0306	153.6121	68.37424	76.81095	83.90153
48.12599	31.65392	24.51279	36.33358	25.6295	22.91475	18.77817	11.8665	16.382
128.5215	31.64711	15.32499	22.58109	36.7271	11.56546	25.39	11.87375	30.85208
0.045313	0	23.40323	0	3.21975	1.531093	11.97222	28.26856	44.66857
12.08387	12.29013	32.66542	23.57164	42.45486	24.14591	83.5078	32.17586	33.77354
30.14634	54.54929	111.2869	156.1706	58.89791	44.21917	62.83838	57.5377	61.72388
21.70841	36.08254	85.74721	95.28607	55.42781	103.2359	63.87469	83.28395	71.39827
67.40331	89.77074	32.68273	48.11648	39.96435	85.4037	23.192	17.62346	45.3188
81.87791	51.87486	158.2698	83.4704	300.2643	300.0742	478.2669	319.1811	306.7216
1328.218	2342.789	615.7232	780.7439	925.8731	1873.063	1038.448	1539.718	689.5782
20.47589	21.10977	2.050611	9.814376	9.751953	7.281022	6.654837	4.197456	10.59692
16.9302	9.631081	163.3713	162.0444	228.7464	539.7808	217.1211	417.8155	198.6907
1.279807	4.350155	71.45462	25.53148	16.08858	66.52657	594.5462	53.15136	33.75766
9.70111	10.51669	192.8691	22.58533	12.91835	8.703838	16.56243	32.45442	79.14069
43.14288	98.96149	6.134723	1.956976	11.30325	8.685797	6.653184	11.74197	4.807969
60.18003	42.20948	110.2691	135.5387	112.6828	55.58287	55.13594	44.28853	108.0329
592.7748	192.7299	51.06326	40.25734	79.75053	68.48919	73.89794	65.4802	65.56914
62.58857	76.56409	94.95761	65.79701	41.53448	41.40645	52.93592	57.61886	52.0729
279.1562	311.5253	191.9714	979.1516	267.3501	333.1145	227.116	184.1618	530.4637
19.33262	34.28349	142.934	91.33717	79.46972	64.07721	96.9773	152.3109	56.89857

TCGA-06-	TCGA-DU	TCGA-P5-	TCGA-DB	TCGA-E1-	TCGA-06-	TCGA-DB	TCGA-P5-	TCGA-HW
32.69626	298.1286	236.6021	172.3092	158.3267	67.6273	186.0633	228.9185	121.2568
41.40785	25.42196	18.04678	13.01376	33.99594	53.37239	29.52026	14.02116	46.01418
89.75062	103.8255	91.23289	140.6507	154.4496	86.69119	79.80317	97.21353	109.1052
1902.59	2518.159	2203.617	2157.999	1746.447	2842.972	2727.508	2647.254	5371.101
138.1862	121.5001	143.3654	142.5019	114.6133	190.5608	213.2694	134.6649	92.17726
0.70812	0.024752	0.000619	0	0	3.809217	1.538546	1.496588	0.049194
38.24489	4.461252	5.013847	10.21689	14.55906	45.73538	7.899432	9.876831	10.99605
88.15743	168.9768	163.4165	139.7009	131.1261	100.0255	170.8791	141.6255	174.5423
5870.273	10124.49	13107.41	4542.328	10837.16	7388.486	6423.524	5652.691	11776.44
37.40956	37.58238	93.23694	56.80566	102.9723	53.34586	90.27298	65.25939	53.37043
27.36697	20.98734	20.05125	13.95476	9.703766	13.32658	23.96812	29.05675	15.79725
510.5208	1043.713	862.1979	1441.792	1598.821	468.7301	1236.816	1111.929	1038.869
145.1859	120.4089	116.297	124.7906	144.7245	140.9981	84.56762	122.2442	117.6334
13.53025	0.026332	0.000656	3.715405	0	0	0.728431	0.09324	0.052505
33.48222	165.6265	130.3318	194.7013	147.6499	40.94969	136.5718	147.0463	100.6264
130.8186	8.879053	26.06703	4.628178	3.873457	42.87575	7.899615	18.17691	41.21015
1178.835	3307.794	1240.162	1853.44	4471.064	2117.936	1652.76	1817.163	3598.917
31.95617	21.02193	45.11518	28.85359	31.0753	29.522	72.72832	33.40434	62.99468
33.4817	25.44681	48.12357	57.72382	16.50028	158.1693	23.86727	37.63425	31.60481
662.0505	131.475	166.4252	176.9334	47.58333	447.7755	114.8895	94.5636	600.0606
779.9114	714.6028	888.2642	783.2799	915.9634	760.2766	1182.53	817.6886	2592.619
1.477813	17.7111	30.07732	22.32961	0.960813	0	18.28814	33.40192	41.24028
1.481761	183.3013	319.8117	13.01012	143.7602	8.555129	94.98117	55.66301	88.53407
60.0212	754.2178	514.3095	448.948	97.12146	49.52188	531.8793	552.2573	220.6655
67.09314	96.09055	84.21493	80.089	163.1982	47.62109	79.81611	72.25996	147.809
42.19585	55.17391	43.1094	50.31873	54.40744	11.41458	60.80442	47.09118	36.35803
289.7015	139.1877	174.445	127.5796	167.064	267.7267	110.9191	91.76164	198.8178
3070.249	450.6524	1136.897	1187.522	962.5894	1036.582	1354.236	852.3713	663.1205
10.05985	44.16582	63.15965	58.70595	15.53103	31.4346	59.97462	23.69545	37.58044
109.3436	177.7278	84.21471	101.5287	39.81472	44.76375	122.2252	94.37373	106.6388
77.32507	119.2089	33.08512	54.9442	8.729761	55.25397	73.47845	59.71255	20.69154
38.97998	75.08839	91.23165	25.12198	85.48485	37.14384	104.7019	77.67202	75.12719
3.81416	29.77828	22.05597	34.50237	2.902329	25.73527	28.01281	23.54017	13.3771
530.0555	666.0035	722.8426	611.9024	1566.776	885.0937	915.0292	824.5947	815.8245
505.3502	187.7712	195.4986	310.1678	176.7775	250.573	382.564	213.8009	157.6332
3434.348	30757.74	9067.109	20337.68	8438.925	2397.071	26108.8	25522.46	14997.28
8.502611	2.250763	4.01128	27.94293	3.873439	24.76833	6.304981	20.88306	17.02217
49.13575	102.6827	57.14606	30.71075	62.16194	44.76623	150.2808	109.5198	100.554
35.08381	11.0885	25.06472	44.69913	7.758465	70.51519	40.68966	18.19549	29.15404
22.57109	4.461137	9.024074	53.09733	5.815897	81.96605	33.49859	51.3255	10.99755
192.0864	48.63876	136.3478	12.07938	30.09889	303.0032	51.81779	8.485352	27.97234
55.42327	100.4452	55.14064	55.88057	25.24451	51.44376	74.29738	127.3327	26.74062
43.67062	5.565582	15.0394	14.87314	1.93174	198.2526	10.2935	14.04548	41.268
74.1066	160.1271	169.4312	209.5944	118.5006	39.043	121.3547	154.0326	71.58275
363.8611	279.43	223.5702	256.1236	129.177	336.322	374.5176	161.1432	140.6863
8.506604	47.40823	15.03893	13.01986	15.53487	29.54351	19.93948	20.85484	9.771355
85.14048	41.99612	41.10544	44.69019	146.6985	47.62709	42.26639	73.5516	35.22012
2390.558	3011.844	6856.472	2147.75	2405.013	746.9272	3293.622	3041.486	2693.53
144.342	725.6055	816.0791	162.9631	82.55075	191.4815	265.8145	187.5592	398.8603
20.30633	15.4765	7.018817	15.82496	11.64872	10.46663	18.35695	18.06509	8.555658
34.48315	26.47925	21.05351	28.89772	5.816946	18.09971	34.45358	37.20679	7.348963
274.0396	336.8373	225.5752	368.8471	407.9712	229.605	282.6601	181.9364	185.512
100.7685	99.37642	66.16889	100.606	47.588	77.1722	135.0644	88.79516	47.32983
2649.73	7202.101	7334.693	9526.213	8405.918	2190.329	10952.55	9362.946	6293.708
11.64175	40.80707	33.08358	51.28972	13.59082	17.14046	42.44145	40.0539	13.3929
79.67395	46.40712	96.24431	50.28403	25.244	57.16048	85.48515	45.88633	48.52443
13.20578	28.70689	34.08625	24.21315	22.33804	10.46345	26.35277	19.49738	31.48213

3051.031	6318.557	3900.943	7879.509	6395.252	3071.616	6884.297	10326.3	9056.232
1235.704	283.8518	125.3204	276.6141	57.29614	387.773	252.2821	70.96269	84.9428
91.63068	34.24178	58.14697	53.11385	16.50302	61.00115	54.37445	37.4698	14.6223
10.05596	37.5744	67.1708	9.284562	32.04595	46.67877	27.8815	5.714285	53.3434
338.5989	0.03349	0.000822	2.766142	0.960623	15.22865	0.723986	4.324595	1.292449
217.8167	159.0629	176.4501	234.7096	222.4345	146.7103	349.8087	235.9781	227.8935
17.13355	30.90332	11.02895	58.75762	42.7558	21.90988	27.96882	30.46638	21.83411
42.18835	49.66963	62.15693	31.66131	16.50306	18.08726	40.74146	40.22249	23.0818
101.5202	96.08025	97.24765	70.77543	55.35873	39.99873	88.62913	47.31487	96.96003
225.6401	50.8492	104.2666	115.4705	234.093	343.0089	95.74361	107.0147	153.9908
9.298051	27.58154	9.023834	22.35919	44.70994	3.791723	8.715321	16.70062	7.349715
13.98502	25.40873	23.05907	89.54386	7.759228	43.84707	65.7001	71.67607	19.43769
138.9066	350.076	365.9316	387.4866	326.3749	95.25602	436.8827	382.9594	273.9384
70.35984	77.25367	56.14264	69.8824	25.24635	32.38382	69.54887	51.32581	23.09821
21.7663	81.74755	85.21758	70.76869	225.3747	21.89272	177.3592	106.8936	89.72912
32.75143	36.45988	38.09735	50.30148	44.68143	31.43103	49.52168	52.69581	31.55874
224.0471	334.6261	288.7357	287.8021	260.3168	188.6331	321.0136	364.9663	226.7016
20.2287	32.04497	13.03422	8.35361	67.0362	19.03898	30.30506	25.08628	31.55108
44.40635	148.0292	117.2999	87.52192	49.52548	756.5439	82.1574	93.1624	180.6699
182.6579	80.66604	235.6004	235.6404	279.7481	133.37	245.9327	259.5519	415.6458
1022.709	2480.544	1876.783	1213.581	692.5497	1704.454	2114.367	2817.697	541.9353
24.11802	101.5688	51.13075	98.75086	25.24376	24.75316	85.47434	128.8151	67.8833
10.84308	547.7195	435.1077	243.0852	88.37967	19.0351	257.8923	383.0093	237.6106
26.46561	43.09672	25.06483	39.10026	90.3431	19.98862	38.27204	150.8642	19.48036
88.28582	48.61365	17.04449	18.59969	84.51277	59.06699	15.88476	27.89974	58.20024
454.2979	725.6568	1338.411	1406.388	546.8491	767.8937	1635.262	1213.288	2115.193
342.3969	32.07149	37.09554	26.04977	18.44295	175.3289	26.26406	1.528701	112.7243
192.8876	144.6864	157.4011	184.4252	64.09756	154.3444	202.0618	138.8395	105.5056
427.0422	91.71475	140.3588	207.6735	56.32487	614.5269	217.9074	137.5892	698.1495
32.70107	7.774288	19.04965	32.5703	13.58635	359.2777	15.08375	4.316294	41.2955
136.5767	216.4689	126.3226	143.4182	483.7561	150.5237	203.6031	256.7384	140.6619
67.18545	57.4259	71.18077	41.90177	65.0839	40.96006	51.89498	27.88259	37.62119
277.0812	643.8942	1080.753	1178.246	389.4965	240.0721	602.0258	1033.986	888.4747
2856.772	1804.68	2019.146	1677.411	2113.62	2453.311	2182.986	1994.791	1693.463
5.570013	0.022409	0.000563	0.911954	0	12.47931	0.738365	0.077758	0.044325
22.56405	30.95269	50.12783	60.54797	32.04659	29.52147	52.6992	95.52397	32.78204
1.479753	68.48377	78.19926	123.908	11.64369	0	63.05286	76.34933	77.57829
729.1828	826.1303	412.051	327.8205	1664.887	706.927	565.2599	524.8243	801.2692
82.73037	49.73415	134.3417	93.13467	59.24343	100.9906	134.9934	113.7896	142.968
61.63781	139.1049	69.17662	111.7838	70.90413	80.98175	95.03694	141.3762	23.11912
20.99085	102.6761	48.1232	60.53304	147.6688	55.25178	40.66502	47.28385	26.74795
18.64435	119.2466	147.374	13.94123	3.873933	9.507276	62.24289	23.75783	72.75245
267.9619	118.1872	151.3856	55.85755	121.414	70.48763	182.8967	63.98824	122.4495
208.4011	150.2414	209.5347	195.5716	290.4259	230.5531	148.4422	194.4486	195.2214
0.698839	22.08655	53.13231	13.02222	2.90228	9.511661	16.73825	20.82964	27.83341
3.812564	146.6521	50.12732	21.40093	24.27569	4.743898	15.09493	41.63949	12.20641
17.11741	60.654	11.02904	25.14063	19.42028	12.36915	35.16235	59.36973	6.148223
43.72818	26.53221	29.07463	42.84474	22.33205	57.1776	52.73763	43.02552	25.51069
15.56015	38.61351	13.03399	27.01355	28.17104	13.32436	23.94642	31.85331	43.51956
20.21014	87.22861	86.21921	88.49745	49.5326	21.89424	98.28109	62.48928	100.5318
16.34277	14.38722	34.08627	8.35605	19.42183	20.95413	7.90573	12.61561	23.04789
1469.308	1841.118	994.5355	1241.517	2587.638	1041.33	1806.103	2282.058	2888.562
20.21515	47.50275	55.14057	49.35734	34.96074	38.09926	91.92486	74.86145	56.97098
837.783	258.48	414.0559	251.4475	81.57944	990.8707	313.7267	112.6099	243.732
400.5063	426.3177	422.0757	312.0001	313.735	322.0177	358.4832	141.7476	210.9917
16.36839	3.355756	8.021336	10.22456	13.59266	119.3055	3.111031	5.702293	3.722268
21.0769	26.48916	24.0611	32.62534	10.6758	46.72881	35.2313	39.99688	8.559924
70.2032	106.0378	90.23044	105.239	258.4007	57.1482	118.9645	104.1553	144.2239

14.76681	5.565455	19.04912	9.287289	8.730739	76.28469	27.94183	11.24475	24.26697
42.89036	148.9856	98.24935	61.46883	19.41507	32.37803	49.47133	79.05917	43.68572
286.7686	81.75418	176.4489	165.8032	547.9208	139.1038	223.6964	119.3958	67.94695
302.1135	537.8307	301.7695	374.4158	469.1591	115.2621	881.7302	420.5752	105.5493
42.85262	120.3913	128.3271	100.578	99.07095	69.53542	138.9402	112.4807	116.3881
57.70575	44.21672	52.13373	73.56343	235.0894	100.9873	72.61201	91.66633	58.25102
30.35461	654.8324	625.592	443.3647	275.8568	79.05749	225.1294	832.2821	164.9216
42.90747	114.7676	52.13295	87.5828	31.07453	62.88615	76.71504	74.85007	26.73702
19.46855	13.28857	38.09655	18.61092	5.816087	15.22938	24.72897	19.5216	29.09565
68.03488	30.94282	22.05698	43.77773	41.76742	46.6886	30.30415	44.40153	20.67455
271.7042	339.0403	263.672	182.537	129.1779	210.5492	295.451	224.9174	76.45558
235.0097	181.1438	290.7404	247.7535	54.38242	236.2815	418.5374	395.3782	105.5328
329.4179	485.96	261.6677	284.9833	759.5938	434.4447	252.2466	304.1096	427.9269
1468.476	2986.447	4093.432	3361.354	3198.594	1207.102	3275.246	4240.78	6136.017
30.46977	68.34249	12.03152	25.14413	15.53355	19.04556	26.346	41.48461	9.776
21.81256	26.52005	28.07179	22.33885	71.90721	10.46176	14.30311	14.01524	41.17222
38.19344	34.27094	50.12821	81.97297	64.10692	41.90857	99.88358	76.31028	117.4556
3.966389	1.128471	0.000527	0	0	5.748619	0.743168	0.072048	0.041256
0	37.47989	29.07323	1.835346	3.873676	0	10.3198	4.319177	2.509586
0	0.026794	0.000667	0.907587	0	5.718016	0.727539	0.095107	6.046784
30.51794	16.58275	37.0931	16.75472	7.760128	17.14285	41.66946	20.83123	8.55992
150.6984	177.7921	123.3145	161.1385	123.3571	175.3144	184.4983	108.3336	100.6514
84.4102	48.60484	72.18337	81.98759	32.04603	38.09957	76.71223	58.28751	25.52773
465.2865	479.344	393.0022	352.0444	1140.368	595.4633	403.9761	419.2983	673.953
88.16377	86.17604	84.21536	70.76216	400.232	135.2862	87.77545	76.47035	90.96939
12.46376	14.36289	17.04325	27.04921	4.845932	12.37941	20.81092	18.00191	4.927276
318.45	2192.226	1116.846	1351.455	600.2755	109.5446	631.5273	2714.648	512.8264
190.4997	181.1332	155.3964	118.268	226.3247	142.9027	174.0511	151.365	140.6531
56.91929	16.61173	9.024059	60.51767	18.44293	138.1523	18.27731	12.65819	46.1437
43.05379	27.60631	21.05398	23.27974	33.03117	51.48534	26.35168	19.49874	10.98285
487.2703	175.6388	162.4148	250.5313	132.0906	413.4998	410.4509	187.5092	252.1684
149.064	302.5976	172.44	171.3608	355.5188	93.35064	236.3412	201.3269	346.6116
116.2943	83.9687	91.23319	76.35142	112.6695	71.43956	119.7393	109.7374	183.0057
135.9595	108.2118	53.13605	123.9005	27.18592	109.5767	91.03824	56.99811	37.6512
29.65911	22.10978	28.07174	20.4746	17.47583	25.71802	37.5566	31.92373	18.24033
67.16966	24.33519	29.07491	65.20328	13.58664	91.48477	38.27973	23.73874	24.31963
58.48496	216.3965	150.3827	160.2188	94.21551	32.37361	181.3422	278.5481	93.36761
21.12932	4.457928	6.016239	3.69834	1.931238	19.06088	3.912513	4.313	6.1354
4.607728	0.031052	0.000766	0	0	3.793518	0	0.112754	3.714468
86.70172	66.27095	68.1738	63.32935	53.41855	69.5498	158.3092	73.55753	75.14432
21.01249	34.24924	39.09971	30.72425	56.34591	12.36684	45.53065	20.94131	32.75745
46.80991	15.50556	33.08507	20.46363	8.72976	93.38907	12.68978	12.65704	10.99995
322.5185	127.042	271.692	270.1115	125.2934	315.3718	386.571	342.7119	138.2456
55.37408	12.19371	25.06494	31.64017	66.04507	146.7448	67.83239	55.62815	44.92049
499.6652	573.2001	578.4741	587.71	656.6269	303.9109	536.5691	691.1959	327.3452
43.68981	36.47049	35.09005	13.01065	51.47933	37.14649	26.28253	41.70927	111.3421
32.74329	27.64033	24.06214	29.78686	4.84469	60.03141	15.89008	16.8066	23.10121
24.89876	53.03131	63.16116	48.41736	82.5671	48.57977	57.46308	74.94079	109.0042
21.78052	75.07113	26.06727	13.94235	237.094	38.10005	24.68347	29.26638	26.73642
41.29039	200.9648	83.21271	476.1014	91.29926	11.41295	370.0098	486.281	90.95913
92.17395	88.33789	96.24463	84.76505	95.19771	64.78288	72.6609	56.97134	38.85382
86.56871	331.3357	347.8866	593.3344	338.9952	34.27835	1034.377	717.3006	788.8908
35.84327	143.4921	102.2598	97.81455	50.50344	31.42381	97.46779	102.5933	55.794
24.93074	57.39354	27.06952	29.79303	58.29088	40.96946	23.9021	40.24847	21.87978
702.6865	186.6948	207.5299	162.963	174.8279	1240.508	226.6835	151.4743	309.178
69.39001	215.4071	321.8209	1627.246	111.6908	100.0171	573.2985	728.6805	215.8506
50.7289	30.95592	30.07744	61.47462	39.81858	99.11225	65.49395	40.33613	27.94978
63.15957	140.2706	192.49	142.4986	197.1873	89.54459	133.3248	131.9107	160.0004

109.2844	94.99966	155.3955	80.08245	71.87059	71.44219	177.3266	95.84418	130.9075
67.96917	62.93979	37.09507	83.85523	18.44421	59.07351	103.9551	44.46728	41.24838
705.0937	61.89459	115.2949	23.25669	27.1855	697.4243	51.01422	27.92309	101.9158
196.716	185.5646	215.5496	302.713	174.8343	135.2752	192.3988	195.7823	169.7509
112.5127	79.51563	57.14607	68.91833	33.98676	86.70491	100.6592	102.6123	23.11696
34.33207	44.16891	24.06198	41.91529	25.24777	49.55152	35.11623	49.89712	13.41573
136.6497	87.27183	161.4107	90.33189	132.1031	100.9848	136.5554	91.68459	84.89751
15.52973	30.94707	20.05199	12.07985	75.77915	47.63866	30.29612	25.09906	19.46956
40.57612	37.56627	52.13267	42.83897	30.10435	11.41323	32.69445	51.33765	35.19101
259.1151	1128.712	1211.085	567.1984	581.8227	307.7169	819.2212	813.4509	386.7606
51.60054	24.32076	23.05938	24.20077	46.63021	23.80773	56.7803	11.25723	7.361456
178.158	77.32646	59.15142	130.412	47.5859	77.16506	163.7959	88.86575	72.76873
182.6803	110.4754	124.3175	98.70432	223.4094	197.2172	129.3024	83.42975	201.2206
60.03414	351.0999	158.4036	111.7529	107.812	19.03443	140.516	169.3244	75.22582
4.606381	13.25125	19.04765	19.58532	11.65422	8.564795	20.04462	15.22113	10.93064
348.9271	713.4579	640.6326	555.1008	1386.124	429.6764	444.7042	473.4172	574.5848
28.87265	28.72298	53.13408	39.12972	14.5604	33.34747	40.76361	41.55665	36.34412
605.7928	605.2653	1165.971	763.7232	596.3905	218.1569	1110.674	968.9339	2071.435
464.6057	5.561119	85.21827	62.37282	163.1743	588.8231	280.2351	18.20442	24.33266
13.95869	75.12008	58.1489	38.16091	431.361	35.23305	125.3996	137.3088	75.19151
34.26631	33.17479	49.12603	19.52959	14.55764	163.8961	52.63625	16.82458	69.1394
459.8871	215.3981	233.596	117.3261	586.6971	489.7186	306.5786	140.3541	603.5726
44.44621	28.75399	21.0547	24.18904	64.10581	69.54907	34.26678	40.36743	40.06358
112.3444	284.9454	274.7001	204.8917	294.3151	121.9339	380.924	150.0382	259.4189
612.798	1916.187	1439.669	1036.617	1098.573	796.4768	1049.935	1199.404	951.6184
94.49039	80.62754	64.16394	78.23294	77.70512	76.21614	143.0397	122.0175	47.33627
40.10954	15.461	13.03346	9.297222	8.734489	32.43521	12.75398	13.90397	4.926771
73.29419	449.4874	406.0345	311.0764	931.5497	160.0438	323.3637	402.5196	387.8841
195.8851	709.0094	538.3717	1709.261	219.5115	24.75189	536.5902	1184.94	858.0765
754.1503	582.0744	793.0215	796.322	2238.951	530.6612	884.6717	1091.064	1414.561
40.55719	89.41015	33.085	53.08651	43.70565	68.60559	85.52018	106.5985	26.73799
35.13355	36.44382	32.08192	54.0492	14.55983	53.37263	40.74571	49.84848	32.73921
60.86009	15.50694	35.09033	113.651	12.61501	97.187	46.25382	112.3202	53.38499
295.8262	54.16259	34.08793	35.36422	14.55899	429.6763	31.05747	84.84671	107.9778
37.39924	0.034569	2.006073	43.75387	10.67239	116.249	59.84524	32.07271	54.59809
43.84475	12.18242	16.0415	7.424025	33.0316	38.12745	2.313287	5.709619	19.42921
262.2371	781.9548	1176.999	820.5475	797.463	369.6462	554.0817	830.1175	590.3817
109.3375	87.25431	83.21223	88.4802	173.8903	87.65093	101.4222	72.23962	105.4345
14.79162	22.08495	26.06598	32.62759	16.50858	21.91459	31.21488	13.96527	14.58843
99.10423	143.5801	67.17208	81.94159	336.1126	177.2171	153.3104	124.9754	127.3006
105.3584	141.3706	81.2077	56.78873	144.7287	21.89255	89.37564	83.39772	89.75612
31.21752	24.31769	18.04675	7.422584	15.53175	37.16029	17.50443	16.78003	23.07668
261.6189	138.0746	154.3938	226.3381	310.8424	237.2428	204.4215	361.9958	124.9031
46.86663	32.04505	57.14488	45.64325	24.27561	33.33962	79.1984	36.12587	35.17543
23.36613	3.356086	23.05943	32.59141	23.30465	30.4811	14.29708	8.489313	35.16541
18.64624	9.984662	5.013824	18.59908	0.960966	182.9883	6.303229	4.320603	7.362409
17.28319	1.143633	0.000749	1.836364	2.903441	29.604	5.534085	0.109707	12.09821
113.9523	124.8141	161.4112	261.7625	123.3559	121.9449	149.3137	190.0889	141.8322
57.68091	240.8106	529.3495	1187.559	262.249	79.05688	553.3068	684.3486	1036.326
13.17952	160.1362	27.07009	1.837748	11.64396	32.37296	34.24795	12.65671	29.18386
46.34398	3.35536	1.003525	0.90543	0.960468	32.42213	0.722817	0.117978	7.346084
56.91667	81.75481	126.322	142.5076	86.44221	78.11228	188.5119	122.1701	93.37874
0	0.027042	0.000673	0	0	3.802416	0	0.096112	0.054
81.23218	62.95808	75.1914	59.60313	37.87379	72.41112	95.08276	76.30772	38.84892
135.9567	67.38601	58.14874	90.348	52.4447	93.37245	101.4359	92.97544	26.75304
312.2212	884.6306	749.9109	841.0565	280.7049	354.4052	919.0963	738.4481	696.9947
442.731	173.4317	381.9732	405.1624	303.0536	430.6487	565.4195	434.4036	252.1737
351.3436	255.1439	346.8839	347.4093	605.1606	229.6005	376.8958	265.1892	482.3711

2603.677	5484.689	3020.699	2329.364	7932.885	2245.59	2709.896	5769.13	6595.495
17.86214	257.2529	419.063	14.87272	24.27093	9.507751	71.0018	142.9534	48.56904
164.6461	58.58098	164.4201	351.1164	70.89477	576.4122	326.5187	435.9218	298.2591
15.54055	57.37627	39.09939	77.37582	43.71584	17.13442	51.98008	60.84188	21.8703
936.0399	1409.285	1261.215	1280.642	1602.699	1360.508	1181.679	1482.566	1847.354
174.0778	131.4557	92.23597	129.4446	315.6949	307.7529	137.2872	136.1314	139.4506
33.54644	5.566129	12.03168	27.92739	37.88082	42.87567	32.71078	25.0828	64.14657
178.7963	322.4218	155.3962	230.0704	112.6679	128.6117	246.7949	184.6001	103.093
16.35526	5.564069	1.003521	3.697279	3.873546	72.49723	2.313171	0.120398	3.723125
36.63378	4.460495	7.018966	7.421943	9.701069	61.92744	5.504987	8.493447	4.936046
45.19936	109.3466	77.19736	37.22759	120.4455	93.36129	84.59594	40.40946	92.16044
292.0801	366.602	265.6764	253.3517	366.2129	110.504	209.2055	292.7485	51.00142
7.720257	4.460987	8.021443	16.74811	5.816217	23.81479	17.52006	8.478112	13.4
9.325438	3.353482	7.018622	11.16868	0.960473	23.84545	2.314958	7.051211	10.93656
222.1023	53.03492	45.11567	54.93865	39.81583	88.61102	167.0834	52.82987	57.00931
52.29562	33.16237	15.03938	22.32776	77.71452	143.9247	16.68575	16.81433	36.41751
49.87153	159.0719	113.2897	262.6454	382.7123	526.8973	166.8202	87.6128	200.0573
13.24664	15.46518	24.06039	22.37277	17.4857	8.561154	19.19073	12.54982	10.95315
150.6255	180.044	256.654	205.8283	227.2905	66.67204	242.7314	302.5417	355.0931
32.77918	52.97363	28.07191	31.66185	37.8834	19.99424	47.15965	34.70994	26.704
24.89472	100.4865	52.13349	69.84659	97.13736	23.79919	62.246	61.14687	49.75544
24.89763	1538.415	1847.706	75.41259	24.27211	116.2136	631.5493	302.7666	194.0417
12.4358	0.032635	0.000803	4.629168	0.960492	13.32746	5.509112	0.119527	7.351018
17.08221	58.55837	51.131	70.77669	4.845057	13.31803	45.45041	91.5977	290.4713
670.0358	466.0509	236.6036	262.6377	360.3648	371.5705	324.9604	298.4958	146.7532
96.77127	163.4382	190.4845	167.664	82.55609	96.218	166.1214	167.8809	77.63806
225.0478	370.8689	129.3292	188.1801	49.52805	41.90274	286.9324	192.7094	67.93668
128.7741	183.3399	226.577	220.7474	222.4394	153.3851	261.9582	259.465	218.1672
1068.997	1266.687	406.0353	551.3785	101.0061	520.1938	302.5541	681.5137	97.06807
42.88332	37.58284	42.108	34.43881	16.50058	48.57975	26.27267	23.75038	12.21257
7.728897	24.2819	27.06833	29.82952	6.78862	16.19047	28.00595	20.81235	10.96938
38.94574	97.21332	173.4415	200.2712	284.6255	71.4405	270.8356	180.3707	111.5517
11.62669	26.52289	31.07937	27.93276	24.2777	8.555309	38.34391	30.56871	36.35624
98.51263	4.460803	5.013839	10.21602	5.815944	118.1848	11.89165	2.929485	3.722474
0.701335	164.3697	43.1102	12.07919	0	4.744051	19.88568	37.55681	13.42178
221.9292	88.36845	90.23024	184.455	24.27113	98.12958	142.1785	81.96345	38.87165
792.0843	55.26737	37.09565	50.26497	9.701969	1535.096	119.7001	29.31838	118.8672
37.3935	114.846	108.2757	277.6681	12.61501	56.19876	221.4009	135.9078	95.76204
8.495483	48.5852	39.09981	22.33223	16.50207	9.507646	25.49714	48.5467	31.55507
21.81855	30.92359	22.05669	47.53023	11.64549	9.508913	36.75977	52.53036	30.30595
33.59444	28.71753	52.13131	45.66705	48.58119	6.649735	36.76655	40.15051	15.82059
80.3457	161.2476	264.6731	132.2485	69.92584	97.16722	150.103	70.93116	111.5647
1621.507	2482.824	1999.096	1763.085	3615.3	3642.325	2677.987	2190.611	3526.271
35.05574	9.984596	13.03431	19.5302	20.386	105.7658	13.48705	30.685	43.70208
170.8952	378.8408	113.2898	196.4966	389.5007	808.9038	326.5254	177.8329	305.5241
103.0563	37.59149	29.07515	58.65743	33.0138	314.4894	54.23245	50.10185	99.40603
92.0499	60.78761	83.21302	60.51165	247.6945	203.8862	98.94139	75.11014	214.5503
49.97302	48.59514	57.14529	34.4478	19.41623	137.27	68.73407	43.06236	29.14798
10.84242	157.9624	241.6158	48.40217	68.95263	453.536	100.5304	55.69268	47.36751
56.92233	400.6796	74.18969	219.8586	24.27104	20.93989	211.7319	126.2922	44.93045
32.74538	14.39947	22.05706	16.73865	13.58707	57.17197	32.69479	29.24882	31.56422
47.57421	81.71871	56.14348	165.8617	28.15798	52.39176	161.4992	55.58823	57.00425
20.46426	1.143592	0.000748	0.905653	0	16.21652	0.723068	0.109445	1.291536
8.628995	0.028152	2.005981	0.906642	0	5.712116	0	0.100647	0.056346
8.517622	11.07051	20.05093	14.89359	7.760957	9.513493	44.95236	38.53138	14.5751
48.40241	13.29613	19.04949	13.94284	12.61548	89.58863	28.68897	7.104341	32.77725
62.68765	34.21945	27.06902	29.80821	19.42098	20.95256	23.93508	20.88625	9.776784
189.723	238.5373	173.4421	225.4063	207.8679	168.6319	216.4083	199.8701	190.3095

74.07616	1296.576	425.0842	1198.714	1423.99	14.2734	1402.202	2227.417	762.4833
37.39318	0.03466	0.000849	1.837226	0	997.9663	0.726881	0.128372	1.288865
0	0.015959	0.000406	0	0	0.946139	0	0.053627	2.379579
3.032349	49.60367	22.05629	15.81933	1.931205	6.65063	24.76288	16.72867	44.69421
426.1873	2238.689	1810.614	1865.566	2927.614	778.372	1439.597	1844.824	2401.279
0	16.59182	50.12591	14.88314	2.902244	3.791372	53.66578	5.710276	37.50971
23262.67	28863.71	24799.2	19732.24	27537.26	20316.2	24634.69	24305.36	34569.39
68.65383	44.21544	71.18197	46.54522	82.55909	120.0508	58.22875	63.95546	44.92659
277.9406	344.5692	251.6415	295.2513	129.1773	208.6415	293.8398	290.1131	282.4393
507.6573	203.2341	261.6668	330.6564	135.9777	296.3076	354.5754	273.4472	203.6797
867.5197	659.333	399.0173	882.06	219.5107	514.4795	819.3135	657.9	395.2099
259.1077	1527.447	3134.987	547.6268	100.0352	1014.658	1239.186	394.3993	540.7199
28.01303	576.446	386.9858	282.205	254.4856	53.33285	511.9206	414.9503	200.0627
82.31034	4.461178	13.03409	1.83558	1.931279	30.48881	15.91011	9.864744	26.68256
82.06817	54.11752	60.15308	44.69822	33.98958	50.49313	55.0972	94.16042	13.42203
13.20404	25.40533	44.1111	12.08521	24.28165	13.32374	30.36312	11.23871	25.4619
113.2362	86.15488	62.15903	72.63611	97.13346	108.617	91.01619	58.40458	126.017
82.68964	240.7255	216.5511	287.8443	66.04019	74.2977	266.7988	206.7399	87.34044
15.52091	61.86387	53.13597	33.5055	34.95808	69.54658	37.46104	48.68203	47.33058
95.16474	154.6512	146.3738	462.942	343.8591	114.312	246.7161	313.6593	953.4747
126.4646	147.9911	149.3806	178.8408	134.0431	68.58178	186.8932	338.2312	112.7625
8.515953	1.144242	1.003524	1.835348	0	29.55509	4.710369	1.537255	2.509414
1353.728	1741.737	2725.947	1688.582	1740.622	1470.064	1984.935	1749.152	3958.913
128.0415	122.5967	86.22031	59.58519	229.2533	70.4888	129.3524	57.04976	121.2267
79.61561	38.69308	58.14882	11.14722	19.41442	172.481	13.48691	15.43614	1.289031
3.812092	32.02322	23.05916	18.61087	86.49523	3.791325	37.56731	12.62765	47.17918
166.2133	660.4142	550.4022	494.5727	438.0715	167.6643	475.0991	476.1024	858.0518
822.7997	2502.698	2917.436	4621.547	2693.501	1937.86	2335.446	3473.136	2733.518
659.0868	364.4682	273.698	343.6774	200.0859	472.5699	360.9009	272.1477	249.7582
33.50193	122.5213	75.19137	98.75319	21.35793	13.31824	79.87734	45.89048	35.21798
17.12042	1.143899	2.006128	0.905711	0.960582	38.12227	4.706498	0.122366	2.509719
76.45498	107.142	75.19231	52.13255	264.2296	100.0311	94.18349	65.3635	92.16511
212.3965	205.4134	205.5235	203.9817	105.8676	161.9633	305.134	173.521	107.9401
145.3762	82.82793	90.22963	108.994	39.81566	81.93781	147.8668	94.32863	40.06836
4.59847	63.81566	18.0459	47.60082	17.4844	5.698881	18.37502	20.76421	13.36495
33.58464	60.66474	25.06422	39.13142	16.50413	14.27537	47.18998	40.17177	18.23947
9.371198	13.22229	8.020655	27.11358	3.876209	3.795752	21.76582	20.47527	4.905654
52.40409	33.13479	94.23627	21.40528	8.730322	7.602358	67.23307	110.2883	27.90586
59.78503	0.032187	1.003525	0.905421	0	117.4331	1.516793	0.117599	2.509089
142.292	50.82316	62.15858	59.60229	39.81673	87.6624	65.4675	73.54986	25.5362
64.01324	82.81484	57.14591	64.26341	79.65338	89.56988	60.66906	62.48585	83.60113
38.21963	43.08408	70.17807	42.83644	31.07537	15.2249	54.30711	25.11008	33.98784
65.73235	37.54681	20.05182	40.05604	34.96768	20.94768	33.52741	23.68444	24.28863
73.34372	189.8955	78.19975	54.93032	166.1114	47.62067	106.9966	113.8032	78.82922
13.98097	38.63308	38.09658	28.87111	8.73062	27.62687	69.68695	33.2847	9.779116
10.87762	17.67053	9.02375	7.427898	6.789232	7.606176	11.93545	16.67099	31.38826
60.85505	74.00725	33.08527	31.64098	114.6253	141.0312	26.26787	39.00024	36.441
53.86392	27.64501	44.11281	35.37551	21.35858	89.57975	23.88195	12.65588	49.71761
6.153557	49.6647	55.13933	95.10408	4.844647	4.743893	55.99134	64.96768	30.32264
155.5493	3.354483	2.006077	22.32511	2.902814	204.9061	1.52175	0.127761	11.00047
29.6604	39.73873	33.08421	34.46646	10.67373	21.90359	32.74414	16.77142	6.148802
66.28456	173.3869	133.3401	108.9584	92.26902	137.1913	128.5296	106.967	179.3745
6.153661	40.89044	30.07752	69.85776	53.41974	12.36546	105.4978	62.47695	54.57237
0.703286	230.7366	95.24301	140.6526	38.84207	0	119.7795	225.938	145.4135
51.48912	12.19359	14.03686	2.767272	0.96095	83.84855	7.898839	1.531282	8.575435
96.79716	55.25504	52.1337	52.13498	58.27144	103.8479	60.62535	48.71846	78.83064
0.700119	56.27705	29.0744	23.26928	31.0804	7.60227	21.50739	31.95138	30.3246
3.845948	1.142087	0.000709	0.906386	1.932535	4.75421	0.724699	0.102448	0.057273

1415.486	76.25019	26.0674	82.86448	290.416	2985.939	470.2115	179.2418	749.1964
1.474377	1.14426	1.003525	0.905421	3.873882	83.99957	2.313518	0.117581	1.293339
244.4116	104.9547	89.22829	167.6417	295.2966	264.8746	126.9058	183.2553	323.5438
309.1983	214.2776	277.7077	257.06	35.92686	277.2488	168.4238	141.7113	107.9639
27.35036	17.68894	20.05137	13.01986	24.2838	24.77275	15.12322	5.707816	20.62639
10.06717	1.143893	5.013837	1.835538	7.759188	81.05782	6.30451	5.711626	8.568214
24.11437	107.1012	81.20689	92.21641	118.5144	30.47006	113.453	110.9242	49.75176
1388.252	1673.159	870.2178	1375.685	553.6523	516.3719	1361.461	1172.883	337.0707
182.8248	117.0624	64.16419	87.54189	87.41651	115.285	118.9906	90.2689	100.6184
589.567	134.7831	123.3153	200.2269	66.03813	988.0632	145.2437	126.4751	134.6353
32.00981	30.9273	12.03161	51.25806	5.816012	19.99586	22.31562	38.80853	21.86455
138.1356	316.9385	279.7123	246.8237	256.4358	138.1355	233.1607	198.5302	115.2239
3.826425	2729.968	1253.193	187.1784	27.18601	31.42155	340.069	1551.648	213.4357
2865.993	6937.043	2895.38	2646.032	9975.595	3874.778	3441.279	6649.264	5890.068
35.05676	111.5148	109.2777	64.25663	27.18613	32.37599	60.65065	117.8391	88.46971
8418.975	3987.042	2101.356	3758.139	3039.298	4034.862	4535.298	4142.164	1155.305
17.87548	6.67084	27.06969	35.38122	5.815899	55.2656	19.89033	16.80462	35.18919
223.2838	135.8764	97.24878	84.72923	182.6061	219.1282	186.8106	111.1828	267.8699
22.5586	51.91916	79.20123	85.71016	141.8476	8.554496	83.9004	45.87273	64.23526
494.4722	27.65645	23.05987	19.52982	267.1232	610.7893	33.44755	18.21012	60.6969
62.37699	33.17831	53.13643	158.3357	59.24035	184.8381	41.43521	83.40832	106.7196
4.072276	0.016045	0.000408	0	0	1.909658	0	0.053938	0.031333
79.81011	13.29399	12.03168	16.74044	0	94.37417	10.29697	1.534705	12.20613
479.9729	3.355851	14.03678	13.94366	16.50194	86.73689	13.49342	4.324288	17.04874
51.52929	49.70257	23.05964	45.63236	31.07516	51.44805	51.10072	27.8776	17.05353
33.49739	50.82572	54.13842	43.75705	48.56027	46.67303	44.66232	38.98408	67.89147
8.500605	262.8352	309.7885	196.52	266.152	20.94005	252.3496	247.0277	552.4413
166.4197	90.5708	49.12603	122.9558	78.67387	103.8506	153.3941	83.33158	88.50542
74.14953	2.248461	0.000847	9.284654	1.931896	111.4819	31.06244	4.318986	35.23056
736.9713	996.2142	1078.749	1057.122	255.4482	879.3743	1615.376	1171.554	615.8508
902.674	520.1927	294.7519	648.2581	362.3014	584.9882	980.6657	652.3414	367.3348
9004.91	20870.77	7815.921	9505.669	28110.37	7984.893	12051.05	19303.66	14694.24
53.98466	24.3158	18.04672	23.27141	21.36279	37.16181	41.56196	22.29195	26.69461
173.3538	175.5915	110.2815	84.73652	216.618	50.47658	134.1282	98.64619	122.4565
81.42279	12.18916	6.016405	6.490826	0.96068	139.2195	2.31429	5.714394	14.62248
24.18107	30.91735	25.06408	29.80706	17.47688	23.8134	33.56351	25.01767	12.19462
7.751829	14.35165	9.023505	13.97352	1.931368	14.2938	15.9995	11.1452	9.733168
46.84621	34.25652	32.08229	24.19497	10.67269	65.75449	47.91276	26.48297	18.26046
185.1035	93.89914	110.2814	88.46582	74.78434	182.9404	153.3284	98.62785	93.382
14.01429	3.355607	2.006133	4.629526	1.931196	44.82973	17.55433	12.57919	10.96654
81.20694	108.2018	147.3736	94.0819	31.07216	55.24988	133.4649	101.2373	83.62614
174.9961	159.0008	94.24041	140.6554	46.61377	141.017	136.5786	73.65591	78.83183
8.502204	33.1181	40.10132	33.54074	16.50521	10.46286	36.77965	30.51011	25.46762
329.4173	441.7892	293.7494	297.092	566.2894	370.6069	364.8551	383.1972	623.0289
39.82054	61.80159	47.1197	109.0771	18.44586	28.57353	39.12458	62.28684	25.50514
57.06768	50.78274	47.11974	60.56797	20.38911	12.36693	51.14519	43.00968	23.08951
117.8016	265.089	470.1978	639.9002	214.6563	376.3324	524.6505	531.5325	298.2302
6.154956	50.83208	59.15127	194.7365	5.816194	4.744566	64.64283	113.7202	59.43985
316.2271	81.77202	76.19525	75.41341	1185.117	426.8499	166.8253	108.4204	82.51586
10.05767	52.9989	45.11496	52.16612	150.6166	18.08496	55.12942	33.38291	20.6783
21.91337	13.26696	11.02865	15.83162	10.67823	8.560831	42.57715	13.92232	10.95518
20.99882	215.0822	18.04699	36.30896	4.844738	7.601814	35.08528	44.46735	23.10619
96.73823	128.1415	187.4779	232.8547	304.0392	162.9124	211.6007	183.2587	236.347
137.5321	22.13214	12.03176	16.7356	20.38598	141.9869	10.29358	12.65946	9.78784
66.3224	25.44526	51.13104	93.14014	5.81625	163.9031	81.42884	141.4142	18.27349
60.04255	272.6976	135.3448	40.02177	43.69868	39.04314	106.1711	40.41242	58.25825
10794.37	8393.853	10328.32	9324.981	17549.06	15166.66	11144.79	10898.52	18083.51
419.2322	392.094	389.9943	408.8691	485.6661	258.1778	425.5587	381.8023	1060.456

39.80648	50.79013	43.10986	107.1963	25.24694	33.33865	71.97041	34.75487	14.628
50.01494	32.04179	30.07705	30.72571	45.65637	30.48064	23.90258	34.7331	20.67067
0	26.41633	13.03313	16.78227	6.791567	1.886943	7.13421	12.49203	3.71447
31.92074	78.4349	98.25054	181.6594	24.27113	100.9887	51.03412	194.0777	82.46248
52.21609	60.78795	31.08032	13.0111	16.50062	198.1676	16.68282	30.7076	36.45787
437.9611	258.4766	308.7877	408.8649	316.6463	847.0068	256.2355	277.7558	344.3164
3.812291	66.13625	23.05899	50.34448	7.759382	11.41671	15.91754	22.25218	14.60617
363.8833	215.3824	218.5574	219.7974	242.8311	350.6195	289.8516	184.7089	180.6649
100.6662	176.6965	58.14916	163.9289	109.7553	59.05225	224.449	127.749	172.1055
163.9619	180.0134	136.3477	209.5793	56.32614	85.73243	362.726	201.1968	107.9309
335.6192	1020.545	2875.327	1635.499	104.8921	1845.454	2186.185	1428.492	2360.114
310.8807	57.47285	83.21292	82.87027	72.83959	201.9874	104.5449	44.58386	141.8524
118.0442	34.26923	50.12814	46.55643	20.38659	104.8259	49.47296	37.58518	14.63543
68.03215	3.355937	9.024065	6.49065	7.758564	40.01354	4.706066	5.714912	31.55303
74.91635	40.90207	72.18441	29.77653	81.58881	108.6169	73.42452	57.01972	121.1769
13.21558	25.39176	44.11044	27.02085	33.03581	11.41863	47.29138	26.32419	19.4138
334.1158	526.8072	109.2796	292.438	412.8148	136.2224	602.8914	249.9813	99.49121
0	1.122141	0.000465	0	0	0.943372	0	0.062462	0.03604
276.5185	104.9463	64.16447	100.5727	170.9565	234.3928	136.5176	163.7883	71.59265
61.60533	35.38564	18.0471	50.26908	37.87024	137.1974	98.17785	20.98972	75.21391
23.41464	19.89373	17.04396	27.01495	27.19949	9.510357	39.21686	34.58635	2.509832
3.04614	0.029738	0.000736	0	0	21.00681	0	0.107217	0.059715
36.70793	35.33917	28.07185	38.19311	8.730311	24.7626	48.77402	31.9437	3.723965
14.74706	120.2444	51.13013	20.46661	53.42643	8.55464	24.69119	59.60849	24.30877
39.72209	397.5405	101.259	266.3833	18.44325	7.603448	550.3752	406.4888	99.47625
109.2974	115.9652	123.3141	66.10916	252.5764	103.8458	130.1715	133.2103	130.8915
21.78548	49.69801	51.1302	47.49971	45.65149	9.507412	33.49319	27.87032	24.31087
289.0735	229.6693	79.20258	252.4515	37.87003	141.0062	296.4225	172.0576	37.66629
104.695	90.54494	57.14602	72.64797	67.02037	65.73586	127.0777	72.18538	27.95971
208.4967	180.0197	133.3402	228.208	64.09704	141.9529	289.1596	161.0382	53.42215
378.6017	302.6527	326.8337	372.5357	773.1874	547.8227	433.5235	456.7663	609.7287
293.6341	166.7852	206.5263	232.8534	91.2952	243.9087	216.3917	252.5707	78.87268
11.6255	30.93436	50.12696	27.93122	11.64484	17.13396	45.55416	34.71176	23.08132
20.39575	17.6429	9.023471	12.10555	1.931398	7.610223	22.47153	13.86021	15.72472
167.0382	5.562124	6.016313	19.52997	0	1240.673	26.26209	29.31907	23.12245
180.3201	329.0856	215.5494	332.531	220.4923	83.82301	155.657	369.04	97.05108
33.5166	47.49991	66.16819	79.19308	37.8762	32.38073	70.31214	72.08206	48.50244
29.61787	16.60595	10.02662	15.80724	0.960769	243.1435	8.697712	1.534062	7.362691
17.10255	15.49927	16.04177	25.13241	5.815925	47.647	12.69768	5.714537	19.4594
235.0017	192.1876	199.5087	222.598	100.0369	229.6096	257.118	198.5486	123.7096
51.06127	4.459242	3.008702	5.56188	3.873768	16.19227	11.93008	2.931256	14.57748
22.55642	64.05821	96.24429	194.7816	42.73213	31.42503	76.68163	116.3491	125.9003
17.13142	11.07938	10.02645	6.492175	1.93122	12.37096	13.51591	7.092481	32.67895
108.4294	427.4192	513.3077	510.4103	296.2507	162.9007	523.8293	599.5432	283.6996
1.474815	0.032917	0.000809	0	0	9.51078	0	0.120749	0.066543
35.08999	26.53787	37.09497	49.36342	51.48158	11.41312	40.6967	63.76814	46.07322
63.24846	87.21434	62.15835	69.86226	30.10217	67.64906	81.4987	92.83848	53.35327
137.3405	98.3361	105.2692	123.8506	334.1444	195.3031	216.3561	219.3744	333.3087
534.0863	557.7111	550.4019	301.757	1746.549	104.7815	413.6095	509.3596	209.7744
72.75946	25.4268	37.09464	44.71347	28.16314	27.61998	44.73456	18.17516	24.29711
26.59657	13.2765	7.018832	20.49115	7.760423	40.05177	15.13717	15.33183	7.350154
1532.727	103.8636	191.4891	57.71705	138.888	1773.092	157.2078	41.80238	197.6789
2.252843	3.355846	2.006134	3.697468	0	19.0523	5.509206	9.839496	24.21746
7.713911	182.061	112.2845	10.21586	27.18721	3.791831	41.47344	48.64632	12.21203
133.454	100.5391	122.3124	103.3615	572.1608	230.5679	135.6916	133.3541	341.72
4.6008	2.250369	1.003525	0.905403	0	12.3794	4.714362	2.92842	22.95638
3.0376	161.1822	173.4401	7.422392	0	21.89311	166.2189	50.08683	20.69704
7.766834	1.143797	1.003518	0	2.903247	20.03604	2.317224	4.293404	2.504356

28.0262	104.88	123.3127	107.139	110.7457	19.03534	68.66626	38.97942	93.28158
235.7571	124.8395	112.2871	192.7815	466.2552	397.3083	121.2931	143.1038	399.9508
67.30389	27.62684	16.04174	39.12357	49.54683	56.23361	31.12308	36.08601	24.28822
34.31212	69.54079	81.20546	38.17783	31.07615	31.42994	66.33708	44.43539	23.09969
7.74361	3.354241	7.018684	11.16544	0.960462	16.19946	11.14545	4.309866	7.337069
20.99907	59.63083	63.16061	56.81783	68.97112	20.94287	55.8996	59.6557	65.42153
89.74682	77.33629	31.08028	32.56989	89.3576	136.2471	63.01607	55.6577	78.84139
113.1797	140.2629	99.25344	188.1597	132.1005	101.9347	117.3527	118.0311	147.8743
128.0872	93.88312	84.21492	136.0009	116.5634	113.3806	150.1892	133.1645	60.66594
213.2019	55.26334	67.17211	45.60947	12.61529	194.3675	69.39445	30.7066	93.3967
159.3484	120.3773	153.3901	102.4488	217.6019	89.55135	147.7691	142.889	81.2554
859.536	743.3248	882.2491	655.6709	2391.44	2680.099	823.1567	584.5512	1573.394
16.40203	1.144247	1.003525	5.563437	0	7.607335	1.516851	2.928752	1.293255
1195.316	1641.172	643.6411	1821.82	491.4839	686.9129	1529.103	1827.996	658.2801
3.115162	0.02317	0.000581	0	0	4.775019	0.736122	0.080713	0.045902
51.52607	23.22953	29.07484	27.92081	44.67832	55.26086	27.08504	22.34731	35.20056
391.0821	405.363	576.4695	596.077	551.7122	852.7094	895.9093	778.7258	516.4352
96.00403	198.7394	97.24813	110.8326	68.95687	102.8922	71.01035	109.6783	58.2533
35.09025	67.34155	49.1252	79.20088	42.73598	26.66273	74.33779	67.90544	24.3118
28.0346	19.92059	16.04193	45.62762	8.729764	37.14548	73.49976	45.86539	69.06402
0	0.023821	2.00566	0	0	0.938346	0	0.083258	1.27118
10.08092	3.355847	12.03133	13.95627	7.760317	20.00653	14.33185	4.319886	10.97122
80.4515	108.1831	52.13325	173.3327	24.27238	59.06619	93.48421	94.26656	26.74577
77.49854	19.91025	14.03671	50.31515	46.63019	47.64831	27.91426	45.73287	26.70616
41.30596	139.1092	101.2577	122.0336	80.61924	36.18707	191.8383	101.2838	63.07358
219.4312	57.47307	70.17981	105.228	34.9556	424.9809	67.79286	44.58428	81.28926
1141.39	1629.06	941.4	689.1985	2006.779	1168.05	954.8989	962.0955	1406.162
31.27319	23.1978	15.03897	13.01907	16.50643	10.46384	19.13241	8.473903	12.18803
268.7365	38.69918	92.23568	10.21667	3.874333	251.5535	35.84555	30.70532	19.48663
18.67858	2.250568	0.000823	1.835667	13.58866	57.19406	6.303371	5.713328	12.19952
15.55233	5.565352	9.023983	8.35541	12.61769	49.57102	19.12029	9.862108	20.64506
10.07967	9.973402	15.03881	5.561064	27.2034	12.37322	15.9362	15.33976	43.46092
129.7376	68.48118	69.17642	85.6957	26.21491	69.54808	121.4705	73.57266	19.48295
70.96033	97.22681	131.3353	221.6734	120.4373	120.9849	127.7015	148.607	242.4086
124.2398	104.8951	87.22208	86.62411	26.21468	58.10866	78.24873	70.8226	30.384
666.0851	523.4838	363.9277	610.0846	239.9113	394.4337	701.968	430.3062	150.3936
92.17985	56.34141	52.13332	43.75767	92.28355	32.37708	27.07209	44.51633	24.32628

TCGA-HT	TCGA-HT	TCGA-HT	TCGA-QH	TCGA-TQ	TCGA-TM	TCGA-FG	TCGA-HT	TCGA-HT
104.6994	232.6023	184.6081	262.6248	179.4395	182.5843	174.4982	239.366	181.5233
40.93684	17.46591	36.22527	24.68572	42.57698	37.89991	36.7822	31.78504	30.98034
46.53894	88.45602	81.43065	75.00328	97.32525	58.51624	130.6913	185.3308	132.8279
1745.085	2468.237	3954.161	3978.089	3096.112	1636.203	2993.63	3858.85	2867.894
118.3685	78.58268	206.2777	178.4265	128.7521	150.7138	201.9444	85.80294	57.51054
3.569126	0.602498	0	0	0.00333	0	0	0.727264	1.739737
15.68193	20.27449	4.052245	3.624582	11.15729	7.692015	17.92051	13.4343	7.034951
144.932	175.6242	132.996	147.2968	169.3022	159.3179	146.303	142.2834	247.0837
9914.874	16190.07	6825.736	13360.59	6431.481	6309.847	7326.771	11248.12	20928.96
63.12978	48.40521	103.2017	42.97962	47.65102	128.4135	73.56015	28.54475	28.29197
26.59636	8.3213	34.96666	30.21688	24.32979	37.97209	40.8368	31.06968	12.36465
1029.71	1059.218	1267.668	1253.444	1331.104	1249.583	1479.648	1374.539	1329.038
83.14256	126.3724	153.8967	72.24695	82.1217	200.6673	103.2206	105.6747	89.39069
1.339875	0	0	0	0.003533	0.81332	0	0	0
57.318	76.49942	276.2084	90.56592	99.35237	168.8497	121.2944	44.44265	46.88634
31.53228	11.11764	12.41671	2.710391	8.116094	2.527695	6.167387	13.43502	7.920641
1137.277	1727.176	3282.051	2713.66	2326.646	1667.22	2449.84	2638.539	2136.528
37.26329	50.57389	65.54095	44.82416	41.56708	61.16596	83.83587	34.93582	27.41428
108.3457	53.98577	17.2815	39.30815	75.02359	22.33103	69.58076	56.37649	106.2459
346.4993	129.8166	63.2531	117.0725	225.0668	67.11282	139.1805	500.7915	334.6523
1100.783	1060.643	756.3489	1038.432	1040.148	919.729	839.5303	899.0934	1835.56
28.62469	18.1496	113.1587	26.507	228.0572	49.94891	196.9747	24.57822	113.4575
229.818	400.9537	54.21908	125.3435	167.2717	122.2847	140.0757	100.9326	424.363
289.8843	654.9098	1352.835	1132.866	597.1119	338.4503	522.0013	531.948	475.5187
156.5593	180.7144	41.6801	119.8669	96.31039	85.24189	103.2871	127.2379	142.5915
38.77503	30.88383	33.42541	29.2683	31.42835	32.71963	54.8712	21.413	16.78623
107.5428	143.2185	68.13372	126.234	81.10849	136.0298	82.07238	104.0712	159.3537
2023.832	495.8051	1545.689	665.1264	880.9839	1472.674	1140.044	441.1376	336.4164
10.64928	52.06183	28.5044	57.6738	56.76697	98.36025	16.35515	29.38163	43.39762
104.0893	87.09711	140.8416	64.02827	72.99448	245.6542	117.4234	73.11303	32.71716
32.20041	32.21522	99.03208	43.89749	94.27744	70.62643	109.664	77.14245	31.83821
37.23692	55.46337	102.5354	107.0923	60.82779	54.23992	70.43748	65.19168	46.02037
12.85468	19.67048	50.54947	35.73749	33.44739	45.79527	57.47537	8.676814	2.60959
457.0088	393.9458	467.2606	550.7582	538.3259	796.5801	719.0327	374.3568	311.6247
158.5233	170.6297	209.6265	122.5726	165.2504	211.8412	435.1942	214.6307	97.35506
3891.339	7872.542	22866.55	14892.14	31600.81	13188.13	29667.33	20969.1	3184.831
24.3779	6.903166	4.748664	7.285208	14.19718	12.0095	5.386761	10.25951	5.265054
29.3162	78.68343	61.25519	99.74882	82.11583	44.74465	186.5037	119.3458	51.33053
51.66288	30.82514	17.29332	24.67442	20.2809	16.30706	10.07926	22.18627	30.07244
35.83748	13.22549	17.99792	16.43623	25.34869	14.58534	19.48444	22.98961	32.73883
174.3835	166.4721	8.931057	138.1425	13.18504	21.46959	49.20462	67.49704	246.1927
26.45341	53.36402	74.58968	67.71386	59.81336	100.8386	57.11424	35.72047	26.52348
14.23174	13.21881	8.228474	10.9426	16.22639	8.552572	17.1233	23.77048	20.31876
78.85901	143.314	151.1791	190.334	166.2585	167.9613	214.5027	146.2939	149.6499
355.9669	238.0766	358.7532	390.692	335.5628	384.1199	458.6095	224.9432	176.1688
17.17018	16.09259	44.79116	29.2922	29.39745	19.79153	51.85091	20.65117	13.24944
63.85103	32.91485	86.42799	23.7548	28.39108	69.75755	21.82159	26.95307	16.77511
4405.317	6516.508	3908.848	5305.654	3689.178	1671.508	3312.864	4976.615	10436.74
993.2935	839.4816	1201.743	2547.397	853.6056	406.4476	657.2713	742.5465	2123.525
18.64968	26.776	17.39123	16.45977	18.24878	82.20533	17.19685	22.28119	7.929075
8.516081	11.86679	23.7297	32.06413	24.32845	40.59502	13.25626	4.687553	3.494946
154.1996	282.3799	254.91	164.6629	265.6129	223.8902	396.7805	279.041	204.514
48.72656	61.06759	144.3992	73.19086	111.5118	168.0703	106.4791	75.5207	33.60601
3233.35	2498.371	12142.53	8119.932	8478.314	7428.584	7603.059	9515.149	2049.718
12.11243	18.21536	41.26764	23.78773	21.2906	50.93695	41.59625	28.64941	25.68372
53.78545	25.8799	68.271	119.914	64.88245	61.13859	72.78815	114.6052	38.0422
12.10703	19.61999	34.90774	21.94949	22.30462	22.37832	37.63453	33.43515	31.00741

2677.206	2593.899	8427.859	8043.074	6552.118	6663.835	6016.991	4704.623	2930.725
109.6787	61.6832	124.5608	94.2016	174.3759	123.9578	208.0785	73.04977	103.5489
25.77844	19.58019	62.16411	32.93182	47.64474	57.7647	38.34985	14.23487	8.807077
80.4672	73.12207	21.47861	44.8193	23.32209	10.27504	8.513919	57.24212	101.901
4.186952	5.494141	11.02887	0	7.102206	0	0	2.301961	5.264557
102.5154	202.9726	474.598	260.761	292.982	190.3044	352.9771	193.9511	169.0955
14.27736	6.90612	34.22422	11.86719	17.23705	34.49188	24.25615	8.668415	6.151862
62.59757	49.96107	41.12716	65.01455	52.71204	46.53505	55.64723	30.98439	53.16839
95.45601	139.2151	54.94711	86.00078	54.74813	68.87309	99.39111	89.03846	218.8459
70.92779	72.22996	49.32234	37.47475	61.84684	293.7032	70.33267	97.71053	62.81807
18.64484	21.085	8.954151	30.22558	11.15548	5.974609	17.98173	36.70391	75.53792
94.59325	12.54336	30.6693	18.27916	20.27829	22.37211	25.80965	15.04479	7.03694
247.5674	205.7713	368.5738	288.2105	239.2545	342.7932	252.7334	253.6011	214.2624
55.29964	26.61044	48.77157	46.66237	51.70186	61.18044	66.586	27.77115	15.89364
145.0251	137.0298	114.2388	117.1144	122.6672	32.66832	223.1876	101.7469	153.2156
35.12077	20.26907	70.48888	31.08997	43.59311	64.63802	45.3855	20.60061	14.12162
399.8131	321.0537	261.19	418.1588	311.2313	295.3986	316.9246	461.2126	270.9434
28.64542	38.61987	22.8977	77.82966	59.8087	5.969551	70.54433	105.2078	67.35034
94.60819	134.0596	113.4177	114.3332	67.92975	39.55407	49.19935	181.9987	210.7108
302.8871	267.6532	144.7961	232.3893	283.8586	285.9369	186.1906	194.7446	330.3024
1678.448	1417.45	2021.345	1768.548	1773.116	1774.912	1845.036	2366.726	999.6193
25.00774	45.59005	133.2738	48.47389	99.34635	49.06068	125.3409	28.54539	16.77516
185.0735	160.0669	644.6255	719.2474	582.9155	195.4606	668.4787	495.4061	32.71132
11.36035	35.03269	23.56578	211.5205	110.4949	28.37069	77.49694	92.28812	21.20512
48.74786	22.36144	30.54513	36.57257	31.43196	26.64644	34.36158	89.09812	52.22488
952.8898	1100.635	1572.052	1533.411	1762.976	967.9388	2254.331	784.5794	1593.781
32.17641	53.2875	13.79949	37.47857	21.29548	14.58029	16.33736	72.29777	65.49206
146.3803	165.0872	151.1423	237.0027	295.0018	342.0381	300.6376	166.9605	97.36909
409.7095	112.2583	212.3273	284.5162	265.6169	179.9335	223.7039	324.3028	276.2203
69.53478	43.43831	19.37115	24.66656	6.08832	8.553897	14.77268	21.37605	99.15929
116.166	72.23126	243.8191	278.1581	225.0603	126.5571	62.50637	197.146	152.272
76.15668	48.44603	69.02151	80.54649	40.55389	63.74733	73.61271	114.6707	18.54962
597.7335	783.2405	515.3744	437.3124	502.8415	993.0041	312.1226	248.7491	714.5496
1155.229	1467.983	2868.326	1900.282	2296.231	2456.974	2601.674	1623.307	1057.164
0.631047	0.610791	0	0	0.003028	0	0	0.73377	0
51.66368	34.34939	60.63281	34.74766	52.71695	93.09511	40.65339	25.37224	27.41315
140.1214	68.81496	87.09564	103.4091	69.9523	93.0306	30.4349	38.09265	111.6053
412.5171	261.1755	323.7612	610.2344	522.105	309.1103	425.579	666.1575	399.2886
168.7888	80.73631	262.3129	134.5157	94.28299	131.7947	130.7194	90.61277	82.33013
55.18864	79.36158	53.55742	56.70625	127.7317	207.7216	108.8105	128.0776	39.80596
33.63268	51.92454	37.51942	55.79771	58.80137	57.68155	68.06761	141.678	63.74304
86.11492	146.9846	66.82074	178.489	203.7549	14.5804	159.7794	153.5683	151.4828
138.4939	179.8869	91.86928	201.3128	98.34032	243.801	133.0092	303.0918	214.3237
99.62847	176.9173	153.1282	189.3641	229.1192	219.5698	95.36815	95.31205	91.15117
15.73745	49.48808	13.15881	38.47982	24.32956	18.93517	16.39749	23.06607	57.71466
48.11599	97.98565	16.60671	73.24774	69.94363	43.06468	103.5658	235.4334	50.48979
10.65828	22.43003	73.51813	36.61276	34.46702	40.51623	65.16236	25.42054	7.03654
19.27967	27.3249	49.49367	41.17031	35.4841	43.06197	46.9663	20.60349	29.1956
7.063249	12.55041	43.35446	14.61581	16.22384	37.08044	39.21501	25.44039	18.57368
89.04885	94.21369	99.01433	86.93574	149.0132	53.37255	119.8495	77.93058	82.36344
41.0307	16.08229	6.842721	13.69863	9.129256	5.971518	3.039775	20.64111	20.34821
1152.369	1599.365	1857.595	2568.205	2134.024	1684.464	2404.505	2722.867	1181.132
24.29838	41.38834	135.4869	95.19905	78.05759	59.42757	152.9001	60.42969	28.29731
426.1816	387.6535	352.3445	330.259	255.48	167.872	283.9507	348.1375	680.9003
213.7483	479.0735	531.4955	541.6501	537.3054	480.5531	552.4561	631.2887	412.6064
4.909555	2.687195	8.953793	2.710052	9.128603	7.700995	6.175804	1.508554	0.84228
15.73662	11.85707	49.07232	38.47903	37.50224	44.90239	21.12345	8.67255	2.609464
110.4867	104.627	76.53824	125.3449	132.8056	87.8128	140.0799	168.5935	108.8978

20.75705	33.754	10.33372	25.61119	15.21094	12.87085	11.65909	10.25762	52.31463
36.51615	47.71021	79.45556	106.1745	59.81429	95.64377	56.3161	96.27123	30.06585
27.14724	52.57283	99.55629	232.4491	94.2848	97.29147	230.9714	86.60826	32.71278
177.1475	181.1229	478.5686	630.4269	761.3436	517.6126	603.3729	654.3904	78.75353
121.2562	189.053	103.735	158.2945	125.7103	137.7944	89.93391	193.2559	172.6866
81.03417	27.97125	75.156	96.97376	71.98232	41.28371	64.8827	67.51895	46.88635
284.1537	383.5838	250.0118	272.6405	566.6978	544.3619	612.0646	373.6783	287.7609
45.17337	35.75371	53.62729	59.47534	52.71739	92.22303	85.38339	55.65105	17.66326
20.75322	23.13494	30.65403	21.02654	16.2246	15.45935	19.51191	12.64829	27.44223
18.56037	16.04554	15.20865	16.43751	22.30746	27.52649	13.21576	15.82336	15.89527
483.8583	174.8346	387.3822	448.3621	370.0282	323.8322	466.4826	294.9583	123.0358
252.6186	103.8551	423.0084	101.5265	269.6657	391.9244	381.1768	161.3402	38.0236
282.6464	407.3599	327.9845	594.71	422.7516	265.2011	345.0112	758.4914	579.9691
4896.87	6210.195	2390.348	4480.373	3107.265	2762.647	2252.599	4550.47	9322.816
10.66219	17.4918	51.76604	66.89391	24.33187	52.6383	16.37606	51.82501	15.90749
29.40223	36.55318	25.02677	38.4382	25.3468	31.8619	35.22356	31.79599	38.08807
81.85097	50.52198	38.91989	29.24741	43.59622	22.33599	58.66324	60.40283	54.88149
0	0	0	0	0.002834	0	0	0	0
19.36911	110.09	1.273348	48.59002	6.088025	3.387874	10.8925	31.09099	148.5526
3.533685	0.596923	1.287379	0	0.003592	0.812643	0	3.943515	2.625653
25.15064	16.10574	25.81575	8.20365	15.20949	25.85605	15.60941	11.06801	11.47726
77.41773	75.77275	178.3786	111.609	118.6146	241.2178	165.9139	75.46184	47.76817
35.09246	39.276	52.22609	35.66154	42.58135	66.33194	53.20022	30.14839	19.43523
378.1001	362.3693	417.1506	500.4526	479.5239	389.223	427.1748	595.4409	431.1826
60.17319	91.94037	177.6586	52.11719	57.79114	98.14303	90.70714	142.2955	67.25288
9.257856	6.927172	12.49892	26.57908	17.2333	28.50905	18.81273	7.089077	7.935155
1979.333	1564.41	1566.592	1631.345	1580.491	739.73	1490.643	1001.668	1803.692
32.16867	72.23521	222.9242	186.6425	183.4962	131.7315	211.2789	169.3136	62.81956
57.31161	26.5644	24.94396	10.9431	60.83178	6.832938	17.11979	60.35159	31.82745
11.38531	4.792927	17.34669	11.86582	4.060862	50.91717	8.523421	14.25108	12.35911
276.2425	145.2957	293.2126	327.5427	419.7053	265.2272	569.7603	263.1106	115.0609
208.0732	265.5373	250.0579	336.7179	222.0205	169.6248	235.5135	396.0072	394.0757
109.7374	120.0702	120.4536	165.607	128.7526	86.07875	105.5852	179.6953	215.1982
61.66146	92.74435	71.01236	112.5585	149.0181	96.46924	112.7323	104.1815	43.35033
13.53607	21.0067	25.03187	28.35662	30.414	43.09828	60.40966	16.63317	14.12812
30.77049	13.2204	22.87284	11.85787	37.51346	30.09845	32.01679	22.18267	15.89067
102.5923	129.2715	235.6863	112.5336	171.325	200.7396	143.2282	152.6872	82.32204
18.68934	7.632616	13.19701	4.541816	22.29952	0.808815	21.96618	3.097213	17.71646
1.323908	0	0	0.882802	1.019007	0	0	2.303968	0.842218
41.54375	39.9534	61.26607	57.62933	84.14236	129.2737	79.83266	48.45034	28.29174
20.00203	71.83117	15.90848	37.50723	75.01091	19.76243	46.1871	50.91843	48.716
42.99962	41.37886	10.31765	9.112843	23.32228	30.95916	10.86123	17.40596	52.22938
289.9681	107.3689	105.7663	166.4998	143.9614	290.2499	183.846	68.2839	78.75799
44.39707	19.54042	34.01318	18.26194	19.26788	23.19373	21.8165	20.58218	15.88837
177.8492	651.1995	707.7461	524.2501	557.5831	1083.499	1068.237	504.8066	386.0263
100.6536	110.5281	20.78138	69.55124	57.78544	25.78908	53.20148	125.8291	124.0811
26.46806	37.8861	35.46704	22.84483	9.129936	26.65638	12.42923	30.158	42.49092
105.5982	68.12799	73.84222	66.78618	88.19692	45.60985	66.49969	79.51823	66.40266
65.34511	25.18677	57.82626	45.73671	38.52669	23.2029	53.98859	52.46404	20.32178
207.5161	94.06538	278.8952	171.1105	232.1489	89.53286	229.391	37.27914	14.11786
96.95018	49.80851	64.7536	99.75181	63.86983	97.35163	133.167	89.87124	34.49368
519.6575	181.8348	1976.743	640.5084	331.5091	328.1084	325.4925	463.5327	156.6814
63.84276	111.8161	54.27632	75.02628	76.03357	89.58943	60.22342	197.4658	81.47104
13.52556	18.86836	30.60097	31.09521	24.33437	21.48928	23.41613	24.59387	21.21659
280.478	244.3284	240.1818	182.0338	182.4877	140.3131	105.5351	162.0884	296.5801
226.6529	665.2353	835.9425	173.8001	990.4626	1564.137	809.1317	269.4287	561.3634
38.68659	27.99915	39.63648	15.5186	20.28109	122.4177	33.58523	9.452022	8.805349
120.5136	163.6809	89.06978	159.1994	104.4232	137.7802	125.1644	170.1442	225.8292

101.1431	202.4489	119.0954	229.7051	111.5178	158.4881	147.9151	147.1004	163.8333
51.65918	38.57574	121.5171	55.8128	68.93462	100.8586	68.90315	38.91535	29.1853
97.46937	76.43317	25.64647	14.60547	17.23987	9.420528	17.12762	47.6104	70.78423
106.8203	117.204	165.7038	118.9115	194.6494	298.8561	95.37665	136.6764	148.722
68.1522	25.87304	51.47674	76.85485	76.03403	116.3207	108.053	37.29768	25.63256
31.53425	29.44949	39.70237	22.84963	19.26643	102.6691	40.69197	17.41871	9.692387
53.00122	80.00597	121.1931	108.8692	99.3531	155.043	124.4132	115.2641	77.00386
15.67773	13.92936	14.5067	22.84609	16.22588	11.13777	5.385249	13.43206	12.34917
81.97101	47.0607	48.06294	48.4923	74.00047	64.62789	56.36747	53.28269	47.81401
1289.647	1728.239	295.9	1091.525	541.3664	492.5606	494.4495	666.1658	2156.191
28.66293	29.46306	69.17879	64.09675	86.15617	42.2152	76.09424	75.70639	22.9935
86.80638	45.55622	111.4761	115.2921	101.3784	164.5642	187.1717	69.12201	42.46006
228.2269	127.7678	123.2053	182.9795	211.8811	109.3292	182.298	207.497	201.8798
162.1867	240.3615	315.0923	313.8922	256.4811	210.1691	238.7432	357.9734	255.065
7.825322	5.521463	18.91083	6.379151	15.20508	20.73136	14.89083	9.505606	6.163027
305.6014	503.598	530.7182	519.6666	523.1163	948.2298	726.9298	719.4865	455.0909
30.12771	22.4179	43.96826	24.68971	38.52185	57.78925	21.07709	6.274062	17.67585
960.1268	1066.988	898.4821	1141.831	1049.271	735.4258	923.2769	902.2858	1591.173
877.2951	23.05543	6.154577	6.372442	10.14326	37.83212	20.25375	21.37931	39.79468
55.89054	147.624	54.23784	101.5579	190.5817	59.38454	118.182	202.1031	18.5447
91.8357	94.11226	18.67507	61.27769	15.21278	23.19309	57.84051	48.42604	114.2333
50.82578	93.99662	279.2436	274.4591	242.299	161.8519	305.9051	373.6352	175.2749
15.66693	39.95134	17.98226	29.24644	29.40489	37.84962	28.87069	35.708	32.72165
215.2314	223.3268	518.4261	354.0926	234.1868	419.4539	343.5347	672.0086	248.7969
1298.1	2000.581	1526.777	1650.528	1161.804	1299.519	1057.047	1318.857	2094.97
36.49711	37.82743	84.97813	82.34182	116.5815	161.1475	144.0976	97.80843	42.46411
5.639926	8.346763	17.44539	15.55513	15.20682	22.43991	6.972451	16.70921	11.49245
224.5352	325.9054	225.5959	449.2446	363.9498	248.8539	413.1618	410.2349	382.5131
61.58941	126.3098	367.7161	473.0159	553.5265	459.8727	465.5586	360.8884	150.4767
922.0809	668.6288	806.5172	724.5996	1054.34	818.9663	933.4459	820.3875	866.8224
39.41074	40.6848	41.7381	62.22201	64.88078	129.3322	68.89455	48.47464	10.57639
17.85193	20.99441	29.91903	20.10476	11.15707	55.17813	22.63985	15.03299	4.379165
45.84531	23.76073	39.60231	19.17746	55.76129	37.84535	41.4013	20.58365	24.74576
98.90344	91.88616	54.19827	26.4984	53.7367	109.3097	35.12025	53.9708	61.04523
4.19186	3.394359	13.10292	0.884394	11.15759	11.99688	0.704891	1.513788	76.13991
2.753769	8.314372	4.052358	2.709933	3.047025	10.28492	10.87908	15.05052	7.037768
991.7457	1521.634	772.415	639.5147	822.183	883.58	549.2253	1029.531	1633.707
93.29253	70.89825	96.83084	81.42163	98.33665	97.32141	108.0094	115.3147	99.1761
10.67825	5.500492	42.73982	10.9539	34.46199	27.59061	29.80235	11.86864	11.47818
100.4005	81.39222	64.66821	147.3023	115.574	108.4837	145.5336	159.8039	69.02451
209.6389	320.6138	73.03676	309.3261	149.0266	52.4786	104.8068	361.9846	644.9656
9.93334	18.1748	8.930896	10.94593	21.29293	12.86657	10.08522	15.03454	11.46656
159.2764	133.4012	86.26388	122.5796	137.8777	281.6657	150.9838	101.696	73.44775
100.0849	20.97946	39.69337	44.83663	26.36181	43.06381	45.39837	10.24993	9.692095
35.14608	8.304332	3.3569	7.283579	17.23908	13.72641	27.34382	7.068271	9.692665
6.339784	13.21815	3.361414	2.71156	26.36378	4.249592	13.20828	27.74728	7.920234
4.93807	1.28875	6.19525	6.384778	8.112199	2.531058	2.263754	3.90492	7.061008
157.1661	133.4387	183.9381	139.9797	152.068	165.3603	168.2497	88.98271	118.6316
378.8047	710.8611	110.6126	429.0769	570.7645	403.8582	1169.896	296.4536	916.4537
11.36285	2.698142	6.147475	10.02882	4.060456	16.30291	5.391317	7.072065	8.807234
43.37606	2.687733	0.58344	0	5.074289	3.388388	2.258881	4.688802	8.81878
193.8651	110.9532	180.4955	140.9043	153.08	251.5776	171.4138	112.8693	72.57221
0	0	0	0	0.003623	0	0	0.722353	0
50.18197	51.93205	99.71876	74.11536	71.97824	99.08866	93.1777	54.03054	53.99553
94.74867	50.49443	67.5195	34.73603	72.99411	136.9985	71.96266	49.23204	33.60395
274.0161	509.2084	974.5526	572.7338	876.9232	1122.21	865.4483	538.1762	404.6122
384.6574	146.698	276.4763	176.5529	203.7753	520.2158	276.1753	123.1416	92.92199
237.4673	233.1422	248.6037	333.0322	321.3712	328.973	377.9481	318.7821	242.5866

1492.459	2303.108	5164.636	2661.474	2743.317	2711.821	3254.918	3903.295	1814.196
135.6313	260.8419	58.40045	248.0061	84.14785	56.78857	75.05288	319.0385	322.44
290.5407	101.0182	116.8834	129.8824	149.0327	142.8983	113.3607	173.2228	124.7964
12.81132	35.83263	80.43828	35.68428	39.53623	124.3315	50.15389	16.62829	21.22152
1034.692	1328.934	1051.662	1440.078	1469.995	1413.191	1150.153	1825.289	1309.534
125.5018	98.23685	75.1051	79.56563	46.6402	235.9782	71.11629	108.8474	100.0149
17.84258	17.45674	15.90898	4.538989	13.18463	27.52848	12.43282	11.84239	15.00949
93.91982	136.9291	282.2493	252.5506	208.8381	337.6977	264.5633	161.3721	50.42231
8.508167	12.56001	6.846755	5.455199	8.115396	20.66021	9.313352	7.873039	3.494467
122.9278	5.494782	13.80155	6.36862	11.15757	5.970026	10.86105	11.83702	11.4616
65.21619	67.34603	111.4278	160.134	134.8325	43.86702	131.4681	180.5442	91.18023
92.47259	177.6901	329.618	133.5595	277.7737	237.709	302.1182	283.0833	102.6738
13.54671	6.903431	4.052093	7.285293	14.19713	1.667435	3.821513	12.65285	10.58396
6.370032	4.809831	4.064455	7.295666	7.100453	4.254371	5.402615	4.695709	4.384973
145.1674	56.14007	93.38777	86.0114	122.6616	143.0585	142.564	77.11594	57.53378
31.49094	17.44059	15.89524	51.22911	38.52704	10.27497	28.09746	20.59084	16.7767
187.9397	207.856	207.5006	138.1242	239.2559	131.7127	197.9112	218.583	271.8214
10.70323	10.4699	4.059148	4.541836	10.14111	38.04329	9.332414	11.08858	3.496023
250.4454	228.9755	353.2434	368.7486	275.7491	143.7796	264.4823	221.7859	234.636
20.01291	22.40487	41.83163	50.34807	30.41504	45.673	61.94281	34.17876	23.88156
124.979	118.1101	87.07893	124.4644	95.29443	97.3339	104.8954	102.5922	123.1205
1377.937	2984.613	572.4728	3048.736	522.1046	210.9311	189.2584	1272.857	4571.993
18.63769	0	3.357161	2.710013	2.033103	0	2.258667	2.301333	7.927709
20.69087	130.07	36.10937	259.9905	60.83009	103.3641	106.4536	140.8176	112.4774
188.6384	253.5131	456.9828	373.2912	351.7848	283.3098	458.5649	514.4251	146.9391
57.30685	93.36203	126.7586	155.5488	136.861	248.1242	182.3778	120.0292	53.08344
55.16381	94.79825	360.0279	161.9738	118.6124	161.9562	60.96745	44.44336	27.40007
144.1895	94.0274	267.5654	150.9502	173.3586	222.2057	233.2111	89.76365	103.5613
602.0739	439.6674	371.8682	291.8328	835.3565	1360.846	795.8235	435.6155	113.2851
46.57847	51.92482	23.56188	33.82393	52.71942	72.34247	39.84271	35.70896	61.97029
10.68176	13.98942	26.54076	11.87199	20.27567	20.67155	30.60252	10.27308	10.59168
172.2727	111.642	289.3281	76.82806	115.5736	215.3568	109.5096	129.5708	80.54174
42.38842	39.36861	36.93298	28.35239	33.45526	54.31839	37.57317	34.98235	86.03237
15.67167	6.195598	9.621424	8.198008	12.17127	5.969724	13.21051	12.6334	11.46206
10.64385	109.843	73.2248	188.6689	111.5041	0.810674	112.878	192.0733	61.10804
148.6292	72.28333	66.7889	67.68313	108.4752	162.8227	104.8457	57.97197	42.45808
172.8764	58.17285	109.9402	24.66649	64.88832	23.19287	63.28579	42.04447	76.98532
70.27219	41.33691	376.9411	126.2807	302.0824	156.8082	337.736	38.08303	27.40124
37.28903	68.98125	78.21284	80.57394	79.06609	72.41616	53.24679	91.61617	66.45702
24.35886	14.65442	32.05088	22.85852	25.34634	50.88263	28.15627	7.86632	18.56477
26.53122	11.12898	36.27137	22.86003	23.31911	68.18431	42.32507	15.83935	8.809054
103.9843	325.4855	135.0923	225.0993	109.4921	55.06125	98.53294	184.4615	404.7972
2886.811	2720.399	2811.075	3299.196	3146.802	2240.762	2832.421	3587.727	3685.155
49.44077	3.394068	8.229292	8.198266	6.088442	20.61042	26.51697	6.274461	35.37696
149.8607	148.088	167.0434	290.0072	453.1641	80.02942	222.1402	509.592	316.0716
44.38928	34.29998	48.65315	16.43184	48.66653	34.39222	22.59801	33.30683	36.25782
82.41745	76.44888	225.6939	103.3585	159.167	81.76061	194.0384	138.28	137.2154
42.31316	11.81709	110.3776	23.76055	34.47151	48.22634	91.70631	22.19004	8.805648
477.4095	273.9634	18.67851	117.0807	21.2954	152.3927	24.94514	67.48693	345.3516
70.96846	106.0485	184.7256	462.2718	122.6677	334.3739	175.3601	153.4848	76.12049
22.15104	16.74391	27.07942	17.35136	24.3352	30.10791	28.11015	16.61579	19.43824
60.96389	58.96454	247.2257	64.03758	110.4972	118.0532	87.66991	27.74732	30.94928
2.043317	0.591512	0	0	0.004032	0	0.701109	0.718293	0.842489
0.614853	2.003748	1.983706	0	0.003764	0	0.703428	0	0.844003
27.37409	11.87396	30.80626	33.90846	16.22169	35.41025	21.15063	7.880691	14.14964
26.46596	18.8541	15.89973	18.26629	23.32172	33.55601	13.99548	26.96881	42.48921
45.34985	31.64072	36.99318	31.11516	43.58676	34.47302	49.43026	30.22327	31.00026
111.15	125.6664	374.9863	174.7463	165.2487	218.7602	177.6091	170.113	106.2188

78.81339	828.8469	2370.029	781.3373	1381.787	564.9047	1395.22	1136.874	184.1194
74.58491	1.295846	1.281931	0	0.004581	0	0	0.722176	0.844578
1.427433	0	0.636341	0	0.002182	0	0	0.758927	1.777657
12.11511	38.79074	11.04705	63.25327	71.95421	12.879	70.01345	87.14955	25.68683
944.261	1623.976	1886.185	1873.764	1609.897	1585.433	1361.427	1658.319	2026.764
8.501908	21.03179	66.55616	15.53124	43.58588	50.04856	39.20423	10.26062	47.88755
24632.46	34294.58	17299.81	40428.45	28409.41	19417.03	24879.33	37913.13	47665.23
49.4187	61.73165	24.94593	35.64843	67.92711	44.73197	44.52153	30.12487	53.97527
158.5048	211.3753	506.5682	145.4454	253.4482	416.0057	386.5941	168.4797	183.2565
407.0054	173.4295	215.1868	169.2402	171.3335	376.3887	229.2396	140.6492	132.7779
869.8751	323.7374	477.0944	371.4408	487.633	964.6266	639.2994	397.4547	146.0478
2811.021	6395.77	263.1484	974.3708	1891.727	1047.173	2053.999	1482.637	5981.414
161.3687	282.353	438.2094	342.1874	213.9124	1191.385	421.0198	702.1904	223.1059
17.86718	9.718811	13.1306	3.624357	23.31912	5.970584	14.01324	6.274738	15.01697
35.09568	23.07442	52.93157	52.14857	50.69002	55.98113	37.51253	42.1027	11.46217
48.98701	55.75805	11.73784	47.62952	30.41217	12.8736	31.32732	31.03009	84.33079
64.51917	83.55929	48.65879	62.19413	125.7065	104.2119	111.1331	98.58088	63.72444
128.4109	167.8921	336.0103	131.7385	236.2073	236.8815	220.719	191.6236	163.8077
24.2845	51.20788	48.68011	31.99133	48.66531	39.57063	42.18736	25.35787	51.32863
322.9683	262.0033	114.1201	113.4197	263.5849	256.6317	127.4669	305.299	296.6312
188.8047	122.8975	206.2846	172.9359	182.4787	117.1052	131.4422	158.2218	89.40036
8.51804	1.287293	8.957951	2.710138	4.060699	0.808921	1.478516	3.891596	5.267792
1327.448	2517.46	2317.98	3030.23	1843.071	1727.504	1945.128	2706.91	3642.697
102.5759	97.58667	155.3738	217.8002	201.7375	111.0799	117.3545	267.3018	126.6158
96.88858	88.4989	8.230078	28.32824	54.74826	5.11078	21.8166	17.40138	71.70272
5.622488	28.08504	44.69495	39.36233	53.72199	23.23121	36.02684	52.59344	18.56585
278.3559	563.4113	523.1338	525.1792	437.9566	353.9292	471.8324	515.1773	689.806
5097.154	4590.382	3274.307	1803.281	3701.342	3052.889	2403.634	1321.968	4375.819
395.4083	223.9921	767.8412	543.4959	478.505	389.2619	745.8632	324.3336	154.0227
263.4026	32.21353	152.1729	68.62129	61.84179	134.4633	56.31298	116.192	27.40684
1.324901	2.687087	1.274757	0.88312	1.018972	1.667529	0	3.095065	3.494184
107.6106	120.8091	86.30328	161.9608	89.21614	70.57714	86.80602	164.6118	160.2882
149.9468	146.7705	181.1017	161.9363	186.5365	306.6674	206.5963	167.734	91.16322
39.37792	35.72188	142.2987	68.61222	50.69266	170.6561	74.32661	22.17534	22.97458
7.801696	18.27724	25.88788	3.625566	17.23435	44.96565	17.21477	12.6848	5.269023
18.58341	16.06466	46.78533	49.4449	26.35997	68.16954	49.39431	19.02751	9.694825
11.52995	3.410689	9.763264	7.308286	8.111383	8.605801	3.842189	5.515962	4.393244
26.5092	21.70464	22.21955	24.68738	10.14335	26.67673	36.78958	27.00101	27.4335
0.612136	0	4.756383	0	3.046928	0.808859	1.478472	0.718008	4.381845
33.63486	54.74688	58.47607	39.31775	58.80112	55.09687	68.07244	66.77479	35.38147
34.35541	44.18344	48.69862	37.48714	76.03277	85.28629	70.42851	46.86136	32.72344
53.11333	96.45446	15.89912	46.65653	44.60782	72.38701	39.87336	70.81801	117.0045
16.41022	19.58182	62.17214	25.60173	29.40152	37.03541	67.44892	33.38048	10.57954
33.61329	99.02878	93.30884	66.76825	66.91345	131.7888	71.93819	186.9514	103.5942
27.25046	11.83337	47.50164	36.61086	24.33272	36.19119	43.10783	23.8205	5.264691
17.94499	21.82871	15.29756	29.32122	7.101276	14.62482	17.21148	26.31175	22.1579
25.00037	38.53052	43.08769	60.368	43.59724	30.94945	53.15207	68.34044	63.73129
73.25941	45.61734	22.17628	39.32405	29.40412	16.3061	41.43003	30.14738	44.25431
33.00078	24.52671	107.1225	88.86074	71.96673	22.3585	70.60687	17.42554	35.41956
3.475498	1.993838	0.588577	0	8.116145	0.811385	5.387421	0.721674	3.496229
52.53652	88.97545	60.82977	32.94061	25.3465	30.13813	28.9395	26.21155	79.83866
96.08635	234.0344	156.7232	182.0823	112.5331	103.3115	136.9129	161.3903	314.4308
30.7621	69.55559	124.9078	63.12792	62.85521	149.1376	87.6938	77.93943	54.88414
65.94057	63.83496	119.1187	188.521	164.2287	160.2277	164.3951	245.856	34.48527
25.72563	50.51772	2.666878	2.711478	6.088487	0.811188	6.168369	11.83665	39.81201
70.97703	47.66047	68.8822	26.49686	33.46059	63.69036	38.2557	57.17623	70.80817
16.41116	46.43386	8.232324	42.09929	41.56341	18.04226	26.56744	74.12133	22.99505
1.329929	0	0	0	1.018789	0.810467	0	1.514569	0

252.4668	54.66865	71.6158	68.5841	591.0438	101.5608	788.6465	402.1732	317.8195
2.038114	0	0	0	2.033089	1.667047	0	0	7.043691
56.57242	86.29125	66.04776	59.43496	79.08066	73.14721	82.07739	87.37506	65.47595
386.8816	269.7303	206.816	111.5892	263.5852	323.8282	477.4348	213.0243	228.4287
5.625643	23.88386	10.34439	10.95117	7.101892	6.834785	17.17385	19.05207	20.35369
38.82999	9.015572	1.967193	14.6129	7.102111	4.247986	6.169621	9.461128	6.150728
30.75127	68.10715	77.31095	56.70894	68.9388	160.3021	80.59734	60.3857	19.4314
1212.809	583.638	1660.019	1464.834	956.002	1631.182	1050.86	750.4364	245.2155
81.76094	56.10244	88.42293	129.0187	116.5846	167.9979	146.385	175.0057	53.08904
449.9802	215.5598	95.29399	84.13613	199.7205	92.0877	230.7674	164.4884	411.7362
80.00587	10.42037	50.28328	16.44239	40.54884	24.08958	41.51792	26.20824	17.67562
134.8335	345.0275	102.284	152.7741	147.0025	174.8009	152.5319	463.6686	238.1887
458.4565	1565.22	1191.852	3730.424	1665.643	260.021	859.9205	1561.497	1952.513
2256.692	2213.183	6554.168	4204.033	3615.176	2480.151	4394.942	4674.411	1810.65
64.54731	117.4204	66.13473	79.59896	69.95251	181.0024	84.5168	108.1775	128.4458
2898.31	2573.602	3853.15	3972.597	4415.047	2987.436	7208.906	3896.21	1031.476
33.67326	11.11441	8.925811	2.710655	11.15741	8.552955	9.297291	6.272786	27.41738
190.8418	103.8525	116.9157	144.5354	158.1541	67.97613	165.835	331.5745	182.3806
122.2322	97.07997	129.8465	92.44325	114.5479	51.65722	69.66406	32.53291	67.30295
179.371	30.77686	12.41219	42.04959	77.05324	24.05328	143.1419	34.88983	48.6497
88.89943	48.3455	43.05722	88.72268	112.5333	111.9262	60.16589	81.8184	54.85161
1.426431	0	0	0	0.002193	0	0	0	0
20.00234	23.09685	12.41647	10.02903	16.22561	26.66519	7.732591	4.683673	34.52009
34.40161	14.63449	11.01844	5.453654	16.22581	5.96952	19.48607	32.55754	17.66696
55.99108	28.71348	50.14404	21.928	166.2342	32.69127	96.39677	75.59882	15.89198
60.96424	94.89966	24.95612	33.82345	32.446	37.84938	34.35527	68.36058	77.92484
65.90418	191.7463	440.4952	198.536	270.6781	247.1824	362.412	346.7272	229.3369
50.14132	58.92296	232.292	78.67107	166.2537	85.24387	125.2374	107.3359	59.29321
1.331021	1.99448	1.28129	0	3.04673	3.390089	0	0.7219	11.46139
468.4859	1064.169	1149.973	1328.485	1229.724	1195.342	1492.99	1140.015	679.0971
230.9647	286.4991	1228.332	396.1474	723.8405	388.3711	854.5561	394.2764	225.7438
4025.527	6873.699	12140.12	14564.59	12009.35	7230.435	10979.21	16471.87	6876.185
12.81392	22.41528	37.64721	43.93882	29.40082	43.09333	61.18484	42.17972	18.56227
101.8376	102.4904	193.009	155.5403	133.821	212.762	238.7568	311.8258	82.31146
8.493782	1.290192	8.231689	0.883409	3.046994	8.554382	24.20747	11.8447	11.46556
15.70692	18.90012	48.93193	16.44572	31.42632	58.68201	26.59441	15.84203	16.79316
9.275306	7.649891	23.86769	9.132626	20.27038	15.51847	14.88607	11.1084	2.611103
32.23324	20.97092	22.18871	32.00442	16.22591	35.28577	21.05205	10.24858	18.55229
83.88756	58.89837	149.0833	73.16848	96.31256	136.9319	115.7826	82.62546	34.48364
10.6855	3.389854	1.966267	12.79053	3.046968	2.526993	6.176961	11.87478	6.154564
109.8886	87.13135	117.8421	97.91498	78.06152	168.9338	70.40796	38.8889	103.627
58.0393	65.94963	93.30406	118.9465	105.4342	225.7587	152.6494	70.70506	44.22955
8.500292	9.721544	47.53581	39.3692	24.33207	43.98438	27.38454	25.42799	6.150876
319.9724	342.7115	400.4491	493.1402	451.1372	256.5877	386.4917	528.666	461.3006
30.09389	18.86839	102.8085	47.58973	22.3072	100.0807	40.6938	28.58156	11.46464
76.27514	33.68411	40.40152	21.93348	44.60547	75.88647	83.92101	58.10685	14.12326
309.2446	189.5558	333.6053	139.95	386.2533	607.2047	355.2224	83.38416	84.95185
5.624307	84.29148	98.94701	72.27036	135.8408	37.84408	357.4517	49.23235	68.16292
198.0014	79.24993	63.94942	49.3687	65.90215	275.5811	51.54699	50.78941	45.10696
31.51735	57.66442	45.97663	59.49149	39.53889	52.55066	44.59937	68.45391	34.51372
5.636224	10.46714	18.83686	35.76036	20.27352	15.49558	42.53116	23.11511	7.045002
17.10995	61.84727	75.31945	81.4628	91.23323	82.73336	166.2807	168.9236	10.57652
142.744	175.5782	99.50251	174.7422	182.4828	197.2101	181.5135	212.2689	207.1938
132.1782	37.12572	8.925296	3.625926	24.33653	19.74879	3.825311	21.37896	77.02791
147.2505	14.6223	55.64311	17.34697	54.74822	97.32231	61.76646	46.04379	42.46202
65.92935	450.9363	162.3531	278.2215	83.13395	51.61984	101.6872	148.6874	619.3325
9572.502	11575	11337.73	13186.72	13182.3	9318.811	11827.39	12077.22	14146.46
230.9667	423.5275	425.5408	829.8814	615.3671	316.8819	640.0944	616.1487	650.82

28.64057	25.20662	58.59323	28.34323	40.55208	80.18818	72.10362	22.99319	10.57774
114.5723	19.57398	15.21196	43.9244	67.91563	21.48956	67.41813	50.92706	9.692518
12.92536	6.234118	26.02921	29.36858	19.25642	15.52618	29.18168	13.5278	4.386487
101.8891	76.50544	126.8105	107.0462	180.4466	186.9631	176.1665	39.67056	49.54505
157.1	54.66268	44.44584	26.49589	40.55753	165.3255	57.80997	110.4372	82.3015
598.4882	321.6232	287.5645	351.3084	324.4163	185.0989	219.7859	206.615	578.1898
14.26973	35.19621	38.41467	32.03577	38.51913	57.83449	43.14262	68.64327	43.44388
197.2833	255.668	361.572	294.6103	269.6678	447.029	427.3191	282.224	160.2312
111.8947	134.1404	200.6796	398.1153	141.9309	67.98554	158.8481	229.8385	176.2167
75.25203	68.02792	479.7574	418.2424	250.3992	97.278	335.8797	263.2505	56.62294
4447.171	3828.378	1431.268	4137.342	2859.894	1017.872	4619.016	2552.708	5474.774
65.19294	60.99393	33.99795	48.45548	63.87399	38.69385	59.38023	80.22279	71.67799
31.4825	27.28849	56.39279	45.72951	45.62319	38.71702	57.10254	62.00435	21.20521
35.85063	2.689005	0.586311	1.796714	0.004488	2.527825	0.702981	4.683734	2.610206
51.58044	38.52263	44.47303	61.27871	77.04991	46.45789	59.40985	69.91894	91.19475
23.69364	26.73588	39.89003	39.39267	25.34335	31.9092	33.73075	30.26435	29.24586
371.6763	300.5664	770.5256	394.3234	764.3887	624.4024	727.005	375.2054	126.5685
0	0	0	0.897447	0.0025	0	0	0	0
35.75864	58.18615	111.3812	64.01308	97.32727	47.30835	61.73176	74.65885	69.0229
95.38964	47.65051	7.537234	22.83624	31.43328	10.27597	13.99086	29.32678	77.88778
15.72039	11.13939	104.6585	26.53748	24.33105	22.38203	23.46636	35.04492	6.151724
0	0	0	0.883243	1.018916	0	0	0.718692	2.614814
20.73812	49.27338	40.44158	34.76855	56.76485	58.64087	64.31797	20.61679	4.379167
36.55468	111.3306	17.29842	60.40399	53.72924	12.86112	51.65912	119.5199	95.72582
13.5181	141.1064	354.6539	236.0505	199.7178	134.3054	316.1705	672.8955	33.59648
68.81386	77.20054	96.78369	88.73425	100.3662	120.5725	86.81587	189.3163	73.46316
16.39458	66.81775	15.90055	50.32321	41.56671	8.552834	29.67727	43.70977	92.16933
100.4082	98.98166	93.96218	189.4132	223.0267	278.28	285.7881	153.4475	43.33962
61.6824	44.87845	85.71305	97.00379	98.33372	89.58807	92.3745	68.35943	31.8352
231.146	180.5369	196.4553	221.4316	269.6612	303.2282	301.3833	147.0518	70.79263
343.6437	230.2787	298.7048	236.0173	154.1018	431.426	230.7388	240.0061	320.4899
108.2687	172.0587	234.7608	182.0626	187.5517	219.6103	148.6238	121.5758	85.84495
25.77992	33.70419	51.6469	61.34817	34.46916	46.53588	43.06824	41.36301	55.83252
4.925253	5.521797	7.585891	12.80901	15.20502	30.29061	14.89177	4.69763	6.163177
185.8206	4.80408	75.79912	5.457026	37.51618	2.533056	11.64819	14.22476	7.038781
108.9798	195.245	188.7217	256.1875	229.116	314.3775	349.0712	189.9777	148.7258
53.81959	23.77872	55.72958	34.74678	42.58113	87.04817	82.24816	32.54042	29.18519
18.55589	28.02233	10.32059	10.94375	27.37585	16.30988	13.99757	22.99046	37.17421
47.41945	31.57712	8.928838	17.35522	15.21177	12.00225	27.34629	11.04772	38.07535
304.3229	155.8674	470.403	198.5282	275.7487	292.8293	280.9316	176.449	111.5253
14.30771	6.209678	18.80731	5.456614	8.114926	2.527046	6.177691	2.301478	4.381272
43.71135	47.71122	75.9621	137.3189	55.75969	81.84171	56.31703	47.66197	42.47429
9.224792	21.04171	1.274144	3.624475	2.033094	1.667292	5.387702	20.64601	24.7921
368.817	465.0265	482.0248	820.7534	537.3049	667.4862	400.6071	344.9949	377.1849
1.32412	0	0	0.882942	0.004364	0.809181	0.70138	0.718511	0
64.65067	51.99103	48.75376	74.14224	67.91983	15.44611	57.14479	78.80024	101.0382
28.60977	30.81306	72.4855	76.87166	53.73184	74.94943	92.42189	39.7019	29.1817
99.63649	71.52152	197.0593	109.7592	109.4943	138.6073	154.0813	131.8994	78.756
80.24727	290.7458	282.0281	401.6516	539.3316	149.7929	443.6695	618.5885	262.0606
131.9051	27.3331	18.70617	19.18595	30.41581	43.06915	25.7708	49.32837	35.40918
12.12711	4.092613	14.57103	6.371963	12.16895	7.700807	7.746411	3.89095	7.040923
284.0505	113.6612	52.11253	94.1999	45.62615	21.47613	51.55129	94.51332	179.6921
7.791206	1.287518	1.966281	4.540148	4.060762	1.667076	6.175073	0	4.380577
105.6406	200.7673	151.5084	71.37253	117.5897	15.44325	133.2196	107.4395	195.0068
115.4501	109.4861	111.3511	150.9464	138.8921	89.51384	117.3054	208.2878	147.8447
6.361808	3.393017	1.96683	0.882731	6.087507	0.808802	2.259509	8.689368	9.712898
58.77535	383.7112	34.01396	335.0706	224.0297	26.64003	414.6436	170.2754	280.0095
2.762347	2.695396	0	1.796793	2.032891	0	3.835163	0.718108	2.612541

34.35298	31.50651	205.9984	46.64204	56.77393	95.63276	121.4125	72.34988	49.5616
107.5306	109.4643	69.5222	37.47477	151.0588	124.8226	186.9548	79.4123	145.174
17.85139	9.713118	22.21411	23.76935	15.21162	17.17901	32.85209	13.43883	11.46593
17.8343	46.3573	57.16479	78.72934	66.90549	78.44242	79.93263	36.53761	26.53046
8.536554	6.221105	4.76191	4.542579	8.114131	5.98062	2.259732	5.491718	7.047643
19.98614	54.08973	92.11861	56.72923	55.7579	68.06317	99.52514	69.21032	24.75347
38.63661	67.3462	34.00385	100.6318	152.0654	55.92993	80.54373	264.1448	211.6907
54.43139	89.13597	132.3304	80.48954	131.7929	116.2449	122.0448	119.2276	89.40118
106.2151	66.66099	107.2785	59.4466	53.73501	165.4197	97.01791	37.28549	38.91581
221.8261	79.97998	72.33262	50.2863	27.37827	105.8943	111.846	48.41007	104.4541
91.09925	78.6118	161.705	63.10543	107.4618	111.9559	115.811	82.64201	63.71882
1082.802	840.7157	1023.824	795.9572	816.1037	749.1889	863.7682	955.5212	846.441
6.360827	1.986839	0	1.795965	1.019027	1.667135	3.042187	0	1.725254
584.7436	736.7621	1331.776	1053.069	1227.697	2022.152	1467.922	1373.755	415.2212
1.359561	0	0	0	0.003126	0.819085	0	0.731498	0
25.02226	36.46403	27.06977	53.06622	75.01573	18.89307	43.79191	36.52667	49.57963
461.3397	245.7266	378.1117	368.6856	383.2165	654.4911	363.7673	297.2456	169.0676
68.09328	72.97691	247.5552	100.6333	116.5856	138.672	133.8248	108.9033	78.77809
45.19182	60.45987	29.1722	42.07768	34.47162	106.0593	43.01533	21.39264	24.75575
45.88463	27.29472	48.02293	73.21085	78.05803	28.37399	152.8843	86.733	39.81993
0	0	0	1.809665	0.00321	0.817744	0.712936	0	0
0	18.94852	7.550331	3.624846	13.18246	9.427553	8.531392	11.07042	10.59072
40.11055	39.95894	116.5043	42.06542	52.71901	250.0914	93.96474	30.13875	27.40682
13.52879	18.87444	14.51688	11.86087	6.088528	48.26144	10.08379	9.455887	12.35195
42.96392	82.87656	179.2778	161.0866	107.459	185.2885	111.9401	92.22795	61.95766
178.6851	75.75465	68.84108	22.8362	114.5614	55.92099	122.7996	54.77028	172.658
798.5809	1283.972	1046.785	1890.237	1628.145	661.3371	1312.134	2582.982	1875.353
41.04883	23.16721	16.65133	46.7213	33.45135	37.9495	30.55398	20.64651	18.57498
147.8325	103.1987	103.7237	35.64598	76.03837	29.22052	42.16293	35.6874	190.3982
2.039276	1.289473	3.356255	1.796277	2.033052	0.809703	0.702193	3.889205	10.58091
29.43112	4.089811	4.748409	6.369498	19.26486	7.694982	5.386482	23.02899	8.809662
30.23081	26.04065	13.16018	23.79572	22.30281	21.53159	20.33816	26.27113	37.25618
63.11874	54.03089	80.81754	68.61475	81.10208	93.89829	133.1592	43.66814	23.861
203.7936	262.0655	168.5174	96.03757	135.8509	124.8349	117.3047	176.4641	186.818
91.88844	77.26284	91.28141	75.93649	85.15737	155.1259	101.7685	86.66893	19.43137
241.7505	187.4425	822.8507	482.176	447.0797	600.3001	595.5259	267.0645	121.2565
33.63288	44.17794	31.93528	27.41595	47.6511	28.36819	21.82105	40.48644	20.31809

TCGA-DU	TCGA-IK-	TCGA-14-	TCGA-06-	TCGA-32-	TCGA-14-	TCGA-12-	TCGA-06-	TCGA-06-
121.7782	165.8529	77.37197	83.60208	313.7301	54.76321	149.9804	52.17329	115.3447
52.9045	33.99486	9.616625	28.30423	18.83956	3.451699	7.331315	22.19544	7.503371
221.5984	92.944	21.48521	72.02075	48.12741	86.70111	84.27721	82.04198	52.34958
3322.957	3527.233	2142.69	1611.235	1659.836	2838.511	2017.308	1959.845	1715.037
77.85787	69.29122	89.01291	61.77165	75.31792	69.15606	95.68154	174.3213	128.1503
0	0	0.337913	25.88208	0.011007	14.96786	0.243912	3.912045	3.211402
10.97929	21.41767	21.24776	306.5932	15.70528	19.4421	20.83688	71.39593	12.84138
169.6915	164.4645	65.63231	66.91365	49.17537	57.96559	43.68464	57.37479	117.4797
12274.68	11222.11	4403.297	7717.867	4767.168	8816.974	5878.751	5939.254	3654.315
80.8532	31.81596	41.82146	34.78049	9.43257	60.99039	25.46532	40.4233	28.85722
10.97948	22.20971	6.622462	10.36894	8.383533	11.40286	24.86479	19.54142	9.633616
1850.635	1462.149	260.5121	787.6501	435.1067	173.4591	186.8109	455.5724	294.7641
91.83242	83.85948	171.2205	87.46135	1069.724	249.9878	479.6999	174.3827	318.186
0	0	3.303754	1.375776	3.146949	15.21176	44.7273	8.961862	1.090537
104.8093	61.66917	50.82982	48.92271	33.48705	93.08167	45.88919	44.35596	37.40265
19.96312	11.6734	52.66079	15.52197	100.3468	93.94046	36.42555	29.98188	121.6186
3183.212	2391.251	1472.955	2748.162	1200.693	1387.336	1531.238	1836.194	1356.228
45.91638	22.79434	30.02531	3.970603	14.66096	17.87044	27.62794	24.82195	27.78474
35.93392	102.6788	91.74213	225.8916	295.9222	133.0257	45.8949	43.05734	135.6108
112.7941	167.1034	222.106	2581.772	3007.832	1444.374	858.389	571.3171	937.5826
1703.902	1147.069	449.2337	819.7519	251.0325	189.4956	223.1487	675.4544	281.9493
24.95404	186.7267	6.69961	0.092668	2.109635	0.19503	0.404799	0.098002	0.022194
117.7857	156.1882	9.650509	29.67124	7.339908	3.436202	11.88933	0.100218	36.33589
114.7905	278.2324	227.6078	74.64052	31.39622	67.614	75.49858	69.09912	134.5778
99.81847	90.8822	18.53183	129.7016	112.9515	13.08701	39.08774	54.745	24.58985
19.9632	24.22548	15.40465	16.79377	10.477	8.264042	14.07619	33.83882	26.70762
174.6821	88.01256	259.4421	313.4036	216.4998	350.929	263.3167	521.4836	173.0078
1217.786	600.1574	525.856	489.6057	412.0964	346.6306	495.5867	1417.026	461.3482
78.85818	89.86295	6.691696	6.543246	2.109884	3.45128	0.397883	7.933136	12.84076
60.88889	64.47776	39.03665	37.36127	27.21156	5.053269	9.61821	79.40106	18.18294
20.96116	52.00172	41.77455	15.53667	69.02627	5.057589	54.65465	57.28241	20.31684
64.88204	70.80583	27.24081	15.53639	5.248445	14.68533	11.89128	36.52102	65.13956
18.96548	10.31975	9.441751	6.529626	3.156293	1.836362	2.760533	13.07676	18.15706
654.8089	759.1103	914.7542	453.6302	666.2388	1213.818	1108.242	892.7038	882.0717
98.81963	174.8133	136.2823	228.7001	82.64305	138.0812	132.1102	177.0298	151.6555
19647.27	10024.29	12963.12	868.6471	474.8508	181.4362	7105.418	3137.589	10078.68
20.9616	11.69104	15.28696	155.5099	57.48226	87.06416	33.84625	35.07015	58.66945
59.89084	102.0957	47.682	29.65529	32.43864	25.89226	39.00343	23.54371	35.26272
18.96482	25.57532	35.82761	428.4258	87.82986	165.6435	56.71282	58.53335	102.4637
22.95768	13.75567	29.95226	262.1152	55.4245	121.0274	54.33374	32.59107	108.8445
59.89038	105.3995	83.25227	826.3474	508.2171	358.6301	131.9285	206.8513	218.8988
35.93418	49.92986	53.24713	20.66638	74.24868	35.43889	101.4745	58.55948	51.26442
20.96116	14.44244	82.26642	244.7054	59.61879	274.0994	166.445	118.2266	95.01818
102.8128	206.2367	36.21212	34.80854	14.66205	11.47871	14.16616	43.06315	20.31907
168.6927	289.3601	180.5125	194.0727	398.4703	123.7022	175.2465	330.505	207.183
14.97234	24.99333	18.01852	23.13157	3.156469	12.99968	9.525299	15.68221	16.03263
32.9395	24.86373	85.2194	37.34385	58.57443	56.2052	115.1535	94.9057	77.94834
3853.992	4411.319	691.3398	553.876	294.9597	351.4627	825.0681	715.851	1056.162
318.4204	726.5878	472.413	341.8392	96.24162	221.538	164.0732	298.0976	523.2675
17.96723	16.6243	9.441522	10.36024	6.292611	0.182657	0.371561	16.94333	2.162973
12.97601	11.719	17.82778	6.529765	4.202011	3.447324	7.260222	11.7871	16.02637
354.356	219.2373	180.446	178.6544	352.453	93.25082	98.16341	200.4564	80.11902
71.86922	66.58795	41.88456	39.91711	23.02808	32.28908	65.99628	74.18133	84.35738
8055.352	7084.967	5857.379	594.988	297.0506	219.9422	1656.395	793.9305	2462.559
25.95292	17.29184	18.02423	14.20642	2.110163	9.83069	7.294248	16.97462	20.29671
28.94673	48.52154	21.41203	19.38679	35.57361	16.28658	47.92635	39.11823	25.65413
34.93696	26.37495	6.6523	9.099436	44.94624	5.055737	13.98916	5.328071	10.70246

6609.98	4384.816	2494.17	2130.322	1814.633	1039.439	1272.546	3080.298	1980.947
145.7343	101.1883	148.2077	561.2802	209.1882	299.9099	385.9296	616.5836	242.4204
34.93643	20.73306	26.89504	16.79669	14.65885	35.29076	34.12287	33.84748	40.57327
21.9594	73.63096	27.1848	97.47423	63.79418	102.2286	56.77115	14.43859	25.65214
0	1.269207	9.595854	10.38769	289.3465	98.32752	5.05752	31.23314	9.637572
230.5807	189.4021	77.49293	128.5637	240.5522	94.83719	132.1013	204.3291	52.35521
16.96878	6.121393	32.01455	6.536597	44.94219	30.35868	22.77069	14.39246	16.03322
43.92043	28.40672	21.1696	10.39187	5.248677	11.45967	11.84125	36.43351	19.24195
107.8043	97.17202	12.6253	78.39616	32.44005	51.48406	20.97429	61.22498	40.60269
113.7925	57.46693	124.4823	478.9343	588.7642	195.6863	220.3034	433.0793	87.59132
81.85607	28.55056	0.553403	1.393927	7.337831	0.183146	2.761303	2.723525	2.162984
28.94741	6.813997	18.14528	2.684403	1.063172	9.846069	18.45151	33.78674	25.63291
402.2694	219.9484	59.8419	64.36016	99.37632	90.03879	107.21	59.98824	131.3703
34.93617	56.95961	29.95297	12.96291	11.52368	22.64296	40.97812	59.78878	24.58141
129.7643	116.5948	12.62299	41.22168	19.89161	8.267695	11.89274	15.74525	20.31894
34.9362	21.41157	24.17222	24.49369	9.43222	60.76905	32.04013	36.47419	24.58086
303.4484	324.1161	98.12376	115.7335	111.9265	157.3222	315.5297	208.2539	148.4552
53.90241	94.00819	35.5969	14.24175	12.56886	36.92822	11.85997	15.72679	10.70658
99.81776	91.4738	98.14535	1595.751	371.2789	919.2895	428.7791	476.1131	277.6502
159.7092	218.571	136.2792	132.4169	43.94713	59.59001	55.05415	152.3246	103.6084
725.6797	1314.944	528.973	294.3446	713.3108	118.9257	822.6459	976.0582	506.2031
95.82632	74.96684	30.17966	52.72182	3.155811	21.08864	9.620266	23.54047	28.85701
228.5841	536.646	77.5286	11.67686	19.89102	14.67815	55.06185	31.3611	84.3903
40.92514	187.8768	30.15352	10.39975	3.155881	5.057724	25.44946	23.53785	28.85614
38.92873	37.38673	38.87178	56.55648	80.52457	45.02701	94.93241	67.65716	44.86512
2075.227	1895.875	481.7574	677.188	625.4566	494.1661	743.1459	369.6992	445.334
44.91766	48.46886	74.17465	273.276	143.274	104.2461	59.42501	215.7577	30.99635
190.6537	304.8905	39.17392	101.5641	25.12115	35.5367	57.26765	126.2629	69.43568
155.7161	141.4329	224.9318	1268.983	928.7123	920.048	431.6683	506.1987	795.5357
33.93755	39.43453	53.75449	205.368	261.4182	102.666	104.5363	57.35189	30.99659
73.86496	181.0847	399.8123	100.3098	170.4855	277.2752	545.6079	152.3095	391.8624
45.91632	84.79972	32.9542	10.39874	32.4345	19.47233	38.85359	32.61087	36.32278
1333.577	293.4214	157.2236	250.6449	116.1134	64.40531	98.22444	169.2974	239.2316
1646.006	1548.776	1048.164	1996.593	912.0297	1573.292	1590.19	2389.164	1606.104
0	0	0.289242	3.81969	19.70366	4.652772	0.212084	6.325735	8.470504
32.93965	40.19817	47.3483	18.09706	12.56976	6.66753	50.01309	33.9051	35.25504
64.88181	65.19627	9.66568	1.388956	7.340499	38.67952	9.61995	0.099269	20.31787
830.4897	734.138	935.2479	813.2897	701.7957	1580.773	1217.029	1312.886	1277.158
76.85993	70.02217	39.08446	56.61338	34.53225	33.91421	77.43168	56.04392	101.4481
44.91777	80.48635	36.09579	19.39219	27.2113	27.50072	48.05605	83.28476	9.639813
85.8442	81.92086	30.19278	33.49952	142.2018	3.444541	36.73331	18.3431	28.85744
40.92498	233.6306	9.66436	16.82393	5.24813	1.826017	11.89547	1.411115	41.66938
61.88682	169.3691	36.2205	54.06865	26.16696	8.263738	50.45967	135.3412	51.28433
257.5314	103.9638	497.8761	200.5047	293.8977	359.1766	960.8993	439.7615	526.432
18.96537	32.03681	3.697096	1.393865	4.202225	1.836578	0.376194	5.324638	14.96369
29.94521	118.4692	9.637534	2.683113	1.062928	0.193204	14.10935	0.097221	16.04301
3.991985	27.74584	23.85557	2.684323	1.063143	9.850213	16.25584	14.40922	39.49367
28.94698	30.47553	18.36816	41.11769	26.15858	27.40139	25.31404	58.46751	32.04742
39.9282	12.39394	15.24335	3.971108	20.92313	6.653048	22.8025	16.98181	17.10053
31.9413	104.2184	15.56113	16.82115	16.75336	3.445041	9.620258	7.935194	20.31717
4.990175	26.37346	37.70898	14.21379	15.69994	44.56506	42.53591	19.5691	25.62961
2047.277	2402.425	1174.905	684.9114	1341.882	1081.036	1767.192	1391.151	967.5215
36.93242	43.66952	24.28337	11.68328	23.0261	1.830191	32.15406	15.73831	34.18982
177.6761	350.3386	516.647	1208.784	427.778	1045.265	731.2399	1010.97	476.2868
342.3771	553.1255	195.4294	354.6408	196.6426	191.0483	222.997	195.3009	216.8017
1.995643	5.430065	17.85664	48.49539	2.110169	49.01038	37.75705	37.53356	30.94061
5.988395	12.40842	6.619414	2.684209	4.202238	3.449245	9.501586	5.324782	18.16117
178.6757	162.4529	56.73159	104.1011	76.36155	67.53356	95.60112	61.25943	57.68864

15.97044	35.44294	21.00828	34.64719	79.41927	22.54061	16.24971	16.99641	21.36942
34.93593	61.05288	18.48729	15.53664	32.43713	19.48619	50.16936	27.43464	32.05828
135.7532	198.6041	118.1194	46.36218	4.201387	22.71083	84.32856	95.04708	22.45479
317.4226	548.3107	157.1035	90.05783	74.27786	41.95504	127.7033	87.31662	51.28779
105.8073	115.1637	77.25547	118.2226	120.2798	81.94001	104.6674	52.16438	66.23042
59.89054	67.22931	91.71554	323.2905	93.08919	196.8681	70.72501	157.3588	159.0909
318.421	431.7274	51.00788	3.958666	20.93687	0.202267	111.7863	58.68931	122.8313
31.94142	35.31713	21.36543	36.03987	19.88929	16.27837	18.65978	32.61291	33.12203
35.93494	13.0799	9.582951	56.32748	17.79197	33.61918	16.26105	23.46663	32.0325
20.96131	10.97711	24.13289	64.11923	54.3753	24.21849	94.07496	102.4317	28.84678
126.7689	396.361	106.9419	251.8266	40.80954	170.1292	136.6771	216.0523	129.2356
122.7762	183.8503	80.42735	256.9278	75.32209	37.14585	43.70436	177.0222	133.5035
604.9003	723.8686	360.3792	689.8542	230.111	216.711	393.1466	551.7307	307.5675
7550.271	5083.055	1546.943	1116.614	587.8086	742.7591	959.0764	1149.18	1791.927
12.97582	31.26283	20.95009	18.04913	15.70057	11.43276	9.551163	23.4507	13.90299
27.949	43.79836	15.37807	25.73674	7.34013	30.49628	49.46648	14.4166	22.43954
102.8138	33.20881	18.49426	54.00161	8.38661	8.2729	14.16117	19.64187	14.979
0.997738	0	2.908865	5.000659	5.204299	3.153184	5.992523	23.17704	5.300826
7.984842	24.33641	0.553596	2.683923	1.063314	0.183186	2.761366	0.092894	7.499564
0	1.281208	3.337698	6.39404	6.270957	16.76139	2.593186	2.680422	19.09713
7.984819	16.60742	15.13041	21.83879	9.428373	34.99386	11.71293	23.40486	26.68507
125.7712	95.00238	59.70474	68.18704	132.8286	89.94633	111.4669	122.3461	128.1489
44.91808	20.00669	18.45541	28.35459	53.34142	16.2791	43.34875	53.36149	40.59193
618.8747	493.345	351.663	639.8092	359.7969	280.842	293.4208	562.171	373.7748
91.83254	96.38264	203.0273	45.08391	431.8728	93.1627	246.8857	134.0543	176.1901
10.97969	7.538974	14.82135	12.87968	2.110016	0.177876	13.72759	14.32955	6.43014
412.2496	1400.429	402.0385	132.459	177.8208	46.75024	611.3445	261.6905	211.4698
184.6641	111.6366	247.4068	160.632	147.4773	198.8322	267.614	248.477	212.5021
49.90858	23.46133	36.19903	1617.808	363.8871	123.473	201.4739	202.8391	118.5358
5.988369	10.2991	15.25897	40.98643	37.63612	47.71171	31.60102	81.43171	55.46711
249.5459	288.6492	77.54868	200.5019	63.81897	107.6857	93.65176	519.0691	103.6118
336.3889	221.3441	59.83828	155.5295	44.99302	82.0253	64.13081	101.611	82.25367
182.6681	190.1976	147.566	159.3027	92.05003	83.56743	36.87763	130.1598	97.1935
118.7846	143.1266	12.62489	79.66672	32.43961	11.48281	11.89546	67.70775	19.25054
10.97935	27.03302	26.76221	30.8394	18.83791	8.259968	9.575638	27.35495	16.03911
32.93959	13.7499	56.11227	415.8625	129.6423	86.36552	27.67359	96.13787	72.60274
124.7732	90.85779	88.8384	30.95376	55.44727	19.50342	111.3311	67.75087	38.47062
2.993828	17.36472	6.54068	67.32144	12.55734	79.72166	2.752791	36.14333	9.628002
0	0	3.623543	53.17542	2.109675	59.02353	11.42787	18.11902	10.68493
118.785	70.78277	15.564	69.38112	23.02763	5.056599	25.46738	67.67515	36.32902
71.87043	44.43312	29.85673	19.36495	20.93184	13.06391	40.87919	11.8321	17.11003
27.94856	21.39357	53.27755	93.66584	168.3164	169.0457	88.12859	75.41811	83.27456
150.7255	103.9826	168.54	105.4511	60.68065	142.876	202.2489	292.7235	213.5787
50.90689	14.44025	53.64824	19.39329	154.7656	370.3667	176.2026	148.1993	97.17401
817.5142	387.1543	213.1734	226.2269	244.753	29.11339	166.3256	407.3538	234.9574
124.775	67.36336	18.45341	39.8824	15.70694	5.059374	7.343031	18.33586	5.367748
23.95586	22.10172	70.14183	129.3549	202.7683	29.0238	54.39499	23.52089	80.05532
90.83523	61.73273	33.10513	56.5702	38.71126	27.48541	27.72323	58.59403	32.05963
12.97564	37.40752	56.0183	20.66283	48.11439	48.15703	130.238	54.65256	48.06001
87.83985	77.63583	161.9369	20.6798	3.155057	8.264754	79.83145	31.35887	91.85171
58.89267	84.0032	38.93952	47.60318	13.61613	48.24507	20.95556	36.53201	36.32956
535.0279	266.4207	180.5923	19.38889	14.66081	53.18328	73.23096	37.86773	104.68
69.8729	71.47305	15.56625	52.72927	39.7571	5.056252	20.95518	49.51356	18.18227
17.96672	29.09111	83.77205	25.76083	16.75023	41.66683	96.12909	31.27318	70.43654
183.6653	192.7854	434.1575	729.7246	701.7846	857.7603	1161.988	2228.403	396.2001
697.7316	665.5214	224.9801	318.7115	36.62515	33.92635	52.78521	138.0666	45.94766
35.9342	12.36083	12.60768	33.48342	79.47395	30.6518	54.56451	110.3862	66.20107
182.6681	124.8676	45.05523	240.1235	91.00444	75.56747	25.53226	79.47331	63.0297

179.6739	136.0319	62.58761	51.49561	121.3235	22.71065	45.91226	59.95978	104.6598
37.93066	36.01611	24.26372	43.71996	17.79803	29.04417	23.15633	39.095	27.78586
23.9557	97.01635	874.9523	755.32	516.6642	1462.976	2572.502	1761.197	782.7186
143.7381	116.4764	288.8812	183.7749	189.3125	442.1933	498.5647	307.0444	334.226
20.96113	58.24154	18.50889	20.67392	13.61617	43.4662	95.13866	80.66398	66.21429
11.9775	11.6738	15.45299	27.04129	16.75033	16.25101	18.58662	19.61509	45.91071
127.7677	111.0087	115.1531	65.61077	47.08214	51.53377	75.28062	78.1512	92.91676
27.94871	21.4098	61.45282	61.59314	25.11457	98.84147	52.10706	31.29478	26.71536
93.83083	47.8834	9.651653	20.65581	13.61507	9.873845	9.611773	15.73289	23.51503
808.5297	819.529	198.5325	214.6821	249.9856	72.42237	120.9391	300.7175	286.2186
47.91332	69.62146	9.621082	16.79684	10.47721	13.05488	7.33284	11.82838	4.299785
94.82752	61.68344	36.1462	101.4898	29.30346	40.31008	39.07983	118.3683	64.08856
202.6316	201.9371	124.4203	199.142	122.3805	211.6484	82.23276	136.701	101.4702
66.87774	252.7484	50.93558	29.67322	23.02937	19.50321	75.3726	31.36085	57.69147
11.97814	6.853274	3.630987	0.083834	3.155256	1.831657	4.962783	6.592953	11.75057
731.6698	591.22	519.5139	280.1843	334.6971	237.5674	316.117	343.6304	462.403
21.95969	15.8636	40.91296	10.38816	11.52193	16.22193	40.6306	23.47726	64.01751
1768.785	1070.743	399.0794	305.8997	167.3619	237.5997	250.3937	313.7373	401.5483
30.94295	26.93359	682.8509	161.98	539.6543	1292.546	843.2609	477.4752	559.5316
122.7771	101.3185	36.15127	24.53081	86.81259	5.051463	59.39445	23.5506	17.11531
61.887	42.91414	71.19996	573.0102	221.6801	109.0043	95.43516	59.94002	67.29147
290.4715	313.613	436.5019	232.625	591.9514	919.7632	435.9699	432.0035	557.4087
24.95392	29.03268	53.45524	152.6216	123.3877	142.2328	99.55572	124.7439	153.7095
288.4755	319.9349	101.0801	337.854	111.9269	136.5091	41.43597	44.37543	68.3732
1325.589	1482.232	782.5256	1097.264	468.5756	550.2822	663.6775	705.4055	1036.924
57.89433	94.40317	53.58561	18.1081	55.44252	75.41482	189.4438	50.82997	115.311
16.96929	8.942763	25.6038	3.963509	8.379642	6.610181	13.71494	18.17675	16.01564
414.2469	446.2578	283.5379	221.0576	107.7451	336.7839	109.5435	100.3257	236.0169
419.2375	156.7046	77.5682	19.38605	27.21198	16.27434	70.96661	186.1985	88.66359
1480.309	768.1194	360.7601	782.4901	386.9956	675.277	343.4877	644.2212	413.295
36.93242	36.01116	38.76228	10.39904	92.01412	46.5759	99.14729	28.72227	51.26215
28.94714	17.94729	3.714129	32.13692	20.92978	28.93904	42.948	59.69306	43.77043
56.89617	29.0268	47.73648	468.9275	94.12384	177.3938	48.03881	118.3068	148.3884
42.92118	44.28217	863.8714	534.4993	575.2388	859.3452	1302.486	1128.007	6222.953
108.8026	2.656966	36.09267	75.82133	57.53344	448.1236	32.27145	41.73822	64.0837
25.95283	8.208752	26.51347	35.88752	25.1018	168.4618	55.59174	55.66848	61.85743
774.5913	997.9059	239.8248	555.1008	410.0028	98.08486	159.5513	302.0209	276.6084
174.6837	89.51326	24.40569	84.81026	23.02871	11.48252	23.24106	59.92955	34.19788
12.97596	15.2109	0.557684	6.532324	5.247707	5.048719	5.034347	19.53378	39.46451
129.7639	160.3173	115.3758	141.3361	56.49522	21.10771	88.92111	144.4485	110.0036
228.5852	271.5502	15.57677	38.66283	13.61599	6.654855	5.032427	30.05924	10.70709
25.95252	19.34506	38.18582	50.00252	52.27486	33.67917	60.54015	67.42605	38.43687
164.7004	141.5097	103.8419	81.04351	85.77841	53.16963	134.2535	212.0782	142.0391
23.95594	13.75923	47.01732	20.64656	7.340549	14.66084	31.99546	53.28668	43.77998
36.9328	4.035362	29.80175	141.9258	67.95395	57.49521	38.60524	45.50252	35.24372
10.97925	5.424351	15.56667	1096.921	40.80269	135.875	101.8081	360.6377	151.5767
2.993891	8.995296	9.02443	65.50953	9.415706	20.4216	15.48017	14.23682	12.80363
169.6916	123.4828	45.04979	39.94751	47.08336	43.54571	27.8022	82.07048	123.8818
1032.124	471.1114	148.378	66.93221	38.71674	45.15435	86.86572	22.24069	80.12052
10.97927	6.123167	47.983	276.0154	179.8836	192.2801	282.9027	327.6012	579.6697
1.995644	1.957515	12.20518	14.17361	51.17906	61.33227	13.82547	40.05021	8.564455
114.7911	120.0349	45.01753	175.9337	34.53337	38.733	23.25864	58.66236	72.63533
0	0	0.391536	101.2649	0.011994	25.67164	0.277785	48.89478	3.219237
31.94131	74.27498	21.41998	24.51958	25.1186	75.31546	52.43468	40.41982	56.6051
46.91416	85.35604	59.40832	111.7052	64.85231	29.10062	88.53493	148.162	88.63131
445.19	422.5276	431.2443	179.9912	396.4027	208.7183	282.1025	233.0508	444.2525
310.4354	129.6469	145.2876	104.1853	51.26843	381.6077	209.2947	430.6662	231.7447
339.3829	314.3386	265.8265	147.8517	145.3944	192.6049	152.6173	218.6878	219.9986

4029.672	4881.727	3411.547	2788.11	2658.659	9045.825	5246.883	4302.153	5696.069
84.84527	101.9584	9.648817	45.08008	25.12105	11.47869	52.71083	4.019896	43.81005
406.2609	140.7376	269.1289	2503.025	695.5014	2151.898	384.0882	876.9024	934.3486
21.95963	17.949	3.714038	12.95292	25.10965	6.663806	9.586601	10.52874	24.5743
1946.461	1036.646	1145.274	1787.131	999.8791	676.9622	1149.566	1327.367	619.3965
119.7817	78.99016	194.844	176.0498	155.8452	472.4245	107.1583	236.8063	264.82
73.8669	20.72323	24.1111	97.30505	51.23883	130.3188	9.601397	44.22194	40.57891
129.7637	174.8743	68.58819	72.05203	44.99237	25.91942	68.612	131.4806	117.4814
1.995643	3.342581	6.629542	115.8607	18.83116	66.37732	5.040709	23.41595	13.89921
11.97744	9.583598	50.43854	179.4166	473.4967	431.2083	23.19287	96.17223	49.13301
106.8056	195.1505	100.5436	27.1019	61.7217	65.92736	45.90464	37.8589	124.9395
281.4887	186.6537	159.603	54.08041	110.8773	30.73088	61.84103	180.8989	48.08343
20.96161	12.38937	15.28027	161.8485	43.90459	80.76098	14.0043	38.93642	56.53696
12.97635	4.746795	6.474334	64.60651	14.6399	34.54905	13.63138	14.30355	17.07366
98.82073	90.25546	24.37217	52.73584	21.98232	32.28743	14.16569	61.20171	29.92575
40.92522	40.88133	70.48458	92.36704	98.28689	64.09291	45.60895	115.5635	99.27277
517.0609	226.8655	368.4288	403.3387	702.7789	213.3973	111.7794	290.1902	150.5925
18.96574	4.042503	3.668161	10.34275	8.380437	8.195383	5.003212	6.608471	9.62795
288.4757	351.9297	89.27314	111.8758	72.18503	213.3376	152.5025	79.50045	131.3698
32.94001	26.31527	12.52422	6.542708	10.47712	16.23757	11.83924	24.78576	26.70845
120.781	107.6332	21.45556	32.22564	33.4851	75.4058	25.49224	17.04509	28.85908
291.4693	1531.724	48.01697	352.1373	76.36941	80.44242	184.5291	39.16166	1250.456
6.986613	1.267285	15.10181	176.5786	42.84249	72.48932	22.63793	33.69101	39.46354
102.8133	131.9778	24.39655	16.82405	12.57034	3.441772	34.53512	2.717969	30.99462
377.314	443.478	312.9391	235.1842	152.7159	126.9242	207.0488	338.3481	216.7975
93.82901	104.739	112.3267	32.2402	166.2863	43.53806	174.5648	78.15956	111.0669
125.7715	49.85694	24.42698	52.77103	34.5327	8.267725	18.71454	115.8019	53.41627
224.5919	97.74733	48.02921	99.01762	50.22176	64.38398	86.74708	134.0936	121.7545
161.7052	528.7582	354.5731	72.07282	160.0397	37.1354	406.8161	184.9063	379.1125
19.96295	34.59658	105.4662	61.69538	132.7941	142.2142	45.72682	43.02104	74.7484
9.981291	11.01647	15.07352	15.46103	8.382813	6.638241	2.76146	5.323044	16.02734
175.6809	124.1881	45.03825	75.88867	36.62525	61.15211	16.44089	66.46756	115.3394
40.92584	24.92481	12.51431	20.63051	9.431458	24.17391	0.394544	0.09639	7.503279
4.990169	12.36146	61.79564	77.00043	74.24563	35.42466	92.44412	226.8753	71.53247
33.93785	146.889	0.61456	3.970367	4.202416	1.83082	2.759235	0.098206	8.5719
74.86349	82.52857	30.30257	118.1777	48.12667	21.10558	41.35901	178.1139	54.48337
44.91753	58.15824	48.05346	60.50707	6566.937	1456.558	663.711	765.9233	119.6256
114.7915	216.1411	24.41227	16.8247	14.66216	1.824827	20.97874	41.74776	14.97954
34.93624	62.56355	6.696505	9.112423	7.340586	16.25927	11.86437	7.934323	33.11487
36.93312	17.26256	35.20217	6.541037	21.97226	9.853412	27.35107	6.630172	17.10517
38.92965	27.74455	18.18389	12.94475	3.156499	8.256549	7.318245	22.1726	23.50276
129.7638	139.453	56.81687	295.2978	213.348	102.7771	61.80265	40.46715	79.04509
3502.63	3790.215	2741.584	4002.132	1545.837	3875.992	2880.296	1850.554	2289.553
32.93944	26.94219	36.07839	1620.834	134.8941	123.2247	52.53855	96.25074	108.9049
426.2248	492.0004	216.0835	556.28	444.5037	386.5592	719.491	739.0032	162.3448
78.8563	24.15727	82.89697	424.4583	303.2291	72.29003	126.9785	174.2085	68.36009
134.7545	169.9738	385.1225	641.8557	194.5365	395.6319	113.9426	234.2015	231.7231
49.90929	22.79777	12.58688	42.42279	10.47812	29.02382	16.38657	67.5745	51.2551
37.93023	101.8932	456.0399	280.0607	1108.492	386.2156	351.6553	48.27878	758.0719
80.8526	197.2539	12.62176	64.32273	8.386131	5.049075	14.16846	44.35759	44.87638
24.95407	17.23195	47.21547	120.3959	173.5073	159.1041	43.23328	58.50403	81.11978
142.7419	50.5936	24.36126	42.47599	18.84501	5.056199	14.16407	37.83053	24.58779
0	1.268554	3.577672	143.0791	14.62849	52.45079	9.174783	72.58156	2.161426
1.99575	0	11.05051	13.92206	1.062288	55.74661	16.86344	42.94044	28.66118
6.986649	11.72625	9.413476	3.967488	4.201827	6.630403	11.64789	10.49042	5.365471
36.93252	40.90442	38.65499	128.0938	34.52365	71.95106	43.2694	48.1539	26.71715
34.93683	32.65582	0.579197	26.98565	2.11011	6.657086	2.76627	23.45498	5.367444
109.7998	129.7	83.30002	125.9732	60.67979	75.59391	95.7979	161.3895	120.6866

1576.135	753.5767	289.9493	20.6662	12.56767	54.77566	34.57903	19.63484	480.5657
6.986542	2.658091	233.6737	21.96221	378.4872	365.6919	144.8552	7.933325	535.7796
0	0.633059	2.57404	1.303824	10.32403	37.30935	0.136423	1.320392	2.125779
37.93192	56.57445	3.702138	5.253805	1.063264	0.185725	5.042306	2.724052	8.567774
1815.698	1593.928	490.6397	1034.34	941.3113	818.0653	472.9824	523.2487	675.9938
6.986566	50.88564	0.575719	2.684453	0.014603	1.836269	2.766034	0.094886	4.299591
34959.43	36299.64	16599.01	31118.01	14702.05	19600.15	12291.13	28974.99	15107.34
63.88336	57.50534	91.64091	164.322	250.9571	195.2094	234.8706	165.1235	134.5374
697.7335	220.6211	98.14024	167.1017	202.9108	170.1474	175.22	200.4607	180.4883
167.6946	144.2462	168.6531	72.06748	66.95609	238.9752	170.6476	251.1568	259.4944
391.2881	377.4359	333.9072	386.7756	130.7553	131.7557	316.0565	448.9783	450.6517
1148.91	3010.527	207.4083	1169.206	1511.304	250.4349	179.9946	135.4654	428.2482
354.3558	308.7966	573.9896	96.47597	19.89086	1.815612	57.3354	18.34218	770.9204
12.97578	6.812854	23.86374	145.4524	29.28507	66.73267	9.566372	18.2928	8.569924
20.96121	40.89054	44.49956	18.09807	48.11423	19.47256	150.2281	46.87455	56.59504
18.96517	50.18142	9.553771	21.87567	6.293965	9.841503	0.385409	16.98737	10.70284
116.7879	66.55158	59.51341	97.64176	78.44749	27.5093	68.39518	56.0397	47.00901
90.8343	169.3346	15.57349	52.78999	97.27931	43.5492	122.8523	53.47271	168.7214
56.8962	37.37069	88.31867	182.1142	205.9736	172.5694	54.76813	49.5227	117.4389
691.7447	289.3929	27.38767	168.3774	70.09363	3.427346	45.97827	76.90102	7.501425
77.85788	83.88278	74.36247	32.24114	15.70795	32.32928	118.249	74.26653	103.5965
1.995643	3.343982	15.02269	25.62207	129.4436	47.37786	20.37949	19.51311	13.89428
4628.584	2534.905	1924.195	1468.607	1016.619	1538.059	1061.2	913.6341	1527.087
111.7965	238.9313	83.08246	30.95557	129.6893	35.52986	61.74714	53.46261	70.50012
22.95749	29.02203	85.65388	109.1641	209.1265	292.2775	30.02842	192.3107	32.06276
44.91903	67.63254	18.1904	2.68429	6.294265	5.058699	14.03676	6.629761	5.367615
851.4532	447.5909	233.6965	186.3983	124.4795	64.40918	120.9128	226.5206	136.7162
4506.805	2756.225	1316.773	1255.361	1637.873	936.7883	836.4162	1016.444	1435.256
230.5802	265.7136	224.7664	282.702	117.1579	207.046	152.6475	555.5303	247.7634
53.90174	71.49228	24.33861	59.12283	7.340609	25.88166	38.95834	67.66309	64.07442
0.997503	8.205388	20.98831	388.2523	28.23881	270.8182	7.313878	102.1118	19.23608
108.802	153.4119	103.5112	105.385	67.99633	109.1021	75.29228	79.45357	74.76981
84.84511	131.7923	136.0402	99.01185	54.40473	94.79757	213.2938	183.4726	140.9695
21.95932	37.37086	36.06168	41.19999	134.8915	38.68073	66.0018	107.9146	83.29063
11.97786	9.631558	0.539241	5.246324	6.291776	1.83564	0.364941	13.06013	11.76039
13.97398	23.54288	32.40743	12.94785	4.202635	6.661223	9.57395	33.81484	32.03491
0.997509	3.364753	3.549031	0.079758	1.062962	3.40535	4.875916	3.997094	7.483247
29.9454	8.896152	12.51003	6.542242	2.109984	16.23051	14.07018	13.12247	21.37398
0	4.040566	3.678518	20.52227	200.3533	89.23727	13.80705	40.03278	3.230922
32.9395	100.0371	47.6147	42.47047	56.48347	8.272844	29.9701	101.3868	13.9113
51.90532	56.17266	9.665648	34.77796	73.20887	17.88839	77.08519	78.04088	83.28205
42.92176	49.26415	12.59264	15.53083	9.432416	14.67504	5.0602	13.13671	22.44885
16.96855	19.34046	24.02339	12.95433	48.09833	11.45877	34.10804	29.9627	28.84156
122.777	92.26547	265.9412	54.05113	102.4976	48.31568	90.97549	59.94542	53.41557
16.96864	33.33378	26.70947	28.27709	4.202622	3.451963	11.8075	31.22238	24.56967
10.9796	28.59901	6.56641	24.33017	11.5148	6.626142	7.236781	7.905247	9.629761
19.96293	62.39759	88.455	98.89244	41.85031	110.5075	68.31251	123.5136	67.28614
33.93781	46.45512	12.6054	79.56631	177.7136	129.2404	23.16861	72.80556	45.92764
27.94894	66.1509	6.685091	1.392523	12.5678	3.451783	7.330007	2.723566	2.162712
8.982891	0	79.63566	234.6116	271.8273	110.4523	269.7557	320.5585	43.80256
20.96149	41.71396	23.91572	9.10614	7.34002	9.854967	18.5029	11.82197	20.30536
121.7783	151.2748	62.67571	101.5638	119.238	115.5639	45.94414	78.17341	96.1261
38.92873	54.09022	6.698007	15.53667	8.386623	5.057591	7.342205	2.719909	35.26019
158.712	159.0105	74.21547	1.387365	2.10883	3.437936	0.417961	0.100025	16.04746
9.981071	17.91435	27.27159	102.6771	212.232	43.45003	65.91853	68.97082	101.4241
112.795	47.77409	82.92186	180.9939	294.8686	190.4427	221.4419	87.22564	129.2022
90.83727	36.09134	15.40887	3.971851	7.340297	1.8346	9.588534	7.931852	4.299789
0	0	0.431806	48.77412	7.321042	109.6937	4.789456	3.979409	13.83946

2752.995	380.1413	517.1215	1130.654	1430.769	3039.831	2020.965	1657.799	1208.844
0.997496	1.266886	9.390677	43.34466	6.291924	12.93194	18.14421	125.8219	99.02934
185.6623	98.43754	250.3996	152.9362	214.4035	453.1583	376.0834	308.271	177.273
190.6529	285.9132	92.24141	953.936	177.8112	202.1629	64.13907	136.733	69.44085
11.97766	19.39185	31.97582	39.68112	27.18818	33.49206	7.293333	40.179	16.03263
4.99017	5.423536	12.44947	32.09217	50.17449	57.2371	22.88064	27.33464	53.34605
53.90159	47.10565	70.94778	25.80844	19.89099	17.89408	27.74412	37.83685	44.87004
508.0755	640.4323	410.8233	507.5773	135.9853	162.2327	200.4296	694.941	277.6766
97.82202	133.9927	62.49102	70.72769	27.21224	48.31599	41.35633	124.8816	51.28056
211.6147	292.0987	168.8618	665.3292	646.328	781.9866	186.6576	537.3221	198.6461
15.97039	16.56037	26.78303	14.22948	32.42215	14.63356	22.95664	19.596	30.97024
310.4361	201.9156	162.6362	179.9073	307.4755	29.1267	123.0216	140.6137	139.9088
133.756	1539.357	24.36459	19.38123	20.93544	5.024981	32.30651	2.707461	136.7184
8093.283	6891.339	6878.21	3005.258	2468.311	12782.27	4538.534	3533.144	9607.698
59.89079	72.84601	70.9546	39.919	15.70788	22.69677	25.48643	27.44429	65.14888
3274.046	3147.623	1452.473	1159.005	982.1066	1117.99	1694.86	3578.565	1638.151
21.95946	11.66967	18.40581	214.902	41.83874	198.6676	23.1158	45.55026	117.3791
90.83413	191.4837	215.5102	253.0868	217.5465	358.9541	141.1633	218.6325	108.947
69.87316	66.64184	6.699209	59.10418	4.202289	9.877582	16.41558	26.13167	21.38367
86.84142	83.85152	379.2798	342.8861	449.6846	405.2314	477.8133	404.4608	349.1579
53.90126	63.03326	141.7717	172.1421	253.0817	256.2736	104.7725	135.3652	147.3704
0	0	4.517352	2.502086	3.117703	2.991027	0.137333	4.910262	4.218455
7.984716	17.24039	24.11444	89.64824	31.38363	125.5843	60.83236	116.6279	29.91265
27.94873	15.14801	61.38485	103.7448	40.79194	73.44823	29.80265	93.41477	28.84839
16.96843	79.9363	6.699821	36.03219	24.0707	3.448608	23.14505	30.01209	29.91923
22.95753	56.85654	33.1161	69.38668	100.3909	143.8308	56.97051	67.68083	83.28657
300.4543	305.4481	12.59599	16.82227	5.246995	9.86431	14.15275	4.016688	17.11472
143.7393	134.014	71.19121	47.63171	20.93732	16.29538	27.77669	83.31646	24.58976
3.992017	4.733181	39.02243	168.0793	23.02851	250.7438	16.43847	301.2148	44.87186
742.6491	1260.915	452.1583	93.91261	381.7663	142.9893	584.1092	412.6262	860.719
505.0815	439.9185	472.2089	177.4149	85.78273	221.5194	472.5044	632.3737	388.7194
20703.35	21209.74	12541.24	5866.534	8897.374	26806.48	22223.07	13653.37	21781.55
25.95255	41.00153	26.79675	37.22924	10.47668	13.04439	20.7436	50.62131	17.10647
170.6898	203.4127	68.5286	47.65172	124.4655	85.16249	77.61836	87.26929	80.11135
16.96854	33.29051	9.621433	132.949	232.9981	278.3973	76.09973	28.67229	27.77579
19.96334	18.66745	15.30848	24.43783	12.56612	6.657937	7.314199	29.91719	14.97041
11.97811	15.30096	3.636134	12.85346	6.28937	6.590903	9.320423	13.01635	7.492953
27.9487	23.49719	35.71665	76.93827	27.20545	125.8025	54.35066	106.38	90.71657
78.85611	69.99173	147.3699	238.7842	104.5954	158.6688	165.5637	253.5248	233.823
20.962	7.524513	9.436035	138.4153	29.26669	27.0809	11.66858	22.09005	24.54744
97.8225	47.80365	30.2217	28.37369	16.75364	16.29178	23.22288	52.11751	30.99335
104.8094	67.92817	47.88942	82.27409	33.48689	48.31908	23.25238	171.6274	65.15862
9.981173	18.67028	0.57914	6.539412	18.83561	1.836073	2.766268	2.724124	8.569438
480.1268	583.6245	301.5107	403.4692	229.0652	303.2545	168.5922	460.6819	242.4318
18.96492	20.72552	29.82483	39.82512	6.294645	9.867531	0.398565	32.56781	19.24354
32.93988	29.08871	12.55213	29.60017	20.9316	6.666239	11.85571	17.02214	6.435877
245.553	141.4426	157.1016	411.1151	234.2903	117.3125	229.7432	402.0861	296.8808
722.6995	276.1158	6.690697	45.05385	5.248139	6.663518	11.89547	0.099472	13.9116
42.92116	60.93368	350.6793	142.7012	328.4005	375.0669	815.3978	539.787	481.5698
64.88265	50.68741	21.28682	32.17428	17.79678	9.87227	11.86885	15.73054	20.31274
10.97964	13.14336	3.671321	11.61756	1.06334	0.17933	9.417347	13.05468	10.69397
11.97745	77.82798	3.708794	6.543874	8.386611	3.448108	14.14477	2.721186	7.503977
250.5447	207.4926	124.4276	147.8037	91.00802	88.41363	193.0957	157.5026	132.4328
7.984713	10.97118	157.8382	97.60145	205.9791	358.9202	119.91	132.5806	107.8387
86.84203	51.26309	24.4045	248.8131	128.6285	139.2545	59.34954	88.49228	165.4747
72.86698	123.5121	33.26784	92.55954	40.80799	25.91692	61.74622	39.16123	89.71576
17425.31	13675.74	25880.52	15429.23	13626.87	18068.4	21219.17	18433	13059.13
498.0942	611.406	224.9324	219.8013	276.1274	152.5934	322.8333	259.0532	318.2446

47.91303	33.26099	18.37647	39.84313	37.65583	22.63272	20.84942	39.05813	14.9763
23.95598	49.33121	29.81911	15.52047	83.62735	22.61477	11.85272	18.31737	17.10945
26.95263	11.08172	9.172348	1.39271	2.109676	0.172372	2.734032	1.41545	4.295623
90.83463	64.45393	39.10138	38.65358	20.93743	32.31743	41.35883	33.9546	69.42823
12.97566	58.16148	513.9891	115.7178	583.5339	72.40331	111.6925	165.3129	152.7202
338.3842	249.7052	439.934	603.8336	375.4832	2312.243	649.4282	834.0078	338.5357
28.94749	48.78202	0.57585	5.255776	2.110131	0.187806	0.385218	2.724157	13.90257
354.3561	298.4119	174.5643	223.5902	146.438	287.0236	175.2009	231.6627	116.4233
159.7097	213.8352	86.11687	73.32684	53.35803	40.34247	77.62266	70.37203	89.72003
147.7313	165.8608	94.94134	19.39453	4.20112	25.91925	131.8854	100.2745	106.803
2193.012	3315.022	157.2175	353.446	504.1371	61.17511	486.6259	1037.239	374.8559
106.8053	64.41772	153.584	600.6379	240.5391	467.3492	468.2403	254.9376	344.8674
20.96117	29.73549	79.31193	125.6821	66.93497	123.0204	97.14572	109.1364	82.21158
7.984714	2.651696	143.4324	14.24246	51.24076	86.07854	89.67372	70.1101	140.8193
99.81853	60.98927	82.81714	128.4061	64.8552	250.9351	81.9056	106.6824	134.5317
33.93901	25.70993	9.497345	1.393808	4.202304	3.449783	11.72508	4.026228	9.633906
257.5312	455.2182	983.5323	169.7028	42.90099	27.51144	145.8802	165.3822	470.9322
0	0.618654	20.10242	4.930328	8.28699	14.00219	9.085449	2.56603	2.133754
148.7295	81.78715	147.6188	201.6497	148.5176	248.2805	364.2489	184.7434	295.7527
86.84168	35.26288	71.38209	255.4352	109.8226	65.93888	50.44191	289.8623	83.31064
20.9617	31.28854	23.66735	9.097543	3.156485	5.054545	5.04667	14.39405	10.70186
0	0	36.76969	0.079531	17.74821	9.602608	0.321715	0.083992	149.6107
15.97036	22.83267	6.683914	7.825725	8.385867	19.40969	9.585065	23.48551	7.503247
52.90398	58.34834	3.711821	5.25803	2.109701	0.194447	29.83153	5.330979	25.64888
289.4739	377.6499	30.34613	2.672816	6.29297	1.817365	141.1622	19.64675	44.88037
114.7913	92.25139	88.81908	46.35713	42.89875	77.10746	151.8454	119.707	84.37463
55.89856	75.77522	12.58719	33.4642	32.43265	32.2083	7.342062	2.721836	12.84239
67.87597	170.0654	45.03665	57.91943	36.62523	14.68954	73.07574	122.3477	79.04249
52.90345	54.07178	41.85177	73.2317	17.79929	14.68861	38.99437	97.51751	26.72286
140.7438	174.8773	83.25731	120.8274	30.35058	80.38528	88.97654	150.974	91.85809
411.2517	275.3811	513.6234	684.7658	603.4759	2318.861	1037.238	559.574	905.5337
68.87403	167.891	124.4424	93.88852	72.18411	46.76354	50.5072	245.8977	65.16809
23.95604	23.52378	9.618916	6.542765	19.88492	13.05364	23.01114	6.632076	22.4421
10.97986	7.556372	3.62996	12.84753	1.063278	3.430647	4.96168	14.29059	16.00622
22.95747	45.66653	487.8698	136.2643	942.2066	499.7038	301.7452	82.09868	1236.311
140.7436	153.9854	312.158	69.49499	150.6179	102.8414	265.5618	221.2229	137.7741
94.82868	36.71208	38.73142	33.47626	19.88916	11.47825	25.40409	41.68865	24.58429
6.986525	18.62623	52.86345	193.151	98.26801	32.18924	23.10398	86.95788	46.9844
6.986532	20.73006	12.53683	90.87763	97.20485	100.167	16.33214	35.14736	28.84328
134.7544	219.9596	98.08451	301.8642	79.50561	126.8715	127.5802	213.4336	222.1217
2.993818	4.735348	17.78151	63.66232	25.08978	84.74264	18.19999	14.36078	3.231096
56.89639	52.0032	21.41042	10.39976	16.75319	17.88635	47.92228	33.92581	32.05816
16.96876	11.69738	15.23079	359.7951	58.51842	22.49046	11.75907	40.1963	26.69323
290.4714	364.2822	210.1323	82.35092	111.9292	327.2358	309.1359	89.92008	231.75
0	0	0.566925	231.4122	14.6535	165.0034	5.043377	80.05655	23.49323
75.86282	75.07003	3.71073	20.65809	5.248645	5.060231	11.8775	14.43545	10.7073
39.92698	51.31887	56.15718	15.53527	27.20876	16.28332	34.42479	52.08051	45.92992
234.5733	137.9948	271.4224	449.5149	252.06	663.113	234.0541	123.7266	289.3904
819.5114	479.5542	118.8292	132.4481	55.45197	144.5622	218.4268	236.918	170.8851
85.84567	45.13929	6.693096	48.77076	11.5231	1.83365	9.59961	53.27162	7.503658
8.983061	5.429845	17.86488	29.45326	13.60625	20.83537	18.25807	34.96626	30.94125
65.87943	206.6574	1780.453	759.341	601.3943	2707.384	2809.325	2845.314	2083.349
5.988403	4.73316	12.29305	190.5046	32.40259	329.5243	0.374526	108.1656	22.42132
20.96117	68.7201	6.698566	14.25244	5.248459	1.829053	34.4427	1.412521	21.38408
247.5501	169.9751	109.768	160.6425	112.9693	158.8549	247.3669	226.4007	116.4183
0	4.741231	6.525733	74.8537	41.77226	67.23464	11.56487	24.59331	17.08115
35.93398	115.2572	0.632008	2.67821	4.201808	0.198849	7.33568	0.099628	16.04737
2.993878	3.356597	9.079821	59.32751	10.46047	15.85501	4.927192	23.17566	10.6803

80.8532	52.68721	12.61975	21.95502	35.57451	16.28902	18.69037	17.04269	26.72235
244.5551	106.7472	168.7156	369.9506	513.4984	667.9992	234.1015	230.3691	234.9415
30.94357	19.34089	43.91843	38.52518	29.29025	36.86536	76.03851	66.1556	46.96971
47.9129	42.30821	18.40816	27.05937	42.88412	19.45857	32.06944	14.4339	24.58193
14.97278	1.266838	17.49164	60.92402	14.64296	33.10621	7.194256	13.03841	18.1435
62.88589	42.28538	27.16089	18.09767	55.43056	16.27668	27.64601	17.03583	23.5169
228.5857	254.2699	44.99678	200.2707	43.94482	6.658075	23.2566	85.94417	13.91152
184.6647	88.05392	30.33338	113.0976	85.77427	110.7335	54.98222	98.95706	61.96062
74.86354	46.38803	77.03868	72.00479	63.8104	45.11181	93.19414	115.7813	76.89856
35.93388	34.56481	106.659	259.3799	140.1524	484.7877	95.73313	175.6495	100.3975
67.8761	99.90486	53.75019	41.22195	89.95213	62.71395	154.053	119.6996	84.37359
1923.503	838.1808	1428.039	1649.629	1649.354	1092.163	759.0065	1649.995	778.5037
1.995647	2.65144	0.530747	95.08178	17.77591	604.4637	18.04997	22.03508	43.68507
547.0047	1117.214	537.6981	204.4082	605.5823	202.3247	1237.8	529.7344	789.1777
0	0	0.304448	11.14618	15.57627	3.224746	2.44449	7.58296	1.086969
16.96843	60.40676	64.58061	93.60963	58.56558	67.22341	34.36336	85.73354	21.38215
389.2915	306.6027	534.4311	797.8353	388.0378	1136.696	1035.286	995.4024	609.7668
123.775	76.95545	97.59146	84.84955	88.90777	29.11884	57.19694	100.2291	74.7679
33.9379	16.53462	27.11763	18.09447	18.843	3.449088	7.342282	24.81965	10.70729
110.7999	24.87285	24.29438	34.76492	23.02634	38.62329	11.88746	22.23426	16.04597
0	0	0.317899	13.61809	4.179769	50.40373	0.230951	12.53369	3.207901
13.97421	4.037739	6.610208	37.0839	115.9018	44.35386	22.63342	16.95345	12.831
24.95394	36.68838	9.665538	56.56099	32.43742	22.68557	9.620164	79.33467	72.61101
24.95424	13.76501	32.6001	42.36283	36.60567	59.03841	42.98478	70.04372	38.44066
93.82949	99.9619	21.46249	33.5112	50.21527	43.49117	45.81429	26.14794	39.53482
54.89943	101.9216	45.07671	244.0214	243.677	529.7096	43.69124	230.2545	165.5238
1772.776	2283.11	1696.587	1460.837	630.687	765.1478	961.1403	1279.226	858.5968
16.96877	52.32238	9.532531	11.65478	3.156485	1.836465	11.7578	19.5619	3.231493
25.95202	48.45365	162.0777	242.6614	175.7003	334.4581	291.8764	201.601	133.4878
5.988351	2.650157	21.09047	123.901	52.27151	258.911	14.05677	63.52447	130.0955
15.97046	11.68897	9.569513	155.5607	45.99621	61.95261	14.01915	23.45775	54.41099
29.94611	27.82955	9.476917	7.811688	3.156386	1.836565	0.375618	15.66722	2.162998
35.93406	74.94756	53.48252	42.47809	36.62083	21.09327	59.23202	100.1167	41.66675
170.6894	208.8751	68.64112	79.76513	300.1524	45.16091	39.16151	50.87851	27.79429
74.8638	53.3653	27.30664	54.02216	19.891	24.29663	41.27351	33.94036	35.26352
245.553	285.8341	292.4968	169.6976	108.7915	142.9619	220.7048	425.513	458.1133
35.93408	25.55783	53.43794	121.8867	171.4689	92.86199	133.0839	184.3635	80.08383

TCGA-19-	TCGA-19-	TCGA-19-	TCGA-12-	TCGA-P5-	TCGA-S9-	TCGA-HT	TCGA-DU	TCGA-HT
85.79931	41.72084	88.50347	167.1675	266.1429	191.3125	158.7504	163.666	127.0847
15.58724	15.14709	17.35936	9.105105	7.108914	25.37323	11.4129	83.685	25.83518
47.89976	18.5184	40.65315	44.28172	68.0714	60.50877	180.3343	183.5863	94.02254
1113.931	1067.881	1350.265	3266.257	1636.158	2912.583	2630.528	2839.32	2256.445
132.0431	53.29955	496.567	93.44887	107.9296	131.7679	88.13687	102.4636	69.23317
21.27529	9.418789	0.440857	0.158877	0.040388	0	0.689538	0.769681	1.043526
25.38319	255.4003	22.61866	24.8352	14.14036	16.58449	37.56488	6.539227	7.246161
85.7986	53.3111	106.6074	32.0039	97.39115	125.909	121.1337	244.7426	178.7352
9045.034	7468.474	4161.873	7611.514	5649.594	6523.05	7333.292	10941.56	11292.52
40.8403	35.02174	48.30944	16.15629	110.1648	60.51402	80.55623	52.04518	34.10529
9.963264	21.55798	26.89386	28.00259	26.9456	18.54282	10.6638	9.861676	15.50384
931.246	368.2988	873.9625	130.5582	693.1929	1223.013	918.74	1226.662	1341.048
119.4767	106.28	186.5966	738.7296	231.0107	126.8838	199.4191	105.7551	84.73104
5.595195	1.849029	10.20232	35.30167	1.229371	0	5.37777	0	0.008468
49.29918	54.92081	73.37171	54.80273	180.529	151.2972	72.80324	100.8323	98.15369
184.0699	136.2135	64.33927	161.6044	37.53843	33.18342	16.02078	14.81352	24.80435
2226.158	2203.241	1544.48	1490.794	2070.069	2144.418	1997.046	2541.767	1574.556
17.01102	13.53024	67.15982	38.83904	112.4159	72.23694	40.61494	28.88189	19.64245
168.3861	310.6715	75.12073	68.83539	30.56559	37.081	19.07236	56.15137	42.37254
1154.298	469.3105	94.8605	2324.977	246.3638	128.8304	487.7131	306.6014	255.2055
636.3507	736.2015	552.6282	128.7995	1073.142	1192.756	774.5495	998.5102	906.0917
1.555971	0.212265	1.010135	0.24465	2.413473	4.870351	19.85472	130.8425	34.10162
106.7871	1.89623	7.409145	7.346675	211.0161	138.6033	95.05828	183.5649	195.2542
84.4406	38.413	207.4754	21.43302	281.4957	380.6696	296.787	217.3538	769.67
73.11101	38.37696	9.735296	47.77027	41.11245	59.53357	128.8942	98.36115	68.1964
25.35427	15.14476	53.23633	9.104095	42.19374	37.09182	46.07451	28.91079	50.61338
440.6898	395.8017	261.272	579.7093	225.184	150.3089	238.5244	122.2827	123.99
1387.528	399.7756	1956.567	575.692	673.2465	1787.194	493.8223	507.4686	1179.873
11.39624	0.209438	58.4822	0.241236	23.50034	42.94917	17.56103	45.48728	35.12958
57.6793	43.31644	158.8731	12.64301	299.8727	250.888	55.92811	61.13109	96.08231
81.38199	20.15543	92.85213	7.357496	27.03725	46.84666	26.75802	23.90513	16.54447
47.8229	35.0062	146.8143	17.90733	78.5491	96.64045	65.1929	61.16263	53.72839
11.34153	6.86919	32.87611	5.56861	24.59619	25.38313	9.128347	4.062523	8.276577
1192.33	707.9853	636.0512	1891.166	1109.474	978.0204	1554.689	649.6623	558.9526
207.8997	69.89712	551.4975	190.1669	224.0169	170.8072	174.0498	232.2702	126.0566
1167.317	64.88556	12452.2	1816.512	31329.78	21755.57	14142.84	13366.73	7956.437
32.2484	41.18695	3.906543	12.56647	15.29485	11.70459	42.28152	9.026033	12.40901
15.62093	21.81765	98.06105	61.6867	58.66728	61.4893	68.24262	70.25312	36.17174
148.2416	171.1063	30.20382	49.31007	39.90512	43.92025	75.23731	19.77299	47.52798
68.66084	98.83288	16.75157	17.87545	17.65618	22.44186	52.17636	25.57477	24.80578
415.2102	482.9705	54.6426	56.61551	22.35849	71.24429	61.26416	66.06226	150.8448
38.01741	11.88135	90.55701	85.95859	124.1604	73.20954	42.90456	31.35798	21.70908
283.8941	186.2948	46.5618	286.3008	18.83583	34.15489	27.52521	57.84565	25.8415
73.15208	51.63659	49.98687	7.345935	76.28228	112.2453	78.93116	169.4905	131.2122
272.4969	202.3944	648.2269	119.9519	190.0414	306.4857	181.7002	164.4398	246.931
28.037	13.44153	48.15884	9.06993	10.613	23.42541	9.88885	14.00657	18.60185
59.02945	89.36824	201.2869	100.0399	76.21986	70.27727	34.43707	10.67125	24.80873
2567.508	522.4939	1557.26	890.8128	1600.979	2500.679	3408.979	3395.62	4823.85
410.2024	368.2004	95.44311	53.11898	95.07501	366.0189	552.1739	385.9747	817.2258
7.167516	3.576398	16.87237	2.053087	3.588239	21.47528	24.58726	28.16263	19.62994
8.560858	3.57651	48.91265	5.568885	33.91412	27.33703	12.21504	9.036379	4.146035
90.04989	71.56139	213.3447	88.298	241.6173	188.3759	338.2593	180.9831	177.7127
59.05381	13.54246	138.4662	26.69184	62.18312	132.7594	38.27132	41.27725	39.2708
1004.399	69.86864	5139.809	626.84	10727.8	5650.451	8981.55	5625.747	7664.032
18.32179	6.885758	41.64313	3.825218	29.29213	33.19331	14.51273	13.17634	29.95563
21.22668	28.41143	111.6141	26.67127	44.60422	70.2784	65.95926	33.00893	41.33475
8.579762	6.89029	20.34879	14.29104	22.29973	39.0522	33.02747	42.24282	22.73236

2281.076	4186.083	5381.788	1459.253	14608.63	5758.794	7320.327	5103.328	4155.409
938.9113	1099.308	1214.472	495.9373	249.8401	228.395	141.7988	95.81294	134.3239
23.96699	16.79478	60.27916	16.0957	35.18818	46.85662	21.412	13.98837	21.70516
110.6302	99.05799	50.11459	26.6477	12.97346	21.46456	49.06314	30.53265	66.11812
7.187735	213.602	0.431589	152.2319	4.763215	0	14.49394	0	3.113102
150.3774	84.80061	133.9923	70.70351	179.4703	182.5211	148.7274	147.9171	157.0487
19.71443	24.85596	49.91917	12.54501	35.12633	45.89211	18.36833	17.32552	26.85976
49.04532	6.903083	31.45022	16.0948	17.64755	28.30246	25.26194	24.76149	23.77032
108.0778	38.36142	49.44076	37.22949	51.65238	73.20212	75.90211	52.02757	120.8711
346.7121	1008.972	126.413	302.4516	167.7423	141.5241	212.4361	124.766	88.8646
1.559963	1.911601	6.840563	0.22868	9.437509	6.823566	15.30247	56.47305	13.43819
7.184385	10.18787	29.75856	10.83065	60.82081	60.53908	10.65181	33.91831	26.86318
165.8232	78.18243	104.2198	81.25768	323.6603	396.295	239.2752	193.3948	279.9851
18.40456	21.76633	75.43529	21.36964	42.23117	61.49864	12.9431	30.54522	46.49207
56.30714	5.23433	19.64459	2.037852	92.67246	111.272	134.2535	93.38662	51.66954
39.3587	31.62648	72.5124	30.0821	35.20984	72.24087	44.48216	46.29448	43.39358
99.87412	40.07243	204.0119	436.0376	209.9622	188.3762	157.1563	329.8429	286.1853
18.3963	23.39246	8.570289	10.86631	3.589253	14.63191	15.251	56.25705	54.74951
1885.431	2106.215	185.3198	121.7043	99.7589	125.9042	170.9614	93.33475	138.4555
111.0921	93.08162	115.8968	37.28636	137.2694	284.0396	175.5853	281.8945	300.6443
828.7832	472.7423	1080.359	218.5545	1276.066	2872.582	1674.21	1325.001	793.4836
52.03302	13.54058	62.95028	2.043983	37.58127	102.4966	81.32868	139.017	28.94052
11.39696	25.14591	230.2688	3.797188	157.2091	636.4229	220.842	262.8497	457.6752
5.7857	10.22563	62.96795	9.120373	60.99332	33.17885	67.49889	216.1065	85.74422
70.19874	94.25721	44.81013	84.29492	29.37992	22.44026	42.12623	28.04292	54.76145
324.6147	333.4989	240.0467	1729.803	1464.864	1581.231	893.4092	2501.367	1581.777
1150.487	144.0453	33.65077	140.5978	20.01243	41.96197	72.80748	23.89719	103.3174
40.89923	41.71781	97.27262	12.63669	17.66665	153.2427	65.87246	197.5956	99.19195
801.7557	419.4858	198.0928	937.4098	367.1268	183.4914	1044.014	227.2523	179.784
60.51408	48.30833	27.22861	35.50063	21.18527	24.39147	117.3502	115.7283	27.90947
77.403	121.2025	251.3753	348.0347	197.0428	120.0495	456.5578	139.6541	187.0058
43.59273	11.87892	77.1097	14.38846	20.00316	57.58927	32.91793	23.91113	38.23372
710.6471	235.6623	345.0554	156.9516	1342.736	878.4673	523.7785	492.6174	1222.213
1925.595	1064.513	2861.306	1392.219	2000.859	1552.919	1735.524	1595.286	1618.979
1.494522	4.786138	0.450436	3.47926	2.365311	0	1.479371	0.775413	0.007246
35.20197	30.02328	95.90788	17.89131	55.11644	171.8361	32.15112	60.36096	35.13507
32.44802	1.901601	19.06659	3.818727	86.7723	182.5528	39.03946	66.11018	92.97966
1474.482	1564.492	538.6622	1785.54	776.4269	547.5678	767.6758	712.4987	593.0453
63.30352	11.88408	129.5382	60.03891	109.0623	117.1311	67.43707	80.15434	92.98687
24.04179	31.74549	107.3783	37.22114	56.33459	58.55908	76.67843	133.9909	46.50223
19.82828	1.902553	25.49543	51.1788	16.4923	25.36856	88.24191	102.5594	26.87481
24.04204	18.51371	6.822356	2.041147	15.32089	44.8918	60.54167	120.7409	185.9365
91.387	56.60634	63.41176	75.90023	116.1307	91.74477	160.3153	155.4145	151.8733
212.1782	540.0946	206.8884	1019.285	214.6698	451.9262	231.5634	153.686	125.026
22.45805	1.911803	10.95238	5.575284	22.27874	17.56632	17.61153	14.84382	16.53571
2.97105	0.210226	29.65923	0.242186	28.18415	20.48986	39.8762	34.69838	23.7719
12.77816	5.242576	37.99463	2.053224	18.80583	42.95555	19.11986	18.14193	9.31137
42.13374	28.32541	57.25991	19.60928	51.57474	69.31317	26.01696	13.98452	43.39219
5.78114	3.581239	20.94224	12.5533	44.46494	45.89057	18.36368	10.68556	19.63518
31.03693	11.88381	22.57555	19.66597	35.23864	68.32482	60.56916	66.12386	63.02494
22.5087	16.72917	7.413943	50.46663	12.95463	29.28431	32.25368	12.34294	24.797
634.9973	613.6485	1361.416	2358.043	1617.354	1873.073	1147.259	951.347	1344.158
24.01932	1.905103	58.90642	16.14507	43.42217	60.51753	39.83368	20.5988	27.90617
1562.696	2105.667	595.8904	642.3395	158.4062	384.564	556	575.2935	776.9363
136.4008	86.48994	301.9504	119.987	337.802	331.859	440.2204	646.4635	336.8177
39.02707	19.89802	43.54251	75.63397	3.588447	8.776598	1.441481	3.235193	2.079629
14.13471	5.232366	57.13442	3.822768	38.59682	33.19618	11.43579	6.545527	13.43917
56.31966	46.66761	41.81679	114.4314	75.10691	104.4365	134.9953	169.5013	104.3536

21.13987	18.39071	12.68522	38.52639	32.82173	14.63364	22.20458	16.48416	13.44205
25.43026	20.1553	41.88276	14.39717	133.5501	67.34938	39.05179	37.97572	28.94016
5.778852	1.896273	44.151	65.3522	56.35636	51.72279	154.1926	749.7157	135.3431
32.47502	98.08708	349.2446	58.40581	191.2274	414.8305	242.2917	290.1045	239.7031
64.73939	63.2148	38.8954	86.41039	52.84147	79.05504	140.3595	117.3622	104.3548
98.34834	211.7036	61.10002	75.83881	59.86669	108.343	111.2047	92.55738	70.26364
66.18541	0.220359	216.2423	21.43474	331.8974	185.4466	495.5591	715.2075	318.214
26.81617	13.53385	70.64665	5.593583	62.14256	96.64548	56.75915	22.2549	39.26678
42.00787	51.02253	17.96842	12.5807	51.50465	35.14168	19.11857	10.68066	33.0586
63.03977	38.16753	100.2039	92.50321	42.21995	47.83	26.78723	13.98473	22.73936
275.2622	147.7313	372.7366	142.7736	133.7576	350.4149	137.2077	187.6023	127.0906
105.4748	68.2377	269.4503	102.3342	151.334	541.7468	196.3125	167.767	87.83172
289.4424	265.4506	223.7558	257.1814	309.6731	285.0047	446.3321	615.8392	360.5821
2487.472	482.7059	1554.338	341.7389	2916.866	2679.299	2683.424	5854.802	5126.569
19.73556	23.27155	42.16512	3.827077	21.13633	35.14387	14.50349	4.060661	13.44151
57.3404	6.900392	19.71758	14.33611	19.98162	22.44459	27.58487	36.38526	24.80138
81.39766	26.76616	102.21	7.357158	49.29112	112.2605	109.0186	114.9946	111.5647
16.8718	4.708496	2.94768	4.939882	1.210787	0	1.490015	0	0.006779
5.773426	0.199055	0.429599	0.228735	0.05365	2.918448	23.80739	56.47211	40.26692
10.96444	5.006228	5.853394	5.295489	0.043839	1.944695	0.684688	0	14.42568
33.53728	5.23236	35.1906	14.24459	36.26662	25.38088	16.06619	9.862248	6.211937
141.8439	132.6384	248.0886	149.535	159.4836	204.9836	68.18059	66.89644	94.02543
22.61808	25.09077	77.68114	37.12582	96.07014	92.73932	46.75689	31.36113	38.23418
515.4849	778.9814	359.07	225.5494	350.7236	529.0269	579.0288	559.5959	433.9363
239.9642	173.9769	174.4003	224.9413	98.5573	91.7442	171.8211	65.23934	90.92734
5.75972	10.09031	33.62442	2.050068	16.41241	16.59437	4.512281	4.065377	12.40234
359.7117	31.75545	311.1943	49.58477	679.1097	652.0071	570.5248	2173.273	703.5942
395.7001	270	210.5269	518.0061	170.074	199.1186	100.395	107.4063	141.5499
200.6291	229.9445	34.81289	14.40272	48.15043	42.93764	168.0278	12.32497	35.14133
30.84312	84.92588	65.25069	64.19462	22.30047	23.42402	19.90358	17.32052	18.60325
210.7705	260.361	401.2424	63.68344	50.50709	348.4576	109.5778	213.2198	367.8039
151.7872	212.2511	165.5059	54.88006	132.5814	208.8762	243.8858	214.0731	195.2731
234.3701	208.6803	70.99482	28.48457	73.94523	133.7193	121.1421	129.7573	162.2053
31.05243	10.2256	109.1371	14.40289	10.62929	53.67794	42.87461	90.10891	64.06175
23.9423	10.19889	65.08635	26.48158	41.00978	51.74366	19.88369	8.195379	14.47537
218.0965	92.52519	40.73109	23.15486	44.59531	95.66707	42.90613	23.90844	20.67607
31.06713	16.86032	72.78095	56.56402	179.3667	174.7259	104.2858	167.8601	142.5711
85.55147	67.83409	9.809368	20.95937	3.586551	7.801141	5.281396	7.383473	10.33922
80.90629	21.21217	0.428595	10.63088	18.70189	0.967018	2.208475	0.761337	2.079286
26.83667	44.91874	116.2556	14.39962	37.58268	76.13482	55.18411	88.48025	47.53296
12.79899	0.210127	26.72679	50.8905	12.96884	41.97153	38.33745	74.51047	34.09821
63.18823	82.70065	38.96716	169.5157	9.456279	15.60716	21.38411	23.0796	29.97251
95.65252	235.3838	1015.35	137.4689	114.9929	325.0388	108.0582	142.1296	206.6355
94.09335	148.8708	40.08534	255.7515	140.6662	28.29629	118.1591	71.05932	18.6112
220.6532	141.1879	598.8775	183.3229	727.0999	408.9686	658.0732	461.2232	389.5099
51.98223	10.22384	12.06829	14.3898	9.456074	30.25109	33.6848	70.2973	58.88877
93.79007	46.43586	48.39738	49.26434	24.6833	29.2758	28.31303	8.192101	34.10114
53.43825	26.77053	37.77479	7.356769	41.09654	66.37163	65.17678	81.02602	82.64894
49.18386	112.1477	67.71811	80.66214	22.34596	20.48845	78.30822	33.84683	23.7744
4.370091	1.895895	166.2664	16.1636	129.0133	107.3643	240.977	123.9817	114.6845
64.63699	20.16211	68.79021	21.42462	55.15144	62.46603	82.08571	52.86971	52.69771
43.71493	11.86275	236.0618	56.64567	369.4292	687.1725	305.221	198.3334	521.7348
28.23963	13.54156	31.33942	0.248165	85.59163	143.5017	92.85084	105.8683	84.71522
42.11255	111.6867	63.17611	61.2607	23.50181	32.20728	39.11339	23.92675	17.57513
1121.914	947.9591	504.8969	2491.527	634.4976	230.3427	447.0738	295.0339	260.3703
97.08703	73.22878	154.3423	21.42001	106.803	316.239	539.1459	476.0983	178.7514
70.15908	97.44764	111.1029	35.39187	76.19157	64.42253	34.44945	35.49886	26.87351
220.3603	221.9	41.80822	39.0353	91.527	126.886	107.3225	157.8841	178.7334

103.9792	18.51856	199.0364	23.20416	34.08386	120.0558	113.4912	116.5417	120.8811
53.37207	62.91007	85.32723	49.3192	30.54222	55.63653	35.22486	60.35757	32.037
2049.283	1412.897	527.7313	3750.183	17.66104	75.14673	183.2072	65.22491	208.7116
125.1283	182.47	163.1695	504.3782	335.3771	213.7567	202.4413	115.6641	198.3724
56.25035	35.03482	122.6606	141.9923	125.3996	111.2808	45.95515	38.7955	27.90812
30.95956	13.51335	37.30074	12.61427	32.8597	61.50266	50.66711	23.92609	12.41108
116.585	45.01144	175.6562	46.0436	141.8904	149.3426	91.22371	112.4069	94.02351
63.08219	167.5247	19.67854	66.60164	41.06109	43.92244	42.93866	12.32789	39.26356
47.7506	5.245537	23.774	10.87325	30.53464	41.96896	17.55144	78.61735	55.7866
924.1102	298.6553	625.5837	46.06915	297.9664	1033.661	772.2898	842.3019	861.6596
11.3939	5.245383	16.18378	3.828117	2.41453	8.774752	11.41213	30.56497	33.0634
89.90985	63.15199	159.9829	28.46697	55.17252	176.6843	53.61609	78.50239	74.39351
282.1658	106.298	60.4703	74.20381	61.05761	81.9809	192.4946	257.1105	128.1207
47.91673	6.892327	83.25488	49.58022	75.11827	178.6233	80.45459	130.5824	110.5554
7.130157	1.905844	18.80084	0.215634	23.35129	18.55295	12.26388	1.584732	16.52399
240.3207	172.683	270.9874	332.8355	641.5293	515.3612	862.8761	591.8369	441.1686
43.42922	29.87029	85.13771	16.07339	48.01881	88.85846	29.12905	18.96587	37.18996
612.4661	306.9597	367.7636	244.9436	533.6915	966.3063	664.1058	1081.192	1352.406
52.14262	564.9302	822.0944	1047.408	69.27187	230.3467	52.81627	25.55129	212.8407
21.23963	5.236061	29.56703	47.76678	18.83948	69.29584	140.4249	100.0189	28.94226
189.3051	193.4452	68.71525	74.04853	76.2623	36.10528	62.83001	66.08746	48.56976
396.0583	258.7621	405.8446	775.4349	249.8547	272.3179	217.7397	252.8956	292.392
117.7685	340.8624	61.76625	248.2225	46.95351	32.20195	50.57019	32.177	38.23724
276.6864	160.9897	66.87659	17.91217	220.5176	215.7071	202.4246	208.2701	288.2527
1346.925	1458.826	905.4257	577.512	1258.462	1301.096	1160.331	1059.657	1108.594
43.66486	15.20069	88.64728	124.6559	71.56069	128.8512	52.09179	67.75695	25.84256
5.758489	11.70292	30.65343	8.989169	14.08254	14.64043	2.974124	7.386087	6.210258
212.1875	106.368	173.6216	44.32519	450.3289	238.1551	179.3862	413.3187	301.6882
178.5246	15.17763	227.8487	111.1946	959.2154	850.1718	987.2656	330.6019	764.5256
590.0039	757.7242	473.3193	301.249	713.118	857.9605	667.938	1003.478	838.9361
18.41674	15.18804	55.39141	63.27822	144.0241	73.21083	32.91483	33.01687	16.54406
11.39237	26.6325	102.7337	10.85494	56.2144	82.993	28.34544	11.50484	10.34487
159.7826	278.7137	26.65653	31.95715	97.31741	108.3499	79.75904	30.5183	38.23827
838.3558	593.4894	13621.24	1123.981	31.73716	50.74532	52.81851	28.0338	97.13225
50.66755	227.7803	38.92554	38.97206	79.75767	28.29656	102.042	18.93802	9.312608
59.9482	84.89698	13.28532	79.59101	17.62781	23.42425	12.96622	9.027541	15.5059
734.5894	298.6601	271.5472	81.27921	494.9833	649.0809	629.6055	884.4578	1124.077
32.45949	36.71214	81.01661	23.19429	44.62398	76.13051	55.92558	120.7268	84.72131
16.90661	11.78363	49.44348	2.053544	37.42516	14.63628	10.66635	4.0619	11.37429
89.99371	109.5311	151.0274	37.27602	121.9956	100.5297	98.11341	209.1853	74.3987
54.92859	3.563633	19.0613	9.109665	3.584155	36.10439	85.83278	214.1547	194.2263
96.27498	129.3255	14.43098	93.89178	42.19002	21.46772	32.97379	18.9625	24.80202
136.3106	159.2124	250.8438	72.43871	204.0589	219.6186	236.2644	148.7621	142.5825
16.99799	13.51683	114.9265	31.79683	46.89563	72.2434	27.55753	18.9532	49.58698
182.591	108.3809	23.80271	57.77366	57.40109	22.44312	29.10529	15.64308	32.03191
647.0995	659.4792	12.64845	58.17898	4.761723	14.63081	7.570438	13.97783	13.44536
16.67982	49.35215	6.911731	17.24518	5.907958	10.73732	1.44314	3.243597	3.111512
84.38489	109.5316	183.1671	40.79134	322.3544	226.458	129.5905	113.2145	136.3799
35.27797	10.19059	64.543	14.36714	499.6566	229.3663	441.696	2382.645	604.4051
98.40325	398.5069	113.0546	349.2781	17.66679	17.55891	14.47217	9.847161	6.212206
33.44386	34.35897	0	107.5126	0.053193	2.918507	2.972473	5.720328	5.178636
130.6119	41.70693	116.5899	39.02472	70.42119	202.0571	97.36056	117.3653	148.772
53.41796	50.54573	0.433939	1.987431	0.044259	0	0	0	0.008687
46.43318	39.96243	96.35279	40.67464	42.26473	78.0884	29.82912	42.11285	59.92621
78.67091	31.74588	178.8181	45.98103	58.67752	98.58594	64.3836	55.34115	37.20564
427.0598	113.0165	453.5546	538.5966	2283.093	898.9659	602.0284	483.5252	341.9886
279.5449	252.1	1084.202	127.0004	241.6377	631.533	235.3975	283.5002	288.2572
161.6503	157.7095	321.274	141.0607	317.8409	341.6244	340.5232	316.5799	281.0249

2820.363	4467.798	1723.419	17748.1	7971.667	4955.493	3811.578	3546.853	4398.198
129.2183	15.20074	12.06776	35.51255	70.42251	78.07889	108.1074	186.8675	91.95877
682.4803	1719.369	76.78469	251.9097	327.2631	434.3473	879.8027	364.4907	313.0585
15.58512	6.902002	53.829	12.60005	70.22341	36.11538	34.51256	23.10657	21.70457
1127.896	1210.294	584.0855	749.9559	1427.35	1154.683	2307.001	1243.99	1490.862
542.9753	433.7401	357.6742	188.3573	265.0237	151.2858	160.2494	123.941	106.4269
53.27071	82.3266	15.0039	47.41467	49.22962	27.32475	48.35232	25.58248	20.67358
105.442	64.90342	299.9646	68.91507	253.2649	233.287	108.0785	65.23465	79.56523
152.3511	60.43185	11.5347	5.578597	3.589076	1.942563	1.441738	3.234764	4.146258
341.0258	432.3015	7.402215	164.3832	2.412568	14.63094	25.991	4.887742	19.64345
94.16136	26.80387	73.36037	53.06094	25.87613	124.9375	104.2811	114.8914	111.5836
81.60896	45.04087	266.0017	63.65964	143.1185	133.7159	173.3054	229.8143	156.0132
28.07713	54.17392	5.07337	16.03453	11.78688	15.61065	48.46521	12.34144	15.50648
16.78747	32.44653	6.281953	3.799271	9.416074	9.756389	20.79913	10.72209	10.33676
15.62115	25.12515	50.63449	23.18264	3.586989	47.82138	31.35956	58.66067	60.9614
132.9778	164.7097	30.19276	51.09447	31.71671	16.58343	37.52519	26.39205	39.26746
220.5745	506.8746	111.7935	167.4003	126.7276	139.5696	337.4775	249.6126	146.7205
15.46121	13.33807	22.87074	15.84926	38.5122	43.95674	19.98072	7.383565	19.62501
78.81459	106.3318	68.63031	148.0269	97.40943	186.425	217.7902	315.7985	255.191
11.39293	13.50279	61.46787	14.34906	42.19761	50.76345	25.26327	22.27537	21.70495
19.83257	13.54307	10.89918	19.6763	52.81888	39.03472	51.32562	95.90717	76.45596
977.4601	41.71336	107.105	178.0729	24.69708	471.4339	743.9072	662.0765	1675.767
14.12888	15.03916	8.601173	2.0535	2.415117	13.65965	50.1202	0.76157	6.211809
4.374621	1.900359	12.64792	14.40317	157.0413	97.60928	12.93734	25.55369	72.32539
259.9073	210.7174	365.6047	199.0837	226.3991	266.4622	269.1521	365.3572	247.9671
59.12939	45.01838	142.8847	231.8192	266.0831	202.0564	149.5763	66.89859	71.29849
158.5627	31.76672	178.0543	7.349741	147.7288	138.6066	65.12595	59.4626	194.2142
126.4949	134.409	269.5316	98.78627	362.2696	302.5929	157.1935	133.0461	179.772
126.5791	36.7449	814.7237	63.68311	689.5927	1395.808	354.2621	225.5942	294.4631
50.63988	105.8362	24.91114	89.59281	46.95227	26.34476	47.49795	29.69506	50.6316
4.374588	5.228708	28.11459	12.50036	15.27417	15.61354	25.35414	14.01788	12.40608
125.0304	35.08999	107.2196	33.75793	158.3144	207.9117	111.9388	146.3129	192.1592
42.06628	23.34838	7.40559	5.59277	8.280601	37.09203	32.20121	39.69691	47.51587
151.0795	269.5097	60.08593	167.5938	5.936637	18.53593	10.63773	15.63448	16.54402
12.80821	0.2128	27.86067	0.245296	10.62847	15.60742	29.84378	37.98969	33.06963
105.3401	73.0837	209.6647	26.71572	51.66164	125.9161	42.86181	67.73765	88.85629
67.58578	306.5823	759.335	1257.192	24.70326	49.76906	107.2851	32.16182	51.67271
5.782001	23.48448	31.90449	2.039595	120.7657	174.7325	71.28342	192.744	81.62359
25.38853	6.905931	12.65991	37.02908	39.88466	26.34775	33.71056	38.83838	23.77238
23.93618	21.67872	27.96366	7.345504	142.5123	60.53516	30.67808	34.73445	26.86513
15.56941	13.47931	39.76096	12.57955	8.278565	42.95537	23.74374	24.77866	34.09069
127.8637	84.74674	38.88977	19.68441	65.74157	87.83904	161.8264	167.8062	132.2493
4064.581	3269.195	1664.553	2210.572	2061.891	2401.12	2948.034	3760.986	2776.127
143.0015	135.5728	13.81427	61.71729	18.83814	6.822472	192.778	75.21178	15.5117
737.1785	691.0517	422.1193	610.4926	314.3592	244.0092	714.872	177.6498	269.6663
309.6841	465.3625	65.20073	387.0391	92.66693	82.96235	65.13024	13.15088	52.7021
542.956	780.7739	135.7617	100.5613	69.26577	125.9064	239.3115	93.34434	160.1461
28.19993	30.00464	92.44003	12.62853	23.5126	52.70929	33.69704	49.59907	37.19928
443.564	605.9399	75.62959	1220.306	51.67926	99.5499	114.9583	81.76256	377.0926
117.9692	64.84262	187.3837	30.23367	99.70698	97.60418	30.57972	110.7587	82.65983
245.6117	156.1552	29.04784	63.15853	15.31459	41.96902	15.24663	13.98221	25.83889
17.02361	39.97834	145.5408	10.8818	15.32012	38.05929	40.58007	59.49295	87.81372
41.14143	55.58313	12.30501	63.524	0.049752	0.967091	0.679833	0	1.045606
57.85989	12.86135	5.191394	5.372169	0.046144	0	2.998672	0	8.260523
15.49152	21.47191	14.51981	32.97482	10.6009	32.22424	19.95534	7.378914	4.145906
70.09413	80.90017	33.72909	61.45568	15.31551	46.85034	66.78591	19.7748	33.06876
15.5617	1.911397	22.68918	2.053506	4.762757	26.35304	6.039125	32.25848	26.863
169.9682	142.6756	237.4073	98.7841	248.5937	269.4033	208.6269	149.589	153.9459

31.05952	30.10032	30.15497	14.36272	662.6765	2530.01	1735.767	698.4467	973.2397
484.4158	140.6692	343.8287	46.00809	0.057305	2.91899	1.44808	2.4138	1.045068
1.421589	7.177602	0	0.093505	0.025745	0	0	0	0.005208
1.559826	3.580066	19.19402	2.053813	8.273483	25.37947	12.97313	30.62622	11.37518
1919.904	1410.849	497.1955	410.3715	2720.882	1615.391	2686.66	1324.996	1852.467
12.76835	0.204172	0	0.234888	1.238454	9.751871	20.67311	110.4299	35.11937
24225.01	27909.09	9972.235	25977.46	25440	28320.58	24986.35	37876.72	29301.68
110.9352	158.8708	66.95363	147.5768	65.72163	74.17636	88.17123	65.25689	76.46051
178.4717	131.1895	342.3419	123.4588	480.7595	258.6565	195.5173	149.5593	223.169
178.4574	112.963	394.9275	128.7157	276.7784	394.3415	174.0402	165.2754	225.2335
535.1024	272.0818	664.2259	97.12235	591.088	1111.764	338.1592	328.9393	491.7889
904.5997	570.5259	113.5238	83.02725	83.3427	939.9471	835.8842	1170.428	4263.823
32.47709	0.220401	173.0538	12.62076	277.9734	451.9282	249.2195	163.611	175.648
79.48193	137.1011	9.165313	22.99251	11.79009	14.63348	23.74348	44.70485	18.60503
44.99072	34.96778	134.644	40.60526	69.15942	47.8254	43.6841	12.32597	25.84009
21.12333	10.18259	21.52072	2.05366	3.589537	14.63422	19.12986	41.40505	42.3445
60.49477	43.33008	75.15341	84.53914	58.68685	44.89097	118.1476	82.64139	59.93214
73.17226	23.49292	286.5761	12.63597	89.18512	265.5037	103.4803	153.7426	301.6596
95.43378	198.0531	41.27204	35.45584	109.018	45.86868	73.61684	29.69207	55.79743
53.54557	6.882783	76.79495	9.096472	42.29715	297.7033	294.5242	483.6796	182.8775
96.9952	16.8594	244.571	84.6728	227.4407	187.4114	167.9942	116.5289	126.0487
59.66542	394.899	41.80593	33.00844	0.053403	0.967	19.17572	0.761442	18.59739
1665.828	1281.677	928.1049	1893.721	2445.357	1893.567	2067.606	2656.668	2639.741
50.71224	25.14883	84.45074	70.61723	51.66833	65.38893	119.6302	243.9756	122.9478
412.9946	157.1001	22.56462	177.1476	28.21758	20.48725	80.50832	23.07179	17.57807
1.55898	5.242894	5.655805	5.589819	10.62012	15.6099	34.5396	38.88995	25.83223
172.9042	76.54451	177.1035	239.5623	769.2634	266.4602	324.37	574.5266	572.3659
1700.978	1503.841	2119.987	392.7804	5324.567	5395.713	3176.606	3377.449	3190.422
341.3127	122.9386	937.5863	160.4167	187.7083	527.0848	328.2274	148.7216	286.1925
64.61427	16.85001	111.5993	63.37868	79.7259	121.0485	19.07797	26.38695	51.66348
127.9288	494.689	18.56498	19.51267	2.415074	0	9.882057	5.714155	2.079574
95.57203	74.77629	40.06515	112.6824	35.25633	80.0317	105.8108	191.0164	133.2768
109.6533	134.3877	321.5689	88.23672	130.2153	258.667	91.19122	71.02254	91.96222
67.45252	51.5398	243.3593	77.43171	94.96897	187.4341	33.66253	24.72843	51.66584
14.09302	3.572314	11.57448	5.557642	55.96836	14.63867	24.61763	9.873235	19.62675
30.90096	15.12874	59.19564	23.01131	70.19125	49.79067	10.64788	18.13826	20.67071
0.11188	0.177319	21.41063	12.16262	15.1686	20.51925	9.982917	10.76517	10.32909
12.78878	3.582296	45.59286	38.6368	28.1678	69.32135	56.10782	52.14899	21.70433
12.71268	6.858169	12.16213	27.82696	0.053041	0	5.279035	0	0.010265
54.83154	31.71956	203.0529	7.357046	15.31972	45.86982	41.35275	40.45596	22.74274
53.42855	31.71667	109.8338	75.60093	93.77278	77.11204	75.94926	27.2141	36.1708
51.95679	1.906335	27.86689	2.047936	12.97233	40.99173	31.38563	68.6559	61.9842
28.14759	5.245233	103.3101	12.6036	45.70292	49.78684	36.81907	8.193982	15.50894
54.90078	53.25809	30.73236	63.55462	119.6103	88.81952	75.88111	70.22095	106.4158
36.45261	20.03724	31.5025	19.53187	55.00841	58.58243	19.88818	21.45825	12.40969
30.66832	13.36476	6.849403	5.559704	12.93072	13.66146	35.46719	23.18589	15.50009
152.8223	660.3731	77.52573	52.98534	23.52778	32.2012	39.03383	40.44522	51.66699
146.9157	107.2714	34.87907	31.89208	25.86088	36.10873	31.37658	32.18822	39.26721
11.39147	3.582244	3.906394	7.350678	160.1052	37.09191	34.51223	64.59628	59.90425
615.2349	286.8546	59.40609	339.0549	28.21417	15.60686	10.63689	2.412911	4.145993
37.85784	15.12932	19.13629	12.58782	12.96192	39.0472	22.96621	26.4325	57.83476
78.7788	64.88951	98.44112	51.33583	90.35497	144.4571	87.36373	176.085	152.9086
4.377384	6.905903	23.16262	5.591649	30.55138	61.49136	45.20044	90.97964	28.94017
4.37177	3.567446	9.736328	0.251083	111.4195	128.8437	151.9171	249.8118	98.15383
1640.67	480.3239	32.51193	269.0428	2.412191	14.63084	12.17081	9.018334	87.81253
54.90231	94.54569	38.32213	290.9818	287.0873	81.98582	118.1267	62.77341	73.36195
5.786281	0.208345	5.071155	3.828204	15.30621	24.39682	18.33568	52.14122	34.09512
4.306196	5.088617	0.430745	2.01328	0.046888	1.943688	11.60015	0.763168	0.009164

466.4539	4512.667	963.174	6733.663	224.087	238.1499	82.73088	70.18879	67.16947
7.160092	48.77771	82.41885	5.559584	1.238854	1.942572	0.67959	0	4.145802
230.2846	309.7	227.4527	549.6299	296.656	137.6205	225.5027	180.1859	152.914
372.0649	160.9809	111.7996	91.81237	131.4141	402.1495	91.93892	135.5049	271.7225
22.48706	49.17351	17.41852	17.72676	16.45454	6.823065	22.23077	9.858621	28.92335
97.45791	67.22672	22.09512	55.72908	2.415067	7.798971	6.03885	11.51065	12.4093
12.81342	30.0856	75.20684	19.67416	128.921	111.2797	114.3578	45.41433	70.25777
903.0826	441.1845	1842.353	192.1523	893.6953	1144.933	509.1733	309.9024	715.9894
103.9358	66.47437	207.3333	24.95791	71.58052	133.7263	59.75369	42.09186	35.14082
344.1174	435.8938	204.5405	346.714	61.06263	90.76408	99.60271	285.1468	187.0139
55.94349	10.20046	45.02655	17.81357	34.00477	38.06993	21.42238	21.45306	19.63859
98.45592	28.4664	47.6334	54.87612	126.7142	133.715	331.4108	279.4259	299.6086
556.2684	1.884374	176.4935	10.83873	342.53	292.8108	641.117	1446.647	672.5959
4939.711	4729.809	3119.339	11874.98	9380.265	7311.721	4359.148	3936.164	4505.652
66.05707	10.22588	54.138	12.64263	73.89578	47.82104	97.44298	84.3218	78.52071
998.7683	701.5319	3752.769	1369.445	2282.369	2271.303	1954.064	2618.609	1730.566
28.19494	52.98182	5.071533	7.357352	38.72381	15.60776	66.8018	44.6308	21.70759
144.7673	448.7492	90.80342	332.3522	193.5382	126.8815	290.7105	276.1058	96.09677
31.02455	1.90436	21.9981	17.90321	49.28082	78.09091	76.74245	70.28244	56.8255
530.3421	1129.431	233.8608	1003.838	56.36809	31.22376	125.7148	32.98817	58.90428
429.1602	390.3889	64.5691	111.0241	158.331	103.4576	132.6526	37.94947	108.4896
12.58307	1.655426	8.59271	3.207435	0.025887	1.95452	0	0	0.005236
50.4858	217.8081	39.64472	26.57046	8.282074	6.822368	16.02048	7.366003	37.19588
43.54251	43.11216	29.64566	54.42051	9.454911	16.58422	39.86475	28.06222	21.70713
17.01172	10.2225	53.65799	9.118434	18.83064	32.20428	78.32329	42.96276	56.82156
45.0454	104.2097	37.1869	87.86505	51.63826	32.20192	102.0778	55.35351	77.48565
8.587717	1.891933	2.761802	2.03094	573.2389	229.3781	180.2081	194.2424	165.3113
26.85281	21.82991	189.8236	23.19795	29.39115	120.0603	34.42069	103.3302	135.3352
218.5563	280.48	59.97251	65.23872	32.90404	15.6068	33.65816	0	3.112422
296.518	104.7243	1534.264	292.4521	899.577	1386.023	494.5894	1242.394	654.0032
120.96	162.7291	1519.102	204.4251	304.9828	627.6159	200.0801	505.8732	728.3712
8519.858	10507.26	4218.404	36623.6	25958.26	11605.43	13964.91	11825.95	15506.82
37.87302	21.69633	44.43111	14.33632	14.13345	21.46808	15.26172	18.96468	21.70386
60.54143	20.17655	193.1037	160.0937	138.4014	138.6006	115.0033	47.87268	55.80365
61.57573	376.9032	59.68765	42.1469	3.589513	9.75096	13.7182	4.886538	7.246036
23.91928	21.65503	48.04406	2.05341	35.15355	43.93333	16.81141	21.46317	26.86348
15.40379	5.193634	33.15691	8.951956	11.7411	17.57488	3.746017	11.55804	7.241192
150.884	105.3991	47.82342	75.29835	27.02221	41.96931	14.47896	15.63777	60.94883
217.4861	210.2262	290.2154	221.3252	130.1863	123.9595	98.89236	95.02382	94.02489
51.41193	89.08465	2.743177	7.306241	11.77071	12.68364	13.7614	4.062642	15.50179
75.84497	44.9394	82.239	12.64241	91.45492	158.1443	49.79527	68.59424	108.4716
116.5476	135.8043	219.6042	16.16442	141.8696	128.8445	44.39668	50.36317	43.40526
8.583263	3.581959	42.15248	0.235825	57.31869	35.1433	52.31801	14.82781	25.83069
359.6341	616.5843	215.0056	283.5591	354.2318	427.5148	433.289	596.8212	497.9872
64.40113	47.96509	46.70789	7.354465	57.40621	29.2779	26.02331	22.26944	43.39035
28.16975	15.16163	50.22881	5.594321	28.18205	42.94838	31.41015	33.04313	24.80426
243.076	223.9795	705.8386	214.9184	673.0916	687.168	487.0535	342.1969	196.3122
7.19075	6.902923	2.172752	3.817136	10.62929	68.32159	31.35542	211.8354	58.89732
766.1961	799.5568	628.4589	601.068	197.0685	132.7373	122.627	108.2185	110.5612
15.60397	20.11662	33.16207	3.826689	48.07639	32.20567	48.3307	47.12272	46.49153
1.559611	11.72718	41.9224	14.1523	11.76045	9.754567	3.741456	9.0442	8.275309
8.598159	3.578498	13.23674	54.54571	57.45951	77.11753	16.01144	11.49898	31.0042
101.2536	48.35473	91.39974	160.2519	161.874	160.0717	204.0068	211.6151	213.8638
108.0435	122.4133	74.60989	86.19883	45.79018	20.4874	15.23844	15.63083	21.71028
179.4419	99.41522	34.82837	47.74771	77.42418	96.632	108.1729	78.51015	48.56886
105.3866	40.05148	24.89262	24.96356	34.0842	58.55582	80.46931	237.3557	70.26518
10275.46	12456.78	7855.697	16336.77	9015.632	10324.83	9377.12	16157.31	13673.98
185.549	94.77962	234.2585	418.9216	273.3209	389.4474	341.2333	621.6322	312.0248

58.87315	10.21696	76.64951	10.86856	24.67719	27.32412	23.70732	27.23542	38.22973
22.58198	11.86306	36.71807	12.61273	14.13892	22.44297	41.42546	33.87452	12.41101
1.557518	5.185888	5.699309	2.044203	9.409234	6.826026	9.940324	6.564042	10.33526
63.31164	74.73481	177.4844	21.44243	86.81204	91.74799	138.1013	109.941	98.15274
112.4849	765.9877	187.7179	58.39151	17.66534	31.22376	43.61414	22.24354	21.71071
445.2801	596.7554	194.0023	1480.642	401.1412	193.2519	450.1563	367.7928	272.7671
7.182537	0.204202	24.4689	2.053675	11.7862	19.51717	5.271816	23.96013	25.82966
165.8365	86.47032	413.0021	121.695	268.5789	227.421	152.5508	180.9837	152.9184
63.34791	26.80812	92.01973	102.2344	106.7616	115.1721	97.34474	169.4695	82.66314
33.88121	8.554753	203.0087	167.148	207.5477	185.4561	91.96657	80.12384	160.1404
1470.592	121.2915	348.4994	1086.065	754.1993	1659.311	1276.872	3147.727	2660.399
143.306	302.9576	57.55929	880.4533	85.67278	101.5045	184.0759	106.5856	170.4727
212.6438	151.7466	133.9102	96.48388	29.37938	40.0128	30.60009	34.66526	25.84128
81.15444	111.7993	61.96271	184.2104	23.50579	58.57136	14.48181	6.539191	9.312345
87.10681	91.20342	71.64477	177.2211	123.1102	92.72575	90.48721	69.39992	56.83327
14.13999	1.911851	33.98746	3.823672	29.28064	25.38017	21.46826	22.31831	21.69711
88.65907	35.0906	1083.888	165.719	252.2092	432.3973	219.2639	57.78579	172.5516
9.057608	1.703383	0.472839	4.782419	0.029726	0	0	0	0.005974
188.1399	127.7355	197.1664	281.0779	160.6745	108.3384	95.80476	164.498	70.26699
304.2927	617.6265	89.12539	14.40221	42.29064	58.55582	246.3654	67.72716	119.8488
2.972329	1.911777	9.175771	2.053812	72.43234	48.82216	16.05242	60.55029	22.73146
4.330739	0.176722	59.12263	2.029584	2.408136	0	3.759497	0	1.045496
19.77194	5.244818	56.1929	17.82564	10.62308	55.6479	20.64734	21.4493	17.57396
11.40283	1.907149	28.46355	30.09669	27.02261	27.32339	43.70451	44.63059	45.45995
5.772087	3.557079	142.7463	93.55408	306.0661	141.5234	80.43522	549.0593	346.0981
29.6633	25.14616	76.29054	67.08084	86.81793	53.67565	101.9845	148.8286	84.72557
19.80823	18.47871	9.151746	5.594406	28.19515	21.46522	31.38921	111.7809	58.88507
66.14682	23.49254	252.76	19.6852	50.49845	208.8882	50.52716	90.0569	78.53026
52.04524	44.92757	122.6805	19.67021	45.78332	84.92095	42.88457	34.65909	36.17153
127.8781	91.37644	326.2695	33.76379	55.19232	124.9323	51.28887	180.2056	167.3733
943.6301	729.3084	410.9834	779.4257	736.5066	550.5006	577.4907	366.9602	501.09
216.2733	68.23072	590.785	33.76617	171.2531	196.1889	167.9279	94.17153	83.69876
19.77858	1.909701	29.10074	55.98153	55.05034	61.50597	23.72254	33.05447	45.45255
12.64434	10.03817	27.18769	5.521394	9.411716	27.35089	0	3.239526	7.240866
522.0102	332.9575	1858.408	191.9014	20.01142	53.6735	37.478	14.80664	10.34501
134.9395	114.5956	382.7491	158.5384	306.0579	201.0682	196.3133	148.7464	84.73238
24.01549	20.13927	26.68787	16.14176	115.9439	100.5519	78.31017	62.84303	39.2665
194.0059	147.8789	63.69207	16.12509	4.763263	26.34734	19.85951	20.60616	19.6417
76.89647	85.50055	25.56776	42.16345	10.62475	6.822412	6.036846	8.193536	8.279117
364.988	147.7084	248.9961	63.67208	163.0603	346.5131	167.9107	176.033	172.5451
65.16263	44.05125	9.199869	17.63022	7.098436	14.63754	11.44638	19.01365	10.34089
35.23138	18.50204	86.99448	23.1674	127.6978	93.71109	62.88454	135.7217	67.15476
41.91873	86.45691	6.244453	16.01284	1.238558	22.44799	20.68067	4.887888	22.73159
209.4014	116.3195	83.78287	366.1127	906.4242	387.4976	423.3472	534.8427	385.3732
257.3419	442.5093	0	14.26821	0.054297	0	19.14422	0	1.045715
11.40415	16.83108	19.67208	19.63411	28.19625	39.03923	21.39227	105.1402	39.26506
54.80071	71.19261	132.1982	51.11553	70.34747	74.18543	56.74351	40.46454	34.10392
272.4619	427.374	128.1395	256.9505	575.6856	287.9416	295.2886	193.3896	169.4476
76.01863	64.94015	289.7159	186.8004	593.3811	302.577	481.672	517.489	322.3525
25.37594	11.86377	19.10678	10.86339	4.763414	43.92513	25.25326	48.80061	25.83676
41.79414	13.40479	49.47519	34.78085	12.94103	27.33615	9.897359	11.52536	10.34162
4373.419	2613.106	936.4612	5312.62	131.433	188.3706	358.8359	83.41091	531.0567
7.171208	24.77351	3.325545	5.572961	9.438905	3.89451	98.95659	14.01573	3.113027
39.42664	0.214215	7.402188	0.247007	9.456326	28.29771	81.34736	153.1316	68.18692
106.8678	198.9439	91.3979	226.9523	131.399	136.644	265.4126	254.6237	178.7409
34.71916	22.97842	3.330623	36.16399	3.586037	2.91865	12.23886	4.065369	2.079524
56.28218	0.216534	6.241184	5.58612	10.62915	189.3791	84.35113	151.3612	85.75407
7.109984	95.05198	1.003123	10.58267	0.050317	0	11.51092	2.412842	1.045663

19.82766	21.8128	41.28698	10.8816	93.77896	76.13505	119.7787	45.42204	52.69705
262.6595	655.7208	168.3986	227.1226	522.9582	94.66899	160.2209	192.5577	119.8594
23.96325	36.45653	77.37823	80.1599	35.18543	14.63252	21.41386	23.9337	15.50891
14.2059	10.22009	55.45088	21.37424	48.08282	48.8041	39.08615	31.37178	22.74046
38.80908	27.75668	4.5057	15.80962	11.75226	10.73243	16.89465	4.065989	7.242299
33.80648	3.578582	27.85985	33.62337	87.87272	54.66025	82.16146	24.73932	34.10251
64.72809	40.04643	18.478	16.16425	18.83991	59.53237	73.56495	243.1692	77.49544
99.79558	48.33181	117.1569	79.40528	151.2814	120.0542	168.7662	132.2502	90.92635
98.32133	54.90249	200.3431	74.0527	84.46362	138.6087	57.45327	67.74159	50.63584
350.7019	718.94	214.1099	221.4961	168.8783	60.50711	48.22137	32.98895	101.2585
68.92221	73.09271	111.3735	173.8697	202.7795	128.8441	95.07645	50.36229	63.0326
835.7796	1733.878	859.946	915.2839	987.5561	741.8041	1289.199	1315.103	776.9513
22.34934	13.3302	15.74398	24.33663	1.238803	3.8949	6.824895	5.723742	3.112702
436.9537	132.909	1750.605	582.7512	1202.144	1799.88	638.0149	650.4762	633.3414
18.44801	19.46192	20.14474	5.078461	0.03773	0	0	0	0.007485
67.328	57.97069	12.06926	40.59509	8.283162	17.55987	15.24403	48.76023	31.00394
839.8344	659.8335	517.697	1152.315	1108.249	854.0626	651.873	629.8461	402.945
71.73282	61.53882	256.4056	37.26011	118.4527	152.2724	78.9427	52.01475	85.75887
21.20897	1.906584	48.97828	10.87469	85.51418	81.02595	30.6186	28.05537	28.93765
26.82167	15.18997	24.34004	10.87928	27.03312	20.48821	25.22676	57.03286	39.26761
1.508315	47.27146	0	3.537719	0.038822	0	3.048398	0	0.007688
19.67659	5.230305	20.99665	50.16191	9.438921	11.70629	19.16171	10.69395	7.244427
95.36727	25.11306	91.08275	14.39801	189.7185	56.60942	42.89236	23.90454	59.926
67.13996	98.51061	65.57709	40.4133	101.7695	26.34939	34.50389	11.50415	20.67256
67.47787	43.31286	71.6691	45.98675	145.3356	87.84639	54.39368	61.96038	51.66725
150.3211	312.8883	88.49501	198.7799	41.12269	42.93682	35.94506	71.02268	50.63888
1355.387	1579.873	812.0983	887.1905	1086.082	1227.889	1641.995	1947.474	1409.243
1.559669	6.887837	20.35822	2.05381	2.415195	12.68163	7.57838	34.77057	18.60249
221.7267	271.3757	122.4068	571.5847	11.80088	47.81781	50.52638	35.47009	77.49747
110.0947	338.6668	12.09039	28.22058	9.45098	1.942655	15.26271	2.409473	2.079517
54.47289	47.71902	10.92685	17.77961	17.63438	11.70443	28.37822	6.541541	9.311199
14.13176	1.911778	10.36492	0.230086	2.415139	9.752896	11.43708	23.15663	30.98336
61.84427	33.3814	153.1432	44.19824	38.7566	78.08645	30.59266	54.52355	36.17167
43.71335	36.75596	41.2185	37.28544	254.4722	117.1212	84.2761	382.8358	174.6088
28.24515	16.85586	103.3091	21.43017	61.01408	89.80085	35.1978	52.86322	53.73184
217.8203	141.1708	1235.747	116.465	303.7935	709.6161	182.447	180.962	324.4192
71.62356	128.8601	48.89213	235.9853	58.6618	45.86955	88.24257	17.28567	36.17124

TCGA-DU	TCGA-DH	TCGA-DU	TCGA-HT	TCGA-S9-	TCGA-DU	TCGA-FG	TCGA-FG	TCGA-E1-
129.5836	157.8104	70.58049	198.0449	103.7586	247.3108	187.9911	135.5409	156.5311
90.60786	17.63721	51.67594	19.87427	22.941	47.80708	26.99871	7.850179	44.65756
113.3575	115.6626	211.3105	200.3752	63.34508	161.0847	124.9941	43.20982	201.0513
4162.933	2800.404	2441.893	3808.586	1405.114	4292.769	2257.893	1861.255	5614.902
109.6573	93.11349	45.96144	41.26554	136.8388	101.8112	190.9909	160.0996	64.55984
0.056572	1.955407	0	0	2.741301	0	0	0	0.071622
21.25179	9.793698	15.00553	13.19563	28.4532	7.714167	3.99979	48.12982	14.58819
261.4645	126.4416	103.1519	233.5563	171.7439	183.1069	389.9815	95.26808	257.6503
14207.94	5667.457	6408.013	21840.07	4805.928	14906.23	6705.681	2694.154	18161.78
102.0525	90.18219	50.76929	25.75899	26.60289	40.52553	74.99643	67.77122	67.08855
8.805431	3.912647	9.456397	13.9765	20.20049	27.12148	14.99928	25.53929	21.05315
1532.105	927.2575	1024.065	1438.498	677.7515	1652.055	1104.947	755.3039	1807.118
67.3599	258.7772	64.22244	70.82706	123.9655	65.42545	256.9878	296.6323	92.17328
0.060439	0	0.722206	0	1.811663	0	0.999936	0	0.07667
168.0636	45.08149	32.46386	89.35933	97.34258	51.19132	121.9942	162.0687	85.54684
18.76617	13.71505	136.1871	2.848715	48.68815	10.56747	4.999742	54.0255	2.7566
4815.515	2249.537	3030.221	2726.956	1382.16	3096.695	1462.93	835.8412	4054.962
26.23461	35.28279	16.59007	34.66806	68.90238	29.12671	66.99682	90.37328	48.67568
32.48826	66.6475	72.19054	123.3972	282.9701	54.75595	88.99576	38.29858	21.18072
637.7979	627.3203	978.1005	192.7179	537.2462	108.1529	165.9921	157.1436	107.9943
1476.034	1202.696	863.6953	1609.224	1066.256	1166.866	1177.944	500.9135	1730.806
7.577875	125.49	0.718997	166.0315	4.558031	84.21769	102.9951	50.09204	63.07197
112.1295	6.853304	607.2492	367.6265	73.44875	251.7015	132.9937	57.9434	136.7741
246.6815	147.0217	138.0579	687.341	68.84436	485.2348	332.9842	189.5588	575.9057
97.16782	137.2323	48.35708	195.2481	43.13535	163.2747	128.9939	25.52961	156.3841
24.95947	27.44252	3.883244	20.61768	15.58508	34.90773	42.99796	82.52499	17.19848
221.7304	122.5179	229.4297	135.8701	163.4585	126.7163	56.99727	207.2422	174.9878
1087.448	589.0891	581.8432	228.1779	505.0901	646.039	1178.944	515.6469	442.0619
110.4647	5.8728	23.76454	59.96039	31.21506	75.74118	26.99871	31.42806	22.44503
196.5588	24.4966	46.77494	33.8868	33.94995	76.90881	77.99628	115.9034	50.07207
11.31562	40.1828	96.13634	25.76187	78.07629	130.6044	35.99827	93.31472	4.073154
27.49114	55.86914	14.20237	63.53695	39.47022	46.24912	74.99644	79.56123	55.27327
14.98534	8.815241	0.716632	8.036484	30.34177	12.04018	17.99914	32.42156	5.383424
620.3953	1476.177	1386.17	310.9321	376.5113	433.7284	1099.948	1314.184	443.3706
180.6608	98.99207	128.5442	122.5597	131.3065	197.2908	180.9914	120.8044	98.7639
13007.24	25154.64	6642.969	7978.258	3017.806	16733.33	34803.34	41604.84	8539.191
6.328009	82.37042	15.82198	4.323613	24.79169	4.866355	5.999695	40.2766	19.78716
29.98583	61.74964	34.86055	70.9166	40.385	143.4049	43.99789	54.99973	82.83841
8.823747	54.89134	14.20482	14.66929	97.40646	10.56323	21.99894	71.70528	23.78336
16.2869	41.16666	18.97906	18.37522	120.4243	11.2783	26.9987	68.76078	30.32321
8.819084	88.21106	130.9563	130.7286	202.0602	71.84527	42.99793	37.31581	75.0834
31.21588	52.9289	14.20324	11.70966	68.89024	34.11378	68.99672	104.1239	23.7904
53.59627	24.49725	22.94187	6.53901	43.14535	9.849599	15.99922	79.56058	19.85692
100.9381	130.3653	69.00237	135.9479	63.34212	179.5991	111.9947	139.4726	151.2248
155.7842	151.9233	143.6206	162.4588	151.509	366.919	202.9903	258.3158	89.5713
27.37722	13.71736	9.453008	8.769623	17.43507	38.60769	25.99877	11.78082	2.757803
26.25082	60.77021	43.61194	22.06011	50.49676	26.96289	50.99756	83.48955	30.3622
3536.431	2888.62	1854.401	8244.633	1257.249	4378.932	5803.724	1063.71	7618.034
142.1106	338.1601	435.7922	1649.422	213.0354	1301.773	506.9759	457.7001	578.7489
11.27556	9.795905	9.461984	3.586965	13.76034	27.15551	5.999696	1.957265	7.999995
12.51299	7.834608	10.2598	1.374239	23.89163	12.75822	19.99905	44.21693	4.071987
300.1841	220.5403	107.0942	282.2317	217.648	453.2191	187.991	167.9508	163.1849
68.52992	49.98507	22.14388	23.53632	77.1456	79.07911	53.99742	74.64653	18.54664
9410.866	5216.573	3219.844	6178.623	534.4702	10020.2	9224.561	9783.657	3944.737
24.9058	8.81446	15.03393	13.22478	16.51451	17.75971	18.99909	53.05621	22.37451
34.9518	66.65344	8.644373	46.49425	63.36807	77.69159	63.99695	63.84255	31.67013
45.93807	28.42705	23.00703	25.85945	22.03341	28.51749	19.99904	29.46776	24.99756

8798.035	8550.209	4128.833	4109.165	3488.954	4905.258	7269.654	7614.964	11689.07
529.2734	178.3889	176.9706	28.70221	202.0263	123.8416	115.9945	370.2899	79.05337
13.79249	12.73496	11.03235	10.97829	27.53857	31.31716	40.99805	18.65626	15.89184
21.26788	84.30436	205.3127	76.17072	51.42546	29.11802	44.99785	25.53102	39.52266
0.07838	11.75497	0.717721	0.642703	0	0	0	7.850301	0.100365
190.616	369.5417	180.97	217.1984	118.447	305.6775	220.9895	248.4966	209.1568
15.00816	12.73661	7.859505	19.17202	19.27497	10.58754	19.99904	57.96989	17.1601
22.4823	19.59805	31.73776	56.26537	23.86	44.21658	62.99703	6.867908	31.59447
103.3583	63.70857	136.6345	149.3847	73.46175	152.6151	76.99633	41.24618	131.4109
134.594	145.0638	107.1043	78.2119	316.8768	106.7598	76.99632	317.2559	118.4731
37.20447	13.71865	48.66845	61.78436	6.397781	24.98644	20.999	7.851281	45.70952
29.88298	13.71643	2.297837	0.642305	45.96121	12.72663	41.99802	94.32765	6.704135
449.5176	260.7316	126.9525	240.8454	87.21542	198.708	349.9834	350.6497	213.114
26.22692	21.55764	36.48906	30.23183	31.208	50.60117	27.99866	34.37382	14.59235
42.4445	374.4763	301.1922	126.3673	78.96625	72.59491	173.9917	82.50138	204.9618
42.36061	39.20599	13.41318	13.93365	55.1186	24.8503	48.99767	141.4702	22.4593
269.0587	194.0741	194.4587	410.8938	80.78534	478.901	261.9875	139.4662	324.8278
44.82767	50.9735	56.42333	71.07742	24.77532	83.58985	19.99903	0.975608	112.6501
56.16112	205.8362	342.9817	141.7651	459.2377	139.5273	78.99622	192.5063	89.57008
443.2714	217.6014	238.9568	239.3779	247.0517	128.1388	191.9909	94.28415	498.2042
1224.524	1206.612	2646.859	1083.77	697.9505	1926.272	1650.921	1795.455	799.8188
59.81309	49.00572	25.32541	11.70878	56.93097	27.67694	80.99615	117.874	67.0849
193.1282	219.5599	151.5666	178.7282	35.78199	682.0994	130.9937	257.3344	221.0183
162.8574	94.10563	8.644365	50.94006	11.90428	227.1881	83.99601	35.35383	112.9233
36.19477	47.04546	53.95886	39.08808	51.41845	35.53351	39.99808	30.44211	21.16935
2532.267	1011.553	857.3175	2021.46	673.1552	1946.966	1591.924	784.7688	4045.411
85.98387	21.5557	76.17045	50.15063	87.23707	54.76107	12.99936	19.63624	29.06771
170.6323	64.68546	150.0387	112.2508	64.2581	339.3865	65.99684	16.6897	176.2144
173.2433	612.6233	450.9038	233.3744	670.4524	122.4036	316.9849	282.8673	117.1985
20.03627	212.7165	264.6024	37.57591	126.7474	20.53433	25.99874	41.24541	36.95388
82.30564	736.169	223.087	177.2853	40.37403	277.8963	159.9924	187.5981	105.3273
61.01221	19.5963	25.335	44.29632	30.28379	88.48215	35.99828	21.60181	15.91058
983.9684	350.9023	273.0139	514.17	852.2905	294.8165	1020.951	690.4843	614.2675
1228.29	1396.767	1617.068	1498.245	1108.476	1821.478	1808.914	1702.142	1281.208
0.0509	0	1.541136	0	1.821319	0	0	0	0.064255
48.59346	34.30209	26.13167	31.70033	25.68865	39.12968	48.99767	62.86321	40.82329
56.10021	48.02439	1.510826	76.83656	72.55006	74.07877	109.9948	94.29392	120.8643
865.7312	1011.56	1031.263	603.5503	371.002	589.7723	544.974	496.9859	947.0056
71.0523	58.80598	54.71499	57.5528	73.45631	74.74648	109.9948	196.4532	55.33663
68.54292	58.80739	40.41888	49.4287	50.49004	122.6317	82.99605	56.96344	44.81647
58.57529	37.2412	146.2563	94.63705	33.03476	89.81863	44.99785	5.885763	69.71436
16.29857	41.16152	23.73102	221.2701	24.76335	221.2361	70.99662	27.49439	78.93404
103.4336	142.128	67.41015	265.4294	91.82054	212.4059	98.99528	57.94306	102.6526
175.7138	228.3802	253.2089	114.4116	296.6355	86.78662	616.9707	425.2947	144.7977
11.2827	2.932477	6.270361	42.31554	16.51869	34.32761	31.99849	19.64291	23.65215
13.79697	33.32394	66.78823	74.79446	3.639898	148.8479	27.99866	10.79687	27.69002
6.329048	9.794328	2.297965	4.323314	22.94784	20.60147	23.99885	44.2058	8.017532
23.73661	27.44077	25.35	13.19509	62.48205	33.44057	41.998	65.81508	14.58897
18.72749	31.36965	10.24724	20.65404	15.59249	14.16682	19.99904	77.62489	27.60104
31.22502	36.26107	57.13506	76.12087	53.25455	122.7155	85.99592	117.8741	40.85844
21.20664	14.69741	79.75354	17.67654	14.67179	14.16464	22.9989	33.3985	15.86259
2310.655	1914.313	1404.309	1806.372	853.1597	2586.669	1889.91	1773.844	2389.872
37.42525	28.41927	14.20381	36.87969	37.63592	54.13183	52.99748	96.26536	39.52356
676.3817	358.7441	502.485	537.082	161.6027	262.0421	374.9821	266.1684	343.3956
409.8301	181.3281	271.4498	526.0758	320.5051	741.7406	343.9836	217.0594	537.904
5.079488	0.972692	22.24464	2.110133	11.91817	2.020162	11.99942	11.78171	0.097169
11.28313	9.795406	3.884423	5.806557	52.44456	20.65067	11.99942	31.43645	4.073455
217.8373	120.5631	193.002	155.2009	50.48171	179.6184	115.9945	57.94349	159.0838

10.05758	22.54146	34.96294	34.76425	24.78877	16.30425	22.9989	8.832791	22.40813
85.88835	27.43828	25.32715	29.46274	63.36806	51.24814	61.99705	190.5763	11.97198
374.5145	189.1848	7.060777	200.3409	4.559972	141.0739	105.9949	27.49386	117.0797
353.7781	238.1814	110.2618	206.0482	48.63946	771.03	333.9841	240.6335	175.0463
99.69451	139.188	93.63481	190.7054	97.335	130.3604	76.99632	71.69494	100.0175
93.45229	172.5224	95.24274	53.10667	85.39745	54.75775	41.99798	36.33413	174.7926
363.6919	179.3696	725.0521	325.0915	59.66028	437.4956	266.9873	478.3378	307.7787
51.08377	38.22338	3.092429	17.62785	54.18475	58.4283	30.99851	127.7055	23.78604
32.37109	21.56055	7.06011	16.17801	45.95443	9.862188	49.99765	48.13622	30.24824
20.01037	15.67568	17.39173	10.97493	17.42054	11.99397	29.99856	24.55012	29.0014
118.4229	70.56545	46.74838	104.8134	146.001	348.4233	192.9908	157.1465	181.5798
174.4308	151.9252	123.7817	100.3859	113.8548	137.4104	142.9932	137.5027	93.50292
572.977	468.5303	583.5161	828.3399	281.0047	907.7404	256.9878	196.4324	727.26
7869.861	1898.623	3173.048	7832.308	2097.588	4193.685	4142.803	1573.47	7769.334
8.813437	16.65834	12.63445	11.73141	23.87179	40.718	9.999507	47.15588	1.435672
42.29684	31.36562	26.96711	35.48043	14.66679	66.495	18.99908	16.69186	52.46204
159.1615	34.30042	84.98468	47.22987	48.65924	43.38476	122.9942	48.12407	99.84544
1.296876	0	3.20071	0	0	0	0	0.977775	0.059645
7.56165	1.952453	48.66751	162.0662	4.559108	53.83149	9.999509	0	45.71082
0.061576	2.935776	3.93767	0	13.86522	2.04786	0.999936	0.976062	0.078158
7.564488	20.58342	7.863007	8.031693	10.9961	12.74778	20.999	30.45361	6.696729
98.45527	107.818	99.19337	38.30948	135.9219	125.3577	77.99627	139.472	48.78955
33.69658	46.06662	19.76751	25.02975	64.29753	40.55154	45.9978	109.0379	30.34489
726.1718	752.7912	527.112	574.7888	354.4786	673.9288	438.9791	98.21177	981.0516
89.75231	100.9554	257.3381	96.72203	58.74685	109.6519	77.99627	104.1091	86.89795
10.01977	8.816473	8.676785	5.074383	26.67838	16.39688	11.99942	25.54582	10.58593
388.74	1212.499	521.503	2034.157	136.8051	2137.356	1817.913	1545.983	2280.426
92.26048	129.3808	58.66197	89.30656	101.9168	137.4299	178.9915	242.6061	135.5412
16.29925	325.4525	42.78823	25.00714	180.9558	18.39686	12.99936	37.31621	33.01401
13.77435	14.69741	3.883475	12.47601	14.67177	14.88122	8.999554	16.69319	8.013664
218.045	454.8152	157.911	143.2361	151.5073	529.437	185.9911	86.42572	207.897
330.0266	260.7321	156.3421	365.0801	135.8988	327.7696	228.9891	134.5556	373.4094
195.5112	125.4621	91.23974	171.4308	120.2981	154.5884	133.9936	69.72978	302.2575
105.8299	25.47696	82.56069	72.37849	29.35695	196.9465	34.99832	4.903751	61.8765
16.26821	19.59893	7.854271	15.43166	31.225	16.29659	16.99918	63.85757	8.019384
42.39779	67.63568	22.94612	18.36607	56.02014	8.425235	16.99917	40.26631	18.5392
173.049	87.2336	55.50184	135.976	34.86575	141.0969	157.9925	138.4923	134.1239
5.074112	15.68267	7.076629	13.26762	19.29769	10.62262	1.999886	17.68071	4.067914
0.072191	1.952623	1.506507	0	0	0	0	18.66826	1.434759
75.96962	30.37893	42.01996	32.41877	35.79169	65.52399	93.99554	45.17673	53.97859
51.02407	48.03211	30.92717	62.91204	22.0175	72.84567	38.99814	57.95546	62.99881
28.73148	32.34034	69.08896	39.83557	90.0344	19.83252	56.99729	24.54841	15.91373
144.5569	105.8539	68.98192	39.04311	190.0984	147.3902	220.9895	221.976	92.18893
21.28003	74.49189	30.87936	22.05383	66.10867	24.10019	65.99685	137.5145	36.94492
350.0853	492.0552	340.5194	396.6957	411.4289	450.1853	501.9761	606.017	660.2223
124.306	39.20375	53.98031	364.1642	19.25482	122.1089	21.99894	14.72561	64.40991
12.5574	36.26368	18.97693	29.48499	51.43415	11.27733	15.99922	37.32064	15.90685
102.0566	96.06439	66.6772	71.66837	54.17227	61.95127	66.99681	30.44178	123.4465
24.99599	25.47836	14.99901	25.03115	25.68799	54.13979	16.99917	49.1086	23.7853
44.94041	60.76507	61.05733	72.32575	40.37624	57.59954	502.9762	155.1894	71.12206
54.84993	54.8874	51.56078	53.88704	30.2777	104.1055	45.9978	26.51249	46.11273
327.6131	463.6363	69.77085	223.7978	52.31292	217.9129	892.9576	428.2411	543.0698
39.93255	72.53428	45.1989	163.5584	51.41412	77.66497	59.99714	42.22954	98.55792
12.55252	39.20767	42.08646	22.83201	14.66391	24.86307	32.99842	29.46286	35.53425
312.7396	773.3747	310.3343	187.5493	355.3956	227.1316	235.9887	692.4496	147.4511
233.0279	346.0024	188.8577	658.316	392.1381	473.691	1159.945	32.40511	464.3441
29.97173	40.18372	42.03628	9.492676	53.26238	29.82966	51.99752	130.6509	19.85192
237.8083	207.8063	153.2177	247.6346	100.086	128.9065	112.9946	92.32169	173.5873

288.713	49.00216	97.61458	141.14	90.90596	132.5147	110.9947	33.38723	105.2595
28.72371	53.9106	16.58873	20.5892	38.55691	41.27114	77.9963	68.75756	15.91051
64.88133	40.17941	160.2786	49.38741	58.7418	47.60446	18.99908	79.55006	56.69553
99.7431	304.8434	138.8676	152.128	272.7712	122.4334	216.9897	477.3616	148.7136
17.54258	44.10317	22.14428	19.83916	78.98518	82.65602	43.99789	107.0653	35.61877
10.0657	16.65625	11.03052	19.12413	66.16805	11.28151	30.99852	186.6778	15.89742
102.1719	60.76544	61.06121	79.72984	81.71884	113.2515	88.99575	66.78402	139.3884
6.331701	42.14718	14.20766	13.93297	12.82441	12.70528	43.9979	157.19	39.48902
65.94554	66.65925	38.07737	41.34976	49.59619	64.90747	30.99851	22.58464	43.42433
631.5859	458.7237	935.9946	2006.945	1068.115	859.1247	747.9644	245.5416	968.0234
33.64519	21.55897	22.97225	31.74613	17.42302	92.989	10.99946	0	43.35938
207.7899	29.39768	35.64479	39.7999	113.8917	66.18666	67.99675	81.52019	77.64871
199.3034	148.0054	364.5291	286.0118	135.9048	286.4731	117.9944	54.01332	276.1175
140.7744	56.84346	106.334	317.1822	23.84289	431.4369	182.9913	60.88947	164.3984
5.065603	5.874915	3.095339	5.082191	21.16216	7.76585	12.99938	31.45052	6.666185
573.0104	816.5049	480.2594	721.8563	475.7166	597.6716	537.9744	490.1133	605.0441
16.26911	19.59883	8.649142	16.1726	37.66515	12.00364	43.99792	42.23878	17.1914
1528.313	639.0797	750.1674	1539.054	850.4255	882.5939	1314.937	882.0103	1540.095
36.22984	44.09998	657.4882	8.765025	599.7656	35.49421	62.99698	335.911	68.53314
273.6658	97.03864	84.12771	141.9408	20.16972	130.4337	72.99652	4.903836	85.5264
33.72945	169.5844	45.97375	101.2202	106.5382	26.23721	20.99898	65.803	85.52735
493.2257	317.5794	305.6081	327.2635	140.4827	493.0187	113.9946	108.0341	536.5505
32.47145	61.7501	18.96719	53.888	37.6291	29.81525	34.99832	63.84134	65.78438
323.8393	220.5401	264.3502	294.0572	179.0672	447.5071	202.9903	228.8493	489.1
1010.278	556.7409	452.4188	2300.781	976.2362	1143.302	1443.931	524.4855	1954.466
54.86646	33.31916	22.93676	35.36876	92.76454	87.63063	122.9942	83.48659	21.17679
5.072121	5.874243	8.677996	8.052168	21.14654	13.51387	13.99933	13.74961	4.066177
672.4669	299.9364	391.3939	520.2213	169.8757	433.1686	330.9842	239.6518	401.1377
764.6992	451.8679	1218.091	367.1497	291.1108	419.5634	445.9788	960.6039	452.4727
1702.678	1085.072	859.733	966.3496	572.136	903.9637	937.9553	429.2129	1918.816
20.02425	39.20363	30.90083	18.36727	78.08905	32.69016	54.99738	91.35328	14.59827
37.35814	9.793935	10.23774	5.800159	75.38554	21.29683	15.99922	115.9349	14.57972
28.74485	149.0029	35.65182	23.53507	104.7161	23.39022	35.99827	159.1301	30.37138
83.5664	24.49685	42.78217	50.1278	770.5517	39.06	60.99708	47.13786	27.74916
10.06986	90.17883	0	5.802519	28.43825	7.003006	99.99525	52.05188	86.80079
43.46383	16.65875	12.63727	5.803243	2.721776	8.438047	9.999507	16.69329	15.86097
632.8378	863.5487	897.8724	1528.77	602.4508	943.9066	717.9658	480.2885	1743.825
194.0876	70.57066	92.88601	105.6823	44.05606	133.3188	62.99699	9.814384	130.1089
13.75673	9.795531	8.660947	2.110129	32.17712	8.447085	11.99942	25.53994	1.43621
227.847	208.7873	80.11847	153.6808	47.72379	215.2396	51.99751	49.1027	311.4332
234.0586	93.11338	147.6633	575.5154	33.02741	352.2675	83.99599	3.921953	488.616
22.4766	29.40392	21.38437	15.42709	25.70168	9.857892	38.99815	32.41193	10.64546
135.8224	179.3745	117.4409	87.09047	98.24311	126.0248	164.9921	200.369	96.11739
33.66416	28.42139	8.645857	12.45532	17.42067	17.70811	29.99856	66.79803	11.96406
15.03714	41.16917	46.0764	8.01724	79.97383	4.15359	10.99946	48.1311	4.075949
25.00866	5.872914	18.96705	5.0639	49.57583	4.157338	6.999647	16.68981	18.54546
8.744295	6.857249	2.302566	1.375838	13.79483	6.343924	7.999612	6.871709	9.229872
149.4762	103.8962	88.06331	121.8709	154.2933	93.96081	256.9878	190.55	115.7959
1091.067	509.696	1699.949	894.0283	540.0053	537.0814	1378.934	321.172	631.3646
12.56003	15.67458	7.061622	6.544496	15.57778	3.452157	6.999648	17.67186	21.18134
2.589498	4.89322	16.66027	3.587798	26.66248	1.310953	1.999886	11.78238	0.096245
120.8388	93.11438	69.00381	53.09872	92.74224	95.4052	95.99542	103.1281	76.37377
0.062188	0	3.934461	0	0	0	0	0	0.078959
39.92688	14.69448	31.6857	35.38277	49.57865	53.38615	80.99615	59.91259	38.23323
25.01295	80.37502	48.36775	16.88063	87.24981	36.94153	91.99562	72.68074	10.65699
592.9459	434.2202	366.7068	548.168	677.7795	618.3238	850.9595	1132.483	502.4999
310.1835	265.6287	100.7359	58.99062	256.2183	149.4938	356.983	225.9009	155.317
555.4225	476.3801	271.4768	371.6473	212.1295	436.0413	248.9881	241.6168	268.3658

9136.757	5874.286	4266.994	2861.314	1800.02	3225.492	5255.75	9901.527	6693.6
21.28232	77.42965	155.627	326.8892	34.86487	130.3609	216.9897	95.26957	97.39061
191.9263	1210.559	246.8225	172.0371	566.6562	74.67226	289.9862	871.2177	382.8149
42.31014	49.01603	9.44296	17.65086	45.02052	19.15174	28.99861	64.8376	22.43294
2678.028	1561.444	2119.674	1917.983	830.2029	1721.083	1139.946	732.7116	1787.491
140.8146	115.6567	190.5145	97.43304	202.9636	58.29376	118.9943	122.7695	86.92642
36.14153	60.77978	42.08224	20.60561	37.65058	18.42445	15.99922	27.4977	34.231
122.1233	136.2437	53.89936	81.18059	163.4718	171.6805	127.9939	290.7401	79.02742
0.076713	6.853662	40.61629	5.805464	27.56504	0.605628	3.99979	3.921467	1.436107
3.835847	41.16336	25.32801	5.063043	10.06742	16.26458	4.999742	13.74321	13.28642
161.8666	155.8551	142.1324	187.778	35.78409	247.45	53.99741	15.70746	194.5063
62.38231	114.6768	123.7909	127.0113	83.54439	234.4094	160.9923	59.90665	180.2182
8.81332	38.23383	5.471634	7.283085	45.96531	5.579601	7.999601	58.94908	26.3111
2.586887	17.6477	6.286044	6.569454	8.245797	10.64532	10.99947	35.3819	6.670596
62.31347	107.8276	119.9706	65.72949	46.81731	140.5384	47.9977	8.832183	42.18164
29.97121	63.71408	128.8225	33.17388	34.87779	38.40327	33.99837	9.814364	35.5932
142.0864	155.8443	254.8127	249.6904	193.7626	176.5894	327.9844	147.3236	465.4679
27.27651	10.77791	14.27382	6.559349	12.84661	7.746296	14.99928	57.00548	9.289564
386.0359	179.3713	184.1418	285.2151	174.4791	304.9489	253.9879	131.6088	520.5588
31.16248	29.40337	15.01094	15.42438	32.13842	37.76904	28.99861	71.71509	23.7452
53.62076	91.15947	102.4487	129.413	49.57182	99.06483	41.99799	55.9813	103.845
46.19075	140.1586	2810.759	3289.189	135.8867	1766.244	631.9699	113.9269	1006.156
8.803033	90.23601	5.475051	2.110127	10.99693	0	1.999886	0.975459	0.09746
82.22055	31.35852	6.265457	102.7341	154.344	149.7717	210.99	102.151	89.43129
398.5864	172.507	331.0324	457.3819	176.3051	503.0249	296.9859	118.8385	331.4748
86.00798	73.50857	51.52409	59.75302	138.6813	122.5129	126.994	229.8448	69.80839
90.96209	99.97829	41.20265	27.96495	44.05285	96.85811	119.9943	206.2742	48.77679
235.3799	148.0059	126.1784	120.3619	184.5967	118.893	218.9896	238.6774	138.1663
295.2975	138.1985	244.4367	134.3508	748.5142	291.2653	655.9688	776.9223	168.4909
66.03073	65.67185	61.10839	64.26005	142.4207	20.54116	52.99747	72.68292	74.95775
12.51545	15.68017	3.884997	8.777887	23.88964	22.10472	17.99914	32.42085	6.69393
164.3931	119.5813	55.49375	99.68715	74.36499	101.1003	150.9928	207.2502	111.8464
23.71808	24.50097	24.5705	42.15752	28.46069	52.11963	44.99787	17.67407	66.83945
16.2925	5.872815	18.97269	5.800192	47.74879	11.98848	8.999551	19.63709	5.392363
15.04749	12.73422	3.092256	75.44544	3.640301	162.9442	7.999599	4.903563	34.27268
56.13033	82.33289	106.3781	27.96539	75.29189	95.43519	70.99661	51.06824	40.89361
67.36884	86.24893	192.8744	61.9485	67.92694	46.1784	187.991	100.1773	244.6575
215.236	44.10184	63.46234	77.54045	30.27429	216.1567	184.9912	74.64416	117.0078
20.01208	27.44062	15.79972	62.15192	26.61303	82.1373	40.99805	45.18173	27.69578
43.5185	17.63805	19.79883	13.20823	68.04368	19.16352	23.99885	100.2192	26.33661
22.46146	11.75519	7.855319	15.43635	10.07128	37.09897	35.99829	10.79754	17.18357
97.22504	130.3629	64.227	430.3823	146.9392	225.2043	194.9907	85.44564	251.0752
4859.249	3074.857	3398.526	4357.52	2194.023	4227.901	1931.908	1078.443	4837.665
11.316	423.5224	34.06232	4.327213	35.78898	3.447617	23.99884	20.61868	27.74457
577.9434	564.593	593.058	228.2033	446.3365	546.4155	75.99636	300.5474	281.5651
97.17252	37.23959	39.61533	25.74811	80.80609	29.08786	12.99936	68.7496	43.51942
169.44	224.4652	157.1514	158.8018	190.1035	108.9026	161.9923	172.8644	156.5756
18.77532	28.42019	15.00131	14.67144	25.69049	31.99106	66.99683	60.89969	25.08785
81.06684	30.37736	35.63427	247.498	183.6637	83.23109	95.99541	109.0173	81.67667
62.35661	60.76575	31.66902	90.83474	25.68025	274.6035	81.99609	75.62491	67.16848
29.9555	43.12719	34.89322	14.67206	26.61028	17.70106	17.99913	35.35597	15.90597
62.30682	27.43789	57.12818	52.40588	35.79104	94.09963	64.9969	2.939602	115.5965
6.284744	2.933328	9.517417	0	0.887797	0.605115	0	0.975517	1.432838
0.064935	0	0	1.382189	0.888946	0	0.999935	0.975799	0.082565
3.834868	8.815526	1.505813	5.069242	5.479038	19.24463	11.99942	27.5082	4.070801
16.28956	62.73629	15.79569	28.00067	24.77074	18.41298	18.99908	17.67272	27.71176
21.21485	12.73594	12.63326	11.73006	5.476561	55.80146	11.99941	3.921394	28.92663
163.2023	140.1639	164.3134	133.6733	172.6543	188.0671	156.9925	169.9188	125.0259

1122.273	778.2705	3.103381	741.7389	50.4779	844.8617	1461.93	359.4771	2381.53
1.334536	4.892993	7.852322	1.381691	89.08009	2.028759	1.999887	5.885904	1.429691
0.035646	0	1.590339	0	0	0	0	0	0.044654
34.78421	2.932459	3.090288	34.82566	9.154946	114.2914	9.999508	6.868432	17.15318
1748.898	1760.424	1658.382	2310.326	933.983	1806.565	1435.932	2745.246	2046.585
2.590293	3.912556	49.36356	25.1112	13.75149	71.65409	92.99568	25.53672	39.34089
47534.43	21663.19	35596.81	47949.91	19807.89	42618.33	29734.58	16666.87	45623.33
74.7874	76.45124	109.5617	45.71381	76.21158	51.19592	183.9913	89.378	55.33984
224.2536	151.9236	200.807	175.7695	212.1352	212.2344	331.9842	415.4756	244.6786
144.5682	139.1811	174.6045	107.7703	196.5229	144.5253	322.9846	270.1045	119.8023
822.0162	213.6743	190.4475	282.1451	235.0805	668.2618	306.9854	400.7329	255.2763
1108.676	140.1587	4696.936	3834.056	2275.822	1258.005	835.9602	486.1796	877.4027
223.0204	197.9937	42.77899	175.0217	218.5614	495.234	222.9894	400.74	200.0045
22.46171	44.11613	90.07035	12.46836	18.34774	8.43331	7.9996	23.57024	22.41193
12.55911	31.36068	17.38348	13.92908	75.33376	34.83755	38.99813	89.38921	9.340824
6.32761	4.892755	18.21623	57.87973	4.558084	47.19393	13.99932	0.975501	28.91642
134.4547	53.90475	109.5751	100.4864	39.46114	121.159	63.99694	45.17498	72.39724
120.8693	43.12014	76.93676	142.5716	34.86404	257.3104	187.9911	152.2399	169.658
25.01078	54.88673	11.02554	41.29376	73.46861	38.37437	51.99751	136.5363	53.99352
763.1643	352.8744	528.1005	414.5973	51.39475	463.2219	231.989	17.67211	471.9858
89.74691	143.1083	127.0053	115.9614	80.79588	145.3314	176.9916	199.3918	115.7861
6.319431	8.815369	6.27342	8.037948	2.721978	3.443928	59.99723	4.903956	0.096659
3281.002	1750.617	1529.723	3794.623	1560.332	2841.643	2161.897	1492.932	3856.375
97.20237	148.0113	228.768	145.5733	73.44855	494.4263	98.99528	56.96111	126.2728
6.329196	44.10206	130.2646	53.1219	135.0356	23.38746	22.99889	21.60078	26.43518
26.17946	57.8463	22.9902	60.05917	5.476344	41.40317	19.99904	0.975527	124.0657
396.133	325.42	380.2446	602.9512	149.6661	494.4133	507.9758	229.8283	908.5848
5519.372	1811.387	6949.697	4269.626	3882.067	2874.352	9293.558	2791.398	3581.594
173.2301	193.0914	197.6096	160.2276	258.0522	351.8987	279.9867	259.296	182.9324
69.74731	28.41851	10.23183	26.5009	28.44129	113.4311	34.99832	93.31426	77.56333
10.05674	6.853273	2.297862	1.375429	10.99124	2.02095	0.999935	1.957303	1.435532
134.5162	68.6075	162.7918	262.5204	62.42449	185.3261	54.99737	18.65402	181.4
93.49962	158.7896	65.81194	62.69676	148.7712	165.9696	115.9945	150.2741	92.17025
38.69412	38.22103	41.21837	10.2312	95.52794	27.67108	83.996	135.5539	21.17494
11.26456	4.893325	14.26656	4.329368	22.98031	12.05484	8.999558	20.62872	14.50115
18.74814	16.65741	3.090288	7.280265	42.26848	25.61007	28.99862	78.59844	11.95252
6.275784	4.8956	3.103202	1.376978	15.65338	3.464455	13.99935	23.5938	1.431437
16.27266	15.67649	8.648222	5.061511	11.90821	11.28659	85.99597	44.20298	9.33351
0.075062	1.952466	13.46427	0	0.887539	1.310913	0	0.975443	0.095948
21.27448	43.1235	107.2656	23.53988	40.38725	44.09759	61.99705	15.70762	11.97233
70.99176	61.75091	35.66127	46.49004	64.28524	70.53516	70.99662	125.7338	60.52673
21.26326	21.55722	23.74739	103.6622	25.68952	69.18287	36.99823	40.26754	70.92725
22.48116	13.71542	15.01093	24.32552	25.69982	27.73898	28.99861	58.94103	8.021806
144.4235	107.8214	74.58218	175.2397	37.6226	94.01043	104.995	54.99744	101.2873
19.98465	20.57988	15.81566	6.540894	13.74877	24.8991	43.99792	46.17064	22.41423
23.60531	10.77729	35.09298	31.93582	17.44935	15.65144	14.99928	9.817106	6.687447
28.74643	75.4732	76.19667	54.60919	57.84129	94.77098	26.9987	40.26408	40.87876
39.91002	51.94902	128.8298	36.87953	74.41019	41.264	61.99705	11.77882	30.34645
28.67874	7.833333	143.483	74.12854	2.721997	60.72611	40.99806	257.4454	17.19807
13.80734	12.73398	21.34919	0	14.65936	0	7.999599	11.7787	0.103898
13.7857	24.50186	11.03617	66.73651	11.90921	50.00123	35.99829	5.885751	40.70301
120.8643	150.9494	119.843	275.0115	94.57385	188.1172	128.9939	52.04934	215.6072
64.77431	40.1828	12.61383	89.47322	55.09502	44.10382	1157.946	30.44209	43.47596
416.6863	98.01734	59.47669	150.0476	14.65919	207.5004	103.995	8.832325	437.1621
10.07007	6.853	764.1834	10.96987	45.90095	9.13747	12.99936	7.850013	13.28739
62.35056	130.3689	69.81194	62.72709	69.77892	66.88992	57.99723	57.94422	123.6008
85.65316	26.46186	89.18259	74.12095	12.82699	61.43691	11.99941	4.903513	79.9
0.066022	3.91567	0	0	0	0	0	4.907601	0.083993

860.8094	129.3767	16.59525	265.856	513.3515	152.3199	30.99851	319.2059	106.6752
1.339685	0	6.275778	0	0.887538	0	0	6.869136	0.095934
216.7256	97.0324	192.1119	107.7875	154.2778	61.85869	89.9957	148.3084	169.7076
274.0397	93.11033	119.8012	105.5512	216.7303	309.2004	216.9897	133.5728	135.5793
19.95729	28.42842	48.5995	17.68687	18.35546	17.04282	13.99932	12.76338	8.010207
13.78011	37.25198	11.03942	5.801864	32.15204	4.153643	2.999838	14.72757	5.391
56.10237	89.19952	42.8077	59.80059	96.4462	75.50355	113.9946	137.5184	76.29382
287.8467	244.0592	552.4777	333.101	495.9093	884.7551	687.9672	853.5276	286.8677
122.0442	53.90424	56.30176	50.15145	75.29236	220.3856	48.99765	58.92657	81.59934
84.8101	227.3992	588.3585	267.3994	279.1786	147.3518	182.9913	78.56796	185.5613
10.06099	10.77449	12.62699	8.760016	59.75196	27.03825	29.99857	52.06503	9.332058
379.7751	236.2273	339.8681	450.9091	85.38001	358.47	121.9942	62.85315	303.7398
41.20707	23.51686	145.9809	1995.855	57.82455	2218.031	1551.926	126.6954	974.6
9910.381	5978.182	4145.502	3153.867	2433.712	3016.74	4753.774	13398.14	9428.132
79.71934	21.55602	60.30031	139.0599	94.60746	59.07639	68.99671	85.4522	102.5205
3066.825	1365.399	2134.652	1606.814	1219.596	6100.159	1733.917	1462.483	1882.317
27.47048	192.1736	15.00215	22.07777	65.23145	5.577106	22.99889	37.32098	38.18389
100.9863	332.2915	258.8176	210.5416	271.8554	202.9963	177.9915	94.28416	284.0554
79.66049	44.10496	28.51054	60.58392	24.76716	87.72464	36.99823	42.23079	91.94051
58.64775	69.58569	144.4409	50.12774	46.80319	14.12746	58.99717	59.90658	157.89
73.57734	291.1308	137.3202	91.53921	120.2963	93.241	179.9914	133.5767	67.19456
1.264709	0	1.589562	0	0	0	0	0	0.044909
3.837902	63.72143	112.2503	16.89972	43.16876	3.442819	11.99941	35.35749	18.52019
20.01364	24.49909	144.9001	13.93394	50.5208	16.27596	14.99927	9.814513	14.59095
13.80275	39.2044	46.02904	36.14882	29.36578	112.1254	63.99696	24.54903	25.09305
12.56155	97.04427	45.99399	79.07174	69.79623	59.08961	55.99733	50.08826	88.07274
341.1758	156.8273	122.1995	266.7721	87.21806	339.9529	240.9885	229.8355	381.2155
113.3267	97.03871	65.84415	87.15769	43.13575	207.5655	68.99671	35.35216	98.64841
0.081192	93.11953	3.887139	1.381122	4.559067	2.737836	4.999743	14.72528	4.071002
726.2689	435.1976	515.9452	586.5372	728.2707	1256.658	1235.941	664.9421	602.5044
335.1393	384.2315	221.4146	381.9178	165.2781	604.8394	492.9765	317.2446	370.9792
21718.06	23604.96	21004.08	12789.39	4107.94	16094.32	12329.41	15703.34	21948.75
37.34323	3.912528	20.59064	13.20492	14.66676	52.13833	8.999552	15.70939	44.63226
97.21823	100.9554	61.84673	104.119	150.6184	205.2508	96.99537	133.5774	93.46563
7.575975	4.892646	55.65655	6.539004	34.89679	0.607258	1.999886	5.885685	4.076036
33.59854	26.46429	18.21085	13.2128	36.75431	18.45579	14.99927	42.2412	25.01621
11.23513	7.836596	8.68834	8.811045	4.562633	7.763182	10.99947	42.2665	4.06141
5.085039	21.55758	23.7518	19.85577	24.77223	15.55981	22.99889	53.04051	17.21719
49.91982	71.54796	62.6458	47.18191	56.91127	88.98051	92.99556	184.6583	39.58834
2.589795	29.41369	7.069884	3.587091	29.4215	8.452188	7.999603	5.886364	31.41579
113.2613	29.3983	54.73638	59.06306	64.27845	55.50981	96.99539	46.15849	92.02328
84.74147	75.47063	34.05322	43.49326	87.23661	120.4155	56.99727	85.44844	56.65602
7.571618	18.61937	30.18101	8.023131	13.75042	15.59185	22.9989	53.05168	13.25403
696.2507	466.5697	414.3703	607.3388	281.923	691.0733	490.9766	291.707	645.7549
10.06551	8.813478	31.72759	19.12486	26.61594	41.32915	23.99885	93.32926	15.89679
48.54013	14.69544	25.35407	22.08911	15.58285	45.62291	18.99908	17.67342	23.75874
547.9989	195.0517	146.787	94.45614	415.1212	104.5968	353.9831	524.4981	117.1921
56.1099	8.813319	8.645007	104.221	19.25174	104.0586	747.9648	21.60087	672.5809
120.9153	211.7182	98.35919	41.25935	179.0681	34.78086	70.9966	285.8197	55.37972
31.19166	49.9915	27.73347	42.10135	20.17687	68.50766	30.99851	55.00607	44.72066
6.314318	7.835318	11.8704	3.58911	8.240889	19.26863	15.99924	23.57832	6.684126
69.70037	8.813283	9.43844	74.6996	22.93102	255.3163	13.99931	80.54708	17.22345
300.1041	198.9788	169.8652	237.1952	117.532	272.9185	108.9948	106.0716	400.9047
10.06991	96.06145	174.8496	19.0985	145.1587	12.69857	6.999647	17.67195	29.05857
17.54432	97.04008	28.49635	13.92465	189.2649	10.56225	87.99581	219.0515	10.65678
41.20504	49.98233	71.38797	487.5521	117.5501	128.9407	212.9899	27.49385	117.0815
23728.53	17417.99	12600.16	17327.92	9449.331	16774.64	11403.46	5511.091	23256.16
472.1109	551.8495	408.022	654.6526	224.0599	641.196	351.9832	107.0516	652.3155

42.35447	25.4797	39.68246	14.67513	16.50094	29.86013	28.99861	66.79712	26.38628
20.00611	19.59761	38.10271	16.16069	42.25142	84.33732	27.99866	11.77927	55.14214
5.063912	3.913516	2.300184	9.566322	4.563459	21.5301	13.99934	9.819575	9.257998
59.86253	45.08166	44.38163	33.88011	120.318	39.78252	107.9949	127.6882	63.22071
23.77306	21.55573	20.55249	43.4767	304.0168	73.97221	10.99946	114.9115	19.86376
214.3464	623.4036	1193.388	490.5476	956.0995	312.6437	386.9816	256.3469	307.8775
23.68496	4.892758	14.22959	16.18835	11.91189	75.97039	16.99918	13.7454	26.30706
188.1461	162.7064	184.9256	151.3789	243.3689	238.6171	201.9904	192.5068	210.5004
173.1154	174.4766	134.9434	228.4041	104.6803	245.9181	88.99575	99.19769	184.0865
105.936	132.3235	35.63578	91.53911	30.27177	160.286	159.9924	152.2399	121.0604
1072.591	1242.877	489.7252	5503.757	1436.352	1553.609	1676.92	452.7844	3999.638
124.6126	148.9872	147.6385	66.3939	117.5369	56.16127	72.99651	110.9838	148.6611
27.49111	15.67478	37.2551	21.32268	40.38933	42.67726	49.99761	47.14219	22.48182
17.52629	16.65603	18.18697	3.585929	7.312642	0	4.999742	2.939377	30.31185
100.8896	102.9211	64.2562	76.79849	75.29585	96.16867	66.9968	73.66173	92.0825
11.28544	10.77585	5.473882	26.63478	9.155494	23.51981	31.99849	23.57344	13.23446
357.539	94.08975	612.9252	177.9542	140.4811	498.6791	300.9857	434.1301	157.9626
0.041287	0	0	0.676651	0	0	0	0	0.051866
130.8231	138.2052	313.7504	75.2709	95.49158	88.96248	102.9951	140.4526	94.78688
235.2572	257.8073	234.3337	44.22584	7.313871	36.21152	18.99908	58.92564	60.61045
10.05125	13.71712	11.84209	5.803828	3.639821	18.47317	25.99876	7.850717	119.9144
0.068887	99.17443	0.717169	0.641742	5.491307	0	0	6.872692	0.087769
21.23647	14.69601	13.42091	5.800331	33.98075	17.72175	18.99908	24.55156	13.26746
43.60943	38.22495	54.80403	91.07732	10.06805	82.8143	32.99842	26.51428	55.2
764.3267	50.96141	1.514535	332.5527	8.23344	362.0095	256.9878	142.4137	345.8058
164.3392	194.0893	84.90744	179.6533	45.88966	155.379	88.99576	41.24535	224.6614
58.50706	55.8732	15.79624	141.5501	2.722533	108.5793	39.99809	39.28549	87.91822
92.23491	121.5419	83.30065	47.18033	66.09706	250.9444	108.9948	84.46414	88.20415
58.57935	65.67144	38.83698	33.15675	67.95748	75.51917	48.99766	49.10585	47.42544
156.9653	104.8754	84.08263	81.1814	67.01235	189.5155	116.9944	34.36917	143.4038
531.9156	583.2133	435.7928	302.0872	505.1081	209.3204	332.9841	433.1448	382.8359
69.85403	78.40777	178.6014	67.12672	179.0799	61.14502	244.9883	313.3281	26.44269
63.37727	42.15087	41.30509	36.20431	25.69957	38.48413	46.99777	55.01045	34.20793
13.69077	6.85584	7.089253	3.594721	11.93553	4.88651	5.9997	57.01974	9.262377
16.29606	62.72397	152.3765	8.76192	864.4246	4.877366	10.99946	95.26653	22.49509
84.79842	223.4836	372.4362	111.4748	220.4142	154.5213	213.9898	624.7048	160.5306
59.77108	61.75425	39.65606	36.14364	53.26642	21.97893	62.99701	137.5311	82.73123
20.01475	22.53813	15.79824	16.15428	41.32315	13.4193	10.99946	23.56727	11.9664
8.820678	11.75451	40.50053	19.86978	14.66458	14.14052	50.99759	15.70886	18.51405
117.172	126.4386	157.9326	87.81757	179.0733	180.1802	203.9903	169.9164	88.24728
13.74507	24.50928	42.28379	5.068683	12.84091	5.587592	6.99965	22.59317	17.1178
97.06239	20.57614	60.32473	45.75435	58.77229	94.85226	93.99554	88.40281	44.78616
8.810608	20.58171	28.60255	14.70791	45.975	7.009262	1.999886	16.69351	4.07493
333.871	448.9287	398.5142	658.401	276.418	649.0929	333.9841	328.0506	409.068
0.07692	1.952462	15.83343	0	0	0	0	1.957272	0.098418
90.76576	34.30259	34.88901	77.69409	27.5284	72.76846	45.99781	26.51397	98.39163
28.73118	43.12457	47.60208	39.8361	34.87647	60.55282	44.99785	91.35154	26.41616
275.2821	267.5924	125.361	114.4214	175.3946	104.6093	122.9941	447.8914	380.016
667.5307	119.5752	150.7558	605.9371	152.4226	687.6087	399.981	270.0999	656.17
60.93153	14.69548	78.76218	30.24978	9.150069	45.62609	15.99922	9.814632	65.59911
16.22608	5.873453	7.06825	9.520654	43.23901	6.300587	3.99979	34.38648	4.072555
108.4795	197.99	309.5264	112.1844	111.0901	90.3455	105.9949	187.5916	94.83973
2.590055	116.7263	12.65081	1.374358	10.99705	2.020169	2.999838	3.921526	8.003463
11.31548	27.4384	139.9332	191.0813	7.312536	144.2122	62.997	15.70771	72.3068
334.9527	347.9793	270.7619	203.9113	77.11402	281.4704	165.9921	82.49783	364.1378
7.548646	2.93267	9.476331	2.849791	6.40111	6.312598	0.999934	8.835053	2.754752
46.16691	7.833222	257.5481	219.0069	36.70588	158.3218	127.9939	20.61856	116.9909
2.583491	13.72601	4.696252	0.641114	3.644369	1.312057	2.999839	4.90537	6.651747

78.45186	95.08429	62.70144	62.04037	57.84938	74.10092	81.9961	100.1902	50.04239
257.8656	365.616	126.1522	93.72394	368.2966	65.41403	148.9929	268.1388	79.05048
13.79155	61.76384	18.99182	10.97894	11.9077	17.71856	27.99866	30.44635	19.81869
24.98685	54.89324	20.56882	30.22903	38.56247	53.45759	30.99851	44.19808	31.6367
19.87197	23.53312	15.08539	4.332902	40.5231	2.021007	7.999606	31.44567	19.65823
52.32072	89.2081	65.13206	45.78015	30.28401	35.55352	52.99748	89.38948	23.7842
190.4668	91.1545	133.3884	201.8425	53.23838	310.2797	42.99793	15.70746	359.8318
149.4617	72.52803	169.1311	156.6561	101.9273	128.2121	159.9924	122.7731	197.1875
67.32105	83.31382	30.87718	25.74862	80.80741	46.91812	78.99623	161.0887	34.32215
100.9584	66.64582	239.8339	151.4481	173.583	30.50716	39.99808	48.12033	54.05248
81.0128	136.2502	61.06715	58.28546	74.37189	107.5623	93.99552	140.4578	57.97129
1728.915	1377.169	916.0754	1200.542	686.0148	1007.942	834.9602	328.0457	1918.938
0.074265	11.75903	0.716387	0	6.400827	0.604836	0.999934	1.957301	2.754989
651.5498	665.5441	662.8196	438.7511	657.5476	1300.807	1426.932	2775.708	630.1321
0.052736	0	0	0	0	0	0	0.97702	0.066635
39.90158	31.36086	58.76492	70.25556	37.63846	50.57065	21.99894	15.70797	21.1595
446.0091	477.3484	446.0991	156.5138	586.8438	222.1387	705.9664	890.8548	278.9678
132.0131	60.76571	69.01063	84.91445	86.31468	93.28006	84.99595	103.1292	101.3076
59.7517	49.00951	19.77151	15.41096	81.78039	22.69846	47.99771	101.1817	23.77839
26.24281	15.67491	34.08018	55.40245	76.24672	32.68653	114.9946	43.21348	35.59442
2.528922	0.97421	0	0	0.893278	0	0	0	0.06868
21.17199	16.66067	17.44335	11.74989	17.4409	8.447863	15.99923	15.71229	9.310521
43.65482	13.71426	35.66193	7.27715	98.29932	31.24747	35.99827	217.1012	6.70869
15.03481	14.69576	42.89713	10.23785	39.4964	5.577215	24.9988	65.81875	18.51177
42.43244	78.41397	34.85423	44.24594	60.59733	85.48157	67.99676	109.0273	52.69501
27.51097	87.23057	175.4455	111.4957	240.6441	54.02245	127.9939	90.35639	85.59948
1626.828	1488.909	1291.589	2290.39	831.1211	2804.034	720.9657	622.7051	1993.966
11.29164	23.52394	17.42483	16.93847	11.91317	75.30191	5.999695	2.939287	23.68483
17.54482	12.73411	100.7796	152.9489	148.7831	40.48565	32.99841	30.44043	63.24582
3.838245	26.46279	22.18596	1.376014	27.54397	0	0	6.867991	1.435054
13.77969	39.2136	17.41399	8.022686	49.64282	1.312454	9.999506	18.65777	15.87117
53.2578	13.71818	19.03887	37.84111	16.51919	22.81242	6.999649	0	35.35112
26.25348	43.12295	32.47621	26.4964	55.08935	61.9428	68.99671	89.38268	21.17372
332.467	110.7556	351.8032	314.1045	323.313	190.1853	296.9859	200.3676	122.4104
78.47618	48.02422	26.91072	43.51326	44.05944	64.07548	61.99704	72.68154	31.68312
211.8388	170.546	190.4574	127.7072	320.5079	384.666	404.9807	390.9135	181.6249
32.47042	46.06436	35.65856	35.37932	69.79766	23.39441	55.99733	73.66532	27.73816

TCGA-DB	TCGA-DU	TCGA-S9-	TCGA-HT	TCGA-HT	TCGA-41-	TCGA-06-	TCGA-19-	TCGA-06-
183.2475	182.6793	126.0343	179.1517	148.9665	85.2002	53.87329	61.47001	57.15486
46.06193	33.82245	78.15845	6.974122	25.39794	13.1389	44.41487	17.40886	1.16441
196.2644	159.8788	144.5136	42.10414	98.21434	30.46069	92.42896	55.65755	46.94683
5764.789	6129.525	2733.402	1532.324	1840.519	1649.499	2574.508	1286.333	1477.021
54.07358	46.45001	139.9572	287.2588	72.85784	88.05958	140.3638	173.822	42.06287
0.000328	0	0	5.290347	0.024511	8.367419	3.521985	6.719977	8.163281
10.01409	22.78847	20.11357	79.12438	13.28141	87.53842	59.5955	44.91465	64.01772
175.2367	182.6801	172.1347	54.3967	174.3324	50.63966	129.8588	39.01099	37.07559
13812.66	16448.31	8684.948	3835.692	14638.92	5833.45	7164.387	3074.971	6055.309
31.04241	40.54971	124.4648	30.69264	41.95675	34.74283	29.30283	39.86689	21.78679
15.02057	17.7395	28.30286	14.02215	36.35955	13.08794	32.68754	18.27534	1.067342
1638.213	1579.748	1642.601	449.5638	1137.62	586.9372	396.5929	323.399	284.7334
103.1398	109.8959	107.1818	204.6203	84.99778	124.086	254.9371	360.1982	62.22444
0.000347	0	0.315788	56.32783	1.137701	4.361426	9.266622	10.94298	0.617249
75.10178	87.08029	125.67	57.04099	77.26046	40.53859	19.95778	49.83384	41.94219
10.01409	9.238095	7.203459	183.0518	8.870321	154.5827	25.77334	170.1304	26.1864
4268.767	6017.129	2564.828	1248.684	1233.647	1760.459	1739.362	1439.354	1547.378
4.005968	25.32089	38.99005	29.82402	166.4064	28.95825	18.77885	25.72487	6.649463
136.1838	48.1474	34.41321	112.4016	55.20474	159.8932	79.5741	414.5126	155.5175
357.4835	205.4329	219.8727	419.7197	402.7754	774.1911	287.8071	1027.845	2050.058
1507.036	1608.522	949.5184	684.9235	856.262	546.565	611.7966	294.2951	309.9509
4.005969	16.85326	20.58934	1.709426	121.2499	0.143233	0.055355	0.770219	1.217532
112.1517	197.9565	45.30329	354.9646	367.262	20.37491	1.230283	4.92272	31.99386
191.2588	447.4059	182.8421	190.5241	729.2425	23.25245	42.18243	79.75643	132.8759
210.2829	142.1222	94.93521	31.56414	97.10286	63.54696	32.81889	48.17324	31.91575
13.01809	24.49397	20.63831	27.20462	29.80213	28.88578	12.9294	8.245878	11.58494
107.1452	131.0352	145.2786	306.5076	119.2004	227.7923	342.6479	307.7195	356.8982
317.4295	353.4278	1213.736	825.441	655.4514	1345.036	1276.096	743.3674	320.0232
34.04611	48.23383	44.0749	10.48832	51.8321	1.60925	8.258363	3.256842	11.62954
30.04112	78.63521	95.48299	85.17728	64.01742	72.14403	88.89516	40.68811	16.84135
20.0276	58.3401	28.50753	60.58641	23.2113	57.70451	94.68333	81.52463	21.75589
40.05446	82.06407	62.30733	163.4991	56.28256	34.73249	15.27693	46.53508	26.68516
10.01393	7.55617	13.24834	22.84731	13.26225	11.64546	3.577554	28.33044	6.437035
329.4457	732.325	649.2552	666.4887	816.5331	775.7121	548.6311	2140.582	441.0298
94.1277	165.7193	237.8903	160.6741	188.6977	164.4511	225.7547	233.663	47.21242
2224.006	31145.2	6865.314	4825.357	6957.049	1356.88	3279.887	946.2236	1052.876
8.011357	0.788171	21.19078	24.58004	5.559589	20.25997	23.41002	65.89109	84.03131
52.07064	76.95982	63.74727	13.11991	120.2207	27.56071	30.47362	41.52827	16.81506
19.02622	9.236587	20.58445	53.56868	17.69486	53.34001	61.97068	164.9621	79.29901
19.02619	10.92945	30.55061	37.74563	14.38518	57.58203	39.78468	121.6461	26.35908
163.2206	34.60592	12.15264	122.918	123.6008	351.373	246.7393	675.6882	409.3301
23.03162	36.32442	45.41997	87.8622	55.17508	39.0273	24.62416	75.7078	21.70276
7.010043	8.390826	14.12699	309.5232	14.38807	44.79116	136.7034	182.3646	41.42997
87.11803	105.6873	280.9075	42.98049	120.2779	36.2267	28.14514	34.85435	26.97118
222.3006	191.9293	300.205	174.7174	193.1217	356.0426	259.6862	330.9691	167.9339
10.014	10.94306	33.2913	23.70998	17.6722	15.95373	7.084021	22.44541	1.086378
35.04778	34.62321	28.99782	301.5852	44.1613	37.61558	19.95371	59.02273	60.97438
4070.5	4733.244	3948.636	808.7192	7318.83	754.228	934.6545	654.3496	845.6936
610.8254	654.5583	1126.001	330.1466	829.7329	202.0212	65.57588	158.7535	475.6167
5.0073	5.012742	42.52318	2.584776	8.8609	18.75387	8.245254	5.755558	6.43679
7.009966	1.629876	22.8132	27.25866	14.36232	15.91406	16.39399	15.77771	6.438746
355.48	391.5988	325.9962	91.27859	276.9545	158.7153	181.3406	134.6547	172.8204
47.06394	29.54054	85.10757	83.43208	28.72782	59.18188	40.99045	74.00322	21.82649
5405.304	10824.64	7598.545	532.9818	2940.627	772.9728	555.6761	369.9658	598.0814
25.03378	10.94282	16.2137	27.23506	16.57202	5.942777	15.24571	14.92691	1.087842
29.03971	32.93234	74.24155	53.55214	83.83562	24.67474	33.97301	19.05709	11.75867
16.02198	32.16418	17.69978	18.41801	18.77665	4.505631	14.08468	4.91944	6.545968

7877.645	6838.918	6828.937	1708.819	2300.654	2530.441	1755.777	2450.489	3109.326
135.1832	34.60592	112.0666	451.4073	193.1246	586.4835	678.2573	860.8986	263.2905
23.03145	11.77921	27.61405	23.68204	26.50056	27.46553	63.06263	37.42397	11.60522
64.08645	23.6237	15.12282	50.92479	67.29204	71.99004	28.12632	66.54789	55.61915
134.1774	1.63072	4.237491	11.37021	4.456675	23.14691	108.4192	59.99388	135.0183
241.326	182.6417	212.1395	131.6871	200.8302	105.4003	67.91182	141.3183	127.5286
8.011339	10.9423	31.77087	25.47077	5.55896	3.061828	25.72573	34.98163	6.52679
35.04738	28.7314	38.10462	21.92252	35.31039	28.89467	10.59464	19.07508	21.2625
76.10297	80.32826	32.94285	30.68751	153.3124	27.57055	70.20598	39.85566	61.48503
177.2397	70.97423	75.96423	418.0933	85.00146	207.6234	426.797	244.4909	406.369
49.06469	37.31488	55.14835	0.83227	18.76222	5.935511	3.577721	2.425934	1.045672
13.01803	4.163954	24.18784	38.67445	12.17274	11.68705	22.2474	32.45079	6.568499
88.1196	356.9262	228.4597	84.25361	244.958	89.56005	69.08236	95.5635	67.37624
25.03422	30.40944	42.50813	50.06319	38.63052	37.54019	49.11734	20.73129	16.63343
117.1582	104.8584	202.1377	50.89104	125.7768	27.57834	11.7691	13.23236	31.94806
23.03154	23.63229	29.06212	50.06594	23.2032	14.58925	33.94853	33.2326	31.13306
331.4476	345.0699	404.7711	156.2766	253.7873	91.00464	79.60845	140.4788	67.3937
108.1443	140.7105	44.04293	15.76153	43.02957	34.65088	21.10689	11.57335	6.636232
93.12638	90.42382	55.17155	621.85	140.1673	461.1377	625.6109	917.5281	662.4705
261.353	327.3226	258.2027	86.01122	265.9111	101.0805	91.29971	88.91081	112.5249
642.8692	1046.055	1192.303	1542.022	1018.474	1179.429	1687.852	915.4806	512.0496
36.04912	28.69736	75.21362	40.36178	54.08208	23.23997	24.6284	59.02354	26.73229
125.1696	435.5921	283.3968	89.52183	135.7536	7.378234	1.228926	46.49305	26.97258
24.03298	44.78861	60.813	13.12037	34.23696	11.72155	12.93857	33.20935	16.77839
46.06251	72.74222	32.97635	129.1885	27.62195	44.78646	100.5182	77.35896	46.26516
3952.339	864.231	2187.675	269.5375	1017.366	824.805	756.8417	536.2799	466.5613
86.11655	86.23744	38.87578	100.1035	41.96812	136.8677	58.53463	166.385	120.8778
166.2245	81.98301	306.0712	25.41342	101.5373	160.0159	128.6844	24.87324	47.10331
214.29	128.4763	252.5615	525.1387	387.3124	423.9151	474.8967	928.9616	711.047
67.09102	13.46441	30.94791	39.46925	55.20403	116.7636	98.26767	98.94064	145.5814
155.21	229.1878	103.6932	230.9626	92.72295	239.2796	87.78831	164.6204	207.2112
57.07706	58.36121	89.29101	25.42577	28.71973	26.09488	32.79504	34.88518	11.73084
728.9849	154.691	490.5196	236.1738	692.9365	281.2996	308.8554	157.9211	173.5225
1363.843	2383.108	2470.414	1839.705	1438.871	1293.364	1846.954	1949.128	1461.318
0.000298	0	0	0	0.022192	4.240243	5.764462	47.53267	7.48574
14.0195	26.16647	48.44035	45.65253	46.35171	51.9058	32.79352	32.38742	16.70395
55.07468	34.61911	321.4697	6.096334	61.80509	21.8066	2.404305	4.920305	6.646189
549.7432	1749.824	499.3668	645.4162	421.5372	1107.158	1274.893	1224.09	914.4577
62.08424	77.77694	98.41086	83.40916	99.30782	64.98445	28.14252	44.84447	31.91456
33.04515	65.09229	116.8741	34.20315	69.52736	66.38487	122.755	58.17265	11.77399
90.12149	137.9662	75.19971	27.17696	41.95737	23.24181	40.98597	34.87032	21.79401
63.08548	82.02672	48.82044	5.219116	107.0095	34.76227	1.231213	9.906548	31.82234
88.11941	84.52611	105.2561	53.52089	165.4963	85.17812	83.09363	140.5324	21.92666
179.2426	103.1086	105.137	544.5157	198.6417	275.4257	557.845	394.177	168.0588
15.02056	17.74124	15.23554	8.738928	53.93799	3.061039	5.914887	0.768406	20.38239
14.01946	117.7985	34.56491	60.63802	65.05313	4.507675	9.427845	5.749543	11.65595
6.008678	16.02322	25.17391	37.78697	16.57983	17.41618	8.255246	31.60854	11.49017
34.04618	25.32983	27.57964	57.11345	30.91407	40.3808	36.27648	39.90831	21.4362
20.02725	24.52124	24.21953	14.01595	14.37267	7.380726	10.58598	6.584537	6.540454
38.05181	52.40681	35.94853	35.96681	86.04486	34.74055	49.15923	19.05634	31.65909
11.01535	9.244723	8.210573	14.01505	19.87761	20.2497	25.73391	15.75669	25.36128
1828.471	2755.244	2438.812	1526.201	975.4483	1028.1	643.397	580.3473	658.5636
39.05305	37.17409	77.29968	58.84005	34.23258	20.35226	25.79069	18.22733	1.232894
387.524	270.5575	275.784	648.9518	781.1966	1225.141	1353.167	1235.806	616.6168
737.9964	804.3431	481.2577	244.9677	314.4915	196.2312	252.6998	161.2545	218.5975
0.000424	2.474053	3.747262	15.78934	5.557422	25.85865	256.5618	81.94624	29.08637
2.003248	10.09825	31.33366	17.54774	12.16577	8.803069	4.747112	5.753857	6.482141
139.188	181.0299	145.9665	56.15914	99.3214	70.771	90.0981	62.31155	56.96215

34.04582	30.45053	9.696766	25.4564	23.18545	21.69984	21.08498	29.94046	25.60028
50.06787	95.61872	60.80979	47.39742	28.72459	31.86115	39.81293	19.88929	21.75498
57.0776	279.2301	121.6503	14.87616	32.04238	21.81656	37.49938	7.414984	21.92213
96.13047	474.4513	885.3656	194.0315	133.5533	111.1969	87.79938	76.42891	87.63454
206.2781	179.3258	120.1474	82.515	195.2637	136.9521	38.66929	45.66872	76.91645
88.11927	111.6289	96.39208	58.79831	61.82126	100.9513	86.5833	93.11341	111.1805
92.12504	313.7484	102.6685	86.00773	415.954	36.2345	45.69172	39.00939	52.28035
24.03295	21.08425	45.93735	80.83517	17.69536	37.57923	40.96853	43.21555	6.651432
15.02071	12.63133	35.68555	21.93218	31.99307	25.98989	22.25282	25.7624	16.28273
14.01947	15.16377	31.5686	40.39234	17.6909	57.53979	65.42602	46.58165	82.75227
135.1831	302.7694	414.6922	197.5625	182.0841	236.474	146.2603	205.3664	32.04854
66.08986	158.9544	279.5268	86.8904	85.00208	236.4272	367.1936	211.2051	67.34101
1347.819	762.8571	535.1428	250.2317	460.1287	284.1526	357.959	232.7738	193.571
7984.788	4810.188	4040.104	1216.182	5078.974	1039.689	1234.092	814.8377	951.8117
11.01536	28.7603	20.6922	33.39124	4.456416	24.5376	17.58396	19.92752	1.113778
49.06577	47.41204	47.16038	13.12852	24.29277	14.56621	15.26158	82.54092	11.55235
61.08263	172.7232	45.88413	42.99943	100.3706	10.28023	62.00339	9.906312	6.650389
1.001685	0.804329	0	1.738049	0.020719	15.90232	7.98462	17.25172	0.400235
96.12531	8.403767	1.772059	0.832272	52.824	1.611659	2.407506	0	6.449938
0.000353	0.791613	0.314532	15.08327	1.138389	8.494488	17.28002	16.03705	0.640665
4.005965	12.64459	24.7852	7.858672	13.26629	10.23205	21.05696	35.84317	15.85889
45.06143	102.2984	136.0024	100.0857	50.79656	161.4155	182.4275	137.2048	146.3528
26.03563	19.39077	33.49764	63.23955	51.86503	79.1488	67.81306	46.54745	21.66581
788.0645	932.848	427.7031	550.593	340.9828	252.4645	380.1894	275.1833	510.7566
84.11405	193.6958	61.14094	336.4635	49.69449	66.47416	101.7992	114.7265	230.6437
14.01903	5.865178	18.8752	13.16013	5.554071	14.43788	8.235849	11.61432	0.979133
1089.472	1546.793	1001.982	208.0676	1215.942	131.3917	77.27117	47.32962	390.6625
122.1654	186.0445	141.354	115.0046	91.61765	158.6446	268.9726	380.9887	251.6576
9.012741	17.69231	32.4298	211.7076	15.4922	197.2956	95.93953	294.5735	254.0405
9.012686	7.550064	21.70467	42.21063	11.06965	52.97595	50.17023	92.67996	29.92604
209.2831	197.8461	286.3127	168.566	239.4586	137.1256	595.2507	237.7906	52.28275
256.3463	492.3161	257.6966	158.0378	208.5544	67.94545	136.9022	77.26551	87.46802
206.2783	214.8521	160.7596	102.7164	125.801	88.06896	86.60497	86.43267	111.9021
90.1217	147.2429	266.0739	30.68855	39.75723	33.32275	66.69611	30.70271	21.85327
14.0194	14.32461	32.65578	58.92106	15.48051	38.85056	43.23038	36.60835	6.596882
15.02086	14.31196	30.50808	63.23489	16.59319	137.8928	65.486	164.916	36.36779
105.1422	115.857	140.0448	57.91836	107.0363	29.02036	36.32871	28.20125	31.96738
2.003246	10.11161	7.754572	12.27492	8.855877	60.96092	26.81861	20.8241	15.34444
1.001865	0	0.308864	7.875883	6.648964	28.39777	34.84594	40.22391	6.148782
58.07864	44.78293	86.64114	55.30527	46.36693	36.18117	64.34276	29.87552	36.60333
57.0767	46.52886	45.54711	6.97364	37.52142	11.71046	12.93308	9.908801	16.55794
12.01681	17.69641	9.674009	63.23098	61.78909	102.1184	104.0032	79.87044	189.5923
49.06688	96.3504	235.9245	500.6702	140.1598	155.8033	300.5657	338.5057	157.5048
22.03034	23.61413	26.99409	71.98962	29.83325	204.2692	141.4587	258.0463	36.84057
432.5847	315.3933	547.0105	400.4152	460.1305	255.3347	166.1646	206.9911	168.3944
61.08243	94.80511	78.30974	16.6358	92.62965	23.22462	22.28566	17.3954	16.72446
28.03826	42.26993	13.64147	73.81523	33.12423	64.76263	23.4488	82.41861	26.42557
80.1081	121.8683	54.32211	70.25183	52.98135	27.55657	42.15387	23.21665	31.68789
23.0316	71.92103	32.0102	177.6318	18.7979	27.53162	52.63929	51.55001	60.32266
22.03035	144.6216	113.2061	219.6038	127.9973	4.496575	5.916067	9.077108	32.00219
39.05317	65.10574	111.9951	35.08592	39.75312	37.6218	62.01038	79.84186	11.76795
215.2912	1112.376	477.4233	65.80807	273.6597	40.55988	14.10404	14.06885	52.28341
51.06928	57.48374	47.36121	17.51178	165.3976	40.49534	10.59973	10.73775	26.76594
20.02748	58.41044	36.07556	76.49348	9.973035	16.01665	49.09434	35.74609	40.36385
196.2658	248.5689	122.9264	1377.943	232.8593	431.1734	2418.45	2114.161	500.8199
1351.825	117.481	790.9849	82.4925	668.6495	165.9828	59.72736	62.29281	148.2506
24.03296	10.0821	32.49638	97.54183	54.07182	56.2364	113.3255	99.88453	74.91638
181.2447	116.6772	151.3365	100.0803	154.4759	90.94861	70.23941	42.33873	67.10841

74.10049	163.2525	105.7841	121.1824	147.8416	85.15785	44.51387	34.02324	31.98804
32.04366	25.3187	70.84902	54.44662	43.04721	53.34595	50.30402	24.88959	31.40334
51.06959	80.27107	14.14499	1126.811	98.24834	635.6836	2450.977	1822.363	931.4976
208.2816	131.0318	113.0821	303.8631	147.8842	322.8344	186.0082	135.4922	102.5284
25.03436	17.69394	46.85596	94.86464	40.85695	43.37538	109.8886	77.33745	6.64703
10.01409	5.854421	27.09379	25.43735	23.19949	16.01752	44.43265	28.23981	1.183651
73.09914	63.37697	155.4036	124.6994	139.0183	88.03085	114.6363	140.5512	71.84483
14.01948	19.39591	5.225748	55.34323	11.07692	157.6296	89.93051	135.8327	50.25244
58.07825	45.66218	146.3286	10.4866	34.22532	13.15455	12.9357	15.73346	6.64655
607.8216	778.8578	754.19	626.9779	1846.991	395.184	222.3121	183.6984	471.0897
49.06593	58.42571	151.3056	8.73118	23.1962	20.30902	9.426495	8.245436	6.621008
75.1017	48.9993	255.913	73.74374	44.17209	54.91312	105.2613	63.98963	41.84771
500.6747	368.823	211.2063	49.12455	160.0085	112.5826	145.0728	183.7627	117.3275
177.2393	280.0211	155.7934	97.44395	86.09618	73.67668	48.02594	29.86279	11.74675
7.009841	6.721369	12.35198	6.991742	12.1414	0.128447	2.405295	0.76811	0.925921
821.1091	761.1349	725.6108	358.2507	504.2702	338.9382	131.0785	382.4683	223.9092
12.01674	9.240756	17.16053	44.82328	43.00575	28.86467	12.92703	8.246829	6.59959
1609.174	1413.166	950.0451	378.4344	1411.226	229.4279	471.4419	211.1388	284.6794
447.6045	8.397444	39.34619	951.2327	207.4679	778.0354	484.1898	119.6763	268.3992
61.08289	536.8029	168.9382	34.19997	61.81713	59.22988	28.14228	30.69903	16.8516
36.04923	19.38405	37.89003	55.28758	59.61185	149.7635	189.3625	223.8644	149.9927
435.5885	950.7302	235.7746	332.8059	156.7226	170.2824	320.5146	667.0007	313.8388
34.04646	36.31479	24.03433	60.57884	27.62389	86.43488	205.5875	110.6686	215.3229
363.4908	301.9142	301.7187	107.0876	209.6658	134.2296	69.08365	157.1129	102.6222
1704.303	3269.533	940.0502	914.9993	1791.916	850.7481	687.8334	823.1806	1006.431
37.05054	63.39896	75.6333	93.09386	70.62979	30.44576	37.48894	77.32426	51.56673
11.01512	7.564534	35.6618	8.749584	3.350856	15.84675	12.8837	14.96628	10.83646
271.3669	973.6071	450.6615	266.0576	254.9076	151.542	100.6637	136.3092	132.9329
30.04116	460.8902	535.185	48.24555	332.1465	26.13282	52.70939	89.73339	47.20817
815.1016	1121.369	942.6132	474.157	624.5576	700.7833	510.0395	259.3696	597.4291
14.01951	16.00491	49.4158	56.2022	33.13001	27.53503	50.3084	52.37999	16.73022
13.01809	10.93289	31.11783	41.28904	7.766687	50.31423	19.93191	25.74745	30.68884
15.02088	13.46431	49.32523	110.6823	29.83143	119.4956	71.36474	171.4579	100.3268
69.09395	59.13044	33.91668	434.659	41.97291	762.5873	364.9878	353.3592	998.3479
7.010037	10.92748	48.32673	27.17411	27.62612	116.642	172.9607	176.4459	36.76633
11.01535	20.27448	2.264622	6.976528	8.866051	30.21788	29.22436	46.67704	105.4849
900.2164	1050.351	652.2496	415.323	1105.591	380.7758	260.9101	211.9716	355.2178
108.1459	126.0518	148.1334	42.10957	44.17041	52.02738	67.8717	31.53283	41.7898
8.01131	12.64602	13.23308	15.78668	15.46566	13.08156	17.56393	29.15356	1.057577
253.3415	294.4223	157.2985	146.6503	62.93154	73.67174	100.6301	53.15346	87.0089
343.4625	379.0802	303.644	16.63243	211.8157	13.16207	29.31516	17.38986	11.75238
11.01541	15.16964	9.687473	30.72728	15.48231	38.87893	35.0861	34.9289	67.61629
56.07633	93.81968	223.1301	172.1107	141.2537	151.44	209.3568	139.6704	77.26484
2.003237	18.55207	43.54324	76.48306	48.53634	13.14839	35.10831	39.07634	16.5696
24.03281	3.319094	16.63937	58.88766	22.09648	90.32712	54.9179	116.7172	40.29503
50.06794	7.545658	17.59142	74.64555	4.455578	106.524	156.572	721.6307	143.3209
13.01735	8.438512	4.789092	14.08112	0.030033	25.46359	23.23483	53.81945	33.81927
135.1827	141.222	161.7605	191.4645	182.0438	53.51518	45.68666	74.78496	52.09625
509.6889	271.4022	207.0153	44.73454	1010.68	62.18416	21.12088	35.68728	188.6636
29.03982	5.858427	26.98211	19.26657	47.48792	280.791	423.1362	195.46	255.1878
6.008623	5.861533	2.758391	25.50353	5.556277	48.41656	11.73489	44.27005	1.020904
38.05197	51.52863	243.2096	82.51631	115.8652	111.0563	80.7509	80.61746	42.01922
0.000356	0	0	0.835308	0.026778	13.96976	6.992996	10.91815	5.291969
42.05717	56.6429	93.13286	64.10095	61.79674	44.79328	46.82247	74.85388	11.76215
29.03977	34.61617	67.18905	111.5556	77.24483	122.39	94.72856	70.66203	36.77591
436.5902	406.7282	547.9263	337.1703	397.2544	315.8875	593.0403	536.3264	153.3323
82.11153	105.6456	471.9752	163.2945	230.6355	212.0484	356.7474	611.2981	82.56909
180.244	293.4355	364.539	463.7022	275.8642	252.3765	155.6259	269.3989	62.386

2332.152	11119.84	2466.211	2304.211	1936.527	1614.941	8293.008	4937.696	3361.555
156.2109	95.53125	41.8341	46.49424	438.9392	29.02326	3.573892	24.04194	42.03094
122.1657	41.3724	248.5963	404.8072	162.2435	608.3522	923.9405	1802.342	1508.994
29.03939	17.71166	43.12491	53.62026	23.19405	23.16381	10.59386	25.74906	16.42735
1832.476	1537.421	1176.969	1167.034	966.6155	970.4125	1267.967	753.3203	1248.816
75.10201	90.43122	126.969	251.1691	118.0949	243.598	253.7939	525.758	212.1956
37.05014	5.854404	23.10452	21.91803	19.89428	48.94714	38.60441	82.4686	45.14714
146.1977	128.5148	176.0772	195.8405	94.92374	149.9802	107.6539	133.8527	37.08568
6.008656	1.630018	3.250726	66.9411	8.864045	4.50345	21.06244	163.131	47.22326
22.03029	13.46522	7.696759	106.3228	4.455916	160.8911	178.6926	459.2052	142.3736
134.1812	246.2278	104.3078	50.88875	76.16066	41.98261	53.86455	53.15977	46.97171
133.1803	131.8892	159.6762	102.7049	166.6264	188.8814	141.5655	146.3213	37.10299
7.010016	2.474052	15.69063	33.39168	17.67776	15.97396	40.87124	50.00829	52.65035
6.008554	5.869093	8.285231	15.82096	7.750816	14.39983	24.46119	36.82068	39.68493
142.1912	55.78608	117.933	28.93305	49.6775	26.12313	43.32623	54.01719	16.81809
62.08381	54.11665	19.58514	45.6463	15.49021	34.71716	64.31728	67.3775	89.23352
659.8902	243.5348	280.4062	245.8677	216.2869	432.3485	274.883	284.3856	293.0629
6.008599	2.47467	19.86921	12.27505	13.25313	5.922959	12.88902	12.44801	15.33981
331.4475	411.9292	419.2001	129.05	171.0498	65.0637	42.18251	47.32495	67.36883
21.02877	16.01544	44.6099	43.92921	35.30947	33.1806	5.919447	21.57689	11.59677
164.2208	222.642	110.9233	40.35511	55.19348	27.56653	31.64505	34.03226	31.80242
1430.933	1149.318	85.83537	118.4964	3110.236	147.2511	52.70678	93.05966	239.1835
0.000425	1.62992	2.264054	20.19516	4.455366	14.50433	33.83925	36.68877	37.99682
58.07878	274.2994	53.78073	35.96007	119.1366	13.16482	3.575751	2.430059	16.83785
208.2818	647.0433	521.96	221.2619	185.4053	276.8827	320.5059	221.1476	293.5876
77.10456	131.9253	135.0286	200.2693	108.147	67.90043	100.6208	138.8756	32.00203
16.02223	29.53417	172.8417	101.8592	39.76256	46.29439	41.00358	21.54768	6.625494
111.1506	131.0466	176.5392	208.1314	133.5332	105.3754	139.2233	109.7171	97.27823
98.13319	140.3152	375.755	1575.594	423.7271	400.8915	431.6419	235.2653	117.9759
24.033	15.15647	31.97279	115.979	61.79993	136.6229	73.68427	37.36817	70.74373
11.01526	12.64847	19.27528	10.50188	18.76258	32.94486	7.080375	14.10351	15.74475
56.07627	109.9138	199.4828	86.90545	182.038	65.02885	32.82335	76.45178	32.01553
27.03673	41.46218	43.12694	3.462133	43.01312	14.57097	11.76156	13.24296	16.42373
3.0046	5.009582	16.11507	38.61355	5.560301	155.0168	317.3931	269.1963	155.4728
90.12101	198.2683	54.4135	9.607746	51.8617	4.506304	0.055461	8.243564	11.72781
54.0735	17.69237	106.828	134.3813	87.18267	112.437	104.1048	73.97201	41.91365
21.02899	36.29661	17.09912	404.8848	116.9972	65.06505	557.7671	121.3487	177.7087
32.04383	22.76781	41.85976	14.87595	79.45768	17.4902	19.95711	12.40075	16.84875
50.06748	85.53888	32.05971	21.03638	38.6265	11.7126	17.60602	13.23775	26.27774
11.01539	10.93569	25.16163	38.66285	13.2764	11.69437	19.92354	9.913212	21.03883
15.02071	31.29681	49.23003	32.50068	26.48915	13.12426	17.5895	11.58068	6.582435
341.4601	193.6855	155.7838	51.76188	293.4333	126.9346	37.50212	76.44604	151.7171
4724.384	6448.322	2910.93	1650.872	2310.568	2593.789	2498.492	5306.973	1921.582
4.005953	5.01095	37.91153	107.1657	4.455294	50.57093	65.52457	69.83402	269.5287
437.5914	515.0225	114.0278	1515.045	178.7923	276.9386	412.9157	471.4847	675.7716
34.04654	43.07296	46.80798	133.5056	12.18172	99.49323	91.25026	106.441	140.3964
210.2841	307.0395	94.78234	165.0747	146.774	292.5236	249.1151	349.3457	401.0442
21.02889	26.17005	48.46967	47.41848	29.81826	54.74211	39.79022	26.56028	16.66389
138.1871	26.99502	29.94962	135.1975	240.5464	298.3756	811.4208	1052.405	422.2231
101.1368	163.2657	422.6047	70.21991	51.89616	27.57961	16.44874	27.36952	6.621767
15.02083	10.08302	18.11053	35.10444	22.10264	100.5098	57.28729	144.994	45.59714
51.06928	74.42253	139.3622	17.51176	48.57305	37.62187	40.98729	20.71967	41.54505
4.005862	0.787869	0.308996	19.40048	2.245736	37.96448	43.98629	74.94546	18.21604
0.00037	0.789753	0	14.14565	0.027877	4.411744	26.52104	37.20308	12.99864
1.001867	1.629859	27.39199	12.26814	12.15982	5.931023	17.55019	15.78371	1.024126
42.05697	21.93401	33.52023	24.54911	38.63505	91.96403	117.9403	125.785	45.7132
8.01136	25.36122	54.30193	6.096855	17.67891	15.97813	14.08814	12.41652	11.45147
119.1613	190.2788	188.4364	175.6248	135.7388	82.33486	170.784	77.27205	47.16896

932.2596	2390.959	1235.118	12.24886	289.1348	17.46843	43.34782	18.22978	57.27356
0.00045	0	1.297606	348.9155	0.034316	46.28195	201.0305	229.7072	110.7181
2.002549	0	2.058174	0	0.015807	0.05996	0.025306	1.66849	15.38012
8.01133	29.63097	7.719993	1.707809	4.455901	8.808573	1.234314	1.59625	1.08023
1618.187	2535.378	1258.08	1217.965	1804.07	899.786	848.0796	738.3497	804.8712
115.1515	11.78751	5.22902	3.462079	22.07965	5.945627	1.234107	2.425939	1.108775
32708.2	38056.7	27734.4	18666.69	31114.02	19155.27	21398.37	11981.61	19483.09
32.04385	53.2282	38.87751	80.77032	39.76182	131.11	215.0708	135.5814	120.8004
163.2209	256.2279	288.8376	151.004	208.5632	111.1799	162.6345	137.9806	167.8557
58.07906	87.04237	280.4529	244.9987	335.4121	184.6305	230.4414	215.35	72.42032
132.1792	272.2569	431.1999	587.493	315.6028	586.7433	458.5316	450.681	183.5109
466.6311	898.0601	1794.875	194.0169	2339.176	839.2196	1014.155	457.2798	1505.744
142.1926	419.4959	196.2166	134.3128	224.0121	36.23431	24.63498	38.17796	32.03729
19.02602	15.17486	19.17103	68.62891	2.248182	31.69524	14.09101	47.47805	30.28328
8.0114	7.545255	29.02815	121.3125	25.41281	53.34742	73.64203	37.38444	21.64629
30.04046	40.65575	59.36946	6.09715	52.88116	20.25465	15.25188	14.92088	6.554674
98.13258	71.00795	136.6675	83.41227	49.68594	56.34978	97.08299	65.65519	41.84011
159.2151	92.98108	91.84778	122.043	130.2153	63.60021	62.05702	68.95825	52.13105
127.1711	109.141	50.82228	57.05732	50.78099	64.92902	73.6962	113.1514	182.3867
115.1561	389.9156	243.3028	22.78009	166.6403	98.2075	28.14544	8.249644	47.22127
72.09785	76.91014	160.7936	195.8659	91.60726	46.31136	35.16212	40.67632	26.97851
12.01655	1.629864	1.771938	22.85029	5.556807	58.34507	33.81792	86.17788	15.64394
4277.78	2827.086	2449.572	1264.491	2724.312	1286.178	1496.078	1268.88	1062.701
134.1813	196.2619	104.2877	51.76572	93.80895	27.58176	67.89387	63.97447	6.613754
19.02629	31.22921	36.41074	167.811	37.55354	415.1984	261.73	55.66963	188.4476
39.05245	79.7117	67.79645	0.832792	18.78291	8.823544	5.91856	15.74914	6.584726
629.8506	693.5263	372.8541	95.66544	563.8168	201.9948	135.7529	123.8299	228.6491
1385.873	1833.363	3457.027	1916.959	3433.809	1179.523	1367.429	510.4927	2502.113
212.2872	164.8511	403.6049	469.8357	183.1998	431.0202	195.3902	340.9245	132.9584
16.02222	30.39141	72.24099	62.34323	49.67143	66.31999	17.61539	27.37994	26.71329
2.003245	2.474117	2.757297	33.38641	2.248235	94.26014	143.2403	173.7731	48.38338
224.3022	290.2401	145.4673	57.03752	93.80777	63.57708	154.3674	104.7554	71.87858
111.1505	99.74465	145.3408	281.0669	133.5299	155.7433	245.5801	184.6098	67.21445
13.01817	10.92743	37.91639	107.1709	40.85782	73.54421	74.86376	78.16673	21.82908
10.01388	10.10838	6.244987	7.864056	7.757329	3.058125	12.8941	6.592726	10.99436
14.0194	15.17274	36.16519	39.54153	9.970777	13.12916	18.7587	26.59248	1.141916
2.003212	4.181089	2.782143	63.8476	6.638249	1.603179	11.66698	0.768777	5.879084
9.012726	12.62774	24.63578	18.40564	56.21971	4.507716	15.26302	2.426463	11.57205
2.003247	0.787548	1.77191	1.707707	2.248159	3.058512	62.79114	140.8021	11.021
30.04107	23.6186	58.80322	35.96662	27.62306	84.98066	36.31187	41.53313	21.78062
41.05583	36.31766	49.36425	43.87886	41.95551	37.61225	50.32593	41.53429	26.7212
42.05699	52.43635	37.99605	43.01673	97.02167	11.71752	17.61049	4.088079	6.649232
15.02077	14.32082	36.11039	46.57074	32.00605	46.03712	26.93076	34.08865	21.24693
220.2964	236.9527	106.3373	29.80676	75.05224	64.99143	41.00249	80.63245	41.9035
15.02072	22.80799	28.66976	55.40584	20.98551	23.13649	28.0801	37.451	16.29309
15.02043	17.76071	16.29748	8.744456	15.45597	8.782842	11.73278	11.60515	15.50284
110.1485	88.80129	43.36233	195.9638	32.03792	174.0597	120.4209	148.1185	124.8356
111.1491	55.81318	41.4514	17.51423	41.94782	50.49379	57.3125	110.7293	41.17251
4.005975	51.64566	5.225678	6.974225	54.02128	13.13732	3.578853	2.426523	6.613355
1.001842	0.790347	5.231298	15.75459	1.140926	290.1389	384.2004	307.2945	322.0047
78.10384	17.71614	57.71513	3.462056	89.21254	63.07327	5.918861	16.58076	11.52917
262.3537	286.7959	127.0444	78.11649	182.0467	89.50936	102.9701	71.45563	57.1156
101.1361	40.55339	14.62265	18.39123	95.95756	23.23693	4.748292	1.599474	26.69743
124.1677	359.7149	158.4273	5.220097	57.41003	18.93277	2.402889	2.431122	6.623635
16.02222	11.77315	7.203561	130.9322	57.3901	458.9801	284.9236	52.35833	70.71197
83.11251	44.76469	79.53609	57.04256	44.17398	100.9381	160.1786	161.3894	135.5948
39.05265	207.0533	41.11572	10.48934	17.68676	1.609797	5.919398	4.08728	11.59008
0.000375	0	0.310757	0.833655	0.028309	11.36924	7.022037	14.25161	16.89887

348.4715	248.5588	160.0179	818.4009	496.5748	729.655	9426.642	3804.598	3279.371
1.001867	1.629867	1.771911	81.17969	0.031865	31.44983	30.31704	41.76596	49.83159
85.1155	87.89524	213.6852	138.7222	107.062	183.1237	397.5482	336.0414	177.1637
174.2358	120.0308	275.9806	165.9384	486.5332	533.0737	394.1198	216.1785	152.7553
25.03377	27.92715	17.21736	24.59135	15.47116	54.32538	16.41008	14.09243	38.6115
35.04712	2.474125	7.70861	13.13207	18.78088	8.821106	75.79048	292.5754	16.22859
52.07067	69.33242	49.82744	50.90376	41.96078	56.31303	43.32799	18.22277	46.55594
577.7811	469.2982	1709.621	835.1132	398.3675	801.6373	539.2709	577.8829	234.1678
134.181	96.39705	169.3914	81.64855	51.89398	95.18502	92.41999	67.31398	56.79396
407.5506	110.7192	212.0264	155.3878	166.6509	386.3656	844.3205	576.3402	418.9523
29.03933	21.10666	26.64709	37.7763	8.868761	24.58027	22.25888	51.63599	6.600868
181.2451	463.5746	184.4173	171.2202	201.9295	92.43138	70.24964	101.3906	132.4549
920.2433	3294.326	963.9846	69.32161	1202.676	102.5564	8.249447	34.02556	148.3146
2420.271	18067.92	3623.501	2920.668	2499.267	2929.795	11370.45	14317.67	3907.538
85.11493	169.2837	92.55278	37.71991	120.2281	24.68625	24.63145	22.38255	31.77447
1870.528	3041.014	3918.508	2220.804	1406.887	1786.459	2140.571	527.1235	744.6272
12.01679	0.78929	39.51162	64.14036	22.1023	24.64276	72.44633	124.9752	88.12066
250.3382	224.0979	162.6056	231.8304	101.5507	266.6734	352.0063	248.6402	287.315
65.08787	38.86405	83.73719	18.39212	71.70651	27.54299	22.2882	9.074913	11.74959
23.0317	38.83383	54.18927	234.4784	67.34951	396.1371	699.104	556.5557	316.6115
43.05875	98.90447	22.03603	198.4864	119.1858	135.567	205.8236	116.3856	211.155
0.000216	0	0	0.858792	0.015892	2.751332	6.74388	10.49257	0.264412
3.004609	9.238034	9.680558	42.15506	16.58812	56.09634	54.92849	26.57092	68.59245
22.0302	19.39698	24.08551	160.1096	18.79431	58.99657	53.7772	59.08408	31.11692
28.03828	28.70838	59.40811	12.24316	62.87677	18.90898	23.45132	18.22934	16.6947
42.0572	43.08785	40.40836	33.3282	73.9264	109.391	65.5135	133.9976	61.08186
458.6183	373.0457	230.5173	29.80405	160.0104	14.60112	4.743127	1.602882	26.99063
79.10708	130.2719	197.2608	43.86474	75.04911	37.65166	57.36178	36.52353	21.8923
1.00184	2.477758	3.75448	78.14779	4.455128	182.6776	348.087	683.8624	46.6656
896.2111	699.3338	857.5163	500.5029	791.1618	346.1958	804.771	513.8388	123.0412
246.3333	534.4695	654.4272	588.3739	453.5093	213.5392	292.4701	169.5673	133.094
11880.05	51121.71	7869.778	6381.434	5111.047	4819.964	19991.15	11213.3	12558.93
35.04728	36.37718	55.17598	18.40701	29.79825	18.86226	68.85724	41.61261	6.603446
130.176	114.9872	169.2004	107.1109	112.5649	70.79249	69.06911	78.94539	26.98349
2.00324	20.25207	9.188106	29.84196	6.66377	83.14964	101.5027	101.7084	26.03343
36.04843	22.81277	25.68588	34.2671	14.37604	15.98116	26.90847	9.914984	25.55697
7.009852	14.38605	15.39212	12.28846	3.349735	15.8	11.70968	11.62838	6.198704
12.01679	13.46837	15.62881	64.14108	36.42783	64.74293	93.43329	147.5067	64.52069
54.07356	79.45142	87.91625	169.509	71.75281	154.2106	191.7682	216.2881	111.6296
19.02572	3.319602	9.23671	16.67393	5.556973	49.88706	18.71802	61.01273	20.13469
43.05857	81.19149	83.1207	46.50991	89.3629	30.43924	51.50394	29.04051	31.7546
122.1649	71.84681	146.5356	129.1064	65.12855	102.3791	102.9377	75.63597	66.7214
12.0167	10.0907	46.26391	57.18529	14.37541	5.946798	7.086391	6.583447	1.118683
743.0035	784.8487	470.7984	434.6699	322.2218	310.092	520.5078	246.9121	329.448
13.01811	20.24864	31.58469	91.46365	18.7916	24.61765	11.76427	18.23789	26.15082
37.05013	22.79021	38.56555	32.47589	41.92549	33.21107	28.10618	16.57094	6.632727
96.13047	80.27177	214.4957	381.9956	288.0071	337.4003	647.9053	227.798	218.4311
255.3426	47.31313	183.0003	3.46449	88.26864	29.00687	8.259313	15.72699	11.77398
40.05471	66.74217	42.31211	853.7959	120.3082	236.4854	433.8702	579.7507	227.8561
47.06354	63.47561	53.48889	18.39733	26.50943	28.93856	15.27137	19.06611	16.62086
17.02297	13.51211	25.93724	18.45133	4.453603	7.351607	9.402593	8.264553	10.9475
52.07039	101.5933	55.90245	32.45958	21.00292	20.34897	12.93718	7.412127	1.226901
248.3353	144.5797	183.4542	106.2183	131.3301	132.7419	147.4117	81.42905	82.32944
24.03303	14.31001	76.13973	57.93348	44.16731	143.8982	150.771	120.6383	216.6096
8.011393	5.856293	65.68225	164.296	72.83903	258.788	238.3738	133.111	61.51346
182.2458	133.6216	77.5137	20.14463	419.0826	82.28542	28.14494	61.47827	66.93613
21667.28	17708.27	11164.61	8661.986	11084.9	9544.523	14773.2	13720.42	13902.94
907.2249	983.6445	549.5093	472.4399	310.0843	127.0606	136.9245	213.6463	188.5149

18.02484	19.39787	46.02276	31.59138	27.60976	58.98423	32.77873	60.75728	21.45747
42.05675	20.24891	65.0596	35.99764	16.58736	31.7745	35.10231	26.57355	35.63086
11.01498	9.27763	86.29072	4.34735	15.43006	4.477961	0.050178	1.596745	0.914862
20.02764	73.54042	168.3901	55.28517	114.7484	56.36339	26.974	78.96643	21.90475
[truncated: 1,926,573 more chars]
