# Supplementary material for: Identification and Verification of an Alternative Polyadenylation-Related lncRNA Prognostic Signature for Glioma
Source: Comput Math Methods Med. 2022 Sep 7;2022:2164229. doi: 10.1155/2022/2164229 (PMC11401696; doi:10.1155/2022/2164229)
Supplement: Supplementary 3 — Table S3: univariate Cox regression analysis of prognosis-related lncRNAs from the CGGA dataset. [file 2164229.f3.pdf]

gene	HR	z	pvalue
AC000068	0.865223	-3.76436	0.000167
AC004447	1.232686	3.559054	0.000372
AC005104	1.0511	3.650183	0.000262
AC005253	1.173751	4.193949	2.74E-05
AC005618	1.132796	3.387021	0.000707
AC007292	0.821725	-4.7453	2.08E-06
AC007308	0.968886	-3.64861	0.000264
AC007461	1.080178	3.735334	0.000187
AC007620	1.089465	4.716724	2.40E-06
AC009227	0.920527	-4.86138	1.17E-06
AC009948	1.219811	4.155434	3.25E-05
AC009961	1.088753	4.018518	5.86E-05
AC010883	0.937278	-4.05948	4.92E-05
AC011899	1.243835	7.193042	6.34E-13
AC012314	1.172073	5.637479	1.73E-08
AC012358	1.066029	3.691429	0.000223
AC027601	1.238741	4.811608	1.50E-06
AC062021	0.881365	-13.4935	1.71E-41
AC068580	1.128966	4.585162	4.54E-06
AC083843	1.192373	3.827459	0.000129
AC084219	1.064076	3.656938	0.000255
AC093390	0.924301	-4.27158	1.94E-05
AC093673	1.24954	5.425938	5.77E-08
AC116366	1.117416	3.487034	0.000488
AC147651	1.241488	7.348183	2.01E-13
AF127936	1.227435	4.365171	1.27E-05
AF131215	0.856583	-3.74959	0.000177
AIRN	0.934301	-3.33919	0.00084
AP000692	1.054139	3.510176	0.000448
AP001062	0.909327	-5.13521	2.82E-07
AP001258	1.242185	5.302106	1.14E-07
AP003068	0.962992	-3.82042	0.000133
AP4B1-AS	1.101518	3.625992	0.000288
C20orf203	0.686459	-10.7263	7.66E-27
C22orf34	1.180805	5.224075	1.75E-07
C6orf3	0.800592	-5.48123	4.22E-08
CACNA1C	0.848753	-5.48016	4.25E-08
CASC15	1.190921	4.438643	9.05E-06
CDIPT-AS	0.927357	-3.48137	0.000499
CIRBP-AS	1.130094	4.469517	7.84E-06
COX10-AS	1.288655	4.856265	1.20E-06
CTA-223H	0.920114	-5.14994	2.61E-07
CTA-268H	0.803655	-10.5398	5.66E-26
CTB-131K	1.150463	4.328378	1.50E-05
CTB-152G	0.805623	-4.15129	3.31E-05
CTB-178M	0.904999	-5.1649	2.41E-07
CTB-31O2	1.161994	4.120806	3.78E-05
CTB-33O1	1.130578	3.698705	0.000217
CTB-36H1	0.854661	-4.3457	1.39E-05
CTB-43P1	0.934431	-3.42318	0.000619
CTC-260E	1.128165	3.767605	0.000165
CTC-273B	0.898136	-5.42508	5.79E-08
CTC-336P	0.940067	-3.89555	9.80E-05
CTC-338M	1.341185	4.446281	8.74E-06
CTC-338M	0.948735	-3.43717	0.000588
CTC-444N	1.381794	4.897718	9.70E-07
CTC-444N	1.213934	4.112668	3.91E-05

CTD-2006	1.277301	4.263627	2.01E-05
CTD-2012	1.155459	3.441071	0.000579
CTD-2012	0.899519	-4.24336	2.20E-05
CTD-2013	1.153416	5.358769	8.38E-08
CTD-2083	1.277163	4.973455	6.58E-07
CTD-2126	0.907673	-5.23907	1.61E-07
CTD-2132	0.905366	-4.3273	1.51E-05
CTD-2231	1.484769	6.933807	4.10E-12
CTD-2287	0.810422	-3.38896	0.000702
CTD-2368	1.265177	5.354637	8.57E-08
CTD-2396	0.95106	-4.55321	5.28E-06
CTD-2537	1.135726	3.924306	8.70E-05
CTD-2561	0.966548	-4.36778	1.26E-05
CTD-2562	0.889697	-6.21525	5.12E-10
CTD-2619	1.293418	5.933995	2.96E-09
CTD-2619	1.389037	6.593677	4.29E-11
CTD-2630	1.246442	4.243648	2.20E-05
CTD-2636	0.862301	-5.77685	7.61E-09
CTD-3099	1.137356	5.066031	4.06E-07
CYB561D2	1.545382	6.793842	1.09E-11
CYP17A1-	0.874219	-9.05487	1.37E-19
DGCR5	0.76846	-8.83184	1.03E-18
DICER1-A	0.764942	-6.92212	4.45E-12
DLEU2L	1.06368	3.815053	0.000136
DNAJC27-	0.878543	-4.53317	5.81E-06
DTX2P1-U	1.142406	4.100005	4.13E-05
ENTPD3-A	0.820059	-3.52857	0.000418
EPB41L4A	0.850297	-4.86485	1.15E-06
ERVK3-1	1.738532	7.4194	1.18E-13
FBXL19-A	1.167883	4.848647	1.24E-06
GNG12-A	1.064648	4.02002	5.82E-05
GS1-124K	1.350426	4.408849	1.04E-05
GSN-AS1	1.122834	4.054297	5.03E-05
GUSBP11	0.810453	-3.43841	0.000585
HCP5	1.270428	7.036147	1.98E-12
HNRNPU-	1.123941	3.4926	0.000478
HOTAIRM	1.228152	9.847584	7.02E-23
IL10RB-AS	1.549765	6.796535	1.07E-11
INE1	1.122559	3.783605	0.000155
ITPK1-AS1	0.921267	-3.60777	0.000309
KB-1125A	0.879564	-5.23069	1.69E-07
LA16c-35	1.278826	5.621979	1.89E-08
LA16c-OS	0.926455	-3.64814	0.000264
LBX2-AS1	1.45449	9.430214	4.09E-21
LIMD1-AS	1.274168	7.418105	1.19E-13
LINC0009	1.20463	3.372158	0.000746
LINC0015	1.29773	10.7633	5.13E-27
LINC0017	0.799862	-7.28401	3.24E-13
LINC0026	1.253592	5.021078	5.14E-07
LINC0033	1.29689	5.385421	7.23E-08
LINC0066	1.356831	6.147745	7.86E-10
LINC0084	0.918918	-5.71487	1.10E-08
LINC0089	1.170975	5.264746	1.40E-07
LINC0090	1.205947	3.769584	0.000164
LINC0092	0.901989	-4.39753	1.09E-05
LINC0099	1.314872	6.167795	6.92E-10
LINC0100	1.228554	3.825952	0.00013
LINC0105	1.362607	9.735478	2.13E-22

LOH12CR2	0.813817	-4.16537	3.11E-05
MAGI1-IT1	1.092109	4.572894	4.81E-06
MAP3K14-	1.115683	3.616215	0.000299
MIR155HC	1.293674	9.947642	2.58E-23
MIR22HG	1.413528	9.092533	9.68E-20
MIR4435-	1.310095	11.63274	2.81E-31
MKNK1-A	1.084244	3.593203	0.000327
MTHFS	1.241528	5.246458	1.55E-07
NDUFA6-A	0.723771	-5.64131	1.69E-08
NOP14-AS	1.318042	4.384367	1.16E-05
OSER1-AS	0.683547	-5.9277	3.07E-09
PAXBP1-A	0.741065	-5.15999	2.47E-07
PAXIP1-A	1.325365	4.335148	1.46E-05
PAXIP1-A	1.34132	8.565418	1.08E-17
PCBP1-AS	1.297327	4.517209	6.27E-06
PCED1B-A	1.186661	5.008197	5.49E-07
PGM5P2	1.14672	6.81633	9.34E-12
PKD1P6	1.112357	3.9813	6.85E-05
PWAR6	0.86772	-4.61001	4.03E-06
RBAKDN	0.949533	-3.69914	0.000216
RMST	1.089711	4.479699	7.47E-06
RNU12	1.035758	3.37371	0.000742
RP1-122P	1.046051	3.560586	0.00037
RP1-193H	0.85964	-3.5882	0.000333
RP1-239B	0.735118	-9.38442	6.33E-21
RP1-257A	0.934188	-3.85189	0.000117
RP1-286D	1.248406	6.140746	8.21E-10
RP1-293L	0.87744	-12.0424	2.13E-33
RP1-315G	0.94298	-3.86085	0.000113
RP1-39G2	1.188498	3.426198	0.000612
RP1-63G5	0.859464	-9.41137	4.90E-21
RP11-101	0.733531	-5.02533	5.03E-07
RP11-101	0.813322	-7.8405	4.49E-15
RP11-106	1.194294	3.579509	0.000344
RP11-110	0.931425	-3.42936	0.000605
RP11-111	0.860398	-4.72402	2.31E-06
RP11-111	0.911251	-3.57598	0.000349
RP11-115	0.913577	-3.62705	0.000287
RP11-118	0.871585	-6.70471	2.02E-11
RP11-120	1.073777	4.295345	1.74E-05
RP11-134	0.918468	-3.43301	0.000597
RP11-134	1.222231	5.721464	1.06E-08
RP11-137	1.12682	4.617947	3.88E-06
RP11-139	0.833199	-3.78181	0.000156
RP11-148	1.392953	6.943673	3.82E-12
RP11-152	0.877042	-4.14326	3.42E-05
RP11-158	1.124352	3.294368	0.000986
RP11-159	1.212506	4.054741	5.02E-05
RP11-166	1.23072	3.88258	0.000103
RP11-167	0.930974	-3.89756	9.72E-05
RP11-178	0.935429	-4.18623	2.84E-05
RP11-178	0.957366	-4.16969	3.05E-05
RP11-179	0.747332	-7.5853	3.32E-14
RP11-196	0.867966	-5.97099	2.36E-09
RP11-196	1.266497	5.338388	9.38E-08
RP11-196	1.202988	5.509654	3.60E-08
RP11-197	1.169356	4.047988	5.17E-05
RP11-21A	0.83495	-10.113	4.84E-24

RP11-227	0.903611	-8.60018	7.96E-18
RP11-234	0.830995	-6.73364	1.65E-11
RP11-23J	1.137416	4.599776	4.23E-06
RP11-259	0.921053	-3.48516	0.000492
RP11-261	0.831954	-9.87156	5.53E-23
RP11-266	0.836178	-4.61928	3.85E-06
RP11-284	0.904467	-4.63727	3.53E-06
RP11-285	0.873095	-7.12188	1.06E-12
RP11-293	0.816723	-3.91559	9.02E-05
RP11-295	1.180388	4.962874	6.95E-07
RP11-295	0.924394	-4.00994	6.07E-05
RP11-298	1.079378	4.135102	3.55E-05
RP11-299	0.835366	-4.67752	2.90E-06
RP11-2B6	1.105791	4.727013	2.28E-06
RP11-303	1.329634	7.128549	1.01E-12
RP11-307	0.883237	-9.71503	2.60E-22
RP11-315	0.912142	-3.71899	0.0002
RP11-320	0.810816	-5.10524	3.30E-07
RP11-325	1.077471	3.595975	0.000323
RP11-332	1.217937	3.31281	0.000924
RP11-345	1.136516	3.874531	0.000107
RP11-345	1.310815	4.642624	3.44E-06
RP11-348	1.478904	5.873055	4.28E-09
RP11-352	0.910074	-4.00518	6.20E-05
RP11-359	0.757289	-5.8694	4.37E-09
RP11-359	0.701362	-5.93756	2.89E-09
RP11-362	0.902627	-4.68165	2.85E-06
RP11-362	1.1297	4.200063	2.67E-05
RP11-379	1.315129	6.063652	1.33E-09
RP11-379	1.110548	3.747931	0.000178
RP11-384	1.173114	4.95144	7.37E-07
RP11-388	0.892099	-4.14422	3.41E-05
RP11-38L	0.827384	-6.25373	4.01E-10
RP11-415	1.22008	7.289932	3.10E-13
RP11-417	1.063201	3.382034	0.00072
RP11-418	1.366986	5.235965	1.64E-07
RP11-420	1.364595	8.246271	1.63E-16
RP11-421	1.372067	5.700501	1.19E-08
RP11-422	1.688365	7.060017	1.66E-12
RP11-423	1.202685	4.199392	2.68E-05
RP11-429	0.886955	-5.18942	2.11E-07
RP11-434	0.942042	-3.50067	0.000464
RP11-441	0.927201	-3.45701	0.000546
RP11-444	1.044011	3.424162	0.000617
RP11-449	1.168203	3.804065	0.000142
RP11-44N	0.903211	-3.79015	0.000151
RP11-458	1.181472	3.617081	0.000298
RP11-464	0.881369	-4.82092	1.43E-06
RP11-473	1.178846	4.345823	1.39E-05
RP11-480	0.915333	-4.52925	5.92E-06
RP11-48B	1.075591	3.523795	0.000425
RP11-48B	1.222696	4.908567	9.17E-07
RP11-490	0.931815	-4.20413	2.62E-05
RP11-491	0.853411	-9.30994	1.28E-20
RP11-500	1.115736	3.482949	0.000496
RP11-503	0.828154	-5.48332	4.17E-08
RP11-506	0.881777	-3.73319	0.000189
RP11-517	1.068484	3.390371	0.000698

RP11-524	1.114588	6.795343	1.08E-11
RP11-53B	1.104189	4.08131	4.48E-05
RP11-546.	0.901083	-3.35373	0.000797
RP11-54A	1.122346	7.43015	1.08E-13
RP11-582.	1.141583	3.960642	7.47E-05
RP11-5C2	1.246516	6.134391	8.55E-10
RP11-638	0.96245	-3.39018	0.000698
RP11-649.	1.060029	3.470257	0.00052
RP11-658	0.822206	-4.15277	3.28E-05
RP11-660	0.90981	-3.74678	0.000179
RP11-661	1.05682	3.608273	0.000308
RP11-686	0.804523	-4.93567	7.99E-07
RP11-690	0.797339	-5.12847	2.92E-07
RP11-6O2	1.096191	5.442142	5.26E-08
RP11-700.	0.943616	-3.36002	0.000779
RP11-712	1.213771	4.448869	8.63E-06
RP11-723	1.173833	4.960667	7.03E-07
RP11-72I8	1.137566	3.887073	0.000101
RP11-770.	0.761015	-8.52701	1.50E-17
RP11-783	0.813865	-10.1596	3.00E-24
RP11-783	1.11187	4.3081	1.65E-05
RP11-798	1.129465	3.391728	0.000695
RP11-806	1.137881	7.166548	7.69E-13
RP11-817	1.254882	4.716021	2.41E-06
RP11-861	0.857213	-3.85193	0.000117
RP11-893	0.932074	-3.4926	0.000478
RP11-967	1.060039	3.404154	0.000664
RP11-98D	0.905553	-4.87379	1.09E-06
RP11-996	1.176681	4.131291	3.61E-05
RP13-39P	0.913901	-3.78397	0.000154
RP13-514	0.84069	-6.22027	4.96E-10
RP13-516	0.859003	-4.91107	9.06E-07
RP3-368A	1.180947	3.991551	6.56E-05
RP3-402G	0.851028	-4.27017	1.95E-05
RP3-412A	1.15774	3.978872	6.92E-05
RP3-428L	1.208891	6.040274	1.54E-09
RP3-510D	1.17079	6.407538	1.48E-10
RP3-525N	0.845352	-6.34114	2.28E-10
RP4-607I7	1.106383	4.90479	9.35E-07
RP4-635E	1.093919	3.637171	0.000276
RP4-665J2	1.125098	4.225803	2.38E-05
RP4-669L	1.110543	3.321193	0.000896
RP4-756G	1.177646	3.791884	0.00015
RP4-761J	1.056911	3.582651	0.00034
RP4-773N	1.486366	6.683425	2.33E-11
RP4-798A	1.227855	3.987689	6.67E-05
RP4-798P	1.156558	5.794312	6.86E-09
RP4-813D	0.834488	-7.55925	4.05E-14
RP5-1021	0.844171	-6.00998	1.86E-09
RP5-1024	1.082163	3.913939	9.08E-05
RP5-1074	1.27593	5.913704	3.34E-09
RP5-1119.	0.852197	-12.3835	3.21E-35
RP5-1142.	0.792629	-4.90857	9.17E-07
RP5-998N	1.058474	4.440474	8.98E-06
RP6-201G	0.879686	-6.14757	7.87E-10
SDCBP2-A	1.243149	3.810313	0.000139
SH3BP5-A	1.297361	5.146307	2.66E-07
SNAI3-AS	0.68161	-7.31777	2.52E-13

SNHG16	1.275474	4.458144	8.27E-06
SNHG17	1.276125	4.144851	3.40E-05
SNHG9	1.159849	4.833153	1.34E-06
SPATA13	1.181459	5.521616	3.36E-08
STARD4-A	1.089905	3.773259	0.000161
STEAP3-A	1.174055	4.745504	2.08E-06
THAP9-AS	1.275988	5.468797	4.53E-08
TMEM9B-	1.175591	4.893034	9.93E-07
TMPO-AS	1.102054	3.440776	0.00058
UBA6-AS1	1.347343	4.354365	1.33E-05
UBL7-AS1	1.269369	4.105984	4.03E-05
VIM-AS1	1.269193	8.642865	5.48E-18
WDFY3-A	0.820234	-5.04918	4.44E-07
XXbac-BP	1.099256	5.84415	5.09E-09
XXbac-BP	0.928509	-4.00106	6.31E-05
YEATS2-A	1.229646	4.821546	1.42E-06
ZNF674-A	1.333352	4.517306	6.26E-06
