# Supplementary material for: Identification and Verification of an Alternative Polyadenylation-Related lncRNA Prognostic Signature for Glioma
Source: Comput Math Methods Med. 2022 Sep 7;2022:2164229. doi: 10.1155/2022/2164229 (PMC11401696; doi:10.1155/2022/2164229)
Supplement: Supplementary 4 — Table S4: univariate Cox regression analysis of prognosis-related lncRNAs from the TCGA dataset. [file 2164229.f4.pdf]

gene	HR	z	pvalue
TOB1-AS1	0.383245	-11.3919	4.59E-30
AC004492	0.718377	-6.00938	1.86E-09
AL122035	0.567769	-10.0107	1.37E-23
NNT-AS1	0.408502	-8.16015	3.35E-16
AC004817	1.121339	7.70798	1.28E-14
LINC0112[illegible]	1.379821	8.25668	1.50E-16
AC005070	0.401323	-13.7824	3.25E-43
OIP5-AS1	0.501118	-6.72542	1.75E-11
AL731563	0.544913	-7.32821	2.33E-13
AC004908	0.687716	-7.02986	2.07E-12
AL157392	0.398637	-16.0628	4.65E-58
KMT2E-AS[illegible]	1.84113	8.28219	1.21E-16
AC020905	1.173667	10.31009	6.34E-25
AC079414	0.369509	-11.7209	9.96E-32
LEF1-AS1	1.753214	13.75094	5.03E-43
LINC0127[illegible]	0.467379	-5.47322	4.42E-08
AC068620	0.638432	-6.68285	2.34E-11
LINC0088[illegible]	1.755561	10.3772	3.15E-25
HCP5	1.617473	11.19843	4.15E-29
AP003486	0.4275	-12.1737	4.29E-34
AC109439	0.849097	-13.7349	6.27E-43
AF106564	0.838068	-12.4777	9.88E-36
DGCR9	0.556628	-14.5388	6.88E-48
AL450326	0.676396	-8.23038	1.87E-16
AC015961	0.679054	-6.7921	1.11E-11
AC020915	2.995071	13.73703	6.09E-43
LINC0131[illegible]	0.798383	-12.968	1.86E-38
AP006623	0.645811	-7.47087	7.97E-14
AP002360	0.640751	-9.18361	4.17E-20
AL008582	0.808043	-6.47339	9.58E-11
SNHG3	1.581241	5.171365	2.32E-07
AC009041	0.618196	-14.2405	5.13E-46
LINC0115[illegible]	1.408587	6.853147	7.22E-12
MANEA-D[illegible]	0.620306	-9.49247	2.26E-21
LINC0261[illegible]	1.737022	9.0578	1.33E-19
L3MBTL4-	1.496388	7.145225	8.98E-13
LINC0090[illegible]	1.72324	11.19218	4.45E-29
AC147651	1.770096	13.85876	1.13E-43
AL117339	0.473186	-15.9899	1.50E-57
AC096642	0.765113	-4.46873	7.87E-06
AL035461	1.828194	8.995484	2.35E-19
PWAR6	0.600417	-15.0057	6.74E-51
AC104083	0.674704	-7.49121	6.82E-14
AC116552	0.860684	-5.66106	1.50E-08
AC027682	0.745255	-5.97094	2.36E-09
AL133367	0.480311	-8.64811	5.24E-18
AC092119	0.752318	-3.83496	0.000126
MIR9-3H[illegible]	0.58635	-15.9053	5.82E-57
AC005899	0.675952	-7.70453	1.31E-14
AL442128	0.634886	-6.58391	4.58E-11
AL358933	0.666303	-11.8376	2.50E-32
AC093010	0.529048	-11.9948	3.78E-33
AC020916	1.628076	9.151917	5.59E-20
LINC0059[illegible]	1.319285	4.715498	2.41E-06
AL512785	1.204019	8.419405	3.78E-17
AC067750	0.361409	-12.5516	3.90E-36
AC090241	0.589913	-8.73879	2.36E-18

AL356019	0.471783	-9.99234	1.65E-23
AC027307	2.261673	12.12773	7.53E-34
AC124312	0.823315	-12.2779	1.19E-34
AC097359	0.783965	-5.94243	2.81E-09
AL353796	0.330477	-14.6769	9.07E-49
AC020558	0.797571	-3.50291	0.00046
AC127070	0.615151	-14.1478	1.93E-45
NDUFA6-	0.31167	-12.819	1.28E-37
AL021068	0.79789	-5.5844	2.35E-08
LINC0150:	2.053314	13.26264	3.81E-40
AL591848	0.43777	-12.7117	5.09E-37
SLC9A3-A	0.569318	-7.86507	3.69E-15
AL391425	0.664469	-10.2618	1.05E-24
GDNF-AS.	0.740978	-14.7809	1.94E-49
AC126407	0.746306	-10.0134	1.33E-23
AL161421	2.071478	9.611981	7.12E-22
WDFY3-A	0.466317	-12.1458	6.04E-34
LINC0034(	1.791962	12.7629	2.64E-37
AC122129	0.700107	-7.90112	2.76E-15
PSMB8-A!	2.103217	10.87969	1.44E-27
TGFB2-AS	1.438432	9.990122	1.68E-23
AL391069	1.380359	4.033951	5.48E-05
MORF4L2	0.632624	-6.61644	3.68E-11
AP003469	0.473272	-12.4086	2.35E-35
AC013451	1.150614	9.413482	4.80E-21
AL355987	0.711392	-4.25471	2.09E-05
AC015540	0.768148	-13.5347	9.76E-42
SNHG16	2.047928	7.637085	2.22E-14
GUSBP11	0.461045	-9.7152	2.60E-22
LINC0178(	0.714133	-5.2056	1.93E-07
AC120114	0.620007	-8.27781	1.25E-16
AL136964	0.779518	-14.0567	7.01E-45
AC114811	0.536013	-8.45286	2.84E-17
SNHG9	1.817452	9.375366	6.89E-21
AC015967	0.655001	-12.5929	2.31E-36
AL121827	0.763773	-14.1433	2.05E-45
AC132938	0.870459	-3.982	6.83E-05
AC025162	0.642313	-11.2827	1.60E-29
AL731533	0.503303	-13.9148	5.15E-44
AC002070	1.66421	7.642127	2.14E-14
AC008124	0.374666	-10.7862	4.00E-27
AL138960	0.566336	-11.3826	5.11E-30
MIR22HG	2.070056	11.21872	3.30E-29
PAXBP1-A	0.294921	-12.1645	4.80E-34
AC010655	1.612046	11.11515	1.06E-28
AC078909	0.889784	-5.54173	3.00E-08
ENTPD1-/	0.380349	-11.9176	9.58E-33
AP001528	1.46961	5.566331	2.60E-08
AC008610	0.606771	-7.5422	4.62E-14
LINC0092{	0.797554	-9.05834	1.32E-19
AL590666	0.695047	-10.3435	4.48E-25
AC009126	0.444862	-6.81291	9.56E-12
AC010186	1.516074	5.418769	6.00E-08
ADGRA1-.	0.71304	-14.7781	2.03E-49
AL355388	0.790633	-3.59146	0.000329
AC245052	2.34169	11.92151	9.14E-33
Z83843.1	0.623909	-6.43687	1.22E-10
AC087741	0.764629	-4.09293	4.26E-05

AC068152	0.623857	-4.35485	1.33E-05
LINC0064:	0.453288	-14.8654	5.53E-50
AC120498	0.848487	-3.3173	0.000909
AL161457	0.716396	-4.89098	1.00E-06
AL445309	0.614496	-12.9488	2.39E-38
AC017002	1.162944	10.24907	1.19E-24
AC061961	0.88195	-8.46138	2.64E-17
LINC0191	1.17439	9.08327	1.05E-19
TMPO-AS	1.641301	6.922498	4.44E-12
AC005696	0.662886	-12.9035	4.30E-38
AP003352	1.407015	3.933282	8.38E-05
AC011899	1.661691	10.74877	6.01E-27
AC005776	2.065711	11.25829	2.11E-29
ANKRD10	0.775963	-3.42121	0.000623
AP000766	0.461015	-12.4447	1.49E-35
AC107027	0.429776	-9.38471	6.31E-21
AC067852	0.772154	-7.07107	1.54E-12
AC138207	1.866788	10.04073	1.01E-23
AC254562	0.651865	-15.5782	1.02E-54
AL157394	1.497119	7.670753	1.71E-14
LINC0215	1.114597	8.209328	2.22E-16
AL160313	0.532005	-14.2622	3.76E-46
AC010226	2.103537	13.64888	2.05E-42
AC002456	2.012084	13.01095	1.06E-38
AC107375	0.475656	-11.8805	1.50E-32
AC011912	0.693112	-11.1144	1.07E-28
BX537318	2.047288	11.31367	1.12E-29
AC073254	0.527405	-8.48581	2.14E-17
DDX11-AS	1.613999	6.46852	9.90E-11
AC007608	0.668929	-10.3937	2.65E-25
ZNF32-AS	0.576559	-7.85435	4.02E-15
AL157700	0.49482	-15.4808	4.68E-54
AC138028	0.664517	-12.144	6.17E-34
POLH-AS:	1.443631	4.474183	7.67E-06
AC093673	2.086896	14.2012	9.01E-46
AL590999	0.735744	-8.62217	6.57E-18
AC010973	1.816349	9.075728	1.13E-19
AL133355	0.434448	-9.74326	1.97E-22
TSC22D1-	0.577842	-8.30011	1.04E-16
CYTOR	1.615754	16.10648	2.30E-58
RAB11B-A	2.045514	7.673868	1.67E-14
RNF139-A	0.659771	-4.13884	3.49E-05
AP001453	1.710441	10.62931	2.18E-26
AL158163	0.487036	-13.5559	7.31E-42
MIR600HC	0.576863	-9.50081	2.08E-21
AC025917	0.83873	-7.81806	5.36E-15
N4BP2L2-	0.657657	-5.21558	1.83E-07
RAP2C-AS	0.73436	-3.45129	0.000558
RUNDC3A	0.564123	-12.9501	2.35E-38
AL121890	0.729924	-9.18163	4.25E-20
AC009113	0.384388	-11.5663	6.11E-31
AP001486	0.431308	-15.138	9.10E-52
CRNDE	1.668111	13.49002	1.79E-41
AL512625	0.690481	-10.2211	1.59E-24
AC093627	1.851098	9.396312	5.65E-21
DLEU1	1.528312	5.323173	1.02E-07
AL445524	2.433985	14.68804	7.69E-49
SGMS1-A:	0.424493	-14.3901	5.97E-47

LINC0029	0.390166	-9.42521	4.29E-21
AC004803	0.380193	-13.7088	8.99E-43
LINC0259	0.618676	-14.5777	3.89E-48
AC007406	0.395662	-9.8323	8.17E-23
AC138207	1.51694	6.776503	1.23E-11
AGAP2-AS	1.532253	16.79463	2.67E-63
AP000924	1.201941	6.588994	4.43E-11
AC112491	1.558897	9.847888	7.00E-23
AL158212	0.394649	-14.5465	6.15E-48
AC010834	0.547856	-9.16204	5.09E-20
AL122125	0.761909	-4.59844	4.26E-06
AC026271	0.741015	-3.97324	7.09E-05
AC124312	0.722793	-12.0694	1.53E-33
AL138724	2.809964	12.43667	1.65E-35
VIM-AS1	1.773879	9.627046	6.15E-22
SMIM25	1.502732	12.11182	9.14E-34
LINC0150	1.413872	7.476272	7.65E-14
AC009118	0.36588	-10.5202	6.97E-26
RNF219-A	0.698558	-12.4955	7.90E-36
FP671120.	1.356397	12.11832	8.45E-34
OSMR-AS	1.549702	11.52839	9.49E-31
FAM13A-A	0.513907	-9.23996	2.47E-20
AC003092	1.155504	11.24959	2.33E-29
AC007038	0.55777	-6.18447	6.23E-10
DICER1-A	0.282975	-12.2789	1.18E-34
AC012360	0.421365	-7.69174	1.45E-14
HAR1A	0.748696	-12.3389	5.59E-35
PCED1B-A	1.550266	8.499846	1.90E-17
AC017083	0.61653	-10.8031	3.33E-27
CTBP1-DT	0.55774	-4.6049	4.13E-06
SLC16A1-	3.278536	13.46315	2.58E-41
AL512353	1.987948	9.064533	1.25E-19
ZKSCAN2-	0.652226	-5.82378	5.75E-09
LINC0060	1.431093	9.840379	7.54E-23
LINC0142	1.630101	14.3892	6.05E-47
LINC0171	0.746291	-4.80507	1.55E-06
AC084125	0.670215	-6.85522	7.12E-12
LINC0228	1.405283	6.832849	8.32E-12
FO393401	1.500937	6.742413	1.56E-11
SEMA6A-A	0.770389	-3.85172	0.000117
AL359643	2.638655	12.30602	8.41E-35
LPP-AS2	1.742865	5.980604	2.22E-09
AL162741	0.797419	-3.74967	0.000177
AC008669	0.408425	-13.9615	2.68E-44
AC009148	0.620942	-7.10946	1.16E-12
FLJ37035	0.371653	-15.3485	3.63E-53
RFPL1S	0.776877	-7.35098	1.97E-13
LINC0114	1.232943	7.76183	8.37E-15
AC027130	0.686484	-12.2413	1.87E-34
MCM3AP-	0.584067	-5.54609	2.92E-08
AC132192	0.641003	-5.21472	1.84E-07
AC010300	0.710084	-7.78339	7.06E-15
AC120053	0.422221	-8.68177	3.90E-18
AC110285	0.733269	-5.99628	2.02E-09
AC026801	1.738261	8.659687	4.73E-18
AL450384	0.462451	-11.2144	3.47E-29
FLJ46284	0.561241	-12.7926	1.80E-37
MIR155HC	1.835122	16.4789	5.20E-61

AC012213	0.842939	-12.4683	1.11E-35
SOCS2-AS	1.611228	13.29555	2.46E-40
AC021739	0.715622	-11.8023	3.80E-32
AL353751	0.501076	-12.1124	9.08E-34
AC009088	0.634736	-7.83733	4.60E-15
KIZ-AS1	0.743789	-6.57461	4.88E-11
AF111167	0.652546	-6.29704	3.03E-10
EBLN3P	0.63615	-3.4998	0.000466
AC008760	1.411717	11.09151	1.38E-28
AL391422	1.611449	7.523808	5.32E-14
LBX2-AS1	2.055143	12.8116	1.41E-37
AC083799	2.462736	9.767888	1.55E-22
AC018638	0.777764	-3.43752	0.000587
LINC0260	1.535572	10.89366	1.24E-27
AC245060	0.626902	-13.4739	2.23E-41
AL441992	2.576362	14.30353	2.08E-46
NCAM1-A	0.835609	-5.72955	1.01E-08
BX640514	1.199878	10.13674	3.80E-24
LINC0163	1.140509	9.334867	1.01E-20
AC253576	0.875107	-4.43344	9.27E-06
AL354740	1.870238	10.21364	1.72E-24
AC053513	0.83589	-6.34681	2.20E-10
AC009283	0.496501	-6.53547	6.34E-11
AL118505	0.709304	-15.181	4.73E-52
HOXA-AS	1.249534	12.64015	1.27E-36
AC099560	1.095122	6.832022	8.37E-12
SMCR5	0.821069	-11.8132	3.34E-32
WAC-AS1	0.27661	-14.7822	1.91E-49
AC012593	0.862445	-10.7052	9.62E-27
NORAD	0.434139	-8.25735	1.49E-16
ZNF236-D	2.428992	12.86468	7.11E-38
AL136295	0.29943	-10.8997	1.16E-27
SLC25A25	0.754876	-3.73133	0.00019
EMX2OS	0.829996	-5.17596	2.27E-07
AC110285	0.676412	-12.8632	7.25E-38
AC060766	1.708173	9.652265	4.81E-22
AC016773	1.282106	3.958977	7.53E-05
ACAP2-IT	0.749793	-6.93474	4.07E-12
ZFHX2-AS	0.472312	-13.6866	1.22E-42
FOXN3-AS	1.761182	7.456269	8.90E-14
AC009102	0.770417	-12.2888	1.04E-34
AC095057	0.438284	-12.8638	7.19E-38
LINC0240	1.408229	10.06844	7.62E-24
OTUD6B-A	0.34153	-10.3725	3.31E-25
AL139274	0.532028	-10.5448	5.37E-26
AC022613	1.728666	13.18797	1.03E-39
ZBTB20-A	0.789059	-12.1012	1.04E-33
AC120036	0.546722	-15.0882	1.94E-51
ZNF710-A	0.473927	-11.0165	3.18E-28
BCRP3	0.649983	-7.3898	1.47E-13
LUCAT1	1.32377	9.880497	5.06E-23
AL583810	0.808002	-6.95798	3.45E-12
AC126118	0.790933	-5.70099	1.19E-08
AC018635	0.893193	-4.71627	2.40E-06
AL021368	0.70195	-9.02613	1.78E-19
AL132711	1.138179	7.305502	2.76E-13
AL136295	0.523549	-6.87981	5.99E-12
LANCL1-A	0.549069	-11.7578	6.44E-32

ACVR2B-/	0.568586	-7.50005	6.38E-14
AC093788	0.784234	-3.64352	0.000269
RNF217-A	0.700728	-9.7879	1.27E-22
AC026356	1.845846	9.193874	3.79E-20
BHLHE40-	1.812674	7.777615	7.39E-15
KCNK4-TE	0.785291	-11.2938	1.41E-29
AL133415	1.459265	14.36053	9.15E-47
AC022400	0.769429	-5.01101	5.41E-07
BCDIN3D-	0.396033	-9.87978	5.09E-23
LINC0046:	0.733127	-11.9775	4.66E-33
AC009227	0.814147	-14.6507	1.33E-48
LOXL1-AS	1.475638	12.60789	1.91E-36
LINC0032	1.864981	9.36841	7.36E-21
AP000721	0.794722	-12.5616	3.43E-36
IL21-AS1	1.148999	9.196591	3.69E-20
AL355974	1.569161	12.96117	2.03E-38
LINC0199:	1.25853	10.59045	3.30E-26
ZNF529-A	2.458536	11.04691	2.27E-28
AC022613	1.996752	8.225284	1.95E-16
SNHG26	1.477055	9.579106	9.79E-22
AC007292	0.470913	-9.27761	1.73E-20
LINC0034:	0.701424	-5.18232	2.19E-07
AC232271	0.684208	-4.43461	9.22E-06
PAXIP1-A:	2.172242	12.836	1.03E-37
LINC0152:	0.566577	-9.64032	5.40E-22
AL022313	0.84343	-13.3281	1.59E-40
AL162171	0.507205	-12.4422	1.54E-35
GABPB1-/	0.56577	-7.30767	2.72E-13
AC098613	1.364158	7.246852	4.27E-13
FAM111A	2.470219	8.615325	6.97E-18
TMEM72-	0.514011	-14.0024	1.51E-44
PVT1	1.834128	13.57976	5.28E-42
LINC0113	1.937824	9.162569	5.07E-20
AC017002	1.151915	9.870093	5.61E-23
AC004067	1.832	12.55747	3.62E-36
AL357060	1.912996	13.66661	1.61E-42
AF111169	0.71393	-7.14371	9.08E-13
AL049597	2.07277	8.065013	7.32E-16
SLC25A21	0.809369	-15.5335	2.06E-54
LINCR-00	0.730509	-6.49643	8.22E-11
AL354919	1.279996	10.44716	1.51E-25
AL391807	0.590784	-10.749	5.99E-27
AC104964	0.748422	-4.66863	3.03E-06
LRRC75A-	0.755694	-3.35574	0.000792
AL035563	0.590161	-6.75395	1.44E-11
LNCTAM3	1.617154	13.10917	2.92E-39
AC106782	0.7701	-3.36134	0.000776
AC073611	2.17339	12.51214	6.41E-36
AC016876	2.326867	9.356715	8.23E-21
AC006033	1.473861	7.290477	3.09E-13
AC022762	0.543384	-10.3925	2.68E-25
AC027348	0.725158	-9.73868	2.06E-22
AC005034	0.61618	-4.38904	1.14E-05
AC103691	0.770323	-4.03265	5.52E-05
AL022328	0.659953	-9.18702	4.04E-20
DNMBP-A	0.798759	-12.3208	7.00E-35
AC026401	1.997231	14.09775	3.92E-45
AL359878	0.607916	-10.8976	1.18E-27

AP000866	0.697489	-9.20142	3.53E-20
AL513217	0.638723	-11.4841	1.59E-30
STX18-AS	0.389343	-7.78217	7.13E-15
TMEM220	1.762671	13.57652	5.52E-42
ITGB2-AS:	1.342369	5.743607	9.27E-09
STXBP5-A	0.741897	-5.37562	7.63E-08
DNAJC3-I	0.396242	-10.7981	3.52E-27
LINC0112	0.829942	-3.50536	0.000456
AL160270	0.831936	-10.5929	3.21E-26
AL159169	0.551855	-9.64523	5.15E-22
SNHG18	1.759957	14.62773	1.87E-48
NRAV	2.587697	12.99841	1.25E-38
AL135999	0.748037	-13.8127	2.14E-43
RAD51-AS	0.524027	-5.77532	7.68E-09
AL035530	0.361831	-14.393	5.72E-47
AP000757	0.581009	-12.5047	7.04E-36
AC107398	0.746343	-9.50246	2.05E-21
CARD8-AS	2.228577	13.45059	3.05E-41
AC009690	2.478876	12.09232	1.16E-33
PIK3CD-A	1.492188	13.04103	7.15E-39
AC018797	3.048431	10.54619	5.29E-26
AP002360	0.702199	-10.202	1.94E-24
AL133325	0.702832	-6.9034	5.08E-12
AL713998	1.179633	12.51114	6.49E-36
ZNF433-A	2.434151	9.107036	8.47E-20
AC125807	1.242153	5.663936	1.48E-08
BX324167	0.699303	-11.5228	1.01E-30
AL606970	1.183666	10.51585	7.30E-26
AC007375	0.775443	-13.5162	1.26E-41
AC053503	0.742268	-13.2136	7.32E-40
AC011477	0.59991	-5.89009	3.86E-09
AL356056	0.580692	-12.18	3.97E-34
AC084018	0.778493	-4.36424	1.28E-05
AC245060	0.722262	-5.47071	4.48E-08
LINC0033	3.249511	12.74419	3.36E-37
AP000350	0.51179	-12.1848	3.75E-34
AP000892	0.82957	-4.15076	3.31E-05
HOTAIRM	1.479312	14.55085	5.77E-48
AC105219	0.564455	-7.66401	1.80E-14
C1RL-AS1	1.840906	13.66205	1.71E-42
LINC0127	1.975121	12.77376	2.30E-37
AC060766	1.647735	9.29674	1.45E-20
AC018648	0.500658	-12.1743	4.26E-34
AL357033	1.821866	10.59074	3.29E-26
TMEM254	0.388647	-12.968	1.86E-38
AC093895	1.177507	12.60708	1.93E-36
AC124312	0.712542	-13.817	2.01E-43
ZNF571-A	0.512385	-10.277	8.95E-25
AC118344	0.823159	-6.14405	8.04E-10
DLGAP4-A	2.148999	11.20234	3.97E-29
MIR4435-	1.630385	15.22816	2.30E-52
AL355922	1.138832	5.121197	3.04E-07
BASP1-AS	0.82737	-11.9508	6.43E-33
USP30-AS	1.460119	10.69904	1.03E-26
TRHDE-AS	0.848462	-12.0138	3.01E-33
LINC0096	1.284216	7.701269	1.35E-14
AC073389	0.482745	-14.1162	3.02E-45
DLGAP1-A	2.201805	10.66771	1.44E-26

AC010319	2.773262	11.37642	5.48E-30
ZNF252P-	0.560017	-7.36996	1.71E-13
AC147067	1.410022	6.337541	2.33E-10
AC010864	0.381297	-11.9176	9.58E-33
AC108449	0.699315	-7.20433	5.83E-13
AL122035	0.453454	-8.65991	4.72E-18
AC015922	1.73105	10.09623	5.74E-24
AC018647	1.793352	5.749649	8.94E-09
AC007344	1.248893	8.767381	1.83E-18
LINC0108	0.549846	-7.97308	1.55E-15
AL590644	1.196963	12.90443	4.25E-38
SUCLG2-A	1.505557	5.054198	4.32E-07
SNHG12	1.879811	7.019815	2.22E-12
AC026471	0.585607	-5.20706	1.92E-07
AL513320	0.704965	-6.15186	7.66E-10
AL589935	0.736556	-6.22149	4.92E-10
AC090796	1.16399	11.93204	8.06E-33
LINC0122	1.65638	8.401639	4.40E-17
AC002398	2.213951	11.46284	2.03E-30
AL731569	0.378787	-12.7723	2.34E-37
AC093726	2.003473	12.21472	2.59E-34
HCG18	0.576231	-4.67567	2.93E-06
AL450263	0.797794	-6.61274	3.77E-11
SBF2-AS1	1.846209	12.22569	2.27E-34
AC061992	1.487155	12.3595	4.33E-35
TRG-AS1	1.62742	8.921947	4.58E-19
AC007619	0.807233	-9.17111	4.68E-20
ID2-AS1	0.479899	-11.5368	8.61E-31
AL022328	0.608345	-7.11017	1.16E-12
AC004148	0.718649	-4.24806	2.16E-05
AL117332	3.065339	14.78134	1.93E-49
