# Supplementary material for: Identification and Verification of an Alternative Polyadenylation-Related lncRNA Prognostic Signature for Glioma
Source: Comput Math Methods Med. 2022 Sep 7;2022:2164229. doi: 10.1155/2022/2164229 (PMC11401696; doi:10.1155/2022/2164229)
Supplement: Supplementary 5 — Table S5: differentially expressed genes in the high- and low-risk groups of the CGGA dataset. [file 2164229.f5.pdf]

logFC	AveExpr	t	P.Value	adj.P.Val	B	
SERPINH1	2.108289	4.656706	21.73628	2.35E-79	3.92E-75	170.0057
IGFBP2	3.29518	6.265065	21.1393	4.46E-76	3.72E-72	162.5106
COL4A2	2.951223	4.546207	20.94537	5.13E-75	2.85E-71	160.084
TNFRSF12	2.746887	4.177909	20.60221	3.83E-73	1.60E-69	155.8008
SRPX2	2.801411	0.616169	20.44337	2.81E-72	9.38E-69	153.8232
COL4A1	3.351609	3.43169	20.15096	1.09E-70	3.03E-67	150.1915
IFI30	2.247164	5.171794	20.11233	1.76E-70	4.20E-67	149.7127
HSPG2	2.087895	3.580707	19.88099	3.15E-69	6.44E-66	146.8493
ESM1	3.610118	-0.23469	19.67844	3.91E-68	5.93E-65	144.349
COL3A1	3.519279	2.396856	19.58809	1.20E-67	1.67E-64	143.2358
ALDOC	-2.15311	8.053794	-19.5293	2.49E-67	3.19E-64	142.5119
ETNPPL	-3.40745	3.360247	-19.0982	5.11E-65	6.10E-62	137.2241
GABRG1	-2.95348	-0.18547	-18.9062	5.42E-64	5.33E-61	134.8793
CD93	2.238032	1.401714	18.78688	2.35E-63	2.17E-60	133.4254
RASL10A	-2.49837	5.070361	-18.6454	1.33E-62	1.16E-59	131.7053
COL1A1	3.148516	2.674028	18.57578	3.11E-62	2.59E-59	130.8603
PLAU	2.440314	1.974978	18.5039	7.47E-62	5.94E-59	129.9889
ANXA2	2.19258	5.807067	18.33035	6.19E-61	4.70E-58	127.8893
SOCS3	2.95373	2.607233	18.22522	2.22E-60	1.61E-57	126.6205
COL6A2	2.477031	4.529312	18.14674	5.76E-60	4.01E-57	125.675
COL1A2	2.468828	3.748576	18.09895	1.03E-59	6.86E-57	125.0999
MMP9	4.03723	0.725998	18.07758	1.33E-59	8.55E-57	124.8428
HSPA6	2.193672	1.25385	17.84554	2.20E-58	1.22E-55	122.0586
FBLIM1	2.10493	2.338397	17.8019	3.72E-58	2.00E-55	121.5363
EXOC3L2	2.753573	-1.35882	17.74312	7.56E-58	3.94E-55	120.8335
FN1	2.008368	6.322847	17.68675	1.49E-57	7.31E-55	120.1604
CTHRC1	2.301345	1.625275	17.62907	2.98E-57	1.38E-54	119.4724
PLAT	2.147234	3.256857	17.5533	7.39E-57	3.33E-54	118.5697
MXRA5	2.197945	-1.11441	17.51992	1.10E-56	4.84E-54	118.1725
STC1	2.701983	-1.73279	17.35381	8.04E-56	3.27E-53	116.2
KIF4A	2.042059	0.16534	17.29704	1.58E-55	6.14E-53	115.5274
FSTL5	-2.54278	-0.86643	-17.2897	1.73E-55	6.55E-53	115.441
MS4A6A	2.06942	4.422574	17.2563	2.57E-55	9.14E-53	115.0453
CD248	2.133141	1.854319	17.1757	6.72E-55	2.34E-52	114.0926
GBP5	2.042937	-1.26806	17.16637	7.51E-55	2.56E-52	113.9826
ADAMDE(	3.21815	-2.44428	17.10495	1.56E-54	5.10E-52	113.2579
GPX8	2.506385	-0.42309	17.09041	1.85E-54	5.94E-52	113.0865
PLEK2	2.444021	-0.86498	17.07264	2.29E-54	7.06E-52	112.8771
MMP14	2.129441	4.622482	16.96568	8.12E-54	2.38E-51	111.6185
RAB42	2.109941	0.037412	16.9297	1.24E-53	3.52E-51	111.1957
HPCAL4	-2.47556	2.391398	-16.8422	3.50E-53	9.41E-51	110.1694
LYZ	2.413913	1.664293	16.7676	8.43E-53	2.13E-50	109.2957
SLCO1A2	-2.23361	1.588004	-16.765	8.70E-53	2.17E-50	109.2652
EMR1	2.204346	-1.87448	16.66472	2.83E-52	6.44E-50	108.0936
IL2RA	2.806793	-3.2281	16.66456	2.84E-52	6.44E-50	108.0917
SERPINE1	2.727798	1.774807	16.6467	3.50E-52	7.68E-50	107.8833
ADAM12	2.255751	-0.22293	16.64452	3.59E-52	7.78E-50	107.8579
IGFBP3	2.494919	4.023099	16.64306	3.65E-52	7.81E-50	107.8408
GDF15	2.497028	1.669022	16.45549	3.29E-51	6.54E-49	105.658
PRLHR	-3.58924	-1.77743	-16.3773	8.21E-51	1.54E-48	104.7515
SLC22A6	-2.68155	-0.73066	-16.3163	1.67E-50	3.04E-48	104.0447
HTRA3	2.073789	0.594253	16.31268	1.75E-50	3.13E-48	104.003
FPR3	2.113529	-0.07711	16.289	2.30E-50	4.04E-48	103.7292
CD163	2.535273	2.610311	16.18377	7.82E-50	1.32E-47	102.5142
TREM1	3.107371	0.017877	16.17539	8.62E-50	1.42E-47	102.4176
SLC25A48	-2.64368	3.274818	-16.169	9.29E-50	1.52E-47	102.3438
SLAMF7	2.057051	-1.96671	16.1578	1.06E-49	1.71E-47	102.215

SPOCD1	3.091215	2.900712	16.14981	1.16E-49	1.86E-47	102.1229
OR51E1	2.317242	-3.66084	16.11318	1.77E-49	2.79E-47	101.7011
MKX	-2.21059	-0.98918	-16.0651	3.10E-49	4.79E-47	101.1481
ANXA1	2.492375	4.564185	16.06093	3.25E-49	4.98E-47	101.1003
COL5A1	2.259888	1.324314	16.03901	4.19E-49	6.24E-47	100.8484
KCNIP2	-2.12728	3.784194	-16.0176	5.37E-49	7.93E-47	100.6027
LIF	2.584376	-0.5216	15.95723	1.08E-48	1.51E-46	99.91025
PDCD1	2.059856	-2.40956	15.93553	1.39E-48	1.93E-46	99.66166
TIMP1	2.903208	6.543323	15.9156	1.74E-48	2.39E-46	99.43352
CFI	2.002477	2.417495	15.8798	2.63E-48	3.55E-46	99.02389
CXCL10	2.570104	-0.4681	15.84177	4.08E-48	5.41E-46	98.58927
SCN3B	-2.17825	2.491022	-15.8318	4.58E-48	6.01E-46	98.47597
GPNMB	2.246187	3.408495	15.82818	4.77E-48	6.22E-46	98.43405
FCGR2B	2.523317	0.211849	15.76984	9.33E-48	1.19E-45	97.76855
PTPRT	-2.28346	0.067737	-15.7479	1.20E-47	1.51E-45	97.51811
IBSP	3.552218	-2.68619	15.61717	5.37E-47	6.36E-45	96.03199
JPH3	-2.4643	3.244524	-15.615	5.51E-47	6.47E-45	96.00706
KSR2	-2.08008	-1.87378	-15.614	5.57E-47	6.50E-45	95.99609
HTR2A	-2.08466	-0.98328	-15.6078	5.98E-47	6.88E-45	95.92567
EMP3	2.268273	4.88483	15.60075	6.48E-47	7.38E-45	95.84567
SERPINA5	2.706213	-1.33953	15.58621	7.65E-47	8.52E-45	95.68076
PTX3	2.401708	0.447085	15.5792	8.29E-47	9.04E-45	95.60129
KCNJ3	-2.18867	-0.71388	-15.5778	8.43E-47	9.13E-45	95.58537
ASPM	2.051789	-0.87757	15.53233	1.42E-46	1.47E-44	95.07027
MELK	2.149516	0.264188	15.52987	1.46E-46	1.49E-44	95.04241
CDH18	-2.75793	0.264027	-15.4826	2.50E-46	2.54E-44	94.50788
HS3ST4	-2.45558	-0.39454	-15.4814	2.53E-46	2.56E-44	94.49394
HPSE2	-2.7237	-1.74884	-15.4246	4.83E-46	4.74E-44	93.85295
COL6A3	2.595055	-0.22244	15.38484	7.59E-46	7.36E-44	93.40426
RRM2	2.098973	1.344781	15.37929	8.08E-46	7.80E-44	93.3417
CEP55	2.029604	-0.82696	15.36523	9.48E-46	9.10E-44	93.18335
CHI3L1	3.600486	6.113236	15.32608	1.48E-45	1.40E-43	92.74252
CLEC12A	2.136949	-1.55344	15.32342	1.52E-45	1.43E-43	92.71259
LRTM2	-2.78117	-0.01932	-15.2447	3.72E-45	3.34E-43	91.82764
RASGRF1	-2.06333	1.035396	-15.2287	4.46E-45	3.93E-43	91.64816
CXCL9	2.17784	-2.25676	15.18281	7.48E-45	6.34E-43	91.13427
KCNK4	-2.2455	0.862851	-15.1789	7.82E-45	6.59E-43	91.09013
GALNT5	2.261918	-4.13578	15.10899	1.72E-44	1.41E-42	90.30837
IDO1	2.496064	-3.79119	15.08336	2.29E-44	1.86E-42	90.02212
SH3GL2	-2.18731	3.138418	-15.0741	2.55E-44	2.04E-42	89.91874
MGAT4C	-2.17041	-0.80326	-15.0681	2.73E-44	2.17E-42	89.8515
PON1	-2.16486	-1.75976	-15.0171	4.83E-44	3.77E-42	89.28323
NTSR2	-2.7925	2.568543	-15.0163	4.88E-44	3.79E-42	89.27426
EN1	3.188845	-1.23052	14.95233	1.00E-43	7.45E-42	88.56217
COL5A2	2.015759	2.033257	14.91299	1.55E-43	1.13E-41	88.12509
ABCC8	-2.38615	4.103466	-14.8161	4.59E-43	3.23E-41	87.05085
PPP1R1A	-2.13346	2.920709	-14.7784	6.98E-43	4.78E-41	86.6336
DLGAP5	2.235864	-0.84537	14.72213	1.31E-42	8.83E-41	86.01255
HTR2C	-2.0763	-3.29848	-14.6228	3.94E-42	2.49E-40	84.91858
SPHKAP	-2.45166	-0.84515	-14.5799	6.33E-42	3.93E-40	84.44742
GJB6	-3.23386	-0.52472	-14.5725	6.87E-42	4.23E-40	84.36597
METTL7B	2.257988	2.789269	14.56834	7.19E-42	4.40E-40	84.32026
SNAP91	-2.08885	3.047524	-14.508	1.40E-41	8.39E-40	83.65837
PAK7	-2.03177	-0.46858	-14.5019	1.50E-41	8.94E-40	83.59195
IGHG2	3.725622	-0.03898	14.49096	1.69E-41	9.87E-40	83.47219
CACNG3	-3.04218	-0.75884	-14.4819	1.87E-41	1.08E-39	83.37341
CACNG2	-2.85997	-0.70526	-14.4603	2.37E-41	1.36E-39	83.13633
NDC80	2.006093	0.545616	14.44792	2.72E-41	1.54E-39	83.00156

DLGAP2	-2.10524	-2.13198	-14.4453	2.80E-41	1.58E-39	82.97333
CABP1	-2.40155	3.239723	-14.3853	5.42E-41	2.98E-39	82.31788
SERPINA3	2.593957	6.366505	14.37382	6.15E-41	3.37E-39	82.19281
ABCC3	2.476931	1.218466	14.36697	6.63E-41	3.63E-39	82.11818
FAM19A1	-2.35566	-0.78725	-14.3349	9.42E-41	5.04E-39	81.76926
ADARB2	-2.06936	2.178215	-14.2566	2.22E-40	1.15E-38	80.9177
TROAP	2.061144	1.433499	14.24102	2.64E-40	1.34E-38	80.74896
VSTM2A	-2.82692	1.343305	-14.2377	2.73E-40	1.38E-38	80.71305
SCRT1	-2.37781	1.779219	-14.1989	4.18E-40	2.06E-38	80.2925
VEGFA	2.357471	5.583258	14.18764	4.72E-40	2.30E-38	80.17063
CLVS2	-2.04597	-1.18824	-14.1709	5.67E-40	2.76E-38	79.98997
IGF2BP2	2.268044	-0.69408	14.12132	9.73E-40	4.65E-38	79.4536
PACSIN1	-2.77221	2.240016	-14.1162	1.03E-39	4.91E-38	79.39839
COL8A1	2.715267	-0.72497	14.10906	1.11E-39	5.29E-38	79.3212
MMP7	2.927052	-2.55868	14.08746	1.41E-39	6.59E-38	79.08821
NEUROD6	-2.81407	-2.44907	-14.0872	1.41E-39	6.60E-38	79.08488
CNNM1	-2.07473	-0.93138	-14.0389	2.38E-39	1.09E-37	78.56475
FCGR2C	2.733411	0.249281	14.03447	2.50E-39	1.14E-37	78.51732
CHRM1	-2.5343	1.022314	-14.0221	2.86E-39	1.30E-37	78.38436
SLC8A2	-2.1538	1.562446	-13.9463	6.50E-39	2.84E-37	77.57036
TRPM8	2.647004	-1.83562	13.9381	7.11E-39	3.07E-37	77.4821
MFAP2	2.147601	1.223083	13.88769	1.22E-38	5.23E-37	76.94211
SIT1	2.017476	-2.14454	13.88545	1.25E-38	5.33E-37	76.91812
KIF20A	2.08934	0.063143	13.87342	1.43E-38	5.99E-37	76.78942
IGFN1	-2.87748	0.360648	-13.8082	2.88E-38	1.17E-36	76.09272
SRRM4	-2.22284	-0.64671	-13.7694	4.38E-38	1.74E-36	75.67914
GRIN1	-3.27276	4.114255	-13.7321	6.53E-38	2.53E-36	75.28258
CACNA1B	-2.19408	-0.26891	-13.669	1.28E-37	4.87E-36	74.61236
HOXD11	3.315618	-3.09895	13.65728	1.46E-37	5.49E-36	74.48771
PRKCG	-2.31079	0.612872	-13.6414	1.73E-37	6.44E-36	74.31922
HOXC9	3.008307	-3.11777	13.59053	2.97E-37	1.07E-35	73.78089
GABRD	-2.13033	3.062626	-13.5876	3.07E-37	1.10E-35	73.74986
GABRB3	-2.1541	1.780475	-13.5705	3.68E-37	1.30E-35	73.56911
DACH2	-2.26204	-0.66961	-13.5264	5.88E-37	2.04E-35	73.10338
WSCD2	-2.42021	-0.27855	-13.5129	6.79E-37	2.33E-35	72.96179
ACTG2	2.227202	0.277631	13.50131	7.68E-37	2.62E-35	72.83918
KHDRBS2	-2.04912	-1.19204	-13.4887	8.78E-37	2.97E-35	72.70657
MMP1	2.359632	-3.9249	13.48326	9.30E-37	3.12E-35	72.64909
GZMK	2.211417	-2.62803	13.45522	1.25E-36	4.13E-35	72.3541
CPLX2	-2.85952	4.12974	-13.4469	1.37E-36	4.48E-35	72.26703
IGLL5	2.927096	-3.34741	13.43309	1.58E-36	5.13E-35	72.12151
HRH3	-2.59244	0.987149	-13.4301	1.64E-36	5.28E-35	72.08972
GABRG2	-2.84982	0.318596	-13.4217	1.79E-36	5.76E-35	72.00174
CBLN2	-2.52268	0.034416	-13.4214	1.79E-36	5.77E-35	71.99841
UBE2C	2.048186	3.373697	13.3767	2.88E-36	9.11E-35	71.52988
SSTR1	-2.34699	-0.75162	-13.3449	4.03E-36	1.27E-34	71.19656
NNMT	2.643917	3.424216	13.33203	4.61E-36	1.44E-34	71.06216
OSR2	2.642897	-2.19354	13.31334	5.62E-36	1.75E-34	70.86674
SYN2	-2.49126	2.09805	-13.296	6.75E-36	2.08E-34	70.68538
GABRA1	-2.8179	-0.23246	-13.2888	7.27E-36	2.24E-34	70.61059
SNCG	-2.33627	5.461899	-13.2552	1.04E-35	3.13E-34	70.26006
AMER3	-2.44492	-0.64456	-13.2201	1.50E-35	4.44E-34	69.89416
CHRNA9	2.639768	-2.62865	13.21421	1.59E-35	4.71E-34	69.83287
HMGA2	2.514641	-3.60352	13.20885	1.69E-35	4.96E-34	69.77709
11-Mar	-2.78695	-1.35627	-13.1737	2.44E-35	7.09E-34	69.41156
LUZP2	-2.22235	2.233674	-13.1292	3.89E-35	1.11E-33	68.94977
SYN1	-2.04895	3.727484	-13.1267	3.99E-35	1.14E-33	68.92361
MYBL2	2.014223	1.695167	13.12434	4.09E-35	1.16E-33	68.8995

SVOP	-2.88421	1.459509	-13.1144	4.54E-35	1.28E-33	68.79607
EIF4E1B	-2.45954	-1.70016	-13.1028	5.12E-35	1.44E-33	68.67628
SYNPR	-2.67033	0.439142	-13.0812	6.42E-35	1.79E-33	68.45261
IL1RN	2.101393	-1.20205	13.07722	6.69E-35	1.86E-33	68.41151
HOXC6	2.536749	-3.27106	13.04734	9.14E-35	2.52E-33	68.10265
PLA2G2A	3.679509	-1.38304	13.04612	9.25E-35	2.54E-33	68.09012
PHLDA2	2.076835	0.769884	13.04514	9.35E-35	2.56E-33	68.07997
IGHG1	3.328014	1.752557	13.01915	1.23E-34	3.31E-33	67.81163
THBS1	2.065481	0.606074	12.98038	1.83E-34	4.87E-33	67.41202
DDN	-2.35379	2.155645	-12.9671	2.10E-34	5.56E-33	67.27582
ALPK2	2.104565	-3.24738	12.93857	2.83E-34	7.38E-33	66.98183
CHI3L2	2.62568	4.935552	12.93244	3.02E-34	7.86E-33	66.91884
TNFSF14	2.112098	-2.42891	12.92799	3.16E-34	8.19E-33	66.87315
MS4A4E	2.361806	-1.38316	12.91516	3.61E-34	9.31E-33	66.74132
POSTN	3.041409	-0.37041	12.90754	3.91E-34	9.97E-33	66.6631
CELF4	-2.04669	2.475429	-12.9049	4.01E-34	1.02E-32	66.63628
CDH9	-2.45274	-1.77339	-12.9027	4.11E-34	1.05E-32	66.61306
USH1C	-2.33666	2.938161	-12.8947	4.46E-34	1.12E-32	66.53153
CKMT1A	-2.36689	1.768589	-12.846	7.38E-34	1.83E-32	66.03245
ACY3	-2.19017	0.946081	-12.8279	8.90E-34	2.19E-32	65.84729
IGHA1	3.187243	0.607692	12.79875	1.20E-33	2.94E-32	65.54929
SYT4	-2.1978	1.034083	-12.7604	1.78E-33	4.31E-32	65.15753
PDPN	2.151697	2.680845	12.7517	1.95E-33	4.69E-32	65.06928
SLC17A7	-2.97507	2.80805	-12.7466	2.06E-33	4.91E-32	65.01763
LUM	2.100683	1.020817	12.74273	2.14E-33	5.09E-32	64.97792
NEUROD2	-2.34175	0.128678	-12.6883	3.74E-33	8.71E-32	64.42425
GABRA5	-2.59702	0.445704	-12.6745	4.31E-33	9.97E-32	64.28376
INA	-2.41616	2.634619	-12.6384	6.24E-33	1.42E-31	63.91828
HOXD10	2.822382	-2.5318	12.61753	7.72E-33	1.75E-31	63.7064
TMEM130	-2.18394	2.842786	-12.6064	8.65E-33	1.95E-31	63.59346
LTF	3.108163	1.747219	12.58185	1.11E-32	2.49E-31	63.34538
RBFOX3	-2.17044	2.07171	-12.5653	1.32E-32	2.92E-31	63.1785
PLEKHS1	2.1098	-3.25444	12.55115	1.52E-32	3.35E-31	63.03535
SOHLH1	-2.91057	-0.39141	-12.5096	2.32E-32	5.04E-31	62.61628
IGHM	2.615207	-1.1657	12.50158	2.52E-32	5.44E-31	62.53563
CKMT1B	-2.16412	2.105244	-12.4864	2.94E-32	6.29E-31	62.38269
CSTA	2.183978	-0.20061	12.48209	3.07E-32	6.57E-31	62.3395
SLC14A1	-2.3244	2.296259	-12.4728	3.37E-32	7.18E-31	62.24613
CCK	-3.03687	3.250096	-12.4706	3.45E-32	7.33E-31	62.22402
IGHA2	3.023069	-1.8704	12.46247	3.75E-32	7.93E-31	62.14224
HOXA5	2.804938	-2.8835	12.43386	5.01E-32	1.05E-30	61.85498
CP	2.029934	2.130812	12.43278	5.06E-32	1.06E-30	61.8441
KLK7	-2.61887	-2.3462	-12.4174	5.91E-32	1.23E-30	61.68981
HOXD9	2.882516	-0.61553	12.4052	6.69E-32	1.39E-30	61.56761
SFRP2	-2.38772	2.429399	-12.3575	1.08E-31	2.19E-30	61.09034
SLC6A17	-2.21243	0.848214	-12.2997	1.94E-31	3.88E-30	60.51289
SLC1A6	-2.4227	0.943711	-12.2705	2.60E-31	5.12E-30	60.22237
KCNC2	-2.55112	0.240201	-12.2397	3.55E-31	6.94E-30	59.91572
UNC13C	-2.31537	-1.68492	-12.2157	4.51E-31	8.76E-30	59.67731
G0S2	2.105229	0.909682	12.17237	6.96E-31	1.33E-29	59.24823
IGLC2	3.512296	-0.93637	12.16485	7.50E-31	1.43E-29	59.17378
HOXA10	2.70498	-1.98151	12.14939	8.75E-31	1.66E-29	59.02082
SYT1	-2.25873	2.730917	-12.1405	9.57E-31	1.80E-29	58.93257
FADS6	-2.24983	-2.80211	-12.1151	1.23E-30	2.30E-29	58.68153
KCNT1	-2.12121	0.980232	-12.1005	1.43E-30	2.63E-29	58.53773
DPEP1	2.318761	0.40592	12.09263	1.54E-30	2.84E-29	58.46024
SV2B	-2.20749	0.231922	-12.0921	1.55E-30	2.85E-29	58.45528
KCNV1	-2.27525	-2.71895	-12.0647	2.04E-30	3.72E-29	58.18465

MAL2	-2.48628	-0.4201	-12.032	2.82E-30	5.06E-29	57.86345
SNAP25	-2.24332	5.285786	-12.0313	2.84E-30	5.09E-29	57.85632
SERTM1	-2.00285	-2.52861	-12.0257	3.00E-30	5.35E-29	57.80183
CCKBR	-2.27184	-0.53406	-12.019	3.21E-30	5.70E-29	57.73579
RBFOX1	-2.1065	1.016325	-12.001	3.83E-30	6.77E-29	57.55891
CLEC2L	-2.40091	1.035279	-11.9951	4.07E-30	7.16E-29	57.50043
MPPED1	-2.17017	0.933941	-11.9866	4.42E-30	7.76E-29	57.41798
MYT1L	-2.358	0.990737	-11.9557	6.00E-30	1.04E-28	57.11452
LPPR3	-2.21023	2.989129	-11.9448	6.69E-30	1.15E-28	57.00783
SHOX2	2.676858	-2.29565	11.91028	9.40E-30	1.59E-28	56.67066
IGLC3	3.380413	0.441434	11.90914	9.51E-30	1.61E-28	56.65947
ACTL6B	-2.21831	3.464608	-11.8964	1.08E-29	1.82E-28	56.5352
ST8SIA3	-2.53926	2.225688	-11.884	1.22E-29	2.05E-28	56.41451
OLFM3	-2.10123	-1.64482	-11.84	1.88E-29	3.09E-28	55.98604
KCNJ4	-2.14656	1.88825	-11.8393	1.89E-29	3.12E-28	55.97832
HOXA4	2.659026	-2.68276	11.7948	2.93E-29	4.75E-28	55.54635
HTR5A	-2.0925	-2.58805	-11.7683	3.79E-29	6.11E-28	55.28907
SYT13	-2.16542	0.884009	-11.7608	4.08E-29	6.54E-28	55.2172
TCERG1L	-2.10622	-1.77884	-11.7305	5.49E-29	8.69E-28	54.92335
ISL2	2.005896	-2.21703	11.7196	6.11E-29	9.61E-28	54.81805
NEFM	-2.3806	2.028511	-11.6984	7.51E-29	1.18E-27	54.61295
NEFL	-2.60898	1.721815	-11.6635	1.06E-28	1.62E-27	54.27658
SYCE1	-2.51482	-0.81443	-11.663	1.06E-28	1.63E-27	54.27165
OPALIN	-2.72309	0.555431	-11.6544	1.15E-28	1.76E-27	54.18938
CALY	-2.54811	4.196056	-11.6241	1.55E-28	2.34E-27	53.89793
GLRA3	-2.0069	-2.32496	-11.6003	1.95E-28	2.92E-27	53.66908
GALNTL5	-2.29119	-2.71601	-11.592	2.11E-28	3.14E-27	53.58928
IL6	2.127197	-1.50074	11.58827	2.19E-28	3.25E-27	53.5535
SNCB	-2.22773	4.793086	-11.5682	2.66E-28	3.92E-27	53.36119
IGF2BP3	2.552909	-1.29511	11.56599	2.72E-28	4.00E-27	53.33994
GABRB2	-2.16493	-0.60805	-11.5312	3.81E-28	5.55E-27	53.00671
CLEC5A	2.127674	-1.35255	11.4934	5.48E-28	7.88E-27	52.64586
CIDEA	-2.26327	-2.73248	-11.4865	5.86E-28	8.41E-27	52.58019
CHGA	-2.33654	3.967933	-11.4605	7.53E-28	1.07E-26	52.3322
SST	-2.80457	2.713699	-11.4416	9.03E-28	1.28E-26	52.15213
IL13RA2	2.156254	0.194757	11.42753	1.03E-27	1.45E-26	52.01854
GPR22	-2.12488	-1.98567	-11.4201	1.11E-27	1.55E-26	51.94757
FAM153B	-2.43164	-1.05229	-11.4059	1.27E-27	1.77E-26	51.81314
HOXD13	2.403464	-3.59547	11.37173	1.76E-27	2.42E-26	51.48903
SLC6A7	-2.42081	-0.75244	-11.3569	2.03E-27	2.76E-26	51.34878
PITX1	2.178065	-0.97003	11.34444	2.29E-27	3.09E-26	51.23079
CPNE6	-2.1128	2.560949	-11.3329	2.56E-27	3.43E-26	51.12171
HOXC10	2.733746	-2.89823	11.32006	2.89E-27	3.87E-26	51.00027
RASAL1	-2.12618	1.515778	-11.3197	2.90E-27	3.88E-26	50.99664
TBR1	-2.01175	-0.41969	-11.3195	2.91E-27	3.88E-26	50.99522
CAMK2A	-2.33006	2.611526	-11.2945	3.69E-27	4.86E-26	50.75888
SAA1	3.421471	-1.52961	11.29191	3.78E-27	4.97E-26	50.73464
IGLC1	3.39849	0.08015	11.2841	4.08E-27	5.34E-26	50.66106
KIF12	-2.01106	-3.20294	-11.2561	5.32E-27	6.90E-26	50.39752
SULT4A1	-2.11661	2.525352	-11.2096	8.28E-27	1.06E-25	49.96013
MYBPH	2.060105	-1.92679	11.14697	1.50E-26	1.87E-25	49.37392
VSNL1	-2.43888	3.267097	-11.1367	1.65E-26	2.05E-25	49.27787
CD70	2.187013	-3.04064	11.10948	2.13E-26	2.63E-25	49.0239
GPR6	-2.10127	-3.30179	-11.0651	3.24E-26	3.95E-25	48.61054
CRYM	-2.43353	2.128838	-11.011	5.38E-26	6.46E-25	48.10843
IGKC	2.723862	2.858324	10.99858	6.05E-26	7.23E-25	47.99329
IGHG3	2.915853	-0.91327	10.9954	6.23E-26	7.45E-25	47.96383
IGJ	2.234013	-1.62469	10.96203	8.51E-26	1.01E-24	47.65516

HTR1A	-2.41335	-2.58983	-10.8752	1.91E-25	2.21E-24	46.85509
SAA2	2.836827	-2.77036	10.76364	5.37E-25	5.99E-24	45.83341
HOXA2	2.14774	-3.60831	10.73795	6.81E-25	7.51E-24	45.59924
PYDC1	-2.37261	-2.10215	-10.6814	1.14E-24	1.24E-23	45.08523
FAM163B	-2.50091	2.322268	-10.6699	1.27E-24	1.37E-23	44.98041
NRGN	-2.34262	6.427742	-10.6697	1.27E-24	1.37E-23	44.97885
CA9	2.070467	0.384458	10.61028	2.20E-24	2.31E-23	44.44119
CTXN3	-2.06198	-3.47249	-10.6038	2.33E-24	2.44E-23	44.38249
SLC32A1	-2.08593	-0.0142	-10.541	4.13E-24	4.25E-23	43.81651
VIPR2	-2.10248	1.960022	-10.5387	4.22E-24	4.33E-23	43.79647
HOXA3	2.419634	-3.01686	10.51556	5.20E-24	5.30E-23	43.58837
HBQ1	-2.36162	0.818668	-10.5151	5.23E-24	5.32E-23	43.58405
HOXA9	2.161509	-3.46237	10.46531	8.21E-24	8.20E-23	43.13811
KLK5	-2.08702	-3.28692	-10.3475	2.37E-23	2.28E-22	42.08834
HIST1H2B	3.704558	-1.96341	10.34677	2.39E-23	2.30E-22	42.08208
MYOD1	-2.02562	-3.47605	-10.2816	4.29E-23	4.05E-22	41.50477
OTP	2.035195	-3.24737	10.27603	4.50E-23	4.24E-22	41.45598
MSMP	2.053583	0.808093	10.15915	1.27E-22	1.16E-21	40.42843
NUPR1L	-2.3118	-2.12177	-10.1547	1.33E-22	1.20E-21	40.38967
TAC1	-2.2534	0.297441	-10.0629	2.98E-22	2.63E-21	39.5886
TEX40	-2.05732	1.192351	-10.0467	3.44E-22	3.02E-21	39.44804
LCN15	-2.0293	-2.02322	-9.91481	1.09E-21	9.19E-21	38.30798
NGB	-2.13681	-0.52729	-9.88666	1.39E-21	1.17E-20	38.0661
CARTPT	-2.35088	-2.86454	-9.87653	1.52E-21	1.27E-20	37.97918
TNNT2	-2.41375	-0.07265	-9.84358	2.03E-21	1.67E-20	37.69702
LOR	-2.00504	-1.6985	-9.77748	3.59E-21	2.89E-20	37.13291
HOXA7	2.414288	-2.32006	9.728647	5.46E-21	4.35E-20	36.71802
NPY	-2.28708	3.272495	-9.67495	8.66E-21	6.79E-20	36.26359
PI3	2.548428	-1.50455	9.663198	9.57E-21	7.47E-20	36.1644
MOBP	-2.12346	3.977587	-9.44385	6.15E-20	4.52E-19	34.3295
CCL18	2.144865	-2.84294	9.088628	1.17E-18	7.84E-18	31.42636
HIST1H3E	2.620322	-1.48104	9.017377	2.09E-18	1.38E-17	30.85439
IGLV1-51	2.16185	-2.59303	9.008687	2.24E-18	1.47E-17	30.78488
IGHG4	2.072816	-2.27773	8.900191	5.38E-18	3.42E-17	29.92135
RETN	2.263132	-2.2974	8.713247	2.39E-17	1.45E-16	28.45275
FXYD4	-2.06756	-2.24701	-8.68135	3.07E-17	1.85E-16	28.20463
PVALB	-2.22849	0.679342	-8.5409	9.24E-17	5.37E-16	27.12077
FAM153C	-2.0094	-1.20407	-8.44232	1.99E-16	1.13E-15	26.36845
HIST1H3H	2.676523	-0.42123	7.77034	3.02E-14	1.46E-13	21.42999
HIST1H3D	2.25428	0.301719	7.526292	1.72E-13	7.90E-13	19.72046
HIST1H2A	2.546328	-0.63962	7.353386	5.77E-13	2.54E-12	18.53707
HIST1H2A	2.398974	-2.5513	6.605373	8.19E-11	3.02E-10	13.68988
HIST1H2B	2.309882	-0.7412	6.312476	5.05E-10	1.73E-09	11.91551
HIST1H2B	2.409644	-1.94897	6.25515	7.15E-10	2.42E-09	11.57655
HIST1H2A	2.27975	-2.7805	6.202989	9.80E-10	3.27E-09	11.2705
HIST1H4B	2.345547	-1.93433	5.044601	5.89E-07	1.52E-06	5.073159
