# Supplementary material for: Identification and Verification of an Alternative Polyadenylation-Related lncRNA Prognostic Signature for Glioma
Source: Comput Math Methods Med. 2022 Sep 7;2022:2164229. doi: 10.1155/2022/2164229 (PMC11401696; doi:10.1155/2022/2164229)
Supplement: Supplementary 1 — Table S1: screening APA-related lncRNAs from the CGGA dataset based on Pearson analysis. [file 2164229.f1.pdf]

id	CGGA_10(	CGGA_10(	CGGA_10:	CGGA_10:	CGGA_10:	CGGA_10:	CGGA_10:	CGGA_10:
AC000068	0.103088	0.205115	1.742734	0.398629	0.07684	0.263658	0.363983	0.998312
AC000120	0.071566	0.498052	1.172872	2.89E-05	0.160202	0.877807	0.523752	0.734524
AC002116	5.788153	2.442987	0.045491	3.031688	7.964315	2.117814	1.916782	0.453218
AC002456	0.390895	5.545729	0.045491	4.302367	1.37531	0.595368	2.705916	1.041652
AC002467	1.761349	1.353608	0.601227	4.365224	2.522684	5.454949	2.569957	6.365065
AC004158	2.002193	10.33476	9.418196	3.269913	1.883535	7.495397	12.63773	4.984488
AC004447	44.27012	32.68313	13.91714	43.49658	46.7056	24.98218	45.1045	32.68313
AC004490	0.080592	0.06715	2.511465	0.034863	0.028333	0.038716	0.005253	0.042771
AC005076	1.988196	1.34798	0.045491	3.5257	4.63189	0.465325	2.248896	1.849654
AC005104	4.33E-05	0.00026	0.045491	0.321746	0.216753	0.166465	1.216768	0.136061
AC005154	46.7056	105.1694	95.22939	44.27012	30.82277	43.49658	126.1556	49.50903
AC005253	0.375094	0.193045	3.55583	1.47842	0.327828	2.180022	2.003723	2.802828
AC005306	10.98644	15.46193	5.591717	16.5625	23.6012	1.736133	14.6058	4.952915
AC005339	28.87506	3.113478	0.045491	14.6058	14.97762	0.444315	1.085224	0.00013
AC005481	0.019784	0.00026	0.796674	0.058442	0.009307	0.038716	0.02189	0.108997
AC005498	4.323045	1.456955	0.956061	11.39744	3.895469	7.38847	3.529466	5.737056
AC005519	0.693571	0.373218	3.008225	2.758088	0.946061	2.312085	4.003117	6.153102
AC005540	0.027056	0.052273	19.06893	2.89E-05	0.020895	0.844156	0.117258	0.07671
AC005606	4.207778	5.180058	0.045491	5.553146	3.726854	0.146999	0.713405	0.998312
AC005618	0.316573	2.261573	6.685087	1.397482	0.402381	0.572987	1.781948	4.620159
AC005775	0.685931	0.843102	0.045491	0.744531	0.889553	0.106522	0.47153	0.289935
AC005785	4.592273	4.72798	2.209141	1.466111	4.814661	0.465325	1.37531	0.515527
AC005944	5.950664	126.1556	0.045491	17.34065	19.99861	224.9555	75.1132	72.69473
AC006019	0.013348	0.029134	4.959358	2.89E-05	0.005173	0.021429	0.009293	0.07671
AC006547	1.286494	1.123016	2.45575	0.940563	0.596111	2.973059	1.159913	3.373983
AC006942	6.900202	2.196017	0.644618	4.170051	3.984747	2.351299	3.061955	1.2214
AC007009	4.655916	0.953506	0.045491	3.172864	2.408882	2.911962	1.270317	0.770188
AC007038	0.291183	0.466068	0.872597	0.321746	0.138831	0.465325	0.523752	0.743694
AC007192	530.1465	72.69473	0.045491	48.53429	332.0776	56.39528	33.4985	13.53913
AC007292	0.359105	0.343629	6.071436	0.754315	0.318499	0.146999	0.402381	1.095657
AC007292	5.9071	4.392316	1.385801	4.620159	2.690823	2.212619	4.370671	4.058514
AC007308	0.035137	0.075339	0.721335	0.050216	0.044322	0.216753	0.233889	0.352597
AC007308	8.780115	33.4985	0.045491	3.418405	17.15902	0.00013	1.980592	0.00013
AC007461	4.33E-05	0.035007	0.601227	0.034863	0.06381	0.021429	0.02189	0.128615
AC007620	0.639495	0.607605	22.52375	7.315512	0.29671	5.195022	1.464214	13.83359
AC008746	2.216162	2.104358	0.681068	4.392316	2.124574	1.886335	0.947677	1.524776
AC009120	2.681169	4.776407	6.297136	6.162446	4.236962	0.763102	2.800332	1.104805
AC009227	4.33E-05	0.058817	8.103925	0.026537	0.020895	0.089149	0.166118	0.229199
AC009506	2.294733	3.233045	8.508514	5.095325	5.530274	2.839351	5.830152	5.238701
AC009948	3.750837	8.333918	14.42661	7.212915	6.38456	11.719	11.719	10.62739
AC009961	0.126429	0.599134	11.77567	0.826407	0.416501	1.71518	0.597417	1.055628
AC010761	1.296905	1.86658	51.52195	0.796674	1.95645	0.558095	0.629098	1.566414
AC010883	0.880498	1.565253	0.045491	0.653968	0.280823	0.499206	3.300664	0.396926
AC011747	4.33E-05	0.00026	0.216025	0.005758	0.002237	0.014791	4.33E-05	0.009293
AC011899	0.348889	0.090584	1.742734	0.692799	0.318499	0.209048	0.101905	0.322684
AC012146	30.49289	20.34063	3.317807	31.55417	33.07147	25.99427	22.52375	37.29684
AC012314	0.071566	0.075339	1.264589	0.163766	0.138831	0.055887	0.1793	0.102165
AC012358	4.33E-05	0.00026	1.046061	0.375743	0.090029	0.209048	0.109466	0.216025
AC012613	4.33E-05	0.00026	0.234358	0.005758	0.002237	0.010274	4.33E-05	0.0121
AC015971	4.33E-05	0.00026	1.127475	2.89E-05	1.44E-05	1.378362	0.145382	0.445996
AC016582	0.019784	0.00026	0.917431	0.365859	1.44E-05	0.047092	4.33E-05	0.229199
AC016700	5.477843	4.227302	2.607035	11.14619	10.48101	3.028629	16.09482	13.64503
AC016722	0.019784	0.260938	2.607035	0.076162	1.44E-05	0.432915	0.087879	0.143218
AC016738	0.26562	0.058817	0.681068	0.034863	0.160202	0.305253	0.382208	0.056782
AC017104	0.846342	1.095657	1.884935	3.87329	1.358189	0.276061	0.912987	0.734524
AC021224	4.33E-05	0.419827	0.601227	0.173059	1.44E-05	0.263658	1.076032	0.985411
AC025165	1.369048	0.922338	3.122915	1.35259	1.041025	0.485577	1.33285	0.940563

AC025171	0.144719	0.679069	11.30987	0.653968	0.274199	0.827273	0.577071	1.865043
AC025335	2.178268	1.731169	0.956061	2.065007	1.935685	3.971061	2.654473	2.371833
AC027601	0.53658	0.256046	1.046061	0.572987	1.29417	0.178629	0.242222	0.579495
AC062021	0.431977	37.81919	0.045491	2.89E-05	1.812114	0.189141	36.26061	8.745238
AC068039	0.545426	0.343629	0.286522	0.477698	0.224921	0.146999	0.152843	0.154719
AC068580	0.375094	0.30176	3.829105	0.872013	0.318499	0.605022	0.493247	1.009625
AC068831	83.489	53.77619	0.045491	37.29684	51.52195	9.121486	42.08807	1.763889
AC074391	0.152843	0.035007	1.884935	0.012136	0.235931	0.263658	0.242222	0.00013
AC079354	0.004214	0.035007	0.045491	2.89E-05	0.005173	0.00013	4.33E-05	0.009293
AC079807	1.770029	1.616385	0.452965	3.163506	1.712706	1.246457	1.776739	2.267157
AC079922	1.753341	0.76	0.601227	2.479221	1.766248	1.795851	1.438672	1.290065
AC083843	0.206061	0.541241	0.359899	0.552749	0.30329	0.73912	0.757273	0.973745
AC083884	4.33E-05	0.419827	4.06303	0.173059	0.224921	0.533874	0.073463	0.951652
AC084219	4.33E-05	0.186465	1.300368	0.0857	1.44E-05	1.042944	0.1793	0.466068
AC090587	0.071566	0.977994	0.555036	0.125859	0.118983	0.641631	0.784372	1.278629
AC091814	4.33E-05	0.023694	0.045491	0.01873	0.005173	0.00013	0.005253	0.031169
AC093390	0.007792	0.018038	0.555036	0.195253	1.44E-05	0.178629	0.005253	0.016472
AC093673	6.028427	3.820758	0.045491	10.7557	12.49452	3.144156	4.082165	3.401126
AC093690	0.088788	0.11368	0.359899	0.183521	0.036566	0.28689	0.26215	0.036631
AC096574	0.027056	0.023694	0.310447	0.050216	0.005173	0.189141	0.087879	0.082727
AC096772	11.14619	9.218514	1.385801	5.576219	6.64254	15.3618	7.519841	4.222583
AC097721	0.027056	0.11368	1.385801	0.076162	1.44E-05	0.106522	0.138326	0.082727
AC099850	0.359105	0.265014	1.551962	0.173059	0.171472	0.73912	0.428694	1.104805
AC108488	4.077403	5.431544	0.335411	8.958225	4.429769	1.418312	5.309264	1.837172
AC110781	0.049278	0.047092	0.26215	0.005758	0.014423	0.117258	0.009293	0.064127
AC116366	0.071566	0.623499	0.644618	0.495584	0.231659	0.296955	0.444538	0.930325
AC132217	16.44768	31.1753	0.045491	14.17388	95.22939	1.553074	7.584502	0.00013
AC142528	0.118528	0.643001	1.706219	0.764315	0.113449	0.106522	0.318694	0.108997
AC144449	0.390895	0.403413	7.456118	0.653968	0.532092	0.763102	0.845887	0.483326
AC147651	0.65798	0.441472	0.045491	2.890245	1.603795	0.00013	0.202987	0.108997
ACVR2B-/	0.16886	0.359697	1.300368	0.259163	0.126335	0.533874	0.72044	0.715108
ADORA2A	0.019784	0.00026	0.359899	0.0857	0.138831	0.106522	0.185642	0.405346
AF038458	1.2214	0.391674	0.045491	0.653968	1.31	0.346494	0.218045	0.593434
AF127577	19.99861	17.51961	0.045491	18.62711	11.83593	0.00013	6.268312	0.143218
AF127936	0.144719	0.30176	1.551962	1.615281	0.183672	1.259747	0.528571	1.229553
AF127936	0.466602	0.769019	0.216025	0.624841	0.243449	0.605022	0.340866	0.681919
AF131215	0.300844	1.388543	10.17029	3.279625	0.337222	2.104358	2.016515	6.036818
AF131215	4.33E-05	0.047092	1.551962	0.042569	1.44E-05	0.00013	4.33E-05	0.428694
AF131215	3.36075	14.70188	1.264589	8.849697	5.165599	1.418312	3.711097	3.747035
AF131216	0.007792	0.12904	1.505678	0.005758	0.020895	0.189141	0.02189	0.190693
AFAP1-AS	0.071566	0.041227	0.998312	0.066616	0.009307	0.127201	0.02189	0.200794
AIRN	0.035137	0.018038	0.26215	0.042569	0.002237	0.071775	0.009293	0.009293
AL136419	0.960866	1.303045	3.747035	1.229553	1.405772	1.361789	1.111241	2.685786
AP000473	1.817828	1.853723	0.045491	4.392316	6.404921	1.378362	2.600014	0.690375
AP000473	0.65798	0.585902	0.045491	2.283752	1.566414	1.754733	4.053506	1.497143
AP000662	0.063225	0.449408	1.300368	0.321746	0.090029	0.614156	0.444538	0.566494
AP000692	0.769365	0.322237	0.831472	0.684033	0.782857	0.263658	0.323535	0.330289
AP000692	4.33E-05	0.802035	0.517612	0.269322	1.44E-05	0.252316	0.390014	0.789423
AP001056	0.316573	0.186465	1.505678	0.269322	0.478182	0.242453	0.318694	0.253074
AP001062	1.712706	2.513752	0.045491	0.916306	1.279452	0.189141	0.728543	0.50803
AP001258	0.324019	0.441472	0.487662	1.189769	0.4971	0.584892	0.500354	0.919827
AP001372	2.092987	1.164257	0.452965	1.991328	1.935685	5.123781	3.993665	3.002576
AP001437	1.783203	3.966623	0.045491	1.65785	2.306277	2.212619	2.263377	0.579495
AP003068	0.886775	0.366912	2.339697	1.339091	1.545657	1.278629	1.386681	1.139221
AP003068	95.22939	26.80355	0.721335	39.53687	91.05541	1.278629	0.53658	0.00013
AP006216	2.312085	0.636688	0.045491	1.150635	3.058802	0.913514	0.698297	0.906126
AP4B1-AS	0.161082	0.047092	0.872597	0.477698	0.183672	0.63114	0.349812	0.884654
ARAP1-AS	38.39042	51.52195	0.045491	7.584502	39.53687	0.00013	1.246457	0.00013

ARAP1-AS	0.251371	0.058817	1.986681	0.163766	0.090029	0.276061	0.26215	0.666097
ARHGAP3	0.049278	0.090584	0.045491	0.614545	0.107626	0.071775	0.437799	0.558427
ASH1L-AS	1.403853	0.98474	20.60009	2.254084	1.438672	2.611797	2.421681	4.251818
ASMTL-AS	14.00414	17.15902	2.914632	5.20267	20.85876	3.104394	12.10289	9.397771
BCYRN1	0.013348	3.734762	8.149307	0.136926	0.020895	0.063911	0.185642	2.047157
BDNF-AS	5.323492	9.031082	5.210317	6.622092	7.795642	6.52899	11.77567	10.53159
BMS1P20	0.418326	0.429567	3.829105	1.63039	0.327828	3.340361	1.474343	3.771349
BOLA3-AS	2.216162	1.605859	1.986681	3.921645	1.255657	8.508514	5.720173	9.101674
BX322557	0.367287	6.011515	59.32013	1.776739	0.337222	16.22108	2.81342	4.644098
C1orf132	1.082179	0.570664	5.950664	0.332395	0.834416	2.229048	0.682799	0.616739
C1orf147	4.33E-05	0.052273	0.045491	0.076162	0.036566	0.00013	0.109466	0.0121
C20orf203	0.916306	0.977994	0.721335	0.507085	0.819048	0.572987	0.810491	0.609177
C22orf34	0.11075	0.023694	1.046061	1.229553	0.216753	0.071775	0.19355	0.20969
C6orf3	3.47531	3.94886	1.602619	8.994791	6.612121	2.630729	10.28573	8.923117
CACNA1C	0.518139	0.186465	0.872597	0.058442	0.06381	0.544877	0.065974	0.593434
CACNA1C	1.308167	1.064242	0.956061	4.745895	3.464315	0.432915	0.745613	0.846797
CAPN10-A	0.624841	1.29267	0.517612	0.90772	0.803665	1.013434	1.321053	2.07658
CARS-AS1	0.004214	0.00026	0.721335	0.058442	1.44E-05	0.00013	0.005253	0.128615
CASC15	3.279625	11.719	2.339697	5.889798	5.500678	5.409437	2.201587	3.343629
CDIPT-AS	0.471746	0.607605	0.045491	0.332395	0.728543	0.960866	0.649668	0.049336
CELF2-AS	4.33E-05	0.00026	5.409437	0.012136	1.44E-05	2.069942	0.051198	0.835498
CHKB-AS1	1.575483	0.743283	0.045491	0.897006	1.8386	0.813261	0.649668	0.453218
CIRBP-AS	0.693571	0.179177	17.71696	2.320072	1.793074	1.042944	1.477388	6.830592
CITF22-1A	3.599329	4.408153	0.452965	3.201457	4.990794	0.178629	1.954993	1.104805
CITF22-92	1.1379	0.315743	0.045491	0.477698	0.793434	0.198788	0.340866	0.4971
CKMT2-AS	1.828781	2.958773	17.34065	3.016984	1.751948	4.039192	4.508196	8.319019
COX10-AS	2.058773	3.256587	9.047532	5.137042	3.201457	3.68381	4.927302	7.468961
CTA-223H	1.901068	4.086926	0.045491	1.776739	0.452965	0.346494	6.191515	1.595729
CTA-268H	1.232078	2.461905	0.517612	0.125859	1.476429	0.533874	1.728615	0.302785
CTA-293F	2.404618	2.223636	0.045491	1.177309	2.944416	0.787136	6.738211	1.581912
CTA-29F1	18.91854	24.73724	3.55583	18.05488	8.508514	9.218514	16.5625	15.07257
CTA-363E	0.799048	0.295671	0.045491	2.89E-05	0.737403	0.00013	4.33E-05	0.00013
CTA-384D	22.52375	22.96463	2.280101	11.65791	24.73724	12.22798	10.1459	4.241905
CTB-113I2	4.039192	9.859719	3.350224	4.557605	5.409437	2.378124	5.266335	2.145137
CTB-113P	4.33E-05	0.00026	0.234358	0.005758	1.44E-05	0.014791	4.33E-05	0.00013
CTB-113P	2.6143	1.551962	0.045491	2.413146	1.989711	1.636118	0.493247	0.200794
CTB-131K	2.858009	2.159004	15.07257	6.433918	2.124574	6.922771	5.687879	8.461703
CTB-152G	1.111241	0.873088	5.11697	0.495584	0.478182	2.212619	1.33285	2.091313
CTB-176F	0.397489	1.471198	0.414473	0.310087	0.171472	0.346494	0.784372	0.726811
CTB-178M	0.799048	1.323131	0.681068	0.365859	5.630815	0.106522	1.216768	1.991328
CTB-191K	7.519841	6.99026	0.045491	1.416407	5.34474	0.00013	0.419827	0.00013
CTB-31O2	105.1694	100.0998	0.045491	31.92385	155.7103	0.00013	6.079596	4.150274
CTB-31O2	1.51215	1.193362	7.795642	1.466111	1.153723	1.179885	2.107922	2.876421
CTB-33O1	0.144719	0.143102	0.601227	0.259163	0.101494	0.323535	0.402381	0.396926
CTB-36H1	2.312085	3.011169	0.796674	1.229553	0.824885	2.990851	3.511263	2.513752
CTB-43P1	4.397792	1.43039	0.045491	0.229048	0.872013	1.401977	1.254704	1.306667
CTB-58E1	0.858297	0.749019	2.398442	0.982128	0.235931	1.378362	1.000173	2.445079
CTB-60E1	4.33E-05	0.00026	12.10289	4.468009	1.44E-05	12.10289	1.216768	4.546443
CTB-89H1	20.51271	25.48179	3.350224	13.19051	19.83727	4.473348	12.49452	5.788153
CTB-92J2	0.579841	1.675722	0.452965	1.216768	0.710722	0.038716	1.246457	1.620058
CTB-96E2	4.33E-05	0.041227	3.479134	0.525325	1.44E-05	1.401977	0.109466	1.837172
CTBP1-AS	1.857677	2.474834	11.36975	15.66023	3.253175	10.82954	9.583463	23.6012
CTC-241N	1.692763	3.119986	0.359899	1.89228	1.734949	1.149978	3.163506	0.673651
CTC-246B	0.11075	0.00026	11.11876	0.846797	1.44E-05	3.340361	0.349812	2.083261
CTC-260E	0.348889	0.550974	11.42512	0.183521	0.183672	0.421025	0.597417	1.337431
CTC-273B	2.584394	3.002576	0.045491	2.567605	3.500808	5.630815	2.984899	3.771349
CTC-281F	0.027056	0.052273	1.090072	0.125859	1.44E-05	0.080837	0.152843	0.036631
CTC-325H	0.175361	0.403413	0.601227	0.248903	0.280823	0.323535	0.728543	0.985411

CTC-332L	2.216162	1.323131	0.045491	0.469711	0.478182	1.179885	0.185642	0.312864
CTC-336P	3.464315	11.19942	15.71851	3.851414	3.687633	7.737807	4.709033	0.474856
CTC-338M	2.826277	4.302367	21.19547	4.721789	3.799885	3.092229	4.23202	9.880779
CTC-338M	23.49249	40.11561	0.045491	1.65785	20.16541	27.96248	38.39042	8.524242
CTC-366B	1.37531	3.370527	0.045491	1.252424	2.214372	1.401977	2.842193	0.586248
CTC-425F	4.029654	0.00026	0.045491	3.220678	8.477662	0.00013	4.33E-05	0.00013
CTC-425C	0.206061	0.466068	2.160649	0.826407	0.235931	2.281926	0.26215	3.36075
CTC-429P	4.241905	10.73084	0.045491	14.97762	7.417359	10.1459	4.563117	3.659668
CTC-429P	0.483045	0.822965	16.22108	1.844098	1.44E-05	7.238023	2.90658	15.56131
CTC-429P	11.09133	14.25954	7.288788	8.164531	11.30987	10.03313	18.62711	13.15079
CTC-429P	0.144719	0.607605	3.775209	0.764315	0.06381	3.695815	0.728543	3.574387
CTC-444N	10.48101	7.710361	4.745895	10.53159	10.62739	7.025599	11.544	7.099913
CTC-444N	2.398442	0.458521	0.045491	1.082915	1.712706	0.614156	0.568369	0.38153
CTC-444N	3.307186	2.104358	0.601227	4.684502	3.606804	1.304877	2.148658	1.124574
CTC-458I2	4.33E-05	0.170808	0.045491	0.298687	1.44E-05	0.6943	4.33E-05	0.803665
CTC-471J1	1.846941	2.173081	0.045491	3.249877	1.819221	1.135887	1.020447	0.558427
CTC-479C	1.330974	0.802035	0.414473	1.35259	1.350772	0.397215	1.171349	0.750866
CTC-487M	1.25886	1.962424	2.558817	1.325743	1.545657	1.623232	2.308203	3.031688
CTC-499B	4.33E-05	0.029134	0.286522	2.89E-05	0.020895	0.047092	4.33E-05	0.064127
CTC-510F	6.653016	3.578038	0.045491	1.808023	6.99026	0.63114	2.349351	0.38153
CTC-524C	8.445743	25.24367	3.122915	10.1028	11.89932	5.630815	15.07257	9.792626
CTC-542B	1.361789	2.77899	0.045491	1.532468	2.727785	0.263658	3.13456	1.399315
CTC-559E	4.33E-05	0.12904	0.644618	2.89E-05	1.44E-05	0.296955	0.331263	0.690375
CTD-2006	1.712706	3.381255	1.809343	4.513622	3.367071	2.396429	7.08733	2.685786
CTD-2006	5.102742	6.355224	0.414473	8.643074	6.760765	2.091313	8.033932	4.376118
CTD-2008	4.33E-05	0.591912	4.745895	0.375743	1.44E-05	0.252316	0.094553	1.131472
CTD-2012	0.103088	0.287821	0.956061	0.183521	0.165657	0.209048	0.081126	0.396926
CTD-2012	2.752893	8.815123	1.300368	1.2214	5.316378	0.813261	2.185556	1.25886
CTD-2013	1.047345	0.970007	2.820974	2.413146	1.1379	2.524892	2.320072	2.335844
CTD-2017	0.251371	0.480794	7.625209	0.421573	0.101494	0.960866	0.301955	1.250058
CTD-2020	1.86658	1.199481	0.045491	1.859084	3.88153	2.893001	2.451645	3.137576
CTD-2047	0.4457	0.643001	2.209141	0.885664	0.68062	1.108947	0.947677	0.877222
CTD-2083	0.056046	0.359697	0.487662	0.351219	0.07684	0.22456	0.318694	0.172453
CTD-2126	20.16541	13.91714	0.045491	2.153535	16.80343	16.34294	6.601833	2.640332
CTD-2132	0.215115	0.330043	18.48514	2.640332	0.581926	2.229048	1.449062	2.371833
CTD-2132	2.413146	4.435159	1.385801	4.33342	4.025267	0.827273	4.978182	1.131472
CTD-2135	0.049278	0.090584	0.452965	0.005758	0.002237	0.178629	0.005253	0.049336
CTD-2154	4.33E-05	0.047092	0.045491	0.0857	0.057518	0.080837	0.340866	0.501905
CTD-2184	4.33E-05	0.219134	1.77404	0.0857	1.44E-05	0.584892	0.19355	0.630823
CTD-2192	1.450693	0.186465	1.340981	0.588954	1.495043	0.751876	0.481414	0.064127
CTD-2196	0.080592	0.850808	44.27012	0.614545	0.028333	18.84404	1.677042	10.93101
CTD-2228	25.99427	45.91841	19.83727	28.23632	36.74522	16.80343	52.56128	26.80355
CTD-2231	12.70768	5.110159	1.172872	26.95429	21.93452	6.162446	7.625209	9.667316
CTD-2260	1.443059	0.83088	10.78056	1.202626	0.824885	4.365224	1.056898	1.669466
CTD-2287	2.181775	4.392316	1.505678	2.351299	2.351299	0.751876	4.863579	1.744055
CTD-2291	0.135606	0.00026	8.940671	0.614545	1.44E-05	0.276061	1.205685	4.456912
CTD-2292	0.631847	0.351789	0.487662	0.195253	0.379791	1.427431	0.500354	0.483326
CTD-2325	35.776	8.677489	0.045491	1.700354	40.11561	34.84299	4.894509	7.315512
CTD-2350	0.826407	0.591912	0.045491	3.566753	2.134545	0.117258	0.218045	1.697792
CTD-2366	1.121703	2.500548	0.681068	1.63039	2.005253	2.000592	3.327619	2.207417
CTD-2368	2.928247	1.650274	15.98892	6.200945	2.55184	11.30987	4.508196	11.42512
CTD-2373	0.789423	0.802035	0.045491	0.288247	0.160202	0.614156	0.35676	0.07671
CTD-2396	24.98218	1.146118	0.045491	1.63039	6.706472	2.468117	0.518139	2.31013
CTD-2396	55.04081	5.366335	0.045491	7.363117	12.22798	8.745238	2.502619	2.104358
CTD-2410	0.027056	0.186465	0.286522	0.156017	0.028333	0.28689	0.138326	0.34619
CTD-2514	100.0998	60.77449	0.045491	118.7028	64.12971	7.417359	24.98218	4.034423
CTD-2517	2.981962	2.404618	5.359134	1.283059	2.453781	2.524892	14.97762	9.989221
CTD-2528	3.008225	3.214545	0.045491	4.034423	3.676032	2.611797	3.201457	3.310758

CTD-2530	0.071566	0.391674	9.179466	2.236407	0.07684	0.774798	1.064242	1.68342
CTD-2537	1.988196	1.452857	0.045491	3.544257	2.351299	0.672857	2.378124	1.776739
CTD-2538	2.392431	0.970007	1.65785	0.916306	2.160649	3.38456	1.111241	0.466068
CTD-2540	26.80355	5.84689	0.045491	5.770996	21.64437	0.672857	0.698297	0.00013
CTD-2540	87.1746	57.84882	0.045491	22.32214	126.1556	17.15902	29.82828	5.266335
CTD-2540	4.33E-05	0.00026	15.41187	0.633853	1.44E-05	4.241905	0.827273	3.807648
CTD-2541	0.902403	1.616385	1.706219	1.700354	1.159149	2.150303	0.96938	1.250058
CTD-2554	0.206061	0.562107	12.77772	3.55583	0.101494	3.249877	4.33E-05	8.958225
CTD-2555	4.33E-05	0.023694	0.831472	0.042569	0.014423	0.106522	4.33E-05	0.026414
CTD-2555	0.291183	0.810491	0.045491	1.94311	0.767569	2.196017	1.65785	3.080317
CTD-2561	0.181797	0.135902	1.046061	0.495584	0.107626	0.189141	0.109466	0.016472
CTD-2561	23.49249	24.50942	0.045491	4.881775	18.76954	0.00013	0.597417	0.00013
CTD-2562	14.51648	7.751869	1.385801	1.270317	10.38202	11.60283	3.119986	4.302367
CTD-2574	0.096371	0.33676	2.209141	0.076162	0.153391	0.346494	0.774798	1.566414
CTD-2574	0.799048	1.688045	5.864084	1.953535	1.316544	2.961768	2.939141	2.781558
CTD-2583	2.127973	1.595729	0.452965	1.833102	0.4971	1.418312	2.003723	2.019618
CTD-2619	2.074906	1.465123	7.570693	5.923838	2.618846	8.923117	2.681169	7.810058
CTD-2619	0.960866	0.599134	3.606804	1.416407	0.603261	1.436623	0.745613	2.278254
CTD-2622	0.466602	1.180613	7.002056	0.982128	0.30329	1.533535	0.233889	1.991328
CTD-2630	0.572626	0.391674	23.82354	1.738456	0.464834	3.317807	1.194221	4.091667
CTD-2636	5.84689	2.185556	0.414473	0.940563	13.53913	2.378124	1.566414	1.881955
CTD-2647	0.215115	0.466068	7.38847	1.189769	1.44E-05	4.392316	0.081126	11.65791
CTD-2647	15.41187	13.15079	0.045491	14.00414	9.418196	0.00013	1.340981	0.00013
CTD-2653	4.33E-05	0.00026	1.172872	2.89E-05	0.044322	0.038716	0.059091	0.322684
CTD-2666	0.27075	2.369841	4.997417	1.150635	0.190844	8.348817	2.396429	13.91714
CTD-3025	7.162698	6.079596	0.045491	3.588369	7.162698	1.64917	5.23176	2.947287
CTD-3099	4.33E-05	0.256046	0.487662	0.095029	1.44E-05	0.397215	0.015491	0.20969
CTD-3184	21.36892	28.87506	0.917431	14.42661	33.4985	9.583463	21.02638	6.39474
CTD-3185	4.012049	1.941703	2.760765	4.193232	4.155339	3.028629	3.188918	2.461905
CTD-3193	4.38684	2.659214	0.045491	0.731732	4.072475	6.25938	7.951371	0.720938
CTD-3222	4.33E-05	0.041227	0.310447	0.0857	0.044322	0.089149	0.202987	0.572626
CYB561D2	11.42512	2.45575	7.737807	18.33909	9.707677	10.19468	9.256537	11.89932
CYP17A1-	3.966623	12.36443	0.045491	1.301169	2.371833	2.312085	3.493362	2.630729
DANCR	224.9555	144.7919	25.73165	135.5245	67.98069	72.69473	167.9996	105.1694
DGCR11	0.338831	0.287821	1.844098	0.387258	0.153391	0.512309	0.301955	0.708124
DGCR5	18.62711	18.26752	11.544	0.562439	8.711905	41.47925	8.677489	9.179466
DICER1-A	13.11646	18.62711	6.297136	9.418196	14.51648	10.70538	31.1753	16.09482
DLEU2L	0.088788	0.00026	0.045491	0.114964	0.036566	0.774798	0.109466	0.322684
DLG1-AS1	0.007792	0.023694	0.26215	0.136926	1.44E-05	0.029769	0.101905	0.00013
DLG5-AS1	1.369048	3.479134	0.045491	0.949827	1.734949	1.093434	0.706984	0.00013
DNAJC27-	13.83359	5.381227	1.65785	10.01009	6.55039	10.2617	13.30906	7.507619
DOCK4-A	0.004214	0.00026	0.045491	0.005758	0.005173	0.146999	0.005253	0.064127
DOCK9-A	1.616385	0.00026	0.045491	0.469711	1.184935	4.328232	0.278802	0.586248
DTX2P1-U	0.259509	0.205115	0.762165	0.229048	0.138831	0.444315	0.349812	1.112641
EAF1-AS1	0.056046	0.157186	1.428398	2.89E-05	0.069942	0.296955	0.131349	0.275873
EIF3J-AS1	5.01088	4.451154	1.551962	12.10289	5.545729	21.19547	10.67991	14.51648
ENTPD1-/	0.088788	0.938413	9.047532	0.959567	0.036566	1.290981	0.597417	4.508196
ENTPD3-/	4.814661	3.621941	0.796674	5.622872	4.857403	4.490801	3.343629	5.638759
EPB41L4A	48.53429	31.55417	13.04605	51.52195	27.96248	91.05541	144.7919	87.1746
ERI3-IT1	0.175361	0.00026	0.917431	2.89E-05	0.050426	0.146999	4.33E-05	0.143218
ERVH48-1	0.004214	0.105022	1.090072	0.076162	0.069942	0.296955	0.202987	0.34619
ERVK3-1	31.55417	11.96574	5.864084	41.47925	35.27608	44.27012	19.68088	21.93452
FAM13A-/	2.890245	1.43039	2.280101	1.270317	1.29417	3.125844	2.281926	2.616573
FAM157A	0.309444	0.411703	0.796674	0.229048	0.386616	1.163579	0.152843	0.543939
FAM66A	0.982128	0.86469	31.1753	0.872013	0.611003	7.911934	4.614618	6.900202
FAM66B	0.466602	0.24184	0.26215	0.375743	0.337222	8.089848	1.677042	0.877222
FAM66C	2.487576	3.214545	28.71146	2.584394	2.034242	14.42661	7.339452	16.85982
FBXL19-A	0.181797	1.072056	1.428398	0.218045	0.235931	0.73912	0.833975	1.595729

FGD5-AS1	24.50942	32.31123	37.81919	34.84299	26.80355	77.74812	65.94551	80.6518
FLNB-AS1	0.007792	0.00026	0.517612	0.104906	1.44E-05	0.252316	0.073463	0.108997
FOXN3-AS	1.901068	0.212172	1.264589	1.35259	2.386248	1.210447	0.464127	0.537518
FOXP1-IT1	4.33E-05	0.06715	0.414473	0.002367	0.002237	0.00013	0.02189	0.102165
FTX	0.676999	2.671299	43.49658	1.211299	0.834416	3.529466	1.226934	3.533211
GABPB1-A	2.74298	7.61176	21.74051	9.218514	3.233045	9.121486	10.01009	28.87506
GMDS-AS	1.419278	3.188918	19.60574	4.540794	1.898153	6.316328	3.833247	4.620159
GNG12-A	0.049278	0.023694	1.986681	0.026537	0.014423	0.00013	0.009293	0.172453
GS1-114I	4.18456	1.805166	0.452965	5.044113	8.445743	0.925801	1.056898	0.373463
GS1-124K	1.070693	0.791854	2.398442	1.09075	1.486551	1.135887	1.637309	2.725498
GS1-124K	0.042713	0.30176	24.98218	0.444315	0.090029	0.90013	0.642395	2.421681
GS1-124K	13.60866	15.07257	1.222958	9.604358	28.23632	9.667316	16.68554	9.707677
GS1-166A	4.927302	4.586753	0.045491	0.469711	1.169091	3.116732	2.876421	0.428694
GS1-393G	2.281926	1.539062	0.045491	0.495584	1.872049	0.346494	0.906126	0.00013
GSN-AS1	0.291183	0.090584	1.172872	0.787987	0.07684	2.860722	0.117258	1.820541
GUSBP11	45.91841	67.98069	12.49452	29.18885	62.33472	15.22851	33.93939	19.68088
H1FX-AS1	6.695887	13.6814	2.45575	5.821804	3.636789	1.64917	6.808369	2.445079
HCG11	0.403651	0.205115	0.234358	0.48583	0.391421	4.198117	0.251371	0.854733
HCG18	3.718716	16.22108	18.84404	4.392316	2.649675	22.52375	8.445743	28.54786
HCG25	52.56128	50.55949	0.762165	42.08807	77.74812	7.08733	40.11561	6.922771
HCP5	1.058896	0.411703	1.172872	2.111299	5.9071	1.604827	0.629098	0.289935
HMGN3-A	0.438831	0.550974	0.335411	0.278651	0.442799	0.844156	0.810491	0.453218
HNRNPU-	2.479221	2.047157	5.068831	7.824473	5.606739	11.83593	12.42965	9.316241
HOTAIRM	0.27075	5.210317	0.045491	1.65785	22.96463	0.178629	0.861429	0.200794
IDH1-AS1	3.24658	1.064242	0.045491	13.19051	9.541645	1.054899	3.263391	4.620159
IL10RB-AS	2.26518	3.855952	27.96248	4.086926	3.94886	2.043795	4.212554	6.622092
INE1	0.807619	1.044235	3.747035	1.63039	0.523211	2.642525	2.81342	6.853608
ITPK1-AS1	0.007792	0.090584	0.310447	0.012136	1.44E-05	0.00013	0.009293	0.042771
JPX	19.68088	28.23632	16.9162	17.92188	12.36443	24.2842	15.98892	45.91841
KANSL1-A	3.730808	2.978968	0.045491	5.22482	6.900202	1.498167	7.212915	4.907929
KB-1125A	5.309264	10.07949	1.340981	1.794531	2.630729	5.560563	6.865	1.977316
KB-1460A	9.377345	6.028427	0.917431	6.116638	8.226219	5.599228	5.056421	5.195022
KB-431C1	3.799885	6.52899	0.487662	4.946587	4.135801	3.885642	6.486104	2.789466
KB-431C1	0.390895	0.205115	1.172872	0.321746	0.138831	0.323535	0.437799	0.268095
KCNQ1OT	0.013348	1.013434	3.122915	0.026537	0.028333	0.063911	0.065974	0.405346
KTN1-AS1	0.331429	0.525678	1.127475	0.66482	0.337222	1.054899	0.977994	1.317475
LA16c-35	0.880498	0.83088	3.008225	1.794531	0.973745	1.45912	2.136335	4.392316
LA16c-39	1.121703	0.219134	4.06303	1.579798	0.268369	0.614156	1.464214	3.002576
LA16c-39	77.74812	40.7932	0.517612	26.95429	31.92385	2.043795	9.141299	2.384141
LA16c-OS	2.685786	1.05153	0.045491	1.738456	2.221775	0.421025	0.637085	0.296234
LBX2-AS1	0.300844	0.226075	0.452965	2.283752	0.489235	0.106522	0.682799	0.181797
LENG8-AS	1.415469	2.600014	26.80355	3.06803	1.243442	6.200945	4.667518	6.125823
LIMD1-AS	0.846342	0.380267	4.997417	1.974228	2.209141	1.795851	3.644343	2.661587
LINC0008	9.989221	3.632973	4.814661	1.915455	6.853608	51.52195	2.003723	6.079596
LINC0009	10.23768	11.25392	30.82277	14.08569	9.218514	45.1045	15.18102	31.55417
LINC0015	13.53913	2.238341	0.045491	144.7919	28.87506	12.63773	2.984899	1.209776
LINC0017	3.377619	9.502294	3.317807	2.735368	2.518124	0.444315	12.16681	1.250058
LINC0020	8.608153	2.893001	0.644618	2.496407	5.295325	3.521934	0.605022	0.770188
LINC0020	4.130794	6.135007	0.917431	5.373781	7.288788	6.497266	9.792626	1.209776
LINC0026	2.230859	1.335743	2.035678	3.672078	3.031688	2.281926	4.160238	5.210317
LINC0029	1.014127	0.623499	0.26215	0.239589	1.935685	0.672857	0.218045	0.335967
LINC0031	0.013348	0.00026	0.385238	0.012136	0.005173	0.010274	0.051198	0.070541
LINC0033	5.172828	1.582915	0.998312	9.297085	6.706472	8.608153	4.997417	3.718716
LINC0034	19.06893	41.47925	7.737807	24.98218	20.68746	14.00414	43.49658	8.780115
LINC0038	4.33E-05	0.00026	0.216025	0.01873	0.009307	0.014791	0.009293	0.009293
LINC0039	0.035137	0.023694	2.209141	0.095029	0.06381	0.421025	0.765606	0.275873
LINC0046	11.19942	43.49658	77.74812	40.11561	19.22139	37.81919	95.22939	83.489
LINC0047	0.243449	0.922338	3.122915	1.35259	0.728543	2.104358	1.484553	2.408882

LINC0048	0.135606	0.06715	1.505678	0.058442	1.44E-05	0.305253	4.33E-05	0.082727
LINC0051	10.28573	25.73165	83.489	29.49693	9.377345	10.78056	62.33472	51.52195
LINC0052	3.1857	5.508131	5.779574	7.737807	3.188918	4.241905	5.500678	7.136537
LINC0061	0.035137	0.018038	1.706219	0.146999	1.44E-05	0.010274	0.028398	0.0121
LINC0063	0.161082	0.186465	5.477843	0.259163	0.050426	1.604827	0.311385	2.474834
LINC0065	0.324019	0.330043	2.035678	0.278651	0.243449	2.768312	0.101905	0.330289
LINC0065	49.50903	44.27012	144.7919	60.77449	32.31123	1360.293	100.0998	184.443
LINC0066	3.581688	7.495397	15.71851	9.541645	3.55583	14.97762	6.210375	19.83727
LINC0066	8.849697	10.67991	29.49693	24.2842	6.922771	31.1753	17.64869	33.93939
LINC0068	1.146118	1.664906	0.045491	0.351219	1.449062	0.614156	1.835743	1.399315
LINC0084	118.7028	167.9996	6.071436	287.197	75.1132	530.1465	287.197	167.9996
LINC0084	8.033932	5.993045	16.74449	16.9777	7.315512	11.19942	14.79742	17.45948
LINC0086	1.201724	0.991631	8.075772	1.89228	1.01544	3.816551	2.240274	6.25938
LINC0089	2.950173	0.480794	0.452965	0.458341	2.236407	3.439437	0.552439	0.998312
LINC0089	1.674401	6.580988	1.706219	1.89228	1.343463	2.630729	3.734762	3.058802
LINC0090	7.08733	3.750837	2.398442	7.625209	5.01088	14.33939	7.013853	10.78056
LINC0092	1.846941	23.38378	105.1694	21.83751	5.993045	11.65791	70.11932	144.7919
LINC0092	0.035137	2.474834	4.445851	0.653968	0.07684	0.421025	1.263752	18.19595
LINC0095	7.339452	0.843102	1.300368	3.144156	2.542605	42.08807	1.194221	5.770996
LINC0096	18.05488	20.68746	184.443	36.74522	13.75978	21.93452	57.84882	40.11561
LINC0099	32.68313	36.74522	8.033932	83.489	60.77449	33.4985	59.32013	59.32013
LINC0100	3.06803	0.511667	2.45575	0.754315	3.346898	7.683766	2.227273	2.320072
LINC0100	8.288874	8.333918	42.08807	19.22139	12.56693	15.22851	25.99427	62.33472
LINC0100	2.011501	4.227302	0.414473	4.667518	5.737056	0.65202	5.950664	4.392316
LINC0102	12.42965	4.029654	11.22667	20.51271	18.05488	6.570433	4.18456	12.70768
LINC0105	0.676999	0.212172	1.046061	1.423506	1.048074	0.800058	0.131349	2.725498
LINC0106	3.807648	5.583968	1.844098	4.277006	4.222583	1.47018	1.36619	1.306667
LIPE-AS1	15.18102	14.33939	1.428398	11.25392	18.91854	27.66779	9.838658	9.751039
LL0XNC01	4.33E-05	0.121494	7.288788	0.125859	1.44E-05	1.663672	0.285253	0.810967
LL0XNC01	0.453463	0.343629	1.385801	0.600887	0.434596	0.533874	0.745613	1.306667
LL21NC02	3.497085	6.30671	0.045491	1.599033	12.63773	8.226219	5.720173	2.225498
LL22NC03	0.730029	34.38341	0.26215	0.581645	0.808983	1.663672	1.198701	6.580988
LMCD1-A	0.206061	0.30176	5.864084	0.358672	0.118983	1.179885	0.637085	3.934986
LOH12CR	2.810678	4.764055	1.602619	3.695815	2.800332	4.758088	4.733824	2.498478
MAGI1-IT	0.004214	0.00026	0.045491	0.012136	1.44E-05	0.00013	4.33E-05	0.026414
MAGI2-AS	12.60233	14.42661	21.02638	20.34063	13.00996	28.54786	27.96248	52.56128
MALAT1	80.6518	1360.293	425.62	252.4251	144.7919	167.9996	155.7103	1360.293
MAN2C1	184.443	203.5795	224.9555	224.9555	530.1465	144.7919	425.62	332.0776
MAP3K14	0.309444	1.224733	7.074329	0.81544	0.687807	1.833102	2.157359	1.799899
MAPKAPK	40.11561	19.68088	14.70188	45.91841	29.49693	26.52537	25.24367	24.04374
MCCC1-A	0.309444	0.403413	0.681068	0.507085	0.352807	0.877807	1.180613	0.522633
MCM3AP-	2.635685	5.01772	8.83241	2.440895	3.077316	2.676392	6.865	3.971061
MIR143H	0.181797	0.11368	2.160649	0.773766	0.014423	0.913514	0.166118	0.0121
MIR155H	0.243449	0.179177	0.045491	0.731732	0.29671	0.127201	0.278802	0.070541
MIR181A1	0.019784	0.00026	1.090072	0.005758	1.44E-05	0.137201	4.33E-05	0.070541
MIR22HG	7.737807	2.978968	2.074906	6.268312	5.796421	13.38418	3.110447	2.818312
MIR4435-	10.19468	1.234444	0.517612	91.05541	12.16681	14.08569	4.586753	4.733824
MKNK1-A	0.181797	0.00026	0.335411	0.058442	0.044322	0.37456	0.094553	0.116392
MMP24-A	91.05541	48.53429	8.412367	55.04081	83.489	105.1694	64.12971	45.1045
MPRIP-AS	4.33E-05	0.00026	1.934271	2.89E-05	1.44E-05	0.00013	0.087879	0.095599
MTHFS	14.17388	9.583463	2.160649	21.54823	21.36892	17.03919	6.632063	15.66023
NADK2-A	4.33E-05	0.00026	2.24562	2.89E-05	1.44E-05	0.90013	4.33E-05	1.418312
NAPA-AS	0.460649	0.373218	2.117814	0.949827	0.458701	1.331919	1.159913	2.8657
NARF-IT1	0.118528	0.00026	2.398442	0.163766	1.44E-05	0.465325	0.242222	0.283882
NAV2-AS	0.004214	0.00026	4.745895	0.002367	0.002237	0.00013	0.015491	0.031169
NCAM1-A	0.259509	1.152165	2.607035	0.458341	0.553059	0.63114	2.214372	2.304452
NCBP2-AS	34.84299	21.19547	35.27608	46.7056	22.52375	46.7056	32.8773	40.7932
NDUFA6-	5.889798	8.445743	35.776	7.149618	7.200505	19.83727	21.54823	22.32214

NNT-AS1	4.575123	12.85504	20.85876	6.922771	4.597792	13.08214	14.08569	17.15902
NOP14-A:	4.23202	2.633232	7.507619	11.30987	5.24562	9.459177	5.447114	11.8058
NPPA-AS:	3.089293	1.186457	3.479134	1.020447	2.911962	0.706494	1.395563	0.648038
NTRK3-AS	4.33E-05	0.018038	0.517612	0.042569	1.44E-05	0.014791	0.005253	0.00013
OIP5-AS1	20.34063	17.28195	27.38726	8.226219	11.09133	95.22939	16.22108	35.776
OSER1-AS	13.23022	9.667316	10.1028	12.46209	12.03267	16.9162	17.51961	17.28195
OXCT1-AS	4.33E-05	0.429567	6.454416	2.092987	0.07684	1.013434	2.058773	3.934986
PAN3-AS:	1.146118	0.938413	0.644618	2.283752	1.386681	1.533535	2.378124	2.019618
PAXBP1-A	1.239423	1.193362	7.683766	0.846797	0.776876	3.028629	2.333867	1.473218
PAXIP1-A:	6.09759	10.1459	15.71851	8.524242	7.683766	9.179466	16.34294	12.77772
PAXIP1-A:	0.597042	1.116609	4.614618	1.325743	1.20653	2.50469	0.583766	0.835498
PCAT6	3.425512	8.256465	1.172872	5.373781	4.170051	1.195794	4.667518	0.854733
PCBP1-AS	6.876392	7.92518	33.4985	11.96574	9.880779	18.33909	13.60866	22.96463
PCED1B-A	12.85504	4.745895	0.644618	30.82277	17.92188	8.048095	4.402973	12.89577
PGM5P2	0.088788	0.00026	1.340981	0.552749	0.101494	0.485577	0.035382	0.438023
PITPNA-A	9.838658	10.98644	0.045491	13.45939	21.02638	7.264502	28.87506	21.36892
PITRM1-A	0.259509	0.06715	1.264589	0.183521	0.126335	0.73912	0.242222	0.24443
PKD1P6	7.683766	14.79742	31.92385	18.62711	9.459177	36.74522	31.55417	41.47925
PPP1R26-	0.53658	0.047092	2.558817	0.288247	0.257143	3.074134	0.698297	0.780952
PRKAG2-A	31.1753	5.679502	1.090072	0.398629	15.276	27.38726	6.404921	3.730808
PROX1-AS	0.383074	0.498052	2.511465	0.218045	0.337222	0.913514	0.251371	0.770188
PRRT3-AS	1.163579	0.850808	0.045491	1.189769	0.898543	0.233175	4.33E-05	0.236089
PSMD5-A	9.218514	19.06893	50.55949	9.838658	4.058514	26.25557	17.78522	60.77449
PSMD6-A	0.027056	0.212172	0.998312	0.229048	0.050426	0.296955	0.117258	0.396926
PWAR6	24.73724	10.43066	7.162698	6.011515	9.646284	67.98069	19.53061	20.34063
RAB11B-A	15.276	3.046551	1.602619	20.68746	13.45939	5.123781	4.140505	4.863579
RAD21-AS	0.027056	0.00026	0.045491	0.066616	0.014423	0.096667	0.065974	0.070541
RAD51-AS	5.477843	4.678846	20.34063	3.606804	4.733824	13.6814	3.236457	16.5625
RAMP2-A	0.4457	2.300678	14.79742	5.295325	1.574517	1.587605	4.016465	10.70538
RAP2C-AS	0.152843	0.083189	0.555036	1.020447	0.512309	0.558095	0.450924	0.750866
RBAKDN	1.458038	2.143261	0.045491	0.949827	1.022929	0.146999	0.833975	0.200794
RBM26-AS	0.81596	2.500548	3.711097	0.525325	0.574993	1.179885	4.776407	1.566414
RGS5	9.502294	8.608153	12.67271	57.84882	5.381227	111.5439	9.924372	22.74797
RMST	7.188095	3.046551	52.56128	0.104906	15.98892	3.651898	0.605022	2.169509
RNF139-A	0.846342	0.705613	0.385238	1.859084	0.793434	1.90798	2.08632	1.306667
RNU12	0.096371	0.00026	203.5795	0.218045	0.095996	0.00013	0.117258	0.00013
RP1-100J:	0.701818	0.351789	8.241342	0.507085	0.523211	0.00013	0.19355	0.136061
RP1-111C	1.549711	4.376118	1.65785	1.510014	1.031284	2.747908	3.921645	3.771349
RP1-117C	4.33E-05	0.00026	0.335411	2.89E-05	1.44E-05	0.029769	4.33E-05	0.190693
RP1-122P	0.042713	0.083189	9.625253	0.288247	1.44E-05	9.316241	0.051198	0.154719
RP1-12G1	4.33E-05	0.00026	9.297085	2.89E-05	1.44E-05	1.054899	1.89228	0.63873
RP1-149A	0.056046	0.029134	0.555036	0.229048	0.07684	0.156017	0.015491	0.016472
RP1-151F:	1.731169	2.947287	0.234358	1.89228	1.235346	2.487576	7.417359	0.998312
RP1-167F:	0.071566	0.518139	4.927302	0.477698	0.280823	0.047092	0.131349	0.268095
RP1-187B	4.33E-05	0.018038	0.286522	0.136926	0.020895	0.00013	0.009293	0.00013
RP1-191J:	0.367287	0.135902	1.428398	0.600887	0.28899	0.37456	0.087879	0.335967
RP1-193H	2.993867	2.404618	0.555036	1.8386	1.971183	3.404372	1.737294	1.889711
RP1-199J:	0.624841	1.842734	0.917431	2.111299	0.687807	0.366436	0.690043	0.421573
RP1-20B2	19.22139	30.82277	0.045491	21.83751	26.25557	0.00013	2.715873	2.002193
RP1-239B	4.667518	11.65791	0.487662	3.4679	2.931068	2.758088	5.993045	1.390476
RP1-257A	155.7103	83.489	1.551962	35.27608	184.443	60.77449	55.04081	23.38378
RP1-265C	0.188586	0.226075	0.601227	0.034863	0.113449	0.146999	0.278802	0.4971
RP1-266L:	0.175361	0.226075	1.884935	0.239589	0.310657	0.454632	0.577071	0.322684
RP1-283E:	0.144719	0.541241	11.22667	1.150635	0.656753	2.117814	0.79487	1.035058
RP1-286D	1.482626	0.636688	8.508514	3.695815	2.579509	2.378124	0.675115	3.002576
RP1-293L(	1.111241	4.614618	0.045491	2.89E-05	1.121017	2.016515	9.031082	5.889798
RP1-29C1	4.33E-05	0.00026	0.216025	0.076162	1.44E-05	0.014791	4.33E-05	0.00013
RP1-30M:	2.939141	1.91811	0.335411	2.375974	3.435988	2.150303	1.425613	0.810967

RP1-313I6	1.146118	1.174315	0.414473	2.792035	0.913514	0.263658	0.977994	1.019134
RP1-315G	1.58645	9.625253	0.045491	0.387258	2.984899	1.361789	3.428788	1.2214
RP1-34H1	0.019784	0.047092	0.917431	0.026537	1.44E-05	5.030866	0.094553	0.522633
RP1-39G2	6.99026	6.653016	1.465123	10.19468	5.942294	13.00996	7.288788	13.64503
RP1-43E1	2.698557	0.910823	0.045491	8.888167	3.750837	3.422237	1.340981	2.964762
RP1-59D1	0.685931	0.667965	2.160649	0.136926	0.97733	0.233175	1.935685	2.630729
RP1-63G5	1.889711	0.930325	1.505678	0.125859	1.495043	2.540462	0.912987	1.515296
RP11-100	0.135606	0.411703	4.673182	0.469711	0.274199	0.558095	0.278802	0.854733
RP11-101	5.123781	2.009906	7.074329	3.5257	3.78798	4.614618	5.195022	3.446732
RP11-101	3.256587	2.947287	1.77404	1.63039	2.630729	1.623232	3.201457	0.835498
RP11-101	41.47925	30.49289	4.502453	47.5018	32.68313	15.46193	49.50903	18.48514
RP11-102	0.495931	0.525678	0.956061	0.773766	0.386616	0.857778	0.493247	1.485483
RP11-106	0.004214	0.00026	0.234358	0.002367	1.44E-05	0.038716	0.02189	0.089473
RP11-106	0.545426	0.753449	1.602619	0.643427	0.694675	0.475137	0.568369	0.579495
RP11-107	9.583463	4.33342	0.045491	3.975498	12.85504	0.00013	0.087879	0.00013
RP11-108	0.858297	1.139885	0.045491	0.90772	1.449062	0.137201	4.33E-05	0.00013
RP11-109	12.77772	12.67271	6.38456	13.72059	11.60283	11.25392	15.56131	14.08569
RP11-109	25.48179	10.48101	0.045491	20.85876	15.18102	1.135887	3.201457	3.55583
RP11-10N	0.367287	1.534603	1.505678	1.1629	0.327828	0.465325	0.658745	1.325743
RP11-110	18.76954	0.504964	0.045491	28.54786	19.68088	0.00013	3.110447	0.00013
RP11-110	14.6058	5.137042	0.045491	0.692799	14.70188	0.00013	0.131349	0.236089
RP11-110	1.599033	1.476429	19.99861	3.94886	2.565556	3.734762	3.846984	8.83241
RP11-110	0.152843	0.650101	9.859719	0.717403	0.146118	1.223846	0.810491	3.807648
RP11-110	0.096371	0.219134	3.266782	0.195253	0.132922	0.595368	0.363983	0.396926
RP11-110	3.679986	8.745238	0.045491	11.719	3.833247	1.390476	7.545584	5.737056
RP11-111	0.316573	2.325931	3.211176	0.93267	0.651631	1.093434	2.378124	2.705916
RP11-111	0.995527	1.146118	0.045491	0.846797	0.913514	0.65202	0.819978	0.413449
RP11-111	0.730029	0.858297	0.286522	0.525325	1.000794	1.121017	0.667201	1.041652
RP11-111	15.88548	20.51271	4.140505	7.991602	9.583463	12.93651	23.16622	9.989221
RP11-111	3.614603	12.93651	0.045491	5.9071	5.704646	2.805382	6.250447	1.849654
RP11-111	1.638499	4.348882	0.045491	1.65785	1.667273	2.250462	1.63039	0.515527
RP11-114	4.535354	0.391674	0.045491	2.89E-05	1.812114	0.00013	0.390014	0.566494
RP11-115	0.438831	0.83697	0.045491	1.370765	0.553059	0.127201	0.706984	0.128615
RP11-118	4.978182	4.145209	0.555036	2.584394	3.35355	4.058514	2.003723	0.835498
RP11-119	1.239423	4.978182	0.831472	1.821861	2.034242	0.584892	3.175916	2.831501
RP11-120	0.027056	0.00026	1.127475	0.173059	0.020895	0.00013	0.051198	0.396926
RP11-121	1.130115	1.072056	0.517612	0.614545	0.710722	1.696494	1.308167	0.750866
RP11-122	0.526566	0.186465	2.339697	0.700418	0.540563	1.636118	1.355527	3.659668
RP11-126	4.696313	19.37139	0.956061	13.91714	15.88548	1.696494	12.03267	12.46209
RP11-126	0.144719	0.00026	16.50509	2.89E-05	0.090029	0.313925	0.051198	0.543939
RP11-127	0.53658	0.900693	10.17029	0.525325	0.458701	1.246457	0.851234	1.349971
RP11-127	2.024488	2.03886	0.045491	2.021068	2.274473	0.683232	0.560325	0.96329
RP11-127	36.26061	17.03919	2.117814	5.238701	18.26752	8.256465	3.343629	5.110159
RP11-129	0.403651	0.275527	0.414473	0.288247	0.171472	1.093434	0.500354	0.438023
RP11-130	1.426522	1.938759	0.917431	3.220678	0.581926	0.572987	2.359264	1.409942
RP11-131	4.33E-05	0.00026	0.045491	0.050216	1.44E-05	0.055887	0.005253	0.009293
RP11-134	0.071566	0.76	0.310447	0.218045	0.090029	0.252316	0.464127	0.657547
RP11-134	2.011501	6.830592	0.045491	1.757244	2.178268	2.703485	2.531515	1.959466
RP11-134	1.793074	0.490375	4.277006	1.728615	1.145426	0.366436	0.53658	0.789423
RP11-137	0.821414	0.525678	3.711097	1.325743	1.064935	4.02088	1.572035	3.636789
RP11-138	4.33E-05	0.035007	0.310447	0.042569	0.028333	0.038716	0.02189	0.021255
RP11-138	287.197	16.09482	4.012049	167.9996	287.197	4.952915	6.171789	24.73724
RP11-139	2.138131	1.898153	0.762165	1.270317	2.141595	1.054899	2.681169	2.292864
RP11-139	0.019784	0.11368	2.074906	0.104906	0.06381	0.021429	0.035382	0.312864
RP11-13J1	2.138131	3.703175	65.94551	7.653391	2.196017	23.6012	10.35839	21.54823
RP11-140	7.013853	3.290101	0.045491	3.78798	5.462475	6.956349	1.799899	2.671299
RP11-140	30.17192	17.39935	1.551962	62.33472	34.84299	29.18885	13.91714	11.03843
RP11-144	4.33E-05	0.287821	12.29473	0.173059	1.44E-05	1.401977	0.615996	1.169791

RP11-145	0.007792	0.047092	0.26215	0.005758	0.020895	0.00013	0.009293	0.00013
RP11-147	0.518139	3.256587	4.445851	0.81544	0.345635	0.584892	0.577071	0.973745
RP11-148	1.410902	1.123016	1.222958	2.092987	1.935685	0.28689	1.205685	0.789423
RP11-151	4.33E-05	1.397482	3.711097	0.136926	1.44E-05	0.28689	0.059091	1.409942
RP11-152	0.035137	0.075339	0.286522	0.01873	0.036566	0.146999	0.109466	0.38153
RP11-152	24.04374	64.12971	0.045491	34.38341	23.275	0.787136	14.88584	3.486436
RP11-152	2.911962	6.287561	3.966623	3.695815	3.521934	1.163579	5.500678	3.8379
RP11-154	0.676999	0.533535	4.105714	1.270317	0.858817	0.533874	0.79487	0.877222
RP11-156	8.381328	6.632063	0.644618	5.754935	5.959033	8.958225	7.363117	5.545729
RP11-156	1.549711	1.595729	2.339697	1.216768	1.073543	1.000173	1.355527	1.095657
RP11-156	8.888167	6.508427	23.6012	7.683766	5.821804	28.23632	8.780115	37.81919
RP11-157	0.970635	1.675722	0.796674	0.90772	2.240274	0.662944	0.605022	0.190693
RP11-158	0.027056	0.029134	0.831472	0.136926	0.014423	0.106522	0.065974	0.261227
RP11-158	0.007792	0.00026	0.045491	0.066616	0.005173	0.00013	0.081126	0.00013
RP11-158	0.552749	1.556392	4.546443	1.202626	0.402381	1.078153	1.644416	1.896667
RP11-159	14.70188	16.68554	11.544	21.36892	14.6058	13.45939	14.25954	21.74051
RP11-159	0.152843	0.373218	3.058802	0.562439	0.251126	5.728615	0.410996	4.881775
RP11-15A	0.196811	6.268312	25.99427	2.92013	0.216753	3.367071	2.078355	5.821804
RP11-15A	1.021746	1.186457	0.286522	0.924329	1.073543	0.706494	0.278802	0.359307
RP11-15H	2.107922	3.172864	67.98069	5.530274	2.011501	10.53159	4.927302	12.1974
RP11-161	15.66023	4.077403	0.045491	18.62711	41.47925	0.198788	9.459177	0.00013
RP11-162	0.152843	0.150743	1.300368	0.421573	0.069942	1.533535	0.47153	0.63873
RP11-164	0.071566	0.047092	0.234358	0.012136	0.005173	0.00013	0.009293	0.0121
RP11-164	1.605859	2.800332	0.555036	2.818312	2.823636	2.180022	3.307186	3.636789
RP11-164	0.007792	0.00026	0.414473	0.095029	0.005173	0.127201	0.043391	0.172453
RP11-166	4.33E-05	1.123016	3.55583	0.034863	0.028333	0.146999	0.172857	0.550072
RP11-166	20.68746	37.29684	36.74522	38.39042	26.52537	32.31123	39.53687	64.12971
RP11-166	0.042713	0.234358	4.359574	0.207713	0.090029	0.127201	0.419827	0.335967
RP11-166	0.004214	0.018038	0.414473	0.012136	0.002237	0.137201	0.009293	0.021255
RP11-167	2.62342	1.253752	1.428398	0.66482	1.37531	0.857778	2.472576	0.726811
RP11-173	4.33E-05	0.00026	0.045491	3.94886	1.44E-05	6.028427	4.952915	15.46193
RP11-178	2.327857	0.24184	0.045491	0.872013	1.672107	0.00013	3.147294	1.095657
RP11-178	11.89932	11.30987	0.045491	0.754315	7.938427	0.827273	5.591717	0.330289
RP11-179	0.367287	1.180613	0.045491	0.269322	0.645036	0.397215	0.774798	0.38153
RP11-17E	1.86658	1.465123	0.045491	3.439437	1.464214	0.512309	0.957915	0.275873
RP11-182	0.11075	0.322237	4.959358	1.550837	0.434596	1.696494	0.96938	4.764055
RP11-182	0.27075	1.723579	0.26215	1.262179	1.243442	1.821861	5.095325	1.977316
RP11-182	4.33E-05	0.00026	0.872597	0.104906	1.44E-05	0.127201	0.226075	0.268095
RP11-182	1.857677	3.783694	11.77567	3.370527	1.612056	2.747908	2.953059	7.276645
RP11-188	0.685931	1.421126	0.045491	1.007244	0.858817	0.706494	0.577071	0.268095
RP11-18H	0.019784	1.314675	4.359574	0.469711	0.113449	0.533874	1.118817	1.776739
RP11-192	6.287561	5.238701	0.872597	2.818312	5.630815	2.860722	3.011169	0.579495
RP11-192	0.035137	0.00026	1.934271	0.066616	1.44E-05	0.242453	0.26215	0.275873
RP11-192	27.66779	21.74051	0.045491	5.158146	13.23022	21.36892	6.52899	3.23987
RP11-196	0.251371	0.06715	0.385238	0.005758	0.268369	0.178629	0.051198	0.056782
RP11-196	4.468009	2.489762	0.045491	3.092229	1.712706	1.90798	0.53658	2.032706
RP11-196	2.112843	0.810491	22.13245	1.68938	2.055289	2.267157	2.050404	3.414574
RP11-196	4.519048	0.76	0.045491	1.385801	4.091667	1.179885	2.092987	0.543939
RP11-196	0.161082	0.105022	1.77404	0.195253	0.216753	0.242453	0.185642	0.253074
RP11-196	2.337821	11.544	0.045491	3.578038	4.33342	0.00013	2.666494	0.865664
RP11-197	1.563045	0.248716	6.911486	3.153485	1.420202	1.488571	2.671299	2.685786
RP11-19P	16.80343	25.99427	0.045491	13.83359	18.26752	3.921645	16.9162	2.705916
RP11-203	29.82828	8.195657	0.831472	19.37139	27.66779	10.33476	4.758088	3.253175
RP11-203	0.088788	0.466068	1.551962	2.302626	0.090029	3.074134	1.118817	1.278629
RP11-206	0.243449	0.135902	4.445851	0.517092	0.095996	2.860722	0.372973	1.443983
RP11-206	0.035137	0.083189	1.809343	0.146999	0.050426	1.361789	0.226075	0.698687
RP11-20B	12.93651	7.468961	0.555036	1.07127	15.3618	0.00013	2.922778	0.07671
RP11-20G	4.445851	1.952027	0.045491	2.792035	1.476429	0.00013	1.662439	0.00013

RP11-20I2	6.653016	15.66023	1.465123	5.351934	8.005685	7.212915	7.38847	7.911934
RP11-20I2	0.300844	0.88316	2.820974	0.517092	0.197727	4.408153	0.987309	4.429769
RP11-212	1.286494	4.575123	18.84404	2.953059	1.145426	1.569553	6.945152	11.09133
RP11-216	2.074906	0.525678	1.884935	1.466111	0.933261	0.877807	1.954993	1.317475
RP11-218	1.702706	2.978968	0.335411	1.370765	1.37531	0.475137	0.79487	1.551962
RP11-21A	1.528975	2.378124	0.045491	0.614545	1.339091	0.584892	3.944084	1.124574
RP11-223	27.24614	7.315512	2.035678	13.60866	30.17192	12.85504	10.43066	21.02638
RP11-225	5.737056	8.539971	0.045491	10.43066	4.715599	4.802655	6.454416	7.238023
RP11-226	4.105714	3.714906	0.310447	4.007583	3.663622	2.981962	5.01772	3.730808
RP11-227	29.49693	16.80343	0.045491	0.050216	18.48514	4.445851	10.23768	0.566494
RP11-228	0.746551	0.953506	4.06303	1.677042	0.889553	1.000173	3.957734	1.799899
RP11-230	2.155447	0.743283	0.045491	0.90772	1.531313	4.003117	1.412771	4.881775
RP11-231	1.504517	2.165851	1.505678	1.794531	2.836717	0.763102	2.003723	0.253074
RP11-234	2.194257	0.86469	0.045491	0.207713	1.325743	1.093434	0.751371	0.283882
RP11-23J	0.135606	0.953506	2.607035	0.846797	0.243449	0.763102	0.318694	0.985411
RP11-23J	0.161082	0.179177	7.977958	0.163766	0.28899	0.960866	0.528571	1.390476
RP11-23N	0.103088	0.351789	1.809343	0.332395	0.050426	1.401977	1.622006	1.25886
RP11-242	0.572626	0.403413	0.917431	1.325743	0.532092	1.34513	1.412771	2.278254
RP11-244	0.206061	0.150743	1.986681	0.207713	0.113449	0.595368	0.410996	0.143218
RP11-248	0.579841	0.248716	2.117814	1.65785	0.834416	0.358211	0.382208	0.797114
RP11-24J	9.438687	1.139885	0.045491	1.036558	9.946364	0.00013	4.33E-05	0.00013
RP11-250	1.978954	1.702706	0.045491	3.738997	5.687879	2.378124	4.997417	1.710173
RP11-250	0.495931	0.930325	0.414473	2.021068	1.724805	0.65202	2.763261	1.085967
RP11-252	0.071566	0.018038	0.956061	0.342381	0.036566	0.209048	0.117258	0.148254
RP11-254	15.41187	4.435159	0.045491	7.482179	10.1459	3.714906	2.157359	0.63873
RP11-258	10.1028	26.52537	0.872597	7.519841	7.38847	6.55039	17.03919	4.927302
RP11-259	0.588312	0.791854	0.045491	0.846797	0.921739	0.198788	0.845887	0.4971
RP11-260	5.238701	2.535974	0.385238	3.895469	3.479134	2.250462	4.222583	2.839351
RP11-261	0.096371	0.162915	2.8657	0.050216	0.197727	0.866717	0.92482	1.095657
RP11-261	0.495931	0.490375	0.310447	0.002367	0.386616	1.029365	1.1407	0.089473
RP11-265	3.66785	7.013853	0.045491	4.814661	3.521934	1.985253	2.600014	1.434055
RP11-265	6.423911	14.00414	0.045491	5.044113	10.28573	0.263658	4.053506	1.047345
RP11-266	4.155339	1.86658	24.50942	6.796566	2.470317	6.404921	8.075772	7.868146
RP11-266	0.224661	0.473016	0.762165	0.218045	0.126335	1.770029	0.477698	1.250058
RP11-267	0.243449	0.504964	1.884935	0.183521	0.036566	0.263658	0.428694	0.405346
RP11-26J	0.126429	0.686299	8.625613	0.173059	0.581926	0.071775	0.26215	0.720938
RP11-274	2.404618	2.584394	0.452965	1.049517	2.600014	0.595368	3.439437	2.567605
RP11-274	5.84689	5.679502	0.681068	3.077316	8.660281	2.162381	4.508196	5.01772
RP11-276	0.007792	0.023694	0.045491	2.89E-05	1.44E-05	0.021429	4.33E-05	0.036631
RP11-276	0.027056	0.090584	0.286522	0.012136	0.009307	0.198788	0.226075	0.095599
RP11-282	0.778326	3.497085	2.45575	1.991328	1.566414	0.800058	3.101371	2.8657
RP11-283	1.4671	1.574517	1.551962	3.286652	1.405772	3.249877	3.457504	4.312251
RP11-284	4.752121	46.7056	287.197	23.82354	16.68554	7.443276	111.5439	135.5245
RP11-284	4.33E-05	0.00026	0.555036	0.398629	0.057518	1.000173	0.79487	0.951652
RP11-285	0.027056	0.00026	0.831472	0.005758	0.014423	0.117258	0.138326	0.222864
RP11-285	7.964315	2.77899	0.045491	1.532468	5.030866	1.449062	2.037114	4.508196
RP11-285	11.96574	5.583968	0.045491	10.85542	8.905642	4.445851	0.667201	5.976724
RP11-285	1.96404	3.672078	0.045491	4.684502	1.783203	0.986797	3.87329	1.009625
RP11-286	0.007792	0.018038	0.452965	0.01873	1.44E-05	0.242453	0.19355	0.12267
RP11-28G	4.33E-05	0.212172	0.286522	0.0857	0.005173	0.014791	0.005253	0.016472
RP11-290	3.33728	2.925426	0.359899	1.020447	2.453781	2.104358	2.174733	2.773644
RP11-293	11.77567	2.978968	0.045491	19.53061	1.978954	23.38378	5.80469	2.225498
RP11-293	0.701818	0.150743	0.414473	0.351219	0.478182	2.50469	0.481414	1.112641
RP11-295	2.800332	0.00026	0.045491	6.796566	2.386248	1.867951	1.031284	3.043528
RP11-295	0.309444	1.193362	0.045491	2.89E-05	0.637994	0.209048	0.285253	0.089473
RP11-297	0.970635	1.565253	7.288788	1.766248	0.560325	1.604827	1.713997	1.250058
RP11-298	0.324019	0.650101	5.662994	1.07127	0.126335	0.166465	0.226075	0.522633
RP11-298	4.33E-05	0.679069	64.12971	0.949827	1.44E-05	7.911934	0.658745	7.570693

RP11-299	5.439278	5.095325	0.917431	3.775209	3.824964	1.278629	2.600014	2.131176
RP11-29G	0.007792	0.029134	0.414473	0.01873	0.009307	2.893001	0.251371	0.070541
RP11-2B6	0.118528	0.00026	6.497266	0.444315	0.118983	1.533535	1.216768	0.818586
RP11-2H3	2.765758	5.976724	0.045491	1.550837	1.581912	1.195794	0.872013	1.349971
RP11-302	2.119596	0.894704	0.045491	0.207713	0.939408	0.263658	0.698297	0.236089
RP11-303	5.34474	3.934986	0.414473	3.507691	5.273362	2.000592	1.713997	2.355289
RP11-303	0.118528	0.097186	1.505678	0.572987	0.176833	0.877807	0.47153	0.070541
RP11-303	4.696313	1.497143	0.045491	4.086926	4.381479	4.586753	1.523824	1.250058
RP11-304	1.37531	1.819221	1.934271	1.423506	1.031284	0.512309	1.713997	1.191905
RP11-305	21.02638	17.78522	0.045491	10.38202	14.08569	4.875527	7.443276	5.915469
RP11-305	0.778326	1.095657	0.045491	0.796674	0.310657	0.533874	0.363983	0.312864
RP11-307	3.820758	4.529913	0.872597	0.534452	2.091313	0.334235	26.80355	0.00013
RP11-307	9.989221	6.210375	15.71851	10.85542	12.77772	2.558817	3.629177	2.661587
RP11-307	0.027056	0.023694	0.796674	0.076162	0.014423	1.135887	0.043391	0.049336
RP11-307	0.418326	0.00026	1.340981	1.122359	0.416501	1.093434	0.728543	0.566494
RP11-311	29.18885	23.6012	0.335411	19.83727	27.10502	9.751039	20.51271	5.044113
RP11-314	2.26518	1.283059	0.045491	2.496407	1.036558	1.268672	0.349812	1.055628
RP11-315	1.247244	1.414574	0.414473	1.510014	1.249221	1.259747	0.833975	0.335967
RP11-316	40.7932	12.56693	0.045491	29.82828	56.39528	1.436623	7.162698	3.058802
RP11-316	0.056046	0.429567	1.046061	2.89E-05	1.44E-05	0.6943	0.073463	0.940563
RP11-318	3.201457	3.077316	0.045491	1.94311	3.188918	0.533874	1.523824	0.50803
RP11-319	1.25886	1.044235	23.16622	1.808023	0.396082	4.445851	1.76873	3.707136
RP11-31F	5.01088	6.796566	4.875527	7.38847	4.580938	7.625209	20.16541	0.460065
RP11-320	0.65798	0.429567	1.264589	0.534452	0.251126	0.276061	0.675115	0.20969
RP11-324	2.882035	5.351934	0.796674	2.187395	2.579509	1.514199	1.883535	1.237879
RP11-325	0.234358	0.157186	7.200505	0.717403	0.28899	1.234444	1.61088	0.558427
RP11-325	0.338831	0.00026	3.775209	0.48583	0.243449	0.960866	0.934488	0.630823
RP11-326	6.355224	10.57984	0.555036	13.00996	9.297085	2.479221	4.241905	18.33909
RP11-326	2.138131	0.391674	0.414473	1.35259	1.438672	0.90013	0.845887	0.835498
RP11-326	1.070693	2.009906	6.071436	1.262179	0.968066	2.579509	2.621133	2.255916
RP11-328	0.056046	0.047092	0.234358	0.050216	0.050426	0.080837	0.226075	0.016472
RP11-328	4.33E-05	0.06715	1.046061	0.269322	1.44E-05	0.216753	2.786898	1.744055
RP11-329	2.31811	1.242641	0.644618	1.749307	2.533745	0.727633	3.930664	1.149978
RP11-329	0.982128	1.539062	1.934271	1.397482	1.766248	0.252316	2.255916	2.755411
RP11-332	0.597042	1.805166	1.884935	2.065007	0.889553	1.304877	1.860491	2.392431
RP11-334	3.022713	2.159004	4.546443	4.145209	4.484892	3.188918	5.123781	5.337547
RP11-334	1.351573	1.565253	0.045491	0.970007	1.113961	0.127201	4.33E-05	0.00013
RP11-334	12.36443	1.765065	16.34294	65.94551	7.074329	287.197	24.50942	70.11932
RP11-337	1.712706	2.24562	2.607035	1.370765	1.405772	4.490801	3.236457	1.763889
RP11-337	0.042713	0.234358	0.414473	0.146999	0.083521	0.827273	0.419827	0.352597
RP11-338	0.027056	0.090584	0.644618	0.005758	0.005173	0.813261	0.450924	0.20969
RP11-33C	2.26518	1.169091	0.831472	3.640606	2.306277	0.499206	2.786898	1.669466
RP11-342	2.252273	2.442987	0.601227	4.745895	3.131544	5.165599	3.87329	4.222583
RP11-342	1.279452	1.986681	0.045491	1.532468	0.620014	1.64917	1.321053	2.556443
RP11-345	0.196811	0.315743	4.277006	1.301169	0.523211	1.331919	0.450924	1.763889
RP11-345	11.51384	6.695887	30.17192	42.75576	12.10289	29.49693	10.1028	27.10502
RP11-347	2.058773	4.207778	0.045491	0.458341	1.264589	2.117814	2.696061	1.041652
RP11-348	0.251371	0.391674	0.26215	0.387258	0.251126	1.34513	0.588954	0.906126
RP11-348	2.294733	2.121198	0.359899	2.496407	2.022778	1.533535	1.580909	2.116032
RP11-350	1.014127	1.731169	3.211176	1.134387	1.195079	1.47018	1.580909	0.666097
RP11-350	3.350224	1.481631	0.045491	0.754315	2.642525	1.013434	0.906126	0.275873
RP11-352	2.461905	2.703485	0.956061	2.845036	2.429957	0.334235	1.980592	0.96329
RP11-353	0.035137	0.00026	0.216025	0.034863	0.002237	0.047092	0.009293	0.036631
RP11-355	2.567605	4.821176	0.998312	4.721789	2.485534	2.378124	5.530274	2.335844
RP11-356	10.05618	15.98892	2.209141	12.22798	23.82354	14.70188	8.134084	13.75978
RP11-359	0.902403	1.695216	10.60361	2.127973	0.373709	3.904517	3.944084	8.226219
RP11-359	0.234358	0.724004	2.820974	0.444315	0.367532	0.313925	0.493247	0.63873
RP11-359	4.33E-05	0.00026	2.558817	0.248903	0.036566	2.611797	0.437799	0.743694

RP11-35G	0.259509	3.909033	5.210317	1.036558	0.352807	0.572987	0.957915	1.919661
RP11-35G	0.118528	0.162915	1.809343	0.163766	0.165657	0.029769	0.117258	0.026414
RP11-360	0.949827	0.938413	0.045491	2.050404	3.131544	0.106522	0.518139	0.4971
RP11-360	8.923117	2.882035	0.045491	9.968355	6.570433	0.00013	2.887489	0.00013
RP11-362.	1.340036	4.990794	0.045491	1.962424	1.222958	0.727633	2.642525	0.877222
RP11-362	0.080592	0.248716	1.046061	0.076162	0.153391	0.397215	0.390014	0.322684
RP11-363	2.92013	0.894704	0.045491	0.835498	1.415469	1.604827	2.270837	0.835498
RP11-365	1.549711	0.843102	0.487662	0.633853	0.921739	0.063911	0.765606	0.275873
RP11-367	1.535722	0.650101	0.045491	2.302626	1.316544	0.485577	0.419827	0.708124
RP11-368	2.011501	2.925426	0.045491	5.942294	2.453781	0.334235	2.99987	2.214372
RP11-368	6.717056	15.276	0.045491	7.911934	10.19468	2.027951	11.14619	2.489762
RP11-374	0.042713	0.035007	1.385801	0.005758	0.005173	0.106522	0.028398	0.543939
RP11-379	1.014127	2.312085	1.934271	2.45772	1.350772	3.842554	4.328232	6.967547
RP11-379	6.063276	4.821176	0.045491	7.002056	4.900996	1.604827	10.53159	3.707136
RP11-37B	0.807619	1.207374	47.5018	2.597792	1.350772	2.630729	2.621133	7.653391
RP11-380	13.30906	5.431544	0.045491	6.830592	11.544	8.711905	3.588369	5.687879
RP11-384	2.419553	5.47	70.11932	5.431544	1.667273	64.12971	6.830592	44.27012
RP11-384	0.403651	0.380267	0.644618	0.358672	0.687807	1.361789	1.321053	1.727323
RP11-385	7.795642	5.530274	0.045491	4.302367	3.131544	3.464315	1.290981	2.705916
RP11-386	1360.293	530.1465	1.340981	105.1694	1360.293	135.5245	105.1694	33.07147
RP11-388	2.216162	0.550974	3.122915	1.35259	0.849841	0.346494	1.118817	0.770188
RP11-388	0.11075	0.162915	0.045491	0.01873	0.044322	0.866717	0.202987	0.302785
RP11-38L	1.91811	1.702706	24.04374	1.738456	1.603795	57.84882	2.970144	33.4985
RP11-390	19.53061	20.16541	1.127475	25.48179	31.1753	6.622092	9.418196	24.2842
RP11-393	0.118528	1.283059	5.11697	0.207713	0.171472	2.337821	1.000173	2.887489
RP11-395.	9.297085	18.05488	2.339697	16.39531	14.17388	9.815642	10.57984	10.19468
RP11-395	16.09482	3.921645	0.045491	0.517092	7.737807	17.64869	5.830152	0.483326
RP11-395	3.648081	15.56131	0.956061	5.330519	4.266753	2.069942	7.238023	4.733824
RP11-396	2.024488	0.977994	0.045491	0.81544	1.502323	0.334235	0.218045	0.24443
RP11-398	1.648066	3.086306	5.359134	4.429769	1.8386	4.821176	3.563182	8.134084
RP11-400	0.11075	1.021746	3.91355	0.321746	0.160202	1.783203	1.954993	2.616573
RP11-400	5.195022	2.649675	27.66779	9.667316	4.814661	12.46209	6.345382	12.89577
RP11-404	4.33E-05	0.00026	3.91355	2.89E-05	1.44E-05	0.558095	0.172857	0.4971
RP11-408.	1.753341	0.749019	0.286522	1.370765	2.189235	2.069942	3.028629	1.620058
RP11-410	17.64869	70.11932	0.045491	24.73724	25.24367	21.74051	29.18885	34.38341
RP11-415.	1.731169	0.12904	9.707677	2.659214	1.863506	2.434444	1.263752	3.497085
RP11-417	0.007792	0.00026	0.045491	0.026537	0.009307	0.038716	0.015491	0.275873
RP11-418.	2.789466	2.600014	28.71146	9.792626	5.01088	4.650007	3.574387	14.17388
RP11-420	1.1379	0.910823	3.211176	1.833102	2.747908	0.90013	0.72044	7.276645
RP11-421	4.077403	11.77567	0.872597	4.067547	6.967547	5.728615	9.297085	4.266753
RP11-421	2.361198	2.978968	0.26215	5.796421	4.365224	4.198117	2.185556	5.872886
RP11-422	0.027056	0.900693	2.209141	0.005758	0.118983	0.63114	0.802395	0.302785
RP11-422	0.933261	0.88316	4.546443	1.931537	0.694675	1.093434	2.037114	1.896667
RP11-423	0.063225	0.295671	0.414473	0.125859	0.153391	0.384394	0.233889	0.720938
RP11-428.	0.488759	0.550974	0.487662	0.744531	0.517612	0.662944	0.47153	0.835498
RP11-428	0.019784	0.023694	1.986681	0.183521	0.014423	0.021429	0.02189	0.136061
RP11-429	1.616385	75.1132	6.570433	11.19942	8.075772	0.925801	14.42661	30.82277
RP11-42C	1.58645	1.819221	0.045491	2.425801	3.058802	0.641631	1.070115	1.566414
RP11-430	1.931537	7.339452	0.045491	3.803766	2.261573	1.636118	4.272165	5.515584
RP11-430	1.692763	3.87329	1.884935	0.076162	4.690159	6.819481	20.85876	0.413449
RP11-434	12.29473	3.68381	0.045491	3.06803	5.864084	19.68088	4.125786	2.104358
RP11-434	13.91714	23.82354	0.045491	9.063983	6.011515	3.921645	13.45939	4.058514
RP11-435	0.567345	3.279625	0.762165	2.8657	1.702706	0.960866	1.254704	0.803665
RP11-435	0.359105	0.029134	19.29639	0.600887	0.126335	0.558095	0.545426	0.818586
RP11-438	0.933261	1.036558	0.045491	0.81544	0.793434	0.029769	0.487128	0.720938
RP11-440	0.390895	2.009906	4.445851	1.870685	0.694675	2.540462	2.078355	4.012049
RP11-441	0.234358	0.248716	2.696061	0.477698	0.165657	2.720996	0.912987	6.653016
RP11-444	4.33E-05	0.00026	3.479134	2.89E-05	1.44E-05	0.90013	0.160462	0.865664

RP11-449	0.701818	0.308788	1.65785	1.229553	0.637994	1.726032	2.410996	3.904517
RP11-44N	16.68554	3.767489	1.172872	6.250447	9.646284	14.17388	13.00996	1.399315
RP11-452	8.711905	6.55039	5.477843	14.51648	6.063276	2.323983	6.900202	11.25392
RP11-452	0.685931	1.329206	27.10502	1.433189	0.442799	2.50469	1.677042	6.580988
RP11-457	3.953297	3.256587	0.286522	4.513622	5.044113	0.717403	2.227273	2.964762
RP11-458	0.616407	1.296905	2.696061	0.780519	0.560325	1.71518	1.580909	0.951652
RP11-458	1.25886	3.156537	0.335411	1.466111	5.500678	0.605022	4.709033	1.278629
RP11-45M	0.056046	0.06715	1.77404	0.387258	0.06381	0.444315	0.015491	0.200794
RP11-45M	0.259509	0.391674	1.602619	0.278651	0.251126	0.410079	0.051198	0.335967
RP11-45P	4.535354	3.521934	1.986681	2.388348	8.905642	5.316378	3.722785	3.8379
RP11-461	0.224661	0.453218	1.884935	0.507085	0.28899	0.936898	0.47153	1.451775
RP11-464	4.33E-05	0.00026	3.397706	2.89E-05	1.44E-05	0.305253	0.043391	1.030678
RP11-464	1.037958	1.62562	7.456118	0.773766	0.280823	1.029365	1.285584	2.773644
RP11-464	1.191198	5.898449	0.335411	1.202626	1.01544	0.421025	2.396429	1.409942
RP11-466	0.056046	0.256046	0.998312	0.058442	0.020895	0.346494	0.172857	0.07671
RP11-46C	2.24562	3.432388	6.011515	3.655714	2.248896	3.754719	6.063276	5.80469
RP11-46J	0.893016	1.456955	0.721335	2.361198	1.640779	0.558095	2.255916	2.725498
RP11-472	4.592273	8.923117	1.844098	10.23768	11.19942	4.529913	7.882222	20.68746
RP11-473	0.259509	0.287821	3.829105	0.398629	0.095996	5.068831	0.390014	4.546443
RP11-473	1.58645	1.164257	10.31025	3.45404	1.29417	14.79742	1.876212	9.063983
RP11-473	4.33E-05	0.00026	0.045491	0.114964	1.44E-05	0.242453	0.218045	0.302785
RP11-474	4.33E-05	0.550974	1.090072	0.173059	0.126335	0.178629	0.577071	0.359307
RP11-477	0.710722	1.314675	0.045491	0.737403	1.000794	0.106522	0.202987	0.616739
RP11-478	0.063225	0.11368	0.487662	0.095029	0.183672	1.135887	0.861429	1.199481
RP11-47A	4.696313	5.84689	38.94854	11.77567	3.414574	13.83359	8.412367	22.52375
RP11-480	0.348889	0.686299	1.706219	0.675115	0.274199	0.683232	1.425613	1.183478
RP11-480	6.570433	2.223636	0.045491	5.00404	7.468961	3.201457	2.227273	9.459177
RP11-483	4.33E-05	0.00026	0.045491	0.050216	1.44E-05	0.055887	0.005253	0.009293
RP11-486	3.88153	4.120815	0.517612	8.256465	2.718434	2.323983	2.359264	2.725498
RP11-488	27.24614	16.9162	11.544	19.68088	48.53429	12.29473	22.32214	23.16622
RP11-488	0.367287	0.419827	1.742734	1.721371	1.531313	3.734762	2.922778	1.662439
RP11-488	7.468961	11.36975	0.045491	10.7557	15.56131	5.821804	9.625253	4.546443
RP11-48B	4.33E-05	0.047092	0.998312	0.042569	0.002237	0.117258	0.059091	0.330289
RP11-48B	1.692763	1.357201	0.487662	1.65785	1.758615	0.572987	1.159913	0.648038
RP11-48F	4.33E-05	0.391674	2.398442	0.050216	1.44E-05	0.055887	1.580909	1.534603
RP11-490	24.2842	14.08569	0.045491	5.00404	12.93651	31.55417	6.55039	26.25557
RP11-490	0.019784	0.029134	0.359899	0.173059	0.020895	0.055887	0.028398	0.031169
RP11-491	0.251371	0.179177	0.26215	0.076162	0.118983	0.305253	0.301955	0.128615
RP11-497	10.73084	20.85876	0.762165	12.93651	15.77678	6.685087	18.48514	10.43066
RP11-498	1.4671	0.170808	2.760765	2.351299	1.581912	0.00013	0.552439	0.770188
RP11-498	10.53159	2.671299	0.045491	3.321284	3.324452	0.973232	1.128788	0.734524
RP11-499	4.557605	6.230245	0.045491	5.599228	7.363117	2.794935	4.644098	4.984488
RP11-499	2.425801	7.38847	5.359134	5.599228	1.962424	3.57057	3.629177	4.266753
RP11-49I1	1.91811	3.514834	1.465123	2.964762	3.002576	0.432915	0.518139	2.207417
RP11-500	0.960866	1.565253	33.93939	3.722785	1.68671	10.93101	1.692763	10.1459
RP11-503	9.101674	6.191515	2.652107	2.143261	8.164531	4.277006	3.439437	2.31404
RP11-504	5.373781	11.14619	1.65785	6.945152	16.5625	2.794935	10.8813	4.456912
RP11-505	14.79742	13.30906	3.397706	14.70188	13.6814	6.464906	13.83359	8.524242
RP11-506	0.196811	0.105022	0.555036	0.614545	0.416501	0.844156	1.263752	1.191905
RP11-506	0.995527	2.415296	0.045491	0.970007	1.010289	0.572987	0.96938	0.780952
RP11-517	2.337821	3.233045	2.280101	2.221775	3.435988	0.877807	1.980592	2.445079
RP11-517	0.206061	0.219134	7.074329	1.134387	0.126335	0.28689	0.509791	1.104805
RP11-517	0.013348	0.035007	0.310447	0.005758	0.020895	0.28689	0.035382	0.154719
RP11-517	0.359105	0.330043	2.074906	0.444315	0.07684	4.56912	0.934488	3.310758
RP11-51J	3.269913	2.855296	0.045491	6.52899	2.784228	5.855281	5.172828	2.661587
RP11-51J	3.552049	1.131472	0.872597	0.90772	1.696494	2.876421	0.331263	1.325743
RP11-521	0.215115	1.021746	2.652107	0.432237	0.251126	1.268672	0.940563	1.604827
RP11-524	4.33E-05	0.083189	0.872597	1.150635	0.216753	0.137201	4.33E-05	0.289935

RP11-525	4.33E-05	0.00026	3.479134	2.89E-05	1.44E-05	0.127201	0.117258	0.34619
RP11-526	5.454949	19.99861	0.335411	3.303925	4.94026	3.66785	6.922771	2.964762
RP11-52A	0.035137	0.256046	2.117814	0.01873	0.014423	0.334235	0.02189	0.302785
RP11-531	3.904517	2.725498	0.762165	5.00404	2.953059	0.866717	1.644416	0.312864
RP11-536	0.738716	0.380267	3.008225	1.150635	0.842193	1.319293	0.751371	1.595729
RP11-536	3.783694	2.173081	1.172872	8.677489	2.151919	1.331919	1.080303	1.160707
RP11-539	8.164531	13.53913	0.998312	12.77772	13.91714	8.149307	9.377345	6.125823
RP11-53B	0.019784	0.035007	2.652107	0.042569	0.002237	0.010274	0.035382	0.24443
RP11-53B	4.33E-05	0.00026	0.681068	0.005758	0.002237	0.029769	4.33E-05	0.042771
RP11-53C	5.508131	7.363117	2.209141	5.180058	4.888023	8.381328	7.991602	5.082063
RP11-540	3.350224	1.335743	9.52197	4.246861	3.290101	7.468961	5.9071	8.994791
RP11-545	2.911962	4.165137	2.558817	4.110779	3.816551	6.287561	7.315512	10.57984
RP11-546	1.2214	1.931537	0.359899	2.584394	1.322092	0.787136	1.189048	0.330289
RP11-548	4.609076	5.508131	0.385238	4.845166	6.25938	0.544877	3.253175	2.104358
RP11-54A	0.096371	0.00026	0.045491	0.552749	0.452965	0.00013	0.278802	0.00013
RP11-552	6.135007	3.957734	1.300368	4.776407	11.719	10.70538	0.588954	8.195657
RP11-552	0.056046	0.052273	0.555036	0.239589	0.06381	0.117258	0.363983	0.365411
RP11-552	0.746551	1.314675	6.38456	4.058514	1.766248	0.276061	2.876421	2.616573
RP11-553	19.37139	155.7103	5.037489	75.1132	25.48179	5.889798	91.05541	31.92385
RP11-563	0.175361	0.281169	1.884935	0.321746	0.176833	0.106522	0.242222	0.322684
RP11-566	2.26518	3.55583	0.045491	1.599033	3.324452	0.146999	4.33E-05	0.483326
RP11-568	0.196811	0.24184	4.198117	0.525325	1.44E-05	0.986797	0.226075	1.443983
RP11-571	3.536955	4.048499	0.831472	1.325743	2.306277	2.134545	2.240274	1.026825
RP11-571	8.643074	8.780115	1.602619	2.8657	7.519841	9.707677	8.288874	2.335844
RP11-572	2.690823	3.8379	1.551962	7.188095	4.63189	0.485577	3.799885	2.019618
RP11-573	0.53658	0.24184	11.96574	0.125859	0.274199	1.163579	0.285253	0.566494
RP11-574	0.438831	0.922338	0.681068	0.458341	0.224921	1.569553	0.810491	2.242092
RP11-579	0.560606	5.679502	0.045491	1.794531	2.408882	0.444315	2.037114	1.515296
RP11-57H	3.300664	1.853723	2.607035	1.497143	2.034242	10.1028	3.36075	4.174964
RP11-582	0.453463	1.986681	8.508514	1.339091	0.611003	2.579509	2.451645	4.702468
RP11-589	7.991602	6.423911	0.045491	3.381255	7.136537	0.00013	0.912987	0.00013
RP11-5C2	2.074906	1.780693	0.045491	4.125786	6.784762	2.950173	0.827273	1.909408
RP11-600	0.397489	0.256046	5.477843	0.995527	0.402381	1.078153	2.065007	2.925426
RP11-603	17.92188	22.13245	0.045491	11.09133	12.29473	6.99026	8.745238	13.30906
RP11-611	17.51961	17.92188	5.295325	17.15902	8.195657	7.363117	11.48368	6.433918
RP11-613	4.33E-05	0.170808	0.414473	0.012136	0.020895	0.00013	4.33E-05	0.216025
RP11-617	18.48514	19.83727	0.998312	13.34662	20.51271	12.16681	8.539971	11.8058
RP11-618	749.7839	77.74812	0.045491	530.1465	749.7839	100.0998	203.5795	100.0998
RP11-61I1	0.949827	1.036558	6.685087	1.497143	1.036558	10.2617	1.180613	11.48368
RP11-621	1.806595	0.322237	0.045491	0.81544	2.755411	0.189141	1.1407	0.474856
RP11-629	5.630815	2.150303	1.222958	1.776739	4.193232	1.663672	2.248896	1.820541
RP11-635	6.760765	3.796075	0.956061	3.921645	6.945152	5.316378	2.489762	4.135801
RP11-638	37.81919	55.04081	1.428398	12.63773	38.94854	1.121017	22.96463	1.075411
RP11-638	3.851414	8.849697	0.045491	3.116732	7.765931	0.614156	4.25658	0.648038
RP11-63M	53.77619	22.52375	0.045491	49.50903	65.94551	0.00013	11.25392	0.00013
RP11-644	4.343838	5.788153	9.946364	9.751039	8.103925	5.123781	8.888167	19.22139
RP11-645	1.806595	0.96329	0.721335	0.826407	0.92816	1.544517	2.078355	1.744055
RP11-646	4.965801	7.545584	0.555036	3.269913	2.061926	1.013434	3.175916	3.263391
RP11-649	0.41026	0.275527	5.25254	1.049517	0.442799	0.558095	1.270317	1.361789
RP11-649	0.088788	0.287821	1.844098	0.114964	0.036566	0.00013	0.051198	0.770188
RP11-658	15.77678	29.49693	6.685087	11.48368	14.00414	7.112496	22.13245	8.608153
RP11-660	5.821804	13.00996	0.045491	11.83593	8.573434	9.377345	17.92188	2.083261
RP11-661	4.33E-05	0.00026	1.986681	0.034863	1.44E-05	0.047092	0.233889	0.042771
RP11-66N	15.98892	6.922771	0.601227	15.3618	11.25392	7.795642	12.56693	4.281847
RP11-672	0.763564	2.710765	0.045491	1.122359	0.782857	0.00013	1.474343	0.770188
RP11-677	0.096371	0.96329	1.602619	0.432237	0.083521	0.913514	0.690043	1.139221
RP11-67L	0.893016	0.359697	1.986681	1.677042	0.661025	3.317807	1.254704	2.802828
RP11-680	0.035137	0.157186	0.644618	0.095029	0.050426	0.683232	0.152843	0.438023

RP11-686	4.370671	10.8813	1.222958	5.654863	4.110779	3.66785	3.606804	1.945916
RP11-690	3.128694	2.337821	1.046061	2.402468	2.579509	6.760765	2.410996	5.316378
RP11-690	4.33E-05	0.058817	0.487662	2.89E-05	1.44E-05	0.6943	0.028398	0.460065
RP11-697	7.570693	10.82954	0.045491	3.45404	8.256465	2.558817	2.74298	0.770188
RP11-69E	28.23632	10.53159	6.8421	8.608153	72.69473	12.46209	53.77619	4.150274
RP11-6N1	7.264502	5.679502	0.045491	6.570433	9.946364	0.00013	1.551962	0.445996
RP11-6O2	0.019784	0.058817	0.721335	0.218045	0.057518	0.055887	0.028398	0.00013
RP11-700	3.128694	6.162446	0.310447	2.375974	5.583968	2.911962	4.468009	2.415296
RP11-707	4.33E-05	0.449408	0.045491	0.982128	1.44E-05	1.093434	0.185642	1.250058
RP11-70L	4.33E-05	0.097186	0.998312	0.01873	0.009307	0.178629	0.19355	0.438023
RP11-712	1.316544	1.380195	0.796674	1.189769	2.005253	2.29873	1.226934	1.473218
RP11-714	4.33E-05	1.029365	0.045491	0.163766	0.057518	0.787136	0.226075	2.789466
RP11-715	0.071566	0.00026	4.198117	0.095029	1.44E-05	1.411861	1.692763	1.290065
RP11-71H	6.784762	3.346898	0.045491	0.995527	4.46267	3.028629	2.472576	0.770188
RP11-722	4.33342	5.788153	1.65785	3.87329	5.075447	1.736133	4.435159	3.68381
RP11-723	0.631847	0.76	2.074906	1.579798	0.352807	1.488571	1.935685	0.951652
RP11-723	8.994791	9.101674	0.359899	8.923117	4.655916	2.337821	5.137042	2.169509
RP11-727	0.126429	1.323131	2.760765	0.278651	0.160202	0.252316	0.35676	0.312864
RP11-727	11.25392	11.89932	0.762165	10.48101	15.46193	6.888297	7.584502	3.957734
RP11-727	5.754935	3.06803	2.117814	5.671126	3.695815	6.125823	2.947287	5.044113
RP11-72I	0.234358	0.248716	1.602619	0.351219	0.190844	0.334235	1.056898	0.413449
RP11-732	16.9162	21.02638	0.045491	8.524242	17.64869	0.00013	4.597792	0.00013
RP11-73K	1.2214	1.587605	0.310447	1.89228	0.824885	0.913514	1.205685	0.854733
RP11-746	2.705916	2.925426	0.045491	0.398629	5.42381	0.866717	2.961768	0.854733
RP11-752	0.096371	0.121494	0.644618	0.01873	0.146118	0.334235	0.202987	0.154719
RP11-761	4.33E-05	0.351789	1.222958	0.005758	1.44E-05	0.038716	4.33E-05	0.572626
RP11-762	0.316573	1.886335	0.644618	1.283059	0.069942	0.096667	0.784372	1.434055
RP11-768	0.916306	0.429567	0.045491	0.872013	0.933261	0.485577	0.457114	0.00013
RP11-769	4.33E-05	0.023694	0.487662	0.173059	1.44E-05	0.047092	0.009293	0.016472
RP11-769	4.33E-05	0.00026	0.335411	0.034863	1.44E-05	0.198788	0.043391	0.089473
RP11-770	2.337821	3.346898	0.045491	1.700354	5.316378	2.069942	3.095166	2.755411
RP11-770	0.606847	0.562107	0.359899	0.248903	0.512309	0.276061	0.363983	0.154719
RP11-774	3.552049	2.544747	1.602619	3.640606	3.962179	2.805382	3.618499	5.316378
RP11-77H	2.789466	0.810491	0.831472	1.007244	1.468131	0.522633	0.947677	0.846797
RP11-783	1.111241	2.019618	0.644618	1.728615	0.757763	0.844156	2.834084	4.034423
RP11-783	1.648066	0.650101	0.045491	4.277006	2.42381	1.223846	1.189048	0.824885
RP11-789	0.042713	0.083189	0.234358	0.0857	0.005173	0.117258	0.101905	0.031169
RP11-78C	9.880779	5.754935	0.045491	2.715873	5.864084	4.165137	2.240274	1.799899
RP11-792	18.19595	36.26061	0.045491	15.18102	25.99427	18.19595	34.84299	17.92188
RP11-793	0.063225	0.029134	0.045491	0.787987	0.138831	1.45912	1.403853	1.691587
RP11-798	0.071566	0.441472	1.385801	0.136926	0.050426	0.166465	0.35676	0.797114
RP11-798	6.355224	11.83593	0.045491	31.1753	6.52899	6.404921	6.674286	3.457504
RP11-79P	3.040505	5.485685	0.045491	1.68938	3.166659	0.857778	1.810664	0.865664
RP11-7F1	0.096371	0.910823	0.310447	1.122359	0.620014	0.397215	0.728543	0.698687
RP11-800	0.291183	0.047092	4.277006	0.195253	0.424848	0.252316	0.19355	0.428694
RP11-802	10.78056	8.477662	0.872597	4.419062	2.973059	6.685087	5.037489	3.68381
RP11-805	7.765931	3.820758	0.045491	17.64869	13.60866	1.770029	1.622006	1.763889
RP11-806	0.080592	0.00026	0.045491	0.469711	1.260635	0.117258	0.331263	0.296234
RP11-810	42.08807	62.33472	0.045491	70.11932	100.0998	5.984885	41.47925	8.019769
RP11-814	0.518139	2.690823	0.045491	1.466111	1.270317	0.544877	0.79487	0.600887
RP11-815	0.181797	0.732193	6.8421	2.006782	0.20746	1.304877	1.754733	3.414574
RP11-817	0.807619	0.562107	7.882222	1.189769	1.108947	1.234444	0.437799	1.710173
RP11-820	13.75978	22.74797	6.200945	8.815123	9.335397	3.116732	7.824473	7.710361
RP11-848	1.146118	0.441472	1.428398	1.974228	1.603795	0.558095	0.690043	0.102165
RP11-849	1.434921	4.914863	1.222958	2.781558	2.993867	1.029365	1.705007	1.710173
RP11-849	0.196811	0.705613	11.03843	0.375743	0.596111	0.465325	0.597417	1.169791
RP11-84C	0.949827	0.910823	1.046061	2.173081	0.954776	0.166465	1.118817	0.715108
RP11-84G	0.063225	0.234358	3.606804	0.458341	0.107626	0.558095	0.481414	1.68342

RP11-861	0.526566	0.667965	0.385238	0.239589	0.808983	0.28689	0.272078	0.365411
RP11-862	252.4251	118.7028	0.045491	203.5795	10.01009	332.0776	5.754935	252.4251
RP11-863	1.014127	0.162915	0.796674	0.298687	0.849841	4.997417	0.382208	0.445996
RP11-864	0.019784	0.00026	0.045491	0.042569	1.44E-05	0.080837	0.340866	0.049336
RP11-867	0.027056	0.511667	0.555036	0.0857	0.06381	0.146999	0.059091	0.515527
RP11-867	3.88153	21.54823	2.696061	3.303925	10.33476	0.572987	0.737857	0.789423
RP11-872	0.309444	0.796234	0.956061	0.432237	0.452965	0.37456	0.713405	0.630823
RP11-875	0.291183	0.322237	5.864084	1.252424	0.345635	0.866717	0.233889	1.497143
RP11-884	2.676392	2.337821	0.601227	2.345527	1.935685	2.579509	3.049603	2.355289
RP11-88I1	4.33E-05	0.052273	0.045491	2.89E-05	1.44E-05	0.263658	0.073463	0.148254
RP11-890	3.730808	2.671299	1.465123	2.939141	3.578038	6.653016	4.875527	6.685087
RP11-893	1.330974	1.445916	0.601227	1.113961	0.68062	0.00013	1.935685	1.462143
RP11-894	0.588312	0.490375	8.195657	1.339091	0.581926	6.316328	2.121198	4.789596
RP11-90L	0.007792	0.041227	1.090072	0.114964	0.07684	0.029769	0.152843	0.537518
RP11-91G	3.904517	5.195022	0.045491	1.65785	2.65684	1.569553	4.292078	1.237879
RP11-91K	1.014127	1.723579	0.045491	0.826407	1.130115	0.00013	1.011486	0.172453
RP11-932	0.359105	0.391674	6.685087	0.572987	0.262525	1.833102	1.36619	2.371833
RP11-932	7.839271	1.754733	0.045491	3.046551	7.200505	5.515584	2.185556	1.654156
RP11-936	0.11075	0.105022	0.601227	0.114964	0.367532	0.014791	0.009293	0.102165
RP11-93H	0.004214	0.029134	0.601227	0.066616	1.44E-05	0.010274	0.02189	0.00013
RP11-95D	2.304452	1.234444	1.090072	3.36075	2.323983	0.827273	0.802395	0.609177
RP11-967	4.33E-05	0.06715	0.045491	0.321746	0.036566	0.499206	0.043391	0.102165
RP11-96D	5.80469	5.295325	0.045491	5.295325	5.366335	0.6943	2.74298	0.042771
RP11-96K	0.309444	0.287821	3.655714	0.684033	0.274199	0.595368	0.301955	0.216025
RP11-977	5.25254	1.91811	0.045491	7.584502	2.964762	1.867951	0.912987	1.055628
RP11-97C	12.10289	15.77678	3.829105	11.89932	13.38418	7.532713	8.940671	6.55039
RP11-981	0.135606	0.226075	6.454416	0.183521	0.095996	0.454632	0.152843	0.50803
RP11-981	3.040505	7.882222	4.546443	5.500678	6.230245	4.845166	11.60283	6.09759
RP11-98D	3.052655	6.796566	0.045491	1.826147	2.81342	0.544877	3.404372	1.055628
RP11-98D	1.674401	1.770029	20.16541	2.735368	1.68671	1.770029	3.563182	2.242092
RP11-98I	0.291183	1.174315	3.829105	0.897006	0.268369	1.268672	0.682799	1.633752
RP11-996	0.161082	0.429567	0.555036	0.780519	0.090029	0.397215	0.84	0.673651
RP11-999	9.335397	13.75978	0.045491	27.96248	10.1028	4.529913	9.707677	15.18102
RP13-103	17.03919	10.78056	13.34662	7.149618	11.42512	3.144156	8.381328	7.212915
RP13-39P	14.88584	39.53687	0.517612	25.99427	21.19547	0.65202	26.52537	0.998312
RP13-487	0.019784	0.411703	1.264589	2.89E-05	0.044322	0.127201	0.015491	0.388579
RP13-514	11.36975	3.058802	0.26215	3.391075	3.869163	26.80355	4.281847	6.727633
RP13-516	7.598312	8.075772	2.8657	8.075772	7.013853	6.956349	9.335397	7.507619
RP13-516	0.916306	1.363557	0.335411	0.066616	0.434596	0.055887	0.912987	0.181797
RP13-582	11.51384	5.01772	1.172872	15.66023	6.210375	4.096407	9.101674	9.502294
RP3-329A	0.359105	0.562107	1.090072	0.924329	0.391421	0.28689	0.92482	0.483326
RP3-355L	0.035137	0.00026	0.286522	0.146999	0.014423	0.28689	0.318694	0.108997
RP3-355L	2.66404	0.490375	0.045491	0.0857	0.793434	1.677042	0.560325	0.190693
RP3-368A	0.834957	1.335743	1.340981	1.510014	0.824885	3.775209	2.429957	3.464315
RP3-368A	2.500548	7.08733	1.986681	1.962424	2.351299	4.56912	5.976724	3.147294
RP3-388M	0.196811	0.275527	0.831472	0.207713	0.216753	0.346494	0.880498	0.973745
RP3-402G	2.800332	1.595729	1.809343	1.370765	2.429957	0.844156	3.722785	1.881955
RP3-405J	0.004214	0.06715	1.934271	0.01873	0.009307	0.063911	0.109466	1.266255
RP3-406A	56.39528	11.42512	0.045491	6.570433	24.50942	17.92188	2.34355	3.080317
RP3-412A	1.152165	0.287821	1.340981	0.534452	0.701818	0.358211	0.372973	1.909408
RP3-418C	4.33E-05	0.429567	0.555036	0.278651	0.262525	0.475137	0.737857	1.317475
RP3-428L	3.66785	5.395584	11.11876	8.075772	4.540794	5.576219	6.345382	6.486104
RP3-508I1	167.9996	27.66779	0.045491	67.98069	43.49658	0.00013	1.677042	1.783203
RP3-508I1	3.767489	5.933066	5.591717	4.207778	7.264502	0.889019	3.36075	3.606804
RP3-510D	0.215115	0.047092	0.045491	0.156017	0.523211	0.029769	0.131349	0.031169
RP3-522D	5.591717	5.720173	0.045491	5.959033	5.770996	2.421681	4.927302	6.30671
RP3-525N	65.94551	87.1746	25.24367	38.94854	135.5245	52.56128	77.74812	65.94551
RP4-530I1	0.004214	0.014935	0.998312	0.005758	1.44E-05	0.021429	0.02189	0.036631

RP4-561L:	0.867814	0.490375	2.607035	0.358672	0.547403	8.849697	0.880498	1.909408
RP4-569M	0.019784	0.00026	1.602619	0.026537	1.44E-05	0.096667	0.059091	0.064127
RP4-597N	4.845166	1.830159	0.956061	1.859084	2.681169	4.614618	0.642395	0.4971
RP4-607I7	0.071566	0.121494	0.045491	0.050216	1.640779	0.00013	0.02189	0.275873
RP4-621B	4.557605	1.397482	0.286522	2.236407	2.533745	2.449509	0.464127	1.409942
RP4-622L!	1.180613	0.873088	0.045491	1.65785	1.744055	0.263658	0.318694	0.095599
RP4-635E	3.008225	0.570664	0.045491	1.240202	2.306277	0.166465	4.33E-05	0.438023
RP4-635E	34.38341	17.64869	2.24562	26.25557	36.26061	19.37139	18.05488	12.1974
RP4-639F:	9.924372	5.01772	3.91355	16.39531	17.03919	5.821804	8.643074	11.60283
RP4-647C	0.056046	0.121494	1.222958	0.48583	0.069942	0.209048	0.081126	0.734524
RP4-665J2	8.815123	1.476429	0.335411	0.731732	6.30671	11.42512	0.642395	0.657547
RP4-669L	12.16681	1.830159	19.60574	8.303947	19.37139	9.902576	7.854069	11.96574
RP4-671G	3.286652	2.180022	0.045491	2.143261	2.453781	1.259747	1.091443	0.128615
RP4-673M	0.902403	1.487619	4.012049	1.104033	0.581926	8.780115	1.449062	7.765931
RP4-740C	9.160382	4.490801	0.045491	1.599033	6.486104	5.01772	1.980592	1.620058
RP4-756G	0.324019	0.630823	0.796674	0.588954	0.416501	0.751876	0.987309	1.035058
RP4-761J1	2.035678	0.769019	0.045491	2.6143	2.363442	0.986797	0.92482	4.091667
RP4-761J1	4.33E-05	0.00026	1.090072	0.259163	0.036566	0.00013	4.33E-05	0.312864
RP4-769N	6.591544	2.136335	8.288874	2.153535	3.921645	16.5625	2.654473	10.70538
RP4-773N	1.877605	1.335743	7.737807	3.842554	2.306277	2.069942	3.404372	8.83241
RP4-794H	1.156869	0.105022	0.045491	0.358672	1.712706	0.522633	0.675115	0.648038
RP4-798A	0.41026	1.123016	2.760765	0.924329	0.28899	1.34513	0.444538	1.620058
RP4-798P	0.431977	0.097186	0.310447	0.469711	0.345635	1.623232	0.428694	0.373463
RP4-800J2	0.056046	0.029134	0.681068	0.076162	0.126335	0.117258	0.051198	0.00013
RP4-813D	2.447294	2.820974	0.414473	1.1629	4.212554	1.013434	1.644416	0.757273
RP5-1021	2.101075	1.465123	0.045491	2.556443	1.434921	2.690823	3.695815	3.864827
RP5-1024	4.33E-05	0.00026	0.359899	0.005758	0.002237	0.021429	4.33E-05	0.021255
RP5-1050	0.063225	0.705613	36.26061	2.203535	0.28899	6.25938	4.402973	9.838658
RP5-1068	1.835743	3.895469	1.340981	2.818312	2.323983	1.45912	4.370671	1.2214
RP5-1074	1.624459	1.064242	1.505678	3.153485	2.494177	1.149978	1.790245	3.002576
RP5-1085	13.00996	13.45939	3.211176	7.112496	12.70768	2.180022	12.32958	3.807648
RP5-1112	2.155447	2.337821	0.045491	0.949827	2.230859	0.089149	0.698297	0.283882
RP5-1112	0.309444	0.599134	3.317807	0.351219	0.434596	0.178629	0.457114	0.489509
RP5-1119	5.696263	7.238023	0.681068	0.005758	2.681169	14.88584	12.93651	12.03267
RP5-1126	0.789423	0.490375	1.046061	0.259163	0.750866	0.432915	0.160462	0.089473
RP5-1136	1.94311	1.519322	0.385238	1.566414	2.165851	1.514199	3.404372	2.498478
RP5-1142	0.16886	0.269892	1.385801	0.218045	0.20746	0.857778	0.292799	0.352597
RP5-1142	0.367287	0.047092	3.266782	1.020447	0.776876	1.959466	1.056898	5.942294
RP5-1159	3.842554	4.02088	3.266782	2.440895	5.395584	3.279625	14.70188	3.514834
RP5-1159	1.91811	1.675722	0.045491	0.959567	1.989711	0.6943	3.816551	1.909408
RP5-1180	22.96463	11.48368	0.045491	22.13245	17.78522	0.00013	4.18456	1.485483
RP5-827C	3.195541	3.566753	0.045491	2.6143	2.5271	1.078153	2.026219	0.743694
RP5-855D	0.995527	1.335743	0.045491	0.858817	0.416501	0.313925	1.152165	0.474856
RP5-855D	1.661328	2.027951	0.045491	6.088593	1.048074	2.104358	2.715873	4.984488
RP5-894A	10.90615	8.958225	0.045491	8.445743	11.96574	4.343838	5.295325	8.319019
RP5-894A	0.495931	0.769019	5.359134	1.036558	0.757763	4.997417	0.605022	3.585029
RP5-902P	5.409437	4.690159	57.84882	2.758088	5.34474	7.839271	35.27608	28.23632
RP5-902P	0.316573	0.429567	1.465123	0.259163	0.367532	0.089149	0.19355	0.268095
RP5-940J5	0.035137	0.083189	7.570693	0.183521	0.153391	0.189141	0.233889	0.388579
RP5-940J5	111.5439	26.25557	1.127475	40.7932	118.7028	30.82277	21.36892	11.19942
RP5-943J3	1.482626	0.712929	0.045491	0.872013	0.505267	0.475137	1.296905	0.537518
RP5-994D	4.077403	0.894704	0.517612	0.744531	2.111299	1.553074	1.180613	0.341241
RP5-998N	4.33E-05	0.00026	4.105714	2.89E-05	0.044322	2.281926	0.331263	0.136061
RP6-201G	3.833247	27.38726	0.26215	3.722785	3.300664	1.770029	32.31123	5.25254
RPPH1	6.808369	13.83359	87331.38	27.38726	9.792626	5.679502	4.508196	36.26061
SACS-AS1	4.33E-05	0.023694	0.234358	0.066616	0.005173	0.021429	0.073463	0.116392
SCARNA2	1.434921	1.952027	530.1465	4.468009	1.036558	1.121017	0.160462	4.684502
SCGB1B2F	4.733824	2.718434	0.045491	6.876392	4.039192	1.108947	1.011486	1.118066

SDCBP2-A	1.482626	2.703485	9.859719	2.735368	2.408882	3.279625	1.76873	4.077403
SFTA1P	1.94311	0.525678	0.045491	1.036558	2.209141	1.71518	0.833975	0.289935
SH3BP5-A	4.18456	2.649675	0.872597	4.323045	4.965801	4.552092	6.38456	3.233045
SH3RF3-A	0.639495	0.373218	6.685087	0.562439	0.620014	2.000592	0.588954	1.896667
SHANK3	22.74797	7.443276	5.591717	11.60283	19.06893	83.489	14.17388	16.22108
SMARCA5	0.056046	0.00026	7.938427	0.114964	1.44E-05	0.774798	0.094553	3.031688
SMC5-AS1	0.096371	0.083189	1.551962	0.207713	0.107626	0.198788	0.349812	0.200794
SNAI3-AS	13.45939	4.869553	17.03919	4.261342	10.82954	16.44768	3.899993	6.622092
SNHG1	30.82277	95.22939	252.4251	111.5439	35.776	23.82354	184.443	118.7028
SNHG11	39.53687	27.10502	0.045491	9.924372	40.7932	14.25954	27.10502	9.625253
SNHG14	51.52195	52.56128	167.9996	17.51961	42.75576	126.1556	36.74522	56.39528
SNHG16	10.38202	7.668579	111.5439	21.19547	6.365065	11.77567	18.76954	38.94854
SNHG17	22.32214	29.82828	1.385801	17.78522	29.82828	16.68554	21.93452	22.13245
SNHG3	4.63189	2.611797	332.0776	10.62739	5.110159	4.508196	5.500678	7.737807
SNHG6	425.62	425.62	4.012049	1360.293	425.62	749.7839	749.7839	530.1465
SNHG8	75.1132	135.5245	42.75576	155.7103	57.84882	155.7103	252.4251	126.1556
SNHG9	144.7919	56.39528	2.45575	95.22939	59.32013	28.87506	48.53429	91.05541
SOX2-OT	72.69473	42.08807	75.1132	126.1556	22.13245	425.62	87.1746	87331.38
SOX21-AS	1.995714	5.150693	4.277006	5.20267	2.274473	2.676392	8.195657	7.188095
SPAG5-AS	1.029365	1.112641	2.696061	1.060188	1.279452	1.71518	1.308167	1.349971
SPATA13	4.048499	4.614618	32.31123	4.445851	4.286962	15.66023	7.037345	18.91854
SRP14-AS	6.250447	3.370527	3.008225	4.227302	10.23768	4.965801	3.47531	5.485685
STARD4-A	0.234358	0.121494	3.008225	0.332395	1.000794	1.514199	1.826147	2.384141
STEAP3-A	0.096371	0.121494	0.045491	0.066616	0.165657	0.276061	0.117258	0.070541
STX18-AS	0.880498	1.547734	3.775209	3.131544	0.849841	4.445851	2.201587	5.838499
SZT2-AS1	0.234358	0.490375	1.385801	0.156017	0.849841	0.071775	0.081126	0.253074
TAPT1-AS	1.070693	8.711905	34.38341	1.974228	3.214545	1.795851	2.451645	4.586753
TERC	0.941104	0.366912	155.7103	0.195253	0.842193	1.029365	0.145382	0.312864
THAP9-AS	9.031082	12.49452	6.297136	14.79742	11.14619	27.10502	20.68746	11.30987
TIPARP-A	3.101371	5.075447	0.045491	0.146999	2.274473	1.378362	2.773644	1.744055
TMEM9B-	0.755253	1.193362	3.169812	6.028427	0.568665	4.863579	0.899055	8.381328
TMLHE-AS	0.019784	0.30176	0.045491	0.156017	1.44E-05	0.029769	0.081126	0.070541
TMPO-AS	0.418326	0.922338	14.00414	0.365859	0.813788	0.233175	1.011486	0.438023
TNKS2-AS	2.043795	3.450577	0.517612	0.744531	1.628009	1.054899	0.477698	1.505678
TOPORS-A	33.4985	16.34294	9.625253	50.55949	21.64437	25.73165	23.38378	19.99861
TP53TG1	21.54823	10.01009	2.8657	72.69473	47.5018	22.13245	13.23022	43.49658
TRAF3IP2-	6.830592	13.08214	16.09482	12.29473	9.751039	9.902576	19.22139	27.66779
TRAM2-A	2.556443	3.144156	1.742734	6.316328	3.227085	4.696313	7.951371	5.737056
TRAPPC12	0.418326	0.429567	1.046061	0.444315	0.190844	0.444315	0.145382	0.630823
TRIM52-A	14.08569	7.824473	3.211176	22.8563	16.22108	18.48514	23.6012	18.69833
TSTD3	1.180613	0.281169	2.209141	1.29267	0.757763	2.378124	0.53658	0.96329
TTN-AS1	1.969733	2.763261	1.742734	0.872013	1.1379	6.853608	5.323492	6.945152
TUG1	12.49452	30.17192	17.34065	20.16541	14.42661	37.29684	40.7932	53.77619
U73166.2	0.65798	3.394286	2.820974	0.995527	0.224921	0.358211	3.676032	5.22482
U91328.19	0.769365	0.480794	0.487662	1.09075	0.478182	2.558817	0.649668	0.593434
UBA6-AS1	4.18456	3.002576	12.39704	9.502294	3.310758	4.673182	5.671126	6.52899
UBL7-AS1	0.438831	0.661443	2.280101	1.370765	0.767569	0.672857	0.658745	1.581912
UBXN7-AS	0.063225	0.695426	3.169812	0.744531	0.176833	0.038716	0.642395	0.985411
USP27X-A	0.152843	0.295671	0.644618	0.517092	0.243449	0.936898	0.682799	0.50803
USP46-AS	1.082179	0.441472	2.209141	0.66482	0.603261	2.676392	1.592395	1.927042
VIM-AS1	0.251371	0.157186	0.045491	0.562439	0.216753	0.089149	0.081126	0.854733
VPS9D1-A	0.755253	2.820974	2.607035	0.90772	0.793434	0.584892	3.314329	3.606804
WDFY3-A	1.846941	6.116638	6.497266	3.401126	1.77404	7.765931	3.220678	6.685087
WEE2-AS1	0.746551	7.061328	26.52537	2.818312	2.911962	0.558095	3.548153	14.33939
WI2-8903	1.247244	0.256046	0.045491	0.543939	0.4971	0.146999	0.1793	0.095599
WWC2-AS	0.152843	0.035007	0.286522	0.675115	0.153391	0.071775	0.043391	0.0121
XXbac-B4	26.52537	29.18885	5.11697	12.16681	24.2842	4.673182	26.25557	6.325945
XXbac-B4	0.453463	1.216768	3.008225	0.717403	0.512309	0.800058	0.784372	1.019134

XXbac-BP	0.013348	0.00026	0.045491	0.012136	0.009307	0.071775	4.33E-05	0.00013
XXbac-BP	0.647345	2.287576	0.045491	0.940563	1.677042	0.252316	0.698297	0.698687
XXbac-BP	6.454416	4.012049	0.045491	1.370765	8.780115	2.524892	1.412771	1.935685
XXbac-BP	2.351299	3.028629	62.33472	30.49289	3.058802	38.39042	4.18456	77.74812
XXbac-BP	12.03267	7.751869	0.917431	6.162446	7.710361	4.894509	4.552092	3.330909
YEATS2-A	2.535974	0.403413	9.179466	2.03886	0.834416	4.012049	1.916782	1.047345
YTHDF3-A	3.521934	1.813564	0.045491	4.965801	4.952915	1.179885	3.588369	2.335844
ZBED3-AS	2.66404	2.216162	12.89577	5.075447	3.253175	4.165137	3.327619	7.443276
ZBTB11-A	0.390895	1.159149	10.45584	1.283059	1.022929	1.846941	1.180613	3.8379
ZBTB20-A	0.096371	0.661443	0.045491	0.125859	1.44E-05	0.276061	0.737857	0.558427
ZEB1-AS1	4.49671	14.88584	46.7056	12.03267	5.137042	3.595758	11.19942	15.77678
ZFAS1	126.1556	184.443	8.728571	425.62	203.5795	80.6518	332.0776	155.7103
ZNF197-A	0.11075	0.00026	0.487662	0.525325	0.050426	0.313925	0.437799	0.810967
ZNF667-A	60.77449	18.76954	2.160649	80.6518	37.29684	65.94551	24.73724	30.17192
ZNF674-A	1.661328	1.731169	2.074906	1.738456	1.364401	1.833102	3.422237	2.547179
ZNRD1-AS	0.710722	0.630823	2.760765	0.982128	0.471313	1.569553	1.226934	1.581912
ZSCAN16-	8.573434	7.417359	0.517612	12.46209	8.573434	4.927302	8.815123	5.855281

CGGA_10:	CGGA_10:	CGGA_10:	CGGA_10:	CGGA_10:	CGGA_104	CGGA_104	CGGA_105	CGGA_105
0.286364	0.505267	0.088788	0.35241	0.190844	0.614156	0.122063	0.07684	1.079697
0.137403	2.126349	0.370462	0.048831	0.390216	0.293038	0.274531	0.506652	1.127475
3.984747	0.34443	5.044113	6.325945	8.711905	1.561912	5.864084	3.718716	1.580909
1.935685	0.035671	2.693442	3.621941	1.881955	0.308672	0.264805	4.609076	3.771349
5.838499	0.942294	3.993665	5.923838	2.676392	5.485685	4.207778	3.944084	9.604358
2.931068	1.710173	0.783939	3.38456	1.487619	12.03267	4.292078	3.330909	14.00414
30.17192	13.60866	26.52537	55.04081	15.88548	35.27608	75.1132	33.93939	30.49289
0.059177	0.620014	0.088788	0.041053	0.149156	0.007395	0.058095	0.021645	0.024964
1.693939	0.321227	5.395584	3.334199	1.196508	2.661587	0.528196	1.870685	0.73583
0.161299	0.035671	0.000967	0.16575	0.004293	1.354567	0.398355	0.000188	0.954776
26.52537	46.7056	15.56131	47.1037	8.849697	64.12971	44.27012	56.39528	43.49658
1.004156	5.068831	0.198405	1.496118	0.299747	3.166659	0.938413	0.994271	1.572035
8.868932	4.217569	8.780115	29.18885	37.29684	3.137576	16.5625	7.495397	4.207778
24.98218	0.035671	56.39528	2.259661	22.32214	3.711097	1.009625	27.66779	2.89E-05
0.000188	0.321227	0.028687	0.041053	0.004293	0.021645	7.22E-05	0.000188	0.062944
5.110159	0.035671	11.30987	1.784755	10.73084	3.435988	1.833102	8.590794	0.232338
1.8386	2.406768	0.318312	3.533211	0.051984	5.560563	3.140866	0.863189	3.8121
0.000188	38.94854	0.000967	0.057128	0.190844	8.66E-05	0.130029	0.041328	2.89E-05
2.755411	0.035671	10.07949	4.684502	8.461703	0.11075	2.850058	1.551962	0.917431
0.3607	1.884935	0.331869	0.91404	0.068896	2.197828	2.270837	0.389574	4.115844
0.322973	0.475433	3.179199	1.219856	4.546443	0.216753	0.327662	0.202294	0.241688
2.417424	6.240346	8.745238	1.937222	42.75576	0.546854	1.202626	4.429769	0.751876
184.443	0.035671	6.52899	29.49693	4.25658	77.74812	95.22939	252.4251	1.823377
0.017215	1.141515	0.046804	0.379192	0.004293	8.66E-05	7.22E-05	0.041328	0.018167
0.575628	14.70188	1.167561	1.697792	0.16868	1.159149	1.191198	0.540563	1.983824
3.944084	5.638759	5.80469	10.01009	5.082063	1.749307	5.330519	3.025671	1.778153
3.651898	3.718716	16.62402	0.91404	9.924372	1.247244	2.398442	5.075447	0.393983
0.269322	1.378362	0.09855	0.307244	0.331097	0.245469	0.406212	0.263146	0.92816
287.197	0.035671	425.62	15.3618	252.4251	39.53687	27.66779	332.0776	4.451154
0.396638	2.511465	0.692381	0.98474	0.780087	0.208254	0.887359	0.350635	0.383889
7.882222	0.88316	5.530274	10.82954	6.497266	1.638499	4.586753	5.638759	3.391075
0.03842	1.670786	0.119683	0.137504	0.079019	0.373939	0.197727	0.027547	0.140765
11.96574	0.035671	15.98892	0.000289	46.7056	8.66E-05	6.191515	28.54786	2.89E-05
0.000188	0.203463	0.036241	0.000289	0.004293	0.011032	0.034863	0.016025	0.155945
1.019134	11.2819	0.000967	2.834084	0.068896	4.348882	1.765065	0.863189	6.09759
1.434921	9.335397	1.47018	1.219856	2.763261	1.623232	4.473348	1.622006	2.063362
1.713997	26.12492	3.005281	4.978182	3.909033	1.484553	4.655916	1.91811	2.604776
0.017215	0.186133	0.000967	0.000289	0.004293	0.037749	0.010519	0.055743	0.188276
2.698557	6.433918	1.228651	4.752121	2.02956	3.303925	2.925426	1.104033	4.715599
5.359134	21.36892	2.112843	10.78056	1.8386	12.93651	12.77772	4.343838	17.64869
0.000188	3.207807	0.057128	0.212172	0.004293	0.985411	0.337222	0.672078	0.319163
0.851234	5.366335	0.861934	1.379278	0.253882	0.669358	0.98956	0.721732	1.542302
0.867814	0.035671	0.000967	1.196508	1.721371	0.467677	1.282208	0.498052	1.49075
0.000188	0.276248	0.028687	0.02316	0.088701	0.011032	0.010519	0.016025	2.89E-05
0.232886	6.433918	0.257684	1.243442	0.058139	0.308672	0.573947	0.320563	0.864199
31.92385	2.055289	42.75576	22.52375	25.24367	32.31123	26.80355	35.776	22.96463
0.082027	0.742417	0.067864	0.065743	0.108016	0.075974	0.106926	0.0843	0.062944
0.000188	0.88316	0.000967	0.147807	0.004293	0.426118	0.114113	0.000188	0.751876
0.000188	0.776349	0.000967	0.02316	0.004293	8.66E-05	7.22E-05	0.012136	0.005303
0.000188	0.242612	0.000967	0.090693	0.004293	0.558095	7.22E-05	0.000188	0.249408
0.000188	0.035671	0.078456	0.048831	0.068896	0.067446	0.027691	0.07684	0.163651
6.560411	0.395859	3.803766	9.502294	11.36975	8.226219	4.519048	1.390476	33.71895
0.017215	0.942294	0.036241	0.090693	0.068896	0.315916	0.043059	0.350635	0.373463
0.332641	0.56145	0.078456	0.041053	0.108016	0.373939	0.735382	0.308903	0.085974
0.710253	0.82772	1.414574	1.575483	5.195022	0.315916	1.107605	0.672078	2.096162
0.612179	0.296234	0.000967	0.5129	0.004293	0.722626	0.168456	0.270491	3.679986
1.418312	3.629177	0.257684	2.936739	0.004293	1.428398	0.584892	1.535722	0.73583

0.174444	7.868146	0.149553	0.777864	0.178442	0.722626	0.251248	0.185909	0.628297
2.710765	2.511465	1.402915	2.124574	0.65101	2.547179	3.249877	2.479221	1.909408
0.256046	2.797835	0.246162	1.145426	0.12925	0.392403	0.832554	0.308903	0.309062
3.767489	0.035671	3.851414	0.429827	19.22139	1.2214	0.251248	1.251659	48.53429
0.753016	0.806227	1.010887	0.379192	2.836717	0.097807	0.299062	0.469358	0.258456
0.487662	30.82277	0.160779	1.985253	0.50254	0.4386	0.772951	0.749019	0.483608
77.74812	0.035671	59.32013	33.93939	15.46193	28.0994	41.47925	67.98069	8.849697
0.181919	0.88316	0.476335	0.065743	0.12925	0.08438	0.337222	0.119423	2.89E-05
0.024553	0.242612	0.000967	0.02316	0.004293	0.007395	0.027691	0.012136	0.002857
3.273045	0.395859	1.010887	1.775325	1.138557	1.609654	1.629235	1.104033	2.911962
2.378124	0.296234	3.640606	1.972706	2.250462	1.341825	1.616385	1.750628	0.891775
0.567619	0.56145	0.119683	1.145426	0.088701	0.809986	0.488759	0.320563	1.11526
0.000188	1.043608	0.046804	0.100101	0.299747	0.043203	0.100483	0.062641	1.341825
0.130714	3.031688	0.000967	0.212172	0.004293	0.245469	0.197727	0.332395	0.070851
0.137403	0.979827	0.119683	0.212172	0.2443	0.747417	0.284863	0.951652	1.315613
0.000188	0.203463	0.028687	0.02316	0.437042	8.66E-05	0.006306	0.012136	2.89E-05
0.012359	0.203463	0.046804	0.000289	0.068896	0.037749	0.010519	0.012136	2.89E-05
6.230245	5.889798	9.968355	9.459177	8.381328	3.046551	7.38847	6.240346	8.780115
0.174444	1.932857	0.046804	0.090693	0.139235	0.264502	0.106926	0.07684	0.232338
0.082027	0.742417	0.000967	0.02316	0.004293	0.124646	0.176833	0.07684	0.039358
18.33909	1.634935	10.43066	5.056421	2.547179	7.162698	11.33981	16.9162	5.662994
0.000188	3.29355	0.000967	0.090693	0.004293	0.067446	0.122063	0.100996	0.107027
0.09772	2.563506	0.465079	1.219856	0.381789	0.534452	0.299062	0.476335	0.763102
4.609076	1.110505	4.907929	3.214545	16.34294	2.506934	3.279625	4.946587	3.57057
0.051335	0.684805	0.208456	0.02316	0.494841	0.016681	0.006306	0.0843	0.009019
0.122785	1.578687	0.057128	0.420707	0.051984	0.928759	1.296905	0.237698	1.236248
4.535354	0.035671	14.00414	8.923117	39.53687	12.10289	0.357013	10.73084	0.763102
0.167532	2.535974	0.057128	0.048831	0.480303	0.343629	0.100483	0.219885	0.093102
0.500722	4.592273	0.217792	0.379192	0.004293	0.283535	0.659163	0.165512	0.43381
0.619365	1.553074	1.078153	1.803918	1.284747	0.655245	1.484553	0.756724	0.874091
0.295837	0.684805	0.279928	0.342381	0.139235	0.293038	0.114113	0.407157	1.159913
0.114437	0.742417	0.057128	0.032251	0.139235	0.160462	0.114113	0.048023	0.115065
2.577157	0.035671	1.748016	0.581378	1.742734	0.230108	0.53658	1.770029	0.652807
10.53159	0.035671	8.134084	17.39935	2.705916	0.693571	15.98892	10.03313	4.23202
0.405786	1.322092	0.088788	0.074978	0.004293	1.266255	0.299062	0.298874	1.869322
0.3607	0.203463	0.235931	0.560866	0.159228	0.40075	0.584892	0.389574	0.474214
0.575628	1.601443	0.128853	1.913997	0.068896	4.519048	1.872049	0.890065	5.959033
0.000188	2.797835	0.057128	0.02316	0.004293	0.011032	0.085426	0.000188	0.055281
3.211176	0.776349	3.279625	6.486104	4.198117	3.273045	7.882222	2.845036	6.045209
0.024553	0.534452	0.028687	0.057128	0.058139	0.049928	0.027691	0.055743	0.055281
0.082027	0.942294	0.160779	0.032251	0.108016	0.091255	0.010519	0.021645	0.013117
0.024553	0.422511	0.067864	0.000289	0.09873	0.016681	0.027691	0.03412	0.002857
1.187229	3.35355	1.253088	2.696061	1.003586	1.729892	1.245671	2.138131	2.178268
0.734524	0.035671	3.207807	5.143867	3.373983	3.230065	5.050267	1.551962	6.612121
1.707576	0.223579	0.189632	4.402973	0.004293	2.703485	4.546443	0.898016	6.876392
0.385512	0.260144	0.138449	0.128369	0.281724	0.183939	0.304387	0.255693	0.816479
0.189755	0.035671	1.123016	0.711046	2.365685	0.40075	0.979149	0.320563	0.107027
0.174444	2.255916	0.000967	0.000289	0.16868	0.150144	0.38153	0.407157	0.298874
0.051335	13.42179	0.000967	0.846342	0.437042	0.176955	0.284863	0.048023	0.43381
1.177309	0.422511	2.066652	1.348975	13.15079	0.684805	2.981962	1.496118	0.309062
0.189755	3.796075	0.149553	1.009625	0.190844	1.017107	2.329784	0.185909	1.566414
2.975974	0.56145	1.460051	1.594675	0.856291	4.764055	2.936739	2.280101	3.457504
1.572035	0.035671	1.380195	1.360022	0.642395	1.428398	1.041652	1.333781	0.393983
0.241688	1.349971	1.02798	1.575483	0.310447	2.290996	2.398442	0.772157	2.178268
2.556443	0.650101	203.5795	9.179466	224.9555	0.546854	24.98218	28.23632	1.52676
2.644885	0.035671	7.099913	1.265426	0.97733	0.634531	4.63189	1.89373	1.675722
0.396638	2.126349	0.057128	0.330635	0.12925	1.104033	0.251248	0.371299	0.456522
21.74051	203.5795	77.74812	5.889798	135.5245	8.66E-05	3.110447	31.92385	2.89E-05

0.017215	7.074329	0.160779	0.448824	0.004293	0.487395	0.528196	0.021645	0.465563
0.000188	0.035671	0.000967	0.083694	0.004293	0.216753	7.22E-05	0.000188	0.444986
0.965714	11.65791	2.451645	1.58645	0.888485	1.729892	2.850058	1.32482	2.474834
15.77678	6.663651	11.09133	12.56693	9.297085	7.519841	38.94854	19.99861	17.51961
0.106118	2.477027	0.067864	0.083694	0.642395	0.104329	0.043059	4.053506	0.763102
6.978903	1.670786	2.868456	4.241905	3.343629	7.625209	3.536955	5.696263	12.10289
1.069033	2.535974	0.444048	1.064242	0.108016	2.109755	0.95101	0.194235	2.941544
2.304452	1.553074	2.367763	5.737056	0.511977	3.672078	4.990794	2.345527	5.238701
0.867814	6.374812	0.839365	1.210447	1.856392	5.881342	2.009906	9.968355	1.369928
1.275325	28.54786	1.438672	0.479401	1.29342	1.762713	2.229048	1.870685	0.319163
0.031234	2.255916	0.000967	0.041053	0.118225	0.056205	0.122063	0.000188	0.024964
1.177309	0.223579	0.856291	0.083694	0.669358	0.392403	0.92482	1.34513	0.490952
0.232886	4.667518	0.270491	1.145426	0.374365	0.634531	0.176833	0.270491	0.258456
6.853608	0.505267	2.27254	4.06303	3.1857	10.28573	14.25954	3.025671	10.05618
0.429827	0.914618	0.000967	0.231659	0.004293	0.237035	0.274531	0.194235	0.140765
0.959062	0.422511	1.648066	0.86518	1.149978	0.315916	3.536955	0.593434	0.73583
0.734524	2.893001	0.257684	0.754784	0.50254	0.992201	0.92482	0.593434	1.019791
0.000188	1.227749	0.000967	0.000289	0.004293	0.007395	0.027691	0.000188	0.024964
2.735368	1.517143	1.870685	6.830592	2.037114	8.319019	2.737944	2.845036	41.47925
1.093434	0.035671	2.638139	0.725267	2.065007	0.043203	0.223824	1.184935	2.89E-05
0.082027	4.990794	0.057128	0.048831	0.004293	0.33329	0.016025	0.000188	0.249408
1.240981	0.88316	2.845036	1.401068	2.468117	0.898543	1.323975	1.296905	0.512641
1.40772	135.5245	0.783939	4.814661	0.159228	0.898543	2.009906	0.453218	4.353925
1.8386	1.578687	2.094574	4.978182	4.408153	0.634531	1.427431	1.373348	1.253088
0.276847	0.914618	0.974906	0.307244	0.234949	1.648066	0.545808	0.548788	0.662237
2.196017	5.068831	1.837172	3.334199	1.997352	4.072475	2.474834	2.157359	4.661717
2.230859	3.35355	1.775325	2.035678	1.068348	6.653016	3.35355	3.008225	9.792626
2.129596	2.445079	1.191905	2.243911	5.568391	0.323319	0.696234	1.667273	2.203535
0.867814	0.035671	2.192496	0.420707	5.323492	0.392403	0.197727	1.094206	0.326219
1.508831	0.035671	2.398442	1.368167	1.390476	0.684805	2.147013	1.353608	0.393983
13.96064	3.672078	8.556703	21.19547	7.162698	7.430317	6.316328	11.03843	5.959033
0.677807	0.035671	5.508131	0.202799	4.101061	8.66E-05	0.176833	0.693925	2.89E-05
18.76954	22.32214	18.91854	16.9162	20.16541	7.868146	8.381328	16.5625	6.486104
3.511263	2.839351	8.477662	6.900202	18.91854	2.810678	3.895469	2.860722	4.839105
0.000188	0.369105	0.000967	0.02316	0.088701	0.043203	7.22E-05	0.000188	2.89E-05
4.115844	0.035671	3.005281	2.066652	1.954993	0.451169	0.979149	4.678846	0.743283
2.621133	4.733824	1.078153	4.626025	0.340556	6.063276	6.967547	2.236407	12.26136
1.381111	2.563506	1.228651	0.36153	0.836537	1.144733	0.938413	1.1629	2.705916
0.946638	1.016494	0.528196	0.174517	0.634192	0.160462	0.631494	1.723579	1.191198
1.508831	0.035671	0.659163	0.956667	13.08214	0.36114	0.641277	0.427403	6.717056
7.212915	0.035671	43.49658	0.832554	65.94551	8.66E-05	1.338261	7.200505	2.89E-05
46.7056	0.035671	167.9996	34.84299	155.7103	1.283911	36.74522	126.1556	2.89E-05
1.177309	6.297136	0.246162	1.859084	0.340556	1.594675	2.515938	1.903636	1.446926
0.145685	0.448557	0.149553	0.147807	0.118225	0.245469	0.284863	0.142698	0.34619
2.129596	0.035671	1.58645	1.436623	1.452857	2.174733	4.770231	2.040606	2.08013
5.24562	0.035671	14.88584	1.749307	7.08733	1.791616	1.685678	2.270837	0.694675
0.648687	1.110505	0.431457	0.941104	0.374365	1.174315	1.528975	0.229199	1.159913
0.000188	5.583968	0.000967	3.401126	0.004293	5.095325	2.600014	0.000188	0.465563
19.37139	6.911486	11.719	13.91714	7.545584	6.028427	25.24367	15.61077	14.88584
0.453463	0.035671	0.119683	0.832554	0.692799	1.296046	0.434596	0.389574	1.748016
0.000188	0.369105	0.057128	0.000289	0.004293	1.017107	7.22E-05	0.000188	0.282583
2.449509	31.92385	1.47018	12.93651	1.515296	9.880779	7.074329	2.073232	7.854069
1.616385	0.296234	0.984069	1.253752	0.617763	1.982208	1.954993	1.729892	1.837172
0.3607	3.153485	0.279928	0.736551	0.004293	2.000592	0.811443	0.048023	1.271876
0.227063	0.776349	0.046804	0.285685	0.263658	0.140765	0.064127	0.288579	1.019791
5.388405	0.035671	7.175397	0.658745	3.792266	3.150433	2.794935	5.821804	2.413146
0.089921	0.296234	0.000967	0.100101	0.004293	0.160462	0.168456	0.048023	0.062944
0.309668	0.035671	0.198405	0.222193	0.004293	0.104329	0.184206	0.142698	0.711912

4.502453	0.035671	6.250447	0.35241	3.842554	1.393571	0.686299	3.077316	0.172453
10.62739	0.035671	7.443276	1.623232	8.573434	2.597792	6.674286	2.094574	2.531515
1.861999	17.15902	1.779423	4.086926	1.160707	3.816551	6.316328	2.805382	7.570693
31.55417	0.979827	45.91841	4.130794	60.77449	24.50942	9.751039	55.04081	4.563117
1.682193	0.242612	1.060188	3.028629	1.395563	0.392403	3.50425	1.226118	2.361198
1.1514	0.035671	4.971991	3.089293	10.62739	8.66E-05	1.477388	0.000188	2.89E-05
0.145685	1.227749	0.444048	0.212172	0.149156	1.37531	0.223824	0.371299	1.159913
7.854069	0.035671	16.28201	6.52899	15.56131	4.043846	7.795642	6.900202	3.391075
1.019134	2.406768	0.444048	2.781558	0.004293	6.011515	3.140866	3.57057	3.864827
9.859719	10.8813	7.911934	10.62739	4.490801	11.09133	10.62739	13.6814	11.22667
0.414473	0.035671	0.000967	0.330635	0.004293	1.283911	0.744531	1.006025	1.959466
13.45939	12.29473	7.363117	6.560411	3.855952	9.256537	24.04374	10.67991	8.958225
0.585592	0.035671	2.178268	0.137504	0.801501	2.109755	0.832554	0.874091	0.964416
3.080317	0.321227	2.192496	3.266782	2.750375	1.471198	3.846984	2.845036	2.042201
0.000188	0.714286	0.000967	0.000289	0.004293	0.693571	7.22E-05	0.000188	1.803918
1.693939	0.035671	1.648066	1.612056	1.012742	1.076652	1.980592	1.817828	1.431414
1.275325	0.979827	1.701616	1.545657	5.591717	1.096335	1.1407	0.908773	0.652807
0.889553	3.153485	0.306176	1.708932	0.263658	1.165873	3.450577	1.8386	3.8121
0.000188	0.186133	0.000967	0.000289	0.004293	8.66E-05	0.010519	0.000188	0.009019
8.608153	0.035671	5.137042	4.72798	1.622006	8.66E-05	3.614603	7.037345	1.431414
9.968355	1.517143	3.917597	18.05488	2.925426	8.780115	14.42661	9.583463	11.60283
2.390447	5.273362	0.50557	1.937222	0.004293	1.017107	2.955916	1.971183	2.755411
0.106118	3.35355	0.10886	0.000289	0.004293	0.40075	0.311046	0.0843	2.89E-05
2.083261	6.760765	2.524892	5.180058	4.272165	0.992201	3.317807	3.482785	8.677489
7.351284	0.035671	2.868456	5.493182	2.410996	2.715873	8.711905	4.946587	9.604358
0.000188	0.035671	0.352006	0.000289	0.004293	0.693571	7.22E-05	0.000188	0.582208
0.059177	1.47842	0.149553	0.137504	0.299747	0.283535	0.264805	0.062641	0.115065
5.158146	1.074827	4.557605	1.886335	8.348817	0.382417	3.025671	8.256465	1.236248
1.883535	2.217951	0.138449	3.548153	0.12925	1.405772	2.489762	1.436623	2.440895
0.309668	4.990794	0.2264	0.35241	0.12925	0.579495	0.274531	0.110188	0.802395
2.858009	0.035671	5.737056	0.692027	8.780115	2.661587	5.770996	5.366335	0.163651
0.745613	5.366335	0.647345	0.941104	0.692799	0.992201	1.566414	0.288579	1.236248
0.205801	0.850808	0.057128	0.342381	0.004293	0.273911	0.130029	0.092302	0.208254
32.68313	0.035671	52.56128	5.056421	25.99427	8.103925	8.319019	37.29684	2.81342
0.781977	2.893001	0.078456	1.33285	0.004293	2.053521	0.932078	0.70404	1.446926
2.500548	0.035671	4.667518	1.130115	6.079596	2.084791	2.16759	1.052208	0.816479
0.114437	0.296234	0.067864	0.02316	0.079019	0.021645	7.22E-05	0.110188	0.047633
0.000188	0.035671	0.000967	0.231659	0.004293	0.558095	7.22E-05	0.000188	0.603737
0.051335	1.553074	0.000967	0.000289	0.204055	0.595714	0.114113	0.150346	0.115065
1.558571	0.035671	2.693442	0.711046	3.747035	0.293038	0.787605	2.558817	0.34619
0.473586	13.72059	0.198405	0.35241	0.079019	6.695887	0.496494	0.721732	5.309264
30.82277	22.52375	11.48368	31.1753	13.23022	49.50903	39.53687	34.84299	29.49693
10.23768	12.60233	6.876392	22.13245	2.565556	6.365065	20.85876	7.08733	25.99427
1.137244	5.454949	1.636118	1.972706	1.012742	1.869322	1.191198	1.146962	1.392511
1.707576	0.276248	1.827403	2.470317	2.42381	1.775325	3.895469	1.277843	2.531515
0.089921	0.035671	0.000967	0.202799	0.004293	0.693571	7.22E-05	0.000188	0.522071
0.859885	0.56145	0.257684	0.35241	0.289935	0.40075	0.765606	1.317475	0.132208
16.04187	0.035671	55.04081	43.49658	67.98069	126.1556	35.776	52.56128	1.559675
1.027403	0.035671	1.906551	1.612056	3.435988	1.144733	0.686299	0.134834	1.474343
2.129596	0.806227	0.908247	1.846941	0.866147	2.394416	2.353247	1.286494	2.81342
2.304452	13.42179	1.748016	3.632973	1.240202	12.85504	9.880779	2.845036	6.433918
1.240981	0.035671	0.752229	0.420707	0.759646	0.293038	1.323975	1.212792	0.77925
4.376118	0.035671	24.50942	0.91404	18.05488	1.492973	1.107605	3.122915	3.57057
36.26061	2.933903	87.1746	5.993045	95.22939	7.868146	4.376118	27.38726	1.159913
0.000188	0.203463	0.088788	0.128369	0.068896	0.117843	0.100483	0.157958	0.232338
100.0998	0.035671	70.11932	23.16622	56.39528	22.32214	26.25557	70.11932	9.541645
3.031688	7.327482	0.752229	3.747035	0.67632	1.549711	3.387864	3.303925	4.125786
4.435159	0.035671	1.078153	2.730072	0.661443	4.814661	5.967879	3.846984	7.08733

0.000188	0.56145	0.000967	2.157359	0.004293	0.875101	0.389336	0.119423	2.08013
3.220678	0.035671	0.908247	2.335844	1.003586	1.667273	7.443276	1.581912	2.432121
2.544747	0.475433	5.576219	2.147013	2.871212	2.380079	3.172864	4.513622	1.502323
4.46267	0.534452	17.15902	4.56912	21.74051	8.66E-05	6.345382	6.079596	1.668369
87.1746	0.035671	252.4251	37.81919	332.0776	20.51271	42.08807	135.5245	8.180094
0.697085	20.85876	0.50557	0.000289	0.004293	1.030022	0.938413	0.850325	2.89E-05
0.463485	2.055289	0.246162	0.296003	0.340556	1.471198	0.521775	2.296732	3.314329
0.276847	3.8379	0.198405	1.401068	0.004293	4.105714	1.517143	0.298874	3.511263
0.000188	2.055289	0.000967	0.100101	0.004293	0.032496	0.034863	0.016025	0.005303
1.034315	0.505267	0.000967	0.439062	0.190844	2.8657	0.811443	1.134387	4.965801
0.122785	1.404812	0.257684	0.202799	0.67632	0.061025	0.144098	0.110188	0.024964
10.28573	5.821804	50.55949	2.625823	144.7919	8.66E-05	5.195022	14.17388	2.89E-05
26.80355	0.035671	20.85876	2.276407	22.74797	9.063983	7.61176	26.80355	1.736133
0.09772	0.589618	0.198405	0.420707	0.521421	0.416219	0.064127	0.229199	0.964416
1.682193	5.638759	1.204228	2.419553	0.932078	2.8657	1.582915	1.126775	2.62342
3.279625	0.395859	2.094574	1.379278	0.253882	0.773766	3.198499	3.482785	1.379278
1.219856	37.81919	2.513752	3.767489	2.496407	5.195022	4.839105	2.289286	1.816436
0.930837	2.839351	0.647345	0.61355	0.412193	1.266255	1.399315	0.672078	1.079697
0.269322	6.663651	1.060188	0.296003	0.458701	0.73127	0.641277	0.142698	0.662237
0.986797	1.932857	1.191905	1.937222	0.320794	2.290996	1.539062	0.882078	2.053521
2.871212	0.203463	12.03267	1.118817	45.1045	2.786898	3.043528	1.884935	1.127475
0.000188	2.640332	0.000967	1.534603	0.004293	4.02088	1.34798	1.126775	2.290996
19.22139	0.035671	19.22139	11.36975	20.34063	0.461234	6.162446	18.48514	2.89E-05
0.000188	0.369105	0.000967	0.057128	0.004293	0.056205	0.144098	0.000188	0.018167
0.753016	9.902576	0.667583	1.232078	0.447085	1.982208	0.887359	2.373824	1.191198
12.77772	0.035671	5.409437	1.913997	3.131544	2.496407	4.188896	9.751039	5.102742
0.044964	1.670786	0.078456	0.128369	0.088701	0.283535	0.043059	0.142698	0.132208
15.66023	1.837172	18.48514	14.79742	21.02638	17.03919	16.44768	21.19547	14.6058
3.632973	1.074827	5.933066	2.335844	4.839105	2.611797	5.095325	3.807648	4.145209
4.764055	0.035671	1.76873	1.636118	2.164113	4.02088	3.980123	2.417424	24.62333
0.106118	2.304452	0.000967	0.074978	0.149156	0.216753	0.389336	0.03412	0.309062
5.591717	126.1556	9.101674	34.38341	6.922771	13.00996	11.42512	5.568391	10.62739
5.500678	0.035671	5.24562	1.026825	21.93452	2.547179	1.89518	8.005685	2.65684
118.7028	15.83113	29.18885	287.197	27.38726	87.1746	70.11932	51.52195	135.5245
0.15456	1.378362	0.288745	0.212172	0.320794	0.4386	0.085426	0.27899	1.236248
16.68554	80.6518	27.10502	2.494177	13.6814	11.03843	1.437648	24.04374	12.56693
15.276	2.59557	22.32214	14.00414	33.4985	14.97762	14.70188	13.45939	13.45939
0.332641	0.979827	0.088788	0.000289	0.004293	0.461234	0.168456	0.21031	0.282583
0.000188	1.227749	0.149553	0.02316	0.299747	8.66E-05	7.22E-05	0.000188	0.018167
1.20824	0.035671	2.944416	1.33285	9.063983	0.712453	1.323975	1.985253	1.030678
38.39042	0.223579	19.53061	4.946587	51.52195	15.66023	6.738211	41.47925	12.26136
0.000188	1.141515	0.057128	0.000289	0.004293	0.011032	7.22E-05	0.016025	0.107027
1.093434	0.035671	4.261342	1.026825	1.189048	1.954993	2.353247	1.995714	0.326219
0.262338	1.25886	0.036241	0.318889	0.004293	0.595714	0.214654	0.03412	0.373463
0.106118	0.776349	0.000967	0.403413	0.004293	0.140765	0.106926	0.134834	0.20127
7.495397	4.733824	4.188896	4.946587	1.960945	15.3618	9.121486	7.683766	14.70188
0.734524	2.893001	0.279928	1.041652	0.2443	1.805166	1.237063	0.672078	2.42381
3.750837	1.517143	7.288788	9.297085	11.48368	5.737056	3.266782	5.287857	5.662994
53.77619	1.837172	51.52195	67.98069	35.776	57.84882	47.5018	42.75576	91.05541
0.000188	3.718716	0.413009	0.174517	0.281724	0.132309	0.214654	0.000188	2.89E-05
0.03842	2.355289	0.036241	0.065743	0.004293	0.230108	0.077915	0.110188	0.115065
34.84299	22.74797	48.53429	40.11561	20.85876	40.7932	50.55949	29.49693	24.62333
2.950173	1.884935	1.271097	2.169509	1.547734	2.538218	3.058802	3.122915	2.511465
0.205801	1.670786	0.208456	0.285685	0.004293	0.848369	0.19039	0.21031	0.282583
0.480303	12.97323	1.156869	0.550664	0.713405	11.77567	3.266782	1.770029	1.171349
0.753016	8.524242	0.279928	0.275527	0.09873	4.965801	3.518384	1.447994	1.483622
2.205476	9.989221	2.183665	3.24658	1.873434	11.14619	4.456912	1.390476	10.31025
0.181919	1.670786	0.046804	0.420707	0.068896	0.634531	0.496494	0.359899	0.864199

32.31123	57.84882	9.751039	50.55949	6.011515	135.5245	40.7932	25.73165	77.74812
0.012359	0.174646	0.046804	0.032251	0.051984	0.169812	0.010519	0.000188	0.039358
1.462143	4.535354	2.893001	2.453781	4.18456	1.27619	1.717641	1.286494	1.959466
0.031234	0.56145	0.000967	0.000289	0.004293	0.007395	0.010519	0.041328	0.078586
1.547734	36.74522	0.487662	1.274481	0.429827	2.053521	1.419278	1.622006	2.666494
4.140505	4.33342	1.748016	3.91355	1.223846	17.39935	5.898449	3.144156	21.74051
1.496118	9.707677	1.47018	2.006782	1.204228	6.345382	2.465916	1.052208	5.068831
0.000188	1.751948	0.046804	0.000289	0.068896	0.747417	0.010519	0.000188	0.483608
1.896667	0.035671	2.048781	7.38847	3.055729	0.858297	4.312251	4.120815	2.203535
0.688182	8.333918	1.136566	1.545657	1.613232	1.174315	0.677403	0.756724	1.61088
0.000188	11.03843	0.160779	0.147807	0.139235	0.783398	7.22E-05	0.150346	0.643853
16.44768	4.863579	13.08214	9.082828	13.00996	9.924372	9.459177	11.48368	11.14619
4.077403	0.035671	2.671299	1.026825	1.395563	0.938911	2.375974	3.926154	0.055281
1.137244	0.035671	3.128694	1.436623	4.077403	0.908247	1.644416	1.639639	0.258456
0.262338	38.39042	0.421342	0.330635	0.253882	1.144733	0.064127	0.672078	0.603737
43.49658	16.09482	29.49693	56.39528	36.26061	31.36473	22.32214	43.49658	20.94257
3.307186	1.110505	6.853608	8.256465	15.77678	7.430317	8.905642	4.739668	4.397792
0.554416	2.355289	0.208456	0.202799	0.108016	1.581912	0.311046	1.212792	0.498355
4.990794	19.22139	3.160022	3.998391	1.856392	10.45584	6.497266	9.082828	13.08214
50.55949	1.634935	72.69473	35.776	38.39042	20.85876	38.39042	37.81919	15.66023
0.377561	4.921082	0.546854	0.956667	0.458701	3.592063	13.38418	0.540563	1.932857
0.593853	0.742417	0.465079	0.267597	0.299747	0.587273	0.870375	0.483608	0.643853
6.153102	1.404812	0.717879	5.662994	0.470289	13.08214	12.29473	3.864827	11.30987
0.000188	0.914618	0.09855	7.417359	2.018167	10.33476	1.566414	0.49127	0.994899
2.03886	7.795642	6.171789	6.601833	8.075772	6.38456	1.119574	2.127973	4.46267
2.871212	6.911486	2.126349	7.751869	1.90798	7.251263	7.013853	2.073232	6.52899
0.799048	10.53159	0.370462	1.924228	0.661443	2.27254	1.629235	1.870685	2.511465
0.03842	1.074827	0.057128	0.041053	0.051984	8.66E-05	0.010519	0.100996	0.024964
14.79742	17.64869	8.888167	7.882222	7.339452	12.36443	19.37139	13.26964	25.24367
3.131544	0.395859	3.442886	3.718716	4.94026	3.909033	2.375974	3.904517	4.188896
8.048095	0.035671	5.838499	2.169509	2.065007	3.263391	0.760433	7.238023	2.132756
17.39935	0.650101	9.141299	6.22031	5.351934	2.987835	9.418196	11.42512	5.395584
5.137042	2.126349	2.227273	4.440548	2.565556	6.240346	7.824473	3.775209	10.78056
0.522367	2.477027	0.067864	0.369567	0.004293	0.367778	0.389336	0.185909	1.079697
0.03842	3.763045	0.078456	0.041053	0.09873	0.043203	0.085426	0.342511	0.531486
0.377561	1.016494	0.361342	1.182771	0.65101	0.898543	0.573947	0.389574	1.675722
0.606847	2.445079	0.119683	3.070952	0.364502	2.463911	1.566414	0.941645	1.661328
0.089921	2.477027	0.387258	2.018167	0.004293	2.037114	1.833102	0.100996	0.628297
27.96248	7.264502	75.1132	30.82277	118.7028	4.696313	29.18885	31.55417	3.391075
0.720938	0.035671	4.597792	2.31811	5.813247	1.017107	2.083261	1.146962	1.271876
0.241688	4.217569	0.246162	1.685678	0.068896	0.36114	1.177309	0.150346	0.444986
3.002576	34.84299	1.856392	1.219856	1.196508	6.8421	4.392316	2.964762	3.679986
1.817828	7.074329	0.605455	19.06893	0.67632	6.808369	3.68381	1.487619	13.38418
13.60866	5.638759	13.30906	2.758088	5.01772	14.6058	9.924372	15.3618	2.243911
14.51648	32.68313	8.164531	15.18102	5.273362	28.87506	15.88548	11.25392	31.92385
9.218514	3.29355	13.6814	59.32013	8.745238	3.47531	25.73165	21.93452	3.640606
1.750628	2.640332	5.599228	5.28039	64.12971	1.165873	15.46193	1.91811	0.73583
5.309264	0.56145	3.314329	3.533211	6.876392	1.017107	4.188896	7.61176	0.163651
6.591544	0.534452	6.956349	5.309264	5.737056	3.24658	9.377345	8.493088	2.361198
1.977316	2.640332	0.591118	2.871212	0.004293	3.909033	6.922771	0.840635	3.179199
1.40772	0.534452	0.404899	0.065743	0.488167	1.609654	0.137403	0.7986	0.628297
0.000188	0.589618	0.000967	0.048831	0.058139	0.011032	7.22E-05	0.055743	2.89E-05
5.088694	0.589618	6.011515	19.37139	1.425613	4.580938	3.471486	4.105714	10.19468
19.68088	4.796126	6.784762	13.15079	3.890556	9.031082	72.69473	20.25302	26.80355
0.012359	0.276248	0.036241	0.000289	0.004293	0.016681	0.006306	0.016025	0.005303
0.051335	0.742417	0.057128	0.041053	0.068896	0.183939	0.093009	0.03412	0.132208
16.5625	2.016515	10.23768	25.73165	7.288788	118.7028	56.39528	18.76954	95.22939
0.429827	3.401126	0.198405	0.318889	0.190844	2.987835	0.595022	0.389574	2.308203

0.000188	21.83751	0.000967	0.000289	0.004293	8.66E-05	7.22E-05	0.000188	0.024964
5.745996	12.03267	4.38684	26.52537	4.620159	34.38341	16.22108	4.053506	56.39528
3.016984	3.092229	2.037114	6.865	0.92342	4.297222	5.259437	3.192229	5.143867
0.000188	2.126349	0.078456	0.083694	1.025613	0.091255	0.100483	0.016025	0.078586
0.286364	1.378362	0.067864	0.420707	0.004293	1.609654	0.151847	0.165512	0.724502
0.473586	3.718716	0.29798	0.065743	0.535657	1.869322	0.832554	0.202294	0.188276
70.11932	155.7103	62.33472	41.47925	15.66023	184.443	167.9996	80.6518	144.7919
4.869553	12.97323	3.418405	10.1028	1.482626	9.377345	4.261342	9.583463	20.51271
9.583463	36.26061	6.063276	14.70188	3.227085	16.68554	23.82354	6.922771	18.62711
0.899055	0.422511	4.752121	2.981962	0.940563	0.467677	0.887359	4.563117	0.373463
252.4251	0.035671	95.22939	19.22139	55.04081	1360.293	46.7056	144.7919	425.62
7.288788	29.49693	5.373781	15.07257	2.701053	18.91854	12.39704	4.921082	23.16622
0.522367	4.130794	1.060188	2.720996	1.102121	4.914863	2.375974	1.126775	4.575123
1.53684	11.51384	1.636118	2.147013	2.547179	3.957734	6.830592	2.040606	0.155945
3.743232	19.99861	1.820541	1.612056	0.692799	6.277937	4.655916	4.678846	1.603795
6.019971	4.096407	1.798535	8.365072	1.812114	16.09482	10.33476	3.877417	14.08569
9.667316	10.23768	1.253088	1.952027	0.899055	26.52537	28.54786	2.715873	118.7028
0.385512	1.601443	0.000967	0.000289	0.004293	2.408882	0.24443	0.069163	8.288874
10.1028	18.05488	8.288874	2.826277	2.577157	15.46193	2.668896	8.711905	0.831472
12.29473	167.9996	21.74051	51.52195	19.37139	40.11561	29.82828	16.80343	33.71895
42.08807	3.153485	9.667316	44.27012	7.737807	65.94551	43.49658	32.68313	155.7103
2.858009	5.068831	0.318312	1.985253	0.583766	7.200505	0.970635	5.00404	5.123781
10.85542	27.38726	5.770996	14.51648	2.236407	24.2842	9.063983	7.710361	34.84299
4.990794	0.505267	0.88417	3.334199	0.351789	1.471198	8.27267	1.938759	12.03267
6.945152	21.02638	10.93101	29.82828	30.82277	12.67271	18.91854	6.116638	5.500678
0.745613	0.82772	0.29798	0.93267	1.213434	0.867287	1.872049	0.684805	1.52676
3.387864	0.035671	2.524892	1.732468	10.8813	0.373939	0.760433	2.903867	1.236248
22.32214	5.500678	30.82277	22.32214	52.56128	20.16541	9.031082	40.7932	9.297085
0.031234	1.016494	0.000967	0.041053	0.004293	0.684805	0.114113	0.178817	0.290693
0.436638	1.404812	0.270491	1.078153	0.281724	1.182771	0.573947	0.165512	1.030678
6.486104	0.684805	11.25392	3.511263	9.707677	1.594675	3.307186	14.33939	0.383889
1.473218	0.035671	0.827273	0.36153	0.67632	0.669358	0.424538	10.1459	5.337547
0.145685	1.438672	0.067864	0.571674	0.149156	1.638499	0.151847	1.333781	1.591299
2.871212	0.806227	3.55961	2.855296	6.116638	4.952915	1.954993	3.233045	3.363911
0.000188	0.296234	0.000967	0.000289	0.004293	0.011032	7.22E-05	0.000188	2.89E-05
20.34063	18.48514	7.049336	20.68746	7.795642	42.75576	10.8813	15.3618	22.52375
135.5245	530.1465	49.50903	167.9996	19.06893	224.9555	224.9555	1360.293	87331.38
167.9996	184.443	83.489	111.5439	87.1746	287.197	425.62	184.443	332.0776
0.585592	3.672078	0.138449	0.744531	0.73912	0.523521	1.717641	0.444762	2.511465
37.81919	7.868146	46.7056	31.92385	25.48179	21.02638	23.6012	24.50942	32.31123
0.83088	1.141515	0.000967	0.658745	0.004293	1.195794	0.363492	0.332395	1.010289
2.461905	2.984899	2.27254	4.776407	6.38456	4.563117	4.808658	2.542605	2.018167
0.06715	8.868932	0.370462	0.941104	0.068896	1.05417	0.53658	0.529423	0.20127
0.220657	0.223579	0.10886	0.777864	0.004293	0.061025	0.713846	0.134834	0.140765
0.000188	0.850808	0.000967	0.065743	0.004293	0.011032	0.070339	0.000188	0.018167
5.9071	24.2842	5.351934	11.719	1.721371	6.978903	11.96574	6.079596	3.479134
6.560411	17.39935	7.625209	49.50903	6.443925	3.930664	27.10502	10.03313	2.65684
0.122785	7.519841	0.028687	0.02316	0.004293	0.11075	0.064127	0.185909	0.078586
65.94551	28.23632	105.1694	70.11932	77.74812	45.91841	62.33472	64.12971	80.6518
0.000188	0.56145	0.057128	0.000289	0.004293	0.11075	7.22E-05	0.069163	0.013117
14.70188	0.620014	7.099913	27.96248	5.671126	16.44768	33.4985	14.51648	11.03843
0.059177	1.25886	0.000967	0.000289	0.004293	0.513752	0.160462	0.000188	0.149156
0.807619	4.096407	0.73583	1.545657	1.947316	2.447294	0.938413	0.453218	2.893001
0.059177	0.589618	0.000967	0.392403	0.004293	0.255209	0.363492	0.246955	0.132208
0.000188	0.88316	0.046804	0.000289	0.058139	8.66E-05	0.010519	0.000188	0.018167
1.093434	0.260144	0.198405	0.439062	0.214964	0.684805	0.932078	0.308903	2.531515
28.23632	22.96463	27.96248	62.33472	17.64869	37.29684	34.84299	24.2842	40.11561
6.830592	10.53159	8.815123	6.045209	9.141299	13.83359	6.900202	4.155339	14.33939

4.661717	7.013853	2.975974	5.967879	2.901032	12.67271	7.61176	4.921082	19.83727
5.71241	13.08214	4.23202	9.968355	4.429769	8.888167	5.01088	3.363911	3.544257
1.1514	0.82772	2.713319	8.288874	2.151919	0.875101	1.765065	2.212619	3.249877
0.000188	0.035671	0.000967	0.000289	0.004293	0.007395	7.22E-05	0.000188	0.009019
29.82828	19.37139	10.33476	4.900996	4.292078	36.74522	16.68554	35.27608	20.94257
15.18102	3.91355	16.28201	15.66023	24.2842	14.42661	14.79742	11.544	24.2842
0.241688	0.650101	0.138449	0.973232	0.004293	3.321284	0.98956	0.12772	0.73583
1.057569	0.321227	1.544517	2.203535	0.811443	3.404372	3.172864	1.146962	2.604776
1.177309	2.640332	1.156869	1.523824	0.899055	2.42381	1.191198	0.890065	1.362713
9.256537	6.063276	4.323045	12.10289	2.02956	10.03313	5.259437	6.404921	31.55417
0.661025	2.126349	0.306176	2.018167	0.331097	2.496407	0.61526	1.019791	1.675722
2.818312	0.979827	2.500548	12.85504	5.788153	1.354567	4.752121	2.616573	3.373983
7.124517	51.52195	4.764055	17.09911	5.515584	24.98218	17.39935	6.38456	21.93452
4.94026	23.82354	4.851227	21.74051	8.923117	15.07257	19.53061	4.802655	8.033932
0.09772	2.08632	0.036241	0.285685	0.16868	0.021645	0.058095	0.142698	0.155945
11.17281	1.016494	5.754935	19.68088	6.325945	13.23022	11.89932	8.493088	15.46193
0.212496	0.914618	0.067864	0.16575	0.149156	0.343629	0.130029	0.219885	0.100281
17.39935	59.32013	3.367071	14.33939	0.599798	31.92385	60.77449	19.83727	17.92188
1.343463	5.821804	0.639892	0.479401	0.780087	0.662944	0.214654	0.593434	0.107027
24.73724	1.884935	28.54786	5.591717	15.3618	8.608153	6.443925	34.38341	7.795642
0.309668	0.223579	0.431457	1.130115	0.535657	0.4386	0.696234	0.63114	0.282583
0.575628	2.165851	0.421342	0.956667	2.08013	0.614156	0.82588	0.483608	0.474214
4.626025	52.56128	2.834084	19.53061	5.062626	46.7056	21.02638	12.77772	22.74797
0.051335	1.404812	0.046804	0.147807	0.204055	0.255209	0.311046	0.185909	0.335786
47.5018	0.534452	18.19595	0.886775	5.646811	28.54786	24.73724	22.74797	16.85982
3.511263	5.728615	17.78522	9.792626	18.33909	7.683766	17.15902	4.845166	7.315512
0.114437	0.035671	0.000967	0.090693	0.088701	0.097807	0.058095	0.03412	0.115065
6.760765	5.889798	2.308203	2.048781	2.325931	10.57984	8.445743	4.261342	6.967547
0.734524	1.167561	0.057128	2.419553	0.458701	4.145209	4.207778	0.721732	1.551962
0.082027	0.534452	0.088788	0.222193	0.004293	0.858297	0.231659	0.055743	0.907273
0.530534	0.174646	4.343838	0.589986	10.38202	0.476551	0.573947	1.146962	2.89E-05
1.1514	0.422511	0.667583	0.767569	0.253882	2.447294	1.1407	0.784372	4.827691
17.09911	25.48179	6.464906	6.171789	3.26	38.94854	2.398442	20.85876	18.19595
6.784762	4.043846	3.711097	30.17192	1.605859	2.987835	8.745238	7.737807	6.55039
1.572035	1.29417	0.915195	2.289286	0.553348	1.384921	1.90798	0.976111	2.789466
0.145685	30.0001	0.088788	0.231659	0.004293	0.117843	0.058095	0.000188	0.039358
0.390418	4.592273	0.331869	0.700837	0.50254	0.293038	0.641277	0.165512	0.115065
1.883535	1.553074	1.191905	0.560866	0.617763	1.572035	1.268672	1.487619	2.496407
0.145685	0.035671	0.000967	0.000289	0.004293	0.075974	0.106926	0.021645	0.107027
0.122785	6.808369	0.000967	0.479401	0.004293	0.255209	0.058095	0.069163	0.172453
0.350837	1.517143	0.000967	2.29873	0.004293	2.018167	1.107605	1.360022	0.265786
0.044964	0.369105	0.000967	0.147807	0.108016	0.216753	0.016025	0.000188	0.155945
3.26	0.035671	1.113304	2.494177	0.703665	2.579509	3.767489	2.337821	2.961768
0.051335	0.56145	0.000967	0.369567	0.004293	0.037749	0.772951	0.237698	0.335786
0.000188	4.440548	0.036241	0.048831	0.088701	0.026876	0.02119	0.110188	0.005303
0.303564	4.130794	0.29798	0.379192	0.50254	0.097807	0.176833	0.110188	0.188276
3.144156	0.035671	2.500548	2.147013	1.301169	2.538218	0.412453	2.355289	1.712706
0.560606	4.990794	0.4971	0.956667	1.938759	0.308672	1.456955	0.270491	0.132208
11.09133	0.035671	19.06893	12.77772	25.73165	1.076652	7.22E-05	9.667316	0.724502
5.266335	0.321227	6.612121	1.109726	7.653391	1.113304	10.48101	5.287857	1.180613
155.7103	0.035671	184.443	38.39042	57.84882	45.1045	48.53429	203.5795	16.34294
0.145685	0.223579	0.119683	0.183052	0.088701	0.293038	0.085426	0.100996	0.282583
0.130714	2.304452	0.057128	0.192381	0.340556	0.33329	0.560325	0.202294	0.554416
0.385512	10.12435	0.536263	1.145426	0.951652	2.136335	0.357013	1.184935	1.712706
1.35259	15.83113	0.692381	6.38456	0.204055	4.261342	2.752893	1.938759	2.335844
1.806595	0.035671	2.538218	0.250476	5.110159	0.83088	1.566414	2.496407	3.679986
0.000188	0.589618	0.046804	0.000289	0.051984	0.007395	7.22E-05	0.000188	0.005303
2.438831	1.978954	2.765758	1.985253	2.065007	1.561912	1.994329	2.792035	3.019848

0.546854	0.035671	1.722475	0.956667	1.557482	1.471198	1.097006	1.938759	0.592857
3.101371	0.035671	4.419062	0.820931	12.03267	0.534452	0.623146	1.052208	2.89E-05
0.198405	0.620014	0.352006	0.500354	0.004293	0.867287	0.137403	0.07684	0.232338
8.226219	0.296234	4.678846	6.22031	1.947316	15.77678	4.808658	8.643074	13.00996
2.961768	0.035671	2.858009	11.30987	2.975974	2.207417	1.035058	0.987821	7.765931
1.337431	0.979827	0.744113	0.275527	0.004293	1.2214	1.261407	0.359899	0.474214
0.781977	0.035671	1.972706	0.090693	1.463304	1.791616	0.765606	2.758088	1.341825
0.189755	14.51648	0.160779	0.275527	0.913514	0.273911	0.299062	0.110188	0.504964
3.286652	12.42965	4.072475	4.702468	4.72798	4.23202	2.681169	3.286652	3.418405
3.307186	0.035671	6.028427	1.182771	4.802655	0.976111	0.696234	3.775209	1.542302
28.54786	1.016494	18.62711	48.53429	5.687879	35.776	53.77619	22.52375	65.94551
0.473586	5.18754	0.62733	0.941104	0.583766	0.885664	0.818066	0.593434	1.341825
0.024553	0.776349	0.036241	0.000289	0.004293	0.104329	0.160462	0.016025	0.018167
0.537807	2.984899	0.331869	0.856782	0.204055	0.595714	0.504964	0.498052	0.763102
0.262338	0.035671	30.17192	3.699495	49.50903	0.075974	6.695887	0.000188	2.89E-05
0.734524	0.035671	1.798535	0.074978	2.81342	8.66E-05	2.270837	1.307417	2.89E-05
9.541645	2.984899	9.625253	12.63773	13.45939	9.815642	15.56131	7.795642	13.91714
13.19051	0.035671	21.54823	10.43066	24.98218	1.717641	7.519841	17.78522	1.283911
1.165065	0.650101	0.612179	0.137504	0.004293	0.273911	0.844747	0.27899	1.283911
2.604776	0.035671	38.94854	27.38726	19.68088	1.667273	21.74051	1.307417	3.195541
2.176501	0.035671	67.98069	2.758088	41.47925	8.66E-05	0.553059	0.359899	2.89E-05
1.693939	20.16541	1.786306	2.35733	2.048781	4.456912	2.061926	1.104033	4.286962
0.000188	9.012937	0.000967	0.489784	0.004293	0.848369	0.481414	0.389574	1.002215
0.241688	1.790245	0.078456	0.420707	0.004293	0.558095	0.214654	0.055743	0.592857
2.604776	0.035671	5.821804	4.06303	6.717056	3.497085	3.703175	6.653016	1.597908
0.493925	0.979827	0.149553	1.118817	0.888485	1.609654	1.655541	1.607143	2.485534
1.252424	0.035671	1.060188	0.35241	0.204055	0.523521	1.035058	0.828167	0.373463
0.840635	0.035671	0.703665	0.296003	0.661443	0.392403	1.566414	0.593434	0.554416
16.9162	4.272165	24.2842	11.01243	59.32013	12.1974	9.838658	18.62711	12.63773
6.325945	0.035671	3.779452	3.482785	3.585029	3.625382	4.881775	3.599329	5.102742
3.563182	0.035671	1.636118	1.803918	0.770188	0.976111	2.936739	2.236407	1.149322
0.174444	0.035671	0.827273	23.71237	0.331097	8.66E-05	4.150274	0.371299	16.22108
0.745613	0.035671	0.520289	0.581378	0.947157	0.353016	0.806652	0.603261	0.241688
5.515584	0.035671	7.710361	1.636118	6.663651	4.179762	14.97762	3.175916	1.244242
1.772756	0.276248	0.797114	1.749307	0.876118	1.234444	4.251818	1.524776	4.286962
0.09772	0.035671	0.000967	0.100101	0.004293	0.192814	7.22E-05	0.000188	0.149156
1.693939	0.369105	0.896522	1.523824	0.470289	0.928759	2.040606	1.447994	0.891775
0.560606	3.031688	0.182597	2.006782	0.159228	2.810678	2.252273	0.672078	1.542302
4.47912	0.035671	2.654473	10.67991	7.964315	6.486104	12.56693	3.343629	15.07257
0.059177	72.69473	0.000967	0.083694	0.004293	0.264502	0.122063	0.062641	0.039358
0.422785	14.21671	1.340036	0.560866	0.97733	0.645916	1.018427	0.712929	0.474214
1.644416	0.035671	3.36075	0.956667	3.672078	0.183939	1.153723	0.756724	0.163651
19.53061	0.035671	26.25557	4.49671	9.459177	3.679986	22.96463	27.96248	6.404921
0.493925	0.260144	0.413009	0.192381	0.139235	0.595714	0.434596	0.407157	0.132208
2.351299	0.035671	3.980123	0.342381	6.772763	0.140765	3.182482	0.577706	0.772157
0.017215	0.395859	0.067864	0.000289	0.088701	0.011032	7.22E-05	0.016025	2.89E-05
0.174444	0.620014	0.10886	0.240346	0.068896	0.230108	0.474856	0.332395	0.522071
2.668896	0.035671	4.990794	1.58645	2.267157	1.708932	1.742734	4.174964	1.736133
0.889553	7.951371	0.413009	2.720996	0.770188	0.693571	1.566414	0.749019	0.793434
1.872049	3.885642	0.257684	1.99899	0.96938	1.405772	1.968283	2.513752	1.513102
0.000188	0.714286	0.036241	0.000289	0.214964	8.66E-05	0.010519	0.000188	2.89E-05
35.27608	0.035671	287.197	425.62	83.489	9.541645	530.1465	24.73724	52.56128
1.53684	0.505267	2.019618	2.035678	3.321284	0.451169	1.655541	2.542605	0.907273
0.017215	0.296234	0.036241	0.016739	0.118225	0.026876	0.122063	0.041328	0.115065
2.845036	4.440548	3.052655	10.23768	1.585296	15.276	6.287561	3.253175	5.993045
10.98644	0.035671	10.73084	1.96404	3.659668	1.888023	8.348817	11.65791	0.603737
27.38726	0.035671	28.23632	53.77619	30.17192	43.49658	22.03348	36.26061	18.91854
0.295837	1.29417	0.000967	0.16575	0.004293	0.634531	0.467951	0.194235	1.458038

0.000188	0.035671	0.342381	0.02316	0.429827	0.016681	7.22E-05	0.062641	2.89E-05
0.703665	3.980123	1.099762	0.5129	1.060188	0.408644	0.53658	6.612121	1.056263
1.010887	2.255916	0.413009	5.606739	1.452857	0.885664	0.168456	0.863189	1.877605
0.295837	1.884935	0.000967	0.000289	0.004293	0.4386	0.327662	0.621688	0.180895
0.000188	0.035671	0.000967	0.032251	0.004293	0.021645	0.077915	0.000188	0.013117
14.97762	0.035671	19.37139	21.93452	37.81919	4.655916	10.17029	12.10289	3.750837
2.533745	0.979827	5.082063	5.259437	4.508196	1.313737	5.42381	3.824964	6.30671
0.877807	2.984899	0.647345	0.787987	0.004293	0.848369	0.82588	0.185909	1.215195
11.89932	0.276248	5.323492	4.540794	1.648066	7.061328	10.93101	7.495397	8.745238
0.986797	3.796075	1.035058	0.797114	0.661443	0.73127	2.229048	1.251659	0.53912
17.09911	53.77619	6.956349	6.09759	4.115844	16.34294	7.188095	8.539971	10.45584
0.703665	0.684805	0.956061	4.034423	3.428788	0.323319	5.462475	0.840635	0.100281
0.059177	1.553074	0.036241	0.147807	0.068896	0.150144	0.05088	0.03412	0.140765
0.000188	0.223579	0.000967	0.032251	0.004293	0.061025	7.22E-05	0.000188	0.047633
0.973745	1.837172	0.599798	0.956667	0.494841	0.534452	0.956667	1.693939	2.227273
22.74797	28.87506	6.079596	10.55571	3.695815	8.815123	25.48179	19.22139	22.32214
0.262338	0.914618	0.09855	0.754784	0.004293	3.022713	0.735382	0.359899	1.559675
0.638362	21.83751	0.279928	2.794935	0.004293	5.037489	2.5271	5.158146	2.018167
0.772626	0.534452	1.89373	1.172208	1.565253	0.595714	1.009625	0.516825	0.582208
3.877417	24.50942	2.911962	4.06303	2.487576	5.130411	1.362713	3.377619	8.103925
1.94311	0.035671	2.212619	52.56128	4.821176	1.36619	7.545584	3.065108	2.89E-05
0.205801	3.629177	0.067864	0.057128	0.004293	1.046703	0.284863	0.27899	0.404704
0.017215	0.276248	0.036241	0.000289	0.004293	8.66E-05	7.22E-05	0.016025	0.005303
2.339697	5.500678	0.09855	2.092987	0.149156	6.591544	4.343838	2.261573	3.796075
0.089921	0.34443	0.000967	0.118983	0.004293	0.160462	0.058095	0.027547	0.078586
0.06715	1.710173	0.000967	0.379192	0.340556	0.323319	0.077915	1.622006	0.628297
35.776	4.535354	19.83727	42.08807	9.583463	56.39528	83.489	25.48179	87.1746
0.212496	3.401126	0.088788	0.083694	0.004293	0.245469	0.311046	0.416219	0.393983
0.000188	0.684805	0.036241	0.016739	0.051984	0.026876	0.02119	0.021645	0.009019
1.418312	0.035671	3.040505	0.98474	4.125786	8.66E-05	3.734762	2.355289	0.456522
0.000188	16.68554	0.000967	1.243442	0.004293	7.025599	2.339697	0.000188	1.11526
0.889553	0.979827	2.259661	1.427431	11.60283	2.683478	1.323975	0.784372	0.582208
12.22798	0.035671	17.64869	3.195541	30.49289	8.66E-05	8.958225	11.14619	1.502323
0.859885	0.035671	0.984069	0.540563	1.092136	0.655245	0.434596	0.772157	0.474214
1.508831	0.035671	1.167561	1.924228	1.402915	0.950418	2.287576	1.085224	1.191198
0.309668	6.335664	0.2264	0.36153	0.351789	1.96404	1.000794	0.359899	0.907273
1.817828	0.589618	0.257684	0.550664	0.004293	3.211176	3.043528	2.138131	1.983824
0.031234	0.242612	0.000967	0.065743	0.088701	0.091255	0.223824	0.194235	0.013117
1.667273	9.141299	1.544517	3.113478	1.765065	3.88153	3.957734	1.469163	6.464906
0.762165	0.035671	1.069033	2.109755	0.569971	1.136566	0.406212	1.040411	0.816479
0.241688	1.634935	0.09855	0.624163	0.004293	1.428398	0.82588	0.263146	1.047345
9.751039	0.260144	4.508196	4.72798	1.068348	2.331825	3.718716	6.99026	1.040411
0.089921	0.806227	0.046804	0.000289	0.190844	0.323319	0.070339	0.185909	0.180895
45.91841	0.714286	16.80343	4.198117	13.91714	8.412367	3.767489	49.50903	1.191198
0.212496	0.035671	0.413009	0.000289	0.661443	0.176955	0.02119	0.416219	0.047633
6.922771	1.349971	4.650007	1.496118	8.994791	2.987835	1.670786	3.585029	0.842193
2.438831	2.563506	0.431457	1.898153	0.634192	2.27254	1.881955	1.469163	7.897078
3.493362	0.035671	1.340036	1.623232	0.661443	2.987835	0.652807	2.737944	6.784762
0.137403	4.165137	0.078456	0.074978	0.004293	0.245469	0.251248	0.041328	0.273434
1.558571	0.035671	2.92013	13.79669	9.838658	8.66E-05	2.760765	2.671299	0.319163
1.1514	5.316378	0.50557	4.272165	0.204055	2.747908	2.770866	0.721732	1.139221
21.28219	0.035671	17.39935	15.98892	11.14619	9.141299	22.03348	19.06893	3.163506
10.43066	7.584502	23.16622	18.33909	7.737807	10.19468	16.09482	16.34294	5.210317
0.537807	0.035671	0.520289	0.429827	0.004293	4.546443	0.818066	0.821898	3.471486
0.220657	10.65365	0.10886	0.500354	0.299747	3.581688	0.231659	0.661025	1.106046
0.106118	6.171789	0.046804	0.074978	0.118225	1.729892	0.027691	0.263146	0.592857
3.676032	0.035671	7.417359	2.802828	16.44768	8.66E-05	5.679502	4.188896	2.89E-05
3.993665	0.174646	2.925426	4.586753	1.443983	1.182771	3.803766	2.681169	2.81342

6.52899	4.38684	3.648081	6.019971	2.114437	4.529913	9.256537	6.144055	6.933961
0.286364	7.795642	0.361342	0.296003	0.263658	1.405772	0.760433	0.672078	1.226118
1.215195	4.165137	1.262965	2.187395	2.42381	4.179762	3.450577	1.379278	14.97762
1.729892	1.751948	1.901068	0.846342	1.938759	1.944517	1.462143	2.254084	0.512641
1.330029	0.448557	0.546854	2.335844	0.617763	2.380079	1.202626	1.1629	1.315613
0.906126	0.34443	4.053506	0.342381	6.268312	0.223939	2.661587	0.359899	0.954776
11.48368	0.035671	22.13245	2.520404	23.6012	3.303925	8.573434	22.13245	7.417359
2.417424	0.035671	6.30671	8.226219	6.55039	3.796075	2.705916	4.650007	5.80469
3.414574	0.035671	2.27254	5.056421	2.81342	3.691724	7.327482	3.122915	4.952915
34.38341	0.035671	38.39042	0.589986	20.68746	3.775209	9.121486	36.74522	4.343838
0.789863	10.73084	0.487662	3.796075	0.577388	1.076652	8.608153	1.134387	0.474214
3.125844	0.34443	1.481631	0.429827	0.836537	4.997417	2.600014	2.447294	2.893001
2.489762	0.035671	1.556392	1.265426	2.131176	1.076652	1.670786	0.63114	1.226118
2.018167	0.620014	1.779423	0.222193	0.692799	2.092987	0.652807	1.639639	0.483608
0.762165	0.34443	0.444048	0.692027	0.004293	1.159149	0.375094	0.641277	0.994899
0.322973	3.153485	0.172612	0.240346	0.088701	1.210447	0.772951	0.444762	0.373463
0.309668	0.035671	0.000967	0.16575	0.004293	1.931537	0.641277	0.07684	4.473348
1.004156	0.942294	0.404899	0.392403	0.190844	2.000592	1.168326	0.444762	3.829105
0.303564	11.11876	0.235931	0.183052	0.748341	0.33329	0.284863	0.359899	0.149156
0.899055	0.035671	1.039798	1.360022	0.470289	0.50469	0.584892	1.286494	1.191198
7.795642	0.035671	10.82954	0.222193	7.839271	8.66E-05	0.19039	3.676032	2.89E-05
3.4679	1.349971	0.946638	1.145426	1.118066	1.27619	2.577157	2.373824	1.690483
0.965714	0.714286	0.421342	1.064242	0.263658	0.595714	0.98956	1.006025	2.335844
0.051335	2.693442	0.149553	0.183052	1.160707	0.11075	0.043059	0.041328	0.024964
7.08733	0.035671	11.65791	11.19942	4.863579	3.869163	5.638759	5.942294	0.954776
10.78056	0.505267	5.71241	7.795642	4.025267	5.47	20.51271	11.89932	9.838658
1.074827	0.242612	0.647345	0.754784	2.773644	0.273911	0.668456	0.661025	0.531486
7.402915	1.196508	4.419062	4.155339	2.911962	3.497085	4.684502	5.796421	3.236457
0.789863	0.850808	0.149553	0.074978	0.004293	0.117843	0.237359	0.219885	1.61088
0.522367	0.34443	0.925801	0.000289	1.8386	0.605455	0.093009	0.577706	0.123853
2.683478	0.369105	2.879228	1.708932	1.369928	2.240274	2.159004	2.616573	1.762713
8.868932	0.035671	4.23202	3.279625	4.597792	0.4386	5.050267	11.19942	1.466111
2.150303	5.984885	1.519322	2.306277	5.238701	8.477662	4.978182	3.314329	5.210317
0.181919	0.82772	0.2264	0.118983	0.09873	0.722626	0.284863	0.27899	0.563175
0.15456	0.203463	0.119683	0.330635	0.068896	0.132309	0.100483	0.07684	1.019791
0.089921	6.911486	0.000967	0.61355	0.253882	0.33329	0.284863	0.416219	0.917431
2.069942	0.534452	0.270491	0.886775	0.412193	0.634531	1.935685	1.019791	7.938427
10.01009	0.035671	4.016465	11.01243	5.872886	1.775325	8.195657	8.590794	17.28195
0.017215	1.29417	0.000967	0.000289	0.004293	8.66E-05	0.010519	0.0843	0.055281
0.06715	0.174646	0.067864	0.100101	0.004293	0.160462	0.027691	0.062641	0.107027
0.938911	2.511465	1.395563	1.924228	6.945152	2.157359	1.323975	0.342511	7.737807
1.935685	1.29417	0.431457	2.259661	0.16868	4.456912	4.914863	1.251659	7.824473
20.85876	11.11876	1.50684	5.024293	7.188095	30.49289	12.03267	3.824964	46.7056
0.181919	1.167561	0.198405	0.601623	0.004293	0.451169	0.424538	0.194235	0.672078
0.000188	0.203463	0.000967	0.000289	0.118225	0.075974	0.070339	0.048023	0.258456
7.288788	0.035671	4.519048	1.47018	12.49452	7.868146	1.595729	3.980123	1.171349
10.19468	0.035671	10.57984	3.877417	15.18102	9.179466	1.338261	14.25954	5.583968
2.116032	0.035671	1.636118	5.395584	0.811443	2.463911	1.765065	1.754733	2.975974
0.024553	0.422511	0.000967	0.041053	0.088701	0.037749	0.02119	0.012136	0.115065
0.000188	0.714286	0.000967	0.016739	0.118225	8.66E-05	7.22E-05	0.07684	0.039358
4.012049	0.321227	2.382035	1.401068	2.174733	1.003016	3.803766	2.944416	1.690483
4.193232	0.035671	13.00996	10.28573	13.75978	2.563506	6.063276	9.707677	2.89E-05
1.057569	0.589618	1.281241	0.137504	0.480303	1.37531	0.595022	0.712929	0.907273
2.048781	0.035671	3.588369	4.096407	2.45575	2.747908	1.547734	0.320563	0.816479
0.429827	0.035671	1.388543	0.469004	1.721371	0.568665	0.460065	0.498052	2.02956
0.537807	15.56131	1.573276	1.078153	1.721371	0.821414	1.228651	1.652771	2.063362
0.269322	5.366335	0.288745	0.711046	0.004293	0.4386	0.319026	0.976111	0.683665
0.369336	4.535354	0.000967	0.524646	0.004293	6.911486	0.100483	0.000188	2.419553

3.563182	0.620014	2.794935	3.169812	4.419062	4.927302	3.50425	2.903867	1.690483
0.31557	0.242612	0.119683	0.032251	0.004293	0.773766	0.214654	0.110188	0.070851
0.656003	6.612121	0.000967	0.736551	0.079019	1.931537	0.806652	0.469358	1.778153
2.621133	0.035671	4.188896	1.427431	2.192496	0.624163	1.107605	3.253175	0.874091
0.656003	0.035671	0.925801	0.5129	2.500548	0.792338	0.92482	1.114618	0.018167
5.295325	1.25886	2.879228	3.939307	2.229048	1.696494	3.025671	5.195022	1.127475
0.444048	0.448557	0.000967	0.183052	0.004293	0.546854	0.231659	0.0843	0.393983
6.414416	0.035671	4.620159	3.089293	0.906825	2.563506	4.770231	6.240346	1.652771
1.275325	1.110505	0.5129	1.389509	3.025671	0.496212	1.372475	1.030022	1.431414
10.35839	0.035671	15.77678	9.141299	20.51271	5.80469	10.05618	16.44768	10.73084
0.31557	1.932857	0.752229	0.231659	0.364502	0.40075	0.98956	0.621688	0.326219
1.485483	0.589618	12.56693	4.198117	62.33472	1.561912	3.397706	2.81342	0.115065
4.043846	11.93253	14.6058	14.6058	19.53061	2.45772	10.2617	4.445851	3.953297
0.031234	0.620014	0.036241	0.137504	0.058139	0.150144	0.168456	0.048023	0.214885
0.9529	0.035671	3.885642	1.232078	1.369928	1.165873	1.282208	0.407157	1.690483
36.74522	0.035671	14.08569	24.98218	5.439278	11.719	37.29684	33.07147	27.38726
1.729892	0.035671	3.269913	0.267597	1.828781	3.507691	1.296905	4.609076	1.171349
1.343463	0.035671	1.046061	0.296003	2.773644	1.174315	0.553059	2.08632	0.724502
10.73084	3.29355	5.110159	13.08214	6.345382	1.017107	18.05488	30.49289	5.044113
0.089921	0.035671	0.000967	0.192381	0.310447	0.36114	0.565714	0.000188	1.748016
2.784228	0.035671	2.353247	3.482785	1.828781	0.273911	1.994329	2.5271	0.981032
0.405786	8.075772	0.476335	2.847547	0.553348	3.065108	2.091313	1.104033	2.31811
8.762677	1.196508	8.711905	7.443276	12.16681	3.909033	7.61176	4.900996	6.250447
0.250007	0.620014	1.451775	0.202799	2.434444	0.124646	0.274531	0.359899	0.20127
4.317648	2.217951	2.625823	2.035678	2.45575	0.558095	4.053506	3.089293	1.369928
0.889553	2.893001	0.000967	1.523824	0.139235	0.762165	0.412453	0.389574	1.572035
0.3607	0.035671	0.000967	1.232078	0.004293	2.069942	0.623146	1.074242	1.466111
4.317648	0.475433	5.195022	4.508196	0.320794	7.025599	21.36892	5.993045	9.218514
1.991328	0.035671	1.827403	1.389509	0.986104	1.561912	2.544747	1.114618	0.592857
1.40772	9.335397	1.228651	1.379278	1.149978	1.808023	1.128788	1.34513	1.379278
0.114437	0.035671	0.000967	0.118983	0.004293	0.075974	0.292597	0.000188	0.498355
0.174444	0.035671	0.000967	0.000289	0.004293	0.83088	0.070339	0.12772	3.917597
1.720144	0.035671	2.429957	1.172208	4.307309	0.451169	1.607143	1.8386	1.618773
1.019134	0.035671	2.009906	0.767569	3.921645	1.152944	1.282208	0.469358	1.253088
0.979827	0.321227	0.5129	1.109726	0.447085	2.661587	0.979149	0.840635	3.019848
1.915455	7.212915	1.497143	5.00404	1.29342	3.944084	5.075447	2.693442	2.941544
0.000188	0.035671	5.508131	0.469004	6.250447	8.66E-05	7.22E-05	0.641277	2.89E-05
57.84882	3.763045	13.53913	19.83727	1.415469	67.98069	45.91841	17.03919	21.36892
0.899055	3.207807	1.123016	1.418312	0.488167	2.053521	2.882035	1.783203	2.02956
0.232886	0.242612	0.10886	0.296003	0.004293	0.534452	0.434596	0.219885	0.047633
0.106118	0.850808	0.046804	0.057128	0.058139	0.416219	0.327662	0.062641	0.078586
0.930837	0.035671	1.994329	1.89228	3.687633	0.83088	0.24443	0.529423	1.846941
2.890245	1.110505	1.786306	3.230065	1.180613	5.01772	3.536955	2.496407	7.495397
0.710253	0.684805	1.519322	1.458038	0.793434	3.734762	0.481414	1.652771	2.255916
0.276847	4.592273	0.208456	0.658745	0.535657	1.982208	0.064127	0.398629	1.304877
9.625253	15.02509	8.958225	15.56131	3.566753	25.99427	11.719	8.780115	16.09482
2.060491	0.035671	2.060491	0.98474	1.634935	2.189235	3.286652	3.330909	5.622872
1.019134	1.29417	0.172612	0.379192	0.16868	0.487395	1.547734	0.398629	1.591299
1.707576	0.88316	0.925801	7.002056	0.422511	1.655541	3.767489	1.930216	3.606804
0.979827	6.135007	1.691587	1.064242	2.996869	0.908247	1.035058	1.80267	0.802395
1.783203	1.074827	4.38684	1.985253	4.33342	1.37531	7.964315	0.721732	2.89E-05
2.845036	0.422511	1.414574	4.424416	1.655541	0.523521	6.125823	1.052208	1.846941
0.024553	0.242612	0.078456	0.032251	0.159228	0.021645	0.02119	0.000188	0.024964
3.435988	0.276248	3.022713	2.652107	3.966623	3.144156	5.130411	3.792266	3.851414
15.46193	2.126349	11.14619	11.14619	8.319019	6.784762	2.375974	11.36975	8.180094
1.283059	1.074827	0.896522	2.31811	0.821898	4.179762	2.009906	0.934488	4.012049
0.295837	0.534452	0.149553	0.231659	0.320794	0.451169	0.631494	0.229199	1.002215
0.369336	2.640332	0.000967	0.000289	0.004293	1.594675	0.496494	0.000188	0.941645

0.405786	8.164531	0.067864	1.041652	0.770188	0.467677	0.686299	0.548788	1.846941
0.06715	0.260144	0.000967	0.524646	0.159228	0.037749	0.223824	0.012136	0.612872
0.973745	0.035671	0.965714	2.652107	0.569971	0.264502	0.424538	0.172294	0.265786
1.925635	0.035671	5.889798	10.73084	0.004293	8.66E-05	3.980123	2.792035	3.037626
1.081544	0.035671	1.02798	1.458038	0.320794	0.821414	1.484553	0.693925	0.232338
0.145685	1.016494	0.088788	0.212172	0.058139	0.245469	0.299062	0.157958	0.43381
1.729892	0.035671	1.736133	1.952027	0.856291	1.283911	2.434444	2.050404	0.816479
0.959062	0.321227	0.870902	0.899567	1.109726	0.08438	0.467951	0.584502	0.582208
2.520404	0.776349	2.212619	4.47912	1.003586	1.41368	2.187395	2.013182	0.672078
1.806595	0.035671	0.81544	2.386248	1.223846	1.775325	0.25733	0.563499	0.572266
5.654863	1.196508	11.42512	7.854069	15.98892	4.776407	6.464906	10.48101	5.662994
0.000188	1.710173	0.046804	0.118983	0.289935	0.043203	0.034863	0.000188	0.078586
1.667273	3.153485	0.620462	2.442987	0.703665	3.984747	1.282208	1.808023	4.990794
9.335397	3.092229	1.167561	8.019769	0.004293	3.086306	15.07257	5.591717	11.09133
1.616385	20.68746	0.774798	3.195541	0.488167	2.579509	1.935685	0.951652	4.365224
9.063983	0.806227	9.924372	4.875527	4.563117	3.192229	14.08569	5.309264	3.796075
2.683478	50.55949	2.569957	9.583463	0.331097	30.82277	7.136537	5.622872	6.433918
0.396638	4.592273	0.128853	0.711046	0.09873	1.931537	0.418023	0.308903	1.066948
2.903867	0.035671	9.335397	4.272165	8.019769	1.708932	2.850058	5.130411	1.89228
87331.38	1.322092	1360.293	155.7103	530.1465	100.0998	87.1746	87331.38	60.77449
1.057569	1.837172	3.767489	1.304877	5.337547	0.792338	1.009625	1.367179	1.002215
0.269322	0.223579	0.000967	0.065743	0.004293	0.255209	0.481414	0.069163	0.107027
2.367763	1.790245	2.713319	0.647691	2.34355	12.42965	2.823636	2.479221	7.025599
19.83727	1.227749	11.96574	15.276	75.1132	30.17192	10.82954	17.51961	8.319019
0.286364	1.141515	0.208456	0.259646	0.09873	1.090072	0.214654	0.840635	1.353608
6.38456	1.553074	6.423911	11.89932	11.719	11.83593	10.53159	9.880779	19.22139
29.49693	1.141515	36.74522	2.847547	31.55417	5.830152	12.85504	13.79669	5.745996
4.435159	0.650101	2.242092	9.751039	3.172864	1.542302	11.22667	4.397792	3.435988
0.429827	0.035671	1.131472	0.876118	2.290996	0.416219	2.770866	0.498052	0.208254
1.99899	2.74544	1.669466	2.668896	0.703665	3.563182	2.489762	2.003723	5.068831
0.31557	3.207807	0.404899	0.846342	0.09873	1.449062	0.832554	1.146962	2.335844
5.22482	29.18885	6.374812	10.05618	5.530274	7.868146	8.539971	3.659668	9.063983
0.000188	16.9777	0.000967	0.222193	0.004293	0.451169	7.22E-05	0.000188	2.89E-05
3.482785	0.242612	1.47018	1.859084	0.713405	1.533535	4.365224	1.80267	2.461905
48.53429	0.035671	21.02638	5.770996	17.15902	38.39042	25.99427	91.05541	75.1132
0.938911	16.28201	0.2264	7.824473	0.118225	2.524892	1.566414	0.7986	5.024293
0.000188	0.369105	0.000967	0.057128	0.004293	0.016681	7.22E-05	0.000188	0.009019
2.831501	40.7932	1.297763	3.895469	1.434921	7.112496	2.690823	2.635685	6.230245
0.514885	12.42965	0.50557	3.279625	0.993521	3.711097	6.125823	0.571342	5.864084
6.900202	1.227749	5.933066	5.788153	4.101061	7.30215	11.60283	3.397706	8.493088
2.390447	1.404812	1.935685	6.125823	1.640779	6.191515	1.484553	2.180022	8.241342
0.082027	0.242612	0.088788	0.032251	0.068896	0.117843	0.327662	0.398629	1.636118
0.946638	2.355289	0.896522	1.784755	0.437042	1.003016	1.91811	1.212792	1.092136
0.106118	0.296234	0.067864	0.222193	0.068896	0.150144	0.24443	0.255693	0.282583
0.585592	0.422511	0.306176	0.128369	0.2443	0.67632	0.853218	0.65241	0.763102
0.000188	0.448557	0.138449	0.000289	0.759646	0.056205	0.058095	0.03412	2.89E-05
6.25938	0.742417	1.565253	0.803665	2.73272	6.423911	6.55039	1.80267	45.1045
3.585029	0.035671	1.827403	1.159913	1.29342	1.533535	0.481414	3.659668	0.816479
8.226219	0.035671	3.877417	1.793074	14.6058	6.760765	2.447294	8.83241	3.195541
5.622872	0.035671	3.552049	1.817828	14.42661	2.27254	3.820758	0.453218	2.831501
22.52375	0.475433	8.226219	1.348975	1.534603	6.570433	6.25938	12.22798	2.673846
23.16622	0.035671	28.87506	5.864084	21.19547	2.933903	13.04605	38.94854	7.025599
1.572035	0.035671	0.827273	3.179199	3.040505	0.416219	0.584892	0.828167	1.409942
0.106118	2.406768	0.444048	0.16575	0.67632	0.755714	0.137403	0.219885	1.458038
0.889553	0.260144	0.639892	1.130115	0.661443	0.323319	0.806652	0.548788	1.379278
1.861999	19.83727	0.752229	0.86518	1.803918	4.261342	1.629235	0.506652	0.864199
0.295837	1.29417	0.182597	0.285685	0.139235	2.586926	0.496494	0.308903	2.238341
0.000188	1.837172	0.000967	0.074978	0.253882	0.255209	0.304387	0.069163	2.89E-05

1.108276	15.41187	0.556126	1.172208	0.787136	2.647244	1.362713	0.814877	1.191198
8.677489	30.49289	35.27608	3.460909	29.49693	8.976508	11.77567	11.96574	0.851659
3.695815	1.438672	5.28039	18.48514	7.061328	4.23202	4.02088	2.410996	5.454949
0.322973	45.1045	0.444048	1.732468	0.759646	1.948918	0.877222	0.732193	2.770866
2.961768	0.321227	1.691587	3.373983	2.468117	0.950418	8.905642	2.737944	3.363911
0.840635	0.296234	0.396082	0.899567	0.088701	1.266255	0.623146	0.63114	3.592063
1.947316	0.620014	0.50557	1.368167	0.888485	1.06355	0.838326	1.040411	4.424416
0.000188	1.884935	0.10886	0.202799	0.12925	0.176955	7.22E-05	0.092302	0.258456
0.000188	4.440548	0.088788	0.074978	0.004293	0.176955	0.05088	0.069163	0.039358
8.019769	0.369105	3.147294	2.470317	4.440548	7.737807	5.967879	7.375794	3.939307
0.276847	0.34443	0.078456	0.110058	0.190844	1.2214	0.184206	0.041328	0.393983
0.000188	1.043608	0.000967	0.057128	0.004293	1.030022	0.130029	0.000188	0.373463
0.546854	0.82772	0.404899	1.052886	0.289935	1.877605	0.605455	0.380649	1.341825
1.50026	0.035671	1.460051	1.232078	0.2443	1.440736	1.595729	1.277843	2.386248
0.06715	0.806227	0.088788	0.057128	0.108016	0.36114	0.034863	0.092302	0.163651
2.461905	1.110505	1.228651	4.272165	0.836537	5.42381	2.287576	1.857677	5.779574
1.321053	0.035671	1.497143	1.89228	0.888485	0.821414	0.375094	1.857677	2.107922
30.49289	0.035671	2.285664	3.921645	5.462475	4.894509	3.614603	5.515584	6.99026
0.06715	3.092229	0.342381	0.147807	0.004293	3.651898	0.223824	0.237698	2.781558
1.485483	13.87537	0.938911	2.936739	0.97733	5.881342	2.187395	1.277843	4.808658
0.000188	0.035671	0.000967	0.100101	0.004293	0.140765	0.151847	0.000188	0.100281
0.000188	0.035671	0.000967	0.524646	0.004293	0.067446	0.151847	0.194235	1.362713
0.762165	0.035671	0.387258	1.210447	1.138557	0.160462	0.504964	0.359899	1.092136
0.198405	0.422511	0.10886	0.183052	0.004293	0.875101	0.53658	0.092302	1.392511
2.941544	33.93939	2.750375	13.45939	1.863506	22.96463	19.83727	3.926154	9.968355
0.567619	4.733824	0.172612	0.91404	0.234949	1.916782	0.95101	0.142698	1.002215
2.359264	1.141515	3.207807	0.128369	4.575123	19.83727	4.959358	5.075447	9.031082
0.017215	0.395859	0.067864	0.000289	0.088701	0.011032	7.22E-05	0.016025	2.89E-05
4.251818	0.369105	2.794935	3.38456	2.119596	4.348882	2.544747	3.040505	3.314329
21.54823	2.304452	14.25954	33.4985	8.677489	11.65791	30.82277	24.98218	59.32013
0.480303	4.165137	0.257684	1.253752	0.951652	1.104033	0.945483	0.12772	4.502453
8.319019	0.475433	2.465916	7.911934	2.296732	5.158146	11.83593	6.760765	10.57984
0.012359	0.850808	0.000967	0.057128	0.068896	0.032496	0.093009	0.048023	0.222698
1.590202	0.035671	0.998831	2.453781	0.940563	0.451169	2.159004	1.418312	2.730072
0.137403	0.035671	0.000967	0.459394	0.004293	8.66E-05	0.176833	0.000188	0.802395
59.32013	0.035671	34.84299	0.000289	48.53429	11.4544	3.833247	62.33472	3.119986
0.130714	3.031688	0.09855	0.048831	0.65101	0.104329	0.027691	0.027547	0.018167
0.396638	0.035671	0.331869	0.02316	0.224921	0.150144	0.027691	0.055743	2.89E-05
11.544	0.34443	5.431544	20.85876	2.151919	8.348817	17.78522	13.79669	19.37139
0.889553	1.751948	1.219019	1.784755	4.392316	0.783398	0.668456	0.529423	2.842193
2.784228	0.650101	27.66779	6.287561	16.80343	0.264502	3.249877	1.410902	0.864199
5.545729	0.369105	4.47912	5.821804	6.464906	3.340361	3.428788	4.709033	4.524481
4.179762	1.29417	0.563499	5.323492	0.447085	3.101371	4.086926	10.1028	6.063276
1.661328	0.035671	1.262965	2.638139	1.547734	0.408644	2.917381	2.236407	1.974228
1.128788	64.12971	0.692381	2.509177	2.092987	4.312251	2.201587	1.543377	2.511465
7.824473	2.74544	12.36443	1.765065	14.17388	2.621133	9.179466	9.316241	3.125844
6.695887	4.33342	3.5257	7.653391	6.570433	7.363117	6.210375	4.261342	8.643074
7.938427	9.902576	20.51271	9.838658	36.74522	6.240346	13.75978	4.650007	8.888167
0.089921	1.349971	0.556126	0.634192	0.004293	0.917994	0.677403	0.092302	2.975974
2.477027	0.035671	2.294733	0.267597	0.821898	0.237035	0.962698	1.667273	0.43381
3.45404	1.553074	1.798535	4.198117	0.599798	0.848369	3.493362	2.953059	2.006782
0.000188	1.932857	0.10886	0.996696	0.004293	0.264502	0.595022	0.453218	0.851659
0.017215	0.505267	0.000967	0.02316	0.058139	0.124646	0.137403	0.016025	0.100281
0.350837	3.718716	0.421342	0.876118	0.004293	5.095325	0.474856	0.621688	1.690483
3.864827	0.035671	6.09759	4.650007	17.03919	3.842554	2.640332	4.468009	0.335786
3.500808	0.395859	5.959033	1.288276	2.676392	0.800058	1.699091	2.327857	0.572266
0.453463	0.942294	0.396082	0.754784	0.488167	0.917994	0.521775	1.052208	1.603795
0.000188	8.075772	0.000967	0.956667	0.004293	0.117843	0.122063	0.000188	0.724502

0.000188	2.217951	0.000967	0.147807	0.004293	0.587273	0.24443	0.069163	0.232338
3.966623	0.56145	1.865043	3.8379	1.025613	2.485534	13.23022	3.980123	2.928247
0.051335	0.395859	0.000967	0.02316	0.004293	0.021645	0.043059	0.092302	0.355051
3.163506	0.035671	2.429957	5.381227	2.547179	0.800058	4.56912	2.873817	3.864827
0.220657	2.016515	0.683232	2.092987	0.811443	0.992201	0.61526	0.332395	0.290693
0.789863	4.637994	2.858009	4.609076	8.119004	3.711097	2.259661	1.89373	3.065108
12.13485	0.035671	9.256537	11.48368	11.30987	9.583463	3.552049	3.57057	4.592273
0.059177	21.54823	0.036241	0.041053	0.09873	0.037749	0.027691	0.03412	0.232338
0.000188	6.028427	0.000967	0.000289	0.004293	0.011032	0.006306	0.021645	0.115065
14.08569	0.534452	3.290101	4.921082	1.118066	6.404921	8.994791	11.09133	5.745996
3.775209	16.44768	1.636118	2.538218	0.960216	7.30215	4.881775	2.094574	5.821804
4.921082	1.932857	1.935685	1.828781	1.082915	6.717056	4.429769	3.676032	7.964315
1.428398	0.035671	0.612179	1.411861	0.470289	0.579495	2.417424	1.884935	0.637994
1.117316	0.589618	3.101371	4.597792	9.541645	0.976111	2.187395	1.729892	1.816436
0.000188	0.534452	0.000967	1.009625	0.149156	8.66E-05	0.705231	0.03412	0.070851
5.42381	3.8379	5.00404	0.000289	16.68554	7.625209	4.914863	6.486104	9.924372
0.137403	0.035671	0.000967	0.250476	0.004293	0.150144	0.114113	0.048023	0.383889
0.656003	6.297136	0.172612	3.460909	0.320794	1.296046	4.827691	0.288579	1.924228
17.64869	1.043608	7.38847	60.77449	11.42512	33.07147	91.05541	12.56693	126.1556
0.082027	2.189235	0.088788	0.174517	0.400534	0.451169	0.231659	0.062641	0.404704
2.621133	0.296234	4.145209	0.956667	3.711097	8.66E-05	0.677403	2.92013	0.208254
0.000188	1.710173	0.000967	0.222193	0.004293	0.783398	0.114113	0.069163	0.652807
4.764055	0.505267	3.66785	1.985253	2.65684	1.136566	1.385801	3.233045	2.928247
8.508514	0.88316	10.01009	20.34063	2.392431	5.614805	8.677489	8.381328	3.314329
1.755988	3.763045	2.911962	4.38684	5.273362	0.67632	4.105714	2.08632	0.941645
1.019134	4.38684	0.288745	0.275527	0.079019	0.821414	0.504964	0.555801	0.415166
0.405786	1.884935	0.138449	0.571674	0.004293	1.484553	0.274531	1.487619	1.11526
1.365296	0.035671	1.349971	2.289286	5.158146	0.117843	1.566414	0.577706	1.369928
3.842554	3.29355	3.080317	2.638139	1.223846	4.978182	4.739668	3.629177	4.72798
1.034315	5.366335	0.306176	2.302626	0.190844	2.157359	2.640332	1.401068	2.62342
7.545584	0.035671	6.570433	6.268312	3.201457	8.66E-05	4.392316	5.22482	0.831472
2.325931	0.276248	1.414574	3.35715	0.703665	2.715873	1.027403	3.144156	1.909408
0.546854	9.082828	0.378889	0.448824	0.190844	1.708932	0.772951	0.320563	2.107922
19.99861	0.035671	25.24367	75.1132	40.7932	8.195657	11.03843	8.888167	10.01009
7.013853	12.81638	5.180058	144.7919	3.529466	6.622092	16.80343	7.061328	3.511263
0.03842	0.979827	0.000967	0.147807	0.004293	0.011032	0.016025	0.548788	0.078586
13.30906	0.035671	15.3618	5.095325	14.25954	18.62711	13.91714	21.54823	10.53159
224.9555	1.47842	530.1465	203.5795	287.197	111.5439	287.197	224.9555	167.9996
1.741414	5.500678	1.094206	0.876118	0.642395	16.80343	1.629235	1.962424	6.374812
2.589185	0.475433	2.308203	1.009625	4.323045	0.73961	0.251248	1.226118	0.149156
5.485685	0.684805	9.541645	1.594675	7.443276	3.563182	0.905426	6.830592	2.453781
6.808369	8.940671	7.570693	11.09133	11.65791	5.754935	5.522929	7.013853	3.065108
55.04081	0.035671	100.0998	11.65791	203.5795	8.66E-05	6.760765	65.94551	6.079596
2.556443	0.395859	3.699495	3.585029	5.485685	1.684646	3.330909	2.327857	0.483608
44.27012	0.035671	40.7932	42.75576	23.16622	2.27254	37.81919	19.53061	6.171789
3.610707	6.53969	3.128694	12.42965	2.236407	5.614805	7.225469	3.286652	13.60866
0.522367	1.167561	0.421342	2.708341	0.331097	2.8657	1.528975	0.976111	2.335844
2.74544	0.035671	1.728615	2.461905	2.602395	1.195794	2.823636	2.236407	4.424416
0.369336	3.672078	0.217792	0.832554	0.770188	0.961486	2.339697	0.320563	1.392511
0.059177	0.650101	0.000967	0.5129	0.004293	0.230108	0.168456	0.000188	0.683665
14.17388	3.153485	11.77567	8.849697	11.544	11.30987	19.99861	12.93651	15.88548
12.13485	0.684805	12.85504	4.684502	3.734762	23.16622	7.225469	15.3618	12.70768
0.114437	2.08632	0.000967	0.183052	0.004293	0.037749	0.251248	0.000188	0.34619
20.16541	2.59557	16.9162	11.25392	29.82828	7.495397	11.48368	10.31025	4.881775
1.074827	0.035671	0.000967	0.876118	0.422511	0.73127	0.92482	0.469358	1.636118
0.396638	0.979827	0.172612	0.259646	0.079019	1.37531	0.504964	0.416219	0.628297
1.122359	1.837172	0.998831	2.408882	0.214964	2.069942	2.823636	0.603261	2.705916
0.051335	0.942294	0.046804	0.032251	0.16868	0.40075	0.016025	0.172294	0.20127

3.711097	3.153485	6.374812	6.945152	4.752121	1.519322	3.91355	3.824964	3.290101
3.91355	0.395859	3.116732	1.47018	1.278629	2.768312	4.343838	2.737944	3.037626
0.051335	0.806227	0.000967	0.02316	0.058139	0.382417	0.034863	0.041328	0.194531
4.535354	0.035671	8.019769	5.024293	8.048095	1.039798	3.655714	6.632063	1.983824
41.47925	7.375794	25.99427	24.50942	34.38341	29.18885	27.96248	42.08807	24.04374
4.597792	0.035671	7.013853	4.839105	14.79742	8.66E-05	8.164531	4.490801	0.941645
0.03842	56.39528	0.036241	0.137504	0.004293	0.192814	0.106926	0.03412	0.093102
2.102626	0.035671	5.110159	3.367071	8.119004	6.210375	6.570433	3.993665	1.542302
0.3607	2.126349	0.000967	0.000289	0.004293	8.66E-05	7.22E-05	0.000188	0.373463
0.074899	1.043608	0.000967	0.000289	0.108016	0.216753	0.02119	0.021645	0.20127
1.35259	5.984885	0.331869	2.203535	0.577388	2.099524	1.595729	1.360022	3.290101
0.089921	0.035671	0.10886	0.192381	0.004293	0.245469	0.231659	0.298874	0.592857
0.106118	0.321227	0.217792	0.240346	0.004293	0.73961	7.22E-05	0.055743	0.34619
10.67991	0.035671	3.230065	3.043528	1.077403	1.609654	18.33909	7.495397	2.361198
2.845036	0.035671	4.297222	4.921082	4.776407	1.775325	3.518384	6.144055	1.995714
0.92342	1.710173	0.000967	1.749307	0.004293	1.519322	1.450693	0.398629	3.195541
7.212915	0.035671	4.827691	10.90615	5.838499	2.380079	6.808369	5.266335	5.515584
0.181919	1.884935	0.067864	0.36153	0.004293	0.061025	0.197727	0.540563	0.363492
10.05618	1.227749	15.46193	7.037345	17.51961	5.881342	19.68088	15.77678	4.933781
4.101061	1.25886	8.412367	6.171789	4.058514	2.380079	4.56912	4.978182	4.808658
0.444048	1.196508	0.217792	0.658745	0.004293	0.382417	0.677403	0.407157	1.551962
7.061328	0.035671	6.55039	21.36892	14.70188	8.66E-05	29.49693	6.171789	15.56131
1.187229	0.035671	0.925801	1.196508	0.82868	1.195794	1.077403	0.840635	1.803918
3.890556	0.035671	6.632063	1.283059	32.31123	0.773766	1.228651	1.34513	1.253088
0.06715	0.395859	0.000967	0.041053	0.004293	0.655245	0.058095	0.110188	0.273434
0.017215	1.141515	0.000967	0.074978	0.004293	0.011032	0.010519	0.398629	0.005303
0.906126	0.534452	0.128853	1.159913	0.004293	8.66E-05	0.565714	1.957958	0.851659
0.840635	0.035671	2.339697	0.98474	2.353247	0.308672	0.553059	0.908773	0.039358
0.000188	0.276248	0.057128	0.065743	0.004293	0.067446	7.22E-05	0.021645	0.085974
0.024553	0.979827	0.000967	0.057128	0.004293	0.097807	0.016025	0.021645	0.018167
0.92342	1.634935	2.073232	2.109755	4.359574	2.119596	3.122915	2.073232	2.006782
1.20824	0.035671	1.380195	0.285685	0.876118	0.192814	1.060823	0.772157	0.039358
4.644098	1.404812	3.24658	2.90658	3.486436	3.435988	5.898449	3.363911	3.5257
0.92342	1.196508	1.728615	1.009625	2.845036	0.523521	1.385801	0.563499	1.215195
1.991328	0.035671	3.334199	0.342381	1.828781	1.37531	0.791371	1.174315	0.743283
1.165065	3.45404	3.962179	9.218514	3.29355	1.003016	1.097006	1.667273	0.981032
0.114437	0.321227	0.028687	0.065743	0.058139	0.08438	0.184206	0.069163	0.093102
9.121486	0.035671	7.868146	4.802655	2.602395	2.933903	7.327482	10.21618	3.314329
25.73165	4.096407	24.04374	25.99427	47.5018	19.99861	23.38378	21.74051	14.42661
0.256046	1.043608	0.000967	0.489784	0.004293	1.857677	0.299062	0.0843	0.53912
0.309668	1.25886	0.10886	0.128369	0.108016	0.392403	0.389336	0.255693	0.662237
10.35839	0.035671	3.68381	22.96463	1.707576	13.60866	7.854069	7.288788	7.264502
3.249877	0.34443	3.080317	4.198117	3.131544	2.890245	1.228651	1.738456	2.461905
1.019134	0.035671	0.160779	0.469004	0.364502	0.353016	0.686299	0.142698	0.490952
0.106118	4.272165	0.10886	0.240346	0.273088	0.323319	0.496494	0.110188	0.009019
13.96064	0.035671	6.325945	2.187395	3.086306	9.218514	4.115844	10.78056	5.47
3.175916	0.035671	8.348817	17.09911	6.135007	1.609654	10.98644	3.204632	3.655714
0.130714	8.27267	0.138449	8.590794	0.412193	0.104329	0.151847	0.194235	2.89E-05
33.4985	0.684805	27.38726	17.64869	29.18885	27.66779	59.32013	30.17192	72.69473
0.612179	0.035671	1.89373	2.884762	0.713405	0.367778	1.806595	0.908773	0.456522
0.342049	1.553074	0.182597	1.078153	0.273088	2.408882	0.504964	0.288579	3.885642
1.137244	3.980123	0.896522	0.820931	0.82868	1.428398	0.970635	1.030022	1.431414
4.33342	0.422511	15.276	4.47912	50.55949	1.354567	11.22667	15.07257	2.08013
0.250007	0.035671	2.329784	0.846342	5.622872	0.534452	3.043528	0.444762	0.724502
0.567619	1.790245	1.648066	1.418312	1.425613	0.992201	0.72044	1.436623	1.89228
0.473586	7.375794	0.421342	0.392403	0.947157	0.416219	0.605455	0.246955	1.236248
1.093434	0.035671	0.965714	0.601623	1.525729	0.669358	1.261407	1.830159	0.77925
0.130714	1.517143	0.000967	0.041053	0.004293	0.684805	0.168456	0.134834	1.296046

0.789863	0.395859	1.297763	0.448824	0.310447	1.182771	1.060823	0.772157	0.43381
749.7839	0.035671	87331.38	36.74522	1360.293	87331.38	0.223824	530.1465	47.5018
0.938911	0.203463	3.334199	0.032251	3.303925	1.833102	7.22E-05	2.304452	0.149156
0.089921	1.25886	0.000967	0.048831	0.004293	0.283535	0.160462	0.000188	0.018167
0.044964	1.016494	0.088788	0.118983	0.310447	0.11075	0.176833	0.298874	0.273434
8.381328	0.035671	39.53687	1.360022	80.6518	0.662944	3.220678	10.43066	1.626782
0.688182	0.296234	0.81544	1.389509	1.697792	0.392403	0.72044	0.469358	0.662237
0.212496	8.868932	0.10886	0.550664	0.289935	0.451169	0.412453	0.416219	0.456522
2.990851	0.776349	1.623232	3.984747	0.73912	2.361198	4.789596	1.903636	4.63189
0.000188	0.448557	0.000967	0.000289	0.159228	0.021645	0.016025	0.000188	0.085974
5.24562	0.684805	3.147294	2.90658	2.040606	5.704646	6.38456	3.303925	7.625209
1.165065	0.035671	0.998831	0.571674	3.486436	0.534452	0.53658	0.350635	1.030678
1.418312	30.0001	0.387258	1.87482	0.004293	5.309264	3.381255	0.976111	3.223882
0.03842	0.505267	0.000967	0.083694	0.004293	0.049928	0.064127	0.000188	0.319163
2.289286	0.475433	4.907929	5.662994	8.625613	3.625382	2.465916	3.095166	1.969733
1.045462	0.035671	3.864827	0.000289	1.68938	8.66E-05	3.23987	1.307417	0.335786
0.600173	1.438672	0.270491	0.98474	0.583766	1.901068	0.659163	0.784372	1.149322
11.03843	0.035671	7.519841	7.002056	7.38847	3.563182	17.28195	8.348817	2.335844
0.082027	2.189235	0.10886	0.110058	0.583766	0.016681	0.070339	0.055743	0.062944
0.000188	0.448557	0.000967	0.041053	0.004293	0.011032	7.22E-05	0.000188	0.002857
1.365296	0.422511	2.252273	2.758088	2.337821	0.985411	2.009906	1.524776	1.466111
0.181919	0.34443	0.000967	0.479401	0.004293	0.408644	0.327662	0.062641	0.383889
3.618499	0.035671	3.820758	7.288788	4.003117	0.976111	3.930664	2.602395	2.673846
0.286364	1.884935	0.396082	0.86518	0.422511	0.301241	0.327662	0.263146	0.373463
1.806595	0.035671	6.268312	20.16541	2.139928	3.011169	2.339697	1.622006	2.107922
14.88584	0.035671	11.83593	17.78522	6.830592	5.266335	17.51961	13.00996	8.445743
0.429827	2.304452	0.09855	0.157468	0.190844	0.223939	0.237359	0.12772	0.282583
6.171789	0.684805	2.201587	1.685678	1.359177	5.323492	8.815123	3.743232	11.544
2.59557	0.035671	1.623232	1.803918	3.214545	1.667273	7.037345	4.72798	1.369928
1.264589	7.443276	0.896522	3.307186	1.127475	2.42381	2.417424	0.693925	3.457504
0.295837	3.45404	0.257684	0.589986	0.139235	1.296046	0.696234	0.555801	2.961768
0.554416	0.56145	0.138449	1.052886	0.12925	0.264502	0.337222	0.255693	1.030678
19.06893	0.035671	10.28573	12.49452	8.539971	4.869553	5.522929	17.15902	12.16681
7.765931	4.990794	24.98218	8.976508	43.49658	5.881342	5.796421	7.854069	6.171789
13.53913	1.47842	13.83359	28.23632	15.276	4.402973	32.31123	7.765931	5.9071
0.000188	1.227749	0.067864	0.000289	0.004293	8.66E-05	7.22E-05	1.317475	0.039358
22.96463	0.223579	13.60866	2.078355	3.227085	10.93101	6.38456	13.53913	5.9071
6.876392	1.578687	7.938427	8.164531	4.789596	5.984885	8.119004	5.606739	8.348817
0.220657	0.321227	0.520289	0.307244	1.068348	0.160462	0.214654	0.255693	0.404704
7.911934	1.016494	31.55417	9.625253	13.83359	22.13245	3.877417	18.33909	26.25557
0.487662	1.074827	0.246162	0.803665	0.458701	0.568665	0.705231	0.219885	0.864199
0.377561	0.620014	0.046804	0.137504	0.088701	0.353016	0.05088	0.027547	0.208254
3.201457	0.035671	4.016465	0.700837	2.644885	0.183939	0.838326	4.120815	0.694675
1.137244	1.932857	0.279928	2.442987	0.299747	2.635685	0.896522	0.721732	5.024293
3.632973	0.742417	1.181342	2.668896	0.422511	3.842554	10.01009	2.666494	3.457504
0.09772	0.505267	0.046804	0.479401	0.004293	0.595714	0.151847	0.12772	0.941645
1.117316	2.477027	1.438672	2.259661	10.53159	2.000592	2.628225	0.641277	0.522071
0.051335	0.276248	0.000967	0.090693	0.058139	0.11075	0.093009	0.021645	0.282583
49.50903	0.035671	33.93939	6.433918	35.27608	8.976508	4.702468	50.55949	1.513102
1.389509	2.673846	1.228651	1.310837	1.223846	1.152944	0.304387	1.052208	1.723579
0.000188	0.035671	0.000967	0.000289	0.004293	0.067446	0.92482	0.000188	0.512641
4.266753	10.98644	1.614257	15.88548	2.160649	8.164531	33.93939	3.687633	6.933961
60.77449	0.035671	332.0776	77.74812	167.9996	8.66E-05	8.412367	53.77619	0.772157
1.604827	2.59557	4.053506	8.539971	16.5625	3.119986	2.131176	3.214545	1.723579
0.03842	0.422511	0.057128	0.5129	0.234949	0.783398	1.872049	0.21031	0.055281
3.36075	0.035671	4.875527	4.56912	6.365065	3.192229	1.655541	3.192229	4.745895
80.6518	2.74544	80.6518	32.31123	27.96248	105.1694	118.7028	95.22939	51.52195
0.024553	1.110505	0.028687	0.041053	0.051984	0.016681	0.010519	0.000188	0.031616

0.965714	1.074827	0.633146	0.448824	0.569971	2.136335	0.95101	0.65241	0.874091
0.122785	0.979827	0.000967	0.083694	0.004293	0.323319	0.214654	0.21031	0.155945
7.697063	0.035671	15.88548	2.955916	10.78056	2.768312	3.636789	5.762965	1.203427
0.734524	0.035671	0.000967	0.074978	0.088701	0.176955	0.521775	0.03412	0.107027
6.09759	0.035671	5.110159	0.658745	1.902352	1.247244	1.616385	5.568391	0.637994
3.016984	4.33342	3.435988	0.369567	5.813247	0.091255	1.1407	0.712929	0.456522
1.656696	0.035671	1.972706	1.265426	2.267157	2.933903	1.060823	2.402468	0.265786
26.25557	1.438672	20.34063	27.10502	10.98644	31.36473	18.19595	28.87506	28.54786
6.414416	0.850808	14.17388	8.711905	13.38418	12.29473	5.500678	6.079596	13.53913
0.03842	4.49671	0.149553	0.183052	0.139235	0.091255	0.375094	0.041328	0.107027
10.85542	0.296234	18.76954	2.217951	8.815123	3.422237	1.539062	14.79742	0.662237
6.622092	42.08807	0.612179	9.418196	2.633232	13.75978	6.09759	13.96064	13.75978
1.827403	1.141515	2.607035	2.018167	0.97733	0.73127	1.338261	2.057056	1.474343
1.010887	8.940671	1.191905	0.479401	1.204228	2.207417	0.553059	1.47842	0.917431
9.179466	0.035671	6.591544	3.648081	1.947316	1.667273	2.252273	12.32958	1.056263
0.585592	6.433918	0.172612	0.36153	0.118225	0.821414	0.251248	0.821898	0.751876
1.224733	0.035671	0.692381	2.839351	0.759646	0.950418	1.89518	0.612179	2.911962
0.059177	1.25886	0.000967	0.137504	0.004293	0.097807	0.389336	0.062641	0.093102
11.30987	1.074827	6.663651	2.574733	3.953297	4.997417	2.489762	7.136537	6.508427
2.770866	6.240346	0.88417	5.638759	0.770188	4.851227	1.639639	1.45912	3.998391
0.648687	1.074827	0.692381	0.420707	1.668369	1.848297	0.389336	0.506652	0.531486
0.677807	2.563506	0.246162	0.439062	0.253882	0.938911	0.504964	1.508831	1.857677
1.177309	0.979827	0.000967	0.318889	0.004293	0.83088	0.61526	0.371299	0.180895
0.137403	0.296234	0.067864	0.048831	0.178442	0.150144	0.251248	0.069163	0.039358
1.524776	0.035671	5.373781	0.560866	10.93101	2.157359	1.367179	2.496407	2.558817
0.405786	0.035671	1.601443	2.520404	4.473348	2.084791	1.728615	2.280101	3.714906
0.000188	0.223579	0.036241	0.032251	0.068896	0.011032	0.006306	0.012136	0.002857
1.027403	18.91854	0.000967	0.91404	0.599798	5.959033	1.107605	0.994271	4.764055
2.320072	0.369105	0.925801	2.432121	0.713405	4.484892	3.297107	1.524776	2.474834
2.500548	0.742417	0.877222	3.058802	0.204055	2.069942	1.742734	1.333781	4.160238
10.1459	4.043846	8.660281	6.653016	5.545729	2.524892	7.765931	8.83241	8.241342
3.002576	1.404812	3.730808	1.636118	3.679986	0.176955	1.644416	2.737944	0.652807
0.50772	2.74544	0.246162	0.157468	0.204055	0.192814	0.496494	0.27899	0.612872
7.124517	0.035671	13.15079	0.876118	10.82954	6.277937	5.130411	8.048095	15.3618
0.575628	0.776349	1.078153	0.403413	0.722179	0.192814	0.264805	0.516825	0.241688
1.977316	0.56145	1.142345	3.043528	0.845887	1.393571	1.202626	0.987821	6.749488
0.606847	3.980123	0.717879	0.212172	0.412193	0.33329	0.274531	0.150346	0.155945
0.762165	3.500808	0.536263	0.647691	0.734113	3.119986	0.713846	0.840635	1.542302
4.952915	2.984899	0.956061	6.250447	0.351789	6.171789	7.468961	3.497085	2.579509
1.861999	0.035671	1.106825	1.092136	0.634192	2.755411	2.212619	1.783203	1.079697
24.50942	0.035671	12.10289	21.54823	5.095325	0.768535	12.93651	21.02638	5.273362
2.917381	0.035671	2.569957	2.109755	3.414574	0.702381	2.187395	2.127973	1.253088
0.913514	0.035671	1.019791	1.159913	0.60886	1.256558	0.496494	0.672078	0.981032
2.304452	0.88316	0.766602	1.99899	1.679596	4.261342	3.098268	2.345527	4.039192
13.19051	2.126349	8.048095	7.351284	5.030866	10.98644	5.359134	7.162698	11.36975
0.818586	3.544257	0.465079	0.658745	0.67632	5.779574	0.357013	0.516825	3.263391
12.70768	23.49249	2.339697	5.720173	4.888023	14.33939	7.61176	14.70188	5.530274
0.269322	0.684805	0.352006	0.275527	2.189235	0.08438	0.337222	0.021645	0.188276
0.205801	1.141515	0.000967	0.137504	0.088701	0.230108	0.197727	0.119423	0.592857
25.24367	0.035671	126.1556	118.7028	184.443	21.36892	111.5439	48.53429	9.667316
0.332641	0.035671	1.039798	0.285685	0.351789	1.869322	1.1407	1.286494	1.191198
1.365296	0.035671	4.188896	1.026825	2.73272	0.546854	1.857677	0.962698	0.490952
0.000188	5.821804	0.000967	0.000289	0.004293	0.702381	7.22E-05	0.000188	0.628297
5.330519	0.035671	1.360866	7.08733	2.42381	3.783694	8.119004	2.933903	10.8813
7.737807	87331.38	10.07949	36.26061	10.48101	4.429769	10.78056	14.6058	4.424416
0.017215	0.203463	0.036241	0.074978	0.004293	8.66E-05	0.144098	0.000188	0.013117
1.524776	425.62	2.08632	9.082828	1.729892	0.382417	0.451429	1.652771	1.49075
4.739668	0.035671	13.38418	2.259661	12.77772	3.031688	3.614603	2.828918	2.54961

2.021068	6.911486	0.571342	1.496118	0.661443	3.88153	2.586926	1.794531	2.432121
1.177309	0.035671	3.195541	6.079596	4.376118	3.651898	1.077403	3.104394	0.694675
5.568391	4.217569	1.691587	8.643074	2.083261	4.155339	5.923838	4.323045	8.923117
0.396638	27.66779	0.270491	2.23267	0.888485	1.821861	0.623146	0.237698	0.619365
22.13245	7.991602	12.42965	8.745238	3.807648	29.49693	14.17388	30.82277	7.456118
0.000188	41.47925	0.000967	0.128369	0.004293	0.091255	0.093009	0.000188	0.039358
0.074899	2.355289	0.036241	0.100101	0.004293	0.132309	0.027691	0.000188	0.355051
14.25954	15.276	20.16541	3.052655	24.50942	4.620159	3.743232	14.42661	2.640332
64.12971	14.42661	9.063983	105.1694	12.22798	55.04081	51.52195	25.99427	252.4251
17.78522	14.70188	41.47925	47.1037	34.84299	22.52375	31.92385	22.32214	6.019971
111.5439	40.11561	25.73165	3.230065	10.67991	91.05541	126.1556	100.0998	37.81919
8.762677	15.41187	6.345382	24.04374	6.612121	13.15079	9.541645	4.678846	38.39042
25.48179	12.70768	19.68088	39.53687	28.87506	17.51961	31.55417	17.92188	3.211176
5.821804	252.4251	4.155339	11.83593	2.584394	5.500678	5.180058	3.644343	14.17388
332.0776	0.035671	224.9555	749.7839	126.1556	425.62	749.7839	287.197	530.1465
144.7919	43.49658	144.7919	224.9555	70.11932	167.9996	77.74812	111.5439	111.5439
72.69473	7.519841	53.77619	135.5245	91.05541	59.32013	135.5245	75.1132	35.27608
83.489	144.7919	23.82354	126.1556	24.73724	749.7839	100.0998	77.74812	184.443
1.224733	2.839351	0.790837	6.808369	1.379278	5.779574	13.60866	1.508831	13.23022
1.330029	2.693442	0.352006	1.411861	0.642395	1.519322	1.162201	0.863189	1.379278
4.207778	95.22939	1.106825	3.585029	0.913514	18.76954	8.643074	4.709033	15.18102
8.075772	1.790245	5.485685	5.9071	3.160022	10.23768	6.784762	6.53969	9.377345
0.799048	0.395859	0.10886	0.832554	0.079019	2.240274	1.217475	0.185909	2.203535
0.082027	1.790245	0.088788	0.090693	0.004293	0.104329	0.160462	0.100996	0.047633
0.600173	2.673846	0.759293	2.217951	0.289935	4.869553	1.935685	0.925801	1.566414
0.000188	0.850808	0.361342	0.571674	0.351789	0.4386	0.137403	0.0843	2.89E-05
1.806595	31.1753	3.511263	4.170051	7.882222	1.749307	2.99987	5.889798	2.604776
0.493925	224.9555	1.718918	0.222193	3.104394	0.183939	7.22E-05	1.019791	2.89E-05
11.36975	8.711905	5.431544	12.16681	3.422237	33.4985	20.68746	14.08569	17.03919
2.755411	0.035671	3.314329	2.367763	4.875527	5.295325	1.323975	4.281847	1.909408
0.332641	4.921082	0.378889	2.92013	0.214964	3.327619	0.434596	0.246955	4.222583
0.03842	0.34443	0.000967	0.174517	0.004293	0.043203	0.027691	0.12772	0.055281
0.444048	4.440548	0.172612	1.196508	0.381789	0.476551	0.292597	0.172294	0.851659
1.644416	0.035671	3.754719	0.996696	4.709033	1.848297	2.243911	1.267085	2.145137
27.66779	8.412367	31.1753	23.71237	22.13245	19.53061	31.1753	22.96463	10.98644
12.89577	2.406768	21.93452	65.94551	27.66779	33.93939	21.54823	17.28195	19.06893
7.288788	9.335397	5.28039	6.125823	5.210317	21.19547	14.51648	6.464906	25.48179
2.339697	1.141515	2.02956	5.123781	1.873434	8.608153	5.462475	2.345527	8.994791
0.789863	0.88316	0.160779	0.147807	0.178442	0.382417	1.734949	0.427403	1.304877
11.42512	1.601443	16.44768	26.80355	14.51648	19.37139	9.604358	8.745238	30.82277
0.500722	1.47842	1.50684	0.379192	0.535657	1.428398	0.753016	0.814877	0.954776
2.544747	7.327482	1.710173	2.55184	0.801501	2.018167	5.830152	2.92013	5.583968
15.3618	23.49249	6.210375	16.34294	9.792626	51.52195	15.66023	13.60866	50.55949
0.429827	5.889798	0.352006	1.480541	0.986104	0.752229	0.806652	1.006025	1.215195
0.973745	2.563506	1.420202	0.725267	0.470289	1.003016	1.765065	1.114618	0.319163
2.477027	3.91355	3.046551	14.97762	1.327525	7.251263	2.737944	2.199639	9.459177
0.500722	1.110505	0.404899	0.856782	0.50254	1.003016	0.573947	0.732193	1.89228
0.051335	3.588369	0.387258	0.540563	1.338261	0.132309	0.284863	0.255693	0.603737
0.405786	1.074827	0.119683	0.439062	0.178442	0.50469	0.7986	0.246955	0.842193
1.027403	0.742417	0.331869	2.048781	0.214964	3.166659	0.914618	0.814877	2.042201
0.436638	287.197	0.09855	1.749307	0.079019	0.237035	0.553059	0.202294	0.465563
1.074827	1.790245	0.870902	2.367763	1.881955	1.791616	1.437648	2.635685	0.879913
2.150303	6.53969	1.240981	0.886775	1.402915	8.288874	2.16759	3.718716	5.439278
1.450693	21.19547	0.487662	1.196508	0.899055	2.42381	0.668456	2.703485	11.22667
0.114437	0.035671	0.974906	0.560866	2.644885	0.192814	1.566414	0.308903	2.89E-05
0.137403	1.634935	0.331869	0.083694	1.742734	0.117843	0.168456	0.048023	0.047633
23.38378	0.620014	9.502294	18.62711	9.502294	11.19942	6.717056	23.6012	17.39935
1.275325	1.141515	0.546854	0.700837	0.866147	0.595714	0.144098	0.882078	0.643853

0.000188	0.203463	0.000967	0.000289	0.004293	0.016681	0.006306	0.03412	0.002857
0.703665	0.035671	0.797114	1.026825	1.605859	0.4386	1.655541	0.288579	0.298874
4.376118	0.035671	7.737807	5.210317	5.044113	2.157359	4.684502	3.049603	3.917597
2.882035	118.7028	0.4971	14.25954	0.390216	9.646284	19.06893	1.927042	3.544257
16.04187	0.242612	11.60283	2.818312	7.519841	1.266255	10.1028	12.16681	3.065108
0.899055	6.335664	0.342381	1.29342	1.487619	2.506934	0.979149	1.848297	2.496407
2.183665	2.74544	7.868146	6.325945	11.25392	2.768312	2.417424	2.758088	1.446926
2.116032	20.42667	2.08632	5.095325	3.548153	5.530274	1.018427	2.116032	4.101061
0.593853	23.16622	0.692381	1.903636	0.876118	1.130115	1.027403	0.427403	1.703795
0.000188	1.043608	0.160779	0.147807	0.004293	0.898543	0.058095	0.229199	1.263752
7.495397	14.70188	2.242092	12.70768	6.09759	22.74797	11.544	3.025671	14.51648
95.22939	0.979827	118.7028	252.4251	111.5439	83.489	155.7103	87.1746	224.9555
0.082027	0.806227	0.000967	0.448824	0.004293	0.487395	0.284863	0.000188	0.612872
62.33472	8.075772	45.1045	17.92188	12.29473	50.55949	42.75576	45.91841	32.68313
1.296046	2.563506	0.850325	2.217951	0.92342	2.240274	2.784228	1.064242	3.493362
0.220657	2.797835	0.4971	0.767569	0.521421	2.308203	1.000794	0.563499	1.877605
6.464906	8.745238	10.38202	5.622872	18.19595	6.978903	6.622092	6.443925	7.363117

CGGA_105	CGGA_105	CGGA_106	CGGA_106	CGGA_106	CGGA_106	CGGA_106	CGGA_107	CGGA_107
0.064372	0.338341	0.218817	1.128788	1.157626	0.557056	0.139567	0.516825	0.111876
0.08557	0.252771	0.068449	1.944517	0.319163	0.706984	1.130115	0.508658	0.241255
6.967547	2.057056	7.276645	0.179177	2.973059	1.865043	3.57057	0.359697	9.880779
3.5257	7.737807	4.086926	0.023997	2.681169	1.173535	2.209141	19.68088	17.39935
6.250447	5.500678	9.160382	0.776349	4.914863	6.784762	4.739668	10.98644	5.872886
1.093434	4.586753	2.08013	5.9071	10.82954	9.838658	2.009906	5.22482	1.525729
31.1753	18.33909	30.17192	6.181652	47.5018	21.74051	29.82828	33.07147	55.04081
0.064372	0.004242	0.020462	1.251659	0.068896	0.086775	0.020346	0.022641	8.66E-05
3.008225	3.307186	2.402468	0.252165	1.839986	1.830159	1.320173	9.792626	3.610707
0.000887	0.681919	0.274365	0.023997	0.182078	0.545808	0.563499	0.544545	0.462092
17.51961	48.53429	43.49658	72.69473	105.1694	40.11561	42.08807	20.68746	77.74812
0.580758	0.968723	0.858817	3.303925	1.783203	1.852338	3.471486	3.330909	0.473319
32.68313	5.366335	8.590794	3.394286	9.707677	5.323492	8.075772	2.421681	53.77619
15.77678	1.34267	2.984899	0.023997	1.421126	1.173535	0.000188	1.007864	17.15902
0.000887	0.01329	0.008283	1.072843	0.086883	0.010707	0.028687	0.205931	0.027922
8.477662	15.18102	8.149307	1.128788	1.34513	5.195022	1.80267	10.38202	2.259661
0.849841	1.594675	1.859084	4.077403	1.068348	3.432388	3.5257	1.613232	0.845887
0.055967	4.33E-05	4.33E-05	10.73084	0.000736	0.094841	0.151688	0.789863	0.034776
7.251263	3.014076	1.064242	0.023997	3.618499	0.153189	2.048781	0.850808	5.180058
0.421342	1.381111	0.304545	2.45575	1.262179	0.631847	0.537807	2.723247	0.749942
3.636789	1.118817	2.449509	0.846797	0.998312	0.047713	0.755253	0.982698	1.320173
5.967879	0.904286	2.735368	3.514834	1.474343	0.756176	0.469004	9.418196	3.909033
0.000887	65.94551	7.897078	0.023997	5.500678	65.94551	8.711905	56.39528	7.363117
0.033745	4.33E-05	0.044805	13.19051	0.113218	0.021645	0.028687	0.102352	8.66E-05
2.091313	2.574733	1.0386	2.542605	1.336587	3.432388	2.419553	1.396494	0.569971
9.031082	2.649675	10.8813	1.588903	4.370671	2.540462	6.808369	3.166659	7.149618
1.96404	0.162915	2.382035	0.513752	0.526299	1.08671	0.395152	0.869365	0.820447
0.178817	0.252771	0.286183	1.511198	0.534214	0.730873	0.41772	0.478182	0.336147
126.1556	42.08807	135.5245	0.023997	9.316241	19.451	11.544	7.61176	40.11561
0.649235	0.446291	0.543665	3.350224	0.614545	0.730873	0.309877	0.447085	0.241255
2.975974	2.065007	3.846984	3.998391	8.660281	1.49075	1.607143	1.369928	7.854069
0.033745	0.025483	0.096811	1.364401	0.289603	0.232338	0.193896	0.116097	0.010519
11.03843	25.99427	8.195657	0.023997	1.754733	1.592395	0.000188	5.388405	8.019769
0.033745	0.007879	0.027879	0.140418	0.235296	0.027237	0.028687	0.041097	0.034776
0.679812	6.162446	0.77925	13.53913	8.445743	12.16681	24.2842	23.16622	0.366147
5.788153	1.135137	3.38456	1.155411	3.507691	1.723579	0.000188	0.493925	2.392431
6.876392	3.119986	4.408153	4.429769	5.720173	3.273045	3.993665	0.92114	6.738211
0.055967	0.057518	4.33E-05	2.294733	0.200519	0.118644	0.028687	0.033874	8.66E-05
1.624459	3.799885	2.823636	13.6814	4.770231	4.115844	6.071436	9.297085	2.715873
3.966623	8.348817	3.407619	3.648081	9.838658	12.85504	15.46193	10.33476	4.644098
0.033745	0.623853	0.534214	8.976508	0.218045	1.340036	4.603434	3.207807	0.98956
0.98474	0.876638	1.384921	9.179466	0.810491	1.434921	1.910974	6.181652	1.65785
1.118066	0.209401	1.596782	0.023997	0.803211	0.19596	0.217316	0.338095	4.46267
0.000887	4.33E-05	4.33E-05	0.196335	0.025274	4.33E-05	0.000188	0.022641	8.66E-05
3.273045	0.302554	2.600014	0.68062	0.153016	0.814877	3.163506	0.782417	1.107605
32.31123	45.1045	50.55949	1.399315	33.07147	59.32013	27.96248	29.82828	15.07257
0.201645	0.041053	0.12324	0.899567	0.086883	0.214401	0.182597	0.128759	0.380649
0.000887	0.033312	4.33E-05	0.776349	0.252619	0.252165	0.193896	0.234949	0.034776
0.000887	4.33E-05	0.004329	0.543939	0.000736	0.033593	0.013867	0.000188	8.66E-05
0.000887	0.131659	4.33E-05	0.252165	0.104149	0.308672	0.182597	0.316126	0.041443
0.000887	0.107626	0.008283	0.196335	0.000736	0.054517	0.099913	0.022641	8.66E-05
7.112496	11.96574	9.751039	1.009625	9.063983	10.23768	4.684502	31.92385	6.210375
0.074177	0.172063	0.053203	0.614545	0.095685	0.205931	0.429827	0.273911	8.66E-05
0.074177	0.24443	0.096811	0.434863	0.162468	0.078586	0.406212	0.50127	0.29798
2.593506	0.399545	1.08671	0.543939	1.329206	0.453218	1.143954	0.596775	3.211176
0.000887	0.841746	0.18324	0.023997	0.765094	0.718355	0.628297	0.740527	8.66E-05
0.193723	0.235296	0.218817	2.944416	1.795851	0.327143	0.790837	0.385238	0.493247

0.542525	0.573947	0.637994	2.263377	0.748341	1.379278	2.009906	2.339697	1.434055
1.252424	5.01088	1.707576	1.43039	3.080317	2.970144	1.4671	2.089639	2.459812
0.679812	0.573947	0.847908	0.776349	0.643853	0.335786	0.976667	0.213218	0.958557
0.000887	1.844098	0.068449	14.17388	1.182771	1.574517	0.000188	0.260584	8.66E-05
0.719942	0.346342	0.479401	0.379495	0.153016	0.645036	0.099913	0.260584	0.292597
2.124574	0.85728	1.242641	10.07949	0.458521	0.718355	2.141595	1.279452	0.504416
72.69473	47.5018	87.1746	0.278499	29.18885	11.89932	11.719	3.446732	29.49693
0.144098	0.018788	0.274365	0.724004	0.078038	0.308672	0.193896	0.338095	0.316126
0.000887	4.33E-05	0.004329	0.128687	0.000736	4.33E-05	0.020346	0.086595	0.021126
0.838846	2.413146	0.57197	0.543939	1.988196	0.891176	0.88158	2.487576	1.65785
2.491948	1.997352	2.248896	0.278499	1.451775	0.93267	1.485483	3.113478	2.955916
0.380851	0.437576	0.191486	0.379495	0.632857	0.318499	1.039199	0.205931	0.366147
0.08557	0.107626	0.15241	2.944416	0.29842	0.439776	0.126659	0.34443	0.091306
0.000887	0.24443	4.33E-05	1.663672	0.000736	0.426118	0.469004	0.227063	0.021126
0.042143	0.191602	0.105498	0.749481	0.440801	0.439776	0.257143	0.461811	0.136061
0.033745	0.007879	4.33E-05	0.128687	0.032799	0.010707	0.020346	0.000188	0.006984
0.000887	0.007879	0.004329	0.379495	0.041782	4.33E-05	0.000188	0.017879	8.66E-05
9.792626	7.251263	10.1459	0.304387	3.820758	9.924372	12.46209	25.24367	14.33939
0.113218	0.252771	0.173723	1.826147	0.227648	0.308672	0.099913	0.10956	0.059091
0.000887	4.33E-05	0.078124	0.230289	0.162468	0.069365	0.068384	0.219307	0.047569
4.198117	6.601833	6.268312	1.04228	5.217569	8.590794	2.227273	2.544747	1.901068
0.000887	0.033312	0.027879	0.379495	0.068896	0.163651	0.339004	0.838846	0.073038
0.499495	0.55215	0.617763	1.547734	0.846342	0.744113	0.429827	0.41026	0.10469
9.141299	3.256587	4.851227	0.802035	4.125786	3.207807	1.638499	4.353925	5.898449
0.000887	0.01329	0.044805	0.304387	0.000736	0.040375	0.037186	0.041097	0.041443
0.113218	0.302554	0.173723	1.883535	0.653203	1.323131	1.21215	0.41026	0.23303
56.39528	49.50903	17.78522	0.023997	3.283139	3.104394	18.33909	2.371833	33.4985
0.103088	0.201789	0.132799	0.929286	0.104149	0.512309	0.182597	1.327525	0.316126
0.868853	0.977994	1.018427	5.088694	0.934488	0.766602	0.707475	0.628658	0.155101
6.116638	1.089394	4.25658	0.463485	0.078038	1.264589	6.695887	1.640779	2.871212
0.042143	0.406912	0.020462	0.981558	0.384798	0.040375	0.113911	0.534452	0.161962
0.042143	0.065094	0.035671	0.86987	0.374365	0.297807	0.068384	0.140924	0.027922
0.620014	0.446291	0.391674	0.023997	0.200519	0.242893	0.406212	0.149423	0.958557
10.43066	12.85504	9.031082	0.023997	5.993045	3.507691	4.508196	0.385238	15.77678
0.283709	1.560779	0.235613	0.701818	0.235296	1.49075	0.578095	0.055671	0.258644
0.380851	0.517092	0.173723	0.776349	0.400354	2.084791	0.86518	2.034242	0.512309
0.266277	1.582915	0.708124	6.8421	7.002056	5.583968	6.09759	3.953297	0.995527
0.000887	4.33E-05	0.008283	2.45575	0.153016	0.327143	0.037186	0.033874	0.015685
2.792035	3.548153	3.113478	2.199639	16.5625	4.580938	4.739668	3.435988	5.788153
0.033745	0.098629	0.008283	0.543939	0.086883	0.118644	0.139567	0.077417	0.006984
0.033745	0.004242	0.027879	2.003723	0.059877	0.040375	0.047468	0.033874	0.015685
0.026609	4.33E-05	0.014084	0.230289	0.025274	0.016104	0.013867	0.000188	8.66E-05
1.531313	1.950519	1.205685	3.077316	0.467677	1.948918	2.24562	2.371833	1.569553
3.4679	3.307186	1.898153	0.023997	3.7586	3.966623	5.22482	4.709033	11.30987
0.299336	2.302626	0.718355	0.981558	3.877417	1.673254	4.25658	1.147807	2.703485
0.103088	0.122944	0.004329	1.399315	0.289603	0.174444	0.737403	0.566104	0.409098
1.816436	0.181068	0.917994	0.543939	0.748341	0.535029	0.395152	0.805801	1.675722
0.103088	0.354798	4.33E-05	0.326912	0.282424	0.354545	0.294711	0.902403	0.065772
0.328737	0.427937	0.499206	2.561162	0.122554	0.956667	2.289286	0.134589	0.415729
3.397706	1.950519	3.034747	0.463485	1.63039	0.163651	0.000188	0.516825	4.796126
0.706053	0.615996	0.654423	0.488167	0.593434	2.150303	3.548153	1.622006	1.107605
1.624459	4.246861	2.145137	1.186457	2.506934	4.72798	1.938759	2.312085	1.270317
1.89373	2.353247	0.687431	0.023997	1.833102	2.531515	1.178939	8.643074	0.958557
0.587965	0.615996	2.276407	2.294733	2.337821	5.24562	4.833398	1.593535	0.845887
80.6518	0.821414	36.26061	0.463485	7.780786	4.33E-05	1.306667	0.000188	46.7056
1.439704	0.784372	0.836537	0.614545	2.234538	0.645036	0.652807	0.000188	1.959466
0.000887	0.226883	0.105498	0.350274	0.041782	0.380649	0.193896	0.157619	0.078528
105.1694	17.15902	23.93364	0.023997	0.000736	4.33E-05	0.000188	0.493925	111.5439

0.095224	0.025483	0.439062	3.303925	0.433131	0.163651	0.676999	0.121818	0.241255
0.000887	0.670519	4.33E-05	1.128788	0.346732	0.766602	0.268961	0.140924	0.059091
1.464214	1.603795	2.535974	37.29684	2.465916	2.786898	1.872049	3.207807	1.542302
17.78522	5.872886	23.6012	5.00404	10.38202	6.250447	16.9162	1.291825	28.87506
0.08557	0.841746	0.014084	4.251818	0.200519	1.660216	0.000188	0.551573	0.034776
6.268312	13.15079	8.445743	17.64869	10.45584	11.48368	7.288788	6.808369	4.261342
0.283709	1.178939	0.647345	2.263377	3.529466	2.713319	3.471486	2.214372	0.467136
1.270317	3.726854	2.174733	2.489762	5.102742	7.795642	4.039192	3.718716	1.204228
0.178817	5.720173	1.02798	56.39528	2.323983	20.85876	5.351934	16.22108	0.352006
0.838846	0.406912	1.458038	9.751039	0.384798	1.173535	0.92816	0.34443	0.813261
0.033745	4.33E-05	0.027879	0.278499	0.086883	0.006825	0.164401	0.173975	0.021126
0.2914	0.543939	0.376782	0.776349	1.250895	0.48583	0.309877	0.048434	0.059091
0.712929	0.640606	0.73127	1.399315	0.095685	0.403175	2.009906	2.500548	0.802395
2.278254	10.95872	0.788369	1.883535	11.544	7.013853	3.195541	4.690159	2.347439
0.133939	0.162915	0.340866	2.396429	0.182078	0.174444	0.664026	0.06912	0.373218
3.182482	0.796234	2.153535	1.155411	2.737944	1.558571	2.628225	0.439062	1.189769
0.550072	0.829192	0.511356	2.489762	1.274481	0.664423	1.542302	1.728615	0.95101
0.000887	0.025483	0.014084	0.379495	0.050577	0.047713	0.037186	0.077417	8.66E-05
2.269134	3.518384	1.677042	2.944416	2.898196	3.446732	3.651898	8.089848	2.630729
0.719942	1.278629	0.873579	0.023997	0.730873	0.581645	0.205382	0.385238	0.882619
0.000887	0.041053	0.096811	6.250447	0.113218	0.287951	0.497424	0.157619	8.66E-05
1.260635	0.573947	3.13456	0.350274	2.947287	1.03189	2.171429	2.600014	1.602619
2.047157	0.302554	1.87899	4.281847	2.92013	1.133622	4.978182	6.135007	3.092229
6.52899	1.535722	3.340361	1.009625	4.946587	1.283911	3.283139	1.613232	4.563117
2.165851	0.743283	2.106089	0.023997	0.408644	0.569625	0.457431	1.405772	1.250895
2.359264	3.38456	2.62342	14.93173	4.272165	6.277937	5.22482	5.993045	4.039192
2.66404	5.560563	2.126349	4.845166	5.788153	8.210938	4.978182	7.854069	3.87329
2.187395	1.059553	1.219019	0.023997	1.222958	2.498478	0.000188	0.000188	0.904286
0.252316	0.181068	0.824885	0.636291	2.278254	0.545808	0.028687	0.027691	0.155101
2.868456	1.670786	1.458038	0.278499	1.684646	0.287951	0.850325	3.792266	7.276645
8.849697	5.273362	12.70768	1.547734	38.39042	11.42512	8.994791	45.1045	5.24562
7.570693	1.214315	1.634935	0.023997	0.000736	4.33E-05	0.000188	0.316126	8.66E-05
12.36443	5.044113	16.68554	2.199639	12.22798	10.53159	21.93452	6.277937	25.48179
7.697063	2.542605	4.140505	0.636291	7.911934	3.286652	2.917381	11.77567	10.23768
0.08557	0.04886	0.035671	0.981558	0.032799	0.027237	0.020346	0.198211	8.66E-05
3.095166	2.205476	1.426522	0.023997	0.998312	1.434921	0.850325	0.583369	2.917381
0.82772	6.808369	2.489762	4.519048	6.443925	9.237525	9.335397	17.34065	3.422237
0.744531	1.34267	0.499206	3.104394	0.89404	1.592395	0.351595	0.575628	0.123853
0.103088	0.561126	0.105498	0.956061	0.252619	0.242893	0.037186	0.359697	0.10469
2.890245	3.783694	3.78798	1.721371	2.417424	2.150303	4.091667	0.992771	4.965801
40.11561	1.865043	4.33E-05	0.023997	0.458521	4.33E-05	0.000188	0.000188	3.511263
70.11932	21.93452	53.77619	0.023997	20.85876	9.179466	4.353925	2.171429	64.12971
0.436017	0.91575	1.368167	3.606804	2.572309	2.002193	5.056421	1.213434	1.626782
0.133939	0.065094	0.078124	0.159639	0.458521	0.222698	0.244589	0.25197	0.010519
0.98474	2.018167	0.931457	0.463485	2.839351	1.643218	0.90013	1.114618	0.409098
3.324452	3.256587	1.96697	0.023997	0.449408	2.644885	0.099913	2.434444	1.708932
0.000887	0.506328	0.246825	1.826147	2.261573	0.308672	0.962698	1.04228	0.820447
0.000887	4.33E-05	3.407619	6.64254	3.24658	1.685678	7.854069	0.838846	8.66E-05
17.39935	27.24614	13.45939	1.283911	8.994791	5.745996	10.53159	5.915469	29.82828
0.649235	1.486551	0.068449	0.159639	0.873579	1.122359	0.244589	1.213434	0.147706
0.000887	0.201789	0.068449	2.396429	1.656696	2.363442	1.757244	0.393983	8.66E-05
2.201587	8.429055	6.454416	15.9372	14.25954	24.2842	25.24367	22.52375	4.170051
1.065584	2.820974	0.917994	0.159639	3.046551	1.323131	1.542302	2.304452	1.954993
0.064372	0.098629	0.200794	3.435988	0.715815	2.518124	0.613211	0.877807	0.111876
0.21645	0.640606	0.675556	5.395584	0.172453	1.323131	0.628297	0.92816	0.065772
2.078355	2.382035	2.970144	0.543939	3.263391	4.025267	0.523983	4.802655	2.331825
0.36114	0.081667	0.027879	0.543939	0.218045	0.163651	0.047468	0.468694	0.181674
0.123535	0.122944	0.078124	0.513752	0.862561	0.591912	0.231984	0.28026	0.053672

1.260635	0.454394	2.351299	0.023997	0.122554	0.48583	0.113911	0.094841	0.709322
3.070952	10.28573	2.735368	0.023997	4.236962	0.631847	0.000188	5.062626	1.636118
3.507691	5.754935	5.210317	7.136537	3.13456	5.351934	7.327482	11.63037	3.13456
15.56131	21.02638	27.10502	0.023997	25.73165	25.99427	12.77772	7.795642	16.34294
1.701616	1.844098	1.08671	0.179177	2.494177	0.906825	1.161501	0.234949	2.132756
12.49452	2.873817	2.502619	0.023997	4.690159	5.137042	5.599228	7.545584	7.598312
0.2914	0.181068	0.499206	3.230065	0.77925	3.031688	1.680895	0.956061	0.155101
7.38847	7.810058	8.288874	0.023997	8.797619	4.007583	2.802828	5.266335	5.309264
0.225823	5.062626	0.888485	8.556703	4.597792	6.518709	3.939307	12.85504	0.336147
9.377345	9.012937	9.562554	3.350224	11.48368	10.01009	8.075772	11.63037	7.6393
0.308442	1.548723	0.376782	2.604776	0.934488	3.286652	1.542302	5.323492	0.23303
10.98644	16.44768	7.598312	5.830152	10.78056	16.68554	13.91714	17.51961	3.107417
1.762713	0.821414	0.560325	0.023997	0.614545	0.545808	0.737403	0.588954	0.632569
4.907929	3.934986	2.66404	0.408175	2.876421	2.294733	2.628225	3.833247	2.579509
0.000887	0.131659	0.191486	3.022713	0.687807	0.674163	0.244589	0.378066	8.66E-05
2.752893	2.160649	0.824885	0.023997	0.954141	3.855952	0.830289	1.303045	1.755988
1.96404	0.373218	1.099762	0.543939	0.90368	0.426118	0.809481	0.102352	2.331825
0.524646	4.086926	1.302107	2.944416	2.138131	4.47912	2.353247	3.486436	0.852172
0.000887	0.025483	4.33E-05	0.543939	0.000736	0.110642	0.028687	0.06912	8.66E-05
7.625209	1.798535	4.077403	0.023997	1.670786	0.143709	0.000188	0.88619	7.558139
6.612121	7.882222	6.497266	1.944517	12.42965	4.312251	5.266335	2.255916	6.230245
1.006623	0.670519	0.352006	0.023997	1.378362	0.591912	0.809481	1.173535	1.614257
0.000887	0.566782	4.33E-05	2.794935	0.400354	0.308672	0.164401	0.425844	8.66E-05
10.53159	6.162446	5.728615	2.858009	2.438831	4.096407	5.11697	5.165599	4.451154
7.443276	7.683766	3.188918	0.023997	10.05618	3.695815	2.304452	2.248896	6.876392
0.000887	0.11531	0.096811	2.638139	0.269141	0.581645	0.268961	0.92816	8.66E-05
0.235931	0.24443	0.078124	0.570664	0.182078	0.766602	0.613211	0.732633	0.435382
2.74544	2.427792	1.677042	1.364401	1.235346	0.34544	1.889711	0.838846	1.306667
0.763564	3.432388	0.523211	2.003723	1.317475	1.763889	2.973059	1.303045	1.739935
0.868853	0.191602	0.162915	5.447114	1.094206	0.86518	0.339004	1.369928	0.084524
3.019848	0.960866	7.697063	0.379495	5.687879	1.434921	3.083319	4.007583	10.48101
0.235931	0.381061	0.391674	3.269913	1.111977	1.215195	1.082179	0.534452	1.243442
0.123535	0.235296	0.087734	0.513752	0.162468	0.318499	0.281068	0.351595	0.188124
6.808369	2.820974	7.697063	0.023997	4.580938	6.054242	0.790837	0.675938	5.28039
0.193723	1.089394	0.439062	12.03267	1.969733	2.970144	2.394416	0.774257	0.223939
5.18754	2.802828	2.174733	0.802035	2.08798	1.982208	1.29267	0.309444	2.703485
0.000887	0.04886	0.044805	0.350274	0.025274	0.094841	0.000188	0.013723	0.027922
0.000887	0.181068	0.060649	0.68062	0.643853	0.222698	0.755253	0.055671	8.66E-05
0.000887	4.33E-05	0.078124	1.456955	0.059877	0.060426	0.099913	0.25197	8.66E-05
0.800058	0.399545	0.760866	0.023997	0.337641	0.414242	0.164401	0.205931	3.243225
0.042143	1.018427	0.256205	48.53429	1.40772	7.737807	3.807648	4.06303	0.059091
18.62711	12.36443	21.74051	25.24367	91.05541	15.46193	31.55417	7.288788	72.69473
20.85876	22.13245	21.19547	2.638139	11.60283	19.22139	30.17192	21.93452	21.02638
1.076032	1.304877	2.091313	16.39531	1.969733	1.938759	4.039192	1.436623	1.008961
2.101075	1.748016	1.189769	1.009625	4.007583	1.240981	1.58645	3.307186	3.68381
0.000887	4.33E-05	4.33E-05	4.758088	0.408644	1.059553	0.193896	1.779423	8.66E-05
0.308442	0.28746	0.18324	0.278499	0.086883	0.308672	0.217316	0.198211	0.20969
45.91841	13.75978	80.6518	0.023997	0.000736	5.838499	2.141595	12.49452	24.2842
2.378124	0.95101	3.286652	0.86987	0.643853	3.807648	1.161501	1.65785	6.316328
1.048802	3.414574	1.535722	1.626782	3.957734	2.581861	1.80267	3.783694	2.068297
1.568564	3.695815	3.166659	22.13245	5.180058	12.77772	10.98644	12.93651	0.890065
0.780519	0.427937	0.488759	0.023997	0.504098	0.263146	0.443543	0.330866	0.493247
26.25557	0.655613	15.3618	0.023997	0.77925	1.229553	0.126659	0.000188	0.188124
51.52195	24.98218	33.93939	0.023997	1.068348	5.195022	0.693925	0.000188	8.66E-05
0.000887	0.28746	0.027879	1.102691	0.068896	0.392944	0.151688	0.173975	0.065772
184.443	167.9996	252.4251	0.023997	52.56128	19.06893	9.52197	3.113478	23.16622
0.649235	0.528196	2.106089	8.075772	4.709033	0.956667	1.948918	0.850808	2.987835
2.337821	5.123781	1.898153	0.023997	3.034747	2.463911	1.178939	2.810678	2.021068

0.350455	1.723579	0.087734	0.776349	2.427792	0.664423	1.559675	0.493925	0.258644
1.563045	1.006623	1.974228	0.379495	4.125786	2.112843	4.626025	1.491977	3.820758
3.489899	1.415469	3.614603	0.023997	1.484553	0.93267	0.578095	1.157626	2.671299
144.7919	11.42512	13.75978	0.023997	0.516061	0.591912	0.000188	0.149423	32.31123
91.05541	16.22108	56.39528	0.023997	13.15079	16.15795	0.000188	7.911934	52.56128
0.000887	3.636789	4.33E-05	6.978903	1.595729	1.898153	5.508131	0.454978	1.175094
0.390216	1.243442	0.418788	1.484553	1.1407	5.671126	1.559675	1.002215	0.478413
0.113218	1.445916	0.617763	2.981962	1.514199	1.541227	1.828781	0.165981	8.66E-05
0.033745	0.007879	0.014084	2.003723	0.086883	0.143709	0.078297	0.041097	0.034776
0.000887	1.89518	0.367778	1.04228	0.922879	4.353925	2.227273	2.136335	0.802395
0.178817	0.107626	0.053203	1.456955	0.542165	0.143709	0.41772	0.128759	0.027922
40.7932	9.604358	3.939307	0.513752	0.000736	4.33E-05	0.000188	0.013723	18.91854
3.340361	1.041025	5.942294	0.434863	1.595729	4.058514	1.623232	1.481631	1.306667
0.055967	0.209401	0.096811	1.511198	0.355051	0.473853	0.151688	1.213434	0.059091
1.085224	1.798535	0.824885	2.333867	2.055289	3.330909	1.92272	1.766248	0.482706
5.266335	3.38456	0.824885	0.023997	2.850058	2.097843	3.8379	5.295325	1.486551
3.381255	3.131544	3.566753	22.42294	3.435988	10.65365	8.180094	22.32214	4.150274
0.726472	0.876638	0.499206	3.350224	1.880375	2.933903	1.518232	4.580938	0.95101
0.868853	0.427937	0.663276	8.134084	0.516061	2.325931	3.414574	2.234538	0.473319
0.726472	0.661443	1.340036	1.511198	1.705007	1.962424	1.306667	2.6143	0.974257
3.820758	0.81228	1.778153	0.179177	3.799885	5.388405	1.436623	3.236457	1.243442
0.299336	1.364401	0.873579	6.978903	1.460051	5.24562	6.39474	2.609293	0.073038
48.53429	40.7932	27.66779	0.023997	2.659214	7.519841	0.850325	1.87482	11.96574
0.000887	0.04886	4.33E-05	1.547734	0.162468	0.242893	0.057857	0.048434	0.015685
0.193723	2.094574	0.418788	24.39681	1.361789	10.65365	2.081696	11.30987	0.117734
3.953297	4.933781	2.261573	0.023997	4.875527	5.00404	1.680895	1.702706	4.072475
0.000887	0.089769	0.008283	0.350274	0.113218	1.046703	0.640289	0.850808	0.117734
26.52537	17.78522	19.06893	0.956061	19.83727	13.45939	9.771833	5.959033	30.82277
6.475505	4.246861	5.993045	1.626782	3.989206	3.266782	2.289286	2.950173	3.022713
0.000887	0.536263	0.816479	0.023997	5.622872	2.818312	0.523983	0.724502	0.117734
0.08557	0.057518	0.020462	2.032706	0.095685	0.276609	0.113911	0.322684	8.66E-05
18.76954	11.19942	31.55417	18.48514	12.56693	18.62711	30.49289	18.91854	11.89932
1.330029	2.263377	2.535974	0.463485	6.443925	1.250058	2.009906	0.385238	1.107605
38.94854	111.5439	42.75576	25.73165	203.5795	100.0998	91.05541	70.11932	26.25557
0.225823	0.311876	0.264048	3.160022	1.235346	0.569625	0.720938	0.468694	0.167273
1.429365	2.205476	6.079596	9.256537	18.48514	10.82954	3.548153	5.728615	1.997352
16.68554	17.39935	19.68088	5.316378	12.63773	9.502294	9.52197	4.655916	20.68746
0.08557	4.33E-05	0.162915	0.023997	0.363492	0.252165	0.88158	0.295671	8.66E-05
0.133939	0.025483	0.044805	0.159639	0.000736	0.054517	0.020346	0.685931	0.059091
4.440548	1.50026	1.253752	0.023997	1.022338	0.591912	1.3829	0.557763	2.978968
9.297085	20.68746	26.80355	3.022713	5.788153	34.84299	6.663651	8.923117	16.9162
0.042143	0.04886	0.020462	0.592532	0.000736	0.060426	0.013867	0.055671	8.66E-05
1.382006	0.73583	1.384921	0.408175	0.068896	0.68557	0.946638	0.330866	1.029365
0.244964	0.269892	0.57197	1.547734	0.319163	0.69583	0.613211	0.213218	0.55645
0.055967	0.122944	4.33E-05	0.956061	0.068896	0.252165	0.990909	0.468694	0.452489
2.535974	9.256537	8.477662	2.604776	14.79742	20.16541	15.56131	15.41187	2.485534
0.33969	1.334762	0.329798	3.695815	1.684646	2.752893	2.955916	1.266255	0.155101
14.08569	8.075772	12.22798	2.944416	5.606739	8.923117	4.875527	2.958773	5.095325
36.26061	59.32013	57.84882	4.563117	95.22939	70.11932	72.69473	39.53687	18.12541
0.000887	0.065094	4.33E-05	6.325945	0.000736	0.054517	0.381789	0.06912	8.66E-05
0.064372	0.033312	0.020462	1.456955	0.132626	0.214401	0.013867	0.086595	0.097547
37.81919	26.80355	46.7056	6.695887	20.68746	55.04081	52.56128	67.98069	19.99861
2.654473	2.755411	2.970144	3.077316	3.034747	2.150303	2.419553	0.583369	1.250895
0.186356	0.172063	0.162915	1.217475	0.374365	1.003016	1.009625	0.402381	0.111876
0.36114	1.34267	1.284747	34.84299	1.724805	1.880375	4.140505	3.930664	0.286522
0.225823	0.763102	0.873579	0.379495	0.707475	1.323131	2.802828	3.256587	0.316126
2.569957	4.135801	3.211176	35.27608	13.75978	10.05618	5.838499	6.508427	2.365685
0.244964	0.329798	0.246825	0.956061	1.578687	1.340036	0.773766	2.003723	0.23303

11.48368	62.33472	22.52375	67.98069	44.27012	105.1694	70.11932	72.69473	17.78522
0.042143	0.073038	0.053203	1.217475	0.041782	0.094841	0.523983	0.055671	0.010519
5.137042	1.445916	5.687879	1.43039	1.052208	1.763889	3.26	2.421681	4.47912
0.000887	0.033312	0.008283	0.899567	0.025274	0.102352	0.020346	0.013723	0.006984
0.487662	1.985253	0.617763	40.11561	1.111977	6.570433	2.65684	8.048095	0.540902
1.746724	6.853608	3.734762	9.52197	12.49452	13.26964	10.80505	30.82277	3.195541
3.116732	3.207807	3.8121	18.62711	4.198117	6.830592	10.43066	7.824473	2.162381
0.08557	0.640606	4.33E-05	14.25954	0.000736	0.730873	0.652807	0.566104	0.010519
10.78056	3.934986	5.373781	0.023997	1.58645	3.052655	3.26	0.288579	16.80343
1.429365	0.81228	1.228651	3.55583	1.190491	2.65684	1.50026	1.514199	2.730072
0.152749	0.488997	0.235613	4.365224	0.516061	1.839986	2.449509	1.934271	0.563499
15.276	12.1974	24.98218	0.379495	16.9162	6.135007	3.711097	14.17388	31.55417
1.05824	0.939408	1.302107	0.326912	1.385801	0.242893	0.294711	0.724502	1.226934
1.737294	1.05088	0.903074	0.023997	0.77925	0.976667	0.55215	0.128759	1.739935
0.607273	1.364401	1.171349	2.45575	0.032799	4.370671	0.809481	0.418557	0.097547
37.29684	14.25954	35.27608	17.92188	49.50903	15.71851	30.82277	6.135007	21.54823
6.570433	3.080317	3.188918	4.034423	31.1753	2.061926	1.607143	0.575628	17.28195
0.471025	1.325743	0.654423	0.179177	0.050577	1.399315	1.268672	1.953535	0.366147
2.378124	10.1028	5.150693	21.02638	6.404921	15.3618	9.707677	17.78522	2.127973
59.32013	42.75576	18.91854	1.456955	35.27608	0.380649	10.57984	0.724502	35.776
2.944416	4.145209	3.201457	2.45575	0.425426	2.394416	15.98892	0.805801	8.103925
0.33969	0.478737	0.286183	0.179177	0.913514	0.276609	0.257143	1.053528	0.323752
1.145426	5.238701	2.106089	1.721371	12.85504	7.315512	7.653391	4.580938	2.400455
4.508196	1.612056	5.095325	0.434863	0.475729	0.392944	6.39474	23.6012	67.98069
11.96574	7.570693	13.60866	0.023997	3.314329	13.26964	8.288874	13.6814	8.923117
7.977958	4.869553	5.485685	15.56131	5.395584	11.60283	8.241342	5.754935	7.200505
0.726472	1.351573	0.769711	7.824473	3.207807	3.540606	3.5257	2.089639	1.260635
0.033745	0.01329	0.008283	0.592532	0.025274	0.102352	0.028687	0.022641	8.66E-05
21.36892	19.37139	17.28195	17.78522	27.38726	40.7932	21.36892	21.02638	12.93651
6.345382	11.96574	5.614805	3.606804	1.861999	4.752121	6.663651	3.4679	4.575123
1.145426	5.082063	1.816436	0.846797	6.717056	0.824416	1.009625	4.429769	0.898016
4.597792	6.570433	4.424416	0.68062	4.990794	6.116638	2.394416	1.462143	2.053521
5.095325	7.710361	4.067547	0.513752	6.663651	4.072475	6.53969	2.810678	5.439278
0.252316	0.354798	0.18324	0.802035	0.392749	0.601227	0.457431	0.316126	0.42202
0.074177	0.155101	0.014084	4.160238	0.059877	0.287951	0.028687	0.198211	0.034776
0.259033	0.960866	0.663276	0.981558	0.653203	0.920483	1.143954	1.421126	0.709322
0.904856	1.144733	0.973745	3.606804	1.957958	2.229048	2.973059	2.171429	1.052208
0.000887	0.181068	0.534214	2.794935	3.66785	1.660216	1.860491	0.493925	0.487915
87.1746	16.68554	42.08807	2.134545	16.68554	4.72798	7.443276	4.034423	50.55949
2.359264	0.302554	1.128131	0.379495	3.373983	0.308672	0.351595	0.086595	0.807619
2.453781	1.883535	2.159004	0.899567	0.534214	0.824416	5.439278	4.46267	2.225498
1.485483	4.353925	2.463911	30.49289	2.602395	7.824473	4.933781	9.502294	1.450693
1.975772	1.997352	2.055289	0.513752	7.327482	6.316328	8.888167	8.256465	2.602395
2.349351	2.556443	6.760765	4.620159	3.233045	9.297085	3.8379	2.320072	3.87329
6.287561	14.97762	11.48368	25.48179	30.82277	38.94854	29.49693	32.31123	11.42512
29.49693	5.560563	23.16622	0.278499	5.01772	13.38418	111.5439	203.5795	75.1132
4.667518	1.144733	2.852677	7.38847	5.302294	0.766602	1.723579	0.740527	3.33728
5.068831	0.73583	0.973745	0.592532	1.061457	0.622092	0.90013	0.149423	5.137042
4.419062	6.52899	6.171789	0.592532	8.033932	7.443276	2.74544	2.693442	6.663651
1.099762	1.817828	1.618773	3.55583	2.898196	3.578038	2.009906	2.703485	3.211176
0.436017	0.338341	0.488759	1.972706	0.142886	0.134942	0.481602	0.048434	0.941645
0.074177	4.33E-05	0.014084	1.251659	0.000736	0.054517	0.057857	0.048434	0.065772
7.532713	5.704646	10.55571	0.023997	4.637994	6.978903	13.53913	6.808369	8.780115
16.80343	10.95872	17.39935	7.468961	33.93939	13.96064	12.46209	5.210317	17.03919
0.042143	4.33E-05	0.008283	0.196335	0.025274	0.004524	0.013867	0.013723	0.027922
0.048889	0.025483	0.035671	1.072843	0.218045	0.185166	0.326017	0.30215	0.141378
7.938427	31.55417	10.19468	91.05541	70.11932	53.77619	23.38378	95.22939	20.34063
0.123535	2.229048	1.472208	4.429769	1.41368	1.046703	8.461703	6.088593	0.303896

0.225823	0.004242	0.246825	1.339091	0.000736	0.134942	0.174444	0.077417	0.065772
8.075772	17.92188	7.625209	64.12971	62.33472	27.10502	32.68313	55.04081	11.48368
2.763261	5.309264	4.739668	4.592273	6.612121	5.704646	6.181652	9.101674	3.107417
0.048889	0.007879	0.014084	4.808658	0.095685	0.102352	0.086991	0.372078	0.181674
0.36114	0.235296	0.12324	11.719	0.922879	0.674163	1.039199	2.124574	0.277417
0.328737	0.381061	0.367778	0.570664	2.005253	1.046703	0.481602	2.911962	0.188124
28.54786	95.22939	49.50903	224.9555	60.77449	144.7919	155.7103	224.9555	18.48514
4.198117	9.141299	5.044113	14.04491	5.553146	14.42661	14.93173	14.6058	3.166659
9.101674	16.5625	15.98892	17.03919	21.02638	24.50942	53.77619	28.54786	8.412367
4.381479	1.291825	2.392431	0.023997	1.397482	0.965714	0.613211	0.838846	2.567605
100.0998	332.0776	287.197	7.037345	77.74812	111.5439	224.9555	20.51271	5.671126
13.23022	18.91854	19.37139	14.04491	14.51648	14.88584	19.99861	15.1268	9.968355
2.337821	2.690823	1.634935	7.037345	4.179762	4.620159	4.272165	1.89373	0.540902
2.337821	1.229553	3.672078	2.890245	10.73084	1.03189	3.046551	0.418557	2.242092
2.065007	3.031688	2.678781	22.42294	4.348882	2.581861	4.783001	1.222179	2.485534
5.439278	10.53159	8.075772	2.23267	10.1459	14.17388	16.68554	13.00996	4.222583
2.625823	18.19595	2.640332	135.5245	64.12971	25.24367	22.96463	8.348817	1.283059
0.000887	0.24443	4.33E-05	7.38847	2.701053	1.215195	0.523983	0.431977	8.66E-05
5.34474	5.273362	11.89932	0.636291	0.319163	14.97762	1.848297	2.234538	1.204228
25.73165	9.707677	25.48179	144.7919	40.7932	28.23632	55.04081	49.50903	33.07147
26.80355	105.1694	38.94854	4.429769	33.4985	80.6518	83.489	80.6518	62.33472
0.868853	2.81342	1.948918	6.088593	4.609076	5.671126	9.377345	4.007583	2.498478
9.541645	17.64869	10.38202	14.93173	15.88548	14.08569	14.25954	45.91841	5.821804
3.651898	5.030866	1.050231	0.023997	4.673182	1.982208	1.699091	1.558571	6.00228
25.99427	6.135007	16.34294	7.264502	12.77772	7.212915	9.625253	75.1132	26.80355
1.672107	4.484892	3.722785	1.102691	0.041782	5.630815	2.917381	3.66785	2.898196
7.162698	11.09133	7.780786	0.981558	4.077403	2.763261	6.210375	3.640606	3.011169
31.92385	29.18885	11.63037	2.032706	5.704646	45.1045	35.27608	52.56128	33.93939
0.000887	0.04886	0.078124	11.86763	0.235296	0.843102	0.676999	1.314675	8.66E-05
0.565426	0.478737	0.460938	2.5271	1.087345	0.814877	1.250058	0.596775	0.458045
4.251818	4.101061	3.754719	0.956061	1.809343	8.745238	2.009906	0.92816	10.78056
0.413449	6.967547	0.352006	0.128687	1.901068	4.145209	0.151688	0.902403	0.161962
0.443781	1.05088	0.550664	19.22139	1.132165	2.294733	0.737403	1.106046	0.111876
1.992944	6.287561	3.957734	0.592532	6.865	2.6143	2.267157	4.900996	1.830159
0.000887	0.007879	0.014084	0.140418	0.000736	0.040375	0.000188	0.017879	0.027922
15.3618	32.68313	21.02638	20.68746	23.16622	31.55417	35.776	59.32013	18.33909
62.33472	1360.293	105.1694	425.62	144.7919	87331.38	332.0776	87331.38	287.197
95.22939	126.1556	126.1556	252.4251	287.197	126.1556	749.7839	118.7028	155.7103
0.939408	0.640606	0.352006	3.198499	0.608543	1.925635	2.538218	0.92114	0.95101
35.776	32.31123	29.18885	8.762677	31.55417	19.99861	24.73724	19.99861	23.38378
0.371299	1.603795	0.264048	0.434863	1.235346	0.214401	0.182597	0.157619	0.223939
2.378124	2.111299	1.867951	5.583968	5.075447	4.424416	2.171429	2.333867	2.955916
0.533874	0.596111	0.637994	0.570664	0.208456	1.910974	0.469004	1.600238	0.258644
1.334762	0.596111	1.110505	0.2129	0.086883	0.040375	1.96404	6.876392	1.795851
0.000887	0.089769	0.027879	0.350274	0.078038	0.060426	0.028687	0.706053	8.66E-05
7.417359	3.971061	12.03267	1.626782	2.990851	11.65791	57.84882	43.49658	7.737807
16.9162	11.48368	21.54823	0.023997	3.783694	11.96574	118.7028	167.9996	83.489
0.000887	0.025483	0.020462	1.155411	0.041782	0.222698	0.537807	0.10956	1.4671
83.489	39.53687	91.05541	11.86763	37.81919	87.1746	62.33472	41.47925	37.81919
0.000887	0.057518	4.33E-05	1.626782	0.000736	0.047713	0.000188	0.033874	8.66E-05
24.50942	26.25557	37.81919	1.04228	7.037345	33.4985	56.39528	17.34065	18.62711
0.000887	0.04886	0.096811	0.023997	0.218045	0.403175	0.830289	0.516825	0.047569
2.449509	1.243442	3.13456	2.097843	2.252273	3.166659	2.502619	1.992944	0.802395
0.074177	0.162915	0.560325	0.252165	0.433131	0.664423	0.406212	0.102352	0.084524
0.026609	4.33E-05	0.004329	5.210317	0.025274	0.004524	0.000188	0.017879	8.66E-05
0.000887	0.623853	0.027879	6.325945	1.809343	0.645036	0.395152	0.869365	0.512309
19.99861	29.49693	40.7932	22.96463	42.75576	32.68313	40.11561	44.27012	21.93452
3.869163	3.095166	5.872886	49.50903	22.74797	12.63773	7.468961	6.325945	3.489899

3.432388	16.80343	4.029654	3.122915	12.10289	17.92188	8.319019	14.79742	3.034747
4.048499	6.876392	8.745238	12.77772	9.418196	12.97323	11.30987	13.79669	4.857403
4.397792	1.10132	2.752893	0.570664	1.286494	1.527792	2.786898	1.229553	12.10289
0.000887	4.33E-05	4.33E-05	0.636291	0.182078	0.016104	0.000188	0.000188	8.66E-05
5.309264	22.32214	7.375794	18.05488	9.625253	30.49289	20.68746	30.17192	5.530274
15.88548	15.276	13.83359	6.808369	28.54786	21.36892	11.77567	5.25254	6.38456
0.133939	1.690483	0.450051	1.251659	2.659214	2.325931	4.353925	2.628225	0.084524
1.452857	1.798535	1.262965	0.023997	2.990851	1.748016	1.783203	2.544747	1.21215
0.949827	1.166717	1.242641	2.489762	1.988196	2.164113	1.21215	1.779423	0.517612
5.889798	8.940671	5.959033	10.73084	11.14619	9.751039	9.082828	28.23632	13.6814
0.698687	1.351573	0.973745	2.604776	0.542165	0.920483	2.628225	5.530274	1.997352
2.909293	2.74298	4.678846	0.701818	8.149307	2.097843	5.576219	2.873817	3.703175
9.880779	13.08214	9.480736	34.38341	13.00996	20.34063	19.06893	20.85876	12.63773
53.77619	31.92385	51.52195	1.128788	3.346898	60.77449	60.77449	21.19547	56.39528
0.064372	0.269892	0.014084	1.186457	0.050577	0.779668	0.139567	1.53684	0.241255
14.70188	10.62739	19.53061	0.463485	25.24367	22.13245	14.93173	14.70188	14.51648
0.055967	0.218045	0.008283	1.663672	0.172453	0.126522	1.009625	0.173975	0.073038
1.762713	9.541645	4.644098	52.56128	21.19547	23.06543	38.39042	12.63773	12.16681
0.2914	0.057518	0.209978	8.033932	0.765094	2.002193	1.3829	0.965065	0.42202
3.779452	1.788874	9.625253	0.023997	2.659214	4.312251	5.409437	1.969733	2.65684
0.659163	0.536263	0.605887	7.38847	0.601623	1.371602	0.309877	0.330866	0.761277
3.214545	0.427937	2.489762	0.023997	1.795851	0.792886	0.497424	1.766248	0.309877
7.468961	30.82277	3.722785	42.75576	6.760765	16.44768	31.1753	25.99427	13.23022
0.000887	0.139841	0.008283	0.899567	0.132626	0.214401	1.082179	0.173975	0.155101
2.229048	13.60866	7.854069	4.323045	13.45939	13.96064	0.88158	5.082063	1.226934
24.98218	8.539971	32.31123	5.158146	20.25302	12.97323	9.19899	6.404921	14.17388
0.000887	0.122944	0.078124	0.140418	0.200519	0.069365	0.086991	0.165981	8.66E-05
3.566753	6.079596	4.155339	12.49452	5.515584	6.191515	6.760765	4.609076	3.269913
0.259033	1.535722	1.08671	6.250447	5.754935	2.922778	3.195541	0.965065	0.359105
0.244964	0.561126	0.209978	0.802035	0.308672	1.173535	0.537807	1.52676	0.181674
0.939408	4.33E-05	1.555289	0.023997	1.008413	3.147294	0.000188	0.173975	1.801284
0.607273	2.531515	0.663276	0.981558	2.068297	0.833975	1.340036	4.739668	0.790303
5.095325	31.1753	6.695887	2.604776	7.327482	18.76954	80.6518	126.1556	11.25392
10.23768	3.904517	3.917597	332.0776	8.608153	6.978903	9.19899	6.900202	47.5018
0.5129	1.030678	0.858817	1.588903	1.578687	1.229553	1.542302	0.61355	1.118066
0.08557	4.33E-05	0.053203	40.7932	0.000736	0.473853	0.151688	0.751371	0.111876
1.099762	0.226883	1.180613	6.760765	0.182078	1.066234	0.809481	2.860722	0.931457
0.565426	2.440895	0.73127	0.488167	3.104394	1.558571	1.320173	0.454978	0.619365
0.000887	0.01329	4.33E-05	0.326912	0.032799	0.134942	0.068384	0.094841	8.66E-05
0.000887	0.506328	1.253752	3.049603	0.000736	5.287857	0.946638	0.902403	0.188124
0.000887	1.670786	0.581926	9.256537	0.954141	2.518124	0.366147	3.917597	8.66E-05
0.08557	0.311876	0.008283	0.65798	0.059877	0.439776	0.294711	0.186017	0.034776
0.753449	2.453781	1.228651	0.159639	3.387864	1.323131	3.471486	2.180022	1.554185
0.235931	0.506328	0.035671	25.99427	1.474343	0.19596	0.457431	0.493925	0.258644
0.133939	0.057518	0.053203	0.2129	0.032799	0.414242	0.057857	0.06912	0.047569
0.186356	0.073038	0.132799	2.134545	0.252619	0.214401	0.429827	0.478182	0.223939
2.129596	3.855952	0.788369	1.186457	3.36075	1.386681	0.664026	0.180368	1.343463
1.230411	0.606421	0.31702	3.606804	1.608427	0.976667	0.139567	0.468694	1.298665
31.55417	12.60233	18.19595	0.023997	2.08798	1.188139	7.136537	1.303045	70.11932
0.904856	1.919661	1.472208	0.278499	9.480736	1.100519	1.320173	0.191602	0.733074
42.75576	28.23632	62.33472	0.488167	65.94551	27.38726	19.83727	5.576219	39.53687
0.042143	0.390895	0.15241	0.824885	0.337641	0.153189	0.078297	0.838846	0.053672
0.178817	0.181068	0.352006	1.251659	0.81596	0.242893	0.596111	0.191602	0.215859
0.380851	1.214315	0.450051	7.61176	0.810491	2.909293	1.082179	1.025613	0.581111
1.543377	2.638139	1.982208	19.06893	2.102626	3.104394	6.25938	5.485685	2.427792
0.095224	0.065094	0.948745	5.088694	10.01009	1.299567	1.656696	0.273911	8.66E-05
0.048889	0.004242	0.004329	0.434863	0.000736	0.033593	0.013867	0.013723	8.66E-05
4.933781	3.307186	1.472208	0.350274	1.839986	1.839986	2.65684	1.779423	2.08632

6.30671	1.748016	7.013853	0.023997	1.957958	2.061926	1.229553	2.911962	2.008312
3.851414	3.172864	3.754719	0.023997	2.438831	4.424416	1.518232	4.101061	1.863506
0.000887	4.33E-05	0.132799	4.758088	0.104149	0.222698	0.720938	0.140924	8.66E-05
5.591717	13.38418	5.614805	2.199639	7.683766	7.417359	9.256537	19.83727	4.241905
6.181652	7.08733	7.938427	0.023997	2.701053	5.388405	10.17029	3.089293	5.638759
0.000887	0.081667	0.329798	1.251659	1.149322	0.569625	0.962698	0.309444	0.95101
0.08557	0.122944	0.534214	3.077316	0.643853	0.34544	0.099913	0.094841	0.010519
0.328737	0.454394	0.617763	5.044113	0.534214	0.730873	1.4671	2.649675	0.415729
4.620159	5.898449	6.063276	5.830152	3.373983	3.401126	2.74544	6.706472	3.55583
2.421681	3.518384	1.652771	1.04228	0.934488	0.612179	0.068384	0.393983	2.021068
24.2842	51.52195	32.68313	1.547734	45.91841	43.49658	27.10502	42.75576	18.76954
0.487662	0.968723	0.499206	1.456955	0.982698	2.481356	1.21215	0.948745	0.258644
0.000887	0.041053	0.020462	0.543939	0.000736	0.102352	0.057857	0.027691	0.015685
1.026825	0.615996	0.73127	2.263377	1.132165	0.664423	0.92816	0.385238	1.068348
22.52375	4.33E-05	6.55039	0.304387	1.91254	4.33E-05	2.438831	0.000188	19.37139
1.762713	0.681919	0.410996	0.023997	2.671299	0.439776	0.000188	0.000188	2.316075
10.38202	15.07257	12.85504	2.032706	11.09133	11.19942	17.15902	18.76954	10.93101
20.34063	16.9162	15.66023	0.023997	5.351934	3.188918	1.723579	2.192496	31.1753
0.000887	0.725267	0.105498	0.899567	0.559221	1.016494	1.009625	0.782417	0.167273
25.24367	5.295325	17.64869	0.023997	19.99861	4.33E-05	8.608153	0.000188	19.53061
13.08214	0.294921	7.136537	0.023997	0.208456	4.33E-05	1.559675	0.116097	0.692381
2.408882	4.029654	2.419553	12.10289	2.586926	5.337547	5.560563	9.646284	2.640332
0.000887	0.585281	0.511356	8.711905	0.355051	2.987835	1.50026	1.240202	0.330043
0.064372	0.089769	0.191486	1.826147	0.218045	1.49075	0.41772	1.604827	0.23303
4.524481	4.933781	6.09759	4.203001	5.855281	3.779452	0.990909	3.373983	3.533211
0.235931	1.05088	0.18324	4.203001	2.234538	2.112843	0.976667	2.114437	0.517612
0.398059	0.606421	0.352006	0.023997	1.028672	0.185166	0.000188	0.28026	0.563499
0.487662	0.661443	0.488759	0.408175	1.451775	0.976667	0.205382	0.309444	0.27075
12.63773	13.00996	17.98838	3.198499	15.3189	9.237525	5.71241	12.56693	22.96463
1.452857	2.74298	1.567576	0.023997	3.514834	0.976667	0.000188	0.706053	4.029654
0.471025	0.543939	0.836537	0.252165	1.484553	0.956667	0.057857	0.173975	0.540902
155.7103	1.43039	12.77772	0.023997	0.374365	4.33E-05	0.000188	7.112496	80.6518
0.452713	0.851234	0.114012	0.023997	0.757273	0.779668	0.164401	0.454978	0.493247
3.4679	4.193232	3.307186	0.023997	3.750837	1.160707	2.59557	1.025613	0.675938
1.485483	5.366335	0.450051	0.776349	4.49671	2.229048	1.412771	0.524986	1.343463
0.113218	0.181068	0.053203	1.751948	0.162468	0.946061	0.000188	0.322684	0.034776
0.318499	1.10132	0.450051	0.252165	1.474343	1.08671	0.755253	0.524986	0.366147
0.413449	0.497749	0.605887	2.060491	2.513752	1.852338	4.900996	0.575628	0.890065
5.210317	16.22108	5.42381	0.326912	10.60361	11.01243	13.30906	8.477662	8.061934
0.000887	4.33E-05	4.33E-05	5.728615	0.319163	0.134942	0.000188	0.338095	8.66E-05
0.436017	0.320931	0.798153	8.976508	0.534214	0.68557	0.578095	4.72798	2.180022
2.438831	0.763102	0.592532	0.023997	0.954141	0.453218	0.217316	0.268528	2.207417
3.592063	6.486104	5.838499	0.023997	7.780786	5.050267	1.50026	2.810678	2.392431
0.201645	0.390895	0.173723	0.179177	0.329091	0.222698	0.193896	0.588954	0.111876
4.644098	1.950519	0.749942	0.023997	1.397482	1.49075	0.164401	0.000188	0.581111
0.033745	0.004242	0.004329	0.023997	0.025274	0.010707	0.020346	0.013723	8.66E-05
0.074177	0.468167	0.191486	0.304387	0.337641	0.545808	0.113911	0.715108	0.198405
2.187395	1.43039	0.581926	0.023997	2.398442	1.477388	0.351595	0.544545	1.542302
5.553146	1.732468	5.560563	3.077316	0.416501	5.745996	1.42456	1.52676	1.889711
0.209401	0.841746	0.760866	3.939307	2.618846	2.27254	4.709033	1.351573	1.107605
0.033745	0.007879	0.044805	0.023997	0.068896	0.033593	0.057857	0.017879	8.66E-05
287.197	15.98892	1360.293	2.23267	332.0776	47.5018	184.443	8.164531	203.5795
0.772157	0.506328	1.08671	1.04228	0.922879	0.327143	2.109755	0.647691	1.1379
0.064372	0.041053	0.020462	3.816551	0.122554	0.134942	0.013867	0.41026	0.047569
2.040606	6.355224	2.984899	65.94551	22.96463	7.482179	9.859719	16.68554	1.623232
4.484892	2.353247	0.858817	0.023997	1.855108	3.422237	0.809481	1.793074	3.464315
29.82828	100.0998	70.11932	0.023997	25.48179	49.50903	38.94854	100.0998	36.26061
0.000887	0.670519	4.33E-05	9.082828	0.243124	0.991631	1.436623	1.04228	0.181674

0.299336	0.01329	0.096811	0.159639	0.104149	4.33E-05	0.000188	0.000188	0.027922
0.550072	1.622006	0.599466	7.697063	0.622496	1.215195	1.3829	1.190491	0.922338
3.792266	0.707799	1.938759	1.944517	1.042944	0.622092	2.941544	0.724502	4.984488
0.000887	0.294921	4.33E-05	3.730808	0.252619	1.016494	0.88158	0.062107	0.167273
0.000887	0.162915	0.035671	0.179177	0.162468	0.094841	0.078297	0.140924	0.047569
34.84299	36.74522	37.29684	0.023997	17.64869	4.33E-05	7.737807	0.000188	95.22939
4.745895	6.55039	4.365224	1.251659	6.830592	4.540794	1.453939	3.128694	6.153102
1.80267	0.468167	0.534214	5.395584	1.772756	0.19596	1.039199	2.673846	0.42202
3.031688	8.288874	3.998391	0.614545	6.345382	6.518709	3.307186	4.839105	3.771349
0.772157	0.939408	2.091313	6.443925	1.111977	0.804675	1.938759	5.18754	1.333781
9.751039	8.573434	17.98838	46.7056	7.6393	30.82277	16.5625	25.48179	5.622872
2.078355	0.427937	1.733766	0.513752	1.008413	0.276609	1.250058	0.243124	2.967453
0.113218	0.122944	0.060649	1.102691	0.095685	0.126522	0.193896	0.575628	0.10469
0.000887	0.01329	4.33E-05	0.023997	0.000736	4.33E-05	0.047468	0.205931	0.021126
0.669769	1.325743	0.560325	1.456955	1.222958	1.299567	0.92816	0.813788	0.482706
5.238701	14.88584	5.754935	9.52197	17.03919	8.815123	10.67991	8.958225	15.18102
0.235931	1.325743	0.162915	1.692763	0.567071	2.481356	2.715873	3.767489	0.123853
0.350455	3.104394	1.064242	15.3618	2.243911	2.713319	6.601833	5.47	0.692381
1.51215	0.649235	1.050231	0.159639	0.289603	0.946061	0.381789	1.014784	0.724885
3.432388	3.350224	3.407619	38.39042	5.898449	13.75978	4.833398	8.573434	0.744531
47.5018	7.938427	16.5625	0.023997	1.501291	0.49974	0.000188	40.11561	224.9555
0.178817	1.006623	0.027879	4.690159	0.346732	0.591912	1.359177	6.22031	0.198405
0.000887	4.33E-05	4.33E-05	0.776349	0.000736	4.33E-05	0.028687	0.077417	0.091306
1.130115	2.502619	1.913997	1.972706	4.25658	1.819221	5.671126	0.992771	4.222583
0.000887	0.025483	0.035671	0.326912	0.200519	0.040375	0.047468	0.041097	8.66E-05
0.064372	0.354798	0.014084	0.776349	0.113218	0.49974	0.182597	0.086595	0.034776
18.91854	33.4985	14.25954	30.82277	32.68313	77.74812	28.54786	36.74522	20.16541
0.161602	1.041025	4.33E-05	10.62739	0.000736	0.453218	0.068384	0.000188	0.20969
0.042143	0.007879	4.33E-05	0.513752	0.032799	0.078586	0.013867	0.048434	0.006984
3.432388	4.33E-05	0.697496	1.283911	0.862561	1.074242	0.596111	0.534452	2.730072
0.000887	2.127973	4.33E-05	3.939307	6.865	21.02638	15.66023	11.89932	8.66E-05
2.053521	1.229553	5.062626	1.43039	2.353247	0.19596	1.095657	0.000188	7.938427
6.345382	3.855952	1.948918	0.023997	3.407619	4.33E-05	0.523983	0.000188	5.942294
0.679812	0.623853	0.961486	0.159639	0.885137	0.49974	0.381789	1.061457	0.467136
2.820974	3.38456	1.555289	0.023997	1.157626	1.51215	1.58645	0.50127	0.915195
0.328737	0.73583	0.647345	6.922771	2.138131	2.752893	2.973059	0.941104	0.034776
0.144098	1.690483	0.096811	0.463485	0.81596	1.574517	2.048781	0.227063	0.303896
0.048889	0.018788	4.33E-05	2.831501	0.182078	0.060426	0.205382	0.086595	0.021126
2.165851	4.502453	1.828781	17.15902	6.277937	5.583968	5.654863	3.276335	0.130498
0.849841	0.585281	0.427937	0.350274	1.204228	0.906825	0.309877	0.675938	0.550072
0.318499	0.55215	0.132799	3.022713	1.381111	0.664423	0.037186	1.953535	0.123853
1.992944	1.575483	2.911962	0.701818	2.839351	1.240981	2.353247	0.378066	2.035678
0.000887	0.107626	4.33E-05	1.155411	0.191703	0.134942	0.139567	0.033874	8.66E-05
5.416623	5.838499	4.833398	0.513752	4.06303	3.486436	3.217612	0.933261	10.28573
0.055967	0.01329	0.12324	0.140418	0.032799	0.021645	0.020346	0.000188	0.097547
12.16681	4.952915	6.653016	0.023997	0.593434	1.299567	0.946638	0.000188	1.916782
0.960216	4.029654	1.171349	3.077316	1.772756	1.264589	1.143954	1.166717	3.286652
0.998312	2.006782	0.439062	0.023997	2.453781	0.153189	1.21215	0.10956	3.464315
0.21645	0.172063	0.15241	1.072843	0.172453	0.453218	0.652807	0.243124	0.504416
8.888167	6.325945	2.234538	0.023997	4.034423	2.701053	0.596111	0.338095	7.074329
1.34267	1.381111	1.755988	8.134084	2.781558	1.016494	2.141595	1.061457	2.250462
22.96463	15.46193	11.96574	0.023997	15.56131	4.370671	1.737294	0.000188	10.82954
10.73084	2.413146	15.77678	0.513752	17.92188	4.193232	17.03919	5.238701	34.38341
0.000887	1.560779	0.903074	0.899567	0.000736	2.031169	4.105714	0.850808	0.403882
0.225823	0.561126	0.173723	6.695887	0.913514	1.925635	1.542302	2.661587	0.241255
0.064372	0.28746	0.053203	3.077316	0.567071	1.147807	1.872049	0.992771	0.117734
7.495397	3.137576	1.707576	0.023997	0.000736	0.163651	0.000188	0.086595	13.53913
4.888023	5.366335	5.309264	0.023997	1.563045	2.463911	0.693925	0.000188	1.421126

3.397706	5.630815	3.394286	1.43039	12.16681	8.539971	3.899993	3.411097	2.083261
0.123535	0.615996	0.256205	4.808658	1.132165	5.195022	1.813564	1.982208	0.161962
2.681169	3.014076	2.392431	7.264502	2.99987	3.953297	1.828781	6.967547	2.068297
0.669769	0.536263	0.961486	2.23267	2.810678	0.68557	1.58645	0.219307	1.428398
0.849841	2.229048	0.599466	0.023997	3.464315	1.422316	1.29267	0.706053	0.983384
7.049336	1.089394	1.08671	0.252165	4.007583	0.645036	0.443543	0.10956	0.241255
13.30906	14.70188	11.25392	2.890245	3.521934	12.36443	3.107417	10.82954	14.6058
5.770996	9.502294	4.058514	0.023997	10.33476	8.134084	4.217569	16.80343	7.339452
5.01772	8.381328	3.497085	0.196335	8.539971	3.855952	3.738997	4.758088	3.125844
0.868853	4.272165	3.78798	0.023997	4.408153	2.914632	1.783203	0.000188	3.718716
1.439704	1.229553	0.917994	7.264502	3.578038	0.906825	4.039192	0.516825	1.863506
1.170491	1.679596	1.384921	1.626782	0.793918	2.210866	1.623232	8.089848	0.716652
1.746724	3.432388	1.733766	0.023997	2.898196	0.991631	0.469004	2.574733	2.884762
0.89404	0.261111	1.162201	0.140418	0.289603	0.557056	0.047468	0.086595	0.147706
0.688975	0.649235	0.499206	2.671299	0.337641	0.645036	1.026219	0.180368	1.144733
0.688975	0.329798	0.235613	3.122915	0.095685	0.557056	0.381789	0.378066	0.498636
0.000887	0.821414	0.439062	1.721371	1.035801	0.414242	1.29267	0.273911	0.053672
0.580758	0.655613	0.29531	2.032706	2.005253	0.86518	1.340036	1.136566	0.426392
0.074177	0.95101	0.647345	10.45584	0.289603	1.323131	0.720938	0.468694	0.117734
1.275325	2.621133	2.013182	0.278499	0.243124	1.865043	1.485483	0.956061	1.250895
27.10502	1.193362	6.967547	0.023997	0.319163	4.33E-05	0.000188	0.000188	2.54961
3.734762	3.24658	1.859084	0.724004	2.055289	1.412771	3.046551	0.628658	2.805382
0.524646	2.382035	0.73127	0.488167	1.697792	1.215195	1.250058	0.493925	0.65241
0.178817	0.007879	0.105498	0.592532	0.172453	0.163651	0.099913	1.369928	0.198405
17.15902	11.60283	10.62739	0.023997	1.052208	1.046703	3.939307	1.481631	5.959033
7.363117	8.226219	7.037345	0.379495	28.23632	3.722785	4.397792	1.846941	4.397792
1.724805	0.269892	0.384798	0.463485	0.698297	0.232338	0.057857	0.000188	0.733074
4.212554	3.080317	3.428788	0.463485	3.714906	2.306277	1.638499	1.846941	2.234538
0.000887	1.25816	0.044805	0.023997	0.308672	0.557056	1.195794	0.620909	0.10469
0.042143	0.04886	0.014084	0.159639	0.29842	0.102352	0.000188	0.033874	8.66E-05
2.359264	1.10132	1.099762	0.023997	3.489899	0.365635	1.069574	0.062107	4.110779
7.188095	13.45939	3.47531	0.023997	2.375974	3.486436	1.009625	2.114437	9.792626
1.614257	2.784228	1.898153	24.98218	4.179762	7.92518	4.091667	2.786898	1.700354
0.095224	0.406912	0.2264	1.339091	0.235296	0.631847	0.395152	0.128759	0.286522
0.033745	0.302554	0.173723	0.252165	0.467677	0.232338	0.217316	0.227063	0.441833
0.244964	1.006623	0.105498	8.556703	0.670144	0.674163	0.55215	1.266255	0.612872
2.053521	1.50026	0.499206	0.196335	1.433189	1.541227	0.268961	0.575628	2.127973
9.063983	4.869553	4.508196	1.04228	4.620159	2.644885	3.493362	0.88619	12.03267
0.000887	0.073038	0.044805	0.408175	0.000736	0.102352	0.000188	0.017879	0.010519
0.042143	0.041053	0.020462	0.023997	0.086883	0.040375	0.047468	0.116097	0.065772
1.21215	1.778153	1.128131	4.946587	4.552092	5.530274	3.098268	4.851227	0.768535
1.085224	4.715599	1.766248	1.547734	3.921645	4.620159	4.484892	3.695815	1.320173
1.170491	20.51271	0.858817	203.5795	80.6518	2.325931	7.977958	6.054242	1.226934
0.103088	0.338341	0.352006	0.350274	0.614545	0.706984	0.720938	0.696234	0.065772
0.064372	0.041053	0.053203	0.408175	0.227648	0.110642	0.047468	0.180368	8.66E-05
7.30215	5.150693	4.900996	0.463485	2.92013	1.323131	0.443543	0.000188	1.21215
21.02638	22.74797	19.22139	0.023997	4.673182	9.924372	4.580938	5.864084	12.46209
3.107417	1.971183	3.150433	0.023997	6.325945	1.100519	1.668369	1.314675	2.506934
0.000887	0.04886	0.035671	0.350274	0.068896	0.047713	0.037186	0.033874	0.047569
0.026609	4.33E-05	4.33E-05	0.434863	0.000736	0.021645	0.000188	0.013723	8.66E-05
1.924228	1.652771	0.581926	0.326912	1.304877	0.833975	0.720938	0.295671	1.916782
13.15079	19.53061	17.51961	0.023997	0.687807	14.33939	5.28039	29.49693	15.56131
0.542525	0.517092	0.663276	0.614545	0.355051	0.535029	0.913514	0.468694	0.323752
6.508427	2.834084	8.508514	0.513752	1.175859	1.51215	7.136537	5.915469	3.479134
1.724805	1.71518	1.618773	0.023997	1.361789	2.996869	0.497424	0.454978	0.931457
1.656696	1.612056	0.858817	4.323045	2.345527	3.256587	0.990909	4.655916	1.3829
0.103088	0.073038	0.2264	2.333867	0.467677	0.060426	0.41772	0.330866	0.657114
0.000887	1.25816	0.511356	17.34065	0.998312	8.412367	5.11697	1.240202	8.66E-05

13.53913	6.663651	8.256465	0.929286	2.522684	2.860722	0.381789	0.34443	1.682193
0.055967	0.55215	0.014084	0.304387	0.122554	0.473853	0.057857	0.534452	0.021126
0.000887	0.707799	0.15241	3.435988	1.076652	1.016494	2.502619	1.070115	0.336147
1.924228	1.243442	0.304545	0.023997	1.052208	1.147807	0.182597	1.717641	0.882619
1.762713	1.455022	1.816436	0.023997	0.737857	0.439776	0.217316	0.219307	0.768535
4.821176	4.869553	2.652107	0.252165	0.678247	3.993665	1.518232	9.063983	5.34474
0.000887	0.073038	0.060649	0.899567	0.104149	0.612179	0.41772	0.149423	0.130498
4.241905	3.493362	2.860722	0.023997	2.365685	3.147294	1.828781	1.090072	3.750837
1.283911	1.018427	0.647345	2.333867	1.244242	0.718355	0.395152	2.024488	1.863506
11.83593	24.73724	13.30906	0.023997	6.518709	22.74797	3.687633	8.868932	13.75978
1.580909	0.841746	2.363442	0.749481	1.149322	0.535029	0.294711	0.048434	0.204278
3.975498	0.478737	1.353608	0.023997	18.76954	4.33E-05	0.151688	0.000188	0.336147
18.48514	4.212554	11.83593	5.830152	8.333918	3.256587	7.977958	6.760765	25.99427
0.048889	0.04886	0.008283	1.883535	0.095685	0.153189	0.113911	0.566104	0.021126
1.522597	0.606421	0.769711	0.023997	1.149322	1.737294	1.412771	1.427431	0.667201
16.22108	24.04374	17.03919	0.023997	17.51961	15.276	16.44768	7.710361	24.04374
2.993867	0.536263	1.801284	0.023997	0.89404	1.910974	1.938759	0.10956	1.019791
2.890245	0.85728	2.043795	0.278499	0.576205	0.34544	0.481602	1.481631	1.682193
167.9996	50.55949	83.489	0.86987	1.63039	1.685678	4.203001	9.141299	135.5245
0.000887	0.772157	0.15241	0.350274	0.757273	0.523983	0.676999	0.000188	8.66E-05
1.580909	1.05088	1.340036	0.776349	1.670786	0.380649	1.542302	0.128759	2.855296
1.085224	1.831631	2.117814	20.85876	4.096407	3.779452	5.158146	4.381479	2.928247
14.33939	7.468961	10.82954	1.78759	7.737807	9.541645	2.289286	5.576219	6.717056
0.390216	0.11531	0.401385	0.326912	0.707475	0.19596	0.429827	0.431977	0.73912
3.156537	2.250462	2.470317	0.749481	1.570794	0.779668	0.676999	0.685931	2.947287
0.55741	2.043795	2.371833	1.663672	0.793918	1.133622	0.990909	0.359697	0.303896
0.000887	2.121198	0.973745	0.379495	0.235296	0.86518	2.690823	0.288579	0.323752
6.911486	21.19547	5.889798	0.981558	7.598312	16.9777	13.00996	6.135007	5.266335
3.019848	1.582915	3.711097	0.023997	0.724004	1.122359	1.121703	1.421126	1.144733
0.462857	0.996696	1.596782	11.77567	1.157626	0.779668	1.268672	1.279452	0.941645
0.000887	0.041053	0.020462	0.278499	0.262785	0.016104	0.174444	0.288579	0.078528
0.000887	0.261111	4.33E-05	0.023997	0.593434	0.086775	0.193896	0.588954	8.66E-05
2.109755	3.518384	0.987309	0.023997	3.636789	4.644098	1.848297	0.478182	3.738997
1.633752	1.089394	0.847908	1.155411	1.34513	2.190866	0.88158	1.014784	4.086926
0.753449	2.084791	1.368167	0.434863	2.068297	1.422316	1.813564	2.765758	1.343463
4.997417	4.029654	3.699495	3.890556	5.381227	1.4671	10.38202	2.765758	2.893001
2.396429	1.883535	0.560325	0.023997	0.000736	4.33E-05	0.000188	0.000188	8.66E-05
0.817547	25.24367	33.07147	19.91794	46.7056	57.84882	105.1694	184.443	6.52899
1.835743	3.014076	2.024488	1.43039	1.563045	5.889798	2.770866	5.143867	1.196508
0.144098	0.373218	0.132799	0.252165	0.337641	1.819221	0.512309	1.351573	0.123853
0.000887	0.302554	0.027879	1.484553	0.200519	0.473853	0.720938	0.351595	0.073038
3.807648	0.977994	1.154567	0.023997	1.94311	1.592395	0.962698	0.941104	2.197828
2.101075	6.830592	2.013182	0.776349	3.422237	4.857403	5.056421	5.622872	1.497143
0.413449	3.493362	0.105498	0.023997	1.744055	2.408882	0.676999	1.327525	1.019791
0.161602	0.468167	0.200794	8.241342	0.346732	2.597792	6.53969	0.566104	0.820447
8.164531	13.6814	15.56131	14.70188	13.91714	22.52375	26.52537	26.80355	5.34474
1.322092	3.046551	2.005253	0.023997	3.726854	0.86518	1.783203	0.774257	1.577569
0.398059	0.707799	0.191486	0.824885	0.282424	0.426118	0.55215	0.25197	0.097547
1.085224	3.767489	1.154567	1.626782	4.302367	2.112843	1.96404	2.77899	1.497143
0.949827	1.111241	3.340361	2.671299	1.820541	1.51215	1.656696	4.952915	2.111299
1.846941	2.382035	1.578687	0.326912	1.533535	0.878932	1.623232	0.666494	3.028629
1.313737	2.339697	1.118817	0.326912	1.235346	0.814877	0.281068	0.149423	2.078355
0.225823	0.033312	0.068449	0.023997	0.025274	0.016104	0.037186	0.033874	0.021126
3.35715	3.869163	1.748016	0.543939	3.860491	2.847547	2.227273	6.38456	3.147294
19.83727	14.12978	13.96064	3.460909	16.44768	15.71851	16.34294	11.83593	18.12541
0.744531	1.950519	1.634935	4.690159	5.447114	3.578038	2.327857	1.793074	1.369048
0.33969	0.725267	0.209978	1.251659	0.988932	0.69583	0.88158	1.570794	0.188124
0.259033	0.302554	4.33E-05	2.542605	0.400354	0.473853	0.790837	0.431977	0.091306

0.790303	1.018427	0.31702	5.395584	1.656696	4.272165	1.903636	1.213434	0.807619
0.443781	0.162915	0.105498	2.981962	0.661861	0.297807	0.164401	0.062107	0.167273
2.852677	0.329798	1.816436	0.023997	0.516061	0.601227	0.523983	0.605887	3.500808
10.60361	2.996869	9.063983	0.023997	3.939307	4.33E-05	1.009625	0.000188	6.171789
0.2914	1.652771	0.439062	0.023997	1.870685	2.27254	0.457431	0.086595	0.915195
0.133939	0.107626	0.053203	1.186457	1.1407	0.047713	0.182597	0.28026	0.508975
1.145426	1.477388	1.913997	0.023997	1.250895	1.198701	1.396494	0.454978	2.996869
1.099762	0.517092	0.329798	0.701818	0.319163	0.276609	0.113911	0.128759	1.532468
1.107605	0.311876	1.118817	0.023997	0.661861	0.956667	0.809481	0.205931	1.169091
0.753449	0.841746	0.73127	0.023997	23.6012	0.535029	0.351595	0.260584	4.776407
10.60361	6.738211	5.704646	0.023997	4.46267	4.072475	2.609293	1.41368	4.203001
0.000887	0.04886	4.33E-05	2.604776	0.113218	0.403175	0.174444	0.055671	0.111876
0.698687	3.457504	1.064242	1.186457	2.55184	3.147294	3.629177	10.67991	2.287576
4.312251	9.335397	6.497266	0.023997	9.968355	4.72798	5.381227	1.514199	4.758088
2.065007	4.125786	1.110505	111.5439	2.453781	3.618499	6.853608	5.545729	1.849654
5.654863	3.734762	6.365065	0.304387	6.945152	6.888297	3.676032	2.248896	1.739935
2.217951	5.942294	9.418196	155.7103	5.123781	46.7056	36.74522	38.94854	1.815
0.123535	0.725267	0.439062	1.588903	0.803211	0.523983	1.447994	1.338261	0.744531
10.48101	6.772763	9.256537	0.023997	1.372475	4.875527	6.956349	9.179466	9.438687
530.1465	749.7839	100.0998	1.826147	28.87506	252.4251	48.53429	530.1465	87331.38
1.701616	0.536263	1.310837	1.91254	0.803211	1.016494	0.707475	0.227063	0.802395
0.000887	0.311876	0.008283	0.350274	0.329091	0.287951	0.068384	0.033874	0.015685
1.118066	2.453781	1.668369	24.73724	2.659214	7.212915	7.625209	2.74544	0.38772
21.74051	13.91714	16.80343	2.294733	39.82624	10.17029	8.711905	8.745238	16.09482
0.123535	0.92816	0.087734	7.136537	0.308672	0.991631	0.244589	3.411097	0.111876
9.19899	12.7427	9.880779	1.78759	12.36443	12.70768	9.141299	10.53159	11.60283
2.839351	4.33E-05	6.99026	0.023997	6.38456	5.720173	0.962698	5.454949	57.84882
2.306277	3.606804	0.858817	0.488167	6.632063	2.210866	1.229553	0.605887	2.939141
1.041025	1.030678	1.384921	0.023997	0.374365	0.242893	1.92272	0.000188	2.094574
1.184935	2.250462	1.766248	3.754719	5.056421	6.853608	3.511263	5.18754	1.219856
0.170938	0.468167	0.401385	4.365224	1.262179	1.371602	1.485483	0.974906	0.215859
5.477843	6.601833	7.482179	18.76954	11.89932	16.5625	14.6058	10.43066	4.758088
0.000887	0.098629	0.18324	3.939307	0.000736	0.354545	0.000188	0.534452	0.155101
1.680895	1.89518	1.677042	0.350274	2.800332	2.408882	1.518232	0.048434	0.915195
13.60866	80.6518	20.85876	0.776349	22.32214	42.08807	21.74051	31.55417	12.70768
1.397482	1.748016	2.203535	10.93101	2.618846	3.464315	11.4544	2.214372	1.486551
0.08557	4.33E-05	4.33E-05	0.278499	0.000736	0.185166	0.020346	0.062107	8.66E-05
4.440548	5.330519	4.38684	20.51271	4.745895	7.545584	8.075772	6.612121	5.454949
3.116732	3.269913	3.340361	2.92013	0.90368	45.91841	13.83359	4.381479	4.49671
4.227302	11.25392	3.518384	0.956061	5.158146	4.207778	5.872886	4.535354	8.625613
2.269134	3.971061	3.243225	0.636291	4.155339	3.521934	7.136537	3.150433	4.933781
0.000887	0.191602	0.035671	0.86987	0.534214	0.040375	0.244589	0.418557	0.123853
1.266255	1.703795	0.888485	0.899567	2.102626	1.188139	2.465916	1.815	1.934271
0.133939	0.346342	0.078124	0.434863	0.122554	0.512309	0.193896	0.393983	0.198405
0.371299	0.725267	0.15241	0.899567	0.698297	1.264589	0.395152	0.61355	0.084524
0.123535	0.007879	0.044805	4.203001	0.000736	0.040375	0.086991	0.28026	0.136061
2.136335	7.112496	1.733766	1.155411	38.94854	3.939307	1.250058	0.92114	1.525729
1.938759	2.784228	0.798153	0.023997	0.440801	1.264589	0.151688	0.61355	3.6029
4.302367	6.011515	1.696494	0.023997	2.308203	1.660216	1.638499	3.089293	2.755411
1.580909	13.23022	1.991328	0.543939	13.08214	3.507691	12.89577	4.965801	5.123781
2.544747	2.638139	4.170051	0.023997	2.417424	2.887489	0.664026	0.524986	0.974257
13.6814	24.50942	6.612121	0.023997	9.141299	6.088593	2.99987	3.397706	5.606739
1.152944	0.269892	0.2264	0.023997	0.687807	0.439776	0.294711	0.116097	1.89373
0.533874	0.364502	0.499206	13.91714	0.449408	0.126522	0.294711	0.88619	0.482706
0.350455	0.478737	0.340866	0.023997	2.055289	0.276609	0.281068	0.322684	0.755253
0.209401	3.1857	1.484553	9.141299	2.181775	5.309264	5.439278	8.288874	0.639892
0.08557	1.404812	0.479401	4.845166	0.810491	5.42381	1.828781	1.061457	0.097547
0.000887	4.33E-05	4.33E-05	8.365072	0.162468	0.276609	0.707475	0.825354	0.161962

1.51215	1.445916	1.118817	5.00404	1.641999	1.685678	2.563506	1.824892	0.595022
8.994791	4.302367	13.08214	1.313737	6.945152	9.583463	8.923117	3.718716	10.01009
11.42512	13.53913	16.22108	1.456955	8.27267	13.83359	21.54823	11.42512	13.08214
0.904856	1.535722	1.426522	18.33909	1.724805	3.895469	2.802828	5.416623	0.575628
3.885642	2.887489	0.376782	0.159639	3.921645	1.299567	1.485483	1.090072	2.294733
0.524646	1.278629	0.256205	0.159639	0.846342	2.150303	0.86518	0.205931	0.241255
2.269134	1.394632	1.546696	0.2129	2.723247	1.198701	1.757244	0.88619	6.695887
0.074177	0.073038	0.035671	2.45575	0.078038	0.414242	0.126659	0.351595	8.66E-05
0.161602	0.497749	0.044805	1.484553	0.086883	0.185166	0.497424	0.487128	0.10469
2.640332	7.810058	1.913997	0.278499	1.296905	4.667518	4.376118	4.408153	5.704646
0.170938	0.606421	0.274365	0.701818	0.614545	0.453218	0.55215	0.732633	0.246955
0.000887	0.065094	4.33E-05	0.408175	0.050577	0.222698	1.436623	0.418557	8.66E-05
1.085224	1.455022	0.873579	3.55583	1.433189	0.853218	2.65684	5.654863	0.783398
0.939408	1.992944	0.460938	0.2129	1.656696	0.766602	0.946638	0.431977	1.343463
0.095224	0.081667	0.068449	0.488167	0.282424	0.232338	0.028687	1.031284	0.198405
1.954993	4.808658	2.868456	2.23267	5.485685	3.7586	3.217612	3.457504	2.094574
1.499214	2.502619	1.707576	0.252165	1.621032	1.323131	1.069574	5.143867	1.008961
23.82354	29.82828	7.519841	0.023997	3.422237	8.381328	6.25938	14.00414	5.00404
0.659163	0.497749	0.410996	0.846797	0.337641	4.802655	4.435159	2.810678	0.097547
1.762713	3.655714	1.778153	8.888167	2.565556	13.15079	9.438687	5.351934	1.237063
0.000887	0.354798	4.33E-05	0.023997	0.059877	0.102352	0.086991	0.077417	0.034776
0.000887	0.585281	0.617763	0.023997	0.585281	0.276609	0.309877	0.077417	0.065772
1.464214	0.784372	1.68671	0.252165	2.114437	0.392944	1.009625	0.330866	1.636118
0.193723	0.089769	4.33E-05	0.023997	0.329091	0.976667	1.054899	0.454978	8.66E-05
2.961768	8.888167	10.67991	45.91841	18.05488	34.38341	46.7056	11.36975	1.947316
0.308442	0.670519	0.592532	2.45575	1.754733	1.240981	1.178939	0.685931	1.019791
0.712929	10.38202	4.655916	0.023997	1.501291	18.12541	1.828781	2.586926	2.522684
0.033745	0.004242	0.004329	0.023997	0.025274	0.010707	0.020346	0.013723	8.66E-05
3.070952	3.636789	2.515938	0.350274	3.324452	1.784755	1.723579	7.037345	3.256587
45.1045	20.34063	34.38341	7.897078	24.2842	12.56693	11.14619	7.737807	38.94854
2.165851	0.630505	0.209978	3.435988	1.608427	0.545808	1.80267	0.92114	0.852172
8.660281	12.49452	6.900202	0.023997	10.23768	7.288788	18.91854	9.751039	13.45939
0.000887	0.01329	0.008283	0.65798	0.078038	0.110642	0.099913	0.219307	0.006984
1.193362	1.43039	0.57197	0.159639	1.542302	1.046703	0.596111	1.558571	1.584105
0.000887	0.04886	4.33E-05	0.278499	0.235296	0.185166	0.000188	0.30215	0.467136
4.174964	7.545584	7.780786	0.023997	1.451775	6.230245	1.009625	10.53159	5.409437
0.042143	0.147807	0.004329	0.824885	0.095685	0.047713	0.000188	0.048434	0.027922
0.000887	4.33E-05	0.020462	0.159639	0.032799	0.047713	0.000188	0.000188	8.66E-05
8.445743	20.85876	10.55571	0.86987	15.77678	11.719	13.38418	12.42965	14.04491
4.277006	1.243442	0.256205	0.434863	1.087345	0.906825	0.676999	0.647691	2.203535
27.66779	0.73583	6.268312	0.023997	2.216162	1.574517	0.946638	1.863506	8.195657
8.573434	5.165599	5.560563	0.023997	1.553074	7.854069	7.238023	1.253088	10.62739
1.883535	4.869553	2.561162	5.395584	3.904517	2.970144	2.304452	0.487128	1.849654
3.273045	3.479134	2.876421	0.614545	1.553074	2.190866	2.048781	1.805166	3.324452
2.091313	1.63039	1.634935	32.31123	1.889711	8.780115	18.05488	12.77772	2.400455
3.500808	4.174964	3.939307	1.313737	3.489899	2.019618	1.320173	1.436623	1.96404
7.697063	3.971061	5.402511	0.570664	8.195657	2.947287	3.511263	0.982698	14.88584
13.83359	10.48101	18.48514	1.155411	10.45584	7.339452	13.23022	23.82354	20.85876
0.000887	0.278651	0.246825	1.313737	0.593434	4.33E-05	0.563499	0.41026	0.974257
1.21215	1.932857	0.114012	0.326912	1.061457	0.718355	0.351595	0.372078	0.015685
2.690823	2.802828	1.273636	4.921082	3.869163	1.264589	0.990909	0.077417	3.489899
0.000887	0.390895	0.162915	3.55583	1.765065	0.403175	1.143954	0.165981	0.286522
0.033745	0.041053	4.33E-05	0.278499	0.032799	0.069365	0.309877	0.094841	0.027922
0.487662	1.325743	0.961486	1.251659	1.304877	4.776407	2.65684	1.053528	0.136061
3.833247	5.416623	3.632973	0.023997	2.417424	2.27254	3.163506	13.15079	3.851414
1.724805	0.55215	1.401068	0.802035	1.928629	1.147807	0.913514	1.070115	0.286522
0.113218	1.638499	0.304545	2.060491	0.516061	1.723579	0.88158	2.574733	0.517612
0.170938	0.139841	0.12324	11.01243	0.095685	3.104394	4.120815	5.993045	0.504416

0.000887	0.201789	4.33E-05	1.251659	0.162468	0.110642	0.41772	0.062107	8.66E-05
1.604827	3.816551	1.816436	0.614545	11.25392	4.827691	1.396494	0.402381	5.500678
0.000887	0.147807	4.33E-05	0.899567	0.132626	0.126522	0.139567	0.180368	8.66E-05
4.174964	5.485685	3.083319	0.023997	1.35259	0.392944	0.913514	0.205931	3.166659
0.998312	0.235296	1.721371	4.921082	1.296905	0.853218	3.13456	0.706053	1.329206
12.10289	9.377345	8.660281	2.45575	1.286494	6.55039	4.764055	2.155447	5.34474
7.339452	17.03919	14.08569	0.023997	7.868146	8.508514	11.83593	7.61176	13.00996
0.042143	0.033312	0.096811	10.8813	0.200519	0.49974	0.309877	0.173975	0.053672
0.033745	0.018788	0.014084	2.638139	0.032799	0.297807	0.057857	0.077417	0.010519
2.763261	8.256465	3.043528	0.304387	5.855281	6.404921	4.120815	5.030866	3.771349
3.243225	4.429769	2.652107	8.365072	4.048499	4.857403	6.508427	4.145209	2.148658
1.975772	7.625209	2.234538	5.180058	3.957734	3.188918	6.601833	2.638139	1.65785
1.006623	1.865043	0.798153	0.543939	1.296905	0.535029	0.652807	0.041097	0.540902
6.22031	3.404372	2.561162	0.023997	2.197828	1.819221	3.070952	4.048499	6.900202
0.113218	0.181068	0.499206	0.023997	0.132626	0.185166	0.850325	2.089639	0.493247
3.842554	13.30906	13.6814	0.023997	0.559221	28.87506	1.109726	3.350224	6.591544
0.113218	0.226883	0.068449	1.721371	0.458521	0.110642	0.443543	0.165981	0.181674
1.297763	0.85728	1.006025	5.25254	5.788153	3.401126	5.316378	3.984747	1.755988
23.6012	87.1746	18.33909	5.830152	135.5245	37.29684	32.31123	21.54823	40.7932
0.350455	0.406912	0.304545	4.077403	0.408644	1.016494	0.55215	0.322684	0.38772
4.540794	1.059553	1.29417	0.023997	0.707475	4.33E-05	0.182597	0.000188	2.522684
0.000887	0.122944	0.105498	2.032706	0.374365	0.439776	0.406212	1.240202	8.66E-05
0.712929	2.302626	1.284747	0.023997	3.207807	1.51215	0.406212	1.50026	0.909856
2.925426	6.99026	3.984747	1.663672	6.900202	5.462475	4.174964	2.487576	1.602619
3.230065	1.732468	1.766248	0.592532	4.236962	1.399315	2.852677	0.813788	6.464906
0.178817	0.329798	0.060649	7.938427	0.289603	0.318499	0.946638	1.622006	0.316126
0.573947	0.390895	0.873579	1.588903	0.601623	1.723579	1.757244	0.911905	0.27075
0.462857	2.961768	0.637994	0.488167	5.24562	1.51215	0.366147	0.461811	3.592063
2.325931	3.934986	2.922778	4.971991	3.080317	4.802655	3.377619	1.691587	0.845887
0.193723	2.873817	0.816479	5.545729	2.375974	4.927302	4.353925	4.94026	0.974257
5.864084	2.542605	4.338629	0.434863	2.884762	0.392944	0.000188	0.000188	5.395584
3.256587	4.614618	1.578687	0.023997	0.873579	1.356364	2.715873	5.366335	10.53159
0.452713	0.821414	0.708124	3.122915	1.209776	1.016494	1.039199	1.462143	0.827273
16.09482	22.96463	10.43066	0.023997	12.03267	11.544	3.493362	22.74797	10.98644
10.19468	8.508514	5.11697	1.313737	11.719	9.335397	8.412367	7.162698	6.079596
0.000887	0.025483	0.105498	0.408175	0.132626	0.102352	0.000188	0.10956	8.66E-05
20.68746	24.2842	14.17388	14.42661	9.377345	15.98892	9.968355	6.355224	21.74051
1360.293	224.9555	87331.38	29.82828	252.4251	530.1465	167.9996	144.7919	184.443
0.706053	8.134084	1.87899	70.11932	1.988196	9.101674	4.875527	2.77899	1.708932
3.13456	2.485534	1.991328	0.023997	0.113218	4.33E-05	0.086991	0.165981	2.498478
7.598312	1.070115	3.592063	0.023997	1.514199	1.147807	3.921645	1.291825	3.243225
6.911486	6.632063	9.667316	2.730072	7.991602	7.099913	7.810058	2.406768	5.923838
75.1132	35.776	20.34063	0.023997	5.646811	3.807648	1.178939	1.547734	59.32013
8.288874	2.593506	4.914863	0.023997	3.397706	0.991631	0.523983	0.813788	15.66023
52.56128	36.26061	45.1045	0.023997	18.62711	0.656753	1.889711	1.122359	32.68313
8.103925	12.42965	10.93101	8.815123	18.19595	14.70188	11.19942	14.29947	6.967547
3.820758	2.150303	1.618773	0.326912	1.35259	0.48583	1.50026	0.34443	1.189769
2.479221	5.671126	1.171349	0.023997	3.207807	0.68557	0.339004	1.002215	2.666494
0.679812	0.749481	0.663276	2.579509	0.873579	1.08671	1.279452	2.325931	0.380649
0.000887	0.181068	0.044805	0.252165	0.086883	0.163651	0.000188	0.583369	0.097547
9.19899	10.82954	10.1028	3.783694	19.06893	8.849697	7.545584	4.49671	20.51271
4.029654	14.12978	5.508131	0.956061	3.799885	11.36975	3.35715	7.188095	3.33728
0.000887	0.041053	4.33E-05	0.434863	0.078038	0.086775	0.057857	0.000188	0.084524
17.03919	10.78056	16.44768	2.199639	9.316241	7.882222	6.316328	11.544	8.319019
1.439704	3.207807	0.873579	0.023997	0.542165	0.252165	0.281068	3.425512	1.439704
0.064372	0.725267	0.173723	1.283911	0.715815	1.016494	0.962698	1.805166	0.23303
1.05824	1.070115	1.368167	1.626782	1.988196	1.910974	2.538218	1.593535	0.60202
0.000887	0.320931	0.035671	1.484553	0.191703	0.354545	0.281068	0.06912	0.041443

4.620159	3.457504	4.678846	1.826147	4.445851	2.27254	2.227273	8.019769	2.138131
0.91575	3.334199	1.511198	0.65798	2.681169	3.855952	1.699091	1.992944	0.478413
0.033745	0.065094	4.33E-05	0.701818	0.068896	0.252165	0.078297	0.048434	8.66E-05
5.704646	4.971991	2.970144	0.179177	3.703175	2.876421	0.790837	1.982208	5.044113
30.49289	18.05488	21.93452	6.570433	34.38341	8.319019	15.1268	7.136537	60.77449
11.09133	5.416623	4.338629	0.023997	3.080317	4.33E-05	1.161501	0.000188	13.30906
0.000887	0.107626	0.027879	4.034423	0.041782	0.078586	0.755253	0.198211	0.198405
8.539971	3.934986	3.829105	0.023997	9.179466	1.386681	1.518232	3.803766	2.676392
0.000887	0.191602	0.15241	0.023997	0.593434	1.925635	0.497424	1.604827	0.130498
0.026609	0.172063	0.035671	0.86987	0.191703	0.163651	0.174444	0.013723	0.053672
0.817547	1.855108	1.578687	9.946364	2.126349	1.016494	1.757244	2.145137	1.8386
0.274877	0.269892	0.060649	2.333867	0.567071	0.252165	0.294711	0.157619	0.136061
0.000887	0.041053	0.035671	3.394286	0.946638	0.569625	0.057857	1.396494	0.041443
0.398059	1.511198	1.790245	0.023997	1.783203	1.852338	1.783203	0.605887	1.182771
4.524481	5.137042	4.193232	2.758088	7.264502	1.323131	2.917381	0.774257	2.768312
0.33969	0.338341	0.77925	1.511198	1.262179	1.215195	1.889711	0.992771	3.521934
8.048095	2.718434	5.915469	0.408175	4.637994	5.088694	4.140505	1.680895	31.92385
0.055967	0.364502	0.15241	0.802035	0.493247	0.205931	0.381789	0.077417	0.20969
8.608153	7.200505	7.375794	0.463485	5.337547	2.540462	3.899993	6.853608	8.508514
5.993045	4.535354	7.558139	1.399315	3.119986	4.764055	3.8379	4.473348	2.347439
0.123535	0.201789	0.141876	0.68062	0.542165	0.354545	0.381789	0.439062	0.286522
9.968355	4.789596	5.976724	0.023997	12.93651	0.174444	0.000188	0.000188	12.46209
0.5129	1.612056	0.741962	0.023997	1.870685	0.523983	1.340036	1.728615	0.697496
1.531313	1.50026	1.384921	0.023997	0.550664	1.003016	0.000188	0.157619	1.1379
0.000887	0.131659	0.020462	1.283911	0.458521	0.464387	0.113911	1.173535	0.167273
0.000887	0.11531	4.33E-05	1.217475	0.122554	0.222698	0.047468	0.295671	8.66E-05
0.123535	0.689509	0.391674	1.102691	0.988932	0.48583	0.381789	0.28026	0.861934
1.283911	1.855108	2.217951	0.023997	0.516061	0.102352	1.082179	0.359697	0.768535
0.000887	0.147807	4.33E-05	0.929286	0.104149	0.110642	0.099913	0.10956	0.047569
0.055967	0.073038	0.044805	1.009625	0.000736	0.094841	0.205382	0.191602	0.015685
2.752893	9.880779	5.00404	0.023997	3.223882	2.325931	1.069574	2.234538	3.29355
0.170938	0.28746	0.256205	0.140418	0.153016	0.094841	0.020346	0.393983	0.215859
3.243225	4.343838	3.321284	1.78759	4.857403	5.24562	3.592063	1.728615	3.363911
2.933903	1.178939	1.02798	1.511198	1.474343	2.27254	1.143954	0.605887	3.13456
0.471025	0.201789	0.675556	3.695815	3.169812	0.744113	0.693925	0.322684	0.097547
4.115844	1.844098	9.160382	0.023997	1.397482	9.031082	22.74797	9.646284	4.796126
0.042143	0.11531	0.035671	0.434863	0.319163	0.047713	0.205382	0.017879	0.021126
7.795642	3.816551	5.28039	0.023997	1.975772	2.735368	3.592063	0.992771	3.783694
25.48179	28.87506	35.776	0.023997	22.52375	18.91854	14.17388	24.04374	51.52195
0.21645	0.346342	0.087734	0.513752	0.550664	1.264589	1.607143	1.702706	0.091306
0.201645	0.24443	0.068449	2.23267	0.319163	0.69583	0.099913	0.28026	0.188124
7.30215	17.28195	10.23768	0.570664	8.745238	9.625253	14.70188	5.044113	12.36443
2.396429	2.205476	2.203535	0.252165	4.034423	1.323131	0.512309	8.412367	2.640332
0.225823	0.615996	0.391674	0.023997	0.384798	0.222698	0.55215	1.640779	0.783398
0.92816	0.28746	0.73127	12.39704	0.078038	0.242893	0.326017	1.360866	0.23303
11.719	10.05618	13.96064	0.023997	5.28039	6.191515	2.786898	2.713319	3.621941
21.93452	6.38456	13.38418	0.023997	6.181652	8.711905	13.45939	4.286962	16.5625
1.633752	1.314675	2.276407	0.023997	0.698297	8.005685	11.4544	1.558571	4.277006
55.04081	67.98069	47.5018	0.981558	51.52195	13.60866	28.23632	19.53061	45.1045
0.477951	0.517092	4.33E-05	0.023997	0.988932	0.392944	0.126659	0.782417	0.625498
0.252316	1.304877	0.304545	8.288874	1.542302	2.190866	1.109726	2.034242	0.366147
0.904856	1.381111	2.402468	7.653391	0.748341	1.819221	1.485483	4.317648	0.868341
14.00414	6.464906	20.51271	1.313737	7.532713	4.145209	7.264502	40.7932	8.573434
1.297763	0.528196	0.987309	0.846797	1.684646	0.094841	0.613211	2.563506	1.283059
2.259661	1.560779	0.760866	0.749481	1.880375	1.160707	0.707475	1.213434	1.636118
0.471025	0.640606	0.31702	6.922771	0.433131	0.523983	0.537807	0.295671	0.852172
1.633752	1.765065	0.235613	0.023997	0.913514	0.252165	0.294711	0.309444	1.068348
0.000887	0.497749	0.200794	2.831501	0.483874	0.174444	1.229553	2.638139	0.174372

0.91575	0.437576	0.816479	0.379495	0.516061	0.380649	0.850325	0.06912	0.744531
87331.38	87331.38	749.7839	0.023997	0.392749	224.9555	1.542302	0.30215	0.097547
0.113218	0.025483	0.3607	0.408175	0.032799	0.645036	0.139567	0.25197	0.053672
0.000887	4.33E-05	0.044805	0.350274	0.032799	0.021645	0.057857	0.077417	0.141378
0.133939	0.172063	0.027879	0.824885	0.104149	0.297807	0.078297	0.062107	0.041443
22.74797	2.367763	3.460909	0.543939	1.533535	0.601227	1.320173	1.680895	9.031082
0.719942	0.585281	0.675556	0.159639	0.483874	0.557056	0.596111	2.013182	0.963853
0.283709	0.585281	0.488759	9.604358	2.02956	0.569625	7.188095	3.330909	1.052208
1.553074	2.413146	1.458038	1.04228	2.868456	2.173081	1.485483	1.582915	1.474343
0.000887	0.04886	4.33E-05	0.434863	0.041782	0.027237	0.099913	0.062107	8.66E-05
1.992944	5.22482	2.802828	0.956061	4.06303	3.446732	4.524481	4.145209	2.380079
0.113218	0.866717	0.173723	0.023997	0.81596	0.718355	0.351595	0.086595	0.671371
0.33969	2.183665	0.824885	10.19468	1.132165	5.967879	2.59557	3.025671	0.761277
0.000887	0.025483	4.33E-05	1.009625	0.282424	0.403175	0.020346	0.033874	0.010519
3.767489	1.817828	3.422237	1.364401	5.950664	1.386681	2.563506	9.924372	6.99026
1.252424	0.427937	0.352006	0.023997	0.601623	0.718355	0.720938	0.000188	2.180022
0.620014	1.364401	0.687431	5.210317	0.632857	1.264589	1.889711	2.468117	0.632569
13.38418	9.418196	8.348817	0.776349	1.861999	2.786898	3.779452	1.631544	13.38418
0.252316	0.098629	0.12324	0.86987	0.032799	0.126522	0.078297	0.774257	0.512309
0.000887	0.007879	4.33E-05	1.692763	0.000736	0.010707	0.000188	0.000188	8.66E-05
2.145137	1.05088	1.384921	0.379495	1.451775	1.133622	1.607143	0.869365	1.219856
0.074177	0.107626	4.33E-05	0.463485	0.218045	0.153189	0.351595	0.675938	0.188124
8.780115	6.404921	6.853608	0.304387	3.446732	2.150303	1.320173	1.369928	9.438687
0.266277	0.596111	0.560325	3.730808	0.433131	0.318499	1.026219	0.877807	0.931457
4.003117	5.630815	5.591717	0.023997	3.479134	5.630815	3.13456	4.115844	4.18456
13.45939	10.1459	5.855281	0.463485	14.08569	10.28573	5.25254	4.557605	9.141299
0.074177	0.147807	0.027879	3.648081	0.104149	0.756176	0.126659	0.213218	0.065772
1.260635	4.907929	0.749942	3.269913	6.153102	2.970144	3.754719	2.214372	2.440895
0.817547	2.150303	0.57197	0.023997	2.55184	0.308672	0.000188	0.000188	0.974257
0.92816	4.174964	1.596782	12.22798	7.964315	1.133622	1.306667	1.405772	1.000794
0.244964	1.765065	0.749942	5.993045	1.385801	1.122359	1.58645	3.089293	0.286522
0.550072	0.92816	0.068449	0.179177	0.172453	0.622092	0.268961	0.894704	0.286522
7.882222	41.47925	7.991602	1.313737	2.973059	8.958225	9.859719	3.629177	1.593535
16.34294	5.454949	8.849697	7.188095	6.518709	3.35715	5.923838	12.29473	13.15079
18.19595	26.52537	9.707677	1.626782	34.84299	4.580938	5.959033	0.140924	19.22139
0.000887	0.329798	4.33E-05	1.186457	0.059877	0.779668	0.000188	0.041097	8.66E-05
1.406746	4.317648	3.113478	0.434863	2.92013	6.316328	2.171429	0.774257	1.130794
5.416623	5.754935	5.402511	2.16759	10.19468	5.500678	7.38847	12.03267	7.315512
0.371299	0.201789	0.352006	0.278499	0.803211	0.078586	0.068384	0.077417	1.250895
9.459177	9.459177	11.14619	2.831501	12.29473	6.653016	10.17029	9.335397	9.667316
0.413449	0.278651	0.218817	1.283911	0.793918	0.464387	0.294711	0.34443	0.724885
0.074177	0.11531	0.068449	0.252165	0.191703	0.19596	0.497424	0.140924	0.021126
1.68938	0.311876	0.479401	0.023997	0.643853	1.356364	0.099913	0.086595	0.396472
1.369048	1.748016	0.858817	1.826147	2.181775	2.248896	2.65684	2.192496	1.465123
2.136335	2.621133	1.790245	1.072843	5.158146	3.077316	1.757244	2.129596	1.3829
0.000887	0.162915	4.33E-05	0.304387	0.534214	0.335786	0.537807	0.696234	0.23303
1.423506	0.81228	1.353608	2.831501	3.588369	2.037114	3.33728	0.911905	1.283059
0.026609	0.107626	4.33E-05	2.097843	0.041782	0.143709	0.268961	0.487128	0.015685
43.49658	23.82354	40.11561	0.023997	0.493247	20.68746	0.563499	0.198211	1.755988
1.499214	0.772157	2.419553	1.399315	0.78526	0.656753	0.676999	1.462143	0.755253
0.000887	0.406912	0.060649	1.283911	0.113218	4.33E-05	0.086991	0.157619	8.66E-05
2.673846	43.49658	2.66404	6.404921	7.570693	64.12971	18.76954	11.14619	1.636118
425.62	30.49289	332.0776	0.023997	17.28195	4.33E-05	2.609293	0.000188	26.52537
10.67991	3.404372	5.238701	0.846797	5.737056	3.310758	8.958225	1.436623	14.42661
1.107605	0.406912	1.535722	0.023997	0.059877	0.174444	0.339004	1.097677	6.107114
5.530274	7.339452	3.497085	1.072843	2.278254	4.959358	5.22482	2.993867	6.287561
36.74522	40.11561	45.91841	19.91794	57.84882	28.54786	27.52753	11.19942	21.19547
0.000887	0.004242	0.004329	0.636291	0.059877	0.118644	0.086991	0.06912	0.015685

0.371299	1.059553	0.427937	2.097843	0.724004	1.723579	1.229553	1.89373	0.396472
0.000887	0.025483	4.33E-05	0.592532	0.095685	0.126522	0.000188	0.28026	8.66E-05
4.557605	2.057056	3.632973	1.78759	1.443059	1.340036	0.381789	1.061457	1.343463
0.550072	0.162915	1.328384	0.023997	0.200519	1.938759	3.592063	1.449877	1.428398
3.930664	2.472576	2.203535	0.023997	1.724805	1.962424	0.693925	0.149423	0.692381
1.762713	0.24443	1.578687	0.023997	0.252619	1.003016	0.182597	0.048434	2.242092
3.169812	1.080909	2.308203	0.570664	0.275058	0.242893	0.946638	0.402381	2.008312
23.38378	18.48514	22.32214	2.003723	14.42661	13.53913	14.79742	9.256537	17.64869
11.19942	21.54823	23.93364	1.217475	4.524481	24.73724	23.16622	14.97762	12.29473
0.487662	0.261111	0.479401	10.82954	0.104149	0.110642	0.457431	0.116097	0.161962
9.418196	2.638139	4.94026	0.023997	0.078038	3.188918	0.720938	2.845036	9.502294
8.348817	7.315512	3.13456	33.4985	4.946587	23.06543	22.52375	16.44768	6.853608
1.34267	1.985253	0.592532	0.023997	1.022338	0.718355	0.257143	0.062107	1.094978
0.92816	1.126775	1.180613	6.508427	1.111977	3.230065	4.174964	11.25392	1.057569
0.868853	3.046551	4.38684	0.023997	1.052208	1.938759	1.026219	0.41026	6.063276
0.587965	1.214315	1.401068	0.570664	0.542165	1.4671	0.830289	1.128788	0.396472
1.364401	2.400455	1.439704	0.543939	1.190491	0.792886	1.21215	2.055289	1.930216
0.152749	0.11531	0.096811	1.102691	0.000736	0.48583	0.281068	0.000188	8.66E-05
1.118066	2.755411	2.08013	10.38202	4.217569	4.580938	3.629177	2.810678	1.343463
1.553074	4.33342	2.351299	11.11876	3.24658	9.141299	7.074329	3.651898	3.640606
1.865043	1.291825	1.219019	0.023997	0.308672	0.34544	0.92816	1.080909	1.614257
0.33969	1.05088	0.173723	3.411097	0.670144	1.962424	0.613211	0.805801	0.441833
0.08557	0.261111	0.209978	0.408175	0.095685	0.365635	0.443543	0.789863	0.563499
0.055967	0.01329	0.044805	0.592532	0.068896	0.185166	0.205382	0.086595	0.073038
3.370527	1.569553	6.011515	2.23267	3.548153	0.365635	1.082179	1.291825	1.175094
2.45772	1.569553	3.540606	0.379495	3.195541	0.523983	2.236407	1.200823	3.860491
0.000887	0.007879	0.004329	0.230289	0.000736	0.010707	0.013867	0.454978	0.006984
0.209401	2.703485	0.888485	13.00996	3.324452	4.894509	8.180094	26.25557	0.795786
2.238341	2.502619	1.511198	0.824885	4.328232	0.991631	1.396494	1.327525	2.021068
1.812114	3.718716	0.858817	0.899567	2.225498	2.481356	1.340036	1.396494	1.666089
10.28573	9.838658	5.024293	0.543939	9.880779	6.738211	5.9071	4.900996	7.276645
2.792035	0.725267	1.491977	0.379495	0.715815	0.426118	0.737403	0.000188	1.094978
0.21645	0.373218	0.286183	2.097843	0.504098	0.69583	0.257143	0.268528	0.241255
0.178817	0.364502	2.463911	3.855952	15.07257	3.540606	0.720938	0.34443	0.010519
0.780519	0.311876	0.675556	1.009625	0.252619	0.242893	0.268961	0.102352	0.595022
2.151919	1.906551	2.890245	0.179177	1.317475	2.481356	1.4671	5.11697	4.376118
0.08557	0.191602	0.470512	1.547734	0.275058	0.464387	0.217316	0.128759	0.181674
0.5129	1.25816	0.488759	2.5271	1.493968	4.696313	3.711097	2.304452	0.530851
8.195657	2.834084	3.263391	6.38456	7.002056	1.613232	2.99987	3.043528	7.507619
1.604827	1.10132	1.484553	0.023997	1.204228	0.976667	1.250058	0.774257	3.387864
27.96248	33.07147	23.38378	0.023997	6.240346	3.35715	10.93101	3.57057	22.32214
2.828918	1.381111	3.166659	0.023997	1.123016	0.779668	0.990909	1.793074	2.683478
0.487662	1.575483	0.858817	0.304387	0.346732	0.756176	0.381789	1.327525	0.482706
1.96404	2.834084	1.511198	1.313737	2.300678	2.043795	3.864827	5.864084	1.401977
12.77772	8.319019	10.33476	2.419553	4.508196	4.096407	4.833398	4.165137	14.97762
0.421342	1.34267	0.460938	4.888023	0.614545	2.363442	2.481356	3.016984	0.671371
5.309264	6.036818	3.435988	59.32013	8.923117	9.924372	5.381227	6.181652	7.6393
0.524646	0.406912	0.329798	2.396429	0.235296	0.153189	0.086991	0.000188	0.27075
0.095224	0.390895	0.18324	2.294733	0.104149	0.403175	0.294711	0.260584	0.117734
111.5439	10.19468	55.04081	0.023997	14.17388	12.10289	45.1045	37.29684	118.7028
0.421342	1.193362	1.384921	0.023997	1.361789	0.252165	0.90013	2.024488	1.123016
3.310758	1.831631	0.961486	0.614545	0.632857	1.477388	1.178939	0.982698	0.70329
0.000887	0.191602	0.200794	4.203001	0.000736	0.756176	0.773766	0.372078	8.66E-05
1.260635	5.80469	1.180613	0.230289	32.31123	1.660216	1.92272	0.431977	0.882619
16.5625	19.06893	6.316328	87331.38	1.921212	27.96248	16.09482	27.96248	24.98218
0.000887	0.007879	4.33E-05	1.009625	0.000736	0.016104	0.000188	0.205931	0.034776
3.993665	3.350224	0.836537	1360.293	2.723247	4.696313	0.406212	15.98892	1.548723
4.851227	4.586753	4.292078	0.023997	1.94311	1.541227	1.938759	0.715108	2.6143

1.553074	2.690823	2.248896	4.468009	2.800332	4.125786	3.236457	1.833102	2.121198
1.96404	1.788874	2.581861	0.023997	0.29842	1.074242	3.807648	0.557763	1.666089
5.22482	4.212554	3.592063	2.981962	3.648081	6.277937	7.882222	1.650274	5.737056
0.533874	0.749481	0.749942	19.37139	1.182771	1.784755	5.11697	0.965065	0.645455
5.754935	5.976724	14.97762	4.650007	10.93101	15.07257	23.82354	6.706472	5.583968
0.000887	4.33E-05	0.078124	7.854069	0.095685	0.297807	4.709033	1.279452	0.065772
0.113218	0.218045	0.12324	0.749481	0.182078	0.163651	0.366147	0.706053	0.396472
3.592063	2.873817	7.417359	16.22108	7.188095	12.49452	3.651898	2.74544	2.212619
23.16622	83.489	20.68746	100.0998	118.7028	48.53429	67.98069	60.77449	30.17192
44.27012	19.83727	31.1753	2.730072	15.18102	11.09133	21.19547	27.10502	48.53429
9.256537	56.39528	25.24367	105.1694	30.49289	42.75576	5.889798	32.68313	7.697063
15.07257	23.38378	11.63037	16.09482	14.6058	26.52537	33.4985	27.66779	7.507619
41.47925	30.17192	41.47925	2.45575	27.96248	31.92385	28.87506	36.26061	37.29684
10.1459	6.423911	6.414416	287.197	6.181652	7.099913	22.13245	14.88584	6.796566
332.0776	530.1465	425.62	1.751948	1360.293	749.7839	1360.293	749.7839	749.7839
77.74812	252.4251	95.22939	13.30906	425.62	135.5245	135.5245	252.4251	65.94551
60.77449	118.7028	167.9996	4.077403	83.489	184.443	65.94551	287.197	144.7919
67.98069	184.443	144.7919	167.9996	111.5439	332.0776	50.55949	135.5245	34.84299
2.026219	3.227085	2.192496	7.136537	17.78522	8.210938	10.1028	10.23768	2.225498
0.350455	0.968723	0.903074	2.638139	1.215195	1.074242	1.340036	0.447085	1.068348
5.18754	8.429055	6.591544	27.66779	4.796126	33.07147	17.92188	15.83113	5.898449
8.940671	14.47154	10.03313	1.186457	10.1028	5.088694	7.327482	1.934271	11.65791
0.026609	0.107626	0.020462	2.419553	1.215195	0.878932	1.306667	2.638139	0.25197
0.033745	0.057518	0.053203	0.159639	0.078038	0.047713	0.126659	0.351595	0.123853
1.048802	3.363911	1.310837	12.16681	2.586926	6.019971	3.307186	4.222583	1.094978
0.36114	4.33E-05	0.2264	0.701818	0.132626	4.33E-05	0.281068	0.06912	1.270317
6.423911	7.765931	1.696494	43.49658	3.346898	4.620159	2.353247	4.900996	2.171429
0.772157	0.155101	0.12324	184.443	0.182078	0.078586	0.217316	11.03843	0.117734
6.475505	21.36892	8.412367	4.323045	13.83359	16.34294	27.52753	13.23022	11.03843
1.594675	4.419062	3.055729	0.570664	0.998312	2.371833	1.371602	1.92272	3.350224
0.763564	2.382035	3.592063	4.281847	2.114437	3.640606	3.966623	3.953297	1.08798
0.074177	0.218045	0.008283	0.252165	0.078038	0.126522	0.037186	0.000188	0.047569
0.328737	0.381061	0.418788	27.10502	0.810491	0.853218	0.497424	3.188918	1.260635
3.397706	1.214315	0.132799	0.023997	0.504098	1.147807	0.676999	1.41368	2.217951
27.38726	52.56128	44.27012	4.845166	24.50942	36.74522	42.75576	15.1268	16.44768
50.55949	53.77619	72.69473	2.263377	23.38378	41.47925	49.50903	42.08807	100.0998
6.39474	15.56131	8.994791	26.25557	16.22108	18.41211	12.22798	13.91714	4.888023
2.026219	8.815123	3.540606	0.614545	5.646811	6.888297	9.438687	4.821176	2.544747
0.161602	0.311876	0.068449	0.956061	0.678247	0.318499	0.755253	0.198211	0.396472
18.05488	14.33939	22.96463	3.350224	26.80355	36.26061	37.29684	17.64869	16.22108
0.949827	2.302626	1.0386	1.364401	0.374365	2.173081	2.267157	1.378362	0.323752
2.165851	1.351573	1.644416	55.04081	0.282424	3.980123	4.174964	6.486104	3.711097
8.815123	33.93939	9.562554	12.39704	42.08807	39.53687	36.26061	48.53429	9.101674
0.421342	0.725267	0.274365	6.612121	1.724805	0.86518	1.026219	2.531515	1.1379
0.630022	0.478737	0.931457	0.196335	0.576205	1.03189	1.161501	1.360866	0.241255
5.366335	6.30671	5.508131	6.153102	7.38847	3.743232	6.956349	5.80469	4.609076
1.070693	1.314675	1.472208	1.547734	1.58645	2.019618	1.757244	2.390447	0.852172
0.462857	0.716234	0.200794	6.508427	0.425426	2.051962	1.985253	0.766602	0.836537
0.36114	0.528196	0.499206	1.217475	0.823983	0.631847	0.990909	1.41368	0.540902
0.92816	1.25816	1.006025	0.929286	2.159004	1.982208	2.109755	3.373983	0.540902
1.111977	0.506328	1.118817	0.023997	0.142886	0.102352	7.074329	1.04228	1.019791
0.421342	0.829192	1.21215	5.830152	1.64917	1.356364	2.502619	2.243911	2.316075
1.34267	6.772763	1.927042	11.36975	3.163506	4.667518	6.144055	2.515938	0.845887
0.82772	7.288788	1.472208	13.79669	1.578687	10.33476	1.903636	7.938427	6.945152
0.462857	0.041053	0.470512	0.023997	0.252619	0.033593	0.113911	0.000188	0.504416
0.649235	0.089769	0.141876	0.488167	0.000736	0.047713	0.41772	0.295671	0.167273
12.85504	15.88548	14.42661	1.883535	36.74522	3.632973	4.039192	2.792035	6.045209
0.63873	1.135137	0.418788	1.944517	0.946638	1.08671	0.55215	1.667273	0.508975

0.000887	0.004242	0.008283	0.179177	0.025274	0.006825	0.000188	0.013723	0.006984
0.992201	0.841746	0.488759	0.023997	0.803211	4.33E-05	0.182597	0.378066	2.474834
8.381328	6.210375	9.316241	0.023997	3.618499	4.927302	2.419553	11.719	15.46193
1.485483	10.23768	2.678781	42.08807	26.25557	155.7103	287.197	83.489	3.885642
8.319019	6.09759	7.339452	0.350274	5.553146	3.188918	2.54961	0.359697	1.157626
5.821804	0.987821	4.72798	3.230065	1.204228	2.42381	0.720938	0.732633	1.50342
7.854069	2.485534	8.905642	1.128788	3.890556	2.42381	5.316378	29.18885	9.297085
4.667518	7.363117	6.22031	80.6518	5.950664	7.697063	3.217612	4.317648	4.33342
0.790303	0.939408	1.075411	8.660281	1.451775	2.554069	3.119986	3.66785	0.686299
0.000887	0.131659	4.33E-05	0.278499	0.873579	0.464387	0.231984	0.724502	0.053672
8.711905	10.95872	3.998391	28.54786	26.52537	18.12541	17.39935	14.42661	5.477843
224.9555	203.5795	184.443	5.950664	224.9555	118.7028	425.62	155.7103	126.1556
0.000887	0.681919	4.33E-05	0.304387	0.516061	0.656753	0.481602	0.140924	8.66E-05
21.54823	37.81919	28.54786	6.045209	27.10502	75.1132	33.93939	105.1694	6.760765
1.485483	1.612056	0.973745	1.102691	2.723247	1.63039	3.098268	5.416623	0.868341
0.462857	0.707799	0.376782	3.939307	1.372475	2.294733	1.453939	1.157626	0.55645
11.65791	11.83593	10.73084	0.570664	6.695887	5.42381	10.2617	7.991602	6.404921

CGGA_108	CGGA_108	CGGA_108	CGGA_108	CGGA_109	CGGA_110	CGGA_110	CGGA_110	CGGA_110
0.57197	0.411169	0.589618	0.55741	0.384394	1.334762	0.856782	2.290996	0.165368
0.414012	0.133939	0.589618	0.266847	0.135902	3.397706	1.783203	0.998831	0.873579
1.50684	5.238701	0.051595	2.153535	3.820758	0.871429	0.018326	0.363983	4.839105
0.979827	3.432388	0.051595	0.583369	10.57984	0.031919	0.018326	0.000418	10.53159
5.993045	5.01772	0.051595	2.847547	6.325945	0.431977	0.734524	3.581688	8.348817
5.976724	1.1379	9.815642	6.695887	4.115844	11.17281	3.507691	12.63773	2.950173
36.26061	21.74051	8.164531	32.68313	62.33472	13.38418	6.808369	14.79742	49.50903
0.06254	0.067547	4.277006	0.035339	0.000895	1.628009	1.128788	0.06715	0.156566
4.105714	3.722785	0.051595	1.06355	3.864827	0.031919	0.018326	1.588903	1.35259
0.325772	0.000895	1.135887	0.359697	0.089235	0.031919	0.018326	0.931457	1.098341
38.94854	16.44768	60.77449	67.98069	100.0998	75.1132	34.84299	32.68313	91.05541
2.638139	0.29404	9.502294	0.439293	1.0386	5.373781	3.446732	1.344257	3.460909
5.942294	50.55949	4.18456	24.50942	22.96463	3.783694	12.70768	1.560779	20.77311
0.00026	17.64869	0.051595	1.859084	1.991328	0.031919	0.018326	0.000418	8.66E-05
0.029928	0.05632	0.333463	0.011962	0.014329	1.473218	0.778326	0.408644	0.036349
0.757763	9.751039	0.514531	3.107417	4.413608	0.275873	0.4357	1.808023	5.259437
1.541227	0.685209	5.402511	1.662439	0.850325	1.527792	0.661443	3.500808	3.921645
0.63114	0.133939	52.56128	0.095685	0	16.80343	11.83593	0.232092	0.036349
0.950418	5.47	0.051595	3.636789	1.492973	0.031919	0.018326	0.159639	2.16759
2.035678	0.393196	11.19942	0.672078	0.595022	0.414012	2.104358	3.606804	3.803766
0.17619	1.20824	0.051595	0.640289	1.441898	0.031919	0.018326	0.000418	0.557763
0.63114	12.70768	7.724084	2.285664	1.177994	5.477843	8.477662	0.33676	2.16759
5.779574	3.803766	0.051595	167.9996	8.381328	0.031919	0.018326	3.125844	45.1045
0.029928	0.209538	2.183665	0.000188	0.014329	26.25557	4.120815	0.077316	8.66E-05
1.556392	0.602287	5.47	0.848369	1.492973	3.314329	1.641999	0.735382	1.125325
1.522597	4.661717	0.839365	4.207778	9.356371	2.74298	4.684502	1.145426	3.147294
3.621941	1.701616	0.051595	1.628009	4.721789	0.031919	2.807937	0.898016	8.66E-05
0.361717	0.303016	0.839365	0.273261	0.290115	1.672107	0.878369	1.123016	0.2914
0.547128	144.7919	0.051595	65.94551	12.70768	0.031919	0.018326	0.000418	13.38418
0.37228	0.872013	6.181652	0.723066	0.322237	4.490801	6.922771	0.433131	1.790245
1.61088	9.924372	4.381479	3.807648	6.738211	6.181652	6.019971	0.745072	3.885642
0.239769	0.088398	1.039199	0.027879	0.056948	3.743232	1.339091	0.387489	0.134589
0.00026	38.94854	0.051595	18.33909	6.443925	0.031919	0.018326	0.000418	3.695815
0.138947	0.000895	0.371075	0.015541	0.129365	0.261558	0.661443	0.243896	0.127128
21.19547	0.157958	42.08807	3.095166	1.415469	11.03843	16.5625	21.36892	9.418196
1.005426	2.789466	0.051595	2.577157	2.26518	2.223636	1.434055	0.609841	1.125325
3.711097	5.646811	8.556703	4.49671	5.720173	11.48368	2.933903	2.633232	3.636789
0.00026	0.045079	0.267597	0.081587	0	4.833398	0.24562	0.470289	8.66E-05
2.941544	2.373824	6.036818	3.921645	4.160238	4.921082	3.263391	6.045209	3.595758
26.52537	0.998312	7.558139	8.573434	5.030866	14.17388	10.43066	34.84299	12.70768
4.145209	0.411169	1.387612	0.574993	0.35676	2.294733	8.019769	1.248232	0.726472
0.92531	0.421573	40.7932	1.806595	0.929286	6.695887	4.914863	3.29355	1.378362
0.00026	2.676392	0.051595	3.618499	2.26518	0.031919	0.500996	1.030678	1.012742
0.016219	0.000895	0.246955	0.011962	0.009062	0.161962	0.516566	0.038918	0.018016
2.616573	0.248716	6.316328	0.111775	0.396638	1.451775	0.125483	0.598795	1.978954
12.32958	33.4985	0.051595	27.38726	21.36892	1.550837	1.339091	20.85876	23.38378
0.149293	0.108268	5.337547	0.011962	0.200916	0.431977	0.878369	0.159639	0.277417
0.231876	0.000895	0.706053	0.300606	0.16316	1.550837	0.358211	0.769019	0.067547
0.037302	0.000895	0.333463	0.000188	0	0.309444	0.450375	0.021602	8.66E-05
0.325772	0.000895	0.051595	0.000188	0	0.031919	0.018326	0.913514	0.337431
0.129553	0.000895	0.051595	0.021558	0.002179	0.290519	0.158521	0.542165	0.193449
0.795339	4.927302	0.051595	8.195657	5.309264	3.014076	3.13456	12.22798	1.608427
0.38285	0.035426	1.794531	0.517864	0.16316	0.535657	0.260404	0.631847	0.547128
0.497424	0.09847	3.500808	0.273261	0.193045	0.348139	0.158521	0.056869	0.547128
3.771349	5.622872	0.992201	0.315916	2.659214	3.68381	2.676392	3.065108	2.489762
0.874603	0.000895	0.758773	2.577157	0	1.123795	0.018326	1.713997	0.448824
1.02487	0.209538	7.507619	0.446551	0.684401	2.453781	4.721789	0.277251	1.070115

0.92531	0.440801	4.105714	0.359697	0.500354	8.573434	4.120815	1.919661	1.608427
1.948918	1.044235	0.706053	1.884935	3.5257	1.550837	2.518124	1.344257	2.647244
0.38285	0.146768	3.687633	0.212395	0.816479	0.63754	1.085224	0.12925	1.318384
0.00026	21.02638	0.051595	52.56128	0.114437	0.431977	0.018326	0.106768	0.076111
0.149293	0.467677	0.550974	0.254019	0.056948	0.992201	1.085224	1.330029	0.419365
2.16759	0.562439	70.11932	0.300606	0.842641	3.314329	6.956349	0.12925	2.860722
29.18885	19.83727	0.051595	46.7056	23.16622	0.031919	0.018326	1.307417	7.532713
0.089921	0.554416	0.91575	0.294502	0.259098	1.123795	0.661443	0.000418	0.142323
0.00026	0.000895	0.267597	0.000188	0.034459	0.031919	0.018326	0.077316	0.052078
1.934271	0.613211	0.051595	3.55961	2.31811	0.031919	0.563175	2.425801	2.119596
3.227085	1.373348	0.66482	0.986797	1.145426	0.554416	0.24562	0.481104	1.445916
2.735368	0.277085	0.414473	0.280823	0.415166	0.672468	0.467951	2.45575	0.997244
0.414012	0.146768	2.055289	0.368658	0.107828	3.640606	1.641999	1.499214	0.409899
0.17619	0.000895	1.794531	0.088398	0.14987	0.742417	0.82772	1.131472	0.460649
0.605022	0.120144	1.877605	0.976111	0.369105	2.74298	1.830159	0.529697	0.479062
0.037302	0.027879	0.414473	0.000188	0.002179	0.161962	0.018326	0.077316	0.023492
0.022727	0.000895	0.051595	0.021558	0	5.720173	0.018326	0.095382	8.66E-05
1.972706	6.230245	4.381479	1.596782	6.268312	0.348139	1.165065	2.725498	18.19595
0.166898	0.000895	0.371075	0.194531	0.049668	1.431414	1.513102	0.25785	0.209538
0.262338	0.035426	0.371075	0.081587	0.049668	0.704848	1.288276	0.213218	0.059654
4.833398	3.034747	0.91575	6.760765	2.62342	3.367071	1.31	5.80469	2.792035
0.205382	0.000895	1.655541	0.294502	0.101659	1.685678	4.292078	0.180079	0.21689
0.816479	0.421573	0.66482	0.879423	0.437799	1.580909	4.251818	1.074827	0.659957
15.77678	10.93101	2.278254	10.45584	7.188095	0.704848	1.39847	1.388543	12.42965
0.022727	0.108268	0.371075	0.011962	0.042367	0.653968	0.84119	0.029928	0.023492
1.205685	0.045079	0.992201	0.409098	0.859351	3.885642	0.943514	0.49127	1.437648
2.987835	47.5018	0.051595	21.02638	6.171789	0.031919	0.018326	3.87329	10.48101
0.442496	0.157958	1.224733	0.510707	0.544877	2.373824	3.775209	0.507403	0.566494
3.110447	0.393196	13.00996	1.06355	0.500354	6.09759	1.736133	0.470289	0.369336
5.28039	0.754784	1.839986	0.34697	0.617763	0.031919	0.337641	0.931457	4.614618
0.352807	0.268369	0.414473	0.266847	0.290115	0.952294	0.661443	1.145426	0.625209
0.149293	0.000895	1.910974	0.081587	0.049668	1.1407	1.736133	0.399784	0.118903
0.454156	2.494177	0.051595	0.687049	0.487128	0.031919	0.018326	0.268095	0.2914
1.310837	2.558817	0.051595	8.923117	7.570693	0.031919	0.018326	0.000418	9.707677
0.885137	0.000895	1.941703	0.453932	0.430837	0.031919	0.204531	0.948745	0.949286
0.861934	0.120144	1.839986	0.368658	0.244033	0.188427	0.24562	1.369048	1.477388
6.876392	0.411169	2.183665	1.616385	2.465916	11.36975	4.251818	4.297222	3.971061
0.100375	0.000895	0.550974	0.000188	0.034459	4.752121	1.601443	0.180079	0.044214
4.796126	9.101674	0.371075	8.849697	6.497266	3.014076	1.549711	3.418405	3.87329
0.072482	0.035426	0.514531	0.057417	0.009062	1.045462	0.376212	0.25785	0.084113
0.072482	0.000895	1.387612	0.011962	0.002179	1.334762	0.390895	0.06715	0.084113
0.022727	0.000895	0.371075	0.011962	0	0.378326	0.531804	0.021602	8.66E-05
1.491977	1.035801	6.036818	2.394416	0.842641	2.839351	2.720996	1.741414	2.300678
10.33476	5.646811	0.874603	3.533211	14.42661	0.031919	0.595022	3.327619	5.770996
7.625209	0.401724	1.039199	1.247244	6.933961	2.77899	0.645916	3.230065	3.651898
0.224372	0.228203	2.141595	0.648687	0.096061	1.527792	0.911905	1.095657	0.2914
0.239769	0.981558	0.051595	0.273261	1.184935	1.094206	1.106046	0.191486	1.428398
0.583766	0.000895	3.083319	0.890577	0.042367	0.687807	2.104358	0.277251	0.378326
0.394329	0.23873	20.16541	0.176955	0.586595	2.524892	1.62562	0.213218	0.884654
1.09075	6.922771	0.051595	2.143261	1.464214	0.031919	0.878369	0.363983	0.643427
2.185556	0.146768	0.839365	0.439293	1.109726	2.203535	2.628225	0.788896	1.865043
1.771392	1.282208	1.224733	1.546696	2.060491	0.60202	0.697908	5.037489	2.975974
4.33342	0.811443	2.107922	2.990851	0.657114	0.031919	0.716234	2.928247	1.660216
2.638139	0.476768	3.500808	0.628658	2.723247	2.050404	3.446732	0.643427	1.300368
0.00026	167.9996	0.051595	0.662237	20.16541	3.435988	0.973745	0.579149	0.92816
0.17619	3.957734	0.051595	2.081696	1.056263	0.031919	0.018326	0.203254	0.142323
0.617071	0.09847	2.625823	0.06824	0.200916	0.378326	0.484423	0.620462	0.82974
0.00026	155.7103	0.051595	18.19595	0	0.031919	26.95429	0.000418	3.080317

0.56145	0.035426	6.433918	0.161299	0.29715	4.490801	4.025267	0.745072	0.250115
0.696659	0.000895	0.051595	0.273261	0.250823	0.36153	0.018326	2.834084	0.504964
5.359134	1.161501	24.39681	2.758088	2.524892	28.54786	20.60009	2.73272	2.518124
5.671126	8.868932	4.845166	4.72798	11.19942	6.653016	9.237525	3.346898	15.66023
0.138947	0.268369	1.794531	1.485483	0.081212	3.989206	5.210317	0.853218	0.134589
8.539971	2.740519	4.552092	7.417359	6.560411	6.181652	2.77899	5.323492	3.536955
3.91355	0.185022	2.99987	0.723066	1.523824	4.365224	3.279625	7.363117	1.772756
5.431544	0.863694	1.655541	3.578038	2.773644	5.165599	2.164113	3.796075	2.189235
3.695815	0.492417	51.52195	10.98644	1.578687	33.4985	62.33472	8.019769	8.905642
0.63114	0.458521	14.97762	0.654423	0.929286	8.195657	6.116638	0.213218	1.456955
0.081587	0.000895	2.531515	0.000188	0.005411	0.309444	0.390895	0.021602	0.111068
0.06254	1.024127	0.448557	1.742734	0.50803	1.723579	0.358211	0.106768	0.361342
0.737857	0.217677	6.580988	0.229928	0.403413	0.805801	2.104358	0.481104	0.2914
7.897078	4.776407	0.589618	11.719	7.668579	2.8657	0.54847	12.10289	3.676032
0.250823	0.078124	3.625382	0.000188	0.268232	1.360022	2.325931	0.268095	0.118903
0.625209	14.08569	2.400455	1.821861	3.025671	0.378326	0.24562	0.268095	0.791854
3.016984	0.458521	2.470317	0.821898	1.770029	2.947287	3.098268	1.417345	1.660216
0.110534	0.000895	0.706053	0.000188	0.020462	0.724885	1.106046	0.021602	0.052078
26.80355	1.357201	5.583968	1.540144	8.119004	0.309444	5.402511	48.53429	3.125844
0.547128	1.328384	0.051595	0.640289	0.064697	0.031919	0.018326	0.06715	0.378326
0.466335	0.045079	2.470317	0.000188	0.027056	2.398442	1.855108	0.724502	0.165368
1.360866	1.761349	0.051595	0.955418	1.249221	0.031919	0.260404	1.435772	1.549711
1.61088	0.602287	37.29684	1.677042	5.044113	77.74812	111.5439	8.445743	12.10289
3.26	3.442886	1.505678	2.683478	3.046551	3.618499	2.223636	1.369048	2.189235
0.296234	0.440801	0.371075	0.168961	1.014127	0.031919	0.260404	0.363983	1.418312
4.468009	2.061926	2.141595	3.201457	2.678781	16.15795	12.93651	7.315512	4.502453
14.6058	2.845036	5.679502	5.47	3.207807	8.461703	12.32958	28.87506	9.179466
0.497424	1.928629	1.135887	6.135007	3.767489	1.815	3.993665	0.713846	0.42715
0.205382	2.604776	0.874603	0.592857	0.625498	0.485014	0.716234	0.118644	0.10404
1.699091	1.849654	0.051595	3.397706	9.418196	0.031919	0.018326	2.593506	2.413146
33.4985	10.23768	3.083319	11.19942	16.5625	3.276335	9.101674	23.38378	8.780115
0.00026	2.936739	0.051595	0.000188	0.384394	0.031919	0.018326	0.000418	0.378326
9.880779	16.34294	17.39935	8.888167	10.78056	4.130794	3.243225	12.7427	15.56131
7.991602	12.49452	1.308167	5.881342	5.447114	0.031919	0.898016	12.03267	7.013853
0.053838	0.000895	0.589618	0.021558	0.014329	0.261558	0.125483	0.029928	0.023492
0.466335	3.35355	0.051595	1.026219	1.42456	0.031919	0.018326	0.000418	0.605022
10.8813	0.708492	2.868456	5.123781	5.095325	9.687496	4.376118	14.42661	11.48368
0.57197	1.50342	1.839986	1.197215	0.268232	4.317648	1.601443	2.234538	0.783398
0.110534	0.700837	0.448557	2.134545	0.14987	1.334762	0.82772	1.066234	0.345238
0.37228	4.839105	0.051595	2.091313	2.789466	0.031919	0.318312	0.518413	1.287403
0.00026	45.91841	0.051595	0.000188	0	0.031919	0.018326	0.000418	8.66E-05
1.89228	111.5439	0.051595	59.32013	25.73165	0.031919	0.018326	0.000418	72.69473
1.61088	0.228203	14.6058	1.319293	1.890996	5.933066	6.663651	1.114618	7.824473
0.361717	0.067547	1.183478	0.095685	0.096061	1.070115	0.125483	0.769019	0.259646
2.252273	1.373348	0.267597	3.636789	1.554185	0.290519	0.147807	1.547734	0.676999
0.250823	4.105714	0.051595	1.405772	0.532092	0.031919	0.018326	0.34583	1.198701
1.556392	0.194098	1.604827	0.534452	1.243442	3.065108	2.073232	0.853218	0.2914
17.71696	0.000895	1.877605	2.341573	3.621941	0.504416	1.759986	0.277251	0.726472
6.181652	13.60866	1.655541	14.00414	11.11876	2.807937	0.734524	8.958225	7.938427
0.57197	0.754784	0.333463	1.87482	0.391962	0.200657	0.018326	3.065108	0.704848
1.434921	0.000895	1.839986	0.000188	0.056948	3.8121	2.197828	0.408644	1.219019
11.57341	1.89373	8.256465	3.046551	9.459177	22.32214	52.56128	18.62711	18.33909
3.588369	1.044235	0.333463	1.932857	1.766248	0.431977	0.137302	1.344257	1.594675
2.335844	0.000895	2.141595	0.229928	0.443543	2.050404	3.394286	3.687633	1.031284
1.628009	0.177143	2.278254	0.728543	0.114437	4.984488	2.577157	8.849697	0.587619
1.310837	5.671126	0.051595	2.069942	1.935685	0.031919	0.018326	0.499206	1.012742
0.239769	0.000895	0.051595	0.000188	0.114437	0.414012	0.856782	0.375281	0.149986
0.528997	0.1686	0.051595	0.503146	0.217792	0.331263	0.018326	1.267879	0.156566

0.34544	1.42456	0.051595	1.162201	0.341053	0.031919	0.018326	0.118644	0.176104
0.484704	3.493362	0.051595	4.620159	1.219019	4.445851	0.018326	1.624459	6.181652
19.37139	2.494177	6.99026	4.317648	5.485685	13.08214	6.663651	5.959033	8.048095
17.15902	31.55417	0.051595	38.94854	24.98218	0.031919	0.018326	0.25785	19.06893
0.795339	2.037114	0.550974	3.227085	1.780693	0.309444	0.147807	0.777403	1.111977
0.00026	3.116732	0.051595	0.000188	1.109726	0.031919	0.018326	0.000418	5.050267
6.622092	0.323535	5.11697	0.185801	0.516566	2.050404	4.227302	1.962424	0.757273
3.314329	17.92188	0.051595	9.751039	4.702468	0.031919	0.018326	3.763045	6.30671
5.130411	0.268369	17.51961	5.337547	4.212554	12.13485	0.578817	4.067547	6.325945
7.495397	6.325945	5.01772	16.5625	17.98838	12.42965	5.359134	6.045209	11.33981
1.491977	0.000895	0.805238	2.950173	0.322237	1.550837	1.034315	5.84689	2.461905
17.39935	7.188095	4.927302	9.335397	10.43066	5.373781	3.548153	15.66023	13.00996
0.938413	1.255657	0.051595	0.965065	1.362713	0.031919	0.018326	1.624459	0.47
3.747035	3.387864	0.477446	1.570794	4.072475	0.200657	0.230606	3.460909	3.442886
2.112843	0.000895	0.589618	0.574993	0.081212	1.3829	0.734524	0.222864	0.052078
1.177994	1.945916	0.051595	1.846941	1.841371	0.331263	0.217496	1.005426	0.726472
0.737857	3.629177	1.387612	1.197215	1.330029	0.85728	0.756176	0.565137	1.89961
3.833247	0.700837	0.051595	0.993521	2.178268	7.558139	3.730808	3.695815	1.125325
0.016219	0.000895	0.66482	0.015541	0.005411	0.414012	0.318312	0.056869	0.059654
0.00026	1.216768	0.051595	1.941703	2.388348	0.031919	0.018326	0.387489	1.498167
6.760765	4.900996	1.604827	20.16541	11.03843	2.8657	1.808023	6.674286	6.784762
0.00026	0.157958	2.703485	3.432388	0.745613	0.031919	0.337641	0.139091	0.968066
1.09075	0.000895	0.051595	0.477107	0	0.826407	0.274365	0.000418	0.090931
5.309264	9.418196	1.991328	7.938427	3.803766	2.050404	2.259661	9.924372	12.16681
1.471198	6.570433	0.051595	8.694697	7.519841	0.290519	0.018326	10.98644	4.212554
0.00026	0.000895	0.051595	0.135902	0.064697	0.031919	4.614618	1.889711	8.66E-05
0.484704	0.108268	0.66482	0.260144	0.415166	0.826407	1.106046	0.326017	0.47
0.92531	4.519048	1.604827	3.26	1.663672	2.398442	3.214545	1.159149	0.625209
2.845036	0.209538	4.277006	1.68938	1.523824	2.554069	0.82772	3.606804	1.531313
0.874603	0.108268	2.107922	0.566104	0.50803	7.668579	2.873817	2.205476	0.487662
2.221775	6.210375	1.444906	2.250462	4.933781	0.031919	1.533535	0.000418	2.040606
0.898543	0.657114	8.849697	0.541241	1.249221	3.089293	15.18102	0.818066	1.511198
0.361717	0.157958	0.589618	0.288081	0.217792	0.805801	0.734524	0.529697	0.685209
6.287561	24.73724	0.051595	7.264502	2.048781	0.724885	0.018326	1.145426	3.722785
3.734762	0.421573	5.302294	0.266847	0.833045	12.63773	5.024293	2.515938	0.939986
2.436768	13.53913	0.051595	1.89961	1.568564	2.361198	0.018326	2.24562	1.549711
0.029928	0.027879	1.039199	0.144228	0.014329	0.60202	0.288867	0.047092	0.044214
0.17619	0.194098	0.051595	0.300606	0.034459	0.466602	0.018326	0.643427	0.193449
0.045693	0.133939	0.051595	0.000188	0.014329	6.888297	2.259661	0.315332	0.185325
0.325772	0.685209	0.051595	0.34697	1.761349	0.031919	0.991631	0.086075	0.651631
5.150693	0.027879	30.82277	0.965065	0.447662	35.27608	11.03843	9.276811	2.106089
12.77772	12.10289	100.0998	48.53429	40.11561	64.12971	105.1694	8.119004	70.11932
16.9162	6.876392	4.02088	10.2617	22.32214	2.243911	4.644098	15.18102	19.53061
1.666089	1.064935	5.679502	0.786667	1.633752	10.23768	8.573434	0.703665	1.031284
9.063983	2.964762	0.839365	3.243225	4.343838	1.007864	1.034315	2.035678	3.179199
1.376378	0.000895	1.308167	0.431198	0.302554	5.813247	1.180613	7.049336	0.142323
0.861934	0.133939	0.66482	0.453932	0.193045	0.261558	0.158521	0.445996	0.635916
37.29684	35.776	0.051595	9.625253	57.84882	0.031919	0.018326	0.000418	31.55417
0.00026	4.33342	0.051595	0.574993	2.013182	1.974228	0.318312	0.139091	1.468131
1.841371	1.035801	0.66482	2.229048	2.944416	0.724885	4.292078	2.392431	1.809343
5.560563	1.909408	13.6814	3.824964	7.38847	21.36892	10.85542	24.04374	10.23768
1.047345	0.805238	0.051595	0.309062	0.20733	0.031919	0.018326	0.609841	0.185325
0.250823	5.915469	0.051595	0.592857	0.244033	0.031919	0.30215	1.215981	8.66E-05
0.00026	19.68088	0.051595	5.037489	1.95645	0.031919	0.018326	0.000418	8.66E-05
0.404899	0.000895	0.514531	0.321746	0.081212	0.233889	0.191602	0.33676	0.097395
6.570433	91.05541	0.051595	95.22939	28.23632	0.031919	0.018326	0.000418	12.93651
0.593434	1.074242	3.855952	9.815642	3.381255	20.16541	12.42965	2.066652	2.760765
3.471486	1.200152	0.051595	3.346898	3.340361	0.031919	0.018326	7.625209	3.425512

1.133622	0.467677	0.051595	1.21215	0.500354	0.031919	1.148564	1.171349	1.87482
3.599329	1.480541	3.563182	1.578687	4.839105	1.550837	0.516566	11.14619	4.721789
1.055628	2.671299	0.051595	1.360022	1.441898	0.031919	0.018326	0.802756	1.846941
1.278629	18.05488	0.051595	0.848369	6.674286	0.031919	0.018326	0.363983	6.808369
0.00026	126.1556	0.051595	44.27012	15.3618	0.031919	0.018326	44.27012	23.82354
2.786898	0.000895	29.82828	1.890996	0.349278	9.141299	20.34063	1.095657	8.66E-05
0.231876	0.657114	1.746724	1.128788	1.014127	2.243911	1.361789	1.962424	0.855801
1.755988	0.217677	0.630022	0.909315	0.777864	1.705007	4.451154	8.508514	0.82974
0.045693	0.000895	2.470317	0.06254	0.034459	2.223636	1.965657	0.191486	0.084113
1.742734	0.120144	0.051595	2.520404	0.734935	1.527792	0.756176	4.990794	1.188139
0.045693	0.157958	3.297107	0.035339	0.042367	1.123795	1.855108	0.047092	0.165368
0.00026	75.1132	0.051595	7.991602	0	10.80505	83.489	0.000418	2.647244
0.767569	8.815123	0.051595	7.570693	1.971183	0.031919	0.018326	0.000418	1.044863
0.605022	0.323535	1.308167	0.763102	0.200916	1.431414	1.339091	1.215981	0.409899
3.94886	0.855801	3.738997	1.654156	2.483492	7.363117	4.424416	3.500808	3.125844
2.252273	1.746724	0.051595	1.753341	2.474834	0.031919	0.230606	6.717056	3.201457
8.164531	2.08013	31.55417	1.712706	5.500678	13.23022	23.38378	4.376118	11.60283
1.92272	0.647345	3.687633	0.838846	2.021068	2.554069	3.939307	3.5257	2.353247
6.830592	0.677403	41.47925	0.315916	0.443543	5.696263	16.80343	0.420289	2.096162
1.384921	0.667965	3.500808	0.640289	0.996111	1.195794	1.288276	2.99987	2.078355
0.56145	1.860491	0.371075	2.715873	2.438831	1.025613	0.018326	1.359177	0.419365
4.814661	0.120144	2.911962	0.687049	0.86987	8.780115	5.638759	12.7427	3.91355
0.00026	13.23022	0.051595	13.26964	4.413608	0.031919	0.018326	0.000418	11.19942
0.053838	0.120144	0.839365	0.095685	0.000895	4.193232	4.338629	0.169971	0.319762
6.518709	0.29404	9.377345	0.753016	1.590202	3.87329	25.99427	5.770996	2.500548
3.486436	6.09759	0.051595	7.443276	4.63189	0.031919	0.018326	1.624459	2.380079
0.38285	0.157958	2.625823	0.161299	0.20733	1.007864	1.052208	1.290981	0.765094
10.53159	26.25557	0.051595	15.56131	16.22108	1.91254	1.905094	9.502294	28.23632
4.894509	5.821804	4.02088	2.727785	6.135007	2.178268	0.898016	1.741414	3.94886
2.199639	1.05088	0.051595	2.143261	1.554185	0.871429	0.217496	14.25954	0.596508
0.224372	0.000895	1.877605	0.111775	0.175714	1.360022	1.39847	0.499206	0.409899
19.60574	4.789596	56.39528	5.80469	13.83359	27.66779	22.32214	5.082063	33.07147
1.190491	7.013853	0.051595	7.751869	1.90798	0.200657	0.018326	0.000418	0.42715
530.1465	52.56128	8.005685	118.7028	95.22939	20.42667	20.60009	1360.293	41.47925
0.647691	0.067547	1.039199	0.237698	0.327662	8.103925	1.641999	1.321053	0.209538
1.396494	7.212915	4.845166	8.780115	3.025671	57.84882	9.838658	8.075772	1.594675
5.654863	42.75576	0.66482	17.92188	10.28573	1.815	3.966623	7.977958	9.276811
0.273261	0.000895	0.051595	0.000188	0.020462	0.031919	0.018326	0.481104	0.337431
0.00026	0.000895	1.224733	0.021558	0	0.188427	0.24562	0.000418	0.044214
0.00026	7.339452	0.051595	1.219019	0.23246	0.031919	0.633853	0.277251	1.693939
13.49926	20.16541	0.333463	3.45404	13.08214	2.666494	4.072475	1.159149	17.78522
0.00026	0.000895	0.758773	0.015541	0.000895	0.309444	0.260404	0.118644	8.66E-05
0.898543	1.110505	0.477446	0.111775	1.102691	0.031919	0.179798	0.106768	1.098341
0.519387	0.194098	2.66404	0.392944	0.244033	1.61088	3.110447	0.756724	0.84119
0.100375	0.000895	0.758773	0.144228	0.056948	1.070115	0.406212	0.288247	0.587619
18.62711	2.823636	1.703795	13.38418	13.6814	4.160238	1.034315	15.77678	7.443276
2.975974	0.323535	1.655541	1.445916	0.702381	2.62342	9.707677	10.1028	1.519322
4.397792	6.632063	0.839365	5.770996	4.140505	1.974228	0.991631	6.433918	5.180058
167.9996	42.08807	1.941703	203.5795	41.47925	8.319019	1.31	33.07147	59.32013
0.528997	0.000895	4.845166	0.000188	0.466335	3.846984	3.993665	0.870902	0.596508
0.081587	0.078124	0.448557	0.260144	0.014329	4.984488	0.878369	0.232092	0.052078
62.33472	24.98218	28.54786	15.77678	53.77619	6.888297	5.889798	32.31123	65.94551
1.415469	2.089639	0.992201	1.712706	0.684401	2.099524	1.68342	2.547179	1.549711
0.776876	0.217677	0.874603	0.144228	0.183672	0.920483	1.085224	0.948745	1.487619
2.406768	0.411169	22.8563	1.921212	1.126075	49.02166	47.5018	1.651522	1.913997
2.068297	0.133939	12.46209	0.280823	0.50803	13.75978	30.17192	0.353492	1.511198
4.702468	1.34798	17.03919	6.560411	5.095325	33.93939	31.1753	13.23022	4.739668
1.290981	0.157958	5.679502	0.592857	0.384394	2.153535	1.513102	3.276335	1.549711

105.1694	7.653391	95.22939	43.49658	33.07147	45.91841	28.54786	83.489	53.77619
0.285556	0.000895	0.874603	0.027879	0.042367	0.161962	0.484423	0.387489	0.310866
0.422511	2.8657	1.308167	1.247244	2.096162	0.222561	0.390895	0.998831	1.978954
0.045693	0.000895	0.051595	0.120216	0.002179	0.200657	0.645916	0.25785	8.66E-05
4.33342	0.401724	24.98218	1.712706	0.833045	47.5018	19.91794	3.26	4.043846
30.17192	3.324452	2.400455	13.53913	8.849697	18.91854	8.429055	29.49693	6.464906
8.103925	0.754784	11.51384	3.217612	5.381227	7.495397	18.05488	6.560411	8.573434
0.56145	0.000895	1.039199	0.000188	0.101659	0.031919	1.68342	1.083651	0.224921
0.898543	2.148658	0.051595	2.153535	2.987835	0.174372	0.018326	3.195541	9.276811
0.979827	1.50342	3.94886	1.017107	2.189235	6.028427	4.614618	1.248232	3.829105
1.742734	0.000895	2.531515	0.229928	0.516566	11.83593	12.53072	3.734762	1.531313
9.218514	13.45939	1.505678	6.674286	31.92385	6.287561	7.238023	6.287561	10.38202
0.361717	0.202799	0.051595	1.445916	2.425801	0.031919	0.018326	0.643427	0.997244
0.00026	5.143867	0.051595	1.335743	1.717641	0.031919	0.406212	0.518413	0.268961
0.660678	0.133939	10.05618	0.185801	0.193045	2.429957	3.548153	0.33676	0.268961
10.21618	29.82828	20.51271	34.84299	27.96248	15.98892	25.24367	13.08214	19.68088
3.343629	9.377345	3.256587	9.047532	4.869553	1.986681	4.575123	3.460909	5.704646
0.484704	0.194098	0.66482	0.368658	1.126075	0.188427	0.108016	0.542165	0.390418
15.46193	2.312085	10.8813	14.6058	4.302367	38.94854	23.6012	53.77619	11.544
5.889798	87.1746	1.444906	50.55949	11.48368	1.123795	1.212792	37.29684	13.30906
6.116638	0.393196	10.57984	0.330043	1.971183	1.761349	0.24562	1.489524	9.121486
1.360866	0.228203	0.630022	0.736977	0.396638	0.901212	0.147807	0.853218	0.613853
8.958225	0.582973	2.183665	5.606739	9.297085	4.946587	0.878369	30.82277	10.7557
21.93452	1.200152	1.039199	0.246825	26.80355	0.031919	0.018326	1.828781	36.74522
1.080303	10.62739	0.051595	1.360022	2.797835	0.031919	2.287576	0.631847	4.678846
11.19942	2.584394	23.49249	3.307186	3.025671	5.25254	3.966623	7.545584	7.570693
2.941544	1.121703	32.49718	1.142345	1.42456	21.93452	17.34065	2.155447	9.646284
0.029928	0.000895	0.414473	0.144228	0.000895	0.687807	0.878369	0.021602	0.018016
23.38378	11.03843	18.48514	21.93452	16.80343	25.48179	14.00414	45.91841	33.4985
1.259747	6.612121	0.051595	3.592063	2.710765	2.26518	1.601443	5.273362	5.447114
2.524892	2.635685	0.051595	4.02088	2.221775	0.031919	0.230606	0.69583	0.968066
3.425512	7.839271	0.333463	2.958773	2.987835	0.826407	0.376212	4.02088	3.442886
2.050404	3.957734	0.371075	6.230245	4.827691	0.901212	0.856782	3.851414	3.536955
0.325772	0.157958	0.051595	0.309062	0.430837	1.451775	0.645916	1.066234	0.448824
0.19544	0.078124	1.308167	0.831472	0.009062	8.256465	4.160238	1.771392	0.036349
1.415469	0.602287	2.278254	0.662237	0.259098	1.025613	1.513102	3.621941	0.749019
2.975974	0.268369	11.06488	1.367179	1.91811	2.807937	3.563182	5.172828	4.888023
4.863579	0.217677	10.2617	0.095685	1.434055	2.223636	5.599228	4.814661	3.404372
1.819221	65.94551	1.941703	28.87506	20.34063	1.195794	3.629177	4.745895	10.93101
0.885137	4.814661	0.805238	2.300678	1.166717	0.783398	0.756176	0.315332	1.087345
0.979827	0.942944	3.083319	0.548788	1.153723	0.805801	0.531804	0.565137	3.387864
7.112496	1.533535	39.53687	4.165137	4.869553	23.16622	18.91854	2.882035	11.03843
5.889798	1.175859	12.13485	0.831472	4.519048	13.87537	8.119004	15.56131	7.625209
3.425512	3.779452	8.461703	3.695815	2.278254	4.408153	12.53072	1.145426	5.923838
33.07147	7.61176	13.49926	9.418196	36.74522	16.34294	59.32013	21.74051	29.18885
184.443	15.46193	29.49693	1.859084	9.625253	1.156869	0.633853	11.544	252.4251
1.278629	29.18885	5.889798	9.141299	1.568564	8.524242	19.22139	0.49127	2.061926
1.216768	8.048095	0.589618	2.069942	2.925426	0.783398	1.31	0.326017	0.277417
2.941544	5.530274	0.630022	7.482179	8.994791	0.826407	0.54847	1.560779	5.830152
4.222583	0.346732	2.939141	2.66404	3.738997	6.727633	6.433918	4.650007	5.123781
0.022727	0.194098	0.448557	0.392944	2.359264	0.174372	0.390895	0.243896	0.165368
0.00026	0.000895	0.371075	0.043297	0.049668	0.588658	1.31	0.095382	0.059654
6.796566	5.030866	1.703795	2.164113	6.52899	0.348139	0.679452	10.05618	8.849697
14.79742	9.880779	5.522929	37.81919	21.93452	21.54823	5.522929	16.22108	19.83727
0.016219	0.000895	0.333463	0.000188	0	0.233889	0.125483	0.038918	0.011962
0.92531	0.000895	0.299747	0.057417	0.458701	5.075447	2.134545	0.948745	0.409899
49.50903	13.91714	19.22139	80.6518	38.94854	24.04374	36.74522	91.05541	50.55949
1.205685	0.133939	2.939141	0.986797	4.212554	4.445851	1.661328	2.465916	1.89961

0.250823	0.027879	1.991328	0.015541	0.000895	1.218182	2.535974	0.459394	0.011962
87.1746	15.88548	44.27012	47.5018	29.49693	118.7028	144.7919	51.52195	51.52195
5.704646	2.008312	1.308167	4.317648	4.508196	2.928247	4.292078	4.881775	8.241342
0.442496	0.000895	0.371075	0.000188	0.121587	3.953297	24.04374	0.021602	8.66E-05
1.0386	0.035426	17.92188	0.763102	0.135902	2.361198	1.564149	1.99899	1.739935
3.536955	0.108268	6.181652	0.793434	0.369105	2.496407	3.718716	5.80469	3.404372
224.9555	21.19547	155.7103	83.489	56.39528	111.5439	135.5245	118.7028	184.443
14.51648	4.193232	5.01772	13.83359	5.889798	6.853608	1.808023	21.93452	7.315512
25.99427	7.037345	22.52375	12.85504	16.9162	34.38341	51.52195	45.1045	32.68313
0.442496	1.477388	0.051595	1.570794	0.822965	1.64917	0.230606	0.684033	1.198701
18.76954	100.0998	0.955418	144.7919	155.7103	2.666494	1.68342	70.11932	8.815123
19.22139	6.760765	8.556703	12.03267	14.08569	11.89932	2.491948	13.45939	17.39935
4.18456	1.51215	2.74298	2.31013	1.166717	4.490801	4.529913	6.945152	0.390418
2.068297	1.42456	5.01772	0.095685	3.321284	1.173535	7.037345	0.029928	21.54823
2.369841	1.194221	62.33472	4.540794	3.253175	13.00996	24.73724	1.852338	18.48514
10.78056	1.692763	4.770231	7.375794	10.67991	11.60283	4.863579	22.74797	12.85504
2.616573	4.155339	0.706053	33.4985	4.160238	155.7103	67.98069	72.69473	14.33939
0.528997	0.000895	0.550974	2.102626	0.35676	13.60866	3.479134	5.416623	0.448824
0.547128	3.201457	5.679502	1.89961	12.36443	1.405772	19.06893	0.25785	2.579509
20.68746	16.22108	203.5795	53.77619	31.1753	167.9996	100.0998	42.75576	60.77449
91.05541	16.9162	5.273362	51.52195	59.32013	1.61088	3.651898	87.1746	80.6518
5.687879	0.892395	5.402511	0.986797	1.238651	2.050404	4.251818	16.80343	18.05488
35.776	3.851414	25.99427	17.51961	30.17192	19.53061	32.31123	9.101674	24.04374
3.578038	0.863694	0.051595	6.853608	5.22482	0.031919	0.288867	3.29355	13.83359
56.39528	26.80355	26.66446	6.365065	32.31123	0.031919	17.15902	6.153102	27.38726
6.99026	0.613211	5.22482	0.111775	1.971183	1.405772	0.30215	0.913514	5.754935
3.227085	3.829105	0.051595	2.964762	2.773644	0.031919	0.018326	0.818066	2.040606
41.47925	60.77449	0.805238	14.33939	37.81919	0.704848	1.830159	5.273362	45.91841
2.005253	0.000895	20.68746	0.06824	0.142244	4.086926	1.577569	2.24562	1.693939
3.529466	0.339913	2.74298	0.528571	0.792886	4.043846	1.212792	2.572309	2.62342
1.819221	12.85504	0.051595	6.325945	8.412367	0.031919	0.697908	0.000418	7.061328
3.807648	1.373348	0.051595	16.09482	0.577706	0.36153	0.114517	5.84689	0.176104
2.221775	0.29404	3.330909	0.780519	0.55215	1.451775	3.855952	1.171349	0.495238
4.125786	3.672078	0.589618	3.890556	3.303925	0.724885	1.212792	1.462143	1.932857
0.016219	0.05632	0.414473	0.015541	0.000895	0.174372	0.450375	0.056869	0.018016
38.39042	5.737056	24.04374	16.22108	33.4985	21.02638	23.16622	33.4985	25.24367
126.1556	45.1045	749.7839	1360.293	224.9555	425.62	425.62	749.7839	224.9555
332.0776	95.22939	252.4251	184.443	332.0776	203.5795	184.443	80.6518	530.1465
1.190491	0.1686	3.330909	0.600548	1.390476	3.367071	2.429957	0.777403	3.008225
25.73165	38.39042	13.00996	28.23632	27.10502	8.643074	20.16541	20.68746	27.66779
0.696659	0.078124	0.874603	0.998831	0.617763	0.901212	0.450375	0.931457	0.643427
2.723247	3.086306	8.905642	6.00228	2.933903	5.576219	8.164531	5.447114	3.899993
0.352807	0.045079	31.92385	0.294502	0.225079	3.618499	1.783203	0.408644	0.277417
0.737857	0.067547	0.299747	0.021558	0.250823	0.397215	0.018326	0.363983	3.233045
0.029928	0.000895	0.448557	0.000188	0.000895	1.508831	0.633853	0.499206	0.21689
10.48101	1.945916	16.68554	2.735368	5.409437	0.724885	3.533211	2.975974	55.04081
80.6518	23.16622	33.93939	0.933261	5.942294	0.414012	1.808023	25.73165	203.5795
0.138947	0.088398	1.387612	0.043297	0.290115	0.031919	1.3829	0.106768	0.319762
83.489	37.81919	6.036818	42.75576	51.52195	3.953297	6.760765	31.1753	57.84882
0.00026	0.000895	0.414473	0.075123	0.027056	0.724885	0.484423	0.077316	0.111068
64.12971	4.637994	4.277006	12.10289	45.1045	3.397706	0.563175	5.273362	31.1753
0.06254	0.000895	0.051595	0.000188	0.114437	1.473218	0.500996	0.268095	0.460649
2.068297	1.746724	5.889798	1.485483	1.014127	2.178268	3.418405	3.851414	0.820931
0.625209	0.000895	1.262179	0.194531	0.056948	1.306667	0.450375	0.232092	0.576205
0.00026	0.000895	1.089394	0.050216	0.000895	3.91355	1.991328	0.029928	0.007749
0.519387	0.067547	0.839365	0.628658	0.50803	6.784762	2.747908	2.961768	0.487662
45.91841	14.21671	53.77619	24.98218	36.26061	40.7932	33.93939	22.96463	43.49658
7.951371	5.143867	25.99427	13.08214	9.256537	50.55949	55.04081	13.53913	3.179199

10.98644	3.975498	5.11697	11.14619	5.646811	14.08569	4.914863	22.13245	4.94026
7.363117	2.882035	16.34294	2.644885	9.541645	7.92518	19.53061	10.73084	10.28573
0.682799	3.57057	0.051595	3.663622	3.122915	0.485014	1.549711	0.840635	2.335844
0.016219	0.000895	0.051595	0.000188	0.014329	0.554416	0.467951	0.021602	8.66E-05
27.66779	4.959358	27.38726	17.15902	9.218514	25.73165	5.485685	27.66779	9.063983
16.44768	44.27012	2.99987	17.03919	15.276	5.838499	18.76954	28.54786	15.77678
4.690159	0.000895	0.91575	0.55741	2.329784	5.439278	1.288276	0.703665	0.6943
4.043846	1.612056	0.514531	1.680895	4.38684	0.36153	0.30215	2.74544	2.153535
1.384921	0.83088	0.589618	0.945483	1.25746	4.286962	3.750837	1.573276	0.849336
17.51961	4.170051	18.84404	9.218514	19.06893	15.66023	15.98892	10.90615	22.96463
4.33342	0.339913	9.707677	0.945483	1.523824	1.741414	1.710173	1.669466	3.585029
2.597792	2.474834	0.051595	4.592273	3.175916	0.783398	3.13456	2.616573	7.225469
75.1132	7.570693	30.17192	8.745238	19.37139	28.23632	70.11932	36.74522	18.91854
22.74797	14.70188	3.397706	3.625382	19.68088	0.88417	0.82772	12.42965	48.53429
1.310837	0.000895	3.256587	0.06824	0.056948	0.331263	0.911905	0.315332	0.884654
19.60574	16.68554	1.505678	20.34063	24.2842	1.527792	1.148564	24.50942	17.51961
0.120216	0.027879	1.703795	0.088398	0.081212	0.88417	0.500996	0.304921	0.460649
50.55949	1.083651	111.5439	12.53072	8.573434	87.1746	118.7028	24.98218	40.11561
2.818312	0.194098	3.563182	0.321746	0.595022	1.405772	9.625253	0.589618	1.994329
4.894509	7.099913	0.874603	6.200945	4.907929	0.85728	2.164113	1.641999	2.685786
1.047345	0.554416	5.889798	0.392944	0.089235	0.233889	0.16925	0.948745	0.241255
0.34544	1.593535	0.051595	0.288081	1.983824	0.031919	0.230606	1.573276	0.791854
42.75576	5.855281	2.236407	23.6012	5.25254	3.243225	10.17029	1.659033	42.08807
0.273261	0.05632	0.514531	0.176955	0.081212	0.841746	1.339091	0.713846	0.378326
10.82954	4.959358	0.66482	11.96574	3.253175	3.68381	0.137302	16.68554	3.107417
7.037345	14.97762	11.77567	3.989206	17.78522	3.128694	3.921645	3.644343	4.082165
0.053838	0.000895	0.051595	0.035339	0.049668	0.174372	0.217496	0.288247	0.090931
15.98892	1.971183	7.882222	3.989206	8.119004	10.1028	3.842554	6.967547	5.976724
1.68342	0.440801	2.236407	1.628009	0.929286	7.737807	8.888167	3.153485	1.328384
1.455988	0.95101	0.514531	0.392944	0.308788	0.309444	0.563175	1.106046	0.997244
1.216768	8.288874	0.051595	2.453781	1.075411	0.031919	1.450693	0.000418	1.712706
1.786306	0.582973	0.589618	2.190866	1.890996	1.334762	0.943514	3.065108	2.831501
144.7919	2.494177	21.11092	30.17192	6.717056	10.62739	9.101674	75.1132	15.02509
0.528997	6.250447	21.11092	3.201457	35.27608	40.11561	203.5795	2.802828	4.188896
1.144733	1.551962	0.758773	1.237063	2.03886	1.815	1.180613	3.432388	2.59557
0.072482	0.000895	91.05541	0.000188	0.175714	32.68313	34.38341	0.000418	8.66E-05
1.216768	0.476768	6.036818	0.706053	0.7257	1.173535	2.628225	0.49127	1.300368
1.055628	0.637085	4.277006	1.308167	0.878932	3.182482	0.484423	2.102626	0.504964
0.00026	0.000895	0.299747	0.043297	0.009062	0.378326	0.24562	0.159639	0.011962
0.639113	0.000895	19.91794	1.387612	0.268232	9.687496	17.03919	5.210317	1.712706
0.337222	0.000895	7.61176	0.424848	0	1.267085	0.018326	0.445996	0.651631
0.34544	0.035426	1.039199	0.144228	0.156941	0.554416	0.260404	0.445996	0.156566
3.563182	0.892395	0.333463	3.324452	2.425801	0.762165	0.018326	0.832013	4.402973
0.296234	0.000895	5.402511	0.057417	0.20733	0.466602	0.179798	0.445996	0.084113
0.06254	0.035426	0.91575	0.015541	0.009062	0.450051	1.661328	0.000418	0.018016
0.484704	0.277085	1.703795	0.120216	0.101659	2.099524	1.450693	0.529697	0.547128
1.50684	1.622006	0.267597	3.016984	2.688304	0.309444	0.318312	1.852338	0.487662
0.231876	2.197828	2.625823	1.06355	0.78526	2.050404	2.845036	1.713997	1.031284
1.989711	64.12971	0.051595	8.048095	8.333918	0.031919	0.018326	0.745072	3.971061
1.02487	2.323983	0.051595	4.894509	2.126349	1.287403	0.337641	3.55583	0.268961
7.443276	72.69473	0.992201	62.33472	46.7056	1.550837	1.361789	17.51961	4.419062
0.497424	0.09847	0.371075	0.057417	0.056948	0.687807	0.390895	0.655245	0.076111
0.273261	0.05632	0.706053	0.288081	0.50803	1.850996	0.716234	0.507403	0.6943
1.326667	0.838846	7.882222	0.933261	0.800512	7.854069	7.737807	1.624459	0.800967
3.397706	0.346732	16.15795	1.662439	8.608153	10.93101	22.13245	1.695216	7.710361
0.00026	0.742417	0.051595	14.79742	0.55215	2.524892	0.018326	0.086075	8.66E-05
0.045693	0.045079	0.246955	0.000188	0.000895	0.261558	0.288867	0.021602	8.66E-05
1.259747	1.238651	0.051595	1.806595	1.808023	0.246955	0.376212	2.205476	2.792035

0.138947	1.988196	0.051595	2.547179	1.616385	0.031919	0.204531	1.435772	2.300678
1.232886	3.014076	0.051595	5.737056	3.489899	0.031919	0.018326	0.000418	4.110779
0.120216	0.05632	0.414473	0.043297	0.363059	0.504416	3.676032	0.315332	8.66E-05
16.34294	3.482785	1.703795	7.854069	10.33476	0.485014	1.148564	12.89577	9.541645
3.153485	11.65791	0.051595	2.190866	10.8813	0.031919	2.287576	3.204632	6.945152
0.605022	0.734935	3.563182	0.574993	0.996111	4.869553	4.802655	0.481104	1.149978
1.259747	0.855801	0.051595	2.474834	0.042367	5.024293	0.623853	0.191486	0.023492
1.055628	0.323535	4.770231	0.176955	0.384394	5.898449	3.750837	0.66482	1.012742
4.764055	2.126349	9.707677	4.077403	1.971183	5.779574	5.337547	2.339697	3.163506
1.556392	3.514834	0.051595	3.061955	0.532092	0.348139	0.018326	0.232092	0.625209
24.04374	9.625253	0.992201	31.55417	44.27012	1.025613	0.358211	50.55949	30.49289
0.825354	0.677403	1.505678	1.044235	0.657114	1.628009	1.255657	1.215981	0.643427
0.06254	0.035426	0.299747	0.043297	0	0.397215	0.125483	0.038918	0.052078
1.005426	0.259033	3.083319	0.856291	0.939986	2.323983	0.812771	0.631847	0.643427
3.548153	28.54786	0.051595	0.000188	2.013182	0.309444	0.716234	0.086075	0.193449
0.037302	2.173081	0.051595	2.242092	0.544877	0.031919	0.018326	0.000418	0.504964
29.82828	12.16681	6.433918	16.44768	18.62711	7.276645	2.90658	14.33939	13.6814
1.831631	28.87506	0.051595	12.16681	10.93101	0.031919	0.018326	0.000418	11.77567
0.617071	0.1686	0.051595	1.155411	0.308788	2.62342	0.24562	2.987835	0.547128
5.606739	27.66779	0.051595	6.071436	11.93253	0.031919	0.018326	2.834084	8.66E-05
0.767569	14.6058	0.051595	0.933261	0	0.031919	0.274365	0.000418	8.66E-05
7.264502	5.599228	4.02088	3.243225	2.737944	12.49452	27.38726	6.738211	5.671126
1.103261	0.146768	5.889798	0.890577	0.415166	12.29473	2.463911	0.598795	0.939986
0.224372	0.217677	0.992201	0.111775	0.244033	1.025613	2.491948	2.254084	0.997244
7.38847	15.56131	2.703485	9.459177	4.468009	1.527792	0.018326	1.771392	3.179199
1.877605	0.458521	1.746724	3.147294	1.20653	5.967879	2.018167	1.87482	1.309084
0.757763	0.378066	0.051595	1.282208	0.544877	2.294733	0.018326	42.08807	0.21689
0.617071	0.313102	0.051595	0.439293	0.466335	0.36153	0.288867	1.043608	0.165368
3.885642	22.52375	1.089394	21.74051	18.76954	4.592273	9.583463	20.16541	8.976508
1.005426	3.207807	0.051595	5.00404	4.721789	0.031919	0.337641	7.430317	0.873579
0.34544	0.935743	0.051595	1.95645	1.793074	0.309444	0.358211	1.588903	0.209538
0.00026	1.247244	0.051595	0.902403	1.184935	0.031919	0.018326	0.33676	2.55184
1.232886	1.541227	0.051595	0.330043	0.568665	0.031919	0.563175	0.077316	0.111068
0.285556	4.721789	0.051595	4.281847	1.109726	0.397215	0.018326	0.375281	1.779423
1.50684	0.787136	0.051595	3.493362	1.296046	0.485014	0.358211	2.65684	0.625209
0.273261	0.05632	0.758773	0.095685	0.107828	0.222561	0.4357	0.445996	0.855801
0.508658	0.637085	0.371075	0.821898	1.049517	0.331263	0.376212	2.74544	0.605022
3.7586	0.000895	11.96574	1.425613	1.20653	2.579509	5.143867	1.307417	4.997417
7.570693	11.89932	1.183478	19.68088	8.923117	0.653968	0.018326	19.83727	5.050267
0.00026	0.000895	14.00414	0.161299	0	37.29684	87.1746	0.06715	0.076111
0.484704	0.512309	6.695887	0.821898	0.763564	10.80505	6.433918	0.655245	0.596508
1.278629	0.768052	0.051595	0.965065	1.492973	0.031919	0.018326	0.777403	1.932857
1.989711	10.57984	0.051595	6.591544	5.00404	0.031919	0.563175	1.931537	1.932857
0.100375	0.157958	0.246955	0.135902	0.290115	0.211501	0.230606	0.433131	0.185325
0.158521	7.683766	1.135887	2.034242	0.833045	1.850996	0.018326	0.180079	8.66E-05
0.016219	0.000895	0.246955	0.021558	0	0.031919	3.699495	0.000418	0.007749
1.259747	0.194098	0.589618	0.856291	0.375743	2.022778	1.014784	0.684033	0.557763
1.269495	1.983824	0.051595	1.87482	1.362713	0.031919	0.018326	0.66482	0.460649
2.068297	1.216768	17.15902	0.496494	0.458701	2.74298	3.279625	0.643427	3.061955
2.887489	0.787136	16.44768	1.729892	3.394286	1.91254	8.849697	1.095657	5.720173
0.00026	0.078124	1.135887	0.000188	0.014329	0.309444	0.125483	0.047092	0.059654
43.49658	32.31123	6.99026	8.089848	87331.38	1.3829	1.930216	111.5439	3.303925
1.0386	2.776421	2.278254	2.975974	2.577157	1.195794	0.661443	0.118644	2.500548
0.029928	0.108268	2.236407	0.075123	0.089235	2.124574	0.991631	0.180079	0.029177
34.38341	1.587605	118.7028	6.071436	8.780115	70.11932	42.75576	13.83359	5.500678
0.979827	5.22482	0.051595	1.546696	2.607035	0.031919	0.018326	1.952027	1.703795
46.7056	55.04081	1.505678	35.776	49.50903	0.309444	0.018326	43.49658	37.81919
1.310837	0.000895	12.56693	0.600548	0.042367	1.508831	0.288867	4.251818	0.42715

0.00026	0.440801	0.051595	0.021558	0.020462	0.031919	0.24562	0.000418	8.66E-05
0.185909	0.538427	3.500808	2.805382	0.971775	4.690159	7.868146	0.445996	1.111977
0.431717	4.277006	6.900202	0.541241	4.02088	0.937655	2.325931	0.898016	5.158146
0.639113	0.09847	3.179199	0.753016	0	3.507691	0.217496	0.987309	0.111068
0.089921	0.000895	0.051595	0.000188	0.020462	0.031919	0.158521	0.853218	0.044214
9.459177	33.93939	0.051595	26.25557	37.29684	0.031919	0.018326	4.445851	2.119596
0.979827	7.710361	0.051595	7.315512	0.42329	0.485014	0.898016	4.47912	3.269913
0.795339	0.268369	4.381479	0.477107	0.78526	6.784762	2.304452	0.518413	0.28443
4.33342	3.1857	0.706053	7.08733	5.923838	0.937655	0.191602	7.697063	5.530274
1.326667	0.657114	11.19942	0.831472	1.663672	5.165599	4.684502	0.507403	1.318384
29.49693	4.46267	38.39042	6.00228	8.815123	55.04081	45.1045	26.25557	17.15902
0.442496	2.225498	3.397706	1.21215	0.487128	0.573947	1.255657	0.288247	2.413146
0.696659	0.088398	0.706053	0.021558	0.020462	0.742417	1.991328	0.33676	0.165368
0.089921	0.000895	0.333463	0.021558	0.009062	0.031919	0.018326	0.149553	0.142323
1.0386	0.872013	1.604827	1.835743	0.481602	3.435988	1.070115	2.556443	1.053528
13.15079	5.788153	16.80343	15.276	15.46193	6.727633	9.335397	14.17388	19.22139
3.514834	0.133939	1.556392	0.744113	0.276847	0.450051	0.484423	4.881775	1.456955
9.968355	0.393196	2.868456	3.651898	2.126349	6.028427	57.84882	4.94026	5.796421
1.064242	1.541227	0.051595	0.424848	0.807136	0.031919	0.204531	1.624459	0.495238
7.697063	4.203001	15.83113	4.690159	5.062626	27.24614	22.52375	10.1459	7.897078
0.00026	17.39935	0.051595	1.556392	7.897078	0.031919	0.018326	0.000418	77.74812
1.169791	0.133939	4.888023	0.111775	0.33329	1.723579	4.863579	0.420289	0.409899
0.00026	0.035426	0.371075	0.000188	0.000895	0.535657	0.358211	0.021602	0.018016
4.397792	0.202799	3.397706	4.851227	2.678781	2.050404	2.35733	1.017107	2.773644
0.129553	0.027879	0.550974	0.000188	0.014329	0.233889	0.516566	0.33676	0.036349
0.149293	0.088398	1.604827	1.128788	0.027056	2.901032	1.052208	0.948745	0.067547
52.56128	15.77678	31.1753	32.31123	29.82828	2.901032	45.91841	184.443	83.489
0.072482	0.088398	2.400455	0.06824	0.212395	3.581688	1.195794	0.69583	0.2914
0.06254	0.035426	0.333463	0.035339	0.002179	0.672468	0.778326	0.056869	0.018016
0.938413	1.186457	0.051595	1.791616	1.944517	1.89228	2.701053	0.000418	1.219019
11.06488	0.000895	0.051595	0.000188	0	1.815	29.18885	0.000418	14.42661
0.885137	2.429957	1.444906	2.214372	5.538001	0.031919	0.018326	1.307417	2.019618
0.00026	24.04374	0.051595	14.70188	0.349278	0.687807	0.018326	0.000418	3.008225
0.325772	0.916306	0.299747	0.848369	0.437799	0.188427	0.204531	0.579149	0.149986
0.404899	3.750837	0.051595	0.102886	1.793074	0.031919	0.018326	0.967446	0.613853
1.156869	0.411169	2.400455	0.418557	1.177994	8.019769	3.110447	0.69583	0.982128
3.471486	0.000895	2.66404	2.203535	0.640606	0.952294	0.018326	1.531313	3.346898
0.361717	0.000895	0.550974	0.095685	0.020462	3.089293	4.739668	0.06715	0.118903
9.707677	2.099524	11.19942	3.022713	2.62342	13.30906	22.74797	9.541645	3.061955
3.771349	0.775844	0.051595	1.090072	0.86987	0.031919	0.018326	0.49127	0.504964
3.195541	0.157958	3.792266	0.808052	0.633146	3.128694	3.966623	4.845166	1.070115
1.133622	2.522684	0.630022	5.337547	2.607035	0.653968	0.018326	0.000418	1.913997
0.205382	0.000895	1.444906	0.06254	0.129365	1.945916	0.531804	0.243896	0.097395
1.652771	6.695887	0.051595	14.17388	6.297136	0.031919	1.085224	0.655245	4.678846
0.00026	0.202799	0.051595	0.111775	0.121587	0.188427	0.204531	0.029928	0.011962
0.296234	7.737807	1.039199	1.753341	0.956667	0.588658	1.070115	0.159639	1.772756
5.359134	1.301169	1.135887	4.096407	1.382006	4.016465	6.433918	7.625209	3.442886
1.556392	2.248896	0.051595	3.962179	1.717641	0.031919	0.018326	3.011169	1.64684
0.454156	0.108268	3.625382	0.088398	0.200916	0.275873	0.337641	0.735382	0.566494
0.537518	17.51961	0.051595	10.82954	1.861999	0.031919	0.697908	0.802756	0.390418
1.80267	0.591118	19.91794	1.219019	2.955916	5.28039	8.288874	0.802756	4.277006
3.227085	15.66023	0.051595	22.52375	8.288874	0.031919	0.018326	0.180079	6.695887
22.13245	10.48101	1.183478	3.548153	18.91854	2.203535	2.429957	2.616573	26.52537
7.495397	0.1686	2.868456	1.270317	1.816436	1.508831	0.018326	0.898016	1.621032
1.232886	0.146768	5.402511	0.400354	0.447662	6.99026	5.102742	3.314329	2.860722
0.508658	0.027879	3.179199	0.050216	0.142244	4.776407	2.018167	1.095657	3.303925
0.089921	5.770996	0.051595	3.921645	0.114437	1.045462	0.260404	0.169971	2.563506
0.00026	1.451775	0.051595	2.577157	1.673254	0.031919	0.018326	0.000418	0.81228

5.728615	3.06803	1.556392	10.57984	4.776407	6.22031	2.845036	8.923117	5.238701
2.701053	0.29404	7.765931	0.679812	0.341053	9.256537	9.101674	6.38456	1.959466
2.917381	2.558817	9.707677	4.207778	2.710765	7.977958	4.451154	1.975772	3.676032
1.248232	2.008312	3.083319	0.924329	0.481602	2.153535	2.491948	0.459394	1.790245
0.784805	0.749481	0.448557	1.006025	1.304877	0.031919	0.16925	2.290996	1.64684
0.964416	6.116638	0.051595	3.824964	0.800512	1.431414	0.337641	0.12925	0.097395
5.050267	18.33909	0.051595	14.51648	14.33939	1.306667	1.533535	3.87329	8.180094
7.037345	3.26	0.051595	5.679502	5.062626	0.031919	0.018326	7.882222	4.440548
8.958225	2.755411	0.414473	5.238701	5.195022	0.414012	0.230606	8.048095	5.137042
0.185909	10.73084	0.051595	11.09133	0.920483	0.031919	0.018326	0.000418	8.66E-05
1.989711	0.554416	15.1268	2.102626	3.909033	4.984488	9.377345	0.408644	4.277006
2.839351	1.612056	0.550974	2.386248	1.354567	0.309444	0.018326	2.73272	0.968066
0.850808	2.950173	0.051595	3.796075	2.230859	0.031919	0.018326	1.852338	1.92272
0.072482	0.313102	0.051595	0.321746	0.544877	0.971205	0.516566	0.029928	0.052078
0.898543	0.313102	0.448557	0.744113	0.611789	0.805801	1.564149	0.931457	1.031284
1.742734	0.045079	1.444906	0.111775	0.78526	4.193232	2.164113	0.49127	1.739935
1.326667	0.000895	0.051595	0.528571	0.7257	2.901032	0.018326	1.560779	0.726472
1.491977	0.339913	0.758773	0.916306	2.542605	0.309444	2.073232	2.90658	0.982128
0.454156	0.613211	4.644098	0.288081	0.516566	2.081696	0.467951	0.34583	0.209538
0.784805	1.200152	0.051595	0.890577	0.276847	0.031919	0.390895	0.445996	1.477388
0.00026	8.019769	0.051595	0.000188	0	0.031919	0.018326	0.000418	8.66E-05
1.09075	0.863694	1.655541	2.683478	2.148658	2.071587	0.204531	2.834084	4.597792
0.431717	0.360101	0.66482	1.319293	1.616385	0.211501	0.54847	1.828781	1.111977
0.081587	0.157958	2.183665	0.144228	0.23246	2.178268	0.812771	0.507403	0.176104
2.240274	8.103925	0.051595	1.765065	2.396429	0.031919	0.018326	3.025671	8.745238
9.179466	5.889798	0.630022	12.7427	13.60866	0.826407	0.406212	11.96574	9.502294
0.06254	3.877417	0.051595	0.728543	1.997352	1.070115	0.633853	0.180079	0.259646
1.786306	1.469163	1.349971	3.377619	3.147294	1.672107	0.191602	2.572309	1.578687
0.304545	0.000895	2.345527	0.309062	0.027056	2.081696	0.018326	1.195794	1.549711
0.037302	0.332395	0.051595	0.55741	0.089235	1.705007	0.318312	0.038918	0.011962
0.149293	3.68381	0.051595	4.894509	1.816436	0.031919	0.018326	3.037626	2.040606
1.666089	4.696313	0.051595	13.6814	1.68342	0.031919	0.018326	1.074827	3.860491
3.083319	0.538427	15.276	2.847547	2.893001	17.92188	16.28201	3.204632	4.473348
0.231876	0.228203	0.630022	0.266847	0.14987	1.195794	6.071436	0.840635	0.176104
0.081587	0.217677	0.299747	0.517864	0.200916	0.36153	0.991631	0.655245	0.165368
0.682799	0.209538	2.939141	0.706053	0.290115	5.28039	1.165065	0.724502	0.390418
0.807136	1.05088	0.805238	2.565556	0.886775	0.450051	0.147807	2.705916	1.244242
0.239769	8.780115	0.706053	11.25392	5.447114	1.007864	0.610224	6.045209	3.829105
0.00026	0.000895	0.706053	0.035339	0	0.992201	0.54847	0.038918	0.011962
0.158521	0.027879	0.246955	0.081587	0.114437	0.031919	0.191602	0.243896	0.47
3.644343	1.960945	2.99987	3.363911	1.330029	5.123781	2.463911	10.53159	2.474834
5.622872	0.742417	2.183665	2.805382	2.955916	2.223636	0.878369	19.06893	3.233045
14.12978	7.765931	24.39681	55.04081	17.28195	252.4251	167.9996	23.6012	9.335397
1.278629	0.378066	0.051595	0.496494	0.302554	0.397215	0.467951	2.515938	0.036349
0.361717	0.000895	0.477446	0.111775	0.096061	0.704848	0.217496	0.106768	0.090931
0.825354	4.586753	2.703485	4.702468	1.094978	0.971205	0.943514	0.139091	0.587619
3.227085	12.22798	0.051595	5.266335	7.519841	0.031919	0.018326	0.818066	10.33476
1.360866	1.928629	0.051595	5.037489	4.552092	0.031919	0.018326	2.572309	2.429957
0.19544	0.000895	0.267597	0.057417	0.020462	0.485014	0.337641	0.25785	0.011962
0.016219	0.05632	0.371075	0.088398	0.020462	0.762165	0.797114	0.000418	0.007749
0.757763	1.919661	0.333463	3.29355	1.902352	0.466602	0.30215	0.818066	0.84119
3.36075	14.88584	0.051595	1.821861	14.17388	0.031919	0.337641	2.31811	6.088593
0.466335	0.23873	1.505678	0.330043	0.595022	0.233889	0.450375	0.213218	0.390418
3.153485	3.172864	0.051595	1.700354	1.166717	0.031919	0.018326	7.112496	6.518709
0.215115	1.667273	0.805238	0.35241	1.62562	0.031919	0.018326	0.000418	0.685209
2.287576	1.52676	6.036818	3.676032	1.91811	10.05618	11.77567	6.900202	1.159149
0.17619	0.108268	9.502294	0.300606	0.50803	1.360022	1.564149	1.095657	0.390418
4.277006	0.000895	9.047532	0.300606	0.646861	5.477843	6.135007	4.580938	2.671299

1.962424	4.072475	0.839365	6.717056	2.308203	1.094206	1.195794	1.624459	0.704848
0.081587	0.027879	0.414473	0.050216	0.020462	0.246955	1.085224	1.588903	0.044214
3.425512	0.157958	1.224733	0.503146	1.075411	0.672468	0.856782	2.290996	4.473348
0.17619	1.417345	0.051595	2.058773	0.939986	0.031919	0.018326	1.531313	2.474834
0.484704	1.801284	0.051595	2.197828	0.833045	0.233889	0.158521	0.203254	0.277417
2.16759	2.685786	1.877605	2.805382	5.576219	0.309444	0.204531	9.729358	5.309264
0.466335	0.000895	1.262179	0.300606	0.101659	1.007864	0.878369	1.171349	0.067547
1.326667	1.223846	0.514531	2.898196	5.538001	0.031919	0.018326	1.975772	2.040606
0.660678	2.973059	3.217612	2.341573	1.401068	2.178268	1.434055	0.459394	1.300368
4.207778	27.10502	0.051595	8.241342	16.34294	0.901212	0.30215	5.923838	7.38847
0.816479	1.255657	0.051595	0.47	0.375743	0.450051	0.54847	0.268095	0.268961
1.455988	22.74797	0.051595	21.36892	0.956667	21.19547	0.318312	0.000418	0.142323
9.141299	18.19595	7.288788	5.110159	9.880779	7.025599	11.14619	3.307186	4.914863
0.38285	0.000895	0.267597	0.000188	0.16316	0.174372	0.147807	0.077316	0.224921
0.605022	2.540462	0.051595	0.392944	1.06355	0.031919	0.018326	1.99899	0.400534
9.838658	9.815642	0.051595	17.64869	18.48514	0.031919	0.018326	17.78522	22.13245
1.568564	1.51215	0.051595	0.294502	0.745613	0.031919	0.018326	0.554026	1.125325
0.138947	2.647244	0.051595	0.763102	1.692763	0.031919	1.236248	0.565137	0.557763
3.330909	27.38726	1.262179	27.66779	12.46209	0.031919	0.018326	1.030678	3.87329
0.682799	0.000895	1.991328	0.095685	0.16316	0.031919	0.467951	0.481104	0.495238
0.394329	2.479221	0.589618	1.846941	1.003586	0.031919	0.217496	0.529697	2.248896
6.088593	0.458521	2.797835	2.173081	2.498478	11.719	32.68313	5.638759	2.019618
77.74812	30.17192	0.051595	20.85876	15.56131	0.971205	1.513102	19.99861	0.487662
0.660678	1.148564	0.299747	0.548788	0.14987	0.431977	1.31	0.315332	0.495238
2.567605	2.037114	0.371075	5.28039	3.147294	0.937655	1.759986	1.865043	1.287403
2.185556	0.228203	6.632063	0.640289	0.822965	1.473218	0.516566	0.987309	0.738312
1.248232	0.000895	7.882222	0.831472	1.268672	1.070115	0.018326	0.998831	1.198701
7.339452	4.072475	0.758773	6.365065	11.60283	0.414012	0.716234	2.858009	4.246861
2.502619	1.770029	0.051595	1.289149	2.139928	0.031919	0.204531	0.213218	0.982128
2.638139	1.451775	12.13485	2.805382	1.861999	5.373781	7.495397	0.445996	2.019618
0.053838	0.067547	1.387612	0.330043	0.101659	0.762165	0.125483	0.213218	0.10404
0.547128	0.000895	0.051595	2.66404	0.516566	0.031919	0.467951	1.235346	8.66E-05
1.080303	6.464906	0.051595	4.440548	3.046551	1.234444	0.288867	1.729892	2.073232
0.639113	4.058514	0.051595	1.498167	1.655541	0.554416	0.358211	1.678319	1.031284
7.737807	0.726133	0.805238	2.410996	1.827403	0.504416	1.255657	6.560411	3.775209
3.122915	2.16759	6.52899	3.722785	2.210866	4.690159	8.365072	2.363442	4.524481
0.00026	3.640606	0.051595	0.583369	0.524986	0.031919	0.018326	0.000418	8.66E-05
118.7028	2.797835	55.04081	37.29684	17.15902	46.7056	7.810058	61.5546	144.7919
4.25658	0.760866	1.505678	2.474834	1.991328	6.071436	3.36075	2.442987	1.959466
1.742734	0.000895	0.371075	0.409098	0.308788	0.397215	0.274365	1.195794	0.715815
0.747879	0.035426	1.262179	0.111775	0.034459	0.952294	0.578817	0.288247	0.268961
2.083261	4.370671	0.051595	0.693925	0.363059	0.031919	0.018326	2.134545	2.882035
6.853608	1.533535	1.444906	3.747035	1.853723	0.704848	0.158521	13.60866	4.058514
1.763889	0.431457	0.051595	2.715873	0.822965	0.031919	0.018326	4.927302	0.651631
0.57197	0.467677	5.47	0.376782	1.268672	14.33939	10.57984	5.80469	1.031284
32.68313	7.238023	10.05618	7.188095	42.08807	7.188095	35.776	26.52537	22.74797
0.138947	3.432388	0.051595	3.939307	1.673254	0.031919	0.018326	3.327619	0.519387
0.737857	0.157958	0.051595	0.566104	0.349278	0.971205	0.450375	2.263377	0.224921
3.227085	1.155411	1.039199	2.386248	2.201587	1.405772	2.388348	8.677489	2.209141
1.344257	1.667273	2.581861	1.296905	0.929286	3.989206	4.575123	0.420289	1.087345
1.290981	3.404372	0.051595	2.818312	1.384921	0.031919	0.018326	0.445996	0.86518
0.352807	4.297222	0.051595	2.081696	0.538117	0.378326	0.018326	0.34583	0.958557
0.016219	0.027879	0.448557	0.035339	0.009062	0.188427	0.125483	0.029928	0.036349
12.53072	5.195022	0.839365	7.625209	5.591717	0.535657	0.217496	4.281847	7.532713
1.819221	20.34063	1.703795	7.013853	7.625209	6.560411	2.325931	4.155339	12.42965
4.429769	1.024127	0.955418	2.975974	2.278254	4.620159	1.549711	9.729358	1.111977
1.641999	0.303016	1.387612	0.487395	0.276847	0.85728	0.484423	1.277843	0.42715
1.89228	0.000895	0.91575	0.12785	0.23246	3.046551	0.484423	3.230065	0.81228

2.369841	1.551962	4.552092	2.011501	0.586595	10.01009	17.92188	2.604776	1.631544
0.185909	0.088398	1.444906	0.221962	0.020462	0.841746	0.484423	1.171349	0.319762
0.00026	1.083651	0.051595	0.715815	1.793074	0.031919	0.018326	1.043608	1.790245
0.00026	0.781465	0.051595	1.109726	2.248896	0.031919	0.217496	0.077316	0.982128
0.089921	1.255657	0.051595	0.872013	0.625498	0.031919	0.018326	0.000418	0.149986
0.129553	0.05632	1.262179	0.203175	0.452266	0.60202	0.318312	0.518413	0.390418
1.50684	0.421573	0.371075	1.405772	0.976111	0.031919	0.018326	1.307417	0.939986
0.053838	1.928629	0.051595	0.687049	0.259098	0.031919	0.018326	0.353492	0.749019
0.583766	1.313737	0.630022	2.625823	1.003586	0.031919	0.018326	0.832013	0.409899
0.158521	1.89961	0.051595	3.47531	4.440548	0.031919	0.018326	2.084791	3.754719
1.742734	19.37139	0.051595	10.1459	1.377446	0.031919	0.018326	0.326017	3.507691
2.159004	0.000895	2.345527	0.015541	0.081212	1.723579	5.273362	0.363983	0.487662
5.754935	0.637085	1.703795	2.683478	3.061955	2.602395	2.690823	11.42512	7.162698
3.984747	0.637085	0.051595	8.958225	8.539971	0.031919	0.108016	5.545729	4.802655
2.863211	2.236407	36.74522	2.92013	2.048781	11.2819	26.25557	4.101061	3.256587
1.693939	10.98644	0.051595	4.38684	3.993665	0.031919	0.230606	5.01772	4.302367
70.11932	1.313737	167.9996	6.653016	4.328232	95.22939	126.1556	11.14619	9.646284
2.270837	0.108268	1.444906	0.088398	0.50803	0.504416	2.720996	12.53072	2.442987
4.429769	7.264502	0.051595	2.121198	3.321284	0.031919	0.878369	0.518413	6.365065
37.81919	749.7839	3.330909	530.1465	77.74812	0.620909	1.128788	16.34294	749.7839
0.053838	5.47	4.552092	1.06355	1.166717	3.783694	3.303925	0.470289	0.353016
0.185909	0.000895	0.333463	0.111775	0.064697	0.233889	0.018326	0.433131	0.067547
6.088593	1.754733	4.381479	2.27254	0.633146	20.42667	11.25392	4.863579	1.865043
9.031082	67.98069	1.444906	12.22798	30.49289	0.031919	9.880779	10.62739	12.29473
1.718918	0.209538	2.055289	1.296905	0.283709	6.560411	4.140505	2.961768	0.537229
9.297085	13.72059	0.630022	13.26964	9.179466	1.850996	3.446732	18.33909	8.477662
21.74051	8.075772	0.051595	1.587605	7.013853	0.031919	0.018326	0.000418	18.62711
1.190491	3.842554	0.051595	4.776407	3.803766	0.031919	0.274365	10.19468	1.188139
0.361717	4.029654	0.051595	1.109726	0.212395	1.64917	0.633853	0.000418	0.800967
5.180058	1.433189	2.236407	2.31013	3.707136	20.68746	13.83359	5.881342	2.413146
1.02487	0.177143	4.105714	1.346991	0.327662	8.905642	1.965657	2.425801	1.456955
13.23022	3.895469	16.15795	4.473348	9.356371	22.74797	19.91794	10.90615	10.03313
0.166898	0.000895	23.82354	0.000188	0	9.19899	9.667316	0.000418	0.625209
0.414012	0.450051	0.051595	2.911962	1.701616	0.031919	0.137302	4.721789	0.958557
14.42661	22.13245	0.051595	87.1746	48.53429	1.783203	0.018326	13.91714	24.73724
2.941544	0.303016	30.49289	0.916306	2.109755	6.135007	4.227302	5.583968	5.351934
0.045693	0.035426	0.448557	0.050216	0.027056	0.331263	0.260404	0.703665	0.029177
10.1459	5.872886	4.770231	3.511263	8.019769	30.17192	44.27012	11.89932	9.968355
8.226219	1.34267	8.728571	1.616385	7.443276	2.26518	6.52899	1.446926	8.119004
3.833247	2.280101	0.706053	10.73084	5.838499	0.620909	0.484423	1.131472	4.56912
2.776421	4.370671	0.448557	2.214372	7.824473	0.36153	3.377619	10.01009	5.095325
0.00026	0.000895	2.107922	0.337222	0.042367	2.74298	0.217496	0.49127	0.369336
2.763261	1.024127	2.99987	0.945483	1.602619	2.124574	3.13456	2.022778	3.283139
0.593434	0.067547	0.589618	0.237698	0.175714	0.504416	0.390895	1.145426	0.28443
0.361717	0.421573	0.630022	0.477107	0.31557	0.397215	0.191602	0.853218	0.390418
0.089921	0.157958	0.589618	0.088398	0.081212	1.685678	1.62562	0.000418	8.66E-05
1.344257	18.76954	0.051595	34.38341	7.339452	3.343629	1.415469	7.225469	2.873817
0.431717	2.382035	0.051595	0.814315	2.169509	0.031919	0.018326	1.290981	0.92816
1.326667	4.170051	0.051595	4.408153	4.63189	0.031919	0.018326	0.000418	6.464906
1.072843	4.557605	0.051595	2.121198	2.157359	0.261558	0.500996	1.889711	16.9162
1.934271	1.024127	0.589618	4.637994	1.780693	0.200657	0.137302	1.828781	0.668456
1.972706	22.32214	0.051595	15.98892	5.180058	0.031919	0.018326	2.681169	7.013853
5.381227	3.418405	0.051595	4.965801	1.663672	0.031919	3.418405	3.66785	2.263377
0.710722	0.313102	1.349971	0.288081	0.33329	6.830592	14.47154	1.903636	2.078355
0.466335	0.538427	0.051595	1.155411	0.920483	0.031919	0.147807	0.931457	0.6943
11.57341	0.726133	22.32214	1.119574	0.964416	9.583463	15.02509	17.28195	4.110779
0.56145	0.05632	1.604827	0.523521	0.403413	5.439278	3.890556	2.351299	0.519387
0.508658	0.000895	2.581861	0.000188	0.169538	0.414012	4.101061	0.470289	0.165368

4.984488	0.29404	2.531515	0.654423	1.344257	4.946587	7.363117	2.109755	4.277006
6.956349	20.85876	0.051595	3.722785	10.1028	2.947287	3.1857	1.359177	4.56912
12.42965	11.36975	1.444906	10.45584	9.667316	0.826407	1.106046	10.33476	13.19051
2.240274	1.100519	29.18885	1.06355	1.084437	37.81919	35.27608	3.5257	2.860722
0.022727	2.914632	0.299747	4.38684	0.568665	0.620909	0.337641	3.404372	1.790245
1.861999	0.209538	0.299747	1.525729	0.384394	1.249221	1.513102	3.899993	0.097395
0.660678	1.516219	0.414473	1.992944	3.29355	0.63754	0.797114	5.037489	4.110779
0.466335	0.000895	1.991328	0.12785	0.169538	2.579509	2.429957	0.326017	0.576205
0.593434	0.228203	5.22482	0.384632	0.073038	3.5257	3.993665	0.000418	0.10404
1.455988	2.890245	0.051595	3.1857	3.1857	0.331263	0.018326	1.106046	4.971991
3.286652	0.045079	0.550974	0.315916	0.657114	1.550837	0.623853	1.940231	0.345238
0.38285	0.000895	2.312085	0.050216	0.129365	0.504416	0.4357	2.765758	0.537229
5.050267	1.68342	1.604827	1.205685	0.816479	4.043846	4.644098	3.346898	1.188139
2.681169	2.160649	0.051595	2.609293	0.709784	0.246955	0.018326	1.547734	0.685209
0.404899	0.035426	0.371075	0.246825	0.056948	1.025613	2.018167	0.433131	0.185325
6.695887	1.870685	0.955418	3.824964	3.632973	2.901032	4.025267	9.583463	5.830152
2.353247	1.579798	0.514531	1.128788	0.734935	0.031919	0.318312	0.433131	1.300368
31.1753	10.35839	0.051595	24.04374	4.251818	0.031919	0.018326	2.221775	8.905642
3.860491	0.000895	0.91575	0.212395	0.064697	1.173535	0.633853	18.91854	3.080317
7.570693	1.100519	15.83113	1.168326	1.877605	7.495397	2.073232	27.10502	5.287857
0.045693	0.000895	0.448557	0.050216	0.135902	0.871429	0.018326	0.139091	0.300606
0.285556	0.000895	0.051595	1.090072	0.042367	0.397215	0.484423	2.234538	0.067547
0.325772	1.997352	0.051595	0.698687	0.800512	0.031919	0.018326	0.315332	0.319762
0.767569	0.000895	0.758773	0.384632	0.586595	0.535657	0.191602	0.802756	1.809343
44.27012	3.367071	50.55949	6.830592	9.968355	39.53687	31.55417	5.477843	5.545729
1.326667	0.209538	1.655541	0.890577	0.886775	4.690159	5.210317	1.369048	1.098341
0.508658	8.643074	0.051595	2.74544	4.140505	0.031919	0.318312	1.359177	6.749488
0.016219	0.000895	0.246955	0.021558	0	0.031919	3.699495	0.000418	0.007749
4.833398	2.647244	0.299747	2.911962	3.156537	0.309444	1.212792	4.02088	4.381479
5.395584	12.42965	1.308167	25.73165	13.75978	18.62711	1.434055	25.99427	23.6012
0.795339	1.945916	8.365072	1.416407	0.500354	8.994791	2.134545	2.442987	2.209141
17.28195	2.8657	0.051595	11.48368	8.333918	0.290519	0.147807	5.273362	13.91714
0.138947	0.027879	0.758773	0.015541	0.034459	0.805801	1.106046	0.554026	0.149986
0.747879	1.386681	0.051595	1.162201	3.098268	0.031919	0.018326	2.351299	1.64684
0.285556	0.000895	0.051595	0.153016	0	0.031919	0.018326	3.217612	0.044214
4.776407	12.93651	0.051595	13.75978	3.122915	0.031919	0.018326	1.729892	2.933903
0.045693	0.027879	0.839365	0.043297	0.169538	1.580909	0.84119	0.000418	0.134589
0.00026	0.303016	0.051595	0.043297	0	0.275873	0.018326	0.277251	8.66E-05
7.570693	5.082063	0.550974	25.99427	11.65791	0.971205	0.973745	6.325945	16.09482
0.795339	1.761349	0.051595	0.993521	1.554185	0.031919	0.30215	4.413608	2.698557
1.114618	25.24367	0.051595	2.925426	1.50684	0.031919	0.358211	0.000418	3.233045
2.353247	8.461703	0.051595	3.47531	3.489899	0.031919	0.610224	6.560411	2.285664
3.297107	1.754733	1.655541	10.01009	1.330029	6.071436	5.84689	7.570693	1.267085
1.861999	2.339697	0.051595	1.921212	1.938759	0.031919	0.4357	0.620462	2.380079
13.38418	1.561912	35.27608	1.226934	2.850058	38.39042	38.39042	6.560411	11.42512
1.423506	6.345382	0.589618	5.165599	2.096162	3.046551	0.734524	1.267879	1.287403
4.096407	5.599228	1.135887	7.225469	7.964315	1.783203	2.388348	7.149618	6.591544
19.83727	24.2842	3.137576	12.36443	15.88548	5.515584	8.643074	3.460909	9.377345
0.816479	0.209538	4.845166	0.528571	1.283911	2.203535	2.933903	0.931457	0.982128
0.508658	0.981558	0.051595	1.95645	0.244033	0.466602	0.137302	0.375281	0.378326
0.776876	1.870685	4.456912	4.281847	1.135137	10.43066	2.197828	1.215981	2.725498
0.724502	0.000895	3.297107	0.86518	0.169538	4.592273	1.710173	0.818066	0.328543
0.313521	0.000895	0.371075	0.015541	0.101659	0.246955	0.137302	0.191486	0.149986
1.125325	0.259033	2.581861	0.462612	0.577706	1.580909	1.052208	0.948745	0.704848
4.661717	19.06893	0.051595	8.643074	2.81342	0.031919	0.018326	0.703665	4.814661
0.231876	3.35355	1.135887	2.164113	1.616385	0.031919	0.018326	1.750628	1.031284
0.325772	0.538427	1.877605	1.472208	0.33329	1.094206	1.212792	2.290996	0.968066
1.841371	0.000895	5.01772	0.000188	0.430837	1.1407	0.390895	2.290996	7.013853

0.454156	0.000895	0.051595	0.000188	0.027056	1.334762	0.898016	0.589618	0.052078
4.546443	1.707576	0.955418	7.824473	6.695887	0.620909	0.578817	1.713997	3.57057
0.149293	0.05632	0.706053	0.221962	0.014329	0.971205	0.484423	0.445996	0.023492
0.129553	2.413146	0.051595	2.31811	2.873817	0.031919	0.204531	2.65684	2.217951
1.047345	0.368882	3.256587	0.541241	2.698557	2.524892	5.210317	0.756724	2.792035
1.861999	8.226219	1.877605	0.879423	2.845036	0.687807	1.034315	2.65684	3.049603
3.26	14.42661	0.051595	7.519841	11.719	0.031919	0.856782	16.44768	16.5625
0.053838	0.146768	2.278254	0.081587	0.156941	2.398442	6.717056	0.353492	0.059654
0.045693	0.05632	0.589618	0.015541	0.056948	0.348139	1.415469	0.149553	0.007749
5.959033	1.336587	0.91575	6.622092	3.540606	0.920483	0.260404	5.508131	3.214545
8.075772	1.357201	11.60283	2.438831	3.005281	16.44768	5.568391	8.256465	5.993045
1.92272	1.091443	1.308167	3.269913	4.272165	8.048095	4.101061	4.02088	2.563506
1.360866	0.476768	0.051595	1.700354	0.833045	0.742417	0.018326	0.420289	0.409899
2.425801	4.637994	1.746724	4.49671	2.29873	1.070115	0.376212	5.062626	6.088593
0.431717	0.088398	0.805238	0.035339	0.322237	0.031919	0.018326	0.222864	1.209776
3.36075	8.539971	0.051595	1.308167	13.91714	0.031919	0.217496	9.667316	6.55039
0.19544	0.000895	2.74298	0.359697	0.42329	1.431414	4.684502	0.470289	0.460649
2.567605	0.323535	27.66779	0.662237	3.621941	11.17281	9.751039	1.184185	3.803766
51.52195	18.62711	3.037626	77.74812	91.05541	2.74298	1.415469	100.0998	31.92385
0.081587	0.401724	1.991328	0.135902	0.474574	1.045462	1.481631	0.34583	0.300606
0.00026	5.210317	0.051595	1.729892	1.283911	0.031919	0.018326	0.000418	8.66E-05
0.825354	0.000895	1.991328	0.309062	0.437799	1.580909	0.018326	2.066652	0.84119
0.850808	1.727323	0.051595	1.753341	1.701616	0.031919	0.16925	1.277843	1.456955
1.360866	6.404921	1.308167	9.924372	5.295325	0.397215	0.467951	57.84882	3.585029
1.718918	4.484892	3.899993	2.197828	7.037345	0.653968	1.128788	2.572309	3.676032
1.190491	0.045079	46.7056	0.12785	0.217792	2.74298	3.581688	0.643427	0.90772
1.429365	0.521775	4.552092	0.384632	0.268232	0.233889	0.633853	2.975974	0.715815
0.508658	3.659668	0.051595	4.739668	3.381255	0.031919	0.661443	0.000418	0.277417
2.392431	1.585296	1.604827	2.776421	1.232078	3.5257	3.13456	3.734762	1.418312
2.019618	0.285844	9.502294	2.500548	2.109755	6.632063	11.544	4.789596	4.381479
0.00026	4.25658	0.051595	5.210317	2.565556	0.031919	0.578817	1.588903	2.153535
4.546443	1.612056	0.051595	1.425613	11.42512	0.031919	0.158521	1.204957	3.625382
2.353247	0.562439	6.784762	0.648687	1.469163	2.124574	1.601443	1.666089	2.269134
8.83241	33.07147	0.051595	18.62711	8.477662	0.031919	0.018326	0.756724	16.39531
22.52375	6.38456	6.8421	15.46193	10.38202	0.031919	1.434055	3.482785	6.967547
0.00026	0.000895	0.051595	0.06824	0.034459	0.466602	0.16925	0.353492	0.023492
6.287561	15.07257	0.051595	13.15079	19.83727	0.031919	0.797114	3.025671	5.889798
30.49289	287.197	15.51162	126.1556	749.7839	0.031919	7.625209	1.056898	167.9996
0.914618	0.667965	6.316328	1.535722	2.839351	5.898449	19.68088	2.181775	0.676999
0.19544	0.657114	0.051595	0.000188	1.757244	0.031919	0.018326	0.713846	0.726472
5.24562	8.711905	0.051595	3.119986	3.895469	0.331263	0.018326	0.353492	3.939307
1.471198	9.141299	1.224733	4.281847	7.238023	2.77899	1.288276	2.887489	4.927302
0.00026	135.5245	0.051595	75.1132	0	0.031919	0.018326	0.000418	10.65365
1.232886	5.560563	0.051595	3.163506	2.157359	0.588658	0.734524	4.557605	2.725498
2.567605	57.84882	0.051595	18.48514	22.74797	0.031919	0.018326	0.000418	13.19051
8.608153	3.014076	10.19468	6.045209	11.83593	9.438687	15.61077	7.049336	8.608153
0.129553	2.073232	0.299747	3.6029	1.91254	0.309444	0.337641	1.99899	2.579509
5.830152	3.585029	0.051595	2.826277	4.667518	0.414012	0.018326	1.659033	1.675722
2.477027	0.781465	1.746724	0.237698	1.068348	4.160238	6.808369	1.017107	1.437648
0.19544	0.000895	0.550974	0.431198	0.156941	0.762165	0.645916	3.595758	0.328543
3.807648	10.28573	0.839365	26.52537	10.82954	6.601833	0.959567	11.03843	9.459177
6.695887	10.78056	0.051595	9.968355	16.44768	1.91254	0.018326	19.37139	3.414574
0.17619	0.000895	1.655541	0.000188	0.135902	0.031919	0.856782	0.288247	0.127128
24.50942	21.93452	7.08733	11.83593	9.583463	1.123795	3.699495	2.765758	11.09133
2.638139	1.451775	0.051595	2.638139	1.983824	0.031919	0.018326	0.713846	2.209141
0.914618	0.067547	1.089394	0.628658	0.447662	1.580909	3.086306	1.828781	1.337431
3.227085	0.647345	1.604827	0.503146	1.50684	1.306667	1.661328	3.104394	2.353247
0.138947	0.045079	2.183665	0.246825	0.114437	0.85728	0.697908	0.139091	0.036349

5.530274	3.743232	1.039199	4.758088	4.096407	1.945916	2.35733	3.55583	2.81342
0.898543	1.667273	0.333463	1.765065	1.42456	0.620909	0.204531	4.101061	1.098341
0.404899	0.000895	0.371075	0.057417	0.009062	0.588658	0.991631	0.243896	0.224921
0.337222	11.14619	0.051595	8.319019	2.438831	0.031919	0.018326	0.387489	2.950173
13.91714	70.11932	19.37139	4.869553	34.84299	0.485014	7.495397	5.416623	36.26061
0.442496	7.911934	0.051595	1.006025	0.833045	0.031919	0.018326	0.000418	0.82974
0.205382	0.045079	17.28195	0.057417	0.156941	0.762165	1.3829	0.470289	0.142323
1.360866	12.77772	0.051595	4.058514	2.329784	0.031919	0.018326	0.000418	2.544747
1.89228	0.000895	0.051595	0.000188	0.101659	1.580909	0.018326	0.000418	0.84119
0.037302	0.000895	0.051595	0.161299	0.005411	0.378326	0.595022	0.769019	0.018016
0.710722	0.303016	7.61176	0.600548	1.997352	0.871429	1.513102	4.188896	2.08632
0.874603	0.000895	0.706053	0.400354	0.396638	2.099524	6.335664	1.68938	8.66E-05
0.110534	0.078124	6.116638	0.057417	0.979149	1.156869	3.507691	0.788896	0.892395
0.979827	1.175859	0.299747	3.407619	2.189235	0.031919	0.018326	1.043608	0.625209
3.095166	13.30906	0.051595	4.456912	4.808658	0.031919	0.018326	2.765758	1.703795
0.850808	0.146768	1.444906	1.498167	2.382035	1.070115	2.236407	2.221775	2.040606
2.903867	16.09482	0.839365	6.365065	6.900202	0.841746	1.106046	3.939307	3.846984
0.185909	0.000895	0.992201	0.55741	0.384394	0.246955	0.16925	0.363983	0.067547
4.661717	15.98892	0.955418	8.461703	18.19595	1.451775	1.601443	0.408644	7.188095
2.306277	3.803766	1.387612	4.222583	3.599329	1.431414	1.710173	2.290996	3.930664
0.422511	0.133939	1.794531	0.541241	0.250823	1.815	1.085224	0.832013	0.916306
0.605022	7.882222	0.051595	1.859084	7.598312	0.031919	0.018326	4.535354	3.269913
1.90798	0.647345	0.299747	0.831472	1.166717	0.031919	0.274365	1.417345	0.81228
0.273261	4.440548	0.051595	1.396494	1.655541	0.920483	0.018326	1.388543	0.28443
0.957273	0.05632	0.414473	0.246825	0.175714	1.218182	3.243225	1.171349	0.537229
0.022727	0.000895	0.758773	0.503146	0	0.535657	0.898016	0.840635	0.011962
0.110534	0.000895	3.792266	2.325931	0.948211	3.128694	0.018326	1.99899	0.277417
0.57197	2.243911	0.051595	0.496494	0.458701	0.031919	0.018326	0.213218	0.319762
0.00026	0.000895	1.941703	0.043297	0.049668	0.233889	0.288867	0.33676	0.127128
0.053838	0.088398	1.444906	0.021558	0	0.309444	1.905094	0.12925	0.310866
0.454156	7.545584	0.051595	4.012049	4.745895	1.741414	0.467951	1.25816	4.650007
0.037302	0.332395	0.051595	0.517864	0.096061	0.174372	0.204531	0.106768	0.084113
3.497085	3.297107	0.514531	2.600014	4.125786	1.451775	1.930216	3.851414	2.961768
0.497424	2.958773	2.345527	1.035058	0.902403	4.921082	3.008225	1.462143	1.40772
0.129553	0.83088	0.333463	1.148564	1.056263	0.397215	0.812771	0.029928	0.165368
0.861934	5.545729	0.051595	0.453932	2.671299	0.031919	0.018326	0.853218	4.758088
0.100375	0.027879	0.514531	0.135902	0.000895	0.431977	0.24562	0.118644	0.029177
2.101075	3.984747	0.051595	5.704646	2.914632	0.031919	0.018326	1.531313	3.147294
19.06893	21.36892	9.160382	36.26061	32.68313	3.743232	6.52899	11.25392	28.54786
1.934271	0.088398	2.66404	0.34697	0.437799	1.508831	0.450375	0.363983	1.34513
0.285556	0.067547	0.371075	0.368658	0.369105	3.089293	1.165065	2.74544	0.528571
6.518709	3.195541	0.299747	5.720173	13.53913	0.397215	0.260404	13.15079	13.45939
5.082063	3.147294	0.91575	1.974228	3.35355	0.290519	0.778326	2.633232	2.225498
3.621941	0.700837	0.051595	2.410996	0.646861	0.331263	0.147807	0.802756	0.90013
0.215115	0.177143	14.383	0.161299	0.238478	2.345527	7.964315	0.077316	0.6943
4.609076	1.765065	0.051595	4.557605	2.308203	1.473218	0.260404	2.408882	2.708341
5.295325	11.719	0.051595	6.028427	12.03267	0.348139	0.376212	7.824473	9.771833
1.641999	0.811443	3.855952	0.000188	0.430837	0.031919	0.467951	0.25785	4.02088
11.36975	51.52195	0.051595	60.77449	47.5018	4.650007	0.318312	2.192496	33.93939
2.335844	1.89373	0.051595	0.706053	0.458701	0.031919	0.018326	0.802756	0.20215
2.019618	0.667965	2.400455	0.55741	0.474574	5.409437	5.210317	8.226219	1.244242
1.232886	0.521775	6.036818	0.693925	1.869322	3.581688	3.163506	0.840635	1.944517
10.93101	20.51271	3.330909	4.990794	7.061328	0.031919	2.073232	0.499206	3.049603
0.660678	3.163506	0.91575	0.574993	1.80267	0.031919	0.318312	0.399784	0.576205
3.06803	3.68381	1.039199	3.610707	1.655541	0.331263	0.716234	0.735382	1.64684
0.158521	1.27619	9.902576	1.525729	0.971775	7.162698	6.171789	1.145426	0.765094
0.776876	3.101371	0.051595	0.600548	0.568665	0.031919	0.018326	1.61088	0.659957
1.593535	0.088398	1.991328	0.384632	0.268232	0.466602	1.930216	0.445996	0.566494

0.19544	0.845887	0.267597	0.733074	0.568665	0.535657	0.623853	0.353492	0.409899
0.00026	87331.38	0.051595	224.9555	1.268672	0.031919	0.260404	0.643427	0.127128
0.037302	0.883665	0.992201	0.111775	0.135902	0.222561	0.24562	0.06715	0.241255
0.081587	0.000895	0.706053	0.043297	0.009062	0.414012	0.24562	0.213218	0.241255
0.072482	0.339913	1.387612	0.392944	0.089235	0.653968	1.106046	0.159639	0.2914
0.158521	48.53429	2.183665	0.462612	1.42456	0.031919	0.734524	2.941544	0.800967
1.532468	0.768052	0.051595	0.890577	0.430837	1.64917	0.318312	1.659033	1.070115
2.477027	1.289149	19.68088	0.337222	1.479452	7.558139	4.758088	1.017107	3.370527
3.182482	0.821898	1.039199	2.74544	2.863211	1.306667	1.180613	4.445851	2.461905
0.158521	0.000895	0.706053	0.050216	0.056948	0.161962	0.114517	0.000418	0.067547
2.567605	2.084791	1.089394	2.887489	3.934986	1.173535	0.358211	6.268312	4.473348
1.125325	1.091443	0.051595	0.933261	0.384394	0.031919	0.018326	0.542165	0.250115
2.863211	0.521775	8.365072	0.772626	1.195079	9.502294	4.529913	6.404921	2.611797
0.325772	0.000895	0.630022	0.212395	0.089235	4.086926	0.406212	0.565137	0.036349
7.238023	5.172828	1.387612	6.287561	5.01772	0.031919	0.716234	3.327619	3.037626
0.84119	3.128694	0.051595	2.591443	1.0386	0.031919	0.018326	1.235346	1.198701
3.330909	0.194098	1.604827	1.753341	0.50803	1.761349	1.661328	1.828781	0.84119
3.011169	3.101371	0.514531	3.201457	4.690159	0.275873	0.179798	1.074827	7.351284
0.072482	0.248716	1.941703	0.057417	0.268232	0.485014	0.147807	0.086075	0.165368
0.016219	0.000895	0.414473	0.015541	0.049668	1.672107	1.014784	0.056869	8.66E-05
0.964416	3.387864	0.414473	1.168326	1.602619	0.85728	0.54847	2.786898	1.012742
0.466335	0.000895	1.349971	0.06254	0.31557	0.031919	0.778326	0.315332	0.310866
0.833535	5.330519	0.758773	4.49671	4.63189	0.161962	0.018326	1.602619	2.579509
0.250823	0.667965	1.089394	0.221962	0.65132	0.841746	0.84119	0.34583	1.012742
2.425801	4.888023	0.051595	2.535974	6.8421	0.031919	0.679452	0.243896	3.610707
14.25954	7.964315	0.051595	17.78522	8.677489	0.937655	0.623853	6.760765	7.710361
0.120216	0.09847	0.550974	0.185801	0.073038	4.086926	2.388348	0.802756	0.21689
5.01772	2.625823	0.514531	5.959033	3.909033	3.581688	1.052208	12.32958	5.447114
1.471198	2.197828	0.051595	5.560563	0.586595	0.431977	0.018326	0.420289	8.66E-05
6.045209	0.872013	4.277006	5.679502	2.201587	3.846984	3.394286	5.9071	0.390418
3.833247	0.108268	2.055289	1.308167	0.702381	4.130794	5.309264	7.519841	0.849336
1.290981	0.248716	0.051595	0.153016	0.403413	0.211501	0.018326	0.375281	0.613853
3.599329	13.15079	0.414473	24.2842	6.8421	0.233889	0.018326	11.77567	8.660281
11.42512	37.29684	8.905642	7.339452	10.17029	7.737807	18.19595	7.765931	10.1028
1.819221	29.49693	2.470317	45.1045	7.08733	0.687807	2.018167	1.095657	2.676392
0.022727	0.000895	0.992201	0.144228	0	2.703485	0.358211	0.086075	0.029177
2.068297	3.233045	0.333463	4.058514	3.578038	0.246955	0.421804	4.978182	0.409899
23.16622	6.011515	2.312085	11.03843	10.01009	2.000592	1.965657	3.926154	13.08214
0.158521	0.692799	0.589618	0.431198	0.415166	0.275873	0.390895	0.139091	0.390418
13.83359	46.7056	0.992201	6.796566	39.53687	0.971205	7.911934	6.116638	15.18102
0.747879	0.547403	0.414473	0.617763	1.523824	2.74298	3.563182	1.759986	1.360022
0.519387	0.000895	0.550974	0.212395	0.042367	0.742417	0.633853	0.788896	0.20215
0.784805	1.121703	0.051595	1.219019	0.121587	0.031919	0.018326	0.222864	0.676999
3.885642	0.512309	1.183478	1.438672	0.538117	2.524892	0.421804	9.218514	11.89932
5.095325	0.667965	0.91575	3.767489	1.126075	1.3829	0.24562	7.288788	6.900202
0.072482	0.045079	0.91575	0.376782	0.78526	1.405772	2.03886	0.832013	0.328543
2.132756	9.541645	2.797835	5.388405	4.328232	1.173535	6.045209	1.903636	2.518124
0.285556	0.05632	1.135887	0.342511	0.027056	2.62342	0.16925	0.507403	0.127128
0.239769	15.276	0.051595	5.508131	8.256465	0.031919	0.595022	0.297345	0.958557
1.666089	0.512309	12.46209	0.075123	1.401068	1.473218	8.119004	0.203254	0.715815
0.914618	0.000895	1.308167	0.628658	0.107828	0.397215	1.948918	0.684033	0.142323
24.73724	4.130794	44.27012	8.524242	4.49671	6.933961	6.580988	17.78522	11.719
2.112843	118.7028	0.051595	19.06893	28.87506	0.031919	0.018326	0.000418	3.55961
2.240274	15.18102	2.703485	10.05618	17.98838	1.431414	2.807937	0.684033	6.268312
0.129553	0.000895	0.66482	0.229928	0.034459	0.031919	0.018326	0.243896	1.012742
4.25658	7.938427	0.477446	3.851414	2.914632	0.60202	0.018326	2.802828	4.586753
16.68554	30.49289	0.333463	105.1694	65.94551	14.79742	14.25954	41.47925	22.52375
0.081587	0.035426	1.039199	0.015541	0.000895	0.653968	0.697908	0.086075	0.118903

2.616573	0.628297	1.604827	0.523521	0.447662	0.901212	1.014784	0.420289	0.92816
0.422511	0.000895	0.91575	0.081587	0	0.920483	0.318312	0.159639	0.118903
0.710722	5.439278	0.051595	1.464214	0.640606	0.653968	0.018326	0.000418	0.635916
0.17619	0.202799	0.051595	0.111775	0.14987	0.222561	0.147807	0.684033	1.739935
0.304545	1.860491	0.371075	0.998831	1.377446	0.031919	0.137302	0.288247	0.460649
0.053838	4.440548	0.051595	0.510707	1.0386	0.762165	0.756176	0.000418	5.287857
0.537518	0.547403	0.051595	0.715815	0.586595	0.031919	0.018326	0.967446	1.772756
8.048095	17.78522	0.758773	22.13245	23.38378	0.414012	2.607035	12.32958	26.25557
10.05618	16.5625	1.556392	5.638759	12.60233	0.971205	1.339091	4.297222	21.93452
0.06254	0.133939	2.911962	0.237698	0.169538	0.519755	0.125483	0.06715	0.400534
3.13456	5.942294	0.706053	1.68938	2.591443	0.031919	1.236248	0.363983	5.583968
8.226219	4.440548	21.36892	5.295325	6.079596	17.28195	21.19547	18.05488	29.82828
0.833535	4.353925	1.135887	1.616385	1.213434	0.031919	0.578817	1.962424	0.250115
4.18456	1.091443	10.8813	0.736977	0.964416	21.74051	7.074329	1.417345	7.264502
0.19544	3.404372	0.051595	2.250462	3.163506	0.414012	0.697908	0.095382	1.219019
0.454156	0.268369	1.941703	1.639639	1.153723	2.361198	0.595022	0.609841	1.252424
0.996111	5.266335	0.051595	0.924329	1.554185	0.348139	0.018326	77.74812	3.998391
0.262338	0.000895	4.721789	0.06824	0.064697	1.195794	2.535974	4.690159	0.319762
4.49671	1.837172	11.96574	2.359264	1.711414	11.42512	3.581688	14.88584	4.145209
4.06303	1.775325	10.48101	2.011501	6.297136	4.130794	11.33981	6.622092	9.031082
0.224372	0.796234	0.371075	0.06254	1.793074	0.211501	0.260404	0.12925	0.81228
0.537518	0.805238	0.589618	1.296905	1.030678	0.783398	3.651898	1.477388	1.070115
2.019618	0.067547	1.703795	0.102886	0.560043	0.174372	0.925801	2.221775	0.765094
0.072482	0.035426	1.556392	0.050216	0.014329	0.573947	0.318312	0.222864	0.224921
0.313521	4.048499	0.371075	1.992944	3.227085	0.535657	1.62562	0.387489	1.111977
0.442496	6.486104	0.051595	2.445079	1.827403	0.031919	0.484423	0.643427	1.578687
0.037302	0.035426	0.051595	0.000188	0.002179	0.161962	0.147807	0.029928	0.023492
6.475505	0.120144	25.48179	2.121198	1.312706	32.31123	24.50942	4.580938	9.838658
6.181652	1.469163	0.758773	3.397706	3.340361	0.724885	0.898016	5.745996	4.888023
3.966623	0.902403	0.448557	1.884935	3.381255	0.222561	0.288867	5.662994	3.324452
3.851414	9.583463	1.505678	14.42661	6.591544	2.26518	0.4357	8.539971	10.1459
0.304545	2.635685	0.758773	1.035058	1.64684	0.762165	1.255657	0.304921	0.92816
0.422511	0.1686	1.308167	0.698687	0.409618	2.022778	1.481631	1.083651	0.576205
0.06254	1.64684	0.051595	13.00996	1.238651	6.345382	0.018326	1.641999	8.66E-05
0.325772	0.935743	1.089394	0.254019	0.250823	0.554416	0.716234	0.213218	0.390418
1.482626	1.520346	0.550974	1.335743	1.692763	0.246955	0.878369	1.771392	3.47531
0.138947	0.217677	5.22482	0.400354	0.20733	0.620909	2.053521	0.159639	0.345238
1.641999	0.538427	11.36975	1.360022	1.056263	1.783203	2.518124	0.518413	3.722785
6.063276	1.997352	4.105714	7.037345	16.09482	4.776407	1.180613	2.533745	16.22108
2.019618	1.142345	0.051595	1.090072	3.993665	0.309444	0.018326	0.987309	2.831501
8.134084	11.30987	0.051595	10.67991	14.79742	0.031919	0.018326	0.000418	7.897078
2.335844	3.885642	0.051595	2.789466	2.322035	0.397215	0.018326	1.043608	1.809343
1.556392	1.34267	0.051595	1.68938	0.568665	0.031919	0.018326	1.235346	0.504964
10.67991	0.467677	0.051595	4.188896	1.890996	1.070115	0.018326	7.188095	6.464906
3.144156	7.519841	0.874603	6.99026	10.98644	0.485014	0.450375	3.66785	8.524242
2.382035	1.42456	5.01772	0.838846	0.684401	1.91254	1.601443	2.765758	2.887489
5.24562	3.128694	126.1556	13.91714	7.938427	72.69473	37.81919	0.518413	25.48179
0.138947	1.40772	5.402511	0.144228	0.107828	0.724885	1.601443	0.06715	0.185325
0.120216	0.078124	6.52899	0.185801	0.212395	1.360022	1.128788	18.19595	0.277417
27.96248	31.92385	1.308167	17.39935	25.48179	0.031919	0.898016	24.73724	27.96248
1.205685	1.301169	0.051595	1.10132	1.014127	0.031919	0.204531	0.948745	0.625209
0.361717	3.324452	0.051595	1.128788	1.766248	0.378326	1.106046	0.967446	0.2914
0.92531	0.000895	11.42512	0.000188	0.375743	2.839351	2.463911	0.304921	2.392431
1.861999	4.317648	0.051595	11.36975	2.465916	0.348139	0.018326	7.737807	1.519322
8.461703	25.73165	87331.38	21.54823	8.508514	87331.38	87331.38	64.12971	4.227302
0.053838	0.000895	0.589618	0.102886	0.027056	0.261558	0.179798	0.243896	0.059654
1.384921	12.03267	530.1465	0.843564	1.126075	530.1465	530.1465	1.729892	4.451154
0.537518	12.56693	0.051595	3.676032	3.599329	0.031919	0.018326	0.34583	1.39847

0.537518	0.83088	5.25254	2.715873	4.043846	5.165599	7.002056	7.697063	2.234538
0.57197	2.280101	0.630022	0.976111	1.344257	0.031919	0.018326	1.235346	0.20215
3.279625	2.465916	7.149618	6.945152	3.885642	3.014076	3.87329	11.30987	12.63773
4.927302	0.088398	14.17388	0.221962	0.740952	30.49289	14.33939	1.017107	1.177309
19.99861	3.707136	6.695887	15.88548	9.031082	14.25954	5.210317	5.172828	4.082165
0.361717	0.000895	14.17388	0.000188	0	12.85504	21.93452	3.899993	8.66E-05
0.724502	0.217677	0.414473	0.237698	0.369105	0.952294	1.339091	0.149553	0.149986
2.425801	9.502294	18.48514	5.068831	4.359574	35.776	9.237525	3.125844	3.57057
35.27608	32.68313	135.5245	155.7103	33.93939	100.0998	14.47154	167.9996	105.1694
24.2842	13.08214	10.2617	18.05488	40.7932	8.608153	11.4544	0.579149	47.5018
30.82277	8.573434	49.50903	36.74522	25.99427	105.1694	0.84119	52.56128	13.53913
72.69473	11.77567	45.91841	21.19547	12.29473	44.27012	46.7056	59.32013	34.38341
27.38726	9.815642	11.83593	49.50903	24.04374	8.728571	16.68554	3.104394	56.39528
15.56131	7.795642	287.197	5.770996	6.99026	287.197	332.0776	9.838658	15.3618
87331.38	1360.293	1.703795	87331.38	1360.293	2.928247	0.450375	252.4251	332.0776
425.62	83.489	67.98069	72.69473	135.5245	42.08807	33.07147	126.1556	95.22939
135.5245	105.1694	23.49249	70.11932	203.5795	3.479134	3.521934	47.5018	75.1132
203.5795	17.28195	144.7919	91.05541	67.98069	126.1556	49.50903	332.0776	87.1746
15.88548	2.255916	8.643074	6.116638	8.075772	10.19468	12.1974	9.276811	7.099913
1.169791	0.501587	4.145209	1.360022	0.777864	5.671126	1.710173	1.828781	1.944517
20.34063	1.52676	83.489	5.9071	13.38418	42.75576	48.53429	30.3324	27.10502
6.028427	9.063983	0.051595	6.900202	12.10289	0.63754	2.491948	4.47912	6.063276
1.541227	0.133939	1.505678	0.359697	1.195079	0.901212	0.797114	4.965801	1.826147
0.129553	0.045079	2.183665	0.088398	0.129365	0.222561	0.84119	0.191486	0.378326
6.622092	1.05088	2.470317	1.237063	2.970144	4.338629	5.967879	5.993045	2.134545
0.072482	0.981558	2.797835	0.300606	0.302554	2.243911	2.518124	0.000418	0.149986
8.573434	3.077316	9.297085	5.622872	1.673254	18.48514	41.47925	5.323492	1.913997
0.710722	2.073232	224.9555	0.111775	0.516566	135.5245	287.197	0.967446	8.66E-05
24.98218	4.25658	4.552092	11.77567	10.48101	2.8657	7.683766	36.26061	25.73165
11.25392	4.18456	1.135887	3.037626	2.565556	0.031919	0.018326	2.290996	2.760765
11.99921	0.323535	2.74298	0.330043	2.03886	2.050404	17.64869	5.381227	7.288788
0.129553	0.035426	0.550974	0.176955	0	0.188427	0.390895	0.507403	0.076111
1.232886	0.685209	17.92188	0.592857	0.673254	7.92518	9.502294	2.45575	0.878932
0.422511	4.473348	0.66482	4.667518	0.684401	0.031919	0.24562	3.387864	8.66E-05
40.11561	25.99427	5.959033	42.08807	16.68554	2.703485	4.292078	9.063983	19.37139
55.04081	43.49658	2.183665	16.68554	60.77449	0.952294	3.8121	14.08569	46.7056
21.54823	12.29473	9.160382	15.07257	15.77678	20.85876	15.46193	31.55417	6.125823
10.40634	1.997352	0.91575	4.110779	4.984488	0.742417	0.578817	6.079596	4.203001
1.577569	0.1686	0.051595	0.260144	0.603737	2.839351	0.376212	1.919661	0.268961
12.53072	25.48179	2.107922	23.82354	21.02638	8.381328	3.279625	27.38726	24.50942
1.406746	0.71956	0.955418	1.319293	0.894704	1.783203	0.54847	1.379278	0.82974
1.114618	1.89961	8.005685	5.770996	9.751039	25.24367	14.79742	0.931457	14.25954
60.77449	11.42512	14.70188	23.16622	27.38726	15.88548	13.11646	105.1694	30.82277
0.625209	0.529423	4.277006	1.64917	1.415469	4.261342	7.112496	0.620462	3.754719
2.802828	0.562439	0.992201	0.763102	1.049517	0.331263	1.31	0.609841	1.758615
6.55039	5.044113	1.941703	2.500548	5.515584	2.839351	7.495397	8.643074	7.765931
4.082165	1.266255	0.514531	1.371602	1.344257	1.1407	0.925801	2.290996	0.90013
1.190491	0.035426	2.74298	0.640289	0.474574	2.000592	5.779574	0.756724	0.193449
0.816479	0.078124	0.955418	0.203175	0.516566	0.85728	0.661443	0.499206	2.096162
2.143261	0.209538	1.135887	1.026219	1.109726	0.60202	0.856782	25.24367	1.608427
1.248232	0.440801	0.333463	0.144228	0.532092	3.207807	13.75978	0.565137	1.87482
1.301169	0.838846	75.1132	5.095325	1.344257	5.075447	4.990794	0.353492	4.328232
1.641999	1.579798	3.446732	4.140505	1.453939	3.68381	4.338629	3.153485	1.177309
0.898543	2.173081	3.500808	6.796566	1.726032	2.74298	12.06778	4.348882	5.47
0.296234	2.789466	0.514531	1.006025	0.20733	0.466602	0.018326	0.118644	0.92816
0.17619	0.067547	1.262179	0.035339	0.20733	0.246955	0.531804	0.06715	0.28443
10.73084	11.25392	1.655541	19.29639	13.45939	5.864084	9.237525	9.179466	5.337547
0.724502	0.547403	4.927302	0.384632	0.430837	0.826407	1.736133	0.756724	0.460649

0.022727	0.000895	0.246955	0.000188	0.000895	0.161962	1.481631	0.000418	0.018016
0.647691	1.541227	0.051595	1.136566	0.693571	0.031919	0.018326	0.095382	3.33728
2.831501	6.079596	0.051595	3.703175	1.983824	0.031919	0.288867	0.931457	3.283139
111.5439	0.981558	42.75576	5.530274	21.54823	36.74522	56.39528	28.23632	44.27012
1.232886	18.48514	0.051595	3.962179	6.612121	0.554416	0.018326	1.713997	0.328543
1.50684	0.501587	4.381479	0.753016	1.153723	3.743232	1.930216	3.049603	1.456955
6.335664	14.33939	0.550974	2.449509	8.958225	1.473218	7.175397	0.987309	5.583968
3.122915	4.739668	8.365072	3.005281	5.696263	36.26061	19.37139	6.784762	3.899993
3.471486	1.255657	13.38418	1.006025	0.740952	15.3618	21.74051	2.31811	2.189235
0.776876	0.000895	0.051595	0.640289	0	0.573947	0.376212	1.477388	0.052078
28.54786	5.11697	80.6518	27.10502	10.53159	23.6012	64.12971	20.51271	7.795642
252.4251	530.1465	1.262179	425.62	167.9996	3.276335	1.481631	135.5245	287.197
0.605022	0.000895	0.051595	0.280823	0.142244	0.031919	0.421804	1.321053	0.059654
39.53687	34.38341	4.381479	25.36273	45.91841	7.363117	3.8121	55.04081	38.39042
8.780115	1.395563	2.531515	2.950173	2.752893	2.579509	1.361789	6.116638	7.653391
1.729892	0.582973	5.180058	0.924329	1.330029	3.314329	4.990794	6.200945	0.715815
13.00996	10.1459	1.703795	8.134084	12.22798	0.901212	1.930216	5.095325	6.125823

CGGA_11(	CGGA_111	CGGA_112	CGGA_112	CGGA_112	CGGA_112	CGGA_112	CGGA_113	CGGA_113
0.342511	0.243124	0.640606	0.380649	0.426645	0.420289	0.272814	0.197908	1.013434
0.418788	0.000736	0.866147	1.183478	0.524986	0.327994	0.315743	0.000144	0.936898
1.477388	7.339452	4.188896	0.05303	4.353925	3.425512	3.442886	8.445743	0.867287
0.50803	3.489899	5.898449	0.05303	7.112496	9.583463	5.560563	3.122915	1.561912
4.72798	3.253175	4.086926	1.401068	5.044113	5.821804	3.35715	4.684502	5.351934
2.990851	1.626782	10.98644	9.667316	1.487619	2.000592	1.1407	0.860924	3.644343
33.4985	48.53429	57.84882	11.19942	21.54823	49.50903	14.70188	33.07147	80.6518
0.013535	0.050519	0.087561	2.74298	0.000577	0.075498	0.073038	0.017937	0.10938
0.334444	3.217612	1.019791	0.514141	4.914863	4.821176	6.287561	4.46267	2.511465
0.973745	0.000736	0.000534	4.170051	0.000577	0.29531	0.354798	0.000144	1.120332
64.12971	18.76954	38.94854	26.80355	27.10502	25.48179	15.3618	17.51961	72.69473
6.019971	0.597417	4.155339	4.702468	0.524986	0.986797	0.204531	0.36267	4.328232
16.34294	38.94854	12.53072	3.803766	10.62739	45.1045	48.53429	37.29684	15.66023
0.944084	13.30906	0.000534	0.05303	4.927302	8.608153	13.53913	21.54823	1.187229
0.116926	0.000736	0.096905	0.714697	0.054863	0.027583	0.06443	0.011486	0.051255
6.25938	7.037345	2.320072	0.05303	2.45575	5.158146	10.78056	13.00996	1.202626
7.339452	0.723066	4.23202	0.563499	0.990188	0.961486	0.331558	0.714697	4.155339
0.212172	0.000736	0.000534	17.51961	0.128088	0.034776	0.00132	0.000144	0.084668
0.520289	1.477388	1.000794	0.05303	6.808369	2.229048	4.292078	1.156869	2.538218
1.014127	0.714286	0.847908	0.05303	0.887359	0.335411	0.544877	0.213218	2.500548
0.194848	0.88158	0.418788	0.05303	0.336147	0.523211	8.994791	0.82772	0.256941
0.584892	4.203001	2.609293	2.836717	2.834084	8.762677	20.34063	10.23768	1.816436
42.08807	28.87506	59.32013	0.05303	3.540606	23.82354	0.914618	1.592395	144.7919
0.000224	0.042027	0.059264	3.754719	0.153391	0.06772	0.00132	1.039199	0.084668
1.857677	1.409942	1.89518	4.222583	0.658362	1.643218	0.677807	0.267013	1.036558
2.569957	6.53969	2.996869	0.917431	4.130794	4.907929	3.493362	7.810058	4.776407
2.55184	2.257749	0.605455	0.05303	1.389509	4.529913	2.042201	6.011515	0.846342
0.368478	0.306811	0.825354	1.820541	0.404408	0.321227	0.413802	0.288247	0.493925
3.334199	52.56128	22.32214	0.05303	48.53429	59.32013	111.5439	184.443	15.276
0.707799	0.520678	0.521068	2.681169	0.404408	1.25886	0.518139	0.479062	0.998831
1.902352	11.19942	6.404921	2.068297	8.590794	14.97762	11.19942	9.792626	9.838658
0.473319	0.042027	0.250296	1.123016	0.031775	0.115801	0.175253	0.048773	0.361717
0.000224	22.13245	9.859719	0.05303	18.48514	20.16541	36.74522	19.83727	6.560411
0.054517	0.000736	0.096905	0.05303	0.054863	0.050844	0.00132	0.000144	0.084668
16.44768	0.393449	7.339452	41.47925	0.658362	0.239048	0.11075	0.28026	5.515584
1.350772	0.565714	3.816551	0.05303	2.396429	3.411097	3.33728	2.884762	2.522684
0.4357	6.107114	3.540606	0.05303	5.923838	4.557605	4.997417	6.508427	4.043846
0.017633	0.000736	0.376407	1.950519	0.06544	0.013348	0.054401	0.000144	0.513456
4.33342	4.783001	4.353925	3.367071	4.684502	3.507691	1.374329	1.985253	3.743232
28.23632	7.363117	7.30215	15.18102	4.016465	4.971991	1.927042	3.885642	5.576219
2.227273	0.597417	0.000534	5.864084	0.880498	8.66E-05	0.43684	0.58259	1.884935
0.349545	0.367532	0.550974	18.05488	1.581912	0.73912	0.639113	1.010289	1.771392
0.494444	3.687633	1.009625	0.05303	1.941703	2.335844	5.037489	5.560563	0.207835
0.000224	0.000736	0.000534	0.251573	0.000577	8.66E-05	0.00132	0.017937	7.22E-05
2.136335	1.055628	0.321227	2.068297	0.141284	0.561775	0.625209	0.341558	0.084668
38.94854	65.94551	33.4985	2.290996	40.7932	31.55417	38.39042	35.776	13.53913
0.494444	0.159228	0.428932	0.970635	0.043391	0.168167	0.054401	0.143102	0.266479
0.290519	0.059264	0.170238	0.05303	0.178442	8.66E-05	0.118983	0.106732	0.476335
0.023492	0.000736	0.000534	0.514141	0.000577	8.66E-05	0.00132	0.000144	0.027431
0.418788	0.000736	0.231768	0.616739	0.000577	0.013348	0.00132	0.000144	0.225671
0.4357	0.000736	0.030534	0.05303	0.031775	8.66E-05	0.043038	0.000144	0.191962
2.24733	19.99861	5.959033	0.616739	8.019769	16.09482	12.29473	16.22108	12.29473
0.614935	0.000736	0.428932	1.727323	0.054863	0.013348	0.00132	0.000144	0.462857
0.247085	0.042027	0.139697	0.341558	0.054863	0.034776	0.034459	0.116284	0.09342
0.944084	1.433189	0.866147	11.68846	1.603795	2.773644	5.47	4.365224	0.962698
1.384921	0.159228	1.684646	0.759293	0.166573	8.66E-05	0.00132	0.064812	1.508831
1.097677	0.269697	2.54961	0.917431	0.222561	0.905426	0.126883	0.073939	0.991631

2.165851	0.620909	0.769019	5.075447	0.373218	0.206263	0.994899	0.238478	0.670519
3.428788	1.003016	4.502453	2.978968	2.117814	1.1629	0.829192	0.846797	1.497143
1.123016	0.679812	1.414574	0.669769	0.285253	0.555036	0.315743	0.28026	0.469358
0.095599	7.08733	17.92188	1.596782	27.52753	9.625253	16.22108	2.540462	59.32013
0.068802	0.338341	0.211335	0.466869	0.275873	0.650556	1.242641	0.507085	0.447085
3.904517	0.819509	1.786306	3.803766	0.190996	0.327994	0.510072	0.267013	0.810491
14.25954	35.27608	14.70188	0.05303	22.13245	40.7932	18.26752	34.38341	22.74797
0.179668	0.042027	0.000534	2.005253	0.000577	0.403175	0.279177	0.09176	7.22E-05
0.000224	0.025274	0.000534	0.05303	0.000577	8.66E-05	0.00132	0.041097	0.027431
2.628225	1.369048	2.160649	0.669769	0.872013	1.283059	1.254704	1.191905	2.35733
1.370765	2.681169	2.08013	0.616739	1.507835	1.094206	2.760765	2.197828	1.502323
1.839986	0.367532	0.935115	1.123016	0.436328	1.267879	0.233752	0.178629	0.476335
0.227244	0.084668	0.106609	1.820541	0.24518	0.075498	0.00132	0.000144	0.409098
2.024488	0.000736	0.356508	1.950519	0.000577	8.66E-05	0.00132	0.026205	0.240491
0.944084	0.050519	1.373348	1.242641	0.054863	0.050844	0.254964	0.125238	0.936898
0.017633	0.032857	0.02316	0.05303	0.02316	8.66E-05	0.00132	0.011486	0.020938
0.017633	0.000736	0.049646	0.917431	0.000577	8.66E-05	0.043038	0.000144	0.010043
5.762965	11.09133	7.683766	2.604776	4.894509	9.792626	9.101674	7.570693	4.524481
0.203254	0.050519	0.192929	1.018427	0.203254	0.042489	0.06443	0.048773	0.564127
0.187121	0.000736	0.067792	0.81228	0.031775	0.013348	0.043038	0.017937	0.084668
2.909293	3.595758	4.419062	3.128694	4.130794	3.544257	1.570794	2.77899	4.971991
0.131991	0.000736	0.118341	0.466869	0.06544	0.034776	0.00132	0.000144	0.216602
1.004791	0.51316	0.892395	3.324452	0.797633	0.514531	0.081667	0.273088	0.642698
7.162698	7.991602	7.175397	1.242641	4.086926	11.14619	16.34294	12.36443	6.888297
0.046724	0.000736	0.000534	0.422237	0.06544	0.007453	0.034459	0.191703	0.006306
1.060188	0.165844	0.466602	0.714697	0.166573	0.156825	0.06443	0.026205	0.775339
203.5795	95.22939	16.34294	0.05303	7.339452	10.78056	57.84882	105.1694	4.614618
0.573326	0.172294	0.356508	1.820541	0.166573	0.187013	0.11075	0.000144	0.439776
0.334444	0.635541	0.449091	7.668579	0.658362	0.168167	0.455325	0.310866	0.34583
8.694697	1.992944	0.749481	2.150303	1.242641	0.483874	2.013182	1.883535	0.184459
0.138009	0.172294	0.345238	1.183478	0.471025	0.605455	0.254964	0.171183	0.256941
0.164993	0.032857	0.376407	0.05303	0.141284	0.034776	0.034459	0.083795	1.195794
0.247085	1.684646	1.337431	0.05303	0.897489	1.350772	0.914618	1.401068	2.157359
0.000224	16.22108	3.425512	0.05303	10.1459	9.256537	2.873817	9.063983	2.981962
2.773644	0.144488	0.648362	0.05303	0.255693	0.187013	0.166118	0.106732	1.288276
0.981032	0.399978	0.550974	0.422237	0.764704	0.168167	0.836537	0.178629	0.504098
4.359574	0.499206	4.155339	7.824473	0.624841	0.393983	0.2129	0.204055	4.914863
0.000224	0.059264	0.030534	1.89373	0.166573	0.027583	0.135606	0.017937	0.10938
2.730072	7.938427	5.754935	2.978968	9.218514	7.238023	5.872886	5.34474	5.80469
0.138009	0.084668	0.096905	0.81228	0.043391	8.66E-05	0.118983	0.017937	0.141876
0.10886	0.025274	0.030534	0.563499	0.031775	0.013348	0.081667	0.011486	0.076392
0.000224	0.032857	0.039993	0.05303	0.031775	0.013348	0.043038	0.011486	0.010043
2.257749	1.055628	1.492973	2.347439	0.862561	1.547734	0.78619	0.650101	2.243911
6.784762	3.324452	4.445851	5.959033	5.788153	2.55184	6.325945	6.772763	0.978571
2.828918	0.209978	3.290101	4.222583	1.487619	0.676999	0.376212	0.68557	0.910823
0.212172	0.359105	0.321227	0.714697	0.24518	0.187013	0.2129	0.064812	0.670519
0.306176	2.162381	0.231768	2.483492	0.415974	0.843102	1.346003	0.947677	0.379495
0.349545	0.000736	0.732633	0.05303	0.113449	8.66E-05	0.118983	0.064812	0.876638
2.08798	0.000736	0.388326	2.229048	0.503781	0.042489	0.254964	0.191703	0.152532
0.678687	2.040606	0.356508	0.05303	2.08798	1.643218	11.36975	6.819481	7.22E-05
2.465916	1.055628	0.935115	1.297763	0.275873	0.462857	0.073038	0.318124	1.176623
5.266335	1.334762	2.243911	0.970635	1.667273	1.505678	0.804675	0.834416	3.169812
1.702706	2.871212	0.892395	0.05303	1.87482	3.125844	1.587605	1.076032	1.37531
1.444906	0.597417	1.145426	3.803766	0.776349	0.445996	0.78619	0.409899	0.876638
0.000224	67.98069	1.700354	1.066234	144.7919	135.5245	425.62	126.1556	1.546696
0.194848	1.404812	0.879913	0.05303	3.058802	2.413146	1.290981	1.839986	0.371075
0.546522	0.209978	1.058896	0.865664	0.153391	0.139235	0.091356	0.09176	0.962698
0.000224	33.93939	0.000534	0.05303	31.1753	53.77619	224.9555	75.1132	21.19547

0.891775	0.000736	0.581645	1.401068	0.233175	0.042489	0.126883	0.017937	0.199582
0.403413	0.000736	0.400173	0.05303	0.2129	0.219885	0.00132	0.125238	0.73619
1.810664	2.871212	2.944416	21.54823	1.89228	1.661328	1.848297	2.209141	3.005281
6.922771	22.96463	10.62739	8.762677	9.751039	8.958225	15.276	16.68554	5.770996
0.039827	0.084668	0.067792	4.529913	0.100996	0.075498	0.376212	0.083795	0.439776
3.518384	4.739668	7.724084	6.8421	7.991602	3.754719	2.839351	3.065108	6.685087
2.270837	0.428694	2.060491	4.272165	0.58259	0.916306	0.00132	0.073939	4.546443
5.545729	1.626782	4.667518	3.367071	2.138131	0.65798	0.53658	1.123016	3.540606
17.15902	1.253752	8.711905	65.94551	0.482078	0.577706	1.418312	0.714697	13.23022
0.914618	0.482367	0.503463	6.663651	0.444762	0.434596	0.708492	1.163579	0.216602
0.10886	0.032857	0.182359	0.272078	0.000577	0.013348	0.00132	0.000144	7.22E-05
0.254365	0.54925	1.31	0.305455	1.135887	1.000173	0.617417	0.310866	1.061457
0.395642	1.521371	0.388326	1.89373	0.744113	0.470512	0.617417	1.114618	0.564127
9.968355	5.720173	14.79742	1.242641	10.98644	7.200505	4.343838	3.404372	22.42294
0.212172	0.186133	0.653968	1.950519	0.166573	0.343629	0.00132	0.033997	1.061457
0.418788	2.483492	5.34474	2.150303	3.648081	26.80355	3.407619	3.334199	15.77678
1.682193	0.572626	0.957915	2.410996	0.909856	1.741414	0.493247	0.657547	0.978571
0.023492	0.000736	0.077547	0.341558	0.02316	8.66E-05	0.00132	0.000144	0.034906
4.94026	1.978954	4.971991	4.620159	1.941703	4.003117	1.115902	2.944416	1.006623
0.788369	0.252922	0.157958	0.05303	0.24518	0.650556	2.415296	0.763564	0.117518
1.028672	0.000736	0.261962	1.820541	0.07632	8.66E-05	0.054401	0.000144	0.917431
1.270317	1.549711	0.231768	0.759293	1.290981	0.483874	1.082915	1.302107	0.833535
2.893001	1.945916	3.290101	25.86296	0.452713	1.801284	0.11075	1.401068	1.699091
1.199481	3.944084	2.174733	1.066234	2.720996	3.107417	3.904517	5.34474	1.176623
0.833045	0.490952	0.118341	0.05303	0.755714	0.561775	0.867814	1.203427	0.117518
4.135801	2.628225	4.402973	12.85504	3.156537	2.040606	1.945916	1.74539	1.816436
7.653391	5.030866	9.19899	9.256537	2.925426	3.350224	1.33285	2.402468	9.397771
0.000224	2.24562	3.599329	1.242641	2.589185	2.363442	12.49452	2.901032	2.065007
0.234949	0.505786	0.866147	1.123016	1.171349	0.856782	9.297085	1.220628	0.705613
1.179885	2.398442	1.700354	0.05303	3.404372	2.92013	3.428788	1.252424	1.533535
19.60574	22.52375	15.77678	3.128694	16.68554	6.922771	5.591717	12.63773	12.70768
0.000224	1.323975	0.000534	0.05303	3.921645	0.547403	3.394286	1.287403	0.199582
24.98218	19.06893	8.195657	6.019971	11.83593	14.88584	16.44768	12.81638	10.98644
5.515584	8.461703	3.917597	3.128694	6.230245	7.737807	27.66779	12.10289	3.377619
0.017633	0.050519	0.000534	0.616739	0.000577	0.013348	0.00132	0.000144	0.076392
1.432302	1.267879	0.661443	0.05303	1.189048	1.241811	1.986681	1.631544	0.332049
11.42512	2.024488	7.38847	8.061934	2.747908	1.213434	0.331558	0.729582	11.83593
0.473319	0.376407	1.244957	5.704646	1.317475	0.723066	1.115902	0.633499	1.469163
0.095599	0.218975	0.559221	0.563499	0.128088	0.420289	0.553059	0.267013	2.104358
0.000224	3.350224	1.429365	0.05303	10.28573	3.324452	5.323492	4.684502	0.610224
0.000224	0.996111	0.000534	0.05303	8.365072	0.213918	59.32013	15.41187	7.22E-05
0.746551	100.0998	29.82828	0.05303	51.52195	167.9996	118.7028	111.5439	28.87506
1.496118	0.384632	4.02088	6.423911	0.404408	0.994271	0.304062	0.28026	2.442987
0.358903	0.067864	0.139697	0.05303	0.06544	0.050844	0.00132	0.000144	0.371075
0.981032	0.98474	2.479221	0.917431	1.803918	1.615281	1.250058	0.752229	0.876638
1.702706	0.419827	1.131472	0.05303	2.834084	1.439704	4.317648	3.625382	1.202626
0.459394	0.505786	1.361789	3.877417	0.559221	1.25886	0.265455	0.213218	1.319293
4.307309	1.043608	1.954993	3.521934	0.000577	8.66E-05	0.00132	0.000144	2.417424
7.882222	22.74797	6.570433	2.229048	16.34294	12.03267	7.710361	14.6058	9.256537
0.273911	1.314675	1.507835	0.05303	0.615584	0.567994	0.204531	0.326017	2.810678
1.409942	0.000736	2.668896	1.655541	0.000577	8.66E-05	0.00132	0.000144	1.362713
11.544	1.673254	12.81638	16.34294	3.334199	2.274473	1.877605	1.652771	12.22798
1.968283	1.827403	2.160649	0.669769	1.834423	0.916306	1.466111	0.909315	1.127475
1.982208	0.067864	2.117814	6.019971	0.373218	0.034776	0.185909	0.041097	0.417063
1.618773	0.847352	0.466602	1.655541	0.166573	0.30158	0.11075	0.253882	1.688045
1.290065	2.003723	2.008312	0.05303	2.984899	1.975772	2.339697	2.032706	1.212792
0.907273	0.000736	0.000534	0.81228	0.190996	0.213918	0.00132	0.213218	0.571674
1.014127	0.178182	0.170238	1.123016	0.088874	0.147807	0.101429	0.083795	1.328384

0.000224	1.253752	0.000534	0.05303	0.624841	0.577706	1.544517	1.685678	0.101609
0.86518	5.42381	2.690823	0.05303	4.376118	1.806595	5.439278	3.775209	3.005281
8.075772	3.324452	6.922771	9.989221	2.485534	1.953535	2.74544	3.150433	2.109755
14.6058	14.88584	29.18885	0.05303	31.92385	16.80343	95.22939	45.91841	2.890245
1.219019	2.327857	2.955916	0.563499	2.216162	2.591443	1.323131	1.660216	3.300664
0.000224	1.1407	0.000534	0.05303	6.717056	4.661717	2.477027	6.900202	1.256558
2.491948	0.172294	1.271097	0.466869	0.314387	0.278651	0.510072	0.185642	0.784372
2.257749	5.754935	3.792266	0.05303	8.048095	18.91854	10.33476	11.25392	13.91714
3.855952	0.73127	5.671126	4.222583	0.776349	0.897006	0.296234	0.545808	12.77772
7.099913	10.8813	10.23768	9.667316	11.77567	15.71851	5.388405	7.668579	21.54823
1.311674	0.209978	1.109726	3.6029	0.000577	0.955418	0.00132	0.371075	5.266335
13.79669	4.997417	15.07257	6.570433	9.968355	4.47912	4.875527	7.495397	21.02638
1.074827	0.572626	0.690043	0.05303	2.022778	1.495043	1.340036	1.797172	0.332049
3.086306	2.967453	2.752893	0.865664	5.770996	3.034747	1.61088	2.65684	3.88153
0.203254	0.092691	0.856291	0.616739	0.190996	8.66E-05	0.00132	0.000144	0.2914
1.618773	1.08671	1.578687	0.05303	2.720996	1.682193	1.199481	1.40772	2.143261
1.199481	1.888023	1.724805	0.341558	1.189048	2.440895	4.125786	2.24733	0.910823
2.148658	0.811861	1.361789	3.128694	0.596775	1.25886	0.324019	0.507085	2.681169
0.194848	0.000736	0.000534	0.514141	0.000577	0.06772	0.00132	0.000144	0.068384
0.000224	1.661328	0.615996	0.05303	2.797835	0.630022	0.404899	3.034747	0.184459
5.630815	10.33476	12.81638	3.253175	9.101674	8.226219	2.73272	8.048095	21.93452
0.546522	0.635541	0.777864	2.150303	0.58259	1.860491	0.00132	0.622092	2.434444
0.241053	0.269697	0.541804	3.754719	0.000577	0.30158	0.272814	0.106732	0.727222
5.165599	10.93101	5.606739	7.061328	8.677489	7.519841	5.165599	6.153102	2.970144
2.569957	5.515584	9.256537	2.681169	7.697063	15.56131	5.137042	5.864084	10.23768
0.241053	0.000736	0.466602	2.068297	0.302785	8.66E-05	0.00132	0.000144	1.127475
1.123016	0.100101	0.211335	0.714697	0.06544	0.2264	0.091356	0.083795	0.225671
0.764315	2.060491	2.267157	0.514141	2.597792	3.387864	5.34474	2.363442	1.89228
2.477027	0.587965	1.544517	3.521934	0.233175	0.723066	0.00132	0.470736	1.061457
1.250058	0.23303	0.568665	4.46267	0.348369	0.30158	0.446811	0.392403	1.100519
3.192229	5.959033	2.392431	0.05303	6.38456	4.179762	11.83593	8.539971	0.679069
1.921212	1.314675	1.544517	1.401068	1.271097	1.1629	0.553059	0.820931	1.187229
0.810491	0.151688	0.148254	0.305455	0.203254	0.386169	0.185909	0.083795	0.417063
1.158384	6.900202	12.06778	0.05303	10.85542	9.101674	11.14619	11.89932	17.51961
1.232886	0.629098	5.056421	14.79742	1.084437	0.650556	0.00132	0.296234	0.819509
2.136335	3.6029	5.872886	0.05303	7.824473	3.324452	8.089848	4.552092	3.031688
0.013535	0.032857	0.049646	0.759293	0.02316	0.013348	0.00132	0.017937	0.034906
0.203254	0.323752	0.211335	0.917431	0.394603	0.139235	0.00132	0.000144	0.867287
0.219683	0.000736	0.182359	0.81228	0.000577	8.66E-05	0.243896	0.000144	0.199582
0.144358	0.393449	0.388326	1.950519	1.000173	1.045462	1.340036	2.074906	0.152532
3.904517	0.059264	3.043528	35.27608	0.264654	0.020606	0.054401	0.033997	2.901032
37.81919	17.03919	118.7028	44.27012	26.25557	27.38726	19.68088	14.08569	33.4985
15.83113	17.92188	9.335397	5.704646	15.66023	17.39935	3.128694	8.381328	7.507619
1.930216	1.033672	1.29417	15.3189	0.787987	0.713846	1.199481	0.558427	0.600173
2.678781	4.451154	3.195541	1.401068	3.350224	4.096407	4.212554	2.836717	2.008312
1.921212	0.114113	0.118341	4.053506	0.128088	0.08438	0.00132	0.073939	1.027403
0.342511	0.172294	0.221443	0.422237	0.302785	0.2264	0.073038	0.064812	0.274704
13.79669	11.25392	0.000534	0.05303	3.574387	1.579798	17.64869	155.7103	7.22E-05
2.386248	2.661587	1.492973	0.05303	4.715599	3.6029	2.08013	2.735368	4.733824
1.702706	1.253752	1.609654	1.297763	1.250058	1.289149	1.340036	1.149322	1.127475
25.99427	1.817828	10.1028	11.83593	3.092229	1.902352	1.449062	1.924228	5.485685
0.299336	0.106926	0.170238	0.05303	0.670519	0.637994	0.722179	0.714697	0.493925
0.754784	1.945916	0.732633	0.05303	3.944084	0.676999	5.959033	4.435159	7.22E-05
1.131472	9.256537	12.36443	0.05303	8.643074	5.11697	60.77449	22.32214	0.757273
0.138009	0.084668	0.309877	0.514141	0.043391	0.013348	0.00132	0.048773	0.162576
6.830592	126.1556	27.66779	0.05303	75.1132	100.0998	80.6518	83.489	26.52537
2.203535	2.180022	2.794935	8.762677	1.974228	6.486104	1.002215	0.545808	3.47531
4.343838	2.847547	3.098268	0.05303	2.925426	1.547734	0.265455	1.323975	5.11697

0.764315	0.456522	1.634935	0.05303	0.275873	0.505786	0.00132	0.000144	1.27619
4.827691	1.637309	4.650007	0.05303	1.308167	1.930216	1.582915	1.02487	5.9071
2.417424	3.489899	1.145426	0.05303	1.823377	1.938759	2.581861	2.685786	2.681169
0.520289	37.81919	3.779452	0.05303	7.013853	21.54823	51.52195	33.93939	1.080909
12.93651	111.5439	23.16622	0.05303	155.7103	47.5018	126.1556	203.5795	44.27012
0.000224	0.000736	2.581861	6.297136	0.000577	0.668947	0.00132	0.000144	7.22E-05
2.325931	0.490952	1.40772	2.604776	0.922338	0.650556	0.196335	0.36267	2.121198
14.00414	0.629098	0.118341	4.272165	0.24518	0.075498	0.00132	0.083795	2.831501
0.081818	0.000736	0.030534	0.917431	0.031775	0.013348	0.043038	0.000144	0.020938
1.290065	0.505786	1.544517	0.05303	0.808485	0.702835	0.00132	0.000144	2.538218
0.034416	0.428694	0.541804	1.820541	0.482078	0.213918	0.346342	0.133781	0.068384
0.000224	14.97762	9.667316	37.29684	23.82354	32.31123	91.05541	42.75576	4.468009
0.000224	2.909293	0.709322	0.05303	8.780115	2.083261	4.039192	5.493182	3.125844
0.299336	0.252922	0.512641	1.123016	0.113449	0.371299	0.376212	0.106732	1.872049
4.435159	1.224733	2.498478	9.52197	1.446926	0.514531	1.186457	1.40772	6.717056
3.290101	0.288081	0.615996	0.05303	1.636118	1.487619	0.455325	0.171183	3.518384
13.91714	1.902352	8.745238	21.36892	2.126349	1.513102	1.784755	2.296732	4.614618
2.296732	0.490952	1.700354	2.547179	0.966825	0.747417	0.393449	0.947677	2.442987
0.899567	0.151688	0.983384	11.4544	0.264654	1.530159	1.41368	0.446291	0.696659
4.63189	1.202626	1.083651	3.754719	0.569625	0.445996	0.393449	0.567345	2.593506
0.342511	0.533874	0.802395	0.380649	8.590794	1.690483	5.545729	2.604776	0.775339
4.63189	0.306811	2.050404	2.547179	0.360281	0.353651	0.00132	0.000144	1.749307
0.000224	10.43066	5.439278	0.05303	7.795642	3.651898	29.18885	14.88584	4.397792
0.06189	0.059264	0.096905	1.950519	0.128088	0.123377	0.043038	0.000144	0.184459
5.454949	0.527215	0.438023	33.07147	0.537518	0.4971	0.00132	0.512641	2.294733
0.882078	5.9071	2.718434	0.05303	6.486104	4.105714	3.699495	4.082165	3.792266
7.653391	0.136926	0.157958	0.759293	0.043391	0.156825	0.175253	0.106732	0.371075
14.42661	25.73165	21.02638	2.347439	16.9162	22.52375	37.81919	28.54786	10.67991
2.828918	3.514834	2.08013	2.483492	5.9071	5.770996	6.116638	5.273362	3.387864
0.068802	1.008961	2.449509	0.05303	0.808485	1.350772	2.882035	0.600887	4.49671
0.194848	0.000736	0.049646	0.05303	0.054863	0.245967	0.06443	0.056782	0.240491
12.16681	16.44768	10.73084	33.4985	12.03267	11.65791	9.418196	9.924372	2.283752
0.334444	2.74298	1.660216	0.05303	8.539971	6.355224	18.48514	6.876392	2.065007
126.1556	167.9996	155.7103	36.26061	126.1556	126.1556	32.31123	100.0998	118.7028
0.403413	0.288081	0.732633	3.253175	0.471025	0.245967	0.233752	0.36267	0.447085
4.63189	5.165599	7.08733	14.25954	8.164531	3.566753	6.674286	6.853608	5.462475
5.9071	19.22139	22.74797	2.068297	20.51271	27.66779	43.49658	21.93452	13.60866
0.389574	0.084668	0.409098	0.05303	0.000577	0.050844	0.00132	0.000144	0.522633
0.023492	0.059264	0.106609	1.018427	0.031775	8.66E-05	0.11075	0.09176	0.015685
1.048074	3.125844	0.777864	0.05303	1.568564	2.463911	6.945152	2.909293	1.884935
40.11561	8.396847	10.05618	2.604776	14.33939	13.6814	45.1045	43.49658	6.922771
0.034416	0.000736	0.030534	0.341558	0.054863	0.042489	0.043038	0.000144	0.043297
1.650274	0.673254	0.321227	0.05303	0.275873	0.092302	0.404899	1.638499	7.22E-05
1.048074	0.159228	0.476551	1.655541	0.233175	0.327994	0.073038	0.334625	0.256941
0.584892	0.000736	0.130029	3.971061	0.000577	0.147807	0.00132	0.000144	0.184459
9.063983	4.690159	11.09133	4.620159	4.875527	3.363911	2.776421	2.529307	11.89932
3.140866	0.27899	2.776421	4.702468	0.897489	0.263463	0.518139	0.608543	1.850996
3.750837	6.53969	4.302367	3.1857	7.625209	4.429769	11.77567	10.05618	1.688045
30.49289	42.75576	111.5439	12.22798	80.6518	34.84299	24.73724	20.94257	64.12971
0.494444	0.172294	0.299553	0.05303	0.569625	0.219885	0.00132	0.000144	0.068384
0.068802	0.114113	0.087561	1.066234	0.043391	8.66E-05	0.034459	0.026205	0.199582
77.74812	25.99427	37.81919	6.967547	38.39042	18.33909	11.89932	31.55417	30.82277
3.086306	0.679812	2.609293	2.483492	1.223846	1.831631	2.174733	1.401068	4.193232
1.088687	0.159228	0.802395	1.242641	0.178442	0.254769	0.204531	0.116284	0.727222
7.765931	0.802756	4.029654	50.55949	0.787987	0.206263	0.889553	0.48583	2.54961
0.981032	0.505786	3.407619	3.971061	0.285253	0.254769	0.146883	0.318124	7.22E-05
3.363911	1.902352	6.695887	40.7932	0.190996	3.377619	3.169812	1.092785	17.64869
1.484553	0.345635	0.261962	2.229048	0.203254	0.139235	0.315743	0.125238	1.771392

135.5245	18.62711	47.5018	64.12971	22.74797	13.53913	6.591544	10.73084	45.1045
0.241053	0.025274	0.299553	0.380649	0.088874	0.042489	0.034459	0.000144	0.015685
2.192496	3.303925	0.892395	1.820541	2.138131	3.672078	3.975498	4.609076	0.827273
0.023492	0.000736	0.000534	0.380649	0.000577	0.007453	0.043038	0.000144	0.051255
1.737294	0.419827	1.578687	35.776	0.58259	0.483874	0.477107	0.552439	2.323983
9.838658	7.013853	11.96574	13.45939	7.653391	5.942294	2.207417	1.90798	16.5625
5.9071	1.921212	5.530274	6.967547	1.867951	1.924228	1.466111	1.491977	4.125786
0.326219	0.000736	0.879913	1.123016	0.06544	0.13114	0.00132	0.017937	0.903074
1.123016	8.195657	2.375974	0.05303	2.300678	9.179466	2.936739	8.888167	2.427792
2.509177	1.281241	1.280274	1.655541	1.018427	1.499214	1.969733	0.998312	1.244242
2.509177	0.000736	0.983384	7.824473	0.415974	0.470512	0.00132	0.163276	0.819509
6.570433	13.45939	6.717056	1.655541	15.56131	23.6012	18.05488	16.34294	5.923838
0.254365	0.904286	0.521068	0.05303	3.230065	1.476429	1.031284	0.876638	1.421126
0.342511	1.507835	1.337431	0.05303	2.898196	4.678846	2.445079	1.645613	0.65132
2.914632	0.12904	0.261962	4.620159	0.2129	0.139235	0.446811	0.191703	0.484141
39.53687	34.38341	40.11561	13.45939	28.87506	42.08807	33.07147	36.74522	45.91841
4.529913	15.61077	3.518384	1.655541	6.404921	8.348817	9.377345	18.48514	1.931537
14.79742	0.121587	0.106609	0.466869	0.153391	0.050844	0.101429	0.204055	0.152532
13.00996	3.058802	6.210375	13.23022	3.574387	1.902352	1.776739	2.55184	9.031082
7.288788	24.04374	24.85971	0.514141	49.50903	111.5439	77.74812	57.12205	55.04081
7.061328	2.408882	1.000794	1.183478	0.636291	1.1629	0.485325	1.491977	0.34583
0.48583	0.428694	0.240101	0.81228	0.348369	0.187013	0.254964	0.253882	0.628297
24.50942	1.532468	14.42661	12.70768	2.604776	1.434055	0.844747	1.455022	17.15902
2.876421	1.385801	0.376407	0.05303	0.559221	24.04374	1.323131	2.852677	0.532857
4.875527	13.60866	5.044113	0.05303	5.889798	7.598312	11.30987	11.83593	5.855281
6.760765	6.404921	5.47	11.68846	4.529913	5.137042	1.940231	5.210317	8.994791
5.082063	0.306811	4.702468	13.64503	0.922338	0.763939	0.78619	0.48583	6.325945
0.023492	0.000736	0.030534	1.349971	0.141284	0.007453	0.126883	0.011486	0.034906
15.46193	18.33909	13.00996	29.18885	24.04374	8.319019	6.230245	12.70768	14.88584
3.125844	9.459177	3.869163	0.616739	6.011515	4.440548	5.366335	5.25254	3.47531
1.881955	1.766248	3.842554	0.05303	2.914632	3.156537	3.7586	1.90798	3.663622
2.933903	2.834084	7.625209	1.596782	4.984488	7.882222	7.363117	6.738211	4.644098
4.408153	3.651898	4.914863	0.616739	5.821804	10.05618	2.65684	3.283139	4.758088
0.696659	0.331263	0.833045	1.401068	0.325317	0.763939	0.00132	0.185642	1.555289
0.10886	0.121587	0.059264	3.061955	0.054863	0.059798	0.11075	0.033997	0.73619
1.660216	0.887359	1.244957	2.229048	1.233665	1.018427	1.300368	1.010289	0.400967
4.776407	1.194221	2.794935	2.604776	1.007244	1.118066	0.185909	0.608543	5.150693
2.569957	0.000736	5.068831	0.05303	0.658362	0.887922	0.493247	0.28026	1.734949
4.435159	50.55949	10.82954	2.290996	35.27608	87.1746	83.489	67.98069	10.43066
0.000224	1.842734	1.337431	0.05303	3.460909	2.243911	9.541645	2.990851	1.340981
5.20267	1.96404	1.019791	2.068297	0.764704	0.874603	0.804675	1.737294	0.743694
21.36892	0.847352	9.946364	7.545584	2.148658	1.695216	1.705007	0.884654	8.762677
2.07658	2.127973	1.464214	42.75576	0.990188	1.341825	0.331558	1.809343	3.989206
3.953297	1.194221	3.493362	11.06488	3.511263	1.523824	1.125325	3.50425	1.699091
32.68313	8.677489	27.38726	34.84299	15.77678	10.1459	4.789596	7.099913	12.42965
34.38341	144.7919	5.195022	2.150303	4.839105	11.60283	3.885642	26.80355	7.507619
1.930216	16.34294	1.672107	6.181652	6.423911	25.24367	75.1132	16.09482	3.989206
0.716652	1.873434	1.096335	0.514141	1.89228	0.794401	1.272756	5.622872	1.187229
6.695887	4.048499	8.319019	1.462143	7.417359	7.839271	5.22482	6.135007	10.01009
5.762965	0.924329	7.013853	1.596782	1.648066	3.233045	0.233752	0.519755	3.043528
0.261227	0.159228	0.030534	1.066234	0.07632	0.08438	0.304062	0.347908	0.010043
0.15127	0.084668	0.000534	0.341558	0.000577	0.013348	0.073038	0.000144	0.027431
12.7427	4.946587	5.671126	0.05303	5.24562	4.614618	1.019134	4.101061	8.348817
14.70188	16.9162	21.36892	5.576219	11.96574	8.033932	4.888023	10.57984	27.96248
0.013535	0.025274	0.000534	0.251573	0.000577	0.013348	0.034459	0.011486	0.006306
0.452713	0.076587	0.139697	0.669769	0.031775	0.013348	0.043038	0.073939	0.191962
25.48179	15.61077	91.05541	37.29684	47.5018	48.53429	6.191515	12.16681	87.1746
1.797172	0.345635	1.930216	2.781558	1.189048	0.505786	0.243896	0.157186	0.949286

0.046724	0.067864	0.000534	0.272078	0.153391	8.66E-05	0.054401	0.000144	7.22E-05
11.14619	18.48514	46.7056	72.69473	33.4985	19.99861	6.00228	11.14619	75.1132
4.048499	6.181652	5.71241	5.864084	3.6029	2.710765	1.002215	1.482626	8.445743
0.418788	0.050519	0.000534	2.068297	0.043391	0.020606	0.091356	0.033997	0.059654
1.839986	0.050519	0.838326	4.959358	0.141284	0.263463	0.00132	0.133781	1.072056
1.326667	0.084668	0.856291	9.902576	0.100996	0.147807	0.135606	0.163276	0.10938
224.9555	21.19547	100.0998	224.9555	41.47925	21.93452	17.15902	21.36892	83.489
17.64869	6.717056	13.15079	4.053506	8.256465	4.644098	0.923874	2.765758	18.05488
36.26061	9.880779	27.10502	28.71146	7.545584	8.508514	4.397792	6.55039	31.1753
0.48583	0.91404	1.259747	0.05303	2.31013	1.953535	3.895469	1.542302	0.803665
30.17192	203.5795	87.1746	3.432388	252.4251	105.1694	184.443	167.9996	126.1556
15.66023	9.541645	18.91854	17.28195	15.07257	5.591717	6.036818	5.9071	9.179466
3.904517	2.882035	3.588369	5.864084	4.053506	1.855108	0.877222	1.455022	2.890245
5.388405	0.419827	2.227273	0.05303	0.373218	0.905426	0.526032	0.53912	0.207835
5.130411	0.580123	2.129596	4.365224	3.230065	0.851234	2.786898	0.417063	0.392403
8.994791	3.895469	5.622872	3.253175	5.560563	2.909293	1.041652	2.276407	10.78056
8.923117	6.345382	80.6518	75.1132	13.53913	33.4985	2.839351	2.124574	57.84882
0.473319	0.172294	3.172864	0.514141	0.38596	0.321227	0.00132	0.000144	4.971991
3.211176	0.98474	1.429365	3.324452	0.776349	0.925801	2.261573	1.960945	0.051255
44.27012	15.07257	60.77449	155.7103	20.85876	15.276	24.98218	25.48179	29.18885
62.33472	42.08807	39.53687	6.423911	42.08807	28.87506	10.43066	22.13245	46.7056
7.238023	0.482367	12.53072	8.625613	0.302785	0.713846	0.947677	0.479062	6.210375
32.31123	17.51961	30.17192	31.73901	13.23022	10.98644	3.588369	5.123781	20.68746
2.136335	3.66785	6.287561	0.05303	3.6029	2.710765	0.677807	2.333867	4.058514
16.74449	35.776	10.01009	1.297763	24.73724	20.85876	39.53687	46.7056	5.217569
6.808369	0.225339	0.400173	0.563499	0.190996	0.353651	0.602287	1.211299	2.642525
0.000224	4.524481	2.81342	0.05303	6.210375	2.092987	10.98644	7.598312	2.121198
37.29684	33.4985	19.99861	0.341558	26.52537	24.73724	42.75576	64.12971	9.583463
0.668947	0.000736	0.605455	10.80505	0.000577	8.66E-05	0.00132	0.000144	0.455974
1.872049	0.467136	1.801284	1.820541	0.686667	0.412193	0.469711	0.318124	0.705613
1.0386	0.159228	2.111299	1.123016	5.416623	5.754935	9.968355	3.930664	5.195022
0.736977	0.847352	1.182056	0.305455	0.524986	0.477446	2.554069	0.657547	11.30987
0.520289	0.12904	1.322092	10.05618	0.302785	0.31456	0.493247	0.171183	0.610224
5.266335	2.250462	1.89518	0.305455	2.581861	2.451645	3.91355	2.066652	3.94886
0.000224	0.000736	0.02316	0.341558	0.000577	0.007453	0.00132	0.056782	7.22E-05
26.52537	14.25954	26.80355	23.71237	12.77772	14.17388	7.598312	11.719	27.10502
252.4251	77.74812	126.1556	425.62	52.56128	51.52195	41.47925	52.56128	87331.38
530.1465	155.7103	749.7839	203.5795	87.1746	203.5795	87.1746	80.6518	224.9555
1.550837	0.121587	1.940231	1.297763	0.203254	0.353651	0.11075	0.106732	1.288276
14.88584	27.66779	25.48179	8.412367	34.84299	29.49693	35.776	30.17192	25.73165
0.452713	0.23303	0.892395	0.970635	0.360281	0.307489	0.632569	0.178629	1.080909
2.958773	2.327857	5.454949	7.188095	2.485534	2.173081	3.993665	2.871212	6.09759
0.4357	0.759646	0.400173	8.27267	0.043391	0.327994	0.175253	0.259784	0.132922
1.179885	0.580123	0.157958	0.305455	0.153391	0.147807	0.054401	0.083795	0.141876
0.017633	0.000736	0.030534	0.759293	0.178442	8.66E-05	0.00132	0.000144	0.152532
16.5625	4.012049	2.852677	10.62739	1.77404	1.915455	1.076032	5.583968	3.330909
27.66779	135.5245	3.263391	2.483492	3.930664	18.19595	3.169812	23.16622	14.70188
0.316335	0.032857	0.182359	0.514141	0.02316	8.66E-05	0.054401	0.017937	0.027431
46.7056	55.04081	17.15902	14.70188	46.7056	26.25557	65.94551	65.94551	33.93939
0.068802	0.050519	0.000534	1.183478	0.000577	8.66E-05	0.06443	0.000144	0.132922
17.98838	28.54786	10.78056	0.669769	17.51961	33.93939	6.853608	11.60283	14.00414
0.891775	0.000736	0.466602	0.563499	0.000577	8.66E-05	0.00132	0.000144	0.051255
0.934488	1.567576	3.028629	9.031082	3.648081	1.180613	1.656696	1.421126	0.541804
0.212172	0.076587	0.356508	0.05303	0.07632	8.66E-05	0.00132	0.000144	0.750404
0.000224	0.032857	0.000534	1.401068	0.07632	0.020606	0.00132	0.000144	0.006306
0.144358	0.136926	1.609654	1.297763	0.153391	0.335411	0.265455	0.000144	3.57057
31.1753	37.29684	44.27012	45.1045	33.93939	23.16622	13.23022	20.34063	19.83727
4.715599	4.546443	20.34063	40.11561	8.103925	4.63189	6.09759	5.18754	8.888167

10.43066	3.980123	9.418196	11.06488	5.614805	3.107417	4.376118	2.884762	7.795642
11.19942	2.483492	6.674286	16.9777	4.580938	4.212554	3.659668	3.36075	5.788153
5.591717	2.169509	1.109726	0.422237	0.732633	5.295325	1.685678	2.917381	2.199639
0.017633	0.000736	0.000534	0.563499	0.043391	0.007453	0.00132	0.000144	0.051255
23.6012	6.653016	14.08569	23.16622	5.462475	5.788153	3.217612	4.752121	20.16541
11.96574	14.00414	15.9372	8.27267	25.73165	18.05488	21.19547	15.56131	25.24367
3.796075	0.706984	4.429769	6.772763	0.686667	0.327994	0.00132	0.000144	1.37531
1.496118	1.334762	2.08013	0.341558	2.08798	1.766248	2.390447	0.96329	1.340981
2.451645	0.620909	1.880375	4.053506	1.603795	0.941104	0.82355	0.68557	0.894704
10.53159	11.14619	20.51271	16.34294	9.031082	7.417359	3.286652	5.082063	11.19942
3.779452	0.904286	0.935115	4.365224	0.615584	0.343629	0.2129	0.392403	0.757273
1.91254	4.429769	7.443276	1.596782	5.030866	5.217569	5.302294	6.653016	2.463911
19.14516	13.08214	13.79669	56.39528	9.541645	11.48368	8.048095	7.162698	35.27608
27.10502	32.68313	6.865	7.951371	19.37139	16.5625	16.68554	24.98218	8.477662
1.650274	0.032857	0.261962	0.81228	0.031775	8.66E-05	0.06443	0.106732	0.191962
17.98838	25.24367	12.70768	1.655541	25.48179	24.98218	13.38418	19.37139	24.04374
0.596111	0.269697	0.221443	1.727323	0.178442	0.147807	0.00132	0.056782	0.727222
75.1132	3.377619	38.39042	70.11932	2.287576	2.953059	0.923874	1.762713	34.38341
1.278629	0.306811	0.049646	0.272078	0.07632	0.219885	0.11075	0.562107	0.610224
1.989711	1.667273	4.529913	1.89373	5.082063	4.039192	3.23987	10.78056	0.661861
0.686299	0.269697	0.157958	1.242641	0.744113	0.2264	0.746551	0.470736	0.2914
0.696659	3.743232	1.096335	0.05303	1.189048	3.521934	2.628225	2.436768	2.417424
56.39528	0.959062	23.38378	15.83113	6.922771	9.063983	5.704646	5.515584	28.23632
0.686299	0.042027	0.118341	1.066234	0.031775	0.075498	0.00132	0.116284	0.043297
9.031082	3.435988	18.69833	5.337547	8.195657	3.869163	3.450577	4.851227	19.99861
6.38456	13.6814	4.292078	4.702468	22.96463	11.89932	29.82828	22.74797	7.200505
0.028745	0.000736	0.087561	0.272078	0.031775	0.013348	0.00132	0.017937	0.010043
10.98644	3.993665	6.116638	5.477843	4.034423	3.172864	1.795851	2.323983	5.068831
8.888167	0.399978	2.111299	4.365224	1.233665	1.392511	0.485325	0.921739	2.369841
0.956061	0.165844	0.409098	0.380649	0.537518	0.4971	0.175253	0.197908	0.225671
0.000224	1.072843	1.209105	0.05303	1.189048	2.011501	5.037489	2.187395	0.885664
1.765065	1.153723	2.019618	1.242641	1.20653	1.018427	0.693218	0.424004	4.563117
15.98892	12.63773	5.71241	42.08807	4.016465	4.851227	2.302626	9.502294	24.50942
18.48514	7.38847	0.487128	184.443	2.939141	3.188918	5.20267	6.685087	1.114618
3.070952	0.98474	2.970144	1.018427	1.87482	3.585029	0.73619	0.729582	1.328384
0.068802	0.000736	0.000534	100.0998	0.100996	8.66E-05	0.101429	0.116284	0.361717
0.227244	2.050404	0.000534	9.141299	0.292251	1.1629	1.002215	1.03189	0.068384
1.139885	0.759646	2.392431	2.290996	1.171349	1.409942	0.223701	0.670144	0.435382
0.034416	0.000736	0.077547	0.305455	0.031775	8.66E-05	0.00132	0.000144	0.068384
3.552049	0.000736	0.847908	1.727323	0.000577	8.66E-05	0.00132	0.056782	1.783203
1.604827	0.000736	1.724805	2.781558	0.000577	8.66E-05	0.376212	0.000144	0.750404
0.891775	0.000736	0.192929	0.05303	0.088874	0.075498	0.00132	0.000144	0.867287
2.725498	0.723066	2.893001	0.341558	1.909408	0.868853	0.477107	1.378362	0.2914
0.426898	0.076587	1.544517	0.514141	1.135887	0.29531	0.00132	0.000144	7.22E-05
0.000224	0.032857	0.030534	0.05303	0.06544	0.020606	0.091356	0.157186	0.059654
0.418788	0.243124	0.118341	1.401068	0.325317	0.271912	0.146883	0.151378	0.216602
1.302107	1.235346	3.343629	0.341558	3.211176	2.31013	1.242641	1.549711	1.975772
0.349545	2.794935	1.473218	3.521934	1.233665	1.877605	3.040505	1.152944	0.476335
3.552049	75.1132	5.23176	0.05303	21.02638	60.77449	21.54823	38.94854	19.37139
0.299336	1.235346	1.634935	0.466869	1.909408	6.22031	14.25954	3.529466	0.757273
14.51648	29.82828	17.28195	0.05303	23.38378	57.84882	46.7056	72.69473	42.75576
0.138009	0.144488	0.428932	0.422237	0.141284	0.092302	0.157706	0.106732	0.170808
0.334444	0.306811	0.466602	0.714697	0.233175	0.278651	0.233752	0.083795	0.636291
1.570794	0.635541	0.732633	5.704646	0.49267	0.596508	0.933874	0.512641	2.723247
2.887489	0.924329	8.348817	3.803766	1.659033	1.58645	1.076032	0.738312	4.307309
0.754784	0.894704	3.028629	0.05303	5.123781	1.25886	4.827691	2.470317	7.751869
0.000224	0.000736	0.02316	0.669769	0.000577	8.66E-05	0.118983	0.048773	7.22E-05
1.881955	2.470317	1.841371	0.05303	2.355289	1.350772	0.889553	1.566414	0.857778

0.799048	4.251818	0.749481	0.05303	3.092229	1.026825	2.229048	3.743232	0.765606
0.596111	8.288874	3.703175	0.05303	2.947287	3.198499	4.508196	5.00404	1.328384
0.219683	0.000736	0.201789	6.088593	0.054863	8.66E-05	0.00132	0.238478	0.068384
13.08214	6.967547	11.77567	0.759293	8.780115	5.9071	2.535974	3.303925	13.00996
1.190491	5.409437	10.57984	0.05303	11.36975	8.075772	4.091667	7.363117	4.277006
0.914618	0.456522	1.609654	6.088593	0.000577	0.630022	0.00132	0.432698	1.487619
0.046724	0.12904	0.732633	1.727323	0.710253	0.353651	0.43684	0.70329	0.819509
0.707799	0.629098	0.333463	5.576219	0.302785	0.439776	0.370101	0.171183	0.936898
1.783203	2.715873	3.816551	7.402915	3.1857	3.307186	2.944416	4.23202	1.232078
0.624841	2.060491	1.348975	0.05303	1.659033	2.427792	5.454949	2.807937	1.469163
24.2842	23.49249	31.92385	0.616739	45.91841	27.10502	6.900202	28.23632	41.47925
1.097677	0.658745	0.718766	1.401068	0.744113	0.439776	0.659163	0.714697	0.743694
0.131991	0.000736	0.067792	0.380649	0.000577	0.007453	0.00132	0.000144	0.051255
1.060188	0.847352	1.155411	2.836717	0.436328	0.532619	0.196335	0.296234	1.58645
0.000224	8.994791	0.170238	0.05303	12.42965	5.395584	36.26061	16.9162	0.124719
0.000224	1.577569	0.924329	0.05303	0.744113	1.227749	1.831631	1.228651	7.22E-05
18.76954	17.64869	12.16681	4.46267	19.22139	12.93651	10.38202	12.42965	16.34294
0.000224	27.38726	13.08214	0.05303	13.45939	24.50942	25.48179	28.87506	14.25954
0.696659	0.306811	0.777864	0.05303	0.325317	0.905426	0.265455	0.223391	1.749307
1.765065	24.98218	0.000534	0.05303	19.99861	24.2842	23.6012	23.6012	2.178268
0.095599	3.192229	0.000534	1.242641	4.241905	3.394286	26.25557	27.96248	0.610224
2.807937	4.241905	3.005281	25.24367	3.944084	3.172864	2.415296	3.87329	4.445851
1.158384	0.225339	0.924329	5.180058	0.275873	0.206263	0.146883	0.09176	1.351573
0.659582	0.042027	0.388326	1.531313	0.24518	0.187013	0.081667	0.041097	0.392403
5.687879	4.323045	3.172864	0.05303	3.91355	3.91355	14.97762	4.839105	6.230245
1.263752	0.744531	1.841371	2.604776	0.787987	0.662944	0.923874	0.213218	4.597792
0.247085	0.323752	0.559221	0.05303	0.755714	1.283059	0.254964	0.777864	1.443983
0.810491	0.419827	0.476551	0.341558	0.647691	0.547403	0.722179	0.58259	1.202626
6.25938	23.82354	14.6058	4.863579	21.19547	14.6058	64.12971	24.2842	9.646284
2.203535	5.454949	2.375974	0.05303	5.330519	3.824964	3.230065	3.230065	2.230859
0.306176	1.360022	0.847908	0.05303	0.849841	2.019618	0.645036	1.065584	1.2214
0.000224	9.751039	0.970007	0.05303	0.178442	5.662994	2.174733	4.241905	0.051255
0.000224	0.367532	1.145426	0.05303	1.559675	1.088687	1.620058	0.930325	0.361717
1.384921	3.014076	2.002193	0.05303	3.381255	4.198117	8.745238	3.464315	2.947287
0.996111	1.921212	3.363911	0.272078	2.597792	2.092987	1.315613	1.211299	3.824964
0.081818	0.100101	0.376407	3.324452	0.24518	8.66E-05	0.00132	0.000144	0.300844
0.846342	0.620909	1.209105	0.341558	0.808485	0.642698	0.501587	0.562107	1.45912
1.47018	0.629098	5.030866	0.669769	0.559221	0.286364	0.42767	0.296234	2.022778
11.65791	20.68746	7.30215	0.714697	12.85504	13.23022	4.978182	8.348817	36.26061
0.389574	0.144488	0.000534	26.52537	0.088874	0.156825	0.00132	0.000144	0.216602
0.395642	0.766118	0.671371	8.625613	0.678687	1.33285	0.716234	0.379192	1.212792
0.799048	1.104805	0.992771	0.05303	1.603795	0.968723	1.766248	2.740519	0.316573
0.50803	4.997417	7.013853	0.05303	11.30987	6.674286	10.01009	9.179466	14.47154
0.187121	0.106926	0.356508	0.272078	0.166573	0.453932	0.196335	0.171183	0.576638
0.164993	1.540144	2.776421	0.05303	2.807937	8.923117	8.149307	3.489899	0.991631
0.023492	0.000736	0.000534	0.05303	0.000577	0.020606	0.043038	0.011486	0.006306
0.418788	0.165844	0.130029	0.616739	0.292251	0.307489	0.196335	0.231183	0.256941
1.199481	2.661587	1.227749	0.05303	1.77404	2.678781	1.277843	2.77899	4.145209
1.190491	1.055628	1.684646	4.222583	0.503781	0.80417	2.797835	1.123016	0.564127
6.99026	0.620909	7.584502	2.547179	1.158384	1.076652	0.204531	0.500722	4.165137
0.000224	0.000736	0.067792	0.466869	0.031775	8.66E-05	0.00132	0.026205	7.22E-05
2.117814	80.6518	6.865	0.05303	203.5795	72.69473	67.98069	135.5245	7.22E-05
0.652807	2.29873	5.082063	0.81228	1.110505	1.476429	3.796075	1.90798	1.487619
0.068802	0.076587	0.049646	1.297763	0.113449	0.050844	0.11075	0.048773	0.216602
25.73165	1.652771	21.19547	29.49693	3.011169	2.463911	0.632569	1.469163	16.9162
0.899567	0.86518	1.872049	0.05303	1.982208	3.095166	2.638139	3.243225	0.917431
23.16622	64.12971	22.52375	1.242641	30.17192	65.94551	38.94854	59.32013	19.53061
2.08798	0.000736	0.793434	15.02509	0.000577	8.66E-05	0.00132	0.000144	1.708932

0.000224	0.000736	0.030534	0.05303	0.203254	0.06772	1.031284	0.048773	7.22E-05
0.788369	0.23303	1.072843	5.704646	0.710253	0.489784	2.567605	0.470736	1.901068
4.091667	2.944416	5.302294	0.563499	1.34267	6.116638	2.042201	2.101075	1.2214
0.000224	0.159228	0.182359	0.669769	0.000577	8.66E-05	0.11075	0.000144	0.679069
0.212172	0.000736	0.299553	0.341558	0.000577	8.66E-05	0.00132	0.000144	0.300844
0.000224	51.52195	20.16541	0.05303	12.49452	55.04081	55.04081	21.74051	13.15079
5.366335	5.515584	4.952915	0.514141	5.150693	6.486104	9.924372	3.217612	3.860491
0.686299	0.428694	1.271097	2.681169	1.242641	1.045462	0.00132	0.000144	0.705613
4.789596	3.57057	6.250447	0.669769	4.783001	4.721789	1.776739	3.065108	6.063276
0.46461	1.072843	1.03189	5.864084	1.061457	1.487619	0.740952	1.065584	0.661861
13.30906	6.325945	15.66023	67.98069	10.67991	6.784762	2.561162	3.78798	9.792626
1.336587	2.111299	0.749481	0.616739	0.897489	2.611797	4.165137	1.985253	0.628297
0.179668	0.050519	0.182359	1.183478	0.043391	0.034776	0.081667	0.026205	0.184459
0.046724	0.000736	0.000534	0.05303	0.000577	8.66E-05	0.00132	0.033997	0.076392
0.872597	0.572626	1.64917	1.401068	0.909856	2.027951	0.659163	0.512641	1.640779
13.23022	10.05618	41.47925	7.951371	6.181652	14.42661	3.464315	9.297085	18.91854
3.767489	0.323752	1.525729	2.74298	0.07632	0.187013	0.054401	0.017937	0.2914
17.78522	0.243124	2.092987	5.864084	0.658362	0.31456	0.584502	0.163276	1.609654
0.882078	2.363442	0.333463	0.466869	1.20653	0.168167	1.394632	1.211299	0.225671
8.445743	5.560563	5.591717	32.68313	4.18456	7.002056	3.824964	2.529307	16.09482
2.750375	30.17192	1.517143	0.05303	3.816551	3.767489	26.80355	24.73724	1.019791
1.004791	0.067864	0.653968	3.68381	0.190996	0.13114	0.196335	0.083795	0.58925
0.000224	0.000736	0.000534	0.05303	0.100996	0.042489	0.00132	0.056782	0.027431
3.606804	2.151919	6.55039	2.547179	1.271097	2.363442	0.223701	0.698687	1.168326
0.102792	0.000736	0.250296	0.422237	0.000577	0.007453	0.00132	0.000144	0.132922
0.06189	0.000736	0.118341	1.349971	0.000577	0.187013	0.233752	0.000144	1.061457
60.77449	15.98892	65.94551	20.25302	53.77619	39.53687	9.256537	13.15079	60.77449
0.403413	0.084668	0.749481	4.529913	0.088874	0.059798	0.00132	0.000144	1.801284
0.013535	0.025274	0.039993	0.81228	0.043391	0.013348	0.101429	0.000144	0.027431
0.846342	1.992944	2.214372	4.365224	2.339697	1.631544	5.34474	3.659668	1.244242
9.541645	0.000736	14.00414	3.1857	0.000577	8.66E-05	0.00132	0.000144	8.075772
0.000224	3.514834	3.172864	3.6029	6.695887	6.153102	30.17192	3.489899	7.22E-05
0.973745	5.439278	0.000534	0.05303	9.418196	4.120815	19.53061	11.51384	1.297763
0.187121	0.706984	0.466602	0.305455	0.404408	0.73912	1.367179	1.01772	0.978571
1.219019	1.705007	0.912446	0.05303	2.6143	2.978968	1.115902	2.306277	0.679069
1.336587	0.541522	2.243911	11.06488	0.686667	0.453932	0.338499	0.083795	0.998831
2.155447	0.144488	4.105714	0.05303	0.000577	0.108016	0.404899	0.09855	3.754719
0.081818	0.000736	0.049646	1.531313	0.000577	8.66E-05	0.091356	0.000144	0.207835
3.377619	3.217612	5.638759	14.6058	6.325945	2.150303	1.853723	1.995714	6.685087
1.0386	0.714286	0.345238	0.05303	1.061457	1.082915	0.902403	0.876638	1.309084
1.400159	0.288081	0.661443	9.418196	0.596775	0.335411	0.073038	0.326017	1.723579
3.57057	3.014076	4.402973	0.05303	2.009906	2.051962	2.112843	3.939307	3.201457
0.459394	0.000736	0.559221	0.422237	0.113449	0.020606	0.091356	0.026205	0.266479
11.89932	5.337547	6.612121	0.05303	2.850058	6.011515	4.49671	9.968355	6.612121
0.046724	0.000736	0.077547	0.251573	0.07632	0.034776	0.166118	0.073939	0.006306
3.640606	6.277937	1.209105	0.05303	2.647244	2.633232	7.911934	4.408153	2.453781
2.807937	2.013182	6.518709	6.772763	2.415296	1.77404	0.354798	0.820931	5.180058
3.107417	3.204632	4.650007	0.05303	4.174964	3.457504	1.056263	2.287576	2.040606
0.882078	0.114113	0.690043	0.616739	0.128088	0.445996	0.101429	0.09176	0.316573
1.409942	8.815123	0.000534	0.05303	8.923117	9.502294	8.711905	5.950664	5.24562
5.993045	1.521371	7.495397	3.1857	1.648066	1.788874	0.363276	0.714697	3.340361
4.575123	13.75978	8.226219	0.05303	24.50942	18.62711	15.07257	15.77678	14.97762
12.10289	14.6058	6.796566	1.462143	13.30906	23.38378	16.80343	19.68088	3.989206
1.669466	0.144488	4.814661	0.05303	0.000577	0.362424	0.00132	0.106732	2.274473
2.990851	0.172294	2.609293	4.053506	0.07632	0.13114	0.265455	0.116284	1.58645
1.953535	0.076587	2.008312	2.410996	0.100996	0.007453	0.101429	0.041097	1.397482
0.000224	8.461703	0.000534	1.462143	3.290101	7.839271	13.6814	7.099913	2.800332
0.290519	3.334199	1.558571	1.655541	1.524776	1.631544	1.269495	1.762713	3.610707

8.608153	5.704646	4.789596	0.917431	5.295325	6.738211	2.873817	4.174964	7.991602
4.519048	0.114113	1.718918	3.6029	0.426645	0.379495	0.446811	0.171183	1.618773
1.941703	2.162381	3.703175	9.256537	3.432388	2.26518	3.659668	1.712706	5.881342
0.726133	1.253752	7.175397	0.865664	0.966825	1.056898	2.404618	0.721335	0.857778
1.464214	0.91404	1.303045	0.759293	3.13456	2.011501	0.584502	1.211299	2.375974
0.290519	2.770866	1.609654	0.563499	7.200505	2.826277	10.19468	2.306277	1.931537
2.538218	31.55417	9.19899	2.229048	24.98218	37.29684	22.32214	20.94257	5.720173
4.863579	6.433918	4.609076	0.05303	7.162698	10.62739	8.288874	4.990794	4.408153
7.417359	7.868146	6.153102	0.380649	4.597792	4.614618	2.644885	4.709033	7.911934
0.247085	7.545584	1.139221	0.05303	2.032706	1.869322	15.66023	8.677489	0.936898
2.009906	1.153723	2.647244	3.521934	1.000173	1.035801	1.594675	1.823377	0.857778
2.698557	1.661328	0.615996	0.563499	0.909856	1.758615	0.814315	0.500722	0.894704
0.584892	2.483492	1.940231	0.05303	2.042201	1.563045	1.290981	2.836717	1.52676
0.125613	0.186133	0.333463	0.714697	0.100996	0.100014	0.763102	0.545808	0.034906
1.278629	0.225339	1.182056	1.596782	0.537518	0.196667	0.501587	0.143102	0.810491
1.89228	0.186133	0.793434	3.68381	0.190996	0.059798	0.11075	0.041097	0.353333
0.85728	0.000736	0.749481	1.242641	0.000577	8.66E-05	0.11075	0.000144	3.397706
3.269913	0.437799	2.498478	0.05303	0.686667	0.540902	0.00132	0.432698	1.901068
0.227244	0.195007	0.201789	4.365224	0.190996	0.245967	0.562439	0.116284	0.191962
0.584892	1.766248	1.019791	0.05303	0.755714	0.620462	0.952294	1.577569	0.424848
0.000224	7.737807	0.000534	0.05303	2.250462	1.465123	14.88584	4.286962	7.22E-05
5.704646	2.661587	4.277006	0.05303	2.181775	0.868853	2.101075	1.087345	4.796126
2.607035	0.499206	3.113478	0.81228	1.281241	0.994271	0.575628	0.318124	1.915455
0.144358	0.23303	0.356508	0.81228	0.444762	0.034776	0.06443	0.213218	0.043297
10.05618	7.456118	1.64917	0.05303	7.363117	7.683766	8.256465	15.276	0.838846
4.150274	7.765931	7.911934	0.669769	8.477662	6.135007	5.80469	5.950664	13.08214
0.164993	1.834423	1.801284	0.341558	1.674401	3.011169	4.952915	1.645613	0.361717
2.176501	2.781558	3.610707	0.05303	2.747908	2.818312	1.883535	2.436768	3.663622
1.074827	0.000736	0.418788	1.066234	0.000577	8.66E-05	0.00132	0.000144	1.801284
1.048074	0.106926	0.289755	0.970635	0.031775	0.050844	0.354798	0.106732	0.059654
1.021097	2.909293	2.944416	0.05303	3.373983	6.045209	2.058773	2.647244	3.497085
1.722475	9.377345	2.565556	0.05303	5.273362	9.141299	3.1857	6.612121	9.480736
6.325945	0.679812	4.875527	24.50942	0.153391	2.65684	4.105714	2.045476	10.93101
0.96627	0.252922	0.709322	1.066234	0.100996	0.343629	0.118983	0.304228	0.152532
0.187121	0.345635	0.449091	0.305455	0.292251	0.948211	0.324019	0.09855	0.687807
0.157958	0.225339	0.321227	5.864084	0.444762	0.278651	0.309668	0.033997	0.522633
0.686299	1.008961	1.713997	0.05303	1.250058	1.401977	0.677807	0.763564	3.807648
1.139885	7.710361	7.854069	1.727323	6.612121	6.191515	3.253175	5.696263	9.218514
0.000224	0.000736	0.030534	0.616739	0.031775	8.66E-05	0.043038	0.000144	0.020938
0.046724	0.092691	0.02316	0.05303	0.031775	0.06772	0.00132	0.011486	0.059654
7.288788	1.736133	1.757244	6.967547	2.768312	2.071587	2.958773	1.645613	4.851227
6.414416	1.360022	8.677489	1.820541	2.194257	3.043528	0.404899	0.500722	11.60283
7.288788	7.964315	332.0776	252.4251	12.56693	15.46193	12.22798	2.978968	65.94551
0.645036	0.106926	0.581645	0.514141	0.336147	0.075498	0.485325	0.073939	0.65132
0.06189	0.032857	0.345238	0.380649	0.128088	0.042489	0.101429	0.083795	0.132922
1.393571	1.469163	1.382006	0.05303	2.964762	1.596782	11.48368	3.816551	0.513456
13.64503	8.134084	9.583463	0.05303	7.765931	18.76954	15.98892	18.19595	5.024293
1.357201	6.045209	5.923838	0.05303	3.092229	3.930664	2.190866	3.435988	1.699091
0.102792	0.025274	0.130029	0.251573	0.000577	8.66E-05	0.101429	0.000144	0.076392
0.000224	0.076587	0.000534	0.422237	0.000577	0.027583	0.054401	0.000144	0.034906
2.671299	1.165065	2.509177	0.341558	0.787987	2.051962	2.207417	1.960945	1.309084
4.468009	7.315512	2.987835	1.018427	4.217569	21.19547	8.445743	20.16541	3.276335
0.652807	0.144488	0.231768	0.380649	0.222561	0.168167	0.279177	0.347908	0.2914
4.451154	8.256465	1.734949	0.05303	3.521934	3.803766	3.35715	5.454949	2.26518
0.316335	2.003723	1.517143	0.05303	1.626782	0.650556	1.726032	4.160238	0.852172
1.857677	1.507835	0.813788	3.68381	1.399315	0.637994	1.695216	2.089639	2.375974
0.882078	0.106926	0.438023	2.290996	0.113449	0.540902	0.00132	0.000144	0.727222
7.519841	0.000736	2.434444	9.667316	0.000577	0.178066	0.243896	0.000144	2.178268

2.359264	10.78056	2.060491	0.759293	7.074329	4.94026	7.977958	4.655916	1.443983
0.125613	0.136926	0.087561	0.380649	0.000577	0.034776	0.28746	0.017937	0.225671
1.722475	0.000736	0.777864	7.824473	0.000577	0.139235	0.296234	0.073939	0.332049
1.400159	0.54925	0.449091	0.05303	0.990188	1.460051	0.877222	0.633499	1.100519
0.956061	1.521371	0.718766	0.05303	1.242641	1.035801	4.552092	5.402511	0.361717
3.290101	4.217569	1.757244	0.05303	1.626782	1.622006	0.693218	2.045476	4.429769
0.538773	0.000736	0.221443	0.714697	0.113449	0.075498	0.118983	0.000144	0.256941
2.325931	2.08013	1.019791	0.05303	3.122915	1.247244	0.530534	2.447294	1.539062
1.565253	2.591443	2.581861	0.514141	1.042944	2.400455	3.540606	2.62342	1.678319
2.524892	11.42512	8.780115	0.05303	15.98892	16.34294	15.77678	18.05488	5.606739
0.638362	0.949827	0.449091	0.05303	1.549711	1.045462	1.995714	1.995714	0.417063
1.014127	0.723066	3.493362	3.521934	5.530274	4.155339	72.69473	19.22139	2.178268
4.067547	20.16541	3.327619	8.164531	11.60283	19.53061	18.91854	26.39047	4.851227
0.290519	0.000736	0.030534	0.563499	0.000577	0.013348	0.00132	0.026205	0.010043
0.914618	0.996111	0.957915	0.05303	1.834423	0.976111	2.607035	1.348975	1.421126
9.707677	11.96574	16.22108	0.05303	13.72059	15.07257	5.416623	10.62739	16.68554
1.219019	2.384141	0.769019	0.05303	0.723066	1.076652	1.135887	1.58645	0.504098
0.326219	0.965065	0.813788	0.05303	1.524776	1.010289	2.174733	3.993665	0.338499
7.162698	83.489	1.218182	1.349971	37.29684	21.74051	9.047532	37.81919	0.679069
0.172612	0.000736	0.777864	0.05303	0.000577	8.66E-05	0.223701	0.000144	0.833535
0.281724	4.039192	1.569553	0.05303	2.057056	2.491948	0.65241	2.363442	6.38456
4.067547	1.673254	3.779452	7.061328	1.537951	0.567994	0.469711	1.634935	2.406768
2.341573	15.77678	0.983384	1.183478	11.19942	2.621133	10.73084	11.19942	95.22939
0.212172	0.490952	0.221443	0.422237	0.797633	1.066234	3.500808	0.650101	0.379495
1.190491	2.725498	2.129596	0.865664	2.678781	2.327857	2.089639	3.101371	2.083261
0.944084	0.701818	0.449091	2.898196	0.088874	0.239048	0.101429	0.432698	1.05088
0.736977	0.000736	0.568665	0.05303	0.113449	0.453932	0.00132	0.133781	0.949286
5.330519	11.60283	6.423911	0.05303	10.73084	3.263391	6.570433	17.39935	3.497085
1.139885	0.605455	1.390476	0.05303	1.727323	0.827273	1.945916	2.124574	0.551861
1.219019	1.202626	1.775325	15.56131	1.846941	0.794401	1.1407	1.080909	0.797114
0.254365	0.092691	0.118341	0.466869	0.031775	0.06772	0.00132	0.041097	0.917431
0.000224	0.000736	0.487128	0.05303	0.325317	0.050844	0.00132	0.000144	2.611797
1.004791	3.807648	2.794935	0.05303	3.895469	1.846941	3.807648	1.781948	3.253175
0.530253	2.74298	1.019791	0.563499	1.960945	1.527792	4.702468	2.032706	1.256558
3.66785	1.104805	1.684646	1.820541	1.143954	1.247244	0.814315	0.758773	3.107417
17.51961	4.508196	5.770996	1.531313	2.898196	4.353925	2.73272	1.455022	4.246861
0.000224	2.180022	0.192929	0.05303	2.068297	2.092987	4.003117	0.769019	7.22E-05
59.32013	26.52537	42.08807	91.05541	17.39935	5.337547	0.404899	0.914618	28.54786
3.363911	1.766248	0.947677	3.877417	1.09075	0.851234	1.041652	1.857677	1.351573
0.981032	0.000736	0.250296	0.05303	0.113449	0.050844	0.073038	0.197908	0.455974
0.4357	0.032857	0.321227	2.150303	0.031775	0.050844	0.043038	0.026205	0.309221
2.763261	3.820758	2.718434	1.183478	2.106089	1.758615	3.204632	3.540606	3.169812
4.25658	2.384141	5.737056	1.297763	3.432388	2.141595	0.859351	2.296732	4.851227
1.393571	0.428694	1.361789	0.05303	1.803918	0.861934	1.826147	1.046061	2.254084
1.370765	0.209978	2.050404	3.877417	0.24518	0.605455	0.632569	0.590303	7.276645
21.74051	6.612121	16.80343	15.3189	21.36892	8.477662	3.930664	6.772763	15.98892
1.028672	1.637309	0.866147	0.05303	5.373781	5.530274	4.02088	2.045476	2.73272
0.944084	0.084668	0.777864	0.305455	0.203254	0.307489	0.135606	0.288247	1.092136
1.749307	1.693939	2.74544	1.401068	1.859084	2.092987	1.1514	2.016515	0.894704
0.96627	1.558571	1.165065	4.170051	1.200152	2.463911	2.261573	2.106089	0.73619
3.192229	1.104805	0.495238	0.05303	0.647691	2.542605	6.036818	2.197828	0.216602
0.624841	4.783001	5.409437	0.05303	2.850058	6.268312	2.901032	2.081696	4.246861
0.039827	0.067864	0.067792	0.422237	0.054863	0.007453	0.054401	0.033997	0.10938
5.568391	4.110779	3.578038	0.466869	3.028629	4.272165	3.540606	3.464315	4.690159
5.737056	12.85504	4.851227	1.349971	17.03919	15.18102	14.33939	14.33939	14.12978
3.993665	0.73127	11.25392	9.667316	2.820974	5.838499	1.554185	1.039199	4.179762
0.86518	0.359105	0.902403	0.917431	0.404408	0.73912	0.469711	0.253882	0.65132
0.85728	0.000736	0.732633	2.068297	0.000577	0.108016	0.00132	0.000144	0.679069

1.810664	0.924329	1.072843	6.181652	0.670519	0.986797	1.510014	0.558427	1.508831
0.403413	0.218975	0.096905	0.714697	0.394603	0.462857	0.06443	0.191703	0.600173
0.000224	3.544257	0.671371	0.05303	0.776349	1.68671	1.685678	1.693939	0.256941
0.000224	7.795642	1.660216	4.702468	5.172828	5.068831	1.346003	6.028427	0.58925
0.85728	0.482367	1.259747	0.05303	2.323983	1.189048	2.261573	1.614257	0.971205
0.267388	0.032857	0.568665	1.950519	0.031775	0.013348	0.06443	0.09855	0.191962
1.357201	0.996111	0.438023	0.380649	0.755714	0.687049	0.530534	1.010289	0.978571
0.046724	1.334762	1.03189	0.05303	1.537951	1.220628	1.125325	1.228651	0.949286
1.953535	0.965065	0.476551	0.669769	1.707576	0.547403	1.191905	1.702706	2.602395
1.587605	2.618846	4.684502	0.05303	3.695815	4.039192	2.839351	5.110159	1.107605
1.541227	5.381227	7.363117	0.05303	10.57984	19.06893	18.26752	10.98644	8.849697
0.395642	0.000736	0.139697	0.563499	0.031775	0.020606	0.043038	0.011486	0.101609
4.839105	1.346003	2.903867	2.836717	1.164257	1.33285	0.722179	0.590303	3.070952
1.712706	3.391075	5.500678	0.05303	4.302367	3.055729	0.00132	1.823377	6.414416
3.397706	1.626782	4.120815	15.02509	3.110447	6.22031	1.290981	1.059553	3.895469
5.454949	5.082063	5.195022	0.05303	7.598312	5.439278	6.210375	9.141299	8.958225
6.153102	2.31404	13.60866	77.74812	3.497085	2.781558	2.058773	0.86987	14.47154
2.863211	0.159228	2.102626	0.917431	0.373218	0.263463	0.126883	0.064812	2.479221
0.000224	5.056421	3.998391	0.05303	3.074134	4.764055	6.25938	10.01009	1.265426
67.98069	1360.293	287.197	5.337547	184.443	530.1465	332.0776	1360.293	1360.293
0.342511	1.445916	1.400159	3.128694	2.701053	2.400455	9.880779	3.659668	1.013434
0.234949	0.000736	0.000534	0.422237	0.043391	0.059798	0.00132	0.000144	0.225671
2.353247	0.949827	9.377345	14.17388	2.445079	1.035801	0.907273	1.771392	5.366335
16.09482	9.924372	28.87506	4.170051	39.53687	46.7056	70.11932	48.53429	3.55961
1.074827	0.201645	1.446926	3.877417	0.203254	0.233434	0.254964	0.163276	2.453781
14.08569	19.53061	5.788153	0.05303	22.32214	13.96064	11.96574	15.41187	15.88548
3.632973	4.261342	0.321227	0.05303	2.240274	15.98892	3.457504	8.319019	7.22E-05
0.96627	4.978182	6.011515	0.380649	5.416623	4.661717	2.190866	3.217612	7.430317
0.157958	2.024488	0.512641	0.05303	1.537951	2.390447	1.449062	1.556392	0.600173
3.140866	2.898196	4.323045	8.27267	2.502619	1.487619	1.056263	1.100519	5.881342
2.117814	0.201645	2.402468	3.6029	0.360281	0.156825	0.584502	0.191703	4.012049
10.82954	5.821804	15.51162	21.74051	6.830592	6.079596	4.115844	4.733824	7.351284
0.764315	0.121587	0.000534	20.51271	0.38596	8.66E-05	0.00132	0.000144	0.216602
1.107605	1.415469	3.290101	0.05303	2.871212	4.012049	0.716234	1.336587	2.713319
23.38378	43.49658	50.55949	1.89373	32.68313	52.56128	22.96463	24.04374	17.39935
3.156537	1.323975	3.966623	5.180058	1.029365	1.045462	0.081667	0.470736	4.796126
0.06189	0.000736	0.000534	0.616739	0.000577	8.66E-05	0.00132	0.000144	0.10938
7.795642	7.653391	7.882222	18.62711	6.876392	6.443925	3.156537	3.851414	7.136537
4.997417	2.068297	2.479221	4.170051	2.194257	2.727785	1.199481	1.660216	3.966623
6.612121	0.696234	9.297085	1.242641	5.373781	10.48101	4.277006	4.49671	6.591544
2.842193	4.343838	4.207778	0.563499	6.365065	11.19942	1.473218	1.753341	5.687879
0.088341	0.000736	0.250296	1.242641	0.07632	0.034776	0.101429	0.026205	0.256941
1.464214	1.594675	1.429365	1.242641	0.872013	1.733766	0.753882	1.087345	2.339697
0.418788	0.359105	0.170238	0.514141	0.113449	0.196667	0.054401	0.157186	0.551861
0.668947	0.456522	0.769019	0.305455	0.636291	0.353651	0.2129	0.204055	0.885664
0.028745	0.067864	0.030534	1.242641	0.031775	0.06772	0.175253	0.116284	0.124719
1.318384	17.22048	23.6012	0.466869	14.17388	35.27608	7.737807	4.869553	47.5018
0.821414	1.484553	1.89518	0.05303	2.009906	2.292864	1.732468	1.393571	1.609654
5.266335	5.687879	11.48368	0.05303	3.877417	3.163506	7.162698	6.404921	1.45912
12.85504	11.30987	4.609076	0.05303	5.959033	6.355224	32.68313	18.62711	17.03919
1.263752	1.637309	1.89518	0.05303	1.446926	1.573276	1.002215	2.066652	2.642525
1.749307	11.48368	13.6814	2.978968	17.78522	8.429055	19.99861	14.51648	12.16681
2.234538	3.065108	1.786306	1.066234	2.185556	3.507691	2.031169	1.414574	1.386681
1.207374	0.86518	0.732633	22.52375	1.281241	1.220628	0.510072	0.409899	0.810491
0.227244	1.055628	0.680173	0.05303	1.581912	1.877605	0.354798	1.1407	2.165851
9.880779	0.51316	4.207778	10.5063	1.89228	1.045462	1.848297	0.492958	4.814661
4.597792	0.172294	4.684502	2.604776	0.471025	0.123377	0.091356	0.133781	2.078355
0.872597	0.000736	0.000534	3.061955	0.000577	0.059798	0.00132	0.000144	0.059654

3.428788	0.338341	2.621133	3.128694	0.49267	0.489784	0.625209	0.53912	1.872049
2.009906	6.486104	8.477662	7.545584	14.6058	7.443276	31.55417	27.38726	2.022778
8.27267	12.29473	8.256465	1.242641	17.92188	17.51961	7.854069	15.98892	11.65791
2.933903	1.484553	2.565556	26.25557	0.744113	2.019618	1.130794	1.02487	2.479221
2.037114	4.058514	3.150433	0.305455	1.846941	2.380079	1.666089	2.153535	1.156869
0.882078	0.316126	2.375974	1.183478	0.314387	0.254769	0.233752	0.326017	1.168326
1.810664	2.216162	1.533535	0.380649	1.3829	4.927302	1.115902	1.505678	3.089293
0.247085	0.076587	0.148254	0.563499	0.2129	0.156825	0.054401	0.000144	0.10938
0.473319	0.084668	0.250296	5.180058	0.190996	0.187013	0.00132	0.253882	0.338499
1.634935	5.01772	2.267157	0.05303	5.20267	3.300664	1.877605	2.50469	3.957734
0.530253	0.100101	0.495238	1.950519	0.292251	0.403175	0.518139	0.288247	1.539062
1.060188	0.000736	0.813788	6.570433	0.000577	8.66E-05	0.00132	0.000144	0.58925
1.550837	1.753341	1.757244	2.836717	0.596775	0.702835	0.518139	1.87899	2.283752
0.914618	1.684646	1.816436	0.422237	2.434444	1.523824	1.549711	1.839986	2.121198
0.376212	0.092691	0.211335	1.89373	0.06544	0.147807	0.196335	0.143102	0.141876
3.046551	1.558571	3.4679	2.836717	2.485534	2.31013	1.481631	1.393571	4.077403
0.907273	1.527792	1.507835	0.05303	2.068297	2.996869	2.138131	2.045476	1.409942
9.141299	12.36443	5.830152	0.05303	9.838658	4.997417	4.053506	5.323492	16.44768
2.308203	0.000736	1.058896	2.150303	0.088874	8.66E-05	0.11075	0.213218	2.860722
3.236457	1.626782	2.449509	19.451	1.20653	1.419278	1.639639	1.358189	5.388405
0.203254	0.000736	0.272951	0.05303	0.000577	8.66E-05	0.00132	0.000144	0.216602
0.000224	0.376407	0.559221	0.05303	0.000577	0.075498	0.00132	0.000144	0.184459
0.426898	1.008961	0.376407	0.05303	2.406768	1.204957	2.107922	1.924228	0.619365
1.464214	0.269697	1.182056	1.462143	0.000577	0.075498	0.00132	0.000144	1.65785
20.42667	3.23987	19.83727	46.7056	7.037345	5.959033	2.607035	2.708341	6.945152
2.216162	0.393449	1.684646	1.242641	0.776349	0.434596	0.233752	0.296234	0.696659
0.652807	9.583463	2.306277	2.005253	12.70768	7.08733	5.720173	11.03843	3.807648
0.023492	0.000736	0.000534	0.05303	0.000577	0.020606	0.043038	0.011486	0.006306
6.365065	4.23202	10.28573	0.05303	4.456912	3.957734	1.935685	2.715873	2.970144
13.38418	45.1045	29.49693	4.115844	29.49693	44.27012	12.93651	20.51271	34.84299
1.824892	4.609076	1.145426	4.365224	3.574387	3.855952	2.117814	0.693571	2.121198
20.16541	8.958225	6.830592	0.05303	6.945152	6.956349	1.570794	5.22482	23.82354
0.131991	0.059264	0.157958	0.865664	0.02316	0.027583	0.034459	0.017937	0.392403
0.563824	1.165065	1.155411	0.305455	1.420202	1.082915	0.544877	1.252424	5.576219
0.000224	0.000736	0.250296	1.066234	0.000577	8.66E-05	0.00132	0.000144	1.469163
2.373824	3.78798	8.288874	2.150303	8.888167	8.868932	7.683766	9.418196	111.5439
0.075339	0.136926	0.067792	2.290996	0.02316	0.007453	0.043038	0.106732	0.010043
0.017633	0.032857	0.250296	0.05303	0.07632	1.518232	0.233752	0.116284	0.910823
48.53429	13.15079	18.05488	0.563499	14.00414	9.707677	2.710765	7.061328	14.12978
1.169791	2.705916	2.375974	0.05303	0.678687	1.123016	1.813564	0.622092	0.435382
0.383348	10.17029	1.801284	0.05303	9.459177	7.002056	27.96248	11.96574	1.662439
3.750837	5.47	2.668896	0.05303	4.894509	5.923838	6.632063	12.03267	1.265426
1.765065	2.465916	3.363911	1.123016	2.630729	2.964762	1.306667	1.444906	13.75978
3.606804	1.978954	2.417424	0.05303	3.022713	1.271097	0.814315	1.947316	1.546696
14.25954	3.192229	6.181652	30.17192	1.614257	1.194221	0.753882	1.336587	1.107605
1.432302	5.150693	4.039192	1.183478	6.153102	4.971991	8.643074	8.976508	1.716364
5.950664	6.674286	7.519841	0.714697	3.540606	9.946364	8.319019	11.65791	8.923117
2.863211	29.18885	5.195022	1.820541	19.06893	29.82828	45.91841	36.26061	4.508196
1.179885	0.288081	1.131472	0.759293	0.24518	0.420289	0.404899	0.213218	1.716364
0.125613	0.894704	1.109726	0.05303	1.096335	0.93267	1.091443	0.834416	1.061457
0.573326	7.570693	3.407619	3.253175	2.355289	3.842554	0.796234	1.220628	11.77567
0.846342	0.565714	0.912446	0.759293	0.24518	0.428456	0.00132	0.000144	3.917597
0.281724	0.032857	0.049646	0.251573	0.000577	8.66E-05	0.00132	0.000144	0.084668
2.477027	0.352807	1.464214	4.959358	0.436328	0.100014	0.2129	0.259784	0.455974
3.236457	6.695887	2.489762	1.596782	4.752121	13.60866	31.92385	17.64869	5.337547
0.219683	0.664423	0.550974	0.669769	1.463304	1.227749	1.737294	2.392431	1.699091
0.652807	0.587965	0.503463	2.229048	0.596775	1.076652	0.376212	0.347908	1.47842
0.138009	0.092691	0.192929	6.8421	0.000577	0.13114	0.11075	0.116284	0.600173

0.06189	0.000736	0.345238	1.123016	0.088874	8.66E-05	0.00132	0.000144	0.353333
1.953535	2.306277	3.816551	1.242641	2.589185	4.039192	2.005253	2.227273	5.42381
0.046724	0.050519	0.139697	0.514141	0.128088	0.059798	0.00132	0.026205	0.447085
0.584892	1.182771	0.690043	0.05303	2.996869	2.371833	1.473218	2.31404	1.168326
1.263752	1.033672	3.730808	2.547179	1.070693	0.968723	0.677807	2.216162	1.288276
3.334199	6.464906	4.06303	0.466869	6.268312	6.30671	13.45939	8.134084	1.988196
3.953297	10.67991	5.25254	1.401068	13.72059	16.9162	9.335397	10.33476	7.697063
0.095599	0.059264	0.049646	2.410996	0.100996	0.08438	0.054401	0.083795	0.141876
0.039827	0.032857	0.000534	0.81228	0.07632	0.013348	0.00132	0.000144	0.020938
4.914863	3.077316	8.149307	0.422237	3.6029	2.597792	1.091443	2.111299	5.24562
7.854069	1.985253	10.1459	5.180058	1.96697	2.150303	1.10132	1.444906	7.625209
4.110779	2.337821	3.387864	3.432388	3.163506	3.286652	1.259747	1.363557	2.150303
0.403413	0.557763	0.628297	0.05303	0.849841	0.532619	0.404899	0.273088	1.156869
1.737294	8.348817	3.860491	0.970635	5.687879	9.418196	6.071436	6.423911	5.754935
0.000224	0.218975	0.049646	0.05303	0.000577	8.66E-05	0.346342	0.304228	0.34583
3.037626	8.849697	2.690823	0.05303	21.74051	7.795642	14.08569	10.1028	4.900996
0.452713	0.067864	0.777864	0.759293	0.24518	0.321227	0.073038	0.000144	0.371075
3.877417	1.736133	6.116638	3.877417	0.325317	2.561162	0.575628	0.440289	1.759986
15.3618	46.7056	72.69473	3.971061	60.77449	50.55949	19.06893	31.1753	62.33472
0.799048	0.323752	0.157958	0.669769	0.373218	0.523211	0.716234	0.492958	0.34583
0.000224	3.687633	0.000534	1.531313	2.567605	2.16759	4.053506	4.914863	7.22E-05
0.799048	0.144488	2.355289	0.05303	0.000577	0.059798	0.00132	0.000144	0.687807
0.678687	0.73127	1.76873	0.05303	1.950519	2.685786	1.250058	1.577569	1.872049
8.005685	2.31013	3.629177	0.759293	3.730808	4.47912	4.317648	3.464315	6.325945
2.373824	5.056421	4.758088	0.917431	3.300664	4.783001	4.644098	4.120815	1.546696
0.716652	0.195007	0.476551	10.62739	0.222561	0.196667	0.126883	0.151378	0.266479
2.053521	0.29767	1.058896	0.05303	0.07632	0.343629	0.518139	0.507085	1.975772
0.349545	4.135801	0.594271	0.05303	2.396429	2.987835	5.933066	1.809343	0.551861
2.465916	1.303961	2.964762	2.978968	2.278254	1.035801	0.907273	1.89228	3.595758
4.029654	0.456522	3.172864	7.668579	0.436328	0.642698	0.223701	0.28026	3.917597
0.000224	5.576219	1.584105	0.05303	9.502294	6.063276	1.705007	4.451154	1.857677
4.696313	0.86518	0.690043	0.05303	1.308167	0.4971	1.023528	3.283139	0.371075
3.253175	0.399978	1.390476	2.229048	0.887359	0.948211	0.814315	0.417063	0.642698
6.876392	19.68088	11.83593	0.05303	25.99427	18.48514	28.23632	15.88548	24.98218
18.19595	25.48179	8.994791	2.068297	9.063983	11.42512	5.830152	10.67991	12.10289
0.081818	0.032857	0.030534	0.05303	0.000577	8.66E-05	0.00132	0.000144	0.216602
5.01772	19.37139	11.60283	0.759293	19.83727	22.74797	14.79742	13.75978	8.256465
144.7919	530.1465	530.1465	21.93452	425.62	287.197	749.7839	530.1465	184.443
4.236962	0.456522	5.123781	7.545584	1.524776	0.439776	1.023528	1.228651	1.997352
1.232886	0.29767	1.390476	0.05303	0.559221	0.755714	2.339697	3.334199	0.084668
3.57057	2.513752	4.814661	1.123016	2.531515	6.760765	4.997417	8.711905	0.775339
7.456118	9.335397	4.251818	1.349971	9.377345	6.325945	8.556703	8.226219	3.217612
9.583463	105.1694	14.17388	0.05303	38.94854	91.05541	252.4251	77.74812	49.50903
1.902352	4.546443	1.930216	0.05303	2.496407	10.19468	11.65791	3.816551	4.667518
0.000224	34.84299	10.67991	0.05303	32.31123	40.11561	33.93939	38.39042	9.968355
7.375794	6.580988	8.033932	14.51648	5.654863	5.508131	4.012049	4.529913	7.200505
1.318384	3.820758	2.701053	0.05303	2.502619	1.670786	1.666089	2.081696	2.584394
0.872597	3.425512	2.214372	0.05303	5.137042	4.227302	4.673182	4.540794	1.801284
1.147807	0.73127	1.757244	4.863579	0.49267	0.532619	0.233752	0.273088	1.080909
0.652807	0.195007	1.072843	1.531313	0.24518	0.233434	0.00132	0.000144	0.833535
8.539971	16.09482	13.23022	5.075447	13.00996	9.335397	14.42661	11.51384	20.34063
1.810664	3.070952	8.524242	0.05303	20.16541	13.08214	6.99026	7.964315	3.779452
0.261227	0.12904	0.272951	0.917431	0.000577	0.100014	0.00132	0.000144	0.101609
7.938427	15.46193	7.545584	4.46267	23.6012	19.22139	26.52537	27.10502	8.195657
0.563824	1.267879	0.935115	0.05303	3.829105	0.353651	0.753882	0.81228	2.494177
0.996111	0.084668	0.615996	1.727323	0.285253	0.115801	0.2129	0.041097	0.757273
1.91254	0.688182	1.271097	3.061955	1.281241	1.123016	0.567994	0.721335	2.022778
0.095599	0.100101	0.201789	0.917431	0.07632	0.139235	0.233752	0.064812	0.266479

2.66404	4.63189	0.838326	1.727323	3.807648	3.457504	6.967547	7.710361	4.207778
1.550837	0.759646	3.06803	0.616739	1.786306	0.955418	1.831631	1.080909	1.850996
0.316335	0.000736	0.201789	1.066234	0.02316	8.66E-05	0.00132	0.011486	0.117518
0.596111	8.319019	2.931068	0.81228	3.460909	6.171789	4.424416	7.911934	8.381328
16.74449	57.84882	13.79669	2.347439	14.51648	30.49289	15.56131	29.49693	53.77619
0.254365	7.288788	0.000534	0.05303	8.134084	4.502453	9.707677	6.250447	0.743694
0.316335	0.114113	0.192929	4.620159	0.000577	0.050844	0.043038	0.073939	0.015685
0.914618	3.917597	3.043528	0.05303	11.48368	5.454949	7.653391	5.671126	3.346898
0.452713	0.000736	1.29417	3.128694	0.404408	8.66E-05	0.00132	0.000144	0.409098
0.882078	0.106926	0.428932	1.066234	0.02316	0.013348	0.073038	0.017937	0.184459
2.148658	0.572626	0.892395	1.727323	0.264654	1.220628	0.324019	1.378362	1.303045
0.772951	0.000736	0.957915	0.05303	0.113449	0.156825	0.00132	0.000144	1.648066
0.668947	0.165844	1.155411	3.6029	0.000577	8.66E-05	0.00132	0.000144	0.541804
0.254365	1.175094	1.165065	0.05303	1.029365	1.513102	0.296234	1.132165	4.667518
1.810664	4.863579	3.799885	1.462143	6.99026	6.404921	9.838658	9.880779	3.137576
0.273911	1.033672	1.271097	0.514141	0.559221	0.843102	0.054401	0.204055	2.768312
0.772951	14.33939	3.243225	0.05303	9.707677	17.64869	6.464906	11.30987	8.762677
0.48583	0.106926	0.333463	1.123016	0.128088	0.034776	0.175253	0.033997	0.819509
2.427792	15.276	12.26136	2.150303	10.43066	11.77567	10.57984	9.707677	6.414416
1.444906	6.011515	2.294733	1.531313	4.33342	3.971061	3.24658	4.927302	4.468009
0.48583	0.114113	0.700418	1.242641	0.128088	0.092302	0.06443	0.064812	1.328384
0.754784	9.687496	0.272951	0.05303	7.200505	2.394416	6.830592	16.44768	3.726854
0.780519	0.404899	0.924329	0.380649	0.930325	0.65798	0.716234	0.608543	1.443983
0.290519	1.445916	1.227749	0.05303	1.977316	2.150303	4.376118	1.839986	1.915455
1.088687	0.121587	0.261962	0.05303	0.000577	0.123377	0.00132	0.056782	0.170808
0.039827	0.032857	0.067792	1.183478	0.031775	8.66E-05	0.00132	0.000144	0.034906
0.334444	0.331263	1.544517	0.865664	0.000577	0.477446	0.00132	0.083795	4.348882
0.247085	3.544257	0.309877	0.05303	0.849841	0.818066	1.108276	1.614257	0.316573
0.054517	0.000736	0.000534	0.05303	0.054863	8.66E-05	0.00132	0.000144	0.17733
0.194848	0.100101	0.039993	0.422237	0.000577	0.050844	0.00132	0.048773	0.051255
1.514199	6.011515	6.116638	0.05303	6.591544	3.783694	7.443276	3.799885	2.199639
0.081818	0.209978	0.221443	0.466869	0.444762	0.168167	0.763102	0.178629	0.424848
2.876421	1.902352	1.479452	1.123016	4.546443	4.752121	1.927042	2.758088	4.086926
1.669466	2.496407	0.983384	0.81228	2.369841	2.134545	2.042201	3.980123	1.669466
0.10886	0.399978	1.757244	0.05303	2.834084	1.247244	2.925426	1.092785	0.371075
2.402468	9.625253	1.009625	0.05303	6.09759	8.381328	1.524776	2.820974	2.652107
0.10886	0.042027	0.039993	0.272078	0.031775	0.020606	0.091356	0.000144	0.132922
2.07658	4.165137	2.863211	0.05303	3.482785	5.545729	1.753341	4.869553	4.02088
19.37139	36.26061	10.19468	3.367071	31.55417	28.54786	62.33472	44.27012	19.68088
2.990851	0.076587	1.672107	0.05303	0.100996	0.034776	0.00132	0.000144	2.511465
0.138009	0.338341	0.106609	1.596782	0.07632	0.042489	0.185909	0.041097	0.455974
10.01009	6.632063	5.42381	0.380649	10.05618	5.381227	2.151919	5.366335	6.325945
1.669466	2.967453	2.852677	0.05303	2.345527	3.651898	4.965801	3.011169	3.005281
0.645036	0.664423	0.289755	0.05303	0.670519	0.477446	0.28746	0.253882	0.827273
0.342511	0.076587	0.376407	9.667316	0.06544	0.108016	0.166118	0.507085	0.101609
2.491948	4.72798	3.425512	0.669769	3.13456	4.039192	3.68381	4.900996	1.734949
2.973059	14.51648	1.244957	0.05303	13.60866	12.77772	7.977958	11.42512	6.25938
3.317807	4.392316	0.157958	0.514141	0.373218	0.335411	1.135887	0.574993	0.247951
4.814661	56.39528	48.53429	1.462143	70.11932	70.11932	40.11561	45.1045	37.29684
1.158384	1.072843	1.322092	0.05303	1.290981	1.132857	2.449509	0.914618	1.654156
2.958773	0.428694	1.786306	9.256537	1.233665	0.827273	0.315743	0.28026	1.915455
0.833045	1.120332	1.109726	6.181652	0.503781	0.880498	0.693218	0.670144	0.991631
2.628225	14.79742	9.031082	0.05303	18.19595	22.13245	35.27608	31.92385	1.618773
0.000224	5.855281	0.761688	2.068297	3.711097	2.792035	5.485685	2.378124	1.432302
0.652807	3.771349	2.538218	0.616739	4.614618	3.68381	2.616573	3.089293	1.816436
0.614935	0.904286	1.096335	3.253175	0.404408	0.412193	0.567994	0.677807	1.362713
0.546522	1.514199	0.947677	0.05303	3.314329	2.805382	1.438672	0.677807	0.532857
0.358903	0.050519	1.019791	2.410996	0.088874	0.075498	0.00132	0.000144	0.571674

0.281724	0.352807	0.690043	0.251573	1.707576	2.034242	2.752893	0.86518	0.628297
25.24367	425.62	144.7919	0.05303	55.04081	10.82954	87331.38	87331.38	0.400967
0.212172	0.032857	0.157958	1.297763	0.000577	0.050844	0.054401	0.246652	0.010043
0.596111	0.000736	0.059264	0.305455	0.000577	8.66E-05	0.00132	0.000144	0.132922
0.316335	0.136926	0.148254	0.865664	0.222561	0.08438	0.28746	0.017937	0.581378
0.349545	2.628225	1.492973	0.05303	4.402973	2.180022	56.39528	10.1459	1.931537
0.92531	1.661328	0.879913	0.305455	0.862561	1.088687	1.115902	1.03189	0.600173
0.138009	0.759646	1.700354	3.68381	1.648066	1.149978	0.510072	0.310866	0.687807
2.66404	2.681169	2.54961	1.349971	2.287576	2.243911	0.78619	1.58645	0.910823
0.095599	0.000736	0.211335	0.272078	0.043391	8.66E-05	0.00132	0.000144	0.084668
3.714906	1.718918	4.575123	1.183478	4.33342	2.628225	2.453781	2.261573	3.321284
0.095599	1.080303	0.970007	0.05303	0.821898	1.369048	3.140866	1.592395	0.09342
6.967547	0.404899	3.68381	8.412367	0.596775	0.861934	0.279177	0.334625	4.067547
0.046724	0.000736	0.118341	1.727323	0.100996	8.66E-05	0.00132	0.000144	0.353333
0.988932	5.395584	0.581645	1.531313	5.395584	5.030866	9.502294	13.53913	1.27619
0.000224	2.644885	0.495238	0.05303	1.223846	3.279625	4.067547	2.216162	1.033672
1.660216	0.894704	1.954993	1.349971	0.264654	0.505786	0.166118	0.000144	1.409942
7.375794	4.715599	1.621032	0.05303	2.355289	2.584394	5.687879	8.019769	2.121198
0.125613	0.29767	0.000534	0.917431	0.07632	0.245967	0.265455	0.288247	0.034906
0.017633	0.025274	0.02316	0.865664	0.07632	8.66E-05	0.081667	0.000144	0.027431
1.370765	2.394416	1.857677	1.297763	2.871212	2.996869	2.561162	4.02088	1.409942
0.358903	0.000736	0.077547	0.563499	0.000577	0.219885	0.00132	0.143102	0.361717
0.179668	8.556703	2.306277	0.05303	5.01088	4.997417	6.30671	8.643074	2.800332
0.395642	0.482367	0.376407	0.714697	1.143954	1.094206	0.469711	0.326017	0.551861
1.158384	6.433918	1.337431	2.347439	5.671126	4.597792	4.343838	7.765931	0.513456
6.486104	11.65791	11.36975	0.05303	13.15079	16.68554	8.815123	10.38202	8.508514
0.102792	0.114113	0.148254	2.483492	0.292251	0.263463	0.101429	0.125238	0.361717
0.891775	2.925426	7.238023	1.462143	3.672078	4.821176	0.914618	1.100519	10.53159
0.520289	2.781558	1.446926	0.05303	1.123016	2.451645	2.359264	1.444906	1.561912
1.749307	1.992944	3.998391	6.967547	1.358189	1.547734	1.360866	2.836717	4.851227
1.881955	0.482367	1.40772	2.410996	0.203254	0.687049	0.2129	0.238478	2.294733
0.445209	0.338341	0.466602	0.272078	0.190996	0.505786	0.233752	0.143102	0.642698
9.625253	6.011515	6.325945	0.05303	7.468961	10.93101	6.171789	7.882222	12.60233
4.959358	19.83727	10.8813	6.900202	12.63773	17.92188	44.27012	27.66779	5.530274
1.179885	30.49289	9.771833	2.068297	42.75576	36.74522	24.50942	30.82277	31.92385
0.023492	0.059264	0.106609	2.068297	0.000577	8.66E-05	0.146883	0.000144	0.846342
2.053521	1.128131	3.885642	0.422237	4.802655	3.089293	1.491977	3.034747	4.445851
7.456118	12.49452	12.42965	2.483492	7.136537	6.38456	6.738211	7.013853	7.013853
0.157958	0.833535	0.749481	0.05303	0.471025	0.59062	1.766248	0.820931	0.23303
5.295325	12.77772	15.51162	2.604776	24.2842	67.98069	22.52375	22.96463	2.879228
0.914618	0.541522	1.120332	0.759293	0.569625	0.856782	0.324019	0.58259	0.719177
0.473319	0.050519	0.118341	0.380649	0.043391	0.050844	0.00132	0.000144	0.400967
1.060188	1.003016	0.503463	0.865664	1.061457	0.187013	0.346342	2.378124	0.949286
3.518384	0.973745	3.363911	1.655541	1.05153	1.573276	0.562439	0.615996	5.704646
3.269913	0.924329	2.882035	0.563499	1.603795	1.301169	0.617417	0.96329	4.997417
0.632208	0.067864	0.640606	2.978968	0.203254	0.100014	0.081667	0.000144	0.240491
1.219019	2.558817	2.81342	2.681169	2.690823	2.668896	13.60866	6.30671	2.750375
0.046724	0.000736	0.272951	1.183478	0.06544	0.042489	0.054401	0.017937	0.936898
6.153102	3.957734	2.81342	0.05303	5.71241	2.572309	17.28195	21.19547	0.256941
0.707799	0.288081	0.221443	1.950519	0.559221	0.286364	2.127973	1.670786	0.015685
0.000224	0.000736	0.118341	0.759293	0.000577	8.66E-05	0.00132	0.000144	0.92531
12.36443	18.91854	3.833247	8.625613	3.363911	12.63773	0.964416	1.533535	20.51271
0.000224	62.33472	2.565556	0.05303	83.489	77.74812	203.5795	144.7919	8.119004
6.088593	12.70768	2.449509	1.018427	4.473348	10.43066	17.51961	14.00414	9.082828
0.261227	0.367532	0.201789	0.05303	0.113449	0.100014	0.157706	0.296234	0.068384
6.191515	10.62739	6.063276	0.422237	12.36443	12.70768	7.013853	5.993045	10.19468
22.13245	29.49693	67.98069	9.52197	50.55949	36.26061	52.56128	35.27608	40.7932
0.15127	0.000736	0.067792	0.616739	0.031775	0.013348	0.00132	0.011486	0.034906

1.941703	0.437799	1.594675	0.563499	0.744113	0.403175	0.376212	0.213218	1.137244
0.530253	0.000736	0.148254	0.422237	0.054863	8.66E-05	0.11075	0.000144	0.34583
0.000224	1.618773	1.446926	1.349971	6.071436	3.489899	8.348817	6.22031	2.022778
0.754784	0.084668	0.000534	0.05303	0.222561	0.386169	0.2129	0.267013	0.162576
0.254365	1.655541	0.568665	0.341558	1.960945	1.198701	1.385801	3.204632	1.202626
0.046724	0.887359	0.077547	0.05303	1.455022	1.128131	3.058802	1.323975	0.705613
1.336587	1.736133	0.309877	0.05303	0.336147	0.523211	0.446811	1.809343	1.061457
11.03843	17.78522	25.24367	0.81228	26.80355	28.23632	9.583463	23.38378	19.06893
9.502294	15.3618	9.707677	1.349971	15.46193	16.22108	10.82954	10.93101	7.276645
0.10886	0.100101	0.661443	2.898196	0.776349	0.187013	0.530534	0.213218	0.015685
2.221775	1.033672	0.825354	0.05303	0.808485	0.747417	3.119986	6.632063	0.455974
10.8813	1.369048	35.27608	27.38726	3.253175	1.447994	0.393449	0.440289	11.48368
0.638362	2.223636	1.841371	0.05303	1.603795	2.640332	0.947677	1.723579	2.786898
5.330519	1.1407	1.930216	30.49289	0.607937	0.994271	0.602287	0.968723	1.857677
0.48583	3.163506	2.752893	0.05303	2.468117	3.904517	1.374329	2.227273	2.768312
0.696659	0.186133	0.594271	1.018427	0.404408	0.29531	0.081667	0.151378	0.797114
4.165137	2.327857	1.954993	0.05303	3.552049	25.99427	1.385801	1.702706	7.276645
2.181775	0.000736	0.231768	0.05303	0.088874	0.327994	0.135606	0.09855	0.827273
1.028672	1.817828	3.599329	16.34294	1.507835	1.741414	0.567994	1.277056	5.654863
4.217569	3.037626	6.653016	2.483492	3.391075	3.363911	0.902403	1.712706	2.26518
0.41026	0.605455	0.594271	0.05303	1.537951	0.708124	1.283911	1.123016	0.207835
1.432302	0.195007	1.058896	0.669769	0.678687	0.731732	0.602287	0.479062	3.083319
3.796075	0.076587	0.531169	0.305455	0.07632	0.06772	0.00132	0.048773	0.191962
0.368478	0.042027	0.118341	2.229048	0.128088	0.059798	0.054401	0.033997	0.034906
0.299336	2.453781	2.434444	0.380649	5.330519	2.511465	14.00414	9.335397	0.117518
0.821414	3.169812	3.618499	2.681169	4.684502	3.022713	4.764055	4.419062	0.750404
0.023492	0.000736	0.02316	0.380649	0.000577	0.007453	0.043038	0.000144	0.034906
8.958225	0.306811	5.302294	22.13245	0.128088	0.702835	0.135606	0.000144	3.934986
3.125844	3.037626	2.197828	0.466869	2.205476	1.902352	1.374329	1.781948	1.997352
2.427792	3.092229	3.387864	0.305455	1.816436	1.962424	0.446811	0.777864	6.045209
1.650274	8.164531	6.230245	3.253175	7.938427	7.765931	4.473348	7.443276	11.36975
1.650274	1.217475	1.29417	0.81228	1.369928	1.460051	1.940231	1.645613	1.072056
0.707799	0.23303	0.400173	0.759293	0.24518	0.371299	0.196335	0.151378	0.885664
0.41026	0.359105	13.49926	5.864084	4.302367	0.961486	4.72798	1.823377	27.66779
0.227244	0.437799	0.709322	0.669769	0.990188	0.948211	0.639113	0.956667	0.712929
1.902352	3.314329	3.128694	0.669769	2.057056	2.65684	1.473218	1.600238	2.283752
0.227244	0.29767	0.261962	1.531313	0.460938	0.156825	0.331558	0.163276	0.2914
5.050267	0.218975	1.446926	1.727323	0.503781	0.483874	0.43684	0.385736	2.831501
3.734762	2.524892	2.509177	1.655541	1.96697	0.630022	4.575123	1.203427	3.153485
1.559675	1.253752	1.429365	0.05303	1.271097	1.319293	1.594675	1.614257	1.061457
0.000224	26.25557	11.14619	0.05303	18.76954	19.83727	6.717056	13.26964	14.33939
1.533535	3.253175	1.775325	0.05303	3.172864	2.040606	7.327482	4.25658	0.838846
1.074827	0.629098	1.724805	0.05303	1.09075	1.004791	0.708492	0.296234	0.810491
10.78056	3.917597	10.38202	1.462143	2.106089	3.227085	1.097677	1.566414	6.454416
8.815123	8.643074	21.93452	0.05303	9.179466	10.73084	7.037345	10.19468	4.227302
6.876392	0.833535	6.30671	0.759293	0.897489	0.532619	0.42767	0.745072	1.314675
29.49693	5.942294	21.74051	9.031082	3.855952	4.907929	5.095325	7.339452	38.94854
0.203254	0.658745	0.628297	0.759293	0.58259	0.80417	1.269495	0.745072	0.379495
0.395642	0.092691	0.321227	4.222583	0.128088	0.994271	0.073038	0.056782	1.340981
31.55417	16.62402	7.765931	1.462143	44.27012	31.92385	47.5018	25.99427	32.31123
1.455988	1.29267	2.092987	0.05303	0.950418	3.211176	0.101429	0.947677	1.202626
0.746551	3.286652	1.348975	0.714697	3.037626	2.944416	2.500548	2.487576	3.552049
2.55184	0.084668	0.970007	7.951371	0.153391	8.66E-05	0.00132	0.000144	0.541804
1.021097	4.012049	7.724084	0.341558	5.500678	2.621133	2.531515	2.958773	10.12435
12.22798	91.05541	2.863211	87331.38	13.91714	14.70188	37.29684	12.29473	7.037345
0.281724	0.000736	0.182359	0.466869	0.031775	0.013348	0.00132	0.017937	3.687633
1.941703	4.678846	2.882035	530.1465	3.314329	8.539971	2.953059	2.681169	1.52676
0.584892	11.89932	2.402468	0.05303	6.518709	4.702468	14.6058	7.417359	5.42381

3.518384	1.718918	2.831501	8.868932	2.300678	1.392511	0.981558	1.173535	1.828781
0.144358	3.750837	1.03189	0.05303	1.518232	2.735368	1.902352	4.007583	0.338499
10.1459	2.944416	8.888167	2.604776	1.925635	4.292078	1.744055	3.334199	5.545729
4.575123	0.668456	1.757244	48.53429	2.380079	0.540902	0.376212	0.492958	0.784372
10.48101	7.507619	11.42512	27.10502	3.6029	3.68381	2.207417	6.99026	11.99921
0.645036	0.136926	0.221443	25.86296	0.000577	8.66E-05	0.00132	0.064812	0.417063
0.116926	0.323752	0.388326	1.531313	0.100996	0.245967	0.126883	0.204055	0.58925
2.165851	4.490801	4.381479	24.98218	4.053506	4.307309	7.288788	6.55039	3.253175
83.489	118.7028	135.5245	167.9996	77.74812	75.1132	15.88548	23.82354	167.9996
17.28195	7.212915	16.09482	15.98892	35.776	3.714906	53.77619	42.08807	10.82954
80.6518	11.36975	45.1045	87.1746	20.68746	10.33476	5.238701	12.22798	52.56128
43.49658	11.77567	30.82277	80.6518	22.52375	13.75978	9.047532	14.25954	35.776
16.9777	22.32214	42.75576	13.23022	45.1045	29.18885	25.99427	62.33472	56.39528
13.64503	13.23022	11.19942	332.0776	14.25954	11.25392	3.743232	7.38847	11.719
287.197	749.7839	425.62	0.669769	749.7839	332.0776	287.197	425.62	749.7839
184.443	60.77449	224.9555	60.04731	167.9996	83.489	105.1694	118.7028	252.4251
50.55949	184.443	43.49658	16.09482	118.7028	184.443	100.0998	55.04081	100.0998
167.9996	49.50903	203.5795	105.1694	56.39528	42.75576	25.73165	33.4985	155.7103
4.745895	2.871212	14.97762	5.576219	3.529466	2.720996	1.784755	1.427431	6.508427
4.193232	0.847352	1.841371	2.898196	0.821898	0.93267	0.59062	0.642013	3.363911
34.84299	2.705916	6.036818	43.49658	2.6143	2.944416	1.1629	1.839986	4.997417
8.508514	8.608153	12.06778	0.272078	7.264502	9.459177	8.089848	9.667316	5.217569
3.536955	0.000736	3.703175	1.727323	0.255693	0.206263	0.101429	0.041097	2.955916
0.088341	0.032857	0.106609	0.305455	0.043391	0.059798	0.054401	0.048773	0.084668
6.088593	1.033672	5.388405	4.776407	2.261573	1.307417	0.728543	0.600887	4.888023
0.068802	0.54925	0.409098	1.596782	0.233175	0.955418	1.1514	0.795786	0.240491
1.570794	3.037626	1.373348	23.71237	3.334199	2.978968	5.788153	4.776407	5.993045
0.164993	0.209978	0.000534	287.197	0.203254	8.66E-05	2.250462	0.36267	0.162576
53.77619	6.853608	24.50942	7.468961	6.560411	9.377345	3.614603	5.143867	27.38726
2.438831	1.602619	0.077547	1.242641	1.786306	1.902352	5.037489	6.325945	1.58645
2.607035	0.949827	3.150433	11.30987	0.452713	0.233434	0.11075	0.40075	0.670519
0.164993	0.114113	0.000534	0.305455	0.06544	8.66E-05	0.00132	0.064812	0.240491
1.25746	2.102626	0.568665	27.96248	0.710253	0.994271	0.632569	0.657547	2.339697
0.46461	4.833398	0.605455	0.05303	3.414574	3.636789	3.55583	3.816551	3.227085
38.39042	40.11561	31.1753	5.477843	37.81919	26.52537	27.38726	32.31123	15.3618
36.74522	53.77619	36.26061	3.521934	57.84882	41.47925	28.54786	47.5018	9.141299
23.82354	9.063983	16.9162	19.99861	9.792626	8.711905	6.876392	6.365065	26.25557
5.388405	2.440895	3.851414	0.917431	3.672078	2.371833	1.960945	2.173081	3.432388
1.455988	0.186133	0.653968	0.466869	0.000577	8.66E-05	0.00132	0.143102	0.903074
20.68746	31.1753	26.25557	5.075447	29.82828	16.44768	20.68746	24.50942	10.12435
0.981032	1.033672	0.749481	5.180058	0.658362	0.642698	0.118983	0.574993	0.417063
8.048095	0.557763	4.563117	22.8563	0.190996	5.638759	1.524776	2.55184	8.319019
87.1746	10.17029	30.49289	25.48179	14.88584	13.45939	11.25392	9.751039	42.08807
0.383348	0.345635	1.096335	6.181652	0.482078	1.149978	4.535354	0.409899	0.379495
3.750837	0.136926	1.044235	1.123016	0.38596	0.371299	0.196335	0.470736	0.34583
4.764055	2.763261	3.998391	3.68381	4.863579	7.061328	2.863211	2.725498	7.882222
1.525729	1.718918	2.347439	2.150303	1.281241	0.986797	1.104805	0.463867	2.66404
0.157958	0.186133	0.503463	5.075447	0.348369	0.420289	0.135606	0.143102	0.361717
0.459394	0.121587	0.628297	1.123016	0.2129	0.278651	0.043038	0.143102	0.379495
2.009906	0.376407	0.661443	0.917431	0.415974	0.420289	0.331558	0.777864	1.555289
1.34513	0.376407	0.096905	0.05303	0.686667	0.532619	0.081667	0.446291	0.316573
1.131472	0.428694	1.492973	1.462143	0.524986	0.968723	1.744055	0.68557	3.442886
2.341573	0.840635	2.871212	3.253175	3.877417	1.643218	2.174733	1.505678	1.828781
3.428788	1.971183	6.345382	6.423911	3.253175	3.107417	3.06803	1.947316	4.758088
0.046724	1.499214	0.409098	0.05303	0.723066	0.925801	2.415296	0.860924	0.216602
0.473319	0.032857	0.449091	0.272078	0.153391	0.020606	0.175253	0.056782	0.051255
5.416623	16.62402	17.64869	10.12435	18.62711	17.78522	9.141299	10.82954	29.49693
0.810491	0.664423	0.802395	2.681169	0.596775	0.453932	0.814315	0.519755	0.316573

0.023492	0.000736	0.000534	0.05303	0.000577	0.013348	0.00132	0.011486	0.015685
0.645036	3.917597	0.732633	0.05303	0.808485	3.080317	0.763102	1.238651	0.971205
2.117814	9.968355	4.546443	0.865664	2.850058	4.888023	3.842554	13.08214	3.217612
18.33909	4.524481	83.489	53.77619	2.042201	4.529913	0.363276	0.479062	23.16622
0.605455	7.625209	14.88584	0.05303	9.667316	12.85504	5.933066	8.195657	21.74051
1.839986	1.827403	2.092987	4.959358	0.373218	0.4971	0.272814	1.182056	1.57645
2.784228	7.507619	3.672078	1.531313	7.697063	5.082063	22.13245	14.79742	2.008312
9.377345	2.802828	3.899993	16.62402	2.261573	3.457504	4.802655	2.820974	5.838499
1.219019	0.91404	1.446926	12.42965	1.135887	0.772157	0.625209	0.795786	1.168326
0.000224	0.151688	0.671371	0.05303	0.000577	8.66E-05	0.383622	0.000144	0.917431
21.19547	9.418196	14.25954	13.23022	10.01009	7.570693	4.227302	4.644098	22.42294
155.7103	224.9555	252.4251	5.337547	530.1465	425.62	167.9996	224.9555	287.197
0.234949	0.100101	0.201789	0.669769	0.394603	0.219885	0.00132	0.000144	0.240491
72.69473	21.54823	55.04081	3.253175	59.32013	22.96463	13.08214	25.24367	33.07147
5.416623	1.799899	2.760765	3.971061	2.270837	1.495043	0.990909	1.05417	3.330909
3.070952	0.771688	1.464214	2.290996	1.000173	1.066234	0.393449	0.223391	1.176623
10.60361	11.544	8.381328	3.432388	11.42512	4.721789	12.77772	11.77567	4.586753

CGGA_113	CGGA_113	CGGA_113	CGGA_113	CGGA_113	CGGA_114	CGGA_114	CGGA_114	CGGA_114
0.243672	0.310866	0.229791	1.498167	0.468384	0.139986	0.18324	0.134517	0.349084
0.307027	0.510072	0.381999	0.896522	0.573326	0.022814	0.087879	0.261342	0.339466
17.15902	2.289286	9.297085	0.041501	5.538001	8.643074	11.36975	3.632973	2.339697
2.461905	3.397706	5.976724	0.041501	7.38847	2.060491	7.112496	0.933874	0.70886
2.145137	9.880779	8.210938	1.498167	5.923838	4.965801	4.49671	3.944084	7.737807
4.402973	5.381227	1.327525	23.16622	1.290981	1.060188	2.051962	3.989206	7.668579
27.66779	51.52195	55.04081	10.95872	91.05541	43.49658	111.5439	29.49693	28.54786
0.048622	0.175361	0.000216	0.956061	0.12267	0.014141	0.096811	0.059582	0.000188
0.81544	1.677042	4.47912	0.041501	3.50425	3.011169	3.921645	1.521371	1.082179
0.001962	1.175094	0.000216	0.041501	0.195253	0.309877	0.5129	2.89E-05	0.000188
34.38341	59.32013	33.4985	82.0704	21.93452	44.27012	26.80355	30.49289	56.39528
0.23246	5.500678	1.327525	1.05153	1.067662	0.657114	2.035678	0.874603	1.037316
35.27608	21.28219	25.24367	0.956061	27.10502	7.327482	40.7932	4.451154	8.694697
4.359574	3.236457	7.653391	0.041501	0.50469	23.82354	13.83359	16.62402	0.70886
0.048622	0.130029	0.022814	0.742872	0.037374	0.002698	0.000952	0.00987	0.000188
4.702468	0.861934	6.011515	0.405786	3.521934	3.489899	2.858009	7.964315	2.290996
0.371299	2.494177	1.599033	0.609177	2.398442	0.814877	2.365685	1.532468	1.386681
0.001962	0.185909	0.000216	36.26061	0	0.049149	0.000952	0.059582	0.000188
1.024127	1.330029	4.359574	0.041501	1.673254	3.921645	3.514834	0.607605	0.863694
0.875101	1.030678	0.619365	0.3414	1.135137	0.342814	1.641999	0.5129	1.261407
0.623499	0.328543	1.246457	0.041501	1.34513	0.987821	0.863189	0.474214	0.000188
5.259437	2.181775	2.863211	2.500548	1.965657	3.7586	6.09759	1.462143	1.43039
44.27012	155.7103	8.381328	0.041501	25.99427	42.08807	50.55949	252.4251	91.05541
0.001962	0.016681	0.148124	3.23987	0.333636	0.014141	0.000952	0.028232	0.000188
0.254365	2.27254	1.40772	2.308203	0.829192	1.143954	0.972504	1.177309	1.29267
14.17388	3.975498	8.958225	0.700058	8.445743	3.953297	11.30987	3.153485	2.961768
0.791371	0.724502	4.025267	0.041501	0.328268	7.964315	4.203001	7.162698	2.112843
0.182482	0.520289	0.229791	1.498167	0.200382	0.251248	0.405094	0.269141	0.73912
75.1132	18.48514	13.23022	0.041501	12.93651	425.62	27.96248	332.0776	46.7056
0.196955	1.044235	1.030022	1.810664	1.118066	0.576205	0.828167	0.292251	0.465079
4.857403	1.906551	4.802655	0.405786	11.544	4.402973	6.191515	3.428788	2.569957
0.001962	0.152749	0.049646	2.676392	0.06381	0.095433	0.061962	0.134517	0.133045
11.06488	6.876392	11.65791	0.041501	5.9071	6.663651	15.276	15.98892	14.00414
0.048622	0.101104	0.058579	0.041501	0.027965	0.002698	0.052915	2.89E-05	0.000188
0.951652	10.80505	0.953506	27.38726	1.236248	0.215115	1.149322	0.7257	5.34474
1.706219	2.558817	5.180058	1.563045	5.302294	2.898196	8.445743	1.620058	2.628225
2.542605	3.8379	7.854069	2.895599	3.588369	2.750375	10.33476	1.301169	3.750837
0.048622	0.000188	0.000216	0.22303	0	0.087403	0.000952	2.89E-05	0.122554
4.927302	3.387864	3.588369	8.940671	6.945152	3.131544	3.829105	1.521371	6.171789
3.243225	14.33939	4.946587	23.93364	9.141299	2.710765	7.402915	4.684502	5.195022
1.823377	4.236962	0.690707	16.34294	0.532619	0.93632	0.457114	0.3514	1.171349
0.595022	2.181775	0.729062	33.28499	0.957273	1.05088	1.860491	0.749481	0.772157
3.514834	0.910823	0.873088	0.041501	3.290101	1.094978	2.281926	2.89E-05	0.92482
0.001962	0.016681	0.022814	0.22303	0.00132	1.44E-05	0.000952	0.002821	0.000188
0.196955	0.510072	0.717403	5.950664	0.361717	0.223701	0.287951	0.134517	0.133045
22.13245	15.07257	21.74051	4.440548	50.55949	57.84882	22.13245	33.4985	29.18885
0.168312	0.328543	0.148124	0.657114	0.440548	0.079466	1.116609	0.068932	0.143333
0.087677	0.447359	0.000216	0.896522	0.068514	0.112641	0.357727	0.181797	0.052208
0.001962	0.000188	0.000216	0.31171	0	0.002698	0.000952	2.89E-05	0.000188
0.001962	0.208867	0.000216	0.31171	0.023442	0.014141	0.000952	0.162107	0.045079
0.001962	0.000188	0.000216	0.252619	0.088788	1.44E-05	0.000952	2.89E-05	0.045079
16.74449	2.147013	4.661717	0.956061	37.81919	3.984747	2.895599	3.750837	5.630815
0.097258	0.824416	0.000216	0.3414	0.068514	0.112641	0.07013	0.214401	0.270837
0.068896	0.837864	0.306176	0.797114	0.291861	0.263304	0.272951	0.874603	0.096905
0.866147	2.673846	2.673846	0.797114	2.225498	1.532468	4.365224	0.483045	0.863694
1.391494	0.352222	0.202799	0.372078	0.291861	1.44E-05	0.431457	0.068932	0.877222
0.108196	0.702381	0.101212	2.500548	0.233434	1.041025	0.981032	0.560325	0.589618

0.971775	0.60886	0.581378	6.355224	0.580123	0.058975	0.726811	0.386854	0.966825
1.668369	2.257749	1.336587	1.563045	3.799885	2.254084	2.828918	2.5271	3.820758
1.044863	0.412453	0.750866	2.752893	0.809481	0.532857	0.771688	0.409899	0.65132
38.94854	0.000188	0.000216	0.041501	4.586753	1.285584	0.340245	1.067662	22.74797
0.492958	1.420202	0.418326	0.609177	0.143709	0.565137	1.209105	0.569293	0.143333
0.359502	1.821861	0.970007	6.297136	0.155224	0.342814	1.149322	0.452965	0.833045
43.49658	29.49693	32.31123	0.041501	29.18885	72.69473	26.25557	62.33472	25.73165
0.068896	0.286681	0.192929	2.421681	0.058615	0.251248	0.22039	0.087165	0.030837
0.001962	0.091616	0.015036	0.041501	0.00132	1.44E-05	0.000952	0.028232	0.01715
2.155447	0.687807	2.427792	1.318384	3.35355	1.041025	3.457504	0.971205	1.965657
0.81544	1.707576	1.553074	1.006025	3.35355	2.254084	1.265426	2.187395	1.037316
0.320563	1.126775	0.376407	0.609177	0.339235	0.182929	1.371602	0.171912	0.598456
0.001962	0.130029	0.041558	1.919661	0.223391	0.069553	0.229791	0.143838	0.070209
0.097258	0.702381	0.041558	1.318384	0.155224	0.095433	0.000952	0.430281	0.556753
0.001962	0.934488	0.148124	1.157626	0.155224	0.079466	0.464834	0.306602	0.281364
0.001962	0.029221	0.015036	0.22303	0.009502	0.002698	0.000952	0.002821	0.01241
0.001962	0.000188	0.000216	0.252619	0.027965	0.058975	0.000952	0.00987	0.000188
2.417424	31.92385	27.38726	3.714906	10.57984	8.83241	18.19595	2.890245	2.31013
0.068896	0.217496	0.0843	0.742872	0.19039	0.049149	0.210505	0.117446	0.209048
0.001962	0.082727	0.041558	0.22303	0.058615	0.087403	0.07013	0.022266	0.191133
5.00404	5.984885	3.529466	3.975498	1.957958	10.82954	3.885642	18.69833	7.417359
0.168312	0.225974	0.000216	0.041501	0.138283	0.022814	0.18324	2.89E-05	0.000188
0.211169	0.458701	0.410079	2.171429	0.274877	0.23303	0.294502	0.24184	0.518716
4.808658	8.477662	7.765931	4.789596	9.838658	3.734762	5.687879	2.6143	2.909293
0.039509	0.000188	0.015036	0.567071	0.00132	0.148557	0.044113	0.171912	0.01715
0.108196	1.075411	0.524986	1.810664	0.554026	0.191804	0.746551	0.530253	0.598456
27.96248	14.08569	3.953297	0.041501	12.16681	30.17192	3.625382	52.56128	4.745895
0.533874	1.175094	0.328737	2.099524	0.554026	0.014141	0.405094	0.275873	0.000188
0.29531	0.225974	0.211501	7.938427	0.037374	0.370657	0.383889	0.420289	0.889019
1.316544	4.323045	2.238341	0.446291	0.223391	1.152944	1.105426	0.269141	0.160851
0.543939	0.152749	0.164618	0.372078	1.079697	0.164877	0.256941	0.181797	0.270837
0.001962	0.175361	0.242222	0.3414	0.032576	0.049149	0.18324	0.101782	0.045079
2.740519	1.519322	0.706053	0.041501	1.876212	1.113961	0.700058	1.019791	0.994899
10.8813	14.17388	8.745238	0.041501	8.573434	8.83241	8.195657	11.30987	4.513622
0.23246	0.152749	0.264654	0.041501	0.155224	0.079466	0.174098	0.420289	1.159913
0.23246	1.566414	0.453709	0.700058	0.405786	0.276429	0.424228	0.398355	0.661861
2.162381	4.096407	0.833975	5.287857	0.933261	0.468694	2.725498	1.133622	6.054242
0.001962	0.016681	0.0843	2.099524	0.04886	1.44E-05	0.078831	0.016104	0.000188
6.443925	3.321284	9.031082	1.980592	2.928247	3.104394	13.15079	2.642525	11.01243
0.039509	0.064848	0.032496	0.797114	0.009502	0.002698	0.061962	0.00987	0.052208
0.048622	0.036869	0.015036	0.488167	0.00267	0.022814	0.000952	0.00987	0.052208
0.001962	0.01189	0.000216	0.22303	0.005729	0.014141	0.028232	0.028232	0.000188
0.778788	3.648081	1.876212	2.752893	1.308167	1.240981	2.23267	1.693939	2.101075
1.447994	2.577157	5.9071	0.742872	21.36892	4.227302	13.75978	1.935685	5.959033
0.384156	1.845519	1.40772	6.454416	7.013853	0.546854	4.978182	1.399315	4.56912
0.307027	0.78619	0.418326	0.041501	0.456818	0.122287	0.174098	0.152749	0.330289
1.808023	0.286681	1.246457	0.609177	0.567345	1.019791	0.625786	0.284863	0.456248
0.001962	0.977994	0.173723	0.041501	0.098211	0.041183	0.000952	0.051595	0.598456
0.182482	0.175361	0.551573	3.394286	0.185642	0.390418	0.137201	0.171912	0.349084
2.6143	2.181775	0.750866	2.500548	1.23127	1.790245	1.947316	1.988196	0.863694
0.457749	1.420202	0.644618	1.05153	0.899055	0.276429	0.482706	0.521775	1.438672
1.89518	1.018427	2.071587	0.797114	1.584105	2.083261	1.410902	2.802828	3.172864
3.57057	0.837864	2.005253	0.956061	3.058802	2.274473	1.505678	2.27254	1.776739
2.02956	1.450693	2.987835	4.721789	1.816436	1.073543	0.482706	0.70404	1.924228
30.82277	0.000188	83.489	3.310758	5.646811	111.5439	48.53429	4.900996	0.000188
1.632648	0.485577	3.66785	0.041501	1.103261	5.923838	1.876212	0.5129	2.276407
0.087677	0.54925	0.349278	0.281068	0.270289	0.032367	0.324502	0.251126	0.293276
18.62711	24.50942	31.1753	0.041501	8.319019	17.78522	17.28195	8.849697	2.223636

0.060173	0.164877	0.138283	1.25816	0.185642	0.002698	0.210505	0.125483	0.143333
0.521775	0.185909	0.242222	0.527518	0.957273	1.44E-05	0.801501	2.89E-05	0.209048
1.602619	2.419553	2.104358	22.03348	2.151919	2.847547	2.621133	2.463911	2.003723
18.76954	7.625209	10.28573	4.307309	9.583463	17.64869	35.27608	18.05488	9.031082
0.108196	0.152749	0.041558	3.394286	0.169394	0.002698	0.247749	4.552092	0.791854
7.162698	2.640332	6.028427	4.959358	3.279625	2.436768	3.334199	6.30671	12.10289
1.159913	3.877417	0.930837	3.166659	0.375094	1.007864	1.105426	0.796234	2.425801
3.107417	8.019769	1.737294	1.620058	2.491948	2.730072	2.24733	5.568391	4.875527
1.034315	8.333918	0.944084	38.39042	2.498478	0.871429	1.240202	2.967453	2.820974
0.395152	1.004156	0.459769	11.30987	0.434863	1.232078	0.609841	1.87482	0.395426
0.001962	0.064848	0.022814	0.527518	0	0.007374	0.03526	0.00987	0.000188
0.89404	0.237359	0.627633	0.3414	0.729582	1.130794	0.551861	0.821414	1.450693
0.254365	0.374827	0.765094	1.810664	0.014026	0.322395	0.801501	0.1986	0.103528
8.660281	2.673846	4.739668	3.089293	3.548153	2.153535	1.853723	3.8121	10.5063
0.048622	0.485577	0.272951	4.043846	0.366912	0.299062	0.000952	0.190224	0.349084
1.763889	0.424538	4.661717	0.527518	6.116638	2.710765	1.341825	0.36153	2.148658
0.641277	1.530159	1.137244	3.394286	1.449062	0.515238	1.89518	0.734935	0.863694
0.001962	0.04557	0.015036	0.31171	0.005729	0.002698	0.000952	0.00987	0.01715
2.08632	4.875527	1.604827	8.461703	12.29473	2.219863	2.465916	3.310758	2.727785
0.221962	0.662237	0.31557	0.041501	0.580123	1.120332	0.808052	1.197922	0.160851
0.097258	0.109466	0.022814	3.714906	0.014026	0.002698	0.044113	0.059582	0.112886
1.365296	1.34513	1.259747	0.041501	1.126075	1.765065	2.060491	0.999502	0.377085
0.492958	4.38684	0.446551	34.84299	5.454949	1.304877	2.703485	1.218182	0.661861
5.493182	5.195022	5.493182	0.527518	1.834423	2.345527	5.560563	1.281241	1.579798
1.000794	0.510072	0.490952	0.281068	4.115844	1.948918	1.062092	0.683232	0.243449
3.028629	8.256465	3.899993	13.38418	2.388348	2.197828	5.576219	2.577157	3.820758
2.306277	11.544	3.833247	6.454416	4.540794	1.600238	4.881775	3.300664	4.101061
1.431414	0.412453	1.246457	2.308203	0.753449	0.954141	3.037626	0.95101	2.223636
1.080303	0.054791	0.551573	0.041501	0.39062	2.071587	0.398355	1.154567	0.661861
1.96404	1.203427	3.450577	0.041501	3.885642	1.336587	3.514834	0.441263	4.203001
8.923117	19.68088	28.54786	2.752893	19.99861	16.44768	22.32214	10.53159	12.85504
0.607937	0.000188	0.65202	0.041501	0	0.490079	0.000952	2.89E-05	0.000188
22.74797	32.68313	14.6058	17.51961	14.51648	12.81638	15.66023	16.62402	6.443925
12.89577	2.794935	8.539971	0.041501	6.433918	4.997417	6.653016	2.925426	5.068831
0.001962	0.082727	0.000216	0.22303	0.00267	0.002698	0.000952	2.89E-05	0.024372
1.823377	0.749942	3.842554	0.041501	0.412193	2.77899	1.770029	1.935685	1.654156
1.706219	7.288788	2.207417	21.45857	4.086926	2.463911	3.975498	3.926154	6.830592
0.939408	0.458701	0.388579	2.831501	0.239408	1.285584	0.349278	2.003723	1.216768
0.437309	1.018427	0.202799	0.31171	0.279452	0.441263	0.471313	1.620058	1.803918
2.382035	0.470512	1.069033	0.041501	2.278254	1.130794	2.438831	0.551861	3.482785
0.756724	0.000188	1.703795	0.041501	0.440548	7.264502	0.174098	0.671371	0.000188
51.52195	29.18885	75.1132	0.041501	44.27012	203.5795	87.1746	77.74812	32.49718
1.068348	1.872049	1.360866	3.23987	3.119986	1.713997	2.54961	2.703485	1.322092
0.143838	0.498918	0.436017	0.405786	0.042929	0.223701	0.157619	0.162107	0.320332
1.49075	0.934488	6.135007	0.527518	0.809481	1.463304	1.520346	2.485534	2.765758
0.568665	0.964416	1.523824	0.041501	0.304062	3.734762	1.116609	2.119596	2.890245
0.855267	0.498918	0.256046	8.396847	3.128694	0.920483	0.954141	1.019791	1.374329
0.001962	2.533745	0.000216	0.200519	6.632063	2.31811	3.411097	1.067662	2.027951
8.256465	17.03919	17.15902	2.308203	7.938427	14.6058	24.73724	11.60283	9.218514
0.982698	0.352222	0.058579	0.041501	1.267085	0.430281	0.828167	0.420289	1.749307
1.25816	1.215195	0.058579	1.734949	0	1.44E-05	0.000952	2.89E-05	1.05417
6.900202	15.46193	2.402468	22.03348	9.792626	3.707136	2.735368	3.659668	8.608153
1.548723	1.707576	1.290981	0.372078	1.245671	1.25886	2.162381	0.960216	1.675722
0.335599	1.34513	0.376407	2.365685	0.350455	0.675938	0.383889	0.314733	0.619365
0.855267	0.675115	0.402381	0.846342	1.062092	0.191804	0.491587	0.409899	0.440029
4.328232	0.384394	5.923838	0.041501	1.597908	2.970144	8.381328	8.089848	1.793074
0.001962	0.164877	0.000216	0.041501	0.018492	1.44E-05	0.124343	0.1986	0.052208
0.120823	0.529971	0.504416	0.041501	0.513752	0.079466	0.287951	0.016104	0.320332

0.521775	1.283059	0.504416	0.041501	0.339235	1.906551	0.972504	3.533211	0.609177
9.256537	0.837864	1.533535	0.041501	3.26	6.045209	2.74544	10.23768	7.212915
10.38202	6.853608	7.251263	15.61077	3.23987	3.104394	5.606739	2.263377	4.907929
18.19595	2.477027	11.25392	0.041501	9.52197	31.1753	17.51961	46.7056	19.53061
0.951652	1.594675	2.347439	0.31171	0.963853	0.852684	3.019848	1.271876	1.374329
0.001962	6.727633	6.443925	0.041501	0.851234	1.240981	3.35715	1.163579	0.000188
0.359502	1.283059	0.551573	2.676392	0.904856	0.139986	0.684401	1.485483	1.006025
5.872886	12.93651	15.276	0.041501	4.614618	9.141299	11.65791	8.539971	7.519841
1.474343	10.31025	0.996111	1.620058	2.398442	1.113961	2.035678	4.47912	11.719
7.697063	14.42661	12.36443	8.625613	9.063983	10.19468	15.88548	11.719	12.42965
0.001962	3.256587	0.202799	7.545584	1.913997	0.191804	0.633146	1.007864	1.986681
5.416623	28.54786	11.77567	7.443276	11.48368	9.047532	17.78522	13.45939	17.28195
1.25816	0.498918	1.593535	0.041501	1.523824	1.232078	2.089639	1.067662	1.147807
2.210866	6.967547	7.710361	0.281068	3.934986	2.421681	8.048095	2.513752	3.581688
0.13347	0.263853	0.000216	0.041501	0.077316	1.44E-05	0.000952	0.04557	0.089062
0.667201	5.599228	4.614618	0.041501	2.194257	0.975556	5.515584	1.133622	1.018427
0.884654	1.628009	1.618773	0.446291	1.050231	1.609654	2.725498	0.890065	0.65132
2.073232	2.367763	0.349278	8.288874	0.696659	0.782417	2.556443	1.532468	3.122915
0.001962	0.064848	0.000216	0.446291	0.042929	1.44E-05	0.000952	0.002821	0.01715
0.469004	9.218514	5.273362	0.041501	2.850058	9.541645	5.855281	11.14619	0.93267
7.375794	7.854069	5.889798	1.05153	11.14619	6.570433	14.29947	6.99026	10.1028
0.791371	0.000188	1.006623	0.797114	1.441898	1.113961	1.290065	2.042201	1.924228
0.196955	0.977994	0.129553	0.609177	0.270289	0.069553	0.000952	0.087165	0.234214
5.22482	8.958225	5.381227	5.395584	1.519322	4.519048	13.30906	1.521371	3.679986
4.563117	2.730072	7.013853	0.041501	10.1459	3.824964	12.22798	3.35355	4.655916
0.406912	0.119141	0.111183	13.87537	0	1.44E-05	0.301241	2.89E-05	0.903074
0.087677	0.622496	0.229791	0.700058	0.456818	0.041183	0.256941	0.143838	0.133045
4.02088	1.359177	4.140505	0.405786	0.456818	2.911962	4.297222	3.533211	2.164113
0.667201	1.722475	0.772626	0.041501	1.220628	0.963853	2.465916	1.497143	2.402468
0.416782	1.159149	0.453709	3.310758	0.385238	0.182929	0.340245	0.275873	0.528196
5.056421	0.910823	6.560411	0.041501	3.975498	4.875527	5.881342	3.175916	2.386248
0.73583	0.582973	2.150303	2.421681	2.633232	0.613853	1.498167	0.784805	1.274481
0.320563	0.364242	0.120433	0.3414	0.328268	0.069553	0.424228	0.117446	0.330289
9.063983	9.707677	5.545729	0.041501	1.33285	29.82828	7.315512	36.26061	9.459177
1.068348	0.60886	1.450693	3.899993	0.912446	0.686299	1.095657	0.596111	1.734949
2.382035	0.837864	3.578038	0.041501	1.816436	2.563506	6.268312	2.873817	3.273045
0.048622	0.04557	0.015036	0.3414	0.005729	0.002698	0.061962	0.051595	0.079531
0.29531	0.250115	0.000216	0.041501	0.053766	0.032367	0.000952	0.059582	0.06088
0.001962	0.910823	0.049646	1.734949	1.012742	0.079466	0.000952	0.068932	0.122554
0.264048	1.371602	6.191515	0.527518	1.374329	2.230859	1.329206	1.085967	0.65132
2.652107	3.511263	0.220657	70.11932	0.25197	0.174372	0.272951	0.596111	3.055729
13.6814	20.34063	20.94257	19.14516	51.52195	52.56128	38.94854	15.07257	32.49718
10.28573	42.42191	24.2842	2.171429	16.22108	12.63773	21.19547	7.238023	9.880779
3.926154	0.737403	0.811861	16.34294	0.963853	1.113961	0.886775	1.910974	2.477027
2.763261	2.289286	3.29355	0.700058	2.016515	1.667273	3.370527	1.243442	2.781558
0.307027	2.201587	0.192929	5.696263	1.325743	1.44E-05	0.000952	2.89E-05	0.661861
0.143838	0.392944	0.349278	0.372078	0.21632	0.920483	0.521775	1.425613	0.395426
11.719	0.000188	42.75576	0.041501	118.7028	24.50942	12.63773	47.5018	11.42512
3.279625	0.687807	4.63189	0.041501	3.147294	0.832013	4.519048	0.582208	2.522684
1.535722	1.566414	2.633232	0.846342	1.356364	0.852684	1.265426	1.935685	3.192229
4.067547	11.17281	5.622872	3.899993	6.325945	2.139928	4.155339	4.145209	7.08733
0.866147	0.000188	0.802395	0.3414	0.19039	0.634531	0.574993	0.890065	0.243449
6.8421	0.000188	0.120433	0.041501	0	10.12435	1.265426	3.253175	0.697085
12.26136	0.000188	0.000216	0.041501	0.462612	21.02638	4.637994	24.2842	6.900202
0.060173	0.225974	0.000216	0.956061	0.032576	0.032367	0.000952	0.232734	0.330289
80.6518	9.256537	47.5018	0.041501	30.82277	67.98069	27.66779	57.84882	52.56128
5.28039	0.824416	0.446551	0.956061	3.266782	3.163506	1.68938	3.533211	3.482785
2.367763	5.195022	5.439278	0.281068	2.269134	3.36075	6.580988	2.90658	3.150433

0.875101	0.78619	1.017107	2.597792	0.35627	0.20733	0.281169	0.190224	0.640606
4.484892	7.225469	2.520404	0.041501	5.082063	1.181342	5.881342	1.666089	2.185556
1.142345	0.63754	2.922778	0.041501	1.050231	3.147294	2.955916	3.153485	2.477027
0.001962	6.297136	9.418196	0.041501	2.485534	8.019769	2.750375	4.921082	0.377085
126.1556	32.31123	40.7932	0.041501	25.73165	184.443	33.93939	100.0998	37.81919
0.982698	5.351934	0.000216	12.85504	0	1.44E-05	0.764315	0.693571	2.290996
0.607937	1.061457	0.120433	5.287857	0.481602	0.769019	0.585281	1.122359	1.852338
2.34355	0.152749	0.551573	0.488167	1.666089	0.370657	0.000952	0.5129	0.640606
0.001962	0.101104	0.058579	0.446291	0.037374	1.44E-05	0.000952	0.00987	0.01715
1.978954	1.50026	0.000216	1.318384	0.518413	0.475729	0.439524	0.784805	2.765758
0.168312	0.109466	0.173723	1.734949	0.117843	0.095433	0.087879	0.036782	0.000188
28.54786	4.174964	13.08214	30.0001	0.886775	10.62739	10.67991	13.15079	9.583463
8.508514	1.203427	1.290981	0.041501	0.796234	26.52537	0.398355	30.82277	6.325945
0.568665	0.286681	0.049646	2.037114	1.026825	0.041183	0.585281	0.171912	0.730476
0.91575	2.597792	2.673846	10.01009	1.460051	0.852684	5.150693	1.595729	1.563045
1.876212	9.121486	1.102691	0.041501	1.298665	1.380195	5.737056	2.07658	2.616573
4.63189	16.22108	6.475505	29.49693	9.968355	2.8657	3.984747	3.253175	3.989206
0.641277	2.703485	2.922778	2.895599	1.947316	0.871429	2.035678	1.048074	1.793074
0.654877	2.147013	0.750866	4.721789	0.426118	0.322395	1.702706	0.569293	0.079531
0.778788	2.434444	0.92531	1.05153	2.928247	1.2214	0.820931	1.416407	1.076032
1.054899	0.250115	2.62342	0.372078	0.762641	6.772763	1.290065	1.728615	2.922778
0.168312	2.026219	1.274481	17.78522	0.180231	0.251248	0.000952	0.551861	1.944517
7.697063	6.945152	14.97762	0.041501	1.135137	4.72798	10.53159	14.88584	7.264502
0.001962	0.064848	0.000216	0.405786	0.077316	1.44E-05	0.104495	0.059582	0.000188
2.229048	5.056421	1.175859	16.74449	2.911962	0.885664	0.848831	2.794935	3.651898
3.324452	1.542302	4.586753	1.05153	6.727633	6.727633	7.61176	6.464906	4.927302
0.143838	1.075411	0.338095	1.318384	0.308672	0.002698	0.137201	0.059582	0.168961
32.31123	8.608153	25.99427	1.498167	16.34294	33.07147	43.49658	11.65791	10.90615
3.065108	3.695815	6.591544	1.620058	7.737807	6.695887	3.644343	4.120815	3.548153
3.310758	1.519322	0.156696	0.041501	2.92013	3.23987	3.227085	4.875527	2.671299
0.182482	0.250115	0.288081	0.488167	0.211025	0.087403	0.000952	0.171912	0.089062
4.281847	17.78522	13.15079	20.08201	15.77678	11.25392	11.77567	7.938427	7.668579
6.30671	0.299336	0.328737	0.797114	0	5.402511	1.492973	2.810678	7.780786
118.7028	64.12971	87.1746	21.74051	40.11561	95.22939	100.0998	55.04081	87.1746
0.182482	0.572266	0.211501	1.318384	0.042929	0.555418	0.272951	0.1986	0.320332
8.412367	4.072475	4.18456	14.65384	1.985253	19.83727	1.250895	27.38726	8.745238
17.64869	4.952915	5.545729	4.125786	9.52197	14.93173	10.01009	11.89932	18.48514
0.120823	0.064848	0.138283	0.041501	0.027965	0.032367	0.000952	0.409899	0.357727
0.001962	0.237359	0.000216	0.527518	0.00267	1.44E-05	0.061962	2.89E-05	0.000188
1.548723	0.910823	2.257749	0.041501	0.4971	1.304877	1.036558	1.449062	1.734949
12.89577	0.401724	8.256465	0.956061	4.667518	9.335397	16.80343	31.1753	1.254704
0.097258	0.016681	0.032496	1.810664	0.018492	0.007374	0.044113	0.068932	0.045079
0.437309	0.109466	0.562439	0.041501	3.089293	3.775209	0.457114	2.477027	0.589618
0.778788	0.412453	0.164618	1.25816	0.440548	0.139986	0.301241	0.332049	0.260938
0.001962	1.45912	0.360462	3.544257	0.279452	1.44E-05	0.124343	0.1986	0.112886
7.212915	13.08214	5.704646	3.714906	3.855952	8.210938	4.592273	12.03267	13.15079
0.991631	1.175094	0.459769	1.810664	0.518413	0.215115	0.717879	0.299199	1.68938
6.508427	2.312085	4.115844	1.006025	2.703485	6.210375	3.574387	4.502453	4.451154
60.77449	70.11932	70.11932	6.749488	18.33909	48.53429	26.52537	87.1746	95.22939
0.001962	0.082727	0.164618	0.527518	0.037374	1.44E-05	0.104495	0.051595	0.000188
0.048622	0.140657	0.068449	1.104033	0.077316	0.022814	0.07013	0.190224	0.096905
24.2842	105.1694	91.05541	5.395584	48.53429	47.5018	70.11932	40.7932	40.7932
1.842734	2.953059	0.944084	3.089293	0.92816	1.720144	0.787136	3.025671	2.890245
0.221962	0.54925	0.220657	1.05153	0.257143	0.122287	0.202641	0.630823	0.42925
0.543939	0.447359	0.562439	10.01009	0.735382	1.846941	0.535657	1.521371	1.520346
0.154848	0.436017	0.735382	18.26752	0.939408	0.712929	0.411436	1.540144	2.5271
10.48101	5.024293	1.4671	22.8563	5.500678	3.618499	2.786898	4.072475	6.200945
0.855267	1.677042	0.220657	1.204957	1.896667	0.087403	0.272951	0.36153	0.518716

50.55949	55.04081	19.68088	111.5439	37.29684	20.34063	18.33909	34.84299	62.33472
0.001962	0.185909	0.032496	0.657114	0.00267	0.014141	0.028232	0.036782	0.070209
2.494177	1.677042	2.150303	1.498167	2.031169	2.518124	1.123795	1.019791	0.749481
0.001962	0.074322	0.015036	0.252619	0.005729	1.44E-05	0.000952	0.028232	0.06088
0.745613	2.847547	0.862561	62.33472	1.523824	0.796674	0.893528	1.883535	1.254704
2.8657	8.573434	8.319019	9.707677	4.227302	2.65684	7.175397	4.94026	13.53913
2.898196	6.200945	3.233045	12.03267	2.993867	1.834423	2.713319	2.24733	5.143867
0.001962	0.225974	0.022814	2.752893	0.083074	0.139986	0.000952	2.89E-05	0.000188
3.279625	11.719	9.792626	0.041501	6.486104	5.044113	4.203001	2.169509	2.223636
0.960216	1.799899	1.940231	1.25816	3.300664	0.963853	1.229553	0.971205	0.845317
0.335599	0.824416	0.211501	9.160382	0.70329	0.079466	0.317229	0.560325	1.147807
11.39744	6.99026	14.42661	4.307309	24.50942	15.3618	6.404921	12.56693	9.502294
1.972706	1.262965	3.119986	0.041501	3.089293	2.638139	0.543665	2.003723	1.41368
1.169791	0.185909	1.533535	0.041501	0.62689	1.886335	1.780693	2.022778	0.916869
0.23246	1.845519	0.328737	4.376118	0.746082	0.139986	0.471313	0.70404	0.673651
41.47925	25.24367	22.74797	5.530274	20.34063	36.26061	42.08807	26.25557	18.91854
13.60866	1.780693	6.52899	2.037114	6.00228	5.515584	12.49452	2.429957	1.909408
0.320563	0.991631	0.173723	0.896522	0.211025	0.657114	0.210505	1.521371	0.489235
5.180058	23.49249	3.052655	16.9777	3.855952	3.307186	3.334199	8.381328	9.101674
37.81919	24.2842	34.38341	0.041501	26.25557	83.489	25.24367	28.23632	27.38726
0.756724	3.57057	2.554069	7.162698	1.896667	1.026825	6.365065	0.671371	0.420498
0.359502	0.529971	0.65202	0.797114	0.4971	0.370657	0.281169	0.492165	0.583766
1.344257	9.968355	6.135007	0.657114	6.945152	2.421681	5.024293	5.359134	5.287857
1.637309	27.96248	29.82828	0.956061	100.0998	1.354567	29.49693	0.109185	0.234214
2.542605	1.983824	1.797172	0.041501	4.655916	4.764055	8.590794	2.104358	4.435159
2.850058	9.792626	5.110159	18.84404	1.700354	2.225498	2.074906	1.87482	5.84689
0.802756	6.054242	0.65202	8.745238	3.679986	0.686299	2.65684	2.685786	1.702706
0.039509	0.054791	0.022814	0.372078	0	0.014141	0.028232	0.036782	0.070209
27.38726	33.07147	10.01009	21.11092	18.48514	8.573434	11.719	15.41187	19.68088
7.882222	5.545729	3.91355	0.041501	2.99987	3.307186	3.930664	2.661587	4.952915
4.082165	1.330029	1.377446	0.041501	2.139928	3.644343	2.190866	7.38847	4.952915
6.38456	3.975498	2.90658	1.919661	1.969733	15.77678	3.046551	11.22667	6.695887
3.87329	3.273045	9.502294	0.372078	3.137576	3.975498	4.101061	3.767489	6.560411
0.13347	0.470512	0.402381	2.500548	0.412193	0.288745	0.317229	0.299199	0.409098
0.060173	0.175361	0.041558	0.956061	0.018492	0.007374	0.078831	0.171912	0.281364
0.843564	1.061457	0.842641	3.544257	0.834416	0.399545	0.814877	0.543939	0.589618
0.23246	5.704646	1.080303	3.471486	1.748016	0.686299	2.060491	0.980505	1.403853
0.001962	0.352222	0.272951	3.820758	0.350455	0.634531	1.881955	1.177309	0.661861
55.04081	9.838658	27.96248	0.797114	27.38726	46.7056	28.54786	19.06893	12.49452
0.791371	0.687807	4.165137	1.378362	1.906551	2.274473	2.148658	0.899567	1.187229
1.602619	2.289286	1.969733	1.318384	5.150693	0.288745	3.533211	0.162107	0.395426
2.250462	6.054242	3.767489	17.98838	5.606739	1.480541	8.590794	2.263377	3.921645
0.507403	7.977958	5.402511	3.899993	4.440548	2.005253	20.34063	2.547179	1.734949
7.938427	2.922778	1.030022	14.04491	5.515584	14.42661	0.853218	23.82354	10.33476
21.54823	21.74051	13.00996	30.49289	18.76954	15.18102	9.792626	14.70188	34.38341
1.718918	252.4251	64.12971	40.11561	4.165137	22.32214	45.1045	23.38378	5.395584
11.96574	2.114437	5.165599	3.899993	31.1753	3.397706	4.281847	1.185685	1.734949
32.68313	0.286681	0.602908	0.527518	1.325743	7.683766	1.290065	6.240346	1.240981
3.904517	6.200945	9.101674	0.041501	9.625253	5.984885	10.57984	3.91355	3.989206
1.291825	3.672078	2.016515	2.831501	4.696313	1.91254	2.955916	1.620058	2.071587
0.283882	0.185909	0.248528	0.797114	0.394877	0.576205	0.087879	1.325743	0.224921
0.001962	0.023492	0.000216	0.22303	0.005729	0.022814	0.000952	2.89E-05	0.030837
1.718918	10.43066	10.78056	0.041501	11.89932	4.188896	5.754935	4.198117	5.606739
14.25954	16.44768	11.544	1.378362	16.44768	14.70188	27.38726	15.18102	19.22139
0.001962	0.016681	0.022814	0.200519	0.00132	0.022814	0.028232	0.005613	0.01241
0.001962	0.119141	0.041558	1.006025	0.270289	0.002698	0.061962	0.028232	0.089062
19.99861	34.38341	23.82354	22.52375	105.1694	10.67991	29.82828	19.37139	50.55949
1.333781	1.018427	1.163579	1.104033	0.580123	0.613853	0.700058	0.662612	1.95645

0.001962	0.01189	0.015036	2.676392	0	0.002698	0.000952	2.89E-05	0.01715
18.33909	42.42191	5.654863	126.1556	10.28573	7.162698	4.739668	4.833398	44.27012
4.789596	5.679502	4.091667	6.570433	6.695887	2.955916	3.869163	3.47531	6.345382
0.087677	0.091616	0.022814	2.597792	0.06381	0.041183	0.000952	0.00987	0.01715
0.457749	0.964416	0.31557	3.089293	0.789863	0.139986	0.364242	0.409899	0.863694
0.283882	0.948745	0.306176	5.01772	0.399545	0.251248	0.22039	0.464387	0.112886
77.74812	203.5795	45.1045	252.4251	30.49289	80.6518	51.52195	105.1694	144.7919
3.592063	25.73165	7.456118	8.348817	3.771349	5.568391	6.250447	6.685087	19.83727
29.82828	23.49249	8.061934	65.94551	12.77772	8.210938	8.745238	9.667316	17.51961
0.283882	0.724502	3.025671	0.041501	0.771176	1.667273	2.281926	0.671371	1.702706
425.62	2.131176	184.443	0.041501	87.1746	56.39528	24.2842	155.7103	530.1465
10.01009	12.16681	11.89932	17.64869	11.36975	6.287561	7.08733	6.464906	13.75978
4.739668	1.004156	0.671371	3.166659	0.037374	1.316544	0.193896	0.831472	3.204632
1.49075	1.780693	0.51039	5.102742	1.482626	2.197828	1.455988	3.869163	1.403853
2.652107	3.648081	1.315613	5.287857	2.644885	1.354567	1.901068	3.687633	2.978968
4.402973	10.17029	6.967547	7.049336	10.67991	5.137042	5.266335	9.179466	11.09133
16.5625	1.64684	4.003117	33.93939	8.019769	4.343838	1.329206	5.295325	40.11561
0.406912	0.485577	0.236089	0.041501	0.649668	1.44E-05	0.000952	0.095029	2.08798
9.356371	1.542302	0.890065	6.570433	1.597908	8.493088	1.123795	17.28195	5.431544
12.49452	38.39042	24.98218	155.7103	28.23632	13.38418	19.99861	15.276	22.32214
33.93939	95.22939	41.47925	2.752893	62.33472	18.12541	30.82277	25.24367	45.1045
1.104805	6.819481	0.897489	1.86658	1.755988	2.765758	2.828918	3.614603	1.749307
15.276	8.888167	22.52375	50.55949	21.19547	9.880779	7.061328	9.729358	13.45939
2.417424	2.828918	10.35839	0.446291	5.872886	2.710765	5.638759	0.734935	4.609076
24.98218	8.089848	49.50903	7.443276	34.84299	16.22108	41.47925	5.889798	11.01243
0.89404	1.697792	1.737294	11.19942	0.963853	1.568564	1.179885	0.95101	0.293276
5.088694	0.000188	6.560411	0.041501	6.853608	2.882035	3.771349	1.344257	3.581688
34.84299	24.98218	57.84882	0.956061	33.28499	20.68746	95.22939	45.1045	16.22108
0.120823	0.374827	0.041558	10.19468	0.093716	0.032367	0.052915	0.261342	0.216602
0.507403	0.875599	0.490952	1.919661	0.946638	0.288745	1.965657	0.24184	0.409098
6.945152	5.22482	11.83593	0.041501	8.745238	15.46193	3.486436	9.968355	5.110159
1.365296	3.511263	0.873088	0.041501	0.19039	0.743283	1.89518	8.643074	11.65791
1.150635	0.622496	0.192929	4.857403	0.211025	0.156407	0.717879	1.384921	1.765065
3.714906	2.162381	4.927302	2.037114	4.003117	2.681169	2.828918	3.236457	3.233045
0.001962	0.016681	0.049646	0.041501	0.00267	0.002698	0.113001	0.002821	0.000188
21.02638	67.98069	7.188095	11.77567	27.66779	10.43066	9.880779	13.91714	35.27608
45.1045	749.7839	95.22939	425.62	64.12971	91.05541	184.443	749.7839	87331.38
252.4251	224.9555	144.7919	184.443	184.443	224.9555	425.62	167.9996	332.0776
0.078124	3.45404	0.236089	1.378362	0.291861	0.370657	0.560866	0.831472	0.629098
16.9162	26.25557	36.74522	9.838658	17.92188	27.66779	23.6012	25.48179	23.82354
0.196955	0.520289	0.182929	0.405786	0.35627	0.515238	0.801501	0.596111	0.640606
1.92272	4.048499	2.187395	3.632973	3.022713	2.345527	3.011169	3.489899	3.330909
0.182482	0.392944	0.970007	3.089293	0.018492	0.370657	0.23873	0.899567	0.168961
0.060173	3.78798	2.74298	0.446291	0.042929	0.182929	2.882035	0.022266	0.024372
0.048622	0.237359	0.022814	1.378362	0	0.007374	0.000952	2.89E-05	0.030837
3.201457	77.74812	7.938427	8.461703	1.748016	11.19942	7.288788	7.443276	3.514834
1.727323	184.443	44.27012	67.98069	5.323492	12.49452	77.74812	12.77772	2.993867
0.048622	0.082727	0.049646	0.896522	0.239408	0.122287	0.061962	0.232734	0.06088
53.77619	56.39528	42.08807	20.85876	32.31123	118.7028	62.33472	65.94551	39.53687
0.001962	0.622496	0.077316	1.006025	0	1.44E-05	0.000952	0.143838	0.143333
12.36443	45.91841	12.03267	4.307309	22.74797	16.80343	17.98838	13.83359	24.73724
0.001962	0.064848	0.000216	0.446291	0.107395	1.44E-05	0.166681	0.214401	0.160851
3.297107	1.359177	2.327857	2.365685	0.385238	0.420498	0.848831	0.569293	1.880375
0.001962	0.773276	0.182929	0.041501	0.128687	0.352807	0.210505	0.409899	0.224921
0.001962	0.000188	0.015036	1.104033	0	1.44E-05	0.000952	2.89E-05	0.024372
0.273261	0.773276	0.516825	1.006025	0.66526	0.215115	0.309062	0.796234	1.817828
19.37139	44.27012	23.16622	60.77449	38.94854	31.92385	37.81919	38.39042	38.94854
5.942294	5.195022	7.570693	20.60009	4.179762	7.991602	6.55039	4.978182	9.141299

8.005685	10.73084	4.540794	10.8813	3.290101	2.970144	4.222583	5.80469	14.42661
7.854069	9.751039	7.061328	17.39935	4.468009	7.363117	4.353925	4.25658	7.288788
3.243225	2.953059	6.876392	3.089293	3.107417	3.031688	4.408153	1.666089	0.955418
0.001962	0.023492	0.015036	0.372078	0	1.44E-05	0.044113	2.89E-05	0.01715
11.14619	19.37139	7.598312	42.75576	4.179762	15.66023	6.727633	45.91841	26.25557
9.418196	17.15902	22.96463	12.1974	13.6814	13.53913	15.98892	14.42661	20.51271
2.633232	0.310866	0.551573	0.742872	0.753449	0.322395	0.521775	0.441263	3.36075
0.802756	2.388348	1.737294	0.527518	1.118066	1.585296	2.339697	1.584105	2.425801
1.104805	0.824416	0.644618	1.563045	1.041652	1.094978	0.535657	1.169791	2.08798
8.745238	14.70188	11.09133	19.75908	36.26061	6.55039	10.73084	7.363117	11.30987
1.054899	6.116638	1.163579	3.166659	2.045476	0.675938	1.34798	1.048074	0.854733
19.75908	2.763261	5.23176	0.041501	5.560563	4.94026	3.743232	2.447294	4.272165
10.33476	13.30906	10.48101	95.22939	16.80343	6.632063	10.8813	8.573434	8.303947
19.06893	52.56128	18.76954	8.815123	4.978182	14.25954	4.758088	8.888167	14.08569
0.097258	0.78619	0.111183	0.372078	0.333636	0.014141	0.229791	0.087165	0.079531
13.00996	13.38418	26.80355	0.657114	29.49693	8.940671	23.38378	6.570433	10.43066
0.060173	0.401724	0.068449	1.157626	0.163276	0.20733	0.137201	0.342215	0.200794
11.39744	26.80355	4.678846	51.52195	18.05488	6.497266	19.53061	24.04374	18.33909
0.039509	1.780693	0.295671	3.310758	0.774798	0.585281	0.49974	1.262179	1.360866
4.145209	2.083261	3.220678	1.318384	2.577157	29.49693	1.380195	35.27608	6.663651
1.474343	0.299336	1.220628	0.252619	0.719177	0.174372	0.294502	0.322395	0.877222
1.876212	0.000188	0.338095	0.041501	0.762641	0.73127	2.382035	0.284863	0.818066
5.056421	12.49452	14.17388	26.95429	28.87506	8.303947	10.93101	13.60866	18.62711
0.048622	0.572266	0.138283	0.797114	0.117843	0.049149	0.03526	0.24184	0.293276
8.923117	6.297136	3.578038	2.964762	2.31404	27.38726	4.814661	37.81919	28.87506
5.545729	7.795642	17.39935	3.544257	3.714906	14.51648	24.50942	5.409437	5.737056
0.048622	0.101104	0.058579	0.22303	0.107395	0.079466	0.03526	0.022266	0.122554
2.511465	6.727633	7.288788	8.694697	2.382035	2.035678	4.297222	4.56912	5.679502
1.738456	1.405772	0.851234	12.85504	4.193232	0.532857	1.809343	0.607605	5.381227
0.320563	1.34513	0.360462	0.252619	0.25197	0.215115	0.193896	0.181797	0.65132
1.622006	0.60886	1.290981	0.041501	1.382006	3.953297	1.396494	1.746724	0.281364
1.519322	2.367763	1.030022	0.797114	1.05417	1.007864	1.186457	0.474214	2.909293
7.495397	4.49671	22.13245	35.27608	2.162381	16.09482	9.297085	34.38341	11.77567
3.201457	4.323045	126.1556	72.69473	749.7839	4.101061	118.7028	4.365224	3.36075
0.654877	1.511198	1.618773	2.421681	1.938759	1.143954	2.043795	1.399315	1.187229
0.001962	0.263853	0.000216	100.0998	0.032576	0.032367	0.265014	0.04557	0.000188
0.623499	0.861934	1.845519	7.162698	0.861934	0.242612	0.639892	0.04557	0.65132
1.49075	1.405772	1.618773	1.498167	0.762641	0.885664	0.775339	1.776739	2.402468
0.001962	0.023492	0.091133	0.446291	0.005729	0.014141	0.044113	0.005613	0.000188
0.426118	1.881955	0.000216	27.81514	0.163276	0.087403	0.324502	2.89E-05	0.93267
0.001962	1.097677	0.000216	2.421681	1.003016	1.44E-05	0.411436	0.693571	1.654156
0.060173	0.237359	0.173723	0.896522	0.265455	0.069553	0.247749	0.051595	0.079531
1.365296	10.01009	1.954993	0.041501	1.207374	1.041025	1.265426	2.858009	2.112843
0.097258	0.119141	0.120433	1.104033	0.12267	0.593146	0.265014	2.89E-05	0.845317
0.001962	0.091616	0.049646	0.405786	0.009502	0.032367	0.000952	0.109185	0.089062
0.371299	0.392944	0.138283	1.734949	0.143709	0.333636	0.174098	0.181797	0.224921
1.660216	0.263853	0.851234	0.609177	2.121198	3.179199	2.99987	2.978968	4.857403
2.210866	0.485577	3.552049	2.752893	0.834416	0.352807	0.944084	0.125483	0.697085
36.74522	13.87537	25.73165	0.041501	15.07257	5.082063	53.77619	4.397792	16.80343
1.391494	0.540563	0.619365	0.252619	0.370657	6.454416	1.835743	4.440548	1.346991
27.10502	10.60361	14.33939	0.846342	95.22939	287.197	9.838658	203.5795	57.84882
0.168312	0.091616	0.0843	0.31171	0.169394	0.049149	0.229791	0.181797	0.365635
0.254365	0.374827	0.264654	0.846342	0.399545	0.191804	0.210505	0.125483	0.440029
1.447994	1.044235	0.913514	2.831501	1.245671	0.852684	1.250895	1.229553	0.697085
0.834416	4.359574	4.198117	6.663651	4.597792	1.823377	6.210375	2.333867	3.095166
2.850058	0.185909	0.000216	0.041501	0	1.684646	0.000952	2.693442	3.820758
0.001962	0.016681	0.058579	0.488167	0	1.44E-05	0.000952	0.002821	0.000188
1.718918	3.422237	3.66785	0.041501	2.08798	2.126349	2.981962	3.025671	1.889711

2.34355	1.612056	2.659214	0.041501	1.420202	1.609654	2.500548	1.325743	1.944517
4.684502	0.60886	3.334199	0.041501	3.471486	1.539062	1.797172	3.581688	3.300664
0.446811	0.185909	0.173723	1.734949	0.088788	0.014141	0.000952	0.162107	0.153016
8.660281	9.316241	13.38418	0.896522	13.79669	6.591544	9.101674	11.03843	12.70768
7.013853	3.877417	8.815123	1.620058	6.250447	1.948918	5.654863	0.796234	4.179762
0.492958	2.367763	0.706053	2.171429	0.696659	0.910339	1.380195	0.683232	1.29267
0.884654	0.016681	0.164618	0.22303	0.072583	4.207778	0.052915	1.436623	1.749307
0.533874	0.687807	0.388579	6.819481	0.658362	0.148557	0.22039	0.1986	0.409098
4.082165	3.397706	3.648081	5.779574	1.225426	4.690159	3.957734	4.802655	3.172864
3.156537	0.948745	2.690823	0.041501	0.684033	2.165851	2.157359	3.904517	2.987835
11.65791	37.29684	65.94551	1.378362	32.68313	24.2842	32.31123	23.16622	33.4985
0.371299	0.885137	1.087345	1.498167	0.63754	0.430281	0.999502	0.860924	1.099762
0.001962	0.074322	0.015036	0.22303	0.00132	1.44E-05	0.028232	0.068932	0.024372
0.581111	2.114437	0.937655	2.308203	0.481602	0.441263	1.149322	0.299199	0.609177
0.001962	0.164877	6.475505	0.041501	2.438831	3.307186	2.425801	2.89E-05	0.000188
0.001962	0.849841	0.851234	0.041501	0	0.899055	0.543665	2.89E-05	0.070209
13.83359	7.977958	22.32214	6.107114	25.48179	11.719	18.91854	10.33476	12.16681
9.771833	18.19595	16.5625	0.041501	7.854069	22.8563	15.77678	11.544	20.68746
0.087677	0.749942	0.306176	0.041501	0.481602	0.164877	0.917994	0.607605	0.791854
0.91575	6.135007	17.78522	0.041501	12.36443	34.84299	29.18885	1.019791	0.000188
0.91575	2.312085	3.497085	0.041501	0	24.98218	1.135137	2.209141	0.000188
3.533211	2.518124	2.863211	18.55613	2.964762	1.499214	5.485685	0.774257	3.330909
0.196955	2.673846	0.000216	6.922771	0.573326	0.288745	0.684401	1.369928	0.303896
0.182482	0.510072	0.111183	1.498167	1.550837	0.122287	0.137201	0.24184	0.209048
5.84689	3.877417	3.68381	0.041501	2.765758	4.715599	12.70768	3.625382	4.058514
0.335599	1.1407	1.889711	2.099524	1.460051	0.309877	1.036558	0.796234	2.600014
1.188139	0.392944	1.030022	0.041501	1.103261	0.910339	0.779668	0.756724	0.65132
0.426118	0.364242	0.661861	0.281068	0.771176	0.602554	0.137201	0.653968	1.47018
23.275	6.335664	23.6012	0.041501	19.06893	13.91714	14.29947	15.41187	9.316241
6.601833	1.3829	4.419062	0.041501	8.994791	3.629177	7.037345	6.268312	4.821176
1.876212	0.401724	1.198701	0.041501	1.429365	1.948918	1.679596	2.07658	1.240981
3.714906	21.54823	20.51271	0.041501	1.449062	2.019618	9.256537	2.89E-05	0.251775
0.469004	0.263853	0.729062	0.488167	0.169394	1.25886	0.340245	0.537229	0.191133
3.962179	0.60886	0.82355	0.041501	1.615281	5.830152	1.173535	4.833398	3.065108
1.909408	0.749942	1.489524	0.281068	1.798535	0.871429	2.438831	1.568564	3.711097
0.001962	0.140657	0.220657	0.609177	0.180231	0.049149	0.07013	2.89E-05	0.112886
0.688182	1.044235	0.65202	0.488167	0.35627	1.181342	1.007244	1.693939	1.486551
0.745613	1.660216	0.873088	1.668369	0.893528	0.657114	0.364242	0.943514	1.76873
25.73165	5.165599	15.61077	0.527518	21.74051	3.734762	18.48514	4.524481	13.38418
1.132857	0.000188	0.148124	12.49452	0.270289	0.032367	0.000952	0.143838	0.000188
0.701818	1.244242	0.897489	9.646284	2.016515	1.369928	0.633146	0.693571	0.661861
0.81544	1.3829	1.835743	0.041501	0.609841	1.198701	1.780693	1.154567	0.576638
5.704646	0.559632	9.141299	0.041501	5.538001	28.23632	8.477662	25.86296	11.544
0.359502	0.263853	0.182929	0.22303	0.128687	0.645916	0.659582	0.662612	0.281364
2.194257	0.675115	2.447294	0.041501	1.737294	2.834084	1.337431	1.666089	0.697085
0.001962	0.016681	0.000216	0.200519	0	0.022814	0.000952	0.002821	0.01241
0.078124	1.075411	0.453709	0.527518	0.63114	0.131768	0.491587	0.269141	0.528196
2.194257	1.190491	0.483608	0.041501	1.290981	4.06303	0.447965	3.310758	2.845036
0.631494	1.044235	0.811861	9.160382	0.233434	0.452266	1.105426	0.543939	0.377085
0.168312	4.789596	1.648066	1.318384	2.438831	2.035678	3.663622	3.8121	4.338629
0.001962	0.064848	0.000216	0.281068	0.032576	1.44E-05	0.000952	0.00987	0.052208
21.36892	18.05488	252.4251	0.041501	111.5439	332.0776	31.55417	27.66779	9.924372
1.333781	2.404618	5.137042	0.31171	3.422237	2.31811	2.981962	1.728615	1.702706
0.108196	0.01189	0.164618	0.567071	0.21632	0.014141	0.096811	0.005613	0.070209
5.923838	7.558139	4.003117	77.74812	5.402511	3.435988	8.319019	5.447114	8.381328
1.557482	4.770231	4.529913	0.041501	1.620058	6.978903	2.126349	9.838658	1.335743
28.23632	18.62711	31.92385	0.041501	39.53687	22.52375	34.38341	28.54786	34.84299
0.371299	0.687807	0.058579	11.14619	0	0.014141	0.000952	0.275873	0.589618

0.001962	0.023492	0.111183	0.252619	0	0.041183	0.044113	0.068932	0.070209
0.395152	0.991631	0.453709	3.975498	1.256558	0.251248	1.229553	1.471198	2.461905
1.718918	5.754935	4.690159	2.243911	11.719	0.634531	3.606804	0.530253	0.854733
0.001962	0.175361	0.077316	2.895599	0	1.44E-05	0.096811	0.796234	0.409098
0.068896	0.63754	0.058579	0.041501	0.163276	0.049149	0.000952	0.04557	0.209048
15.88548	16.34294	77.74812	0.041501	24.73724	17.51961	72.69473	12.16681	14.25954
4.222583	3.373983	1.074827	0.041501	5.366335	3.06803	2.500548	1.436623	5.082063
0.211169	1.734949	0.229791	13.38418	0.886775	0.148557	0.157619	0.452965	0.420498
3.163506	4.193232	3.599329	1.05153	5.044113	6.454416	8.905642	10.57984	7.037345
2.210866	0.33969	1.069033	3.820758	1.212792	1.524776	0.85728	1.040411	1.335743
24.04374	15.18102	3.78798	48.53429	5.622872	12.10289	6.486104	14.79742	24.98218
0.866147	5.330519	1.855108	1.980592	2.162381	1.539062	5.110159	0.441263	0.281364
0.078124	0.164877	0.091133	1.437648	0.088788	0.022814	0.193896	0.00987	0.133045
0.196955	0.091616	0.000216	0.3414	0.00267	1.44E-05	0.07013	0.00987	0.000188
0.416782	1.330029	0.980505	3.632973	0.468384	0.602554	0.98956	0.860924	1.240981
7.276645	14.6058	4.783001	11.77567	14.79742	12.42965	36.26061	15.77678	14.79742
0.701818	3.816551	0.164618	1.919661	0.200382	0.112641	0.471313	0.502843	0.903074
1.244957	0.470512	0.627633	6.355224	2.104358	0.885664	0.746551	2.750375	4.203001
1.61088	0.800512	1.128131	0.281068	0.820931	0.782417	2.190866	0.398355	0.715462
5.796421	3.975498	4.575123	18.55613	10.38202	2.287576	4.535354	3.464315	6.808369
1.188139	118.7028	33.93939	0.041501	34.38341	14.33939	126.1556	1.133622	0.802756
0.264048	0.749942	0.111183	3.899993	0.279452	0.223701	0.07013	0.24184	0.409098
0.001962	0.029221	0.032496	0.041501	0	1.44E-05	0.000952	2.89E-05	0.000188
2.323983	1.108276	0.78526	3.394286	4.552092	0.743283	1.240202	2.263377	2.112843
0.001962	0.064848	0.041558	0.041501	0.023442	0.049149	0.000952	0.036782	0.079531
0.001962	0.687807	0.032496	1.104033	0.018492	0.022814	0.281169	0.322395	0.420498
16.34294	91.05541	26.25557	15.3189	16.09482	21.19547	57.84882	31.92385	41.47925
0.349084	0.074322	0.281068	2.500548	0.25197	0.370657	0.332395	0.3514	0.465079
0.001962	0.101104	0.022814	0.3414	0.009502	0.002698	0.03526	0.002821	0.030837
1.273636	0.000188	0.970007	0.041501	2.257749	2.373824	1.219019	0.971205	0.877222
6.570433	11.65791	0.000216	28.87506	0	1.44E-05	0.000952	0.88316	10.62739
3.125844	0.000188	2.380079	1.378362	2.828918	1.697792	4.965801	0.378066	2.365685
6.808369	1.175094	3.807648	0.041501	0	9.297085	7.570693	9.625253	3.820758
0.480548	0.109466	0.349278	0.041501	0.291861	0.381061	0.447965	0.618658	0.609177
2.02956	1.244242	6.135007	0.041501	2.410996	0.645916	3.314329	0.642698	2.185556
3.446732	1.030678	0.619365	6.181652	0.149654	0.200382	0.193896	0.662612	2.671299
0.939408	3.482785	0.360462	0.041501	0.481602	0.675938	0.899055	3.110447	2.402468
0.001962	0.458701	0.041558	2.597792	0.117843	1.44E-05	0.157619	0.117446	0.000188
8.288874	2.229048	1.489524	12.32958	1.803918	1.480541	2.190866	1.776739	3.095166
1.548723	0.761688	0.833975	3.771349	0.538773	1.198701	0.828167	0.910823	0.93267
0.211169	1.030678	0.129553	5.395584	0.66526	0.276429	0.202641	0.261342	0.556753
4.990794	2.434444	2.758088	0.956061	8.849697	5.622872	2.802828	7.037345	1.335743
0.078124	0.130029	0.000216	0.041501	0.009502	0.007374	0.087879	0.109185	0.070209
8.797619	7.911934	4.702468	0.041501	11.96574	16.5625	6.30671	42.08807	8.412367
0.078124	0.04557	0.041558	0.22303	0.005729	0.565137	0.028232	0.24184	0.030837
3.002576	4.468009	2.468117	0.041501	2.181775	1.557482	3.8121	3.025671	1.070115
1.934271	1.845519	1.315613	1.980592	3.040505	1.732468	1.419278	1.384921	2.638139
4.058514	0.582973	3.68381	0.041501	3.008225	3.188918	1.072056	2.447294	1.579798
0.196955	0.263853	0.138283	1.204957	0.328268	0.087403	0.061962	0.117446	0.386378
5.821804	0.225974	4.272165	0.041501	2.045476	0.420498	3.037626	1.416407	1.817828
0.688182	3.625382	2.574733	8.048095	2.876421	1.396494	3.78798	1.253752	2.185556
8.608153	7.468961	8.660281	0.041501	6.30671	12.22798	12.56693	12.70768	9.377345
7.417359	18.33909	10.1459	4.857403	15.98892	33.93939	11.09133	11.09133	6.967547
0.001962	3.8379	0.376407	0.041501	0.897006	1.480541	1.357201	2.463911	2.477027
0.371299	0.824416	0.436017	3.632973	1.031284	0.420498	0.301241	0.492165	0.465079
0.078124	1.061457	0.120433	1.05153	0.163276	0.139986	0.147706	0.36153	0.133045
2.752893	1.763889	4.614618	2.964762	1.153723	6.355224	11.86763	2.831501	0.270837
2.461905	1.945916	2.720996	0.041501	2.445079	1.872049	4.165137	2.429957	1.71518

8.134084	5.84689	7.737807	2.308203	3.574387	5.762965	2.939141	5.737056	7.315512
1.068348	1.677042	0.4971	3.975498	0.532619	0.23303	0.383889	0.7257	1.322092
3.816551	5.088694	3.204632	5.530274	4.857403	1.411861	1.197922	1.972706	2.941544
2.250462	0.761688	0.504416	4.125786	0.820931	1.99899	1.471198	1.776739	1.76873
1.391494	1.108276	0.980505	0.041501	1.05824	1.455988	2.408882	0.98956	2.185556
1.679596	0.064848	0.589618	0.041501	0	2.242092	0.229791	1.040411	1.834423
10.82954	2.477027	30.49289	2.597792	19.68088	30.82277	22.96463	12.36443	10.82954
1.447994	11.09133	9.063983	0.041501	2.676392	4.007583	7.710361	5.959033	5.591717
2.312085	3.944084	6.191515	0.252619	5.337547	2.219863	4.592273	2.363442	4.227302
4.445851	0.197338	0.272951	0.041501	0.820931	38.94854	0.229791	29.82828	13.23022
0.875101	2.068297	1.220628	4.535354	3.877417	0.686299	1.072056	0.874603	1.159913
1.948918	0.401724	1.76873	0.041501	1.50684	1.810664	0.23873	2.280101	2.242092
1.602619	2.349351	3.253175	0.041501	0.893528	0.899055	3.718716	1.056263	0.966825
0.23246	0.130029	0.717403	0.31171	0.228918	1.600238	0.424228	6.653016	0.357727
1.150635	0.064848	0.220657	0.956061	0.707475	0.399545	0.332395	0.569293	0.761688
0.196955	0.299336	0.164618	3.632973	0.713405	0.309877	0.391169	0.560325	0.200794
0.001962	0.392944	0.000216	1.006025	0	0.148557	0.104495	1.085967	0.889019
1.012114	0.662237	0.466335	0.846342	1.597908	0.769019	0.853218	0.662612	1.486551
0.264048	1.030678	0.220657	5.950664	0.361717	0.805238	0.439524	0.342215	0.697085
0.884654	3.043528	0.671371	0.041501	0.4971	0.634531	0.899055	0.910823	1.702706
1.177994	0.175361	0.426392	0.041501	0.624163	4.650007	0.954141	1.085967	0.191133
1.535722	5.238701	3.636789	0.041501	1.50026	1.960945	4.012049	2.280101	2.961768
0.595022	0.559632	2.371833	0.609177	0.809481	0.490079	1.316544	0.88316	1.43039
0.048622	0.263853	0.182929	0.405786	0.12267	0.014141	0.104495	0.028232	0.160851
2.975974	5.679502	12.63773	0.041501	17.28195	11.96574	4.145209	8.319019	0.465079
9.771833	5.515584	5.095325	0.252619	13.38418	8.164531	8.508514	9.377345	13.30906
0.688182	1.215195	1.786306	0.041501	1.389509	0.506003	0.317229	0.378066	0.818066
1.024127	2.404618	3.214545	2.243911	2.792035	4.125786	4.338629	4.626025	4.402973
0.001962	0.63754	0.000216	0.041501	0.159639	1.44E-05	0.000952	0.430281	0.863694
0.802756	0.000188	0.473853	0.041501	0.037374	0.546854	0.18324	0.831472	0.293276
2.08632	1.845519	2.644885	0.041501	3.330909	2.477027	1.980592	2.5271	5.770996
8.319019	4.863579	7.456118	0.041501	2.136335	5.687879	5.309264	6.404921	4.261342
9.667316	1.34513	1.182771	6.181652	15.88548	1.863506	3.35715	2.676392	5.030866
0.273261	0.328543	0.211501	3.471486	0.107395	0.182929	0.247749	0.095029	0.089062
0.533874	0.119141	0.51039	0.609177	0.35627	0.069553	0.324502	0.171912	0.096905
0.437309	0.773276	0.360462	7.724084	0.322973	0.223701	0.787136	0.223485	0.877222
2.112843	2.210866	2.705916	0.041501	1.952027	0.515238	0.83697	0.899567	0.721335
9.583463	3.45404	6.287561	0.405786	7.162698	4.752121	5.454949	3.695815	4.546443
0.001962	0.064848	0.000216	0.488167	0.009502	1.44E-05	0.07013	0.028232	0.000188
0.120823	0.082727	0.173723	0.22303	0.042929	0.032367	0.028232	0.078211	0.070209
3.659668	3.747035	3.718716	5.950664	3.414574	1.336587	2.021068	0.492165	4.38684
1.632648	3.55583	1.533535	3.310758	2.449509	1.491977	2.210866	2.169509	4.857403
18.91854	2.367763	4.984488	32.49718	11.03843	2.8657	13.08214	6.325945	48.53429
0.437309	0.175361	0.000216	0.742872	0	0.112641	0.124343	2.89E-05	0.576638
0.097258	0.091616	0.058579	0.31171	0.445996	0.049149	0.113001	2.89E-05	0.089062
5.622872	0.510072	2.248896	0.797114	1.087345	3.273045	0.301241	2.939141	5.84689
9.707677	8.288874	8.923117	0.041501	3.314329	4.419062	23.82354	7.824473	5.34474
2.781558	2.229048	4.23202	0.041501	4.203001	1.805166	6.071436	1.693939	2.027951
0.001962	0.082727	0.000216	0.281068	0.00267	1.44E-05	0.052915	0.04557	0.112886
0.068896	0.036869	0.068449	0.527518	0.009502	0.002698	0.028232	0.00987	0.079531
1.034315	1.594675	3.497085	0.700058	3.381255	2.236407	2.296732	2.703485	2.042201
4.145209	0.328543	5.316378	0.041501	3.113478	15.276	5.538001	18.69833	4.484892
0.335599	0.225974	0.120433	1.620058	0.228918	0.885664	0.087879	1.369928	0.386378
3.621941	6.560411	5.47	0.041501	3.679986	3.163506	4.827691	0.98956	2.535974
3.738997	0.54925	1.860491	0.041501	0.842641	0.058975	0.935743	0.068932	0.589618
0.335599	0.977994	1.210447	8.694697	0.957273	1.548723	1.921212	1.644416	0.903074
0.283882	0.875599	0.581378	1.86658	0.333636	1.44E-05	0.639892	0.306602	0.357727
2.145137	1.482626	0.120433	20.08201	0.228918	0.200382	0.137201	0.306602	3.629177

3.57057	1.283059	2.939141	1.318384	4.338629	2.394416	1.797172	1.410902	3.110447
0.048622	0.299336	0.015036	0.567071	0.027965	0.032367	0.03526	0.398355	0.191133
0.001962	1.799899	0.446551	0.041501	0.580123	0.215115	0.908247	0.642698	0.730476
1.622006	1.075411	0.328737	0.041501	0.73961	5.259437	2.842193	4.029654	0.50772
1.291825	0.299336	1.344257	0.041501	1.183478	1.396494	0.595714	2.07658	0.449408
4.110779	4.145209	3.869163	0.041501	2.194257	4.667518	8.075772	4.227302	1.216768
0.154848	0.447359	0.091133	0.567071	0.098211	0.164877	0.000952	0.569293	0.482706
1.679596	4.524481	4.217569	0.041501	0.998312	7.327482	3.730808	5.210317	4.317648
2.210866	1.283059	0.873088	0.405786	2.607035	1.437648	2.008312	0.386854	0.536905
20.16541	10.38202	15.18102	0.041501	11.65791	20.16541	17.39935	10.93101	19.37139
1.123016	0.250115	0.644618	0.372078	1.175094	1.296905	0.357727	0.582208	0.209048
1.273636	0.208867	0.793434	0.041501	1.853723	3.574387	0.886775	1.333781	2.638139
15.98892	9.031082	16.68554	5.614805	22.32214	12.16681	10.82954	3.35355	5.25254
0.001962	0.016681	0.164618	0.22303	0.032576	0.002698	0.000952	0.00987	0.038182
0.371299	0.447359	3.324452	0.041501	1.05417	2.535974	0.281169	1.290065	0.311876
12.10289	40.7932	15.07257	0.041501	8.134084	26.25557	22.74797	32.31123	23.38378
0.791371	1.317475	0.000216	0.041501	0.939408	3.767489	0.301241	1.436623	2.136335
1.738456	0.237359	1.182771	0.041501	1.046703	0.370657	0.247749	0.933874	1.082179
47.5018	14.00414	27.10502	0.567071	0.81544	8.677489	28.87506	5.42381	8.573434
0.29531	0.352222	0.000216	0.609177	0	0.112641	0.124343	0.409899	0.781977
3.691724	0.800512	2.089639	0.956061	2.758088	1.746724	2.327857	1.559675	2.690823
1.718918	1.579798	0.961486	14.84163	2.147013	1.491977	1.666089	1.693939	4.130794
6.674286	0.875599	4.376118	2.171429	0.554026	4.125786	27.10502	5.359134	1.545657
0.211169	0.082727	0.129553	0.527518	0.195253	0.686299	0.574993	0.715462	0.377085
4.592273	1.004156	2.169509	0.896522	5.576219	2.361198	1.290065	2.022778	1.776739
0.001962	1.359177	0.961486	1.378362	0.50469	1.463304	1.290065	1.243442	1.070115
0.143838	0.687807	0.272951	0.567071	0.205382	0.657114	0.49974	1.449062	0.357727
4.661717	5.309264	20.25302	1.25816	7.824473	5.638759	15.56131	5.591717	24.04374
0.855267	0.948745	0.811861	0.896522	0.713405	1.87899	2.417424	2.221775	2.425801
0.81544	1.482626	1.210447	14.29947	1.108947	1.585296	2.068297	1.762713	3.249877
0.078124	0.237359	0.058579	0.405786	0.113146	0.002698	0.157619	0.068932	0.179798
0.834416	0.000188	0.418326	0.041501	0.601623	1.44E-05	0.000952	0.059582	2.101075
1.986681	1.297763	1.91254	2.421681	2.690823	1.732468	2.339697	1.462143	3.330909
1.706219	1.34513	0.683232	3.166659	0.904856	1.094978	0.737403	0.551861	1.47018
0.91575	3.169812	0.970007	0.896522	2.365685	0.546854	2.31811	1.197922	1.889711
5.889798	2.229048	3.497085	3.025671	2.474834	2.304452	4.261342	1.737294	1.87482
0.001962	0.185909	2.207417	0.041501	0.279452	1.060188	0.265014	2.89E-05	0.260938
0.855267	111.5439	17.92188	82.0704	3.008225	53.77619	25.48179	126.1556	47.5018
3.040505	1.227749	0.690707	3.310758	1.196508	1.354567	0.908247	1.122359	2.242092
0.320563	0.237359	0.111183	0.041501	0.107395	0.148557	0.07013	0.1986	0.697085
0.068896	0.33969	0.000216	0.446291	0.102662	0.032367	0.087879	0.342215	0.160851
0.469004	1.845519	2.005253	0.041501	0.809481	1.515296	4.473348	0.874603	1.322092
3.387864	6.39474	1.604827	3.025671	1.584105	2.126349	2.786898	2.818312	6.054242
1.068348	1.482626	0.862561	0.041501	0.854242	0.842193	1.135137	0.371075	1.486551
0.457749	0.458701	0.742872	4.535354	1.788874	0.215115	0.439524	1.521371	0.810967
23.6012	7.417359	24.73724	26.95429	15.3618	19.53061	14.79742	12.93651	27.10502
1.948918	0.812771	4.519048	0.041501	2.586926	1.639639	2.768312	1.608427	3.548153
0.349084	0.197338	0.306176	0.446291	0.143709	0.36153	0.471313	0.831472	0.440029
1.92272	2.730072	2.659214	0.896522	2.703485	1.411861	3.799885	1.716364	3.50425
3.636789	0.436017	1.422316	1.437648	1.277056	1.667273	1.505678	1.154567	1.274481
0.701818	0.861934	3.618499	0.041501	0.319026	2.083261	1.396494	2.042201	0.377085
2.939141	0.675115	0.551573	0.041501	2.737944	2.29873	0.535657	2.447294	1.106825
0.048622	0.036869	0.032496	0.3414	0.042929	0.041183	0.052915	0.005613	0.024372
6.00228	4.952915	8.210938	0.488167	6.888297	2.373824	5.80469	2.561162	2.554069
7.276645	10.31025	14.70188	0.3414	7.598312	12.70768	4.702468	11.4544	12.29473
2.922778	0.594646	2.893001	5.01772	2.509177	1.073543	1.364401	1.344257	4.513622
0.533874	0.392944	0.248528	0.956061	0.233434	0.215115	0.726811	0.214401	0.684805
0.001962	0.299336	0.068449	2.243911	0.297807	0.032367	0.000952	0.036782	0.243449

1.132857	3.169812	0.77925	6.454416	1.389509	0.342814	1.135137	0.502843	1.062092
0.120823	0.424538	0.211501	1.25816	0.205382	0.139986	0.210505	0.005613	0.052208
0.384156	2.201587	2.427792	0.041501	1.666089	1.05088	6.230245	0.1986	0.409098
2.81342	2.388348	2.74298	0.041501	1.798535	6.978903	2.465916	2.676392	0.000188
1.591299	0.63754	0.192929	0.041501	0.829192	1.316544	1.440736	0.839365	2.509177
0.168312	0.412453	0.192929	0.252619	0.399545	0.112641	0.23873	0.232734	0.160851
0.426118	2.477027	0.906126	0.22303	0.614935	1.504517	1.149322	2.289286	0.640606
1.215195	0.152749	0.980505	0.041501	1.026825	0.721732	0.726811	0.537229	0.465079
0.623499	0.991631	1.220628	1.006025	0.239408	3.33728	1.479452	1.356364	1.386681
1.391494	0.000188	1.666089	0.041501	6.171789	0.191804	3.214545	0.764704	0.000188
3.726854	6.268312	4.814661	6.922771	5.889798	8.134084	3.128694	5.923838	8.780115
0.001962	0.064848	0.058579	6.181652	0.200382	1.44E-05	0.03526	0.00987	0.038182
2.382035	7.288788	3.074134	2.037114	2.008312	0.796674	1.828781	2.351299	3.820758
3.514834	7.225469	6.508427	0.041501	4.776407	5.568391	7.545584	6.55039	7.363117
1.753341	3.482785	1.438672	39.53687	1.816436	1.163579	3.128694	1.122359	3.330909
5.030866	6.560411	9.625253	0.041501	12.10289	13.60866	1.036558	9.082828	5.266335
3.533211	14.51648	2.847547	135.5245	1.810664	6.268312	0.528571	4.952915	29.49693
0.120823	2.312085	0.338095	2.964762	0.440548	0.164877	1.179885	0.693571	0.730476
1.142345	3.625382	24.04374	0.041501	3.581688	11.30987	7.653391	7.724084	3.13456
224.9555	87331.38	224.9555	3.820758	252.4251	749.7839	1360.293	87331.38	749.7839
1.012114	0.910823	2.257749	4.857403	2.339697	2.477027	1.905094	1.122359	1.037316
0.001962	0.04557	0.041558	0.31171	0.027965	0.007374	0.000952	0.420289	0.024372
5.583968	0.364242	0.627633	15.77678	0.676659	2.083261	0.65132	2.996869	8.445743
13.75978	36.26061	37.81919	1.810664	47.5018	11.89932	19.06893	7.264502	15.3618
0.273261	0.749942	0.360462	3.714906	0.297807	0.095433	0.166681	0.847908	1.267085
16.22108	7.38847	6.760765	1.05153	6.268312	8.711905	8.226219	6.345382	17.78522
1.808023	22.74797	0.635036	1.668369	0.713405	28.54786	0.317229	21.93452	4.048499
7.625209	0.737403	6.250447	0.041501	1.584105	3.536955	4.667518	2.24733	4.46267
3.243225	0.436017	2.327857	0.041501	2.820974	1.380195	2.281926	0.474214	0.349084
3.092229	4.614618	2.057056	5.395584	3.869163	2.126349	1.085224	2.661587	4.984488
0.060173	1.45912	0.173723	2.831501	0.489509	0.215115	0.551861	0.642698	0.833045
9.012937	9.121486	5.123781	16.34294	5.788153	7.288788	8.711905	6.853608	7.112496
0.001962	0.119141	0.000216	2.597792	0.058615	1.44E-05	0.000952	2.89E-05	0.303896
1.273636	0.737403	0.295671	0.742872	1.374329	2.882035	1.240202	2.652107	1.309084
72.69473	11.89932	14.79742	1.563045	30.17192	27.10502	28.23632	64.12971	65.94551
0.307027	4.72798	2.922778	10.62739	1.245671	1.210447	2.666494	1.716364	1.880375
0.060173	0.023492	0.032496	0.372078	0	1.44E-05	0.000952	2.89E-05	0.000188
3.799885	5.462475	11.48368	15.9372	12.67271	2.451645	11.03843	2.773644	9.625253
1.948918	10.07949	5.062626	14.42661	5.165599	1.775325	1.741414	0.95101	1.216768
5.638759	6.819481	5.821804	0.372078	3.618499	0.625209	6.464906	6.071436	8.303947
3.201457	4.586753	8.348817	1.104033	7.264502	1.886335	4.667518	2.755411	5.754935
0.039509	0.724502	0.022814	0.567071	0.018492	0.156407	0.07013	0.582208	0.216602
0.492958	2.794935	1.50026	1.204957	1.196508	1.198701	2.531515	0.734935	1.322092
0.221962	0.559632	0.120433	0.446291	0.169394	0.112641	0.398355	0.299199	0.409098
0.607937	0.572266	0.349278	0.31171	0.274877	0.333636	0.340245	0.596111	1.091443
0.078124	0.101104	0.058579	0.797114	0.072583	0.069553	0.000952	0.016104	0.200794
22.32214	1.097677	5.638759	0.372078	11.30987	1.163579	2.449509	1.857677	12.22798
1.244957	1.018427	3.750837	0.041501	1.584105	1.130794	2.876421	0.530253	3.037626
4.833398	1.307417	8.994791	0.041501	7.288788	2.6143	9.031082	5.050267	9.418196
1.679596	0.54925	7.519841	0.041501	5.754935	3.33728	3.574387	8.164531	3.432388
2.633232	1.511198	0.473853	0.041501	1.05824	9.218514	1.872049	19.99861	4.155339
13.91714	20.16541	10.1028	0.041501	5.671126	21.36892	11.96574	28.87506	22.52375
2.834084	0.33969	2.31811	0.041501	8.608153	0.452266	1.780693	0.933874	1.187229
0.581111	0.447359	0.369336	9.397771	0.422511	0.410079	0.482706	0.134517	0.598456
1.034315	0.761688	1.489524	0.041501	1.146962	0.721732	1.717641	0.683232	0.916869
0.91575	4.921082	1.563045	9.52197	1.938759	0.46153	2.513752	1.462143	0.916869
1.323975	0.197338	0.306176	1.318384	0.274877	0.322395	0.210505	0.502843	2.164113
0.349084	0.164877	0.000216	32.49718	0.434863	0.032367	0.096811	0.322395	0.000188

0.73583	3.944084	0.82355	4.043846	3.397706	0.666097	0.872013	1.133622	0.697085
5.416623	1.924228	4.397792	10.53159	8.089848	32.68313	11.544	13.75978	6.38456
8.381328	9.502294	11.96574	0.405786	16.5625	4.207778	9.335397	2.119596	14.17388
2.145137	2.114437	0.619365	23.38378	9.418196	0.712929	1.471198	0.980505	2.257749
1.763889	4.49671	2.289286	0.041501	3.55961	2.730072	4.535354	2.725498	3.037626
0.756724	0.197338	0.31557	0.372078	0.418326	0.532857	0.229791	0.980505	0.545426
3.659668	2.181775	0.811861	0.252619	3.435988	0.743283	1.419278	0.922879	1.082179
0.001962	0.447359	0.068449	1.734949	0.175144	0.007374	0.000952	2.89E-05	0.038182
0.23246	0.63754	0.138283	0.896522	0	0.174372	0.174098	0.232734	0.000188
3.679986	3.35715	3.78798	0.896522	2.895599	9.838658	6.071436	9.335397	2.671299
0.939408	0.401724	0.192929	2.597792	0.474574	0.191804	0.265014	0.190224	0.966825
0.001962	0.286681	0.000216	0.041501	0	1.44E-05	0.000952	0.087165	0.330289
1.323975	0.485577	0.862561	1.104033	1.382006	0.410079	1.938759	0.275873	1.450693
3.125844	0.319163	0.589618	0.041501	1.634935	1.05088	1.062092	0.749481	2.31013
0.283882	0.04557	0.256046	0.281068	0.285844	0.049149	0.210505	0.059582	0.160851
2.752893	3.169812	2.633232	1.980592	2.453781	1.629235	2.500548	1.313737	5.522929
0.756724	0.299336	1.89518	0.797114	1.196508	1.834423	1.965657	1.007864	0.761688
9.356371	7.099913	5.606739	12.03267	1.608427	4.251818	7.443276	5.704646	6.933961
1.080303	4.402973	0.402381	4.18456	0.304062	0.032367	0.700058	0.378066	1.006025
1.591299	8.815123	1.238651	29.18885	1.282208	1.568564	3.387864	2.628225	3.521934
0.001962	0.208867	0.000216	0.041501	0	1.44E-05	0.000952	0.059582	0.168961
0.001962	0.000188	0.091133	0.041501	0	0.049149	0.669358	0.059582	0.000188
0.778788	0.948745	0.851234	0.041501	1.441898	0.468694	1.159149	0.206616	0.598456
0.001962	0.800512	0.321385	0.488167	0.274877	0.131768	0.405094	0.117446	0.000188
30.3324	9.583463	8.780115	53.16874	5.591717	6.663651	2.574733	6.071436	15.88548
0.254365	0.485577	0.562439	1.104033	1.626782	0.452266	0.645455	0.483045	0.772157
9.859719	0.65101	2.413146	0.742872	1.094206	5.47	1.085224	6.808369	7.625209
0.001962	0.016681	0.000216	0.200519	0	0.022814	0.000952	0.002821	0.01241
5.720173	2.794935	6.796566	0.3414	6.210375	2.898196	7.683766	3.253175	3.548153
24.50942	15.56131	37.29684	4.376118	23.16622	21.93452	20.68746	19.83727	20.85876
2.740519	0.622496	1.718918	5.195022	2.257749	0.191804	0.398355	0.693571	1.309084
8.103925	18.91854	11.60283	0.041501	10.05618	5.402511	10.23768	5.447114	9.541645
0.048622	0.164877	0.022814	0.657114	0.00267	0.007374	0.104495	0.016104	0.103528
1.738456	1.530159	2.531515	0.527518	1.51215	1.316544	2.993867	0.860924	1.588903
0.437309	0.529971	0.077316	0.041501	0.072583	1.44E-05	0.087879	0.036782	0.661861
12.56693	15.66023	4.025267	6.922771	0.762641	17.39935	6.144055	60.77449	29.82828
0.120823	0.082727	0.032496	1.25816	0.06381	0.014141	0.044113	0.078211	0.045079
0.13347	0.000188	0.000216	0.041501	0	0.087403	0.000952	0.284863	0.143333
11.60283	10.67991	15.46193	1.810664	12.85504	5.9071	17.03919	10.48101	13.6814
0.001962	2.893001	3.860491	1.668369	0.399545	1.25886	2.327857	0.386854	0.567619
6.486104	2.335844	6.922771	0.041501	1.23127	5.123781	5.24562	1.857677	0.000188
4.684502	3.147294	3.711097	1.104033	2.037114	5.855281	5.821804	1.63039	4.888023
2.964762	3.074134	3.137576	1.563045	5.123781	1.600238	3.188918	7.200505	4.655916
2.02956	4.343838	1.636118	2.500548	11.83593	1.667273	2.162381	1.085967	1.099762
4.752121	12.77772	3.394286	37.81919	1.537951	1.568564	2.102626	1.919661	5.493182
8.195657	1.511198	2.419553	5.01772	2.257749	6.900202	3.592063	8.958225	6.153102
13.49926	3.092229	4.419062	0.446291	6.580988	4.188896	8.256465	3.074134	3.953297
20.51271	3.944084	39.53687	5.872886	14.6058	17.92188	33.4985	9.502294	8.888167
0.001962	1.519322	0.516825	1.980592	0.861934	0.112641	1.023528	0.813261	0.518716
0.81544	0.812771	0.562439	0.041501	0.418326	0.954141	0.863189	1.644416	0.640606
3.87329	3.895469	2.496407	3.089293	1.167561	2.178268	3.592063	2.90658	3.300664
0.001962	0.498918	0.101212	3.899993	0.107395	0.309877	0.935743	0.322395	0.810967
0.001962	0.130029	0.022814	0.405786	0.005729	1.44E-05	0.03526	0.059582	0.045079
0.73583	1.262965	0.349278	2.171429	0.63114	0.482367	0.157619	1.776739	2.509177
5.01772	3.860491	8.711905	0.041501	6.116638	4.839105	7.897078	4.739668	5.287857
1.044863	0.485577	3.377619	1.006025	0.322973	3.131544	1.440736	2.831501	2.554069
1.068348	1.579798	0.338095	2.243911	0.784805	0.399545	0.340245	1.177309	1.617489
0.13347	9.179466	0.742872	5.102742	0.032576	1.44E-05	2.417424	2.89E-05	0.200794

0.001962	0.447359	0.000216	0.041501	0	0.131768	0.000952	0.171912	0.000188
4.808658	2.367763	3.514834	0.657114	8.348817	3.50425	3.55583	3.220678	8.849697
0.001962	0.054791	0.022814	0.700058	0.163276	0.014141	0.096811	0.24184	0.191133
1.978954	1.175094	2.187395	0.846342	1.673254	2.730072	4.034423	1.693939	1.817828
1.235346	2.747908	1.274481	1.05153	2.427792	1.47018	0.726811	0.683232	0.609177
6.210375	1.707576	2.644885	3.310758	1.245671	4.101061	3.019848	1.817828	4.246861
9.625253	8.711905	9.880779	0.041501	10.01009	10.48101	13.38418	10.05618	5.889798
0.097258	0.286681	0.111183	6.922771	0.375094	0.069553	0.210505	0.059582	0.160851
0.039509	0.04557	0.022814	1.668369	0.072583	1.44E-05	0.044113	0.002821	0.01715
3.703175	8.333918	5.210317	1.437648	2.289286	5.150693	6.674286	11.36975	7.238023
2.710765	7.737807	2.359264	7.938427	2.08013	2.681169	2.339697	3.989206	3.730808
2.59557	2.577157	3.074134	2.895599	2.531515	2.502619	1.570794	4.165137	4.655916
1.20653	0.470512	0.4971	0.372078	0.658362	1.240981	1.007244	1.281241	0.583766
4.900996	6.911486	6.325945	2.171429	5.195022	1.396494	8.134084	1.369928	2.616573
0.001962	0.837864	0.148124	0.041501	0.117843	0.069553	0.137201	2.89E-05	0.038182
26.66446	1.159149	7.683766	2.171429	4.839105	19.06893	1.105426	7.327482	11.36975
0.243672	0.33969	0.148124	1.25816	0.366912	0.174372	0.07013	0.275873	0.179798
1.291825	2.730072	1.137244	4.789596	0.753449	0.743283	1.828781	0.251126	0.863694
31.92385	34.84299	51.52195	4.043846	42.08807	8.608153	91.05541	13.34662	64.12971
0.320563	0.328543	0.402381	0.700058	0.313521	0.182929	0.23873	0.117446	0.395426
0.960216	0.000188	1.954993	0.041501	1.356364	2.053521	1.396494	0.95101	0.474856
0.001962	1.061457	0.077316	0.446291	0.113146	1.44E-05	0.174098	0.162107	0.179798
2.155447	1.393571	2.823636	0.372078	0.939408	4.007583	2.068297	3.428788	2.003723
4.468009	4.990794	155.7103	1.204957	4.614618	7.570693	80.6518	8.780115	8.033932
5.165599	1.1407	4.440548	0.372078	8.381328	2.361198	6.760765	1.63039	1.607143
0.120823	0.540563	0.242222	13.23022	0.21632	0.410079	0.281169	0.756724	0.133045
0.196955	0.991631	0.120433	0.609177	0.274877	0.523983	0.843564	0.342215	0.4971
3.842554	0.424538	0.833975	0.041501	2.964762	0.782417	0.999502	0.284863	2.018167
2.404618	2.068297	1.041652	2.964762	1.225426	5.259437	1.41368	5.538001	4.952915
0.492958	4.637994	1.40772	5.872886	1.298665	0.70329	1.607143	2.925426	2.750375
3.070952	3.944084	5.515584	0.041501	2.786898	4.776407	3.78798	3.707136	4.246861
0.901732	9.418196	5.062626	1.104033	3.233045	1.578687	6.956349	2.139928	2.061926
0.715108	0.861934	1.119574	4.721789	0.839365	0.645916	0.79487	0.630823	0.833045
13.23022	16.5625	10.8813	0.041501	6.518709	10.38202	12.42965	17.51961	16.5625
9.502294	6.028427	332.0776	4.914863	10.73084	7.737807	30.49289	4.921082	6.99026
0.001962	0.164877	0.091133	0.041501	0	0.014141	0.044113	2.89E-05	0.052208
24.73724	6.508427	13.60866	0.041501	12.67271	13.30906	9.418196	16.44768	24.50942
144.7919	47.5018	530.1465	0.041501	1360.293	1360.293	224.9555	287.197	287.197
0.89404	1.159149	2.238341	6.454416	3.829105	0.657114	1.023528	4.739668	10.5063
1.61088	0.225974	10.35839	0.041501	5.25254	1.336587	1.357201	1.677042	0.122554
1.244957	3.588369	7.037345	0.896522	3.058802	9.751039	5.210317	4.429769	2.554069
10.55571	3.482785	5.195022	2.500548	6.191515	5.323492	3.754719	4.776407	6.200945
48.53429	28.23632	27.66779	0.041501	8.164531	40.11561	23.16622	24.98218	31.92385
10.73084	1.604827	2.296732	0.041501	4.451154	1.05088	3.655714	1.608427	0.93267
0.866147	9.459177	38.94854	0.041501	17.15902	33.4985	59.32013	21.54823	2.267157
15.66023	5.088694	5.42381	26.52537	3.953297	3.33728	5.770996	4.56912	11.96574
1.024127	1.722475	5.295325	0.567071	3.435988	1.986681	2.621133	1.143954	0.697085
3.610707	0.65101	3.636789	0.041501	4.086926	1.746724	7.468961	1.056263	2.831501
0.688182	1.437648	0.65202	2.099524	0.66526	0.342814	0.692799	0.261342	1.037316
0.001962	0.140657	0.000216	0.446291	0.474574	0.032367	0.23873	0.152749	0.000188
11.06488	12.22798	17.51961	3.310758	19.37139	10.7557	13.91714	8.134084	12.63773
14.97762	3.35715	15.98892	0.896522	1.913997	11.544	20.51271	14.17388	4.029654
0.001962	0.352222	0.000216	0.405786	0.023442	1.44E-05	0.147706	0.028232	0.000188
19.53061	2.005253	21.36892	8.539971	23.38378	15.07257	16.09482	8.477662	10.90615
4.609076	1.799899	1.648066	0.041501	6.230245	0.242612	1.455988	0.618658	0.545426
0.521775	0.63754	0.281068	0.405786	0.762641	0.156407	0.349278	0.332049	0.749481
0.243672	1.283059	1.171349	2.676392	1.691587	1.618773	0.917994	1.425613	1.68938
0.068896	0.217496	0.058579	0.896522	0.088788	0.041183	0.174098	0.1986	0.168961

4.72798	2.577157	2.713319	6.297136	9.924372	4.63189	3.885642	6.423911	3.877417
3.188918	1.075411	2.554069	0.742872	1.139885	3.88153	1.867951	3.581688	4.155339
0.048622	0.082727	0.041558	1.05153	0.037374	0.002698	0.000952	0.036782	0.103528
6.64254	3.917597	4.984488	2.037114	3.192229	6.497266	11.19942	5.500678	7.037345
87.1746	24.73724	46.7056	1.86658	26.52537	24.73724	55.04081	27.96248	30.82277
4.715599	0.000188	1.604827	0.041501	0.789863	6.52899	4.802655	2.382035	0.889019
0.23246	0.582973	0.091133	10.19468	0.068514	0.002698	0.202641	0.036782	0.079531
2.59557	2.903867	3.182482	0.041501	0.729582	2.574733	4.424416	4.661717	2.201587
0.001962	0.299336	0.000216	7.162698	0	0.10381	0.000952	0.152749	0.000188
0.060173	0.01189	0.049646	0.22303	0.027965	0.022814	0.137201	0.00987	0.079531
1.092136	1.845519	1.259747	3.025671	1.938759	1.42456	1.316544	2.022778	1.240981
0.29531	0.000188	0.264654	0.041501	0.385238	1.44E-05	0.645455	0.223485	0.365635
0.349084	0.675115	0.757763	9.838658	0.963853	0.022814	0.078831	0.398355	0.474856
2.058773	1.691587	3.394286	0.041501	4.408153	6.876392	1.749307	8.994791	2.31013
1.244957	1.677042	3.418405	0.041501	2.151919	4.546443	4.135801	3.404372	3.548153
0.802756	1.437648	1.156869	1.157626	2.320072	0.490079	1.410902	1.056263	1.006025
9.297085	3.33728	14.51648	0.700058	13.15079	3.792266	9.141299	6.760765	5.71241
0.154848	0.934488	0.446551	0.041501	0.018492	0.139986	0.317229	0.452965	0.4971
15.56131	10.53159	5.545729	0.797114	10.48101	14.79742	8.797619	9.792626	7.584502
2.981962	4.323045	7.08733	1.919661	2.398442	5.110159	4.38684	6.240346	4.353925
0.108196	0.824416	0.256046	2.500548	0.35627	0.309877	0.521775	1.154567	0.409098
14.08569	7.824473	8.477662	0.041501	14.88584	7.653391	8.412367	3.128694	5.110159
0.715108	0.800512	0.360462	0.31171	0.606847	0.743283	1.304877	0.642698	1.159913
0.568665	1.579798	0.697908	0.742872	1.622006	2.987835	2.351299	2.24733	0.191133
0.182482	0.436017	0.041558	1.734949	0.25197	0.10381	0.22039	0.04557	0.089062
0.048622	0.250115	0.000216	1.734949	0	0.007374	0.03526	0.464387	0.556753
0.991631	1.283059	0.683232	2.365685	0.405786	0.058975	1.085224	0.378066	1.634935
0.82588	0.773276	1.797172	0.041501	0.027965	1.568564	0.411436	0.899567	0.889019
0.001962	0.263853	0.022814	0.3414	0.06381	1.44E-05	0.000952	0.016104	0.000188
0.060173	0.064848	0.000216	1.157626	0.107395	1.44E-05	0.113001	0.022266	0.024372
12.26136	4.376118	3.966623	0.041501	3.743232	0.910339	2.31811	0.749481	7.710361
0.196955	0.352222	0.349278	0.446291	0.133658	0.675938	0.124343	1.210447	0.143333
3.107417	3.019848	2.879228	0.700058	2.895599	3.574387	3.439437	3.877417	5.217569
1.431414	1.612056	2.961768	0.527518	2.23267	0.824416	2.678781	0.398355	1.360866
1.391494	0.197338	0.4971	0.252619	2.294733	4.150274	0.521775	1.197922	2.327857
1.092136	12.39704	0.697908	0.041501	0.724004	23.49249	3.754719	1.497143	0.629098
0.001962	0.054791	0.032496	0.742872	0.032576	0.095433	0.000952	0.051595	0.089062
4.519048	4.145209	4.115844	0.488167	4.927302	7.545584	4.18456	8.745238	4.370671
38.39042	15.77678	38.39042	4.959358	20.51271	18.48514	16.34294	20.51271	23.16622
0.001962	1.34513	0.041558	0.041501	0.481602	0.139986	0.332395	0.292251	0.545426
0.087677	0.33969	0.129553	1.104033	0.169394	0.049149	0.22039	0.261342	0.209048
10.43066	30.82277	6.653016	5.779574	6.55039	4.796126	10.1028	7.288788	11.14619
2.511465	2.24562	3.877417	0.041501	1.99899	2.099524	3.321284	2.042201	1.130115
1.150635	0.299336	0.802395	0.041501	0.513752	0.381061	0.999502	0.134517	1.091443
0.623499	0.152749	0.173723	3.544257	0.072583	0.634531	0.157619	0.068932	0.052208
10.93101	1.962424	6.230245	0.041501	2.320072	7.061328	3.144156	9.297085	3.679986
3.493362	9.924372	29.18885	0.041501	6.888297	11.39744	13.60866	3.414574	4.592273
0.001962	13.45939	1.230411	2.243911	0.462612	8.745238	0.229791	2.89E-05	0.000188
26.25557	43.49658	50.55949	4.125786	56.39528	32.31123	65.94551	27.10502	43.49658
1.113304	0.140657	1.797172	0.041501	0.562439	0.565137	2.531515	0.715462	1.47018
0.654877	2.922778	0.101212	4.23202	1.035801	0.122287	0.726811	0.24184	1.533535
0.568665	1.677042	0.944084	6.922771	0.689509	0.613853	0.999502	0.831472	0.556753
91.05541	0.662237	10.43066	1.919661	21.54823	19.22139	12.16681	7.653391	6.268312
1.316544	0.319163	0.57197	0.041501	2.964762	0.93632	1.396494	0.582208	0.349084
2.59557	3.110447	2.257749	0.896522	3.214545	1.568564	4.034423	1.797172	2.124574
0.778788	0.662237	1.017107	7.443276	1.072056	0.200382	0.79487	0.232734	0.661861
0.834416	0.328543	2.08013	0.041501	0.159639	0.223701	1.594675	0.582208	1.037316
0.843564	1.030678	0.418326	2.365685	0.285844	0.007374	0.193896	0.275873	1.386681

0.307027	0.263853	0.541241	0.567071	2.194257	0.420498	0.383889	0.409899	0.420498
749.7839	7.162698	0.032496	1.25816	1.067662	59.32013	9.179466	530.1465	252.4251
0.143838	0.000188	0.049646	1.25816	0.018492	2.066652	0.03526	2.953059	0.583766
0.001962	0.101104	0.022814	0.3414	0.169394	1.44E-05	0.000952	0.152749	0.000188
0.048622	0.458701	0.264654	0.252619	0.185642	0.032367	0.294502	0.269141	0.224921
2.290996	1.371602	2.973059	3.632973	0.951652	2.65684	1.797172	2.092987	0.877222
0.843564	0.540563	1.511198	0.372078	1.012742	0.613853	0.999502	0.784805	0.749481
1.519322	0.498918	0.802395	3.089293	0.405786	0.164877	0.491587	0.214401	1.450693
2.270837	2.993867	2.834084	2.365685	2.351299	1.886335	2.169509	1.883535	2.652107
0.048622	0.119141	0.032496	0.446291	0.00132	1.44E-05	0.000952	0.036782	0.01715
2.511465	3.310758	2.805382	1.104033	2.214372	3.514834	3.070952	4.789596	5.821804
1.20653	0.250115	0.295671	0.041501	0.66526	0.899055	0.256941	0.464387	0.567619
0.221962	4.115844	0.793434	10.01009	1.067662	1.455988	1.304877	2.199639	1.301169
0.13347	0.036869	0.022814	0.609177	0.279452	0.002698	0.03526	0.016104	0.168961
6.240346	1.437648	5.351934	2.099524	7.112496	4.043846	6.52899	2.199639	2.531515
2.127973	0.54925	2.296732	0.041501	2.325931	1.746724	1.672107	1.253752	0.270837
0.154848	1.175094	0.833975	7.724084	0.405786	0.370657	0.814877	0.420289	1.675722
5.20267	11.48368	7.339452	0.405786	6.580988	5.366335	1.947316	5.654863	1.282208
0.264048	0.016681	0.295671	2.597792	0.058615	0.069553	0.405094	0.016104	0.030837
0.001962	0.064848	0.000216	0.446291	0.00267	1.44E-05	0.000952	2.89E-05	0.000188
1.849654	1.371602	2.31404	1.05153	3.450577	2.053521	1.848297	1.449062	1.508831
0.492958	0.54925	0.000216	0.041501	0.451169	0.032367	0.087879	0.087165	0.772157
2.947287	6.695887	8.590794	0.041501	3.679986	3.663622	8.019769	3.074134	3.651898
0.492958	1.018427	0.996111	1.563045	0.796234	0.532857	0.759646	0.171912	0.545426
1.788874	4.563117	8.412367	0.041501	5.068831	5.402511	3.346898	1.540144	1.599033
6.601833	7.363117	16.85982	0.041501	15.46193	9.792626	14.51648	9.418196	8.033932
0.13347	0.04557	0.295671	1.919661	0.098211	0.215115	0.210505	0.152749	0.179798
4.502453	2.62342	2.633232	0.896522	1.834423	4.456912	2.081696	6.900202	6.727633
3.6029	0.529971	1.869322	0.041501	0.939408	3.131544	2.134545	2.042201	2.339697
1.948918	0.63754	1.390476	0.609177	2.950173	1.232078	3.116732	0.831472	2.042201
0.745613	2.026219	0.729062	4.637994	0.886775	0.322395	0.398355	0.292251	1.43039
0.243672	0.374827	0.562439	1.318384	0.322973	0.276429	0.585281	0.386854	0.270837
6.240346	3.432388	1.928629	0.041501	10.98644	9.179466	4.317648	23.6012	4.745895
10.67991	4.770231	17.03919	5.696263	13.79669	8.075772	14.08569	6.760765	5.165599
1.876212	6.230245	18.05488	0.31171	20.85876	16.68554	22.52375	8.815123	3.877417
0.001962	0.091616	0.000216	1.378362	0.005729	1.44E-05	0.000952	1.067662	0.224921
2.013182	0.412453	2.134545	0.3414	1.353608	11.48368	1.498167	19.60574	8.524242
5.330519	11.39744	13.30906	0.041501	10.8813	8.303947	17.64869	6.210375	8.180094
0.715108	0.036869	0.381999	0.281068	0.223391	0.322395	0.981032	0.152749	0.270837
31.1753	16.09482	12.13485	0.896522	33.93939	13.08214	13.6814	14.00414	22.96463
0.641277	1.1407	0.697908	0.567071	1.691587	0.309877	0.872013	0.306602	0.365635
0.068896	0.263853	0.041558	0.041501	0	0.049149	0.124343	0.143838	0.377085
0.182482	0.702381	2.392431	0.041501	0.25197	2.77899	0.645455	4.029654	0.730476
1.273636	4.702468	2.308203	1.25816	3.521934	0.482367	1.732468	1.666089	2.08798
1.692763	3.78798	1.876212	1.204957	4.292078	1.720144	3.249877	3.644343	2.355289
0.29531	0.299336	0.483608	1.318384	0.322973	0.263304	0.309062	0.630823	0.395426
6.443925	1.872049	2.483492	0.527518	8.089848	1.609654	1.913997	1.497143	2.242092
0.001962	0.849841	0.058579	1.498167	0.005729	0.002698	0.061962	0.00987	0.143333
35.776	12.29473	9.924372	1.668369	9.751039	40.7932	2.740519	31.55417	7.339452
0.767085	1.1407	0.504416	2.831501	0.573326	1.143954	1.013434	0.784805	0.456248
0.595022	0.000188	0.091133	4.637994	0.426118	1.44E-05	0.124343	2.89E-05	0.000188
6.054242	31.1753	3.407619	20.60009	11.19942	4.508196	2.047157	4.586753	4.789596
39.53687	16.80343	105.1694	0.041501	15.66023	155.7103	37.29684	43.49658	17.64869
6.335664	17.92188	10.93101	4.125786	9.880779	2.353247	11.60283	2.333867	2.708341
0.273261	1.579798	0.402381	0.041501	0.85728	0.299062	0.061962	0.117446	0.000188
6.717056	1.437648	2.725498	0.041501	3.214545	5.485685	2.640332	2.781558	5.462475
45.91841	8.745238	20.94257	10.78056	18.62711	87.1746	16.68554	59.32013	49.50903
0.001962	0.152749	0.058579	0.372078	0.00267	0.007374	0.07013	0.016104	0.01715

1.132857	0.401724	0.723066	0.896522	0.532619	0.975556	0.659582	1.369928	1.644416
0.001962	0.286681	0.000216	0.31171	0	1.44E-05	0.281169	0.152749	0.000188
2.34355	3.236457	2.939141	0.041501	0.440548	12.03267	1.197922	6.464906	2.941544
0.406912	1.924228	0.697908	0.041501	0.426118	0.032367	0.096811	2.89E-05	0.234214
1.823377	0.352222	1.940231	0.041501	0.73961	5.796421	0.779668	3.799885	1.974228
2.058773	1.482626	2.169509	0.657114	0.375094	3.909033	0.5129	2.925426	0.42925
1.25816	0.702381	1.422316	0.797114	1.103261	2.898196	0.669358	2.561162	1.205685
16.74449	19.22139	59.32013	4.043846	45.1045	31.55417	42.75576	24.73724	16.68554
13.08214	8.539971	11.03843	3.714906	12.42965	25.99427	5.075447	5.923838	12.56693
0.307027	0.800512	0.376407	0.657114	0	0.23303	0.157619	0.206616	0.070209
2.59557	4.990794	5.576219	0.488167	4.721789	17.15902	5.838499	13.34662	2.242092
8.348817	16.68554	2.365685	11.60283	6.036818	4.150274	15.18102	6.036818	11.19942
3.243225	0.594646	2.520404	0.041501	2.882035	1.697792	3.711097	1.405772	2.071587
2.725498	8.048095	1.148564	16.5625	1.079697	1.173535	0.954141	1.03189	2.339697
3.944084	1.227749	3.899993	0.609177	5.095325	7.112496	2.895599	7.897078	3.279625
0.855267	4.174964	0.173723	0.896522	0.25197	0.482367	0.370657	0.464387	0.70886
0.802756	2.162381	6.796566	0.041501	2.375974	0.852684	4.120815	0.314733	1.41368
0.108196	0.572266	0.192929	0.446291	0.068514	1.44E-05	0.000952	0.036782	0.160851
1.99899	0.164877	1.210447	15.18102	3.7586	6.30671	1.169091	10.67991	4.302367
2.953059	6.591544	6.99026	8.048095	7.495397	1.504517	7.854069	2.234538	3.966623
0.641277	0.78619	0.77925	0.446291	1.126075	1.05088	0.287951	0.715462	1.450693
0.283882	1.450693	0.644618	0.797114	1.216768	0.390418	0.574993	1.111241	1.734949
0.243672	0.529971	0.173723	0.567071	0.601623	0.430281	0.603261	1.085967	0.209048
0.060173	0.208867	0.101212	0.3414	0.143709	0.032367	0.113001	0.022266	0.052208
7.212915	0.101104	1.327525	0.446291	0.820931	2.563506	0.301241	1.584105	2.845036
3.548153	1.482626	0.930837	0.041501	1.859084	1.272756	1.888023	1.776739	2.148658
0.001962	0.016681	0.032496	0.31171	0.00132	0.002698	0.000952	0.005613	0.000188
1.823377	7.099913	0.381999	25.99427	0.85728	0.251248	1.304877	1.218182	3.192229
1.61088	1.227749	2.834084	0.488167	1.39847	1.198701	5.967879	1.559675	2.212619
0.834416	4.424416	2.993867	0.3414	6.404921	1.437648	5.130411	1.919661	3.377619
7.570693	20.85876	13.6814	1.563045	6.760765	10.28573	12.85504	10.28573	10.2617
1.323975	1.763889	1.069033	1.318384	0.846342	2.126349	1.479452	1.705007	0.122554
0.273261	0.424538	0.459769	3.544257	0.573326	0.410079	0.411436	0.430281	0.440029
2.181775	0.384394	0.111183	1.006025	0.270289	12.56693	0.18324	17.78522	9.838658
0.492958	0.374827	0.862561	0.797114	0.265455	1.304877	0.779668	0.715462	0.440029
2.429957	2.419553	1.954993	0.281068	7.991602	1.790245	1.265426	1.243442	1.486551
0.395152	0.130029	0.101212	0.567071	0.042929	0.871429	0.193896	0.398355	0.191133
0.521775	5.42381	0.459769	3.025671	0.128687	0.645916	0.700058	1.067662	1.834423
4.043846	11.60283	8.590794	1.05153	4.468009	1.639639	14.42661	3.35355	6.028427
1.177994	1.542302	5.855281	0.446291	3.273045	1.104805	2.465916	1.644416	1.803918
11.30987	10.07949	25.48179	0.041501	13.53913	13.45939	17.98838	12.85504	10.73084
4.302367	0.761688	7.149618	0.041501	0.601623	2.421681	5.366335	0.7257	1.520346
0.631494	1.1407	2.696061	0.041501	0.394877	0.814877	1.905094	0.275873	0.449408
3.592063	4.048499	4.039192	0.041501	2.45772	0.885664	10.78056	0.618658	4.101061
9.218514	8.923117	17.28195	1.157626	9.063983	6.933961	12.10289	5.388405	10.2617
0.701818	2.794935	1.041652	5.47	0.4971	0.390418	0.754315	0.386854	0.945483
7.653391	8.780115	3.083319	8.180094	2.807937	3.179199	7.991602	12.63773	20.34063
0.855267	0.392944	0.410079	0.896522	0.580123	0.309877	0.431457	0.181797	0.209048
0.108196	0.594646	0.264654	1.25816	0.228918	0.002698	0.124343	0.117446	0.234214
20.85876	65.94551	53.77619	0.041501	7.653391	60.77449	25.99427	26.80355	9.316241
1.431414	0.064848	1.119574	0.041501	2.54961	0.975556	1.988196	1.056263	1.076032
2.398442	0.837864	3.005281	0.041501	2.068297	2.987835	1.123795	1.143954	0.863694
0.982698	1.359177	0.077316	3.23987	0.322973	1.44E-05	0.000952	0.181797	0.270837
8.711905	0.374827	1.563045	0.041501	4.490801	1.790245	5.9071	2.312085	15.46193
4.23202	25.48179	15.3618	87331.38	14.17388	10.23768	20.85876	8.412367	11.86763
0.048622	0.04557	0.041558	1.204957	0.077316	0.014141	0.18324	0.016104	0.000188
1.333781	4.402973	2.071587	530.1465	8.256465	1.336587	10.98644	2.058773	1.595729
7.532713	0.662237	1.928629	0.527518	2.750375	7.112496	3.098268	2.763261	3.110447

2.359264	4.72798	2.483492	8.940671	1.570794	1.120332	2.453781	1.666089	2.698557
2.210866	1.215195	1.450693	1.980592	0.313521	8.539971	0.954141	1.51215	0.365635
5.20267	9.667316	3.227085	4.440548	5.402511	2.847547	3.829105	4.272165	6.079596
1.291825	0.824416	0.120433	12.32958	0.285844	0.482367	0.585281	0.582208	1.301169
21.93452	5.821804	5.493182	16.09482	3.783694	37.81919	3.169812	42.75576	12.03267
4.978182	0.208867	0.000216	40.7932	0	0.014141	0.000952	0.028232	0.000188
0.283882	0.237359	0.418326	2.243911	0.233434	0.156407	0.301241	0.04557	0.200794
4.927302	2.201587	7.251263	6.355224	0.904856	21.74051	4.053506	22.96463	8.643074
111.5439	80.6518	67.98069	75.1132	75.1132	18.12541	60.77449	21.19547	77.74812
23.275	37.81919	40.11561	16.74449	57.84882	34.38341	33.07147	18.33909	14.88584
42.75576	27.38726	6.612121	118.7028	9.063983	62.33472	18.76954	80.6518	67.98069
7.061328	45.1045	17.64869	57.84882	6.116638	9.256537	14.70188	7.327482	13.83359
40.7932	75.1132	72.69473	6.250447	49.50903	20.85876	56.39528	15.88548	17.92188
4.661717	13.87537	30.17192	287.197	7.238023	5.337547	12.29473	3.276335	5.143867
530.1465	530.1465	287.197	1.919661	87331.38	530.1465	749.7839	425.62	1360.293
155.7103	167.9996	167.9996	36.74522	60.77449	105.1694	144.7919	118.7028	184.443
105.1694	100.0998	203.5795	9.047532	31.92385	135.5245	203.5795	33.93939	118.7028
95.22939	87.1746	34.84299	203.5795	35.27608	23.16622	34.84299	95.22939	167.9996
5.560563	5.545729	4.115844	8.940671	3.829105	1.618773	4.174964	1.369928	6.876392
1.034315	2.312085	0.611789	3.025671	1.803918	0.885664	1.173535	1.085967	1.817828
3.334199	20.68746	2.758088	105.1694	3.50425	2.502619	3.450577	5.180058	8.524242
6.99026	3.297107	10.05618	0.446291	7.188095	3.909033	6.956349	5.993045	8.923117
0.039509	1.579798	0.120433	0.488167	0.834416	0.131768	0.863189	0.596111	0.661861
0.108196	0.65101	0.138283	0.3414	0.037374	0.156407	0.078831	0.284863	0.096905
2.127973	2.312085	0.944084	9.397771	1.420202	1.25886	2.111299	1.644416	4.592273
0.395152	0.000188	0.173723	0.657114	1.267085	0.223701	0.543665	0.04557	0.209048
2.417424	4.851227	1.353608	15.46193	2.214372	1.285584	1.429365	5.165599	8.089848
0.521775	0.000188	0.295671	332.0776	0.093716	0.998831	0.000952	0.821414	0.000188
7.780786	20.51271	6.423911	13.00996	13.30906	9.667316	12.77772	17.03919	15.07257
4.959358	2.975974	2.169509	0.041501	7.443276	1.765065	1.754733	2.07658	2.349351
1.738456	3.536955	3.204632	5.950664	3.249877	1.181342	0.981032	0.3514	2.164113
0.001962	0.064848	0.000216	0.797114	0.014026	0.007374	0.000952	0.016104	0.024372
0.446811	0.54925	0.953506	25.24367	2.725498	0.576205	0.908247	0.125483	0.4971
2.569957	0.436017	1.636118	0.041501	1.550837	3.321284	0.872013	0.734935	2.451645
22.96463	15.88548	19.06893	6.454416	10.23768	35.776	16.5625	35.776	23.6012
25.24367	57.84882	20.68746	3.166659	40.7932	30.49289	15.07257	21.74051	42.08807
6.125823	12.56693	8.888167	31.36473	8.195657	7.468961	5.933066	8.608153	21.02638
6.738211	5.770996	2.371833	4.23202	2.950173	3.676032	1.607143	2.642525	8.180094
0.971775	1.004156	0.148124	0.609177	0.155224	0.223701	0.398355	0.223485	0.697085
17.39935	19.99861	28.23632	1.498167	17.78522	18.91854	19.22139	10.73084	25.48179
0.469004	1.450693	1.274481	1.104033	0.328268	1.2214	0.560866	1.313737	1.274481
1.012114	1.203427	3.36075	34.38341	5.22482	2.65684	9.946364	2.382035	1.171349
16.44768	41.47925	11.33981	14.84163	14.97762	7.710361	14.88584	14.51648	28.23632
0.82588	0.964416	0.436017	2.421681	5.821804	0.251248	0.726811	0.430281	0.697085
0.437309	1.553074	0.683232	0.567071	0.753449	1.732468	0.491587	1.344257	0.761688
3.014076	5.42381	2.863211	6.454416	2.121198	2.210866	4.241905	2.042201	5.309264
1.188139	1.845519	1.095657	6.107114	1.245671	0.852684	1.486551	0.299199	1.486551
0.23246	0.328543	0.202799	2.597792	0.4971	0.139986	1.540144	0.36153	0.556753
0.426118	0.849841	0.229791	0.527518	0.762641	0.352807	0.521775	0.261342	0.545426
1.226118	1.821861	0.581378	1.006025	0.361717	0.613853	0.535657	0.860924	1.423506
0.182482	1.962424	1.809343	0.041501	1.453939	0.191804	0.609841	0.051595	0.518716
1.474343	5.330519	0.426392	3.632973	1.074827	0.452266	1.780693	2.369841	1.944517
6.335664	1.203427	0.980505	6.663651	0.802756	1.026825	1.036558	2.914632	12.77772
2.781558	9.063983	3.253175	8.288874	3.35355	1.060188	1.062092	2.485534	14.97762
0.615996	0.559632	0.524986	0.041501	2.359264	0.788369	1.471198	0.284863	0.06088
0.182482	0.130029	0.295671	0.797114	0.009502	0.333636	0.157619	0.101782	0.096905
11.77567	10.8813	6.738211	0.527518	6.433918	19.37139	11.25392	16.22108	13.60866
1.344257	0.319163	0.473853	1.668369	0.350455	0.753882	0.899055	0.806227	0.730476

0.001962	0.029221	0.015036	0.657114	0.00267	0.032367	0.028232	0.036782	0.000188
2.145137	1.420202	0.717403	0.567071	0.684033	0.288745	2.24733	0.051595	1.006025
3.046551	7.558139	3.552049	0.041501	2.08798	4.907929	3.625382	5.095325	2.290996
7.08733	135.5245	1.89518	59.32013	126.1556	5.599228	6.784762	5.110159	35.776
6.55039	3.128694	4.764055	0.041501	4.392316	18.62711	4.952915	10.82954	10.38202
0.543939	1.297763	0.862561	4.23202	1.584105	1.354567	0.814877	2.304452	0.833045
4.959358	0.977994	9.968355	0.446291	8.508514	5.671126	10.05618	2.593506	3.014076
2.569957	4.217569	2.730072	7.276645	2.895599	2.329784	3.055729	2.119596	5.366335
0.89404	2.893001	0.873088	20.34063	0.462612	0.694675	1.209105	0.398355	0.761688
0.875101	0.074322	0.000216	0.488167	0.163276	0.087403	0.18324	0.101782	1.119574
16.09482	11.17281	5.821804	55.04081	13.08214	3.864827	3.8121	4.875527	16.39531
135.5245	425.62	1360.293	1.86658	530.1465	167.9996	332.0776	72.69473	203.5795
0.143838	0.082727	0.31557	0.527518	0.088788	1.44E-05	0.737403	0.1986	0.330289
26.66446	62.33472	62.33472	2.099524	55.04081	75.1132	46.7056	50.55949	36.74522
2.02956	4.661717	4.38684	10.19468	4.990794	0.954141	3.236457	1.056263	2.148658
0.960216	1.307417	0.757763	3.166659	0.50469	0.441263	0.779668	0.839365	0.73912
10.19468	8.677489	8.849697	1.25816	7.780786	7.238023	6.443925	6.036818	7.468961

CGGA_114	CGGA_115	CGGA_115	CGGA_115	CGGA_115	CGGA_115	CGGA_115	CGGA_115	CGGA_116
0.128088	0.319762	0.368882	0.458521	0.271912	0.088341	0.55215	0.149019	0.254964
0.212496	0.075772	0.382626	0.277417	0.279315	0.254964	0.446811	0.149019	0.000924
4.965801	9.179466	1.550837	3.767489	7.519841	8.923117	2.011501	6.162446	7.188095
10.23768	7.025599	0.534452	1.391494	3.971061	9.418196	4.297222	0.577071	1.003586
3.188918	2.903867	4.709033	5.195022	6.116638	5.295325	3.160022	4.188896	6.55039
0.578485	0.964416	7.558139	2.306277	2.117814	1.169091	8.075772	1.992944	1.587605
44.27012	32.68313	39.24271	28.23632	59.32013	46.7056	24.2842	43.49658	27.66779
0.067446	0.075772	0.022727	0.114841	0.046962	0.047504	0.009185	0.084906	0.038088
4.592273	1.215981	2.45575	3.147294	3.160022	7.824473	2.011501	2.758088	7.38847
0.121147	0.000736	0.360938	0.225505	7.22E-05	0.000534	5.77E-05	0.000433	0.18324
60.77449	12.49452	56.39528	15.07257	72.69473	26.25557	48.53429	41.47925	19.37139
0.92816	0.319762	6.09759	0.369336	1.68342	0.459048	0.695051	0.55645	0.493586
28.54786	35.27608	14.08569	45.1045	24.2842	26.52537	23.82354	8.994791	23.38378
7.991602	25.48179	0.000115	9.792626	1.692763	3.953297	1.860491	13.26964	20.16541
0.014906	0.034603	0.164993	0.125354	0.011631	0.000534	5.77E-05	0.065974	0.057994
36.26061	6.967547	6.443925	6.622092	5.447114	8.226219	1.846941	11.89932	12.85504
1.716364	0.420707	6.011515	0.658362	2.203535	0.514141	1.021097	0.642395	0.293882
0.184055	0.064488	0.000115	0.084113	0.250115	0.000534	0.155945	0.000433	0.000924
3.676032	1.91254	0.683232	7.911934	1.465123	0.954776	2.718434	0.776876	2.630729
1.948918	0.274199	3.855952	0.509791	0.692027	0.612525	2.638139	0.239769	0.293882
0.837864	0.247583	0.057583	7.545584	0.15127	0.598456	0.86469	0.065974	1.438672
4.043846	10.48101	1.187229	21.54823	2.950173	6.738211	2.750375	3.324452	5.923838
42.75576	0.000736	40.7932	1.507835	22.32214	24.2842	36.74522	118.7028	87.1746
0.022475	0.044214	0.015765	0.141876	0.027879	0.039358	0.009185	0.025483	0.028867
0.50254	1.541227	1.218182	0.66526	1.771392	1.149978	0.927561	1.588903	0.000924
7.402915	16.68554	4.359574	3.824964	15.3618	24.73724	2.02956	5.082063	5.238701
1.762713	7.025599	1.022929	5.855281	1.058896	2.518124	5.77E-05	3.253175	8.923117
0.408427	0.193723	0.444986	0.225505	0.468384	0.235296	0.503781	0.202799	0.254964
16.5625	87.1746	3.489899	111.5439	32.31123	144.7919	22.74797	425.62	425.62
0.204964	0.68557	1.102121	0.518716	1.215981	0.780519	0.56443	0.79487	0.330635
1.905094	5.9071	2.953059	16.09482	15.46193	7.697063	6.632063	6.230245	12.16681
0.036696	0.095772	0.413009	0.114841	0.176104	0.065253	0.09381	0.13824	0.048225
27.38726	27.38726	0.000115	26.80355	7.38847	15.77678	33.07147	10.1028	19.83727
0.014906	0.000736	0.189394	0.001811	7.22E-05	0.065253	0.101782	0.025483	0.000924
2.815866	0.140657	16.09482	0.176955	1.353608	0.080202	2.8657	0.310866	0.095108
3.632973	4.067547	1.175859	4.381479	1.516219	1.791616	0.754315	2.053521	2.659214
3.019848	6.171789	1.776739	8.888167	3.68381	4.429769	2.990851	3.540606	5.737056
0.022475	0.02596	0.042266	0.001811	0.083983	0.000534	0.073362	0.025483	0.000924
3.131544	2.440895	5.351934	3.166659	3.450577	3.367071	2.81342	2.160649	1.934271
2.901032	3.944084	17.45948	3.06803	2.890245	2.223636	8.711905	3.855952	2.451645
0.022475	1.046061	1.077403	0.651631	0.810967	0.039358	1.482626	0.032915	0.450678
0.600173	1.029365	1.117316	0.375743	1.215981	1.085224	1.656696	0.726811	0.471746
2.027951	1.720144	0.240851	3.926154	5.583968	2.021068	3.24658	0.784805	1.003586
8.66E-05	0.02596	0.015765	0.001811	0.006407	0.023059	0.005577	0.000433	0.000924
0.408427	1.87899	0.737403	0.533535	0.692027	0.196436	0.12904	0.259964	0.363276
91.05541	33.93939	42.08807	29.18885	41.47925	28.87506	28.54786	37.29684	48.53429
0.143218	0.044214	0.307734	0.133297	0.279315	0.196436	0.079921	0.032915	0.038088
0.09873	0.000736	0.035382	0.084113	0.100996	0.000534	5.77E-05	0.000433	0.000924
0.008254	0.000736	0.000115	0.001811	0.006407	0.029769	0.009185	0.025483	0.000924
0.06057	0.044214	0.435382	0.001811	7.22E-05	0.000534	0.101782	0.000433	0.000924
0.151176	0.000736	0.087504	0.056869	7.22E-05	0.000534	5.77E-05	0.000433	0.000924
8.994791	10.1459	10.05618	15.66023	7.964315	10.67991	9.902576	6.486104	9.297085
0.067446	0.086443	0.468167	0.001811	7.22E-05	0.000534	0.370281	0.000433	0.000924
1.016494	0.075772	0.517612	0.103434	0.10956	0.172294	0.46153	0.276609	0.048225
2.535974	4.513622	0.697496	3.243225	0.976111	2.065007	1.677042	1.601443	2.621133
0.394603	0.075772	2.294733	0.001811	7.22E-05	0.088341	0.713846	0.000433	0.000924
0.128088	0.130823	2.227273	0.176955	0.629098	0.155224	0.584502	0.585902	0.264242

0.711371	0.645036	2.24733	0.981558	0.656003	0.661025	0.498355	0.13824	0.321227
1.184185	0.895368	1.89228	0.658362	1.267879	2.223636	2.214372	1.68342	0.897489
0.250007	0.274199	0.911905	0.141876	1.136566	0.371876	0.335786	0.927561	0.193449
8.66E-05	4.297222	0.758254	60.77449	28.54786	1.356364	4.609076	9.792626	28.87506
0.258644	0.442193	0.035382	0.298874	0.374365	0.266032	0.446811	0.449408	0.65132
0.444315	0.411948	0.692381	0.259163	0.884654	0.447965	0.329798	0.310866	0.11329
24.04374	23.6012	10.43066	23.6012	21.36892	23.6012	42.75576	80.6518	34.84299
0.114517	0.075772	0.049531	0.001811	0.232215	0.320144	0.042078	0.566494	0.483326
0.036696	0.034603	0.029221	0.038535	7.22E-05	0.000534	0.014488	0.025483	0.038088
2.060491	1.455988	2.953059	0.416219	1.677042	1.751948	2.180022	2.810678	2.035678
2.396429	1.948918	1.550837	1.460981	1.52676	1.11526	1.591299	2.160649	2.673846
0.687049	0.350635	0.789863	0.33676	0.365036	0.235296	1.021097	0.230606	0.154098
8.66E-05	0.044214	0.240851	0.001811	0.304062	0.047504	0.193196	0.000433	0.000924
0.114517	0.000736	1.091443	0.046724	0.15127	0.000534	0.162179	0.065974	0.048225
0.06057	0.034603	1.004156	0.216025	0.232215	0.235296	0.851659	0.166573	0.095108
0.008254	0.034603	0.000115	0.093203	0.027879	0.000534	0.009185	0.000433	0.000924
8.66E-05	0.02596	0.000115	0.074711	7.22E-05	0.000534	0.005577	0.020051	0.000924
8.164531	11.60283	3.730808	6.135007	6.135007	11.14619	3.321284	5.395584	6.125823
0.268369	0.000736	0.224661	0.001811	0.242222	0.000534	0.22456	0.276609	0.067937
0.121147	0.000736	0.205382	0.065519	0.055967	0.029769	0.086775	0.093009	0.000924
3.763045	2.815866	1.988196	2.359264	4.140505	4.029654	4.971991	8.849697	4.783001
8.66E-05	0.064488	0.232886	0.216025	0.073939	0.065253	0.188276	0.149019	0.000924
0.349812	0.342511	0.497749	0.205931	0.328737	0.328543	1.061457	0.211501	0.281724
8.849697	12.03267	3.592063	19.22139	4.739668	3.695815	5.068831	3.792266	4.302367
0.008254	0.000736	0.01013	0.038535	0.019408	0.039358	0.035065	0.041183	0.264242
0.305808	0.064488	0.435382	0.065519	0.539466	0.235296	0.773276	0.108124	0.000924
18.62711	57.84882	7.30215	51.52195	7.991602	21.93452	8.815123	22.13245	18.62711
0.496494	0.000736	0.413009	0.277417	0.311876	0.534214	0.613853	0.276609	0.146623
0.437309	0.1686	0.181674	0.587965	0.533535	0.335786	0.821414	0.620909	0.281724
1.230411	2.197828	0.649668	1.149322	0.328737	0.534214	0.408427	1.68342	1.358189
0.551861	0.203463	0.707799	0.351219	0.28746	0.039358	0.335786	0.449408	0.363276
0.06057	0.02596	0.205382	0.056869	0.197128	0.065253	0.014488	0.075772	0.076775
1.634935	0.265014	0.737403	1.169091	2.417424	0.894704	0.35627	1.185685	2.797835
6.39474	15.98892	0.497749	6.423911	8.728571	7.112496	8.660281	9.667316	9.418196
0.305808	0.086443	2.325931	0.001811	0.526299	0.293499	0.162179	0.000433	0.154098
0.694675	0.319762	0.692381	0.187893	0.311876	0.245967	0.487128	0.220505	0.2443
1.52676	0.282424	3.730808	0.298874	1.453939	0.293499	2.474834	0.285079	0.126335
8.66E-05	0.000736	0.000115	0.074711	7.22E-05	0.000534	0.014488	0.049387	0.000924
5.515584	7.061328	2.061926	7.264502	5.9071	3.397706	8.348817	3.511263	4.145209
0.022475	0.044214	0.096999	0.056869	0.019408	0.029769	0.12904	0.025483	0.048225
0.008254	0.044214	0.070967	0.038535	0.027879	0.023059	0.009185	0.057352	0.126335
0.008254	0.000736	0.01013	0.001811	7.22E-05	0.023059	5.77E-05	0.084906	0.000924
1.497143	1.587605	3.144156	0.610606	2.225498	0.701255	1.771392	1.35259	0.706984
4.043846	10.57984	5.25254	9.880779	3.128694	8.348817	3.544257	2.565556	3.718716
1.076032	0.553348	3.253175	0.578485	1.226934	1.678319	2.842193	0.819509	0.826407
0.114517	0.226623	0.674163	0.40075	0.744113	0.088341	0.35627	0.194098	0.281724
1.310837	1.720144	0.173456	1.351573	1.030678	1.036558	0.432237	0.760866	1.685678
0.134942	0.000736	1.048802	0.001811	0.133117	0.000534	1.102691	0.000433	0.000924
0.509242	0.442193	0.649668	0.001811	0.675115	0.224661	0.079921	0.118644	0.126335
2.640332	4.125786	0.477446	16.34294	1.74539	2.447294	1.392511	1.614257	2.515938
0.962698	1.039199	2.647244	0.33676	0.710253	0.954776	0.670945	1.458038	0.403175
1.369928	1.256558	6.210375	0.810491	1.648066	2.390447	1.50026	1.859084	1.027403
12.49452	0.822965	3.679986	2.069942	1.050231	2.134545	4.776407	2.572309	2.609293
1.986681	1.074242	3.55961	0.216025	2.08798	1.569553	1.130794	1.309084	0.614156
23.38378	287.197	1.326667	224.9555	12.85504	95.22939	2.474834	50.55949	135.5245
0.559221	0.843564	0.000115	0.242612	3.098268	2.092987	0.911364	2.987835	4.376118
0.258644	0.000736	1.290981	0.125354	0.29671	0.029769	0.169105	0.211501	0.057994
38.39042	332.0776	0.000115	135.5245	7.22E-05	34.84299	21.93452	15.276	38.94854

0.052814	0.053766	0.629538	0.216025	0.160707	0.029769	0.055397	0.065974	0.057994
1.330974	0.000736	0.597792	0.001811	0.683232	0.000534	0.258268	0.184459	0.000924
2.136335	2.276407	3.144156	1.331919	2.558817	1.159149	1.64684	1.573276	2.361198
15.66023	22.96463	9.583463	14.97762	18.33909	47.5018	11.30987	19.99861	15.98892
0.029221	0.119553	0.173456	0.637994	0.16925	0.039358	0.974257	0.025483	0.138449
2.698557	3.095166	4.839105	1.693939	6.423911	11.09133	13.38418	5.210317	4.952915
0.662944	0.489509	1.567576	0.205931	2.203535	0.534214	0.695051	0.413009	0.264242
2.34355	1.304877	5.462475	0.773276	2.145137	1.806595	3.113478	2.781558	1.395563
2.229048	0.203463	12.7427	1.024127	1.465123	2.750375	1.991328	1.960945	1.027403
0.586248	0.784805	1.361789	0.829192	0.496494	0.856782	0.370281	1.373348	0.566104
8.66E-05	0.034603	0.029221	0.001811	0.046962	0.000534	5.77E-05	0.000433	0.048225
0.143218	0.371075	1.306667	0.981558	0.757763	1.043608	0.760433	1.373348	1.387612
0.371587	1.830159	0.767085	0.328737	0.338658	0.277417	0.035065	0.184459	0.0857
1.78759	3.350224	12.85504	7.570693	8.958225	4.802655	5.950664	2.040606	4.413608
1.052886	0.053766	0.232886	0.046724	0.118225	0.235296	0.12904	0.000433	0.000924
1.41368	5.309264	1.004156	2.740519	4.077403	7.363117	2.398442	1.728615	4.207778
1.210447	0.299062	1.594675	0.70886	0.781977	0.567345	1.009625	0.699192	0.411436
8.66E-05	0.000736	0.042266	0.001811	0.006407	0.000534	0.009185	0.000433	0.000924
1.216768	2.876421	7.112496	2.621133	0.757763	2.730072	8.477662	2.016515	1.675722
0.559221	1.614257	0.000115	2.254084	0.242222	0.277417	0.515238	1.20653	1.01772
0.008254	0.000736	0.368882	0.001811	7.22E-05	0.000534	0.048889	0.000433	0.048225
1.244957	3.236457	0.48684	4.058514	2.584394	1.962424	0.304387	1.799899	3.330909
0.531804	0.095772	4.007583	0.578485	2.693442	1.460051	1.136566	1.025613	1.65785
3.243225	7.854069	0.747879	4.012049	3.077316	3.418405	1.950519	2.189235	1.884935
0.430476	1.596782	0.173456	0.836017	0.723506	1.508831	0.381999	0.666847	0.817547
5.165599	2.496407	2.584394	2.375974	2.713319	2.162381	4.067547	2.858009	1.775325
5.030866	1.928629	14.65384	1.317475	3.266782	2.234538	3.418405	1.65785	1.790245
7.264502	1.736133	0.933874	13.15079	2.898196	0.831472	5.22482	0.954141	2.5271
0.250007	0.497424	0.070967	5.530274	1.994329	1.284747	1.884935	1.827403	2.434444
2.834084	0.964416	1.463304	3.595758	1.968283	5.381227	1.195794	3.726854	3.460909
17.28195	10.33476	11.65791	15.3618	25.48179	17.03919	13.45939	18.62711	17.51961
0.114517	0.92531	0.000115	3.971061	0.250115	0.224661	0.350455	0.310866	0.945483
12.93651	15.77678	5.728615	15.276	20.16541	10.82954	10.8813	16.5625	17.09911
14.08569	13.08214	4.921082	15.9372	7.625209	4.513622	4.72798	5.287857	9.924372
0.06057	0.000736	0.000115	0.001811	0.011631	0.047504	5.77E-05	0.000433	0.000924
1.731169	1.87899	0.981032	0.92482	2.155447	2.119596	2.089639	1.548723	1.325743
2.561162	0.590303	10.1459	0.62689	3.5257	1.139221	5.266335	1.563045	0.723506
0.948745	0.364769	0.968723	1.112641	0.675115	0.661025	0.641277	1.049517	0.905426
0.329798	0.214885	0.326219	0.369336	0.348629	0.266032	0.918557	0.522367	0.420289
4.105714	5.80469	0.808983	6.345382	1.401068	11.83593	3.055729	0.426645	5.431544
9.625253	15.66023	0.215729	67.98069	1.409942	4.179762	4.702468	2.248896	7.162698
30.17192	105.1694	2.236407	126.1556	62.33472	100.0998	51.52195	105.1694	167.9996
0.78619	0.379495	2.967453	0.441472	3.5257	1.052208	1.406746	1.614257	0.303896
0.412713	0.106926	0.240851	0.084113	0.221962	0.056046	0.155945	0.065974	0.048225
0.488463	0.765606	1.25816	1.133622	1.579798	1.052208	2.327857	1.248232	1.074827
2.243911	1.810664	0.597792	4.49671	0.487662	2.234538	1.693939	1.039199	1.65785
0.339466	0.68557	1.860491	0.416219	1.307417	0.155224	0.911364	0.948211	0.172958
8.66E-05	0.000736	4.188896	0.001811	4.684502	0.000534	0.244747	1.163579	0.000924
21.19547	14.51648	6.830592	10.57984	15.07257	13.23022	12.22798	12.89577	10.82954
0.746984	0.327994	1.624459	0.205931	1.010289	0.362864	0.730476	0.342814	0.450678
0.067446	0.000736	0.918557	0.001811	0.011631	0.000534	5.77E-05	0.000433	0.000924
1.603795	2.111299	17.78522	2.050404	9.667316	3.407619	4.49671	2.975974	1.727323
2.74544	0.525678	2.524892	1.435772	2.800332	1.522597	3.125844	1.248232	1.368167
0.036696	0.119553	1.870685	0.216025	1.050231	0.164509	0.073362	0.230606	0.11329
0.408427	0.1686	2.116032	0.298874	0.403175	0.254964	0.174517	0.310866	0.471746
8.573434	3.663622	2.498478	3.842554	3.629177	1.317475	4.135801	4.227302	5.431544
0.036696	0.000736	0.92482	0.084113	0.10956	0.065253	0.029062	0.000433	0.154098
0.075339	0.000736	1.153723	0.001811	0.394329	0.188124	0.169105	0.158124	0.11329

0.496494	0.705613	0.000115	1.992944	1.692763	0.353175	0.362864	2.081696	1.972706
2.08632	6.591544	4.105714	4.203001	4.039192	5.238701	11.77567	5.266335	3.803766
8.226219	2.953059	8.226219	2.016515	4.900996	3.113478	4.626025	3.418405	2.194257
111.5439	13.45939	10.7557	53.77619	13.08214	36.74522	56.39528	49.50903	67.98069
2.127973	0.790837	1.268672	1.767431	1.757244	1.09075	1.909408	1.813564	1.102691
5.25254	5.838499	0.000115	15.18102	1.89373	6.717056	3.037626	1.670786	3.220678
0.143218	0.335411	2.975974	0.268095	0.394329	0.245967	0.41482	0.292107	0.162915
6.365065	12.16681	3.037626	11.19942	3.442886	3.500808	5.095325	11.42512	9.625253
0.936898	1.132165	3.381255	0.001811	1.985253	0.523983	4.673182	2.586926	0.000924
9.101674	6.865	7.882222	4.650007	9.751039	12.36443	18.48514	9.751039	7.964315
0.157958	0.119553	3.080317	0.001811	0.561126	0.641631	1.449062	0.118644	0.126335
10.67991	9.063983	10.67991	4.63189	10.60361	8.164531	9.751039	7.737807	4.739668
1.857677	0.545152	0.88158	0.560043	2.331825	1.338261	1.240981	1.014127	1.523824
2.496407	3.993665	1.695216	2.463911	4.029654	4.603434	1.965657	3.128694	2.109755
0.052814	0.086443	0.454156	0.001811	0.055967	0.000534	0.232215	0.000433	0.000924
2.520404	1.688045	1.519322	1.549711	2.097843	1.666089	1.406746	1.62562	2.171429
1.202626	2.104358	1.198701	2.784228	1.898153	1.728615	0.956061	1.238651	2.019618
1.846941	0.469711	1.708932	0.942944	1.909408	1.405772	2.459812	1.135137	0.855267
0.008254	0.000736	0.049531	0.046724	0.011631	0.000534	0.014488	0.000433	0.000924
1.615281	6.374812	0.164993	0.883665	0.629098	5.165599	3.095166	6.601833	4.23202
13.30906	3.521934	11.51384	4.365224	11.03843	6.028427	14.42661	6.00228	4.655916
2.815866	0.371075	1.708932	1.543377	0.986104	0.285844	1.392511	0.620909	0.146623
0.285426	0.000736	1.410902	0.001811	7.22E-05	0.097092	5.77E-05	0.000433	0.000924
13.53913	9.418196	7.737807	8.83241	8.445743	5.485685	3.211176	3.486436	3.679986
6.570433	4.709033	4.490801	6.52899	7.149618	4.696313	7.737807	3.869163	4.484892
0.759293	0.000736	0.000115	0.001811	0.100996	0.000534	0.101782	0.000433	0.000924
0.157958	0.053766	0.435382	0.305253	0.279315	0.097092	0.342814	0.075772	0.057994
2.593506	2.941544	1.932857	7.238023	2.31404	0.88619	2.642525	4.135801	4.49671
1.202626	0.075772	3.23987	0.509791	1.495043	0.760433	1.823377	1.006025	0.138449
0.313312	0.158766	0.649668	0.375743	0.54619	0.080202	0.432237	0.166573	0.0857
5.217569	5.821804	2.879228	5.273362	4.140505	17.15902	1.823377	4.997417	7.558139
0.623499	0.625498	1.039798	0.509791	1.041025	1.578687	0.874091	0.611789	0.67632
1.09075	0.106926	0.382626	0.084113	0.10956	0.172294	0.232215	0.065974	0.103528
1.872049	19.29639	3.899993	11.544	17.39935	11.30987	5.687879	31.92385	35.27608
0.430476	0.386169	3.172864	0.509791	2.396429	0.379495	0.280469	0.268687	0.254964
4.56912	4.207778	3.672078	11.01243	3.489899	2.681169	2.696061	2.50469	4.06303
0.022475	0.02596	0.035382	0.038535	0.019408	0.000534	0.145815	0.084906	0.057994
0.091255	0.000736	0.261111	0.001811	0.083983	0.000534	0.086775	0.118644	0.000924
0.036696	0.064488	0.087504	0.001811	7.22E-05	0.000534	0.232215	0.118644	0.000924
0.855267	1.465123	0.070967	1.195794	0.884654	2.750375	0.833045	1.238651	2.008312
0.901212	0.064488	4.451154	0.114841	0.183052	0.088341	0.858817	0.093009	0.000924
19.99861	24.2842	65.94551	13.53913	67.98069	105.1694	41.47925	29.82828	46.7056
10.35839	15.22851	13.45939	4.165137	16.22108	18.05488	15.46193	9.968355	7.456118
1.016494	0.817013	1.846941	1.249221	2.011501	0.842193	1.991328	1.482626	0.716234
3.518384	2.710765	2.922778	5.302294	3.119986	2.34355	3.518384	1.859084	2.42381
0.067446	0.193723	0.403175	0.141876	0.073939	0.000534	0.317576	0.000433	0.11329
0.358672	0.086443	0.215729	0.103434	0.25733	0.277417	0.217908	0.611789	0.38127
0.759293	203.5795	0.000115	23.82354	0.789423	425.62	1.634935	70.11932	53.77619
1.270317	4.580938	0.578485	1.865043	0.872597	4.764055	0.724885	1.634935	1.409942
2.981962	0.895368	2.35733	0.889553	1.909408	1.546696	3.497085	1.363557	1.224733
7.061328	2.283752	10.8813	2.287576	13.75978	1.848297	1.761349	3.8379	1.368167
0.388326	0.430837	0.468167	0.726472	0.926926	0.680173	0.746984	1.135137	0.340079
0.177879	0.948211	0.554026	7.162698	0.434596	1.129452	0.613853	5.976724	5.180058
0.540188	1.434921	1.102121	35.776	1.136566	4.419062	6.830592	3.8121	19.68088
0.143218	0.000736	0.382626	0.056869	0.125981	0.145216	0.422511	0.084906	0.038088
51.52195	55.04081	15.3189	72.69473	65.94551	30.17192	70.11932	55.04081	77.74812
0.868341	0.750404	1.643218	2.016515	17.92188	1.296905	3.585029	3.57057	0.801501
3.407619	2.027951	3.36075	0.560043	2.890245	3.188918	2.676392	3.363911	2.300678

0.593853	0.130823	1.634935	0.389336	0.450051	0.254964	0.381999	0.128687	0.000924
3.651898	1.387612	6.830592	1.118817	3.098268	1.139221	4.155339	2.361198	1.114618
1.098341	2.250462	1.452857	2.396429	1.813564	2.955916	1.262179	2.524892	3.518384
10.19468	77.74812	0.000115	26.52537	2.349351	6.464906	5.872886	7.315512	10.98644
50.55949	184.443	7.200505	118.7028	20.34063	118.7028	44.27012	155.7103	252.4251
8.66E-05	0.68557	5.9071	0.001811	0.593853	0.000534	1.240981	0.000433	0.000924
1.082915	0.442193	2.148658	0.328737	0.25733	0.567345	1.307417	0.535368	0.566104
2.214372	0.150065	3.55961	0.074711	0.320794	0.000534	0.258268	0.470736	0.11329
8.66E-05	0.044214	0.015765	0.001811	0.006407	0.000534	0.009185	0.041183	0.000924
3.578038	0.000736	2.544747	0.001811	0.710253	0.941645	1.741414	1.53684	0.000924
0.052814	0.1686	0.049531	0.114841	0.125981	0.065253	0.183413	0.249719	0.293882
36.74522	43.49658	0.000115	77.74812	7.22E-05	16.44768	9.707677	2.828918	23.16622
0.802035	1.91254	1.870685	8.134084	1.74539	4.863579	3.160022	12.89577	12.63773
0.349812	0.106926	0.444986	0.518716	0.160707	0.402381	0.808052	0.285079	0.233304
1.383911	0.730476	2.81342	1.235346	1.703795	1.238651	1.915455	1.20653	0.801501
1.310837	1.233665	1.437648	0.394603	1.566414	0.661025	2.520404	1.085967	0.629538
4.292078	2.953059	9.141299	1.753341	5.788153	3.046551	1.672107	2.845036	1.472208
0.883665	0.730476	1.248232	0.407922	1.215981	0.88619	1.260635	0.73912	0.509791
0.313312	0.450375	0.948211	0.918557	0.374365	0.371876	0.400354	0.402063	0.493586
1.47842	0.800512	2.673846	0.441472	1.092785	1.149978	0.498355	0.806227	0.321227
1.036558	1.473218	0.597792	9.924372	1.507835	2.584394	0.641277	1.298665	5.050267
0.075339	0.119553	3.330909	0.357013	0.311876	0.328543	0.740527	0.352006	0.000924
21.02638	20.51271	0.224661	28.87506	1.330974	11.544	19.37139	2.489762	10.43066
8.66E-05	0.02596	0.105606	0.074711	0.055967	0.056046	0.035065	0.025483	0.028867
1.036558	0.695426	6.071436	0.458521	1.004156	0.4357	1.162201	1.195794	0.330635
4.272165	4.770231	0.683232	1.351573	4.110779	3.47531	6.784762	5.23176	7.683766
0.128088	0.247583	0.345238	0.093203	0.027879	0.047504	0.101782	0.020051	0.028867
32.31123	22.32214	14.79742	32.68313	22.52375	25.99427	22.13245	23.16622	28.23632
3.566753	5.28039	2.747908	4.524481	5.123781	5.409437	3.989206	5.044113	5.959033
0.164401	0.309221	1.187229	1.537951	1.806595	0.560043	3.23987	3.8379	0.751876
0.157958	0.119553	0.353175	0.001811	0.29671	0.164509	0.22456	0.000433	0.057994
12.22798	14.33939	13.11646	7.737807	11.14619	11.65791	3.211176	8.348817	9.101674
1.798535	3.88153	1.374329	17.39935	2.27254	2.162381	26.52537	3.606804	5.622872
203.5795	31.55417	100.0998	42.75576	55.04081	75.1132	167.9996	87.1746	80.6518
0.143218	0.186017	0.249719	0.187893	0.28746	0.196436	0.155945	0.249719	0.193449
0.276847	4.690159	1.604827	10.33476	6.38456	8.319019	7.951371	18.76954	16.80343
17.15902	13.53913	33.07147	36.26061	12.77772	13.08214	11.93253	15.18102	22.74797
0.052814	0.000736	0.261111	0.001811	0.100996	0.145216	5.77E-05	0.075772	0.000924
0.107287	0.000736	0.000115	0.001811	7.22E-05	0.029769	5.77E-05	0.000433	0.000924
2.723247	4.179762	0.271912	9.502294	1.409942	2.987835	2.581861	1.827403	2.884762
83.489	13.00996	15.3189	48.53429	9.541645	15.3618	15.56131	15.46193	23.71237
8.66E-05	0.000736	0.042266	0.001811	0.006407	0.000534	0.066696	0.000433	0.000924
8.66E-05	2.404618	0.497749	0.426898	0.575916	1.980592	0.258268	2.248896	2.316075
0.339466	0.238225	0.737403	0.205931	0.264372	0.088341	0.288745	0.382417	0.38127
0.559221	0.193723	0.423535	0.216025	0.027879	0.205801	0.280469	0.057352	0.000924
9.583463	3.578038	20.34063	2.944416	9.218514	4.451154	6.933961	8.694697	5.195022
0.458521	0.226623	3.233045	0.587965	0.803665	0.335786	1.561912	0.149019	0.684401
9.377345	8.019769	8.477662	11.96574	4.833398	4.33342	5.606739	7.013853	7.339452
11.36975	19.29639	77.74812	40.7932	111.5439	45.91841	80.6518	72.69473	60.77449
8.66E-05	0.000736	0.335967	0.001811	7.22E-05	0.180368	5.77E-05	0.000433	0.000924
0.022475	0.02596	0.215729	0.001811	0.027879	0.023059	0.135051	0.041183	0.038088
31.55417	30.82277	40.11561	16.68554	48.53429	33.4985	13.75978	31.55417	25.73165
1.449062	1.830159	2.281926	2.069942	1.191905	0.856782	1.370765	2.449509	1.395563
0.151176	0.319762	1.624459	0.251126	0.29671	0.188124	0.446811	0.259964	0.095108
1.052886	1.039199	4.217569	0.54847	1.298665	0.245967	1.156869	1.65785	0.471746
0.572626	0.669358	2.194257	0.125354	0.772951	0.145216	0.525678	0.577071	0.193449
1.64917	3.198499	6.045209	2.554069	3.91355	3.442886	7.991602	2.223636	3.370527
0.292395	0.203463	0.747879	0.292395	0.242222	0.136356	0.86469	0.239769	0.233304

41.47925	11.48368	91.05541	7.468961	20.51271	14.88584	55.04081	19.53061	10.38202
0.067446	0.02596	0.156407	0.001811	0.011631	0.000534	0.014488	0.049387	0.000924
2.616573	5.22482	0.578485	4.105714	1.924228	3.070952	0.613853	1.813564	3.917597
0.008254	0.000736	0.022727	0.001811	0.006407	0.000534	0.042078	0.000433	0.000924
1.278629	0.392554	4.302367	1.007864	0.569293	0.807619	1.156869	0.885137	0.2443
5.485685	1.941703	28.54786	2.630729	9.418196	2.600014	8.888167	2.533745	2.922778
3.387864	2.126349	9.256537	1.062092	2.950173	1.640779	2.316075	1.319293	1.236248
0.313312	0.034603	0.554026	0.001811	7.22E-05	0.029769	5.77E-05	0.025483	0.000924
5.110159	10.28573	2.341573	4.193232	10.43066	5.515584	2.055289	3.944084	2.329784
1.340036	1.446926	1.482626	1.767431	1.968283	1.491977	1.033672	0.962107	1.069033
0.450924	0.150065	1.335743	0.001811	0.205649	0.661025	0.388831	0.000433	0.0857
15.07257	10.23768	10.7557	13.45939	20.85876	35.27608	13.23022	17.78522	15.3618
2.944416	1.233665	0.261111	0.242612	1.477388	2.092987	2.257749	3.904517	2.388348
0.724502	1.278629	0.360938	2.388348	0.996111	2.302626	1.333781	1.700354	1.348975
0.388326	0.226623	0.789863	0.277417	0.487662	0.353175	0.252468	0.535368	0.103528
31.1753	42.75576	30.49289	41.47925	39.53687	22.96463	37.81919	45.1045	30.49289
6.674286	8.83241	3.052655	16.9162	7.897078	19.99861	9.418196	4.546443	8.412367
0.164401	0.635541	0.799553	0.153983	0.133117	0.469004	0.145815	0.522367	0.213355
5.25254	2.159004	9.179466	2.572309	3.394286	1.972706	8.923117	3.921645	2.400455
77.74812	51.52195	24.2842	46.7056	38.94854	32.68313	45.1045	45.91841	55.04081
0.915195	3.482785	4.155339	0.426898	3.137576	1.338261	0.121941	1.064935	0.420289
0.746984	0.392554	0.524986	0.259163	0.365036	0.353175	0.376212	0.322078	0.450678
3.422237	1.278629	17.45948	1.36619	3.820758	1.422316	5.770996	2.818312	0.836537
35.27608	6.945152	13.11646	2.845036	1.902352	3.588369	1.054899	1.992944	0.330635
11.14619	11.09133	8.780115	12.29473	5.530274	9.502294	2.547179	4.365224	7.519841
2.625823	7.683766	6.622092	3.300664	5.500678	2.696061	4.086926	3.153485	2.290996
0.791854	0.599798	3.869163	0.242612	1.89373	0.894704	0.956061	0.726811	0.430837
0.008254	0.02596	0.000115	0.046724	0.006407	0.029769	0.055397	0.025483	0.000924
14.17388	12.36443	25.99427	15.77678	11.83593	9.335397	21.36892	10.57984	11.89932
11.25392	4.490801	12.42965	4.451154	6.079596	4.552092	6.00228	4.529913	5.068831
3.367071	0.68557	3.975498	4.077403	2.911962	2.104358	4.690159	5.754935	2.720996
5.044113	3.944084	2.544747	6.045209	3.381255	7.443276	4.38684	10.8813	8.164531
3.450577	2.784228	3.659668	2.710765	3.807648	3.23987	6.738211	4.155339	2.515938
0.50254	0.086443	0.554026	0.153983	0.133117	0.088341	0.55215	0.13824	0.146623
0.036696	0.106926	0.042266	0.176955	0.027879	0.029769	0.342814	0.032915	0.067937
0.901212	2.147013	1.346991	0.681919	1.27619	1.247244	0.317576	0.707799	0.566104
0.680173	0.379495	3.799885	0.793918	1.267879	0.716652	1.240981	0.632569	0.233304
0.143218	0.106926	3.855952	0.141876	0.796674	0.412453	0.394877	0.438023	0.392251
48.53429	91.05541	4.359574	95.22939	42.75576	45.1045	20.85876	47.5018	56.39528
0.458521	2.014863	0.096999	6.39474	2.752893	1.706219	0.837403	1.775325	3.833247
1.705007	1.36619	2.24733	0.687049	1.298665	0.266032	0.821414	0.535368	0.430837
5.28039	2.066652	11.25392	2.190866	4.222583	3.47531	1.68671	2.50469	0.293882
2.652107	0.653586	2.073232	0.651631	2.531515	2.502619	1.591299	1.68342	0.629538
0.418788	2.45772	3.273045	1.092785	2.429957	7.200505	2.043795	25.73165	12.10289
10.43066	7.264502	27.66779	4.978182	11.09133	15.98892	21.54823	13.08214	10.03313
14.51648	111.5439	6.653016	5.720173	7.417359	5.881342	1.449062	28.54786	14.6058
3.160022	13.38418	6.52899	83.489	10.05618	3.747035	8.412367	9.459177	8.333918
3.640606	2.483492	0.544877	2.863211	1.075411	1.744055	1.002215	9.541645	1.727323
7.037345	5.056421	6.250447	5.095325	6.695887	4.281847	6.079596	13.6814	8.508514
1.505678	1.132165	5.687879	0.70886	4.402973	1.972706	1.991328	2.121198	0.471746
0.797633	0.226623	0.140087	0.065519	0.125981	0.680173	0.311046	0.850325	0.450678
8.66E-05	0.044214	0.022727	0.001811	0.046962	0.029769	0.086775	0.065974	0.028867
4.502453	4.851227	8.711905	1.518232	5.560563	3.55961	2.139928	3.511263	2.884762
21.74051	9.141299	16.34294	11.01243	19.99861	11.36975	30.82277	16.9162	7.824473
0.029221	0.053766	0.01013	0.038535	0.006407	0.023059	5.77E-05	0.020051	0.038088
0.067446	0.000736	0.758254	0.001811	0.083983	0.065253	0.101782	0.065974	0.000924
7.570693	6.784762	126.1556	12.63773	18.76954	15.88548	83.489	15.98892	18.19595
2.710765	0.299062	2.59557	0.328737	0.656003	0.879913	2.117814	0.413009	0.629538

0.036696	0.053766	0.01013	0.001811	0.036934	0.000534	0.005577	0.000433	0.028867
15.98892	14.79742	36.26061	14.70188	23.6012	4.933781	30.49289	6.717056	10.67991
3.91355	2.055289	4.155339	2.134545	5.889798	3.128694	4.424416	3.711097	2.236407
0.022475	0.075772	0.070967	0.046724	0.036934	0.029769	0.019408	0.041183	0.126335
0.258644	0.000736	1.218182	0.001811	0.221962	0.065253	0.525678	0.100188	0.038088
0.091255	0.957273	0.614545	0.31171	0.190058	0.164509	1.002215	0.230606	0.193449
45.91841	32.31123	135.5245	23.16622	38.39042	40.7932	105.1694	64.12971	37.81919
5.00404	2.752893	16.5625	1.703795	7.037345	5.01772	7.868146	4.365224	3.610707
15.83113	7.238023	23.38378	5.110159	8.780115	9.101674	13.08214	8.303947	5.217569
1.540144	0.868341	0.823983	2.50469	1.61088	3.083319	6.345382	1.588903	2.609293
224.9555	67.98069	155.7103	91.05541	184.443	287.197	425.62	144.7919	184.443
11.30987	7.519841	30.17192	5.821804	10.23768	9.687496	8.019769	6.250447	7.049336
0.972504	2.159004	4.25658	1.317475	3.707136	1.192633	1.47018	1.399315	1.179885
0.724502	1.896667	0.959567	0.465801	2.404618	1.626782	0.09381	2.710765	1.429365
2.365685	1.085224	8.445743	2.101075	1.692763	2.375974	7.495397	2.032706	0.826407
10.93101	2.815866	8.815123	2.016515	5.323492	3.367071	4.802655	5.309264	1.972706
4.764055	4.029654	111.5439	5.477843	49.50903	2.807937	53.77619	4.796126	3.153485
0.083983	0.226623	8.745238	0.187893	0.213615	0.000534	2.509177	0.000433	0.000924
0.388326	7.417359	1.318384	1.435772	0.996111	3.343629	0.188276	19.68088	7.212915
20.34063	30.49289	59.32013	30.82277	19.68088	20.16541	28.23632	17.15902	11.09133
67.98069	19.83727	72.69473	9.667316	24.73724	28.54786	52.56128	22.52375	21.19547
1.817828	0.750404	13.83359	0.416219	4.424416	0.224661	5.889798	2.647244	0.213355
7.697063	8.524242	31.1753	4.397792	33.93939	12.42965	14.88584	12.16681	8.048095
4.814661	1.473218	3.290101	0.70886	5.088694	3.500808	3.792266	2.333867	1.685678
32.68313	44.27012	15.46193	36.74522	16.85982	50.55949	15.07257	11.48368	24.04374
2.243911	5.599228	0.289221	0.369336	3.471486	1.666089	0.035065	1.363557	0.321227
9.031082	4.888023	3.699495	10.62739	4.402973	5.915469	9.459177	5.137042	8.445743
100.0998	45.1045	10.98644	38.39042	13.60866	77.74812	18.26752	29.49693	39.53687
0.128088	0.000736	1.187229	0.001811	0.25733	0.116926	0.162179	0.184459	0.000924
1.196508	0.238225	2.148658	0.441472	0.487662	0.430281	0.538773	0.220505	0.254964
2.127973	3.691724	1.226118	5.838499	7.897078	10.90615	3.307186	15.3618	10.57984
0.855267	0.255887	0.48684	0.788369	0.952294	0.320144	15.88548	1.741414	1.102691
0.47974	0.238225	1.117316	0.133297	0.183052	0.116926	2.413146	0.373709	0.162915
6.028427	2.931068	3.606804	3.816551	2.102626	3.930664	5.402511	3.163506	3.153485
0.008254	0.086443	0.01013	0.046724	0.019408	0.000534	5.77E-05	0.000433	0.057994
16.9162	10.70538	47.5018	6.116638	16.44768	20.60009	23.16622	9.838658	6.956349
252.4251	45.91841	530.1465	31.1753	53.77619	49.50903	87331.38	95.22939	33.93939
287.197	126.1556	1360.293	105.1694	425.62	184.443	184.443	184.443	91.05541
0.371587	0.226623	4.752121	0.166118	0.738312	0.155224	0.730476	0.184459	0.000924
10.48101	29.49693	33.4985	29.49693	27.10502	19.53061	15.66023	26.52537	33.07147
0.47974	0.130823	1.187229	0.001811	0.264372	0.371876	0.713846	0.789423	0.076775
2.164113	2.388348	4.25658	3.695815	2.868456	1.833102	3.7586	2.053521	2.388348
0.258644	0.420707	0.468167	0.114841	0.504416	0.749481	0.055397	0.73912	0.172958
0.221111	0.430837	0.568665	0.133297	0.118225	0.172294	0.066696	0.032915	0.103528
0.029221	0.000736	0.131991	0.001811	0.036934	0.000534	5.77E-05	0.000433	0.000924
12.85504	6.09759	7.991602	1.030678	4.814661	3.457504	2.581861	7.495397	3.691724
37.29684	167.9996	8.048095	6.865	5.295325	3.533211	0.670945	10.62739	8.256465
0.052814	0.000736	0.261111	0.001811	0.027879	0.390418	0.048889	0.118644	0.103528
42.08807	83.489	24.04374	56.39528	50.55949	83.489	62.33472	111.5439	83.489
0.083983	0.053766	0.000115	0.001811	7.22E-05	0.000534	0.055397	0.000433	0.000924
3.763045	26.25557	7.795642	6.028427	10.78056	21.54823	18.76954	18.05488	15.56131
0.044762	0.000736	0.181674	0.001811	0.100996	0.000534	0.155945	0.000433	0.000924
1.230411	3.198499	1.437648	1.460981	2.903867	2.375974	1.765065	1.102691	1.499214
0.091255	0.000736	0.345238	0.176955	0.176104	0.073672	0.140188	0.000433	0.000924
8.66E-05	0.000736	0.000115	0.001811	7.22E-05	0.000534	5.77E-05	0.000433	0.000924
1.762713	0.186017	1.776739	0.001811	0.478182	0.311227	1.195794	0.276609	0.126335
19.83727	27.10502	39.24271	16.44768	45.91841	31.1753	27.38726	33.93939	30.82277
4.952915	7.188095	7.417359	8.103925	10.38202	7.315512	9.101674	8.625613	6.454416

5.568391	3.450577	20.85876	3.767489	5.044113	4.952915	17.03919	3.269913	2.542605
5.366335	4.193232	7.351284	3.334199	6.171789	4.49671	2.967453	4.007583	3.23987
3.055729	3.022713	0.88158	2.579509	3.930664	1.613232	1.1514	2.312085	2.941544
0.008254	0.034603	0.000115	0.001811	0.019408	0.000534	0.042078	0.000433	0.000924
9.063983	3.851414	27.38726	3.47531	6.365065	4.833398	15.98892	15.88548	6.365065
9.838658	12.70768	19.53061	19.99861	14.33939	19.83727	14.17388	12.10289	17.92188
1.237879	0.000736	5.309264	0.176955	0.946061	0.136356	2.117814	0.149019	0.146623
1.779423	1.326667	3.771349	2.509177	2.429957	0.709322	1.518232	2.08798	1.758615
0.488463	0.645036	2.81342	1.034315	1.626782	0.842193	1.417345	1.409942	1.064242
7.765931	3.691724	13.91714	4.827691	13.45939	6.365065	9.377345	6.876392	5.024293
0.846342	0.459394	1.99899	0.196955	0.781977	1.356364	0.719942	0.373709	0.233304
3.544257	6.116638	5.180058	5.477843	14.6058	6.230245	8.226219	7.049336	4.413608
6.99026	7.795642	30.82277	12.85504	8.573434	8.608153	10.19468	7.264502	6.570433
27.96248	80.6518	15.66023	14.42661	30.82277	14.33939	7.112496	10.43066	13.30906
0.469711	0.000736	0.382626	0.205931	0.125981	0.000534	0.035065	0.057352	0.000924
13.75978	11.01243	19.14516	13.30906	14.79742	16.80343	11.42512	11.30987	14.08569
0.052814	0.02596	0.360938	0.001811	0.213615	0.088341	0.09381	0.084906	0.126335
5.068831	1.314675	48.53429	1.975772	16.34294	2.852677	14.33939	11.06488	1.20653
0.329798	0.265014	0.66526	0.056869	0.055967	0.224661	0.267597	1.064935	0.660678
2.561162	2.173081	1.030678	1.716364	2.470317	3.91355	1.741414	23.6012	15.18102
0.394603	0.186017	0.435382	0.596775	0.434596	0.447965	0.800512	0.73912	0.392251
1.626782	1.402915	1.077403	3.36075	1.659033	0.701255	0.740527	0.595022	1.445916
7.737807	3.457504	29.82828	3.989206	13.34662	5.560563	35.776	5.770996	2.760765
0.171371	0.119553	0.403175	0.065519	0.064372	0.065253	0.193196	0.049387	0.028867
10.98644	2.688304	20.16541	3.767489	11.96574	3.548153	7.911934	22.32214	8.643074
4.429769	21.02638	4.620159	18.19595	6.011515	23.82354	4.115844	5.01772	11.14619
0.052814	0.000736	0.042266	0.001811	0.011631	0.047504	0.06088	0.093009	0.000924
5.976724	2.31404	13.38418	0.957273	5.088694	1.678319	7.351284	3.204632	1.305772
3.279625	0.193723	2.165851	0.328737	1.298665	0.974257	2.668896	0.760866	0.35627
0.329798	0.536905	0.737403	0.125354	0.15127	0.771176	0.453463	0.158124	0.254964
1.120332	2.337821	0.403175	8.608153	2.225498	2.281926	0.670945	0.566494	6.612121
1.749307	0.47974	1.948918	0.578485	1.784755	1.290981	1.846941	0.449408	0.836537
5.366335	10.70538	32.31123	3.182482	3.314329	6.088593	4.655916	14.51648	2.818312
5.439278	3.074134	9.356371	4.338629	1.330974	18.91854	20.68746	7.363117	4.586753
2.207417	0.570664	2.281926	0.676999	1.155411	0.412453	1.385801	1.439704	0.519755
0.212496	0.358672	0.140087	0.001811	0.10956	0.235296	0.055397	0.000433	0.000924
0.521421	1.05417	0.578485	0.503781	0.593853	0.965714	0.252468	0.449408	0.330635
1.190491	0.868341	1.776739	0.745072	1.61088	0.670144	1.805166	1.025613	0.606154
0.044762	0.000736	0.015765	0.056869	0.011631	0.000534	5.77E-05	0.000433	0.000924
0.365411	0.000736	0.065152	0.001811	0.046962	0.000534	0.048889	0.000433	0.000924
0.285426	0.000736	1.580909	0.001811	7.22E-05	0.000534	0.599798	0.000433	0.000924
0.067446	0.193723	0.629538	0.001811	0.064372	0.000534	0.288745	0.000433	0.000924
1.971183	0.616739	3.422237	0.441472	1.968283	0.732193	3.201457	2.024488	1.128788
0.767569	0.130823	0.326219	0.001811	0.328737	0.180368	0.538773	0.000433	0.233304
0.029221	0.140657	0.049531	0.065519	7.22E-05	0.056046	0.042078	0.041183	0.000924
0.044762	0.203463	0.215729	0.317576	0.264372	0.039358	0.1086	0.259964	0.363276
0.640289	1.36619	1.502323	1.968283	1.314675	1.815	5.737056	2.602395	2.008312
0.92816	2.294733	1.237063	2.800332	1.579798	1.123016	1.111977	0.632569	1.313737
23.82354	35.776	3.381255	50.55949	12.03267	26.80355	4.72798	17.03919	22.13245
1.036558	2.055289	0.639495	2.274473	1.977316	1.25746	6.695887	4.508196	4.222583
13.00996	19.06893	7.351284	59.32013	31.92385	51.52195	50.55949	287.197	144.7919
0.164401	0.282424	0.454156	0.166118	0.197128	0.073672	0.204812	0.065974	0.126335
0.285426	0.247583	0.392944	0.216025	0.338658	0.285844	0.478413	0.166573	0.076775
1.026825	1.01544	1.614257	0.98956	1.539062	0.215267	0.46153	0.832013	0.621688
1.098341	1.240981	5.381227	0.836017	4.764055	2.931068	0.882078	1.515296	0.637994
8.66E-05	0.319762	0.000115	14.08569	6.808369	1.909408	1.615281	2.890245	5.770996
8.66E-05	0.000736	0.01013	0.046724	0.011631	0.023059	0.009185	0.000433	0.000924
3.266782	1.514199	1.077403	1.267879	2.483492	2.730072	1.291825	1.329206	2.071587

2.871212	0.214885	0.497749	5.416623	7.22E-05	2.944416	1.370765	1.928629	2.855296
14.6058	1.95645	0.607937	10.19468	2.078355	4.193232	4.376118	4.603434	3.404372
8.66E-05	0.000736	0.148384	0.125354	0.055967	0.088341	0.288745	0.13824	0.057994
7.468961	2.696061	17.92188	2.644885	9.859719	4.207778	6.028427	6.560411	5.373781
8.319019	7.882222	5.553146	4.650007	6.486104	17.78522	2.337821	4.227302	10.48101
0.339466	0.653586	1.346991	0.321746	1.147807	0.495931	0.350455	0.285079	0.18324
8.66E-05	0.386169	0.156407	0.450051	1.244242	0.440029	0.323752	2.659214	2.096162
0.298687	0.1686	0.607937	0.587965	0.419596	0.277417	0.193196	0.249719	0.392251
5.754935	5.493182	4.408153	5.062626	6.153102	2.621133	1.701616	6.162446	4.609076
3.718716	1.889711	2.269134	8.288874	3.314329	1.338261	2.483492	3.486436	4.655916
25.99427	19.99861	25.73165	10.19468	28.87506	30.82277	38.39042	23.38378	16.09482
0.450924	0.60824	0.991631	0.54847	0.575916	0.459048	0.808052	0.683665	0.614156
0.014906	0.02596	0.01013	0.001811	7.22E-05	0.000534	0.005577	0.000433	0.000924
0.78619	0.469711	0.933874	0.560043	1.226934	0.277417	0.487128	0.577071	0.37228
8.66E-05	16.5625	0.087504	40.11561	0.617417	9.031082	5.77E-05	0.390895	6.287561
1.008413	2.396429	0.000115	2.190866	0.320794	0.749481	0.56443	1.110505	1.613232
16.44768	9.707677	14.25954	12.70768	22.74797	13.60866	17.92188	10.23768	11.544
24.2842	31.1753	2.922778	24.04374	13.00996	12.63773	9.902576	20.68746	21.02638
0.221111	0.150065	0.92482	0.362864	0.617417	0.145216	1.050231	0.995527	0.392251
3.599329	52.56128	0.000115	22.96463	9.101674	24.04374	3.387864	21.74051	34.38341
1.026825	21.19547	0.289221	31.55417	1.383911	6.063276	5.77E-05	4.286962	14.97762
5.056421	2.852677	5.25254	4.586753	1.994329	2.192496	2.171429	0.832013	1.553074
8.66E-05	0.000736	1.724805	0.292395	0.348629	0.245967	0.267597	0.128687	0.138449
0.157958	0.000736	0.758254	0.133297	0.125981	0.412453	0.432237	0.118644	0.067937
3.266782	6.250447	3.699495	8.241342	5.545729	4.851227	11.03843	6.830592	7.882222
1.09075	0.335411	1.507835	0.465801	1.566414	0.447965	3.086306	0.438023	0.293882
0.655245	0.536905	0.249719	0.617763	0.83088	0.771176	0.851659	0.776876	1.074827
0.402063	0.442193	0.66526	0.700418	1.104033	1.043608	0.754315	0.917431	0.766118
25.24367	12.42965	14.00414	34.84299	17.03919	17.92188	24.50942	14.6058	21.93452
2.29873	0.843564	4.217569	3.500808	5.062626	1.175859	6.38456	2.898196	3.877417
0.531804	0.430837	0.88158	1.386681	0.926926	0.422785	1.346003	2.040606	1.093434
7.697063	24.98218	0.000115	2.341573	2.386248	1.935685	1.021097	0.995527	0.945483
1.643218	1.224733	0.375094	0.54847	1.233665	0.612525	0.54619	0.603737	1.409942
1.330974	3.691724	3.080317	6.079596	7.061328	2.567605	7.824473	7.08733	7.795642
0.92816	1.01544	1.87899	1.100519	3.743232	1.369928	4.914863	1.064935	1.368167
0.014906	0.000736	0.423535	0.001811	7.22E-05	0.000534	0.114964	0.000433	0.000924
0.680173	0.420707	0.774798	0.60202	0.692027	0.362864	0.713846	1.174315	0.529697
0.778326	0.525678	3.514834	0.73619	1.659033	0.514141	1.074827	0.73912	0.172958
7.339452	9.625253	12.56693	9.707677	18.48514	15.276	15.276	5.454949	9.031082
0.09873	0.000736	0.000115	0.001811	0.160707	0.254964	0.048889	0.158124	0.000924
0.540188	0.430837	0.534452	0.803211	1.924228	0.680173	0.381999	1.174315	0.987309
0.546522	1.587605	0.799553	1.36619	0.16925	0.440029	1.477388	2.096162	3.414574
2.815866	3.8379	1.015967	4.845166	2.063362	10.33476	8.780115	22.96463	15.07257
0.305808	0.075772	0.66526	0.187893	0.176104	0.155224	0.114964	0.259964	0.172958
1.634935	10.1028	0.568665	6.464906	2.752893	2.192496	1.111977	1.580909	3.816551
8.66E-05	0.034603	0.000115	0.038535	7.22E-05	0.039358	0.005577	0.000433	0.000924
0.298687	0.282424	0.307734	0.268095	0.320794	0.328543	0.837403	0.158124	0.223701
1.443059	3.119986	0.900693	2.199639	0.946061	1.414574	2.581861	5.030866	2.90658
0.531804	1.000173	0.823983	2.498478	0.675115	0.4357	0.244747	0.438023	0.629538
0.70329	0.514141	5.967879	0.478413	2.037114	1.129452	0.73619	2.635685	0.223701
0.022475	0.000736	0.035382	0.001811	0.055967	0.088341	0.029062	0.084906	0.000924
6.045209	252.4251	6.011515	75.1132	80.6518	167.9996	24.98218	126.1556	111.5439
0.694675	1.074242	0.816479	4.473348	4.927302	1.271097	1.64684	3.283139	2.955916
0.014906	0.064488	0.189394	0.074711	0.019408	0.039358	0.066696	0.032915	0.076775
6.622092	3.4679	26.52537	3.47531	12.29473	3.939307	6.230245	4.317648	0.905426
3.031688	3.563182	0.578485	1.055628	0.803665	2.621133	1.718918	6.933961	4.082165
144.7919	40.11561	51.52195	31.92385	22.13245	42.08807	23.6012	52.56128	37.29684
0.143218	0.000736	2.053521	0.001811	0.118225	0.293499	0.740527	0.084906	0.000924

0.014906	0.290317	0.000115	0.4971	0.011631	0.056046	5.77E-05	0.065974	0.138449
1.322092	0.489509	0.948211	0.205931	0.403175	1.65785	4.863579	0.158124	0.598124
2.723247	2.223636	1.25816	1.380195	4.012049	4.626025	2.280101	0.620909	1.286494
8.66E-05	0.000736	0.382626	0.001811	0.176104	0.245967	0.730476	0.259964	0.000924
0.09873	0.000736	0.317229	0.001811	7.22E-05	0.039358	0.048889	0.041183	0.000924
18.05488	62.33472	6.956349	25.99427	36.26061	59.32013	24.04374	35.05953	42.08807
4.419062	1.633752	2.026219	5.351934	7.149618	2.057056	7.351284	5.137042	6.38456
0.450924	0.247583	1.077403	0.646378	0.63873	0.328543	0.403882	0.065974	0.202987
3.150433	2.581861	5.409437	2.092987	4.292078	3.500808	6.900202	6.036818	3.544257
1.971183	0.705613	1.660216	0.503781	1.244242	1.184185	1.002215	1.992944	0.962698
12.63773	10.62739	23.16622	3.387864	6.250447	6.25938	10.1028	8.923117	10.28573
2.449509	3.909033	0.88158	9.141299	2.735368	0.661025	1.162201	1.418312	1.114618
0.184055	0.1686	0.164993	0.103434	0.046962	0.056046	0.055397	0.032915	0.067937
0.036696	0.000736	0.042266	0.001811	0.055967	0.000534	0.014488	0.000433	0.000924
0.981032	0.342511	0.789863	0.970007	0.533535	0.320144	1.08671	0.438023	0.254964
17.78522	5.654863	21.36892	3.089293	17.64869	4.900996	14.56114	11.25392	5.84689
0.606421	0.214885	3.829105	0.426898	0.160707	0.039358	0.641277	0.149019	0.172958
2.768312	0.044214	5.84689	0.298874	1.173535	0.514141	3.975498	0.666847	0.303896
1.615281	3.286652	0.477446	0.935115	1.075411	1.422316	0.662944	0.632569	1.445916
3.975498	4.053506	13.53913	4.307309	3.394286	3.730808	2.327857	3.521934	3.188918
13.23022	47.5018	0.298153	12.36443	1.852338	21.74051	14.70188	4.888023	4.875527
1.244957	0.119553	1.660216	0.093203	0.403175	0.000534	0.381999	0.057352	0.000924
0.029221	0.000736	0.000115	0.001811	7.22E-05	0.039358	0.009185	0.000433	0.000924
2.901032	0.60824	5.030866	0.62689	1.813564	1.338261	4.365224	1.373348	0.293882
0.044762	0.000736	0.057583	0.001811	7.22E-05	0.000534	0.042078	0.108124	0.000924
0.091255	0.140657	0.240851	0.54847	0.073939	0.080202	1.354567	0.128687	0.067937
19.53061	23.16622	95.22939	17.28195	34.84299	15.07257	31.92385	24.73724	17.64869
0.731732	0.000736	0.123535	0.001811	0.374365	0.155224	0.511667	0.300606	0.223701
8.66E-05	0.034603	0.022727	0.065519	0.006407	0.023059	0.009185	0.000433	0.000924
1.47842	1.99899	0.981032	6.784762	1.445916	1.909408	1.732468	1.728615	2.673846
0.339466	0.000736	5.123781	0.001811	1.824892	0.000534	1.771392	0.000433	0.000924
5.679502	8.461703	0.534452	10.93101	5.416623	7.765931	1.621032	3.310758	5.165599
14.88584	10.05618	0.607937	18.76954	3.334199	7.545584	5.22482	9.297085	15.276
0.640289	0.514141	0.48684	1.460981	0.384156	0.390418	0.977994	0.566494	0.994271
2.993867	3.043528	1.187229	1.728615	1.092785	1.617489	1.360022	1.928629	1.837172
0.236638	0.000736	3.172864	0.317576	2.178268	0.353175	0.754315	0.699192	0.162915
2.698557	0.140657	2.693442	0.001811	1.771392	0.000534	1.793074	0.149019	0.000924
0.022475	0.1686	0.423535	0.065519	0.064372	0.047504	0.066696	0.000433	0.000924
2.150303	3.632973	6.153102	2.727785	3.043528	3.083319	3.691724	2.369841	2.089639
2.561162	0.525678	1.153723	0.141876	0.515238	0.402381	0.304387	1.064935	0.637994
0.358672	0.309221	1.141515	0.233752	0.183052	0.088341	0.840635	0.13824	0.000924
2.114437	1.514199	4.429769	2.236407	4.490801	2.820974	6.365065	6.09759	3.803766
0.022475	0.000736	0.392944	0.001811	0.019408	0.000534	0.019408	0.065974	0.000924
9.502294	1.465123	7.92518	2.544747	5.00404	6.55039	25.99427	24.50942	12.49452
0.022475	0.064488	0.022727	0.074711	0.019408	0.544545	0.238889	0.490079	0.172958
3.610707	2.337821	1.816436	7.653391	2.109755	2.427792	2.398442	2.121198	2.797835
2.03886	0.379495	4.25658	0.47246	2.306277	0.650556	3.439437	1.049517	0.542525
2.815866	0.750404	2.35733	1.240202	2.371833	0.680173	2.531515	2.618846	2.035678
0.190693	0.034603	0.517612	0.084113	0.205649	0.097092	0.232215	0.108124	0.057994
6.079596	9.989221	0.587965	12.22798	3.5257	8.508514	10.43066	0.892395	6.230245
1.497143	1.149322	4.490801	0.824416	3.432388	2.212619	1.185685	1.690483	0.897489
7.136537	12.26136	7.482179	19.06893	10.33476	8.780115	14.08569	12.42965	18.41211
7.545584	17.51961	9.031082	6.39474	12.63773	16.5625	4.029654	29.18885	27.38726
0.305808	0.282424	2.953059	0.001811	1.363557	0.000534	0.773276	0.158124	0.000924
0.204964	0.000736	3.899993	0.362864	0.600548	0.277417	0.408427	0.481104	0.000924
0.09873	0.140657	2.236407	0.103434	0.328737	0.039358	0.183413	0.149019	0.048225
3.169812	7.964315	0.000115	8.319019	0.539466	2.621133	2.917381	4.575123	7.288788
1.322092	1.196508	0.000115	1.909408	2.494177	1.139221	1.209105	2.053521	1.387612

3.346898	3.397706	4.302367	2.101075	5.662994	3.297107	12.03267	5.654863	4.451154
0.229928	0.214885	1.978954	0.34443	0.504416	0.164509	1.074827	0.292107	0.138449
1.741414	2.08632	2.81342	3.06803	5.030866	2.021068	2.975974	1.905094	3.718716
1.026825	1.000173	2.148658	0.560043	2.723247	1.123016	1.215981	2.618846	1.753341
1.184185	1.480541	1.004156	1.380195	0.328737	0.188124	0.605455	0.500722	0.403175
0.458521	6.749488	0.707799	8.075772	3.286652	2.973059	2.225498	0.707799	3.779452
5.395584	53.77619	12.1974	14.00414	36.74522	37.29684	9.792626	26.80355	38.39042
3.486436	4.614618	5.25254	5.591717	3.851414	2.931068	10.62739	8.381328	6.30671
6.277937	2.513752	5.337547	2.621133	4.63189	3.095166	4.776407	2.171429	2.496407
8.66E-05	9.880779	1.030678	13.83359	2.784228	2.911962	13.30906	25.24367	16.68554
1.082915	1.000173	4.188896	2.893001	2.760765	0.749481	4.626025	1.389509	0.723506
1.705007	0.653586	0.872597	0.870375	1.164257	0.670144	1.267879	1.674401	1.154567
0.70329	1.91254	1.077403	1.386681	1.136566	1.303045	2.547179	1.093434	1.236248
0.036696	0.226623	0.215729	0.752229	0.328737	1.11526	0.519019	1.992944	1.057569
0.855267	0.177879	1.737294	0.587965	0.675115	0.50469	0.56443	0.490079	0.440801
0.212496	0.392554	1.141515	0.141876	0.073939	0.224661	0.140188	0.230606	0.038088
0.177879	0.000736	0.578485	0.001811	0.055967	0.000534	0.12904	0.000433	0.000924
0.496494	0.342511	4.039192	0.103434	0.434596	1.069574	1.267879	0.470736	0.509791
3.008225	0.469711	0.240851	0.375743	0.29671	0.293499	0.135051	0.352006	0.2443
2.351299	1.91254	0.368882	1.267879	0.976111	0.534214	0.874091	0.910823	0.629538
8.256465	7.61176	0.000115	11.42512	0.744113	19.68088	1.170491	4.251818	5.088694
18.76954	8.195657	5.530274	2.451645	2.666494	8.048095	1.701616	1.677042	1.1407
3.651898	1.099004	3.214545	0.54847	1.183478	1.338261	2.011501	0.832013	0.509791
0.044762	0.064488	0.114358	0.225505	0.183052	0.039358	0.101782	0.065974	0.18324
5.855281	18.62711	4.359574	7.112496	7.351284	9.968355	2.489762	6.116638	7.765931
9.792626	6.191515	6.695887	6.191515	8.849697	7.991602	29.18885	9.924372	5.84689
0.988932	1.799899	0.900693	2.758088	2.547179	1.47018	0.780952	0.577071	2.216162
2.449509	2.638139	3.172864	1.617489	3.147294	1.379278	4.575123	3.984747	2.914632
0.052814	0.000736	1.909408	0.114841	0.046962	0.000534	0.280469	0.000433	0.000924
0.008254	0.075772	0.131991	1.426522	1.136566	4.392316	0.22456	1.014127	0.37228
1.278629	2.216162	3.156537	2.26518	3.507691	3.663622	3.632973	2.477027	6.784762
10.62739	7.099913	1.463304	4.484892	4.381479	5.20267	8.958225	4.869553	3.460909
3.4679	5.137042	6.760765	4.25658	6.55039	3.327619	4.251818	3.407619	1.753341
0.546522	0.203463	0.968723	0.33676	0.311876	0.145216	0.21088	0.118644	0.213355
0.412713	0.106926	0.114358	0.074711	0.118225	0.155224	0.376212	0.128687	0.162915
0.586248	0.459394	0.123535	0.251126	0.41026	0.245967	0.760433	0.049387	0.2443
0.948745	0.47974	1.423506	0.31171	1.495043	0.724004	1.870685	0.776876	1.224733
5.366335	6.830592	1.519322	4.715599	13.23022	8.888167	5.462475	5.381227	7.737807
8.66E-05	0.000736	0.000115	0.046724	0.011631	0.000534	0.1086	0.000433	0.038088
0.044762	0.044214	0.096999	0.074711	0.09241	0.029769	0.048889	0.128687	0.057994
2.259661	1.810664	2.50469	2.638139	1.839986	2.147013	4.207778	1.123016	1.481631
2.860722	0.790837	4.563117	0.407922	2.055289	1.204957	2.941544	1.275325	0.73619
2.197828	2.104358	184.443	19.83727	60.77449	10.5063	37.29684	8.075772	5.323492
0.746984	0.000736	1.502323	0.001811	0.213615	0.301955	5.77E-05	0.100188	0.103528
0.221111	0.034603	0.240851	0.001811	0.304062	0.000534	0.262785	0.025483	0.000924
0.394603	1.855108	1.708932	9.297085	1.092785	5.037489	5.796421	3.606804	5.11697
30.49289	17.39935	9.838658	17.15902	3.899993	10.5063	7.08733	10.82954	9.335397
2.871212	2.903867	2.216162	1.935685	4.557605	1.590202	3.648081	2.150303	2.842193
0.008254	0.044214	0.131991	0.001811	0.006407	0.000534	0.019408	0.000433	0.000924
8.66E-05	0.000736	0.000115	0.001811	0.011631	0.000534	0.121941	0.000433	0.000924
0.516061	1.266255	0.726472	1.124574	2.355289	2.390447	4.02088	2.723247	1.972706
33.93939	16.09482	16.68554	14.51648	4.715599	13.15079	11.25392	7.225469	14.33939
0.298687	0.238225	0.335967	0.141876	0.221962	0.631494	0.252468	0.648362	0.392251
5.591717	7.443276	2.511465	5.22482	2.048781	3.899993	0.35627	1.275325	3.518384
0.855267	2.08632	0.096999	1.485483	5.933066	1.86658	1.582915	0.976667	1.064242
1.64917	1.188139	2.723247	2.415296	1.712706	1.308167	2.019618	1.248232	1.702706
0.402063	0.106926	0.271912	0.001811	0.064372	0.097092	0.453463	0.000433	0.213355
0.578485	0.119553	5.654863	0.141876	0.264372	0.224661	0.844747	0.268687	0.000924

1.615281	2.014863	2.901032	6.99026	3.077316	1.47018	2.659214	2.781558	3.566753
0.022475	0.064488	0.123535	0.038535	0.133117	0.073672	0.024315	0.118644	0.0857
0.753449	0.000736	2.723247	0.187893	0.552749	0.000534	0.280469	0.334625	0.340079
1.555289	0.193723	0.649668	0.394603	1.004156	1.097677	1.805166	2.287576	1.934271
3.921645	1.855108	1.948918	3.711097	1.824892	2.621133	0.262785	1.977316	0.598124
1.336587	2.31404	2.390447	2.000592	1.164257	1.230411	1.927042	2.723247	1.601443
0.067446	0.106926	0.078167	0.001811	0.064372	0.000534	5.77E-05	0.819509	0.000924
1.884935	2.845036	2.341573	0.518716	3.077316	5.123781	2.496407	2.928247	3.35355
1.533535	1.969733	1.502323	2.016515	1.833102	1.422316	1.965657	0.585902	2.353247
64.12971	19.53061	6.560411	13.23022	6.591544	18.62711	21.02638	21.19547	20.51271
0.81544	1.678319	0.368882	1.639639	0.986104	0.831472	0.440548	0.995527	1.438672
4.673182	9.276811	1.567576	155.7103	2.27254	3.57057	11.48368	8.888167	8.797619
10.55571	33.07147	3.699495	21.74051	9.859719	15.66023	5.044113	10.48101	13.83359
0.022475	0.000736	0.164993	0.001811	0.006407	0.000534	0.055397	0.084906	0.000924
0.919827	1.046061	0.639495	1.502323	1.25816	1.264589	0.487128	0.55645	2.685786
22.74797	12.10289	10.1028	7.188095	17.51961	10.23768	15.3618	23.82354	10.73084
1.960945	0.713846	1.022929	1.041025	1.393571	2.871212	0.204812	3.089293	3.043528
1.256558	0.853218	0.707799	1.901068	0.617417	3.833247	2.81342	2.429957	1.816436
22.13245	5.409437	4.490801	21.93452	8.195657	40.11561	33.4985	21.02638	30.17192
0.067446	0.000736	1.965657	0.001811	0.356508	0.106118	0.217908	0.100188	0.000924
1.919661	3.428788	0.544877	1.149322	1.924228	1.546696	1.921212	1.093434	2.151919
3.098268	1.465123	3.310758	2.169509	1.422316	1.414574	2.089639	0.901732	0.53658
3.518384	4.739668	0.345238	29.82828	3.077316	6.486104	2.834084	6.967547	5.638759
0.855267	0.834416	0.423535	3.110447	0.320794	0.277417	0.311046	0.577071	1.093434
6.116638	1.64684	0.948211	2.257749	3.23987	1.479452	3.563182	1.601443	2.673846
0.496494	0.497424	1.153723	0.382417	0.205649	0.362864	0.388831	0.300606	0.000924
0.640289	0.000736	0.28026	0.001811	0.133117	0.000534	5.77E-05	0.611789	0.000924
26.80355	6.808369	8.677489	5.143867	10.28573	14.25954	10.98644	5.287857	11.19942
1.428398	2.126349	0.948211	1.634935	0.539466	1.247244	1.634935	3.401126	2.630729
3.266782	1.132165	0.597792	2.611797	0.731732	0.842193	1.235346	1.717641	1.523824
0.151176	0.000736	0.196335	0.046724	0.118225	0.000534	0.1086	0.041183	0.057994
0.177879	0.000736	0.799553	0.216025	0.374365	0.196436	1.041652	0.065974	0.000924
3.934986	2.243911	2.24733	3.581688	1.852338	2.477027	4.827691	1.402915	4.63189
2.434444	3.317807	1.1629	5.210317	1.058896	1.590202	2.225498	0.96938	1.685678
2.08632	0.47974	3.975498	1.085967	1.018427	0.650556	2.043795	0.653968	0.730873
5.737056	1.278629	10.53159	2.750375	4.990794	0.691674	4.317648	2.197828	2.129596
8.66E-05	2.074906	0.000115	1.793074	7.22E-05	1.848297	5.77E-05	0.000433	0.970007
1.210447	16.80343	18.76954	0.918557	42.08807	6.52899	17.78522	35.776	29.49693
2.893001	1.304877	2.417424	1.085967	1.11526	1.431414	3.35715	1.185685	1.325743
0.516061	0.106926	0.948211	0.001811	0.118225	0.000534	0.629538	0.065974	0.000924
0.083983	0.053766	0.423535	0.046724	0.141825	0.000534	0.019408	0.041183	0.038088
1.09075	3.984747	0.683232	3.125844	1.050231	1.460051	1.602619	0.175144	0.723506
4.869553	2.678781	5.728615	2.375974	3.993665	2.547179	3.747035	2.586926	2.058773
0.92816	0.661025	0.148384	0.817547	1.771392	0.311227	1.761349	1.35259	1.4671
0.874603	0.342511	1.708932	0.740952	1.004156	0.196436	0.487128	0.292107	0.629538
21.93452	6.045209	29.34289	3.450577	19.83727	10.57984	6.153102	12.03267	9.160382
1.779423	3.179199	1.795851	1.596782	1.712706	2.538218	4.529913	3.025671	3.286652
0.298687	0.140657	0.656753	0.166118	0.403175	0.301955	0.432237	0.535368	0.202987
2.509177	1.412771	1.89228	0.863694	2.178268	1.097677	2.879228	1.373348	1.179885
1.555289	3.711097	1.346991	1.889711	3.147294	1.02487	1.666089	1.799899	2.735368
0.981032	4.297222	0.799553	5.180058	1.307417	2.104358	1.111977	2.556443	1.248232
4.140505	2.31404	4.72798	1.628009	3.363911	0.965714	1.406746	2.005253	4.135801
0.091255	0.064488	0.029221	0.205931	0.011631	0.029769	0.005577	0.000433	0.028867
5.923838	2.197828	5.942294	3.518384	4.94026	1.962424	5.439278	1.89228	2.831501
19.22139	14.97762	6.116638	9.583463	6.760765	37.81919	21.74051	11.544	24.73724
2.425801	1.701616	5.210317	1.259747	1.712706	1.379278	1.938759	0.73912	1.523824
0.632208	0.350635	0.850808	0.362864	0.803665	0.266032	0.869365	0.239769	0.18324
8.66E-05	0.075772	2.099524	0.103434	0.190058	0.000534	5.77E-05	0.211501	0.000924

1.828781	1.766248	0.968723	0.963853	0.926926	0.534214	2.563506	0.352006	0.330635
0.221111	0.203463	0.049531	0.153983	0.561126	0.000534	0.204812	0.025483	0.067937
1.434921	1.969733	0.454156	2.280101	1.226934	1.763889	0.690043	0.382417	0.781465
3.019848	9.792626	0.000115	4.667518	6.464906	5.266335	1.815	0.910823	2.388348
1.41368	1.188139	0.726472	0.842193	0.952294	1.87899	2.633232	0.892395	1.559675
0.022475	0.106926	0.28026	0.056869	0.036934	0.328543	0.662944	0.093009	0.048225
1.126775	1.256558	1.306667	1.195794	0.394329	0.552439	0.684033	2.150303	0.519755
0.559221	0.790837	0.403175	2.050404	0.561126	0.819978	0.773276	0.850325	1.069033
1.343463	1.609654	0.148384	1.446926	0.365036	0.581645	0.323752	0.648362	2.216162
2.561162	4.644098	2.438831	3.35355	1.37531	3.163506	3.767489	3.699495	1.027403
6.922771	10.53159	2.763261	13.91714	4.851227	4.696313	8.508514	8.226219	12.77772
0.06057	0.034603	0.368882	0.065519	0.019408	0.088341	0.042078	0.000433	0.000924
2.768312	0.514141	5.082063	0.518716	2.417424	1.139221	3.464315	0.995527	0.826407
4.933781	1.514199	5.110159	0.658362	6.325945	2.584394	7.598312	4.451154	1.224733
4.077403	1.455988	4.546443	1.081544	2.127973	1.405772	1.837172	2.349351	1.418312
1.78759	5.779574	2.165851	4.927302	3.544257	4.739668	5.923838	10.33476	9.880779
5.568391	3.387864	16.80343	1.558571	6.028427	4.365224	5.295325	3.080317	2.316075
0.89404	0.095772	2.216162	0.317576	0.29671	0.097092	0.400354	0.352006	0.067937
14.42661	5.01772	3.36075	4.25658	1.341825	8.461703	4.519048	8.958225	6.045209
1360.293	1360.293	67.98069	203.5795	52.56128	1360.293	749.7839	530.1465	1360.293
0.718766	3.36075	0.629538	2.990851	1.041025	2.248896	0.833045	1.87482	3.618499
0.014906	0.000736	0.148384	0.001811	0.027879	0.000534	5.77E-05	0.202799	0.103528
1.178939	2.931068	8.075772	0.942944	2.164113	0.965714	1.437648	2.369841	2.236407
16.80343	17.03919	19.83727	64.12971	25.24367	21.02638	40.7932	15.07257	44.27012
0.204964	0.106926	1.361789	0.153983	0.320794	0.116926	1.195794	0.202799	0.0857
17.39935	14.17388	14.51648	14.6058	8.728571	9.583463	9.297085	7.402915	12.22798
8.66E-05	10.19468	0.326219	4.338629	3.233045	4.312251	2.792035	13.75978	14.00414
1.749307	2.763261	1.077403	3.548153	5.71241	2.681169	3.144156	3.606804	3.533211
1.173535	2.063362	1.493968	0.389336	3.663622	0.974257	0.911364	1.473218	2.776421
2.496407	1.046061	4.802655	1.58645	1.865043	1.751948	2.474834	2.269134	1.890996
0.134942	0.226623	1.685678	0.31171	0.847352	0.196436	0.613853	0.239769	0.11329
4.18456	8.573434	12.1974	4.524481	9.625253	4.392316	3.397706	6.52899	5.259437
0.184055	0.000736	0.105606	0.001811	0.083983	0.000534	5.77E-05	0.000433	0.233304
1.45912	2.074906	0.587965	0.539466	2.558817	1.640779	1.142345	2.797835	0.791854
126.1556	15.56131	32.68313	20.68746	26.80355	31.92385	118.7028	48.53429	40.7932
1.523824	0.983384	2.763261	0.166118	3.055729	0.701255	0.851659	0.901732	0.126335
8.66E-05	0.000736	0.078167	0.001811	0.011631	0.056046	0.014488	0.000433	0.000924
6.135007	7.136537	10.93101	3.204632	8.660281	3.317807	3.783694	2.477027	4.990794
2.520404	7.938427	1.557482	1.502323	5.47	2.440895	1.170491	2.066652	0.994271
4.546443	5.123781	7.92518	4.609076	6.945152	5.704646	9.625253	4.733824	2.652107
3.94886	3.511263	4.789596	1.784755	5.416623	6.99026	2.459812	1.992944	2.735368
0.014906	0.000736	0.096999	0.001811	0.213615	0.047504	0.467951	0.108124	0.038088
1.286494	0.750404	2.216162	0.817547	1.771392	0.749481	0.90368	0.832013	0.668947
0.285426	0.095772	0.403175	0.166118	0.118225	0.136356	0.400354	0.041183	0.028867
0.731732	0.309221	0.597792	0.176955	0.487662	0.285844	0.323752	0.322078	0.202987
8.66E-05	0.119553	0.070967	0.465801	0.036934	0.065253	0.024315	0.075772	0.000924
9.541645	6.685087	25.48179	18.48514	23.38378	2.234538	46.7056	2.74298	12.03267
0.746984	1.412771	1.948918	0.918557	1.104033	1.02487	0.821414	1.87482	3.330909
16.22108	4.440548	5.84689	6.162446	4.312251	7.897078	7.443276	3.052655	9.541645
3.984747	5.195022	1.759986	5.238701	14.88584	7.697063	9.335397	1.960945	5.500678
2.852677	0.722626	1.1629	0.856291	4.174964	1.284747	2.955916	8.103925	4.016465
12.03267	14.25954	2.325931	18.62711	10.12435	14.79742	14.56114	18.48514	24.2842
2.351299	1.099004	3.450577	3.460909	0.744113	1.608427	2.597792	1.757244	2.139928
0.613853	0.843564	1.039798	0.637994	0.569293	0.362864	0.244747	0.470736	0.751876
0.47974	1.01544	0.423535	0.407922	1.267879	0.788369	0.579495	0.490079	0.363276
1.471198	0.450375	11.30987	0.793918	1.401068	1.491977	2.147013	0.620909	0.493586
0.171371	0.095772	4.978182	0.103434	0.348629	0.116926	0.370281	0.285079	0.038088
0.313312	0.000736	0.435382	0.001811	7.22E-05	0.000534	5.77E-05	0.000433	0.000924

0.380087	0.203463	4.007583	0.762641	1.634935	0.320144	0.888485	0.892395	0.440801
40.11561	12.93651	6.365065	19.68088	15.56131	16.09482	3.614603	26.25557	31.1753
17.03919	18.19595	7.697063	11.89932	18.91854	11.719	10.57984	5.323492	10.93101
0.671371	1.578687	3.019848	0.998312	3.363911	1.39847	2.089639	1.65785	0.855267
3.394286	2.382035	1.437648	2.909293	4.684502	2.083261	2.413146	3.651898	1.898153
0.578485	0.379495	1.580909	0.277417	0.419596	0.371876	0.938413	0.220505	0.223701
4.72798	1.064242	1.965657	1.249221	2.494177	2.281926	2.990851	1.123016	0.801501
8.66E-05	0.000736	0.224661	0.187893	0.064372	0.136356	0.09381	0.166573	0.000924
0.052814	0.000736	0.196335	0.001811	0.160707	0.155224	0.272814	0.075772	0.000924
4.63189	1.982208	3.330909	2.5271	5.638759	3.381255	1.602619	5.530274	6.749488
0.236638	0.02596	0.758254	0.103434	0.213615	0.422785	0.605455	0.268687	0.154098
0.036696	0.000736	1.374329	0.001811	0.083983	0.000534	0.09381	0.000433	0.000924
2.378124	0.800512	2.308203	1.801284	1.950519	0.866147	1.539062	1.039199	0.501905
1.471198	0.645036	1.437648	1.948918	2.255916	0.691674	4.297222	0.653968	1.540144
0.236638	0.075772	0.326219	0.065519	0.141825	0.088341	0.204812	0.041183	0.095108
2.388348	1.304877	7.519841	1.578687	3.767489	1.219019	5.500678	1.87482	1.803918
0.718766	1.402915	2.569957	1.549711	1.477388	0.701255	0.589986	1.298665	1.481631
5.295325	5.976724	27.10502	8.381328	6.632063	2.162381	5.560563	1.68342	6.830592
0.424848	0.086443	2.132756	0.153983	0.046962	0.235296	0.22456	0.511667	0.172958
1.581912	1.115902	6.287561	1.703795	1.648066	0.807619	1.406746	1.573276	1.587605
0.09873	0.000736	0.205382	0.001811	0.027879	0.000534	0.029062	0.000433	0.000924
0.694675	0.000736	1.391494	0.141876	0.781977	0.106118	0.467951	0.211501	0.000924
1.762713	3.034747	0.307734	1.085967	0.926926	1.546696	1.185685	0.653968	1.057569
0.190693	0.000736	1.391494	0.001811	0.271912	0.056046	0.169105	0.000433	0.000924
3.253175	3.26	22.52375	3.548153	16.09482	3.779452	7.545584	11.14619	3.011169
0.724502	0.158766	2.472576	0.357013	0.629098	0.155224	1.094978	0.470736	0.223701
4.827691	3.738997	6.784762	7.938427	4.207778	5.855281	2.786898	7.136537	11.25392
8.66E-05	0.034603	0.000115	0.038535	7.22E-05	0.039358	0.005577	0.000433	0.000924
6.39474	2.784228	9.502294	2.826277	6.063276	3.35355	2.380079	3.381255	2.720996
14.25954	37.81919	22.74797	21.02638	87.1746	20.85876	22.52375	27.38726	22.52375
0.941645	0.450375	3.253175	1.244242	6.345382	0.328543	2.066652	0.481104	0.684401
7.225469	6.685087	11.14619	1.865043	6.876392	6.171789	11.719	4.188896	2.373824
0.036696	0.000736	0.049531	0.038535	0.006407	0.000534	0.005577	0.020051	0.038088
2.579509	1.551962	0.568665	0.672078	1.603795	2.192496	0.503781	1.261407	0.692799
0.052814	0.000736	0.000115	0.001811	7.22E-05	0.000534	0.048889	0.000433	0.000924
4.212554	9.012937	4.546443	7.824473	3.629177	5.728615	11.14619	18.91854	29.18885
0.107287	0.1686	0.022727	0.093203	0.036934	0.056046	0.079921	0.075772	0.057994
8.66E-05	0.034603	0.015765	0.259163	0.160707	0.073672	0.055397	0.166573	0.450678
11.96574	6.287561	14.383	3.640606	12.46209	12.29473	21.19547	5.872886	5.654863
2.060491	2.436768	0.823983	1.827403	0.526299	0.402381	0.793918	0.158124	0.845887
3.726854	22.74797	0.774798	38.94854	3.195541	10.1459	1.443059	1.717641	14.79742
9.459177	8.643074	4.575123	7.854069	3.110447	4.833398	1.431414	3.310758	6.632063
4.246861	0.669358	3.381255	0.949286	4.524481	1.651522	10.1459	1.770029	1.057569
3.043528	4.678846	0.808983	2.316075	2.203535	2.212619	2.701053	1.248232	1.921212
2.496407	2.410996	9.541645	1.15614	2.470317	1.962424	1.732468	1.185685	1.952027
5.872886	6.011515	1.117316	6.570433	5.933066	3.711097	2.90658	14.00414	13.15079
14.97762	5.679502	10.60361	7.697063	7.315512	6.888297	12.85504	4.827691	3.391075
45.1045	25.24367	11.65791	33.07147	32.68313	25.73165	8.994791	16.80343	32.68313
0.067446	0.106926	0.607937	0.001811	1.445916	0.311227	0.765094	0.000433	0.392251
0.70329	0.740527	0.196335	0.92482	0.810967	0.916869	1.938759	0.632569	0.363276
1.434921	1.587605	1.130794	1.856392	4.281847	1.89228	3.714906	3.052655	2.574733
0.285426	0.000736	0.621299	0.001811	0.221962	0.106118	0.55215	0.108124	0.000924
0.052814	0.000736	0.140087	0.046724	7.22E-05	0.047504	0.042078	0.025483	0.000924
1.058896	0.075772	2.922778	0.093203	0.356508	0.254964	0.493925	0.55645	0.193449
33.07147	8.119004	4.359574	17.51961	7.013853	4.752121	8.103925	5.485685	11.48368
1.301169	1.205685	0.56145	1.821861	0.86469	1.149978	0.684033	4.02088	2.337821
0.50254	0.570664	1.391494	0.369336	0.772951	0.788369	0.982698	0.769019	0.519755
0.509242	0.561775	0.215729	0.114841	0.055967	0.402381	0.055397	0.342814	0.000924

0.157958	0.000736	0.205382	0.001811	7.22E-05	0.000534	5.77E-05	0.000433	0.000924
6.433918	2.74298	1.057569	1.161501	4.789596	5.728615	4.673182	2.928247	2.747908
0.029221	0.034603	0.105606	0.046724	0.197128	0.029769	0.145815	0.239769	0.038088
2.993867	2.234538	1.248232	2.106089	1.283059	2.34355	2.066652	0.760866	2.776421
0.567345	0.599798	1.89228	0.587965	2.225498	0.732193	0.90368	1.185685	0.845887
9.316241	21.54823	2.922778	4.907929	5.01772	1.763889	2.81342	3.500808	7.315512
12.77772	11.544	3.195541	5.872886	5.259437	20.60009	6.508427	14.88584	8.195657
0.177879	0.034603	0.070967	0.114841	0.027879	0.073672	0.06088	0.032915	0.057994
8.66E-05	0.000736	0.035382	0.056869	7.22E-05	0.000534	0.014488	0.000433	0.000924
3.4679	1.660216	6.365065	1.317475	3.68381	3.297107	8.381328	6.784762	2.561162
3.877417	1.86658	12.7427	1.317475	3.373983	2.308203	2.489762	2.876421	1.297763
2.781558	1.278629	5.42381	1.076032	2.836717	2.021068	3.775209	3.182482	1.638499
1.45912	0.778788	0.726472	0.73619	1.314675	0.447965	1.508831	1.215195	0.340079
6.063276	9.792626	1.015967	7.882222	3.663622	4.165137	3.904517	1.941703	3.747035
0.236638	0.158766	0.000115	0.205931	0.600548	0.000534	5.77E-05	0.000433	0.000924
9.751039	21.74051	5.881342	17.78522	3.644343	14.42661	1.991328	8.461703	16.5625
0.036696	0.000736	0.718355	0.084113	0.772951	0.000534	0.217908	0.158124	0.000924
0.687049	0.91404	5.195022	0.817547	7.558139	2.361198	4.402973	0.927561	0.363276
62.33472	24.04374	44.27012	24.98218	83.489	24.98218	135.5245	14.79742	19.99861
0.444315	0.868341	0.368882	0.637994	0.365036	0.560043	0.446811	0.100188	0.330635
1.165873	4.863579	0.000115	3.989206	1.253088	3.269913	1.566414	2.45575	3.533211
8.66E-05	0.000736	1.482626	0.001811	0.311876	0.000534	0.217908	0.000433	0.000924
1.126775	1.36619	1.130794	1.283059	1.634935	1.53684	1.267879	3.107417	2.755411
1.190491	3.300664	4.359574	6.55039	2.964762	4.971991	9.141299	8.303947	6.079596
3.895469	4.946587	3.346898	8.164531	5.137042	0.954776	2.925426	2.797835	2.941544
0.349812	0.095772	0.850808	0.205931	0.141825	0.127273	0.323752	0.300606	0.095108
0.171371	0.695426	1.972706	0.610606	0.926926	0.552439	0.342814	0.566494	0.095108
1.402915	3.548153	2.073232	9.751039	0.54619	4.077403	3.939307	1.757244	4.752121
0.92816	1.326667	2.939141	1.016494	2.145137	1.962424	2.043795	4.46267	2.621133
1.310837	0.290317	2.723247	0.47246	1.924228	0.544545	3.921645	0.666847	0.281724
2.607035	5.493182	0.000115	2.5271	3.820758	3.457504	3.890556	3.8121	3.917597
1.932857	4.338629	1.902352	1.055628	0.478182	7.251263	1.711414	1.844098	2.08632
1.340036	0.402063	4.02088	0.856291	1.164257	0.598456	0.362864	0.683665	0.330635
6.717056	15.46193	5.923838	34.38341	6.191515	12.56693	19.99861	10.28573	17.28195
7.507619	5.150693	12.97323	10.19468	19.53061	8.711905	16.68554	12.29473	8.994791
8.66E-05	0.000736	0.022727	0.001811	0.100996	0.000534	0.21088	0.000433	0.000924
4.990794	17.64869	11.19942	10.8813	10.98644	16.68554	16.9162	17.28195	16.44768
7.911934	749.7839	60.77449	530.1465	1360.293	87331.38	126.1556	1360.293	87331.38
1.210447	2.223636	20.51271	1.543377	0.872597	0.691674	1.945916	0.342814	0.340079
1.731169	1.115902	0.189394	4.292078	0.221962	2.361198	0.558824	0.585902	2.180022
10.01009	4.429769	2.009906	3.734762	2.693442	2.415296	2.765758	5.671126	4.802655
8.508514	6.922771	4.921082	5.323492	5.395584	13.6814	6.570433	5.830152	8.728571
184.443	100.0998	21.19547	184.443	8.994791	27.38726	19.53061	30.82277	75.1132
6.888297	3.621941	2.544747	8.923117	1.771392	2.558817	5.538001	2.586926	4.888023
7.013853	36.26061	0.029221	37.29684	25.73165	22.32214	11.93253	33.4985	35.776
5.416623	8.288874	9.625253	3.563182	4.620159	12.49452	7.212915	5.180058	5.050267
0.531804	1.029365	1.410902	1.165065	2.50469	1.364401	2.947287	0.659957	0.606154
21.54823	1.91254	2.683478	3.614603	1.667273	4.888023	4.424416	2.081696	2.019618
0.418788	0.47974	1.20824	0.4357	1.790245	0.487662	0.55215	0.585902	0.37228
8.66E-05	0.000736	0.933874	0.321746	0.100996	0.000534	0.198961	0.000433	0.000924
9.316241	13.30906	19.14516	16.22108	14.00414	10.38202	17.39935	13.53913	11.77567
14.00414	6.423911	14.383	5.366335	15.77678	5.295325	16.34294	13.45939	13.38418
0.044762	0.000736	0.000115	0.001811	0.133117	0.000534	5.77E-05	0.000433	0.000924
23.16622	17.28195	6.591544	37.81919	16.5625	29.82828	8.849697	14.42661	17.78522
4.02088	1.29417	0.737403	0.672078	0.723506	1.069574	2.165851	0.784805	1.348975
0.358672	0.034603	3.019848	0.187893	0.279315	0.245967	1.1514	0.259964	0.154098
0.687049	0.853218	1.542302	0.362864	1.25816	1.02487	0.81544	0.901732	0.712453
0.083983	0.150065	0.196335	0.056869	0.16925	0.000534	0.019408	0.093009	0.000924

9.179466	4.770231	2.630729	11.09133	3.930664	3.334199	7.697063	4.715599	6.486104
1.343463	0.948211	3.993665	0.98956	1.61088	1.39847	2.257749	2.955916	1.638499
0.022475	0.000736	0.28026	0.001811	7.22E-05	0.000534	0.06088	0.025483	0.000924
5.889798	5.959033	1.580909	7.765931	6.277937	6.674286	5.830152	3.651898	4.783001
80.6518	56.39528	43.49658	100.0998	70.11932	29.49693	47.5018	33.07147	52.56128
8.923117	9.667316	0.861934	8.694697	4.251818	6.967547	3.66785	4.38684	6.325945
0.524986	0.119553	0.224661	0.065519	0.183052	0.047504	0.101782	0.057352	0.000924
1.643218	2.014863	2.836717	7.339452	5.238701	3.695815	7.288788	5.942294	7.964315
8.66E-05	0.000736	1.268672	0.001811	7.22E-05	0.000534	0.169105	0.000433	0.000924
8.66E-05	0.075772	0.298153	0.065519	0.046962	0.023059	0.1086	0.041183	0.000924
2.097843	0.274199	3.273045	0.426898	0.884654	1.798535	2.129596	0.850325	0.574228
0.339466	0.000736	0.477446	0.001811	0.064372	0.000534	0.370281	0.202799	0.000924
0.221111	0.000736	1.519322	0.153983	0.328737	0.000534	0.432237	0.118644	0.076775
3.707136	1.176623	0.614545	0.416219	1.865043	1.678319	0.96329	5.80469	1.685678
1.505678	6.52899	1.759986	2.421681	5.754935	4.678846	5.295325	6.38456	8.075772
1.362713	0.203463	1.685678	0.196955	0.701818	0.631494	0.800512	0.075772	0.000924
12.29473	8.119004	3.450577	9.218514	4.875527	12.85504	4.207778	4.927302	6.674286
0.268369	0.053766	0.173456	0.001811	0.205649	0.196436	0.519019	0.202799	0.000924
5.217569	7.162698	6.486104	10.78056	14.08569	11.48368	10.23768	11.83593	13.75978
7.188095	4.661717	2.763261	3.083319	3.88153	2.031169	2.106089	4.752121	4.990794
0.321068	0.140657	0.568665	0.176955	0.29671	0.065253	0.29715	0.268687	0.146623
3.599329	16.44768	0.000115	3.640606	4.365224	8.539971	6.054242	7.225469	7.653391
3.182482	0.309221	2.45575	0.700418	0.41026	0.620462	0.86469	0.806227	0.589618
1.286494	3.767489	0.621299	4.281847	1.244242	0.390418	0.613853	2.565556	3.233045
0.402063	0.000736	0.758254	0.001811	0.10956	0.116926	0.169105	0.084906	0.0857
0.036696	0.000736	0.000115	0.001811	0.006407	0.088341	0.56443	0.057352	0.000924
0.808052	0.000736	0.468167	0.001811	0.083983	0.000534	2.066652	0.128687	0.000924
0.808052	3.563182	0.629538	1.20824	0.588312	0.523983	0.311046	1.2214	2.818312
0.044762	0.053766	0.035382	0.001811	7.22E-05	0.000534	0.024315	0.000433	0.000924
0.014906	0.000736	0.181674	0.056869	0.011631	0.000534	0.014488	0.093009	0.000924
7.136537	4.053506	1.737294	1.744055	6.674286	6.772763	4.586753	1.287403	4.508196
0.212496	0.335411	0.173456	0.4971	0.242222	0.301955	0.29715	0.73912	0.868341
3.008225	2.593506	1.673254	1.608427	3.585029	3.214545	3.150433	2.944416	3.29355
2.150303	2.638139	0.850808	2.563506	3.460909	1.559675	1.124574	1.102691	2.442987
0.339466	0.226623	1.218182	1.915455	2.784228	1.204957	3.851414	4.946587	5.47
9.256537	2.74298	1.99899	1.254704	4.914863	2.718434	0.140188	4.086926	6.717056
0.157958	0.02596	0.114358	0.001811	0.006407	0.000534	0.073362	0.032915	0.000924
4.484892	5.075447	1.218182	1.793074	5.351934	3.618499	3.169812	8.048095	4.429769
53.77619	30.17192	11.09133	47.5018	24.04374	52.56128	36.26061	18.19595	43.49658
0.190693	0.034603	2.429957	0.001811	7.22E-05	0.155224	0.193196	0.041183	0.057994
0.151176	0.130823	0.289221	0.196955	0.141825	0.097092	0.244747	0.065974	0.103528
28.87506	4.971991	21.74051	2.630729	2.984899	8.019769	6.760765	5.855281	3.625382
2.797835	1.166717	3.606804	2.715873	3.851414	1.744055	4.900996	1.89228	2.941544
1.1514	0.545152	0.477446	0.369336	0.593853	0.116926	0.808052	0.390895	0.519755
0.114517	1.901068	0.360938	0.216025	0.205649	0.254964	0.029062	0.402063	0.213355
2.413146	2.419553	1.624459	5.80469	4.609076	1.821861	4.597792	9.063983	2.797835
5.323492	20.34063	2.794935	8.019769	5.180058	13.38418	2.917381	5.889798	8.608153
1.264589	2.8657	0.105606	0.001811	1.409942	0.402381	0.198961	0.065974	0.461234
59.32013	72.69473	12.49452	45.91841	46.7056	42.75576	67.98069	32.31123	42.75576
2.150303	1.104033	0.831472	2.944416	1.884935	0.266032	1.62562	0.877807	0.614156
0.243016	0.411948	3.640606	0.970007	0.459394	0.311227	0.96938	0.166573	0.321227
1.374329	1.314675	1.335743	0.637994	0.757763	0.760433	0.515238	0.648362	0.684401
47.5018	26.52537	3.195541	33.4985	30.49289	15.18102	24.73724	7.780786	29.82828
0.47974	2.234538	0.413009	7.136537	2.026219	1.491977	0.622496	1.329206	5.217569
1.567576	2.600014	3.080317	4.096407	2.597792	2.447294	3.703175	2.066652	3.404372
0.834957	0.882078	1.391494	0.863694	0.986104	0.50469	0.82684	0.149019	0.509791
1.932857	0.661025	0.861934	1.200152	1.082915	1.02487	0.657114	0.927561	2.589185
0.593853	0.044214	1.280274	0.187893	0.141825	0.047504	0.388831	0.000433	0.233304

1.196508	0.430837	0.461811	1.277843	0.692027	1.11526	0.453463	0.184459	0.637994
1.905094	75.1132	34.84299	87331.38	2.026219	70.11932	252.4251	135.5245	332.0776
0.171371	0.053766	0.164993	0.233752	0.304062	0.560043	5.77E-05	2.287576	0.660678
8.66E-05	0.000736	0.392944	0.001811	0.15127	0.000534	0.066696	0.049387	0.000924
0.052814	0.1686	0.307734	0.34443	0.083983	0.088341	0.422511	0.075772	0.067937
7.964315	4.789596	1.015967	42.08807	1.121703	3.387864	1.136566	1.093434	3.422237
0.883665	1.247244	0.823983	1.20824	0.898543	0.771176	0.342814	0.481104	1.010887
4.348882	0.497424	2.018167	0.458521	1.484553	0.362864	1.215981	0.726811	0.660678
1.695216	1.156869	2.647244	1.235346	2.386248	1.569553	3.037626	1.799899	1.236248
0.06057	0.000736	0.049531	0.001811	7.22E-05	0.000534	0.014488	0.000433	0.000924
3.119986	1.766248	6.900202	1.722475	2.693442	2.708341	3.188918	4.115844	2.597792
1.330974	0.570664	1.437648	3.220678	1.341825	0.328543	0.754315	0.611789	2.035678
0.546522	0.086443	6.414416	0.351219	0.908773	0.335786	0.335786	1.163579	0.440801
0.540188	0.034603	0.049531	0.046724	0.055967	0.047504	0.178997	0.065974	0.000924
9.141299	3.869163	2.561162	7.38847	4.490801	2.640332	4.563117	4.043846	5.976724
1.202626	0.938911	0.000115	4.776407	4.833398	1.204957	0.342814	3.636789	2.373824
0.846342	0.469711	3.310758	0.298874	0.884654	0.188124	1.256558	0.276609	0.420289
7.824473	5.704646	1.988196	5.500678	2.361198	10.78056	3.381255	6.99026	5.993045
0.909315	0.327994	0.105606	0.458521	0.10956	0.215267	0.162179	0.100188	0.146623
8.66E-05	0.02596	0.000115	0.001811	7.22E-05	0.023059	0.014488	0.032915	0.000924
1.406746	3.220678	1.004156	1.68671	1.729892	2.640332	2.625823	1.439704	2.561162
8.66E-05	0.075772	0.368882	0.103434	0.046962	0.000534	0.042078	0.075772	0.076775
6.153102	9.377345	1.472208	5.560563	4.012049	4.914863	5.500678	3.4679	4.397792
0.632208	0.459394	0.861934	0.478413	0.961486	1.449877	0.12904	0.032915	0.303896
4.193232	5.576219	4.086926	4.678846	1.215981	9.924372	0.65241	6.277937	8.677489
8.797619	10.43066	7.002056	9.625253	13.34662	6.922771	16.44768	10.78056	11.36975
0.190693	0.075772	0.140087	0.251126	0.118225	0.127273	0.140188	0.093009	0.095108
8.019769	1.64684	5.630815	3.307186	4.776407	1.945916	3.94886	1.977316	1.128788
1.173535	1.720144	1.795851	2.855296	3.585029	1.390476	3.890556	2.810678	2.194257
1.674401	0.514141	2.763261	1.373348	5.195022	1.219019	2.936739	1.482626	0.879913
0.778326	0.299062	1.921212	0.518716	0.878932	0.097092	1.455022	0.458701	0.202987
1.256558	0.442193	1.064935	0.298874	0.28746	0.277417	0.155945	0.13824	0.18324
5.47	10.78056	10.2617	8.83241	3.984747	12.77772	1.62562	21.36892	11.96574
14.79742	20.68746	7.417359	39.53687	9.989221	8.134084	7.519841	8.573434	15.77678
11.89932	25.73165	4.408153	44.27012	44.27012	17.64869	38.94854	16.09482	23.71237
0.044762	0.000736	0.057583	0.001811	7.22E-05	0.000534	0.029062	0.000433	0.000924
2.045476	2.259661	1.673254	2.221775	2.048781	2.931068	1.855108	10.01009	6.210375
11.19942	4.990794	10.82954	7.795642	12.10289	4.72798	8.134084	9.335397	5.942294
1.045462	0.379495	0.454156	1.660216	0.539466	0.245967	0.700837	0.595022	0.493586
12.16681	10.93101	7.049336	15.56131	12.46209	8.288874	12.7427	6.063276	9.707677
0.981032	0.392554	0.816479	0.717879	0.857778	0.412453	0.629538	0.300606	0.293882
8.66E-05	0.044214	0.140087	0.065519	0.019408	0.073672	5.77E-05	0.041183	0.000924
0.313312	0.843564	0.000115	0.889553	0.952294	1.326667	0.432237	2.647244	2.259661
1.115902	0.853218	5.821804	0.489509	0.884654	0.402381	1.431414	0.632569	0.440801
1.41368	0.790837	4.894509	0.70886	1.718918	0.894704	3.104394	1.779423	0.598124
0.268369	0.119553	0.468167	0.084113	0.459394	0.235296	0.647345	0.249719	0.233304
2.308203	4.490801	1.187229	14.79742	5.158146	2.511465	3.418405	2.668896	3.763045
0.014906	0.044214	0.181674	0.056869	0.011631	0.000534	0.042078	0.025483	0.000924
1.674401	29.18885	1.391494	24.73724	0.819509	17.51961	4.317648	25.99427	13.23022
0.171371	2.496407	0.345238	1.931537	0.884654	0.612525	0.244747	0.691674	1.401068
8.66E-05	0.000736	0.382626	0.141876	0.213615	0.000534	5.77E-05	0.100188	0.000924
24.73724	1.455988	24.98218	1.51215	7.212915	1.65785	2.019618	3.435988	1.921212
29.82828	118.7028	0.000115	332.0776	8.048095	65.94551	13.15079	35.05953	95.22939
6.210375	17.15902	1.673254	14.17388	6.570433	5.614805	5.030866	4.429769	6.125823
0.298687	2.42381	0.148384	0.277417	0.338658	1.613232	0.024315	0.000433	0.000924
1.828781	8.226219	4.586753	6.967547	9.297085	3.034747	4.46267	5.959033	7.911934
24.50942	29.82828	75.1132	33.93939	35.27608	39.53687	91.05541	100.0998	49.50903
0.008254	0.02596	0.035382	0.001811	0.006407	0.000534	0.009185	0.000433	0.028867

0.767569	0.580758	1.921212	0.153983	0.937655	0.544545	0.487128	1.439704	1.154567
0.029221	0.000736	0.105606	0.001811	7.22E-05	0.000534	0.09381	0.000433	0.000924
2.914632	9.480736	0.181674	8.048095	3.128694	3.718716	1.021097	4.241905	7.238023
0.711371	1.633752	0.707799	0.001811	0.54619	0.980505	0.589986	0.118644	0.000924
1.202626	3.588369	0.181674	1.297763	1.34798	2.666494	0.808052	3.783694	2.638139
4.46267	1.379278	0.096999	2.863211	0.781977	1.806595	0.695051	1.87482	2.216162
1.443059	2.586926	0.568665	0.66526	1.267879	0.933874	0.47246	0.675556	3.092229
16.68554	18.05488	22.03348	10.38202	26.52537	20.34063	19.22139	27.66779	21.54823
17.92188	28.54786	15.83113	17.03919	25.99427	21.36892	4.281847	11.65791	18.41211
0.197338	0.553348	0.249719	0.286183	0.141825	0.029769	0.335786	0.108124	0.264242
0.600173	9.989221	1.077403	3.043528	0.515238	8.256465	0.232215	12.22798	9.256537
7.795642	3.691724	41.47925	5.704646	5.259437	1.728615	5.172828	3.57057	2.993867
2.768312	1.434921	0.307734	1.118817	1.52676	1.602619	1.977316	2.150303	1.401068
2.579509	1.156869	4.524481	0.518716	0.893016	0.905426	1.185685	0.841746	1.305772
2.106089	1.36619	2.939141	1.124574	5.515584	4.155339	1.797172	10.19468	5.872886
0.221111	0.255887	0.554026	0.125354	0.338658	0.402381	0.493925	0.490079	0.223701
4.058514	2.035678	1.423506	1.297763	1.353608	3.253175	1.47018	1.174315	1.944517
0.044762	0.000736	0.454156	0.001811	0.141825	0.000534	0.101782	0.000433	0.000924
1.548723	1.256558	6.071436	1.849654	2.190866	1.952027	1.267879	6.749488	4.715599
1.971183	1.988196	4.965801	1.277843	6.09759	2.147013	1.971183	1.87482	1.273636
1.286494	1.609654	0.497749	0.426898	0.29671	2.8657	0.579495	1.064935	1.76873
0.250007	0.590303	1.057569	0.458521	0.744113	0.760433	1.203427	0.438023	0.450678
0.276847	0.053766	0.444986	0.103434	0.100996	0.205801	0.101782	0.490079	0.057994
0.083983	0.319762	0.096999	0.056869	0.064372	0.180368	0.086775	0.075772	0.067937
1.278629	7.468961	0.718355	11.48368	2.800332	25.48179	5.402511	4.965801	5.150693
2.320072	2.035678	5.754935	3.034747	1.985253	1.308167	2.257749	2.586926	4.655916
0.014906	0.000736	0.049531	0.001811	0.006407	0.000534	5.77E-05	0.000433	0.000924
2.388348	0.309221	12.36443	0.268095	1.566414	0.116926	2.735368	0.352006	0.000924
2.396429	0.553348	3.699495	1.343463	3.016984	1.783203	3.585029	2.257749	1.128788
1.884935	1.132165	4.94026	0.856291	2.371833	1.159149	1.111977	1.728615	1.190491
8.119004	9.218514	2.487576	8.958225	11.42512	13.75978	10.48101	8.694697	7.264502
0.808052	1.499214	0.413009	3.588369	1.298665	0.430281	0.730476	1.844098	2.5271
0.184055	0.203463	0.326219	0.268095	0.320794	0.320144	0.487128	0.184459	0.138449
0.014906	0.392554	0.078167	9.418196	8.888167	2.890245	3.330909	8.005685	8.539971
0.469711	0.895368	0.224661	0.416219	0.328737	0.560043	0.232215	0.901732	0.868341
1.278629	1.338261	1.902352	1.572035	2.984899	3.055729	2.055289	1.78759	1.934271
0.107287	0.371075	0.205382	0.587965	0.526299	0.328543	0.169105	0.585902	0.411436
0.92816	0.47974	3.214545	0.382417	0.749942	0.235296	0.262785	0.760866	0.392251
5.545729	4.580938	6.365065	1.435772	6.508427	3.207807	10.05618	2.406768	1.313737
2.183665	0.882078	1.846941	1.485483	2.386248	3.486436	2.572309	1.215195	1.154567
18.48514	27.66779	0.000115	9.179466	33.4985	17.28195	10.78056	11.96574	11.60283
4.408153	1.499214	0.497749	5.302294	2.461905	3.651898	2.625823	2.987835	1.853723
1.362713	0.450375	0.683232	0.672078	0.434596	0.343629	1.146118	0.750866	0.743694
8.797619	1.499214	3.899993	1.351573	2.417424	0.691674	2.990851	1.396494	2.071587
13.91714	9.583463	6.900202	6.22031	7.710361	10.43066	9.52197	5.622872	8.103925
0.606421	0.379495	2.993867	0.40075	1.058896	0.831472	0.280469	0.511667	0.801501
8.477662	5.728615	26.80355	3.833247	18.19595	7.897078	5.671126	4.814661	3.877417
0.134942	0.625498	0.517612	1.190491	0.441833	0.788369	0.193196	0.548788	0.913514
0.298687	0.106926	0.289221	0.065519	0.271912	0.180368	0.135051	0.057352	0.138449
20.51271	11.89932	15.07257	62.33472	9.335397	9.880779	12.42965	19.37139	16.34294
1.158384	1.91254	2.08632	0.803211	1.283059	0.631494	0.719942	0.850325	0.975556
1.45912	1.402915	0.747879	3.077316	1.852338	2.031169	1.021097	2.518124	6.404921
8.66E-05	0.000736	2.034242	0.001811	7.22E-05	0.000534	0.155945	0.000433	0.000924
6.277937	2.31404	5.062626	4.086926	12.93651	2.789466	17.15902	3.747035	2.876421
56.39528	19.68088	0.000115	70.11932	6.653016	29.18885	6.09759	11.719	13.08214
0.052814	0.034603	0.057583	0.046724	0.006407	0.000534	0.019408	0.025483	0.000924
1.383911	4.900996	4.451154	6.162446	3.207807	2.876421	1.884935	2.533745	2.274473
7.6393	4.359574	2.269134	11.14619	2.365685	5.323492	2.23267	4.155339	8.288874

3.763045	1.176623	1.932857	0.998312	2.836717	3.279625	3.233045	1.287403	1.826147
0.450924	4.359574	1.20824	3.243225	1.200823	0.631494	0.46153	5.351934	1.989711
10.73084	3.644343	7.049336	1.581912	5.959033	2.259661	2.781558	2.287576	2.248896
0.70329	0.678247	2.396429	0.47246	1.52676	0.514141	0.81544	0.666847	0.751876
5.150693	5.337547	10.2617	4.796126	17.15902	7.598312	4.402973	53.77619	9.751039
0.091255	0.000736	1.102121	0.001811	7.22E-05	0.000534	5.77E-05	0.000433	0.000924
0.600173	0.106926	0.477446	0.001811	0.320794	0.047504	0.323752	0.211501	0.076775
2.243911	7.288788	2.673846	7.212915	6.967547	8.849697	3.263391	19.06893	19.22139
43.49658	15.07257	167.9996	22.74797	126.1556	27.96248	100.0998	25.48179	27.96248
2.834084	41.47925	2.747908	43.49658	3.851414	60.77449	32.68313	22.74797	26.25557
52.56128	12.85504	53.77619	6.727633	30.17192	14.08569	49.50903	67.98069	17.09911
7.6393	14.88584	31.55417	8.195657	12.22798	11.89932	9.583463	6.036818	10.03313
12.36443	31.92385	15.18102	25.48179	27.38726	38.39042	31.55417	17.39935	22.96463
7.315512	5.679502	9.924372	3.147294	13.15079	6.508427	4.46267	4.365224	4.965801
425.62	530.1465	425.62	425.62	332.0776	749.7839	1360.293	749.7839	530.1465
155.7103	64.12971	224.9555	80.6518	95.22939	111.5439	144.7919	167.9996	155.7103
87331.38	27.96248	80.6518	52.56128	75.1132	126.1556	95.22939	37.81919	105.1694
65.94551	15.3618	203.5795	27.66779	40.7932	13.30906	111.5439	38.94854	33.4985
2.74544	2.547179	10.33476	1.975772	6.853608	2.212619	6.230245	2.171429	1.429365
1.383911	0.817013	1.493968	0.478413	0.810967	0.724004	2.292864	0.648362	0.751876
2.333867	2.074906	10.43066	1.351573	3.718716	2.807937	5.830152	2.876421	1.339091
13.83359	7.570693	9.356371	8.241342	5.28039	11.03843	16.09482	5.102742	6.191515
0.243016	0.064488	3.005281	0.176955	0.42715	0.056046	0.446811	0.084906	0.048225
0.143218	0.000736	0.087504	0.056869	0.073939	0.056046	0.22456	0.158124	0.048225
1.066948	1.074242	3.807648	1.262179	1.703795	1.317475	1.50026	0.892395	1.675722
0.623499	0.489509	0.674163	1.277843	0.434596	0.612525	5.77E-05	0.249719	1.198701
2.970144	3.909033	2.879228	5.662994	4.012049	4.145209	11.09133	2.333867	5.599228
0.50254	0.000736	0.000115	0.369336	0.083983	1.284747	0.267597	0.194098	1.401068
15.3618	3.493362	57.84882	2.693442	4.323045	4.47912	6.653016	11.60283	3.659668
6.486104	3.796075	3.529466	3.939307	2.109755	4.105714	7.037345	2.197828	3.279625
0.846342	0.342511	4.894509	0.298874	0.731732	1.02487	1.431414	0.184459	0.349084
0.06057	0.000736	0.065152	0.001811	7.22E-05	0.097092	0.114964	0.000433	0.000924
0.444315	0.274199	1.198701	0.692799	0.54619	0.390418	0.525678	0.259964	0.233304
2.797835	3.754719	0.900693	3.486436	1.465123	7.037345	3.370527	1.700354	4.096407
35.776	33.4985	17.15902	27.10502	24.98218	21.19547	30.17192	36.26061	25.24367
38.94854	50.55949	45.1045	25.24367	22.96463	91.05541	19.68088	28.23632	45.91841
6.508427	5.42381	18.62711	9.377345	10.19468	7.795642	8.745238	7.6393	4.914863
3.869163	2.893001	8.195657	1.558571	3.707136	2.973059	4.241905	2.223636	1.613232
0.067446	0.000736	0.508658	0.084113	0.176104	0.106118	0.558824	0.413009	0.067937
22.52375	18.48514	18.33909	19.53061	43.49658	32.31123	17.28195	15.56131	27.10502
0.948745	1.166717	0.850808	0.426898	0.961486	0.447965	0.487128	1.573276	0.542525
4.140505	2.752893	2.257749	0.717879	0.533535	0.612525	4.952915	1.135137	1.297763
20.16541	11.36975	38.39042	12.42965	12.70768	8.573434	32.31123	9.418196	7.625209
0.962698	0.186017	2.396429	3.909033	0.976111	0.311227	2.380079	0.500722	0.589618
0.578485	1.36619	1.795851	0.389336	0.872597	0.856782	0.746984	1.123016	0.574228
2.884762	4.086926	8.319019	2.909293	2.65684	2.361198	2.367763	2.547179	1.87899
0.731732	0.91404	1.972706	0.894704	1.030678	0.807619	0.65241	0.55645	0.621688
1.749307	0.514141	0.497749	0.242612	0.617417	0.000534	0.573947	0.352006	0.202987
0.632208	0.1686	0.948211	0.125354	0.348629	0.459048	0.46153	0.363276	0.138449
2.005253	0.514141	2.860722	0.426898	0.16925	1.069574	0.947677	0.566494	0.202987
1.165873	1.01544	0.477446	0.277417	0.384156	0.379495	0.41482	0.419827	0.330635
0.802035	1.188139	1.948918	1.816436	1.495043	0.691674	3.253175	0.683665	0.483326
1.655541	1.080909	6.830592	1.100519	0.701818	1.25746	4.933781	2.445079	2.019618
5.180058	2.024488	4.125786	1.572035	3.207807	5.143867	16.22108	0.603737	2.461905
0.808052	0.705613	0.096999	1.271876	0.468384	0.749481	0.54619	0.438023	1.190491
0.128088	0.335411	0.123535	0.483608	0.160707	0.106118	0.009185	0.239769	0.154098
15.83113	7.653391	6.508427	11.83593	14.70188	9.838658	26.80355	17.64869	8.797619
2.320072	0.247583	0.649668	0.292395	0.459394	0.301955	1.339091	0.691674	0.264242

8.66E-05	0.044214	0.01013	0.084113	7.22E-05	0.000534	5.77E-05	0.041183	0.000924
0.941645	0.868341	1.519322	1.596782	1.283059	0.379495	1.455022	0.806227	1.102691
4.900996	17.78522	2.524892	5.898449	3.971061	4.440548	3.552049	10.53159	6.454416
3.286652	1.205685	50.55949	0.60202	155.7103	3.514834	5.9071	31.1753	1.20653
6.622092	6.230245	2.132756	5.737056	7.351284	3.435988	3.679986	12.56693	13.53913
0.469711	0.625498	1.423506	1.842734	1.11526	0.620462	0.947677	1.174315	1.128788
13.38418	13.60866	3.578038	10.73084	6.738211	11.99921	4.971991	6.560411	10.23768
10.78056	3.926154	4.821176	4.381479	2.855296	8.677489	3.614603	1.458038	1.65785
1.779423	1.326667	1.835743	0.700418	1.050231	0.926291	0.596111	0.806227	0.845887
0.107287	0.000736	1.057569	0.001811	0.54619	0.155224	1.291825	0.000433	0.000924
15.56131	8.048095	29.34289	8.412367	10.53159	5.821804	20.34063	10.98644	5.351934
73.90397	135.5245	287.197	167.9996	224.9555	332.0776	155.7103	203.5795	203.5795
0.121147	0.000736	0.683232	0.125354	0.504416	0.000534	0.135051	0.000433	0.000924
73.90397	22.13245	49.50903	14.88584	40.11561	72.69473	18.99374	51.52195	57.84882
3.8379	0.778788	2.544747	1.175859	2.323983	2.000592	1.945916	1.548723	0.962698
0.78619	0.442193	1.472208	0.292395	1.363557	0.371876	1.050231	0.620909	0.363276
18.19595	10.38202	8.573434	12.49452	8.319019	9.459177	6.464906	7.882222	8.134084

CGGA_11[illegible]	CGGA_11[illegible]	CGGA_11[illegible]	CGGA_11[illegible]	CGGA_11[illegible]	CGGA_11[illegible]	CGGA_11[illegible]	CGGA_11[illegible]	CGGA_11[illegible]
0.009452	0.756724	0.524646	0.268824	1.025613	0.165108	0.524646	0.218355	0.274055
0.009452	0.070909	0.223485	0.412193	0.747417	0.579841	0.288745	0.194235	0.353492
5.381227	3.536955	5.28039	7.327482	3.57057	7.558139	5.545729	4.802655	4.188896
2.768312	2.984899	1.710173	8.075772	1.271097	2.03886	1.89373	0.450527	9.276811
3.137576	6.365065	1.746724	6.325945	1.95645	3.829105	3.065108	5.638759	2.461905
0.683232	6.55039	3.632973	0.909856	3.50425	1.693939	1.65785	3.083319	6.045209
10.82954	45.91841	33.07147	36.74522	53.77619	40.11561	72.69473	46.7056	27.38726
0.28728	0.007734	0.001053	0.046962	0.07632	0.050844	0.030491	0.174646	0.020938
2.261573	1.89518	1.393571	3.962179	3.06803	2.792035	0.941104	1.87482	0.765606
0.009452	0.411948	0.103341	4.33E-05	0.402381	0.000433	0.155224	0.116659	0.394156
8.573434	34.84299	28.54786	29.18885	30.82277	45.1045	38.39042	20.51271	20.85876
0.343824	0.80417	1.005426	0.535945	2.304452	0.535657	1.455022	1.123016	0.689509
52.56128	21.02638	16.80343	20.51271	8.445743	20.85876	13.6814	3.939307	52.56128
22.74797	0.627937	2.685786	14.51648	1.816436	4.484892	1.64917	7.38847	5.056421
0.009452	8.66E-05	0.002655	0.023304	0.028196	0.000433	0.030491	0.000101	0.000274
10.33476	4.016465	2.668896	8.005685	4.789596	3.687633	8.762677	5.110159	1.50342
0.441833	2.459812	1.828781	0.420058	6.144055	0.686667	3.028629	0.762165	0.736977
0.009452	8.66E-05	0	4.33E-05	2.89E-05	0.000433	0.000375	0.000101	0.054026
6.612121	1.338261	1.499214	1.648066	0.65798	3.783694	2.160649	3.083319	2.190866
0.129971	0.71956	1.256558	0.504416	3.028629	0.662612	0.832013	0.400967	0.487662
5.545729	1.57645	0.243232	0.757273	0.305253	1.219856	1.401977	0.778326	0.069784
67.98069	3.346898	3.373983	6.063276	1.169091	5.143867	3.314329	2.459812	6.945152
4.827691	35.27608	17.03919	4.33E-05	22.52375	29.49693	29.18885	30.49289	100.0998
0.11811	0.018492	0	0.023304	0.028196	0.060087	0.000375	0.000101	0.000274
0.610606	0.691674	2.647244	1.05417	1.839986	1.517143	1.556392	2.159004	0.252316
7.099913	10.73084	3.718716	8.573434	2.283752	4.776407	14.51648	5.821804	3.217612
5.180058	1.988196	0.233593	2.540462	0.354242	1.313737	0.166465	9.502294	1.288276
0.296234	0.305253	0.454978	0.400354	0.585592	0.420289	0.438831	0.084069	0.689509
167.9996	135.5245	51.52195	65.94551	49.50903	65.94551	30.82277	425.62	23.6012
0.31785	0.82974	0.372078	0.619365	0.489235	0.545808	0.695051	0.441472	0.410541
12.85504	13.83359	2.096162	6.53969	3.816551	8.780115	10.01009	3.381255	3.269913
0.204278	0.095303	0.024632	0.108997	0.159105	0.080722	0.120361	0.108124	0.020938
50.55949	3.303925	17.51961	20.34063	0.727633	21.02638	6.423911	0.12772	23.38378
0.009452	0.011962	0.064488	0.009163	0.165657	0.031818	0.276429	0.046544	0.000274
0.129971	0.528571	4.47912	0.393449	7.519841	0.308442	1.112641	0.540563	1.216768
4.927302	2.666494	1.155411	2.474834	2.55184	2.713319	3.381255	1.136566	0.274055
2.975974	3.899993	5.323492	4.33342	2.831501	5.622872	5.591717	3.479134	4.626025
0.129971	8.66E-05	0.064488	4.33E-05	0.116825	0.000433	0.023492	0.064848	0.034199
1.241811	5.130411	3.621941	4.752121	3.747035	3.909033	5.745996	2.955916	2.990851
1.679596	8.164531	2.970144	7.037345	10.98644	4.125786	3.730808	3.334199	6.414416
0.009452	0.049336	0.70404	0.039589	0.72044	0.395426	1.055628	0.490079	2.659214
0.431977	0.73583	1.436623	0.393449	1.1407	1.241811	0.490952	0.720938	1.463304
4.661717	1.315613	2.129596	1.137244	0.747417	1.546696	1.957958	0.155945	1.055628
0.07583	8.66E-05	0	0.004949	0.003175	0.000433	0.000375	0.441472	0.015714
0.450375	0.12075	0.15127	1.108947	0.214885	0.308442	0.467403	0.293276	0.441263
28.23632	25.24367	19.68088	57.84882	26.52537	27.38726	40.11561	34.38341	25.48179
0.192929	0.182821	0.064488	0.087619	0.134942	0.112121	0.312475	0.100483	0.04062
0.009452	0.108124	0.017172	0.023304	0.134942	0.000433	0.048341	0.108124	0.054026
0.009452	8.66E-05	0	4.33E-05	2.89E-05	0.000433	0.000375	0.014964	0.015714
0.009452	0.189278	0.032092	0.015599	0.273672	0.000433	0.000375	0.055671	0.069784
0.009452	0.011962	0.032092	4.33E-05	0.01697	0.000433	0.212698	0.014964	0.000274
17.28195	9.902576	4.914863	15.3618	14.21671	8.711905	3.864827	5.838499	4.833398
0.009452	0.079466	0.277417	0.031046	0.15241	0.031818	0.041616	0.055671	0.798153
0.141775	0.419596	0.31456	0.023304	0.140657	0.050844	0.695051	0.340866	0.096905
5.056421	0.71956	0.951652	1.41368	0.632208	2.876421	1.971183	1.421126	1.428398
0.009452	0.370101	2.058773	0.116176	0.288081	0.000433	0.400173	0.209048	0.60886
0.009452	0.821898	0.686667	0.130714	1.184935	0.60886	0.561775	0.391962	0.468384

0.810967	0.2129	0.410823	0.570664	1.224733	0.37228	1.013434	0.116659	0.591118
0.648687	1.377446	2.037114	1.209776	1.971183	1.464214	1.64917	1.80267	2.236407
0.214964	0.684401	0.732193	0.619365	0.679812	0.413009	0.84119	0.561126	0.370281
8.745238	23.16622	12.42965	15.98892	0.146479	10.01009	0.000375	3.691724	0.60886
0.601227	0.221111	0.223485	0.255087	0.220808	0.328268	0.190541	0.460065	0.600548
1.189048	0.558095	1.042944	0.275873	0.637085	0.353651	0.533196	0.720938	0.181919
4.94026	35.776	28.87506	28.23632	21.93452	30.49289	56.39528	56.39528	70.11932
0.009452	0.141082	0.055967	0.183052	0.134942	0.000433	0.400173	0.470736	0.353492
0.009452	8.66E-05	0.001053	0.009163	2.89E-05	0.042078	0.048341	0.023059	0.020938
1.386681	1.833102	1.731169	1.179885	1.016494	0.771688	1.118817	1.661328	2.236407
3.648081	1.482626	1.549711	2.727785	2.661587	1.975772	1.146118	1.779423	0.699192
0.089553	0.237035	0.36114	0.299062	1.451775	0.37228	0.653968	0.381999	1.312706
0.009452	0.018492	0.189632	0.06031	0.227244	0.112121	0.097843	0.100483	0.274055
0.009452	8.66E-05	0.465563	4.33E-05	0.372504	0.112121	0.089726	0.000101	0.458341
0.2264	0.283535	0.260765	0.009163	0.649668	0.069668	0.226883	0.100483	0.719942
0.336328	8.66E-05	0.010786	0.004949	0.003175	0.000433	0.055671	0.000101	0.015714
0.009452	8.66E-05	0	0.015599	0.003175	0.000433	0.000375	0.055671	0.000274
7.683766	4.429769	3.851414	6.580988	2.647244	7.765931	17.28195	5.302294	6.612121
0.141775	0.141082	0.141876	0.176299	0.056638	0.000433	0.305657	0.075231	0.131443
0.009452	0.056869	0.010786	0.053485	0.028196	0.091133	0.023492	0.229502	0.054026
0.752229	5.720173	3.307186	3.91355	4.370671	3.482785	2.65684	10.01009	8.728571
0.009452	0.042713	0.017172	4.33E-05	0.06912	0.000433	0.106696	0.000101	0.113218
0.385736	0.221111	0.543939	0.163766	0.305253	0.404113	0.576205	0.36267	0.447965
26.52537	5.273362	4.557605	5.295325	2.058773	8.103925	17.51961	1.227749	11.63037
0.106926	0.007734	0.010786	0.015599	0.028196	0.000433	0.089726	0.240101	0.000274
0.009452	0.237035	0.620014	0.130714	0.402381	0.133535	0.352222	0.250115	0.60886
24.04374	5.439278	12.49452	11.83593	2.89E-05	37.81919	11.48368	19.68088	31.92385
0.129971	0.133045	0.189632	0.163766	0.092049	0.353651	0.582973	0.000101	0.299062
0.11811	1.012114	0.57531	0.49127	0.472713	0.471025	0.384964	0.798153	1.089394
2.178268	0.204964	1.124574	2.858009	0.140657	1.008961	1.066948	0.78619	11.42512
0.2264	0.426392	0.294921	0.202294	0.523752	0.653586	0.155224	0.41026	0.062381
0.009452	0.079466	0.095382	0.004949	0.129733	0.022266	0.131768	0.174646	0.020938
2.129596	0.442496	1.132165	2.353247	1.708932	4.348882	0.897489	1.20824	0.954141
1.679596	13.00996	12.60233	13.75978	2.876421	13.91714	21.19547	7.938427	18.19595
0.009452	1.177994	1.350772	1.080909	1.184935	0.526566	0.47974	0.046544	0.487662
0.237359	0.261111	0.432468	0.235043	0.252165	0.345238	0.416782	0.283535	1.245671
0.11811	2.681169	2.355289	0.393449	3.404372	0.328268	1.222179	0.658362	1.685678
0.009452	0.011962	0.032092	0.039589	0.035505	0.000433	0.030491	0.000101	0.028081
4.155339	13.23022	5.485685	5.599228	4.150274	8.573434	5.671126	3.66785	8.677489
0.106926	0.034502	0.017172	0.039589	0.097879	0.042078	0.069365	0.014964	0.076241
0.07583	0.018492	0.006421	0.031046	0.056638	0.031818	0.048341	0.030613	0.020938
0.081667	0.018492	0.001053	0.009163	0.01697	0.000433	0.000375	0.038139	0.000274
1.720144	1.281241	2.400455	0.814877	1.95645	1.05417	1.640779	1.074827	1.245671
4.130794	5.779574	6.796566	6.508427	5.068831	5.123781	7.037345	1.191198	2.178268
0.180368	1.931537	8.728571	1.118817	2.903867	0.753449	3.116732	1.136566	2.325931
0.2264	0.403651	0.277417	0.217172	0.423038	0.287951	0.113911	0.420707	0.104329
3.113478	1.245671	0.372078	3.373983	0.32925	0.437799	0.832013	0.596775	0.68062
0.009452	0.289452	0.198059	0.06031	0.288081	0.000433	0.000375	0.000101	1.171349
0.402937	0.2129	0.36114	0.593434	0.07632	0.27899	0.288745	0.038139	0.121818
17.39935	0.764704	1.074827	1.867951	0.3414	2.901032	5.923838	0.78619	1.194221
0.141775	0.596111	1.872049	0.611789	0.625209	0.249408	0.643853	0.508658	1.245671
0.431977	1.2214	3.125844	1.554185	2.050404	1.629235	2.302626	2.807937	1.614257
1.406746	4.47912	2.255916	4.667518	0.965714	1.950519	3.771349	1.05824	4.603434
0.353795	1.32482	0.543939	2.089639	1.618773	0.413009	1.250058	1.826147	0.736977
530.1465	14.29947	3.585029	126.1556	3.391075	155.7103	46.7056	32.68313	8.119004
0.336328	3.428788	1.690483	1.852338	2.445079	3.011169	1.693939	3.890556	0.23246
0.421804	0.355556	0.465563	0.053485	0.372504	0.143333	0.490952	0.091833	0.121818
144.7919	8.66E-05	19.99861	155.7103	2.89E-05	70.11932	29.82828	5.530274	167.9996

0.376407	0.24562	0.087734	0.1379	0.180613	0.069668	0.233434	0.108124	0.069784
0.009452	0.550368	0.338499	0.053485	0.188009	0.176104	0.226883	0.000101	0.090772
0.970635	2.207417	2.194257	1.948918	2.752893	2.171429	1.513102	3.691724	0.882078
12.56693	21.74051	8.677489	28.54786	25.99427	12.56693	10.33476	11.19942	14.51648
0.995527	0.204964	1.78759	0.087619	0.06912	0.123102	0.174776	0.008975	2.453781
1.087345	7.264502	12.16681	3.540606	6.335664	5.923838	9.541645	4.900996	8.429055
0.089553	0.670519	1.991328	0.611789	2.283752	0.599466	0.498052	1.191198	0.68062
0.296234	3.479134	2.323983	1.315613	3.016984	2.021068	1.388543	4.160238	0.765606
1.932857	1.093434	5.42381	0.619365	7.61176	0.196667	0.561775	1.931537	3.820758
0.752229	0.82974	0.404408	0.579149	0.841746	0.599466	0.473586	1.693939	0.945483
0.009452	8.66E-05	0.002655	0.009163	0.021696	0.000433	0.000375	0.000101	0.000274
0.421804	0.926926	1.746724	0.757273	1.372475	1.464214	0.366674	0.798153	0.5129
0.328268	0.221111	0.286681	0.483608	0.335786	0.479401	0.406912	0.240101	0.535029
2.563506	8.210938	17.64869	5.770996	5.654863	7.519841	18.12541	2.638139	6.808369
0.009452	0.268369	0.189632	0.023304	1.184935	0.042078	0.023492	0.572987	0.04062
5.25254	1.700354	3.944084	2.676392	2.981962	3.391075	1.419278	1.165873	1.227749
0.395152	0.845317	1.005426	0.579149	0.841746	0.852172	1.238651	0.481414	0.773276
0.009452	8.66E-05	0.001053	0.004949	0.035505	0.000433	0.055671	0.000101	0.020938
1.060823	10.28573	5.889798	1.428398	11.03843	1.000173	7.495397	3.714906	4.776407
1.765065	0.475137	0.25197	0.498355	0.092049	0.672468	0.341241	1.191198	0.542828
0.009452	0.034502	0.047323	0.015599	0.347655	0.000433	0.000375	0.038139	0.034199
1.706219	1.20824	0.925801	1.584105	1.409942	1.710173	0.568369	3.022713	0.784372
1.084437	1.700354	1.969733	0.053485	2.351299	1.06355	1.572035	1.80267	1.153723
3.35715	2.961768	1.585296	3.829105	0.747417	3.5257	2.815866	1.473218	7.495397
1.158384	0.895945	0.57531	1.259747	0.116825	0.771688	0.233434	1.237879	0.224762
1.302107	3.536955	3.957734	1.692763	3.618499	3.23987	2.378124	2.07658	1.720144
1.076032	3.140866	4.684502	2.640332	5.25254	2.398442	5.150693	2.681169	3.7586
10.98644	3.585029	0.70404	1.575483	0.569293	4.155339	2.160649	0.976111	1.412771
4.353925	3.629177	0.585902	0.471313	0.372504	1.736133	0.166465	0.78619	0.468384
2.81342	2.607035	1.302107	0.882619	1.169091	2.474834	2.210866	2.009906	1.171349
3.266782	24.98218	20.51271	11.03843	21.02638	23.06543	27.96248	17.03919	9.063983
3.904517	0.596111	0.861934	0.202294	0.140657	0.672468	0.000375	0.561126	0.000274
17.03919	16.62402	19.06893	22.96463	6.011515	12.36443	10.12435	11.03843	21.02638
21.19547	5.388405	7.264502	15.88548	4.94026	10.93101	3.290101	3.820758	4.626025
0.11811	0.011962	0.047323	4.33E-05	2.89E-05	0.000433	0.023492	0.000101	0.121818
1.602619	1.539062	3.273045	1.179885	0.605887	2.628225	0.299747	1.326667	3.290101
0.450375	5.323492	3.687633	1.554185	6.464906	1.190491	2.958773	4.105714	2.928247
2.097843	0.656392	1.103261	0.384798	0.688975	0.793918	0.504098	1.083651	1.395563
0.353795	0.434329	0.995527	0.176299	0.784372	0.591616	0.467403	0.209048	1.204957
11.83593	7.991602	2.058773	6.674286	0.793918	1.34798	0.728543	0.730873	1.035058
111.5439	0.204964	0	3.640606	2.89E-05	5.337547	0.000375	0.000101	8.643074
126.1556	52.56128	29.82828	91.05541	19.29639	91.05541	44.27012	65.94551	111.5439
0.569293	2.180022	2.003723	0.91575	3.718716	0.567345	2.281926	0.956667	1.121703
0.009452	0.169675	0.268824	0.217172	0.260144	0.060087	0.113911	0.165108	0.090772
0.395152	1.594675	2.189235	0.822965	3.404372	1.241811	0.453463	1.533535	2.696061
8.958225	0.564127	5.210317	0.922338	0.841746	3.418405	0.61969	1.421126	1.833102
0.246955	1.716364	0.655245	0.412193	2.069942	0.287951	1.431414	2.860722	0.23246
0.009452	2.180022	0.475433	4.33E-05	6.853608	0.000433	3.679986	4.072475	0.000274
2.941544	14.17388	11.19942	19.22139	7.339452	24.2842	14.79742	13.42179	29.49693
0.097633	0.50557	1.224733	1.040411	0.347655	1.259747	1.267879	0.420707	0.924329
0.009452	8.66E-05	0	4.33E-05	0.747417	0.000433	0.000375	0.000101	0.000274
1.32482	6.772763	7.765931	2.382035	13.75978	2.81342	6.287561	5.599228	3.703175
0.760866	2.060491	1.465123	1.247244	2.132756	2.005253	1.250058	1.095657	1.587605
0.141775	0.2129	0.498052	0.031046	1.15614	0.220657	0.055671	2.176501	0.000274
0.773276	0.24562	0.768535	0.261775	0.850808	0.269509	0.406912	0.13824	0.370281
5.337547	5.9071	1.839986	4.365224	2.185556	6.345382	0.902403	2.847547	0.370281
0.009452	0.034502	0.017172	0.094733	0.110289	0.000433	0.190541	0.283535	0.054026
0.009452	0.314387	0.15127	0.163766	0.10381	0.196667	0.373218	0.023059	0.24518

1.502323	0.275873	0.585902	0.642698	0.480548	0.526566	0.389812	2.243911	0.131443
2.834084	5.576219	6.404921	4.984488	1.850996	6.028427	6.738211	4.419062	9.968355
0.891775	3.807648	11.30987	3.307186	9.047532	3.842554	2.776421	3.574387	4.715599
70.11932	38.39042	19.83727	13.83359	33.07147	27.10502	7.037345	36.74522	12.1974
0.719942	2.031169	1.224733	1.130794	1.518232	2.696061	2.834084	1.29417	2.139928
25.48179	1.94311	1.575483	2.323983	0.766602	8.745238	1.755988	2.159004	1.480541
0.493247	0.305253	0.338499	0.315094	3.269913	0.362179	1.593535	0.229502	0.436017
9.667316	5.560563	7.315512	5.493182	3.214545	11.30987	3.011169	7.724084	10.07949
0.009452	1.997352	3.711097	0.619365	4.678846	0.249408	3.540606	0.798153	1.245671
2.654473	14.29947	12.93651	8.348817	9.179466	7.417359	11.06488	8.288874	16.80343
0.009452	1.256558	2.058773	0.144935	1.701616	0.112121	1.189769	0.000101	1.25886
2.941544	7.495397	8.27267	5.984885	15.66023	7.882222	15.07257	8.019769	6.784762
0.83697	0.82974	0.31456	1.721371	0.46461	1.618773	1.330029	0.82868	0.954141
2.345527	2.267157	2.181775	3.125844	3.256587	4.039192	4.160238	2.300678	2.802828
0.009452	8.66E-05	0.095382	4.33E-05	1.546696	0.080722	0.145382	0.116659	0.000274
0.697908	2.124574	0.850325	1.124574	1.169091	1.392511	0.859885	1.123016	2.554069
9.397771	1.482626	1.014784	1.560779	0.977994	1.680895	1.89373	1.779423	0.699192
0.502222	0.485014	3.101371	0.767085	2.069942	0.873088	0.744113	0.778326	1.857677
0.009452	8.66E-05	0.006421	4.33E-05	0.07632	0.000433	0.000375	0.000101	0.046667
0.009452	3.407619	1.215981	3.179199	0.850808	1.381111	3.799885	5.704646	5.095325
2.157359	10.8813	9.541645	7.417359	8.923117	12.29473	19.83727	6.8421	18.91854
0.009452	2.666494	3.971061	1.033672	1.280274	1.89961	2.50469	0.908773	2.236407
0.009452	0.204964	0.25197	0.067547	0.097879	0.000433	0.201789	0.000101	0.24518
5.71241	3.691724	6.250447	9.751039	3.747035	6.230245	4.086926	2.117814	4.758088
1.502323	6.063276	6.560411	5.553146	4.323045	9.459177	19.06893	3.1857	6.22031
0.009452	0.321746	0.666847	4.33E-05	0.195685	0.000433	0.341241	0.304062	0.000274
0.214964	0.350837	0.213355	0.195007	0.392403	0.185909	0.411169	0.174646	0.173189
8.256465	2.230859	1.701616	2.535974	2.24562	3.317807	1.721371	2.718434	4.077403
0.009452	1.2214	1.717641	0.299062	1.794531	0.487662	1.848297	1.011486	1.233665
0.376407	0.16145	0.543939	0.183052	0.569293	0.345238	0.151176	0.293276	0.215599
19.53061	4.47912	2.08013	6.088593	1.477388	1.89373	0.744113	2.489762	4.508196
0.867287	1.363557	0.939408	0.948745	1.262179	0.852172	2.045476	0.674163	0.934488
0.097633	0.182821	0.338499	0.376017	0.243124	0.176104	0.425137	0.185022	0.333463
19.06893	11.09133	4.302367	11.544	3.346898	7.162698	0.000375	16.9162	7.456118
0.009452	1.533535	1.487619	0.123023	1.600238	0.259163	1.013434	0.518139	0.282799
4.609076	4.016465	1.224733	4.580938	1.326667	7.08733	4.990794	1.931537	1.77404
0.089553	0.011962	0.032092	0.06031	0.028196	0.022266	0.069365	0.000101	0.113218
0.009452	0.268369	0.123853	0.116176	0.084228	0.000433	0.06246	0.000101	0.000274
0.129971	8.66E-05	0.017172	0.031046	0.243124	0.000433	0.145382	0.209048	0.054026
4.419062	0.727633	0.31456	0.729582	0.252165	0.672468	0.61969	0.771176	1.35259
0.11811	0.141082	1.910974	0.087619	2.394416	0.165108	0.196436	0.584134	0.441263
8.477662	83.489	60.77449	23.38378	64.12971	41.47925	93.1424	35.27608	14.6058
0.560606	12.63773	24.50942	19.37139	7.839271	10.82954	9.792626	10.38202	7.136537
2.009906	1.57645	0.962107	1.340981	1.867951	0.639113	0.334625	2.283752	1.121703
2.740519	2.703485	2.815866	3.489899	2.967453	4.236962	2.472576	1.496118	2.975974
0.009452	8.66E-05	0.132468	0.06031	1.016494	0.000433	0.000375	0.064848	0.113218
0.129971	0.268369	0.57531	0.195007	0.541804	0.185909	0.438831	0.498636	0.274055
26.80355	27.66779	6.38456	15.66023	19.53061	9.880779	4.789596	105.1694	20.16541
4.508196	1.2214	1.78759	2.216162	0.711371	2.066652	1.618773	1.191198	1.881955
0.450375	3.676032	2.970144	1.664906	2.339697	1.423506	1.772756	1.455988	1.035058
1.369048	3.747035	3.687633	2.922778	15.41187	2.294733	5.745996	4.586753	1.921212
0.431977	0.426392	0.696234	0.586248	0.354242	0.832554	0.687431	0.584134	0.960866
34.84299	1.416407	7.810058	0.929812	0.82772	1.935685	0.254156	9.459177	0.164401
75.1132	6.632063	8.643074	5.180058	11.60283	13.00996	0.000375	24.73724	1.711414
0.009452	0.018492	0.350274	0.039589	0.140657	0.080722	0.048341	0.165108	0.083521
38.39042	87.1746	75.1132	135.5245	31.1753	203.5795	83.489	59.32013	40.11561
1.585296	6.945152	2.08013	1.420202	8.075772	0.885664	5.25254	2.361198	1.335743
0.493247	2.569957	3.077316	1.948918	4.236962	2.820974	7.08733	2.402468	3.446732

0.009452	1.32482	0.722626	0.336508	2.436768	0.556126	1.957958	0.185022	0.274055
2.768312	1.63039	3.799885	2.850058	6.181652	1.451775	5.030866	1.959466	2.276407
3.68381	1.848297	1.238651	3.655714	0.411169	3.340361	2.096162	3.201457	3.588369
25.99427	1.799899	16.9162	47.5018	2.89E-05	16.22108	17.15902	1.89518	10.28573
332.0776	40.7932	77.74812	105.1694	8.288874	135.5245	21.93452	80.6518	41.47925
0.009452	8.66E-05	1.585296	4.33E-05	1.097677	0.000433	0.576205	0.000101	0.901212
0.402937	0.751371	2.442987	0.240346	2.283752	0.446811	1.476429	1.191198	1.711414
0.009452	1.104805	2.207417	0.102251	0.757273	0.353651	2.08798	0.304062	0.560606
0.141775	8.66E-05	0.002655	0.053485	0.056638	0.000433	0.041616	0.023059	0.000274
0.009452	1.03189	3.321284	0.368658	2.267157	0.112121	2.126349	0.329798	0.993521
0.278304	0.049336	0.006421	0.06031	0.084228	1.05417	0.138009	0.091833	0.020938
155.7103	1.281241	25.24367	49.50903	2.89E-05	26.52537	6.153102	4.49671	28.87506
14.88584	8.573434	7.668579	2.584394	8.149307	4.110779	0.822965	15.66023	8.348817
0.601227	0.461811	0.383074	0.169675	0.188009	0.567345	0.416782	0.12772	0.113218
1.121703	1.2214	1.233665	0.987821	1.495043	0.809986	0.91575	1.779423	1.159913
0.296234	2.139928	1.510014	1.803918	4.174964	2.882035	2.828918	0.13824	0.993521
2.871212	1.618773	2.425801	2.048781	9.297085	1.766248	3.829105	4.125786	1.90798
0.995527	0.614935	0.79487	0.579149	1.472208	0.723506	1.214315	0.73961	0.487662
1.476429	1.054899	0.416782	0.291183	3.632973	0.153867	0.432698	0.174646	0.668456
0.719942	1.03189	1.163579	0.944084	3.425512	1.06355	2.442987	1.713997	0.625209
37.29684	2.320072	3.606804	1.809343	5.679502	6.287561	1.902352	5.720173	0.755253
0.009452	0.253687	0.732193	0.662612	1.435772	0.220657	0.524646	0.329798	1.867951
20.16541	3.324452	10.82954	3.851414	1.1407	15.18102	9.377345	7.795642	65.94551
0.009452	0.034502	0.072482	0.004949	0.042713	0.022266	0.06246	0.000101	0.090772
0.663651	1.61088	2.903867	0.757273	1.451775	0.917431	0.995527	2.02956	1.587605
1.720144	3.303925	2.148658	5.984885	2.151919	3.588369	10.62739	5.082063	11.96574
0.15562	0.056869	0.064488	0.176299	0.035505	0.031818	0.106696	0.014964	0.458341
23.38378	20.51271	20.34063	27.10502	17.15902	23.38378	19.99861	25.48179	21.93452
4.18456	4.907929	3.763045	5.00404	4.302367	3.699495	5.137042	6.297136	3.606804
1.288276	1.472208	5.754935	0.962107	2.08013	1.416407	0.217677	3.730808	0.181919
0.180368	0.355556	0.338499	0.046962	0.234358	0.050844	0.29404	0.084069	0.083521
6.933961	7.964315	10.60361	9.297085	10.67991	10.07949	8.923117	10.1028	6.830592
15.98892	5.388405	5.9071	3.648081	2.107922	2.850058	0.952294	3.283139	6.674286
15.276	203.5795	95.22939	87.1746	155.7103	87.1746	224.9555	57.84882	64.12971
0.1686	0.196667	0.213355	0.1379	0.314156	0.249408	0.113911	0.561126	0.198059
5.872886	14.6058	7.363117	3.230065	6.653016	6.632063	0.352222	20.85876	0.113218
38.94854	20.34063	21.74051	16.34294	18.76954	28.23632	42.08807	9.625253	7.264502
0.009452	0.16145	0.032092	0.046962	0.411169	0.000433	0.000375	0.000101	0.076241
0.1686	0.011962	0	0.108997	0.01697	0.000433	0.030491	0.084069	0.028081
8.319019	1.826147	1.176623	1.62562	0.585592	3.256587	0.774257	1.421126	3.363911
59.32013	6.99026	10.1459	13.15079	11.22667	12.63773	14.88584	6.64254	11.83593
0.009452	8.66E-05	0.047323	0.004949	0.005512	0.000433	0.000375	0.000101	0.020938
0.648687	0.910339	0.423781	1.007864	0.784372	0.123102	0.247583	1.959466	0.706984
0.246955	0.289452	0.57531	0.188276	0.314156	0.27899	0.268232	0.055671	0.190541
0.296234	0.088066	0.017172	0.102251	0.134942	0.100779	0.138009	0.038139	0.149423
2.097843	9.667316	12.03267	4.959358	14.33939	5.110159	8.524242	10.8813	3.249877
0.106926	0.485014	2.096162	0.299062	2.380079	0.715815	0.628297	0.540563	0.402698
11.77567	2.917381	6.22031	6.011515	4.429769	6.717056	2.621133	6.738211	2.652107
35.776	64.12971	91.05541	22.74797	91.05541	111.5439	12.93651	111.5439	24.50942
0.009452	0.133045	0.181674	4.33E-05	0.267388	0.000433	0.000375	0.064848	0.000274
0.141775	0.034502	0.223485	0.009163	0.288081	0.060087	0.089726	0.023059	0.04062
21.54823	21.19547	23.16622	27.38726	41.47925	20.68746	22.96463	53.77619	14.00414
1.775325	1.447994	2.355289	1.369048	3.486436	1.975772	4.241905	1.632648	2.644885
0.336328	0.370101	0.338499	0.453218	0.939408	0.060087	0.260274	0.146999	0.062381
2.238341	1.416407	2.400455	0.227063	3.008225	0.153867	0.406912	3.040505	0.82974
0.31785	0.169675	2.037114	0.227063	2.831501	0.112121	0.467403	1.410902	1.245671
0.31785	4.715599	4.650007	1.066948	6.900202	1.50684	1.072843	6.365065	2.058773
0.237359	0.426392	0.404408	0.169675	0.159105	0.080722	0.400173	0.185022	0.82974

4.49671	28.87506	48.53429	18.76954	72.69473	17.64869	25.99427	26.39047	23.82354
0.106926	0.007734	0.010786	0.004949	0.195685	0.042078	0.000375	0.064848	0.015714
5.889798	1.89518	1.176623	3.877417	2.432121	1.803918	1.618773	2.31811	0.615584
0.009452	8.66E-05	0.080281	4.33E-05	2.89E-05	0.000433	0.041616	0.008975	0.062381
0.457749	0.727633	2.225498	0.471313	1.794531	0.535657	1.27619	0.381999	1.896667
1.096335	8.210938	8.412367	5.796421	14.70188	6.674286	11.65791	4.597792	4.429769
1.319293	1.447994	3.904517	1.92272	3.57057	2.132756	2.642525	3.599329	4.894509
0.009452	0.018492	0.404408	0.015599	0.227244	0.000433	0.160462	0.014964	0.000274
2.511465	3.377619	4.667518	8.164531	1.303961	6.865	10.53159	3.533211	7.315512
2.683478	0.947157	0.976667	1.061457	1.15614	1.299567	1.112641	1.136566	1.035058
0.009452	0.528571	0.620014	0.094733	1.244957	0.420289	0.92482	0.218355	0.323319
8.923117	13.34662	7.315512	16.68554	10.7557	19.37139	23.16622	11.89932	10.01009
0.819509	1.841371	0.976667	0.938413	0.46461	1.148564	0.832013	2.349351	2.903867
7.188095	1.256558	2.08013	1.325743	1.580909	2.683478	1.734949	2.02956	1.395563
1.006025	0.15456	0.722626	0.261775	0.569293	0.259163	0.460065	0.313723	0.343434
28.54786	33.07147	29.49693	31.1753	32.68313	40.7932	33.93939	31.92385	44.27012
12.77772	10.55571	3.687633	4.048499	2.459812	6.695887	7.824473	4.49671	7.911934
0.07583	0.305253	0.294921	0.315094	0.615584	0.165108	0.190541	1.112641	0.173189
1.790245	2.873817	14.42661	3.457504	7.037345	3.45404	2.449509	7.251263	8.048095
41.47925	14.70188	29.18885	77.74812	10.53159	75.1132	21.54823	31.1753	35.27608
0.31785	0.275873	3.971061	3.169812	0.775844	1.182056	0.98474	1.669466	0.65202
0.192929	0.614935	0.498052	0.3514	0.418023	0.337222	0.425137	0.518139	0.430281
1.311674	2.873817	1.903636	1.02798	6.250447	1.8386	6.230245	4.519048	2.529307
3.5257	1.61088	1.3829	6.717056	3.536955	1.975772	0.504098	1.304877	1.452857
11.06488	3.993665	2.323983	7.724084	3.330909	13.72059	5.477843	3.283139	2.139928
0.995527	4.408153	3.659668	6.784762	3.486436	5.454949	2.720996	2.763261	11.36975
0.535029	2.485534	1.585296	0.858817	2.688304	0.599466	1.902352	1.191198	1.245671
0.204278	0.034502	0.006421	0.015599	0.003175	0.022266	0.048341	0.023059	0.083521
6.933961	16.62402	17.78522	19.83727	14.00414	15.3618	9.583463	12.36443	24.73724
4.456912	3.367071	6.135007	8.134084	5.9071	6.464906	9.667316	3.263391	4.174964
1.23127	4.575123	3.381255	1.179885	2.294733	5.395584	3.131544	4.188896	3.169812
3.957734	5.044113	7.810058	3.411097	5.704646	6.738211	11.60283	6.028427	5.821804
2.270837	6.063276	4.513622	4.684502	3.50425	4.348882	6.028427	3.803766	5.351934
0.431977	0.204964	0.383074	0.570664	0.841746	0.29798	0.576205	0.174646	0.26562
0.106926	0.049336	0.404408	0.039589	0.042713	0.060087	0.048341	0.030613	0.524646
2.178268	0.976667	0.605022	0.509242	0.990909	0.873088	1.089394	0.658362	0.305253
0.353795	1.054899	1.465123	0.483608	2.74544	0.479401	1.678319	1.165873	1.534603
0.009452	1.661328	0.404408	0.268824	4.007583	0.000433	1.688045	0.481414	0.113218
91.05541	27.38726	14.51648	64.12971	4.851227	56.39528	50.55949	22.96463	47.5018
6.55039	1.429365	0.543939	1.548723	1.351573	2.196017	2.666494	1.011486	1.395563
0.62645	0.550368	0.465563	1.29267	0.990909	0.941645	2.390447	0.849841	0.726472
2.776421	1.997352	1.921212	2.290996	7.013853	2.740519	5.770996	1.519322	1.815
0.141775	1.692763	0.775844	1.92272	5.395584	0.963853	13.83359	2.577157	0.625209
2.333867	8.134084	4.365224	1.821861	8.149307	1.766248	0.780519	19.83727	6.922771
3.8121	16.44768	22.52375	7.882222	21.54823	10.73084	9.418196	16.80343	9.101674
6.853608	8.815123	3.993665	12.22798	5.737056	10.62739	2.425801	27.66779	51.52195
40.11561	19.53061	3.687633	8.048095	1.944517	14.00414	8.677489	2.107922	5.493182
2.425801	6.250447	0.567619	4.626025	2.807937	3.046551	0.561775	5.447114	9.031082
3.034747	9.902576	6.325945	5.439278	7.264502	7.013853	6.374812	7.288788	2.432121
0.28728	3.070952	2.242092	1.171349	5.22482	1.190491	1.957958	1.680895	1.833102
0.683232	0.856782	0.711912	0.67632	0.917431	0.196667	0.653968	1.041025	0.252316
0.081667	0.011962	0.006421	0.004949	0.012785	0.042078	0.000375	0.055671	0.020938
0.984069	3.198499	3.659668	7.683766	6.30671	5.606739	5.095325	6.268312	5.583968
2.333867	25.48179	22.74797	16.9777	23.6012	15.88548	31.92385	12.93651	38.39042
0.089553	8.66E-05	0	4.33E-05	0.009163	0.000433	0.018102	0.008975	0.000274
0.009452	0.064127	0.002655	4.33E-05	0.440029	0.000433	0.201789	0.108124	0.000274
6.116638	44.27012	57.84882	18.91854	77.74812	12.77772	19.22139	17.92188	22.74797
0.569293	1.692763	1.80267	0.894704	1.15614	0.318312	0.891176	2.187395	0.198059

0.097633	0.018492	0	0.009163	0.035505	0.000433	0.120361	0.038139	0.000274
5.515584	15.46193	35.27608	9.541645	36.26061	19.22139	7.938427	11.51384	17.28195
0.441833	5.409437	5.044113	3.23987	6.580988	4.586753	5.889798	4.451154	2.765758
0.411436	8.66E-05	0.002655	0.023304	0.012785	0.042078	0.160462	0.046544	0.054026
0.009452	0.335108	0.350274	0.094733	0.532857	0.165108	0.226883	0.460065	0.274055
0.15562	0.577071	0.850325	0.255087	0.220808	0.176104	0.312475	0.165108	0.121818
10.38202	57.84882	64.12971	22.13245	83.489	31.55417	33.4985	91.05541	60.77449
1.334762	3.286652	17.28195	3.743232	19.83727	5.485685	4.120815	7.868146	17.39935
2.112843	10.48101	17.15902	6.423911	17.39935	8.958225	10.19468	18.33909	5.381227
3.829105	0.895945	1.163579	0.737857	2.107922	0.859351	1.045462	1.559675	1.298665
55.04081	425.62	203.5795	287.197	252.4251	184.443	75.1132	155.7103	21.36892
2.55184	8.868932	21.36892	8.643074	15.98892	11.48368	7.112496	9.179466	4.468009
0.476551	3.574387	4.193232	1.617489	4.302367	1.50684	0.797114	1.693939	2.270837
0.683232	1.57645	1.090072	1.209776	1.495043	0.556126	0.597042	1.559675	5.447114
2.417424	3.070952	2.013182	1.50342	3.679986	1.131472	2.879228	0.956667	4.519048
1.096335	6.345382	7.519841	3.632973	7.238023	3.169812	7.212915	7.099913	5.622872
2.147013	26.52537	45.1045	8.103925	65.94551	7.212915	0.804675	10.19468	4.603434
0.009452	0.355556	0.596508	0.322078	4.343838	0.176104	0.000375	0.12772	0.198059
3.718716	4.456912	2.609293	1.867951	6.695887	1.96697	1.884935	14.42661	1.045462
27.10502	20.85876	22.32214	10.19468	40.7932	20.16541	17.78522	12.67271	16.22108
5.366335	41.47925	42.08807	34.84299	33.4985	30.82277	55.04081	25.24367	77.74812
0.610606	2.65684	5.030866	1.821861	8.745238	0.723506	3.363911	0.730873	2.544747
2.463911	19.22139	24.73724	10.01009	18.33909	9.751039	15.66023	16.68554	9.141299
0.009452	3.651898	3.144156	2.531515	3.439437	2.758088	7.251263	1.594675	4.397792
49.50903	7.882222	12.85504	55.04081	15.41187	17.28195	18.48514	13.23022	15.66023
1.251659	0.229199	1.287403	2.011501	0.72044	0.27899	2.035678	0.861429	1.534603
5.599228	6.019971	1.074827	6.738211	1.484553	3.4679	4.597792	1.888023	3.321284
83.489	15.77678	13.60866	26.80355	2.953059	33.93939	18.76954	23.38378	35.776
0.009452	0.025483	0.181674	0.046962	0.252165	0.080722	0.000375	0.023059	0.36153
0.365859	0.550368	0.432468	0.757273	1.03246	0.526566	1.376378	0.240101	0.82974
13.49926	6.153102	2.931068	4.684502	1.937222	4.655916	4.38684	5.030866	0.181919
0.431977	1.555289	6.22031	0.767085	1.082179	1.480541	0.679812	0.771176	22.32214
0.2264	0.403651	1.80267	0.116176	1.593535	0.230108	0.097843	0.26562	1.111977
5.28039	4.456912	1.675722	4.23202	3.679986	2.784228	3.1857	3.217612	2.74544
0.009452	0.018492	0.006421	0.009163	0.005512	0.000433	0.069365	0.000101	0.000274
6.079596	16.80343	21.02638	11.36975	22.74797	11.77567	26.52537	10.70538	37.29684
14.33939	95.22939	87331.38	118.7028	203.5795	118.7028	332.0776	95.22939	87331.38
87.1746	224.9555	144.7919	203.5795	425.62	224.9555	749.7839	118.7028	287.197
1.406746	0.751371	0.962107	0.080895	0.46461	0.413009	1.01772	0.36267	1.055628
31.55417	21.54823	30.82277	18.05488	27.66779	38.39042	20.16541	27.38726	14.21671
0.009452	0.442496	0.585902	0.080895	1.385801	0.308442	0.659582	1.463304	0.353492
6.028427	3.442886	3.101371	2.347439	4.789596	2.304452	4.328232	1.992944	4.125786
0.106926	0.011962	0.213355	0.261775	0.595022	0.143333	0.389812	0.658362	0.131443
0.129971	0.095303	0.072482	0.116176	0.084228	0.220657	1.540144	0.185022	2.150303
0.009452	0.034502	0	4.33E-05	0.035505	0.022266	0.000375	0.008975	0.083521
0.690043	2.078355	2.807937	4.198117	4.47912	2.196017	2.126349	21.54823	9.356371
5.195022	3.585029	2.225498	7.38847	3.632973	7.824473	7.854069	15.46193	37.81919
0.081667	0.018492	0.047323	0.004949	0.10381	0.080722	0.083074	0.023059	0.020938
48.53429	80.6518	65.94551	35.27608	48.53429	16.62402	24.04374	167.9996	67.98069
0.192929	0.056869	0.039747	4.33E-05	2.89E-05	0.000433	0.000375	0.000101	0.046667
3.324452	8.288874	17.39935	18.48514	22.32214	9.256537	39.53687	18.19595	26.80355
0.009452	8.66E-05	0.189632	4.33E-05	0.201898	0.000433	0.069365	0.046544	0.076241
3.119986	1.790245	1.53684	0.987821	1.559675	1.259747	0.92482	2.257749	0.798153
0.009452	0.38285	0.072482	4.33E-05	0.784372	0.133535	0.000375	0.638362	0.069784
0.009452	8.66E-05	0.002655	4.33E-05	2.89E-05	0.000433	0.000375	0.000101	0.034199
0.141775	0.229199	2.323983	0.217172	0.766602	0.165108	0.870902	0.313723	0.784372
9.625253	42.08807	36.74522	26.52537	28.23632	31.1753	22.74797	40.11561	15.77678
6.38456	15.18102	6.00228	2.826277	11.42512	8.164531	9.880779	11.60283	2.961768

2.941544	6.200945	10.05618	4.519048	11.65791	5.737056	7.697063	3.55583	11.19942
3.137576	5.538001	4.990794	3.6029	8.780115	4.02088	4.721789	8.477662	3.192229
3.500808	0.926926	1.626782	3.707136	1.004156	1.340036	0.882619	2.681169	5.493182
0.009452	8.66E-05	0	4.33E-05	0.01697	0.000433	0.030491	0.038139	0.000274
2.302626	11.544	13.79669	6.922771	16.9777	6.830592	10.73084	22.52375	12.56693
62.33472	22.13245	20.68746	15.46193	12.63773	16.80343	9.502294	14.97762	6.52899
0.259098	2.159004	1.36619	0.551284	4.603434	0.259163	2.066652	0.987821	0.299062
1.132165	1.232078	1.651522	0.868341	2.831501	2.091313	2.08798	2.577157	1.573276
0.551861	1.524776	1.401068	0.962107	1.518232	0.852172	0.865664	1.473218	0.554026
1.846941	11.36975	10.43066	5.84689	6.518709	5.976724	8.849697	7.061328	5.704646
0.089553	0.919192	1.061457	0.570664	0.335786	0.328268	1.250058	1.259747	1.80267
3.921645	10.33476	7.025599	9.397771	4.419062	6.423911	4.328232	4.637994	2.529307
9.792626	11.63037	11.60283	8.677489	24.2842	9.031082	11.74734	9.583463	15.88548
19.68088	7.468961	38.94854	70.11932	4.997417	26.80355	9.218514	10.43066	24.2842
0.246955	0.056869	0.243232	0.031046	0.116825	0.27899	0.166465	0.000101	0.402698
6.695887	23.6012	16.68554	22.52375	15.56131	18.76954	32.68313	7.724084	7.991602
0.106926	0.064127	0.277417	0.06031	0.440029	0.112121	0.239228	0.064848	0.020938
1.846941	15.3618	12.10289	2.964762	37.29684	2.386248	16.09482	8.149307	18.48514
0.343824	0.856782	0.338499	0.400354	1.727323	0.287951	0.524646	0.956667	0.131443
8.180094	8.868932	3.025671	5.110159	6.978903	2.964762	1.055628	37.29684	3.78798
0.543131	0.520678	0.543939	0.376017	0.585592	0.395426	0.498052	0.259033	0.090772
2.285664	0.856782	0.517359	2.382035	0.625209	1.495043	0.787987	1.237879	0.504098
2.355289	16.22108	13.91714	2.011501	13.83359	1.288276	14.08569	9.141299	2.021068
0.009452	0.079466	0.243232	0.039589	0.201898	0.022266	0.183521	0.100483	0.23246
3.150433	13.45939	13.6814	3.256587	23.16622	7.653391	4.63189	33.07147	12.10289
18.76954	7.430317	5.872886	7.013853	6.695887	14.70188	6.268312	17.39935	7.188095
0.009452	0.011962	0.110642	0.031046	0.056638	0.000433	0.041616	0.108124	0.113218
2.408882	4.888023	4.684502	3.578038	9.838658	3.944084	6.967547	3.35355	4.894509
0.535029	2.763261	1.664906	0.767085	0.766602	0.821414	0.239228	0.771176	2.047157
0.259098	0.289452	0.394877	0.094733	0.429827	0.362179	0.283709	0.100483	0.062381
13.91714	0.784372	1.626782	2.153535	0.288081	3.327619	1.146118	0.26562	1.135887
0.1686	1.003586	2.194257	0.683665	2.24562	0.686667	1.132857	0.36267	1.266255
1.20653	5.638759	11.36975	4.802655	6.830592	3.699495	6.933961	14.88584	14.97762
1.076032	3.166659	5.395584	7.765931	1.409942	3.66785	2.581861	3.621941	184.443
0.742872	1.555289	1.327525	1.230411	2.470317	1.092136	2.472576	1.705007	0.714697
0.180368	0.176558	0.095382	4.33E-05	0.305253	0.000433	0.212698	0.116659	0.173189
0.31785	0.670519	0.103341	0.509242	0.32925	0.535657	0.485014	0.638362	0.468384
0.009452	1.455988	2.096162	1.087345	1.636118	0.672468	0.524646	1.548723	1.335743
0.009452	0.034502	0.002655	4.33E-05	0.009163	0.031818	0.000375	0.038139	0.000274
2.207417	0.114358	1.921212	4.33E-05	0.10381	0.196667	0.000375	0.000101	0.380649
0.511667	8.66E-05	2.129596	4.33E-05	0.252165	0.000433	0.602287	0.000101	0.891775
0.009452	8.66E-05	0.017172	0.009163	0.65798	0.031818	0.07684	0.000101	0.299062
0.269697	1.377446	2.987835	0.997244	4.696313	2.227273	4.529913	1.421126	1.121703
0.009452	0.275873	0.666847	0.116176	0.440029	0.069668	0.634531	0.481414	0.26562
0.129971	0.011962	0.002655	0.015599	0.005512	0.000433	0.023492	0.008975	0.04062
0.246955	0.24562	0.055967	0.169675	0.456522	0.123102	0.155224	0.391962	0.305253
1.020447	2.134545	2.181775	1.594675	2.5271	2.157359	3.730808	1.99899	1.755988
2.698557	0.80417	0.907273	1.776739	1.253088	2.474834	7.136537	0.259033	1.363557
23.6012	7.351284	10.35839	38.39042	1.533535	42.08807	12.03267	16.34294	80.6518
2.59557	5.754935	1.596782	1.594675	6.181652	5.095325	0.174776	5.545729	1.896667
30.17192	126.1556	43.49658	40.11561	44.27012	42.75576	5.050267	184.443	45.91841
0.15562	0.314387	0.213355	0.053485	0.214885	0.404113	0.276429	0.108124	0.181919
0.129971	0.419596	0.141876	0.291183	0.15241	0.143333	0.602287	0.201789	0.305253
2.034242	0.475137	1.256558	0.744531	1.628009	0.626118	1.1629	0.540563	1.01544
0.009452	1.905094	1.549711	1.809343	9.502294	1.629235	3.578038	1.632648	1.734949
3.095166	13.08214	1.962424	0.509242	0.499986	1.672107	0.000375	3.980123	0.000274
0.009452	0.011962	0	4.33E-05	0.003175	0.000433	0.018102	0.000101	0.000274
1.288276	1.363557	1.634935	3.877417	2.096162	1.594675	2.434444	2.257749	2.316075

4.802655	1.133622	0.819509	3.707136	1.656696	2.451645	0.717027	1.826147	2.710765
15.88548	1.080303	3.204632	3.008225	1.876212	2.157359	4.888023	0.942944	4.808658
0.009452	0.064127	0.047323	4.33E-05	0.092049	0.000433	0.000375	0.240101	0.24518
1.783203	7.751869	12.32958	8.477662	12.36443	7.339452	10.57984	8.608153	5.788153
5.500678	3.921645	7.854069	6.191515	3.975498	7.598312	5.381227	4.46267	3.425512
0.009452	2.230859	1.246457	0.749942	2.628225	0.545808	1.388543	0.528196	0.5129
0.402937	1.159149	0.454978	0.799048	1.197922	0.520678	0.023492	1.496118	0.062381
0.742872	0.361948	0.223485	0.261775	0.585592	0.487662	0.668947	0.431717	0.477698
3.5257	6.423911	2.903867	3.799885	3.214545	4.684502	3.140866	4.814661	2.432121
5.030866	3.676032	2.058773	2.365685	0.802395	4.317648	1.560779	2.047157	1.663672
2.882035	37.29684	18.62711	25.99427	32.31123	30.17192	43.49658	25.99427	25.99427
0.970635	0.677403	0.83088	0.629538	1.082179	0.437799	0.568369	0.572987	0.487662
0.009452	0.011962	0.010786	0.015599	0.009163	0.000433	0.069365	0.008975	0.028081
0.129971	0.558095	0.84119	0.804675	0.757273	0.520678	0.561775	0.313723	0.82974
42.08807	0.355556	0.508658	5.530274	3.116732	22.74797	4.06303	4.016465	3.116732
0.962107	8.66E-05	0	0.440548	2.89E-05	0.901212	0.347403	1.237879	0.784372
14.51648	16.34294	18.48514	17.78522	13.6814	13.45939	18.12541	17.64869	11.48368
25.73165	6.808369	9.625253	22.32214	1.766248	39.53687	10.82954	10.1459	17.51961
0.009452	0.764704	0.896522	0.116176	1.177309	0.328268	0.832013	0.185022	0.784372
18.48514	20.68746	6.717056	16.9777	12.10289	21.93452	4.764055	42.08807	7.737807
60.77449	0.305253	0.132468	1.275325	0.097879	5.143867	0.673254	20.68746	0.000274
2.538218	2.367763	3.779452	3.707136	4.029654	3.87329	4.212554	2.99987	2.81342
0.009452	0.182821	0.596508	0.080895	1.828781	0.239885	0.865664	0.26562	0.548788
0.493247	0.169675	0.223485	0.3514	0.642698	0.842193	0.514885	0.084069	0.000274
8.677489	6.99026	1.36619	11.09133	6.210375	10.5063	4.365224	2.955916	11.14619
1.679596	1.315613	1.977316	0.336508	1.295108	0.60886	0.882619	0.778326	1.982208
0.402937	0.305253	0.198059	0.412193	0.418023	0.567345	0.947157	0.720938	0.380649
0.246955	0.108124	0.543939	0.611789	0.296234	0.763939	0.588954	0.849841	0.580123
105.1694	20.16541	9.297085	21.02638	19.29639	16.09482	16.80343	15.77678	8.319019
0.962107	6.876392	2.563506	8.608153	3.290101	5.47	2.109755	4.292078	5.42381
0.995527	1.482626	1.176623	2.147013	1.766248	0.686667	1.06355	1.381111	1.927042
0.900693	0.839365	1.143175	32.68313	0.214885	5.872886	1.74539	1.713997	4.468009
1.20653	0.877807	0.740101	0.477446	0.042713	0.821414	0.947157	0.730873	1.363557
2.752893	6.663651	4.034423	4.348882	3.57057	7.288788	1.401977	5.210317	1.931537
0.940563	3.33728	3.944084	1.749307	1.043608	1.727323	4.424416	0.809481	3.743232
0.009452	8.66E-05	0.002655	0.268824	0.097879	0.000433	0.030491	0.250115	0.054026
0.246955	0.821898	0.925801	0.515238	0.880498	0.686667	1.177309	1.041025	1.35259
0.779668	1.267879	1.872049	0.284048	2.351299	0.686667	0.90013	0.876638	0.290519
1.96404	29.18885	13.00996	25.24367	6.443925	11.65791	7.162698	9.838658	14.21671
0.009452	0.12075	0.103341	0.046962	2.89E-05	0.000433	0.151176	0.13824	0.000274
1.027403	0.82974	0.454978	0.570664	1.097677	0.753449	0.733074	2.107922	0.554026
0.683232	2.114437	1.256558	1.266255	0.679812	2.150303	0.460065	0.942944	1.153723
5.838499	12.56693	9.031082	5.462475	8.888167	10.8813	11.19942	19.99861	13.91714
0.081667	0.350837	0.243232	0.074358	0.361717	0.123102	0.668947	0.490079	0.113218
6.900202	1.734949	2.618846	3.217612	0.736551	4.188896	1.382006	2.187395	1.434921
0.15562	0.007734	0.001053	4.33E-05	2.89E-05	0.000433	0.000375	0.000101	0.000274
0.278304	0.536263	0.326681	0.183052	0.273672	0.308442	0.797114	0.038139	0.416782
0.385736	2.096162	1.962424	1.87482	1.477388	2.060491	2.925426	4.125786	5.22482
0.83697	0.812771	0.939408	1.087345	0.578817	1.131472	1.935685	0.798153	1.335743
0.141775	1.716364	2.281926	0.471313	2.27254	1.06355	5.165599	2.31013	0.882078
0.278304	8.66E-05	0	0.004949	2.89E-05	0.000433	0.023492	0.038139	0.020938
35.27608	77.74812	34.38341	14.97762	144.7919	36.74522	135.5245	749.7839	27.96248
3.618499	2.586926	1.596782	0.780952	3.610707	3.101371	2.678781	3.070952	0.639892
0.343824	0.088066	0.080281	0.1379	0.084228	0.022266	0.069365	0.023059	0.131443
2.043795	6.830592	2.586926	3.921645	11.30987	3.201457	7.625209	5.864084	2.400455
1.41368	2.254084	2.225498	2.836717	1.572035	2.489762	3.394286	2.834084	11.89932
21.74051	43.49658	32.68313	83.489	47.5018	46.7056	65.94551	30.82277	29.82828
0.009452	0.343629	0.394877	0.023304	0.757273	0.091133	0.120361	0.038139	0.353492

0.619012	0.034502	0	0.108997	0.009163	0.050844	0.069365	0.209048	0.000274
1.006025	0.705231	2.242092	0.360462	0.456522	0.287951	1.382006	0.046544	3.022713
2.964762	1.734949	0.939408	1.428398	2.42381	1.651522	3.011169	0.707475	1.204957
0.009452	0.12075	0.233593	0.053485	0.392403	0.080722	0.000375	0.055671	1.275325
0.009452	0.025483	0.198059	0.06031	0.123535	0.069668	0.06246	0.023059	0.113218
27.66779	28.54786	37.29684	18.33909	13.00996	59.32013	111.5439	3.201457	118.7028
3.457504	4.959358	3.340361	11.65791	5.606739	3.116732	2.281926	1.559675	5.11697
0.15562	0.253687	0.243232	0.235043	0.736551	0.395426	0.853218	0.283535	0.290519
0.683232	5.158146	6.063276	3.588369	3.877417	4.277006	5.654863	7.824473	5.872886
0.787605	2.145137	1.155411	1.303961	2.688304	0.885664	1.250058	1.632648	0.65202
4.277006	7.697063	8.445743	9.459177	10.7557	4.72798	8.195657	8.319019	6.486104
3.26	1.354567	0.635916	1.199481	0.585592	1.431414	2.990851	0.550974	1.80267
0.237359	0.042713	0.064488	0.053485	0.056638	0.112121	0.131768	0.046544	0.04062
0.009452	8.66E-05	0.087734	4.33E-05	2.89E-05	0.000433	0.035758	0.038139	0.000274
0.810967	1.594675	1.061457	0.426898	1.351573	0.645455	1.455022	0.666847	1.994329
2.402468	13.75978	18.19595	11.30987	35.27608	15.98892	30.49289	10.93101	17.92188
0.204278	0.558095	0.83088	0.188276	1.420202	0.413009	0.206768	0.293276	0.812771
0.106926	0.461811	2.602395	0.793918	2.99987	0.185909	1.668369	0.898016	3.718716
1.132165	0.392554	0.532381	1.857677	0.523752	1.92272	2.210866	0.273434	0.591118
5.687879	5.591717	4.702468	5.150693	8.412367	5.195022	3.783694	3.544257	3.404372
2.760765	4.043846	3.005281	31.92385	2.89E-05	8.477662	3.588369	2.07658	5.00404
0.141775	0.127049	0.15127	0.144935	0.965714	0.176104	0.329091	0.174646	0.190541
0.009452	0.018492	0.002655	0.004949	2.89E-05	0.000433	0.023492	0.008975	0.000274
0.328268	5.217569	1.53684	1.821861	2.903867	0.925801	6.632063	1.191198	3.321284
0.009452	0.025483	0.017172	4.33E-05	0.062381	0.000433	0.106696	0.000101	0.020938
0.180368	0.034502	0.294921	0.046962	0.06912	0.133535	0.226883	0.014964	1.711414
5.654863	25.73165	37.81919	33.4985	60.77449	23.82354	93.1424	24.04374	49.50903
0.009452	0.114358	0.15127	0.046962	0.509242	0.153867	0.98474	0.611789	0.000274
0.081667	0.018492	0.001053	0.004949	0.003175	0.031818	0.018102	0.000101	0.028081
8.815123	1.190491	0.655245	0.729582	1.91254	2.121198	1.060188	0.861429	0.746551
0.009452	8.66E-05	2.425801	4.33E-05	8.694697	0.000433	0.000375	0.000101	0.000274
13.49926	1.919661	0.925801	5.037489	4.508196	3.217612	10.38202	0.898016	0.806227
30.49289	7.061328	4.246861	10.43066	0.859351	14.25954	4.160238	3.803766	21.19547
0.875101	0.543398	0.666847	0.67632	0.516825	1.036558	0.400173	0.329798	0.849336
1.65785	0.903074	1.754733	2.752893	0.917431	4.140505	3.089293	0.155945	5.545729
0.683232	1.338261	1.746724	0.515238	2.16759	0.437799	1.021746	0.996111	0.131443
0.009452	1.2214	2.304452	0.188276	2.257749	0.133535	1.581912	0.329798	3.061955
0.1686	8.66E-05	0.006421	4.33E-05	0.165657	0.000433	0.035758	0.12772	0.224762
0.663651	3.775209	5.576219	3.687633	7.188095	3.975498	3.330909	2.789466	4.529913
0.760866	0.392554	0.48619	1.394632	1.435772	1.423506	1.513102	0.391962	0.568665
0.009452	0.237035	0.443543	0.108997	1.219019	0.133535	0.712453	0.100483	0.524646
0.180368	4.702468	2.826277	6.036818	4.47912	5.266335	4.025267	5.47	3.163506
0.009452	0.070909	0.032092	4.33E-05	0.361717	0.000433	0.06246	0.023059	0.156825
4.292078	6.853608	4.376118	7.570693	2.860722	6.653016	4.839105	14.6058	15.3618
0.779668	0.127049	0.095382	0.080895	0.267388	0.050844	0.018102	0.283535	0.015714
11.36975	1.29417	4.592273	2.442987	1.1407	3.975498	1.491977	1.023528	3.885642
1.052208	1.499214	4.535354	1.29267	3.651898	2.196017	1.612056	2.150303	0.882078
1.273636	0.034502	7.545584	2.705916	0.314156	6.612121	2.143261	4.673182	1.587605
0.259098	0.070909	0.338499	0.227063	0.347655	0.091133	0.106696	0.064848	0.113218
9.063983	4.673182	4.789596	3.743232	0.775844	11.03843	2.65684	1.519322	5.238701
0.11811	1.905094	1.762713	1.711414	3.7586	1.219856	2.727785	1.259747	1.130115
6.653016	12.85504	15.88548	15.56131	3.909033	17.92188	11.25392	13.6814	31.55417
2.820974	11.03843	9.792626	18.62711	19.99861	14.08569	10.28573	21.93452	13.60866
0.009452	2.459812	0.83088	4.33E-05	4.552092	0.143333	1.037958	1.074827	0.164401
0.719942	0.643427	0.465563	0.217172	2.609293	0.591616	0.701818	0.259033	0.388326
0.180368	0.141082	0.110642	0.108997	1.435772	0.050844	0.460065	0.046544	0.096905
8.539971	2.107922	4.312251	10.57984	2.89E-05	5.366335	6.808369	0.720938	34.84299
0.719942	1.555289	2.461905	1.711414	0.872597	1.641999	2.671299	3.058802	5.671126

0.93267	11.89932	8.381328	5.359134	7.456118	5.266335	5.576219	6.8421	6.079596
0.543131	0.411948	1.302107	0.247251	1.983824	0.29798	0.876118	0.283535	0.504098
3.334199	3.166659	3.077316	1.766248	2.579509	3.061955	2.08798	1.741414	1.439704
1.580909	1.239423	0.475433	1.348975	4.198117	2.506934	1.214315	1.34267	0.714697
0.796674	1.618773	0.819509	1.692763	0.859351	1.229553	1.028672	0.420707	2.496407
9.335397	2.31013	2.16759	1.436623	1.360022	7.112496	4.468009	1.175859	1.386681
24.98218	13.91714	67.98069	67.98069	16.22108	52.56128	144.7919	32.31123	7.08733
5.295325	5.337547	3.204632	7.112496	4.508196	11.42512	12.63773	4.429769	6.153102
2.425801	3.101371	9.063983	3.50425	5.923838	6.250447	8.103925	2.31811	5.447114
10.1028	10.01009	4.110779	8.005685	2.329784	5.044113	0.174776	17.51961	0.23873
0.742872	4.502453	1.31	0.975556	8.608153	1.680895	5.855281	0.889553	1.452857
1.397482	2.540462	2.618846	1.477388	1.608427	0.89404	1.169791	2.659214	1.492973
1.302107	1.02487	0.585902	1.073543	0.736551	2.600014	1.688045	1.20824	2.496407
0.343824	0.485014	0.543939	0.152114	0.890065	0.153867	0.299747	2.933903	0.140548
0.365859	0.528571	0.976667	0.3514	0.65798	0.37228	0.597042	0.381999	1.171349
0.009452	0.12075	0.25197	0.163766	0.372504	0.123102	0.174776	0.218355	0.843102
0.009452	0.327662	0.635916	0.108997	0.977994	0.000433	0.485014	0.12772	0.380649
0.1686	1.01772	1.499214	0.169675	2.002193	0.308442	1.250058	1.496118	0.535029
0.543131	0.411948	0.243232	0.299062	0.314156	0.437799	0.113911	0.431717	0.882078
0.502222	0.296955	0.861934	0.814877	0.372504	0.975556	0.687431	0.36267	1.055628
7.061328	0.327662	0.303564	4.048499	0.234358	4.851227	0.000375	2.219863	1.024127
0.569293	2.710765	3.921645	4.007583	1.559675	1.556392	4.440548	0.658362	3.655714
0.141775	1.57645	2.207417	0.868341	1.069574	0.981558	2.964762	0.329798	1.982208
0.15562	0.018492	0.072482	0.074358	0.092049	0.080722	0.206768	0.313723	0.034199
2.417424	2.625823	3.606804	14.00414	0.296234	9.297085	3.975498	7.964315	6.191515
2.572309	14.51648	12.22798	7.363117	10.1028	7.737807	12.16681	7.430317	19.451
2.524892	0.710722	1.436623	0.954776	0.977994	2.649675	2.078355	0.185022	0.259163
1.096335	2.145137	3.077316	1.491977	5.050267	4.758088	3.230065	2.42381	3.855952
0.009452	0.056869	0.432468	4.33E-05	0.220808	0.000433	0.000375	0.000101	0.323319
0.296234	0.376407	0.103341	0.217172	0.32925	0.362179	0.069365	0.470736	0.000274
1.076032	2.540462	8.994791	2.611797	1.082179	6.325945	3.104394	2.625823	3.290101
0.365859	4.125786	2.668896	6.230245	1.930216	9.687496	5.22482	3.644343	18.33909
10.73084	3.407619	4.435159	6.036818	4.125786	2.051962	3.471486	3.107417	1.596782
0.31785	0.335108	0.25197	0.299062	0.595022	0.185909	0.366674	0.055671	0.259163
0.089553	0.335108	0.326681	0.163766	0.499986	0.29798	0.347403	0.12772	0.416782
0.353795	0.614935	0.57531	1.066948	0.305253	0.196667	0.366674	0.108124	0.65202
0.551861	0.622496	1.3829	0.624473	1.465123	1.819221	2.078355	0.908773	2.676392
3.877417	5.959033	5.180058	4.272165	3.052655	6.911486	4.025267	3.497085	9.459177
0.11811	8.66E-05	0.095382	4.33E-05	2.89E-05	0.000433	0.023492	0.000101	0.000274
0.009452	0.141082	0.047323	0.046962	0.049834	0.031818	0.106696	0.023059	0.000274
9.541645	2.417424	3.23987	1.420202	2.058773	1.495043	1.267879	0.976111	3.204632
0.278304	2.197828	2.148658	1.449062	5.050267	1.277056	2.057056	2.03886	1.755988
6.153102	40.11561	45.91841	7.824473	105.1694	14.42661	7.780786	4.281847	4.003117
0.009452	0.221111	0.31456	4.33E-05	0.957273	0.196667	0.183521	0.611789	0.000274
0.180368	0.127049	0.072482	0.015599	0.123535	0.080722	0.305657	0.014964	0.062381
8.048095	5.273362	4.814661	2.174733	0.833975	2.600014	1.540144	5.082063	2.400455
11.48368	6.287561	8.83241	10.1459	5.942294	13.53913	22.52375	3.367071	5.323492
0.773276	2.74298	3.025671	3.083319	7.61176	4.365224	3.303925	1.191198	1.881955
0.009452	0.034502	0.072482	4.33E-05	0.012785	0.000433	0.000375	0.008975	0.069784
0.1686	0.007734	0.017172	0.023304	0.003175	0.000433	0.035758	0.000101	0.190541
1.511198	2.254084	3.290101	1.130794	2.944416	2.250462	3.66785	2.42381	2.561162
9.179466	22.74797	4.94026	12.39704	7.162698	6.443925	13.91714	21.19547	10.19468
0.560606	0.434329	0.383074	0.275873	0.679812	0.27899	0.222193	1.669466	0.784372
3.536955	0.434329	1.163579	4.297222	1.645613	1.556392	1.455022	2.668896	3.192229
3.204632	1.682193	2.16759	3.877417	0.159105	0.788369	0.733074	0.165108	0.773276
1.109726	1.177994	1.132165	1.124574	1.744055	1.306667	1.29267	1.281241	2.021068
0.009452	0.449091	0.123853	0.648961	0.288081	0.000433	0.797114	0.064848	0.333463
0.009452	0.289452	2.037114	4.33E-05	2.42381	0.239885	0.000375	0.666847	0.402698

4.473348	5.933066	6.423911	5.654863	2.698557	8.539971	4.174964	2.59557	2.447294
0.009452	0.034502	0.055967	0.023304	0.116825	0.022266	0.06246	0.064848	0.062381
0.009452	0.314387	0.557763	0.080895	0.819048	0.000433	0.509242	0.596775	0.206263
0.009452	0.627937	1.29417	1.845519	0.129733	0.809986	0.639892	1.104805	1.824892
3.204632	1.037958	0.84119	2.196017	0.159105	1.651522	1.734949	0.82868	2.011501
1.543377	2.55184	1.762713	7.200505	0.32925	2.696061	2.850058	3.022713	8.888167
0.009452	0.296955	0.277417	4.33E-05	0.129733	0.100779	0.000375	0.209048	0.215599
0.431977	3.028629	4.451154	1.575483	3.236457	2.03886	4.312251	6.772763	3.446732
11.65791	3.55961	1.005426	1.471198	0.926926	2.109755	2.805382	1.34267	1.298665
15.3618	9.141299	24.98218	19.99861	6.230245	19.68088	16.5625	10.62739	53.77619
1.397482	0.635541	0.524646	0.809986	0.207835	1.282208	1.250058	1.153723	1.434921
46.7056	9.047532	1.690483	2.797835	5.788153	5.01772	6.325945	0.707475	0.560606
18.62711	13.5739	11.11876	14.17388	7.558139	15.77678	12.22798	9.924372	8.923117
0.089553	0.127049	0.123853	0.031046	0.035505	0.042078	0.069365	0.201789	0.054026
1.980592	1.63039	0.508658	0.767085	1.533535	0.93267	0.797114	1.041025	0.282799
3.489899	18.91854	18.33909	15.77678	19.68088	16.34294	34.84299	22.74797	33.4985
0.269697	0.321746	1.436623	1.962424	0.578817	1.108276	1.303045	2.529307	0.309062
4.914863	0.926926	2.304452	0.814877	0.784372	2.947287	2.201587	0.73961	0.323319
12.49452	2.531515	1.675722	3.273045	3.330909	55.04081	10.93101	23.82354	8.195657
0.009452	0.550368	0.205931	0.06031	0.793918	0.384964	0.183521	0.075231	0.909856
1.006025	1.069574	1.664906	2.554069	1.372475	3.648081	1.01772	1.175859	4.246861
0.009452	2.171429	1.327525	1.788874	2.768312	0.901212	1.35259	3.122915	1.921212
4.644098	15.98892	3.763045	6.922771	4.101061	5.993045	15.77678	14.25954	16.68554
2.223636	0.283535	0.16575	0.656003	0.32925	0.821414	0.602287	0.116659	0.299062
1.476429	2.018167	2.535974	2.034242	2.386248	1.758615	1.884935	2.129596	3.179199
0.009452	0.376407	0.198059	0.445996	1.484553	0.249408	0.47974	1.559675	0.31456
0.009452	0.684401	0.116595	0.611789	0.615584	0.328268	0.891176	1.281241	0.000274
1.263752	10.38202	15.77678	14.84163	4.839105	10.1459	1.799899	4.952915	6.727633
2.257749	1.950519	1.889711	1.05417	0.72044	1.381111	1.947316	1.023528	1.513102
1.027403	3.101371	0.722626	1.788874	1.420202	2.171429	0.576205	1.983824	2.159004
0.009452	0.064127	0.181674	0.015599	0.012785	0.050844	0.131768	0.13824	0.104329
0.009452	0.691674	0.123853	0.080895	0.890065	0.069668	0.276429	0.000101	0.000274
2.642525	3.50425	5.123781	4.135801	3.24658	4.827691	2.250462	0.674163	1.216768
5.068831	1.01772	1.202626	1.584105	0.698297	2.66404	1.037958	0.460065	0.719942
0.828167	1.177994	1.327525	1.315613	1.994329	1.099762	1.618773	0.798153	1.275325
1.994329	2.618846	6.464906	3.156537	11.544	3.407619	3.217612	2.243911	3.179199
4.684502	0.411948	0.303564	1.39847	2.89E-05	0.653586	0.000375	0.798153	0.542828
0.246955	48.53429	4.160238	0.683665	62.33472	9.968355	1.05153	252.4251	2.099524
0.83697	2.524892	1.861999	1.456955	0.926926	1.190491	1.484553	2.540462	1.663672
0.009452	0.088066	0.383074	0.087619	0.116825	0.060087	0.166465	0.146999	0.23873
0.009452	0.025483	0.173189	0.015599	0.146479	0.031818	0.113911	0.12772	0.164401
2.112843	0.513456	1.215981	0.901212	0.361717	2.398442	1.528975	1.34267	0.726472
0.569293	2.807937	2.380079	2.205476	2.713319	2.978968	2.223636	2.089639	3.269913
0.673254	1.104805	1.030678	0.744531	2.223636	1.108276	1.581912	0.596775	1.940231
0.93267	0.261111	0.416782	0.483608	0.965714	0.645455	1.484553	0.373939	0.198059
4.338629	17.58415	15.276	11.96574	36.74522	8.677489	17.92188	29.6626	9.751039
3.1857	2.642525	1.475469	1.163579	1.126775	1.778153	1.971183	0.611789	4.222583
0.31785	0.283535	0.925801	0.157792	1.05824	0.123102	0.438831	0.611789	0.954141
0.593146	1.548723	2.765758	1.377446	4.272165	1.950519	2.50469	1.632648	2.758088
2.996869	2.347439	0.754784	2.382035	0.90013	1.89373	3.829105	0.965714	1.153723
2.925426	2.459812	1.449877	1.355527	0.990909	0.901212	3.743232	2.563506	3.640606
2.345527	1.641999	4.513622	3.008225	2.16759	6.570433	0.733074	0.93267	4.933781
0.237359	0.018492	0.017172	0.067547	0.003175	0.042078	0.048341	0.038139	0.028081
3.829105	5.130411	4.067547	4.94026	4.236962	5.42381	4.863579	1.29417	6.019971
7.991602	25.99427	11.89932	17.15902	9.583463	7.264502	9.179466	7.668579	4.49671
1.189048	1.399315	1.817828	0.929812	8.508514	1.464214	1.605859	2.138131	0.370281
0.2264	0.622496	0.605022	0.49127	0.354242	0.404113	0.393983	0.209048	0.394156
0.009452	0.196667	0.557763	4.33E-05	0.252165	0.209841	0.126429	0.100483	0.149423

3.286652	1.416407	1.074827	0.593434	0.65798	1.367179	0.737857	0.391962	2.388348
0.296234	0.189278	0.047323	0.116176	0.372504	0.112121	0.239228	0.038139	0.164401
0.638362	1.524776	0.896522	5.088694	0.850808	1.083651	2.453781	0.304062	0.755253
0.009452	1.594675	8.888167	6.760765	2.89E-05	4.808658	3.198499	7.765931	3.552049
0.502222	2.124574	0.508658	1.092785	1.177309	2.371833	0.995527	0.490079	3.217612
0.141775	0.025483	0.294921	0.06031	0.129733	0.000433	0.206768	0.030613	0.113218
0.246955	1.143175	0.70404	0.780952	1.016494	0.579841	0.519019	1.616385	1.525729
0.881082	0.305253	0.277417	0.669358	0.347655	1.602619	1.261407	0.293276	1.159913
0.192929	0.868341	1.111241	1.266255	0.578817	1.367179	0.485014	3.1857	2.432121
0.511667	2.727785	0.394877	3.895469	4.63189	4.620159	3.457504	1.112641	17.64869
18.33909	4.752121	8.048095	4.281847	6.632063	12.42965	7.251263	5.00404	15.18102
0.089553	8.66E-05	0.006421	0.009163	0.234358	0.031818	0.145382	0.014964	0.020938
0.601227	1.746724	2.815866	1.253752	2.567605	1.036558	2.947287	1.095657	1.96697
0.365859	6.464906	7.238023	3.767489	5.679502	3.418405	7.911934	5.123781	5.687879
2.59557	1.455988	3.101371	3.137576	3.370527	2.496407	1.947316	0.685209	2.116032
2.792035	8.048095	5.671126	7.200505	6.268312	5.23176	0.908247	13.08214	5.165599
1.288276	5.068831	3.921645	3.944084	10.82954	1.219856	0.609841	8.745238	5.788153
0.106926	0.596111	1.739935	0.169675	1.618773	0.209841	2.481356	0.185022	0.504098
7.854069	4.752121	1.664906	5.137042	0.642698	4.456912	5.28039	1.575483	10.38202
287.197	184.443	749.7839	1360.293	50.55949	425.62	1360.293	100.0998	1360.293
15.18102	0.877807	1.634935	1.692763	0.736551	2.802828	4.484892	1.603795	1.50342
0.009452	0.079466	0.047323	4.33E-05	0.180613	0.031818	0.055671	0.091833	0.034199
1.189048	2.961768	3.457504	1.303961	7.683766	1.517143	0.416782	3.533211	1.266255
57.84882	17.92188	22.13245	40.7932	42.75576	43.49658	41.47925	20.34063	19.83727
0.296234	0.24562	1.150635	0.163766	0.819048	0.153867	0.379495	0.13824	0.65202
6.570433	14.79742	11.65791	14.70188	5.493182	17.39935	18.91854	11.30987	21.54823
17.51961	3.984747	0.173189	2.323983	3.890556	0.579841	0.447965	28.23632	8.815123
1.369048	5.01772	3.204632	3.043528	3.450577	4.424416	9.924372	2.73272	7.288788
0.697908	1.96697	3.179199	5.273362	0.129733	4.207778	1.214315	0.441472	0.206263
1.144733	3.4679	3.457504	1.385801	3.370527	1.701616	2.021068	2.453781	3.350224
0.269697	0.614935	0.754784	0.240346	1.680895	0.404113	0.498052	0.313723	0.333463
2.668896	6.922771	7.964315	4.580938	8.241342	5.576219	5.821804	9.335397	3.820758
0.385736	0.268369	0.36114	4.33E-05	0.06912	0.000433	0.120361	0.174646	0.000274
1.613232	1.416407	1.189769	1.548723	4.174964	1.53684	3.140866	2.117814	0.924329
9.397771	46.7056	53.77619	56.39528	28.71146	35.776	34.38341	29.6626	57.84882
0.009452	0.419596	2.509177	0.948745	3.300664	0.455649	2.990851	2.021068	1.163579
0.009452	0.034502	0.055967	4.33E-05	0.021696	0.000433	0.030491	0.000101	0.000274
5.110159	4.637994	7.136537	3.442886	9.335397	2.802828	7.443276	3.169812	2.666494
0.601227	1.080303	1.641999	4.49671	1.409942	1.960945	1.826147	2.474834	3.373983
1.434921	7.112496	5.056421	0.702835	3.926154	7.037345	4.198117	4.758088	15.98892
1.64684	2.569957	9.237525	4.160238	5.309264	4.609076	4.690159	2.59557	1.833102
0.141775	0.38285	0.326681	0.009163	0.273672	0.000433	0.000375	0.075231	0.215599
0.551861	0.947157	1.327525	1.130794	1.311674	1.566414	3.7586	1.237879	0.699192
0.07583	0.148846	0.394877	0.06031	0.234358	0.153867	0.247583	0.116659	0.765606
0.141775	0.403651	0.939408	0.217172	0.280823	0.37228	0.379495	0.313723	0.806227
0.376407	0.025483	0.141876	0.116176	0.028196	0.153867	0.041616	0.023059	0.000274
4.715599	24.73724	24.2842	14.6058	18.48514	24.04374	4.302367	4.783001	7.598312
1.910974	0.848831	2.727785	0.744531	2.882035	3.87329	2.413146	2.402468	2.842193
17.71696	3.747035	8.195657	5.923838	3.104394	7.443276	6.695887	2.776421	7.339452
4.039192	2.55184	4.47912	4.894509	6.365065	3.391075	7.38847	6.922771	1.306667
0.259098	3.807648	2.836717	1.29267	4.881775	1.431414	1.4671	13.00996	1.216768
16.80343	18.05488	16.44768	9.625253	8.103925	25.24367	10.8813	16.09482	19.451
2.563506	0.812771	2.024488	3.058802	2.740519	1.688045	1.501291	0.956667	2.868456
0.476551	0.550368	0.383074	0.793918	1.1407	0.308442	0.780519	0.36267	0.062381
0.683232	0.577071	0.48619	0.445996	0.489235	1.070693	2.294733	0.798153	1.363557
4.776407	0.947157	1.962424	0.315094	4.236962	0.556126	3.94886	0.250115	0.689509
0.129971	0.577071	2.727785	0.102251	2.752893	0.123102	0.453463	0.561126	0.206263
0.009452	0.056869	0.213355	4.33E-05	0.418023	0.000433	0.000375	0.000101	0.68062

0.336328	1.03189	1.401068	0.619365	3.307186	0.545808	1.491977	1.083651	0.198059
27.38726	8.780115	1.615281	15.276	9.418196	9.101674	1.924228	12.77772	2.621133
11.544	8.508514	4.246861	30.3324	11.36975	14.6058	7.780786	4.758088	13.00996
0.673254	2.347439	1.3829	1.385801	2.647244	2.03886	5.180058	1.041025	0.798153
1.860491	3.002576	3.227085	3.401126	2.917381	1.950519	6.063276	1.741414	1.614257
0.089553	0.2129	0.907273	0.453218	0.516825	0.328268	0.366674	0.750866	0.934488
1.369048	1.563045	2.442987	1.605859	2.725498	1.172208	3.540606	0.518139	2.217951
0.365859	0.148846	0.158766	0.015599	0.280823	0.100779	0.069365	0.075231	0.04062
0.009452	0.056869	0.095382	0.046962	0.159105	0.000433	0.000375	0.165108	0.323319
1.799899	6.325945	3.411097	5.273362	3.008225	4.524481	5.967879	5.493182	8.780115
0.421804	0.079466	0.732193	0.053485	0.595022	0.209841	0.400173	0.340866	0.173189
0.009452	0.042713	0.103341	4.33E-05	0.267388	0.060087	0.000375	0.000101	0.000274
0.581926	2.477027	0.995527	1.655541	1.692763	2.227273	1.560779	0.596775	0.945483
0.673254	3.089293	1.132165	3.552049	1.271097	4.082165	2.390447	0.942944	1.216768
0.009452	0.189278	0.123853	0.053485	0.214885	0.031818	0.312475	0.075231	0.054026
1.052208	3.775209	4.007583	1.283059	5.416623	2.196017	5.439278	2.474834	1.648066
0.62645	2.078355	2.003723	3.192229	3.439437	3.391075	3.636789	1.011486	0.882078
8.780115	1.746724	8.958225	39.53687	22.96463	7.136537	5.395584	5.381227	91.05541
0.009452	0.2129	0.233593	0.039589	1.289149	0.000433	0.29404	0.381999	0.299062
1.109726	1.790245	1.393571	2.089639	3.588369	1.989711	1.910974	2.176501	1.579798
0.009452	8.66E-05	0.087734	4.33E-05	0.10381	0.000433	0.000375	0.000101	0.000274
0.009452	0.467136	0.055967	4.33E-05	0.605887	0.384964	0.254156	0.146999	0.23246
1.360022	0.467136	0.508658	1.033672	0.372504	1.89961	0.756724	0.373939	1.596782
0.009452	0.12075	0.372078	0.087619	0.667583	0.209841	0.201789	0.000101	0.299062
5.351934	14.88584	10.1028	6.486104	34.84299	4.933781	2.066652	15.18102	6.365065
0.385736	0.756724	0.740101	0.299062	1.971183	0.556126	1.572035	0.250115	0.524646
10.43066	4.984488	14.17388	9.218514	3.518384	7.625209	1.554185	4.343838	2.850058
0.15562	0.007734	0.001053	4.33E-05	2.89E-05	0.000433	0.000375	0.000101	0.000274
1.560779	3.91355	3.799885	4.927302	9.141299	6.760765	9.838658	2.243911	4.077403
9.502294	30.17192	31.1753	23.16622	15.18102	21.19547	38.94854	22.32214	25.24367
0.619012	1.267879	0.896522	1.766248	1.184935	0.479401	0.98474	1.259747	0.290519
1.311674	5.671126	5.500678	6.464906	5.295325	8.381328	7.882222	4.673182	18.76954
0.1686	0.034502	0.002655	0.004949	0.092049	0.069668	0.097843	0.000101	0.04062
0.828167	0.965065	1.014784	1.41368	0.866717	1.259747	3.163506	0.707475	3.533211
0.009452	0.056869	0	4.33E-05	2.89E-05	0.000433	0.247583	0.000101	0.496797
28.87506	19.37139	23.6012	10.28573	11.89932	6.200945	1.118817	15.56131	33.07147
0.097633	0.056869	0.006421	0.094733	0.049834	0.050844	0.023492	0.008975	0.062381
0.259098	0.196667	0.055967	0.195007	0.146479	0.185909	0.000375	0.174646	0.000274
1.865043	8.745238	13.53913	12.39704	10.35839	10.35839	23.71237	4.978182	18.62711
3.632973	0.2129	0.896522	0.6943	0.354242	1.359177	0.628297	0.849841	0.843102
20.34063	4.365224	4.179762	4.863579	3.391075	17.03919	1.74539	4.039192	3.005281
4.990794	7.824473	4.222583	11.42512	5.515584	4.914863	4.06303	3.754719	4.484892
0.551861	3.493362	4.72798	3.944084	5.01772	2.978968	4.546443	2.515938	7.824473
0.204278	2.750375	2.225498	2.752893	1.289149	4.524481	3.269913	1.87482	2.496407
0.954776	1.96697	2.111299	1.377446	3.824964	2.893001	2.050404	1.473218	2.970144
10.23768	8.477662	6.00228	6.088593	3.890556	8.643074	4.251818	6.39474	2.961768
4.586753	7.545584	5.302294	8.226219	7.558139	7.363117	17.64869	3.122915	9.418196
51.52195	38.94854	9.397771	53.77619	13.60866	23.6012	25.24367	15.276	8.019769
0.62645	0.392554	0.596508	0.336508	0.615584	0.100779	0.416782	0.229502	0.104329
0.995527	0.196667	1.030678	0.570664	0.692799	1.590202	0.341241	1.314675	1.547734
0.962107	2.329784	3.125844	1.798535	3.172864	3.493362	2.789466	2.884762	2.367763
0.009452	0.656392	0.775844	4.33E-05	1.680895	0.100779	0.504098	0.229502	0.458341
0.009452	8.66E-05	0.032092	0.046962	0.084228	0.000433	0.000375	0.008975	0.046667
0.610606	0.494841	0.646378	0.420058	0.667583	0.196667	0.664026	0.518139	0.568665
22.13245	6.900202	1.074827	19.06893	2.967453	8.195657	6.443925	3.514834	10.43066
2.453781	0.976667	1.074827	0.710253	0.480548	1.282208	1.141515	3.846984	1.685678
0.852684	0.965065	1.061457	1.209776	0.747417	0.695051	1.806595	0.625498	0.916869
0.192929	8.66E-05	0.613211	0.157792	0.173059	0.249408	0.276429	0.000101	3.005281

0.009452	8.66E-05	0.039747	4.33E-05	0.220808	0.080722	0.000375	0.000101	0.000274
0.431977	8.994791	6.796566	7.162698	2.502619	3.169812	10.05618	3.022713	7.964315
0.009452	0.025483	0.213355	0.009163	0.110289	0.080722	0.174776	0.014964	0.04062
1.632648	1.197922	3.016984	2.685786	1.608427	2.673846	2.263377	3.864827	0.580123
0.493247	0.910339	0.31456	0.838846	1.708932	1.000173	1.755988	1.34267	0.477698
13.38418	1.758615	9.502294	23.82354	1.994329	10.28573	1.65785	2.459812	3.984747
5.530274	12.29473	5.704646	14.33939	10.05618	10.78056	16.9162	8.849697	6.268312
0.269697	0.070909	0.072482	0.144935	0.06912	0.050844	0.089726	0.014964	0.069784
0.097633	8.66E-05	0.001053	0.023304	0.028196	0.022266	0.048341	0.014964	0.015714
0.569293	3.944084	5.454949	2.498478	6.028427	4.758088	9.335397	6.717056	6.09759
1.476429	2.438831	4.348882	2.29873	6.518709	1.803918	3.957734	3.411097	4.145209
0.984069	3.610707	4.776407	1.677042	3.330909	2.649675	3.771349	2.750375	3.249877
0.009452	0.883665	0.173189	0.757273	1.326667	0.556126	0.628297	0.976111	1.558571
7.570693	2.763261	5.821804	5.062626	1.645613	7.854069	5.704646	1.754733	8.226219
0.009452	8.66E-05	0	4.33E-05	2.89E-05	0.123102	0.000375	0.528196	1.428398
27.96248	7.92518	15.18102	42.08807	3.851414	19.53061	6.52899	11.36975	10.53159
0.009452	0.079466	0.213355	4.33E-05	0.273672	0.060087	0.341241	0.108124	0.000274
0.523752	6.171789	1.499214	1.449062	1.766248	0.963853	0.882619	0.36267	0.591118
11.14619	72.69473	40.7932	30.82277	40.11561	49.50903	118.7028	14.33939	45.1045
0.760866	0.296955	0.260765	0.291183	0.173059	0.471025	0.247583	0.165108	0.424004
2.871212	2.254084	0	3.799885	0.180613	5.165599	1.985253	0.194235	2.29873
0.009452	8.66E-05	0.15127	0.046962	0.499986	0.080722	0.769365	0.000101	0.000274
1.466111	2.347439	2.349351	1.179885	1.004156	1.855108	2.117814	2.789466	3.040505
1.96404	8.348817	7.495397	4.413608	6.922771	5.788153	3.507691	8.195657	11.77567
7.238023	4.145209	2.950173	5.576219	3.6029	5.062626	9.101674	1.826147	1.867951
0.180368	0.376407	0.181674	0.1379	0.335786	0.249408	0.160462	0.400967	0.458341
0.259098	0.743283	0.746984	0.858817	1.303961	0.328268	0.411169	0.155945	0.82974
3.204632	4.241905	2.341573	4.484892	0.625209	2.628225	0.967446	1.652771	4.445851
0.601227	2.781558	2.882035	1.584105	3.35355	1.241811	1.230411	4.381479	2.223636
0.246955	1.267879	3.644343	0.586248	2.394416	0.362179	2.035678	1.281241	5.11697
0.796674	3.855952	2.341573	3.283139	2.609293	5.508131	4.222583	2.690823	5.560563
0.819509	2.939141	2.522684	2.290996	0.595022	1.340036	5.295325	2.884762	9.179466
0.467403	1.281241	1.090072	0.67632	2.034242	0.507085	1.250058	0.611789	0.31456
44.27012	8.089848	27.52753	6.30671	9.101674	28.54786	6.30671	12.10289	46.7056
1.623232	18.19595	4.814661	10.62739	13.38418	12.22798	12.36443	8.381328	25.73165
0.009452	8.66E-05	0	4.33E-05	0.084228	0.000433	0.083074	0.000101	0.31456
9.751039	21.36892	26.25557	20.68746	11.48368	18.62711	19.68088	10.53159	14.42661
224.9555	530.1465	252.4251	224.9555	224.9555	252.4251	530.1465	1360.293	29.18885
1.096335	2.842193	31.92385	0.929812	3.279625	0.793918	1.87899	0.720938	2.333867
4.540794	0.743283	0.31456	1.040411	0.523752	0.963853	4.620159	1.259747	0.600548
7.519841	4.33342	2.74298	5.654863	4.343838	3.829105	3.842554	6.475505	3.080317
11.719	8.711905	6.560411	12.70768	6.09759	7.964315	5.622872	5.864084	3.046551
184.443	1.01772	9.751039	50.55949	4.921082	95.22939	13.38418	7.598312	155.7103
3.511263	4.241905	4.286962	6.830592	0.841746	2.628225	7.545584	0.861429	5.18754
20.51271	28.23632	5.84689	43.49658	9.667316	57.84882	126.1556	34.84299	30.17192
1.282208	9.707677	19.53061	2.174733	7.456118	5.889798	5.082063	6.612121	8.288874
1.020447	1.267879	3.799885	1.124574	4.752121	3.45404	1.349971	1.381111	1.815
3.984747	5.158146	4.272165	5.123781	2.993867	3.35355	13.6814	1.074827	3.68381
0.421804	0.455649	0.80417	0.543939	1.004156	0.732193	1.42456	0.508658	0.380649
0.009452	0.108124	0.141876	0.176299	0.605887	0.000433	0.000375	0.046544	0.156825
8.134084	11.14619	13.34662	21.19547	9.218514	16.44768	7.570693	8.608153	16.09482
10.8813	18.48514	26.80355	24.73724	35.776	21.74051	8.608153	8.608153	5.402511
0.009452	8.66E-05	0.024632	4.33E-05	0.084228	0.000433	0.06246	0.000101	0.131443
34.38341	22.96463	10.48101	24.98218	12.42965	16.9162	10.67991	16.22108	10.82954
0.638362	1.143175	1.074827	2.238341	0.990909	1.036558	1.013434	0.100483	1.626782
0.097633	0.392554	0.976667	0.336508	1.472208	0.27899	0.460065	0.12772	0.798153
0.129971	1.044863	1.014784	0.831472	1.850996	0.753449	1.27619	1.693939	0.458341
0.15562	0.042713	0.16575	0.087619	0.195685	0.091133	0.190541	0.000101	0.353492

4.456912	9.418196	5.088694	6.653016	3.8379	4.508196	4.145209	4.039192	2.953059
0.852684	1.472208	5.180058	1.266255	3.147294	1.641999	1.230411	3.156537	1.96697
0.009452	8.66E-05	0.064488	0.004949	0.097879	0.000433	0.048341	0.055671	0.034199
8.103925	2.500548	6.028427	4.519048	1.860491	8.319019	5.560563	5.150693	13.08214
23.16622	11.25392	30.17192	42.75576	6.760765	67.98069	77.74812	17.15902	62.33472
12.70768	4.058514	6.365065	4.644098	2.89E-05	8.994791	2.210866	2.59557	3.767489
0.009452	0.034502	0.141876	0.074358	0.049834	0.080722	0.438831	0.038139	0.140548
7.38847	4.907929	6.508427	3.043528	2.410996	2.489762	3.6029	3.169812	8.477662
0.009452	0.314387	0	4.33E-05	0.559221	0.000433	0.174776	0.155945	0.000274
0.009452	0.095303	0.064488	0.046962	0.042713	0.069668	0.089726	0.008975	0.140548
0.246955	1.377446	1.675722	1.369048	1.994329	0.781465	0.504098	1.726032	2.006782
0.009452	0.221111	0.567619	4.33E-05	0.480548	0.37228	0.174776	0.000101	1.204957
0.009452	0.088066	0.268824	0.039589	0.429827	0.000433	0.389812	0.165108	0.000274
0.411436	2.917381	1.772756	1.915455	2.083261	0.86518	0.138009	6.325945	3.116732
4.338629	5.217569	6.622092	5.23176	4.603434	8.075772	10.48101	2.884762	5.47
0.009452	0.910339	1.014784	0.528196	2.735368	0.069668	1.028672	0.720938	0.98474
8.711905	7.61176	4.752121	9.256537	8.694697	10.07949	11.42512	5.515584	9.667316
0.62645	0.070909	0.173189	0.123023	0.402381	0.153867	0.389812	0.313723	2.250462
12.10289	12.7427	7.938427	13.00996	7.30215	11.83593	7.737807	12.29473	13.6814
3.632973	4.135801	4.650007	6.580988	3.864827	5.704646	6.045209	8.888167	2.868456
0.081667	0.361948	0.443543	0.108997	0.82772	0.209841	0.634531	0.321068	0.299062
6.830592	7.037345	11.719	8.27267	0.766602	5.950664	15.98892	5.915469	21.74051
0.543131	0.895945	1.215981	0.656003	1.533535	1.11526	1.581912	0.685209	1.144733
22.96463	0.558095	5.381227	1.436623	0.418023	1.848297	0.797114	1.191198	3.926154
0.009452	0.169675	0.095382	0.067547	0.411169	0.080722	0.737857	0.174646	0.083521
0.009452	8.66E-05	0.260765	4.33E-05	2.89E-05	0.042078	0.000375	0.023059	0.945483
0.009452	1.245671	0.686667	0.074358	0.260144	0.000433	1.29267	0.771176	4.012049
0.610606	0.751371	1.023528	1.594675	1.262179	1.387612	0.126429	1.974228	1.078153
0.009452	0.011962	0	0.023304	0.01697	0.050844	0.069365	0.000101	0.028081
0.009452	0.034502	0	4.33E-05	0.009163	0.080722	0.030491	0.000101	0.076241
11.30987	1.416407	11.11876	3.707136	5.238701	5.00404	6.079596	4.851227	2.349351
0.411436	0.221111	0.48619	0.393449	0.321566	0.287951	0.083074	0.498636	0.215599
2.470317	4.18456	6.297136	2.563506	3.953297	3.002576	6.404921	3.980123	3.022713
2.312085	1.054899	0.732193	2.048781	0.704444	1.873434	1.303045	0.720938	1.74539
1.799899	4.875527	1.189769	1.837172	1.233665	2.31404	4.277006	2.474834	0.156825
5.560563	1.544517	0.557763	9.583463	2.768312	2.060491	0.393983	6.8421	1.321053
0.009452	0.108124	0.087734	0.023304	0.097879	0.022266	0.023492	0.008975	0.096905
2.097843	2.494177	3.23987	3.962179	2.16759	3.895469	6.591544	6.191515	11.09133
53.77619	39.53687	11.51384	28.87506	16.44768	29.18885	20.34063	19.37139	15.56131
0.009452	0.361948	0.36114	4.33E-05	1.39847	0.080722	0.283709	0.304062	0.353492
0.081667	0.169675	0.25197	0.087619	0.092049	0.091133	0.299747	0.209048	0.333463
0.92114	7.751869	7.625209	12.29473	7.112496	6.865	12.10289	3.754719	10.23768
2.365685	4.673182	2.755411	2.868456	3.926154	3.061955	6.653016	1.533535	1.335743
0.180368	0.784372	0.746984	0.669358	0.402381	0.645455	2.045476	0.36267	0.798153
0.28728	0.283535	0.116595	0.094733	0.321566	0.209841	0.089726	0.431717	0.096905
0.88619	2.569957	5.110159	3.381255	4.370671	3.7586	2.747908	7.025599	2.914632
2.996869	6.404921	2.713319	14.84163	4.821176	14.17388	8.381328	5.762965	4.155339
0.15562	0.108124	0	1.464214	0.084228	0.133535	0.756724	0.091833	1.089394
23.82354	59.32013	70.11932	72.69473	23.38378	83.489	105.1694	38.39042	59.32013
0.441833	1.515296	1.132165	1.156869	1.91254	2.723247	1.476429	0.750866	2.776421
0.192929	0.656392	1.061457	0.543939	1.15614	0.672468	0.425137	0.329798	0.496797
0.457749	0.995527	1.340981	0.831472	0.793918	0.909315	1.64917	1.136566	0.843102
40.7932	27.10502	5.068831	33.93939	14.21671	20.34063	45.1045	8.958225	5.273362
4.077403	2.124574	0.677807	1.861999	1.533535	2.773644	2.117814	1.259747	0.625209
2.802828	2.278254	1.80267	1.749307	2.040606	4.003117	2.035678	0.908773	4.165137
3.414574	0.419596	0.475433	1.118817	0.90013	0.487662	0.659582	0.13824	0.477698
0.845317	0.756724	3.125844	0.901212	0.766602	3.083319	1.189769	0.976111	3.404372
0.328268	0.108124	1.090072	0.015599	0.615584	0.000433	0.217677	0.250115	0.305253

1.873434	0.883665	0.567619	0.997244	1.280274	1.883535	0.774257	0.666847	0.580123
1360.293	14.97762	425.62	80.6518	25.24367	287.197	60.77449	75.1132	32.31123
0.891775	0.361948	0.080281	0.202294	1.082179	0.000433	0.030491	1.888023	0.034199
0.009452	8.66E-05	0	4.33E-05	0.140657	0.000433	0.000375	0.014964	0.113218
0.511667	0.114358	0.158766	0.053485	0.07632	0.069668	0.131768	0.165108	0.806227
80.6518	0.596111	3.321284	4.105714	1.680895	1.803918	0.920483	1.693939	22.52375
1.132165	1.406746	0.887922	1.143954	0.637085	1.05417	0.797114	0.720938	0.82974
0.365859	0.392554	1.023528	1.046703	0.392403	0.771688	0.959567	0.420707	0.849336
0.655613	1.833102	2.647244	1.513102	2.257749	2.234538	1.705007	2.102626	1.824892
0.009452	8.66E-05	0	4.33E-05	0.005512	0.259163	0.083074	0.12772	0.000274
1.737294	2.826277	4.053506	3.489899	4.863579	2.5271	4.18456	3.497085	3.061955
3.94886	0.169675	0.543939	0.889019	0.3414	1.076652	0.106696	1.227749	1.245671
0.237359	1.093434	1.150635	0.498355	4.067547	0.455649	1.200152	1.87482	0.773276
0.009452	0.011962	0.158766	4.33E-05	0.084228	0.031818	0.097843	0.014964	0.206263
8.381328	6.99026	2.281926	9.838658	4.563117	5.788153	3.621941	3.754719	4.077403
5.630815	0.699192	0.696234	2.227273	1.772756	2.547179	1.560779	0.674163	0.252316
0.129971	1.037958	1.953535	0.477446	2.151919	0.556126	1.303045	0.250115	0.96627
4.402973	3.263391	3.730808	6.53969	2.540462	5.337547	9.297085	6.011515	12.36443
1.20653	0.148846	0.039747	0.152114	0.028196	0.27899	0.597042	0.116659	0.156825
0.097633	0.018492	0	4.33E-05	0.005512	0.000433	0.000375	0.000101	0.020938
3.890556	1.464214	2.250462	2.216162	1.106825	2.280101	1.549711	1.434055	1.565253
0.009452	0.253687	0.181674	4.33E-05	0.569293	0.133535	0.183521	0.046544	0.000274
3.860491	3.88153	5.515584	6.865	1.828781	6.911486	8.048095	3.367071	8.164531
0.457749	0.699192	0.613211	0.322078	0.3414	0.318312	0.797114	0.508658	0.26562
3.930664	1.682193	1.855108	6.345382	3.314329	6.125823	4.519048	3.227085	2.131176
7.136537	6.772763	9.667316	9.397771	8.556703	13.15079	8.149307	8.226219	18.05488
0.237359	0.176558	0.16575	0.080895	0.116825	0.123102	0.329091	0.209048	0.156825
1.177994	4.408153	5.84689	2.710765	8.477662	3.7586	3.904517	3.820758	7.38847
1.04228	1.604827	4.535354	1.905094	1.566414	2.026219	1.177309	1.314675	3.855952
0.796674	1.877605	3.061955	1.648066	3.131544	2.547179	0.737857	1.87482	1.186457
0.129971	0.691674	0.666847	0.426898	2.096162	0.446811	1.320173	0.584134	1.098341
0.237359	0.296955	0.095382	0.412193	0.3414	0.37228	0.524646	0.259033	1.233665
3.993665	24.50942	9.101674	2.515938	16.9777	11.25392	7.495397	12.22798	14.79742
42.75576	9.625253	7.456118	16.09482	7.417359	15.66023	14.6058	4.875527	9.859719
8.994791	36.74522	6.967547	29.49693	8.195657	37.29684	11.30987	15.98892	39.53687
0.009452	8.66E-05	0.223485	4.33E-05	0.07632	0.000433	0.030491	0.000101	0.181919
2.489762	5.976724	4.135801	2.909293	5.439278	2.917381	0.701818	12.42965	3.500808
4.661717	10.1028	5.959033	8.27267	7.30215	9.687496	14.97762	9.418196	3.909033
1.846941	0.335108	0.181674	0.602287	0.180613	0.487662	0.687431	0.12772	0.402698
32.31123	7.61176	9.141299	21.93452	12.70768	21.36892	80.6518	6.591544	6.55039
0.441833	0.650101	0.585902	0.434069	0.679812	0.633853	1.195794	0.240101	0.5129
0.009452	0.127049	0.189632	4.33E-05	0.392403	0.112121	0.155224	0.12772	0.259163
1.219019	1.746724	0.338499	0.689509	0.679812	0.86518	0.723066	1.793074	0.639892
0.296234	1.127475	1.810664	0.938413	3.486436	0.981558	2.201587	0.638362	2.259661
0.11811	2.192496	1.817828	1.007864	5.967879	1.566414	1.285584	3.263391	7.625209
0.192929	0.229199	0.326681	4.33E-05	0.532857	0.112121	0.151176	0.116659	0.149423
12.63773	3.314329	2.903867	3.058802	4.077403	2.217951	4.644098	2.337821	1.815
0.097633	0.056869	0.055967	0.004949	0.110289	0.050844	0.041616	0.014964	0.259163
17.71696	7.162698	12.60233	13.53913	13.23022	6.784762	2.92013	20.16541	7.570693
1.924228	0.494841	0.198059	1.156869	0.559221	0.591616	1.320173	1.245671	2.069942
0.009452	0.148846	0.394877	4.33E-05	0.305253	0.196667	0.097843	0.304062	0.000274
1.144733	4.637994	8.019769	4.007583	9.256537	5.638759	3.125844	7.625209	48.53429
203.5795	16.09482	31.55417	38.94854	4.997417	100.0998	42.75576	144.7919	24.04374
20.85876	2.735368	5.976724	9.179466	3.414574	9.502294	7.653391	3.236457	17.78522
0.11811	0.101609	0.260765	0.535945	0.056638	0.176104	0.160462	0.13824	7.519841
6.25938	5.993045	9.707677	14.25954	2.903867	9.838658	2.242092	3.147294	4.266753
22.52375	65.94551	59.32013	62.33472	67.98069	36.26061	59.32013	40.7932	42.75576
0.009452	0.007734	0.002655	0.004949	0.028196	0.000433	0.06246	0.008975	0.000274

0.535029	0.684401	0.754784	0.838846	1.572035	0.545808	0.539812	0.674163	0.380649
0.009452	8.66E-05	0.080281	4.33E-05	0.049834	0.050844	0.048341	0.091833	0.046667
11.96574	0.727633	1.739935	4.217569	1.351573	5.210317	0.891176	5.762965	1.275325
0.009452	0.056869	0.181674	0.477446	2.89E-05	0.626118	0.47974	0.000101	0.252316
1.425613	1.700354	1.465123	1.007864	0.819048	2.132756	3.23987	4.637994	1.171349
8.180094	1.116609	0.585902	2.498478	0.07632	0.287951	0.254156	0.889553	2.487576
1.679596	0.461811	0.080281	0.563175	1.600238	0.771688	0.131768	2.388348	1.80267
8.445743	15.61077	36.26061	20.16541	29.82828	32.31123	35.776	21.02638	50.55949
16.09482	8.625613	14.00414	23.6012	14.42661	25.73165	13.6814	16.5625	2.597792
0.655613	0.455649	0.047323	0.426898	0.234358	0.269509	0.305657	0.229502	0.046667
4.620159	2.050404	2.111299	1.130794	2.481356	1.306667	6.250447	3.66785	8.849697
5.788153	13.34662	6.695887	6.365065	13.30906	4.207778	3.714906	1.931537	7.765931
0.192929	0.715462	2.380079	2.227273	0.810491	2.363442	5.238701	0.908773	2.349351
0.62645	1.190491	0.83088	0.543939	0.541804	0.832554	0.61969	2.283752	1.335743
0.214964	4.110779	1.839986	3.829105	3.104394	3.432388	5.266335	7.339452	4.105714
0.009452	0.606154	0.596508	0.209264	0.909315	0.520678	0.545152	0.908773	1.130115
1.910974	1.239423	2.522684	2.941544	3.095166	1.693939	4.397792	0.956667	2.842193
0.009452	8.66E-05	0.087734	0.087619	0.321566	0.000433	0.190541	0.000101	0.23246
1.456955	3.8379	2.970144	0.938413	3.8379	0.93267	1.346003	5.050267	1.626782
1.241811	2.254084	4.875527	3.104394	3.953297	2.099524	6.011515	1.669466	1.685678
1.311674	0.812771	1.436623	1.477388	0.383348	0.209841	0.035758	0.596775	0.524646
0.787605	0.536263	1.27619	0.528196	0.859351	0.941645	0.908247	0.373939	1.700354
0.009452	0.289452	0.70404	0.116176	0.499986	0.031818	1.64917	0.340866	0.190541
0.009452	0.034502	0.116595	0.144935	0.123535	0.080722	0.07684	0.075231	0.140548
17.15902	7.238023	1.977316	5.23176	1.667273	4.188896	3.792266	3.953297	0.82974
4.845166	6.38456	2.755411	2.404618	1.850996	4.667518	1.540144	2.03886	0.121818
0.009452	8.66E-05	0.002655	4.33E-05	0.042713	0.050844	0.041616	0.000101	0.000274
0.009452	0.088066	1.549711	0.144935	4.451154	0.672468	1.045462	0.625498	0.726472
0.476551	2.285664	2.755411	2.153535	2.304452	2.34355	4.739668	0.898016	1.734949
0.31785	0.987821	3.049603	2.132756	5.576219	2.171429	4.048499	1.421126	1.381111
3.107417	5.42381	9.459177	5.872886	3.807648	8.888167	6.876392	7.162698	19.06893
3.303925	0.677403	1.287403	1.648066	0.736551	2.425801	0.930325	1.587605	2.207417
0.180368	0.370101	0.394877	0.217172	0.625209	0.328268	0.425137	0.201789	0.714697
5.933066	10.82954	9.179466	0.656003	2.871212	0.842193	0.023492	18.05488	0.140548
1.353608	0.558095	0.350274	0.729582	0.440029	0.771688	0.443543	0.611789	0.36153
0.88619	1.563045	1.991328	2.42381	2.16759	1.11526	2.242092	1.29417	1.720144
0.457749	0.622496	0.303564	0.528196	0.411169	0.437799	0.120361	0.441472	0.274055
1.169091	0.550368	1.224733	0.412193	1.876212	0.579841	0.701818	0.540563	0.600548
0.697908	6.738211	6.297136	2.727785	12.16681	3.201457	11.14619	1.841371	3.350224
0.733074	0.957915	1.246457	1.930216	1.95645	1.480541	2.850058	1.395563	1.692763
3.233045	8.412367	14.08569	19.68088	4.63189	17.78522	23.71237	15.3618	19.99861
7.495397	3.211176	1.132165	4.02088	2.502619	4.082165	2.947287	1.959466	2.776421
0.551861	0.196667	0.498052	0.91575	0.841746	0.950418	1.601443	0.41026	0.198059
0.867287	5.295325	1.410902	4.082165	5.088694	2.696061	3.070952	0.750866	1.727323
6.30671	5.515584	16.15795	9.667316	6.876392	9.562554	10.98644	8.815123	10.67991
0.353795	0.485014	0.475433	0.799048	1.766248	0.567345	0.393983	0.470736	0.104329
12.42965	9.502294	6.738211	4.519048	19.06893	3.391075	13.23022	3.877417	13.38418
3.172864	0.204964	0.711912	0.426898	1.420202	1.015967	0.659582	0.174646	0.24518
0.2264	0.196667	0.16575	0.102251	0.372504	0.069668	0.366674	0.038139	0.173189
252.4251	12.49452	35.776	12.10289	26.25557	32.8773	5.351934	44.27012	28.54786
0.009452	0.919192	1.27619	1.420202	0.280823	0.763939	0.930325	1.003016	0.568665
2.802828	0.596111	1.690483	1.303961	1.340036	3.256587	0.967446	2.978968	2.367763
0.009452	8.66E-05	0.646378	4.33E-05	0.165657	0.000433	0.069365	0.000101	0.104329
2.388348	6.695887	7.991602	7.08733	4.965801	7.938427	3.703175	1.860491	4.408153
14.70188	19.83727	15.66023	9.502294	2.944416	22.13245	14.70188	9.063983	10.57984
0.009452	8.66E-05	0.047323	0.009163	0.056638	0.091133	0.041616	0.023059	0.028081
5.976724	1.302107	5.439278	2.584394	1.608427	2.758088	13.45939	0.13824	1.373348
29.82828	2.718434	4.160238	5.737056	4.752121	8.445743	6.200945	4.188896	5.044113

0.593146	2.242092	3.204632	1.541227	1.867951	2.005253	2.708341	1.850996	1.171349
3.414574	1.482626	0.784805	0.962107	1.744055	1.02487	1.028672	2.327857	1.404812
1.511198	4.067547	6.876392	4.029654	9.625253	3.574387	5.110159	1.860491	4.650007
0.129971	0.50557	0.896522	0.981558	1.420202	1.045462	1.1514	1.445916	0.290519
1.888023	17.28195	8.075772	5.165599	21.19547	4.575123	5.872886	45.91841	8.573434
0.009452	0.042713	0.039747	4.33E-05	0.267388	0.000433	0.048341	0.000101	0.000274
0.089553	0.182821	0.243232	0.116176	0.201898	0.176104	0.190541	0.055671	0.090772
15.46193	11.719	5.25254	3.860491	6.38456	6.028427	3.55961	14.00414	0.819978
9.838658	111.5439	105.1694	111.5439	184.443	64.12971	48.53429	38.94854	42.08807
30.82277	23.82354	21.54823	45.1045	18.62711	24.50942	47.5018	19.06893	33.93939
5.123781	31.36473	55.04081	17.64869	51.52195	20.51271	29.49693	50.55949	144.7919
7.911934	18.69833	12.32958	9.063983	18.05488	13.23022	8.762677	12.53072	6.900202
29.49693	19.68088	39.53687	41.47925	22.13245	45.91841	36.74522	27.10502	30.49289
3.610707	6.09759	10.01009	8.508514	20.16541	10.23768	5.195022	7.315512	4.650007
135.5245	1360.293	530.1465	87331.38	87331.38	87331.38	87331.38	530.1465	425.62
77.74812	100.0998	155.7103	75.1132	111.5439	167.9996	13.00996	87.1746	203.5795
95.22939	118.7028	83.489	144.7919	118.7028	105.1694	40.7932	126.1556	83.489
19.37139	47.5018	126.1556	31.55417	135.5245	32.8773	26.80355	48.53429	87.1746
1.219019	2.006782	5.591717	2.461905	12.03267	2.773644	1.74539	2.445079	3.984747
0.467403	1.338261	1.475469	0.975556	1.169091	0.86518	1.957958	0.898016	1.035058
1.096335	1.767431	8.477662	3.930664	5.172828	1.935685	5.050267	3.300664	56.39528
6.508427	6.570433	8.728571	12.93651	6.695887	14.97762	27.66779	3.833247	5.788153
0.009452	0.275873	0.620014	0.039589	0.793918	0.060087	0.648362	0.273434	0.181919
0.009452	0.15456	0.31456	0.067547	0.097879	0.143333	0.145382	0.146999	0.069784
1.096335	2.789466	2.255916	1.143954	4.236962	1.241811	2.398442	2.047157	1.111977
1.808023	0.434329	0.141876	0.528196	0.129733	0.487662	1.303045	0.146999	0.402698
10.19468	4.839105	7.456118	2.515938	2.223636	4.702468	7.188095	1.153723	4.552092
1.948918	0.275873	0.243232	0.123023	0.585592	0.000433	0.212698	0.165108	0.206263
1.020447	9.751039	18.91854	8.445743	16.34294	7.558139	27.38726	6.922771	14.08569
4.875527	0.821898	2.129596	4.473348	0.977994	3.726854	2.425801	1.726032	3.606804
0.009452	1.852338	1.953535	0.729582	5.337547	0.653586	1.4671	0.155945	0.131443
0.009452	0.049336	0.223485	0.053485	0.123535	0.000433	0.069365	0.000101	0.343434
0.523752	0.957915	0.350274	1.098341	0.532857	0.653586	0.897489	0.481414	0.699192
10.28573	3.166659	1.163579	7.061328	2.143261	3.201457	5.366335	3.273045	2.190866
14.17388	33.4985	13.15079	29.82828	25.48179	27.66779	28.23632	37.81919	13.53913
25.24367	34.38341	42.75576	33.07147	23.82354	77.74812	52.56128	26.80355	22.13245
3.890556	8.019769	13.23022	10.67991	18.91854	7.38847	13.30906	7.099913	13.23022
1.543377	4.016465	8.319019	3.340361	5.172828	2.294733	4.343838	3.574387	5.273362
0.009452	0.148846	1.264589	0.123023	1.280274	0.060087	0.555036	0.572987	0.323319
18.05488	29.82828	25.73165	24.04374	30.17192	23.06543	8.573434	23.16622	8.075772
0.708124	0.743283	1.287403	1.404812	0.990909	0.686667	0.341241	1.34267	0.615584
1.446926	0.392554	0.754784	4.179762	0.747417	2.860722	11.96574	2.955916	6.135007
7.468961	17.58415	30.49289	9.924372	31.55417	14.33939	19.37139	11.14619	26.25557
3.074134	2.329784	0.655245	0.412193	1.05824	1.162201	1.783203	0.229502	0.625209
0.31785	0.888485	0.916306	0.729582	0.578817	0.496494	0.787987	1.983824	0.746551
2.122799	2.597792	6.674286	2.24562	6.144055	4.317648	6.99026	3.083319	4.353925
0.328268	0.82974	1.315613	0.831472	1.303961	0.885664	1.156869	0.481414	1.06355
1.466111	0.596111	0.79487	0.400354	0.411169	0.710253	0.832013	0.498636	0.872013
0.2264	0.305253	0.57531	0.275873	0.449408	0.123102	0.545152	0.313723	0.309062
0.946638	1.499214	0.666847	0.420058	1.211299	0.599466	0.935743	0.720938	1.045462
0.421804	0.314387	0.260765	0.522071	0.146479	0.345238	1.910974	0.13824	0.675115
3.574387	2.513752	1.401068	1.012742	2.807937	0.992771	2.151919	0.720938	2.349351
0.875101	4.408153	8.103925	1.677042	3.632973	2.121198	3.401126	1.616385	1.547734
1.20653	5.493182	17.92188	1.703795	2.55184	2.784228	3.91355	0.730873	7.653391
2.563506	0.275873	0.260765	0.3514	0.173059	1.000173	1.4671	0.540563	0.542828
1.006025	0.034502	0.095382	0.183052	0.012785	0.069668	0.035758	0.185022	0.04062
5.599228	17.78522	32.31123	10.93101	13.15079	12.70768	28.87506	21.36892	15.46193
0.328268	0.606154	0.84119	0.858817	0.625209	1.008961	0.648362	0.528196	0.812771

0.089553	0.025483	0	0.015599	2.89E-05	0.000433	0.041616	0.000101	0.034199
0.593146	0.606154	0.277417	0.636291	0.65798	1.848297	2.715873	0.064848	2.281926
9.218514	4.155339	4.348882	10.07949	0.140657	6.028427	3.330909	5.976724	4.286962
0.15562	70.11932	3.489899	3.672078	100.0998	1.778153	4.563117	24.98218	3.895469
4.888023	4.597792	15.07257	7.264502	9.880779	18.19595	24.73724	5.915469	5.024293
0.441833	0.976667	1.499214	0.579149	3.147294	0.723506	1.303045	1.931537	1.121703
20.68746	9.237525	5.359134	17.51961	5.821804	8.412367	13.08214	3.610707	2.461905
3.061955	1.975772	5.337547	2.269134	2.647244	2.181775	1.89373	1.34267	2.058773
0.80417	0.910339	0.646378	0.787987	1.043608	0.800967	0.550072	0.987821	1.204957
0.180368	0.056869	0.36114	0.405786	0.220808	0.000433	0.145382	0.000101	0.000274
4.323045	15.88548	10.19468	12.63773	20.60009	4.789596	8.958225	6.153102	16.9162
100.0998	252.4251	332.0776	252.4251	332.0776	530.1465	203.5795	224.9555	95.22939
0.009452	0.064127	0.110642	0.284048	0.188009	0.176104	0.432698	0.064848	0.198059
7.288788	29.49693	49.50903	36.0183	52.56128	22.52375	31.1753	49.50903	15.276
0.962107	1.833102	2.16759	1.664906	2.16759	1.57645	2.515938	1.616385	1.306667
0.214964	0.939986	1.103261	0.483608	1.580909	0.420289	0.91575	0.381999	0.554026
19.91794	8.923117	10.2617	13.91714	6.404921	8.815123	8.524242	11.09133	12.81638

CGGA_11	CGGA_11	CGGA_11	CGGA_11	CGGA_12	CGGA_12	CGGA_12	CGGA_12	CGGA_12
0.604214	0.225079	0.220808	0.709322	0.112706	1.150635	0.745613	0.197338	0.188009
0.252165	0.62733	0.000101	0.709322	11.22667	0.340245	1.066234	0.00075	0.91404
2.513752	5.500678	10.98644	0.061962	17.64869	0.951652	0.388326	5.323492	2.839351
1.570794	5.583968	6.580988	0.061962	3.993665	1.901068	0.034545	0.917431	2.540462
3.373983	2.194257	2.810678	0.953506	3.404372	5.454949	1.066234	2.31404	7.697063
8.333918	2.535974	1.113304	6.738211	1.267085	23.16622	5.984885	1.251659	8.445743
44.27012	44.27012	36.26061	5.976724	100.0998	34.84299	9.297085	41.47925	33.07147
0.299553	0.043297	0.052814	1.558571	0.148016	0.022958	0.981032	0.143838	0.000216
2.828918	1.126075	7.570693	0.580758	3.065108	2.616573	0.034545	1.633752	2.540462
0.000693	0.656753	0.000101	0.061962	0.19127	0.580758	0.034545	0.00075	0.352597
33.07147	45.1045	24.50942	83.489	42.75576	52.56128	75.1132	21.93452	39.53687
2.089639	0.780087	0.342381	6.414416	3.610707	1.603795	2.685786	0.41772	7.225469
30.17192	22.13245	48.53429	1.863506	20.16541	6.888297	1.603795	21.36892	5.056421
0.000693	0	5.143867	0.061962	0.000476	1.283059	0.034545	40.11561	0.000216
0.06772	0.009113	0.000101	0.479062	0.021948	0.009733	1.240202	0.026068	0.123535
1.580909	5.889798	7.417359	0.061962	7.124517	2.66404	0.034545	6.738211	7.188095
4.086926	1.691587	0.195823	3.095166	4.715599	1.867951	1.556392	0.299336	3.217612
0.288247	0	0.000101	11.30987	0.080519	0.319026	17.78522	0.00075	0.245967
1.623232	1.960945	3.425512	0.061962	0.000476	1.867951	0.034545	3.058802	0.428932
2.378124	0.601227	0.866717	0.882619	1.333781	1.306667	1.102691	0.290866	3.217612
0.643001	0.563824	2.673846	1.175859	1.135137	0.390895	0.034545	1.340036	0.07482
1.477388	2.77899	7.964315	2.66404	1.475469	0.697085	3.169812	10.53159	0.879913
91.05541	41.47925	16.34294	0.061962	37.29684	105.1694	0.034545	29.49693	40.11561
0.126883	0	0.059582	4.94026	0.000476	4.33E-05	11.57341	0.053579	0.039293
1.237063	1.239423	0.330462	5.976724	4.174964	1.651522	6.64254	0.434069	1.530159
3.125844	5.855281	6.830592	1.02487	9.179466	2.432121	0.600548	4.091667	3.738997
0.610606	1.978954	1.845519	0.061962	4.145209	0.468384	0.034545	4.789596	0.153651
0.321068	0.334444	0.230108	1.863506	0.309668	0.3607	0.717403	0.133781	0.278499
3.614603	23.6012	184.443	0.061962	9.541645	30.17192	0.034545	425.62	2.524892
1.060823	1.29267	0.253492	5.316378	1.033672	0.613211	4.140505	0.425844	0.941645
8.958225	3.23987	23.82354	2.92013	3.829105	4.468009	1.980592	7.964315	1.371602
0.145556	0.049185	0.023608	1.02487	0.331097	0.181068	2.784228	0.152222	0.188009
4.827691	12.93651	22.74797	0.061962	15.56131	10.1459	0.034545	19.22139	0.000216
0.000693	0.006219	0.023608	0.341558	0.178182	0.022958	0.547403	0.034863	0.091306
3.57057	2.461905	0.153521	15.71851	3.286652	7.991602	21.19547	0.103716	21.36892
0.000693	3.651898	3.207807	0.061962	6.063276	0.602908	0.034545	3.414574	0.530253
2.201587	1.746724	6.404921	1.175859	4.888023	3.552049	8.849697	6.011515	1.748016
0.380267	0.001039	0.000101	1.479452	0.000476	0.109466	0.388326	0.103716	0.000216
4.370671	2.219863	3.233045	4.94026	4.281847	6.888297	7.482179	1.609654	3.7586
6.760765	4.563117	1.439704	12.22798	8.643074	15.77678	27.10502	2.773644	22.96463
0.894704	0.487915	0.789863	1.863506	1.588903	1.135137	3.540606	0.053579	3.486436
2.533745	1.126075	0.460361	12.39704	2.254084	0.468384	14.97762	0.214654	1.279452
5.606739	0.512641	3.659668	0.061962	1.102121	0.580758	0.034545	3.065108	0.474856
0.000693	0.002128	0.032092	0.061962	0.039885	4.33E-05	4.424416	0.00075	0.030577
0.299553	0.467136	0.29404	1.02487	0.564784	0.173059	5.864084	0.233175	2.556443
27.10502	20.85876	19.37139	2.602395	25.48179	36.26061	1.435772	31.55417	31.1753
0.195007	0.118983	0.144293	1.921212	0.527215	0.038139	1.370765	0.044719	0.174906
0.359899	0.009113	0.000101	0.819048	0.112706	0.347186	0.717403	0.00075	0.340866
0.024423	0.000476	0.000101	0.430281	0.000476	0.005382	0.451169	0.00075	0.014545
0.106732	0.002128	0.000101	0.430281	0.000476	0.227446	0.196667	0.00075	0.766602
0.000693	0.002128	0.000101	0.061962	0.080519	0.009733	0.196667	0.00075	0.245967
18.05488	12.16681	8.923117	1.33285	2.320072	15.98892	0.420289	6.900202	1.613232
0.518139	0.375743	0.015945	0.341558	0.178182	0.118341	1.102691	0.00075	0.657114
0.201789	0.409899	0.039827	0.953506	0.790837	0.149755	0.451169	0.083521	0.188009
0.92531	2.410996	3.442886	0.430281	4.715599	0.885137	1.772756	2.55184	1.896667
0.49127	0.237035	0.046609	0.953506	0.843102	2.845036	0.547403	0.00075	1.05153
0.933874	0.380447	0.153521	3.019848	0.432237	0.383074	2.136335	0.290866	0.319163

1.128788	0.265455	0.466602	3.179199	0.514885	1.23127	6.978903	0.034863	4.086926
2.083261	0.726811	1.721371	4.451154	2.820974	4.110779	0.745613	1.481631	6.045209
0.933874	0.912987	0.174235	2.730072	0.68557	0.16575	1.980592	0.233175	0.245967
25.73165	0.125238	1.481631	21.19547	0.000476	56.39528	0.034545	21.02638	0.214098
0.252165	0.992201	0.411169	0.061962	0.393716	0.061176	0.451169	0.911905	0.392403
1.354567	0.942944	0.247951	28.87506	0.790837	0.450924	15.88548	0.352222	4.170051
13.30906	43.49658	23.16622	0.061962	27.96248	16.34294	0.034545	56.39528	10.28573
0.270924	0.231508	0.338341	3.179199	0.203463	0.118341	4.959358	0.290866	0.225339
0.000693	0.027655	0.039827	0.061962	0.021948	0.005382	0.17733	0.00075	0.201789
2.513752	4.597792	1.925635	0.061962	1.403853	3.8379	0.034545	0.751371	1.381111
1.386681	1.833102	2.134545	0.819048	1.65785	1.668369	0.306602	1.778153	1.703795
0.463485	0.225079	0.247951	0.479062	0.640289	0.648961	0.220274	0.290866	1.417345
0.106732	0.022814	0.000101	1.175859	0.759293	0.29531	2.102626	0.00075	0.06544
0.06772	0.163276	0.000101	0.061962	0.600173	0.29531	1.165873	0.034863	1.417345
1.082915	0.285556	0.100014	3.45404	0.625209	0.587619	0.948211	0.093622	0.646378
0.059726	0.017446	0.009978	0.30329	0.039885	4.33E-05	0.306602	0.026068	0.056652
0.045281	0.00404	0.000101	0.819048	0.000476	0.009733	5.323492	0.026068	0.000216
5.560563	9.880779	7.315512	1.408831	8.539971	4.552092	4.179762	7.188095	29.18885
0.162799	0.152843	0.059582	0.061962	0.420498	0.083896	0.577388	0.034863	0.123535
0.170938	0.060729	0.000101	0.341558	0.068384	0.187532	0.242222	0.034863	0.153651
5.337547	3.730808	2.524892	1.699091	5.137042	6.830592	1.772756	11.30987	2.417424
0.116595	0.049185	0.107157	0.061962	0.319567	0.074978	0.547403	0.062857	0.51316
1.030678	0.480022	0.114437	0.640289	0.309668	0.938911	1.370765	0.265339	0.91404
3.35355	3.214545	12.16681	1.699091	9.047532	4.419062	0.511667	11.19942	3.851414
0.024423	0.001039	0.07767	0.341558	0.068384	4.33E-05	1.326667	0.072583	0.047965
0.633853	0.494841	0.086443	0.580758	1.565253	0.800512	3.13456	0.044719	1.110505
2.327857	6.580988	24.04374	0.061962	5.745996	4.519048	0.034545	18.33909	2.29873
1.992944	0.375743	0.093312	3.592063	0.843102	0.173059	0.451169	0.062857	0.720938
0.724885	0.430837	0.279177	2.66404	0.27759	1.142345	0.830289	0.290866	0.153651
0.49127	1.554185	1.320173	0.061962	3.156537	0.101429	0.034545	0.619012	8.348817
0.454632	0.285556	0.207713	1.408831	0.289603	0.657547	0.630505	0.224214	0.635036
0.279177	0.081587	0.160779	0.769019	0.239769	0.173059	2.185556	0.053579	0.329798
0.774798	0.968066	1.556392	0.061962	0.112706	0.218975	0.034545	1.819221	1.371602
4.034423	11.96574	5.47	0.061962	10.40634	5.630815	0.034545	7.625209	0.000216
0.162799	0.430837	0.188427	1.253088	0.454978	0.173059	0.388326	0.00075	0.74868
0.279177	0.303564	0.114437	0.30329	0.527215	0.885137	0.263853	0.325772	2.217951
3.691724	1.539062	0.355303	8.164531	2.681169	6.653016	6.796566	0.152222	2.556443
0.098211	0	0.032092	0.640289	0.080519	0.009733	1.663672	0.00075	0.039293
7.683766	5.71241	11.03843	0.709322	4.524481	7.653391	1.009625	3.91355	2.248896
0.052143	0.00404	0.039827	0.882619	0.068384	0.038139	0.630505	0.044719	0.164084
0.000693	0.001039	0.015945	0.430281	0.029502	0.038139	1.511198	0.093622	0.091306
0.075231	0.000476	0.000101	0.061962	0.000476	0.005382	0.388326	0.026068	0.000216
3.404372	2.402468	0.561775	3.676032	2.009906	1.546696	1.723579	0.819048	2.506934
1.128788	4.63189	5.044113	0.061962	4.145209	5.530274	1.240202	4.875527	6.632063
0.709784	2.944416	0.74868	0.061962	3.691724	9.989221	2.600014	0.668947	3.966623
1.060823	0.311385	0.07767	1.253088	0.123535	1.079697	0.511667	0.143838	0.234502
0.78619	1.250058	2.016515	0.529697	0.19127	0.781977	0.511667	1.251659	1.05153
1.354567	0.3414	0.000101	0.061962	0.342641	0.375743	2.54961	0.00075	1.397482
0.186861	0.795786	0.188427	4.277006	0.899567	0.038139	3.864827	0.00075	1.568564
0.633853	2.143261	15.98892	0.061962	1.360022	0.623499	0.034545	5.500678	0.392403
0.454632	0.409899	0.627633	1.175859	1.65785	1.006025	0.948211	0.214654	2.794935
1.201724	1.720144	1.387612	0.061962	3.321284	3.610707	1.066234	1.117316	5.638759
3.57057	7.212915	2.470317	1.33285	1.395563	2.394416	0.034545	3.738997	1.381111
2.23267	2.07658	0.505267	4.277006	0.443052	1.161501	2.071587	0.516061	2.073232
3.799885	16.68554	91.05541	0.061962	0.745072	4.33E-05	0.717403	144.7919	0.000216
0.877807	1.699091	1.535722	0.061962	1.859084	0.16575	0.034545	1.362713	0.188009
0.345036	0.178817	0.059582	0.709322	0.454978	0.520289	0.451169	0.034863	1.237879
0.000693	11.14619	167.9996	0.061962	0.000476	4.33E-05	0.034545	64.12971	0.000216

0.500722	0.088788	0.015945	5.18754	0.309668	0.127403	5.323492	0.053579	0.352597
0.556753	0.595022	0.059582	0.061962	0.539466	0.276847	0.034545	0.00075	0.268369
3.926154	1.932857	1.245671	14.65384	1.618773	4.155339	11.22667	1.739935	3.116732
21.74051	28.54786	20.34063	1.558571	9.968355	2.538218	8.762677	13.75978	2.909293
0.836537	1.115902	0.135144	11.09133	0.080519	1.360866	5.864084	0.072583	0.153651
4.620159	4.563117	4.49671	19.99861	7.468961	11.89932	7.482179	3.214545	3.899993
2.644885	1.354567	0.120433	2.408882	6.727633	2.216162	4.067547	0.133781	2.000592
2.784228	2.611797	0.654423	3.29355	5.23176	4.451154	0.388326	1.024127	4.745895
13.15079	8.288874	1.008961	57.84882	3.065108	16.09482	24.50942	0.6114	19.68088
0.508658	0.368882	1.137244	19.29639	0.300606	0.687431	12.93651	1.242641	1.126775
0.235043	0.07013	0.000101	0.341558	0.049531	0.022958	1.277056	0.00075	0.188009
1.041652	0.548153	0.355303	0.640289	0.454978	0.587619	0.263853	1.20653	0.307973
0.098211	0.334444	0.046609	1.863506	1.003016	0.243896	5.381227	0.053579	1.358189
9.687496	7.136537	1.297763	1.02487	2.491948	13.00996	8.677489	5.720173	1.859084
0.2443	0.251775	0.07767	2.820974	0.178182	0.141284	3.279625	0.122179	0.500722
2.442987	1.842734	3.279625	0.819048	1.532468	1.317475	0.034545	1.986681	0.377323
2.805382	0.803665	0.549661	1.980592	1.741414	1.915455	1.468131	0.997792	1.49075
0.038088	0	0.015945	0.479062	0.039885	0.005382	0.480794	0.00075	0.047965
1.965657	1.307417	1.729892	2.511465	7.38847	22.13245	17.78522	2.061926	4.110779
0.299553	0.163276	3.286652	0.061962	0.600173	0.267951	0.034545	1.242641	0.51316
0.038088	0	0.000101	1.408831	0.058773	0.067792	5.576219	0.00075	0.530253
1.632648	0.368882	1.405772	0.882619	0.112706	1.595729	0.034545	1.596782	0.810491
4.413608	2.839351	0.494444	27.10502	8.164531	0.895368	44.27012	0.553059	1.431414
2.280101	3.695815	2.544747	0.38596	1.49075	1.031284	0.745613	2.903867	1.092136
1.11526	0.557056	1.819221	0.061962	2.465916	0.500996	0.511667	0.687431	0.377323
4.984488	2.855296	1.877605	7.08733	5.788153	6.738211	7.532713	1.639639	3.273045
3.869163	2.470317	1.472208	6.316328	4.644098	7.200505	0.911905	1.539062	13.53913
2.876421	3.47531	3.107417	1.479452	2.069942	2.649675	0.830289	3.450577	0.810491
2.153535	0.480022	1.033672	0.580758	0.080519	0.556753	0.329798	3.334199	0.030577
1.727323	2.442987	4.94026	0.061962	8.048095	3.796075	0.034545	2.345527	1.49075
33.93939	17.51961	6.268312	1.980592	5.195022	13.83359	1.556392	12.16681	10.57984
0.218528	0.487915	1.414574	0.061962	0.539466	4.33E-05	0.034545	0.596775	0.000216
6.464906	21.02638	9.141299	7.570693	10.40634	10.1028	6.52899	21.74051	10.85542
5.25254	6.784762	14.33939	0.061962	6.22031	6.230245	2.032706	6.876392	6.335664
0.030375	0.012626	0.000101	0.341558	0.068384	0.022958	0.263853	0.00075	0.039293
0.894704	1.517143	4.029654	0.061962	0.899567	0.997244	0.034545	2.950173	0.364242
5.976724	2.725498	0.796234	4.575123	8.412367	11.03843	2.600014	0.751371	13.75978
1.108947	0.537229	0.460361	2.92013	0.564784	0.773276	2.784228	0.711912	0.51316
1.80267	1.011486	0.272244	0.341558	0.721732	0.82974	0.69119	0.50127	0.319163
0.145556	4.007583	2.101075	0.061962	2.99987	0.757273	0.034545	3.655714	0.123535
0.321068	0	8.226219	0.061962	0.000476	4.33E-05	0.034545	12.70768	0.000216
32.68313	59.32013	75.1132	0.061962	6.772763	12.03267	0.034545	167.9996	0.377323
3.909033	3.877417	1.008961	6.414416	4.984488	1.455022	5.864084	0.341241	0.720938
0.209538	0.094949	0.052814	0.061962	0.514885	0.218975	0.451169	0.072583	0.153651
2.513752	0.543665	1.33285	0.430281	3.5257	3.993665	0.17733	1.580909	0.720938
1.613232	1.29267	3.917597	0.061962	2.725498	1.534603	0.034545	2.705916	0.783939
2.593506	0.769711	0.135144	4.451154	0.539466	3.300664	0.717403	0.161962	0.694675
2.468117	0	0.000101	6.414416	1.833102	2.683478	0.35627	0.00075	3.055729
8.573434	13.08214	15.88548	1.408831	14.6058	5.309264	3.599329	16.9162	10.19468
1.128788	0.380447	0.24184	0.38596	0.356032	0.951652	0.306602	0.308903	0.90368
0.91575	0	0.000101	0.38596	1.135137	1.697792	1.191905	0.00075	1.064935
8.477662	6.374812	1.603795	11.86763	13.53913	14.79742	26.52537	1.596782	19.37139
2.644885	1.406746	1.42456	1.863506	1.360022	1.880375	0.420289	1.251659	0.657114
0.574993	0.055397	0.000101	4.174964	2.285664	0.730029	2.500548	0.00075	1.733766
1.212792	0.265455	0.166898	3.957734	1.066948	0.773276	4.563117	0.333831	0.268369
4.733824	6.853608	4.207778	1.408831	6.622092	5.050267	2.600014	2.873817	0.955418
0.059726	0	0.000101	0.061962	0.000476	0.030433	0.034545	0.062857	0.25785
0.279177	0.157706	0.195823	0.061962	0.368658	0.738312	0.034545	0.00075	0.474856

0.000693	0.055397	1.591299	0.061962	0.000476	0.187532	0.034545	1.977316	0.500722
2.540462	9.562554	7.507619	0.061962	4.217569	7.653391	0.034545	9.625253	1.397482
13.38418	6.695887	3.147294	3.45404	11.89932	4.419062	6.30671	3.089293	5.796421
19.22139	19.83727	49.50903	0.061962	7.468961	23.38378	0.034545	50.55949	9.751039
1.913997	0.835498	2.03886	0.430281	1.798535	1.729892	0.034545	2.011501	0.635036
0.000693	0.906126	5.056421	0.061962	11.09133	4.33E-05	0.034545	1.422316	5.409437
0.518139	0.956667	0.160779	4.370671	1.235346	1.079697	4.739668	0.152222	1.064935
5.662994	5.28039	8.958225	0.061962	12.77772	9.063983	0.034545	15.07257	10.05618
5.576219	4.207778	0.310267	26.66446	4.502453	15.61077	9.438687	0.00075	5.409437
11.60283	13.23022	11.65791	2.300678	16.34294	18.33909	7.751869	10.78056	12.7427
2.031169	1.259747	0.376595	1.921212	0.856782	5.050267	3.66785	0.00075	2.996869
6.181652	14.25954	3.711097	2.408882	22.52375	11.544	9.121486	6.760765	13.34662
0.999502	0.842193	1.762713	0.061962	2.150303	0.687431	0.034545	1.588903	0.278499
1.326667	1.57645	1.51215	0.580758	6.853608	1.603795	0.220274	2.828918	4.277006
0.000693	0.081587	0.000101	2.820974	0.000476	0.375743	1.980592	0.00075	0.926926
1.091443	1.048074	0.622092	0.061962	3.493362	1.668369	0.745613	1.501291	2.635685
1.758615	1.652771	1.663672	1.629235	1.246457	0.489784	1.690483	1.707576	0.646378
1.819221	1.595729	0.602287	1.33285	2.642525	2.538218	1.556392	0.768052	2.212619
0.000693	0	0.009978	1.10132	0.039885	0.005382	0.874603	0.00075	0.056652
0.235043	0.803665	1.903636	0.061962	3.404372	0.304921	0.034545	5.638759	1.012114
10.1459	9.989221	5.864084	2.602395	7.61176	9.562554	0.745613	9.583463	4.49671
1.449062	0.999502	0.093312	1.175859	2.196017	1.668369	2.973059	0.782417	0.622821
0.500722	0.398903	0.000101	2.92013	0.319567	0.187532	1.326667	0.00075	0.377323
1.995714	3.904517	3.179199	0.953506	9.707677	4.323045	3.104394	2.432121	10.78056
6.601833	4.222583	3.718716	0.640289	12.22798	5.704646	0.35627	5.950664	7.013853
0.442799	0.07013	0.000101	1.175859	0.000476	2.005253	1.326667	0.00075	0.000216
0.405786	0.136061	0.266847	1.175859	0.551861	0.406212	0.911905	0.214654	0.377323
1.924228	2.630729	3.820758	3.851414	1.184935	2.267157	2.243911	6.443925	0.164084
1.11526	1.283059	0.348629	7.570693	1.578687	2.196017	0.35627	0.083521	3.514834
1.341825	0.251775	0.207713	1.558571	0.342641	0.821898	4.424416	0.152222	0.720938
1.78759	3.381255	15.18102	0.061962	3.457504	1.25816	0.034545	9.218514	5.838499
0.886775	0.992201	0.990909	1.863506	1.383911	0.866147	7.225469	0.462857	1.308167
0.353175	0.735382	0.07767	0.30329	0.970635	0.574228	0.662237	0.083521	0.694675
7.200505	3.080317	6.171789	0.061962	3.286652	3.779452	0.034545	46.7056	1.639639
2.432121	1.307417	0.000101	10.31025	4.597792	2.243911	8.994791	0.143838	0.994271
1.765065	0.699192	2.834084	0.061962	0.805238	3.223882	1.240202	7.315512	0.40645
0.106732	0.07013	0.023608	0.580758	0.102352	0.022958	0.511667	0.034863	0.056652
0.604214	0.07013	0.039827	0.061962	0.215433	0.566782	0.034545	0.133781	0.364242
0.225823	0	0.000101	2.511465	0.203463	0.319026	5.056421	0.00075	0.102886
0.442799	1.329206	2.31404	0.769019	0.994899	0.406212	0.830289	2.524892	1.896667
0.405786	0.088788	0.000101	47.5018	1.045462	3.767489	64.12971	0.072583	6.52899
65.94551	37.81919	37.81919	72.69473	38.94854	47.5018	35.776	30.49289	18.19595
9.459177	18.48514	9.667316	3.179199	30.82277	9.667316	4.302367	5.302294	32.31123
1.226118	1.15614	0.875101	16.5625	1.277843	3.286652	4.959358	0.981558	1.012114
3.707136	1.968283	3.672078	0.819048	1.607143	4.003117	0.600548	2.773644	2.440895
0.336328	0.049185	0.068023	5.737056	0.000476	1.629235	3.279625	0.00075	1.919661
0.54847	0.199134	0.07767	0.529697	0.823983	0.398059	0.329798	0.425844	0.547778
0.000693	7.08733	87.1746	0.061962	29.82828	6.423911	0.034545	33.93939	19.06893
0.154978	1.755988	4.776407	1.253088	1.833102	1.039199	0.034545	0.971775	2.429957
2.876421	1.530159	2.860722	2.209141	1.618773	3.585029	1.468131	2.171429	2.394416
6.088593	5.381227	1.316544	6.240346	13.23022	4.927302	18.26752	1.132165	11.03843
0.373939	0.147323	0.648961	0.061962	0.239769	0.193997	0.034545	0.793434	0.047965
1.364401	3.300664	4.115844	0.061962	1.146962	0.101429	0.034545	8.643074	0.000216
0.858817	7.288788	11.25392	0.061962	4.302367	2.129596	0.034545	36.26061	0.000216
0.116595	0.081587	0.000101	0.479062	0.102352	0.304921	0.285556	0.034863	0.201789
5.508131	167.9996	60.77449	0.061962	35.27608	18.69833	0.034545	77.74812	3.166659
24.04374	4.597792	1.046061	11.86763	1.898153	6.717056	11.96574	0.92531	0.892395
1.553074	3.137576	1.387612	0.061962	7.795642	3.599329	0.717403	2.26518	3.367071

1.277843	0.999502	0.160779	4.709033	2.681169	1.80267	0.600548	0.00075	3.1857
7.653391	5.788153	0.951652	0.953506	13.79669	1.2214	0.034545	1.685678	3.851414
1.052886	1.250058	4.053506	0.882619	3.381255	0.669358	0.034545	3.36075	2.569957
0.052143	9.815642	19.99861	0.061962	0.000476	0.536905	0.034545	19.99861	1.453939
23.82354	65.94551	55.04081	0.061962	65.94551	6.045209	0.034545	155.7103	12.89577
0.000693	2.611797	0.387258	0.061962	0.712453	0.705231	35.27608	0.00075	3.116732
0.78619	1.096335	0.220808	1.921212	0.98474	2.035678	1.690483	0.425844	1.876212
0.116595	0.906126	0.068023	0.882619	1.289149	1.360866	0.034545	0.224214	6.632063
0.059726	0	0.023608	1.02487	0.000476	0.009733	2.136335	0.00075	0.225339
1.47018	1.147807	0.144293	0.061962	0.668947	1.845519	1.066234	0.133781	1.343463
0.116595	0.163276	0.059582	1.699091	0.112706	0.074978	1.216768	0.205498	0.102886
0.000693	2.685786	44.27012	0.061962	0.000476	4.343838	0.034545	52.56128	0.000216
0.279177	1.919661	3.223882	2.209141	3.137576	4.575123	0.034545	12.49452	0.123535
1.201724	0.320144	0.505267	2.511465	0.215433	1.204228	0.451169	0.257518	0.392403
3.829105	1.623232	0.780087	1.253088	2.882035	2.805382	5.23176	1.093434	2.909293
2.333867	2.90658	1.046061	0.061962	7.61176	0.450924	0.034545	1.379278	3.283139
3.089293	2.597792	1.094206	14.17388	12.93651	2.55184	11.83593	0.981558	20.68746
1.060823	0.827273	0.445209	3.676032	3.993665	1.369048	4.067547	0.50127	6.374812
0.396082	1.283059	1.046061	11.99921	0.98474	0.118341	8.994791	0.380851	2.820974
1.226118	1.436623	0.597042	1.699091	2.950173	1.079697	2.642525	0.717027	2.31013
0.530851	0.581111	2.65684	0.529697	0.476768	2.331825	0.242222	3.533211	0.766602
0.454632	0.76	0.000101	7.495397	0.823983	2.939141	6.153102	0.341241	7.339452
1.191198	31.92385	22.32214	0.061962	21.54823	1.507835	0.034545	8.075772	0.000216
0.098211	0.017446	0.023608	1.558571	0.092612	0.093009	1.216768	0.112771	0.102886
0.448824	2.394416	0.450527	4.709033	6.287561	5.630815	11.09133	0.248716	8.958225
2.432121	3.514834	4.46267	0.061962	2.611797	2.792035	0.034545	5.381227	1.974228
0.059726	0.027655	0.009978	0.882619	0.123535	0.218975	1.772756	0.044719	2.037114
22.52375	19.68088	32.31123	3.29355	21.19547	18.19595	0.329798	34.38341	14.33939
1.77404	2.518124	4.067547	0.882619	5.568391	4.789596	1.102691	6.171789	3.851414
13.23022	5.745996	0.407157	0.061962	4.302367	5.351934	0.35627	2.820974	0.450924
0.288247	0.163276	0.052814	1.02487	0.393716	0.368658	0.263853	0.053579	0.708492
3.300664	9.989221	8.677489	25.24367	24.2842	11.30987	57.84882	5.337547	24.50942
1.654156	2.805382	5.500678	0.061962	1.523824	9.356371	0.034545	19.68088	0.734524
100.0998	184.443	38.39042	12.77772	203.5795	749.7839	19.06893	100.0998	36.74522
0.405786	0.157706	0.052814	2.139928	0.289603	0.173059	3.66785	0.205498	0.428932
12.10289	5.560563	2.406768	40.11561	2.66404	6.580988	20.16541	16.68554	0.994271
23.38378	8.958225	31.1753	5.316378	22.13245	17.15902	2.032706	22.32214	9.101674
0.000693	0.022814	0.000101	0.061962	0.266479	0.16575	0.034545	0.00075	0.364242
0.000693	0.002128	0.059582	0.640289	0.000476	0.030433	0.196667	0.00075	0.039293
0.740527	3.783694	2.681169	0.061962	0.000476	0.697085	0.034545	3.276335	0.439524
20.34063	17.78522	45.1045	7.200505	13.91714	13.42179	2.461905	15.3618	11.96574
0.038088	0.009113	0.000101	0.580758	0.029502	0.022958	0.630505	0.00075	0.030577
0.186861	0.334444	1.309084	0.061962	1.082915	0.135144	0.034545	2.289286	0.487662
0.421804	0.311385	0.235613	2.300678	1.016494	0.193997	0.745613	0.034863	0.428932
0.261962	0.152843	0.259509	1.253088	0.239769	0.235296	0.034545	0.112771	0.708492
11.83593	10.82954	2.08013	4.808658	10.95872	19.99861	3.019848	3.738997	8.256465
2.173081	1.436623	0.100014	5.030866	1.016494	2.604776	2.243911	0.272951	2.050404
2.031169	5.23176	8.412367	2.92013	4.927302	7.519841	2.54961	4.348882	4.673182
224.9555	40.7932	14.88584	10.82954	80.6518	167.9996	1.191905	20.34063	72.69473
0.098211	0.032915	0.114437	1.558571	0.319567	0.061176	5.493182	0.00075	0.941645
0.463485	0.055397	0.032092	1.558571	0.123535	0.202294	1.009625	0.053579	0.278499
18.76954	34.84299	14.70188	9.687496	126.1556	22.74797	18.26752	17.39935	91.05541
1.303961	2.037114	1.069033	0.529697	1.202626	1.517143	1.872049	1.748016	1.279452
0.252165	0.494841	0.120433	0.430281	0.454978	0.218975	1.408831	0.053579	1.530159
2.74544	2.529307	0.833535	42.75576	1.102121	5.00404	30.82277	0.507403	3.775209
0.000693	1.088687	0.015945	15.46193	0.640289	2.763261	19.06893	0.122179	2.263377
6.433918	2.696061	0.849841	8.524242	3.975498	14.42661	28.54786	1.837172	3.397706
0.811443	0.66482	0.266847	1.10132	0.254156	1.697792	2.8657	0.133781	1.876212

26.80355	34.38341	10.23768	50.55949	42.08807	91.05541	80.6518	13.6814	100.0998
0.000693	0.006219	0.000101	0.640289	0.112706	0.022958	0.511667	0.00075	0.530253
1.099762	1.267085	5.576219	1.02487	1.31	1.578687	0.981032	2.23267	2.349351
0.059726	0.125238	0.000101	0.30329	0.000476	4.33E-05	0.285556	0.00075	0.014545
2.784228	1.164257	0.483045	31.1753	1.452857	2.432121	62.33472	0.567994	6.063276
25.48179	7.653391	1.33285	9.880779	11.30987	24.50942	4.424416	1.850996	8.303947
2.978968	2.254084	1.237063	20.68746	5.23176	4.72798	6.900202	1.262965	11.83593
0.178997	0.130823	0.000101	0.061962	0.049531	0.141284	10.82954	0.053579	1.110505
0.380267	4.096407	3.792266	0.061962	7.839271	0.566782	0.034545	2.860722	3.618499
2.574733	1.699091	1.046061	2.820974	2.542605	0.917994	6.900202	0.933874	1.474343
0.846797	0.186133	0.100014	7.495397	0.759293	0.595368	8.288874	0.00075	2.429957
17.15902	7.037345	26.52537	0.061962	14.33939	6.019971	3.78798	17.64869	10.85542
2.013182	0.414012	0.904286	0.061962	1.4671	1.019134	0.034545	2.134545	0.500722
0.565137	0.519019	2.666494	4.875527	1.846941	0.135144	0.830289	1.596782	0.000216
0.082222	0.418557	0.153521	3.391075	3.156537	0.135144	12.32958	0.152222	0.474856
19.37139	22.32214	21.93452	20.34063	14.79742	14.04491	22.74797	47.5018	11.60283
8.888167	13.38418	14.51648	1.253088	5.47	2.609293	2.74298	7.653391	3.803766
0.136147	0.368882	0.366147	0.819048	0.564784	0.210693	0.388326	0.179798	1.545657
10.19468	5.9071	1.177309	14.65384	7.765931	16.80343	31.55417	2.600014	18.48514
15.77678	30.82277	10.67991	0.061962	6.475505	27.38726	0.034545	87.1746	8.643074
0.235043	1.048074	0.376595	0.640289	2.304452	0.227446	0.948211	0.434069	18.76954
0.744531	0.404408	0.259509	0.38596	0.490079	0.997244	0.220274	0.560043	1.143954
10.2617	2.964762	0.568369	1.10132	8.256465	10.67991	5.130411	0.793434	15.88548
0.145556	5.679502	4.02088	0.061962	1.350772	4.33E-05	0.034545	4.207778	20.85876
2.863211	4.752121	23.38378	1.175859	2.380079	3.714906	0.034545	4.609076	4.053506
5.462475	3.750837	1.965657	7.200505	4.952915	3.536955	13.04605	3.779452	11.22667
2.073232	3.411097	0.533535	8.815123	2.701053	2.159004	14.84163	0.371075	9.179466
0.059726	0.009113	0.009978	0.38596	0.029502	0.005382	1.408831	0.034863	0.014545
17.28195	24.04374	7.037345	6.414416	14.70188	29.18885	20.94257	8.815123	33.93939
3.163506	5.606739	8.075772	3.518384	10.03313	8.508514	0.420289	5.654863	4.540794
4.317648	3.005281	2.445079	0.061962	0.215433	3.4679	0.451169	6.570433	0.364242
4.155339	4.47912	3.536955	0.709322	2.911962	2.449509	0.388326	10.03313	1.05153
3.317807	3.548153	2.860722	2.060491	6.622092	5.545729	0.388326	3.588369	2.586926
0.448824	0.360101	0.235613	0.769019	0.239769	0.383074	1.102691	0.480794	0.364242
0.846797	0.678247	0.023608	1.408831	0.058773	0.3607	10.45584	0.062857	0.188009
1.45912	0.529971	0.817013	3.851414	1.532468	1.317475	0.420289	0.371075	1.064935
3.019848	2.185556	0.259509	5.646811	2.23267	1.49075	4.857403	0.308903	4.802655
0.966825	0.285556	0.355303	4.875527	1.808023	0.332395	8.477662	0.122179	0.974257
7.710361	20.34063	52.56128	1.921212	22.32214	5.273362	0.69119	67.98069	3.691724
1.249221	1.623232	3.775209	0.061962	0.393716	1.590202	0.034545	3.327619	0.462352
0.672078	0.811861	0.382208	0.580758	4.115844	0.267951	1.326667	0.170938	6.135007
6.277937	5.959033	0.899567	15.07257	16.80343	2.159004	36.74522	0.805238	12.36443
7.375794	1.954993	0.684033	7.495397	7.938427	6.853608	36.26061	0.447359	13.23022
3.993665	2.705916	2.784228	17.15902	3.065108	5.889798	1.723579	10.28573	4.207778
14.70188	12.70768	10.05618	18.99374	19.53061	26.25557	38.94854	9.751039	30.82277
6.054242	20.51271	6.230245	0.061962	57.84882	3.726854	15.56131	17.28195	224.9555
12.36443	3.47531	14.25954	10.82954	3.404372	8.048095	9.771833	14.79742	0.734524
0.7986	0.726811	20.85876	0.479062	0.878932	0.109466	7.897078	4.207778	0.225339
8.164531	5.454949	4.655916	1.479452	8.075772	9.356371	0.306602	4.709033	8.815123
4.302367	3.599329	0.74868	3.29355	12.63773	1.935685	1.511198	0.534452	2.794935
0.082222	2.037114	1.405772	4.277006	0.611789	0.158766	0.242222	0.308903	0.635036
0.030375	0.017446	0.023608	0.709322	0.039885	0.009733	0.874603	0.034863	0.102886
2.845036	3.095166	5.671126	0.479062	15.88548	4.440548	0.263853	1.739935	15.98892
30.82277	22.96463	9.751039	4.451154	19.22139	16.68554	3.340361	11.544	10.05618
0.000693	0.001039	0.000101	0.30329	0.021948	4.33E-05	0.220274	0.026068	0.000216
0.052143	0.07013	0.052814	0.580758	0.380447	0.390895	0.911905	0.00075	0.174906
42.75576	35.27608	8.780115	37.29684	50.55949	135.5245	29.82828	7.570693	75.1132
1.372475	1.679596	0.366147	6.144055	0.668947	3.679986	1.772756	0.352222	1.110505

0.038088	0.001039	0.000101	0.640289	0.068384	0.022958	3.340361	0.00075	0.234502
52.56128	24.50942	6.808369	27.38726	23.82354	67.98069	118.7028	8.319019	44.27012
5.402511	3.632973	1.890996	3.45404	4.053506	9.562554	0.830289	2.171429	6.30671
0.075231	0	0.07767	2.300678	0.058773	0.045916	5.056421	0.044719	0.000216
0.617763	0.168456	0.039827	5.976724	0.167273	0.556753	4.796126	0.152222	3.618499
0.024423	0.118983	0.316335	11.09133	1.246457	0.061176	1.89961	0.170938	6.475505
56.39528	77.74812	16.74449	155.7103	167.9996	126.1556	184.443	24.73724	252.4251
13.00996	12.36443	2.470317	4.072475	10.82954	7.737807	3.074134	2.183665	21.19547
19.99861	11.19942	4.952915	6.612121	26.80355	24.85971	3.78798	4.914863	35.27608
4.650007	4.261342	4.207778	0.061962	1.277843	0.390895	0.034545	1.96697	0.278499
126.1556	203.5795	64.12971	2.511465	29.49693	155.7103	0.034545	203.5795	11.89932
11.77567	6.663651	5.704646	13.23022	25.24367	17.03919	9.646284	5.576219	19.22139
4.603434	2.289286	1.033672	6.99026	1.4671	4.971991	4.365224	1.175859	1.816436
0.126883	1.623232	1.983824	3.179199	3.5257	0.149755	3.971061	1.432302	3.966623
3.975498	5.745996	2.784228	7.795642	1.944517	2.584394	20.94257	1.96697	4.650007
8.381328	5.25254	1.971183	7.200505	14.25954	15.61077	5.23176	2.151919	14.08569
80.6518	36.26061	0.528196	126.1556	3.621941	87.1746	83.489	1.432302	4.946587
0.430476	1.707576	0.000101	3.767489	0.70329	1.880375	1.723579	0.00075	0.578485
0.345036	2.176501	3.457504	8.905642	5.830152	2.207417	3.24658	2.45575	6.107114
57.84882	21.19547	27.38726	49.50903	21.93452	20.34063	95.22939	9.667316	51.52195
29.18885	48.53429	21.36892	5.646811	56.39528	45.91841	3.921645	15.66023	135.5245
5.720173	9.562554	0.505267	12.97323	12.06778	1.668369	1.408831	0.493247	10.05618
42.08807	16.34294	3.435988	21.54823	15.07257	49.50903	37.81919	4.739668	20.51271
5.864084	2.725498	1.348975	0.061962	4.359574	4.614618	0.034545	2.119596	2.794935
0.765606	26.25557	59.32013	5.030866	18.76954	19.53061	2.836717	15.98892	17.64869
0.045281	2.451645	2.500548	2.730072	2.009906	0.16575	10.95872	0.133781	5.720173
5.323492	4.145209	13.60866	0.061962	0.759293	10.62739	3.78798	7.112496	2.933903
9.276811	29.03196	31.55417	0.341558	46.7056	11.77567	1.036558	53.77619	53.77619
0.311227	0.049185	0.000101	31.55417	0.178182	0.319026	1.872049	0.00075	3.618499
1.010289	0.512641	0.361342	1.479452	1.003016	0.63873	0.830289	0.272951	1.224733
8.103925	5.622872	19.68088	0.061962	6.663651	4.170051	0.034545	18.91854	1.594675
27.96248	10.43066	0.376595	0.061962	1.235346	5.143867	0.034545	2.715873	0.562439
1.277843	1.329206	0.742417	4.94026	0.856782	1.612056	5.638759	0.233175	0.678687
1.89518	2.964762	5.9071	0.882619	3.636789	4.086926	0.285556	3.266782	2.073232
0.000693	0.001039	0.039827	0.061962	0.039885	4.33E-05	0.511667	0.00075	0.000216
17.92188	16.44768	10.48101	18.99374	15.46193	37.81919	31.92385	8.780115	47.5018
530.1465	87331.38	50.55949	530.1465	118.7028	1360.293	530.1465	75.1132	87331.38
184.443	252.4251	45.91841	224.9555	749.7839	144.7919	252.4251	105.1694	155.7103
1.021097	1.496118	0.000101	0.430281	0.823983	0.500996	3.211176	0.00075	0.974257
21.36892	14.97762	28.23632	15.56131	44.27012	25.48179	3.78798	37.81919	26.80355
1.082915	0.524646	0.394156	0.061962	1.267085	1.150635	0.034545	0.00075	1.160707
5.273362	2.542605	2.101075	10.07949	3.425512	6.365065	6.900202	2.800332	4.170051
0.556753	0.081587	0.180895	10.19468	1.452857	0.045916	12.85504	0.425844	2.094574
0.098211	0.157706	0.299747	0.061962	0.823983	0.030433	0.220274	0.00075	8.849697
0.288247	0.017446	0.000101	0.580758	0.000476	0.067792	0.874603	0.034863	0.234502
1.364401	6.464906	3.957734	6.240346	12.36443	3.211176	6.30671	3.493362	62.33472
8.134084	18.62711	6.967547	1.699091	135.5245	1.710173	23.82354	9.297085	184.443
0.000693	0.118983	0.401385	0.341558	0.112706	0.022958	2.74298	0.161962	0.268369
87.1746	91.05541	57.84882	17.64869	95.22939	35.27608	8.164531	118.7028	65.94551
0.000693	0.027655	0.000101	1.02487	0.000476	0.118341	0.420289	0.00075	0.113766
0.643001	25.48179	12.85504	2.139928	23.6012	31.55417	4.039192	9.880779	70.11932
0.000693	0	0.000101	0.640289	0.514885	0.101429	0.034545	0.00075	1.262965
2.815866	0.685931	0.84119	8.365072	1.016494	3.175916	3.104394	0.48684	1.974228
0.270924	0.060729	0.000101	0.061962	0.380447	0.443781	4.857403	0.00075	1.064935
0.045281	0	0.009978	1.175859	0.000476	0.005382	0.242222	0.00075	0.000216
2.092987	0.956667	0.000101	3.019848	1.360022	2.143261	2.302626	0.00075	0.392403
45.91841	25.99427	21.19547	38.94854	34.38341	42.08807	16.15795	22.74797	48.53429
14.17388	8.048095	2.453781	48.53429	5.439278	11.14619	40.7932	4.150274	3.217612

10.33476	7.162698	2.860722	2.139928	12.56693	26.80355	14.12978	3.317807	12.42965
2.987835	5.770996	4.46267	12.39704	8.119004	7.495397	7.897078	3.128694	9.377345
0.959567	2.359264	8.381328	0.819048	1.4671	1.019134	0.420289	2.491948	1.012114
0.059726	0.001039	0.000101	0.709322	0.000476	0.005382	1.408831	0.026068	0.000216
15.56131	8.539971	2.425801	33.07147	18.62711	33.4985	20.34063	10.65365	25.99427
17.64869	14.6058	10.57984	9.583463	20.51271	29.82828	5.864084	13.83359	13.96064
3.317807	1.059553	0.086443	4.072475	1.833102	2.955916	1.277056	0.161962	1.308167
3.66785	1.784755	0.909856	0.061962	0.775339	3.343629	0.874603	1.235346	2.145137
2.153535	0.811861	0.962698	3.095166	0.577071	2.092987	1.191905	1.006025	1.077403
27.38726	7.264502	4.429769	26.66446	11.48368	12.10289	5.638759	5.351934	19.53061
0.662237	1.15614	0.401385	5.316378	2.209141	0.63873	2.973059	0.380851	4.946587
16.9162	8.677489	3.807648	0.061962	4.217569	6.153102	0.547403	4.323045	5.102742
12.1974	10.23768	5.295325	23.16622	17.03919	13.23022	52.56128	4.888023	25.24367
0.858817	56.39528	10.28573	0.061962	13.30906	2.876421	3.414574	8.677489	42.75576
0.06772	0.065519	0.039827	0.061962	0.490079	0.015657	2.642525	0.034863	0.678687
20.16541	10.1459	19.22139	0.061962	17.15902	17.92188	0.034545	9.256537	26.52537
0.045281	0.147323	0.032092	1.980592	0.254156	0.109466	2.102626	0.122179	0.657114
45.1045	16.09482	1.25816	55.04081	35.776	24.04374	105.1694	1.33285	49.50903
0.235043	0.157706	0.135144	17.64869	0.514885	0.432698	2.54961	0.453709	2.230859
5.030866	1.734949	3.137576	2.060491	2.304452	3.663622	0.034545	19.83727	9.687496
0.235043	0.292799	0.401385	5.646811	1.267085	0.81228	0.511667	0.410079	0.646378
1.108947	0.938911	0.698297	0.061962	0.551861	0.285685	0.034545	1.416407	3.055729
18.19595	12.29473	2.257749	27.81514	30.49289	6.580988	28.87506	7.683766	27.96248
0.178997	0.237035	0.086443	0.38596	0.123535	0.340245	0.783939	0.034863	0.487662
8.508514	6.028427	3.058802	3.767489	7.351284	21.54823	2.54961	12.29473	3.343629
3.150433	5.172828	31.92385	4.451154	9.377345	2.630729	2.784228	7.468961	8.381328
0.136147	0.049185	0.015945	0.061962	0.068384	0.149755	0.034545	0.053579	0.133658
5.508131	4.456912	1.456955	11.30987	8.256465	8.994791	9.19899	2.483492	7.697063
3.953297	0.487915	0.638362	9.063983	1.65785	5.180058	0.717403	0.833045	3.722785
0.421804	0.360101	0.272244	0.529697	0.625209	0.747417	0.717403	0.272951	1.279452
0.78619	1.307417	2.425801	0.061962	0.454978	0.724502	0.034545	3.595758	2.281926
2.65684	0.537229	0.528196	1.10132	2.254084	1.710173	0.17733	0.687431	5.024293
22.13245	5.337547	1.690483	14.47154	62.33472	12.36443	3.864827	4.752121	23.38378
0.279177	11.25392	24.73724	252.4251	14.51648	1.161501	100.0998	6.653016	5.770996
2.419553	1.15614	0.944784	0.769019	2.104358	1.507835	0.420289	0.651631	2.394416
0.421804	0.081587	0.107157	75.1132	0.000476	4.33E-05	13.34662	0.093622	0.500722
0.442799	0.438023	0.98474	3.851414	0.407662	0.406212	2.185556	0.625209	0.892395
2.398442	1.011486	0.287641	1.629235	1.2214	3.317807	0.285556	0.596775	0.392403
0.075231	0.002128	0.000101	0.061962	0.029502	0.045916	0.196667	0.00075	0.082569
0.448824	0	0.000101	1.33285	0.775339	0.481602	1.511198	0.00075	3.327619
1.713997	0.906126	0.230108	10.98644	1.678319	1.422316	7.402915	0.00075	3.263391
0.209538	0.147323	0.015945	0.529697	0.215433	0.149755	0.034545	0.00075	0.164084
3.993665	1.365296	1.028672	0.341558	3.899993	1.867951	0.17733	1.067662	3.249877
0.000693	0.346732	0.07767	5.737056	0.215433	0.235296	2.600014	0.00075	0.593853
0.000693	0.075693	0.100014	0.341558	0.000476	0.045916	1.277056	0.00075	0.056652
0.288247	0.186133	0.07767	0.709322	0.226883	0.218975	1.408831	0.214654	0.530253
1.341825	1.259747	1.64917	0.479062	3.195541	2.984899	0.263853	2.26518	0.578485
1.701616	1.239423	1.290065	1.253088	2.852677	0.852172	1.723579	1.588903	0.174906
1.654156	9.218514	34.84299	0.061962	8.888167	3.714906	0.034545	13.00996	8.134084
2.162381	0.851659	3.192229	1.253088	2.491948	4.490801	0.220274	8.994791	0.164084
24.98218	9.751039	41.47925	1.253088	6.433918	64.12971	0.034545	184.443	10.62739
0.201789	0.088788	0.032092	1.175859	0.167273	0.304921	1.277056	0.133781	0.091306
0.195007	0.353175	0.180895	1.408831	0.625209	0.347186	1.408831	0.062857	0.562439
0.867814	0.835498	0.702835	5.560563	1.299567	1.269495	5.762965	0.625209	1.21215
4.125786	2.735368	2.513752	3.179199	6.39474	1.306667	16.85982	0.933874	3.57057
5.150693	0.094949	0.742417	2.209141	0.000476	14.17388	0.451169	4.770231	0.000216
0.000693	0.001039	0.000101	0.341558	0.021948	4.33E-05	0.662237	0.00075	0.000216
1.314675	4.764055	0.916306	1.33285	3.256587	1.483622	0.034545	1.731169	1.160707

0.990188	2.763261	2.157359	0.709322	1.4671	1.558571	0.388326	1.527792	2.794935
4.673182	8.815123	1.595729	0.061962	3.917597	3.06803	0.034545	6.632063	3.166659
0.052143	0	0.195823	4.709033	0.102352	0.443781	1.556392	0.00075	0.082569
5.545729	12.42965	3.500808	1.33285	7.188095	9.101674	0.874603	3.718716	10.43066
2.061926	5.646811	12.77772	0.061962	10.28573	1.651522	2.185556	3.070952	6.277937
1.139885	1.115902	0.355303	2.92013	0.476768	0.304921	2.461905	0.462857	1.092136
2.491948	2.597792	0.195823	5.821804	0.000476	2.59557	4.796126	1.443983	0.000216
0.437576	0.320144	0.07767	3.019848	0.289603	0.450924	0.69119	0.240851	1.68342
4.965801	4.690159	2.740519	19.83727	2.950173	3.211176	2.54961	4.348882	0.810491
2.725498	2.489762	2.607035	0.769019	1.289149	1.23127	0.034545	5.030866	0.329798
32.31123	26.95429	18.76954	0.709322	36.26061	38.39042	0.783939	16.5625	34.84299
1.052886	0.835498	0.483045	0.709322	1.146962	1.174315	1.935685	0.493247	1.237879
0.000693	0.006219	0.000101	0.819048	0.049531	0.038139	0.17733	0.00075	0.021558
0.585281	0.334444	0.188427	1.33285	0.938911	0.613211	0.511667	0.282266	1.49075
0.336328	0.178817	29.49693	0.061962	0.254156	4.33E-05	0.034545	8.445743	0.091306
0.47153	0.430837	1.909408	0.061962	1.246457	4.33E-05	0.034545	2.792035	0.000216
18.48514	12.03267	11.48368	4.072475	20.77311	28.87506	2.243911	11.09133	14.51648
6.181652	13.15079	30.49289	0.061962	8.643074	5.165599	0.034545	26.80355	8.103925
0.824416	0.251775	0.259509	1.629235	0.878932	1.121017	0.285556	0.447359	1.516219
3.276335	8.590794	14.42661	0.061962	6.335664	4.33E-05	0.034545	20.68746	0.000216
0.000693	0.103088	3.984747	0.061962	0.70329	0.340245	0.034545	4.833398	0.000216
5.402511	3.464315	2.65684	22.52375	5.591717	7.315512	13.23022	1.286494	7.519841
0.643001	0.060729	0.287641	9.583463	0.136061	1.34267	8.256465	0.00075	1.330029
0.982698	0.199134	0.059582	1.78759	0.407662	0.545808	0.577388	0.103716	1.184935
15.98892	10.67991	8.256465	3.957734	2.396429	4.990794	2.302626	10.1028	2.494177
4.155339	1.919661	0.247951	2.139928	2.304452	3.144156	1.980592	0.380851	2.652107
0.540902	0.751371	0.188427	0.061962	0.823983	0.494841	0.034545	1.220628	0.487662
0.311227	0.445455	0.796234	0.061962	1.172208	0.218975	0.034545	0.575628	0.578485
37.29684	17.39935	27.96248	6.8421	16.9162	19.83727	2.419553	25.24367	13.15079
7.061328	2.153535	4.863579	0.061962	2.270837	3.651898	0.034545	4.484892	0.450924
1.637309	0.587619	1.172208	0.640289	0.254156	0.687431	0.034545	2.468117	0.28689
1.052886	1.382006	1.198701	0.061962	11.36975	4.33E-05	0.034545	1.311674	0.000216
1.45912	1.307417	1.615281	0.061962	0.490079	0.63873	0.420289	1.024127	0.319163
4.086926	3.563182	2.031169	0.061962	2.254084	2.855296	0.511667	10.57984	0.547778
2.565556	2.421681	0.78619	0.580758	2.701053	6.335664	0.69119	1.96697	0.500722
0.345036	0	0.023608	0.061962	0.000476	0.29531	2.136335	0.00075	1.110505
0.91575	0.524646	0.577388	0.529697	0.611789	1.039199	0.220274	1.041652	0.646378
2.31013	1.910974	0.259509	5.030866	2.185556	0.773276	3.971061	0.352222	8.888167
5.485685	16.9162	7.443276	4.174964	11.83593	25.99427	0.034545	7.288788	4.034423
0.090108	0.027655	0.100014	5.560563	0.123535	0.840635	16.34294	0.00075	0.214098
2.322035	1.184185	0.477951	13.87537	1.003016	0.800512	10.45584	0.730029	0.74868
0.000693	0.512641	3.738997	0.061962	1.003016	0.738312	0.034545	1.458038	0.340866
3.101371	3.411097	7.911934	0.061962	4.145209	4.490801	0.034545	37.29684	1.040411
0.126883	0.19355	0.127201	0.30329	0.092612	0.406212	0.242222	0.248716	0.123535
0.585281	0.529971	2.591443	0.061962	2.593506	1.064242	0.874603	3.533211	0.82868
0.030375	0.000476	0.023608	0.061962	0.000476	0.015657	0.034545	0.026068	0.014545
1.594675	0.543665	0.174235	1.175859	0.867814	0.687431	0.874603	0.214654	0.547778
2.459812	1.080303	1.372475	0.061962	0.70329	2.410996	0.034545	3.107417	0.102886
0.396082	0.726811	0.445209	5.316378	2.820974	0.312323	4.959358	0.971775	1.766248
3.446732	2.367763	0.411169	0.061962	4.445851	1.283059	15.3618	0.189509	3.343629
0.030375	0.00404	0.052814	1.253088	0.000476	4.33E-05	0.196667	0.00075	0.030577
14.08569	33.4985	126.1556	7.327482	48.53429	4.814661	5.23176	25.48179	11.544
6.433918	1.919661	1.898153	3.29355	2.335844	1.587605	0.783939	3.089293	0.974257
0.098211	0.173319	0.07767	1.78759	0.215433	0.030433	12.67271	0.034863	0.056652
22.96463	8.019769	2.489762	167.9996	3.864827	12.46209	51.52195	0.959062	6.570433
1.603795	1.978954	2.747908	0.061962	3.227085	2.325931	0.034545	10.43066	1.199481
10.73084	39.53687	62.33472	0.061962	33.4985	15.276	0.034545	51.52195	38.10481
0.824416	0.178817	0.000101	21.83751	0.080519	0.520289	6.900202	0.00075	0.245967

0.000693	0	0.214964	0.061962	0.000476	0.030433	0.034545	0.034863	0.000216
2.051962	4.02088	2.531515	4.277006	1.925635	0.82974	15.18102	0.864199	1.68342
2.153535	2.964762	2.192496	2.300678	1.507835	1.150635	2.243911	0.864199	1.876212
0.672078	0.678247	0.000101	7.200505	0.19127	0.383074	2.071587	0.179798	0.07482
0.000693	0.006219	0.000101	0.061962	0.123535	0.045916	0.034545	0.00075	0.352597
2.666494	36.74522	65.94551	0.061962	11.42512	6.088593	0.034545	45.91841	0.000216
4.563117	1.867951	4.709033	0.061962	2.725498	3.529466	0.034545	6.036818	2.196017
0.321068	0.620462	0.127201	4.451154	0.239769	0.968066	1.009625	0.189509	0.635036
5.745996	2.826277	2.579509	1.479452	5.696263	8.711905	0.242222	6.591544	6.911486
1.748016	1.546696	1.237063	6.99026	1.507835	1.187229	3.414574	0.898543	0.60886
5.359134	7.810058	4.745895	42.08807	6.508427	19.68088	22.32214	6.088593	17.92188
3.217612	1.029365	1.633752	0.061962	1.565253	0.929286	0.388326	1.262965	0.892395
0.06772	0.094949	0.023608	0.529697	0.167273	0.061176	1.102691	0.044719	0.174906
0.075231	0.032915	0.000101	0.061962	0.039885	0.074978	0.196667	0.00075	0.143333
1.07127	0.827273	0.279177	3.851414	1.678319	0.917994	1.216768	0.67632	0.841746
6.30671	9.667316	7.288788	8.005685	19.37139	14.04491	13.83359	8.711905	11.22667
0.311227	0.243672	0.201501	0.580758	0.178182	2.449509	0.451169	0.00075	10.23768
2.061926	2.860722	0.166898	3.518384	8.365072	1.944517	11.74734	0.093622	12.29473
0.405786	0.620462	0.698297	0.061962	1.340981	0.375743	0.451169	1.860491	0.392403
8.815123	3.695815	2.325931	36.26061	7.710361	8.815123	26.25557	2.558817	7.443276
1.152944	3.116732	77.74812	0.061962	1.452857	2.515938	0.034545	7.037345	111.5439
0.126883	0.360101	0.000101	0.061962	0.000476	0.218975	4.179762	0.00075	0.530253
0.030375	0.001039	0.023608	0.430281	0.039885	4.33E-05	0.242222	0.00075	0.047965
2.6143	2.367763	0.505267	4.370671	3.113478	3.107417	3.722785	0.596775	1.184935
0.06772	0.001039	0.009978	0.341558	0.029502	0.030433	0.034545	0.00075	0.113766
1.010289	0.460065	0.068023	0.709322	0.092612	0.406212	2.136335	0.00075	0.201789
29.82828	53.77619	17.51961	22.32214	31.1753	60.77449	27.38726	19.37139	31.92385
0.617763	0.251775	0.000101	1.980592	0.27759	0.173059	6.153102	0.093622	0.766602
0.000693	0.00404	0.039827	0.430281	0.039885	0.015657	0.577388	0.034863	0.021558
1.80267	1.607143	3.125844	1.10132	0.640289	0.773276	7.049336	3.028629	0.419365
5.18754	0.472713	0.000101	0.061962	9.880779	6.443925	23.16622	0.00075	9.625253
2.947287	2.066652	7.002056	0.061962	0.527215	1.651522	0.034545	13.60866	0.487662
2.104358	2.895599	11.19942	0.061962	2.797835	1.839986	0.034545	27.66779	0.000216
0.54847	0.292799	0.757273	0.38596	0.092612	0.63873	0.196667	0.553059	0.201789
0.442799	2.90658	2.197828	0.061962	1.475469	0.252165	0.034545	1.95645	1.568564
1.819221	3.179199	0.07767	4.451154	1.925635	2.515938	1.663672	0.233175	1.594675
0.990188	1.374329	0.323535	0.061962	0.843102	1.566414	0.034545	0.425844	0.74868
0.145556	0.022814	0.000101	4.174964	0.000476	0.390895	0.35627	0.00075	0.030577
2.533745	3.930664	1.251659	14.08569	2.042201	7.868146	11.89932	1.567576	3.300664
1.041652	0.780087	1.089394	0.061962	0.239769	0.657547	0.034545	0.822965	0.307973
1.303961	0.678247	0.032092	6.144055	0.823983	1.850996	2.365685	0.00075	0.530253
3.330909	5.47	3.489899	0.529697	3.269913	3.188918	2.461905	5.180058	1.545657
0.000693	0.017446	0.059582	0.479062	0.136061	0.29531	1.191905	0.00075	0.329798
7.570693	5.20267	8.539971	0.061962	5.137042	4.745895	0.034545	23.6012	1.371602
0.059726	0.043297	0.382208	0.341558	0.068384	0.030433	0.263853	0.233175	0.014545
0.990188	3.8121	4.272165	0.061962	1.754733	0.657547	3.340361	4.563117	2.600014
4.580938	1.250058	0.523211	2.209141	0.490079	2.485534	3.414574	0.660678	3.014076
2.347439	1.633752	1.856392	1.175859	1.323975	2.119596	0.034545	3.089293	3.166659
0.106732	0.393196	0.466602	0.580758	0.021948	0.158766	0.830289	0.026068	0.307973
1.47018	2.747908	6.210375	0.061962	0.19127	1.974228	0.745613	5.9071	1.766248
3.26	1.053528	1.017107	13.11646	3.137576	1.52676	7.288788	0.738312	2.248896
6.365065	7.977958	9.101674	0.061962	2.852677	16.5625	0.034545	23.16622	4.323045
5.439278	9.031082	35.776	2.820974	3.089293	3.754719	4.424416	38.39042	13.6814
4.086926	0.424004	0.093312	6.99026	1.102121	2.016515	2.243911	0.00075	2.394416
0.811443	0.574993	0.144293	3.518384	1.433189	0.630022	2.836717	0.083521	4.110779
0.49127	0.334444	0.07767	1.479452	1.082915	0.319026	4.227302	0.026068	2.145137
0.116595	1.919661	5.522929	0.061962	1.944517	4.33E-05	0.034545	10.19468	1.733766
2.408882	2.132756	1.808023	0.38596	1.542302	1.839986	0.034545	1.919661	0.000216

10.67991	7.810058	1.396494	1.479452	5.259437	10.23768	2.071587	4.978182	6.277937
0.977994	0.66482	0.174235	4.277006	0.856782	2.394416	7.149618	0.214654	3.486436
7.61176	3.314329	1.372475	14.00414	2.173081	6.287561	15.46193	1.286494	5.704646
1.889711	0.898016	1.128131	5.316378	1.146962	0.669358	2.715873	1.481631	1.50342
0.846797	0.827273	0.990909	0.061962	1.578687	0.765094	0.034545	0.721732	0.766602
8.333918	1.684646	0.803665	2.408882	0.775339	2.955916	0.35627	1.901068	0.06544
4.863579	60.77449	13.26964	0.819048	75.1132	9.256537	1.89961	33.4985	3.691724
5.359134	6.09759	6.922771	0.061962	4.338629	5.745996	0.034545	6.036818	6.99026
5.530274	3.223882	3.147294	0.061962	5.942294	4.946587	0.196667	2.725498	3.899993
3.6029	2.794935	2.174733	0.061962	0.112706	9.880779	0.034545	72.69473	0.000216
3.7586	1.867951	2.673846	7.200505	2.150303	1.40772	6.796566	0.282266	1.320173
2.805382	3.381255	0.394156	0.061962	1.102121	3.343629	0.35627	1.515296	1.092136
1.501291	1.164257	1.181342	0.061962	2.304452	2.378124	0.69119	1.432302	2.160649
0.359899	0.186133	0.440029	0.529697	0.148016	0.252165	1.723579	0.839365	0.039293
1.021097	0.404408	0.702835	3.019848	0.625209	0.630022	1.102691	0.233175	0.657114
0.098211	0.251775	0.323535	3.179199	0.539466	0.520289	2.74298	0.083521	0.941645
0.366674	0.032915	0.000101	0.769019	0.514885	1.566414	0.830289	0.00075	1.143954
1.226118	0.842193	0.498918	0.430281	2.451645	1.806595	0.35627	0.272951	1.662439
0.261962	0.574993	0.135144	6.518709	0.577071	0.210693	2.500548	0.352222	0.462352
0.252165	0.787605	1.283911	0.061962	1.235346	0.312323	0.034545	0.58925	1.703795
0.299553	1.382006	4.105714	0.061962	0.000476	0.415729	0.034545	13.15079	0.000216
2.061926	2.518124	0.587619	0.061962	10.67991	2.252273	0.717403	0.575628	0.855801
1.738456	1.105426	0.611789	0.430281	5.622872	1.638499	0.242222	0.852684	0.841746
0.329091	0.107749	0.188427	2.139928	0.226883	0.243896	2.685786	0.053579	0.329798
1.082915	3.022713	7.710361	0.061962	1.728615	0.181068	0.034545	7.882222	5.545729
14.00414	7.625209	4.72798	0.479062	6.911486	18.05488	0.196667	10.48101	5.024293
1.440736	1.283059	1.414574	0.430281	0.331097	0.951652	0.830289	1.685678	0.307973
3.13456	2.069942	1.444906	0.061962	5.068831	3.439437	0.034545	2.890245	2.349351
0.617763	0.438023	0.000101	0.709322	0.167273	0.678247	0.662237	0.00075	1.733766
3.276335	0.19355	0.120433	1.175859	0.021948	1.187229	0.451169	0.394877	0.021558
3.66785	2.219863	1.524776	0.061962	2.642525	3.518384	0.034545	2.45575	1.040411
3.851414	6.200945	3.35355	0.061962	4.419062	4.266753	0.034545	10.65365	1.431414
6.570433	2.630729	1.133622	6.911486	3.899993	6.335664	40.11561	1.633752	1.748016
0.106732	0.217496	0.214964	0.882619	0.502843	0.276847	2.032706	0.093622	0.25785
0.430476	0.334444	0.153521	0.341558	0.342641	0.602908	0.329798	0.122179	0.578485
0.261962	0.393196	0.55645	8.164531	1.172208	0.383074	4.140505	0.50127	0.91404
2.061926	0.898016	0.488759	0.061962	1.542302	0.997244	0.263853	1.876212	0.474856
5.662994	8.780115	6.738211	0.061962	3.648081	2.349351	1.240202	6.287561	2.281926
0.145556	0.049185	0.000101	0.709322	0.049531	0.009733	1.690483	0.00075	0.039293
0.261962	0.017446	0.009978	0.341558	0.102352	0.267951	0.167532	0.034863	0.25785
2.958773	1.096335	1.754733	17.64869	1.754733	6.956349	8.089848	0.619012	3.78798
3.26	1.436623	0.833535	0.640289	3.089293	5.654863	2.365685	0.774257	3.851414
111.5439	29.82828	1.748016	287.197	14.00414	34.38341	155.7103	4.644098	4.802655
0.592208	0.537229	0.259509	0.061962	0.745072	0.840635	0.511667	0.00075	0.245967
0.145556	0.118983	0.052814	0.061962	0.112706	0.218975	0.196667	0.00075	0.102886
2.126349	1.823377	15.3618	0.061962	1.246457	5.959033	0.034545	18.62711	0.133658
0.405786	11.719	18.62711	0.061962	4.888023	2.993867	0.034545	15.88548	2.933903
5.745996	2.584394	2.180022	0.061962	1.780693	3.370527	0.874603	1.805166	1.173535
0.045281	0.00404	0.023608	0.479062	0.029502	0.022958	0.451169	0.00075	0.030577
0.06772	0.032915	0.009978	0.479062	0.021948	0.005382	0.874603	0.034863	0.014545
2.31013	0.671371	2.197828	0.061962	1.452857	2.616573	0.220274	2.984899	1.143954
3.651898	12.63773	16.22108	0.061962	8.849697	0.353333	0.034545	5.395584	14.97762
0.530851	0.346732	0.710253	0.769019	0.454978	0.687431	0.306602	0.687431	0.955418
1.060823	1.662439	2.561162	0.061962	4.783001	1.749307	0.034545	1.994329	15.77678
0.000693	2.136335	1.974228	0.061962	1.033672	0.566782	0.034545	1.014127	0.352597
3.851414	1.662439	1.057569	7.570693	3.599329	2.210866	8.994791	1.422316	1.397482
0.518139	0.107749	0.235613	2.511465	1.360022	0.468384	2.600014	0.299336	0.547778
0.396082	0.285556	0.000101	20.85876	1.369928	4.048499	3.540606	0.00075	5.679502

1.643218	8.256465	2.281926	1.175859	2.66404	2.005253	0.196667	3.028629	1.613232
0.059726	0.000476	0.009978	0.709322	0.102352	0.187532	0.196667	0.072583	0.25785
0.944784	0.353175	0.000101	3.019848	0.476768	0.917994	2.898196	0.224214	2.621133
0.218528	1.275325	0.978571	0.061962	0.70329	0.840635	0.034545	5.25254	0.188009
0.000693	2.421681	0.401385	0.479062	2.009906	4.33E-05	0.285556	0.640924	0.646378
1.416407	1.746724	1.548723	0.061962	2.967453	1.578687	0.35627	1.876212	7.482179
0.106732	0.50127	0.000101	0.061962	0.466335	0.536905	2.600014	0.00075	0.000216
0.886775	0.819978	3.738997	0.061962	3.179199	2.181775	0.329798	3.544257	2.481356
2.703485	1.477388	1.600238	1.558571	1.65785	1.039199	4.609076	1.49075	0.60886
3.23987	19.06893	28.87506	0.061962	19.06893	5.075447	0.034545	35.776	4.508196
0.836537	1.105426	0.82868	0.953506	0.656392	0.227446	0.034545	1.673254	0.419365
31.55417	2.917381	11.719	0.061962	2.380079	12.22798	0.034545	8.348817	0.000216
6.653016	10.98644	23.6012	9.583463	5.788153	7.710361	3.864827	10.73084	4.365224
0.030375	0	0.000101	0.341558	0.029502	0.009733	0.306602	0.00075	0.419365
1.158384	0.360101	1.353608	0.061962	3.031688	2.267157	0.034545	1.416407	0.392403
16.34294	21.93452	12.03267	0.061962	23.38378	15.88548	0.034545	17.03919	9.687496
0.442799	1.2214	0.780087	0.061962	0.867814	1.056263	0.034545	2.491948	0.000216
0.387056	1.652771	9.377345	0.709322	0.266479	0.468384	0.034545	1.701616	0.06544
1.953535	7.854069	37.29684	0.061962	3.66785	1.880375	0.874603	21.19547	5.976724
1.632648	0.438023	0.000101	0.061962	0.356032	0.235296	1.009625	0.00075	0.810491
0.662237	1.096335	1.712706	0.061962	0.823983	1.064242	0.034545	3.002576	1.064935
2.390447	1.623232	1.615281	8.164531	1.565253	3.387864	16.68554	0.299336	4.888023
11.719	8.365072	5.720173	2.92013	2.083261	12.46209	0.034545	13.45939	2.371833
0.270924	0.452489	1.34513	0.769019	0.443052	0.181068	0.451169	0.971775	0.188009
3.877417	3.750837	2.145137	0.709322	1.323975	2.931068	1.723579	2.673846	0.578485
0.396082	0.125238	0.046609	9.218514	3.930664	0.800512	2.302626	0.161962	3.140866
0.279177	0.62733	0.000101	2.66404	1.578687	0.398059	0.034545	0.00075	1.568564
4.198117	14.70188	10.78056	0.882619	3.55961	5.273362	0.511667	5.062626	3.464315
0.329091	2.261573	1.102121	0.061962	1.082915	0.917994	0.034545	1.400159	1.092136
2.914632	0.656753	0.771688	5.976724	0.734113	1.269495	4.227302	2.151919	2.773644
0.405786	0.038139	0.023608	0.430281	0.080519	0.390895	0.480794	0.062857	0.000216
2.55184	0.258456	0.235613	0.061962	0.112706	3.052655	0.480794	0.00075	0.056652
1.11526	1.517143	4.597792	0.061962	2.554069	3.629177	0.034545	1.805166	0.841746
0.909315	1.623232	3.327619	0.061962	1.507835	1.931537	0.034545	1.301169	0.941645
2.369841	1.848297	1.008961	0.580758	1.369928	2.042201	0.263853	0.596775	2.459812
6.717056	4.007583	1.784755	3.676032	15.98892	1.994329	1.468131	0.751371	2.394416
0.366674	0.103088	2.941544	0.061962	1.618773	0.127403	0.034545	1.673254	0.000216
75.1132	13.6814	1.113304	37.81919	43.49658	65.94551	77.74812	4.927302	42.08807
1.364401	1.707576	1.633752	2.300678	2.628225	5.838499	4.959358	2.289286	6.210375
0.136147	0.178817	0.000101	0.061962	0.899567	0.908247	0.35627	0.044719	1.160707
0.162799	0.113074	0.000101	0.529697	0.049531	0.415729	0.451169	0.062857	0.474856
0.689509	0.346732	2.219863	0.061962	0.790837	0.529697	0.034545	2.119596	1.919661
3.300664	2.77899	1.947316	0.640289	4.468009	4.207778	0.745613	1.701616	4.770231
1.603795	0.851659	0.996696	0.061962	1.716364	2.290996	0.034545	1.544517	0.487662
0.944784	1.420202	1.128131	3.29355	1.255657	0.424538	4.140505	0.044719	1.662439
12.70768	24.2842	5.923838	9.989221	26.25557	21.02638	14.70188	7.824473	23.16622
1.481631	1.455022	3.984747	1.33285	1.741414	2.649675	0.034545	4.18456	2.349351
0.366674	0.519019	0.120433	0.479062	0.266479	0.747417	0.285556	0.214654	0.29671
0.92531	2.236407	0.978571	0.580758	3.864827	3.040505	1.326667	1.791616	1.594675
2.225498	2.747908	2.413146	5.976724	1.126075	1.722475	1.935685	1.15614	0.439524
2.112843	2.320072	2.572309	0.819048	1.277843	1.546696	0.034545	3.975498	0.143333
1.405772	2.205476	1.578687	0.061962	1.542302	1.327525	0.480794	4.661717	0.174906
0.052143	0.022814	0.009978	0.30329	0.021948	0.005382	0.242222	0.044719	0.056652
8.256465	5.679502	4.690159	0.430281	4.684502	8.849697	0.263853	3.930664	5.00404
10.90615	9.707677	14.08569	2.209141	3.214545	12.70768	1.511198	13.91714	4.429769
4.222583	0.96329	1.61088	6.8421	2.911962	3.860491	6.153102	0.868341	1.876212
1.226118	0.693218	0.207713	1.175859	0.136061	0.781977	0.745613	0.352222	0.340866
0.000693	0.022814	0.127201	2.300678	0.157792	0.63873	2.8657	0.00075	0.500722

1.425613	1.080303	0.803665	7.417359	1.433189	1.638499	7.824473	0.462857	2.727785
0.201789	0.19355	0.023608	5.560563	0.215433	0.332395	0.69119	0.00075	0.164084
0.075231	1.20824	1.069033	0.061962	1.988196	4.33E-05	0.034545	0.440801	1.224733
2.784228	2.461905	0.605455	0.061962	9.047532	4.33E-05	0.034545	2.24733	3.125844
0.653968	0.917994	1.036558	0.882619	0.928759	1.334762	0.034545	1.661328	0.000216
0.106732	0.006219	0.541241	0.953506	0.356032	0.613211	1.370765	0.170938	0.319163
1.553074	0.55215	0.348629	0.061962	1.49075	1.283059	0.034545	1.460981	1.896667
0.617763	0.529971	0.659957	0.580758	0.432237	0.259784	0.600548	1.142345	0.278499
0.894704	1.096335	0.305455	0.061962	1.114618	1.135137	0.034545	1.901068	0.164084
1.440736	1.232886	1.487619	0.061962	1.277843	3.06803	0.034545	1.301169	0.562439
9.418196	3.435988	5.80469	2.511465	3.065108	5.030866	0.034545	14.04491	4.338629
0.136147	0.07013	0.100014	3.518384	0.092612	0.101429	14.51648	0.034863	0.06544
3.909033	2.871212	0.72044	1.253088	4.752121	4.058514	2.419553	0.687431	11.42512
7.013853	8.923117	1.607143	0.061962	7.013853	8.539971	0.745613	1.379278	4.875527
1.091443	3.256587	1.022338	45.51146	3.78798	2.159004	19.75908	1.024127	8.005685
4.397792	4.508196	4.38684	0.061962	1.877605	2.181775	0.034545	8.019769	3.691724
4.125786	3.411097	1.678319	56.39528	15.77678	26.52537	111.5439	1.4671	23.82354
0.909315	1.447994	0.046609	0.430281	2.150303	0.340245	2.302626	0.062857	2.094574
3.829105	7.977958	8.103925	0.061962	2.773644	8.745238	0.034545	5.095325	3.014076
67.98069	749.7839	1360.293	0.061962	111.5439	184.443	4.484892	1360.293	287.197
1.010289	0.975556	2.810678	0.580758	0.790837	0.800512	2.365685	3.796075	0.278499
0.136147	0.075693	0.000101	0.061962	0.039885	0.450924	0.034545	0.00075	0.000216
6.277937	2.747908	0.921739	67.98069	0.092612	6.772763	9.583463	0.88158	0.91404
0.894704	18.19595	9.541645	1.558571	59.32013	18.48514	0.285556	29.18885	17.51961
0.999502	1.275325	0.214964	3.851414	0.136061	1.644416	3.864827	0.197338	0.657114
9.924372	9.502294	12.49452	0.769019	6.53969	13.08214	0.451169	11.48368	7.584502
4.222583	0	16.9162	0.061962	0.300606	3.370527	0.034545	14.70188	13.60866
2.513752	3.632973	2.234538	0.061962	7.882222	5.238701	0.388326	4.193232	0.82868
1.720144	3.730808	5.238701	0.061962	0.970635	0.304921	0.480794	2.194257	0.377323
7.765931	2.794935	0.561775	3.019848	3.442886	4.586753	13.34662	1.20653	3.514834
2.933903	0.487915	0.114437	3.45404	1.146962	1.427431	6.52899	0.103716	1.568564
11.65791	4.424416	3.514834	15.98892	18.05488	8.195657	6.153102	4.072475	14.70188
0.000693	0.648038	0.153521	13.6814	0.000476	0.141284	2.071587	0.00075	0.955418
1.953535	1.539062	0.577388	0.061962	1.395563	1.794531	0.329798	2.445079	1.237879
41.47925	75.1132	32.68313	2.139928	27.66779	75.1132	0.034545	45.1045	19.83727
1.433189	3.223882	0.323535	6.738211	3.807648	0.895368	17.78522	0.072583	9.256537
0.000693	0.012626	0.015945	0.061962	0.092612	0.015657	0.911905	0.00075	0.047965
3.691724	4.789596	5.838499	5.030866	12.29473	4.614618	19.37139	1.34798	11.65791
1.139885	4.990794	0.648961	3.391075	5.568391	1.463304	13.53913	1.041652	9.583463
7.938427	5.266335	7.545584	0.769019	5.515584	6.685087	0.745613	6.268312	5.447114
1.819221	5.050267	3.028629	0.529697	7.124517	2.649675	1.066234	1.544517	6.865
0.846797	0.032915	0.039827	2.92013	0.049531	0.730029	0.630505	0.072583	0.091306
2.248896	0.581111	0.483045	3.29355	1.839986	1.174315	0.830289	0.959062	2.586926
0.585281	0.524646	0.059582	0.479062	0.19127	0.500996	0.420289	0.122179	0.720938
0.47153	0.353175	0.180895	0.38596	0.443052	0.285685	0.242222	0.388023	0.593853
0.045281	0.027655	0.144293	2.139928	0.000476	0.053932	1.102691	0.072583	0.021558
5.993045	21.54823	3.57057	0.529697	2.026219	5.337547	0.420289	8.849697	1.453939
0.836537	0.574993	0.996696	0.061962	4.272165	1.198701	0.034545	2.633232	2.556443
4.25658	19.22139	5.959033	1.558571	4.484892	3.957734	0.034545	5.062626	4.863579
14.42661	3.058802	15.61077	0.061962	5.967879	5.295325	0.034545	3.7586	11.48368
2.468117	2.839351	1.133622	0.061962	1.340981	4.745895	0.034545	6.191515	0.530253
12.85504	13.60866	14.79742	0.061962	9.751039	9.179466	0.034545	31.1753	8.303947
2.097843	1.486551	3.273045	0.061962	1.114618	1.770029	0.034545	2.807937	1.249221
0.92531	0.430837	0.424538	8.728571	0.539466	0.500996	3.500808	0.282266	0.593853
0.78619	0.648038	0.632857	0.061962	0.878932	0.875599	0.034545	0.833045	0.143333
2.633232	1.806595	0.956667	5.976724	6.287561	3.107417	22.96463	0.933874	7.584502
1.834423	1.164257	0.059582	3.851414	1.944517	1.210447	7.991602	0.083521	0.810491
0.000693	0.081587	0.000101	5.560563	0.000476	0.375743	3.921645	0.00075	1.568564

3.083319	1.341825	0.466602	1.33285	3.493362	0.997244	5.762965	0.493247	1.440736
5.788153	9.356371	25.73165	1.78759	7.991602	3.743232	0.220274	26.25557	8.923117
5.704646	13.53913	10.82954	4.174964	5.366335	7.824473	1.603795	3.846984	15.56131
3.57057	1.201724	1.219856	29.49693	2.882035	3.243225	26.80355	0.782417	4.484892
0.717027	4.348882	3.107417	0.640289	1.599033	4.678846	0.196667	2.477027	1.184935
0.625209	0.438023	0.376595	0.640289	0.157792	2.565556	1.277056	0.462857	0.720938
6.39474	4.110779	1.852338	0.430281	3.957734	0.648961	0.196667	1.076032	1.94311
0.178997	0.136061	0.023608	0.640289	0.420498	0.093009	1.468131	0.00075	0.667201
0.396082	0.186133	0.000101	2.66404	0.368658	0.304921	8.849697	0.170938	0.474856
2.112843	6.486104	3.895469	1.253088	2.572309	4.888023	1.468131	4.135801	5.950664
0.413593	0.487915	0.086443	0.529697	0.539466	1.019134	0.662237	0.026068	0.766602
0.090108	0.032915	0.023608	0.769019	0.000476	0.252165	2.185556	0.00075	1.224733
2.65684	2.351299	0.904286	14.93173	0.514885	2.708341	1.820541	0.744531	0.91404
2.442987	2.185556	1.198701	0.061962	0.226883	2.815866	0.034545	2.08013	0.578485
0.345036	0.173319	0.032092	1.175859	0.029502	0.218975	0.242222	0.044719	0.214098
5.309264	2.489762	1.51215	2.820974	3.011169	7.598312	1.468131	1.76873	3.428788
0.744531	3.131544	1.799899	0.640289	1.045462	1.327525	0.35627	1.639639	0.329798
5.056421	11.77567	3.536955	0.061962	1.202626	8.477662	0.034545	3.829105	12.16681
0.154978	0.032915	0.059582	2.408882	1.728615	1.161501	5.493182	0.00075	9.297085
1.165065	1.607143	0.978571	6.738211	4.445851	3.8379	25.48179	1.03246	16.28201
0.162799	0.043297	0.000101	0.061962	0.000476	0.030433	0.034545	0.00075	0.245967
0.740527	0.275873	0.000101	0.882619	0.112706	1.006025	0.034545	0.122179	0.091306
0.699697	0.507085	3.303925	0.061962	1.588903	0.536905	0.034545	1.667273	0.201789
0.421804	0.231508	0.000101	0.061962	0.266479	0.545808	0.034545	0.00075	0.622821
26.52537	11.544	1.520346	62.33472	7.417359	21.74051	67.98069	2.26518	13.34662
1.701616	0.529971	0.348629	3.391075	1.333781	1.019134	1.216768	0.205498	1.199481
0.288247	5.830152	1.615281	0.061962	1.790245	2.252273	1.240202	8.119004	0.000216
0.030375	0.000476	0.023608	0.061962	0.000476	0.015657	0.034545	0.026068	0.014545
5.923838	3.8121	2.770866	0.38596	4.198117	2.898196	0.034545	2.101075	2.263377
43.49658	55.04081	12.10289	10.55571	51.52195	38.94854	1.326667	15.56131	10.73084
2.363442	4.039192	1.028672	14.25954	0.856782	1.957958	3.599329	0.660678	0.657114
8.019769	6.663651	2.941544	0.061962	10.23768	7.558139	0.511667	4.440548	9.101674
0.024423	0.009113	0.000101	0.30329	0.080519	0.083896	1.468131	0.026068	0.174906
1.433189	2.171429	0.568369	0.061962	1.728615	1.23127	0.034545	1.443983	1.21215
0.757273	0.027655	0.000101	1.921212	0.000476	1.722475	0.034545	0.00075	3.055729
2.845036	15.3618	7.765931	0.061962	6.580988	17.39935	0.034545	29.82828	1.919661
0.06772	0.032915	0.174235	0.430281	0.068384	0.038139	0.306602	0.044719	0.06544
0.154978	0.032915	0.039827	0.061962	0.000476	0.083896	0.034545	0.472713	0.000216
14.6058	11.48368	5.687879	0.640289	17.39935	13.15079	0.69119	8.508514	13.83359
0.170938	0.587619	1.128131	0.061962	3.113478	1.006025	0.034545	0.942294	4.365224
1.89518	2.199639	12.63773	0.061962	1.770029	0.697085	0.034545	11.03843	2.073232
1.030678	5.150693	12.42965	0.061962	1.975772	2.016515	0.451169	9.121486	2.281926
4.764055	6.287561	1.748016	0.061962	2.990851	6.52899	4.673182	1.132165	1.624459
1.664906	1.250058	2.425801	0.061962	2.836717	1.2214	0.034545	1.909408	2.524892
1.701616	3.192229	1.309084	21.19547	4.039192	5.515584	32.68313	0.997792	22.22729
1.537951	4.814661	8.477662	0.709322	0.712453	2.538218	2.032706	14.04491	1.173535
8.256465	7.188095	10.53159	0.709322	9.625253	3.544257	1.165873	6.240346	2.248896
15.46193	30.49289	40.7932	4.575123	12.70768	15.07257	1.191905	32.68313	5.864084
0.959567	0.795786	0.135144	1.78759	1.898153	0.406212	3.24658	0.00075	0.841746
0.279177	0.975556	0.247951	0.580758	0.420498	0.976111	0.329798	0.681494	0.268369
5.18754	3.489899	0.858297	3.851414	1.716364	2.683478	4.609076	2.236407	0.694675
1.701616	0.912987	0.000101	5.976724	0.775339	1.135137	1.556392	0.00075	0.855801
0.045281	0.00404	0.000101	0.38596	0.102352	0.109466	0.263853	0.00075	0.234502
0.724885	0.574993	0.046609	2.820974	0.915195	1.334762	5.606739	0.00075	2.556443
7.824473	10.07949	14.97762	0.061962	3.754719	4.907929	0.034545	28.54786	4.519048
0.518139	0.557056	2.061926	0.061962	0.407662	0.595368	0.420289	2.81342	0.879913
2.031169	1.884935	0.460361	1.175859	0.899567	2.565556	1.191905	0.394877	1.330029
0.430476	0.275873	0.335108	3.676032	3.66785	0.127403	8.412367	0.00075	23.6012

0.2443	0	0.153521	0.709322	0.000476	0.118341	0.388326	0.00075	0.593853
4.49671	4.397792	1.947316	0.061962	1.780693	4.376118	0.480794	2.119596	2.820974
0.186861	0.055397	0.023608	0.341558	0.215433	0.181068	0.69119	0.00075	0.214098
2.581861	2.31013	1.145426	0.061962	1.65785	1.612056	0.034545	2.26518	0.164084
0.78619	1.607143	0.810491	4.94026	1.289149	1.306667	2.302626	0.380851	1.371602
0.999502	5.23176	2.635685	1.479452	7.737807	1.240981	1.326667	6.945152	2.703485
2.763261	8.412367	17.39935	0.061962	9.792626	6.99026	1.036558	16.44768	22.74797
0.059726	0.081587	0.07767	1.253088	0.226883	0.061176	2.784228	0.083521	0.268369
0.038088	0.001039	0.000101	0.953506	0.080519	0.022958	0.948211	0.00075	0.091306
6.601833	2.518124	1.450693	0.479062	11.77567	6.612121	0.451169	3.767489	4.721789
3.070952	4.508196	1.401977	20.16541	4.984488	4.833398	11.42512	1.754733	5.295325
2.593506	3.040505	1.133622	4.875527	4.597792	4.365224	0.911905	1.95645	2.794935
2.6143	1.523824	0.716652	0.061962	0.254156	2.225498	0.306602	1.242641	0.562439
3.179199	3.283139	6.404921	0.061962	5.137042	4.875527	1.240202	5.062626	3.397706
0.082222	0.049185	0.07767	0.061962	0.049531	0.083896	0.034545	0.00075	1.795851
0.000693	10.8813	4.575123	0.061962	4.145209	0.259784	0.034545	11.96574	0.734524
0.625209	0.326681	0.100014	1.78759	0.380447	0.276847	3.24658	0.00075	0.678687
4.851227	6.135007	0.505267	16.80343	2.396429	1.770029	15.98892	0.179798	1.959466
62.33472	51.04072	34.38341	1.558571	77.74812	100.0998	3.049603	30.17192	28.54786
0.178997	0.519019	0.230108	2.92013	0.226883	0.375743	7.61176	0.257518	0.352597
0.000693	0.587619	4.165137	0.882619	0.000476	4.33E-05	0.034545	4.38684	0.000216
0.54847	0.320144	0.000101	1.921212	1.452857	0.415729	1.102691	0.00075	0.974257
1.372475	1.139885	2.002193	0.529697	1.383911	1.249221	0.306602	3.179199	2.196017
7.136537	3.989206	4.709033	0.061962	2.396429	5.439278	3.211176	9.335397	4.839105
2.003723	3.895469	6.612121	1.02487	3.636789	2.773644	0.874603	3.207807	3.66785
0.540902	0.519019	0.046609	16.68554	0.148016	0.697085	3.722785	0.103716	0.795339
0.47153	1.048074	0.153521	0.479062	1.146962	0.730029	1.511198	0.205498	1.262965
1.553074	1.486551	6.784762	0.061962	0.342641	1.974228	0.034545	3.156537	0.164084
2.893001	2.013182	0.927561	2.602395	1.369928	4.323045	2.81342	2.647244	2.117814
4.484892	5.583968	0.305455	8.365072	2.380079	3.640606	6.071436	0.265339	5.679502
1.314675	3.23987	2.386248	0.061962	2.425801	0.319026	0.034545	6.240346	0.000216
0.2443	1.767431	5.330519	0.061962	3.404372	0.840635	0.480794	2.950173	4.365224
3.070952	1.259747	0.849841	5.737056	2.752893	1.566414	9.297085	0.516061	2.050404
12.29473	8.745238	15.61077	0.061962	10.03313	19.29639	0.034545	12.63773	9.541645
12.03267	7.697063	7.507619	1.253088	4.524481	23.82354	1.935685	11.60283	7.417359
0.480548	0	0.000101	0.529697	0.058773	0.181068	0.263853	0.00075	0.153651
9.335397	24.98218	12.93651	9.218514	20.77311	8.381328	1.603795	24.04374	7.225469
135.5245	332.0776	530.1465	0.061962	287.197	118.7028	3.464315	287.197	52.56128
1.903636	4.802655	0.921739	6.612121	2.23267	2.367763	1.326667	0.189509	2.481356
0.225823	0.92482	5.454949	0.061962	5.745996	1.150635	0.034545	1.609654	0.869365
9.377345	4.236962	3.086306	2.730072	2.335844	3.107417	0.388326	6.423911	1.308167
3.227085	6.287561	18.05488	0.061962	6.191515	5.923838	0.034545	7.854069	5.638759
12.56693	4.650007	105.1694	0.061962	3.227085	18.69833	0.034545	126.1556	14.6058
1.841371	6.063276	3.47531	0.061962	7.049336	2.290996	0.034545	7.495397	1.358189
5.889798	29.03196	38.94854	0.061962	20.34063	2.693442	0.034545	49.50903	0.60886
10.01009	6.876392	9.19899	15.276	7.653391	10.53159	12.77772	3.687633	6.717056
0.717027	2.701053	0.692381	0.479062	2.196017	1.198701	0.034545	1.317475	2.117814
1.416407	3.842554	4.130794	0.061962	3.321284	2.59557	0.547403	5.530274	0.720938
2.083261	1.496118	0.195823	3.592063	0.938911	2.143261	2.973059	0.333831	0.708492
0.437576	0.07013	0.000101	3.179199	0.000476	0.332395	3.414574	0.00075	0.352597
16.22108	11.33981	13.83359	2.408882	10.53159	20.68746	3.864827	19.53061	5.165599
7.468961	21.74051	11.42512	0.061962	9.237525	31.92385	0.717403	15.46193	1.639639
0.075231	0.017446	0.000101	0.580758	0.080519	0.187532	0.034545	0.00075	0.245967
10.48101	17.15902	18.33909	0.953506	11.68846	12.63773	1.638499	18.05488	11.30987
1.33285	1.02487	2.274473	0.061962	0.889553	1.830159	0.034545	0.567994	1.029365
0.689509	0.671371	0.107157	1.33285	0.490079	0.800512	0.451169	0.161962	0.562439
1.297763	0.795786	0.803665	0.882619	2.114437	2.053521	1.772756	0.6114	1.594675
0.098211	0.258456	0.059582	1.558571	0.368658	0.158766	0.874603	0.044719	0.201789

4.914863	7.417359	7.795642	0.709322	1.507835	7.911934	1.137244	5.439278	1.68342
1.77404	1.530159	0.736551	0.479062	3.621941	2.538218	0.196667	2.101075	0.500722
0.000693	0.00404	0.000101	0.38596	0.039885	0.149755	0.451169	0.00075	0.102886
2.893001	3.651898	5.395584	0.061962	1.780693	3.493362	0.034545	12.77772	4.086926
28.54786	9.179466	51.52195	18.41211	45.1045	1.019134	14.84163	70.11932	27.66779
0.480548	1.561912	15.46193	0.061962	0.000476	4.33E-05	0.034545	5.210317	0.879913
0.090108	0.049185	0.07767	1.408831	0.226883	0.045916	21.54823	0.034863	0.082569
1.201724	1.239423	7.339452	0.061962	2.511465	2.565556	0.034545	8.412367	3.899993
0.261962	0	0.000101	3.179199	0.000476	1.091443	3.722785	0.00075	1.613232
0.136147	0.038139	0.009978	0.529697	0.068384	0.045916	2.365685	0.026068	0.25785
0.662237	1.50684	1.193362	2.92013	0.775339	1.483622	2.185556	0.380851	0.74868
0.209538	0.178817	0.29404	0.061962	0.226883	0.545808	1.435772	0.122179	0.082569
0.811443	0.529971	0.000101	3.676032	1.692763	1.135137	0.329798	0.00075	1.126775
1.89518	2.07658	1.690483	0.529697	3.156537	3.327619	0.034545	4.105714	0.462352
2.713319	2.839351	6.674286	1.78759	5.030866	2.826277	1.009625	9.968355	0.307973
2.74544	1.011486	0.42925	2.060491	0.342641	1.382006	0.69119	0.161962	1.94311
7.112496	5.050267	33.07147	0.061962	2.725498	4.241905	0.577388	6.695887	1.199481
0.209538	0.438023	0.068023	1.253088	0.368658	0.657547	0.034545	0.00075	0.245967
7.162698	7.737807	25.24367	3.767489	2.042201	5.9071	2.973059	15.18102	2.961768
4.105714	5.366335	3.644343	1.699091	1.728615	3.175916	0.981032	4.376118	3.418405
0.824416	0.587619	0.093312	1.699091	0.970635	0.520289	1.277056	0.122179	0.657114
1.701616	8.164531	4.927302	0.061962	25.73165	0.038139	0.034545	8.226219	0.000216
0.7986	1.196508	1.433189	0.341558	1.235346	0.976111	0.306602	1.110505	0.926926
1.128788	0.751371	3.263391	0.061962	0.805238	1.901068	0.034545	3.179199	0.869365
0.518139	0.081587	0.000101	1.33285	0.239769	0.149755	0.745613	0.053579	0.487662
0.540902	0.022814	0.015945	0.529697	0.029502	0.458045	1.277056	0.053579	0.06544
2.257749	1.041652	0.342381	1.980592	0.420498	1.099762	2.500548	0.859885	0.667201
0.604214	0.398903	0.972504	0.061962	2.114437	0.173059	0.034545	1.948918	0.214098
0.098211	0.009113	0.07767	0.38596	0.136061	0.022958	0.285556	0.00075	0.102886
0.082222	0.055397	0.000101	0.640289	0.000476	0.015657	1.240202	0.034863	0.245967
3.019848	3.548153	9.063983	0.061962	3.47531	5.20267	0.511667	3.464315	1.224733
0.592208	0.243672	0.401385	0.640289	0.215433	0.227446	0.196667	0.619012	0.021558
1.794531	4.94026	2.267157	0.479062	2.737944	4.776407	0.547403	3.566753	2.839351
3.217612	1.184185	1.639639	1.699091	2.285664	0.976111	2.185556	1.34798	1.624459
0.480548	1.57645	1.008961	0.341558	0.551861	1.215981	0.285556	2.355289	0.25785
0.480548	3.324452	2.73272	0.061962	1.988196	0.613211	0.911905	3.169812	21.02638
0.225823	0.103088	0.000101	0.529697	0.112706	0.101429	0.17733	0.00075	0.113766
3.885642	4.370671	2.302626	0.061962	3.548153	3.566753	0.034545	5.366335	2.160649
34.38341	37.29684	42.75576	7.92518	25.99427	32.68313	8.412367	34.84299	12.22798
1.277843	0.075693	0.059582	0.061962	0.878932	0.959567	0.167532	0.034863	1.237879
0.454632	0.275873	0.059582	0.640289	0.239769	0.325317	1.638499	0.170938	0.578485
2.269134	8.319019	16.09482	0.061962	3.585029	4.650007	0.630505	8.119004	25.73165
3.414574	2.058773	3.904517	0.061962	2.477027	5.576219	0.35627	2.993867	1.474343
0.757273	0.787605	0.450527	0.061962	1.055628	1.23127	0.034545	0.425844	0.82868
0.178997	0.173319	0.407157	4.072475	0.123535	0.015657	3.66785	0.189509	0.694675
2.408882	6.325945	2.898196	0.882619	5.030866	5.745996	0.034545	5.737056	2.325931
3.404372	7.570693	12.29473	0.061962	6.287561	3.300664	0.034545	8.608153	9.459177
0.288247	1.201724	0.180895	1.699091	0.938911	0.038139	2.74298	0.161962	2.117814
19.83727	62.33472	46.7056	0.061962	45.91841	40.7932	0.034545	57.84882	17.15902
0.709784	1.321053	1.057569	0.061962	1.016494	1.710173	0.034545	1.644416	1.143954
1.128788	0.398903	0.207713	1.78759	0.823983	1.781948	6.071436	0.371075	2.727785
0.610606	1.623232	0.858297	3.29355	0.823983	0.747417	3.340361	0.402698	1.68342
14.97762	38.94854	28.54786	0.061962	7.545584	11.83593	0.034545	21.54823	1.816436
0.592208	0.735382	2.470317	0.061962	0.656392	0.678247	0.034545	1.098341	0.974257
2.419553	1.652771	2.207417	0.061962	2.820974	1.006025	0.745613	2.563506	1.126775
0.681068	1.126075	0.996696	11.51384	0.790837	0.773276	7.402915	0.257518	0.578485
0.508658	0.751371	0.927561	1.699091	1.826147	0.390895	0.034545	1.62562	0.28689
1.277843	0.50127	0.046609	1.78759	0.092612	1.629235	1.165873	0.00075	0.428932

0.540902	0.445455	0.716652	0.341558	0.420498	0.202294	0.263853	0.6114	0.113766
0.038088	155.7103	87331.38	0.061962	70.11932	4.297222	0.034545	332.0776	1.064935
0.106732	0.055397	0.466602	9.218514	0.136061	0.243896	0.783939	0.553059	0.562439
0.000693	0	0.000101	0.061962	0.000476	0.158766	0.451169	0.00075	0.102886
0.366674	0.311385	0.220808	0.819048	0.112706	0.193997	1.408831	0.093622	0.225339
0.396082	1.707576	25.48179	2.820974	1.877605	0.285685	0.034545	16.80343	0.869365
2.633232	0.705231	0.335108	0.061962	0.734113	1.566414	0.388326	0.965065	0.562439
0.774798	1.033672	0.608543	7.683766	1.944517	0.712453	11.30987	0.371075	3.101371
1.654156	1.321053	1.387612	0.819048	2.773644	3.599329	1.850996	1.762713	3.464315
0.106732	0.002128	0.000101	0.061962	0.039885	0.135144	0.577388	0.103716	0.225339
1.808023	2.805382	2.688304	1.408831	4.752121	3.91355	1.102691	3.425512	3.926154
1.924228	0.671371	0.951652	0.061962	0.843102	0.821898	0.034545	1.262965	0.329798
1.191198	0.956667	0.342381	2.730072	2.967453	1.49075	18.62711	0.233175	5.515584
0.480548	0.147323	0.032092	0.769019	0.123535	0.202294	1.036558	0.00075	0.153651
3.595758	4.125786	9.459177	5.030866	1.235346	4.110779	1.036558	7.911934	2.000592
1.175094	1.232886	4.18456	0.061962	0.775339	4.33E-05	2.243911	2.939141	0.462352
1.580909	1.096335	0.100014	3.592063	1.578687	1.449062	2.642525	0.189509	0.766602
0.617763	8.103925	8.445743	0.580758	5.330519	1.546696	0.600548	6.978903	4.540794
0.030375	0.186133	1.155411	1.408831	0.092612	0.030433	2.136335	0.161962	0.174906
0.030375	0.00404	0.009978	0.061962	0.000476	0.005382	1.820541	0.026068	0.014545
1.326667	1.080303	2.884762	0.38596	2.026219	1.046061	0.388326	2.502619	1.279452
0.421804	0.157706	0.046609	0.061962	0.239769	0.202294	0.034545	0.00075	0.593853
3.373983	4.921082	7.468961	0.30329	5.110159	2.304452	0.034545	5.560563	1.639639
0.709784	0.705231	0.98474	0.580758	0.215433	0.545808	0.329798	0.651631	0.810491
0.774798	1.669466	11.14619	6.414416	10.82954	1.684646	0.034545	2.850058	5.195022
14.51648	8.508514	8.849697	0.061962	10.60361	10.78056	3.722785	13.38418	7.882222
0.225823	0.107749	0.188427	0.640289	0.102352	0.181068	4.302367	0.214654	0.133658
4.733824	3.614603	1.629235	0.529697	1.819221	7.938427	2.365685	2.491948	2.600014
3.201457	1.932857	1.128131	0.061962	1.277843	2.984899	0.547403	2.600014	0.102886
6.240346	1.50684	0.488759	13.6814	2.23267	7.351284	3.66785	1.110505	0.268369
2.162381	1.041652	0.166898	2.511465	1.066948	3.343629	2.685786	0.083521	2.230859
0.209538	0.173319	0.272244	0.430281	0.226883	0.602908	0.329798	0.233175	0.622821
6.956349	26.52537	4.096407	0.061962	15.18102	13.30906	0.034545	12.10289	4.207778
11.42512	14.00414	20.51271	8.524242	9.335397	8.256465	8.333918	31.92385	6.508427
13.79669	17.92188	9.838658	0.882619	5.915469	8.348817	1.820541	28.87506	1.05153
0.454632	0.022814	0.000101	1.699091	0.058773	0.127403	2.243911	0.062857	0.06544
1.713997	2.928247	4.174964	1.253088	1.31	3.131544	0.600548	7.225469	0.82868
15.3618	15.88548	7.363117	2.060491	6.967547	11.09133	6.738211	9.121486	8.994791
0.709784	0.512641	0.796234	0.061962	0.790837	0.259784	0.329798	0.603737	0.174906
9.141299	8.888167	35.27608	7.327482	14.08569	31.1753	1.935685	13.08214	8.556703
1.513102	0.885137	0.287641	0.38596	0.745072	0.866147	2.302626	0.282266	0.855801
0.045281	0.00404	0.000101	0.061962	0.167273	0.210693	1.723579	0.00075	0.307973
0.480548	0.217496	0.272244	0.061962	0.266479	0.738312	0.034545	2.24733	1.092136
1.45912	1.406746	0.394156	2.060491	2.572309	3.895469	2.642525	0.425844	11.719
4.63189	1.374329	0.938413	0.819048	1.988196	5.409437	0.783939	1.501291	7.112496
1.481631	0.742872	0.000101	2.602395	0.068384	1.400159	0.547403	0.093622	0.364242
6.144055	5.20267	6.250447	9.063983	2.23267	2.449509	0.420289	4.684502	0.547778
0.225823	0.088788	0.023608	0.819048	0.029502	0.368658	1.370765	0.026068	0.268369
0.617763	13.91714	19.83727	0.061962	2.931068	3.816551	0.034545	62.33472	0.60886
0.380267	1.728615	1.42456	4.277006	0.68557	0.285685	3.540606	0.582208	0.364242
0.218528	0	0.361342	0.061962	0.356032	1.174315	0.034545	0.00075	0.000216
3.179199	30.17192	1.138557	4.277006	5.259437	7.251263	7.897078	1.986681	18.91854
0.000693	17.28195	144.7919	0.061962	11.14619	8.780115	0.034545	111.5439	2.281926
3.816551	7.443276	16.74449	3.676032	9.667316	2.267157	4.857403	3.514834	4.297222
0.329091	0.62733	0.454394	0.061962	2.425801	0.135144	0.285556	0.526032	0.462352
7.507619	3.864827	3.625382	2.060491	2.542605	6.55039	0.662237	4.63189	2.073232
48.01804	29.49693	33.93939	11.09133	27.10502	72.69473	19.06893	95.22939	7.653391
0.030375	0.002128	0.009978	0.580758	0.080519	0.005382	1.277056	0.00075	0.201789

0.500722	0.773766	0.424538	3.767489	0.915195	1.249221	0.981032	0.660678	1.191198
0.000693	0	0.000101	1.479452	0.157792	0.193997	0.874603	0.00075	0.29671
1.380195	1.57645	2.294733	0.061962	0.656392	1.781948	4.140505	18.76954	2.263377
0.052143	0.217496	0.796234	0.061962	0.98474	0.648961	0.263853	0.240851	3.300664
0.329091	1.815	2.518124	0.061962	0.432237	0.4386	0.034545	3.672078	0.278499
0.463485	3.300664	6.09759	1.78759	1.826147	0.141284	0.329798	3.297107	1.343463
0.311227	2.710765	1.42456	0.061962	0.790837	0.787136	0.034545	1.762713	2.145137
11.03843	27.38726	26.25557	0.529697	39.53687	13.75978	1.603795	22.13245	18.62711
4.709033	33.07147	15.07257	1.863506	7.570693	6.956349	1.137244	11.8058	12.49452
0.038088	0.113074	0.093312	4.072475	0.454978	0.101429	0.451169	0.034863	0.869365
0.075231	0.76	7.854069	1.10132	7.991602	0.895368	0.630505	8.573434	4.673182
4.875527	25.24367	3.381255	44.27012	13.79669	8.923117	33.93939	5.11697	30.49289
0.877807	1.554185	1.501291	0.061962	0.867814	0.959567	0.420289	3.049603	0.245967
3.500808	1.275325	0.602287	5.976724	1.565253	3.807648	7.61176	0.668947	16.9162
0.970635	1.903636	4.077403	0.061962	2.335844	1.039199	0.034545	8.539971	2.073232
1.149322	0.563824	0.472208	2.730072	1.692763	1.031284	0.948211	0.265339	0.91404
1.47018	1.662439	2.725498	0.709322	3.917597	1.483622	0.034545	1.510014	4.135801
0.154978	0.022814	0.000101	1.78759	0.514885	0.045916	2.973059	0.00075	0.352597
5.056421	1.341825	0.968066	16.34294	1.202626	7.251263	4.302367	4.397792	9.815642
5.123781	8.226219	2.839351	9.880779	7.351284	2.66404	10.1459	1.05824	3.7586
0.178997	1.382006	4.540794	0.061962	0.640289	0.520289	0.511667	1.05824	1.224733
1.041652	1.640779	0.549661	0.709322	1.082915	0.738312	2.243911	0.380851	1.262965
0.06772	0.141183	0.032092	0.38596	0.420498	0.127403	1.408831	0.072583	1.516219
0.082222	0.060729	0.127201	1.558571	0.123535	0.030433	0.285556	0.044719	0.091306
4.519048	4.851227	8.048095	0.882619	1.925635	2.201587	0.69119	5.687879	0.091306
1.314675	1.429365	6.028427	0.953506	2.981962	1.835743	0.034545	2.101075	0.678687
0.024423	0.000476	0.032092	0.061962	0.039885	0.009733	0.196667	0.00075	0.056652
8.711905	1.587605	0.144293	25.99427	2.162381	7.149618	10.45584	0.133781	10.53159
3.750837	1.126075	2.122799	1.33285	2.150303	3.88153	0.034545	1.919661	3.514834
1.314675	1.767431	0.875101	0.30329	2.797835	2.119596	0.388326	0.805238	1.639639
10.53159	9.438687	6.695887	0.953506	6.433918	11.22667	0.874603	12.22798	4.978182
0.574993	0.842193	1.983824	0.882619	0.805238	1.546696	0.547403	2.390447	0.593853
0.463485	0.265455	0.207713	1.78759	0.656392	0.458045	1.872049	0.257518	0.225339
25.24367	0.368882	0.72044	2.408882	0.289603	22.32214	0.783939	6.088593	0.091306
0.106732	0.76	0.764704	0.479062	0.380447	0.101429	0.874603	0.890577	0.214098
3.195541	1.720144	1.767431	0.529697	1.523824	2.394416	0.285556	1.356364	4.802655
0.329091	0.60824	0.230108	1.78759	0.136061	0.319026	0.981032	0.325772	0.174906
0.235043	1.283059	0.207713	3.518384	1.944517	0.987309	3.169812	0.394877	2.635685
6.738211	5.71241	0.810491	5.976724	4.783001	11.65791	10.73084	1.093434	2.773644
1.341825	2.219863	2.129596	0.061962	4.468009	4.18456	0.034545	1.661328	0.82868
3.651898	23.16622	11.83593	0.061962	14.17388	2.280101	0.034545	8.048095	0.000216
1.449062	2.434444	5.082063	0.061962	2.425801	2.410996	0.034545	3.975498	2.178268
1.846941	0.906126	0.568369	0.769019	1.016494	1.759986	0.034545	0.651631	1.397482
2.565556	3.94886	0.445209	1.699091	2.701053	5.323492	1.009625	3.340361	12.56693
4.338629	10.07949	7.38847	1.10132	7.315512	8.134084	0.630505	7.225469	5.606739
0.448824	0.699192	0.382208	0.819048	1.016494	1.355527	1.435772	0.92531	6.865
13.79669	15.07257	7.238023	59.32013	8.445743	10.85542	47.5018	4.302367	14.42661
0.387056	0.557056	0.505267	0.529697	0.309668	0.083896	1.772756	0.88158	0.188009
0.235043	0.094949	0.07767	2.300678	0.420498	0.29531	2.461905	0.083521	0.40645
29.49693	6.250447	47.5018	0.061962	41.47925	12.85504	3.13456	83.489	40.7932
1.11526	3.35715	1.241811	0.061962	0.454978	2.410996	0.034545	1.169091	0.377323
0.78619	0.983384	2.436768	0.061962	0.98474	0.529697	0.034545	4.241905	0.500722
0.162799	0.633853	0.000101	3.518384	0.148016	0.987309	1.165873	0.00075	2.417424
12.93651	10.78056	0.98474	0.061962	2.511465	9.792626	0.034545	5.622872	0.783939
144.7919	83.489	13.15079	87331.38	8.539971	7.868146	87331.38	26.52537	34.38341
0.195007	0	0.000101	0.061962	0.049531	0.030433	0.306602	0.00075	0.164084
15.66023	9.815642	3.327619	749.7839	1.433189	1.019134	425.62	2.419553	10.48101
0.811443	5.515584	7.625209	0.061962	12.16681	0.902403	0.034545	7.519841	1.199481

2.681169	1.228651	2.635685	3.095166	5.210317	3.651898	2.898196	1.277843	4.207778
2.173081	0.217496	0.771688	0.061962	2.380079	0.81228	0.745613	4.072475	3.217612
2.474834	7.061328	3.625382	5.500678	4.419062	2.378124	2.898196	2.755411	8.780115
1.416407	1.064935	0.259509	15.71851	0.356032	0.895368	29.18885	0.651631	1.816436
35.776	10.19468	2.489762	23.82354	12.85504	14.70188	13.91714	9.541645	5.576219
0.000693	0	0.000101	0.061962	0.000476	0.29531	12.22798	0.00075	0.855801
0.329091	0.118983	0.174235	1.10132	0.203463	0.285685	1.980592	0.00075	0.268369
5.082063	3.885642	4.808658	41.47925	4.94026	7.037345	6.30671	14.42661	1.841371
118.7028	72.69473	11.96574	203.5795	49.50903	111.5439	24.85971	42.75576	118.7028
20.85876	14.79742	43.49658	5.821804	64.12971	17.28195	9.924372	17.78522	30.17192
27.66779	33.93939	13.38418	118.7028	27.38726	55.04081	65.94551	39.53687	20.34063
49.50903	14.17388	5.123781	64.12971	22.8563	22.96463	59.32013	4.575123	55.04081
48.01804	15.46193	17.78522	8.365072	87.1746	21.36892	11.57341	20.51271	59.32013
21.19547	9.063983	3.327619	332.0776	8.195657	7.468961	287.197	3.514834	20.08201
332.0776	425.62	425.62	10.82954	530.1465	87331.38	0.830289	530.1465	425.62
203.5795	144.7919	56.39528	24.85971	425.62	224.9555	38.39042	80.6518	167.9996
95.22939	111.5439	111.5439	2.602395	252.4251	51.04072	1.468131	43.49658	83.489
72.69473	95.22939	24.2842	70.11932	40.7932	287.197	126.1556	22.96463	87.1746
6.674286	4.193232	1.729892	1.175859	6.99026	10.38202	1.326667	1.452857	6.019971
1.416407	1.429365	1.363557	4.072475	1.126075	1.198701	2.784228	0.668947	1.21215
3.373983	10.33476	2.363442	34.6132	8.319019	9.297085	72.69473	1.62562	43.49658
7.238023	5.439278	12.70768	0.709322	8.797619	5.388405	3.049603	7.136537	4.603434
0.218528	0.081587	0.07767	0.640289	1.033672	2.815866	1.277056	0.083521	4.02088
0.136147	0.118983	0.032092	0.30329	0.19127	0.135144	0.388326	0.083521	1.191198
1.501291	1.486551	0.887359	5.409437	3.227085	4.419062	6.153102	1.006025	2.586926
0.098211	0.678247	3.065108	1.10132	0.102352	4.33E-05	5.696263	0.380851	0.082569
6.116638	6.808369	5.165599	22.8563	1.599033	9.838658	7.991602	3.904517	1.453939
0.846797	0.09855	0.335108	184.443	0.000476	4.33E-05	167.9996	2.302626	0.000216
4.764055	10.53159	3.917597	1.980592	19.99861	10.28573	4.609076	6.39474	67.98069
4.048499	2.261573	6.945152	1.479452	1.2214	2.898196	0.034545	4.007583	0.994271
4.709033	0.795786	0.259509	2.92013	5.088694	2.280101	12.67271	0.410079	7.061328
0.145556	0.065519	0.059582	0.819048	0.000476	0.061176	0.577388	0.034863	0.06544
1.226118	0.311385	0.299747	3.957734	0.454978	1.507835	18.26752	0.472713	0.91404
0.604214	3.204632	5.654863	0.061962	0.443052	1.679596	0.034545	2.800332	0.841746
22.74797	19.37139	17.92188	2.209141	22.8563	16.22108	4.101061	27.96248	35.776
26.25557	31.55417	70.11932	4.451154	47.5018	27.10502	1.89961	27.10502	31.55417
13.60866	13.83359	6.486104	15.98892	12.06778	30.82277	32.31123	6.30671	9.459177
3.57057	5.110159	1.501291	0.709322	4.808658	6.30671	0.717403	1.89373	4.397792
0.749481	0.480022	0.032092	0.882619	0.759293	1.174315	0.285556	0.161962	0.188009
35.27608	27.96248	13.75978	9.989221	29.18885	41.47925	2.365685	13.53913	16.28201
0.518139	0.656753	0.472208	0.819048	0.970635	0.840635	1.603795	0.362179	1.397482
7.443276	5.80469	0.440029	24.85971	4.359574	0.529697	0.783939	2.003723	17.39935
17.78522	18.05488	6.508427	13.11646	16.44768	62.33472	20.68746	9.838658	45.91841
0.944784	2.003723	1.703795	9.218514	0.889553	2.316075	9.707677	1.567576	1.453939
0.867814	0.851659	0.29404	1.558571	0.668947	1.187229	0.547403	0.703665	2.506934
2.693442	3.750837	3.726854	6.99026	4.557605	4.23202	3.104394	2.673846	12.10289
1.580909	0.494841	0.382208	2.060491	1.159149	1.29417	1.556392	0.738312	2.117814
0.311227	4.086926	0.107157	2.060491	0.331097	0.353333	6.028427	0.534452	0.352597
0.454632	0.275873	0.342381	0.640289	0.899567	0.875599	0.511667	0.240851	1.766248
0.500722	1.554185	0.577388	0.769019	1.202626	3.300664	1.191905	0.325772	2.758088
0.201789	0.648038	0.505267	0.580758	0.938911	0.181068	0.034545	0.440801	2.863211
8.643074	4.739668	0.944784	11.63037	1.016494	1.974228	14.00414	0.453709	1.662439
0.662237	5.530274	1.716364	3.179199	2.628225	3.223882	3.279625	2.710765	1.568564
3.446732	7.598312	1.531313	3.179199	3.425512	6.116638	3.78798	1.553074	15.46193
0.617763	0.055397	1.965657	0.061962	0.289603	0.481602	0.329798	1.474343	0.113766
0.030375	0.081587	0.174235	0.061962	0.539466	0.045916	0.600548	0.083521	0.153651
20.68746	18.33909	8.288874	2.408882	11.03843	17.64869	3.464315	14.51648	2.022778
1.060823	0.851659	0.515527	2.209141	0.454978	0.81228	2.302626	0.88158	0.40645

0.024423	0	0.000101	0.061962	0.021948	0.005382	0.034545	0.026068	0.039293
0.7986	0.569625	1.353608	0.061962	0.712453	0.227446	0.034545	0.782417	0.892395
2.257749	4.715599	5.28039	1.02487	5.855281	1.455022	0.034545	4.661717	6.414416
105.1694	22.52375	0.323535	46.7056	18.33909	40.11561	91.05541	0.782417	14.17388
4.222583	3.975498	2.958773	0.061962	2.285664	4.907929	0.511667	14.17388	0.783939
1.727323	1.341825	1.072843	5.316378	1.523824	2.171429	5.638759	0.793434	1.21215
3.679986	8.477662	26.80355	0.640289	4.091667	3.418405	1.370765	7.38847	1.703795
4.058514	2.335844	3.610707	23.38378	5.967879	4.863579	13.64503	1.34798	5.821804
0.836537	0.648038	1.172208	6.144055	0.805238	1.441898	12.16681	0.668947	2.863211
0.886775	0.19355	0.153521	0.061962	0.27759	2.725498	0.388326	0.240851	0.000216
16.80343	15.77678	6.325945	18.19595	7.288788	36.74522	42.08807	2.842193	6.162446
287.197	126.1556	95.22939	10.07949	87331.38	252.4251	3.211176	135.5245	332.0776
0.098211	0.157706	0.434863	0.061962	0.539466	0.353333	1.772756	0.00075	0.164084
19.60574	57.84882	16.44768	10.48101	105.1694	39.53687	0.220274	33.07147	77.74812
4.802655	2.558817	1.226118	2.820974	3.179199	5.591717	1.066234	0.890577	7.751869
1.405772	0.932078	0.160779	4.709033	1.255657	1.434055	1.935685	0.493247	0.841746
8.429055	15.276	4.764055	0.38596	13.45939	12.77772	2.136335	7.710361	5.477843

CGGA_121	CGGA_121	CGGA_122	CGGA_122	CGGA_122	CGGA_123	CGGA_123	CGGA_123	CGGA_123
0.379791	0.868341	0.642395	0.204156	0.281558	0.405599	0.15456	0.19039	0.228788
0.387489	0.656392	0.372973	0.489784	0.430087	0.148254	0.263304	0.974906	0.394603
0.92531	1.392511	10.01009	3.651898	2.322035	15.3618	3.57057	2.839351	6.464906
5.889798	3.68381	4.217569	9.667316	0.683232	6.054242	0.465079	1.957958	7.882222
4.644098	6.019971	4.120815	5.068831	2.618846	3.38456	3.38456	3.679986	2.834084
7.38847	5.855281	2.577157	4.764055	3.984747	2.151919	4.125786	15.07257	3.343629
22.32214	38.94854	126.1556	33.07147	37.29684	83.489	28.23632	30.17192	60.77449
0.038918	0.063405	0.028139	0.001255	0.014675	0.057208	0.018182	0.007417	0.000303
3.824964	2.009906	1.069574	2.489762	1.474343	1.790245	1.726032	1.156869	1.492973
0.105996	0.664423	0.451169	0.079675	0.227648	0.617071	0.140483	0.700418	0.000303
29.82828	40.7932	15.276	37.81919	36.26061	34.84299	25.99427	44.27012	118.7028
1.092785	2.720996	0.686667	0.819978	1.082915	1.465123	0.878369	1.717641	2.157359
4.965801	11.544	29.18885	15.77678	12.49452	18.48514	20.68746	7.882222	27.10502
1.271097	0.732633	0.00062	2.50469	13.30906	1.44E-05	1.717641	7.149618	0.000303
0.00904	0.032367	0.057352	0.000534	0.008175	0.01062	0.008882	8.66E-05	0.027006
2.481356	6.99026	3.137576	9.838658	11.14619	6.55039	3.119986	4.402973	4.721789
0.96627	6.601833	2.805382	0.687049	1.623232	3.581688	1.282208	3.089293	4.110779
0.051595	0.040159	0.070462	0.010072	0.036133	0.06355	2.89E-05	8.66E-05	0.178066
1.371602	0.952294	0.415729	2.890245	1.595729	0.697085	0.352222	1.200152	0.643001
0.775339	0.629538	0.489784	0.562439	0.345635	0.382417	0.36153	0.81544	0.802395
1.218182	0.346494	0.166465	0.675938	0.259033	0.558427	0.600173	0.789863	0.074069
0.862561	0.901212	4.348882	1.870685	3.397706	3.24658	2.593506	1.476429	2.540462
80.6518	53.77619	105.1694	87.1746	203.5795	35.27608	87.1746	111.5439	80.6518
0.002121	0.024199	0.057352	0.000534	0.008175	0.027431	0.063362	8.66E-05	0.046962
1.201724	1.604827	1.89961	1.386681	1.8386	1.50684	1.672107	1.938759	1.358189
1.539062	2.216162	6.39474	6.153102	5.779574	5.830152	5.80469	5.337547	6.144055
0.184206	0.506652	0.679069	0.843564	4.125786	0.86469	0.338831	2.515938	0.709784
0.225505	0.768052	0.542525	0.313312	0.259033	0.734935	0.237359	0.411948	0.474856
36.74522	20.34063	15.56131	100.0998	252.4251	19.22139	59.32013	144.7919	13.83359
0.184206	0.542828	0.664423	0.399545	0.642698	0.796234	0.800058	0.463485	0.394603
3.425512	5.088694	2.136335	3.792266	3.726854	9.121486	8.019769	3.921645	2.390447
0.067027	0.193997	0.141623	0.014906	0.080837	0.090772	0.089553	0.07013	0.164747
7.911934	3.286652	13.15079	18.05488	13.00996	7.417359	19.83727	11.42512	13.15079
0.099704	0.202388	0.049646	0.010072	0	0.020267	0.018182	8.66E-05	0.039214
3.207807	12.63773	0.948745	1.870685	0.578817	3.695815	1.062092	0.935115	4.140505
0.368882	0.236089	3.002576	1.708932	1.3829	2.944416	2.479221	1.180613	2.212619
3.45404	4.007583	2.281926	3.581688	2.474834	2.051962	4.33342	2.406768	5.050267
0.059264	0.183824	0.00062	0.000534	0.041782	0.116724	2.89E-05	0.115188	0.020577
2.964762	4.802655	5.439278	3.91355	1.716364	3.747035	2.415296	1.77404	2.574733
9.121486	7.38847	4.752121	4.312251	3.625382	5.110159	3.820758	4.115844	7.162698
0.323535	0.211025	0.772626	1.311674	0.31456	0.445996	0.317403	0.911364	0.620014
0.786667	0.995527	3.401126	0.539812	0.786667	1.36619	0.66526	0.496797	2.032706
2.173081	0.901212	3.324452	2.302626	0.705613	1.748016	3.890556	0.998831	1.903636
0.002121	0.008095	0.03518	0.000534	0.001342	1.44E-05	0.003001	8.66E-05	0.000303
0.147323	0.193997	0.291616	0.235296	0.326681	0.734935	0.547778	0.122554	0.709784
41.47925	27.38726	23.16622	29.49693	34.84299	30.17192	34.38341	31.1753	24.2842
0.031977	0.174646	0.282799	0.10254	0.036133	0.116724	0.052525	0.089812	0.178066
0.161962	0.130202	0.063745	0.319026	0	0.020267	2.89E-05	0.122554	0.054574
0.000996	0.012222	0.022958	0	0	0.00298	0.003001	0.010707	0.000303
0.045491	0.484704	0.00062	0.000534	0.00246	1.44E-05	0.008882	0.175036	0.000303
0.014084	0.032367	0.132626	0	0	0.015491	2.89E-05	0.010707	0.000303
5.770996	2.975974	10.73084	4.468009	2.666494	21.74051	9.667316	6.570433	4.796126
0.286522	0.236089	0.439293	0.084459	0.265455	0.51316	0.399307	0.20733	0.425844
0.323535	0.099488	0.633499	0.319026	0.504964	0.196436	0.376212	0.215859	0.566782
0.516825	1.019134	2.45772	1.810664	0.728088	1.36619	1.085967	0.679452	0.744531
0.806652	1.423506	0.31456	0.360101	0.281558	0.453932	0.249899	0.690707	0.102626
0.589618	1.809343	1.142345	0.115014	0.948211	1.327525	0.537807	0.266032	0.612872

0.589618	0.971205	0.451169	0.474214	0.307973	0.460938	0.42329	0.845317	0.511032
2.909293	2.572309	1.986681	4.814661	2.531515	2.210866	2.491948	2.248896	2.882035
0.323535	0.410541	0.932078	0.416501	0.423535	1.516219	1.019791	1.022929	2.021068
8.493088	4.292078	0.00062	0.041717	0.786667	5.770996	2.294733	0.496797	7.495397
0.092193	0.395859	1.311674	0.39062	1.872049	1.880375	0.700058	1.357201	0.242893
0.618658	0.61969	2.740519	0.571674	0.558824	1.664906	0.591616	1.119574	0.89404
29.18885	33.07147	25.48179	53.77619	67.98069	10.38202	52.56128	95.22939	30.49289
0	0.174646	0.273088	0.129971	0.086595	0.189906	0.119791	0.07013	0.178066
0.000996	9.38E-05	0.028139	0.000534	0.008175	0.027431	0.003001	0.010707	0.020577
2.964762	2.427792	1.274481	1.200823	1.616385	1.755988	1.672107	1.577569	0.669769
1.736133	1.268672	1.594675	1.540144	1.186457	1.64917	1.651522	1.815	1.079697
0.891176	1.259747	0.372973	0.21689	0.24518	0.189906	0.168961	0.198788	0.669769
0.184206	0.174646	0.256797	0.074358	0.036133	0.070209	2.89E-05	0.397215	0.10886
0.254365	0.550974	0.106926	0.343434	0.104906	0.123456	0.168961	0.535368	0.039214
0.264805	0.740527	1.579798	0.424848	1.338261	1.152165	0.708492	0.690707	0.943514
0.000996	9.38E-05	0.093009	0	0.001342	0.116724	0.005123	8.66E-05	0.046962
0.019221	0.008095	0.00062	0	0	1.44E-05	0.008882	0.007417	0.016537
11.01243	2.355289	20.16541	10.57984	5.959033	6.153102	4.353925	1.456955	2.451645
0.184206	0.228398	0.20759	0.145909	0.203593	0.303564	0.168961	0.607937	0.287951
0.002121	0.024199	0.049646	0.00303	0.01101	0.03518	0.057857	0.036869	0.178066
5.195022	6.653016	3.065108	8.429055	12.16681	4.739668	4.115844	10.57984	6.230245
0.085974	0.040159	0.256797	0.041717	0.185909	0.020267	0.161602	0.131768	0.054574
0.494185	0.312475	0.429827	0.196667	0.173853	0.504098	0.36153	0.721335	0.687431
4.402973	4.007583	9.541645	3.083319	2.936739	4.343838	7.08733	4.203001	3.824964
0.004935	9.38E-05	0.00062	0.00303	0.014675	0.001674	0.035916	0.010707	0.020577
0.753882	0.61969	1.142345	0.33785	0.829192	0.749942	0.674675	0.721335	1.074827
7.824473	5.923838	22.13245	30.49289	167.9996	6.345382	80.6518	8.048095	42.75576
0.360938	0.346494	0.50557	0.595714	0.063362	0.706984	0.47153	0.232734	0.35241
0.542165	0.24417	0.473853	0.416501	0.196667	0.196436	0.399307	0.899567	0.734113
0.6114	0.38596	3.536955	0.730476	1.608427	1.211299	1.215981	0.299942	0.474856
0.278304	0.513456	0.132626	0.079675	0.173853	0.310267	0.132799	0.166681	0.157186
0.002121	0.29842	0.384632	0.00303	0.097843	0.296955	0.075418	0.045079	0.342814
0.045491	0.825354	0.00062	0.496797	0.591118	1.873434	0.231356	0.344733	0.20733
7.737807	4.851227	7.545584	8.226219	14.17388	4.529913	10.48101	5.373781	11.83593
0.741962	0.936898	0.093009	0.489784	0.742417	1.180613	0.73583	0.472208	0.986104
0.572626	0.775339	0.970635	0.39062	0.289452	0.203593	0.338831	0.361717	0.95101
4.165137	5.238701	2.479221	3.57057	0.814315	1.744055	0.957915	0.956667	2.863211
0.059264	0.017879	0.106926	0.041717	0	0.049913	0.008882	8.66E-05	0.027006
7.653391	6.738211	7.037345	6.784762	1.821861	6.99026	3.236457	2.238341	5.84689
0.085974	0.048023	0.042078	0.019726	0.008175	0.006118	0.082973	0.010707	0.060491
0.00904	0.024199	0.03518	0.029639	0.052143	0.006118	0.003001	0.007417	0.020577
0.000996	0.012222	0.00062	0.001255	0.014675	1.44E-05	2.89E-05	8.66E-05	0.000303
1.305772	1.974228	4.072475	1.776739	2.061926	3.092229	3.330909	2.789466	2.863211
7.188095	9.160382	8.164531	5.71241	3.87329	3.346898	3.350224	1.938759	5.872886
9.218514	7.991602	8.573434	3.730808	2.306277	2.909293	2.967453	1.267085	6.30671
0.254365	0.417532	0.042078	0.089639	0.57197	0.39062	0.17671	0.141284	0.425844
0.542165	0.090851	1.374329	0.787605	0.376407	0.148254	0.300606	0.131768	0.410079
0.342987	0.629538	0.932078	0.145909	0.758773	0.382417	0.510707	0.763564	0.687431
0.067027	0.32925	0.686667	0.441833	0.289452	1.102691	0.231356	0.247417	1.321053
1.793074	1.100519	0.657114	5.123781	0.545808	0.413593	2.925426	1.446926	1.048074
1.439704	1.111241	0.772626	0.780519	0.765094	0.909315	0.682359	0.266032	1.883535
2.698557	3.851414	2.122799	2.715873	2.506934	1.394632	1.778153	2.644885	2.07658
3.425512	1.498167	0.808983	2.781558	1.758615	0.886775	3.91355	1.737294	1.710173
2.021068	1.100519	1.453939	1.415469	0.735382	1.729892	1.842734	0.411948	1.370765
0.763939	1.122359	2.600014	10.19468	0.926291	5.030866	1.816436	2.893001	0.000303
1.859084	1.21215	1.01772	0.903074	2.432121	0.921739	0.566104	1.070693	0.768535
0.130202	0.768052	0.355051	0.157316	0.173853	0.413593	0.082973	0.325527	0.325108
5.788153	3.077316	1.810664	27.10502	29.49693	5.316378	6.956349	3.820758	0.000303

0.147323	0.312475	0.239228	0.014906	0.450527	0.154978	0.42329	0.353175	0.435382
0.387489	0.581111	0.542525	0.313312	0	0.343824	0.383622	0.07013	0.000303
2.713319	2.515938	3.055729	0.798153	1.915455	2.509177	1.304877	1.77404	1.608427
8.033932	8.164531	33.07147	24.04374	28.87506	33.07147	23.6012	10.01009	42.08807
1.139885	0.070209	0.256797	1.361789	4.644098	0.310267	1.330029	2.031169	0.802395
20.16541	14.51648	7.136537	8.643074	9.218514	6.784762	9.377345	17.58415	7.200505
0.96627	3.286652	2.584394	1.147807	0.808485	1.767431	1.633752	1.022929	1.535722
3.330909	4.597792	1.392511	3.016984	2.871212	3.113478	2.727785	4.251818	4.490801
4.814661	7.519841	2.967453	11.544	9.751039	0.899567	1.99899	3.529466	6.404921
0.839365	0.79487	0.372973	1.517143	1.42456	0.428694	1.062092	1.097006	0.845317
0	9.38E-05	0.028139	0.010072	0.03088	0.077027	0.041667	8.66E-05	0.039214
0.37417	1.056898	0.114618	0.196667	1.106046	0.834957	0.445996	0.690707	0.636291
0.249278	0.174646	1.197215	0.324502	0.141378	0.437042	0.439062	0.159004	1.102691
14.97762	17.78522	9.687496	1.938759	4.946587	6.297136	10.05618	3.6029	7.315512
0.105996	0.22039	0.415729	0.079675	0.332222	0.310267	0.052525	0.079755	0.566782
3.367071	3.055729	2.281926	0.716234	0.948211	7.276645	4.251818	0.935115	3.23987
0.564127	1.185685	1.029365	0.700418	0.416782	0.758773	0.600173	0.721335	0.643001
0.000996	9.38E-05	0.042078	0	0.004899	1.44E-05	2.89E-05	0.01588	0.000303
7.112496	1.486551	5.576219	1.977316	7.697063	1.588903	1.801284	2.917381	2.928247
0.403413	0.121587	0.329524	0.620909	0.515815	0.318124	2.351299	1.267085	0.000303
0.051595	0.492165	0.00062	0.006162	0.008175	0.00298	0.024423	0.010707	0.020577
0.884654	0.784805	1.50026	1.318384	1.07127	1.278629	0.452965	0.565714	1.102691
1.249221	3.172864	3.324452	3.266782	2.474834	6.414416	2.415296	3.089293	5.976724
1.371602	1.435772	5.889798	1.797172	2.078355	3.192229	3.019848	1.852338	2.540462
0.502843	0.320144	2.696061	1.050231	0.435123	0.999502	0.531804	0.866147	1.056898
5.137042	4.851227	5.530274	4.402973	3.397706	3.722785	4.519048	4.63189	3.303925
3.286652	5.165599	4.261342	3.166659	2.577157	3.55961	3.179199	3.47531	3.703175
3.648081	1.604827	0.657114	3.414574	2.747908	2.08798	6.22031	1.112641	2.181775
1.487619	0.435123	0.329524	0.183304	0.642698	0.413593	0.908247	0.20733	0.171277
8.103925	2.31404	3.659668	10.8813	1.193362	2.018167	3.846984	1.209105	1.296905
32.68313	7.339452	8.815123	7.570693	4.709033	9.121486	11.03843	13.6814	3.799885
0.37417	0.166465	0.497749	0	0.154329	1.44E-05	2.89E-05	8.66E-05	0.612872
8.556703	8.643074	22.96463	12.22798	17.64869	12.22798	16.68554	9.141299	11.48368
5.266335	4.424416	4.034423	3.089293	3.290101	3.070952	8.940671	1.535722	7.037345
0.002121	0.048023	0.00062	0.000534	0.001342	0.00298	0.005123	0.022576	0.082027
0.634192	1.749307	0.594646	3.077316	2.768312	1.428398	1.633752	1.877605	2.283752
5.030866	8.815123	6.365065	3.775209	3.005281	6.297136	3.507691	6.365065	5.24562
1.513102	1.344257	0.910823	0.982128	1.314675	0.688182	0.931457	2.708341	0.794401
0.679069	0.359307	1.765065	0.787605	1.937222	1.248232	1.052208	1.418312	1.505678
2.698557	1.344257	1.302107	2.879228	1.259747	3.703175	3.037626	0.956667	2.138131
0.395426	9.38E-05	0.26562	1.104033	4.907929	0.688182	2.89E-05	8.66E-05	0.303016
26.52537	6.297136	46.7056	45.91841	45.91841	51.52195	23.16622	34.38341	15.88548
1.353608	2.126349	7.710361	1.096335	1.809343	6.019971	3.317807	2.178268	4.764055
0.259336	0.460938	0.497749	0.019726	0.430087	0.256652	0.089553	0.131768	0.171277
1.90798	2.690823	0.299199	1.386681	1.391494	0.758773	1.158384	1.901068	0.863694
1.156869	2.922778	4.739668	2.388348	0.926291	1.989711	0.752229	1.401977	2.847547
0.444986	0.982128	0.808983	0.539812	0.721732	1.755988	0.369336	0.036869	0.963853
1.400159	0.723506	0.521775	0	0	1.44E-05	2.89E-05	0.131768	0.000303
8.849697	9.52197	16.5625	18.19595	16.15795	11.48368	12.10289	10.1459	18.76954
0.360938	0.995527	1.403853	0.163983	0.126219	1.439704	0.271746	0.565714	0.832554
0.549019	0.79487	0.00062	0.00303	0.004899	0.55215	0.024423	0.627633	0.252165
10.1459	11.8058	9.459177	6.695887	2.24562	12.56693	5.351934	10.28573	13.75978
2.868456	2.216162	1.249221	2.752893	1.34798	1.748016	1.726032	1.209105	1.235346
0.37417	0.506652	0.34544	0.145909	0.086595	0.600173	0.17671	0.089812	0.612872
0.184206	0.901212	1.100519	0.33785	0.27075	0.617071	0.224921	0.397215	0.274365
4.402973	4.881775	2.122799	4.135801	4.990794	3.599329	3.57057	3.249877	2.270837
0.16886	0.139336	0.181227	0	0.076111	0.020267	0.068629	0.079755	0.10886
0.147323	0.673254	0.224372	0.183304	0.025844	0.343824	0.140483	0.122554	0.082027

0.439293	0.451977	0.00062	1.446926	1.886335	0.504098	0.658362	2.014863	0.135354
7.482179	4.034423	4.140505	7.710361	11.89932	4.946587	11.60283	7.225469	10.12435
4.552092	3.428788	7.795642	2.975974	2.169509	7.037345	3.672078	2.638139	5.591717
65.94551	12.42965	13.53913	72.69473	26.52537	18.91854	49.50903	36.26061	16.22108
2.217951	2.31404	3.089293	1.905094	1.251659	1.945916	1.828781	1.691587	3.227085
2.944416	1.226934	0.00062	0	4.281847	1.227749	3.439437	8.66E-05	1.744055
0.259336	1.176623	1.883535	0.331688	0.430087	0.574228	2.520404	1.323131	2.248896
4.307309	14.08569	5.22482	7.188095	4.552092	1.89373	5.654863	11.14619	3.930664
10.01009	6.922771	7.824473	3.860491	3.256587	5.195022	4.796126	6.888297	7.964315
20.34063	13.91714	18.05488	16.44768	14.70188	12.29473	14.97762	11.719	15.98892
2.944416	2.148658	2.398442	1.988196	0.386169	1.102691	1.863506	2.320072	1.930216
11.01243	22.96463	14.08569	12.03267	13.75978	8.288874	8.780115	22.96463	23.82354
0.534214	0.901212	1.382006	0.843564	0.887922	0.663276	0.974257	1.022929	0.794401
2.02956	3.796075	2.683478	2.805382	2.013182	1.561912	1.672107	2.581861	2.489762
0	0.682799	0.00062	0.02482	0	0.239589	2.89E-05	0.508975	0.089921
0.862561	2.45772	2.616573	0.999502	1.449877	1.456955	0.768052	2.225498	2.032706
0.473853	1.344257	2.542605	0.765606	1.179885	2.210866	1.443983	0.899567	1.315613
2.02956	3.307186	1.798535	1.330029	2.45575	1.394632	2.574733	3.175916	2.911962
0	0.032367	0.028139	0.000534	0.001342	0.049913	2.89E-05	0.029553	0.046962
3.070952	0.346494	3.869163	2.701053	5.696263	0.203593	2.847547	3.962179	3.718716
6.443925	13.75978	11.11876	8.381328	9.335397	18.62711	10.98644	9.459177	10.05618
2.061926	2.876421	3.57057	1.181342	2.026219	1.145426	2.114437	1.613232	2.518124
0.241991	0.435123	0.00062	0.176017	0	0.170808	0.057857	0.587965	0.394603
1.104033	5.560563	4.236962	2.429957	3.718716	4.875527	3.156537	1.565253	3.040505
6.230245	7.839271	7.417359	4.678846	3.8121	5.622872	4.739668	5.130411	6.250447
0.870902	0.656392	0.00062	0	0	0.445996	2.89E-05	0.371075	0.157186
0.270837	0.22039	0.439293	0.10254	0.076111	0.196436	0.132799	0.299942	0.149156
1.126775	2.975974	0.997792	2.955916	2.518124	2.392431	2.693442	1.754733	1.696494
1.439704	2.887489	1.100519	1.50684	0.786667	1.439704	1.121703	1.786306	1.991328
0.218355	0.600173	0.617071	0.21689	0.3514	0.617071	0.24417	0.299942	0.252165
7.780786	2.584394	2.81342	10.33476	2.855296	7.824473	4.343838	2.789466	0.602908
0.891176	1.344257	1.302107	1.215195	0.578817	1.394632	0.774798	0.480548	0.444762
0.154444	0.289755	0.50557	0.145909	0.220079	0.248341	0.212395	0.198788	0.266479
4.827691	4.900996	3.182482	8.019769	22.96463	8.075772	5.316378	6.287561	8.381328
0.799553	1.634935	0.384632	0.30215	0.62645	2.836717	0.931457	1.446926	1.608427
2.600014	3.457504	1.483622	0.999502	2.234538	2.496407	2.294733	2.320072	1.136566
0.038918	0.024199	0.106926	0.084459	0.036133	0.084069	0.068629	0.089812	0.096999
0.589618	0.38596	0.00062	0	0	0.090772	0.089553	0.299942	0.370281
0.099704	0.157316	0.256797	0.010072	0.196667	0.077027	2.89E-05	8.66E-05	0.295491
0.799553	0.564127	0.307734	1.977316	1.783203	0.899567	0.941104	0.855801	0.502843
1.971183	3.533211	0.407157	0.687049	0.802035	0.042489	0.192121	1.142345	0.234726
56.39528	60.77449	45.91841	56.39528	12.85504	52.56128	22.32214	7.964315	49.50903
11.74734	9.604358	18.76954	14.04491	11.719	11.42512	18.76954	14.6058	17.28195
0.975556	1.240202	2.221775	1.830159	1.027403	1.456955	1.304877	2.581861	1.471198
2.45575	2.270837	2.227273	1.600238	1.193362	2.217951	2.101075	1.483622	2.212619
0	0.193997	0.396472	0	0	1.44E-05	2.89E-05	8.66E-05	0.000303
0.775339	0.53469	0.396472	0.235296	0.662237	0.170808	0.309062	0.25733	0.370281
10.8813	6.876392	16.34294	40.11561	75.1132	1.44E-05	33.4985	51.52195	55.04081
0.753882	2.413146	3.953297	0.930325	0.585902	0.453932	0.630022	1.180613	1.593535
4.709033	2.283752	1.240202	2.990851	1.74539	2.027951	3.019848	1.837172	1.223846
3.439437	11.8058	4.696313	2.367763	5.25254	3.45404	2.903867	6.09759	7.288788
0.891176	0.784805	0.239228	0.129971	0.289452	0.221847	1.019791	1.180613	0.721732
0.644235	0.926291	0.00062	1.147807	1.52676	0.382417	3.799885	2.143261	0.000303
8.677489	7.099913	0.00062	14.04491	25.73165	1.483622	7.061328	20.34063	0.993521
0.286522	0.183824	0.239228	0.089639	0.191133	0.162576	0.192121	0.299942	0.333088
47.5018	64.12971	37.29684	135.5245	45.1045	111.5439	77.74812	167.9996	20.25302
3.276335	6.22031	5.366335	0.765606	2.696061	8.905642	2.520404	1.382006	6.978903
2.649675	4.540794	4.198117	3.122915	3.588369	4.105714	2.225498	4.174964	6.365065

0.992201	1.401068	0.924329	0.496797	0.735382	0.537229	1.341825	0.107828	1.261407
1.240202	3.397706	13.08214	3.314329	3.340361	7.175397	3.544257	3.648081	14.97762
0.379791	1.035801	0.896522	2.217951	4.317648	0.734935	1.411861	2.406768	0.62733
4.597792	4.508196	1.91811	12.36443	9.968355	0.921739	11.11876	9.667316	4.198117
27.38726	21.54823	45.1045	32.31123	100.0998	47.5018	27.38726	31.55417	45.1045
1.859084	9.38E-05	1.040411	1.729892	8.303947	1.44E-05	7.443276	2.99987	0.000303
1.417345	1.989711	2.019618	1.226118	1.484553	1.614257	1.452857	0.946638	1.850996
1.699091	1.401068	0.617071	0.10254	0.939408	0.483874	0.787987	0.508975	0.178066
0.00904	0.077417	0.063745	0	0.001342	0.00298	0.018182	0.098918	0.039214
3.172864	2.329784	0.671371	0.710253	1.106046	1.394632	3.486436	4.359574	2.574733
0.092193	0.024199	0.100188	0.010072	0.307973	0.437042	0.041667	0.166681	0.074069
11.60283	0.768052	0.00062	20.51271	8.149307	1.135137	7.737807	4.655916	0.000303
7.482179	2.496407	1.127475	15.66023	16.68554	1.428398	3.002576	10.93101	0.963853
0.625786	0.320144	0.174235	0.313312	0.070065	0.398059	0.219307	0.325527	0.280967
1.76873	2.427792	2.263377	1.720144	1.016494	0.98956	1.304877	1.793074	1.434055
1.111977	2.236407	1.706219	1.685678	3.450577	2.479221	1.90798	3.283139	2.740519
2.776421	6.674286	8.348817	3.984747	2.683478	4.673182	2.148658	5.409437	5.923838
1.147807	2.009906	1.543377	1.464214	0.786667	0.504098	0.81228	1.628009	0.916869
0.204661	0.564127	0.560325	0.687049	0.025844	0.646378	0.752229	0.734935	0.533874
0.799553	1.344257	1.197215	0.857778	0.926291	1.211299	1.002215	1.938759	1.370765
1.487619	2.037114	2.091313	1.568564	0.848369	0.582208	0.392403	0.518716	0.612872
0.957273	1.845519	0.407157	0.251775	0.62645	0.256652	0.700058	1.483622	0.126883
19.83727	7.938427	11.77567	21.36892	14.79742	5.217569	29.82828	53.77619	36.26061
0.002121	0.032367	0.070462	0.001255	0.008175	0.177879	0.005123	0.01588	0.032915
3.207807	1.857677	2.933903	1.965657	1.410902	2.730072	1.602619	1.803918	2.789466
7.430317	3.820758	5.25254	3.002576	4.397792	4.212554	6.335664	7.363117	5.923838
0.025274	0.157316	0.078211	0.014906	0.036133	0.049913	0.047323	0.122554	0.096999
15.276	15.22851	17.92188	23.16622	20.85876	18.33909	25.24367	12.63773	14.6058
4.165137	3.450577	4.217569	5.439278	3.38456	3.909033	3.518384	2.770866	3.917597
2.666494	2.671299	2.34355	1.035058	6.967547	5.508131	2.708341	0.325527	5.976724
0.067027	0.183824	0.141623	0.163983	0.296003	0.359307	0.616407	0.232734	0.171277
7.964315	9.667316	13.79669	8.849697	7.854069	10.17029	10.57984	17.58415	8.994791
13.23022	0.712929	1.266255	2.725498	4.212554	2.106089	9.838658	2.192496	2.335844
135.5245	95.22939	70.11932	70.11932	42.08807	95.22939	126.1556	52.56128	62.33472
0.323535	0.542828	0.396472	0.204156	0.265455	0.326017	0.148124	0.150144	0.266479
6.830592	2.909293	4.626025	4.776407	18.19595	2.676392	4.419062	6.181652	0.661443
27.66779	17.28195	10.33476	17.71696	11.30987	21.93452	30.17192	23.82354	11.36975
0	0.130202	0.00062	0.204156	0	1.44E-05	0.101609	0.122554	0.164747
0.014084	9.38E-05	0.03518	0.006162	0	0.015491	0.024423	0.010707	0.027006
1.76873	1.944517	0.692799	2.248896	1.34798	2.961768	0.931457	1.483622	0.318889
19.37139	10.1459	10.57984	57.84882	10.43066	17.15902	22.52375	37.81919	13.30906
0.117446	0.012222	0.070462	0.006162	0.019784	0.03518	0.041667	0.036869	0.096999
0.105996	0.917431	0.573326	1.111241	0.880498	0.271912	0.345036	1.022929	0.92531
0.241991	0.289755	0.896522	0.331688	0.227648	0.154978	0.42329	0.700418	0.234726
0.403413	0.252771	0.299199	0.601623	0.366912	0.39062	2.89E-05	0.480548	0.171277
13.30906	16.09482	8.412367	10.62739	9.012937	11.01243	7.363117	15.276	8.348817
1.434921	2.116032	1.594675	0.843564	0.409098	0.493247	0.504964	1.112641	0.594646
5.044113	3.659668	3.904517	5.477843	3.703175	5.01088	6.171789	9.968355	4.524481
62.33472	80.6518	29.82828	40.7932	80.6518	75.1132	45.1045	70.11932	46.7056
0	9.38E-05	0.106926	0.196667	0.091977	1.44E-05	2.89E-05	8.66E-05	0.096999
0.204661	0.032367	0.093009	0.074358	0.13114	0.027431	0.075418	0.19039	0.096999
18.76954	37.81919	53.77619	33.4985	29.82828	16.22108	19.06893	42.08807	35.27608
2.400455	4.821176	0.88619	1.340036	2.157359	1.938759	2.207417	5.485685	2.654473
0.313723	0.723506	0.337013	0.246652	0.154329	0.493247	0.331097	0.451688	0.435382
1.132857	1.64917	1.127475	0.936898	1.214315	1.828781	1.595729	3.156537	5.47
2.925426	3.128694	0.772626	1.856392	0.538117	0.970007	0.413304	3.464315	3.464315
10.31025	6.443925	3.659668	4.833398	3.083319	4.586753	4.007583	3.993665	4.312251
0.634192	0.557056	0.852684	0.765606	0.753016	0.467677	0.524646	1.493968	0.744531

83.489	70.11932	31.1753	35.27608	31.1753	31.92385	46.7056	57.84882	51.52195
0.014084	0.083983	0.070462	0.010072	0.036133	0.006118	0.018182	0.089812	0.089921
0.661443	0.520289	1.040411	1.662439	1.734949	0.852172	3.002576	0.763564	1.079697
0.031977	0.012222	0.063745	0.00303	0.112569	0.077027	0.108341	0.022576	0.102626
0.852172	1.277843	1.01772	1.238651	1.117316	1.035801	1.661328	1.92272	1.21215
6.287561	15.56131	9.012937	3.750837	4.048499	7.737807	3.621941	7.92518	5.728615
3.909033	4.212554	5.295325	3.55583	3.110447	3.414574	2.308203	2.216162	5.560563
0.249278	0.492165	0.049646	0.006162	0.080837	0.39062	2.89E-05	8.66E-05	0.221587
1.603795	2.45772	2.50469	2.735368	5.923838	4.63189	7.765931	2.445079	5.158146
0.786667	1.094206	3.016984	1.181342	1.886335	3.101371	2.08632	1.938759	2.126349
0.618658	0.425844	1.443983	0.360101	0.982698	0.290317	0.309062	1.012114	0.986104
22.52375	5.123781	9.397771	28.54786	10.93101	7.710361	12.63773	6.268312	6.612121
2.236407	0.275238	1.050231	0.424848	3.043528	0.921739	1.739935	1.677042	2.157359
0.65202	1.226934	0.868341	0.745613	1.410902	0.608543	1.019791	0.159004	0.881082
0.117446	1.311674	0.307734	0.456522	0.47153	0.326017	0.856782	4.251818	0.721732
24.04374	34.38341	26.80355	26.52537	35.27608	30.82277	40.7932	33.93939	41.47925
4.927302	2.000592	11.48368	8.256465	3.529466	5.662994	9.707677	2.320072	4.776407
0.218355	0.513456	0.123377	0.474214	0.954141	0.239589	0.331097	1.277843	1.223846
8.075772	10.48101	6.570433	7.625209	7.653391	5.576219	6.011515	9.541645	8.075772
33.07147	9.707677	30.82277	0.737403	36.74522	20.85876	32.68313	40.7932	11.57341
2.31013	0.723506	4.580938	1.40772	0.998831	2.818312	2.069942	0.911364	5.095325
0.37417	0.29842	0.384632	0.416501	0.485325	0.27811	0.237359	0.434069	0.425844
7.991602	15.22851	6.591544	4.160238	3.407619	4.165137	3.217612	8.625613	5.560563
6.706472	0.723506	100.0998	12.93651	1.159149	2.451645	3.019848	2.296732	3.153485
3.350224	2.671299	6.8421	7.532713	3.687633	5.044113	6.760765	3.679986	1.403853
3.070952	4.745895	5.210317	3.921645	3.35355	3.370527	3.94886	3.156537	7.683766
1.120332	3.163506	3.435988	1.568564	1.903636	4.125786	1.317475	3.110447	2.939141
0.00904	0.017879	0.03518	0.006162	0.025844	0.001674	0.035916	0.122554	0.082027
16.09482	9.063983	10.8813	31.1753	10.98644	9.063983	16.9162	35.52604	21.54823
6.153102	3.077316	5.813247	3.511263	4.907929	4.552092	9.459177	3.377619	1.519322
3.5257	2.270837	0.98474	3.198499	5.515584	1.492973	2.225498	3.343629	1.535722
11.544	7.882222	4.845166	6.865	9.625253	5.530274	12.29473	17.78522	5.068831
7.08733	8.288874	4.140505	4.381479	4.160238	3.663622	5.11697	5.788153	5.439278
0.360938	0.591118	0.132626	0.157316	0.332222	0.359307	0.498052	0.597042	0.474856
0.067027	0.056638	0.181227	0.047431	0.320332	0.221847	0.24417	0.313102	0.342814
0.618658	0.581111	0.384632	0.176017	0.653203	0.437042	0.47153	0.576205	0.721732
1.477388	1.927042	3.486436	1.088687	1.443059	2.378124	1.507835	1.901068	2.378124
0.286522	1.578687	1.403853	0.474214	0.829192	1.953535	0.856782	0.834416	2.091313
9.667316	8.134084	18.62711	24.2842	25.24367	22.96463	28.54786	15.18102	13.23022
1.232886	1.240202	1.553074	1.426522	1.410902	1.806595	1.411861	1.859084	1.252424
0.352807	0.802756	3.7586	0.351595	0.220079	1.343463	1.076652	0.150144	1.048074
1.232886	3.266782	7.013853	3.6029	3.414574	3.346898	4.096407	7.288788	6.978903
1.371602	19.83727	4.552092	1.988196	1.146118	2.944416	1.548723	1.677042	4.56912
4.217569	2.800332	1.23127	4.678846	13.42179	2.807937	2.993867	8.195657	2.708341
35.27608	19.68088	17.45948	22.96463	13.42179	13.6814	15.98892	9.012937	15.07257
5.34474	2.08632	155.7103	9.335397	18.48514	2.323983	1.87899	5.287857	35.776
2.45575	2.496407	2.122799	0.65798	1.186457	1.860491	5.056421	0.876638	2.402468
0.403413	0.70329	0.307734	2.009906	9.179466	2.165851	0.863694	2.470317	0.709784
4.845166	11.14619	4.552092	8.608153	7.251263	2.66404	2.836717	2.604776	5.082063
2.359264	3.939307	5.560563	2.470317	2.81342	7.124517	1.801284	3.249877	7.417359
0.747879	0.183824	0.858817	1.607143	0.914618	0.170808	0.760433	0.371075	0.185166
0.004935	9.38E-05	0.028139	0.000534	0.008175	0.027431	0.005123	8.66E-05	0.000303
3.648081	5.71241	7.765931	5.024293	2.725498	3.540606	3.750837	5.950664	4.286962
15.3618	25.99427	21.74051	19.68088	24.04374	21.19547	18.19595	24.50942	27.96248
0	0.008095	0.022958	0	0.001342	0.001674	0.003001	0.007417	0.016537
0.138326	0.166465	0.337013	0.189632	0.119683	0.109185	0.063362	0.175036	0.178066
87.1746	57.84882	48.53429	25.48179	13.96064	24.50942	37.29684	16.80343	27.38726
4.770231	2.176501	1.05824	0.765606	0.642698	2.009906	1.828781	0.956667	0.709784

0.000996	9.38E-05	0.028139	0.001255	0.004899	1.44E-05	0.293038	0.022576	0.054574
23.6012	32.31123	65.94551	7.013853	14.08569	39.53687	12.46209	4.145209	47.5018
2.802828	3.578038	3.57057	2.794935	2.384141	3.629177	3.201457	7.149618	4.272165
0.025274	9.38E-05	0.00062	0.02482	0.025844	0.03518	2.89E-05	0.029553	0.228788
0.618658	1.007244	0.123377	0.21689	0.112569	0.06355	0.082973	0.25733	0.266479
0.598456	0.32925	0.429827	0.519387	0.609177	0.042489	0.439062	0.224762	1.136566
287.197	111.5439	57.84882	64.12971	126.1556	46.7056	57.84882	105.1694	155.7103
5.373781	13.6814	9.968355	9.625253	7.625209	12.70768	4.557605	7.315512	9.880779
10.23768	20.16541	8.923117	8.508514	8.958225	12.16681	9.968355	18.91854	17.92188
1.456955	0.834416	2.683478	2.652107	3.6029	2.713319	6.486104	3.377619	3.529466
749.7839	287.197	51.52195	203.5795	135.5245	144.7919	332.0776	332.0776	167.9996
8.556703	16.44768	8.539971	7.938427	6.508427	11.14619	11.89932	19.68088	10.57984
1.859084	3.614603	1.553074	0.843564	0.970007	1.472208	1.516219	4.174964	1.666089
1.168326	1.156869	2.083261	2.647244	3.917597	1.664906	1.896667	1.554185	2.540462
2.119596	2.720996	3.172864	5.238701	2.823636	2.355289	5.210317	6.250447	2.410996
4.246861	13.53913	8.958225	6.414416	3.428788	6.956349	6.287561	20.68746	6.423911
28.23632	59.32013	8.477662	4.484892	10.1459	65.94551	11.96574	3.962179	26.80355
0.687049	1.111241	0.0857	0.151688	0.301082	1.343463	0.184358	0.045079	0.403651
1.371602	3.975498	3.78798	5.662994	10.1459	0.653586	5.788153	24.73724	10.23768
29.49693	52.56128	60.77449	27.66779	21.36892	34.38341	33.07147	26.52537	34.84299
77.74812	46.7056	49.50903	65.94551	32.31123	48.53429	42.08807	38.94854	56.39528
5.830152	4.174964	8.728571	3.66785	5.01088	13.83359	11.719	2.730072	13.53913
34.84299	12.29473	25.73165	7.468961	8.303947	15.88548	11.39744	10.5063	33.07147
4.261342	5.47	6.749488	3.122915	1.809343	3.113478	4.557605	3.771349	5.560563
9.335397	8.381328	13.00996	17.15902	11.83593	15.77678	13.42179	5.082063	12.77772
0.313723	1.259747	1.29267	1.426522	1.793074	0.955418	0.095303	0.690707	6.796566
1.762713	4.46267	0.932078	5.71241	3.8121	1.37531	8.381328	4.513622	3.895469
30.49289	31.1753	42.08807	43.88335	47.5018	23.82354	24.2842	18.48514	38.39042
0.254365	0.550974	0.093009	0.204156	0.086595	0.109185	0.101609	0.535368	0.164747
0.286522	1.385801	0.88619	0.223211	0.46153	0.493247	0.47153	0.488759	0.902403
13.75978	1.857677	12.16681	3.703175	10.78056	0.55215	2.747908	4.338629	2.190866
5.316378	0.926291	11.42512	5.302294	10.38202	8.256465	5.9071	7.519841	10.73084
3.297107	2.176501	0.761688	1.411861	0.485325	0.582208	1.112641	3.188918	1.113304
3.944084	3.23987	2.654473	3.703175	3.734762	1.945916	7.315512	4.827691	2.805382
0	0.012222	0.057352	0.000534	0	0.006118	0.005123	0.022576	0.000303
42.75576	30.49289	17.78522	23.82354	24.2842	13.15079	21.36892	50.55949	21.93452
87331.38	144.7919	87331.38	749.7839	87331.38	1360.293	87331.38	749.7839	87331.38
224.9555	425.62	530.1465	95.22939	184.443	530.1465	224.9555	118.7028	749.7839
1.447994	0.936898	1.100519	0.450051	0.926291	2.126349	1.158384	0.480548	0.709784
23.38378	25.24367	19.22139	18.33909	23.16622	16.09482	25.73165	26.25557	16.34294
0.42767	0.732633	0.384632	0.33785	0.57197	0.834957	0.445996	1.554185	1.074827
3.147294	5.889798	3.966623	3.38456	4.251818	1.839986	2.747908	3.6029	2.928247
0.111068	0.183824	0.247085	0.068896	0.345635	0.090772	0.161602	0.107828	0.164747
0.051595	0.040159	0.679069	0.235296	0.019784	0.230606	0.119791	0.07013	0.234726
0	9.38E-05	0.028139	0	0	0.03518	2.89E-05	0.01588	0.000303
9.838658	4.302367	4.165137	13.08214	6.454416	3.842554	4.160238	5.351934	9.297085
2.815866	2.094574	55.04081	6.591544	12.32958	6.760765	2.887489	2.958773	23.6012
0.211905	0.157316	0.057352	0.223211	0.076111	0.027431	0.035916	0.079755	0.096999
57.84882	45.91841	35.776	48.53429	70.11932	33.93939	70.11932	34.84299	126.1556
0.00904	0.032367	0.141623	0.00303	0.025844	1.44E-05	0.052525	0.062857	0.000303
12.03267	13.45939	30.49289	28.0994	24.73724	14.6058	19.68088	16.22108	25.24367
0.352807	0.460938	0.00062	0.10254	0.063362	0.03518	0.101609	0.313102	0.000303
1.040411	1.553074	0.705231	0.675938	0.376407	1.227749	2.789466	1.565253	1.608427
0.306176	0.174646	0.473853	0.163983	0.141378	0.183672	0.035916	0.215859	0.066789
0	0.008095	0.028139	0	0.004899	1.44E-05	0.003001	8.66E-05	0.000303
1.329206	3.707136	1.863506	0.650101	1.037958	2.5271	0.908247	1.837172	1.808023
34.38341	27.96248	36.74522	20.68746	27.96248	33.4985	21.74051	28.54786	22.52375
9.121486	15.98892	9.218514	2.818312	7.375794	17.39935	5.872886	4.286962	11.42512

11.2819	12.10289	8.241342	7.737807	11.36975	5.395584	11.30987	10.40634	7.765931
6.181652	8.319019	9.625253	8.976508	4.179762	6.717056	5.00404	5.872886	6.8421
0.870902	1.523824	3.370527	1.699091	1.702706	2.361198	1.87899	0.922879	2.708341
0.004935	9.38E-05	0.00062	0.001255	0.008175	1.44E-05	0.008882	0.007417	0.032915
24.73724	16.68554	14.33939	14.42661	33.93939	6.738211	11.83593	29.49693	16.44768
20.68746	15.66023	12.85504	6.830592	15.46193	11.09133	20.51271	14.97762	13.6814
1.971183	3.227085	1.718918	1.010887	0.16145	2.074906	1.578687	0.380447	1.629235
2.909293	2.000592	1.677042	1.165873	0.765094	0.886775	1.516219	1.43039	2.99987
2.187395	2.597792	0.750404	1.261407	1.170491	0.950418	1.002215	2.048781	1.150635
19.06893	11.42512	16.68554	10.38202	5.654863	11.544	7.991602	7.264502	6.945152
2.600014	0.647345	4.802655	1.330029	1.511198	1.248232	1.595729	1.105426	1.130115
3.330909	3.227085	2.828918	4.338629	2.565556	7.6393	7.911934	1.082915	4.881775
13.91714	15.88548	21.54823	10.1028	13.6814	10.1028	11.48368	16.5625	12.49452
10.67991	15.77678	4.174964	25.99427	10.62739	45.1045	31.1753	12.10289	53.77619
0.059264	1.435772	0.049646	0.135051	0.01101	0.084069	0.029978	0.576205	0.126883
15.98892	13.30906	11.19942	15.46193	6.760765	13.30906	14.79742	8.888167	6.079596
0.241991	0.682799	0.100188	0.047431	0.154329	0.256652	0.063362	0.20733	0.126883
15.18102	22.52375	27.10502	14.25954	23.38378	37.81919	18.05488	18.62711	29.49693
0.564127	0.048023	0.808983	0.319026	2.092987	0.148254	0.119791	0.380447	0.303016
5.34474	2.616573	0.671371	7.149618	31.55417	2.097843	2.693442	6.53969	3.511263
1.428398	0.629538	0.26562	0.519387	0.376407	0.214199	0.73583	1.992944	0.046962
0.542165	0.425844	1.718918	0.62689	0.289452	1.382006	1.389509	0.496797	0.199264
33.93939	36.26061	8.728571	21.74051	17.51961	27.66779	7.288788	13.53913	13.91714
0.33329	0.166465	0.247085	0.068896	0.13114	0.096739	0.431198	0.285079	0.252165
14.17388	47.5018	4.120815	9.459177	25.99427	10.60361	5.959033	12.77772	15.46193
4.770231	6.900202	5.130411	10.23768	6.653016	4.053506	9.256537	14.08569	7.013853
0.111068	0.048023	0.049646	0.06381	0.019784	0.020267	0.005123	0.159004	0.14202
6.404921	10.57984	5.993045	6.079596	6.922771	5.745996	5.889798	11.60283	3.799885
2.600014	3.820758	1.443983	0.866717	0.393983	1.713997	3.425512	1.357201	0.577706
0.724502	0.740527	0.307734	0.308903	0.147121	0.154978	0.237359	0.380447	0.474856
0.342987	0.901212	0.808983	0.196667	1.128131	0.148254	0.57101	0.876638	0.000303
4.02088	2.209141	1.069574	2.513752	0.854733	1.64917	0.616407	2.096162	1.1629
21.36892	15.3618	9.792626	11.77567	14.25954	2.520404	19.99861	20.16541	14.70188
15.56131	14.42661	25.99427	42.75576	5.416623	29.49693	26.80355	2.107922	1.593535
1.316544	2.720996	0.730873	0.910823	0.747879	0.955418	0.609177	1.277843	1.062821
0.038918	9.38E-05	0.00062	0.02482	0.025844	0.109185	0.052525	0.062857	0.089921
0.510072	0.157316	0.821898	0.246652	0.086595	0.049913	0.28443	0.55645	0.096999
2.316075	2.413146	0.843102	0.319026	1.519322	2.750375	0.404704	1.446926	0.486551
0	0.017879	0.03518	0.006162	0.104906	0.006118	2.89E-05	8.66E-05	0.096999
0.092193	0.995527	0.256797	1.129452	0.765094	0.981558	1.253752	1.446926	1.903636
0.917431	2.474834	1.01772	0	0	1.44E-05	0.383622	0.388831	1.030022
0.031977	0.656392	0.26562	0.06381	0.00246	0.154978	0.082973	0.029553	0.295491
2.831501	3.989206	2.136335	2.347439	2.276407	0.981558	1.569553	1.915455	1.252424
0.395426	0.759646	0.355051	0.014906	0	0.663276	0.082973	8.66E-05	0.266479
0.002121	0.012222	0.00062	0.135051	0.036133	0.049913	2.89E-05	0.089812	0.074069
0.051595	0.121587	0.188276	0.06381	0.112569	0.096739	0.089553	0.183175	0.14202
3.26	5.80469	1.365296	1.761349	2.106089	1.545657	2.681169	2.618846	1.278629
1.710173	0.70329	2.148658	0.465325	0.861429	1.299567	1.019791	0.690707	0.453218
0.16886	8.923117	8.608153	9.297085	3.747035	1.44E-05	2.708341	8.66E-05	6.760765
3.699495	1.664906	0.714286	2.078355	6.876392	1.060823	7.037345	1.220628	1.519322
72.69473	17.92188	21.02638	75.1132	144.7919	15.276	39.53687	75.1132	16.5625
0.197547	0.202388	0.0857	0.014906	0.091977	0.196436	0.108341	0.388831	0.117446
0.48583	0.723506	0.463867	0.047431	0.386169	0.445996	0.205382	0.653586	0.453218
0.524315	1.344257	0.962698	0.866717	1.082915	1.04228	0.716652	0.81544	1.183478
1.550837	3.726854	5.720173	2.542605	0.867814	3.518384	2.967453	4.814661	6.497266
2.730072	0.656392	0.00062	0.246652	1.727323	0.663276	1.492973	0.397215	0.000303
0	0.008095	0.042078	0.001255	0	1.44E-05	2.89E-05	0.007417	0.039214
2.047157	1.91811	2.852677	2.035678	1.656696	1.806595	2.329784	1.754733	2.371833

2.400455	1.456955	0.560325	2.078355	2.911962	2.873817	1.888023	1.613232	3.153485
1.073543	0.893016	4.490801	7.037345	2.178268	5.266335	2.101075	1.05088	3.625382
0.241991	0.077417	0.057352	0.079675	0.24518	0.049913	0.089553	0.029553	0.096999
5.553146	14.97762	5.165599	5.9071	4.709033	7.276645	4.160238	11.25392	6.922771
3.971061	3.172864	6.011515	8.303947	2.602395	8.048095	2.681169	3.869163	3.864827
0.944084	1.664906	2.45772	0.424848	1.367179	0.909315	1.540144	0.480548	1.546696
0.360938	0.740527	0.093009	0.183304	1.8386	1.04228	0.237359	0.353175	0.821898
0.473853	0.236089	0.100188	0.196667	0.057518	0.248341	0.101609	0.275058	0.417532
2.898196	1.64917	2.173081	3.016984	4.025267	3.687633	6.153102	7.668579	3.314329
1.995714	0.712929	1.311674	1.20653	2.531515	3.031688	2.860722	4.513622	1.962424
37.29684	43.49658	34.38341	26.80355	21.74051	15.46193	27.96248	35.52604	38.94854
0.775339	0.61969	1.20824	0.539812	0.566104	0.82588	1.190491	1.012114	0.821898
0.014084	0.048023	0.028139	0	0.008175	0.020267	0.005123	0.054156	0.089921
0.419596	0.910339	0.800967	0.595714	0.400967	0.574228	0.623146	0.597042	0.92531
0	9.38E-05	0.429827	0	0	0.318124	0.168961	0.166681	0.000303
0.147323	9.38E-05	0.473853	1.708932	0	0.310267	0.459394	0.518716	0.000303
16.85982	14.84163	16.22108	10.78056	10.01009	12.81638	13.23022	10.98644	12.42965
5.295325	7.049336	14.51648	18.76954	18.33909	14.17388	17.28195	11.96574	18.33909
0.644235	0.926291	0.224372	0.21088	0.416782	0.453932	0.36153	0.607937	0.734113
7.136537	9.38E-05	5.180058	8.119004	4.440548	1.416407	7.854069	4.440548	2.556443
1.566414	9.38E-05	0.868341	0.196667	1.082915	0.248341	0.184358	0.175036	0.000303
2.45575	3.640606	2.24733	1.729892	1.680895	3.166659	2.380079	2.542605	3.040505
1.603795	2.07658	0.463867	0.368658	0.60202	0.183672	0.908247	1.401977	0.14202
0.387489	0.572266	0.20759	0.287951	0.289452	0.264654	0.28443	0.587965	0.252165
12.56693	11.19942	4.529913	4.72798	5.295325	5.881342	12.03267	5.42381	1.261407
1.793074	2.323983	2.852677	1.157626	1.460981	2.500548	2.887489	0.998831	2.323983
0.467136	0.435123	0.624473	0.539812	0.55215	0.405599	0.716652	1.290065	0.252165
1.201724	0.520289	0.141623	0.798153	0.670144	0.776349	0.716652	1.082915	0.832554
20.85876	11.03843	10.5063	19.22139	20.34063	8.210938	21.93452	10.73084	7.532713
2.129596	3.137576	3.460909	2.693442	4.770231	1.189769	1.274481	1.677042	1.278629
0.917431	0.607605	0.601623	1.261407	2.400455	0.672468	0.98474	0.946638	0.403651
1.592395	9.38E-05	4.034423	0.526299	6.019971	0.845887	2.160649	8.66E-05	4.490801
0.671371	0.692799	0.291616	0.666847	0.867814	0.310267	0.646861	0.518716	0.774798
5.864084	3.771349	0.730873	4.120815	4.655916	3.442886	6.830592	0.922879	1.666089
4.952915	3.489899	4.016465	1.446926	1.968283	4.696313	3.691724	3.273045	2.261573
0.147323	9.38E-05	0.256797	0.035584	0.097843	0.06355	0.008882	0.19039	0.000303
0.96627	1.32482	0.821898	0.810967	1.052208	0.768535	0.55741	1.130115	0.774798
1.111977	1.722475	1.65785	0.843564	1.299567	2.018167	1.682193	2.143261	2.77899
9.179466	8.195657	8.508514	9.880779	5.9071	16.5625	8.048095	3.377619	9.502294
0.147323	0.063405	0.20759	0.135051	0.409098	0.049913	0.309062	8.66E-05	0.082027
0.862561	0.395859	0.705231	1.035058	1.008413	0.981558	0.708492	0.496797	0.669769
0.323535	0.425844	0.282799	0.183304	0.926291	0.697085	1.197922	0.198788	0.687431
17.92188	7.288788	5.316378	5.993045	16.34294	5.485685	6.125823	13.30906	5.638759
0.197547	0.404704	0.174235	0.441833	0.173853	0.189906	0.205382	1.237879	0.054574
0.534214	0.682799	0.224372	0.196667	1.460981	1.492973	1.242641	1.334762	1.370765
0	0.017879	0.028139	0.00026	0.000837	0.006118	0.003001	0.029553	0.000303
0.444986	0.275238	1.197215	0.666847	0.376407	0.724004	0.856782	0.411948	0.721732
3.350224	2.531515	1.750628	1.306667	5.11697	1.678319	1.085967	1.77404	2.640332
0.323535	0.29842	1.382006	0.765606	0.393983	0.672468	1.522597	2.259661	1.085224
1.400159	4.402973	4.490801	1.446926	2.644885	3.588369	0.413304	2.124574	2.593506
0.031977	9.38E-05	0.00062	0.035584	0.001342	0.03518	0.041667	0.022576	0.054574
22.74797	14.6058	44.27012	29.18885	28.54786	12.46209	50.55949	12.49452	32.68313
0.929812	0.982128	2.616573	1.121703	1.391494	3.953297	1.975772	0.667583	1.947316
0.184206	0.090851	0.181227	0.053579	0.112569	0.183672	0.17671	0.054156	0.074069
6.99026	10.90615	5.654863	2.847547	8.477662	5.287857	3.640606	13.15079	3.303925
1.781948	2.116032	2.554069	2.522684	6.019971	0.617071	4.007583	5.095325	2.116032
53.77619	28.87506	24.50942	49.50903	57.84882	31.55417	64.12971	64.12971	40.7932
0.671371	1.701616	0.00062	0.241255	0.16145	0.852172	0.140483	0.442193	0.594646

0	9.38E-05	0.00062	0.000534	0	1.44E-05	2.89E-05	8.66E-05	0.000303
0.494185	0.775339	6.454416	8.164531	6.22031	5.462475	15.18102	2.459812	2.690823
1.29267	1.385801	4.888023	1.298665	0.786667	3.966623	2.283752	1.628009	1.458038
0.099704	0.312475	0.299199	0.176017	1.037958	0.706984	0.577388	0.215859	0.993521
0.138326	0.252771	0.132626	0	0.019784	1.44E-05	0.018182	0.022576	0.157186
36.26061	20.51271	50.55949	30.82277	43.49658	3.175916	12.46209	12.36443	1.198701
4.456912	2.737944	2.238341	2.263377	2.577157	3.767489	3.47531	3.450577	2.911962
0.233434	1.411861	0.407157	0.504416	0.400967	0.221847	0.376212	0.855801	0.333088
7.363117	7.519841	4.297222	6.570433	6.181652	3.070952	4.212554	6.8421	5.508131
1.498167	1.240202	0.781977	0.982128	0.728088	1.561912	0.793434	0.777864	1.085224
31.92385	10.43066	6.808369	17.03919	8.445743	6.956349	9.082828	7.824473	7.443276
0.451688	1.156869	0.560325	0.579495	0.642698	1.579798	0.609177	0.748341	0.832554
0.014084	0.077417	0.0857	0.014906	0.036133	0.10254	0.057857	0.215859	0.046962
0.031977	0.139336	0.00062	0.001255	0	1.44E-05	0.008882	0.036869	0.027006
1.016494	1.906551	1.050231	0.903074	0.829192	0.758773	0.92342	1.901068	1.102691
10.57984	36.74522	28.23632	11.03843	17.28195	24.2842	13.83359	12.22798	17.58415
0.644235	1.779423	0.528571	0.595714	0.27075	0.183672	0.452965	1.391494	0.577706
4.125786	4.821176	1.453939	6.663651	3.38456	2.165851	0.524646	2.248896	0.518716
0.524315	1.02798	0.551284	0.650101	0.5229	0.633853	1.492973	0.911364	0.543131
4.286962	4.261342	4.323045	2.176501	4.376118	3.223882	3.037626	3.317807	4.424416
18.33909	0.664423	18.91854	10.28573	6.454416	1.839986	3.317807	3.771349	0.463485
0.852172	1.056898	0.273088	0.183304	0.376407	0.483874	0.212395	0.463485	0.126883
0	0.012222	0.028139	0	0	0.00298	0.005123	8.66E-05	0.000303
3.043528	2.936739	5.409437	3.279625	2.306277	3.734762	5.462475	5.485685	3.651898
0.019221	0.099488	0.00062	0.006162	0	0.00298	0.013146	0.107828	0.039214
0.700837	0.22039	0.772626	0.343434	0.5229	0.784372	0.781977	0.653586	0.577706
55.04081	77.74812	20.34063	26.25557	50.55949	27.38726	26.25557	27.38726	33.4985
0.138326	0.732633	0.00062	0.151688	0.070065	0.359307	0.591616	0.335599	0.000303
0.00904	0.024199	0.00062	0.000534	0.004899	0.006118	0.003001	0.036869	0.016537
0.502843	0.784805	0.51316	1.34513	0.898543	1.343463	2.207417	1.277843	1.358189
2.02956	4.365224	7.30215	0	0	3.648081	2.89E-05	1.972706	1.393571
13.6814	0.228398	4.402973	16.22108	0.326681	2.199639	3.397706	0.721335	2.335844
3.207807	0.506652	6.99026	6.316328	10.1459	2.151919	8.164531	2.259661	2.953059
0.661443	0.638362	0.224372	0.21088	0.259033	0.239589	0.199488	0.576205	0.394603
0.826407	1.392511	2.382035	0.866717	1.027403	1.716364	1.104805	1.082915	2.425801
1.362713	1.066234	0.679069	0.531486	0.289452	1.327525	0.653586	2.432121	1.519322
2.964762	2.685786	1.889711	1.40772	1.616385	1.204228	1.429365	0.876638	2.586926
0.123968	0.083983	0.00062	0.058773	0.080837	0.090772	2.89E-05	8.66E-05	0.259646
2.936739	6.019971	1.579798	1.614257	1.539062	4.655916	3.236457	1.130115	3.182482
0.37417	1.122359	0.123377	0.551861	0.492417	1.285584	0.542165	0.175036	0.881082
0.661443	1.64917	0.714286	0.257684	0.3514	0.641631	0.293038	0.291025	0.774798
1.672107	2.027951	2.642525	4.563117	5.889798	2.563506	2.294733	3.404372	4.451154
0.051595	0.32925	0.049646	0	0.014675	0.01062	0.035916	0.24	0.000303
7.162698	6.125823	9.397771	8.677489	38.39042	6.116638	9.418196	13.83359	5.330519
0.233434	0.183824	0.03518	0.21689	0.259033	0.10254	0.075418	0.062857	0.020577
1.261407	3.256587	1.182771	3.497085	4.451154	1.767431	4.609076	12.93651	1.198701
1.156869	4.086926	1.23127	2.212619	1.29342	1.408831	0.870902	1.502323	0.832554
0.763939	2.386248	0.917994	2.131176	1.758615	1.159149	0.992771	8.66E-05	0.266479
0.038918	0.520289	0.239228	0.407157	0.063362	0.326017	0.101609	0.141284	0.185166
3.714906	0.395859	5.967879	5.302294	2.618846	1.664906	2.018167	1.957958	3.578038
2.016515	2.81342	5.266335	1.40772	1.519322	4.827691	1.952027	1.737294	2.805382
9.541645	10.1028	11.03843	9.792626	14.88584	4.802655	16.39531	13.75978	10.82954
3.734762	5.062626	14.6058	11.83593	8.888167	6.695887	8.608153	6.181652	15.276
2.400455	0.70329	3.536955	0.482367	1.410902	0.790837	0.399307	0.266032	5.00404
0.775339	2.037114	0.407157	0.095931	0.320332	0.566782	0.674675	0.823983	0.687431
0.395426	1.100519	0.852684	0.068896	0.46153	0.566782	0.338831	0.480548	0.698687
2.296732	0.550974	0.00062	1.473218	4.353925	1.44E-05	4.397792	1.706219	1.010887
1.447994	3.592063	1.25816	2.453781	2.189235	1.237879	3.363911	10.62739	1.721371

7.339452	4.94026	5.545729	4.02088	6.738211	6.601833	9.179466	6.404921	7.991602
1.305772	1.411861	0.649235	0.368658	0.530851	0.537229	0.674675	1.220628	0.734113
3.381255	2.671299	3.160022	2.078355	1.68671	5.172828	3.074134	3.019848	2.628225
0.598456	2.116032	0.940563	0.551861	1.855108	1.25886	0.863694	1.156869	1.062821
0.42767	1.311674	1.20824	0.551861	0.786667	1.394632	1.011486	0.690707	1.337431
2.061926	1.456955	0.320931	1.035058	2.061926	3.346898	0.92342	0.640289	1.434055
9.707677	22.32214	38.94854	7.824473	17.15902	25.73165	36.26061	10.23768	18.55613
8.994791	6.945152	7.061328	6.53969	4.802655	6.486104	3.767489	4.733824	8.694697
3.775209	10.53159	3.514834	2.92013	2.290996	3.529466	5.42381	5.591717	3.754719
11.89932	1.906551	0.00062	13.6814	22.13245	9.880779	23.38378	6.888297	3.279625
1.566414	1.793074	3.137576	2.221775	0.642698	2.378124	4.292078	1.220628	2.306277
1.487619	1.837172	0.750404	1.880375	2.947287	1.358189	1.801284	0.667583	1.480541
1.845519	1.492973	2.765758	1.111241	2.35733	2.243911	1.726032	1.070693	3.88153
0.294921	0.353333	0.057352	1.415469	3.110447	0.133658	0.577388	2.048781	0.089921
0.191602	1.701616	0.50557	0.819978	0.57197	0.672468	0.510707	0.508975	0.81228
0.117446	0.591118	0.917994	0.504416	0.55215	0.109185	0.132799	0.640289	0.753016
0.724502	1.89373	0.100188	0.129971	0.147121	0.049913	0.126335	0.763564	0.502843
2.187395	1.927042	1.412771	1.848297	0.386169	0.776349	0.73583	0.721335	0.474856
0.111068	0.712929	0.042078	0.324502	0.180505	0.421804	0.113911	0.036869	0.533874
1.066948	1.185685	1.150635	1.415469	0.906126	0.672468	0.708492	4.145209	1.821861
0.161962	0.852684	0.00062	10.01009	3.034747	1.44E-05	2.89E-05	2.320072	0.266479
4.086926	6.706472	3.7586	2.529307	2.978968	3.083319	3.236457	3.57057	3.944084
3.147294	2.750375	3.722785	0.843564	1.094206	2.009906	2.642525	1.824892	1.744055
0.00904	0.040159	0.063745	0.00303	0.03088	0.070209	0.095303	0.098918	0.311227
3.122915	3.953297	2.083261	4.875527	5.638759	2.8657	6.464906	9.012937	1.445916
15.88548	13.15079	7.598312	8.711905	11.65791	12.81638	14.47154	12.03267	17.78522
0.700837	1.007244	0.896522	0.88619	0.275382	1.89373	0.369336	0.198788	0.463485
2.367763	5.302294	3.391075	3.207807	6.632063	2.975974	2.789466	6.054242	4.227302
0.556126	0.425844	0.821898	0.38153	0.126219	0.221847	0.24417	1.972706	0.648961
0.038918	0.404704	0.028139	0.145909	0.705613	0.090772	0.212395	0.335599	1.235346
1.710173	2.768312	1.443983	1.797172	3.57057	3.011169	2.487576	1.52676	1.696494
6.063276	2.134545	13.60866	7.061328	5.454949	4.46267	6.38456	7.854069	6.011515
4.502453	3.150433	3.198499	1.880375	3.276335	4.927302	4.317648	4.888023	11.03843
0.249278	0.520289	0.188276	0.229654	0.259033	0.230606	0.184358	0.480548	0.234726
0.352807	0.28189	0.149755	0.21088	0.211631	0.351595	0.15456	0.335599	0.164747
0.43381	0.600173	0.535029	0.92342	0.136356	0.566782	0.101609	0.299942	0.385512
1.971183	2.153535	3.104394	0.562439	0.880498	1.189769	1.548723	1.060823	0.986104
4.125786	6.508427	9.880779	7.327482	9.101674	17.64869	4.715599	4.563117	7.795642
0.019221	9.38E-05	0.157468	0.041717	0.036133	0.084069	0.192121	0.054156	0.126883
0.197547	0.107749	0.157468	0.019726	0.014675	0.03518	0.095303	0.07013	0.096999
5.23176	1.89373	2.792035	1.699091	1.435772	1.953535	2.365685	1.251659	1.261407
4.029654	6.268312	3.043528	2.535974	1.738456	3.324452	2.045476	3.418405	3.370527
32.31123	91.05541	27.96248	4.012049	5.696263	80.6518	12.16681	3.629177	15.56131
1.434921	1.168326	0.196955	0.294921	0.447085	0.230606	0.465079	0.535368	0.417532
0.225505	0.139336	0.114618	0.163983	0.004899	0.042489	0.108341	0.022576	0.149156
5.71241	3.533211	1.20824	4.402973	4.072475	0.467677	17.92188	13.60866	2.970144
9.047532	12.49452	6.632063	13.60866	3.8121	13.75978	15.3618	23.38378	5.030866
1.757244	2.29873	2.50469	2.498478	1.74539	3.269913	2.947287	3.037626	2.212619
0.092193	0.083983	0.00062	0.010072	0.057518	0.015491	0.068629	0.107828	0.082027
0.019221	9.38E-05	0.03518	0.006162	0.014675	0.096739	0.024423	0.036869	0.027006
1.672107	2.063362	1.959466	1.977316	2.845036	1.902352	2.255916	1.628009	1.434055
9.792626	2.323983	0.98474	8.815123	21.54823	4.900996	6.22031	21.93452	3.511263
1.225426	0.320144	0.149755	0.810967	0.829192	0.318124	0.271746	0.442193	0.92531
1.111977	1.360866	2.398442	4.739668	1.279452	0.248341	2.92013	2.143261	1.883535
1.29267	1.29267	0.141623	0.745613	0.220079	1.267879	0.745613	1.938759	0.698687
1.68342	0.600173	3.953297	2.429957	2.139928	5.976724	2.225498	2.225498	4.655916
0.763939	0.542828	1.33285	0.519387	0.400967	0.382417	0.331097	0.451688	0.594646
2.329784	3.077316	0.948745	0.287951	0.441053	0.189906	0.439062	1.992944	0.734113

1.016494	4.198117	0.740952	3.775209	2.630729	3.346898	4.502453	9.218514	0.566782
0.051595	0.090851	0.157468	0.068896	0.326681	0.027431	0.013146	0.20733	0.074069
1.030678	1.360866	0.88619	0.368658	0.455325	0.310267	0.498052	0.700418	0.643001
1.447994	1.89373	0.247085	1.050231	2.257749	0.326017	2.339697	5.150693	2.229048
0.494185	0.775339	0.157468	0.465325	0.820447	0.382417	1.763889	0.887922	2.096162
0.634192	0.893016	9.838658	3.497085	3.428788	1.189769	2.584394	4.971991	1.492973
0.42767	0.363983	0.114618	0.21689	0.24518	1.44E-05	2.89E-05	0.079755	0.000303
3.536955	4.053506	2.415296	6.717056	5.976724	1.860491	2.207417	4.23202	1.986681
0.806652	1.02798	2.238341	0.675938	1.272756	1.828781	2.114437	0.986797	1.175859
19.68088	13.04605	11.719	13.83359	24.98218	17.78522	28.87506	20.85876	24.50942
0.278304	0.542828	0.567619	0.638362	0.435123	0.633853	0.885137	0.998831	1.593535
2.993867	1.476429	1.197215	0.723066	0.653203	0.133658	4.914863	2.238341	2.5271
5.195022	3.188918	7.653391	3.930664	3.890556	5.915469	6.335664	4.690159	5.130411
0.00904	0.193997	0.049646	0.068896	0.004899	0.00298	0.005123	0.098918	0.054574
1.550837	1.141515	0.932078	0.843564	0.441053	0.413593	0.331097	0.353175	0.444762
17.78522	30.82277	22.52375	22.52375	32.68313	17.92188	17.64869	21.36892	45.91841
0.753882	1.734949	0.337013	2.040606	1.460981	1.135137	0.781977	1.604827	0.502843
2.565556	1.193362	0.705231	3.66785	0.504964	1.382006	7.545584	2.479221	0.526032
0.852172	14.84163	2.45772	3.110447	49.50903	2.259661	2.392431	19.53061	12.93651
1.048074	0.564127	0.363737	0.229654	0.485325	0.318124	0.452965	0.463485	0.873088
0.747879	2.221775	1.750628	1.172872	2.061926	1.794531	0.806227	0.808983	0.974906
1.299567	1.883535	2.055289	2.185556	0.609177	0.834957	1.739935	1.323131	0.35241
1.710173	5.25254	6.39474	12.29473	3.552049	2.236407	4.535354	4.251818	7.38847
0.278304	0.157316	0.20759	0.135051	0.203593	0.084069	0.219307	0.045079	0.074069
1.417345	2.209141	2.62342	4.353925	3.703175	1.828781	2.826277	2.285664	2.181775
0.715815	1.883535	0.34544	0.482367	0.595368	0.886775	0.101609	0.946638	1.569553
0.917431	1.935685	0.578095	0.089639	0.898543	1.285584	0.231356	0.371075	2.126349
25.99427	11.60283	5.095325	14.97762	3.971061	19.22139	19.22139	29.82828	9.815642
2.531515	1.050231	0.997792	1.481631	0.634531	0.857778	2.708341	1.471198	1.850996
0.661443	2.355289	0.482706	1.181342	1.117316	1.428398	0.842641	0.607937	1.261407
0	0.24417	0.063745	0.000534	0.014675	0.116724	0.047323	0.010707	0.185166
1.126775	2.640332	0.157468	0.519387	0.036133	0.857778	0.263304	0.166681	0.577706
4.286962	3.128694	4.667518	1.737294	1.644416	3.926154	8.348817	1.251659	1.710173
1.634935	1.007244	2.009906	0.930325	1.027403	1.877605	2.427792	0.535368	0.789423
1.558571	2.369841	1.050231	1.473218	0.653203	1.25886	1.429365	1.717641	0.511032
1.4671	5.560563	3.722785	3.94886	3.78798	9.237525	5.485685	2.65684	7.264502
0.117446	9.38E-05	0.00062	0.115014	0.185909	1.44E-05	0.140483	8.66E-05	0.191602
27.10502	62.33472	18.19595	7.288788	51.52195	49.50903	3.820758	25.73165	91.05541
3.163506	2.189235	0.877222	1.464214	2.659214	0.950418	1.215981	2.789466	3.016984
0.715815	0.647345	0.141623	0.331688	0.545808	0.027431	0.168961	0.224762	0.494841
0.184206	0.275238	0.063745	0.074358	0.185909	0.020267	0.041667	0.55645	0.295491
0.907273	0.460938	1.01772	0.987821	0.998831	0.734935	0.931457	1.382006	0.602908
3.824964	5.165599	4.490801	3.807648	2.331825	3.497085	2.243911	3.211176	4.620159
1.371602	2.485534	0.384632	0.519387	0.939408	1.860491	0.726472	1.142345	1.3829
0.379791	0.893016	0.761688	0.953506	1.203427	1.358189	0.716652	0.627633	0.709784
12.49452	25.48179	26.25557	7.683766	10.73084	19.83727	10.82954	17.92188	19.37139
2.187395	3.771349	1.177309	2.834084	2.589185	1.313737	0.585281	2.048781	2.654473
0.891176	0.656392	0.633499	0.407157	0.530851	0.296955	1.052208	0.547778	0.360462
2.281926	3.55583	3.263391	1.898153	1.391494	4.053506	2.045476	2.320072	3.104394
1.316544	0.612525	1.007864	1.74539	0.808485	1.089394	2.148658	1.544517	1.930216
1.831631	9.38E-05	1.816436	2.057056	2.331825	0.970007	3.179199	0.587965	1.947316
0.884654	1.596782	0.463867	2.90658	2.805382	5.545729	3.217612	1.042944	0.661443
0	0.012222	0.049646	0.010072	0.001342	0.027431	0.029978	0.107828	0.020577
3.507691	5.388405	5.493182	2.574733	3.025671	2.933903	3.439437	1.985253	4.012049
40.7932	7.363117	7.868146	11.544	20.68746	8.643074	14.88584	8.780115	5.728615
1.959466	3.38456	1.443983	2.394416	1.170491	1.780693	1.264589	2.048781	1.480541
0.687049	0.712929	0.256797	0.17013	0.220079	0.724004	0.42329	0.371075	0.394603
0.556126	1.64917	0.00062	0.058773	0.019784	1.44E-05	2.89E-05	0.371075	0.000303

0.58259	1.094206	1.428398	0.539812	1.135137	0.944084	1.633752	0.834416	2.283752
0.059264	0.268095	0.106926	0.035584	0.014675	0.27811	0.035916	0.062857	0.199264
0.516825	0.359307	4.18456	0.38153	0.320332	0.445996	0.331097	0.451688	0.474856
0	9.38E-05	3.486436	2.498478	4.946587	2.693442	6.674286	2.65684	13.38418
1.660216	1.596782	0.90368	1.361789	1.783203	0.633853	1.197922	0.974906	1.458038
0.099704	0.174646	0.256797	0.39062	0.104906	0.049913	0.047323	0.183175	0.117446
1.477388	0.784805	0.551284	0.571674	2.263377	0.591616	1.205685	2.882035	2.051962
0.278304	0.404704	0.617071	0.183304	0.515815	0.804675	0.781977	0.496797	0.566782
0.891176	1.21215	0.535029	0.730476	1.401068	0.909315	0.369336	1.323131	0.511032
3.747035	1.809343	5.838499	2.380079	0.091977	1.767431	0.821898	0.150144	6.443925
5.056421	8.033932	6.325945	2.280101	7.558139	8.103925	5.381227	6.433918	6.888297
0.045491	0.040159	0.20759	0.035584	0.063362	0.090772	0.041667	0.098918	0.10886
4.63189	3.743232	3.236457	2.786898	1.656696	3.404372	1.507835	2.855296	2.157359
9.502294	10.05618	7.112496	6.287561	7.456118	7.225469	6.55039	9.792626	12.03267
2.353247	5.687879	2.296732	1.437648	1.595729	2.365685	1.121703	2.27254	1.883535
5.754935	4.02088	1.889711	4.338629	9.012937	5.704646	7.61176	3.860491	5.923838
10.45584	18.91854	4.490801	3.13456	11.77567	3.939307	6.028427	5.915469	14.17388
0.715815	1.95645	2.353247	0.324502	1.052208	2.531515	0.600173	1.200152	1.252424
2.584394	2.800332	0.00062	2.693442	1.574517	3.011169	1.460051	6.8421	8.226219
1360.293	224.9555	1360.293	1360.293	1360.293	287.197	425.62	1360.293	287.197
1.513102	0.692799	1.826147	0.765606	1.623232	1.449062	2.556443	1.717641	0.802395
0.138326	0.148254	0.049646	0.115014	0.185909	1.44E-05	0.101609	1.717641	0.164747
3.957734	6.125823	1.302107	0.700418	2.990851	4.519048	1.121703	1.717641	3.984747
15.66023	35.27608	22.74797	8.888167	6.612121	10.17029	32.31123	7.780786	22.13245
0.929812	1.130115	0.821898	0.189632	0.689509	0.582208	0.42329	0.789863	0.242893
11.74734	12.85504	7.238023	10.93101	7.08733	11.60283	10.62739	9.335397	7.112496
6.784762	0.825354	2.628225	22.74797	26.25557	0.796234	0.249899	5.024293	0.000303
3.796075	2.842193	5.316378	1.489524	3.307186	4.978182	2.498478	1.267085	3.592063
0.259336	0.077417	0.337013	0.643427	0.591118	4.317648	1.062092	0.845317	1.143954
3.330909	4.402973	3.303925	1.298665	3.957734	3.45404	2.529307	2.27254	2.708341
0.661443	0.982128	1.323131	0.407157	0.970007	1.169791	0.726472	1.220628	0.587619
5.993045	7.099913	8.075772	3.233045	4.971991	7.795642	6.508427	5.720173	9.031082
0.117446	0.107749	0.384632	0.120823	0.393983	0.077027	0.082973	8.66E-05	0.000303
1.746724	3.35355	0.609509	1.27619	2.162381	3.043528	1.492973	1.837172	3.26
167.9996	56.39528	77.74812	155.7103	60.77449	44.27012	72.69473	60.77449	33.93939
0.47974	4.034423	4.597792	1.104033	2.199639	4.286962	1.205685	1.650274	7.136537
0.019221	0.017879	0.042078	0.035584	0	1.44E-05	0.008882	0.01588	0.032915
2.425801	8.539971	4.33342	3.890556	3.703175	7.375794	3.201457	5.84689	3.182482
2.92013	2.126349	4.365224	1.590202	1.886335	3.767489	2.609293	0.986797	23.275
4.690159	7.964315	6.345382	4.227302	7.938427	5.942294	3.425512	3.799885	14.79742
2.6143	5.522929	2.296732	4.678846	1.634935	5.687879	1.096335	1.307417	2.789466
1.189769	0.289755	0.093009	0.06381	0.455325	0.256652	0.15456	0.079755	0.199264
1.104033	1.722475	3.172864	0.780519	0.296003	1.37531	0.760433	1.373348	1.31
0.43381	0.378066	0.521775	0.489784	0.326681	0.230606	0.352222	0.361717	0.425844
0.444986	1.094206	0.705231	0.424848	0.515815	0.768535	0.277763	0.525325	0.916869
0.00904	0.048023	0.00062	0.053579	0.001342	0.01062	0.029978	0.022576	0.039214
4.067547	10.82954	4.261342	2.429957	3.201457	17.51961	2.078355	0.565714	4.833398
0.459394	2.842193	3.188918	2.243911	1.799899	0.845887	3.101371	0.434069	1.620058
4.435159	4.580938	3.722785	7.765931	6.591544	7.882222	4.47912	2.593506	6.287561
7.570693	13.04605	14.00414	3.511263	7.212915	2.887489	15.56131	1.251659	0.916869
2.713319	4.63189	1.927042	3.94886	12.56693	3.711097	3.065108	8.888167	2.489762
31.55417	14.70188	14.17388	21.02638	15.07257	15.56131	18.33909	18.19595	14.51648
0.679069	1.193362	1.483622	1.25746	0.880498	0.504098	0.992771	0.748341	0.533874
0.342987	0.451977	0.439293	0.145909	0.01101	0.221847	0.323535	0.721335	0.687431
0.33329	0.79487	0.372973	0.246652	0.386169	0.878369	0.28443	0.451688	0.342814
4.419062	4.684502	3.220678	2.602395	1.821861	1.516219	0.941104	1.290065	2.345527
0.753882	2.050404	0.451169	0.319026	0.289452	1.927042	0.482367	2.084791	1.102691
0.211905	9.38E-05	0.00062	0.360101	0.126219	0.042489	2.89E-05	8.66E-05	0.000303

0.786667	1.411861	1.935685	1.600238	0.961486	2.535974	1.383911	4.869553	1.580909
5.082063	3.771349	3.428788	11.544	16.44768	4.440548	5.576219	4.359574	1.321053
6.967547	12.03267	6.028427	5.638759	5.439278	10.28573	7.951371	2.35733	11.25392
1.859084	1.722475	1.29267	0.765606	0.796674	1.902352	1.029365	2.192496	1.685678
1.612056	3.820758	2.194257	1.720144	2.644885	2.451645	1.002215	4.312251	4.978182
1.173535	1.219856	0.415729	0.39062	0.31456	0.749942	0.42329	0.690707	0.394603
1.249221	1.64917	6.922771	2.442987	2.863211	11.77567	3.655714	1.737294	3.35715
0.051595	0.083983	0.114618	0.00303	0.057518	0.133658	0.063362	0.107828	0.074069
0.031977	9.38E-05	0.363737	0.06381	0.126219	0.10254	0.047323	0.411948	0.333088
7.545584	5.454949	7.683766	1.691587	8.089848	3.926154	5.366335	4.222583	7.61176
1.622006	0.607605	0.396472	0.324502	0.320332	0.421804	0.263304	0.442193	0.444762
0.138326	0.843102	0.132626	0.014906	0.047143	0.154978	0.047323	0.299942	0.117446
2.755411	1.201724	0.714286	0.780519	0.3514	1.551962	1.975772	1.156869	1.010887
1.809343	1.456955	0.624473	0.857778	0.585902	3.663622	2.180022	1.156869	0.963853
0.16886	0.338341	0.063745	0.079675	0.070065	0.154978	0.063362	0.232734	0.10886
4.869553	3.877417	1.99899	2.939141	1.146118	4.839105	3.47531	5.130411	3.327619
2.931068	1.974228	0.705231	0.360101	0.595368	1.816436	1.043608	0.789863	0.62733
3.679986	11.30987	10.43066	6.345382	3.87329	11.83593	6.706472	8.005685	18.19595
0.516825	1.056898	0.256797	0.019726	0.27075	1.44E-05	0.15456	0.58259	0.178066
3.236457	4.150274	2.164113	1.995714	1.595729	1.64917	1.253752	3.249877	1.535722
0.117446	9.38E-05	0.00062	0.089639	0.036133	0.096739	2.89E-05	0.442193	0.054574
0.278304	0.893016	0.00062	0.095931	0.112569	1.44E-05	0.352222	0.175036	0.228788
1.371602	0.572266	0.730873	1.080303	0.307973	0.768535	1.612056	0.565714	1.143954
0.175714	0.404704	0.50557	0.145909	0.510072	0.162576	0.119791	0.266032	0.287951
12.93651	8.694697	7.212915	3.842554	4.709033	7.339452	9.880779	10.1028	9.667316
0.806652	1.749307	0.609509	0.687049	0.430087	1.008961	0.674675	0.690707	0.518716
6.355224	4.667518	0.320931	8.195657	3.043528	4.429769	3.984747	9.880779	16.09482
0	0.017879	0.028139	0.00026	0.000837	0.006118	0.003001	0.029553	0.000303
1.040411	4.921082	1.770029	1.915455	2.831501	3.269913	1.816436	6.808369	3.414574
18.91854	37.29684	91.05541	15.56131	35.776	40.11561	45.91841	25.99427	43.49658
0.826407	6.570433	1.553074	0.053579	0.492417	5.082063	0.465079	0.922879	2.261573
8.348817	10.67991	19.53061	12.42965	5.671126	11.68846	7.251263	8.493088	14.42661
0.00904	0.083983	0.070462	0.00303	0.004899	0.001674	0.005123	0.010707	0.000303
0.753882	1.284747	3.78798	0.456522	1.634935	1.664906	0.806227	0.653586	1.554185
0.564127	0.130202	0.093009	0	0.025844	0.162576	2.89E-05	8.66E-05	0.000303
13.11646	15.46193	10.05618	9.729358	59.32013	5.508131	10.67991	15.3618	16.9162
0.002121	9.38E-05	0.123377	0.035584	0.047143	0.042489	0.035916	0.036869	0.096999
0.342987	0.121587	0.00062	0.00303	0.147121	0.049913	2.89E-05	8.66E-05	0.000303
9.751039	21.02638	19.37139	11.11876	9.792626	14.08569	17.03919	11.09133	18.55613
0.352807	0.638362	0.542525	0.068896	0.400967	0.10254	0.205382	0.285079	2.390447
1.498167	1.688045	0.528571	1.679596	1.554185	1.927042	2.380079	0.935115	0.551284
6.181652	2.094574	3.610707	5.195022	2.863211	4.614618	6.52899	2.192496	2.283752
2.676392	5.388405	1.182771	2.955916	6.486104	5.409437	6.922771	5.024293	4.752121
0.878932	1.201724	2.281926	2.322035	1.443059	0.970007	3.179199	2.157359	2.229048
1.809343	3.68381	3.442886	0.745613	0.906126	2.181775	1.492973	2.143261	2.474834
4.586753	3.518384	2.032706	5.737056	9.459177	5.881342	2.860722	4.597792	4.468009
4.888023	7.013853	16.44768	8.048095	7.037345	16.34294	17.39935	7.92518	11.14619
12.70768	6.045209	19.83727	13.91714	7.854069	13.91714	12.36443	4.456912	9.237525
0.899055	0.581111	1.835743	0.375094	1.286494	1.089394	0.255498	0.079755	0.620014
1.080909	1.369928	0.542525	0.780519	0.970007	0.852172	0.489509	0.547778	1.136566
1.859084	3.367071	3.618499	2.043795	2.035678	4.575123	2.276407	3.581688	3.529466
0.549019	1.591299	0.642395	0.38153	0.585902	0.421804	0.161602	1.412771	0.35241
0.045491	0.063405	0.028139	0.019726	0.001342	0.01062	0.013146	0.054156	0.046962
1.592395	2.16759	0.772626	0.30215	0.926291	1.483622	1.076652	1.640779	0.677807
5.830152	2.659214	4.451154	3.890556	2.768312	2.925426	14.25954	6.601833	2.898196
0.862561	1.447994	0.609509	1.340036	3.243225	0.398059	0.517092	2.630729	1.223846
1.539062	0.564127	2.895599	0.754315	1.027403	2.122799	1.90798	0.721335	1.069574
0.6114	0.148254	0.100188	0.074358	0.566104	0.116724	0.741457	8.66E-05	4.440548

0	0.139336	0.00062	0	0	1.44E-05	2.89E-05	8.66E-05	0.000303
5.095325	4.328232	5.622872	7.532713	6.30671	6.414416	12.56693	3.435988	8.905642
0.154444	0.121587	0.20759	0.010072	0.13114	0.015491	0.018182	0.054156	0.000303
1.925635	3.771349	2.967453	1.165873	0.808485	1.916782	1.443983	3.404372	0.802395
1.281241	0.775339	2.363442	0.687049	0.441053	2.003723	0.524646	0.653586	0.594646
1.120332	1.809343	1.569553	4.286962	2.221775	7.468961	4.135801	3.418405	4.227302
5.373781	7.765931	8.134084	7.327482	13.60866	6.443925	6.365065	7.668579	4.381479
0.000996	0.063405	0.078211	0.010072	0.041782	0.123456	0.029978	0.054156	0.046962
0	0.012222	0.022958	0.000534	0.001342	0.00298	0.003001	8.66E-05	0.027006
7.225469	9.880779	6.063276	5.80469	8.033932	5.42381	4.959358	8.319019	4.857403
3.663622	7.049336	5.082063	4.06303	3.217612	3.767489	4.212554	8.256465	4.592273
6.088593	6.297136	2.967453	2.489762	3.290101	4.18456	3.544257	5.25254	3.703175
0.671371	0.435123	0.679069	1.050231	1.186457	0.230606	0.638362	0.690707	0.342814
2.730072	3.851414	6.967547	2.863211	2.758088	3.975498	3.975498	2.084791	2.763261
0	0.032367	0.273088	0.115014	0.025844	0.955418	0.018182	8.66E-05	0.046962
3.618499	5.959033	0.896522	19.53061	8.348817	15.66023	13.15079	8.089848	19.68088
0.249278	0.040159	0.671371	0.129971	0.326681	0.749942	0.445996	0.029553	0.060491
6.423911	0.404704	2.993867	1.559675	0.24518	4.302367	3.730808	0.834416	1.972706
126.1556	83.489	43.49658	45.1045	27.38726	53.77619	60.77449	39.53687	65.94551
0.473853	0.252771	0.21632	0.095931	0.104906	0.264654	0.338831	0.107828	0.333088
1.098341	0.166465	0.320931	1.925635	1.656696	0.467677	1.104805	0.480548	0.234726
0.204661	0.417532	0.273088	0.06381	0.234026	0.421804	0.317403	0.25733	0.425844
1.940231	1.100519	1.473218	1.401977	2.659214	1.102691	1.595729	1.344257	1.422316
5.864084	2.597792	5.754935	7.911934	7.697063	3.909033	5.638759	4.302367	6.045209
1.218182	1.872049	4.72798	1.531313	2.148658	2.917381	2.727785	1.565253	1.930216
0.33329	0.520289	0.439293	0.223211	0.558824	0.405599	0.113911	0.55645	0.185166
0.578817	1.447994	0.750404	0.351595	0.765094	0.467677	0.577388	1.493968	0.453218
2.993867	0.682799	1.531313	2.565556	0.423535	1.977316	2.131176	8.66E-05	1.122359
2.755411	4.650007	1.189048	2.021068	4.058514	1.080303	2.609293	5.195022	2.489762
3.807648	3.089293	8.241342	3.6029	4.424416	5.34474	5.25254	3.317807	5.110159
1.746724	9.38E-05	4.609076	2.763261	5.337547	1.614257	3.300664	3.230065	2.451645
11.96574	1.240202	9.335397	8.119004	4.281847	1.113304	5.287857	2.014863	3.625382
1.68342	1.301169	2.50469	0.519387	0.814315	1.50684	0.916306	0.55645	1.492973
21.11092	19.37139	11.89932	12.16681	7.175397	9.377345	13.72059	8.711905	10.98644
8.643074	4.353925	5.00404	10.5063	7.175397	7.061328	7.162698	8.745238	5.395584
0.004935	0.107749	0.03518	0.029639	0	0.154978	0.168961	0.19039	0.318889
19.37139	10.98644	14.70188	17.71696	15.88548	16.68554	21.54823	15.77678	19.22139
332.0776	126.1556	332.0776	252.4251	287.197	332.0776	530.1465	252.4251	135.5245
11.39744	13.23022	1.935685	4.715599	1.716364	5.095325	8.745238	22.32214	1.198701
1.006623	0.202388	0.396472	2.197828	0.595368	0.428694	6.622092	2.002193	0.117446
2.187395	0.818066	2.842193	2.6143	2.630729	1.072056	2.207417	1.565253	1.422316
5.01088	6.09759	5.599228	9.541645	4.881775	4.696313	10.1459	5.762965	4.900996
12.85504	31.92385	23.6012	21.54823	44.27012	4.540794	24.73724	17.15902	15.3618
4.435159	2.355289	8.103925	3.33728	5.515584	5.830152	9.082828	3.037626	3.38456
5.9071	8.994791	31.55417	13.38418	9.667316	16.44768	29.18885	11.30987	24.73724
16.5625	7.683766	6.900202	10.82954	8.643074	9.771833	15.07257	13.42179	12.29473
1.504517	3.55583	0.877222	1.517143	1.272756	1.299567	0.941104	0.215859	2.002193
1.799899	3.214545	1.624459	2.939141	1.266255	2.483492	2.42381	2.014863	1.183478
0.852172	1.02798	1.428398	0.163983	0.366912	2.554069	0.57101	1.483622	0.935743
0.080361	0.202388	0.337013	0.10254	0.063362	0.271912	0.101609	8.66E-05	0.157186
8.780115	14.33939	10.1459	14.70188	23.6012	12.63773	11.11876	7.584502	8.303947
6.38456	8.694697	16.80343	5.330519	8.780115	8.210938	13.72059	10.40634	6.171789
0	0.048023	0.149755	0.084459	0.227648	0.027431	0.082973	0.098918	0.000303
6.888297	10.19468	10.93101	7.964315	11.22667	6.64254	13.30906	8.573434	7.92518
1.030678	0.542828	0.896522	2.31404	0.070065	0.343824	1.852338	1.05088	0.325108
0.839365	1.435772	0.521775	0.745613	0.954141	0.445996	1.569553	1.391494	0.761688
1.456955	2.176501	1.594675	1.006623	0.837403	1.449062	1.052208	1.323131	1.794531
0.175714	0.28189	0.247085	0.176017	0.147121	0.049913	0.126335	0.597042	0.032915

4.47912	3.101371	3.29355	2.322035	4.881775	2.470317	5.030866	4.620159	3.401126
3.147294	3.921645	1.069574	1.626782	3.243225	1.633752	3.330909	3.820758	1.962424
0.092193	0.236089	0.078211	0.019726	0.019784	1.44E-05	0.013146	0.010707	0.046962
3.24658	3.011169	6.717056	4.984488	6.063276	4.261342	3.926154	5.950664	3.104394
30.17192	33.93939	67.98069	23.6012	40.7932	60.77449	9.52197	25.24367	29.18885
3.699495	1.845519	2.097843	5.25254	3.497085	1.806595	2.308203	0.667583	1.403853
0.019221	0.121587	0.372973	0.06381	0.057518	0.170808	0.075418	0.098918	0.102626
8.412367	3.411097	1.563045	3.651898	2.708341	1.916782	3.074134	2.392431	3.529466
0.130202	9.38E-05	0.282799	0.730476	0.112569	0.154978	2.89E-05	0.411948	0.000303
0.059264	0.346494	0.028139	0.019726	0.014675	0.10254	0.008882	0.175036	0.066789
3.479134	1.156869	2.425801	2.131176	2.365685	1.701616	1.633752	2.285664	3.425512
0.43381	0.460938	0.239228	0.089639	0.47153	0.141082	0.148124	0.451688	0.221587
1.132857	0.647345	0.750404	0.120823	0.281558	0.332395	0.300606	0.089812	0.370281
2.24733	1.201724	3.500808	6.374812	6.922771	1.909408	0.741457	2.967453	1.244242
3.101371	4.424416	2.941544	2.625823	3.13456	3.497085	8.508514	4.145209	4.914863
1.132857	2.402468	3.552049	1.786306	0.854733	2.392431	0.908247	0.956667	1.677042
4.802655	5.942294	17.28195	13.45939	5.172828	9.459177	5.942294	2.496407	6.888297
0.775339	0.417532	0.114618	0.287951	0.211631	0.177879	0.842641	0.496797	0.533874
6.674286	7.417359	11.63037	10.98644	16.15795	8.019769	7.339452	3.442886	7.854069
3.610707	3.489899	3.803766	4.082165	5.606739	5.959033	3.691724	6.116638	3.479134
0.306176	0.581111	1.563045	0.531486	0.670144	0.734935	0.726472	0.734935	0.783398
3.122915	2.8657	28.87506	2.453781	10.48101	5.855281	7.519841	1.691587	15.18102
1.337431	1.301169	0.489784	1.298665	0.376407	1.066234	0.768052	1.097006	0.774798
0.459394	1.085967	2.045476	1.172872	2.895599	1.806595	2.308203	0.404704	1.535722
0.33329	0.139336	0.093009	0.511667	0.097843	0.10254	0.212395	0.748341	0.171277
0.323535	0.063405	0.166465	0.135051	0.196667	0.049913	0.205382	0.313102	0.117446
0.732633	2.386248	2.116032	0.360101	1.519322	1.739935	1.389509	1.748016	3.651898
0.286522	3.411097	0.329524	1.147807	0.906126	0.203593	0.101609	0.380447	0.082027
0	0.090851	0.078211	0.041717	0.008175	0.006118	0.041667	0.122554	0.027006
0	0.099488	0.100188	0.029639	0.014675	0.077027	0.008882	0.029553	0.032915
5.447114	3.273045	1.569553	2.380079	1.322092	5.745996	3.860491	4.990794	2.436768
0.419596	0.338341	0.106926	0.264805	0.914618	0.189906	0.452965	0.380447	0.20733
3.763045	5.745996	3.691724	3.414574	3.095166	4.392316	3.497085	4.49671	5.295325
0.342987	0.572266	2.129596	0.745613	0.753016	1.780693	1.224733	0.518716	1.403853
2.322035	1.401068	1.775325	0.343434	1.772756	2.770866	0.98474	0.607937	0.228788
0.154444	1.492973	1.798535	2.776421	1.855108	0.413593	1.312706	0.763564	0.935743
0.092193	0.139336	0.063745	0.068896	0.080837	0.116724	0.192121	0.054156	0.135354
3.101371	2.8657	6.695887	4.952915	6.355224	4.053506	4.188896	6.717056	4.592273
60.77449	18.33909	15.18102	31.55417	17.39935	27.96248	36.74522	26.80355	19.99861
0.403413	1.664906	1.594675	0.135051	0.141378	0.617071	0.277763	0.81544	1.048074
0.154444	0.157316	0.355051	0.313312	0.211631	0.133658	0.184358	0.442193	0.242893
11.11876	17.03919	4.900996	21.19547	8.256465	8.445743	13.91714	12.70768	11.719
2.776421	2.45772	1.283059	1.568564	2.285664	2.199639	2.131176	2.565556	1.21215
0.534214	0.211025	0.415729	0.235296	0.259033	0.453932	0.237359	0.19039	0.425844
0.074358	0.166465	0.282799	0.108506	0.558824	0.077027	0.148124	0.141284	1.867951
3.439437	7.6393	4.236962	5.454949	6.570433	2.254084	8.241342	48.53429	2.970144
3.367071	1.809343	6.287561	6.922771	4.203001	1.307417	3.833247	3.820758	3.500808
0.671371	0.252771	0.50557	1.104033	0.780087	0.91575	0.231356	0.247417	0.502843
75.1132	50.55949	64.12971	36.74522	40.11561	56.39528	100.0998	46.7056	83.489
0.605455	0.378066	0.329524	0.229654	0.16145	1.62562	0.885137	0.508975	0.845317
0.625786	1.837172	0.997792	0.308903	0.211631	0.608543	0.616407	0.653586	0.444762
0.644235	1.51215	1.464214	1.157626	1.094206	1.884935	0.658362	1.886335	1.122359
5.662994	3.307186	10.38202	6.107114	5.11697	13.45939	10.28573	7.584502	6.717056
0.403413	0.268095	1.029365	0.441833	0.27075	0.98956	0.638362	0.388831	1.198701
1.831631	1.085967	3.833247	1.116609	2.116032	2.451645	2.339697	1.507835	2.50469
0.379791	0.825354	0.692799	0.324502	0.914618	0.834957	1.172872	0.607937	0.821898
0.43381	2.506934	0.00062	0.441833	0.47153	1.227749	0.898543	0.653586	0.453218
0.852172	1.141515	0.291616	0.019726	0.112569	1.023528	0.101609	0.344733	0.199264

0.204661	0.268095	0.843102	0.257684	0.578817	0.54619	1.253752	0.965065	0.577706
24.2842	87331.38	0.363737	87331.38	425.62	3.869163	1360.293	87331.38	0.117446
0.410996	0.077417	0.106926	0.151688	1.37531	0.03518	0.249899	0.175036	0.027006
0.059264	0.032367	0.247085	0	0	0.006118	0.008882	0.285079	0.000303
0.204661	0.130202	0.521775	0.496797	0.416782	0.359307	0.600173	0.353175	0.318889
0.270837	1.675722	3.104394	2.676392	3.747035	1.47842	0.674675	0.789863	0.832554
0.473853	0.527821	1.249221	0.399545	0.345635	1.135137	0.392403	0.700418	0.602908
1.498167	2.758088	2.238341	0.196667	0.510072	2.406768	0.638362	0.122554	0.620014
2.65684	2.922778	2.718434	1.889711	2.078355	1.992944	2.351299	2.419553	2.31404
0.031977	0.099488	0.00062	0.00026	0.001342	1.44E-05	2.89E-05	0.175036	0.046962
5.409437	11.09133	3.198499	4.552092	4.620159	4.419062	5.395584	5.024293	3.26
1.066948	0.759646	3.253175	0.331688	0.545808	0.893016	0.504964	0.411948	0.821898
1.781948	4.620159	2.752893	2.035678	2.306277	1.145426	1.411861	4.174964	2.199639
0.085974	0.032367	0.224372	0.006162	0.025844	0.096739	0.132799	0.115188	0.066789
3.944084	1.91811	1.750628	2.203535	3.35355	3.796075	3.300664	1.446926	1.480541
0.138326	0.211025	1.142345	1.192633	1.286494	2.489762	1.370765	0.956667	0.259646
1.212792	1.620058	1.33285	0.539812	0.3514	1.237879	0.537807	1.748016	1.972706
3.019848	5.195022	5.337547	11.25392	7.315512	2.628225	6.423911	7.780786	13.45939
0.123968	0.032367	0.239228	0.10254	0.03088	0.10254	0.255498	0.010707	0.066789
0.000996	0.012222	0.00062	0	0.001342	0.00298	2.89E-05	0.01588	0.000303
2.141595	1.392511	2.363442	1.776739	1.076652	1.343463	2.267157	1.237879	1.1629
0.080361	9.38E-05	0.157468	0.014906	0.019784	0.141082	0.15456	0.045079	0.074069
3.147294	2.823636	5.915469	4.796126	3.6029	3.816551	6.191515	6.325945	3.904517
0.952294	0.305455	0.00062	0.432698	0.080837	0.625498	0.231356	0.175036	0.242893
0.644235	2.063362	4.650007	1.311674	2.127973	1.678319	1.896667	3.703175	3.195541
5.80469	9.397771	8.888167	10.5063	8.226219	4.317648	7.710361	6.738211	9.459177
0.184206	0.261111	0.093009	0.204156	0.154329	0.042489	0.041667	0.07013	0.082027
4.348882	10.33476	3.998391	4.424416	4.508196	5.788153	4.47912	1.786306	3.425512
1.139885	2.126349	0.551284	0.810967	2.500548	1.307417	1.282208	1.737294	0.81228
1.809343	2.31404	0.686667	1.157626	1.322092	2.283752	0.974257	3.885642	2.50469
1.4671	1.141515	1.220628	0.432698	0.670144	1.102691	1.184935	0.421342	1.235346
0.184206	0.759646	0.372973	0.331688	0.265455	0.133658	0.413304	1.070693	0.577706
7.430317	7.911934	5.942294	6.054242	16.5625	7.315512	6.956349	6.601833	6.653016
4.739668	5.662994	7.868146	5.165599	7.251263	7.443276	7.443276	4.529913	6.497266
17.51961	18.76954	8.381328	6.632063	13.83359	11.96574	17.51961	4.971991	12.10289
0.300844	0.017879	0.063745	0.324502	1.539062	0.332395	0.271746	0.313102	0.157186
6.486104	5.638759	1.142345	5.576219	12.42965	1.906551	3.655714	5.638759	3.88153
10.53159	6.853608	5.439278	6.663651	6.853608	9.924372	10.7557	5.737056	7.08733
0.241991	0.236089	0.521775	0.183304	0.338831	0.672468	0.591616	0.098918	0.280967
8.940671	10.62739	8.677489	23.38378	3.544257	24.73724	6.591544	6.433918	4.540794
0.410996	0.759646	1.007864	0.343434	0.423535	0.663276	0.489509	0.224762	0.81228
0.105996	0.581111	0.049646	0.120823	0.13114	0.06355	2.89E-05	0.371075	0.259646
0.233434	0.53469	0.567619	0.041717	3.217612	0.359307	0.623146	0.535368	0.687431
0.975556	4.198117	1.50026	1.192633	0.926291	1.323975	0.836537	1.972706	2.065007
2.685786	4.120815	1.418312	2.640332	2.725498	1.29267	1.661328	3.747035	3.687633
0.342987	0.600173	0.282799	0.151688	0.275382	0.776349	0.309062	0.299942	0.20733
2.061926	1.766248	4.072475	0.88619	1.460981	3.486436	3.621941	1.401977	5.195022
0.175714	0.268095	0.239228	0.001255	0.03088	0.10254	0.018182	0.115188	0.149156
13.60866	11.48368	8.195657	34.38341	23.82354	7.495397	34.84299	80.6518	9.237525
0.661443	0.395859	1.344257	0.974257	0.961486	0.86469	1.563045	2.35733	2.725498
0.123968	0.083983	0.363737	0.035584	0.104906	0.06355	0.068629	0.175036	0.10886
4.535354	9.335397	3.921645	2.633232	6.277937	9.838658	1.896667	2.676392	6.210375
8.381328	20.68746	2.77899	34.84299	55.04081	4.563117	43.49658	87.1746	10.35839
2.831501	2.199639	8.319019	6.028427	2.116032	6.808369	6.900202	4.203001	8.303947
0.306176	0.211025	0.90368	1.786306	3.6029	0.154978	0.537807	0.19039	6.107114
1.392511	1.793074	3.895469	3.544257	3.651898	7.519841	4.419062	3.230065	3.29355
252.4251	72.69473	30.17192	47.5018	65.94551	38.94854	91.05541	67.98069	77.74812
0.002121	0.040159	0.03518	0	0.001342	0.03518	0.013146	0.007417	0.032915

0.65202	1.268672	0.247085	0.416501	0.758773	0.804675	0.399307	1.267085	1.328384
0.014084	0.032367	0.100188	0.053579	0.141378	1.44E-05	0.024423	0.183175	0.046962
1.353608	1.553074	1.959466	3.303925	2.925426	3.599329	2.148658	2.807937	2.359264
0.510072	0.435123	0.51316	0.551861	2.127973	0.566782	1.029365	0.022576	0.761688
1.417345	0.945483	0.594646	3.214545	3.939307	0.714286	3.086306	3.514834	4.003117
0.379791	0.166465	0.821898	2.400455	2.018167	1.05417	0.205382	8.66E-05	3.26
0.899055	0.305455	0.962698	0.551861	1.50026	0.467677	2.89E-05	0.291025	3.211176
12.26136	29.82828	37.81919	22.32214	29.18885	19.99861	19.53061	30.49289	25.73165
7.049336	7.519841	6.876392	11.11876	7.964315	18.76954	6.423911	10.78056	12.36443
0.184206	0.29842	0.132626	0.432698	0.104906	0.483874	0.293038	0.232734	0.295491
1.845519	1.168326	2.019618	6.054242	7.519841	0.170808	1.612056	7.363117	1.903636
25.73165	24.73724	26.52537	7.38847	21.19547	41.47925	51.52195	14.33939	67.98069
1.428398	1.620058	1.435772	1.673254	2.078355	2.984899	2.255916	2.369841	1.837172
1.925635	1.435772	1.374329	1.096335	0.742417	1.048802	0.271746	1.471198	1.020447
1.643218	1.035801	2.097843	5.00404	9.880779	3.394286	1.612056	3.984747	2.882035
0.278304	0.749481	1.543377	1.06355	0.538117	0.834957	1.394632	2.944416	1.278629
0.826407	1.974228	3.486436	0.843564	0.62645	5.915469	1.778153	0.597042	1.535722
0.080361	0.32925	0.00062	0.019726	0	0.03518	2.89E-05	8.66E-05	0.074069
2.373824	4.776407	2.642525	3.016984	6.784762	1.492973	1.507835	4.690159	2.882035
2.468117	5.302294	4.440548	2.879228	1.634935	8.319019	4.764055	4.900996	4.468009
0.793434	0.476335	0.962698	1.767431	1.338261	0.845887	1.460051	0.866147	1.223846
1.016494	0.673254	1.986681	1.080303	1.608427	1.729892	1.47018	1.156869	1.505678
0.099704	0.395859	0.781977	0.246652	0.578817	0.230606	0.168961	0.488759	0.783398
0.249278	0.083983	0.463867	0.053579	0.104906	0.084069	0.126335	0.054156	0.10886
4.678846	1.094206	1.666089	4.609076	1.322092	2.181775	7.795642	2.285664	1.434055
6.749488	2.442987	0.98474	2.542605	3.005281	3.166659	5.462475	1.554185	0.993521
0.000996	0.032367	0.03518	0	0	0.00298	0.005123	0.045079	0.000303
2.730072	4.241905	1.826147	1.268672	1.094206	6.570433	2.506934	5.25254	6.695887
2.34355	2.485534	1.382006	2.453781	1.50026	2.259661	2.556443	2.031169	1.328384
0.65202	3.464315	1.798535	1.938759	2.269134	2.415296	0.870902	1.577569	3.227085
10.82954	5.266335	10.23768	7.363117	11.48368	10.78056	11.77567	9.377345	9.815642
0.65202	0.952294	1.06355	1.720144	1.74539	0.653586	0.891775	0.607937	1.189048
0.313723	0.527821	0.664423	0.287951	0.409098	0.520022	0.323535	0.361717	0.636291
13.38418	2.189235	0.070462	2.975974	9.707677	2.635685	2.114437	2.931068	1.629235
0.191602	0.268095	0.601623	0.287951	0.530851	0.566782	0.331097	0.411948	0.602908
3.544257	1.927042	2.425801	2.347439	0.820447	1.775325	2.013182	1.209105	1.837172
0.249278	0.236089	0.26562	0.176017	0.423535	0.332395	0.439062	0.150144	0.417532
0.899055	4.261342	4.048499	0.595714	0.814315	2.333867	1.394632	0.998831	8.905642
5.737056	3.966623	7.570693	5.28039	5.062626	2.784228	3.529466	7.417359	3.966623
4.47912	2.355289	2.5271	2.834084	2.234538	1.331919	1.801284	3.128694	1.867951
8.608153	11.65791	8.780115	20.16541	19.37139	10.60361	12.89577	23.6012	20.85876
2.203535	2.063362	1.820541	1.870685	2.210866	1.189769	1.270317	0.690707	1.261407
0.826407	1.240202	0.740952	0.60824	0.423535	0.51316	0.383622	0.679452	1.069574
3.172864	4.72798	1.543377	2.109755	1.944517	3.640606	3.655714	2.855296	2.032706
6.933961	13.60866	10.28573	15.07257	9.256537	14.00414	10.19468	12.85504	8.019769
1.111977	2.45772	0.586248	0.601623	0.455325	0.256652	0.369336	1.03246	0.378889
5.942294	33.4985	15.98892	12.49452	24.50942	14.51648	7.951371	11.42512	25.48179
0.085974	0.53469	0.567619	0.189632	0.203593	1.113304	0.300606	0.299942	0.164747
0.379791	0.425844	0.299199	0.10254	0.076111	0.203593	0.140483	0.183175	0.178066
17.28195	5.71241	24.73724	24.98218	42.75576	20.51271	20.34063	17.39935	48.53429
0.306176	0.28189	1.156869	0.551861	1.50026	1.180613	0.517092	0.887922	0.242893
0.419596	1.360866	1.403853	0.754315	0.990188	0.758773	0.600173	0.627633	0.444762
0	0.852684	0.497749	0.30215	0	2.283752	0.063362	0.480548	0.35241
7.264502	10.2617	2.871212	1.797172	2.410996	12.03267	4.034423	2.238341	4.609076
33.4985	20.85876	52.56128	19.83727	64.12971	87331.38	33.93939	31.92385	37.55802
0.00904	9.38E-05	0.123377	0.019726	0.019784	0.057208	2.89E-05	0.036869	0.000303
2.24733	0.982128	1.835743	1.817828	11.60283	5.34474	7.188095	3.55961	12.85504
3.585029	3.592063	5.056421	3.625382	3.122915	4.272165	4.365224	4.072475	1.685678

3.011169	2.81342	1.877605	3.57057	1.994329	3.722785	1.771392	2.369841	1.3829
1.249221	0.591118	1.150635	1.06355	1.799899	0.123456	1.002215	1.595729	4.246861
2.07658	18.48514	4.096407	3.397706	4.072475	4.814661	3.544257	5.447114	4.857403
0.700837	1.53684	0.551284	0.264805	0.683232	1.343463	0.293038	0.344733	1.030022
6.135007	7.136537	12.36443	8.573434	28.23632	9.968355	12.22798	24.04374	44.27012
0.038918	0.077417	0.00062	0	0	1.44E-05	2.89E-05	0.131768	0.000303
0.211905	0.353333	0.123377	0.456522	0.104906	0.248341	0.035916	0.022576	0.089921
3.45404	2.54961	5.47	6.99026	16.80343	6.054242	3.799885	8.089848	4.408153
50.55949	135.5245	59.32013	25.73165	31.92385	105.1694	37.81919	45.1045	30.17192
22.13245	14.17388	33.93939	30.17192	18.62711	38.39042	35.27608	27.10502	28.23632
67.98069	87.1746	16.09482	50.55949	111.5439	62.33472	44.27012	56.39528	105.1694
6.55039	16.80343	15.77678	8.429055	5.560563	11.30987	8.134084	7.025599	15.77678
17.03919	29.49693	40.11561	15.3618	16.9162	37.29684	27.10502	19.06893	30.82277
2.852677	11.96574	4.984488	3.098268	4.179762	28.87506	3.703175	2.868456	6.53969
530.1465	749.7839	287.197	425.62	332.0776	252.4251	749.7839	425.62	332.0776
203.5795	167.9996	184.443	83.489	105.1694	167.9996	62.33472	184.443	100.0998
95.22939	118.7028	203.5795	167.9996	30.3324	100.0998	95.22939	83.489	75.1132
184.443	332.0776	72.69473	144.7919	56.39528	87.1746	135.5245	530.1465	72.69473
12.10289	9.604358	7.38847	2.263377	2.078355	5.137042	4.077403	3.06803	8.539971
1.281241	1.311674	2.567605	1.34798	2.099524	1.828781	2.131176	1.382006	1.744055
9.256537	8.348817	8.019769	12.77772	4.592273	6.088593	9.141299	19.99861	7.824473
10.62739	12.22798	7.175397	19.99861	7.911934	9.771833	15.77678	14.70188	6.38456
0.634192	0.775339	1.040411	0.343434	0.289452	0.493247	0.199488	0.247417	0.266479
0.161962	0.289755	0.273088	0.02482	0.070065	0.141082	0.063362	0.079755	0.10886
2.173081	4.312251	1.106825	2.35733	1.50026	1.985253	1.771392	2.107922	1.445916
0	9.38E-05	0.106926	0.620909	0.03088	0.338095	0.383622	0.159004	0.636291
8.711905	2.572309	5.779574	6.900202	5.309264	11.01243	15.88548	9.924372	9.179466
0.249278	9.38E-05	0.224372	0	0.376407	1.44E-05	2.89E-05	0.313102	0.20733
16.85982	22.13245	7.911934	13.23022	15.3618	9.297085	8.643074	19.22139	11.65791
3.043528	1.019134	2.375974	2.90658	2.092987	1.408831	2.987835	5.056421	1.130115
2.217951	2.922778	2.727785	1.464214	0.829192	4.236962	2.513752	2.917381	0.518716
0.002121	0.032367	0.0857	0.084459	0.008175	0.03518	0.192121	0.045079	0.126883
0.33329	0.359307	0.781977	0.308903	0.154329	0.768535	0.392403	0.25733	0.311227
0.992201	1.385801	0.473853	1.251659	1.117316	0.653586	1.896667	1.180613	1.710173
42.08807	29.18885	13.30906	20.85876	34.38341	21.36892	30.82277	33.07147	22.74797
26.80355	35.776	35.27608	42.08807	26.80355	67.98069	24.98218	29.18885	31.1753
17.15902	23.6012	15.46193	4.914863	11.42512	9.667316	12.70768	8.256465	16.68554
10.31025	5.337547	4.696313	3.55583	3.13456	5.238701	6.054242	5.180058	4.667518
0.360938	0.557056	0.422237	0.360101	0.705613	0.574228	0.383622	0.70886	0.643001
24.98218	17.39935	23.82354	8.976508	9.838658	23.38378	23.82354	15.56131	17.03919
0.510072	1.252424	1.197215	0.754315	1.330029	0.653586	0.55741	1.022929	1.048074
5.662994	0.759646	0.730873	4.851227	0.926291	3.201457	3.411097	1.737294	5.180058
40.11561	39.53687	19.68088	16.9162	12.93651	11.19942	20.16541	28.87506	20.25302
2.666494	1.219856	1.99899	1.614257	1.401068	5.576219	2.339697	0.855801	0.360462
0.753882	0.520289	0.396472	0.496797	0.970007	0.343824	0.700058	0.434069	1.772756
2.625823	4.067547	4.626025	2.708341	3.276335	4.150274	4.034423	4.058514	3.842554
0.917431	0.884654	1.220628	0.416501	0.220079	0.871429	1.112641	1.130115	1.102691
0.342987	0.378066	1.810664	0.562439	0.220079	4.473348	0.726472	0.463485	1.772756
0.862561	0.425844	0.407157	0.716234	0.296003	0.537229	0.383622	0.291025	0.734113
2.359264	1.779423	0.781977	1.226118	1.511198	0.453932	0.682359	1.290065	1.458038
0.175714	0.38596	0.551284	0.614545	0.386169	0.688182	0.57101	0.122554	0.474856
1.577569	1.793074	6.135007	2.442987	2.531515	5.662994	3.26	2.65684	5.42381
12.36443	8.033932	1.050231	2.602395	3.78798	2.063362	9.031082	5.522929	4.833398
13.83359	2.786898	13.79669	6.967547	7.112496	26.52537	12.89577	2.893001	4.739668
0.352807	0.289755	0.078211	0.120823	0.409098	0.128687	0.585281	0.325527	0.333088
0.051595	0.114719	0.100188	0.129971	0.019784	0.116724	0.119791	0.098918	0.221587
12.63773	17.15902	12.42965	15.18102	25.48179	27.10502	29.49693	24.2842	10.12435
0.952294	0.460938	0.463867	0.843564	0.820447	0.672468	1.112641	0.285079	0.494841

0.000996	0.008095	0.042078	0.00303	0.01101	0.01062	0.008882	8.66E-05	0.016537
0.741962	1.476429	3.137576	0.511667	1.094206	1.204228	0.878369	0.640289	0.935743
4.667518	1.141515	3.703175	2.735368	6.250447	2.392431	3.926154	2.216162	2.051962
7.30215	3.904517	12.49452	3.397706	2.432121	70.11932	5.696263	14.08569	20.51271
4.145209	13.38418	2.567605	1.691587	8.849697	10.93101	2.967453	4.424416	4.63189
0.534214	2.402468	1.365296	0.987821	1.8386	0.930325	1.784755	6.345382	2.171429
3.297107	2.283752	6.191515	5.838499	1.783203	5.80469	5.22482	3.211176	3.799885
3.101371	6.830592	4.155339	5.266335	2.35733	5.150693	2.62342	6.054242	2.628225
1.249221	0.61969	1.311674	0.587273	0.676999	0.845887	0.566104	0.834416	0.551284
1.056263	0.656392	0.093009	0.271544	0.13114	0.367532	2.89E-05	0.353175	0.089921
11.83593	9.924372	14.88584	3.5257	4.524481	9.335397	9.52197	4.789596	9.625253
111.5439	184.443	144.7919	80.6518	91.05541	184.443	155.7103	91.05541	95.22939
0.105996	0.157316	0.106926	0.079675	0	0.343824	2.89E-05	0.150144	0.199264
38.94854	49.50903	27.66779	43.88335	37.81919	16.80343	18.48514	55.04081	31.55417
1.634935	3.188918	2.136335	1.531313	0.906126	1.96404	1.233665	1.313737	1.554185
1.383911	0.852684	1.142345	0.450051	1.027403	1.25886	1.370765	1.52676	0.587619
10.07949	8.089848	11.25392	5.330519	6.55039	6.8421	9.924372	7.625209	8.256465

CGGA_123	CGGA_124	CGGA_125	CGGA_125	CGGA_125	CGGA_126	CGGA_126	CGGA_126	CGGA_126
0.763564	0.176407	0.322395	0.028333	0.198405	0.068932	0.573326	0.613853	0.970635
0.10531	0.169971	0.50127	0.000231	0.28728	0.825354	2.589185	0.134444	0.416219
1.351573	3.014076	5.591717	4.473348	2.300678	10.19468	0.573326	2.715873	2.240274
1.928629	2.524892	0.242771	30.49289	4.33E-05	7.683766	0.51316	0.628658	0.620462
2.690823	3.014076	0.768535	2.216162	1.346991	3.256587	4.709033	5.195022	3.128694
7.991602	3.110447	2.498478	0.893016	3.820758	1.056263	2.496407	8.333918	10.33476
47.5018	36.26061	30.49289	23.06543	25.99427	26.52537	8.677489	40.11561	29.82828
0.092691	2.16E-05	0.047431	0.052338	0.163983	0.021429	1.670786	0.030375	0.088283
1.373348	1.603795	0.457431	6.945152	1.15614	4.125786	0.024141	0.98474	0.73583
0.318499	0.800967	0.688579	0.1379	0.353795	5.77E-05	0.024141	0.522367	0.416219
49.50903	31.55417	252.4251	12.49452	144.7919	22.96463	60.77449	51.52195	55.04081
1.621032	1.113304	0.905426	0.662944	0.919827	0.642395	4.307309	1.8386	1.736133
17.78522	5.34474	20.16541	52.56128	5.522929	33.4985	2.119596	15.66023	5.545729
1.608427	9.751039	0.990909	26.52537	4.33E-05	14.6058	0.024141	2.061926	1.47018
7.22E-05	0.000188	0.077417	0.014545	0.005007	0.087807	0.719942	0.020267	0.023492
2.941544	2.696061	0.002338	7.653391	0.382626	9.218514	3.7586	2.800332	3.718716
1.429365	1.495043	1.156869	0.57531	1.443983	0.272078	1.549711	2.371833	1.755988
0.07767	0	0.002338	0.000231	4.33E-05	5.77E-05	38.94854	8.66E-05	0.044805
1.863506	1.660216	0.231984	14.51648	0.538773	8.745238	0.024141	1.118066	0.366147
1.734949	0.245469	2.151919	0.333088	0.440029	0.363059	4.307309	0.709322	2.106089
0.891775	0.006061	0.242771	13.15079	0.163983	2.77899	0.024141	0.635916	0.058694
1.172872	0.656003	1.416407	59.32013	0.588954	14.70188	7.363117	2.434444	1.210447
56.39528	135.5245	0.809481	1.378362	11.60283	2.34355	0.024141	18.41211	39.53687
0.05189	0.003016	0.002338	0.216025	0.032092	0.013117	2.091313	0.077027	0.16145
2.487576	2.069942	0.434069	0.341053	1.011486	0.522367	3.718716	1.527792	0.832013
3.156537	1.256558	4.952915	9.541645	3.536955	7.545584	3.266782	2.400455	2.400455
0.533535	1.468131	0.276609	3.91355	1.766248	1.588903	0.024141	1.212792	0.208254
0.388579	0.372973	1.416407	0.254156	0.474214	0.343211	0.41026	0.420058	0.373939
20.68746	155.7103	15.22851	70.11932	224.9555	91.05541	0.024141	40.7932	12.77772
1.064935	0.333831	0.425426	1.01544	0.365036	0.56443	2.160649	0.672857	0.628297
4.451154	1.095657	2.043795	10.67991	1.049517	4.424416	3.563182	3.851414	5.923838
0.037525	0.152655	0.061025	0.145556	0.069026	0.031046	2.119596	0.167417	0.322973
16.80343	9.335397	0.002338	48.53429	4.33E-05	38.94854	0.024141	18.91854	3.980123
0.022886	7.22E-05	0.203254	0.000231	4.33E-05	0.021429	0.383074	0.009394	0.012266
1.651522	2.104358	1.093434	0.043391	2.089639	0.105022	18.99374	1.207374	12.49452
2.107922	2.248896	3.47531	5.01772	1.859084	4.597792	2.708341	0.734524	1.794531
2.306277	5.788153	8.019769	6.153102	1.64917	5.351934	4.145209	6.036818	3.779452
0.05189	0	0.002338	0.020087	0.024473	5.77E-05	0.180231	0.160072	0.270837
3.4679	2.312085	1.744055	1.703795	2.922778	2.009906	8.556703	4.978182	6.171789
6.079596	9.792626	8.226219	2.021068	9.946364	2.642525	10.98644	6.116638	6.967547
0.679812	0.645916	0.802035	0.028333	0.31456	0.35676	11.48368	0.593434	0.511356
1.129452	3.110447	2.74298	0.88417	1.750628	0.981032	28.87506	1.599033	1.537951
3.734762	0.499206	1.244242	1.930216	0.789423	2.654473	0.024141	0.47	1.273636
0.827273	7.22E-05	0.047431	0.014545	0.005007	5.77E-05	0.180231	8.66E-05	0.008882
0.122179	0.227446	0.809481	0.384632	0.260404	0.545152	7.162698	0.390216	0.250007
32.31123	29.82828	2.823636	23.38378	25.24367	20.16541	3.846984	55.04081	19.99861
0.070909	0.046667	0.362179	0.233175	0.078737	0.13039	2.119596	0.090303	0.088283
7.22E-05	0.16119	0.188745	0.000231	0.11039	0.021429	0.217677	8.66E-05	0.304387
0.008369	0.000433	0.002338	0.014545	0.024473	0.006912	0.88158	0.009394	0.008882
0.013175	0.000433	0.002338	0.000231	0.175144	5.77E-05	0.383074	8.66E-05	0.392251
7.22E-05	0.00119	0.002338	0.000231	0.024473	5.77E-05	0.024141	0.030375	0.072583
7.002056	1.568564	0.674163	5.366335	1.57645	7.584502	1.95645	11.25392	18.05488
0.54847	0.342814	1.145426	0.000231	0.342987	0.021429	0.82772	0.484423	0.263853
0.240202	0.169971	0.978571	0.1379	0.382626	0.040909	0.51316	0.398629	0.126797
0.507403	0.840635	2.710765	10.43066	0.871429	6.55039	0.598456	1.396494	4.508196
0.74868	0	0.844747	0.000231	0.718766	0.105022	1.282208	0.449726	0.73583
0.793918	0.474574	0.777864	0.548153	0.428932	0.304387	3.083319	0.690043	0.705231

0.609509	0.16119	1.89518	0.171472	0.448261	0.516566	5.606739	0.96329	0.749019
1.226934	2.863211	4.207778	0.956061	3.521934	1.099762	1.131472	1.751948	2.45575
0.381999	0.268961	0.446551	0.583369	0.146999	0.304387	1.312706	0.530253	0.610606
30.82277	0.312626	0.002338	3.397706	0.555036	1.174315	0.024141	19.99861	43.49658
0.270087	0.474574	1.199481	0.670519	0.324913	1.034315	0.217677	0.167417	0.183824
0.915195	0.43347	1.010887	0.297439	0.28728	0.62645	18.26752	1.033672	0.722179
32.68313	60.77449	67.98069	18.76954	167.9996	67.98069	0.024141	42.08807	11.83593
0.270087	0.397489	0.066696	0.254156	0.198405	0.158521	0.437576	0.020267	0.192251
7.22E-05	0.003016	0.306176	0.062107	0.007821	0.006912	0.024141	8.66E-05	0.000115
1.471198	1.553074	1.244242	1.385801	2.219863	0.923874	1.073543	2.223636	1.182056
1.288276	1.064935	1.568564	1.810664	0.836537	1.359177	1.417345	1.710173	0.832013
0.443781	0.260144	1.191905	0.347908	0.670144	0.401176	0.383074	0.484423	0.638362
0.07767	0.260144	0.261775	0.086025	0.409618	0.040909	4.307309	0.205801	0.468694
0.065519	0.312626	1.358189	0.020087	0.501905	5.77E-05	1.255657	0.175036	0.140087
0.858297	0.552439	1.651522	0.297439	0.456522	0.139697	0.942944	1.340036	1.182056
0.008369	7.22E-05	0.090476	0.000231	0.005007	0.006912	0.142698	0.006558	0.012266
0.005916	0.006061	0.002338	0.035981	0.017063	0.006912	0.024141	0.030375	0.044805
2.470317	9.179466	12.56693	21.19547	5.082063	16.80343	3.7586	4.445851	6.268312
0.122179	0.027965	0.173189	0.020087	0.334805	0.078081	0.573326	0.338341	0.216602
0.022886	0.091616	0.269697	0.078081	0.007821	0.040909	0.598456	0.096905	0.113521
4.821176	13.53913	1.662439	2.269134	15.3618	4.667518	0.327143	5.150693	5.576219
0.085426	0.027965	0.132107	0.000231	0.024473	5.77E-05	1.89373	0.083983	0.132626
0.518139	0.43347	0.124791	0.106847	0.474214	0.458341	2.92013	0.407403	0.839365
5.687879	3.243225	6.045209	27.96248	2.169509	14.00414	0.327143	6.144055	1.617489
0.008369	0.071861	0.083521	0.127626	0.296465	0.097258	0.773766	8.66E-05	0.018038
1.05417	0.630022	1.244242	0.171472	0.566494	0.087807	2.496407	0.753882	1.47018
11.65791	16.74449	0.579841	30.82277	24.50942	10.33476	9.256537	30.82277	65.94551
0.470736	0.10531	0.483045	0.117734	0.28728	0.021429	2.192496	0.315094	0.225339
0.29798	0.333831	0.002338	0.333088	0.353795	0.423535	7.519841	0.780519	0.98956
0.617071	1.455988	6.52899	2.765758	1.272756	12.77772	1.131472	0.299444	0.979149
0.20215	0.10531	0.666097	0.22456	0.233752	0.343211	0.35241	0.273434	0.348139
0.154444	0.066472	0.002338	0.052338	0.198405	0.006912	0.265786	0.114719	0.140087
0.600548	1.297763	0.472713	1.613232	0.687049	1.323975	0.024141	0.249091	0.800058
9.297085	16.34294	51.52195	3.058802	21.74051	9.377345	0.024141	8.089848	5.485685
0.381999	0.144719	0.290866	0.000231	0.186017	5.77E-05	0.971205	0.544214	0.313312
0.3671	0.416219	1.835743	0.273434	0.809481	0.13039	0.28728	0.299444	0.518413
3.125844	2.735368	2.068297	0.273434	1.111977	0.212698	4.56912	3.877417	5.395584
7.22E-05	0.059459	0.054574	0.216025	0.078737	5.77E-05	1.776739	0.030375	0.192251
9.179466	5.745996	11.719	6.8421	3.016984	4.47912	0.805238	7.08733	7.363117
0.044495	0.006061	0.156089	0.052338	0.054711	5.77E-05	0.51316	0.061602	0.065801
0.022886	0.00119	0.066696	0.052338	0.078737	0.006912	1.282208	0.006558	0.032727
7.22E-05	0.010592	0.002338	0.014545	0.032092	0.021429	0.41026	8.66E-05	0.008882
3.754719	2.138131	2.434444	1.168326	0.990188	1.026219	4.251818	3.691724	2.26518
5.165599	6.52899	9.031082	3.625382	1.890996	4.863579	0.41026	4.715599	1.905094
3.147294	6.560411	2.895599	0.640289	2.996869	0.390418	0.598456	2.649675	3.192229
0.381999	0.43347	0.261775	0.1379	0.095772	0.149293	1.628009	0.47	2.331825
0.403651	0.342814	1.017107	3.253175	0.566494	1.111977	2.496407	0.567619	0.713846
1.486551	0.672468	0.132107	0.152532	0.154444	0.049834	0.327143	0.838846	0.088283
0.092691	0.236638	0.990909	0.197128	0.198405	0.238889	2.818312	0.818066	0.16145
2.45575	0.870375	4.814661	6.876392	0.209978	6.933961	0.024141	1.243442	0.348139
0.630505	0.736551	0.722626	0.347908	0.919827	0.35676	1.89373	0.657547	1.128788
1.734949	2.970144	0.92816	0.654423	5.568391	1.295108	0.630022	1.673254	2.805382
1.384921	2.058773	4.715599	0.74868	1.916782	1.898153	0.024141	3.500808	2.210866
0.784805	1.04228	0.203254	0.518139	0.742872	0.263146	4.086926	1.146118	1.688045
0.318499	14.17388	0.002338	530.1465	1.252424	144.7919	3.944084	0.285844	0.705231
0.169675	4.597792	0.002338	1.286494	5.110159	0.911905	0.024141	0.189141	0.225339
0.224762	0.253074	1.059553	0.171472	0.809481	0.021429	0.160779	0.537807	0.477446
12.16681	28.87506	0.002338	184.443	4.33E-05	155.7103	0.024141	8.66E-05	0.000115

0.247085	0.144719	0.071775	0.355556	0.242771	0.078081	3.479134	0.377561	0.550664
7.22E-05	0.066472	0.231984	0.216025	0.06088	0.049834	0.573326	0.148124	0.278802
2.150303	1.650274	1.387612	1.168326	1.954993	0.911905	31.55417	2.371833	3.160022
8.493088	7.751869	29.03196	12.93651	5.28039	31.1753	5.454949	19.53061	7.558139
2.34355	10.48101	0.863189	0.152532	3.8379	0.087807	2.160649	1.609654	0.832013
8.780115	5.638759	2.683478	1.997352	10.73084	3.703175	1.708932	8.958225	14.12978
1.094206	1.514199	0.551573	0.384632	0.809481	0.188427	1.549711	1.416407	1.884935
3.36075	2.871212	1.302107	1.135887	7.683766	0.903074	1.708932	2.240274	5.830152
10.01009	8.994791	6.316328	2.828918	10.40634	0.737403	57.84882	6.706472	10.1028
0.617071	2.045476	0.561126	0.493925	1.612056	0.56443	14.21671	0.649235	0.590303
7.22E-05	0.016133	0.002338	0.062107	0.069026	5.77E-05	1.282208	0.025736	0.012266
0.793918	0.656003	0.077417	0.583369	0.809481	0.380851	0.024141	0.559221	0.357013
0.070909	0.202294	0.763939	0.273434	0.27759	0.204531	12.36443	0.213059	0.153983
15.77678	5.696263	5.28039	2.839351	6.063276	4.348882	0.971205	10.62739	9.946364
0.338499	0.542165	0.588954	0.000231	0.198405	0.049834	4.402973	0.009394	0.401724
1.845519	0.571674	0.252619	2.112843	0.260404	1.499214	0.306602	1.819221	0.638362
1.267085	0.757763	1.805166	0.478182	0.465563	0.923874	2.773644	1.133622	0.921739
0.022886	0	0.061025	0.000231	0.017063	5.77E-05	0.383074	0.009394	0.051595
5.530274	3.820758	2.544747	1.405772	4.18456	2.855296	8.728571	2.668896	6.808369
0.416219	0.723066	1.050231	1.070693	0.324913	1.609654	0.024141	0.442496	0.208254
7.22E-05	0.016133	0.071775	0.000231	0.11039	5.77E-05	2.773644	8.66E-05	0.241991
0.255087	1.285584	0.394603	2.565556	0.516825	1.826147	0.024141	0.57531	2.210866
5.855281	2.807937	4.921082	0.807136	3.711097	1.131472	33.71895	1.819221	9.012937
2.08013	1.195794	4.419062	5.720173	1.022929	5.889798	1.104805	2.390447	2.27254
0.375281	0.571674	1.265426	1.113304	0.538773	2.116032	0.46461	0.354798	0.270837
4.170051	2.789466	6.612121	1.378362	3.610707	1.852338	11.09133	4.445851	6.287561
2.538218	3.428788	4.02088	1.346991	1.890996	1.679596	2.022778	3.464315	5.373781
7.315512	0.93632	2.002193	8.226219	0.555036	3.350224	1.708932	8.711905	4.875527
1.363557	0.305657	0.164509	4.451154	0.652807	0.763102	0.024141	0.890577	0.780087
3.711097	1.082915	0.666097	2.805382	1.290065	1.985253	0.28728	2.975974	2.964762
16.9162	9.141299	19.06893	4.575123	6.601833	9.924372	2.955916	16.22108	17.39935
7.22E-05	1.240981	0.002338	5.855281	4.33E-05	5.42381	0.024141	8.66E-05	0.000115
11.77567	18.62711	38.94854	14.88584	21.54823	14.93173	15.276	9.880779	13.26964
6.268312	1.41368	19.68088	15.98892	4.673182	6.423911	6.019971	5.813247	7.911934
0.022886	7.22E-05	0.002338	0.000231	4.33E-05	5.77E-05	0.630022	0.055339	0.590303
0.667965	2.386248	2.683478	1.118817	2.936739	1.174315	0.024141	1.108276	0.559221
4.709033	5.130411	8.048095	0.548153	7.795642	0.954141	5.395584	5.638759	8.905642
1.070115	1.669466	0.802035	2.477027	1.930216	0.675556	2.361198	0.763939	0.949827
1.695216	2.355289	0.853752	0.392944	1.710173	0.49974	0.573326	2.000592	0.941104
2.730072	2.970144	5.165599	11.17281	0.998312	2.169509	0.024141	2.589185	0.628297
7.22E-05	0.040678	2.243911	749.7839	4.33E-05	70.11932	0.024141	8.66E-05	0.000115
19.53061	15.71851	0.002338	135.5245	4.33E-05	135.5245	0.024141	15.77678	10.98644
1.567576	1.188139	1.554185	1.462143	0.742872	0.56443	7.162698	2.839351	2.950173
0.127532	0.112121	0.077417	0.086025	0.087677	0.059459	0.437576	0.299444	0.468694
2.02956	1.941703	0.331429	0.893016	2.703485	0.684033	0.51316	1.356364	1.755988
0.820931	3.363911	2.858009	4.424416	2.02956	4.424416	0.024141	0.838846	0.270837
0.803211	0.681494	1.145426	0.086025	0.606154	0.149293	2.320072	1.146118	2.280101
0.149423	0	0.002338	0.000231	4.33E-05	5.77E-05	4.456912	8.66E-05	0.897006
14.79742	27.66779	32.49718	9.707677	23.16622	21.93452	2.192496	17.03919	6.316328
0.735382	0.388326	0.740952	0.731732	0.260404	0.659582	0.024141	0.818066	0.769365
0.149423	0.027965	0.002338	0.000231	0.032092	5.77E-05	0.630022	0.014964	1.374329
5.606739	3.921645	4.580938	1.877605	3.864827	1.997352	23.16622	6.09759	8.573434
2.652107	1.204228	1.517143	0.740101	1.272756	0.598456	0.160779	2.400455	2.898196
0.318499	0.236638	0.002338	0.043391	0.382626	5.77E-05	0.91404	0.273434	0.1686
0.255087	0.236638	0.386378	0.556753	0.222193	0.139697	1.104805	0.587273	1.046703
3.566753	1.445916	0.231984	3.034747	0.516825	3.703175	0.024141	1.910974	1.060823
0.164509	0.00119	0.386378	0.000231	0.069026	5.77E-05	0.217677	0.266681	0.097186
0.526566	0.135354	0.331429	0.162107	0.428932	0.204531	0.28728	0.121147	1.007864

0.099286	1.179885	0.002338	1.77404	1.194221	1.124574	0.024141	0.219481	0.000115
6.956349	3.820758	0.002338	1.600238	2.628225	4.317648	0.024141	10.19468	8.608153
4.667518	1.888023	4.272165	2.341573	2.451645	3.581688	18.26752	5.130411	7.08733
28.87506	7.507619	2.572309	51.52195	1.502323	20.94257	0.024141	45.1045	18.76954
3.011169	1.113304	2.99987	1.971183	1.569553	1.267085	0.024141	3.321284	1.158384
1.486551	1.204228	0.002338	21.74051	4.33E-05	17.39935	0.024141	1.852338	0.000115
0.592208	0.532381	0.203254	0.53912	0.670144	0.245325	1.746724	2.104358	1.703795
6.853608	3.632973	7.200505	11.09133	6.011515	10.82954	0.024141	3.869163	4.557605
10.1459	5.439278	0.777864	0.000231	0.61969	5.77E-05	2.708341	6.55039	6.144055
16.44768	15.56131	5.889798	6.404921	10.67991	9.541645	6.717056	15.88548	17.58415
2.373824	2.121198	2.600014	0.000231	3.479134	5.77E-05	2.361198	2.394416	1.431414
11.42512	17.34065	8.83241	6.695887	16.44768	10.1028	5.395584	10.35839	10.19468
0.705231	1.04228	0.520678	1.31	1.557482	2.270837	0.024141	0.734524	0.216602
2.659214	4.145209	10.38202	4.203001	8.226219	5.388405	0.024141	2.114437	1.47018
0.059408	0.052431	0.002338	0.062107	4.33E-05	0.188427	2.234538	0.213059	0.33969
2.708341	1.433189	2.750375	2.353247	0.718766	4.222583	0.51316	1.684646	1.112641
1.724805	0.91404	1.581912	5.687879	0.697496	2.333867	0.630022	1.269495	0.570317
1.853723	5.47	3.414574	0.712929	2.02956	0.371299	0.51316	2.696061	2.931068
0.008369	0.00119	0.061025	0.028333	0.007821	0.006912	0.41026	8.66E-05	0.012266
2.018167	9.101674	3.188918	2.060491	8.923117	2.069942	0.024141	1.579798	2.173081
18.62711	9.502294	17.15902	5.416623	8.590794	7.795642	0.805238	17.51961	8.288874
2.797835	2.845036	4.739668	0.444986	2.124574	0.763102	0.437576	2.053521	2.053521
7.22E-05	0.059459	0.002338	0.000231	4.33E-05	5.77E-05	0.024141	0.383622	0.590303
3.070952	3.574387	5.388405	10.05618	2.392431	8.815123	1.670786	3.851414	4.63189
9.459177	4.900996	13.15079	3.984747	5.409437	5.770996	0.265786	6.622092	4.959358
7.22E-05	0.112121	0.002338	0.000231	4.33E-05	0.068932	0.437576	0.114719	0.485325
0.263146	0.021696	0.362179	0.117734	0.007821	0.254574	0.630022	0.127128	0.518413
2.157359	2.180022	0.630823	9.459177	2.183665	4.165137	0.971205	2.31404	1.103261
1.281241	3.807648	8.608153	0.106847	6.738211	0.272078	1.455988	2.143261	1.460051
0.443781	0.895368	0.472713	0.617763	0.409618	0.615996	4.752121	0.509791	1.197922
2.114437	0.60886	0.967446	6.054242	0.652807	2.855296	4.678846	1.8386	2.053521
0.974257	0.700058	2.032706	1.368167	0.836537	0.903074	8.780115	0.722626	1.03303
0.247085	0.236638	1.335743	0.052338	0.119322	0.087807	0.437576	0.354798	0.296955
4.508196	7.212915	0.508658	16.5625	9.256537	6.30671	0.024141	4.490801	4.702468
1.688045	0.078038	0.002338	0.106847	0.501905	0.078081	2.320072	0.922338	2.673846
5.056421	1.308167	0.967446	8.888167	2.312085	4.821176	0.024141	2.950173	2.091313
0.143838	0.010592	0.371587	0.000231	0.119322	0.006912	0.35241	0.096905	0.032727
0.194848	0.135354	0.353333	0.000231	0.087677	5.77E-05	0.850808	0.425635	0.931457
0.037525	0.305657	0.002338	0.043391	0.296465	0.031046	0.35241	8.66E-05	0.539812
0.289603	2.069942	1.805166	4.484892	1.930216	2.022778	0.024141	0.559221	0.373939
0.438023	1.022929	0.353333	0.028333	1.095657	0.006912	18.99374	0.685209	3.435988
95.22939	9.604358	144.7919	10.98644	27.96248	12.29473	59.32013	30.49289	51.52195
15.98892	23.6012	26.25557	5.24562	17.28195	11.14619	6.560411	10.23768	12.93651
1.116609	0.800967	0.639892	0.43684	1.674401	0.819509	5.395584	1.233665	2.673846
3.514834	1.137244	6.876392	2.388348	0.998312	3.227085	1.708932	2.300678	2.644885
7.22E-05	0.016133	0.002338	0.000231	4.33E-05	5.77E-05	1.312706	0.181919	1.42456
0.187532	0.780087	0.181385	0.095851	0.730873	0.139697	0.160779	0.291861	0.225339
3.679986	32.31123	0.002338	36.74522	52.56128	40.7932	0.024141	3.122915	5.923838
0.644235	0.645916	0.195685	2.216162	0.578817	2.936739	0.024141	0.530253	2.600014
2.150303	2.027951	0.809481	0.859351	1.995714	0.932078	1.225426	2.800332	2.931068
3.367071	5.062626	2.304452	1.41368	3.966623	1.775325	16.5625	2.649675	5.024293
0.483874	0.700058	0.261775	0.478182	1.194221	0.494841	0.024141	0.550664	0.437576
0.784805	37.29684	0.002338	8.103925	12.70768	13.38418	0.024141	2.708341	0.278802
4.435159	34.84299	0.002338	22.52375	7.037345	36.26061	0.024141	9.838658	0.000115
0.232215	0.280094	0.526032	0.035981	0.390895	0.031046	0.217677	0.249091	0.233889
126.1556	52.56128	3.953297	38.94854	31.55417	80.6518	0.024141	135.5245	40.11561
3.890556	0.542165	3.585029	1.378362	1.545657	0.719177	4.946587	4.863579	13.00996
3.095166	3.163506	3.207807	0.726133	9.335397	1.950519	0.160779	2.909293	2.173081

0.696234	1.022929	0.777864	0.095851	0.373939	0.068932	1.670786	1.403853	0.270837
3.993665	5.500678	17.39935	6.772763	6.433918	2.463911	0.383074	3.783694	2.584394
0.57197	3.211176	0.148254	3.489899	2.591443	3.198499	0.6657	1.05088	1.602619
6.876392	7.751869	12.42965	24.98218	9.418196	37.81919	0.024141	11.48368	1.905094
36.74522	50.55949	6.210375	87331.38	105.1694	111.5439	0.024141	25.24367	20.34063
1.741414	0.856782	4.644098	0.000231	4.881775	5.77E-05	20.51271	8.66E-05	0.000115
1.806595	0.820931	2.084791	0.1379	0.998312	0.668947	1.159913	1.285584	1.560779
0.832554	1.495043	0.002338	0.035981	0.555036	0.059459	2.234538	0.345635	0.856782
0.022886	0	0.002338	0.086025	0.017063	0.031046	2.396429	0.025736	0.027922
1.033672	2.713319	0.551573	0.000231	2.640332	0.505267	1.925635	2.255916	2.417424
0.022886	0.091616	0.083521	0.376017	0.078737	0.430281	2.192496	0.041443	0.216602
12.29473	1.308167	3.775209	100.0998	4.33E-05	55.04081	53.77619	6.900202	0.000115
3.754719	95.22939	0.002338	5.395584	4.222583	7.584502	0.024141	5.395584	2.240274
0.20215	0.10531	0.290866	0.117734	0.095772	0.068932	0.437576	0.291861	0.664026
1.384921	1.553074	0.763939	1.081544	1.213434	1.244957	2.320072	1.356364	2.533745
2.863211	2.863211	3.36075	0.712929	4.758088	1.788874	0.024141	2.708341	0.147807
1.688045	1.799899	1.821861	3.028629	1.930216	2.797835	18.48514	2.50469	2.533745
1.246457	1.590202	0.978571	0.851234	1.502323	0.554416	3.144156	1.126775	1.225426
0.326219	0.397489	0.298153	2.554069	0.119322	0.730476	10.28573	0.790837	0.416219
1.172872	0.883665	0.508658	0.617763	0.891176	0.995527	3.230065	1.369928	1.084437
1.599033	3.500808	0.066696	6.772763	4.251818	4.277006	0.142698	2.990851	2.584394
0.705231	0.416219	0.543939	0.180079	0.428932	0.13039	2.818312	1.002215	2.400455
11.25392	8.412367	5.9071	19.37139	3.204632	33.93939	0.024141	21.54823	0.73583
0.07767	7.22E-05	0.173189	0.000231	0.007821	5.77E-05	1.073543	0.127128	0.366147
1.027403	1.982208	1.059553	1.330029	2.045476	0.179545	7.751869	1.645613	2.644885
5.439278	3.536955	4.94026	2.101075	8.888167	5.266335	0.024141	6.079596	2.35733
0.116659	0.194372	0.434069	0.233175	0.175144	0.049834	2.061926	0.014964	0.097186
27.66779	16.5625	35.776	33.93939	13.30906	28.23632	2.955916	25.99427	18.91854
4.376118	4.614618	3.172864	5.309264	3.895469	6.191515	1.073543	4.323045	2.320072
9.335397	4.715599	0.895945	0.640289	30.17192	0.295837	0.024141	2.50469	12.22798
0.180895	0.010592	0.156089	0.000231	0.209978	0.121494	1.195079	0.197338	0.278802
7.363117	8.677489	6.374812	10.1459	6.772763	10.28573	45.91841	14.17388	14.42661
7.737807	1.697792	0.195685	5.082063	2.524892	5.959033	0.024141	5.238701	4.212554
144.7919	57.84882	47.5018	24.04374	87.1746	31.55417	8.210938	144.7919	95.22939
0.175036	0.227446	0.532092	0.189141	0.269141	0.43684	1.455988	0.383622	0.392251
13.75978	9.063983	0.188745	3.227085	16.68554	3.479134	7.238023	8.477662	16.15795
25.24367	7.417359	13.96064	18.48514	9.297085	20.94257	3.373983	32.68313	15.276
7.22E-05	0.046667	0.188745	0.062107	0.186017	0.059459	0.024141	8.66E-05	0.140087
0.013175	7.22E-05	0.002338	0.095851	4.33E-05	0.113074	0.82772	0.066407	0.032727
2.119596	2.487576	0.717027	3.87329	1.596782	4.06303	0.024141	2.187395	0.493925
25.99427	4.49671	0.132107	55.04081	0.428932	20.34063	1.670786	18.05488	4.900996
0.017778	0.00119	0.071775	0.000231	0.069026	0.040909	0.437576	0.055339	0.058694
0.240202	1.364401	0.090476	1.05824	0.982698	1.066948	0.971205	0.205801	0.113521
0.57197	0.227446	1.767431	0.996696	0.1379	0.323319	2.625823	0.189141	0.270837
0.11075	0.372973	0.261775	0.171472	0.27759	0.031046	2.589185	0.299444	0.769365
7.854069	5.671126	7.443276	2.207417	11.83593	2.134545	4.359574	11.89932	14.88584
1.135137	0.820931	1.041025	0.22456	0.899567	0.196436	3.083319	1.133622	0.839365
6.116638	6.019971	4.053506	6.591544	6.52899	6.268312	1.670786	6.325945	8.849697
83.489	53.77619	10.98644	26.80355	25.73165	22.52375	3.718716	87.1746	167.9996
0.070909	0	0.242771	0.000231	0.146999	0.158521	1.159913	8.66E-05	0.175592
0.20215	0.188124	0.090476	0.014545	0.250823	0.013117	1.282208	0.254769	0.278802
15.66023	72.69473	29.03196	37.29684	36.26061	34.84299	11.42512	18.76954	30.49289
2.08013	3.128694	3.864827	1.05824	4.907929	2.561162	1.046061	2.248896	1.947316
0.194848	0.43347	0.93632	0.127626	0.260404	0.013117	3.599329	0.225209	0.459769
1.486551	1.022929	0.090476	0.323535	0.324913	0.212698	22.42294	1.172872	5.024293
0.492165	0.93632	0.002338	0.243341	0.933261	0.139697	8.83241	0.702835	0.105873
5.143867	3.317807	0.648038	5.351934	2.524892	1.146962	13.79669	3.939307	13.45939
0.667965	0.736551	4.502453	0.189141	0.670144	0.113074	1.708932	1.233665	1.439704

37.81919	40.11561	15.88548	7.625209	46.7056	10.43066	83.489	43.49658	57.84882
7.22E-05	0.006061	0.148254	0.070736	0.163983	5.77E-05	0.598456	0.020267	0.088283
0.81228	2.831501	0.465325	4.609076	0.891176	4.086926	3.899993	1.43039	1.42456
0.187532	0.021696	0.269697	0.000231	0.087677	5.77E-05	0.573326	0.121147	0.044805
1.451775	2.355289	2.984899	1.237879	2.392431	0.581111	64.12971	1.869322	2.842193
10.57984	4.281847	6.268312	1.971183	3.122915	2.498478	5.942294	8.677489	14.12978
2.640332	2.511465	5.409437	2.365685	1.465123	1.65785	7.625209	3.207807	4.312251
0.1379	0.118903	0.054574	0.000231	0.146999	0.087807	0.746551	0.160072	0.183824
6.653016	6.784762	53.77619	0.654423	8.381328	8.319019	0.024141	3.529466	1.518232
1.419278	1.152165	2.068297	2.8657	1.036558	1.443059	4.359574	1.77404	1.374329
0.318499	0.388326	0.789423	0.837864	0.61969	0.350274	21.36892	0.319957	0.675938
12.85504	8.573434	9.459177	15.88548	12.36443	11.96574	13.96064	13.42179	7.38847
1.667273	1.87482	2.842193	1.135887	3.930664	2.134545	0.024141	1.609654	2.189235
0.312323	2.500548	0.465325	6.09759	0.555036	1.841371	0.024141	1.29342	1.632648
0.1379	0.416219	0.630823	0.285253	0.588954	0.314156	7.099913	0.722626	0.1686
24.73724	30.17192	26.80355	22.74797	28.87506	41.47925	10.98644	25.48179	21.93452
8.103925	4.440548	9.377345	14.6058	2.408882	7.30215	1.312706	4.241905	2.210866
0.159351	1.41368	0.276609	0.145556	1.290065	0.363059	1.389509	0.319957	0.241991
8.711905	17.78522	17.03919	1.997352	14.79742	3.55961	14.21671	6.297136	16.9162
40.7932	45.91841	167.9996	47.5018	91.05541	87.1746	1.708932	41.47925	15.07257
0.57197	2.62342	9.218514	0.683665	1.873434	0.939986	24.98218	1.269495	1.932857
0.416219	0.63873	0.622496	0.216025	1.036558	0.371299	0.746551	0.420058	0.949827
6.830592	10.43066	7.795642	0.963853	19.22139	0.840635	1.417345	5.728615	5.530274
3.019848	3.771349	18.05488	14.79742	1.733766	6.52899	3.083319	1.660216	1.947316
3.659668	0.949827	0.425426	9.625253	0.859351	7.625209	2.496407	4.160238	5.500678
3.824964	5.545729	8.319019	2.953059	2.727785	3.147294	12.06778	4.990794	6.738211
2.08013	1.364401	2.477027	1.330029	0.88158	0.684033	15.66023	2.683478	5.830152
0.070909	0.021696	0.066696	0.264048	0.024473	0.087807	0.485577	0.077027	0.051595
18.76954	25.24367	38.39042	13.00996	20.16541	21.74051	26.25557	23.6012	33.4985
7.099913	2.855296	3.807648	2.327857	2.55184	7.824473	0.024141	8.888167	8.164531
2.306277	2.261573	0.666097	2.417424	1.690483	1.64917	0.024141	4.381479	1.87482
6.240346	16.9162	4.563117	2.485534	13.08214	6.760765	0.51316	7.991602	2.106089
4.597792	5.01772	3.885642	1.78759	5.238701	4.552092	0.88158	4.06303	5.095325
0.342987	0.28899	0.515815	0.043391	0.566494	0.231876	0.6657	0.43347	0.503463
0.483874	0.16119	1.651522	0.070736	0.324913	0.049834	2.74544	0.621688	0.790303
0.696234	0.388326	0.283882	0.355556	0.755253	0.413009	0.91404	0.425635	1.602619
2.445079	1.378362	4.207778	1.613232	1.011486	0.451429	4.851227	2.765758	2.026219
0.858297	0.219134	1.856392	0.347908	0.399784	0.304387	1.355527	1.243442	0.000115
18.48514	11.83593	28.23632	65.94551	6.99026	75.1132	1.549711	16.9162	12.85504
2.931068	0.16119	0.688579	5.622872	4.33E-05	4.150274	0.024141	4.678846	0.960216
0.609509	0.656003	3.824964	0.693218	0.687049	2.73272	4.359574	0.890577	0.664026
3.457504	3.279625	3.493362	2.388348	2.611797	1.618773	34.84299	2.223636	2.898196
1.591299	3.137576	4.839105	0.233175	4.067547	0.487662	2.818312	3.820758	7.495397
2.773644	8.075772	0.362179	1.930216	14.33939	1.997352	4.193232	2.147013	3.606804
16.22108	16.22108	7.136537	4.965801	17.03919	8.256465	16.09482	13.30906	20.51271
1.429365	46.7056	42.75576	41.47925	12.06778	45.91841	36.74522	2.784228	2.621133
7.061328	0.93632	0.622496	22.13245	0.516825	7.468961	1.588903	7.6393	6.404921
0.600548	4.575123	1.135137	2.259661	4.039192	5.704646	0.437576	0.628658	0.257518
10.80505	13.60866	6.063276	4.869553	7.977958	4.086926	0.6657	6.365065	9.397771
2.238341	2.59557	8.075772	0.925801	2.828918	0.505267	2.92013	3.008225	2.252273
0.974257	4.317648	1.327525	0.731732	2.55184	0.615996	0.28728	0.530253	0.257518
0.037525	7.22E-05	0.061025	0.078081	0.011696	0.021429	0.850808	0.020267	0.097186
3.253175	5.515584	20.68746	2.296732	6.079596	4.043846	0.573326	4.678846	2.494177
27.38726	26.80355	13.83359	7.795642	16.22108	15.56131	1.988196	31.92385	32.68313
0.005916	0.000188	0.042266	0.014545	4.33E-05	0.006912	0.180231	8.66E-05	0.008882
0.143838	0.040678	0.002338	0.052338	0.186017	0.021429	0.746551	0.127128	0.65101
87.1746	42.75576	21.02638	9.256537	30.82277	6.900202	8.643074	75.1132	105.1694
0.579841	0.949827	0.083521	0.461811	0.448261	0.413009	9.562554	0.825354	3.842554

0.092691	0.000433	0.054574	0.306176	0.011696	0.031046	1.417345	0.009394	0.044805
34.84299	13.6814	22.52375	6.486104	4.907929	6.8421	35.776	27.38726	42.08807
4.150274	1.761349	1.468131	1.179885	1.750628	1.909408	4.034423	4.614618	5.770996
7.22E-05	7.22E-05	0.116494	0.117734	0.017063	0.105022	1.853723	8.66E-05	0.132626
0.381999	0.245469	1.030678	0.062107	0.428932	0.13039	4.251818	0.153867	0.664026
0.507403	0.723066	0.402698	0.485325	0.209978	0.323319	3.266782	0.544214	2.106089
64.12971	126.1556	18.62711	23.82354	135.5245	21.19547	167.9996	100.0998	111.5439
7.519841	19.22139	37.81919	2.269134	22.96463	1.554185	8.303947	5.545729	6.486104
9.792626	11.36975	8.888167	4.702468	12.49452	6.933961	26.52537	10.5063	19.68088
6.922771	1.637309	6.706472	5.068831	0.730873	2.593506	0.850808	7.795642	3.273045
332.0776	47.5018	1.629235	29.82828	59.32013	60.77449	1.417345	203.5795	80.6518
13.08214	8.461703	6.240346	3.55961	9.063983	5.485685	14.33939	15.56131	9.459177
1.880375	0.949827	0.520678	0.629098	1.272756	0.668947	2.274473	2.961768	4.025267
1.188139	2.386248	6.00228	0.818066	1.519322	1.609654	11.17281	0.855267	0.493925
3.422237	2.928247	7.08733	0.74868	2.068297	2.77899	17.03919	4.281847	4.277006
5.095325	10.1459	5.00404	2.051962	9.729358	1.809343	2.547179	7.264502	11.25392
45.91841	11.89932	0.22303	3.008225	6.230245	5.095325	15.07257	53.77619	135.5245
2.127973	0.152655	0.002338	0.052338	0.087677	0.204531	1.159913	0.593434	2.600014
0.905426	5.28039	0.945483	1.558571	6.601833	1.801284	9.377345	3.45404	0.518413
23.38378	28.54786	24.98218	33.4985	16.5625	19.53061	155.7103	32.31123	83.489
41.47925	67.98069	111.5439	11.60283	64.12971	17.92188	8.210938	47.5018	33.93939
5.180058	1.495043	14.6058	0.693218	12.06778	0.62645	2.66404	3.66785	6.55039
12.77772	7.351284	3.397706	6.079596	7.710361	6.612121	48.53429	13.23022	12.16681
4.323045	3.47531	12.22798	0.968723	5.068831	1.527792	0.598456	7.175397	4.609076
13.60866	3.730808	13.30906	75.1132	1.94311	21.54823	29.82828	15.18102	7.938427
0.232215	3.150433	12.16681	1.35259	0.990188	4.821176	7.443276	0.398629	1.29417
1.827403	1.925635	0.002338	7.495397	0.859351	3.411097	2.160649	4.563117	7.882222
24.50942	9.031082	8.539971	425.62	9.838658	59.32013	3.899993	38.39042	4.978182
0.085426	0.16119	0.322395	0.035981	0.305087	0.021429	2.818312	0.121147	0.140087
0.663651	0.194372	1.615281	0.285253	0.529971	0.522367	1.073543	1.163579	1.112641
3.599329	3.043528	1.805166	9.316241	0.128283	2.66404	0.485577	7.951371	6.210375
9.667316	12.42965	14.51648	0.875599	14.70188	0.730476	0.024141	7.112496	4.429769
0.986104	0.816479	1.31	0.171472	1.111977	0.634531	1.009625	0.810967	1.076652
3.796075	2.408882	0.717027	3.144156	3.223882	2.515938	1.809343	3.738997	6.64254
7.22E-05	0.003016	0.083521	0.000231	0.011696	0.013117	0.630022	0.020267	0.000115
19.68088	22.32214	70.11932	5.522929	37.81919	7.964315	23.6012	22.74797	20.16541
87331.38	87331.38	87331.38	30.17192	87331.38	100.0998	425.62	87331.38	749.7839
155.7103	118.7028	749.7839	155.7103	203.5795	126.1556	224.9555	184.443	184.443
0.540188	0.757763	1.776739	0.947677	0.305087	0.401176	5.217569	0.57531	1.000794
31.1753	30.82277	18.33909	27.66779	31.92385	34.38341	9.101674	29.18885	31.92385
1.256558	0.723066	0.605022	0.000231	1.532468	0.304387	1.514199	1.308167	0.82355
4.23202	3.599329	4.984488	6.181652	4.408153	1.930216	9.792626	4.281847	6.355224
0.11075	0.425635	1.427431	0.791371	0.324913	0.087807	27.10502	0.377561	0.140087
0.013175	0.260144	3.095166	0.254156	0.697496	0.572987	0.024141	0.114719	0.208254
0.031818	0	0.173189	0.000231	0.024473	0.059459	0.850808	0.035339	0.079921
1.543377	32.68313	62.33472	2.688304	26.52537	5.9071	38.39042	3.043528	3.672078
0.763564	25.73165	155.7103	28.23632	14.6058	33.07147	105.1694	2.223636	1.61088
0.05189	0.118903	0.098124	0.028333	0.222193	0.013117	1.455988	0.090303	0.044805
44.27012	91.05541	36.26061	62.33472	83.489	65.94551	17.22048	49.50903	77.74812
0.187532	0.182929	0.394603	0.000231	0.209978	0.021429	0.544545	0.273434	0.032727
7.136537	22.96463	16.80343	9.063983	13.15079	25.24367	1.159913	7.38847	9.121486
7.22E-05	0.245469	0.002338	0.000231	0.047504	5.77E-05	0.265786	8.66E-05	0.459769
1.401068	0.832013	0.298153	2.092987	0.373939	1.618773	2.440895	1.828781	2.252273
0.630505	0.21088	0.394603	0.052338	0.154444	5.77E-05	0.241688	0.398629	0.051595
0.013175	0	0.002338	0.000231	0.007821	0.006912	1.588903	0.014964	0.058694
1.429365	0.91404	0.002338	0.152532	1.381111	5.77E-05	0.198694	1.51215	3.040505
29.82828	26.25557	14.88584	15.66023	29.49693	17.64869	47.5018	26.25557	38.39042
9.968355	8.149307	3.300664	14.97762	7.013853	3.163506	9.377345	12.49452	14.79742

13.38418	8.103925	5.102742	3.783694	10.53159	3.877417	6.717056	12.22798	17.92188
4.408153	6.019971	4.23202	6.054242	5.024293	3.975498	21.54823	5.638759	4.673182
1.307417	3.552049	14.33939	3.204632	1.812114	5.00404	0.485577	2.696061	3.754719
0.017778	2.16E-05	0.002338	0.014545	0.011696	5.77E-05	0.383074	0.009394	0.079921
12.70768	35.27608	10.65365	3.340361	77.74812	5.068831	19.22139	14.00414	21.19547
24.2842	11.42512	6.653016	25.48179	15.66023	15.66023	3.011169	18.41211	18.62711
1.960945	0	0.002338	0.000231	4.33E-05	5.77E-05	8.412367	1.099762	2.181775
2.668896	0.92531	0.838326	2.234538	1.213434	1.182056	1.549711	1.819221	1.405772
1.591299	1.137244	0.339235	0.444986	1.612056	0.659582	2.192496	1.609654	2.153535
8.994791	6.019971	7.868146	4.323045	6.853608	7.765931	25.73165	7.854069	8.711905
1.045462	1.338261	3.055729	0.740101	1.834423	1.211299	4.946587	1.303045	0.69583
3.869163	0.571674	8.288874	1.300368	4.203001	3.080317	2.320072	6.365065	6.486104
8.923117	13.34662	20.51271	12.63773	8.745238	8.958225	39.53687	14.33939	17.58415
11.36975	17.64869	29.49693	12.39704	15.77678	18.33909	13.5739	14.97762	5.754935
0.037525	0.000188	2.084791	0.035981	0.250823	0.021429	2.440895	0.102886	0.000115
14.00414	7.443276	12.36443	5.788153	11.06488	11.25392	0.383074	19.37139	20.68746
0.11075	0.118903	0.371587	0.000231	0.48619	0.113074	1.225426	0.225209	0.147807
13.00996	18.33909	34.38341	4.077403	19.37139	3.207807	77.74812	26.52537	32.31123
0.57197	1.799899	0.905426	0.670519	1.080909	0.263146	3.083319	0.912446	1.238651
1.837172	20.68746	5.439278	4.94026	40.11561	3.055729	0.805238	0.832013	2.145137
0.326219	0.169971	0.042266	0.285253	0.409618	0.709784	0.241688	0.390216	0.348139
0.741457	0.91404	1.68938	2.148658	0.250823	1.841371	0.573326	0.685209	0.287641
15.18102	10.33476	27.38726	3.750837	4.18456	5.583968	33.71895	15.276	6.063276
0.276429	0.425635	0.895945	0.020087	0.409618	0.031046	1.628009	0.219481	0.416219
9.101674	38.94854	3.182482	3.846984	67.98069	4.529913	0.630022	8.256465	15.18102
3.599329	3.243225	1.94311	43.49658	5.770996	22.32214	8.905642	8.556703	4.419062
0.013175	0.021696	0.148254	0.000231	0.186017	0.021429	0.142698	0.066407	0.044805
7.795642	3.404372	11.83593	1.473218	6.674286	3.860491	8.033932	6.25938	4.851227
2.755411	1.009625	0.140303	0.392944	0.767085	0.812771	1.746724	2.162381	2.567605
0.431457	0.236638	0.195685	0.206465	0.198405	0.836017	0.241688	0.390216	0.437576
0.318499	1.179885	0.959062	6.570433	4.33E-05	3.321284	0.024141	0.607273	0.579841
1.793074	2.427792	0.990909	0.755253	2.252273	0.620462	1.131472	1.482626	3.160022
4.096407	24.04374	1.581912	2.577157	36.74522	1.349971	49.50903	14.25954	22.13245
11.06488	16.04187	46.7056	5.050267	12.93651	6.287561	184.443	5.84689	12.29473
1.331919	1.669466	2.572309	0.591912	2.375974	0.825354	0.942944	1.599033	1.305772
0.127532	0.16119	0.116494	0.062107	4.33E-05	0.231876	111.5439	8.66E-05	0.444315
0.533535	0.176407	0.817547	0.566782	0.054711	0.719177	14.70188	0.390216	0.072583
1.32482	1.353608	2.165851	0.755253	2.419553	0.581111	2.320072	1.04228	2.373824
7.22E-05	0.010592	0.002338	0.020087	0.007821	5.77E-05	0.160779	8.66E-05	0.126797
7.22E-05	0.710722	1.346003	0.062107	0.054711	5.77E-05	6.153102	0.061602	2.269134
7.22E-05	1.527792	0.002338	0.000231	4.33E-05	5.77E-05	3.7586	0.912446	0.000115
0.154444	0.021696	0.306176	0.062107	0.11039	0.013117	0.217677	0.160072	0.216602
1.172872	1.834423	2.895599	0.647345	5.25254	1.026219	0.142698	1.108276	3.552049
0.187532	0.112121	0.002338	0.000231	0.146999	0.031046	1.670786	0.213059	0.175592
7.22E-05	0.091616	0.164509	0.086025	0.040043	0.006912	4.007583	8.66E-05	0.027922
0.132468	0.052431	0.203254	0.106847	0.233752	0.139697	2.209141	0.219481	0.241991
3.544257	1.297763	0.622496	0.901212	4.46267	1.554185	0.41026	3.163506	2.071587
1.116609	0.21088	1.867951	1.572035	0.538773	1.974228	1.312706	0.838846	0.600548
6.028427	1.327525	0.002338	40.11561	4.33E-05	57.84882	0.024141	8.164531	5.123781
6.622092	4.857403	0.863189	1.959466	6.30671	2.022778	0.160779	2.000592	3.45404
62.33472	252.4251	41.47925	20.34063	252.4251	30.17192	0.024141	62.33472	47.5018
0.187532	0.027965	0.116494	0.070736	0.186017	0.121494	0.46461	0.160072	0.270837
0.416219	0.118903	1.030678	0.22456	0.233752	0.105022	1.131472	0.544214	0.477446
0.774798	1.072843	2.386248	0.968723	0.632857	0.554416	4.402973	0.743283	1.147807
1.761349	2.654473	1.68938	0.837864	0.222193	1.420202	21.02638	2.290996	1.835743
2.955916	2.45575	0.124791	2.649675	1.418312	0.780087	0.024141	6.570433	6.433918
0.005916	2.16E-05	0.061025	0.206465	4.33E-05	0.013117	2.547179	0.006558	0.032727
2.35733	3.771349	4.125786	1.717641	3.276335	3.860491	1.489524	2.361198	1.61088

2.127973	0.856782	0.386378	3.722785	0.697496	1.873434	0.024141	3.266782	1.997352
6.784762	1.163579	0.002338	3.722785	4.33E-05	1.890996	1.255657	2.975974	6.079596
0.29798	0.135354	0.002338	0.000231	0.1379	0.078081	1.046061	0.030375	0.638362
4.965801	7.30215	8.923117	3.043528	9.541645	3.147294	1.588903	4.959358	5.679502
3.227085	1.732468	1.076032	9.031082	0.908247	5.515584	0.024141	3.792266	1.092785
2.091313	2.807937	3.080317	0.323535	0.501905	0.13039	1.282208	2.300678	0.278802
0.793918	3.921645	0.054574	0.323535	1.399315	0.221587	0.142698	0.790837	1.389509
0.143838	0.10531	0.394603	0.171472	0.087677	0.371299	9.562554	0.291861	0.38153
3.211176	1.941703	1.615281	2.828918	1.644416	6.045209	17.85355	4.959358	3.578038
2.597792	2.081696	1.731169	2.618846	2.259661	5.24562	0.024141	3.253175	1.210447
40.11561	44.27012	17.51961	11.25392	50.55949	17.09911	0.719942	37.81919	34.84299
1.172872	1.317475	1.102691	1.081544	0.845887	0.528997	1.073543	0.931457	1.092785
0.013175	0.00119	0.047431	0.000231	0.011696	5.77E-05	0.241688	0.061602	0.018038
0.756176	0.388326	3.113478	0.254156	0.456522	0.522367	2.160649	0.890577	0.846342
0.477446	0.091616	0.140303	80.6518	0.296465	47.5018	0.6657	8.66E-05	0.088283
0.974257	0.227446	0.002338	1.450693	4.33E-05	2.047157	0.024141	8.66E-05	0.000115
13.45939	8.711905	10.85542	14.42661	10.57984	9.418196	3.011169	15.07257	16.34294
11.83593	8.048095	11.93253	29.49693	0.718766	32.31123	0.024141	12.85504	3.993665
0.592208	0.388326	1.093434	0.043391	0.845887	0.059459	0.630022	0.559221	0.518413
6.240346	15.88548	9.141299	19.99861	17.15902	19.83727	0.024141	2.975974	6.433918
1.604827	12.26136	0.88158	17.28195	4.38684	7.443276	0.024141	0.354798	0.780087
3.446732	1.853723	2.055289	2.124574	1.399315	1.542302	13.26964	2.153535	6.486104
1.718918	1.317475	0.465325	0.197128	0.789423	0.139697	5.158146	0.847908	0.906126
0.208355	0.227446	0.148254	0.127626	0.28728	0.196436	2.547179	0.47	0.897006
6.297136	1.170491	2.234538	5.704646	0.566494	10.73084	0.024141	3.058802	7.795642
4.312251	2.104358	1.835743	1.248232	1.164257	0.43684	1.159913	3.310758	2.429957
0.774798	1.455988	0.681068	0.640289	0.899567	0.868341	0.024141	0.47	0.366147
0.827273	0.870375	0.483045	0.285253	0.919827	0.371299	0.241688	1.146118	0.686299
24.04374	8.319019	25.48179	77.74812	13.91714	24.98218	4.193232	22.13245	25.99427
3.514834	2.638139	9.541645	1.547734	7.507619	2.481356	0.024141	3.588369	3.273045
1.340036	1.995714	1.91811	0.306176	2.688304	1.085224	0.024141	1.05088	0.931457
0.715462	43.49658	16.34294	6.181652	2.671299	13.91714	0.024141	0.398629	0.357013
0.949286	1.104033	2.351299	0.53912	0.933261	1.04228	0.024141	0.134444	0.638362
7.238023	2.823636	0.394603	2.259661	4.165137	4.402973	0.024141	8.745238	2.941544
5.030866	1.308167	1.252424	1.070693	2.855296	1.754733	0.180231	4.592273	3.363911
0.164509	0.021696	0.002338	0.000231	0.024473	0.059459	0.024141	8.66E-05	0.250007
0.974257	1.682193	0.853752	0.461811	2.611797	0.668947	0.180231	0.581645	0.816479
1.124574	0.689509	4.739668	0.509242	1.022929	0.423535	4.851227	1.645613	1.771392
10.53159	5.599228	3.5257	7.08733	5.024293	3.94886	0.573326	23.16622	29.49693
0.187532	0.188124	0.002338	0.000231	0.163983	0.13039	70.11932	0.04785	0.33969
0.592208	0.689509	0.612525	1.624459	0.566494	0.620462	19.37139	0.681068	0.873579
1.351573	1.896667	3.095166	2.341573	0.982698	1.763889	0.024141	1.04228	0.000115
10.73084	31.1753	8.994791	5.295325	80.6518	5.646811	0.024141	7.570693	3.169812
0.270087	0.43347	0.261775	0.106847	0.718766	0.172734	0.28728	0.338341	0.263853
1.869322	1.256558	1.156869	2.730072	3.110447	4.914863	0.544545	1.146118	1.169791
7.22E-05	0	0.002338	0.000231	0.017063	5.77E-05	0.160779	8.66E-05	0.000115
0.644235	0.586595	1.209776	0.233175	0.399784	0.049834	0.573326	0.657547	0.493925
2.509177	3.730808	1.496118	1.070693	7.212915	2.116032	0.024141	2.174733	0.979149
0.540188	0.450924	2.217951	2.101075	0.529971	3.975498	8.728571	1.527792	0.427403
1.10132	2.058773	2.609293	0.937655	1.252424	1.012114	3.846984	0.98474	2.189235
0.008369	0.006061	0.002338	0.062107	4.33E-05	0.031046	1.549711	0.025736	0.018038
6.808369	87.1746	42.08807	45.1045	425.62	118.7028	0.024141	15.46193	0.000115
2.421681	2.331825	1.581912	2.630729	0.501905	2.24733	1.588903	1.883535	3.098268
0.070909	0.003016	0.002338	0.095851	0.078737	0.031046	0.805238	0.134444	0.113521
7.200505	0.92531	2.718434	2.008312	2.577157	1.950519	40.11561	7.175397	28.23632
4.034423	6.486104	1.931537	1.959466	21.02638	5.22482	0.024141	2.898196	0.313312
39.53687	14.25954	30.82277	25.24367	39.53687	49.50903	2.061926	105.1694	24.73724
1.554185	0.28899	1.36619	0.000231	1.290065	5.77E-05	2.625823	0.600173	0.98956

7.22E-05	0	0.071775	1.65785	4.33E-05	0.510707	0.198694	8.66E-05	0.000115
3.962179	2.463911	4.609076	1.31	1.721371	1.075411	15.56131	4.034423	1.374329
1.780693	0.870375	9.335397	7.911934	0.48619	5.679502	2.061926	1.323131	1.060823
1.188139	0.499206	2.786898	0.000231	0.305087	0.097258	3.401126	0.370657	0.257518
0.213218	0.052431	0.371587	0.095851	0.017063	5.77E-05	0.024141	0.04785	0.241991
34.38341	23.16622	57.84882	45.91841	14.93173	72.69473	0.024141	50.55949	7.710361
4.519048	3.128694	3.610707	2.124574	1.095657	3.182482	0.241688	3.043528	3.223882
0.289603	0.380851	0.339235	0.117734	0.163983	0.401176	6.784762	0.345635	0.686299
5.195022	15.46193	2.99987	2.31404	18.55613	4.18456	0.217677	5.01772	7.443276
0.381999	0.43347	0.346342	0.413449	0.516825	0.528997	9.968355	0.802395	1.262965
7.653391	6.865	7.264502	4.33342	8.677489	7.507619	42.08807	7.061328	10.01009
1.384921	1.568564	5.933066	6.210375	0.365036	2.280101	0.746551	1.133622	0.322973
0.05189	0.027965	0.116494	0.014545	0.054711	0.040909	0.805238	0.035339	0.079921
0.031818	0	0.116494	0.000231	4.33E-05	5.77E-05	0.327143	0.030375	0.038139
1.890996	1.137244	2.81342	1.106825	1.337431	0.572987	1.455988	1.609654	0.686299
17.15902	27.10502	31.1753	6.674286	26.80355	7.938427	5.00404	12.10289	11.74734
0.1379	0.963853	0.838326	0.000231	0.501905	0.068932	0.773766	0.243016	1.723579
2.561162	3.975498	6.945152	0.216025	5.210317	0.149293	9.502294	3.340361	3.29355
0.784805	0.810967	0.579841	1.508831	0.718766	3.172864	0.51316	1.008413	0.33969
4.292078	4.614618	0.945483	3.966623	3.29355	1.801284	16.68554	4.086926	4.348882
2.839351	21.74051	287.197	21.93452	3.401126	38.39042	0.024141	7.765931	5.180058
0.092691	0.066472	0.22303	0.000231	0.078737	0.068932	3.563182	0.141825	0.225339
7.22E-05	7.22E-05	0.002338	0.000231	4.33E-05	5.77E-05	0.024141	8.66E-05	0.000115
3.890556	1.240981	4.155339	0.629098	0.885664	0.753449	1.670786	3.57057	2.477027
7.22E-05	0.066472	0.188745	0.000231	0.069026	0.006912	0.160779	0.009394	0.072583
1.080909	0.723066	1.68938	0.162107	0.28728	0.13039	2.440895	0.890577	0.590303
53.77619	45.1045	27.96248	12.77772	70.11932	28.87506	44.27012	27.10502	33.07147
0.735382	0.176407	1.713997	0.127626	1.472208	0.040909	1.549711	0.847908	0.610606
0.008369	0.000188	0.061025	0.095851	0.040043	0.021429	0.306602	0.009394	0.012266
0.891775	1.445916	1.835743	4.821176	0.88158	3.273045	0.024141	1.959466	1.197922
7.22E-05	0	0.002338	0.000231	4.33E-05	5.77E-05	8.477662	1.493968	4.082165
1.837172	0.194372	0.655613	4.246861	0.516825	3.957734	0.692381	5.638759	1.305772
5.01088	4.317648	0.472713	18.26752	4.833398	14.33939	0.024141	4.839105	1.975772
0.663651	0.227446	0.231984	0.740101	0.632857	1.099762	0.217677	0.567619	0.503463
2.400455	0.571674	2.5271	1.499214	1.011486	3.080317	0.024141	0.496212	0.216602
1.688045	0.268961	0.666097	0.285253	0.365036	5.77E-05	6.853608	1.590202	3.324452
1.869322	2.654473	7.200505	0.216025	2.879228	0.158521	1.853723	2.577157	1.632648
0.11075	0.046667	0.066696	0.000231	4.33E-05	5.77E-05	1.046061	8.66E-05	0.233889
3.980123	1.812114	0.919827	1.237879	1.011486	1.554185	6.287561	2.683478	5.068831
0.55645	2.027951	1.821861	0.254156	0.399784	0.581111	0.024141	0.175036	0.208254
1.267085	0.757763	0.418557	0.035981	0.800058	5.77E-05	4.678846	0.98474	0.437576
3.846984	6.063276	1.358189	1.070693	3.493362	2.417424	0.265786	3.895469	4.160238
7.22E-05	0.059459	0.098124	0.000231	0.186017	0.013117	0.485577	0.04785	0.058694
9.880779	18.76954	6.162446	2.975974	21.36892	4.952915	0.024141	7.683766	4.673182
0.013175	0.182929	0.002338	0.095851	0.409618	0.139697	0.024141	8.66E-05	0.038139
2.890245	2.331825	2.638139	4.927302	0.48619	8.288874	0.024141	6.475505	0.808485
1.213434	4.933781	3.864827	0.471313	3.188918	0.545152	0.692381	1.629235	14.70188
1.950519	8.226219	1.755988	1.728615	3.792266	2.065007	0.024141	1.910974	8.103925
0.044495	0.466602	0.543939	0.206465	0.28728	0.097258	1.809343	0.090303	0.749019
4.292078	1.389509	0.579841	17.39935	0.146999	14.17388	0.024141	4.397792	2.083261
2.34355	0.746551	1.776739	0.968723	0.652807	1.217475	6.09759	2.479221	1.703795
23.82354	12.85504	0.93632	10.78056	7.507619	21.36892	0.024141	21.19547	5.788153
9.031082	7.882222	40.11561	6.612121	10.05618	18.48514	5.813247	6.210375	3.792266
0.986104	2.671299	0.002338	0.000231	4.33E-05	5.77E-05	0.024141	0.420058	1.546696
0.431457	0.312626	0.543939	0.233175	0.501905	0.196436	5.704646	0.484423	1.305772
0.431457	0.12697	0.181385	0.078081	0.342987	0.049834	5.088694	0.291861	0.675938
3.407619	5.439278	15.22851	11.99921	5.646811	20.51271	0.971205	1.8386	1.015967
1.351573	2.27254	1.615281	1.624459	2.124574	3.23987	0.024141	2.563506	1.147807

12.42965	9.838658	20.34063	2.630729	9.141299	6.079596	1.670786	7.824473	11.60283
0.784805	0.406212	1.289149	0.43684	0.474214	0.350274	4.193232	0.690043	2.696061
4.94026	1.568564	2.331825	2.642525	0.956061	1.775325	17.22048	4.927302	2.950173
1.056898	0.481414	1.260635	1.277056	0.742872	1.196508	3.317807	0.613853	1.219019
3.211176	1.308167	1.480541	0.988377	1.066948	1.763889	0.024141	1.568564	0.960216
1.235346	0.904856	0.002338	2.468117	0.632857	3.266782	0.024141	1.078153	2.842193
38.94854	11.77567	0.261775	17.64869	27.10502	25.48179	1.988196	70.11932	16.68554
5.622872	4.833398	6.922771	5.22482	6.125823	15.77678	0.024141	6.653016	7.519841
3.618499	3.47531	3.493362	3.442886	5.646811	4.46267	0.241688	6.230245	4.914863
9.502294	16.74449	1.183478	3.377619	13.38418	1.826147	0.024141	18.19595	8.958225
3.269913	0.350837	1.126075	0.528571	0.546854	0.581111	8.033932	2.174733	2.558817
3.679986	2.365685	0.441263	0.74868	1.455988	0.836017	0.692381	1.099762	2.438831
3.055729	1.982208	0.561126	1.39847	0.61969	1.873434	0.024141	1.803918	2.331825
0.289603	1.240981	0.164509	0.323535	2.715873	0.554416	0.41026	0.600173	0.620462
0.624163	0.466602	0.802035	0.189141	0.919827	0.323319	0.35241	0.763939	0.331558
0.031818	0.458701	0.967446	0.243341	0.687049	0.105022	3.899993	0.141825	0.153983
0.065519	0.474574	0.231984	0.000231	0.61969	5.77E-05	0.306602	0.308672	0.921739
1.05417	1.216768	0.833045	0.062107	0.823983	0.204531	0.180231	0.657547	2.197828
0.276429	0.466602	0.083521	0.376017	0.260404	0.430281	12.81638	0.04785	0.270837
0.483874	0.552439	0.402698	0.1379	0.908247	2.77899	0.773766	1.323131	1.137244
0.65132	1.553074	0.002338	6.011515	0.222193	16.44768	0.024141	1.78759	0.000115
1.288276	0.825354	4.451154	0.731732	3.699495	4.586753	0.28728	2.50469	2.842193
1.288276	0.689509	0.853752	0.403651	1.431414	0.642395	0.306602	2.000592	2.037114
0.05189	0.09855	0.298153	0.180079	0.047504	0.158521	2.625823	0.090303	0.140087
2.16759	4.338629	7.570693	7.188095	12.77772	9.707677	0.024141	4.281847	3.885642
20.42667	12.53072	23.38378	3.204632	17.78522	7.363117	0.142698	14.88584	15.3618
1.821861	1.064935	0.483045	3.273045	0.342987	1.308167	0.180231	0.613853	0.192251
3.493362	4.673182	3.33728	1.982208	6.717056	2.984899	0.265786	4.365224	4.236962
0.175036	0.61969	1.085224	0.062107	0.566494	5.77E-05	1.455988	0.266681	0.416219
0.507403	0.236638	0.002338	0.106847	0.373939	0.221587	0.024141	0.587273	0.960216
4.49671	3.418405	5.366335	1.462143	4.580938	1.898153	0.024141	3.939307	1.677042
4.586753	4.302367	8.508514	4.23202	5.110159	11.65791	0.024141	6.475505	6.695887
2.797835	1.834423	2.506934	7.545584	1.690483	5.082063	2.978968	5.606739	8.134084
0.20215	0.219134	0.132107	0.206465	0.209978	0.179545	2.589185	0.285844	0.559221
0.318499	0.333831	0.740952	0.070736	0.678247	0.245325	0.241688	0.213059	0.270837
0.617071	0.118903	0.844747	0.078081	0.373939	0.333636	0.971205	0.160072	0.477446
3.33728	2.197828	3.36075	1.035058	2.591443	2.236407	0.180231	1.883535	0.686299
7.363117	7.38847	14.70188	5.950664	6.819481	13.75978	1.195079	6.297136	3.435988
0.149423	0.021696	0.188745	0.000231	0.047504	0.078081	0.88158	0.189141	0.058694
0.031818	0.059459	0.242771	0.020087	0.040043	5.77E-05	0.024141	0.025736	0.051595
3.394286	1.821861	1.856392	2.773644	1.372475	1.464214	5.889798	2.800332	4.082165
2.708341	3.211176	0.941104	0.485325	4.429769	0.675556	1.195079	2.939141	3.851414
51.52195	1.660216	2.322035	3.691724	1.337431	2.227273	3.899993	111.5439	100.0998
0.348139	0.21088	0.173189	0.162107	0.28728	5.77E-05	1.312706	0.266681	0.65101
0.194848	0.000433	0.213355	0.052338	0.032092	0.021429	0.198694	0.055339	0.140087
12.22798	1.50342	0.877222	3.592063	4.881775	6.717056	0.024141	17.64869	8.381328
7.037345	3.243225	3.971061	12.39704	2.895599	40.11561	0.024141	9.141299	3.885642
3.55961	2.477027	3.172864	2.438831	1.545657	2.855296	0.306602	4.217569	1.87482
0.008369	0.021696	0.066696	0.000231	0.11039	5.77E-05	0.180231	0.102886	0.027922
0.031818	0.006061	0.132107	0.127626	0.024473	0.013117	1.009625	0.04785	0.027922
2.509177	4.637994	2.600014	2.185556	3.944084	2.417424	0.024141	2.382035	2.225498
8.573434	2.563506	0.002338	6.808369	2.628225	16.34294	0.46461	14.79742	4.145209
0.224762	0.522367	0.331429	0.243341	1.123795	0.158521	0.383074	0.291861	0.886775
0.827273	2.62342	4.38684	3.904517	2.329784	5.110159	0.024141	0.613853	1.280274
2.048781	0.021696	0.164509	4.38684	0.334805	0.831472	0.383074	2.939141	1.492973
3.137576	1.553074	3.659668	2.668896	1.954993	1.429365	10.07949	5.259437	2.083261
0.338499	0.071861	0.709784	0.162107	0.373939	0.113074	2.396429	0.462352	0.686299
0.728543	0	0.22303	0.000231	0.242771	5.77E-05	4.034423	0.398629	1.273636

8.493088	1.941703	1.931537	5.976724	4.353925	9.625253	0.327143	6.365065	1.158384
0.065519	0.260144	0.077417	0.028333	0.353795	0.006912	0.160779	0.006558	0.038139
0.416219	0.60886	0.213355	0.095851	0.597042	0.196436	8.303947	0.690043	0.579841
1.928629	3.687633	2.649675	1.158384	2.752893	1.575483	0.024141	1.29342	0.780087
0.54847	1.982208	0.283882	3.052655	0.382626	0.581111	0.198694	1.720144	1.688045
1.793074	10.82954	11.77567	2.855296	6.335664	4.914863	0.46461	1.727323	1.57645
0.332641	0.202294	0.140303	0.000231	0.163983	5.77E-05	0.91404	0.167417	0.192251
2.581861	8.461703	0.750866	0.837864	3.743232	3.233045	0.241688	3.711097	2.520404
2.24733	1.481631	1.402915	3.008225	0.409618	2.092987	1.455988	1.471198	1.158384
26.25557	22.52375	33.4985	18.05488	24.98218	20.68746	0.024141	17.39935	9.707677
0.624163	0.442193	0.22303	1.135887	0.1379	1.323975	0.241688	0.530253	0.233889
18.33909	0.084524	0.213355	19.68088	0.11039	5.366335	0.024141	21.93452	30.17192
5.638759	1.925635	3.307186	31.1753	2.150303	24.50942	12.70768	3.962179	3.885642
0.031818	0.046667	0.066696	0.020087	0.024473	5.77E-05	0.850808	8.66E-05	0.012266
0.450051	1.364401	0.181385	3.581688	0.373939	0.43684	0.024141	0.484423	1.103261
17.92188	55.04081	45.91841	8.381328	75.1132	14.51648	0.024141	19.83727	13.79669
0.375281	1.624459	0.002338	1.68671	1.272756	3.172864	0.024141	1.43039	0.304387
1.761349	1.308167	1.135137	1.531313	1.381111	1.618773	0.485577	2.577157	0.880498
2.690823	3.975498	105.1694	24.2842	15.56131	83.489	0.6657	8.333918	9.012937
0.443781	0.630022	0.612525	0.078081	0.175144	5.77E-05	0.024141	0.266681	1.405772
0.891775	2.586926	2.487576	2.112843	1.612056	3.807648	0.51316	0.522367	0.451169
1.997352	1.163579	0.709784	0.82684	2.27254	0.730476	11.544	1.261407	3.939307
28.23632	5.381227	2.055289	28.54786	3.055729	8.994791	1.046061	24.50942	7.765931
0.247085	0.091616	0.339235	2.092987	0.250823	1.162201	1.046061	0.559221	0.270837
3.019848	2.533745	5.068831	1.921212	2.24733	3.885642	0.024141	1.445916	1.845519
0.375281	0.60886	0.697496	0.180079	0.61969	0.13039	5.056421	8.66E-05	0.401724
0.232215	0.458701	0.002338	0.152532	0.88158	5.77E-05	2.66404	0.338341	0.192251
17.28195	6.019971	2.002193	2.708341	6.125823	5.47	1.389509	8.923117	7.417359
1.235346	1.327525	0.290866	1.360866	1.225426	1.018427	0.28728	1.118066	0.808485
1.08798	1.022929	0.817547	0.909856	0.823983	0.684033	14.88584	1.002215	1.147807
0.099286	0.003016	0.231984	0.000231	0.095772	5.77E-05	0.241688	0.04785	0.216602
0.450051	0.071861	0.002338	0.000231	0.529971	5.77E-05	0.024141	8.66E-05	1.103261
6.423911	0.757763	2.176501	2.618846	0.48619	2.337821	0.024141	5.359134	3.098268
1.307417	0.312626	1.513102	4.839105	0.588954	4.277006	0.485577	1.94311	0.769365
1.941703	1.216768	4.502453	0.782417	1.252424	0.634531	0.46461	1.546696	1.137244
6.900202	2.895599	5.959033	1.286494	1.63039	1.754733	1.776739	7.882222	2.584394
7.22E-05	1.170491	0.002338	9.502294	4.33E-05	4.49671	0.024141	0.167417	0.000115
26.52537	64.12971	0.561126	0.996696	19.68088	1.420202	118.7028	30.17192	87.1746
1.519322	1.905094	1.327525	0.937655	2.509177	1.700354	2.74544	1.243442	3.659668
0.416219	0.268961	0.825354	0.070736	0.365036	0.013117	0.35241	0.225209	0.610606
0.092691	0.084524	0.231984	0.000231	0.087677	5.77E-05	2.92013	0.077027	0.539812
1.384921	1.022929	2.043795	3.738997	1.317475	6.967547	0.024141	2.068297	2.611797
3.310758	3.5257	1.793074	1.645613	5.137042	1.489524	0.88158	3.909033	5.830152
0.882078	1.995714	0.597792	0.485325	1.40772	1.320173	0.024141	1.609654	1.643218
0.450051	0.397489	2.786898	0.693218	0.964416	0.333636	4.086926	0.484423	1.007864
14.33939	13.45939	8.365072	5.11697	15.88548	5.560563	14.97762	10.5063	8.319019
3.040505	2.322035	1.395563	1.94311	0.871429	2.642525	0.024141	1.645613	1.47018
0.65798	1.317475	0.493247	0.180079	1.859084	0.333636	0.46461	0.407403	0.705231
2.987835	3.391075	1.520346	1.35259	3.223882	1.575483	0.383074	3.711097	2.911962
1.471198	0.656003	1.387612	2.112843	0.529971	2.280101	8.608153	1.195794	1.326667
2.436768	2.289286	1.489524	2.74298	1.612056	5.316378	0.024141	1.932857	2.106089
2.283752	3.043528	1.877605	1.224733	1.545657	3.640606	0.024141	1.660216	0.524986
0.008369	7.22E-05	0.047431	0.035981	0.005007	0.059459	0.160779	0.014964	0.018038
5.560563	3.014076	7.545584	4.508196	2.74298	3.783694	0.51316	3.939307	4.222583
18.19595	14.42661	1.722475	8.134084	14.04491	10.67991	0.88158	11.19942	10.48101
2.34355	0.825354	0.919827	1.267085	1.834423	0.88158	2.496407	1.636118	3.50425
0.961486	0.28899	0.173189	0.273434	0.296465	0.363059	0.82772	0.621688	0.713846
0.044495	0.458701	0.002338	0.000231	0.382626	5.77E-05	1.628009	0.108124	0.873579

2.380079	1.152165	3.754719	0.518139	0.678247	0.343211	6.876392	1.751948	1.147807
0.116659	0.182929	0.213355	0.1379	0.047504	0.049834	0.630022	0.096905	0.088283
0.644235	0.840635	2.776421	2.327857	0.836537	2.402468	0.024141	0.649235	0.079921
5.485685	2.524892	16.5625	1.25746	6.560411	1.691587	0.024141	4.086926	4.888023
2.02956	1.96697	0.283882	0.677403	1.011486	1.861999	0.024141	1.471198	0.761688
0.05189	0.372973	0.188745	0.052338	0.28728	0.021429	0.544545	0.121147	0.065801
1.205685	2.248896	2.032706	0.583369	4.140505	0.505267	0.383074	1.118066	1.305772
0.728543	0.202294	2.117814	0.866147	0.823983	1.679596	0.024141	0.922338	0.270837
0.450051	2.069942	0.722626	0.901212	1.690483	1.308167	0.024141	0.734524	0.072583
1.980592	0.91404	0.465325	1.024127	0.342987	0.970007	0.024141	1.450693	4.529913
3.718716	7.507619	3.540606	13.26964	5.210317	11.42512	0.024141	4.875527	8.797619
0.008369	0	0.116494	0.106847	0.032092	0.049834	2.320072	0.066407	0.147807
2.048781	3.034747	5.056421	1.190491	2.02956	1.489524	3.1857	2.269134	2.931068
7.625209	10.05618	13.6814	1.214315	16.80343	1.429365	0.024141	6.808369	6.772763
1.746724	1.853723	4.188896	1.145426	1.545657	1.780693	27.66779	1.163579	2.292864
6.622092	21.02638	1.886335	4.644098	10.1459	3.930664	0.306602	5.130411	3.381255
6.135007	4.440548	0.919827	1.405772	4.292078	0.554416	31.1753	8.429055	18.26752
1.070115	0.92531	17.92188	0.347908	2.600014	0.172734	0.942944	1.921212	1.197922
4.888023	0.832013	3.599329	14.25954	1.690483	5.388405	0.024141	4.034423	1.374329
252.4251	749.7839	118.7028	287.197	749.7839	749.7839	6.230245	425.62	144.7919
1.631544	0.522367	1.877605	7.737807	0.823983	4.043846	4.251818	1.557482	1.000794
0.164509	0.312626	0.132107	0.000231	0.809481	0.040909	0.024141	0.285844	0.200519
2.181775	1.216768	0.346342	1.823377	1.564149	0.097258	8.958225	3.511263	5.704646
46.7056	15.18102	21.19547	40.7932	10.23768	26.25557	1.195079	37.29684	24.2842
0.74868	0.372973	0.386378	0.171472	0.48619	0.139697	1.355527	0.871429	1.664906
12.03267	7.30215	9.646284	7.443276	7.061328	8.677489	2.92013	15.3618	8.539971
7.22E-05	8.256465	9.502294	11.42512	5.796421	5.583968	0.024141	0.952294	3.324452
5.143867	4.012049	10.17029	2.296732	8.958225	3.783694	0.180231	4.546443	3.324452
1.567576	0.066472	0.252619	0.617763	0.154444	1.516219	0.327143	1.493968	0.296955
3.310758	2.563506	7.162698	1.434921	3.29355	0.70404	3.435988	3.175916	3.779452
0.986104	1.227749	0.817547	0.1379	0.809481	0.068932	2.773644	1.828781	1.812114
5.671126	4.833398	3.566753	4.115844	4.370671	4.376118	16.9162	7.417359	10.7557
7.22E-05	0	0.002338	0.000231	4.33E-05	5.77E-05	9.625253	8.66E-05	0.000115
3.94886	4.281847	1.867951	1.006623	5.830152	1.217475	0.180231	1.791616	1.947316
67.98069	23.82354	2.586926	10.8813	21.93452	15.3618	0.024141	83.489	56.39528
1.05088	1.455988	8.365072	0.243341	1.972706	0.263146	16.22108	1.710173	1.915455
7.22E-05	0.010592	0.261775	0.000231	4.33E-05	0.013117	0.773766	0.077027	0.088283
3.890556	3.599329	11.544	5.553146	2.408882	2.022778	20.51271	3.610707	4.312251
1.816436	1.697792	5.180058	1.77404	1.272756	5.24562	5.515584	2.329784	1.643218
4.212554	7.911934	4.789596	3.6029	5.976724	6.570433	0.573326	7.519841	5.34474
4.007583	6.738211	5.796421	2.758088	2.628225	2.720996	0.544545	1.684646	1.617489
0.169675	0.372973	0.750866	0.086025	0.597042	0.006912	0.437576	0.496212	1.947316
1.980592	0.949827	4.597792	1.277056	0.908247	0.998312	1.549711	1.396494	1.50342
0.624163	0.397489	1.593535	0.052338	0.809481	0.105022	0.35241	0.127128	0.216602
0.424004	0.532381	0.322395	0.233175	0.501905	0.158521	0.28728	0.537807	0.808485
7.22E-05	0.003016	0.002338	0.1379	0.06088	0.105022	1.455988	0.014964	0.044805
42.75576	2.081696	0.140303	5.47	1.31	5.959033	0.383074	14.51648	21.36892
0.882078	0.342814	0.22303	2.884762	0.474214	6.808369	0.485577	1.146118	0.790303
5.80469	3.904517	0.757763	13.08214	0.163983	7.112496	0.024141	3.297107	2.126349
9.751039	1.716364	0.322395	13.38418	1.766248	3.217612	0.35241	12.93651	1.42456
1.928629	11.719	0.967446	1.046061	28.54786	1.554185	0.160779	1.684646	2.54961
14.88584	19.06893	6.433918	12.10289	11.17281	12.22798	0.024141	16.68554	16.5625
1.116609	0.672468	2.398442	4.557605	0.809481	3.659668	0.46461	1.527792	1.755988
0.30596	0.006061	0.148254	0.444986	0.054711	0.423535	7.037345	0.370657	0.579841
0.803211	0.825354	0.853752	1.035058	0.718766	1.111977	0.024141	0.299444	0.65101
2.205476	4.077403	1.327525	3.043528	0.146999	1.111977	17.85355	2.522684	1.896667
1.094206	0.36114	0.002338	0.297439	0.662944	0.087807	0.544545	0.86469	1.389509
0.127532	0.052431	0.002338	0.000231	4.33E-05	5.77E-05	0.51316	8.66E-05	0.000115

1.288276	1.481631	0.967446	0.74868	1.049517	1.691587	4.752121	1.852338	1.57645
4.115844	1.277056	2.27254	44.27012	3.588369	11.77567	5.295325	2.765758	4.043846
7.964315	5.576219	9.418196	6.978903	4.484892	10.78056	1.853723	10.1028	10.38202
1.296905	0.895368	1.317475	0.693218	0.529971	0.684033	24.04374	1.216768	3.324452
3.034747	2.909293	3.432388	2.784228	3.588369	3.807648	0.306602	4.764055	3.273045
0.949286	1.163579	0.322395	0.233175	1.620058	0.28443	0.28728	0.383622	1.128788
3.125844	1.87482	35.27608	1.499214	3.36075	3.040505	0.46461	4.115844	3.578038
0.022886	0.091616	0.132107	0.347908	0.163983	0.013117	1.925635	0.077027	0.153983
0.247085	0.09855	0.002338	0.000231	0.119322	5.77E-05	5.11697	0.484423	0.322973
3.401126	6.945152	5.545729	3.625382	11.06488	3.536955	0.265786	6.706472	3.864827
0.276429	0.202294	0.002338	0.106847	0.175144	0.113074	0.383074	0.722626	0.705231
0.159351	0.059459	0.457431	0.000231	0.428932	5.77E-05	0.217677	0.077027	0.313312
1.531313	0.194372	0.306176	0.461811	0.465563	0.825354	7.162698	1.056898	1.855108
2.826277	0.656003	2.386248	0.57531	1.301169	1.004791	0.217677	2.607035	1.932857
0.159351	0.059459	0.173189	0.028333	0.054711	0.031046	1.417345	0.090303	0.304387
4.46267	1.888023	3.432388	1.006623	1.930216	1.168326	2.091313	3.909033	4.376118
1.915455	0.769365	0.92816	1.207374	0.899567	0.7986	0.265786	2.73272	0.590303
6.191515	1.941703	1.085224	8.780115	4.33E-05	6.028427	0.024141	8.089848	7.468961
0.122179	1.009625	0.188745	0.000231	0.859351	0.121494	1.708932	0.061602	0.73583
1.116609	5.165599	1.068348	1.846941	2.784228	1.679596	15.46193	1.227749	3.4679
0.031818	0	0.077417	0.000231	0.11039	5.77E-05	0.217677	0.114719	0.038139
0.255087	0	0.140303	0.000231	4.33E-05	5.77E-05	0.024141	0.273434	0.73583
0.617071	0.789863	1.030678	1.547734	0.933261	1.708932	0.024141	0.628658	0.451169
0.180895	0.36114	0.083521	0.127626	0.087677	5.77E-05	0.024141	0.442496	0.241991
11.06488	3.317807	1.289149	3.536955	2.045476	2.329784	36.26061	7.951371	21.02638
0.845887	0.710722	1.030678	0.306176	0.642698	0.423535	1.809343	0.753882	0.846342
5.056421	1.913997	0.188745	1.190491	0.697496	0.970007	0.024141	5.47	0.98956
7.22E-05	0	0.002338	0.000231	0.017063	5.77E-05	0.160779	8.66E-05	0.000115
1.734949	1.786306	2.561162	2.513752	2.058773	3.829105	0.265786	1.501291	1.137244
45.1045	31.92385	45.1045	12.29473	35.776	11.03843	1.925635	36.74522	37.29684
1.621032	0.832013	0.990909	0.556753	1.131472	0.314156	3.518384	0.484423	8.075772
8.288874	8.625613	24.73724	3.514834	7.099913	7.417359	0.024141	12.70768	11.39744
0.008369	0.052431	0.090476	0.020087	0.024473	0.021429	0.28728	8.66E-05	0.065801
1.10132	2.427792	1.927042	1.31	2.451645	1.489524	0.142698	0.780519	0.856782
0.247085	0.322078	0.106696	0.000231	0.047504	5.77E-05	1.255657	8.66E-05	0.886775
13.6814	19.68088	3.025671	17.92188	8.019769	8.849697	0.024141	13.53913	4.933781
0.008369	0.010592	0.098124	0.014545	0.007821	5.77E-05	1.746724	0.009394	0.032727
0.059408	0.16119	0.002338	0.035981	0.27759	0.021429	0.024141	0.43347	0.263853
12.36443	22.13245	6.00228	3.926154	7.6393	6.64254	0.024141	8.019769	12.42965
1.116609	4.353925	6.632063	12.56693	3.771349	5.646811	0.437576	1.323131	1.280274
1.543377	2.750375	0.181385	83.489	4.33E-05	46.7056	0.024141	0.931457	1.29417
4.272165	2.337821	3.540606	4.586753	4.971991	6.345382	0.51316	5.762965	4.827691
6.171789	6.268312	2.487576	1.817828	12.63773	2.031169	0.544545	3.529466	3.799885
2.432121	1.527792	4.900996	0.610224	3.479134	3.904517	0.598456	4.130794	1.742734
0.872013	1.227749	2.421681	1.863506	0.919827	2.474834	52.56128	0.672857	4.115844
5.993045	6.423911	2.470317	6.717056	4.814661	5.137042	2.274473	3.962179	3.113478
13.15079	5.838499	50.55949	6.464906	8.149307	9.502294	1.159913	12.63773	4.47912
9.838658	2.533745	9.063983	50.55949	5.024293	18.62711	15.88548	11.03843	10.28573
0.353795	1.124574	0.871429	1.91254	0.529971	0.238889	2.589185	1.146118	1.007864
0.769019	1.03303	0.269697	0.629098	0.456522	1.420202	0.024141	0.231356	0.518413
6.674286	1.338261	3.055729	1.558571	1.476429	2.438831	2.160649	5.976724	2.661587
1.060188	0.746551	1.260635	0.078081	0.409618	0.430281	0.35241	1.163579	0.477446
0.017778	0.010592	0.261775	0.028333	0.054711	0.006912	0.142698	0.055339	0.032727
1.160707	0.322078	0.090476	0.052338	0.095772	5.77E-05	5.687879	1.202626	1.147807
6.717056	0.481414	3.36075	15.3618	0.222193	5.796421	0.024141	10.93101	6.09759
0.518139	2.685786	0.425426	1.94311	4.802655	1.554185	0.544545	1.163579	0.357013
1.08798	1.137244	1.335743	0.384632	1.301169	0.254574	2.361198	1.527792	2.225498
0.348139	0.36114	2.201587	0.1379	0.678247	0.487662	4.145209	0.455974	0.257518

0.059408	0.09855	0.106696	0.000231	4.33E-05	5.77E-05	1.009625	0.061602	0.000115
8.539971	6.560411	10.85542	1.902352	2.895599	2.74544	0.51316	5.959033	3.4679
0.059408	0.091616	0.276609	0.000231	0.087677	5.77E-05	0.241688	0.061602	0.183824
1.394632	4.857403	1.209776	1.755988	2.219863	3.381255	0.024141	2.55184	1.374329
0.687807	1.481631	0.588954	0.82684	0.28728	0.88158	4.429769	0.818066	1.588903
2.45575	1.433189	3.792266	6.423911	3.771349	2.463911	3.1857	5.210317	2.400455
10.43066	6.760765	15.98892	15.07257	18.05488	14.93173	0.024141	7.6393	5.25254
0.013175	0.027965	0.173189	0.086025	0.06088	0.006912	6.622092	0.025736	0.120433
0.017778	0.00119	0.042266	0.014545	0.017063	0.013117	1.708932	0.009394	0.097186
5.728615	8.005685	4.519048	1.492973	15.46193	2.62342	0.306602	5.671126	6.717056
3.629177	3.442886	5.030866	2.197828	4.292078	2.50469	6.497266	4.94026	4.160238
3.23987	3.266782	6.404921	2.513752	7.558139	1.919661	1.355527	4.413608	2.823636
0.735382	1.613232	1.468131	0.355556	1.095657	0.868341	0.024141	1.252424	1.439704
5.654863	3.66785	11.42512	8.711905	2.159004	9.838658	0.024141	5.295325	6.144055
7.22E-05	0.333831	0.434069	0.095851	0.48619	0.049834	1.225426	8.66E-05	0.113521
3.529466	4.203001	2.786898	11.544	5.388405	8.429055	0.024141	2.928247	0.873579
0.492165	0.00119	0.543939	0.403651	0.032092	0.059459	0.51316	0.462352	0.769365
3.446732	0.769365	1.233665	0.866147	0.456522	0.528997	27.10502	5.172828	6.210375
75.1132	30.49289	77.74812	12.22798	38.39042	19.06893	2.818312	59.32013	70.11932
0.312323	0.312626	0.269697	0.189141	0.222193	0.516566	0.544545	0.254769	0.444315
0.526566	0.800967	0.002338	3.425512	1.036558	5.123781	0.024141	2.045476	0.000115
0.318499	0.09855	0.290866	0.000231	0.353795	5.77E-05	0.971205	0.780519	0.408175
1.235346	3.163506	3.236457	1.126775	5.366335	1.244957	0.35241	1.519322	1.481631
8.134084	8.508514	3.236457	7.212915	10.78056	6.171789	1.312706	9.256537	6.911486
2.931068	1.03303	7.625209	5.576219	1.213434	5.165599	0.773766	3.350224	1.828781
0.092691	0.442193	0.520678	0.062107	0.61969	0.204531	19.99861	0.066407	0.357013
0.263146	1.812114	1.846941	0.333088	1.204228	0.13039	1.809343	0.780519	0.790303
2.214372	0.450924	0.483045	4.440548	0.566494	1.667273	0.024141	2.283752	3.91355
2.283752	6.09759	0.451169	0.693218	4.758088	1.644416	2.160649	2.255916	2.964762
4.419062	3.8379	8.745238	0.654423	2.55184	0.451429	8.508514	3.851414	3.738997
3.482785	7.697063	2.572309	2.893001	7.417359	4.690159	0.024141	2.544747	1.784755
3.91355	4.338629	6.570433	1.959466	5.889798	4.626025	0.198694	3.479134	1.50342
1.296905	0.630022	0.639892	0.528571	0.28728	0.371299	4.650007	1.314675	0.931457
15.56131	12.77772	1.427431	32.31123	1.036558	28.54786	0.024141	10.43066	12.10289
13.91714	4.655916	6.374812	4.408153	5.351934	7.112496	1.628009	7.351284	9.335397
0.255087	0.135354	0.106696	0.000231	0.069026	5.77E-05	1.104805	0.231356	0.225339
22.32214	16.44768	8.134084	8.539971	9.101674	17.28195	0.805238	21.36892	12.56693
167.9996	332.0776	0.612525	91.05541	184.443	287.197	0.024141	287.197	287.197
9.063983	1.568564	4.096407	3.321284	2.27254	0.819509	3.052655	6.967547	3.726854
0.684033	0.664423	0.777864	2.341573	2.950173	4.881775	0.024141	0.952294	0.000115
2.496407	3.953297	3.236457	8.445743	1.780693	4.125786	1.009625	2.274473	2.485534
6.063276	5.238701	1.209776	5.993045	3.975498	6.210375	2.440895	6.191515	6.591544
31.55417	12.26136	23.6012	95.22939	9.792626	167.9996	0.024141	48.53429	13.11646
4.359574	1.400159	22.13245	6.900202	2.474834	7.724084	0.024141	3.921645	2.773644
30.17192	33.4985	31.55417	27.38726	37.29684	52.56128	0.024141	34.38341	5.830152
10.38202	5.295325	4.241905	2.283752	7.149618	4.644098	20.16541	9.946364	10.55571
0.470736	2.408882	1.168326	1.224733	0.718766	0.62645	0.160779	2.445079	1.03303
3.107417	1.455988	4.307309	2.248896	1.418312	3.807648	0.024141	3.070952	1.632648
0.820931	0.425635	0.796234	0.347908	0.31456	0.371299	3.011169	1.027403	0.979149
0.29798	0.36114	0.353333	0.000231	0.11039	0.040909	0.46461	0.517092	0.638362
14.6058	11.30987	9.646284	8.677489	9.583463	10.01009	0.850808	19.68088	24.04374
15.41187	5.864084	2.911962	4.524481	2.911962	3.747035	0.024141	7.238023	9.218514
0.044495	0.034416	0.090476	0.000231	4.33E-05	5.77E-05	0.598456	0.04785	0.175592
13.30906	6.674286	8.180094	32.68313	4.609076	12.10289	2.773644	9.101674	7.991602
1.477388	0.895368	0.203254	0.566782	0.606154	0.970007	0.024141	0.86469	0.713846
1.116609	0.442193	0.526032	0.162107	0.670144	0.049834	1.225426	0.825354	1.651522
1.197215	2.217951	0.314913	0.314387	1.690483	0.430281	1.355527	0.890577	1.182056
0.29798	0.071861	0.002338	0.000231	0.1379	5.77E-05	1.009625	0.197338	0.1686

6.486104	2.234538	1.604827	4.376118	2.654473	3.24658	2.92013	6.508427	3.536955
2.421681	4.261342	0.520678	0.774257	5.745996	1.25816	0.198694	1.94311	2.708341
0.085426	0.000188	0.047431	0.000231	0.054711	5.77E-05	0.485577	0.020267	0.120433
3.482785	5.959033	4.965801	8.958225	2.34355	8.348817	0.130202	4.048499	3.659668
2.760765	14.6058	55.04081	64.12971	16.9162	64.12971	4.251818	27.66779	21.74051
0.705231	7.545584	1.017107	4.715599	4.33E-05	9.335397	0.024141	1.974228	0.000115
0.092691	0.071861	0.451169	0.028333	0.032092	0.068932	7.625209	0.04785	0.000115
3.310758	0.840635	7.013853	3.984747	3.464315	3.842554	0.024141	3.428788	2.465916
7.22E-05	0.066472	0.002338	0.000231	4.33E-05	5.77E-05	4.881775	0.197338	0.000115
0.037525	0.000433	0.181385	0.020087	0.032092	0.021429	0.719942	0.061602	0.16145
1.772756	3.5257	3.137576	0.693218	2.715873	0.684033	6.497266	1.51215	2.400455
0.07767	0.36114	1.183478	0.000231	0.48619	0.615996	0.024141	0.077027	0.313312
0.143838	0.071861	0.306176	0.000231	0.222193	0.068932	1.282208	0.763939	1.069574
1.793074	14.79742	0.612525	1.046061	18.33909	1.012114	0.142698	1.269495	1.361789
4.540794	2.554069	2.063362	3.080317	3.917597	7.991602	0.327143	5.522929	4.292078
1.706219	0.93632	3.47531	0.106847	1.213434	0.097258	0.51316	1.222179	1.492973
8.608153	4.473348	12.03267	12.70768	7.751869	12.93651	0.241688	6.443925	3.150433
0.579841	0.416219	0.579841	0.000231	0.708124	0.087807	0.82772	0.496212	0.427403
8.256465	4.673182	3.450577	12.85504	4.46267	7.013853	0.88158	5.42381	9.838658
2.847547	6.125823	0.612525	2.953059	5.266335	4.261342	1.628009	2.489762	3.578038
0.500354	1.905094	0.717027	0.062107	0.933261	0.087807	1.588903	1.195794	0.906126
22.52375	7.570693	13.53913	5.671126	6.171789	9.031082	0.024141	8.608153	2.447294
1.235346	0.586595	0.838326	0.471313	1.443983	0.510707	0.180231	1.29342	0.485325
2.101075	6.830592	0.717027	9.19899	1.972706	3.350224	0.024141	3.297107	2.126349
0.122179	0.059459	0.425426	0.062107	4.33E-05	5.77E-05	2.234538	0.197338	0.313312
0.255087	0.260144	0.605022	0.000231	0.448261	0.006912	1.159913	0.43347	0.304387
2.316075	0.840635	5.25254	0.095851	1.346991	0.272078	0.383074	1.953535	1.190491
0.116659	0.60886	0.188745	2.659214	0.88158	1.244957	0.91404	0.249091	0.225339
0.027619	0.059459	0.071775	0.117734	0.024473	5.77E-05	0.51316	0.066407	0.032727
0.031818	0.040678	0.002338	0.043391	0.011696	5.77E-05	1.225426	8.66E-05	0.113521
2.365685	3.29355	1.128788	5.165599	4.343838	3.188918	0.327143	3.479134	4.193232
0.194848	0.36114	0.116494	0.197128	0.606154	0.333636	0.265786	0.496212	0.664026
3.743232	6.591544	1.402915	2.333867	5.454949	2.868456	0.630022	3.095166	2.477027
0.905426	1.216768	1.805166	3.57057	0.687049	3.703175	2.022778	0.940563	2.696061
2.065007	0.980505	0.077417	1.198701	1.272756	0.28443	0.024141	3.286652	2.805382
0.961486	3.486436	2.378124	5.638759	3.055729	6.443925	0.024141	2.421681	1.723579
0.044495	0.071861	0.231984	0.020087	0.242771	5.77E-05	0.241688	0.148124	0.113521
3.101371	10.55571	4.789596	4.012049	10.90615	10.62739	0.024141	3.144156	3.140866
37.29684	15.276	15.46193	38.39042	17.39935	32.68313	11.25392	34.84299	25.48179
0.353795	0.219134	0.231984	0.052338	0.078737	5.77E-05	0.024141	0.672857	0.722179
0.540188	0.305657	0.709784	0.020087	0.163983	0.087807	0.485577	0.254769	0.579841
8.745238	5.150693	75.1132	3.767489	6.945152	11.89932	0.024141	8.089848	4.758088
3.182482	1.553074	3.324452	2.223636	2.089639	3.031688	3.011169	3.122915	2.320072
1.172872	0.10531	1.168326	0.518139	0.31456	0.231876	0.024141	0.363276	0.531486
0.11075	0.152655	0.697496	0.376017	0.06088	0.380851	13.6814	0.127128	0.16145
5.25254	5.095325	7.598312	2.081696	11.77567	9.968355	0.024141	8.381328	3.324452
4.709033	7.468961	2.831501	9.377345	5.295325	16.9162	0.024141	4.888023	3.06803
0.116659	0.689509	2.760765	1.237879	0.11039	4.764055	6.497266	8.66E-05	0.278802
105.1694	59.32013	28.54786	29.18885	33.07147	44.27012	4.086926	91.05541	50.55949
2.392431	0.406212	0.188745	1.31	0.399784	1.873434	0.024141	0.517092	0.132626
0.793918	0.36114	0.688579	0.233175	0.390895	0.371299	0.91404	1.033672	1.481631
0.579841	1.41368	0.871429	1.624459	0.501905	0.620462	5.754935	1.146118	1.238651
3.381255	0.92531	0.579841	18.26752	0.718766	13.83359	12.53072	5.439278	9.397771
1.419278	0.522367	0.532092	3.536955	0.11039	1.004791	0.024141	0.254769	0.866147
2.325931	0.60886	3.8379	2.668896	0.823983	4.297222	0.51316	2.765758	2.341573
0.56443	0.397489	1.317475	2.884762	0.777864	0.981032	5.942294	0.871429	2.126349
2.160649	0.689509	1.867951	1.422316	0.27759	2.259661	0.327143	0.753882	0.69583
0.438023	0.12697	0.002338	0.028333	0.069026	5.77E-05	2.496407	0.273434	1.238651

0.803211	0.268961	1.267085	0.925801	0.546854	2.047157	0.180231	0.702835	0.620462
203.5795	80.6518	0.148254	252.4251	9.880779	87331.38	0.024141	1360.293	1.121017
0.037525	1.240981	0.042266	0.376017	0.353795	0.469358	0.773766	0.020267	0.140087
0.027619	0.016133	0.283882	0.000231	0.007821	0.021429	0.6657	8.66E-05	0.032727
0.687807	0.21088	0.697496	0.206465	0.250823	0.139697	0.6657	0.69583	0.408175
2.538218	6.900202	7.112496	72.69473	4.857403	62.33472	1.046061	1.146118	1.997352
0.705231	0.333831	0.457431	1.728615	0.260404	0.895368	0.773766	1.099762	0.65101
0.255087	0.169971	1.755988	0.428932	0.390895	0.88158	5.553146	0.231356	0.906126
2.628225	4.473348	1.877605	1.286494	4.508196	1.631544	0.35241	2.413146	2.898196
7.22E-05	0.176407	0.002338	0.014545	0.10254	5.77E-05	0.46461	0.009394	0.023492
4.783001	4.802655	1.416407	1.434921	7.149618	2.214372	0.719942	3.767489	4.592273
0.395859	0.895368	0.002338	3.13456	0.198405	1.066948	0.024141	0.299444	0.664026
1.020447	3.317807	0.579841	0.53912	1.982208	0.204531	16.34294	2.136335	2.153535
0.099286	7.22E-05	0.140303	0.162107	0.087677	0.021429	0.437576	0.354798	0.33969
4.907929	1.353608	5.044113	5.737056	2.671299	5.180058	2.708341	4.839105	1.492973
1.531313	1.338261	0.605022	2.157359	1.326667	1.639639	0.024141	0.96329	1.210447
0.735382	0.586595	1.118817	0.341053	0.767085	0.238889	2.022778	0.567619	1.326667
2.27254	10.90615	16.9162	9.316241	13.60866	11.36975	0.024141	4.490801	1.632648
0.127532	0.003016	0.339235	1.663672	0.011696	0.49974	2.873817	0.090303	0.058694
0.008369	2.16E-05	0.002338	0.014545	0.005007	5.77E-05	0.217677	8.66E-05	0.023492
1.591299	2.008312	1.175859	2.176501	2.451645	2.842193	0.35241	1.660216	1.158384
0.044495	0.006061	0.002338	0.000231	0.040043	5.77E-05	0.024141	0.04785	0.000115
5.923838	5.180058	7.288788	5.9071	5.309264	9.751039	0.024141	4.519048	3.031688
0.353795	0.397489	0.465325	0.583369	0.269141	0.371299	1.776739	0.331869	0.357013
3.086306	1.845519	2.410996	8.019769	0.845887	4.678846	0.024141	0.544214	1.03303
7.456118	10.1028	9.924372	9.751039	10.98644	8.508514	0.437576	9.031082	4.012049
0.175036	0.236638	0.047431	0.052338	0.365036	0.059459	1.355527	0.066407	0.140087
4.33342	11.25392	4.620159	1.214315	13.6814	1.974228	0.719942	3.479134	3.629177
3.233045	4.38684	0.002338	0.712929	1.890996	1.826147	0.024141	3.095166	2.011501
2.24733	1.240981	1.156869	0.509242	1.390476	1.404812	0.198694	4.105714	6.00228
1.012114	0.388326	2.254084	0.162107	1.066948	0.172734	3.1857	1.191905	2.417424
0.164509	0.499206	0.757763	0.22456	0.27759	0.28443	0.719942	0.291861	0.468694
6.956349	21.54823	0.919827	4.058514	32.31123	8.381328	0.024141	11.30987	7.653391
7.882222	3.092229	10.05618	37.81919	2.996869	30.49289	7.625209	6.830592	5.679502
33.93939	14.97762	8.477662	13.75978	10.19468	16.22108	1.073543	28.54786	13.11646
0.070909	2.895599	0.276609	0.020087	2.219863	5.77E-05	0.485577	0.285844	0.287641
4.952915	10.90615	0.515815	1.703795	33.93939	1.464214	0.024141	3.163506	2.50469
11.719	7.938427	14.17388	4.63189	3.851414	4.402973	2.773644	10.57984	7.339452
0.673254	0.027965	0.887922	1.443059	0.040043	0.70404	0.773766	0.613853	0.357013
9.256537	4.217569	3.511263	33.07147	3.037626	12.56693	1.159913	8.780115	5.00404
1.004156	0.333831	0.825354	0.485325	0.409618	0.737403	0.942944	0.607273	0.664026
0.1379	0.176407	0.261775	0.086025	0.163983	5.77E-05	0.573326	0.04785	0.401724
0.187532	1.03303	0.156089	1.78759	4.546443	0.697496	0.024141	0.657547	0.38153
1.508831	2.138131	9.838658	0.548153	3.080317	0.911905	1.988196	0.890577	3.049603
3.283139	4.077403	1.910974	0.556753	5.830152	0.939986	0.6657	1.660216	3.397706
0.477446	0.182929	0.242771	0.000231	0.269141	0.113074	1.809343	0.325772	0.257518
2.16759	1.000794	1.985253	5.330519	0.697496	3.373983	5.00404	3.144156	4.003117
0.154444	0.016133	0.195685	0.028333	0.017063	5.77E-05	0.971205	0.219481	0.468694
11.89932	21.93452	3.207807	11.77567	34.84299	48.53429	0.024141	23.82354	4.575123
0.533535	0.397489	1.222958	2.287576	1.123795	1.343463	4.56912	0.449726	0.348139
0.255087	0	0.140303	0.000231	4.33E-05	5.77E-05	0.327143	0.077027	0.208254
2.911962	7.964315	6.486104	2.845036	4.155339	2.975974	4.620159	2.577157	8.905642
18.91854	24.98218	2.66404	118.7028	18.55613	203.5795	0.024141	36.26061	2.132756
4.821176	3.391075	12.93651	24.73724	2.104358	14.25954	21.93452	6.738211	3.711097
0.456818	1.104033	1.445916	1.190491	1.011486	0.873579	1.046061	0.141825	0.1686
2.369841	1.799899	1.713997	5.553146	3.730808	3.29355	0.024141	4.307309	8.445743
50.55949	48.53429	13.96064	21.54823	95.22939	36.74522	1.104805	60.77449	34.38341
0.017778	0.003016	0.042266	0.095851	0.024473	0.006912	1.489524	0.035339	0.027922

0.318499	0.723066	0.290866	0.403651	1.337431	0.590303	1.159913	0.254769	0.477446
7.22E-05	0.194372	0.071775	0.000231	0.175144	5.77E-05	0.573326	8.66E-05	0.072583
1.988196	2.164113	0.967446	4.597792	2.815866	5.959033	0.573326	2.421681	1.537951
0.263146	0.562807	4.484892	0.035981	0.078737	0.314156	5.813247	0.325772	0.313312
1.62562	5.788153	0.242771	0.947677	7.751869	2.74544	0.024141	1.146118	0.749019
7.22E-05	1.799899	6.55039	4.888023	0.61969	4.519048	0.598456	8.66E-05	0.000115
1.108276	1.423506	1.041025	2.021068	1.557482	1.066948	0.024141	8.66E-05	0.600548
19.22139	33.93939	44.27012	15.77678	40.7932	19.37139	0.692381	16.34294	10.62739
7.002056	36.74522	10.01009	11.99921	8.780115	18.19595	0.91404	8.429055	9.604358
0.342987	0.066472	0.231984	0.617763	0.024473	0.221587	9.101674	0.030375	0.192251
0.483874	15.3618	6.028427	5.095325	8.226219	4.851227	0.265786	1.845519	0.192251
7.288788	14.00414	23.82354	5.770996	11.30987	10.1459	32.8773	16.5625	11.96574
1.543377	3.796075	3.137576	1.581912	6.335664	3.005281	0.28728	0.996111	0.468694
0.483874	3.077316	1.175859	1.224733	0.474214	1.919661	28.54786	0.331869	1.518232
1.145426	7.697063	10.1028	1.202626	5.500678	2.876421	0.024141	0.69583	1.147807
0.851659	0.895368	0.50127	0.180079	0.871429	0.444538	1.95645	0.972504	0.949827
1.992944	1.761349	3.953297	3.4679	1.797172	4.508196	0.51316	1.04228	0.970635
0.164509	0.135354	0.098124	0.052338	0.047504	5.77E-05	1.417345	0.108124	0.373939
1.477388	11.09133	1.076032	0.731732	12.42965	1.026219	8.134084	1.438672	5.302294
3.796075	1.514199	2.032706	1.434921	1.750628	1.763889	5.754935	2.8657	3.223882
0.3671	0.598456	1.327525	0.528571	1.182771	1.092136	0.573326	0.420058	0.051595
1.567576	1.553074	1.776739	0.323535	1.532468	0.295837	1.225426	1.645613	1.103261
0.263146	0.645916	1.604827	0.028333	0.718766	0.031046	0.88158	0.219481	0.270837
0.05189	0.091616	0.106696	0.062107	0.186017	0.078081	0.217677	0.121147	0.038139
2.065007	0.532381	0.002338	4.620159	1.131472	4.575123	0.35241	1.519322	2.138131
2.27254	0.630022	0.402698	1.863506	1.143175	1.909408	0.971205	3.043528	2.026219
7.22E-05	2.16E-05	0.054574	0.000231	0.007821	0.006912	0.485577	8.66E-05	0.008882
1.419278	0.322078	0.93632	0.273434	0.198405	0.13039	13.96064	1.77404	6.116638
3.29355	1.697792	4.921082	1.202626	1.890996	2.169509	0.437576	2.522684	2.781558
1.941703	2.59557	2.322035	0.807136	1.596782	1.363557	0.692381	1.636118	1.305772
10.28573	11.19942	18.19595	4.524481	19.99861	8.643074	0.88158	15.98892	10.93101
1.012114	0.672468	1.050231	3.188918	0.305087	2.705916	1.046061	0.509791	0.357013
0.483874	0.372973	0.817547	0.297439	0.418788	0.231876	1.104805	0.455974	0.579841
8.381328	13.11646	0.054574	1.78759	15.07257	0.771688	0.024141	13.83359	18.48514
0.30596	0.542165	1.31	0.774257	0.465563	1.168326	0.773766	0.219481	0.175592
1.499214	1.637309	1.674401	0.943514	2.183665	2.604776	0.544545	1.89961	1.374329
0.29798	0.16119	0.156089	0.421025	0.163983	0.245325	2.773644	0.377561	0.38153
1.706219	1.637309	0.681068	1.248232	0.27759	0.413009	3.563182	1.285584	1.546696
2.479221	3.211176	21.74051	1.099762	7.598312	2.690823	5.180058	9.459177	4.18456
1.734949	1.009625	2.359264	1.77404	2.842193	2.304452	0.265786	2.019618	1.273636
9.218514	27.38726	9.583463	8.508514	9.502294	17.09911	0.024141	11.83593	4.529913
1.499214	5.745996	2.312085	7.824473	2.219863	4.203001	0.024141	1.736133	1.821861
1.213434	0.245469	2.055289	0.365635	1.143175	1.244957	0.573326	0.66482	0.38153
2.847547	2.027951	5.545729	0.501905	1.359177	2.498478	0.327143	2.463911	2.465916
8.412367	12.06778	12.10289	18.91854	12.1974	16.5625	1.255657	9.707677	4.557605
0.3671	0.710722	0.729582	0.851234	0.859351	0.649668	4.251818	1.126775	0.357013
10.8813	3.29355	14.79742	15.56131	5.568391	9.583463	31.92385	27.96248	31.1753
0.424004	0.059459	0.362179	2.157359	0.069026	0.846342	1.389509	0.181919	0.241991
0.180895	0.10531	0.402698	0.117734	0.233752	0.087807	2.091313	0.425635	0.357013
11.14619	530.1465	39.53687	1360.293	3.68381	1360.293	0.598456	19.22139	24.98218
0.05189	0.789863	0.871429	0.556753	0.859351	0.598456	0.024141	0.855267	1.262965
1.761349	1.682193	0.697496	2.953059	3.401126	2.74544	1.255657	0.838846	1.046703
0.180895	0.406212	0.002338	0.000231	0.305087	5.77E-05	5.217569	0.070909	1.137244
8.348817	8.005685	0.276609	1.855108	3.998391	1.535722	0.024141	5.560563	23.82354
23.16622	9.377345	332.0776	49.50903	18.76954	27.66779	87331.38	17.28195	87331.38
0.099286	0.010592	0.124791	0.000231	0.007821	0.021429	0.142698	0.066407	0.097186
4.684502	1.896667	1.050231	7.598312	0.175144	3.610707	530.1465	3.691724	60.77449
2.773644	2.609293	5.485685	16.68554	3.803766	11.09133	0.024141	3.227085	0.921739

1.960945	3.119986	3.934986	1.482626	2.688304	2.214372	11.77567	2.31404	3.008225
0.858297	13.11646	0.290866	3.122915	3.401126	1.801284	0.024141	0.952294	0.897006
1.679596	8.923117	31.92385	2.485534	7.938427	4.440548	7.443276	2.434444	4.733824
0.276429	0.305657	0.213355	0.189141	0.409618	0.121494	37.29684	0.509791	1.103261
6.045209	24.73724	1.744055	4.281847	20.51271	3.450577	10.82954	11.65791	13.38418
0.037525	0.00119	0.148254	0.043391	4.33E-05	5.77E-05	25.99427	8.66E-05	0.000115
0.116659	0.066472	0.116494	0.106847	0.128283	0.059459	2.708341	0.096905	0.153983
5.056421	8.888167	1.421126	9.880779	8.048095	4.297222	11.60283	6.772763	7.061328
118.7028	51.52195	87.1746	15.276	55.04081	30.82277	144.7919	118.7028	155.7103
26.80355	13.75978	21.36892	53.77619	13.45939	42.08807	8.905642	33.07147	13.5739
35.776	144.7919	29.82828	8.075772	332.0776	15.98892	72.69473	28.23632	38.94854
16.34294	20.34063	10.17029	10.53159	9.377345	7.162698	51.52195	13.60866	26.25557
28.54786	13.34662	91.05541	42.08807	19.53061	39.53687	13.79669	25.73165	22.52375
6.011515	10.98644	9.968355	6.38456	4.524481	3.895469	252.4251	3.767489	37.81919
1360.293	425.62	184.443	144.7919	530.1465	530.1465	0.942944	749.7839	425.62
425.62	83.489	22.74797	67.98069	45.1045	50.55949	46.7056	167.9996	118.7028
65.94551	42.08807	126.1556	60.77449	18.91854	77.74812	10.19468	64.12971	27.10502
91.05541	75.1132	83.489	20.68746	126.1556	51.52195	91.05541	126.1556	91.05541
4.222583	3.8379	1.581912	1.849654	2.703485	2.760765	2.873817	4.783001	8.745238
1.401068	2.408882	1.427431	0.341053	1.367179	0.56443	1.312706	1.609654	1.518232
10.33476	6.632063	65.94551	2.417424	17.51961	3.648081	67.98069	5.788153	7.212915
8.033932	5.720173	17.64869	4.914863	8.711905	12.42965	0.024141	10.98644	7.824473
0.348139	1.732468	0.895945	0.070736	1.96697	0.006912	1.628009	0.522367	0.761688
0.1379	0.059459	0.515815	0.014545	0.1379	0.113074	1.131472	0.070909	0.065801
2.08013	0.963853	0.952294	0.548153	2.159004	0.825354	2.92013	1.910974	2.037114
0.164509	0.091616	0.630823	1.592395	4.33E-05	0.469358	0.024141	0.236421	0.000115
14.08569	4.077403	9.101674	7.882222	3.401126	3.350224	32.8773	14.6058	5.180058
7.22E-05	0.397489	0.483045	0.937655	0.154444	0.401176	287.197	8.66E-05	2.210866
10.65365	25.48179	95.22939	4.365224	38.94854	6.144055	8.303947	11.42512	12.03267
2.538218	0.416219	3.163506	4.540794	0.642698	5.679502	0.024141	1.74539	2.696061
0.872013	0.43347	1.662439	0.376017	0.516825	0.204531	10.62739	0.550664	0.800058
0.175036	0.010592	1.010887	0.062107	0.222193	0.068932	0.383074	0.083983	0.051595
0.637085	0.322078	0.809481	0.478182	0.260404	0.469358	50.55949	0.420058	0.65101
3.188918	0.769365	0.132107	8.994791	0.448261	3.031688	0.598456	1.795851	1.219019
29.18885	21.19547	6.079596	14.33939	10.01009	22.13245	5.266335	52.56128	28.54786
25.48179	20.16541	16.09482	35.776	34.38341	29.49693	5.477843	31.1753	13.5739
14.97762	11.544	5.01772	5.381227	6.508427	7.037345	10.07949	12.77772	25.24367
5.337547	4.715599	3.324452	1.450693	4.733824	2.54961	1.159913	4.546443	5.050267
0.845887	0.870375	1.145426	0.043391	1.225426	0.179545	0.82772	1.482626	0.485325
20.85876	12.67271	11.03843	25.99427	14.25954	14.42661	3.317807	13.42179	27.96248
0.941645	1.527792	0.697496	0.285253	1.225426	1.066948	0.46461	1.065584	1.510014
2.863211	4.933781	2.66404	3.521934	3.55961	2.259661	15.77678	2.99987	0.263853
23.6012	28.23632	56.39528	11.719	43.49658	13.53913	13.15079	24.04374	46.7056
2.136335	1.317475	4.292078	1.091443	1.194221	0.28443	6.967547	2.45575	4.655916
0.949286	1.240981	0.140303	0.273434	1.690483	0.401176	1.009625	0.477107	1.835743
4.096407	3.514834	6.808369	3.957734	4.039192	4.609076	3.317807	4.63189	3.521934
0.930325	0.488997	0.434069	1.081544	0.871429	1.385801	2.061926	1.021746	1.225426
1.599033	0.202294	1.427431	0.333088	0.529971	0.323319	7.625209	1.243442	0.511356
0.667965	0.219134	0.648038	0.086025	0.501905	0.087807	1.628009	0.363276	0.960216
0.687807	0.757763	1.252424	0.461811	1.022929	0.371299	2.209141	1.065584	1.210447
0.159351	0.28899	1.886335	0.471313	0.269141	1.473218	6.230245	0.020267	0.105873
6.570433	1.716364	1.502323	8.573434	0.260404	1.724805	9.924372	7.545584	5.409437
7.417359	2.735368	1.903636	1.091443	5.796421	1.162201	6.019971	5.889798	3.182482
7.570693	6.335664	8.677489	2.600014	6.250447	2.169509	8.412367	7.037345	4.348882
0.65798	0.305657	1.387612	1.035058	1.164257	1.473218	0.024141	0.550664	0.531486
0.065519	0.00119	0.148254	0.297439	0.040043	0.212698	2.66404	0.035339	0.012266
21.54823	25.99427	15.3618	6.55039	29.18885	12.49452	1.255657	26.80355	19.22139
0.56443	0.372973	0.446551	0.1379	0.538773	0.528997	4.145209	1.027403	0.468694

0.013175	0.016133	0.002338	0.000231	0.007821	0.006912	0.180231	8.66E-05	0.000115
0.986104	1.137244	2.520404	1.341825	0.269141	2.227273	0.024141	2.630729	0.427403
2.871212	11.65791	1.135137	7.468961	3.29355	10.98644	0.024141	2.174733	2.876421
5.9071	5.082063	13.00996	1.65785	2.045476	2.54961	100.0998	10.28573	53.77619
4.72798	10.67991	2.263377	3.179199	13.23022	3.610707	0.024141	4.783001	1.076652
1.070115	2.164113	2.129596	0.963853	3.098268	3.29355	3.113478	1.579798	1.865043
4.529913	1.650274	1.604827	11.65791	0.474214	6.685087	4.456912	5.500678	2.018167
3.310758	2.058773	3.26	3.23987	1.502323	5.923838	10.73084	2.928247	3.618499
0.974257	0.542165	1.093434	0.53912	0.708124	1.048802	34.38341	0.587273	1.664906
0.55645	0.16119	0.106696	0.000231	0.054711	0.097258	0.51316	0.345635	0.832013
10.48101	3.640606	1.968283	6.653016	4.650007	3.401126	65.94551	11.77567	15.66023
184.443	111.5439	30.17192	105.1694	72.69473	252.4251	3.846984	155.7103	203.5795
0.30596	0.066472	0.124791	0.070736	0.250823	0.049834	0.024141	0.148124	0.392251
25.73165	184.443	11.65791	19.22139	62.33472	22.74797	4.251818	23.38378	26.80355
2.815866	1.87482	1.985253	1.118817	0.964416	1.048802	3.899993	1.94311	2.018167
1.315613	0.60886	1.156869	0.376017	1.204228	0.939986	1.746724	1.277056	1.664906
8.164531	8.780115	7.375794	25.73165	5.923838	11.30987	1.046061	9.218514	7.598312

CGGA_127	CGGA_128	CGGA_128	CGGA_129	CGGA_129	CGGA_130	CGGA_130	CGGA_130	CGGA_130
1.913997	0.377085	0.546522	0.625786	0.249899	0.646378	0.585281	0.46461	0.282266
0.374365	0.000216	0.000245	0.312864	0.508975	0.27062	1.609654	0.415447	0.011696
2.487576	7.737807	4.053506	1.175859	4.863579	3.659668	0.193348	6.25938	2.5271
2.625823	15.56131	2.002193	4.376118	2.914632	3.300664	0.019978	7.938427	3.521934
3.500808	2.061926	5.323492	2.633232	2.547179	6.125823	1.531313	4.702468	5.01772
7.375794	1.786306	2.417424	1.564149	4.33342	7.288788	6.830592	1.411861	1.962424
39.53687	75.1132	22.74797	13.53913	55.04081	23.16622	7.200505	27.38726	18.19595
0.057583	0.106926	0.032915	0.281558	0.078297	0.019574	0.747879	0.071097	0.011696
0.250649	1.717641	4.508196	9.625253	3.150433	1.873434	0.019978	2.810678	4.764055
0.301955	0.159942	0.000245	0.019574	0.692027	0.292597	0.558095	0.154444	0.011696
75.1132	24.2842	13.45939	8.994791	95.22939	30.17192	80.6518	26.80355	9.141299
2.234538	0.508975	0.48619	0.562439	0.749019	0.97733	2.487576	0.315916	0.313312
8.994791	67.98069	20.51271	64.12971	23.38378	13.91714	4.212554	26.52537	25.73165
4.33E-05	14.6058	3.820758	17.64869	6.268312	0.254365	0.019978	10.62739	7.780786
0.011602	0.020216	0.032915	0.375281	0.026291	0.019574	0.558095	0.040794	0.189394
1.830159	3.080317	9.583463	2.831501	2.190866	2.353247	0.85728	12.42965	9.047532
2.203535	0.709784	0.555418	0.019574	1.006623	2.009906	1.277843	0.218975	0.383889
0.374365	0.052431	0.049719	0.019574	4.33E-05	0	25.24367	0.06088	0.011696
0.578485	3.034747	9.707677	12.16681	1.974228	0.655613	0.019978	11.65791	12.03267
1.991328	0.667965	0.436638	0.596775	0.478413	0.866717	2.577157	0.483874	0.448557
0.516306	1.541227	2.300678	10.48101	0.162915	0.093009	0.019978	1.624459	4.261342
1.239423	5.068831	6.125823	224.9555	1.624459	0.615996	4.402973	8.888167	19.53061
80.6518	83.489	0.889019	0.019574	2.290996	0	0.019978	0.728543	0.011696
0.027965	0.020216	0.146118	0.723506	4.33E-05	0.002619	2.693442	0.080079	0.011696
1.872049	0.098384	0.480303	0.019574	1.613232	1.850996	6.580988	0.38127	0.468167
2.296732	3.150433	2.554069	2.451645	6.876392	6.443925	6.978903	5.942294	2.511465
1.574517	1.628009	1.710173	2.713319	0.437309	0.857778	0.019978	1.363557	1.765065
0.333636	0.221111	0.455325	0.1686	0.672468	0.603737	1.015967	0.227648	0.232092
27.66779	24.50942	100.0998	87.1746	21.02638	29.49693	0.019978	42.75576	70.11932
0.589986	0.28189	0.223701	0.758773	0.548788	0.520678	2.378124	0.291183	0.574993
4.626025	6.135007	12.85504	14.97762	5.545729	3.230065	2.378124	9.356371	7.991602
0.323319	0.044026	0.153759	0.116659	0.111544	0.081746	1.758615	0.040794	0.143709
4.33E-05	20.51271	23.38378	31.92385	11.83593	10.1459	0.019978	24.04374	42.08807
0.039668	0.077172	0.000245	0.019574	0.019408	0.009322	0.388579	0.000404	0.011696
6.230245	0.448557	0.069206	0.019574	4.789596	1.713997	9.989221	0.049834	0.173723
1.066234	3.869163	2.90658	1.631544	1.662439	1.248232	0.418326	3.253175	3.36075
2.760765	5.210317	7.507619	7.013853	6.653016	3.578038	3.578038	5.737056	6.508427
0.1986	0.000216	0.000245	0.019574	0.019408	0.019574	1.049517	0.020765	0.011696
6.414416	4.292078	2.996869	1.387612	3.78798	3.314329	6.240346	2.820974	2.472576
16.85982	6.116638	2.882035	2.621133	7.882222	6.706472	8.005685	1.87899	2.511465
1.149322	0.349278	0.339004	0.155945	0.692027	0.400354	12.77772	0.475433	0.634531
0.843102	1.724805	1.186457	0.019574	1.600238	1.741414	16.22108	0.742417	0.313312
0.1986	3.464315	3.363911	2.287576	0.896522	0.412193	0.019978	10.1459	8.711905
4.33E-05	0.014545	0.000245	0.139091	0.019408	0	0.175592	0.000404	0.011696
0.139986	0.276061	0.297439	0.60886	1.863506	0.161962	2.449509	0.483874	0.409098
18.19595	17.28195	35.27608	20.68746	31.92385	33.93939	2.183665	46.7056	34.38341
0.093102	0.401385	0.059957	0.116659	0.124719	0.069784	1.082179	0.089315	0.086443
0.099488	0.199488	0.049719	0.019574	0.033261	0.204156	0.193348	0.000404	0.011696
4.33E-05	0.000216	0.01588	0.019574	4.33E-05	0.000361	0.279452	0.000404	0.011696
0.093102	0.000216	0.041328	0.019574	0.026291	0.001111	0.330635	0.000404	0.011696
0.021818	0.000216	0.000245	0.019574	0.013088	0.026364	0.019978	0.000404	0.011696
48.53429	2.42381	19.06893	11.19942	12.29473	6.30671	0.525325	11.96574	13.75978
0.236421	0.020216	0.000245	0.019574	0.07114	0.368658	0.525325	0.000404	0.011696
0.267597	0.86987	0.049719	0.019574	0.362424	0.009322	0.607273	0.000404	0.011696
2.931068	3.414574	4.115844	9.101674	2.306277	1.062092	0.156566	4.678846	2.681169
0.506003	0.114618	0.000245	0.019574	0.328932	1.118817	1.2214	0.000404	0.157958
1.278629	2.34355	0.186861	0.019574	0.658362	0.187713	1.428398	0.040794	0.214964

0.441472	0.580404	0.909315	0.176558	0.766118	0.585281	3.738997	0.165512	0.480022
3.401126	2.329784	1.172872	1.028672	1.744055	1.623232	1.995714	1.111241	1.05824
0.762165	0.42715	0.16145	0.176558	2.07658	0.419365	0.959567	0.337222	0.112886
22.74797	0.098384	46.7056	15.88548	4.33E-05	3.253175	1.497143	28.54786	31.1753
0.399307	0.337431	0.69583	1.387612	0.517359	0.359899	0.885664	0.227648	0.373218
0.843102	0.443052	0.233889	0.701818	0.918557	0.520678	4.519048	0.218975	0.342049
26.80355	40.7932	52.56128	7.112496	32.31123	57.84882	0.716234	23.6012	10.33476
0.585281	0.632569	0.347186	0.307973	0.201789	0.032857	3.618499	0.040794	0.42329
0.011602	0.014545	0.000245	0.289603	0.004257	0.005303	0.114841	0.000404	0.011696
1.138557	2.730072	1.179885	2.081696	2.554069	2.353247	0.279452	1.388543	0.789423
0.875599	1.628009	1.78759	1.669466	2.003723	0.784372	0.986104	2.084791	3.156537
1.416407	1.870685	0.310657	0.144098	0.996111	0.344127	0.330635	0.262965	0.297807
0.119062	0.027879	0.000245	0.23246	4.33E-05	0.223701	1.644416	0.218975	0.011696
0.016133	0.000216	0.000245	0.019574	0.078297	0.299942	0.470512	0.000404	0.011696
1.819221	0.098384	0.108124	0.894704	0.07114	0.514531	0.773766	0.06088	0.362179
0.005512	0.020216	0.01588	0.281558	0.040909	0.000361	0.114841	0.040794	0.011696
0.011602	0.014545	0.01588	0.281558	4.33E-05	0	0.279452	0.020765	0.011696
3.36075	7.162698	6.011515	5.050267	5.24562	5.395584	0.85728	8.348817	16.9162
0.229199	0.098384	0.09746	0.019574	0.162915	0.299942	0.558095	0.029928	0.011696
0.267597	0.184877	0.000245	0.019574	0.033261	0.069784	0.304228	0.000404	0.128853
9.625253	5.137042	3.726854	1.590202	5.309264	6.632063	9.625253	3.94886	2.647244
0.021818	0.000216	0.049719	0.019574	0.033261	0.299942	0.558095	0.06088	0.011696
0.648362	0.152222	0.242893	0.752229	0.231659	0.846797	2.487576	0.330289	0.214964
2.221775	5.923838	12.10289	19.68088	7.339452	3.796075	0.019978	10.8813	12.42965
0.016133	0.000216	0.01588	0.019574	0.013088	0	0.304228	0.020765	0.086443
1.239423	0.137201	0.069206	0.12697	0.709322	0.392403	1.400159	0.118225	0.097633
6.784762	18.05488	38.39042	20.85876	20.51271	155.7103	0.019978	19.68088	24.98218
0.405974	0.221111	0.041328	0.240202	0.335599	0.278802	0.805238	0.000404	0.011696
1.89518	0.235296	1.136566	0.368297	0.749019	1.044863	8.976508	0.337222	0.656392
0.460938	1.532468	0.785714	0.161818	2.604776	1.603795	0.019978	1.775325	1.201724
0.802035	0.027879	0.400534	0.463485	0.572266	0.204156	0.959567	0.46461	0.551284
0.323319	0.027879	0.01588	0.019574	0.084906	0.049834	0.304228	0.06088	0.011696
0.229199	1.049517	1.549711	2.388348	0.840635	0.527215	0.019978	3.233045	2.281926
5.638759	14.25954	7.468961	2.129596	11.14619	11.42512	0.019978	8.994791	4.529913
0.516306	0.184877	0.000245	0.1686	1.750628	2.047157	0.019978	0.080079	0.011696
0.267597	0.574993	0.252165	0.240202	0.282583	0.763564	0.832554	0.218975	0.574993
2.863211	2.649675	0.146118	0.139091	1.570794	1.869322	7.625209	0.19039	0.097633
0.07013	0.000216	0.059957	0.019574	0.013088	0.001111	1.11526	0.000404	0.097633
3.110447	13.53913	8.477662	4.875527	5.770996	6.036818	1.2214	6.079596	9.880779
0.011602	0.027879	0.069206	0.12697	0.026291	0.043824	0.63754	0.040794	0.112886
0.113146	0.020216	0.078124	0.214199	0.00785	0.049834	0.805238	0.020765	0.112886
0.008586	0.027879	0.01588	0.260144	0.004257	0.005303	0.114841	0.020765	0.011696
4.557605	0.587965	0.648362	0.856291	1.96404	2.392431	2.378124	0.636688	0.914618
2.060491	11.14619	5.788153	2.190866	6.738211	3.172864	2.331825	9.459177	3.230065
5.316378	1.819221	0.684033	0.289603	5.933066	2.259661	3.676032	0.790837	0.189394
1.641999	0.106926	0.353939	0.350274	0.38153	0.147706	0.446551	0.098384	0.313312
0.542525	1.401068	1.595729	4.552092	0.780519	0.127273	0.358211	1.333781	2.269134
0.405974	0.000216	0.000245	0.019574	0.171183	1.053528	0.805238	0.000404	0.011696
0.275058	0.47246	0.049719	0.019574	1.377446	0.543131	0.986104	0.330289	0.249538
0.992201	5.056421	6.404921	24.04374	0.867287	1.64917	0.019978	4.105714	9.377345
2.333867	0.819509	0.455325	0.133045	0.787987	0.585281	0.304228	0.299747	0.128853
3.211176	1.153723	1.283059	0.403175	0.563175	3.150433	0.805238	0.996696	1.139885
0.604214	9.625253	2.227273	0.249091	1.392511	4.745895	1.049517	4.007583	0.685209
1.515296	0.847352	0.574993	0.019574	1.64684	0.773766	1.497143	0.490664	0.249538
4.33E-05	15.276	118.7028	530.1465	53.77619	6.738211	0.525325	224.9555	87331.38
0.159791	1.034315	1.668369	1.190491	4.174964	0.351789	0.019978	3.904517	2.635685
0.333636	0.3121	0.000245	0.40886	0.539466	0.621688	0.214401	0.000404	0.143709
4.33E-05	28.23632	111.5439	144.7919	59.32013	9.968355	0.019978	144.7919	126.1556

0.648362	0.035426	0.09746	0.443543	0.138009	0.120433	1.015967	0.089315	0.097633
0.374365	0.000216	0.223701	0.019574	0.124719	0.299942	0.304228	0.165512	0.011696
4.752121	1.706219	3.086306	1.055628	0.867287	2.74544	29.82828	2.084791	2.763261
15.1268	26.52537	12.16681	7.519841	26.80355	7.6393	3.676032	18.48514	7.964315
2.378124	0.098384	0.252165	1.724805	0.057128	1.592395	20.51271	0.080079	0.574993
4.863579	4.764055	3.418405	0.662237	4.145209	9.459177	3.35715	3.350224	2.069942
2.327857	0.855801	0.310657	0.139091	1.433189	1.656696	6.772763	0.34601	0.342049
6.674286	1.169791	1.397482	0.614156	0.973745	1.978954	2.693442	0.820931	1.33285
15.77678	0.498636	0.53658	0.184055	0.494185	3.644343	27.66779	0.46461	0.987821
0.741962	0.304733	1.330974	1.651522	0.766118	0.400354	13.87537	0.756176	0.724004
0.011602	0.035426	0.032915	0.225339	0.048528	0.001111	0.304228	0.000404	0.011696
0.353175	0.184877	0.598456	0.639892	0.387056	0.570317	0.716234	0.630505	0.761688
0.145909	0.265224	0.117374	0.249091	0.60886	0.026364	0.63754	0.315916	0.143709
7.839271	1.477388	7.507619	5.838499	9.815642	7.765931	1.186457	6.486104	7.112496
0.236421	0.326017	0.203333	0.710722	0.185642	0.13386	1.082179	0.029928	0.157958
0.829192	1.143175	2.703485	4.563117	4.821176	3.028629	0.019978	3.464315	1.934271
1.602619	0.569625	0.655245	0.518716	0.98474	0.962698	0.986104	0.630505	0.740952
0.005512	0.000216	0.032915	0.019574	0.00785	0	0.214401	0.000404	0.011696
3.595758	2.109755	3.321284	4.673182	5.855281	1.928629	6.171789	1.484553	0.436638
0.341804	0.27075	1.230411	1.938759	0.048528	0.216753	0.019978	0.475433	1.602619
0.005512	0.000216	0.01588	0.019574	4.33E-05	0.002619	3.070952	0.020765	0.097633
2.150303	0.393983	1.930216	2.045476	1.815	0.210693	0.676659	1.68671	2.24733
3.119986	2.925426	0.528571	0.019574	3.040505	1.472208	26.52537	0.330289	0.448557
1.89518	7.136537	3.743232	3.55961	7.38847	1.578687	0.716234	4.535354	3.909033
2.404618	0.28189	0.901212	0.201501	1.262965	0.82355	0.156566	0.653586	1.012742
5.366335	2.398442	2.353247	3.034747	2.035678	3.50425	18.55613	1.228651	2.647244
4.667518	3.816551	2.361198	0.332641	3.824964	4.46267	5.704646	2.375974	2.834084
8.195657	1.628009	8.365072	16.9162	0.556753	0.6657	2.652107	4.272165	10.48101
0.323319	0.349278	2.565556	21.36892	0.019408	0.120433	0.330635	2.084791	6.443925
0.301955	4.086926	5.395584	1.705007	0.362424	0.986797	0.279452	2.485534	3.457504
18.48514	4.863579	9.625253	5.671126	12.70768	18.33909	2.050404	16.09482	12.49452
4.33E-05	0.265224	3.655714	3.730808	0.193896	0.120433	0.019978	3.404372	4.408153
8.694697	13.6814	13.15079	17.15902	24.2842	9.377345	1.049517	14.08569	12.85504
5.821804	7.697063	12.22798	21.02638	3.718716	7.037345	0.019978	17.03919	31.55417
4.33E-05	0.035426	0.023232	0.019574	0.00785	0.019574	0.585281	0.000404	0.011696
0.667201	2.758088	1.779423	1.387612	2.893001	0.351789	0.019978	2.053521	2.069942
8.493088	3.377619	0.740527	0.019574	4.49671	4.552092	8.005685	0.608543	0.32925
0.762165	0.206768	0.546522	1.997352	0.500996	1.044863	2.74298	0.659163	0.942294
0.851659	0.377085	0.153759	0.718766	0.185642	0.563499	0.234726	0.299747	0.265786
1.149322	3.175916	4.715599	4.456912	0.840635	3.253175	0.019978	16.34294	2.116032
0.229199	14.08569	43.49658	77.74812	1.236248	0.317576	0.019978	21.36892	77.74812
21.74051	36.26061	80.6518	111.5439	39.53687	0	0.019978	118.7028	80.6518
7.264502	1.753341	0.347186	1.721371	2.263377	1.018427	4.783001	0.277763	0.491876
0.093102	0.221111	0.049719	0.260144	0.787987	0.197908	0.358211	0.000404	0.011696
1.249221	1.160707	0.92531	1.810664	1.377446	1.374329	0.525325	0.92342	2.051962
2.950173	1.972706	4.067547	5.959033	0.209841	2.438831	0.019978	3.57057	6.475505
3.595758	0.27075	0.369105	0.312864	0.153651	0.527215	2.818312	0.154444	0.112886
2.003723	0.000216	0.000245	0.019574	1.299567	0	11.22667	0.000404	0.011696
6.55039	28.87506	15.3189	5.210317	22.32214	21.74051	1.339091	13.91714	10.62739
0.360281	0.86987	0.297439	0.019574	1.115902	1.083651	0.019978	0.721335	0.588954
1.087345	0.000216	0.032915	0.019574	4.33E-05	0	3.014076	0.000404	0.011696
20.16541	1.588903	2.173081	1.069574	5.165599	4.875527	22.32214	1.972706	2.151919
1.326667	1.753341	1.043608	0.562439	1.495043	1.89373	0.388579	2.413146	1.581912
0.93267	0.052431	0.09746	0.019574	0.290115	0.230808	6.171789	0.154444	0.011696
0.275058	0.027879	0.36153	0.483874	0.632208	0.327143	4.338629	0.315916	0.297807
1.779423	5.576219	3.327619	1.724805	0.929286	3.290101	0.525325	7.765931	5.180058
0.204964	0.000216	0.000245	0.019574	0.153651	0.161962	0.193348	0.000404	0.011696
0.3671	0.159942	0.171061	0.019574	0.084906	0.245772	0.63754	0.181227	0.373218

0.411948	0.367532	1.152165	0.214199	0.177879	0.97733	0.019978	0.373709	0.42329
7.545584	12.70768	5.409437	0.019574	3.101371	4.188896	0.019978	3.370527	7.824473
4.376118	3.993665	3.160022	1.277056	4.914863	5.431544	13.6814	5.993045	2.367763
16.68554	34.84299	45.1045	41.47925	11.89932	11.96574	0.019978	53.77619	47.5018
1.497143	2.984899	1.326667	2.925426	2.132756	2.169509	0.019978	1.303045	1.47018
4.33E-05	8.381328	4.348882	19.06893	13.75978	0	0.019978	4.241905	7.315512
0.893016	0.28189	0.242893	0.710722	0.267807	0.464127	0.358211	0.427937	0.342049
6.135007	8.412367	13.30906	14.88584	6.967547	4.435159	0.019978	5.143867	29.18885
6.830592	0.000216	0.213218	0.019574	1.310837	2.365685	5.745996	0.218975	0.011696
12.22798	12.16681	8.319019	2.74544	11.19942	9.902576	8.396847	8.164531	5.42381
1.763889	0.000216	0.36153	0.019574	0.267807	1.721371	3.953297	0.000404	0.011696
8.590794	14.17388	6.153102	4.343838	7.991602	12.10289	5.889798	5.439278	4.709033
0.418326	1.247244	1.549711	1.856392	0.882619	0.97733	0.832554	1.896667	0.802756
0.93267	6.830592	2.427792	1.948918	2.922778	2.950173	0.304228	2.901032	2.069942
0.113146	0.077172	0.000245	0.260144	4.33E-05	0.54925	3.953297	0.000404	0.32925
0.388831	2.329784	1.304877	0.639892	1.974228	1.592395	0.019978	2.494177	1.613232
0.981032	1.673254	1.330974	8.660281	1.675722	1.118817	0.585281	2.447294	3.703175
2.150303	1.329206	0.53658	0.019574	2.77899	2.50469	6.38456	0.323535	0.779668
0.016133	0.014545	0.000245	0.019574	0.026291	0.002619	0.175592	0.020765	0.112886
0.113146	6.55039	1.057569	0.019574	4.468009	1.995714	0.019978	0.80417	0.011696
9.562554	13.75978	6.125823	3.198499	9.141299	7.519841	1.644416	6.22031	3.975498
2.131176	1.381111	0.520678	0.019574	2.947287	1.062092	0.019978	0.842641	0.622496
0.236421	0.000216	0.153759	0.375281	0.209841	0.03842	1.310837	0.000404	0.824416
0.843102	6.171789	4.739668	3.610707	5.431544	6.063276	1.400159	9.297085	9.256537
3.595758	6.045209	4.965801	3.373983	5.431544	7.468961	0.019978	5.687879	3.730808
1.055628	0.121703	0.000245	0.019574	0.096263	0.506869	2.652107	0.000404	0.011696
0.250649	0.393983	0.273434	0.639892	0.177879	0.286364	0.558095	0.049834	0.265786
1.268672	3.730808	3.91355	16.34294	1.043608	0.888485	0.607273	5.500678	4.881775
1.731169	2.600014	0.331869	0.019574	4.839105	2.400455	1.609654	0.000404	0.011696
0.843102	0.544545	0.475137	0.690707	0.153651	0.425635	5.984885	0.165512	0.610606
0.704444	4.684502	2.628225	22.32214	0.929286	1.143954	1.11526	6.653016	20.51271
1.209105	1.275325	0.436638	1.062821	0.802035	0.595368	2.331825	0.907273	0.88316
0.399307	0.462857	0.126429	0.625786	0.444762	0.629098	0.525325	0.080079	0.173723
13.30906	9.541645	6.454416	11.544	1.068348	3.726854	1.11526	20.34063	10.8813
3.651898	0.159942	0.059957	0.019574	1.099004	0.70886	12.63773	0.27062	0.458701
2.131176	2.367763	6.945152	5.720173	1.624459	4.227302	0.019978	9.101674	12.60233
0.057583	0.014545	0.000245	0.019574	0.013088	0.009322	0.114841	0.020765	0.086443
0.382626	0.192929	0.223701	0.019574	0.153651	0.019574	0.330635	0.000404	0.011696
0.204964	0.052431	0.108124	0.019574	4.33E-05	0	1.400159	0.000404	0.249538
0.609841	3.91355	0.793434	2.08798	0.342641	0.230808	0.279452	0.968723	0.92342
0.250649	0.332395	0.078124	0.116659	0.308788	0.773766	53.77619	0.071097	0.173723
91.05541	48.53429	27.10502	7.683766	25.99427	19.22139	24.50942	25.24367	15.98892
9.121486	5.638759	9.335397	3.394286	22.96463	20.16541	4.519048	11.77567	6.345382
3.5257	0.688182	0.706984	4.207778	0.717879	1.008413	33.4985	0.653586	0.772157
2.579509	3.243225	4.758088	4.845166	2.567605	3.065108	1.11526	4.222583	4.673182
0.557763	0.000216	0.000245	0.019574	0.078297	0	1.883535	0.000404	0.011696
0.441472	0.172294	0.087403	0.019574	0.216753	0.182482	0.935115	0.165512	0.112886
12.03267	12.77772	41.47925	13.38418	5.485685	12.56693	0.019978	5.838499	4.802655
1.215195	3.895469	1.065584	2.451645	0.772951	2.925426	0.019978	4.039192	8.477662
3.273045	2.153535	1.870685	0.473016	2.567605	2.776421	2.909293	1.314675	1.191905
4.984488	1.460051	2.190866	1.931537	3.070952	8.888167	12.85504	2.31404	2.408882
0.145909	0.767569	1.274481	0.680173	0.462857	0.857778	0.019978	0.337222	1.219856
1.96404	0.000216	29.49693	18.91854	0.342641	27.38726	0.019978	5.576219	3.214545
5.088694	0.000216	75.1132	42.75576	4.241905	13.60866	0.019978	8.797619	21.36892
0.260404	0.06886	0.032915	0.019574	0.033261	0.327143	1.186457	0.06088	0.011696
6.717056	40.11561	135.5245	27.10502	100.0998	105.1694	0.019978	80.6518	52.56128
15.46193	3.377619	1.023528	0.986104	2.725498	0.998831	8.643074	0.981032	0.814315
2.078355	3.644343	0.956061	0.218817	5.130411	4.359574	0.156566	1.831631	0.112886

0.809986	0.486551	0.310657	0.019574	1.043608	0.838326	2.973059	0.543398	0.265786
4.592273	5.500678	0.785714	0.888485	3.500808	5.71241	0.019978	1.277843	1.012742
1.835743	2.417424	3.687633	2.871212	1.236248	1.260635	0.716234	1.484553	2.353247
4.33E-05	7.339452	13.91714	29.18885	35.776	29.18885	0.019978	31.1753	9.541645
15.3618	100.0998	87.1746	126.1556	42.08807	33.07147	0.019978	184.443	167.9996
4.33E-05	0.000216	0.000245	0.019574	4.33E-05	0.400354	10.80505	0.000404	0.011696
1.128788	0.296955	0.117374	0.1686	0.453463	1.859084	5.381227	0.19039	0.189394
0.609841	0.035426	0.1793	0.019574	1.50684	1.511198	4.402973	0.38127	0.011696
4.33E-05	0.000216	0.049719	0.190844	0.00785	0.019574	0.935115	0.06088	0.011696
0.475729	0.265224	0.186861	0.019574	0.909315	2.630729	0.358211	0.199488	0.011696
0.333636	0.121703	0.223701	0.599466	0.040909	0.009322	0.676659	0.118225	1.170491
0.119062	26.80355	44.27012	118.7028	9.667316	0.577706	0.019978	55.04081	118.7028
8.164531	1.275325	5.439278	11.48368	2.887489	1.813564	4.783001	4.557605	3.002576
1.564149	0.276061	0.153759	0.647345	0.201789	0.204156	1.339091	0.154444	0.789423
3.486436	1.460051	1.123795	1.268672	1.755988	1.568564	7.402915	0.945483	1.201724
1.757244	4.227302	0.855801	0.176558	1.080303	1.850996	0.418326	2.50469	0.772157
3.869163	2.388348	1.549711	1.78759	2.522684	1.948918	17.09911	1.449062	2.116032
0.609841	2.136335	0.53658	0.350274	1.146962	1.394632	4.338629	0.434596	0.733074
2.150303	0.610224	0.706984	1.960945	0.692027	0.570317	3.676032	0.366912	0.685209
1.971183	0.326017	0.710722	0.680173	1.458038	1.092785	3.500808	0.524646	0.789423
1.239423	1.575483	13.38418	17.28195	2.306277	0.527215	0.156566	2.958773	13.53913
0.597042	0.098384	0.09746	0.019574	0.437309	1.421126	6.171789	0.108124	0.011696
3.803766	9.792626	27.96248	17.39935	7.737807	10.57984	0.019978	11.42512	21.02638
0.175469	0.000216	0.01588	0.116659	0.162915	0.000361	0.805238	0.080079	0.011696
3.089293	0.235296	0.126429	1.944517	1.600238	1.364401	31.1753	0.440289	0.740952
2.274473	6.153102	3.26	1.997352	4.965801	2.427792	0.019978	2.174733	2.101075
0.051472	0.152222	0.023232	0.249091	0.10404	0.063319	1.277843	0.049834	0.173723
14.00414	45.1045	26.80355	27.66779	13.6814	17.78522	2.378124	36.74522	30.82277
2.604776	3.06803	6.945152	4.207778	4.02088	4.203001	1.400159	6.045209	6.028427
8.412367	0.077172	1.298665	2.055289	1.931537	4.397792	0.019978	0.800058	2.29873
0.418326	0.296955	0.041328	0.019574	0.019408	0.140303	0.832554	0.049834	0.011696
7.625209	4.408153	13.00996	4.145209	8.539971	14.33939	19.22139	5.855281	10.57984
3.747035	1.673254	15.56131	22.13245	4.072475	1.928629	0.019978	7.363117	17.78522
67.98069	144.7919	77.74812	24.98218	126.1556	118.7028	18.55613	91.05541	37.29684
0.609841	0.114618	0.283709	0.671371	0.453463	0.175866	2.378124	0.307734	0.342049
7.339452	0.367532	10.78056	6.045209	2.255916	6.475505	95.22939	4.978182	12.93651
8.288874	7.882222	35.776	35.776	10.01009	16.9162	2.449509	23.16622	40.7932
0.099488	0.000216	0.000245	0.019574	0.048528	0.056869	0.255887	0.000404	0.011696
0.008586	0.044026	0.108124	0.680173	0.064848	0.014286	0.193348	0.000404	0.143709
1.80267	0.647345	4.440548	8.333918	3.249877	1.96697	0.446551	3.775209	5.821804
11.42512	25.73165	45.91841	91.05541	6.153102	18.91854	4.907929	15.88548	45.1045
0.039668	0.000216	0.000245	0.019574	4.33E-05	0.009322	0.773766	0.020765	0.097633
0.506003	0.596508	0.428456	0.019574	0.393716	0.471025	0.019978	1.453939	0.500996
0.679452	0.86987	0.297439	0.631847	0.231659	0.498918	1.609654	0.569971	0.011696
0.099488	0.0843	0.049719	0.710722	0.393716	0.763564	1.277843	0.06088	0.011696
19.68088	1.724805	4.46267	2.276407	9.541645	11.30987	2.050404	2.978968	3.625382
1.3829	0.995527	0.262338	0.176558	0.572266	1.734949	5.217569	0.543398	0.750404
9.502294	3.394286	13.53913	10.82954	9.256537	7.824473	3.618499	6.464906	12.60233
135.5245	10.38202	40.7932	65.94551	38.94854	64.12971	3.676032	30.49289	95.22939
4.33E-05	0.152222	0.153759	0.019574	4.33E-05	0	2.909293	0.000404	0.011696
0.145909	0.06886	0.059957	0.176558	0.040909	0.069784	2.050404	0.06088	0.202294
23.6012	22.52375	23.16622	14.6058	30.82277	41.47925	10.48101	25.48179	18.05488
1.941703	2.201587	1.360022	0.463485	3.718716	2.961768	1.186457	1.32482	1.068348
0.426118	0.177143	0.194531	0.501905	0.241255	0.223701	0.358211	0.256797	0.143709
6.107114	0.393983	0.639495	1.675722	0.231659	1.394632	37.81919	0.353795	0.675556
0.726472	0.241255	0.252165	0.42925	0.453463	0.344127	7.351284	0.165512	0.157958
12.10289	2.77899	3.497085	5.409437	2.290996	3.367071	38.66948	2.276407	1.21215
1.316544	0.569625	0.16145	0.144098	0.259033	0.998831	1.580909	0.098384	0.480022

62.33472	17.51961	11.60283	3.921645	111.5439	50.55949	77.74812	9.256537	6.632063
0.087619	0.014545	0.023232	0.116659	0.033261	0.03842	1.015967	0.000404	0.143709
0.418326	1.132857	2.445079	4.933781	1.578687	0.921739	1.277843	2.703485	3.8121
0.046212	0.000216	0.000245	0.019574	0.026291	0.093009	0.279452	0.000404	0.011696
2.625823	0.603737	0.901212	1.087345	0.996111	1.453939	23.16622	0.497749	0.789423
12.63773	1.96404	5.838499	2.378124	5.337547	12.42965	9.838658	3.286652	3.644343
7.099913	3.028629	2.417424	0.973232	1.476429	3.35715	16.50509	1.058896	1.793074
0.275058	0.14482	0.023232	0.019574	0.00785	0.014286	1.049517	0.000404	0.157958
2.035678	9.335397	3.529466	1.157626	6.632063	5.493182	0.019978	4.188896	1.68671
1.89518	1.89228	1.615281	1.468131	1.420202	1.092785	2.183665	1.634935	1.546696
0.731732	0.28189	0.136479	0.019574	0.048528	0.155844	6.900202	0.205931	0.011696
8.348817	16.9162	18.05488	12.77772	15.276	9.256537	4.082165	16.68554	15.276
0.597042	1.96404	1.626782	0.29715	0.700418	0.26215	0.019978	1.340036	0.987821
2.347439	3.175916	2.831501	3.984747	2.718434	1.225426	0.019978	3.687633	3.034747
0.360281	0.0843	0.583766	0.375281	0.177879	1.323975	6.38456	0.098384	0.189394
16.5625	39.53687	29.18885	26.80355	46.7056	24.04374	15.46193	38.39042	21.54823
5.088694	16.5625	16.22108	30.82277	10.67991	4.821176	1.925635	13.38418	16.68554
0.62645	0.077172	0.049719	0.116659	0.462857	1.062092	1.150635	0.089315	0.086443
14.97762	2.472576	3.227085	1.985253	3.699495	12.93651	19.53061	2.210866	2.269134
13.15079	32.31123	91.05541	51.52195	34.38341	36.26061	0.494444	72.69473	111.5439
2.365685	2.337821	0.446291	0.307973	3.514834	2.481356	1.688045	2.196017	0.362179
0.907273	0.557056	0.400534	0.133045	0.35241	0.432698	0.255887	0.283882	0.409098
5.696263	2.259661	0.92531	0.814877	9.297085	9.502294	2.74298	1.058896	1.048074
0.506003	95.22939	0.901212	1.901068	14.00414	1.225426	0.019978	3.163506	0.533196
2.810678	1.102691	10.93101	2.964762	3.87329	3.201457	0.019978	4.655916	10.67991
6.674286	3.730808	3.435988	1.287403	8.711905	4.435159	2.652107	3.303925	3.489899
3.343629	2.593506	0.318124	0.019574	2.035678	1.159913	9.667316	0.440289	0.656392
0.021818	0.035426	0.126429	0.12697	0.00785	0.009322	0.234726	0.071097	0.112886
14.33939	16.44768	24.04374	6.922771	10.23768	22.74797	51.52195	10.98644	12.77772
2.45772	4.900996	5.295325	3.87329	4.888023	4.227302	4.155339	7.38847	5.687879
2.187395	1.628009	2.855296	0.557056	2.210866	0.97733	0.716234	2.845036	2.290996
4.212554	3.35355	4.990794	3.595758	8.762677	7.443276	1.797172	4.978182	4.188896
4.376118	5.024293	2.339697	3.098268	5.993045	4.900996	0.585281	3.080317	2.850058
0.74868	0.288579	0.108124	0.307973	0.342641	0.419365	1.082179	0.118225	0.112886
0.341804	0.035426	0.16145	0.877222	0.033261	2.276407	2.183665	0.089315	0.232092
0.516306	0.455649	0.446291	0.483874	0.35241	1.131472	0.773766	0.483874	1.33285
3.061955	1.47018	0.388831	1.551962	1.853723	1.826147	2.296732	0.353795	0.265786
4.261342	0.326017	0.273434	0.019574	0.766118	0.514531	1.150635	0.19039	0.011696
8.493088	65.94551	60.77449	83.489	37.29684	12.49452	2.183665	64.12971	67.98069
0.718355	1.762713	4.72798	9.179466	2.181775	0.813788	1.557482	6.153102	8.508514
0.648362	0.933874	0.475137	0.23246	2.126349	0.943514	0.304228	0.700418	0.551284
5.737056	3.775209	1.310837	1.997352	3.13456	2.380079	45.91841	1.727323	1.74539
3.046551	1.123016	0.625498	0.625786	6.486104	2.616573	7.468961	0.558824	0.143709
11.57341	2.016515	1.531313	1.916782	0.645455	2.671299	37.29684	1.700354	1.569553
22.32214	7.991602	7.653391	3.6029	10.5063	14.97762	42.75576	9.418196	6.287561
10.53159	16.09482	3.699495	11.14619	11.96574	4.02088	1.644416	3.253175	6.423911
17.28195	4.881775	28.23632	332.0776	3.249877	1.453939	2.860722	10.82954	33.93939
1.701616	14.33939	1.898153	3.435988	4.575123	0.646378	2.74298	2.652107	2.304452
10.98644	10.33476	4.603434	3.214545	4.198117	4.959358	0.832554	6.900202	6.612121
3.140866	2.993867	0.613853	0.557056	2.243911	0.962698	3.35715	0.690707	0.352807
0.884654	5.150693	0.146118	0.161818	1.420202	0.943514	6.171789	0.415447	0.143709
0.021818	0.000216	0.049719	0.214199	0.00785	0.000361	0.193348	0.127403	0.173723
2.55184	8.477662	3.049603	0.523211	7.136537	10.1028	0.019978	2.941544	1.992944
38.94854	25.24367	14.70188	4.272165	21.54823	29.82828	5.47	11.19942	5.976724
0.005512	0.014545	0.01588	0.122121	0.004257	0	0.156566	0.020765	0.086443
0.468167	0.106926	0.023232	0.019574	0.10404	0.001111	1.497143	0.071097	0.011696
59.32013	18.62711	14.08569	5.855281	42.75576	47.5018	30.49289	15.56131	4.978182
2.909293	0.740527	0.468384	0.281558	1.006623	1.159913	3.792266	0.558824	0.448557

0.027965	0.000216	0.023232	0.019574	0.064848	0.175866	0.255887	0.000404	0.011696
52.56128	6.365065	14.79742	5.158146	29.18885	11.60283	57.84882	14.70188	12.16681
5.696263	2.084791	2.90658	0.518716	5.295325	3.610707	8.868932	1.959466	1.867951
0.192251	0.098384	0.032915	0.1686	0.07114	0.001111	2.616573	0.000404	0.097633
0.570664	0.14482	0.117374	0.116659	0.275382	0.368658	14.00414	0.108124	0.011696
0.843102	0.367532	0.480303	0.184055	0.10404	0.197908	1.428398	0.049834	0.112886
118.7028	12.10289	24.98218	18.48514	38.39042	91.05541	167.9996	17.28195	25.24367
6.622092	2.491948	1.772756	1.453939	4.978182	31.55417	5.838499	2.45575	1.650274
18.91854	9.377345	6.508427	3.730808	15.88548	15.07257	25.99427	5.545729	6.55039
2.958773	1.510014	3.94886	4.272165	0.517359	0.888485	0.304228	2.725498	1.962424
32.68313	19.37139	126.1556	37.81919	332.0776	332.0776	7.468961	749.7839	56.39528
11.77567	4.881775	8.573434	3.35355	8.940671	17.98838	14.29947	8.643074	8.445743
3.101371	0.367532	2.607035	0.723506	1.355527	4.003117	8.868932	2.210866	1.89228
1.602619	4.02088	0.684033	0.42202	2.139928	1.550837	4.907929	0.766118	0.761688
2.710765	7.653391	1.844098	2.355289	2.014863	2.290996	11.14619	1.425613	1.513102
8.319019	3.002576	3.028629	1.599033	6.116638	10.73084	3.953297	2.42381	3.052655
126.1556	1.149978	4.63189	3.38456	32.68313	26.25557	105.1694	8.195657	4.397792
1.076652	0.000216	0.16145	0.019574	0.231659	1.214315	4.519048	0.199488	0.157958
5.522929	0.3121	1.937222	1.651522	0.780519	6.853608	30.82277	0.394877	0.32925
40.11561	15.77678	22.13245	33.93939	16.22108	16.22108	118.7028	27.96248	23.82354
30.49289	47.5018	15.3189	4.764055	70.11932	65.94551	2.786898	17.78522	8.573434
6.00228	9.459177	0.620462	1.328384	3.113478	2.225498	12.16681	0.586595	0.32925
13.23022	7.795642	5.515584	2.498478	16.34294	11.544	17.64869	6.423911	3.125844
2.77899	5.485685	1.50342	0.88316	3.899993	4.655916	0.019978	1.266255	0.189394
1.779423	31.55417	20.85876	70.11932	15.18102	26.52537	1.839986	40.7932	22.32214
0.532619	3.06803	0.436638	0.535657	2.876421	1.131472	6.717056	0.622821	0.011696
5.01088	4.251818	5.576219	8.256465	4.597792	2.021068	1.015967	6.622092	8.608153
5.855281	77.74812	19.37139	62.33472	10.60361	9.729358	1.460051	34.38341	28.87506
0.07013	0.177143	0.069206	0.019574	0.019408	0.27062	14.93173	0.000404	0.011696
1.249221	0.709784	0.822965	0.38772	0.733593	0.73912	1.2214	0.5129	0.622496
12.7427	8.976508	12.36443	5.47	0.241255	2.009906	1.049517	8.493088	5.238701
11.30987	1.067662	0.965065	2.470317	0.599466	10.98644	0.114841	0.490664	1.441898
0.704444	0.508975	0.513456	0.019574	0.918557	2.092987	11.60283	0.227648	0.588954
2.673846	3.011169	3.122915	3.775209	2.567605	6.345382	0.716234	3.422237	4.145209
0.005512	0.060729	0.000245	0.019574	0.013088	0.005303	0.330635	0.000404	0.143709
23.82354	22.32214	11.36975	3.327619	15.3618	19.68088	19.37139	7.839271	5.259437
749.7839	287.197	42.08807	22.96463	184.443	87331.38	425.62	29.18885	32.68313
425.62	203.5795	83.489	105.1694	749.7839	167.9996	252.4251	126.1556	75.1132
1.826147	0.177143	0.493925	0.614156	1.028672	0.677403	2.102626	0.127403	0.011696
25.48179	11.48368	33.4985	39.53687	28.23632	37.29684	7.124517	27.10502	53.77619
2.296732	0.386378	0.171061	0.019574	0.581378	1.008413	3.230065	0.530253	0.011696
6.079596	3.283139	2.802828	8.888167	3.188918	3.428788	3.738997	3.175916	5.560563
0.081818	0.121703	0.059957	0.184055	0.185642	0.27062	6.240346	0.34601	0.011696
0.039668	0.617417	0.108124	0.019574	1.115902	0.254365	0.138571	0.145556	0.011696
0.15241	0.060729	0.000245	0.019574	4.33E-05	0	0.418326	0.000404	0.011696
5.770996	12.03267	2.390447	0.986104	7.315512	3.592063	5.704646	2.129596	1.139885
9.418196	36.74522	2.542605	4.690159	15.56131	5.337547	2.652107	3.330909	5.210317
0.039668	0.044026	0.01588	0.190844	0.185642	0.026364	1.369928	0.000404	0.097633
100.0998	35.776	59.32013	48.53429	43.49658	67.98069	13.53913	47.5018	65.94551
0.081818	0.000216	0.078124	0.019574	4.33E-05	0.069784	0.279452	0.000404	0.011696
14.51648	21.02638	10.23768	5.351934	15.66023	25.48179	0.279452	8.923117	6.365065
0.039668	0.000216	0.000245	0.019574	0.153651	0.140303	2.487576	0.080079	0.011696
1.341825	0.152222	2.084791	0.368297	0.418557	2.713319	5.745996	1.641999	3.346898
0.671724	0.000216	0.059957	0.557056	0.138009	0.237893	0.418326	0.000404	0.448557
0.008586	0.014545	0.000245	0.019574	4.33E-05	0.056869	2.102626	0.020765	0.011696
0.679452	1.034315	0.108124	0.019574	1.052886	0.693571	2.421681	0.071097	0.011696
42.75576	11.65791	20.16541	12.36443	31.55417	25.99427	47.5018	18.76954	21.93452
24.98218	4.203001	6.830592	9.335397	5.337547	2.524892	35.776	5.25254	6.663651

15.56131	4.833398	4.758088	4.845166	6.423911	10.38202	13.04605	1.925635	6.107114
10.1028	2.839351	4.827691	3.346898	4.435159	6.570433	11.74734	3.201457	4.048499
6.054242	6.653016	2.5271	1.25746	3.655714	1.865043	0.019978	4.096407	1.376378
0.016133	0.035426	0.01588	0.019574	0.00785	0	0.330635	0.000404	0.011696
19.99861	7.697063	3.890556	3.317807	7.795642	16.44768	43.49658	3.957734	3.276335
12.36443	6.497266	16.5625	43.49658	13.91714	27.10502	9.377345	17.92188	30.17192
1.857677	0.42715	0.000245	0.019574	0.532092	0.998831	2.693442	0.000404	0.011696
2.917381	1.247244	2.820974	1.771392	1.636118	1.702706	0.019978	1.74539	3.442886
1.78759	0.855801	1.636118	0.980505	0.772951	1.727323	2.531515	0.870375	1.237063
13.42179	8.711905	4.690159	2.893001	7.710361	8.149307	13.34662	7.570693	3.851414
1.128788	1.256558	0.369105	0.396082	1.225426	1.374329	1.925635	0.483874	0.214964
9.179466	4.490801	6.365065	0.568665	3.679986	2.973059	0.358211	8.797619	4.323045
21.54823	11.83593	9.667316	15.276	19.83727	12.63773	18.76954	7.456118	9.047532
3.718716	4.776407	9.459177	8.412367	72.69473	33.4985	4.783001	23.82354	5.788153
4.33E-05	0.949827	0.000245	1.514199	0.241255	0.087165	0.388579	0.000404	0.011696
12.42965	11.77567	19.53061	6.38456	16.44768	23.38378	0.019978	17.15902	18.91854
0.093102	0.436017	0.049719	0.019574	0.335599	0.16868	0.959567	0.080079	0.112886
56.39528	25.99427	2.173081	3.588369	15.98892	8.365072	100.0998	1.582915	1.25746
0.609841	0.184877	0.049719	0.746551	0.321068	0.076162	8.195657	0.181227	0.128853
9.459177	1.432302	2.970144	7.839271	1.089394	1.578687	4.212554	2.540462	4.025267
0.087619	0.587965	0.574993	0.41482	0.22303	0.6657	2.860722	0.241255	0.562439
0.741962	1.786306	1.96697	0.746551	2.703485	1.592395	0.304228	2.696061	1.481631
17.51961	9.667316	9.19899	7.443276	2.210866	8.061934	38.66948	6.865	5.959033
0.113146	0.213615	0.023232	0.019574	0.321068	0.204156	0.676659	0.136667	0.143709
28.23632	4.179762	4.927302	3.625382	13.38418	10.93101	8.256465	4.900996	5.515584
3.310758	6.019971	30.17192	32.31123	14.17388	12.77772	5.923838	14.6058	35.27608
0.07013	0.06886	0.117374	0.116659	0.048528	0.127273	0.019978	0.000404	0.011696
3.166659	4.802655	3.279625	1.599033	5.266335	8.226219	15.98892	3.052655	1.084437
2.831501	1.516219	0.53658	0.961486	1.520346	1.260635	2.74298	0.622821	0.562439
0.315743	0.137201	0.807619	0.149986	0.209841	0.480022	0.358211	0.315916	0.458701
0.353175	0.760433	2.842193	5.942294	4.33E-05	0.278802	0.019978	2.556443	8.348817
1.028672	0.883665	0.822965	0.207713	1.107605	2.084791	0.773766	0.549661	0.714697
31.92385	11.36975	2.647244	0.710722	7.443276	15.77678	41.78366	1.277843	1.68342
3.595758	51.52195	2.628225	0.671371	6.036818	10.05618	332.0776	5.959033	0.987821
1.991328	1.263752	0.940563	1.000794	2.003723	2.009906	1.186457	0.711046	0.824416
0.051472	0.3121	0.000245	0.586595	0.177879	0.161962	87.1746	0.21176	0.189394
0.051472	0.917431	0.400534	1.302107	0.342641	0.535657	3.166659	1.119574	0.824416
1.435772	0.953506	0.598456	0.671371	0.563175	1.633752	0.832554	0.859885	1.674401
0.021818	0.000216	0.000245	0.019574	4.33E-05	0.026364	0.138571	0.000404	0.011696
1.349971	0.455649	0.000245	0.737403	4.33E-05	1.248232	1.049517	0.181227	0.313312
4.33E-05	0.337431	0.000245	0.019574	0.275382	0.70886	2.577157	0.000404	0.011696
0.360281	0.14482	0.023232	0.019574	0.308788	0.155844	0.525325	0.029928	0.011696
2.487576	1.08671	0.730029	0.602908	1.272756	1.106046	0.214401	0.870375	0.856291
4.33E-05	0.152222	0.000245	0.019574	0.548788	0.570317	9.751039	0.071097	0.011696
0.021818	0.060729	0.078124	0.116659	0.048528	0.026364	0.63754	0.108124	0.097633
0.260404	0.035426	0.171061	1.268672	0.308788	0.155844	1.925635	0.049834	0.157958
1.507835	2.792035	2.190866	1.202626	1.440736	2.987835	0.446551	1.49075	2.160649
0.783939	0.979827	1.468131	3.87329	2.190866	0.693571	2.693442	1.656696	3.599329
1.935685	41.47925	28.54786	24.50942	36.26061	5.881342	0.019978	65.94551	19.99861
3.486436	4.513622	6.250447	2.776421	0.599466	0.351789	0.304228	2.302626	4.814661
55.04081	15.07257	72.69473	59.32013	33.4985	24.73724	2.183665	29.82828	50.55949
0.192251	0.027879	0.186861	0.019574	0.145051	0.13386	0.607273	0.165512	0.282266
0.516306	0.25197	0.126429	0.877222	0.412193	0.245772	0.773766	0.165512	0.342049
1.497143	1.199481	0.48619	1.080303	0.882619	1.535722	8.762677	0.670945	0.959062
2.996869	2.442987	0.607605	0.214199	3.052655	3.632973	13.34662	0.677403	0.249538
3.439437	1.022338	8.643074	8.019769	0.064848	0.076162	0.585281	5.22482	15.3618
4.33E-05	0.027879	0.000245	0.161818	0.048528	0.000361	0.193348	0.000404	0.011696
1.360866	2.037114	1.379278	0.58259	4.637994	2.491948	0.193348	2.45575	1.541227

1.668369	0.610224	5.062626	5.082063	2.417424	2.212619	0.019978	3.621941	5.576219
2.887489	4.227302	5.821804	13.91714	2.850058	4.721789	0.019978	6.171789	9.792626
0.204964	0.114618	0.16145	0.289603	4.33E-05	0.005303	10.43066	0.049834	0.011696
4.94026	6.830592	2.981962	2.094574	8.348817	18.62711	1.369928	5.28039	2.367763
0.769365	4.468009	2.922778	1.117316	5.704646	8.103925	0.019978	5.654863	2.90658
1.545657	1.389509	0.194531	0.019574	0.82684	0.728088	0.773766	0.579495	0.011696
0.426118	0.000216	0.579149	1.015967	0.193896	0.299942	1.531313	0.778326	0.373218
0.570664	0.420498	0.455325	1.168326	0.308788	0.155844	2.693442	0.353795	0.588954
5.923838	1.548723	4.272165	4.067547	1.335743	3.754719	8.149307	4.752121	4.207778
2.417424	1.717641	6.230245	2.725498	1.060188	1.865043	0.279452	6.107114	7.037345
53.77619	30.49289	18.19595	4.620159	48.53429	75.1132	1.015967	12.10289	10.73084
1.223846	0.624841	0.555418	0.443543	1.458038	0.957273	1.688045	0.299747	0.970635
4.33E-05	0.000216	0.000245	0.019574	4.33E-05	0.002619	0.494444	0.020765	0.011696
0.783939	1.329206	0.613853	0.207713	0.645455	0.6657	1.460051	0.53658	0.352807
4.33E-05	14.70188	31.55417	40.7932	2.150303	0	1.797172	33.4985	59.32013
4.33E-05	2.483492	1.96697	1.763889	0.508975	0	0.019978	1.988196	3.077316
13.75978	14.88584	15.18102	17.51961	12.63773	18.76954	5.538001	14.42661	13.91714
2.604776	20.68746	17.92188	19.99861	21.93452	6.230245	0.019978	31.55417	22.13245
0.532619	0.326017	0.041328	0.019574	0.267807	0.677403	0.330635	0.181227	0.214964
4.33E-05	0.000216	25.48179	12.10289	23.82354	14.12978	0.019978	13.45939	45.91841
0.217908	0.834416	10.67991	23.6012	0.589986	0.857778	0.019978	22.74797	16.34294
5.123781	3.321284	4.246861	1.737294	2.331825	3.266782	16.9162	4.115844	3.489899
1.455988	0.106926	0.000245	0.019574	1.124574	0.27062	10.05618	0.21176	0.265786
1.25816	0.000216	0.186861	0.41482	0.342641	0.327143	2.050404	0.108124	0.232092
1.631544	5.110159	11.77567	5.395584	6.772763	0.543131	0.019978	7.136537	8.412367
1.620058	1.132857	0.297439	1.819221	1.542302	1.928629	3.230065	0.277763	0.600173
0.175469	1.06355	0.53658	0.019574	0.572266	1.187229	0.019978	0.653586	0.352807
0.382626	0.717879	0.721335	0.41482	0.658362	0.629098	0.304228	0.53658	0.772157
22.13245	19.60574	37.29684	67.98069	8.994791	18.19595	1.959466	23.38378	55.04081
6.518709	6.268312	5.024293	3.618499	4.614618	4.33342	0.019978	2.477027	2.494177
2.515938	1.256558	1.014127	0.1686	1.828781	2.124574	0.470512	1.129452	0.959062
4.33E-05	3.618499	1.002215	1.972706	13.45939	1.131472	0.019978	3.592063	14.97762
0.475729	0.533535	0.855801	1.328384	1.433189	0.610606	0.279452	1.515296	1.842734
5.662994	2.227273	8.288874	7.162698	0.749019	1.776739	0.019978	4.140505	7.882222
2.683478	1.870685	1.250058	1.146962	2.964762	4.188896	0.330635	2.210866	1.084437
0.087619	0.060729	0.000245	0.019574	0.241255	0.113369	1.049517	0.277763	0.011696
1.23127	0.746082	0.555418	0.58259	1.089394	0.722179	0.494444	0.594271	0.436638
2.250462	0.892395	0.353939	0.463485	1.744055	0.73912	1.925635	0.165512	0.656392
25.24367	19.99861	11.19942	2.491948	27.10502	14.00414	0.304228	10.1028	6.853608
0.399307	0.221111	0.000245	0.019574	0.048528	0.063319	13.04605	0.000404	0.011696
1.022338	0.493247	0.630505	1.420202	0.315094	0.700418	7.08733	0.636688	0.541522
1.076652	2.07658	1.364401	4.655916	2.139928	0.336328	0.019978	2.169509	2.647244
4.456912	10.19468	7.149618	8.333918	8.164531	6.171789	2.531515	8.412367	8.677489
0.484704	0.159942	0.213218	0.116659	0.090108	0.383889	0.193348	0.165512	0.128853
0.484704	0.551284	3.574387	12.63773	2.331825	0.962698	1.839986	3.799885	8.815123
0.005512	0.000216	0.000245	0.240202	4.33E-05	0.001111	0.138571	0.000404	0.011696
0.884654	0.603737	0.273434	0.396082	0.249899	0.655613	0.470512	0.19039	0.297807
2.487576	0.968066	1.710173	1.110505	0.938911	1.664906	0.019978	3.065108	2.535974
0.617071	1.29267	3.663622	0.697908	0.589986	0.794401	1.2214	0.579495	0.92342
3.016984	1.350772	0.09746	0.019574	1.433189	1.248232	2.531515	0.000404	0.297807
0.005512	0.020216	0.032915	0.923874	4.33E-05	0.002619	0.358211	0.000404	0.011696
20.68746	6.443925	224.9555	16.09482	287.197	56.39528	0.019978	167.9996	87.1746
3.899993	3.307186	2.491948	4.397792	1.204957	0.230808	0.832554	1.700354	3.065108
0.192251	0.114618	0.049719	0.190844	0.064848	0.076162	1.310837	0.154444	0.112886
28.54786	5.522929	1.952027	2.171429	9.101674	5.11697	65.94551	1.988196	3.195541
1.971183	2.353247	2.126349	4.508196	3.521934	2.616573	0.019978	3.182482	1.569553
14.79742	27.10502	32.68313	25.24367	36.74522	43.49658	0.558095	27.66779	33.07147
0.656753	0.326017	0.108124	0.019574	0.10404	0.846797	17.28195	0.000404	0.011696

4.33E-05	0.044026	0.203333	4.115844	4.33E-05	0	0.255887	0.227648	1.376378
2.445079	1.87899	0.506328	2.586926	0.896522	2.868456	4.281847	0.677403	0.282266
1.405772	5.330519	1.152165	1.130794	3.998391	1.910974	0.935115	2.73272	0.740952
4.33E-05	0.14482	0.078124	0.019574	4.33E-05	0.451977	0.558095	0.154444	0.011696
0.016133	0.090584	0.117374	0.019574	0.078297	0.002619	0.214401	0.000404	0.011696
11.93253	80.6518	53.77619	29.82828	47.5018	17.39935	0.019978	41.47925	33.4985
5.568391	1.848297	6.171789	5.123781	2.474834	2.131176	1.609654	10.19468	5.770996
0.769365	0.740527	0.462092	0.710722	0.581378	0.368658	4.978182	0.490664	0.541522
8.134084	5.024293	2.868456	0.710722	4.764055	7.737807	0.63754	2.776421	2.586926
1.008413	0.654423	0.740527	1.240202	1.287403	0.813788	3.397706	1.048074	1.001414
11.68846	3.092229	7.697063	2.547179	5.993045	5.359134	64.12971	4.251818	5.720173
0.453218	5.622872	1.662439	15.77678	0.965065	0.299942	1.339091	2.931068	2.823636
0.076465	0.090584	0.049719	0.403175	0.057128	0.087165	0.525325	0.029928	0.097633
0.06368	0.060729	0.000245	0.019574	0.064848	0.005303	0.019978	0.000404	0.011696
0.922879	0.688182	0.579149	0.782417	1.287403	1.187229	1.310837	0.373709	0.600173
5.9071	23.6012	4.413608	1.651522	20.34063	11.77567	11.06488	8.27267	3.144156
0.516306	0.436017	0.032915	0.190844	0.328932	0.527215	0.494444	0.23412	0.157958
0.907273	0.793918	0.213218	0.019574	0.487128	4.745895	15.88548	0.299747	0.282266
0.175469	0.922338	1.737294	0.894704	0.418557	0.621688	0.214401	1.515296	1.721371
4.174964	0.855801	3.648081	6.632063	2.8657	6.796566	34.6132	4.833398	4.673182
4.33E-05	33.07147	8.103925	25.99427	12.10289	5.24562	0.019978	5.123781	13.08214
0.710253	0.14482	0.000245	0.019574	0.10404	0.182482	3.116732	0.000404	0.157958
4.33E-05	0.027879	0.023232	0.326912	0.040909	0	0.330635	0.000404	0.011696
6.749488	3.377619	0.822965	0.184055	4.802655	1.948918	1.082179	0.520289	0.373218
0.132713	0.000216	0.000245	0.019574	0.026291	0.03842	0.234726	0.000404	0.097633
0.604214	0.114618	0.049719	0.551284	0.078297	0.204156	1.015967	0.040794	0.685209
44.27012	20.85876	17.39935	18.76954	45.91841	55.04081	26.25557	20.16541	14.33939
4.33E-05	0.241255	0.000245	0.019574	0.973745	0.82355	0.525325	0.000404	0.011696
0.003413	0.027879	0.023232	0.375281	0.004257	0	0.193348	0.000404	0.112886
0.875599	1.319293	4.397792	12.56693	0.760433	0.210693	3.297107	3.031688	4.783001
4.802655	0.000216	0.000245	0.019574	0.064848	0	5.576219	0.000404	0.011696
0.434329	1.561912	10.1028	14.17388	1.786306	2.073232	0.747879	7.339452	13.23022
0.309062	10.78056	18.91854	36.74522	8.256465	0.26215	0.019978	10.43066	26.52537
0.360281	0.580404	1.023528	1.335743	0.393716	0.292597	0.019978	0.887922	1.721371
0.453218	5.447114	1.163579	1.43039	1.89518	3.065108	0.019978	0.968723	1.170491
1.316544	0.377085	0.194531	0.501905	1.043608	0.832013	9.031082	0.283882	0.342049
2.259661	4.302367	0.513456	0.019574	0.572266	1.835743	1.959466	0.622821	0.011696
0.769365	0.000216	0.000245	0.350274	0.019408	0	2.331825	0.000404	0.011696
5.720173	1.361789	3.175916	2.34355	3.540606	2.925426	10.98644	2.970144	2.361198
0.07013	1.673254	0.262338	0.019574	0.581378	0.899055	0.019978	1.296046	0.373218
1.3829	0.098384	0.233889	1.000794	0.867287	0.773766	3.500808	0.291183	0.32925
3.816551	9.502294	2.126349	0.019574	5.044113	3.939307	1.644416	4.02088	0.6657
0.011602	0.000216	0.000245	0.201501	0.048528	0.002619	0.63754	0.000404	0.011696
5.210317	22.13245	2.300678	4.745895	5.500678	3.984747	0.019978	4.292078	6.045209
0.07013	0.000216	0.032915	0.535657	0.00785	0.026364	0.304228	0.098384	0.097633
0.418326	3.217612	6.591544	3.842554	1.947316	5.381227	1.609654	4.007583	1.43039
1.845519	1.628009	0.520678	0.273672	2.371833	5.431544	6.287561	1.048074	1.227749
0.382626	2.243911	1.549711	0.478413	3.070952	5.754935	0.019978	1.820541	1.556392
0.22303	0.296955	0.069206	0.139091	0.599466	0.309062	0.832554	0.181227	0.468167
4.33E-05	5.606739	10.48101	9.377345	3.87329	2.148658	0.019978	6.019971	10.95872
3.718716	1.29267	0.948211	0.144098	2.404618	1.279452	4.46267	0.790837	0.42329
4.678846	45.91841	21.19547	8.477662	10.1028	13.75978	0.019978	21.19547	10.82954
7.839271	13.08214	16.34294	1.869322	18.48514	6.653016	3.297107	13.83359	7.288788
4.456912	0.000216	0.000245	0.019574	1.799899	0.773766	1.497143	0.241255	0.011696
1.555289	0.28189	0.297439	0.403175	0.153651	0.344127	7.810058	0.040794	0.282266
0.301955	0.060729	0.126429	0.326912	0.201789	0.140303	4.46267	0.118225	0.143709
0.281364	15.66023	6.079596	11.03843	4.397792	3.011169	0.019978	8.27267	14.88584
1.341825	3.679986	2.558817	0.176558	2.77899	10.78056	0.019978	0.896522	0.949827

12.53072	8.134084	3.984747	1.485483	7.443276	6.99026	2.248896	3.404372	4.096407
2.173081	0.448557	0.339004	1.335743	0.664026	0.26215	5.788153	0.27062	0.533196
5.351934	0.839365	2.765758	2.911962	1.1407	2.335844	5.323492	3.201457	4.039192
1.104805	1.834423	0.750866	3.676032	0.682799	0.327143	19.99861	1.158384	1.289149
1.845519	1.319293	1.821861	0.825354	0.733593	0.577706	0.193348	2.104358	1.450693
1.809343	0.221111	10.62739	7.795642	1.587605	0.514531	0.019978	6.695887	17.15902
6.200945	23.82354	11.14619	4.540794	14.97762	21.93452	1.688045	32.31123	13.15079
9.335397	5.366335	8.711905	2.190866	7.532713	3.939307	0.019978	4.348882	7.854069
3.5257	5.47	3.807648	2.445079	5.130411	8.694697	0.019978	2.635685	4.222583
6.674286	2.506934	14.6058	18.62711	1.405772	5.942294	0.019978	10.38202	15.18102
3.595758	1.284747	1.129452	2.506934	1.636118	0.646378	1.400159	0.987821	0.959062
1.590202	1.06355	1.163579	1.397482	1.50684	1.487619	0.019978	1.040411	1.219856
1.872049	2.696061	2.099524	4.246861	2.547179	1.702706	0.019978	2.243911	2.723247
0.783939	0.25197	0.630505	1.044235	0.209841	0.406681	6.088593	0.27062	0.69583
0.648362	0.304733	0.598456	0.443543	0.98474	0.677403	3.230065	0.127403	0.143709
0.267597	0.569625	0.283709	0.019574	0.162915	0.344127	2.102626	0.071097	0.232092
0.93267	0.332395	0.000245	0.019574	0.487128	0.471025	0.019978	0.000404	0.011696
1.094206	1.132857	0.568961	0.133045	0.840635	1.374329	0.470512	0.27062	0.011696
0.119062	0.229199	0.339004	0.473016	0.162915	0.155844	1.925635	0.181227	0.342049
0.323319	0.754784	0.96938	0.42202	0.589986	1.297763	0.019978	0.594271	1.026219
4.33E-05	4.003117	17.78522	2.713319	2.909293	0	0.019978	7.315512	3.743232
0.679452	2.498478	1.853723	1.310837	3.233045	3.334199	0.676659	3.23987	0.834957
0.726472	0.617417	0.468384	0.133045	1.533535	2.047157	0.304228	1.09075	0.249538
0.132713	0.0843	0.194531	0.888485	0.193896	0.081746	1.049517	0.098384	0.011696
0.792886	6.580988	6.900202	4.72798	9.880779	6.612121	0.019978	8.048095	4.297222
15.1268	10.73084	7.430317	4.307309	11.68846	10.8813	0.019978	6.345382	3.909033
0.418326	0.827273	1.648066	4.23202	3.775209	0.70886	0.019978	2.666494	3.137576
3.179199	3.544257	3.340361	2.121198	2.084791	3.028629	0.234726	1.791616	2.701053
0.388831	0.696234	0.000245	0.019574	0.82684	0.677403	0.470512	0.000404	0.011696
2.579509	0.052431	1.92272	2.911962	0.026291	0.093009	0.959567	0.34601	0.901212
0.775844	3.110447	2.153535	1.370765	3.310758	3.101371	0.019978	4.302367	2.312085
4.655916	12.56693	6.423911	1.539062	6.612121	5.095325	0.019978	3.869163	4.839105
10.19468	1.724805	2.922778	6.30671	0.909315	3.043528	40.7932	2.404618	0.436638
0.315743	0.533535	0.126429	0.319567	0.299444	0.254365	1.995714	0.249408	0.362179
0.192251	0.393983	0.153759	0.019574	0.40645	0.442799	0.234726	0.307734	0.282266
0.275058	1.06355	0.262338	0.254769	0.373463	0.784372	2.331825	0.218975	0.313312
0.697085	1.771392	0.994899	1.157626	2.097843	0.621688	0.63754	0.770664	0.856291
1.426522	15.18102	5.00404	1.397482	10.05618	5.576219	0.676659	8.849697	2.863211
0.057583	0.000216	0.000245	0.281558	0.00785	0.093009	0.676659	0.000404	0.011696
0.106522	0.304733	0.023232	0.122121	0.07114	0.056869	0.175592	0.029928	0.011696
1.308167	0.912446	1.989711	2.802828	2.511465	1.433189	3.953297	2.463911	4.281847
3.300664	2.143261	0.855801	0.40886	2.604776	5.024293	3.88153	0.878369	0.588954
530.1465	9.297085	8.745238	0.923874	26.52537	30.82277	126.1556	8.381328	3.521934
0.405974	0.090584	0.394877	0.019574	0.393716	0.376212	0.85728	0.256797	0.232092
0.15241	0.06886	0.087403	0.019574	0.162915	0.009322	0.279452	0.029928	0.011696
5.316378	3.283139	11.96574	7.38847	4.614618	4.609076	0.446551	4.424416	8.780115
1.128788	8.815123	18.76954	7.188095	6.464906	16.34294	0.019978	11.03843	10.53159
4.338629	1.779423	4.034423	2.308203	5.796421	4.914863	0.019978	2.720996	3.110447
0.021818	0.06886	0.023232	0.1686	0.064848	0.043824	0.156566	0.000404	0.011696
4.33E-05	0.000216	0.01588	0.190844	0.004257	0.019574	0.234726	0.020765	0.011696
1.209105	2.329784	1.449877	1.731169	1.480541	0.957273	0.156566	1.340036	1.867951
5.80469	13.23022	4.603434	14.51648	6.325945	1.078925	1.460051	7.212915	7.443276
0.637085	0.393983	0.310657	0.483874	0.393716	0.278802	0.525325	0.21176	0.6657
0.578485	1.89961	4.130794	2.470317	3.300664	2.597792	0.019978	2.150303	4.025267
0.851659	0.70329	1.737294	1.799899	1.158384	1.421126	0.019978	5.110159	5.165599
6.365065	1.806595	1.656696	2.713319	0.362424	2.021068	8.149307	0.878369	1.620058
0.418326	0.386378	0.000245	0.019574	0.795786	0.888485	1.339091	0.5129	0.011696
1.286494	0.000216	0.000245	0.019574	4.33E-05	0.299942	32.68313	0.000404	0.011696

1.198701	3.307186	11.719	6.945152	3.223882	12.29473	1.369928	6.135007	3.625382
0.125123	0.027879	0.01588	0.019574	0.048528	0.03842	0.585281	0.000404	0.086443
0.570664	0.152222	0.000245	0.019574	0.849336	0.480022	2.183665	0.118225	0.011696
1.991328	2.270837	2.209141	0.834416	0.462857	2.644885	0.019978	1.100519	1.501291
0.671724	4.429769	1.693939	1.146962	1.985253	0.442799	0.193348	1.441898	1.779423
1.028672	14.51648	3.29355	0.019574	4.266753	2.561162	0.019978	1.839986	1.628009
0.145909	0.000216	0.000245	0.019574	0.138009	0.087165	0.63754	0.000404	0.011696
0.56443	2.306277	2.23267	0.019574	3.26	2.882035	0.019978	2.210866	0.297807
1.120332	3.110447	1.320173	6.443925	1.724805	1.053528	0.214401	1.209776	2.961768
7.991602	31.92385	16.80343	11.36975	45.1045	20.34063	0.85728	21.74051	10.38202
0.667201	0.880498	1.90798	2.094574	1.043608	1.333781	0.214401	1.460051	0.352807
51.52195	3.663622	67.98069	52.56128	0.462857	1.062092	0.019978	6.107114	105.1694
6.865	11.30987	19.68088	26.25557	8.573434	7.061328	8.333918	18.62711	19.68088
4.33E-05	0.000216	0.023232	0.1686	0.033261	0.026364	0.525325	0.029928	0.157958
1.360866	0.486551	1.421126	1.523824	0.616739	0.376212	0.019978	1.937222	5.137042
30.17192	33.93939	10.28573	3.939307	27.96248	32.68313	0.019978	9.968355	8.164531
0.656753	0.533535	2.45575	0.625786	0.909315	1.053528	0.019978	1.616385	1.546696
0.736551	1.027403	2.180022	1.175859	0.299444	1.995714	0.019978	1.656696	2.169509
1.468131	91.05541	70.11932	11.60283	33.93939	7.188095	1.644416	5.195022	19.37139
0.648362	0.090584	0.000245	0.019574	0.658362	1.214315	0.019978	0.000404	0.011696
0.578485	6.497266	1.310837	0.766602	2.8657	1.402915	0.019978	2.703485	1.43039
2.850058	1.219019	1.253088	2.261573	0.996111	2.060491	18.91854	0.968723	1.201724
10.1459	3.644343	32.31123	29.49693	24.50942	6.210375	0.019978	6.508427	19.06893
0.275058	0.304733	1.565253	3.049603	0.275382	0.230808	0.446551	1.64684	3.457504
4.251818	5.309264	2.831501	4.145209	3.921645	3.5257	0.805238	2.190866	2.635685
1.371602	0.647345	0.252165	0.019574	0.849336	0.451977	0.255887	0.000404	0.011696
0.992201	0.000216	0.000245	0.019574	0.60886	0.032857	0.304228	0.098384	0.011696
2.121198	12.36443	8.195657	0.019574	8.888167	15.3618	0.716234	14.88584	5.351934
0.532619	2.069942	1.844098	0.625786	0.956667	0.73912	1.049517	0.96329	1.001414
1.602619	2.150303	1.876212	0.79487	1.64684	0.432698	8.976508	0.569971	1.122359
0.125123	0.27075	0.049719	0.019574	0.35241	0.026364	0.255887	0.029928	0.011696
0.145909	0.000216	0.000245	0.019574	0.193896	0.432698	0.019978	0.000404	0.011696
0.884654	2.973059	3.536955	2.59557	4.684502	2.194257	0.019978	4.281847	4.881775
0.915195	0.892395	4.165137	4.003117	1.346991	1.187229	0.255887	2.922778	4.445851
1.3829	2.289286	0.876638	0.774257	1.705007	2.031169	0.418326	1.022338	1.018427
6.967547	4.155339	2.868456	1.624459	8.508514	5.454949	0.832554	1.204228	1.391494
0.125123	0.486551	4.38684	6.967547	1.449062	0.093009	0.019978	4.535354	6.268312
155.7103	0.000216	4.198117	0.019574	3.310758	3.346898	60.77449	0.490664	0.634531
4.839105	1.724805	1.957958	1.434921	1.215195	2.763261	1.049517	0.932078	0.866147
0.388831	0.563175	0.041328	0.019574	0.335599	0.192814	0.019978	0.000404	0.097633
0.399307	0.090584	0.000245	0.133045	0.096263	0.113369	1.797172	0.000404	0.011696
0.434329	5.23176	2.671299	4.02088	2.964762	0.506869	0.019978	3.29355	7.188095
3.227085	3.404372	1.865043	1.272756	2.752893	4.408153	0.525325	1.903636	2.436768
0.405974	1.012114	1.859084	0.850808	1.299567	1.813564	0.019978	0.82772	0.824416
2.727785	1.102691	0.339004	0.184055	0.623146	1.237879	2.818312	0.475433	0.551284
11.48368	12.63773	7.824473	2.234538	16.5625	31.1753	20.68746	12.36443	3.334199
1.066234	2.823636	3.24658	2.367763	2.210866	1.237879	0.019978	3.011169	4.312251
0.532619	0.401385	0.126429	0.019574	0.682799	0.752229	0.138571	0.315916	0.189394
0.875599	4.586753	1.197922	0.814877	5.687879	2.839351	0.747879	1.596782	1.661328
2.558817	1.190491	2.132756	2.335844	1.433189	1.297763	2.693442	2.121198	1.989711
1.076652	1.65785	2.045476	5.439278	2.652107	0.73912	0.494444	0.907273	2.807937
1.028672	1.582915	0.956061	0.190844	4.655916	0.646378	0.304228	5.01772	2.417424
0.011602	0.044026	0.059957	0.110289	0.057128	0.032857	0.214401	0.080079	0.086443
3.55961	5.576219	3.621941	7.08733	3.414574	5.11697	0.716234	6.22031	3.381255
6.414416	6.325945	10.82954	5.754935	17.15902	7.938427	1.580909	17.39935	15.66023
2.727785	1.303045	2.300678	2.207417	1.035058	1.910974	3.166659	1.395563	2.427792
0.597042	0.213615	0.546522	0.514141	0.581378	0.637994	0.418326	0.711046	0.588954
4.33E-05	0.000216	0.000245	0.019574	0.096263	0	3.070952	0.000404	0.011696

1.468131	1.06355	0.347186	2.602395	0.733593	1.498167	4.338629	0.46461	0.942294
0.113146	0.14482	0.213218	0.207713	0.124719	0.081746	0.85728	0.299747	0.157958
4.33E-05	2.483492	1.549711	0.019574	1.763889	0.278802	0.019978	1.218182	2.02956
5.568391	1.594675	7.938427	6.808369	7.08733	7.112496	0.019978	4.814661	9.480736
2.654473	0.717879	2.126349	0.729582	1.985253	1.092785	0.019978	1.402915	1.460051
0.250649	0.349278	0.000245	0.019574	0.096263	0.093009	0.388579	0.136667	0.265786
1.104805	1.561912	0.932078	1.310837	2.069942	1.266255	0.279452	0.690707	0.634531
0.260404	0.709784	0.986797	1.080303	0.548788	0.376212	0.156566	1.727323	1.535722
0.704444	1.532468	0.895368	1.310837	0.563175	0.655613	0.019978	0.932078	0.656392
0.460938	2.442987	2.939141	0.260144	3.807648	3.314329	0.019978	0.389084	2.914632
1.685678	5.522929	12.56693	21.74051	3.718716	3.552049	0.885664	14.51648	17.51961
0.184206	0.000216	0.078124	0.266681	0.090108	0.026364	1.2214	0.06088	0.011696
3.179199	2.77899	0.762641	0.214199	1.974228	3.479134	1.758615	0.932078	0.706053
9.031082	6.876392	1.779423	0.29715	9.063983	9.667316	0.019978	1.314675	0.574993
2.727785	1.673254	2.281926	0.850808	3.092229	2.828918	75.1132	1.839986	1.43039
7.430317	2.618846	4.907929	5.337547	6.297136	5.056421	0.304228	5.165599	6.876392
14.42661	2.31404	2.209141	5.9071	3.540606	2.987835	144.7919	1.850996	1.779423
0.762165	3.606804	0.233889	0.319567	1.204957	0.96938	0.607273	0.136667	0.143709
3.980123	10.48101	3.26	1.925635	3.425512	4.160238	0.019978	7.456118	3.55961
144.7919	87331.38	530.1465	203.5795	530.1465	224.9555	12.29473	252.4251	203.5795
0.22303	1.96404	2.807937	18.05488	1.115902	0.451977	3.792266	3.640606	8.048095
0.119062	0.319026	0.000245	0.019574	4.33E-05	0.223701	0.175592	0.000404	0.011696
8.940671	0.386378	0.940563	1.716364	2.126349	3.279625	25.48179	0.925801	1.026219
5.056421	32.68313	34.84299	56.39528	18.76954	6.760765	5.259437	36.26061	27.38726
0.382626	0.355051	0.318124	0.312864	0.362424	1.139221	4.440548	0.205931	0.265786
7.519841	11.19942	18.62711	12.29473	9.502294	13.45939	2.786898	17.51961	18.33909
17.39935	1.268672	3.204632	35.27608	1.099004	1.801284	1.797172	1.582915	4.586753
5.416623	3.57057	4.484892	4.429769	6.900202	4.140505	0.019978	4.072475	3.263391
0.399307	1.797172	1.008413	1.328384	3.493362	0.728088	0.019978	2.868456	0.480022
5.01088	0.839365	1.409942	1.310837	2.792035	3.1857	5.323492	1.224733	1.637309
1.590202	0.044026	0.353939	0.914618	0.145051	0.471025	1.727323	0.19039	0.802756
13.6814	2.544747	5.493182	4.397792	5.366335	5.71241	16.68554	4.376118	6.967547
0.081818	0.000216	0.108124	0.019574	4.33E-05	0.113369	4.281847	0.118225	0.011696
0.578485	1.199481	1.143954	0.307973	2.933903	2.31404	0.156566	1.277843	0.656392
24.2842	34.38341	25.99427	3.457504	21.74051	59.32013	1.688045	35.776	11.09133
1.78759	2.174733	0.468384	0.019574	4.370671	3.195541	6.717056	0.299747	0.011696
0.008586	0.000216	0.000245	0.019574	0.078297	0.019574	0.446551	0.000404	0.011696
2.950173	7.765931	3.086306	0.774257	10.17029	5.9071	21.19547	5.302294	2.818312
1.128788	1.008413	0.669769	1.015967	3.404372	1.776739	5.323492	1.253752	0.714697
7.225469	7.188095	3.160022	2.217951	8.849697	8.508514	0.716234	3.432388	2.939141
2.131176	12.42965	1.994329	1.048802	11.42512	9.218514	1.557482	5.095325	1.848297
0.731732	0.060729	0.01588	0.144098	0.057128	0.100281	0.558095	0.000404	0.011696
1.857677	2.847547	1.464214	0.214199	1.995714	1.511198	0.585281	0.91575	1.133622
0.353175	0.332395	0.146118	0.116659	0.275382	0.595368	0.279452	0.154444	0.086443
0.426118	0.296955	0.213218	0.225339	0.637994	0.458521	0.418326	0.23412	0.265786
0.057583	0.000216	0.041328	2.425801	0.033261	0.002619	0.805238	0.181227	0.157958
16.09482	2.581861	12.77772	4.101061	17.28195	11.719	1.277843	15.66023	11.83593
1.047345	6.191515	1.262179	0.240202	2.047157	1.323975	0.019978	2.053521	1.535722
2.873817	25.48179	2.638139	11.77567	3.144156	9.063983	0.019978	9.880779	3.644343
2.45772	1.826147	4.907929	4.586753	2.351299	4.978182	0.019978	4.125786	11.19942
5.545729	1.924228	1.351573	0.342381	3.687633	3.68381	0.255887	1.504517	0.802756
5.416623	10.90615	14.88584	16.68554	6.135007	8.694697	0.019978	12.70768	23.6012
2.31013	1.762713	2.221775	1.810664	1.690483	1.623232	0.959567	3.869163	1.496118
0.863189	0.121703	0.88417	1.25746	0.35241	0.286364	8.923117	1.022338	0.600173
0.315743	0.717879	0.493925	0.631847	1.533535	0.784372	0.175592	1.184185	0.789423
2.404618	3.058802	0.446291	2.815866	1.89518	3.479134	7.532713	0.735382	0.874603
1.545657	0.172294	0.203333	0.122121	0.599466	0.93267	4.63189	0.218975	0.265786
4.33E-05	0.246162	0.000245	0.019574	0.057128	0	10.53159	0.000404	0.011696

3.944084	0.730476	0.785714	0.266681	0.672468	1.355527	1.644416	0.38127	0.32925
5.959033	8.608153	15.88548	26.52537	2.489762	6.036818	6.454416	21.93452	9.625253
10.01009	2.737944	13.6814	2.531515	14.33939	9.031082	1.609654	12.85504	7.507619
4.419062	0.624841	0.710722	1.378362	1.021097	1.271876	15.66023	0.497749	1.591299
2.436768	3.464315	5.545729	3.077316	5.22482	3.885642	0.019978	3.930664	3.926154
2.091313	0.393983	0.242893	0.019574	0.508975	1.143954	0.470512	0.241255	0.313312
6.171789	6.717056	1.510014	1.263752	4.198117	2.828918	0.156566	2.882035	1.298665
0.175469	0.0843	0.297439	0.929812	4.33E-05	0.014286	1.727323	0.136667	0.232092
0.106522	0.06886	0.000245	0.019574	4.33E-05	0	1.959466	0.000404	0.011696
2.187395	3.687633	4.307309	3.718716	2.014863	3.002576	0.019978	3.080317	3.019848
1.120332	0.781977	0.455325	1.387612	0.362424	0.230808	2.050404	0.21176	0.157958
4.33E-05	0.035426	0.000245	0.019574	4.33E-05	0.03842	0.234726	0.000404	0.011696
1.037958	0.927561	1.163579	1.551962	1.322092	1.491977	3.792266	1.303045	1.25746
0.93267	1.37531	2.628225	0.95101	0.98474	2.078355	0.019978	3.350224	2.126349
0.175469	0.044026	0.078124	0.019574	0.048528	0.049834	0.911905	0.080079	0.011696
5.030866	3.544257	2.713319	1.426522	2.836717	3.68381	2.531515	1.539062	2.126349
0.516306	0.775339	2.045476	1.000794	2.371833	1.751948	0.019978	3.201457	1.833102
28.87506	23.38378	3.303925	0.662237	22.52375	15.66023	0.019978	7.897078	0.834957
0.281364	0.14482	0.078124	0.019574	0.471025	0.336328	2.183665	0.080079	0.373218
2.296732	1.190491	1.621032	1.268672	2.190866	2.084791	17.09911	0.953506	1.321053
0.229199	0.000216	0.000245	0.019574	0.07114	0.419365	0.019978	0.000404	0.143709
1.170491	0.000216	0.000245	0.019574	0.07114	0.286364	0.676659	0.000404	0.011696
0.15241	1.992944	1.092136	0.88316	1.533535	0.763564	0.234726	1.997352	2.794935
0.275058	0.137201	0.000245	0.019574	0.795786	0.16868	0.019978	0.000404	0.011696
23.16622	1.23127	3.864827	3.286652	8.762677	5.923838	56.39528	4.94026	3.002576
1.522597	0.905426	0.446291	0.225339	0.82684	1.197922	1.369928	0.407157	0.409098
3.089293	0.000216	4.012049	2.190866	6.39474	13.30906	4.082165	8.019769	1.820541
0.005512	0.000216	0.000245	0.240202	4.33E-05	0.001111	0.138571	0.000404	0.011696
2.091313	3.625382	2.574733	1.230411	3.552049	5.679502	0.330635	3.824964	1.833102
18.33909	22.96463	20.34063	10.38202	35.27608	72.69473	8.524242	26.25557	13.6814
4.140505	1.284747	2.483492	0.019574	2.306277	3.984747	1.644416	3.223882	0.901212
13.53913	14.79742	4.222583	0.518716	14.70188	8.256465	0.156566	3.137576	2.269134
0.027965	0.014545	0.000245	0.190844	0.057128	0.000361	1.186457	0.000404	0.086443
0.907273	2.055289	0.598456	0.527821	1.750628	2.101075	0.019978	1.596782	0.574993
3.074134	0.000216	0.000245	0.019574	4.33E-05	0	0.255887	0.000404	0.011696
9.562554	23.16622	6.045209	16.22108	3.984747	6.900202	0.019978	15.77678	8.923117
0.021818	0.192929	0.01588	0.894704	0.138009	0.002619	1.11526	0.020765	0.128853
0.192251	0.000216	0.36153	0.38772	0.057128	0.005303	0.019978	0.27062	0.856291
9.968355	27.38726	7.991602	1.828781	17.78522	16.09482	0.255887	7.683766	3.659668
0.382626	2.911962	2.472576	6.325945	2.139928	2.169509	0.019978	1.624459	4.130794
0.333636	3.703175	26.52537	57.84882	5.402511	2.068297	0.019978	19.22139	51.52195
6.38456	4.096407	6.325945	7.911934	10.43066	5.493182	0.019978	5.044113	4.827691
3.629177	5.737056	1.474343	1.328384	5.993045	7.212915	2.74298	1.515296	0.874603
0.468167	2.432121	3.028629	0.019574	1.828781	2.119596	0.019978	2.593506	0.685209
1.795851	2.254084	0.876638	2.513752	2.171429	1.083651	29.18885	1.314675	1.721371
1.913997	6.632063	4.165137	5.737056	4.637994	2.31404	2.577157	9.356371	6.325945
7.724084	9.751039	7.545584	4.077403	11.30987	11.19942	1.015967	5.238701	5.754935
7.225469	37.29684	22.52375	44.27012	12.03267	13.38418	7.468961	45.1045	41.47925
1.416407	0.401385	0.087403	0.019574	0.412193	0.432698	5.323492	0.098384	0.232092
0.818586	0.696234	0.771176	0.488463	0.760433	0.419365	0.156566	0.883665	0.789423
5.28039	3.57057	1.772756	0.61969	4.091667	2.616573	2.183665	1.515296	1.156869
1.198701	0.090584	0.000245	0.019574	1.236248	0.998831	0.330635	0.000404	0.011696
0.051472	0.044026	0.000245	0.019574	0.040909	0.032857	0.558095	0.000404	0.097633
2.050404	0.166789	0.059957	0.90772	0.589986	1.187229	7.200505	0.000404	0.610606
4.863579	19.83727	11.65791	18.33909	3.425512	3.536955	0.019978	12.56693	15.77678
1.087345	1.247244	1.604827	1.881955	1.136566	1.453939	0.85728	2.164113	5.462475
1.104805	0.296955	0.233889	0.468167	0.185642	1.225426	1.727323	0.38127	0.779668
0.051472	0.259033	0.283709	0.019574	1.363557	0.216753	3.014076	0.000404	0.202294

0.184206	0.000216	0.000245	0.019574	4.33E-05	0.204156	0.019978	0.000404	0.011696
2.114437	10.01009	2.939141	1.748016	8.075772	6.191515	0.63754	3.921645	1.178939
0.093102	0.052431	0.023232	0.019574	0.040909	0.081746	0.911905	0.049834	0.011696
1.138557	3.092229	3.363911	1.184935	3.290101	3.695815	0.019978	2.034242	2.681169
2.021068	0.979827	0.977994	1.044235	2.363442	0.383889	3.500808	0.82772	0.480022
2.003723	1.834423	5.630815	9.968355	11.25392	10.23768	2.296732	10.5063	2.472576
3.829105	20.34063	12.63773	3.008225	13.00996	15.88548	0.019978	12.77772	12.22798
0.159791	0.106926	0.032915	0.307973	0.07114	0.056869	3.500808	0.071097	0.173723
0.008586	0.014545	0.01588	0.155945	0.004257	0.009322	1.277843	0.020765	0.011696
4.110779	4.733824	1.230411	0.746551	4.739668	6.508427	0.885664	0.834416	1.418312
5.485685	4.603434	1.710173	0.523211	4.863579	4.696313	8.089848	2.013182	1.460051
3.851414	2.289286	1.662439	1.505678	3.394286	5.493182	3.014076	1.504517	1.581912
2.060491	1.08671	0.750866	0.586595	1.322092	0.921739	0.358211	0.842641	0.750404
2.531515	7.570693	6.52899	6.011515	5.622872	3.137576	1.310837	6.287561	13.38418
0.184206	0.276061	0.000245	0.019574	0.019408	0	0.019978	0.000404	0.011696
0.736551	2.593506	4.586753	7.765931	5.796421	26.80355	3.500808	11.83593	1.910974
0.532619	0.206768	0.09746	0.019574	0.508975	0.254365	0.193348	0.262965	0.157958
1.189769	1.369928	0.730029	0.312864	3.214545	0.943514	9.256537	0.883665	0.622496
64.12971	56.39528	39.53687	19.83727	91.05541	62.33472	2.909293	39.53687	25.99427
0.119062	0.259033	0.620462	0.535657	0.412193	0.230808	1.497143	0.415447	0.523983
4.33E-05	2.143261	4.198117	3.957734	1.310837	0.646378	0.019978	3.592063	7.238023
0.523983	0.000216	0.136479	0.019574	0.171183	0.197908	1.959466	0.080079	0.011696
1.935685	3.26	1.673254	1.491977	2.752893	1.364401	0.193348	1.886335	2.511465
6.325945	37.81919	7.854069	1.302107	4.715599	5.959033	0.330635	2.855296	3.796075
2.327857	5.770996	5.172828	8.288874	2.953059	2.676392	0.607273	7.188095	5.662994
0.484704	0.367532	0.069206	0.562439	0.231659	0.458521	8.762677	0.277763	0.32925
0.792886	0.587965	0.36153	0.273672	1.068348	0.912446	2.050404	0.415447	0.6657
3.38456	2.823636	8.608153	4.222583	1.014127	1.543377	0.019978	2.755411	7.653391
3.128694	0.995527	1.859084	0.774257	2.500548	2.925426	6.674286	1.522597	1.346003
3.046551	2.289286	0.213218	0.602908	2.417424	2.925426	7.264502	0.241255	0.436638
0.204964	3.198499	4.789596	3.150433	2.903867	4.292078	0.019978	5.671126	2.240274
1.326667	8.256465	2.143261	0.144098	2.964762	2.068297	0.304228	1.530159	0.362179
1.515296	0.760433	0.822965	0.463485	0.418557	0.838326	0.911905	0.579495	0.866147
7.598312	17.92188	24.73724	46.7056	13.15079	7.495397	0.585281	14.25954	64.12971
8.381328	7.964315	8.134084	6.423911	12.93651	9.838658	0.019978	4.875527	3.065108
0.033954	0.000216	0.126429	0.019574	0.07114	0.019574	0.63754	0.000404	0.011696
7.002056	13.83359	12.93651	5.493182	24.04374	12.16681	7.351284	19.53061	10.19468
167.9996	19.60574	1360.293	167.9996	144.7919	530.1465	21.45857	1360.293	749.7839
2.996869	4.110779	1.431414	1.215981	3.699495	7.315512	17.51961	1.022338	1.037316
0.07013	1.169791	0.986797	3.125844	0.342641	1.204957	0.019978	1.812114	0.970635
1.731169	5.165599	3.06803	9.063983	2.850058	2.644885	0.019978	4.029654	8.195657
3.5257	5.381227	7.911934	8.608153	7.598312	4.508196	1.797172	10.93101	13.45939
7.061328	42.08807	105.1694	184.443	9.625253	20.85876	0.019978	135.5245	144.7919
2.77899	10.57984	10.38202	9.218514	5.754935	3.50425	0.019978	2.828918	5.500678
4.626025	60.77449	51.52195	25.60672	80.6518	28.23632	0.019978	31.92385	36.74522
11.83593	2.941544	5.71241	1.669466	11.77567	9.335397	5.646811	2.438831	3.711097
1.308167	6.250447	1.468131	0.929812	4.110779	0.962698	0.138571	1.340036	2.138131
3.192229	5.351934	4.376118	2.129596	3.160022	4.821176	0.019978	5.337547	3.201457
1.360866	0.436017	0.252165	1.044235	0.755253	0.595368	2.183665	0.558824	0.733074
0.353175	0.06886	0.000245	0.019574	0.10404	0.056869	0.747879	0.136667	0.011696
14.08569	9.880779	18.48514	8.445743	13.83359	8.745238	2.378124	16.9162	17.64869
10.33476	2.353247	17.15902	8.728571	10.98644	4.933781	0.255887	22.32214	6.727633
4.33E-05	0.000216	0.000245	0.019574	0.249899	0.014286	0.85728	0.000404	0.011696
7.964315	26.25557	21.93452	31.55417	13.60866	19.53061	5.024293	25.73165	29.49693
0.106522	4.179762	1.980592	0.514141	0.692027	1.323975	0.019978	1.010887	1.068348
0.93267	0.25197	0.223701	0.403175	0.393716	0.677403	1.015967	0.098384	0.282266
1.533535	0.754784	0.663276	0.40886	1.724805	1.558571	1.557482	0.677403	0.856291
0.033954	0.192929	0.059957	0.29715	0.111544	0.187713	2.421681	0.040794	0.011696

5.462475	3.122915	5.606739	6.508427	2.171429	8.019769	1.369928	6.945152	4.620159
2.563506	0.905426	1.481631	0.801501	2.084791	2.131176	0.418326	0.987821	1.33285
0.027965	0.000216	0.000245	0.116659	0.026291	0.019574	0.747879	0.000404	0.011696
2.55184	15.3618	5.591717	6.663651	7.532713	4.637994	0.019978	9.502294	10.05618
35.27608	155.7103	11.83593	49.50903	30.17192	35.27608	4.715599	87.1746	44.27012
4.33E-05	4.644098	6.876392	9.541645	6.560411	3.065108	0.019978	8.319019	12.10289
0.021818	0.265224	0.049719	0.019574	0.138009	0.056869	10.67991	0.029928	0.011696
0.992201	4.94026	5.22482	5.704646	8.643074	5.28039	0.019978	8.103925	15.56131
1.205685	0.000216	0.000245	0.019574	0.308788	0.093009	23.6012	0.000404	0.011696
0.027965	0.184877	0.032915	0.112323	0.07114	0.063319	0.607273	0.029928	0.202294
2.863211	2.31404	0.562107	0.869365	2.633232	2.533745	4.082165	0.46461	0.342049
4.33E-05	0.580404	0.000245	0.396082	0.145051	0.557056	2.248896	0.098384	0.011696
1.426522	0.276061	0.186861	0.019574	0.078297	0.254365	3.578038	0.108124	0.011696
3.464315	6.287561	0.562107	0.596775	4.821176	2.259661	0.019978	0.96329	0.468167
3.330909	6.738211	4.94026	1.977316	6.250447	3.984747	0.676659	4.888023	4.586753
2.429957	3.78798	0.117374	0.019574	3.346898	1.677042	0.773766	0.071097	0.112886
4.715599	18.76954	7.38847	5.137042	12.42965	8.849697	0.214401	10.33476	5.068831
0.453218	0.044026	0.136479	0.36153	0.412193	0.646378	0.330635	0.000404	0.011696
4.952915	15.88548	10.73084	9.297085	10.38202	3.253175	5.143867	20.51271	9.667316
4.827691	2.723247	4.888023	3.040505	4.266753	4.048499	1.082179	6.25938	4.353925
1.716364	0.624841	0.069206	0.38772	0.616739	0.412193	1.369928	0.020765	0.112886
2.498478	13.15079	6.454416	9.687496	6.674286	9.583463	0.019978	4.502453	1.346003
1.296905	1.414574	0.855801	1.816436	1.1407	1.092785	0.019978	0.728543	1.037316
1.481631	1.954993	2.671299	28.23632	4.33E-05	2.470317	0.019978	7.162698	21.74051
0.382626	0.326017	0.146118	0.019574	0.117734	0.113369	0.388579	0.080079	0.128853
0.309062	0.020216	0.023232	0.133045	4.33E-05	0.049834	1.580909	0.029928	0.097633
1.008413	0.725267	0.000245	0.814877	1.195079	0.603737	1.644416	0.000404	0.011696
1.655541	0.973745	0.506328	0.710722	0.362424	0.351789	0.019978	0.497749	0.890065
0.008586	0.098384	0.000245	0.019574	0.048528	0.001111	0.234726	0.000404	0.011696
4.33E-05	0.020216	0.000245	0.019574	0.048528	0.005303	0.773766	0.000404	0.011696
0.688579	1.582915	3.563182	3.188918	4.684502	3.095166	0.676659	4.502453	9.924372
0.648362	0.349278	0.546522	0.861934	0.131768	0.03842	0.255887	0.299747	0.533196
3.5257	3.55961	3.233045	2.257749	4.110779	5.165599	1.428398	2.914632	2.151919
1.198701	3.94886	2.868456	2.08798	1.696494	1.333781	1.609654	2.800332	3.36075
1.149322	0.467136	3.095166	1.705007	0.138009	0.076162	1.531313	1.3829	1.556392
6.922771	2.016515	1.262179	1.810664	6.39474	1.464214	0.747879	1.870685	2.417424
0.192251	0.06886	0.032915	0.019574	0.057128	0.100281	0.279452	0.000404	0.011696
3.422237	7.363117	2.727785	2.74544	4.468009	2.148658	0.019978	4.365224	2.447294
17.03919	27.96248	48.53429	36.26061	24.98218	28.54786	1.644416	49.50903	46.7056
0.974257	0.184877	0.023232	0.019574	0.548788	0.514531	1.688045	0.029928	0.097633
0.741962	0.304733	0.136479	0.326912	0.111544	0.204156	0.805238	0.06088	0.097633
6.443925	12.29473	5.088694	3.119986	8.940671	15.98892	2.786898	6.978903	2.210866
1.564149	5.959033	4.557605	4.429769	2.941544	4.120815	0.234726	3.5257	4.174964
0.399307	1.002215	0.655245	0.42202	0.342641	0.570317	0.388579	0.790837	0.600173
0.287951	0.184877	0.36153	0.602908	0.124719	0.076162	3.428788	0.181227	0.265786
4.715599	2.941544	9.751039	1.78759	5.720173	13.23022	0.558095	2.990851	2.320072
1.978954	3.040505	13.23022	8.539971	15.46193	3.457504	0.019978	10.05618	16.22108
4.33E-05	0.533535	0.598456	0.368297	0.7257	0.140303	0.234726	0.415447	2.210866
23.38378	64.12971	65.94551	33.07147	77.74812	80.6518	5.671126	67.98069	62.33472
0.617071	1.695216	1.876212	1.146962	1.248232	1.131472	0.019978	1.43039	1.391494
1.395563	0.455649	0.669769	0.396082	0.766118	1.008413	2.050404	0.360938	0.574993
1.198701	0.905426	1.065584	1.310837	1.060188	1.044863	9.459177	0.5129	0.979149
3.783694	18.33909	25.73165	28.87506	8.226219	3.824964	1.248232	51.52195	21.19547
0.484704	1.160707	4.863579	11.30987	1.440736	0.82355	0.019978	2.901032	10.28573
0.460938	1.947316	2.727785	1.353608	1.755988	2.283752	0.525325	3.738997	5.591717
0.523983	1.149978	0.428456	2.171429	0.692027	0.73912	6.38456	1.175859	0.987821
0.236421	2.398442	1.320173	1.901068	1.559675	1.260635	0.019978	2.820974	2.02956
0.802035	0.060729	0.069206	0.019574	0.249899	0.383889	2.947287	0.136667	0.189394

0.29531	0.968066	0.92531	5.770996	1.705007	0.392403	0.255887	1.680895	3.695815
9.859719	0.12904	87331.38	135.5245	1.107605	1360.293	0.330635	111.5439	83.489
1.668369	0.221111	0.01588	0.436638	0.078297	0.03842	9.335397	0.020765	0.086443
0.021818	0.000216	0.000245	0.019574	0.013088	0.032857	0.019978	0.029928	0.011696
0.275058	0.027879	0.069206	1.353608	0.064848	0.299942	0.63754	0.080079	0.202294
0.851659	12.93651	22.32214	80.6518	2.540462	0.876118	1.082179	29.49693	91.05541
0.399307	0.647345	1.035801	1.699091	0.342641	1.214315	0.279452	1.43039	2.178268
0.426118	0.503781	0.648362	0.350274	0.772951	0.317576	5.143867	1.713997	0.856291
1.481631	2.533745	1.492973	0.869365	1.905094	3.214545	0.388579	1.504517	1.367179
0.087619	0.000216	0.000245	0.019574	4.33E-05	0	0.175592	0.000404	0.202294
4.739668	2.069942	2.136335	1.539062	6.297136	3.214545	1.688045	2.404618	2.341573
0.818586	0.508975	2.180022	8.195657	0.162915	0.637994	0.019978	2.104358	4.990794
2.221775	0.527821	0.153759	0.312864	1.028672	1.64917	6.088593	0.21176	0.202294
0.260404	0.077172	0.023232	0.019574	0.111544	0.076162	0.959567	0.049834	0.011696
3.166659	7.854069	7.238023	14.08569	3.47531	4.435159	0.358211	7.495397	11.14619
0.648362	1.612056	2.300678	6.570433	0.858297	0.100281	0.019978	5.323492	5.309264
1.779423	0.467136	0.428456	0.240202	0.682799	1.139221	2.74298	0.249408	0.297807
2.091313	19.22139	6.64254	6.900202	5.654863	6.475505	0.019978	5.030866	3.034747
0.016133	0.503781	0.223701	1.532468	0.131768	0.056869	0.607273	0.291183	1.418312
0.005512	0.000216	0.01588	0.249091	0.013088	0	0.607273	0.020765	0.011696
0.907273	1.712706	1.797172	2.114437	3.7586	1.948918	0.63754	3.101371	2.659214
0.039668	0.166789	0.000245	0.019574	0.145051	0	0.019978	0.000404	0.011696
3.993665	8.868932	6.028427	5.309264	6.99026	9.902576	0.019978	7.991602	5.704646
0.542525	0.12904	0.799553	0.690707	0.275382	0.351789	2.378124	0.549661	0.468167
0.204964	5.447114	4.33342	7.417359	4.012049	2.159004	0.019978	2.715873	7.625209
9.859719	15.98892	8.780115	6.784762	18.05488	7.363117	0.911905	9.031082	11.36975
0.016133	0.166789	0.032915	0.281558	0.124719	0.13386	3.297107	0.145556	0.214964
3.55961	5.687879	1.710173	1.157626	7.112496	6.287561	2.183665	2.35733	1.770029
2.931068	1.08671	2.099524	4.33342	1.215195	3.754719	0.019978	1.083651	2.479221
4.484892	0.927561	2.216162	1.753341	2.351299	2.574733	6.287561	1.218182	1.867951
1.89518	0.27075	0.252165	0.436638	1.080303	0.912446	3.953297	0.199488	0.112886
0.516306	0.617417	0.233889	0.019574	0.231659	0.838326	0.418326	0.020765	0.097633
10.05618	2.461905	7.212915	6.486104	6.706472	8.923117	0.304228	10.57984	5.28039
7.495397	16.34294	24.50942	45.91841	7.495397	9.297085	4.212554	25.99427	49.50903
7.653391	9.418196	34.38341	14.79742	24.73724	14.88584	1.150635	37.81919	40.11561
0.3671	0.000216	0.000245	0.019574	4.33E-05	0.100281	2.050404	0.029928	0.112886
4.557605	1.702706	2.050404	3.198499	3.846984	3.457504	3.014076	3.005281	2.109755
7.002056	6.404921	9.418196	3.340361	5.898449	11.03843	2.183665	10.5063	5.662994
0.550664	0.827273	1.283059	1.618773	0.10404	0.106847	0.418326	1.346991	1.283059
3.921645	21.19547	18.33909	24.2842	17.39935	10.43066	2.487576	16.50509	57.84882
1.533535	0.610224	0.528571	0.856291	0.882619	0.73912	0.773766	0.614156	0.394877
0.333636	0.035426	0.213218	0.019574	0.153651	0.076162	0.358211	0.040794	0.011696
0.836537	0.192929	0.446291	1.008413	0.556753	0.498918	0.019978	1.370765	2.185556
1.948918	2.382035	0.475137	0.653203	2.752893	1.995714	0.959567	0.690707	0.533196
5.23176	4.914863	1.680895	0.508341	3.939307	3.052655	0.470512	1.058896	0.761688
1.060188	0.114618	0.283709	0.019574	0.231659	0.100281	1.150635	0.06088	0.313312
3.290101	3.101371	3.636789	27.38726	3.507691	1.421126	2.577157	4.764055	9.968355
0.139986	0.0843	0.01588	0.161818	0.057128	0.359899	0.676659	0.020765	0.128853
1.160707	7.002056	29.82828	18.19595	5.210317	34.84299	0.019978	10.01009	4.907929
0.287951	0.415974	1.728615	0.551284	0.275382	0.986797	0.911905	0.766118	0.901212
4.33E-05	0.090584	0.000245	0.019574	0.241255	0.032857	1.015967	0.000404	0.011696
7.315512	4.540794	1.930216	1.612056	18.62711	16.5625	7.625209	1.204228	2.659214
1.835743	8.677489	252.4251	425.62	64.12971	18.48514	0.019978	75.1132	135.5245
4.23202	18.91854	9.19899	22.74797	8.381328	3.1857	2.652107	7.417359	13.00996
0.617071	3.198499	0.273434	0.144098	0.290115	0.081746	0.019978	0.394877	0.409098
4.592273	2.143261	6.612121	8.075772	6.945152	5.788153	1.925635	11.14619	10.01009
49.50903	70.11932	57.84882	24.73724	49.50903	42.75576	12.63773	57.84882	27.96248
0.021818	0.014545	0.01588	0.019574	0.013088	0.005303	0.494444	0.020765	0.011696

0.915195	0.462857	0.171061	0.307973	0.462857	0.6657	4.715599	0.766118	0.214964
0.087619	0.265224	0.000245	0.332641	4.33E-05	0.076162	1.049517	0.000404	0.011696
2.078355	1.093434	6.287561	7.598312	0.882619	2.31404	1.925635	4.38684	11.77567
0.046212	0.06886	0.778326	0.176558	0.145051	0.204156	0.019978	0.400173	0.011696
1.335743	1.664906	2.116032	0.90772	3.578038	2.925426	0.019978	1.240202	0.88316
1.022338	7.238023	1.578687	6.345382	0.373463	1.511198	1.049517	3.552049	3.394286
2.221775	1.753341	1.320173	0.766602	2.50469	1.713997	0.019978	1.530159	1.569553
14.70188	57.84882	13.83359	7.545584	31.1753	40.11561	2.296732	20.85876	9.418196
13.42179	3.703175	14.97762	7.882222	9.729358	27.96248	3.014076	18.33909	15.46193
0.125123	0.020216	0.087403	0.144098	0.096263	0.13386	2.531515	0.118225	0.143709
1.927042	4.733824	3.26	1.763889	0.623146	4.082165	0.446551	1.158384	1.184935
20.34063	55.04081	4.307309	1.215981	13.53913	7.264502	59.32013	1.667273	1.273636
0.597042	5.095325	1.455022	0.019574	2.933903	2.789466	0.019978	2.533745	1.692763
2.683478	1.937222	0.428456	1.036558	1.021097	0.425635	12.10289	0.690707	0.970635
4.397792	5.402511	1.980592	1.246457	5.044113	1.880375	0.019978	2.882035	0.511667
0.542525	0.88619	0.419076	0.116659	0.153651	1.083651	0.676659	0.136667	0.143709
0.484704	4.272165	1.223846	2.009906	2.996869	2.259661	0.019978	1.95645	1.661328
0.039668	0.060729	0.126429	0.019574	0.153651	0.056869	0.234726	0.000404	0.011696
3.401126	1.329206	1.94311	1.215981	0.548788	2.725498	13.75978	0.742417	2.402468
3.061955	3.175916	0.965065	0.596775	4.535354	5.359134	5.788153	1.624459	1.048074
0.323319	0.905426	0.771176	0.42925	0.882619	1.287403	1.2214	0.91575	0.436638
1.296905	0.455649	0.283709	0.41482	0.909315	1.741414	1.758615	0.323535	0.342049
0.287951	0.897489	0.000245	0.149986	0.259033	0.309062	0.986104	0.000404	0.086443
0.046212	0.25197	0.049719	0.342381	0.057128	0.076162	0.607273	0.029928	0.011696
1.416407	1.153723	8.048095	9.687496	1.052886	1.511198	0.986104	5.71241	6.99026
1.349971	0.863189	3.397706	3.363911	1.204957	2.290996	0.773766	3.38456	4.637994
0.011602	0.027879	0.000245	0.019574	0.019408	0.001111	0.124228	0.029928	0.011696
10.23768	0.819509	0.428456	0.019574	1.880375	0.621688	11.39744	0.000404	0.011696
3.310758	2.77899	2.472576	0.342381	3.172864	3.659668	0.193348	1.791616	1.346003
2.77899	2.289286	0.730029	0.273672	3.621941	4.06303	0.470512	1.434921	0.685209
8.089848	11.25392	12.03267	2.823636	14.08569	10.48101	3.953297	9.838658	12.36443
0.468167	0.880498	1.693939	4.490801	1.420202	0.700418	0.358211	2.181775	4.709033
0.3671	0.486551	0.146118	0.319567	0.373463	0.344127	1.797172	0.227648	0.202294
16.44768	0.265224	6.200945	3.581688	0.033261	0.26215	2.134545	2.022778	7.911934
0.353175	0.827273	0.506328	0.662237	0.532092	0.412193	1.758615	0.945483	0.32925
2.337821	1.002215	1.680895	0.680173	1.578687	2.371833	0.234726	2.061926	1.037316
0.393449	0.184877	0.568961	0.457749	0.10404	0.210693	0.676659	0.50469	0.740952
1.022338	0.401385	0.36153	0.337641	1.96404	0.595368	2.577157	0.227648	0.979149
4.053506	5.287857	1.626782	1.168326	4.292078	6.125823	1.248232	1.473218	0.622496
0.863189	3.175916	1.693939	1.236248	0.589986	2.31404	0.019978	1.656696	1.391494
2.164113	16.22108	10.57984	2.758088	25.48179	30.49289	0.019978	13.6814	6.153102
0.836537	2.661587	3.939307	7.737807	2.535974	2.868456	1.015967	6.978903	6.900202
0.3671	1.857677	1.102121	0.825354	1.39847	1.677042	0.019978	1.100519	1.441898
3.921645	7.276645	1.136566	0.60886	3.23987	8.149307	0.019978	2.893001	0.979149
7.765931	21.74051	8.412367	2.323983	40.11561	13.53913	0.234726	16.80343	3.521934
0.858297	0.632569	0.895368	0.249091	1.043608	1.159913	1.580909	0.852172	0.143709
34.84299	12.22798	3.428788	8.573434	8.412367	6.38456	55.04081	4.914863	3.50425
0.081818	0.569625	0.528571	3.028629	0.241255	0.223701	1.049517	0.475433	1.358189
0.099488	0.206768	0.059957	0.019574	0.342641	0.27062	3.397706	0.108124	0.128853
26.25557	184.443	64.12971	287.197	21.36892	38.39042	0.019978	50.55949	155.7103
0.792886	1.555289	0.740527	0.019574	1.006623	1.633752	0.019978	2.511465	0.313312
1.268672	2.858009	2.116032	3.493362	2.230859	1.297763	1.688045	2.285664	2.939141
0.281364	0.106926	0.000245	0.019574	0.145051	0.278802	5.259437	0.000404	0.011696
10.8813	3.175916	7.08733	1.263752	9.179466	7.897078	0.138571	5.143867	4.445851
87331.38	59.32013	14.25954	30.49289	10.93101	12.85504	87331.38	33.93939	18.62711
0.039668	0.020216	0.000245	0.478413	0.019408	0.026364	0.446551	0.000404	0.011696
9.377345	6.38456	2.498478	8.728571	2.474834	5.28039	530.1465	4.155339	2.194257
2.404618	3.718716	9.541645	7.468961	2.710765	4.933781	0.019978	12.93651	4.47912

1.819221	1.779423	1.035801	0.326912	2.429957	2.805382	7.037345	1.603795	1.40772
4.644098	0.696234	2.026219	0.690707	0.478413	0.451977	0.494444	1.111241	1.242641
3.699495	10.53159	2.703485	1.661328	29.82828	7.854069	1.339091	3.279625	2.081696
0.718355	0.709784	0.546522	0.591912	0.929286	0.359899	27.38726	0.323535	0.588954
47.5018	8.539971	4.875527	2.973059	8.134084	4.721789	35.27608	1.549711	1.321053
0.125123	0.000216	0.000245	0.019574	0.162915	0.009322	3.618499	0.06088	0.011696
0.255693	0.25197	0.203333	0.207713	0.090108	0.120433	0.832554	0.049834	0.394877
10.38202	5.330519	6.853608	13.6814	3.599329	2.427792	62.33472	5.381227	10.43066
65.94551	35.27608	36.26061	8.508514	155.7103	83.489	52.56128	59.32013	31.92385
29.82828	30.17192	42.75576	37.29684	21.19547	16.68554	5.788153	43.49658	27.10502
43.49658	33.4985	8.923117	5.25254	41.47925	51.52195	155.7103	13.00996	7.545584
17.15902	11.719	9.101674	8.780115	14.79742	20.68746	22.52375	9.924372	17.03919
18.05488	27.66779	30.82277	32.68313	34.84299	25.24367	5.431544	30.17192	38.94854
19.53061	4.272165	4.451154	4.272165	17.64869	13.08214	203.5795	9.667316	6.240346
287.197	167.9996	425.62	252.4251	87331.38	287.197	2.378124	530.1465	287.197
203.5795	43.49658	144.7919	95.22939	105.1694	135.5245	36.26061	70.11932	224.9555
70.11932	425.62	47.5018	45.1045	118.7028	87.1746	8.643074	155.7103	72.69473
87.1746	24.04374	49.50903	34.38341	51.52195	425.62	184.443	22.96463	20.68746
9.297085	2.174733	2.339697	1.044235	4.408153	2.009906	10.1459	2.325931	5.80469
0.992201	3.137576	0.583766	0.260144	1.675722	1.078925	6.036818	0.756176	0.69583
4.376118	5.976724	2.591443	1.044235	7.061328	11.48368	31.92385	1.74539	0.932078
4.429769	8.976508	7.751869	1.560779	9.218514	8.195657	0.959567	6.622092	8.849697
0.965065	0.326017	0.049719	0.019574	0.508975	0.70886	0.716234	0.020765	0.011696
0.106522	0.213615	0.023232	0.019574	0.064848	0.056869	0.214401	0.040794	0.011696
2.429957	1.275325	0.932078	2.518124	1.310837	0.857778	11.65791	0.932078	1.298665
4.33E-05	0.647345	0.562107	1.797172	0.453463	0.392403	1.015967	1.549711	0.600173
8.745238	2.109755	2.531515	8.134084	2.474834	6.09759	20.94257	3.714906	4.609076
0.662237	0.436017	0.721335	2.993867	4.33E-05	0.043824	287.197	0.820931	1.546696
13.00996	17.03919	5.337547	2.229048	16.80343	14.79742	9.063983	4.058514	2.681169
0.475729	3.833247	4.473348	7.570693	1.204957	0.54925	0.234726	3.314329	2.834084
1.795851	0.337431	0.353939	0.019574	2.210866	2.427792	7.882222	0.622821	0.128853
0.039668	0.044026	0.023232	0.019574	0.171183	0.216753	0.716234	0.029928	0.011696
0.697085	0.410079	0.932078	0.463485	0.741457	0.686667	12.93651	0.690707	1.49075
1.435772	2.353247	1.812114	5.273362	1.886335	2.828918	0.607273	5.976724	3.150433
24.50942	4.06303	28.87506	21.19547	27.38726	21.36892	4.082165	28.23632	32.31123
41.47925	15.46193	27.52753	20.34063	67.98069	38.94854	0.214401	34.84299	24.73724
18.62711	4.984488	10.33476	7.6393	11.03843	11.89932	21.93452	7.112496	11.96574
6.749488	1.256558	3.101371	2.061926	4.125786	6.796566	0.470512	2.676392	2.210866
0.783939	0.754784	0.087403	0.019574	0.787987	0.677403	1.248232	0.145556	0.011696
20.85876	6.784762	23.6012	13.60866	19.99861	32.31123	4.46267	28.87506	26.25557
0.851659	0.596508	0.730029	0.019574	0.453463	1.688045	1.688045	0.878369	0.588954
5.23176	10.43066	0.807619	0.671371	2.147013	1.928629	11.06488	0.878369	2.763261
27.96248	28.54786	15.07257	10.19468	25.73165	22.13245	15.56131	12.03267	14.51648
2.388348	0.503781	1.474343	3.533211	0.282583	0.899055	4.63189	1.624459	1.674401
1.564149	0.241255	0.436638	0.196551	0.795786	1.312706	1.277843	0.5129	0.480022
6.486104	2.955916	5.351934	3.113478	4.764055	5.309264	6.64254	3.394286	3.1857
1.716364	0.827273	1.002215	0.019574	1.533535	1.727323	8.256465	1.143954	0.942294
0.434329	0.206768	0.528571	1.110505	0.444762	0.480022	2.487576	0.353795	0.706053
0.971205	0.415974	0.146118	0.019574	0.508975	0.327143	0.805238	0.136667	0.011696
1.809343	0.979827	0.863694	0.935115	0.819048	0.752229	0.85728	0.422237	0.646861
0.175469	1.072843	0.108124	0.38772	1.559675	0.309062	13.87537	0.366912	0.265786
7.683766	1.016494	1.520346	1.691587	0.717879	1.801284	1.995714	1.022338	0.970635
2.131176	1.381111	2.190866	1.184935	1.724805	3.457504	9.707677	1.32482	1.49075
5.881342	1.786306	1.092136	2.034242	4.557605	13.15079	15.3618	3.672078	2.101075
0.244033	1.551962	1.421126	5.872886	0.500996	0.359899	0.418326	0.95101	2.781558
0.06368	0.137201	0.032915	1.985253	0.138009	0.063319	0.805238	0.06088	0.189394
27.38726	5.545729	11.06488	5.606739	23.16622	19.06893	1.400159	12.63773	11.25392
0.532619	0.522071	0.339004	0.176558	0.500996	0.327143	4.338629	0.602287	1.099762

0.003413	0.000216	0.01588	0.139091	4.33E-05	0	0.193348	0.000404	0.097633
1.405772	9.256537	1.648066	0.38772	1.335743	0.471025	0.019978	1.720144	1.637309
2.604776	2.099524	7.264502	1.901068	3.599329	4.317648	0.019978	21.54823	5.082063
77.74812	1.109726	0.381061	0.019574	10.8813	2.683478	72.69473	0.848831	0.69583
4.338629	1.937222	7.188095	3.273045	12.77772	9.792626	0.330635	13.60866	6.591544
1.286494	1.982208	1.831631	0.312864	1.495043	3.592063	1.959466	0.249408	0.214964
4.096407	6.345382	10.1459	10.53159	6.088593	3.128694	3.297107	11.36975	14.42661
4.927302	3.137576	5.754935	2.802828	5.095325	8.061934	15.276	2.845036	3.36075
1.978954	0.800512	1.152165	1.123016	0.896522	0.906825	17.78522	0.622821	1.721371
0.697085	0.137201	0.000245	1.062821	4.33E-05	1.07127	1.049517	0.000404	0.011696
27.10502	7.08733	7.751869	4.043846	19.22139	10.53159	36.74522	6.922771	6.52899
95.22939	52.56128	155.7103	155.7103	425.62	203.5795	5.381227	203.5795	1360.293
0.212395	0.152222	0.000245	0.019574	0.282583	0.557056	0.019978	0.000404	0.011696
30.82277	29.49693	21.74051	7.264502	16.09482	53.77619	8.643074	33.07147	14.6058
2.203535	1.612056	1.460051	1.638499	2.210866	2.54961	1.497143	1.762713	1.170491
1.507835	1.002215	0.981032	0.196551	1.172872	0.986797	3.738997	0.790837	0.480022
8.539971	5.80469	13.60866	20.51271	8.477662	13.6814	1.150635	15.46193	19.22139

CGGA_131	CGGA_131	CGGA_131	CGGA_131	CGGA_132	CGGA_132	CGGA_132	CGGA_133	CGGA_133
0.79487	1.692763	0.191804	0.596111	0.821414	0.7257	0.173059	0.259098	0.340866
0.203333	2.638139	0.09176	0.105108	0.469358	1.434055	0.078831	0.34619	0.09847
1.348975	0.019978	7.315512	3.446732	3.397706	0.676999	7.653391	8.888167	2.996869
0.726133	0.019978	3.885642	1.692763	1.10132	0.018016	4.715599	4.150274	1.863506
2.531515	0.257518	3.061955	2.276407	1.659033	1.006025	4.620159	2.673846	8.210938
6.508427	8.033932	3.052655	0.631494	1.350772	6.045209	1.041025	2.673846	3.489899
13.6814	9.459177	49.50903	25.24367	64.12971	6.135007	38.94854	62.33472	28.87506
0.041782	0.932078	0.016104	0.159942	0.055671	1.96697	0.04456	0.083608	0.000115
1.815	0.019978	0.588954	2.114437	0.800512	0.384156	6.508427	0.396306	3.655714
0.244033	0.522071	0.09176	0.001126	0.622092	0.510707	8.66E-05	3.61E-05	0.000115
184.443	45.1045	41.47925	12.77772	43.49658	57.84882	16.34294	50.55949	12.67271
0.36267	4.007583	0.882078	0.441053	2.936739	4.721789	0.349084	1.605859	0.545426
9.541645	1.880375	48.53429	55.04081	27.10502	0.247417	40.11561	27.66779	8.677489
1.174315	0.019978	3.482785	14.79742	2.89E-05	0.018016	17.28195	3.61E-05	12.42965
0.056782	1.056898	0.01153	0.057691	0.249278	0.54925	0.04456	0.003535	0.010014
2.013182	0.019978	5.493182	9.418196	1.296905	0.384156	5.959033	3.119986	6.475505
0.916869	1.742734	1.753341	0.347186	2.823636	3.169812	0.728088	3.387864	0.872013
0.000895	13.83359	0.016104	0.001126	0.024733	25.60672	8.66E-05	0.122475	0.174906
1.000794	0.019978	1.978954	12.93651	1.499214	0.018016	8.103925	0.764704	1.176623
1.242641	0.337222	0.596111	0.396638	2.895599	2.23267	0.569293	0.249091	0.603261
1.189769	0.382208	0.341558	1.255657	0.197727	3.699495	0.944084	0.148932	0.405786
0.505267	3.104394	6.8421	28.87506	2.558817	5.967879	8.241342	2.718434	0.702835
12.42965	0.019978	50.55949	0.001126	67.98069	0.018016	12.16681	111.5439	25.99427
0.056782	13.15079	0	0.132107	0.042807	3.363911	0.052525	0.017576	0.010014
0.41026	2.089639	0.82868	0.587965	1.523824	4.907929	0.365224	2.127973	1.443059
1.867951	2.203535	7.175397	5.530274	2.459812	3.363911	5.606739	8.958225	4.207778
0.000895	0.019978	1.584105	3.738997	5.044113	0.018016	5.056421	1.370765	3.606804
0.695426	1.010289	0.244589	0.402381	0.39062	0.842193	0.250476	0.417063	0.174906
28.87506	0.019978	42.75576	83.489	23.82354	0.018016	83.489	8.994791	155.7103
0.630505	4.048499	0.31702	0.616407	1.350772	4.696313	0.262655	0.755714	0.231768
1.228651	2.763261	6.900202	9.707677	8.508514	3.269913	4.508196	3.640606	5.395584
0.033874	4.297222	0.01153	0.105108	0.080664	1.082915	0.008312	0.20733	0.034906
0.000895	0.019978	18.76954	22.13245	7.558139	0.018016	21.93452	21.74051	11.57341
0.048889	0.337222	0.002143	0.001126	0.055671	0.018016	0.012864	0.007395	0.016133
3.266782	16.68554	1.159149	0.040469	2.654473	26.80355	0.188009	1.833102	0.277085
2.248896	1.152165	3.763045	8.888167	3.91355	0.7257	3.566753	0.992201	1.677042
2.453781	4.881775	7.363117	7.417359	5.599228	7.443276	7.468961	2.429957	3.536955
0.352597	2.518124	0	0.057691	0.142244	0.384156	8.66E-05	0.007395	0.010014
5.062626	3.049603	2.506934	2.363442	2.477027	6.900202	3.220678	4.733824	5.500678
8.319019	8.303947	4.965801	1.548723	5.720173	12.29473	5.34474	5.050267	5.295325
0.708124	4.077403	1.056898	0.001126	1.68342	10.62739	0.650556	0.806227	0.350635
3.734762	4.637994	0.996111	1.47018	2.737944	23.16622	0.980505	2.325931	0.958557
1.055628	0.019978	2.203535	4.667518	0.420498	0.018016	4.323045	1.43039	1.791616
0.026876	0.162576	0.100483	0.001126	0.00303	0.149293	8.66E-05	0.003535	0.010014
0.064127	2.045476	0.612525	0.205382	0.414242	2.941544	0.863189	0.788896	0.138644
13.00996	0.463867	20.34063	24.73724	17.92188	1.877605	30.82277	26.25557	45.91841
0.071183	0.585592	0.175253	0.159942	0.086443	1.845519	0.116825	0.233175	0.010014
0.478737	0.463867	0.016104	0.001126	0.103716	0.311227	0.026364	0.025339	0.000115
0.033874	0.768535	0	0.032431	2.89E-05	0.202388	0.026364	3.61E-05	0.000115
0.033874	0.257518	0.075418	0.066883	2.89E-05	0.288413	0.026364	0.049769	0.000115
0.041782	0.134517	0.143333	0.076587	0.186017	0.133045	8.66E-05	0.007395	0.000115
8.994791	0.019978	4.927302	6.423911	8.643074	0.485325	4.49671	5.745996	11.719
0.867814	1.030022	0.01153	0.001126	0.73912	1.217475	0.086991	0.579495	0.016133
0.108124	0.337222	0.257143	0.001126	0.055671	1.604827	0.102792	0.508658	0.034906
0.419365	1.010289	1.614257	4.067547	2.099524	6.99026	7.795642	1.106825	0.702835
1.215981	0.019978	0.166118	0.001126	0.648038	0.938911	8.66E-05	0.041328	0.174906
0.531169	4.881775	0.797114	0.149654	1.329206	10.48101	1.360022	0.620909	0.322237

0.531169	5.295325	0.328543	0.971775	0.672857	6.268312	0.711046	0.488167	0.498355
2.925426	1.944517	1.863506	0.631494	1.629235	1.402915	0.872013	1.244957	1.530159
0.117626	0.906825	1.046703	0.179177	0.573326	1.779423	0.28443	1.618773	0.350635
3.008225	2.143261	4.863579	57.84882	7.339452	0.334805	0.488759	0.177734	20.85876
0.341241	1.23127	0.415166	0.359697	0.581378	0.117518	0.466869	0.9529	0.174906
0.129553	7.668579	0.322395	0.228203	0.274055	20.34063	0.365224	1.954993	0.254769
59.32013	0.019978	56.39528	16.74449	35.27608	0.018016	29.82828	21.54823	33.93939
0.048889	0.403413	0.150346	0.001126	0.086443	2.153535	0.139697	0.091977	0.148846
0.041782	0.019978	0.001017	0.032431	0.019942	0.018016	0.019048	0.012063	0.000115
2.581861	0.019978	3.119986	0.692027	2.922778	0.485325	1.618773	1.201724	2.037114
0.976667	0.162576	0.807136	2.483492	1.10132	0.410541	2.261573	1.568564	1.690483
0.708124	0.502843	0.534214	0.132107	0.469358	0.224214	0.156941	0.682359	0.201789
0.162468	3.207807	0.05303	0.095433	0.117843	2.513752	0.094553	0.091977	0.053391
0.579495	0.175592	0.086667	0.040469	0.242453	1.469163	8.66E-05	0.292597	0.016133
1.000794	2.690823	0.05303	0.179177	0.639892	0.806227	0.139697	0.935743	0.09847
0.026876	0.114012	0.001017	0.032431	0.00811	0.149293	0.012864	0.033261	0.010014
0.026876	2.600014	0	0.001126	2.89E-05	0.133045	0.019048	0.012063	0.000115
3.860491	0.257518	6.191515	6.079596	4.952915	0.485325	19.99861	5.9071	5.80469
0.152114	1.410902	0.086667	0.001126	0.179423	0.410541	8.66E-05	0.158766	0.09847
0.033874	0.979149	0.030613	0.001126	0.080664	0.247417	0.035079	0.025339	0.000115
6.144055	9.082828	4.058514	2.586926	4.47912	0.906126	4.170051	6.250447	7.545584
0.000895	1.34513	0.016104	0.001126	0.097633	1.934271	0.035079	0.074358	0.000115
0.602554	1.65785	0.28689	0.411436	0.179423	2.312085	0.164877	0.20733	0.592208
1.691587	0.187532	8.573434	16.74449	5.493182	1.252424	15.3618	5.123781	4.348882
0.071183	0.362179	0.001017	0.076587	0.00303	0.485325	0.026364	3.61E-05	0.044214
0.467951	1.842734	0.299199	0.066883	0.965714	1.497143	0.078831	1.106825	0.148846
31.55417	0.019978	80.6518	12.42965	32.68313	0.806227	14.97762	6.52899	13.6814
1.139221	0.663276	0.133297	0.132107	0.847908	1.006025	0.094553	0.098831	0.116659
0.319762	2.763261	0.120361	0.086075	0.019942	10.01009	0.533535	1.064935	1.186457
0.719177	0.019978	2.187395	0.655613	0.274055	1.082915	2.489762	2.535974	0.231768
0.152114	0.746551	0.244589	0.376407	0.384156	0.806227	0.130714	0.140188	0.466335
0.033874	1.106825	0.065152	0.032431	0.130606	0.617071	0.06886	0.567994	0.034906
0.36267	0.019978	3.363911	6.52899	2.045476	0.018016	1.092785	3.61E-05	0.933261
14.33939	0.019978	19.53061	4.914863	5.599228	0.018016	7.417359	13.19051	14.51648
0.087973	0.238889	1.108276	0.001126	0.229199	0.906126	8.66E-05	0.240851	0.06254
1.327525	0.187532	0.299199	0.196955	0.346494	1.378362	0.217792	0.385736	0.277085
1.336587	12.93651	1.433189	0.040469	1.690483	4.557605	0.28443	4.082165	0.635541
0.056782	4.971991	0.004964	0.001126	0.074286	1.315613	8.66E-05	0.007395	0.000115
6.191515	1.813564	8.677489	6.486104	3.750837	0.876118	8.677489	9.687496	6.956349
0.162468	0.663276	0.004964	0.057691	0.055671	0.457114	0.008312	0.007395	0.025087
0.056782	2.384141	0.004964	0.032431	0.029639	0.906126	8.66E-05	0.003535	0.025087
0.033874	0.203773	0.002143	0.057691	0.015714	0.384156	8.66E-05	3.61E-05	0.025087
2.34355	1.593535	0.92482	0.926291	3.842554	2.153535	0.897489	3.397706	0.772157
1.585296	1.916782	5.130411	8.625613	3.998391	1.753341	8.539971	5.500678	4.082165
1.426522	3.703175	2.477027	0.277417	2.415296	0.972504	0.679812	4.927302	1.03246
0.708124	1.106825	0.450924	0.353492	0.473319	0.676999	0.102792	0.187229	0.284863
0.319762	0.337222	0.218975	0.939986	0.197727	1.152165	0.78619	1.370765	1.574517
1.043608	0.873579	0.224372	0.474856	0.82772	1.845519	0.060022	1.037316	0.000115
0.142244	1.469163	0.836017	0.49974	0.414242	3.734762	0.232734	1.064935	0.201789
0.505267	0.019978	1.978954	10.57984	0.214502	0.54925	4.029654	0.839365	2.59557
0.781977	0.382208	0.882078	0.286364	0.745072	1.061457	0.173059	1.226934	0.391169
4.696313	0.956667	1.567576	1.017107	2.710765	1.315613	0.872013	1.855108	2.238341
4.18456	0.019978	4.626025	2.831501	3.089293	0.018016	1.305772	6.808369	2.698557
0.885137	2.432121	0.684033	0.179177	0.69583	2.839351	0.28443	4.203001	1.443059
1.555289	5.165599	24.2842	425.62	0.813788	0.018016	111.5439	2.871212	118.7028
4.650007	0.019978	0.639113	1.514199	0.527821	0.018016	2.216162	1.244957	3.816551
0.467951	0.312864	0.161082	0.245772	0.61526	0.842193	8.66E-05	0.34619	0.078211
5.217569	0.019978	67.98069	118.7028	3.24658	0.018016	95.22939	4.614618	29.49693

0.056782	3.175916	0.161082	0.169971	0.340556	3.098268	0.130714	0.476335	0.034906
0.968723	1.384921	0.91404	0.001126	0.430476	0.018016	0.060022	3.61E-05	0.157056
0.808983	26.80355	1.192633	2.276407	4.875527	37.29684	1.64684	1.477388	2.898196
3.479134	12.63773	25.99427	7.625209	11.48368	7.38847	10.40634	70.11932	3.536955
1.29417	10.1459	0.042655	0.310447	0.95101	7.38847	0.139697	1.510014	0.116659
16.68554	3.104394	4.091667	1.450693	2.485534	3.763045	3.610707	11.74734	6.900202
0.684401	3.340361	1.040411	0.205382	1.827403	2.758088	0.633499	3.324452	0.702835
5.933066	6.784762	1.614257	0.698297	1.697792	3.065108	0.944084	2.763261	2.834084
11.14619	31.1753	0.381999	1.186457	10.73084	64.12971	0.324286	3.340361	0.746984
0.401385	19.06893	0.381999	0.63754	0.573326	7.653391	0.633499	0.850808	0.592208
0.000895	0.812771	0.016104	0.048889	2.89E-05	1.082915	8.66E-05	0.074358	0.016133
1.595729	1.010289	0.907273	1.296905	1.179885	0.311227	0.203954	0.375281	0.545426
0.224055	1.152165	0.28689	0.465801	0.208867	6.622092	0.657547	0.550072	0.107027
23.82354	2.384141	5.915469	8.119004	9.335397	0.592532	5.22482	4.863579	8.429055
0.033874	2.871212	0.588954	0.057691	0.024733	1.434055	0.225209	0.488167	0.025087
2.74298	0.542828	6.230245	11.48368	2.609293	1.779423	3.279625	1.278629	1.359177
0.352597	2.270837	1.122359	0.971775	1.791616	1.521371	0.83697	0.579495	0.786667
0.000895	0.382208	0.004964	0.032431	0.011573	0.533196	8.66E-05	3.61E-05	0.000115
2.6143	2.143261	3.730808	1.07127	11.30987	8.677489	3.33728	2.984899	4.63189
0.630505	0.019978	0.025483	0.324719	0.442496	0.018016	0.409336	0.579495	1.374329
0.071183	4.140505	0	0.048889	0.019942	1.129452	8.66E-05	0.003535	0.000115
0.129553	0.956667	0.522367	1.241811	0.242453	0.311227	0.918557	0.670144	1.310837
4.212554	23.6012	2.55184	0.269892	4.851227	25.60672	1.381111	4.338629	0.82772
1.644416	1.443059	3.147294	3.058802	1.262179	0.972504	4.888023	4.667518	2.529307
0.551573	0.312864	0.445209	0.610224	0.067403	1.182771	0.690707	1.121017	0.702835
3.507691	9.625253	2.024488	2.018167	4.451154	8.888167	1.867951	3.711097	2.727785
3.450577	5.295325	6.8421	1.622006	3.799885	7.288788	3.33728	3.563182	2.569957
4.003117	11.11876	1.74539	9.625253	2.196017	0.510707	2.250462	3.877417	2.225498
0.741457	2.203535	0.20759	1.968283	0.080664	0.647345	0.674675	0.466602	1.982208
1.771392	0.019978	0.843102	2.597792	1.705007	0.018016	1.957958	1.662439	3.407619
14.79742	4.952915	8.048095	10.1028	14.88584	6.622092	7.136537	10.05618	18.91854
0.000895	0.019978	0.434069	4.408153	2.89E-05	0.018016	3.6029	3.61E-05	0.000115
15.66023	2.845036	14.00414	16.34294	7.188095	4.402973	23.38378	15.56131	10.82954
18.76954	1.23127	5.34474	28.54786	13.00996	1.182771	8.075772	3.877417	10.8813
0.000895	0.257518	0.002143	0.066883	0.015714	0.567994	0.019048	0.033261	0.000115
0.162468	0.362179	1.883535	1.202626	2.589185	0.018016	3.137576	2.465916	1.387612
5.165599	6.52899	3.324452	0.30158	7.964315	7.288788	0.314913	3.028629	3.131544
2.647244	2.953059	0.381999	0.698297	0.813788	1.182771	0.473853	0.890065	0.854242
2.522684	1.443059	0.31702	0.169971	2.786898	0.224214	0.139697	0.922338	0.201789
1.29417	0.019978	2.678781	2.752893	0.442496	0.018016	2.192496	1.449877	3.743232
1.159913	0.019978	2.073232	56.39528	2.89E-05	0.018016	14.08569	0.747879	3.350224
0.000895	0.019978	64.12971	95.22939	25.24367	0.018016	126.1556	32.11754	56.39528
0.675115	6.663651	1.724805	0.376407	2.31811	6.09759	0.526299	7.795642	0.635541
0.064127	0.2757	0.180895	0.115014	0.221587	0.457114	0.026364	0.316811	0.016133
4.155339	0.019978	0.558427	1.092785	0.965714	0.149293	1.748016	0.645455	1.551962
6.30671	0.019978	1.515296	5.754935	1.253752	0.018016	3.536955	2.696061	1.776739
0.214502	3.514834	0.797114	0.255209	1.262179	2.890245	0.102792	0.326017	1.218182
0.000895	2.089639	0	0.001126	1.037958	9.377345	8.66E-05	3.61E-05	2.280101
22.32214	3.425512	13.6814	11.09133	14.97762	1.217475	17.92188	13.08214	18.76954
1.507835	0.148701	1.737294	0.519387	2.29873	0.133045	0.396472	0.365859	0.37417
0.000895	2.203535	0	0.001126	0.420498	2.513752	8.66E-05	0.620909	0.000115
4.376118	28.87506	2.760765	1.556392	8.461703	17.22048	1.212792	8.711905	4.38684
1.660216	0.502843	1.148564	1.171349	2.285664	0.202388	1.131472	0.90772	2.099524
0.142244	2.723247	0	0.188276	0.6657	5.302294	0.035079	0.292597	0.332886
0.244033	1.384921	0.541522	0.076587	2.027951	3.66785	0.210505	0.305087	0.053391
1.189769	0.019978	2.860722	10.98644	1.731169	0.972504	2.74298	1.006025	2.59557
0.516825	0.337222	0.120361	0.001126	0.197727	0.311227	0.06886	0.025339	0.044214
0.564127	0.812771	0.080895	0.255209	0.265224	0.018016	0.019048	0.177734	0.09847

0.310866	0.019978	0.980505	1.312706	0.306392	0.018016	1.078153	0.091977	2.382035
2.278254	0.019978	4.833398	2.518124	2.89E-05	0.018016	3.934986	9.687496	7.288788
3.540606	7.507619	4.348882	3.722785	5.515584	10.98644	5.302294	5.976724	3.479134
0.352597	0.019978	9.667316	24.50942	6.933961	0.647345	21.19547	27.38726	43.49658
3.230065	0.337222	1.877605	1.085224	2.993867	0.018016	1.443059	1.968283	1.648066
0.000895	0.019978	1.414574	8.27267	2.89E-05	0.018016	9.418196	2.800332	0.854242
0.821414	3.266782	0.143333	0.384156	4.165137	3.548153	0.365224	2.283752	0.025087
6.365065	0.019978	9.335397	14.70188	6.622092	0.018016	12.63773	5.395584	15.276
0.849841	9.583463	0.310866	0.001126	3.829105	3.442886	0.481414	7.977958	1.889711
9.101674	7.625209	9.256537	4.696313	8.923117	4.535354	7.854069	16.80343	11.22667
4.984488	0.69119	0.05303	0.001126	0.885664	1.779423	8.66E-05	1.355527	1.008413
9.063983	5.210317	11.63037	8.994791	10.62739	4.47912	11.65791	14.70188	5.720173
0.162468	0.382208	1.872049	2.496407	0.507403	0.35798	1.102121	0.316811	0.883665
5.838499	0.019978	3.249877	3.144156	2.045476	0.202388	4.575123	2.815866	2.984899
0.000895	2.763261	0.086667	0.086075	0.330635	0.699192	8.66E-05	0.114235	0.000115
0.821414	0.019978	2.574733	2.333867	1.296905	0.018016	2.102626	1.074827	0.82772
0.505267	0.663276	1.542302	3.548153	2.238341	0.485325	1.739935	1.209776	0.723506
4.47912	4.971991	1.056898	0.457431	1.869322	4.764055	0.357727	2.037114	2.270837
0.041782	0.203773	0.004964	0.001126	0.005007	0.410541	8.66E-05	3.61E-05	0.010014
4.292078	0.019978	1.272756	0.662237	0.893528	0.018016	3.934986	2.355289	3.223882
14.00414	2.953059	14.79742	6.230245	17.64869	1.315613	8.134084	7.938427	6.475505
4.003117	0.843102	1.991328	0.001126	2.993867	1.283911	0.262655	1.585296	1.128788
0.000895	4.484892	0.042655	0.001126	0.465079	0.906126	8.66E-05	0.158766	0.398355
5.993045	1.410902	5.967879	3.930664	7.668579	4.851227	7.175397	6.738211	4.029654
17.78522	0.019978	9.792626	6.011515	12.56693	0.018016	6.865	3.4679	5.439278
0.000895	0.019978	0.836017	0.001126	0.542525	2.312085	8.66E-05	3.61E-05	0.116659
0.191804	1.742734	0.558427	0.076587	0.479062	0.567994	0.210505	0.431198	0.284863
0.833975	2.491948	7.737807	7.061328	3.450577	2.006782	5.388405	0.729062	2.276407
4.402973	1.692763	3.005281	0.115014	1.68342	1.753341	0.04456	1.341825	0.488167
0.543398	6.135007	0.490375	0.800058	0.793918	5.395584	0.295491	0.365859	0.254769
0.000895	0.019978	3.644343	7.911934	2.89E-05	0.647345	4.212554	6.475505	6.107114
0.79487	4.684502	1.244242	1.061457	1.139885	3.363911	0.547778	0.691674	0.746984
0.753016	0.956667	0.534214	0.235613	0.426118	0.288413	0.06886	0.20733	0.09847
2.248896	0.312864	10.70538	11.83593	2.647244	0.018016	10.93101	1.438672	15.98892
0.244033	12.93651	0.604618	0.220274	2.31013	7.175397	0.376595	2.037114	0.523521
3.163506	0.403413	2.789466	9.063983	2.895599	0.753882	7.061328	1.341825	3.743232
0.244033	0.148701	0.001017	0.086075	0.086443	0.224214	0.008312	0.058139	0.010014
0.000895	0.019978	0.213615	0.001126	0.029639	0.202388	8.66E-05	0.16886	0.183672
0.117626	8.075772	0.05303	0.001126	0.124646	1.497143	8.66E-05	0.326017	0.000115
0.224055	0.2757	0.439776	1.815	1.613232	0.018016	2.74298	0.292597	0.847352
1.828781	45.91841	0.630823	0.048889	0.454632	25.99427	0.102792	0.259098	0.116659
17.15902	95.22939	31.1753	10.62739	53.77619	15.98892	14.25954	36.74522	28.23632
20.51271	1.410902	11.48368	4.789596	12.77772	4.597792	12.29473	11.30987	12.93651
0.833975	15.77678	0.73619	0.426645	0.686299	9.082828	0.357727	2.944416	1.74539
3.428788	0.812771	2.339697	5.037489	2.922778	0.7257	3.5257	3.249877	3.536955
0.000895	1.983824	0	0.001126	0.507403	6.727633	0.06886	3.61E-05	0.000115
0.41026	0.403413	0.244589	0.086075	0.426118	0.117518	0.180368	0.439524	0.262965
10.03313	0.019978	8.256465	6.760765	2.89E-05	2.62342	77.74812	11.65791	14.88584
1.910974	0.019978	2.449509	3.019848	2.823636	0.018016	4.353925	2.101075	2.628225
2.628225	1.278629	1.198701	0.449091	1.373348	1.106825	0.83697	1.919661	3.223882
1.916782	15.66023	4.776407	4.012049	4.546443	13.26964	2.434444	5.679502	1.74539
0.505267	0.019978	0.353175	0.533535	0.286522	0.018016	0.49974	0.508658	0.79487
2.483492	0.019978	1.29342	5.967879	0.286522	0.018016	3.763045	0.922338	3.065108
1.507835	0.019978	5.576219	22.74797	3.957734	0.018016	8.195657	3.4679	13.15079
0.654423	0.542828	0.070065	0.057691	0.235613	0.510707	0.026364	0.140188	0.157056
53.77619	0.019978	144.7919	62.33472	72.69473	0.018016	67.98069	67.98069	95.22939
0.885137	12.7427	4.193232	0.811861	2.950173	4.174964	0.481414	6.135007	1.176623
6.404921	0.019978	3.307186	0.596111	6.475505	0.018016	1.271876	2.718434	2.011501

0.885137	1.568564	0.946638	0.001126	1.623232	3.002576	8.66E-05	0.34619	1.113961
3.55583	0.69119	6.508427	1.794531	5.130411	0.647345	1.305772	11.36975	0.909856
2.119596	0.312864	1.007244	1.889711	2.08632	0.018016	2.77899	1.145426	2.967453
2.263377	0.019978	22.32214	19.22139	5.545729	0.018016	37.81919	5.024293	7.991602
56.39528	0.019978	40.11561	224.9555	50.55949	0.018016	100.0998	135.5245	75.1132
3.507691	11.89932	0	0.001126	3.025671	4.091667	8.66E-05	3.61E-05	0.000115
2.270837	1.539062	0.310866	0.324719	1.986681	0.676999	0.349084	1.50026	0.715815
1.867951	0.746551	1.326667	0.001126	1.890996	5.796421	0.06886	0.406681	0.37417
0.000895	1.842734	0.002143	0.076587	0.036089	1.129452	0.019048	0.017576	0.044214
1.96697	1.384921	0.907273	0.001126	1.002215	0.647345	0.217792	3.169812	1.078153
0.256652	0.956667	0.299199	0.310447	0.639892	1.521371	0.06886	0.025339	0.12772
0.000895	2.871212	26.52537	77.74812	2.89E-05	1.812114	50.55949	7.136537	21.93452
0.916869	0.768535	3.095166	12.56693	1.578687	0.018016	5.366335	1.191198	12.36443
0.36267	1.056898	0.065152	0.457431	0.208867	1.106825	0.164877	0.476335	0.451429
1.139221	9.968355	1.040411	1.340036	1.219856	1.845519	1.381111	1.738456	0.968066
5.976724	0.019978	5.967879	2.212619	3.137576	0.018016	2.349351	0.922338	2.489762
0.438831	14.17388	4.039192	1.68671	2.31013	45.91841	1.889711	5.959033	2.199639
1.03303	3.23987	0.630823	0.668947	0.69583	5.030866	0.669358	0.806227	0.79487
0.933261	6.853608	0.328543	0.580123	1.271097	17.85355	0.449726	1.121017	0.554726
0.614935	1.944517	1.229553	0.474856	1.633752	2.513752	0.334444	0.567994	0.909856
0.959567	0.2757	1.421126	17.92188	1.162201	0.18324	4.188896	0.96627	9.625253
2.666494	9.316241	0.822965	0.001126	0.172612	4.130794	0.217792	1.209776	0.088514
4.302367	0.019978	34.84299	13.83359	3.91355	0.018016	16.22108	16.34294	5.530274
0.033874	1.624459	0.00811	0.220274	0.042807	0.906126	0.026364	0.017576	0.034906
3.052655	31.55417	0.843102	0.132107	0.573326	11.14619	0.130714	3.016984	1.584105
9.968355	0.019978	6.079596	2.040606	4.921082	0.018016	3.618499	4.058514	4.312251
0.071183	1.365296	0.120361	0.228203	0.130606	2.153535	0.156941	0.041328	0.034906
21.64437	1.539062	33.4985	32.68313	20.85876	1.96697	26.80355	17.92188	18.62711
5.704646	0.906825	2.715873	4.851227	2.834084	0.876118	7.112496	3.971061	6.036818
5.728615	0.019978	1.696494	0.616407	6.772763	0.018016	0.82772	0.798153	1.708932
0.056782	1.127475	0.106732	0.001126	0.489235	1.554185	0.078831	0.34619	0.138644
2.292864	16.5625	5.545729	5.838499	6.191515	24.73724	9.924372	10.1459	11.96574
1.547734	0.019978	4.575123	7.710361	0.648038	0.018016	2.973059	3.743232	11.30987
203.5795	32.68313	135.5245	48.53429	105.1694	252.4251	37.29684	83.489	224.9555
0.224055	7.668579	0.09176	0.159942	0.061025	1.434055	0.094553	0.269697	0.311876
6.464906	75.1132	2.449509	4.978182	2.037114	10.48101	4.261342	1.006025	14.97762
14.08569	1.692763	21.36892	29.18885	20.68746	4.402973	18.48514	10.5063	27.10502
0.000895	0.019978	0.127273	0.076587	0.042807	0.018016	8.66E-05	0.041328	0.12772
0.000895	0.238889	0.025483	0.001126	0.036089	0.54925	8.66E-05	0.007395	0.000115
3.450577	0.019978	3.195541	4.029654	1.724805	0.018016	2.174733	0.979149	1.791616
1.284747	2.577157	16.80343	34.84299	3.195541	0.592532	19.83727	22.96463	21.19547
0.000895	0.312864	0.004964	0.032431	0.011573	0.617071	8.66E-05	0.033261	0.000115
0.000895	0.019978	0.499206	0.713405	0.61526	0.018016	1.062821	0.656392	1.21215
0.428456	1.842734	0.684033	0.523983	0.371075	2.365685	0.086991	0.326017	0.208867
0.959567	1.34513	0.080895	0.066883	0.311227	0.876118	0.156941	0.158766	0.025087
9.031082	2.554069	5.037489	2.378124	10.67991	1.082915	2.947287	5.238701	8.381328
1.447994	3.934986	0.304733	0.465801	1.603795	3.548153	0.210505	1.422316	0.498355
5.515584	3.885642	5.493182	10.43066	1.510014	1.96697	6.99026	5.366335	6.365065
38.94854	2.600014	23.82354	33.93939	87.1746	3.125844	24.73724	25.48179	111.5439
0.233175	4.557605	0	0.001126	2.89E-05	1.728615	0.060022	0.058139	0.000115
0.152114	4.808658	0.002143	0.140188	0.136479	1.434055	0.019048	0.106205	0.034906
17.39935	11.42512	31.55417	23.82354	24.73724	10.85542	43.49658	35.27608	26.25557
5.34474	1.65785	2.084791	2.160649	2.621133	1.315613	1.164257	2.339697	0.883665
0.181797	0.292597	0.120361	0.169971	0.317229	1.061457	0.052525	0.305087	0.053391
0.286522	53.77619	1.069574	0.384156	1.415469	1.554185	0.409336	2.535974	1.222958
0.172179	35.776	0.31702	0.220274	1.156869	8.461703	0.116825	0.357475	0.459769
1.605859	40.11561	6.622092	7.363117	1.839986	22.13245	1.541227	6.355224	4.365224
0.516825	2.230859	0.360462	0.076587	0.335786	3.303925	0.180368	0.532092	0.302785

45.91841	48.53429	34.38341	8.119004	33.93939	65.94551	11.48368	53.77619	25.11292
0.129553	0.382208	0.001017	0.124098	0.015714	0.7257	0.008312	0.066205	0.034906
0.602554	0.611003	1.505678	2.223636	0.590303	1.697792	3.377619	2.373824	2.59557
0.319762	0.563824	0	0.001126	0.124646	0.18324	0.008312	0.033261	0.025087
3.792266	59.32013	0.788896	0.842641	2.27254	30.49289	0.295491	1.064935	0.573326
15.66023	19.451	5.942294	2.964762	22.74797	6.365065	3.66785	4.115844	6.956349
2.730072	7.92518	2.365685	0.934488	2.254084	15.77678	1.351573	5.454949	2.099524
0.087973	2.800332	0.106732	0.095433	0.565714	0.7257	0.04456	0.041328	0.016133
8.711905	0.019978	10.70538	2.895599	8.849697	0.018016	12.03267	5.923838	2.382035
0.726133	3.207807	1.056898	1.872049	0.917431	2.006782	1.819221	2.554069	0.803665
0.916869	17.03919	0.69583	0.076587	0.679812	12.13485	0.052525	0.620909	0.12772
15.88548	2.463911	9.160382	10.73084	2.511465	2.312085	13.96064	11.74734	28.54786
2.868456	0.019978	1.614257	0.587965	1.179885	0.018016	2.77899	1.226934	2.947287
1.013434	0.019978	1.34798	5.337547	0.454632	1.037958	2.524892	3.61E-05	2.050404
0.117626	8.608153	0.280967	0.179177	0.197727	7.38847	0.28443	1.02487	0.208867
41.47925	24.98218	65.94551	29.49693	21.02638	18.76954	33.4985	37.81919	24.04374
3.303925	3.738997	10.93101	12.85504	3.008225	3.207807	10.33476	6.772763	4.764055
0.446551	0.713846	0.20215	0.066883	0.165981	2.54961	0.148846	0.396306	0.284863
12.89577	30.65783	5.062626	2.540462	9.968355	19.68088	4.048499	4.927302	3.450577
83.489	0.663276	37.29684	75.1132	25.73165	0.247417	42.75576	23.82354	65.94551
0.695426	0.585592	2.477027	0.196955	1.179885	4.130794	2.696061	3.944084	1.03246
0.899567	0.238889	0.427937	0.353492	0.358442	0.457114	0.250476	0.521068	0.582973
13.23022	2.331825	3.588369	0.541522	16.22108	3.330909	1.031284	4.990794	2.529307
0.09746	0.019978	6.045209	27.66779	8.048095	4.261342	70.11932	56.39528	0.246162
1.737294	0.019978	1.414574	7.162698	4.626025	0.018016	5.515584	10.28573	5.056421
5.01088	5.431544	6.674286	2.386248	5.889798	12.42965	3.783694	3.028629	2.544747
0.781977	13.91714	1.198701	0.724004	3.695815	10.35839	0.473853	2.153535	1.015967
0.071183	1.106825	0.016104	0.132107	0.015714	0.806227	0.04456	0.017576	0.010014
38.39042	14.25954	14.6058	13.23022	9.179466	11.65791	9.502294	17.78522	21.02638
11.36975	0.257518	7.668579	5.381227	2.876421	0.485325	5.238701	5.872886	9.101674
2.427792	0.292597	3.540606	3.533211	1.405772	0.018016	1.455988	3.500808	6.316328
8.573434	1.256558	4.048499	3.411097	9.815642	0.384156	4.135801	7.545584	5.770996
8.608153	0.585592	4.473348	2.715873	3.442886	0.510707	4.370671	4.012049	4.529913
0.329798	1.916782	0.558427	0.179177	0.203593	1.632648	0.130714	0.466602	0.21632
1.798535	3.799885	0.080895	0.159942	1.139885	2.483492	0.094553	0.091977	0.044214
0.564127	1.181342	0.499206	1.146962	0.405346	1.90798	0.49974	0.567994	1.463304
1.067662	3.207807	1.326667	0.888485	4.370671	4.016465	0.619012	3.005281	0.603261
0.543398	2.871212	0.534214	0.105108	1.396494	2.006782	0.139697	3.303925	0.340866
5.728615	2.065007	37.81919	65.94551	20.16541	0.384156	57.84882	15.276	31.92385
1.085224	0.337222	1.451775	6.738211	3.5257	0.018016	6.230245	2.037114	1.880375
1.253088	0.403413	1.852338	0.513752	2.083261	1.469163	1.229553	1.145426	0.466335
0.341241	27.10502	4.217569	2.993867	3.552049	42.75576	1.528975	3.771349	1.387612
0.764704	2.895599	1.466111	0.124098	2.073232	9.459177	0.760866	4.266753	2.065007
4.23202	17.78522	1.229553	1.860491	2.164113	8.994791	1.728615	7.824473	8.477662
25.48179	43.49658	10.23768	4.851227	10.1459	29.18885	6.210375	19.29639	13.83359
2.54961	0.257518	6.153102	9.19899	4.586753	8.303947	55.04081	9.968355	12.1974
1.043608	10.62739	4.715599	31.55417	7.38847	4.990794	6.423911	3.201457	10.73084
1.845519	2.089639	3.6029	2.50469	5.704646	0.676999	1.53684	1.145426	1.280274
6.99026	0.522071	4.575123	5.993045	7.468961	0.699192	5.538001	2.373824	5.01772
0.849841	2.554069	2.890245	0.549661	5.662994	3.065108	1.041025	6.079596	0.917431
0.341241	1.944517	0.569293	0.159942	0.39062	1.728615	0.466869	4.519048	0.192929
0.087973	0.956667	0.016104	0.057691	0.029639	0.906126	0.04456	0.007395	0.044214
4.857403	0.663276	4.067547	2.74298	6.325945	0.224214	5.821804	3.397706	3.450577
12.63773	9.52197	32.68313	9.946364	26.52537	4.739668	10.82954	21.93452	9.968355
0.026876	0.382208	0.016104	0.032431	0.00303	0.117518	8.66E-05	0.003535	0.010014
0.071183	2.638139	0.047908	0.001126	0.00811	0.617071	0.102792	0.747879	0.000115
65.94551	33.4985	33.07147	15.77678	83.489	40.11561	12.70768	26.66446	40.7932
0.401385	1.568564	0.996111	0.179177	0.172612	2.312085	0.607273	1.037316	0.76

0.064127	3.544257	0.020678	0.057691	0.029639	2.312085	8.66E-05	0.033261	0.053391
30.49289	67.98069	10.8813	13.53913	30.17192	43.49658	8.477662	11.03843	29.18885
2.292864	3.340361	3.730808	1.983824	5.950664	3.980123	2.296732	2.217951	5.309264
0.033874	1.783203	0.070065	0.048889	0.036089	3.66785	0.06886	0.091977	0.025087
0.968723	3.13456	0.150346	0.124098	0.405346	10.85542	0.116825	0.177734	0.107027
0.09746	5.508131	0.427937	0.095433	0.124646	2.807937	0.303896	0.280967	0.498355
60.77449	167.9996	33.93939	18.76954	57.84882	105.1694	24.50942	118.7028	57.84882
52.56128	4.519048	2.947287	2.597792	21.54823	4.952915	2.003723	7.375794	3.632973
16.04187	22.52375	9.924372	5.056421	15.41187	24.2842	6.706472	13.19051	8.83241
0.531169	0.542828	0.743283	1.733766	2.353247	0.018016	3.150433	1.903636	1.927042
77.74812	0.4457	105.1694	100.0998	30.82277	10.35839	91.05541	287.197	203.5795
16.04187	9.707677	5.704646	6.268312	19.53061	9.082828	6.601833	9.082828	13.00996
1.228651	7.854069	1.688045	1.483622	2.185556	4.535354	0.813261	2.630729	3.55583
0.753016	2.638139	1.578687	0.115014	0.859351	8.923117	0.777864	4.236962	0.262965
1.386681	28.23632	2.74298	1.137244	1.992944	13.00996	1.29342	3.957734	0.382626
9.838658	8.381328	4.524481	1.712706	7.495397	1.877605	3.734762	7.013853	4.419062
42.75576	144.7919	16.22108	5.123781	33.07147	91.05541	1.561912	10.57984	9.335397
4.614618	10.55571	0.05886	0.196955	1.551962	1.106825	8.66E-05	0.140188	0.116659
1.414574	12.49452	1.306667	1.582915	0.565714	11.544	1.069574	5.110159	7.737807
26.80355	111.5439	10.1459	27.96248	21.36892	95.22939	17.78522	64.12971	21.36892
135.5245	1.127475	46.7056	8.625613	47.5018	6.99026	27.38726	77.74812	32.31123
3.792266	8.477662	2.114437	1.104805	10.43066	5.337547	1.248232	11.09133	0.999502
11.57341	20.68746	8.119004	4.353925	16.85982	22.96463	6.967547	15.18102	16.44768
8.27267	0.019978	4.678846	0.396638	2.737944	0.018016	1.528975	2.169509	1.889711
5.395584	1.692763	18.62711	47.5018	17.15902	9.946364	28.54786	14.17388	12.49452
1.067662	0.019978	2.210866	0.196955	0.847908	1.96697	3.188918	6.622092	0.803665
0.000895	0.019978	4.575123	5.788153	1.128131	0.018016	7.175397	2.31013	6.695887
4.758088	1.181342	35.776	35.27608	20.51271	1.581912	65.94551	31.1753	20.51271
0.224055	4.241905	0.05303	0.140188	0.136479	9.52197	8.66E-05	0.041328	0.034906
0.916869	1.880375	0.763102	0.706494	0.867814	1.497143	0.547778	0.598456	0.382626
0.933261	1.486551	3.23987	8.849697	4.23202	0.457114	8.573434	7.099913	5.123781
23.6012	0.175592	0.710722	0.63754	4.419062	0.133045	0.547778	7.417359	0.36267
1.585296	2.763261	0.304733	0.095433	0.942294	13.15079	0.309062	1.477388	0.332886
5.599228	1.20824	4.376118	2.287576	2.380079	0.617071	3.439437	3.269913	3.574387
0.000895	0.362179	0.002143	0.032431	0.024733	0.384156	8.66E-05	0.033261	0.000115
55.04081	23.06543	14.51648	5.150693	13.53913	27.10502	6.079596	19.06893	11.11876
87331.38	530.1465	287.197	35.776	87331.38	332.0776	34.38341	87331.38	48.53429
167.9996	203.5795	530.1465	105.1694	252.4251	203.5795	155.7103	749.7839	72.69473
0.643427	5.01772	0.817013	0.159942	1.373348	2.269134	0.274877	0.816479	0.293882
34.84299	14.79742	13.30906	40.7932	27.66779	6.464906	31.1753	19.99861	44.27012
0.741457	0.403413	0.31702	0.220274	1.334762	0.567994	0.04456	0.90772	0.107027
5.11697	7.810058	2.563506	4.397792	5.351934	7.339452	2.408882	2.047157	1.997352
0.352597	4.580938	0.517359	0.179177	0.599134	17.64869	0.156941	0.233175	0.116659
0.152114	0.238889	0.639113	0.057691	0.214502	0.133045	0.148846	1.014784	0.078211
0.033874	1.030022	0.002143	0.001126	0.049336	1.521371	8.66E-05	3.61E-05	0.016133
4.715599	3.104394	5.34474	0.774257	2.150303	6.135007	2.961768	14.88584	3.310758
6.632063	0.221962	14.42661	5.576219	14.00414	28.23632	35.776	15.46193	5.381227
0.048889	1.443059	0.086667	0.001126	0.019942	0.842193	0.019048	0.017576	0.078211
44.27012	8.149307	43.49658	36.74522	25.48179	19.37139	31.92385	155.7103	60.77449
0.492165	0.746551	0.01153	0.001126	0.015714	0.384156	8.66E-05	0.083608	0.034906
11.4544	0.019978	19.68088	5.704646	19.68088	0.806227	14.33939	42.08807	22.74797
0.000895	0.873579	0.070065	0.001126	0.042807	0.018016	8.66E-05	0.16886	0.06254
1.067662	1.152165	0.957273	1.661328	0.893528	7.037345	1.202626	0.816479	1.997352
0.000895	2.112843	0.100483	0.001126	0.500722	0.567994	0.035079	0.521068	0.107027
0.041782	1.624459	0.004964	0.040469	0.011573	1.037958	8.66E-05	0.003535	0.000115
0.531169	4.733824	1.108276	0.066883	1.050231	3.47531	0.130714	3.5257	0.208867
16.5625	30.65783	13.60866	15.07257	16.09482	32.31123	20.51271	34.84299	36.74522
4.758088	87.1746	6.404921	5.485685	5.030866	24.98218	6.365065	5.606739	7.443276

19.83727	14.08569	5.082063	3.55961	10.1028	4.796126	3.310758	13.87537	6.749488
3.640606	11.36975	4.251818	2.661587	4.286962	17.39935	3.367071	6.171789	5.754935
2.292864	0.019978	5.622872	1.896667	1.545657	0.430837	4.47912	3.500808	1.152165
0.033874	0.426392	0.025483	0.001126	0.005007	0.533196	8.66E-05	0.012063	0.000115
35.27608	36.74522	8.539971	3.877417	13.45939	14.08569	5.591717	14.97762	9.377345
19.06893	10.78056	12.85504	30.82277	17.03919	7.977958	12.22798	7.570693	23.6012
0.181797	7.264502	0.907273	0.001126	2.27254	5.447114	0.086991	1.477388	0.224372
2.278254	0.463867	0.773766	2.417424	2.061926	0.54925	2.45772	1.550837	2.143261
1.595729	6.036818	1.150635	0.888485	0.885664	1.521371	0.533535	0.521068	1.359177
11.86763	10.1028	11.14619	3.7586	12.16681	15.02509	5.485685	9.19899	6.632063
1.516219	1.084437	1.159149	0.169971	1.072843	2.758088	0.760866	2.185556	0.231768
2.483492	1.056898	6.784762	1.614257	3.435988	3.442886	2.511465	11.25392	8.888167
11.4544	49.50903	10.01009	11.25392	15.18102	56.39528	10.19468	19.53061	8.83241
10.57984	0.542828	55.04081	13.6814	7.558139	7.897078	21.02638	15.07257	18.05488
0.162468	0.019978	0.328543	0.086075	0.055671	10.1028	8.66E-05	0.041328	0.016133
21.64437	1.127475	10.82954	10.38202	6.171789	1.217475	13.45939	10.85542	30.49289
0.181797	1.256558	0.180895	0.066883	0.286522	0.842193	0.060022	0.083608	0.034906
14.6058	126.1556	13.00996	2.363442	31.55417	77.74812	1.901068	42.75576	2.099524
0.551573	3.91355	0.180895	0.179177	0.511977	4.402973	0.250476	0.187229	0.340866
4.808658	0.746551	2.131176	4.323045	1.518232	1.90798	7.765931	2.429957	14.33939
1.076652	1.084437	0.404408	0.507085	0.365224	5.137042	0.365224	0.106205	0.573326
1.228651	0.019978	4.160238	2.547179	1.848297	0.54925	0.890065	1.662439	0.746984
2.483492	20.16541	24.04374	4.46267	4.56912	38.39042	5.9071	13.6814	7.625209
0.478737	1.842734	0.20215	0.066883	0.136479	0.35798	0.094553	0.280967	0.025087
30.82277	7.238023	4.236962	4.614618	9.418196	0.202388	6.55039	20.85876	16.22108
3.38456	4.241905	5.273362	17.03919	3.799885	4.47912	16.68554	5.855281	19.37139
0.129553	0.019978	0.042655	0.048889	0.015714	0.018016	8.66E-05	0.106205	0.078211
9.377345	15.46193	5.273362	2.138131	7.013853	4.796126	3.422237	4.529913	2.502619
1.215981	9.082828	1.326667	0.831472	1.705007	6.830592	0.607273	1.632648	1.32482
0.505267	0.257518	0.20759	0.124098	0.117843	0.334805	0.706053	0.292597	0.238478
0.968723	0.019978	2.477027	11.544	0.454632	0.018016	1.988196	0.21632	2.931068
2.320072	0.873579	1.644416	0.30158	0.679812	0.334805	0.365224	0.90772	1.863506
29.82828	25.24367	3.779452	0.876118	4.546443	35.05953	5.172828	7.765931	6.717056
4.125786	80.6518	14.17388	1.061457	3.971061	287.197	13.96064	28.87506	2.513752
1.845519	1.23127	1.103261	0.596111	1.489524	1.252424	0.728088	1.081544	1.049517
0.000895	44.27012	0.030613	0.507085	0.049336	72.69473	0.210505	0.049769	0.000115
0.319762	2.638139	1.007244	0.774257	0.352222	6.922771	0.116825	0.13114	0.702835
1.798535	0.426392	2.807937	0.391421	1.479452	2.269134	0.396472	0.53912	0.942294
0.041782	0.175592	0.004964	0.040469	0.019942	0.133045	0.012864	0.13114	0.06254
0.602554	3.464315	0.630823	0.001126	1.36619	0.938911	8.66E-05	0.922338	0.201789
0.654423	2.408882	0.20759	0.449091	0.813788	3.47531	0.217792	1.270317	0.582973
0.302352	0.148701	0.004964	0.086075	0.442496	0.18324	0.026364	0.083608	0.000115
8.075772	0.238889	1.633752	0.262525	1.323975	0.117518	0.901212	1.226934	1.261407
0.152114	0.956667	1.175859	0.066883	0.186017	7.225469	8.66E-05	0.747879	0.293882
0.048889	0.463867	0.00811	0.048889	0.061025	0.592532	0.019048	0.007395	0.044214
0.129553	1.322092	0.143333	0.149654	0.256941	4.851227	0.139697	0.158766	0.350635
2.628225	0.403413	1.819221	1.423506	2.380079	0.202388	1.919661	2.879228	2.740519
2.483492	1.842734	1.414574	2.770866	0.672857	1.434055	3.489899	0.715462	1.204228
2.765758	0.019978	44.27012	72.69473	3.855952	0.018016	62.33472	9.751039	23.82354
2.569957	0.728543	0.65798	1.497143	3.273045	0.133045	2.270837	0.620909	7.315512
50.55949	1.384921	26.25557	52.56128	51.52195	0.876118	46.7056	13.45939	144.7919
0.392251	0.502843	0.106732	0.095433	0.371075	0.224214	0.019048	0.114235	0.138644
0.203333	1.758615	0.310866	0.228203	0.559632	0.806227	0.196551	0.645455	0.254769
0.630505	7.507619	1.272756	1.909408	2.392431	5.9071	0.61355	1.370765	0.473319
0.456248	5.295325	2.477027	0.915195	3.06803	18.41211	0.457431	6.911486	1.346003
0.224055	1.365296	0.505786	9.141299	0.286522	0.018016	0.953506	0.13114	8.210938
0.033874	0.426392	0	0.001126	2.89E-05	0.247417	0.012864	3.61E-05	0.010014
2.826277	0.337222	2.306277	0.851659	1.697792	0.972504	2.565556	2.572309	1.165065

0.591616	0.019978	1.314675	4.003117	0.908773	0.018016	1.78759	0.729062	3.962179
0.000895	0.019978	3.663622	4.222583	2.89E-05	0.018016	2.876421	14.08569	3.471486
0.108124	4.808658	0.037186	0.196955	2.89E-05	1.129452	0.094553	0.091977	0.000115
11.96574	0.713846	9.297085	3.980123	10.78056	4.174964	3.842554	6.8421	5.137042
2.54961	0.019978	3.796075	4.770231	6.011515	0.018016	10.1459	5.720173	4.277006
0.684401	3.370527	1.244242	0.441053	1.359177	3.877417	0.256429	1.25816	0.37417
1.857677	1.278629	0.239885	1.742734	0.752583	1.152165	0.342215	0.177734	1.453939
0.079531	8.033932	0.370657	0.420707	0.186017	6.727633	0.148846	0.53912	0.340866
1.139221	6.738211	3.034747	2.429957	1.751948	5.030866	2.425801	2.718434	4.900996
1.127475	0.019978	3.8121	5.137042	1.396494	0.018016	2.003723	1.386681	2.847547
87.1746	0.69119	17.78522	8.711905	42.08807	2.106089	20.68746	32.11754	36.26061
0.899567	1.758615	0.415166	0.698297	1.167561	1.217475	0.633499	1.050231	0.523521
0.026876	0.134517	0.016104	0.040469	0.015714	0.311227	0.008312	0.017576	0.000115
0.79487	2.518124	1.408831	0.318124	0.500722	2.196017	0.595368	0.729062	0.513752
0.684401	0.663276	0	41.47925	0.426118	0.288413	36.26061	3.61E-05	4.277006
0.000895	0.019978	0.250476	2.050404	0.639892	0.334805	4.203001	3.61E-05	1.040411
10.62739	2.723247	13.83359	15.56131	11.83593	3.034747	18.33909	10.19468	16.80343
16.9162	0.019978	27.66779	36.26061	13.6814	0.018016	24.98218	14.51648	13.53913
1.307417	2.463911	0.360462	0.095433	0.917431	0.288413	0.060022	0.521068	0.311876
18.05488	0.019978	1.969733	19.53061	8.590794	0.018016	26.25557	5.591717	27.66779
0.564127	0.019978	2.216162	12.22798	0.229199	0.018016	5.34474	3.824964	1.409942
1.139221	9.082828	3.011169	4.490801	3.256587	33.07147	3.450577	3.005281	4.053506
1.189769	4.933781	0.1379	0.286364	1.23127	4.650007	0.156941	0.839365	0.293882
0.267597	2.331825	0.20759	0.269892	0.249278	2.868456	0.078831	0.292597	0.340866
1.644416	2.914632	7.061328	14.08569	7.112496	1.182771	2.040606	3.799885	10.57984
2.765758	4.007583	0.505786	0.568369	3.425512	1.604827	0.633499	1.855108	0.672468
0.675115	0.019978	0.484423	0.724004	0.724004	0.018016	1.143954	0.417063	0.473319
0.808983	0.812771	0.397763	0.718355	1.219856	0.288413	0.309062	0.670144	0.854242
27.38726	3.610707	19.37139	33.4985	15.88548	5.745996	17.03919	13.87537	29.82828
7.6393	0.4457	6.316328	2.810678	9.751039	0.842193	3.06803	0.715462	5.037489
1.992944	0.663276	0.890065	0.956667	2.327857	0.430837	0.711046	0.447085	1.113961
0.753016	0.019978	8.508514	0.549661	1.805166	0.018016	24.2842	3.269913	1.622006
0.741457	0.019978	1.360022	1.700354	0.256941	0.018016	1.25886	0.357475	0.657547
6.116638	0.019978	1.702706	3.422237	3.610707	0.018016	2.66404	1.940231	9.256537
3.655714	0.257518	3.6029	1.497143	2.567605	0.410541	1.152165	2.611797	2.863211
0.129553	1.298665	0.004964	0.001126	0.00811	1.217475	0.052525	0.025339	0.06254
1.886335	0.362179	0.604618	0.558095	0.847908	0.18324	0.669358	0.715462	0.73961
0.579495	5.295325	1.192633	0.698297	1.323975	3.065108	0.060022	4.150274	1.218182
21.02638	0.542828	21.02638	7.136537	27.96248	1.106825	8.711905	21.02638	19.99861
0.256652	51.52195	0	0.001126	2.89E-05	29.49693	0.094553	0.058139	0.138644
0.543398	8.780115	0.625209	1.104805	0.752583	5.704646	0.518413	0.406681	0.968066
1.265426	0.019978	0.434069	2.197828	0.565714	0.018016	2.823636	0.788896	2.720996
32.31123	0.019978	3.50425	6.404921	5.273362	0.018016	15.56131	4.696313	10.23768
0.467951	0.175592	0.264242	0.205382	0.148016	0.247417	0.04456	0.177734	0.21632
1.043608	0.019978	1.991328	7.443276	1.026219	0.510707	5.095325	0.140188	1.817828
0.000895	0.019978	0.004964	0.001126	0.00303	0.018016	0.008312	3.61E-05	0.000115
0.753016	0.843102	0.150346	0.235613	0.733593	0.384156	0.376595	0.466602	0.322237
5.088694	0.019978	0.933261	1.860491	2.08013	0.018016	3.022713	1.462143	2.259661
0.172179	2.800332	1.219019	0.338658	1.219856	3.404372	1.248232	2.094574	0.82772
0.579495	6.945152	3.034747	0.596111	1.109726	6.674286	0.82772	2.325931	0.36267
0.000895	0.162576	0	0.001126	2.89E-05	1.753341	8.66E-05	0.025339	0.025087
332.0776	0.019978	24.98218	42.75576	2.89E-05	0.018016	184.443	41.47925	749.7839
0.885137	0.728543	0.933261	2.863211	1.87899	0.647345	3.043528	1.90798	1.969733
0.172179	0.611003	0.080895	0.310447	0.607605	2.807937	0.123102	0.292597	0.016133
0.467951	77.74812	6.423911	1.601443	7.668579	87.1746	0.912987	4.38684	5.180058
6.079596	0.019978	1.831631	2.895599	2.453781	0.018016	5.47	2.494177	3.989206
64.12971	0.238889	47.5018	21.19547	34.38341	0.676999	33.07147	25.99427	41.47925
0.833975	3.207807	0.180895	0.001126	0.653586	9.946364	8.66E-05	0.280967	0.078211

0.000895	0.175592	0	0.971775	0.00811	0.133045	0.256429	3.61E-05	0.107027
1.901068	3.207807	0.274365	0.706494	4.863579	10.70538	0.547778	4.715599	0.36267
2.159004	0.522071	4.429769	1.365296	2.500548	1.779423	3.775209	2.579509	0.723506
2.765758	4.484892	0	0.001126	0.599134	0.267951	8.66E-05	0.280967	0.000115
0.056782	0.162576	0.01153	0.001126	0.011573	0.247417	8.66E-05	0.017576	0.025087
51.52195	0.019978	51.52195	42.08807	6.99026	0.018016	87.1746	4.863579	42.75576
7.188095	0.663276	7.839271	6.784762	8.303947	0.384156	5.630815	0.944784	4.49671
0.341241	5.165599	0.360462	0.720938	0.265224	1.697792	8.66E-05	0.041328	0.772157
10.03313	0.221962	3.35355	1.757244	6.268312	0.384156	2.828918	3.614603	5.838499
0.244033	5.553146	1.29342	1.171349	0.69583	10.1459	0.457431	2.269134	1.247244
4.277006	50.55949	10.78056	3.640606	6.695887	60.77449	4.338629	8.493088	11.39744
0.382208	0.257518	2.661587	1.666089	1.010289	2.365685	4.150274	1.355527	0.554726
0.071183	1.181342	0.086667	0.105108	0.091833	1.378362	0.052525	0.033261	0.034906
0.000895	0.019978	0	0.001126	0.00811	0.018016	8.66E-05	3.61E-05	0.000115
1.96697	2.723247	0.630823	0.368038	1.559675	1.182771	0.737403	0.992201	0.603261
17.64869	7.037345	16.68554	5.720173	44.27012	5.881342	8.625613	19.29639	5.42381
0.41026	0.812771	0.65798	0.568369	0.522071	0.533196	0.180368	0.670144	0.224372
10.7557	20.51271	1.629235	0.457431	6.153102	14.42661	0.06886	3.574387	0.473319
0.867814	0.019978	0.817013	1.700354	1.043608	0.133045	1.842734	0.598456	1.204228
2.406768	29.18885	3.083319	5.409437	5.9071	27.66779	2.597792	3.442886	5.082063
7.136537	0.019978	3.750837	3.019848	0.500722	0.018016	53.77619	2.953059	6.876392
0.000895	1.842734	0.639113	0.205382	0.323146	2.62342	8.66E-05	0.21632	0.116659
0.048889	0.746551	0	0.076587	2.89E-05	0.117518	0.019048	3.61E-05	0.016133
1.307417	2.203535	4.94026	0.212698	3.283139	1.350772	0.619012	4.552092	1.236248
0.000895	0.932078	0.042655	0.001126	0.005007	0.18324	0.019048	0.058139	0.044214
0.630505	1.723579	0.1379	0.105108	0.793918	1.934271	0.123102	0.521068	0.044214
118.7028	20.34063	52.56128	18.62711	80.6518	50.55949	18.62711	20.34063	31.55417
0.286522	3.13456	0.479401	0.196955	0.235613	3.23987	8.66E-05	0.187229	0.428932
0.026876	0.585592	0	0.032431	0.00811	0.334805	0.012864	0.007395	0.000115
1.876212	1.692763	1.905094	4.473348	0.992771	1.697792	2.320072	1.50026	0.76
0.000895	5.950664	0.353175	0.001126	3.514834	0.018016	8.66E-05	7.854069	0.000115
0.000895	0.69119	2.114437	5.614805	2.89E-05	0.617071	2.90658	2.520404	11.57341
4.875527	0.019978	7.882222	14.51648	4.597792	0.018016	8.348817	0.025339	8.348817
1.242641	0.019978	0.375743	0.929812	0.420498	0.133045	0.679812	0.456248	0.681494
0.27759	0.019978	2.789466	2.784228	2.214372	0.018016	1.849654	2.325931	1.927042
0.419365	6.443925	0.569293	0.001126	1.080909	5.967879	0.156941	2.153535	0.582973
3.629177	0.812771	3.227085	0.001126	1.938759	0.410541	0.349084	3.182482	0.116659
0.000895	3.829105	0.00811	0.076587	0.091833	0.617071	8.66E-05	0.187229	0.000115
2.647244	21.93452	4.193232	2.628225	5.622872	9.646284	1.455988	3.169812	3.775209
1.253088	0.019978	2.345527	1.723579	1.484553	0.018016	0.324286	0.882078	0.84
1.043608	2.463911	0.381999	0.001126	1.253752	6.045209	8.66E-05	0.806227	0.350635
2.205476	1.181342	7.339452	2.936739	11.03843	0.107157	3.718716	2.737944	1.261407
0.000895	0.482706	0.00811	0.105108	0.036089	0.430837	8.66E-05	0.066205	0.025087
7.136537	0.019978	13.38418	4.637994	25.99427	0.018016	9.541645	6.30671	6.423911
0.117626	0.403413	0.004964	0.115014	0.005007	0.018016	0.078831	0.007395	0.053391
0.916869	2.065007	7.025599	4.130794	3.599329	1.469163	5.80469	1.844098	0.76
8.149307	2.463911	2.216162	0.527821	12.46209	2.269134	0.872013	0.9529	1.040411
10.33476	0.019978	3.829105	1.296905	13.60866	0.018016	1.493968	0.620909	1.514199
0.591616	0.337222	0.257143	0.124098	0.214502	2.051962	0.110058	0.292597	0.09847
7.037345	0.019978	7.08733	10.28573	6.560411	0.018016	6.00228	1.748016	5.110159
0.684401	9.707677	1.122359	0.277417	4.012049	5.137042	0.88158	5.381227	1.03246
7.868146	0.019978	27.96248	22.52375	19.37139	0.018016	18.05488	3.743232	21.54823
12.70768	3.077316	9.751039	10.48101	7.225469	0.938911	11.544	15.98892	15.07257
0.203333	2.432121	0.257143	0.001126	2.229048	0.842193	0.188009	1.700354	0.473319
0.382208	9.397771	0.191804	0.245772	1.437648	3.169812	0.533535	0.861934	0.231768
0.172179	5.614805	0.030613	0.115014	0.821414	1.934271	0.188009	0.63114	0.09847
10.53159	0.019978	2.638139	9.880779	1.906551	1.434055	9.459177	1.711414	4.484892
0.666097	0.019978	2.852677	1.017107	1.71518	0.018016	2.119596	2.355289	1.889711

16.22108	3.425512	21.54823	1.974228	19.06893	1.604827	5.01772	6.287561	6.853608
0.456248	7.124517	1.015967	0.541522	1.648066	5.137042	0.365224	1.330029	0.254769
5.47	6.39474	1.491977	3.8379	3.207807	5.745996	1.712706	1.884935	3.55583
2.406768	1.593535	0.946638	1.374329	1.890996	8.195657	0.962698	0.897489	0.603261
1.085224	0.312864	1.515296	0.741962	4.067547	0.7257	1.718918	1.081544	0.847352
0.630505	0.843102	1.175859	5.838499	0.489235	0.224214	1.595729	0.90772	5.351934
22.13245	0.873579	16.44768	27.38726	65.94551	3.303925	52.56128	105.1694	18.48514
8.849697	0.4457	3.286652	5.088694	10.57984	0.018016	8.625613	3.930664	11.48368
7.315512	0.221962	5.462475	2.823636	5.454949	0.165844	3.820758	4.115844	4.25658
1.950519	0.019978	1.797172	4.281847	1.296905	0.018016	3.890556	0.9529	22.96463
0.392251	7.315512	1.784755	0.859351	1.848297	5.337547	0.547778	3.703175	1.799899
0.959567	0.426392	3.779452	1.445916	0.837403	0.224214	1.798535	1.320173	1.74539
2.047157	0.019978	0.988377	1.828781	2.367763	0.018016	3.5257	1.477388	2.216162
0.591616	3.019848	0.280967	0.30158	0.469358	0.592532	0.396472	0.63114	0.573326
2.073232	0.585592	1.235346	0.433131	0.917431	2.051962	0.052525	0.828167	0.311876
0.392251	3.763045	0.250476	0.001126	0.165981	5.18754	0.130714	0.439524	0.06254
0.224055	0.2757	0.150346	0.149654	0.352222	0.267951	8.66E-05	1.278629	0.488167
1.215981	1.322092	1.007244	0.286364	1.427431	0.430837	0.349084	0.788896	0.603261
0.233175	2.914632	0.534214	0.149654	0.124646	3.578038	0.232734	0.73912	0.148846
1.680895	0.019978	0.511356	0.310447	1.389509	0.018016	0.642698	1.765065	1.099004
0.000895	0.019978	3.730808	5.238701	0.358442	0.018016	5.630815	0.656392	3.381255
2.647244	0.019978	3.934986	1.303961	2.723247	0.018016	1.87899	5.180058	2.114437
2.903867	0.585592	1.338261	0.533535	1.772756	0.18324	0.890065	2.922778	0.984069
0.064127	1.486551	0.037186	0.159942	0.091833	2.586926	0.173059	0.049769	0.174906
8.348817	0.019978	3.618499	5.259437	5.395584	0.018016	12.77772	2.081696	5.696263
24.50942	0.337222	14.88584	4.23202	13.38418	0.267951	5.42381	8.780115	8.780115
0.492165	0.426392	1.272756	4.933781	1.302107	0.311227	2.628225	0.424538	1.015967
3.529466	0.221962	1.831631	1.637309	3.655714	0.592532	4.851227	3.169812	3.223882
0.382208	1.152165	0.91404	0.001126	0.235613	1.663672	8.66E-05	0.406681	0.06254
0.087973	0.382208	0.185909	0.188276	0.142244	0.384156	0.433131	0.066205	0.391169
13.91714	0.019978	3.016984	4.130794	3.089293	0.018016	3.877417	2.355289	2.024488
9.880779	0.019978	12.29473	2.770866	6.591544	0.018016	7.625209	6.045209	7.363117
4.115844	19.68088	10.05618	13.75978	3.314329	5.18754	1.666089	9.377345	2.773644
0.142244	2.671299	0.517359	0.441053	0.435382	2.713319	0.110058	0.148932	0.208867
1.013434	0.221962	0.229199	0.286364	0.280469	0.457114	0.04456	0.140188	0.231768
0.976667	4.115844	0.375743	0.001126	0.535657	3.842554	0.542165	0.106205	0.284863
4.312251	0.362179	0.988377	0.624163	0.917431	0.457114	1.514199	1.414574	1.543377
6.464906	0.786667	13.08214	5.037489	4.626025	0.676999	9.968355	7.049336	2.529307
0.033874	0.522071	0	0.066883	0.015714	0.410541	0.012864	0.058139	0.000115
0.191804	0.019978	0.030613	0.001126	0.042807	0.018016	8.66E-05	0.114235	0.044214
1.828781	3.544257	1.584105	3.26	0.859351	4.029654	2.642525	2.116032	1.763889
3.104394	2.600014	4.077403	0.713405	4.508196	0.54925	0.533535	3.35715	1.833102
8.958225	332.0776	22.52375	6.335664	55.04081	40.7932	2.081696	14.00414	7.882222
0.319762	0.403413	0.370657	0.001126	0.435382	2.402468	0.06886	0.249091	0.165844
0.033874	0.312864	0.080895	0.001126	0.153651	0.202388	0.06886	0.098831	0.053391
5.143867	0.019978	1.824892	5.515584	1.71518	0.018016	5.037489	2.283752	8.573434
0.564127	0.019978	25.24367	14.00414	10.28573	0.018016	16.09482	12.03267	5.515584
2.852677	0.019978	5.395584	3.807648	2.823636	0.018016	2.850058	0.846342	3.144156
0.026876	0.134517	0.001017	0.001126	0.024733	0.202388	8.66E-05	0.033261	0.000115
0.09746	0.482706	0	0.076587	0.036089	0.457114	0.035079	0.007395	0.016133
3.687633	0.203773	1.184935	1.775325	1.238651	0.149293	2.730072	1.349971	1.846941
0.191804	0.019978	10.33476	10.1459	3.040505	0.018016	12.85504	10.95872	4.540794
0.352597	1.256558	0.370657	0.159942	0.265224	0.457114	0.232734	0.259098	0.488167
2.205476	0.019978	2.524892	3.172864	1.834423	0.457114	3.984747	1.370765	2.461905
0.000895	0.362179	4.745895	3.101371	0.479062	0.018016	2.021068	0.798153	1.863506
4.808658	13.5739	0.710722	0.836017	2.765758	10.48101	1.092785	2.922778	2.08632
1.067662	0.906825	0.366147	0.001126	0.559632	2.312085	0.196551	0.639113	0.000115
0.614935	4.637994	0.574228	0.001126	0.581378	18.19595	8.66E-05	0.579495	0.09847

4.888023	1.443059	8.477662	6.335664	7.430317	0.753882	2.928247	1.393571	8.728571
0.152114	0.611003	0.025483	0.048889	0.015714	0.133045	0.012864	0.025339	0.088514
0.630505	2.270837	0.229199	0.179177	1.518232	2.659214	0.173059	0.992201	0.148846
1.751948	0.019978	2.084791	1.434921	0.522071	0.018016	0.690707	0.16886	0.958557
0.224055	0.019978	1.737294	0.057691	0.901732	0.676999	2.040606	1.037316	0.702835
8.815123	0.019978	3.022713	1.608427	8.797619	0.224214	5.210317	1.618773	2.544747
0.492165	1.880375	0.042655	0.001126	0.148016	1.182771	8.66E-05	0.148932	0.000115
1.568564	0.019978	2.688304	1.312706	2.345527	0.018016	2.003723	2.269134	4.101061
0.302352	0.746551	2.993867	6.685087	2.810678	2.337821	2.127973	1.729892	0.958557
24.2842	0.019978	17.64869	14.29947	19.99861	0.384156	22.74797	45.1045	13.38418
1.228651	0.362179	1.022338	1.670786	0.420498	0.202388	1.651522	1.014784	0.66482
2.730072	11.57341	2.225498	31.92385	3.581688	0.430837	3.962179	1.53684	17.28195
1.845519	6.316328	6.443925	21.02638	6.345382	14.21671	22.32214	10.1028	17.03919
0.000895	0.162576	0.016104	0.001126	0.103716	1.90798	8.66E-05	0.012063	0.016133
0.885137	0.019978	0	2.316075	0.901732	0.018016	3.962179	3.61E-05	2.225498
43.49658	0.019978	25.48179	8.019769	18.19595	0.018016	13.23022	20.51271	15.77678
1.189769	0.019978	1.069574	1.475469	1.569553	0.018016	1.372475	1.585296	3.195541
1.203427	0.019978	1.652771	1.637309	0.752583	0.457114	2.216162	2.073232	0.92482
3.150433	0.663276	45.91841	17.15902	12.46209	0.334805	24.04374	18.76954	31.1753
0.531169	0.611003	0	0.001126	0.686299	0.592532	8.66E-05	0.567994	0.078211
2.868456	0.019978	4.833398	3.034747	3.107417	0.018016	2.238341	1.521371	0.862561
1.435772	18.48514	1.152944	1.592395	1.010289	11.14619	1.121703	1.310837	1.543377
9.792626	4.140505	7.112496	14.29947	3.984747	1.697792	19.37139	6.674286	24.2842
0.419365	0.2757	0.450924	1.400159	0.330635	0.288413	0.642698	0.224661	0.603261
4.733824	1.20824	5.599228	3.230065	4.174964	0.806227	1.78759	1.757244	1.563045
0.456248	0.4457	0.604618	0.001126	0.6657	0.842193	0.148846	1.121017	0.582973
0.753016	0.292597	0.143333	0.001126	0.535657	1.217475	8.66E-05	2.638139	0.183672
14.97762	0.019978	10.57984	5.614805	7.598312	0.311227	11.60283	13.00996	7.08733
0.79487	0.019978	1.130794	2.08013	0.69583	0.018016	1.578687	3.303925	2.07658
0.36267	5.210317	0.583369	0.816479	0.659163	4.650007	0.669358	0.682359	1.543377
0.162468	0.611003	0.191804	0.048889	0.124646	0.384156	0.008312	0.007395	0.06254
0.849841	0.019978	0.042655	0.001126	0.197727	0.018016	8.66E-05	0.158766	0.000115
6.888297	0.019978	2.406768	4.02088	2.993867	0.35798	1.628009	2.31013	3.606804
1.925635	0.663276	2.173081	4.155339	1.283911	0.247417	2.34355	0.992201	2.065007
3.734762	1.056898	1.542302	0.904286	3.407619	1.497143	0.737403	0.53912	1.247244
4.003117	5.553146	6.365065	1.94311	5.082063	3.147294	0.990909	12.77772	1.690483
0.000895	0.019978	0.807136	5.439278	2.89E-05	0.018016	2.863211	0.177734	0.000115
2.792035	60.77449	2.058773	1.061457	24.50942	49.50903	6.171789	27.10502	49.50903
1.318384	2.384141	2.225498	0.732193	0.976111	3.207807	1.041025	0.715462	2.07658
0.329798	0.019978	0.150346	0.001126	0.346494	0.567994	8.66E-05	0.269697	0.025087
0.064127	0.482706	0.037186	0.095433	0.172612	1.283911	8.66E-05	0.476335	0.025087
2.08798	0.482706	0.511356	3.083319	4.130794	0.018016	3.595758	1.729892	1.176623
7.417359	1.880375	2.005253	1.525729	3.695815	0.972504	2.157359	5.195022	3.334199
1.547734	0.019978	0.604618	0.76	2.214372	0.906126	1.521371	0.922338	0.883665
0.630505	2.953059	0.788896	1.137244	1.415469	6.727633	0.38772	1.050231	0.284863
12.56693	11.83593	10.28573	7.037345	19.22139	25.24367	9.335397	18.19595	14.70188
4.733824	0.019978	0.96329	2.535974	2.099524	0.938911	2.939141	1.662439	1.997352
1.426522	0.979149	0.490375	0.169971	0.208867	0.288413	0.164877	0.550072	0.148846
2.060491	1.030022	3.422237	1.779423	1.71518	1.877605	2.939141	3.201457	1.661328
1.055628	2.065007	2.406768	2.174733	0.867814	3.636789	1.041025	1.738456	1.471198
0.000895	0.932078	1.287403	1.518232	0.752583	0.018016	6.00228	0.598456	2.752893
5.431544	0.019978	7.468961	2.847547	1.072843	0.018016	1.131472	1.462143	1.290981
0.033874	0.1214	0.047908	0.140188	0.011573	0.35798	0.026364	0.012063	0.025087
7.519841	0.403413	8.994791	8.445743	4.764055	0.288413	3.734762	3.324452	2.178268
10.28573	2.723247	14.08569	12.67271	8.226219	1.469163	7.519841	10.33476	20.16541
2.805382	7.037345	3.119986	3.864827	3.236457	1.152165	0.718766	0.700418	2.59557
0.516825	1.181342	0.620909	0.580123	0.500722	0.224214	0.225209	0.34619	0.746984
0.256652	2.270837	0.381999	0.001126	0.148016	0.806227	8.66E-05	0.158766	0.000115

2.531515	4.580938	0.547778	0.766602	2.018167	6.560411	0.419596	1.050231	0.76
0.162468	0.563824	0.05886	0.124098	0.117843	2.429957	0.04456	0.280967	0.459769
2.083261	0.019978	1.451775	1.137244	1.827403	0.018016	3.055729	0.532092	1.040411
8.539971	0.019978	3.307186	9.751039	6.717056	0.018016	4.690159	5.942294	5.950664
1.57645	0.019978	2.187395	0.732193	0.82772	0.018016	1.948918	0.828167	1.387612
0.079531	0.636291	0.127273	0.040469	0.221587	0.842193	0.094553	0.148932	0.06254
1.189769	0.134517	0.604618	0.519387	0.69583	0.165844	0.953506	1.02487	1.113961
2.073232	0.148701	2.283752	2.050404	0.942294	0.288413	0.934488	0.406681	0.340866
0.947677	0.019978	0.150346	1.312706	0.280469	0.267951	2.760765	0.90772	1.409942
4.696313	0.502843	1.338261	1.255657	1.306667	0.018016	2.876421	0.682359	1.628009
6.760765	0.019978	2.384141	17.28195	8.461703	0.77925	7.911934	6.706472	6.316328
0.000895	3.425512	0.037186	0.095433	0.165981	3.065108	0.060022	0.098831	0.025087
2.975974	1.880375	2.331825	0.616407	2.567605	1.434055	1.171349	2.283752	1.301169
13.30906	0.019978	5.754935	0.610224	7.625209	0.699192	1.78759	7.911934	3.676032
2.148658	19.22139	2.107922	2.018167	2.214372	35.776	1.143954	1.43039	0.894704
8.381328	0.812771	2.236407	6.653016	4.678846	0.267951	9.583463	10.62739	7.911934
5.838499	105.1694	2.638139	2.978968	8.103925	75.1132	0.918557	12.29473	3.144156
1.356364	3.175916	0.973232	0.245772	2.538218	0.485325	0.239589	4.174964	0.12772
0.000895	0.019978	6.967547	4.381479	4.193232	0.018016	4.94026	4.508196	4.25658
425.62	0.502843	749.7839	287.197	332.0776	6.38456	87331.38	332.0776	425.62
1.982208	3.66785	1.403853	7.495397	2.654473	1.663672	5.273362	1.711414	0.933261
0.041782	0.611003	0.09176	0.001126	0.015714	0.165844	8.66E-05	3.61E-05	0.016133
1.277056	27.38726	1.272756	1.434921	3.150433	18.99374	0.357727	2.08798	1.941703
36.26061	3.829105	12.22798	80.6518	34.84299	4.650007	42.08807	21.19547	26.52537
1.076652	5.462475	0.334625	0.331558	0.715108	2.713319	0.196551	0.567994	0.302785
15.66023	0.2757	13.23022	15.276	14.17388	3.930664	14.70188	10.73084	20.68746
0.000895	1.692763	2.506934	6.210375	2.89E-05	0.018016	7.212915	0.567994	10.67991
20.25302	0.337222	4.323045	3.29355	6.240346	0.247417	6.250447	1.985253	4.72798
1.03303	0.292597	3.966623	2.6143	2.776421	0.018016	1.08671	0.729062	0.613853
4.353925	7.737807	1.466111	1.582915	4.370671	6.464906	1.305772	2.429957	2.967453
0.98956	8.958225	0.143333	0.596111	0.95101	3.877417	0.295491	0.871429	0.488167
3.034747	14.33939	3.482785	3.588369	8.888167	18.99374	5.082063	9.19899	7.765931
0.000895	4.101061	0.05303	0.001126	0.086443	15.41187	8.66E-05	0.20733	0.000115
4.946587	0.585592	2.703485	1.078925	2.29873	0.149293	1.505678	3.324452	1.622006
19.99861	0.019978	57.84882	13.42179	30.49289	0.699192	19.53061	100.0998	45.1045
1.672107	7.456118	3.104394	0.169971	3.169812	11.2819	0.576205	2.554069	0.702835
0.087973	0.019978	0.016104	0.001126	0.208867	1.434055	0.012864	0.049769	0.016133
7.991602	18.33909	5.788153	6.717056	9.815642	19.22139	6.865	4.38684	3.049603
2.102626	2.306277	1.969733	0.687431	3.457504	7.724084	1.605859	7.710361	2.659214
10.48101	0.906825	13.75978	3.771349	2.037114	0.533196	3.422237	8.539971	6.277937
7.545584	0.187532	8.164531	3.346898	5.42381	1.152165	3.66785	4.827691	2.698557
0.579495	4.241905	0.00811	0.048889	0.091833	0.567994	8.66E-05	0.122475	0.070065
1.159913	1.256558	1.835743	0.568369	2.285664	1.96697	1.343463	1.884935	1.634935
0.98956	0.69119	0.328543	0.115014	0.3014	0.247417	0.078831	0.396306	0.078211
0.781977	0.337222	0.334625	0.169971	0.599134	0.247417	0.262655	0.882078	0.36267
0.033874	1.723579	0.030613	0.040469	0.005007	3.363911	0.052525	0.007395	0.12772
95.22939	3.763045	26.80355	11.60283	14.51648	0.267951	10.28573	1.618773	7.584502
1.982208	0.019978	0.710722	4.490801	1.617489	0.018016	2.388348	0.776876	1.236248
0.543398	0.019978	18.19595	11.36975	10.23768	0.533196	3.934986	6.978903	1.643218
7.6393	0.019978	1.935685	21.93452	2.970144	0.018016	14.88584	1.748016	22.32214
4.914863	0.148701	3.172864	1.312706	3.330909	0.018016	1.389509	1.201724	4.888023
5.295325	0.019978	8.608153	18.33909	29.18885	0.018016	18.19595	9.625253	18.19595
3.711097	3.049603	2.406768	5.068831	4.145209	0.430837	2.928247	0.567994	2.794935
0.602554	29.49693	0.185909	0.751371	0.867814	10.2617	0.493925	0.34619	0.332886
0.753016	0.162576	0.547778	0.806227	0.813788	0.018016	1.25886	0.34619	0.480022
0.947677	29.82828	4.952915	1.475469	2.018167	15.88548	0.856782	2.644885	0.803665
0.654423	5.813247	0.274365	0.338658	1.205685	2.967453	0.164877	2.073232	0.183672
0.191804	2.671299	0.229199	0.001126	2.89E-05	5.24562	0.130714	3.61E-05	0.000115

0.531169	5.864084	1.360022	0.449091	2.415296	2.051962	0.376595	2.190866	0.382626
1.265426	9.19899	4.597792	16.5625	1.197215	2.153535	13.53913	6.079596	15.18102
13.15079	0.019978	11.83593	12.16681	6.30671	2.659214	7.08733	7.61176	9.160382
0.79487	32.31123	0.788896	1.104805	1.948918	18.62711	0.555418	1.833102	2.996869
4.091667	0.134517	5.560563	2.240274	6.240346	0.753882	3.796075	1.370765	2.369841
2.483492	0.337222	0.860924	0.105108	0.880498	0.510707	0.28443	0.508658	0.428932
5.538001	0.362179	6.738211	1.803918	3.128694	0.334805	1.351573	2.65684	0.82772
0.162468	2.270837	0.00811	0.277417	0.036089	0.510707	0.180368	0.34619	0.000115
0.000895	4.101061	0.127273	0.245772	0.172612	3.47531	0.052525	0.249091	0.12772
4.650007	0.019978	6.922771	2.964762	5.813247	0.457114	5.00404	5.515584	5.323492
0.614935	1.181342	0.213615	0.205382	0.274055	0.7257	0.139697	0.334625	0.620909
0.056782	1.084437	0	0.001126	0.117843	0.018016	8.66E-05	0.13114	0.000115
1.115902	2.953059	0.646861	1.078925	0.992771	3.442886	0.441833	0.197042	1.186457
6.784762	0.019978	3.276335	2.243911	4.900996	0.018016	1.041025	0.670144	2.153535
0.142244	2.022778	0.133297	0.048889	0.086443	1.728615	0.019048	0.083608	0.044214
4.23202	2.384141	4.764055	1.644416	4.23202	2.402468	0.83697	1.919661	2.628225
1.327525	0.403413	0.973232	2.941544	1.927042	0.018016	0.78619	1.812114	1.703795
7.276645	0.019978	22.96463	12.67271	21.93452	0.018016	6.674286	8.677489	6.00228
0.516825	2.577157	0.353175	0.086075	0.527821	2.365685	8.66E-05	0.240851	0.350635
1.507835	11.30987	0.867287	1.277056	2.214372	7.570693	2.642525	1.855108	2.037114
0.000895	0.019978	0.047908	0.001126	0.061025	0.18324	8.66E-05	0.106205	0.000115
0.531169	0.312864	0.175253	0.001126	0.493925	0.018016	8.66E-05	0.158766	0.000115
0.630505	0.019978	1.069574	2.250462	0.274055	0.018016	3.243225	0.431198	1.290981
0.000895	0.585592	0.127273	0.001126	0.442496	0.311227	0.026364	0.305087	0.000115
2.453781	46.7056	2.589185	2.647244	9.256537	26.25557	1.853723	18.33909	10.1028
0.808983	6.240346	0.980505	0.692027	0.908773	0.972504	0.466869	0.871429	0.523521
3.953297	0.786667	6.116638	13.00996	2.895599	0.384156	2.270837	1.064935	6.99026
0.000895	0.019978	0.004964	0.001126	0.00303	0.018016	0.008312	3.61E-05	0.000115
3.266782	0.134517	7.136537	3.35355	6.560411	1.434055	2.66404	2.415296	1.889711
32.68313	2.022778	39.53687	18.19595	60.77449	2.513752	26.52537	46.7056	27.38726
0.492165	1.692763	2.463911	1.700354	5.337547	8.590794	0.819509	4.292078	1.236248
10.43066	0.019978	24.73724	1.824892	9.459177	0.224214	6.125823	7.315512	4.690159
0.041782	0.4457	0.004964	0.057691	0.005007	0.77925	0.012864	0.033261	0.010014
2.35733	0.134517	1.74539	1.68671	0.847908	0.018016	2.468117	1.618773	1.310837
0.467951	0.238889	0.030613	0.001126	0.172612	0.018016	0.04456	0.249091	0.12772
3.655714	0.019978	12.85504	43.49658	7.991602	0.018016	10.73084	18.48514	19.68088
0.079531	0.482706	0.05886	0.124098	0.061025	0.876118	8.66E-05	0.140188	0.010014
0.319762	0.134517	0.01153	0.568369	0.117843	0.018016	8.66E-05	0.033261	0.428932
6.345382	0.636291	11.93253	3.574387	18.41211	0.842193	12.36443	10.95872	10.78056
6.230245	0.019978	0.980505	2.693442	1.484553	0.018016	2.066652	1.037316	1.426522
0.214502	0.019978	1.614257	45.1045	1.427431	0.018016	31.55417	2.392431	3.640606
5.01088	0.786667	4.217569	4.241905	5.195022	0.334805	4.029654	3.119986	6.922771
12.49452	3.640606	6.52899	0.904286	6.52899	3.980123	1.078153	3.663622	2.727785
2.887489	0.542828	2.563506	1.400159	2.696061	0.018016	3.06803	1.784755	1.204228
0.382208	25.73165	1.673254	0.527821	2.337821	47.5018	1.143954	2.936739	1.947316
1.307417	1.813564	6.945152	11.30987	2.640332	0.699192	6.632063	6.706472	5.22482
17.51961	2.112843	15.77678	6.685087	13.23022	1.812114	6.297136	12.85504	4.397792
4.198117	0.746551	20.68746	51.52195	9.667316	10.85542	19.68088	22.74797	20.34063
0.543398	2.845036	0.456248	0.245772	1.499214	1.632648	0.130714	0.806227	0.000115
0.907273	0.292597	1.515296	0.706494	0.581378	0.149293	0.986104	1.106825	0.82772
0.821414	3.300664	7.175397	2.65684	7.225469	5.082063	1.628009	3.486436	1.810664
0.401385	1.298665	0.31702	0.001126	1.037958	4.323045	8.66E-05	0.476335	0.183672
0.000895	0.2757	0.00811	0.057691	0.080664	0.117518	8.66E-05	0.025339	0.016133
0.244033	6.591544	0.322395	0.220274	0.69583	2.45772	0.173059	0.656392	1.059553
1.343463	0.019978	5.409437	11.89932	7.765931	0.018016	8.288874	5.22482	6.00228
1.886335	0.502843	0.366147	2.339697	0.780087	0.018016	4.851227	0.456248	2.518124
1.253088	0.843102	0.445209	0.751371	0.837403	2.196017	0.225209	1.53684	1.670786
0.09746	2.518124	0.143333	0.001126	2.89E-05	0.485325	0.425844	1.662439	0.070065

0.09746	3.885642	0.030613	0.001126	2.89E-05	2.402468	8.66E-05	0.114235	0.254769
2.903867	0.463867	11.03843	2.323983	2.577157	0.334805	0.819509	11.19942	3.707136
0.244033	0.728543	0.020678	0.066883	0.042807	0.430837	0.012864	0.033261	0.034906
1.585296	0.238889	2.55184	3.156537	1.415469	0.018016	2.984899	1.360866	3.080317
0.244033	4.140505	1.360022	0.648687	0.780087	6.560411	1.006623	1.006025	0.942294
1.992944	1.20824	3.975498	6.900202	4.353925	1.082915	6.601833	2.254084	3.962179
34.38341	0.019978	8.119004	9.52197	6.738211	0.54925	12.49452	7.653391	11.65791
0.033874	5.993045	0.042655	0.086075	0.117843	2.914632	0.148846	0.066205	0.010014
0.033874	1.624459	0.004964	0.040469	0.019942	0.567994	0.035079	0.003535	0.000115
10.82954	0.4457	4.907929	0.896522	7.854069	0.247417	2.396429	6.591544	3.414574
2.026219	8.149307	4.789596	1.569553	6.622092	7.570693	1.285584	7.61176	1.833102
8.103925	3.207807	3.711097	1.176623	4.789596	1.934271	1.372475	2.591443	3.207807
0.675115	0.019978	1.770029	0.682359	0.449408	0.018016	0.974906	0.521068	1.374329
11.77567	0.812771	5.037489	11.42512	8.797619	0.018016	13.6814	6.230245	3.939307
0.142244	0.019978	0.133297	0.001126	0.172612	0.018016	0.148846	0.083608	0.034906
2.292864	0.019978	7.288788	13.08214	2.668896	0.485325	14.79742	5.082063	14.33939
0.000895	0.463867	0.150346	0.140188	0.559632	1.497143	0.06886	0.670144	0.21632
0.419365	8.477662	2.242092	0.596111	1.670786	8.348817	1.006623	8.075772	1.616385
126.1556	3.175916	70.11932	24.98218	52.56128	1.521371	22.96463	36.26061	55.04081
0.372727	1.181342	0.310866	0.465801	0.172612	0.972504	0.38772	0.375281	0.322237
1.356364	0.238889	1.056898	4.419062	1.791616	0.018016	3.750837	0.375281	3.796075
0.000895	0.69119	0.127273	0.001126	2.89E-05	0.457114	8.66E-05	0.559632	0.000115
3.479134	0.812771	1.314675	2.000592	1.741414	0.18324	3.55961	1.711414	3.279625
5.500678	0.312864	3.207807	2.160649	10.82954	24.50942	135.5245	5.47	10.43066
1.399315	0.542828	7.625209	6.612121	2.911962	1.96697	5.323492	1.798535	2.294733
0.516825	2.953059	0.269322	0.169971	0.142244	12.85504	0.008312	0.240851	0.034906
0.36267	0.768535	0.479401	0.245772	1.23127	1.845519	0.547778	0.620909	0.12772
0.630505	0.019978	1.235346	1.692763	1.179885	0.018016	1.222179	0.715462	3.807648
2.563506	5.431544	0.957273	0.859351	1.99899	1.753341	1.131472	2.879228	2.786898
2.138131	5.662994	1.593535	0.426645	5.838499	5.515584	0.371075	4.473348	0.473319
12.77772	0.019978	3.172864	2.339697	3.895469	0.35798	5.71241	0.598456	5.337547
1.265426	0.019978	1.778153	0.788369	0.535657	0.430837	5.500678	8.019769	1.634935
0.36267	2.143261	0.755253	0.904286	1.389509	2.868456	0.650556	1.798535	0.968066
1.595729	0.019978	6.268312	26.80355	19.83727	0.510707	27.96248	5.273362	17.51961
5.933066	3.156537	11.77567	3.738997	5.950664	155.7103	6.297136	6.423911	9.667316
0.142244	1.322092	0	0.032431	0.011573	0.35798	8.66E-05	0.007395	0.000115
9.335397	3.425512	20.51271	13.60866	16.44768	1.497143	15.98892	30.17192	19.22139
19.53061	13.42179	155.7103	1360.293	144.7919	28.54786	530.1465	425.62	1360.293
0.808983	24.50942	2.828918	1.406746	4.203001	11.77567	0.728088	14.33939	0.746984
1.727323	0.019978	1.030022	1.104805	1.927042	0.018016	3.864827	0.385736	0.603261
0.808983	0.019978	3.340361	6.99026	3.370527	0.247417	5.662994	2.116032	3.425512
5.754935	2.800332	3.046551	9.256537	5.993045	4.091667	7.964315	6.772763	9.707677
7.737807	0.019978	95.22939	135.5245	38.39042	1.182771	105.1694	4.456912	24.73724
8.149307	0.257518	7.025599	5.110159	7.288788	0.018016	4.370671	4.484892	2.065007
67.98069	0.019978	10.53159	37.29684	26.25557	0.018016	56.39528	16.44768	39.53687
5.576219	7.810058	5.01772	1.434921	4.105714	11.89932	3.125844	14.25954	8.539971
1.925635	0.292597	3.346898	1.629235	1.834423	0.224214	1.092785	0.897489	0.473319
2.792035	0.019978	4.110779	6.125823	4.33342	3.734762	4.058514	3.614603	2.294733
0.36267	2.518124	0.817013	0.49974	1.128131	2.312085	0.607273	1.195794	0.62733
0.732193	0.873579	0.120361	0.001126	0.982698	0.842193	0.094553	0.375281	0.06254
11.65791	3.934986	16.5625	13.30906	22.32214	0.938911	11.36975	7.882222	17.92188
21.19547	0.463867	13.15079	6.191515	21.74051	0.938911	4.241905	5.25254	22.13245
0.079531	0.221962	0.020678	0.066883	0.029639	1.402915	8.66E-05	0.091977	0.053391
7.964315	1.742734	15.3618	45.91841	8.195657	6.268312	15.66023	11.14619	15.88548
0.79487	0.019978	3.730808	1.514199	1.859084	0.018016	0.934488	2.836717	1.690483
2.183665	1.322092	0.375743	0.196955	0.553059	1.554185	0.139697	0.521068	0.302785
0.695426	1.278629	0.534214	0.751371	2.203535	0.906126	0.794401	1.477388	1.049517
0.256652	0.636291	0.05886	0.228203	0.249278	0.617071	8.66E-05	0.21632	0.09847

2.581861	1.742734	3.324452	6.580988	4.38684	1.497143	4.120815	6.622092	7.251263
4.402973	0.4457	1.253088	0.821898	1.289149	0.133045	1.285584	2.781558	2.461905
0.071183	0.611003	0	0.001126	0.061025	0.18324	0.008312	0.148932	0.000115
3.792266	0.019978	6.38456	10.03313	6.423911	0.485325	10.62739	2.855296	5.646811
40.11561	16.9162	100.0998	21.36892	38.94854	9.179466	49.50903	25.24367	4.23202
2.941544	0.019978	4.236962	16.22108	2.89E-05	0.018016	10.23768	1.49075	5.788153
0.079531	9.52197	0.133297	0.032431	0.086443	1.182771	0.102792	0.058139	0.053391
7.938427	0.257518	2.069942	7.683766	0.917431	0.018016	8.958225	2.217951	11.77567
0.000895	2.331825	0.106732	0.001126	2.89E-05	12.7427	8.66E-05	0.197042	0.000115
0.09746	1.127475	0.185909	0.048889	0.091833	1.434055	0.019048	0.017576	0.000115
2.509177	1.758615	1.810664	0.402381	0.976111	5.638759	0.49974	2.836717	1.32482
1.716364	5.095325	0.106732	0.001126	0.405346	1.217475	0.196551	0.148932	0.000115
0.000895	3.91355	0.292597	0.169971	0.346494	4.091667	0.078831	0.316811	0.498355
3.953297	0.019978	3.540606	0.876118	0.926291	0.149293	1.716364	4.776407	2.579509
4.091667	0.312864	2.434444	4.715599	3.500808	0.592532	9.121486	3.799885	5.366335
1.356364	1.983824	1.664906	0.294502	0.706494	1.812114	0.148846	1.632648	0.473319
11.719	0.019978	10.48101	15.98892	3.536955	0.533196	9.880779	4.49671	7.251263
0.492165	0.019978	0.080895	0.001126	0.648038	0.18324	0.130714	0.197042	0.332886
4.402973	0.906825	11.36975	9.946364	7.911934	0.806227	11.719	12.49452	9.792626
4.678846	0.238889	3.394286	5.671126	4.429769	1.554185	3.422237	3.098268	3.989206
0.329798	1.20824	0.347186	0.076587	0.69583	1.812114	0.116825	1.25816	0.070065
18.19595	0.019978	6.99026	14.88584	10.35839	0.224214	32.31123	17.64869	8.319019
1.343463	0.203773	1.081544	1.130115	0.976111	0.149293	0.457431	0.897489	1.176623
1.622006	0.403413	1.466111	24.2842	4.266753	0.77925	3.101371	0.828167	3.119986
0.087973	1.758615	0.1379	0.149654	0.235613	0.35798	0.052525	0.334625	0.06254
0.087973	2.554069	0.002143	0.001126	0.208867	0.647345	8.66E-05	0.083608	0.044214
4.072475	2.408882	0.710722	0.115014	2.345527	1.497143	0.303896	1.568564	0.201789
0.821414	0.019978	0.427937	0.310447	0.221587	0.018016	0.906825	1.145426	1.092136
0.041782	0.292597	0.042655	0.076587	0.00811	0.676999	8.66E-05	0.058139	0.000115
0.000895	0.746551	0.004964	0.095433	0.061025	2.051962	8.66E-05	0.025339	0.000115
8.643074	0.019978	3.993665	3.672078	0.581378	0.018016	3.699495	2.238341	4.115844
0.256652	0.482706	0.274365	0.507085	0.473319	0.288413	0.217792	0.158766	0.76
3.711097	1.23127	4.348882	2.810678	3.763045	0.906126	4.082165	4.424416	3.518384
1.023528	0.979149	1.376378	2.5271	4.025267	0.753882	3.220678	1.145426	0.723506
0.191804	0.843102	0.588954	1.156869	0.454632	0.224214	0.769019	0.839365	4.445851
0.516825	0.019978	5.165599	3.373983	1.026219	0.018016	2.556443	3.4679	3.585029
0.064127	0.482706	0.075418	0.001126	0.042807	0.224214	8.66E-05	0.140188	0.000115
3.195541	0.019978	7.495397	2.895599	6.079596	0.018016	6.945152	4.758088	3.401126
28.54786	1.410902	30.49289	32.31123	10.95872	2.713319	34.84299	27.96248	42.08807
0.286522	0.426392	0.106732	0.057691	0.317229	0.202388	8.66E-05	0.598456	0.034906
0.531169	2.408882	0.143333	0.124098	0.581378	2.365685	0.04456	0.177734	0.070065
22.74797	0.203773	7.519841	3.610707	11.719	0.288413	6.464906	8.83241	5.037489
3.450577	0.019978	1.477388	4.535354	5.180058	0.410541	2.805382	1.191198	4.155339
0.98956	0.019978	0.907273	0.95101	0.442496	0.018016	0.699697	0.439524	1.152165
0.142244	6.784762	0.150346	0.095433	0.061025	9.859719	0.268961	0.488167	0.157056
8.429055	0.611003	9.031082	1.475469	3.346898	1.252424	3.796075	3.201457	4.170051
7.339452	0.019978	5.309264	10.67991	5.210317	0.018016	13.30906	1.968283	14.08569
0.181797	0.019978	1.94311	0.549661	0.153651	1.934271	1.712706	2.565556	0.174906
39.53687	2.045476	53.77619	59.32013	49.50903	0.117518	72.69473	39.53687	59.32013
1.149322	0.019978	3.131544	3.980123	1.633752	0.384156	1.121703	0.098831	0.883665
1.085224	4.557605	0.381999	0.396638	1.243442	2.868456	0.232734	0.508658	0.480022
1.043608	4.808658	1.192633	0.580123	1.510014	6.830592	0.890065	1.865043	0.79487
0.000895	1.593535	15.88548	39.53687	5.062626	3.065108	7.824473	30.49289	18.33909
0.181797	0.426392	0.434069	6.508427	0.581378	0.018016	1.901068	0.521068	4.101061
3.655714	0.713846	1.604827	3.6029	5.309264	0.457114	3.975498	2.016515	2.740519
0.602554	6.784762	1.421126	1.359177	1.05824	7.570693	1.164257	0.456248	0.157056
4.043846	0.019978	1.499214	2.685786	1.948918	0.288413	1.514199	0.9529	1.113961
0.129553	1.813564	0.127273	0.124098	0.330635	2.807937	8.66E-05	0.224661	0.238478

1.253088	0.187532	1.215195	1.707576	0.542525	0.18324	0.409336	0.73912	0.573326
5.195022	0.019978	87331.38	144.7919	135.5245	1.96697	332.0776	184.443	77.74812
0.071183	5.838499	0.106732	0.359697	0.049336	0.35798	0.173059	0.158766	0.208867
0.000895	0.636291	0.113766	0.169971	0.159639	0.18324	8.66E-05	3.61E-05	0.000115
0.438831	0.542828	0.030613	0.196955	0.442496	0.842193	0.019048	0.305087	0.053391
2.593506	0.482706	8.210938	67.98069	1.116609	1.581912	20.25302	2.823636	2.333867
0.719177	0.482706	0.616407	1.340036	0.659163	1.152165	0.890065	0.588658	1.261407
0.401385	3.610707	1.515296	0.896522	0.294502	3.034747	0.268961	0.747879	0.174906
4.025267	1.384921	2.250462	1.541227	1.306667	0.384156	1.948918	2.404618	2.898196
0.041782	0.148701	0.037186	0.032431	0.005007	0.202388	8.66E-05	3.61E-05	0.000115
4.365224	1.20824	2.518124	1.959466	2.621133	0.617071	1.971183	3.971061	4.348882
1.537951	0.019978	2.099524	3.824964	2.529307	0.334805	2.203535	0.177734	0.999502
0.602554	8.477662	0.82868	0.324719	1.510014	8.780115	0.970007	3.005281	0.672468
0.129553	3.300664	0.01153	0.001126	0.080664	0.938911	0.04456	0.017576	0.016133
7.061328	0.337222	4.490801	7.737807	2.037114	0.018016	4.952915	3.290101	5.872886
1.660216	0.019978	2.463911	8.164531	2.229048	0.018016	1.471198	3.61E-05	1.90798
0.885137	2.022778	0.843102	0.220274	1.314675	1.252424	0.110058	0.992201	0.513752
3.8379	0.019978	12.85504	4.058514	2.500548	0.018016	10.8813	9.256537	4.135801
0.203333	0.713846	0.191804	0.420707	0.130606	1.663672	0.357727	0.305087	0.088514
0.000895	2.143261	0	0.066883	0.011573	0.806227	8.66E-05	3.61E-05	0.000115
2.263377	1.030022	1.338261	2.106089	1.927042	0.410541	3.391075	1.438672	2.037114
0.000895	0.019978	0.381999	0.001126	0.384156	0.018016	0.094553	0.158766	0.000115
8.745238	0.337222	5.431544	6.853608	6.107114	0.018016	7.570693	2.506934	4.875527
0.329798	0.382208	0.353175	0.347186	0.405346	2.483492	0.376595	0.417063	0.532092
0.708124	0.019978	2.005253	2.369841	2.737944	0.753882	11.19942	3.381255	4.597792
13.45939	0.426392	16.34294	8.745238	13.08214	2.269134	16.9162	4.863579	12.29473
0.267597	6.423911	0.175253	0.359697	0.3014	0.938911	0.173059	0.041328	0.078211
6.028427	1.568564	5.323492	3.595758	4.529913	0.77925	2.327857	4.456912	2.024488
1.96697	0.019978	3.55583	1.455988	3.829105	0.018016	1.728615	0.861934	2.558817
2.868456	8.958225	1.326667	0.888485	6.663651	2.45772	0.88158	0.424538	3.080317
2.205476	4.540794	0.397763	0.149654	1.072843	2.23267	0.225209	0.764704	0.942294
0.36267	0.148701	2.203535	0.368038	0.435382	0.202388	0.052525	0.439524	0.053391
12.36443	0.292597	12.63773	10.8813	8.412367	0.018016	7.938427	22.32214	16.5625
4.47912	5.485685	12.36443	38.39042	11.19942	11.39744	21.74051	6.560411	7.468961
33.4985	2.022778	22.74797	29.82828	42.75576	0.7257	19.22139	12.10289	37.81919
1.479452	1.813564	0	0.001126	0.599134	0.457114	8.66E-05	0.187229	0.078211
8.27267	1.486551	1.207374	3.464315	1.670786	0.133045	2.447294	2.895599	5.821804
6.25938	3.66785	8.728571	7.519841	11.25392	0.876118	7.495397	5.431544	6.591544
0.224055	0.257518	0.6657	1.123795	0.358442	0.384156	0.926291	0.292597	0.459769
19.22139	1.514199	5.855281	15.66023	9.924372	3.8121	19.06893	28.54786	11.11876
0.543398	1.983824	1.114618	1.035058	1.026219	0.457114	0.542165	0.396306	0.36267
0.129553	1.084437	0.070065	0.001126	0.091833	0.224214	0.019048	0.122475	0.025087
0.773276	0.019978	0.274365	1.733766	0.384156	0.018016	2.31013	0.882078	0.933261
2.35733	2.463911	2.487576	0.964416	1.623232	1.061457	1.078153	1.310837	0.437799
3.008225	0.906825	3.070952	0.821898	1.389509	0.7257	1.152165	1.438672	1.574517
0.614935	0.426392	0.086667	0.105108	0.352222	1.217475	0.180368	0.114235	0.437799
1.149322	2.600014	3.147294	10.82954	0.965714	2.62342	2.250462	5.302294	2.447294
0.244033	0.873579	0.004964	0.048889	0.197727	1.129452	0.052525	0.025339	0.016133
1.318384	1.65785	23.16622	11.19942	6.287561	0.7257	15.18102	4.650007	10.93101
0.203333	1.34513	0.73619	0.376407	0.352222	5.553146	0.906825	3.074134	0.786667
0.129553	2.828918	0	0.001126	2.89E-05	0.311227	8.66E-05	3.61E-05	0.078211
9.218514	5.898449	15.56131	1.950519	12.93651	14.70188	1.578687	8.445743	4.053506
17.03919	0.019978	45.1045	252.4251	16.34294	0.018016	252.4251	4.789596	52.56128
0.372727	0.843102	11.09133	17.78522	4.921082	5.28039	25.48179	6.475505	4.135801
0.382208	0.019978	0.596111	0.228203	0.165981	0.410541	0.728088	2.08798	0.070065
6.933961	0.019978	9.502294	8.573434	7.824473	1.006025	10.40634	2.447294	2.6143
91.05541	47.5018	38.94854	49.50903	41.47925	7.08733	33.93939	29.82828	38.94854
0.033874	0.502843	0.004964	0.048889	0.005007	1.350772	0.012864	0.007395	0.010014

0.849841	2.384141	0.690707	0.648687	0.599134	1.402915	0.210505	0.764704	0.428932
0.000895	2.045476	0.065152	0.001126	0.086443	1.252424	0.060022	3.61E-05	0.034906
0.267597	1.742734	2.351299	9.377345	2.425801	0.018016	3.998391	2.355289	5.545729
0.214502	0.019978	0.728088	0.140188	0.136479	0.77925	0.334444	0.406681	0.165844
1.828781	0.292597	2.351299	1.123795	0.766118	0.149293	2.021068	3.771349	2.308203
1.095657	0.221962	3.50425	6.30671	1.334762	2.269134	3.022713	4.696313	0.657547
0.244033	0.522071	0.52689	0.751371	0.630022	0.018016	0.901212	1.064935	1.471198
45.1045	0.636291	27.10502	20.34063	33.4985	0.485325	38.39042	23.38378	10.98644
7.824473	1.030022	15.18102	18.48514	15.276	1.90798	11.14619	15.77678	13.91714
0.162468	2.993867	0.166118	0.220274	0.019942	0.842193	0.173059	0.357475	0.157056
1.242641	0.403413	1.167561	2.479221	0.465079	0.384156	5.71241	0.755714	4.419062
9.459177	24.2842	10.19468	3.230065	37.81919	17.51961	1.701616	18.05488	1.443059
5.485685	0.019978	2.964762	1.852338	2.114437	0.753882	2.688304	2.447294	1.498167
0.602554	6.355224	1.076032	0.997244	1.03189	7.765931	1.006623	1.550837	1.280274
2.852677	0.019978	5.23176	2.50469	2.597792	0.018016	3.711097	1.729892	2.818312
0.719177	1.278629	0.788896	0.140188	0.493925	0.647345	0.256429	1.201724	0.36267
2.427792	0.019978	6.153102	5.646811	4.307309	0.485325	7.882222	1.954993	1.247244
0.000895	0.636291	0.070065	0.235613	2.89E-05	0.842193	0.094553	0.439524	0.116659
3.614603	7.964315	1.731169	1.733766	1.640779	13.79669	1.68342	1.014784	4.312251
3.340361	3.578038	5.515584	1.614257	4.47912	7.175397	2.863211	4.048499	1.310837
0.654423	0.312864	0.82868	1.078925	0.61526	0.224214	0.777864	1.244957	0.37417
2.730072	1.056898	0.450924	0.751371	2.438831	1.283911	0.334444	1.449877	0.322237
0.392251	0.502843	0.479401	0.040469	0.399978	1.812114	0.04456	0.944784	0.09847
0.000895	0.463867	0.065152	0.188276	0.024733	0.972504	0.06886	0.292597	0.06254
1.159913	0.542828	1.223846	3.771349	0.365224	0.753882	3.166659	1.320173	6.277937
4.597792	0.362179	1.688045	2.151919	3.695815	1.061457	1.988196	0.53912	3.518384
0.041782	0.187532	0.004964	0.040469	0.029639	0.18324	0.008312	0.007395	0.000115
0.916869	24.04374	1.532468	0.001126	3.046551	13.45939	0.274877	5.068831	0.36267
3.034747	0.873579	3.407619	1.115902	3.025671	0.384156	1.521371	1.684646	2.461905
1.815	0.403413	2.506934	1.423506	6.045209	0.35798	1.758615	3.422237	0.894704
18.48514	5.770996	9.625253	6.153102	5.662994	0.753882	8.780115	7.288788	11.83593
0.732193	0.337222	1.731169	5.180058	1.072843	1.350772	2.720996	0.499495	0.968066
0.630505	0.996696	0.397763	0.220274	0.553059	1.350772	0.28443	0.249091	0.246162
1.343463	6.853608	0.52689	7.570693	0.214502	0.334805	1.758615	0.197042	9.751039
0.392251	0.843102	0.939986	1.160707	0.6657	0.77925	0.996696	0.550072	0.405786
2.003723	0.69119	1.056898	0.987821	2.150303	0.311227	1.701616	1.037316	2.225498
0.233175	1.65785	0.341558	0.662237	0.352222	2.106089	0.180368	0.249091	0.554726
0.087973	2.143261	1.097006	1.044863	1.03189	4.130794	0.28443	1.977316	0.382626
3.303925	2.463911	9.541645	0.732193	5.855281	3.125844	1.158384	3.771349	1.863506
3.266782	0.019978	3.663622	0.882078	2.538218	0.133045	2.500548	1.449877	1.165065
6.99026	0.019978	32.31123	7.938427	16.85982	0.018016	18.91854	17.51961	10.28573
3.324452	1.278629	3.581688	4.752121	1.09075	0.018016	3.625382	1.684646	2.681169
0.329798	0.019978	2.225498	1.202626	0.565714	0.267951	1.410902	0.579495	0.437799
0.531169	0.956667	7.911934	2.08013	5.568391	0.018016	1.634935	1.865043	1.165065
10.12435	1.593535	21.74051	6.045209	10.8813	0.384156	8.994791	4.758088	6.107114
0.968723	2.518124	0.728088	0.465801	0.565714	1.252424	0.562439	0.280967	0.672468
3.820758	100.0998	19.06893	4.990794	18.76954	44.27012	4.965801	26.66446	1.359177
0.591616	0.611003	0.224372	3.137576	0.465079	1.037958	0.396472	0.106205	0.311876
0.27759	1.056898	0.690707	0.245772	0.153651	2.513752	0.123102	0.21632	0.088514
6.560411	0.019978	30.17192	155.7103	20.34063	0.018016	80.6518	57.84882	26.80355
0.684401	0.019978	2.151919	1.171349	2.567605	0.018016	0.737403	0.466602	0.603261
1.555289	1.842734	1.306667	4.440548	0.469358	0.842193	3.125844	0.240851	2.07658
0.000895	1.084437	0.625209	0.001126	0.214502	5.745996	0.04456	0.776876	0.000115
4.614618	0.585592	9.063983	4.888023	5.737056	0.018016	1.570794	5.024293	7.417359
111.5439	87331.38	2.164113	64.12971	203.5795	87331.38	75.1132	87.1746	6.144055
0.048889	0.563824	0.042655	0.001126	0.110534	0.288413	0.019048	0.025339	0.044214
1.47018	425.62	1.991328	5.295325	36.74522	530.1465	1.236248	4.808658	0.322237
8.048095	0.019978	6.738211	13.91714	3.283139	0.018016	8.048095	4.012049	1.726032

2.693442	9.19899	3.842554	0.842641	2.061926	3.511263	1.171349	2.29873	1.846941
5.143867	0.238889	0.616407	1.416407	0.527821	0.288413	2.335844	2.591443	3.269913
16.44768	4.484892	12.16681	3.122915	3.734762	1.521371	2.973059	5.273362	1.32482
0.564127	26.52537	0.511356	0.359697	1.128131	22.42294	0.441833	1.985253	0.779668
6.99026	30.17192	5.295325	1.799899	5.295325	11.2819	5.959033	22.13245	9.031082
0.000895	17.39935	0.025483	0.001126	0.091833	20.16541	0.026364	3.61E-05	0.044214
0.09746	1.127475	0.150346	0.057691	0.049336	0.567994	0.130714	0.177734	0.12772
2.765758	28.54786	2.931068	12.10289	3.169812	12.56693	3.890556	3.588369	12.85504
252.4251	56.39528	62.33472	25.48179	155.7103	70.11932	21.54823	25.73165	67.98069
18.91854	7.375794	17.15902	50.55949	27.38726	9.031082	30.49289	33.4985	35.776
75.1132	184.443	29.82828	7.212915	37.29684	12.7427	10.1028	144.7919	23.16622
28.23632	21.19547	8.019769	7.991602	29.82828	39.53687	17.15902	10.43066	17.15902
25.73165	10.67991	29.49693	30.17192	45.1045	16.9162	39.53687	24.73724	19.06893
11.30987	155.7103	6.876392	7.264502	13.91714	184.443	8.319019	4.222583	10.62739
1360.293	0.746551	425.62	203.5795	1360.293	4.575123	287.197	252.4251	87331.38
80.6518	35.27608	126.1556	126.1556	167.9996	22.42294	118.7028	65.94551	184.443
5.838499	0.932078	203.5795	70.11932	70.11932	5.838499	60.77449	167.9996	126.1556
72.69473	118.7028	87.1746	30.49289	95.22939	167.9996	25.73165	43.49658	51.52195
7.683766	7.288788	2.607035	2.438831	2.061926	6.830592	1.819221	7.212915	4.419062
1.615281	3.779452	1.130794	0.866717	2.164113	3.47531	0.493925	1.821861	0.854242
18.62711	27.96248	7.417359	1.296905	9.031082	41.47925	2.81342	8.256465	2.502619
31.1753	0.69119	14.70188	4.614618	9.101674	1.96697	9.218514	11.51384	4.971991
0.310866	2.763261	0.20759	0.032431	0.553059	1.106825	0.04456	0.269697	0.06254
0.129553	0.134517	0.100483	0.048889	0.024733	0.288413	0.086991	0.13114	0.088514
2.34355	7.124517	3.796075	1.287403	2.710765	6.045209	0.980505	1.711414	1.218182
0.551573	1.783203	1.300368	1.629235	0.203593	0.842193	0.28443	0.13114	0.148846
15.46193	19.99861	2.531515	7.188095	8.958225	36.26061	1.798535	8.643074	3.842554
0.000895	224.9555	0	1.556392	0.847908	224.9555	0.116825	1.113304	1.824892
33.07147	5.993045	15.07257	3.058802	48.53429	11.96574	4.900996	9.52197	7.519841
0.319762	0.019978	1.433189	2.640332	2.438831	0.018016	1.505678	1.684646	5.950664
0.531169	1.723579	0.39062	0.376407	1.23127	5.137042	0.357727	1.954993	0.564127
0.630505	0.542828	0.004964	0.040469	0.280469	0.133045	0.026364	0.007395	0.000115
0.976667	10.93101	0.558427	1.143175	0.837403	55.04081	0.737403	0.049769	0.66482
4.25658	0.019978	2.196017	4.900996	4.965801	0.018016	2.320072	0.53912	3.169812
13.53913	2.845036	16.9162	19.37139	16.68554	8.83241	23.16622	28.23632	47.5018
20.85876	0.019978	17.92188	24.04374	36.26061	3.269913	48.53429	38.94854	32.68313
12.29473	25.48179	10.1028	11.96574	10.53159	16.68554	7.339452	9.418196	9.562554
5.638759	0.996696	3.214545	1.574517	3.314329	0.7257	2.157359	5.485685	4.082165
1.174315	2.800332	0.946638	0.001126	0.469358	0.592532	0.026364	0.550072	0.174906
37.81919	8.888167	8.923117	19.99861	17.51961	3.511263	20.85876	16.5625	27.96248
1.771392	1.322092	0.445209	0.482706	0.249278	2.586926	0.569293	0.828167	0.883665
1.336587	10.85542	2.449509	0.788369	3.599329	1.728615	1.078153	4.135801	2.681169
47.5018	27.66779	25.73165	12.36443	14.08569	15.18102	9.707677	14.79742	17.39935
0.392251	9.316241	1.770029	1.903636	1.607143	4.053506	0.633499	2.197828	1.165065
0.933261	1.030022	0.334625	0.149654	0.454632	0.842193	0.49974	0.96627	1.152165
6.25938	5.462475	2.776421	2.993867	6.423911	3.842554	2.893001	3.563182	5.238701
0.564127	1.593535	0.427937	1.160707	0.926291	3.877417	1.29342	0.645455	1.409942
0.438831	2.518124	0.578817	0.368038	0.733593	2.713319	0.250476	5.165599	0.224372
0.319762	0.522071	0.257143	0.269892	0.274055	0.906126	0.012864	0.499495	0.451429
1.023528	1.880375	0.788896	0.331558	0.813788	34.38341	0.409336	0.816479	0.208867
0.428456	0.257518	0.52689	0.331558	0.607605	0.018016	0.342215	0.456248	0.12772
0.531169	14.6058	1.015967	1.115902	3.998391	13.08214	0.728088	4.592273	0.582973
9.707677	7.212915	1.207374	1.115902	2.196017	6.464906	0.980505	6.404921	3.297107
37.29684	1.443059	4.546443	0.997244	16.5625	9.52197	1.798535	6.116638	1.434055
1.209105	0.019978	0.468694	2.45575	0.706494	0.018016	1.771392	0.375281	0.647691
0.000895	0.1214	0.113766	0.048889	0.024733	0.533196	0.139697	0.148932	0.116659
25.99427	4.241905	28.87506	9.335397	9.541645	2.006782	7.683766	8.288874	13.23022
0.79487	1.813564	0.890065	0.774257	0.256941	1.845519	0.203954	0.488167	0.76

0.033874	0.257518	0.001017	0.001126	2.89E-05	0.510707	0.008312	0.003535	0.010014
0.773276	0.019978	3.266782	1.349971	1.648066	0.018016	1.587605	0.935743	0.779668
2.518124	0.019978	5.838499	4.165137	2.438831	0.018016	6.404921	3.536955	5.095325
1.937222	64.12971	8.780115	0.908773	22.13245	67.98069	1.236248	33.93939	3.489899
14.88584	0.312864	8.288874	17.39935	8.539971	0.018016	9.063983	1.244957	6.191515
1.460051	4.241905	2.269134	0.675115	1.36619	6.464906	0.441833	1.477388	0.538427
2.331825	1.539062	3.869163	14.6058	3.300664	6.230245	8.508514	4.408153	9.297085
2.094574	23.82354	3.4679	4.597792	3.500808	13.91714	3.439437	2.481356	3.019848
0.933261	14.00414	0.517359	1.035058	0.535657	13.5739	0.619012	0.788896	1.03246
0.392251	0.463867	0.505786	0.086075	0.384156	0.247417	8.66E-05	0.259098	0.000115
16.34294	24.73724	14.97762	7.339452	13.30906	62.33472	5.923838	9.082828	10.38202
287.197	2.671299	111.5439	167.9996	287.197	4.323045	167.9996	126.1556	287.197
0.108124	0.2757	0.39062	0.001126	0.294502	0.267951	8.66E-05	0.21632	0.070065
27.10502	6.181652	28.54786	28.23632	56.39528	12.13485	29.49693	24.50942	34.6132
1.356364	1.539062	1.810664	1.491977	2.863211	4.091667	1.372475	1.510014	1.32482
1.228651	4.881775	1.253088	0.376407	0.965714	3.269913	0.538117	1.094978	0.635541
7.795642	1.152165	8.319019	19.68088	5.165599	0.906126	10.98644	14.42661	8.728571

CGGA_13[illegible]	CGGA_13[illegible]	CGGA_13[illegible]	CGGA_13[illegible]	CGGA_13[illegible]	CGGA_13[illegible]	CGGA_13[illegible]	CGGA_13[illegible]	CGGA_13[illegible]
0.057352	0.390216	0.257143	0.290317	1.521371	0.225339	0.175469	0.240346	0.207157
0.097547	0.432698	0.113911	0.690375	1.744055	0.693925	0.465801	0.408644	0.24518
6.632063	1.29417	3.860491	2.447294	1.102691	3.043528	5.062626	4.952915	5.195022
0.717879	1.05088	4.302367	3.846984	0.023694	5.622872	12.56693	0.571674	4.63189
2.947287	8.226219	3.754719	12.81638	1.983824	3.330909	7.951371	3.726854	5.500678
1.217475	26.12492	1.243442	6.900202	13.91714	5.030866	2.438831	4.770231	4.160238
21.54823	21.19547	17.28195	41.47925	6.22031	60.77449	52.56128	24.2842	118.7028
0.057352	0.039214	0.087229	0.090772	0.810491	0.005382	0.116494	0.204156	0.050519
5.351934	4.473348	1.597908	0.89404	0.023694	1.985253	3.792266	1.682193	2.984899
0.000354	0.152114	0.001118	0.184877	0.023694	0.682799	0.000685	0.313312	0.522367
31.92385	29.18885	9.687496	111.5439	59.32013	57.84882	28.54786	60.77449	43.49658
0.464387	1.514199	0.408175	6.171789	5.323492	2.826277	1.953535	1.519322	1.031284
18.33909	7.468961	23.38378	11.42512	3.236457	3.521934	12.85504	3.536955	19.99861
8.226219	4.33E-05	11.19942	0.000606	0.023694	2.901032	22.32214	1.44E-05	7.339452
0.028622	0.005541	0.032172	0.145685	0.636688	0.04114	0.105498	0.002424	0.031818
14.33939	1.844098	7.212915	0.440801	1.244242	1.081544	4.222583	9.063983	2.335844
0.182237	1.794531	0.281068	4.715599	1.491977	3.314329	1.837172	1.153723	2.489762
0.000354	0.167835	0.001118	1.711414	10.40634	0.065974	0.318889	0.19544	0.168961
5.728615	1.070115	6.22031	0.203254	0.023694	1.01772	3.370527	0.508975	1.409942
0.353016	0.764315	0.393196	1.578687	1.87899	5.976724	0.490079	1.139221	0.196097
1.338261	0.195823	3.743232	0.090772	0.023694	0.232734	0.837403	0.541804	0.080592
8.412367	0.764315	8.365072	1.438672	4.644098	0.660332	1.244957	1.631544	3.166659
3.065108	64.12971	0.343824	30.82277	0.023694	47.5018	4.808658	35.27608	34.84299
0.019848	4.33E-05	0.152532	0.184877	3.47531	0.04114	0.026291	0.002424	0.000476
0.464387	2.058773	0.480303	4.626025	5.515584	0.919192	1.277843	2.66404	1.183478
9.276811	3.055729	2.765758	2.155447	2.114437	3.975498	3.6029	3.679986	5.01088
6.55039	0.559221	2.554069	0.194235	0.023694	0.550664	3.350224	6.622092	1.29342
0.291183	0.269697	0.269322	0.717027	0.810491	0.671724	0.318889	0.395642	0.469711
332.0776	33.4985	49.50903	4.984488	0.023694	5.864084	15.56131	184.443	23.38378
0.168023	0.319163	0.336328	0.89404	4.783001	0.262655	0.700058	0.363492	0.750866
4.435159	5.821804	6.52899	0.376407	2.604776	1.277843	3.249877	1.412771	2.465916
0.047792	0.039214	0.077316	0.249538	1.382006	0.147554	0.026291	0.248903	0.156089
28.54786	1.919661	38.39042	0.000606	0.023694	6.38456	6.632063	2.701053	7.417359
0.000354	0.010043	0.041097	0.04062	0.227063	0.082973	0.116494	0.02311	0.000476
0.047792	27.96248	0.067446	24.50942	16.34294	8.288874	4.557605	0.618211	1.890996
5.704646	0.753882	2.540462	1.514199	0.414473	2.574733	3.391075	1.924228	2.678781
3.754719	2.178268	5.788153	1.591299	2.836717	6.181652	5.779574	0.946061	5.696263
0.000354	0.160584	0.001118	0.080281	0.982128	4.33E-05	0.000685	0.037908	0.022958
2.121198	5.654863	2.207417	6.622092	6.00228	3.220678	6.443925	3.401126	3.807648
1.953535	12.56693	2.518124	22.13245	14.65384	16.44768	6.443925	4.120815	5.359134
0.36114	1.615281	0.077316	1.455988	3.028629	0.531804	0.228009	1.950519	0.432468
0.530534	0.374365	0.871429	0.988932	10.17029	6.464906	1.624459	0.852172	3.008225
1.288276	0.205931	3.169812	3.011169	0.023694	0.56145	1.429365	1.44E-05	1.367179
0.000354	0.005541	0.001118	0.022511	0.227063	4.33E-05	0.026291	0.002424	0.022958
0.589618	0.269697	1.546696	7.212915	0.308442	0.599466	0.465801	0.33329	0.957273
67.98069	31.1753	45.91841	21.36892	1.465123	18.48514	33.07147	42.75576	31.92385
0.047792	0.058139	0.049769	0.70404	0.88417	0.147554	0.175469	0.090476	0.168961
0.000354	0.07316	0.159942	0.280823	0.515527	0.531804	0.432915	0.222193	0.060426
0.028622	4.33E-05	0.041097	0.022511	0.253492	4.33E-05	0.000685	1.44E-05	0.000476
0.000354	0.329798	0.001118	0.31702	0.586595	0.750404	0.000685	0.213615	0.000476
0.000354	0.030613	0.001118	0.440801	0.203593	4.33E-05	0.059177	0.005224	0.050519
7.625209	20.51271	10.98644	3.166659	0.541241	0.717027	3.321284	3.78798	3.796075
0.028622	0.573947	0.041097	0.50557	0.390014	0.457749	0.079755	0.351789	0.070909
0.028622	0.082973	0.032172	1.360022	1.132165	0.283882	0.743694	0.323319	0.311227
2.221775	1.033672	1.853723	1.438672	1.382006	2.659214	5.158146	0.256941	1.082179
0.000354	2.591443	0.001118	1.360022	0.702381	0.730029	1.429365	1.046061	0.291183
0.038535	0.497749	0.067446	1.002215	3.077316	0.96938	0.465801	0.483326	0.824416

0.311227	0.885664	0.281068	4.179762	4.990794	2.201587	0.630022	0.186356	0.340245
1.400159	3.249877	1.655541	2.308203	1.305772	8.089848	2.134545	1.988196	1.395563
0.136147	0.390216	0.20215	0.646378	1.491977	0.541804	0.277763	0.408644	1.574517
0.274055	7.112496	7.545584	0.119899	8.477662	4.33E-05	0.12904	0.375281	0.227828
0.636688	0.233304	0.184719	0.781977	0.541241	0.342987	0.164084	0.204156	0.254574
0.19132	0.805801	0.175866	2.522684	10.55571	0.333463	0.597417	0.675115	0.571674
80.6518	21.54823	21.02638	1.320173	0.023694	17.03919	37.29684	45.1045	80.6518
0.327143	0.07316	0.303896	0.573326	2.353247	0.099812	0.477951	0.459582	0.35798
0.019848	0.010043	0.001118	0.055498	0.130029	0.010014	0.026291	0.002424	0.000476
0.636688	1.983824	1.741414	1.191905	0.308442	0.945483	1.497143	1.557482	2.148658
2.513752	1.061457	2.384141	2.327857	1.102691	0.671724	1.703795	1.180613	1.776739
0.230606	0.442193	0.43347	0.807136	0.144293	1.673254	0.866717	0.408644	0.571674
0.000354	0.251371	0.001118	0.080281	2.234538	1.039798	0.197908	0.186356	0.143579
0.028622	0.319163	0.001118	1.288276	0.203593	2.102626	0.12904	0.351789	0.080592
0.038535	0.453463	0.105873	0.690375	1.521371	0.50254	0.265014	0.675115	0.143579
0.000354	0.005541	0.049769	0.137201	0.144293	0.010014	0.036696	0.005224	0.022958
0.047792	0.049062	0.032172	0.000606	0.363276	4.33E-05	0.000685	0.029553	0.000476
5.295325	1.935685	14.97762	16.5625	0.541241	11.83593	21.74051	7.124517	5.583968
0.136147	0.298687	0.001118	0.421804	0.702381	0.252165	0.206984	0.384394	0.090245
0.000354	0.065354	0.041097	0.228593	0.515527	0.04114	0.04886	0.118759	0.227828
6.374812	7.315512	2.540462	3.618499	1.557482	5.266335	4.236962	9.141299	5.671126
0.000354	0.030613	0.067446	1.455988	0.023694	4.33E-05	0.069163	0.155101	0.060426
0.182237	1.197922	0.192929	1.275325	1.436623	0.50254	0.432915	0.675115	0.133189
9.729358	2.449509	11.77567	2.50469	1.34798	5.050267	6.200945	3.578038	7.519841
0.097547	4.33E-05	0.05824	0.027619	0.334625	0.005382	0.04886	0.072381	0.000476
0.087302	0.432698	0.077316	1.898153	1.244242	1.298665	0.288247	0.375281	0.478182
64.12971	7.6393	34.84299	26.25557	0.023694	17.78522	11.96574	6.011515	155.7103
0.087302	0.485577	0.152532	0.536263	0.810491	0.116825	0.455974	0.016681	0.186587
0.320563	1.008961	1.039199	0.536263	5.259437	0.609509	0.90772	0.794401	1.751948
2.826277	0.467403	5.34474	5.180058	0.023694	1.615281	16.09482	0.606154	5.23176
0.19132	0.688975	0.263463	0.145685	1.465123	0.541804	0.30596	0.222193	0.156089
0.047792	0.160584	0.001118	0.080281	0.227063	0.057417	0.092511	0.037908	0.178442
1.472208	0.160584	1.006025	0.100101	0.023694	0.405094	1.537951	0.137063	0.469711
6.153102	3.404372	7.013853	0.000606	0.023694	21.74051	14.97762	6.922771	21.74051
0.077027	1.357201	0.096667	1.288276	1.010289	1.030678	0.252468	0.675115	0.432468
0.221277	0.733593	0.425635	1.643218	0.515527	1.384921	1.497143	0.408644	0.668456
0.047792	5.881342	0.257143	4.875527	6.685087	7.683766	2.000592	1.615281	1.43039
0.000354	0.010043	0.122944	0.203254	2.081696	0.082973	0.079755	0.016681	0.000476
2.936739	5.447114	7.363117	3.13456	1.92272	7.951371	5.9071	2.461905	7.938427
0.028622	0.065354	0.067446	0.228593	0.414473	0.082973	0.036696	0.010202	0.022958
0.097547	0.017063	0.113911	0.145685	0.949827	0.010014	0.036696	0.081587	0.022958
0.019848	4.33E-05	0.001118	0.034416	0.203593	0.005382	0.026291	0.037908	0.000476
0.605455	2.290996	1.121017	5.309264	2.90658	2.026219	2.164113	1.519322	1.977316
3.104394	2.531515	9.141299	4.048499	0.702381	21.02638	7.339452	2.984899	5.165599
0.182237	2.531515	0.512309	1.643218	0.982128	23.16622	2.671299	1.022338	1.688045
0.057352	0.390216	0.281068	0.624473	1.305772	0.180368	0.66526	0.118759	0.300094
0.945483	0.620909	3.156537	0.547778	0.227063	0.30373	1.850996	0.541804	1.056898
0.000354	0.329798	0.001118	0.690375	2.31404	0.405094	0.116494	0.508975	0.099192
0.108506	0.251371	0.408175	1.798535	1.192633	1.05153	0.000685	0.384394	0.616739
7.710361	1.717641	5.056421	0.536263	0.702381	0.682799	0.891775	0.852172	1.584105
0.521421	1.522597	0.541804	3.718716	2.269134	2.361198	1.085224	0.395642	0.824416
1.326667	4.603434	0.753449	1.944517	0.636688	4.397792	2.589185	4.721789	1.273636
1.68342	0.298687	4.125786	1.016494	1.044235	0.541804	1.555289	1.112641	3.783694
0.642395	1.997352	0.648687	1.159913	2.77899	0.65101	2.209141	2.018167	0.442799
144.7919	0.904286	252.4251	0.753882	0.023694	1.102121	42.08807	1.44E-05	56.39528
3.391075	0.611003	3.195541	0.000606	0.023694	2.586926	2.786898	1.463304	0.606154
0.028622	0.374365	0.001118	1.591299	0.253492	1.006025	0.092511	0.473016	0.262965
95.22939	4.33E-05	155.7103	0.000606	0.023694	4.845166	14.88584	6.433918	55.04081

0.028622	0.390216	0.077316	0.483874	1.845519	0.147554	0.079755	0.323319	0.217172
0.000354	0.522633	0.001118	0.090772	0.023694	0.377085	0.206984	0.204156	0.000476
1.749307	3.363911	2.238341	3.394286	30.82277	3.083319	2.659214	2.941544	1.766248
14.08569	2.81342	8.643074	13.15079	3.236457	2.640332	5.737056	9.377345	24.73724
0.108506	1.591299	0.122944	0.646378	16.80343	0.091306	0.092511	2.248896	0.031818
3.707136	15.88548	3.679986	3.411097	3.47531	6.497266	4.46267	9.625253	5.614805
0.160332	3.214545	0.152532	4.307309	2.604776	2.876421	1.791616	2.831501	1.367179
1.096335	4.281847	0.716234	5.530274	1.712706	3.144156	3.618499	6.277937	0.971205
0.763102	10.78056	0.710722	18.91854	33.93939	5.01088	1.165065	6.945152	0.322078
1.728615	0.597417	1.285584	2.219863	10.82954	0.437576	0.240346	2.287576	0.935115
0.038535	0.082973	0.001118	0.154978	0.636688	0.057417	0.04886	0.010202	0.108413
0.605455	0.141623	0.257143	0.137201	0.515527	0.313723	0.141825	0.618211	0.347908
0.388023	0.260144	0.63114	3.4679	1.675722	0.580404	0.687049	0.33329	0.534214
2.438831	9.101674	5.366335	1.678319	2.520404	11.60283	2.90658	3.726854	7.149618
0.028622	0.130115	0.041097	0.602287	1.436623	0.07482	0.036696	0.606154	0.554416
1.906551	1.008961	3.297107	0.413449	1.2214	1.195079	0.687049	1.602619	1.766248
0.70329	0.81596	0.490079	2.489762	2.565556	0.82684	1.429365	0.351789	0.866717
0.000354	0.017063	0.001118	0.047792	0.227063	0.010014	0.026291	1.44E-05	0.041782
1.385801	8.412367	1.285584	2.390447	13.6814	7.468961	0.925801	3.629177	3.763045
1.434921	0.401724	2.715873	0.145685	0.438831	0.522633	0.240346	1.969733	0.080592
0.038535	0.120144	0.049769	1.191905	1.491977	0.232734	0.059177	0.363492	0.000476
2.635685	2.267157	3.113478	0.73912	1.010289	0.342987	1.404812	0.844156	2.274473
0.221277	1.957958	1.449062	2.540462	19.83727	2.489762	4.207778	2.349351	3.930664
3.754719	1.264589	5.915469	3.24658	0.88417	1.133622	3.47531	1.009625	5.993045
2.270837	0.467403	1.569553	0.428932	0.18153	0.49127	1.116609	1.192633	0.143579
1.721371	5.123781	2.574733	3.457504	14.08569	6.497266	4.424416	3.807648	2.089639
2.31811	7.570693	2.116032	3.340361	4.990794	8.005685	4.317648	3.037626	4.745895
5.123781	0.319163	5.044113	0.184877	0.541241	4.33E-05	0.719177	0.693218	0.186587
2.489762	0.41026	1.685678	0.100101	0.736977	4.33E-05	0.152749	0.289935	0.168961
2.396429	3.78798	3.028629	1.044235	0.023694	3.116732	3.037626	1.749307	0.935115
14.25954	14.17388	12.63773	4.875527	2.180022	11.42512	13.23022	19.22139	10.45584
1.400159	0.160584	4.552092	0.249538	0.023694	0.216753	0.000685	1.44E-05	0.442799
13.00996	6.772763	15.3618	24.2842	1.92272	9.276811	8.958225	17.22048	12.93651
6.876392	3.330909	16.68554	2.289286	0.60886	2.296732	7.08733	2.261573	6.622092
0.000354	0.092193	0.077316	0.027619	0.848369	0.016277	0.036696	0.010202	0.000476
5.068831	1.10132	2.037114	0.12904	0.023694	1.644416	1.154567	1.139221	6.414416
0.664423	12.29473	0.469711	10.62739	3.236457	17.98838	3.939307	4.502453	3.363911
0.945483	1.393571	0.587273	0.675938	2.114437	0.793918	0.421025	1.295108	0.456818
0.379192	0.919827	0.067446	0.913514	0.308442	1.481631	0.421025	0.606154	0.331558
2.417424	0.522633	12.49452	0.807136	0.023694	1.355527	2.074906	1.421126	0.000476
39.53687	4.33E-05	29.18885	0.000606	0.023694	0.108413	0.277763	0.342381	1.521371
135.5245	8.677489	55.04081	26.80355	0.023694	0.159639	56.39528	23.6012	64.12971
0.300606	1.335743	0.336328	3.984747	5.720173	2.750375	1.925635	2.051962	1.916782
0.087302	0.215599	0.077316	0.308672	0.023694	0.483045	0.12904	0.256941	0.060426
1.994329	2.394416	1.165065	0.73912	0.281364	4.272165	1.443983	1.935685	1.395563
2.104358	1.867951	6.374812	0.145685	0.023694	1.081544	0.966825	2.165851	0.704444
0.160332	1.828781	0.067446	0.23873	2.269134	0.377085	0.676659	2.871212	0.494841
0.000354	1.29417	0.001118	5.037489	2.565556	4.33E-05	6.09759	3.230065	2.159004
14.17388	3.442886	14.70188	11.48368	1.160707	36.26061	29.49693	6.355224	26.80355
0.182237	1.033672	0.263463	0.636291	0.161962	1.370765	2.121198	0.19544	0.584134
0.000354	3.031688	0.001118	2.019618	1.382006	4.33E-05	0.30596	1.44E-05	0.000476
1.422316	16.09482	2.101075	22.8563	20.08201	10.35839	6.663651	7.38847	4.535354
0.861429	2.367763	1.252424	1.058896	0.636688	3.016984	2.031169	0.684805	1.260635
0.000354	0.374365	0.001118	0.913514	2.014863	0.475137	0.588954	1.068348	0.060426
0.221277	0.81596	0.001118	1.607143	1.010289	1.195079	0.277763	0.363492	0.254574
4.894509	3.236457	3.726854	0.794401	0.023694	2.600014	2.958773	7.238023	0.340245
0.274055	0.082973	0.067446	0.000606	0.390014	0.448824	0.240346	0.062381	0.262965
0.077027	0.308788	0.096667	0.753882	0.60886	0.417063	0.141825	0.256941	0.236638

1.659033	0.715462	1.243442	0.403175	0.023694	0.313723	0.392251	2.66404	0.24518
8.103925	8.180094	6.171789	5.037489	0.023694	1.072843	3.659668	7.074329	2.8657
2.465916	4.921082	3.88153	9.924372	15.1268	6.036818	6.601833	3.363911	2.92013
60.77449	25.73165	44.27012	6.508427	0.023694	3.820758	8.711905	46.7056	19.22139
1.510014	2.02956	1.664906	0.988932	0.023694	2.727785	1.820541	0.483326	2.581861
5.646811	4.33E-05	10.28573	1.179885	0.023694	3.26	3.119986	1.44E-05	6.210375
0.404408	0.863694	0.175866	3.269913	1.590202	1.309084	0.640289	1.232078	0.668456
9.418196	0.340866	13.30906	4.715599	0.023694	5.409437	6.663651	8.412367	8.134084
0.42715	13.53913	0.001118	17.28195	21.54823	14.65384	2.644885	5.110159	1.354567
7.882222	14.42661	8.195657	13.00996	5.259437	20.16541	17.51961	11.01243	17.39935
0.000354	4.067547	0.23246	2.678781	2.740519	4.946587	0.953506	1.545657	0.659957
7.251263	11.96574	5.395584	17.45948	3.377619	40.11561	20.51271	8.134084	12.36443
2.026219	0.215599	1.196508	0.070339	0.810491	0.945483	1.759986	0.571674	0.900693
2.703485	1.155411	2.421681	3.966623	0.161962	7.882222	8.019769	1.668369	4.552092
0.000354	0.798153	0.001118	1.934271	1.629235	0.693925	0.277763	0.045346	0.168961
2.579509	0.994899	0.956667	2.839351	0.023694	2.807937	3.163506	0.176104	1.903636
1.415469	0.453463	1.883535	0.839365	0.586595	0.56145	0.562439	1.060188	1.592395
0.505267	4.586753	0.607273	3.300664	4.086926	4.56912	1.244957	2.871212	2.792035
0.000354	0.017063	0.001118	0.062035	0.586595	0.091306	0.000685	1.44E-05	0.000476
4.802655	0.185909	1.546696	0.000606	0.023694	9.880779	3.439437	2.461905	7.037345
6.210375	6.653016	5.606739	3.88153	1.557482	11.36975	7.443276	3.55961	11.14619
0.353016	1.591299	0.152532	0.646378	0.463867	2.99987	1.331919	0.803665	1.493968
0.000354	0.298687	0.001118	0.164509	0.023694	0.750404	0.000685	0.155101	0.000476
4.408153	2.531515	5.024293	4.921082	5.720173	6.325945	12.22798	2.996869	3.855952
4.752121	5.037489	4.47912	3.269913	0.023694	7.824473	8.608153	2.131176	6.063276
0.000354	2.102626	0.001118	0.000606	9.063983	4.33E-05	0.000685	1.44E-05	0.000476
0.168023	0.242893	0.447359	0.753882	0.982128	0.333463	0.092511	0.186356	0.217172
4.135801	1.651522	3.207807	0.660332	3.119986	0.813788	2.259661	0.863694	1.395563
0.000354	1.89518	0.077316	3.411097	1.382006	5.309264	2.351299	1.068348	2.209141
0.411948	0.707475	0.05824	1.621032	3.29355	0.838846	0.4457	0.618211	0.311227
11.57341	1.226118	10.90615	0.839365	0.023694	0.730029	3.648081	3.921645	5.165599
0.61355	0.659582	0.497749	1.016494	2.604776	1.786306	1.313737	1.167561	0.984069
0.136147	0.110642	0.096667	0.228593	0.438831	0.49127	0.277763	0.186356	0.099192
17.03919	2.941544	4.802655	1.418312	0.023694	4.696313	2.90658	38.39042	3.300664
0.066537	2.394416	0.20215	1.217475	10.8813	0.858817	1.043608	1.728615	0.949827
4.733824	2.831501	7.519841	0.000606	1.778153	1.030678	2.236407	3.160022	3.6029
0.038535	0.101104	0.032172	0.04062	0.281364	0.065974	0.059177	0.052338	0.041782
0.000354	0.850808	0.077316	1.944517	0.253492	0.241688	0.141825	0.108997	0.121147
0.000354	4.33E-05	0.077316	0.184877	1.305772	0.065974	0.000685	0.256941	0.121147
3.610707	0.597417	1.285584	1.002215	0.848369	1.419278	0.55215	0.529697	0.584134
0.047792	4.696313	0.001118	7.456118	31.92385	3.672078	0.455974	2.781558	0.24518
17.15902	22.96463	13.38418	49.50903	67.98069	10.67991	5.545729	19.83727	13.30906
7.991602	11.60283	11.14619	36.26061	1.382006	37.29684	35.27608	10.1459	13.00996
0.832554	3.249877	0.384798	1.607143	26.12492	1.01772	0.805238	2.852677	1.142345
2.673846	3.011169	3.529466	3.439437	0.636688	2.547179	2.737944	1.153723	3.672078
0.000354	2.635685	0.001118	0.335411	4.165137	0.671724	0.228009	0.248903	0.000476
0.505267	0.195823	0.175866	0.393449	0.463867	1.150635	0.288247	1.260635	0.291183
111.5439	19.68088	13.23022	8.288874	0.023694	4.808658	14.79742	100.0998	20.34063
2.066652	2.122799	2.111299	2.701053	0.463867	2.928247	2.850058	1.557482	2.970144
1.121703	6.079596	1.992944	2.390447	2.710765	2.316075	1.415469	1.859084	1.01772
2.390447	6.454416	1.770029	14.88584	4.927302	14.51648	9.377345	5.614805	2.479221
0.779668	0.650101	0.692799	0.164509	0.023694	1.435772	0.991631	0.909315	0.596508
6.760765	2.415296	2.879228	0.145685	0.023694	0.572987	0.465801	13.91714	0.000476
51.52195	5.737056	10.82954	1.736133	0.023694	4.33E-05	0.000685	24.62333	0.000476
0.077027	0.269697	0.001118	0.194235	0.636688	0.262655	0.252468	0.23101	0.099192
118.7028	53.77619	105.1694	0.636291	0.023694	3.68381	48.53429	30.49289	77.74812
0.485325	1.50342	0.350455	3.201457	6.933961	0.879913	1.455022	1.89228	2.640332
1.23127	3.201457	0.768052	2.327857	0.023694	12.77772	7.417359	1.247244	3.629177

0.000354	0.897489	0.001118	4.096407	2.145137	1.91254	0.925801	0.323319	0.133189
1.637309	0.678687	1.972706	6.464906	0.390014	7.951371	4.473348	2.688304	3.957734
4.614618	1.603795	3.4679	1.240981	0.023694	2.531515	2.765758	3.8379	4.644098
31.1753	1.957958	118.7028	0.000606	0.023694	5.42381	8.019769	4.297222	28.23632
184.443	1.662439	59.32013	0.000606	0.023694	11.25392	105.1694	53.77619	46.7056
0.000354	4.33E-05	0.001118	2.730072	16.04187	1.985253	0.000685	1.44E-05	0.000476
0.379192	3.343629	0.512309	1.834423	0.982128	1.888023	0.676659	1.930216	0.512641
0.247085	4.696313	0.001118	1.523824	4.524481	4.33E-05	0.849841	1.112641	0.108413
0.038535	4.33E-05	0.087229	0.055498	1.102691	0.082973	0.092511	0.005224	0.060426
0.108506	3.939307	0.400354	2.261573	0.702381	3.45404	0.371075	2.141595	0.340245
0.127201	0.269697	0.242453	0.070339	0.490664	0.031775	0.197908	0.062381	0.060426
42.75576	2.459812	87.1746	0.000606	7.938427	4.33E-05	4.140505	1.44E-05	22.32214
18.76954	4.429769	7.327482	2.970144	0.023694	2.091313	1.382006	13.30906	5.821804
0.444538	1.560779	0.384798	0.346732	2.77899	0.262655	0.503463	0.342381	0.000476
1.015967	2.02956	1.121017	4.292078	1.712706	3.387864	2.826277	1.859084	1.615281
1.94311	0.697908	1.34513	7.456118	0.023694	5.864084	3.618499	1.572035	1.861999
1.495043	6.454416	1.096335	17.15902	16.5625	10.80505	9.751039	4.091667	1.534603
0.353016	1.10132	0.384798	2.363442	2.31404	5.330519	2.164113	1.615281	0.935115
1.510014	0.839365	0.648687	9.989221	12.63773	0.879913	0.719177	0.313312	0.311227
0.787987	1.190491	0.455325	2.192496	1.845519	1.757244	1.555289	2.078355	0.340245
2.212619	1.662439	4.529913	0.174776	0.144293	3.116732	3.921645	1.969733	0.730476
0.19132	4.540794	0.744113	6.978903	4.451154	3.425512	0.700058	0.606154	0.331558
35.27608	11.65791	24.50942	3.002576	0.023694	20.51271	8.745238	8.445743	8.412367
0.000354	0.065354	0.067446	0.080281	2.269134	0.005382	0.026291	1.44E-05	0.041782
0.370657	4.946587	0.28899	10.48101	28.87506	3.314329	4.597792	6.580988	1.154567
7.765931	4.048499	3.993665	1.031284	0.334625	9.031082	6.404921	5.172828	7.495397
0.066537	0.340866	0.184719	1.300368	0.566104	1.139221	0.29671	0.029553	0.022958
19.99861	13.15079	28.54786	4.875527	1.87899	12.70768	19.83727	10.93101	18.48514
6.570433	2.196017	8.608153	2.238341	1.87899	2.676392	5.323492	4.091667	4.603434
0.81596	1.888023	0.249899	1.179885	0.023694	8.005685	1.517143	10.67991	0.388326
0.077027	0.092193	0.001118	0.853218	0.636688	0.294271	0.265014	1.44E-05	0.000476
9.968355	14.25954	11.30987	38.94854	30.49289	16.68554	19.22139	16.9777	15.66023
12.22798	2.941544	18.91854	1.399315	0.023694	4.33E-05	0.562439	3.743232	2.102626
50.55949	105.1694	70.11932	67.98069	37.29684	64.12971	72.69473	75.1132	105.1694
0.127201	0.279452	0.221732	0.826407	1.92272	0.405094	0.465801	0.27759	0.300094
12.60233	6.162446	2.925426	0.690375	24.73724	1.355527	2.884762	24.04374	0.156089
20.51271	20.16541	24.73724	4.440548	7.276645	8.089848	10.23768	10.62739	7.149618
0.000354	0.120144	0.001118	0.413449	0.023694	0.96329	0.000685	0.222193	0.000476
0.047792	0.030613	0.133939	0.034416	0.281364	4.33E-05	0.000685	1.44E-05	0.031818
2.109755	0.863694	8.412367	0.308672	0.023694	0.671724	0.866717	0.813261	1.230411
48.53429	10.03313	33.07147	15.66023	2.950173	2.121198	9.256537	28.54786	4.096407
0.000354	0.039214	0.032172	0.100101	0.203593	0.023651	0.059177	0.052338	0.022958
2.236407	0.319163	0.425635	0.335411	0.023694	1.742734	0.630022	1.87482	2.823636
0.388023	0.308788	0.077316	1.202626	2.404618	1.05153	0.597417	0.541804	0.442799
0.057352	0.319163	0.001118	0.624473	1.192633	0.475137	0.000685	0.204156	0.000476
2.396429	14.79742	3.310758	8.256465	1.778153	7.327482	9.418196	11.60283	4.821176
0.311227	2.768312	0.63114	1.749307	6.865	1.408831	0.465801	0.938911	0.24518
11.22667	6.591544	13.83359	5.42381	1.521371	4.644098	4.535354	7.653391	7.315512
43.49658	111.5439	23.6012	59.32013	9.480736	28.23632	83.489	95.22939	14.79742
0.160332	0.242893	0.105873	0.090772	2.180022	0.180368	0.105498	0.052338	0.000476
0.019848	0.141623	0.049769	0.23873	0.772157	0.091306	0.141825	0.213615	0.050519
32.31123	26.52537	19.99861	77.74812	7.882222	118.7028	95.22939	59.32013	41.47925
2.535974	1.977316	1.072056	1.678319	1.815	1.806595	0.542525	2.099524	2.425801
0.066537	0.850808	0.122944	1.607143	0.586595	0.283882	0.265014	0.342381	0.156089
0.238889	4.808658	0.192929	2.597792	34.6132	0.879913	1.791616	7.625209	0.999502
0.000354	1.369048	0.23246	2.019618	18.91854	1.062821	0.04886	1.924228	0.378889
1.240202	10.93101	1.433189	7.025599	50.55949	2.887489	3.217612	4.016465	3.363911
0.11803	3.7586	0.192929	3.78798	1.675722	0.96938	0.630022	0.72044	0.108413

12.49452	75.1132	12.36443	91.05541	87.1746	144.7919	31.55417	45.91841	31.55417
0.000354	0.023954	0.032172	0.260584	0.702381	0.108413	0.092511	0.204156	0.041782
4.058514	2.229048	6.933961	3.816551	1.2214	0.50254	0.763939	1.321053	1.997352
0.057352	0.030613	0.001118	0.04062	0.227063	0.049336	0.000685	0.016681	0.022958
0.471746	2.727785	0.376595	5.34474	40.7932	2.500548	1.331919	1.444906	0.696234
1.188139	9.583463	1.477388	12.93651	9.604358	11.14619	14.12978	4.863579	6.297136
0.877807	6.591544	1.096335	7.162698	15.66023	6.181652	2.826277	4.502453	4.419062
0.000354	0.160584	0.077316	0.393449	1.2214	1.20824	0.265014	0.005224	0.031818
4.814661	1.514199	6.135007	2.638139	0.023694	7.061328	6.830592	1.859084	8.994791
1.163579	1.591299	1.39847	2.941544	2.565556	1.230411	1.313737	1.094978	1.483622
0.136147	1.433189	0.001118	1.418312	7.61176	1.090072	0.34443	1.482626	0.080592
21.02638	7.238023	22.32214	3.35715	3.47531	5.485685	15.77678	14.51648	10.01009
2.489762	1.164257	0.724885	0.100101	0.023694	0.933874	1.02798	4.039192	2.230859
1.978954	1.061457	1.632648	0.589618	0.541241	0.572987	0.381999	2.461905	0.347908
0.485325	2.122799	0.275058	3.411097	2.950173	0.136356	0.392251	2.55184	1.070693
37.81919	11.719	37.81919	30.49289	14.79742	21.36892	20.34063	21.02638	23.6012
6.508427	4.02088	7.495397	2.178268	2.710765	7.570693	7.188095	1.498167	6.967547
0.379192	1.10132	0.167273	2.955916	0.363276	0.50254	0.30596	1.498167	0.217172
2.270837	16.5625	2.581861	18.33909	17.71696	15.88548	5.037489	9.459177	3.034747
37.29684	4.397792	65.94551	5.530274	0.736977	14.93173	32.31123	21.36892	38.94854
1.217475	1.087345	2.828918	13.23022	3.644343	13.23022	0.588954	1.232078	1.29342
0.256046	0.611003	0.376595	0.23873	0.253492	1.05153	0.5229	0.459582	0.616739
0.63114	7.08733	0.816479	8.103925	2.77899	31.55417	5.646811	4.424416	3.381255
2.063362	0.82772	9.179466	4.096407	0.023694	11.96574	5.80469	1.89228	5.855281
4.048499	5.993045	14.51648	2.447294	0.515527	1.20824	7.854069	5.037489	4.365224
2.750375	4.851227	3.175916	12.70768	4.927302	6.978903	7.136537	3.26	7.710361
0.471746	1.757244	0.686667	9.687496	7.991602	4.25658	2.074906	2.361198	1.661328
0.077027	0.023954	0.067446	0.070339	0.438831	0.005382	0.059177	0.037908	0.041782
10.48101	30.49289	8.888167	28.54786	14.97762	28.54786	27.52753	15.07257	13.42179
4.690159	5.993045	6.967547	2.155447	1.409942	4.535354	3.566753	3.38456	3.131544
5.309264	4.02088	2.479221	0.308672	0.334625	1.090072	1.072843	4.212554	1.070693
9.838658	5.762965	2.597792	2.839351	0.566104	3.8379	2.836717	11.14619	3.230065
3.939307	3.460909	4.715599	3.439437	0.253492	8.149307	5.821804	3.052655	5.137042
0.152316	0.659582	0.20215	0.215115	0.848369	0.813788	0.252468	0.439776	0.378889
0.077027	0.160584	0.05824	0.280823	2.114437	0.241688	0.105498	0.146003	0.060426
0.221277	1.615281	0.945483	0.624473	1.92272	1.298665	1.331919	0.529697	0.721335
0.283709	1.615281	0.242453	5.568391	2.081696	2.185556	1.759986	1.273636	1.733766
0.097547	0.542828	0.455325	2.111299	5.599228	1.541227	1.043608	0.563499	1.409942
57.84882	5.855281	83.489	3.816551	0.636688	5.821804	23.82354	3.679986	34.38341
3.610707	0.432698	4.827691	0.249538	0.390014	0.717027	1.072843	0.833975	1.507835
0.63114	0.421342	1.816436	4.003117	0.949827	1.435772	4.012049	0.240346	1.114618
0.787987	3.899993	1.64917	9.480736	20.94257	14.00414	4.971991	6.162446	2.016515
0.688182	13.38418	1.055628	10.19468	0.414473	16.9162	11.09133	1.022338	2.139928
6.250447	5.316378	1.670786	4.965801	12.46209	2.384141	1.154567	19.37139	6.760765
6.135007	27.66779	6.760765	37.81919	27.96248	26.80355	16.68554	26.52537	9.541645
15.56131	2.08632	10.38202	155.7103	1.629235	72.69473	184.443	30.17192	75.1132
6.876392	1.443983	13.91714	0.933874	11.25392	0.609509	5.337547	1.034315	3.144156
5.728615	0.242893	1.308167	0.536263	2.353247	0.475137	0.891775	3.095166	6.191515
2.535974	3.864827	3.640606	4.227302	0.490664	6.888297	8.888167	4.353925	4.160238
0.42715	3.055729	0.20215	3.644343	2.950173	5.454949	3.855952	1.545657	2.316075
0.221277	0.224466	0.376595	0.080281	1.983824	0.099812	0.216602	1.421126	0.108413
0.038535	0.030613	0.041097	0.194235	0.490664	0.065974	0.036696	0.072381	0.060426
2.479221	4.894509	2.802828	8.319019	0.848369	12.22798	6.945152	6.622092	8.164531
11.57341	16.34294	6.09759	19.99861	4.292078	19.83727	14.42661	13.53913	15.98892
0.108506	4.33E-05	0.001118	0.022511	0.023694	0.010014	0.000685	1.44E-05	0.022958
0.000354	0.215599	0.096667	0.137201	1.010289	0.159639	0.105498	0.072381	0.196097
6.591544	70.11932	10.01009	22.52375	72.69473	203.5795	42.75576	30.82277	17.09911
0.353016	1.378362	0.639495	0.753882	5.679502	1.995714	0.503463	0.606154	0.62689

0.000354	0.030613	0.001118	0.184877	1.436623	4.33E-05	0.000685	0.02311	0.000476
3.734762	55.04081	7.417359	29.18885	64.12971	26.52537	39.53687	9.792626	59.32013
2.057056	8.524242	1.757244	5.830152	3.47531	5.075447	4.086926	4.575123	4.077403
0.000354	0.030613	0.041097	0.12904	3.442886	0.016277	0.04886	0.002424	0.070909
0.066537	0.994899	0.041097	1.479452	14.17388	1.030678	0.381999	0.518139	0.080592
0.136147	2.018167	0.320563	2.621133	7.683766	0.954776	1.085224	0.666097	0.424004
29.49693	126.1556	21.36892	224.9555	135.5245	332.0776	91.05541	126.1556	51.52195
2.281926	10.82954	1.527792	21.02638	3.6029	27.38726	11.33981	12.26136	6.230245
6.268312	35.27608	6.717056	45.91841	19.53061	37.81919	15.18102	28.23632	9.583463
2.404618	1.252424	2.174733	1.781948	0.023694	1.286494	0.784805	1.482626	0.857778
224.9555	224.9555	167.9996	7.854069	6.933961	24.50942	9.751039	252.4251	14.97762
6.717056	24.98218	8.256465	21.64437	13.75978	20.34063	13.6814	12.63773	8.923117
1.47842	6.853608	1.416407	3.223882	7.200505	1.179885	2.164113	2.489762	2.572309
1.262965	0.962698	0.350455	3.24658	1.491977	1.795851	1.288276	2.755411	4.251818
2.591443	2.238341	1.626782	6.116638	12.93651	2.547179	1.001414	2.394416	1.766248
2.043795	23.93364	1.953535	22.8563	3.944084	17.15902	10.33476	8.643074	8.762677
0.311227	23.6012	2.773644	7.212915	155.7103	1.898153	6.508427	11.544	3.55961
0.057352	1.544517	0.001118	0.988932	13.04605	0.108413	0.392251	0.23101	0.000476
3.008225	13.60866	0.425635	10.73084	9.838658	2.333867	2.589185	24.62333	2.35733
13.23022	22.32214	19.06893	37.29684	184.443	12.03267	18.19595	25.99427	16.68554
17.92188	48.53429	22.96463	75.1132	2.871212	91.05541	59.32013	34.84299	72.69473
0.570317	1.451775	0.269322	14.79742	11.86763	8.429055	1.682193	2.569957	1.377446
5.366335	25.48179	6.153102	19.68088	21.93452	10.93101	16.5625	14.6058	15.88548
0.994899	1.757244	1.597908	6.865	0.023694	5.287857	4.039192	1.192633	4.096407
19.83727	8.849697	32.31123	28.23632	3.163506	11.719	19.06893	6.240346	25.24367
0.853218	0.697908	1.615281	2.871212	1.815	2.316075	2.752893	2.234538	2.696061
7.212915	5.150693	26.80355	1.766248	0.023694	0.76	5.24562	3.464315	3.394286
56.39528	15.3618	27.38726	27.81514	7.532713	31.1753	22.96463	39.53687	40.7932
0.000354	0.764315	0.001118	1.884935	13.23022	1.65785	0.092511	0.19544	0.121147
0.327143	0.650101	0.20215	1.191905	2.90658	1.401068	0.755253	0.289935	1.130794
6.443925	3.442886	1.266255	4.408153	0.023694	1.603795	4.292078	3.188918	5.359134
1.070115	3.644343	0.400354	3.058802	0.363276	3.659668	0.81544	3.363911	1.203427
0.576638	5.447114	0.212049	0.454156	8.365072	0.793918	0.579495	0.92114	1.114618
3.025671	3.957734	6.30671	1.857677	0.88417	3.439437	2.461905	4.907929	2.261573
0.000354	4.33E-05	0.001118	0.027619	0.281364	0.005382	0.000685	0.010202	0.000476
10.28573	51.52195	9.335397	55.04081	24.2842	24.04374	20.16541	18.62711	14.70188
47.5018	287.197	64.12971	749.7839	425.62	87331.38	135.5245	1360.293	167.9996
75.1132	60.77449	75.1132	252.4251	224.9555	167.9996	111.5439	155.7103	287.197
0.108506	2.178268	0.105873	0.913514	2.048781	3.792266	0.866717	1.220628	0.721335
41.47925	17.78522	37.29684	20.16541	6.772763	33.93939	33.93939	33.07147	26.52537
0.127201	1.544517	0.144228	2.192496	1.102691	1.553074	0.288247	0.498636	0.432468
2.092987	5.395584	2.468117	2.597792	7.149618	4.56912	2.810678	2.642525	2.758088
0.182237	0.650101	0.343824	1.591299	3.833247	0.232734	1.165065	0.267201	0.254574
0.274055	0.049062	0.469711	3.521934	0.023694	2.165851	4.058514	0.090476	1.011486
0.000354	4.33E-05	0.001118	0.055498	0.363276	0.099812	0.079755	0.052338	0.070909
3.077316	2.349351	3.552049	38.39042	3.78798	18.19595	6.727633	21.93452	10.80505
7.112496	1.906551	10.45584	144.7919	0.848369	75.1132	167.9996	15.18102	37.29684
0.174776	0.120144	0.001118	0.547778	0.949827	0.272814	0.04886	0.256941	0.050519
70.11932	49.50903	50.55949	100.0998	14.47154	59.32013	35.776	135.5245	126.1556
0.000354	4.33E-05	0.001118	0.000606	0.308442	0.049336	0.000685	0.016681	0.000476
14.00414	20.85876	14.33939	47.5018	2.048781	15.77678	43.49658	29.49693	33.07147
0.000354	0.319163	0.001118	0.675938	1.957958	2.102626	0.000685	0.090476	0.000476
0.979149	2.591443	1.360022	2.474834	3.78798	0.693925	1.837172	1.915455	0.522367
0.000354	0.224466	0.087229	0.000606	1.491977	0.377085	0.079755	0.081587	0.262965
0.019848	4.33E-05	0.001118	0.034416	0.490664	0.005382	0.026291	0.002424	0.000476
0.000354	1.493968	0.001118	0.271133	2.114437	1.081544	0.318889	0.70886	0.584134
23.49249	43.49658	20.85876	42.08807	31.55417	27.10502	45.1045	56.39528	31.1753
3.57057	8.103925	4.965801	4.130794	43.49658	6.760765	3.552049	12.85504	7.188095

3.125844	18.91854	5.18754	10.67991	8.303947	15.98892	6.38456	7.315512	4.776407
3.286652	12.67271	3.625382	14.42661	12.46209	5.359134	7.625209	8.958225	6.036818
3.422237	0.611003	2.022778	1.058896	0.023694	2.611797	0.700058	1.64917	3.1857
0.000354	4.33E-05	0.001118	0.000606	0.669358	0.005382	0.026291	1.44E-05	0.000476
8.780115	29.49693	3.842554	29.82828	25.48179	17.51961	13.53913	50.55949	8.319019
9.646284	21.93452	18.19595	11.36975	9.989221	21.93452	15.88548	16.80343	8.888167
0.000354	4.222583	0.152532	1.337431	20.08201	1.776739	1.260635	1.321053	0.27241
1.485483	2.635685	1.055628	1.217475	0.414473	1.720144	2.850058	1.365296	3.820758
0.806652	3.113478	0.775339	2.019618	3.78798	1.577569	0.60824	1.71518	0.534214
3.87329	13.75978	3.672078	15.276	12.22798	12.13485	9.583463	6.191515	5.381227
0.379192	1.215195	0.615584	6.171789	3.707136	6.738211	1.277843	1.20824	0.919192
4.827691	2.145137	2.855296	2.447294	2.269134	1.706219	4.519048	3.144156	3.486436
5.881342	20.68746	6.830592	24.98218	46.7056	11.89932	10.82954	12.70768	19.68088
19.06893	5.210317	19.53061	126.1556	1.044235	44.27012	20.68746	18.91854	29.18885
0.057352	0.251371	0.041097	4.348882	0.023694	1.01772	0.66526	0.165657	0.857778
10.8813	17.22048	16.9162	7.545584	0.023694	8.815123	16.44768	13.75978	13.42179
0.047792	0.160584	0.067446	0.376407	1.712706	0.180368	0.116494	0.323319	0.108413
0.583369	26.12492	0.648687	70.11932	65.94551	36.74522	9.902576	24.98218	7.964315
0.077027	0.329798	0.242453	2.08798	3.676032	0.39062	0.277763	1.749307	0.456818
13.91714	3.68381	3.754719	2.701053	0.772157	1.286494	2.417424	28.87506	1.097677
0.283709	1.264589	0.88316	0.636291	3.529466	1.757244	0.55215	0.459582	0.156089
1.426522	0.329798	1.640779	1.072056	0.586595	0.39062	4.18456	0.571674	1.74539
5.28039	7.839271	8.226219	33.07147	38.94854	30.82277	8.994791	20.68746	7.653391
0.019848	0.17619	0.049769	0.728088	1.044235	0.908773	0.152749	0.081587	0.156089
15.276	15.98892	3.695815	2.987835	1.957958	9.989221	8.256465	48.53429	9.502294
16.5625	9.625253	22.52375	6.945152	4.814661	8.288874	18.55613	7.991602	12.77772
0.000354	0.17619	0.001118	0.100101	0.144293	0.031775	0.116494	0.176104	0.000476
2.196017	8.608153	1.569553	7.991602	9.838658	4.72798	5.11697	6.464906	3.544257
0.11803	6.045209	0.863189	0.839365	10.05618	4.110779	1.968283	1.331919	1.212792
0.605455	0.925801	0.535657	0.70404	0.203593	0.750404	0.175469	0.27759	0.156089
5.088694	0.329798	4.626025	0.346732	0.023694	4.33E-05	0.000685	0.582973	0.432468
0.749481	4.456912	0.497749	1.498167	1.010289	3.471486	2.107922	2.099524	1.377446
8.048095	22.13245	6.287561	64.12971	11.19942	135.5245	36.74522	27.38726	4.821176
3.407619	0.904286	5.583968	8.445743	287.197	530.1465	8.226219	5.439278	33.93939
0.583369	0.733593	0.63114	1.217475	1.010289	2.531515	1.277843	1.432302	1.273636
0.000354	4.33E-05	0.363276	1.169791	83.489	0.159639	0.187229	0.186356	0.090245
1.173535	0.678687	0.593853	0.184877	14.47154	0.620014	1.013434	0.27759	0.503781
0.538427	2.434444	0.463102	0.933874	0.982128	1.631544	1.02798	1.232078	0.606154
0.028622	0.010043	0.001118	0.000606	0.203593	0.023651	0.000685	0.016681	0.050519
0.583369	1.859084	0.700058	1.798535	4.72798	2.051962	0.942944	0.108997	0.000476
0.000354	2.958773	0.001118	4.597792	6.727633	2.227273	1.555289	2.331825	0.000476
0.000354	0.101104	0.001118	0.194235	0.161962	0.159639	0.252468	0.222193	0.031818
1.180613	1.175859	0.753449	2.66404	0.281364	4.241905	2.723247	1.765065	0.875101
0.000354	0.039214	0.001118	0.194235	6.433918	1.05153	0.277763	0.029553	0.196097
0.038535	0.010043	0.001118	0.10938	0.227063	0.016277	0.079755	0.029553	0.060426
0.152316	0.110642	0.087229	0.863694	1.268672	0.108413	0.55215	0.439776	0.311227
1.593535	2.500548	2.174733	0.31702	0.636688	4.869553	1.154567	2.461905	1.699091
1.022338	0.251371	3.230065	0.290317	2.014863	0.241688	2.031169	0.165657	0.432468
29.18885	3.137576	31.1753	8.780115	0.023694	6.210375	24.04374	1.44E-05	95.22939
3.975498	2.925426	6.876392	0.290317	0.490664	0.353795	0.532857	3.230065	1.916782
167.9996	28.23632	67.98069	5.606739	0.982128	11.65791	5.646811	91.05541	32.68313
0.057352	0.260144	0.096667	0.027619	0.566104	0.225339	0.175469	0.146003	0.133189
0.077027	0.532857	0.297532	0.271133	1.675722	0.82684	0.216602	0.289935	0.616739
0.570317	0.764315	0.463102	1.736133	5.80469	2.278254	0.743694	1.444906	0.635916
1.046061	2.621133	0.001118	7.6393	6.063276	2.547179	3.321284	2.489762	3.381255
1.621032	3.182482	5.704646	0.000606	0.334625	4.33E-05	0.000685	2.461905	0.000476
0.000354	0.017063	0.049769	0.022511	0.18153	0.005382	0.000685	1.44E-05	0.000476
1.728615	1.070115	2.436768	1.360022	0.949827	4.72798	2.810678	1.905094	4.302367

3.55583	2.333867	5.560563	2.238341	0.023694	1.466111	1.382006	2.114437	1.521371
4.592273	4.440548	3.957734	0.440801	0.023694	1.150635	2.984899	4.827691	1.842734
0.000354	0.152114	0.001118	0.100101	4.851227	4.33E-05	0.105498	0.363492	0.000476
5.056421	9.256537	2.642525	7.710361	0.772157	16.34294	13.60866	8.075772	6.717056
5.485685	3.663622	3.895469	9.179466	2.404618	4.776407	4.927302	3.022713	9.459177
0.160332	0.039214	0.001118	0.249538	0.736977	1.945916	0.65202	0.606154	0.677403
0.972504	0.160584	0.23246	0.000606	0.917431	0.010014	0.092511	1.696494	0.031818
0.174776	0.775339	0.311544	1.132165	4.783001	0.252165	0.4457	0.267201	0.178442
5.195022	7.136537	4.609076	2.611797	18.19595	1.165065	2.274473	3.921645	3.648081
4.38684	2.750375	5.024293	1.320173	0.023694	0.609509	1.331919	1.783203	0.721335
14.42661	33.93939	14.88584	31.55417	0.281364	105.1694	55.04081	20.85876	39.53687
0.622821	1.205685	0.716234	1.455988	0.669358	1.329206	0.755253	1.076652	0.522367
0.000354	0.039214	0.001118	0.080281	0.363276	0.010014	0.04886	0.016681	0.000476
0.212172	0.195823	0.257143	1.217475	0.949827	1.435772	1.313737	0.342381	1.169791
27.66779	4.33E-05	40.7932	0.000606	0.023694	4.33E-05	10.19468	1.44E-05	8.573434
1.163579	0.049062	3.350224	0.000606	0.023694	4.33E-05	0.000685	1.44E-05	0.730476
10.23768	11.83593	15.18102	6.486104	3.904517	18.62711	18.91854	10.80505	11.89932
25.99427	5.881342	21.74051	7.112496	0.023694	3.144156	11.19942	8.539971	23.16622
0.097547	0.753882	0.067446	0.495584	0.810491	1.91254	0.60824	0.489509	0.554416
27.10502	4.33E-05	27.10502	0.000606	0.023694	6.591544	26.80355	0.186356	30.49289
4.105714	4.33E-05	11.89932	0.000606	0.023694	0.717027	1.043608	1.192633	0.773766
3.113478	5.704646	1.906551	6.819481	15.276	3.404372	4.376118	1.351573	3.340361
0.11803	0.723066	0.144228	3.4679	6.25938	1.370765	0.403413	0.803665	0.606154
0.396472	1.087345	0.122944	0.959062	2.673846	0.313723	0.357727	0.498636	0.236638
8.888167	5.18754	9.418196	1.031284	1.305772	1.435772	4.125786	4.150274	6.345382
0.202294	2.069942	0.184719	0.974257	2.710765	3.098268	1.724805	1.060188	0.799048
0.711371	0.092193	0.548153	0.393449	0.227063	0.448824	1.578687	0.82355	0.503781
0.563824	0.744113	0.692799	0.440801	0.161962	0.353795	0.719177	0.459582	0.696234
25.24367	15.276	27.96248	5.872886	1.629235	4.33342	17.92188	13.83359	9.297085
3.236457	1.184185	1.582915	0.31702	0.023694	3.083319	3.687633	1.295108	4.135801
1.360022	0.41026	0.724885	0.70404	0.023694	0.30373	0.240346	1.180613	0.561775
10.05618	0.010043	45.1045	0.000606	0.023694	29.82828	2.939141	0.240346	8.195657
0.212172	0.215599	1.096335	0.558824	0.023694	0.099812	0.105498	0.33329	0.866717
3.726854	2.267157	8.134084	0.228593	0.023694	0.116825	0.966825	1.815	1.661328
0.965065	3.166659	1.553074	1.288276	0.414473	6.116638	2.361198	0.863694	4.678846
0.000354	0.329798	0.001118	0.228593	0.308442	0.283882	0.12904	0.02311	0.000476
0.688182	1.205685	0.490079	0.646378	0.203593	2.440895	1.06355	1.304877	0.810967
0.230606	1.236248	0.535657	5.454949	5.950664	1.826147	0.826407	1.321053	1.105426
3.35355	9.792626	15.276	10.05618	0.586595	13.75978	18.55613	5.454949	18.05488
0.066537	4.33E-05	0.001118	0.547778	18.76954	0.205931	0.187229	0.313312	0.000476
0.5129	0.511667	0.905426	1.058896	8.643074	0.136356	0.81544	1.87482	0.616739
1.673254	0.442193	6.191515	2.345527	0.390014	2.255916	2.485534	1.20824	1.937222
22.32214	5.47	7.854069	0.454156	0.023694	11.03843	9.667316	12.06778	6.38456
0.144488	0.974906	0.05824	0.12904	0.334625	0.426645	0.331688	1.390476	0.080592
4.376118	0.329798	7.288788	0.184877	0.023694	0.466335	0.925801	0.885664	2.373824
0.038535	4.33E-05	0.032172	0.04062	0.023694	0.010014	0.026291	0.002424	0.060426
0.202294	0.550974	0.269322	0.421804	1.044235	0.890065	0.514885	0.186356	0.186587
4.188896	2.786898	1.608427	0.249538	0.023694	1.277843	1.591299	2.349351	3.211176
2.104358	0.994899	2.90658	7.911934	4.402973	2.398442	0.588954	1.076652	1.640779
0.19132	1.888023	0.311544	4.307309	2.673846	5.530274	3.204632	1.331919	1.688045
0.000354	0.049062	0.041097	0.000606	0.334625	0.016277	0.000685	0.02311	0.031818
155.7103	7.288788	77.74812	6.25938	5.80469	53.77619	144.7919	27.96248	87.1746
1.434921	0.952294	1.695216	1.002215	0.669358	0.632857	1.288276	0.803665	0.773766
0.028622	0.039214	0.144228	0.062035	1.491977	0.082973	0.116494	0.010202	0.060426
1.371602	19.99861	1.640779	15.02509	111.5439	2.296732	5.088694	6.900202	5.737056
5.687879	1.306667	3.939307	1.514199	0.023694	6.036818	1.806595	5.687879	20.68746
62.33472	34.84299	51.52195	23.38378	0.88417	13.15079	30.82277	62.33472	36.74522
0.000354	0.805801	0.001118	1.962424	27.10502	0.550664	0.105498	0.70886	0.108413

0.311227	4.33E-05	0.384798	0.000606	0.023694	4.33E-05	0.116494	1.44E-05	0.000476
0.496494	1.433189	1.296905	1.438672	4.348882	2.384141	0.542525	1.340981	0.168961
1.205685	0.659582	2.058773	2.638139	1.521371	1.20824	1.691587	0.529697	2.132756
0.000354	0.141623	0.001118	0.366674	1.983824	4.33E-05	0.000685	0.118759	0.178442
0.000354	0.120144	0.001118	0.10938	0.281364	0.091306	0.079755	0.016681	0.041782
30.17192	10.73084	36.26061	1.288276	0.023694	4.007583	24.98218	0.954776	135.5245
5.942294	2.378124	5.493182	1.103261	0.88417	1.68938	3.128694	1.950519	5.266335
0.097547	0.485577	0.087229	1.249221	9.031082	0.919192	0.55215	0.582973	0.763102
3.975498	6.508427	2.818312	4.203001	0.438831	11.77567	10.57984	8.888167	6.011515
0.832554	0.952294	0.905426	0.853218	8.643074	0.781977	1.085224	0.946061	0.866717
6.52899	40.7932	4.519048	25.24367	60.77449	13.34662	4.650007	14.25954	4.048499
1.763889	0.130115	2.207417	1.857677	0.736977	0.996696	1.881955	0.313312	5.23176
0.066537	0.130115	0.041097	0.290317	0.810491	0.39062	0.12904	0.045346	0.060426
0.000354	0.141623	0.001118	0.31702	0.308442	0.016277	0.036696	0.005224	0.031818
0.61355	1.472208	0.563499	1.117316	2.114437	1.242641	1.116609	0.82355	0.891176
6.335664	10.38202	4.888023	17.64869	3.29355	38.39042	29.18885	10.19468	15.77678
0.230606	3.404372	0.184719	6.55039	0.334625	2.463911	0.891775	0.946061	0.090245
0.127201	3.493362	0.041097	0.516825	8.573434	5.762965	0.796674	4.63189	0.442799
2.290996	0.707475	2.037114	0.974257	0.281364	1.20824	2.000592	0.632208	0.396638
3.025671	7.964315	4.105714	7.683766	32.31123	5.25254	5.42381	5.950664	3.080317
10.55571	2.178268	36.74522	32.31123	0.023694	55.04081	87.1746	1.815	22.96463
0.057352	0.764315	0.077316	0.184877	3.833247	0.483045	0.069163	0.204156	0.186587
0.000354	0.010043	0.087229	0.027619	0.18153	0.005382	0.000685	1.44E-05	0.041782
0.576638	2.155447	0.563499	4.328232	2.404618	1.516219	1.211299	2.234538	2.180022
0.000354	0.039214	0.001118	0.145685	0.281364	0.116825	0.059177	0.099913	0.000476
0.057352	0.361717	0.144228	0.602287	1.557482	0.342987	0.105498	0.165657	0.060426
15.46193	22.74797	14.79742	40.11561	33.07147	65.94551	29.82828	22.96463	24.2842
0.000354	0.269697	0.001118	0.000606	3.163506	0.353795	0.357727	0.165657	0.236638
0.019848	0.010043	0.032172	0.022511	0.363276	0.005382	0.000685	0.029553	0.000476
2.673846	0.390216	1.387612	0.145685	3.377619	0.171061	0.000685	0.644618	0.935115
0.000354	5.560563	0.001118	46.7056	0.023694	4.33E-05	0.000685	4.655916	0.000476
4.150274	1.685678	8.83241	1.884935	0.023694	0.116825	3.632973	3.052655	4.188896
11.77567	1.935685	30.82277	0.000606	0.023694	0.781977	5.923838	1.304877	4.067547
0.694675	0.668456	0.822381	0.300245	0.281364	0.225339	0.371075	0.582973	0.730476
2.145137	0.733593	2.243911	0.357244	0.023694	3.269913	3.747035	0.473016	0.935115
0.19132	4.894509	0.077316	1.058896	13.30906	0.56145	0.772626	0.98956	0.282078
0.411948	0.110642	0.376595	0.393449	1.845519	2.765758	0.000685	0.27759	1.169791
0.038535	0.017063	0.049769	0.000606	2.436768	0.769711	0.079755	0.118759	0.000476
1.739935	7.175397	1.883535	3.663622	14.65384	2.696061	3.407619	2.5271	3.095166
1.052886	0.81596	0.455325	0.10938	0.023694	1.577569	2.060491	0.553059	0.799048
0.000354	1.603795	0.23246	0.913514	4.644098	0.717027	0.4457	0.27759	0.217172
5.560563	0.597417	1.685678	0.646378	0.023694	2.026219	1.837172	3.346898	4.473348
0.000354	0.082973	0.001118	0.215115	0.848369	0.342987	0.079755	0.108997	0.041782
22.52375	1.844098	3.457504	2.871212	0.023694	5.976724	2.477027	13.15079	3.116732
0.343629	0.039214	0.077316	0.034416	0.281364	0.108413	0.000685	0.23101	0.022958
11.14619	1.794531	5.18754	2.556443	0.023694	1.355527	0.66526	5.898449	2.807937
0.979149	5.591717	0.384798	1.798535	0.982128	1.65785	1.806595	3.211176	1.169791
2.760765	3.6029	1.670786	0.428932	0.023694	1.564149	2.259661	1.232078	2.037114
0.077027	0.850808	0.041097	0.863694	0.669358	0.39062	0.079755	0.23101	0.396638
5.00404	0.994899	7.737807	0.000606	0.023694	0.609509	1.476429	0.002424	5.898449
0.490375	1.10132	0.408175	3.507691	7.315512	3.116732	1.980592	1.765065	3.718716
21.93452	5.381227	14.00414	0.280823	0.023694	9.179466	12.10289	5.71241	17.78522
18.91854	5.150693	14.17388	11.719	1.102691	7.375794	11.42512	17.92188	16.34294
0.247085	1.197922	0.159942	3.699495	0.949827	10.48101	0.925801	1.311674	0.432468
0.168023	1.033672	0.105873	1.262965	5.259437	1.006025	0.676659	0.782857	0.27241
0.028622	0.260144	0.041097	3.439437	3.377619	1.123016	0.331688	0.395642	0.121147
9.179466	4.33E-05	8.445743	0.000606	1.010289	9.924372	7.598312	0.052338	24.50942
5.165599	2.563506	1.232078	0.000606	0.023694	1.928629	2.609293	2.871212	3.019848

4.086926	6.900202	3.578038	4.546443	2.269134	4.990794	4.557605	6.653016	4.365224
0.283709	2.349351	0.384798	3.058802	4.292078	1.370765	0.477951	1.20824	0.347908
1.415469	6.297136	1.834423	5.25254	9.377345	2.361198	2.659214	1.340981	1.475469
0.945483	0.798153	0.555418	1.360022	6.144055	0.592208	0.5229	0.852172	0.48583
0.681068	1.215195	0.587273	1.711414	0.203593	1.408831	1.476429	0.351789	0.848369
4.292078	2.459812	3.507691	0.145685	0.917431	0.188745	0.73127	0.794401	5.01088
13.6814	5.545729	40.11561	2.410996	2.673846	15.276	37.81919	14.08569	7.276645
5.515584	12.93651	9.792626	8.381328	0.023694	10.8813	9.968355	4.203001	3.457504
2.073232	5.500678	2.735368	2.914632	0.253492	8.958225	5.872886	2.181775	5.638759
21.74051	10.1028	25.24367	0.000606	0.023694	4.33E-05	0.000685	15.276	0.412193
0.965065	1.631544	0.587273	2.248896	7.013853	0.717027	0.925801	0.675115	2.621133
1.57645	1.776739	1.857677	1.660216	0.949827	0.813788	1.537951	3.432388	1.283911
1.530159	1.34513	0.849336	1.132165	0.023694	4.592273	5.158146	1.304877	1.944517
1.23127	0.532857	0.281068	0.070339	0.949827	0.147554	0.026291	5.80469	0.196097
0.411948	0.573947	0.067446	1.217475	3.077316	1.123016	0.265014	0.595368	0.643853
0.202294	0.744113	0.096667	2.556443	4.086926	1.030678	0.392251	0.439776	0.949827
0.000354	0.764315	0.001118	0.384632	0.308442	1.266255	0.105498	0.118759	0.584134
0.300606	1.794531	0.184719	1.320173	0.702381	4.946587	0.772626	1.009625	0.432468
0.274055	0.130115	1.437648	0.690375	2.180022	0.125859	0.206984	0.473016	0.882619
2.162381	0.874091	1.25886	1.159913	0.023694	1.845519	1.185685	0.323319	1.070693
21.19547	4.33E-05	12.03267	0.000606	0.023694	4.33E-05	0.000685	1.44E-05	0.984069
1.400159	1.813564	2.276407	1.300368	0.917431	10.43066	3.273045	1.572035	4.445851
0.5129	1.155411	0.658745	0.516825	0.390014	5.143867	2.227273	0.70886	1.385801
0.077027	0.101104	0.077316	0.249538	2.269134	0.125859	0.228009	0.062381	0.156089
10.19468	1.560779	11.06488	0.818066	0.023694	4.212554	10.28573	3.080317	4.016465
5.770996	8.256465	5.679502	3.743232	0.438831	9.541645	5.671126	5.864084	9.256537
0.81596	0.467403	1.516219	0.145685	0.023694	0.272814	0.381999	0.118759	0.824416
3.119986	1.631544	2.618846	1.578687	0.023694	3.877417	3.425512	2.740519	4.203001
0.077027	1.403853	0.001118	0.959062	0.281364	2.078355	0.252468	0.684805	0.442799
0.247085	0.049062	0.113911	0.022511	0.848369	0.016277	0.092511	0.563499	0.000476
2.270837	2.860722	1.711414	0.357244	0.772157	2.520404	2.185556	2.197828	2.180022
7.315512	2.320072	7.991602	0.308672	0.023694	2.131176	13.75978	2.5271	8.539971
1.845519	7.037345	1.801284	5.150693	33.4985	4.46267	2.485534	3.998391	2.308203
0.388023	0.650101	0.152532	0.326017	1.521371	0.252165	0.206984	0.222193	0.546522
0.108506	0.478413	0.159942	0.164509	0.253492	0.39062	0.503463	0.240346	0.35798
0.256046	0.573947	0.363276	0.807136	4.33342	0.457749	0.277763	0.052338	0.571674
0.81596	1.10132	0.841746	1.660216	0.18153	1.01772	2.045476	0.833975	1.621032
3.94886	2.609293	3.711097	2.461905	0.18153	1.945916	2.369841	3.243225	5.477843
0.000354	0.039214	0.001118	0.027619	0.363276	0.031775	0.000685	0.005224	0.000476
0.028622	0.269697	0.077316	0.249538	0.130029	0.171061	0.079755	0.016681	0.000476
0.90772	4.150274	2.384141	3.78798	6.00228	2.463911	1.837172	1.20824	2.792035
0.799048	5.416623	0.571342	4.096407	0.982128	9.751039	2.873817	2.701053	1.985253
0.333636	36.74522	0.775339	2.474834	77.74812	2.928247	3.521934	6.695887	6.622092
0.097547	1.147807	0.28899	0.260584	0.023694	0.353795	0.116494	0.289935	0.217172
0.028622	0.224466	0.05824	0.027619	0.636688	0.188745	0.116494	0.005224	0.070909
16.22108	12.85504	11.96574	0.145685	0.917431	0.365859	1.043608	5.493182	3.750837
38.39042	8.888167	12.10289	1.766248	0.023694	7.519841	5.24562	5.515584	12.03267
2.800332	2.018167	1.252424	0.876118	0.88417	2.227273	2.24562	1.321053	1.755988
0.057352	0.058139	0.001118	0.090772	0.334625	0.07482	0.000685	0.081587	0.022958
0.019848	0.017063	0.041097	0.034416	0.363276	0.010014	0.036696	0.037908	0.031818
2.451645	1.403853	1.159149	0.440801	0.023694	1.524776	1.415469	2.141595	1.808023
30.49289	2.727785	13.53913	4.365224	0.023694	0.908773	9.838658	9.924372	8.048095
0.832554	0.251371	0.469711	0.602287	0.414473	0.252165	0.277763	2.065007	0.369834
4.733824	4.603434	4.261342	5.287857	0.023694	3.953297	6.945152	2.003723	4.058514
1.022338	0.453463	3.340361	0.863694	0.414473	1.786306	0.849841	0.529697	0.909315
1.039798	2.250462	2.073232	2.068297	11.719	1.553074	2.984899	1.557482	1.688045
0.097547	1.11526	0.001118	0.573326	2.014863	0.580404	0.228009	0.529697	0.291183
0.000354	5.576219	0.001118	4.502453	20.51271	0.39062	0.000685	2.410996	0.35798

9.218514	3.88153	4.424416	0.959062	1.192633	0.159639	1.104033	2.31811	1.123795
0.066537	0.017063	0.001118	0.047792	0.253492	0.56145	0.152749	0.675115	0.168961
0.000354	1.05088	0.175866	1.925635	1.305772	1.68938	1.196508	1.102691	1.082179
1.757244	0.597417	0.528196	0.690375	0.023694	3.06803	3.507691	2.165851	1.377446
1.400159	0.195823	3.493362	0.794401	0.023694	0.417063	1.528975	0.204156	0.432468
3.321284	2.474834	1.039199	3.966623	0.023694	9.335397	8.348817	2.941544	6.900202
0.000354	0.167835	0.001118	0.215115	1.629235	0.705231	0.116494	0.419827	0.207157
2.735368	2.871212	2.554069	0.944784	0.023694	6.55039	4.165137	4.424416	4.46267
0.92482	0.215599	2.101075	0.346732	1.244242	0.813788	1.599033	0.656003	2.089639
24.50942	8.303947	21.19547	8.412367	0.023694	28.87506	17.03919	11.42512	29.49693
1.217475	0.522633	2.668896	0.194235	0.023694	4.33E-05	0.392251	0.384394	0.962698
7.468961	5.416623	15.07257	0.119899	0.023694	0.196335	0.805238	1.519322	2.117814
15.07257	5.18754	17.51961	6.772763	7.238023	2.887489	16.9162	5.654863	16.9162
0.000354	0.224466	0.001118	0.215115	0.281364	0.065974	0.04886	0.23101	0.060426
1.262965	1.416407	1.296905	0.137201	0.023694	1.50684	1.348975	1.643218	0.999502
14.88584	11.544	11.25392	9.583463	0.023694	31.92385	27.10502	18.76954	30.17192
4.49671	1.279452	2.421681	0.145685	0.023694	0.262655	0.30596	1.380195	1.367179
2.893001	1.433189	1.808023	0.249538	0.515527	0.377085	0.331688	1.022338	0.080592
87.1746	19.22139	35.776	20.34063	0.363276	7.288788	40.7932	6.162446	3.5257
0.000354	0.707475	0.001118	0.454156	1.010289	0.740527	0.000685	0.553059	0.596508
1.495043	0.215599	1.360022	0.807136	0.308442	1.341825	1.578687	0.885664	2.498478
0.787987	5.110159	1.582915	5.898449	20.94257	2.185556	2.398442	1.915455	1.985253
24.2842	11.22667	30.49289	2.410996	3.47531	0.671724	50.55949	2.331825	224.9555
1.338261	0.167835	2.129596	0.228593	0.203593	0.405094	0.514885	0.176104	0.522367
3.500808	1.640779	2.292864	0.589618	0.772157	1.898153	1.791616	1.380195	1.890996
0.256046	0.453463	0.23246	3.223882	1.778153	2.421681	1.260635	1.444906	1.123795
0.396472	0.81596	0.001118	5.630815	0.334625	0.660332	0.503463	1.247244	0.217172
10.33476	14.33939	11.06488	3.370527	1.044235	18.76954	6.116638	4.312251	6.486104
2.043795	0.974906	2.607035	1.812114	1.044235	0.869365	1.555289	0.529697	1.230411
0.538427	1.357201	2.31013	1.498167	6.00228	0.705231	1.599033	1.827403	1.989711
0.000354	0.030613	0.049769	0.000606	0.586595	0.116825	0.059177	0.02311	0.254574
0.000354	4.419062	0.001118	0.31702	0.636688	0.262655	0.216602	0.256941	0.000476
1.89228	2.978968	2.468117	0.12904	0.023694	1.757244	5.606739	1.988196	4.212554
1.163579	0.885664	2.955916	1.058896	0.281364	1.995714	3.552049	0.508975	4.302367
0.694675	2.308203	1.030678	2.345527	1.102691	2.727785	1.806595	0.885664	0.686667
0.972504	1.575483	1.711414	4.067547	3.904517	2.640332	1.703795	2.461905	4.56912
2.429957	0.152114	4.650007	0.203254	0.023694	0.294271	0.981032	0.12798	0.254574
3.846984	65.94551	0.469711	118.7028	24.50942	45.91841	38.39042	77.74812	45.91841
1.127475	4.236962	1.308167	1.91254	3.57057	2.165851	1.313737	2.234538	1.070693
0.057352	1.184185	0.144228	0.483874	0.363276	1.453939	0.206984	0.204156	0.090245
0.000354	0.141623	0.05824	0.807136	1.629235	0.225339	0.141825	0.419827	0.196097
2.404618	2.600014	3.703175	0.164509	0.023694	1.384921	4.888023	0.96627	1.997352
1.673254	6.063276	1.546696	5.00404	0.736977	6.011515	4.376118	2.349351	3.1857
0.916869	2.045476	1.360022	2.23267	0.023694	1.945916	1.476429	1.950519	0.919192
0.221277	0.374365	0.328268	2.66404	2.474834	2.489762	0.73127	1.20824	0.616739
8.711905	24.50942	4.338629	27.10502	11.30987	22.74797	26.25557	26.80355	12.70768
3.824964	3.089293	3.779452	0.346732	0.515527	3.885642	2.339697	1.273636	2.898196
0.320563	0.640606	0.152532	0.624473	0.281364	1.006025	0.197908	0.595368	0.546522
1.010289	2.058773	1.906551	1.275325	0.949827	4.696313	3.204632	1.482626	3.201457
2.6143	1.700354	2.08632	1.016494	3.944084	0.632857	1.176623	0.844156	1.544517
2.429957	1.1379	5.316378	0.558824	0.023694	1.298665	2.361198	1.739935	3.975498
1.271876	0.453463	2.939141	0.31702	0.023694	0.531804	0.30596	0.763102	0.635916
0.108506	0.082973	0.001118	0.080281	0.253492	0.010014	0.036696	0.081587	0.050519
2.335844	3.201457	5.381227	4.827691	0.334625	4.46267	4.110779	1.631544	3.966623
17.64869	10.45584	18.76954	4.789596	1.712706	17.64869	4.535354	7.710361	2.725498
1.022338	5.316378	1.05153	3.022713	7.013853	0.82684	0.743694	2.55184	0.875101
0.212172	0.640606	0.623499	0.300245	2.014863	0.541804	0.381999	0.19544	0.412193
0.127201	0.374365	0.001118	0.384632	1.629235	1.298665	0.228009	0.267201	0.143579

0.264372	2.178268	0.615584	3.080317	8.849697	2.121198	1.393571	0.439776	0.866717
0.028622	0.390216	0.096667	0.100101	2.90658	0.437576	0.252468	0.108997	0.196097
1.146118	0.215599	1.96404	3.223882	0.023694	0.838846	4.888023	0.384394	1.237879
3.256587	4.33E-05	8.477662	0.000606	0.023694	0.417063	9.541645	0.844156	4.445851
0.945483	1.700354	3.327619	0.000606	0.023694	0.76	0.562439	2.384141	0.512641
0.127201	0.352222	0.067446	0.421804	0.88417	0.82684	0.277763	0.289935	0.121147
0.994899	1.164257	0.849336	3.13456	0.023694	0.890065	1.925635	1.631544	1.799899
1.152944	0.185909	0.648687	0.062035	0.60886	0.082973	0.288247	0.146003	0.512641
1.953535	1.1379	0.793918	0.73912	0.023694	0.572987	2.723247	1.749307	1.661328
1.110505	4.33E-05	4.397792	0.174776	0.023694	1.732468	5.01772	1.402915	7.08733
9.031082	6.240346	27.66779	1.058896	0.023694	4.212554	6.772763	6.433918	11.19942
0.038535	0.092193	0.041097	0.925801	2.710765	0.108413	0.105498	0.072381	0.000476
0.688182	4.484892	0.724885	5.485685	1.436623	7.375794	3.763045	2.852677	1.337431
2.145137	4.328232	1.20653	2.425801	0.363276	10.21618	8.815123	4.266753	5.80469
0.70329	2.102626	1.821861	6.036818	55.71804	3.750837	2.609293	1.682193	1.592395
12.10289	1.813564	11.719	5.266335	0.023694	1.230411	3.695815	12.49452	11.03843
1.468131	15.77678	2.839351	51.52195	126.1556	3.497085	8.164531	22.32214	9.968355
0.077027	1.416407	0.113911	3.182482	1.268672	3.855952	0.755253	0.72044	0.24518
6.784762	3.820758	4.752121	3.679986	0.023694	1.384921	1.260635	5.754935	6.876392
749.7839	118.7028	530.1465	95.22939	2.520404	1360.293	332.0776	530.1465	749.7839
2.212619	0.361717	4.94026	0.944784	2.404618	0.848831	1.043608	1.167561	1.615281
0.000354	0.668456	0.001118	0.12904	0.023694	0.241688	0.000685	0.33329	0.168961
0.92482	2.978968	1.177994	2.66404	29.49693	0.954776	0.805238	10.33476	0.686667
38.94854	3.442886	48.53429	19.06893	1.2214	15.56131	34.38341	5.123781	25.99427
0.168023	1.651522	0.20215	1.660216	5.381227	1.673254	0.265014	0.448824	0.156089
10.43066	17.03919	12.77772	9.179466	1.160707	6.365065	13.00996	6.09759	8.508514
4.174964	1.969733	0.275058	1.834423	0.023694	2.563506	12.63773	40.45441	18.76954
2.600014	3.404372	2.939141	0.769365	0.18153	8.923117	3.957734	2.349351	3.300664
1.288276	0.07316	4.18456	0.100101	0.023694	0.108413	0.597417	0.595368	0.291183
0.986797	5.23176	1.006025	2.730072	4.990794	3.659668	2.621133	3.13456	2.230859
0.108506	1.480541	0.328268	0.675938	5.082063	2.011501	0.55215	1.572035	0.596508
3.792266	9.397771	4.597792	15.02509	17.15902	7.625209	10.40634	13.08214	7.212915
0.000354	0.885664	0.001118	2.308203	2.048781	4.33E-05	0.000685	0.137063	0.000476
1.306667	1.126775	1.632648	0.260584	0.253492	1.898153	1.364401	1.87482	2.640332
34.84299	59.32013	23.82354	5.485685	3.529466	39.53687	18.05488	52.56128	30.82277
0.212172	1.019791	0.343824	13.83359	5.462475	7.264502	3.833247	1.192633	4.620159
0.000354	0.058139	0.001118	0.174776	0.463867	0.023651	0.000685	0.005224	0.080592
2.092987	4.946587	2.238341	8.849697	17.92188	8.226219	10.05618	4.150274	4.513622
0.843564	2.847547	2.276407	7.212915	2.642525	2.727785	7.911934	0.897006	5.088694
6.230245	4.49671	4.927302	5.959033	0.772157	4.135801	4.863579	4.473348	13.60866
1.749307	2.178268	1.757244	8.745238	0.438831	8.288874	7.238023	2.197828	3.486436
0.028622	0.260144	0.032172	0.440801	1.629235	0.196335	0.187229	0.27759	0.108413
0.247085	1.813564	0.724885	1.621032	2.673846	2.765758	3.037626	0.98956	2.792035
0.087302	0.195823	0.041097	0.602287	0.438831	0.717027	0.216602	0.12798	0.331558
0.182237	0.688975	0.23246	0.413449	0.203593	0.80417	1.132857	0.773276	0.606154
0.077027	0.058139	0.096667	0.047792	1.744055	0.031775	0.059177	1.44E-05	0.031818
0.770664	5.515584	5.316378	1.103261	2.145137	0.919192	4.833398	0.632208	3.101371
1.89228	0.130115	2.022778	1.202626	0.023694	1.706219	3.407619	1.765065	1.070693
4.990794	2.145137	2.839351	3.166659	0.023694	3.917597	1.196508	3.87329	3.909033
22.13245	9.838658	10.33476	0.119899	0.023694	1.706219	15.07257	4.067547	1.493968
4.072475	2.349351	1.20653	0.260584	0.227063	1.329206	1.806595	12.93651	1.306667
17.78522	12.1974	16.09482	4.160238	0.023694	8.780115	7.653391	15.83113	8.103925
1.544517	1.05088	2.263377	0.154978	0.023694	1.845519	1.578687	0.108997	2.621133
0.570317	0.584502	0.863189	0.366674	6.55039	0.457749	0.953506	0.165657	0.388326
0.589618	0.242893	0.328268	0.31702	0.308442	0.682799	2.008312	0.267201	1.385801
0.740952	3.851414	1.113304	8.134084	16.9162	5.095325	2.850058	1.87482	0.668456
0.097547	1.615281	0.113911	0.807136	4.56912	0.620014	0.318889	1.402915	0.156089
0.000354	0.185909	0.001118	1.232078	1.305772	0.205931	0.000685	0.240346	0.24518

1.485483	1.906551	0.303896	8.210938	1.629235	1.030678	1.196508	2.065007	0.721335
28.23632	2.693442	20.16541	0.839365	2.950173	0.945483	7.049336	17.51961	3.707136
8.256465	6.268312	13.60866	8.888167	0.848369	11.30987	11.544	3.552049	11.39744
0.70329	3.775209	1.335743	4.502453	22.96463	1.516219	1.980592	1.592395	1.273636
3.089293	3.031688	1.561912	0.516825	0.203593	3.144156	3.163506	1.682193	4.135801
0.274055	2.415296	0.175866	0.260584	1.491977	0.457749	0.331688	0.740101	0.196097
0.986797	0.938911	1.405772	1.872049	0.541241	0.919192	2.287576	1.167561	1.560779
0.174776	0.110642	0.049769	0.346732	0.023694	0.171061	0.04886	0.27759	0.000476
0.000354	0.522633	0.096667	0.080281	6.772763	0.04114	0.175469	0.240346	0.000476
4.338629	2.08632	5.068831	1.399315	0.736977	10.62739	6.945152	4.952915	5.044113
0.127201	1.325743	0.257143	0.357244	1.192633	0.550664	0.432915	0.213615	0.369834
0.000354	0.733593	0.001118	1.660216	1.491977	0.405094	0.059177	0.541804	0.000476
0.490375	3.747035	2.513752	1.132165	8.412367	1.102121	1.043608	0.863694	0.824416
0.90772	1.640779	2.404618	0.280823	0.203593	0.793918	0.81544	0.763102	1.056898
0.144488	0.361717	0.087229	0.04062	1.92272	0.147554	0.105498	0.108997	0.121147
1.121703	7.6393	1.770029	2.08798	2.710765	5.165599	3.679986	2.394416	2.349351
0.90772	1.416407	3.425512	0.454156	0.023694	1.631544	1.443983	0.909315	0.456818
2.961768	3.166659	3.869163	11.19942	0.023694	4.397792	10.01009	3.998391	53.77619
0.000354	3.137576	0.184719	4.272165	2.710765	2.750375	0.318889	1.656696	0.156089
1.146118	6.162446	2.396429	15.51162	6.933961	8.745238	3.140866	4.353925	2.678781
0.000354	0.07316	0.001118	0.164509	0.023694	0.283882	0.069163	0.137063	0.000476
0.000354	0.81596	0.001118	0.346732	1.010289	0.353795	0.000685	1.44E-05	0.000476
1.338261	1.019791	4.408153	0.070339	0.023694	1.072843	1.555289	0.213615	1.306667
0.000354	0.744113	0.001118	0.728088	0.023694	1.341825	0.152749	0.582973	0.35798
2.579509	25.24367	2.710765	35.776	57.84882	3.820758	7.570693	10.23768	7.545584
0.221277	0.650101	0.192929	1.749307	2.114437	0.80417	0.4457	0.699697	0.35798
1.880375	2.831501	1.449062	1.202626	1.778153	4.286962	3.286652	6.717056	1.890996
0.038535	4.33E-05	0.032172	0.04062	0.023694	0.010014	0.026291	0.002424	0.060426
3.592063	2.760765	1.177994	3.300664	0.281364	5.864084	5.217569	2.261573	3.6029
15.88548	19.37139	12.29473	15.3618	5.599228	42.08807	24.2842	13.6814	25.73165
0.622821	1.813564	0.518716	1.551962	7.38847	1.553074	0.700058	0.384394	1.260635
4.120815	6.819481	3.975498	20.68746	0.18153	29.18885	14.51648	2.895599	8.381328
0.038535	0.141623	0.001118	0.10938	0.253492	0.426645	0.141825	0.052338	0.050519
0.806652	0.374365	0.607273	0.89404	0.203593	5.80469	5.137042	0.582973	3.1857
0.000354	2.111299	0.001118	0.335411	0.736977	4.33E-05	0.000685	1.44E-05	0.080592
45.91841	8.815123	15.46193	11.06488	0.88417	2.250462	6.772763	31.92385	14.42661
0.019848	0.039214	0.067446	0.100101	1.074242	0.023651	0.04886	0.037908	0.041782
0.182237	0.058139	0.212049	0.000606	0.023694	4.33E-05	0.000685	0.072381	0.000476
7.570693	9.902576	5.545729	14.00414	0.669358	49.50903	17.78522	6.853608	12.56693
1.031284	0.251371	2.145137	1.275325	0.023694	6.64254	0.784805	0.240346	1.842734
22.96463	1.462143	18.33909	5.959033	0.023694	1.603795	6.560411	1.615281	10.8813
6.945152	3.842554	11.83593	2.289286	0.023694	4.110779	4.193232	3.273045	2.415296
1.544517	2.898196	0.548153	2.261573	1.465123	4.110779	1.663672	4.251818	2.540462
3.993665	2.035678	1.318384	3.718716	0.023694	2.600014	5.704646	1.220628	2.498478
0.92482	7.061328	1.582915	15.98892	32.68313	2.26518	3.895469	2.984899	2.159004
8.677489	2.212619	5.295325	0.675938	1.629235	0.933874	2.339697	4.721789	2.958773
5.967879	4.863579	6.653016	3.763045	1.010289	4.946587	3.779452	4.907929	6.591544
22.74797	8.711905	42.08807	3.507691	4.524481	4.535354	16.22108	12.56693	15.07257
0.000354	0.390216	0.122944	0.913514	2.520404	0.660332	0.12904	0.863694	0.000476
1.360022	0.904286	0.73583	1.044235	0.023694	0.682799	0.849841	0.782857	2.230859
2.567605	1.493968	1.441898	0.974257	3.730808	1.102121	2.417424	0.803665	3.687633
0.000354	0.839365	0.001118	0.228593	4.241905	0.671724	0.240346	0.155101	0.322078
0.019848	0.049062	0.001118	0.228593	0.227063	0.180368	0.105498	0.016681	0.099192
0.221277	2.871212	0.087229	1.514199	2.520404	1.123016	0.152749	1.935685	0.262965
9.646284	4.281847	22.74797	1.399315	0.669358	1.959466	5.704646	5.993045	8.256465
2.928247	1.685678	1.640779	1.337431	0.023694	1.68938	2.055289	2.781558	2.171429
0.300606	2.164113	0.480303	1.085967	2.710765	0.890065	0.477951	1.482626	0.546522
0.000354	0.390216	0.571342	16.74449	3.644343	22.96463	2.29873	0.118759	2.540462

0.000354	0.453463	0.001118	0.000606	1.305772	0.205931	0.092511	1.44E-05	0.090245
2.139928	3.57057	2.292864	1.262965	1.010289	5.662994	2.24562	3.022713	4.721789
0.000354	0.07316	0.001118	0.280823	0.810491	0.333463	0.164084	0.090476	0.070909
3.391075	1.126775	2.276407	0.465325	0.023694	3.536955	4.690159	1.505678	0.763102
1.217475	1.078925	0.571342	1.300368	6.38456	0.437576	1.132857	1.668369	0.857778
6.287561	1.935685	10.62739	4.650007	0.772157	2.165851	5.500678	4.297222	3.8379
18.19595	9.968355	18.48514	8.539971	1.92272	5.20267	16.80343	7.938427	12.63773
0.11803	0.017063	0.167273	0.335411	2.380079	0.065974	0.164084	0.016681	0.133189
0.019848	0.005541	0.032172	0.100101	0.566104	0.031775	0.036696	0.005224	0.031818
3.629177	8.048095	1.308167	7.08733	0.203593	18.33909	7.482179	8.573434	5.062626
1.930216	4.048499	1.741414	7.810058	11.11876	4.46267	3.266782	4.535354	4.33342
1.808023	5.638759	1.597908	3.57057	4.053506	4.673182	2.8657	3.707136	2.436768
1.371602	0.559221	1.014784	0.055498	0.227063	0.136356	0.187229	0.930325	0.857778
3.930664	2.045476	8.103925	1.249221	0.490664	5.864084	7.737807	1.192633	5.25254
0.077027	0.092193	0.001118	0.839365	0.023694	0.39062	1.244957	0.016681	0.561775
7.737807	3.182482	7.683766	0.818066	0.308442	1.20824	4.833398	8.780115	10.05618
0.182237	0.390216	0.067446	0.413449	1.2214	0.099812	0.34443	0.02311	0.262965
0.291183	1.11526	0.369834	1.320173	18.19595	0.954776	4.25658	0.553059	4.490801
16.09482	87.1746	23.16622	31.1753	5.20267	224.9555	40.11561	7.443276	27.66779
0.411948	0.167835	0.490079	0.280823	3.904517	0.225339	0.069163	0.062381	0.616739
3.846984	4.33E-05	5.34474	0.215115	0.023694	4.33E-05	1.691587	0.498636	1.248232
0.077027	0.340866	0.001118	0.335411	1.521371	0.448824	0.412453	0.23101	0.300094
2.535974	2.069942	1.416407	1.217475	0.541241	2.99987	1.827403	2.461905	2.29873
5.044113	2.547179	5.217569	3.595758	0.636688	7.327482	3.984747	4.678846	7.468961
3.450577	1.147807	4.376118	2.839351	1.2214	1.68938	3.507691	1.905094	3.230065
0.144488	0.421342	0.192929	0.589618	11.544	0.550664	0.432915	0.803665	0.824416
0.353016	0.885664	0.144228	3.101371	2.114437	2.440895	0.925801	1.273636	0.668456
1.585296	1.033672	6.900202	0.000606	0.023694	0.693925	1.517143	0.875599	1.056898
2.647244	5.25254	1.35259	2.461905	2.871212	2.440895	1.132857	4.927302	2.659214
0.212172	3.011169	0.221732	6.591544	5.80469	4.946587	1.528975	2.181775	1.604827
4.56912	0.152114	2.789466	0.000606	0.023694	1.826147	5.366335	1.034315	3.909033
2.727785	2.652107	3.651898	7.375794	0.203593	7.710361	4.667518	1.260635	6.99026
0.496494	1.34513	0.871429	1.781948	6.508427	2.165851	1.055628	0.684805	0.402698
19.37139	18.48514	33.4985	6.30671	0.023694	14.79742	25.24367	19.99861	19.37139
8.849697	4.752121	8.319019	4.348882	1.87899	12.85504	10.67991	5.821804	23.82354
0.000354	4.33E-05	0.001118	0.000606	1.160707	0.30373	0.000685	0.081587	0.060426
19.22139	7.780786	16.34294	4.827691	3.29355	13.91714	10.78056	10.53159	13.75978
1360.293	155.7103	749.7839	167.9996	17.51961	38.94854	203.5795	425.62	48.53429
0.965065	16.68554	1.582915	2.701053	44.68731	0.900693	1.196508	5.493182	0.27241
3.921645	1.403853	3.052655	0.10938	0.390014	0.945483	0.784805	0.794401	0.837403
3.227085	1.794531	4.678846	2.274473	1.557482	2.091313	2.860722	6.355224	5.337547
7.964315	6.945152	13.6814	4.764055	0.60886	2.947287	5.439278	6.967547	5.762965
83.489	3.730808	203.5795	0.000606	0.023694	3.144156	15.66023	11.48368	47.5018
5.576219	2.178268	10.05618	1.812114	0.023694	2.727785	3.425512	1.535722	1.306667
55.04081	1.070115	42.75576	0.000606	0.023694	27.66779	49.50903	2.678781	50.55949
3.610707	18.19595	4.23202	12.06778	17.39935	5.933066	7.162698	9.19899	10.19468
1.09075	1.433189	0.753449	0.403175	0.203593	4.508196	2.259661	0.351789	2.065007
1.566414	1.306667	5.654863	0.483874	0.023694	2.611797	4.808658	1.003586	2.518124
0.264372	1.34513	0.249899	2.776421	2.604776	1.370765	1.759986	0.82355	1.044235
0.000354	0.319163	0.001118	0.573326	1.192633	0.04114	0.542525	0.037908	0.000476
14.51648	9.297085	10.55571	5.287857	2.081696	11.544	8.134084	6.99026	16.5625
8.958225	10.60361	6.570433	1.117316	0.736977	6.796566	3.730808	7.124517	6.135007
0.000354	0.101104	0.001118	0.062035	0.60886	0.171061	0.000685	0.029553	0.000476
25.73165	4.370671	30.17192	1.834423	1.845519	6.433918	11.719	9.968355	14.08569
0.464387	1.175859	1.801284	0.876118	0.023694	1.928629	2.417424	0.529697	1.409942
0.11803	1.522597	0.184719	0.602287	1.521371	1.266255	0.288247	0.595368	0.178442
0.546854	1.560779	0.607273	2.474834	0.636688	3.497085	1.578687	1.950519	1.367179
0.000354	0.152114	0.001118	0.23873	1.675722	0.313723	0.079755	0.222193	0.041782

7.443276	5.037489	6.414416	3.411097	0.772157	2.051962	3.058802	3.754719	2.725498
2.026219	3.833247	1.20653	0.913514	0.636688	4.029654	1.476429	4.105714	1.507835
0.019848	0.374365	0.001118	0.215115	0.586595	0.252165	0.000685	0.23101	0.121147
5.530274	2.671299	9.459177	1.249221	0.023694	5.180058	8.508514	2.31811	5.959033
40.11561	6.210375	33.93939	19.83727	5.80469	67.98069	26.52537	18.33909	67.98069
11.22667	4.33E-05	17.64869	2.111299	0.023694	10.1459	3.998391	0.302785	6.653016
0.038535	0.17619	0.032172	0.280823	4.644098	0.513752	0.164084	0.176104	0.262965
7.519841	2.155447	5.622872	0.000606	0.023694	3.855952	2.540462	1.696494	5.545729
0.000354	2.609293	0.001118	2.178268	2.474834	4.33E-05	0.000685	0.763102	0.207157
0.028622	0.110642	0.001118	0.184877	0.566104	0.272814	0.000685	0.010202	0.178442
0.136147	2.178268	0.563499	2.74298	3.028629	1.68938	1.691587	1.969733	1.447994
0.000354	0.787987	0.001118	0.440801	6.727633	0.793918	0.532857	0.146003	0.108413
0.000354	1.019791	0.001118	0.536263	6.653016	0.417063	0.630022	0.23101	0.000476
1.92272	1.019791	1.096335	0.717027	0.161962	1.815	3.47531	4.519048	4.343838
8.477662	3.113478	7.964315	1.232078	0.023694	4.535354	6.325945	1.842734	3.428788
0.136147	1.591299	0.221732	0.839365	1.268672	1.355527	0.796674	0.875599	0.571674
6.423911	2.786898	5.679502	1.944517	0.253492	2.410996	4.709033	3.122915	5.416623
0.057352	0.640606	0.212049	0.290317	1.044235	0.426645	0.700058	0.02311	0.254574
12.93651	5.287857	7.810058	9.179466	0.023694	2.765758	9.751039	6.55039	12.46209
6.830592	4.620159	4.165137	1.962424	0.917431	3.24658	4.650007	7.175397	3.763045
0.019848	0.453463	0.001118	0.974257	1.778153	1.706219	0.240346	0.313312	0.424004
5.770996	4.33E-05	5.838499	0.000606	0.023694	10.57984	30.17192	3.578038	21.54823
0.832554	1.717641	2.08632	1.262965	0.144293	1.865043	1.298665	0.863694	0.740952
4.894509	0.269697	4.575123	0.690375	0.023694	3.031688	1.682193	2.165851	1.097677
0.047792	0.319163	0.096667	0.215115	1.778153	0.353795	0.092511	0.099913	0.080592
0.000354	0.167835	0.001118	0.203254	0.702381	0.216753	0.04886	0.176104	0.031818
0.202294	0.715462	0.249899	0.393449	0.023694	1.631544	0.392251	0.782857	1.273636
1.262965	0.242893	2.540462	1.191905	0.023694	0.632857	1.497143	0.518139	1.460981
0.000354	0.030613	0.067446	0.145685	0.334625	0.196335	0.079755	0.005224	0.070909
0.000354	0.233304	0.001118	0.137201	0.334625	0.405094	0.092511	0.037908	0.000476
2.153535	4.563117	4.575123	0.624473	1.491977	2.676392	3.439437	2.538218	2.823636
0.353016	0.167835	0.343824	0.027619	0.541241	0.057417	0.105498	0.82355	0.168961
2.800332	6.028427	3.718716	2.597792	1.34798	7.238023	2.786898	3.464315	2.436768
2.236407	1.008961	2.449509	1.962424	0.60886	0.82684	0.966825	1.153723	1.604827
1.083651	0.542828	0.91404	0.194235	1.957958	4.33E-05	0.187229	2.621133	0.784805
2.355289	0.925801	9.880779	16.09482	0.023694	0.82684	5.351934	7.074329	10.98644
0.019848	0.07316	0.001118	0.174776	0.144293	0.049336	0.04886	0.072381	0.156089
6.116638	2.367763	2.111299	3.13456	0.023694	7.08733	3.711097	6.580988	7.061328
49.50903	36.26061	56.39528	17.45948	4.348882	9.502294	21.19547	18.19595	21.36892
0.038535	1.522597	0.001118	4.94026	0.023694	1.384921	0.331688	1.068348	0.340245
0.066537	0.467403	0.096667	0.280823	1.305772	0.781977	0.105498	0.363492	0.060426
6.967547	14.88584	5.915469	18.62711	0.023694	17.98838	6.695887	5.395584	4.135801
3.089293	2.978968	3.486436	1.159913	0.463867	2.185556	3.877417	2.887489	1.409942
0.230606	0.478413	0.615584	0.260584	0.414473	1.309084	1.260635	0.165657	1.154567
0.605455	0.215599	0.175866	1.058896	12.70768	0.049336	0.514885	0.12798	0.196097
13.15079	12.67271	4.039192	3.439437	0.463867	6.287561	1.827403	11.01243	3.8379
12.36443	2.693442	14.6058	3.644343	0.023694	5.388405	21.02638	2.506934	12.29473
1.468131	4.33E-05	6.063276	5.591717	0.023694	0.262655	6.250447	0.099913	3.663622
45.1045	31.92385	95.22939	8.923117	0.023694	51.52195	41.47925	19.06893	91.05541
0.530534	2.394416	2.895599	0.454156	0.023694	1.150635	2.29873	1.321053	0.534214
0.160332	1.776739	0.249899	2.048781	3.730808	1.87899	0.763939	0.473016	0.810967
0.740952	0.946638	0.93267	2.621133	2.048781	1.090072	1.443983	1.615281	1.343463
20.16541	7.175397	26.52537	0.558824	7.08733	0.550664	6.250447	3.38456	9.687496
2.073232	4.33E-05	2.597792	1.254704	1.845519	0.171061	1.176623	0.23101	1.203427
1.446926	1.591299	2.252273	1.479452	1.044235	3.043528	2.148658	0.037908	2.465916
0.404408	0.573947	0.528196	0.393449	7.08733	0.592208	0.34443	0.12798	0.616739
1.463304	1.29417	1.252424	0.000606	0.023694	0.793918	0.619365	0.730873	0.962698
0.000354	1.700354	0.001118	1.085967	1.778153	0.365859	0.503463	0.553059	0.156089

0.411948	0.467403	0.809481	0.357244	1.305772	0.580404	0.455974	0.363492	0.388326
87331.38	87331.38	87331.38	16.34294	0.023694	4.33E-05	0.12904	87331.38	6.414416
0.681068	0.017063	0.032172	0.300245	2.212619	0.031775	0.092511	2.500548	0.050519
0.000354	0.082973	0.001118	0.034416	0.023694	0.07482	0.036696	0.062381	0.000476
0.097547	0.215599	0.122944	0.215115	0.463867	0.091306	0.187229	0.267201	0.133189
15.77678	0.723066	52.56128	0.174776	0.541241	2.384141	4.025267	1.842734	3.327619
0.740952	1.019791	1.387612	0.516825	0.463867	0.333463	1.703795	0.571674	0.686667
0.291183	0.573947	0.356508	1.418312	5.515584	1.91254	1.364401	0.395642	0.35798
1.434921	2.693442	1.45912	2.131176	0.566104	5.737056	3.493362	2.287576	3.648081
0.019848	0.049062	0.001118	0.326017	0.203593	0.171061	0.000685	0.155101	0.000476
2.773644	4.397792	2.145137	2.701053	1.192633	6.325945	2.958773	5.056421	3.253175
1.916782	0.308788	1.076652	0.119899	0.023694	0.057417	2.019618	0.27759	0.763102
0.108506	2.333867	0.320563	10.98644	9.480736	3.24658	1.901068	3.726854	2.171429
0.028622	0.160584	0.041097	0.366674	1.160707	0.065974	0.000685	0.108997	0.070909
7.013853	2.941544	15.56131	1.202626	1.409942	1.062821	4.758088	2.740519	4.661717
1.78759	0.432698	2.818312	0.23873	0.023694	4.33E-05	0.465801	1.44E-05	0.236638
0.343629	1.50342	0.400354	1.834423	1.590202	1.102121	0.676659	0.730873	0.799048
17.39935	4.130794	7.443276	3.13456	0.023694	12.29473	9.179466	3.629177	13.08214
0.238889	0.030613	1.135887	0.215115	2.081696	0.07482	0.357727	0.02311	0.108413
0.000354	0.023954	0.001118	0.027619	0.636688	4.33E-05	0.000685	1.44E-05	0.000476
1.808023	1.29417	2.371833	1.103261	0.60886	1.776739	2.074906	1.321053	2.406768
0.066537	0.298687	0.087229	0.646378	0.023694	0.56145	0.228009	0.090476	0.080592
7.037345	2.349351	6.045209	0.495584	0.023694	9.792626	7.951371	2.018167	6.819481
0.238889	0.288745	0.678687	0.290317	2.145137	0.225339	0.29671	0.342381	0.534214
3.803766	1.033672	10.78056	4.429769	0.023694	2.201587	10.1028	1.668369	8.348817
10.55571	6.727633	9.276811	5.830152	0.023694	10.35839	14.6058	12.36443	14.88584
0.097547	0.152114	0.087229	0.174776	1.744055	0.125859	0.092511	0.23101	0.108413
1.306667	2.045476	0.841746	1.044235	1.675722	6.063276	3.236457	5.172828	6.738211
1.906551	2.727785	0.998312	0.818066	0.023694	0.717027	0.490079	2.051962	1.980592
1.065584	4.397792	0.793918	1.381111	8.103925	0.740527	1.566414	2.302626	3.166659
0.11803	2.652107	0.328268	0.959062	9.316241	1.706219	1.462143	0.675115	0.561775
0.291183	0.885664	0.28899	1.217475	0.144293	1.242641	0.152749	0.473016	1.183478
10.1028	11.14619	6.268312	2.970144	0.203593	16.80343	4.317648	7.584502	16.44768
19.53061	5.337547	29.49693	5.754935	10.78056	7.136537	11.65791	4.557605	12.1974
9.880779	0.885664	31.55417	0.465325	1.160707	8.149307	19.53061	3.610707	35.27608
0.11803	0.07316	0.001118	0.249538	1.590202	0.023651	0.069163	1.365296	0.24518
4.353925	4.096407	2.292864	0.974257	0.636688	3.640606	1.393571	19.60574	3.57057
4.839105	6.365065	6.250447	8.994791	2.380079	8.694697	6.945152	5.788153	8.643074
0.264372	0.160584	0.724885	0.062035	0.308442	0.272814	0.197908	0.037908	0.27241
26.80355	7.507619	17.85355	4.096407	2.212619	14.93173	10.53159	5.01088	45.1045
0.419827	0.497749	0.336328	0.794401	0.60886	0.632857	0.532857	0.27759	0.469711
0.038535	0.110642	0.001118	0.675938	0.586595	0.342987	0.12904	0.33329	0.080592
1.146118	0.205931	0.408175	1.202626	0.023694	0.572987	1.220628	1.842734	2.024488
0.656003	2.871212	0.599798	7.061328	1.557482	9.418196	1.517143	1.584105	1.819221
1.92272	3.699495	0.993521	5.395584	1.160707	5.583968	2.185556	2.197828	3.5257
0.077027	0.81596	0.122944	0.090772	2.269134	0.108413	0.216602	0.176104	0.24518
3.35355	2.727785	5.165599	3.78798	6.09759	1.286494	0.55215	1.180613	2.373824
0.019848	0.101104	0.001118	0.308672	0.541241	0.159639	0.116494	0.010202	0.050519
77.74812	19.53061	26.25557	7.6393	0.023694	0.136356	1.415469	23.82354	1.661328
1.978954	0.744113	2.698557	1.535722	1.815	0.205931	0.805238	1.060188	2.024488
0.000354	0.269697	0.001118	0.10938	2.436768	0.342987	0.116494	0.155101	0.000476
1.452857	6.55039	3.080317	23.16622	4.165137	3.803766	11.77567	5.42381	36.26061
530.1465	6.632063	184.443	0.000606	0.023694	2.978968	33.4985	33.93939	42.75576
8.923117	2.996869	16.22108	10.57984	3.47531	4.067547	7.545584	2.349351	10.23768
0.861429	0.233304	1.664906	2.522684	0.023694	0.900693	1.211299	0.045346	2.390447
5.395584	2.958773	10.1459	2.556443	1.382006	4.212554	4.888023	5.950664	6.297136
35.776	42.75576	39.53687	6.570433	17.71696	22.32214	9.502294	47.5018	28.87506
0.000354	0.030613	0.001118	0.271133	0.390014	0.07482	0.04886	0.02311	0.041782

0.853218	0.82772	0.144228	0.376407	1.268672	1.165065	0.65202	1.969733	0.35798
0.000354	0.101104	0.001118	0.346732	0.702381	0.147554	0.105498	0.19544	0.090245
11.42512	1.919661	4.914863	1.749307	0.023694	0.730029	1.759986	9.687496	3.116732
0.388023	0.352222	1.36619	0.89404	0.023694	0.283882	0.092511	1.44E-05	0.236638
4.965801	1.591299	1.680895	0.602287	0.023694	0.793918	0.676659	3.87329	1.724805
3.334199	0.352222	1.39847	0.280823	0.810491	0.417063	0.000685	2.410996	0.311227
2.815866	1.155411	1.34513	0.959062	0.023694	0.262655	2.581861	1.815	3.230065
21.36892	11.09133	11.65791	7.751869	1.465123	42.75576	27.52753	20.51271	38.39042
10.67991	8.643074	20.68746	10.8813	2.404618	6.888297	14.00414	13.38418	8.849697
0.168023	0.049062	0.555418	0.925801	7.532713	0.136356	0.187229	0.062381	0.000476
3.707136	1.859084	1.495043	2.914632	0.130029	3.934986	5.28039	9.418196	6.784762
1.757244	9.335397	1.232078	34.38341	27.52753	21.54823	7.482179	14.97762	2.192496
2.29873	0.497749	0.905426	0.717027	0.023694	2.347439	1.546696	1.340981	2.349351
1.065584	10.23768	0.73583	13.45939	13.04605	0.900693	1.901068	3.363911	1.720144
6.09759	0.279452	2.436768	0.473016	0.363276	1.87899	2.958773	3.113478	5.395584
0.530534	1.700354	0.350455	1.159913	1.34798	0.252165	0.710253	0.82355	0.837403
2.013182	1.226118	2.449509	0.558824	0.023694	2.509177	2.644885	0.342381	2.335844
0.000354	0.049062	0.001118	0.573326	0.463867	0.241688	0.30596	1.44E-05	0.000476
5.855281	5.614805	1.335743	10.1028	8.643074	5.454949	3.058802	11.89932	2.274473
1.217475	4.519048	1.189048	8.075772	3.077316	5.788153	3.578038	2.720996	3.1857
1.380195	0.390216	0.400354	0.769365	0.18153	0.272814	0.455974	1.022338	0.824416
0.477698	0.753882	0.275058	0.717027	1.044235	1.845519	0.532857	0.863694	0.478182
0.028622	0.260144	0.032172	1.962424	0.772157	1.68938	0.381999	0.70886	0.424004
0.028622	0.120144	0.001118	0.473016	0.586595	0.125859	0.152749	0.072381	0.108413
7.795642	2.333867	9.031082	0.326017	1.491977	0.04114	0.866717	1.765065	0.824416
3.025671	3.663622	3.851414	1.360022	2.987835	0.49127	0.925801	1.463304	1.011486
0.000354	0.005541	0.001118	0.027619	0.161962	0.016277	0.000685	0.010202	0.000476
0.19132	11.01243	0.122944	8.815123	23.6012	3.917597	1.415469	3.500808	1.971183
1.566414	2.349351	1.495043	0.646378	0.772157	7.417359	3.011169	0.978571	2.037114
0.717879	0.994899	0.571342	3.182482	0.253492	6.230245	3.217612	1.351573	3.381255
7.061328	6.240346	9.377345	5.696263	0.669358	19.22139	14.25954	6.404921	13.91714
2.513752	0.288745	2.5271	0.794401	0.702381	1.039798	1.613232	0.595368	0.730476
0.230606	0.269697	0.23246	0.547778	0.848369	0.96329	0.73127	0.222193	0.412193
1.994329	6.727633	2.489762	0.137201	1.675722	4.33E-05	0.000685	22.74797	0.031818
0.93267	0.092193	0.555418	0.31702	1.192633	0.283882	0.477951	0.582973	0.635916
1.495043	2.459812	1.779423	1.240981	0.463867	1.985253	2.074906	1.615281	1.604827
0.212172	0.288745	0.440029	0.602287	1.34798	0.065974	0.059177	0.176104	0.322078
0.538427	0.912446	0.393196	5.082063	2.77899	2.185556	1.055628	0.518139	0.730476
1.400159	2.925426	1.834423	2.489762	10.17029	8.625613	1.989711	2.621133	5.614805
1.519322	1.033672	0.716234	0.184877	0.023694	3.934986	1.298665	1.009625	2.048781
13.08214	4.33E-05	10.67991	4.272165	0.023694	16.09482	13.83359	2.410996	21.02638
4.338629	1.1379	6.345382	0.000606	0.023694	2.371833	3.578038	1.71518	2.290996
0.870375	0.697908	1.128788	0.753882	0.023694	1.91254	0.942944	0.289935	0.522367
1.771392	4.207778	1.741414	5.830152	0.363276	9.459177	1.724805	1.71518	2.285664
9.335397	6.772763	7.037345	7.964315	1.074242	7.911934	7.013853	7.175397	8.677489
0.444538	1.575483	0.393196	4.673182	2.836717	1.845519	0.579495	1.421126	0.561775
3.089293	11.22667	2.90658	14.6058	29.82828	14.33939	4.397792	10.73084	8.445743
0.546854	0.215599	0.639495	0.465325	1.044235	0.196335	0.421025	0.289935	0.156089
0.000354	0.185909	0.133939	0.547778	1.675722	0.333463	0.187229	0.27759	0.207157
33.93939	8.745238	100.0998	60.77449	0.586595	155.7103	21.93452	118.7028	25.48179
0.733593	0.511667	0.400354	0.249538	0.023694	0.858817	0.710253	0.302785	0.65132
2.287576	0.260144	3.1857	0.000606	0.60886	0.483045	0.90772	1.799899	1.574517
0.000354	2.500548	0.001118	2.941544	2.642525	0.333463	0.277763	0.473016	0.168961
0.986797	13.45939	1.793074	0.290317	0.023694	2.574733	1.925635	1.592395	10.53159
13.45939	184.443	425.62	39.53687	87331.38	15.3618	1360.293	35.776	8.762677
0.000354	4.33E-05	0.001118	0.000606	0.144293	0.171061	0.000685	0.010202	0.000476
3.35355	8.019769	3.425512	3.540606	530.1465	2.928247	8.780115	4.978182	2.285664
8.134084	1.11526	8.048095	0.194235	0.023694	2.727785	5.942294	4.888023	3.511263

0.81596	3.460909	1.159149	3.340361	8.711905	4.174964	3.321284	2.461905	1.09075
2.445079	0.983384	3.521934	5.568391	0.023694	1.466111	4.025267	4.150274	7.824473
2.625823	2.925426	3.011169	8.508514	0.88417	6.933961	2.621133	2.66404	2.970144
0.174776	3.022713	0.20215	3.763045	18.62711	1.179885	1.636118	0.930325	0.584134
9.276811	15.66023	2.219863	16.44768	11.11876	4.620159	4.851227	44.27012	9.101674
0.000354	0.850808	0.001118	10.1459	3.236457	4.33E-05	0.079755	0.395642	0.000476
0.108506	0.141623	0.159942	0.290317	1.382006	0.216753	0.197908	0.165657	0.108413
10.82954	3.930664	4.286962	1.872049	24.98218	1.564149	3.921645	16.15795	2.936739
14.79742	72.69473	22.13245	56.39528	144.7919	56.39528	80.6518	32.31123	32.31123
36.74522	28.87506	41.47925	27.81514	8.477662	24.98218	57.84882	22.13245	28.54786
26.52537	45.91841	8.780115	22.32214	118.7028	29.49693	16.34294	65.94551	44.27012
6.922771	34.38341	9.792626	53.77619	31.1753	25.99427	23.6012	10.43066	17.64869
28.87506	21.74051	29.82828	31.92385	7.532713	27.96248	100.0998	20.16541	29.82828
4.23202	40.11561	4.077403	18.05488	203.5795	15.46193	12.70768	5.238701	7.38847
425.62	425.62	332.0776	425.62	3.163506	749.7839	425.62	332.0776	530.1465
91.05541	749.7839	91.05541	530.1465	19.06893	111.5439	155.7103	203.5795	52.56128
100.0998	80.6518	80.6518	184.443	2.565556	70.11932	75.1132	111.5439	332.0776
59.32013	1360.293	25.99427	332.0776	252.4251	184.443	77.74812	224.9555	35.776
1.50342	19.06893	2.022778	12.36443	9.316241	14.65384	6.019971	2.781558	10.33476
0.300606	0.697908	1.030678	1.535722	1.521371	2.347439	0.34443	1.112641	1.097677
4.529913	14.6058	1.537951	57.84882	47.5018	35.27608	10.1459	6.045209	9.218514
9.583463	6.325945	11.60283	3.88153	0.88417	2.03886	5.062626	5.409437	2.768312
0.028622	0.723066	0.001118	1.925635	0.949827	7.780786	1.013434	0.763102	1.447994
0.000354	0.058139	0.049769	0.346732	0.023694	0.188745	0.105498	0.204156	0.156089
0.681068	4.971991	0.923874	2.914632	4.814661	3.370527	2.24562	3.107417	1.193362
0.521421	4.33E-05	0.297532	0.000606	0.463867	0.065974	0.000685	0.118759	0.442799
5.110159	6.345382	6.00228	4.546443	39.82624	2.410996	2.984899	2.018167	1.883535
1.039798	0.340866	1.023528	0.119899	332.0776	0.091306	0.925801	0.186356	0.534214
4.678846	15.02509	4.851227	26.52537	4.990794	43.49658	8.195657	14.42661	11.83593
3.334199	4.048499	6.443925	2.839351	0.541241	1.253752	2.685786	0.498636	2.248896
0.152316	3.68381	0.001118	5.830152	3.377619	1.435772	2.92013	3.008225	0.571674
0.000354	0.049062	0.001118	0.12904	0.161962	0.057417	0.000685	0.029553	0.000476
0.388023	0.611003	0.692799	0.959062	28.54786	0.986104	1.650274	0.176104	1.385801
2.873817	0.925801	5.150693	1.91254	0.023694	0.272814	1.850996	1.783203	3.401126
42.08807	28.54786	34.38341	16.9162	4.292078	13.08214	17.22048	51.52195	49.50903
40.7932	38.94854	57.84882	65.94551	3.529466	23.38378	45.91841	31.1753	83.489
6.374812	17.92188	7.074329	8.994791	35.776	13.00996	10.98644	7.737807	7.882222
2.034242	7.417359	2.868456	6.772763	2.180022	5.075447	3.779452	4.067547	2.996869
0.057352	0.640606	0.113911	0.346732	0.515527	0.609509	0.331688	0.666097	0.388326
13.83359	16.80343	20.34063	8.164531	6.601833	23.6012	30.49289	16.9777	14.17388
0.90772	1.34513	0.593853	1.607143	1.778153	1.50684	2.164113	2.165851	1.105426
1.697792	0.912446	0.82974	2.755411	20.34063	5.330519	1.393571	2.55184	4.174964
7.495397	47.5018	12.42965	72.69473	11.719	80.6518	22.52375	16.5625	16.80343
0.546854	0.962698	1.039199	2.192496	12.77772	1.123016	0.331688	0.483326	0.291183
0.343629	0.659582	0.548153	0.675938	1.2214	0.996696	0.755253	1.463304	0.984069
2.909293	9.012937	4.575123	9.256537	6.685087	8.539971	6.063276	5.950664	4.513622
0.576638	1.279452	1.370765	1.217475	3.676032	1.564149	2.494177	0.763102	1.203427
0.152316	0.839365	0.249899	0.260584	4.72798	0.294271	0.503463	0.204156	0.763102
0.11803	0.744113	0.242453	1.103261	0.949827	1.090072	0.619365	0.632208	0.347908
0.726133	3.279625	0.88316	2.890245	1.268672	2.26518	0.925801	0.693218	0.432468
0.396472	0.288745	0.993521	2.800332	0.161962	1.298665	0.981032	0.090476	1.154567
0.546854	2.018167	0.541804	10.28573	5.889798	1.133622	1.476429	0.852172	1.560779
1.644416	9.101674	3.207807	2.955916	9.707677	1.945916	1.116609	3.340361	1.056898
0.787987	2.776421	1.25886	5.530274	9.541645	14.25954	2.786898	0.833975	0.784805
1.110505	0.092193	1.516219	0.062035	0.253492	0.031775	0.465801	0.289935	0.070909
0.087302	0.141623	0.041097	0.646378	0.541241	0.07482	0.105498	0.045346	0.217172
13.38418	12.10289	8.677489	1.798535	3.198499	9.583463	9.063983	11.83593	13.83359
0.740952	1.279452	1.135887	2.131176	1.557482	0.437576	0.866717	0.92114	0.424004

0.019848	0.023954	0.001118	0.04062	0.161962	0.023651	0.000685	1.44E-05	0.031818
0.733593	1.462143	1.370765	0.326017	0.023694	1.329206	1.850996	0.419827	1.584105
7.683766	2.442987	6.325945	9.063983	0.023694	9.063983	4.424416	9.859719	5.28039
1.131472	41.47925	0.376595	105.1694	37.81919	5.287857	17.39935	11.19942	13.53913
8.288874	2.459812	2.917381	0.10938	0.023694	3.711097	4.343838	6.819481	2.361198
4.188896	3.310758	1.296905	2.019618	5.302294	1.481631	1.104033	2.410996	2.451645
8.445743	3.279625	17.39935	2.678781	1.192633	2.440895	5.515584	2.384141	3.993665
3.201457	7.598312	3.930664	5.737056	41.47925	7.495397	2.563506	2.973059	2.858009
0.605455	1.859084	1.196508	4.203001	13.83359	0.879913	1.37531	0.885664	1.154567
0.000354	3.074134	0.001118	0.260584	0.515527	0.262655	0.265014	0.176104	0.090245
2.236407	8.180094	7.112496	14.33939	44.68731	7.598312	7.375794	4.863579	10.67991
126.1556	135.5245	224.9555	203.5795	4.02088	252.4251	530.1465	105.1694	203.5795
0.000354	0.233304	0.184719	0.300245	0.334625	0.522633	0.105498	1.44E-05	0.000476
33.4985	32.68313	17.15902	32.68313	2.353247	100.0998	67.98069	70.11932	21.93452
0.994899	2.652107	0.716234	2.914632	2.31404	4.673182	3.086306	0.897006	1.640779
0.404408	2.554069	0.802395	1.551962	4.644098	1.419278	0.5229	1.545657	0.677403
15.18102	7.911934	15.98892	7.136537	1.305772	9.141299	13.91714	8.745238	12.10289

CGGA_136	CGGA_136	CGGA_136	CGGA_136	CGGA_136	CGGA_137	CGGA_137	CGGA_137	CGGA_138
0.705613	0.78619	0.343434	0.987821	0.425426	0.112222	1.465123	1.169791	0.339235
0.200794	0.733593	1.52676	1.928629	0.283709	0.095851	0.375094	0.659957	1.27619
0.690375	1.412771	0.049105	1.167561	2.720996	6.784762	1.359177	0.038182	9.101674
1.295108	7.668579	0.049105	0.024553	6.508427	5.606739	2.597792	0.038182	3.074134
4.752121	3.691724	3.263391	0.72044	8.958225	5.110159	4.150274	1.695216	3.644343
7.683766	8.849697	4.456912	18.41211	1.705007	0.480303	4.277006	6.200945	4.921082
19.68088	21.36892	7.839271	5.9071	25.24367	24.73724	36.74522	6.045209	33.07147
0.041053	0.000231	2.281926	2.016515	0.101429	0.058543	0.073261	5.762965	0.115065
2.331825	1.539062	1.211299	0.25785	3.775209	9.667316	3.025671	0.986104	1.678319
0.833045	0.484423	6.116638	3.726854	0.001277	0.18153	1.066234	3.939307	0.370462
72.69473	23.38378	57.84882	80.6518	10.82954	17.64869	91.05541	46.7056	47.5018
1.465123	0.968723	6.978903	5.23176	0.330289	0.441053	2.506934	7.363117	2.644885
3.137576	3.370527	2.197828	1.572035	30.49289	15.46193	4.557605	4.946587	8.677489
1.220628	5.638759	0.049105	0.024553	14.97762	13.30906	0.000188	0.038182	2.89E-05
0.013442	0.004329	1.057569	0.312323	0.034776	0.000938	0.036241	0.477698	0.002749
8.348817	4.170051	1.159913	0.454632	7.882222	11.65791	2.257749	0.659957	3.87329
1.91811	2.45575	2.805382	2.212619	0.453463	0.314387	4.997417	3.939307	2.630729
0.134517	0.035505	6.819481	11.83593	0.001277	0.086443	0.249278	9.792626	2.89E-05
0.177143	0.119553	0.049105	0.024553	7.136537	1.662439	0.861934	0.038182	0.319957
3.037626	2.329784	1.755988	6.685087	0.321385	0.796674	0.789863	1.695216	2.014863
8.66E-05	0.113218	0.049105	0.024553	0.761688	1.982208	0.22456	0.038182	0.578817
0.299942	0.607273	3.5257	6.135007	5.646811	4.709033	1.037958	7.61176	0.86469
135.5245	105.1694	0.049105	0.024553	0.001277	6.210375	55.04081	0.038182	36.26061
8.66E-05	0.490952	1.369928	4.016465	0.079885	0.000938	0.000188	2.145137	0.051443
2.050404	1.040411	2.104358	1.601443	1.229553	0.340866	2.5271	6.316328	2.031169
2.388348	2.673846	0.793434	2.16759	2.572309	19.68088	2.014863	1.255657	6.25938
1.707576	0.858297	0.049105	0.024553	4.096407	0.532092	0.000188	0.038182	0.601227
0.515815	0.329091	2.026219	1.307417	0.240202	0.10404	0.92816	0.742872	0.359307
51.52195	87.1746	0.049105	0.024553	80.6518	28.54786	9.625253	0.038182	21.36892
0.335967	0.352006	4.193232	4.592273	0.515815	0.258838	0.469711	7.61176	0.721335
4.266753	1.563045	1.618773	3.025671	10.1028	3.993665	4.025267	3.939307	4.312251
0.031378	0.106926	0.382626	0.918557	0.113074	0.037605	0.256941	1.543377	0.430087
8.164531	6.632063	0.049105	0.024553	36.74522	16.09482	4.965801	0.038182	2.89E-05
0.021602	0.066407	0.267013	0.336508	0.001277	0.037605	0.162251	0.038182	0.130202
13.19051	4.451154	30.49289	21.36892	0.303564	0.048225	12.42965	25.73165	7.911934
0.267201	1.645613	0.049105	0.838846	2.828918	2.933903	0.565137	0.659957	1.343463
1.000794	1.855108	4.043846	8.303947	5.993045	3.493362	4.165137	14.51648	1.995714
0.111876	0.253882	0.049105	2.068297	0.044964	0.000938	0.345635	0.262785	0.089473
4.408153	3.625382	4.535354	9.101674	3.043528	3.150433	5.737056	2.981962	5.431544
4.997417	10.48101	30.17192	10.48101	2.421681	3.750837	6.570433	10.35839	8.134084
2.214372	0.650556	5.110159	3.855952	0.249899	0.42202	0.418023	2.278254	0.840635
0.783939	0.944784	126.1556	16.5625	0.600173	0.594271	1.169091	80.6518	0.804675
0.858297	1.855108	0.049105	0.024553	5.80469	0.796674	1.202626	0.038182	0.21689
8.66E-05	0.000231	0.049105	0.024553	0.001277	0.037605	0.019942	0.185909	0.020548
0.08044	0.162576	1.473218	0.950418	0.090108	0.805801	0.22456	0.659957	0.59062
34.84299	49.50903	4.292078	2.068297	41.47925	32.68313	21.19547	4.46267	41.47925
0.049625	0.029596	2.468117	1.367179	0.057821	0.18153	0.143709	2.609293	0.288867
0.36114	0.171061	1.211299	0.867287	0.001277	0.068629	0.40645	0.321227	0.169538
8.66E-05	0.000231	0.306811	0.196097	0.001277	0.000938	0.013405	0.038182	0.004654
0.443543	0.154848	0.267013	0.178182	0.001277	0.000938	0.40645	0.038182	0.488997
0.122554	0.066407	0.733593	0.336508	0.044964	0.086443	0.178997	0.235296	0.057893
5.485685	5.71241	1.872049	1.129452	12.56693	11.19942	3.414574	3.179199	10.80505
0.40886	0.467677	1.755988	0.893016	0.044964	0.000938	0.325108	0.916306	0.319957
0.111876	0.196551	2.333867	0.751876	0.001277	0.000938	0.028232	0.874603	0.185642
0.833045	0.656753	2.333867	0.024553	1.668369	2.678781	0.546522	2.037114	1.331919
2.331825	0.337431	0.847352	0.867287	0.001277	0.000938	1.331919	0.742872	0.232886
0.543665	0.253882	3.211176	3.614603	0.444048	0.112222	1.443983	24.50942	0.505267

0.795339	0.852684	2.805382	6.316328	0.425426	0.457431	0.769365	6.475505	1.634935
4.484892	2.518124	1.573276	2.124574	0.843564	1.580909	4.821176	0.619012	2.151919
0.335967	0.585902	1.264589	1.329206	0.101429	0.218975	0.335967	1.95645	1.861999
2.763261	3.128694	0.049105	18.99374	47.5018	0.000938	14.383	0.038182	0.804675
0.090188	0.235137	0.425844	0.395426	0.303564	0.163983	0.124957	0.206984	0.051443
0.874603	0.313521	21.11092	10.05618	0.069459	0.287951	0.833975	22.13245	1.351573
23.6012	31.55417	0.049105	0.024553	20.16541	21.74051	6.55039	0.038182	8.711905
0.049625	0.046883	8.745238	3.286652	0.313102	0.258838	0.000188	2.981962	0.097633
0.021602	0.000476	0.049105	0.024553	0.034776	0.048225	0.013405	0.038182	0.002749
0.724502	2.901032	0.997792	0.553348	0.936898	0.556126	1.149322	0.038182	2.320072
1.604827	1.330974	0.733593	0.336508	1.464214	3.074134	1.25886	0.701255	1.331919
0.648961	0.219885	0.425844	0.196097	0.313102	0.18153	0.494185	0.357013	0.232886
0.15329	0.459582	4.043846	2.287576	0.101429	0.18153	0.315743	4.592273	0.789423
0.36114	0.209401	0.049105	0.690707	0.001277	0.000938	0.657547	1.505678	0.663276
0.247251	0.384964	1.211299	2.359264	0.123456	0.058543	0.874603	1.913997	1.142345
8.66E-05	0.000476	0.049105	0.024553	0.034776	0.0771	0.000188	0.038182	0.002749
0.013442	0.004329	0.235613	1.329206	0.001277	0.000938	0.000188	0.185909	0.002749
2.797835	3.005281	2.805382	0.024553	8.573434	22.13245	0.867814	1.754733	6.497266
0.08044	0.18324	0.049105	0.284646	0.001277	0.058543	0.426118	0.206984	0.391962
0.212172	0.046883	1.057569	0.641631	0.001277	0.000938	0.178997	0.701255	0.025685
10.28573	11.25392	1.057569	1.261407	3.521934	2.489762	4.881775	1.020447	4.776407
0.134517	0.004329	1.928629	0.918557	0.001277	0.172453	0.240346	1.11526	0.073939
0.990909	0.733593	1.111977	1.828781	0.584134	0.360281	0.842641	1.632648	0.514885
1.137244	4.091667	4.392316	2.16759	10.57984	8.445743	3.738997	3.566753	6.144055
0.041053	0.001025	0.306811	0.312323	0.034776	0.048225	0.019942	0.394156	0.065801
0.396638	0.92531	0.343434	1.286494	0.057821	0.230808	0.882078	0.986104	0.804675
9.502294	11.19942	0.049105	0.024553	6.423911	19.22139	3.263391	0.038182	29.82828
0.588658	0.510072	0.997792	1.261407	0.123456	0.112222	0.325108	1.695216	0.106407
1.066234	0.209401	4.392316	8.089848	0.434329	0.50772	0.657547	11.36975	0.982698
0.36114	0.467677	3.5257	0.25785	0.515815	2.347439	0.435123	1.468131	1.252424
0.624163	0.235137	0.997792	0.480794	0.270837	0.163983	0.630823	0.578095	0.207157
0.031378	0.055397	0.382626	0.613853	0.001277	0.037605	0.4457	0.357013	0.051443
0.74868	0.088788	0.049105	0.024553	1.449062	0.720938	2.02956	0.038182	0.057893
1.178939	6.612121	0.049105	0.024553	9.031082	9.968355	6.39474	0.038182	2.852677
0.74868	1.744055	0.948211	0.751876	0.101429	0.095851	1.521371	0.436017	1.962424
0.599134	0.476551	1.663672	0.161962	0.345238	0.586595	0.554416	0.578095	0.207157
5.28039	3.330909	2.654473	5.11697	0.228788	0.42202	5.638759	1.695216	2.296732
0.013442	0.113218	0.624473	1.064242	0.001277	0.068629	0.116595	0.235296	0.081212
4.690159	4.952915	0.531169	1.684646	7.188095	5.638759	8.923117	0.619012	1.634935
0.008225	0.024423	0.476551	0.641631	0.034776	0.000938	0.080592	1.169791	0.03829
0.070541	0.019466	1.52676	3.166659	0.001277	0.028398	0.073261	1.543377	0.073939
0.021602	0.000137	0.049105	0.216025	0.034776	0.000938	0.013405	0.290115	0.004654
1.578687	1.419278	2.145137	3.327619	1.098341	1.249221	1.932857	4.179762	1.703795
3.310758	6.374812	0.049105	0.024553	4.440548	3.695815	3.738997	0.701255	4.540794
2.850058	5.993045	0.847352	0.363059	0.283709	0.360281	4.857403	1.95645	2.90658
0.467403	0.162576	1.418312	1.167561	0.330289	0.121818	0.856782	1.387612	0.057893
0.090188	0.219885	0.672857	0.553348	1.449062	2.337821	0.000188	1.11526	1.226118
0.299942	0.62733	1.159913	1.644416	0.240202	0.112222	0.088701	4.733824	0.065801
0.489784	0.676999	1.418312	1.404812	0.090108	0.466335	0.615584	2.981962	2.109755
0.543665	0.887359	0.049105	0.504416	4.563117	1.574517	0.639495	0.038182	0.90368
0.874603	0.78619	1.52676	0.867287	0.337431	0.759646	1.111977	0.916306	1.204957
5.402511	3.414574	0.343434	0.454632	1.177994	1.876212	4.292078	0.290115	2.567605
0.769365	1.759986	0.049105	0.024553	1.8386	1.249221	1.128131	0.038182	1.070115
2.515938	2.329784	3.68381	1.545657	0.218045	1.637309	2.312085	1.95645	2.151919
0.543665	0.592208	0.049105	0.553348	203.5795	75.1132	0.692381	0.038182	3.35715
1.308167	1.125325	0.049105	0.024553	2.406768	2.489762	1.99899	0.038182	0.440548
0.443543	0.2914	0.049105	0.426645	0.001277	0.000938	0.960216	0.786667	0.467136
8.66E-05	12.42965	0.049105	0.024553	105.1694	43.49658	0.000188	0.038182	2.89E-05

0.070541	0.313521	1.982208	2.287576	0.069459	0.10404	0.256941	3.028629	2.415296
0.08044	0.024423	0.049105	1.877605	0.228788	0.190058	0.91404	0.508658	0.477446
1.992944	2.189235	12.1974	29.49693	1.953535	1.595729	3.998391	22.74797	2.238341
2.259661	5.654863	3.110447	4.408153	7.545584	4.348882	6.945152	2.190866	35.27608
0.908247	6.011515	5.770996	23.38378	0.147323	0.048225	0.240346	5.545729	0.176955
10.98644	5.813247	1.701616	4.160238	3.089293	6.570433	13.15079	3.26	9.52197
2.642525	2.408882	3.855952	2.097843	0.321385	0.441053	3.767489	1.543377	2.567605
8.608153	4.678846	2.917381	1.877605	0.788896	1.595729	3.926154	0.832554	2.936739
15.56131	2.730072	28.54786	45.91841	0.470512	1.167561	5.01772	47.5018	5.630815
0.624163	0.585902	3.166659	9.276811	0.408427	0.547403	0.335967	6.045209	0.874091
0.021602	0.000231	0.531169	0.336508	0.001277	0.000938	0.019942	1.255657	0.089473
0.577388	0.421342	0.382626	1.167561	0.610224	0.112222	2.718434	0.235296	0.176955
0.25733	0.545426	1.52676	0.72044	0.181674	0.759646	0.363276	1.387612	1.716364
14.00414	13.00996	0.476551	2.287576	7.519841	1.580909	19.83727	0.916306	2.474834
0.049625	0.25785	3.917597	3.236457	0.044964	0.239885	0.554416	1.306667	0.319957
1.220628	0.669769	1.618773	0.336508	3.35355	1.088687	3.055729	0.508658	0.789423
1.000794	1.158384	1.819221	2.287576	0.684805	0.466335	2.040606	1.426522	0.90368
8.66E-05	0.004329	0.476551	0.918557	0.001277	0.000938	0.045491	0.742872	0.025685
4.667518	11.06488	16.62402	3.763045	3.460909	1.486551	3.163506	23.16622	4.082165
0.530851	0.359502	0.049105	0.25785	0.761688	2.365685	0.064372	0.038182	0.451169
0.40886	0.228788	0.531169	1.261407	0.001277	0.000938	0.385238	0.742872	0.578817
1.220628	1.158384	1.755988	0.024553	2.322035	6.404921	0.460361	0.038182	1.985253
0.658362	2.392431	11.01243	7.765931	0.462092	1.260635	4.06303	18.33909	15.02509
0.705613	1.238651	0.733593	1.261407	3.930664	4.105714	1.392511	0.916306	2.252273
0.348889	0.806227	0.049105	0.024553	0.525325	1.149322	0.240346	0.262785	2.661587
6.685087	3.266782	1.755988	9.438687	2.092987	3.775209	7.288788	13.53913	3.387864
5.646811	5.23176	9.438687	7.124517	3.169812	2.35733	5.855281	8.940671	5.583968
1.094206	0.944784	0.049105	2.463911	5.671126	0.942294	1.720144	1.805166	0.370462
0.530851	0.661025	0.476551	0.553348	1.919661	0.829192	0.300844	0.038182	0.185642
3.077316	1.759986	0.049105	0.024553	3.266782	11.25392	2.909293	0.038182	0.804675
12.93651	14.88584	11.17281	1.644416	12.03267	11.48368	15.18102	4.946587	7.710361
8.66E-05	0	0.049105	0.024553	1.852338	1.046061	0.170678	0.038182	0.138009
7.188095	13.72059	4.796126	3.065108	15.66023	13.6814	9.480736	3.722785	19.06893
3.172864	2.871212	0.049105	0.641631	17.39935	10.23768	5.821804	0.986104	4.603434
0.013442	0.000231	0.531169	0.284646	0.001277	0.037605	0.019942	0.619012	0.004654
2.302626	1.855108	0.049105	0.024553	1.420202	1.780693	1.383911	0.619012	0.381789
9.625253	5.500678	2.468117	4.592273	0.667583	1.876212	11.60283	11.719	4.312251
1.330974	1.02487	2.243911	2.406768	0.882619	0.230808	0.994271	2.666494	0.734113
0.50254	0.876638	0.049105	0.690707	0.159351	0.086443	0.576638	0.206984	0.176955
0.769365	0.799048	0.049105	0.867287	2.850058	4.445851	0.692381	0.038182	2.063362
0.396638	0	0.049105	0.024553	21.93452	0.6114	0.000188	0.038182	2.89E-05
3.66785	23.16622	0.049105	0.024553	83.489	44.27012	11.09133	0.038182	2.89E-05
1.052208	1.405772	5.402511	7.002056	0.303564	0.676999	1.678319	21.54823	7.443276
0.059654	0.352006	0.049105	0.196097	0.057821	0.258838	0.281558	0.235296	0.081212
2.415296	2.45575	0.343434	0.504416	1.797172	0.933261	2.871212	0.262785	0.751371
2.347439	5.47	0.049105	0.024553	7.363117	2.893001	1.136566	0.038182	0.997244
1.877605	0.577071	1.982208	4.984488	0.259033	0.490375	1.455022	2.768312	0.789423
4.789596	0	5.770996	14.12978	0.001277	0.000938	4.644098	7.162698	2.89E-05
3.514834	13.72059	1.418312	0.950418	16.34294	27.10502	5.614805	1.169791	2.996869
1.220628	0.267388	0.306811	0.024553	0.772626	0.340866	1.47842	0.038182	0.154329
2.331825	0.421342	4.796126	1.877605	0.001277	0.000938	0.904856	0.986104	1.29342
17.64869	5.737056	6.508427	16.9162	2.084791	2.45772	11.65791	9.459177	19.53061
1.844098	1.645613	0.049105	0.312323	1.376378	2.178268	2.281926	0.357013	0.624473
2.398442	0.17746	3.6029	3.966623	0.20733	0.18153	0.92816	1.11526	0.514885
0.479062	0.119553	8.590794	1.026825	0.462092	0.172453	1.159913	1.95645	0.549019
3.606804	2.967453	0.049105	0.024553	2.063362	8.149307	3.055729	0.038182	1.080909
0.624163	0	0.049105	0.395426	0.001277	0.000938	0.281558	0.832554	2.89E-05
0.530851	0.305253	0.531169	0.526299	0.001277	0.058543	0.576638	0.038182	0.207157

0.990909	0.215433	0.049105	0.426645	1.261407	0.572987	0.215729	0.038182	0.90368
2.797835	2.638139	0.049105	0.024553	3.38456	2.718434	2.169509	0.038182	3.7586
5.71241	6.591544	12.36443	9.880779	3.500808	4.058514	7.6393	9.583463	5.553146
28.54786	37.29684	0.049105	0.024553	24.2842	80.6518	6.632063	0.038182	19.68088
0.908247	1.371602	0.343434	0.024553	1.680895	0.987309	2.380079	0.262785	0.505267
8.66E-05	0	0.049105	0.024553	7.061328	7.112496	1.075411	0.038182	2.89E-05
1.242641	0.799048	2.984899	2.359264	0.270837	0.132309	0.994271	2.278254	2.394416
3.718716	1.978954	1.418312	0.024553	20.68746	15.77678	5.522929	0.832554	1.555289
3.998391	6.071436	43.49658	17.15902	0.74868	1.297763	11.03843	4.592273	8.412367
9.218514	9.604358	5.522929	5.967879	7.468961	10.98644	13.23022	4.821176	5.696263
2.565556	1.692763	3.917597	3.726854	0.48619	0.000938	2.474834	2.432121	1.007244
11.03843	14.42661	1.819221	3.150433	4.667518	6.632063	16.9162	2.666494	6.876392
0.609841	0.52689	0.049105	0.426645	0.959062	2.136335	1.300368	0.038182	0.185642
1.446926	2.502619	0.580758	0.235137	2.31404	3.962179	4.241905	0.038182	1.070115
1.242641	0.577071	0.049105	3.55961	0.293499	0.10404	0.895945	3.792266	0.319957
0.990909	1.1379	0.049105	0.024553	1.404812	1.766248	2.800332	0.038182	0.751371
0.267201	0.743283	0.343434	1.026825	1.621032	0.821898	0.874603	0.477698	1.380195
2.654473	1.667273	2.243911	0.024553	0.593853	0.6114	2.451645	3.722785	2.394416
0.049625	0.000476	0.267013	0.235137	0.001277	0.000938	0.064372	0.578095	0.051443
0.690375	4.484892	0.049105	0.024553	1.389509	2.316075	1.05153	0.038182	2.89E-05
4.557605	10.12435	1.872049	2.730072	5.130411	2.765758	14.25954	4.02088	3.008225
1.220628	0.614156	4.043846	1.404812	0.584134	0.155945	3.283139	5.915469	0.269141
0.454394	0.095108	4.456912	0.024553	0.001277	0.000938	0.921739	0.038182	0.391962
3.87329	2.558817	1.473218	2.24733	9.418196	5.950664	3.086306	3.179199	2.176501
4.94026	6.414416	0.476551	0.024553	7.264502	2.765758	13.08214	0.038182	1.542302
0.467403	0.599466	1.618773	1.928629	0.001277	0.000938	0.615584	2.190866	0.636688
0.224466	0.476551	1.321053	0.72044	0.079885	0.258838	0.143709	0.742872	0.106407
1.540144	2.329784	0.425844	0.641631	4.145209	0.539812	1.965657	1.58645	1.409942
1.511198	1.759986	6.045209	1.877605	0.171277	0.351219	2.77899	7.025599	1.380195
0.530851	0.421342	2.197828	4.115844	0.303564	0.396638	0.426118	5.337547	0.329524
0.908247	1.148564	0.049105	0.751876	5.095325	95.22939	0.639495	0.578095	1.661328
0.624163	1.71518	1.819221	3.192229	0.619012	0.6943	1.189048	6.404921	3.273045
0.38772	0.415166	0.847352	0.838846	0.181674	0.18153	0.281558	0.290115	0.400534
9.297085	6.497266	0.049105	0.690707	16.74449	7.074329	5.195022	0.038182	2.485534
3.273045	0.78619	4.603434	11.11876	0.313102	0.208687	2.538218	1.695216	1.87482
3.233045	0.799048	0.049105	0.024553	8.319019	2.644885	3.45404	0.038182	1.018427
0.031378	0.010851	0.672857	0.613853	0.001277	0.028398	0.028232	0.916306	0.015714
0.348889	0.007554	0.049105	0.363059	0.159351	0.000938	0.511032	0.53912	0.097633
0.278499	0.041183	1.111977	1.759986	0.079885	0.141472	0.045491	0.578095	0.185642
0.165108	0.62733	0.049105	0.584134	1.111977	1.343463	0.385238	0.916306	0.242612
7.598312	2.92013	15.3189	33.4985	0.001277	0.086443	5.416623	24.04374	1.947316
32.31123	8.319019	33.93939	62.33472	11.25392	17.92188	28.54786	7.507619	48.53429
13.75978	11.36975	4.343838	2.826277	10.65365	20.34063	8.590794	2.609293	17.15902
2.565556	1.989711	5.838499	17.92188	0.478182	1.508831	1.119574	15.22851	4.120815
2.141595	2.136335	0.793434	0.918557	2.477027	4.881775	3.086306	1.543377	1.888023
0.443543	0.415166	19.68088	12.63773	0.001277	0.000938	0.483045	6.945152	0.59062
0.658362	0.329091	0.624473	0.504416	0.181674	0.239885	0.501905	0.290115	0.232886
15.07257	118.7028	5.110159	0.024553	11.36975	2.429957	0.000188	5.050267	24.16397
1.753341	1.092785	0.847352	0.024553	6.028427	2.019618	3.489899	0.038182	2.176501
2.006782	3.031688	0.733593	0.72044	1.098341	4.067547	1.637309	0.394156	2.607035
5.515584	6.717056	5.933066	8.508514	2.230859	1.233665	17.51961	5.493182	11.77567
0.432915	0.635036	0.049105	0.024553	0.977994	0.218975	0.315743	0.038182	0.430087
1.525729	1.200152	0.049105	0.024553	2.758088	0.50772	0.124957	0.038182	5.295325
1.094206	7.238023	0.049105	0.024553	12.49452	1.061457	1.287403	0.038182	15.3618
0.144488	0.235137	0.049105	1.129452	0.044964	0.121818	0.231984	1.212792	0.081212
38.94854	56.39528	0.049105	0.024553	87.1746	20.51271	28.23632	0.038182	15.66023
2.676392	1.29342	8.461703	7.584502	0.667583	0.314387	7.212915	3.028629	7.625209
4.851227	4.413608	0.049105	0.024553	0.852172	2.014863	4.513622	0.262785	1.409942

1.242641	0.752229	2.805382	2.212619	0.417345	0.000938	2.800332	2.768312	0.578817
1.242641	2.292864	0.049105	0.024553	1.498167	1.188139	4.535354	0.038182	13.60866
1.799899	1.209105	2.333867	1.064242	3.921645	2.690823	0.824885	2.236407	1.555289
2.522684	2.23267	0.049105	0.024553	28.87506	21.93452	2.755411	0.038182	2.89E-05
10.67991	34.84299	0.049105	0.024553	167.9996	72.69473	18.33909	0.038182	30.82277
5.302294	0.490952	0.049105	4.592273	0.001277	0.000938	1.34513	12.60233	2.89E-05
1.831631	1.05417	1.418312	2.327857	0.069459	0.480303	2.227273	1.212792	0.919192
4.297222	1.590202	9.707677	5.01088	0.218045	0.218975	0.833975	1.632648	1.191905
0.021602	0.004329	0.343434	1.167561	0.044964	0.0771	0.064372	0.659957	0.106407
5.150693	2.043795	0.049105	2.406768	0.270837	0.230808	2.333867	0.578095	0.829192
0.335967	0.095108	0.997792	1.026825	0.159351	0.086443	0.054935	1.863506	0.207157
8.66E-05	8.976508	0.049105	9.541645	75.1132	29.18885	4.839105	0.038182	2.89E-05
10.8813	27.96248	0.049105	0.454632	6.374812	1.156869	1.744055	0.038182	7.037345
0.956667	0.450924	1.111977	1.828781	0.293499	0.258838	0.657547	1.58645	0.549019
1.930216	2.533745	0.343434	0.987821	1.330974	1.009625	1.902352	2.278254	3.144156
1.578687	3.083319	0.049105	0.024553	1.355527	0.996111	3.57057	0.038182	0.932078
6.508427	3.220678	9.815642	22.74797	1.202626	4.281847	6.8421	13.72059	9.438687
1.525729	1.480541	2.654473	2.826277	0.584134	1.077403	2.014863	3.792266	1.323131
0.844747	0.113218	22.13245	14.29947	0.889553	0.095851	1.05153	16.68554	2.014863
3.290101	0.908247	6.423911	2.652107	0.727222	1.429365	1.31	2.931068	3.052655
2.073232	4.978182	0.049105	0.454632	9.256537	3.55961	0.744113	1.020447	1.02798
3.87329	2.373824	0.049105	0.72044	0.37417	0.132309	3.514834	1.11526	2.74544
0.811443	11.42512	0.049105	0.024553	13.00996	10.1459	3.824964	0.038182	4.946587
0.134517	0.088788	2.730072	2.574733	0.090108	0.000938	0.345635	0.578095	0.020548
3.008225	2.740519	21.74051	40.7932	0.714697	0.457431	0.96938	24.73724	11.09133
2.073232	4.072475	0.580758	0.024553	3.195541	3.625382	4.721789	0.038182	1.018427
0.15329	0.125123	3.68381	0.6657	0.001277	0.141472	0.22456	2.432121	0.459048
5.737056	13.34662	1.755988	1.455988	22.96463	12.42965	14.17388	1.543377	17.51961
3.962179	4.261342	1.418312	2.359264	5.545729	6.365065	3.13456	2.713319	3.223882
6.570433	2.81342	0.049105	0.553348	0.727222	1.041025	4.012049	0.038182	4.984488
0.070541	0.149019	0.580758	1.684646	0.069459	0.000938	0.249278	0.659957	0.259033
11.33981	10.33476	42.08807	24.2842	10.53159	13.60866	10.23768	53.77619	23.82354
2.676392	4.012049	0.049105	0.480794	7.212915	2.380079	1.881955	0.916306	0.242612
105.1694	144.7919	30.99903	24.50942	65.94551	91.05541	135.5245	118.7028	51.52195
0.122554	0.384964	1.701616	2.097843	0.321385	0.121818	0.565137	3.028629	0.381789
9.838658	9.218514	8.412367	18.19595	6.063276	5.662994	3.718716	0.619012	10.28573
9.541645	8.241342	2.145137	5.082063	31.1753	15.3618	11.719	0.954776	14.51648
8.66E-05	0.162576	0.049105	0.284646	0.001277	0.000938	0.395152	0.038182	0.195823
8.66E-05	0.002035	3.6029	0.426645	0.101429	0.048225	0.000188	1.754733	0.03829
1.427431	1.29342	0.049105	0.480794	4.272165	2.947287	0.256941	0.038182	1.532468
9.031082	30.82277	3.055729	3.676032	31.92385	36.74522	9.377345	0.262785	3.7586
0.021602	0.019466	0.306811	0.426645	0.034776	0.000938	0.013405	0.436017	0.259033
0.942294	1.082915	0.049105	0.024553	0.819978	2.219863	0.264242	0.659957	0.967446
0.267201	0.253882	5.20267	0.690707	0.193348	0.743283	0.483045	1.468131	1.76873
0.38772	0.119553	1.321053	1.097677	0.159351	0.068629	0.335967	1.349971	0.329524
19.06893	8.396847	2.868456	2.327857	3.533211	4.135801	14.6058	1.426522	9.52197
3.061955	1.828781	5.889798	3.363911	0.330289	0.112222	1.881955	6.404921	1.204957
6.162446	7.519841	2.145137	3.214545	10.65365	7.795642	4.082165	0.619012	10.48101
126.1556	62.33472	4.456912	10.48101	67.98069	48.53429	126.1556	9.968355	56.39528
0.08044	0.142698	2.513752	0.024553	0.001277	0.112222	0.162251	2.432121	0.154329
0.267201	0.142698	0.049105	2.406768	0.057821	0.058543	0.162251	1.020447	0.138009
30.82277	34.38341	5.00404	10.60361	18.33909	53.77619	37.29684	14.74965	64.12971
1.330974	3.1857	0.624473	1.759986	1.537951	0.480303	2.561162	1.064935	1.962424
0.278499	0.752229	1.159913	0.553348	0.101429	0.095851	0.418023	0.578095	1.441898
5.646811	2.45575	9.924372	49.50903	0.619012	1.233665	2.269134	26.52537	5.395584
2.715873	2.901032	4.043846	3.422237	0.101429	0.331097	0.196955	10.21618	6.433918
10.1028	7.136537	24.73724	37.81919	1.191198	3.529466	7.495397	11.48368	7.417359
0.467403	0.778326	3.68381	2.730072	0.171277	0.141472	0.385238	5.984885	2.119596

95.22939	57.84882	87.1746	65.94551	9.667316	10.53159	80.6518	55.04081	67.98069
0.15329	0.066407	0.624473	0.363059	0.069459	0.028398	0.206616	0.578095	0.319957
1.242641	0.743283	2.730072	0.838846	3.6029	11.544	0.511032	1.306667	2.474834
0.031378	0.060022	0.235613	0.144163	0.034776	0.000938	0.019942	0.038182	2.89E-05
2.398442	2.760765	13.49926	37.02103	0.337431	0.405974	2.351299	22.52375	4.33342
12.53072	10.62739	1.618773	13.08214	7.430317	3.644343	19.68088	9.880779	12.32958
3.533211	3.962179	21.54823	25.48179	1.339091	2.644885	5.71241	10.62739	7.737807
8.66E-05	0.228788	0.997792	0.584134	0.001277	0.0771	0.143709	0.832554	0.549019
0.891775	2.628225	0.049105	0.024553	3.314329	6.011515	2.333867	0.038182	1.451775
0.769365	1.39847	2.600014	1.959466	1.1407	1.389509	1.546696	1.468131	2.90658
0.577388	0.389574	25.24367	8.164531	0.113074	0.457431	1.295108	13.15079	1.962424
7.824473	7.495397	6.819481	3.507691	13.15079	45.91841	3.207807	2.500548	14.42661
0.858297	1.275325	0.049105	0.024553	0.240202	0.47246	0.598124	0.038182	0.16145
0.396638	0.62733	1.057569	0.024553	4.155339	1.662439	0.308442	0.038182	1.142345
0.822381	0.908247	2.069942	1.097677	0.159351	0.322395	0.300844	6.107114	9.583463
11.96574	13.23022	23.06543	20.85876	28.23632	24.04374	27.96248	19.06893	18.05488
2.373824	4.091667	1.701616	2.359264	8.923117	6.486104	4.557605	5.42381	3.35715
2.449509	1.330974	0.425844	0.178182	0.147323	0.086443	0.325108	0.53912	1.364401
17.92188	20.85876	7.049336	22.96463	2.800332	3.300664	10.38202	14.74965	13.15079
0.648961	22.8563	0.049105	0.284646	77.74812	46.7056	20.85876	0.038182	3.540606
4.016465	1.573276	3.6029	2.922778	0.534452	4.424416	1.007864	3.722785	5.273362
1.029365	0.78619	0.382626	0.284646	0.453463	0.367287	0.570317	0.436017	0.370462
6.8421	9.707677	7.724084	1.991328	0.810491	0.466335	5.302294	4.529913	4.519048
0.323752	3.962179	0.049105	0.454632	0.944784	59.32013	1.287403	2.609293	8.019769
5.42381	2.316075	0.049105	0.024553	6.374812	12.56693	1.373348	0.038182	8.608153
6.200945	4.927302	15.46193	11.93253	6.653016	4.709033	5.068831	11.09133	5.630815
1.137244	4.043846	11.93253	20.68746	0.515815	0.565426	2.939141	21.36892	3.435988
0.041053	0.035505	0.235613	0.526299	0.044964	0.037605	0.045491	0.262785	0.045281
17.15902	35.27608	13.04605	24.85971	13.83359	9.583463	16.44768	12.10289	37.81919
7.938427	6.071436	3.68381	2.6143	6.784762	5.158146	5.477843	0.659957	1.925635
5.568391	3.471486	0.049105	0.024553	3.327619	1.297763	3.45404	0.954776	0.776349
12.85504	8.319019	0.425844	0.584134	5.22482	2.964762	6.011515	1.020447	1.605859
6.230245	5.316378	1.057569	0.867287	3.442886	4.348882	8.210938	1.064935	2.860722
0.467403	0.235137	1.211299	1.217475	0.090108	0.10404	0.904856	0.235296	0.195823
0.122554	0.837403	1.819221	2.509177	0.147323	0.028398	0.064372	2.432121	0.065801
1.121703	0.380649	3.775209	1.759986	1.360866	1.106046	0.938911	2.092987	0.650556
1.109726	2.153535	1.755988	3.286652	0.494841	0.268961	2.847547	4.297222	1.678319
0.705613	0.502843	1.264589	3.855952	0.001277	0.322395	0.639495	4.376118	3.074134
6.414416	6.945152	3.917597	1.199481	53.77619	33.07147	5.366335	1.754733	6.144055
0.50254	0.76	0.733593	0.395426	5.095325	0.539812	1.269495	0.038182	0.840635
1.014784	0.428694	1.057569	0.526299	0.434329	1.439704	0.751371	1.064935	0.804675
2.449509	4.115844	26.80355	26.80355	1.382006	1.876212	5.591717	16.80343	7.188095
1.974228	2.720996	8.103925	4.875527	0.86469	1.876212	1.953535	15.61077	1.925635
23.38378	10.98644	6.560411	13.83359	1.947316	6.277937	3.816551	6.560411	9.989221
34.38341	18.41211	25.48179	31.1753	8.849697	13.23022	25.48179	15.61077	38.39042
6.632063	27.38726	17.39935	0.690707	4.586753	49.50903	3.220678	9.459177	13.75978
0.122554	1.112641	2.984899	10.17029	12.22798	4.673182	1.169091	3.394286	1.43039
1.427431	1.504517	0.531169	1.428398	0.74868	0.796674	0.178997	0.874603	1.041652
9.438687	3.743232	0.580758	0.426645	7.938427	5.075447	5.22482	1.064935	2.807937
2.236407	1.194221	2.388348	2.212619	0.337431	1.05088	3.909033	2.092987	4.900996
0.165108	2.106089	1.618773	5.993045	0.113074	0.322395	0.300844	2.278254	0.763939
8.66E-05	0.004329	0.049105	0.363059	0.001277	0.121818	0.028232	0.262785	0.045281
10.05618	3.895469	0.425844	0.235137	3.743232	8.573434	8.445743	0.954776	5.583968
8.319019	12.10289	2.069942	5.409437	8.815123	6.230245	24.98218	3.792266	16.5625
8.66E-05	0.000231	0.049105	0.178182	0.034776	0.000938	0.000188	0.206984	0.002749
0.224466	0.000231	0.580758	1.129452	0.044964	0.086443	0.345635	0.916306	0.349812
80.6518	47.5018	7.92518	56.39528	14.51648	16.80343	100.0998	52.56128	19.99861
1.456955	1.87482	2.145137	4.072475	0.345238	1.580909	2.040606	2.145137	4.188896

0.122554	0.095108	0.306811	3.422237	0.079885	0.000938	0.013405	0.916306	0.03829
45.1045	17.51961	18.84404	72.69473	17.78522	9.297085	64.12971	72.69473	15.02509
6.922771	4.745895	0.793434	5.044113	2.345527	3.29355	7.991602	2.609293	4.921082
0.049625	0.001025	2.145137	5.295325	0.101429	0.058543	0.045491	1.863506	0.115065
0.858297	0.565714	23.93364	7.443276	0.171277	0.172453	1.025613	7.810058	1.622006
1.066234	0.614156	6.116638	3.676032	0.293499	0.441053	0.315743	1.913997	1.532468
287.197	167.9996	111.5439	100.0998	24.04374	17.51961	144.7919	126.1556	118.7028
13.60866	8.815123	9.297085	5.477843	5.409437	5.330519	17.92188	5.762965	8.780115
22.13245	12.93651	11.51384	24.04374	5.881342	11.89932	19.06893	19.83727	55.04081
0.566782	1.260635	1.618773	0.024553	4.802655	1.389509	0.664423	0.038182	0.982698
203.5795	749.7839	0.049105	1.455988	184.443	184.443	224.9555	0.038182	144.7919
21.74051	13.83359	10.60361	16.09482	9.924372	14.88584	19.22139	10.35839	18.76954
4.814661	2.203535	5.770996	7.061328	2.538218	1.86658	3.971061	8.677489	5.130411
0.908247	1.209105	6.045209	1.237063	0.50469	0.622092	0.895945	2.609293	5.9071
2.776421	3.595758	1.701616	15.02509	0.362864	0.622092	2.320072	6.475505	9.179466
9.792626	6.268312	1.159913	3.422237	2.917381	3.962179	9.082828	6.250447	10.23768
41.47925	28.54786	49.50903	144.7919	6.830592	0.163983	62.33472	67.98069	23.38378
1.330974	1.29342	0.901732	3.286652	0.001277	0.000938	2.54961	0.394156	0.440548
16.62402	6.230245	2.868456	7.710361	1.365296	3.150433	0.598124	9.179466	22.13245
22.32214	33.93939	135.5245	126.1556	14.25954	8.539971	40.7932	111.5439	42.75576
53.77619	60.77449	12.85504	6.956349	14.17388	35.776	37.81919	37.81919	62.33472
4.082165	7.025599	5.591717	16.28201	1.048074	0.547403	14.70188	5.42381	17.78522
21.54823	11.719	36.74522	29.18885	6.632063	7.363117	16.68554	27.10502	15.46193
1.622006	4.451154	0.049105	0.024553	1.222179	2.136335	5.34474	0.038182	1.191905
4.626025	15.3618	9.815642	4.802655	25.73165	155.7103	9.838658	4.179762	11.48368
0.421804	2.593506	8.005685	0.641631	0.48619	3.055729	0.814315	1.913997	2.524892
5.037489	3.707136	0.049105	2.16759	6.99026	11.33981	2.65684	0.038182	1.472208
35.27608	45.91841	1.057569	0.641631	22.74797	203.5795	10.95872	1.387612	42.08807
0.769365	0.366674	13.83359	14.47154	0.069459	0.000938	0.716234	14.17388	0.751371
0.658362	0.806227	0.624473	2.124574	0.444048	0.367287	0.778326	1.58645	1.263752
3.895469	9.179466	0.049105	1.572035	1.881955	12.70768	2.958773	0.038182	3.7586
3.122915	7.38847	0.382626	0.024553	0.57531	0.095851	2.138131	0.038182	0.259033
2.642525	1.228651	2.654473	4.212554	0.249899	0.415166	2.066652	2.190866	2.151919
4.508196	5.23176	1.982208	0.987821	1.881955	4.959358	1.373348	0.235296	3.783694
0.008225	0.002035	0.049105	0.024553	0.001277	0.0771	0.013405	0.038182	0.03829
29.18885	21.93452	31.55417	21.19547	8.381328	5.762965	27.66779	14.97762	32.31123
1360.293	87331.38	425.62	425.62	32.31123	38.94854	111.5439	530.1465	203.5795
100.0998	155.7103	155.7103	287.197	70.11932	52.56128	287.197	224.9555	425.62
1.109726	0.819048	1.928629	2.359264	0.240202	0.155945	1.568564	2.768312	2.644885
30.49289	16.62402	19.99861	10.95872	49.50903	30.17192	25.73165	19.37139	22.32214
0.189141	0.778326	0.476551	0.6657	0.090108	0.148932	1.217475	2.092987	0.578817
3.851414	3.614603	5.287857	15.71851	3.011169	1.637309	4.419062	16.39531	2.361198
0.822381	0.18324	7.584502	2.287576	0.425426	0.466335	0.345635	21.02638	1.43039
0.059654	0.261775	0.049105	0.284646	0.001277	1.952027	0.170678	0.436017	0.410823
8.66E-05	0.101212	0.733593	1.167561	0.001277	0.000938	0.088701	1.95645	0.097633
4.484892	3.824964	29.18885	4.592273	1.023528	3.993665	3.895469	15.07257	9.377345
5.23176	14.08569	23.38378	0.613853	7.765931	50.55949	1.795851	11.99921	14.12978
0.15329	0.142698	0.672857	0.584134	0.044964	0.095851	0.240346	0.954776	0.440548
40.11561	77.74812	11.2819	5.737056	60.77449	37.81919	20.68746	5.950664	126.1556
0.070541	0.066407	1.264589	1.286494	0.001277	0.058543	0.143709	0.659957	0.015714
24.98218	16.09482	6.240346	0.641631	10.38202	33.93939	6.200945	8.033932	18.33909
0.543665	0.305253	0.049105	0.950418	0.001277	0.000938	1.136566	1.11526	1.191905
2.155447	0.935743	9.562554	6.268312	2.378124	4.323045	2.151919	5.493182	3.435988
0.25733	0.088788	0.476551	0.454632	0.001277	0.000938	0.231984	0.986104	0.477446
0.008225	0.000137	0.793434	2.967453	0.057821	0.000938	0.028232	0.986104	0.002749
0.924329	1.082915	1.473218	6.760765	0.001277	0.068629	2.333867	2.713319	1.460981
42.08807	29.82828	33.28499	41.47925	30.17192	33.4985	30.49289	39.53687	29.49693
11.48368	6.99026	18.48514	47.5018	4.644098	5.591717	15.98892	7.737807	12.70768

18.91854	8.976508	3.425512	10.60361	4.18456	5.395584	12.10289	13.60866	6.497266
10.73084	5.18754	13.49926	18.05488	3.370527	7.188095	5.143867	20.51271	16.22108
0.489784	0.935743	0.049105	0.216025	2.027951	1.856392	1.359177	0.038182	2.238341
8.66E-05	0.004329	0.235613	1.237063	0.001277	0.000938	0.000188	0.53912	2.89E-05
43.49658	22.8563	18.05488	27.96248	5.024293	3.514834	15.07257	35.27608	18.19595
23.06543	31.1753	13.23022	7.175397	33.07147	12.63773	23.6012	4.667518	12.56693
3.334199	1.629235	0.847352	3.327619	0.001277	0.148932	4.609076	5.646811	1.648066
2.099524	2.547179	0.306811	0.336508	3.058802	0.780952	3.391075	0.508658	0.742872
2.259661	1.427431	0.672857	3.025671	0.944784	0.711371	1.373348	1.349971	1.738456
9.880779	9.256537	28.87506	20.16541	4.952915	8.745238	12.85504	9.729358	10.73084
0.609841	1.563045	19.29639	4.875527	0.525325	0.780952	0.950418	7.288788	2.415296
3.233045	2.479221	1.321053	1.488571	8.745238	7.162698	4.150274	0.578095	4.188896
11.25392	18.91854	39.24271	38.94854	9.541645	5.18754	14.08569	38.39042	21.54823
8.762677	38.39042	2.730072	2.652107	29.82828	16.5625	29.82828	6.365065	87.1746
0.165108	0.149019	1.264589	0.553348	0.044964	0.058543	0.40645	0.508658	0.440548
13.87537	15.61077	0.847352	0.454632	22.13245	25.24367	11.48368	0.742872	12.03267
0.312864	0.060022	2.243911	1.877605	0.057821	0.068629	0.162251	2.190866	0.612872
18.62711	24.50942	45.1045	64.12971	1.034315	2.111299	22.13245	29.82828	77.74812
1.152944	0.898543	3.263391	7.061328	0.852172	0.441053	0.645916	0.477698	1.380195
9.377345	17.03919	1.701616	1.545657	3.998391	1.447994	1.909408	0.290115	5.337547
0.624163	0.2914	21.11092	5.638759	0.337431	0.734113	0.681494	1.169791	1.041652
0.288579	0.502843	1.111977	0.024553	2.164113	1.399315	1.025613	0.038182	1.191905
20.94257	21.54823	15.66023	12.16681	2.31404	5.056421	41.47925	42.42191	44.27012
0.090188	0.261775	0.531169	0.480794	0.079885	0.037605	0.264242	0.742872	0.269141
45.91841	32.31123	0.672857	3.422237	7.061328	2.879228	25.24367	1.064935	8.319019
6.162446	5.030866	1.573276	6.830592	19.53061	11.719	3.767489	6.107114	16.34294
0.08044	0.024423	0.531169	0.196097	0.044964	0.058543	0.099004	0.290115	0.057893
5.34474	7.570693	7.507619	11.03843	2.26518	2.392431	9.179466	10.28573	5.351934
2.155447	0.981558	6.423911	7.507619	0.397489	1.217475	7.037345	1.426522	1.605859
0.705613	0.565714	0.306811	0.426645	0.556126	0.383348	0.692381	0.290115	1.232886
1.909408	5.770996	0.049105	0.426645	5.18754	4.990794	0.744113	0.038182	0.288867
2.331825	1.905094	0.476551	2.509177	0.65202	0.958557	2.236407	1.255657	1.555289
29.49693	11.30987	1.755988	4.715599	1.498167	8.923117	10.62739	29.49693	52.56128
3.110447	8.119004	203.5795	203.5795	1.287403	14.97762	9.751039	105.1694	9.297085
2.119596	1.313737	1.755988	1.545657	1.090072	0.866147	2.451645	1.020447	0.982698
0.15329	0.066407	287.197	87.1746	0.362864	0.172453	0.000188	144.7919	0.051443
0.267201	0.25785	17.15902	13.45939	0.556126	2.126349	0.385238	17.85355	0.459048
3.592063	1.209105	1.573276	2.967453	0.353651	0.92482	2.461905	2.325931	1.29342
0.059654	0	0.049105	0.161962	0.001277	0.000938	0.045491	0.235296	0.007821
2.961768	0.819048	14.6058	4.984488	0.001277	0.000938	2.221775	2.092987	4.881775
2.270837	1.645613	4.101061	2.406768	0.397489	0.351219	1.018427	1.805166	1.018427
0.588658	0.046883	0.382626	0.216025	0.113074	0.000938	0.528997	1.064935	2.89E-05
2.31404	1.573276	0.267013	0.178182	0.91404	1.937222	1.323975	0.038182	1.070115
0.177143	0.29767	3.47531	13.38418	0.228788	0.258838	0.938911	2.325931	0.154329
0.013442	0.060022	0.476551	0.526299	0.057821	0.199264	0.045491	0.832554	0.011227
0.467403	0.07767	0.901732	3.025671	0.249899	0.367287	0.088701	3.842554	0.421573
2.961768	2.831501	0.267013	0.426645	2.487576	1.133622	3.750837	0.290115	1.331919
0.555036	0.819048	0.049105	2.6143	1.569553	1.124574	0.800967	1.754733	0.663276
1.220628	8.677489	0.049105	0.024553	46.7056	39.53687	7.598312	0.038182	1.588903
2.214372	1.169791	0.235613	0.613853	2.203535	1.285584	0.760433	0.235296	0.319957
47.5018	111.5439	0.049105	2.068297	57.84882	28.87506	14.79742	0.786667	28.23632
0.299942	0.083413	0.343434	0.751876	0.057821	0.058543	0.435123	0.321227	0.097633
0.212172	0.2914	0.306811	1.518232	0.044964	0.258838	0.722626	0.038182	0.329524
0.637994	2.238341	0.343434	9.880779	1.1407	0.480303	3.055729	5.050267	1.09075
1.152944	2.770866	7.724084	6.685087	1.004156	1.652771	5.500678	22.96463	3.214545
1.669466	2.150303	0.049105	2.893001	8.164531	0.000938	1.919661	0.038182	0.734113
8.66E-05	0.000231	0.049105	0.178182	0.001277	0.028398	0.000188	0.185909	0.002749
1.63039	4.043846	2.281926	0.235137	1.191198	2.178268	1.429365	0.832554	0.624473

1.718918	1.96404	0.049105	0.526299	3.482785	3.578038	1.557482	1.212792	1.310837
0.624163	3.414574	0.049105	0.024553	4.952915	6.508427	2.091313	0.038182	5.373781
1.000794	0.142698	1.111977	2.327857	0.001277	0.000938	0.692381	1.169791	0.359307
15.18102	16.62402	1.418312	2.287576	4.764055	4.978182	17.28195	1.805166	7.507619
6.808369	5.287857	0.049105	0.024553	8.643074	7.683766	3.851414	0.038182	4.188896
0.101212	0.467677	1.473218	4.272165	0.001277	0.351219	1.248232	5.259437	1.142345
1.718918	1.916782	0.901732	2.035678	0.65202	0.000938	1.007864	1.11526	0.269141
0.335967	0.25785	3.363911	3.966623	0.123456	0.29531	0.345635	7.025599	0.523983
4.140505	2.384141	11.51384	15.88548	3.877417	3.711097	2.572309	10.95872	3.621941
1.41368	2.063362	0.580758	0.312323	3.414574	1.588903	1.136566	1.754733	1.821861
31.55417	45.1045	1.057569	1.877605	19.06893	23.6012	53.77619	1.306667	14.12978
1.41368	0.991631	1.418312	1.404812	0.600173	0.287951	1.277843	1.632648	0.829192
0.013442	0.007554	0.343434	0.312323	0.001277	0.000938	0.019942	0.874603	0.031775
0.443543	0.421342	1.52676	1.928629	0.259033	0.457431	0.716234	2.879228	0.941104
8.66E-05	0.095108	0.049105	0.024553	28.54786	32.31123	0.000188	0.038182	0.146234
8.66E-05	0.17746	0.049105	0.024553	5.25254	1.826147	0.028232	0.038182	2.89E-05
13.30906	11.57341	2.654473	2.544747	12.77772	17.78522	20.34063	2.092987	14.70188
6.135007	9.459177	0.049105	0.024553	27.38726	12.93651	9.031082	0.038182	4.47912
1.029365	0.876638	2.730072	0.6657	0.293499	0.30373	1.429365	0.53912	0.21689
2.498478	0	0.049105	0.024553	19.22139	25.99427	0.736551	0.038182	3.679986
8.66E-05	0	0.049105	0.024553	5.381227	4.552092	0.215729	0.038182	0.122395
3.648081	2.628225	38.39042	15.51162	5.309264	4.540794	7.363117	18.12541	4.359574
1.355527	1.466111	2.654473	5.330519	0.515815	0.141472	1.402915	6.663651	1.29342
0.50254	0.190058	4.845166	3.166659	0.090108	0.631494	0.325108	3.321284	2.131176
5.984885	3.457504	4.845166	0.024553	7.162698	6.717056	10.33476	2.145137	1.252424
1.556392	2.035678	0.997792	2.826277	0.37417	0.360281	2.461905	2.373824	0.86469
0.36114	0.837403	0.049105	0.024553	0.610224	0.622092	0.751371	0.038182	0.207157
0.637994	0.366674	0.049105	0.613853	0.641631	0.141472	0.789863	0.262785	0.505267
13.00996	12.77772	2.730072	1.644416	27.96248	31.55417	11.544	3.061955	9.838658
2.03886	3.198499	0.901732	0.024553	2.818312	1.888023	5.180058	0.038182	0.477446
0.891775	0.752229	0.531169	0.024553	1.277843	0.163983	0.814315	0.038182	0.115065
6.011515	0.806227	0.049105	0.024553	0.567994	18.05488	0.736551	0.038182	2.89E-05
0.247251	0.717027	0.049105	0.284646	1.510014	1.766248	0.4457	0.038182	0.051443
8.288874	2.373824	0.049105	0.690707	3.095166	2.157359	3.303925	0.038182	1.041652
3.334199	3.207807	0.267013	0.312323	1.668369	1.041025	5.454949	0.394156	0.339235
8.66E-05	0.035505	7.049336	0.363059	0.069459	0.058543	0.188009	2.325931	0.329524
1.152944	2.022778	0.476551	0.395426	0.619012	0.239885	1.47842	0.436017	0.349812
1.696494	0.876638	3.5257	6.079596	0.228788	0.278802	2.597792	4.946587	5.923838
10.1459	17.28195	0.624473	0.336508	13.30906	14.00414	12.70768	1.95645	9.335397
8.66E-05	0.083413	19.83727	11.74734	0.001277	0.10404	0.000188	6.663651	0.269141
1.094206	0.484423	10.21618	8.381328	0.86469	1.303961	0.598124	6.865	0.410823
1.094206	1.87482	0.049105	0.024553	1.531313	4.096407	1.087345	0.038182	0.081212
4.894509	10.85542	0.049105	0.024553	9.880779	2.178268	10.57984	0.038182	1.505678
0.489784	0.228788	0.267013	0.312323	0.20733	0.121818	0.22456	0.206984	0.391962
0.40886	0.490952	0.049105	0.024553	5.366335	2.000592	1.087345	0.038182	0.636688
0.013442	0.001025	0.580758	0.024553	0.034776	0.037605	0.000188	0.185909	0.007821
0.588658	0.717027	0.733593	0.918557	0.193348	0.268961	0.528997	0.659957	0.279452
1.669466	1.844098	0.049105	0.024553	2.947287	2.371833	1.824892	0.038182	0.853218
0.50254	0.450924	4.72798	10.70538	1.048074	2.243911	0.385238	26.12492	2.884762
2.415296	1.330974	1.928629	5.583968	0.181674	0.340866	6.250447	1.695216	4.145209
0.021602	0.004329	0.948211	0.024553	0.001277	0.000938	0.019942	0.53912	0.004654
5.889798	44.27012	3.775209	0.024553	111.5439	105.1694	16.5625	10.85542	22.96463
0.811443	0.467677	0.425844	1.329206	2.126349	1.035801	2.77899	0.701255	0.817547
0.049625	0.171061	0.476551	1.488571	0.113074	0.028398	0.143709	0.954776	0.03829
20.34063	8.048095	55.04081	118.7028	1.813564	0.499986	12.16681	95.22939	3.820758
3.486436	3.726854	0.049105	0.024553	3.343629	1.842734	1.770029	0.038182	0.467136
35.776	50.55949	1.618773	0.838846	36.26061	83.489	15.77678	7.025599	49.50903
1.637309	0.402937	16.22108	20.51271	0.001277	0.000938	1.037958	27.38726	1.156869

0.013442	0	0.049105	0.024553	0.444048	0.141472	0.000188	0.038182	0.007821
1.201724	1.940231	9.924372	3.763045	0.876118	0.631494	0.851234	6.760765	1.716364
0.515815	0.778326	5.993045	1.717641	1.320173	2.243911	1.373348	3.87329	2.089639
0.25733	0.305253	1.928629	2.406768	0.453463	0.000938	0.153016	2.768312	0.138009
0.070541	0.024423	0.306811	0.024553	0.001277	0.000938	0.231984	0.290115	0.089473
14.88584	19.06893	0.049105	0.024553	24.73724	27.38726	6.454416	0.038182	8.27267
1.799899	5.259437	1.573276	0.454632	4.312251	3.816551	4.323045	2.931068	1.753341
0.38772	1.074242	8.868932	13.91714	0.37417	0.218975	0.353175	2.609293	0.308788
11.09133	8.164531	0.672857	0.395426	3.080317	2.941544	7.737807	0.874603	3.799885
0.979149	0.769019	24.50942	8.429055	0.386378	1.046061	0.375094	15.98892	2.048781
23.82354	9.880779	23.93364	39.53687	6.287561	7.570693	13.6814	45.91841	22.74797
0.235931	0.402937	4.535354	1.572035	2.727785	1.061457	0.134444	4.179762	0.742872
0.177143	0.07767	0.382626	0.918557	0.057821	0.058543	0.153016	0.394156	0.138009
0.021602	0	1.211299	0.196097	0.001277	0.000938	0.143709	0.038182	2.89E-05
1.959466	1.02487	5.522929	1.601443	0.727222	0.322395	1.373348	4.46267	0.601227
11.42512	7.991602	2.868456	6.365065	5.337547	3.993665	34.38341	7.363117	6.706472
2.961768	0.577071	3.166659	0.526299	0.159351	0.163983	1.455022	1.754733	1.080909
4.034423	2.887489	3.055729	8.256465	0.057821	0.132309	1.416407	3.792266	2.151919
0.690375	0.428694	0.267013	0.024553	1.234444	2.597792	0.418023	0.262785	0.400534
6.464906	5.622872	65.03761	50.55949	5.150693	5.696263	7.882222	40.7932	6.933961
1.604827	3.6029	0.049105	0.024553	4.814661	118.7028	2.871212	0.038182	0.742872
0.543665	0.518413	0.793434	3.934986	0.001277	0.190058	0.692381	1.805166	0.497424
8.66E-05	0	0.267013	0.178182	0.079885	0.121818	0.019942	0.321227	0.011227
1.242641	1.539062	0.476551	2.652107	0.171277	1.020447	0.807136	1.306667	8.905642
0.070541	0.071861	0.306811	0.363059	0.001277	0.037605	0.036241	0.206984	0.051443
0.40886	0.510072	1.473218	1.959466	0.345238	0.132309	0.325108	0.874603	0.308788
40.7932	53.77619	23.06543	44.27012	15.98892	15.07257	49.50903	13.87537	14.6058
0.224466	0.717027	7.112496	3.286652	0.001277	0.095851	0.554416	1.212792	0.122395
0.008225	0.07767	0.235613	0.216025	0.044964	0.037605	0.019942	0.290115	0.031775
0.624163	0.6943	0.847352	1.877605	1.298665	0.523752	1.443983	1.387612	0.259033
13.19051	2.254084	0.049105	0.024553	0.001277	0.000938	13.45939	0.038182	5.477843
0.577388	1.125325	1.369928	0.024553	4.353925	1.328384	2.400455	0.038182	0.90368
0.376017	0.819048	0.049105	0.024553	16.22108	5.788153	0.000188	0.038182	2.89E-05
0.278499	0.510072	0.049105	0.216025	1.498167	0.163983	0.639495	0.235296	0.298153
1.137244	3.005281	0.049105	0.024553	2.037114	1.133622	1.095657	0.038182	0.073939
2.776421	1.539062	4.236962	9.792626	0.218045	0.132309	2.628225	5.259437	2.90658
1.437648	0.959062	0.847352	0.480794	0.001277	0.000938	2.858009	0.038182	1.393571
0.348889	0.029596	2.388348	3.327619	0.001277	0.095851	0.064372	1.863506	0.176955
5.515584	4.978182	11.68846	23.6012	3.482785	2.197828	6.144055	6.404921	2.871212
0.454394	0.769019	0.049105	0.024553	0.626118	0.586595	1.059553	0.038182	0.065801
0.666847	1.330974	10.82954	7.545584	0.079885	0.155945	1.018427	6.011515	0.763939
2.6143	2.292864	0.733593	0.526299	2.073232	1.041025	4.101061	1.169791	1.87482
0.134517	0.029596	0.997792	2.124574	0.001277	0.000938	0.188009	3.566753	0.578817
11.544	4.563117	0.049105	0.024553	3.397706	3.66785	5.143867	0.038182	7.288788
0.031378	0.041183	0.235613	0.196097	0.090108	0.155945	0.088701	0.185909	0.065801
0.967446	1.692763	0.049105	0.526299	4.597792	1.117316	0.706984	0.832554	4.012049
5.813247	3.989206	1.872049	2.789466	1.705007	0.322395	7.112496	2.432121	1.821861
2.472576	3.632973	0.997792	0.024553	3.533211	0.958557	3.699495	0.038182	0.675556
0.312864	0.281169	0.580758	0.454632	0.044964	0.068629	0.639495	1.95645	0.919192
1.381111	1.667273	0.049105	0.024553	13.49926	8.319019	1.059553	0.038182	2.89E-05
1.718918	0.887359	7.375794	8.923117	0.470512	0.6114	2.820974	13.08214	3.962179
10.93101	9.502294	0.049105	0.024553	16.9162	5.855281	11.39744	0.038182	0.451169
4.266753	3.36075	2.654473	2.858009	10.33476	13.15079	7.339452	1.169791	11.65791
1.484553	1.692763	0.049105	2.6143	0.001277	0.000938	2.281926	0.038182	3.659668
0.858297	1.158384	1.928629	3.55961	0.134711	0.148932	2.157359	1.863506	3.457504
0.40886	1.1379	2.197828	1.991328	0.069459	0.10404	1.159913	2.373824	2.773644
0.101212	1.283059	1.264589	1.644416	7.737807	9.063983	0.000188	1.064935	1.142345
8.66E-05	1.916782	0.049105	0.024553	1.692763	1.343463	1.05153	0.038182	0.650556

8.089848	7.468961	0.531169	1.167561	6.079596	1.722475	8.348817	1.58645	4.312251
3.457504	2.225498	3.363911	4.160238	0.321385	0.155945	2.6143	4.376118	3.113478
4.524481	1.953535	16.9162	11.57341	3.058802	3.110447	3.55961	10.43066	3.926154
0.715462	0.29767	5.287857	9.604358	0.987309	0.6943	1.594675	4.667518	2.031169
1.590202	0.202641	0.049105	0.024553	2.432121	0.268961	1.402915	0.038182	0.279452
1.399315	0.778326	0.049105	0.25785	10.78056	0.155945	1.31	0.038182	0.259033
20.51271	6.325945	0.624473	1.644416	29.18885	5.447114	44.27012	1.468131	7.839271
12.53072	4.261342	0.049105	0.024553	9.792626	10.93101	10.1028	0.786667	2.440895
5.172828	2.666494	0.267013	0.312323	3.877417	4.115844	10.78056	0.290115	3.253175
6.653016	15.276	0.049105	0.024553	15.77678	0.601623	3.799885	0.038182	2.225498
0.874603	0.726133	4.72798	6.464906	0.852172	1.020447	1.89228	5.259437	3.327619
3.606804	2.584394	0.049105	2.327857	1.202626	0.30373	3.621941	0.038182	2.214372
0.858297	1.497143	0.049105	0.024553	2.901032	1.531313	0.887922	1.468131	0.288867
0.956667	0.476551	0.306811	0.395426	0.240202	0.190058	0.134444	0.235296	2.970144
0.690375	0.536905	2.468117	1.717641	0.147323	0.728543	1.443983	0.874603	0.982698
0.38772	0.599466	2.868456	6.079596	0.079885	0.218975	0.315743	2.981962	1.057569
0.990909	1.209105	0.580758	1.828781	0.001277	0.000938	0.96938	0.477698	0.86469
1.295108	1.539062	0.343434	0.72044	0.470512	0.658362	2.666494	0.262785	2.269134
0.267201	0.29767	4.845166	4.272165	0.20733	0.622092	0.134444	8.815123	0.601227
0.891775	1.065584	0.049105	0.553348	1.040411	1.734949	0.570317	0.038182	1.364401
8.66E-05	0.81228	0.049105	0.024553	4.353925	14.25954	0.000188	0.038182	2.89E-05
2.591443	1.839986	0.049105	0.284646	1.778153	2.740519	3.588369	0.038182	2.607035
0.858297	0.968723	0.049105	0.395426	0.761688	0.841746	3.104394	0.038182	1.172208
0.090188	0.136573	1.264589	1.572035	0.079885	0.268961	0.045491	2.554069	0.176955
1.651522	6.784762	0.049105	0.024553	5.530274	9.031082	3.489899	0.038182	0.982698
7.558139	11.96574	0.382626	0.454632	6.601833	2.654473	6.116638	0.290115	7.112496
0.432915	0.435382	0.343434	0.363059	3.500808	0.331097	1.359177	0.038182	0.011227
2.831501	1.931537	0.049105	0.024553	2.66404	2.353247	4.082165	1.064935	0.932078
1.094206	0.635036	0.948211	1.026825	0.193348	0.000938	1.195794	9.012937	1.622006
0.454394	0.051335	0.425844	1.286494	0.65202	0.000938	0.099004	0.357013	0.106407
7.443276	0.635036	0.049105	0.024553	2.688304	1.149322	9.667316	0.038182	0.319957
3.407619	5.898449	0.049105	0.024553	6.853608	5.889798	1.169091	0.038182	3.300664
6.287561	1.439704	6.64254	14.12978	0.889553	1.96697	5.993045	4.297222	7.964315
0.50254	0.29767	0.343434	1.428398	0.321385	0.037605	0.895945	1.306667	0.742872
0.443543	0.136573	1.663672	0.751876	0.270837	0.208687	0.546522	0.701255	0.185642
0.588658	0.305253	3.425512	2.6143	0.193348	1.030678	0.645916	2.826277	0.477446
1.029365	0.876638	0.049105	0.426645	0.57531	0.396638	2.194257	0.038182	1.007244
2.178268	3.055729	0.672857	0.363059	5.130411	2.92013	3.971061	0.262785	1.91254
8.66E-05	0.051335	0.267013	0.284646	0.001277	0.000938	0.019942	0.290115	0.015714
0.059654	0.029596	0.235613	0.131443	0.090108	0.068629	0.099004	0.038182	0.169538
3.233045	2.584394	2.281926	4.408153	2.477027	1.722475	3.944084	3.566753	1.935685
6.200945	3.640606	1.573276	0.72044	0.707475	0.933261	8.103925	0.262785	2.151919
56.39528	19.99861	26.52537	167.9996	6.717056	1.522597	70.11932	135.5245	28.87506
1.137244	0.550368	0.672857	1.367179	0.134711	0.000938	0.395152	3.450577	1.634935
0.101212	0.066407	0.343434	0.613853	0.159351	0.058543	0.325108	0.206984	0.007821
2.141595	4.02088	0.049105	0.480794	9.297085	2.269134	1.511198	0.038182	3.47531
5.037489	10.05618	0.049105	0.024553	6.045209	5.762965	10.19468	0.038182	3.962179
1.381111	2.887489	0.049105	0.613853	3.904517	1.826147	4.997417	0.038182	0.601227
0.08044	0.060022	0.267013	0.336508	0.001277	0.000938	0.064372	0.038182	0.020548
0.021602	0.007554	0.049105	0.526299	0.001277	0.000938	0.013405	0.262785	0.073939
1.473218	1.427431	0.049105	0.178182	2.111299	0.702835	3.414574	0.038182	0.477446
12.26136	10.19468	0.049105	0.024553	11.60283	17.28195	3.909033	1.468131	0.949827
0.858297	0.599466	0.580758	0.336508	0.283709	0.515527	0.469711	0.321227	0.467136
0.555036	2.145137	0.049105	0.024553	6.967547	5.22482	0.960216	0.038182	7.570693
1.446926	1.260635	0.049105	0.024553	2.882035	5.18754	0.623146	0.038182	1.057569
2.178268	4.451154	4.236962	5.11697	1.510014	1.808023	1.881955	4.376118	1.753341
0.090188	0.162576	7.6393	5.044113	0.134711	0.208687	0.615584	7.225469	0.288867
6.760765	1.96404	72.69473	42.75576	0.001277	0.000938	4.690159	51.52195	2.953059

3.172864	4.745895	3.166659	0.867287	4.620159	1.178939	2.973059	1.426522	2.337821
0.432915	0.274704	0.476551	0.336508	0.001277	0.048225	0.170678	0.578095	1.112641
1.078153	0.476551	3.317807	0.024553	0.001277	0.18153	0.842641	3.644343	1.216768
1.295108	1.795851	0.049105	0.024553	1.738456	0.556126	1.217475	0.038182	1.041652
0.376017	0.421342	0.049105	0.780087	0.584134	0.743283	0.124957	0.038182	0.690043
2.628225	3.581688	0.901732	0.024553	1.821861	3.092229	1.295108	0.436017	5.872886
0.421804	0.029596	0.624473	0.024553	0.001277	0.000938	0.178997	0.986104	2.89E-05
3.310758	3.493362	0.049105	0.25785	1.778153	3.726854	2.506934	0.394156	2.361198
0.74868	0.726133	1.473218	1.064242	1.63039	0.866147	1.455022	0.394156	0.459048
11.19942	29.49693	0.049105	0.454632	23.16622	10.48101	15.276	0.038182	3.606804
0.049625	0.484423	0.425844	0.024553	1.004156	0.572987	0.814315	0.916306	0.505267
1.427431	0.876638	0.049105	1.237063	27.66779	6.99026	2.216162	0.701255	0.523983
3.7586	5.530274	3.47531	5.788153	18.48514	26.52537	5.477843	8.134084	6.355224
0.38772	0.125123	0.425844	0.024553	0.001277	0.000938	0.064372	0.742872	0.370462
1.578687	0.536905	0.049105	0.024553	3.070952	2.257749	0.778326	0.038182	0.097633
16.22108	30.17192	0.049105	0.024553	10.28573	14.17388	21.93452	0.038182	9.667316
1.201724	1.125325	0.049105	0.024553	2.554069	2.666494	0.856782	0.038182	2.884762
0.666847	1.405772	0.049105	0.024553	1.268672	4.312251	0.722626	0.786667	1.409942
7.351284	12.85504	1.321053	0.336508	5.821804	27.96248	7.188095	1.020447	15.56131
0.323752	0.095108	0.049105	0.024553	0.001277	0.000938	0.554416	0.038182	0.298153
0.637994	1.839986	0.997792	0.553348	1.971183	1.223846	1.728615	0.477698	0.430087
4.236962	2.81342	7.92518	21.74051	1.575483	2.868456	3.833247	12.42965	2.763261
4.997417	6.443925	0.049105	0.024553	29.49693	8.976508	0.606421	1.387612	9.989221
0.235931	0.106926	2.197828	0.336508	1.753341	1.030678	0.215729	0.321227	0.269141
0.942294	3.083319	0.049105	0.867287	2.26518	0.774798	2.597792	1.212792	0.675556
0.844747	1.554185	11.51384	1.026825	0.001277	0.314387	0.53658	0.954776	1.91254
1.446926	0.76	3.211176	0.950418	0.001277	0.199264	0.824885	1.255657	0.563824
16.39531	10.73084	1.264589	1.828781	3.957734	4.381479	50.55949	1.064935	5.515584
1.052208	2.106089	0.049105	0.284646	1.821861	1.266255	0.91404	0.436017	0.734113
1.540144	0.643427	4.535354	7.6393	1.555289	1.35259	1.240202	9.729358	0.675556
0.090188	0.041183	1.111977	1.064242	0.034776	0.000938	0.385238	0.53912	0.011227
1.896667	0.051335	0.049105	0.024553	0.001277	0.000938	3.303925	0.038182	0.065801
1.496118	2.607035	0.049105	0.024553	3.783694	3.207807	2.893001	0.038182	1.27619
1.484553	0.968723	0.049105	0.024553	3.043528	3.7586	0.760433	0.786667	1.130794
2.03886	1.573276	0.793434	0.526299	0.876118	0.642395	2.673846	0.477698	1.588903
1.669466	3.031688	0.997792	4.212554	1.650274	0.796674	5.381227	1.543377	4.188896
0.15329	0.149019	0.049105	0.024553	3.621941	1.178939	0.335967	0.038182	2.89E-05
91.05541	80.6518	95.22939	29.82828	4.696313	4.212554	40.11561	24.2842	43.49658
2.755411	3.394286	4.343838	2.287576	1.123016	0.523752	2.207417	1.064935	3.699495
0.690375	0.778326	0.425844	0.584134	0.044964	0.000938	0.469711	0.038182	0.982698
0.267201	0.202641	2.388348	0.950418	0.069459	0.000938	0.453932	1.805166	0.319957
2.119596	0.866147	0.049105	0.526299	5.622872	4.082165	1.287403	0.038182	0.339235
8.381328	4.043846	0.997792	0.918557	1.928629	1.982208	4.101061	0.290115	3.166659
1.779423	0.676999	0.049105	0.024553	1.420202	0.450375	1.668369	0.038182	0.514885
0.979149	0.733593	2.145137	6.760765	0.619012	0.565426	5.210317	5.050267	1.821861
31.1753	26.25557	10.82954	14.47154	11.14619	10.85542	31.92385	9.924372	24.50942
2.735368	1.34513	3.5257	1.064242	2.984899	1.652771	3.095166	1.58645	1.513102
0.515815	0.858297	0.049105	0.584134	0.218045	0.112222	1.179885	0.235296	0.232886
2.850058	2.162381	0.267013	1.237063	1.519322	0.979827	3.025671	2.373824	1.555289
1.308167	1.260635	8.524242	4.592273	1.621032	3.083319	0.92816	8.396847	2.252273
0.530851	1.158384	0.049105	0.144163	2.254084	1.156869	0.483045	0.038182	2.89E-05
1.465123	1.34513	0.624473	0.024553	2.522684	1.030678	2.390447	0.038182	0.057893
0.008225	0.000137	0.235613	0.161962	0.090108	0.028398	0.099004	0.508658	0.031775
2.565556	4.33342	0.531169	0.780087	3.722785	3.166659	4.323045	0.578095	1.89961
21.19547	19.22139	2.069942	2.359264	14.79742	12.16681	10.43066	0.659957	19.83727
3.439437	3.1857	6.335664	3.934986	2.005253	1.009625	5.553146	2.190866	2.276407
0.609841	0.435382	1.264589	1.759986	0.37417	0.42202	1.128131	0.436017	0.232886
0.599134	0.826407	0.476551	1.644416	0.001277	0.000938	1.568564	0.742872	0.514885

1.696494	0.778326	8.195657	7.839271	1.396494	0.658362	0.938911	5.646811	1.252424
0.031378	0.060022	4.193232	3.327619	0.65202	0.248528	0.124957	2.092987	0.176955
0.323752	0.2914	0.049105	0.024553	2.29873	2.535974	0.651631	0.038182	1.216768
8.66E-05	0	0.049105	0.950418	7.061328	6.945152	0.824885	0.038182	1.232886
1.427431	1.667273	0.049105	0.024553	0.91404	0.6943	0.833975	0.038182	1.240202
0.36114	0.321566	0.997792	0.454632	0.001277	0.068629	0.325108	0.786667	0.115065
0.769365	0.944784	0.343434	0.144163	0.567994	1.856392	0.453932	0.357013	0.889553
0.144488	0.313521	0.624473	1.097677	1.537951	0.942294	0.769365	0.394156	0.459048
1.456955	0.78619	0.049105	0.024553	2.842193	1.458038	0.673651	0.038182	0.982698
1.066234	4.145209	0.049105	0.024553	0.675938	1.183478	0.895945	0.038182	1.409942
4.484892	4.072475	0.049105	0.024553	13.60866	4.733824	6.200945	0.038182	1.191905
0.070541	0.101212	2.333867	1.877605	0.069459	0.037605	0.290866	2.713319	0.59062
4.297222	2.487576	1.663672	1.129452	0.819978	1.702706	4.661717	1.468131	4.821176
6.079596	8.019769	0.901732	0.024553	1.762713	1.928629	8.210938	0.206984	2.073232
3.47531	2.683478	100.0998	59.32013	2.073232	2.048781	6.497266	34.84299	2.459812
5.984885	6.028427	0.049105	0.024553	7.854069	3.428788	4.721789	0.038182	3.8379
48.53429	6.464906	37.29684	111.5439	1.71518	8.005685	15.46193	62.33472	10.1459
1.052208	1.427431	0.847352	0.867287	0.057821	0.121818	1.62562	1.212792	3.734762
2.642525	6.738211	0.049105	0.024553	6.53969	16.68554	4.535354	0.038182	7.780786
332.0776	1360.293	18.33909	4.212554	224.9555	87331.38	184.443	5.84689	224.9555
0.74868	0.769019	3.263391	1.367179	4.833398	2.106089	1.656696	2.879228	0.663276
0.08044	0.07767	0.343434	0.024553	0.001277	0.30373	0.028232	0.038182	0.349812
25.24367	3.116732	4.661717	26.25557	2.465916	3.192229	7.683766	9.179466	9.141299
18.76954	14.17388	3.775209	2.652107	17.92188	23.82354	24.04374	0.038182	8.226219
1.137244	1.645613	0.948211	6.268312	0.303564	0.208687	1.465123	1.255657	0.817547
19.22139	8.711905	4.603434	0.584134	17.28195	13.53913	8.958225	0.508658	9.625253
5.454949	7.175397	2.984899	0.024553	4.888023	5.366335	0.000188	0.038182	0.690043
3.110447	3.083319	0.049105	0.024553	5.568391	1.539062	5.082063	0.038182	0.889553
0.74868	1.645613	0.049105	0.024553	1.971183	2.257749	1.678319	0.038182	0.16145
4.626025	4.650007	3.263391	7.584502	1.983824	1.919661	5.614805	4.46267	4.261342
0.783939	0.959062	0.997792	4.468009	0.228788	0.314387	1.53684	1.805166	2.089639
12.16681	5.337547	15.77678	18.62711	5.754935	5.915469	10.01009	17.28195	12.77772
0.802395	0.29767	5.110159	2.124574	0.159351	0.141472	0.272612	5.950664	0.451169
1.63039	1.89373	0.476551	0.178182	1.902352	0.278802	2.925426	0.262785	1.351573
55.04081	72.69473	2.805382	1.644416	21.54823	23.16622	52.56128	0.038182	57.84882
1.052208	3.691724	10.48101	5.838499	0.478182	0.383348	3.500808	10.7557	5.180058
0.059654	0.046883	0.425844	0.395426	0.001277	0.000938	0.028232	0.235296	0.025685
7.683766	5.872886	8.005685	12.36443	5.687879	5.622872	10.5063	23.82354	6.933961
1.525729	2.831501	4.236962	3.614603	1.287403	2.710765	1.037958	23.6012	3.904517
7.112496	8.445743	0.948211	0.480794	6.171789	3.370527	4.451154	1.306667	4.758088
5.075447	4.484892	1.369928	0.196097	4.241905	3.307186	9.924372	1.543377	3.47531
0.189141	0.113218	0.997792	1.545657	0.001277	0.000938	0.170678	0.357013	0.051443
2.259661	0.81228	1.057569	2.212619	0.83697	0.996111	2.040606	2.190866	0.982698
0.323752	0.34583	0.624473	0.363059	0.101429	0.141472	0.469711	0.53912	0.21689
0.769365	0.442193	0.235613	0.235137	0.353651	0.0771	0.824885	0.659957	0.523983
0.08044	0.002035	0.531169	0.918557	0.134711	0.112222	0.153016	1.632648	0.045281
7.351284	18.62711	0.049105	0.235137	19.37139	1.162201	11.96574	0.235296	1.263752
1.381111	2.000592	0.049105	0.024553	2.74544	1.200823	1.668369	0.038182	0.130202
3.26	5.158146	0.049105	0.024553	4.552092	3.031688	7.315512	0.038182	2.544747
13.87537	3.66785	0.049105	0.216025	5.915469	31.92385	11.77567	0.357013	0.279452
6.464906	4.821176	0.049105	0.161962	1.056263	0.676999	1.813564	0.038182	2.238341
18.48514	15.61077	0.049105	0.024553	26.80355	5.447114	22.52375	0.038182	1.09075
1.094206	1.522597	0.049105	0.950418	3.763045	0.429827	1.720144	0.038182	0.949827
0.543665	0.396082	6.738211	8.780115	1.222179	0.490375	0.736551	11.83593	1.130794
0.189141	0.321566	0.049105	0.024553	1.277843	0.218975	1.087345	0.038182	0.081212
4.667518	4.540794	14.84163	8.849697	0.819978	0.780952	5.302294	10.55571	4.881775
5.545729	1.945916	0.624473	3.614603	0.134711	0.048225	8.849697	0.436017	2.496407
0.15329	0.25785	6.738211	3.422237	0.001277	0.172453	0.206616	4.236962	0.21689

1.295108	0.81228	2.026219	2.463911	0.453463	0.258838	2.057056	2.145137	6.028427
7.238023	5.950664	4.603434	1.545657	26.25557	47.5018	2.925426	0.206984	5.409437
6.570433	7.339452	6.162446	1.518232	11.544	18.33909	9.141299	1.695216	10.19468
3.370527	2.000592	14.25954	24.85971	1.261407	1.003586	3.273045	20.85876	3.435988
0.956667	1.302107	0.382626	0.235137	3.722785	1.041025	4.473348	0.321227	0.889553
2.099524	1.89373	0.580758	1.286494	0.283709	0.121818	1.025613	1.913997	0.701255
0.705613	0.826407	0.267013	0.336508	1.449062	1.722475	1.701616	0.262785	3.926154
0.090188	0.029596	1.264589	2.097843	0.001277	0.000938	0.178997	0.742872	0.549019
0.070541	0.171061	4.94026	0.780087	0.001277	0.000938	0.483045	9.316241	0.207157
7.351284	5.110159	0.948211	0.024553	3.24658	6.09759	2.6143	0.436017	3.659668
0.479062	0.490952	1.264589	0.751876	0.259033	0.0771	0.842641	0.235296	1.041652
0.15329	0.428694	0.672857	1.572035	0.044964	0.000938	0.435123	0.038182	0.578817
1.437648	1.645613	6.240346	6.210375	1.298665	1.328384	1.435772	4.02088	1.728615
0.979149	1.629235	0.049105	0.216025	2.164113	0.702835	1.909408	0.038182	0.400534
0.177143	0.235137	0.793434	1.129452	0.123456	0.132309	0.215729	1.426522	0.138009
5.439278	3.679986	2.145137	3.855952	1.983824	2.000592	6.365065	1.632648	4.029654
2.178268	1.1379	0.476551	0.25785	1.251659	2.034242	1.701616	0.701255	0.721335
8.83241	14.00414	0.049105	0.024553	7.315512	2.597792	10.70538	0.038182	12.03267
3.648081	1.183478	4.150274	2.24733	0.001277	0.095851	0.978571	3.107417	2.720996
9.335397	4.003117	16.62402	6.865	1.401068	1.722475	4.251818	9.838658	5.821804
0.090188	0.095108	0.049105	0.363059	0.001277	0.000938	0.40645	0.321227	0.065801
1.753341	0.496797	1.264589	0.024553	0.001277	0.121818	0.882078	0.038182	2.89E-05
0.942294	0.778326	0.049105	0.024553	1.947316	2.500548	0.501905	0.038182	0.636688
0.609841	0.18324	0.049105	1.367179	0.001277	0.000938	0.706984	4.529913	0.650556
29.82828	13.91714	33.28499	43.49658	3.253175	1.86658	13.38418	30.49289	20.85876
0.833045	1.125325	1.159913	1.261407	0.345238	0.405974	1.491977	0.786667	1.252424
3.414574	16.22108	0.049105	0.024553	15.18102	1.734949	7.037345	0.701255	5.180058
0.013442	0.001025	0.580758	0.024553	0.034776	0.037605	0.000188	0.185909	0.007821
3.851414	2.074906	0.793434	0.613853	3.966623	3.220678	5.662994	0.786667	2.607035
10.40634	17.15902	1.111977	4.016465	37.29684	16.22108	28.87506	2.432121	26.25557
0.348889	1.554185	4.292078	5.477843	2.66404	0.287951	0.96938	3.028629	1.80267
5.515584	8.524242	0.476551	0.25785	3.034747	3.276335	11.25392	0.394156	9.438687
0.122554	0.071861	0.580758	0.553348	0.034776	0.028398	0.196955	0.321227	0.051443
1.000794	0.893016	0.049105	0.161962	1.371602	0.933261	2.644885	0.235296	0.497424
0.224466	0.171061	0.049105	0.284646	0.193348	0.000938	0.000188	0.436017	2.89E-05
36.26061	25.48179	0.049105	0.024553	21.02638	7.495397	17.39935	1.426522	7.315512
0.031378	0.119553	0.847352	1.217475	0.001277	0.112222	0.036241	0.53912	0.020548
0.070541	0.046883	0.049105	0.024553	0.240202	0.000938	0.153016	0.038182	2.89E-05
17.03919	10.01009	0.049105	0.024553	7.598312	8.780115	26.80355	1.11526	6.116638
0.858297	0.669769	1.663672	0.024553	2.628225	2.901032	0.576638	0.742872	0.505267
0.200794	0.717027	0.049105	0.024553	33.4985	11.96574	0.778326	0.038182	0.578817
2.24733	5.088694	0.049105	0.024553	4.609076	11.03843	1.521371	0.038182	5.373781
3.334199	4.286962	1.52676	2.463911	1.86658	0.866147	7.710361	1.805166	0.949827
1.078153	1.603795	0.049105	0.641631	3.104394	8.412367	1.803918	0.038182	1.622006
7.625209	2.345527	46.7056	33.07147	1.650274	3.588369	4.348882	34.38341	7.339452
3.962179	4.529913	0.580758	2.035678	7.911934	3.179199	2.6143	1.695216	1.27619
2.763261	4.23202	1.573276	1.367179	5.500678	6.345382	5.754935	1.468131	9.063983
4.266753	7.668579	5.110159	7.363117	25.99427	62.33472	7.417359	3.450577	11.42512
0.101212	0.599466	1.819221	0.690707	0.001277	0.322395	0.722626	2.236407	0.176955
0.74868	0.219885	0.306811	0.336508	1.371602	0.753016	1.813564	0.038182	0.057893
0.924329	3.87329	1.819221	5.788153	2.421681	0.774798	3.220678	6.316328	1.364401
0.715462	0.337431	0.049105	4.468009	0.001277	0.121818	1.873434	3.566753	0.932078
0.122554	0.019466	0.343434	0.235137	0.057821	0.048225	0.064372	0.321227	0.146234
4.851227	1.082915	3.363911	1.877605	0.37417	0.208687	1.953535	1.754733	2.607035
3.775209	8.524242	0.049105	0.024553	9.218514	12.49452	1.383911	0.038182	5.976724
1.511198	1.513102	0.049105	0.584134	2.367763	0.720938	1.087345	0.038182	0.232886
1.974228	0.799048	1.982208	1.601443	1.123016	0.689509	0.760433	0.394156	1.057569
0.25733	0.130823	0.049105	1.167561	0.113074	0.340866	0.994271	0.038182	0.549019

8.66E-05	0.014935	0.049105	1.601443	0.001277	0.000938	0.073261	2.092987	0.045281
2.602395	6.171789	0.267013	0.480794	0.788896	1.041025	6.045209	0.321227	3.807648
0.101212	0.125123	0.049105	0.504416	0.044964	0.000938	0.375094	0.53912	0.169538
1.355527	2.502619	0.049105	0.284646	3.514834	3.253175	2.227273	0.038182	0.279452
1.178939	0.935743	1.755988	7.839271	0.303564	0.90772	1.392511	4.101061	2.509177
0.40886	5.454949	2.145137	1.064242	10.1459	6.591544	6.171789	4.02088	13.23022
10.48101	18.19595	0.049105	0.024553	12.63773	14.6058	4.222583	0.038182	15.18102
0.031378	0.095108	0.901732	2.893001	0.079885	0.155945	0.073261	1.426522	0.339235
0.013442	0.035505	0.235613	0.838846	0.034776	0.037605	0.045491	0.262785	0.065801
11.77567	5.485685	0.672857	0.480794	1.833102	3.407619	8.445743	0.619012	3.327619
5.34474	3.771349	6.240346	14.29947	1.650274	1.429365	5.967879	13.72059	8.815123
6.738211	5.553146	1.473218	0.553348	1.431414	1.835743	5.933066	1.543377	5.01088
0.50254	0.733593	0.425844	1.877605	0.91404	0.278802	0.729062	0.038182	0.675556
1.779423	1.244957	0.049105	1.404812	5.942294	4.839105	1.712706	0.394156	0.789423
8.66E-05	0	0.049105	0.024553	0.001277	0.743283	0.000188	0.619012	0.349812
4.540794	13.53913	0.049105	0.024553	33.93939	5.00404	11.83593	0.477698	32.68313
0.090188	0.136573	2.104358	1.428398	0.001277	0.172453	0.315743	2.278254	0.889553
0.73583	1.29342	19.53061	25.99427	0.337431	0.767085	1.594675	8.815123	2.871212
52.56128	65.94551	1.418312	4.946587	37.81919	22.52375	95.22939	0.508658	16.68554
0.267201	0.999502	1.057569	1.217475	0.444048	0.30373	0.570317	0.832554	0.242612
0.25733	0	0.049105	0.918557	4.696313	4.034423	0.099004	0.038182	1.393571
8.66E-05	0.599466	1.369928	4.875527	0.001277	0.095851	0.789863	0.436017	0.721335
2.747908	2.316075	0.049105	0.553348	2.126349	1.605859	2.207417	0.038182	1.007244
9.625253	8.396847	0.049105	1.026825	4.429769	2.468117	6.760765	8.241342	4.709033
0.795339	1.244957	0.049105	0.808485	3.588369	2.868456	3.414574	0.659957	3.435988
0.530851	0.484423	30.99903	13.23022	0.249899	0.068629	0.453932	59.32013	0.391962
0.715462	0.769019	2.104358	1.286494	0.171277	0.148932	0.842641	0.874603	1.678319
0.189141	0.968723	0.049105	0.024553	2.647244	7.938427	0.800967	0.038182	0.90368
7.558139	4.193232	0.948211	3.095166	1.488571	0.572987	3.350224	1.805166	3.851414
2.797835	3.78798	4.392316	6.464906	0.259033	0.351219	3.195541	15.3618	3.223882
8.66E-05	2.189235	0.049105	0.024553	8.195657	3.327619	1.881955	0.038182	0.742872
1.165873	4.429769	0.382626	0.024553	1.023528	8.849697	0.546522	0.321227	2.661587
1.220628	1.03303	2.069942	3.726854	0.626118	0.622092	2.109755	1.349971	1.995714
12.10289	19.83727	0.049105	0.024553	45.91841	16.9162	11.30987	0.038182	7.882222
5.872886	4.894509	2.197828	1.329206	6.53969	5.485685	7.570693	6.911486	3.50425
0.122554	0.029596	0.049105	0.690707	0.001277	0.000938	0.134444	0.53912	0.057893
18.05488	21.74051	0.948211	0.024553	13.6814	9.179466	12.63773	0.038182	14.79742
144.7919	203.5795	0.049105	9.667316	425.62	749.7839	118.7028	12.81638	749.7839
8.905642	6.830592	1.159913	16.44768	0.696234	0.728543	25.99427	9.459177	46.7056
0.599134	0.421342	0.049105	0.024553	0.567994	1.212792	0.196955	0.038182	2.285664
1.651522	2.607035	4.535354	1.064242	6.240346	4.802655	2.470317	0.832554	0.410823
5.933066	4.413608	2.805382	2.758088	13.49926	15.88548	3.314329	3.179199	5.720173
5.779574	9.968355	0.049105	5.150693	91.05541	55.04081	13.53913	0.038182	0.763939
0.530851	1.364401	0.049105	0.553348	7.013853	5.416623	1.416407	0.038182	5.210317
2.701053	33.4985	0.049105	0.024553	43.49658	26.25557	8.677489	0.038182	0.259033
15.3618	7.751869	29.49693	16.80343	5.439278	7.443276	8.762677	21.19547	10.62739
0.942294	0.676999	0.049105	0.161962	1.680895	0.199264	1.103261	0.235296	0.232886
2.285664	5.130411	0.672857	0.363059	3.655714	3.566753	2.461905	1.11526	1.947316
1.094206	0.585902	0.733593	2.016515	0.408427	0.390014	1.744055	2.236407	1.441898
0.212172	0.305253	0.901732	1.759986	0.001277	0.095851	0.91404	1.505678	0.03829
12.63773	7.315512	2.388348	4.802655	15.276	9.924372	17.15902	2.145137	7.363117
19.53061	16.9162	0.948211	0.780087	14.42661	7.288788	13.75978	1.212792	6.25938
8.66E-05	0.083413	0.476551	1.518232	0.001277	0.000938	0.272612	3.644343	0.391962
7.468961	11.83593	1.321053	3.55961	23.38378	25.73165	6.978903	1.11526	18.62711
1.456955	0.496797	0.049105	0.480794	0.714697	0.155945	0.395152	0.038182	0.279452
2.132756	0.435382	1.573276	1.367179	0.147323	0.095851	0.985411	1.020447	0.663276
2.270837	2.091313	1.057569	0.918557	0.889553	1.253752	2.294733	0.742872	1.204957
0.165108	0.467677	1.159913	1.717641	0.001277	0.048225	0.40645	1.995714	0.612872

4.140505	6.570433	0.531169	2.893001	9.141299	3.904517	3.002576	1.255657	2.238341
7.911934	3.310758	0.476551	1.828781	1.47842	0.734113	4.130794	0.290115	1.480541
0.299942	0.07767	0.306811	0.867287	0.001277	0.000938	0.53658	0.206984	0.065801
2.119596	3.989206	0.049105	0.838846	11.74734	3.407619	7.08733	1.387612	0.650556
17.51961	30.49289	0.267013	1.717641	56.39528	22.96463	6.674286	3.26	23.6012
8.66E-05	0	0.049105	0.024553	12.36443	12.10289	0.000188	0.038182	0.370462
0.059654	0.024423	15.07257	0.395426	0.001277	0.058543	0.064372	7.868146	0.122395
3.895469	7.175397	0.049105	0.024553	9.377345	2.489762	6.717056	0.038182	0.051443
1.220628	0	0.049105	6.191515	0.001277	0.000938	0.000188	0.038182	1.252424
0.08044	0.010851	1.211299	0.363059	0.034776	0.000938	0.453932	1.913997	0.22456
1.496118	2.162381	3.775209	1.877605	0.171277	0.759646	2.216162	2.768312	2.607035
0.555036	0.556126	0.049105	6.019971	0.001277	0.000938	1.383911	0.038182	2.89E-05
0.609841	0.614156	4.456912	5.530274	0.001277	0.086443	0.546522	2.236407	1.080909
2.006782	3.962179	0.049105	0.024553	0.660678	1.188139	0.760433	0.038182	0.549019
3.521934	2.263377	0.049105	0.690707	8.226219	3.846984	4.978182	0.038182	2.685786
1.014784	1.219019	2.917381	1.518232	0.057821	0.239885	1.759986	1.169791	2.703485
4.105714	6.153102	0.425844	0.613853	10.43066	6.911486	5.933066	0.357013	6.663651
0.041053	0.274704	1.819221	0.526299	0.079885	0.000938	0.453932	3.061955	2.89E-05
5.137042	3.544257	0.049105	0.918557	15.56131	8.348817	9.480736	0.742872	6.433918
6.38456	3.29355	0.049105	1.428398	3.711097	4.620159	3.327619	1.805166	2.703485
0.122554	1.074242	1.52676	2.068297	0.057821	0.0771	0.375094	2.278254	1.122359
1.137244	2.42381	0.049105	0.024553	8.348817	12.26136	3.074134	0.038182	2.214372
1.974228	1.219019	0.343434	0.024553	1.11526	0.851659	1.095657	0.038182	1.156869
3.172864	2.000592	0.049105	0.024553	3.816551	1.009625	0.281558	0.038182	1.331919
0.070541	0.321566	1.418312	1.367179	0.069459	0.000938	0.206616	1.020447	0.624473
0.165108	0.442193	0.425844	0.641631	0.001277	0.058543	0.064372	0.206984	0.03829
0.690375	2.091313	0.793434	1.601443	0.397489	0.000938	1.037958	0.619012	0.721335
1.109726	0.733593	0.049105	0.024553	1.155411	2.597792	0.000188	0.038182	0.339235
0.013442	0.010851	0.382626	0.480794	0.001277	0.000938	0.000188	0.508658	0.03829
0.013442	0.004329	0.382626	0.426645	0.001277	0.000938	0.196955	0.786667	0.065801
4.971991	2.035678	0.049105	1.488571	5.47	13.38418	4.429769	0.038182	4.451154
0.278499	0.267388	0.531169	0.336508	0.193348	0.148932	0.249278	0.508658	0.03829
3.921645	4.609076	0.624473	1.797172	2.494177	2.063362	4.881775	0.786667	3.327619
0.783939	0.518413	0.672857	1.307417	2.421681	1.944517	1.359177	0.786667	1.263752
2.225498	1.480541	0.733593	0.72044	0.819978	0.29531	3.179199	0.394156	0.535368
2.155447	2.92013	2.561162	0.024553	2.823636	19.06893	0.729062	1.212792	3.113478
0.212172	0.07767	0.049105	0.024553	0.001277	0.037605	0.143709	0.262785	0.057893
1.859084	4.261342	0.049105	0.024553	2.572309	2.386248	3.672078	0.038182	2.196017
16.62402	17.64869	16.34294	7.061328	35.27608	60.77449	12.56693	5.599228	40.7932
0.942294	0.76	1.473218	3.119986	0.044964	0.058543	2.183665	0.436017	1.634935
0.165108	0.359502	0.672857	1.717641	0.218045	0.037605	0.300844	0.701255	0.488997
6.612121	8.905642	1.321053	0.196097	5.024293	9.707677	9.562554	0.53912	7.507619
2.485534	2.106089	0.847352	0.480794	4.067547	4.863579	1.402915	0.701255	1.728615
0.348889	0.373939	0.997792	0.024553	0.734935	0.539812	1.044235	0.619012	0.298153
0.224466	0.171061	1.618773	6.685087	0.044964	0.230808	0.196955	1.863506	0.391962
2.911962	6.30671	0.049105	0.024553	4.029654	1.744055	4.752121	0.038182	6.622092
2.141595	4.312251	0.049105	0.024553	16.09482	29.82828	1.803918	0.508658	3.407619
8.66E-05	0.253882	34.38341	0.024553	0.584134	5.381227	0.000188	0.659957	0.535368
30.17192	59.32013	1.573276	0.024553	59.32013	51.52195	31.1753	0.038182	19.37139
1.355527	3.227085	0.049105	0.024553	1.298665	1.369048	1.169091	0.038182	0.440548
3.037626	0.743283	2.427792	2.574733	0.675938	0.499986	2.014863	5.143867	0.853218
1.473218	1.183478	4.603434	8.608153	0.641631	2.858009	0.692381	4.101061	1.89961
4.603434	4.381479	10.03313	0.553348	21.74051	25.48179	2.169509	0.786667	8.381328
0.299942	0.359502	0.049105	0.024553	5.309264	2.810678	0.124957	0.038182	0.650556
1.094206	0.550368	0.049105	0.808485	5.855281	2.280101	3.163506	0.038182	0.410823
0.348889	0.428694	6.423911	15.276	0.425426	0.239885	1.501291	15.61077	0.763939
0.783939	0.726133	0.049105	0.024553	2.378124	1.275325	2.436768	0.038182	0.065801
1.25816	0.014935	1.928629	3.855952	0.069459	0.30373	0.824885	1.11526	0.459048

0.212172	0.337431	0.306811	0.454632	0.876118	0.90772	0.554416	0.874603	0.16145
111.5439	95.22939	0.049105	0.024553	749.7839	9.335397	1.770029	0.619012	87331.38
1.737294	1.228651	0.793434	2.406768	0.675938	0.028398	0.073261	0.954776	0.919192
0.031378	0	0.672857	0.584134	0.101429	0.000938	0.108701	0.321227	0.073939
0.090188	0.125123	0.306811	1.167561	0.313102	0.121818	0.170678	1.169791	0.031775
1.484553	0.78619	1.057569	0.553348	17.64869	7.212915	0.904856	1.695216	0.624473
0.724502	0.235137	0.306811	0.641631	1.472208	1.447994	0.4457	0.321227	0.535368
2.347439	0.476551	1.473218	8.226219	0.641631	0.702835	1.701616	6.200945	1.480541
3.037626	2.558817	1.264589	0.838846	2.494177	1.156869	3.249877	1.064935	1.678319
0.212172	0.002035	0.235613	0.144163	0.001277	0.10404	0.064372	0.206984	0.015714
7.002056	5.576219	0.531169	0.987821	1.650274	2.126349	6.632063	0.53912	3.35715
0.769365	0.366674	0.733593	0.024553	3.621941	1.260635	0.40645	0.038182	0.339235
2.776421	2.950173	2.026219	6.956349	0.218045	0.30373	2.400455	4.236962	3.679986
0.134517	0.162576	0.847352	1.877605	0.001277	0.037605	0.335967	0.394156	0.250115
2.35733	3.394286	1.321053	1.367179	7.683766	13.75978	1.62562	0.038182	4.155339
0.543665	0.442193	0.049105	0.024553	2.834084	1.550837	0.789863	0.038182	2.89E-05
3.037626	1.744055	0.901732	2.685786	0.193348	0.000938	1.604827	1.995714	1.605859
2.050404	7.882222	0.049105	0.235137	3.125844	7.545584	1.953535	0.357013	5.515584
0.08044	0.041183	2.281926	0.584134	0.240202	0.996111	0.045491	1.995714	0.130202
0.008225	0	0.306811	1.064242	0.034776	0.037605	0.013405	0.357013	0.004654
1.511198	1.778153	0.793434	0.690707	2.436768	2.577157	1.656696	0.619012	0.675556
0.666847	0.051335	0.049105	0.25785	0.249899	0.000938	0.178997	0.038182	0.339235
1.651522	3.279625	0.049105	0.024553	8.033932	6.612121	3.13456	0.206984	1.204957
0.648961	0.066407	2.984899	2.406768	0.515815	1.3829	0.300844	1.913997	0.488997
2.515938	3.842554	1.321053	0.024553	5.568391	15.22851	1.846941	0.038182	1.492973
8.256465	12.36443	0.049105	2.463911	12.93651	6.695887	9.082828	2.190866	4.821176
0.122554	0.228788	0.343434	2.016515	0.228788	0.148932	0.143709	1.064935	0.146234
4.72798	2.958773	0.476551	2.858009	2.164113	0.753016	3.163506	1.020447	1.78759
0.908247	1.953535	0.049105	0.024553	4.281847	0.642395	2.121198	0.038182	1.172208
2.591443	2.074906	1.928629	11.36975	2.104358	0.601623	2.365685	4.990794	0.959062
1.763889	0.536905	1.057569	4.524481	0.240202	0.415166	1.34513	4.236962	1.472208
0.588658	0.752229	0.793434	0.454632	0.259033	0.218975	0.651631	0.477698	0.535368
14.25954	14.33939	0.049105	0.196097	11.30987	3.934986	10.28573	0.321227	1.76873
3.921645	9.924372	5.351934	8.508514	22.52375	9.141299	8.164531	7.868146	8.27267
3.796075	15.77678	5.110159	1.991328	34.38341	16.34294	2.584394	2.236407	1.232886
0.312864	0.991631	0.049105	1.367179	0.001277	0.048225	0.088701	0.659957	0.089473
11.14619	8.643074	0.049105	0.454632	3.975498	1.702706	3.944084	0.038182	2.440895
6.945152	8.573434	2.069942	1.488571	6.404921	4.381479	11.14619	0.954776	4.222583
0.049625	0.055397	0.382626	0.504416	0.619012	0.208687	0.231984	0.321227	0.381789
8.210938	18.76954	2.145137	3.803766	35.776	40.11561	45.1045	0.436017	5.130411
0.278499	0.752229	0.267013	0.584134	0.408427	0.390014	1.240202	0.619012	0.709322
0.059654	0.07767	0.531169	0.395426	0.001277	0.058543	0.188009	1.468131	0.106407
0.783939	0.577071	0.049105	0.024553	1.308167	0.841746	0.108701	0.038182	0.690043
2.931068	3.022713	0.476551	1.828781	0.74868	0.490375	4.261342	0.986104	3.35715
2.429957	3.614603	0.476551	1.064242	1.048074	0.466335	3.971061	0.619012	2.031169
0.323752	0.421342	2.026219	2.16759	0.181674	0.10404	0.851234	0.986104	0.477446
1.540144	2.316075	1.321053	4.524481	5.454949	1.088687	3.340361	3.450577	4.029654
0.25733	0.142698	0.793434	2.097843	0.034776	0.028398	0.4457	1.632648	0.051443
4.580938	7.443276	0.049105	1.717641	15.88548	6.045209	3.373983	0.038182	13.53913
0.376017	0.536905	21.11092	0.690707	0.362864	0.866147	0.073261	14.51648	2.089639
0.212172	0.029596	0.049105	5.788153	0.001277	0.000938	0.722626	0.038182	0.176955
11.33981	12.03267	60.77449	4.655916	2.196017	2.554069	4.586753	12.26136	4.965801
8.66E-05	9.297085	0.049105	0.024553	135.5245	65.94551	4.490801	0.038182	2.89E-05
2.31404	2.871212	9.082828	3.025671	7.339452	11.42512	3.672078	7.61176	3.87329
0.08044	0.34583	0.049105	0.024553	0.001277	2.554069	0.116595	0.038182	0.690043
4.667518	5.591717	0.049105	2.967453	16.44768	2.535974	6.978903	1.169791	12.22798
44.27012	41.47925	2.333867	19.53061	40.11561	6.30671	87.1746	2.713319	40.11561
0.049625	0.007554	0.624473	0.918557	0.001277	0.048225	0.028232	1.169791	0.106407

3.621941	1.419278	1.111977	1.928629	0.37417	0.720938	1.179885	1.632648	1.172208
0.122554	0.07767	0.382626	0.751876	0.001277	0.000938	0.080592	0.508658	0.020548
1.696494	1.620058	0.049105	0.024553	10.19468	1.492973	1.846941	0.954776	0.675556
0.031378	0.014935	0.531169	0.024553	0.249899	2.740519	0.272612	0.038182	0.081212
1.220628	2.091313	0.049105	0.024553	1.597908	0.933261	0.546522	0.53912	1.460981
0.021602	0.726133	0.049105	0.235137	3.566753	0.405974	0.108701	0.832554	0.86469
0.189141	0.305253	0.049105	0.024553	1.107605	3.407619	0.000188	0.038182	1.492973
12.77772	25.73165	0.997792	0.395426	18.76954	11.77567	39.53687	2.037114	13.08214
15.46193	20.68746	1.418312	1.717641	19.68088	21.02638	7.238023	2.037114	26.80355
0.224466	0.18324	3.47531	6.760765	0.181674	0.155945	0.188009	1.913997	0.459048
2.472576	6.191515	1.473218	0.024553	1.32482	11.33981	0.300844	0.321227	2.936739
9.438687	10.28573	12.42965	30.17192	0.556126	2.449509	47.5018	13.23022	53.77619
0.844747	0.991631	0.049105	0.312323	2.216162	1.020447	1.323975	0.038182	0.339235
4.557605	1.29342	15.98892	7.339452	1.004156	1.560779	1.861999	6.911486	3.644343
3.370527	2.740519	0.049105	0.024553	2.230859	1.481631	3.529466	0.578095	2.063362
1.014784	0.281169	1.321053	1.488571	0.20733	0.331097	0.494185	4.101061	1.505678
0.924329	0.585902	0.049105	0.024553	2.701053	2.718434	1.594675	0.477698	1.172208
8.66E-05	0.101212	3.6029	0.25785	0.001277	0.000938	0.308442	4.821176	0.269141
8.677489	6.045209	10.21618	13.15079	2.050404	1.233665	3.588369	13.42179	5.273362
5.037489	3.55583	3.855952	3.676032	1.983824	1.458038	7.38847	5.050267	3.300664
0.891775	1.219019	0.672857	0.024553	0.610224	3.479134	0.335967	0.262785	1.142345
1.25816	1.71518	1.321053	1.097677	0.610224	0.230808	1.831631	1.020447	0.853218
0.443543	0.113218	1.321053	0.196097	0.001277	0.058543	0.231984	0.578095	1.09075
0.090188	0.130823	2.104358	1.064242	0.057821	0.0771	0.264242	1.505678	0.207157
1.330974	1.786306	1.52676	1.026825	3.699495	8.815123	1.491977	1.543377	3.87329
2.591443	1.466111	0.049105	0.395426	3.38456	6.808369	1.075411	0.619012	2.807937
0.008225	0.002035	0.343434	0.235137	0.001277	0.000938	0.036241	0.262785	0.051443
7.495397	3.055729	16.9162	15.3618	0.584134	0.340866	5.576219	10.7557	4.984488
1.844098	1.169791	0.531169	0.893016	1.191198	1.162201	2.871212	0.832554	2.394416
1.604827	2.831501	0.343434	0.196097	1.147807	0.50772	6.900202	0.701255	1.422316
8.556703	10.12435	1.159913	0.867287	10.23768	10.19468	9.707677	1.212792	3.606804
8.66E-05	1.484553	1.321053	1.129452	1.852338	1.223846	0.92816	1.11526	0.804675
0.288579	0.565714	1.057569	1.428398	0.147323	0.268961	0.554416	6.560411	0.308788
10.62739	9.541645	0.267013	2.967453	10.98644	0.037605	3.864827	0.290115	2.496407
0.165108	0.442193	0.847352	0.584134	0.714697	0.258838	0.363276	1.255657	0.440548
2.022778	1.87482	0.425844	0.25785	1.555289	2.654473	1.68671	0.508658	3.034747
0.421804	0.095108	1.872049	1.097677	0.417345	0.29531	0.300844	1.11526	0.488997
1.36619	1.330974	5.20267	1.797172	0.707475	0.086443	2.091313	3.939307	2.996869
3.273045	2.392431	3.055729	8.958225	0.626118	0.767085	4.18456	3.179199	12.03267
0.979149	1.014784	0.049105	0.024553	1.013434	1.100519	2.109755	0.038182	1.204957
11.65791	11.57341	0.049105	0.024553	15.3618	12.36443	10.1459	0.038182	6.760765
0.956667	1.39847	0.049105	0.024553	2.522684	3.66785	1.648066	0.038182	1.057569
0.795339	0.650556	0.049105	0.024553	0.929812	0.594271	1.136566	0.038182	0.370462
2.472576	3.243225	0.049105	0.690707	2.547179	1.702706	6.876392	0.038182	1.678319
3.820758	8.075772	2.197828	0.6657	10.01009	7.824473	9.297085	2.432121	5.788153
0.924329	0.599466	3.110447	5.23176	1.1629	0.829192	1.604827	3.792266	3.57057
5.779574	6.345382	32.68313	91.05541	4.053506	1.712706	17.64869	30.82277	11.719
0.165108	0.046883	1.52676	1.261407	0.843564	0.375281	0.673651	0.53912	0.319957
0.36114	0.253882	3.6029	2.287576	0.079885	0.30373	0.606421	8.333918	0.21689
25.99427	7.545584	1.211299	0.641631	45.1045	100.0998	7.519841	0.038182	20.16541
0.924329	1.313737	0.049105	0.024553	0.929812	1.297763	1.435772	0.436017	0.488997
1.622006	3.016984	0.049105	0.336508	3.574387	1.447994	2.269134	0.038182	1.422316
1.525729	0.837403	2.197828	0.024553	0.001277	0.000938	3.29355	10.67991	1.565253
5.47	9.141299	0.049105	0.312323	2.933903	1.003586	18.19595	0.038182	1.76873
77.74812	11.65791	87331.38	87331.38	52.56128	530.1465	8.643074	87331.38	4.789596
0.013442	0.014935	0.267013	0.235137	0.069459	0.058543	0.080592	0.436017	0.004654
4.203001	3.439437	749.7839	749.7839	3.297107	4.016465	1.546696	425.62	5.606739
3.493362	1.563045	0.049105	0.024553	3.734762	7.315512	3.195541	0.038182	2.524892

2.735368	2.472576	6.978903	5.180058	1.197922	1.722475	4.039192	7.456118	4.23202
0.515815	3.116732	0.624473	1.026825	0.72044	1.372475	0.206616	0.038182	1.156869
2.006782	4.927302	2.243911	3.614603	2.502619	2.402468	6.760765	1.543377	7.839271
3.310758	0.599466	12.1974	9.989221	0.675938	0.499986	2.294733	12.97323	3.131544
33.93939	14.79742	5.00404	12.93651	2.66404	2.703485	4.752121	4.990794	59.32013
0.111876	0.171061	12.93651	5.583968	0.079885	0.068629	0.143709	45.1045	5.662994
0.177143	0.18324	1.663672	1.329206	0.147323	0.314387	0.170678	0.038182	0.232886
10.78056	9.604358	10.48101	30.82277	9.707677	4.752121	3.435988	6.560411	9.792626
87.1746	39.53687	167.9996	224.9555	48.53429	15.98892	252.4251	203.5795	38.94854
11.60283	26.80355	21.11092	11.93253	8.461703	37.29684	21.74051	12.36443	36.74522
60.77449	67.98069	39.24271	135.5245	8.075772	14.79742	65.94551	60.77449	33.4985
25.99427	19.68088	83.489	46.7056	18.62711	9.838658	24.50942	100.0998	13.00996
6.784762	21.02638	13.91714	8.303947	64.12971	18.91854	38.39042	31.1753	33.93939
7.074329	5.287857	252.4251	332.0776	9.968355	5.47	13.30906	252.4251	3.962179
87331.38	425.62	41.47925	3.422237	332.0776	425.62	749.7839	12.60233	252.4251
530.1465	530.1465	62.33472	35.776	118.7028	126.1556	332.0776	75.1132	111.5439
59.32013	91.05541	40.7932	9.19899	144.7919	111.5439	67.98069	11.83593	184.443
252.4251	252.4251	48.53429	184.443	34.84299	287.197	155.7103	155.7103	530.1465
12.36443	3.743232	5.687879	6.612121	3.207807	2.597792	14.97762	6.663651	3.699495
1.029365	1.02487	3.263391	4.758088	0.65202	0.466335	1.813564	3.87329	1.974228
7.683766	15.98892	25.73165	23.16622	1.563045	1.194221	8.288874	57.84882	26.52537
8.508514	6.374812	1.418312	0.312323	8.103925	13.45939	6.52899	0.038182	10.05618
0.942294	0.267388	0.847352	1.129452	0.069459	0.068629	2.333867	1.255657	1.142345
0.165108	0.060022	0.531169	0.131443	0.001277	0.058543	0.249278	0.321227	0.089473
6.028427	2.345527	2.984899	9.880779	0.906825	1.412771	5.238701	3.107417	3.166659
0.070541	0.321566	0.049105	1.545657	0.50469	0.30373	0.521421	0.786667	0.563824
5.366335	4.894509	20.68746	31.92385	8.256465	2.440895	2.351299	16.9162	2.807937
0.648961	0.650556	224.9555	252.4251	0.556126	0.18153	0.308442	287.197	0.207157
19.83727	16.80343	9.562554	4.408153	4.174964	5.696263	16.80343	8.333918	14.00414
2.331825	3.807648	0.049105	0.024553	3.201457	3.110447	0.778326	0.394156	0.763939
4.370671	1.047345	18.19595	6.414416	0.330289	3.655714	6.088593	3.939307	13.42179
0.059654	0.041183	0.476551	0.161962	0.001277	0.000938	0.045491	0.235296	0.081212
0.467403	0.190058	50.55949	36.26061	0.780087	0.91575	0.851234	56.39528	1.393571
1.190491	2.136335	0.624473	0.024553	4.312251	3.370527	0.325108	0.038182	0.232886
28.23632	37.81919	10.1459	4.359574	30.82277	38.39042	19.37139	24.98218	27.38726
33.07147	32.68313	3.980123	1.329206	31.55417	252.4251	16.34294	3.566753	50.55949
16.9162	17.92188	15.18102	32.68313	8.677489	7.238023	18.05488	26.80355	7.212915
8.643074	5.679502	0.793434	0.780087	2.776421	2.784228	5.439278	3.792266	6.191515
1.220628	0.981558	0.672857	0.867287	0.079885	0.000938	2.057056	1.064935	0.421573
28.87506	21.19547	14.08569	6.135007	25.48179	42.75576	23.38378	4.776407	30.49289
1.41368	1.02487	0.624473	2.789466	0.788896	1.644416	0.375094	0.742872	1.692763
2.285664	6.53969	15.3189	5.330519	2.589185	3.286652	0.657547	48.53429	4.603434
37.29684	36.26061	10.1028	13.00996	11.48368	8.976508	43.49658	12.16681	34.84299
0.715462	0.78619	8.643074	8.849697	1.314675	0.547403	1.568564	3.842554	1.480541
1.330974	0.704848	0.531169	0.395426	0.567994	0.556126	0.583766	0.578095	0.636688
4.048499	5.788153	13.15079	8.164531	4.789596	6.911486	4.952915	10.05618	4.678846
1.121703	0.717027	3.055729	2.858009	1.314675	1.072056	1.34513	1.255657	1.542302
0.588658	1.02487	2.197828	2.685786	0.171277	0.112222	0.290866	2.826277	0.195823
0.637994	0.352006	1.819221	0.363059	0.159351	0.278802	0.325108	1.11526	0.804675
2.498478	1.89373	6.045209	3.55961	0.714697	1.009625	1.359177	8.396847	1.861999
0.467403	0.518413	0.624473	0.178182	0.259033	0.490375	0.134444	0.038182	0.089473
1.220628	1.39847	3.055729	7.710361	0.525325	0.405974	2.127973	5.18754	2.922778
14.17388	7.363117	2.281926	7.443276	1.519322	1.412771	6.423911	4.297222	2.74544
8.556703	6.853608	5.00404	10.70538	4.029654	3.783694	1.637309	12.60233	3.327619
0.111876	0.046883	0.049105	0.235137	1.082179	0.258838	0.4457	0.038182	0.298153
0.090188	0.088788	0.343434	0.25785	0.069459	0.121818	0.170678	0.262785	0.232886
10.53159	11.89932	1.473218	2.406768	11.09133	6.749488	18.62711	3.792266	3.711097
1.000794	0.577071	4.343838	2.652107	0.283709	0.457431	0.882078	5.259437	0.410823

0.021602	0.001025	0.267013	0.131443	0.001277	0.000938	0.000188	0.038182	0.025685
0.421804	0.887359	0.049105	0.024553	0.515815	0.30373	1.159913	0.038182	0.410823
2.415296	2.81342	0.049105	0.024553	4.519048	14.70188	1.373348	0.038182	1.364401
118.7028	48.53429	75.1132	53.77619	0.83697	1.399315	30.17192	87.1746	72.69473
4.971991	4.650007	0.049105	0.480794	16.74449	3.253175	21.54823	0.038182	0.650556
1.220628	1.731169	5.402511	5.409437	0.57531	0.199264	2.415296	11.99921	7.507619
2.893001	3.26	1.321053	1.759986	12.42965	24.98218	2.77899	1.11526	5.102742
5.102742	2.647244	15.88548	34.84299	2.970144	11.14619	3.767489	18.76954	9.012937
0.908247	0.806227	19.06893	14.84163	1.396494	1.423506	0.789863	19.22139	3.052655
0.990909	0.286681	0.531169	1.601443	0.001277	0.000938	0.938911	0.436017	0.410823
16.39531	12.56693	80.6518	77.74812	6.135007	2.509177	19.99861	37.29684	9.256537
425.62	184.443	6.685087	5.872886	252.4251	332.0776	425.62	5.110159	100.0998
0.278499	0.409098	2.069942	0.024553	0.123456	0.000938	0.162251	0.038182	1.156869
49.50903	51.52195	4.236962	4.016465	20.85876	36.26061	57.84882	3.179199	27.10502
2.214372	2.013182	3.363911	3.763045	1.251659	1.253752	2.24733	1.913997	2.485534
1.201724	1.731169	2.468117	4.016465	0.515815	0.631494	1.813564	2.325931	1.393571
6.464906	13.60866	0.531169	1.545657	12.10289	24.2842	5.302294	0.477698	8.195657

CGGA_138	CGGA_138	CGGA_138	CGGA_138	CGGA_138	CGGA_139	CGGA_139	CGGA_139	CGGA_139
1.11526	0.305087	0.344733	0.277763	0.471313	0.276248	0.097807	0.260144	0.287821
1.263752	0.110642	0.532381	1.772756	0.141284	0.534452	0.064812	0.087504	0.002056
2.032706	3.387864	2.119596	1.691587	4.179762	1.884935	10.07949	4.413608	3.592063
11.14619	2.922778	4.007583	4.429769	0.151082	0.000101	2.189235	7.911934	6.30671
8.745238	3.783694	2.690823	2.970144	5.295325	7.897078	5.591717	7.037345	5.01772
4.251818	2.800332	1.290981	8.365072	2.171429	6.144055	1.120332	1.078153	0.861934
30.82277	35.27608	14.6058	62.33472	31.1753	31.1753	49.50903	41.47925	16.9162
0.002367	0.083795	0.107749	0.038139	0.067547	0.026609	0.073211	0.070339	0.061385
2.828918	3.672078	7.339452	1.497143	3.644343	2.174733	1.069033	4.783001	7.482179
1.570794	0.002857	0.010346	1.25816	0.000173	0.156486	0.000188	4.33E-05	0.002056
65.94551	18.33909	6.830592	155.7103	31.55417	35.27608	18.33909	18.48514	8.524242
2.419553	0.859885	0.463102	5.24562	0.453709	2.124574	0.853752	1.112641	0.337013
2.136335	31.92385	16.22108	8.048095	23.38378	1.734949	18.05488	16.44768	10.98644
1.44E-05	4.946587	17.28195	0.000245	9.604358	0.000101	10.23768	4.33342	19.06893
0.017063	0.110642	0.107749	0.154329	0.000173	0.073211	0.064812	0.040469	0.002056
2.850058	2.6143	5.950664	2.673846	7.519841	2.014863	6.64254	7.964315	8.348817
1.409942	0.925801	0.243124	5.576219	0.38127	3.993665	1.821861	1.104033	0.10886
1.44E-05	0.002857	0.010346	0.743694	0.048528	0.403413	0.3514	0.167013	0.002056
0.230108	13.00996	14.00414	0.55741	10.01009	0.316126	6.144055	4.101061	6.695887
6.181652	0.348889	0.282078	1.931537	0.480022	0.883665	0.231356	0.578485	0.355794
0.520289	4.875527	10.01009	0.297532	3.663622	0.632857	0.48684	0.333831	2.116032
0.369567	4.058514	17.64869	0.788896	4.145209	0.581926	4.135801	1.544517	7.264502
26.80355	6.454416	0.010346	67.98069	4.408153	118.7028	55.04081	21.36892	0.933261
1.44E-05	0.148701	0.010346	0.038139	0.096263	0.000101	0.000188	0.017345	0.002056
0.899567	0.65241	0.518413	1.376378	0.462092	2.459812	0.844747	0.918557	1.240202
4.508196	3.57057	5.950664	1.568564	3.574387	1.211299	3.695815	6.685087	5.654863
0.497749	0.428932	6.240346	3.407619	4.827691	4.272165	5.754935	1.321053	9.541645
0.411703	0.527518	0.257042	0.800058	0.517092	0.35676	0.271746	0.144293	0.171183
5.889798	39.53687	49.50903	3.442886	144.7919	77.74812	287.197	23.16622	29.49693
0.617763	0.819048	0.929286	0.319957	0.639113	0.467403	0.598795	0.478737	0.66526
3.160022	12.85504	4.952915	2.045476	11.74734	2.990851	3.160022	4.188896	6.876392
0.059459	0.110642	0.092915	0.258059	0.048528	0.251573	0.056623	0.04785	0.050577
1.44E-05	29.18885	28.54786	0.000245	18.62711	0.000101	17.92188	9.012937	32.68313
0.347655	0.002857	0.12132	0.248153	0.000173	0.033997	0.000188	0.009978	0.002056
10.1459	0.359697	0.209401	21.54823	0.067547	7.363117	1.363557	0.57197	0.160332
1.301169	2.160649	2.522684	1.113961	2.259661	1.064242	1.175094	1.248232	1.288276
1.223846	8.134084	4.272165	2.337821	6.808369	1.335743	4.029654	4.468009	3.314329
0.011551	0.058773	0.163766	0.045714	0.048528	0.008882	0.247417	0.040469	0.002056
7.495397	2.337821	2.45772	4.524481	2.323983	3.55961	2.014863	2.294733	2.489762
24.73724	5.402511	2.31811	22.96463	3.500808	10.57984	4.236962	4.626025	3.290101
3.083319	0.002857	0.010346	1.801284	0.000173	0.73961	0.000188	0.464127	1.176623
0.781977	0.69583	0.695426	1.758615	0.390216	0.457431	0.694675	1.050231	0.616739
0.171472	2.818312	5.821804	0.000245	1.968283	0.000101	0.478413	1.759986	2.621133
0.030433	0.002857	0.010346	0.015685	0.000173	0.000101	0.000188	0.005411	0.002056
0.520289	0.305087	0.419076	1.187229	0.16925	0.185909	0.406681	0.517359	1.481631
75.1132	19.99861	38.39042	31.1753	28.23632	24.73724	35.27608	55.04081	30.3324
0.076465	0.24632	0.092915	0.20215	0.077417	0.156486	0.081212	0.281364	0.10886
0.681494	0.002857	0.010346	0.384798	0.048528	0.073211	0.000188	0.281364	0.002056
0.002367	0.002857	0.010346	0.030577	0.019048	0.000101	0.012944	0.005411	0.002056
0.359105	0.002857	0.010346	1.034315	0.000173	0.77925	0.000188	0.033146	0.002056
1.44E-05	0.002857	0.010346	0.143968	0.000173	0.057027	0.018167	4.33E-05	0.002056
4.23202	13.6814	10.05618	2.600014	21.74051	2.914632	4.490801	3.57057	11.60283
0.475433	0.058773	0.010346	0.674675	0.000173	0.534452	0.117843	0.056205	0.050577
0.067864	0.047792	0.010346	0.849841	0.019048	0.390895	1.224733	0.215115	0.041472
0.738312	3.993665	3.966623	1.337431	2.371833	0.365635	1.097677	3.095166	7.824473
0.385736	0.002857	0.010346	1.048074	0.000173	0.5229	0.209538	4.33E-05	0.002056
3.726854	0.270924	0.243124	0.940563	0.16925	1.144733	0.471746	0.699697	0.160332

2.162381	0.387258	0.431977	1.636118	0.250823	0.294921	0.048124	0.564127	1.047345
5.662994	1.465123	0.706494	3.4679	1.163579	3.536955	2.136335	3.57057	1.250895
0.136573	0.581111	0.107749	0.34697	0.309221	0.346342	0.546522	0.532381	0.087807
1.44E-05	18.19595	23.82354	0.000245	24.98218	0.185909	1.519322	0.070339	0.148701
0.011551	0.500354	0.382208	0.106609	0.273672	0.377085	0.802035	0.108997	0.443781
0.855801	0.538117	0.463102	0.54847	0.29767	0.859351	0.984069	0.532381	0.220505
6.784762	30.17192	10.1459	7.112496	33.4985	18.19595	53.77619	52.56128	13.45939
0.106522	0.24632	0.209401	0.428694	0.372504	0.019942	0.088968	0.281364	0.07039
1.44E-05	0.002857	0.010346	0.000245	0.000173	0.013867	0.032172	0.025411	0.002056
6.063276	0.925801	0.82868	1.426522	1.423506	2.895599	0.239228	1.959466	1.748016
1.052886	2.481356	3.175916	0.764315	2.136335	1.869322	1.580909	1.90798	2.513752
0.520289	0.772157	0.195685	0.967446	0.265339	1.064242	0.263304	0.31702	0.220505
0.421804	0.002857	0.010346	0.55741	0.123853	0.04096	0.073211	0.167013	0.002056
0.738312	0.058773	0.010346	1.746724	0.000173	0.305253	0.000188	4.33E-05	0.002056
0.803211	0.282583	0.179798	1.580909	0.141284	0.752229	0.200382	0.167013	0.07039
0.006046	0.002857	0.092915	0.022814	0.011962	0.000101	0.018167	0.005411	0.050577
0.002367	0.083795	0.092915	0.088283	0.019048	0.057027	0.000188	4.33E-05	0.002056
7.598312	5.770996	7.136537	7.327482	5.950664	3.263391	17.09911	22.13245	18.62711
0.19544	0.148701	0.010346	0.297532	0.058117	0.118644	0.135606	0.009978	0.002056
0.076465	0.058773	0.010346	0.064012	0.048528	0.423781	0.182929	0.009978	0.002056
3.799885	4.752121	1.89961	4.413608	4.914863	16.28201	10.62739	3.877417	2.254084
0.144163	0.175592	0.010346	0.469358	0.000173	0.033997	0.000188	0.070339	0.002056
0.727633	0.258838	0.474574	0.365859	0.273672	0.632857	0.190693	0.206616	0.547403
2.996869	10.43066	11.83593	3.266782	7.545584	1.187229	2.147013	3.610707	7.200505
0.030433	0.058773	0.107749	0.064012	0.087165	0.231876	0.117843	0.009978	0.041472
1.244957	0.235137	0.08044	0.705231	0.058117	0.377085	0.155224	0.432468	0.041472
3.387864	27.96248	36.74522	6.808369	13.23022	6.250447	27.96248	5.137042	35.776
0.468167	0.096371	0.010346	0.308788	0.16925	0.10132	0.081212	0.333831	0.002056
0.561775	0.500354	0.753449	0.469358	0.438312	0.717403	0.330866	0.3414	0.489235
1.341825	0.849336	1.716364	2.035678	1.387612	0.231876	2.323983	1.178939	3.7586
0.421804	0.570317	0.393196	0.384798	0.38127	0.500722	0.231356	0.333831	0.160332
0.017063	0.071429	0.010346	0.319957	0.011962	0.166291	0.048124	0.079387	0.050577
0.129365	1.636118	1.163579	0.393983	0.399307	0.336147	1.162201	0.727222	1.030022
1.44E-05	7.598312	3.243225	0.000245	6.853608	0.73961	9.751039	12.10289	4.077403
0.271912	0.002857	0.179798	0.764315	0.000173	0.118644	0.000188	0.206616	0.002056
0.82588	0.258838	0.505786	1.96697	0.25785	0.701818	0.280678	0.267807	0.220505
6.365065	0.500354	0.12132	10.1028	0.282424	2.640332	1.191198	0.727222	0.210505
0.030433	0.175592	0.092915	0.211335	0.087165	0.026609	0.081212	0.025411	0.002056
3.450577	12.29473	5.950664	6.171789	10.57984	2.349351	6.191515	5.024293	5.560563
0.106522	0.083795	0.092915	0.248153	0.019048	0.092302	0.000188	0.017345	0.041472
0.011551	0.096371	0.092915	0.143968	0.048528	0.156486	0.220079	0.04785	0.050577
1.44E-05	0.058773	0.010346	0.000245	0.011962	0.10132	0.032172	0.009978	0.002056
1.062092	1.161501	0.443052	2.842193	0.809481	1.853723	1.203427	0.937655	0.743694
13.91714	3.217612	3.989206	11.51384	2.178268	2.524892	2.436768	7.417359	2.502619
7.162698	0.54619	0.344733	8.195657	0.316573	2.047157	1.519322	3.486436	0.061385
0.421804	0.235137	0.315916	0.248153	0.38127	0.73961	0.387056	0.325527	0.210505
0.22039	1.47842	1.880375	0.248153	1.284747	0.118644	1.097677	1.855108	3.330909
0.548788	0.002857	0.010346	0.297532	0.000173	0.073211	0.000188	4.33E-05	0.002056
0.561775	0.24632	0.149019	0.520678	0.000173	0.413449	0.163038	0.2443	0.160332
1.082179	4.715599	13.15079	0.096905	3.303925	0.717403	2.690823	1.889711	3.243225
5.500678	0.62645	0.243124	2.396429	0.414012	1.403853	0.231356	1.218182	0.540563
6.853608	0.849336	1.119574	4.160238	1.440736	6.287561	1.401977	2.855296	1.691587
1.600238	1.149978	1.334762	2.07658	0.984069	1.080303	2.479221	1.732468	2.008312
1.007864	0.86987	0.195685	1.426522	0.445996	2.014863	1.175094	1.667273	0.606154
1.44E-05	224.9555	749.7839	0.000245	167.9996	0.847908	91.05541	31.55417	155.7103
1.44E-05	3.895469	2.936739	0.705231	4.067547	1.144733	3.655714	1.699091	1.412771
1.017107	0.124646	0.092915	1.034315	0.019048	0.643853	0.163038	0.274704	0.002056
1.44E-05	95.22939	126.1556	0.000245	80.6518	0.000101	30.82277	29.82828	144.7919

0.203954	0.378066	0.243124	0.23101	0.160779	0.413449	0.097807	0.117446	0.050577
0.504964	0.002857	0.010346	0.54847	0.000173	0.000101	0.000188	0.445209	0.002056
1.905094	2.417424	1.980592	2.126349	2.607035	2.03886	2.35733	3.037626	3.446732
6.38456	16.68554	5.295325	3.592063	11.25392	7.212915	22.13245	12.93651	7.149618
0.144163	0.2129	0.474574	0.173319	0.187381	0.082872	0.012944	0.033146	0.119553
11.83593	3.595758	1.205685	7.724084	6.107114	5.933066	2.776421	4.821176	2.398442
1.727323	0.490079	0.135144	3.792266	0.250823	2.283752	1.097677	1.050231	0.49974
5.915469	2.873817	0.532381	7.188095	1.727323	10.43066	2.890245	3.172864	1.094978
9.377345	0.614545	0.257042	10.17029	1.061457	31.55417	1.880375	0.891775	1.422316
0.964416	0.509242	0.648362	0.469358	0.724004	1.360022	2.676392	0.744531	0.443781
0.011551	0.002857	0.010346	0.064012	0.036934	0.073211	0.018167	0.04785	0.002056
0.19544	1.058896	0.393196	0.154329	1.131472	0.444762	0.502843	0.333831	0.061385
0.271912	0.639495	0.329798	0.567994	0.250823	0.222864	0.55741	0.509791	0.261227
4.592273	5.302294	7.737807	7.882222	3.256587	2.941544	2.126349	2.850058	1.721371
0.1214	0.058773	0.163766	0.193348	0.141284	0.611003	0.163038	0.308672	0.050577
0.765606	3.672078	2.640332	0.674675	2.207417	0.632857	0.748341	2.842193	1.23127
1.128788	1.045462	0.505786	1.481631	0.666097	0.596508	0.835498	0.718766	0.466335
0.030433	0.002857	0.010346	0.045714	0.000173	0.033997	0.000188	0.017345	0.002056
21.93452	0.997792	2.234538	14.08569	4.120815	9.377345	1.569553	1.931537	1.612056
0.309444	0.570317	1.299567	0.705231	1.163579	0.444762	0.55741	0.835498	1.691587
0.369567	0.110642	0.092915	0.734935	0.027547	1.172208	0.018167	0.070339	0.050577
0.584502	1.25886	2.931068	1.600238	2.676392	3.450577	2.051962	2.003723	3.243225
2.375974	2.071587	0.566782	1.723579	0.34697	0.475729	1.412771	2.730072	1.282208
0.561775	4.445851	4.978182	1.857677	2.652107	0.534452	4.115844	2.485534	4.302367
0.369567	2.708341	0.606154	0.459394	0.909315	0.490664	1.175094	1.831631	3.47531
5.110159	1.697792	2.203535	4.46267	2.119596	4.02088	1.68342	1.387612	2.363442
7.002056	3.125844	2.119596	15.56131	2.24733	3.236457	2.063362	2.794935	1.250895
0.271912	3.140866	8.226219	0.599134	3.734762	0.752229	1.097677	0.380087	0.871429
0.1214	2.183665	3.734762	0.11531	3.428788	0.413449	0.862561	0.371876	0.272078
5.20267	1.045462	5.583968	1.731169	2.197828	0.73961	1.330974	1.968283	3.201457
2.419553	17.39935	9.792626	5.330519	15.3189	10.95872	9.583463	18.91854	8.019769
1.44E-05	2.792035	10.48101	0.000245	1.916782	0.000101	0.200382	0.844156	8.226219
5.47	11.2819	12.42965	13.6814	13.75978	13.45939	27.38726	10.38202	8.381328
4.292078	8.780115	22.74797	4.140505	12.42965	3.107417	3.726854	7.238023	15.77678
0.051595	0.002857	0.149019	0.073867	0.019048	0.000101	0.000188	0.040469	0.050577
1.139885	1.569553	1.278629	1.059553	1.989711	2.027951	3.618499	2.302626	0.871429
14.25954	0.742417	0.431977	11.83593	0.900693	7.683766	3.214545	4.115844	0.762165
1.161501	0.795339	2.099524	1.072843	0.690375	0.918557	0.623853	0.420058	0.315743
0.090188	0.318312	0.315916	0.599134	0.372504	0.206768	0.387056	0.215115	0.07039
0.548788	1.326667	5.950664	1.362713	2.818312	0.000101	2.294733	0.918557	3.446732
1.44E-05	52.56128	80.6518	0.000245	28.54786	0.000101	8.573434	2.609293	21.74051
1.369048	100.0998	87.1746	0.000245	105.1694	0.980505	135.5245	36.26061	56.39528
1.149978	0.65241	0.135144	3.192229	0.543939	1.910974	2.776421	1.351573	0.097879
0.22039	0.175592	0.010346	0.599134	0.036934	0.500722	0.040375	0.033146	0.002056
1.488571	2.642525	1.100519	2.187395	2.283752	2.327857	1.01772	0.861429	0.939986
1.322092	7.951371	7.251263	0.777403	5.259437	2.384141	2.644885	2.230859	9.256537
2.225498	0.9529	0.107749	0.656392	0.42925	3.052655	0.440548	0.891775	0.7257
2.917381	0.002857	0.010346	3.763045	0.000173	11.19942	2.494177	0.425844	0.002056
6.601833	22.74797	8.445743	13.15079	14.79742	3.095166	28.54786	23.6012	15.98892
0.178297	1.008961	0.257042	1.839986	0.96938	0.467403	0.361948	0.48684	0.540563
2.375974	0.002857	0.010346	1.708932	0.000173	2.124574	0.081212	4.33E-05	0.119553
17.15902	1.713997	1.960945	12.85504	2.148658	10.82954	1.89518	6.210375	1.481631
1.205685	0.946061	1.435772	2.126349	1.284747	1.296046	1.330974	3.45404	1.203427
1.873434	0.305087	0.010346	4.733824	0.000173	4.637994	0.609509	0.260144	0.087807
1.289149	0.24632	0.257042	3.750837	0.282424	0.643853	0.387056	0.380087	0.595022
2.485534	4.661717	3.370527	1.600238	4.33342	6.071436	1.790245	7.854069	5.687879
0.136573	0.002857	0.010346	0.308788	0.048528	0.5229	0.000188	0.108997	0.002056
0.672468	0.083795	0.179798	1.45912	0.106118	0.185909	0.064812	0.160072	0.002056

0.076465	0.175592	0.382208	0.000245	0.4971	1.853723	3.070952	1.631544	1.1407
1.744055	2.65684	4.863579	6.674286	4.614618	2.668896	7.251263	1.869322	2.089639
8.195657	5.28039	1.305772	8.288874	3.394286	5.696263	3.975498	6.945152	2.106089
43.49658	29.82828	65.94551	9.377345	32.68313	17.64869	17.78522	32.31123	31.1753
0.944084	1.816436	1.175094	2.652107	1.297763	0.632857	1.77404	1.578687	0.563499
1.263752	1.697792	17.39935	0.000245	4.160238	0.000101	3.016984	7.08733	13.60866
1.149978	0.329798	0.370462	1.99899	0.462092	0.980505	0.457749	0.464127	0.148701
1.369048	15.46193	25.99427	7.724084	15.3189	7.683766	2.29873	8.27267	15.66023
10.28573	0.002857	0.010346	6.772763	0.616739	7.061328	2.126349	1.699091	0.002056
16.44768	9.604358	5.044113	18.48514	10.23768	8.556703	10.43066	8.508514	4.397792
4.661717	0.293276	0.358211	4.381479	0.203175	1.853723	0.1086	0.40645	0.002056
7.668579	6.09759	4.039192	20.94257	5.130411	8.195657	11.03843	8.958225	4.338629
0.964416	0.728088	1.880375	0.656392	1.256558	0.980505	0.430281	1.759986	0.698687
1.819221	2.752893	2.45772	4.115844	2.259661	1.144733	2.574733	4.875527	2.805382
0.106522	0.161962	0.282078	0.240671	0.000173	0.35676	0.000188	0.056205	0.119553
0.634531	1.734949	0.86987	3.722785	1.556392	1.629235	1.3829	1.690483	0.743694
0.309444	2.577157	3.588369	0.376212	2.165851	0.980505	1.759986	1.125325	1.422316
2.436768	0.396306	0.876638	5.662994	0.584134	1.778153	0.308788	0.718766	0.68557
0.076465	0.002857	0.010346	0.173319	0.000173	0.019942	0.000188	0.056205	0.002056
0.774257	3.394286	0.452965	0.088283	1.320173	0.365635	8.608153	3.629177	3.367071
5.704646	10.19468	2.967453	8.711905	9.082828	4.927302	8.348817	7.443276	2.535974
0.203954	0.762165	0.010346	1.286494	0.250823	0.559221	2.447294	0.59062	0.466335
0.803211	0.002857	0.292799	1.216768	0.000173	0.092302	0.190693	0.603737	0.770664
5.762965	8.643074	8.608153	11.51384	4.012049	2.593506	3.993665	5.217569	8.195657
2.089639	6.717056	2.668896	3.851414	6.443925	2.770866	3.695815	4.845166	4.413608
0.090188	0.002857	0.010346	0.143968	0.000173	0.110188	0.000188	4.33E-05	0.002056
0.548788	0.187013	0.209401	0.996696	0.25785	0.185909	0.280678	0.391421	0.240671
0.1214	3.953297	5.515584	1.678319	4.946587	0.883665	1.564149	0.853752	1.886335
2.690823	0.460938	0.010346	3.350224	0.290693	1.828781	1.3829	1.094206	0.097879
0.645036	0.781465	0.344733	1.195079	0.324502	0.611003	0.263304	0.193997	0.389574
9.047532	9.541645	5.323492	1.351573	5.779574	1.119574	2.45575	3.687633	37.29684
1.433189	0.889553	0.595022	0.894704	0.607273	0.838326	1.401977	1.884935	0.851234
0.33785	0.199394	0.010346	0.645916	0.036934	0.231876	0.097807	0.413802	0.23101
1.713997	13.75978	8.888167	3.407619	15.56131	17.15902	29.18885	4.067547	7.710361
1.029365	0.602554	0.010346	2.229048	0.232338	2.219863	0.056623	0.555418	0.002056
1.029365	3.939307	11.42512	2.31404	9.968355	2.459812	0.808485	5.158146	4.927302
0.017063	0.047792	0.010346	0.064012	0.000173	0.10132	0.1086	0.005411	0.061385
0.144163	0.110642	0.209401	0.540902	0.000173	0.10132	0.064812	0.174646	0.002056
0.46153	0.24632	0.010346	0.419365	0.000173	0.611003	0.048124	4.33E-05	0.200382
0.486551	0.930837	0.780952	0.173319	1.003586	0.390895	1.460051	2.312085	1.240202
10.07949	0.161962	0.010346	5.330519	0.027547	9.438687	0.126335	0.137063	0.119553
19.83727	14.88584	7.375794	15.46193	20.85876	35.776	40.11561	53.77619	6.325945
17.78522	13.42179	4.261342	25.48179	9.924372	8.226219	9.377345	15.88548	13.00996
2.089639	0.527518	0.876638	1.012742	0.724004	3.381255	0.598795	0.683232	0.563499
2.006782	3.966623	4.47912	2.419553	4.468009	1.144733	1.363557	2.459812	3.747035
1.35259	0.161962	0.22096	4.827691	0.096263	0.377085	0.000188	4.33E-05	0.148701
0.441053	0.124646	0.107749	0.636291	0.232338	1.391494	0.319365	0.181919	0.087807
19.22139	2.083261	7.977958	12.36443	19.99861	19.99861	70.11932	42.08807	21.02638
4.096407	3.514834	2.071587	0.599134	2.227273	0.390895	1.693939	4.353925	7.443276
7.417359	0.937655	1.290981	2.419553	1.759986	3.314329	1.718918	1.631544	1.602619
10.01009	1.215195	1.403853	33.4985	1.655541	9.297085	4.33342	3.207807	0.733593
1.44E-05	0.438831	0.648362	0.449091	1.212792	0.413449	0.457749	0.333831	1.121703
0.090188	4.519048	10.33476	0.419365	10.38202	2.810678	9.418196	1.480541	4.359574
0.699697	11.89932	25.48179	0.000245	29.49693	20.85876	25.48179	3.095166	12.7427
0.203954	0.096371	0.010346	0.248153	0.000173	0.176104	0.097807	0.130714	0.061385
6.230245	46.7056	45.91841	3.707136	83.489	6.784762	60.77449	30.49289	34.1614
1.062092	2.515938	0.606154	0.777403	0.700837	5.614805	1.718918	1.770029	0.23101
3.514834	1.008961	0.231659	7.212915	1.414574	2.786898	2.494177	2.558817	0.808052

1.205685	0.570317	0.010346	0.967446	0.000173	0.632857	0.247417	0.532381	0.002056
1.667273	1.465123	1.948918	5.855281	0.878369	1.997352	3.432388	2.157359	0.002056
1.905094	2.671299	4.758088	1.404812	3.851414	3.034747	1.449877	2.434444	6.784762
4.440548	13.53913	53.77619	1.048074	7.264502	0.727633	14.97762	17.78522	34.1614
7.570693	80.6518	118.7028	17.28195	118.7028	11.96574	118.7028	27.66779	105.1694
6.738211	0.002857	0.010346	2.947287	0.000173	1.109726	0.000188	0.92816	0.002056
1.856392	0.47153	0.092915	3.227085	0.390216	0.611003	0.175714	0.908247	0.240671
0.33785	0.002857	0.179798	3.885642	0.282424	2.427792	0.430281	4.33E-05	0.002056
0.006046	0.002857	0.010346	0.23101	0.067547	0.033997	0.064812	4.33E-05	0.07039
1.636118	0.175592	0.489509	1.978954	0.000173	2.078355	0.1086	0.398059	0.002056
0.113838	0.096371	0.740101	0.134076	0.851234	0.082872	0.430281	0.117446	0.96329
1.44E-05	18.91854	95.22939	0.000245	47.5018	0.000101	15.66023	7.351284	100.0998
1.61088	2.671299	8.288874	1.172872	7.598312	11.77567	17.39935	2.380079	3.290101
0.634531	0.329798	0.617417	0.520678	0.242453	0.390895	0.056623	0.093622	0.365859
5.024293	1.824892	1.262965	7.519841	1.1407	2.855296	1.035058	1.178939	1.358189
2.136335	0.339235	0.463102	5.959033	1.440736	1.477388	1.08798	1.437648	0.002056
7.882222	2.013182	1.226934	7.545584	1.34267	6.674286	1.974228	3.917597	1.701616
4.130794	0.877807	0.304062	5.439278	0.50557	1.211299	1.027403	1.561912	0.616739
1.501291	0.387258	0.790837	1.888023	1.050231	0.6657	0.763102	0.274704	0.272078
1.713997	0.509242	0.452965	1.857677	0.809481	3.618499	0.568369	1.387612	0.649235
1.007864	4.802655	27.10502	1.545657	11.36975	3.993665	6.738211	2.633232	5.210317
3.256587	0.728088	0.010346	7.061328	0.195346	1.296046	0.209538	0.152316	0.002056
0.468167	9.604358	14.42661	0.000245	11.03843	0.000101	3.585029	6.153102	15.07257
0.002367	0.2129	0.135144	0.211335	0.027547	0.019942	0.040375	0.04785	0.002056
6.601833	0.742417	0.753449	7.795642	0.273672	4.739668	0.957273	0.070339	0.389574
2.148658	5.462475	2.165851	3.037626	5.47	2.500548	6.414416	4.281847	1.805166
0.710722	0.096371	0.163766	1.523824	0.027547	0.082872	0.088968	0.093622	0.119553
8.608153	21.74051	27.38726	8.075772	27.66779	6.365065	38.39042	25.48179	25.48179
5.409437	4.029654	7.482179	3.266782	5.454949	3.91355	3.877417	5.745996	7.112496
1.934271	4.203001	1.761349	3.037626	2.802828	6.978903	2.661587	1.595729	0.784372
0.32925	0.083795	0.010346	0.183304	0.036934	0.082872	0.000188	0.056205	0.07039
18.33909	14.17388	10.8813	18.69833	9.707677	11.719	10.35839	11.36975	16.80343
0.369567	12.63773	20.68746	0.449091	9.418196	2.027951	2.845036	1.567576	1.945916
91.05541	57.84882	44.27012	91.05541	72.69473	60.77449	40.7932	184.443	36.26061
0.520289	0.460938	0.195685	0.705231	0.512641	0.622496	0.535945	0.234502	0.401176
7.625209	7.737807	4.397792	8.849697	12.10289	42.08807	5.855281	2.371833	3.223882
7.710361	12.70768	40.11561	6.580988	30.82277	9.160382	7.824473	16.34294	14.21671
0.71956	0.002857	0.010346	0.437576	0.132922	0.632857	0.440548	4.33E-05	0.002056
0.030433	0.002857	0.010346	0.000245	0.048528	0.000101	0.000188	0.079387	0.129913
0.090188	1.396494	6.063276	0.143968	2.640332	0.559221	2.45575	2.08013	6.287561
27.10502	14.51648	50.55949	29.49693	36.74522	8.048095	9.19899	11.96574	50.55949
0.017063	0.047792	0.010346	0.015685	0.000173	0.008882	0.025007	4.33E-05	0.002056
1.205685	0.24632	0.231659	0.2871	1.675722	2.810678	2.860722	0.968066	1.449062
0.878369	0.348889	0.626118	0.975556	0.212496	0.596508	0.064812	0.380087	0.531169
1.072843	0.258838	0.135144	0.726133	0.151082	1.080303	0.000188	0.160072	0.220505
17.34065	3.552049	3.046551	17.09911	4.408153	16.85982	5.47	7.710361	3.636789
2.178268	0.781465	0.668456	2.873817	0.82355	1.335743	0.449726	0.130714	0.68557
8.508514	10.5063	18.12541	2.887489	8.888167	4.348882	3.101371	7.212915	17.39935
39.53687	167.9996	62.33472	50.55949	100.0998	105.1694	95.22939	49.50903	21.93452
0.584502	0.002857	0.304062	0.459394	0.000173	0.146768	0.000188	4.33E-05	0.002056
0.163521	0.161962	0.257042	0.106609	0.160779	0.185909	0.025007	0.025411	0.041472
60.77449	24.73724	21.54823	95.22939	21.36892	48.53429	57.84882	37.29684	27.38726
0.796674	1.307417	1.236248	2.109755	1.230411	2.089639	1.330974	0.92816	0.389574
0.393716	0.282583	0.27075	0.868853	0.11531	1.242641	0.387056	0.199582	0.171183
3.629177	0.751371	0.010346	8.493088	0.817547	9.707677	1.348975	0.791371	0.62645
2.607035	0.110642	0.431977	4.115844	0.160779	7.136537	0.738312	1.078153	0.220505
10.23768	4.921082	3.707136	9.335397	3.750837	12.36443	2.909293	3.599329	2.270837
2.794935	0.369105	0.195685	1.468131	0.290693	0.980505	0.231356	0.380087	0.325317

100.0998	10.62739	6.00228	135.5245	11.42512	70.11932	15.77678	18.19595	8.608153
0.1214	0.047792	0.010346	0.393983	0.019048	0.35676	0.012944	0.025411	0.041472
2.577157	1.499214	3.330909	0.578817	2.542605	1.048802	2.820974	1.869322	5.123781
0.011551	0.002857	0.010346	0.000245	0.000173	0.000101	0.018167	0.033146	0.002056
2.347439	0.961486	0.659163	2.901032	0.700837	2.531515	0.308788	0.754784	0.247951
17.92188	5.821804	3.529466	29.18885	5.855281	8.461703	2.23267	6.09759	4.94026
12.93651	1.47842	0.929286	9.792626	1.819221	4.490801	1.759986	1.968283	1.733766
0.609509	0.002857	0.010346	0.777403	0.067547	0.156486	0.000188	0.009978	0.002056
2.267157	2.349351	2.953059	3.172864	2.506934	0.316126	9.625253	2.90658	5.47
2.051962	1.499214	1.644416	1.772756	1.010289	0.847908	1.392511	0.953506	0.892395
2.194257	0.449091	0.282078	3.172864	0.067547	0.955418	0.370657	0.199582	0.002056
22.13245	9.335397	12.77772	12.26136	15.66023	10.95872	14.6058	23.82354	25.99427
0.548788	1.89518	0.557056	0.393983	0.772951	1.763889	2.031169	2.434444	1.458038
0.911905	3.207807	4.552092	0.193348	2.845036	0.326421	1.300368	1.218182	2.129596
1.463304	0.282583	0.405346	1.51215	0.187381	0.688975	0.568369	0.070339	0.171183
11.36975	32.68313	22.96463	23.82354	31.92385	25.48179	30.17192	18.76954	14.00414
2.642525	14.08569	12.29473	3.160022	8.412367	2.398442	2.987835	13.38418	10.05618
0.790303	0.047792	0.092915	0.777403	0.11531	2.487576	0.387056	0.124459	0.191371
14.17388	4.592273	3.518384	22.52375	2.81342	14.33939	7.868146	2.278254	1.772756
5.630815	87.1746	72.69473	9.502294	95.22939	11.48368	42.75576	25.73165	3.50425
3.563182	0.527518	0.626118	2.635685	0.690375	1.468131	1.483622	0.555418	3.767489
0.656753	0.639495	0.518413	0.764315	0.399307	0.6657	0.449726	0.289603	0.272078
9.924372	2.207417	0.790837	13.00996	1.204228	7.839271	3.334199	4.715599	0.563499
33.93939	1.225426	2.359264	30.17192	0.674675	1.492973	2.701053	59.32013	3.975498
4.338629	3.169812	20.16541	1.664906	5.704646	2.365685	1.519322	5.217569	20.68746
7.327482	7.765931	2.221775	5.872886	3.058802	3.87329	4.016465	4.783001	5.704646
2.784228	1.537951	0.617417	5.075447	0.480022	2.914632	1.664906	1.464214	0.138644
1.44E-05	0.047792	0.092915	0.015685	0.077417	0.008882	0.018167	0.005411	0.041472
24.98218	28.54786	9.968355	14.51648	16.68554	16.5625	10.35839	10.19468	11.83593
6.019971	3.711097	5.28039	3.792266	5.838499	2.283752	4.33342	13.87537	7.315512
0.781977	1.111241	1.834423	0.882078	4.251818	7.238023	2.871212	2.131176	2.03886
1.369048	3.833247	2.860722	1.404812	4.508196	5.095325	6.268312	4.821176	1.91811
5.80469	4.419062	2.55184	4.115844	3.890556	3.676032	3.846984	3.8121	2.604776
0.520289	0.110642	0.329798	0.319957	0.203175	0.377085	0.097807	0.350455	0.160332
0.038485	0.124646	0.149019	0.143968	0.123853	0.04096	0.025007	0.033146	0.07039
0.855801	0.781465	1.480541	2.81342	0.512641	1.097677	0.406681	0.371876	1.094978
2.723247	0.986104	0.257042	4.592273	0.745613	2.174733	1.191198	1.057569	0.23101
0.595714	0.490079	0.010346	1.801284	0.273672	2.593506	1.175094	1.33285	0.002056
2.225498	62.33472	56.39528	2.292864	57.84882	6.433918	52.56128	28.23632	65.94551
0.153759	7.868146	6.900202	0.469358	4.222583	0.196097	1.01772	3.074134	2.66404
0.656753	0.69583	0.405346	0.365859	0.909315	0.346342	0.771176	1.475469	1.261407
9.751039	1.919661	1.443059	9.031082	0.932078	5.323492	2.531515	2.805382	0.473586
6.019971	5.538001	0.243124	11.09133	0.941645	3.574387	2.043795	4.312251	0.698687
6.414416	1.86658	1.152944	1.678319	3.921645	39.53687	18.76954	2.5271	2.03886
70.11932	8.849697	5.583968	36.74522	8.994791	40.11561	9.792626	11.19942	9.502294
19.37139	7.991602	7.824473	167.9996	6.045209	14.42661	50.55949	37.81919	75.1132
0.548788	34.84299	27.96248	0.836017	26.25557	1.602619	4.18456	2.698557	3.529466
0.178297	1.452857	0.82868	0.248153	3.101371	1.452857	9.502294	1.65785	1.399315
5.42381	5.614805	5.373781	6.967547	5.9071	13.83359	15.88548	8.103925	3.846984
4.376118	1.47842	0.257042	4.684502	0.724004	1.898153	1.162201	2.069942	0.401176
0.634531	0.096371	0.010346	0.11531	0.16925	0.596508	2.276407	0.88316	0.07039
0.1214	0.258838	0.010346	0.022814	0.019048	0.04096	0.117843	0.100887	0.079091
8.780115	3.373983	0.780952	2.81342	3.256587	10.23768	3.122915	11.42512	6.036818
13.64503	23.16622	3.707136	19.37139	12.56693	8.381328	19.99861	12.56693	4.575123
1.44E-05	0.058773	0.08044	0.022814	0.019048	0.026609	0.025007	4.33E-05	0.041472
1.139885	0.058773	0.010346	0.532092	0.036934	0.156486	0.040375	0.087504	0.050577
144.7919	11.83593	11.25392	126.1556	11.89932	41.47925	15.276	14.88584	6.622092
5.704646	0.509242	0.344733	2.515938	0.242453	1.556392	0.239228	0.350455	0.171183

0.006046	0.137201	0.107749	0.015685	0.000173	0.008882	0.1086	4.33E-05	0.087807
20.68746	34.38341	10.53159	75.1132	17.39935	13.30906	5.838499	35.776	16.5625
5.337547	1.47842	1.51215	5.838499	3.672078	4.661717	3.137576	3.198499	2.544747
0.011551	0.002857	0.163766	0.022814	0.027547	0.013867	0.169538	0.033146	0.10886
2.102626	0.148701	0.12132	1.390476	0.151082	1.516219	0.231356	0.325527	0.002056
4.609076	0.937655	0.282078	6.52899	0.38127	1.391494	0.319365	0.371876	0.531169
530.1465	25.48179	24.04374	749.7839	33.07147	1360.293	45.1045	29.18885	18.48514
14.6058	3.266782	2.058773	27.96248	2.579509	12.56693	4.381479	6.570433	5.25254
36.26061	6.900202	5.309264	34.38341	6.967547	25.24367	9.19899	11.719	7.545584
0.385736	1.644416	3.460909	0.656392	2.119596	0.268961	1.68342	2.434444	1.905094
1360.293	135.5245	135.5245	37.29684	91.05541	100.0998	37.81919	21.19547	18.91854
16.9162	10.38202	6.443925	12.26136	9.751039	8.288874	6.365065	11.09133	6.99026
2.895599	1.856392	1.334762	1.376378	2.197828	2.978968	1.348975	1.240202	1.449062
4.174964	0.838846	0.358211	1.953535	0.405974	5.195022	3.394286	0.82868	0.978571
2.242092	2.199639	1.269495	2.060491	1.617489	3.803766	2.345527	3.998391	1.176623
15.88548	2.31013	1.205685	20.34063	3.370527	10.38202	6.853608	4.678846	3.179199
11.74734	22.96463	4.135801	19.53061	5.950664	15.56131	5.606739	5.530274	3.953297
1.102691	0.614545	0.010346	3.192229	0.067547	0.546522	0.064812	4.33E-05	0.181674
5.050267	0.670519	0.505786	2.823636	3.037626	32.31123	5.28039	1.125325	4.323045
40.11561	24.98218	26.25557	47.5018	34.38341	43.49658	13.75978	21.02638	22.52375
224.9555	14.97762	6.653016	87.1746	15.07257	32.68313	35.776	33.07147	22.96463
3.387864	0.751371	1.278629	14.70188	0.666097	3.428788	2.944416	2.453781	0.49974
11.74734	4.307309	3.442886	28.23632	6.560411	12.49452	6.784762	13.45939	5.515584
1.139885	1.905094	0.27075	2.584394	2.802828	1.819221	1.542302	2.609293	0.355794
18.19595	15.77678	30.49289	18.19595	17.78522	6.853608	8.164531	33.93939	55.04081
4.312251	0.2129	0.950418	3.734762	0.324502	0.766602	2.104358	2.673846	2.129596
9.792626	7.061328	14.79742	2.786898	5.976724	0.390895	4.614618	7.795642	14.79742
67.98069	18.05488	32.31123	51.52195	13.60866	10.48101	41.47925	29.49693	46.7056
2.225498	0.002857	0.010346	1.839986	0.036934	0.955418	0.064812	4.33E-05	0.002056
0.609509	0.509242	0.505786	1.568564	0.616739	0.596508	0.271746	0.613211	0.443781
5.967879	6.685087	3.648081	3.518384	7.482179	6.749488	11.544	8.134084	10.1459
0.561775	2.59557	0.884654	0.800058	1.644416	1.006025	1.316544	0.308672	0.401176
2.461905	0.137201	0.315916	0.645916	0.316573	1.688045	0.419076	0.350455	0.191371
6.560411	1.396494	4.789596	5.522929	3.394286	3.169812	2.479221	4.94026	5.295325
0.002367	0.002857	0.010346	0.030577	0.027547	0.000101	0.025007	0.017345	0.061385
80.6518	10.33476	4.47912	12.93651	8.319019	21.93452	6.297136	13.23022	6.079596
118.7028	50.55949	21.02638	224.9555	64.12971	167.9996	203.5795	111.5439	27.66779
155.7103	126.1556	70.11932	252.4251	126.1556	749.7839	224.9555	135.5245	33.4985
1.754733	0.071429	0.695426	0.800058	0.096263	1.788874	0.994899	1.650274	0.443781
15.77678	45.1045	41.47925	20.51271	45.91841	34.38341	45.91841	48.53429	35.27608
0.672468	0.47153	0.010346	1.127475	0.178066	0.937655	0.694675	0.325527	0.002056
4.845166	3.150433	4.965801	5.180058	2.083261	3.407619	2.621133	2.396429	2.306277
0.530851	0.339235	0.344733	0.857778	0.096263	0.064812	0.1086	0.358211	0.079091
0.781977	0.359697	0.179798	2.652107	0.222698	0.176104	0.182929	0.791371	0.7257
0.051595	0.002857	0.010346	0.11531	0.000173	0.033997	0.040375	0.033146	0.079091
13.53913	1.359177	1.08671	16.09482	1.886335	18.91854	26.80355	9.063983	3.672078
25.48179	6.486104	5.439278	111.5439	3.29355	10.05618	46.7056	50.55949	67.98069
0.178297	0.071429	0.010346	0.163983	0.027547	0.166291	0.056623	0.017345	0.171183
37.81919	77.74812	45.1045	45.91841	55.04081	95.22939	155.7103	22.32214	53.77619
1.44E-05	0.083795	0.010346	0.000245	0.000173	0.000101	0.040375	4.33E-05	0.002056
29.82828	10.28573	7.251263	22.13245	13.08214	14.6058	24.98218	35.27608	16.34294
1.934271	0.002857	0.010346	1.012742	0.067547	0.241053	0.064812	4.33E-05	0.002056
2.051962	4.067547	2.304452	0.996696	1.491977	0.67632	0.280678	1.400159	3.321284
0.230108	0.527518	0.010346	0.636291	0.058117	0.937655	0.378326	0.130714	0.002056
1.44E-05	0.002857	0.092915	0.022814	0.019048	0.008882	0.000188	0.009978	0.002056
1.052886	0.348889	0.12132	1.468131	0.187381	0.829192	0.280678	0.137063	0.002056
32.68313	33.93939	19.53061	40.7932	25.48179	50.55949	33.93939	31.1753	39.53687
4.424416	8.075772	6.653016	13.45939	6.560411	23.38378	7.519841	4.965801	3.025671

14.88584	5.123781	6.268312	17.39935	5.030866	8.958225	3.192229	4.644098	4.440548
21.54823	3.35355	3.211176	9.297085	3.743232	9.924372	8.958225	5.638759	7.061328
2.652107	1.805166	1.771392	0.788896	2.083261	1.348975	4.003117	4.752121	2.836717
0.002367	0.002857	0.107749	0.000245	0.000173	0.026609	0.000188	4.33E-05	0.002056
28.54786	5.720173	4.007583	34.84299	5.687879	62.33472	10.73084	5.679502	4.894509
15.276	17.78522	29.82828	15.66023	22.13245	15.46193	6.876392	14.51648	17.78522
2.089639	0.002857	0.010346	2.419553	0.333636	2.698557	0.263304	0.744531	0.002056
2.225498	3.125844	2.628225	2.574733	2.101075	1.335743	1.530159	1.493968	1.304877
3.131544	1.111241	0.790837	2.060491	1.256558	2.542605	0.748341	0.699697	0.886775
13.38418	5.662994	2.740519	16.22108	4.888023	7.839271	5.770996	10.73084	4.381479
14.70188	0.517612	0.405346	12.70768	0.212496	1.296046	0.835498	1.609654	0.743694
2.577157	6.52899	4.839105	2.337821	6.64254	1.925635	3.566753	6.38456	2.858009
22.74797	9.297085	9.101674	35.776	7.08733	14.88584	6.922771	12.03267	11.30987
16.5625	8.677489	15.98892	17.64869	3.273045	5.530274	16.09482	15.46193	32.31123
0.69119	0.002857	0.010346	3.160022	0.000173	0.206768	0.000188	0.193997	0.050577
20.34063	19.06893	13.45939	24.85971	19.37139	7.162698	8.019769	24.50942	22.32214
0.045693	0.083795	0.135144	0.297532	0.036934	0.766602	0.527821	0.100887	0.041472
18.76954	3.767489	1.357201	59.32013	1.989711	37.81919	20.85876	9.377345	0.473586
1.905094	0.460938	0.107749	1.415469	0.488463	2.365685	1.143175	0.650101	0.138644
2.290996	2.558817	2.359264	5.9071	11.19942	28.54786	23.16622	2.589185	4.359574
0.32925	0.69583	0.393196	0.520678	0.390216	0.285079	0.200382	0.800967	0.455649
0.1214	2.445079	1.451775	0.956667	0.918557	0.064812	1.108947	1.502323	1.176623
42.08807	8.226219	6.200945	18.33909	8.075772	15.276	2.329784	3.860491	4.875527
0.385736	0.071429	0.010346	0.913514	0.019048	0.196097	0.073211	0.033146	0.002056
9.276811	8.195657	3.394286	14.97762	9.031082	91.05541	19.53061	4.025267	1.468131
8.019769	11.719	33.07147	10.57984	26.52537	5.056421	12.36443	19.83727	42.75576
0.144163	0.002857	0.010346	0.096905	0.067547	0.110188	0.097807	0.04785	0.002056
10.80505	3.533211	1.019134	11.03843	2.616573	7.495397	1.498167	5.123781	1.779423
0.584502	0.838846	0.358211	4.160238	1.284747	1.296046	0.209538	1.02487	0.365859
1.533535	0.359697	1.152944	0.777403	0.123853	0.5229	0.048124	0.167013	0.160332
0.246479	1.881955	11.48368	0.015685	4.586753	0.10132	11.17281	0.848831	2.238341
4.72798	0.877807	0.474574	4.783001	0.626118	2.964762	1.434921	0.876638	0.563499
58.58447	4.105714	1.734949	105.1694	4.307309	51.52195	4.914863	4.048499	2.701053
46.7056	1.27619	1.692763	10.38202	2.101075	5.872886	8.745238	21.74051	1.779423
1.215981	1.672107	0.82868	1.896667	1.171349	3.363911	0.896522	1.650274	0.819048
0.045693	0.235137	0.282078	0.240671	0.077417	0.000101	0.000188	0.108997	0.119553
0.486551	0.680173	0.195685	0.811861	0.160779	0.294921	0.340245	1.099004	1.094978
1.244957	1.569553	0.358211	2.014863	0.9529	2.698557	1.162201	0.215115	0.253492
1.44E-05	0.002857	0.010346	0.106609	0.000173	0.013867	0.000188	4.33E-05	0.002056
2.032706	0.002857	0.010346	0.393983	0.000173	0.000101	0.000188	0.04785	0.002056
1.474343	0.407662	0.799553	1.746724	0.000173	0.444762	0.419076	4.33E-05	0.002056
0.076465	0.002857	0.010346	0.449091	0.000173	0.156486	0.000188	0.108997	0.119553
7.363117	3.052655	0.912446	3.824964	0.616739	4.721789	3.807648	1.218182	0.401176
0.263853	0.110642	0.010346	0.173319	0.000173	0.206768	0.000188	0.260144	0.002056
0.006046	0.124646	0.010346	0.030577	0.019048	0.033997	0.1086	0.299444	0.346342
0.163521	0.187013	0.149019	0.297532	0.087165	0.326421	0.319365	0.325527	0.261227
1.560779	1.525729	1.489524	2.045476	1.61088	3.962179	0.3514	1.578687	2.820974
0.163521	2.124574	2.147013	0.449091	1.810664	0.444762	0.220079	1.104033	1.157626
1.474343	55.04081	23.6012	4.091667	18.76954	1.006025	21.93452	17.03919	52.56128
0.755714	4.839105	4.49671	1.022929	10.82954	2.190866	5.889798	1.149322	5.337547
26.52537	51.52195	47.5018	6.135007	87.1746	75.1132	332.0776	27.38726	26.52537
0.186465	0.161962	0.209401	0.193348	0.123853	0.138326	0.064812	0.063131	0.061385
0.309444	0.2129	0.243124	0.682359	0.048528	0.166291	0.200382	0.167013	0.061385
1.39847	0.808983	1.007864	1.443059	0.399307	3.618499	0.568369	1.240202	0.927561
4.016465	0.428932	0.370462	4.207778	0.399307	2.104358	2.436768	4.212554	1.035058
0.113838	8.319019	8.075772	0.000245	11.96574	2.294733	3.179199	0.124459	0.819048
1.44E-05	0.002857	0.08044	0.015685	0.000173	0.000101	0.000188	4.33E-05	0.002056
1.244957	1.198701	0.895945	1.51215	1.531313	1.211299	3.939307	1.776739	1.643218

0.548788	2.417424	3.655714	0.143968	2.708341	1.080303	2.758088	0.108997	0.801501
5.993045	2.531515	7.653391	4.851227	3.230065	1.898153	2.092987	4.203001	4.272165
1.831631	0.083795	0.010346	0.000245	0.087165	1.801284	0.209538	4.33E-05	0.181674
8.573434	2.901032	1.797172	16.80343	3.552049	13.60866	5.395584	6.125823	3.214545
12.63773	3.217612	3.220678	1.600238	3.984747	0.77925	2.676392	8.923117	6.900202
0.475433	0.47153	0.292799	0.726133	0.048528	0.185909	0.706984	0.918557	0.002056
0.153759	1.396494	0.474574	0.449091	1.050231	1.543377	0.632857	0.04785	0.210505
0.625786	0.449091	0.532381	0.500996	0.34697	0.444762	0.299553	0.358211	0.220505
1.667273	1.582915	3.195541	1.534603	3.78798	4.077403	5.11697	1.724805	3.061955
0.171472	1.359177	3.885642	1.148564	5.671126	1.869322	3.334199	0.59062	1.623232
33.4985	30.82277	7.212915	39.53687	22.96463	21.36892	24.2842	65.94551	18.76954
0.964416	0.919827	0.895945	1.172872	0.543939	0.874091	0.440548	0.780519	0.753882
0.038485	0.002857	0.010346	0.022814	0.011962	0.064812	0.025007	0.005411	0.002056
0.347655	0.889553	0.163766	1.072843	0.390216	0.92816	0.3514	0.699697	0.389574
0.076465	45.91841	67.98069	0.000245	30.49289	0.082872	3.137576	3.327619	72.69473
1.44E-05	0.002857	1.889711	0.000245	2.270837	0.000101	0.802035	0.82868	4.039192
18.48514	16.22108	15.77678	19.75908	12.26136	8.994791	10.01009	20.85876	13.79669
2.194257	12.49452	19.68088	3.68381	12.63773	0.467403	18.62711	17.64869	22.74797
0.911905	0.002857	0.010346	0.956667	0.372504	0.423781	0.756724	0.420058	0.002056
1.44E-05	33.4985	36.26061	0.11531	35.27608	0.000101	34.84299	26.80355	43.49658
1.44E-05	13.42179	33.4985	0.11531	14.33939	0.475729	4.048499	0.193997	13.08214
7.737807	3.101371	5.416623	5.662994	3.807648	4.302367	2.644885	2.485534	5.28039
1.600238	0.187013	0.231659	1.545657	0.309221	1.187229	0.494841	0.848831	0.522367
0.899567	0.509242	0.149019	0.540902	0.096263	0.444762	0.200382	0.252922	0.097879
3.585029	10.1028	4.609076	0.674675	11.60283	0.559221	4.408153	8.745238	4.863579
3.640606	0.538117	1.06355	3.636789	0.36267	1.020447	0.220079	0.540902	0.210505
0.300606	0.910339	0.010346	0.22039	0.488463	1.109726	0.684033	0.73583	0.698687
0.656753	0.55741	0.636688	1.452857	0.480022	0.346342	0.406681	0.709784	0.23101
15.3618	26.52537	55.04081	14.33939	35.776	11.42512	15.98892	11.48368	37.81919
3.47531	5.687879	1.820541	3.266782	6.99026	4.839105	7.149618	3.35355	2.850058
1.652771	1.742734	0.684401	1.286494	1.511198	1.265426	2.871212	0.810491	0.553636
0.402698	2.207417	2.807937	0.319957	1.219856	0.128759	2.276407	2.818312	77.74812
0.451977	1.028672	1.315613	0.081126	1.163579	0.35676	0.729062	1.149322	1.112641
0.497749	2.398442	7.532713	0.975556	9.502294	4.789596	11.544	2.003723	1.167561
0.803211	1.440736	0.920483	1.857677	0.932078	0.57101	0.927561	1.869322	0.315743
0.153759	0.282583	0.010346	0.636291	0.000173	0.276248	0.000188	4.33E-05	0.061385
0.738312	0.449091	0.405346	1.426522	0.65101	1.616385	0.748341	0.640606	0.49974
1.441898	1.27619	0.304062	1.246457	0.65101	1.810664	0.3514	0.31702	0.002056
28.23632	9.141299	5.493182	10.31025	8.524242	5.24562	10.98644	14.42661	10.38202
0.186465	0.002857	0.010346	0.437576	0.067547	0.048716	0.406681	0.215115	0.253492
0.530851	1.01772	0.841746	0.480794	0.709784	1.602619	2.092987	0.861429	0.808052
0.571674	1.374329	2.823636	0.355556	1.655541	0.092302	2.465916	3.953297	4.312251
1.359177	7.697063	7.061328	2.673846	17.64869	8.348817	7.495397	6.717056	3.153485
0.431457	0.137201	0.08044	0.173319	0.178066	1.374329	0.794401	0.087504	0.061385
1.44E-05	3.169812	4.519048	0.193348	5.80469	0.766602	1.77404	3.747035	2.341573
0.002367	0.002857	0.010346	0.015685	0.000173	0.082872	0.012944	0.017345	0.002056
0.300606	0.369105	0.489509	0.437576	0.151082	0.285079	0.182929	0.398059	0.281169
1.580909	2.461905	1.889711	0.355556	3.944084	3.536955	1.594675	1.125325	1.008413
0.595714	0.970635	2.263377	1.497143	0.799553	0.231876	0.588658	0.503146	1.592395
5.568391	0.438831	0.179798	4.266753	0.626118	4.927302	1.801284	3.223882	0.200382
0.011551	0.002857	0.195685	0.022814	0.000173	0.000101	0.000188	4.33E-05	0.002056
22.32214	184.443	57.84882	3.377619	203.5795	7.188095	105.1694	224.9555	118.7028
0.814315	3.286652	2.898196	0.674675	2.685786	0.766602	3.286652	1.820541	1.176623
0.038485	0.161962	0.282078	0.053802	0.058117	0.033997	0.012944	0.124459	0.079091
11.09133	5.485685	2.339697	9.667316	2.876421	14.17388	6.230245	5.9071	1.261407
1.744055	2.768312	1.556392	3.428788	3.217612	3.283139	6.325945	1.510014	2.901032
52.56128	22.52375	32.68313	18.91854	45.1045	27.38726	30.49289	56.39528	64.12971
0.451977	0.002857	0.010346	0.836017	0.000173	1.468131	0.000188	0.025411	0.002056

1.44E-05	0.407662	2.058773	0.022814	0.106118	0.000101	0.025007	0.009978	0.466335
1.600238	0.270924	0.86987	0.726133	0.462092	0.305253	0.387056	1.341825	1.188139
1.237879	1.51215	1.462143	1.678319	0.759646	0.559221	1.027403	3.939307	1.985253
0.106522	0.002857	0.010346	0.081126	0.000173	0.285079	0.073211	4.33E-05	0.119553
0.246479	0.002857	0.010346	0.338658	0.067547	0.138326	0.081212	0.017345	0.002056
1.44E-05	23.71237	37.81919	1.127475	42.75576	2.104358	42.08807	39.53687	40.7932
1.017107	11.36975	7.710361	1.618773	10.53159	3.514834	2.209141	1.737294	5.993045
0.359105	0.318312	0.010346	0.500996	0.532857	0.829192	0.319365	0.800967	0.355794
6.706472	4.888023	1.812114	6.345382	3.346898	11.30987	6.99026	5.71241	3.747035
0.834416	0.527518	0.860404	0.54847	0.924329	0.980505	0.748341	1.33285	0.416782
25.73165	4.557605	3.614603	16.34294	5.088694	26.25557	8.461703	5.599228	5.737056
0.282424	3.439437	2.442987	1.232078	1.372475	0.581926	1.633752	1.711414	1.650274
0.19544	0.047792	0.163766	0.22039	0.019048	0.110188	0.048124	0.079387	0.07039
0.098001	0.047792	0.010346	0.124343	0.000173	0.092302	0.000188	0.063131	0.002056
0.834416	0.781465	0.636688	1.376378	0.759646	0.937655	0.430281	0.380087	0.191371
6.784762	6.365065	3.340361	42.08807	7.112496	10.62739	12.81638	12.22798	4.673182
4.47912	0.282583	0.382208	5.00404	0.160779	2.964762	0.220079	0.517359	0.315743
4.312251	0.137201	0.092915	0.258059	0.067547	2.24562	0.609509	1.699091	0.129913
0.774257	1.615281	1.688045	1.034315	1.363557	0.688975	1.175094	1.134387	2.363442
13.79669	6.601833	5.238701	13.83359	4.003117	6.210375	3.266782	2.106089	3.904517
3.404372	13.60866	8.643074	16.5625	3.511263	0.546522	16.5625	87.1746	111.5439
0.584502	0.002857	0.304062	0.532092	0.000173	0.92816	0.512641	0.199582	0.002056
0.023608	0.002857	0.092915	0.000245	0.019048	0.000101	0.000188	0.009978	0.087807
5.915469	0.900693	0.22096	3.037626	0.421804	2.472576	1.120332	2.212619	0.220505
0.017063	0.071429	0.010346	0.096905	0.000173	0.118644	0.032172	0.009978	0.002056
0.238059	0.137201	0.706494	0.22039	0.077417	0.118644	0.048124	4.33E-05	0.087807
38.94854	11.14619	10.62739	57.84882	16.22108	46.7056	31.92385	43.49658	13.53913
0.098001	0.002857	0.010346	0.073867	0.067547	0.483326	0.220079	4.33E-05	0.002056
0.011551	0.002857	0.092915	0.022814	0.027547	0.026609	0.000188	0.017345	0.002056
0.441053	2.768312	3.944084	0.437576	3.095166	0.847908	1.120332	1.351573	1.596782
31.55417	0.002857	0.010346	9.541645	0.000173	6.464906	0.000188	4.33E-05	0.002056
1.44E-05	4.016465	17.15902	0.567994	6.497266	0.231876	1.834423	9.297085	8.728571
1.44E-05	17.58415	19.37139	0.000245	17.15902	0.000101	10.07949	5.942294	12.22798
0.153759	0.369105	1.451775	0.173319	0.674675	0.483326	0.640924	0.308672	0.939986
0.699697	0.997792	1.350772	0.54847	1.399315	1.242641	0.200382	0.524986	1.491977
3.370527	0.527518	0.22096	1.497143	0.212496	1.712706	0.3514	0.299444	0.002056
3.691724	0.199394	0.772626	5.416623	0.766118	2.542605	1.300368	0.188124	0.002056
0.300606	0.071429	0.010346	0.258059	0.096263	0.073211	0.032172	0.144293	0.07039
3.113478	2.873817	1.53684	4.012049	2.955916	3.198499	1.330974	1.770029	2.713319
0.584502	1.553074	0.684401	0.753016	1.230411	0.829192	1.759986	1.087345	0.455649
1.102691	0.396306	0.010346	1.272756	0.16925	1.187229	0.378326	0.308672	0.079091
1.618773	2.671299	0.753449	2.337821	2.558817	4.125786	5.737056	2.831501	0.210505
0.1214	0.175592	0.010346	0.338658	0.000173	0.581926	0.025007	4.33E-05	0.002056
2.267157	5.583968	2.876421	4.227302	8.381328	21.02638	5.923838	6.045209	3.025671
0.113838	0.047792	0.149019	0.015685	0.250823	0.251573	0.220079	0.188124	0.079091
1.551962	2.230859	1.578687	0.34697	1.76873	2.219863	5.316378	1.573276	1.678319
2.300678	1.452857	0.566782	1.96697	0.932078	2.472576	1.25886	2.347439	4.796126
0.129365	3.450577	1.443059	1.113961	2.676392	1.592395	3.101371	3.824964	8.149307
1.102691	0.224661	0.12132	0.088283	0.123853	0.241053	0.126335	0.299444	0.808052
0.656753	16.44768	12.60233	0.211335	7.482179	0.000101	2.758088	3.799885	9.418196
2.032706	1.205685	0.179798	2.500548	0.729582	2.487576	1.423506	3.792266	0.455649
1.419278	15.276	11.17281	2.139928	18.05488	3.128694	20.16541	11.30987	7.938427
3.767489	11.544	5.454949	3.885642	21.54823	12.22798	39.53687	13.15079	4.63189
2.337821	0.002857	0.010346	3.086306	0.25785	4.696313	0.914618	0.100887	0.002056
1.819221	0.680173	0.443052	4.338629	0.445996	2.24562	0.175714	0.299444	0.473586
0.878369	0.161962	0.12132	2.996869	0.067547	1.211299	0.1086	0.2443	0.129913
1.44E-05	7.795642	11.36975	0.000245	6.876392	0.000101	6.518709	11.65791	21.36892
0.230108	2.173081	0.753449	0.338658	1.102121	0.000101	1.530159	2.194257	1.491977

6.967547	9.179466	2.058773	4.502453	6.079596	5.976724	9.838658	2.419553	1.748016
3.842554	1.27619	0.304062	3.885642	0.414012	2.895599	0.419076	0.289603	0.23101
3.754719	2.810678	3.726854	3.792266	1.830159	1.778153	0.844747	1.412771	3.50425
0.32925	1.47842	0.860404	0.705231	2.008312	2.03886	4.150274	1.376378	0.718355
0.790303	1.928629	1.036558	2.060491	1.701616	0.285079	1.412771	2.032706	1.468131
1.44E-05	19.22139	5.638759	0.000245	13.00996	0.874091	3.179199	0.628297	1.795851
5.568391	17.03919	60.77449	8.493088	20.68746	6.019971	15.56131	70.11932	14.6058
2.941544	10.70538	7.162698	6.210375	10.33476	1.712706	5.687879	6.250447	10.62739
4.709033	5.614805	2.421681	6.088593	2.765758	3.005281	1.783203	4.429769	3.188918
0.548788	5.838499	7.112496	0.000245	20.34063	11.14619	14.17388	2.558817	11.77567
1.310837	2.922778	1.784755	0.54847	1.230411	1.484553	0.706984	1.200152	0.401176
0.944084	1.058896	1.315613	1.896667	0.799553	5.158146	0.771176	0.100887	0.753882
1.017107	2.828918	4.419062	3.058802	1.906551	0.596508	1.412771	3.011169	1.584105
0.765606	0.407662	0.606154	0.030577	0.878369	4.863579	1.224733	0.517359	0.200382
1.253088	0.751371	0.010346	1.113961	0.438312	0.847908	0.220079	0.908247	0.272078
1.831631	0.148701	0.12132	1.204228	0.222698	1.233665	0.478413	0.167013	0.23101
1.322092	0.24632	0.010346	1.746724	0.000173	1.048802	0.081212	0.056205	0.002056
3.290101	0.438831	0.12132	2.035678	0.50557	1.572035	0.862561	1.567576	0.707475
0.393716	0.480303	0.405346	0.480794	0.333636	1.272756	0.478413	0.215115	0.389574
0.645036	0.795339	0.382208	1.172872	0.924329	0.937655	0.706984	0.613211	0.68557
1.44E-05	5.102742	4.365224	0.000245	8.524242	0.000101	7.200505	9.792626	8.256465
1.426522	1.396494	0.884654	7.824473	2.101075	1.222179	2.845036	1.178939	0.409336
0.727633	0.407662	0.54619	3.220678	0.690375	0.581926	0.319365	0.555418	0.148701
0.106522	0.096371	0.243124	0.096905	0.151082	0.146768	0.319365	0.056205	0.171183
3.346898	3.486436	8.134084	1.816436	7.765931	2.058773	13.38418	2.842193	15.46193
5.259437	11.60283	3.290101	5.553146	6.325945	4.620159	7.964315	5.337547	2.08013
0.211905	2.558817	2.953059	0.124343	0.924329	0.196097	0.289755	0.380087	0.443781
1.779423	4.997417	1.750628	2.770866	4.039192	3.803766	2.574733	1.815	1.544517
0.843564	0.002857	0.010346	1.691587	0.000173	0.859351	0.299553	4.33E-05	0.002056
0.203954	0.359697	0.179798	0.23101	1.016494	1.006025	0.546522	0.040469	0.002056
1.072843	3.086306	0.730476	1.99899	2.887489	2.349351	1.31	1.896667	0.831472
0.992201	9.859719	3.691724	1.99899	6.210375	0.955418	9.541645	6.685087	4.764055
5.00404	3.57057	5.662994	2.281926	2.836717	3.718716	1.620058	0.848831	0.718355
0.727633	0.137201	0.149019	0.656392	0.222698	0.795786	0.209538	0.193997	0.220505
0.497749	0.199394	0.010346	0.277763	0.178066	0.128759	0.040375	0.274704	0.247951
1.072843	0.096371	0.010346	0.588658	0.141284	0.073211	0.588658	0.628297	0.851234
0.699697	1.919661	0.231659	1.072843	0.996111	0.838326	1.143175	0.464127	0.119553
1.341825	4.18456	1.994329	3.110447	2.978968	1.080303	4.745895	3.418405	0.743694
1.44E-05	0.058773	0.010346	0.000245	0.000173	0.000101	0.000188	0.009978	0.002056
0.038485	0.110642	0.092915	0.088283	0.019048	0.10132	0.000188	0.033146	0.041472
6.153102	4.082165	3.588369	3.636789	2.371833	0.654877	0.117843	1.609654	2.207417
4.770231	1.525729	0.370462	5.779574	0.976667	3.514834	1.449877	2.24562	0.514531
31.1753	12.93651	6.808369	30.49289	9.838658	16.28201	1.143175	6.784762	0.389574
0.855801	0.235137	0.163766	0.376212	0.265339	0.817013	0.000188	0.070339	0.210505
0.083896	0.110642	0.12132	0.124343	0.058117	0.118644	0.126335	0.079387	0.002056
2.194257	5.238701	8.849697	0.459394	8.134084	2.472576	2.607035	1.790245	2.008312
3.404372	5.302294	9.625253	2.160649	8.195657	4.110779	6.00228	3.4679	6.738211
1.35259	7.339452	2.723247	0.836017	3.450577	1.391494	1.277056	4.655916	2.051962
0.083896	0.002857	0.08044	0.106609	0.011962	0.185909	0.018167	0.005411	0.002056
0.002367	0.002857	0.010346	0.022814	0.027547	0.000101	0.000188	0.009978	0.061385
0.911905	2.417424	2.035678	1.468131	2.518124	1.688045	2.470317	1.8386	1.277056
8.477662	1.290981	8.677489	3.23987	6.612121	4.174964	20.34063	12.77772	28.23632
0.765606	0.318312	0.243124	0.449091	0.309221	2.308203	0.419076	0.167013	0.240671
2.117814	2.642525	2.909293	3.548153	2.758088	2.327857	2.063362	3.074134	9.687496
0.369567	5.462475	4.174964	0.365859	2.270837	0.000101	1.212792	2.666494	2.735368
1.274481	1.856392	2.27254	2.203535	1.304877	1.74539	0.794401	1.005426	3.016984
0.834416	0.002857	0.010346	0.54847	0.087165	0.688975	0.088968	0.063131	0.002056
2.678781	0.161962	0.474574	3.885642	0.195346	9.583463	0.000188	0.193997	0.002056

0.602287	2.59557	5.493182	0.459394	4.739668	1.080303	2.415296	2.201587	2.933903
0.203954	0.058773	0.010346	0.329798	0.000173	2.267157	0.135606	0.040469	0.050577
1.89373	0.175592	0.195685	3.610707	0.077417	0.546522	0.319365	0.968066	0.002056
1.801284	1.537951	0.895945	2.718434	3.944084	1.391494	5.530274	1.544517	0.416782
0.083896	1.759986	3.493362	0.124343	0.16925	0.10132	4.150274	0.225079	0.784372
1.819221	1.724805	1.50342	8.226219	1.989711	1.734949	5.568391	2.981962	3.38456
0.292799	0.002857	0.010346	0.297532	0.000173	0.346342	0.000188	4.33E-05	0.002056
2.436768	1.127475	0.443052	2.477027	2.386248	4.490801	5.316378	4.990794	2.993867
0.699697	3.632973	2.278254	1.072843	0.716652	0.377085	1.483622	1.321053	1.850996
5.630815	7.519841	14.51648	10.8813	12.03267	3.080317	12.29473	21.93452	11.14619
0.203954	1.237063	0.304062	0.106609	1.204228	0.196097	0.465801	0.289603	0.522367
0.393716	42.08807	111.5439	0.000245	34.84299	1.323131	3.464315	1.544517	3.610707
3.207807	19.53061	23.38378	2.529307	18.33909	2.786898	7.765931	16.5625	26.80355
0.163521	0.002857	0.092915	0.682359	0.019048	1.144733	0.048124	0.070339	0.050577
0.625786	5.195022	3.574387	1.978954	3.077316	1.374329	0.387056	1.855108	2.649675
3.726854	14.25954	4.990794	11.77567	12.36443	14.51648	32.31123	19.68088	8.075772
1.232078	0.62645	1.315613	1.100519	2.909293	3.592063	4.067547	0.398059	1.176623
1.844098	0.762165	2.31811	0.073867	1.839986	0.185909	0.521421	1.471198	1.748016
2.178268	26.25557	15.07257	1.873434	25.24367	5.530274	2.384141	19.37139	57.84882
0.402698	0.581111	0.010346	0.868853	0.000173	0.817013	0.190693	0.137063	0.002056
0.595714	3.095166	1.253088	1.51215	1.171349	0.457431	2.23267	2.365685	0.998312
9.179466	1.525729	1.451775	3.310758	1.039798	4.203001	1.25886	3.13456	2.419553
1.96697	27.66779	52.56128	24.04374	27.10502	2.752893	4.473348	7.38847	14.08569
0.402698	0.500354	5.195022	0.11531	1.573276	0.214805	0.675115	0.663276	1.974228
1.488571	2.071587	1.872049	1.034315	1.693939	1.348975	3.533211	1.387612	1.288276
0.271912	0.002857	0.010346	1.896667	0.067547	3.718716	0.659163	0.40645	0.425426
0.1214	0.002857	0.010346	0.996696	0.000173	3.107417	0.000188	0.819978	0.002056
5.381227	4.921082	1.613232	4.067547	10.19468	6.268312	2.987835	6.853608	2.221775
0.617763	0.527518	1.909408	0.308788	1.573276	0.895945	0.763102	1.884935	1.188139
0.843564	0.407662	0.740101	1.157626	2.925426	2.174733	2.786898	1.406746	0.939986
0.023608	0.071429	0.08044	0.124343	0.011962	0.057027	0.032172	0.025411	0.002056
0.378326	0.002857	0.010346	0.619365	0.000173	0.156486	0.000188	0.063131	0.002056
3.192229	3.8121	2.540462	2.887489	2.955916	0.467403	1.120332	3.297107	5.18754
2.392431	2.860722	4.802655	1.157626	2.065007	0.937655	0.649235	1.200152	5.530274
1.950519	0.772157	0.764315	3.610707	1.016494	0.992771	0.640924	1.192633	0.903074
4.096407	2.263377	1.498167	2.642525	1.957958	2.014863	4.18456	2.131176	1.188139
1.44E-05	4.661717	11.96574	0.000245	3.011169	0.110188	0.667583	0.908247	4.609076
48.53429	25.73165	0.912446	53.77619	8.288874	287.197	33.07147	23.38378	3.125844
8.149307	0.961486	0.715462	3.989206	1.563045	2.941544	1.374329	1.437648	1.288276
3.610707	0.083795	0.12132	0.940563	0.000173	0.490664	0.081212	0.144293	0.129913
0.378326	0.002857	0.092915	0.428694	0.048528	0.444762	0.1086	0.009978	0.050577
0.246479	8.256465	7.795642	1.351573	3.909033	0.867814	1.212792	2.278254	6.036818
6.316328	3.363911	1.425613	6.508427	2.148658	3.644343	2.255916	2.558817	2.66404
1.640779	1.248232	2.157359	2.442987	1.34267	0.285079	0.000188	1.455988	0.639113
2.917381	0.175592	0.505786	1.953535	0.340866	0.92816	0.659163	0.650101	0.443781
25.24367	4.965801	3.921645	26.52537	7.795642	30.82277	8.210938	13.60866	8.149307
1.545657	3.632973	2.290996	2.914632	2.628225	0.838326	2.23267	1.690483	1.678319
0.347655	0.096371	0.092915	0.376212	0.242453	0.475729	0.465801	0.364242	0.079091
3.098268	1.600238	0.950418	4.709033	1.284747	1.670786	1.801284	1.855108	1.390476
0.681494	1.600238	1.556392	0.567994	1.819221	1.452857	2.147013	1.667273	2.820974
0.421804	1.058896	6.738211	2.718434	3.644343	0.859351	2.820974	5.351934	2.270837
0.774257	3.980123	2.078355	0.480794	1.901068	0.643853	1.594675	2.112843	2.398442
0.076465	0.047792	0.107749	0.038139	0.019048	0.019942	0.000188	0.056205	0.087807
4.644098	3.909033	3.747035	5.984885	3.824964	1.898153	3.137576	2.181775	3.578038
23.6012	15.56131	7.037345	9.141299	9.541645	22.32214	13.53913	17.28195	21.54823
3.829105	2.112843	1.425613	1.896667	1.989711	3.934986	0.48684	1.493968	1.886335
0.468167	0.449091	0.626118	0.355556	0.532857	0.35676	0.289755	0.260144	0.253492
1.383911	0.002857	0.163766	2.887489	0.000173	2.190866	0.000188	0.04785	0.002056

1.545657	0.527518	1.299567	2.187395	0.34697	0.413449	0.280678	0.564127	0.656392
0.106522	0.161962	0.010346	0.134076	0.067547	0.206768	0.025007	0.57197	0.041472
1.44E-05	1.36619	1.578687	0.248153	1.545657	0.110188	1.143175	1.913997	2.882035
1.44E-05	5.662994	6.717056	0.000245	8.445743	0.000101	0.957273	4.690159	14.88584
1.177994	1.78759	1.253088	1.148564	2.148658	1.841371	1.316544	0.215115	1.121703
1.000794	0.096371	0.010346	0.822381	0.25785	0.214805	0.126335	0.174646	0.148701
0.504964	1.881955	0.576638	2.092987	0.709784	1.828781	3.101371	0.891775	1.767431
0.076465	0.997792	1.197215	0.23101	1.03189	0.222864	0.535945	0.540902	0.547403
0.113838	1.179885	1.236248	0.923874	2.725498	1.172208	3.160022	1.233665	1.240202
1.062092	3.334199	2.058773	1.232078	1.707576	0.727633	1.519322	4.266753	1.661328
1.383911	9.502294	22.13245	4.227302	7.964315	0.688975	6.250447	6.063276	7.482179
0.163521	0.058773	0.092915	0.329798	0.036934	0.073211	0.032172	0.100887	0.07039
9.604358	1.037958	0.557056	7.625209	0.839365	3.198499	0.771176	1.364401	0.95101
2.705916	1.905094	0.010346	4.783001	1.863506	3.35355	4.851227	6.287561	0.808052
5.359134	2.043795	1.163579	3.953297	1.256558	2.190866	1.006025	2.047157	1.146962
4.067547	6.028427	3.869163	3.037626	7.854069	6.978903	15.46193	8.711905	6.663651
12.77772	1.432302	1.100519	20.68746	1.675722	45.91841	10.5063	3.714906	1.995714
1.826147	0.199394	0.382208	3.917597	0.132922	1.315613	0.457749	0.517359	0.041472
5.050267	2.112843	5.01772	2.109755	7.037345	4.529913	2.494177	2.805382	6.933961
83.489	287.197	184.443	118.7028	1360.293	203.5795	1360.293	287.197	87331.38
0.738312	3.425512	7.598312	0.868853	3.303925	0.643853	0.729062	1.680895	2.556443
0.059459	0.002857	0.010346	0.2871	0.212496	0.346342	0.3514	0.033146	0.061385
5.158146	1.881955	1.969733	1.99899	1.759986	28.23632	0.706984	0.564127	0.914618
7.327482	11.65791	77.74812	17.98838	51.52195	19.06893	27.10502	18.05488	11.42512
0.974257	0.235137	0.370462	3.128694	0.232338	0.980505	0.169538	0.215115	0.138644
10.93101	16.80343	12.70768	9.031082	17.51961	8.103925	7.112496	10.23768	17.64869
7.124517	18.48514	3.679986	11.96574	13.53913	19.22139	59.32013	8.461703	48.53429
2.850058	2.890245	2.45772	3.407619	4.135801	1.639639	2.384141	2.941544	1.667273
0.451977	2.280101	0.648362	0.2871	1.469163	0.156486	1.392511	0.92816	0.240671
7.911934	2.468117	0.950418	5.606739	1.779423	3.993665	2.415296	1.200152	0.831472
0.878369	1.335743	0.452965	2.500548	0.543939	1.31	0.271746	0.445209	0.061385
10.07949	6.316328	5.704646	10.31025	6.760765	12.13485	5.967879	7.882222	10.57984
0.300606	0.002857	0.010346	0.666494	0.106118	0.336147	0.040375	0.087504	0.160332
0.504964	0.742417	0.586595	2.673846	1.414574	1.584105	3.046551	1.447994	0.247951
72.69473	16.9162	16.80343	29.82828	15.18102	25.73165	22.52375	38.94854	18.19595
2.630729	0.387258	0.010346	4.246861	0.414012	2.640332	1.569553	2.337821	0.23101
0.006046	0.071429	0.010346	0.000245	0.000173	0.013867	0.000188	0.033146	0.002056
11.22667	3.486436	1.941703	7.002056	3.529466	5.110159	2.255916	8.461703	6.162446
2.978968	0.65241	1.019134	5.485685	1.491977	1.296046	3.500808	4.145209	1.449062
11.22667	8.539971	2.633232	5.80469	3.726854	4.900996	3.679986	7.288788	6.52899
4.914863	3.610707	1.052886	6.365065	2.306277	2.500548	2.607035	3.373983	3.101371
0.129365	0.058773	0.010346	0.599134	0.048528	0.413449	0.145909	0.079387	0.041472
1.133622	1.190491	0.648362	2.673846	1.219856	1.144733	1.25886	3.160022	1.399315
0.431457	0.137201	0.092915	0.726133	0.058117	0.206768	0.064812	0.193997	0.07039
0.369567	0.359697	0.27075	1.404812	0.242453	0.795786	0.494841	0.160072	0.07039
0.006046	0.002857	0.179798	0.022814	0.123853	0.128759	0.081212	0.079387	0.050577
3.618499	42.75576	4.302367	6.404921	23.16622	2.124574	2.003723	3.022713	5.80469
0.944084	2.445079	2.696061	0.894704	2.008312	0.992771	3.370527	0.853752	0.639113
3.691724	2.947287	3.921645	0.913514	4.353925	2.836717	2.316075	3.518384	3.179199
18.91854	3.651898	28.87506	2.092987	4.203001	10.1459	7.570693	3.695815	6.443925
1.533535	2.214372	0.282078	1.953535	2.463911	20.16541	10.67991	1.02487	0.62645
2.485534	20.60009	25.24367	5.606739	14.42661	5.646811	11.719	10.78056	11.09133
0.22039	2.092987	2.304452	1.390476	1.596782	1.144733	0.922879	2.468117	1.837172
0.738312	0.849336	0.532381	0.636291	0.674675	0.483326	0.073211	0.683232	1.01544
0.098001	1.225426	0.179798	0.578817	1.277843	0.534452	0.471746	3.846984	0.376782
3.807648	0.889553	3.78798	5.704646	0.724004	3.55961	0.478413	1.387612	1.277056
0.992201	0.517612	0.292799	2.515938	0.195346	2.513752	0.667583	0.130714	0.079091
1.580909	0.002857	0.010346	0.894704	0.141284	0.316126	0.155224	0.108997	0.002056

3.340361	0.480303	0.27075	3.743232	0.471313	1.919661	1.702706	0.645036	0.148701
13.00996	15.66023	21.36892	5.302294	12.26136	12.03267	14.70188	17.51961	38.94854
6.632063	14.33939	9.502294	10.01009	6.345382	2.735368	6.116638	16.09482	17.03919
2.300678	1.382006	0.895945	2.139928	0.878369	1.629235	0.976667	0.663276	1.584105
4.277006	3.783694	1.980592	2.187395	5.576219	1.971183	2.447294	1.950519	2.106089
1.369048	0.148701	0.195685	0.734935	0.543939	1.428398	0.579149	0.464127	0.23101
1.177994	1.759986	0.961486	1.390476	1.604827	0.500722	1.153723	1.87899	1.134387
0.359105	0.199394	0.12132	0.619365	0.160779	0.128759	0.032172	0.017345	0.07039
0.098001	0.305087	0.010346	0.384798	0.273672	0.196097	0.289755	0.333831	0.002056
2.768312	0.961486	2.842193	7.402915	2.212619	3.718716	4.965801	2.964762	2.306277
0.867814	0.096371	0.163766	1.172872	0.048528	0.559221	0.256046	0.130714	0.181674
0.983384	0.002857	0.010346	0.857778	0.000173	0.261111	0.000188	4.33E-05	0.002056
1.161501	1.905094	0.54619	0.540902	1.617489	0.883665	0.169538	1.290065	0.903074
0.282424	1.316544	1.63039	0.619365	2.640332	0.196097	0.659163	0.988377	0.96329
0.421804	0.124646	0.135144	0.193348	0.048528	0.146768	0.064812	0.199582	0.002056
2.723247	2.207417	1.47018	5.095325	1.852338	3.034747	2.189235	3.022713	2.398442
0.954141	0.614545	2.723247	1.691587	1.399315	0.786667	0.873579	1.641999	2.254084
1.000794	2.642525	5.553146	19.06893	1.830159	7.598312	1.633752	3.846984	2.676392
3.864827	0.270924	0.010346	6.632063	0.087165	3.767489	0.209538	0.04785	0.563499
9.459177	1.697792	2.481356	14.17388	1.185685	7.288788	3.824964	2.647244	2.642525
0.051595	0.002857	0.010346	0.193348	0.000173	0.110188	0.000188	4.33E-05	0.002056
0.765606	0.002857	0.010346	0.419365	0.273672	0.268961	0.000188	0.144293	0.002056
0.983384	2.515938	3.040505	0.338658	1.256558	0.231876	0.675115	2.337821	1.741414
1.519322	0.096371	0.010346	1.481631	0.000173	0.752229	0.048124	0.234502	0.002056
5.024293	5.560563	2.373824	5.24562	4.552092	18.62711	5.654863	6.784762	0.933261
1.570794	0.65241	0.474574	1.481631	0.372504	0.895945	0.271746	0.671724	0.49974
0.911905	2.577157	9.031082	0.716652	1.76873	1.296046	1.852338	2.212619	2.145137
0.002367	0.002857	0.010346	0.015685	0.000173	0.082872	0.012944	0.017345	0.002056
1.51215	2.882035	1.299567	3.578038	2.708341	3.080317	3.300664	6.52899	2.509177
7.225469	17.58415	17.51961	13.60866	13.83359	11.36975	13.19051	16.9162	10.8813
0.878369	2.933903	0.961486	1.910974	1.531313	1.639639	0.598795	0.57197	0.306811
15.66023	7.6393	1.652771	17.51961	4.451154	5.056421	12.16681	8.348817	3.422237
0.153759	0.058773	0.010346	0.682359	0.000173	0.073211	0.018167	0.017345	0.041472
0.710722	1.34267	0.576638	3.816551	0.976667	0.500722	1.460051	1.831631	1.067662
0.211905	0.124646	0.010346	0.297532	0.000173	0.000101	0.000188	4.33E-05	0.002056
4.096407	6.749488	11.544	4.661717	11.09133	29.82828	14.25954	2.773644	3.648081
0.059459	0.002857	0.010346	0.053802	0.027547	0.073211	0.040375	0.093622	0.07039
0.045693	0.058773	0.304062	0.000245	0.38127	0.222864	0.175714	4.33E-05	0.002056
15.07257	8.348817	2.887489	22.74797	7.710361	6.09759	7.037345	6.497266	4.971991
0.230108	3.074134	6.653016	1.004791	1.372475	0.43381	1.056898	1.815	9.218514
0.561775	49.50903	75.1132	1.443059	11.30987	0.6657	7.598312	5.9071	49.50903
5.568391	1.537951	8.539971	3.636789	3.552049	2.219863	3.975498	9.751039	11.89932
2.136335	1.179885	1.652771	2.547179	1.34267	4.02088	3.160022	2.207417	0.443781
1.677042	1.074242	0.81544	0.469358	1.930216	1.265426	0.934488	2.941544	5.050267
12.22798	1.78759	0.81544	13.91714	2.051962	5.530274	2.165851	1.623232	1.1407
1.810664	4.241905	5.80469	1.204228	10.1028	4.23202	7.868146	3.588369	3.394286
3.895469	5.959033	6.365065	4.614618	6.674286	3.514834	6.210375	7.162698	5.082063
6.135007	20.34063	34.84299	6.900202	25.99427	6.486104	14.00414	19.99861	70.11932
0.411703	0.002857	0.010346	2.229048	0.273672	0.717403	0.729062	1.02487	0.247951
0.090188	1.396494	0.518413	0.34697	0.690375	0.512309	0.906825	0.946061	0.578095
0.359105	6.063276	0.939408	0.490952	1.711414	0.908247	1.90798	1.406746	0.939986
0.421804	0.808983	0.010346	0.106609	0.000173	0.390895	0.000188	0.070339	0.002056
0.136573	0.002857	0.010346	0.500996	0.027547	0.185909	0.000188	0.017345	0.002056
1.263752	0.002857	0.163766	1.1379	0.058117	4.603434	0.182929	0.509791	0.002056
5.110159	6.64254	18.33909	4.875527	8.745238	2.810678	6.570433	4.529913	10.28573
0.609509	4.343838	2.132756	1.691587	2.802828	1.801284	1.412771	1.005426	1.643218
1.383911	0.175592	0.532381	1.022929	0.282424	1.584105	0.623853	0.174646	0.7257
0.106522	0.002857	0.010346	2.229048	0.000173	0.000101	0.163038	0.524986	0.466335

0.098001	0.002857	0.010346	0.154329	0.077417	0.057027	0.220079	4.33E-05	0.002056
4.959358	2.289286	1.187229	6.30671	2.083261	1.950519	3.070952	4.397792	1.121703
0.051595	0.002857	0.010346	0.666494	0.000173	0.013867	0.032172	0.137063	0.002056
0.814315	3.699495	2.390447	0.894704	2.725498	0.611003	3.640606	3.002576	6.230245
1.289149	1.084437	0.505786	0.520678	0.626118	1.688045	1.551962	1.364401	0.23101
2.451645	2.941544	9.902576	3.266782	2.481356	1.468131	5.622872	3.223882	9.687496
12.13485	1.51215	10.7557	9.583463	14.97762	12.29473	14.88584	12.85504	25.24367
0.113838	0.071429	0.010346	0.096905	0.067547	0.026609	0.097807	0.087504	0.07039
0.045693	0.047792	0.092915	0.015685	0.036934	0.026609	0.025007	0.005411	0.002056
2.850058	1.615281	1.06355	6.900202	2.008312	9.541645	5.351934	4.370671	1.368167
7.854069	1.499214	1.391494	10.53159	1.194221	5.855281	2.531515	3.966623	1.634935
2.784228	2.432121	1.32482	5.351934	1.819221	4.808658	2.718434	1.667273	0.595022
0.538773	1.569553	0.474574	0.674675	0.941645	1.222179	1.594675	0.699697	0.365859
2.941544	7.545584	9.256537	5.037489	4.690159	0.622496	3.016984	4.575123	9.815642
0.602287	0.329798	0.179798	0.692799	0.106118	0.000101	0.502843	0.325527	0.002056
2.737944	3.726854	4.120815	3.507691	5.638759	6.116638	4.33342	5.770996	3.636789
0.263853	0.602554	0.135144	0.143968	0.048528	0.033997	0.048124	0.215115	0.002056
1.501291	0.970635	0.489509	1.736133	0.488463	0.444762	1.027403	4.667518	0.892395
49.50903	48.53429	13.23022	42.75576	42.08807	14.25954	12.93651	30.17192	17.51961
0.211905	0.460938	0.636688	0.20215	0.203175	0.110188	0.361948	0.174646	0.240671
1.44E-05	4.043846	4.702468	0.000245	4.789596	0.000101	2.794935	2.024488	4.644098
0.282424	0.002857	0.179798	0.692799	0.000173	0.261111	0.000188	0.267807	0.002056
1.950519	1.553074	1.052886	2.250462	3.077316	3.33728	4.016465	1.65785	1.815
51.52195	6.454416	4.535354	3.792266	5.381227	6.653016	12.81638	6.876392	3.824964
2.410996	3.926154	4.241905	1.100519	5.381227	0.546522	1.434921	7.002056	4.012049
0.300606	0.071429	0.209401	1.246457	0.11531	1.484553	0.256046	0.225079	0.148701
1.102691	0.591912	0.405346	1.468131	0.50557	0.955418	0.370657	0.613211	0.261227
3.743232	6.64254	5.9071	0.193348	4.875527	0.346342	0.738312	4.413608	9.141299
2.607035	1.945916	0.848369	3.058802	1.737294	7.519841	3.663622	1.259747	0.770664
2.461905	0.581111	0.419076	4.297222	0.290693	2.365685	1.650274	1.005426	0.191371
1.44E-05	3.346898	3.182482	0.000245	4.978182	0.000101	5.351934	3.629177	3.6029
16.34294	0.591912	0.790837	17.09911	1.102121	1.452857	2.574733	5.576219	3.659668
1.463304	0.639495	0.648362	1.267085	0.791854	1.468131	0.430281	1.014127	0.261227
8.660281	53.77619	59.32013	8.119004	28.87506	5.696263	8.288874	18.62711	33.07147
6.911486	9.031082	3.944084	11.14619	8.815123	5.821804	7.710361	13.87537	6.191515
0.33785	0.002857	0.010346	0.183304	0.000173	0.013867	0.440548	4.33E-05	0.07039
13.79669	9.218514	14.88584	6.135007	15.88548	11.60283	14.08569	14.08569	11.19942
203.5795	252.4251	530.1465	38.94854	87331.38	155.7103	749.7839	87331.38	1360.293
35.27608	0.639495	0.974906	2.477027	0.543939	6.433918	0.675115	1.845519	0.097879
0.32925	0.925801	2.74544	0.106609	1.194221	0.185909	0.967446	0.663276	3.116732
1.177994	5.754935	7.897078	1.910974	4.946587	5.485685	5.01772	2.447294	2.483492
8.319019	3.363911	9.218514	3.086306	10.8813	5.024293	7.149618	13.53913	15.56131
4.261342	56.39528	105.1694	8.762677	77.74812	3.829105	21.36892	20.42667	83.489
1.017107	4.997417	5.622872	1.195079	3.585029	0.622496	1.542302	3.223882	3.043528
1.44E-05	35.776	26.52537	0.000245	40.11561	2.104358	47.5018	62.33472	29.82828
16.80343	6.287561	2.339697	3.722785	4.655916	5.409437	4.236962	6.355224	7.149618
1.274481	2.27254	2.019618	2.529307	1.989711	1.360022	1.191198	0.918557	1.368167
1.39847	3.672078	2.755411	1.172872	4.266753	1.256558	1.498167	4.702468	3.895469
0.954141	0.329798	0.532381	1.497143	0.462092	0.654877	0.706984	0.509791	0.649235
0.755714	0.002857	0.010346	1.816436	0.000173	0.176104	0.000188	0.04785	0.002056
10.57984	21.93452	13.30906	15.98892	22.74797	5.889798	17.64869	14.70188	10.48101
12.13485	7.868146	4.174964	2.396429	12.85504	4.077403	16.34294	17.39935	8.815123
0.22039	0.002857	0.010346	0.064012	0.000173	0.092302	0.000188	4.33E-05	0.002056
7.443276	9.667316	30.82277	10.73084	22.52375	7.795642	6.830592	15.07257	24.50942
3.107417	1.028672	0.431977	1.1379	1.644416	0.57101	0.738312	0.791371	2.320072
2.267157	0.124646	0.12132	1.216768	0.232338	0.581926	0.056623	0.188124	0.119553
3.442886	1.139221	0.532381	2.375974	0.674675	2.718434	0.675115	1.724805	0.992201
0.230108	0.148701	0.010346	0.692799	0.027547	0.251573	0.081212	0.087504	0.002056

2.485534	3.381255	6.345382	3.011169	5.779574	3.128694	2.217951	2.898196	7.08733
1.905094	1.121017	1.137244	3.147294	1.797172	6.518709	2.531515	1.290065	0.540563
0.359105	0.002857	0.092915	0.20215	0.011962	0.268961	0.018167	0.005411	0.002056
1.139885	8.381328	8.412367	3.975498	6.404921	2.150303	9.880779	8.573434	10.33476
14.51648	7.162698	37.29684	1.708932	65.94551	17.03919	23.38378	47.5018	26.25557
0.475433	7.911934	19.06893	0.000245	6.30671	0.423781	6.028427	3.297107	21.19547
0.319365	0.083795	0.010346	0.355556	0.000173	0.222864	0.040375	0.108997	0.050577
2.252273	2.618846	15.3618	4.297222	8.241342	0.966825	4.875527	5.47	2.524892
0.790303	0.002857	0.010346	0.248153	0.000173	1.788874	0.000188	4.33E-05	0.002056
0.067864	0.096371	0.107749	0.064012	0.048528	0.082872	0.000188	4.33E-05	0.002056
3.233045	0.002857	0.12132	1.216768	0.187381	2.616573	2.607035	1.428398	0.191371
0.645036	0.148701	0.010346	1.048074	0.000173	0.688975	0.000188	0.063131	0.002056
1.992944	0.002857	0.149019	0.913514	0.106118	0.908247	0.000188	0.188124	0.097879
2.162381	0.961486	0.243124	1.758615	1.194221	5.195022	10.1459	2.633232	0.43381
2.242092	5.915469	7.482179	1.772756	5.522929	1.524776	2.776421	5.84689	6.39474
3.425512	0.449091	0.107749	2.698557	0.141284	2.628225	0.145909	2.047157	0.365859
6.116638	9.063983	5.762965	6.135007	8.643074	5.056421	2.547179	15.56131	12.7427
0.211905	0.002857	0.010346	0.437576	0.000173	0.073211	0.088968	0.663276	0.002056
9.667316	14.65384	9.063983	5.889798	13.38418	8.134084	13.00996	9.218514	15.18102
1.282208	4.863579	3.016984	2.529307	5.28039	4.927302	5.351934	5.323492	6.063276
0.32925	0.318312	0.08044	0.319957	0.106118	0.622496	0.55741	0.451977	0.160332
1.44E-05	1.759986	6.30671	0.636291	8.048095	0.423781	4.557605	9.707677	14.97762
1.68938	0.614545	0.684401	0.936898	0.887359	1.200823	0.598795	1.104033	1.224733
0.929286	3.909033	16.44768	0.894704	5.745996	1.048802	0.844747	1.595729	13.15079
1.161501	0.187013	0.010346	0.490952	0.000173	0.413449	0.073211	0.056205	0.002056
1.44E-05	0.083795	0.010346	0.000245	0.019048	0.013867	0.000188	4.33E-05	0.002056
0.378326	0.175592	0.010346	1.497143	0.106118	0.000101	0.000188	0.252922	0.002056
0.814315	0.490079	1.613232	0.931457	1.624459	0.766602	5.238701	0.762641	2.789466
0.163521	0.002857	0.010346	0.124343	0.000173	0.013867	0.000188	0.009978	0.002056
0.255887	0.002857	0.179798	0.459394	0.019048	0.013867	0.163038	0.009978	0.002056
4.808658	4.696313	3.899993	2.212619	4.203001	0.166291	0.835498	6.443925	5.591717
0.038485	0.581111	0.987821	0.038139	0.532857	0.403413	0.419076	0.167013	0.10886
3.147294	2.124574	1.937222	3.086306	3.113478	3.236457	4.048499	2.312085	2.419553
0.69119	6.210375	3.460909	0.393983	2.54961	0.403413	1.675722	1.716364	4.101061
1.474343	1.283059	1.636118	0.124343	3.321284	2.205476	1.985253	0.800967	0.171183
4.678846	1.283059	2.868456	4.592273	1.886335	1.452857	24.73724	15.77678	19.37139
0.059459	0.096371	0.179798	0.124343	0.019048	0.268961	0.056623	0.009978	0.050577
1.035058	3.279625	1.403853	3.533211	4.096407	3.816551	6.38456	5.075447	2.221775
41.47925	27.38726	39.53687	15.3618	38.39042	11.83593	16.22108	19.53061	42.08807
1.463304	0.002857	0.010346	2.07658	0.019048	1.265426	0.000188	0.206616	0.002056
0.359105	0.137201	0.257042	0.319957	0.212496	0.166291	0.308788	0.174646	0.087807
15.98892	1.805166	1.315613	16.44768	5.720173	6.945152	3.747035	26.25557	5.493182
0.765606	2.378124	4.317648	1.434921	4.667518	1.910974	0.659163	3.037626	3.043528
0.634531	0.581111	0.474574	0.836017	1.039798	0.326421	0.419076	0.464127	0.639113
0.171472	0.270924	0.092915	0.134076	0.302554	0.10132	0.457749	0.079387	0.10886
1.072843	4.043846	1.53684	3.310758	4.839105	3.217612	4.557605	3.885642	1.491977
4.770231	19.68088	11.89932	3.493362	14.88584	1.556392	12.03267	17.92188	34.84299
0.634531	0.828167	1.205685	0.143968	0.250823	0.251573	1.519322	4.468009	10.73084
8.348817	65.94551	46.7056	15.88548	50.55949	4.997417	26.25557	80.6518	51.52195
1.177994	0.602554	2.380079	1.059553	1.803918	0.701818	0.82772	0.57197	1.377446
1.767431	0.396306	0.805238	5.123781	0.598795	0.752229	0.135606	0.445209	0.7257
1.837172	0.65241	0.740101	0.578817	0.799553	1.233665	0.494841	1.485483	2.238341
1.000794	5.515584	18.48514	2.187395	16.44768	3.91355	6.345382	20.68746	23.16622
1.082179	3.034747	10.67991	0.000245	3.23987	0.546522	0.430281	3.404372	5.788153
0.992201	5.881342	4.155339	1.443059	5.34474	0.118644	0.471746	2.468117	4.053506
0.497749	1.198701	0.706494	0.403413	0.57461	0.346342	0.220079	0.532381	0.295837
0.561775	1.307417	2.421681	0.619365	1.644416	0.166291	0.794401	0.555418	1.710173
0.765606	0.002857	0.010346	0.777403	0.067547	0.955418	0.032172	0.225079	0.002056

0.46153	0.55741	3.957734	0.106609	0.700837	0.214805	0.340245	0.84	0.914618
2.642525	144.7919	332.0776	0.081126	332.0776	67.98069	62.33472	17.15902	425.62
0.46153	0.096371	0.010346	0.277763	0.309221	5.28039	1.268672	0.070339	0.138644
0.144163	0.058773	0.010346	0.045714	0.048528	0.138326	0.040375	4.33E-05	0.002056
0.067864	0.235137	0.489509	0.106609	0.096263	0.092302	0.145909	0.144293	0.160332
0.781977	19.83727	83.489	1.664906	19.53061	1.109726	1.727323	2.589185	60.77449
0.238059	0.889553	1.425613	1.022929	1.511198	0.457431	0.319365	0.876638	1.346991
2.252273	0.639495	0.27075	1.888023	0.290693	0.895945	0.190693	1.057569	0.315743
3.019848	2.955916	1.146118	4.091667	1.717641	2.652107	2.515938	2.388348	2.129596
0.163521	0.002857	0.08044	0.143968	0.011962	0.176104	0.000188	0.005411	0.002056
5.967879	1.881955	2.02956	5.462475	2.148658	7.315512	3.695815	3.310758	2.461905
0.246479	1.465123	4.212554	0.355556	1.707576	0.000101	0.1086	0.628297	2.66404
2.690823	0.428932	0.382208	8.958225	0.372504	4.348882	2.043795	0.744531	0.129913
0.271912	0.224661	0.12132	0.134076	0.036934	0.057027	0.040375	4.33E-05	0.097879
4.174964	7.288788	10.38202	2.375974	9.218514	1.997352	2.165851	4.739668	17.92188
1.44E-05	1.664906	3.975498	0.000245	2.735368	0.214805	1.191198	1.826147	0.851234
2.102626	0.55741	0.163766	1.323131	0.36267	1.452857	0.419076	0.451977	0.831472
3.953297	1.525729	3.775209	5.381227	3.407619	5.195022	6.706472	2.681169	6.967547
0.038485	0.378066	0.27075	0.022814	0.16925	0.019942	0.155224	0.478737	1.434921
0.002367	0.002857	0.107749	0.000245	0.036934	0.008882	0.018167	4.33E-05	0.002056
1.39847	2.29873	2.6143	1.313737	1.310837	0.709784	1.869322	2.882035	3.921645
0.656753	0.002857	0.010346	0.268687	0.000173	0.231876	0.256046	0.04785	0.002056
0.520289	6.191515	5.416623	1.337431	4.440548	0.483326	2.845036	6.028427	8.524242
0.645036	0.002857	0.315916	0.610224	0.709784	0.241053	0.706984	0.540902	0.819048
12.49452	8.048095	11.30987	2.690823	6.135007	2.174733	1.483622	7.737807	12.10289
5.259437	21.02638	6.956349	12.63773	10.28573	7.112496	11.30987	10.1028	6.717056
0.255887	0.175592	0.149019	0.419365	0.187381	0.326421	0.155224	0.108997	0.160332
3.851414	2.378124	1.242641	8.319019	1.399315	4.292078	3.779452	1.300368	1.05824
0.347655	3.011169	2.577157	1.376378	2.860722	0.908247	1.284747	1.186457	1.721371
2.225498	4.016465	1.219856	1.618773	1.978954	1.934271	2.189235	1.400159	1.074827
1.713997	0.2129	0.675938	3.327619	0.242453	1.572035	0.387056	0.386854	0.138644
1.102691	0.24632	0.010346	1.953535	0.151082	0.512309	0.182929	0.364242	0.210505
2.281926	1.928629	9.751039	4.397792	4.343838	10.78056	10.82954	7.112496	1.850996
3.233045	21.19547	42.08807	7.468961	24.73724	3.842554	7.315512	10.43066	31.55417
0.113838	41.47925	24.50942	0.882078	39.53687	3.33728	31.55417	22.52375	18.05488
0.067864	0.096371	0.010346	0.064012	0.000173	0.064812	0.025007	4.33E-05	0.002056
4.752121	1.989711	2.329784	1.826147	4.644098	27.66779	6.171789	2.032706	1.422316
4.359574	7.013853	6.028427	6.612121	7.911934	6.632063	8.677489	10.82954	6.663651
0.083896	0.714286	2.203535	0.258059	0.591912	0.10132	0.263304	1.186457	0.743694
56.39528	12.03267	51.52195	6.464906	15.46193	3.144156	9.459177	20.16541	62.33472
0.710722	0.500354	0.231659	0.510072	0.34697	0.268961	0.220079	0.3414	0.160332
0.271912	0.002857	0.010346	0.500996	0.027547	0.423781	0.064812	0.017345	0.002056
0.163521	2.29873	0.939408	2.060491	1.855108	1.484553	1.842734	0.48684	0.49974
3.536955	0.970635	0.405346	6.287561	0.512641	3.107417	0.967446	0.937655	0.425426
2.506934	2.320072	0.463102	5.056421	1.573276	5.530274	3.55583	1.218182	0.306811
0.431457	0.224661	0.010346	0.258059	0.160779	0.241053	0.667583	0.281364	0.002056
0.944084	5.044113	5.195022	1.277843	4.02088	1.03303	1.810664	1.770029	1.085967
0.067864	0.047792	0.12132	0.996696	0.019048	0.082872	0.018167	0.009978	0.002056
2.136335	3.754719	8.019769	3.8379	14.17388	8.075772	19.22139	4.38684	5.42381
0.561775	1.045462	1.578687	0.682359	0.9529	0.73961	0.494841	0.621688	2.199639
0.402698	0.002857	0.010346	2.996869	0.000173	0.268961	0.000188	4.33E-05	0.002056
2.737944	3.236457	2.242092	9.179466	2.567605	9.101674	11.17281	2.558817	0.831472
1.44E-05	111.5439	155.7103	0.000245	135.5245	0.000101	33.4985	27.96248	184.443
2.325931	13.15079	11.74734	2.509177	5.210317	1.712706	5.80469	6.404921	12.63773
0.33785	0.083795	1.108947	0.567994	0.036934	0.064812	1.392511	2.141595	1.282208
2.917381	7.824473	10.82954	4.188896	6.591544	1.734949	3.276335	4.490801	8.319019
37.29684	40.7932	14.08569	25.24367	52.56128	72.69473	100.0998	25.99427	12.16681
0.017063	0.002857	0.010346	0.143968	0.000173	0.057027	0.018167	0.025411	0.002056

1.501291	0.148701	0.22096	1.351573	0.354242	9.502294	0.934488	0.564127	0.365859
0.076465	0.002857	0.195685	0.836017	0.000173	0.176104	0.040375	4.33E-05	0.002056
0.814315	6.240346	7.443276	1.376378	6.497266	3.581688	5.20267	0.640606	6.011515
0.186465	0.002857	0.382208	0.449091	0.036934	0.026609	0.073211	0.413802	1.094978
1.04228	1.759986	1.08671	0.268687	1.759986	2.855296	4.990794	2.254084	2.287576
0.790303	2.365685	3.833247	0.682359	1.930216	0.316126	2.805382	2.673846	2.621133
1.39847	3.074134	1.489524	0.355556	2.187395	3.034747	5.788153	2.544747	7.288788
19.99861	16.34294	8.711905	24.50942	14.6058	17.28195	22.96463	25.24367	10.43066
6.830592	10.1459	21.19547	7.911934	11.74734	9.256537	65.94551	22.8563	23.6012
0.129365	0.002857	0.231659	0.022814	0.087165	0.206768	0.064812	0.093622	0.649235
2.810678	0.55741	1.334762	5.180058	3.414574	13.19051	8.412367	15.66023	9.179466
15.18102	2.481356	2.043795	35.27608	2.417424	5.754935	3.370527	5.25254	2.341573
0.69119	1.636118	0.987821	1.376378	1.085967	0.654877	1.960945	1.968283	1.080303
2.941544	1.560779	0.489509	6.945152	0.700837	2.876421	1.921212	0.607605	0.819048
1.161501	1.582915	0.884654	0.329798	2.593506	4.222583	11.25392	4.508196	1.634935
0.6657	0.538117	0.195685	1.157626	0.42925	0.423781	0.527821	0.274704	0.061385
2.281926	1.335743	1.027403	1.216768	1.519322	0.457431	0.48684	4.266753	1.815
0.090188	0.002857	0.010346	0.34697	0.000173	0.110188	0.000188	4.33E-05	0.002056
6.464906	2.628225	0.876638	9.418196	2.747908	18.33909	6.956349	1.087345	0.95101
5.500678	1.432302	1.197215	5.779574	1.1407	2.341573	1.790245	5.075447	0.578095
0.992201	0.65241	0.532381	0.2871	0.517092	0.73961	0.844747	0.773276	2.755411
1.455022	0.270924	0.358211	0.904856	0.242453	0.895945	0.190693	0.663276	0.261227
1.177994	0.124646	0.010346	1.25816	0.000173	1.374329	0.299553	0.093622	0.002056
0.090188	0.096371	0.135144	0.073867	0.000173	0.231876	0.145909	0.260144	0.002056
1.600238	5.102742	6.088593	0.268687	6.107114	0.859351	1.620058	1.869322	4.025267
3.926154	2.043795	4.814661	0.240671	3.192229	1.080303	2.335844	1.623232	8.923117
0.006046	0.002857	0.010346	0.030577	0.000173	0.057027	0.018167	0.005411	0.050577
12.29473	1.248232	0.22096	4.959358	0.106118	5.821804	1.006025	1.014127	0.376782
1.919661	1.805166	1.253088	3.4679	2.323983	1.222179	1.873434	3.002576	1.346991
2.337821	1.074242	0.431977	3.68381	0.817547	1.616385	0.82772	2.35733	0.595022
2.078355	14.00414	6.486104	7.468961	10.48101	2.078355	11.89932	7.598312	6.162446
0.144163	2.65684	2.002193	1.313737	1.950519	0.512309	2.189235	0.671724	1.047345
0.136573	0.224661	0.22096	0.777403	0.203175	0.222864	0.271746	0.215115	0.200382
0.814315	10.05618	6.325945	0.376212	9.141299	21.19547	5.967879	0.398059	0.698687
0.203954	0.369105	0.419076	0.23101	0.523521	0.546522	0.896522	0.40645	0.507085
3.754719	1.096335	1.357201	3.29355	1.50342	1.296046	2.415296	2.5271	2.461905
0.090188	0.419365	0.257042	0.268687	0.340866	0.377085	0.535945	0.079387	0.087807
0.656753	0.639495	0.431977	6.028427	0.512641	1.533535	0.976667	0.835498	0.200382
9.968355	0.742417	0.566782	8.660281	0.9529	3.169812	3.101371	8.164531	0.443781
4.338629	1.411861	0.715462	3.966623	1.540144	1.159149	1.143175	1.80267	0.540563
1.767431	11.42512	3.450577	0.000245	12.77772	0.000101	19.83727	21.54823	12.56693
0.954141	3.980123	7.765931	1.758615	5.130411	1.200823	1.945916	2.230859	4.101061
0.727633	1.350772	0.419076	2.31404	1.267879	0.305253	0.449726	0.800967	0.129913
3.043528	1.881955	0.209401	5.273362	1.556392	4.077403	0.896522	1.759986	1.020447
7.938427	7.363117	5.47	9.838658	7.061328	3.792266	8.508514	11.60283	6.591544
4.38684	0.828167	0.730476	7.061328	0.65101	3.016984	1.069033	0.564127	0.794401
7.38847	4.535354	4.72798	12.16681	2.109755	23.82354	6.956349	7.683766	2.755411
0.059459	0.47153	1.852338	0.163983	0.73619	0.092302	0.163038	0.451977	0.794401
0.710722	0.058773	0.179798	0.297532	0.067547	0.026609	0.056623	0.2443	0.325317
3.493362	91.05541	203.5795	55.04081	60.77449	24.2842	77.74812	44.27012	287.197
0.6657	0.602554	0.370462	1.826147	0.438312	1.533535	1.035058	0.946061	1.167561
0.378326	3.533211	2.513752	1.25816	2.676392	1.869322	4.557605	1.162201	1.534603
0.144163	0.2129	0.010346	0.62733	0.000173	1.048802	0.000188	0.167013	0.002056
0.855801	16.09482	2.654473	1.426522	10.1459	2.104358	3.276335	2.148658	1.510014
2.577157	17.92188	167.9996	6.486104	22.32214	3.691724	8.780115	10.1459	167.9996
0.023608	0.058773	0.010346	0.038139	0.000173	0.013867	0.018167	0.025411	0.002056
1.441898	5.323492	5.553146	1.216768	3.13456	0.403413	1.542302	2.069942	1.905094
0.911905	2.558817	12.16681	1.415469	4.266753	1.670786	4.236962	6.830592	6.622092

2.879228	2.361198	0.772626	3.192229	1.406746	2.628225	1.580909	2.217951	1.643218
0.645036	2.398442	2.432121	1.172872	1.357201	0.717403	25.73165	0.650101	3.086306
6.210375	3.249877	0.884654	11.68846	2.051962	7.08733	3.418405	3.629177	3.201457
0.738312	0.889553	0.586595	3.500808	0.340866	1.616385	2.676392	0.683232	0.531169
4.984488	5.439278	1.596782	11.2819	8.164531	64.12971	32.68313	4.603434	1.188139
1.744055	0.096371	0.010346	0.55741	0.048528	0.611003	0.000188	0.033146	0.002056
0.504964	0.224661	0.092915	0.393983	0.077417	0.261111	0.073211	0.478737	0.306811
3.083319	5.976724	7.938427	1.25816	6.019971	10.8813	29.82828	4.130794	4.519048
32.31123	83.489	20.34063	100.0998	62.33472	37.29684	27.66779	46.7056	11.48368
23.06543	40.11561	42.75576	45.1045	48.53429	19.68088	22.32214	36.74522	45.91841
38.39042	11.09133	3.300664	48.53429	16.85982	87.1746	48.53429	13.30906	2.890245
27.38726	31.1753	12.49452	32.31123	16.09482	12.13485	6.601833	14.25954	15.88548
23.82354	64.12971	33.93939	60.77449	33.93939	13.08214	24.50942	51.52195	25.73165
6.706472	12.77772	6.162446	9.880779	6.171789	5.409437	5.056421	10.53159	6.830592
749.7839	749.7839	252.4251	87331.38	749.7839	530.1465	530.1465	530.1465	203.5795
111.5439	203.5795	144.7919	332.0776	184.443	252.4251	144.7919	252.4251	87.1746
332.0776	59.32013	100.0998	70.11932	29.18885	55.04081	80.6518	203.5795	224.9555
425.62	25.99427	23.16622	203.5795	29.82828	144.7919	23.6012	45.91841	41.47925
6.560411	5.062626	3.070952	7.002056	1.601443	3.128694	1.702706	3.052655	2.556443
1.253088	0.930837	0.405346	1.337431	0.607273	2.384141	1.035058	0.876638	0.455649
21.74051	1.553074	0.939408	23.6012	1.727323	7.468961	2.933903	4.160238	1.886335
11.544	7.697063	4.198117	3.377619	7.653391	2.593506	4.105714	15.3618	11.65791
4.63189	0.083795	0.08044	9.101674	0.019048	1.516219	0.396638	0.174646	0.07039
0.163521	0.071429	0.010346	0.258059	0.058117	0.305253	0.018167	0.017345	0.002056
7.188095	1.489524	0.996696	5.217569	1.549711	4.18456	0.623853	1.544517	1.217475
1.44E-05	0.24632	0.474574	0.000245	0.709784	0.064812	0.190693	0.564127	0.346342
1.68938	1.600238	6.423911	2.584394	3.711097	1.428398	1.277056	1.650274	2.868456
0.163521	0.925801	0.695426	0.20215	1.414574	0.559221	0.527821	0.252922	0.540563
29.49693	4.170051	3.230065	46.7056	4.857403	22.96463	8.815123	13.75978	3.8121
1.734949	7.315512	10.28573	1.497143	3.24658	1.187229	1.097677	6.55039	5.545729
11.36975	0.704848	0.636688	4.440548	0.38127	1.997352	0.55741	2.157359	1.047345
0.22039	0.058773	0.010346	0.081126	0.048528	0.013867	0.000188	0.040469	0.061385
0.617763	0.859885	0.81544	1.187229	0.785714	0.316126	0.169538	0.819978	1.01544
2.436768	3.266782	5.662994	0.532092	2.747908	2.413146	1.162201	2.925426	2.836717
10.62739	26.80355	28.23632	26.80355	37.29684	24.98218	38.94854	40.7932	30.82277
55.04081	32.31123	31.1753	28.54786	21.02638	15.77678	25.99427	67.98069	80.6518
9.604358	6.784762	12.03267	19.75908	8.711905	9.335397	8.075772	7.6393	5.821804
15.46193	2.542605	2.402468	5.395584	2.371833	4.165137	2.644885	2.773644	1.602619
1.089394	0.096371	0.010346	0.923874	0.187381	2.24562	0.396638	0.108997	0.002056
28.87506	23.71237	29.49693	21.19547	18.91854	8.461703	11.65791	30.82277	19.68088
0.548788	0.838846	0.557056	1.127475	1.016494	1.048802	1.006025	0.299444	0.480022
8.940671	6.508427	1.948918	17.98838	2.826277	6.749488	4.715599	0.754784	4.952915
34.38341	14.65384	14.33939	80.6518	13.45939	29.18885	11.96574	9.880779	10.53159
2.810678	0.961486	1.119574	0.500996	0.900693	0.786667	0.512641	0.918557	1.104805
1.322092	0.55741	0.532381	1.195079	0.809481	1.997352	1.25886	0.471025	0.253492
14.08569	2.341573	4.715599	14.25954	5.238701	5.24562	3.394286	5.044113	4.745895
2.607035	2.494177	1.108947	2.442987	1.119574	0.346342	0.209538	1.02487	2.063362
0.186465	0.058773	0.179798	0.480794	0.036934	0.305253	0.190693	0.3414	0.181674
1.017107	0.224661	0.257042	0.836017	0.067547	0.643853	0.330866	0.613211	0.171183
4.130794	0.419365	0.243124	1.99899	0.324502	2.089639	0.684033	0.613211	0.346342
0.954141	0.071429	0.010346	0.308788	0.222698	0.138326	0.280678	0.800967	0.656392
0.595714	2.29873	1.073543	2.901032	0.91404	1.751948	1.633752	0.613211	1.623232
2.941544	1.149978	2.647244	1.801284	1.906551	3.29355	1.056898	1.170491	0.616739
14.79742	2.280101	2.678781	13.53913	2.083261	2.718434	0.190693	2.755411	2.890245
0.1214	0.986104	2.922778	0.000245	0.871429	0.146768	0.430281	1.493968	1.003016
0.006046	0.096371	0.195685	0.124343	0.067547	0.10132	0.073211	0.087504	0.002056
6.663651	10.93101	8.256465	7.264502	11.544	8.288874	15.07257	9.502294	8.677489
0.475433	0.688579	0.648362	0.599134	0.833975	0.622496	0.771176	0.621688	0.522367

0.011551	0.002857	0.010346	0.096905	0.000173	0.048716	0.018167	4.33E-05	0.002056
0.292799	2.240274	1.834423	0.931457	1.297763	0.546522	1.078925	1.896667	0.718355
5.84689	4.445851	6.240346	1.323131	4.029654	2.440895	13.91714	3.518384	14.42661
58.58447	1.421126	0.489509	27.66779	1.230411	38.39042	4.72798	1.412771	0.939986
3.47531	9.707677	4.827691	3.885642	7.824473	2.561162	3.394286	1.989711	1.412771
0.974257	0.910339	0.22096	2.035678	0.844156	2.384141	1.330974	0.744531	0.66526
4.456912	8.412367	17.78522	2.014863	9.377345	2.104358	2.23267	11.83593	24.73724
11.65791	4.145209	4.077403	8.445743	2.898196	1.50342	1.613232	3.283139	7.991602
2.547179	1.411861	0.981032	2.024488	1.113304	1.280274	0.808485	1.272756	1.815
0.634531	0.137201	0.179798	1.148564	0.000173	0.196097	0.000188	0.160072	0.200382
7.765931	6.819481	6.956349	7.854069	4.529913	6.706472	4.614618	6.125823	3.707136
87.1746	1360.293	425.62	425.62	287.197	80.6518	184.443	425.62	135.5245
0.186465	0.002857	0.010346	0.449091	0.000173	0.064812	0.000188	0.124459	0.129913
77.74812	17.28195	13.38418	64.12971	20.16541	65.94551	56.39528	45.1045	13.79669
3.276335	1.742734	0.96938	5.075447	1.34267	2.078355	1.078925	1.820541	1.05824
2.895599	0.670519	0.675938	2.337821	0.65101	2.24562	0.419076	0.458045	0.355794
6.464906	9.792626	29.18885	6.772763	9.335397	4.408153	6.00228	5.266335	15.276

CGGA_13	CGGA_14	CGGA_14	CGGA_14	CGGA_14	CGGA_14	CGGA_14	CGGA_14	CGGA_14
0.339235	0.341053	0.343824	0.198694	0.355051	0.742417	0.082323	0.824885	1.348975
0.339235	0.020722	0.841746	0.900693	0.591616	4.33E-05	0.780519	0.659163	0.722626
2.367763	1.953535	1.480541	3.703175	0.037302	3.659668	2.131176	0.05824	0.744531
4.251818	1.581912	6.55039	10.43066	2.345527	3.211176	4.174964	0.802756	0.412193
5.095325	2.792035	4.096407	7.839271	1.809343	8.677489	3.140866	2.173081	4.552092
7.08733	1.842734	8.226219	2.947287	6.796566	3.971061	1.303961	7.049336	18.19595
32.31123	18.91854	31.92385	65.94551	25.73165	31.1753	17.28195	29.82828	25.99427
0.035007	0.2129	0.049769	0.121147	0.094733	0.005072	0.003413	0.068175	0.004964
5.150693	3.273045	3.276335	6.116638	0.948211	4.198117	8.477662	0.78619	0.368038
0.147908	0.020722	0.477698	1.111977	2.461905	4.33E-05	0.003413	1.748016	1.065584
18.48514	9.583463	42.08807	33.93939	30.49289	16.09482	10.78056	57.84882	126.1556
1.50684	0.341053	2.600014	2.609293	3.166659	0.525678	0.21619	3.276335	3.340361
9.256537	33.93939	6.591544	15.56131	2.628225	11.42512	31.92385	2.563506	2.261573
4.33E-05	14.70188	2.89E-05	8.643074	0.00026	10.05618	30.49289	0	1.44E-05
0.013766	0.2129	0.049769	0.040563	0.146768	0.005072	0.092121	0.086522	0.035007
3.846984	7.495397	1.661328	3.676032	0.700837	6.784762	5.942294	0.680173	2.392431
1.950519	0.409618	4.198117	2.019618	5.22482	0.650556	0.257518	4.323045	3.482785
0.035007	0.020722	0.049769	0.112323	0.050426	4.33E-05	0.003413	0.017778	0.074286
0.560325	15.77678	0.217908	1.111977	0.294921	2.261573	15.66023	1.160707	1.44E-05
4.101061	0.400354	3.391075	0.525678	0.413449	0.323146	0.583369	1.485483	1.505678
0.283535	7.911934	0.469358	0.494185	0.00026	0.489235	6.38456	0.291861	0.130029
2.485534	53.77619	1.092136	1.130115	0.43381	1.357201	35.776	0.224762	0.256205
56.39528	4.644098	51.52195	16.80343	37.29684	25.48179	0.003413	45.91841	53.77619
0.06772	0.23412	0.104149	0.023009	0.156089	0.044719	0.152532	0.001147	0.026724
1.446926	0.862561	2.92013	1.446926	2.481356	1.64917	0.100375	2.109755	1.318384
5.830152	3.518384	2.987835	6.727633	0.43381	3.91355	1.780693	0.597792	1.1629
0.147908	9.218514	0.916869	3.833247	0.294921	5.25254	8.381328	0.05824	1.113961
0.347403	0.66482	1.009625	0.34544	1.541227	0.191133	0.305657	0.78619	0.438831
19.68088	70.11932	18.91854	14.97762	1.044235	70.11932	87.1746	0	16.04187
1.289149	1.184185	0.883665	0.595368	0.413449	0.396638	1.094978	0.474214	0.31171
4.292078	16.80343	3.450577	1.209776	1.274481	6.738211	10.01009	1.805166	1.662439
0.195563	0.409618	0.296234	0.070736	0.591616	0.044719	0.226623	0.261111	0.209841
9.771833	37.81919	6.011515	5.679502	0.00026	6.570433	46.7056	0	1.44E-05
0.007965	0.020722	0.057352	0.014545	0.183939	0.026537	0.003413	0.371876	0.066205
4.376118	0.293723	9.751039	4.655916	57.84882	0.396638	0.003413	40.11561	9.063983
4.540794	1.256558	3.540606	3.482785	1.044235	1.880375	6.55039	0.597792	0.165108
4.277006	6.876392	4.155339	4.077403	3.47531	3.552049	4.312251	3.851414	0.912446
0.162576	0.020722	0.469358	7.22E-05	0.00026	4.33E-05	0.003413	0	0.567619
2.961768	2.392431	6.443925	4.802655	1.412771	5.830152	1.935685	4.563117	3.521934
4.946587	3.659668	8.539971	8.958225	24.73724	4.302367	3.150433	5.923838	14.25954
0.331263	0.020722	0.811861	1.89518	1.39847	0.176104	0.970007	1.222179	3.192229
1.938759	0.895945	1.272756	1.819221	2.823636	0.659957	0.826407	3.457504	0.965714
0.468694	4.702468	2.89E-05	0.595368	0.165512	1.120332	3.783694	0	0.165108
4.33E-05	0.020722	2.89E-05	0.007864	0.016609	4.33E-05	0.003413	0.006948	0.004964
0.268095	0.28189	0.137201	3.016984	0.365411	0.46153	0.395642	0.891176	0.165108
45.1045	24.50942	29.18885	30.82277	11.60283	62.33472	21.54823	11.25392	33.07147
0.213059	0.162576	0.137201	0.26562	0.183939	0.119423	0.053838	0.139697	0.05767
0.026364	0.020722	0.287821	0.079957	1.183478	4.33E-05	0.100375	0.465079	0.31171
4.33E-05	0.020722	0.027056	0.007864	0.00026	4.33E-05	0.072193	0.001147	0.004964
0.007965	0.020722	0.200382	0.090476	0.81596	0.010945	0.003413	0.648961	2.618846
4.33E-05	0.020722	0.071696	0.014545	0.610606	4.33E-05	0.092121	0.391421	0.081212
13.00996	17.15902	4.789596	3.450577	1.482626	19.06893	14.79742	1.872049	4.115844
0.339235	0.270924	0.320563	0.177973	1.511198	0.010945	0.003413	0.586934	0.474214
0.06772	0.020722	0.232583	0.227446	0.267388	0.110642	0.003413	0.269892	0.20127
3.747035	18.62711	0.883665	1.191905	1.183478	1.002215	4.33342	0.659163	0.155743
0.355556	0.020722	0.492417	0.525678	1.194221	0.533535	0.21619	0.982698	1.035058
1.432302	0.270924	0.894704	0.984069	1.145426	0.332886	0.082323	0.329091	0.70886

0.88417	1.073543	0.62689	1.191905	1.560779	0.396638	0.713405	2.597792	1.449877
1.738456	0.888485	2.223636	4.077403	2.584394	2.106089	0.899567	2.597792	3.119986
0.681919	0.23412	0.327403	0.763102	0.355051	0.248341	0.226623	0.310267	0.05767
34.38341	26.25557	9.418196	7.22E-05	0.00026	31.55417	52.56128	0	21.74051
0.310657	0.377814	0.313102	0.456522	0.335967	0.101991	0.50469	0.168167	0.147554
0.42925	1.005426	1.077403	3.36075	1.118817	0.208571	0.21619	0.484704	0.294271
19.22139	10.1459	10.33476	39.53687	16.5625	35.776	8.643074	8.745238	6.717056
0.183939	0.368038	2.89E-05	0.198694	0.05886	0.388023	0.305657	0.041443	0.209841
0.004387	0.11803	0.003066	7.22E-05	0.016609	0.005072	0.082323	0.001147	0.002157
1.301169	0.862561	1.860491	1.748016	1.605859	1.624459	1.376378	1.416407	1.960945
1.069574	2.032706	1.37531	2.609293	0.844747	2.097843	2.061926	0.41026	1.000173
1.03189	0.28189	0.402381	1.031284	3.644343	0.302352	0.188586	1.089394	0.836537
0.195563	0.020722	0.873088	0.286681	0.483326	0.148016	0.206061	0.586934	0.182929
0.392554	0.020722	0.579495	0.366147	1.130115	4.33E-05	0.003413	0.553348	0.358903
1.267085	0.66482	0.62689	0.29715	0.725267	0.119423	0.4971	0.360462	0.688975
0.043499	0.020722	0.003066	0.014545	0.037302	0.005072	0.053838	0	0.002157
4.33E-05	0.140418	2.89E-05	7.22E-05	0.016609	0.005072	0.003413	0.011602	0.05767
3.540606	6.738211	4.120815	17.28195	38.39042	14.79742	12.22798	7.724084	2.74544
0.355556	0.020722	0.217908	0.112323	0.454632	0.010945	0.003413	0.196811	0.338095
0.007965	0.128283	0.015541	0.112323	0.022958	0.03526	0.053838	0.02482	0.165108
4.586753	2.23267	6.900202	5.599228	7.991602	4.328232	1.604827	4.900996	8.048095
0.035007	0.020722	0.119683	0.090476	0.413449	0.03526	0.003413	0.251573	0.182929
0.633853	0.223701	0.615996	0.237035	0.833975	0.599466	0.003413	0.853218	0.752229
6.30671	22.96463	5.273362	6.210375	12.03267	4.397792	19.99861	12.10289	2.638139
4.33E-05	0.020722	0.020462	0.014545	0.05886	0.03526	0.092121	0.02482	0.013766
0.522633	0.223701	0.763564	0.535368	1.232886	0.199134	0.082323	0.876638	0.868853
20.51271	36.26061	7.212915	167.9996	32.31123	7.468961	35.27608	3.957734	5.696263
0.392554	0.020722	0.638362	0.276429	0.394877	0.2757	0.543939	0.280678	0.104791
0.365224	0.400354	0.296234	0.584134	0.700837	1.403853	0.206061	0.495931	0.752229
0.42925	0.997792	0.287821	17.39935	1.330029	0.845887	1.499214	0.434329	0.229048
0.741962	0.198788	0.305253	0.26562	0.385238	0.259336	0.247951	0.508341	0.528997
0.506328	0.162576	0.155361	0.049531	0.873088	0.044719	0.082323	0.224762	0.218817
2.511465	0.617417	0.424228	7.22E-05	0.898543	0.489235	1.185685	0.032915	0.049719
7.112496	4.392316	4.096407	9.924372	4.096407	9.751039	2.805382	1.132165	1.44E-05
0.977994	0.020722	0.841746	0.254964	0.635541	0.130115	0.003413	3.236457	0.567619
0.870375	0.787136	1.020447	0.763102	0.534452	0.22456	0.38596	1.196508	0.926926
4.47912	0.303564	4.990794	1.541227	4.535354	0.754315	0.109654	0.41026	7.625209
0.043499	0.020722	0.264805	0.049531	0.124791	4.33E-05	0.092121	0.003102	0.004964
7.264502	8.608153	4.451154	2.459812	2.903867	8.780115	5.606739	0.269892	4.096407
0.051414	0.020722	0.145216	0.070736	0.174444	0.062244	0.053838	0.319365	0.209841
0.007965	0.128283	0.034242	0.049531	0.076587	0.110642	0.053838	0.101905	0.081212
4.33E-05	0.020722	0.010101	7.22E-05	0.00026	0.010945	0.21619	0.003102	0.008759
3.172864	1.044863	2.083261	1.916782	4.058514	1.336587	1.360866	2.225498	1.348975
4.978182	5.576219	14.33939	8.868932	15.18102	8.075772	4.888023	11.83593	2.834084
2.223636	0.427937	11.89932	4.150274	10.28573	0.958557	0.275527	8.381328	2.410996
1.053528	0.293723	0.734524	0.159495	0.294921	0.302352	0.206061	0.352222	0.338095
0.162576	3.005281	0.16575	1.329206	0.085887	1.300368	3.269913	0.196811	0.20127
0.250476	0.331869	0.424228	7.22E-05	1.673254	0.070967	0.286681	0.959062	0.049719
0.400173	0.020722	0.343824	0.787987	0.375743	0.044719	0.003413	0.760433	0.319762
0.419076	23.6012	0.690043	0.481104	1.07127	2.071587	18.62711	0	0.089235
0.384156	0.427937	0.774257	1.359177	2.958773	0.55741	0.152532	2.540462	1.492973
1.717641	1.190491	3.464315	2.569957	1.383911	1.388543	1.185685	5.00404	5.095325
1.927042	0.020722	1.32482	0.858297	0.681919	3.606804	0.427937	0.771688	1.481631
1.301169	0.562107	3.373983	3.414574	1.009625	1.532468	0.072193	0.597792	1.471198
4.33E-05	1360.293	2.89E-05	35.776	0.326912	105.1694	1360.293	0.215115	0.120823
0.183939	1.880375	0.155361	0.867814	2.563506	2.242092	2.515938	1.269495	0.677403
0.324019	0.184877	0.560325	0.584134	1.685678	0.053297	0.188586	0.508341	0.492165
2.386248	203.5795	2.89E-05	36.74522	0.00026	28.54786	184.443	0	1.44E-05

0.365224	0.573665	0.513752	0.314913	0.998312	0.062244	0.257518	0.352222	0.265339
0.484423	0.020722	0.296234	0.070736	0.394877	0.148016	0.003413	0.495931	0.174372
2.524892	2.50469	2.382035	1.475469	2.276407	3.092229	0.713405	2.540462	3.672078
21.74051	11.60283	10.05618	11.86763	1.578687	5.25254	9.838658	3.610707	2.518124
0.506328	0.787136	0.68062	0.040563	0.236089	0.167013	0.583369	0.434329	1.471198
9.667316	2.269134	13.38418	4.077403	6.171789	6.028427	1.600238	9.968355	11.19942
3.391075	0.303564	2.893001	1.763889	3.028629	0.633853	0.130714	5.068831	4.174964
2.640332	0.960866	5.630815	2.515938	8.608153	2.565556	0.26562	3.566753	9.438687
5.439278	2.404618	21.74051	3.6029	5.80469	0.541241	3.163506	15.98892	13.83359
0.324019	0.543939	0.554416	0.697085	0.473853	0.845887	0.737857	0.530851	1.426522
0.051414	0.020722	0.104149	0.139567	0.094733	0.053297	0.003413	0.02482	0.066205
1.525729	0.511032	0.841746	0.06031	0.236089	0.302352	0.833535	0.352222	0.501905
0.355556	0.341053	0.256205	1.541227	0.591616	0.51039	0.247951	0.17733	0.245772
10.57984	6.404921	24.04374	4.535354	30.82277	6.162446	5.273362	17.51961	11.83593
0.195563	0.020722	0.240202	0.070736	0.076587	0.208571	0.003413	0.111068	0.285079
8.348817	1.953535	2.820974	1.236248	0.413449	2.143261	3.962179	0.521068	0.483045
0.784372	0.409618	0.513752	1.07127	1.700354	0.81596	0.358672	1.307417	0.912446
0.013766	0.020722	0.027056	0.040563	0.094733	4.33E-05	0.142698	0.086522	0.004964
12.39704	3.917597	3.489899	2.050404	29.18885	2.060491	1.444906	36.74522	15.66023
4.33E-05	0.895945	0.095996	0.567345	0.183939	0.790303	1.820541	0.068175	0.623853
0.051414	0.23412	0.469358	0.040563	0.712929	0.019048	0.003413	1.222179	1.386681
0.268095	2.90658	1.33285	2.569957	0.355051	3.1857	5.813247	0.738716	0.804675
4.869553	1.548723	4.338629	6.153102	1.312706	1.21215	0.960866	2.685786	4.130794
2.394416	7.136537	0.925801	4.875527	1.355527	2.524892	5.00404	0.63873	0.349812
0.259033	1.0386	0.16575	1.446926	0.029928	0.888485	0.635541	0.205931	0.174372
3.552049	2.470317	4.752121	1.934271	2.903867	2.635685	2.337821	11.48368	8.445743
3.699495	3.160022	8.164531	4.965801	21.02638	2.474834	2.267157	16.34294	11.14619
2.300678	6.830592	2.011501	0.770664	0.276061	2.122799	8.888167	0.077244	0.113074
0.42925	3.334199	0.209156	0.049531	0.00026	1.880375	5.737056	0.006948	0.319762
1.927042	2.993867	1.21215	3.738997	0.246825	2.607035	2.410996	1.828781	1.772756
6.39474	4.451154	8.508514	5.330519	8.288874	15.66023	6.088593	7.049336	8.288874
0.140548	8.477662	0.894704	7.22E-05	0.00026	0.692381	5.687879	0.280678	1.44E-05
12.22798	10.43066	7.264502	11.09133	8.061934	12.49452	22.32214	6.53969	5.796421
7.570693	26.52537	2.914632	4.397792	0.655245	9.141299	27.10502	0.802756	3.297107
0.035007	0.020722	0.015541	0.014545	0.085887	4.33E-05	0.063478	0.003102	0.019726
1.480541	1.128131	2.42381	2.863211	1.685678	1.613232	1.329206	0.771688	1.379278
6.695887	0.473586	10.43066	7.417359	26.52537	2.565556	0.38596	19.22139	7.558139
2.034242	2.011501	2.097843	0.237035	1.90798	0.692381	3.588369	1.03189	2.089639
1.557482	0.341053	1.558571	0.219481	0.833975	0.156486	0.645036	0.876638	0.403175
3.279625	1.298665	1.035058	0.707799	0.103622	3.381255	5.373781	0.319365	1.126775
4.33E-05	126.1556	2.89E-05	0.504964	0.00026	12.16681	100.0998	0	0.237035
26.80355	95.22939	11.65791	30.17192	0.00026	64.12971	118.7028	0	1.44E-05
2.59557	0.768052	1.62562	3.36075	1.022929	0.742417	0.516306	1.633752	1.379278
0.522633	0.020722	0.320563	0.177973	0.929286	0.019048	0.063478	0.565426	0.386378
1.602619	1.331919	2.834084	0.943514	3.895469	1.237879	1.438672	2.096162	3.890556
1.691587	4.207778	4.857403	0.606154	1.641999	0.702835	5.024293	1.066948	2.463911
1.41368	0.162576	1.197215	0.752583	0.43381	1.57645	0.305657	0.521068	1.318384
0.204531	0.020722	0.744113	4.328232	0.00026	1.067662	0.003413	0	2.021068
7.882222	7.468961	6.64254	26.52537	20.16541	18.91854	8.677489	8.643074	5.238701
1.172208	0.2129	0.672078	0.545426	1.022929	0.341241	0.176558	0.78619	0.616739
1.727323	0.020722	0.68062	0.254964	0.473853	4.33E-05	0.003413	1.748016	0.642698
9.179466	2.290996	14.70188	11.86763	7.519841	4.46267	1.666089	13.30906	14.65384
1.393571	1.024127	1.391494	1.370765	0.599134	2.024488	0.944784	2.449509	2.24733
1.118066	0.173059	2.363442	0.943514	1.624459	0.240671	0.003413	0.391421	1.906551
0.838326	0.2129	1.305772	0.504964	1.252424	0.363492	0.899567	2.392431	0.926926
3.792266	4.029654	5.591717	0.276429	0.404704	5.25254	7.264502	0.399307	4.130794
0.162576	0.020722	0.16575	0.129553	0.493247	0.140087	0.003413	0.982698	0.089235
0.118644	0.020722	0.615996	0.26562	2.252273	0.026537	0.082323	0.824885	0.956667

0.213059	0.377814	0.079921	0.101429	0.305455	0.517359	1.921212	0.474214	1.113961
3.625382	3.552049	7.025599	1.50684	2.703485	12.36443	2.993867	0.508341	4.620159
4.082165	1.524776	6.25938	6.52899	2.640332	3.435988	1.162201	5.606739	8.164531
38.94854	32.31123	33.93939	11.96574	1.07127	40.7932	80.6518	2.723247	19.14516
1.717641	1.505678	1.650274	1.692763	4.179762	1.995714	1.930216	1.307417	0.876118
3.482785	16.44768	2.89E-05	5.25254	3.754719	4.552092	11.719	0.876638	1.44E-05
1.289149	0.28189	2.349351	0.889019	2.845036	0.2757	0.427937	1.828781	1.278629
4.198117	24.73724	4.563117	5.737056	7.653391	11.14619	10.98644	10.38202	5.47
6.39474	0.644235	8.365072	6.071436	12.67271	1.954993	0.627937	16.9162	5.381227
11.83593	3.975498	12.16681	11.48368	7.38847	8.888167	5.508131	11.60283	11.4544
1.783203	0.360101	3.644343	3.243225	3.521934	1.148564	0.176558	5.485685	2.65684
17.39935	3.8379	15.77678	11.544	22.03348	5.373781	3.037626	14.6058	5.9071
0.757273	0.360101	1.009625	0.37417	0.465563	0.878369	0.944784	0.802756	0.653586
3.763045	2.000592	2.382035	4.614618	4.927302	2.355289	1.739935	2.597792	1.006025
0.283535	0.778326	1.471198	0.139567	1.89373	0.140087	0.003413	1.341825	0.349812
1.856392	1.638499	1.849654	1.995714	3.058802	1.591299	1.401068	1.434921	0.265339
1.253088	5.754935	0.645455	0.837403	0.803211	0.824416	7.625209	0.565426	0.403175
1.37531	1.174315	2.489762	1.680895	5.302294	2.034242	1.260635	4.814661	3.457504
4.33E-05	0.020722	0.00544	0.031299	0.114113	0.005072	0.072193	0.121941	0.035007
0.004387	1.51215	0.12904	6.287561	5.172828	2.635685	0.627937	1.974228	0.049719
12.77772	2.914632	9.459177	7.037345	12.77772	6.162446	2.494177	11.03843	7.112496
2.238341	0.020722	2.54961	1.90798	2.481356	0.951652	0.003413	1.553074	1.662439
0.811861	0.020722	1.21215	0.187381	0.521068	0.140087	0.583369	0.251573	0.70886
7.839271	5.259437	6.191515	10.33476	2.903867	5.050267	4.381479	4.921082	4.370671
10.70538	3.707136	3.391075	4.927302	10.8813	4.655916	3.877417	7.225469	4.292078
0.384156	0.020722	0.232583	0.112323	0.103622	4.33E-05	0.003413	1.372475	0.773276
0.162576	0.258268	0.287821	0.416219	0.344733	0.183413	0.226623	0.906825	0.303564
1.359177	7.532713	1.263752	0.955418	1.118817	2.164113	8.888167	1.119574	0.926926
0.659163	0.020722	0.873088	1.541227	7.598312	0.888485	0.003413	1.697792	2.483492
0.551284	0.617417	0.975556	0.567345	0.655245	0.313312	0.675938	1.731169	0.698687
3.984747	7.795642	0.925801	5.737056	0.00026	5.599228	15.07257	0.565426	1.724805
1.075411	0.602908	1.064242	0.912446	0.976667	0.824416	0.516306	1.842734	0.847352
0.331263	0.23412	0.104149	0.384632	0.473853	0.176104	0.003413	0.891176	0.285079
10.23768	10.53159	7.938427	1.995714	0.670945	11.60283	19.68088	0.553348	4.739668
2.451645	0.53469	2.725498	1.236248	0.81596	1.310837	0.109654	1.816436	1.781948
4.644098	8.573434	3.592063	1.797172	1.685678	2.970144	5.71241	1.916782	2.955916
0.082027	0.2129	0.057352	0.06031	0.146768	0.005072	0.003413	0.011602	0.019726
0.147908	0.020722	0.434863	0.416219	0.662237	4.33E-05	0.003413	0.02482	0.965714
0.082027	0.020722	0.174098	0.040563	0.183939	0.09176	0.003413	0.474214	0.377323
0.977994	1.031284	0.376017	0.29715	0.146768	0.293276	3.486436	0.269892	0.377323
0.88417	0.128283	6.325945	0.889019	8.976508	0.217172	0.072193	14.33939	18.05488
21.54823	13.15079	25.99427	35.27608	1.927042	38.39042	7.737807	16.80343	43.49658
15.3618	3.618499	12.49452	33.4985	17.28195	15.56131	3.833247	20.34063	4.997417
0.622496	0.466602	1.705007	1.525729	0.305455	1.57645	0.723066	1.553074	2.572309
2.485534	4.557605	2.181775	2.162381	1.171349	3.640606	3.330909	3.975498	2.820974
1.009625	0.020722	0.353016	0.35676	0.276061	0.080779	0.003413	0.041443	1.772756
0.484423	0.020722	0.402381	0.428196	1.29267	0.167013	0.100375	0.553348	0.623853
1.329206	2.353247	4.297222	21.36892	0.988377	35.27608	17.51961	0.329091	27.96248
1.315613	6.443925	0.959567	3.540606	1.261407	1.256558	5.075447	1.616385	0.303564
2.491948	0.854733	2.121198	1.145426	0.670945	2.509177	0.960866	2.440895	3.307186
6.653016	1.499214	11.09133	9.335397	15.41187	2.715873	1.080303	22.32214	10.19468
0.162576	0.400354	0.296234	0.456522	1.842734	0.979827	0.770188	1.007244	0.358903
0.140548	12.70768	1.048074	0.35676	1.083651	3.36075	13.08214	1.697792	1.615281
0.941104	33.4985	2.89E-05	7.22E-05	1.330029	11.09133	39.53687	1.01772	14.88584
0.355556	0.020722	0.191804	0.139567	0.560866	0.019048	0.003413	0.291861	0.319762
70.11932	28.23632	20.51271	23.6012	0.00026	111.5439	25.99427	1.160707	4.435159
7.911934	2.24562	2.789466	2.66404	0.547778	2.723247	0.528997	1.713997	3.414574
3.807648	0.223701	4.048499	3.869163	9.52197	2.153535	0.237215	2.895599	2.561162

0.988377	0.020722	2.54961	0.668456	5.872886	0.958557	0.003413	2.828918	0.377323
8.134084	1.581912	3.35355	4.586753	16.44768	0.770664	1.041652	5.454949	0.513456
1.994329	4.764055	0.568369	2.66404	1.700354	2.164113	4.016465	1.32482	1.02487
1.969733	11.65791	1.351573	25.48179	10.1459	10.1028	41.47925	1.660216	1.44E-05
46.7056	167.9996	9.377345	31.1753	0.610606	80.6518	167.9996	3.043528	16.68554
1.938759	0.020722	2.320072	7.22E-05	0.00026	1.599033	0.003413	1.395563	5.044113
2.23267	0.184877	2.463911	0.955418	2.8657	0.650556	0.537518	3.327619	3.414574
0.58925	0.020722	2.382035	0.079957	4.077403	0.499986	0.003413	7.570693	4.397792
0.019351	0.198788	0.057352	0.070736	0.05886	0.026537	0.152532	0.05824	0.002157
0.859885	0.020722	3.606804	1.50684	3.201457	0.2757	0.176558	4.644098	5.323492
0.035007	1.094206	0.034242	0.149156	0.547778	0.471977	0.833535	0.049221	0.066205
8.815123	111.5439	2.89E-05	7.327482	0.00026	12.63773	144.7919	0	1.44E-05
3.223882	4.012049	4.370671	4.217569	1.216768	15.07257	6.268312	2.427792	3.566753
0.408427	0.63114	1.136566	0.535368	0.257042	0.481602	0.324019	1.222179	0.599466
1.432302	0.96938	2.847547	6.268312	7.315512	1.550837	1.952027	3.629177	2.373824
3.718716	0.981558	5.976724	3.286652	4.626025	0.419076	0.50469	4.721789	3.192229
3.975498	2.000592	8.573434	7.212915	8.429055	2.132756	1.791616	10.28573	4.644098
2.165851	0.701818	3.110447	2.089639	3.407619	0.624841	0.286681	3.655714	1.553074
0.941104	1.488571	1.806595	1.574517	0.976667	0.803211	1.084437	0.553348	0.386378
2.470317	0.53469	1.401068	0.955418	1.780693	0.867287	0.305657	2.967453	2.903867
1.795851	31.1753	1.224733	0.514531	2.740519	16.9162	21.36892	4.261342	3.022713
0.400173	0.020722	7.339452	1.25816	13.83359	0.754315	0.003413	24.50942	13.23022
2.440895	15.276	4.241905	22.96463	4.796126	12.03267	28.54786	0	0.130029
0.162576	0.312626	0.137201	0.023009	0.044113	0.070967	0.092121	0.006948	0.081212
1.545657	0.740527	2.181775	3.188918	2.845036	1.954993	0.749481	4.261342	7.795642
4.603434	1.805166	3.55961	2.755411	4.655916	3.533211	3.297107	3.327619	3.243225
0.082027	0.511032	0.209156	0.254964	3.116732	0.03526	0.296955	1.355527	0.386378
14.51648	24.04374	10.23768	21.74051	4.978182	22.32214	33.4985	6.191515	13.15079
3.763045	6.695887	3.944084	2.917381	1.624459	5.967879	3.089293	3.7586	3.022713
4.814661	2.898196	3.489899	1.273636	1.927042	1.517143	0.960866	7.795642	10.73084
0.118644	0.020722	0.334235	0.031299	0.267388	0.101991	0.247951	0.30158	0.294271
7.351284	12.42965	10.82954	30.49289	8.319019	12.70768	9.218514	7.653391	9.815642
10.8813	20.34063	1.948918	0.697085	0.204661	5.395584	22.74797	0.310267	1.706219
155.7103	31.55417	167.9996	50.55949	1360.293	167.9996	22.13245	111.5439	135.5245
0.2757	0.749019	0.801501	0.707799	0.81596	0.363492	0.142698	0.360462	0.58925
8.089848	5.923838	12.93651	0.29715	0.394877	20.34063	3.049603	3.147294	25.48179
24.2842	23.16622	11.544	8.210938	3.493362	18.62711	29.82828	10.03313	18.48514
0.051414	0.020722	0.240202	0.384632	0.624841	4.33E-05	0.003413	0.853218	0.483045
4.33E-05	0.162576	2.89E-05	0.014545	0.00026	4.33E-05	0.119322	0.003102	1.44E-05
1.226934	5.855281	0.506003	0.878369	0.236089	3.128694	11.89932	0.669769	0.191962
9.880779	29.82828	7.765931	18.19595	1.641999	50.55949	67.98069	4.261342	17.34065
0.060173	0.020722	0.155361	0.031299	0.076587	0.019048	0.092121	0.05824	0.035007
0.089553	0.951652	0.705231	1.748016	0.214805	0.780952	0.810967	0.371876	0.992201
0.259033	0.020722	0.62689	0.384632	1.107605	0.217172	0.469004	1.007244	0.303564
0.408427	0.245325	0.200382	0.040563	1.216768	4.33E-05	0.003413	0.78619	0.327403
8.164531	3.037626	11.19942	9.968355	6.454416	7.710361	1.669466	22.74797	20.16541
0.821414	0.685209	2.063362	0.72044	5.075447	0.633853	0.38596	2.993867	3.077316
3.101371	19.37139	5.630815	7.074329	5.044113	12.26136	13.15079	3.432388	4.927302
77.74812	67.98069	67.98069	33.07147	27.96248	57.84882	18.76954	42.08807	100.0998
4.33E-05	0.020722	0.392749	7.22E-05	0.483326	4.33E-05	0.003413	1.222179	0.395426
0.147908	0.140418	0.402381	0.06031	0.493247	0.03526	0.130714	0.434329	0.294271
35.27608	16.9162	46.7056	75.1132	50.55949	36.74522	16.22108	65.94551	34.84299
2.255916	1.90798	2.477027	1.191905	4.667518	0.860404	1.545657	2.270837	2.322035
0.339235	0.151587	0.568369	0.481104	0.624841	0.119423	0.286681	0.771688	0.73583
2.08798	1.915455	5.25254	1.475469	1.541227	1.094978	0.645036	1.828781	10.23768
0.060173	0.198788	0.320563	0.818586	1.29267	0.355556	0.188586	2.671299	3.401126
5.788153	4.997417	7.897078	2.442987	3.621941	4.222583	0.583369	5.959033	17.34065
0.821414	0.787136	0.256205	0.770664	3.632973	0.208571	0.163038	1.283911	0.847352

60.77449	4.978182	87.1746	40.7932	118.7028	21.02638	7.061328	91.05541	111.5439
0.074603	0.020722	0.200382	0.121147	0.276061	0.010945	0.003413	0.121941	0.551573
0.88417	5.287857	0.774257	5.088694	0.225079	1.880375	6.591544	0.802756	0.616739
0.132799	0.020722	0.071696	0.014545	0.022958	4.33E-05	0.003413	0.003102	0.008759
1.423506	0.913514	2.513752	1.779423	3.201457	0.341241	0.600173	5.030866	5.165599
17.15902	2.925426	16.68554	13.60866	23.38378	8.134084	1.105426	44.27012	27.66779
1.660216	3.428788	5.462475	5.28039	4.292078	2.230859	0.553059	9.335397	9.297085
0.026364	0.020722	0.434863	0.34544	1.86658	4.33E-05	0.003413	0.78619	0.139235
3.192229	3.581688	1.21215	7.795642	2.252273	2.474834	1.244957	2.199639	0.501905
1.401977	2.535974	1.902352	2.417424	1.252424	1.21215	2.101075	1.518232	1.01544
0.543131	0.740527	0.857778	0.867814	1.541227	0.191133	0.488997	1.974228	1.541227
9.968355	12.56693	12.85504	15.66023	1.877605	27.38726	13.72059	7.417359	5.553146
1.50684	0.523211	0.240202	0.595368	1.972706	1.067662	0.723066	0.959062	0.836537
2.112843	8.134084	1.020447	1.097006	0.898543	2.384141	4.292078	0.553348	0.113074
0.250476	0.23412	0.952294	1.721371	0.988377	0.248341	0.176558	0.802756	0.789423
21.93452	25.73165	19.68088	20.16541	27.66779	24.73724	27.66779	23.38378	23.6012
2.577157	15.46193	1.732468	6.865	3.957734	5.42381	13.38418	0.78619	1.348975
0.302554	0.128283	0.544877	1.359177	0.493247	0.313312	0.072193	0.919192	2.032706
9.707677	3.457504	17.78522	5.165599	24.2842	4.048499	3.614603	29.49693	29.82828
37.81919	72.69473	15.276	32.68313	14.17388	67.98069	70.11932	9.502294	10.5063
0.58925	0.903074	0.916869	2.23267	1.750628	1.890996	0.275527	6.297136	1.318384
0.355556	0.511032	0.313102	0.525678	0.700837	0.396638	0.618658	0.669769	0.688975
1.401977	1.404812	12.36443	5.150693	26.80355	1.779423	0.600173	28.23632	13.08214
1.147807	0.466602	0.952294	7.112496	2.129596	6.200945	2.329784	5.42381	0.796674
4.359574	10.33476	5.462475	5.28039	0.114113	10.21618	16.9162	1.713997	4.484892
4.38684	2.681169	6.144055	9.101674	14.65384	3.944084	2.574733	4.971991	4.978182
3.214545	1.360022	3.676032	2.961768	4.702468	0.702835	0.458341	4.105714	2.139928
0.007965	0.020722	0.027056	0.023009	0.044113	0.005072	0.142698	0.011602	0.002157
11.89932	15.88548	23.38378	15.46193	20.51271	20.51271	12.77772	64.12971	24.04374
5.030866	4.165137	4.789596	6.99026	1.750628	5.662994	6.738211	3.877417	3.077316
2.349351	1.1629	1.315613	0.932078	0.670945	3.699495	2.781558	1.948918	2.089639
11.42512	3.256587	9.707677	1.680895	27.38726	5.095325	4.160238	4.764055	2.8657
7.737807	2.053521	5.993045	6.045209	6.967547	4.484892	2.925426	6.622092	5.130411
0.458701	0.020722	0.774257	0.314913	1.232886	0.148016	0.072193	0.876638	0.58925
0.534214	0.28189	0.939408	0.040563	0.681919	0.167013	0.247951	0.340866	0.155743
0.293276	1.146962	0.925801	1.733766	2.290996	1.21215	1.340036	1.974228	2.628225
2.209141	1.51215	2.5271	2.529307	4.039192	0.541241	0.86987	2.489762	1.986681
1.557482	0.846797	1.593535	2.217951	2.016515	4.33E-05	0.003413	0.41026	1.758615
12.63773	80.6518	2.820974	27.66779	1.721371	30.49289	111.5439	2.139928	3.144156
1.267085	9.397771	1.112641	0.697085	0.948211	1.223846	8.134084	0.540563	0.81544
0.534214	0.491876	0.744113	6.38456	2.217951	0.702835	0.516306	1.143954	0.20127
4.058514	1.106046	10.19468	5.560563	6.250447	1.300368	1.340036	8.849697	6.900202
1.135137	0.020722	1.37531	11.14619	21.19547	1.718918	2.254084	15.276	3.763045
3.122915	1.581912	6.612121	3.450577	0.493247	4.965801	2.638139	3.957734	14.97762
19.37139	3.792266	36.74522	17.92188	17.39935	13.53913	4.933781	38.39042	37.81919
3.428788	10.67991	3.695815	287.197	59.32013	15.18102	10.1028	10.21618	5.01772
4.435159	38.94854	1.235346	2.035678	0.700837	9.541645	53.77619	0.280678	2.347439
1.082915	2.369841	0.492417	0.514531	0.443304	1.491977	1.172208	0.669769	0.81544
4.317648	4.863579	9.121486	7.653391	4.508196	5.20267	4.145209	11.30987	5.553146
6.09759	0.990909	2.725498	3.966623	6.454416	0.834416	0.257518	2.013182	2.990851
0.132799	0.020722	0.327403	0.34544	0.085887	0.549019	0.206061	0.891176	0.57531
0.060173	0.184877	2.89E-05	0.112323	0.05886	0.03526	0.053838	0	1.44E-05
5.316378	1.842734	3.750837	14.56114	11.09133	4.06303	1.739935	9.377345	2.800332
17.92188	6.55039	19.29639	22.13245	30.0001	14.42661	3.807648	24.16397	18.33909
0.004387	0.11803	0.00544	7.22E-05	0.022958	0.005072	0.063478	0	1.44E-05
0.2757	0.128283	0.137201	0.159495	0.156089	0.080779	0.206061	0.929286	0.513456
64.12971	3.8121	126.1556	48.53429	62.33472	34.84299	8.508514	144.7919	155.7103
1.423506	0.360101	2.207417	1.748016	0.276061	1.054899	0.342215	2.597792	3.703175

0.043499	0.020722	0.049769	0.101429	0.029928	4.33E-05	0.003413	0.139697	0.002157
42.75576	16.09482	49.50903	10.8813	52.56128	19.83727	6.674286	75.1132	45.91841
7.697063	1.267085	3.507691	5.770996	2.978968	5.130411	1.143954	4.575123	4.33342
0.019351	0.258268	0.015541	0.210505	0.225079	0.053297	0.176558	0.130924	0.035007
0.310657	0.151587	0.857778	0.139567	2.203535	0.259336	0.003413	3.78798	2.223636
0.235455	0.66482	0.155361	0.405346	3.944084	0.156486	0.247951	1.682193	0.826407
87.1746	24.2842	332.0776	105.1694	224.9555	48.53429	24.73724	332.0776	1360.293
20.85876	2.159004	13.83359	6.423911	45.1045	3.144156	1.489524	16.09482	11.4544
22.13245	6.30671	26.95429	20.51271	33.07147	11.48368	4.783001	34.84299	36.26061
5.889798	1.821861	2.404618	0.752583	0.624841	1.169091	5.25254	0.205931	0.722626
203.5795	30.49289	1360.293	13.23022	24.04374	184.443	65.94551	41.47925	287.197
13.6814	5.553146	25.24367	23.16622	10.5063	12.26136	4.241905	17.39935	17.64869
2.800332	1.565253	4.266753	2.145137	4.317648	3.230065	0.882619	5.622872	6.191515
0.648362	0.020722	0.579495	2.201587	1.044235	0.355556	0.450678	3.588369	0.428932
2.367763	2.325931	4.575123	1.165065	1.591299	0.860404	0.666097	2.993867	5.287857
6.784762	3.404372	9.502294	10.23768	18.33909	3.944084	1.700354	14.25954	12.42965
95.22939	6.663651	100.0998	2.683478	10.43066	14.33939	0.863694	25.73165	83.489
2.647244	0.184877	5.737056	0.237035	2.703485	4.33E-05	0.003413	3.711097	2.638139
1.539062	0.96938	1.989711	4.484892	2.048781	4.145209	0.656392	3.276335	9.583463
12.39704	40.7932	44.27012	18.33909	39.53687	21.19547	27.38726	30.49289	30.82277
62.33472	5.942294	70.11932	87.1746	95.22939	42.08807	5.11697	105.1694	44.27012
3.163506	0.81228	4.965801	6.612121	15.88548	0.268369	0.846797	8.958225	6.079596
18.05488	2.434444	24.2842	13.30906	12.93651	19.53061	3.68381	21.93452	18.91854
3.55961	0.198788	3.792266	3.993665	6.30671	2.847547	0.592532	3.204632	1.348975
16.5625	48.53429	8.195657	19.83727	1.465123	19.22139	38.39042	5.9071	4.203001
0.534214	0.020722	0.296234	1.65785	2.683478	0.929286	1.014127	6.749488	0.677403
1.969733	11.96574	4.323045	1.246457	1.29267	7.339452	12.85504	0.340866	1.01544
30.49289	39.53687	18.33909	37.81919	4.207778	26.80355	45.1045	9.902576	11.57341
0.250476	0.020722	1.351573	0.227446	3.058802	0.130115	0.003413	3.363911	1.270317
0.622496	0.728088	0.82974	0.92342	1.927042	0.499986	0.351595	3.107417	1.426522
5.9071	8.256465	2.789466	4.552092	1.145426	3.659668	2.735368	1.872049	3.592063
10.19468	1.968283	6.853608	0.395152	4.317648	0.824416	1.750628	2.90658	2.192496
2.176501	0.140418	2.5271	0.72044	0.413449	0.906126	0.275527	0.41026	3.340361
3.66785	3.046551	3.179199	2.019618	1.90798	3.855952	4.397792	2.373824	6.268312
0.007965	0.020722	2.89E-05	0.040563	0.029928	4.33E-05	0.003413	0.011602	1.44E-05
23.6012	6.475505	40.7932	16.09482	38.94854	14.00414	6.784762	60.77449	38.94854
87331.38	17.03919	87331.38	126.1556	87331.38	83.489	26.80355	87331.38	530.1465
287.197	105.1694	252.4251	252.4251	167.9996	126.1556	75.1132	224.9555	144.7919
1.573276	0.491876	1.37531	1.50684	0.763102	0.489235	0.176558	1.616385	1.603795
22.32214	45.1045	19.99861	31.55417	22.96463	33.4985	44.27012	37.81919	28.54786
0.392554	0.020722	1.29267	1.154567	0.547778	0.2757	0.003413	0.78619	1.652771
1.557482	7.854069	3.779452	2.802828	5.416623	2.048781	9.541645	4.678846	9.335397
0.339235	0.81228	0.320563	1.062821	0.988377	0.633853	0.286681	0.30158	0.599466
0.089553	0.020722	0.174098	2.34355	7.013853	0.191133	0.130714	1.32482	0.130029
0.043499	0.020722	0.015541	0.159495	0.124791	4.33E-05	0.003413	0.068175	0.074286
2.068297	0.293723	3.962179	21.02638	11.2819	6.09759	0.944784	4.702468	9.968355
4.557605	5.477843	3.767489	224.9555	70.11932	8.958225	9.792626	17.64869	3.26
0.035007	0.020722	0.209156	0.129553	0.246825	0.005072	0.003413	0.17733	0.147554
33.28499	51.52195	22.52375	95.22939	19.99861	100.0998	64.12971	16.22108	46.7056
0.204531	0.020722	0.232583	0.031299	0.037302	4.33E-05	0.082323	0.086522	1.44E-05
10.33476	7.264502	17.03919	60.77449	15.276	18.33909	4.519048	26.52537	10.62739
0.051414	0.020722	0.568369	0.643427	0.521068	0.053297	0.003413	1.842734	0.465325
2.705916	3.640606	1.136566	1.236248	1.017107	2.042201	2.873817	0.760433	1.189769
0.213059	0.020722	0.087619	0.276429	0.294921	4.33E-05	0.109654	0.391421	0.066205
4.33E-05	0.020722	0.020462	7.22E-05	0.016609	4.33E-05	0.063478	0.001147	0.004964
1.253088	0.020722	3.089293	0.149156	4.408153	0.080779	0.003413	0.574993	1.603795
16.34294	21.02638	33.4985	41.47925	19.22139	38.94854	14.6058	25.48179	35.776
12.03267	7.188095	17.15902	3.6029	10.82954	9.335397	6.695887	14.51648	25.73165

11.48368	6.045209	21.19547	4.900996	16.68554	6.277937	5.11697	31.92385	27.38726
5.22482	5.959033	6.297136	8.539971	5.630815	6.64254	5.195022	5.123781	10.03313
1.983824	2.74298	0.615996	3.435988	0.725267	1.517143	2.673846	0.891176	1.189769
0.035007	0.151587	2.89E-05	0.007864	0.00026	4.33E-05	0.072193	0	0.002157
11.544	4.484892	28.54786	16.44768	48.53429	8.226219	2.289286	70.11932	87.1746
23.82354	42.75576	18.62711	10.38202	21.54823	18.19595	34.1614	19.83727	21.19547
2.267157	0.020722	3.046551	0.467951	0.465563	1.367179	0.801501	2.845036	6.749488
2.121198	2.280101	2.50469	2.035678	0.91575	3.518384	1.207374	1.32482	1.906551
1.525729	0.927561	1.934271	0.847908	1.412771	1.200152	1.041652	2.45575	3.829105
10.07949	3.179199	13.45939	13.38418	9.356371	8.923117	3.269913	10.82954	11.25392
0.957915	0.184877	0.841746	2.331825	5.454949	0.471977	0.275527	5.366335	2.005253
3.407619	2.524892	4.096407	3.644343	0.385238	5.737056	5.872886	1.380195	1.471198
15.77678	11.719	14.08569	18.91854	36.26061	9.880779	5.075447	23.82354	28.87506
22.52375	2.802828	26.95429	67.98069	18.62711	15.276	10.48101	12.77772	2.970144
0.007965	0.128283	0.095996	0.37417	6.374812	4.33E-05	0.100375	0.715815	0.165108
9.459177	17.39935	16.80343	19.68088	6.876392	28.87506	10.8813	9.541645	10.35839
0.400173	0.020722	0.327403	0.070736	0.385238	0.010945	0.063478	0.540563	0.446551
24.04374	2.101075	24.98218	18.62711	36.74522	1.972706	1.409942	20.16541	31.92385
0.283535	0.11803	1.035058	0.305253	0.050426	1.1407	0.332049	0.62689	1.1629
2.74298	5.838499	2.685786	7.710361	0.560866	6.830592	4.557605	1.697792	8.319019
0.543131	0.523211	0.506003	0.96627	1.659033	0.617071	0.675938	2.758088	0.58925
1.612056	3.24658	0.225823	1.965657	0.858297	1.830159	1.641999	0.434329	0.256205
6.464906	5.976724	17.64869	8.319019	46.7056	1.267879	1.692763	45.1045	14.00414
0.10381	0.020722	0.256205	0.112323	1.482626	0.010945	0.053838	0.615584	0.762165
10.48101	3.06803	25.48179	3.016984	22.32214	13.72059	0.592532	18.69833	47.5018
7.697063	36.74522	4.821176	14.17388	1.29267	23.38378	33.07147	3.7586	3.640606
0.051414	0.020722	0.041472	0.079957	0.194372	0.09176	0.063478	0.121941	0.104791
5.18754	1.867951	7.443276	6.475505	8.660281	2.410996	0.57461	9.218514	14.51648
2.947287	0.427937	6.144055	2.933903	0.787987	1.21215	0.583369	1.468131	5.696263
0.355556	0.173059	0.811861	0.334026	1.145426	0.217172	0.21619	1.731169	1.035058
0.659163	10.28573	0.453932	7.22E-05	0.037302	1.317475	3.860491	0.17733	1.44E-05
1.126075	0.322684	3.022713	1.380195	2.361198	1.624459	0.50469	3.175916	3.993665
7.951371	2.705916	16.5625	40.11561	126.1556	9.297085	3.140866	62.33472	70.11932
1.25886	0.020722	4.392316	72.69473	0.204661	5.447114	1.77404	29.03196	2.842193
1.660216	0.360101	0.916869	1.634935	6.622092	0.81596	0.450678	3.471486	1.449877
0.2757	0.377814	0.119683	7.22E-05	0.591616	4.33E-05	0.003413	0.139697	0.089235
0.302554	0.67632	0.12904	0.456522	0.067864	1.169091	0.003413	0.553348	0.513456
2.005253	0.951652	1.765065	0.633146	1.367179	1.357201	0.675938	2.373824	4.644098
0.043499	0.020722	0.010101	7.22E-05	0.214805	0.03526	0.003413	0.049221	0.074286
4.814661	0.020722	2.725498	0.37417	1.927042	4.33E-05	1.505678	1.890996	1.348975
1.717641	0.020722	1.159913	1.706219	3.621941	4.33E-05	0.003413	2.472576	2.089639
0.317403	0.020722	0.334235	0.079957	1.058896	4.33E-05	0.163038	0.251573	0.026724
0.478182	0.562107	1.948918	1.130115	2.481356	1.739935	0.226623	5.646811	1.837172
0.259033	0.020722	0.607605	0.494185	0.509791	0.313312	0.003413	1.160707	0.104791
0.026364	0.020722	2.89E-05	0.023009	0.103622	0.080779	0.163038	0.041443	0.002157
0.468694	0.322684	0.240202	0.584134	0.225079	0.208571	0.3671	0.233752	0.218817
3.391075	1.239423	2.770866	1.273636	3.381255	2.153535	1.674401	4.18456	3.457504
0.543131	2.925426	0.68062	0.210505	1.232886	1.079697	2.035678	1.089394	0.826407
6.345382	50.55949	8.445743	16.9162	1.721371	7.558139	37.81919	2.339697	4.609076
1.486551	6.99026	1.545657	1.008413	0.423781	5.515584	2.229048	1.066948	2.74544
34.84299	30.82277	24.50942	24.50942	3.236457	118.7028	36.50291	7.443276	42.75576
0.302554	0.258268	0.376017	0.023009	0.473853	0.176104	0.142698	0.727222	0.474214
0.498918	0.511032	0.232583	3.966623	0.305455	0.167013	0.406681	0.63873	0.664026
0.800967	1.944517	1.077403	1.111977	1.89373	0.906126	0.645036	1.416407	2.518124
1.239423	0.827273	4.857403	6.508427	5.381227	1.763889	0.846797	2.323983	0.981558
3.156537	4.678846	1.315613	7.22E-05	0.00026	11.01243	7.288788	0	6.55039
0.004387	0.128283	0.003066	7.22E-05	0.029928	4.33E-05	0.003413	0.003102	1.44E-05
2.616573	1.073543	3.110447	3.414574	4.003117	2.143261	1.434921	4.02088	1.303961

2.068297	0.602908	1.235346	3.993665	0.413449	2.289286	2.465916	0.540563	1.175859
5.47	7.339452	4.016465	2.019618	0.276061	4.49671	8.815123	1.852338	1.439704
4.33E-05	0.020722	0.384394	7.22E-05	0.00026	4.33E-05	0.152532	0.196811	2.173081
6.717056	1.720144	12.56693	6.99026	9.179466	4.978182	2.101075	22.96463	11.06488
8.745238	1.76873	7.558139	3.6029	4.236962	2.668896	2.367763	3.061955	1.706219
1.821861	0.020722	1.535722	0.818586	2.740519	0.672857	0.003413	1.212792	0.412193
0.932078	1.154567	1.272756	7.22E-05	0.413449	0.55741	0.723066	0.280678	2.392431
0.250476	0.258268	0.444762	0.707799	0.404704	0.285253	0.436017	0.41026	0.492165
3.122915	2.981962	4.821176	3.869163	0.183939	6.250447	6.063276	0.946061	4.016465
1.748016	2.586926	1.340036	1.559675	0.067864	4.115844	4.63189	0.530851	1.256558
33.93939	8.103925	47.5018	34.38341	72.69473	36.26061	8.958225	59.32013	26.80355
1.876212	1.371602	1.650274	0.72044	2.176501	0.499986	1.049517	1.048802	1.084437
0.004387	0.020722	0.015541	0.014545	0.214805	4.33E-05	0.003413	0.02482	0.013766
0.773766	0.322684	0.690043	2.112843	0.88619	0.541241	0.142698	0.648961	0.581645
0.096573	56.39528	0.087619	3.122915	0.103622	5.28039	83.489	0.05824	1.44E-05
4.33E-05	2.858009	0.820931	0.254964	0.00026	2.961768	3.91355	0	1.44E-05
15.98892	11.25392	15.56131	13.45939	6.830592	16.34294	13.83359	18.69833	11.74734
15.88548	27.66779	6.738211	9.256537	0.236089	10.48101	21.93452	0	0.349812
1.246457	0.28189	1.608427	0.787987	3.644343	0.03526	0.003413	3.691724	1.564149
2.333867	41.47925	2.89E-05	17.51961	2.715873	26.25557	20.68746	2.515938	1.44E-05
4.33E-05	31.92385	0.607605	0.34544	0.225079	2.65684	15.276	0	0.130029
5.273362	5.9071	4.165137	5.42381	2.304452	4.039192	3.188918	11.89932	7.653391
0.458701	0.846797	2.453781	1.130115	2.584394	0.408175	0.342215	1.890996	1.516219
0.490664	0.020722	0.320563	0.416219	0.670945	0.208571	0.196667	0.906825	0.483045
3.330909	11.30987	4.06303	3.909033	4.758088	8.412367	11.25392	1.872049	2.045476
3.002576	0.827273	3.855952	0.818586	3.307186	0.388023	0.951652	4.174964	2.29873
0.302554	0.020722	0.240202	0.305253	1.450693	0.396638	0.188586	0.659163	0.275238
0.741962	0.377814	0.724004	0.101429	0.763102	0.541241	0.553059	0.749481	0.599466
11.03843	49.50903	9.792626	9.218514	3.279625	22.96463	59.32013	7.765931	15.18102
3.464315	2.579509	2.668896	2.62342	3.321284	4.025267	2.116032	2.540462	2.990851
1.37531	0.501905	0.588312	0.37417	1.07127	1.460981	0.478737	0.63873	1.672107
1.024127	2.060491	0.663276	135.5245	6.200945	3.984747	2.789466	0	1.44E-05
0.671724	1.05824	0.506003	0.139567	0.136147	0.979827	1.370765	0.139697	0.218817
4.039192	3.493362	3.909033	0.254964	0.267388	9.792626	4.72798	0.251573	3.521934
4.519048	1.013434	3.422237	1.460981	3.926154	2.034242	0.979827	6.876392	1.860491
0.375094	0.020722	0.071696	0.112323	0.581378	4.33E-05	0.003413	1.670786	0.209841
1.226934	0.617417	0.959567	0.584134	2.382035	0.780952	0.592532	1.842734	1.303961
2.08798	1.044863	2.011501	3.122915	1.209105	0.293276	0.003413	0.876638	0.926926
26.25557	4.043846	12.10289	8.815123	5.123781	26.52537	5.770996	15.18102	6.674286
0.118644	0.020722	0.049769	0.079957	0.00026	4.33E-05	0.003413	0.495931	1.44E-05
0.821414	1.204228	0.841746	0.467951	0.194372	1.388543	1.003586	0.484704	0.773276
0.999502	4.193232	0.402381	0.620014	0.501905	1.079697	1.468131	0.738716	0.237035
15.56131	5.638759	4.888023	4.217569	13.53913	11.86763	10.93101	3.404372	6.830592
0.111876	0.140418	0.279315	0.334026	0.753016	0.439524	0.196667	0.959062	0.513456
2.190866	2.16759	1.471198	0.943514	1.436623	2.447294	11.65791	0.853218	0.421342
0.007965	0.11803	0.003066	0.023009	0.022958	0.010945	0.130714	0.001147	1.44E-05
0.765094	0.303564	0.499206	0.428196	0.599134	0.259336	0.462857	0.692381	0.421342
3.110447	3.962179	0.873088	0.606154	2.159004	1.707576	1.57645	1.57645	1.146118
0.551284	1.581912	0.544877	3.273045	1.183478	0.541241	1.340036	1.731169	0.20127
3.192229	0.553059	2.860722	4.376118	4.468009	0.396638	0.003413	3.300664	2.502619
0.035007	0.128283	2.89E-05	0.014545	0.037302	0.010945	0.003413	0.001147	0.013766
4.946587	57.84882	5.606739	530.1465	42.75576	287.197	49.50903	53.77619	8.195657
0.88417	2.781558	1.362713	2.31811	1.009625	2.365685	3.122915	0.866717	0.744531
0.400173	0.151587	0.225823	0.06031	0.284646	0.010945	0.100375	0.291861	0.042713
4.770231	2.815866	11.83593	3.188918	12.56693	2.252273	2.013182	5.838499	21.36892
1.118066	3.373983	2.051962	2.544747	5.855281	2.668896	2.941544	3.869163	2.173081
42.08807	15.18102	27.38726	43.49658	7.456118	75.1132	40.11561	8.241342	25.24367
0.61355	0.020722	1.184185	0.595368	2.602395	0.070967	0.003413	2.90658	3.321284

4.33E-05	2.101075	2.89E-05	7.22E-05	0.00026	0.09176	1.014127	0	1.44E-05
6.171789	0.198788	2.151919	0.456522	0.873088	0.332886	1.525729	1.633752	0.653586
2.470317	1.488571	1.558571	2.24733	2.320072	1.153723	2.289286	1.03189	0.688975
0.283535	0.020722	0.049769	0.286681	1.312706	0.2757	0.003413	0.261111	0.421342
0.019351	0.020722	0.376017	0.023009	0.00026	0.019048	0.003413	0.261111	0.066205
3.6029	24.98218	11.14619	24.2842	2.506934	44.27012	37.29684	0	1.44E-05
2.755411	3.877417	1.99899	2.08013	2.141595	2.876421	7.327482	3.204632	1.906551
1.009625	0.270924	0.894704	0.787987	1.216768	0.979827	0.38596	0.456522	1.200823
5.351934	1.727323	6.876392	5.330519	14.79742	4.609076	1.89961	8.075772	7.315512
0.235455	0.418326	1.136566	1.446926	0.326912	1.388543	0.645036	0.727222	0.722626
8.958225	8.728571	20.85876	11.30987	19.53061	5.754935	5.959033	27.96248	35.27608
1.141515	2.091313	0.402381	2.683478	0.655245	2.114437	17.39935	0.553348	0.089235
0.118644	0.020722	0.217908	0.112323	0.236089	0.044719	0.053838	0.233752	0.174372
0.026364	0.020722	0.010101	0.040563	0.443304	4.33E-05	0.003413	0.159495	0.004964
2.312085	0.778326	1.020447	1.019134	6.853608	0.617071	1.041652	2.373824	1.213434
15.66023	2.159004	15.18102	15.3618	35.776	5.855281	3.230065	24.16397	13.00996
0.859885	0.258268	2.54961	0.697085	8.905642	0.2757	0.565137	3.300664	4.907929
8.412367	0.151587	3.88153	0.324019	7.061328	0.642013	0.706053	8.445743	5.696263
0.659163	1.712706	0.811861	0.395152	0.645455	1.02798	1.593535	0.83697	0.165108
5.638759	4.776407	8.994791	6.808369	4.965801	5.933066	3.625382	12.85504	7.697063
2.026219	6.632063	2.463911	100.0998	17.15902	17.92188	23.16622	0	1.575483
0.317403	0.020722	0.672078	0.149156	0.43381	4.33E-05	0.003413	0.446551	1.035058
0.013766	0.020722	0.00544	0.06031	0.022958	4.33E-05	0.003413	0.011602	1.44E-05
1.691587	0.331869	1.705007	3.581688	4.424416	1.739935	0.092121	2.400455	2.683478
0.051414	0.020722	0.00544	0.014545	0.094733	0.026537	0.003413	0.121941	0.155743
0.773766	1.019791	0.544877	0.159495	0.521068	0.019048	0.537518	0.340866	0.139235
41.47925	5.439278	37.29684	24.98218	184.443	29.18885	12.16681	184.443	50.55949
0.259033	0.020722	1.49075	0.139567	0.712929	0.379791	0.003413	0.399307	0.089235
0.013766	0.151587	0.027056	0.031299	0.05886	0.019048	0.092121	0.02482	0.019726
0.648362	6.135007	0.225823	0.227446	0.591616	1.237879	3.783694	0.150245	0.898016
3.860491	0.020722	11.719	2.802828	0.00026	4.33E-05	0.003413	0	3.926154
0.478182	5.068831	1.705007	1.748016	0.00026	9.707677	16.80343	0.101905	1.056898
4.690159	34.38341	0.444762	2.99987	1.261407	8.195657	29.49693	0.280678	0.513456
0.671724	1.267085	0.384394	0.72044	0.294921	0.935115	1.731169	0.340866	0.483045
2.524892	0.458701	1.035058	1.64684	3.703175	1.161501	0.666097	0.760433	0.474214
2.349351	0.368038	3.88153	0.912446	0.670945	0.968723	0.003413	1.313737	1.837172
5.454949	0.020722	4.546443	0.545426	2.276407	0.233175	0.003413	2.440895	2.173081
0.132799	0.020722	0.041472	7.22E-05	0.534452	4.33E-05	0.003413	0.02482	0.275238
5.614805	3.028629	2.99987	3.703175	4.468009	3.718716	1.226118	5.20267	4.473348
0.478182	0.020722	0.883665	0.92342	0.174444	1.130794	0.163038	0.251573	0.454632
0.606154	0.820447	1.112641	0.878369	1.927042	0.581645	0.092121	0.615584	1.772756
2.540462	0.020722	2.770866	2.302626	1.842734	2.487576	0.003413	3.414574	0.57531
0.096573	0.020722	0.041472	0.139567	0.326912	4.33E-05	0.003413	0.446551	0.174372
7.037345	1.616385	7.238023	3.783694	3.47531	4.38684	5.22482	6.706472	9.541645
0.118644	0.140418	0.041472	0.007864	0.00026	0.140087	0.21619	0.006948	0.104791
3.816551	2.955916	4.63189	1.008413	0.257042	2.197828	2.400455	1.174315	0.956667
3.381255	0.951652	5.217569	1.565253	3.926154	0.720938	1.600238	12.63773	2.270837
1.673254	1.890996	2.873817	0.456522	2.083261	1.090072	0.50469	4.053506	1.074827
0.15456	0.473586	0.485325	0.237035	0.858297	0.053297	0.296955	0.310267	0.358903
4.557605	12.93651	0.663276	2.515938	3.095166	6.888297	10.53159	0.553348	1.44E-05
2.901032	0.351219	2.337821	3.464315	1.736133	1.517143	0.488997	1.341825	1.553074
22.96463	7.339452	5.273362	11.25392	9.141299	16.5625	13.53913	4.323045	8.365072
11.25392	5.821804	4.888023	9.771833	6.335664	11.19942	13.26964	7.625209	3.618499
1.41368	0.020722	4.609076	1.680895	11.19942	0.499986	0.003413	6.99026	2.502619
0.895945	0.501905	0.993521	1.541227	3.058802	0.130115	0.450678	2.810678	1.938759
0.659163	0.020722	0.663276	0.878369	2.740519	0.070967	0.082323	2.225498	1.23127
4.33E-05	15.07257	2.89E-05	0.237035	4.077403	4.312251	16.34294	1.948918	0.074286
2.078355	1.119574	0.392749	1.591299	1.824892	2.176501	1.238651	0.224762	1.44E-05

8.226219	2.337821	6.365065	3.387864	8.149307	5.47	1.593535	8.288874	6.876392
0.977994	0.820447	2.296732	1.031284	3.754719	0.408175	0.478737	3.166659	3.703175
5.295325	4.207778	6.64254	4.130794	2.176501	4.796126	3.55583	3.016984	4.222583
1.329206	0.701818	1.480541	0.26562	0.787987	0.754315	1.499214	1.269495	0.358903
2.415296	0.740527	0.993521	1.488571	0.509791	1.099004	0.351595	1.007244	1.742734
0.162576	28.54786	0.82974	0.112323	0.443304	5.373781	9.751039	0.111068	0.446551
20.34063	14.88584	7.112496	5.20267	8.573434	23.16622	14.51648	33.93939	10.43066
6.191515	11.19942	4.752121	3.428788	18.91854	11.719	3.131544	10.62739	9.160382
4.508196	3.632973	6.079596	5.309264	9.667316	2.735368	3.074134	10.67991	5.150693
0.268095	16.34294	8.728571	0.254964	0.00026	18.76954	17.15902	0.251573	6.414416
1.135137	0.981558	1.723579	2.99987	0.873088	1.412771	0.723066	1.452857	1.742734
2.434444	0.96938	2.642525	1.130115	0.898543	1.850996	0.940563	2.013182	2.941544
1.41368	3.917597	1.661328	1.837172	3.179199	2.269134	2.789466	2.950173	0.858817
0.268095	0.449408	0.607605	0.079957	0.236089	1.819221	0.163038	0.150245	1.724805
0.895945	0.656003	0.763564	0.467951	1.605859	0.396638	0.351595	0.814315	1.303961
0.089553	0.020722	0.597417	1.475469	0.509791	0.09176	0.003413	0.456522	1.175859
0.22039	0.020722	1.112641	0.77925	2.725498	0.062244	0.142698	2.304452	2.445079
0.800967	0.28189	2.705916	0.770664	5.056421	0.60886	0.226623	4.456912	3.279625
0.15456	0.389574	0.232583	0.689509	0.394877	0.191133	0.488997	0.205931	0.403175
0.347403	0.846797	0.568369	1.209776	0.743283	1.274481	0.706053	0.704848	0.541804
4.33E-05	5.606739	2.89E-05	7.22E-05	0.00026	3.767489	3.435988	0	1.44E-05
1.0386	1.668369	6.107114	3.36075	1.842734	2.189235	0.469004	3.566753	1.639639
0.659163	0.481789	3.750837	0.900693	1.809343	0.834416	0.376934	3.432388	1.590202
0.10381	0.377814	0.010101	0.305253	0.136147	0.148016	0.770188	0.011602	0.191962
3.223882	8.365072	1.002215	18.48514	1.700354	7.288788	6.325945	0	1.126775
7.625209	3.418405	9.218514	4.130794	6.760765	7.162698	2.858009	9.667316	8.643074
2.720996	3.303925	1.419278	0.324019	0.963853	0.888485	4.702468	0.17733	0.026724
3.249877	1.51215	3.110447	4.025267	3.028629	3.273045	2.509177	2.323983	2.107922
0.060173	0.020722	1.765065	0.334026	3.131544	0.053297	0.003413	1.682193	1.516219
0.043499	1.073543	0.182713	7.22E-05	0.016609	0.313312	0.810967	0.032915	0.438831
5.553146	1.34798	2.618846	1.209776	2.703485	2.647244	1.555289	1.553074	3.482785
3.718716	1.925635	1.672107	7.519841	0.294921	10.67991	2.180022	0.391421	1.175859
6.277937	10.8813	7.08733	1.616385	2.003723	2.474834	1.312706	7.964315	8.728571
0.268095	0.23412	0.68062	0.26562	1.202626	0.313312	0.305657	1.01772	1.968283
1.024127	0.020722	0.638362	0.276429	0.560866	0.240671	0.063478	0.78619	0.403175
0.671724	0.020722	0.62689	1.177994	0.225079	0.428694	0.163038	1.174315	1.528975
2.281926	0.341053	1.62562	1.65785	4.207778	0.566104	0.376934	1.731169	0.847352
5.056421	2.602395	8.303947	2.162381	10.05618	4.06303	4.833398	1.994329	1.791616
0.082027	0.020722	0.027056	0.079957	0.067864	0.010945	0.003413	0.006948	1.44E-05
0.013766	0.020722	0.027056	0.101429	0.05886	0.010945	0.053838	0.095029	0.074286
2.511465	3.6029	4.667518	3.303925	3.35355	1.696494	2.730072	5.143867	3.860491
3.747035	0.562107	6.933961	3.266782	9.880779	1.21215	0.237215	7.598312	5.47
83.489	10.80505	91.05541	1.329206	12.13485	8.477662	4.678846	9.815642	55.04081
0.923874	0.020722	1.035058	7.22E-05	0.773766	0.348629	0.3671	0.329091	1.213434
0.162576	0.020722	0.095996	0.129553	0.146768	0.026537	0.109654	0.205931	0.191962
1.738456	17.78522	3.089293	1.177994	0.610606	5.933066	14.25954	1.03189	4.222583
5.728615	5.500678	6.760765	6.591544	7.363117	3.407619	8.019769	4.946587	2.322035
3.35715	2.31404	1.99899	1.982208	5.095325	2.060491	1.722475	1.616385	1.778153
0.007965	0.020722	0.104149	0.031299	0.454632	0.010945	0.053838	0.05824	0.096999
0.10381	0.11803	0.027056	0.06031	0.022958	0.010945	0.072193	0.017778	0.002157
2.121198	1.286494	2.073232	1.082179	7.795642	2.060491	1.604827	1.597908	0.885137
3.921645	9.707677	6.25938	0.818586	1.465123	4.222583	11.77567	0.83697	1.672107
0.195563	0.501905	0.392749	0.366147	0.165512	0.60886	0.4971	0.291861	1.652771
3.014076	4.952915	0.334235	6.911486	4.535354	3.346898	3.005281	2.304452	0.623853
0.800967	5.259437	1.020447	1.460981	0.305455	1.460981	3.373983	0.553348	0.245772
1.480541	3.55961	2.668896	2.802828	1.794531	2.341573	2.035678	2.373824	2.903867
0.773766	0.020722	0.82974	0.72044	2.668896	0.248341	0.003413	1.187229	0.567619
2.223636	0.020722	6.819481	0.237035	8.149307	0.191133	0.003413	3.489899	8.958225

2.138131	2.970144	3.373983	1.43039	1.511198	5.530274	1.995714	1.587605	2.021068
0.324019	0.020722	0.079921	0.428196	0.044113	0.026537	0.003413	0.715815	1.035058
0.400173	0.020722	0.939408	1.87899	2.252273	4.33E-05	0.142698	2.597792	1.906551
2.034242	1.184185	0.744113	0.159495	0.645455	2.396429	1.722475	0.946061	1.056898
0.226883	2.477027	0.020462	0.070736	0.165512	1.550837	2.380079	0.422237	0.20127
1.557482	0.573665	1.831631	8.508514	8.429055	3.26	2.355289	5.172828	0.898016
0.250476	0.020722	0.145216	0.079957	0.624841	4.33E-05	0.003413	0.540563	0.349812
1.757244	0.427937	5.062626	1.692763	2.189235	3.172864	0.003413	2.723247	2.77899
1.274481	5.654863	1.362713	0.889019	0.763102	1.357201	2.068297	1.174315	0.698687
16.68554	11.36975	8.923117	15.18102	18.12541	12.56693	18.33909	8.164531	5.34474
0.195563	1.031284	0.638362	0.900693	0.267388	0.713846	1.105426	0.508341	0.05767
0.681919	224.9555	0.907273	0.384632	0.183939	25.73165	57.84882	0	3.156537
6.865	22.32214	2.909293	20.85876	1.641999	13.83359	26.52537	2.540462	2.703485
0.060173	0.020722	0.363983	0.314913	0.103622	0.010945	0.003413	0.521068	0.191962
1.568564	5.381227	0.984069	0.438312	1.877605	1.902352	2.593506	0.936898	1.318384
17.28195	8.958225	16.15795	20.68746	23.16622	14.70188	5.993045	15.61077	6.497266
2.630729	1.763889	0.104149	0.620014	0.473853	1.850996	0.206061	1.03189	1.370765
1.239423	1.867951	0.607605	0.129553	0.194372	1.532468	2.386248	1.01772	1.45912
9.838658	11.89932	4.690159	47.5018	15.66023	13.72059	21.19547	6.591544	2.124574
0.970635	0.020722	0.453932	0.643427	2.628225	0.176104	0.003413	0.738716	1.492973
1.267085	0.787136	1.136566	1.401068	2.048781	1.237879	1.593535	1.255657	1.056898
2.59557	1.428398	4.408153	2.776421	1.412771	1.491977	0.516306	2.800332	6.153102
14.21671	17.64869	4.241905	42.75576	5.762965	17.51961	14.88584	4.678846	29.49693
0.283535	4.25658	0.095996	0.494185	0.05886	0.780952	6.911486	0.187229	0.174372
1.795851	1.794531	1.49075	1.154567	1.29267	1.223846	0.899567	1.341825	1.492973
0.61355	0.438312	0.384394	2.31811	1.482626	0.233175	0.003413	1.504517	0.81544
0.61355	0.020722	0.145216	2.285664	1.145426	0.101991	0.003413	0.692381	0.567619
10.38202	0.943514	14.51648	3.944084	3.563182	6.277937	1.391494	6.819481	3.957734
1.135137	1.1379	0.873088	1.329206	0.483326	2.289286	1.432302	0.474214	0.57531
0.988377	0.749019	0.894704	1.052208	0.963853	1.479452	0.286681	0.606421	2.057056
0.118644	0.140418	0.137201	0.040563	1.183478	0.044719	0.092121	0.121941	1.44E-05
0.283535	0.020722	1.849654	7.22E-05	2.304452	0.22456	0.003413	0.310267	1.23127
2.823636	4.145209	3.540606	0.416219	2.400455	4.63189	3.089293	3.276335	1.379278
0.606154	8.677489	1.573276	0.384632	0.547778	2.396429	10.73084	3.363911	0.677403
2.498478	0.701818	1.68938	1.359177	2.928247	1.130794	0.732633	3.820758	2.903867
4.174964	1.679596	7.402915	14.00414	1.794531	1.176623	1.370765	3.489899	1.303961
0.140548	8.005685	2.89E-05	0.535368	0.534452	0.2757	11.19942	0	0.209841
43.49658	0.757763	48.53429	80.6518	77.74812	37.29684	0.315094	10.78056	52.56128
0.633853	1.05824	1.962424	1.826147	2.382035	1.336587	1.267085	1.928629	5.062626
0.204531	0.322684	0.320563	0.014545	1.770029	0.062244	0.315094	0.906825	0.876118
0.10381	0.020722	0.272244	0.101429	0.174444	0.010945	0.119322	0.233752	0.973232
1.602619	9.751039	0.434863	2.050404	4.424416	1.613232	5.024293	1.776739	0.664026
3.775209	2.369841	8.075772	4.241905	10.1028	3.334199	0.899567	7.468961	8.226219
1.50684	1.794531	5.238701	0.82868	6.153102	1.664906	1.700354	5.23176	2.710765
1.876212	0.368038	0.939408	0.867814	4.039192	0.419076	0.3671	6.53969	4.456912
18.62711	2.735368	28.87506	18.05488	12.13485	13.38418	3.632973	32.8773	22.74797
5.696263	3.367071	2.151919	1.34798	3.846984	1.805166	3.816551	3.489899	1.426522
0.904856	0.020722	1.29267	0.286681	4.130794	0.240671	0.196667	0.971775	1.706219
2.058773	1.286494	3.074134	1.062821	5.889798	1.460981	1.100519	1.948918	1.528975
1.311674	3.233045	0.638362	0.96627	0.610606	1.805166	3.581688	0.319365	0.412193
0.741962	2.306277	1.064242	1.052208	0.670945	2.207417	9.667316	4.302367	1.360866
2.567605	1.184185	3.726854	0.070736	0.773766	1.111977	1.162201	1.03189	0.57531
0.035007	0.020722	0.010101	0.014545	0.029928	0.019048	0.072193	0.068175	0.002157
3.792266	3.052655	3.195541	2.544747	3.35355	3.50425	4.802655	6.028427	3.993665
13.87537	8.319019	6.967547	3.256587	5.515584	22.74797	8.608153	8.888167	7.162698
0.895945	1.886335	2.99987	1.019134	1.972706	2.547179	0.931457	2.978968	5.993045
0.310657	0.2129	1.092136	0.237035	0.591616	0.742417	0.163038	0.971775	1.006025
0.051414	0.020722	1.584105	0.481104	1.083651	0.053297	0.003413	2.427792	1.628009

2.765758	6.591544	1.535722	1.525729	2.928247	0.958557	1.105426	2.109755	1.680895
0.082027	0.63114	0.174098	0.101429	0.521068	0.130115	0.142698	0.352222	0.120823
0.250476	0.602908	0.137201	5.704646	0.246825	0.581645	0.951652	0	0.20127
3.414574	7.570693	2.89E-05	4.575123	0.00026	3.824964	3.540606	0	1.44E-05
0.384156	2.150303	1.172872	0.079957	0.375743	1.161501	1.474343	0.508341	1.906551
0.019351	0.293723	0.095996	0.34544	0.581378	0.03526	0.063478	0.187229	0.541804
1.097677	0.997792	0.939408	1.721371	1.183478	1.47018	1.143954	1.616385	1.23127
1.089394	0.787136	0.363983	0.159495	0.365411	0.332886	1.329206	0.319365	0.066205
1.203427	2.768312	0.713405	0.943514	1.721371	1.542302	1.881955	0.340866	0.377323
1.044235	2.021068	1.009625	2.201587	2.101075	6.717056	1.132165	4.359574	1.599033
3.869163	14.51648	6.036818	6.830592	2.703485	7.558139	19.53061	5.20267	4.808658
0.183939	0.360101	0.232583	0.049531	0.365411	0.09176	0.119322	0.224762	0.074286
2.138131	0.466602	5.416623	4.484892	9.924372	1.169091	0.618658	7.162698	4.673182
6.250447	0.418326	5.485685	7.495397	6.945152	3.606804	0.21619	4.261342	2.322035
7.443276	1.668369	5.560563	1.312706	3.563182	1.591299	1.080303	4.552092	3.846984
8.923117	3.283139	3.55961	2.640332	7.339452	8.745238	4.160238	5.704646	3.91355
13.08214	1.465123	35.27608	2.909293	12.36443	5.409437	1.514199	3.432388	65.94551
1.126075	0.162576	1.197215	1.415469	3.460909	0.156486	0.176558	5.082063	2.502619
4.029654	4.429769	2.834084	12.60233	0.00026	2.242092	9.924372	0.648961	0.836537
749.7839	135.5245	144.7919	425.62	144.7919	332.0776	287.197	155.7103	60.77449
1.772756	7.964315	0.790303	0.620014	0.833975	1.728615	12.03267	0.540563	0.631847
0.082027	0.020722	2.89E-05	0.040563	0.00026	0.044719	0.003413	0.215115	0.412193
14.00414	2.955916	14.17388	0.912446	1.29267	1.613232	2.254084	3.930664	29.18885
44.27012	18.19595	9.838658	46.7056	8.365072	27.10502	77.74812	20.68746	22.52375
0.988377	0.389574	1.948918	0.37417	2.069942	0.259336	0.305657	1.132165	2.8657
9.179466	16.5625	10.78056	15.07257	14.65384	22.52375	11.03843	11.14619	12.10289
4.33E-05	15.56131	1.672107	28.23632	0.844747	9.256537	15.77678	0.121941	6.830592
4.145209	3.381255	3.236457	1.87899	9.297085	4.563117	3.899993	4.145209	3.092229
0.988377	0.717027	0.597417	0.237035	0.076587	1.120332	1.275325	0.168167	0.042713
3.953297	1.51215	3.921645	2.981962	4.003117	2.947287	1.080303	6.876392	5.366335
1.573276	0.418326	2.181775	0.305253	1.343463	0.363492	0.565137	1.697792	3.166659
8.849697	7.162698	9.583463	10.28573	7.200505	8.445743	5.42381	11.65791	10.1028
0.419076	0.020722	0.523211	0.198694	0.344733	0.09176	0.003413	0.484704	0.209841
1.50684	0.223701	1.910974	1.165065	3.971061	1.37531	0.645036	2.563506	1.696494
167.9996	2.065007	57.84882	15.276	3.726854	45.91841	5.150693	42.75576	41.47925
3.172864	0.258268	3.803766	6.171789	10.7557	0.713846	0.003413	5.34474	3.058802
0.013766	0.020722	0.027056	0.014545	0.534452	4.33E-05	0.130714	0.121941	0.049719
6.760765	4.814661	6.508427	8.048095	7.251263	3.428788	1.623232	12.03267	4.261342
0.870375	0.617417	1.89373	10.73084	3.544257	1.099004	0.931457	2.579509	3.566753
3.636789	3.23987	8.365072	4.997417	2.382035	7.430317	3.507691	5.687879	5.9071
8.573434	1.068348	7.558139	5.898449	5.538001	3.211176	1.119574	12.93651	2.990851
0.569971	0.173059	0.264805	0.023009	0.156089	0.101991	0.092121	0.017778	0.58925
1.369048	0.617417	1.820541	2.263377	4.003117	1.256558	0.565137	2.199639	1.528975
0.310657	0.020722	0.568369	0.334026	1.541227	0.080779	0.047965	0.704848	0.474214
1.301169	0.162576	0.653203	0.494185	0.998312	0.323146	0.247951	1.269495	0.804675
0.035007	0.128283	0.00544	0.079957	0.00026	0.044719	0.742417	0.003102	1.44E-05
16.22108	8.365072	18.19595	1.591299	5.351934	7.61176	0.882619	8.923117	3.566753
2.190866	1.452857	1.593535	3.330909	0.803211	0.790303	2.242092	4.02088	0.623853
4.667518	4.272165	5.560563	2.005253	1.541227	2.735368	3.022713	1.364401	1.403853
21.02638	7.443276	7.402915	8.868932	2.252273	6.55039	8.256465	1.089394	4.292078
1.393571	0.481789	1.8386	1.177994	0.712929	3.55961	0.723066	1.948918	9.707677
28.23632	19.06893	10.93101	13.53913	6.268312	6.922771	15.3618	2.283752	11.99921
1.432302	2.786898	0.533196	1.574517	2.616573	2.304452	1.427431	1.341825	1.938759
0.681919	0.553059	0.533196	0.405346	0.394877	0.599466	0.26562	0.802756	0.492165
1.446926	1.34798	0.763564	0.900693	1.89373	0.233175	0.723066	0.606421	0.20127
1.821861	2.325931	4.174964	2.210866	6.580988	0.659957	1.489524	10.48101	4.827691
2.901032	0.389574	7.162698	0.139567	0.00026	0.119423	0.226623	4.946587	4.039192
0.124646	0.020722	0.049769	0.090476	1.223846	4.33E-05	0.003413	0.565426	0.858817

2.078355	0.466602	3.518384	1.925635	2.413146	0.790303	0.324019	2.199639	2.29873
3.037626	19.22139	6.036818	7.443276	0.00026	21.74051	45.91841	0.111068	2.445079
8.019769	9.792626	11.30987	14.70188	4.946587	10.57984	6.632063	9.063983	2.410996
1.919661	1.77404	3.518384	2.895599	1.343463	2.261573	0.910339	3.807648	4.715599
3.824964	4.575123	2.136335	4.179762	5.24562	2.438831	1.863506	4.359574	1.924228
0.522633	0.223701	1.159913	0.070736	2.978968	0.371587	0.053838	2.440895	1.291825
2.852677	2.217951	1.147807	1.574517	0.844747	1.284747	0.931457	2.6143	1.074827
0.043499	0.23412	0.506003	0.049531	0.635541	4.33E-05	0.163038	0.565426	0.319762
0.484423	0.449408	0.544877	0.324019	0.124791	0.130115	0.237215	0.187229	0.265339
4.145209	0.740527	2.834084	3.644343	1.107605	6.52899	3.726854	4.47912	4.039192
0.355556	0.710722	1.064242	0.227446	0.029928	0.302352	0.469004	0.597792	0.956667
0.051414	0.020722	0.434863	0.070736	1.972706	4.33E-05	0.003413	1.283911	1.135887
1.475469	0.862561	1.742734	0.660332	1.29267	1.388543	1.197922	2.339697	1.824892
1.20653	1.224733	1.035058	0.525678	1.145426	1.403853	0.692381	1.731169	0.688975
0.060173	0.2129	0.287821	0.210505	0.194372	0.167013	0.053838	0.205931	0.327403
2.961768	1.659033	4.392316	1.733766	3.703175	2.635685	0.826407	5.754935	6.250447
2.375974	0.981558	1.419278	1.291825	0.833975	2.304452	1.53684	1.485483	1.986681
24.50942	6.325945	8.888167	6.653016	8.711905	4.907929	7.08733	3.404372	7.951371
0.302554	0.854733	3.300664	0.96627	5.838499	0.348629	0.4971	6.297136	7.402915
2.005253	2.053521	6.685087	6.135007	11.74734	2.876421	1.427431	14.00414	11.65791
0.074603	0.020722	0.020462	0.169538	0.276061	4.33E-05	0.003413	0.224762	0.265339
0.082027	0.020722	0.16575	0.26562	0.267388	0.080779	0.003413	0.041443	0.912446
0.904856	2.011501	0.424228	0.808052	0.581378	1.002215	3.68381	0.371876	0.229048
0.7257	0.020722	0.939408	0.237035	1.412771	0.09176	0.003413	1.089394	0.868853
14.79742	4.241905	23.16622	9.583463	15.56131	7.049336	3.414574	5.881342	17.92188
0.2757	0.389574	1.125325	0.29715	0.787987	0.428694	0.296955	1.485483	1.590202
6.612121	1.488571	10.1028	0.818586	0.454632	5.687879	3.703175	2.723247	2.77899
0.007965	0.11803	0.003066	0.023009	0.022958	0.010945	0.130714	0.001147	1.44E-05
2.005253	1.638499	2.404618	2.873817	4.077403	2.304452	0.770188	4.408153	1.791616
27.38726	13.6814	30.82277	25.24367	10.01009	28.23632	12.56693	15.46193	22.13245
1.320173	3.188918	2.223636	1.273636	1.700354	0.921739	0.528997	2.353247	1.516219
6.079596	2.24562	9.256537	19.06893	19.06893	4.764055	1.312706	9.418196	6.365065
0.096573	0.020722	0.240202	0.121147	0.534452	0.005072	0.072193	0.553348	0.049719
3.566753	1.044863	1.419278	3.387864	2.304452	0.854733	0.543939	2.413146	0.599466
4.33E-05	0.020722	0.041472	7.22E-05	5.337547	0.053297	0.003413	0	0.965714
21.19547	3.930664	16.34294	5.381227	0.645455	13.60866	14.70188	2.758088	11.30987
0.035007	0.128283	0.057352	0.129553	0.029928	0.019048	0.692381	0.003102	0.026724
0.043499	1.05824	0.119683	0.014545	0.00026	0.101991	1.003586	0	0.066205
11.65791	2.725498	14.25954	14.42661	24.98218	10.73084	2.494177	37.29684	13.60866
0.916869	6.116638	1.112641	3.783694	3.944084	1.985253	5.508131	2.392431	1.006025
0.7257	64.12971	1.035058	8.019769	1.412771	11.65791	56.39528	0.692381	0.744531
4.174964	10.93101	3.24658	1.554185	2.101075	8.524242	6.355224	2.031169	5.745996
12.93651	1.174315	4.338629	1.25816	7.545584	1.684646	1.633752	5.591717	3.792266
2.74298	0.862561	1.723579	5.42381	2.276407	1.479452	0.478737	0.83697	1.615281
2.845036	1.247244	6.52899	5.654863	4.597792	1.707576	1.525729	6.355224	6.497266
6.028427	3.4679	2.083261	1.145426	1.03189	4.827691	10.38202	1.587605	2.124574
8.195657	7.938427	5.143867	3.833247	3.595758	4.508196	6.116638	5.23176	3.521934
11.09133	34.84299	6.443925	12.70768	1.721371	25.99427	51.52195	2.045476	7.598312
0.999502	0.66482	0.663276	1.50684	1.202626	0.348629	0.003413	1.222179	0.560325
0.392554	1.05824	0.499206	0.159495	1.194221	0.913514	0.770188	1.03189	0.528997
3.307186	3.334199	1.68938	2.24733	1.312706	1.550837	1.143954	0.540563	0.528997
1.37531	0.020722	1.37531	0.366147	1.824892	0.080779	0.003413	0.495931	0.567619
0.026364	0.020722	0.145216	0.177973	0.305455	4.33E-05	0.003413	0.187229	0.218817
3.35715	0.020722	2.453781	0.584134	2.602395	0.51039	0.516306	3.610707	3.548153
5.728615	13.75978	1.849654	4.833398	1.107605	6.045209	19.83727	1.587605	2.8657
2.058773	4.789596	0.453932	0.286681	3.450577	2.316075	1.974228	1.372475	1.516219
1.387612	0.81228	1.672107	1.329206	0.404704	1.47018	1.058896	0.760433	0.81544
0.448557	0.020722	0.272244	1.779423	21.74051	4.33E-05	0.003413	0.456522	0.782417

0.060173	0.020722	0.256205	0.129553	0.501905	4.33E-05	0.130714	0.187229	0.089235
3.6029	0.449408	3.980123	4.297222	3.846984	0.921739	0.305657	6.09759	4.413608
0.15456	0.258268	0.568369	0.06031	0.315916	0.062244	0.063478	0.62689	0.386378
0.999502	2.887489	1.184185	4.297222	4.684502	3.195541	1.416407	1.395563	1.303961
1.453939	1.390476	1.224733	2.066652	0.284646	0.951652	0.839365	0.484704	0.912446
6.819481	3.984747	3.621941	6.028427	1.209105	3.321284	5.295325	1.902352	1.046061
8.677489	6.784762	7.025599	5.20267	2.159004	10.8813	13.45939	5.737056	8.556703
0.060173	0.11803	0.240202	0.219481	0.067864	0.019048	0.196667	0.130924	0.042713
0.007965	0.020722	0.057352	0.06031	0.094733	0.010945	0.047965	0.032915	0.035007
5.273362	1.1629	8.134084	4.272165	12.22798	2.860722	0.723066	11.77567	7.443276
3.521934	1.216768	6.685087	5.238701	8.905642	2.003723	0.592532	9.667316	7.868146
3.35715	1.204228	5.309264	2.08013	3.095166	2.635685	0.979827	6.622092	8.728571
0.408427	0.511032	0.413304	0.314913	0.394877	1.420202	0.684805	0.446551	0.492165
5.373781	13.60866	0.713405	3.303925	7.882222	3.895469	5.788153	3.381255	1.200823
0.060173	0.020722	0.079921	0.535368	0.547778	0.062244	0.003413	0.006948	1.44E-05
4.343838	0.723066	10.48101	3.243225	11.96574	6.695887	11.544	7.08733	0.567619
0.259033	0.020722	0.16575	0.219481	0.136147	4.33E-05	0.100375	0.446551	0.155743
2.153535	0.481789	1.934271	2.112843	1.673254	1.867951	0.142698	1.395563	1.845519
37.02103	19.83727	83.489	44.27012	56.39528	45.1045	22.52375	52.56128	38.39042
0.513456	0.322684	0.469358	0.198694	0.094733	0.633853	0.488997	0.508341	0.245772
0.697085	6.518709	2.89E-05	1.209776	0.00026	2.114437	6.045209	0.05824	1.44E-05
0.622496	0.020722	1.29267	0.752583	0.963853	4.33E-05	0.003413	1.104805	0.898016
2.349351	1.474343	1.68938	1.392511	4.376118	2.289286	1.94311	2.031169	1.881955
7.288788	1.082915	4.63189	3.55961	91.05541	8.256465	3.962179	6.063276	4.702468
1.50684	4.365224	1.125325	4.077403	1.160707	4.082165	5.273362	0.727222	0.946638
0.235455	0.128283	0.296234	0.24632	2.091313	0.22456	0.092121	0.669769	0.698687
1.097677	0.67632	1.962424	1.89518	4.003117	0.217172	0.3671	4.721789	2.483492
1.226934	8.048095	1.056263	1.591299	0.00026	3.407619	6.464906	0.196811	1.860491
2.668896	0.888485	4.524481	1.603795	5.500678	2.781558	0.855801	3.711097	6.38456
4.978182	0.151587	4.721789	2.361198	4.236962	0.566104	0.351595	5.25254	3.718716
0.916869	3.06803	2.89E-05	3.993665	0.645455	1.985253	1.692763	0.101905	0.155743
0.852684	0.331869	2.042201	7.468961	4.875527	3.243225	1.014127	3.734762	1.924228
1.634935	0.685209	1.705007	1.616385	0.355051	1.037316	0.478737	1.32482	1.706219
13.53913	65.94551	19.06893	13.6814	12.67271	17.78522	42.75576	11.36975	6.612121
4.702468	4.222583	5.024293	12.29473	67.98069	12.85504	3.230065	6.355224	8.608153
0.013766	0.020722	0.240202	7.22E-05	0.124791	0.119423	0.003413	0.086522	0.338095
23.38378	6.423911	26.25557	12.03267	7.456118	15.3618	5.638759	19.68088	14.33939
224.9555	749.7839	287.197	155.7103	1.659033	87331.38	252.4251	21.74051	56.39528
8.089848	1.1379	11.48368	0.984069	0.787987	0.780952	0.855801	3.089293	7.697063
0.490664	0.768052	0.071696	0.567345	0.085887	0.633853	2.887489	1.57645	0.664026
6.443925	6.374812	1.159913	3.783694	2.903867	5.788153	7.911934	2.770866	0.858817
4.626025	7.765931	5.560563	3.034747	2.683478	7.751869	14.17388	2.65684	5.42381
40.11561	91.05541	15.66023	10.5063	4.160238	30.17192	203.5795	2.723247	4.776407
5.662994	6.345382	0.744113	2.895599	0.509791	2.823636	4.077403	2.283752	0.349812
9.502294	27.38726	2.276407	27.38726	8.149307	59.32013	29.18885	0	1.44E-05
8.711905	2.561162	13.75978	7.074329	3.381255	13.00996	2.820974	9.902576	6.153102
0.419076	2.174733	1.241811	2.386248	1.624459	0.692381	1.172208	0.959062	3.058802
2.190866	3.266782	1.305772	2.609293	2.382035	2.410996	3.599329	2.083261	1.394632
1.289149	0.438312	0.883665	5.351934	0.681919	0.692381	0.855801	1.948918	1.590202
0.310657	0.020722	0.713405	0.121147	1.274481	0.053297	0.21619	1.648066	0.642698
14.88584	11.544	9.924372	10.01009	11.2819	9.459177	12.70768	10.8813	15.07257
20.68746	4.302367	19.53061	1.50684	3.895469	19.99861	7.991602	15.3618	13.30906
0.096573	0.020722	0.034242	0.049531	0.465563	4.33E-05	0.003413	0.150245	1.44E-05
12.16681	23.38378	9.335397	10.62739	3.179199	15.77678	34.1614	4.392316	4.952915
0.400173	1.703795	1.159913	0.889019	0.483326	1.427431	0.988377	1.828781	1.096335
0.711912	0.258268	1.092136	0.467951	1.044235	0.285253	0.247951	1.78759	1.23127
1.579798	0.951652	2.618846	1.329206	4.914863	0.770664	0.450678	1.974228	2.192496
0.111876	0.258268	0.384394	0.129553	0.257042	0.019048	0.072193	0.241991	0.465325

4.145209	6.518709	3.33728	3.36075	1.641999	6.475505	6.945152	3.236457	4.519048
2.640332	0.854733	4.419062	1.154567	4.266753	1.707576	1.275325	3.895469	5.395584
0.118644	0.020722	0.264805	0.014545	1.202626	0.005072	0.109654	0.474214	0.528997
7.839271	11.83593	4.198117	2.005253	3.895469	5.068831	10.28573	2.225498	3.640606
47.5018	87.1746	27.66779	24.04374	13.60866	24.50942	60.77449	4.990794	16.44768
4.33E-05	9.179466	2.89E-05	4.764055	1.765065	5.872886	11.39744	0	1.44E-05
0.111876	0.020722	0.12904	0.227446	0.385238	0.010945	0.092121	0.41026	0.089235
6.39474	11.77567	3.214545	5.599228	2.003723	3.799885	6.135007	0	0.319762
0.204531	0.020722	0.753449	0.545426	0.803211	4.33E-05	0.003413	0.704848	0.147554
0.026364	0.162576	0.071696	0.079957	0.076587	0.005072	0.063478	0.906825	0.245772
1.440736	0.151587	1.419278	1.246457	1.465123	1.079697	0.257518	1.948918	1.986681
0.259033	0.020722	0.724004	0.079957	0.344733	4.33E-05	0.003413	0.715815	3.227085
0.140548	0.020722	1.888023	0.210505	0.225079	0.355556	0.003413	0.329091	1.074827
1.89373	0.351219	2.296732	1.052208	1.412771	1.850996	0.919192	0.508341	1.860491
4.082165	6.967547	3.300664	2.755411	4.535354	4.082165	3.754719	6.663651	2.735368
2.540462	0.020722	1.419278	1.246457	1.750628	0.499986	0.003413	0.919192	1.360866
10.62739	5.210317	3.930664	5.737056	2.903867	7.430317	3.655714	1.597908	2.683478
0.250476	0.020722	0.544877	0.169538	0.645455	0.080779	0.100375	0.111068	0.191962
15.276	14.08569	5.00404	9.625253	1.022929	9.218514	15.56131	4.198117	3.738997
3.110447	3.350224	3.089293	3.482785	6.125823	5.295325	3.767489	4.739668	3.092229
0.800967	0.140418	0.638362	0.405346	2.060491	0.080779	0.26562	1.066948	0.492165
13.60866	2.489762	1.056263	12.22798	6.374812	3.321284	3.182482	1.341825	1.44E-05
0.697085	0.787136	1.077403	1.359177	1.436623	1.223846	0.875101	2.139928	1.096335
1.50684	21.36892	1.112641	0.35676	1.641999	1.284747	16.09482	0.659163	2.209141
0.331263	0.020722	0.191804	0.567345	0.146768	0.140087	0.130714	0.456522	0.631847
0.019351	0.198788	0.256205	7.22E-05	0.022958	4.33E-05	0.082323	0.003102	0.120823
1.044235	0.020722	1.471198	0.643427	1.118817	0.60886	0.332049	1.395563	0.256205
0.859885	0.409618	0.313102	1.446926	0.662237	1.850996	4.198117	0.111068	0.303564
0.026364	0.020722	0.057352	0.014545	0.165512	4.33E-05	0.152532	0.139697	0.026724
0.035007	0.341053	0.010101	0.149156	0.204661	4.33E-05	0.196667	0.261111	0.165108
9.031082	5.316378	3.33728	2.62342	2.159004	2.895599	7.545584	4.440548	1.881955
0.375094	1.031284	0.119683	0.06031	0.204661	0.2757	1.162201	0.095029	0.245772
6.738211	2.269134	4.752121	0.943514	3.253175	2.797835	2.24733	3.820758	3.792266
1.579798	3.939307	0.734524	1.34267	1.482626	1.244957	3.340361	1.119574	0.616739
3.792266	0.427937	2.196017	0.149156	0.05886	3.824964	1.77404	0.521068	2.593506
1.246457	5.351934	0.615996	6.475505	1.578687	7.795642	3.175916	0.586934	0.858817
0.172734	0.020722	0.087619	0.079957	0.067864	0.010945	0.003413	0.086522	0.147554
3.058802	1.390476	4.451154	3.926154	6.900202	3.172864	3.249877	2.879228	3.022713
27.66779	44.27012	22.74797	21.19547	9.438687	46.7056	72.69473	6.125823	11.99921
1.190491	0.020722	0.883665	1.145426	2.345527	0.03526	0.003413	1.372475	0.70886
0.310657	0.020722	0.256205	0.237035	1.261407	0.080779	0.119322	0.574993	0.664026
6.325945	1.835743	8.103925	6.443925	6.414416	4.251818	1.722475	6.125823	6.64254
2.558817	2.644885	0.975556	1.692763	1.524776	3.391075	2.805382	3.640606	3.150433
1.044235	0.151587	0.506003	0.633146	0.929286	0.666097	0.286681	3.107417	0.642698
0.074603	0.223701	0.155361	0.77925	0.183939	0.156486	0.257518	0.121941	0.035007
1.717641	0.427937	2.975974	3.077316	6.497266	5.00404	0.656392	2.031169	3.975498
5.25254	13.38418	2.5271	15.98892	2.413146	14.6058	8.573434	2.540462	0.688975
0.162576	0.409618	0.232583	2.24733	0.00026	0.110642	1.267085	0	0.191962
50.55949	35.776	32.68313	38.94854	37.81919	72.69473	30.82277	10.1459	7.468961
0.375094	1.224733	0.485325	0.227446	0.833975	1.1407	1.765065	0.995527	0.642698
1.651522	0.602908	2.250462	0.984069	4.56912	0.51039	0.60824	6.876392	3.432388
1.274481	1.256558	1.282208	1.634935	0.493247	1.079697	0.618658	1.452857	0.358903
9.356371	18.33909	5.439278	7.598312	0.00026	17.39935	36.50291	0.597792	2.723247
0.384156	7.824473	0.638362	0.998831	0.725267	3.750837	8.288874	0.05824	0.303564
5.01088	4.49671	2.349351	1.359177	2.797835	1.317475	2.898196	2.858009	0.528997
1.126075	1.867951	1.391494	0.535368	1.274481	0.199134	1.100519	1.066948	0.722626
1.821861	1.1379	0.62689	0.984069	2.810678	1.094978	0.50469	1.078925	0.528997
0.560325	0.020722	1.002215	0.219481	0.725267	0.019048	0.801501	0.866717	0.465325

1.539062	1.867951	0.402381	0.366147	0.236089	0.208571	2.479221	0.876638	0.104791
65.94551	100.0998	28.23632	0.620014	0.00026	135.5245	530.1465	39.53687	167.9996
0.051414	0.020722	0.353016	0.049531	0.029928	0.713846	0.790303	0.168167	1.291825
0.007965	0.020722	0.041472	0.079957	0.267388	4.33E-05	0.003413	0.329091	0.05767
0.61355	0.733074	0.16575	0.159495	0.355051	0.130115	0.863694	0.139697	0.081212
1.03189	77.74812	0.857778	4.614618	0.423781	2.242092	126.1556	1.364401	0.560325
0.852684	0.878932	0.506003	0.878369	0.326912	1.256558	2.400455	0.565426	0.438831
1.069574	0.400354	2.442987	1.65785	1.130115	0.130115	0.247951	2.013182	1.335743
1.301169	1.267085	3.014076	2.31811	9.356371	3.034747	1.329206	2.671299	2.547179
0.035007	0.020722	0.003066	7.22E-05	0.146768	4.33E-05	0.003413	0.139697	0.066205
3.446732	1.286494	7.795642	2.640332	6.685087	2.981962	2.196017	6.423911	7.951371
0.089553	2.939141	0.724004	0.286681	0.81596	1.763889	4.655916	0.121941	0.139235
2.026219	0.020722	4.47912	3.676032	8.365072	0.599466	0.305657	6.819481	4.272165
0.204531	0.020722	0.279315	0.070736	0.225079	0.044719	0.063478	0.130924	0.120823
2.901032	7.532713	0.939408	3.450577	1.118817	7.683766	10.43066	0.383622	2.518124
0.932078	5.095325	0.841746	0.37417	0.00026	0.355556	5.316378	0.101905	1.44E-05
1.346991	0.11803	1.440736	1.312706	1.962424	0.867287	0.315094	2.828918	2.903867
1.708932	5.591717	2.250462	9.859719	1.90798	4.419062	2.652107	3.939307	2.618846
0.019351	0.644235	0.041472	0.090476	0.114113	0.053297	1.03189	0.068175	0.013766
4.33E-05	0.020722	0.010101	0.007864	0.016609	0.005072	0.053838	0.003102	1.44E-05
3.236457	2.839351	1.750628	1.603795	2.786898	1.902352	3.005281	2.225498	0.885137
0.226883	0.020722	0.343824	0.237035	0.174444	4.33E-05	0.003413	0.474214	0.229048
4.23202	7.697063	1.788874	7.363117	4.266753	4.597792	7.188095	0.241991	0.275238
0.339235	0.173059	0.155361	0.438312	0.156089	0.672857	0.970007	0.095029	0.327403
2.223636	5.720173	6.486104	7.570693	1.374329	8.164531	2.322035	2.045476	1.824892
11.60283	9.838658	7.625209	11.42512	10.93101	13.38418	9.880779	4.851227	6.591544
0.096573	0.128283	0.469358	0.049531	0.787987	0.101991	0.109654	0.715815	0.327403
3.002576	1.286494	6.443925	0.96627	5.9071	2.197828	1.474343	21.36892	7.507619
2.451645	2.096162	1.102121	0.494185	1.171349	4.025267	3.55583	1.01772	0.804675
2.300678	1.331919	2.363442	1.209776	1.145426	2.836717	1.233665	1.03189	4.808658
1.197922	0.258268	1.776739	1.130115	2.069942	0.51039	0.196667	2.931068	3.201457
0.419076	0.128283	0.413304	0.334026	1.312706	0.148016	0.053838	0.946061	0.722626
3.6029	2.858009	19.83727	1.401068	0.803211	9.989221	8.412367	10.53159	12.70768
9.356371	47.5018	4.888023	8.745238	2.345527	8.048095	50.55949	5.34474	6.345382
18.76954	22.13245	1.197215	9.771833	0.734524	33.93939	23.38378	1.504517	2.392431
0.111876	0.020722	1.035058	0.014545	0.05886	0.010945	0.003413	0.150245	0.275238
4.715599	2.257749	7.30215	0.955418	9.031082	3.718716	2.547179	3.957734	16.80343
5.976724	4.468009	6.933961	8.694697	6.922771	4.851227	5.34474	10.1028	9.256537
0.392554	1.423506	0.111299	0.24632	0.050426	0.293276	1.143954	0.241991	0.229048
105.1694	25.48179	10.33476	36.26061	3.595758	16.22108	30.17192	23.16622	7.013853
0.408427	0.617417	0.820931	0.494185	1.524776	0.419076	0.376934	0.615584	0.368038
0.10381	0.020722	0.063745	0.334026	0.929286	4.33E-05	0.003413	0.771688	0.395426
0.784372	1.616385	0.287821	0.620014	0.501905	0.581645	1.150635	0.83697	1.154567
1.545657	0.553059	3.998391	2.097843	14.33939	0.259336	0.275527	10.21618	3.8121
1.668369	0.400354	3.779452	2.417424	7.112496	1.624459	0.4971	6.250447	4.739668
0.22039	0.360101	0.645455	0.06031	0.335967	0.348629	0.072193	0.280678	0.349812
3.578038	12.85504	0.801501	1.763889	1.450693	1.664906	7.598312	1.816436	1.975772
0.204531	0.23412	0.554416	0.040563	1.058896	0.010945	0.047965	0.738716	0.412193
3.022713	5.024293	3.031688	7.22E-05	0.610606	10.33476	9.707677	0.456522	5.950664
0.468694	1.601443	0.071696	1.165065	0.763102	0.617071	0.882619	0.446551	0.560325
0.468694	0.020722	0.264805	0.446551	0.00026	0.080779	0.003413	2.160649	1.000173
4.47912	1.146962	12.63773	4.721789	17.51961	4.251818	1.216768	18.05488	4.644098
13.45939	332.0776	5.821804	25.99427	2.447294	87.1746	224.9555	0	1.44E-05
7.061328	37.29684	2.725498	6.38456	2.602395	3.058802	14.08569	2.828918	1.481631
0.183939	0.553059	0.264805	1.634935	0.963853	0.302352	0.38596	0.101905	0.074286
6.900202	5.316378	5.737056	3.387864	1.058896	6.853608	7.938427	5.402511	2.24733
55.04081	20.68746	41.47925	14.56114	9.968355	49.50903	24.98218	46.7056	77.74812
0.026364	0.020722	0.015541	0.040563	0.114113	4.33E-05	0.003413	0.077244	0.05767

0.599798	0.128283	1.682193	0.679452	0.635541	0.591912	0.4971	1.553074	3.993665
4.33E-05	0.020722	0.087619	7.22E-05	0.375743	4.33E-05	0.003413	0.032915	0.096999
2.882035	14.33939	2.165851	1.401068	1.145426	2.703485	10.23768	0.319365	0.796674
0.392554	0.020722	0.111299	0.121147	0.037302	0.659957	0.082323	0.086522	0.139235
0.61355	1.113304	1.391494	0.837403	0.948211	1.274481	1.384921	1.089394	0.73583
0.711912	7.697063	0.034242	0.101429	0.225079	0.419076	3.899993	0.196811	1.44E-05
1.0386	0.951652	0.544877	1.763889	0.581378	2.003723	0.995527	0.291861	0.551573
24.98218	6.570433	20.68746	19.53061	34.38341	11.25392	8.745238	25.24367	14.42661
6.210375	18.76954	12.03267	8.477662	8.976508	27.66779	11.39744	27.10502	6.153102
0.026364	0.162576	0.182713	0.652807	0.284646	0.053297	0.553059	0.02482	0.349812
0.628658	1.609654	0.820931	2.201587	2.939141	2.931068	2.139928	7.375794	3.763045
28.54786	3.507691	32.31123	7.911934	29.49693	2.597792	0.003413	30.17192	19.53061
2.394416	0.2129	2.083261	1.008413	2.810678	0.581645	0.645036	1.805166	0.368038
0.870375	1.77404	4.145209	1.90798	1.03189	0.942944	0.537518	1.852338	4.739668
0.811861	0.449408	0.782857	2.802828	0.763102	3.957734	0.427937	0.946061	1.02487
0.579495	0.198788	0.62689	0.446551	0.581378	0.591912	0.342215	0.692381	0.847352
3.934986	2.644885	0.841746	1.668369	3.131544	0.770664	0.882619	1.758615	1.056898
0.051414	0.020722	0.041472	7.22E-05	1.252424	4.33E-05	0.003413	0.121941	0.349812
2.74298	2.74298	5.737056	2.263377	7.288788	3.574387	1.132165	16.50509	10.28573
3.263391	0.757763	6.717056	3.783694	8.849697	1.532468	1.03189	5.881342	2.089639
0.88417	0.960866	0.513752	0.808052	0.156089	0.682799	1.841371	0.150245	0.303564
2.59557	0.778326	2.097843	0.620014	2.773644	0.428694	0.537518	2.914632	0.762165
0.339235	0.020722	0.579495	0.584134	0.948211	0.019048	0.003413	2.472576	0.327403
0.074603	0.351219	0.137201	0.198694	0.114113	4.33E-05	0.130714	0.241991	0.155743
2.800332	5.024293	1.962424	0.286681	0.05886	6.760765	14.97762	0.540563	0.377323
1.267085	2.145137	3.88153	1.446926	0.294921	2.325931	5.671126	0.553348	2.427792
4.33E-05	0.020722	0.010101	0.023009	0.174444	0.005072	0.003413	0.049221	0.004964
3.428788	0.585592	9.063983	3.098268	1.799899	0.2757	0.315094	1.395563	6.30671
1.927042	1.233665	1.608427	2.820974	2.668896	2.447294	1.207374	4.067547	4.039192
2.765758	0.341053	3.422237	2.882035	3.8121	1.054899	0.450678	4.49671	0.981558
12.29473	7.112496	9.625253	10.78056	11.89932	8.994791	4.959358	3.629177	2.223636
0.293276	2.465916	0.588312	0.984069	0.581378	1.451775	2.419553	0.982698	0.31171
0.302554	0.293723	0.597417	0.481104	1.171349	0.233175	0.109654	0.63873	0.338095
4.451154	10.1028	3.606804	0.06031	0.00026	10.38202	6.967547	0.553348	21.93452
0.88417	0.473586	0.320563	0.24632	0.246825	0.293276	0.3671	0.139697	0.113074
2.153535	1.195794	1.788874	3.077316	1.083651	2.703485	1.185685	2.024488	1.824892
0.347403	0.360101	0.248528	0.139567	0.165512	0.355556	0.247951	0.215115	0.358903
3.279625	0.573665	1.732468	3.286652	6.622092	0.302352	0.761277	2.124574	1.590202
7.545584	0.692381	5.737056	3.895469	5.01088	1.584105	0.332049	2.096162	5.266335
1.464214	1.195794	1.33285	2.050404	1.118817	0.713846	0.749481	0.971775	2.223636
14.21671	5.137042	3.074134	20.34063	3.058802	8.608153	5.165599	1.670786	1.44E-05
0.569971	4.524481	0.653203	1.488571	0.454632	3.107417	7.468961	0.83697	1.200823
0.392554	1.524776	0.734524	0.878369	1.312706	0.148016	0.469004	2.151919	0.609509
3.29355	0.66482	1.798535	2.459812	10.23768	1.367179	0.528997	8.241342	3.944084
9.418196	2.547179	10.01009	12.77772	10.38202	11.01243	6.250447	4.921082	5.872886
0.773766	0.997792	3.529466	2.302626	5.788153	1.176623	0.528997	3.998391	5.210317
7.765931	9.502294	12.42965	6.250447	3.8121	3.566753	8.780115	5.855281	20.34063
0.711912	2.109755	0.560325	0.129553	0.344733	0.130115	0.305657	0.196811	0.066205
0.162576	0.184877	0.597417	0.198694	0.571674	0.140087	0.176558	0.62689	0.275238
16.80343	118.7028	7.854069	22.74797	100.0998	32.31123	749.7839	51.52195	13.42179
0.543131	0.020722	1.682193	0.668456	0.194372	0.642013	0.324019	1.434921	0.688975
1.700354	2.083261	0.811861	0.689509	0.907273	1.850996	2.500548	0.692381	0.762165
0.534214	0.020722	0.724004	0.730873	0.267388	0.191133	0.003413	0.760433	1.084437
7.795642	4.888023	15.98892	1.042944	10.65365	3.26	2.522684	12.22798	4.456912
126.1556	19.99861	34.84299	10.67991	25.48179	23.6012	28.23632	5.454949	32.31123
0.007965	0.020722	0.104149	0.06031	0.067864	0.026537	0.003413	0.360462	0.026724
16.9162	5.515584	4.721789	3.211176	2.101075	0.834416	4.323045	2.283752	2.581861
4.23202	12.36443	3.407619	4.696313	0.681919	4.796126	8.888167	2.642525	1.135887

2.8657	0.67632	3.726854	1.779423	4.468009	1.451775	0.618658	5.720173	3.432388
0.648362	4.592273	0.533196	9.101674	0.165512	7.495397	3.163506	0.78619	12.49452
2.668896	1.565253	5.500678	4.752121	7.200505	1.670786	1.906551	4.971991	3.279625
1.118066	1.174315	1.89373	1.130115	2.203535	0.672857	0.38596	1.468131	1.781948
7.417359	3.214545	7.964315	9.179466	12.85504	11.544	2.432121	7.495397	37.29684
0.268095	0.020722	0.145216	7.22E-05	0.335967	0.03526	0.092121	0.224762	0.492165
0.183939	0.020722	0.119683	0.121147	0.174444	0.110642	0.063478	0.329091	0.395426
9.101674	14.6058	5.737056	5.123781	2.345527	13.38418	11.09133	2.472576	5.599228
100.0998	20.85876	95.22939	57.84882	135.5245	56.39528	11.14619	126.1556	118.7028
40.7932	59.32013	2.207417	56.39528	13.15079	20.85876	22.96463	17.15902	12.29473
29.49693	3.695815	60.77449	13.83359	49.50903	23.82354	1.03189	77.74812	91.05541
37.02103	19.53061	20.16541	24.73724	75.1132	9.625253	6.404921	67.98069	26.25557
31.55417	40.11561	12.29473	51.52195	31.92385	47.5018	27.96248	35.27608	39.53687
13.15079	5.454949	36.26061	12.93651	26.25557	5.180058	2.07658	13.00996	4.592273
1360.293	252.4251	530.1465	1360.293	287.197	1360.293	155.7103	425.62	87331.38
252.4251	530.1465	111.5439	144.7919	252.4251	155.7103	43.49658	167.9996	252.4251
144.7919	75.1132	203.5795	184.443	25.99427	144.7919	47.5018	18.91854	59.32013
91.05541	20.51271	224.9555	29.18885	530.1465	40.11561	34.84299	252.4251	332.0776
8.608153	4.120815	14.42661	3.052655	9.707677	3.1857	3.391075	11.719	6.045209
1.89373	0.553059	1.989711	1.097006	2.290996	0.481602	0.488997	2.472576	0.81544
7.315512	1.154567	7.683766	11.65791	11.42512	3.005281	0.995527	48.53429	17.51961
4.752121	4.468009	8.780115	2.361198	3.507691	5.337547	4.18456	7.724084	5.630815
0.628658	0.128283	1.52676	1.634935	3.279625	0.080779	0.047965	8.134084	3.457504
0.074603	0.020722	0.137201	0.149156	0.534452	0.053297	0.092121	0.041443	0.155743
3.446732	1.524776	2.99987	1.25816	1.685678	0.581645	0.226623	4.198117	6.414416
4.33E-05	1.835743	0.363983	0.139567	0.165512	0.285253	0.656392	0	0.174372
5.933066	6.230245	4.241905	2.755411	1.560779	2.770866	7.013853	2.858009	2.8657
0.339235	4.715599	0.079921	7.22E-05	0.00026	0.148016	1.155411	0	0.237035
9.063983	2.419553	13.30906	12.85504	83.489	7.751869	2.820974	43.49658	32.68313
1.795851	1.082915	1.048074	3.55961	1.232886	6.443925	4.419062	0.484704	2.800332
1.545657	0.2129	1.806595	2.257749	1.673254	1.954993	0.826407	6.706472	3.104394
0.007965	0.020722	0.041472	7.22E-05	0.844747	4.33E-05	0.003413	0.261111	0.05767
0.61355	0.617417	0.363983	1.34267	0.493247	0.834416	0.469004	1.452857	0.446551
1.89373	1.749307	0.672078	1.525729	0.599134	4.18456	5.530274	1.132165	1.135887
28.87506	12.03267	37.81919	37.29684	5.993045	60.77449	20.34063	6.784762	23.38378
59.32013	33.07147	45.1045	49.50903	20.34063	42.75576	31.55417	28.54786	19.83727
14.42661	8.412367	16.44768	8.288874	40.11561	9.101674	6.028427	29.03196	20.68746
4.739668	2.690823	8.643074	4.513622	8.149307	4.702468	1.68342	11.96574	9.031082
1.440736	0.020722	1.092136	0.305253	3.131544	0.080779	0.469004	3.128694	1.652771
25.24367	16.68554	38.39042	27.96248	14.25954	27.96248	18.05488	16.68554	21.02638
0.448557	0.377814	2.083261	1.797172	0.825354	1.120332	0.21619	1.872049	1.45912
0.697085	0.778326	0.763564	1.225426	16.9162	1.267879	2.380079	9.179466	18.62711
19.83727	15.66023	45.91841	17.09911	80.6518	13.08214	13.26964	135.5245	80.6518
0.569971	3.783694	1.21215	0.314913	0.624841	1.915455	1.666089	0.422237	1.791616
0.375094	0.562107	0.883665	0.889019	0.670945	0.854733	0.226623	0.959062	1.881955
6.230245	4.165137	2.327857	7.765931	2.481356	6.230245	3.163506	14.17388	7.868146
1.568564	0.960866	0.907273	2.112843	1.39847	1.244957	0.60824	2.62342	1.175859
0.490664	0.409618	0.894704	1.111977	0.91575	0.439524	0.951652	0.319365	0.349812
0.534214	0.28189	0.663276	0.606154	0.454632	0.341241	0.119322	0.853218	0.981558
1.983824	0.438312	1.401068	1.291825	8.815123	0.682799	0.780519	2.173081	3.26
0.688579	0.140418	0.217908	1.668369	0.948211	0.419076	0.003413	0.853218	0.081212
3.373983	2.174733	2.302626	1.856392	1.560779	0.713846	1.80267	0.62689	1.628009
3.192229	0.644235	11.96574	0.847908	3.334199	2.586926	2.16759	8.556703	13.75978
15.07257	0.913514	7.598312	5.515584	6.52899	4.096407	1.312706	6.933961	4.839105
0.365224	1.90798	0.079921	0.159495	0.103622	0.742417	1.863506	0.586934	1.44E-05
0.043499	0.303564	0.015541	0.584134	0.037302	0.148016	1.505678	0.02482	0.013766
19.99861	11.03843	14.88584	4.012049	13.34662	13.91714	7.795642	10.98644	5.238701
0.639495	0.151587	0.506003	0.467951	0.645455	0.566104	0.516306	0.465079	0.599466

4.33E-05	0.140418	2.89E-05	0.031299	0.022958	4.33E-05	0.003413	0.006948	0.004964
1.017107	2.209141	0.424228	1.50684	1.216768	1.223846	1.460981	0.78619	0.191962
1.757244	4.392316	2.320072	6.727633	2.823636	5.130411	7.882222	1.504517	2.069942
53.77619	0.020722	33.07147	32.31123	11.74734	2.747908	0.119322	8.643074	4.06303
6.956349	5.788153	8.303947	2.502619	2.616573	2.710765	7.710361	2.173081	2.955916
2.101075	0.162576	2.151919	1.209776	4.003117	1.037316	0.635541	2.323983	2.322035
3.514834	13.45939	2.642525	4.456912	0.454632	10.21618	17.92188	2.879228	2.445079
2.652107	9.063983	4.029654	2.515938	2.23267	4.160238	4.764055	16.50509	5.508131
0.916869	2.053521	1.637309	1.591299	1.145426	1.850996	0.931457	0.540563	2.29873
0.339235	0.020722	1.172872	7.22E-05	0.355051	0.062244	0.427937	0.187229	1.541227
27.96248	9.297085	10.33476	7.327482	9.815642	12.77772	2.593506	15.88548	16.34294
184.443	184.443	155.7103	332.0776	203.5795	252.4251	105.1694	203.5795	184.443
0.06772	0.020722	0.653203	7.22E-05	0.571674	0.070967	0.003413	0.802756	0.609509
52.56128	10.01009	52.56128	27.10502	32.68313	29.49693	12.36443	80.6518	72.69473
2.415296	1.423506	2.860722	1.965657	2.026219	1.47018	0.899567	10.73084	3.763045
0.870375	0.270924	1.682193	0.818586	1.367179	0.913514	0.395642	3.204632	1.986681
6.956349	14.79742	10.55571	12.49452	4.56912	11.36975	12.29473	7.112496	7.339452

CGGA_141	CGGA_141	CGGA_141	CGGA_141	CGGA_141	CGGA_142	CGGA_142	CGGA_142	CGGA_142
0.167128	0.844156	2.210866	0.953506	0.441833	0.010101	0.123535	0.283709	1.168326
0.560325	0.182237	0.723506	1.446926	0.339466	0.150974	0.351789	0.773766	0.319567
2.109755	3.163506	0.292597	0.015	11.09133	0.540902	3.363911	0.283709	0.029062
10.82954	0.929812	0.381789	0.015	9.687496	0.010101	2.604776	0.05088	0.029062
9.276811	6.760765	3.540606	1.263752	3.993665	6.414416	6.749488	2.597792	1.801284
1.812114	4.435159	33.07147	3.095166	2.132756	1.45912	0.952294	2.597792	4.863579
21.54823	26.25557	22.13245	7.162698	135.5245	22.96463	17.78522	8.149307	3.829105
0.000996	0.035758	0.029704	4.667518	0.199134	0.12132	0.088788	3.065108	1.801284
1.767431	3.648081	1.177994	0.015	0.826407	4.067547	7.112496	1.213434	0.029062
0.482078	0.0014	0.283276	0.015	1.779423	0.010101	0.001248	2.102626	2.725498
64.12971	15.46193	83.489	59.32013	28.87506	27.66779	10.82954	95.22939	77.74812
3.792266	0.3671	2.723247	6.019971	3.869163	0.509509	0.460649	5.821804	6.374812
3.944084	18.48514	2.970144	3.175916	40.11561	4.667518	14.88584	4.155339	1.168326
0.357727	13.38418	0.003333	0.015	0.587273	0.010101	62.33472	0.05088	0.029062
0.039214	0.0014	0.029704	1.056898	0	0.176407	0.001248	0.655245	1.290065
7.238023	7.443276	2.178268	1.684646	5.671126	16.50509	14.79742	0.05088	0.029062
1.436623	0.377814	2.831501	1.214315	4.802655	0.609177	0.29671	2.248896	2.625823
0.311046	0.0014	0.469004	11.89932	0	0.010101	0.001248	19.37139	10.82954
2.737944	6.900202	0.96938	0.015	0.279177	0.186861	7.002056	0.05088	0.029062
0.625786	0.446291	1.072843	7.810058	2.820974	0.307973	0.123535	4.277006	0.773276
0.357727	2.522684	0.469004	0.015	0.573665	0.010101	0.673651	0.05088	1.082179
1.247244	2.678781	0.222561	5.560563	3.489899	9.418196	8.888167	7.188095	5.568391
47.5018	0.0014	50.55949	0.015	144.7919	184.443	1.278629	0.05088	0.029062
0.039214	0.0014	2.89E-05	1.87899	0.002107	0.299062	0.048124	7.375794	1.801284
2.898196	1.222958	0.526566	4.212554	2.625823	3.750837	1.886335	2.92013	4.33342
2.496407	3.014076	1.351573	4.125786	8.089848	8.745238	6.153102	0.773766	2.944416
0.259336	3.119986	0.625209	0.885137	1.513102	3.381255	15.56131	0.05088	0.029062
0.259336	0.208571	0.634192	0.885137	0.441833	0.352006	0.245772	1.213434	1.416407
15.276	77.74812	5.082063	0.015	80.6518	287.197	425.62	0.05088	0.029062
0.722626	0.595714	0.359502	4.212554	0.895368	0.967446	0.480022	7.92518	4.424416
1.620058	5.838499	1.952027	3.425512	3.771349	6.808369	6.570433	4.839105	2.725498
0.077482	0.195563	0.181797	0.710722	0.231768	0.186861	0.113521	0.606847	0.499986
0.000996	35.27608	2.89E-05	0.015	9.101674	1.309084	29.82828	0.05088	0.029062
0.058817	0.047143	0.045411	0.19596	0.016356	0.010101	0.001248	0.506003	0.266032
30.3324	0.08044	9.179466	83.489	2.060491	1.449062	0.066616	14.79742	7.839271
0.516825	2.308203	0.906825	0.015	5.606739	8.381328	1.449062	3.137576	0.029062
1.944517	4.135801	1.771392	2.219863	3.101371	2.975974	2.703485	10.67991	3.926154
0.039214	0.092121	0.240851	0.543398	0.004293	0.078925	0.001248	0.05088	0.319567
7.61176	6.09759	3.8121	3.676032	2.584394	3.052655	1.411861	4.007583	5.864084
29.82828	4.875527	18.48514	12.81638	6.760765	1.45912	1.554185	4.661717	7.327482
3.35355	0.059408	1.631544	2.628225	0.946061	1.780693	0.256797	5.923838	1.501291
1.283059	0.498918	0.418788	34.84299	1.443059	0.010101	0.480022	21.28219	13.53913
0.974257	2.290996	0.625209	0.015	0.173723	2.852677	0.48684	0.05088	0.029062
0.000996	0.0014	2.89E-05	5.062626	0	0.101429	0.001248	0.05088	0.178182
15.88548	0.356508	0.231876	8.103925	0.968066	0.472713	0.331429	2.763261	5.23176
32.31123	32.68313	27.96248	2.693442	26.52537	47.5018	39.53687	1.795851	0.029062
0.12324	0.059408	0.022958	2.509177	0.34619	0.398059	0.169105	4.007583	2.009906
0.12324	0.0014	0.675556	1.313737	0.307973	0.010101	0.066616	0.407403	0.029062
0.039214	0.0014	2.89E-05	0.10132	0	0.186861	0.057208	0.249899	0.266032
0.311046	0.0014	1.046061	0.409336	0	0.010101	0.001248	0.05088	0.178182
0.000996	0.0014	0.100592	0.458521	0.062107	0.010101	0.001248	0.05088	0.029062
0.910339	20.16541	3.921645	2.308203	4.519048	2.569957	6.38456	1.50684	1.168326
0.529971	0.047143	0.350043	1.684646	0.369336	0.078925	0.001248	1.50684	0.178182
0.370657	0.092121	0.181797	0.953506	0.28899	0.327828	0.001248	0.606847	1.501291
0.609509	2.763261	2.283752	0.543398	1.548723	1.492973	3.442886	1.079697	2.361198
1.447994	0.105253	2.114437	0.582208	0.441833	0.010101	0.001248	1.010887	0.62689
0.311046	0.11803	0.469004	3.175916	1.300368	1.042944	0.113521	4.007583	2.725498

1.232078	1.39847	2.126349	6.933961	0.732193	0.237893	0.319163	4.007583	4.863579
2.765758	2.308203	4.077403	2.308203	2.337821	1.400159	0.565714	1.388543	2.440895
0.609509	0.286522	0.165108	1.313737	1.592395	0.262655	0.077547	1.977316	1.290065
0.000996	37.81919	14.97762	0.015	0	0.010101	2.406768	0.05088	29.49693
0.409899	0.105253	0.022958	0.345635	0.587273	2.415296	1.118066	0.05088	0.370462
3.722785	0.347403	0.703665	14.47154	2.855296	0.442193	0.204531	22.52375	14.29947
8.477662	16.09482	2.139928	0.015	17.15902	23.38378	31.92385	0.05088	0.029062
0.437309	0.965065	0.310866	1.363557	0.007417	1.20653	0.541804	10.55571	18.91854
0.039214	0.0014	0.011364	0.246825	0	0.010101	0.001248	0.05088	0.178182
1.472208	1.574517	1.801284	1.263752	1.763889	1.780693	1.831631	0.05088	0.029062
1.296905	3.363911	1.097677	0.458521	0.881082	2.65684	2.597792	0.606847	0.499986
0.876638	0.259964	0.862561	1.000794	0.279177	0.114235	0.144719	0.365859	0.370462
0.088175	0.105253	0.20127	5.409437	0.140765	0.010101	0.144719	6.560411	0.926926
0.370657	0.0014	0.553348	3.256587	0.316126	0.010101	0.001248	0.713846	0.370462
0.609509	0.182237	1.165873	0.50469	1.954993	0.134444	0.307027	1.388543	1.290065
0.287641	0.0014	0.003333	0.10132	0.007417	0.107626	0.048124	0.249899	0.151847
0.000996	0.08044	0.126219	0.246825	0.002107	0.204661	0.157056	0.05088	0.266032
15.66023	9.031082	1.856392	0.015	2.465916	8.888167	10.38202	9.646284	1.168326
0.167128	0.0014	0.327403	0.31557	0.208254	0.176407	0.29671	0.895945	0.697496
0.077482	0.0014	0.231876	0.27969	0.041847	0.24632	0.039134	0.05088	0.697496
4.261342	4.914863	6.153102	1.160707	5.366335	5.591717	6.443925	1.267085	0.218182
0.804675	0.0014	0.126219	0.19596	0.016356	0.010101	0.001248	1.320173	0.319567
0.86469	0.560325	0.539466	1.056898	0.387056	0.010101	0.157056	2.713319	1.591299
4.297222	7.188095	1.699091	0.409336	8.677489	0.472713	11.19942	1.861999	0.029062
0.000996	0.0014	0.016609	0.458521	0.149423	0.352006	0.394156	0.9529	0.432237
1.136566	0.105253	0.581645	1.591299	1.091443	0.271342	0.048124	1.148564	0.499986
3.179199	25.99427	8.195657	4.381479	29.82828	1.322092	19.22139	0.05088	0.029062
0.437309	0.246652	0.418788	1.731169	0.114719	0.010101	0.066616	0.895945	2.944416
0.529971	0.475729	0.862561	11.25392	0.190693	0.010101	0.532619	10.07949	5.088694
11.83593	1.732468	0.772951	1.446926	0.710722	0.010101	2.384141	0.895945	0.432237
0.193723	0.299747	1.030022	1.214315	0.173723	0.078925	0.133297	0.655245	0.697496
0.137302	0.0014	0.292597	0.409336	0.199134	0.307973	0.048124	0.455974	0.218182
0.12324	0.46461	2.89E-05	0.015	0.208254	1.154567	1.554185	0.05088	0.029062
0.000996	9.480736	2.89E-05	0.015	6.900202	2.876421	5.614805	0.05088	0.029062
6.475505	0.299747	1.362713	0.671371	0.353795	0.010101	0.278802	0.05088	0.029062
1.749307	0.454394	0.871429	0.627633	0.215433	0.262655	0.394156	1.861999	2.440895
5.754935	0.486551	7.737807	2.509177	2.642525	0.12132	0.057208	1.010887	2.531515
0.068514	0.0014	0.069834	0.458521	0	0.271342	0.001248	0.283709	0.562807
4.140505	8.019769	4.47912	1.108276	4.323045	1.759986	2.445079	0.05088	1.801284
0.182078	0.047143	0.136356	1.87899	0.022511	0.078925	0.048124	1.669466	0.62689
0.077482	0.069459	0.045411	2.823636	0.004293	0.085498	0.066616	1.010887	1.693939
0.000996	0.0014	0.016609	0.10132	0.001126	0.142698	0.088788	0.326421	0.178182
2.472576	1.129452	1.732468	2.882035	3.5257	0.996111	0.85728	5.123781	3.025671
1.612056	2.936739	3.957734	0.582208	9.792626	1.351573	2.703485	0.05088	0.851659
0.86469	0.246652	2.470317	0.844747	7.653391	0.089769	0.268095	0.558095	0.851659
0.625786	0.221443	1.711414	1.214315	0.332395	0.262655	0.256797	1.267085	0.62689
0.573665	0.997792	0.240851	0.409336	0.772626	2.996869	0.91575	1.320173	0.697496
1.121017	0.0014	0.4971	2.882035	0.307973	0.010101	0.001248	0.655245	0.029062
1.788874	0.092121	0.069834	0.844747	0.632857	0.010101	0.001248	2.92013	1.23127
0.259336	3.266782	0.069834	0.015	1.02798	0.12132	7.08733	1.621032	1.168326
5.591717	0.617071	1.39847	1.160707	0.643853	0.57531	0.100996	2.713319	2.107922
4.222583	1.290065	6.796566	0.345635	2.280101	2.747908	1.417345	0.365859	0.997244
0.666097	2.104358	2.283752	1.781948	0.754315	0.010101	1.272756	1.010887	0.029062
0.370657	0.987821	1.78759	2.164113	1.644416	1.154567	0.443052	2.449509	2.194257
0.33785	287.197	1.305772	0.015	0.834416	530.1465	224.9555	0.05088	2.440895
0.837403	3.803766	0.147244	0.015	1.161501	4.519048	4.323045	0.05088	0.029062
0.560325	0.246652	0.406212	0.752583	0.622496	0.12132	0.066616	0.455974	0.029062
0.000996	62.33472	2.89E-05	0.015	0	0.010101	83.489	0.05088	0.029062

0.822381	0.0014	0.381789	1.87899	0.597417	0.142698	0.001248	2.369841	0.997244
0.245772	0.129553	0.450051	0.015	0.269892	0.010101	0.001248	0.606847	0.029062
2.369841	4.120815	4.580938	25.86296	2.432121	4.47912	2.337821	23.82354	19.53061
4.402973	3.820758	2.410996	5.323492	26.80355	17.39935	4.067547	4.323045	5.568391
0.584134	0.299747	1.128131	4.048499	1.330974	0.459394	0.342049	1.267085	20.94257
5.515584	6.191515	13.00996	3.899993	6.738211	5.720173	2.647244	3.734762	7.911934
2.472576	0.595714	3.043528	2.628225	3.679986	1.062092	0.180079	2.5271	0.218182
8.149307	0.965065	6.508427	0.543398	3.119986	4.783001	1.128131	3.303925	2.944416
10.38202	1.053528	35.776	56.39528	16.09482	0.299062	1.729892	52.56128	52.56128
1.96697	0.3671	1.085967	5.23176	1.02798	1.449062	0.988932	8.149307	7.584502
0.245772	0.0014	0.078297	1.214315	0.120556	0.142698	0.001248	0.606847	0.432237
0.04886	0.338658	0.936898	0.174444	0.532092	1.052208	0.565714	0.249899	0.562807
1.283059	0.259964	0.393449	2.263377	0.694675	0.167273	0.872013	1.079697	4.33342
0.910339	9.377345	3.588369	2.367763	2.373824	2.24562	2.941544	1.795851	2.531515
0.505267	0.0014	0.100592	1.634935	0.279177	0.459394	0.039134	0.407403	1.898153
0.370657	0.746551	0.212496	0.37228	2.625823	0.550974	1.368167	0.05088	2.194257
1.247244	0.768535	1.445916	2.053521	1.394632	0.647345	0.256797	1.727323	1.693939
0.000996	0.0014	0.022958	0.31557	0.002107	0.114235	0.039134	0.655245	0.218182
1.004791	4.863579	3.833247	15.83113	3.885642	2.290996	1.484553	14.21671	4.770231
0.287641	0.910339	0.581645	0.015	0.394156	0.996111	2.406768	0.05088	0.432237
0.299336	0.0014	2.269134	1.000794	0.002107	0.230108	0.039134	0.326421	0.562807
0.696659	5.266335	1.152944	0.409336	2.084791	1.863506	3.998391	1.861999	1.416407
4.003117	0.853752	5.150693	31.55417	5.28039	3.450577	0.474574	16.09482	28.39209
1.296905	5.42381	0.581645	0.918557	3.411097	0.550974	3.266782	0.455974	0.62689
1.217475	0.547128	0.136356	0.015	1.404812	0.620909	1.006623	0.05088	0.697496
4.053506	2.408882	7.92518	4.997417	4.502453	3.367071	2.540462	12.16681	13.83359
8.745238	2.104358	11.48368	12.70768	2.820974	0.299062	1.299567	4.978182	5.864084
0.722626	4.02088	2.922778	0.015	1.962424	0.874091	3.179199	0.05088	0.029062
0.088175	1.742734	0.20127	0.246825	0.279177	3.286652	2.26518	0.326421	1.591299
1.34513	3.663622	1.321053	0.752583	0.895368	2.876421	1.89518	0.895945	0.432237
10.21618	27.96248	20.51271	5.638759	4.802655	12.63773	7.162698	3.486436	2.531515
0.722626	1.932857	0.11811	0.015	0	0.010101	6.933961	0.05088	0.029062
24.73724	17.92188	5.00404	8.728571	17.71696	31.92385	19.99861	5.123781	5.568391
3.233045	11.719	5.259437	2.308203	0.772626	0.010101	9.377345	2.449509	3.175916
0.058817	0.0014	0.006537	0.015	0.002107	0.010101	0.057208	0.05088	1.501291
3.283139	1.099004	0.745613	0.015	1.102691	2.23267	3.070952	0.05088	0.029062
15.3618	1.215195	11.11876	6.933961	6.30671	1.440736	0.144719	4.661717	1.591299
0.33785	0.835498	1.189048	3.425512	0.754315	0.947157	1.803918	4.082165	0.562807
0.088175	0.425137	0.3014	0.345635	1.146962	0.598456	0.256797	0.326421	0.266032
0.926291	1.075411	2.252273	0.015	2.238341	0.934488	1.309084	0.05088	0.029062
0.000996	24.2842	2.89E-05	0.015	0	2.917381	77.74812	0.05088	0.029062
7.92518	95.22939	2.89E-05	0.015	40.7932	56.39528	135.5245	0.05088	0.029062
1.382006	0.11803	0.923874	5.062626	10.57984	1.132857	0.351789	13.60866	6.570433
0.182078	0.0014	0.274704	0.174444	0.402698	0.789423	0.144719	1.213434	0.319567
2.005253	2.290996	2.882035	0.015	0.754315	1.799899	1.465123	0.407403	0.029062
2.392431	4.165137	0.936898	0.015	3.269913	4.38684	14.70188	0.05088	0.029062
0.974257	0.844156	1.534603	3.425512	0.91404	1.237063	0.351789	0.834416	4.241905
5.00404	0.0014	3.779452	2.931068	0.607273	8.815123	0.001248	9.179466	3.025671
7.037345	19.99861	6.00228	2.164113	9.297085	10.8813	11.77567	1.92272	0.319567
0.137302	0.853752	1.058896	0.19596	0.34619	0.398059	0.113521	0.326421	0.218182
2.690823	0.0014	2.382035	0.246825	0.06811	0.010101	0.001248	2.304452	7.417359
24.2842	3.214545	13.23022	10.70538	9.541645	2.688304	1.484553	19.68088	13.08214
1.121017	1.850996	3.022713	0.458521	0.91404	1.186457	0.85728	0.455974	0.218182
0.749481	0.155945	1.771392	1.108276	1.763889	2.018167	0.077547	2.92013	4.978182
0.193723	0.486551	2.831501	3.425512	0.597417	1.154567	0.38285	1.079697	0.926926
0.682359	4.06303	1.905094	0.015	5.351934	7.315512	7.188095	0.05088	0.029062
0.219481	0.0014	0.19127	0.710722	0.096999	0.22096	0.144719	0.407403	1.082179
0.205267	0.155945	1.340981	0.153016	0.035584	0.010101	0.100996	0.9529	0.029062

0.492958	0.893528	0.283276	0.015	0.622496	6.268312	5.47	0.05088	0.029062
1.716364	7.315512	2.016515	3.792266	2.018167	3.367071	6.404921	0.05088	9.218514
5.942294	3.957734	8.849697	13.75978	4.236962	1.94311	1.153723	12.29473	8.412367
27.38726	50.55949	19.99861	0.015	17.92188	14.51648	52.56128	0.05088	0.029062
0.696659	1.783203	1.275325	0.015	1.097006	0.158521	0.846797	0.05088	0.266032
3.621941	3.080317	2.89E-05	0.015	0	0.010101	5.720173	0.05088	0.029062
0.640606	0.195563	1.072843	1.263752	1.921212	0.57531	0.307027	2.248896	1.591299
4.328232	22.32214	6.695887	0.015	2.901032	8.195657	19.37139	0.05088	0.029062
9.707677	0.826407	11.03843	1.405772	12.63773	0.789423	0.001248	9.179466	6.819481
7.737807	9.880779	12.63773	2.308203	20.34063	6.200945	5.560563	5.323492	7.327482
2.126349	1.229553	4.38684	0.458521	5.088694	0.010101	0.001248	2.031169	2.725498
7.964315	5.993045	4.451154	4.667518	22.52375	9.880779	6.865	7.074329	1.082179
0.299336	1.63039	0.33785	0.015	1.320173	0.290866	1.600238	0.05088	0.432237
2.243911	3.175916	0.96938	0.015	2.45575	1.03246	2.337821	0.455974	0.029062
1.311674	0.314156	1.112641	1.781948	0	0.010101	0.001248	0.05088	0.499986
1.266255	2.290996	0.240851	0.31557	1.87482	0.107626	1.816436	0.05088	0.029062
0.452965	0.910339	0.319762	0.918557	2.65684	1.309084	3.025671	1.566414	0.697496
1.555289	2.174733	3.128694	0.710722	1.483622	0.010101	0.619365	1.010887	7.124517
0.100996	0.0014	0.052619	0.27969	0.002107	0.158521	0.001248	0.655245	0.319567
0.000996	1.234444	2.89E-05	0.015	1.268672	9.924372	2.230859	0.05088	0.029062
3.058802	5.538001	8.762677	3.095166	8.180094	5.745996	1.994329	3.263391	0.370462
0.193723	0.644618	0.612872	0.015	1.824892	3.57057	0.60824	0.895945	2.194257
0.452965	0.272814	0.370462	0.409336	0.772626	0.010101	0.001248	6.674286	0.773276
6.967547	6.071436	5.599228	0.953506	3.343629	6.09759	2.520404	1.320173	1.591299
2.784228	6.853608	4.101061	0.246825	3.971061	2.269134	2.950173	0.05088	0.029062
0.000996	0.0014	0.33785	4.603434	0.089769	0.010101	0.001248	1.669466	2.107922
0.370657	0.314156	0.165108	0.37228	0.504416	0.22096	0.001248	1.621032	0.029062
0.86469	3.957734	0.418788	2.102626	1.798535	1.391494	3.037626	0.773766	0.370462
2.021068	0.272814	2.765758	16.22108	1.429365	0.898016	0.077547	4.49671	2.107922
0.850808	0.413449	1.487619	1.363557	0.834416	0.290866	0.361342	2.369841	2.944416
9.082828	3.29355	1.487619	2.628225	5.500678	7.443276	11.06488	2.836717	0.697496
0.205267	0.3671	0.906825	5.754935	4.02088	0.658745	0.443052	2.449509	4.770231
0.452965	0.208571	0.222561	0.115584	0.299553	0.24632	0.088788	1.010887	0.432237
2.425801	10.90615	3.414574	0.015	14.33939	4.02088	43.49658	0.05088	0.029062
1.690483	0.338658	2.339697	5.560563	4.845166	0.388326	0.394156	2.449509	21.93452
0.357727	8.477662	1.605859	2.263377	1.700354	3.511263	4.789596	0.05088	2.009906
0.058817	0.092121	0.037374	1.544517	0.074978	0.010101	0.077547	1.010887	0.151847
1.059553	0.259964	0.612872	0.219885	0.131768	0.010101	0.001248	0.05088	0.029062
0.077482	0.0014	0.581645	0.797114	0.597417	0.010101	0.001248	0.407403	1.168326
0.112771	0.23303	2.89E-05	0.015	1.455988	3.435988	1.638499	0.606847	0.029062
5.80469	0.105253	17.78522	38.39042	0.208254	0.176407	0.088788	43.88335	37.81919
14.29947	16.22108	47.5018	37.81919	48.53429	18.19595	8.815123	53.77619	56.39528
25.73165	13.53913	3.792266	10.85542	14.51648	6.00228	6.486104	5.737056	1.290065
3.699495	0.617071	2.178268	7.162698	2.084791	1.092136	1.286494	6.900202	27.24614
2.472576	2.750375	2.427792	0.671371	1.418312	1.104033	1.753341	1.267085	0.997244
0.287641	0.272814	5.654863	2.440895	0	0.010101	0.001248	5.522929	14.17388
0.100996	0.182237	0.458701	0.19596	0.451169	0.846342	0.191602	0.249899	0.178182
21.19547	15.18102	41.47925	0.015	31.1753	26.25557	75.1132	0.05088	0.029062
0.765606	2.481356	0.33785	0.627633	1.798535	0.828167	4.048499	0.05088	0.029062
4.971991	2.973059	6.508427	1.108276	2.31404	1.369928	0.766118	0.655245	1.23127
5.9071	2.438831	10.1028	18.12541	19.37139	2.296732	1.191905	15.56131	6.374812
0.068514	1.242641	0.231876	0.015	0.055815	0.567619	0.846797	0.05088	0.029062
0.193723	1.209105	2.89E-05	0.015	3.411097	4.02088	47.5018	0.05088	0.029062
2.209141	9.792626	4.540794	0.015	1.656696	60.77449	80.6518	0.05088	0.029062
0.245772	0.0014	0.100592	0.752583	0.165657	0.319026	0.039134	0.606847	0.499986
13.23022	45.1045	8.381328	0.015	6.22031	8.412367	95.22939	0.05088	0.029062
1.86658	0.572987	2.470317	4.933781	5.770996	2.453781	0.216025	6.830592	8.412367
1.217475	1.63039	1.952027	0.015	4.473348	1.415469	0.746082	0.05088	0.029062

1.520346	0.717879	2.868456	1.313737	0.182359	0.010101	0.001248	0.773766	15.46193
1.734949	0.944084	0.581645	0.015	18.76954	0.010101	1.054899	0.05088	0.773276
1.767431	2.973059	0.450051	0.015	1.044863	2.535974	4.888023	0.05088	0.697496
1.889711	10.31025	0.370462	0.015	0	5.591717	33.93939	0.05088	0.029062
6.808369	144.7919	9.583463	0.015	42.08807	332.0776	203.5795	0.05088	0.029062
2.209141	0.0014	3.842554	5.409437	0.803665	0.010101	0.001248	4.402973	25.11292
1.581912	0.676999	3.718716	3.57057	0.722179	0.167273	1.144733	3.904517	3.926154
0.822381	0.272814	5.359134	0.015	1.193362	0.290866	0.001248	4.007583	6.911486
0.287641	0.0014	0.011364	0.409336	0.041847	0.128283	0.039134	2.102626	1.416407
1.136566	0.286522	3.253175	2.628225	1.239423	0.010101	0.144719	0.05088	0.029062
0.039214	0.08044	0.037374	1.495043	0.089769	0.496212	0.191602	1.50684	1.501291
0.000996	91.05541	2.89E-05	0.015	0.784372	10.57984	72.69473	27.96248	0.029062
4.776407	4.552092	3.128694	0.015	7.495397	6.760765	21.19547	0.05088	0.851659
0.955418	0.486551	1.198701	0.710722	0.131768	0.212698	0.225671	1.148564	1.801284
2.765758	2.069942	3.91355	1.056898	1.839986	1.910974	1.335743	2.181775	0.562807
3.864827	0.835498	2.789466	0.50469	1.118817	0.212698	1.729892	0.05088	0.029062
14.6058	1.527792	4.914863	19.83727	11.36975	3.625382	2.219863	26.80355	17.22048
2.947287	0.572987	1.211299	5.923838	1.937222	1.251659	0.766118	4.839105	0.773276
1.232078	0.388023	0.581645	8.958225	4.802655	0.010101	0.755714	14.00414	6.144055
2.640332	0.800058	2.043795	3.57057	1.833102	1.380195	0.708124	1.388543	4.029654
0.039214	27.10502	2.219863	0.10132	0.402698	3.989206	12.16681	0.05088	0.370462
6.808369	0.413449	11.65791	3.57057	0.361717	0.010101	0.268095	3.401126	2.625823
1.716364	9.838658	2.89E-05	0.015	3.243225	19.99861	45.1045	0.05088	0.029062
0.000996	0.059408	0.060729	1.056898	0.022511	0.459394	0.001248	0.455974	2.009906
2.520404	0.707475	2.572309	24.2842	5.024293	0.496212	0.965714	34.84299	30.49289
0.734935	6.355224	2.858009	0.015	2.685786	6.738211	5.287857	0.05088	0.029062
2.047157	0.035758	0.181797	2.053521	0.089769	0.089769	0.077547	4.082165	0.319567
10.33476	27.66779	12.10289	0.015	15.46193	11.48368	17.03919	1.861999	2.361198
4.552092	2.903867	4.115844	3.175916	5.068831	7.795642	5.500678	4.007583	0.370462
0.452965	1.664906	9.751039	0.752583	10.7557	1.237063	1.378362	0.05088	0.499986
0.000996	0.11803	0.3014	0.153016	0.480022	0.352006	0.001248	0.773766	0.499986
35.27608	13.30906	10.82954	38.94854	19.53061	10.12435	11.65791	42.08807	37.29684
0.370657	10.67991	3.747035	0.015	1.213434	3.7586	8.348817	0.05088	0.62689
41.47925	184.443	118.7028	24.85971	67.98069	34.84299	24.2842	11.22667	20.16541
0.625786	0.182237	0.319762	1.544517	0.28899	0.010101	0.113521	1.388543	0.697496
0.765606	7.570693	8.888167	15.1268	12.03267	22.74797	18.05488	3.137576	15.83113
6.518709	30.82277	11.83593	2.219863	10.5063	24.2842	21.74051	1.213434	4.978182
0.393983	0.0014	0.283276	0.671371	0.089769	0.230108	0.001248	0.05088	0.029062
0.000996	0.0014	2.89E-05	0.752583	0	0.010101	0.180079	2.449509	0.432237
1.247244	2.678781	0.612872	0.015	0.487662	1.863506	4.440548	0.05088	2.194257
33.93939	21.54823	3.377619	0.710722	26.25557	30.0001	37.29684	1.795851	3.107417
0.04886	0.0014	0.037374	0.752583	0.041847	0.078925	0.048124	0.606847	0.218182
1.328384	0.644618	0.772951	0.015	0.587273	1.45912	3.025671	0.05088	0.029062
0.492958	0.0014	0.310866	0.671371	0.679452	0.370657	0.331429	6.335664	5.23176
0.955418	0.0014	0.592532	9.218514	0.65241	0.340866	0.100996	0.407403	0.370462
19.83727	3.128694	21.02638	3.899993	6.30671	10.5063	4.091667	3.548153	4.678846
2.569957	0.498918	3.377619	2.567605	1.172208	0.911364	0.38285	3.548153	5.088694
8.412367	8.994791	5.560563	2.74298	4.217569	7.854069	17.64869	2.102626	3.175916
50.55949	155.7103	135.5245	6.25938	60.77449	91.05541	40.7932	7.558139	8.780115
0.112771	0.0014	0.20127	2.006782	0	0.010101	0.225671	3.401126	0.562807
0.04886	0.0014	0.393449	1.544517	0.10938	0.254365	0.057208	1.079697	1.349971
80.6518	20.34063	25.48179	14.88584	55.04081	44.27012	30.49289	18.69833	2.194257
1.581912	1.089394	1.305772	1.214315	1.468131	1.268672	1.025613	0.834416	1.591299
1.652771	0.11803	0.892395	0.246825	0.560043	0.290866	0.307027	1.50684	0.851659
7.276645	0.533535	8.508514	8.83241	4.637994	4.978182	0.886775	20.85876	48.53429
5.984885	0.195563	0.96938	8.381328	2.210866	0.388326	0.144719	10.67991	0.218182
3.381255	5.606739	17.58415	7.897078	3.581688	6.443925	1.137244	13.19051	28.87506
3.890556	0.208571	0.993521	3.962179	1.193362	0.114235	0.278802	3.065108	5.088694

126.1556	15.88548	126.1556	100.0998	35.27608	14.70188	6.830592	64.12971	72.69473
0.33785	0.047143	0.156017	0.797114	0.114719	0.509509	0.077547	0.506003	0.62689
4.125786	2.506934	0.713405	0.31557	2.210866	4.155339	3.921645	1.010887	0.266032
0.000996	0.0014	0.022958	0.219885	0.035584	0.010101	0.001248	0.05088	0.178182
5.056421	0.454394	2.35733	21.02638	1.102691	0.509509	0.523521	30.49289	7.668579
13.42179	3.750837	38.94854	10.31025	8.226219	8.940671	1.575483	2.031169	7.124517
6.967547	2.018167	8.762677	14.17388	3.644343	0.679452	1.841371	16.9777	38.39042
0.469711	0.0014	0.418788	0.797114	0.016356	0.12132	0.001248	2.031169	0.62689
3.031688	2.465916	2.89E-05	0.015	1.608427	3.217612	1.341825	0.05088	0.029062
0.974257	1.195794	1.112641	1.634935	3.101371	0.925801	1.29342	6.414416	0.997244
2.925426	0.208571	1.905094	6.09759	0.803665	0.609177	0.191602	9.276811	9.859719
14.00414	12.10289	8.241342	9.459177	4.990794	20.68746	17.39935	7.276645	7.124517
0.790837	2.27254	0.838846	0.918557	1.348975	0.658745	1.767431	1.213434	0.029062
0.23246	2.489762	0.612872	0.015	1.937222	1.546696	3.005281	0.05088	0.029062
2.07658	0.059408	0.488167	2.628225	1.118817	0.459394	0.541804	2.597792	0.697496
13.91714	17.64869	16.34294	16.09482	38.39042	21.54823	22.52375	6.749488	17.22048
0.77925	5.959033	4.23202	1.544517	9.160382	9.707677	5.165599	0.606847	2.531515
3.921645	0.105253	1.771392	0.153016	0.710722	0.996111	0.191602	0.506003	0.266032
10.67991	4.839105	44.27012	9.771833	8.381328	3.722785	3.816551	12.03267	9.859719
0.393983	118.7028	8.477662	0.27969	9.838658	27.38726	66.9631	0.9529	0.997244
15.07257	1.222958	2.685786	6.663651	0.607273	0.472713	0.755714	6.560411	1.082179
0.272951	0.666097	1.020447	0.458521	0.254574	0.398059	0.494444	0.455974	0.773276
6.153102	0.876638	11.19942	4.381479	5.696263	1.449062	0.532619	4.007583	1.082179
8.226219	0.314156	0.310866	0.31557	8.27267	0.73583	0.307027	0.05088	0.029062
9.946364	12.22798	1.198701	0.015	7.061328	5.838499	11.48368	0.9529	0.029062
18.76954	3.851414	6.09759	11.74734	4.484892	1.471198	2.008312	18.12541	6.911486
6.663651	0.314156	2.065007	19.60574	4.140505	0.620909	0.416501	21.64437	7.911934
0.000996	0.0014	0.011364	0.27969	0.004293	0.204661	0.077547	0.407403	0.370462
37.81919	12.36443	30.49289	28.87506	13.53913	4.140505	7.002056	29.03196	39.53687
7.683766	14.00414	6.335664	0.918557	4.921082	5.266335	3.442886	0.05088	1.290065
0.765606	5.309264	4.821176	0.015	1.182056	5.150693	3.895469	0.05088	0.029062
1.50684	3.585029	1.641999	0.627633	5.838499	9.792626	8.256465	0.655245	0.319567
4.188896	4.667518	5.500678	0.918557	4.261342	1.531313	2.057056	0.606847	1.082179
0.573665	0.454394	0.406212	0.627633	0.394156	0.442193	0.001248	0.895945	0.218182
0.077482	0.11803	0.33785	4.170051	0.074978	0.134444	0.113521	1.388543	1.082179
0.804675	1.880375	1.888023	1.000794	0.607273	0.775339	0.965714	2.248896	1.591299
2.276407	0.533535	2.065007	4.802655	2.698557	0.898016	0.245772	6.335664	3.548153
1.059553	0.0014	1.189048	1.000794	3.799885	0.724502	0.001248	5.923838	5.366335
3.422237	59.32013	2.485534	0.246825	7.417359	53.77619	70.11932	0.558095	0.851659
0.152114	2.367763	0.265786	0.015	1.502323	0.230108	5.530274	0.05088	0.029062
5.754935	0.893528	0.479062	0.953506	1.005426	0.63873	0.216025	1.148564	0.851659
12.85504	2.018167	9.418196	19.99861	10.1459	3.414574	1.389509	36.74522	16.85982
10.21618	2.453781	2.219863	30.0001	2.31404	1.434055	1.335743	2.5271	2.107922
16.09482	3.851414	7.570693	5.143867	11.14619	33.93939	7.780786	5.737056	12.46209
33.4985	9.625253	27.66779	34.38341	43.49658	22.13245	5.654863	33.07147	26.39047
75.1132	8.745238	3.989206	8.728571	28.54786	38.39042	20.85876	35.27608	0.773276
0.272951	13.6814	2.410996	2.628225	2.887489	6.345382	5.779574	2.449509	7.911934
0.259336	0.585281	0.790303	0.543398	3.479134	5.770996	4.580938	0.365859	1.693939
3.8379	5.34474	10.73084	0.132799	7.964315	7.074329	5.044113	0.9529	1.416407
1.266255	0.935743	3.043528	0.50469	12.1974	0.759646	0.144719	1.795851	11.2819
0.424004	0.286522	0.505786	1.160707	0.968066	0.352006	0.157056	1.669466	5.864084
0.000996	0.0014	0.052619	0.174444	0.055815	0.142698	0.001248	0.407403	0.62689
22.32214	2.538218	4.33342	0.246825	9.160382	4.888023	2.114437	0.455974	0.562807
9.459177	6.808369	18.91854	1.405772	22.32214	7.625209	4.23202	3.137576	2.725498
0.000996	0.0014	2.89E-05	0.246825	0.002107	0.22096	0.057208	0.326421	0.266032
0.167128	0.0014	0.283276	0.31557	0.199134	0.158521	0.113521	1.320173	0.266032
36.74522	33.07147	184.443	28.23632	33.07147	5.366335	9.646284	32.31123	38.94854
0.765606	0.533535	3.877417	2.367763	1.502323	0.911364	0.100996	5.42381	8.149307

0.077482	0.155945	0.029704	0.10132	0.001126	0.12132	0.066616	1.452857	1.082179
25.99427	22.13245	72.69473	77.74812	24.04374	6.508427	4.067547	45.1045	49.50903
7.61176	4.978182	5.20267	6.727633	3.581688	2.349351	1.791616	2.181775	6.011515
0.205267	0.047143	0.022958	1.405772	0.048225	0.128283	0.268095	0.506003	4.029654
2.898196	0.246652	2.178268	6.819481	0.369336	0.176407	0.057208	9.646284	16.09482
3.980123	0.155945	0.693218	1.731169	0.316126	0.582208	0.268095	4.839105	0.319567
184.443	44.27012	252.4251	111.5439	184.443	67.98069	36.26061	126.1556	118.7028
17.03919	3.098268	11.60283	12.81638	14.00414	3.034747	3.276335	4.49671	5.23176
43.49658	7.964315	30.82277	22.96463	20.16541	13.91714	6.30671	33.93939	19.14516
0.381999	0.347403	0.871429	0.37228	2.053521	2.890245	2.325931	0.05088	0.029062
6.287561	135.5245	167.9996	0.671371	83.489	70.11932	184.443	1.795851	4.863579
21.93452	14.25954	18.62711	13.38418	14.84163	9.101674	6.423911	25.24367	15.61077
2.765758	2.630729	5.123781	4.484892	1.227749	4.650007	1.335743	2.369841	6.454416
4.115844	0.129553	1.007864	6.019971	2.174733	0.703665	0.331429	7.074329	2.285664
2.147013	0.400173	2.203535	3.095166	3.172864	3.150433	1.037316	7.92518	4.33342
16.68554	3.243225	16.9162	2.509177	8.815123	5.9071	2.114437	13.45939	6.819481
17.39935	2.750375	105.1694	52.56128	6.30671	2.607035	0.532619	38.39042	126.1556
1.022929	0.0014	9.583463	1.263752	0.028939	0.010101	0.001248	3.065108	0.926926
10.1028	2.27254	3.330909	20.68746	12.85504	64.12971	8.780115	7.558139	7.765931
27.66779	16.9162	38.39042	167.9996	37.81919	12.03267	12.22798	135.5245	252.4251
100.0998	18.19595	62.33472	30.49289	44.27012	14.17388	10.05618	17.78522	0.926926
4.721789	0.400173	6.153102	4.125786	6.443925	0.262655	0.802395	11.42512	24.73724
13.53913	13.83359	15.3618	29.18885	13.91714	17.28195	3.957734	55.04081	35.27608
1.690483	2.308203	3.080317	0.015	5.568391	1.546696	0.662237	1.452857	0.029062
28.39209	7.264502	4.155339	13.96064	12.42965	21.02638	21.93452	12.89577	9.218514
4.851227	0.259964	1.812114	3.676032	3.119986	0.819978	0.278802	6.414416	2.361198
2.164113	6.045209	1.046061	1.924228	1.069574	1.682193	5.18754	0.05088	0.029062
91.05541	36.74522	17.15902	0.37228	95.22939	77.74812	49.50903	0.9529	0.997244
1.652771	0.0014	1.78759	10.01009	0.114719	0.010101	0.001248	17.51961	19.75908
0.955418	0.585281	1.305772	1.313737	0.580123	0.262655	0.191602	1.452857	2.873817
5.630815	7.683766	3.192229	2.882035	0.410823	20.16541	14.17388	0.05088	0.029062
0.822381	1.169091	2.139928	0.094278	10.05618	1.062092	0.468694	0.05088	0.151847
1.121017	0.560325	1.935685	6.518709	0.387056	0.299062	0.216025	2.102626	8.524242
3.33728	3.439437	4.272165	1.000794	2.723247	2.429957	4.067547	1.148564	0.319567
0.029978	0.0014	2.89E-05	0.543398	0	0.082323	0.001248	0.326421	0.218182
42.08807	8.164531	43.49658	5.143867	16.22108	8.849697	7.430317	20.42667	17.92188
749.7839	57.84882	287.197	530.1465	1360.293	43.49658	24.73724	425.62	425.62
167.9996	34.84299	111.5439	135.5245	425.62	87.1746	45.91841	224.9555	184.443
1.078153	0.129553	0.734524	5.062626	3.726854	0.484423	0.331429	0.773766	1.290065
19.68088	52.56128	30.17192	16.9777	23.275	45.91841	40.11561	16.9777	15.61077
0.437309	0.0014	1.198701	1.363557	0.417532	0.010101	0.001248	1.621032	1.349971
3.327619	2.349351	8.288874	7.456118	2.855296	1.828781	2.673846	19.83727	4.029654
0.469711	0.314156	0.745613	14.65384	0.299553	0.703665	0.157056	5.323492	10.98644
3.058802	0.23303	0.069834	0.27969	0.487662	0.089769	0.048124	0.407403	0.218182
0.12324	0.0014	0.060729	2.567605	0.048225	0.010101	0.039134	0.455974	0.029062
48.53429	1.960945	4.188896	3.256587	8.762677	7.710361	3.548153	23.6012	8.303947
57.84882	5.388405	4.49671	20.51271	17.71696	18.48514	9.792626	34.38341	3.175916
0.469711	0.0014	0.069834	0.671371	0.261775	0.459394	0.088788	1.079697	0.926926
77.74812	80.6518	51.52195	10.70538	77.74812	203.5795	66.9631	12.16681	12.89577
0.058817	0.0014	0.016609	2.053521	0	0.142698	0.001248	1.010887	0.266032
135.5245	12.42965	8.923117	0.19596	20.68746	10.98644	8.461703	5.323492	0.029062
0.891176	0.0014	0.592532	1.056898	0.028939	0.010101	0.001248	0.895945	0.029062
4.776407	2.174733	1.558571	4.381479	2.567605	0.307973	2.089639	2.304452	6.011515
0.259336	0.475729	0.772951	2.693442	0.710722	0.010101	0.001248	1.566414	0.029062
0.000996	0.035758	0.003333	0.797114	0.001126	0.010101	0.001248	1.566414	0.997244
0.370657	0.0014	1.416407	1.446926	3.06803	0.281169	0.123535	1.320173	0.029062
55.04081	33.4985	31.92385	55.04081	37.29684	55.04081	23.82354	56.39528	64.12971
4.49671	7.795642	18.19595	15.98892	8.994791	10.82954	6.508427	8.477662	40.11561

11.719	6.55039	32.68313	6.09759	7.136537	3.350224	4.413608	13.60866	14.97762
15.56131	5.662994	7.92518	26.95429	9.880779	7.112496	3.55961	24.16397	20.34063
1.153723	2.713319	1.097677	0.19596	7.882222	2.174733	1.638499	0.05088	6.011515
0.000996	0.0014	0.003333	0.132799	0.002107	0.095772	0.077547	0.249899	0.697496
46.7056	5.462475	48.53429	25.48179	15.07257	12.39704	8.745238	45.91841	20.68746
15.46193	38.39042	19.06893	7.938427	13.6814	15.66023	21.36892	5.123781	9.859719
0.543939	0.756176	4.302367	0.582208	1.290981	1.222958	0.846797	2.763261	10.01009
0.696659	2.162381	3.471486	0.219885	1.468131	1.251659	1.77404	0.455974	0.851659
1.944517	1.374329	4.609076	1.833102	0.922338	1.434055	1.006623	1.079697	2.361198
7.443276	5.80469	12.36443	24.50942	13.23022	6.808369	2.54961	37.81919	15.22851
7.212915	0.338658	2.369841	5.923838	2.981962	0.911364	0.191602	14.51648	5.366335
3.750837	7.136537	2.043795	2.628225	3.317807	1.492973	1.016494	0.05088	1.591299
19.451	5.210317	21.19547	45.1045	16.80343	5.351934	6.933961	21.02638	32.31123
155.7103	8.226219	6.8421	2.164113	19.22139	5.622872	13.6814	5.821804	2.194257
1.175094	0.047143	0.060729	3.734762	0.035584	0.010101	0.001248	3.401126	0.370462
16.44768	29.49693	20.34063	0.015	11.30987	8.573434	7.991602	2.5271	0.029062
0.205267	0.035758	0.222561	1.87899	0.131768	0.010101	0.057208	1.148564	0.997244
26.52537	0.51039	26.25557	91.05541	46.7056	4.56912	0.809986	87.1746	43.49658
0.804675	0.069459	1.374329	4.048499	1.710173	0.819978	0.673651	18.69833	4.678846
2.176501	8.319019	5.704646	1.313737	8.888167	32.31123	23.6012	0.05088	0.266032
0.409899	0.377814	0.274704	6.819481	0.062107	0.352006	0.775339	14.383	31.1753
6.345382	0.893528	0.797114	0.015	0.895368	0.010101	1.857677	0.05088	0.029062
22.52375	0.436638	2.339697	5.84689	12.42965	5.704646	4.764055	17.22048	22.13245
0.370657	0.047143	0.381789	2.219863	0.279177	0.237893	0.001248	0.455974	0.562807
2.961768	6.830592	26.52537	1.833102	12.10289	19.68088	11.96574	0.655245	1.23127
12.67271	15.07257	4.23202	3.629177	5.545729	9.179466	25.99427	5.22482	4.33342
0.077482	0.069459	0.136356	0.153016	0.055815	0.271342	0.001248	0.05088	0.178182
9.160382	1.302107	7.598312	3.734762	5.737056	2.23267	1.272756	8.905642	11.11876
0.347186	1.474343	8.677489	3.425512	2.181775	0.996111	0.077547	1.213434	8.089848
1.612056	0.617071	1.8386	0.132799	0.410823	0.150974	0.066616	0.249899	0.151847
0.955418	1.863506	0.862561	0.543398	0	3.877417	4.667518	0.05088	0.029062
2.327857	1.053528	8.958225	0.627633	0.803665	0.57531	0.319163	0.773766	1.898153
6.808369	7.013853	144.7919	11.57341	5.238701	7.339452	5.00404	8.365072	31.55417
2.243911	0.79487	3.747035	332.0776	6.967547	0.724502	1.554185	184.443	287.197
1.247244	0.902403	2.252273	1.405772	1.127475	0.271342	0.718355	1.213434	1.290065
0.100996	0.0014	2.89E-05	95.22939	0.238478	0.724502	0.001248	70.11932	80.6518
0.584134	0.987821	0.265786	11.96574	0.182359	0.967446	0.225671	18.41211	32.68313
0.640606	0.780087	3.022713	0.627633	1.889711	0.158521	0.85728	1.010887	0.773276
0.000996	0.0014	0.052619	0.50469	0.011551	0.010101	0.001248	0.283709	0.029062
4.46267	0.208571	0.458701	1.684646	0.035584	2.050404	0.001248	1.92272	10.60361
1.716364	0.780087	1.679596	0.015	1.203427	0.703665	0.001248	4.580938	2.873817
0.205267	0.059408	0.265786	0.115584	0.441833	0.010101	0.001248	0.558095	0.029062
1.620058	0.987821	2.685786	0.015	2.292864	0.703665	0.982128	0.05088	0.178182
0.077482	0.092121	0.078297	5.062626	0.22303	0.010101	0.001248	3.904517	5.23176
0.039214	0.0014	2.89E-05	0.246825	0.022511	0.254365	0.133297	0.834416	0.029062
0.765606	0.092121	0.181797	2.219863	0.361717	0.837403	0.319163	1.977316	2.285664
0.492958	2.08798	1.416407	0.844747	1.824892	1.652771	2.621133	0.05088	0.178182
0.505267	0.876638	0.370462	2.628225	0.5229	0.254365	0.898543	1.669466	1.082179
0.000996	25.24367	2.89E-05	0.015	2.74298	1.019134	24.50942	0.05088	0.029062
0.272951	8.677489	3.618499	0.10132	1.049517	4.217569	4.392316	0.326421	0.499986
14.29947	75.1132	24.04374	2.693442	57.84882	100.0998	105.1694	1.795851	0.997244
0.039214	0.182237	0.327403	0.458521	0.096999	0.010101	0.100996	0.558095	0.319567
0.167128	0.105253	0.381789	0.37228	0.238478	0.319026	0.048124	0.407403	0.499986
1.247244	0.560325	1.247244	6.404921	1.394632	1.639639	0.685931	6.125823	0.432237
4.096407	1.180613	1.699091	33.07147	5.150693	1.092136	1.389509	22.96463	13.30906
0.112771	9.297085	11.36975	0.015	0.431977	1.33285	5.01772	0.05088	1.23127
0.000996	0.047143	0.016609	0.19596	0.001126	0.142698	0.066616	0.365859	0.499986
1.812114	2.786898	1.222179	0.797114	2.388348	0.134444	1.465123	0.05088	0.029062

3.179199	3.7586	1.848297	0.015	2.047157	2.216162	4.286962	0.558095	0.029062
0.595714	4.120815	2.065007	0.015	2.047157	0.010101	7.037345	0.05088	0.029062
0.543939	0.0014	1.618773	0.582208	0.10938	0.010101	0.474574	0.365859	3.243225
6.028427	4.419062	11.30987	2.567605	6.423911	5.439278	4.23202	0.9529	0.697496
5.530274	5.770996	1.262179	0.015	4.251818	2.589185	3.934986	0.05088	1.898153
0.23246	0.221443	1.831631	1.108276	1.615281	0.230108	0.216025	2.449509	0.370462
0.000996	1.129452	2.810678	0.153016	0.861429	4.251818	0.662237	0.05088	4.33342
0.86469	0.129553	0.981032	2.978968	0.114719	0.167273	0.426645	2.304452	4.863579
2.673846	4.620159	4.869553	8.019769	3.930664	7.200505	4.72798	7.697063	17.58415
0.23246	4.292078	2.459812	0.31557	1.592395	0.582208	6.52899	0.05088	1.898153
21.02638	32.31123	27.38726	2.102626	32.68313	17.15902	14.33939	0.9529	0.499986
1.382006	0.730476	1.362713	1.160707	1.044863	0.509509	0.879423	0.9529	1.23127
0.000996	0.0014	0.109293	0.19596	0.001126	0.010101	0.001248	0.506003	0.218182
0.822381	0.129553	0.532619	2.102626	0.459394	0.529697	0.001248	2.369841	0.697496
0.000996	48.53429	0.052619	0.31557	0.10381	0.010101	56.39528	0.506003	0.029062
0.000996	3.401126	2.89E-05	0.015	0.361717	0.010101	2.760765	0.05088	0.029062
6.663651	21.02638	13.91714	4.745895	10.82954	13.00996	8.134084	1.566414	2.285664
7.112496	9.179466	0.993521	0.015	9.377345	14.08569	26.80355	0.05088	0.029062
0.722626	0.475729	0.981032	1.263752	0.246162	0.669358	0.245772	0.407403	0.319567
0.000996	33.93939	2.89E-05	0.015	6.518709	0.010101	60.77449	0.05088	0.029062
0.000996	13.08214	0.147244	0.015	0	0.114235	167.9996	0.05088	0.029062
8.539971	3.207807	8.333918	30.0001	3.083319	3.921645	2.647244	12.36443	14.6058
1.472208	0.696659	1.605859	4.048499	0.881082	0.472713	0.157056	4.72798	6.454416
1.944517	0.625786	1.321053	4.667518	0.560043	0.327828	0.088788	2.763261	1.501291
1.328384	10.73084	7.882222	0.015	2.698557	1.702706	9.256537	2.661587	0.029062
1.103261	0.533535	2.621133	1.363557	2.676392	1.222958	0.180079	2.661587	1.898153
0.23246	0.893528	0.265786	0.219885	0.316126	0.010101	0.941104	0.05088	0.029062
0.437309	0.585281	0.693218	0.132799	0.125354	0.713405	0.60824	0.05088	0.029062
17.92188	49.50903	25.73165	4.857403	11.19942	28.23632	31.55417	6.475505	2.107922
2.047157	5.560563	7.08733	0.015	2.914632	1.910974	2.197828	0.05088	0.029062
0.193723	1.482626	1.139221	0.671371	0.410823	0.352006	1.426522	0.05088	0.029062
1.040411	11.14619	2.89E-05	0.015	1.039199	1.702706	7.38847	0.05088	0.029062
1.059553	1.222958	0.319762	0.015	0.165657	0.010101	0.952294	0.05088	1.801284
1.022929	5.22482	3.175916	0.409336	1.763889	1.251659	4.413608	0.05088	0.029062
0.505267	1.770029	1.487619	0.19596	2.221775	0.299062	0.576638	0.05088	0.029062
0.182078	0.182237	0.651631	1.960945	0.055815	0.010101	0.001248	0.606847	0.029062
0.822381	0.730476	0.923874	0.174444	0.732193	0.647345	0.619365	0.365859	0.266032
2.569957	0.347403	1.046061	6.518709	1.889711	0.809986	0.225671	7.495397	4.424416
17.78522	19.68088	12.03267	0.345635	8.849697	0.352006	1.916782	0.05088	0.029062
0.000996	0.105253	0.136356	8.83241	0.041847	0.560325	0.001248	19.06893	11.4544
1.217475	0.666097	0.571342	11.48368	0.881082	3.008225	0.802395	15.1268	6.685087
0.974257	2.953059	0.592532	0.015	0.269892	2.415296	2.797835	0.05088	0.029062
0.543939	12.03267	1.39847	0.752583	7.519841	22.52375	16.62402	0.05088	0.029062
0.469711	0.246652	0.359502	0.37228	0.261775	0.398059	0.38285	0.05088	0.266032
0.000996	3.606804	0.283276	3.33728	0.643853	0.340866	3.330909	1.079697	0.029062
0.058817	0.035758	2.89E-05	0.115584	0.041847	0.114235	0.113521	0.05088	0.218182
0.529971	0.377814	0.488167	0.627633	0.451169	0.262655	0.066616	0.895945	1.082179
0.152114	1.850996	0.693218	0.015	0.378066	5.522929	3.618499	0.05088	0.029062
1.986681	1.412771	0.772951	16.5625	1.742734	0.262655	2.29873	21.28219	3.243225
2.109755	0.413449	1.962424	14.08569	12.93651	0.230108	0.001248	6.830592	4.49671
0.000996	0.0014	2.89E-05	0.153016	0.016356	0.095772	0.225671	0.455974	1.23127
4.188896	43.49658	11.11876	0.015	30.49289	62.33472	155.7103	6.674286	13.30906
0.926291	1.252424	1.112641	0.10132	4.690159	2.747908	1.32482	1.079697	0.499986
0.058817	0.059408	0.022958	3.031688	0.011551	0.204661	0.100996	4.155339	2.009906
7.375794	3.283139	31.1753	51.52195	4.845166	0.010101	1.600238	91.05541	155.7103
2.209141	2.996869	1.404812	0.015	4.307309	4.821176	7.824473	0.05088	0.029062
56.39528	38.94854	33.93939	1.591299	30.17192	37.81919	51.52195	0.05088	7.584502
0.86469	0.0014	2.554069	21.83751	0.208254	0.010101	0.039134	8.694697	45.51146

0.000996	0.129553	0.016609	0.627633	0	0.089769	0.685931	0.05088	0.178182
1.175094	0.606421	0.651631	6.663651	14.6058	1.277056	1.359177	9.989221	13.83359
0.696659	0.756176	0.20127	2.823636	3.993665	0.22096	0.952294	8.556703	1.801284
0.560325	0.11803	0.406212	4.381479	0.551284	0.010101	0.001248	4.978182	2.625823
0.000996	0.0014	0.11811	0.015	0.007417	0.010101	0.001248	0.05088	0.029062
0.000996	17.51961	1.112641	0.015	5.933066	7.653391	18.48514	0.05088	0.029062
1.652771	7.288788	1.848297	2.367763	0.732193	2.950173	5.614805	0.05088	1.349971
0.696659	0.23303	0.782857	0.627633	0.784372	0.63873	0.169105	9.082828	2.725498
6.028427	5.687879	7.977958	1.214315	5.439278	7.683766	3.566753	0.606847	0.499986
0.560325	0.676999	1.152944	8.019769	1.279452	0.863189	0.673651	5.671126	8.958225
34.38341	10.1028	28.23632	44.27012	14.84163	24.98218	12.49452	46.7056	62.33472
0.409899	1.031284	0.458701	1.160707	1.097006	0.692027	1.054899	4.49671	3.726854
0.219481	0.08044	0.173456	0.582208	0.074978	0.12132	0.169105	0.506003	0.432237
0.182078	0.0014	0.052619	0.015	0.004293	0.010101	0.001248	0.558095	0.562807
0.850808	0.617071	1.262179	5.923838	0.65241	0.567619	0.416501	1.861999	1.349971
12.42965	6.876392	9.968355	13.96064	27.81514	5.976724	2.445079	6.475505	0.926926
9.541645	0.129553	5.84689	2.164113	0.10938	0.010101	0.048124	2.369841	0.370462
5.889798	0.168312	3.35355	2.978968	2.410996	1.734949	0.319163	9.276811	2.009906
0.749481	1.169091	0.592532	0.153016	0.679452	1.546696	2.114437	0.05088	0.697496
5.165599	5.704646	19.60574	36.74522	5.395584	6.365065	3.548153	36.26061	34.38341
23.16622	9.480736	0.086219	0.015	0	7.402915	6.784762	0.05088	0.029062
1.040411	0.314156	0.871429	3.175916	0.089769	0.947157	0.157056	2.248896	0.029062
0.000996	0.0014	2.89E-05	0.458521	0.002107	0.281169	0.001248	0.283709	0.178182
3.422237	0.129553	2.091313	0.582208	3.101371	0.852684	0.100996	1.452857	3.321284
0.167128	0.0014	0.173456	0.409336	0.055815	0.010101	0.039134	0.283709	0.218182
0.393983	0.155945	0.526566	0.582208	0.394156	0.370657	0.216025	1.669466	0.697496
67.98069	12.56693	57.84882	65.94551	18.33909	30.0001	14.51648	43.88335	22.96463
0.100996	0.0014	0.231876	2.882035	0.254574	0.010101	0.001248	0.9529	0.029062
0.100996	0.047143	0.006537	0.31557	0	0.010101	0.048124	0.283709	0.178182
0.182078	0.86518	0.212496	3.425512	0.732193	1.925635	2.990851	5.42381	4.424416
16.22108	0.0014	8.033932	6.612121	11.68846	0.010101	0.001248	7.92518	5.864084
1.788874	2.713319	1.460981	4.745895	0.182359	1.601443	7.545584	0.05088	0.029062
0.000996	19.53061	1.247244	0.015	0	9.459177	25.24367	0.05088	0.029062
0.193723	1.652771	0.957273	0.797114	0.199134	0.101429	1.232886	0.05088	0.319567
1.078153	0.730476	0.957273	0.015	1.279452	1.083651	2.131176	0.05088	0.029062
1.86658	0.0014	2.382035	2.77899	2.668896	0.809986	0.216025	3.734762	11.86763
1.059553	0.0014	3.098268	2.367763	0.361717	0.010101	0.169105	0.05088	4.33342
0.137302	0.0014	0.231876	0.797114	0.028939	0.775339	0.001248	0.326421	1.23127
2.07658	2.567605	7.456118	10.07949	1.78759	2.62342	1.600238	6.028427	14.29947
0.560325	1.149322	0.439293	0.015	0.332395	0.934488	0.37228	0.05088	0.029062
0.876638	0.520678	2.847547	10.07949	0.441833	0.010101	0.342049	3.401126	9.082828
1.672107	1.640779	2.445079	0.015	5.462475	1.322092	1.626782	1.388543	0.029062
0.152114	0.059408	0.126219	0.953506	0.007417	0.010101	0.001248	0.407403	0.319567
3.058802	5.591717	4.058514	0.015	16.34294	19.06893	14.25954	0.05088	2.531515
0.029978	0.059408	0.022958	0.27969	0.299553	0.290866	0.361342	0.05088	0.178182
3.310758	1.605859	0.249278	0.710722	1.213434	0.459394	9.880779	0.05088	0.029062
3.35355	6.454416	3.957734	3.425512	1.146962	1.006025	0.4357	1.92272	0.697496
0.939408	11.30987	0.029704	0.015	0.732193	0.703665	2.069942	0.05088	0.029062
2.496407	0.347403	0.222561	1.781948	0.182359	0.078925	0.133297	2.181775	0.851659
0.000996	11.65791	0.212496	0.710722	0.784372	0.010101	4.626025	0.05088	0.029062
2.164113	0.666097	1.234444	7.339452	5.976724	1.570794	0.216025	11.09133	8.958225
3.699495	16.44768	4.435159	0.015	4.02088	10.38202	16.09482	0.05088	0.029062
5.381227	7.710361	3.651898	2.823636	25.11292	2.269134	15.77678	1.92272	1.349971
0.822381	0.0014	3.632973	9.082828	2.069942	0.010101	0.001248	0.895945	3.4679
2.091313	0.338658	3.45404	2.978968	1.513102	0.010101	0.361342	4.839105	3.107417
1.749307	0.059408	0.923874	2.693442	0.643853	0.378737	0.100996	3.599329	2.009906
0.000996	8.711905	2.89E-05	0.409336	0.614156	7.751869	7.339452	0.05088	0.029062
0.000996	2.621133	2.89E-05	0.015	1.87482	3.24658	2.973059	0.05088	0.029062

3.058802	5.01772	10.23768	0.37228	6.171789	5.745996	1.865043	1.452857	1.693939
2.496407	0.314156	5.359134	3.734762	0.968066	0.658745	0.38285	5.923838	5.23176
2.708341	4.370671	5.024293	11.17281	2.802828	1.842734	1.954993	20.42667	10.17029
0.482078	0.446291	0.562107	6.335664	1.360022	2.705916	1.404812	6.335664	20.94257
0.891176	1.574517	1.007864	0.015	0.513752	2.396429	0.245772	0.05088	0.029062
0.287641	10.43066	1.631544	0.015	0.968066	0.010101	8.103925	0.05088	0.926926
0.790837	13.00996	3.401126	2.882035	15.3618	10.93101	18.19595	2.99987	2.194257
3.367071	11.48368	4.914863	1.634935	5.654863	0.158521	7.854069	0.05088	0.029062
4.312251	4.307309	7.402915	0.174444	3.052655	1.910974	2.432121	0.365859	0.029062
0.077482	12.93651	1.662439	0.671371	6.622092	17.03919	26.52537	0.05088	0.029062
2.873817	0.876638	1.718918	2.440895	4.435159	0.898016	0.65241	5.671126	10.60361
0.790837	1.664906	5.22482	0.27969	1.523824	2.556443	2.6143	1.727323	0.029062
0.804675	2.255916	1.980592	0.015	0.946061	0.724502	1.513102	0.05088	0.029062
0.058817	0.327994	0.625209	0.132799	0.762641	0.863189	0.766118	1.010887	0.62689
1.103261	0.413449	1.007864	0.345635	1.118817	0.010101	0.048124	1.621032	6.011515
1.555289	0.105253	1.518232	2.164113	0.532092	0.078925	0.113521	1.669466	1.501291
0.584134	0.0014	4.620159	0.797114	0.34619	0.010101	0.001248	1.079697	0.029062
1.600238	0.23303	3.895469	0.409336	1.548723	0.863189	0.268095	0.05088	0.029062
2.316075	0.208571	0.806227	3.500808	0.316126	0.957273	0.225671	3.340361	2.944416
2.54961	0.486551	0.923874	0.015	1.062821	0.63114	2.760765	0.558095	0.029062
0.000996	2.145137	0.359502	0.015	0	3.119986	28.23632	0.05088	0.029062
0.926291	1.099004	2.312085	0.710722	4.863579	0.378737	1.729892	0.05088	0.029062
0.167128	0.606421	0.675556	0.345635	2.254084	0.352006	0.191602	0.407403	0.266032
0.33785	0.259964	0.109293	1.056898	0.215433	0.598456	0.204531	2.92013	2.625823
3.944084	11.60283	0.469004	0.015	2.421681	14.97762	9.012937	0.05088	0.029062
2.408882	6.250447	5.330519	0.115584	8.508514	5.210317	3.198499	0.05088	0.178182
0.137302	1.261407	0.086219	0.015	0.5229	0.996111	1.39847	0.506003	0.029062
0.955418	3.471486	2.554069	1.000794	3.726854	3.327619	3.011169	0.05088	0.432237
0.609509	0.105253	1.020447	3.846984	0.361717	0.010101	0.001248	3.303925	3.548153
0.039214	0.46461	0.450051	0.115584	0.513752	0.496212	0.468694	0.05088	0.926926
0.219481	3.028629	1.935685	0.31557	1.592395	2.705916	2.308203	0.558095	0.029062
0.543939	5.287857	2.89E-05	0.015	4.946587	2.138131	4.468009	0.05088	0.029062
3.089293	4.174964	6.423911	3.962179	4.655916	3.536955	3.533211	3.904517	135.5245
0.666097	0.272814	1.679596	0.409336	0.10938	0.176407	0.307027	1.148564	1.290065
0.23246	0.314156	0.517359	0.246825	0.190693	0.540902	0.088788	0.455974	0.266032
0.910339	0.327994	0.581645	5.754935	0.394156	0.340866	0.066616	1.861999	1.898153
0.469711	1.160707	0.713405	0.153016	1.172208	0.167273	0.38285	0.05088	0.029062
0.974257	3.098268	1.534603	0.37228	6.054242	3.707136	2.369841	0.326421	0.029062
0.000996	0.0014	2.89E-05	0.27969	0.089769	0.167273	0.001248	0.558095	0.266032
0.058817	0.155945	0.078297	0.132799	0.035584	0.082323	0.001248	0.283709	0.178182
1.50684	3.930664	10.62739	1.108276	2.567605	2.008312	1.025613	1.50684	2.944416
3.599329	1.358189	4.678846	0.458521	2.483492	0.828167	0.37228	0.606847	1.168326
8.061934	5.158146	80.6518	20.16541	29.49693	2.925426	0.256797	13.19051	91.05541
0.876638	0.182237	1.888023	0.015	0.632857	0.010101	0.256797	0.655245	0.029062
0.039214	0.0014	0.109293	0.015	0.10938	0.107626	0.088788	0.283709	0.266032
1.004791	11.83593	2.252273	0.015	0.772626	6.933961	11.36975	0.05088	0.029062
10.1028	1.972706	2.394416	0.015	10.65365	1.682193	14.6058	0.05088	0.029062
0.86469	2.472576	2.666494	0.015	1.203427	0.010101	2.243911	0.05088	0.029062
0.100996	0.0014	0.165108	0.31557	0.007417	0.095772	0.048124	0.05088	0.029062
0.000996	0.047143	2.89E-05	0.174444	0.022511	0.134444	0.048124	0.713846	0.432237
0.790837	1.566414	1.699091	0.844747	1.626782	2.647244	2.683478	0.455974	0.029062
20.68746	9.256537	6.796566	0.015	13.45939	17.78522	10.23768	0.895945	0.029062
0.926291	0.876638	0.479062	0.31557	0.441833	1.20653	1.006623	0.326421	0.773276
10.93101	4.959358	0.946638	0.015	2.647244	2.465916	6.277937	0.05088	0.029062
0.482078	0.788369	1.085967	0.015	0.954776	0.010101	2.292864	0.713846	0.773276
2.611797	2.914632	2.810678	14.47154	3.536955	2.296732	2.252273	8.905642	7.327482
0.393983	0.0014	0.592532	4.667518	0.324913	0.010101	0.001248	1.795851	2.285664
7.92518	0.0014	8.677489	67.98069	1.017107	0.010101	0.144719	19.37139	50.55949

2.109755	2.061926	1.487619	0.844747	1.213434	0.947157	7.882222	0.773766	1.23127
0.058817	0.0014	1.416407	0.153016	0.016356	0.101429	0.088788	0.365859	0.029062
2.737944	0.246652	2.178268	4.048499	1.005426	0.281169	0.077547	2.369841	0.029062
0.272951	1.32482	0.936898	0.015	0.946061	0.57531	0.443052	0.05088	0.029062
0.000996	0.400173	0.310866	0.582208	0.47246	0.010101	4.212554	0.05088	0.029062
3.031688	4.524481	1.618773	0.27969	1.455988	1.391494	2.727785	0.895945	0.432237
0.259336	0.0014	0.052619	2.219863	0.048225	0.010101	0.001248	1.213434	0.62689
2.834084	2.698557	3.23987	0.015	2.520404	8.940671	1.191905	0.05088	0.029062
0.245772	1.770029	0.33785	1.684646	0.560043	0.361717	0.819048	3.904517	2.944416
5.439278	10.62739	2.970144	0.015	17.03919	6.38456	15.46193	0.05088	0.029062
0.393983	0.168312	0.069834	0.015	0.157619	1.639639	1.351573	0.05088	0.029062
0.000996	21.74051	10.5063	0.015	0.532092	12.56693	21.54823	0.05088	0.62689
4.721789	17.28195	2.6143	8.590794	6.591544	11.719	17.28195	9.480736	9.707677
0.609509	0.047143	0.539466	0.345635	0.048225	0.114235	0.100996	0.283709	0.029062
0.516825	3.343629	0.54619	0.543398	0.671371	3.263391	3.703175	0.05088	0.029062
12.29473	12.49452	5.295325	0.015	20.51271	13.38418	9.924372	0.05088	0.029062
1.232078	1.118066	0.612872	0.015	0.614156	0.692027	3.38456	0.05088	0.029062
0.311046	0.780087	1.460981	0.27969	0.710722	3.482785	4.597792	0.05088	1.082179
5.500678	31.92385	5.538001	0.015	5.864084	41.47925	11.719	0.655245	0.029062
0.272951	0.142511	1.387612	0.409336	0.332395	0.010101	0.001248	0.05088	0.029062
0.560325	1.622006	0.249278	0.31557	1.091443	0.010101	0.474574	0.558095	0.029062
5.28039	1.489524	7.710361	14.65384	1.968283	3.68381	0.268095	7.824473	18.48514
3.283139	17.39935	19.60574	1.544517	3.750837	10.19468	11.544	1.669466	0.029062
0.370657	1.776739	0.20127	0.246825	0.120556	1.20653	1.600238	0.365859	1.082179
0.749481	2.740519	1.518232	0.27969	1.947316	2.607035	1.535722	0.365859	0.266032
3.548153	0.105253	1.631544	1.591299	0.299553	0.010101	0.001248	1.795851	1.591299
3.233045	0.0014	2.485534	2.102626	1.193362	0.010101	0.001248	5.737056	1.290065
6.663651	4.261342	5.813247	1.056898	3.699495	3.625382	5.779574	0.05088	0.697496
2.860722	2.308203	0.571342	0.015	1.080909	1.251659	2.114437	0.05088	0.029062
3.655714	1.019134	3.540606	3.095166	0.754315	1.287403	0.503146	5.522929	7.519841
0.100996	0.105253	0.022958	0.582208	0.048225	0.010101	0.001248	0.773766	0.029062
0.000996	0.0014	0.871429	0.015	0.22303	0.010101	0.001248	0.05088	0.029062
0.167128	5.238701	1.007864	0.015	1.250058	2.834084	1.118066	0.05088	0.029062
0.709784	5.082063	0.993521	0.27969	0.279177	1.669466	2.893001	0.05088	0.499986
2.673846	1.272756	2.02956	1.056898	1.069574	0.24632	0.541804	0.455974	0.562807
2.925426	1.580909	3.747035	0.752583	11.89932	1.369928	0.697085	1.566414	2.285664
0.492958	4.012049	0.109293	0.015	0.120556	0.010101	11.25392	0.05088	0.029062
24.98218	12.70768	67.98069	46.7056	87.1746	135.5245	3.016984	40.7932	36.26061
6.570433	1.004791	3.175916	2.102626	1.182056	0.846342	0.930837	2.304452	2.531515
2.447294	0.142511	0.458701	0.19596	0.096999	0.010101	0.001248	0.05088	0.029062
0.666097	0.0014	0.539466	1.781948	0.299553	0.101429	0.144719	2.369841	2.531515
1.472208	5.409437	0.892395	0.015	0.10938	0.319026	3.346898	0.05088	0.029062
5.671126	2.887489	6.876392	1.405772	2.388348	2.535974	1.455988	1.669466	0.926926
2.640332	1.215195	2.160649	0.710722	1.017107	0.010101	1.341825	0.05088	0.029062
1.533535	0.314156	1.020447	7.625209	1.02798	0.58925	0.113521	2.597792	0.029062
13.30906	11.03843	15.46193	36.0183	23.6012	27.10502	6.230245	22.13245	18.33909
0.219481	4.94026	1.631544	8.477662	0.560043	0.450375	2.099524	0.9529	0.029062
0.452965	0.129553	1.78759	0.37228	0.215433	0.262655	0.191602	0.283709	0.029062
1.153723	1.732468	1.139221	2.053521	2.768312	0.550974	1.054899	1.320173	0.926926
2.737944	2.481356	0.734524	6.663651	0.906126	4.47912	2.259661	3.263391	6.685087
2.304452	3.904517	1.699091	0.409336	1.813564	1.995714	3.290101	0.05088	0.029062
0.259336	1.722475	0.126219	0.37228	1.300368	1.33285	0.809986	0.05088	0.62689
0.04886	0.047143	0.003333	0.174444	0.055815	0.204661	0.235296	0.249899	0.218182
1.555289	4.888023	4.47912	0.918557	4.46267	1.94311	1.886335	0.05088	0.997244
15.18102	7.545584	8.333918	1.160707	15.18102	15.98892	12.56693	0.713846	0.370462
2.882035	2.375974	8.164531	0.458521	1.502323	1.006025	1.253752	1.727323	2.194257
0.325317	0.46461	1.058896	0.582208	0.34619	0.254365	0.133297	0.506003	1.168326
0.370657	0.0014	3.192229	0.246825	0.028939	0.010101	0.001248	0.05088	0.029062

2.973059	1.479452	1.944517	2.263377	0.679452	0.496212	0.523521	4.155339	4.678846
0.167128	0.286522	0.20127	1.160707	0.007417	0.089769	0.066616	2.304452	3.926154
0.000996	2.104358	0.109293	0.015	0.208254	0.010101	0.48684	0.05088	0.029062
0.000996	7.049336	0.265786	0.132799	3.256587	2.671299	4.690159	0.05088	0.029062
0.137302	1.901068	1.597908	0.015	1.161501	0.010101	1.099762	0.05088	0.029062
0.393983	0.0014	0.359502	2.567605	0.480022	0.176407	0.048124	1.079697	0.151847
4.529913	1.195794	0.936898	0.115584	1.376378	1.546696	1.499214	0.365859	0.178182
0.167128	0.853752	0.147244	0.219885	0.246162	0.57531	1.286494	0.05088	0.851659
0.584134	1.449877	0.517359	0.015	0.424004	3.877417	2.760765	0.05088	0.562807
0.709784	3.022713	0.96938	0.015	2.112843	0.010101	0.394156	0.05088	0.029062
1.296905	10.03313	4.597792	0.015	0.871429	9.335397	15.276	0.05088	0.029062
0.219481	0.0014	0.147244	1.313737	0.208254	0.254365	0.001248	1.977316	2.107922
7.013853	0.965065	4.46267	3.57057	2.876421	0.920483	0.416501	3.401126	1.290065
1.153723	1.945916	1.112641	1.495043	6.706472	5.381227	0.204531	0.773766	1.23127
4.353925	0.696659	2.586926	57.84882	1.991328	0.692027	0.755714	42.75576	60.77449
1.944517	5.976724	2.23267	0.015	6.011515	3.799885	7.264502	0.606847	0.029062
44.27012	2.408882	53.77619	64.12971	14.17388	6.55039	1.215195	118.7028	95.22939
1.402915	0.142511	1.823377	0.797114	3.457504	0.352006	0.048124	1.267085	1.168326
4.881775	5.388405	0.826407	2.509177	2.465916	2.498478	14.42661	0.05088	0.029062
530.1465	87331.38	77.74812	1.924228	252.4251	1360.293	1360.293	4.661717	0.562807
0.000996	1.261407	0.165108	1.544517	0.983384	2.62342	3.457504	2.836717	0.773276
0.152114	0.0014	0.359502	0.27969	0.096999	0.082323	0.001248	0.05088	0.029062
3.207807	3.037626	16.09482	5.638759	6.011515	3.169812	2.243911	6.028427	25.73165
8.888167	39.53687	14.17388	1.214315	11.544	59.32013	32.68313	8.033932	0.029062
0.804675	0.246652	2.666494	1.160707	0.622496	0.319026	0.29671	5.056421	3.321284
8.940671	14.97762	14.51648	1.405772	2.981962	5.210317	10.60361	0.05088	0.029062
29.18885	7.991602	2.531515	1.495043	2.855296	72.69473	59.32013	1.621032	0.029062
0.492958	4.997417	3.45404	0.27969	2.50469	1.287403	2.014863	0.05088	0.029062
0.000996	1.32482	0.052619	0.37228	0.754315	0.010101	1.162201	0.05088	0.62689
1.456955	2.255916	6.922771	8.412367	2.855296	4.323045	1.505678	5.056421	4.770231
1.091443	0.520678	3.8121	3.899993	2.950173	0.319026	0.123535	1.795851	3.243225
19.99861	9.924372	13.83359	13.38418	15.66023	10.05618	5.409437	13.08214	27.24614
2.219863	0.155945	0.359502	4.048499	0	0.010101	0.001248	7.276645	3.632973
0.482078	1.252424	1.007864	0.582208	1.300368	0.809986	0.864199	0.05088	0.218182
40.7932	23.06543	42.42191	1.363557	17.28195	20.51271	15.88548	0.05088	0.029062
4.650007	0.0014	1.416407	27.66779	2.74298	1.780693	0.001248	40.11561	1.290065
0.04886	0.059408	0.037374	0.19596	0.016356	0.167273	0.001248	0.326421	0.319567
12.67271	1.722475	6.297136	17.45948	7.212915	4.783001	2.040606	26.12492	2.810678
3.263391	5.158146	2.369841	3.33728	2.349351	0.529697	0.416501	8.905642	7.013853
11.19942	4.644098	5.576219	0.582208	4.709033	5.838499	3.330909	0.326421	0.370462
4.096407	2.489762	3.640606	2.931068	3.514834	0.713405	1.575483	2.248896	0.432237
0.077482	0.0014	1.305772	0.409336	0.096999	0.212698	0.032727	0.558095	0.773276
1.34513	1.691587	1.8386	1.87899	1.644416	1.734949	0.516306	4.227302	3.175916
0.424004	0.182237	0.418788	1.000794	0.254574	0.142698	0.057208	0.407403	0.432237
0.245772	0.595714	0.553348	0.345635	0.339466	0.417345	0.256797	0.606847	0.62689
0.077482	0.0014	0.016609	0.27969	0	0.12132	0.523521	1.148564	0.926926
1.672107	1.820541	18.76954	0.458521	1.005426	0.967446	2.014863	1.079697	0.562807
0.86469	2.770866	0.100592	0.015	0.542525	2.216162	3.321284	0.05088	0.029062
2.327857	1.833102	1.097677	0.015	2.901032	22.32214	3.691724	0.05088	1.290065
1.296905	3.975498	4.170051	0.015	18.48514	3.536955	17.15902	0.05088	0.029062
0.734935	1.45912	3.253175	0.19596	4.043846	9.604358	3.606804	0.05088	0.029062
6.125823	20.68746	3.957734	0.015	8.134084	9.502294	26.25557	2.304452	0.029062
1.078153	3.544257	2.016515	0.015	0.662237	1.052208	1.411861	0.895945	0.997244
0.393983	1.031284	0.756724	3.734762	0.662237	0.920483	0.836537	4.277006	5.23176
0.325317	0.746551	0.274704	0.153016	0.190693	0.398059	0.586934	0.05088	0.029062
7.868146	1.138557	7.264502	16.9777	1.742734	0.658745	0.4357	12.46209	12.26136
0.287641	0.08044	4.63189	1.833102	2.210866	0.425635	0.066616	1.079697	6.250447
0.469711	0.0014	0.532619	11.74734	0	0.010101	0.001248	2.92013	4.49671

2.005253	0.314156	1.50026	2.823636	5.11697	0.669358	0.82868	9.356371	1.801284
12.93651	21.36892	4.419062	1.544517	3.820758	50.55949	42.75576	3.137576	3.243225
18.48514	6.612121	5.454949	13.75978	10.7557	6.486104	3.714906	0.05088	2.725498
4.650007	1.913997	7.519841	20.85876	3.279625	0.388326	1.062821	23.275	17.22048
0.609509	4.043846	1.558571	0.844747	1.721371	1.019134	3.223882	0.05088	0.432237
0.609509	0.987821	2.518124	0.543398	0.269892	0.775339	0.268095	0.895945	0.997244
0.722626	0.46461	1.007864	0.31557	4.609076	0.692027	0.732633	0.606847	0.851659
0.137302	0.0014	0.19127	2.567605	0.10381	0.370657	0.225671	3.401126	0.266032
0.311046	0.299747	0.370462	8.905642	0.22303	0.852684	0.100996	8.27267	2.361198
1.716364	3.046551	2.065007	0.015	4.563117	4.272165	2.308203	1.388543	0.029062
0.424004	0.221443	1.072843	0.458521	0.513752	0.186861	0.066616	0.506003	0.697496
0.357727	0.0014	0.562107	0.409336	0	0.010101	0.001248	0.05088	0.851659
2.520404	1.751948	2.638139	5.638759	0.607273	1.072843	0.864199	3.486436	10.17029
0.452965	2.061926	1.152944	0.19596	0.299553	0.472713	0.416501	0.05088	0.029062
0.347186	0.221443	0.517359	0.50469	0.074978	0.010101	0.001248	0.606847	0.851659
4.650007	2.506934	5.238701	2.628225	1.89961	2.569957	1.226118	2.597792	2.944416
0.837403	3.220678	0.136356	0.246825	0.496494	0.669358	0.996111	0.05088	0.029062
23.6012	11.89932	1.058896	4.802655	2.388348	4.72798	5.030866	0.05088	0.029062
8.103925	0.0014	4.869553	3.57057	0.082323	0.010101	0.001248	0.895945	1.416407
20.34063	1.711414	6.738211	26.52537	1.483622	0.911364	1.449062	15.77678	14.08569
0.682359	0.0014	0.3014	0.37228	0.048225	0.010101	0.001248	0.407403	0.029062
0.409899	0.0014	0.327403	0.015	0.131768	0.010101	0.001248	0.05088	0.029062
0.088175	2.212619	0.265786	0.627633	0.324913	0.692027	1.994329	0.05088	0.029062
0.357727	0.0014	1.165873	0.015	0.826407	0.010101	0.001248	0.05088	1.416407
27.10502	4.198117	19.37139	32.31123	9.418196	4.02088	1.513102	24.16397	40.7932
0.469711	0.413449	3.330909	1.108276	1.182056	0.658745	0.268095	1.50684	2.194257
1.382006	3.140866	2.091313	1.405772	2.600014	8.256465	9.968355	0.05088	0.029062
0.058817	0.035758	2.89E-05	0.115584	0.041847	0.114235	0.113521	0.05088	0.218182
1.632648	1.790245	3.080317	1.731169	2.359264	2.786898	2.572309	0.655245	0.62689
4.440548	19.06893	10.33476	3.792266	24.50942	24.73724	14.00414	5.614805	4.678846
1.059553	0.635036	0.946638	3.33728	1.193362	0.212698	0.001248	5.323492	11.74734
18.05488	4.752121	6.028427	0.153016	11.03843	3.833247	2.718434	0.05088	0.029062
0.100996	0.059408	0.136356	1.313737	0.011551	0.150974	0.001248	0.606847	0.151847
0.347186	1.374329	0.292597	0.246825	1.592395	0.679452	0.632569	0.283709	0.029062
0.409899	0.299747	1.475469	3.962179	0.035584	0.010101	0.001248	0.05088	0.499986
20.85876	19.83727	5.915469	1.591299	12.70768	30.49289	34.84299	0.05088	4.575123
0.137302	0.035758	0.011364	1.495043	0.022511	0.433131	0.29671	1.795851	1.168326
0.000996	0.86518	0.006537	0.174444	0.055815	0.433131	0.29671	0.05088	0.178182
9.625253	7.225469	17.28195	1.495043	12.42965	4.696313	3.223882	3.548153	0.029062
3.890556	2.27254	1.331919	1.313737	0.929812	0.142698	2.089639	0.05088	0.029062
3.422237	28.54786	0.11811	0.015	0.976667	1.995714	53.77619	0.05088	0.029062
4.297222	10.98644	2.160649	0.015	3.243225	5.477843	5.9071	0.05088	0.029062
1.96697	1.175094	2.091313	1.544517	2.520404	0.837403	1.241811	1.320173	1.801284
2.898196	2.845036	1.234444	1.495043	0.542525	1.322092	2.205476	0.05088	0.62689
16.85982	2.438831	10.01009	30.82277	2.410996	2.312085	0.982128	67.98069	23.82354
0.790837	3.930664	0.782857	3.676032	3.489899	6.063276	7.938427	3.065108	3.926154
[truncated: 596,739 more chars]
